# Structure and variation of the mitochondrial genome of fishes

**DOI:** 10.1186/s12864-016-3054-y

**Published:** 2016-09-07

**Authors:** Takashi P. Satoh, Masaki Miya, Kohji Mabuchi, Mutsumi Nishida

**Affiliations:** 1 Atmosphere and Ocean Research Institute, The University of Tokyo, 5-1-5 Kashiwanoha, Kashiwa City, Chiba 277-8654 Japan; 2 Collection Center, National Museum of Nature and Science, 4-1-1 Amakubo, Tsukuba City, Ibaraki 305-0005 Japan; 3 Natural History Museum and Institute, 955-2 Aoba-cho, Chuo-ku, Chiba City, Chiba 260-8682 Japan; 4 Present address: Seto Marine Biological Laboratory, Field Science Education and Research Center, Kyoto University, 459 Shirahama, Nishimuro, Wakayama 649-2211 Japan; 5 Present address: University of the Ryukyus, 1 Senbaru, Nishihara-cho, Okinawa 908-0213 Japan

**Keywords:** Mitochondrial genome, Fish, Comparative genomics, Gene rearrangement

## Abstract

**Background:**

The mitochondrial (mt) genome has been used as an effective tool for phylogenetic and population genetic analyses in vertebrates. However, the structure and variability of the vertebrate mt genome are not well understood. A potential strategy for improving our understanding is to conduct a comprehensive comparative study of large mt genome data. The aim of this study was to characterize the structure and variability of the fish mt genome through comparative analysis of large datasets.

**Results:**

An analysis of the secondary structure of proteins for 250 fish species (248 ray-finned and 2 cartilaginous fishes) illustrated that cytochrome c oxidase subunits (COI, COII, and COIII) and a cytochrome bc1 complex subunit (Cyt b) had substantial amino acid conservation. Among the four proteins, COI was the most conserved, as more than half of all amino acid sites were invariable among the 250 species. Our models identified 43 and 58 stems within 12S rRNA and 16S rRNA, respectively, with larger numbers than proposed previously for vertebrates. The models also identified 149 and 319 invariable sites in 12S rRNA and 16S rRNA, respectively, in all fishes. In particular, the present result verified that a region corresponding to the peptidyl transferase center in prokaryotic 23S rRNA, which is homologous to mt 16S rRNA, is also conserved in fish mt 16S rRNA. Concerning the gene order, we found 35 variations (in 32 families) that deviated from the common gene order in vertebrates. These gene rearrangements were mostly observed in the area spanning the ND5 gene to the control region as well as two tRNA gene cluster regions (IQM and WANCY regions). Although many of such gene rearrangements were unique to a specific taxon, some were shared polyphyletically between distantly related species.

**Conclusions:**

Through a large-scale comparative analysis of 250 fish species mt genomes, we elucidated various structural aspects of the fish mt genome and the encoded genes. The present results will be important for understanding functions of the mt genome and developing programs for nucleotide sequence analysis. This study demonstrated the significance of extensive comparisons for understanding the structure of the mt genome.

**Electronic supplementary material:**

The online version of this article (doi:10.1186/s12864-016-3054-y) contains supplementary material, which is available to authorized users.

## Background

The mitochondrial (mt) genome is essential for life in almost all eukaryotes. Mt genomes in vertebrates are compact, generally spanning 16–17 kbp in size, and the repertoire of encoded genes is extremely conserved. Genes typically encoded in the vertebrate mt genome are those for 13 proteins, two ribosomal RNAs (rRNAs), and 22 transfer RNAs (tRNAs) with two noncoding regions, the control region (CR), and the origin of L-strand replication (O_L_). The gene order of the mt genome also tends to be conserved among vertebrates for 37 genes and two noncoding regions, which are basically arranged in the same order from hagfish to eutherian mammals [1–4].

As whole mt genome sequence data from various vertebrates have been accumulated, various variations have been revealed concerning the features of genes on these genomes such as the start/stop codons of protein-coding genes and tRNA gene structure [5–10]. In addition, many cases of variation in gene order have also been found in vertebrate mt genomes [10–16]. However, most of previous studies have just reported specific variations without thorough comparisons so that the overall picture of the structural variability of the vertebrate mt genome is not well understood. Meanwhile, mt genomes have been used as effective tools for phylogenetic and population genetic analyses in vertebrates [17–21]. Moreover, some mt genome mutations are known to be related to human serious disease (eg MELAS and MERRF) [22–25]. It is therefore important to clarify details regarding variability in the vertebrate mt genome.

An effective method toward this goal is to conduct comprehensive comparisons of mt genome data from many representative species belonging to various phylogenetic groups within a group of a higher taxonomic rank such as subclass. In this study, we report the results of our comprehensive characterization of variability of structure of the mt genomes of ray-finned fishes (Class Actinopterygii, *sense* Nelson [26]). This group comprises nearly 99 % of the 30,000 known species of fishes and more than half of all vertebrates. The reason why we have focused on ray-finned fishes is twofold. First, the available mt genome data are abundant for ray-finned fishes. Although whole mt genomes had been sequenced for only 15 species of the group 15 years ago, since then, the number of genomes has increased to 1,847 species (2 coelacanths, 11 cyclostomes, 5 lungfishes, 100 cartilaginous fishes, and 1,729 ray-finned fishes: 01 August 2015; in the NCBI Organelle Genome Resources, http://www.ncbi.nlm.nih.gov/genomes/OrganelleResource.cgi?opt=organelle&taxid=7742) due to the development of experimental methods [27]. Second, robust phylogeny, which is indispensable for comparative analyses, is established for this group. Currently, we have comprehensive molecular phylogenies with a broad consensus [20, 21, 28–30]. Using mt genome sequence data from a total of 250 species (248 ray-finned fishes and 2 cartilaginous fishes), we conducted a detailed observation and comparative analysis of the basic structure and arrangement of genes. The mt genomic sequences of 28 of the 250 species are reported for the first time in this study.

## Methods

### Taxonomic sampling of fish mt genome data

Fish comprise a paraphyletic group consisting of three major groups: jawless fishes (approximately 100 species), cartilaginous fishes (approximately 970), and bony fishes (approximately 27,000), the latter of which is divided into two classes, namely lobe-finned fishes (such as coelacanths and lungfishes) and ray-finned fishes (such as gars, sturgeons, and teleosts). In particular, ray-finned fishes comprise an extremely diverse and abundant group consisting of 44 orders, more than 453 families, and nearly 27,000 species, representing half of all living vertebrates [26].

To cover the entirety of ray-finned fishes, we performed taxon sampling mainly using data registered in the database under the following conditions: (1) cover half of the total number of families in each order and (2) when half of the families cannot be covered, two or more species are added to the analysis from the family that can be obtained easily. Consequently, 42 orders (approximately 95.5 %), 208 families (approximately 45.9 %), and 248 species (approximately 0.9 %) of all ray-finned fishes were chosen (Additional file 1: Table S1). A dataset composed of 250 fish mt genomes (2 cartilage fish and 248 ray-finned fishes) was used for comparison analysis.

### Materials, DNA extraction, sequencing, and editing data

Of the aforementioned 250 species, data for 28 ray-finned fishes are reported in this study for the first time at the family level (Additional file 1: Table S1). A portion of the epaxial musculature (ca. 0.25 g) was excised from fresh specimens of each species and immediately preserved in 99.5 % ethanol. Total genomic DNA was extracted using a DNeasy tissue kit (Qiagen) or Gentra Puregene tissue kit (Qiagen) following the manufacturer’s protocol.

The mt genomes were amplified in their entirety using a long PCR technique [31]. Eight fish-versatile long PCR primers were used in various combinations to amplify the entire mt genome in two reactions. The long-PCR products were diluted with TE buffer (1:19) for subsequent uses as PCR templates.

A total of 178 fish-versatile PCR primers were used in various combinations to amplify contiguous, overlapping segments of the entire mt genome, and 32 species-specific primers were designed for several species. A list of PCR primers used in this study is available from TPS upon request. Long PCR and subsequent short PCR were conducted as previously described in the literature (eg, Miya and Nishida [27]; Inoue et al. [32]). Double-stranded PCR products, purified using ExoSAP-IT enzyme (USB), were subsequently used for direct cycle sequencing with dye-labeled terminators (Applied Biosystems). Primers used were the same as those for PCR. All sequencing reactions were performed according to the manufacturer’s instructions. Labeled fragments were analyzed on a Model 3100/3130xl DNA sequencer (Applied Biosystems).

The DNA sequences were edited and analyzed with AutoAssembler version 2.1 (Applied Biosystems) and DNASIS-Mac version 3.7 (Hitachi Software Engineering Co. Ltd.). The locations of the 13 protein-coding genes were determined by comparisons of the DNA or amino acid sequences of fish mt genomes. The 22 tRNA genes were identified by their cloverleaf secondary structures and anticodon sequences. These secondary structures were assessed with DNASIS-Mac ver. 3.7 (Hitachi Software Engineering). The two rRNA genes were identified by sequence similarity and secondary structure [33].

### Comparative analysis of mt genome data

#### Protein-coding gene

For 13 protein genes obtained from 250 fishes, we compared genetic features (eg, start/stop codons and gene length) based on the nucleotide sequences. The structural characteristics of 13 proteins were also compared on the basis of the amino acid sequences as follows. First, the amino acid sequences of the 13 proteins for 250 fishes were aligned using CLUSTAL X [34]. We next compared the secondary structures of the 13 proteins as estimated by the SOSUI program [35] using alignment data of the amino acid sequence and information from UniProt database [36], which is the world’s most comprehensive catalog of information on proteins. In the comparative analysis, we regarded a mode of amino acid sequence length of each protein (modal length) as a standard value to specify the positions of variation sites. As cytochrome *c* oxidase subunit I (COI) and a cytochrome *bc*1 complex subunit (Cyt *b*) were advanced in structural analysis among the 13 proteins, we discussed structural features in the fish mt genome with reference to the UniProt for both genes, especially Esposti et al. [37] for Cyt *b*.

#### tRNA gene

The sequences of the tRNA genes were aligned visually for every stem and loop regions, and their structural features (eg, base frequency in each nucleotide position) were surveyed. The typical secondary structure for nuclear tRNA genes was estimated, and each nucleotide position of the structure was numbered 1–73 from the 5’- to 3’-ends [38]. We followed this numbering method with some modification. Because of large length variations in loop regions of mt tRNA genes, we used numbers up to 83 (instead of 73), which was the maximum total number of nucleotides obtained by summing the largest number of nucleotide of each loop region in all tRNAs. The base frequencies in each nucleotide position were obtained on the basis of this positioning. The occurrence of nucleotide pair types in 21 pair sites common to all 22 tRNAs (clarified in a subsequent section) was also summarized. The standard secondary structures of fish mt tRNAs were estimated by integrating the obtained results.

#### rRNA gene

We used Japanese whiting (*Sillago japonica*) as a representative species for depicting the core secondary structure of fish rRNAs. The rRNA sequences of 250 fishes were aligned using CLUSTAL X. The secondary structures of 12S rRNA and 16S rRNA were estimated with reference to Wang and Lee [39] and Burk et al. [40], respectively. We considered that a potential base pairing property must occur in at least 75 % of examined fishes (75 % rule) according to Springer and Douzery [41]. In identifying potential base pairing to recognize stems, we allowed noncanonical G–U interactions (wobble base pairs) in addition to standard Watson-Crick base pairs (A–U and G–C). Stems were delimited in our proposed model by bilateral bulges of two or more base pairs (unilateral bulges were allowed in the context of a single stem) according to Wang and Lee [39].

#### Noncoding regions

The sequences of the CR were aligned using CLUSTAL X. CR includes the regulation and initiation sites of mt genome replication and transcription [42]. We focused on four conserved sequence blocks (CSB-I, CSB-II, CSB-III, and CSB-D), which were reported to be conserved in the CRs of mammals and fishes [43–45]. The sequence of CSBs was identified via alignment and comparison with the human CR sequence.

The sequences of the O_L_, which is related to L-strand replication, were aligned visually. O_L_ has a stem-loop secondary structure within a tRNA gene cluster (WANCY region). The secondary structure was assessed using DNASIS-Mac ver. 3.7 (Hitachi Software Engineering). The characteristics of the O_L_ (eg, base composition and conserved sequence motifs) were surveyed.

#### Phylogenetic framework for gene rearrangement analysis

To discuss the evolutionary features of gene rearrangements of fish mt genomes, we created an order-level consensus tree from recent results of molecular phylogenetic studies of ray-finned fishes [20, 21, 28–30, 32]. Because there was not big difference between the nuclear genome tree and the mt genome tree at the order level, we created the consensus tree which covered all the orders of fishes by combining those topologies (Fig. 10). Nodes inconsistent among them were indicated with multiple branching in the tree.

## Results and discussion

### Organization of the fish mt genome

**Fig. 1:**
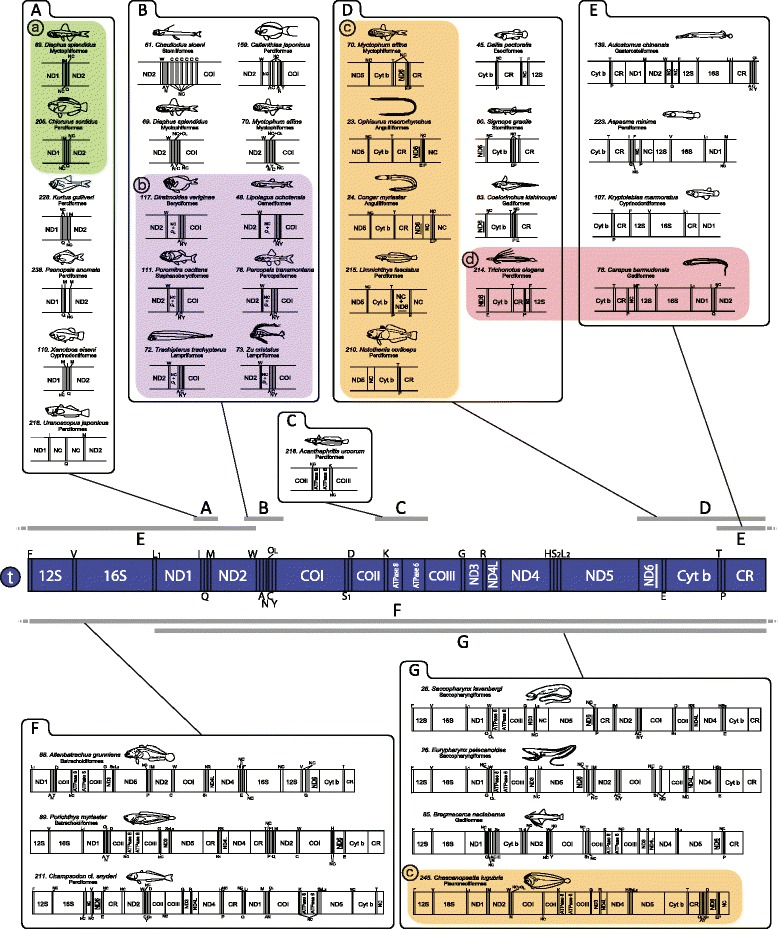
Linearized representation of the typical vertebrate gene order (circled t) and rearranged gene orders observed in fishes. All protein-coding genes are encoded on the H-strand with the exception of ND6 (underlined), which is encoded on the L-strand. Transfer RNA (tRNA) genes are designated by single-letter amino acid codes, and those encoded on the H- and L-strands are presented above and below the gene map, respectively. Capitalized **A**–**G** denote mt genome regions involved in major gene rearrangements. Coloured shadings (circled a-d) indicate fish groups that share the same unique gene order at least in part, and are related to Fig. 10. Numerals in front of the species name are the same as those in Additional file 1. 12S and 16S, 12S and 16S ribosomal RNA genes, respectively; ATPase 6 and 8, ATPase subunit 6 and 8 genes, respectively; COI–III, cytochrome *c* oxidase subunits I–III genes, respectively; CR, putative control region; Cyt *b*, cytochrome b gene; L1, L2, S1, and S2, tRNA Leu (UUR), tRNA Leu (CUN), tRNA Ser (UCN), and tRNA Ser (AGY) genes, respectively; NC, noncoding sequences of ≥50 bp; ND1–6 and 4 L, NADH dehydrogenase subunit 1–6 and 4 L genes, respectively

The 250 fish mt genomes compared in this study contained 37 genes (13 protein coding, 22 tRNA, and 2 rRNA genes) and 2 noncoding regions (CR and O_L_), as typically found in other vertebrates, with the exception of *Limnichthys fasciatus* (Barred sand burrower), the ND6 gene of which was not identified (Fig. 1: D: 215). The gene may be sandwiched between two CR-like regions as found in the mt genome of notothenioids fishes, whose ND6 gene was first missed and then found in the sandwiched region [46]. The ND6 gene in *Limnichthys fasciatus* may also have been missed during the PCR or sequence assembly. This possibility should be examined by genomic hybridization analysis. In addition, as observed in other vertebrates, most genes were encoded on the H-strand, excluding the ND6 gene and eight tRNA genes on the L-strand. In the following cases, sequences were not completely determined owing to existence of a long homopolymer (eg, TTTTTT…) in the mt genome that prevented sequencing reactions: the tRNA-Pro gene of *Lampris guttatus* (opah), the 12S rRNA gene of *Brama japonica* (Pacific pomfret), the ND1 gene of *Synbranchus marmoratus* (Marbled swamp eel), and the CR of 68 fishes.

The 37 genes were arranged in the same order as in the typical vertebrate mt genome in 214 fishes (Fig. 1), whereas gene rearrangements were found in the remaining 36 fishes (clarified later in the text). We observed intergenic regions with a few bases to a few dozens of bases as well as genes in which a few bases overlapped, as in the typical vertebrate mt genome (clarified later in the text).

The base composition of the mt genes of 250 fishes is shown in Tables 1 and 2. The mean base composition of the L-strand was as follows: A, 28.3 %; C, 28.7 %; G, 16.6 %; and T, 26.5 % (C ≈ A > T > G). This composition was similar to that of other vertebrate mt genomes (eg, Asakawa et al. [47]). In the protein-coding genes, although the first codon positions did not display deflection of the base composition, the second codon positions exhibited a proportion of lower G residues and higher proportion of T residues. There was an anti-G bias in the third codon positions, as noted in typical vertebrates. The RNA genes were generally GC rich in the stem regions but A rich in the loop regions (Table 2). It is thought that the high content of GC in the tRNA and rRNA stem regions stabilizes the secondary structure of these RNAs, as the hydrogen bonding strength of G–C is higher than that of A–T. The CR was found to be AT rich, as reported in other vertebrates [48, 49] (Table 2). In the O_L_ region, the stem was GC rich, whereas that of the loop was A rich. This base composition bias was similar to that of H-strand coding tRNAs.

**Table 1:** Average base composition (%) of the 13 protein-coding genes in mt genomes of 250 fishes

	Total				1st position				2nd position				3rd position			
	A	T	G	C	A	T	G	C	A	T	G	C	A	T	G	C
H-strand coded																
ND1	25.0	28.3	15.5	31.3	23.8	20.8	26.3	29.1	17.1	41.7	11.6	29.7	33.8	22.4	8.5	35.3
ND2	27.2	25.9	13.2	33.7	30.4	18.1	20.6	31.0	15.6	39.0	11.1	34.4	35.7	20.4	8.1	35.8
COI	24.7	29.7	18.4	27.2	24.9	22.0	30.9	22.2	18.1	40.5	15.0	26.3	30.8	26.7	9.3	33.2
COII	28.6	27.3	16.6	27.5	23.8	18.7	31.2	26.3	27.6	37.8	11.0	23.6	34.3	25.4	7.3	33.0
ATP8	30.6	26.2	11.5	31.7	29.8	23.9	15.2	31.0	22.7	31.8	11.4	34.1	37.4	22.6	8.3	31.8
ATP6	25.6	29.4	13.5	31.5	28.0	16.0	21.1	34.9	14.2	47.1	11.4	27.3	34.4	24.9	8.2	32.5
COIII	24.9	28.0	17.3	29.9	20.0	25.0	28.6	26.5	20.8	36.5	16.7	26.0	33.7	22.2	6.6	37.4
ND3	22.3	31.0	15.5	31.3	19.1	24.4	25.2	31.3	15.7	44.9	12.6	26.8	32.2	23.0	8.8	36.0
ND4L	22.7	28.4	15.6	33.3	19.8	24.1	24.5	31.5	13.5	40.1	14.7	31.8	33.5	20.8	8.2	37.5
ND4	26.6	27.7	15.0	30.8	28.8	20.1	21.0	30.1	16.1	41.1	15.0	27.9	34.8	21.7	9.1	34.4
ND5	28.0	27.5	14.0	30.5	32.6	19.8	22.4	25.2	19.5	39.7	12.2	28.6	31.7	22.9	7.8	37.6
Cyt b	24.8	29.5	15.3	30.5	24.1	24.2	25.8	25.9	20.0	41.2	13.5	25.3	31.1	22.1	6.7	40.1
Average	25.9	28.2	15.1	30.8	25.4	21.4	24.4	28.8	18.4	40.1	13.0	28.5	33.6	22.9	8.1	35.4
L-strand coded																
ND6	15.4	37.8	32.8	14.0	12.8	32.0	42.8	12.4	12.0	44.1	23.1	20.9	21.3	37.1	32.3	9.4

**Table 2:** Average base composition (%) of RNA genes and non-coding regions in mt genomes of 250 fishes

	tRNAs (H-strand coded)			tRNAs (L-strand coded)			12S rRNA (H-strand coded)			16S rRNA (H-strand coded)		
	Stem	Loop	Total	Stem	Loop	Total	Stem	Loop	Total	Stem	Loop	Total
A	23.7	40.7	30.4	20.4	30.2	24.2	20.1	40.4	31.0	21.1	41.5	33.7
T	23.0	27.8	24.8	26.4	37.7	30.8	21.6	20.4	21.0	21.9	21.1	21.3
G	26.4	13.3	21.2	30.1	23.6	27.6	30.3	14.6	21.9	29.0	14.7	20.3
C	26.9	18.2	23.5	23.1	8.5	17.4	28.0	24.6	26.2	28.0	22.7	24.8
	Control region			Origin of L-strand replication								
		Total		Stem	Loop	Total						
A		32.5		21.1	41.5	14.1						
T		31.1		21.9	21.1	21.4						
G		15.1		29.0	14.7	30.1						
C		21.4		28.0	22.7	34.4						

### Features of protein-coding genes

#### Start codon

**Fig. 2:**
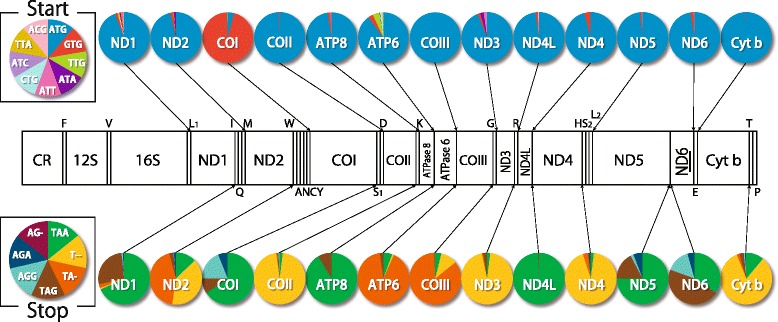
Use frequency of start and stop codons for 13 protein-coding genes in the 250 fish mt genomes. Gene abbreviations are the same as those in Fig. 1 Additional files 2 and 3 for detailed information)

The 13 protein-coding genes of 250 fishes were found to use nine types of start codons (Fig. 2; Additional file 2: Table S2). Among the nine codons, ATG was predominant as found in other vertebrates, and it was used exclusively in the COIII gene. GTG was heavily used only in the COI gene as a start codon in more than 95 % species, whereas this codon was rarely used in the other genes in less than 4 % species.

Of the nine codons, ACG was identified as a start codon in the ND4L gene of the black ghost knifefish *Apteronotus albifrons* (Gymnotiformes). This is the first example in cellular organisms, although this codon is reported as a start codon in the genome of adenoviruses and the Sendai virus [50–52]. TCG, which has a one-nucleotide difference from ACG, has been reported as a start codon in the COI gene in the mt genome of two mosquitoes [53].

#### Stop codon

Seven types of stop codons were recognized in 250 fish mt genomes (Fig. 2, Additional file 3: Table S3). Four of these were complete stop codons (TAA, TAG, AGA, and AGG), and the other three were incomplete stop codons (TA-, T--, and AG-). According to Ojala et al. [54], these incomplete stop codons may be completed to TAA or AGA by the addition of a poly A tail during RNA processing. Stop codons of the TAA series (including TA- and T--) were most frequently used in all genes excluding the ND6 gene, which is coded on the opposite strand.

The incomplete stop codons TA-, T--, and AG- were used mostly when the 3’-end of the protein-coding genes (ie, ND2, COII, ATP6, COIII, ND3, ND4, Cyt *b*) was followed by a tRNA gene encoded on the same strand. The existence of a tRNA gene, which functions as punctuation marker, may allow transcription to terminate without complete stop codons. On the contrary, only complete stop codons were used in the ND5, ND6, and COI genes. These genes were followed by a gene encoded on the opposite strand, which causes transcription without punctuation and may explain the exclusive use of complete stop codons by these genes.

#### Variation in gene length

**Fig. 3:**
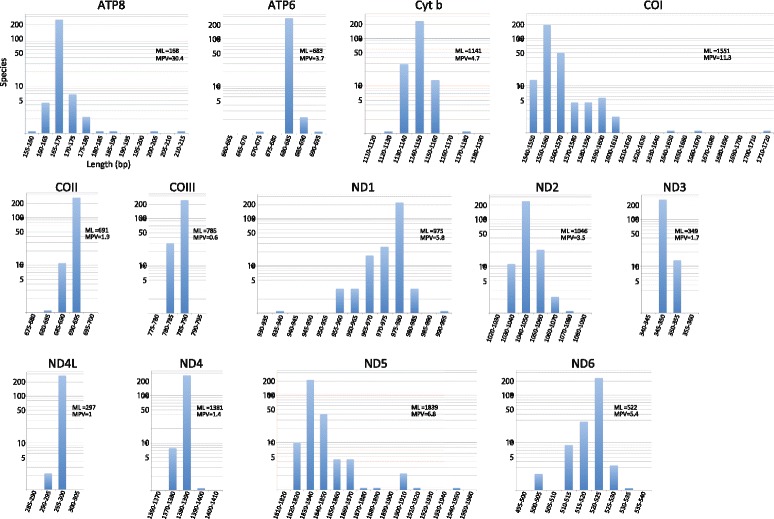
Histogram of the length of protein-coding genes in the 250 fish mt genomes. The X and Y axes are the range of gene length (bp) and number of species (log), respectively. ML indicates the modal length of the gene. Maximum percentage variation (MPV, %) = [Largest − Smallest]/Modal * 100

Each of the 13 protein-coding genes had length variation (Fig. 3, Additional file 4: Table S4). The variation in the COI, ND2, ND5, and Cyt *b* genes was large (only 39.2–74.4 % of species used in this study displayed the modal length of each gene), whereas that of the remaining nine genes was relatively small (>79 % of species possessed the modal length of each gene). The former four genes are relatively large (>1,000 bp) among the 13 protein-coding genes in the mt genome. This indicates that the level of length variation is somehow related with gene size. Although the ND4 gene is similar in length to the four genes, its length variation was small, as the same modal length is retained in 90.4 % of the species (Fig. 3, Additional file 4: Table S4). This suggests that length variation occurs as a byproduct of gene rearrangement because there are few instances of gene rearrangement around the ND4 gene but many instances around the four genes (see below).

#### Overlap between protein-coding genes

Among four pairs of protein-coding genes located directly adjacent to each other (ATP8-ATP6, ATP6-COIII, ND4L-ND4, and ND5-ND6), all but ATP6-COIII had some overlap between adjacent genes. Two cases (ATP8-ATP6 and ND4L-ND4) involved overlap between genes encoded on the same strand, and the other (ND5-ND6) involved overlap between genes encoded on the opposite strand. The former two cases had overlaps of the reading frame in all fish mt genomes (with two exceptions in ATP8-ATP6). Such overlaps in the two regions were previously identified in vertebrate mt genomes. The length of overlap was generally 10 nucleotides in ATP8-ATP6 and exclusively 7 nucleotides in ND4L-ND4 (Additional file 5: Table S5). Overlap was also found in almost all species between the ND5 and ND6 genes, which were encoded on opposite strands (Additional file 5: Table S5). The length of overlap was mainly four nucleotides in this region, although there was some variation.

#### Secondary structure of proteins

Common features found for all 13 proteins were as follows: (1) several hydrophobic amino acids were observed in transmembrane regions as well as general membrane proteins, (2) conserved amino acid sites were concentrated in some specific areas in and around transmembrane regions, and (3) insertions and deletions (indels) of amino acids occurred intensively near the C- and N-termini (Additional file 6: Figure S1). In addition, COI, COII, COIII, and Cyt *b* were found to have more conserved amino acid sites than the other genes. Among these genes, COI was the most conserved, in which more than half of all amino acid sites were invariable throughout the 250 species (Additional file 7: Table S6).

#### Tertiary structure of proteins

We next performed more detailed analysis of the structure of mt proteins focusing on COI and Cyt *b* by estimating structural models (eg, Esposti et al. [37]; Tsukihara et al. [55]) (Additional file 6: Figure S1-c and S1-f, respectively). As a result, it became clear that both proteins had several metal prosthetic sites with a central role in a redox reaction, and those sites were invariable in all 250 fishes, as reported for the mammalian mt genome [55, 56]. Specifically, in COI, the ligand-binding sites for heme-a (positions 61 and 378), heme-a3 (376), and CuB (240, 244, 290, and 291), which are the active centers of respiratory complex IV [55, 57], had histidine or tyrosine exclusively in all fish mt genomes (Additional file 6: Figure S1-c). In Cyt *b*, two pairs of ligand-binding sites with heme (positions 83:182 and 97:196), which is the center of the redox reaction [37], also had histidine exclusively. In addition, two pairs of heme pocket sites (34:116 and 48:130) had glycine in this position in all 250 fishes (Additional file 6: Figure S1-f).

### Features of tRNAs

#### Secondary structure

**Fig. 4:**
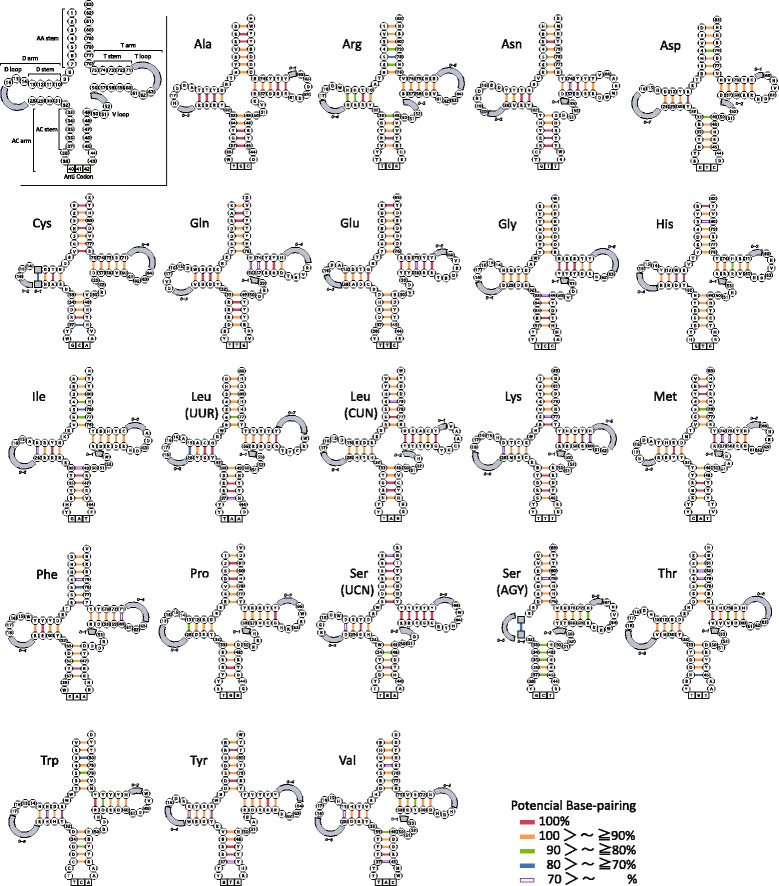
Secondary structure model of 22 mitochondrial transfer RNAs (tRNAs) displaying variation in the 250 fishes. In the inset, nucleotide positions common to all tRNAs are numbered from 1 to 83. Stem and loop regions with length variation are denoted by a shaded square and arc, respectively. Conserved nucleotides are shown in the figure by IUB code [89] as follows: K (G, T), M (A, C), N (A, C, G, T), R (A, G), S (G, C), W (A, T), and Y (C, T). Frequencies of Watson–Crick and wobble base pairing observed in the 250 fishes are shown with color-coded bars (see Additional files 8 and 9 for detailed information)

We estimated and compared the secondary structure of mt 22 tRNAs in the 250 fishes. The sequences of all tRNA genes were folded into a canonical cloverleaf secondary structure basically composed of four domains and a short variable loop: the amino acid or acceptor (AA) stem, the dihydrouridine (D) arm (D stem + D loop), the anticodon (AC) arm (AC stem + AC loop), the thymidine (T) arm (T stem + T loop), and the variable (V) loop (Fig. 4; Additional files 8 and 9: Tables S7 and S8, respectively). Regarding the stems except the D stem, the length was fixed (AA stem = 7 bp, AC stem = 5 bp, and T stem = 5 bp). Although D stem of most tRNAs was 4 bp in length, tRNA-Ser (AGY) and tRNA-Cys displayed length variation (3 or 4 bp), and the tRNA-Ser gene (AGY) of 19 fishes had only a small loop without making the stem (Table 3). Regarding the four loops, whereas the length of the AC loop was fixed (seven nucleotides), the other three loops were rather variable in length (V loop, mostly four or five nucleotides with a range of three to six nucleotides; T loop, mostly seven nucleotides with a range of 3–10 nucleotides; and D loop, extremely variable with a range of 3–14 nucleotides) (Table 3).

**Table 3:** Size of D stem and D, V and T loops of 22 tRNA genes in mt genomes of 250 fishes

	D stem (bp)			D loop (nt)												V loop (nt)				T loop (nt)							
tRNA	0	3	4	3	4	5	6	7	8	9	10	11	12	13	14	3	4	5	6	3	4	5	6	7	8	9	10
H-strand coded																											
Arg			250		6	153	86	4	1							1	248	1			1	2	1	244		2	
Asp			250	3	13	29	20	32	86	53	14					1	248	1					2	248			
Gly			250			17	41	61	112	19							249	1		1	1		3	243	2		
His			250	2	6	194	42	6									247	3					5	244	1		
Ile			250	3	18	116	54	53	6								248	2				1		247	2		
Leu (UUR)^a^			250			1		2		18	209	19	1				186	64				1	1	244	4		
Leu (CUN)^a^			250		1		1	29	219								4	245	1				1	249			
Lys			250			1	3	6	24	137	65	14					7	243		1			11	238			
Met^b^			252			182	58	12									251	1					6	243	3		
Phe			250			172	54	18	5	1							244	6		1	1	5	208	35			
Ser (AGY)^a^	19^c^	207	24	81	144	6											180	67	3					2	4	244	
Thr			250			9	27	145	47	15	6				1		14	236		1		2	7	238	1	1	
Trp			250		1	17	29	94	76	31	1		1				250					2	2	246			
Val			250			4	10	35	165	26	10						245	5		1		1		248			
L-strand coded																											
Ala			250			248	1	1									250						2	248			
Asn			250					2	242	6							2	248						250			
Cys		116	134	140	105	5											250				1	3	109	130	5	1	1
Gln			250				1	236	12	1							247	3			1	1	3	244	1		
Glu^d^			249	1	27	201	20										249						1	247	1		
Pro^e^			248		4	29	170	32	7	5	1						247	1					1	244	2	1	
Ser (UCN)^a^			250			1	2	234	13							1	247	2					1	247	1	1	
Tyr			250	30	39	14	91	76									250				1		3	243	3		

^a^DNA degeneracies are represented by IUB (International Union of Biochemistry) code: N(A C G T), R(A G), Y(C T)

^b^252 genes compared due to gene duplication

^c^D-arm replacement loop

^d^249 genes compared

^e^248 genes compared

#### Wobble base pairs in the stem regions

Figure 4 illustrates the frequencies of Watson-Crick and wobble pairing in each of 21 base pair sites (7 in AA stem + 4 in D stem + 5 in AC stem + 5 in T stem) of each tRNA in 250 fishes. There were nine sites with Watson-Crick and wobble pairing (hydrogen bonding) at a high rate of more than 95 % in each stem (2–81 and 7–76 pair sites in AA stem; 10–31 and 11–30 in D stem; 34–48, 35–47, and 36–46 in AC stem; 56–75 and 59–72 in T stem). Those sites may play an important role in maintaining stem structure. Among tRNAs, tRNA-Ala and tRNA-Pro had higher frequencies (>98 % overall average) of hydrogen bonding than the others (Additional file 9: Table S8). The hydrogen bonding frequency was higher in L-strand-coding tRNAs than in H-strand-coding tRNAs (Mann-Whitney *U*-test, *p* < 0.02).

Table 4 shows the number of G–U wobble base pairs in each stem of the mt tRNAs for the 250 fishes. Although there were rather large variations of frequency of wobble base pairs in individual stems and tRNAs, wobble base pairs comprised approximately 7 % of base pairs in the four stems in the fish mt tRNAs on average, with no big difference among the four stems (6.0 % in the T stem to 9.8 % in the D stem). However, there was a big difference among the tRNAs (1.0 % in the tRNA-Leu (CUN) gene to 20.9 % in the tRNA-Glu gene). Furthermore, there was a significant difference in the frequency of wobble pairs between tRNAs coded by different strands (13.3 % in the L-strand-coded tRNAs vs. 3.5 % in the H-strand-coded tRNAs; Mann-Whitney *U*-test, *p* < 0.01). This may reflect the difference in base composition between the L- and H-strands, as transcripts from the L-strand contain greater numbers of guanine residues than those from the H-strand (G ≈ T > A > > C in L-strand transcripts vs. C ≈ A > T > > G in H-strand transcripts).

**Table 4:** Occurrence of wobble pairings in the stem regions of 22 tRNA genes in mt genome of 250 fishes

		AA stem (7 bp)	D stem (4 bp)	AC stem (5 bp)	T stem (5 bp)	Total	(Percentage)
Base pairs × 250 species		1750	1000	1250	1250	5250	
	Ala	280	210	241	345	1076	20.50
	Asn	116	112	12	71	311	5.92
	Cys	288	146	47	68	549	10.46
L-strand	Gln	210	289	32	36	567	10.80
code	Glu	420	2	547	128	1097	20.90
	Pro	123	435	167	175	900	17.14
	Ser (UCN)^a^	24	64	159	319	566	10.78
	Tyr	257	46	185	27	515	9.81
	Average (%)	12.27	9.31	9.93	8.35	13.29	
	Arg	90	42	21	63	216	4.11
	Asp	78	18	80	131	307	5.85
	Gly	36	296	55	8	395	7.52
	His	55	67	34	38	194	3.70
	Ile	44	20	11	11	86	1.64
	Leu (UUR)^a^	78	139	13	19	249	4.74
H-strand	Leu (CUN)^a^	39	1	10	1	51	0.97
code	Lys	68	6	15	5	94	1.79
	Met	83	3	27	10	123	2.34
	Phe	113	14	44	54	225	4.29
	Ser (AGY)^a^	26	38	61	77	202	3.85
	Thr	26	4	67	16	113	2.15
	Trp	20	216	17	34	287	5.47
	Val	43	16	4	21	84	1.60
	Average (%)	3.26	6.29	2.62	2.79	3.52	
Coefficient of variation		0.944	1.209	1.458	1.258	0.842	
Grand average (%)		6.54	9.93	6.72	6.03	7.07	

^a^DNA degeneracies are represented by IUB (International Union of Biochemistry) code: N(A C G T), R(A G), Y(C T)

#### Mitochondrial transcription termination factor (mTERF) binding site

**Fig. 5:**
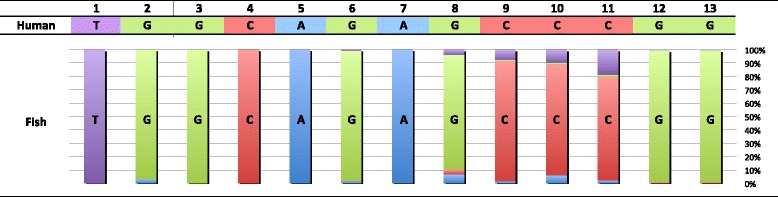
Base frequencies (%) of the mitochondrial transcription termination factor binding site in the tRNA-Leu (UUR) gene in the mt genomes of 250 fishes (see Additional file 8 for detailed information)

To examine the presence or absence of the mt transcription termination factor (mTERF) binding site reported for the mammalian mt genome, we compared the fish mt tRNA-Leu (UUR), which contains the target binding site for mTERF [58–60]. This site is involved in the regulation of the level of transcription from the two rRNA genes and the remaining downstream genes coded on the H-strand [61]. Christianson and Clayton [58] and Kruse et al. [59] reported a tridecamer sequence (5’-TGGCAGAGCCCGG-3’) in the tRNA-Leu (UUR) gene as a key sequence of the mTERF binding site in the human mt genome [58, 59]. As a result of the present comparative analysis, the fish tRNA-Leu (UUR) gene also had this tridecamer motif in the same region corresponding to a part of the D arm as found in the human mt genome (Fig. 5 and Additional file 8: Table S7). The sequence of the motif in fishes was nearly identical to the human tridecamer sequence. The predominant base in all 13 nucleotide sites was completely identical to that in humans, and in particular, four bases were completely invariable in all 250 fishes and in humans (Fig. 5: positions 3–5 and 7). This conservation implies that this region also functions as the mTERF binding site in the fish mt genome.

### Features of rRNAs

#### Secondary structure

**Fig. 6:**
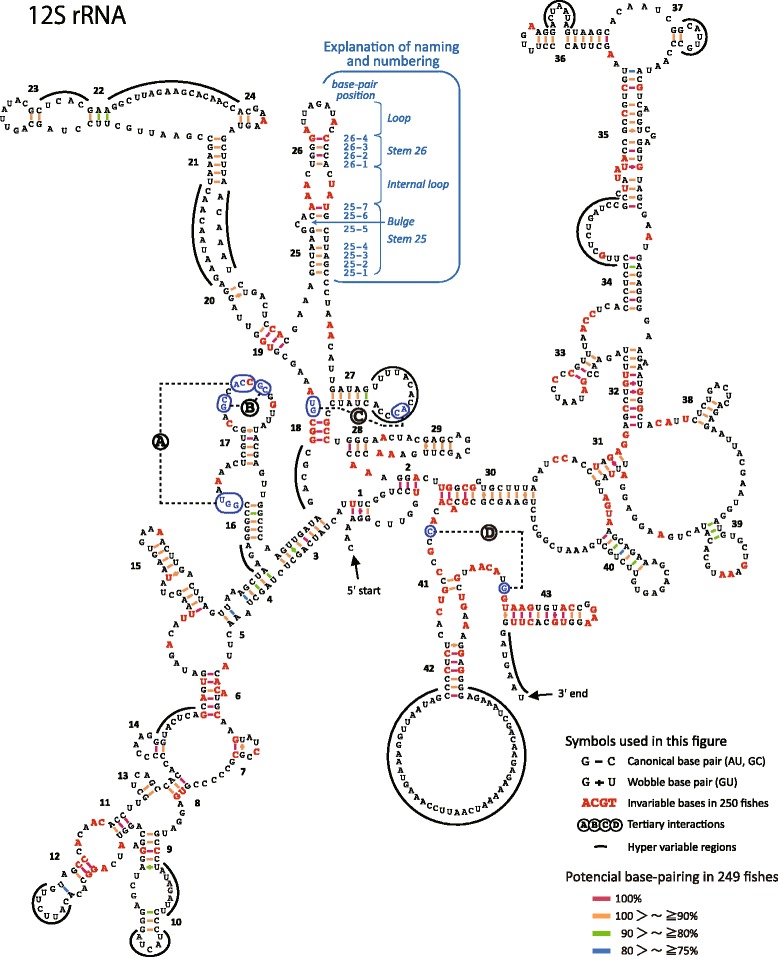
Secondary structure model of 12S ribosomal RNA genes exhibiting variation among the 250 fishes. Standard sequence is represented by that of *Sillago japonica*. Numerals refer to the stem code number. *Bold red letters* indicate invariable bases in the 250 fishes. Frequencies of Watson-Crick and wobble base pairing observed in 250 fishes are shown with color-coded bars and crosses, respectively. *Dotted lines* named **A**–**D** refer to tertiary interactions. Hypervariable regions are indicated by *thick lines* around the sequences

**Fig. 7:**
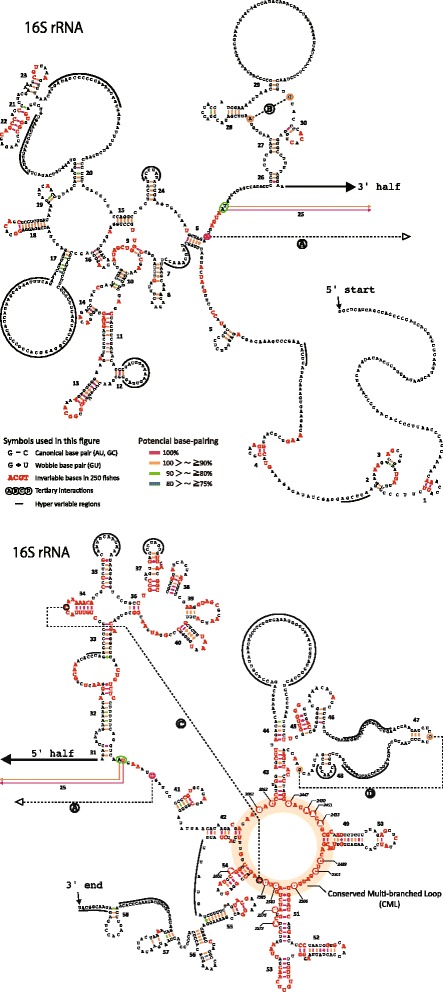
Secondary structure model of 16S ribosomal RNA (rRNA) gene exhibiting variation among the 250 fishes. See the legend of Fig. 6 for details. Figure 7a and 7b show the *left half* and *right half* of the 16S rRNA model, respectively. *Highlighted letters* in the CML region show the core sites of activity in the peptidyl transferase center. Italicized numerals of four digits in and around the CML region indicate nucleotide positions for 23S rRNA of *Escherichia coli*

We estimated representative secondary structure models for fish 12S rRNA and 16S rRNA based on the comprehensive fish mt genome data (Figs. 6 and 7). Among various features of the secondary structures of rRNAs, we mainly focused on stem structure, which plays a central role for forming the skeleton of the rRNAs. Original data used to estimate the rRNA models can be found in Additional files 10, 11, 12 and 13: Figs. S2 and S3; Tables S9 and S10, respectively.

Our fish mt rRNA models identified 149 and 319 invariable sites in 12S rRNA and 16S rRNA, respectively, in all compared fishes (Figs. 6 and 7, bold types). The models also revealed that both rRNAs had some regions with large variation in length and sequence (hypervariable regions) (Figs. 6 and 7, solid curved lines). We recognized 43 and 58 stems within 12S rRNA and 16S rRNA, respectively. These numbers were larger than those in most other vertebrate models of 12S rRNA (carp = 38, Van de Peer et al. [62]; cow = 40, Springer and Douzery [41]; mouse = 38, Van de Peer et al. [63]; and the same as that in gobioid fishes, Wang and Lee [39]) and 16S rRNA (cow = 54, Gutell et al. [33] and De Rijk et al. [64]; mammals = 53, Burk et al. [40]). A description on the details of differences between the present and previous studies is given in Additional file 14: Table S11. The main reason for the higher number of stems in the present study is that, through comparing 250 fish mt genomes (249 for 12S rRNA), we were able to identify with certainty new stem structures on the basis of the 75 % rule employed for the regions, which was not performed in prior studies.

The stems identified generally appeared to be extremely stable; specifically, 91.4 % (201/220) of all base pairs in 12S rRNA and 92.9 % (276/297) in 16S rRNA had Watson-Crick or wobble base pairing in more than 90 % of species (Figs. 6 and 7, red and orange bars). Among a total of 43 stems of 12S rRNA, 14 had a perfect stem structure in more than 98 % of species (Fig. 6 and Additional file 12: Table S9; Stems 1, 2, 6, 8, 11, 13, 14, 18, 19, 24, 26, 31, 41, and 43). Similarly, 19 of the 58 stems of 16S rRNA had a perfect stem structure in more than 98 % of species (Fig. 7 and Additional file 13: Table S10; Stems 1, 4, 5, 6, 9, 11, 13, 16, 22, 34, 38, 39, 42, 45, 48, 50, 51, 53, and 54). Those stable areas may have important roles to maintain the higher-order structure of rRNAs.

#### Conserved multibranched loop in mt 16S rRNA

Four nucleotide pairs for tertiary interactions in both of the fish mt rRNAs were identified as found in previous studies on mammal mt rRNAs (Figs. 6 and 7A–D). Those sites were highly conserved although not invariable (Additional file 10: Figure S2 and Additional file 11: Figure S3). It should be noted that among four areas in which those sites are located, one area including a site for tertiary interaction ‘C’ in 16S rRNA was extremely conserved, being composed of a highly conservative loop and five conserved stems (Fig. 7). The conserved multibranched loop (CML, orange donut shape in Fig. 7) is known as the peptidyl transferase center in prokaryotic 23S ribosomal large subunit RNA (23S rRNA; Polacek and Mankin [65]; Sato et al. [66]), which is homologous rRNA to mt 16S rRNA [65, 66]. The peptidyl transferase center catalyzes two reactions in protein synthesis: (1) peptide bond formation during protein elongation and (2) peptide release of nascent polypeptide from tRNA during the termination of protein synthesis [65]. The present result verified that the structure of the CML is conserved even in fish mt 16S rRNA as the core of the peptidyl transferase center, and nucleotides known as functionally important in prokaryotic 23S rRNA, such as A2451, U2506, U2585, C2452, and A2602, were easily identified as being invariable (Fig. 7: highlighted characters in the CML) [65, 67]. Although the structure and function of 23S rRNA have been studied in detail using *Escherichia coli*, the eukaryotic and mt large ribosomal subunits (28S and 16S) have not been studied until recently [68, 69], probably because it is much difficult to study them experimentally. The present results may contribute to deepen our structural and functional understanding of the mt large ribosomal subunit.

### Features of noncoding regions

#### Control region

**Fig. 8:**
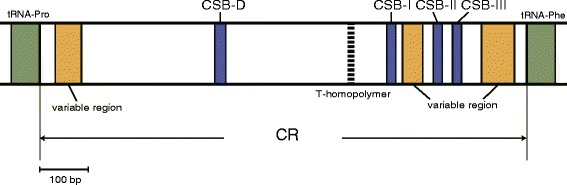
Schematic diagram of the control region of the fish mt genome. Locations of conserved sequence block domains and variable regions are mapped. The location of the T-homopolymer region is represented by *broken lines*

Full-length CR sequences were obtained from 182 fishes. Those sequences displayed large length variation (ranging from 724 nucleotides in *Pempheris schwenkii* to 1,401 nucleotides in *Paralichthys olivaceus*). The relative position of the four CSBs was the same as that reported in some vertebrates (Fig. 8) [70–73]. Continuous poly-T chains spanning more than eight nucleotides, presumably functioning as a hindrance to the sequencing reaction, were observed in the upper region of CSB-I in almost all fishes examined. The mode values of the lengths of CSB-D, CSB-I, CSB-II, and CSB-III were 18, 22, 17, and 19 nucleotides, respectively (Table 5, Additional file 15: Figure S4). Base composition was extremely specific to each CSB as follows: CSB-D, T rich; CSB-I, AT rich; CSB-II, C rich; and CSB-III, AC rich (Table 5, Additional file 15: Figure S4). CSB-D and CSB-I were identified in all fishes compared in this study; however, CSB-II and CSB-III were partially or completely missing in some fishes. The same condition was also reported in other vertebrates (eg, Sbisà et al. [70]; Roques et al. [71]; Nilsson [72]; Wang et al. [73]) . Although their function is not yet clear, the common occurrence of CSB-D and CSB-I in vertebrate mt genomes suggests that they have important roles in replication and transcription of the genome.

**Table 5:** Features of the four CSBs of the 250 fishes

		CSB-D	CSB-I	CSB-II	CSB-III
Mode [range] of length (nt)		18 [16–22]	22 [20–24]	17 [16–21]	19 [17–21]
Base composition (%)	A	7.4	40.7	23.5	40.7
	T	42.6	31.1	9.8	9.6
	G	21.6	14.7	0.4	12.5
	C	28.4	13.4	66.2	37.2

#### Origin of L-strand replication

**Fig. 9:**
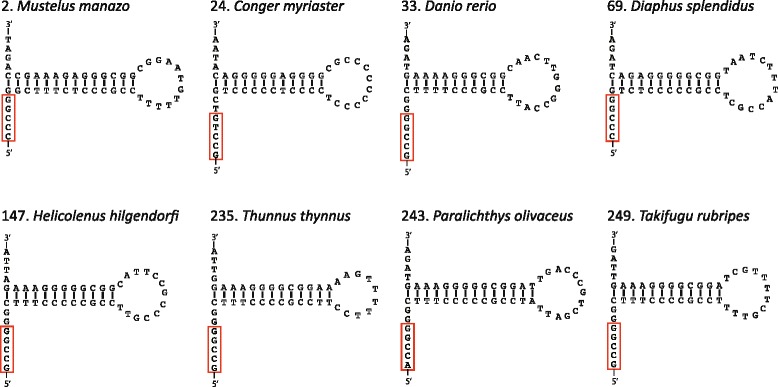
Representative stem-loop structures of the origin of L-strand replication in the fish mt genome. Red box represents a conserved sequence motif, which is necessary for in vitro replication of the L-strand in mammals. Numerals in front of the species name are the same as those in Additional file 1

O_L_ was identified in 245 fishes, whereas it was not found in the remaining five fishes (Additional file 16: Figure S5). The obtained 245 O_L_ sequences displayed considerable length variation, ranging from 22 to 87 nucleotides, but they exclusively had the potential to form a stable stem-loop structure (Table 6; Fig. 9). Stem and loop lengths ranged from 8 to 39 bp and from 3 to 23 nucleotides, respectively. The stem was moderately GC rich similarly as the RNA stems, whereas the loop was A rich similarly as the RNA loops (Table 2). The 5’-end (tRNA-Cys side) of the loop was T rich (Additional file 16: Figure S5). A conserved sequence motif (5’-GCCGG-3’) that was reported as necessary for in vitro replication of the L-strand in mammals [74, 75] was observed in nearly 70 % of species (Table 6).

**Table 6:** Features for origin of L-strand replication (O_L_) in fishes

Stem length			Loop length			Conserved sequence motif		
(bp)	Species	(%)	(nt)	Species	(%)	5’ → 3’	Species	(%)
8	3	1.22	3	5	2.04	GCCGG	165	67.35
9	2	0.82	4	6	2.45	ACCGG	32	13.06
10	8	3.27	5	14	5.71	GCTGG	6	2.45
11	23	9.39	6	15	6.12	GCCGA	6	2.45
12	33	13.47	7	9	3.67	CCCGG	5	2.04
13	58	23.67	8	5	2.04	GCCTG	4	1.63
14	54	22.04	9	7	2.86	GCCAG	3	1.22
15	33	13.47	10	23	9.39	GCCCG	3	1.22
16	18	7.35	11	38	15.51	GCCTA	2	0.82
17	11	4.49	12	43	17.55	GCTAG	2	0.82
19	1	0.41	13	42	17.14	GCCAA	2	0.82
39	1	0.41	14	26	10.61	CCCCC	2	0.82
			15	8	3.27	TCCGG	1	0.41
			16	1	0.41	GCAGG	1	0.41
			17	1	0.41	TCCCG	1	0.41
			21	1	0.41	ACCTG	1	0.41
			23	1	0.41	ACGGG	1	0.41
						GTCGT	1	0.41
						GGGCT	1	0.41
						CTCGC	1	0.41
						TCCGA	1	0.41
						CTATC	1	0.41
						TCCCT	1	0.41
						CCCTC	1	0.41
						GGGGG	1	0.41

### Gene rearrangements in the fish mt genome

#### Type and scale of gene rearrangements

Most of the fishes compared in the present study had the typical gene order widely shared among vertebrate mt genomes (Fig. 1). However, deviations from the conserved gene order were found in 35 species from 32 families (35/250 species = 14 %). Among them, the gene orders of 22 species were explicitly reported in detail for the first time in this study. All of the gene rearrangements involved local position changes (shuffling) or displacement to the separated location (translocation) of genes on the same encoded strand. Although switching of the encoded strand (inversion) was not found in this study, there have been reported five cases of inversion in fish mt genomes thus far [76–78]. This suggests that inversion is rare in the fish mt genome.

Local shuffling was observed within two tRNA gene clusters (IQM and WANCY) (Fig. 1A and B, respectively) and within the tRNA-Lys-ATPase 8-ATPase 6 gene region (Fig. 1C). Transfer of the O_L_ within the WANCY region was also observed (b: in Fig. 1). Some of these shufflings involved gene duplication (Fig. 1A: 110, 206, 238; and B: 61, 159). The translocations were observed at various sites in the fish mt genome (Fig. 1D–G). Among these translocations, those observed in some fishes were massive and complicated (Fig. 1F and G). Some translocations involved duplicated CRs (Fig. 1E: 107, 139; F: 89, 211; and G: 25).

#### Implication of hotspots and mechanism for gene rearrangement

The rearranged mt genomes had exclusively noncoding (NC) sequences in and around the region in which rearrangement was observed (Fig. 1); for example, *Kurtus gulliveri* with a gene rearrangement in IQM region had three NC sequences (50, 88, and 17 bp, respectively) between each gene in and around the region (Fig. 1A: 228).

The existence of these NC sequences implies their relevance to the mechanisms of gene rearrangement. Among the several mechanisms proposed to explain mt gene rearrangements, the tandem duplication-random loss (TDRL) model [79–81] is commonly considered the most plausible in vertebrates [10, 12, 15, 16, 27]. This model assumes tandem duplication of a block of multiple genes arising from the failure of DNA replication, such as strand slippage and mispairing [82] and incorrect initiation or termination [10], followed by random deletion of one of each of the pairs of the redundant genes. Because partial sequences of the original genes were usually observed in the NC regions in this study (data not shown; see also Mabuchi et al. [83]), they appear to be vestiges of duplication of a gene block. The existence of the NC sequences found in this study may be evidence in support of the TDRL model.

We were able to clarify some regions in which the gene rearrangements were observed with high frequency in the fish mt genome, namely IQM and WANCY clusters and the region from the ND5 gene to the CR. These hot spots include replication origins of both the L- and H-strands, or they are located in their vicinity [84, 85]. Therefore, it is possible to believe that gene duplication, which is assumed to be a trigger of gene rearrangement in the TDRL model [10, 86, 87], can easily occur there, and consequently, unique gene orders are frequently observed in those regions (see the next section).

#### Phylogenetic aspect of gene rearrangement

**Fig. 10:**
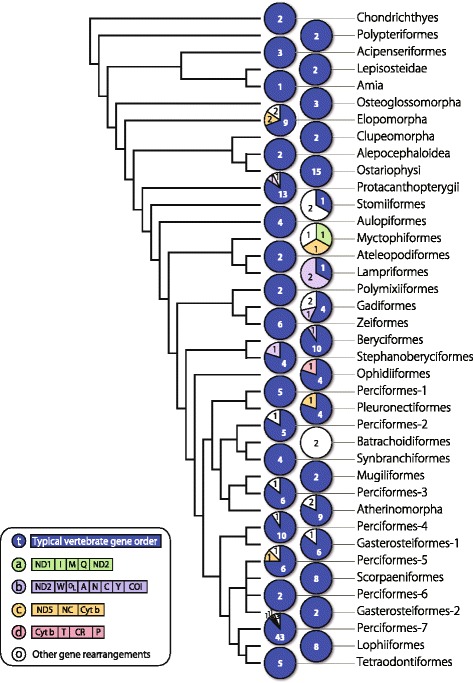
Frequencies of the typical (t), major rearranged gene orders (a–d), and other gene rearrangements (o) mapped on a consensus phylogenetic tree. See Fig. 1 for abbreviation of gene names

The mt gene rearrangements were observed to have occurred in various groups among fishes from the series Elopomorpha to the order Perciformes (Figs. 1 and 10). In the fish rearrangements compared in this study, we discovered some cases in which a specific gene order was shared between species that were distantly related phylogenetically. For example, the gene orders of I-M-NC-Q-NC (a: in Figs. 1 and 10) were shared in two distantly related fishes (b: in Figs. 1 and 10). Similarly, shifting of the O_L_ (b: in Figs. 1 and 10), ND5-NC-Cyt *b* (c: in Figs. 1 and 10), and Cyt *b*-T-CR-P (d: in Figs. 1 and 10) was also shared among nonclosely related species. These facts indicate that similar gene orders sometimes occurred independently in the evolutionary history of fishes. Therefore, we should be careful to use a variant gene order as a marker for phylogenetic analysis. Evolution of gene rearrangements will be discussed in detail elsewhere.

## Conclusions

Through large-scaled comparative analysis of the mt genomes of 250 fish species, we elucidated various structural aspects of fish mt genomes and encoded genes. For the first time, we quantitatively described variation of start and stop codon usage among protein-coding genes and the secondary structures of tRNAs and rRNAs. Such empirical data would be important for understanding the functions of the mt genome and its genes. Furthermore, these data also appear useful for the development of programs for nucleotide sequence comparison and structural estimation of fish mt genes and their products. In fact, the preliminary data of this study have already contributed the successful development of a much-used automatic sequence annotation system for fish mt genomes (MitoAnnotator) [88]. These empirical data were only obtained through large-scaled comparative analysis of mt genomes from many species. This study demonstrated the significance of extensive comparisons for understanding the structure and function of the mt genome.

## Additional files

Additional file 1: Table S1. List of species examined in this study with DDBJ/EMBL/GenBank accession numbers. (XLS 83 kb) Additional file 2: Table S2. Start codons in 13 protein-coding genes in the mt genomes of 250 fishes. (XLSX 64 kb) Additional file 3: Table S3. Stop codons in 13 protein-coding genes in the mt genomes of 250 fishes. (XLSX 53 kb) Additional file 4: Table S4. Length variation in 13 protein-coding genes in the mt genomes of 250 fishes. (XLSX 53 kb) Additional file 5: Table S5. Variation in overlap length between protein-coding genes in the mt genomes of 250 fishes. (XLSX 41 kb) Additional file 6: Figure S1-a. Aligned amino acid sequences of the ATP8 gene in mt genomes of 250 fishes. **Figure S1-b.** Aligned amino acid sequences of the ATP6 gene in mt genomes of 250 fishes. **Figure S1-c.** Aligned amino acid sequences of the COI gene in mt genomes of 250 fishes. **Figure S1-d.** Aligned amino acid sequences of the COII gene in mt genomes of 250 fishes. **Figure S1-e.** Aligned amino acid sequences of the COIII gene in mt genomes of 250 fishes. **Figure S1-f.** Aligned amino acid sequences of the Cyt b gene in mt genomes of 250 fishes. **Figure S1-g.** Aligned amino acid sequences of the ND1 gene in mt genomes of 249 fishes. **Figure S1-h.** Aligned amino acid sequences of the ND2 gene in mt genomes of 250 fishes. **Figure S1-i.** Aligned amino acid sequences of the ND3 gene in mt genomes of 250 fishes. **Figure S1-j.** Aligned amino acid sequences of the ND4L gene in mt genomes of 250 fishes. **Figure S1-k.** Aligned amino acid sequences of the ND4 gene in mt genomes of 250 fishes. **Figure S1-l.** Aligned amino acid sequences of the ND5 gene in mt genomes of 250 fishes. **Figure S1-m.** Aligned amino acid sequences of the ND6 gene in mt genomes of 249 fishes. (ZIP 3250 kb) Additional file 7: Table S6. Proportion of invariable amino acid sites in 13 protein-coding genes in the mt genomes of 250 fishes. (XLSX 41 kb) Additional file 8: Table S7. Base frequencies for 83 nucleotide positions of 22 tRNA genes in the mt genomes of 250 fishes. (XLS 13112 kb) Additional file 9: Table S8. Occurrence of nucleotide pair types in 21 pair sites common to all 22 tRNAs in the mt genomes of 250 fishes. (XLSX 107 kb) Additional file 10: Figure S2. Aligned nucleotide sequences of the 12S rRNA gene in the mt genomes of 249 fishes. (PDF 537 kb) Additional file 11: Figure S3. .Aligned nucleotide sequences of the 16S rRNA gene in the mt genomes of 250 fishes. (PDF 893 kb) Additional file 12: Table S9. Potential base pairing properties of stem regions in the 12S rRNA gene in the mt genomes of 250 fishes. (XLSX 76 kb) Additional file 13: Table S10. Potential base pairing properties of stem regions in the 16S rRNA gene in the mt genomes of 250 fishes. (XLSX 86 kb) Additional file 14: Table S11. Differences between previous and present rRNA secondary structure models. (XLSX 59 kb) Additional file 15: Figure S4-a. Aligned nucleotide sequences of the conserved sequence blocks-D and -I in the control region (CR) in mt genomes of 182 fishes. Figure S4-b. Aligned nucleotide sequences of the conserved sequence blocks-II and -III in the control region (CR) in mt genomes of 182 fishes. (ZIP 87 kb) Additional file 16: Figure S5. Aligned nucleotide sequences of the origin of L-strand replication (blue and magenta letters) in the mt genomes of 250 fishes. (PDF 38 kb)

## Abbreviations

12S rRNA and 16S rRNA: 12S and 16S ribosomal RNA, respectively
ATP6 and ATP8: ATPase subunit 6 and 8, respectively
CML: Conserved multibranched loop
COI–III: Cytochrome *c* oxidase subunits I–III, respectively
CR: Putative control region
CSB: Conserved sequence block
Cyt *b*: Cytochrome b
mt genome: Mitochondrial genome
NC: Noncoding sequences
NCBI: National Center for Biotechnology Information
ND1–6 and 4 L: NADH dehydrogenase subunit 1–6 and 4 L
O_L_: Origin of L-strand replication
tRNA: Transfer RNA
