# Supplementary material for: Structure and variation of the mitochondrial genome of fishes
Source: BMC Genomics. 2016 Sep 7;17(1):719. doi: 10.1186/s12864-016-3054-y (PMC5015259; doi:10.1186/s12864-016-3054-y)
Supplement: Additional file 6: Figure S1-a. — Aligned amino acid sequences of the ATP8 gene in mt genomes of 250 fishes. Figure S1-b. Aligned amino acid sequences of the ATP6 gene in mt genomes of 250 fishes. Figure S1-c. Aligned amino acid sequences of the COI gene in mt genomes of 250 fishes. Figure S1-d. Aligned amino acid sequences of the COII gene in mt genomes of 250 fishes. Figure S1-e. Aligned amino acid sequences of the COIII gene in mt genomes of 250 fishes. Figure S1-f. Aligned amino acid sequences of the Cyt b gene in mt genomes of 250 fishes. Figure S1-g. Aligned amino acid sequences of the ND1 gene in mt genomes of 249 fishes. Figure S1-h. Aligned amino acid sequences of the ND2 gene in mt genomes of 250 fishes. Figure S1-i. Aligned amino acid sequences of the ND3 gene in mt genomes of 250 fishes. Figure S1-j. Aligned amino acid sequences of the ND4L gene in mt genomes of 250 fishes. Figure S1-k. Aligned amino acid sequences of the ND4 gene in mt genomes of 250 fishes. Figure S1-l. Aligned amino acid sequences of the ND5 gene in mt genomes of 250 fishes. Figure S1-m. Aligned amino acid sequences of the ND6 gene in mt genomes of 249 fishes. (ZIP 3250 kb) [file 12864_2016_3054_MOESM6_ESM.zip › Additional file 6 prot align/AF6a-ATP8.pdf]

Additional file 6: Figure S1-a. Aligned amino acid sequences of the ATP8 gene in mt genomes of 250 fishes.

Species name abbreviation followed by aligned amino acid sequences shown by one letter abbreviation. See Additional file 1 for abbreviation of species name. Amino acids shown by magenta letter denote hydrophobic residues. Bold type A with yellow background indicates putative transmembrane regions. Asterisk '*' indicates a fully conserved residue. Colon ':' and period '.' indicate 'strong' and 'weak' groups in the level of conservativeness, respectively, in the Gonnet Pam250 matrix, in which the strong and weak groups are defined as strong score >0.5 and weak score =<0.5, respectively (Thompson et al., 1997).

ATP8

[1/2 of aligned sequences]

To be continued on page 6.

```
                             A
Scca    ----------MPQLNPSPWFIILLFSWVIFMVILPNKVMNHLFNNEPALKSTE-------
Muma    ----------MPQLNPNPWFLILLFSWIVFLTILPNKVMSHLFNNDPTLKSTE-------
Erca    ----------MPQLNPDPWFIILIFTWTVFLTILPNKITFYKIPNEPLNS----------
Pose    ----------MPQLNPDPWFIILIFTWTVFLTILPNKVIMHKTPNEPLTK----------
Actr    ----------MPQLNPSPWFMILIFSWLIFLIILPPKVLGHTFTNEPTHKNAE-------
Scal    ----------MPQLNPSPWFMILIFSWLIFLIVLPPKVLGHTFTNEPTHKNAE-------
Posp    ----------MPQLNPSPWFMILVFSWLVFLIILPPKVLSHTFTNEPTHKNAE-------
Atsp    ----------MPQLNPTPWFFILILSWLTFLIIIPSKVMGHSFATEPTAQNVE-------
Leoc    ----------MPQLNPTPWFFILFLSWLTFLIILPSKIMEHSFTMEPTTQSVE-------
Amca    ----------MPQLDLAPWVLYLLYSWMILLVVLTPKILGHTFPNEPTTQ----------
Osbi    ----------MPQLNPAPWLLIFLFSWLVLLTMIPPKILKHHFTNEPTSQTTE-------
Pabu    ----------MPQLNPTPWFLMLLFSWLVFLM-IPQKIMKHHFSTDPSTSN---------
Hial    ----------MPQLNPAPWFLILLFSWLVFLTIIPTKIMGHSFTNEPTTRTAK-------
Elha    ----------MPQLNPAPWFAILVFSWLVFLTTIPTKVMAHSFMNEPSTQSAE-------
Mlcy    ----------MPQLNPSPWFAILMFSWLMFLTTIPPKIMAHHFTNDPNTQSTE-------
Algl    ----------MPQLNPAPWFFIFVLSWFIYLTVIHTKVTGHVFANEPTVSV---------
Ptgi    ----------MPQLNPAPWFAILVFTWLVFLTIIPTKVMAHTFTNEPTTQSAE-------
Alaf    ----------MPQLNPAPWLAILLFSWLMFLIIIPTKVMGHLFTNEPSVQSAE-------
Nock    ----------MPQLNPAPWLAILLFSWLMFLIIIPTKVMGHLYSNEPSVQSAE-------
Anja    ----------MPQLNPAPWFTILVFSWAVFLAILPTKVMAHTFNNEPNLQTAK-------
Gyki    ----------MPQLNPNPWFMILVFSWLVFIFIAPTKVMSHTFNNEPNPRTTE-------
Syka    ----------MPQLNPAPWFAILVFSWAVFLTVIPTKIMAHTFNNEPNPQATK-------
Opma    ----------MPQLNPTPWFAILVFSWVIFLAIIPTKVMGHIFNNEPNPQTAK-------
Comy    ----------MPQLNPTPWLAILVFSWAAFLIILPTKVISHIFTNDPNPQKAK-------
Sasp    ----------MPQLNPDPWFMILIVAWVAFLVIIPVKILTHVINNEIAQQ----------
Eupe    ----------MPQLNPSPWFMILVFSWAVFLIIIPTKIMAHATSNQPTSLQ---------
Enja    ----------MPQLNPAPWFFILILSWLTFLIILPPKVLAHEFTNEPTVMGAE-------
Same    ----------MPQLNPAPWFAILVFSWLIFLTVIPPKVLAHNFNNEPSTIGAE-------
Chch    ----------MPQLNPAPWFAILIFSWLVFLTVIPPKVLGHSFTNEPTPLNVK-------
Grgr    ----------MPQLDPAIWFPILIFSWILFTVAIPHKVATLASPNTPKDPNTEEANVPPS
Caau    ----------MPQLNPGPWFAILVFSWLVFLTIIPTKILSHISPNEPTPVSAE-------
Cyca    ----------MPQLNPGPWFAILVFSWLIFLTIIPTKILSHISPNEPTPVSAE-------
Dare    ----------MPQLNPKPWFMILFFSWVIFLTIIPTKIINHIQPNDPTQVD---------
Cost    ----------MPQLNPAPWFMILLFSWLIFLTVIPTKVLNHISPNEPTPFSTD-------
Leec    ----------MPQLNPAPWFAILVFSWLIFLTIIPTKVMNHITPNEPAILDSE-------
Fola    ----------MPQLNPAPWFTILVFSWMIFLAIIPTKVMGHTSPNDSSPLSTE-------
Clmc    ----------MPQLNPAPWFAILVFSWLIFLTIVPSKVLKHTFNNEPTTLSVE-------
Phin    ----------MPQLNPSPWFLILLFSWMIFLLIIPSKVLKHVFPNEPEVLNAK-------
Icpu    ----------MPQLNPAPWFAILVFSWLIFLTVIPNKVLNHTFTNEVTALSAE-------
Psto    ----------MPQLNPAPWFAILVFSWLIFLTVIPHKVLSHTFTNEITALSAK-------
Cora    ----------MPQLNPAPWFFILVFTWLIFLTIIPYKILNHTFTNDMNPISTE-------
Eisp    ----------MPQLNPAPWLAILLFSWFIFLAVLPAKVMKHTFTNEPTALSAK-------
Apal    ----------VPQLNPAPWFTILLFSWVIFLTVIPPKMLKHLFISPPTALSTK-------
Eslu    ----------MPQLNPAPWLSILVFSWFVFLFVIPPKVLNHSFMNEPAPQSAE-------
Dape    ----------MPQLNPAPWFSILIFSWLVFLIVIPPKMLNHTFINEPAPQNTE-------
                                   1
```

[1/2 of aligned sequences]

```
Glse      ----------MPQLNPTPWFPILVFSWLVLLTIIPPKVTSHTFSNEPDTLSTQ-------
Naar      ----------MPQLNPAPWFAILVFSWLVLLTIIPPKILNHTFPNEPSTLSAQ-------
Lioc      ----------MPQLNPAPWFAILVFSWLVLLTIIPPKVLNHTFPNEPSTLSAQ-------
Opso      ----------MPQLNPAPWFIILVFSWLVFLVVLPPKVLSHTPTNQPDALNTQ-------
Alte      ----------MPQLNPAPWFAILVFTWLVFLTMIPPKVLGHTFTNEPTALSTE-------
Plap      ----------MPQLNPAPWFAILVFSWLVFLTVIPPKVLAHTFTNEPTTTSTE-------
Plal      ----------MPQLNPAPWFAILVFSWLVFLTVIPPKVLGHVFSNEPTVQSAE-------
Sami      ----------MPQLNPAPWFAILVFSWLVFLTVIPPKVLGHTFPNEPTVQSAE-------
Rere      ----------MPQLNPAPWFAILVFSWVIFLTVIPPKVLGHIFTHEPTVSSSE-------
Gama      ----------MPQLNPAPWFAILTFSWLIFLTIIPPKVLGHTFPNEPTAQSTE-------
Onmy      ----------MPQLNPAPWFAILVFSWLVFLTVIPPKVLGHTFTNEPTSQSTE-------
Sasa      ----------MPQLNPAPWFAILVFSWLVFLTVIPPKVLGHTFTNEPTSQSTE-------
Cola      ----------MPQLNPAPWFAILVFSWLVFLTVIPPKVLGHTFTNEPTSQSTE-------
Dita      ----------MPQLNPSPWFAILLFSWLVFLIILPPKIIAHTFPGEPAL-STE-------
Gogr      ----------MPQLNPAPWLSISLLAWLILLTLIPPKILAHFTHNKL-TP----------
Chsl      ---------MLPHLEPTHWFYTLICAWLMFLVLIYPYITSLTFPNEPSFVPE--------
Atja      ----------MPQLNPAPWFSILIFSWLVFLLIIPSKVIKHTFPHDPASHDTK-------
Iido      ----------MPQLNPAPWFSILVFSWLVFLLIIPSKVIKHTFPNEPASHSTK-------
Auja      ----------MPQLNPAPWFAILVFSWLVFLAVIPPKILSHSFPNDPTTQSTK-------
Chag      ----------MPQLNPNPWFAILVFSWLVFLIVIPPKIVNHTFPYDPTATSVE-------
Hami      ----------MPQLNPAPWFAILVFSWLVFLVILPSKVLNHIFPNDPTTKSTE-------
Saun      ----------MPQLNPAPWFAILVFSWLVFLTVLPPKILNHSFPNDPTTQSTE-------
Nema      ----------MPQLNPAPWFTILVLSWLVFLVTIPPKVLSHTYPHDPATQSAE-------
Disp      ----------MPQLNPAPWFAILVFSWLVFLITIPPKVLSHTFPHEPTSQSTE-------
Myaf      ----------MPQLNPAPWFAILIFSWLVFLITIPPKVLSHTFPNETTSRSTE-------
Lagu      ----------MPQLDPAPWFYILAYSWLVFLTILPAKTTTHIFPNNPAPEDPK-------
Trtr      ----------MPQLNPAPWFLIFIFSWFVFLSILPPKIMTHSFPNEPLVKSSE-------
Zucr      ----------MPQLNPSPWFLIFIFSWFVFLTILPPKVMTHNFPNEPLAKDVK-------
Pxja      ----------MPQLNPSPWFAIMVFSWLIFLTVIPPKVLAHNFPNDPALHSTE-------
Pxlo      ----------MPQLNPSPWFAIMVFSWLIFLTVIPPKVLAHNFPNDPALHSTE-------
Pctr      ----------MPQLNPSPWFMVLVFSWLVFLTILPPKIVAHTFPNDPAAQSTE-------
Apsa      ----------MPQLNPAPWFMILVFSWCIFLSVLPPKVMAHSFPNDPNPQSTK-------
Cabe      ----------VPQLIPGPWLMMLVLSWTTLLFVIPPKVLAYKYPAKPDSQ----------
Bzze      ----------MPQLNPGPWLMILIFAWTVLLVVIPPKILAHTYPNEPTAQ----------
Siim      ----------MPHLELTSWLLNCWVSWIMILVVIPFKIIELRYVETGAPLLTG-------
Ctru      ----------MPQLNPAPWLAILIFSWLVFTTVMPPKILAHTFPNEPTTQSTE-------
Dpbr      ----------MPQLNPAPWFAILIFSWLVFTTVIPPKILAHTFPNEPTAQSTE-------
Caki      ----------MPQLNPNPWFMIMAYTWTIFLLIVMPATLLFIIPNETTTQ----------
Phja      ---------VNKEKEESRVLWIFLVGWCCFLAMIPLTLAKVLYPNLPNPD----------
Brsp      ----------MPQLNPIPWFSIAVFTWMIFLFIMLPKVYSHQFPNNPSFY----------
Gamo      ----------MPQLNPAPWFMIFMFTWAIFLTILPPKVMAHTFPNEPSPQGMT-------
Lolo      ----------MPQLNPAPWFMIFMFTWVIFLTILPPKVLAHTFPNEPSPQSMT-------
Batr      ----------MPQLDPHPWLFNHVFTCFILFIPL-MALITFTLTNKTMPK----------
Prmy      ----------MPQLNPSPWLKFFLLSWMVFIIPMAVISMGTY-TSAPSST----------
Lose      ----------MPQLDLSPWFLVLFLTWSVFLLIVPQKVLAHSFPFKPNSR----------
Loam      ----------MPQLNPTPWFAILVFSWLVFLTILPSKVMAHAFPNEPTPQSTE-------
Chab      ----------MPQLNPTPWLYILLLTWLIFLVVLPPKIMAHVFPNMPHLP----------
Chto      ----------MPQLNPTPWLYILLLTWLIFLVVLPPKIMAHVFPNMPHLH----------
Majo      ----------MPQLAPTPWLAILLFTWLIFLIILPPKILAHNLPNEPAPQAAT-------
Hlst      ----------MPQLSPGPWFAILLFSWFILLTILVPKVLTHSLVNEPMPN----------
Clpe      ----------MPQLNPAPWFTILLSTWLILLVVLTPKVLTHTFPYAPDPA----------
Mlmr      ----------MPQLNPLPWFTILFYSWLVFLVILPPKVLAHSFPNEPNPEATE-------
                             2
```

To be continued on page 6.

To be continued on page 7.

[1/2 of aligned sequences]

```
Crcr     ----------MPQLNPAPWLLILLFSWLIFLTIIPPKILSHTSPNEPAHQSAE-------
Muce     ----------MPQLNPAPWLLILIFSWLIFLTIIPPKILSHTSPNEPAHQSAE-------
Bege     ----------MPQLNPSPWFAILVFSWLIFLIFIPPKVLAHIFPNEPTPQSTE-------
Mela     ----------MPQLKLKPWFPILALAWLIFMALAPRKVIEYSYPNRFTPQ----------
Hats     ----------MPQLNPAPWFAILVFSWLVFLIVIPPKVLAHIFPNEPTPQSTE-------
Orla     ----------MPQLNPAPWFAIMVFSWLVFLAVLPPKVLAHYFPNDPTPQSVK-------
Cosa     ----------MPQLNPAPWFAILMFTWLIFLTIIPPKIMAHTFPNEPNTMSTQ-------
Exsp     ----------MPQLDPAPWFAILVFSWFIFLTIIPPKVLAHSFPNEPTPQSTK-------
Depa     ----------MPQLNPSPWFAILAFTWLIFLTVIPQKIKAHTFPNSPTSQSVK-------
Rima     ----------MPQLNPAPWFLILVFSWLIFLALMPPKILTHQFPHEPSTLTAH-------
Fuol     ----------MPQLMPEPWFMTFLLTWSVLLTVIPMQIMALTFPYEPTLQ----------
Gmaf     ----------MPQLTPTPWLAYLLFSWLIFSVIVLPKVSTHTFMESPAPL----------
Xeei     ----------MPQLDPAPWFLILVFSWLVFLTLIPSKVLAHNFSNEPASHTKQ-------
Pros     ----------MPQLNPSPWLAILIFSWLIFLVFLPPKVLAHTFPNEPSAQSAE-------
Scmi     ----------MPQLATGPWFYILVFSWLIFLTVLPPKVFSHKFPNEPSPQSTE-------
Rolo     ----------MPQLNPAPWLMILIFSWLIFLTLIPPKILAHTFPNEPTTQSAE-------
Cere     ----------MPQLNPAPWFIILIFSWAMFLTIIPPKILAHTFPNEPSTQSTK-------
Daga     ----------MPQLNPSPWLAILVFSWLVFLSFVPFKVLAHTFPNEHQAQDEK-------
Anco     ----------MPQLNPAPWFLILIFSWTIFLALLPSKVLAHTFPNEPTTQSTE-------
Dmve     ----------MPQLNPAPWFATLVFSWFIFLVVIPPKILAHTYPNEPTTQSTE-------
Dmar     ----------MPQLNPAPWFATLVFSWFIFLVVIPPKILAHTYPNEPTAQSTE-------
Anka     ----------MPQLNPAPWFLILVFSWFIFLTLLPPKVLAHSFPNEPTAQSTE-------
Moja     ----------MPQLNPAPWFLILVFSWFIFLALLPSKVLAHTFPNEPTTQSTE-------
Hoja     ----------MPQLNPAPWFLILVFSWFIFLALLPSKVLAHTFPNEPTTQSTK-------
Bede     ----------MPQLNPAPWFAILIFSWLIFLTFLPPKVLAHTFPNELTTQSTE-------
Besp     ----------MPQLNPAPWFAILIFSWLIFLTFLPPKVLAHTFPNELTTQSTE-------
Mysp     ----------MPQLDPAPWFMTLVFSWAVFLVVIPPKVTAHVFPNEPSARTAE-------
Osja     ----------MPQLNPAPWFMILVFSWFIFLTLIPPKVMAHNFPNEPSPQAAE-------
Sgro     ----------MPQLNPAPWFMILVFSWFIFLTLIPPKVLAHTFPNEPSAQSAE-------
Pzpa     ----------MPQLDPNPWLSIFLLTWSVFLVVLPTKILAHNFPNELILQ----------
Zeja     ----------MPQLNPAPWLMIFMFSWLVFLTVVVPKALAHSFPNEPNPQATE-------
Znne     ----------MPQLNPTPWLMILLFSWMVFLTFVPPKILAHTLPNESVPQ----------
Zefa     ----------MPQLNPAPWLMILLFSWMVFLTMVPPKVLAHNFPNEPTVQDSK-------
Acni     ----------MPQLNPAPWLMIFVFSWLVFLAVIPPKILAHNFTNEPTTQTTE-------
Ncrh     ----------MPQLNPAPWLMIFVFSWLVFLAVIPPKILAHNFTNEPTPQTTE-------
Agca     ----------MPQLNPTPWFAILTFSWLIFLMVLPSKVLAHTFPNEPASQSTE-------
Hydy     ----------MPQLDPSPWFAMLVFSWLVFLAIIPPKVMAHTFSNEPTLQSTD-------
Gsac     ----------MPQLDPSPWFAMLIFSWLVFLVVIPPKVMAHIFSNEPAMQSAE-------
Pevo     ----------MPQLNPSPWFLIMLFSWLVLLTLVFPKIMQHESTNEPVHQ----------
Hiku     ----------MPQLNPSPWLMILLFTWLVFTTIIPPKIMAHKYPNEPNVLSTK-------
Inpa     ----------VPQLTPNPWLYILLASWLVFLTVLPTKTLTYVFPNNPALK----------
Auch     ---------MRP--PLPNWLISLLVAWATITGKLPYGFSHTNL---FAPP----------
Fico     ----------MPQLNPSPWFAILIFSWLVFLTVIPPKILAHTFPNEPTAQSTE-------
Macs     ----------MPQLDPSPWFAILVFSWVIFITIIPPKIMAHIFPNEPTSQSAE-------
Moal     ----------MPQLNPAPWFYILIFSWLVFILIIPHKVVAHTYPNPPTSKTTE-------
Syma     ----------MPQLNPSPWFTILIFSWFIFLALLPSKITAHTFPNEPQLKTSK-------
Mafr     ----------MPQLNPAPWFAILTFSWLIFLTILPSKTTAHPFPNEPAPQSTM-------
Dcpe     ----------MPQLNPSPWFLILIFSWLVFLTVIPPKVLSHSLLNDPNPS----------
Dcti     ----------MPQLNPSPWFAILIFSWLVFLTVIPPKVLSHTLLNDPNVSDSL-------
Hehi     ----------MPQLNPAPWFAILVFSWMVFLAVIPAKVTAHTFPNTPTLQSAK-------
Stam     ----------MPQLNPAPWFAILAFSWLVFLTLLPAKITAHTFPNEPTLQSTE-------
Hogi     ----------MPQLNPTPWLAMLVFSWMVYMSILPIKVMAHSFVHKPTPKT---------
                                    3
```

To be continued on page 8.

[1/2 of aligned sequences]

```
Erzo    ----------MPQLNPAPWLAILVFSWLVFLVVIPPKVIAHTFPNEPTLQSAE-------
Hxot    ----------MPQLNPAPWFAILIFSWLIFLTVIPPKVMAHTFPNEPTLQSAE-------
Core    ----------MPQLNPAPWFAILVFSWLVFLAIIPPKVMAHTFPNEPTLQSAE-------
Apve    ----------MPQLNPTPWFTILIFSWLIFLTVIPPKVMAHTFPYEPTLQSTE-------
Latj    ----------MPQLNPAPWLTILVFSWLIFLTMIPPKILAHSFPNEPTPHSAQ-------
Laja    ----------MPQLNPGPWFAILIFSWLVFLTVVPPKVMAHLFPSDPSMQNTE-------
Syja    ----------MPQLDLAPWFYILIVSWLLLATILPTKLLAHVFTKTPVDS----------
Epme    ----------MPQLLPLPWFGTLLFAWVVFLAFFPKKVMAHTFPYQPAPL----------
Grse    ----------MPQLNPAPWFIILAFTWVIFLTILPPKILAHTTPNSPSLLDKK-------
Clja    ----------MPQLNPTPWFSFLALAWLVLLVILPVKILSHTFPNAIALL----------
Ogcy    ----------MPQLDPTPWFAIFIFSWAVFLTVLPPKIASHTFPNDPAPQGTT-------
Plna    ----------IPQLDPSPWLFIMATSWLTFLLLIPSKLMAHQYPHDPSPQ----------
Lema    ----------MPQLNPAPWFAILVFSWLIFLTIIPPKVLAHTFPNEPTLQSAE-------
Etzo    ----------MPQLNPAPWFAILVFTWLIFLVIVPTKILAHTYPNEPTSQSTE-------
Apse    ----------MPQLNPAPWFMILVFSWLVFLTIIPPKIIAHTFPNEPTSQAAQ-------
Epde    ----------MPQLNPSPWFAILVFSWLILLTVVPPKVMAHTFLNEPNPQSAK-------
Slja    ----------MPQLNPGPWFMAFVFSWLVFLTIIPSKVLANSFPNEPASHAVE-------
Bsja    ----------MPQLNPLCWFNMLAFSWVAFTTIIPTKIKNVSFPNFPELKE---------
Ecna    ----------MPQLNPAPWFAILIFTWLVFLTIIPPKVLAHQFPNDPTLQSTQ-------
Cohi    ----------IPQLNPSPWLMTLVMLWLVFLIFLPPKILPHTLPNKPVII----------
Caar    ----------MPQLNPAPWFAILTFSWLIFLTVIPPKVMAHTFPNEPTPQSTE-------
Came    ----------MPQLNPAPWFAILTFSWLIFLTVLPSKVMSHTYPNEPTPQSTE-------
Mema    ----------MPQLNPAPWFSILIFSWLVFLIMIPPKVLAHTYPNEPTPQSTE-------
Lenu    ----------MPQLNPSPWLSILVFSWLVFLTILPLKVTAHIYPNEPAPQTAQ-------
Brja    ----------MPQLNPSPWLAILVFSWLIFLIIIPPKVMAHTFPNEPSPQSTE-------
Plma    ----------MPQLNPAPWLAILVFSWLVFLVIIPPKVMAHTFPNEPTPQSTE-------
Emst    ----------MPQLNPAPWFAILVFSWLVFLTILPPKVMAHTFPNEPTPQSTQ-------
Ptti    ----------MPQLNPAPWFAILVFSWLVFLTILPPKVMAHTFPNEPTSQSTE-------
Losu    ----------MPQLNPTPWLLTFIFSWVTFLTILTAKVLAHQFPTEPSLQ----------
Geoy    ----------MPQLNPTPWLAIFMFSWFVFLATIPSKMMAHDFPNDPTLQAAL-------
Dipi    ----------MPQLNPSPWFAILVFSWLVFLTILPPKVMAHTYPNDPDPQTSE-------
Pama    ----------MPQLNPAPWFNILVFSWVVFLTILLPKVLAHTFPNEPTLQSTE-------
Leob    ----------MPQLNPSPWFLILVFTWLVFLYFLPPKVLGHKYPNDPNPTTAE-------
Neba    ----------MPQLNPAPWFAILVFSWTVFLAILPPKVMAHSFPNEPAPQNTQ-------
Pdpl    ----------MPQLNPSPWFYIFIVSWLVLLTVAFPKTLDYTFPNEATLQ----------
Nimi    ----------MPQLNPAPWLAIMVFSWLTFLIILPPKVMAHLFPNEPALQSTQ-------
Uptr    ----------MPQLDPAPWFNIFALSWAILLTVMPPKVLAHTFPAEPTAQSAE-------
Pesc    ----------MPQLNPTPWLLTFLFSWTIVIALLPLKVAKYITPNLPSQK----------
Baar    ----------MPQLIPAPWFAILIFSWATFLILLPPKILAHVFFNEPSPLNTK-------
Moar    ----------MPQLNPAPWFAILVFSWLVFLTVLPPKVMAHTFPNEPTSQSTE-------
Toja    ----------MPQLNPAPWFTILIFSWLIFLTVLPPKIMAHSYPNVLTHQNTK-------
Chau    ----------MPQLDPAPWFNILVFSWLVFLVIIPPKIMAHTFPNELNPQGTH-------
Chse    ----------MPQLNPRPWMAILMFTWVVFLTVIPTKIIEHCFPNEQIDS----------
Enar    ----------MPQLNPTPWFATLIFSWLIFLTVIVPKVLSHTFPNEPTPQSTE-------
Hpty    ----------MPQLNPAPWFAILIFSWLIFLTVVPPKVMAHTFPNEPSPQSTE-------
Nana    ----------MPQLNPAPWFSTTTFSWFIFMTLIPPKVIEYIFPKKPAPK----------
Mcst    ----------MPQLDPAPWFTILFFSWTVFLVVIPPKVMAHTFPNEPAHQTAE-------
Rhox    ----------MPQLNPAPWFAILVFSWLVLLVVIPPKVVAHTFPKEPTLQSAE-------
Opfa    ----------MPQLNPAPWFTILIFSWMIFLIVIPPKVMAHTFPYEPTPQSAE-------
Paar    ----------MPQLDPAPWFAIFFFSWTVFLIVLPPKVLSHTFPNEPAAQSTK-------
Gozo    ----------MPQLNPTPWCAILIFSWLVFLTIIPPKVLAHTFPNEPTSQSTE-------
Ackr    ----------MPQLNPAPWLAILIFAWLTFLLILPPKIMAHTFPKEPTPQDTK-------
```

To be continued on page 9.

[1/2 of aligned sequences]

```
Elev    ----------MPQLNPAPWLMILIFSWFVFLTLIPPKVLAHTYPNEPSPRDTK-------
Trdu    ----------MPQLNPAPWFAILVFSWLVFLTVIPPKVLAHTFPNDPTLQSTE-------
Amoc    ----------MPQLNPAPWFTILMFSWLIFLTVIPPKILAHTFPNEPTHQSTE-------
Hame    ----------MPQLNPSPWLAIFLFSWLVFLTVIPPKVLAHTFSNEPTSQSTE-------
Chso    ----------MPQLNPRPWFWIFMFAWVIFLGVLPKKVLSHKFLNDPASE----------
Lyto    ----------MPQLNPTPWFAILVFSWLVFLAVLPPKVLAHTFPNEPTLQSTD-------
Encr    ----------MPQLNPAPWFAILVFSWLVFLTIIPPKVLAHTFPNEPTLQSTE-------
Bvar    ----------MPQNIFSGPLTMMFLGLQLFFTAGHAKVMNHVSSPTPNLTLP--------
Noco    ----------MPVNYHTSCFTMLALSLFLYFTMGHAKIVGHATTLKPS---P--------
Chsp    ----------MPQLNPAPWFLILIFTWTIFLAMMLPKIMAQRAPLNPLPH----------
Arja    ----------MPQLNPAPWFAILVFSWLVFLAVIPPKVMAHTFPNEPTLQSAA-------
Pase    ----------MPQLNPAPWLAIFMFTWIVFLVVIPPKVMAHMFPNPPLSKETK-------
Trel    ----------MPQLNPLPWFLTLTTGWVTLTVFVFPKVLKLTFPNEPAPK----------
Lifa    ----------MPQLNPEPWFSTACLVWVTFLVVLMPKFVDLVLPSSPLAGPSA----PGT
Acur    ----------MPQLMTNIWFNVLILAWVVFLVILIPKMMNFKFLNSITPSQH--------
Ampe    ----------MPQLNPTPWFAILAFSWLVFLTVIPPKVMAHTFPNTPTSKSTE-------
Urja    ----------MPQLNPSPWFYILVTSWMIFLTIIPPKLLTHAFPNKPAPKT---------
Enet    ----------MPQLNPSPWFAILVFSWLVFLAIIPSKILAYKFPNDFSTPSTE-------
Ptbr    ----------MPQLDPAPWLMISTFAWINLLVFLPPKIATHIYPKEPSMP----------
Safa    ----------MPQLNPAPWFMILFFSWLVFLTVIPPKILAHKFPNEPSLTTAE-------
Icae    ----------MPQLNPAPWLAILVFSWLVFLIVLPPKVMAHTFPNEPTPQSTQ-------
Asmi    ----------MPQLDPTPWLITLLFSWFTLLTIVPLKLLPHLVPNEFTRK----------
Foal    ----------MPQLDPAPWFLIMTFSWTIFLGFMPFKIMSHAFPNKPDSS----------
Drze    ----------MPQLNPAPWLLTLLFSWLVFLIIMPQKVSSHIFPNGPNTL----------
Rhas    ----------MPQLNPAPWFAILVFSWLVFLTILPPKVLAHTFPNEPTPQSTQ-------
Elac    ----------MPQLNPAPWFAILVFSWLVFLTILPPKILAHTFPNEPTPQSTQ-------
Kugu    ----------MPQLNPTPWFAILIFSWLVFLTIIPTKVLSHTFPNDPTPQSTQ-------
Plor    ----------MPQLNPAPWFAILVFSWLVFLITIPPKITAHTFPNEPTLQSTE-------
Sgun    ----------MPQLNPAPWFAILVFSWLVFLTILPPKVMAHTFPNEPTLQSTE-------
Zaco    ----------MPQLNPSPWFAILVFSWLVFLTVLPPKVMAHTFPNDPSPQSTE-------
Zbfl    ----------MPQLNPAPWFAILVFSWLVFLTVLPPKVMAHTFPNEPTPQSTE-------
Spba    ----------MPQLDPAPWFPILILCWTVFLIFIPPKVMAHTQPNQPTPQSAE-------
Game    ----------MPQLNPAPWLAILVFSWLVFLIIIPPKVMAHTFPNEPTPQSTE-------
Thth    ----------MPQLNPAPWLAILVFSWLVFLIIIPPKVMAHSFPNEPTPQSTE-------
Xigl    ----------MPQLNPAPWFAILAFSWLVFLTVIPPKVLAHSFPNEPSSHSAE-------
Hyja    ----------MPQTMPAPWFYSLACIWCVFLCAIVPKLIIQKFPNKFPAKVMKKD-----
Psan    ----------MPQTMPTPWFYSFLCTWCMFLAIVVPLLMAQKFPNKFPVKLEKKT-----
Cupa    ----------MPQLNPAPWLAILVFSWLIFLIIVIPKVMAHTFPNEPTPQSTQ-------
Mpch    ----------MPQLNPAPWLAILFFSWFIFLTIIPPKIMAHTFPNESALKDSK-------
Char    ----------MPQLNPSPWFAILVFSWLIFLLIIPQKILAHNFPNEPTPLSTE-------
Pser    ----------MPQLDPAPWLAILVFSWLVFLIVIPPKVMAHSFPNEPTSQSTE-------
Prol    ----------MPQLNPAPWFMILVFSWMVFLTIIPPKVLAHTFPNEPTPQSTQ-------
Plbi    ----------MPQLDPAPWFAILVFSWMILLTIIPPKVLAHIFPNEPTSQSTQ-------
Calu    ----------MPQLNPAPWLMILVFSWLIFLTVVPIKVTAHITPSHPEPG----------
Papa    ----------MPQLDVKPWVPVLAVTWLAFLFLLPPKVISHRHHTNPTNP----------
Sufr    ----------MPQLNPAPWFAILVFSWLVFLFFLPPKVMAHVFPNESSPQNTE-------
Stci    ----------MPQLDPAPWLMILVLSWSILLTVIPPKVLSHTFPNTPNAQSAE-------
Taru    ----------MPQLNPAPWFLIMVFSWCVFLIFLPPKIMAHLFPNEPSSQNTC-------
Rala    ----------MPQLNPAPWFAILVFSWLVFLTVLPPKVSAHSFPNEPTPQSTE-------
```

To be continued on page 10.

[2/2 of aligned sequences]

```
Scca    ----KSKPDPWNWPWL*-------
Muma    ----KPKPNPWNWPWL*-------
Erca    -DPLGSNMDTWSWPWY*-------
Pose    -DPSSLLTEIWSWPWH*-------
Actr    ----KIKPEPWTWPWS*-------
Scal    ----KIKPEPWTWPWS*-------
Posp    ----KIKPEPWTWPWS*-------
Atsp    ----KPNPEPLNWPWP*-------
Leoc    ----KPNPEPLNWPWP*-------
Amca    -SAKKPETQSWAWPWS*-------
Osbi    ----KQPLAPWNWPWH*-------
Pabu    ---AKEQTSPWTWPWH*-------
Hial    ----ETHLTSWTWPWH*-------
Elha    ----KPKPEPWNWPWH*-------
Mlcy    ----KPKPEPWNWPWY*-------
Algl    -DKLESKPEPWNWPWH*-------
Ptgi    ----KPKPEPWNWPWH*-------
Alaf    ----KPKPEPWNWPWH*-------
Nock    ----KPKPEPWNWPWY*-------
Anja    ----KPKTDSWNWPWY*-------
Gyki    ----TTTTNPWNWPWH*-------
Syka    ----TPKTDPWNWPWH*-------
Opma    ----KPQLSSWTWPWY*-------
Comy    ----KPELETWNWPWY*-------
Sasp    -TTDKLKTNPWGWPWA*-------
Eupe    -APTALTPDPWNWPWH*-------
Enja    ----KPKPESWNWPWY*-------
Same    ----KAKPEPWTWPWY*-------
Chch    ----KAKPEPWNWPWH*-------
Grgr    QHPSPAPLQPWNWPWH*-------
Caau    ----KHKTESWDWPW*--------
Cyca    ----KHKTESWDWPW*--------
Dare    --AKEHKNDTWNWPW*--------
Cost    ----EHKAQPWDWPWQ*-------
Leec    ----KHKTEPWNWPWQ*-------
Fola    ----KHKTESWDWPWQ*-------
Clmc    ----KPKTEPWNWPWH*-------
Phin    ----DSKPEPWNWPWH*-------
Icpu    ----KLKSDTWNWPWH*-------
Psto    ----TLKSDTWNWPWH*-------
Cora    ----SLKTDTWNWPWH*-------
Eisp    ----APKTEPWNWPWH*-------
Apal    ----DSKTQPWNWP*---------
Eslu    ----KPKFNPWNWPWY*-------
Dape    ----KPKTEPWSWPWY*-------
Glse    ----KTKPEPWNWPWH*-------
Naar    ----KTKPEPWNWPWH*-------
Lioc    ----KTKPEPWNWPWH*-------
Opso    ----KPKPEPWDWPWY*-------
Alte    ----KTKPEPWNWPWH*-------
Plap    ----KTKPEPWNWPWH*-------
Plal    ----KTNPESWNWPWH*-------
```

[2/2 of aligned sequences]

```
Sami    ----KSSPESWNWPWH*-------
Rere    ----ETNPVSWTWPWL*-------
Gama    ----KAKPESWPWPWH*-------
Onmy    ----KAKPEPWNWPWH*-------
Sasa    ----KAKPEPWNWPWH*-------
Cola    ----KAKPEPWNWPWH*-------
Dita    ----KTKTEPWNWPWH*-------
Gogr    -GTEKTQTKPWDWSWY*-------
Chsl    -EKAPSTLQSWSWPWE*-------
Atja    ----PPTPNPWNLPWH*-------
Iido    ----PPAPSPWNLPWY*-------
Auja    ----TPKSQPWNWPWH*-------
Chag    ----KPAHTPWHWPWY*-------
Hami    ----KTKPEPWNWPWH*-------
Saun    ----KTKPEPWNWPWH*-------
Nema    ----KPKTDPWNWPWH*-------
Disp    ----KPKTNPWNWPWH*-------
Myaf    ----KSKTNPWNWPWA*-------
Lagu    ----KPTTQPWAWPWH*-------
Trtr    ----DIKTNPWNWPWH*-------
Zucr    ----DTKTNSWNWPWH*-------
Pxja    ----MTKTDSWNWPWY*-------
Pxlo    ----MTKTDSWNWPWY*-------
Pctr    ----TTKTEPWSWPWL*-------
Apsa    ----TTKTTPWTWPWY*-------
Cabe    -KSHTPTTPSWVLPWT*-------
Bzze    -NNKAPESQPWNLPWS*-------
Siim    YDVSPVPTLPWT*-----------
Ctru    ----KPKTEPWNWPWH--------
Dpbr    ----KPKTEPWNWPWH*-------
Caki    -NASTPKTEPWAWPWH*-------
Phja    -STKLPKSTPWHWPWR*-------
Brsp    -KTSSQQSTSWNWPWH*-------
Gamo    ----TPKTAPWNWPWH*-------
Lolo    ----APKTTPWNWPWH*-------
Batr    -QAQQ-SKTHTHWPWN*-------
Prmy    --MAKKASTFWSWTWT*-------
Lose    -HTKTTNLNNWNWSWH*-------
Loam    ----KSKTNPWTWPWH*-------
Chab    -HIFNFQTNNWYWPWR*-------
Chto    -HIFNFQTNNWYWPWR*-------
Majo    ----KPKTESWSWPWH*-------
Hlst    -TVKKTKKEAWPWPWQ*-------
Clpe    -TASTSKTQPWPWQWH*-------
Mlmr    ----ASQPQTWVWPWY*-------
Crcr    ----KPKTEVWTWPWY*-------
Muce    ----KPKTEVWTWPWY*-------
Bege    ----KPKTESWNWPWH*-------
Mela    -GSKTPKSLPWTWLWH*-------
Hats    ----KSKAESWNWPWH*-------
Orla    ----KSKSETWTWPWL*-------
Cosa    ----KPKTESWNWPWH*-------
```

[2/2 of aligned sequences]

```
Exsp    ----LPKTESWNWPW*--------
Depa    ----KHKTEPWNWPWY*-------
Rima    ----KPKANSWTWAWQ*-------
Fuol    -SDNALTTQTWNWQWL*-------
Gmaf    -TKQKPDTEPWNWPWH*-------
Xeei    ----KPQTESWNWPWH*-------
Pros    ----STRTQSWNWPWS*-------
Scmi    ----STRTQAWNWPWS*-------
Rolo    ----KPKTEPWNWPWH*-------
Cere    ----ETMTKPWNWPWH*-------
Daga    ----ESVTEPWNWPWH*-------
Anco    ----KPKTEPWNWTWH*-------
Dmve    ----KPPTTPWAWPWH*-------
Dmar    ----KPPTTPWAWPWH*-------
Anka    ----KPKTEPWNWTWH*-------
Moja    ----KPKTEPWNWTWH*-------
Hoja    ----KPKTEPWNWTWH*-------
Bede    ----SPNTQSWNWPWS*-------
Besp    ----SPNTQAWNWPWS*-------
Mysp    ----TLEHKPWHWPWH*-------
Osja    ----TLKTKSWNWPWH*-------
Sgro    ----VHKTKAWDWPWH*-------
Pzpa    -ITEATKANSWNWPWL*-------
Zeja    ----TMKATPWNWPWP*-------
Znne    -DSKTDLAHSWNWPWY*-------
Zefa    ----TTETSPWTWPWQ*-------
Acni    ----TMKPNPWNWPWH*-------
Ncrh    ----TMKPSPWNWPWH*-------
Agca    ----KPKTEPWTWPWH*-------
Hydy    ----KRLANPWIWPWP*-------
Gsac    ----KRFTDPWTWPWP*-------
Pevo    -SAKALLTTPWTHQWQ*-------
Hiku    ----TLETTPWNWQWH*-------
Inpa    -GLEKYKMTPWSWPWH*-------
Auch    -RKKKPSLFSRIWKFFRGR*----
Fico    ----TPKTDPWNWPWQ*-------
Macs    ----APKTEPWNWPWH*-------
Moal    ----SPKTESWSWPW*--------
Syma    ----PIKTEPWNWPWS*-------
Mafr    ----TPKTETWNWPWY*-------
Dcpe    -DSPSYQAEAWSWPWS*-------
Dcti    ----SYLAEPWNWPWS*-------
Hehi    ----KAETDPWTWPWH*-------
Stam    ----KSKTEPWTWPWH*-------
Hogi    --AKEFKTQPWTWPW*--------
Erzo    ----KPKADSWIWPWY*-------
Hxot    ----KPKTESWTWPWQ*-------
Core    ----KPKTEPWTWPWQ*-------
Apve    ----NPKTAPWAWPWQ*-------
Latj    ----MPKTDPWVWPWH*-------
Laja    ----KSSTESWNWPWY*-------
Syja    -STAQPKTNNWQWPWFPASSTNS*
```

[2/2 of aligned sequences]

```
Epme    -KPQKLEKTPWNWPWA*-------
Grse    ----KQKMTPWSWPWH*-------
Clja    -VAGKLNMNTWNWPWH*-------
Ogcy    ----KPTTQPWNWPWP*-------
Plna    -NAEKPKTTSWDWPWH*-------
Lema    ----TPKTEPWNWPWH*-------
Etzo    ----KPKTEPWTWPWH*-------
Apse    ----KPKTDSWNWPWH*-------
Epde    ----TPKTESWNWPWH*-------
Slja    ----KMKSDPWSWPWH*-------
Bsja    --TDKPKTKSWTWPWL*-------
Ecna    ----KPTTDPWIWPWH*-------
Cohi    -DNKKSMANPWNWLWH*-------
Caar    ----KPKTEPWNWPWQ*-------
Came    ----KPKTEAWSWPWQ*-------
Mema    ----KPATDAWNWPWH*-------
Lenu    -TTDTLKTESWTWPWH*-------
Brja    ----KPKGEPWNWPWY*-------
Plma    ----KPKGEPWNWPWY*-------
Emst    ----KPKTEPWTWPWH*-------
Ptti    ----KPKTEPWTWPWH*-------
Losu    -NKKTLDLESWTWPWH*-------
Geoy    ----SPITEPWSWPWF*-------
Dipi    ----KSKTDPWNWPWY*-------
Pama    ----KPKTEPWTWPWY*-------
Leob    ----KPKTASWAWPWN*-------
Neba    ----KSQANSWSWPWL*-------
Pdpl    -DTQEAKRGTWLWPWT*-------
Nimi    ----ESKADTWNWPWL*-------
Uptr    ----APKTTPWNWSCP*-------
Pesc    -STAKPHTSSWLWPWR*-------
Baar    ----KSKQEPWYWPWH*-------
Moar    ----KPKTEPWTWPWH*-------
Toja    ----KTKTEAWPWPWH*-------
Chau    ----KPKMSSWAWPWH*-------
Chse    -APMFSEPDTWLWPWL*-------
Enar    ----KPKTEPWNWPWH*-------
Hpty    ----KPKTEPWNWPWH*-------
Nana    -TTQKPKTEPWGWPWH*-------
Mcst    ----KIKTSPWNWPWY*-------
Rhox    ----KPKTEPWNWPWH*-------
Opfa    ----KPKTDSWNWPWH*-------
Paar    ----TSKITPWNWPWY*-------
Gozo    ----KPQTNSWNWPWY*-------
Ackr    ----TNKHSSWAWSWP*-------
Elev    ----TFETKPWNWPWY*-------
Trdu    ----KPKTEPWSWPWY*-------
Amoc    ----KPKTDPWTWPWH*-------
Hame    ----APRTTPWAWPWH*-------
Chso    -TKETPVSAPWSWRWH*-------
Lyto    ----KPKTDPWTWPWH*-------
Encr    ----KPKTEPWTWPWH*-------
```

[2/2 of aligned sequences]

```
Bvar      QHSGGTTHQPWLWPWL*-------
Noco      WPAKFLTEQKWSWPWS*-------
Chsp      -NTKAPVTKTWTWPWR*-------
Arja      ----KPKTESWTWPWQ*-------
Pase      ----KAETTAWAWPWY*-------
Trel      -NAEEIKKLSWHWTW*--------
Lifa      SHNHPSNLTYWNWPWSQASSTNS*
Acur      -AGSQPSTTPWDWTWQ*-------
Ampe      ----KPKATTWNWPWH*-------
Urja      --PKERSVFNWNWPWR*-------
Enet      ----KVKSHPWNWPWH*-------
Ptbr      -QAKDYITEPWTW*----------
Safa      ----KTKTKSWTWPWY*-------
Icae      ----KPKGEPWNWPWH*-------
Asmi      -DTNKLKNTTWTWPWQ*-------
Foal      -AKEVSTKNSWTWPW*--------
Drze      -NTTAFKNNMWNWPWL*-------
Rhas      ----KSKPEPWNWPWH*-------
Elac      ----KPKTESWNWPWH*-------
Kugu      ----KSKTDPWNWPWH*-------
Plor      ----KSKTEPWTWPWH*-------
Sgun      ----KPKTEPWTWPWH*-------
Zaco      ----KPKTESWTWPWH*-------
Zbfl      ----KPMSEPWTWPWH*-------
Spba      ----KSKETPWTWPWY*-------
Game      ----KPKGEPWNWPWH*-------
Thth      ----KPKGEPWNWPWH*-------
Xigl      ----QPKTESWNWPWH*-------
Hyja      ----TMKPGDSEWPWH*-------
Psan      ----NLKPGDSEWSWH*-------
Cupa      ----KPKGEPWNWPWY*-------
Mpch      ----KPKTEPWNWPWH*-------
Char      ----ISKTEPWNWPWH*-------
Pser      ----KLEADSWAWPWY*-------
Prol      ----KPKTESWNWPWY*-------
Plbi      ----KSKTETWNWPWY*-------
Calu      -LTSTSDKAMWTWPWQ*-------
Papa      -AKNKQTKNPWNWPWH*-------
Sufr      ----KPKTESWNWPWH*-------
Stci      ----KLETNPWTWLWH*-------
Taru      ----PKEIKPWPWSWH*-------
Rala      ----KPQTESWSWPWH*-------
```
