# Supplementary material for: Structure and variation of the mitochondrial genome of fishes
Source: BMC Genomics. 2016 Sep 7;17(1):719. doi: 10.1186/s12864-016-3054-y (PMC5015259; doi:10.1186/s12864-016-3054-y)
Supplement: Additional file 6: Figure S1-a. — Aligned amino acid sequences of the ATP8 gene in mt genomes of 250 fishes. Figure S1-b. Aligned amino acid sequences of the ATP6 gene in mt genomes of 250 fishes. Figure S1-c. Aligned amino acid sequences of the COI gene in mt genomes of 250 fishes. Figure S1-d. Aligned amino acid sequences of the COII gene in mt genomes of 250 fishes. Figure S1-e. Aligned amino acid sequences of the COIII gene in mt genomes of 250 fishes. Figure S1-f. Aligned amino acid sequences of the Cyt b gene in mt genomes of 250 fishes. Figure S1-g. Aligned amino acid sequences of the ND1 gene in mt genomes of 249 fishes. Figure S1-h. Aligned amino acid sequences of the ND2 gene in mt genomes of 250 fishes. Figure S1-i. Aligned amino acid sequences of the ND3 gene in mt genomes of 250 fishes. Figure S1-j. Aligned amino acid sequences of the ND4L gene in mt genomes of 250 fishes. Figure S1-k. Aligned amino acid sequences of the ND4 gene in mt genomes of 250 fishes. Figure S1-l. Aligned amino acid sequences of the ND5 gene in mt genomes of 250 fishes. Figure S1-m. Aligned amino acid sequences of the ND6 gene in mt genomes of 249 fishes. (ZIP 3250 kb) [file 12864_2016_3054_MOESM6_ESM.zip › Additional file 6 prot align/AF6b-ATP6.pdf]

Additional file 6: Figure S1–b. Aligned amino acid sequences of the ATP6 gene in mt genomes of 250 fishes.

Species name abbreviation followed by aligned amino acid sequences shown by one letter abbreviation. See Additional file 1 for abbreviation of species name. Amino acids shown by magenta letter denote hydrophobic residues. A-F in bold types with yellow background indicate putative transmembrane regions. Highlighted 'H' and 'Y' letters indicate metal binding sites. Numerals on the amino acid sequences correspond to position number of amino acid residues in the human sequence. Asterisk '*' indicates a fully conserved residue. Colon ':' and period '.' indicate 'strong' and 'weak' groups in the level of conservativeness, respectively, in the Gonnet Pam250 matrix, in which the strong and weak groups are defined as strong score >0.5 and weak score =<0.5, respectively (Thompson et al., 1997).

ATP6

[1/4 of aligned sequences] **A**

```
Scca   --MIMSFFDQFLSPSFLGI-PLIALAISIPWLMFPTPTNRWLNNRLLTLQAWFINRFIYQ
Muma   --MIMSFFDQFLSPSLIGI-PLIALAILIPWLTFPTPSSRWLNNRLITLQAWFINRFIYQ
Erca   --MMLNFFDQFASQSLLGI-PLIAIAMLVPWLLFPSPYKRWLNNRLITFQYWFISRATNQ
Pose   --MTLTFFDQFASQTFLGI-PLIAIALLLPWLLFPTPNKRWMNNRLITIQSWFITRATNQ
Actr   --MILSFFDQFMSPTHLGI-PLIALALSLPWVLIPTPTNRWLNNRLLTLQGWFINRFTQQ
Scal   --MILSFFDQFMSPTHLGI-PLIALALSLPWVLIPTPTNRWLNNRLLTLQGWFINRFTQQ
Posp   --MILSFFDQFMSPTHLGI-PLIALALSLPWILIPTPTNRWLNNRLLTLQAWFINRFTQQ
Atsp   --MTLSLFDQFAIPTYLGI-PLIIIALTFPWILYPTPTSRWLNNRLLTLQGWFVNRFTQQ
Leoc   --MTLSLFDQFAIPTYLGI-PLIIIALTFPWILYPTPTNRWLNNRLLTLQGWFINRFTQQ
Amca   --MILSFFDQFDSPIICYI-PLMILAMTLPWILYPTPTDRWLNNRLLTLQGWFMNRFTQQ
Osbi   --MTLSFFDQFSITSFLGI-PLIALALTLPWILYPTPQNRCMNNRLLTLQSWFIRQFTHQ
Pabu   --MTLSFFDQFSLTSFMGV-PLVALALTLPWILWPTPQNRCLNNRLITLQSWFIRQLTNQ
Hial   --MTLSFFDQFSLSSYMGI-PLIVLALTLPWVLYPTPQNRYSNNRLLTLQAWFIRQFTHQ
Elha   --MALSFFDQFASPTYLKV-PLIALAIALPWVLYPAPTSRWLNNRLLTLQGWFINRFTQQ
Mlcy   --MMLSFFDQFASPTYLGI-PLIALALSLPWILYPTPSNRWLNNRLLTLQAWFINRFTQQ
Algl   --MALSFFDQFMSPTYLGV-PLVALALSLPWVLYPTPSSRWLNNRLLTLQGWFLNRFTQQ
Ptgi   --MALNFFDQFMSPTYLGV-PLIALALTLPWVLYPTPSARWLNNRMLTLQGWFINRFTQQ
Alaf   --MTLNFFDQFMSPTHLGI-PLIALALSLPWVLFPTPSARWLNNRLLTLQGWFINRFTQQ
Nock   --MVLNFFDQFMSPTHLGI-PLIVLALSLPWVLFPTPSARWVNNRLLTIQGWFINRFTQQ
Anja   --MMLSFFDQFMSPTFMGI-SLITLALTLPWILYPTPTSRWLNNRVLTLQGWFVNRFTQQ
Gyki   --MALNFFDQFMSPTLMGI-PLMGLALLLPWILYPTCTPRWLNNRLLTLQSWFIGLFTQQ
Syka   --MALNFFDQFMSPTYMGI-PLMALALTLPWLLYPTPTTRWLNNRLLTLQSWFINRFTQQ
Opma   --MMLNFFDQFMSPTYMGI-PLMAVALVLPWTLYPTPSLRWLNNRLLTLQGWFINRFTQQ
Comy   --MVLGFFDQFMSPVLMWI-PLMALALTLPWLLFPSPSMRWINNRLLTLQGWFINRFTQQ
Sasp   --MSLGLFDQFMSPSLMGL-PLIVPALILPWMLFFPPHSRWLNGRFSTLQGWFISQFARQ
Eupe   --MALNLFDQFMSPAFMGI-PLMALALTLPWVLLPTPSARWLDSRVSTLQNWFMCQFTRQ
Enja   --MMLSFFDQFASPNYMGI-PLIALAIVLPWILFPTPTSRWLNNRLLTLQGWFINRVSQQ
Same   --MMLSFFDQFMSPSYLGI-PLIAVAIALPWALYPTPTSRWLNNRVLTLQGWFINRFTQQ
Chch   --MALNFFDQFMSPTYLGI-PLIALALSLPWVLFPTPSSRWLNNRLLTLQGWFINRFTQQ
Grgr   --MALSLFDQFTSPVLLGI-PLIAIAVAMPWILLRSALNRFLTSRFLEIQSWLLHQFARQ
Caau   --MMVSFFDQFASPSYLGI-PLIAIAIALPWVLYPTSSSRWINNRLITIQGWFINRFTNQ
Cyca   --MMVSFFDQFASPSYLGI-PLIAIAIALPWVLYPTPPARWINNRLITIQGWFINRFTNQ
Dare   --MMTSFFDQFASPYLLGI-PLILVAMLLPWLLFPAPTSRWINNRLITVQTWLTGRFTNQ
Cost   --MAMSFFDQFASPSCLGI-PLIAVAIALPWVMYPTPSARWINNRLITIQGWLINRFTNQ
Leec   --MAMSFFDQFASPSYLGI-PLIAIAIALPWMLFPTPSSRWINNRLITVQGWLISRFTNQ
Fola   --MAMSFFDQFASPSYLGI-PLIAIAIALPWVLFPLPSLRWVNNRLITIQGWLINRFTNQ
Clmc   --MTLSFFDQFMSPTYLGI-PLIAIALTLPWILYPSPSNRWLNNRLITLQGWFINRFTQQ
Phin   --MTLSFFDQFMSPTYLGI-PLIAIALTFPWIMLPAPSSRWLNNRMITLQSWFINRFTQQ
Icpu   --MALNLFDQFMSPTHLGI-PLIAIALTLPWILIPSPTSRYQNNRLISLQSWFIKTFTQQ
Psto   --MALNLFDQFMSPTHLGI-PLIAIALTLPWILVPAPTNRYQSNRLVSLQSWFIKTFTQQ
Cora   --MALNMFDQFMSTAYLGI-PLITIALTLPWILVPSPSNRWQNNRLVTLQSWFIKNFTSQ
Eisp   --LALNFFDQFMSPTHLGI-PLIAIALSLPWILYPSPSDRWLNNRLITLQAWFMNRFTQQ
Apal   --MAPNFFDQFSSPTHFGV-PLIMIALTFPWILYPTPSNRWLENRLVTLQNWFINRFTQQ
Eslu   --MMLNFFDQFMSPVLLGI-PLIALALLLPWILFPNPAARWINNRLITLQGWSISQATHQ
Dape   --MMLNFFDQFMSPVFLGF-PLIALALCLPWMLFPSPTTRWINNRFITLQAWSINRATYQ
                                    1
```

To be continued on page 6.

[1/4 of aligned sequences]

```
Glse   --MTLSFFDQFMSPVHLGI-PLMALALTLPWILFPTPSARWLNNRLITLQAWFINRFTQQ
Naar   --MTLSFFDQFSSPTHFGV-PLMALALTLPWILFPTPSARWLNNRLITLQGWFINRFTQQ
Lioc   --MTLSFFDQFSSPTYLGI-PLMALALTLPWILFPTPSARWLNNRLITLQGWFINRFTQQ
Opso   --MMLNFFDQFMSPTHLGV-PLMALALTLPWLLLPAPSTRWLNSRLITLQSWFINQFTRQ
Alte   --MTLSFFDQFMSPTYFGI-PLMALALTLPWILYPTPSARWLNNRLLTLQGWFINRFTQQ
Plap   --MTLSFFDQFMSPTYLGI-PLMALALTLPWLLYPTPSTRWLNNRLVTLQGWFLNRFTQQ
Plal   --MTLSFFDQFMSPVFLGV-PLIALALSLPWILFPTPTARWLNNRLLTLQGWFINRFTQQ
Sami   --MTLSFFDQFMSPVFLGI-PLMALALSLPWILFPTPTDRWLNNRLLTLQGWFINRFTQQ
Rere   --MTLSFFDQFMSPSLFGI-PLIALALSLPWILFPTPSARWLGNRLLALQSWSINRFTQQ
Gama   --MTLSFFDQFMSPTFLGV-PLMALALTLPWMLFPTPSSRWLNNRLLTLQGWFINRFTQQ
Onmy   --MTLSFFDQFMSPTYLGI-PLIAVALTLPWILFPTPSARWLNNRLITLQGWFINRFTQQ
Sasa   --MTLSFFDQFMSPTYLGI-PLIAVALTLPWILFPTPSTRWLNNRLITLQGWFINRFTQQ
Cola   --MTLSFFDQFMSPTYLGI-PLIAVALTLPWILFPTPSARWLNNRLITLQGWFINRFTQQ
Dita   --MALSFFDQFLSPTHLGI-PLIALALLLPWALLPTPSGRWLNNRLLTLQGWFVSRFAQQ
Gogr   --MMLNLFDQFASPMHLNI-PLISLALVLPWALLPTPSHQWQNSRLLTLQGWFISKLAKQ
Chsl   --MGMGIFAQFASPFLLGI-PLLALAVTFPWLILPPTSRQLVDNRFRTLVDWFAGSVTKH
Atja   --MTLSFFDQFMSPTYMGI-PLMALALTLPWILYPTPSSRWLNNRLMSLQSWFIGQFTHQ
Iido   --MMLSFFDQFMSPTYMGI-PLMALALTLPWILYPTPSSRWLNNRLVSLQSWFIGQFTHQ
Auja   --MTLSFFDQFMSPTYLGI-PLIALALTLPWILFPSSSPRWLENRLLTLQNWFIGRFTQQ
Chag   --MVLNFFDQFMSPTLFGI-PLIGLAMTLPWILFPLPSRRWQDSRLLALQNWFINRFTQQ
Hami   --VALNFFDQFMSPSLMGI-PLIGLAMTLPWILFTSPTPRWISNRFLTLQGWFINRFTQQ
Saun   --MALNFFDQFMSPTLMGI-PLIGLAMTLPWILFTSPTPRWLTNRFLTLQGWFINRFTQQ
Nema   --MALNFFDQFMSPTLIGI-PLIALALTLPWILYPTPSARWLNNRVLTLQGWFLNRFTQQ
Disp   --MALNFFDQFASPVLYGI-PLMALALTLPWMLYPTATNRWLNSRLLTLQSWFMNRFTQQ
Myaf   --MSLSMFDQFMSPTLLGI-PLIALALTLPWILYPTVTSRWLNNRLITLQGWFMSSFSQQ
Lagu   --MTLSLFDQFMSPFLLGV-PLIIIALTLPWSLFPTATRRWLNNRVLTLQGWFINRFAQQ
Trtr   --MTLSFFDQFMSPSLLGI-PLIAIALTLPWTLLPTVTRRWLNNRLLTLQGWFIGRFTQQ
Zucr   --MTLSFFDQFMSPTLLGI-PLIAVALALPWTLFPSATRRWLNNRLLTLQGWFISRFTQQ
Pxja   --MMLSFFDQFMSPTYLGI-PLIALAISLPWILYPTPSNRWLNNRLLTLQGWFINRFTQQ
Pxlo   --MMLSFFDQFMSPTYLGI-PLIALAISLPWILYPTPSNRWLNNRLLTLQGWFINRFTQQ
Pctr   --MTLSLFDQFMSPTLLGV-PLILAAMILPWALLPTPTTRWLENRMLTLQSWFINRFTQQ
Apsa   --MMLSLFDQFMSPTLLGI-PLIIIALSLPWIMLPKPSTRWLENRLLTLQNWFINRFTQQ
Cabe   --MNLTLFDQFASPTLMGI-PLVVIATLAPWTLYPALPGHWLKTRLGALLSSAFKSVAQQ
Bzze   --MILNLFDQFASPTLLAI-PLVVFAMLAPWDMYPAPGKQWLGNRMLAIQAWTFKTLTQQ
Siim   --MDMDLFSQFTSPILFGV-PLWVIAMTYPVYCLPNTLQNTVKPRLTTALMWTLLQAMMQ
Ctru   --MTLSFFDQFMSPTFLGI-PLMALALSLPWILFPAPTTRWLNNRLLTLQGWFINRFTQQ
Dpbr   --MTLSFFDQFMSPTFLGL-PLIALALSLPWLLFPAPTTRWLNNRLLTLQGWFINRFTQQ
Caki   --MTLSLFNQFSSPSLLGI-PLIIAALTLPWLLFSLPSGRWLNGRMTTLHEWFIARFTNQ
Phja   --LTLSLFSQFFSPVWLGI-PLIFISLIMPWVLFPSPSSRWFSNRLVSIQGWMLANFTKQ
Brsp   --MTLSLFSQFESTTLFGI-SLIFIAITFPALLLPSPTLRWLENRTLTLQNWFMGQFTNQ
Gamo   --MTLSLFDQFSSPSFLGI-PMILMALALPWLLIPTPTSRWLSNRVVSLQGWFIARFTNQ
Lolo   --MTLSLFDQFSSPSYLGI-PLILIALSLPWVLFPAPSTRWLNNRMLTLQGWFIARFTNQ
Batr   --LLVNLFNQFEPPIMLNL-PLISLAVIFPLILLTLKQARWLNNRISQLQMSSIKHFITH
Prmy   --VTMNLFGQFESPTTLGI-PLMVVAIILPVLLLKFNSSRWSKNRMASVQAFWTKQFTSQ
Lose   --MTLSLFEQFEINSTFGV-PVIILAFTLPWLLIATPSRKLLTNRLVSVQKRFIRWVVKE
Loam   --MTLSLFDQFMSPWLLGV-PLIALALALPWTLFPTPSTRWLNNRLLTLQGWFINRFTQQ
Chab   --MTLSLFDQFMSPTLLGV-PLLPIALLLPWALFPNPACRWLLARLLTLQNWFIKQYAKH
Chto   --MTLSLFDQFMSPTFLGV-PLLPIALLLPWALFPNPACRWLLARLLTLQNWFIKQYAKH
Majo   --MTLSFFDQFMSPFYLGV-PLMALALSLPWVLLPAPRTRWLNNRLITIQSWFISRFVQQ
Hlst   --MTMNLFGQFMSPILLGI-PLMAIALALPWILFPNPHNRWLNNRLVTLQGWFISRFTQQ
Clpe   --MTLSFFDQFSSPSYLGI-PLIALALTLPLILFPTPSSKWLSNRLLAMQNWFINQLSKQ
Mlmr   --MVLNFFDQFESPSLLGI-PLVVLALSLPLLLFPTPSPRWLNSRLLTLQNWFINRFTQQ
```

To be continued on page 7.

[1/4 of aligned sequences]

```
Crcr   --MMLSFFDQFMSPVFLGI-PLIALALTLPWTLFPKLTTRWLNNRLLTLQSWFINNFTQQ
Muce   --MMLSFFDQFMSPVFLGI-PLIALALTLPWTLFPKLTTRWLNNRLLTLQSWFINNFTQQ
Bege   --MTLSFFDQFMSPTLLGI-PLMALALSLPWILFPKPTTRWLNNRLLTIQGWFINSFTQQ
Mela   --VTLGLFDQFLSPVFLGV-PLIALALSLPWILYPQPTSRWVNNRLLALQGCFINRFTQQ
Hats   --MTLSFFDQFMSPTLLGI-PLMALALSLPWILYPKPTARWLNSRLLTLQGWFINRFTQQ
Orla   --MTLSLFDQFMSPTLLGV-PLIGLALALPWVLFFQPGARWMNNRLVTLQAVFMNWFVKQ
Cosa   --MTLSFFDQFMSPTFLGI-PLIALALSLPWILYPQPSARWLNNRPLTLQNWFIGRFTQQ
Exsp   --MMVSFFDQFMSPVFLGI-PLIALALSLPWTLFPRPSARWLHNRTLTLQNWFMNRFTQQ
Depa   --MVLSFFDQFMSPSFLGI-PLIALALSLPWILYPQPTTRWLNNRPLTLQNWFIGRFTQQ
Rima   --MTMNLFNQFLSPTFLGL-PLIAIALMAPCLLYPSPAPGWQSNRWTTLQNWFIGSFIKQ
Fuol   --MTVTLFDQFLSPTLFKL-PLIVIALTTPWLLHHVTTTRFSSERLLSIKKISVSALTKH
Gmaf   --MTLSLFDQFLSPALMGV-PLAAAALALPWALFPRPTLRWVNNRLLTLQNWFFGQFAQQ
Xeei   --MTLSLFDQFISPTLLGV-PLIAIALTLPWVMFPQPTLRWVNNRLLTLQGWFISQFTQQ
Pros   --MILSFFDQFMSPTYMGI-PLIALALSLPWLMYPAPSGRWLNNRLLTLQGWFVNRFTQQ
Scmi   --MILSFFDQFMSPMLLGV-PLIILALSLPWLLFPAPSGRWLNNRLLTLQAWFINRLTRQ
Rolo   --MTLSFFDQFMSPTYLGI-PLIALALSLPWILYPTPSARWLNNRLLTLQGWFINRFTQQ
Cere   --VTLSMFDQFASPVYLGI-PLMALALSLPWILLPTPSTRWLGNRLLTLQAHSINRFTQQ
Daga   --MTLNMFEQFMSPTYLGL-PLIGLALVLPLLLYPTPSRRWLGNRLLSLQAHLINRFVQQ
Anco   --MTLSLFDQFMSPTLLGI-PLIAISLSLPWVLFPTPSLRWLNNRLLSLQGWFINRFAQQ
Dmve   --MTLSLFDHFISPVFLGI-PLVALATALPWLLFPAPTTRWLNNRLLSVQNWFIGTFAKH
Dmar   --MTLSLFDHFISPIFLGV-PLVALATALPWLLFPAPTTRWLNNRLLSVQNWFIGTFAKH
Anka   --MTLSLFDQFMSPTLLGI-PLIAISLSLPWVLFPTPTTRWLNNRLLSLQGWFINRFAQQ
Moja   --MTLSLFDQFMSPTLLGI-PLIALSLTLPWILFPTPTLRWLNNRLLSLQSWFINRFAQQ
Hoja   --MTLSLFDQFMSPTLLGI-PLIAVALTLPWILFPTPSLRWLNNRLLSLQGWFINRFAQQ
Bede   --MILSFFDQFMSPTYLGI-PLIALALSLPWILYPAPTGRWLNNRLLALQGWFINRFTQQ
Besp   --MILSFFDQFMSPTYLGI-PLIALALSLPWILYPAPTSRWLNNRLLALQGWFINRFTQQ
Mysp   --MTLSFFDQFASPTHLGI-PLVLLALVLPWTLYPTPSSRWLGNRLIALQSWFFNRFTQQ
Osja   --MTLSFFDQFMSPTYLGV-PLIALALALPWVLYPAPSARWLNNRLLTLQGWFINRFTQQ
Sgro   --MTLSFFDQFMSPTYLGI-PLIALALALPWVLYPTPSARWLNNRLLTLQGWFINRFTQQ
Pzpa   --MTLSFFDQFSSPMLMGI-PLILVALLLPWLLLPTPTRRWLSNRLLTIQGWFISQFSQQ
Zeja   --LTVSLFDQFLSPSYLGI-PLILVALTLPWLLFPAPSPRWLNNRLLTIQNRFINQFVQQ
Znne   --MMLSFFDQFAAPVYLGI-PLIVLALALPWILLPTPSARWKNNRFVNLEGWFIRNFAKE
Zefa   --MTMNLFNQFLSPQYLGI-PLILVALALPWLLFPPSSSRWLNNRLLTLQSWFINRLTQQ
Acni   --MTLSFFDQFLSPTYLGI-PLILVALTLPWLLFPTPSPRWLNNRLLTIQGWFINRFTQQ
Ncrh   --MTLSFFDQFLSPTYLGI-PLILVALTLPWLLFPAPSPRWLNNRLLTIQGWFISRFTQQ
Agca   --MTLSFFDQFMSPTYLGI-PLMALALILPWVFFPRPSSRWLENRLLTLQSWFINRFTQQ
Hydy   --MTLSFFDQFMTPIFLGI-PLAAIAIALPWVLFPTPTTRWVSNRFLALQGWFINRFTQQ
Gsac   --MTLSFFDQFMSPVFMGI-PLAAIAIALPWVLLPTPTARWTSNRFLGLQGWFINRFTQQ
Pevo   --MTVNLFEQFSSPSLLGI-PLIVLAFLAPASLFPSLPNRWIQGRLIAIQSWSINQFTQQ
Hiku   --MTLSFFDQFISPMFLGI-PLMALAIMIPWIMFPTPSSRWMNNRMLTLQNWFINLFTKQ
Inpa   --MTLSLFDQFMSPTLLGI-PLILLALTLPWTLLPAATSRLLSSRMVTTQSWLIGQITRQ
Auch   --VAGNVLAHITPPSKFGV-SLMFLALATPILLVTLPTNRYLAGRYLAVKNWALAHFVHH
Fico   --MTMSFFDQFMSPTLLGI-PLIALALALPWILFPTPSPRWINNRLLTLQNWFINRFTQQ
Macs   --MTLSFFDQFMSPTLLGI-PLIALALALPWILFPTPTSRWLNNRLLTVQGWFISRFSQQ
Moal   --MMVNSFSQFESPVFLTI-PLILLALILPWAFYPSPSPRWLSNRTLTLQNWFIGRFAKQ
Syma   --MILNLFDQFATPSLLGI-PLIALALTLPWVLYPSPSPRWLNNRTLTLQNWFINNFTKQ
Mafr   --MVLNFFDQFMSPTYLGI-PLIALALTLPWIFYPTHSTRWLNNRLLTLQNWFIGQFTKQ
Dcpe   --MILSLFDQFETPWYMGV-PLIAIAIASPWLFLPVPTPRWLSNRLLSLQAWFVTNATQQ
Dcti   --MILSLFDQFESPWYIGI-PLIAIALVSPWLFLPTPTPRWLSNRLVSLQGWFVTRATHQ
Hehi   --MTLSFFDQFMSPTYLGI-PLMALALTLPWLLYPMPTTRWLNNRFLALQGWFINRFTQQ
Stam   --MTLSFFDQFMSPTYLGI-PLMALALTLPWILYPTPTTRWLNNRFLALQGWFINRFTQQ
Hogi   --MMTSLFDQFASPVLFGI-PLAAIALVLPWLLYPKPSNRWLNSRYLTLQAWAINRFTHQ
```

To be continued on page 8.

[1/4 of aligned sequences]

```
Erzo    --MMLSFFDQFMSPTLLGI-PLIAIALTIPWVMFPTPTTRWLNNRFLSLQGWFINRFTLQ
Hxot    --MTVSLFDQFMSPTLLGI-PLIALAITLPWIMYPAPTTRWLNSRFLALQGWFINRFTQQ
Core    --MTVSLFDQFMSPTLLGI-PLIALAITLPWIMYPAPTTRWLNSRFLALQGWFINRFTQQ
Apve    --MTVSLFDQFMSPTLLGL-PLIVLATTLPWIMYPTPTTRWLNSRLLALQGWFINRFTQQ
Latj    --MALNFFDQFMSPNFLGV-PLIVVAMTLPWVLYPAPSARWLNNRLLTLQGWSINRFTQQ
Laja    --MMLSFFDQFMSPMFFGV-PLMAAAILFPWLFYPTPTTRWIDSRLLAAQNWFLNRFAKQ
Syja    MTMIPSLFDQFMSPVLFGV-PLIIIALALPWLLFPTPSNRLLNNRLLTLQNWFVGTFVKQ
Epme    --LSVSFFDQFMSTTYLGI-PLIALALIFPSILYPTLTTRWLNNRLLTLQSAFINRFTHQ
Grse    --MTLSFFDQFMSPTYLGI-PLMALALTLPWILYPNPTTRWLNNRSLALQGWFITRIVQQ
Clja    --MTLSFFDQFMSPTYLGI-PLMMMSLSLPWCLLPSRSTRWMGSRIIVMQAWFIVGFMYQ
Ogcy    --LALNFFDQFMSPVYLGI-PLITLALTLPWILFPKSSTRWLKNRFMTLQDWFLARFTQQ
Plna    --VTLSFFDQFMSPTFLGV-PLIVLALTLPWTLYPKPTDQWLNNRLLTLQNWFINRFTQQ
Lema    --MTLSFFDQFMSPTYLGI-PLIALALSLPWTLFPTPSTRWLNNRLLTLQGWFINRFIQQ
Etzo    --MTLSFFDQFMSPTFLGV-PLMALALSLPWILYPTPSTRWLNNRFLALQSWFINRFTQQ
Apse    --MTLSFFDQFMSPVYLGI-PLMALALTLPWLLFPTPSTRWMNNRLLTLQSWFINWFTSQ
Epde    --MTLSFFDQFMSPSLLGI-PLMALALTLPWLLFPTPTDRWLNNRLLTLQNWFINRFTQQ
Slja    --LALGFFDQFESPIYLGI-PLMGLAICLPWVLFPTPSRRWLTNRVLTLQNWLINRFTYQ
Bsja    --MALSFFDQFASPTYLGI-PLVAIALSLPWVLYPTPCERWQNNRLLTVQNWSIARFTQQ
Ecna    --MTLSFFDQFMSPTMLGI-PLMALALLLPWILFPTPSTRWLNNRLLTLQNSTIGQYTQQ
Cohi    --MALGFFDQFMSPVLMGI-PLNFLSFILPWILYPKPPSAWSKSRTSTLQTNAINKFTQQ
Caar    --MAMSFFDQFMSPVYLGI-PLIALALTLPWILYPTPSARWLNNRLLTLQGWFINRFTQQ
Came    --LAMSFFDQFMSPVYLGV-PLMALALTLPWVLYPTPSARWLNNRLLTLQGWFINRFTQQ
Mema    --MTLSLFDQFMSPVYFGV-PLIALALTLPWVLYPTPSNRWLNNRLLTLQGWAMNRYTQQ
Lenu    --MALNLFDQFMSPSFMGI-PLMALALTLPWLLFPQPTSRWLNNPLLTLQNWFIGQFTSQ
Brja    --MMLSFFDQFMSPVYLGI-PLIALALTLPWLLFPAPTTRWLNNRLLTLQNWFIGRFAHE
Plma    --MMLSFFDQFMSPVYLGI-PLMALALTLPWLLFPTPTARWLNNRLLTLQNWFIGRFAHE
Emst    --MTLSFFDQFMSPSYLGI-PLMALALSLPWILFPTPSARWLNNRMLTLQNWFINRFTQQ
Ptti    --MTLSFFDQFMSPNYLGI-PLMALALSLPWTLYPTPSTRWLNNRLLTLQGWFINRFTQQ
Losu    --LALNFFDQFISPSFLGI-PLMALSMILPWALFPNPSTRWVNNRLMTLQNWAMAQFMRQ
Geoy    --MILSFFDQFMSPVYMGI-PLMALALTLPWILYPAPTSRWLNNRLLTLQGWFINRFTQQ
Dipi    --MMLSFFDQFMSPTYLGI-PLITLALVLPWILFPTPTARWLNNRLLTLQGWFISRFTNQ
Pama    --MMLSFFDQFSSPFFMGI-PLMALALVLPWILFPTPSSHWLNNRLTTLQSWFVSSFARQ
Leob    --MTLSFFDQFSSPSLLGI-PLMALALSLPWVLYPTPSARWLNNRVLTLQGWFINRFTQQ
Neba    --MTLSLFDQFMSPVYLGI-PLMALALTLPWILFPAPSSRWISNRLTTVQNWGISQYTRQ
Pdpl    --MNLSLFDQFMSPTLMCI-PLIVVALIIPWLFFPNPLPRWQNNRLLSLQNWFIGRFTQQ
Nimi    --LTVSLFDQFMSPTYLGI-PLLALALTLPWIFFPSPTSRWLNNRLLTLQAWFINRFTQQ
Uptr    --MSLGFFEQFMSPTFLGI-PLIAIALAMPWLFISAPGSRWVTNRQLTLQAWSINRFLYQ
Pesc    --MAVGFFDQFMSPVFHGI-PLMAIAILAPWALILSPPPKWLTNRFITLKSSFINQFTKQ
Baar    --LTLSFFDQFMSPSYLGI-PLMALALTLPWILYPTPSSRWLHSRLLSLQSWLINQLTKQ
Moar    --MTLSFFDQFMSPTYLGI-PLMALALSLPWILFPTPSTRWLNNRLLTLQSWFINRFTQQ
Toja    --VTLSFFDQFMSPTYLGI-PLMALALTLPWVLFPTPSARWLNNRVLTLQGWFINRFTYQ
Chau    --MALSFFDQFLSPVLYGI-SLTALALTLPWVLFPTPTSRWLNNRLLSLQSWFISRFTQQ
Chse    --MTLSLFDQFMSPWFLGT-PLLAISLVLPWILLPTPSLRWLNNRLITTQAWFINRFTQQ
Enar    --MTLSFFDQFMSPVCLGI-PLIVLALTLPWILFPSFASRWLPNRLLGLQNWFLSQFTQQ
Hpty    --MTLSFFDQFMSPSYLGI-PLMALALTLPWVLYPTPSHRWLNNRLLTLQGWFIGQFTKQ
Nana    --MTLSLFDQFASPTILGI-SLIILALALPWILFPTPSLRWSGNRLLTVQGWFINRFTQQ
Mcst    --MMLSFFDQFMSPVFMGI-PLIALALTLPWILFPTPSTQWLNNRLLTLQNWFIGRFTQQ
Rhox    --MTLSFFDQFMSPSLLGI-PLMALALTLPWTLFPAPTPRWLHNRMLTLQNWFIGRFSQE
Opfa    --MTLSFFDQFMSPVFLGI-PLIALALTLPWILFPTPTSRWLNNRLLALQSWFINRFTQQ
Paar    --MVLNFFDQFMSPSFLGV-PLIAVALTLPWTFFPNASPRWLNNRTLALQGWFINRFINQ
Gozo    --MMLSFFDQFMSPLFLGI-PLIGLALTLPWLLFPTPTPRWLGNRLLTAQNWFINRFVSQ
Ackr    --MALNFFNQFESPSFMWI-PLMALALTLPWILLPNPTLRWANNRTLTVQNWTIGRFVHQ
```

To be continued on page 9.

[1/4 of aligned sequences]

```
Elev      --MMLSFFDQFASPTFLGI-PLITLALTLPWILYPSPSSRWLNNRLLTLQGWFINRFTQQ
Trdu      --MMLSFFDQFMSPTYLGI-PLIALALSLPWILYPKPTPRWLNNRLVTLQGWFINRFTQQ
Amoc      --VTLSFFDQFMSPTLLGI-PLIALALTLPWVLFPKPSSRWLNNRVLTLQSWFINGFAQQ
Hame      --MTLSFFDQFMSPSFMGV-PLMALALVLPWILFPKPSPQWVTNRVLTLQGWFINRFTQQ
Chso      --MALSFFDQFLSPTFLGV-PLMVIALTLPWVFFPEAPSQWLTNRYMTLQGWFINRFTQQ
Lyto      --MTLSFFDQFMSPTHLGV-PLMALALTLPWILYPTPTARWLNNRFLALQGWFINRFTQQ
Encr      --MTLSFFDQFMSPTYLGI-PLMALALTLPWILYPTPTARWLNNRFLALQGWFINRFTQQ
Bvar      --LTLDMFDQFFPPLFLGV-PLMIPALAFPWLLLPSPSGRWLTSRLLTAQSWFIFSFTKQ
Noco      --LILGLFDQFFSPYFLGV-PLLAIAIAAPWVLFPKPSNRVINGRSLVAQNSFVLNFAKQ
Chsp      --MTLSLFDQFEAPTRFGV-TLFALAVVLPLLLYPAPSRRWLSNRLLSTQKWAIHAYIKQ
Arja      --MTVSLFDQFMSPTLLGI-PLIALALTLPWIMYPAPSTRWLNSRFLALQGWFINRFTQQ
Pase      --LVLSFFDQFQSPVYLGI-PLMALALTLPWALYPTPTHRWVNNRLITLQSWSINRFTQQ
Trel      --MMDHLFDQFDSPLFYGV-PLIVPALVLPWAFYFSPSDRWAAHRAGTLQTWFINRFSYQ
Lifa      --MITSIFDQFLSPVLMGI-SLTTVATLSPAMLIVVSNNRWNKTRMLATQGWFFTRFIKE
Acur      --MAMSYFSQFASPYFLGI-PLFALAVILPWLLFPHPPNNWSSNRAFAIQALAIKNTTKV
Ampe      --MTLSLFDQFMSPVFLGI-PLIVLALTLPWTLFPTPSTRWLNSRLLALQGWFLNRFTQQ
Urja      --MTVSLFNQFLSPTMYGL-PLILLALTLPWVLFSAPSTRWLSNRFVTFKNWLIKDFMQQ
Enet      --MTLTFFDQFMSPTMLGI-PLIALALTLPWTLFPSPTSRWLNNRLITVQAWFISRFTHQ
Ptbr      MMTSVSLFDQFESPVLMGL-PLVIISLSVPLILYPQLTPQWLTNAYSAIQSWFINRATHQ
Safa      --MMLNFFDQFLSPTLMGI-PLILVSLTIPWALYPQPTARWLNNRLLTLQGWFINRFTQQ
Icae      --MTLSFFDQFMSPVYLGI-PLIALALTLPWLLFPTPTTRWLNNRLLTLQNWFIGRFAHE
Asmi      --MTVNFFDQFMSPNFLGI-PMILLSLLLPWFLFPSPSSLWIKNRLVTLQSWALNQFVKQ
Foal      --MMANYFSQFASPVLWGVLPLIIIALAYPLFIMSSASTKEMTHRMRTLEGWFIGQLVYQ
Drze      --MTLSIFDQFASPFLLGL-PLIGLALILPWILFPSASARWVNSRWTTLQSWFIRNFTNH
Rhas      --MTLSFFDQFMSPTYLGI-PLIALALLLPWLLFPTPSSRWLNNRLLTLQGWFVNRFTYQ
Elac      --MALNFFDQFMSPTYLGI-PLIALALTLPWTLFPTPSSRWLNNRVLTLQGWFINRFTYQ
Kugu      --MTLSFFDQFMSPTYLGI-PLIALAITLPWILFPASSTRWLNNRLLTLQSWFINRTTSQ
Plor      --MTLSFFDQFMSPTFMGI-PLMALALTLPWILYPTPSARWLNNRLLTLQGWFINRFTHQ
Sgun      --MTLSFFDQFMSPTYLGI-PLMALAIALPWILYPTPSSRWLNNRLLTLQSWFINRFTQQ
Zaco      --MTLSFFDQFMSPSYLGI-PLMALALSLPWILFPTPSARWLNNRSLTLQNWFINRFTQQ
Zbfl      --MTLSFFDQFMSPTYLGI-PLMALALSLPWILYPTPSSRWLNNRLLTLQGWFINRFSQQ
Spba      --MMLSFFDQFMSPTFLGI-PLIVLAVALPWVLYPTPAQRWLNNRLLTLQNWFIFRFTQQ
Game      --MTLSFFDQFMSPVYLGI-PLIALALTLPWLLFPTPTTRWLNNRLLTLQSWFIGRFAHE
Thth      --MTLSFFDQFMSPVFLGI-PLMALALTLPWVLFPTPTSRWLNNRLLTLQNWFIGRFAHE
Xigl      --MTLSFFDQFMSPTYLGI-PLIALAIALPWILYPTPSSRWLNNRLLTLQGWFINRFTQQ
Hyja      --LALDLFDQFQSPVWWSI-PLVVVAVIVPALILPSPTARWLENRVLAAQNWSIGQFTLQ
Psan      VILALGLFDQFASPVRWGV-PLVVLALAFPITTLPLYTARWVCNRLSMVQNWFIARFTHQ
Cupa      --MMLSFFDQFMSPVYLGI-PLIALALTLPWLLFPTPTTRWLNNRLLTLQNWFIGRLTRE
Mpch      --MALNFFDQFASPTLFGI-PLIVLALTLPWVLFPAPSSRWLGNRLTTLQNWFVNRFTQQ
Char      --MTLSFFNQFASPTLLGI-PLIAIALALPWILLPAPSSRWLNNRLSTLQGWFINRFTQQ
Pser      --MMLSFFDQFISPSYLGI-PLIFVSLALPWILFPTPSPRWLNNRLLSLQGWFMSSFTKQ
Prol      --MMLSFFDQFMSPVYLGI-PLIALAIILPWALFPTPSSRWMNNRLLTLQGWFINRFTSQ
Plbi      --MMLSLFDQFLSPALLGI-PLIALAITLPWTLFPAPVSRWLNNRLISLQGWFINGFTSQ
Calu      --MTVSFFDQFASPSYLGI-PLIALAIALPWLFFPAPTARWAVNRLVALQTWSLNGFTSH
Papa      --MTLTYFDQFTISSHLGI-PLFLLAVTLPWALYPKTLSTLVANRTLALQGQLIFKFLKQ
Sufr      --MTLSFFDQFMSPFFLGI-PLMALALLLPWILFPTPTARWQTNRFLTLQNWFINRFTQQ
Stci      --MTLSFFDQFISPVFLGI-PLIALALLVPWLLFPAATSRWVNSRLLALQGWFISNFTNQ
Taru      --MTLSFFDQFLSPTAFGI-PLIALALLLPWTLFPTPTNRWTNNRLLTLQSQFINRFTQQ
Rala      --MTLSFFDQFMSPTLMGI-PLMALALSLPWILYPTPTARWLNNRLLTLQNWFINRFTQQ
                 : ::          :   :   *
```

To be continued on page 10.

[2/4 of aligned sequences]

```
                            B                              C
Scca  LMQPMNLGGHKWAILFTALMLFLITINLLGLLPYTFTPTTQLSLNMAFALPLWLTTVLIG
Muma  LMQPMNLGGHKWAVLFTALMLFLITINLLGLLPYTFTPTTQLSLNMAFALPLWLTTVLIG
Erca  LMLPLNINAHKWALILVALLLFLITLNLLGLLPYTFTPTTQLSMNMALAVPLWLATVLIG
Pose  LMLPLNIGAHKWAVVLIALLLFLITLNLLGLLPYTFTPTTQLSMNMALAVPLWLATVLIG
Actr  LMLPINLGGHKWAVLLTALMLLLITLNLLGLLPYTFTPTTQLSLNMGLAVPLWLATVIIG
Scal  LMLPINLGGHKWAVLLTALMLLLITLNLLGLLPYTFTPTTQLSLNMGLAVPLWLATVIIG
Posp  LMLPINLGGHKWAVLLTALMLLLITLNLLGLLPYTFTPTTQLSLNMGFAVPLWLATVIIG
Atsp  LLLPLNVGGHKWALILTSLMLYLITLNLLGLLPYTFTPTTQLSLNMGFAVPLWLATVIIG
Leoc  LLLPLNLGGHKWALILTSLMLYLITLNLLGLLPYTFTPTTQLSLNMGFAVPLWLATVIIG
Amca  LLTPLNVGGHKWALLLTSVMVFLITMNLLSLLPYTFTPTAQLSMSLGLAVPLWLATVLIG
Osbi  LFLPINQGGHKWALLLASVLIFLITLNLLGILPYTFTPTTQLSMNMGFAIPLWLATILIG
Pabu  LFMPINQNGHKWALLLASLLVFLISLNLLGILPYTFTPTTQLSMNMGLAVPLWLATVLIG
Hial  LFLPINPGGHKWALLLASFLIFLITLNLLGILPYTFTPTTQLSLNMGLAVPLWLAAVLIG
Elha  LLLPLNVGGHKWALLLASLMIFLLTQNLLGLLPYTFTPTTQLSLNMGFAVPLWLATVIIG
Mlcy  LLLPLNIGGHKWAMLFASLMIFLMTLNLLGLLPYTFTPTTQLSLNMGLAVPLWLATVIIG
Algl  LFLPLNVGGHKWAVLLASLMVFLITLNMLGLLPYTFTPTTQLSLNMGLAVPLWLATVAIG
Ptgi  LLLPLNVGGHKWALLFASLMIFLLTLNLMGLLPYTFTPTTQLSLNMALAVPLWLATVIIG
Alaf  LLLPLNVGGHKWALLFTSLMVFLITLNLLGLLPYTFTPTTQLSLNMGFAVPLWLATVIIG
Nock  LLLPLNVGGHKWALMFTSLMVFLIMLNLLGLLPYTFTPTTQLSLNMGFAVPLWLATVIIG
Anja  LLLPLNVGGHKWAIMLTSLMLFLLTTNLLGLLPYTFTPTTQLSLNMGFAVPLWLATVIIG
Gyki  IFMPLSPAGHKWATLFASLMLFLISMNLLGLLPYTFTPTTQLSLNMAFAVPLWLATVIIG
Syka  LLMPLNVGGHKWALMLTSLMMFLFTINLLGLLPYTFTPTTQLSLNMGLAVPLWLATVIIG
Opma  LFLPINTGGHKWAILLTSLMLFLLTINLLGLLPYTFTPTTQLSLNMGFAVPLWLATVIIG
Comy  IFTPINNPGHKWATLLTALMLFLISINMLGLLPYTYTPTTQLSLNLGFAVPLWLATVIIG
Sasp  LLMPLNKGGHKWAVVLTSLMLYLLTMNMLGLLPYTFTPTTQLSFNLGFAVPLWMATMIIG
Eupe  LLTPLNMGAHKWAVIFASLMSFLLVINILGLLPYTFTPTTQLSLNLGLAVPLWMATVIIG
Enja  IFLPINPGGHKWAMLFTSLLVFLITINMLGLLPYTFTPTTQLSLNMAFAVPLWLATVIIG
Same  LLLPINPGGHKWAVLLTSLMLFLITINMLGLLPYTFTPTTQLSLNMGLAVPLWLATVIIG
Chch  MLLPLNLGGHKWAVLLTSMMIFLVTLKMLGLLPYTFTPTTQLSLNMGFAVPLWLATVVIG
Grgr  IFSPLNPEGHKWALLITSLMVFLFTLNVLGLLPYTFTPTTQLSMNMGFAVPFWLATVIVG
Caau  LMLPLNVGGHKWALLLASLMIFLITINMLGLLPYTFTPTTQLSLNMGFAVPLWLATVIIG
Cyca  LMLPLNVGGHKWALLLASLMIFLITINMLGLLPYTFTPTTQLSLNMGFAVPLWLATVIIG
Dare  LMTPLNFSGHKWALLFASLMVFLITINLLGLLPYTFTPTTQLSLNMGFAVPLWLATVIIG
Cost  LMLPLNVGGHKWALLLASLMIFLITINMLGLLPYTFTPTTQLSLNMGFAVPLWLATVLIG
Leec  LMLPLNLGGHKWALLLASLMVFLITINMLGLLPYTFTPTTQLSLNMGFAVPLWLATVLIG
Fola  LMLPLNTGGHKWALLLASLMVFLITINMLGLLPYTFTPTTQLSLNMGLAIPLWLATVIIG
Clmc  LLLPLNPAGHKWALMLASLMIFLLSLNMLGLLPYTFTPTTQLSLNMGLAVPLWLATVIIG
Phin  LLLPLNLPGHKWALMLTSLMILLLSLNMLGLLPYTFTPTTQLSLNMAIATPFWLATVIIG
Icpu  LLTPLNQAGHKWALILASLMIFILTLNVLGLLPYTFTPTTQLSLNMGLAVPLWLATVIIG
Psto  LLLPINQAGHKWAMILASLMIFILTLNVLGLLPYTFTPTTQLSLNMGLAVPLWLATVIIG
Cora  LLTPLNKSGHKWALMLASLMIFILSLNMLGLLPYTFTPTTQLSLNMGLAVPFWLATVLIG
Eisp  LLLPLNPGGHKWALIFTSLMVFLLSINLLGLLPYTFTPTTQLSLNMGLAVPFWLATVIIG
Apal  LLLPLNPEGHKWALMLSSLMIFLISLNLLGLLPYTFTPTTQLSLNLGLAVPFWLATVLIG
Eslu  LFTPLNQGGHKWATLLISLMIFLISLNLLGLLPYTFTPTTQLSLNMAFAVPLWLATVIVG
Dape  LFSPLSVGGHKWALLLISLMIFLITLNLLGLLPYTFTPTTQLSLNMAFAIPLWLATVVIG
Glse  LLLPLNLAGHKWATMLTSLMLFLITLNMLGLLPYTFTPTTQLSLNLGLAVPLWLATVIIG
Naar  LLLPLNLAGHKWAAILASLMLFLISLNMLGLLPYTFTPTTQLSLNLGLAVPLWLATVIIG
Lioc  LLLPLNLGGHKWATMLASLMLFLISLNMLGLLPYTFTPTTQLSLNLGLAVPLWLATVIIG
Opso  LLLPLNLAGHKWAAVLTSLMLFLITLNMLGLLPYTFTPTTQLSLNLGFAFPLWFATVLIG
Alte  LLLPLNLGGHKWAALLTSLMIFLITLNMLGLLPYTFTPTTQLSLNMGLAVPLWLATIIIG
Plap  LLLPLNLGGHKWAALLTSLMIFLITLNMLGLLPYTFTPTTQLSLNMGLAVPLWLATVVIG
```

To be continued on page 11.

[2/4 of aligned sequences]

To be continued on page 12.

```
Plal  LLLPLNVGGHKWATILTSLMLFLITLNMLGLLPYTFTPTTQLSLNMGLAVPLWLATVIIG
Sami  LLLPLNPGGHKWALILTSLMLFLITINMLGLLPYTFTPTTQLSLNMGLAVPLWLATVIIG
Rere  LLLPLNLGGHKWAAILTSLMLFLITMNMLGLLPYTFTPTTQLSLNMGLAVPLWLATVIIG
Gama  LLLPLNLGGHKWALVLTSLMVFLITLNMLGLLPYTFTPTTQLSLNMGLAVPLWLATVIIG
Onmy  LLLPLNLGGHKWAALLTSLMLFLITLNMLGLLPYTFTPTTQLSLNMGLAVPLWLATVIIG
Sasa  LLLPLNLGGHKWAVLLTSLMLFLITLNMLGLLPYTFTPTTQLSLNMGLAVPLWLATVIIG
Cola  LLLPLNLGGHKWAVMLTSLMLFLITLNMLGLLPYTFTPTTQLSLNMGLAVPLWLATVIIG
Dita  LLTPLNPAGHKWAAIFTCLIVFLMTLNILGLLPYTFTPTTQLSLNLGLAVPLWLATVIIG
Gogr  LLLPLNAGGHKWATILMSLLTFLITLNILGLLPYTFTPTTQLSLNLSLAIPLWLSTVALG
Chsl  LLGPVGPKGHVWAALLVSIIVFIAAHNILGLLPYTFVPTCLLSFNLALAVPIWLATVLMG
Atja  LMLPLGLGGHKWATMLTSLMIFLITINMLGLLPYTFTPTTQLSLNMGFALTLWMATVIIG
Iido  LMLPLNIGGHKWATMLTSLMIFLITINMLGLLPYTFTPTTQLSLNMGFALTLWMATVIIG
Auja  LFQPVNLGGHKWALLLASLMIFLISLNMLGLLPYTFTPTTQLSLNMGLAVPLWLATVIIG
Chag  LLLPLNFGGHKWAALLSSLMVFLMALNMLGLLPYTYTPTTQLSLNLGLAVPLWLATVLIG
Hami  LFQPLNAGGHKWALLFTSLMMFLFSLNMLGLLPYTFTPTTQLSLNMGLAVPFWLATVIIG
Saun  LFQPLNPGGHKWALLFTSLMMFLFSLNMLGLLPYTFTPTTQLSLNMGLAVPFWLATVIIG
Nema  LLLPVNLGGHKWAALFTSLMIFLITLNMLGLLPYTFTPTTQLSLNMGLAVPLWLATVIIG
Disp  LLLPINLGGHKWATLFTSLMIFLITLNMLGLLPYTFTPTTQLSLNMGLAVPLWLATVIIG
Myaf  LLLPLNPGGHKWAALFTSLMMFLITLNMLGLLPYTFTPTTQLSLNLGLAVPLWLATVVLG
Lagu  LLLPLSTAGHKWAVLLVSLMMYLITLNMLGLLPYTFTPTTQLSLNLALAVPLWLATVITG
Trtr  LLLPLNLPGHKWALLFASLMIFLISLNMLGLLPYTFTPTTQLSLNMAFAVPLWLATVIIG
Zucr  LLLPLNSAGHKWAVLFASLMIFLITLNMLGLLPYTFTPTTQLSLNMAFAVPLWLATVIIG
Pxja  LLLPLNVGGHKWATMFTSLMIFLITINMLGLLPYTFTPTTQLSLNMGFAVPLWLATVIIG
Pxlo  LLLPLNVGGHKWATMFTSLMIFLITINMLGLLPYTFTPTTQLSLNMGFAVPLWLATVIIG
Pctr  LLLPLSLAGHKWATILTSLMIFLITINMLGLLPYTFTPTTQLSLNMGLAVPMWLATVIIG
Apsa  LLLPLNPGGHKWATMLTSLMIFLITINMLGLLPYTFTPTTQLSLNMGLAVPMWLATVLIG
Cabe  IVLPIPEKGHKWSALFVSLLVLLITLNTLGTLPYTFTPTTQLSMSLGLALPLWLATVIIG
Bzze  IVMPANVMGHKWALLLMSLLVFLITLNMMGTLPYTFTPTTQLSLSLGLAFPLWLATVIIG
Siim  IMLKISATAQKWVVILTSTVTLLLLYNLFGTLPYVYPPTTQLSIAFGLAFPLWLGTLVKG
Ctru  LLLPLNLGGHKWATLLTSLMIFLITLNMLGLLPYTFTPTTQLSLNMGFAVPLWLATVIIG
Dpbr  LLLPLNLGGHKWATLLTSLMIFLITLNMLGLLPYTFTPTTQLSLNMGFAVPLWLATVIIG
Caki  LFLPLSIKGHKWAPLFASLMMFLFSINLLGLLPYTFTPTTQLSLNLAIAIPLWLATVLIG
Phja  ILLPLNEEGHKWAMLFVSLMIFLFGSNLLGLLPYTFTSTTQLSLNLALAVPLWMATILVG
Brsp  IFQPLNTPGHKWALLLTSLAIFLMTSNVLGLLPYTFTPTTQLSLNMALALPLWLATVILG
Gamo  LFLPLNVGGHKWAPLLASLMMFLLTLNMLGLMPYIFTPTTQLSLNLGLAVPLWLATVLIG
Lolo  LFLPLNVGGHKWAPLLASLMIFLLTLNMLGLMPYTFTPTTQLSLNMGLAVPLWLATVIIG
Batr  LMTPVAPTGHKWSLILASLMTLLVVMNSTGLLPYTFTPTTHLSINWGLAFSFWLATLITG
Prmy  LMQPINKPGHKWALLLTSLFLFIITTNTLGLLPYTFTPTTQLSMNLGLAIPLWLATVITG
Lose  LIRPMSLGGHKWALLLTSLLLFIMTLNIFGLLPHSFTPTTQLAMNLGLSVPLWMATVIVG
Loam  LLQPMSLGGHKWALLLTSLMLYLITLNMLGLLPYTFTPTTQLSLNIGFAVPLWLATVIIG
Chab  LFTPLDMGGHKWALLFTSLMVFIMTLNLLGLLPYTFTPTTQLALTLGLAMPLWLMTIIIG
Chto  LFTPLDMGGHKWALLFTSLMVFIMTLNLLGLLPYTFTPTTQLALTLGLAMPLWLMTIIIG
Majo  LMSPLNPGAHKWALLFASLMLFLITLNMLGLLPYTFTPTTQLSLNMGLAVPLWLATVIIG
Hlst  LMLPLSPNAHKWSLIFTALVLFLMSLNMLGLLPYTFTPTTQLSLSLGLAVPLWLATVIIG
Clpe  LLSPLSLGGHKWALLLIPLMLFLITLNALGLLPYTFTPTTQLSLNIGLAVPLWLATVLIG
Mlmr  LLLPLSLGGHKWALLLTSLMLFLITLNMLGLLPYTYTPTTQLSLNMGLAVPLWLATVIIG
Crcr  IFQPMNLPGHKWALLFASLMVFLMSLNLLGLLPYTFTPTTQLSLNMALAVPLWLATIIIG
Muce  IFQPMNLPGHKWALLFASLMVFLMSLNLLGLLPYTFTPTTQLSLNMALAVPLWLATIIIG
Bege  IFQPINLGGHKWATLLTSLMLFLITLNMLGLLPYTFTPTTQLSLNMAFAVPLWLATVIIG
Mela  IFQPINPKGHKWAALFTALMLFLVTLNTLGLLPYTFTPTTQLSLNMAFAVPLWLATVLIG
Hats  IFQPISLGGHKWAAILTSLMIFLITLNMLGLLPYTFTPTTQLSLNMAFAVPLWLATVIIG
Orla  IFQPMSLGGHKWAALLMSLMLFLITLNMLGLLPYTFTPTTQLSLNMAFAVPLWLATVIIG
```

[2/4 of aligned sequences]

To be continued on page 13.

```
Cosa IFQPLSPAGHHWALILASLMMFLFTLNVLGLLPYTFTPTTQLSMNMGLAVPLWLATVIIG
Exsp IFMPMPALGHRWALILMSLMIYLLSLNMLGLLPYTFTPTTQLSLSMGLATPLWLATVITG
Depa ILQPLSPTGHHWALLLATLMTFLFTLNVLGLLPYTFTPTTQLSMNMGLAVPLWLATVIIG
Rima TLSPINTKGHSWALLLATLMVFLITLNTLGLLPYTFTPTTQLSLNMAFAVPLWLATVIMG
Fuol IFQPLGPLGHKWALLFVSLFMFLITLNVLGLLPYTFTPTTQLSLTFSLALPLWLATVLIG
Gmaf IFLPINIAGHKWAVLLTSLMLFLITLNTAGLLPYTFTPTTQLSMNMGFAVPLWLATVILG
Xeei IFQPINIPGHKWALLLTSLMLFLISINMLGLLPYTYTPTTQLSLNMAFAVPLWLATVIIG
Pros LLLPINMGGHKWAALFTSLMIFLITLNMLGLLPYTFTPTTQLSLNMGLAVPLWLATVIIG
Scmi LFVLINFTGHKWASLFTALMLFLITLNMLGLLPYTFTPTTQLSLNLGFAVPLWLATVIIG
Rolo LLLPLNLGGHKWATLLTSLMIFLITLNMLGLLPYTFTPTTQLSLNMGLAVPLWLATVIIG
Cere LLLPLNLMGHKWAILLTSLMVFLITLNMLGLLPYTFTPTTQLSLNLGLAVPLWMATIIIG
Daga IFLPMNLSGHKWATLLTSVMIFLITLNLLGLLPYTFTPTTQLSLNLGLAVPLWLATVTIG
Anco LMLPLNPGGHKWALLLTALMIFLITINLLGLLPYTFTPTTQLSLNMGFAVPLWLATVVIG
Dmve ISLPLNVGGHKWALLLTSLLIFLITINMLGLIAYSYTPTTQLSFSLGFAVPLWLATVITG
Dmar ISLPLNVGGHKWALLLTSLLIFLITINMLGLIAYSYTPTTQLSFSLGFAVPLWLATVITG
Anka LMLPLNPGGHKWALLLTALMIFLITVNLLGLLPYTFTPTTQLSLNMGLAAPLWLTTVIIG
Moja LMLPLNPGGHKWALLLTALMIFLITVNLLGLLPYTFTPTTQLSLNMGLAAPLWLTTVIIG
Hoja LMLPLNPGGHKWALLLTALMIFLISINLLGLLPYTFTPTTQLSLNMGLAAPLWLTTVIIG
Bede LLLPMSLGGHKWAALFTSLMLFLITLNMLGLLPYTFTPTTQLSLNMGLAVPLWLATVIIG
Besp LLLPMSLGGHKWAALFTSLMLFLITLNMLGLLPYTFTPTTQLSLNMGLAVPLWLATVIIG
Mysp LFLPLNREGHKWATILMSLMIFLITLNMLGLLPYTYTPTTQLSMNMGFAVPLWLATVLIG
Osja LLLPLNLGGHKWATILTSLMIFLITLNMLGLLPYTFTPTTQLSLNMGLAVPLWLATVIIG
Sgro LLLPLNLGGHKWATLFTSLMIFLITLNMLGLLPYTFTPTTQLSLNMGLAVPLWLATVIIG
Pzpa LLHPLSPAGHKWALLFASLMLFLISINMLGLLPYTFTPTTQLSLNLGLAVPLWLATVIVG
Zeja LLLPINVGGHKWALILASLMIFLITINMLGLLPYTFTPTTQLSLNMGFAVPLWLATVIIG
Znne LLLPIKQGGHKWALLFTSLVVFLITMNVLGLLPYIFTPTTQLSLSLGFAVPLWLATVITG
Zefa LLLPINVGGHKWALLFASLMIFLITINMLGLIPYTFTPTTQLSLNMGFAVPLWLATVIIG
Acni LLLPINVGGHKWALIFASLMIFLITINMLGLLPYTFTPTTQLSLNMGFAVPLWLATVIIG
Ncrh LLLPINVGGHKWALIFASLMIFLITINMLGLLPYTFTPTTQLSLNMGFAVPLWLATVIIG
Agca LLLPLNMGGHKWALLLTSLMLFLISLNMLGLLPYTFTPTTQLSLNLGLAVPLWLATVIIG
Hydy LLLPVNLGGHKWAALLTSLMIFLITLNMLGLLPYTFTPTTQLSINLGLATPLWLATVIIG
Gsac LLLPVNLGGHKWAALLTSLMIFLITLNMLGLLPYTFTPTTQLSINLGLATPLWLATVIIG
Pevo LLTPLNMAGHKWALMFACLGAYLVTLNLLGLLPFTFTPTTQLSVSLGIALPLWLSTVIIG
Hiku LLLPLNTTGHKWALIFASLMIFLISLNMLGLLPYTFTPTTQLSLNMGLAVPLWLATVIIG
Inpa LLLPLSPAGHKWAIIFASLMVYLITLNLLGLLPYTFTPTTQLSLNLGLAFPLWLATVLIG
Auch IMLPLTPPMHKWAIYFTSIMTYLLLLNLMGLMPYIYAPTSNMLITLGLAIPATIATISLG
Fico ILLPLTLGGHKWALLLTSLMIFLITINMLGLLPYTYTPTTQLSLNLGLAVPLWLATVIIG
Macs LLLPINPGGHKWALIFASLMIFLITSNMLGLLPYTFTPTTQLSLNMGFAVPLWLATVIIG
Moal LLASSTPKVHLWATLLVSLMLYIISLNLLGMLPYTFTPTTQLSMNMGLAVPLWLATVLVG
Syma LMTPLNINGHKWATILTSLMIFLITLNLLGLLPYTFTPTTQLSLNMGFAIPLWLATVIIG
Mafr LFLPLNVPGHKWATILISLMIFLITLNMLGLLPYTFTPTTQLSLNMGLAVPLWLATVLLG
Dcpe IMLPLSPAGQKWALLFTSLMMYIISLNLLGLLPYTFTPTTQLSMSLGLAVPLWLATVLIG
Dcti IMLPLSPAGHKWALLFTSLMVYLISLNLLGLLPYTFTPTTQLSMSLGLAVPLWLATVLIG
Hehi LLLPLNIGGHKWAALLTSLMIFLITLNMLGLLPYTFTPTTQLSLNLGLAVPLWLATVIIG
Stam LLLPVNLGGHKWAALLASLMIFLITLNMLGLLPYTFTPTTQLSLNLGLAVPLWLATVIIG
Hogi LLLPLKEPAHRWAVLLVSLMIFVLTLNVLGLLPYTFTPTTQLSLNLGLATPLWLATVLIG
Erzo LLLPLNLGGHKWATLFASLMIYLISLNMLGLLPYTFTPTTQLSLNLGLATPLWLATVIIG
Hxot LLLPLSLGGHKWAALLTSLMIFLITMNMLGLLPYTFTPTTQLSLNLGLATPLWLATVIVG
Core LLLPLSLGGHKWAALLTSLMIFLITMNMLGLLPYTFTPTTQLSLNLGLATPLWLATVIIG
Apve LLLPLNLSGHKWAALLTSLMIFLITMNLLGLLPYTFTPTTQLSLNLGLATPLWLATVIIG
Latj LLLPLNQEGHKWAALLASLMVFLLSLNMLGLLPYTFTPTTQLSLNLGLAVPLWLATVIIG
Laja LLEPVNMAGHDWALLLTSLMLFIMSLNMLGLLPYTFTPTTQLSLNLGLAVPLWLATVIIG
```

[2/4 of aligned sequences]

```
Syja LLLPVNMGGHKWALLLASLMLFLITLNMLGLLPYTFTPTTQLSLNLGLAIPLWLATVIIG
Epme LLLPLNVGGHKWATLLASLMLFLISLNMLGLLPYTFTPTAQLSLNLGFAVPLWLATVIIG
Grse LLLPLNREGHKWATLLASLMIFLITLNMLGLLPYTFTPTTQLSLNLGLAVPLWLATVIIG
Clja LFIPLNREGHKWALILTSLMMFLLGLNVLGLLPYTFTPTTQLSHNLGIAVPFWLATVIIG
Ogcy MFQPVNPTGHKWALLLATLMTFLLALNMLGLLPYTFTPTTQLSLNMAMAVPLWLSTVIIG
Plna IFQPLNVTGHKWAMLLTSLMVFLITLNMLGLLPYTFTPTTQLSLNMAFAIPLWLASVIIG
Lema LLLPLNPAGHKWAVLFMSLMLFLITLNMLGLLPYTFTPTTQLSLNMALAVPLWLATVIIG
Etzo LLLPLNVGGHKWAALLASLMIFLITLNMLGLLPYTFTPTTQLSLNLGLAVPLWLATVIIG
Apse LFLPINLGGHKWATILASLMIFLISLNMLGLLPYTFTPTTQLSLNMGMAVPLWLATVIIG
Epde LLLPLSLGGHKWAVLLTSLMLFLITLNMLGLLPYTFTPTTQLSLNLGLAVPLWLATLILG
Slja LMMPISPGGHKWALMLGSLMLFLITMNMLGLLPYTFTPTTQLSINMGLAVPLWLSTVLIG
Bsja LLLPLNVGAHKWALLLTSLMVLLLTLNLLGLLPYTFTPTTQLSLNLGFAVPLWLATVIIG
Ecna LLLPINQGGHKWATLLTSLMIFLITINMLGLLPYTFTPTTQLSMNMGLAVPLWLATVIIG
Cohi LLLPLDKGAHKWALLFAALMMFLITLNTLGLLPYTFTPTTQLSLNLGLAVPLWLATVIIG
Caar LLLPLNPGGHKWATLFTSLMIFLISLNMLGLLPYTFTPTTQLSLNMGLAVPLWLATVIIG
Came LLLPLNPGGHKWATLLTSLMIFLITLNMLGLLPYTFTPTTQLSLNMGLAVPLWLATVIIG
Mema LLQPLNLGGHKWAALFTSLMLYLITLNMLGLLPYTFTPTTQLSMNMGLAVPLWLATVIIG
Lenu LLLPINQKGHKWAMMFASLMLFLISLNMLGLMPYTFTPTTQLSMNMGFAVPLWLATVIIG
Brja LFLPVNLPGHKWATLLTSLMLFLISLNMLGLLPYTFTPTTQLSLNMGLAFPLWLATVIIG
Plma LFLPVNLPGHKWAVLMTSLMLFLISLNMLGLLPYTFTPTTQLSLNMGLAFPLWLATVIIG
Emst LLLPLNPGGHKWALILTSLMLFLITLNMLGLLPYTFTPTTQLSLNMGLAVPLWLATVIIG
Ptti LLLPLNPGGHKWALILTSLMLFLITLNMLGLLPYTFTPTTQLSLNMGLAVPLWLATVIIG
Losu LFLPINQEGHKWALLFTALMIYLATFNLLGLLPYTFTPTTQLSLNMGIAIPLWLGTVIIG
Geoy LLMPLNFGGHKWALLFTSLMVYLITLNMLGLLPYTFTPTTQLSLNLGLAVPLWLATVIIG
Dipi LLLPMSPAGHKWALLFASLMLFLISLNMLGLLPYTFTPTTQLSLNMGLAVPLWLATVIIG
Pama LLMPVNQPGHKWALILTALMVFLLGLNLLGLLPYTFTPTTQLSINLGFAVPLWLATVLIG
Leob LLLPLNIGGHKWALILASLMLLLLTQNMLGLLPYTFTPTTQLSLNMGLAVPLWLATVIIG
Neba LLQPLNPGGHKWALLFTSLMVFIMTLNMLGLLPYTFTPTTQLSLNMGIAVVLWLATVIIG
Pdpl LLLPLSMPGHKWAAFFASLMIFILSLNMGGLLPYTFTPTTQLSLNMGLAVPLWLATVLIG
Nimi ILTPLSLGGHKWALILTSLMIFLITLNILGLLPYTFTPTTQLSLNLALAFPLWLATVLIG
Uptr LLMPLKPGGHKWATLFTALMIFLVSLNVLGLFPYTFTPTTQLSMNLGLAVPLWLATVIIG
Pesc LFLPISLAGHKWALLFTSLMLFLITLNMLGLLPYTFTPTTQLSMNLGLALPLWLMTVTIG
Baar LLLPVSLGGHKWAALLTSLLLFLITLNMLGLLPYTFTPTTQLSMNIGFAVPLWLATVIIG
Moar LLLPLNPGGHKWALLFSSLMLFLITLNMLGLLPYTFTPTTQLSLNMGLAVPLWLATVIIG
Toja LLLPLNPGGHKWATLLASLMVFLLSLNMLGLLPYTFTPTTQLSLNMGLAVPLWLATVIIG
Chau LLLPLNPAGHKWALIFCSLMVFLISLNSLGLLPYTFTPTTQLALNMGFAVPFWLATVLTG
Chse TFLPLNVEGHKWALLLTSLMMFLVFLNMLGLLPYTFTPTTQLSLNLGLAIPLWLATVIIG
Enar LLLPLSPAGHKWALLFGSLMIYLISLNMLGLLPYTFTPTTQLSLNLGLAVPLWLATVLIG
Hpty LLLPVNLGGHKWAVLLTSLMLFLITLNMLGLLPYTFTPTTQLSLNMGLAVPLWMATVVIG
Nana LLLPLNLRGHKWAVMLTSLMIFLITLNMLGLLPYTFTPTTQLSINLGLAAPLWLATVIIG
Mcst LMLPLNPGGHKWAMLLASLMLFLISLNMLGLLPYTFTPTTQLSLNMGLAVPLWLATVLIG
Rhox LLLPLSPAGHKWAMLFASLMLYLISLNMLGLLPYTFTPTTQLSLNLGLAVPLWLATVIIG
Opfa LLLPLSLGGHKWAILLTSLMLFLISLNMLGLLPYTFTPTTQLSLNMGLAVPLWLATVLIG
Paar LLLPLNQGGHKWAALFASLMLFLISINMLGLLPYTYTPTTQLSLNLGLAFPLWLMTVIIG
Gozo LLLPLNPGGHKWAVLLASLMLFLITLNMLGLLPYTFTPTTQLSLNLGLAVPLWLATVIIG
Ackr LMSPLNMAGHKWALLLTSLMAFIVTLNLLGLLPYTFTPTTQLSLNLGLAVPLWLATIIIG
Elev LLLPLNVGGHKWAVMLTSLMLFLITLNMLGLLPYTFTPTTQLSMNMALAVPLWLATVIIG
Trdu IFQPLSLGGHKWAALLASLMLFLITLNMLGLLPYTFTPTTQLSLNMAFAVPLWLATVIIG
Amoc IFQPINQGGHKWAALLTSLMIFLITLNMLGLLPYTFTPTTQLSMNMAFAVPLWLATVIIG
Hame LLLPLNLPGHKWAALLTSLMLFLLTLNMLGLLPYTFTPTTQLSLNMGLAVPFWLATVLMG
Chso LLLPLNVGGHKWAVLLTSLMLFILSLNMMGLLPYTFTPTTQLSLNMGLAVPLWLATVLIG
Lyto LLLPLSLGGHKWAALLTSLMIFLITLNMLGLLPYTFTPTTQLSLNLGLATPLWLATVIIG
```

To be continued on page 14.

[2/4 of aligned sequences]

To be continued on page 15.

```
Encr  LLLPLSLGGHKWAALLTSLMIFLITLNMVGLLPYTFTPTTQLSLNLGLATPLWLATVIIG
Bvar  IFSPLNFGGHKWALLLTSLMVYLISLNMLGLLPYTFTPTTQLALNLGIAIPLWLGTVLIG
Noco  ILLPLNVGAHKWALLLTVLMVNLITLNLLGLLPYTFTPTTQLPLNMGFAIPLWLATVVIG
Chsp  LLIPIATTTHRWAALLGALMIFIITLNVMGLLPYTFTPTAQLSFNMALAFPIWLATVIIG
Arja  LLLPLSLGGHKWAALLTSLMIFLITMNMMGLLPYTFTPTTQLSLNLGLATPLWLATVIIG
Pase  LLLPVNIGSHKWALMLTSLMVLLFTLNMLGLLPYTFTPTTQLSLNMGLAVPLWMATVIIG
Trel  IFLPLNSGAHKWAPMLAAMALLLLAMNLLGLLPYTFTPTTQLCLNLGFALPLWLATVLTG
Lifa  VATPLSTAGQKWTLLFMALLICLMSLNLLGLLPYTFTPTTQLSLNLAMALPLWITTIYAG
Acur  LVVSLDKPAHKWALMIIALFTYLITMNTLGLLPYTFTPTTQLSLNLGLAFPLWLTTVIIG
Ampe  LLLPINLGGHKWALLFASLMLYIISLNMLGLLPYTFTPTTQLSLNLGLAVPLWLATVIIG
Urja  LLLPINLGGHKWALLLTSLMLFLISLNTLGLLPYTFTPTTQLSLNLGFAAPLWMATVIIG
Enet  IFQPLGLPGHKWALLLTSLMLFLITLNMLGLLPYTFTPTTQLSLNMALAVPLWLATVIIG
Ptbr  ILLPLSPAGHKWALLLMSLLVYLITINMLGLLPYSFTPTTQLSMNMALAVPLWLATVILG
Safa  IFLPLSPSGHKWALMLASLMLFLITLNMLGLLPYTFTPTTQLSLNMALAVPLWLATVIIG
Icae  LFMPVNLPGHKWAVLLTSLMLFLISMNMLGLLPYTFTPTTQLSLNMGLAFPLWLATVIIG
Asmi  ICSPLHPAGHKWALMFTSLMLFLVSLNTLGLLPYTFTPTTQLSLNLGLAIPLWLATVVIG
Foal  LFSPLNKQAQKWALLYTTLAVYLLLLNLLGLLPYVFTPTTQLSMNLALAVPLWLATVTIG
Drze  IIQPLNMAGHKWALLITSLMMIIMSLNMLGHIPYSYTPTTQLSLNLGLAVPLWLMTVILG
Rhas  LFMPLNQGGHKWATILTSLMLFLISLNMLGLLPYTFTPTTQLSLNMGLAVPLWLATVLIG
Elac  LMMPINAGGHKWATIFASLMLFLISLNMLGLLPYTFTPTTQLSMNMGLAVPLWMATVLIG
Kugu  LFMPMNQAGHKWATILMSLMIFLISLNMLGLLPYTFTPTTQLSLNLGLAVPLWLATVLIG
Plor  LLTPLNLGGHKWALLLTSLMLFLVTLNMLGLLPYTFTPTTQLSLNLGLAVPLWLATVIIG
Sgun  LLLPLSPGGHKWALLLASLMLFLITINMLGLLPYTFTPTTQLSLNMGLAVPLWLATVIIG
Zaco  LLLPLNPGGHKWALILTSLMLFLITLNMLGLLPYTFTPTTQLSLNMGLAVPLWLATVIIG
Zbfl  LLLPLNPGGHKWALIFSSLMLYLITLNMLGLLPYTFTPTTQLSLNMGLAVPLWLATVIIG
Spba  LLLPINPGGHKWATILASLMVFLLTMNILGLLPYTFTPTTQLSLNMGLAVPFWLATVIIG
Game  LFMPVNLPGHKWAVLLTSLMLFLISLNMLGLLPYTFTPTTQLSLNMGLAVPLWLATVIIG
Thth  LFMPVNLPGHKWAVLLTSLMLFLISLNMLGLLPYTFTPTTQLSLNMGLAFPLWLATVIIG
Xigl  LLLPLNPGGHKWALLFTSLMIFLISLNMLGLLPYTFTPTTQLSLNMGLAVPLWLATVIIG
Hyja  VFLPVGIPGHKWALILASLMMYLTTLNLLGLLPYTFSPTAQLSINLALAFPLWLATVIIG
Psan  VFTPVSHPGHKWALILSSLMLYLTSLNTLGLLPYTFTPTTQLSVNLALAFPLWLATVIIG
Cupa  LFLPVNLPGHKWAVLLASLMLFLISLNMLGLLPYTFTPTTQLSLNMGLAFPLWMATVIIG
Mpch  LFLPLNQEGHKWALMFSSLMIFLLSLNMLGLLPYTFTPTTQLSMNMGLALPFWLATVIIG
Char  LLLPLNVGGHKWALLLTSLMIFLITLNTLGLLPYTFTPTTQLSMNMGLAVPLWLATVIIG
Pser  LLLPIKPAGHKWAILFTSLVLFLIMSNMLGLLPYTFTPTTQLSLSMGFAAPLWLATVLTG
Prol  LLLPLNLGGHKWATLFASLMIFLLSINMLGLLPYTFTPTTQLSLNMGLAVPLWLATVIIG
Plbi  LLLPLGPRGHKWALLFVSLVLYLISINMLGLLPYTYTPTTQLSLNLGLAVPLWLATVIIG
Calu  LLLPMNTPGHKWAALFAALLTYLITINVLGLLPYTFTPTTQLSLSMGLAAPLWLGTVLTG
Papa  LLSPLPESAHKWAVMFASLLIYLITLNMLGLLPYTFTPTTQLSLNLGLALPLWLATVIIG
Sufr  LLLPMNLGGHKWALILTSLMLYLITLNMLGLLPYTFTPTTQLSLNMGLAVPLWLATVIIG
Stci  LLLPINFAGHKWALMLMSLMLFLITINMLGLLPYTFTPTTQLSMNMGLAVPLWLATVIIG
Taru  LLLPLNMGGHKWALMFASLMVFLITINMLGLLPYTFTPTTQLSVNMALAVPLWLATVIIG
Rala  LLLPLNLGGHKWALIFTSLMLFLITLNMLGLLPYTFTPTTQLSLNMGLAVPLWLATVIIG
              : *           :   :  . : . :  *  :    .::    : ::  *
```

[3/4 of aligned sequences]

```
                                        D                E
Scca   MFNQPTIALGHLLPEGTPTPLVPVLIIIETISLFIRPLALGVRLTANLTAGHLLMQLIAT
Muma   MLNQPTIALGHLLPEGTPTPLVPILIIIKTISLFIRPLALGVRLTANLTAGHLLMQLIAT
Erca   MRNQPTHSTAHLLPEGTPTLLIPILIIIETISLFIRPLALGVRLTANLTAGHLLIQLIST
Pose   MRNQPTHSLAHLLPEGTPTLLIPILIIIETISLFIRPLALGVRLTANLTAGHLLIQLIST
Actr   MRNQPTAALGHLLPEGTPIPLIPVLIIIETISLFIRPLALGVRLTANLTAGHLLIQLIAT
Scal   MRNQPTAALGHLLPEGTPIPLIPVLIIIETISLFIRPLALGVRLTANLTAGHLLIQLIAT
Posp   MRNQPTAALGHLLPEGTPVPLIPVLIVIETISLFIRPLALGVRLTANLTAGHLLIQLIAT
Atsp   MRNQPTAALGHLLPEGTPAPLIPVLIIIETISLMIRPLALGVRLTANLTAGHLLIQLLST
Leoc   MRNQPTAALGHLLPEGTPAPLIPVLIIIETISLMIRPLALGVRLTANLTAGHLLIQLLST
Amca   MRNQPTAALGHLLPEGTPTPLIPVLIIIETISLFIRPVALGVRITANLTAGHLLIQLIAT
Osbi   ARYQPTHTLAHLLPVGTPAPLIPILIMIETISLFIRPIALGVRLTANLTAGHLLIQLIST
Pabu   ARNQPTHTLAHMLPTGTPGPLIPVLIIIETISLFIRPIALGVRLTANLTAGHLLIQLISM
Hial   IRKQLTHTLAHMLPVGTPGPLIPVLIIIETISLFIRPIALGVRLTANLTAGHLLIQLIST
Elha   MRNQPTAALGHLLPEGTPVPLIPVLIIIETISLFIRPLALGVRLTANLTAGHLLIQLIAT
Mlcy   MRNQPTIALGHLLPEGTPAPLIPVLIIIETISLFIRPLALGVRLTANLTAGHLLIQLIAT
Algl   MRNQPTAALGHLLPEGTPTPLIPVLIIIETISLFIRPLALGVRLTANLTAGHLLIQLIAT
Ptgi   MRNQPTAALGHLLPEGTPVPLIPVLIIIETISLFIRPLALGVRLTANLTAGHLLIQLIAT
Alaf   MRNQPTAALGHLLPEGTPVPLIPVLIIIETISLFIRPLALGVRLTANLTAGHLLIQLIAT
Nock   MRNQPTAALGHLLPEGTPILLIPVLIIIETISLFIRPLALGVRLTANLTAGHLLIQLIAT
Anja   MRNQPTVALGHLLPEGTPVPLIPVLIIIETISLFIRPLALGVRLTANLTAGHLLIQLIAT
Gyki   MRNQPTVALGHLLPEGTPVPLIPVLIIIETISLFIRPLALGVRLTANLTAGHLLIQLIAT
Syka   MRNQPTAALGHLLPEGTPALLIPVLIIIETISLFIRPLALGVRLTANLTAGHLLIQLIAT
Opma   MRNQPTVALGHLLPEGTPLPLIPVLIIIETISLFIRPLALGVRLTANLTAGHLLIQLIAT
Comy   LRNQPTISLGHLLPEGTPIPLIPVLIIIETISLFIRPLALGVRLTANLTAGHLLIQLIAT
Sasp   LRDRPTTTLGHLLPEGTPQPLIPMLIIIETISMWIRPLALGVRLTANLTAGHLLIQLVST
Eupe   LRNQPTATLGHLLPEGTPSPLIPMLIIIETISLCIRPLALGVRLTANLTAGHLLIQLIAT
Enja   MRNQPTAALGHLLPEGTPGPLIPVLIVIETISLFIRPLALGVRLTANLTAGHLLIQLIAT
Same   MRNQPTAALGHLLPEGTPVPLIPVLIIIETISLFIRPLALGVRLTANLTAGHLLIQLIAT
Chch   LRNQPTVALGHLLPEGTPVVLIPFLIIVETFSLLIRPLALGVRLTANLTAGHLLIQLIAT
Grgr   AQKQPNIALAHLLPEGTPLALIPVLIIIETISLFIRPLALGVRLTANLTAGHLLIYLISM
Caau   MRNQPTVALGHLLPEGTPIPLIPVLIIIETISLFIRPLALGVRLTANLTAGHLLIQLIAT
Cyca   MRNQPTVALGHLLPEGTPIPLIPVLIIIETISLFIRPLALGVRLTANLTAGHLLIQLIAT
Dare   MKNQPTIALGHLLPEGTPIPLIPALIIIETISLFIRPLALGVRLTANLTAGHLLIQLIAT
Cost   MRNQPTVALGHLLPEGTPIPLIPVLIIIETISLFIRPLALGVRLTANLTAGHLLIQLIAT
Leec   MRNQPTVALGHLLPEGTPIPLIPVLIIIETISLFIRPLALGVRLTANLTAGHLLIQLIAT
Fola   LRNQPTVALGDLLPEGTPLPLIPVLIIIETISLFIRPLALGVRLTANLTAGHLLIQLIAT
Clmc   MRNQPTASLGHLLPEGTPVPLIPVLIIIETISLFIRPLALGVRLTANLTAGHLLIQLIAT
Phin   MRNQPTAALGHLLPEGTPIPLIPILIIIETISLFIRPLALGVRLTANLTAGHLLIQLIAT
Icpu   LRNQPTAALGHLLPEGTPALLIPILIIIETISLFIRPLALGVRLTANLTAGHLLIQLIST
Psto   LRNQPTAALGHLLPEGTPVPLIPVLIIIETISLFIRPLALGVRLTANLTAGHLLIQLIST
Cora   LRNQPTAALGHLLPEGTPVPLIPVLIIIETISLFIRPLALGVRLTANLTAGHLLIQLISS
Eisp   MRNQPTAALGHLLPEGTPVLLIPVLIIIETISLFIRPLALGVRLTANLTAGHLLIQLIAT
Apal   MRNRPTAALAHLLPEGTPPLLIPILIMIETVSLFIRPLALGVRLTANLTAGHLLIQLIAT
Eslu   LRNQPTIALGHLLPEGTPLPLIPVLIIIETISLFIRPLALGVRLTANLTAGHLLIQLIAT
Dape   LRNQPTIALGHLLPEGTPLLLIPVLIIIETISLFIRPLALGVRLTANLTAGHLLIQLIAT
Glse   MRNQPTAALGHLLPEGTPTPLIPILIIIETISLLIRPLALGVRLTANLTAGHLLIQLIAT
Naar   MRNQPTAALGHLLPEGTPTPLIPILIVIETISLFIRPLALGVRLTANLTAGHLLIQLIAT
Lioc   MRNQPTAALGHLLPEGTPTPLIPILIVIETISLFIRPLALGVRLTANLTAGHLLIQLIAT
Opso   MRNQPTTALGHLLPEGTPTPLIPILIIIETVSLLIRPLALGVRLTANLTAGHLLIQLIAT
Alte   MRNQPTAALGHLLPEGTPTPLIPVLIVIETISLFIRPLALGVRLTANLTAGHLLIQLIAT
Plap   MRNQPTAALGHLLPEGTPVPLIPVLIIIETISLFIRPLALGVRLTANLTAGHLLIQLIAT
```

To be continued on page 16.

[3/4 of aligned sequences]

```
Plal  MRNQPTAALGHLLPEGTPVPLIPVLIIIETISLFIRPLALGVRLTANLTAGHLLIQLIAT
Sami  MRTQPTAALGHLLPEGTPGPLIPVLIIIETISLFIRPLALGVRLTANLTAGHLLIQLIAT
Rere  MRNQPTAALGHLLPEGTPVPLIPVLIIIETISLFIRPLALGVRLTANLTAGHLLIQLIAT
Gama  MRNQPTAALGHLLPEGTPVPLIPVLIIIETISLFIRPLALGVRLTANLTAGHLLIQLIAT
Onmy  MRNQPTAALGHLLPEGTPVPLIPVLIIIETISLFIRP-ALGVRLTANLTAGHQ---LIAT
Sasa  MRNQPTAALGHLLPEGTPVPLIPVLIIIETISLFIRPLALGVRLTANLTAGHLLIQLIAT
Cola  MRNQPTAALGHLLPEGTPVPLIPVLIIIETISLFIRPLALGVRLTANLTAGHLLIQLIAT
Dita  MRNQPTAALGHLLPEGTPTLLIPVLIVIETISLLIRPLALGVRLTANLTAGHLLIGLIST
Gogr  LQNQPTVSLGHLLPEGTPSLLIPVLITIETISLLIRPLALGVRLTANLTAGHLLIGLVSM
Chsl  LRHQPTITFGHLLPEGTPIPLIPLMIVIESFSLLIRPVSLAVRLTANMTAGHLLLKLIST
Atja  MRNQPTMVLGHLLPEGTPVPLIPVLIIIETISLFIRPIALGVRLTANLTAGHLLIQLIAT
Iido  MRNQPTMVLGHLLPEGTPAPLIPVLIIIETISLFIRPIALGVRLTANLTAGHLLIQLIAT
Auja  MRNQPTAALGHLLPEGTPTPLIPILIIIETISLFIRPLALGVRLTANLTAGHLLIQLIAT
Chag  FRNQPTHALAHLLPEGTPTLLIPILIIIETISLFIRPLALGVRLTANLTAGHLLMQLIAT
Hami  MRNQPTVALGHLLPEGTPTPLIPILIIIETISLFIRPIALGVRLTANLTAGHLLIQLIAT
Saun  MRNQPTIALGHLLPEGTPTPLIPVLIIIETISLFIRPIALGVRLTANLTAGHLLIQLIAT
Nema  MRNQPTHALGHLLPEGTPTPLIPILIIIETISLFIRPLALGVRLTANLTAGHLLIQLIAT
Disp  MRNQPTHALGHLLPEGTPTPLIPVLIIIETISLFIRPLALGVRLTANLTAGHLLIQLIAT
Myaf  MRNDPTHALGHLLPEGTPTPLIPVLIIIETISLFIRPLALGVRLTANLTAGHLLILLIAK
Lagu  MRNQPTHALGHLLPEGTPTPLIPALIIIETISLFIRPLALGVRLTANLTAGHLLIQLTST
Trtr  MRNQPTHALGHLLPEGTPTALIPILIIIETISLFIRPLALGVRLTANLTAGHLLIQLIAT
Zucr  MRNQPTHALGHLLPEGTPTALIPILIIIETISLFIRPLALGVRLTANLTAGHLLIQLIAT
Pxja  MRNQPTHALGHLLPEGTPTLLIPVLIIIETISLFIRPLALGVRLTANLTAGHLLIQLIAT
Pxlo  MRNQPTHALGHLLPEGTPTLLIPVLIIIETISLFIRPLALGVRLTANLTAGHLLIQLIAT
Pctr  MRNQPTHSLAHLLPEGTPTPLIPILIIIETISLFIRPLALGVRLTANLTAGHLLIQLIAT
Apsa  MRNQPTHSLGHLLPEGTPTPLIPVLIVIETISLFIRPLALGVRLTANLTAGHLLIQLIAT
Cabe  LRYNPTHSFAHLLPQSTPVALIPALIMIETASLLVRPLALGVRLAANLTAGHMILQMIAS
Bzze  LRYHPVRSIGHLLPESTPTLLIPVLIIIETISLLVRPLALGVRLAANLTAGHLILQMIAS
Siim  VKLSVTNTLAHFLPPSTPAALVPVLVIIETTSMLVRPVALGMRLAANLTSGHMIMHAVSS
Ctru  MRNQPTHALGHLLPEGTPTPLIPVLIIIETISLFIRPLALGVRLTANLTAGHLLIQLIAT
Dpbr  MRNQPTHALGHLLPEGAPTPLIPVLIIIETISLFIRPLALGVRLTANLTAGHLLIQLIAT
Caki  LRNQPTHTFGHFLPEGTPTLLIPILIIIENISLIIRPIALGVRLTANLTAGHLLIHLVSS
Phja  ARNRPVHVLGHLLPEGTPTLLIPVLIIIETISLFIRPLALAVRLTANLTAGHLLIHLISS
Brsp  LRHHTNHAFAHLLPEGTPTLLIPILIVIESISLIIRPLALAVRLTANLTAGHLLIHLIGA
Gamo  MRNQPTHALGHFLPEGTPTALIPILIIMQTISLFIRPLALGVRLTANLTAGHLLIHLISS
Lolo  MRNQPTHALGHFLPEGTPTPLIPVLIIIETISLFIRPLALGVRLTANLTAGHLLIHLISS
Batr  FYNNPTQAFGHMLPKNTPNFLIPLLIIIETASQLVRPLALGVRLTANLTAGHLLIHLISS
Prmy  FRNDSTHALGHLLPESTPTLLIPILVVIETASLFIRPLALGVRLTANLTAGHLLIQLIAT
Lose  FRKHRSAALAHFLPLGAPNALIPILVIIELISLIVRPLALAVRLTANITAGHLMMQIVAT
Loam  MRNQPTVALGHLLPEGTPTPLIPILIVIETISLFIRPLALGVRLTANLTAGHLLIQLIAT
Chab  LRNKPNAVIVHFLPKGTPRPLIPVLIIIETTSVIIRPLALAVRLMANLTAGHLMMQLIAM
Chto  LRNKPNAVIIHFLPKGTPRPLIPVLIIIETTSVIIRPLALAVRLMANLTAGHLMMQLIAM
Majo  MRNQPTIALGHLLPEGTPTPLIPVLIIIETISLFIRPLALGVRLTANLTAGHLLIQLIAT
Hlst  LRYQPTMALGHLLPEGTPIPLIPVLVIIETISLLIRPLALGVRLTANLTAGHLLIHLISI
Clpe  MRNQPTIALGHLLPEGTPTPLIPVLVIIETISLLIRPLALGVRLTANLTAGHLLIQLIAT
Mlmr  MRNQPTIALGHLLPEGTPALLVPVLIIIETISLFIRPLALGVRLTANLTAGHLLIQLIAT
Crcr  MRNQPTHALGHLLPEGTPTLLIPVLIIIETISLFIRPLALGVRLTANLTAGHLLIQLIAT
Muce  MRNQPTHALGHLLPEGTPTLLIPVLIIIETISLFIRPLALGVRLTANLTAGHLLIQLIAT
Bege  MRNQPTHALGHLLPEGTPTLLIPILIIIETISLFIRPLALGVRLTANLTAGHLLIQLIAT
Mela  MRYQPTHALGHLLPEGTPAPLIPLLIIIETASLFIRPLALGVRLTANLTAGHLLIQLTST
Hats  MRNQPTHALGHLLPEGTPTLLIPVLIIIETISLFIRPLALGVRLTANLTAGHLLIQLIAT
Orla  MRNQPTHALGHLLPEGTPTALIPVLIVIETISLFIRPLALGVRLTANLTAGHLLIQLIAT
```

To be continued on page 17.

[3/4 of aligned sequences]

To be continued on page 18.

```
Cosa  MRNQPTHALGHLLPEGTPTLLIPVLIIIETISLFIRPLALGVRLTANLTAGHLLIQLIAT
Exsp  MRNQPTHALGHLLPEGTPTLLIPVLIIIETISLFIRPIALGVRLTANLTAGHLLIQLIAT
Depa  MRNQPTHALGHLLPEGTPTPLIPVLIIIETISLVIRPLALGVRLTANLTAGHLLIQLIAS
Rima  FRNSPTHALGHLLPEGTPTPLVPILIIIESISLLIRPLALGVRLTANLTAGHLLIQLVSS
Fuol  FRLDPTRALAHMLPQGTPPLLIPILIIIETISVLIRPLALAVRLMANLTAGHLLIHLIST
Gmaf  MRTQPTHSLGHLLPEGTPTLLIPVLIIIETISLFIRPLALGVRLTANLTAGHLLIQLVST
Xeei  ARNQPTHALGHLLPEGTPTPLIPVLIIIETISLFIRPLALGVRLTANLTAGHLLIQLIAT
Pros  MRNQPTHALGHLLPEGTPTLLIPVLIIIETISLFIRPLALGVRLTANLTAGHLLIQLIAT
Scmi  MRNQPTHALGHLLPEGTPILLIPLLIVIETISLFIRPLALGVRLTANLTAGHLLIQLIAT
Rolo  MRNQPTHALGHLLPEGTPTPLIPILIIIETISLFIRPLALGVRLTANLTAGHLLIQLIAT
Cere  MRNQPTHALAHLLPEGTPTLLIPVLIIIETISLFIRPLALGVRLTANLTAGHLLIQLIAT
Daga  MRNQPTHALGHLLPEGTPTPLIPVLIIIETISLFIRPLALGVRLTANLTAGHLLIQLIST
Anco  MRNQPTHALGHLLPEGTPTALIPTLIIIETISLFIRPLALGVRLTANLTAGHLLIQLIAM
Dmve  LRTQPTHAFAHLLPEGTPVLLIPVLVIIETISLMIRPIALGVRLTANLTAGHLLIHLLST
Dmar  LRTQPTHAFAHLLPEGTPVLLIPVLVIIETISLMIRPIALGVRLTANLTAGHLLIHLLST
Anka  MRNQPTHALGHLLPEGTPTPLIPVLIIIETISLFIRPLALGVRLTANLTAGHLLIQLIAM
Moja  MRNQPTHALGHLLPEGTPTPLIPVLIIIETISLFIRPLALGVRLTANLTAGHLLIQLIAM
Hoja  MRNQPTHALGHLLPEGTPTLLIPVLIIIETISLFIRPLALGVRLTANLTAGHLLIQLIAM
Bede  MRNQPTHALGHLLPEGTPTLLIPVLIIIETISLFIRPLALGVRLTANLTAGHLLIQLIAT
Besp  MRNQPTHALGHLLPEGTPTLLIPVLIIIETISLFIRPLALGVRLTANLTAGHLLIQLIAT
Mysp  MRNQPTHALGHLLPEGTPVLLIPVLVIIETISLFIRPLALGVRLTANLTAGHLLMQLIAT
Osja  MRNQPTHALGHLLPEGTPSLLIPILIIIETISLFIRPLALGVRLTANLTAGHLLIQLIAT
Sgro  MRNQPTHALGHLLPEGTPTLLIPVLIIIETISLFIRPLALGVRLTANLTAGHLLIQLIAT
Pzpa  MRNQPTQSLAHLLPEGTPTTLIPILIIIETISLFIRPLALGVRLTANLTAGHLLIQLIST
Zeja  MRNQPTHALGHLLPEGTPTALIPVLIIIETISLFIRPLALGVRLTANLTAGHLLIQLIAT
Znne  MRNQPKIAFAHLLPEGTPTLLIPILIIIETVSLLVRPLALGLRLTANLTAGHLLIELIAS
Zefa  MRNQPTHTLGHLLPEGTPTALIPILIIIETISLFIRPLALGVRLTANLTAGHLLIHLIST
Acni  MHNQPTHALGHLLPEGTPTALIPILIIIETISLFIRPLALGVRLTANLTAGHLLIQLIAT
Ncrh  MHNQPTHALGHLLPEGTPTALIPILIIIETISLFIRPLALGVRLTANLTAGHLLIQLIAT
Agca  MRNQPTAALGHLLPEGTPVLLIPILIVIETISLFIRPLALGVRLTANLTAGHLLMQLIAT
Hydy  MRNQPTHALGHLLPEGTPGPLIPVLIIIETISLFIRPLALGVRLTANLTAGHLLIQLIAT
Gsac  MRNQPTHALGHLLPEGTPGPLIPVLIIIETISLFIRPLALGVRLTANLTAGHLLIQLIAT
Pevo  MRNQPTHALAHFLPEGTPPALVPILIIIETISLFIRPLALGVRMTANLTAGHLLMNLVAS
Hiku  MRNQPTHSLGHLLPEGTPVPLIPVLIIIETISLFIRPIALGVRLTANLTAGHLLIQLIAT
Inpa  LRNQPTHALGHLLPEGTPTPLIPILIIIETISLFIRPLALGVRLTANLTAGHLLMQLIAT
Auch  MRTQPAHALAHLVPEGSPMPLVPFLILIETMSLLIRPLALGIRLAANLTAGHLLIKLLSS
Fico  MRNQPTHALGHLLPEGTPTLLIPILIIIETISLFIRPIALGVRLTANLTAGHLLIQLIAT
Macs  MRNQPTHALGHLLPEGTPTLLIPILIIIETISLFIRPLALGVRLTANLTAGHLLIQLIAT
Moal  FRNQPTLSLAHLLPEGTPTLLVPVLIIIETISLLIRPFALGVRLTANLTAGHLLIQLIAT
Syma  FRNQPTHAFAHLLPEGTPTLLVPILIVIETISLFIRPLALGVRLTANLTAGHLLIQLIAT
Mafr  MLTQPTHSLGHLLPEGTPTPLIPILIIIETISLLIRPLALGVRLTANLTAGHLLIQLIAT
Dcpe  MRNQPSDSLAHLLPEGTPTLLIPVLIIIETISLFIRPLALGVRLTANLTAGHLLIQLVSM
Dcti  MRNQPSHSLAHLLPEGTPTPLIPVLIIIETISLFIRPLALGVRLTANLTAGHLLIQLVST
Hehi  MRNQPTHALGHLLPEGTPGPLIPVLIIIETISLFIRPLALGVRLTANLTAGHLLIQLIAT
Stam  MRNQPTHALGHLLPEGTPGPLIPVLIIIETISLFIRPLALGVRLTANLTAGHLLIQLIAT
Hogi  MRNQPTHSLGHLLPEGTPGPLIPVLIIIETISLFIRPLALGVRLTANLTAGHLLIQLIAT
Erzo  MRNQPTHALGHLLPEGTPGPLIPILIIIETISLFIRPLALGVRLTANLTAGHLLMQLIAT
Hxot  MRNQPTHALGHLLPEGTPGPLIPVLIVIETISLFIRPLALGVRLTANLTAGHLLIQLIST
Core  MRNQPTHALGHLLPEGTPGPLIPVLIVIETISLFIRPLALGVRLTANLTAGHLLIQLIST
Apve  VRNQPTHALGHLLPEGTPGPLIPVLIIIETISLFIRPLALGVRLTANLTAGHLLIQLIST
Latj  MRNRPTHALGHLLPEGTPVLLIPILIIIETISLFIRPLALGVRLTANLTAGHLLIQLIAT
Laja  FRNKPTVALGHLLPEGTPILLIPVLIIIETISLLIRPLALGVRLTANLTAGHLLIQLISS
```

[3/4 of aligned sequences]

```
Syja FRNNPTMALGHLLPEGTPAPLIPVLIVIETISLFIRPLALGVRLTANLTAGHLLIQLSAT
Epme MRNQPNHALGHLLPEGTPNLLIPMLIIIETISLFIRPLALGVRLTANLTAGHLLIQLIST
Grse MRNQPTHALGHLLPEGTPGALIPVLIMIETISLFIRPLALGVRLTANLTAGHLLIQLIAT
Clja MRNRPTIALGHLLPEGTPWALIPVLIIIETISLFIRPLALGVRLTANLTAGHLLIQLIST
Ogcy MRNQPTLALAHLLPEGTPVMLIPVLIVIETISLFIRPLALGVRLTANLTAGHLLIQLIAT
Plna MRNQPTHALGHLLPEGTPTLLIPVLIIIETISLFIRPLALGVRLTANLTAGHLLIQLIAT
Lema LRNQPTIALGHLLPEGTPTPLIPVLIVIETISLLIRPLALGVRLTANLTAGHLLIQLIAT
Etzo MRNQPTHALGHLLPEGTPGPLIPVLIVIETISLFIRPLALGVRLTANLTAGHLLIQLIAT
Apse MRNQPTHALGHLLPEGTPTLLIPVLIIIETISLFIRPLALGVRLTANLTAGHLLIQLIAT
Epde MRNQPTLALGHLLPEGTPTLLIPVLIIIETISLFIRPIALGVRLTANLTAGHLLIQLIAT
Slja MRNQPTVALGHLLPEGTPTPLIPILIIIETISLFIRPLALGVRLTANLTAGHLLIQLIST
Bsja MWNKPTDALGHLLPEGTPVPLIPVLIIIETISLFIRPLALGVRLTANLTAGHLLIQLIST
Ecna MRNQPTHSLGHLLPEGTPPLLIPVLIIIETISLFIRPLALGVRLTANLTAGHLLMQLIAT
Cohi MRNKMSHTLAHLLPEGTPVLLIPVLIVIETISLLIRPLALGVRLTANLTAGHLLIQLTAT
Caar MRNQPTHALGHLLPEGTPTPLIPVLIIIETISLFIRPLALGVRLTANLTAGHLLIQLIAT
Came MRNQPTHALGHLLPEGTPTLLIPVLIIIETISLFIRPLALGVRLTANLTAGHLLIQLIAT
Mema MRNQPTHALGHLLPEGTPVPLIPALIIIETISLFIRPLALGVRLTANLTAGHLLIQLIST
Lenu MRNRPTETLGHLLPEGTPNALIPMLVIIETISLFIRPLALGVRLTANLTAGHLLIQLIAS
Brja MRNQPTEALGHLLPEGTPTLLIPILIVIETISLFIRPLTLGVRLTANLTAGHLLIQLIAT
Plma MRNQPTEALGHLLPEGTPTPLIPVLIIIETISLFIRPLALGVRLTANLTAGHLLIQLIAT
Emst MRNQPTIALGHLLPEGTPTLLIPVLIIIETISLFIRPLALGVRLTANLTAGHLLIQLIAT
Ptti MRNQPTIALGHLLPEGTPTLLIPVLIIIETISLFIRPLALGVRLTANLTAGHLLIQLIAT
Losu LRNQPTISLGHLLPEGTPIPLIPILIIIETISLIIRPLALGVRLTANLTAGHLLIQLIST
Geoy LRNQPTVALGHLLPEGTPTLLIPVLIIIETISLFIRPLALGVRLTANLTAGHLLIQLIAT
Dipi MRYQPTASLGHLLPEGTPVPLIPVLIIIETISLFIRPLALGVRLTANLTAGHLLIQLIAT
Pama FRYQPNFSLAHLLPEGTPLLLIPVLIIIETISLMIRPLALGVRLTANLTAGHLLIQLIST
Leob LRNQPTIALGHLLPEGTPTPLIPVLIIIETISLFIRPLALGVRLTANLTAGHLLIQLIAT
Neba LRNQPTVALGHLLPEGTPTPLIPVLIIIETISLFIRPVALGVRLTANLTAGHLLIQLIAT
Pdpl MRNQPTPTLAHLLPEGTPTPLIPVLIIIETISLFIRPLALGVRLTANLTAGHLLIHLIAT
Nimi LRNQPTAALGHLLPEGTPTPLIPVLVIIETISLLIRPLALGVRLTANLTAGHLLMQLTST
Uptr MRNQPTESLGHLLPEGTPTPLIPVLIIIETISLFIRPLALGVRLTANLTAGHLLIQLIST
Pesc MRNQPTVVLGHLLPEGTPTPLIPVLIIIETISLLIRPLALGVRLTANLTAGHLLIQLIAT
Baar FRNQPTIALGHLLPEGTPTPLIPVLIIIETISLLIRPLALGVRLTANLTAGHLLIQLIAT
Moar MRNQPTVALGHLLPEGTPTPLIPVLIIIETISLFIRPLALGVRLTANLTAGHLLIQLIAT
Toja MRNQPTHALGHLLPEGTPTLLIPVLIIIETISLFIRPLALGVRLTANLTAGHLLIQLIAT
Chau LRNQPNVAIAHILPEATPGPLVPVLIMIETASLFIRPLALGVRLTANLTAGHLLIQLIST
Chse MRSQPTAALGHLLPEGTPTPLIPILIIIETISLFIRPFALGVRLTANLTAGHLLIQLVSM
Enar MRNQPTIALGHLLPEGTPTPLIPVLIIIETISLFIRPLALGVRLTANLTAGHLLIQLIAT
Hpty FRNQPTIALGHLLPEGTPTPLIPVLIIIETISLFIRPLALGVRLTANLTAGHLLIQLIAT
Nana MRNQPTHALGHLLPEGTPTPLIPILIIIETISLFIRPLALGVRLTANLTAGHLLMQLIAT
Mcst MRNQPTIALGHLLPEGTPTPLIPVLIIIETISLFIRPLALGVRLTANLTAGHLLIQLIAT
Rhox MRNQPTIALGHLLPEGTPTLLIPILIIIETISLFIRPVALGVRLTANLTAGHLLIQLIAT
Opfa LRNQPTIALGHLLPEGTPTPLIPVLIIIETISLFIRPLALGVRLTANLTAGHLLIQLIAT
Paar MRNQPNATLGHLLPEGTPTPLIPILIVIETISLLIRPLALGVRLTANLTAGHLLIQLIAT
Gozo MRNQPTHALGHLLPEGTPTLLIPILIIIETISLFIRPLALGVRLTANLTAGHLLIQLIAT
Ackr LLNQPTHALGHLLPEGTPTLLIPALIVIETISLFIRPLALGVRLTANLTAGHLLIQLIAT
Elev MRNQPTHALGHLLPEGTPTLLIPVLIIIETISLFIRPLALGVRLTANLTAGHLLIQLIAT
Trdu MRNQPTHALGHLLPEGTPTLLIPVLIIIETISLFIRPLALGVRLTANLTAGHLLIQLIAT
Amoc MRNQPTHALAHLLPEGTPTPLIPILIMIETISLFIRPLALGVRLTANLTAGHLLIQLIAT
Hame LRNQPTAALGHLLPEGTPPLLIPVLIVIETISLFIRPLALGVRLTANLTAGHLLIQLIAT
Chso MRNQPTVALGHLLPEGTPTLLIPILIIIETLSLFIRPLALGVRLTANLTAGHLLIQLIAT
Lyto MRNQPTHALGHLLPEGTPGPLIPVLIIIETISLFIRPLALGVRLTANLTAGHLLIQLIAT
```

To be continued on page 19.

[3/4 of aligned sequences]

```
Encr  MRNQPTHALGHLLPEGTPGPLIPVLIIIETISLFIRPLALGVRLTANLTAGHLLIQLIAT
Bvar  LRNQPTHALGHLLPEGTPTPLIPILVIIETISLLVRPLALGVRLTANLTAGHLLIQLIAT
Noco  LRNQPTIALGHLLPEGTPAPLIPVLIIIETISLFVRPFALGVRLTANLAAGHLLIQLLSS
Chsp  FRNQPTPSLAHFLPEGTPPPLIPVLIIIETISLFIRPIALAVRLTANLTAGHLLIHLLSL
Arja  MRNQPTHALGHLLPEGTPGPLIPVLIVIETISLFIRPLALGVRLTANLTAGHLLIQLIST
Pase  LRNQPTIALGHLLPEGTPTPLIPVLIIIETISLFIRPLALGVRLTANLTAGHLLIQLIAT
Trel  LRFKFNDSMAHLLPEGTPTALVPILIIVETISLAIRPFALAVRIFANLTAGHLLMALGSA
Lifa  LRHAPKDMIAHVLPQGTPNALIPMLILVETMSLLIRPIALAMRITANLTAGHLLMHLISA
Acur  FFYHTNRSLAHMAPQGTPPYLVPAVILIETISLFIRPISLAVRLTANLTAGHLLIQLLAT
Ampe  MRNQPTVALGHLLPEGTPTPLIPVLIIIETISLFIRPLALGVRLTANLTAGHLLIQLIAT
Urja  FRNQPNTALAHLLPEGTPPPLTPVLIIIETVSLFIRPLALGVRLTANLTAGHLLMHLIAS
Enet  MRTQPTHALGHLLPEGTPVLLIPVLIIIETISLFIRPLALGVRLTANLTAGHLLIQLIAT
Ptbr  MRNFTTRSLAHLVPEGSPTALIPILIIIETISLFIRPIALGVRLTANLTAGHLLIQLIAT
Safa  MRNQPTHALGHLLPEGTPTPLIPILIIIETISLFIRPLALGVRLTANLTAGHLLIQLIAT
Icae  MRNQPTEALGHLLPEGTPTLLIPILIIIETISLFIRPLALGVRLTANLTAGHLLIQLIAT
Asmi  LRNHTTRSFAHLLPEGTPALLVPVLIIIETISLFIRPFALGVRLTANLTAGHLLIHLIST
Foal  MRKYPTRSLAHFLPEGTPPALIPMLVLIETASLLIRPIALAVRLTANLTAGHLLMQLIGA
Drze  MRRQPIPSLAHFLPEGTPILLIPLLILIELISLMIRPLALGVRLTANLTAGHLLLRLIAY
Rhas  LRNQTTAALGHLLPEGTPVPLIPVLIIIETISLFIRPLALGVRLTANLTAGHLLIQLIAT
Elac  MRNQPTAALGHLLPEGTPAPLIPVLIIIETISVFIRPLALGVRLTANLTAGHLLIQLIAT
Kugu  LRNQWTATLGHLLPEGTPTPLIPALIIIETISLFIRPLALGVRLTANLTAGHLLIQLTAT
Plor  MRNQPTVALGHFLPEGTPTLLIPVLIVIETISLFIRPLALGVRLTANLTAGHLLIQLIAT
Sgun  MRNQPTIALGHLLPEGTPTPLIPVLIIIETISLFIRPLALGVRLTANLTAGHLLIQLIAT
Zaco  MRNQPTIALGHLLPEGTPTPLIPVLIIIETISLFIRPLALGVRLTANLTAGHLLIQLIAT
Zbfl  MRNQPTVALGHLLPEGTPTPLIPVLIIIETISLFIRPLALGVRLTANLTAGHLLIQLIAT
Spba  MRNQPTHALGHLLPEGTPTPLIPVLIIIETISLFIRPLALGVRLTANLTAGHLLIQLIAT
Game  MRNQPTEALGHLLPEGTPTLLIPVLIIIETISLFIRPLALGVRLTANLTAGHLLIQLIAT
Thth  MRNQPTEALGHLLPEGTPTLLIPVLIVIETISLFIRPLALGVRLTANLTAGHLLIQLIAT
Xigl  MRNQPTHALGHLLPEGTPTLLIPILIIIETISLFIRPLALGVRLTANLTAGHLLIQLIAT
Hyja  LRNQPTQTFGHLLPEGTPMLLIPVLIIIETISLLIRPIALSVRLTANLTAGHLLIHLIAM
Psan  LRNQPTQSLGHLLPEGTPTPLIPILIIIETISLLIRPVALSVRLTANLTAGHLLIHLISM
Cupa  MRNQPTEALGHLLPEGTPTPLIPVLIIIETISLFIRPLALGVRLTANLTAGHLLIQLIAT
Mpch  MRNQPTHALGHLLPEGTPTLLIPILIIIETISLLIRPLALGVRLTANLTAGHLLIQLIAT
Char  LRTQPTHALGHLLPEGTPTLLIPVLIIIETISLFIRPLALGVRLTANLTAGHLLIQLIAT
Pser  MRTQITHALAHLLPEGTPVPLIPVLIIIETASLFIRPLALGVRLTANLTAGHLLIQLIAT
Prol  MRNQPTHALGHLLPEGTPTALIPVLIIIETISLFIRPLALGVRLTANLTAGHLLIQLIAT
Plbi  MWNQPTIALGHLLPEGTPTPLIPVLIIIETISLFIRPLALGVRLTANLTAGHLLIQLIAM
Calu  LRNQPNHALAHLLPEGTPTLLIPILIIIETISLFIRPVALGVRLTANLTAGHLLMQLIST
Papa  MRDYPARSLAHFLPPGTPLYLIPLLIIIETISLLIRPLSLAIRLTANLTAGHLMIQLIAT
Sufr  MRNQPTHALGHLLPEGTPTLLIPVLIIIETISLFIRPLALGVRLTANLTAGHLLIQLIAT
Stci  LRGQPTVSLAHLLPEGTPPALIPVLIIIETLSLFIRPFALGVRLTANLTAGHLLLQLVST
Taru  MRNNPTAALGHLLPEGTPNALIPILIIIETVSLFIRPLALGVRLTANLTAGHLLIQLIAT
Rala  MRNQPTVALGHLLPEGTPTLLIPVLIIIETISLFIRPLALGVRLTANLTAGHLLIQLIAT
                   .. *  :*  * * :: ::   *   :** :*.:*: **:::**        .
```

To be continued on page 20.

[4/4 of aligned sequences]

F

```
Scca AAFVLLTMMPTVA----LLTSLVLFLLTILEVAVAMIQAYVFVLLLSLYLQENV--
Muma AAFVLLTIMPTVA----LLTSLILFLLTILEVAVAMIQAYVFVLLLSLYLQENM--
Erca ATFVMLSFMPTIA----MLTFIVLALLTLLEIAVAMIQAYVFVLLLSLYLQENV--
Pose ATFVMLPIMPTIA----MLTLTVLALLTILEIAVAMIQAYVFVLLLSLYLQENV--
Actr AAFVLLPMMPTVA----ILTSMVLFLLTLLEVAVAMIQAYVFVLLLSLYLQENV*-
Scal AAFVLLPMMPTVA----ILTSTVLFLLTLLEVAVAMIQAYVFVLLLSLYLQENV--
Posp AAFVLLPMMPTVA----ILTSVVLFLLTLLEVAVAMIQAYVFVLLLSLYLQENV--
Atsp ATFVLLQLMPTVA----TLTAIVMLLLTLLEVAVAMIQAYVFVLLLSLYLQENV--
Leoc ATFVLLQLMPTVA----TLTAMVMLLLTLLEVAVAMIQAYVFVLLLSLYLQENV--
Amca AAFVLIPMMPTVA----LLTSIVLFLLTLLEIAVAMIQAYVFVLLLSLYLQENV--
Osbi AAFHMFFMMPTVA----TLTMILLLLLSVLELAVAVIQAYVFVLLVSLYLQESV--
Pabu ATFVMLFMSPAVS----AITMILLFLLSILELAVAIIQAYVFVLLLSLYLQESV--
Hial AAFNLFFMMPAVS----ALTMVLLLLLSVLELAVAVIQAYVFVLLVSLYLQESV--
Elha AVFVLLPMMPTVA----ILTAVVLFLLTLLEVAVAMIQAYVFVLLLSLYLQENV*-
Mlcy AAFVLFPMMPTVA----ILTSTVLFLLTLLEVAVAMIQAYVFVLLLSLYLQENV--
Algl AAFVLAPMMPAVA----ISAAVVLFLLTLLEVAVAMIQAYVFVLLLSLYLQENV--
Ptgi AAFVLFPMMPTVA----ILTATVLFLLTLLEVAVAMIQAYVFVLLLSLYLQENV--
Alaf AAFVLAPMMPTVA----ILTTTVLFLLTLLEVAVAMIQAYVFVLLLSLYLQENV--
Nock AAFVLTPMMPTVA----ILTTTVLFLLTLLEVAVAMIQAYVFVLLLSLYLQENV--
Anja AVFVLLPMMPTVA----ILTATVLFLLTLLEVAVAMIQAYVFVLLLSLYLQENV*-
Gyki AVFVLLPLMPTVA----ILTALVLFLLTLLEVAVAMIQAYVFVLLLSLYLQENV--
Syka AVYVLLPMMTTVA----LLTATVLFLLTLLEVAVAMIQAYVFVLLLSLYLQENV--
Opma AAFVLLPMMPTVA----LLTTTVLFLLTLLEVAVAMIQAYVFVLLLSLYLQENV--
Comy AAFVLLPLMPTVA----ALTFTVLFLLTLLEVAVAMIQAYVFVLLLSLYLQENV*-
Sasp AVFTLLTTASLTA----VLALVALLLLTTLEVAVAMIQAYVFILLLSLYLQENV--
Eupe AIFALMPTMMLTA----ALTMVVLFLLTLLEVAVAMIQAYVFVLLLSLYLQENT--
Enja GAFVLLPIMPTVA----ILTATVLFLLTLLEVAVAMIQAYVFVLLLSLYLQENV--
Same AVFVLLPLMPTVA----ILTATVLFLLTLLEVAVAMIQAYVFVLLLSLYLQENV*-
Chch AAFVLLPMMPTVA----ILTATVLFLLTLLEVAVAMIQAYVFVLLLTLYLQENT--
Grgr AAFVLTPMMPAVG----LLTAAVLFLLTLLEVAVAMIQAYVFVLLLSLYLQENV--
Caau AVFVLMPMMPTVA----ILTATVLFLLTLLEVAVAMIQAYVFVLLLSLYLQENV--
Cyca AVFVLLPMMPTVA----ILTAAVLFLLTLLEVAVAMIQAYVFVLLLSLYLQENV--
Dare AVFVLLPMMPAVA----ILTASVLFLLTLLEVAVAMIQAYVFILLLSLYLQENI--
Cost AVFVLLPMMPTVA----ILTATVLFLLTLLEVAVAMIQAYVFVLLLSLYLQENV--
Leec AVFVLLPLMPTVA----ILTAAVLFLLTLLEVAVAMIQAYVFVLLLSLYLQENV--
Fola AVFVLLPMMPTVA----ILTATVLFLLTLLEVAVAMIQAYVFVLLLSLYLQENV*-
Clmc AAFVLLPLMTTVA----LLTTTILFLLTLLEVAVAMIQAYVFVLLLSLYLQENV--
Phin ATLVLLPKITTVA----LLTALVLLLLTLLEVAVAMIQAYVFVLLLSLYLQENT--
Icpu ATITLMPMMTTVA----TLTAILLVLLTLLEVAVAIIQAYVFVLLLSLYLQENV--
Psto ATITLMPMMTTVA----TLTAILLVLLTLLEVAVAVIQAYVFVLLLSLYLQENV--
Cora ATFVMLPMMATAA----VFTATLLVLLTLLEVAVAMIQAYVFVLLVSLYLQENI*-
Eisp AAFVMLPTMTTLA----VLTAIVLLLLTLLEVAVAMIQAYVFVLLLSLYLQENV--
Apal ATAVLLPMTVTLA----TLTMTVLFLLTLLEIAVAMIQAFVFILLLSLYLQENI--
Eslu AVFVLLPLMPTVA----ILTASVLFLLTLLEVAVAMIQAYVFILLLSLYLQENV--
Dape AVLVLLPIMPAVA----ILTASILFLLTLLEVAVAMIQAYVFVLLLSLYLQENV--
Glse AAFVLLPIMPTVA----ILTSTVLFLLTLLEVAVAMIQAYVFVLLLSLYLQENV*-
Naar AAFVLLPIMPTVA----ILTATVLFLLTLLEVAVAMIQAYVFVLLLSLYLQENV--
Lioc AAFVLLPIMPTVA----ILTASVLFLLTLLEVAVAMIQAYVFVLLLSLYLQENV--
Opso AAFVLLPIMPAVA----ILTAIILFLLTLLEVAVAMIQAYVFVLLLSLYLQENI--
Alte AAFVLLPMMPTVA----ILTATVLFLLTLLEVAVAMIQAYVFVLLLSLYLQENV--
Plap AAFVLLPMMPTVA----ILTATVLFLLTLLEVAVAMIQAYVFVLLLSLYLQENV--
```

[4/4 of aligned sequences]

```
Plal AAFVLLPLMPTVA----ILTSAVLFLLTLLEVAVAMIQAYVFVLLMSLYLQENV*-
Sami AAFVLLPLMPTVA----ILTSIVLFLLTLLEVAVAMIQAYVFVLLMSLYLQENV--
Rere AAFVLLPIMPTVA----IMTSAVLFLLTLLEVAVAMIQAYVFVLLLSLYLQENV--
Gama AAFVLLPLMPAVA----LLTSAVLFLLTLLEVAVAMIQAYVFVLLLSLYLQENV--
Onmy AAFVLLPMMPTVA----ILTSIVLFLLTLLEIAVAMIQAYVFVLLLSLYLQENV*-
Sasa AAFVLMPIMPTVA----ILTSIVLFLLTLLEIAVAMIQAYVFVLLLSLYLQENV--
Cola AAFVLLPLMPTVA----ILTAIVLFLLTLLEIAVAMIQAYVFVLLLSLYLQENV*-
Dita AAVVLLPLMPAVA----ALTAVILFLLTLLEVAVAMIQAYVFVLLLSLYLQENI*-
Gogr AAFTLLPTAPLLA----ILSIIPLFLLTLLEIAVAMIQAFVFVLLLSLYLQENT*-
Chsl ATGAALAKAPELG----LLAFPAVVGFSILEVAVAMIQAYVFVLLVTLYMQENIS*
Atja GAYVLLPMMPTVA----TLTAVLLLLLTLLETAVAMIQAYVFVLLLSLYLQENV*-
Iido GAYVLLPMMPTIA----TLTAILLLLLTLLETAVAMIQAYVFVLLLSLYLQENV*-
Auja AAFVLLPLMPTVA----ILTTLLLFLLTLLEVAVAMIQAYVFVLLLSLYLQENV--
Chag AAFVLLPMMPAVA----ILTGIVLFLLTLLEVAVAMIQAYVFVLLLSLYLQENV*-
Hami AAFVLLPLMPSVA----ILTALLLFLLTLLEVAVAMIQAYVFVLLLSLYLQENV*-
Saun AAFVLLPLMPSVA----ILTALLLFLLTLLEVAVAMIQAYVFVLLLSLYLQENV*-
Nema AAFVLLPIMPTVA----ILTATLLFLLTLLEIAVAMIQAYVFVLLLSLYLQENV*-
Disp AAFVLLPLMPTVA----ILTATLLFLLTLLEVAVAMIQAYVFVLLLSLYLQENV*-
Myaf GAFVLLSIMPAVA----ILTSIVLVLLTLLEVAVAMIQAYVFVLLLSLYLQENV*-
Lagu ATFVLLPLMPAVA----ISTGALLFLLTLLEIAVAMIQAYVFVLLLSLYLQENV--
Trtr AAFVLLPLMPSVA----ILTTALLFFLTLLEIAVAMIQAYVFVLLLSLYLQENI*-
Zucr AAFVLLPLMPSVA----VLTATLLLLLTLLEIAVAMIQAYVFVLLLSLYLQENI*-
Pxja AAFVLLPLMPTVA----ILTATLLFLLTLLEVAVAMIQAYVFVLLLSLYLQENV*-
Pxlo AAFVLLPLMPTVA----ILTATLLFLLTLLEVAVAMIQAYVFVLLLSLYLQENV--
Pctr AVFVLLPLMPTIA----ILTSVLLFLLTLLEVAVAMIQAYVFVLLLSLYLQENV*-
Apsa AVFVLLPLMPTIA----IITSVLLFLLTLLEVAVAMIQAYVFVLLLSLYLQENV*-
Cabe TGITMLYALSPAG----IFGVIILYLLTGLEIMVAAIQAYVLVLLLSLYLQENV--
Bzze TTIGLLCLMNPAG----LLGAVVLYLLACLELAVAAIQAYVFVLLLSLYLQENV--
Siim VSLVLIQSASLSA----ALSLTLLGALTCLEMAIAGIQAYVFVLLTTLYLQENT*-
Ctru AAFVLLPLMPTVA----ILTAALLFLLTLLEIAVAMIQAYVFVLLLSLYLQENI--
Dpbr AAFVLLPLMPTVA----LLTTTLLFLLTLLEIAVAMIQAYVFVLLLSLYLQENI--
Caki AVFTLLPSMTTVA----LLTAILLFLLTLLEVAVAMIQAYVFVLLLSLYLQENC*-
Phja GTFIMLSMMPSVA----ILTGALLLVLTLLEVAVAMIQAYVFVLLLSLYLQENI--
Brsp AFFTLI-SMPLVA----SFTGILLFLLTLLEIAVAMIQAYVFVLLLSLYLQENV*-
Gamo AVFVLMPMMPVVA----ILTAVLLLLLTMLEVAVAMIQAYVFILLLSLYLQENV*-
Lolo AAFVLMPMMPTVA----LLTAILLLMLTLLEVAVAMIQAYVFILLLSLYLQENV*-
Batr TLMQSATSIPLLF----ICLFPLLPLLSLLEVAMAIIQAYVFVLLLSLYLQENI--
Prmy TITKLLIMSISAA----LITMALLVLLTILEIAVAMIQAYVFVLLLSLYLQENV--
Lose GAQVLIPLMPVTA----FITLLTLILFFILEAAMALVQAYVFVLLLSLYLEESQ*-
Loam AAFVLLPLMPTVA----LLTATLLFLLTLLEVAVAMIQAYVFVLLLSLYLQENV--
Chab ATLALIPYLMPIW----ILTWTLLSLLLVLEVAVALIQAYVFVLLLSLYLQENI--
Chto ATLTLIPYLMPLW----ILTWTLLSLLLVLEVAVALIQAYVFVLLLSLYLQENI--
Majo AAFVLLPLMPTVA----LLTSTLLLLLTLLEVAVAMIQAYVFVLLLSLYLQENV--
Hlst ASIVLLPLMPTVS----LLTMIMLLLLTLLEIAVAMIQAYVFVLLLSLYLQENT--
Clpe AAFVLVPLMPAVA----ILTATVLLLLTLLEVAVALIQAYVFVLLLTLYLQENV*-
Mlmr AAFVLLPLMTTVA----ILTSTVLLLLTLLEVGVAMIQAYVFVLLLSLYLQENV--
Crcr ATFVLLPLMPPVA----FLTAILLLLLSLLEVAVALIQAYVFVLLLSLYLQENI*-
Muce ATFVLLPLMPPVA----FLTAILLLLLSLLEVAVALIQAYVFVLLLSLYLQENI*-
Bege AAFVLLPLMPTVA----LLTSILLFLLTLLEVAVAMIQAYVFVLLLSLYLQENV--
Mela AVFTLMFLMPTLA----FLTSILLLLLTLLEVAVALIQAYVFVLLLSLYLQENL--
Hats AAFVLLPLMPAVA----VLTSALLFLLTLLEVAVAMIQAYVFVLLLSLYLQENV--
Orla AAFVLFPMMPSVA----ILTSILLFLLTLLEIAVAMIQAYVFVLLLSLYLQENV--
```

[4/4 of aligned sequences]

```
Cosa    AAFVLASTAPTLA----ILTTMLLFLLTLLEIAVAMIQAYVFVLLLSLYLQENI*-
Exsp    AAFQLLPIMPTVA----LTTTVLLFLLTLLEVAVAMIQAYVFVLLLSLYLQENV*-
Depa    AAFVLALMSPSLA----LLTAALLFLLTLLEIAVAMIQAYVFVLLLSLYLQENI--
Rima    AAFSLLLSQTTAA----SLAVGLLLLLTILEVAVAMIQAYVFVLLLSLYLQENV--
Fuol    GVFLLTSMMPMTA----VLALMLLGLLTLLEIAVAVIQAYVFVLLLSLYLLENV--
Gmaf    ATSVLLMTMPVLA----VLTATLLFLLTLLEVAVAMIQAYVFVLLLSLYLQENV--
Xeei    AAFVLLPIMPMTA----LLTSILLLLLTLLEIAVAMIQAYVFVLLLSLYLQENV--
Pros    ATAVLAPLMPTVA----FLTGTLLLLLTLLEVAVAMIQAYVFVLLLSLYLQENV*-
Scmi    TTAVLTPLMPAVA----FLTGTLLLLLTLLEIAVAMIQAYVFVLLLSLYLQENT*-
Rolo    AAFVLLPLMPTVA----ILTATLLFLLTLLEIAVAMIQAYVFVLLLSLYLQENV*-
Cere    AAFVLLPLMPAVA----ILTTILLFLLTLLEIAVAMIQAYVFVLLLSLYLQENV--
Daga    ATFVLLPLMPTVA----ILTTALLFLLTLLEIAVAMIQAYVFILLLSLYLQENV*-
Anco    GINALVSVMPTTA----LIMSVLLLLMTLLEVAVAMIQAYVFVLLLSLYLQENV--
Dmve    GAYVLYPTMPAVA----LLPTIFMFALTLLEVAVAMIQAYVFVLLLSLYLQENV--
Dmar    GAYVLYPTMPAVA----LLPTIFMFALTLLEVAVAMIQAYVFVLLLSLYLQENV--
Anka    GISALVSMMPTTA----LIMMVLLLLMTLLEVAVAMIQAYVFVLLLSLYLQENV--
Moja    GICALVSLMPTTA----LIMSILLLLMTLLEVAVAMIQAYVFVLLLSLYLQENV--
Hoja    GISALVSVMPVTA----LIMMVLLLLMTLLEVAVAMIQAYVFVLLLSLYLQENV--
Bede    ATAVLAPLMPTVA----ILTGTLLLMLTLLEVAVAMIQAYVFVLLLSLYLQENV--
Besp    ATAVLAPLMPTVA----ILTGTLLLLLTLLEVAVAMIQAYVFVLLLSLYLQENV*-
Mysp    ATFVLLSIMPGVA----VLTAILLLMLSLLEVAVAMIQAYVFILLLSLYLQENV*-
Osja    AAFVLLPLMPTVA----ILTATLLFLLTLLEVAVAMIQAYVFVLLLSLYLQENV--
Sgro    AAFVLLPLMPTVA----ILTATLLFLLTLLEVAVAMIQAYVFVLLLSLYLQENV--
Pzpa    AAFLLLPLMPAVA----ALTTLLLFFLTLLEVAVAMIQAYVFVLLLSLYLQENI*-
Zeja    AAFVLLPLMPTVA----IFTSVLLFLLTLLEVAVAMIQAYVFILLLSLYLQENV*-
Znne    AATTLLPLMPVVA----FLTAVILFLLTLLEIAVAMIQAYVFVLLLSLYLQENV*-
Zefa    AAFVLLPLMPTVA----LLTSLLLFLLTLLEIAVAMIQAYVFVLLLTLYLQENV*-
Acni    AAFVLLPLMPTVA----LLTSLLLFLLTLLEVAVAMIQAYVFVLLLSLYLQENV--
Ncrh    AAFVLLPLMPTVA----LLTSLLLFLLTLLEVAVAMIQAYVFVLLLSLYLQENV--
Agca    AAFVLLPLMPTVA----ILTTVLLFLLTLLEIAVAMIQAYVFVLLLSLYLQENV*-
Hydy    ASFVLLPLMPAVA----FTITLVLFLLTLLEVAVAMIQAYVFVLLLTLYLQENV--
Gsac    ASFVLLSLMPAVA----ITVTVVLFLLTLLEVAVAMIQAYVFVLLLTLYLQENV*-
Pevo    GAFFIFPHSMLAG----ATISLFLLMLSALEVAVAMIQAYVFVLLLSLYLQENT--
Hiku    AAFVLMPLMPTVA----LLTTILLFMLTLLEVAVAMIQAYVFVLLLSLYLQENV--
Inpa    ASFVLLPLMPVVA----LTTTALLFLLTLLEIAVAMIQAYVFVLLLSLYLQENT*-
Auch    ATKILISTLPLLA----LFTLTALTVMTLLELAVAVIQAYVFTLLLALYTQENV--
Fico    GAFVLLPLMPTVA----ILTSILLFLLTLLEVAVAMIQAYVFVLLLSLYLQENV--
Macs    AAFVLLPLMPTVA----ILTTTLLFLLTLLEVAVAMIQAYVFVLLLSLYLQENV--
Moal    ATLAFMSIMPAIA----TLTSILLLLLTLLEIAVAMIQAYVFVLLLSLYLQENS*-
Syma    ATFVLLPLMPMIS----LLTAILLFLLTILEIAVAMIQAYVFVLLLSLYLQENV--
Mafr    AAFVLMPMMTSVA----ILTTALLFLLTLLEVAVAMIQAYVFVLLLSLYLQENI*-
Dcpe    ASLAMASVLPAAS----LLTSALLLMLTLLEVAVAMIQAYVFVLLLSLYLQENV*-
Dcti    ASFVLLSVMPTVA----VLTSALLLMLTLLEVAVAMIQAYVFVLLLSLYLQENV--
Hehi    GAFVLLPLMPTVA----IITTTVLVLLTLLEVAVAMIQAYVFVLLLTLYLQENV*-
Stam    AAFVLLPLMPTVA----IVTSTVLVLLTLLEVAVAMIQAYVFVLLLTLYLQENV--
Hogi    ATTVLMSMMPAVA----VLTASVLFLLTLLEIAVAMIQAYVFVLLLTLYLQENV--
Erzo    AAFTLAPSMPTVA----IVTSGVLVLLTLLEIAVAMIQAYVFVLLLSLYLQENV--
Hxot    AAFVMMPLMPAVA----LLTSTVLVLLTLLEVAVAMIQAYVFVLLLTLYLQENV--
Core    AAFVLISSMPAVA----LLTSTVLVLLTLLEVAVAMIQAYVFVLLLTLYLQENV*-
Apve    AAFVLLSSMPAIA----LLTSTVLVLLTLLEVAVAMIQAYVFVLLLTLYLQENV--
Latj    ASFVLLPLMPVVA----VMTTILLLLLTLLEVAVAMIQAYVFVLLLSLYLQENV--
Laja    AVFVLLPLMPMVA----IATSLLLFMLSLLEVAVAMIQAYVFVLLLTLYLQENV--
```

[4/4 of aligned sequences]

Syja	AVFVLLPLMPAVA----ASTLVLMALLNFLEIAVAMIQAYVFVLLLTLYLQENV*-
Epme	AAFVLLPLMPTVA----ILTATVLVLLTLLEVAVAMIQAYVFVLLLTLYLQENI--
Grse	AAFVLIPLMPTVA----LLTSTVLVLLTLLEIAVAMIQAYVFVLLLTLYLQENV--
Clja	AVLVLTPMMTTVA----ILTATVLFLLTILEIAVAMIQAYVFVLLLSLYLQENV--
Ogcy	ASFVLAPTMPAVA----ISTTMLLLLLTLLEVAVAMIQAYVFVLLLSLYLQENV--
Plna	AAFVLFPLMPPVA----LLTAILLFLLTLLEIAVAMIQAYVFVLLLSLYLQENL--
Lema	AAFVLLPMMPTVA----VLTAILLLLLTLLEVAVAMIQAYVFVLLLSLYLQENV--
Etzo	AAFVLLPLMPTVA----ILTSTVLVLLTLLEVAVAMIQAYVFVLLLTLYLQENV--
Apse	AAFVLLPLMPTVA----GLTAALLFLLTLLEVAVAMIQAYVFVLLLSLYLQENV*-
Epde	GAFVLLPLMPTVA----ILTTILLFLLTLLEVAVAMIQAYVFVLLLSLYLQENV--
Slja	AAFVLLPLMPAVA----ILTTILLFLLTLLEVAVAMIQAYVFVLLLSLYLQENV--
Bsja	AVFVLVPLMPLVA----VLTATLLFLLTLLEVAVAMIQAYVFVLLLSLYLQENV--
Ecna	AAFVLLPLMPTVA----ILTSILLVLLTLLEIAVAMIQAYVFVLLLSLYLQENV--
Cohi	ATFVLLPYMPLVA----IPTAALLFLLTILEMAVAMIQAYVFVLLLSLYLQENV*-
Caar	AAFVLLPLMPTVA----ILTAILLFLLTLLEVAVAMIQAYVFVLLLSLYLQENV*-
Came	AAFVLLPLMPTVA----ILTTILLFLLTLLEVAVAMIQAYVFVLLLSLYLQENV--
Mema	AAFTLLSLMPTVA----ALTATLLFLLTLLEVAVAMIQAYVFVLLLSLYLQENV*-
Lenu	AVFVLASSMPPVA----LITAVLLILLTILEVAVAMIQAYVFVLLLSLYLQENV--
Brja	AATVLLPLMPTVA----ILTATLLFLLTLLEVAVAMIQAYVFVLLLSLYLQENV--
Plma	AATVLLPLMPTVA----ILTATVLFLLTLLEVAVAMIQAYVFVLLLSLYLQENV--
Emst	AAFVLLPLMPTVA----ILTATLLFLLTLLEVAVAMIQAYVFVLLLSLYLQENV--
Ptti	AAFVLLPLMPTVA----ILTATLLFLLTLLEVAVAMIQAYVFVLLLSLYLQENV--
Losu	ATMVLLPLMPLVG----VMTATLLLLLTLLEIAVAMIQAYVFILLVSLYLQENI--
Geoy	AAFVLLPLMPTVA----ILTATLLFLLTLLEVAVAMIQAYVFVLLLSLYLQENV--
Dipi	AAFVLLPLMPTVA----ILTTTLLFLLTLLEVAVAMIQAYVFVLLLSLYLQENV--
Pama	GMFVLLPLQPTVA----ILTGILLLMLSMLEIAVAVIQAYVFILLLSLYLQENV*-
Leob	AAFVLLPLMPAVA----ILTGALLFLLTLLEVAVAMIQAYVFVLLLSLYLQENV--
Neba	ASFTLLPLMPAVS----ILTMVLLLLLTLLEVAVAMIQAYVFVLLLSLYLQENV--
Pdpl	AVFVLAQTLPVVA----ILTAALLFLLTLLEVAVAAIQAYVFVLLLSLYLQENI--
Nimi	AAFVLLSMMPAVA----ILTTILLFLLTLLEVAVAMIQAYVFVLLLSLYLQENV--
Uptr	AAFVLTTSMPTVA----LLTMVVLFLLTLLEVAVAMIQAYVFVLLLSLYLQENV--
Pesc	ATLVLTPLMPLVG----ALTAGLLLLLSLLEIAVAMIQAYVFVLLLSLYLEENL--
Baar	AAFVLLPLMPTVA----AITAVLLFLLSLLEVAVAMIQAYVFVLLLTLYLQENV--
Moar	AAFVLLPLMPTVA----ILTATLLFLLTLLEVAVAMIQAYVFVLLLSLYLQENV--
Toja	AAFVLLPLMPTVA----VLTAILLLLLTLLEVAVAMIQAYVFVLLLSLYLQENV--
Chau	ATFVLTPLMPGVA----LLTMILLFLLSLLEVAVAMIQAYVFVLLLSLYLQENL--
Chse	AVMVLLPLMPAVA----IPTAVLLLMLTLLEVAVAMIQAYVFVLLLSLYLQENV--
Enar	AVFVLLPLMPAVS----AITALLLLLLTLLEIAVAMIQAYVFVLLLSLYLQENV--
Hpty	AAFVLLPLMPTVA----IVTSMLLFLLSLLEVAVAMIQAYVFVLLLTLYLQENV--
Nana	AVFVLLPLIPAVA----ILTTALLLLLTLLEVAVAMIQAYVFVLLLSLYLQENV--
Mcst	AAFVLMPLMPAVA----ILTGTLLFLLTLLEIAVAMIQAYVFVLLLSLYLQENV*-
Rhox	AAFVLLPLMPTVA----ILTGILLFLLTLLEVAVAMIQAYVFVLLLSLYLQENV--
Opfa	AAFVLMPLMPTVA----ILTGTLLFLLTLLEVAVAMIQAYVFVLLLSLYLQENV--
Paar	GAFVLLPIMPMVA----IPTMALLFLLTLLEVAVAMIQAYVFVLLLSLYLQENV--
Gozo	AAFVLLPLMPAVA----ALTGALLFLLTLLEIAVAMIQAYVFVLLLSLYLQENV--
Ackr	ATFVLFPLMPVVA----VLTGVLLFLLSLLEVAVAVIQAYVFILLISLYLQENL--
Elev	AAFVLLPLMPTVA----VLTATLLFLLTLLEVAVAMIQAYVFVLLISLYLQENI*-
Trdu	AAFVLTPLMPTVA----ILTAALLLLLTLLEVAVAMIQAYVFVLLLSLYLQENV*-
Amoc	AALVLLPLMPTVA----ILTGALLLMLTLLEVAVAMIQAYVFVLLLSLYLQENV--
Hame	AAFVLASSMPIVS----LLTASVLFLLTLLEVAVAMIQAYVFVLLLSLYLQENV--
Chso	AVFVLTPLMPTVA----LLTAILLVLLTILEVAVAMIQAYVFVLLLSLYLQENV--
Lyto	AAFVLMPLMPAVA----LLTSAVLVLLTLLEIAVAMIQAYVFVLLLTLYLQENV--

[4/4 of aligned sequences]

```
Encr      AAFVLLPLMPAVA----LLTSTVLVLLTLLEIAVAMIQAYVFVLLLTLYLQENV--
Bvar      GTFLMLSLMPPVA----LLTGTLLVLLSLLEIAVAAIQAYVFVLLLSLYLQENI--
Noco      ATLFLIDQMWPVA----ILTGLVMALLTLLELAVAAIQAYVFVLLLSLYLQENT*-
Chsp      ATYLLIFIMPLAS----LLAYVVLFLLTLLEMAVAMIQAYVFVLLISLYLQENS*-
Arja      AAFVLLSSMPAVA----LLTSTVLVLLTLLEVAVAMIQAYVFVLLLTLYLQENV*-
Pase      AAFVLLPLMPTVA----ILTATLLFLLTLLEVAVAMIQAYVFVLLLSLYLQENV*-
Trel      TLTYLISSASIAA----IPAVLAIFALTILEIAVAIIQAYIFVLLLSLYLQENV--
Lifa      GIFFLMSALPPVA----LITSLALAALTVLEMLVGVLQAYVFVLLLSMYLKEAL--
Acur      AALKMAMKMSCLGITMSILAGMSMAPLILLEIAVAAIQAYVFVLLFALYLQENV*-
Ampe      AAFILLPMMPTVA----ILTLVLMFLLTLLEIAVAMIQAYVFVLLLSLYLQENV--
Urja      ATFGLMTTLPPLA----AILMILLYLLTILEVAVAVIQAYVFVLLLSLYLQENV*-
Enet      AAFVLLPLMPTVA----ILTSSLLLLLTLLEVAVAMIQAYVFVLLLSLYLQENV*-
Ptbr      ATFALMFSSPPTA----MITVILFVLMTILEIAVAMIQAYVFVLLLTLYLQENV--
Safa      AAFVLLPLMPTVA----LLTTALLFLLTLLEVAVAMIQAYVFVLLLSLYLQENV*-
Icae      AATVLLPLMPTVA----ILTATLLFLLTLLEVAVAMIQAYVFVLLLSLYLQENV--
Asmi      GVFVLSSLSMPAA----ILTSLLLLLLTLLEIAVAMIQAYVFVLLLSLYLQENI--
Foal      ATFVLAPMMPVVS----LLTGTLLLLLTLLEVAVAMIQAFVFVLLLTLYLQENT--
Drze      ATHALVAFMPMLT----TLLVTLLILLMLLEIAVAIIQSYVFVMLLTLYLQENV--
Rhas      AAFVLLPLMPTVA----ILTASILFLLTILEVAVAMIQAYVFVLLLSLYLQENV--
Elac      AAFVLIPLMPTVA----LLTGVVLFLLTILEVAVAMIQAYVFVLLLSLYLQENV--
Kugu      AAFVLAPLMPSIA----LLTTAILLLLTLLEVAVAMIQAYVFVLLLTLYLNEVI*-
Plor      AAFTLLPLMPTVA----ILTATLLFLLTLLEVAVAMIQAYVFVLLLSLYLQENV--
Sgun      AAFVLLPLMPTVA----ILTTTLLFLLTLLEVAVAMIQAYVFVLLLSLYLQENV--
Zaco      AAFVLLPLMPTVA----ILTAALLFLLTLLEVAVAMIQAYVFVLLLSLYLQENV--
Zbfl      AAFVLLPLMPTVA----ILTTTLLFLLTLLEVAVAMIQAYVFVLLLSLYLQENV--
Spba      AAFVLLPLMPTVA----ILTATLLFLLTLLEVAVAMIQAYVFVLLLSLYLQENV--
Game      AATVLLPLMPTVA----ILTATLLFLLTLLEVAVAMIQAYVFVLLLSLYLQENV--
Thth      AATVLLPLMPTVA----ILTATLLFLLTLLEVAVAMIQAYVFVLLLSLYLQENV--
Xigl      AAFVLLPLMPTVA----ILTATLLFLLTLLEVAVAMIQAYVFVLLLSLYLQENV--
Hyja      AVAVLLPVMTVVA----IPTAILLLLLMLLELAVAVIQAYVFVLLLSLYLQENV--
Psan      AVAVLLPLMPLVA----IPTSVLLLLLMLLEIAVAVIQAYVFVLLLSLYLQENV--
Cupa      AATVLLPLMPTVA----ILTATVLFLLTLLEVAVAMIQAYVFVLLLSLYLQENV--
Mpch      AVFVLLPLMPTIA----ILTGLLLFLLTLLEVAVALIQAYVFVLLLSLYLQENV--
Char      AAFVLLPLMPTVA----ILTATLLFLLTLLEVAVAMIQAYVFVLLLSLYLQENV--
Pser      AAFVLLPLMPSVA----ILTTVLLFLLTLLEVAVAMIQAYVFVLLLSLYLQENV--
Prol      AAFVLLPIMPMIA----ISTATLLFLLTLLEVAVAMIQAYVFVLLLSLYLQENV--
Plbi      GAFALLPLMPLIG----LTTMTVLYLLTLLEVAVAVIQAYVFVLLLSLYLQENV--
Calu      AACVLLPIMPTVA----ILSAAVLFLLTLLEVAVAMIQAYVFVLLLSLYLQENV--
Papa      AALSLAPLMPALA----LSSFTLLALLTLLEIAVAMIQAYVFVLLLSLYLQENT--
Sufr      AAYVLLPLMPTVA----ILTATLLLLLTLLEVAVAMIQAYVFVLLLSLYLQENV--
Stci      AVYVLMSFLPAVA----LITTALLFLLTLLEIAVALIQAHVFVLLLSLYLQENV--
Taru      AAFVLLPLMPTVA----ILTSTLLFLLTLLEVAVAMIQAYVFVLLLSLYLQENV--
Rala      AAFVLLPLMPTVA----ILTATLLFLLTLLEVAVAMIQAYVFVLLLSLYLQENV--
                                 .  :  **  :. :*..:: :* ::*  *
```
