# Supplementary material for: Structure and variation of the mitochondrial genome of fishes
Source: BMC Genomics. 2016 Sep 7;17(1):719. doi: 10.1186/s12864-016-3054-y (PMC5015259; doi:10.1186/s12864-016-3054-y)
Supplement: Additional file 6: Figure S1-a. — Aligned amino acid sequences of the ATP8 gene in mt genomes of 250 fishes. Figure S1-b. Aligned amino acid sequences of the ATP6 gene in mt genomes of 250 fishes. Figure S1-c. Aligned amino acid sequences of the COI gene in mt genomes of 250 fishes. Figure S1-d. Aligned amino acid sequences of the COII gene in mt genomes of 250 fishes. Figure S1-e. Aligned amino acid sequences of the COIII gene in mt genomes of 250 fishes. Figure S1-f. Aligned amino acid sequences of the Cyt b gene in mt genomes of 250 fishes. Figure S1-g. Aligned amino acid sequences of the ND1 gene in mt genomes of 249 fishes. Figure S1-h. Aligned amino acid sequences of the ND2 gene in mt genomes of 250 fishes. Figure S1-i. Aligned amino acid sequences of the ND3 gene in mt genomes of 250 fishes. Figure S1-j. Aligned amino acid sequences of the ND4L gene in mt genomes of 250 fishes. Figure S1-k. Aligned amino acid sequences of the ND4 gene in mt genomes of 250 fishes. Figure S1-l. Aligned amino acid sequences of the ND5 gene in mt genomes of 250 fishes. Figure S1-m. Aligned amino acid sequences of the ND6 gene in mt genomes of 249 fishes. (ZIP 3250 kb) [file 12864_2016_3054_MOESM6_ESM.zip › Additional file 6 prot align/AF6c-COI.pdf]

Additional file 6: Figure S1-c. Aligned amino acid sequences of the COI gene in mt genomes of 250 fishes.

Species name abbreviation followed by aligned amino acid sequences shown by one letter abbreviation. See Additional file 1 for abbreviation of species name. Amino acids shown by magenta letter denote hydrophobic residues. A-L in bold types with yellow background indicate putative transmembrane regions. Highlighted 'H' and 'Y' letters indicate metal binding sites. Numerals on the amino acid sequences correspond to position number of amino acid residues in the human sequence. Asterisk '*' indicates a fully conserved residue. Colon ':' and period '.' indicate 'strong' and 'weak' groups in the level of conservativeness, respectively, in the Gonnet Pam250 matrix, in which the strong and weak groups are defined as strong score >0.5 and weak score =<0.5, respectively (Thompson et al., 1997).

COI

[1/10 of aligned sequences]

A

Scca VAINRWLFSTNHKDIGTLY**LIFGAWAGMVGTALSLLIRAELG**QPGSLLGDDQIYNV**IVTA**
Muma VAINRWLFSTNHKDIGTLY**LIFGAWAGMVGTALSLLIRAELG**QPGSLLGDDQIYNV**IVTA**
Erca VTITRWLFSTNHKDIGTLY**LIFGAWAGMVGTALSLLIRAELG**QPGALMGDDQIYNV**IVTA**
Pose VTITRWLFSTNHKDIGTLY**LIFGAWAGMVGTALSLLIRAELG**QPGALMGDDQIYNV**IVTA**
Actr MAITRWFFSTNHKDIGTLY**LVFGAWAGMVGTALSLLIRAELS**QPGALLGDDQIYNV**IVTA**
Scal VAITRGFFSTNHKDIGTLY**LVFGAWAGMVGTALSLLIRAELS**QPGALLGDDQIYNV**IVTG**
Posp VAITRWFFSTNHKDIGTLY**LVFGAWAGMVGTALSLLIRAELS**QPGALLGDDQIYNV**IVTA**
Atsp VAITRWFFSTNHKDIGTLY**MVFGAWAGMVGTALSLLIRAELS**QPGTLLGDDQIYNV**IVTA**
Leoc MAITRWFFSTNHKDIGTLY**MVFGAWAGMVGTALSLLIRAELS**QPGTLLGDDQIYNV**IVTA**
Amca MTITRWFFSTNHKDIGTLY**LVFGAWAGMVGTALSLLIRAELS**QPGALLGDDQIYNV**IVTA**
Osbi VKITRWFFSTNHKDIGTLY**LIFGAWAGMVGTALSLLIRAELS**QPGSLLGDDQIYNV**IVTA**
Pabu VTMTRWLFSTNHKDIGTLY**LVFGAWAGMVGTALSLLIRAELS**QPGALLGDDQIYNV**IVTA**
Hial VAITRWFFSTNHKDIGTLY**LVFGAWAGMVGTALSLLIRAELS**QPGALLGDDQIYNV**IVTA**
Elha VAITRWFFSTNHKDIGTLY**LIFGAWAGMVGTALSLLIRAELS**QPGALLGDDQIYNV**IVTA**
Mlcy MAITRWFFSTNHKDIGTLY**LVFGAWAGMVGTALSLLIRAELS**QPGALLGDDQIYNV**IVTA**
Algl MAITRWFFSTNHKDIGTLY**LVFGAWAGMVGTALSLLIRAELS**QPGALLGDDQIYNV**IVTA**
Ptgi MAITRWFFSTNHKDIGTLY**LMFGAWAGMVGTALSLLIRAELS**QPGALLGDDQIYNV**IVTA**
Alaf MAITRWFFSTNHKDIGTLY**LVFGAWAGMVGTALSLLIRAELS**QPGALLGDDQIYNV**IVTA**
Nock VAITRWFFSTNHKDIGTLY**FVFGAWAGMVGTALSLLIRAELS**QPGAFLGDDQIYNV**IVTA**
Anja MAITRWFFSTNHKDIGTLY**LVFGAWAGMVGTALSLLIRAELS**QPGALLGDDQIYNV**IVTA**
Gyki VAITRWFFSTNHKDIGTLY**LVFGAWAGMVGTALSLLIRAELS**QPGALLGDDQIYNV**IVTA**
Syka VTITRWFFSTNHKDIGTLY**LVFGAWAGMVGTALSLLIRAELS**QPGALLGDDQIYNV**IVTA**
Opma VTITRWFFSTNHKDIGTLY**LVFGAWAGMVGTALSLLIRAELS**QPGALLGDDQIYNV**IVTA**
Comy VAINRWFFSTNHKDIGTLY**LVFGAWAGMVGTALSLLIRAELS**QPGALLGDDQIYNV**IVTA**
Sasp VMITRWLFSTNHKDIGTLY**LVFGAWAGLVGTALSMLIRTELS**QPGTMLEDDQIYNV**IVTA**
Eupe MTITRWFFSTNHKDIGTLY**LVFGAWAGMVGTALSLLIRAELT**QPGALLGDDQIYNV**IVTA**
Enja VAITRWFFSTNHKDIGTLY**LIFGAWAGMVGTALSLLIRAELS**QPGALLGDDQIYNV**IVTA**
Same VAITRWFFSTNHKDIGTLY**LVFGAWAGMVGTALSLLIRAELS**QPGALLGDDQIYNV**IVTA**
Chch VAITRWFFSTNHKDIGTLY**LVFGAWAGMVGTALSLLIRAELS**QPGSLLGDDQIYNV**IVTA**
Grgr VTITRWFFSTNHKDIGTLY**LVFGAWAGMVGTALSLLIRAELS**QPGSLLGDDQIYNV**IVTA**
Caau VAITRWFFSTNHKDIGTLY**LVFGAWAGMVGTALSLLIRAELS**QPGSLLGDDQIYNV**IVTA**
Cyca VAITRWFFSTNHKDIGTLY**LVFGAWAGMVGTALSLLIRAELS**QPGSLLGDDQIYNV**IVTA**
Dare VTITRWFFSTNHKDIGTLY**LVFGAWAGMVGTALSLLIRAELS**QPGALLGDDQIYNV**IVTA**
Cost VAITRWFFSTNHKDIGTLY**LVFGAWAGMVGTALSLLIRAELS**QPGSLLGDDQIYNV**IVTA**
Leec VAITRWFFSTNHKDIGTLY**LVFGAWAGMVGTALSLLIRAELS**QPGSLLGDDQIYNV**IVTA**
Fola VAITRWFFSTNHKDIGTLY**LVFGAWAGMVGTALSLLIRAELN**QPGALLGDDQIYNV**IVTA**
Clmc VAITRWFFSTNHKDIGTLY**LVFGAWAGMVGTALSLLIRAELS**QPGALMGDDQIYNV**IVTA**
Phin VAITRWFFSTNHKDIGTLY**LVFGAWAGMVGTALSLLIRAELN**QPGSLLGDDQIYNV**IVTA**
Icpu VTITRWFFSTNHKDIGTLY**LVFGAWAGMVGTALSLLIRAELA**QPGALLGDDQIYNV**IDTA**
Psto VTITRWFFSTNHKDIGTLY**LVFGAWAGMVGTALSLLIRAELA**QPGALLGDDQSYNV**IVTA**
Cora VTITRWFFSTNHKDIGTLY**LVFGAWAGMVGTALSLLIRAELN**QPGSLLGDDQIYNV**IVTA**
Eisp VAITRWFFSTNHKDIGTLY**MVFGAWAGMVGTALSLLIRAELS**QPGALLGDDQIYNV**IVTA**
Apal VAFTRWFFSTNHKDIGTLY**MVFGAWAGMIGTALSLLIRAELN**QPGTLLEDDQIYNV**AVTA**
Eslu VAITRWFFSTNHKDIGTLY**LVFGAWAGMVGTALSLLIRAELS**QPGALLGDDQIYNV**IVTA**

1

To be continued on page 6.

[1/10 of aligned sequences]

```
Dape      VAITRWFFSTNHKDIGTLYLVFGAWAGMVGTALSLLIRAELSQPGALLGDDQIYNVIVTA
Glse      VAITRWFFSTNHKDIGTLYLVFGAWAGMVGTALSLLIRAELSQPGALLGDDQIYNVIVTA
Naar      VAITRWFFSTNHKDIGTLYLVFGAWAGMVGTALSLLIRAELSQPGALLGDDQIYNVIVTA
Lioc      VAITRWFFSTNHKDIGTLYLVFGAWAGMVGTALSLLIRAELSQPGALLGDDQIYNVIVTA
Opso      VAITRWFFSTNHKDIGTLYLVFGAWAGMVGTALSLLIRAELSQPGALLGDDQIYNVIVTA
Alte      VAITRWFFSTNHKDIGTLYLVFGAWAGMVGTALSLLIRAELSQPGALLGDDQIYNVIVTA
Plap      VAITRWFFSTNHKDIGTLYLVFGAWAGMVGTALSLLIRAELSQPGALLGDDQIYNVIVTA
Plal      VAITRWFFSTNHKDIGTLYLIFGAWAGMVGTALSLLIRAELSQPGALLGDDQIYNVIVTA
Sami      VAITRWFFSTNHKDIGTLYLIFGAWAGMVGTALSLLIRAELSQPGALLGDDQIYNVIVTA
Rere      VAITRWFFSTNHKDIGTLYLVFGAWAGMVGTALSLLIRAELSQPGALLGDDQVYNVIVTA
Gama      VAITRWFFSTNHKDIGTLYLVFGAWAGMVGTALSLLIRAELSQPGALLGDDQIYNVIVTA
Onmy      VAITRWFFSTNHKDIGTLYLVFGAWAGMVGTALSLLIRAELSQPGALLGDDQIYNVIVTA
Sasa      VAITRWFFSTNHKDIGTLYLVFGAWAGMVGTALSLLIRAELSQPGALLGDDQIYNVIVTA
Cola      VAITRWFFSTNHKDIGTLYLVFGAWAGMVGTALSLLIRAELSQPGALLGDDQIYNVIVTA
Dita      VAITRWFFSTNHKDIGTLYLVFGAWAGMVGTALSLLIRAELSQPGALLGDDQIYNVIVTA
Gogr      VMVTRWFFSTNHKDIGTLYLMFGAWAGMVGTALSLLIRAELSQPGALLGDDQIFNVIVTA
Chsl      VAITRWFFSTNHKDIGTLYLIFGAWAGMVGTALSLLIRAELSQPGAFMGDDQIYNVIVTA
Atja      VAITRWFFSTNHKDIGTLYLVFGAWAGMVGTALSLLIRAELNQPGALLGDDQIYNVIVTA
Iido      VAITRWFFSTNHKDIGTLYLVFGAWAGMVGTALSLLIRAELNQPGALLGDDQIYNVIVTA
Auja      VTITRWFFSTNHKDIGTLYLVFGAWAGMVGTALSLLIRAELSQPGALLGDDQIYNVIVTA
Chag      VMITRWLFSTNHKDIGTLYLLFGAWAGMVGTALSLLIRAELSQPGALLGDDQIYNVIVTA
Hami      VTITRWFFSTNHKDIGTLYLVFGAWAGMVGTALSLLIRAELSQPGALLGDDQIYNVIVTA
Saun      VTVTRWFFSTNHKDIGTLYLVFGAWAGMVGTALSLLIRAELSQPGALLGDDQIYNVIVTA
Nema      VAITRWFFSTNHKDIGTLYLVFGAWAGMVGTALSLLIRAELSQPGALLGDDQIYNVIVTA
Disp      VAITRWFFSTNHKDIGTLYLIFGAWAGMVGTALSLLIRAELSQPGALLGDDQIYNVIVTA
Myaf      -VTTRWLFSTNHKDIGTLYLIFGAWAGMVGTALSLLIRAELSQPGALLGDDQIYNVIVTA
Lagu      VAITRWFFSTNHKDIGTLYLVFGAWAGMVGTALSLLIRAELSQPGALLGDDQIYNVIVTA
Trtr      VAITRWFFSTNHKDIGTLYLIFGAWAGMVGTALSLLIRAELSQPGALLGDDQIYNVIVTA
Zucr      VAITRWFFSTNHKDIGTLYLIFGAWAGMVGTALSLLIRAELSQPGALLGDDQIYNVIVTA
Pxja      VAITRWFFSTNHKDIGTLYLVFGAWAGMVGTALSLLIRAELSQPGALLGDDQIYNVIVTA
Pxlo      VAITRWFFSTNHKDIGTLYLVFGAWAGMVGTALSLLIRAELSQPGALLGDDQIYNVIVTA
Pctr      VAITRWFFSTNHKDIGTLYLVFGAWAGMVGTALSLLIRAELSQPGALLGDDQIYNVVVTA
Apsa      VAITRWFFSTNHKDIGTLYLVFGAWAGMVGTALSLLIRAELSQPGALLGDDQIYNVIVTA
Cabe      VMITRWLFSTNHKDIGTLYLIFGAWAGMVGTALSLLIRAELGQPGALLGDDQIYNVIVTA
Bzze      VAITRWFFSTNHKDIGTLYLVFGAWAGMVGTALSLLIRAELSQPGALLGDDQIYNVIVTA
Siim      VISARWLFSTNHKDIGTLYLVFGAWAGMVGTALSLLIRAELSQPGSLLGDDQIYNVIVTA
Ctru      VAITRWFFSTNHKDIGTLYLVFGAWAGMVGTALSLLIRAELSQPGSLLGDDQIYNVIVTA
Dpbr      VAITRWFFSTNHKDIGTLYLVFGAWAGMVGTALSLLIRAELSQPGSLLGDDQIYNVIVTA
Caki      VVITRWFFSTNHKDIGTLYLVFGAWAGMVGTALSLLIRAELSQPGALLGDDQIYNVIVTA
Phja      VISPRWFFSTNHKDIGTLYFIFGAWAGMVGTALSLLIRAELSQPGALFGDDQIYNVIVTA
Brsp      MAITRWMFSTNHKDIGTLYLLFGTWAGMVGTALSILIRTELAQPGALLGDDQIYNVIVTA
Gamo      VAITRWFFSTNHKDIGTLYLVFGAWAGMVGTALSLLIRAELSQPGALLGDDQIYNVIVTA
Lolo      VAITRWFFSTNHKDIGTLYLVFGAWAGMVGTALSLLIRAELSQPGALLGDDQIYNVIVTA
Batr      MWITRWLYSTNHKDIGTLYLVFGTWAAMVGAALSLLLRTELSQPGPFLGNDQIYNVIVTA
Prmy      VSLTRWMFSTNHKDIGTLYLIFGAWAAMVGMALSLLIRTELTQPGSLLGNDQIYNVIVTA
Lose      MTVTRWFFSTNHKDIGTLYLIFGAWAGMVGTGLSLLIRAELSQPGALLGDDQIYNVIVTA
Loam      VAITRWFFSTNHKDIGTLYLIFGAWAGMVGTALSLLIRAELSQPGALLGDDQIYNVIVTA
Chab      VAITRWFFSTNHKDIGTLYLVFGAWAGMVGTALSLLIRAELSQPGSLLGDDQIYNVIVTA
Chto      VAITRWFFSTNHKDIGTLYLVFGAWAGMVGTALSLLIRAELSQPGSLLGDDQIYNVIVTA
Majo      VAITRWFFSTNHKDIGTLYLVFGAWAGMVGTALSLLIRAELSQPGALLGDDQIYNVIVTA
Hlst      VTITRWFFSTNHKDIGTLYLIFGAWAGMVGTALSLLIRAELSQPGALLGDDQIYNVIVTA
Clpe      VITTRWLFSTNHKDIGTLYLIFGAWAGMVGTALSLLIRAELSQPGALLGDDQVYNVIVTA
                                      2
```

To be continued on page 7.

[1/10 of aligned sequences]

```
Mlmr    VAITRWFFSTNHKDIGTLYLIFGAWAGMVGTALSLLIRAELSQPGALLGDDQIYNVIVTA
Crcr    VAVTRWFFSTNHKDIGTLYLVFGAWAGMVGTALSLLIRAELSQPGALLGDDQIYNVIVTA
Muce    VAVTRWFFSTNHKDIGTLYLVFGAWAGMVGTALSLLIRAELSQPGALLGDDQIYNVIVTA
Bege    VAITRWFFSTNHKDIGTLYLVFGAWAGMVGTALSLLIRAELSQPGSLLGDDQIYNVIVTA
Mela    VMITRWFFSTNHKDIGTLYLVFGAWAGMVGTALSLLIRAELSQPGSLLGDDQIYNVIVTA
Hats    VAITRWFFSTNHKDIGTLYLVFGAWAGMVGTALSLLIRAELSQPGSLLGDDQIYNVIVTA
Orla    VAITRWFFSTNHKDIGTLYLIFGAWAGMVGTALSLLIRAELSQPGSLLGDDQIYNVIVTA
Cosa    VAITRWFFSTNHKDIGTLYLVFGAWAGMVGTALSLLIRAELSQPGSLLGDDQIYNVIVTA
Exsp    VAITRWFFSTNHKDIGTLYLVFGAWAGMVGTALSLLIRAELSQPGSLLGDDQIYNVIVTA
Depa    VAITRWFFSTNHKDIGTLYLVFGAWAGMVGTALSLLIRAELSQPGSLLGDDQIYNVIVTA
Rima    VAITRWLFSTNHKDIGTLYLVFGAWAGMVGTALSLLIRAELSQPGSLLGDDQIYNVIVTA
Fuol    VVITRWFFSTNHKDIGTLYLVFGAWAGMVGTALSLLIRAELSQPGSLLGDDQIYNVIVTA
Gmaf    VATTRWLFSTNHKDIGTLYLLFGAWAGMVGTALSLLIRAELSQPGTLLGDDQIYNVIVTA
Xeei    VAITRWFFSTNHKDIGTLYLVFGAWAGMVGTALSLLIRAELSQPGSLLGDDQIYNVIVTA
Pros    VAITRWFFSTNHKDIGTLYLVFGAWAGMVGTALSLLIRAELSQPGALLGDDQIYNVIVTA
Scmi    VAITRWLFSTNHKDIGTLYLVFGAWAGMVGTALSLLIRAELSQPGALLGDDQIYNVIVTA
Rolo    VAITRWFFSTNHKDIGTLYLVFGAWAGMVGTALSLLIRAELSQPGALLGDDQIYNVIVTA
Cere    VAITRWFFSTNHKDIGTLYLLFGAWAGMVGTALSLLIRAELSQPGALLGDDQIYNVIVTA
Daga    VAITRWFFSTNHKDIGTLYLVFGAWAGMVGTALSLLIRAELSQPGALLGDDQIYNVIVTA
Anco    VAITRWFFSTNHKDIGTLYLVFGAWAGMVGTALSLLIRAELSQPGALLGDDQIYNVIVTA
Dmve    MMITRWFFSTNHKDIGTLYLIFGAWAGMVGTALSLLIRAELSQPGALLGDDQIYNVIVTA
Dmar    MMITRWFFSTNHKDIGTLYLIFGAWAGMVGTALSLLIRAELSQPGALLGDDQIYNVIVTA
Anka    VAITRWFFSTNHKDIGTLYLVFGAWAGMVGTALSLLIRAELSQPGALLGDDQIYNVIVTA
Moja    VAITRWFFSTNHKDIGTLYLVFGAWAGMVGTALSLLIRAELSQPGALLGDDQIYNVIVTA
Hoja    VAITRWFFSTNHKDIGTLYLVFGAWAGMVGTALSLLIRAELSQPGALLGDDQIYNVIVTA
Bede    VAITRWFFSTNHKDIGTLYLVFGAWAGMVGTALSLLIRAELSQPGALLGDDQIYNVIVTA
Besp    VAITRWFFSTNHKDIGTLYLVFGAWAGMVGTALSLLIRAELSQPGALLGDDQIYNVIVTA
Mysp    VAITRWFFSTNHKDIGTLYLVFGAWAGMVGTALSLLIRAELSQPGALLGDDQIYNVIVTA
Osja    VAITRWFFSTNHKDIGTLYLVFGAWAGMVGTALSLLIRAELSQPGALLGDDQIYNVIVTA
Sgro    VAITRWFFSTNHKDIGTLYLVFGAWAGMVGTALSLLIRAELSQPGALLGDDQIYNVIVTA
Pzpa    VAITRWFFSTNHKDIGTLYLVFGAWAGMVGTALSLLIRAELSQPGALLGDDQIYNVIVTA
Zeja    VAITRWFFSTNHKDIGTLYLVFGAWAGMVGTALSLLIRAELSQPGALLGDDQIYNVIVTA
Znne    VTITRWFFSTNHKDIGTLYLVFGAWAGMVGTALSLLIRAELSQPGALLGDDQIYNVIVTA
Zefa    VAITRWFFSTNHKDIGTLYLVFGAWAGMVGTALSLLIRAELSQPGALLGDDQIYNVIVTA
Acni    VAITRWFFSTNHKDIGTLYLVFGAWAGMVGTALSLLIRAELSQPGALLGDDQIYNVIVTA
Ncrh    VAITRWFFSTNHKDIGTLYLVFGAWAGMVGTALSLLIRAELSQPGALLGDDQIYNVIVTA
Agca    VAITRWFFSTNHKDIGTLYLVFGAWAAMVGTALSLLIRAELSQPGALLGDDQIYNVVVTA
Hydy    VAITRWFFSTNHKDIGTLYLVFGAWAGMVGTALSLLIRAELSQPGALLGDDQIYNVIVTA
Gsac    VAITRWFFSTNHKDIGTLYLVFGAWAGMVGTALSLLIRAELSQPGALLGDDQIYNVIVTA
Pevo    VAITRWFFSTNHKDIGTLYLIFGAWAGMVGTALSLLIRAELSQPGALLGDDQIYNVIVTA
Hiku    VAITRWFFSTNHKDIGTLYLVFGAWAGMVGTALSLLIRAELSQPGALLGDDQIYNVIVTA
Inpa    VAITRWLFSTNHKDIGTLYLVFGAWAGMVGTALSLLIRAELSQPGALLGDDQIYNVIVTA
Auch    VMITRWLFSTNHKDIGTLYLIFGAWAAMVGTALSLIIRAELSQPGSLLGNDQLYNVVVTA
Fico    VAITRWFFSTNHKDIGTLYLIFGAWAGMVGTALSLLIRAELSQPGALLGDDQIYNVIVTA
Macs    VAITRWFFSTNHKDIGTLYLVFGAWAGMVGTALSLLIRAELSQPGALLGDDQIYNVIVTA
Moal    VTITRWFFSTNHKDIGTLYLVFGAWAGMVGMALSLLIRAELCQPGSLLGDDQIYNVIVTA
Syma    VAITRWFFSTNHKDIGTLYLVFGAWAGILGSGLSLLIRTELCQPGTVIGDDQIYNVIVTA
Mafr    VAITRWFFSTNHKDIGTLYLVFGAWAGMVGTALSLLIRAELSQPGALLGDDQIYNVIVTA
Dcpe    VAITRWFFSTNHKDIGTLYLVFGAWAGMVGTALSLLIRAELSQPGALLGDDQIYNVIVTA
Dcti    VAITRWFFSTNHKDIGTLYLVFGAWAGMVGTALSLLIRAELSQPGALLGDDQIYNVIVTA
Hehi    VAITRWFFSTNHKDIGTLYLVFGAWAGMVGTALSLLIRAELSQPGALLGDDQIYNVIVTA
Stam    VAITRWFFSTNHKDIGTLYLVFGAWAGMVGTALSLLIRAELSQPGALLGDDQIYNVIVTA
```

To be continued on page 8.

[1/10 of aligned sequences]

```
Hogi    VAITRWFFSTNHKDIGTLYLVFGAWAGMVGTALSLLIRAELSQPGALLGDDQIYNVIVTA
Erzo    VATTRWFFSTNHKDIGTLYLVFGAWAGMVGTALSLLIRAELSQPGALLGDDQIYNVIVTA
Hxot    VAITRWFFSTNHKDIGTLYLVFGAWAGMVGTALSLLIRAELSQPGALLGDDQIYNVIVTA
Core    VAITRWFFSTNHKDIGTLYLVFGAWAGMVGTALSLLIRAELSQPGALLGDDQIYNVIVTA
Apve    VAITRWFFSTNHKDIGTLYLVFGAWAGMVGTGLSLLIRAELSQPGALLGDDQIYNVIVTA
Latj    VAITRWFFSTNHKDIGTLYLVFGAWAGMVGTALSLLIRAELSQPGALLGDDQIYNVIVTA
Laja    VAITRWFFSTNHKDIGTLYLVFGAWAGMVGTALSLLIRAELSQPGALLGSDQIYNVIVTA
Syja    VAITRWFFSTNHKDIGTLYLIFGAWAGMVGTALSLLIRAELSQPGALLGDDQIYNVIVTA
Epme    VAITRWFFSTNHKDIGTLYLVFGAWAGMVGTALSLLIRAELSQPGALLGDDQIYNVIVTA
Grse    VAITRWFFSTNHKDIGTLYLVFGAWAGMVGTALSLLIRAELSQPGALLGDDQIYNVIVTA
Clja    VAITRWFFSTNHKDIGTLYLVFGAWAGMVGTALSLLIRAELSQPGALLGDDQIYNVIVTA
Ogcy    VAITRWFFSTNHKDIGTLYLVFGAWAGMVGTALSLLIRAELSQPGALLGDDQIYNVIVTA
Plna    VSITRWFFSTNHKDIGTLYLLFGAWAGMVGTALSLLIRAELCQPGALLGDDQIYNVIVTA
Lema    VAITRWFFSTNHKDIGTLYLVFGAWAGMVGTALSLLIRAELSQPGALLGDDQIYNVIVTA
Etzo    VATTRWFFSTNHKDIGTLYLVFGAWAGMVGTALSLLIRAELSQPGALLGDDQIYNVIVTA
Apse    VAITRWFFSTNHKDIGTLYLVFGAWAGMVGTALSLLIRAELSQPGALLGDDQIYNVIVTA
Epde    VAITRWFFSTNHKDIGTLYLVFGAWAGMVGTALSLLIRAELSQPGALLGDDQIYNVIVTA
Slja    VTVTRWFFSTNHKDIGTLYLVFGAWAGMVGTALSLLIRAELSQPGALLGDDQIYNVIVTA
Bsja    VATTRWFFSTNHKDIGTLYLVFGAWAGMVGTALSLLIRAELSQPGALLGDDQIYNVIVTA
Ecna    VAITRWFFSTNHKDIGTLYLVFGAWAGMVGTALSLLIRAELSQPGSLLGDDQIYNVIVTA
Cohi    VTTTRWFYSTNHKDIGTLYLIFGVLAGMTGTGLSLLIRAELSQPGSLLGDDQTYNVIVTA
Caar    VAITRWFFSTNHKDIGTLYLVFGAWAGMVGTALSLLIRAELSQPGALLGDDQIYNVIVTA
Came    VAITRWFFSTNHKDIGTLYLVFGAWAGMVGTALSLLIRAELSQPGALLGDDQIYNVIVTA
Mema    VAITRWLFSTNHKDIGTLYLLFGAWAGMVGTALSLLIRAELNQPGTLLGDDQIYNVIVTA
Lenu    VAITRWFFSTNHKDIGTLYMVFGAWAGMVGTALSLLIRAELSQPGALLGDDHIYNVIVTA
Brja    VAITRWFFSTNHKDIGTLYLVFGAWAGMVGTALSLLIRAELSQPGALLGDDQIYNVIVTA
Plma    VAITRWFFSTNHKDIGTLYLVFGAWAGMVGTALSLLIRAELSQPGALLGDDQIYNVIVTA
Emst    VAITRWFFSTNHKDIGTLYLVFGAWAGMVGTALSLLIRAELSQPGALLGDDQIYNVIVTA
Ptti    VAITRWFFSTNHKDIGTLYLVFGAWAGMVGTALSLLIRAELSQPGALLGDDQIYNVIVTA
Losu    VMITRWLFSTNHKDIGTLYLVFGAWAGMVGTALSLLIRAELNQPGALLGDDQTYNVIVTA
Geoy    VAITRWFFSTNHKDIGTLYLVFGAWAGMVGTALSLLIRAELSQPGSLLGDDQIYNVIVTA
Dipi    VAITRWFFSTNHKDIGTLYLVFGAWAGMVGTALSLLIRAELSQPGALLGDDQIYNVIVTA
Pama    VAITRWFFSTNHKDIGTLYLVFGAWAGMVGTALSLLIRAELSQPGALLGDDQIYNVIVTA
Leob    VAITRWFFSTNHKDIGTLYLVFGAWAGMVGTALSLLIRAELSQPGALLGDDQIYNVIVTA
Neba    VAITRWFFSTNHKDIGTLYLLFGAWAGMVGTALSLLIRAELSQPGALLGDDQIYNVIVTA
Pdpl    VAITRWFFSTNHKDIGTLYLIFGAWAGMVGTALSLLIRAELSQPGALLGDDQIYNVIVTA
Nimi    MAITRWFFSTNHKDIGTLYLIFGAWAGMVGTALSLLIRAELSQPGSLLGDDQVYNVIVTA
Uptr    VAITRWFFSTNHKDIGTLYLVFGAWAGMVGTALSLLIRAELAQPGALLGDDQIYNVIVTA
Pesc    LTATRWFFSTNHKDIGTLYLVFGAWAGMVGTALSLLIRAELSQPGSLLGDDQIYNVIVTA
Baar    VAITRWFFSTNHKDIGTLYLVFGAWAGMVGTALSLLIRAELSQPGALLGDDHIYNVIVTA
Moar    MAITRWFFSTNHKDIGTLYLVFGAWAGMVGTALSLLIRAELSQPGALLGDDQIYNVIVTA
Toja    VAITRWFFSTNHKDIGTLYLVFGAWAGMVGTALSLLIRAELSQPGALLGDDQIYNVIVTA
Chau    VAITRWFFSTNHKDIGTLYLVFGAWAGMVGTALSLLIRAELSQPGSLLGDDQIYNVIVTA
Chse    VAITRWLFSTNHKDIGTLYLLFGAWAGMVGTALSLLIRAELNQPGSLLGDDQIYNVIVTA
Enar    VAITRWFFSTNHKDIGTLYLVFGAWAGMVGTALSLLIRAELSQPGALLGDDQIYNVIVTA
Hpty    VAITRWFFSTNHKDIGTLYLVFGAWAGMVGTALSLLIRAELSQPGALLGDDQIYNVIVTA
Nana    VTITRWFFSTNHKDIGTLYLVFGAWAGMVGTALSLLIRAELSQPGALLGDDQIYNVIVTA
Mcst    VAITRWFFSTNHKDIGTLYLVFGAWAGMVGTALSLLIRAELSQPGALLGDDQIYNVIVTA
Rhox    VAVTRWFFSTNHKDIGTLYLIFGAWAGMVGTALSLLIRAELSQPGALLGDDQIYNVIVTA
Opfa    VAITRWFFSTNHKDIGTLYLVFGAWAGMVGTALSLLIRAELSQPGAFLGDDQIYNVIVTA
Paar    VAITRWFFSTNHKDIGTLYLVFGAWAGMVGTALSLLIRAELSQPGALLGDDQIYNVIVTA
Gozo    VAITRWFFSTNHKDIGTLYLVFGAWAGMVGTALSLLIRAELSQPGALLGDDQIYNVIVTA
```

To be continued on page 9.

[1/10 of aligned sequences]

```
Ackr    VMITRWFFSTNHKDIGTLYLVFGAWAGMVGTALSLLIRAELSQPGPFLGDDQIYNVIVTA
Elev    VAITRWFFSTNHKDIGTLYLVFGAWAGMVGTALSLLIRAELSQPGALLGDDQIYNVIVTA
Trdu    VAITRWFFSTNHKDIGTLYLVFGAWAGMVGTALSLLIRAELSQPGALLGDDQIYNVIVTA
Amoc    VAITRWFFSTNHKDIGTLYLVFGAWAGMVGTALSLLIRAELSQPGALLGDDQIYNVIVTA
Hame    VAITRWLFSTNHKDIGTLYLVFGAWAGMVGTALSLLIRAELSQPGALLGDDQIYNVIVTA
Chso    MAITRWFFSTNHKDIGTLYLVFGAWAGMVGTALSLLIRAELSQPGALLGDDQIYNVIVTA
Lyto    VAITRWFFSTNHKDIGTLYLVFGAWAGMVGTALSLLIRAELSQPGALLGDDQIYNVLVTA
Encr    VAITRWFFSTNHKDIGTLYLVFGAWAGMVGTALSLLIRAELSQPGALLGDDQIYNVIVTA
Bvar    VTTTRWFFSTNHKDIGTLYMVFGAWAGMVGTALSLLIRAELSQPGALLGDDQIYNVIVTA
Noco    VAITRWFFSTNHKDIGTLYLVFGAWAGMVGTALSLLIRAELSQPGSLLGDDQIYNVIVTA
Chsp    MTITRWLFSTNHKDIGTLYLIFGACAGMIGTALSLIIRTELAQPGALLGNDQIYNVIVTA
Arja    VAITRWFFSTNHKDIGTLYLVFGAWPGMVGTALSLLIRAELSQPGALLGDDQIYNVIVTA
Pase    VSITRWFFSTNHKDIGTLYLIFGAWAAMVGTALSLLIRAELSQPGALLGDDQIYNVIVTA
Trel    VAITRWLFSTNHKDIGTLYLIFGAWAGMIGTALSLLIRAELSQPGALLGDDQIYNVIVTA
Lifa    VAITRWFFSTNHKDIGTLYLVFGAWAGMVGTALSLLIRAELSQPGALLGDDQIYNVIVTA
Acur    VAITRWFFSTNHKDIGTLYMVFGAWAGMVGTALSLLIRAELSQPGALLGDDQIYNVIVTA
Ampe    VAITRWFFSTNHKDIGTLYLVFGAWAAMVGTALSLLIRAELSQPGALLGDDQIYNVIVTA
Urja    VAITRWLYSTNHKDIGTLYLVFGAWAAMVGTALSLLIRAELSQPGALLGDDQIYNVIVTA
Enet    VAITRWFFSTNHKDIGTLYLIFGAWAGMVGTALSLLIRAELSQPGALLGDDQIYNVIVTA
Ptbr    VAITRWFFSTNHKDIGTLYLVFGAWAGMVGTALSLLIRAELSQPGSLLGDDQIYNVIVTA
Safa    VAITRWFFSTNHKDIGTLYLVFGAWAGMVGTALSLLIRAELSQPGALLGDDQIYNVIVTA
Icae    VAITRWFFSTNHKDIGTLYLVFGAWAGMVGTALSLLIRAELSQPGALLGDDQIYNVIVTA
Asmi    VETTRWLFSTNHKDIGTLYMVFGAFAGMIGTALSLIIRAELSQPGTLLGDDQLYNVIVTA
Foal    MMITRWFFSTNHKDIGTLYLIFGAWAGMVGTALSLLIRAELSQPGALLGDDQIYNVIVTA
Drze    VANSRWFFSTNHKDIGTLYLVFGAWAGMVGTALSLLIRAELSQPGALLGDDQIYNVIVTA
Rhas    VAITRWFFSTNHKDIGTLYLVFGAWAGMVGTALSLLIRAELSQPGALLGDDQIYNVIVTA
Elac    VAITRWFFSTNHKDIGTLYLVFGAWAGMVGTALSLLIRAELSQPGALLGDDQIYNVIVTA
Kugu    VAITRWFFSTNHKDIGTLYMVFGAWAGMVGTALSLLIRAELSQPGALLGDDQIYNVIVTA
Plor    VAITRWFFSTNHKDIGTLYLVFGAWAGMVGTALSLLIRAELNQPGALLGDDQIYNVIVTA
Sgun    VATTRWLFSTNHKDIGTLYLVFGAWAGMVGTALSLLIRAELSQPGALLGDDQIYNVIVTA
Zaco    MAITRWFFSTNHKDIGTLYLVFGAWAGMVGTALSLLIRAELSQPGALLGDDQIYNVIVTA
Zbfl    VAITRWFFSTNHKDIGTLYLVFGAWAGMVGTALSLLIRAELSQPGALLGDDQIYNVIVTA
Spba    VAITRWFFSTNHKDIGTLYLLFGAWAGMVGTALSLLIRAELSQPGSLLGDDQIYNVIVTA
Game    VAITRWFFSTNHKDIGTLYLVFGAWAGMVGTALSLLIRAELSQPGALLGDDQVYNVIVTA
Thth    VAITRWFFSTNHKDIGTLYLVFGAWAGMVGTALSLLIRAELSQPGALLGDDQIYNVIVTA
Xigl    VAITRWFFSTNHKDIGTLYLVFGAWAGMVGTALSLLIRAELSQPGALLGDDQIYNVIVTA
Hyja    VAITRWFFSTNHKDIGTLYLVFGAWAGMVGTALSLLIRAELSQPGALLGDDQIYNVIVTA
Psan    VVSSRWLFSTNHKDIGTLYLVFGAWAGMVGTALSLLIRAELSQPGALLGDDQIYNVIVTA
Cupa    VAITRWFFSTNHKDIGTLYLVFGAWAGMVGTALSLLIRAELNQPGALLGDDQIYNVIVTA
Mpch    VAITRWFFSTNHKDIGTLYLIFGAWAGMVGTALSLLIRAELSQPGSLLGDDQIYNVIVTA
Char    VAITRWFFSTNHKDIGTLYLVFGAWAGMVGTALSLLIRAELSQPGALLGDDQIYNVVVTA
Pser    VAITRWFFSTNHKDIGTLYLVFGAWAGMVGTALSLLIRAELSQPGTLLGDDQIYNVIVTA
Prol    VAITRWFFSTNHKDIGTLYLVFGAWAGMVGTALSLLIRAELSQPGALLGDDQIYNVIVTA
Plbi    VAITRWFFSTNHKDIGTLYLVFGAWAGMVGTGLSLLIRAELSQPGALLGDDQIYNVIVTA
Calu    VATTRWLFSTNHKDIGTLYLVFGAWAAMVGTALSLLIRAELSQPGALLGDDQIYNVIVTA
Papa    VTITRWLFSTNHKDIGTLYLVFGAWAGMVGTALSLLIRAELNQPGTLLGDDQIYNVIVTA
Sufr    VAITRWLFSTNHKDIGTLYLIFGAWAGMVGTALSLLIRAELSQPGALLGDDQIYNVIVTA
Stci    VATTRWLFSTNHKDIGTLYMIFGAWAGMVGTALSLLIRAELSQPGALLGDDQMYNVVVTA
Taru    VAITRWFFSTNHKDIGTLYLVFGAWAGMVGTALSLLIRAELSQPGALLGDDQIYNVIVTA
Rala    VAITRWFFSTNHKDIGTLYLVFGAWAGMVGTALSLLIRAELSQPGALLGDDQIYNVIVTA
            * ::***********::**.   .: *  .**:::*:** ***  .:  .*: :**  *.
```

To be continued on page 10.

[2/10 of aligned sequences]

```
     61     B                                      C
Scca HAFVMIFFMVMPVMIGGFGNWLVPLMIGAPDMAFPRMNNMSFWLLPPSFLLLLASAGVEA
Muma HAFVMIFFMVMPIMIGGFGNWLVPLMIGAPDMAFPRMNNMSFWLLPPSFLLLLASAGVEA
Erca HAFVMIFFMVMPIMIGGFGNWLVPLMIGAPDMAFPRMNNMSFWLLPPSFLLLLTSSAVEA
Pose HAFVMIFFMVMPIMIGGFGNWLVPLMIGAPDMAFPRMNNMSFWLLPPSLLLLLTSSAVEA
Actr HAFVMIFFMVMPIMIGGFGNWLVPLMIGAPDMAFPRMNNMSFWLLPPSFLLLLASSGVEA
Scal HAFVMIFFMVMPIMIGGFGNWLVPLMIGAPDMAFPRMNNMSFWLLPPSFLLLLASSGVEA
Posp HAFVMIFFMVMPIMIGGFGNWLVPLMIGAPDMAFPRMNNMSFWLLPPSFLLLLASSGVEA
Atsp HAFVMIFFMVMPVMIGGFGNWLVPLMIGAPDMAFPRMNNMSFWLLPPSFLLLLASSGIEA
Leoc HAFVMIFFMVMPVMIGGFGNWLVPLMIGAPDMAFPRMNNMSFWLLPPSFLLLLASSGIEA
Amca HAFVMIFFMVMPVMIGGFGNWLVPLMIGAPDMAFPRMNNMSFWLLPPSFLLLLASSGVEA
Osbi HAFVMIFFMVMPIMIGGFGNWLIPLMIGAPDMAFPRMNNMSFWLLPPSFLLLLASSGVEA
Pabu HAFVMIFFMVMPIMIGGFGNWLVPLMIGAPDMAFPRMNNMSFWLLPPSFLLLLASSGVEA
Hial HAFVMIFFMVMPIMIGGFGNWLVPLMIGAPDMAFPRMNNMSFWLLPPSFLLLLASSGVEA
Elha HAFVMIFFMVMPIMIGGFGNWLIPLMIGAPDMAFPRMNNMSFWLLPPSFLLLLASSGVEA
Mlcy HAFVMIFFMVMPILIGGFGNWLVPLMIGAPDMAFPRMNNMSFWLLPPSFLLLLASSGVEA
Algl HAFVMIFFMVMPILIGGFGNWLVPLMIGAPDMAFPRMNNMSFWLLPPSFLLLLASSGVEA
Ptgi HAFVMIFFMVMPILIGGFGNWLVPLMIGAPDMAFPRMNNMVFDYC-PLLLLLSSSGVEA
Alaf HAFVMIFFMVMPVMIGGFGNWLVPLMIGAPDMAFPRMNNMSFWLLPPSFLLLLSSSGVEA
Nock HAFVMIFFMVMPIMIGGFGNWLVPLMIGAPDMAFPRMNNMSFWLLPPSFLLLLSSSGVEA
Anja HAFVMIFFMVMPVMIGGFGNWLVPLMIGAPDMAFPRMNNMSFWLLPPSFLLLLASSGVEA
Gyki HAFVMIFFMVMPIMIGGFGNWLIPLMIGAPDMAFPRMNNMSFWLLPPSFLLLLASSGVEA
Syka HAFVMIFFMVMPVMIGGFGNWLVPLMIGAPDMAFPRMNNMSFWLLPPSFLLLLASSGVEA
Opma HAFVMIFFMVMPVMIGGFGNWLVPLMIGAPDMAFPRMNNMSFWLLPPSFLLLLASSGVEA
Comy HAFVMIFFMVMPVMIGGFGNWLVPLMIGAPDMAFPRMNNMSFWLLPPSFLLLLTSSGVEA
Sasp HAFVMIFFMVMPVMIGGFGNWLIPLMIGAPDMAFPRMNNMSFWLLPPSLLLLLASSGIEA
Eupe HAFVMIFFMVMPIMIGGFGNWLIPLMIGAPDMAFPRMNNMSFWLLPPSFLLLLASSGVEA
Enja HAFVMIFFMVMPILIGGFGNWLVPLMLGAPDMAFPRMNNMSFWLLPPSFLLLLASSGVEA
Same HAFVMIFFMVMPILIGGFGNWLVPLMIGAPDMAFPRMNNMSFWLLPPSFLLLLASSGVEA
Chch HAFVMIFFMVMPILIGGFGNWLVPLMIGAPDMAFPRMNNMSFWLLPPSFLLLLASSGVEA
Grgr HAFVMIFFMVMPILIGGFGNWLIPLMIGAPDMAFPRMNNMSFWLLPPSFLLLLASSGVEA
Caau HAFVMIFFMVMPILIGGFGNWLVPLMIGAPDMAFPRMNNMSFWLLPPSFLLLLASSGVEA
Cyca HAFVMIFFMVMPILIGGFGNWLVPLMIGAPDMAFPRMNNMSFWLLPPSFLLLLASSGVEA
Dare HAFVMIFFMVMPILIGGFGNWLVPLMIGAPDMAFPRMNNMSFWLLPPSFLLLLASSGVEA
Cost HAFVMIFFMVMPILIGGFGNWLIPLMIGAPDMAFPRMNNMSFWLLPPSFLLLLASSGVEA
Leec HAFVMIFFMVMPILIGGFGNWLVPLMIGAPDMAFPRMNNMSFWLLPPSFLLLLASSGVEA
Fola HAFVMIFFMVMPILIGGFGNWLVPLMIGAPHMAFPRMNNMSFWLLPPSFLLLLASSGVEA
Clmc HAFVMIFFMVMPVMIGGFGNWLVPLMIGAPDMAFPRMNNMSFWLLPPSFLLLLASSGVEA
Phin HAFVMIFFMVMPIMIGGFGNWLVPLMIGAPDMAFPRMNNMSFWLLPPSFLLLLASSGVEA
Icpu HAFVMIFFMVMPIMIGGFGNWLVPLMIGAPDMAFPRMNNMSFWLLPPSFLLLLASSGVEA
Psto HAFIMIFFMVMPIMIGGFGNWLVPLMIGAPDMAFPRMNNMSFWLLPPSFLLLLASSGVEA
Cora HAFIMIFFMVMPIMIGGFGNWLIPLMIGAPDMAFPRMNNMSFWLLPPSFLLLLASSGVEA
Eisp HAFVMIFFMVMPIMIGGFGNWLIPLMIGAPDMAFPRMNNMSFWLLPPSFLLLLASSGVEA
Apal HAFVMIFFMVMPIMIGGFGNWLIPLMIAAPDMAFPRMNNMSFWLLPPSFFLLLASAGLEA
Eslu HAFVMIFFMVMPVMIGGFGNWLIPLMIGAPDMAFPRMNNMSFWLLPPSFLLLLASSGVEA
Dape HAFVMIFFMVMPIMIGGFGNWLIPLMIGAPDMAFPRMNNMSFWLLPPSFLLLLASSGVEA
Glse HAFVMIFFMVMPIMIGGFGNWLIPLMIGAPDMAFPRMNNMSFWLLPPSFLLLLASSGVEA
Naar HAFVMIFFMVMPIMIGGFGNWLIPLMIGAPDMAFPRMNNMSFWLLPPSFLLLLASSGVEA
Lioc HAFVMIFFMVMPIMIGGFGNWLIPLMIGAPDMAFPRMNNMSFWLLPPSFLLLLASSGVEA
Opso HAFVMIFFMVMPIMIGGFGNWLIPLMIGAPDMAFPRMNNMSFWLLPPSFLLLLASSGVEA
Alte HAFVMIFFMVMPIMIGGFGNWLLPLMLGAPDMAFPRMNNMSFWLLPPSLLLLLSSSGVEA
Plap HAFVMIFFMVMPIMIGGFGNWLLPLMLGAPDMAFPRMNNMSFWLLPPSLLLLLSSSGVEA
```

To be continued on page 11.

[2/10 of aligned sequences]

To be continued on page 12.

Plal	HAFVMIFFMVMPIMIGGFGNWLIPLMIGAPDMAFPRMNNMSFWLLPPSFLLLLASSGVEA
Sami	HAFVMIFFMVMPILIGGFGNWLIPLMIGAPDMAFPRMNNMSFWLLPPSFLLLLASSGVEA
Rere	HAFVMIFFMVMPIMIGGFGNWLIPLMIGAPDMAFPRMNNMSFWLLPPSFLLLLASSGVEA
Gama	HAFVMIFFMVMPIMIGGFGNWLIPLMIGAPDMAFPRMNNMSFWLLPPSFLLLLASSGVEA
Onmy	HAFVMIFFMVMPIMIGGFGNWLIPLMIGAPDMAFPRMNNMSFWLLPPSFLLLLSSSGVEA
Sasa	HAFVMIFFMVMPIMIGGFGNWLIPLMIGAPDMAFPRMNNMSFWLLPPSFLLLLASSGVEA
Cola	HAFVMIFFMVMPIMIGGFGNWLIPLMIGAPDMAFPRMNNMSFWLLPPSFLLLLASSGVEA
Dita	HAFVMIFFMVMPIMIGGFGNWLIPLMIGAPDMAFPRMNNMSFWLLPPSFLLLLASSGVEA
Gogr	HAFVMIFFMVMPIMIGGFGNWLIPLMIGAPDMAFPRMNNMSFWLLPPSFLLLLASSGVEA
Chsl	HAFVMIFFMVMPIMIGGFGNWLVPLMIGAPDMAFPRMNNMSFWLLPPSFLLLLASSGVEA
Atja	HAFVMIFFMVMPIMIGGFGNWLIPLMIGAPDMAFPRMNNMSFWLLPPSFLLLLASSGVEA
Iido	HAFVMIFFMVMPIMIGGFGNWLIPLMIGAPDMAFPRMNNMSFWLLPPSFLLLLASSGVEA
Auja	HAFVMIFFMVMPIMIGGFGNWLIPLMIGAPDMAFPRMNNMSFWLLPPSFLLLLASSGVEA
Chag	HAFVMIFFMVMPIMIGGFGNWLIPLMIGAPDMAFPRMNNMSFWLLPPSFLLLLASSAVEA
Hami	HAFVMIFFMVMPIMIGGFGNWLIPLMIGAPDMAFPRMNNMSFWLLPPSFLLLLASSGVEA
Saun	HAFVMIFFMVMPIMIGGFGNWLIPLMIGAPDMAFPRMNNMSFWLLPPSFLLLLASSGVEA
Nema	HAFVMIFFMVMPIMIGGFGNWLIPLMIGTPDMAFPRMNNMSFWLLPPSFLLLLASSGVEA
Disp	HAFVMIFFMVMPIMIGGFGNWLIPLMIGAPDMAFPRMNNMSFWLLPPSFLLLLASSGVEA
Myaf	HAFVMIFFMVMPIMIGGFGNWLIPLMIGAPDMAFPRMNNMSFWLLPPSFLLLLASSGVEA
Lagu	HAFVMIFFMVMPIMIGGFGNWLIPLMIGAPDMAFPRMNNMSFWLLPPSFLLLLASSGVEA
Trtr	HAFVMIFFMVMPIMIGGFGNWLIPLMIGAPDMAFPRMNNMSFWLLPPSFLLLLASSGVEA
Zucr	HAFVMIFFMVMPIMIGGFGNWLIPLMIGAPDMAFPRMNNMSFWLLPPSFLLLLASSGVEA
Pxja	HAFVMIFFMVMPIMIGGFGNWLIPLMIGAPDMAFPRMNNMSFWLLPPSFLLLLASSGVEA
Pxlo	HAFVMIFFMVMPIMIGGFGNWLIPLMIGAPDMAFPRMNNMSFWLLPPSFLLLLASSGVEA
Pctr	HAFVMIFFMVMPIMIGGFGNWLVPLMIGAPDMAFPRMNNMSFWLLPPSFLLLLASSGVEA
Apsa	HAFVMIFFMVMPVMIGGFGNWLVPLMIGAPDMAFPRMNNMSFWLLPPSFLLLLASSGVEA
Cabe	HAFVMIFFMVMPIMIGGFGNWLIPLMIGAPDMAFPRMNNMSFWLLPPSFLLLLASSGVEA
Bzze	HAFVMIFFMVMPIMIGGFGNWLIPLMIGAPDMAFPRMNNMSFWLLPPSFLLLLASSGVEA
Siim	HAFVMIFFMVMPIMIGGFGNWLVPLMIGAPDMAFPRMNNMSFWLLPPSFLLLLASSGVEA
Ctru	HAFVMIFFMVMPIMIGGFGNWLVPLMIGAPDMAFPRMNNMSFWLLPPSFLLLLASSGVEA
Dpbr	HAFVMIFFMVMPIMIGGFGNWLIPLMIGAPDMAFPRMNNMSFWLLPPSFLLLLASSGVEA
Caki	HAFVMIFFMVMPLMIGGFGNWLVPLMIGAPDMAFPRMNNMSFWLLPPSFLLLLASSGVEA
Phja	HAFVMIFFMVMPVMIGGFGNWLVPLMIGAPDMAFPRMNNMSFWLLPPSFLLLLASSGVEA
Brsp	HAFVMIFFMVMPVMIGGFGNWLVPLMIGAPDMAFPRMNNMSFWLLPPSLLLLLTSMTVES
Gamo	HAFVMIFFMVMPLMIGGFGNWLIPLMIGAPDMAFPRMNNMSFWLLPPSFLLLLASSGVEA
Lolo	HAFVMIFFMVMPLMIGGFGNWLVPLMIGAPDMAFPRMNNMSFWLLPPSFLLLLASSGVEA
Batr	HAFVMIFFMVMPIMIGGFGNWLIPLMIGAPDMAFPRMNNMSFWLLPPSFFLLLASSTVEA
Prmy	HAFVMIFFMVMPIMIGGFGNWLIPLMIGAPDMAFPRMNNMSFWLLPPSFLLLLASSVVET
Lose	HAFVMIFFMVMPIMIGGFGNWLIPLMIGAPDMAFPRMNNMSFWLLPPSFLLLLASSGVEA
Loam	HAFVMIFFMVMPIMIGGFGNWLIPLMIGAPDMAFPRMNNMSFWLLPPSFLLLLASSGVEA
Chab	HAFVMIFFMVMPVMIGGFGNWLIPLMIGAPDMAFPRMNNMSFWLLPPSFLLLLASSGVEA
Chto	HAFVMIFFMVMPVMIGGFGNWLIPLMIGAPDMAFPRMNNMSFWLLPPSFLLLLASSGVEA
Majo	HAFVMIFFMVMPIMIGGFGNWLVPLMIGAPDMAFPRMNNMSFWLLPPSFLLLLASSGVEA
Hlst	HAFVMIFFMVMPIMIGGFGNWLVPLMIGAPDMAFPRMNNMSFWLLPPSFLLLLASSGVEA
Clpe	HAFVMIFFMVMPIMIGGFGNWLIPLMIGAPDMAFPRMNNMSFWLLPPSFLLLLASSGVEA
Mlmr	HAFVMIFFMVMPIMIGGFGNWLVPLMIGAPDMAFPRMNNMSFWLLPPSFLLLLASSGVES
Crcr	HAFVMIFFMVMPIMIGGFGNWLVPLMIGAPDMAFPRMNNMSFWLLPPSFLLLLASSGVEA
Muce	HAFVMIFFMVMPIMIGGFGNWLIPLMIGAPDMAFPRMNNMSFWLLPPSFLLLLASSGVEA
Bege	HAFVMIFFMVMPVMIGGFGNWLVPLMIGAPDMAFPRMNNMSFWLLPPSFLLLLASSGVEA
Mela	HAFVMIFFMVMPIMIGGFGNWLVPLMIGAPDMAFPRMNNMSFWLLPPSFLLLLASSGVEA
Hats	HAFVMIFFMVMPIMIGGFGNWLIPLMIGAPDMAFPRMNNMSFWLLPPSFLLLTSSGVEA
Orla	HAFVMIFFMVMPIMIGGFGNWLIPLMIGAPDMAFPRMNNMSFWLLPPSFLLLLASSGVEA

To be continued on page 13.

Cosa	HAFVMIFFMVMPIMIGGFGNWLIPLMIGAPDMAFPRMNNMSFWLLPPSFLLLLASSGVEA
Exsp	HAFVMIFFMVMPIMIGGFGNWLIPLMIGAPDMAFPRMNNMSFWLLPPSFLLLLASSGVEA
Depa	HAFVMIFFMVMPIMIGGFGNWLVPLMIGAPDMAFPRMNNMSFWLLPPSFLLLLASSGVEA
Rima	HAFVMIFFMVMPIMIGGFGNWLVPLMIGAPDMAFPRMNNMSFWLLPPSFLLLLASSGVEA
Fuol	HAFVMIFFMVMPIMIGGFGNWLIPLMIGAPDMAFPRMNNMSFWLLPPSFLLLLASSGVEA
Gmaf	HAFVMIFFMVMPIMIGGFGNWLVPLMIGAPDMAFPRMNNMSFWLLPPSFLLLLASSGVEA
Xeei	HAFVMIFFMVMPIMIGGFGNWLIPLMIGAPDMAFPRMNNMSFWLLPPSFLLLLASSGVEA
Pros	HAFVMIFFMVMPIMIGGFGNWLVPLMIGAPDMAFPRMNNMSFWLLPPSFLLLLSSSGVEA
Scmi	HAFVMIFFMVMPVMIGGFGNWLVPLMIGAPDMAFPRMNNMSFWLLPPSFLLLLSSSAVEA
Rolo	HAFVMIFFMVMPIMIGGFGNWLVPLMIGAPDMAFPRMNNMSFWLLPPSFLLLLASSGVEA
Cere	HAFVMIFFMVMPIMIGGFGNWLIPLMIGAPDMAFPRMNNMSFWLLPPSFLLLLASSGIEA
Daga	HAFVMIFFMVMPIMIGGFGNWLIPLMIGAPDMAFPRMNNMSFWLLPPSFLLLLASSGVEA
Anco	HAFVMIFFMVMPVMIGGFGNWLVPLMIGAPDMAFPRMNNMSFWLLPPSFLLLLSSSGVEA
Dmve	HAFVMIFFMVMPMMIGGFGNWLIPLMIEAPDMAFPRMNNMSFWLLPPSFLLLLASSGVEA
Dmar	HAFVMIFFMVMPMMIGGFGNWLIPLMIEAPDMAFPRMNNMSFWLLPPSFLLLLASSGVEA
Anka	HAFVMIFFMVMPIMIGGFGNWLVPLMIGAPDMAFPRMNNMSFWLLPPSFLLLLASSGVEA
Moja	HAFVMIFFMVMPIMIGGFGNWLVPLMIGAPDMAFPRMNNMSFWLLPPSFLLLLASSGVEA
Hoja	HAFVMIFFMVMPIMIGGFGNWLIPLMIGAPDMAFPRMNNMSFWLLPPSFLLLLASSGVEA
Bede	HAFVMIFFMVMPIMIGGFGNWLIPLMIGAPDMAFPRMNNMSFWLLPPSFLLLLASSGVEA
Besp	HAFVMIFFMVMPIMIGGFGNWLIPLMIGAPDMAFPRMNNMSFWLLPPSFLLLLASSGVEA
Mysp	HAFVMIFFMVMPIMIGGFGNWLIPLMIGAPDMAFPRMNNMSFWLLPPSFLLLLASSGVEA
Osja	HAFVMIFFMVMPIMIGGFGNWLIPLMIGAPDMAFPRMNNMSFWLLPPSFLLLLASSGVEA
Sgro	HAFVMIFFMVMPIMIGGFGNWLIPLMIGAPDMAFPRMNNMSFWLLPPSFLLLLASSGVEA
Pzpa	HAFVMIFFMVMPVMIGGFGNWLIPLMIGAPDMAFPRMNNMSFWLLPPSFLLLLASSGVEA
Zeja	HAFVMIFFMVMPIMIGGFGNWLIPLMIGAPDMAFPRMNNMSFWLLPPSFLLLLASSGVEA
Znne	HAFVMIFFMVMPIMIGGFGNWLIPLMIGAPDMAFPRMNNMSFWLLPPSFLLLLASSGVEA
Zefa	HAFVMIFFMVMPIMIGGFGNWLIPLMIGAPDMAFPRMNNMSFWLLPPSFLLLLASSGVEA
Acni	HAFVMIFFMVMPIMIGGFGNWLVPLMIGAPDMAFPRMNNMSFWLLPPSFLLLLASSGVEA
Ncrh	HAFVMIFFMVMPIMIGGFGNWLVPLMIGAPDMAFPRMNNMSFWLLPPSFLLLLASSGVEA
Agca	HAFVMIFFMVMPIMIGGFGNWLIPLMIGAPDMAFPRMNNMSFWLLPPSFLLLLASSMVEA
Hydy	HAFVMIFFMVMPIMIGGFGNWLIPLMIGAPDMAFPRMNNMSFWLLPPSFLLLLASSGVEA
Gsac	HAFVMIFFMVMPIMIGGFGNWLIPLMIGAPDMAFPRMNNMSFWLLPPSFLLLLASSGVEA
Pevo	HAFVMIFFMVMPIMIGGFGNWLIPLMIGAPDMAFPRMNNMSFWLLPPSFLLLLASSGVEA
Hiku	HAFVMIFFMVMPIMIGGFGNWLVPLMIGAPDMAFPRMNNMSFWLLPPSFLLLLASSGVEA
Inpa	HAFVMIFFMVMPIMIGGFGNWLIPLMLGAPDMAFPRMNNMSFWLLPPSLLLLLASSGVEA
Auch	HAFVMIFFMVMPIMIGGFGNWLIPLMIGAPDMAFPRMNNMSFWLLPPSFLLLLTSSAVEA
Fico	HAFVMIFFMVMPIMIGGFGNWLIPLMIGAPDMAFPRMNNMSFWLLPPSFLLLLASSGVEA
Macs	HAFVMIFFMVMPIMIGGFGNWLIPLMIGAPDMAFPRMNNMSFWLLPPSFLLLLASSGVEA
Moal	HAFIMIFFMVMPIMIGGFGNWLVPLMIGAPDMAFPRMNNMSFWLLPPSFLLLLASAGIEA
Syma	HAFVMIFFMVMPIMIGGFGNWLVPLMIGSPDMAFPRMNNMSFWLLPPSFLLLLASAGVEA
Mafr	HAFVMIFFMVMPIMIGGFGNWLIPLMIGAPDMAFPRMNNMSFWLLPPSFLLLLASSAVES
Dcpe	HAFVMIFFMVMPIMIGGFGNWLIPLMIGAPDMAFPRMNNMSFWLLPPSFLLLLASSGVEA
Dcti	HAFVMIFFMVMPIMIGGFGNWLIPLMIGAPDMAFPRMNNMSFWLLPPSFLLLLASSGVEA
Hehi	HAFVMIFFMVMPIMIGGFGNWLIPLMIGAPDMAFPRMNNMSFWLLPPSFLLLLASSGVEA
Stam	HAFVMIFFMVMPIMIGGFGNWLIPLMIGAPDMAFPRMNNMSFWLLPPSFLLLLASSGVEA
Hogi	HAFVMIFFMVMPIMIGGFGNWLIPLMIGAPDMAFPRMNNMSFWLLPPSFLLLLASSGVEA
Erzo	HAFVMIFFMVMPIMIGGFGNWLIPLMIGAPDMAFPRMNNMSFWLLPPSFLLLLASSGVEA
Hxot	HAFVMIFFMVMPIMIGGFGNWLIPLMIGAPDMAFPRMNNMSFWLLPPSFLLLLASSGVEA
Core	HAFVMIFFMVMPIMIGGFGNWLIPLMIGAPDMAFPRMNNMSFWLLPPSFLLLLASSGVEA
Apve	HAFVMIFFMVMPIMIGGFGNWLIPLMIGAPDMAFPRMNNMSFWLLPPSFLLLLASSGVEA
Latj	HAFVMIFFMVMPIMIGGFGNWLIPLMIGAPDMAFPRMNNMSFWLLPPSFLLLLASSGVEA
Laja	HAFVMIFFMVMPIMIGGFGNWLIPLMIGAPDMAFPRMNNMSFWLLPPSLLLLLSSSAVEA

[2/10 of aligned sequences]

```
Syja  HAFVMIFFMVMPIMIGGFGNWLLPLMIGAPDMAFPRMNNMSFWLLPPSFLLLLASSGVEA
Epme  HAFVMIFFMVMPIMIGGFGNWLIPLMIGAPDMAFPRMNNMSFWLLPPSFLLLLASSGVEA
Grse  HAFVMIFFMVMPIMIGGFGNWLIPLMIGAPDMAFPRMNNMSFWLLPPSFLLLLASSGVEA
Clja  HAFVMIFFMVMPIMIGGFGNWLIPLMIGAPDMAFPRMNNMSFWLLPPSFLLLLASSGVEA
Ogcy  HALCNNFFMVMPIMIGGFGNWLIPLMIGGPDMAFPRMNNMSFWLLPPPHPPPMASSGVEA
Plna  HAFVMIFFMVMPILIGGFGNWLVPLMIGAPDMAFPRMNNMSFWLLPPSFLLLLASSGVEA
Lema  HAFVMIFFMVMPIMIGGFGNWLVPLMIGAPDMAFPRMNNMSFWLLPPSFLLLLASSGVEA
Etzo  HAFVMIFFMVMPIMIGGFGNWLIPLMIGAPDMAFPRMNNMSFWLLPPSFLLLLASSGVEA
Apse  HAFVMIFFMVMPIMIGGFGNWLIPLMIGAPDMAFPRMNNMSFWLLPPSFLLLLASSGVEA
Epde  HAFVMIFFMVMPIMIGGFGNWLIPLMIGAPDMAFPRMNNMSFWLLPPSFLLLLASSGVEA
Slja  HAFVMIFFMVMPILIGGFGNWLVPLMIGAPDMAFPRMNNMSFWLLPPSFLLLLASSGVEA
Bsja  HAFVMIFFMVMPIMIGGFGNWLIPLMIGAPDMAFPRMNNMSFWLLPPSFLLLLASSGVEA
Ecna  HAFVMIFFMVMPVMIGGFGNWLVPLMIGAPDMAFPRMNNMSFWLLPPSFLLLLTSSGVEA
Cohi  HAFVMIFFMVMPIMIGGFGNWLIPLMLGAPDMAFPRMNNMSFWLLPPSFLLLLASSGVEA
Caar  HAFVMIFFMVMPIMIGGFGNWLIPLMIGAPDMAFPRMNNMSFWLLPPSFLLLLASSGVEA
Came  HAFVMIFFMVMPIMIGGFGNWLIPLMIGAPDMAFPRMNNMSFWLLPPSFLLLLASSGVEA
Mema  HAFVMIFFMVMPIMIGGFGNWLIPLMVGAPDMAFPRMNNMSFWLLPPSFLLLLASSGVEA
Lenu  HAFVMIFFMVMPIMIGGFGNWLIPLMIGAPDMAFPRMNNMSFWLLPPSFLLLLASSGIEA
Brja  HAFVMIFFMVMPIMIGGFGNWLIPLMIGAPDMAFPRMNNMSFWLLPPSFLLLLASSGVEA
Plma  HAFVMIFFMVMPIMIGGFGNWLVPLMIGAPDMAFPRMNNMSFWLLPPSFLLLLASSGVEA
Emst  HAFVMIFFMVMPIMIGGFGNWLIPLMIGAPDMAFPRMNNMSFWLLPPSFLLLLASSGVEA
Ptti  HAFVMIFFMVMPIMIGGFGNWLIPLMIGAPDMAFPRMNNMSFWLLPPSFLLLLASSGVEA
Losu  HAFVMIFFMVMPIMIGGFGNWLIPLMIGAPDMAFPRMNNMSFWLLPPSFLLLLASSGVEA
Geoy  HAFVMIFFMVMPIMIGGFGNWLIPLMIGAPDMAFPRMNNMSFWLLPPSFLLLLASSGVEA
Dipi  HAFVMIFFMVMPILIGGFGNWLVPLMIGAPDMAFPRMNNMSFWLLPPSFLLLLASSGVEA
Pama  HAFVMIFFMVMPIMIGGFGNWLIPLMIGAPDMAFPRMNNMSFWLLPPSFLLLLASSGVEA
Leob  HAFVMIFFMVMPIMIGGFGNWLIPLMIGAPDMAFPRMNNMSFWLLPPSFLLLLASSGVEA
Neba  HAFVMIFFMVMPIMIGGFGNWLIPLMIGAPDMAFPRMNNMSFWLLPPSFLLLLASSGIEA
Pdpl  HAFVMIFFMVMPIMIGGFGNWLVPLMIGAPDMAFPRMNNMSFWLLPPSFLLLLASSGVEA
Nimi  HAFVMIFFMVMPVMIGGFGNWLIPLMIGAPDMAFPRMNNMSFWLLPPSFLLLLTSSGVEA
Uptr  HAFVMIFFMVMPIMIGGFGNWLIPLMIGAPDMAFPRMNNMSFWLLPPSFLLLLASSGVEA
Pesc  HAFVMIFFMVMPIMIGGFGNWLIPLMIGAPDMAFPRMNNMSFWLLPPSFLLLLASSGVEA
Baar  HAFVMIFFMVMPIMIGGFGNWLVPLMIGAPDMAFPRMNNMSFWLLPPSFFLLLTSSALEA
Moar  HAFVMIFFMVMPIMIGGFGNWLIPLMIGAPDMAFPRMNNMSFWLLPPSFLLLLASSGVEA
Toja  HAFVMIFFMVMPIMIGGFGNWLIPLMIGAPDMAFPRMNNMSFWLLPPSFLLLLASSGVEA
Chau  HAFVMIFFMVMPIMIGGFGNWLIPLMIGAPDMAFPRMNNMSFWLLPPSFFLLLASSGVES
Chse  HAFVMIFFMVMPAMIGGFGNWLVPLMIGAPDMAFPRMNNMSFWLLPPSLLLLLASAGVEA
Enar  HAFVMIFFMVMPIMIGGFGNWLIPLMIGAPDMAFPRMNNMSFWLLPPSFLLLLASSGVEA
Hpty  HAFVMIFFMVMPIMIGGFGNWLLPLMIGAPDMAFPRMNNMSFWLLPPSFLLLLASSGVEA
Nana  HAFVMIFFMVMPIMIGGFGNWLIPLMIGAPDMAFPRMNNMSFWLLPPSFLLLLASSGVEA
Mcst  HAFVMIFFMVMPIMIGGFGNWLIPLMIGAPDMAFPRMNNMSFWLLPPSFLLLLASSGVEA
Rhox  HAFVMIFFMVMPIMIGGFGNWLIPLMIGAPDMAFPRMNNMSFWLLPPSFLLLLASSGVEA
Opfa  HAFVMIFFMVMPIMIGGFGNWLIPLMIGAPDMAFPRMNNMSFWLLPPSFLLLLASSGVEA
Paar  HAFVMIFFMVMPIMIGGFGNWLIPLMIGAPDMAFPRMNNMSFWLLPPSFLLLLASSGVEA
Gozo  HAFVMIFFMVMPIMIGGFGNWLIPLMIGAPDMAFPRMNNMSFWLLPPSFLLLLASSGVEA
Ackr  HAFVMIFFMVMPIMIGGFGNWLVPLMIGAPDMAFPRMNNMSFWLLPPSLLLLLASSGVEA
Elev  HAFVMIFFMVMPIMIGGFGNWLVPLMIGAPDMAFPRMNNMSFWLLPPSFLLLLASSGVEA
Trdu  HAFVMIFFMVMPIMIGGFGNWLVPLMIGAPDMAFPRMNNMSFWLLPPSFLLLLASSGVEA
Amoc  HAFVMIFFMVMPIMIGGFGNWLVPLMLGAPDMAFPRMNNMSFWLLPPSFLLLLASSGVEA
Hame  HAFVMIFFMVMPIMIGGFGNWLIPLMIGAPDMAFPRMNNMSFWLLPPSFLLLLASSGVEA
Chso  HAFVMIFFMVMPIMIGGFGNWLIPLMIGAPDMAFPRMNNMSFWLLPPSFLLLLASSGVEA
Lyto  HAFVMIFFMVMPIMIGGFGNWLVPLMIGAPDMAFPRMNNMSFWLLPPSFLLLLASSGVEA
```

To be continued on page 14.

[2/10 of aligned sequences]

```
Encr  HAFVMIFFMVMPIMIGGFGNWLIPLMIGAPDMAFPRMNNMSFWLLPPSFLLLLASSGVEA
Bvar  HAFVMIFFMVMPLMIGGFGNWLIPLMIGAPDMAFPRMNNMSFWLLPPSFVLLLASSGVEA
Noco  HAFVMIFFMVMPIMIGGFGNWLIPLMIGAPDMAFPRMNNMSFWLLPPSFLLLLASSGVEA
Chsp  HAFVMIFFMVMPIMMGGFGNWLIPMMIGAPDMAFPRMNNMSFWLLPPALLLLLASSGVEQ
Arja  HAFVMIFFMVMPIMIGGFGNWLIPLMIGAPDMAFPRMNNMSFWLLPPSFLLLLASSGVEA
Pase  HAFVMIFFMVMPIMIGGFGNWLIPLMIGAPDMAFPRMNNMSFWLLPPSLLLLLASSGVEA
Trel  HAFVMIFFMVMPIMIGGFGNWLIPLMIGAPDMAFPRMNNMSFWLLPPSFLLLLASSGVEA
Lifa  HAFVMIFFMVMPIMIGGFGNWLIPLMIGAPDMAFPRMNNMSFWLLPPSFLLLLTSSGVEA
Acur  HAFVMIFFMVMPVMIGGFGNWLIPLMIGAPDMAFPRMNNMSFWLLPPSFLLLLASSGVEA
Ampe  HAFVMIFFMVMPIMIGGFGNWLIPLMIGAPDMAFPRMNNMSFWLLPPSLLLLLASSGVEA
Urja  HAFVMIFFMVMPVMIGGFGNWLVPLMIGAPDMAFPRMNNMSFWLLPPSLILLLASSGVEA
Enet  HAFVMIFFMVMPIMIGGFGNWLIPLMIGAPDMAFPRMNNMSFWLLPPSFLLLLASSGVEA
Ptbr  HAFVMIFFMVMPIMIGGFGNWLIPLMIGAPDMAFPRMNNMSFWLLPPSFLLLLASSGVEA
Safa  HAFVMIFFMVMPIMIGGFGNWLIPLMIGAPDMAFPRMNNMSFWLLPPSFLLLLASSGVEA
Icae  HAFVMIFFMVMPIMIGGFGNWLIPLMIGAPDMAFPRMNNMSFWLLPPSFLLLLASSGVEA
Asmi  HAFVMIFFMVMPIMIGGFGNWLIPLMIGAPDMAFPRMNNMSFWLLPPSFLLLLASSSVEA
Foal  HAFVMIFFMVMPIMIGGFGNWLVPLMIGAPDMAFPRMNNMSFWLLPPSFLLLLASSGVEA
Drze  HAFVMIFFMVMPVMIGGFGNWLIPLMIGAPDMAFPRMNNMSFWLLPPSFLLLLASSAVEA
Rhas  HAFVMIFFMVMPILIGGFGNWLVPLMIGAPDMAFPRMNNMSFWLLPPSFLLLLASSGVEA
Elac  HAFVMIFFMVMPIMIGGFGNWLIPLMIGAPDMAFPRMNNMSFWLLPPSFLLLLASSGVEA
Kugu  HAFVMIFFMVMPIMIGGFGNWLVPLMIGAPDMAFPRMNNMSFWLLPPSFLLLLASSGVEA
Plor  HAFVMIFFMVMPVMIGGFGNWLIPLMIGAPDMAFPRMNNMSFWLLPPSFLLLLASSGVEA
Sgun  HAFVMIFFMVMPIMIGGFGNWLIPLMIGAPDMAFPRMNNMSFWLLPPSFLLLLASSGVEA
Zaco  HAFVMIFFMVMPIMIGGFGNWLIPLMIGAPDMAFPRMNNMSFWLLPPSFLLLLASSGVEA
Zbfl  HAFVMIFFMVMPIMIGGFGNWLIPLMIGAPDMAFPRMNNMSFWLLPPSFLLLLASSGVEA
Spba  HAFVMIFFMVMPIMIGGFGNWLIPLMIGAPDMAFPRMNNMSFWLLPPSFLLLLSSSAVEA
Game  HAFVMIFFMVMPIMIGGFGNWLIPLMIGAPDMAFPRMNNMSFWLLPPSFLLLLASSGVEA
Thth  HAFVMIFFMVMPIMIGGFGNWLIPLMIGAPDMAFPRMNNMSFWLLPPSFLLLLASSGVEA
Xigl  HAFVMIFFMVMPIMIGGFGNWLIPLMIGAPDMAFPRMNNMSFWLLPPSFLLLLASSGVEA
Hyja  HAFVMIFFMVMPIMIGGFGNWLIPLMIGAPDMAFPRMNNMSFWLLPPSFLLLLASSGVEA
Psan  HAFVMIFFMVMPIMIGGFGNWLIPLMLGAPDMAFPRMNNMSFWLLPPSFLLLLASSGVEA
Cupa  HAFVMIFFMVMPIMIGGFGNWLIPLMIGAPDMAFPRMNNMSFWLLPPSFLLLLASSGVEA
Mpch  HAFVMIFFMVMPVMIGGFGNWLIPLMIGAPDMAFPRMNNMSFWLLPPSFLLLLASSGVEA
Char  HAFVMIFFMVMPMMIGGFGNWLVPLMIGAPDMAFPRMNNMSFWLLPPSFLLLLASSAVEA
Pser  HAFVMIFFMVMPIMIGGFGNWLIPLMIGAPDMAFPRMNNMSFWLLPPSFLLLLASSGVEA
Prol  HAFVMIFFMVMPIMIGGFGNWLIPLMIGAPDMAFPRMNNMSFWLLPPSFLLLLASSGVEA
Plbi  HAFVMIFFMVMPIMIGGFGNWLIPLMIGAPDMAFPRMNNMSFWLLPPSFLLLLASSGVEA
Calu  HAFVMIFFMVMPIMIGGFGNWLVPLMVGAPDMAFPRMNNMSFWLLPPSFLLLLCSSGVEA
Papa  HAFVMIFFMVMPIMIGGFGNWLVPLMIGAPDMAFPRMNNMSFWLLPPAFLLLLASSGVEA
Sufr  HAFVMIFFMVMPIMIGGFGNWLIPLMIGAPDMAFPRMNNMSFWLLPPSLLLLLASSSVEA
Stci  HAFVMIFFMVMPIMIGGFGNWLIPLMIGAPDMAFPRMNNMSFWLLPPSFLLLLASSGVEA
Taru  HAFVMIFFMVMPIMIGGFGNWLIPLMIGAPDMAFPRMNNMSFWLLPPSFLLLLASSGVEA
Rala  HAFVMIFFMVMPIMIGGFGNWLIPLMIGAPDMAFPRMNNMSFWLLPPSFLLLLASSGVEA
      **:   ****** ::*******:*:*:  *.********* *    *     : *   :*
```

To be continued on page 15.

D

```
Scca    GAGTGWTVYPPLAGNMAHAGRSVDLTIFSLHLAGISSILASINFITTIINMKPPAVSQYQ
Muma    GAGTGWTVYPPLASNLAHAGPSVDLAIFSLHLAGISSILASINFITTIINMKPPAISQYQ
Erca    GVGTGWTVYPPLAGNLAHAGASVDLAIFSLHLAGVSSILGAINFITTIINMKPPATSQYQ
Pose    GVGTGWTVYPPLAGNLAHAGASVDLAIFSLHLAGVSSILGAINFITTIINMKPPSTSQYQ
Actr    GAGTGWTVYPPLAGNLAHAGASVDLTIFSLHLAGVSSILGAINFITTIINMKPPAVSQYQ
Scal    GAGTGWTVYPPLAGNLAHAGASVDLTIFSLHLAGISSILGAINFITTIINMKPPAVSQYQ
Posp    GAGTGWTVYPPLAGNLAHAGASVDLTIFSLHLAGVSSILGAINFITTIINMKPPAVFQYQ
Atsp    GAGTGWTVYPPLASNLAHAGASVDLTIFSLHLAGISSILGAINFITTILNMKPPAASQYQ
Leoc    GAGTGWTVYPPLASNLAHAGASVDLTIFSLHLAGISSILGAINFITTILNMKPPAASQYQ
Amca    GAGTGWTVYPPLASNLAHAGASVDLTIFSLHLAGVSSILGAINFITTIINMKPPAASQYQ
Osbi    GAGTGWTVYPPLAGNLAHAGASVDLTIFSLHLAGVSSILGAINFITTIINMKPPAITQYQ
Pabu    GAGTGWTVYPPLAGNLAHAGASVDLTIFSLHLAGVSSILGAINFITTIINMKPPAISQYQ
Hial    GAGTGWTVYPPLAGNLAHAGASVDLTIFSLHLAGVSSILGAINFITTIINMKPPAISQYQ
Elha    GAGTGWTVYPPLAGNLAHAGASVDLTIFSLHLAGVSSILGAINFITTIINMKPPAMTQYQ
Mlcy    GAGTGWTVYPPLAGNLAHAGASVDLTIFSLHLAGVSSILGAINFITTIINMKPPAMSQYQ
Algl    GAGTGWTVYPPLSGNLAHAGASVDLTIFSLHLAGVSSILGAINFITTIINMKPPAISQYQ
Ptgi    GAGTGWTVYPPLAGNLAHAGASVDLTIFSLHLAGVSSILGAINFITTIINMKPPAISQYQ
Alaf    GAGTGWTVYPPLASNLAHAGASVDLTIFSLHLAGISSILGAINFITTIINMKPPAISQYQ
Nock    GAGTGWTVYPPLAGNLAHAGASVDLTIFSLHLAGVSSILGAINFITTIINMKPPAISQYQ
Anja    GAGTGWTVYPPLAGNLAHAGASVDLTIFSLHLAGISSILGAINFITTIINMKPPAITQYQ
Gyki    GAGTGWTVYPPLAGNLAHAGASVDLTIFSLHLAGVSSILGAINFITTIINMKPPAITQYQ
Syka    GAGTGWTVYPPLAGNLAHAGASVDLTIFSLHLAGISSILGAINFITTIINMKPPAISQYQ
Opma    GAGTGWTVYPPLAGNLAHAGASVDLTIFSLHLAGVSSILGAINFITTIINMKPPAITQYQ
Comy    GAGTGWTVYPPLSGNLAHAGASVDLTIFSLHLAGVSSILGAINFITTIINMKPPATTQYQ
Sasp    GAGTGWTVYPPLAGNLAHAGASVDLTIFSLHLAGISSILGAINFITTIINMKPPAISQYQ
Eupe    GAGTGWTVYPPLAGNLAHAGASVDLTIFSLHLAGISSILGAINFITTIINMKPPAISQYQ
Enja    GAGTGWTVYPPLAGNLAHAGASVDLTIFSLHLAGISSILGAINFITTIINMKPPAISQYQ
Same    GAGTGWTVYPPLAGNLAHAGASVDLTIFSLHLAGISSILGAINFITTIINMKPPAISQYQ
Chch    GAGTGWTVYPPLAGNLAHAGASVDLTIFSLHLAGVSSILGAINFITTIINMKPPAISQYQ
Grgr    GAGTGWTVYPPLAGNLAHAGASVDLTIFSLHLAGVSSILGAINFITTIINMKPPAISQYQ
Caau    GAGTGWTVYPPLAGNLAHAGASVDLTIFSLHLAGVSSILGAINFITTTINMKPPAISQYQ
Cyca    GAGTGWTVYPPLAGNLAHAGASVDLTIFSLHLAGVSSILGAINFITTTINMKPPAISQYQ
Dare    GAGTGWTVYPPLAGNLAHAGASVDLTIFSLHLAGVSSILGAINFITTTINMKPPTISQYQ
Cost    GAGTGWTVYPPLAGNLAHAGASVDLTIFSLHLAGVSSILGAINFITTTINMKPPAISQYQ
Leec    GAGTGWTVYPPLAGNLAHAGASVDLTIFSLHLAGASSILGAINFITTTINMKPPAISQYQ
Fola    GAGTGWTVYPPLAGNLAHAGASVDLTIFSLHLAGVSSILGAINFITTTINMKPPALSQYQ
Clmc    GAGTGWTVYPPLAGNLAHAGASVDLTIFSLHLAGVSSILGAINFITTIINMKPPAISQYQ
Phin    GAGTGWTVYPPLAGNLAHAGASVDLTIFSLHLAGVSSILGAINFITTIINMKPPAISQYQ
Icpu    GAGTGWTVYPPLAGNLAHAGASVDLTIFSLHLAGVSSILGAINFITTIINMKPPAISQYQ
Psto    GAGTGWTVYPPLAGNLAHAGASVDLAIFSLHLAGASSILGAINFITTIINMKPPAISQYQ
Cora    GAGTGWTVYPPLAGNLAHAGASVDLTIFSLHLAGVSSILGAINFITTIINMKPPAISQYQ
Eisp    GAGTGWTVYPPLAGNLAHAGASVDLTIFSLHLAGVSSILGSINFITTIINMKPPAISQYQ
Apal    GVGTGWTLYPPLAGNAAHAGASVDLTIFSLHLAGVSSILGSINFITTIINMKPPTMSQYQ
Eslu    GAGTGWTVYPPLAGNLAHAGASVDLTIFSLHLAGISSILGAINFITTIINMKPPAISQYQ
Dape    GAGTGWTVYPPLAGNLAHAGASVDLTIFSLHLAGISSILGAINFITTIINMKPPAISQYQ
Glse    GAGTGWTVYPPLAGNLAHAGASVDLTIFSLHLAGISSILGAINFITTIINMKPPAISQYQ
Naar    GAGTGWTVYPPLAGNLAHAGASVDLTIFSLHLAGISSILGAINFITTIINMKPPTISQYQ
Lioc    GAGTGWTVYPPLAGNLAHAGASVDLTIFSLHLAGISSILGAINFITTIINMKPPAISQYQ
Opso    GAGTGWTVYPPLAGNLAHAGASVDLTIFSLHLAGISSILGSINFITTIVNMKPPAISQYQ
Alte    GVGTGWTVYPPLAGNLAHAGASVDLAIFSLHLAGVSSILGSINFITTITNMKPPAISQYQ
Plap    GAGTGWTVYPPLAGNLAHAGASVDLAIFSLHLAGVSSILGSINFITTITNMKPPAISQYQ
```

To be continued on page 16.

[3/10 of aligned sequences]

```
Plal  GAGTGWTVYPPLAGNLAHAGASVDLTIFSLHLAGISSILGAINFITTIINMKPPAISQYQ
Sami  GAGTGWTVYPPLAGNLAHAGASVDLTIFSLHLAGISSILGAINFITTIINMKPPAISQYQ
Rere  GAGTGWTVYPPLSGNLAHAGASVDLTIFSLHLAGISSILGAINFITTIINMKPPAISQYQ
Gama  GAGTGWTVYPPLAGNLAHAGASVDLTIFSLHLAGISSILGAINFITTIINMKPPAISQYQ
Onmy  GAGTGWTVYPPLAGNLAHAGASVDLTIFSLHLAGISSILGAINFITTIINMKPPAISQYQ
Sasa  GAGTGWTVYPPLAGNLAHAGASVDLTIFSLHLAGISSILGAINFITTIINMKPPAISQYQ
Cola  GAGTGWTVYPPLAGNLAHAGASVDLTIFSLHLAGISSILGAVNFITTIINMKPPAISQYQ
Dita  GAGTGWTVYPPLAGNLAHAGASVDLTIFSLHLAGISSILGAINFITTIINMKPPAISQYQ
Gogr  GAGTGWTVYPPLAGNLAHAGASVDLTIFSLHLAGISSILGAINFITTITNMKPPAASQYQ
Chsl  GAGTGWTVYPPLSGNLAHAGASVDLTIFSLHLAGISSILGAINFITTIYNMKPAGMSQYQ
Atja  GAGTGWTVYPPLAGNLAHAGASVDLTIFSLHLAGVSSILGAINFITTIINMKPPAVSQYQ
Iido  GAGTGWTVYPPLAGNLAHAGASVDLTIFSLHLAGVSSILGAINFITTIINMKPPAISQYQ
Auja  GAGTGWTVYPPLAGNLAHAGASVDLTIFSLHLAGISSILGAINFITTIINMKPPAISQYQ
Chag  GAGTGWTVYPPLASNLAHAGASVDLTIFSLHLAGISSILGAINFITTIINMKPPAITQYQ
Hami  GAGTGWTVYPPLAGNLAHAGASVDLTIFSLHLAGISSILGAINFITTIINMKPPAISQYQ
Saun  GAGTGWTVYPPLAGNLAHAGASVDLTIFSLHLAGISSILGAINFITTIINMKPPAISQYQ
Nema  GAGTGWTVYPPLAGNLAHAGASVDLTIFSLHLAGISSILGAINFITTIINMKPPAISQYQ
Disp  GAGTGWTVYPPLAGNLAHAGASVDLTIFSLHLAGVSSILGAINFITTIINMKPPAITQYQ
Myaf  GAGTGWTVYPPLAGNLAHAGASVDLTIFSLHLAGVSSILGAINFITTIINMKAPAVSQYQ
Lagu  GVGTGWTVYPPLAGNLAHAGASVDLAIFSLHLAGVSSILGAINFITTIINMKPPAISQYQ
Trtr  GAGTGWTVYPPLSGNLAHAGASVDLTIFSLHLAGVSSILGAINFITTIINMKPPAISQYQ
Zucr  GAGTGWTVYPPLAGNLAHAGASVDLTIFSLHLAGVSSILGAINFITTIINMKPPAISQYQ
Pxja  GAGTGWTVYPPLAGNLAHAGASVDLTIFSLHLAGVSSILGAINFITTIINMKPPAISQYQ
Pxlo  GAGTGWTVYPPLAGNLAHAGASVDLTIFSLHLAGVSSILGAINFITTIINMKPPAISQYQ
Pctr  GAGTGWTVYPPLAGNLAHAGASVDLTIFSLHLAGASSILGAVNFITTIINMKPPAISQYQ
Apsa  GAGTGWTVYPPLAGNLAHAGASVDLTIFSLHLAGASSILGAINFITTIINMKPPAISQYQ
Cabe  GAGTGWTVYPPLAGNLAHAGASVDLTIFSLHLAGVSSILGAINFITTIINMKPPAASQYQ
Bzze  GAGTGWTVYPPLAGNLAHAGASVDLTIFSLHLAGVSSILGAINFITTIINMKPPAISQYQ
Siim  GAGTGWTVYPPLSGNLAHAGASVDLTIFSLHLAGISSILGAINFITTIINMKPPAITQYQ
Ctru  GAGTGWTVYPPLAGNLAHAGASVDLTIFSLHLAGVSSILGAINFITTIINMKPPAISQYQ
Dpbr  GAGTGWTVYPPLAGNLAHAGASVDLTIFSLHLAGISSILGAINFITTIINMKPPAISQYQ
Caki  GAGTGWTVYPPLAGNLAHAGASVDLTIFSLHLAGISSILGAINFITTIINMKPPAITQYQ
Phja  GAGTGWTVYPPLAGNLAHAGASVDLTIFSLHLAGISSILGAINFITTIINMKPPAVLQYQ
Brsp  GVGTGWTVYPPLASNCAHSGASVDLAIFSLHLAGISSILGAINFITTIINMKPPAMDNFR
Gamo  GAGTGWTLYPPLAGNLAHAGASVDLTIFSLHLAGISSILGAINFITTIINMKPPAISQYQ
Lolo  GAGTGWTVYPPLAGNLAHAGASVDLTIFSLHLAGVSSILGAINFITTIINMKPPAISQYQ
Batr  EAGTGWTIYPPLANNIAHAGASVDLTIFSLHLAGVSSILGAINFITTIINMKPKASTQYQ
Prmy  GVGTGWTVYPPLAGNLAHAGASVDLAIFSLHLAGISSILGAINFITTIINMKTPASTQYQ
Lose  GAGTGWTVYPPLAGNLAHAGASVDLTIFSLHLAGVSSILGAINFITTIINMKPPAISQYQ
Loam  GAGTGWTVYPPLAGNLAHAGASVDLTIFSLHLAGISSILGAINFITTIINMKPPTISQYQ
Chab  GAGTGWTVYPPLSGNLAHAGASVDLTIFSLHLAGISSILGAINFITTIINMKPPAISQYQ
Chto  GAGTGWTVYPPLAGNLAHAGASVDLTIFSLHLAGISSILGAINFITTIINMKPPAISQYQ
Majo  GAGTGWTVYPPLAGNLAHAGASVDLTIFSLHLAGVSSILGAINFITTIINMKPPATSQYQ
Hlst  GAGTGWTVYPPLAGNLAHAGASVDLTIFSLHLAGVSSILGAINFITTIFNMKPPSTSQYQ
Clpe  GAGTGWTVYPPLAGNLAHAGASVDLTIFSLHLAGVSSILGAINFITTIINMKPPAISQYQ
Mlmr  GAGTGWTVYPPLAGNLAHAGASVDLTIFSLHLAGVSSILGAINFITTIINMKPPAISQYQ
Crcr  GAGTGWTVYPPLASNLAHAGASVDLTIFSLHLAGVSSILGAINFITTIINMKPPATSQYQ
Muce  GAGTGWTVYPPLASNLAHAGASVDLTIFSLHLAGVSSILGAINFITTIINMKPPATSQYQ
Bege  GAGTGWTVYPPLAGNLAHAGASVDLTIFSLHLAGISSILGAINFITTIINMKPPAISQYQ
Mela  GAGTGWTVYPPLAGNLAHAGASVDLTIFSLHLAGVSSILGAINFITTIINMKPPAISQYQ
Hats  GAGTGWTVYPPLAGNLAHAGASVDLTIFSLHLAGVSSILGAINFITTIINMKPPAISQYQ
Orla  GAGTGWTVYPPLSGNLAHAGASVDLTIFSLHLAGISSILGAINFITTIINMKPPAISQYQ
```

To be continued on page 17.

[3/10 of aligned sequences]

Cosa	GAGTGWTVYPPLAGNLAHAGASVDLTIFSLHLAGVSSILGAINFITTIINMKPPVISQYQ
Exsp	GAGTGWTVYPPLAGNLAHAGASVDLTIFSLHLAGVSSILGAINFITTIINMKPPAISQYQ
Depa	GAGTGWTVYPPLAGNLAHAGASVDLTIFSLHLAGISSILGAINFITTIINMKPPAISQYQ
Rima	GAGTGWTVYPPLAGNLAHAGASVDLTIFSLHLAGVSSILGAINFITTGLNMKPPALSQYQ
Fuol	GAGTGWTVYPPLAGNLAHAGASVDLTIFSLHLAGISSILGAINFITTIINMKPPAISQYQ
Gmaf	GAGTGWTVYPPLAGNLAHAGPSVDLTIFSLHLAGISSILGAINFITTIINMKPPAASQYQ
Xeei	GAGTGWTVYPPLASNLAHAGASVDLTIFSLHLAGVSSILGAINFITTIINMKPPATSQYQ
Pros	GAGTGWTVYPPLASNLAHAGASVDLTIFSLHLAGVSSILGAINFITTIINMKPPAISQYQ
Scmi	GAGTGWTVYPPLASNLAHAGASVDLTIFSLHLAGVSSILGAINFITTIINMKPPATTQHQ
Rolo	GAGTGWTVYPPLAGNLAHAGASVDLTIFSLHLAGISSILGAINFITTIINMKPPAISQYQ
Cere	GAGTGWTVYPPLAGNLAHAGASVDLTIFSLHLAGVSSILGAINFITTIINMKPPAISQYQ
Daga	GAGTGWTVYPPLAGNLAHAGASVDLTIFSLHLAGISSILGAINFITTIINMKPPAISQYQ
Anco	GAGTGWTVYPPLAGNLAHAGASVDLTIFSLHLAGISSILGAINFITTIINMKPPAISQYQ
Dmve	GAGTGWTVYPPLAGNLAHAGASVDLTIFSLHLAGISSILGAINFITTIFNMKPPGVSIYQ
Dmar	GAGTGWTVYPPLAGNLAHAGASVDLTIFSLHLAGISSILGAINFITTIFNMKPPGVSIYQ
Anka	GAGTGWTVYPPLAGNLAHAGASVDLTIFSLHLAGVSSILGAINFITTIINMKPPAISQYQ
Moja	GAGTGWTVYPPLAGNLAHAGASVDLTIFSLHLAGISSILGAINFITTIINMKPPAISQYQ
Hoja	GAGTGWTVYPPLAGNLAHAGASVDLTIFSLHLAGVSSILGAINFITTIINMKPPAISQYQ
Bede	GAGTGWTVYPPLAGNLAHAGASVDLTIFSLHLAGVSSILGAINFITTIINMKPPAISQYQ
Besp	GAGTGWTVYPPLAGNLAHAGASVDLTIFSLHLAGVSSILGAINFITTIINMKPPAISQYQ
Mysp	GAGTGWTVYPPLAGNLAHAGASVDLTIFSLHLAGISSILGAINFITTIINMKPPAISQYQ
Osja	GAGTGWTVYPPLAGNLAHAGASVDLTIFSLHLAGISSILGAINFITTIINMKPPAISQYQ
Sgro	GAGTGWTVYPPLAGNLAHAGASVDLTIFSLHLAGISSILGAINFITTIINMKPPAISQYQ
Pzpa	GAGTGWTVYPPLAGNLAHAGASVDLTIFSLHLAGISSILGAINFITTIINMKPPAISQYQ
Zeja	GAGTGWTVYPPLAGNLAHAGASVDLTIFSLHLAGISSILGAINFITTIINMKPPAISQYQ
Znne	GAGTGWTVYPPLSGNLAHAGASVDLTIFSLHLAGISSILGAINFITTIINMKPPAISQYQ
Zefa	GAGTGWTVYPPLAGNLAHAGASVDLTIFSLHLAGISSILGAINFITTIVNMKPPAISQYQ
Acni	GAGTGWTVYPPLAGNLAHAGASVDLTIFSLHLAGISSILGAINFITTIINMKPPAISQYQ
Ncrh	GAGTGWTVYPPLAGNLAHAGASVDLTIFSLHLAGISSILGAINFITTIINMKPPAISQYQ
Agca	GAGTGWTVYPPLAGNLAHAGASVDLTIFSLHLAGISSILGAINFITTIINMKPPAISQYQ
Hydy	GAGTGWTVYPPLSGNLAHAGASVDLTIFSLHLAGISSILGAINFITTIINMKPPAISQYQ
Gsac	GAGTGWTVYPPLSGNLAHAGASVDLTIFSLHLAGISSILGAINFITTIINMKPPAISQYQ
Pevo	GAGTGWTVYPPLAGNLAHAGASVDLTIFSLHLAGISSILGAINFITTIINMKPPAISQYQ
Hiku	GAGTGWTVYPPLAGNLAHAGASVDLTIFSLHLAGVSSILGAINFITTIINMKPPSISQYQ
Inpa	GVGTGWTVYPPLAGNLAHAGASVDLAIFSLHLAGVSSILGSINFITTIINMKPPAISQYQ
Auch	GSGTGWTVYPPLAGNLAHAGASVDLTIFSLHLAGISSILGAINFITTIINMKPPAASPYQ
Fico	GAGTGWTVYPPLAGNLAHAGASVDLTIFSLHLAGVSSILGAINFITTIINMKPPAISQYQ
Macs	GAGTGWTVYPPLSGNLAHAGASVDLTIFSLHLAGISSILGAINFITTIINMKPPAISQYQ
Moal	GAGTGWTVYPPLAGNLAHAGASVDLTIFSLHLAGVSSILGAINFITTIINMKPPAISQYQ
Syma	GAGTGWTVYPPLAGNLTHAGASVDLTIFSLHLAGASSILGAINFITTIINMKHPTVTQYQ
Mafr	GAGTGWTVYPPLASNLAHAGASVDLTIFSLHLAGVSSILGAINFITTIINMKPPAISQYQ
Dcpe	GAGTGWTVYPPLAGNLAHAGASVDLTIFSLHLAGVSSILGAINFITTIINMKPPAISQYQ
Dcti	GAGTGWTVYPPLAGNLAHAGASVDLTIFSLHLAGVSSILGAINFITTIINMKPPAISQYQ
Hehi	GAGTGWTVYPPLAGNLAHAGASVDLTIFSLHLAGISSILGAINFITTIINMKPPAISQYQ
Stam	GAGTGWTVYPPLAGNLAHAGASVDLTIFSLHLAGISSILGAINFITTIINMKPPAISQYQ
Hogi	GAGTGWTVYPPLAGNLAHAGASVDLTIFSLHLAGISSILGAINFITTIINMKPPAISQYQ
Erzo	GAGTGWTVYPPLASNLAHAGASVDLTIFSLHLAGISSILGAINFITTIINMKPPAISQYQ
Hxot	GAGTGWTVYPPLSGNLAHAGASVDLTIFSLHLAGISSILGAINFITTIINMKPPAISQYQ
Core	GAGTGWTVYPPLAGNLAHAGASVDLTIFSLHLAGISSILGAINFITTIINMKPPAISQYQ
Apve	GAGTGWTVYPPLAGNLAHAGASVDLTIFSLHLAGISSILGAINFITTIINMKPPAMSQYQ
Latj	GAGTGWTVYPPLASNLAHAGASVDLTIFSLHLAGISSILGAINFITTIINMKPATVSMYQ
Laja	GAGTGWTVYPPLASNLAHAGASVDLTIFSLHLAGVSSILGAINFITTIINMKPPAISQYQ

To be continued on page 18.

[3/10 of aligned sequences]

Syja	GAGTGWTVYPPLASNLAHAGASVDLTIFSLHLAGISSILGAINFITTTINMKPPAISQYQ
Epme	GAGTGWTVYPPLAGNLAHAGASVDLTIFSLHLAGVSSILGAINFITTIINMKPPAISQYQ
Grse	GAGTGWTVYPPLAGNLAHAGASVDLTIFSLHLAGISSILGAINFITTIINMKPPAISQYQ
Clja	GAGTGWTVYPPLAGNLAHAGASVDLTIFSLHLAGISSILGAINFITTIINMKPPAISQYQ
Ogcy	GAGTGWTVYPPLSGNLAHAGASVDLTIFSLHLAGISSILGAINFITTIINMKPPTISQYQ
Plna	GAGTGWTVYPPLAGNLAHAGASVDLTIFSLHLAGISSILGAINFITTIINMKPPMMTQYQ
Lema	GAGTGWTVYPPLAGNLAHAGASVDLTIFSLHLAGVSSILGAINFITTIINMKPPAISQYQ
Etzo	GAGTGWTVYPPLAGNLAHAGASVDLTIFSLHLAGISSILGAINFITTIINMKPPAISQYQ
Apse	GAGTGWTVYPPLAGNLAHAGASVDLTIFSLHLAGVSSILGAINFITTIINMKPPAITQYQ
Epde	GAGTGWTVYPPLAGNLAHAGASVDLTIFSLHLAGISSILGAINFITTIINMKPPAISQYQ
Slja	GAGTGWTVYPPLAGNLAHAGASVDLTIFSLHLAGISSILGAINFITTIINMKPPATSQYQ
Bsja	GAGTGWTVYPPLAGNLAHAGPSVDLTIFSLHLAGVSSILGAINFITTIINMKPPATTQYQ
Ecna	GAGTGWTVYPPLAGNLAHAGASVDLTIFSLHLAGISSILGAINFITTIINMKPAAASMYQ
Cohi	GAGTGWTVYPPLAGNLAHAGASVDLTIFSLHLAGVSSILGAINFITTIINMKPPTVTMYQ
Caar	GAGTGWTVYPPLAGNLAHAGASVDLTIFSLHLAGVSSILGAINFITTIINMKPPAVSMYQ
Came	GAGTGWTVYPPLAGNLAHAGASVDLTIFSLHLAGVSSILGAINFITTIINMKPPAVSMYQ
Mema	GAGTGWTVYPPLAGNLAHAGASVDLTIFSLHLAGVSSILGAINFITTIINMKPPTVSMYQ
Lenu	GAGTGWTVYPPLAGNLAHAGASVDLTIFSLHLAGISSILGAINFITTIINMKPPAITQFQ
Brja	GAGTGWTVYPPLAGNLAHAGASVDLTIFSLHLAGVSSILGAINFITTIINMKPSAISQYQ
Plma	GAGTGWTVYPPLAGNLAHAGASVDLTIFSLHLAGVSSILGAINFITTIINMKPAAISQYQ
Emst	GAGTGWTVYPPLAGNLAHAGASVDLTIFSLHLAGVSSILGAINFITTIINMKPPAISQYQ
Ptti	GAGTGWTVYPPLAGNLAHAGASVDLTIFSLHLAGVSSILGAINFITTIINMKPPAISQYQ
Losu	GAGTGWTVYPPLASNLAHAGASVDLTIFSLHLAGISSILGAINFITTIINMKPPAVSQYQ
Geoy	GAGTGWTVYPPLSGNLAHAGASVDLTIFSLHLAGISSILGAINFITTIINMKPPAISQYQ
Dipi	GAGTGWTVYPPLAGNLAHAGASVDLTIFSLHLAGISSILGAINFITTIINMKPPAISQYQ
Pama	GAGTGWTVYPPLAGNLAHAGASVDLTIFSLHLAGISSILGAINFITTIINMKPPAISQYQ
Leob	GAGTGWTVYPPLAGNLAHAGASVDLTIFSLHLAGVSSILGAINFITTIINMKPPAISQYQ
Neba	GAGTGWTVYPPLAGNLAHAGASVDLTIFSLHLAGISSILGAINFITTIFNMKPPAISQYQ
Pdpl	GAGTGWTVYPPLAGNLAHAGASVDLTIFSLHLAGVSSILGAINFITTIFNMKPASASMYQ
Nimi	GAGTGWTVYPPLASNLAHAGASVDLAIFSLHLAGVSSILGAINFITTIINMKPPAITQYQ
Uptr	GAGTGWTVYPPLAGNLAHAGASVDLTIFSLHLAGISSILGAINFITTIINMKPPAISQYQ
Pesc	GAGTGWTVYPPLAGNLAHAGASVDLTIFSLHLAGVSSILGAINFITTIVNMKPPAMIPYN
Baar	GAGTGWTVYPPLAGNLAHAGASVDLTIFSLHLAGVSSILGAINFITTIINMKPPAVSQYQ
Moar	GAGTGWTVYPPLAGNLAHAGASVDLTIFSLHLAGVSSILGAINFITTIINMKPPAISQYQ
Toja	GAGTGWTVYPPLAGNLAHAGASVDLTIFSLHLAGVSSILGAINFITTIINMKPTVVTMYQ
Chau	GAGTGWTVYPPLAGNLAHAGASVDLTIFSLHLAGVSSILGAINFITTILNMKPPAMSQYQ
Chse	GAGTGWTVYPPLAGNLAHAGASVDLTIFSLHLAGISSILGAINFITTIINMKPPATSQYQ
Enar	GAGTGWTVYPPLSGNLAHAGASVDLTIFSLHLAGISSILGAINFITTIINMKPPAISQYQ
Hpty	GAGTGWTVYPPLAGNLAHAGASVDLTIFSLHLAGVSSILGAINFITTIINMKPPAISQYQ
Nana	GVGTGWTVYPPLASNLAHAGASVDLAIFSLHLAGISSILGSINFITTIINMKPPAMTMYQ
Mcst	GAGTGWTVYPPLAGNLAHAGASVDLTIFSLHLAGISSILGAINFITTIINMKPPAISQYQ
Rhox	GAGTGWTVYPPLAGNLAHAGASVDLTIFSLHLAGVSSILGAINFITTIINMKPPAISQYQ
Opfa	GAGTGWTVYPPLAGNLAHAGASVDLTIFSLHLAGISSILGAINFITTIINMKPPAISQYQ
Paar	GAGTGWTVYPPLAGNLAHAGASVDLTIFSLHLAGISSILGAINFITTIINMKPPAISQYQ
Gozo	GAGTGWTVYPPLAGNLAHAGASVDLTIFSLHLAGVSSILGAINFITTIINMKPPAISQYQ
Ackr	GAGTGWTVYPPLSGNLAHAGASVDLTIFSLHLAGISSILGAINFITTIINMKPPAASPYQ
Elev	GAGTGWTVYPPLAGNLAHAGASVDLTIFSLHLAGVSSILGAINFITTIINMKPPALSQYQ
Trdu	GAGTGWTVYPPLSGNLAHAGPSVDLTIFSLHLAGVSSILGAINFITTIINMKPPAISQYQ
Amoc	GAGTGWTVYPPLSGNLAHAGASVDLTIFSLHLAGVSSILGAINFITTIINMKPPAITQYQ
Hame	GAGTGWTVYPPLAGNLAHAGASVDLTIFSLHLAGISSILGAINFITTIINMKPPAISQYQ
Chso	GAGTGWTVYPPLAGNLAHAGASVDLTIFSLHLAGISSILGAINFITTIINMKPPAISQYQ
Lyto	GAGTGWTVYPPLSGNLAHAGASVDLTIFSLHLAGISSILGAINFITTIINMKPPAISQYQ

To be continued on page 19.

[3/10 of aligned sequences]

```
Encr    GAGTGWTVYPPLSGNLAHAGASVDLTIFSLHLAGISSILGAINFITTIINMKPPAISQYQ
Bvar    GAGTGWTVYPPLAGNLAHAGASVDLTIFSLHLAGISSILGAINFITTIINMKPPAISQYQ
Noco    GAGTGWTVYPPLSGNLAHAGASVDLTIFSLHLAGISSILGAINFITTIINMKPPAISQYQ
Chsp    GAGTGWTVYPPLASNIAHQGASVDLTIFSLHLAGVSSILGAINFITTIINMKPPAMTMYM
Arja    GAGTGWTVYPPLAGNLAHAGASVDLTIFSLHLAGISSILGAINFITTIINMKPPAISQYQ
Pase    GAGTGWTVYPPLAGNLAHAGASVDLTIFSLHLAGISSILGAINFITTILNMKPPAVSQYQ
Trel    GAGTGWTVYPPLAGNLAHAGASVDLTIFSLHLAGVSSILGAINFITTIINMKPPAVSQYQ
Lifa    GAGTGWTVYPPLAGNLAHAGASVDLTIFSLHLAGISSILGAINFITTIINMKPAAISQYQ
Acur    GAGTGWTVYPPLAGNLAHAGASVDLTIFSLHLAGISSILGAINFITTIINMKPPSMSQYQ
Ampe    GAGTGWTVYPPLAGNLAHAGASVDLTIFSLHLAGISSILGAINFITTIINMKPPAISQYQ
Urja    GAGTGWTVYPPLAGNLAHAGASVDLTIFSLHLAGISSILGAINFITTIINMKPPGASQYQ
Enet    GAGTGWTVYPPLSGNLAHAGASVDLTIFSLHLAGISSILGAINFITTIINMKPPAISQYQ
Ptbr    GAGTGWTVYPPLSGNLAHAGASVDLTIFSLHLAGISSILGAINFITTIINMKPPAISQYQ
Safa    GAGTGWTVYPPLSGNLAHAGASVDLTIFSLHLAGVSSILGAINFITTIINMKPPAISQYQ
Icae    GAGTGWTVYPPLSGNLAHAGASVDLTIFSLHLAGVSSILGAINFITTIINMKPAAISQYQ
Asmi    GAGTGWTVYPPLSANLAHAGASVDLAIFSLHLAGISSILGAINFITTIINMKPPAATQYH
Foal    GAGTGWTVYPPLSSNLAHAGASVDLTIFSLHLAGVSSILGAINFITTITNMKPPALTQYQ
Drze    GAGTGWTVYPPLAGNLAHAGPSVDLTIFSLHLAGVSSILGAINFITTITNMKPPGMNQYQ
Rhas    GAGTGWTVYPPLAGNLAHAGASVDLTIFSLHLAGISSILGAINFITTIINMKPPAISQYQ
Elac    GAGTGWTVYPPLAGNLAHAGASVDLTIFSLHLAGVSSILGAINFITTIINMKPPAISQYQ
Kugu    GAGTGWTVYPPLAGNLAHAGASVDLTIFSLHLAGVSSILGAINFITTIINMKPPAVSQYQ
Plor    GAGTGWTVYPPLASNLAHAGASVDLTIFSLHLAGISSILGAINFITTIINMKPPAISQYQ
Sgun    GAGTGWTVYPPLAGNLAHAGASVDLTIFSLHLAGISSILGAINFITTIINMKPPAISQYQ
Zaco    GAGTGWTVYPPLAGNLAHAGASVDLTIFSLHLAGISSILGAINFITTIINMKPPAISQYQ
Zbfl    GAGTGWTVYPPLAGNLAHAGASVDLTIFSLHLAGISSILGAINFITTIINMKPPAISQYQ
Spba    GAGTGWTVYPPLAGNLAHAGASVDLTIFSLHLAGISSILGAINFITTIINMKPAATSMYQ
Game    GAGTGWTVYPPLAGNLAHAGASVDLTIFSLHLAGISSILGAINFITTIINMKPAAISQYQ
Thth    GAGTGWTVYPPLAGNLAHAGASVDLTIFSLHLAGVSSILGAINFITTIINMKPAAISQYQ
Xigl    GAGTGWTVYPPLAGNLAHAGASVDLTIFSLHLAGISSILGAINFITTIINMKPAAVSMYQ
Hyja    GAGTGWTVYPPLAGNLAHAGASVDLTIFSLHLAGISSILGAINFITTIINMKPAAISQYQ
Psan    GAGTGWTVYPPLAGNLAHAGASVDLTIFSLHLAGISSILGAINFITTIINMKPAAVSQYQ
Cupa    GAGTGWTVYPPLAGNLAHAGASVDLTIFSLHLAGVSSILGAINFITTIINMKPAAISQYQ
Mpch    GVGTGWTVYPPLASNLAHAGASVDLAIFSLHLAGVSSILGAINFITTIINMKPPAISQYQ
Char    GAGTGWTVYPPLASNLAHAGASVDLTIFSLHLAGVSSILGAINFITTIINMKPPAISQYQ
Pser    GAGTGWTVYPPLAGNLAHAGASVDLAIFSLHLAGISSILGAINFITTIINMKPPTVSMYQ
Prol    GAGTGWTVYPPLASNLAHAGASVDLTIFSLHLAGISSILGAINFITTIINMKPTTVTMYQ
Plbi    GAGTGWTVYPPLAGNLAHAGASVDLTIFSLHLAGISSILGAINFITTIINMKPTAVTMYQ
Calu    GAGTGWTVYPPLAGNLAHAGASVDLTIFSLHLAGVSSILGAINFITTIINMKPAPMTMYQ
Papa    GAGTGWTVYPPLAGNLAHAGASVDLTIFSLHLAGISSILGAINFITTIINMKPAAMSMYQ
Sufr    GAGTGWTVYPPLAGNLAHAGASVDLTIFSLHLAGISSILGAINFITTIINMKPPAISQYQ
Stci    GAGTGWTVYPPLAGNLAHAGASVDLTIFSLHLAGISSILGAINFITTIMNMKPPAMTQYQ
Taru    GAGTGWTVYPPLAGNLAHAGASVDLTIFSLHLAGVSSILGAINFITTIINMKPPAISQYQ
Rala    GAGTGWTVYPPLAGNLAHAGASVDLTIFSLHLAGISSILGAINFITTIINMKPPAISQYQ
          *****:****: * :* * ****:******** **** .::*****  ***      .
```

To be continued on page 20.

[4/10 of aligned sequences]

E — 240

Scca	TPLFVWSILVTTVLLLLSLPVLAAGITMLLTDRNLNTTFFDPAGGGDPILYQHLFWFFGH
Muma	TPLFVWSILVTTILLLLSLPVLAAGITMLLTDRNLNTTFFDPAGGGDPILYQHLFWFFGH
Erca	TPLFVWSVLVTAVLLLLSLPVLAAGITMLLTDRNLNTTFFDPAGGGDPILYQHLFWFFGH
Pose	TPLFVWSVLITAVLLLLSLPVLAAGITMLLTDRNLNTTFFDPAGGGDPILYQHLFWFFGH
Actr	TPLFVWSVLITAVLLLLSLPVLAAGITMLLTDRNLNTTFFDPAGGGDPILYQHLFWFFGH
Scal	TPLFVWSVLVTAVLLLLSLPVLAAGITMLLTDRNLNTTFFDPAGGGDPILYQHLFWFFGH
Posp	TPLFVWSVLVTAVLLLLSLPVLAAGITMLLTDRNLNTTFFDPAGGGDPILYQHLFWFFGH
Atsp	TPLFVWSVLITAVLLLLSLPVLAAGITMLLTDRNLNTTFFDPAGGGDPILYQHLFWFFGH
Leoc	TPLFVWSVLITAVLLLLSLPVLAAGITMLLTDRNLNTTFFDPAGGGDPILYQHLFWFFGH
Amca	TPLFVWSVLITAVLLLLSLPVLAAGITMLLTDRNLNTTFFDPAGGGDPILYQHLFWFFGH
Osbi	TPLFIWSVLVTAVLLLLSLPVLAAGITMLLTDRNLNTTFFDPAGGGDPILYQHLFWFFGH
Pabu	TPLFVWSVLVTAVLLLLSLPVLAAGITMLLTDRNLNTTFFDPAGGGDPILYQHLFWFFGH
Hial	TPLFVWAVLITAVLLLLSLPVLAAGITMLLTDRNLNTTFFDPAGGGDPILYQHLFWFFGH
Elha	TPLFVWAVLITAVLLLLSLPVLAAGITMLLTDRNLNTTFFDPAGGGDPILYQHLFWFFGH
Mlcy	TPLFVWSVLVTAVLLLLSLPVLAAGITMLLTDRNLNTTFFDPAGGGDPILYQHLFWFFGH
Algl	TPLFVWSVLVTAVLLLLSLPVLAAGITMLLTDRNLNTTFFDPAGGGDPILYQHLFWFFGH
Ptgi	TPLFVWSVLITAVLLLLSLPVLAAGITMLLTDRNLNTTFFDPAGGGDPILYQHLFWFFGH
Alaf	TPLFVWSVLVTAVLLLLSLPVLAAGITMLLTDRNLNTTFFDPAGGGDPILYQHLFWFFGH
Nock	TPLFVWSVLVTAVLLLLSLPVLAAGITMLLTDRNLNTTFFDPAGGGDPILYQHLFWFFGH
Anja	TPLFVWAVLVTAVLLLLSLPVLAAGITMLLTDRNLNTTFFDPAGGGDPILYQHLFWFFGH
Gyki	TPLFVWAVLVTAVLLLLSLPVLAAGITMLLTDRNLNTTFFDPAGGGDPILYQHLFWFFGH
Syka	TPLFVWAVLVTAVLLLLSLPVLAAGITMLLTDRNLNTTFFDPAGGGDPILYQHLFWFFGH
Opma	TPLFVWSVLVTAVLLLLSLPVLAAGITMLLTDRNLNTTFFDPAGGGDPILYQHLFWFFGH
Comy	TPLFVWSVLITAVLLLLSLPVLAAGITMLLTDRNLNTTFFDPAGGGDPILYQHLFWFFGH
Sasp	TPLFIWAVLVTAVLLLLSLPVLAAGITMLLTDRNLNTTFFDPAGGGDPILYQHLFWFFGH
Eupe	TPLFVWSVLVTAVLLLLSLPVLAAGITMLLTDRNLNTTFFDPAGGGDPILYQHLFWFFGH
Enja	TPLFVWAVLITAVLLLLSLPVLAAGITMLLTDRNLNTTFFDPAGGGDPILYQHLFWFFGH
Same	TPLFVWSVLVTAVLLLLSLPVLAAGITMLLTDRNLNTTFFDPAGGGDPILYQHLFWFFGH
Chch	TPLFVWAVLVTAVLLLLSLPVLAAGITMLLTDRNLNTTFFDPAGGGDPILYQHLFWFFGH
Grgr	TPLFVWAVLITAVLLLLSLPVLAAGITMLLTDRNLNTTFFDPAGGGDPILYQHLFWFFGH
Caau	TPLFVWSVLVTAVLLLLSLPVLAAGITMLLTDRNLNTTFFDPAGGGDPILYQHLFWFFGH
Cyca	TPLFVWSVLVTAVLLLLSLPVLAAGITMLLTDRNLNTTFFDPAGGGDPILYQHLFWFFGH
Dare	TPLFVWAVLVTAVLLLLSLPVLAAGITMLLTDRNLNTTFFDPAGGGDPILYQHLFWFFGH
Cost	TPLFVWAVLVTAVLLLLSLPVLAAGITMLLTDRNLNTTFFDPAGGGDPILYQHLFWFFGH
Leec	TPLFVWAVLVTAVLLLLSLPVLAAGITMLLTDRNLNTTFFDPAGGGDPILYQHLFWFFGH
Fola	TPLFVWAVLITAVLLLLSLPVLAAGITMLLTDRNLNTTFFDPAGGGDPILYQHLFWFFGH
Clmc	TPLFVWAVLITAVLLLLSLPVLAAGITMLLTDRNLNTTFFDPAGGGDPILYQHLFWFFGH
Phin	TPLFVWAVLITAVLLLLSLPVLAAGITMLLTDRNLNTTFFDPAGGGDPILYQHLFWFFGH
Icpu	TPLFVWAVLITAVLLLLSLPVLAAGITMLLTDRNLNTTFFDPAGGGDPILYQHLFWFFGH
Psto	TPLFVWAVLITAVLLLLSLPVLAAGITMLLTDRNLNTTFFDPAGGGDPILYQHLFWFFGH
Cora	TPLFVWAVLITAVLLLLSLPVLAAGITMLLTDRNLNTTFFDPAGGGDPILYQHLFWFFGH
Eisp	TPLFIWATLVTTVLLLLSLPVLAAGITMLLTDRNLNTTFFDPAGGGDPILYQHLFWFFGH
Apal	LPLFIWSLLVTTVLLLLSLPVLAAGITMLLTDRNLNTAFFDPTGGGDPILYQHLFWFFGH
Eslu	TPLFVWAVLITAVLLLLSLPVLAAGITMLLTDRNLNTTFFDPAGGGDPILYQHLFWFFGH
Dape	TPLFIWAVLITAVLLLLSLPVLAAGITMLLTDRNLNTTFFDPAGGGDPILYQHLFWFFGH
Glse	TPLFVWAVLITAVLLLLSLPVLAAGITMLLTDRNLNTTFFDPAGGGDPILYQHLFWFFGH
Naar	TPMFVWAVLITAVLLLLSLPVLAAGITMLLTDRNLNTTFFDPAGGGDPILYQHLFWFFGH
Lioc	TPLFVWAVLITAVLLLLSLPVLAAGITMLLTDRNLNTTFFDPAGGGDPILYQHLFWFFGH
Opso	TPLFVWALLITTVLLLLSLPVLAAGITMLLTDRNLNTTFFDPAGGGDPILYQHLFWFFGH
Alte	TPLFVWAVLITAVLLLLSLPVLAAGITMLLTDRNLNTTFFDPAGGGDPILYQHLFWFFGH
Plap	TPLFIWSVLVTTVLLLLSLPVLAAAITMLLTDRNLNTTFFDPAGGGDPILYQHLFWFFGH

To be continued on page 21.

[4/10 of aligned sequences]

To be continued on page 22.

```
Plal  TPLFVWAVLITAVLLLLSLPVLAAGITMLLTDRNLNTTFFDPAGGGDPILYQHLFWFFGH
Sami  TPLFVWSVLITAVLLLLSLPVLAAGITMLLTDRNLNTTFFDPAGGGDPILYQHLFWFFGH
Rere  TPLFVWSVLITAVLLLLSLPVLAAGITMLLTDRNLNTTFFDPAGGGDPILYQHLFWFFGH
Gama  TPLFVWAVLITAVLLLLSLPVLAAGITMLLTDRNLNTTFFDPAGGGDPILYQHLFWFFGH
Onmy  TPLFVWAVLVTAVLLLLSLPVLAAGITMLLTDRNLNTTFFDPAGGGDPILYQHLFWFFSH
Sasa  TPLFVWAVLVTAVLLLLSLPVLAAGITMLLTDRNLNTTFFDPAGGGDPILYQHLFWFFGH
Cola  TPLFVWAVLITAVLLLLSLPVLAAGITMLLTDRNLNTTFFDPAGGGDPILYQHLFWFFGH
Dita  TPLFVWAVLVTAVLLLLSLPVLAAGITMLLTDRNLNTTFFDPAGGGDPILYQHLFWFFGH
Gogr  TPLFIWAVLVTAILLLLSLPVLAAGITMLLTDRNLNTSFFDPSGGGDPILYQHLFWFFGH
Chsl  APLFVWSVLITAVLLLLSLPVLAAGITMLLTDRNLNTTFFDPAGGGDPILYQHLFWFFGH
Atja  TPLFVWSVLITAVLLLLSLPVLAAGITMLLTDRNLNTTFFDPAGGGDPILYQHLFWFFGH
Iido  TPLFVWSVLITAVLLLLSLPVLAAGITMLLTDRNLNTTFFDPAGGGDPILYQHLFWFFGH
Auja  TPLLVWAVLITAVLLLLSLPVLAAGITMLLTDRNLNTTFFDPAGGGDPILYQHLFWFFGH
Chag  TPLFVWAVLITAVLLLLSLPVLAAGITMLLTDRNLNTTFFDPAGGGDPILYQHLFWFFGH
Hami  TPLFVWAVLITAVLLLLSLPVLAAGITMLLTDRNLNTTFFDPAGGGDPILYQHLFWFFGH
Saun  TPLFVWAVLITAVLLLLSLPVLAAGITMLLTDRNLNTTFFDPAGGGDPILYQHLFWFFGH
Nema  TPLFVWAVLITAVLLLLSLPVLAAGITMLLTDRNLNTTFFDPAGGGDPILYQHLFWFFGH
Disp  TPLFVWAVLITAVLLLLSLPVLAAGITMLLTDRNLNTTFFDPAGGGDPILYQHLFWFFGH
Myaf  TPLFVWAVLITAVLLLLSLPVLAAGITMLLTDRNLNTTFFDPAGGGDPILYQHLFWFFGH
Lagu  TPLFVWATLITAVLLLLSLPVLAAGITMLLTDRNLNTTFFDPSGGGDPILYQHLFWFFGH
Trtr  TPLFVWAVLITAVLLLLSLPVLAAGITMLLTDRNLNTTFFDPSGGGDPILYQHLFWFFGH
Zucr  TPLFVWAVLITAVLLLLSLPVLAAGITMLLTDRNLNTTFFDPSGGGDPILYQHLFWFFGH
Pxja  TPLFVWSVLITAVLLLLSLPVLAAGITMLLTDRNLNTTFFDPAGGGDPILYQHLFWFFGH
Pxlo  TPLFVWSVLITAVLLLLSLPVLAAGITMLLTDRNLNTTFFDPAGGGDPILYQHLFWFFGH
Pctr  TPLFVWSVLITAVLLLLSLPVLAAGITMLLTDRNLNTTFFDPAGGGDPILYQHLFWFFGH
Apsa  TPLFVWSVLITAVLLLLSLPVLAAGITMLLTDRNLNTTFFDPAGGGDPILYQHLFWFFGH
Cabe  TPLFVWAVLITAVLLLLSLPVLAAGITMLLTDRNLNTTFFDPAGGGDPILYQHLFWFFGH
Bzze  TPLFVWSVLITAVLLLLSLPVLAAGITMLLTDRNLNTTFFDPAGGGDPILYQHLFWFFGH
Siim  TPLFVWSVLITAVLLLLSLPVLAAGITMLLTDRNLNTTFFDPAGGGDPILYQHLFWFFGH
Ctru  TPLFVWSVLITAVLLLLSLPVLAAGITMLLTDRNLNTTFFDPAGGGDPILYQHLFWFFGH
Dpbr  TPLFVWSVLITAVLLLLSLPVLAAGITMLLTDRNLNTTFFDPSGGGDPILYQHLFWFFGH
Caki  TPLFVWAVLITAVLLLLSLPVLAAGITMLLTDRNLNTSFFDPAGGGDPILYQHLFWFFGH
Phja  TPLFVWSVLITAILLLLALPVLAAGITMLLTDRNLNTSFFDPAGGGDPILYQHLFWFFGH
Brsp  TPLFVWSVLVTAVLLLLSLPVLAAGITMLLTDRNLNTSFFDPAGGGDPVLYQHLFWFFGH
Gamo  TPLFVWAVLITAVLLLLSLPVLAAGITMLLTDRNLNTSFFDPAGGGDPILYQHLFWFFGH
Lolo  TPLFVWAVLITAVLLLLSLPVLAAGITMLLTDRNLNTSFFDPAGGGDPILYQHLFWFFGH
Batr  TPLFIWALMITAVLLLLSLPVLAAGITMLLTDRNLNTTFFDPGGGGDPILFQHLFWFFGH
Prmy  TPLFIWATLVTAVLLLLSLPVLAAGITMLLTDRNLNTTFFDPAGGGDPILYQHLFWFFGH
Lose  TPLFVWAVLVTAVLLLLSLPVLAAGITMLLTDRNLNTTFFDPTGGGDPILYQHLFWFFGH
Loam  TPLFVWAVLITAVLLLLSLPVLAAGITMLLTDRNLNTTFFDPTGGGDPILYQHLFWFFGH
Chab  TPLFVWAVLITAVLLLLSLPVLAAGITMLLTDRNLNTTFFDPAGGGDPILYQHLFWFFGH
Chto  TPLFVWAVLITAVLLLLSLPVLAAGITMLLTDRNLNTTFFDPAGGGDPILYQHLFWFFGH
Majo  TPLFVWAVLITAVLLLLALPVLAAGITMLLTDRNLNTTFFDPAGGGDPILYQHLFWFFGH
Hlst  TPLFVWSVLITAVLLLLALPVLAAGITMLLTDRNLNTTFFDPAGGGDPILYQHLFWFFGH
Clpe  TPLFVWSVLITAVLLLLSLPVLAAGITMLLTDRNLNTTFFDPAGGGDPILYQHLFWFFGH
Mlmr  TPLFVWSVLITAVLLLLSLPVLAAGITMLLTDRNLNTTFFDPAGGGDPILYQHLFWFFGH
Crcr  TPLFVWAVLITAVLLLLSLPVLAAGITMLLTDRNLNTSFFDPAGGGDPILYQHLFWFFGH
Muce  TPLFVWAVLITAVLLLLSLPVLAAGITMLLTDRNLNTSFFDPAGGGDPILYQHLFWFFGH
Bege  TPLFVWAVLITAVLLLLSLPVLAAGITMLLTDRNLNTTFFDPAGGGDPILYQHLFWFFGH
Mela  TPLFVWAVLITAVLLLLSLPVLAAGITMLLTDRNLNTTFFDPAGGGDPILYQHLFWFFGH
Hats  TPLFVWAVLITAVLLLLSLPVLAAGITMLLTDRNLNTTFFDPAGGGDPILYQHLFWFFGH
Orla  TPLFVWAVLITAVLLLLSLPVLAAGITMLLTDRNLNTTFFDPAGGGDPILYQHLFWFFGH
```

Cosa TPLFVWAVLITAVLLLLSLPVLAAGITMLLTDRNLNTTFFDPAGGGDPILYQHLFWFFGH
Exsp TPLFVWAVLITAVLLLLSLPVLAAGITMLLTDRNLNTTFFDPAGGGDPILYQHLFWFFGH
Depa TPLFVWAVLITAVLLLLSLPVLAAGITMLLTDRNLNTTFFDPAGGGDPILYQHLFWFFGH
Rima TPLFVWAVMITAVLLLLSLPVLAAGITMLLTDRNLNTTFFDPAGGGDPILYQHLFWFFGH
Fuol TPLFVWAVLITAVLLLLSLPVLAAGITMLLTDRNLNTTFFDPAGGGDPILYQHLFWFFGH
Gmaf TPLFVWAVLITAVLLLLSLPVLAAGITMLLTDRNLNTTFFDPAGGGDPILYQHLFWFFGH
Xeei TPLFVWAVLITAVLLLLSLPVLAAGITMLLTDRNLNTTFFDPAGGGDPILYQHLFWFFGH
Pros TPLFVWSVMITAVLLLLSLPVLAAGITMLLTDRNLNTTFFDPAGGGDPILYQHLFWFFGH
Scmi TPLFVWSVLITAILLLLSLPVLAAGITMLLTDRNLNTTFFDPAGGGDPILYQHLFWFFGH
Rolo TPLFVWSVLVTAVLLLLSLPVLAAGITMLLTDRNLNTTFFDPSGGGDPILYQHLFWFFGH
Cere TPLFVWSVLVTAVLLLLSLPVLAAGITMLLTDRNLNTTFFDPAGGGDPILYQHLFWFFGH
Daga TPLFVWSVLITAVLLLLSLPVLAAGITMLLTDRNLNTTFFDPAGGGDPILYQHLFWFFGH
Anco TPLFVWSVLITAVLLLLSLPVLAAGITMLLTDRNLNTTFFDPAGGGDPILYQHLFWFFGH
Dmve IPLFVWAVLITAVLLLLSLPVLAAGITMLLTDRNLNTTFFDPAGGGDPILYQHLFWFFGH
Dmar IPLFVWAVLITAVLLLLSLPVLAAGITMLLTDRNLNTTFFDPAGGGDPILYQHLFWFFGH
Anka TPLFVWAVLITAVLLLLSLPVLAAGITMLLTDRNLNTTFFDPAGGGDPILYQHLFWFFGH
Moja TPLFVWAVLITAVLLLLSLPVLAAGITMLLTDRNLNTTFFDPAGGGDPILYQHLFWFFGH
Hoja TPLFVWAVLITAVLLLLSLPVLAAGITMLLTDRNLNTTFFDPAGGGDPILYQHLFWFFGH
Bede TPLFVWAVLITAVLLLLSLPVLAAGITMLLTDRNLNTTFFDPAGGGDPILYQHLFWFFGH
Besp TPLFVWAVLITAVLLLLSLPVLAAGITMLLTDRNLNTTFFDPAGGGDPILYQHLFWFFGH
Mysp TPLFVWAVLITAVLLLLSLPVLAAGITMLLTDRNLNTTFFDPAGGGDPILYQHLFWFFGH
Osja TPLFVWAVLITAVLLLLSLPVLAAGITMLLTDRNLNTTFFDPSGGGDPILYQHLFWFFGH
Sgro TPLFVWAVLITAVLLLLSLPVLAAGITMLLTDRNLNTTFFDPAGGGDPILYQHLFWFFGH
Pzpa TPLFVWAVLITAVLLLLSLPVLAAGITMLLTDRNLNTSFFDPAGGGDPILYQHLFWFFGH
Zeja TPLFVWAVLITAVLLLLSLPVLAAGITMLLTDRNLNTSFFDPAGGGDPILYQHLFWFFGH
Znne TPLFVWAVLITAVLLLLSLPVLAAGITMLLTDRNLNTSFFDPAGGGDPILYQHLFWFFGH
Zefa TPLFVWSVLITAVLLLLSLPVLAAGITMLLTDRNLNTSFFDPAGGGDPILYQHLFWFFGH
Acni TPLFVWAVLITAVLLLLSLPVLAAGITMLLTDRNLNTSFFDPAGGGDPILYQHLFWFFGH
Ncrh TPLFVWAVLITAVLLLLSLPVLAAGITMLLTDRNLNTSFFDPAGGGDPILYQHLFWFFGH
Agca TPLFVWAVLITAVLLLLSLPVLAAGITMLLTDRNLNTTFFDPAGGGDPILYQHLFWFFGH
Hydy TPLFVWSVLITAVLLLLSLPVLAAGITMLLTDRNLNTTFFDPAGGGDPILYQHLFWFFGH
Gsac TPLFVWSVLITAVLLLLSLPVLAAGITMLLTDRNLNTTFFDPAGGGDPILYQHLFWFFGH
Pevo TPLFVWSVLITAVLLLLSLPVLAAGITMLLTDRNLNTTFFDPAGGGDPILYQHLFWFFGH
Hiku TPLFVWAVLVTAVLLLLSLPVLAAGITMLLTDRNLNTTFFDPSGGGDPILYQHLFWFFGH
Inpa TPLFIWALLITTVLLLLSLPVLAAGITMLLTDRNLNTTFFDPAGGGDPILYQHLFWFFGH
Auch LPLFVWAVLVTAVLLLLSLPVLAAGITMLLTDRNLNTTFFDPAGGGDPILYQHLFWFFGH
Fico TPLFVWAVLITAVLLLLSLPVLAAGITMLLTDRNLNTTFFDPAGGGDPILYQHLFWFFGH
Macs TPLFVWAVLITAVLLLLSLPVLAAGITMLLTDRNLNTTFFDPAGGGDPILYQHLFWFFGH
Moal TPLFVWSVMITAILLLLSLPVLAAGITMLLTDRNLNTTFFDPAGGGDPILYQHLFWFFGH
Syma TPLFVWSIMITAVLLLLSLPVLAAGITMLLTDRNLNTTFFDPAGGGDPILYQHLFWFFGH
Mafr TPLFVWALLITAVLLLLSLPVLAAGITMLLTDRNLNTTFFDPAGGGDPILYQHLFWFFGH
Dcpe TPLFVWAVLVTAVLLLLSLPVLAAGITMLLTDRNLNTTFFDPAGGGDPILYQHLFWFFGH
Dcti TPLFVWAVLVTAVLLLLSLPVLAAGITMLLTDRNLNTTFFDPAGGGDPILYQHLFWFFGH
Hehi TPLFVWAVLITAVLLLLSLPVLAAGITMLLTDRNLNTTFFDPAGGGDPILYQHLFWFFGH
Stam TPLFVWAVLITAVLLLLSLPVLAAGITMLLTDRNLNTTFFDPAGGGDPILYQHLFWFFGH
Hogi TPLFVWAVLITAVLLLLSLPVLAAGITMLLTDRNLNTTFFDPAGGGDPILYQHLFWFFGH
Erzo TPLFVWAVLITAVLLLLSLPVLAAGITMLLTDRNLNTTFFDPAGGGDPILYQHLFWFFGH
Hxot TPLFVWSVLITAVLLLLSLPVLAAGITMLLTDRNLNTTFFDPAGGGDPILYQHLFWFFGH
Core TPLFVWSVLITAVLLLLSLPVLAAGITMLLTDRNLNTTFFDPAGGGDPILYQHLFWFFGH
Apve TPLFVWSVLITAVLLLLSLPVLAAGITMLLTDRNLNTTFFDPAGGGDPILYQHLFWFFGH
Latj IPLFVWAVLITAVLLLLSLPVLAAGITMLLTDRNLNTAFFDPAGGGDPILYQHLFWFFGH
Laja TPLFVWAVLITAVLLLLSLPVLAAGITMLLTDRNLNTTFFDPAGGGDPILYQHLFWFFGH

To be continued on page 23.

[4/10 of aligned sequences]

To be continued on page 24.

```
Syja TPLFVWAVLITAVLLLLSLPVLAAGITMLLTDRNLNTTFFDPAGGGDPILYQHLFWFFGH
Epme TPLFVWAVLITAVLLLLSLPVLAAGITMLLTDRNLNTTFFDPAGGGDPILYQHLFWFFGH
Grse TPLFVWAVLITAVLLLLSLPVLAAGITMLLTDRNLNTTFFDPAGGGDPILYQHLFWFFGH
Clja TPLFVWAVLITAVLLLLSLPVLAAGITMLLTDRNLNTTFFDPAGGGDPILYQHLFWFFGH
Ogcy TPLFVWAVLITAVLLLLSLPVLAAGITMLLTDRNLNTTFFDPAGGGDPILYQHLFWFFGH
Plna TPLFVWAVLITAVLLLLSLPVLAAGITMLLTDRNLNTTFFDPAGGGDPILYQHLFWFFGH
Lema TPLFVWSVLITAVLLLLSLPVLAAGITMLLTDRNLNTTFFDPAGGGDPILYQHLFWFFGH
Etzo TPLFVWAVLITAVLLLLSLPVLAAGITMLLTDRNLNTTFFDPAGGGDPILYQHLFWFFGH
Apse TPLFVWAVLITAVLLLLSLPVLAAGITMLLTDRNLNTTFFDPAGGGDPILYQHLFWFFGH
Epde TPLFVWAVLITAVLLLLSLPVLAAGITMLLTDRNLNTTFFDPAGGGDPILYQHLFWFFGH
Slja TPLFVWSVLITAVLLLLSLPVLAAGITMLLTDRNLNTTFFDPAGGGDPILYQHLFWFFGH
Bsja TPLFVWSVLITAVLLLLSLPVLAAGITMLLTDRNLNTTFFDPAGGGDPILYQHLFWFFGH
Ecna LPLFVWAVLITAVLLLLSLPVLAAGITMLLTDRNLNTAFFDPAGGGDPILYQHLFWFFGH
Cohi IPLFVWAVLITAVLLLLSLPVLAAGITMLLTDRNLNTAFFDPAGGGDPILYQHLFWFFGH
Caar IPLFVWAVLITAVLLLLSLPVLAAGITMLLTDRNLNTAFFDPAGGGDPILYQHLFWFFGH
Came IPLFVWAVLITAVLLLLSLPVLAAGITMLLTDRNLNTAFFDPAGGGDPILYQHLFWFFGH
Mema IPLFVWAVLITAVLLLLSLPVLAAGITMLLTDRNLNTAFFDPTGGGDPILYQHLFWFFGH
Lenu TPLFVWAVLITAVLLLLSLPVLAAGITMLLTDRNLNTTFFDPAGGGDPILYQHLFWFFGH
Brja TPLFVWSVLITAVLLLLSLPVLAAGITMLLTDRNLNTTFFDPAGGGDPILYQHLFWFFGH
Plma TPLFVWSVLITAVLLLLSLPVLAAGITMLLTDRNLNTTFFDPAGGGDPILYQHLFWFFGH
Emst TPLFVWAVLITAVLLLLSLPVLAAGITMLLTDRNLNTTFFDPAGGGDPILYQHLFWFFGH
Ptti TPLFVWAVLITAVLLLLSLPVLAAGITMLLTDRNLNTTFFDPAGGGDPILYQHLFWFFGH
Losu TPLFVWAVLITAVLLLLSLPVLAAGITMLLTDRNLNTTFFDPAGGGDPILYQHLFWFFGH
Geoy TPLFVWAVLITAVLLLLSLPVLAAGITMLLTDRNLNTTFFDPAGGGDPILYQHLFWFFGH
Dipi TPLFVWSVLVTAVLLLLSLPVLAAGITMLLTDRNLNTTFFDPAGGGDPILYQHLFWFFGH
Pama TPLFVWAVLITAVLLLLSLPVLAAGITMLLTDRNLNTTFFDPAGGGDPILYQHLFWFFGH
Leob TPLFVWAVLITAVLLLLSLPVLAAGITMLLTDRNLNTTFFDPAGGGDPILYQHLFWFFGH
Neba TPLFVWAVLITAVLLLLSLPVLAAGITMLLTDRNLNTTFFDPAGGGDPILYQHLFWFFGH
Pdpl LPLFVWAVLVTAVLLLLSLPVLAAGITMLLTDRNLNTAFFDPAGGGDPVLYQHLFWFFGH
Nimi TPLFVWAVLITAVLLLLSLPVLAAGITMLLTDRNLNTTFFDPAGGGDPILYQHLFWFFGH
Uptr TPLFVWAVLITAVLLLLSLPVLAAGITMLLTDRNLNTTFFDPAGGGDPILYQHLFWFFGH
Pesc LPLFVWAVLITAVLLLLSLPVLAAGITMLLTDRNLNTTFFDPAGGGDPILYQHLFWFFGH
Baar TPLFVWSVLVTAVLLLLSLPVLAAGITMLLTDRNLNTTFFDPAGGGDPILYQHLFWFFGH
Moar TPLFVWAVLITAVLLLLSLPVLAAGITMLLTDRNLNTTFFDPAGGGDPILYQHLFWFFGH
Toja IPLFVWAVLITAVLLLLSLPVLAAGITMLLTDRNLNTAFFDPAGGGDPILYQHLFWFFGH
Chau TPLFVWSVLITAVLLLLSLPVLAAGITMLLTDRNLNTTFFDPAGGGDPILYQHLFWFFGH
Chse TPLFVWAVLITAVLLLLSLPVLAAGITMLLTDRNLNTTFFDPAGGGDPILYQHLFWFFGH
Enar TPLFVWAVLITAVLLLLSLPVLAAGITMLLTDRNLNTTFFDPAGGGDPILYQHLFWFFGH
Hpty TPLFVWAVLITAVLLLLSLPVLAAGITMLLTDRNLNTTFFDPAGGGDPILYQHLFWFFGH
Nana IPLFVWALLITTVLLLLSLPVLAAGITMLLTDRNLNTSFFDPAGGGDPILYQHLFWFFGH
Mcst TPLFVWAVLITAVLLLLSLPVLAAGITMLLTDRNLNTTFFDPAGGGDPILYQHLFWFFGH
Rhox TPLFVWAVLITAVLLLLSLPVLAAGITMLLTDRNLNTTFFDPAGGGDPILYQHLFWFFGH
Opfa TPLFVWAVLITAVLLLLSLPVLAAGITMLLTDRNLNTTFFDPAGGGDPILYQHLFWFFGH
Paar TPLFVWAVLITAVLLLLSLPVLAAGITMLLTDRNLNTTFFDPAGGGDPILYQHLFWFFGH
Gozo TPLFVWAVLITAVLLLLSLPVLAAGITMLLTDRNLNTTFFDPAGGGDPILYQHLFWFFGH
Ackr LPLFVWAVLITAVLLLLSLPVLAAGITMLLTDRNLNTTFFDPAGGGDPILYQHLFWFFGH
Elev TPLFVWSVLITAVLLLLSLPVLAAGITMLLTDRNLNTTFFDPAGGGDPILYQHLFWFFGH
Trdu TPLFVWSVLITAVLLLLSLPVLAAGITMLLTDRNLNTTFFDPAGGGDPILYQHLFWFFGH
Amoc TPLFVWAVLITAVLLLLSLPVLAAGITMLLTDRNLNTTFFDPAGGGDPILYQHLFWFFGH
Hame TPLFVWAVLITAVLLLLSLPVLAAGITMLLTDRNLNTTFFDPAGGGDPILYQHLFWFFGH
Chso TPLFVWAVLITAVLLLLSLPVLAAGITMLLTDRNLNTTFFDPAGGGDPILYQHLFWFFGH
Lyto TPLFVWSVLITAVLLLLSLPVLAAGITMLLTDRNLNTTFFDPAGGGDPILYQHLFWFFGH
```

To be continued on page 25.

```
Encr   TPLFVWSVLITAVLLLLSLPVLAAGITMLLTDRNLNTTFFDPAGGGDPILYQHLFWFFGH
Bvar   TPLFVWAVLVTAVLLLLSLPVLAAGITMLLTDRNLNTTFFDPAGGGDPILYQHLFWFFGH
Noco   TPLFVWAVLITAVLLLLSLPVLAAGITMLLTDRNLNTTFFDPAGGGDPILYQHLFWFFGH
Chsp   VPLFVWSVLITAILLLLALPVLAAGITMLLTDRNLNTTFFDPAGGGDPILYQHLFWFFGH
Arja   TPLFVWSVLITAVLLLLSLPVLAAGITMLLTDRNLNTTFFDPAGGGDPILYQHLFWFFGH
Pase   TPLFVWAVLITAVLLLLSLPVLAAGITMLLTDRNLNTTFFDPAGGGDPILYQHLFWFFGH
Trel   TPLFVWAVLITAVLLLLSLPVLAAGITMLLTDRNLNTTFFDPAGGGDPILYQHLFWFFGH
Lifa   TPLFVWAVLITAVLLLLSLPVLAAGITMLLTDRNLNTTFFDPAGGGDPILYQHLFWFFGH
Acur   TPLFVWAVLITAVLLLLSLPVLAAGITMLLTDRNLNTTFFDPAGGGDPILYQHLFWFFGH
Ampe   TPLFVWAVLITAVLLLLSLPVLAAGITMLLTDRNLNTTFFDPAGGGDPILYQHLFWFFGH
Urja   MPLFVWAVLVTAILLLLSLPVLAAAITMLLTDRNLNTTFFDPAGGGDPILYQHLFWFFGH
Enet   TPLFVWAVLITAVLLLLSLPVLAAGITMLLTDRNLNTTFFDPAGGGDPILYQHLFWFFGH
Ptbr   TPLFVWAVLITAVLLLLSLPVLAAGITMLLTDRNLNTTFFDPAGGGDPILYQHLFWFFGH
Safa   TPLFVWAVLITAVLLLLSLPVLAAGITMLLTDRNLNTTFFDPAGGGDPILYQHLFWFFGH
Icae   TPLFVWSVLITAVLLLLSLPVLAAGITMLLTDRNLNTTFFDPAGGGDPILYQHLFWFFGH
Asmi   TPLFVWAVLITAVLLLLSLPVLAAGITMLLTDRNLNTTFFDPAGGGDPILYQHLFWFFGH
Foal   TPLFVWSVLITAVLLLLSLPVLAAGITMLLTDRNLNTTFFDPAGGGDPILYQHLFWFFGH
Drze   TPLFVWAVLITAVLLLLSLPVLAAGITMLLTDRNLNTTFFDPAGGGDPILYQHLFWFFGH
Rhas   TPLFVWSVLITAVLLLLSLPVLAAGITMLLTDRNLNTTFFDPAGGGDPILYQHLFWFFGH
Elac   TPLFVWAVLITAVLLLLSLPVLAAGITMLLTDRNLNTTFFDPAGGGDPILYQHLFWFFGH
Kugu   TPLFVWSVLITAVLLLLSLPVLAAGITMLLTDRNLNTSFFDPAGGGDPILYQHLFWFFGH
Plor   TPLFVWAVLITAVLLLLSLPVLAAGITMLLTDRNLNTTFFDPAGGGDPILYQHLFWFFGH
Sgun   TPLFVWAVLITAVLLLLSLPVLAAGITMLLTDRNLNTTFFDPAGGGDPILYQHLFWFFGH
Zaco   TPLFVWAVLITAVLLLLSLPVLAAGITMLLTDRNLNTTFFDPAGGGDPILYQHLFWFFGH
Zbfl   TPLFVWAVLITAVLLLLSLPVLAAGITMLLTDRNLNTTFFDPAGGGDPILYQHLFWFFGH
Spba   IPLFVWAVLITAVLLLLSLPVLAAGITMLLTDRNLNTAFFDPAGGGDPILYQHLFWFFGH
Game   TPLFVWAVLITAVLLLLSLPVLAAGITMLLTDRNLNTTFFDPAGGGDPILYQHLFWFFGH
Thth   TPLFVWAVLITAVLLLLSLPVLAAGITMLLTDRNLNTTFFDPAGGGDPILYQHLFWFFGH
Xigl   IPLFVWAVLITAVLLLLSLPVLAAGITMLLTDRNLNTAFFDPAGGGDPILYQHLFWFFGH
Hyja   TPLFVWAVLITAVLLLLSLPVLAAGITMLLTDRNLNTTFFDPAGGGDPILYQHLFWFFGH
Psan   TPLFVWAVLITAVLLLLSLPVLAAGITMLLTDRNLNTTFFDPAGGGDPILYQHLFWFFGH
Cupa   TPLFVWSVLITAVLLLLSLPVLAAGITMLLTDRNLNTTFFDPAGGGDPILYQHLFWFFGH
Mpch   TPLFVWAVLITAVLLLLSLPVLAAGITMLLTDRNLNTTFFDPAGGGDPILYQHLFWFFGH
Char   TPLFVWAILITAVLLLLSLPVLAAGITMLLTDRNLNTTFFDPAGGGDPILYQHLFWFFGH
Pser   IPLFVWAVLITAVLLLLSLPVLAAGITMLLTDRNLNTTFFDPAGGGDPILYQHLFWFFGH
Prol   IPLFVWAVLITAVLLLLSLPVLAAGITMLLTDRNLNTTFFDPAGGGDPILYQHLFWFFGH
Plbi   IPLFVWAVLITAVLLLLSLPVLAAGITMLLTDRNLNTTFFDPAGGGDPILYQHLFWFFGH
Calu   MPLFVWAVLITAVLLLLSLPVLAAGITMLLTDRNLNTAFFDPAGGGDPILYQHLFWFFGH
Papa   IPLFVWSVLVTAVLLLLSLPVLAAGITMLLTDRNLNTTFFDPAGGGDPILYQHLFWFFGH
Sufr   TPLFVWAVLITAVLLLLSLPVLAAGITMLLTDRNLNTTFFDPAGGGDPILYQHLFWFFGH
Stci   MPLFVWAVLVTAVLLLLSLPVLAAGITMLLTDRNLNTTFFDPAGGGDPILYQHLFWFFGH
Taru   TPLFVWAVLITAVLLLLSLPVLAAGITMLLTDRNLNTTFFDPAGGGDPILYQHLFWFFGH
Rala   TPLFVWAVLITAVLLLLSLPVLAAGITMLLTDRNLNTTFFDPAGGGDPILYQHLFWFFGH
        *:::*: ::*::****:****** .************.**** *****.*.*******.*
```

[5/10 of aligned sequences]

```
     244 F                                   G         290 291
Scca PEVYILILPGFGMISHVVAYYSGKKEPFGYMGMVWAMMAIGLLGFIVWAHHMFTVGMDVD
Muma PEVYILILPGFGMISHVVAYYSGKKEPFGYMGMVWAMMAIGLLGFIVWAHHMFTVGMDVD
Erca PEVYILILPGFGMISHIVAYYSGKNEPFGYMGMVWAMMAIGLLGFIVWAHHMFTVGMDVD
Pose PEVYILILPGFGMISHIVAYYSGKNEPFGYMGMVWAMMAIGLLGFIVWAHHMFTVGMDVD
Actr PEVYILILPGFGMISHIVAYYAGKKEPFGYMGMVWAMMAIGLLGFIVWAHHMFTVGMDVD
Scal PEVYILILPGFGMISHIVAYYAGKKEPFGYMGMVWAMMAIGLLGFIVWAHHMFTVGMDVD
Posp PEVYILILPGFGMISHIVAYYAGKKEPFGYMGMVWAMMAIGLLGFIVWAHHMFTVGMDVD
Atsp PEVYILILPGFGMISHIVAYYAGKKEPFGYMGMVWAMMAIGLLGFIVWAHHMFTVGMDVD
Leoc PEVYILILPGFGMISHIVAYYAGKKEPFGYMGMVWAMMAIGLLGFIVWAHHMFTVGMDVD
Amca PEVYILILPGFGMVSHIVAYYAGKKEPFGYMGMVWAMMAIGLLGFIVWAHHMFTVGMDVD
Osbi PEVYILILPGFGMISHIVAYYSGKKEPFGYMGMVWAMMAIGLLGFIVWAHHMFTVGMDVD
Pabu PEVYILILPGFGMISHIVAYYSGKKEPFGYMGMVWAMMAIGLLGFIVWAHHMFTVGMDVD
Hial PEVYILILPGFGMISHIVAYYAGKKEPFGYMGMVWAMMAIGLLGFIVWAHHMFTVGMDVD
Elha PEVYILILPGFGMISHIVAYYAGKKEPFGYMGMVWAMMAIGLLGFIVWAHHMFTVGMDVD
Mlcy PEVYILILPGFGMISHIVAYYAGKKEPFGYMGMVWAMMAIGLLGFIVWAHHMFTVGMDVD
Algl PEVYILILPGFGMISHIVAYYSGKKEPFGYMGMVWAMMAIGLLGFIVWAHHMFTVGMDVD
Ptgi PEVYILILPGFGMISHIVAYYAGKKEPFGYMGMAWAMMAIGLLGFIVWAHHMFTVGMDVD
Alaf PEVYILILPGFGMISHIVAYYAGKKEPFGYMGMVWAMMAIGLLGFIVWAHHMFTVGMDVD
Nock PEVYILILPGFGMISHIVAYYAGKKEPFGYMGMVWAMMAIGLLGFIVWAHHMFTVGMDVD
Anja PEVYILILPGFGMISHIVAYYSGKKEPFGYMGMVWAMMAIGLLGFIVWAHHMFTVGMDVD
Gyki PEVYILILPGFGMISHIVAYYAGKPQPFGYMGMVWAMMAIGLLGFIVWAHHMFTVGMDVD
Syka PEVYILILPGFGMISHIVAYYAGKKEPFGYMGMVWAMMAIGLLGFIVWAHHMFTVGMDVD
Opma PEVYILILPGFGMISHVVTYYAGKKEPFGYMGMVWAMMAIGLLGFIVWAHHMFTVGMDVD
Comy PEVYILILPGFGMISHIVAYYSGKKEPFGYMGMVWAMMAIGLLGFIVWAHHMFTVGMDVD
Sasp PEVYILILPGFGMISHIVTYYSGKKEPFGYMGMVWAMMAIGLLGFIVWAHHMFTVGMDVD
Eupe PEVYILILPGFGMISHIITYYSGKKEPFGYMGMVWAMMAIGLLGFIVWAHHMFTVGMDVD
Enja PEVYILILPGFGMISHIVAYYAGKKEPFGYMGMVWAMMAIGLLGFIVWAHHMFTVGMDVD
Same PEVYILILPGFGMISHIVAYHAGKKEPFGYMGMVWAMMAIGLLGFIVWAHHMFTVGMDVD
Chch PEVYILILPGFGMISHIVAYYAGKKEPFGYMGMVWAMMAIGLLGFIVWAHHMFTVGMDVD
Grgr PEVYILILPGFGMISHIVAYYSGKKEPFGYMGMVWAMMAIGLLGFIVWAHHMFTVGMDVD
Caau PEVYILILPGFGIISHVVAYYSGKKEPFGYMGMVWAMMAIGLLGFIVWAHHMFTVGMDVD
Cyca PEVYILILPGFGIISHVVAYYSGKKEPFGYMGMVWAMMAIGLLGFIVWAHHMFTVGMDVD
Dare PEVYILILPGFGIISHVVAYYAGKKEPFGYMGMVWAMMAIGLLGFIVWAHHMFTVGMDVD
Cost PEVYILILPGFGIISHVVAYYAGKKEPFGYMGMVWAMMAIGLLGFIVWAHHMFTVGMDVD
Leec PEVYILILPGFGIISHVVAYYAGKKEPFGYMGMVWAMMAIGLLGFIVWAHHMFTVGMDVD
Fola PEVYILILPGFGIISHVVAYYAGKKEPFGYMGMVWAMMAIGLLGFIVWAHHMFTVGMDVD
Clmc PEVYILILPGFGMISHIVAYYSGKKEPFGYMGMVWAMMAIGLLGFIVWAHHMFTVGMDVD
Phin PEVYILILPGFGMISHIVAYYSGKKEPFGYMGMVWAMMAIGLLGFIVWAHHMFTVGMDVD
Icpu PEVYILILPGFGMISHIVAYYAGKKEPFGYMGMVWAMMAIGLLGFIVWAHHMFTVGMDVD
Psto PEVYILILPGFGMISHIVAYYSGKKEPFGYMGMVWAMMAIGLLGFIVWAHHMFTVGMDVD
Cora PEVYILILPGFGMISHIVAYYAGKKEPFGYMGMVWAMMAIGLLGFIVWAHHMFTVGMDVD
Eisp PEVYILILPGFGMISHIVAYYSGKKEPFGYMGMVWAMMAIGLLGFIVWAHHMFTVGMDVD
Apal PEVYILILPGFGMVSHIVAYYSGKKEPFGYLGMVWAMMAIGLLGFIVWAHHMFTVGMDVD
Eslu PEVYILILPGFGMISHIVAYYSGKKEPFGYMGMVWAMMAIGLLGFIVWAHHMFTVGMDVD
Dape PEVYILILPGFGMISHIVAYYSGKKEPFGYMGMVWAMMAIGLLGFIVWAHHMFTVGMDVD
Glse PEVYILILPGFGMISHIVAYYSGKKEPFGYMGMVWAMMAIGLLGFIVWAHHMFTVGMDVD
Naar PEVYILILPGFGMISHIVAYYSGKKEPFGYMGMVWAMMAIGLLGFIVWAHHMFTVGMDVD
Lioc PEVYILILPGFGMISHIVAYYSGKKEPFGYMGMVWAMMAIGLLGFIVWAHHMFTVGMDVD
Opso PEVYILILPGFGMISHIVAYYSGKKEPFGYMGMVWAMMAIGLLGFIVWAHHMFTVGMDVD
Alte PEVYILILPGFGIISHIVAYYSGKKEPFGYMGMVWAMMAIGLLGFIVWAHHMFTVGMDVD
Plap PEVYILILPGFGIISHVVAYYAGKKEPFGYMGMVWAMMAIGLLGFIVWAHHMFTVGMDVD
```

To be continued on page 26.

[5/10 of aligned sequences]

```
Plal  PEVYILILPGFGMISHIVAYYSGKKEPFGYMGMVWAMMAIGLLGFIVWAHHMFTVGMDVD
Sami  PEVYILILPGFGMISHIVAYYSGKKEPFGYMGMVWAMMAIGLLGFIVWAHHMFTVGMDVD
Rere  PEVYILILPGFGMISHIVAYYSGKKEPFGYMGMVWAMMAIGLLGFIVWAHHMFTVGMDVD
Gama  PEVYILILPGFGMISHIVAYYSGKKEPFGYMGMVWAMMAIGLLGFIVWAHHMFTVGMDVD
Onmy  PEVYILILPGFGMISHIVAYYSGKKEPFGYMGMVWAMMAIGLLGFIVWAHHMFTVGMDVD
Sasa  PEVYILILPGFGMISHIVAYYSGKKEPFGYMGMVWAMMAIGLLGFIVWAHHMFTVGMDVD
Cola  PEVYILILPGFGMISHIVAYYSGKKEPFGYMGMVWAMMAIGLLGFIVWAHHMFTVGMDVD
Dita  PEVYILILPGFGMISHIVAYYAGKKEPFGYMGMVWAMMAIGLLGFIVWAHHMFTVGMDVD
Gogr  PEVYILILPGFGMISHIVAYYSGKKEPFGHMGMVWAMMAIGLLGFIVWAHHMFTVGMDVD
Chsl  PEVYILILPGFGMISHIIAYYAGKKEPFGYMGMAWAMMAIGLLGFIVWAHHMFTVGMDVD
Atja  PEVYILILPGFGMISHIVAYYSGKKEPFGYMGMVWAMMAIGLLGFIVWAHHMFTVGMDVD
Iido  PEVYILILPGFGMISHIVAYYSGKKEPFGYMGMVWAMMAIGLLGFIVWAHHMFTVGMDVD
Auja  PEVYILILPGFGMISHIVAYYAGKKEPFGYMGMVWAMMAIGLLGFIVWAHHMFTVGMDVD
Chag  PEVYILILPGFGMISHIVAYYSGKKEPFGYMGMVWAMMAIGLLGFIVWAHHMFTVGMDVD
Hami  PEVYILILPGFGMISHIVAYYAGKKEPFGYMGMVWAMMAIGLLGFIVWAHHMFTVGMDVD
Saun  PEVYILILPGFGMISHIVAYYAGKKEPFGYMGMVWAMMAIGLLGFIVWAHHMFTVGMDVD
Nema  PEVYILILPGFGMISHIVAYYSGKKEPFGYMGMVWAMMAIGLLGFIVWAHHMFTVGMDVD
Disp  PEVYILILPGFGMISHIVAYYSGKKEPFGYMGMVWAMMAIGFLGFIVWAHHMFTVGMDVD
Myaf  PEVYILILPGFGMISHIVAYYSGKKEPFGYMGMVWAMMAIGFLGFIVWAHHMFTVGMDVD
Lagu  PEVYILILPGFGMISHIVAYYSGKKEPFGYMGMVWAMMAIGLLGFIVWAHHMFTVGMDVD
Trtr  PEVYILILPGFGMISHIVAYYSGKKEPFGYMGMVWAMMAIGLLGFIVWAHHMFTVGMDVD
Zucr  PEVYILILPGFGMISHIVAYYAGKKEPFGYMGMVWAMMAIGLLGFIVWAHHMFTVGMDVD
Pxja  PEVYILILPGFGMISHIVAYYSGKKEPFGYMGMVWAMMAIGLLGFIVWAHHMFTVGMDVD
Pxlo  PEVYILILPGFGMISHIVAYYSGKKEPFGYMGMVWAMMAIGLLGFIVWAHHMFTVGMDVD
Pctr  PEVYILILPGFGMISHIVAYYSGKKEPFGYMGMVWAMMAIGLLGFIVWAHHMFTVGMDVD
Apsa  PEVYILILPGFGMISHIVAYYSGKKEPFGYMGMVWAMMAIGLLGFIVWAHHMFTVGMDVD
Cabe  PEVYILILPGFGMISHIVAYYSGKKEPFGYMGMVWAMMAIGLLGFIVWAHHMFTVGMDVD
Bzze  PEVYILILPGFGMISHIVAYYSGKKEPFGYMGMVWAMMAIGLLGFIVWAHHMFTVGMDVD
Siim  PEVYILILPGFGMISHIVAYYAGKKEPFGYMGMVWAMMAIGLLGFIVWAHHMFTVGMDVD
Ctru  PEVYILILPGFGMISHVVAYYSGKKEPFGYMGMVWAMMAIGLLGFIVWAHHMFTVGMDVD
Dpbr  PEVYILILPGFGMISHIVAYYSGKKEPFGYMGMVWAMMAIGLLGFIVWAHHMFTVGMDVD
Caki  PEVYILILPGFGMISHIVAYYSGKKEPFGHMGMVWAMMAIGLLGFIVWAHHMFTVGMDVD
Phja  PEVYILILPGFGMISHIVAYYSGKKEPFGHMGMVWAMMAIGFLGFIVWAHHMFTVGMDVD
Brsp  PEVYILILPGFGIISHVVTFHAGKKTPFGSMGMIWAMVSIGYLGFIVWAHHMFTVGLDVD
Gamo  PEVYILILPGFGMISHIVAYYSGKKEPFGYMGMVWAMMAIGLLGFIVWAHHMFTVGMDVD
Lolo  PEVYILILPGFGMISHIVAYYSGKKEPFGYMGMVWAMMAIGLLGFIVWAHHMFTVGMDVD
Batr  PEVYILILPGFGMISHIVAFYSGKKEPFGYMGMVWAMMTIGLLGFIVWAHHMFTVGMDVD
Prmy  PEVYILILPGFGMISHVVTFYSGKAEPFGYMGMVWAMVAIGFLGFIVWAHHMFTVGMDVD
Lose  PEVYILILPGFGMVSHIVAYYAGKKEPFGYLGMVWAMMAIGLLGFIVWAHHMFTVGMDVD
Loam  PEVYILILPGFGMVSHIVAYYAGKKEPFGYLGMVWAMMAIGLLGFIVWAHHMFTVGMDVD
Chab  PEVYILILPGFGMVSHIVAYYSGKKEPFGHMGMVWAMMAIGLLGFIVWAHHMFTVGMDVD
Chto  PEVYILILPGFGMVSHIVAYYSGKKEPFGHMGMVWAMMAIGLLGFIVWAHHMFTVGMDVD
Majo  PEVYILILPGFGMISHIVAYYSGKKEPFGYMGMVWAMMAIGLLGFIVWAHHMFTVGMDVD
Hlst  PEVYILILPGFGMISHIVAYYSGKKEPFGYMGMVWAMMAIGLLGFIVWAHHMFTVGMDVD
Clpe  PEVYILILPGFGMISHIVAYYSGKKEPFGYMGMVWAMMAIGLLGFIVWAHHMFTVGMDVD
Mlmr  PEVYILILPGFGMISHIVAYYSGKKEPFGYMGMVWAMMAIGLLGFIVWAHHMFTVGMDVD
Crcr  PEVYILILPGFGMISHIVAYYSGKKEPFGYMGMVWAMMAIGLLGFIVWAHHMFTVGMDVD
Muce  PEVYILILPGFGMISHIVAYYSGKKEPFGYMGMVWAMMAIGLLGFIVWAHHMFTVGMDVD
Bege  PEVYILILPGFGMISHIVAYYSGKKEPFGYMGMVWAMMAIGLLGFIVWAHHMFTVGMDVD
Mela  PEVYILILPGFGMISHIVAYYAGKKEPFGYMGMVWAMMAIGLLGFIVWAHHMFTVGMDVD
Hats  PEVYILILPGFGMISHIVAYYSGKKEPFGYMGMVWAMMAIGLLGFIVWAHHMFTVGMDVD
Orla  PEVYILILPGFGMISHIVAYYSGKKEPFGYMGMVWAMMAIGLLGFIVWAHHMFTVGMDVD
```

To be continued on page 27.

[5/10 of aligned sequences]

To be continued on page 28.

```
Cosa  PEVYILILPGFGMISHIVAYYSGKKEPFGYMGMVWAMMAIGLLGFIVWAHHMFTVGMDVD
Exsp  PEVYILILPGFGMISHIVAYYSGKKEPFGYMGMVWAMMAIGLLGFIVWAHHMFTVGMDVD
Depa  PEVYILILPGFGMISHIVAYYSGKKEPFGYMGMVWAMMAIGLLGFIVWAHHMFTVGMDVD
Rima  PEVYILILPGFGMISHIVAYYSGKKEPFGYMGMVWAMMAIGLLGFIVWAHHMFTVGMDVD
Fuol  PEVYILILPGFGMISHIVAYYSGKKEPFGYMGMVWAMMAIGLLGFIVWAHHMFTVGMDVD
Gmaf  PEVYILILPGFGMISHIVAYYAGKKEPFGYMGMVWAMMAIGLLGFIVWAHHMFTVGMDVD
Xeei  PEVYILILPGFGMISHIVAYYSGKKEPFGYMGMVWAMMAIGLLGFIVWAHHMFTVGMDVD
Pros  PEVYILILPGFGMISHIVAYYSGKKEPFGYMGMVWAMMAIGLLGFIVWAHHMFTVGMDVD
Scmi  PEVYILILPGFGIVSHIIAYYSGKKETFGYGGMVWAMSAIGFLGFVVWAHHMFTVGMDVD
Rolo  PEVYILILPGFGMISHIVAYYSGKKEPFGYMGMVWAMMAIGLLGFIVWAHHMFTVGMDVD
Cere  PEVYILILPGFGMISHIVAYYSGKKEPFGYMGMVWAMMAIGLLGFIVWAHHMFTVGMDVD
Daga  PEVYILILPGFGMISHIVAYYSGKKEPFGYMGMVWAMMAIGLLGFIVWAHHMFTVGMDVD
Anco  PEVYILILPGFGMISHIVAYYSGKKEPFGYMGMVWAMMAIGLLGFIVWAHHMFTVGMDVD
Dmve  PEVYILILPGFGMISHIVAFYAGKKEPFGYMGMVWAMMAIGFLGFIVWAHHMFTVGMDVD
Dmar  PEVYILILPGFGMISHIVAFYAGKKEPFGYMGMVWAMMAIGFLGFIVWAHHMFTVGMDVD
Anka  PEVYILILPGFGMISHIVAYYSGKKEPFGYMGMVWAMMAIGLLGFIVWAHHMFTVGMDVD
Moja  PEVYILILPGFGMISHIVAYYSGKKEPFGYMGMVWAMMAIGLLGFIVWAHHMFTVGMDVD
Hoja  PEVYILILPGFGMISHIVAYYSGKKEPFGYMGMVWAMMAIGLLGFIVWAHHMFTVGMDVD
Bede  PEVYILILPGFGMISHIVAYYSGKKEPFGYMGMVWAMMAIGLLGFIVWAHHMFTVGMDVD
Besp  PEVYILILPGFGMISHIVAYYSGKKEPFGYMGMVWAMMAIGLLGFIVWAHHMFTVGMDVD
Mysp  PEVYILILPGFGMISHIVAYYSGKKEPFGYMGMVWAMMAIGLLGFIVWAHHMFTVGMDVD
Osja  PEVYILILPGFGMISHIVAYYSGKKEPFGYMGMVWAMMAIGLLGFIVWAHHMFTVGMDVD
Sgro  PEVYILILPGFGMISHIVAYYSGKKEPFGYMGMVWAMMAIGLLGFIVWAHHMFTVGMDVD
Pzpa  PEVYILILPGFGMISHIVAYYSGKKEPFGYMGMVWAMMAIGLLGFIVWAHHMFTVGMDVD
Zeja  PEVYILILPGFGMISHIVAYYSGKKEPFGYMGMVWAMMAIGLLGFIVWAHHMFTVGMDVD
Znne  PEVYILILPGFGMISHIVAYYSGKKEPFGYMGMVWAMMAIGLLGFIVWAHHMFTVGMDVD
Zefa  PEVYILILPGFGMISHIVAYYSGKKEPFGYMGMVWAMMAIGLLGFIVWAHHMFTVGMDVD
Acni  PEVYILILPGFGMISHIVAYYSGKKEPFGYMGMVWAMMAIGLLGFIVWAHHMFTVGMDVD
Ncrh  PEVYILILPGFGMISHIVAYYSGKKEPFGYMGMVWAMMAIGLLGFIVWAHHMFTVGMDVD
Agca  PEVYILILPGFGMISHIVAYYSGKKEPFGYMGMVWAMMAIGLLGFIVWAHHMFTVGMDVD
Hydy  PEVYILILPGFGMISHIVAYYSGKKEPFGYMGMVWAMMAIGLLGFIVWAHHMFTVGMDVD
Gsac  PEVYILILPGFGMISHIVAYYSGKKEPFGYMGMVWAMMAIGLLGFIVWAHHMFTVGMDVD
Pevo  PEVYILILPGFGMISHIVAYYAGKKEPFGYMGMVWAMMAIGLLGFIVWAHHMFTVGMDVD
Hiku  PEVYILILPGFGMISHIVAYYSGKKEPFGYMGMVWAMMAIGLLGFIVWAHHMFTVGMDVD
Inpa  PEVYILILPGFGMISHVVAFYTGKKEPFGYMGMVWAMMAIGLLGFIVWAHHMFTVGMDVD
Auch  PEVYILILPGFGMVSHVVAYYAGKKEPFGYMGMVWAMMAIGLLGFIVWAHHMFTVGMDVD
Fico  PEVYILILPGFGMISHIVAYYAGKKEPFGYMGMVWAMMAIGLLGFIVWAHHMFTVGMDVD
Macs  PEVYILILPGFGMISHIVAYYAGKKEPFGYMGMVWAMMAIGLLGFIVWAHHMFTVGMDVD
Moal  PEVYILILPGFGMISHIVTFYAGKKEPFGYMGMVWAMMAIGLLGFIVWAHHMFTVGMDVD
Syma  PEVYILILPGFGMISHVVAYYSGKKEPFGYMGMVWAMMAIGLLGFIVWAHHMFTVGMDVD
Mafr  PEVYILILPGFGMISHIVAYYAGKKEPFGYMGMVWAMMAIGLLGFIVWAHHMFTVGMDVD
Dcpe  PEVYILILPGFGMISHIVAYYAGKKEPFGYMGMVWAMMAIGLLGFIVWAHHMFTVGMDVD
Dcti  PEVYILILPGFGMISHIVAYYAGKKEPFGYMGMVWAMMAIGLLGFIVWAHHMFTVGMDVD
Hehi  PEVYILILPGFGMISHIVAYYSGKKEPFGYMGMVWAMMAIGLLGFIVWAHHMFTVGMDVD
Stam  PEVYILILPGFGMISHIVAYYSGKKEPFGYMGMVWAMVAIGLLGFIVWAHHMFTVGMDVD
Hogi  PEVYILILPGFGMISHIVAYYSGKKEPFGYMGMVWAMMAIGLLGFIVWAHHMFTVGMDVD
Erzo  PEVYILILPGFGMISHIVAYYSGKKEPFGYMGMVWAMMAIGLLGFIVWAHHMFTVGMDVD
Hxot  PEVYILILPGFGMVSHIVAYYSGKKEPFGYMGMVWAMMAIGLLGFIVWAHHMFTVGMDVD
Core  PEVYILILPGFGMVSHIVAYYSGKKEPFGYMGMVWAMMAIGLLGFIVWAHHMFTVGMDVD
Apve  PEVYILILPGFGMVSHIVAYYSGKKEPFGHMGMVWAMMAIGFLGFIVWAHHMFTVGMDVD
Latj  PEVYILILPGFGMISHVVAYYCGKKEPFGYMGMVWAMMAIGLLGFIVWAHHMFTVGMDVD
Laja  PEVYILILPGFGMISHIVAYYCGKEEPFGYMGMVWAMMAIGLLGFIVWAHHMFTVGMDVD
```

[5/10 of aligned sequences]

```
Syja  PEVYILILPGFGMISHIVAYYAGKKEPFGYMGMVWAMMAIGLLGFIVWAHHMFTVGMDVD
Epme  PEVYILILPGFGMISHIVAYYSGKKEPFGYMGMVWAMMAIGLLGFIVWAHHMFTVGMDVD
Grse  PEVYILILPGFGMISHIVAYYAGKKEPFGYMGMVWAMMAIGLLGFIVWAHHMFTVGMDVD
Clja  PEVYILILPGFGMISHIVAYYSGKKEPFGYMGMVWAMMAIGLLGFIVWAHHMFTVGMDVD
Ogcy  PEVYILILPGFGMISHIVAYYSGKKEPFGYMGMVWAMMAIGLLGFIVWAHHMFTVGMDVD
Plna  PEVYILILPGFGMISHIVAYYAGKKEPFGYMGMVWAMMAIGLLGFIVWAHHMFTVGMDVD
Lema  PEVYILILPGFGMISHIVAYYSGKKEPFGYMGMVWAMMAIGLLGFIVWAHHMFTVGMDVD
Etzo  PEVYILILPGFGMISHIVAYYAGKKEPFGYMGMVWAMMAIGLLGFIVWAHHMFTVGMDVD
Apse  PEVYILILPGFGMISHIVAYYAGKKEPFGYMGMVWAMMAIGLLGFIVWAHHMFTVGMDVD
Epde  PEVYILILPGFGMISHIVAYYAGKKEPFGYMGMVWAMMAIGLLGFIVWAHHMFTVGMDVD
Slja  PEVYILILPGFGMISHIVAYYAGKKEPFGYMGMVWAMMAIGLLGFIVWAHHMFTVGMDVD
Bsja  PEVYILILPGFGMISHIVAYYSGKKEPFGYMGMVWAMMAIGLLGFIVWAHHMFTVGMDVD
Ecna  PEVYILILPGFGMISHIVAYYCGKKEPFGYMGMVWAMMAIGLLGFIVWAHHMFTVGMDVD
Cohi  PEVYILILPGFGMISHIVAYYAGKKEPFGYMGMVWAMMAIGLLGFIVWAHHMFTVGMDVD
Caar  PEVYILILPGFGMISHIVAYYAGKKEPFGYMGMVWAMMAIGLLGFIVWAHHMFTVGMDVD
Came  PEVYILILPGFGMISHIVAYYSGKKEPFGYMGMVWAMMAIGLLGFIVWAHHMFTVGMDVD
Mema  PEVYILILPGFGMISHIVAYYAGKKEPFGYMGMVWAMMAIGLLGFIVWAHHMFTVGMDVD
Lenu  PEVYILILPGFGMISHIVAYYTGKKEPFGYMGMVWAMMAIGLLGFIVWAHHMFTVGMDVD
Brja  PEVYILILPGFGMISHIVAYYSGKKEPFGYMGMVWAMMAIGLLGFIVWAHHMFTVGMDVD
Plma  PEVYILILPGFGMISHIVAYYSGKKEPFGYMGMVWAMMAIGLLGFIVWAHHMFTVGMDVD
Emst  PEVYILILPGFGMISHIVAYYSGKKEPFGYMGMVWAMMAIGLLGFIVWAHHMFTVGMDVD
Ptti  PEVYILILPGFGMISHIVAYYAGKKEPFGYMGMVWAMMAIGLLGFIVWAHHMFTVGMDVD
Losu  PEVYILILPGFGMISHIVAYYSGKKEPFGYMGMVWAMMAIGLLGFIVWAHHMFTVGMDVD
Geoy  PEVYILILPGFGMVSHIVAYYSGKKEPFGYMGMVWAMMAIGLLGFIVWAHHMFTVGMDVD
Dipi  PEVYILILPGFGIISHIVAYYAGKKEPFGYMGMVWAMMAIGLLGFIVWAHHMFTVGMDVD
Pama  PEVYILILPGFGMISHIVAYYSGKKEPFGYMGMVWAMMAIGLLGFIVWAHHMFTVGMDVD
Leob  PEVYILILPGFGMISHIVAYYSGKKEPFGYMGMVWAMMAIGLLGFIVWAHHMFTVGMDVD
Neba  PEVYILILPGFGIISHVVAYYAGKKEPFGYMGMVWAMMAIGLLGFIVWAHHMFTVGMDVD
Pdpl  PEVYILILPGFGIISHVVAYYCGKKEPFGYMGMVWAMMAIGLLGFIVWAHHMFTVGMDVD
Nimi  PEVYILILPGFGMVSHIVAYYSGKKEPFGYMGMVWAMMAIGLLGFIVWAHHMFTVGMDVD
Uptr  PEVYILILPGFGMISHIVAYYAGKKEPFGYMGMVWAMMAIGLLGFIVWAHHMFTVGMDVD
Pesc  PEVYILILPGFGMISHIVAYYAGKKEPFGYMGMVWAMLAIGLLGFIVWAHHMFTVGMDVD
Baar  PEVYILILPGFGMISHIVAYYSGKKEPFGHMGMVWAMMAIGLLGFIVWAHHMFTVGMDID
Moar  PEVYILILPGFGMISHIVAYYSGKKEPFGYMGMVWAMMAIGLLGFIVWAHHMFTVGMDVD
Toja  PEVYILILPGFGMISHIVAYYSGKKEPFGYMGMVWAMMAIGLLGFIVWAHHMFTVGMDVD
Chau  PEVYILILPGFGMISHIVAYYSGKKEPFGYMGMVWAMMAIGLLGFIVWAHHMFTVGMDVD
Chse  PEVYILILPGFGMISHIVAYYSGKKEPFGYMGMVWAMMAIGLLGFIVWAHHMFTVGMDVD
Enar  PEVYILILPGFGMISHIVAYYSGKKEPFGYMGMVWAMMAIGLLGFIVWAHHMFTVGMDVD
Hpty  PEVYILILPGFGMISHIVAYYSGKKEPFGYMGMVWAMMAIGLLGFIVWAHHMFTVGMDVD
Nana  PEVYILILPGFGMVSHIVAYYCGKKEPFGYMGMVWAMMAIGLLGFIVWAHHMFTVGMDVD
Mcst  PEVYILILPGFGMISHIVAYYSGKKEPFGYMGMVWAMMAIGLLGFIVWAHHMFTVGMDVD
Rhox  PEVYILILPGFGMISHIVAYYSGKKEPFGYMGMVWAMMAIGLLGFIVWAHHMFTVGMDVD
Opfa  PEVYILILPGFGMISHIVAYYSGKKEPFGYMGMVWAMMAIGLLGFIVWAHHMFTVGMDVD
Paar  PEVYILILPGFGMISHIVAYYSGKKEPFGYMGMVWAMMAIGLLGFIVWAHHMFTVGMDVD
Gozo  PEVYILILPGFGMISHIVAYYSGKKEPFGYMGMVWAMMAIGLLGFIVWAHHMFTVGMDVD
Ackr  PEVYILILPGFGMISHIITYYSGKTEPFGYMGMVWAMVAIGLLGFIVWAHHMFTVGMDVD
Elev  PEVYILILPGFGMISHIVAYYSGKKEPFGYMGMVWAMMAIGLLGFIVWAHHMFTVGMDVD
Trdu  PEVYILILPGFGMISHIVAYYAGKKEPFGYMGMVWAMMAIGLLGFIVWAHHMFTVGMDVD
Amoc  PEVYILILPGFGMISHIVAYYSGKKEPFGYMGMVWAMMAIGLLGFIVWAHHMFTVGMDVD
Hame  PEVYILILPGFGMISHIVAYYSGKKEPFGYMGMVWAMMAIGLLGFIVWAHHMFTVGMDVD
Chso  PEVYILILPGFGMISHIVAYYSGKKEPFGYMGMVWAMMAIGLLGFIVWAHHMFTVGMDVD
Lyto  PEVYILILPGFGMISHIVAYYSGKKEPFGYMGMVWAMMAIGLLGFIVWAHHMFTVGMDVD
```

To be continued on page 29.

[5/10 of aligned sequences]

```
Encr   PEVYILILPGFGMISHIVAYYSGKKEPFGYMGMVWAMMAIGLLGFIVWAHHMFTVGMDVD
Bvar   PEVYILILPGFGMISHIVAYYAGKKEPFGYMGMVWAMMAIGLLGFIVWAHHMFTVGMDVD
Noco   PEVYILILPGFGMISHIVAYYAGKQEPFGYMGMVWAMMAIGLLGFIVWAHHMFTVGMDVD
Chsp   PEVYILIIPGFGMVSHVVTYYAGKKEPFGYMGMAWAMMSIGLLGFIVWAHHMFTIGMDVD
Arja   PEVYILILPGFGMVSHIVAYYSGKKEPFGYMGMVWAMMAIGLLGFIVWAHHMFTVGMDVD
Pase   PEVYILILPGFGMISHIVAYYSGKKEPFGYMGMVWAMMAIGLLGFIVWAHHMFTVGMDVD
Trel   PEVYILILPGFGMISHIVAYYSGKKEPFGYMGMVWAMMAIGLLGFIVWAHHMFTVGMDVD
Lifa   PEVYILILPGFGMISHIVAYYSGKKEPFGYMGMVWAMMAIGLLGFIVWAHHMFTVGMDVD
Acur   PEVYILILPGFGMISHIVAYYSGKKEPFGYMGMVWAMMAIGLLGFIVWAHHMFTVGMDVD
Ampe   PEVYILILPGFGMISHIVAYYSGKKEPFGYMGMVWAMMAIGLLGFIVWAHHMFTVGMDVD
Urja   PEVYILILPGFGMVSHIVAYYSDKKEPFGYMGMVWAMLAIGLLGFIVWAHHMFTVGMDVD
Enet   PEVYILILPGFGMISHIVAYYSGKKEPFGYMGMVWAMMAIGLLGFIVWAHHMFTVGMDVD
Ptbr   PEVYILILPGFGMISHIVAFYSGKKEPFGYMGMVWAMMAIGLLGFIVWAHHMFTVGMDVD
Safa   PEVYILILPGFGMISHIVAYYSGKKEPFGYMGMVWAMMAIGLLGFIVWAHHMFTVGMDVD
Icae   PEVYILILPGFGMISHIVAYYSGKKEPFGYMGMVWAMMAIGLLGFIVWAHHMFTVGMDVD
Asmi   PEVYILILPGFGMISHIVTHYSGKKEPFGYMGMVWAMVAIGLLGFIVWAHHMFTVGMDVD
Foal   PEVYILILPGFGMISHIVAYYAGKKEPFGYMGMVWAMMAIGLLGFIVWAHHMFTVGMDVD
Drze   PEVYILILPGFGMISHIVAYYAGKKEPFGYMGMVWAMMAIGLLGFIVWAHHMFTVGMDVD
Rhas   PEVYILILPGFGMISHIVAYYSGKKEPFGYMGMVWAMMAIGLLGFIVWAHHMFTVGMDVD
Elac   PEVYILILPGFGMISHIVAYYAGKKEPFGYMGMVWAMMAIGLLGFIVWAHHMFTVGMDVD
Kugu   PEVYILILPGFGMISHIVAYYAGKKEPFGYMGMVWAMMAIGLLGFIVWAHHMFTVGMDVD
Plor   PEVYILILPGFGMISHIVAYYSGKKEPFGYMGMVWAMMAIGLLGFIVWAHHMFTVGMDVD
Sgun   PEVYILILPGFGMISHIVAYYSGKKEPFGYMGMVWAMMAIGLLGFIVWAHHMFTVGMDVD
Zaco   PEVYILILPGFGMISHIVAYYAGKKEPFGYMGMVWAMMAIGLLGFIVWAHHMFTVGMDVD
Zbfl   PEVYILILPGFGMISHIVAYYSGKKEPFGYMGMVWAMMAIGLLGFIVWAHHMFTVGMDVD
Spba   PEVYILILPGFGMISHIVAYYSGKKEPFGYMGMVWAMMAIGLLGFIVWAHHMFTVGMDVD
Game   PEVYILILPGFGMISHIVAYYSGKKEPFGYMGMVWAMMAIGLLGFIVWAHHMFTVGMDVD
Thth   PEVYILILPGFGMISHIVAYYSGKKEPFGYMGMVWAMMAIGLLGFIVWAHHMFTVGMDVD
Xigl   PEVYILILPGFGMISHIVAYYSGKKEPFGYMGMVWAMMAIGLLGFIVWAHHMFTVGMDVD
Hyja   PEVYILILPGFGMISHIVAYYSGKKEPFGYMGMVWAMMAIGLLGFIVWAHHMFTVGMDVD
Psan   PEVYILILPGFGMISHIVAYYSGKKEPFGYMGMVWAMMAIGLLGFIVWAHHMFTVGMDVD
Cupa   PEVYILILPGFGMISHIVAYYAGKKEPFGYMGMVWAMMAIGLLGFIVWAHHMFTVGMDVD
Mpch   PEVYILILPGFGMISHIVAYYAGKKEPFGYMGMVWAMMAIGLLGFIVWAHHMFTVGMDVD
Char   PEVYILILPGFGMISHIVAYYSGKKEPFGYMGMVWAMMAIGLLGFIVWAHHMFTVGLDVD
Pser   PEVYILILPGFGMISHIVAYYSGKKEPFGYMGMVWAMMAIGLLGFIVWAHHMFTVGMDVD
Prol   PEVYILILPGFGMISHIVAYYSGKKEPFGYMGMVWAMMAIGLLGFIVWAHHMFTVGMDVD
Plbi   PEVYILILPGFGMISHIVAYYAGKKEPFGYMGMVWAMMAIGLLGFIVWAHHMFTVGMDVD
Calu   PEVYILILPGFGMISHIVAYYAGKKVPFGYMGMVWAMMAIGLLGFIVWAHHMFTVGMDVD
Papa   PEVYILILPGFGMISHIVAYYAGKKEPFGYMGMVWAMMAIGLLGFIVWAHHMFTVGMDVD
Sufr   PEVYILILPGFGMISHIVAYYSGKKEPFGYMGMVWAMMAIGLLGFIVWAHHMFTVGMDVD
Stci   PEVYILILPGFGMISHIVAYYSGKKEPFGYMGMVWAMMAIGLLGFIVWAHHMFTVGMDVD
Taru   PEVYILILPGFGMISHIVAYYSGKKEPFGYMGMVWAMMAIGLLGFIVWAHHMFTVGMDVD
Rala   PEVYILILPGFGMISHIVAYYSGKKEPFGYMGMVWAMMAIGLLGFIVWAHHMFTVGMDVD
       *******:****::**::::  *   **  ** *** :** ***:********:*:*:*
```

To be continued on page 30.

[6/10 of aligned sequences]

```
                    H                                   I
Scca  TRAYFTSATMIIAIPTGVKVFSWLATLHGGSIKWETPLLWALGFIFLFTVGGLTGIVLAN
Muma  TRAYFTSATMIIAIPTGVKVFSWLATLHGGSIKWDTPLLWALGFIFLFTVGGLTGIVLAN
Erca  TRAYFTSATMIIAIPTGVKVFSWLATLHGGAIKWETPMLWALGFIFLFTVGGLTGIILAN
Pose  TRAYFTSATMIIAIPTGVKVFSWLATLHGGAIKWETPMLWALGFIFLFTVGGLTGIILAN
Actr  TRAYFTSATMIIAIPTGVKVFSWLATLHGGSIKWDTPLLWALGFIFLFTVGGLTGIVLAN
Scal  TRAYFTSATMIIAIPTGVKVFSWLATLHGGSIKWDTPLLWALGFIFLFTVGGLTGIVLAN
Posp  TRAYFTSATMIIAIPTGVKVFSWLATLHGGSIKWDTPLLWALGFIFLFTVGGLTGIVLAN
Atsp  TRAYFTSATMIIAIPTGVKVFSWLATLHGGSIKWDTPLLWALGFIFLFTVGGLTGIVLAN
Leoc  TRAYFTSATMIIAIPTGVKVFSWLATLHGGSIKWDTPLLWALGFIFLFTVGGLTGIVLAN
Amca  TRAYFTSATMVIAIPTGVKVFSWLATLHGGAIKWETPLLWALGFIFLFTVGGLTGIVLAN
Osbi  TRAYFTSATMIIAIPTGVKVFSWLATLYGGSIKWEAPFLWALGFIFLFTVGGLTGIILAN
Pabu  TRAYFTSATMIIAIPTGVKVFSWLATLHGGSIKWDTPMLWALGFIFLFTVGGLTGIILAN
Hial  TRAYFTSATMIIAIPTGVKVFSWLATLHGGSIKWDTPLLWALGFIFLFTVGGLTGIVLAN
Elha  TRAYFTSATMIIAIPTGVKVFSWLATLHGGSIKWDTPLLWALGFIFLFTVGGLTGIVLAN
Mlcy  TRAYFTSATMIIAIPTGVKVFSWLATLHGGSIKWDTPLLWALGFIFLFTVGGLTGIVLAN
Algl  TRAYFTSATMIIAIPTGVKVFSWLATLHGGSIKWDTPLLWALGFIFLFTVGGLTGIILSN
Ptgi  TRAYFTSATMIIAIPTGVKVFSWLATLHGGSIKWDTPLLWALGFIFLFTVGGLTGIVLAN
Alaf  TRAYFTSATMIIAIPTGVKVFSWLATLHGGSIKWDTPLLWALGFIFLFTVGGLTGIVLAN
Nock  TRAYFTSATMIIAIPTGVKVFSWLATLHGGSIKWDTPLLWALGFIFLFTVGGLTGIVLAN
Anja  TRAYFTSATMIIAIPTGVKVFSWLATLHGGVIKWETPLLWALGFIFLFTVGGLTGIVLAN
Gyki  TRAYFTSATMIIAIPTGVKVFSWLATLHGGQIKWETPLLWALGFIFLFTVGGLTGIVLAN
Syka  TRAYFTSATMIIAIPTGVKVFSWLATLHGGAIKWETPLLWALGFIFLFTVGGLTGIVLAN
Opma  TRAYFTSATMIIAIPTGVKVFSWLATLHGGAIKWETPMLWALGFIFLFTVGGLTGIVLAN
Comy  TRAYFTSATMIIAIPTGVKVFSWLATLHGGTIKWDTPLLWALGFIFLFTVGGLTGIVLAN
Sasp  TRAYFTSATMIIAIPTGVKVFSWLATLHGGAIKWEAPLLWALGFIFLFTEGGLTGVVLAN
Eupe  TRAYFTSATMIIAIPTGVKVFSWLATLHGGAIKWETPLLWALGFIFLFTVGGLTGIVLAN
Enja  TRAYFTSATMIIAIPTGVKVFSWLATLHGGAIKWETPMLWALGFIFLFTVGGLTGIVLAN
Same  TRAYFTSATMIIAIPTGVKVFSWLATLHGGSIKWDTPLLWALGFIFLFTVGGLTGIVLAN
Chch  TRAYFTSATMIIAIPTGVKVFSWLATLHGGSIKWETPLLWALGFIFLFTVGGLTGIVLAN
Grgr  TRAYFTSATMIIAIPTGVKVFSWLATLHGGSIKWETPMLWALGFIFLFTVGGLTGIVLAN
Caau  TRAYFTSATMIIAIPTGVKVFSWLATLHGGSIKWETPMLWALGFIFLFTVGGLTGIVLSN
Cyca  TRAYFTSATMIIAIPTGVKVFSWLATLHGGSIKWETPMLWALGFIFLFTVGGLTGIVLSN
Dare  TRAYFTSATMIIAIPTGVKVFSWLATLHGGAIKWETPMLWALGFIFLFTVGGLTGIVLAN
Cost  TRAYFTSATMIIAIPTGVKVFSWLATLHGGSIKWETPLLWALGFIFLFTVGGLTGIVLAN
Leec  TRAYFTSATMIIAIPTGVKVFSWLATLHGGSIKWETPMLWALGFIFLFTVGGLTGIVLAN
Fola  TRAYFTSATMIIAIPTGVKVFSWLATLHGGTIKWDTPMLWALGFIFLFTVGGLTGIVLSN
Clmc  TRAYFTSATMIIAIPTGVKVFSWLATLHGGSIKWETPLLWALGFIFLFTVGGLTGIVLAN
Phin  TRAYFTSATMIIAIPTGVKVFSWLATLHGGLIKWDTPMLWALGFIFLFTVGGLTGIVLAN
Icpu  NRAYFTSATMIIAIPTGVKVFSWLATLHGGSIKWETPLLWALGFIFLFTVGGLTGIVLAN
Psto  TRAYFTSATMIIAIPTGVKVFSWLATLHGGSIKWETPMLWALGFIFLFTVGGLTGIVLAN
Cora  TRAYFTSATMIIAIPTGVKVFSWLATLHGGSIKWETPLLWALGFIFLFTVGGLTGIVLSN
Eisp  TRAYFTSATMIIAIPTGVKVFSWLATLHGGSIKWETPLLWALGFIFLFTVGGLTGIVLAN
Apal  TRAYFTSATMIIAIPTGVKVFSWLATLHGGSIKWETPLLWALGFIFLFTVGGLTGIILSN
Eslu  TRAYFTSATMIIAIPTGVKVFSWLATLHGGSIKWETPLLWALGFIFLFTVGGLTGIVLAN
Dape  TRAYFTSATMIIAIPTGVKVFSWLATLHGGSIKWETPLLWALGFIFLFTVGGLTGIVLAN
Glse  TRAYFTSATMIIAIPTGVKVFSWLATLHGGSIKWETPLLWALGFIFLFTVGGLTGIVLAN
Naar  TRAYFTSATMIIAIPTGVKVFSWLATLHGGSIKWETPLLWALGFIFLFTVGGLTGIVLAN
Lioc  TRAYFTSATMIIAIPTGVKVFSWLATLHGGSIKWETPLLWALGFIFLFTVGGLTGIVLAN
Opso  TRAYFTSATMIIAIPTGVKVFSWLATLHGGSIKWETPLLWALGFIFLFTVGGLTGIVLAN
Alte  TRAYFTSATMIIAIPTGVKVFSWLATLHGGSIKWETPLLWALGFIFLFTVGGLTGIVLSN
Plap  TRAYFTSATMIIAIPTGVKVFSWLATLHGGSIKWETPLLWALGFIFLFTVGGLTGIVLSN
```

To be continued on page 31.

Plal	TRAYFTSATMIIAIPTGVKVFSWLATLHGGSIKWETPLLWALGFIFLFTVGGLTGIVLAN
Sami	TRAYFTSATMIIAIPTGVKVFSWLATLHGGSIKWETPLLWALGFIFLFTVGGLTGIVLAN
Rere	TRAYFTSATMIIAIPTGVKVFSWLATLHGGSIKWETPLLWALGFIFLFTVGGLTGIVLAN
Gama	TRAYFTSATMIIAIPTGVKVFSWLATLHGGSIKWETPLLWALGFIFLFTVGGLTGIVLAN
Onmy	TRAYFTSATMIIAIPTGVKVFSWLATLHGGSIKWETPLLWALGFIFLFTVGGLTGIVLAN
Sasa	TRAYFTSATMIIAIPTGVKVFSWLATLHGGSIKWETPLLWALGFIFLFTVGGLTGIVLAN
Cola	TRAYFTSATMIIAIPTGVKVFSWLATLHGGSIKWETPLLWALGFIFLFTVGGLTGIVLAN
Dita	TRAYFTSATMIIAIPTGVKVFSWLATLHGGSIKWETPLLWALGFIFLFTVGGLTGIVLAN
Gogr	TRAYFTSATMIIAIPTGVKVFSWLATLHGGSIKWETPLLWALGFIFLFTVGGLTGIILAN
Chsl	TRAYFTSATMIIAIPTGVKVFSWLATLNGGAIKWETPMLWSLGFVFLFTVGGLTGIVLAN
Atja	TRAYFTSATMIIAIPTGVKVFSWLATLHGGSIKWETPMLWALGFIFLFTVGGLTGIVLAN
Iido	TRAYFTSATMIIAIPTGVKVFSWLATLHGGSIKWETPMLWALGFIFLFTVGGLTGIVLAN
Auja	TRAYFTSATMIIAIPTGVKVFSWLATLHGGSIKWETPLLWALGFIFLFTVGGLTGIILAN
Chag	TRAYFTSATMIIAIPTGVKVFSWLATLHGGTIKWETPMLWALGFIFLFTIGGLTGIVLSN
Hami	TRAYFTSATMIIAIPTGVKVFSWLATLHGGSIKWETPLLWALGFIFLFTVGGLTGIVLAN
Saun	TRAYFTSATMIIAIPTGVKVFSWLATLHGGAIKWETPLLWALGFIFLFTVGGLTGIVLAN
Nema	TRAYFTSATMIIAIPTGVKVFSWLATLHGGSIKWETPLLWALGFIFLFTVGGLTGIVLAN
Disp	TRAYFTSATMIIAIPTGVKVFSWLATLHGGTIKWETPMLWALGFIFLFTVGGLTGIVLAN
Myaf	TRAYFTSATMIIAIPTGVKVFSWLATLHGGTIKWDTPMLWALGFIFLFTVGGLTGIVLAN
Lagu	TRAYFTSATMIIAIPTGVKVFSWLATLHGGTIKWETPLLWALGFIFLFTVGGLTGIVLAN
Trtr	TRAYFTSATMIIAIPTGVKVFSWLATLHGGSIKWETPLLWALGFIFLFTVGGLTGIVLAN
Zucr	TRAYFTSATMIIAIPTGVKVFSWLATLHGGSIKWETPLLWALGFIFLFTVGGLTGIVLAN
Pxja	TRAYFTSATMIIAIPTGVKVFSWLATLHGGSIKWETPLLWALGFIFLFTVGGLTGIVLAN
Pxlo	TRAYFTSATMIIAIPTGVKVFSWLATLHGGSIKWETPLLWALGFIFLFTVGGLTGIVLAN
Pctr	TRAYFTSATMIIAIPTGVKVFSWLATLHGGSIKWETPLLWALGFIFLFTVGGLTGIVLAN
Apsa	TRAYFTSATMIIAIPTGVKVFSWLATLHGGSIKWETPMLWALGFIFLFTVGGLTGIVLAN
Cabe	TRAYFTSATMIIAIPTGVKVFSWLATLHGGAIKWDTPMLWSLGFIFLFTVGGLTGIVLAN
Bzze	TRAYFTSATMIIAIPTGVKVFSWLATLHGGSIKWETPLLWALGFIFLFTVGGLTGIVLAN
Siim	TRAYFTSATMIIAIPTGVKVFSWLATLHGSFIKWDAPMLWALGFIFLFTVGGLTGIILAN
Ctru	TRAYFTSATMIIAIPTGVKVFSWLATLHGGNIKWDTPLLWALGFIFLFTVGGLTGIVLAN
Dpbr	TRAYFTSATMIIAIPTGVKVFSWLATLHGGSIKWDTPLLWALGFIFLFTVGGLTGIVLAN
Caki	TRAYFTSATMIIAIPTGVKVFSWLATLHGGSIKWDTPLLWALGFIFLFTVGGLTGVVLAN
Phja	TRAYFTSATMVIAIPTGVKVFSWLATLHGSTIKWDAAFLWALGFIFLFTVGGLTGIILAN
Brsp	TRAYFTSATMIIAIPTGVKVFSWLATLYGGTMKWNTPILWSLGFIFLFTVGGLTGVILAN
Gamo	TRAYFTSATMIIAIPTGVKVFSWLATLHGGSIKWETPLLWALGFIFLFTVGGLTGIVLAN
Lolo	TRAYFTSATMIIAIPTGVKVFSWLATLHGGSIKWETPLLWALGFIFLFTVGGLTGIVLAN
Batr	TRAYFTSATMIIAIPTGIKIFSWLATLYGGTLKWEAPLLWALGFIFLFTVGGLTGIVLAN
Prmy	TRAYFTSATMVIAIPTGVKVFSWLATLHGGTISWEPPLLWALGFIFLFTVGGLTGIILAN
Lose	TRAYFTSATMIIAIPTGVKVFSWLATLHGGAIKWETPLLWALGFIFLFTVGGLTGIVLSN
Loam	TRAYFTSATMIIAIPTGVKVFSWLATLHGGAIKWETPLLWALGFIFLFTVGGLTGIVLAN
Chab	TRAYFTSATMIIAIPTGVKVLSWLATLHGAKIKWEALLLWALGFIFLFTVGGLTGIVLAN
Chto	TRAYFTSATMIIAIPTGVKVFSWLATLHGAKIKWEAPLLWALGFIFLFTVGGLTGIVLAN
Majo	TRAYFTSATMIIAIPTGVKVFSWLATLHGGFIKWETPLLWALGFIFLFTVGGLTGIVLAN
Hlst	TRAYFTSATMIIAIPTGVKVFSWLATLHGGHIKWETPLLWALGFIFLFTVGGLTGIVLAN
Clpe	TRAYFTSATMIIAIPTGVKVFTWLATLHGGSIKSETPMLWALGFIFLFTVGGLTGIVLAN
Mlmr	TRAYFTSATMIIAIPTGVKVFSWLATLHGGAIKWETPLLWALGFIFLFTVGGLTGIVLAN
Crcr	TRAYFTSATMIIAIPTGVKVFSWLATLHGGSIKWETPLLWALGFIFLFTVGGLTGIVLAN
Muce	TRAYFTSATMIIAIPTGVKVFSWLATLHGGSIKWETPLLWALGFIFLFTVGGLTGIVLAN
Bege	TRAYFTSATMIIAIPTGVKVFSWLATLHGGAIKWETPLLWALGFIFLFTVGGLTGIVLAN
Mela	TRAYFTSATMIIAIPTGVKVFSWLATLHGGAIKWETPLLWALGFIFLFTVGGLTGIVLAN
Hats	TRAYFTSATMIIAIPTGVKVFSWLATLHGGAVKWETPLLWALGFIFLFTVGGLTGIILAN
Orla	TRAYFTSATMIIAIPTGVKVFSWLATLHGGSIKWETPLLWALGFIFLFTVGGLTGIVLAN

To be continued on page 32.

[6/10 of aligned sequences]

```
Cosa    TRAYFTSATMIIAIPTGVKVFSWLATLHGGSIKWETPLLWALGFIFLFTVGGLTGIVLAN
Exsp    TRAYFTSATMIIAIPTGVKVFSWLATLHGGTIKWETPLLWALGFIFLFTVGGLTGIVLAN
Depa    TRAYFTSATMIIAIPTGVKVFSWLATLHGGAIKWETPLLWALGFIFLFTVGGLTGIVLAN
Rima    TRAYFTSATMIIAIPTGVKVFSWLATLHGGAIKWETPLLWALGFIFLFTVGGLTGIVLAN
Fuol    TRAYFTSATMIIAIPTGVKVFSWLATLHGGSIKWETPLLWALGFIFLFTVGGLTGIVLAN
Gmaf    TRAYFTSATMIIAIPTGVKVFSWLATLHGGALKWDTPLLWALGFIFLFTVGGLTGIILAN
Xeei    TRAYFTSATMIIAIPTGVKVFSWLATLHGGAIKWETPLLWALGFIFLFTVGGLTGIVLAN
Pros    TRAYFTSATMIIAIPTGVKVFSWLATLHGGTIKWETPLLWALGFIFLFTVGGLTGIVLSN
Scmi    TRSYFTSATMIIAIPTGMKVFSWLATLHGGSIKWETPLLWALGFIFLFTVGGLTGIILSN
Rolo    TRAYFTSATMIIAIPTGVKVFSWLATLHGGAIKWETPLLWALGFIFLFTVGGLTGIVLAN
Cere    TRAYFTSATMIIAIPTGVKVFSWLATLHGGSIKWETPLLWALGFIFLFTVGGLTGIVLAN
Daga    TRAYFTSATMIIAIPTGVKVFSWLATLHGGSIKWETPLLWALGFIFLFTVGGLTGIVLAN
Anco    TRAYFTSATMIIAIPTGVKVFSWLATLHGGSIKWETPLLWALGFIFLFTVGGLTGIVLAN
Dmve    TRAYFTSATMIIAIPTGVKVFSWLATLHGGHIKWETPLLWALGFIFLFTVGGLTGIILAN
Dmar    TRAYFTSATMIIAIPTGVKVFSWLATLHGGHIKWETPLLWALGFIFLFTVGGLTGIILAN
Anka    TRAYFTSATMIIAIPTGVKVFSWLATLHGGAIKWETPLLWALGFIFLFTVGGLTGIVLAN
Moja    TRAYFTSATMIIAIPTGVKVFSWLATLHGGAIKWETPLLWALGFIFLFTVGGLTGIVLAN
Hoja    TRAYFTSATMIIAIPTGVKVFSWLATLHGGAIKWETPLLWALGFIFLFTVGGLTGIVLAN
Bede    TRAYFTSATMIIAIPTGVKVFSWLATLHGGSIKWETPLLWALGFIFLFTVGGLTGIVLAN
Besp    TRAYFTSATMIIAIPTGVKVFSWLATLHGGSIKWETPLLWALGFIFLFTVGGLTGIVLAN
Mysp    TRAYFTSATMIIAIPTGVKVFSWLATLHGGSIKWETPLLWALGFIFLFTVGGLTGIVLAN
Osja    TRAYFTSATMIIAIPTGVKVFSWLATLHGGSIKWETPLLWALGFIFLFTVGGLTGIVLAN
Sgro    TRAYFTSATMIIAIPTGVKVFSWLATLHGGAIKWETPLLWALGFIFLFTVGGLTGIVLAN
Pzpa    TRAYFTSATMIIAIPTGVKVFSWLATLHGGSVKWETPLLWALGFIFLFTVGGLTGIVLAN
Zeja    TRAYFTSATMIIAIPTGVKVFSWLATLHGGSIKWETPLLWALGFIFLFTVGGLTGIVLAN
Znne    TRAYFTSATMIIAIPTGVKVFSWLATLHGGSIKWETPLLWALGFIFLFTVGGLTGIVLAN
Zefa    TRAYFTSATMIIAIPTGVKVFSWLATLHGGSIKWETPLLWALGFIFLFTVGGLTGIVLAN
Acni    TRAYFTSATMIIAIPTGVKVFSWLATLHGGSIKWETPLLWALGFIFLFTVGGLTGIVLAN
Ncrh    TRAYFTSATMIIAIPTGVKVFSWLATLHGGSIKWETPLLWALGFIFLFTVGGLTGIVLAN
Agca    TRAYFTSATMIIAIPTGVKVFSWLATLHGGNVKWDAPLLWALGFIFLFTVGGLTGIVLAN
Hydy    TRAYFTSATMIIAIPTGVKVFSWLATLHGGSIKWETPLLWALGFIFLFTVGGLTGIILAN
Gsac    TRAYFTSATMIIAIPTGVKVFSWLATLHGGSIKWETPLLWALGFIFLFTVGGLTGIVLAN
Pevo    TRAYFTSATMIIAIPTGVKVFSWLATLHGGSIKWETPMLWALGFIFLFTVGGLTGIVLAN
Hiku    TRAYFTSATMIIAIPTGVKVFSWLATLHGGSIKWETPLLWALGFIFLFTVGGLTGIVLAN
Inpa    TRAYFTSATMIIAIPTGVKVFSWLATLHGGTIKWETPMLWALGFIFLFTLGGLTGIVLAN
Auch    TRAYFTSATMIIAIPTGVKVFSWLATLHGGNIKWDTPLLWALGFIFLFTVGGLTGIVLAN
Fico    TRAYFTSATMIIAIPTGVKVFSWLATLHGGSIKWDTPLLWALGFIFLFTVGGLTGIVLAN
Macs    TRAYFTSATMIIAIPTGVKVFSWLATLHGGVIKWETPLLWALGFIFLFTVGGLTGIVLAN
Moal    TRAYFTSATMIIAIPTGVKVFSWLATLHGGQIKWETPLLWALGFIFLFTMGGLTGVVLAN
Syma    TRAYFTSATMIIAIPTGVKIFSWLATLHGGTIKWETPLLWALGFIFLFTMGGLTGVVLAN
Mafr    TRAYFTSATMIIAIPTGVKVFSWLATLHGGVIKWETPLLWALGFIFLFTVGGLTGIVLAN
Dcpe    TRAYFTSATMIIAIPTGVKVFSWLATLHGGSIKWETPLLWALGFIFLFTVGGLTGIVLAN
Dcti    TRAYFTSATMIIAIPTGVKVFSWLATLHGGAIKWETPLLWALGFIFLFTVGGLTGIVLAN
Hehi    TRAYFTSATMIIAIPTGVKVFSWLATLHGGSIKWETPLLWALGFIFLFTVGGLTGIVLAN
Stam    TRAYFTSATMIIAIPTGVKVFSWLATLHGGSIKWETPLLWALGFIFLFTVGGLTGIVLAN
Hogi    TRAYFTSATMIIAIPTGVKVFSWLATLHGGTVKWETPLLWALGFIFLFTVGGLTGIVLAN
Erzo    TRAYFTSATMIIAIPTGVKVFSWLATLHGGSIKWEAPLLWALGFIFLFTVGGLTGIILAN
Hxot    TRAYFTSATMIIAIPTGVKVFSWLATLHGGSIKWETPLLWALGFIFLFTVGGLTGIVLAN
Core    TRAYFTSATMIIAIPTGVKVFSWLATLHGGSIKWETPLLWALGFIFLFTVGGLTGIVLAN
Apve    TRAYFTSATMIIAIPTGVKVFSWLATLHGGSIKWETPLLWALGFIFLFTVGGLTGIILAN
Latj    TRAYFTSATMIIAIPTGVKVFSWLATIHGGNIKWDTPMLWALGFIFLFTVGGLTGIILAN
Laja    TRAYFTSATMIIAIPTGVKVFSWLATLHGGNIKWETPFLWALGFIFLFTVGGLTGIILAN
```

To be continued on page 33.

To be continued on page 34.

```
Syja TRAYFTSATMIIAIPTGVKVFSWLATLHGGNIKWETPLLWALGFIFLFTVGGLTGIILAN
Epme TRAYFTSATMIIAIPTGVKVFSWLATLHGGAIKWETPLLWALGFIFLFTVGGLTGIVLAN
Grse TRAYFTSATMIIAIPTGVKVFSWLATLHGGSIKWETPLLWALGFIFLFTVGGLTGIVLAN
Clja TRAYFTSATMIIAIPTGVKVFSWLATLHGGVIKWETPLLWALGFIFLFTVGGLTGIVLAN
Ogcy TRAYFTSATMIIAIPTGVKVFSWLATLHGGSIKWETPLLWALGFIFLFTVGGLTGIVLAN
Plna TRAYFTSATMIIAIPTGVKVFSWLATLHGGAIKWETPLLWALGFIFLFTVGGLTGIVLAN
Lema TRAYFTSATMIIAIPTGVKVFSWLATLHGASIKWETPLLWALGFIFLFTVGGLTGIVLAN
Etzo TRAYFTSATMIIAIPTGVKVFSWLATLHGGSIKWETPLLWALGFIFLFTVGGLTGIVLAN
Apse TRAYFTSATMIIAIPTGVKVFSWLATLHGGAIKWETPLLWALGFIFLFTVGGLTGIVLAN
Epde TRAYFTSATMIIAIPTGVKVFSWLATLHGGSVKWEAPLLWALGFIFLFTVGGLTGIILAN
Slja TRAYFTSATMIIAIPTGVKVFSWLATLHGGSIKWETPLLWALGFIFLFTVGGLTGIVLAN
Bsja TRAYFTSATMIIAIPTGVKVFSWLATLHGGALKWETPLLWALGFIFLFTVGGLTGIVLAN
Ecna TRAYFTSATMIIAIPTGVKVFSWLATIHGGDIKWETPFLWALGFIFLFTVGGLTGIVLAN
Cohi TRAYFTSATMIIAIPTGVKVFSWLATLHGGNLKWETPLLWALGFIFLFTVGGLTGIILAN
Caar TRAYFTSATMIIAIPTGVKVFSWLATLHGGSIKWETPMLWALGFIFLFTVGGLTGIVLAN
Came TRAYFTSATMIIAIPTGVKVFSWLATLHGGSIKWETPMLWALGFIFLFTVGGLTGIVLAN
Mema TRAYFTSATMIIAIPTGVKVFSWLATLHGGAIKWETPMLWALGFIFLFTVGGLTGIILAN
Lenu TRAYFTSATMIIAIPTGVKVFSWLATLHGGNIKWSAPLLWALGFIFLFTVGGLTGIVLSN
Brja TRAYFTSATMIIAIPTGVKVFSWLATLHGGTIKWDTPLLWAIGFIFLFTVGGLTGIVLAN
Plma TRAYFTSATMIIAIPTGVKVFSWLATLHGGSIKWETPLLWALGFIFLFTVGGLTGIVLAN
Emst TRAYFTSATMIIAIPTGVKVFSWLATLHGGSIKWETPLLWALGFIFLFTVGGLTGIVLAN
Ptti TRAYFTSATMIIAIPTGVKVFSWLATLHGGSIKWETPLLWALGFIFLFTVGGLTGIVLAN
Losu TRAYFTSATMIIAIPTGVKVFSWLATLHGGSIKWDTPLLWALGFIFLFTVGGLTGIVLAN
Geoy TRAYFTSATMIIAIPTGVKVFSWLATIHGGNLKWDTPLLWALGFIFLFTVGGLTGIVLAN
Dipi TRAYFTSATMIIAIPTGVKVFSWLATLHGGVIKWDTPLLWALGFIFLFTVGGLTGIVLAN
Pama TRAYFTSATMIIAIPTGVKVFSWLATLHGGTIKWDTPMLWALGFIFLFTVGGLTGIVLAN
Leob TRAYFTSATMIIAIPTGVKVFSWLATLHGGSIKWETPLLWALGFIFLFTVGGLTGIVLAN
Neba TRAYFTSATMIIAIPTGVKVFSWLATLHGASLKWDAPLLWALGFIFLFTVGGLTGIVLAN
Pdpl TRAYFTSATMIIAIPTGVKVFSWLATIHGGDIKWETPLLWALGFIFLFTVGGLTGIILAN
Nimi TRAYFTSATMIIAIPTGVKVFSWLATLHGGILKWEAAFLWALGFIFLFTVGGLTGIVLAN
Uptr TRAYFTSATMIIAIPTGVKVFSWLATLHGGSIKWDTPLLWALGFIFLFTVGGLTGIVLAN
Pesc TRAYFTSATMVIAIPTGVKVFSWLATFYGGRITWDAPLLWASGFIFLFTVGGLTGIILAN
Baar TRAYFTSATMIIAIPTGVKVFSWLATLHGGSIKWETPLLWALGFIFLFTVGGLTGIILAN
Moar TRAYFTSATMIIAIPTGVKVFSWLATLHGGAIKWETPLLWALGFIFLFTVGGLTGIVLAN
Toja TRAYFTSATMIIAIPTGVKVFSWLATLHGGSIKWETPMLWALGFIFLFTVGGLTGIVLAN
Chau TRAYFTSATMIIAIPTGVKVFSWLATLHGGAIKWETPLLWALGFIFLFTVGGLTGIVLAN
Chse TRAYFTSATMIIAIPTGVKVFSWLATLHGANIKWEAPLLWALGFIFLFTVGGLTGIILAN
Enar TRAYFTSATMIIAIPTGVKVFSWLATLHGGAVKWEAPLLWALGFIFLFTVGGLTGIILAN
Hpty TRAYFTSATMIIAIPTGVKVFSWLATLHGGSIKWETPLLWALGFIFLFTVGGLTGIILAN
Nana TRAYFTSATMIIAIPTGVKVFSWLATLHGGIIKWDTPFLWALGFIFLFTVGGLTGIVLAN
Mcst TRAYFTSATMIIAIPTGVKVFSWLATLHGESIKWETPLLWALGFIFLFTVGGLTGIVLAN
Rhox TRAYFTSATMIIAIPTGVKVFSWLATLHGGAVKWETPLLWALGFIFLFTVGGLTGIVLAN
Opfa TRAYFTSATMIIAIPTGVKVFSWLATLHGGAIKWETPLLWALGFIFLFTVGGLTGIVLAN
Paar TRAYFTSATMIIAIPTGVKVFSWLATLHGGVIKWETPLLWALGFIFLFTVGGLTGIVLAN
Gozo TRAYFTSATMIIAIPTGVKVFSWLATLHGGSIKWETPLLWALGFIFLFTVGGLTGIVLAN
Ackr TRAYFTSATMVIAIPTGVKVFSWLATLHGGALKWDAPLWWALGFIFLFTVGGLTGIILAN
Elev TRAYFTSATMIIAIPTGVKVFSWLATLHGASIKWETPLLWALGFIFLFTVGGLTGIVLAN
Trdu TRAYFTSATMIIAIPTGVKVFSWLATLHGGSIKWETPLLWALGFIFLFTVGGLTGIVLAN
Amoc TRAYFTSATMIIAIPTGVKVFSWLATLHGGTVKWDTPLLWALGFIFLFTVGGLTGIVLAN
Hame TRAYFTSATMIIAIPTGVKVFSWLATLHGGSIKWETPLLWALGFIFLFTVGGLTGIVLAN
Chso TRAYFTSATMIIAIPTGVKVFSWLATLHGGSIKWETPLLWALGFIFLFTVGGLTGIVLAN
Lyto TRAYFTSATMIIAIPTGVKVFSWLATLHGGSIKWETPLLWALGFIFLFTVGGLTGIILAN
```

[6/10 of aligned sequences]

To be continued on page 35.

```
Encr   TRAYFTSATMIIAIPTGVKVFSWLATLHGGSIKWETPLLWALGFIFLFTVGGLTGIILAN
Bvar   TRAYFTSATMIIAIPTGVKVFSWLATLHGGSIKWETPLLWALGFIFLFTVGGLTGIILAN
Noco   TRAYFTSATMIIAIPTGVKVFSWLATLHGGTIKWEAPLLWALGFIFLFTVGGLTGIVLAN
Chsp   TRAYFTSATMIIAIPTGIKVFSWLATLHGGNITWNTSLLWALGFIFLFTVGGLTGVILSN
Arja   TRAYFTSATMIIAIPTGVKVFSWLATLHGGSIKWETPLLWALGFIFLFTVGGLTGIVLAN
Pase   TRAYFTSATMIIAIPTGVKVFSWLATLHGGTVKWETPLLWALGFIFLFTVGGLTGIVLAN
Trel   TRAYFTSATMIIAIPTGVKVFSWLATLHGGTIKWETPMLWALGFIFLFTVGGLTGIVLAN
Lifa   TRAYFTSATMIIAIPTGVKVFSWLATLHGGNINWDTPLLWALGFIFLFTVGGLTGIILAN
Acur   TRAYFTSATMIIAIPTGVKVFSWLATLHGGSIKWETPLLWALGFIFLFTVGGLTGIILAN
Ampe   TRAYFTSATMIIAIPTGVKVFSWLATLHGGSIKWETPLLWALGFIFLFTVGGLTGIVLAN
Urja   TRAYFTSATMIIAIPTGVKVFSWLATLHGANIKWEAPLLWALGFIFLFTVGGLTGIILAN
Enet   TRAYFTSATMIIAIPTGVKVFSWLATLHGGAIKWETPLLWALGFIFLFTVGGLTGIVLAN
Ptbr   TRAYFTSATMIIAIPTGIKVFSWLATLHGASIKWETPLLWALGFIFLFTVGGLTGIVLAN
Safa   TRAYFTSATMIIAIPTGVKVFSWLATLHGGSIKWETPLLWALGFIFLFTVGGLTGIVLAN
Icae   TRAYFTSATMIIAIPTGVKVFSWLATLHGGVVKWETPLLWAIGFIFLFTVGGLTGIVLAN
Asmi   TRAYFTSATMVIAIPTGVKVFSWLATLHGGVIKWETPLLWALGFIFLFTVGGLTGIILAN
Foal   TRAYFTSATMIIAIPTGVKVFSWLATLHGGAIKWEAALLWALGFIFLFTVGGLTGIVLAN
Drze   TRAYFTSATMIIAIPTGVKVFSWLATLHGGTLKWDAPLMWALGFIFLFTVGGLTGIVLAN
Rhas   TRAYFTSATMIIAIPTGVKVFSWLATLHGGAIKWETPLLWALGFIFLFTVGGLTGIVLAN
Elac   TRAYFTSATMIIAIPTGVKVFSWLATLHGGAIKWETPLLWALGFIFLFTVGGLTGIVLAN
Kugu   TRAYFTSATMIIAIPTGVKVFSWLATLHGGNLKWETPLLWALGFIFLFTVGGLTGIVLAN
Plor   TRAYFTSATMIIAIPTGVKVFSWLATLHGGSIKWETPLLWALGFIFLFTVGGLTGIILAN
Sgun   TRAYFTSATMIIAIPTGVKVFSWLATLHGGAIKWETPLLWALGFIFLFTVGGLTGIVLAN
Zaco   TRAYFTSATMIIAIPTGVKVFSWLATLHGGSIKWETPLLWALGFIFLFTVGGLTGIVLAN
Zbfl   TRAYFTSATMIIAIPTGVKVFSWLATLHGGSIKWETPLLWALGFIFLFTVGGLTGIVLAN
Spba   TRAYFTSATMIIAIPTGVKVFSWLATLHGGSIKWETPMLWALGFIFLFTVGGLTGIVLAN
Game   TRAYFTSATMIIAIPTGVKVFSWLATLHGGAVKWETPLLWAIGFIFLFTVGGLTGIVLAN
Thth   TRAYFTSATMIIAIPTGVKVFSWLATLHGGAVKWETPLLWAIGFIFLFTVGGLTGIVLAN
Xigl   TRAYFTSATMIIAIPTGVKVFSWLATLHGGAIKWETPLLWALGFIFLFTVGGLTGIVLAN
Hyja   TRAYFTSATMIIAIPTGVKVFSWLATLHGGAIKWETPLLWALGFIFLFTVGGLTGIILAN
Psan   TRAYFTSATMIIAIPTGVKVFSWLATLHGGTIKWETPLLWALGFIFLFTVGGLTGIILAN
Cupa   TRAYFTSATMIIAIPTGVKVFSWLATLHGGAIKWETPLLWAIGFIFLFTVGGLTGIVLAN
Mpch   TRAYFTSATMIIAIPTGVKVFSWLATLHGGTIKWDTPLLWALGFIFLFTVGGLTGIVLAN
Char   TRAYFTSATMIIAIPTGVKVFSWLATLHGGAIKWETPLLWALGFIFLFTIGGLTGIVLAN
Pser   TRAYFTSATMIIAIPTGVKVFSWLATLHGGNIKWETPLLWALGFIFLFTVGGLTGIVLAN
Prol   TRAYFTSATMIIAIPTGVKVFSWLATLHGGNIKWETPLLWALGFIFLFTVGGLTGIVLAN
Plbi   TRAYFTSATMIIAIPTGVKVFSWLATLHGGSIKWETPLLWALGFIFLFTVGGLTGIVLAN
Calu   TRAYFTSATMIIAIPTGVKVFSWLATLHGGKIKWETPMLWALGFIFLFTVGGLTGVVLSN
Papa   TRAYFTSATMIIAVPTGVKVFSWLATLHGGNTKWETPLLWALGFIFLFTVGGLTGIVLSN
Sufr   TRAYFTSATMIIAIPTGVKVFSWLATLHGGTIKWETPLLWALGFIFLFTVGGLTGIVLAN
Stci   TRAYFTSATMIIAIPTGVKVFSWLATLHGGAIKWETPLLWALGFIFLFTVGGLTGIVLAN
Taru   TRAYFTSATMIIAIPTGVKVFSWLATLHGGSIKWETPMLWALGFIFLFTVGGLTGIVLAN
Rala   TRAYFTSATMIIAIPTGVKVFSWLATLHGGSIKWETPLLWALGFIFLFTVGGLTGIVLAN
       .*:*******:**:***:*:::****: *   . .  : *: **:**** *****::*:*
```

[7/10 of aligned sequences]

```
                              376 378     J                                       K
Scca   SSLDIVLHDTYYVVAHFHYVQTMGAVFAIMAGFIHWFPLMSGFTLHSTWTKIQFVLMFIG
Muma   SSLDIVLHDTYYVVAHFHYVLSMGAVFAIMAGFIHWFPLMSGFTLHQTWTKIQFAVMFIG
Erca   SSLDIMLHDTYYVVAHFHYVLSMGAVFAIMGGFVHWFPLFSGYTLHPTWTKIHFGVMFIG
Pose   SSLDIMLHDTYYVVAHFHYVLSMGAVFAIMGGFVHWFPLFSGYTLHSTWTKIHFGVMFIG
Actr   SSLDIVLHDTYYVVAHFHYVLSMGAVFAIMGAFVHWFPLFTGYTLHGTWSKIHFAVMFVG
Scal   SSLDIVLHDTYYVVAHFHYVLSMGAVFAIMGAFVHWFPLFTGYTLHSTWSKIHFAVMFVG
Posp   SSLDIVLHDTYYVVAHFHYVLSMGAVFAIMGAFVHWFPLFTGYTLHSTWSKIHFAVMFVG
Atsp   SSLDIMLHDTYYVVAHFHYVLSMGAVFAIMGAFVHWFPLFTGYTLHNTWSKIHFGVMFAG
Leoc   SSLDIMLHDTYYVVAHFHYVLSMGAVFAIMGAFVHWFPLFTGYTLHNTWSKIHFGVMFAG
Amca   SSLDIVLHDTYYVVAHFHYVLSMGAVFAIMGGFVHWFPLFSGYTLHPTWSKIHFGVMFVG
Osbi   SSLDIVLHDTYYVVAHFHYVLSMGAVFAIMGGFVHWFPLFSGYTLHGTWTKIHFGVMFIG
Pabu   SSLDIVLHDTYYVVAHFHYVLSMGAVFAIMGGFVHWFPLFSGYTLHNTWTKIHFGVMFIG
Hial   SSLDIVLHDTYYVVAHFHYVLSMGAVFAIMGAFVHWFPLFSGYTLHSTWTKIHFGVMFLG
Elha   SSLDIVLHDTYYVVAHFHYVLSMGAVFAIMGAFVHWFPLFTGYTLHSTWTKIHFGVMFVG
Mlcy   SSLDIVLHDTYYVVAHFHYVLSMGAVFAIMGAFVHWFPLFTGYTLHSTWTKIHFGVMFVG
Algl   SSLDIVLHDTYYVVAHFHYVLSMGAVFAIMGGFVHWFPLFTGYTLHSTWTKIHFGVMFVG
Ptgi   SSLDIVLHDTYYVVAHFHYVLSMGAVFAIMGGFVHWFPLFSGYTLHSTWTKIHFGVMFVG
Alaf   SSLDIVLHDTYYVVAHFHYVLSMGAVFAIMGAFVHWFPLFTGYTLHSTWTKIHFGVMFLG
Nock   SSLDIVLHDTYYVVAHFHYVLSMGAVFAIMGAFVHWFPLFSGYTLHSTWTKIHFGVMFLG
Anja   SSIDIVLHDTYYVVAHFHYVLSMGAVFAIMGGFVHWFPLFSGYTLHSTWTKVHFGIMFVG
Gyki   SSIDIVLHDTYYVVAHFHYVLSMGAVFAIMGGFVHWFPLFTGYTLHDTWTKIHFGIMFIG
Syka   SSLDIVLHDTYYVVAHFHYVLSMGAVFAIMGGFVHWFPLFTGYTLHSTWTKIHFGIMFVG
Opma   SSIDIVLHDTYYVVAHFHYVLSMGAVFAIMAGFVHWFPLFTGYTLHDTWTKIHFGVMFIG
Comy   SSIDIVLHDTYYVVAHFHYVLSMGAVFAIMGGFVHWFPLFSGYTLHSLWTKIHFGVMFAG
Sasp   SSIDIVLHDTYYVVAHFHYVLSMGAVFAIMGGFVHWFPLFTGYTLHQKWTKVHFGIMFLG
Eupe   SSIDIVLHDTYYVVAHFHYVLSMGAVFAIMGGFIHWFPLFTGYTLHQKWTKVHFGIMFLG
Enja   SSLDIVLHDTYYVVAHFHYVLSMGAVFAIVAGFVHWFPLFTGYTLHSTWTKIHFGVMFVG
Same   SSLDIVLHDTYYVVAHFHYVLSMGAVFAIMAAFVHWFPLFTGYTLHSTWTKIHFGVMFVG
Chch   SSLDIVLHDTYYVVAHFHYVLSMGAVFAIMGAFVHWFPLFTGYTLHSTWTKIHFGVMFVG
Grgr   SSLDIVLHDTYYVVAHFHYVLSMGAVFAIMAAFVHWFPLFTGYTLHDTWTKVHFGVMFAG
Caau   SSLDIVLHDTYYVVAHFHYVLSMGAVFAIMAAFVHWFPLLTGYTLHSAWTKIHFGVMFIG
Cyca   SSLDIVLHDTYYVVAHFHYVLSMGAVFAIMAAFVHWFPLLTGYTLHSTWTKIHFGVMFIG
Dare   SSLDIVLHDTYYVVAHFHYVLSMGAVFAIMAGFVHWFPLFTGYTLNSVWTKIHFGVMFIG
Cost   SSIDIVLHDTYYVVAHFHYVLSMGAVFAIMAGFVHWFPLFTGYTLHSTWTKIHFGVMFLG
Leec   SSLDIVLHDTYYVVAHFHYVLSMGAVFAIMAAFVHWFPLFSGYTLHSTWTKIHFGVMFIG
Fola   SSLDIVLHDTYYVVAHFHYVLSMGAVFAIMAGFVHWFPLFTGFSLHDTWTKIHFGVMFIG
Clmc   SSLDIVLHDTYYVVAHFHYVLSMGAVFAIMGAFVHWFPLLSGYTLHDTWTKIHFGVMFAG
Phin   SSLDIVLHDTYYVVAHFHYVLSMGAVFAIMGAFVHWFPLFTGFTLHSTWTKIHFGVMFIG
Icpu   SSLDIVLHDTYYVVAHFHYVLSMGAVFAIMGAFVHWFPLFTGYTMHDTWTKIHFGTMFVG
Psto   SSLDIVLHDTYYVVAHFHYVLSMGAVFAIMGAFVHWFPLFTGYTMHDTWTKIHFGTMFVG
Cora   SSLDIVLHDTYYVVAHFHYVLSMGAVFAIMGAFVHWFPLFTGYTLHDTWTKIHFGVMFAG
Eisp   SSIDISLHDTYYVVAHFHYVLSMGAVFAIMGAFIHWFPLFSGYTLHSTWTKIHFGVMFVG
Apal   SSIDVSLHDTYYVVAHFHYVLSMGAVFAIMGAFVHWFPLFSGYTLHNTWTKIHFGVMFVG
Eslu   SSLDIVLHDTYYVVAHFHYVLSMGAVFAIMGAFVHWFPLFSGYTMHTTWTKIHFGIMFVG
Dape   SSLDIVLHDTYYVVAHFHYVLSMGAVFAIMGAFVHWFPLFSGYTLHSTWTKIHFGIMFVG
Glse   SSLDIVLHDTYYVVAHFHYVLSMGAVFAIVAAFVHWFPLFTGFTLHSTWTKIHFGVMFIG
Naar   SSLDIVLHDTYYVVAHFHYVLSMGAVFAIVAAFVHWFPLFTGYTLHSTWTKIHFGVMFIG
Lioc   SSLDIVLHDTYYVVAHFHYVLSMGAVFAIVAAFVHWFPLFSGYTLHSTWSKIHFGVMFIG
Opso   SSLDIVLHDTYYVVAHFHYVLSMGAVFAIVAAFVHWFPLFTGFTLHSTWTKVHFGVMFIG
Alte   SSLDIVLHDTYYVVAHFHYVLSMGAVFAIMAGFVHWFPLFSGYTLHSTWSKIHFGVMFLG
Plap   SSLDIVLHDTYYVVAHFHYVLSMGAVFAIMAGFVHWFPLFSGYTLHSTWSKIHFGVMFLG
```

To be continued on page 36.

[7/10 of aligned sequences]

Plal	SSLDIVLHDTYYVVAHFHYVLSMGAVFAILAAFVHWFPLFSGYTLHSTWTKIHFGIMFVG
Sami	SSLDIVLHDTYYVVAHFHYVLSMGAVFAILAAFVHWFPLFSGYTLHSTWTKIHFGIMFLG
Rere	SSLDIVLHDTYYVVAHFHYVLSMGAVFAIVGGFVHWFPLFTGYTLHSTWTKIHFGIMFLG
Gama	SSLDIVLHDTYYVVAHFHYVLSMGAVFAILAGFVHWFPLFSGYTLHSTWTKIHFGVMFIG
Onmy	SSLDIVLHDTYYVVAHFHYVLSMGAVFAIMGAFVHWFPLFTGYTLHSTWTKIHFGIMFIG
Sasa	SSLDIVLHDTYYVVAHFHYVLSMGAVFAIMGAFVHWFPLFTGYTLHSTWTKIHFGIMFIG
Cola	SSLDIVLHDTYYVVAHFHYVLSMGAVFAIMGAFVHWFPLFTGYTLHSTWTKIHFGIMFIG
Dita	SSLDIILHDTYYVVAHFHYVLSMGAVFAIMAAFVHWFPLFSGYTLHSTWTKIHFGIMFLG
Gogr	SSLDIILHDTYYVVAHFHYVLSMGAVFAIMAGLVHWFPLFSGYTLHDTWTKTHFGIMFLG
Chsl	SSLDIVLHDTYYVVAHFHYVLSMGAVFAIMAGFVHWFPLFSGYTLHDTWTKAHFTIMFVG
Atja	SSLDIMLHDTYYVVAHFHYVLSMGAVFAIMAAFVHWFPLFSGYTLHSTWTKIHFGVMFVG
Iido	SSLDIMLHDTYYVVAHFHYVLSMGAVFAIMAAFVHWFPLFSGYTLHNTWTKIHFGVMFVG
Auja	SSLDIVLHDTYYVVAHFHYVLSMGAVFAIVAAFVHWFPLFTGYTLHGTWTKIHFGVMFVG
Chag	SSLDIVLHDTYYVVAHFHYVLSMGAVFAIMAGFVHWFPLFSGYTLHGTWTKIHFAVMFVG
Hami	SSLDIVLHDTYYVVAHFHYVLSMGAVFAIVAAFVHWFPLFSGYTLHSTWTKIHFGVMFVG
Saun	SSLDIVLHDTYYVVAHFHYVLSMGAVFAIVAAFVHWFPLFSGYTLHSTWTKIHFGVMFVG
Nema	SSLDIVLHDTYYVVAHFHYVLSMGAVFAIVAAFVHWFPLFTGYTLHSTWTKIHFGVMFVG
Disp	SSLDIVLHDTYYVVAHFHYVLSMGAVFAILAAFVHWFPLFSGYTLHGTWTKIHFAVMFVG
Myaf	SSLDIVLHDTYYVVAHFHYVLSMGAVFAIVAGFVHWFPLFSGYTLHSTWTKIHFGVMFVG
Lagu	SSLDIVLHDTYYVVAHFHYVLSMGAVFAIVAAFVHWFPLFSGYTLHETWTKVHFGVMFVG
Trtr	SSLDIVLHDTYYVVAHFHYVLSMGAVFAIMAAFVHWFPLFTGYTLQSTWTKIHFGVMFVG
Zucr	SSLDIVLHDTYYVVAHFHYVLSMGAVFAIMAAFVHWFPLFTGYTLQSTWTKIHFGVMFVG
Pxja	SSLDIVLHDTYYVVAHFHYVLSMGAVFAIMAAFVHWFPLFSGYTLHSTWTKIHFGVMFLG
Pxlo	SSLDIVLHDTYYVVAHFHYVLSMGAVFAIMAAFVHWFPLFSGYTLHSTWTKIHFGIMFLG
Pctr	SSLDIILHDTYYVVAHFHYVLSMGAVFAIMAAFVHWFPLFSGYTLHSTWTKVHFGVMFAG
Apsa	SSLDIILHDTYYVVAHFHYVLSMGAVFAIMAAFVHWFPLFSGYTLHTTWTKIHFGVMFAG
Cabe	SSLDIVLHDTYYVVAHFHYVLSMGAVFAIMAGFVHWFPLFTGYTLHSTWTKAHFGVMFAG
Bzze	SSLDIVLHDTYYVVAHFHYVLSMGAVFAIMAAFVHWFPLFTGYTLHSTWTKAHFGVMFAG
Siim	SSLDIVLHDTYYVVAHFHYVLSMGAVFAIMAGLMHWFPLFTGYTLNETWTKAHFAVMFAG
Ctru	SSLDIVLHDTYYVVAHFHYVLSMGAVFAIMAAFVHWFPLFTGYTLHSAWTKIHFGVMFAG
Dpbr	SSLDIVLHDTYYVVAHFHYVLSMGAVFAIVAAFVHWFPLFSGYTLHSTWTKIHFGVMFVG
Caki	SSLDIVLHDTYYVVAHFHYVLSMGAVFAIMAAFVHWFPLFSGYTLHDTWTKVHFGVMFLG
Phja	SSLDIILHDTYYVVAHFHYVLSMGAVFAIMAGFVHWFPLFTGYTLHASWSKAHFGVMFAG
Brsp	SSLDIILHDTYYVVAHFHYVLSMGAVFAIMAGFVHWFPLFSGYTLHEGASKIQFFIMFTG
Gamo	SSLDIVLHDTYYVVAHFHYVLSMGAVFAIMAAFVHWFPLFTGYTLHDTWTKIHFGVMFVG
Lolo	SSLDIVLHDTYYVVAHFHYVLSMGAVFAIMAAFVHWFPLFTGYTLHDTWTKIHFGVMFVG
Batr	SSLDVVLHDTYYVVAHFHYVLSMGAVFAIMAGFVHWFPLFTGFTLNNKLAKTHFGLTFVG
Prmy	SSLDIVLHDTYYVVAHFHYVLSMGAVFAIMAGFTHWFPLFTGYTLNSAFSKAQFYTMFLG
Lose	SSLDIMLHDTYYVVAHFHYVLSMGAVFAIMGAFVHWFPLFSGYTLHSTWTKIHFGVMFVG
Loam	SSLDIILHDTYYVVAHFHYVLSMGAVFAIMGAFVHWFPLFSGYTLHSTWTKIHFGIMFAG
Chab	SSLDIVLDDTYYVVAHFHYVLSMGAVFAIVGAFVHWFPLFSGYTLYNTWTKVHFGVMFAG
Chto	SSLDIVLHDTYYVVAHFHYVLSMGAVFAIVGAFVHWFPLFSGYTLHNTWTKVHFGVMFAG
Majo	SSLDIVLHDTYYVVAHFHYVLSMGAVFAIVAGFVHWFPLFTGYTLHETWTKMHFGVMFVG
Hlst	SSLDIVLHDTYYVVAHFHYVLSMGAVFAIVAGFVHWFPLFTGYTLHETWTKMHFGVMFIG
Clpe	SSLDVVLHDT*YVVAHFHYVLSMGAVFAIVGAFVHWFPLFSGYTLHDTWTKVHFGVMFIG
Mlmr	SSLDIVLHDTYYVVAHFHYVLSMGAVFAIIAAFVHWFPLFSGYTLHSTWTKIQFGVMFLG
Crcr	SSLDIVLHDTYYVVAHFHYVLSMGAVFAIMGGFVHWFPLFSGYTLHKTWTKIHFGVMFIG
Muce	SSLDIVLHDTYYVVAHFHYVLSMGAVFAIMGGFVHWFPLFSGYTLHKTWTKIHFGVMFIG
Bege	SSLDIVLHDTYYVVAHFHYVLSMGAVFAIVAAFVHWFPLFSGYTLHSTWTKIHFGVMFIG
Mela	SSLDIVLHDTYYVVAHFHYVLSMGAVFAIIAAFVHWFPLFSGYTLHSTWTKIHFGIMFLG
Hats	SSLDIVLHDTYYVVAHFHYVLSMGAVFAIMAAFVHWFPLFSGYTLHSTWTKIHFGVMFVG
Orla	SSLDIMLHDTYYVVAHFHYVLSMGAVFAIMGAFVHWFPLFSGYTLHGTWTKIHFGVMFVG

To be continued on page 37.

To be continued on page 38.

```
Cosa  SSLDIVLHDTYYVVAHFHYVLSMGAVFAIVGAFIHWFPLFSGYTLHNTWTKIHFGVMFIG
Exsp  SSLDIVLHDTYYVVAHFHYVLSMGAVFAIMAAFVHWFPLFSGYTLHETWTKIHFGIMFIG
Depa  SSLDIVLHDTYYVVAHFHYVLSMGAVFAIVGAFVHWFPLFSGYTLHDTWTKIHFGVMFIG
Rima  SSLDIVLHDTYYVVAHFHYVLSMGAVFAIIAAFVHWFPLFSGYTLHDTWTKIHFGAMFLG
Fuol  SSLDIVLHDTYYVVAHFHYVLSMGAVFAIIAAFVHWFPLFSGYTLHSTWTKIHFGIMFVG
Gmaf  SSLDIVLHDTYYVVAHFHYVLSMGAVFAIFAGFVHWFPLFSGYTLHSTWTKIHFGIMFVG
Xeei  SSLDIVLHDTYYVVAHFHYVLSMGAVFAIIAAFVHWFPLFSGYTLHTTWTKIHFGIMFFG
Pros  SSLDIVLHDTYYVVAHFHYVLSMGAVFAIMAAFVHWFPLFSGYTLHDTWTKIHFAVMFLG
Scmi  SSLDIVLHDTYYVVAHFHYVLSMGAVFAIMGAFVHWFPLFSGYTLHNTWTKIHFAVMFVG
Rolo  SSLDIVLHDTYYVVAHFHYVLSMGAVFAIMAAFVHWFPLFSGYTLHSTWTKIHFGVMFLG
Cere  SSLDIVLHDTYYVVAHFHYVLSMGAVFAIMAAFVHWFPLFSGYTLHSTWTKAQFVTMFVG
Daga  SSLDIVLHDTYYVVAHFHYVLSMGAVFAIIAGFVHWFPLFTGYVLHKTWTKIQFALMFVG
Anco  SSLDIVLHDTYYVVAHFHYVLSMGAVFAIMAAFVHWFPLFSGYTLHSTWTKIHFGVMFFG
Dmve  SSLDIILHDTYYVVAHFHYVLSMGAVFAIMAAFVHWFPLFTGYSLHSNLTKIHFCIMFLG
Dmar  SSLDIILHDTYYVVAHFHYVLSMGAVFAIMAAFVHWFPLFTGYSLHSSLTKIHFCIMFLG
Anka  SSLDIVLHDTYYVVAHFHYVLSMGAVFAIMAAFVHWFPLFTGYTLHSTWTKIHFGVMFFG
Moja  SSLDIVLHDTYYVVAHFHYVLSMGAVFAIMAAFVHWFPLFTGYTLHSTWTKIHFGVMFFG
Hoja  SSLDIVLHDTYYVVAHFHYVLSMGAVFAIMAAFVHWFPLFSGYTLHSTWTKIHFGVMFFG
Bede  SSLDIVLHDTYYVVAHFHYVLSMGAVFAIVAAFVHWFPLFTGYTLHDTWTKIHFAVMFLG
Besp  SSLDIVLHDTYYVVAHFHYVLSMGAVFAIVAAFVHWFPLFTGYTLHDTWTKIHFAVMFLG
Mysp  SSLDVVLHDTYYVVAHFHYVLSMGAVFAIVAAFVHWFPLFTGYTLHSTWTKIHFGVMFLG
Osja  SSLDIVLHDTYYVVAHFHYVLSMGAVFAIVAAFVHWFPLFTGYTLHSTWTKIHFGVMFLG
Sgro  SSLDIVLHDTYYVVAHFHYVLSMGAVFAIVAAFVHWFPLFTGYTLHNTWTKIHFGVMFLG
Pzpa  SSLDIVLHDTYYVVAHFHYVLSMGAVFAIVAAFVHWFPLFTGYTLHNTWTKIHFGVMFLG
Zeja  SSLDIVLHDTYYVVAHFHYVLSMGAVFAIVAAFVHWFPLFSGYTLHDTWTKIHFGIMFLG
Znne  SSLDVVLHDTYYVVAHFHYVLSMGAVFAIVAAFVHWFPLLSGYTLHSTWTKAHFGIMFLG
Zefa  SSLDIVLHDTYYVVAHFHYVLSMGAVFAIVAAFVHWFPLFSGYTLHSTWTKIHFGVMFLG
Acni  SSLDIVLHDTYYVVAHFHYVLSMGAVFAIVAAFVHWFPLFSGYTLHSTWTKIHFGVMFLG
Ncrh  SSLDIVLHDTYYVVAHFHYVLSMGAVFAIVAAFVHWFPLFSGYTLHSTWTKIHFFVMFLG
Agca  SSLDIVLHDTYYVVAHFHYVLSMGAVFAIVGGFIHWFPLFTGYTLHETWAKIHFGIMFVG
Hydy  SSLDIVLHDTYYVVAHFHYVLSMGAVFAIVAGFVHWFPLFSGYTLHSTWTKVHFGVMFAG
Gsac  SSLDIVLHDTYYVVAHFHYVLSMGAVFAIIAGFVHWFPLFSGYTLHSTWTKVHFGVMFAG
Pevo  SSLDIVLHDTYYVVAHFHYVLSMGAVFAIMAGFVHWFPLFTGYTLHSVWTKLHFGVMFVG
Hiku  SSLDIVLHDTYYVVAHFHYVLSMGAVFAIMAGFVHWFPLFTGYTLHSSWTKIHFGVMFVG
Inpa  SSLDIVLHDTYYVVAHFHYVLSMGAVFAIMAAFVHWFPLFSGYALHSTWTKVHFGVMFAG
Auch  SSLDIILHDTYYVVAHFHYVLSMGAVFAIMAGFVHWFPLFSGYTLHPTWTKVHFGVMFTG
Fico  SSIDIVLHDTYYVVAHFHYVLSMGAVFAIMAGFVHWFPLFSGYTLHSTWTKVHFGVMFIG
Macs  SSLDIVLHDTYYVVAHFHYVLSMGAVFAIMAGFVHWFPLFTGYTLHDTWTKIHFGVMFAG
Moal  SSLDIMLHDTYYVVAHFHYVLSMGAVFAIFAAFAHWFPLFSGYTLHNVWTKIHFGVMFVG
Syma  SSLDIMLHDTYYVVAHFHYVLSMGAVFAIFAAFAHWFPLFTGYTLHSTLMKMHFGVMFVG
Mafr  SSLDIVLHDTYYVVAHFHYVLSMGAVFAIMAAFVHWFPLFSGYTLHDTWTKIHFGVMFVG
Dcpe  SSLDIVLHDTYYVVAHFHYVLSMGAVFAIVAGFVHWFPLFTGYTLHDTWTKIHFGVMFVG
Dcti  SSLDIVLHDTYYVVAHFHYVLSMGAVFAIVAGFVHWFPLFTGYTLHDTWTKIHFGVMFVG
Hehi  SSLDIVLHDTYYVVAHFHYVLSMGAVFAIVAAFVHWFPLFSGYTLHSTWTKIHFGIMFLG
Stam  SSLDIVLHDTYYVVAHFHYVLSMGAVFAIVAAFVHWFPLFSGYTLHSTWTKIHFGVMFIG
Hogi  SSLDIVLHDTYYVVAHFHYVLSMGAVFAIVAAFVHWFPLFSGYTLHSTWTKIHFGIMFVG
Erzo  SSLDIVLHDTYYVVAHFHYVLSMGAVFAIVGGFVHWFPLFSGYTLHSTWTKIHFAIMFAG
Hxot  SSLDIVLHDTYYVVAHFHYVLSMGAVFAIVAGFVHWFPLFSGYTLHSTWTKIHFGVMFVG
Core  SSLDIVLHDTYYVVAHFHYVLSMGAVFAIVAGFVHWFPLFSGYTLHSTWTKIHFGVMFLG
Apve  SSLDIVLHDTYYVVAHFHYVLSMGAVFAIVAGFVHWFPLFSGYTMHSTWTKIHFGVMFLG
Latj  SSLDIVLHDTYYVVAHFHYVLSMGAVFAIVAGFVHWFPLFTGYTLHTTWTKIHFGIMFAG
Laja  SSLDIVLHDTYYVVAHFHYVLSMGAVFAIMAGFVHWFPLFTGYTMHSTWTKVHFAIMFAG
```

[7/10 of aligned sequences]

To be continued on page 39.

```
Syja SSLDIVLHDTYYVVAHFHYVLSMGAVFAIVAGFVHWFPLFTGYTLHSTWTKIHFGIMFAG
Epme SSLDIVLHDTYYVVAHFHYVLSMGAVFAIVAAFVHWFPLFTGYTLHSTWTKIHFGIMFVG
Grse SSLDIVLHDTYYVVAHFHYVLSMGAVFAIVAAFVHWFPLFSGYTLHSTWTKIHFGVMFIG
Clja SSLDIVLHDTYYVVAHFHYVLSMGAVFAIVAGFVHWFPLFTGYTLHPTWTKIHFSIMFLG
Ogcy SSLDIVLHDTYYVVAHFHYVLSMGAVFAIVAAFVHWFPLFSGYTLHETWTKIHFGVMFAG
Plna SSLDIVLHDTYYVVAHFHYVLSMGAVFAIVAGFVHWFPLFSGYTLHDTWTKIHFGVMFVG
Lema SSLDIVLHDTYYVVAHFHYVLSMGAVFAIVAAFVHWFPLFSGYTLHTTWTKIHFGIMFIG
Etzo SSLDIVLHDTYYVVAHFHYVLSMGAVFAIVAAFVHWFPLFSGYTLHSTWTKIHFGIMFAG
Apse SSLYIVLHDTYYVVAHFHYVLSMGAVFAIVAAFVHWFPLFTGYTLHSTWSKVHFAVMFVG
Epde SSLDIVLHDTYYVVAHFHYVLSMGAVFAIIAAFVHWFPLFTGYTLHSVWTKVHFGIMFIG
Slja SSLDIVLHDTYYVVAHFHYVLSMGAVFAIMAAFVHWFPLFTGYTLHDVWTKIHFGVMFVG
Bsja SSLDIVLHDTYYVVAHFHYVLSMGAVFAIVAGFMHWFPLFSGYTLHPTWTKIHFTVMFLG
Ecna SSLDIVLHDTYYVVAHFHYVLSMGAVFAIAGGFVHWFPLFTGYTLHETWTKVHFGVMFVG
Cohi SSLDIVLHDTYYVVAHFHYVLSMGAVFAIVAGFIHWFPLFTGYTLHDTWTKIHFGVMFVG
Caar SSLDIVLHDTYYVVAHFHYVLSMGAVFAIVAGFVHWFPLFTGYTLHDTWTKIHFGVMFVG
Came SSLDIVLHDTYYVVAHFHYVLSMGAVFAIVAGFVHWFPLFTGYTLHDTWTKVHFGVMFAG
Mema SSLDIVLHDTYYVVAHFHYVLSMGAVFAIIAAFVHWFPLFSGYTLHDTWTKIHFGIMFAG
Lenu SSLDIVLHDTYYVVAHFHYVLSMGAVFAIIAGFVHWFPLLSGYTLHETWAKVHFGVMFAG
Brja SSLDIVLHDTYYVVAHFHYVLSMGAVFAIVAAFVHWFPLFSGYTLHSTWTKIHFGVMFVG
Plma SSLDIVLHDTYYVVAHFHYVLSMGAVFAIVAGFVHWFPLFSGYTLHSTWTKIHFGVMFLG
Emst SSLDIVLHDTYYVVAHFHYVLSMGAVFAIVAAFVHWFPLFSGYTLHSTWTKIHFGIMFVG
Ptti SSLDIVLHDTYYVVAHFHYVLSMGAVFAIVAAFVHWFPLFSGYTLHSTWTKIHFGVMFVG
Losu SSLDIVLHDTYYVVAHFHYVLSMGAVFAIMGAFVHWFPLFSGYTLHNVWTKIHFGIMFIG
Geoy SSLDIVLHDTYYVVAHFHYVLSMGAVFAIMGGFVHWFPLFTGYTLHETWTKVHFGIMFTG
Dipi SSLDIVLHDTYYVVAHFHYVLSMGAVFAIVAAFVHWFPLFSGYTLHSTWTKIHFGVMFVG
Pama SSLDIVLHDTYYVVAHFHYVLSMGAVFAIMAGFVHWFPLFSGYTLHSTWTKIHFGVMFLG
Leob SSLDIVLHDTYYVVAHFHYVLSMGAVFAIVAGFVHWFPLFSGYTLHSTWTKVHFAVMFAG
Neba SSLDVVLHDTYYVVAHFHYVLSMGAVFAIVAGFVHWFPLFTGYTLHQTWTKVHFGIMFVG
Pdpl SSLDIVLHDTYYVVAHFHYVLSMGAVFAIVAGFVHWFPLFTGYTLHETWTKVHFGVMFTG
Nimi SSLDIVLHDTYYVVAHFHYVLSMGAVFAIMGGFVHWFPLFTGYTLHPTWSKTHFAIMFTG
Uptr SSLDIVLHDTYYVVAHFHYVLSMGAVFAIVAGFVHWFPLFTGYTLHSTWTKIHFGVMFVG
Pesc SSLDIVLHDTYYVVAHFHYVLSMGAVFAIVAGFLHWFPLFTGYSLHGTWAKVHFCIMFAG
Baar SSLDIVLHDTYYVVAHFHYVLSMGAVFAIVAGFVHWFPLFSGYTLHDTWTKIHFGIMFAG
Moar SSLDIVLHDTYYVVAHFHYVLSMGAVFAIVAAFVHWFPLFSGYTLHSTWTKIHFGIMFVG
Toja SSLDIVLHDTYYVVAHFHYVLSMGAVFAIVGGFVHWFPLFTGYVLHSTWTKIHFGVMFVG
Chau SSLDIVLHDTYYVVAHFHYVLSMGAVFAILAAFVHWFPLFTGYTLHTTWTKIHFGIMFVG
Chse SSLDIVLHDTYYVVAHFHYVLSMGAVFAIIAGFVHWFPLFTGYTLHKTWTKIHFGLMFTG
Enar SSLDIVLHDTYYVVAHFHYVLSMGAVFAIVGGFVHWFPLFTGYTLHETWTKVHFGIMFAG
Hpty SSLDIVLHDTYYVVAHFHYVLSMGAVFAIVAGFVHWFPLFTGYTLHSTWTKIHFGIMFAG
Nana SSLDIVLHDTYYVVAHFHYVLSMGAVFAIMGGFMHWFPLFSGYTMHSTWSKAHFGVMFFG
Mcst SSLDIVLHDTYYVVAHFHYVLSMGAVFAIVAAFVHWFPLFTGYTLHSTWTKIHFAIMFVG
Rhox SSLDIVLHDTYYVVAHFHYVLSMGAVFAIVAAFVHWFPLFTGYTLHEVWTKVHFGIMFAG
Opfa SSLDIVLHDTYYVVAHFHYVLSMGAVFAIVAAFVHWFPLFTGYTLHSTWTKIHFGIMFIG
Paar SSLDIVLHDTYYVVAHFHYVLSMGAVFAIVAAFVHWFPLFTGYTLHSTWTKIHFGVMFVG
Gozo SSLDIVLHDTYYVVAHFHYVLSMGAVFAIVAAFVHWFPLFTGYTLHSTWTKIHFGIMFAG
Ackr SSLDIILHDTYYVVAHFHYVLSMGAVFAIAAGIVHWFPLFTGYSLNEIWTKAHFAVMFTG
Elev SSLDIVLHDTYYVVAHFHYVLSMGAVFAIVAAFVHWFPLFTGYTLHSTWTKIHFALMFTG
Trdu SSLDIMLHDTYYVVAHFHYVLSMGAVFAIVAGFVHWFPLFSGYTLHDTWTKIHFGVMFAG
Amoc SSLDIVLHDTYYVVAHFHYVLSMGAVFAIVAGFVHWFPLFSGYTLHTTWTKIHFGVMFVG
Hame SSLDIVLHDTYYVVAHFHYVLSMGAVFAIVAAFVHWFPLFTGYTLHSTWTKIHFGVMFIG
Chso SSLDIVLHDTYYVVAHFHYVLSMGAVFAIVAAFVHWFPLFTGYTLHPTWTKIHFGVMFVG
Lyto SSLDIVLHDTYYVVAHFHYVLSMGAVFAIVAGFVHWFPLFSGYTLHSTWTKIHFGVMFAG
```

[7/10 of aligned sequences]

```
Encr        SSLDIVLHDTYYVVAHFHYVLSMGAVFAIVAGFVHWFPLFSGYTLHSTWTKIHFGVMFAG
Bvar        SSLDIVLHDTYYVVAHFHYVLSMGAVFAIVAAFIHWFPLFTGYTLHSTWTKIHFGIMFVG
Noco        SSLDIVLHDTYYVVAHFHYVLSMGAVFAIVAAFVHWFPLFTGYTMHSVWTKIHFTVMFIG
Chsp        SSMDTALHDTYYVVAHFHYVLSMGAVFAILAGFVHWFPLFSGYTLHETWSKIHFALMFMG
Arja        SSLDIVLHDTYYVVAHFHYVLSMGAVFAIVAGFVHWFPLFSGYTLHSTWTKIHFGVMFLG
Pase        SSLDIVLHDTYYVVAHFHYVLSMGAVFAIIAGFIHWFPLFTGYTLHSAWTKVHFGVMFLG
Trel        SSLDIVLHDTYYVVAHFHYVLSMGAVFAIIAAFIHWFPLFTGYTLNSTWSKMHFAVMFTG
Lifa        SSLDIVLHDTYYVVAHFHYVLSMGAVFAIMGGFVHWFPLFTGYTLHTFWTKVHFGIMFAG
Acur        SSLDIVLHDTYYVVAHFHYVLSMGAVFAIVAGFVHWFPLFTGYTLHSTWTKVHFGVMFLG
Ampe        SSLDIVLHDTYYVVAHFHYVLSMGAVFAIIAGFVHWFPLFSGYTLHSTWTKVHFGIMFLG
Urja        SSLDIILHDTYYVVAHFHYVLSMGAVFAIMGALVHWFPLFTGYTLNAAWTKIHFAIMFAG
Enet        SSLDIVLHDTYYVVAHFHYVLSMGAVFAIVAGFVHWFPLFSGYTLHPTWTKVHFGVMFLG
Ptbr        SSLDIVLHDTYYVVAHFHYVLSMGAVFAIVAAFVHWFPLFTGYTLHDTWTKIHFGIMFAG
Safa        SSLDIVLHDTYYVVAHFHYVLSMGAVFAIVAAFVHWFPLFSGYTLHSTWTKIHFGVMFVG
Icae        SSLDIVLHDTYYVVAHFHYVLSMGAVFAIVAAFVHWFPLFTGYTLHSTWTKIHFGVMFVG
Asmi        SSLDIVLHDTYYVVAHFHYVLSMGAVFAIIAGFVHWFPLFTGYTLHSTWTKIHFSVMFAG
Foal        SSLDIVLHDTYYVVAHFHYVLSMGAVFAIMGGFVHWFPLFSGYTLHKLWTKIHFAVMFLG
Drze        SSLDIVLHDTYYVVAHFHYVLSMGAVFAIMGGFVHWFPLFTGYTLHQSWTKIHFAVMFVG
Rhas        SSLDITLHDTYYVVAHFHYVLSMGAVFAIMAAFVHWFPLFTGYTLHSTWSKVHFGVMFVG
Elac        SSLDIMLHDTYYVVAHFHYVLSMGAVFAIMAGFVHWFPLLTGYTLHSTWSKIHFGVMFVG
Kugu        SSLDIVLHDTYYVVAHFHYVLSMGAVFAIVAAFVHWFPLFTGYTLHPTWSKVHFGVMFTG
Plor        SSLDIVLHDTYYVVAHFHYVLSMGAVFAIVAAFVHWFPLLTGYTLHSTWTKIHFGVMFAG
Sgun        SSLDIVLHDTYYVVAHFHYVLSMGAVFAIVAAFVHWFPLFSGYTLHSTWTKIHFGIMFVG
Zaco        SSLDIVLHDTYYVVAHFHYVLSMGAVFAIVAAFVHWFPLFSGYTLHSTWTKIHFGVMFVG
Zbfl        SSLDIVLHDTYYVVAHFHYVLSMGAVFAIVAAFVHWFPLFSGYTLHSTWTKIHFGIMFVG
Spba        SSLDIVLHDTYYVVAHFHYVLSMGAVFAIVAGFVHWFPLFTGFTLHNTWTKIHFALMFTG
Game        SSLDIVLHDTYYVVAHFHYVLSMGAVFAIVAAFVHWFPLFTGYTLHSTWTKIHFGVMFIG
Thth        SSLDIVLHDTYYVVAHFHYVLSMGAVFAIVAAFVHWFPLFTGYTLHSTWTKIHFGVMFVG
Xigl        SSLDIVLHDTYYVVAHFHYVLSMGAVFAIVAAFVHWFPLFTGYTLHSTWTKIHFGVMFVG
Hyja        SSLDIVLHDTYYVVAHFHYVLSMGAVFAIVAGFVHWFPLFSGYTLHDTWTKIHFGIMFAG
Psan        SSLDIVLHDTYYVVAHFHYVLSMGAVFAIVAGFVHWFPLFTGYTLHNMWTKIHFGIMFLG
Cupa        SSLDIVLHDTYYVVAHFHYVLSMGAVFAIVAGFVHWFPLFTGYTLHSTWTKIHFGVMFVG
Mpch        SSLDIVLHDTYYVVAHFHYVLSMGAVFAIMGAFVHWFPLFTGYTFHETLSKIHFGVMFIG
Char        SSLDIILHDTYYVVAHFHYVLSMGAVFAIMGAFIHWFPLFSGYTLHSTWTKIHFGVMFVG
Pser        SSIDIVLHDTYYVVAHFHYVLSMGAVFAIVGAFVHWFPLFTGYTLHSTWTKIHFAIMFAG
Prol        SSLDIVLHDTYYVVAHFHYVLSMGAVFAIVAAFVHWFPLFTGYTLHSTWTKVHFGVMFIG
Plbi        SSLDIVLHDTYYVVAHFHYVLSMGAVFAIVAAFVHWFPLFTGYTLHSTWTKVHFGLMFVG
Calu        SSLDIVLHDTYYVVAHFHYVLSMGAVFAIVAGFVHWFPLFSGYTLHPSWTKVHFGVMFTG
Papa        SSLDIVLHDTYYVVAHFHYVLSMGAVFAIMAGFVHWFPLFTGYTLHQGWTKVHFGVMFLG
Sufr        SSLDIVLHDTYYVVAHFHYVLSMGAVFAIVAAFVHWFPLFSGYTLHDTWTKIHFGVMFVG
Stci        SSLDIILHDTYYVVAHFHYVLSMGAVFAIVAAFVHWFPLFSGYTLHDTWTKVHFGVMFAG
Taru        SSLDIVLHDTYYVVAHFHYVLSMGAVFAIMGAFVHWFPLFSGYTLHSTWTKIHFGVMFIG
Rala        SSLDIVLHDTYYVVAHFHYVLSMGAVFAIMGAFVHWFPLFSGYTLHSTWTKIHFGVMFIG
            **:   *.** ********* :*******  ..: *****::*: :     *  :*   * *
```

To be continued on page 40.

[8/10 of aligned sequences]

L

Scca	VNLTFFPQHFLGLAGMPRRYSDYPDAYALWNTVSSIGSLISLVAVIMLLFIIWEAFSSKR
Muma	VNLTFFPQHFLGLAGMPRRYSDYPDAYTLWNTVSSIGSLISLVAVIMLLFIIWEAFASKR
Erca	VNLTFFPQHFLGLAGMPRRYSDYPDAYTLWNSLSSIGSMISLTAVIMFLFILWEAFAAKR
Pose	VNLTFFPQHFLGLAGMPRRYSDYPDAYTLWNSLSSIGSMISLTAVIMFLFILWEAFAAKR
Actr	VNLTFFPQHFLGLAGMPRRYSDYPDAYALWNTVSSIGSLISLVAVIMFLFILWEAFAAKR
Scal	VNLTFFPQHFLGLAGMPRRYSDYPDAYALWNTISSIGSLISLVAVIMFLFILWEAFAAKR
Posp	VNLTFFPQHFLGLAGMPRRYSDYPDAYALWNTVSSVGSLISLIAVIMFLFILWEAFAAKR
Atsp	VNLTFFPQHFLGLAGMPRRYSDYPDAYALWNTISSIGSLVSLVAGIMFLFIVWEAFSAKR
Leoc	VNLTFFPQHFLGLAGMPRRYSDYPDAYALWNTISSIGSLISLVAVIMFLFIVWEAFSAKR
Amca	VNLTFFPQHFLGLAGMPRRYSDYPDAYTLWNTVSSIGSLVSLIAVVMFLFMLWEAFAAKR
Osbi	VNMTFFPQHFLGLAGMPRRYSDYPDAYTLWNAISSIGSLVSLVAVAMFLFILWEAFAAKR
Pabu	VNLTFFPQHFLGLAGMPRRYSDYPDAYTLWNTVSSIGSLISLVAVIMFLFILWEAFVAKR
Hial	VNLTFFPQHFLGLAGMPRRYSDYPDAYTLWNTVSSIGSLISLVAVIMFLFILWEAFAAKR
Elha	VNLTFFPQHFLGLAGMPRRYSDYPDAYTLWNTVSSIGSMVSLEAVIMFLFILWEAFAAKR
Mlcy	VNLTFFPQHFLGLAGMPRRYSDYPDAYTLWNTISSIGSLISLVAVIMFLFILWEAFAAKR
Algl	VNVTFFPQHFLGLAGMPRRYSDYPDAYTLWNTVSSIGSLISLVAVIMFLFILWEAFAAKR
Ptgi	VNLTFFPQHFLGLAGMPRRYSDYPDAYTLWNTVSSIGSLISLVGVIMFLFILWEAFAAKR
Alaf	VNLTFFPQHFLGLAGMPRRYSDYPDAYTLWNTVSSIGSLISLMAVIMFLFILWEAFAAKR
Nock	VNLTFFPQHFLGLAGMPRRYSDYPDAYTLWNTVSSIGSLVSLLAVIMFLFILWEAFAAKR
Anja	VNLTFFPQHFLGLAGMPRRYSDYPDAYTLWNTISSIGSLISLTAVILFLFILWEAFTAKR
Gyki	VNLTFFPQHFLGLAGMPRRYSDYPDAYTLWNTISSIGSLVSLTAVILFLFILWEAFASKR
Syka	VNLTFFPQHFLGLAGMPRRYSDYPDAYTLWNTISSIGSLISLTAVILFLFILWEAFTAKR
Opma	VNLTFFPQHFLGLAGMPRRYSDYPDAYTLWNTVSSIGSLVSLTAVVLFLFILWEAFSAKR
Comy	VNMTFFPQHFLGLAGMPRRYSDYPDAYTLWNTISSIGSLVSLTAVVLFLFILWEAFAAKR
Sasp	VNLTFFPQHFLGLAGMPRRYSDYPDAYTLWNVISSIGSLISIVAVALFMFILWEAFAVKR
Eupe	VNLTFFPQHFLGLAGMPRRYSDYPDAYTLWNFISSIGSLISLTAVILFLFILWEAFAAKR
Enja	VNLTFFPQHFLGLAGMPRRYSDYPDAYTLWNTVSSIGSLISLVAVIMFLFIIWEAFAAKR
Same	VNLTFFPQHFLGLAGMPRRYSDYPDAYTLWNTVSSIGSLISLVAVIMFLFILWEAFAAKR
Chch	VNLTFFPQHFLGLAGMPRRYSDYPDAYTLWNTLSSIGSLISLVAVIMFLFILWEAFAAKR
Grgr	VNLTFFPQHFLGLAGMPRRYSDYPDAYALWNTVSSIGSLVSLVAVIMFLFILWEAFTAKR
Caau	VNLTFFPQHFLGLAGMPRRYSDYPDAYALWNTVSSIGSLISLVAVIMFLFILWEAFAAKR
Cyca	VNLTFFPQHFLGLAGMPRRYSDYPDAYALWNTVSSIGSLISLVAVIMFLFILWEAFAAKR
Dare	VNLTFFPQHFLGLAGMPRRYSDYPDAYALWNTVSSIGSLISLVAVIMFLFILWEAFTAKR
Cost	VNLTFFPQHFLGLAGMPRRYSDYPDAYTLWNTVSSIGSMISLVAVIMFLFILWEAFASKR
Leec	VNLTFFPQHFLGLAGMPRRYSDYPDAYTLWNTVSSIGSLISLVAVIMFLFILWEAFAAKR
Fola	VNLTFFPQHFLGLAGMPRRYSDYPDAYTLWNTVSSIGSLISLVAVIIFLFILWEAFASKR
Clmc	VNLTFFPQHFLGLAGMPRRYSDYPDAYTLWNTISSIGSLVSLVAVIMFLFILWEAFTAKR
Phin	VNLTFFPQHFLGLAGMPRRYSDYPDAYTLWNTISSIGSLVSLIAVVMFLFILWEAFAAKR
Icpu	VNLTFFPQHFLGLAGMPRRYSDYPDAYSLWNIISSIGSLVSLVAVVMFLYILWEAFTAKR
Psto	VNLTFFPQHFLGLAGMPRRYSDYPDAYSLWNIISSVGSLISLVAVVMFLYILWEAFTAKR
Cora	VNITFFPQHFLGLAGMPRRYSDYPDAYTLWNTVSSIGSLMSLVAVIMFLFILWEAFAAKR
Eisp	VNMTFFPQHFLGLAGMPRRYSDYPDAYTLWNTVSSIGSLISLIAVIMFLFILWEAFSAKR
Apal	VNLTFFPQHFLGLAGMPRRYSDYPDAYTLWNTVSSIGSMISLVAVIMFLFILWEAFSAKR
Eslu	VNLTFFPQHFLGLAGMPRRYSDYPDAYTLWNTISSIGSLISLVAVIMFLFILWEAFTAKR
Dape	VNLTFFPQHFLGLAGMPRRYSDYPDAYTLWNTVSSIGSLISLVAVILFLFILWEAFASKR
Glse	VNLTFFPQHFLGLAGMPRRYSDYPDAYTLWNTISSIGSLISLVAVIMFLFIIWEAFAAKR
Naar	VNLTFFPQHFLGLAGMPRRYSDYPDAYTLWNTVSSIGSLVSLVAVIMFLFILWEAFAAKR
Lioc	VNLTFFPQHFLGLAGMPRRYSDYPDAYTLWNTVSSIGSLISLVAVIMFIFILWEAFSAKR
Opso	VNLTFFPQHFLGLAGMPRRYSDYPDAYTLWNTVSSIGSLISLVAVIMFLFILWEAFAAKR
Alte	VNLTFFPQHFLGLAGMPRRYSDYPDAYALWNTVSSIGSMISMVAVIMFLFILWEAFAAKR
Plap	VNLTFFPQHFLGLAGMPRRYSDYPDAYALWNTVSSIGSMISLVAVIMFLFILWEAFAAKR

To be continued on page 41.

Plal	VNLTFFPQHFLGLAGMPRRYSDYPDAYTLWNTVSSIGSLISLVAVIMFLFILWEAFAAKR
Sami	VNLTFFPQHFLGLAGMPRRYSDYPDAYTLWNTISSIGSLISLVAVIMFLFILWEAFVAKR
Rere	VNLTFFPQHFLGLAGMPRRYSDYPDAYTLWNTVSSIGSLISLVAVIMFLFILWEAFAAKR
Gama	VNLTFFPQHFLGLAGMPRRYSDYPDAYTLWNTVSSIGSLISLVAVIMFLFILWEAFAAKR
Onmy	VNLTFFPQHFLGLAGMPRRYSDYPDAYTLWNTVSSIGSLVSLVAVIMFLFILWEAFAAKR
Sasa	VNLTFFPQHFLGLAGMPRRYSDYPDAYTLWNTISSIGSLISLVAVIMFLFILWEAFAAKR
Cola	VNLTFFPQHFLGLAGMPRRYSDYPDAYTLWNTVSSIGSLISLVAVIMFLFILWEAFAAKR
Dita	VNLTFFPQHFLGLAGMPRRYSDYPDAYTLWNTVSSTGSLISLVAVVMFLFILWEAFAAKR
Gogr	VNLTFFPQHFLGLAGMPRRYSDYPDAYTLWNTVSSAGSLISLIAVVMLLFIVWEAFTAKR
Chsl	VNLTFFPQHFLGLAGMPRRYSDYPDAYTLWNTVSSIGSLISLVAVIMFLYILWEAFASKR
Atja	VNLTFFPQHFLGLAGMPRRYSDYPDAYTLWNTVSSIGSMISLIAVIMFLFIIWEAFAAKR
Iido	VNLTFFPQHFLGLAGMPRRYSDYPDAYTLWNTVSSIGSMISLIAVIMFLFIIWEAFAAKR
Auja	VNLTFFPQHFLGLAGMPRRYSDYPDAYTLWNTVSSIGSLISLVAVIMFLFIIWEAFAAKR
Chag	VNVTFFPQHFLGLAGMPRRYSDYPDAYTLWNTVSSVGSMISLVAVIMFLFIIWEAFAAKR
Hami	VNLTFFPQHFLGLAGMPRRYSDYPDAYTLWNTVSSIGSLISLVAVIMFLFIIWEAFAAKR
Saun	VNLTFFPQHFLGLAGMPRRYSDYPDAYTLWNTVSSIGSLISLVAVIMFLFIIWEAFAAKR
Nema	VNLTFFPQHFLGLAGMPRRYSDYPDAYTLWNTVSSIGSLISLVAVIMFLFIVWEAFAAKR
Disp	VNLTFFPQHFLGLAGMPRRYSDYPDAYTLWNTVSSIGSLISLVAVIMFLFIIWEAFASKR
Myaf	VNLTFFPQHFLGLAGMPRRYSDYPDAYTMWNTVSSIGSLISLVAVIMFLFILWEAFSAKR
Lagu	VNLTFFPQHFLGLAGMPRRYSDYPDAYTLWNTVSSIGSLVSLVAVVMFLFIIWEAFAAKR
Trtr	VNLTFFPQHFLGLAGMPRRYSDYPDAYTLWNTVSSAGSLISLVAVIMFLFIIWEAFAAKR
Zucr	VNLTFFPQHFLGLAGMPRRYSDYPDAYTLWNTVSSAGSLISLVAVIMFLFIIWEAFAAKR
Pxja	VNVTFFPQHFLGLAGMPRRYSDYPDAYTLWNTVSSMGSLVSLIAVIMFLFIIWEAFAAKR
Pxlo	VNLTFFPQHFLGLAGMPRRYSDYPDAYTLWNTVSSMGSLVSLIAVIMFLFILWEAFAAKR
Pctr	VNLTFFPQHFLGLAGMPRRYSDYPDAYTLWNTVSSMGSLVSLVAVIMFLFIIWEAFAAKR
Apsa	VNLTFFPQHFLGLAGMPRRYSDYPDAYTLWNTVSSIGSLISLVAVIMFLFILWEAFAAKR
Cabe	VNLTFFPQHFLGLAGMPRRYSDYPDAYTLWNTVSSIGSLISLVAVIMFLFIIWEAFAAKR
Bzze	VNLTFFPQHFLGLAGMPRRYSDYPDAYTLWNTVSSLGSLISLVAVIMFLFIIWEAFAAKR
Siim	VNLTFFPQHFLGLAGMPRRYSDYPDAYTMWNTVSSLGSLVSLIAVTMLLYILWEAFTAKR
Ctru	VNLTFFPQHFLGLAGMPRRYSDYPDAYTLWNTVSSIGSLISLLAVIMFLFILWEAFAAKR
Dpbr	VNLTFFPQHFLGLAGMPRRYSDYPDAYTLWNTVSSIGSLISLLAVIMFLFIIWEAFAAKR
Caki	VNLTFFPQHFLGLAGMPRRYSDYPDAYTLWNSVSSIGSLMSLTAVIMFLFILWEAFAAKR
Phja	VNLTFFPQHFLGLAGMPRRYSDYPDAYALWNTISSIGSLISLIAVIMFLFIVWEAFTAKR
Brsp	VNMTFFPQHFLGLAGMPRRYSDYPDAFAFWNLLSSIGSLISLIAVILFMFIVWEAFVSKR
Gamo	VNLTFFPQHFLGLAGMPRRYSDYPDAYTLWNTVSSIGSLISLMAVIMFLFILWEAFAAKR
Lolo	VNLTFFPQHFLGLAGMPRRYSDYPDAYTLWNTVSSIGSLISLMAVIMFLFILWEAFAAKR
Batr	VNLTFFPQHFLGLAGMPRRYSDYPDAYSLWNFVSSTGSIISQVATILLLYMIWEAFSSKR
Prmy	VNLTFFPQHFLGLAGMPRRYSDYPDAYTAWNLVSSLGSTISLMAGIMLLFIIWEAFSAKR
Lose	VNLTFFPQHFLGLAGMPRRYSDYPDAYALWNVISSIGSLISLIAVIIFLFILWEAFASKR
Loam	VNLTFFPQHFLGLAGMPRRYSDYPDAYTLWNTVSSIGSLISLIAVIMFLFILWEAFTAKR
Chab	VNLTFFPQHFLGLAGMPRRYSDYPDAYTLWNTVSSIGSLVSLVAVIMFLFIIWEAFAAKR
Chto	VNLTFFPQHFLGLAGMPRRYSDYPDAYTLWNTVSSIGSLVSLVAVIMFLFIIWEAFAAKR
Majo	VNLTFFPQHFLGLAGMPRRYSDYPDAYTLWNTVSSAGSLISLVAVIMFLFIIWEAFAAKR
Hlst	VNLTFFPQHFLGLAGMPRRYSDYPDAYTLWNAVSSLGSLISLVAVIMFLFIIWEAFAAKR
Clpe	VNLTFFPQHFLGLAGMPRRYSDYPDAYTLWNTVSSVGALISLVAVIMFLFILWEAFASKR
Mlmr	VNLTFFPQHFLGLAGMPRRYSDYPDAYTLWNTVSSIGSLISLVAVIMFLFIIWEAFAAKR
Crcr	VNLTFFPQHFLGLAGMPRRYSDYPDAYTLWNSISSMGSLVSLVAVILFLFIIWEAFVAKR
Muce	VNLTFFPQHFLGLAGMPRRYSDYPDAYTLWNSISSMGSLVSLVAVILFLFIIWEAFVAKR
Bege	VNLTFFPQHFLGLAGMPRRYSDYPDAYTLWNTVSSIGSLISLVAVIMFLFIIWEAFAAKR
Mela	VNLTFFPQHFLGLAGMPRRYSDYPDAYTLWNTVSSFGSLISLIAVIMFLFIIWEAFSAKR
Hats	VNLTFFPQHFLGLAGMPRRYSDYPDAYTLWNTVSSIGSLISLVAVIMFLFIIWEAFAAKR
Orla	VNLTFFPQHFLGLAGMPRRYSDYPDAYTLWNTISSLGSLISLIAVIMFLFILWEAFAAKR

To be continued on page 42.

To be continued on page 43.

Cosa	VNLTFFPQHFLGLAGMPRRYSDYPDAYTLWNTVSSIGSLISLIAVIMFLFIIWEAFAAKR
Exsp	VNLTFFPQHFLGLAGMPRRYSDYPDAYTLWNTISSIGSLISLIAVIMFLFIIWEAFAAKR
Depa	VNLTFFPQHFLGLAGMPRRYSDYPDAYTLWNTVSSIGSLISLVAVIMFLFIIWEAFAAKR
Rima	VNLTFFPQHFLGLAGMPRRYSDYPDAYALWNTISSIGSLISLVAVIMFLFIVWEAFAAKR
Fuol	VNLTFFPQHFLGLAGMPRRYSDYPDAYTLWNTVSSIGSLISLVAVIMFLFIIWEAFAAKR
Gmaf	VNLTFFPQHFLGLAGMPRRYSDYPDAYTLWNTVSSIGSLISLTAVVLFLFILWEAFTAKR
Xeei	VNLTFFPQHFLGLAGMPRRYSDYPDAYTLWNTVSSIGSMVSLIAVIMFLFIIWEAFAAKR
Pros	VNLTFFPQHFLGLAGMPRRYSDYPDAFTLWNTVSSIGSLISLVAVIMFLFILWEAFAAKR
Scmi	VNLTFFPQHFLGLAGMPRRYSDYPDAFTLWNAVSTVGAFISLLAVILFLFILWEAFTAKR
Rolo	VNLTFFPQHFLGLAGMPRRYSDYPDAYTLWNTVSSVGSLISLVAVIMFLFILWEAFAAKR
Cere	VNLTFFPQHFLGLAGMPRRYSDYPDAYTLWNTVSSIGSLISLVAVIMFLFILWEAFAAKR
Daga	VNITFFPQHFLGLAGMPRRYSDYPDAYTLWNSISSLGSLISLIAVLMLLFIVWEAFAAKR
Anco	VNLTFFPQHFLGLAGMPRRYSDYPDAYTLWNTVSSIGSLVSLLAVIMFLFIIWEAFAAKR
Dmve	VNVTFFPQHFLGLAGMPRRYSDYPDAYTLWNTVSSIGSLVSLVAVILFLYIIWEAFTSKR
Dmar	VNVTFFPQHFLGLAGMPRRYSDYPDAYTLWNTVSSIGSLVSLVAVILFLYIIWEAFTSKR
Anka	VNLTFFPQHFLGLAGMPRRYSDYPDAYTLWNTVSSIGSLVSLLAVIMFLFIIWEAFAAKR
Moja	VNLTFFPQHFLGLAGMPRRYSDYPDAYTLWNTVSSIGSLVSLLAVIMFLFIIWEAFAAKR
Hoja	VNLTFFPQHFLGLAGMPRRYSDYPDAYTLWNTVSSIGSLVSLLAVIMFLFIIWEAFAAKR
Bede	VNLTFFPQHFLGLAGMPRRYSDYPDAYTLWNTVSSMGSLVSLVAVIMFLFIIWEAFAAKR
Besp	VNLTFFPQHFLGLAGMPRRYSDYPDAYTLWNTVSSMGSLVSLVAVIMFLFIIWEAFAAKR
Mysp	VNITFFPQHFLGLAGMPRRYSDYPDAYTLWNTVSSIGSLISLVAVIMFLFIIWEAFAAKR
Osja	VNLTFFPQHFLGLAGMPRRYSDYPDAYTLWNTISSIGSLISLVAVIMFLFIIWEAFAAKR
Sgro	VNLTFFPQHFLGLAGMPRRYSDYPDAYTLWNTISSIGSLISLVAVIMFLFIIWEAFTAKR
Pzpa	VNLTFFPQHFLGLAGMPRRYSDYPDAYTLWNTVSSLGSLVSLVAVVMFLFIIWEAFAAKR
Zeja	VNLTFFPQHFLGLAGMPRRYSDYPDAYALWNTVSSIGSLVSLVAVVMFLFIIWEAFAAKR
Znne	VNLTFFPQHFLGLAGMPRRYSDYPDAYALWNTVSSLGSLISLVAVIMFLFIIWEAFAAKR
Zefa	VNLTFFPQHFLGLAGMPRRYSDYPDAYTLWNTVSSMGSLVSLVAVIMFLFIIWEAFAAKR
Acni	VNLTFFPQHFLGLAGMPRRYSDYPDAYTLWNTVSSIGSLVSLVAVIMFLFIIWEAFAAKR
Ncrh	VNLTFFPQHFLGLAGMPRRYSDYPDAYTLWNTVSSIGSLVSLVAVIMFLFIIWEAFAAKR
Agca	VNLTFFPQHFLGLAGMPRRYSDYPDAYTLWNTISSIGSLVSLTAVILFLFIIWEAFAAKR
Hydy	VNLTFFPQHFLGLAGMPRRYSDYPDAYTLWNTVSSIGSLVSLVAVIMFLFIIWEAFAAKR
Gsac	VNLTFFPQHFLGLAGMPRRYSDYPDAYTLWNTVSSIGSLVSLVAVIMFLFIIWEAFAAKR
Pevo	VNLTFFPQHFLGLAGMPRRYSDYPDAYALWNLVSSAGSLVSLVAVIMFLFIVWEAFAAKR
Hiku	VNLTFFPQHFLGLAGMPRRYSDYPDAYSLWNTVSSIGSLVSLIAVIMFLFIIWEAFAAKR
Inpa	VNLTFFPQHFLGLAGMPRRYSDYPDAYTLWNTVSSIGSMISLIAVILFLFILWEAFAAKR
Auch	VNMTFFPQHFLGLAGMPRRYSDYPDAYTLWNTLSSLGSLVSLIAVIMFLFILWEAFTSKR
Fico	VNLTFFPQHFLGLAGMPRRYSDYPDAYTLWNTVSSIGSLISLVAVIMFLFILWEAFAAKR
Macs	VNLTFFPQHFLGLAGMPRRYSDYPDAYTLWNTVSSFGSLVSLVAVIMFLFIIWEAFAAKR
Moal	VNLTFFPQHFLGLAGMPRRYSDYPDAYTFWNTISSFGSLISLVAVIMFLLIIWEAFAAKR
Syma	VNLTFFPQHFLGLAGMPRRYSDYPDAYTFWNVISTIGTAITLMAVLLYMLIIWEAFAAKR
Mafr	VNLTFFPQHFLGLAGMPRRYSDYPDAYTLWNTISSIGSLISLTAVIMFLFIIWEAFATKR
Dcpe	VNVTFFPQHFLGLAGMPRRYSDYPDAYTLWNTVSSIGSLISLVAVIMFLFIIWEAFSAKR
Dcti	VNITFFPQHFLGLAGMPRRYSDYPDAYTLWNTVSSIGSLISLVAVIMFLFIIWEAFAAKR
Hehi	VNLTFFPQHFLGLAGMPRRYSDYPDAYTLWNTVSSIGSLISLVAVIMFLFIIWEAFAAKR
Stam	VNLTFFPQHFLGLAGMPRRYSDYPDAYTLWNTVSSIGSLISLIAVIMFLFIIWEAFAAKR
Hogi	VNLTFFPQHFLGLAGMPRRYSDYPDAYTLWNTVSSIGSLISLTAVIMFLFIIWEAFAAKR
Erzo	VNLTFFPQHFLGLAGMPRRYSDYPDAYTLWNTVSSIGSLVSLVAVVLFLFIIWEAFAAKR
Hxot	VNLTFFPQHFLGLAGMPRRYSDYPDAYTLWNTVSSIGSLVSLVAVIMFLFIIWEAFAAKR
Core	VNLTFFPQHFLGLAGMPRRYSDYPDAYTLWNTVSSIGSLISLVAVIMFLFIIWEAFAAKR
Apve	VNLTFFPQHFLGLAGMPRRYSDYPDAYALWNTISSIGSLVSLVAVIMFLFIIWEAFSAKR
Latj	VNLTFFPQHFLGLAGMPRRYSDYPDAYALWNTMSSIGSLVSLVAVILFLFIIWEAFIAKR
Laja	VNLTFFPQHFLGLAGMPRRYSDYPDAYAMWNTISSIGSLVSLVAVILFLFIIWEAFAAKR

[8/10 of aligned sequences]

To be continued on page 44.

```
Syja  VNLTFFPQHFLGLAGMPRRYSDYPDAYTLWNTVSSIGSLVSLVAVILFLFIIWEAFAAKR
Epme  VNLTFFPQHFLGLAGMPRRYSDYPDAYTLWNTVSSIGSMISLVAVIMFLFIIWEAFASKR
Grse  VNLTFFPQHFLGLAGMPRRYSDYPDAYTLWNTISSIGSLISLVAVIMFLFIIWEAFAAKR
Clja  VNLTFFPQHFLGLAGMPRRYSDYPDAYTLWNTVSSIGSLISLLAVIMFLFIIWEAFAAKR
Ogcy  VNLTFFPQHFLGLAGMPRRYSDYPDAYTLWNTVSSVGSLVSLVAVIMFLFIIWEAFASKR
Plna  VNLTFFPQHFLGLAGMPRRYSDYPDAYTLWNTISSIGSLVSLLAVIMFLFIIWEAFVAKR
Lema  VNLTFFPQHFLGLAGMPRRYSDYPDAYTLWNTISSIGSLVSLVAVIMFLFIIWEAFAAKR
Etzo  VNLTFFPQHFLGLAGMPRRYSDYPDAYTLWNTVSSIGSLISLVAVIMFLFIIWEAFAAKR
Apse  VNLTFFPQHFLGLAGMPRRYSDYPDAYTLWNTVSSLGSLVSLVAVIMFLFIIWEAFSAKR
Epde  VNLTFFPQHFLGLAGMPRRYSDYPDAYTLWNTVSSFGSLISLVAVIVFLFIIWEAFAAKR
Slja  VNLTFFPQHFLGLAGMPRRYSNYPDAYTLWKTVSSIGSLISLVAVIMFLFVIWEAFAAKR
Bsja  VNLTFFPQHFLGLAGMPRRYSDYPDAYTLWNTVSSLGSLISLTAVILFLFIIWEAFAAKR
Ecna  VNLTFFPQHFLGLAGMPRRYSDYPDAYALWNTVSSIGSLVSLVAVVLLLFIIWEAFVAKR
Cohi  VNLTFFPQHFLGLAGMPRRYSDYPDAYTLWNTVSSIGSLVSLIAVVMFLFIIWEALTAKR
Caar  VNLTFFPQHFLGLAGMPRRYSDYPDAYTLWNTISSIGSLVSLVAVIMFLFIIWEAFAAKR
Came  VNLTFFPQHFLGLAGMPRRYSDYPDAYTLWNTVSSLGSLVSLVAVIMFLFIIWEAFAAKR
Mema  VNLTFFPQHFLGLAGMPRRYSDYPDAYTLWNTISSIGSLVSLVAVILFLFIIWEAFAAKR
Lenu  VNLTFFPQHFLGLAGMPRRYSDYPDAYTLWNTISSIGSLVSLVAVIMFLYIIWEAFSAKR
Brja  VNLTFFPQHFLGLAGMPRRYSDYPDAYTLWNSVSSIGSLISLVAVIMFLFIIWEAFAAKR
Plma  VNLTFFPQHFLGLAGMPRRYSDYPDAYTLWNTVSSIGSLISLVAVIMFLFIIWEAFAAKR
Emst  VNLTFFPQHFLGLAGMPRRYSDYPDAYTLWNTVSSIGSLISLVAVIMFLFIIWEAFAAKR
Ptti  VNLTFFPQHFLGLAGMPRRYSDYPDAYTLWNTVSSIGSLISLVAVIMFLFIIWEAFAAKR
Losu  VNLTFFPQHFLGLAGMPRRYSDYPDAYTLWNTVSSIGSLISLVAVIMLLFIIWEAFAAKR
Geoy  VNLTFFPQHFLGLAGMPRRYSDYPDAYTLWNTISSIGSLVSLVAVVLLLFIIWEAFAAKR
Dipi  VNLTFFPQHFLGLAGMPRRYSDYPDAYTLWNTVSSIGSMISLVAVILFLFIIWEAFAAKR
Pama  VNLTFFPQHFLGLAGMPRRYSDYPDAYTLWNTISSIGSLISLVAVILFLFILWEAFASKR
Leob  VNLTFFPQHFLGLAGMPRRYSDYPDAYTLWNTVSSIGSMISLVAVIMFLFIIWEAFAAKR
Neba  VNLTFFPQHFLGLAGMPRRYSDYPDAYTLWNTVSSIGSMVSLVAVILFLFIIWEAFAAKR
Pdpl  VNLTFFPQHFLGLAGMPRRYSDYPDAYSLWNTISSIGSLVSLVAVIMFLFIIWEAFAVKR
Nimi  VNLTFFPQHFLGLAGMPRRYSDYPDAYALWNTVSSMGSLISLVAVVLLLFIIWEAFTAKR
Uptr  VNLTFFPQHFLGLAGMPRRYSDYPDAYTLWNTVSSIGSLISLVAVIMFLFIIWEAFAAKR
Pesc  VNLTFFPQHFLGLAGMPRRYSDYPDAYALWNTVSSIGSLISLTAVVLFLFIIWEAFSAKR
Baar  VNLTFFPQHFLGLAGMPRRYSDYPDAYTLWNTVSSIGSLVSLVAVILFLFIIWEAFAAKR
Moar  VNLTFFPQHFLGLAGMPRRYSDYPDAYTLWNTVSSIGSLISLVAVIMFLFIIWEAFAAKR
Toja  VNLTFFPQHFLGLAGMPRRYSDYPDAYTLWNSISSIGSLISLVAVIMFLFIIWEAFIAKR
Chau  VNLTFFPQHFLGLAGMPRRYSDYPDAYTLWNTMSSFGSLISLTAVIMFLFIIWEAFAAKR
Chse  VNLTFFPQHFLGLAGMPRRYSDYPDAYTLWNSISSMGSLVSLLAVSLFMFIIWEAFSTKR
Enar  VNLTFFPQHFLGLAGMPRRYSDYPDAYTLWNSISSIGSLVSLVAVVLFLYIIWEAFAAKR
Hpty  VNLTFFPQHFLGLAGMPRRYSDYPDAYTLWNTTSSIGSLISLVAVILFLFIIWEAFAAKR
Nana  VNLTFFPQHFLGLAGMPRRYSDFPDAYALWNTVSSIGSLVSLVAVIMFLFILWEAFAAKR
Mcst  VNLTFFPQHFLGLAGMPRRYSDYPDAYTLWNTVSSIGSLISLVAVIMFLFIIWEAFAAKR
Rhox  VNLTFFPQHFLGLAGMPRRYSDYPDAYTLWNTVSSIGSLVSLVAVILFLFIIWEAFAAKR
Opfa  VNLTFFPQHFLGLAGMPRRYSDYPDAYTLWNTVSSIGSLISLVAVIMFLFIIWEAFAAKR
Paar  VNLTFFPQHFLGLAGMPRRYSDYPDAYTLWNTVSSIGSLISLMAVVMFLFIIWEAFSAKR
Gozo  VNLTFFPQHFLGLAGMPRRYSDYPDAYTLWNTVSSIGSLISLVAVIMFLFIVWEAFAAKR
Ackr  VNLTFFPQHFLGLAGMPRRYSDYPDAYALWNSISSFGSLVSMVGVAMFLFIIWEAFASKR
Elev  VNLTFFPQHFLGLAGMPRRYSDYPDAYTLWNTVSSLGSLVSLVAVIMFLFIIWEAFVAKR
Trdu  VNMTFFPQHFLGLAGMPRRYSDYPDAYTLWNTVSSIGSMISLIAVIMFLFIIWEAFATKR
Amoc  VNLTFFPQHFLGLAGMPRRYSDYPDAYTMWNTVSSIGSLVSLVAVIMFLFIIWEAFAAKR
Hame  VNLTFFPQHFLGLAGMPRRYSDYPDAYTLWNTVSSIGSLISLVAVIMFLFIIWEAFASKR
Chso  VNLTFFPQHFLGLAGMPRRYSDYPDAYTLWNTISSIGSLISLVAVIMFLFIIWEAFTAKR
Lyto  VNLTFFPQHFLGLAGMPRRYSDYPDAYTLWNTVSSVGSLVSLIAVIMFLFIIWEAFAAKR
```

[8/10 of aligned sequences]

To be continued on page 45.

```
Encr    VNLTFFPQHFLGLAGMPRRYSDYPDAYTLWNTVSSIGSLISLVAVIMFLFIIWEAFAAKR
Bvar    VNLTFFPQHFLGLAGMPRRYSDYPDAYTLWNTVSSIGSLISLVAVIMFLFIIWEAFAAKR
Noco    VNLTFFPQHFLGLAGMPRRYSDYPDAYTLWNTVSSIGSLVSLVAVIMFLFIIWEAFAAKR
Chsp    VNMTFFPQHFLGIAGMPRRYSDYPDAYTLWNSVSSLGALISLVAVVLFLFIIWEAFTAKR
Arja    VNLTFFPQHFLGLAGMPRRYSDYPDAYTLWNTVSSIGSLISLAAVIMFLFIIWEAFAAKR
Pase    VNLTFFPSTFLGLAGMPRRYSDYPDAYTLWNTISSIGSLISLIGVILFLCIIWEAFSAKR
Trel    VNLTFFPQHFLGLAGMPRRYSDYPDAYTLWNTVSSIGSLISLIAVIMFLFIIWEAFAAKR
Lifa    VNLTFFPQHFLGLAGMPRRYSDYPDAYTLWNTISSMGSLVSLVAVVLFLFIIWEAFATKR
Acur    VNLTFFPQHFLGLAGMPRRYSDYPDAYALWNTISSIGSLVSLVAVVIFLFIIWEAFAAKR
Ampe    VNLTFFPQHFLGLAGMPRRYSDYPDAYTLWNTVSSIGSMISLVGVILFLCIIWEAFAAKR
Urja    VNLTFFPQHFLGLAGMPRRYSDYPDAYALWNTVSSIGSMISLVGVVLFLCITWEAFAAKR
Enet    VNLTFFPQHFLGLAGMPRRYSDYPDAYTLWNTVSSIGSLISLVAVIMFLFIIWEAFAAKR
Ptbr    VNLTFFPQHFLGLAGMPRRYSDYPDAYTLWNTVSSIGSLVSLLAVILFLFIIWEAFAAKR
Safa    VNLTFFPQHFLGLAGMPRRYSDYPDAYTLWNTISSIGSLVSLVAVIMFLFIIWEAFAAKR
Icae    VNLTFFPQHFLGLAGMPRRYSDYPDAYTLWNTVSSIGSLISLVAVIMFLFIIWEAFAAKR
Asmi    VNITFFPQHFLGLAGMPRRYSDYPDAYTLWNTISSIGSLVSLIAVIILLFIIWEAFVAKR
Foal    VNLTFFPQHFLGLAGMPRRYSDYPDAYALWNTVSSIGSMISLVAVIMFLFILWEAFSAKR
Drze    VNITFFPQHFLGLAGMPRRYSDYPDAYTLWNMVSSIGSIISLIAVIMFLFIIWEAFATKR
Rhas    VNLTFFPQHFLGLAGMPRRYSDYPDAYTLWNTVSSLGSLVSLTAVIMFLFIIWEAFASKR
Elac    VNLTFFPQHFLGLAGMPRRYSDYPDAYTLWNTVSSLGSLVSLTAVIMFLFIIWEAFAAKR
Kugu    VNLTFFPQHFLGLAGMPRRYSDYPDAYTLWNTISSMGSLVSLVAVIMFLFIIWEAFTAKR
Plor    VNLTFFPQHFLGLAGMPRRYSDYPDAYTLWNTVSSIGSLVSLVAVIMFLFIIWEAFVAKR
Sgun    VNLTFFPQHFLGLAGMPRRYSDYPDAYTLWNTVSSIGSLISLVAVIMFLFIIWEAFAAKR
Zaco    VNLTFFPQHFLGLAGMPRRYSDYPDAYTLWNTVSSIGSLISLVAVIMFLFIIWEAFAAKR
Zbfl    VNLTFFPQHFLGLAGMPRRYSDYPDAYTLWNTVSSIGSLISLVAVIMFLFIIWEAFAAKR
Spba    VNLTFFPQHFLGLAGMPRRYSDYPDAYTVWNTISSIGSMVSLVAVILFLFIIWEAFAVKR
Game    VNLTFFPQHFLGLAGMPRRYSDYPDAYTLWNTISSIGSLISLVAVIMFLFIIWEAFAAKR
Thth    VNLTFFPQHFLGLAGMPRRYSDYPDAYTLWNTISSIGSLISLVAVIMFLFIIWEAFAAKR
Xigl    VNLTFFPQHFLGLAGMPRRYSDYPDAYALWNTVSSIGSLVSLVAVIMFLFIIWEAFTAKR
Hyja    VNLTFFPQHFLGLAGMPRRYSDYPDAYAVWNTVSSIGSLVSLVAVIMFLFIIWEAFAAKR
Psan    VNLTFFPQHFLGLAGMPRRYSDYPDAYTLWNTISSIGSLVSLTGVIMFLFIIWEAFTAKR
Cupa    VNLTFFPQHFLGLAGMPRRYSDYPDAYTLWNTVSSIGSLISLVAVIMFLFIIWEAFAAKR
Mpch    VNLTFFPQHFLGLAGMPRRYSDYPDAYTLWNTMSSIGSLISLVAVILFLYIIWEAFVSKR
Char    VNLTFFPQHFLGLAGMPRRYSDYPDAYTLWNTISSIGSMISLVAVTMFLYIIWEAFVTKR
Pser    VNLTFFPQHFLGLAGMPRRYSDYPDAYTLWNTISSIGSMVSLVAVIMFLFIIWEAFAAKR
Prol    VNLTFFPQHFLGLAGMPRRYSDYPDAYALWNTVSSIGSLMSLVAVIMFLFIIWEAFSAKR
Plbi    VNLTFFPQHFLGLAGMPRRYSDYPDAYTLWNTISSIGSLMSLVAVILFLFIIWEAFTAKR
Calu    VNLTFFPQHFLGLAGMPRRYSDYPDAYTLWNGVSSIGSLVSLVAVIMFLFIIWEAFSAKR
Papa    VNLTFFPQHFLGLAGMPRRYSDYPDAYTLWNTISSVGSMVSLVAVIMFLFIIWEAFSTKR
Sufr    VNLTFFPQHFLGLAGMPRRYSDYPDAYTLWNTVSSIGSLVSLMAVILFLFIIWEAFAAKR
Stci    VNLTFFPQHFLGLAGMPRRYSDYPDAYTLWNTVSSFGSLISLVAVIMFLFIIWEAFAAKR
Taru    VNLTFFPQHFLGLAGMPRRYSDYPDAYALWNSVSSIGSMVSLVAVIMFLFILWEAFTAKR
Rala    VNLTFFPQHFLGLAGMPRRYSDYPDAYTLWNTVSSIGSLISLVAVIMFLFIIWEAFAAKR
        **:****. ***:********::***:: *:  *: *: ::  .  : : : ***:  **
```

[9/10 of aligned sequences]

```
Scca   EVLSIELPNTNVEWLHGCPPPYHTYEEPAFVQVQRSF*----------------------
Muma   EVLSVELPHTNVEWLHGCPPPYHTYEEPAFVQVQQPSF*---------------------
Erca   EVQTVNLTYTNVEWLHGCPPPYHTYEEPAFVQSPNSRE*---------------------
Pose   EVQTVELTYTNVEWLHGCPPPYHTYEEPAFVQSPQARE*---------------------
Actr   EVMSVELTTTNVEWLHGCPPPYHTYEEPAFVQVQSTN*----------------------
Scal   EVMSVELTTTNVEWLHGCPPPYHTYEEPAFVQVQSTN*----------------------
Posp   EVLSVELTATNVEWLHGCPPPYHTYEEPAFVQVQSTN*----------------------
Atsp   EVLSVELTSTNVEWLHECPPPYHTYEEPAFVQVQAQQE*---------------------
Leoc   EVLSVELTSTNVEWLHECPPPYHTYEEPAFVQVQVTTT*---------------------
Amca   EVLAVEYAATNVEWLHGCPPPYHTYEEPAYVQAHRA*-----------------------
Osbi   EVQTAELTTTNAEWLHGCPPPYHTFEEPAFVQIQLPLTEK-G*-----------------
Pabu   EVLSVELTHTNVEWLNGCPPPYHTFEEPAFVQVQTKRE*---------------------
Hial   EVMSVELTATNVEWLHGCPPPYHTFEEPAFVQVQSGKRE*--------------------
Elha   EVLAVELTATNIEWLHGCPPPYHTFEEPAFVQMQYTFTHPDLLNPAYYWRG*--------
Mlcy   EVMAVELTTTNVEWLHGCPPPYHTFEEPAFVQMQYTFTHPDLLNPNYYWRG*--------
Algl   EVLSVELAENNVEWLHGCPPPYHTFEEPAFVQVQSNY*----------------------
Ptgi   EVLSVELTAMNVEWLHGCPPPYHTFEEPAFVQVQSNWRE*--------------------
Alaf   EVLSVELTAMNVEWLHGCPPPYHTFEEPAFVQVQSAFEKG*-------------------
Nock   EVLSVELTAMNVEWLHGCPPPYHTFEEPAFVQVQPAFEKG*-------------------
Anja   EVKWVELTETNVEWLHGCPPPYHTFEEPAYVRVQPPSEDQKSEAKAHIQE*---------
Gyki   TVKWVELTPTNVEWLHGCPPPYHTFEEPGYVRVHPAWDYK-FWDTNPLYGKVVKYS*---
Syka   EVKWVELTTMNVEWLHGCPPPYHTFEEPAYVRVQPAWAGPHSNEITQPQKG*--------
Opma   EVKWVELTTSNIEWLNGCPPPYHTFEEPAYVRVHPAWAYK-FWDNNPLFKKGYYTPKYYN
Comy   EVKWVELTTSNVEWLHGCPPPYHTFEEPAYVQVQPY-KFK-YY*----------------
Sasp   TVVGAELTRANIEWMHGCPPPHHTFEEPAYVQIQLYWGCPELWVPYIPWKEPPLQEEGYK
Eupe   EVLGAELTMTNVEWINGCPPPYHTFEEPAYVQIQLYWGAPELWVPMIPFKEDPTCP--FK
Enja   EVASVELTITNVEWLHGCPPPYHTFEEPAFVQVK*-------------------------
Same   EVSSVELTMTNVEWLHGCPPPYHTFEEPAFVQVQAK*-----------------------
Chch   EVLSVELTATNVEWLHGCPPPYHTFEEPAFVQVQSN*-----------------------
Grgr   EVLSVEMASTNVEWLHGCPPPYHTFEEPAFVLVQE*------------------------
Caau   EVLSVELTMTNVEWLHGCPPPYHTYEEPAFVQIQSN*-----------------------
Cyca   EVLSVELTATNVEWLHGCPPPYHTYEEPAFVQIQSN*-----------------------
Dare   EVLSVELTATNVEWLHGCPPPYHTFEEPAFVQIQSN*-----------------------
Cost   EVLSVELTATNAEWLHGCPPPYHTFEEPAFVQVQSN*-----------------------
Leec   EVSSVELTMTNVEWLHGCPPPYHTFEEPAFVQVQSN*-----------------------
Fola   QVMSVELTMTNVEWLHGCPPPYHTFEEPAFVQVRSN*-----------------------
Clmc   EVLSVEFTSTNVEWLHGCPPPYHTFEEPAFVQVQPWRE*---------------------
Phin   EVLSIELTPTNVEWLHGCPPPYHTFEEPAFVQVQSYEKG*--------------------
Icpu   EVLSVELTSTNVEWLHGCPPPYHTFEEPAFVQVQTN*-----------------------
Psto   EVLSVELTSTNVEWLHGCPPPYHTFEEPAFVQVRTN*-----------------------
Cora   EVLSVELTPTNAEWLHGCPPPYHTFEEPAFVQIQEIEKG*--------------------
Eisp   EVLSVELTATNPEWLHGCPPPYHTFEEPAFVQVQSHKSSSS*------------------
Apal   EVLFTELTSTNVEWLHGCPPPYHTCEEPAYVQPQPYKFA*KEGI---------------E
Eslu   EVMSVEMTSTNVEWLHGCPPPHHTFEEPAFVQVQTN*-----------------------
Dape   EVLSIEMTSTNAEWLHGCPPPYHTFEEPAFVQVQSN*-----------------------
Glse   EVMQVELTSTNVEWLHGCPPPYHTFEEPAFVQVRTN*-----------------------
Naar   EVMSVELTSTNVEWLHGCPPPYHTFEEPAFVQVQTN*-----------------------
Lioc   EVLSVALTSTNVEWLHGCPPPYHTFEEPAFVQVQTK*-----------------------
Opso   EVMSIELTSTNVEWLHGCPPPYHTFEEPAFVQVQTS*-----------------------
Alte   EVLSVELTATNVEWLHGCPPPYHTFEEPAFVQVQAN*-----------------------
Plap   EVLSVELTATNVEWLHGCPPPYHTFEEPAFVQVQAN*-----------------------
```

To be continued on page 46.

[9/10 of aligned sequences]

To be continued on page 47.

```
Plal  EVMSVELTSTNVEWLHGCPPPYHTFEEPAFVQVQAN*-----------------------
Sami  EVMSVELTSTNVEWLHGCPPPYHTFEEPAYVQVQAH*-----------------------
Rere  EVMSVELTSTNVEWLHGCPPPYHTFEEPAFVQVQAH*-----------------------
Gama  EVLSVELTATNVEWLHGCPPPYHTFEEPAFVQVQAH*-----------------------
Onmy  EVASIELTSTNVEWLHGCPPPYHTFEEPAFVQVQAN*-----------------------
Sasa  EVASIEMTSTNVEWLHGCPPPYHTFEEPAFVQVQAS*-----------------------
Cola  EVASIELTSTNVEWLHGCPPPYHTFEEPAFVQVQAA*-----------------------
Dita  EVLSVELTTTNVEWLHGCPPPYHTFEEPAFVQVQITDEKG*-------------------
Gogr  EVHSIELTATNVEWLHGCPPPYHTFEEPAFVQHCS*------------------------
Chsl  EVLTVEMATTNVEWLHGCPPPYHTFEEPAFVRIRD*------------------------
Atja  EVASIELTSTNIEWLHGCPPPYHTFEEPAFVLVKAG*-----------------------
Iido  EVASIELTSTNIEWLHGCPPPYHTFEEPAFVLVKTA*-----------------------
Auja  EVMSVELTSTNVEWLHGCPPPYHTFEEPAFVQVQTN*-----------------------
Chag  EVGSVDMTSTNVEWLHGCPPPYHTFEEPAFVQVKSH*-----------------------
Hami  EVAAVELTATNVEWLHGCPPPYHTFEEPAFVQVQGK*-----------------------
Saun  EVAAVELTATNVEWLHGCPPPYHTFEEPAFVQVQGK*-----------------------
Nema  EVLSVELTTTNVEWLHGCPPPYHTFEEPAFVQVQTT*-----------------------
Disp  EVLSVELTTTNVEWLHGCPPPYHTFEEPAFVQIRTN*-----------------------
Myaf  EVLSVEMTTTNVEWLHGCPPPYHTFEEPAFVQIRKG*-----------------------
Lagu  EVLSVELASTNVEWLHGCPPPHHTFEEPAFVQPNKLREK*--------------------
Trtr  EVLSVEMASTNVEWLHGCPPPYHTFEEPAFVQVQTN*-----------------------
Zucr  EVMSVEMASTNVEWLHGCPPPYHTFEEPAYVQVQTN*-----------------------
Pxja  EVLSVELTTTNVEWLHGCPPPYHTFEEPAFVQVRSN*-----------------------
Pxlo  EVLSVELTATNVEWLHGCPPPYHTFEEPAFVQVRSN*-----------------------
Pctr  EVASVEMTTTNVEWLHGCPPPYHTFEEPAFVLVRNT*-----------------------
Apsa  EVLSVEMTSTNAEWLHGCPPPYHTFEEPAFVQVQTRG*----------------------
Cabe  EVLSVEMTTTNVEWLHGCPPPYHTFEEPAFVQIQAGSEK*--------------------
Bzze  EVLSVELTSTNVEWLHGCPPPYHTFEEPAFVRVRLD*-----------------------
Siim  EVLFVEYADTNVEWLHGCPPPYHTFEEPAFVQTRLY*-----------------------
Ctru  EVMSVELTSTNVEWLHGCPPPYHTFEEPAFVQVQTN------------------------
Dpbr  EVMSVELTSTNVEWLHGCPPPYHTFEEPAFVQVQTN*-----------------------
Caki  EVKAVELTLTNVEWLHGCPPPYHTFEEPAFVQSHSS*-----------------------
Phja  EVLLLELTSTNIEWLHGCPPPAHTFEEPAFVQTQIPWYEKG*------------------
Brsp  EVAYVDMASSNLEWLHGTPPPYHTFEEPPFMLSPRRND*---------------------
Gamo  EVMAVEMTMTNVEWLHGCPPPYHTFEEPAFVQIQTR*-----------------------
Lolo  EVLAVELTMTNVEWLHGCPPPYHTFEEPAFVQVQTR*-----------------------
Batr  TPSNLELMPTNLEWLNNTPPPYHTFEEPTFTTLPNKK*----------------------
Prmy  KISTIEFMSTNMEWLNNSPPAYHTFEEPPFVITPIKKS*---------------------
Lose  EVQAVDMTTTNVEWLHGCPPPYHTFEEPAFVQTQLGAHK*--------------------
Loam  EVLSVELAATNVEWLHGCPPPYHTFEEPAFVQVQTN*-----------------------
Chab  EVLSVELTTTNVEWLHGCPPPYHTFEEPAFVQVQTNW-----------------------
Chto  EVLSVELTTTNVEWLHGCPPPYHTFEEPAFVQVQTNWS*---------------------
Majo  EVLSVELTTTNVEWLHGCPPPYHTFEEPAFVQVQAN------------------------
Hlst  EVLSVELTTTNVEWLHGCPPPYHTFEEPAFVQVQMTYNRE*-------------------
Clpe  EVVAVELTITNVEWLHGCPPPYHTFEEPAFVQVQAN*-----------------------
Mlmr  EVLSVELAMTNVEWLNGCPPPYHTFEEPAFVQVQTN*-----------------------
Crcr  EIRAVELTSTNVEWLHGCPPPYHTFEEPAFVLVQRPLPTKKS*-----------------
Muce  EIRAVELTSTNVEWLHGCPPPYHTFEEPAFVLVQRPLPTKKS*-----------------
Bege  EVFSVELTATNVEWLHGCPPPYHTFEEPAFVQVQQD*-----------------------
Mela  EVLSVEFTATNLEWLHGCPPPYHTFEEPAFVQIPQA*-----------------------
Hats  EVLSVELTATNVEWLYGCPPPYHTFEEPAFVQIQQG*-----------------------
Orla  EVLSVELTATNVEWLHGCPPPYHTFEEPAFVQIQQPKF*---------------------
```

[9/10 of aligned sequences]

```
Cosa   EVASVELTSTNVEWLHGCPPPYHTFEEPAFVQIQQTN*----------------------
Exsp   EVLSVELTSTNVEWLHGCPPPYHTFEEPAFVQIKQTI*----------------------
Depa   EVSSVELVTTNVEWLHGCPPPYHTFEEPAFVQTQQVN*----------------------
Rima   EVLSVELTATNAEWLHGCPPPYHTFEEPAFIQFNTSDL*---------------------
Fuol   EVLSVEMTATNVEWLHGCPPPYHTFEEPAFVQIQHNS*----------------------
Gmaf   EVLSTHLITTNVEWLHGCPPPYHTFEEPAFVQLQQTSK*---------------------
Xeei   EVLSVEMTPTNVEWLHGCPPPYHTFEEPAFVQIQHN*-----------------------
Pros   EVLSVELTTTNVEWLHGCPPPYHTFEEPAFVLVRAD*-----------------------
Scmi   EFLYADLIETNVEWLHGCPPPYHTFEEPAFVLCTRTL*----------------------
Rolo   EVLSVEMTTTNVEWLHGCPPPYHTFEEPAFVQVRTS*-----------------------
Cere   EVLSVELTSTNVEWLHGCPPPYHTFEEPAFVQVQTT*-----------------------
Daga   EVTSVELTSTNVEWLHGCPPPYHTFEEPAFVRA*--------------------------
Anco   EVLSVELTATNVEWLHGCPPPYHTFEEPAFVQVQTN*-----------------------
Dmve   EVIMVDLVSTNVEWLHGCPPPYHTFEEPAFVRV*--------------------------
Dmar   EVIMVDLVSTNVEWLHGCPPPYHTFEEPAFVRI*--------------------------
Anka   EVLSVELTSTNVEWLHGCPPPYHTFEEPAFVQVQTN*-----------------------
Moja   EVLSVELTSTNVEWLHGCPPPYHTFEEPAFVQVQAN*-----------------------
Hoja   EVLSVELTSTNVEWLHGCPPPYHTFEEPAFVQVQTN*-----------------------
Bede   EVLSVELTATNVEWLHGCPPPYHTFEEPAFVLVQAD*-----------------------
Besp   EVLSVELTATNVEWLHGCPPPYHTFEEPAFVLVQAD*-----------------------
Mysp   EVASVELTMTNVEWLHGCPPPYHTFEEPAFVQVQKKLT*---------------------
Osja   EVMSVELTMTNVEWLHGCPPPYHTFEEPAFVQVQTN*-----------------------
Sgro   EVLSVELTTTNVEWLHGCPPPYHTFEEPAFVQVQTN*-----------------------
Pzpa   EVLLVEMTSTNIEWLHGCPPPYHTFEEPAFVQVNQPYEEG*-------------------
Zeja   EVLSVKLTSTNIEWLHGCPPPYHTFEEPAFVQINAAKWE*--------------------
Znne   EVLSVELTSMNVEWLHGCPPPYHTFEEPAFVQISSSKRE*--------------------
Zefa   EVSSVEMTSTNVEWLHGCPPPYHTFEEPAFVQINLSAEKG*-------------------
Acni   EVLSVEMTSTNVEWLHGCPPPYHTFEEPAFVQISLANEKG*-------------------
Ncrh   EVLSVEMTSTNVEWLHGCPPPYHTFEEPAFVQISLANEKG*-------------------
Agca   EVLSVQLTTTNVEWLHGCPPPYHTFEEPAFVQVNSKHRE*--------------------
Hydy   EVLAVELTTTNVEWLHGCPPPYHTFEEPAFVQVQSN*-----------------------
Gsac   EVLAVEMTTTNVEWLHGCPPPYHTFEEPAFVQVQSN*-----------------------
Pevo   EVAMVELTTTNVEWLHGCPPPYHTFEEPAYVQVQAN*-----------------------
Hiku   EVLMVELTSTNIEWLHGCPPPYHTFEEPAFVQVQTNN*----------------------
Inpa   EVCSVEMTTTNVEWLHGCPPPYHTFEEPAFVQVHTR*-----------------------
Auch   EVLSTEMTTTNMEWLHGCPPPYHTLEEPTFVLPQQE*-----------------------
Fico   EVMSVELTTTNVEWLHGCPPPYHTFEEPAFVQVQAN*-----------------------
Macs   EVMSVELASTNVEWLHGCPPPYHTFEEPAFVQVQSN*-----------------------
Moal   EVLSISHTTTNLEWLHGCPPPYHTFEEPAFVQVNHHRE*---------------------
Syma   EVCAVELTSSNVEWLHGCPPPYHTFEEPAFIQIQTRATRQPYQMYY-------------R
Mafr   EVLSIELTPTNAEWLHGCPPPYHTFEEPAFVQVHSN*-----------------------
Dcpe   EVLSVELTTTNVEWLHGCPPPYHTFEEPAFVLVKAD*-----------------------
Dcti   EVLSVELTTTNVEWLHGCPPPYHTFEEPAFVLVKAD*-----------------------
Hehi   EVLATDLTTTNVEWLHGCPPPYHTFEEPAFVQVQAD*-----------------------
Stam   EVLAVELTATNVEWLHGCPPPYHTFEEPAFVQVQSN*-----------------------
Hogi   EVSAVELTTTNVEWLHGCPPPYHTFEEPAFVQVRSN*-----------------------
Erzo   EVLAVELTATNVEWLHGCPPPYHTFEEPAFVQVQPN*-----------------------
Hxot   EVLAVELTTTNVEWLHGCPPPYHTFEEPAFVLVQSN*-----------------------
Core   EVLAVELTTTNVEWLHGCPPPYHTFEEPAFVLVQSN*-----------------------
Apve   EVLAVELTTTNVEWLHGCPPPYHTFEEPAFVLAQSN*-----------------------
Latj   EVLAVEFTATNVEWLHGCPPPYHTFEQPAFVQTRNS*-----------------------
Laja   EVLAVEMAATNVEWLHGCPPPYHTFEEPAYVRVRLD*-----------------------
```

To be continued on page 48.

[9/10 of aligned sequences]

To be continued on page 49.

```
Syja EVLSVEMSTTNVEWLHGCPPPYHTFEEPAFVQVQWTYEKG*-------------------
Epme EVLAVELTMTNVEWLHGCPPPYHTFEEPAFVQIRSH*-----------------------
Grse EVSVVALTTTNVEWLHGCPPPYHTFEEPAFVQVRSN*-----------------------
Clja EVSAVELTSTNVEWLHGCPPPYHTFEEPAFVQVQSK*-----------------------
Ogcy EVLFVEMTTTNVEWLHGCPPPYHTFEEPAFVQIQVYNLEPTPTQT---------------
Plna EVLSVEMASANVEWLHGCPPPYHTFEEPAFVLLYVPIAMRIIQVFVASRKM-------FR
Lema EVLAVELTTTNVEWLHGCPPPYHTFEEPAFVQVQSN*-----------------------
Etzo EVLAVELTMTNVEWLHGCPPPYHTFEEPAFVQVQSN*-----------------------
Apse EVLSVELTETNVEWLHGCPPPYHTFEEPAFVQVQLN*-----------------------
Epde EVLSVELTTTNIEWLYGCPPPYHTFEEPAFVQVQSANEKG*-------------------
Slja EVQSVELTMMNVEWLHGCPPPYHTFEEPAFVQVQSK*-----------------------
Bsja EVKAVELTTTNVEWLHGCPPPYHTFEEPAFVQVRSKTRRE*-------------------
Ecna EVLSVEMTHLNVEWLHGCPPPYHTFEEPAFVQIRSN*-----------------------
Cohi EVLSVEMTMTNVEWLHGCPPPYHTFEEPAFVQARTTT*----------------------
Caar EVLSVELTATNVEWLHGCPPPYHTFEEPAFVQIRSN*-----------------------
Came EVLSVELTATNVEWLHGCPPPYHTFEEPAFVQIRSN*-----------------------
Mema EVLSVQLTATNVEWLHGCPPPYHTFEEPAFVQIRLH*-----------------------
Lenu EVLSVEMAMMNVEWLHGCPPPYHTFEEPAFVQVSHK*-----------------------
Brja EVLSVDMTTTNVEWLHGCPPPYHTFEEPAFVLVQSD*-----------------------
Plma EVLSVELTTTNVEWLHGCPPPYHTFEEPAFVLVQSD*-----------------------
Emst EVLSVELTMTNVEWLHGCPPPYHTFEEPAFVQVQSN*-----------------------
Ptti EVMSVELTMTNVEWLHGCPPPYHTFEEPAFVQVQSN*-----------------------
Losu EVLSVELTSTNVEWLHGCPPPYHTFEEPAFVQTQTTLVT*--------------------
Geoy EVLSVELTATNIEWLHGCPPPYHTFEEPAFVQVQSN*-----------------------
Dipi EVSSVELTTTNVEWLHGCPPPYHTFEEPAFVQVQAYYEKG*-------------------
Pama EVRAADMTSTNVEWLHGCPPPYHTFEEPAFVQVKVRTSRE*-------------------
Leob EVLSVDLTMTNVEWLHGCPPPYHTFEEPAFVQVQSK*-----------------------
Neba EVLSVQHTATNVEWLHGCPPPYHTFEEPAFVQVQTK*-----------------------
Pdpl EVLALELTHTNVEWLHGCPPPYHTFEQPAFVQIRTN*-----------------------
Nimi EVLGVRFTTTNVEWLHGCPPPYHTFEEPPFVQVQYSRT*---------------------
Uptr EVLAVELTATNVEWLHGCPPPYHTFEEPAFVQVQSH*-----------------------
Pesc EVLYVEFASTNVEWLHGCPPPHHTFEEPAFVRAQST*-----------------------
Baar EVLAVEMTPTNVEWLHGCPPPYHTFEEPAFVRVQSY*-----------------------
Moar EVLSVELTATNVEWLHGCPPPYHTFEEPAFVQVQSN*-----------------------
Toja EVLSVEMTSTNVEWLHGCPPPYHTFEQPAFVQTRLY*-----------------------
Chau EVRSVELTTMNVEWLHGCPPPYHTFEEPAFVQVN*-------------------------
Chse EVLSVEHTVTNVEWLHGCPPPYHTFEEPAFVQVQSR*-----------------------
Enar EVLAVELASTNVEWLHGCPPPYHTFEEPAFVQVQAY*-----------------------
Hpty EVLAVELTTTNVEWLHGCPPPYHTFEEPAFVQVQSN*-----------------------
Nana EVLSVEMVYTNVEWLHGCPPPYHTFEEPAFVQAQEN*-----------------------
Mcst EVLAVELTMTNVEWLHGCPPPYHTFEEPAFVQVQTN*-----------------------
Rhox EVLSVEMTATNVEWLHGCPPPYHTFEEPAFVQVQVSSY*---------------------
Opfa EVLSVELTTTNVEWLHGCPPPYHTFEEPAFVQIQSN*-----------------------
Paar EVLAVELTMTNVEWLHGCPPPYHTFEEPAFVQVQSN*-----------------------
Gozo EVLAVELTETNVEWLHGCPPPYHTFEEPAFVQVRTN*-----------------------
Ackr EVLGTDLTATNVEWLHGCPPPYHTFEEPAFVLMHSK*-----------------------
Elev EVFAVEMTMTNVEWLHGCPPPYHTFEEPAFVQIQHN*-----------------------
Trdu EVSSVELTATNVEWLHGCPPPYHTFEEPAFVQVQQAWLNYEKPASAPSNPH*--------
Amoc EVLWVELTSTNVEWLHGCPPPYHTFEEPAFVQIQQTSLEHK*------------------
Hame EVISVELTITNVEWLHGCPPPYHTFEEPAFVQVQGKKRE*--------------------
Chso EVMSVELTSTNIEWLHGCPPPYHTFEEPAFVQVQTNSRE*--------------------
Lyto EVLAVELTTTNVEWLHGCPPPYHTFEEPAFVQVQSN*-----------------------
```

To be continued on page 50.

```
Encr      EVLAVELTTTNVEWLHGCPPPYHTFEEPAFVQVQSN*-----------------------
Bvar      EVLAVGLTTTNVEWLHGCPPPYHTFEEPAFVQIRTT*-----------------------
Noco      EVSSVELTTTNVEWLHGCPPPYHTFEEPAFVQIHYN*-----------------------
Chsp      VVETVMATTTNIEWLMGCPPPSHTFEEPVFVQVQTYHTHKLLPTSL*-------------
Arja      EVLAVELTTTNVEWLHGCPPPYHTFEEPAFVLAQSN*-----------------------
Pase      EVQAVALTTTNLEWLYGCPPPYHTFEEPVFVQIQSTPEKG*-------------------
Trel      EVLNVHMTSTNVEWLHGCPPPYHTFEEPAYVQIQSA*-----------------------
Lifa      EVLAVEMTSTNVEWLHGCPPPYHTFEEPAFVQVQVD*-----------------------
Acur      IVSAVALTSTNIEWLNGCPPPYHTFEEPAFVQVQTS*-----------------------
Ampe      EVLSVELTATNLEWLYGCPPPYHTFEEPVFVQVQTGLRE*--------------------
Urja      EISLIKHTTTNLEWLYGCPPPYHTFEEPVFVQIQAYNEKG*-------------------
Enet      EVLAVELAMTNVEWLHGCPPPYHTFEEPAFVQVQSAPLS*--------------------
Ptbr      EITTLAPNLVHVEWLHGCPPPYHTFEEPAFVQVQHLNPQS*-------------------
Safa      EVSAVELTLTNVEWLHGCPPPYHTFEEPAFVQIQQTSPIYTS--P*--------------
Icae      EVLSVELTATNVEWLHGCPPPYHTFEEPAFVLVRSD*-----------------------
Asmi      VVTSVEMTASNVEWLHGYPPPYHTFEEPVFFQTQQPWLQNHQTV*---------------
Foal      EVLSVEQTATNVEWLYGCPPPYHTFEEPAFVQVQCRE*----------------------
Drze      EVMSVQLASTNIEWLYGCPPPYHTFEEPVFMRTHYNLT*---------------------
Rhas      EVLSVELTATNIEWLHGCPPPYHTFEEPAFVQVQASH*----------------------
Elac      EVLSVELTATNVEWLHGCPPPYHTFEEPAFVQVQPAR*----------------------
Kugu      EVLSVDLTTTNIEWLYGCPPPYHTFEEPAFVQVQDYPRK*--------------------
Plor      EVLWVEFTATNVEWLHGCPPPYHTFEEPAFVQVQTN*-----------------------
Sgun      EVLSVELTMTNVEWLHGCPPPYHTFEEPAFVQVQAH*-----------------------
Zaco      EVLSVELTATNVEWLHGCPPPYHTFEEPAFVQVQAG*-----------------------
Zbfl      EVSSVELTMTNVEWLHGCPPPYHTFEEPAFVQVQSN*-----------------------
Spba      LVHSVEFTATNVEWLHGCPPPYHTFEEPAFVQIQSHWREG*-------------------
Game      EVLSVELTATNVEWLHGCPPPYHTFEEPAFVLVQSD*-----------------------
Thth      EVMSVELTSTNIEWLHGCPPPYHTFEEPAFVLVQSD*-----------------------
Xigl      EVLSVELTATNVEWLHGCPPPYHTFEQPAFVQVRSH*-----------------------
Hyja      EVLSVEMTATNVEWLHGCPPPYHTFEEPAFVLVRSD*-----------------------
Psan      EVLSVELTTTNVEWLHGCPPPFHTFEQPTFTMTQED*-----------------------
Cupa      EVLSVELTTTNVEWLHGCPPPYHTFEEPAFVLVKSD*-----------------------
Mpch      EVLSTEMTMTNVEWLHGCPPPYHTFEEPAFVQVKS*------------------------
Char      EVAATELTSTNAEWLHGCPPPYHTFEEPAFVQVRPN*-----------------------
Pser      EVLSVTMTSTNVEWLHGCPPPYHTFEQPAFVQIRAD*-----------------------
Prol      EVLSVLMTATNVEWLHGCPPPYHTFEEPAFVRAPLN*-----------------------
Plbi      EVGAVELTSTNIEWLYGCPPPYHTFEEPAFVQVRMNSNG*--------------------
Calu      EVLSVELSMSNIEWLHGCPPPYHTFEEPAFVQNRQLPGRVT*------------------
Papa      EVLAVDLSSTNIEWLHGCPPPYHTFEEPAFVQIRP*------------------------
Sufr      EVLSVELTSTNVEWLHGCPPPYHTFEEPAFVQVQAT*-----------------------
Stci      EVSHVELTSTNIEWLHGCPPPYHTFEEPAFVQVQWR*-----------------------
Taru      EVQSVELTMTNVEWLHGCPPPYHTFEEPAFVQTQTSIRE*--------------------
Rala      EVLSVELTMTNVEWLHGCPPPYHTFEEPAFVQVQSTN*----------------------
                    . **:   **  ** *:* :
```

[10/10 of aligned sequences]

```
Scca    --------------------------------
Muma    --------------------------------
Erca    --------------------------------
Pose    --------------------------------
Actr    --------------------------------
Scal    --------------------------------
Posp    --------------------------------
Atsp    --------------------------------
Leoc    --------------------------------
Amca    --------------------------------
Osbi    --------------------------------
Pabu    --------------------------------
Hial    --------------------------------
Elha    --------------------------------
Mlcy    --------------------------------
Algl    --------------------------------
Ptgi    --------------------------------
Alaf    --------------------------------
Nock    --------------------------------
Anja    --------------------------------
Gyki    --------------------------------
Syka    --------------------------------
Opma    PKYYGPV-----*-------------------
Comy    --------------------------------
Sasp    YPEVPQNWNLPEWEMPKKPDAEMPKKPDIEK*
Eupe    EDSMAPL-----KESPHKSA*-----------
Enja    --------------------------------
Same    --------------------------------
Chch    --------------------------------
Grgr    --------------------------------
Caau    --------------------------------
Cyca    --------------------------------
Dare    --------------------------------
Cost    --------------------------------
Leec    --------------------------------
Fola    --------------------------------
Clmc    --------------------------------
Phin    --------------------------------
Icpu    --------------------------------
Psto    --------------------------------
Cora    --------------------------------
Eisp    --------------------------------
Apal    PP*-----------------------------
Eslu    --------------------------------
Dape    --------------------------------
Glse    --------------------------------
Naar    --------------------------------
Lioc    --------------------------------
Opso    --------------------------------
Alte    --------------------------------
Plap    --------------------------------
```

[10/10 of aligned sequences]

```
Plal  --------------------------------
Sami  --------------------------------
Rere  --------------------------------
Gama  --------------------------------
Onmy  --------------------------------
Sasa  --------------------------------
Cola  --------------------------------
Dita  --------------------------------
Gogr  --------------------------------
Chsl  --------------------------------
Atja  --------------------------------
Iido  --------------------------------
Auja  --------------------------------
Chag  --------------------------------
Hami  --------------------------------
Saun  --------------------------------
Nema  --------------------------------
Disp  --------------------------------
Myaf  --------------------------------
Lagu  --------------------------------
Trtr  --------------------------------
Zucr  --------------------------------
Pxja  --------------------------------
Pxlo  --------------------------------
Pctr  --------------------------------
Apsa  --------------------------------
Cabe  --------------------------------
Bzze  --------------------------------
Siim  --------------------------------
Ctru  --------------------------------
Dpbr  --------------------------------
Caki  --------------------------------
Phja  --------------------------------
Brsp  --------------------------------
Gamo  --------------------------------
Lolo  --------------------------------
Batr  --------------------------------
Prmy  --------------------------------
Lose  --------------------------------
Loam  --------------------------------
Chab  --------------------------------
Chto  --------------------------------
Majo  --------------------------------
Hlst  --------------------------------
Clpe  --------------------------------
Mlmr  --------------------------------
Crcr  --------------------------------
Muce  --------------------------------
Bege  --------------------------------
Mela  --------------------------------
Hats  --------------------------------
Orla  --------------------------------
```

[10/10 of aligned sequences]

```
Cosa    --------------------------------
Exsp    --------------------------------
Depa    --------------------------------
Rima    --------------------------------
Fuol    --------------------------------
Gmaf    --------------------------------
Xeei    --------------------------------
Pros    --------------------------------
Scmi    --------------------------------
Rolo    --------------------------------
Cere    --------------------------------
Daga    --------------------------------
Anco    --------------------------------
Dmve    --------------------------------
Dmar    --------------------------------
Anka    --------------------------------
Moja    --------------------------------
Hoja    --------------------------------
Bede    --------------------------------
Besp    --------------------------------
Mysp    --------------------------------
Osja    --------------------------------
Sgro    --------------------------------
Pzpa    --------------------------------
Zeja    --------------------------------
Znne    --------------------------------
Zefa    --------------------------------
Acni    --------------------------------
Ncrh    --------------------------------
Agca    --------------------------------
Hydy    --------------------------------
Gsac    --------------------------------
Pevo    --------------------------------
Hiku    --------------------------------
Inpa    --------------------------------
Auch    --------------------------------
Fico    --------------------------------
Macs    --------------------------------
Moal    --------------------------------
Syma    P*------------------------------
Mafr    --------------------------------
Dcpe    --------------------------------
Dcti    --------------------------------
Hehi    --------------------------------
Stam    --------------------------------
Hogi    --------------------------------
Erzo    --------------------------------
Hxot    --------------------------------
Core    --------------------------------
Apve    --------------------------------
Latj    --------------------------------
Laja    --------------------------------
```

[10/10 of aligned sequences]

```
Syja  --------------------------------
Epme  --------------------------------
Grse  --------------------------------
Clja  --------------------------------
Ogcy  --------------------------------
Plna  *-------------------------------
Lema  --------------------------------
Etzo  --------------------------------
Apse  --------------------------------
Epde  --------------------------------
Slja  --------------------------------
Bsja  --------------------------------
Ecna  --------------------------------
Cohi  --------------------------------
Caar  --------------------------------
Came  --------------------------------
Mema  --------------------------------
Lenu  --------------------------------
Brja  --------------------------------
Plma  --------------------------------
Emst  --------------------------------
Ptti  --------------------------------
Losu  --------------------------------
Geoy  --------------------------------
Dipi  --------------------------------
Pama  --------------------------------
Leob  --------------------------------
Neba  --------------------------------
Pdpl  --------------------------------
Nimi  --------------------------------
Uptr  --------------------------------
Pesc  --------------------------------
Baar  --------------------------------
Moar  --------------------------------
Toja  --------------------------------
Chau  --------------------------------
Chse  --------------------------------
Enar  --------------------------------
Hpty  --------------------------------
Nana  --------------------------------
Mcst  --------------------------------
Rhox  --------------------------------
Opfa  --------------------------------
Paar  --------------------------------
Gozo  --------------------------------
Ackr  --------------------------------
Elev  --------------------------------
Trdu  --------------------------------
Amoc  --------------------------------
Hame  --------------------------------
Chso  --------------------------------
Lyto  --------------------------------
```

[10/10 of aligned sequences]

```
Encr       --------------------------------
Bvar       --------------------------------
Noco       --------------------------------
Chsp       --------------------------------
Arja       --------------------------------
Pase       --------------------------------
Trel       --------------------------------
Lifa       --------------------------------
Acur       --------------------------------
Ampe       --------------------------------
Urja       --------------------------------
Enet       --------------------------------
Ptbr       --------------------------------
Safa       --------------------------------
Icae       --------------------------------
Asmi       --------------------------------
Foal       --------------------------------
Drze       --------------------------------
Rhas       --------------------------------
Elac       --------------------------------
Kugu       --------------------------------
Plor       --------------------------------
Sgun       --------------------------------
Zaco       --------------------------------
Zbfl       --------------------------------
Spba       --------------------------------
Game       --------------------------------
Thth       --------------------------------
Xigl       --------------------------------
Hyja       --------------------------------
Psan       --------------------------------
Cupa       --------------------------------
Mpch       --------------------------------
Char       --------------------------------
Pser       --------------------------------
Prol       --------------------------------
Plbi       --------------------------------
Calu       --------------------------------
Papa       --------------------------------
Sufr       --------------------------------
Stci       --------------------------------
Taru       --------------------------------
Rala       --------------------------------
```
