# Supplementary material for: Structure and variation of the mitochondrial genome of fishes
Source: BMC Genomics. 2016 Sep 7;17(1):719. doi: 10.1186/s12864-016-3054-y (PMC5015259; doi:10.1186/s12864-016-3054-y)
Supplement: Additional file 6: Figure S1-a. — Aligned amino acid sequences of the ATP8 gene in mt genomes of 250 fishes. Figure S1-b. Aligned amino acid sequences of the ATP6 gene in mt genomes of 250 fishes. Figure S1-c. Aligned amino acid sequences of the COI gene in mt genomes of 250 fishes. Figure S1-d. Aligned amino acid sequences of the COII gene in mt genomes of 250 fishes. Figure S1-e. Aligned amino acid sequences of the COIII gene in mt genomes of 250 fishes. Figure S1-f. Aligned amino acid sequences of the Cyt b gene in mt genomes of 250 fishes. Figure S1-g. Aligned amino acid sequences of the ND1 gene in mt genomes of 249 fishes. Figure S1-h. Aligned amino acid sequences of the ND2 gene in mt genomes of 250 fishes. Figure S1-i. Aligned amino acid sequences of the ND3 gene in mt genomes of 250 fishes. Figure S1-j. Aligned amino acid sequences of the ND4L gene in mt genomes of 250 fishes. Figure S1-k. Aligned amino acid sequences of the ND4 gene in mt genomes of 250 fishes. Figure S1-l. Aligned amino acid sequences of the ND5 gene in mt genomes of 250 fishes. Figure S1-m. Aligned amino acid sequences of the ND6 gene in mt genomes of 249 fishes. (ZIP 3250 kb) [file 12864_2016_3054_MOESM6_ESM.zip › Additional file 6 prot align/AF6d-COII.pdf]

Additional file 6: Figure S1–d. Aligned amino acid sequences of the COII gene in mt genomes of 250 fishes.

Species name abbreviation followed by aligned amino acid sequences shown by one letter abbreviation. See Additional file 1 for abbreviation of species name. Amino acids shown by magenta letter denote hydrophobic residues. A and B in bold types with yellow background indicate putative transmembrane regions. Highlighted 'C' and 'H' letters indicate metal binding sites. Numerals on the amino acid sequences correspond to position number of amino acid residues in the human sequence. Asterisk '*' indicates a fully conserved residue. Colon ':' and period '.' indicate 'strong' and 'weak' groups in the level of conservativeness, respectively, in the Gonnet Pam250 matrix, in which the strong and weak groups are defined as strong score >0.5 and weak score =<0.5, respectively (Thompson et al., 1997).

COII

[1/4 of aligned sequences]

A

```
Scca    MAHPSQLGFQDAASPVMEELIHFHDHTLMIVFLISTLVLYIITAMVSTKLTNKYILDSQE
Muma    MAHPSQLGFQDAASPVMEELIHFHDHTLMIVFLISTLVLYIITAMVTTKLTNKYILDSQE
Erca    MAHPAQLGLQDASSPIMEELMHFHDHALMIVFLISTLVLYIITTTVSTKLTNKHLLDAQE
Pose    MAHPAQLGLQDASSPIMEELLHFHDHALMIVFLISTLVLYIITTTVSTKLTNKHLLDAQE
Actr    MAHPSQLGFQDAASPVMEELLHFHDHTLMIVFLISTLVLYIIVAMVSTKLTNKYVLDSQE
Scal    MAHPSQLGFQDAASPVMEELLHFHDHTLMIVFLISTLVLYIIVAMVSTKLTNKYVLDSQE
Posp    MAHPSQLGFQDAASPVMEELLHFHDHTLMIVFLISTLVLYIIVAMVSTKLTNKYVLDSQE
Atsp    MAHPSQLGFQDAASPVMEELLHFHDHALMIVFLISTLVLYIIVAMVSTKLTNKHILDSQE
Leoc    MAHPSQLGFQDAASPVMEELLHFHDHALMIVFLISTLVLYIIVAMVSTKLTNKHILDSQE
Amca    MAHPMQLGFQDAASPVMEELLHFHDHALMIVFLISTAVLYIIVVTVTTKLTDKYVLDAQE
Osbi    MATPLQLGFQDAASPVMEELIHFHDHALMVVYLISSFVLYIIITLVMTKLTNKHAHDSQE
Pabu    MAYPSQLGLQNAASPAMEELVHFHDHTLMIAYLISNLVLYIIFAVVTTKLTNKHALDSQE
Hial    MAHPSQLGFQDAASPVMEELLHFHDHALMIVFLISTLVLYIIVAMVSTKLTNKYILDSQE
Elha    MAHPSQLGFQDAASPVMEELLHFHDHALMIVFLISTLVLYIIVAMVSTKLTDKFTIDSQE
Mlcy    MAHPSQLGFQDAASPVMEELLHFHDHALMIVFLISTLVLYIIVAMVSTKLTDKYTIDSQE
Algl    MAHPSQLGFQNAASPVMEELLSFHDHTLMIVSMISIMVLYIIVAVVSAKLTDKHILDSQG
Ptgi    MAHPSQLGFQDAASPVMEELLHFHDHALMIVFLISTLVLYIIVAMVSTKLTNKYILDSQE
Alaf    MAHPSQLGFQDAASPVMEELLHFHDHALMIVFLISTLVLYIIVAMVTTKLTNKYILDSQE
Nock    MAHPSQLGFQDAASPVMEELLHFHDHALMIVFLISTLVVYIIVAMVTTKLTNKYILDSQE
Anja    MAHPSQLGFQDAASPLMEELLHFHDHALMIVFLISVLVLYIIVAMVTAKVTNMFILDSQE
Gyki    MAYPAQLGFQDAASPVMEELLHFHDHALMIVFLISTLVLYIIVAMVTTTLTDTYILDSQE
Syka    MAHPSQLGFQDAASPVMEEMLHFHDHALMIVFLISTLVLYIIVAMVSTKLTNMYILDSQE
Opma    MAHPSQLGFQDAASPVMEEMIHFHDHALMIVFLISTLVLYIIVAMVTTNLINLYILDSQG
Comy    MAQPSQLGFQDAASPVMEEMIHFHDHALMIVFLISTLVLYIIVAMVSTNLTNTYILDSQE
Sasp    MATPMQVGFQDAASPLMEELIHFHDHALMIIFLISTLVLYIITMMMTAKVIDLYILDSQE
Eupe    MANPSQLGFQDSASPLMEELLHFHDHALMIVFLISTLVLYVITAMVTAKVTNMFILDSQE
Enja    MAHPSQLGLQDAASPVMEELIHFHDHALMIVFLISTLVLYVIVAMVSIKLTNKYILDSQE
Same    MAHPSQLGLQDAASPVMEELLHFHDHALMIVLLISTLVLYIIVSMVSTKLTDKYILDSQE
Chch    MAHPSQLGFQDAASPVMEELLHFHDHALMIVFLISTLVLYIIVAMVTAKVTNKYIIDSQE
Grgr    MAYPTQLGFQDAASPVMEELLHFHDHALMIVFLISTLVLYIIVAMVSTKLTNKFVLDSQE
Caau    MAHPTQLGFQDAASPVMEELLHFHDHALMIVFLISTLVLYIIIAMVSTKLTNKYILDSQE
Cyca    MAHPTQLGFQDAASPVMEELLHFHDHALMIVLLISTLVLYIITAMVSTKLTNKYILDSQE
Dare    MAHPAQLGFQDAASPVMEELLCFHDHALMIVFLISTLVLYIIIAMVSTKLTNKFILDSQE
Cost    MAHPTQLGFQDAASPVMEELLHFHDHALMIVFLISTLVLYIIVAMVSTKLTNKYILDSQE
Leec    MAHPTQLGFQDAASPVMEELLHFHDHALMIVFLISTLVLYIIIAMASTKLTNKYILDSQE
Fola    MAHPTQLGFQDAASPVMEELLHFHDHALMIVFLISALVLYVIITTVSTKLTNMYILDSQE
Clmc    MAHPSQLGFQDAASPVMEELLHFHDHALMIVFLISTLVLYIIVAMVSTKLTNKYILDSQE
Phin    MAHPSQLGFQDAASPVMEELLHFHDHALMIVFLISTLVLYIIVAMVSTKLTNKYILDSQE
Icpu    MAHPSQLGFQDAASPVMEELLHFHDHALMIVFLISTLVLYIIVVMVTTKLTNKFILDSQE
Psto    MAHPSQLGFQDAASPVMEELLHFHDHALMIVFLISTLVLYIIVVMVTTKLTNKYILDSQE
Cora    MAHPSQLGLQDAASPVMEELLHFHDHALMIVFLISTLVLYIIVVMVSTKLTNKYILDSQE
Eisp    MAHPSQLGFQDAASPVMEELLHFHDHALMIVFMISTLVLYIMVAMVSTKLTNKYILDSQE
Apal    MTSPMQLGFQDAASPIMEELLLFHDHTLMIIFAISTLILYIMTATVTTNLTNKYMLDAQE
Eslu    MAHPSQLGFQDAASPVMEELLHFHDHALMIVLLISTLVLYIIVAMVSTKLTNKYILDSQE
                                  1
```

To be continued on page 6.

[1/4 of aligned sequences]

Dape	MAHPSQLGFQDAASPVMEELLHFHDHALMIVLLISTLVLYIIVAMVSTKLTNKYILDSQE
Glse	MAHPSQLGFQDAASPVMEELLHFHDHALMIVFLISTLVLYIIVAMVSTKLTNKYILDSQE
Naar	MAHPSQLGFQDAASPVMEELLHFHDHALMIVFLISTLVLYIIVAMVSTKLTNKYILDSQE
Lioc	MAHPSQLGFQDAASPVMEELLHFHDHALMIVILISTLVFYIIIAMVTTKLTNKFILDSQE
Opso	MAHPSQLGFQDAASPVMEELLHFHDHALMIVFLISTLVLYIIMAMVSTKLTNKYILDSQE
Alte	MAHPSQLGFQDAASPVMEELLHFHDHALMIVLLISTFVLYIIVAMVSTKLTNKYILDSQE
Plap	MAHPSQLGFQDAASPVMEELLHFHDHALMIVLLISTFVLYIIMAMVSTKLTNKYILDSQE
Plal	MAHPSQLGFQDAASPVMEELLHFHDHALMIVLLISTLVLYIIVAMVSTKLTNKYILDSQE
Sami	MAHPSQLGFQDAASPVMEELLHFHDHALMIVLLISTLVLYIIVAMVSTKLTNKYILDSQE
Rere	MAHPSQLGFQDAASPVMEELLHFHDHALMIVLLISTLVVYIIVAMVSTKLTNKYILDSQE
Gama	MAHPSQLGFQDAASPVMEELLHFHDHALMIVFLISTLVLYIIVAMVSTKLTNKYILDSQE
Onmy	MAHPSQLGFQDAASPVMEELLHFHDHALMIVLLISTLVLYIIVAMVSTKLTNMYILDSQE
Sasa	MAHPSQLGFQDAASPVMEELLHFHDHALMIVLLISTLVLYIIVAMVSTKLTNKYILDSQE
Cola	MAHPSQLGFQDAASPVMEELLHFHDHALMIVLLISTLVLYIIVAMVSTKLTNKYILDSQE
Dita	MAHPSQLGFQDAASPVMEELLHFHDHALMIVILISTLVLYIIVAMVSTKLTNKYILDSQE
Gogr	MAHPSQLGFQDAASPVMEELIHFHDHALMVVILISTLVLYIILAMVSTTLTNKFILDSQE
Chsl	MAHPTQLGFQDAASPVMEELLHFHDHSLMVVTIISIFVLYIISAMATTVLTDKLLIDAQE
Atja	MAHPSQLGFQDAASPVMEELLHFHDHALMIVFLISTLVLYIIVAMVSTKLTNKYILDSQE
Iido	MAHPSQLGFQDAASPVMEELLHFHDHALMIVFLISTLVLYIIVAMVSTKLTNKYILDSQE
Auja	MAHPSQLGFQDAASPVMEELLHFHDHALMIVFLISTLVLYIIVAMVSTKLTNKYILDSQE
Chag	MAHPSQLGFQDAASPVMEELLHFHDHALMIVLLISTLVLYLLVAMISTKLTDKYILDSQE
Hami	MAHPSQLGFQDAASPVMEELLHFHDHALMIVFLISTLVLYIIVAMVSTKLTNKYILDSQE
Saun	MAHPSQLGFQDAASPVMEELLHFHDHALMIVFLISTLVLYIIVAMVSTKLTNKYILDSQE
Nema	MAHPSQLGFQDAASPVMEELLHFHDHALMIVFLISTLVLYIIVAMVSTKLTNKYILDSQE
Disp	MAHPSQLGFQDAASPVMEELLHFHDHALMVVILISSLVFYIIIAMVSTKLTNKYILDSQE
Myaf	MAHPSQLGFQDAASPVMEELLHFHDHALMVVLLISTLVFYIIIAMVTTKLTNKYILDSQE
Lagu	MAHPSQLGFQDAASPVMEELLHFHDHALMIVFLISTLVLYIILTTVSTKLTDKYILDSQE
Trtr	MAYPSQFGFQDAASPVMEELLHFHDHALMIVFLISTLVLYIIIAMVSTKLTNKYILDSQE
Zucr	MAYPSQLGFQDAASPVMEELLHFHDHALMIVFLISTLVLYIIVAMVSTKLTNKFILDSQE
Pxja	MAHPSQLGFQDAASPVMEELLHFHDHALMIVFLISTLVLYIIVAMVSTKLTNKYILDSQE
Pxlo	MAHPSQLGFQDAASPVMEELLHFHDHALMIVLLISTLVLYIIVAMVSTKLTNKYILDSQE
Pctr	MAHPSQLGFQDAASPLMEELLHFHDHALMIVLLISTLVLYIIIAMVSTKLTNKFILDSQE
Apsa	MAHPSQLGFQDAASPLMEELLHFHDHALMIVFLISTLVLYIIVAMVSTKLTNKYILDSQE
Cabe	MAHPSQLGFQDATSPVMEELIHFHDHALMIVFLISTLVLYIIVAMVSTKLTNKLILDSQE
Bzze	MAHPSQLGFQDATSPVMEELIHFHDHALMIVFLISTLVLYIIVAMVSTKLTNKYILDSQE
Siim	MAHPAQLGFQDATSPLMEELLHFHDHVLMVVFLISVLVLYIIVSMVTSNLLDKNTLDSQE
Ctru	MAHPSQLGFQDAASPVMEELLHFHDHALMIVFLISTLVLYIIVATVSTKLTNKYILDSQE
Dpbr	MAHPTQLGFQDAASPVMEELLHFHDHALMIVFLISTLVLYIIVAMVSTKLTNKYILDSQE
Caki	MAHPSQLGFQDAASPLMEELLHFHDHTLMVVFLISTLVLYIIVAMVSTKLTDKLISDSQE
Phja	MAHPSQLGFQDAASPLMEELLHFHDHTLMVVFLISAFVLYIISAMISASVYDKLVLDSPE
Brsp	MAYPFQIMFQDAASPLMENFLHFHDHTMMMVFLISALVLYIMTTVAFTKLYDLGKNDSQE
Gamo	MAHPSQLGFQDAASPVMEELLHFHDHALMIVFLISTLVLYIIVAMVSTKLTNKYILDSQE
Lolo	MAHPSQLGFQDAASPVMEELLHFHDHALMIVFLISTLVLYIIVAMVSTKLTNKYILDSQE
Batr	MAHPSQYTFQDACSPTMEELIHFHDHTLMVVILISTFVLYLLCAFSTTKLYSTSTFSHHS
Prmy	MPLAAQLNFQDASSPHMKELLQFHDSGLVVIIFISVFVLYVLFILTTTKLYNKLILDSHT
Lose	MAYPTQLGFQDAASPVMEELLHFHDHVLMIVFLISTLVLYIMAAMVSTKLTSKFLLDSQE
Loam	MAHPSQLGFQDAASPVMEELLHFHDHVLMIVFLISTLVLYIIVAMVSTKLTSKYLLDSQE
Chab	MAHPTQLGFQDAASPIMEELLHFHDHTLMIVFLISTLVLYIIIAMISTKLTNKYILDSQE
Chto	MAHPTQLGFQDAASPIMEELLHFHDHTLMIVFLISTLVLYIIVAMISTKLTNKYILDSQE
Majo	MAHHAQLGFQDAASPVMEELIHFHDHALMVLFLISTLVLYIIVAMVSTKLTNKYILDSQE
Hlst	MAHHAQLGFQDAASPVMEELIHFHDHALMILFLISTLVLYIIVAMVTTKLTNKYILDSQE
Clpe	MAHPSQLGFQDAASPVMEELLHFHDHALMIVFLISTLVLYIIVATVSTKLTNKYILDSQE

2

To be continued on page 7.

[1/4 of aligned sequences]

```
Mlmr    MAHPSQLGFQDAASPVMEELLHFHDHTLMIVFLISTLVLYIIVAMVSTKLTNKYILDSQE
Crcr    MAYPSQLGFQDAASPLMEELLHFHDHALMIIFLISTFVLYIIVAMVTTKLTNSFILDSQE
Muce    MAYPSQLGFQDAASPLMEELLHFHDHALMIIFLISTFVLYIIVAMVTTKLTNSFILDSQE
Bege    MAHPSQLGFQDAASPVMEELLHFHDHALMIVFLISTLVLYIIVAMVTTKLTNKFILDSQE
Mela    MAHPSQLGFQDAASPMMEELLHFHDHALMIIFLISTLVLYIIVAMVTTKLTNKFILDSQE
Hats    MAHPSQLGFQDAASPVMEELLHFHDHALMVIFLISALVLYIIVAMVTTKLTNKLILDSQE
Orla    MAHPSQLGFQDAASPVMEELLHFHDHALMIVFLISTLVLYIIVAMVTTKLTNKFILDSQE
Cosa    MAHPSQLGFQDAASPVMEELLHFHDHALMIVFLISTLVLYIIVAMVTTKLTNKFILDSQE
Exsp    MSHPSQLGFQDAASPVMEELLHFHDHALMIVFLISTLVLYIIVAMVTTKLTNKFILDSQE
Depa    MAHPSQLGFQDAASPVMEELLHFHDHALMIVFLISTLVLYIIVAMVTTKLTNKFILDSQE
Rima    MAHPSQLGFQDAASPVMEELLHFHDHTLMIVFLISTLVLYIIMAMVTTKLTNKFILDSQE
Fuol    MAHPSQLGFQDAASPVMEELLHFHDHTLMIVFLISTLVLYIIVAMVTTKLTNKFILDSQE
Gmaf    MAHPSQLGFQDAASPVMEELLHFHDHALMIVFLISAQVLYIIVALITTKLTDKFILDSQE
Xeei    MAHPSQLGFQDAASPVMEELLHFHDHTLMIVFLISTLVLYIIVAMVTTKFTNKFILDSQE
Pros    MAHPSQLGFQDAASPVMEELLHFHDHALMIVFLISTFVLYIIVAMVTTKLTNKYVLDSQE
Scmi    MACPSQLGFQDAASPVMRELTSFHDHSLMVLFLVSAFVLYLIAAMVVTKLYCHFITDAYE
Rolo    MAHPSQLGFQDAASPVMEELLHFHDHALMIVFLISTLVLYIIVAMVSTKLTNKYILDSQE
Cere    MAHPLQLGFQDAASPIMEEFIHFHDHTLMIVFLISSLVLYIIMAMVSTKLTNKYILDSQE
Daga    MAHPSQLGFQDAASPVMEELIHFHDHTLMVVFLISTLVLYIIVAMVSTNLTNKYILDSQE
Anco    MAHPSQLGFQDAASPVMEELLHFHDHALMIVFLISTLVLYIIVAMVTTKLTNKYILDSQE
Dmve    MARPSQLGFQDAASPVMEELLNFHDYAMVIVFLISTLVLYIIVVTASTKLTDKKLTQSQG
Dmar    MARPSQLGFQDAASPVMEELLNFHDYAMVIVFLISTLVLYIIVVTASTKLTDKKLTQSQG
Anka    MAHPSQLGFQDAASPVMEELLHFHDHALMIVFLISTLVLYIIVAMVTTKLTNKYILDSQE
Moja    MAHPSQLGFQDAASPVMEELLHFHDHALMIVFLISTLVLYIIVAMVTTKLTNKYILDSQE
Hoja    MAHPSQLGFQDAASPVMEELLHFHDHALMIVFLISTLVLYIIVAMVTTKLTNKYILDSQE
Bede    MAHPSQLGFQDAASPVMEELLHFHDHALMIVFLISTLVLYIIVAMVSTKLTNKFILDSQE
Besp    MAHPSQLGFQDAASPVMEELLHFHDHALMIVFLISTLVLYIIVAMVSTKLTNKFILDSQE
Mysp    MAHPSQLGFQDAASPVMEELLHFHDHALMIVFLISTLVLYIIVAMVSTKLTNKYILDSQE
Osja    MAHPSQLGFQDAASPVMEELLHFHDHALMIVFLISTLVLYIIVAMVSTKLTNKYILDSQE
Sgro    MAHPSQLGFQDAASPVMEELLHFHDHALMIVFLISTLVLYIIVAMVSTKLTNKYILDSQE
Pzpa    MAHPTQLGFQDAASPVMEELLHFHDHALMIVFLISTLVLYIIVAMVSTKLTYKHILDSQE
Zeja    MAHPSQLGFQDAASPVMEELLHFHDHALMIVFLISTLVLYIIIAMVSTKLTNKYILDSQE
Znne    MAHPTQLGFQDAASPVMEELLHFHDHALMIVFLISTLVLYIIVAMVSTKLTNKHILDSQE
Zefa    MAHPSQLGFQDAASPVMEELLHFHDHALMIVFLISTLVLYIIVAMVSTKLTNKHILDSQE
Acni    MAHPSQLGFQDAASPVMEELLHFHDHALMIVFLISTLVLYIIVAMVSTKLTNKHILDSQE
Ncrh    MAHPSQLGFQDAASPVMEELLHFHDHALMIVFLISTLVLYIIVAMVSTKLTNKHILDSQE
Agca    MAHPSQLGFQDAASPVMEELLHFHDHALMIVFMISTFVLYIIVAMVSTKLTNKYILDSQE
Hydy    MANPSQLGFQDAASPVMEELLHFHDHALMIVFLISTLVLYIIVAMVSTKLTNKYILDSQE
Gsac    MANPSQLGFQDAASPVMEELLHFHDHTLMIVFLISTLVLYIIVAMVSTKLTNKYILDSQE
Pevo    MAHPSQLGFQDAASPVMEELLHFHDHALMIVFLISTLVLYIIVVTVSTKLTNKFLLDSQE
Hiku    MAYPSQLGFQDAASPVMEELLHFHDHALMIVFLISTLVLYIIVAMVTTKLTNKFLLDSQE
Inpa    MAHPAQLGFQDAASPIMEELLHFHDHALMILFLISTLVLYIILAMVTAKLTNKNNIDAQE
Auch    MSSPTQIGFQDAGAPMMEELLHFHDHALMVVFLISAFVLYIITTMVTTSLTNKSLMDSQE
Fico    MAHPSQLGFQDAASPVMEELLHFHDHALMIVFLISTLVLYIIVAMVSTKLTNKFILDSQE
Macs    MAHPSQLGFQDAASPVMEELLHFHDHALMIVFLISTLVLYIIVAMVTTKLTNKFLLDSQE
Moal    MAYPLQLGLQDATSPVMEELIQFHDHTLMIALLVSTMVFYTILTTVTTKLTNKNMLDSQG
Syma    MAYPLQLGLQDAASPVMEELIQFHDHTMMIAFAISITVLYLIVAMVTTHMTDKQLLDSHV
Mafr    MAHPSQLGFQDAASPVMEELLHFHDHALMIVFLISTLVLYIIVAMVTTKLTNKNIIDSQE
Dcpe    MAHPSQLGFQDAASPVMEELLHFHDHALMIVFLISALVLYIIVAMVTTKLTNKFLLDSQE
Dcti    MAHPSQLGFQDAASPVMEELLHFHDHALMIVFLISALVLYIIAAMVTTKLTNKFLLDSQE
Hehi    MAHPSQLGFQDAASPVMEELLHFHDHALMIVFLISALVLYIILAMVTTKLTNKYILDSQE
Stam    MAHPSQLGFQDAASPVMEELLHFHDHALMIVFLISTLVLYIIVAMVSTKLTNKYILDSQE
```

3

To be continued
on page 8.

[1/4 of aligned sequences]

```
Hogi    MAHPSQLGFQDAASPVMEELLHFHDHALMIVFLISTLVLYIIVAMVTTKLTNKYILDSQE
Erzo    MAHPSQLGFQDAASPVMEELLHFHDHALMVVLLISAFVLYIIVAMISTKLTNKYVLDSQE
Hxot    MAHPSQLGFQDAASPVMEELLHFHDHALMIVFLISTLVLYIIVAMVSTKLTNKYILDSQE
Core    MAHPSQLGFQDAASPVMEELLHFHDHALMIVFLISTLVLYIIVAMVSTKLTNKYILDSQE
Apve    MAHPSQLGFQDAASPVMEELLHFHDHALMIVFLISTLVLYIIVAMVSTKLTNKFILDSQE
Latj    MAHPSQLGFQDAASPLMEELLHFHDHALMIVLLISVFVLYIIVTMITAKLTDKLILDSQE
Laja    MAHPSQLGFQDAASPVMEELLHFHDHALMIIFLISAMVLYIIVAMITTKLTDKYILDSQE
Syja    MAHPSQLGFQDAASPVMEELLHFHDHALMIAFLISTLVLYIIVAMVTTKLTNKYILDSQE
Epme    MAHPTQLGLQDAASPVMEELLHFHDHALMIVFLISTLVLYIIVAMVSTKLTNKYILDSQE
Grse    MAHPSQLGFQDAASPVMEELLHFHDHALMIVFLISTLVLYIIVAMVTTKLTNKFILDSQE
Clja    MAHPSQLGFQDAASPVMEELLHFHDHALMIVLLISTFVLYIMIAMVTTKLTNKYILDSQE
Ogcy    MSQPSQMGFQDATSPLMEELLHFHDHALMIVFLISTLVLYIIVAMVSTELTNKLILDSQE
Plna    MAYPSQIGFQDAASPVMEELLYFHDHALMIVFLISTLVLYIIVAMVTAKVTNKFILDSQE
Lema    MAHPSQLGFQDAASPVMEELLHFHDHALMIVFLISTLVLYIIVAMVTTKLTNKYILDSQE
Etzo    MAHPSQLGFQDAASPVMEELLHFHDHALMIVFLISTLVLYIIVAMVSTKLTNKYILDSQE
Apse    MAHPSQLGFQDAASPVMEELLHFHDHALMIVFLISTLVLYIIVAMVTTKLTNKNILDSQE
Epde    MAHPSQLGFQDAASPVMEELLHFHDHALMIVFLISTLVLYIIVAMVSTKLTNKYILDSQE
Slja    MAHPSQLGFQDAASPVMEELLHFHDHALMIVFLISTLVLYIIVAMVSTKLTNKYILDSQE
Bsja    MAHPSQLGFQDAASPIMEELLHFHDHALMIVLLISTLVLYIIIAMVSTKLVNLYILDSQE
Ecna    MAHPAQLGFQDAASPLMEELLRFHDHALMVIFLISAFVFYVIIAMITAKTTDMLVLDSQV
Cohi    MAHPSQLGLQDAASPLMEELLYFYDHASMIVFLICAFVLYILTVVITSSFSNKYTLDSQE
Caar    MAHPSQLGFQDAASPLMEELLHFHDHALMIVFLISTLVLYIIVAMVTAKFTDKLILDSQE
Came    MAHPSQLGFQDAASPLMEELLHFHDHALMIVFLISTLVLYIIVAMVTAKFTDKLILDSQE
Mema    MAHPAQLGFQDAASPLMEELLHFHDHALMIVLLISTIVLYIITAMVTAKFSDKLILDSQE
Lenu    MAHPSQLGFQDAASPVMEELVHFHDHALMIAFLISTVVLYIMVAMISTKLTNTRILDSQE
Brja    MAHPSQLGFQDAASPVMEELLHFHDHALMIVFLISTLVLYIIVAMVSTKLTNKYILDSQE
Plma    MAHPSQLGFQDAASPVMEELLHFHDHALMIVFLISTLVLYIIVAMVSTKLTNKYILDSQE
Emst    MAHPSQLGFQDAASPVMEELLHFHDHALMIVFLISTLVLYIIVAMVSTKLTNKYILDSQE
Ptti    MAHPSQLGFQDAASPVMEELLHFHDHALMIVFLISTLVLYIIVAMVSTKLTNKYILDSQE
Losu    MAYPTQLGFQDAASPVMEELLHFHDHALMIVFLISTFVLYIIIAMVSTKLTNKFILDSQE
Geoy    MAHPSQLGFQDAASPVMEELLHFHDHALMIIFLISVFVFYIILNMVTSKLTNLNLLDSQE
Dipi    MAHPSQLGFQDAASPVMEELLHFHDHALMIVFLISTLVLYIILAMVSTKLTNKYILDSQE
Pama    MAHPSQLGFQDAASPVMEELLHFHDHALMIVFLISTLVLYIIVATVSTKLTNKYILDSQE
Leob    MAHPSQLGFQDAASPVMEELLHFHDHALMIVFLISTLVLYIIVAMVSTKLTNMYILDSQE
Neba    MAHPAQLGFQDAASPVMEELLHFHDHAMMIVLLISILVLYVIVAMISTKLTDKYILDSQE
Pdpl    MAHPSQLGLQDAASPFMEELIYFHDHAMVIIFIISAFVLYIIVAMVATNLTDKRTLDSQE
Nimi    MAHPAQVGFQDATSPLMEELLYFHDHALMIVFLISVMVLYIIVCMITTNLSDKLILDSQE
Uptr    MAHPSQLGFQDAASPVMEELLHFHDHALMIVFLISTFVLYIIVAMVTTKLTNKFLLDSQE
Pesc    MAHPAQVGFQDAASPLMEELLYFHDHALLILTLVSVFVLYIIIAMLSTKLYDSYTLDAQM
Baar    MAYPLQLGFQDAASPIMEELLHFHDHALMITFLISTLVLYIIVAMVVTKITNKYILDSQE
Moar    MAHPSQLGFQDAASPVMEELLHFHDHALMIVFLISTLVLYIIVAMVSTKLTNKYILDSQE
Toja    MAHPTQLGFQDAASPLMEELLHFHDHALMIVFLISTMVLYIIVAMVTAKLTDKLILDSQE
Chau    MAHPTQLGFQDAASPIMEELLHFHDHALMIVFMISTLVLYIIVAMVTTKITNKHILDSQE
Chse    MAHPQQLGFQDATSPLMEELLHFHDHALMIVFLISAFVLYIMVAMVTTKIYDKYILDSQE
Enar    MAHPSQLGFQDAASPVMEELLHFHDHALMIVFMISTMVLYIIVAMISTKFTDKYILDSQE
Hpty    MAHPSQLGFQDAASPVMEELLHFHDHALMITFLISTLVLYIIVAMVSTKLTNKYILDSQE
Nana    MAHPLQLGFQDAASPVMEELLHFHDHALMTVFLISALVLYIIVAMVTTKLTNKNILDSQE
Mcst    MAHPSQLGFQDAASPVMEELLHFHDHALMIVFLISTLVLYIIVAMVSTKLTNKYILDSQE
Rhox    MAHPSQLGFQDAASPVMEELLHFHDHALMIVFLISTLVLYIILAMVSTKLTNKYILDSQE
Opfa    MAHPSQLGFQDAASPVMEELLHFHDHALMIVFLISTLVLYIIVAMVTTKLTNKFILDSQE
Paar    MAHPSQLGFQDAASPVMEELLHFHDHALMIVFLISTLVLYIIVAMVSTKLTNKYILDSQE
Gozo    MAHPLQLGFQDAASPVMEELLHFHDHALMIVFLISTLVLYIIVAMVSTKLTNKYILDSQE
```

To be continued on page 9.

[1/4 of aligned sequences]

```
Ackr    MAHPAQLGLQDAASPMMEELLFFHDHAMMVVFLISVFVIYIITSMLVTNLSDKYVLDSPE
Elev    MAHPSQLGFQDAASPVMEELLHFHDHALMIVFLISTLVLYIIVAMITTKLTNKYILDSQE
Trdu    MAHPSQLGFQDAASPVMEELLHFHDHALMIVFLISTFVLYIIVAMVTTKLTNKFILDSQE
Amoc    MAHPTQLGFQDAASPVMEELLHFHDHALMIVFMISTLVLYIIVAMVTTKLTNKYILDSQE
Hame    MAHPSQLGFQDAASPVMEELLHFHDHALMIVFLISTLVLYIMVAMVSTKLTNKYILDSQE
Chso    MAHPSQLGFQDAASPVMEELLHFHDHALMIVFLISTFVLYIIVAMVSTKLTNSYILDSQE
Lyto    MAHPSQLGFQDAASPVMEELLHFHDHALMIVFLISTLVLYIIVAMVSTKLTNKYILDSQE
Encr    MAHPSQLGFQDAASPVMEELLHFHDHALMIVFLISTLVLYIIVAMVSTKLTNKYILDSQE
Bvar    MAHPSQLGFQDAASPVMEELIHFHDHALMIALLISTLVLYFIVAMVSTKLTNKYILDSQE
Noco    MAFPSQLGFQDAASPVMEELLHFHDHAMMIVFMISTLVLYFIVALVTSSLTNKYILDSQE
Chsp    MSHASQLGFQDAASPMMEEFIHFHDHLLMVTIAISTFILYMLVTTVTTKLTDNFTQDSQE
Arja    MAHPSQLGFQDAASPVMEELLHFHDHALMIVFLISTLVLYIIVAMVSTKLTNKYILDSQE
Pase    MAHTNQFGFQDATSPLMEELLKFHDHATMLVFVISTFVIYIMVFVTSCKLTNKFVLDYQT
Trel    MAHPSQLGFQDAASPVMEELIHFHDHALMIVFLISTLVIYSIATTVSTKLSNQSIIDSQE
Lifa    MAHASQLGFQDATSPLMEELLHFHDHALMITFLISTFVLYIIVAMVSTKLTNKYILDSQE
Acur    MAHPSQLGFQDAASPVMEELLHFHDHALMITFLISTLVLYILIAMVSTKLTDKFILDSQE
Ampe    MAHPSQLGFQDAASPVMEELLHFHDHALMIVFLISTLVMYIIVAMVSTKLTNKYILDSQE
Urja    MAHPTQLGFQDAASPVMEELLHFHDHALMIIFLISTFVLYIIATMISTKLTNKLILDSQE
Enet    MAHPSQLGLQDAASPVMEELLHFHDHALMIVFLISTLVLYIIVAMVSTKLTNKYILDSQE
Ptbr    MAHPSQLGLQDAASPVMEELHHFHDHAMMVVFMISAFVLYIIVAMVSTKLTNKYILDSQE
Safa    MAHPSQLGLQDAASPVMEELLHFHDHALMIVFLISTLVLYIIVAMVSTKLTNKYILDSQE
Icae    MAHPSQLGFQDAASPVMEELLHFHDHALMIVFLISTLVLYIIVAMVSTKLTNKYILDSQE
Asmi    MSLPAQLGFSDAVSPVMEELLHFHDHVLMIVFAISTFVFYIITVTVTAKTTNKFIVDSQE
Foal    MSNASQLGFQDAASPIMEELLHFHDHSLMIVFLISTFVLYIIVAMVTTTLTNKLILDSQE
Drze    MAHPSQLGFQDAASPVMEELLHFHDHALMTLFLISTFVLYIIVTMVTTMLTDKLLVDAQE
Rhas    MAHPSQLGFQDAASPVMEELLHFHDHALMIVFLISTLVLYIIVAMVTTKLTNKNILDSQE
Elac    MAHPSQLGFQDAASPVMEELLHFHDHALMIVFLISTLVLYIIVAMVSTKLTNKNILDSQE
Kugu    MAHPSQLGFQDAASPVMEELLHFHDHALMIIFLISTFVLYIITTMITSKLTCKNILDSQE
Plor    MAHPAQLGFQDAASPVMEELLHFHDHALMIVFLISTTVLYIIVAMVTTKLTDKYVLDSQE
Sgun    MAHPSQLGFQDAASPVMEELLHFHDHALMIVFLISTLVLYIIVAMVSTKLTNKYILDSQE
Zaco    MAHPSQLGFQDAASPVMEELLHFHDHALMIVFLISTLVLYIIVAMVSTKLTNKYILDSQE
Zbfl    MAHPSQLGFQDAASPVMEELLHFHDHALMIVFLISTLVLYIIVAMVSTKLTNKYILDSQE
Spba    MAHPSQLGLQDAASPLMEELLHFHDHAMMIILLISVFVLYIITAMATAKFTDKLILDSQE
Game    MAHPSQLGFQDAASPVMEELLHFHDHALMIVFLISTLVLYIIVAMVSTKLTNKYILDSQE
Thth    MAHPSQLGFQDAASPVMEELLHFHDHALMIVFLISTLVLYIIVAMVSTKLTNKYILDSQE
Xigl    MAHPSQLGFQDAASPLMEELLHFHDHALMIVFLISTMVLYIIVAMVTAKFTDKLVLDSQE
Hyja    MAQPMQLGFQDAASPLMEELLHFHDHALMIIFLISVVVLYIIVLMISTKLTDTYILDSQE
Psan    MAHPSQLGFQDAASPLMEELLHFHDHALMIIFLISTMVLYIIVAMISTKLSNMYILDSQE
Cupa    MAHPSQLGFQDAASPVMEELLHFHDHALMIVFLISTLVLYIIVAMISTKLTNKYILDSQE
Mpch    MAHPSLLGFQDAASPVMEELLQFHDHALVILFLISAFVLYIIVAMVTTKLTNKYILDSQE
Char    MAHPSQLGLQDAASPVMEELLHFHDHTLMIVFLISTLVLYIIVAMVTTKLTNKNILDSQE
Pser    MAHPTQLGFQDAASPLMEELLHFHDHALMIVFLISTLVLYTIVAMVTAKLSDKLILDSQE
Prol    MAHPSQLGFQDAASPLMEELLHFHDHALMIVILISTMVLYIIVAMVTAKLTDKLVLDSQE
Plbi    MAHPSQLGFQDAASPLMEELLHFHDHALMIVILISTMVLYIIVAMVTAQLTDKLVLDSQE
Calu    VAHPGQLGLQDAASPLMEELLHFHDYTLMVIILISIMVAYIMTTTVTAKVTNKLVLDSQE
Papa    MAQPSQMGLQDAASPVMEELLHFHDHALMIVLFISTLVLYIIIAMVTTELTDKLILDSQE
Sufr    MAHPSQLGFQDAASPVMEELLHFHDHALMIVFLISTLVLYIIVAMVSTKLTNKYILDSQE
Stci    MAHPSQLGLQDAASPLMEELLRFHDHALMIVFLISVLVLYIIVAMVTTKLTNKYILDSQE
Taru    MAHPSQLGFQDAASPVMEELLHFHDHALMIVFLISTLVLYIIVAMVSTKLTNKYILDSQE
Rala    MAHPAQLGFQDAASPVMEELLHFHDHALMIVFLISTLVLYIIVAMVSTKLTNKYILDSQE
        :         :.:: :* *.::  *:*    :     :.  : * :           .
```

To be continued on page 10.

[2/4 of aligned sequences]

```
                B
Scca    IEIVWTILPAIILIMIALPSLRILYLMDEINDPHLTIKAMGHQWYWSYEYTDYEDLGFDS
Muma    IEIVWTILPAIILIMIALPSLRILYLMDEINDPHLTIKAMGHQWYWSYEYTDYEDLGFDS
Erca    IEMVWTVMPALVLITIALPSLRILYLMDEINDPHLTIKATGHQWYWSYEYTDYSTLNFDS
Pose    IEMVWTVMPALVLITIALPSLRILYLMDEINDPHLTIKATGHQWYWSYEYTDYETLNFDS
Actr    IEIVWTVLPAVILILIALPSLRILYLMDEINDPHLTIKAMGHQWYWSYEYTDYEDLGFDS
Scal    IEIVWTVLPAVILILIALPSLRILYLMDEINDPHLTIKAMGHQWYWSYEYTDYEDLGFDS
Posp    IEIVWTVLPAVILILIALPSLRILYLMDEINDPHLTIKAMGHQWYWSYEYTDYENLGFDS
Atsp    VEIVWTILPAVILIMIALPSLRILYLMDEINDPHLTIKAMGHQWYWSYELTDYEDLNFDS
Leoc    VEIVWTILPAVILIMIALPSLRILYLMDEINDPHLTIKAMGHQWYWSYELTDYEDLNFDS
Amca    IEMVWTIMPAVVLILIALPSLRILYLMDEINDPHLTIKAIGHQWYWSYEYTDFTDLEFDS
Osbi    IEIVWTILPAIILIVVALPSLRILYLMDEINNPHLTVKAIGHQWYWSYEYTDYKDLAFDS
Pabu    IEIIWTVLPAVILILIALPSLRILYLMDEINDPHLTVKAIGHQWYWSYEYTDYKDLNFDS
Hial    IEIVWTILPAVILILIALPSLRILYLMDEINDPHLTVKAIGHQWYWSYEYTDYKDLGFDS
Elha    IEIVWTVLPAVILILIALPSLRILYLMDEINDPHLTIKAMGHQWYWSYEYTDYQDLGFDS
Mlcy    IEIVWTVLPAVILILIALPSLRILYLMDEINDPHLTIKAMGHQWYWSYEYTDYQDLGFDS
Algl    VEIVWTVLPAVILVLIALPSLRALYLMDEVDNPHLTVKTMGHQWYWSYEYTDYEDLEFDS
Ptgi    IEIVWTILPAVILILIALPSLRILYLMDEINDPHLTIKAMGHQWYWSYEYTDYEDLGFDS
Alaf    IEIVWTILPAVILILIALPSLRILYLMDEINDPHLTIKAMGHQWYWSYEYTDYEDLGFDS
Nock    IEIIWTVLPAVILILIALPSLRILYLMDEINDPHLTIKAMGHQWYWSYEYTDYEDLGFDS
Anja    IEIVWTVLPAAILILIALPSLRILYLMDEINDPHLTIKAIGHQWYWSYEYTDYEDLGFDS
Gyki    IEIVWTVLPAVILILIALPSLRILYLMDEINDPHLTIKAIGHQWYWSYEYTDYEDLSFDS
Syka    IEIVWTILPAVILILIALPSLRILYLMDEINDPHLTVKAIGHQWYWSYEYTDYEDLGFDS
Opma    IEIVWTVLPAIILILIALPSLRILYLMDEINDPHLTVKAIGHQWYWSYEYTDYEDLGFDS
Comy    IEIVWTILPAVILILIALPSLRILYLMDEINDPHLTIKAVGHQWYWSYEYTDYEDLGFDS
Sasp    VEIIWTILPAAFLILMALPSLRLLYLMDEINSPHLTIKAIGHQWYWSYEYTDYHNLEFDS
Eupe    IEIVWTILPAAILVLIALPSLRLLYLMDEINNPHLTIKAIGHQWYWSYEYTDYEGLEFDS
Enja    IEIVWTILPAVILIMIALPSLRILYLMDEINDPHLTIKAVGHQWYWSYEYTDYEDLGFDS
Same    IEIVWTVLPAVILILIALPSLRILYLMDEINDPHLTIKAMGHQWYWSYEYTDYEDLGFDS
Chch    IEIVWTVLPAVILTLIALPSLRILYLMDEINDPHLTVKAMGHQWYWSYEYTDYENLGFDS
Grgr    VEIVWTILPAIILILIALPSLRILYLMDEINDPHLTIKAVGHQWYWSYEYTDYENLGFDA
Caau    IEIVWTILPAVILVLIALPSLRILYLMDEINDPHLTIKAMGHQWYWSYEYTDYENLGFDS
Cyca    IEIVWTILPAVILVLIALPSLRILYLMDEINDPHLTIKAMGHQWYWSYEYTDYENLGFDS
Dare    IEIVWTVLPAIILILIALPSLRILYLMDEINDPHVTIKAVGHQWYWSYEYTDYENLEFDS
Cost    IEIVWTVLPAVILVLIALPSLRILYLMDEINDPHLTIKAMGHQWYWSYEYTDYEDLGFDS
Leec    IEIVWTVLPAVILILIALPSLRILYLMDEINDPHLTIKAMGHQWYWSYEYTDYENLGFDS
Fola    IEIVWTVLPALILILIALPSLRILYLMDEINDPHLTIKAMGHQWYWSYEYTDYENLSFDS
Clmc    IEIVWTVLPAVILILIALPSLRILYLMDEVNDPHLTVKAMGHQWYWSYEYTDYEDLGFDS
Phin    IEIIWTILPAVILIMIALPSLRILYLMDEINNPHLTVKAIGHQWYWSYEYTDYENLGFDS
Icpu    IEIVWTILPAVILILIALPSLRILYLMDEVNDPHLTVKAMGHQWYWSYEYTDYENLAFDS
Psto    IEIVWTVLPAVILILIALPSLRILYLMDEVNDPHLTVKAMGHQWYWSYEYTDYENLAFDS
Cora    IEIIWTILPAVILVMIALPSLRILYLMDEVNDPHLTVKAMGHQWYWSYEYTDYENLAFDS
Eisp    IEIIWTILPAVILIMIALPSLRILYLMDEINNPHLTVKAIGHQWYWSYEYTDYENLNFDS
Apal    MEIIWTALPAVILIMIAFPSLRTLYLMDEMQDPHLTIKAVGHQWYWHYEYTDFSEMEFDS
Eslu    IEIVWTILPAVILILIALPSLRILYLMDEVNDPHLTIKAVGHQWYWSYEYTDYEELAFDS
Dape    IEIVWTILPAVILILIALPSLRILYLMDEVNDPHLTIKAMGHQWYWSYEYTDYEELTFDS
Glse    IEIVWTILPAVILILIALPSLRILYLMDEVNDPHLTIKAMGHQWYWSYEYTDYEDLGFDS
Naar    IEIVWTILPAVILILIALPSLRILYLMDEINDPHLTIKAMGHQWYWSYVYTDYEDLGFDS
Lioc    IEVVWTVLPALILILIALPSLRILYLMDEINDPHLTVKAIGHQWYWSYEYTDYEDLGFDS
Opso    IEIIWTILPAVILILIALPSLRILYLMDEINDPHLTIKAMGHQWYWSYEYTDYEDLGFDS
Alte    IEIVWTILPSVILILIALPSLRILYLMDEINDPHLTIKAMGHQWYWSYEYTDYEDLAFDS
Plap    IEIVWTILPSVILILIALPSLRILYLMDEINDPHLTIKAMGHQWYWSYEYTDYEDLGFDS
```

To be continued on page 11.

[2/4 of aligned sequences]

To be continued on page 12.

Plal	IEIIWTVLPAVILILIALPSLRILYLMDEINDPHLTIKAMGHQWYWSYEYTDYEDLGFDS
Sami	IEIIWTVLPAVILILIALPSLRILYLMDEINDPHLTIKAMGHQWYWSYEYTDYEDLGFDS
Rere	IEIIWTVLPAVILILIALPSLRILYLMDEINDPHLTIKAMGHQWYWSYEYTDYEDLGFDS
Gama	IEIVWTVLPAVILILIALPSLRILYLMDEINDPHLTIKAMGHQWYWSYEYTDYEDLGFDS
Onmy	IEIVWTVLPAVILILIALPSLRILYLMDEINDPHLTIKAMGHQWYWSYEYTDYEDLGFDS
Sasa	IEIVWTVLPAVILILIALPSLRILYLMDEINDPHLTIKAMGHQWYWSYEYTDYEDLGFDS
Cola	IEIVWTVLPAVILILIALPSLRILYLMDEINDPHLTIKAMGHQWYWSYEYTDYEDLGFDS
Dita	IEIIWTILPAVILILIALPSLRILYLMDEINDPHVTVKAMGHQWYWSYEYTDYKDLGFDS
Gogr	VEIIWTILPALILILIALPSLRILYLMDESNNPHLTIKAVGHQWFWSYEFTDYKDLTFDS
Chsl	LEVVWTVLPALVLILIGFPSLLILYVMDEIDDPLLTVKAVGHQWYWSYEYTDFSDLGFDS
Atja	IEIIWTVLPAVILILIALPSLRILYLMDEINDPHLTVKAMGHQWYWSYEYTDYEDLGFDS
Iido	IEIIWTILPAVILILIALPSLRILYLMDEINDPHLTVKAMGHQWYWSYEYTDYEDLGFDS
Auja	IEIIWTILPAVILILIALPSLRILYLMDEINDPHLTIKAMGHQWYWSYEYTDYEDLGFDS
Chag	IEIIWTILPAVILILIALPSLRILYLMDEISDPHLTIKAMGHQWYWSYEYTDYEDLGFDS
Hami	IEIIWTILPAVILILIALPSLRILYLMDEINDPHLTIKAMGHQWYWSYEYTDYEDLGFDS
Saun	IEIIWTILPAVILILIALPSLRILYLMDEINDPHLTIKAMGHQWYWSYEYTDYEDLGFDS
Nema	IEIIWTVLPAVILILIALPSLRILYLMDEINDPHLTIKAMGHQWYWSYEYTDYEDLGFDS
Disp	IEIIWTILPAVILILIALPSLRILYLMDEINDPHLTIKAMGHQWYWSYEYTDYEDLGFDA
Myaf	IEIIWTVLPAVILILIALPSLRILYLMDEIEDPHLTIKAVGHQWYWSYEYTDYQDLNFDA
Lagu	IEIIWTILPAVILILIALPSLRILYLMDEINDPHLTIKTLGHQWYWSYEYTDYEDLGFDS
Trtr	IEIIWTLLPAVILILIALPSLRILYLMDEINDPHLTIKAVGHQWYWSYEYTDYEDLGFDS
Zucr	IEIIWTVLPAVILILIALPSLRILYLMDEVNDPHLTIKAMGHQWYWSYEYTDYEDLGFDS
Pxja	IEIIWTVLPAVILILIALPSLRILYLMDEINDPHLTIKAMGHQWYWSYEYTDYEDLGFDS
Pxlo	IEIIWTVLPAVILILIALPSLRILYLMDEINDPHLTIKAMGHQWYWSYEYTDYEDLGFDS
Pctr	IEIIWTVLPAVILILIALPSLRILYLMDEINDPHLTIKAMGHQWYWSYEYTDYEDLGFDS
Apsa	IEIIWTILPAVILILIALPSLRILYLMDEINDPHLTIKAVGHQWYWSYEYTDYENLGFDS
Cabe	VEIIWTVLPAAILIMIALPSLRILYLMDEINAPHLTIKAVGHQWYWSYEYTDYEDLAFDS
Bzze	VEIIWTVLPAVILIMIALPSLRILYLMDEINAPHLTIKAMGHQWYWSYEYTDYEDLGFDS
Siim	IETIWTILPAFTLTMVALPSLRILYLMDEINDPHLTLKTVGHQWYWAYEYTDYVDVEFDS
Ctru	IEIIWTILPAVILIMIALPSLRILYLMDEINDPHLTIKAMGHQWYWSYEYTDYEDLGFDS
Dpbr	IEIIWTVLPAVILIMIALPSLRILYLMDEINDPHLTIKAMGHQWYWSYEYTDYEDLAFDS
Caki	IEIIWTVLPAVILILIALPSLRILYLMDEINNPHLTVKAVGHQWYWSYEYTDYNDLGFDA
Phja	IEIVWTVLPAVILVAIALPSLRILYIMDEINSPFITIKAMGHQWYWSYEYTDYKELAFDS
Brsp	VEIIWTISPAFILVIIAVPSIRILYLSDEILHPCLTIKALGHQWYWSYEYSDYSDIDFDS
Gamo	IEIIWTVLPAVILILIALPSLRILYLMDEINDPHLTIKAMGHQWYWSYEYTDYEDLGFDS
Lolo	IEIIWTVLPAVILILIALPSLRILYLMDEINDPHLTIKAMGHQWYWSYEYTDYEDLGFDS
Batr	IEMVWTILPALILIWTALPSVHILYTADEVNNPSTTVKAIGHQWYWTYQYTDSNPTEITS
Prmy	TEMVWTMIPALILIALALPSLRILYLMEELNFPSVTIKAIGHQWYWSYEYTDHDKLEFSS
Lose	VEIIWTILPAVILILIALPSLRILYLMDEIDNPHLTVKALGHQWYWSYEYTDYQDLEFDS
Loam	VEIIWTILPAIILILIALPSLRILYLMDEVDDPHLTIKAMGHQWYWSYEYADYVDLEFDS
Chab	VEIIWTVLPAIILTLIALPSLRILYLMDEINDPHLTIKAIGHQWYWSYEYTDYTDLGFDS
Chto	VEIIWTVLPAIILTLIALPSLRILYLMDEINDPHLTIKAIGHQWYWSYEYTDYTDLGFDS
Majo	IEIIWTIIPAIILILVALPSLRILYLMDEVNNPHITIKALGHQWYWSYEYTDYEDLGFDS
Hlst	IEVIWTIIPAIILIMVALPSLRILYLMDEVNNPHITIKAMGHQWYWSYEYTDYENLEFDS
Clpe	IEIIWTILPAVILVLIALPSLRILYLMDEVNSPHLTIKALGHQWYWSYEYSDYQDLSFDS
Mlmr	IEIIWTILPAIILILIALPSLRILYLMDEINDPHLTIKAIGHQWYWSYEYTDYESLGFDS
Crcr	IEIIWTVLPAVILILIALPSLRILYLMDEINDPHLTIKAVGHQWYWSYEYTDYEDLEFDS
Muce	IEIIWTVLPAVILILIALPSLRILYLMDEINDPHLTIKAVGHQWYWSYEYTDYEDLEFDS
Bege	IEIIWTILPAIILILIALPSLRILYLMDEINDPHLTIKAMGHQWYWSYEYTDYEDLGFDS
Mela	IEIIWTILPAIILILIALPSLRILYLMDEINDPHLTIKAMGHQWYWSYEYTDYEDLMFDS
Hats	IEIIWTILPAIILILIALPSLRILYLMDEINDPHLTIKAMGHQWYWSYEYTDYEDLGFDS
Orla	IEIIWTLLPAIILILIALPSLRILYLMDEINDPHLTIKAMGHQWYWSYEYTDYEDLGFDS

[2/4 of aligned sequences]

To be continued on page 13.

Cosa	IEIIWTILPAIILILIALPSLRILYLMDEINDPHLTIKAMGHQWYWSYEYTDYEDLNFDS
Exsp	IEIIWTVLPAIILILIALPSLRILYLMDEINDPHLTIKAMGHQWYWSYEYTDYEDLGFDS
Depa	IEIIWTILPAVILILIALPSLRILYLMDEINDPHLTIKAVGHQWYWSYEYTDYEDLGFDS
Rima	IEIIWTLLPALILILIALPSLRILYLMDEVNDPHLTVKALGHQWYWSYEYTDYNNFGFDS
Fuol	IEIIWTLLPAMILILIALPSLRILYLMDEINDPHLTIKAMGHQWYWSYEYTDYEDLGFDS
Gmaf	IEIVWTLLPAMILILIALPSLRILYLMDEINDPHLTIKAMGHQWYWSYEYTDYEDLNFDS
Xeei	IEIIWTLLPAMILILIALPSLRILYLMDEINDPHLTIKAVGHQWYWSYEYTDYEDLGFDS
Pros	IEIIWTILPAVVLIMIALPSLRILYLMDEINDPHLTIKAMGHQWYWSYEYTDYEDLGFDS
Scmi	LEVIWTVLPLMIIVGVGLPSLRLLYLTDELRNPYLTIKAMGHQWYWSYEYSDIEELGFDS
Rolo	IEIIWTVLPAVILILIALPSLRILYLMDEINDPHLTIKAMGHQWYWSYEYTDYEDLGFDS
Cere	IEIIWTVLPAVILILIALPSLRILYLMDEINDPHLTIKAIGHQWYWSYEYSDYEDLGFDS
Daga	IEVIWTILPAVILILIALPSLRILYLMDEINDPHLTIKAVGHQWYWSYEYTDYEELEFDS
Anco	IEIIWTVLPAVILILIALPSLRILYLMDEINDPHLTIKAMGHQWYWSYEYTDYEDLGFDS
Dmve	AEMMWTVAPAILLIMVALPSLRILYIMDEINNPHLTVKAVGHQWFWSYEYSDHESIEFES
Dmar	AEMIWTVAPAILLIMVALPSLRILYIMDEINNPHLTVKAVGHQWFWSYEYSDHENIEFES
Anka	IEIIWTILPAVILIMIALPSLRILYLMDEINDPHLTIKAMGHQWYWSYEYTDYEDLGFDS
Moja	IEIIWTVLPAVILILIALPSLRILYLMDEINDPHLTIKAMGHQWYWSYEYTDYEDLGFDS
Hoja	IEIIWTVLPAVILILIALPSLRILYLMDEINDPHLTIKAMGHQWYWSYEYTDYEDLGFDS
Bede	IEIIWTVLPAVILILIALPSLRILYLMDEINDPHLTIKAMGHQWYWSYEYTDYEDLGFDS
Besp	IEIIWTVLPAVILILIALPSLRILYLMDEINDPHLTIKAMGHQWYWSYEYTDYEDLGFDS
Mysp	IEIIWTVLPAVILILIALPSLRILYLMDEINDPHLTIKAMGHQWYWSYEYTDYEDLGFDS
Osja	IEIIWTVLPAVILILIALPSLRILYLMDEINDPHLTIKAMGHQWYWSYEYTDYEDLGFDS
Sgro	IEIIWTVLPAVILILIALPSLRILYLMDEINDPHLTIKAMGHQWYWSYEYTDYEDLGFDS
Pzpa	IEIIWTVLPAVILILIALPSLRILYLMDEINDPHLTIKAMGHQWYWSYEYTDYKDLEFDS
Zeja	IEIIWTVLPAAVLILIALPSLRILYLMDEINDPHLTIKAMGHQWYWSYEYTDYEDLGFDS
Znne	IEIIWTVLPAVILILIALPSLRILYLVDEVNDPHLTIKAMGHQWYWSYEYTDYEDLGFDS
Zefa	IEIIWTVLPAVILILIALPSLRILYLVDEVNDPHLTIKAVGHQWYWSYEYTDYADLGFDS
Acni	IEIIWTVLPAVILILIALPSLRILYLMDEINDPHLTIKAMGHQWYWSYEYTDYEDLGFDS
Ncrh	IEIIWTVLPAVILILIALPSLRILYLMDEINDPHLTIKAMGHQWYWSYEYTDYEDLGFDS
Agca	IEVIWTVLPAVILILIALPSLRILYLMDEINDPHLTVKAIGHQWYWSYEYTDYEDLGFDS
Hydy	IEIIWTVLPAIILILIALPSLRILYLMDEINNPLLTIKAVGHQWYWSYEYTDYEDLGFDA
Gsac	IEIIWTILPAIILILIALPSLRILYLMDEVNNPLLTIKAVGHQWYWSYEYTDYEDLGFDA
Pevo	IEIVWTILPALVLVLIALPSLRILYLMDEVNDPHLTIKALGHQWYWSYEYTDYNDLSFDA
Hiku	IEIIWTVLPAIILILIALPSLRILYLMDEVNDPHLTIKAVGHQWYWSYEYTDYEDLAFDS
Inpa	IEIIWTVLPAIILILIALPSLRILYLMDEINEPHLTIKAIGHQWYWSYEYTDYEDLAFDS
Auch	MEIIWTVLPAIVLVLIALPSLRILYLADEINDPHLTIKAVGHQWYWTYEYTDYQNISFDS
Fico	IEIIWTVLPAIILILIALPSLRILYLMDEINDPHLTIKAVGHQWYWSYEYTDYEDLGFDS
Macs	IEIIWTVLPAIILILIALPSLRILYLMDEINDPHLTIKAVGHQWYWSYEYTDYEDLGFDS
Moal	IETIWTIAPALILILIAMPSLRVLYMMDEVNNPHLTVKTIGHQWYWSYEYTDYKNLEFDS
Syma	IETLWTILPAFILVAIALPSLRVLYIMDELNNPHLTIKTIGHQWYWSYEYTDYDDLCFDS
Mafr	IEIIWTILPAIILVLIALPSLRILYLMDEINDPHLTIKAMGHQWYWSYEYTDYEDLGFDS
Dcpe	IEIIWTVLPAIILILIALPSLRILYLMDEINDPHLTIKAIGHQWYWSYEYTDYEDLGFDS
Dcti	IEIIWTVLPAIILILIALPSLRILYLMDEINDPHLTIKAIGHQWYWSYEYTDYEDLGFDS
Hehi	IEIIWTILPAIILILIALPSLRILYLMDEINNPLLTIKAVGHQWYWSYEYTDYEDLGFDS
Stam	IEIIWTVLPAVILILIALPSLRILYLMDEINNPLLTIKAVGHQWYWSYEYTDYEDLGFDS
Hogi	IEIIWTILPAIILILIALPSLRILYLMDEINNPLLTIKAVGHQWYWSYEYTDYEDLGFDS
Erzo	IEIIWTVLPAVILILIALPSLRILYLMDEINSPLLTIKAVGHQWYWSYEYTDYEDLGFDS
Hxot	IEIIWTVLPAIILILIALPSLRILYLMDEINSPLLTIKAVGHQWYWSYEYTDYEDLGFDA
Core	IEIIWTVLPAIILILIALPSLRILYLMDEINSPLLTIKAVGHQWYWSYEYTDYEDLGFDA
Apve	IEIIWTVLPAIILILIALPSLRILYLMEEINSPLLTIKAVGHQWYWSYEYTDYENLEFDA
Latj	IEIIWTVLPAIVLIMIALPSLRILYLMDEINDPHLTIKAMGHQWYWSYEYTDYDDLGFDS
Laja	IEIIWTVLPAIVLIMIALPSLRILYLMDEVNDPHLTIKAVGHQWYWSYEYTDFEDLGFDS

[2/4 of aligned sequences]

To be continued on page 14.

```
Syja IEIIWTVLPAIVLILIALPSLRILYLMDEVNDPHLTIKAMGHQWYWSYEYTDFEDLAFDS
Epme IEIIWTILPAVVLILIALPSLRILYLMDEINDPHITIKAMGHQWYWSYEYTDYEDLGFDS
Grse IEIIWTILPAIILILIALPSLRILYLMDEINNPHLTIKAMGHQWYWSYEYTDYEELGFDS
Clja IEIIWTVLPAVILILIALPSLRILYLMDEINNPHLTIKAMGHQWYWSYEYTDYDDLGFDS
Ogcy IEVIWTVLPALILILIALPSLKILYLMDEINDPHLTIKTVGHQWYWSYEYTDYEELGFDS
Plna IEIIWTILPAAILILIALPSLRILYLMDEINDPHLTVKAIGHQWYWSYEYTDYDDLTFDS
Lema IEIIWTILPAIILILIALPSLRILYLMDEINDPHLTIKAMGHQWYWSYEYTDYEDLGFDS
Etzo IEIIWTVLPAIILILIALPSLRILYLMDEINDPHLTIKAMGHQWYWSYEYTDYEDLGFDS
Apse IEIIWTILPAIILILIALPSLRILYLMDEVNNPHLTVKAIGHQWYWSYEYTDYKELGFDS
Epde IEIIWTVLPAIILILIALPSLRILYLMDEINDPHLTIKAMGHQWYWSYEYTDYEDLGFDS
Slja IEIIWTVLPAVVLILIALPSLRILYLMDEINDPHLTIKAMGHQWYWSYEYTDYEELGFDS
Bsja IEVIWTVLPAVILILIALPSLRILYLMDEINDPHLTVKAIGHQWYWSYEYTDYQDLGFDS
Ecna VEIIWTVLPAVILILIALPSLRILYLMDEINDPHLTIKAMGHQWYWSYEYTDYEDLGFDS
Cohi VETVWTVVPAVVLIAIAIPSLRTLYLMDENDSPHLTIKAVGHQWYWSYEYTDYQELAFDS
Caar IEIIWTILPAIILILIALPSLRILYLMDEINDPHLTIKAMGHQWYWSYEYTDYEDLGFDS
Came IEIIWTILPAVILILIALPSLRILYLMDEINDPHLTIKAMGHQWYWSYEYTDYQDLGFDS
Mema IEIIWTLLPAIILVLIALPSLRILYLMDEIDDPHLTIKAMGHQWYWSYEYTDYENLEFDS
Lenu VEIIWTILPAIILILIALPSLRILYLMDEINNPHLTVKAIGHQWYWSYEYTDYGELGFDS
Brja IEIIWTILPAIILILIALPSLRILYLMDEINDPHLTIKAVGHQWYWSYEYTDYEDLGFDS
Plma IEIIWTILPAIILILIALPSLRILYLMDEINDPHLTIKAVGHQWYWSYEYTDYEDLGFDS
Emst IEIIWTVLPAVILILIALPSLRILYLMDEINDPHLTIKAMGHQWYWSYEYTDYEDLGFDS
Ptti IEIIWTILPAIILILIALPSLRILYLMDEINDPHLTIKAMGHQWYWSYEYTDYEDLGFDS
Losu IEIIWTILPAIILILIALPSLRILYLMDEINEPHLTIKAVGHQWYWSYEYTDYEDLGFDS
Geoy IEIIWTVLPAMILILIALPSLRILYLMDEINDPHLTIKAMGHQWYWSYEYTDYEDLGFDS
Dipi IEIIWTILPAIILILIALPSLRILYLMDEINDPHLTIKAVGHQWYWSYEYTDYEDLGFDS
Pama IEVIWTILPAVILILIALPSLRILYLMDEINDPHLTIKAVGHQWYWSYEYTDYEELGFDS
Leob IEIIWTILPAVILILIALPSLRILYLMDEINDPHLTIKAIGHQWYWSYEYTDYQDLGFDS
Neba IEIIWTVLPAITLIMIALPSLRILYLMDEVNNPHLTVKAIGHQWYWSYEYTDYMDLGFDS
Pdpl VEIIWTVLPAVILIILALPSLRLLYLMDEVNHPHITLKAIGHQWYWSYEYSDFQDIAFDS
Nimi IETIWTVLPAITLILIALPSLRILYLMDEINDPHLTIKAMGHQWYWSYEYTDYEDLGFDS
Uptr IEIIWTVLPAVILIMIALPSLRILYLMDEINDPHLTIKAVGHQWYWSYEYTDYEDLGFDS
Pesc IEIVWTALPAFILIMIALPSLRILYLMDEIDDPLLTVKAMGHQWYWSYEYTDFEDLQFDS
Baar VEIIWTVLPAIILIMIALPSLRILYLMDEINDPHLTVKAMGHQWYWSYEYTDFEALGFDS
Moar IEIIWTILPAIILILIALPSLRILYLMDEINDPHLTIKAMGHQWYWSYEYTDYEDLGFDS
Toja IEIIWTLLPAIILILIALPSLRILYLMDEINDPHLTIKAMGHQWYWSYEYTDYEDLGFDS
Chau IEIVWTVLPAVILVLIALPSLRILYLMDEVNNPHLTVKAIGHQWYWTYEYTDYGELSFDS
Chse IEIIWTILPAIILIMIALPSLRILYIMDEVNDPHLTVKAMGHQWYWSYEYTDYEELGFDS
Enar IEIIWTVLPAIVLILIALPSLRILYLMDEINDPHLTIKAMGHQWYWSYEYTDYEDLGFDS
Hpty IEIIWTILPAIILILIALPSLRILYLMDEINDPHLTIKAMGHQWYWSYEYTDFEDLGFDS
Nana IEIIWTVLPAIILMMIALPSLRILYLMDEINDPHLTVKAVGHQWYWSYEYTDYEDCEFDS
Mcst IEIIWTILPAIILILIALPSLRILYLMDEINDPHLTIKAMGHQWYWSYEYTDYEDLGFDS
Rhox IEIIWTVLPAIILILIALPSLRILYLMDEINDPHLTIKAMGHQWYWSYEYTDYEDLGFDA
Opfa IEIIWTVLPAVILILIALPSLRILYLMDEINDPHLTVKAMGHQWYWSYEYTDYEALGFDS
Paar IEIIWTILPAVILILIALPSLRILYLMDEINDPHLTIKAMGHQWYWSYEYTDYNELGFDS
Gozo IEIIWTVLPAVILILIALPSLRILYLMDEINDPHLTIKAMGHQWYWSYEYTDYEDLGFDS
Ackr IEIVWTVLPAFTLIAVALPSLRILYLMDEINAPHLTIKAMGHQWYWTYEYTDFENFEFDS
Elev VEIIWTLLPAIILILIALPSLRILYLMDEVNNPHLTVKAMGHQWYWSYEYTDYTDLGFDS
Trdu IEIIWTLLPAIILILIALPSLRILYLMDEINDPHLTIKAMGHQWYWSYEYTDYEDLGFDS
Amoc IEIIWTVLPALILILIALPSLRILYLMDEINDPHLTIKAIGHQWYWSYEYTDYEELGFDS
Hame IEVIWTILPAVILILIALPSLRILYLMDEINDPHLTIKAIGHQWYWSYEYTDYEELGFDS
Chso IEIIWTILPAVILILIALPSLRILYLMDEINDPHLTIKAMGHQWYWSYEYTDYEELGFDS
Lyto IEIIWTVLPAIILILIALPSLRILYLMDELNSPLLTIKAVGHQWYWSYEYTDYEDLGFDA
```

[2/4 of aligned sequences]

To be continued on page 15.

```
Encr  IEIIWTVLPAIILILIALPSLRILYLMDEINNPLLTIKAVGHQWYWSYEYTDYEDLGFDA
Bvar  IEIIWTILPGFILILIALPSLRILYLMDEINDPHLTVKALGHQWYWSYEYTDYQTLEFDS
Noco  IEIIWTVLPAIILILIALPSLRILYLMDEINDPHLTIKAMGHQWYWSYEYTDYDALEFDS
Chsp  LEIVWTLLPSLILLFIAIPSVRILYLTEEHNDIMLTIKAIGHQWYWNYEYTDFGDLQFDS
Arja  IEIIWTVLPAIILILIALPSLRILYLMDEINSPLLTIKAVGHQWYWSYEYTDYEDLGFDA
Pase  IEFIWTVLPAVILLLLALPSLRILYLLDELVDTHMSIKAMGHQWYWSYEYTDILDLEFEA
Trel  IEVVWTVLPALILILIALPSLRVLYLMDEISNPHLTIKTVGHQWYWSYEYTDYEELEFDA
Lifa  IETIWTVLPAIILILIALPSLQILYLMDEINDPHLTIKAMGHQWYWSYEYTDFENLGFDS
Acur  VEIIWTVLPAVILILIALPSLRILYLMDEINDPHLTIKAMGHQWYWSYEYTDFEELGFDS
Ampe  IEIIWTVLPAVILILIALPSLRILYLMDEVNDPHLTIKAMGHQWYWSYEYTDYEDLGFDS
Urja  IEIIWTVLPAIILILIALPSLRILYLMDEVNSPHLTIKAVGHQWYWSYEYTDYQDLEFDS
Enet  IEIIWTVLPAMILILIALPSLRILYLMDEVNDPHLTIKAVGHQWYWSYEYTDYEDLGFDS
Ptbr  VEIIWTVLPALILILIALPSLRILYLMDEINSPHLTIKAVGHQWYWSYEYTDYEDLSFDS
Safa  IEIIWTLLPALILILIALPSLRILYLMDEINDPHLTIKAVGHQWYWSYEYTDYEELGFDS
Icae  IEIIWTILPAIILILIALPSLRILYLMDEINDPHLTIKAVGHQWYWSYEYTDYEDLGFDS
Asmi  IEIIWTLLPAIILVLIALPSLRILYLMDEVNDPHLTIKAVGHQWYWSYEYTDFQDLTFDS
Foal  VEIIWTVFPALILILIALPSLRILYLIEEVNNPHLTVKAIGHQWYWSYEYTDYNELCFDS
Drze  IEIVWTILPAAVLILIALPSLRILYLMEEINEPHLTIKAVGHQWYWSYEYTDYTELNFDA
Rhas  IEIIWTILPAIILILIALPSLRILYLMDEINDPHLTIKAMGHQWYWSYEYTDYEDLGFDS
Elac  IEIIWTILPAIILILIALPSLRILYLMDEINDPHLTIKAMGHQWYWSYEYTDYEDLGFDS
Kugu  IEIIWTVLPAIVLVLIALPSLRILYLMDEINNPHLTIKAVGHQWYWSYEYTDYEDLGFDS
Plor  IEIIWTILPAIILIMIALPSLRILYLMDEINDPHLTIKAMGHQWYWSYEYTDYEDLGFDS
Sgun  IEIIWTILPAIILVLIALPSLRILYLMDEINDPHLTIKAMGHQWYWSYEYTDYEDLGFDS
Zaco  IEIIWTILPAIILILIALPSLRILYLMDEINDPHLTIKAMGHQWYWSYEYTDYEDLGFDS
Zbfl  IEIIWTILPAIILILIALPSLRILYLMDEINDPHLTIKAMGHQWYWSYEYTDYEDLGFDS
Spba  VEIIWTVLPAVILILIALPSLRLLYLMDEINHPHLTIKAMGHQWYWSYEYTDYEDLAFDS
Game  IEIIWTILPAIILILIALPSLRILYLMDEINDPHLTIKAVGHQWYWSYEYTDYEDLGFDS
Thth  IEIIWTILPAIILILIALPSLRILYLMDEINDPHLTIKAVGHQWYWSYEYTDYEDLGFDS
Xigl  IEIIWTLLPAIILILIALPSLRILYLMDEINDPHLTIKALGHQWYWSYEYTDYEDLGFDS
Hyja  IEIIWTVLPAIILILIALPSLRILYLMDEINDPHLTIKAVGHQWYWSYEYTDYEDLGFDS
Psan  IEIIWTVLPAIVLILIALPSIRILYLMDEINDPHLTIKAMAHQWYWSYEYTDYEDLGFDS
Cupa  IEIIWTVLPAIILILIALPSLRILYLMDEINDPHLTIKAMGHQWYWSYEYTDYEDLGFDS
Mpch  IEIIWTILPAIILISIALPSLRILYMMDEINNPHLTVKAVGHQWYWSYEYTDYENLEFDS
Char  IEIVWTILPAIILILIALPSLRILYLMDEINDPHLTIKTMGHQWYWSYEYTDYEDLGFDS
Pser  IEIIWTLLPAMILVLIALPSLRILYLMDETNDPHLTLKALGRQWYWSYEYSDYGDLGFDA
Prol  IEIIWTVLPAIILILIALPSLRILYLMDEINDPHLTIKAMGHQWYWSYEYTDYEDLGFDS
Plbi  IEVIWTVLPAIILILIALPSLRILYLMDEINDPHLTIKALGHQWYWSYEYTDYQDLGFDS
Calu  IEIVWTVLPAVVLVTIALPSLRLLYMMDEVNTPHMTVKAIGHQWYWTYEYTDFEDLEFDA
Papa  IEIIWTVMPAAVLILIALPSLRILYLMDEVNDPHLTIKAVGHQWYWSYEYTDYMDLTFDS
Sufr  IEIIWTILPAVILILIALPSLRILYLMDEINDPHLTIKAMGHQWYWSYEYTDYEDLGFDS
Stci  IEIIWTILPAIVLILIAFPSIRILYMMDEINSPHLTIKAIGHQWYWSYEYTDYEDLAFDS
Taru  IEIIWTILPAIILILIALPSLRILYLMDEINDPHLTIKAMGHQWYWSYEYTDYSDLAFDS
Rala  IEIIWTVLPAVILILIALPSLRILYLMDEINDPHLTIKAMGHQWYWSYEYTDYEDLGFDS
       * :**  *    :  ..**:  **  :*      ::*: ..**:* *  :*     : :
```

[3/4 of aligned sequences]

```
                                                     161
Scca   YMIQTQDLTPGQFRLLETDHRMVVPMESPIRVLVSAEDVLHAWAVPALGVKMDAVPGRLN
Muma   YMIQTQDLAPGQFRLLETDHRMVVPMESPIRVLVSAEDVLHSWAVPALGVKMDAVPGRLN
Erca   YMMPTQDLMPGQFRLLDTDNRMVVPMESPVRMLITAEDVLHSWAVPSLGLKMDAVPGRLN
Pose   YMVPTQDLLPGQFRLLDTDNRMVVPTNSPVRMLITAEDVLHSWAVPSLGLKMDAVPGRLN
Actr   YMIPTQDLAPGQFRLLETDHRMVVPMESPIRVLVSAEDVLHSWAVPALGIKMDAVPGRLN
Scal   YMIPTQDLIPGQFRLLEADHRMVVPMESPIRVLVSAEDVLHSWAVPALGIKMDAVPGRLN
Posp   YMIPTQDLTPGQFRLLETDHRMVVPMESPIRVLVSAEDVLHAWAVPALGIKMDAVPGRLN
Atsp   YMIPTSDLTPGQFRLLETDHRMIVPMESPIRMLISAEDVLHSWAVPSLGMKMDAVPGRLN
Leoc   YMIPTSDLSPGQFRLLETDHRMVVPMESPIRMLISAEDVLHSWAVPSLGMKMDAVPGRLN
Amca   YMVPTQDLSNGQFRLLETDHRMVVPMDSPVRVLITAEDVLHSWAVPSLGIKMDAVPGRLN
Osbi   YMVPTQDLAPGQFRLLEVDHRMVVPTESPIRVLITADDVLHSWAVPALGVKMDAVPGRLN
Pabu   YMTPTQELTPGQFRLLEVDHRMVIPMESPIRVLITADDVLHSWAVPALGVKMDAVPGRLN
Hial   YMVPTQDLTPGQFRLLEVDHRMVVPMESPVRVLVSAEDVLHSWAVPALGVKMDAVPGRLN
Elha   YMIPTQDLTPGQFRLLETDHRMVVPMESPVRVLVSAEDVLHSWAVPALGVKMDAVPGRLN
Mlcy   YMIPTQDLAPGQFRLLETDHRMVVPMESPVRVLVSAEDVLHSWAVPALGVKMDAVPGRLN
Algl   YMVPTQDLAPGHFRLLETDHRMVVPVESPVRVLVSAEDVLHSWAVPSLGVKVDAVPGRLN
Ptgi   YMVPTQDLTPGQFRLLETDHRMVVPMESPVRVLVSAEDVLHSWAVPALGVKMDAVPGRLN
Alaf   YMVPTQELNPGQFRLLETDHRMVVPMEAPVRVLVSAEDVLHSWAVPALGVKMDAVPGRLN
Nock   YMIPTQDLTPGQFRLLETDHRMVVPMEAPVRMLISAEDVLHSWAVPALGVKMDAVPGRLN
Anja   YMIPTQDLTPGQFRLLETDHRMVVPMESPVRVLVTAEDVLHSWAVPSLGVKMDAVPGRLN
Gyki   YMVPTQDLAPGQFRLLETDHRMVVPMESPVRVLVTAEDVLHSWAVPALGVKMDAVPGRLN
Syka   YMIPTQDLSPGQFRLLEADHRMVVPMEAPVRVLVTAEDVLHSWAVPALGVKMDAVPGRLN
Opma   YMIPTQDLSPGQFRLLETDHRMVVPMESPVRVLVTAEDVLHSWAVPAMGVKMDAVPGRLN
Comy   YMIPTQDLTPGQFRLLEADHRMVIPMESPIRVLVTAEDVLHSWAVPSLGVKMDAVPGRLN
Sasp   YMIPTQELPQGHFRLLEVDHRMVIPTNAPIRMLITAEDVLHSWAVPALGTKMDAVPGRLN
Eupe   YMIPSQDLAPGQFRLLEADHRMVIPLDSPVRMLITAEDVLHSWAVPALGLKMDAVPGRLN
Enja   YMVPTQDLIPGQFRLLETDHRMVVPMESPVRVLVTAEDVLHSWAVPALGVKMDAVPGRLN
Same   YMVPTQDLVPGQFRLLETDHRMVVPMESPIRVLVSAEDVLHSWAVPALGVKMDAVPGRLN
Chch   YMIPTQDLSPGQFRLLETDHRMVVPMESPIRVLVSAEDVLHSWAVPALGVKMDGVPGRLN
Grgr   YMVPTQDLDPGQFRLLETDHRMVIPMESPIRILVSAEDVLHSWAVPALGIKMDAVPGRLN
Caau   YMVPTQDLAPGQFRLLETDHRMVVPMESPVRILVSAEDVLHSWAVPSLGVKMDAVPGRLN
Cyca   YMVPTQDLAPGQFRLLETDHRMVVPMESPVRVLVSAEDVLHSWAVPSLGVKMDAVPGRLN
Dare   YMVPTQDLTPGGFRLLETDHRMVVPKESPIRILVSAEDVLHSWAVPSLGIKMDAVPGRLN
Cost   YMIPTQDLTPGQFRLLETDHRMVVPMESPIRVLVSAEDVLHSWAVPSLGIKMDGVPGRLN
Leec   YMIPTQDLSPGQFRLLETDHRMVVPMESPIRVLVSAEDVLHSWAVPSLGIKMDAVPGRLN
Fola   YMIPTQDLTPGQFRLLETDHRMVVPMESPIRILVSAEDVLHSWALPAMGVKMDAVPGRLN
Clmc   YMIPTQDLTPGQFRLLETDHRMVIPMESPIRVLVSAEDVIHSWAVPALGVKMDAIPGRLN
Phin   YMIPTQDLSPGQFRLLEADHRMVVPMEAPVRVLISAEDVLHSWAVPALGVKMDAVPGRLN
Icpu   YMIPTQDLVPGQFRLLETDHRMVIPMESPIRVLVSAEDVLHSWAVPALGIKMDAVPGRLN
Psto   YMIPTQDLIPGQFRLLETDHRMVIPMESPIRVLVSAEDVLHSWAVPALGIKMDAVPGRLN
Cora   YMVPTQDLTPGQFRLLETDHRMVVPMESPIRVLVSAEDVLHSWAVPALGIKMDAVPGRLN
Eisp   YMVPTQDLAPGQFRLLETDHRMVVPMESPIRILVTAEDVLHSWAVPALGVKMDAVPGRLN
Apal   YLVPTNDLAPGEFRMLETDNRVVLPINTPIRVLATATDVLHSWAVPALGVKADAVPGRLN
Eslu   YMVPTQDLLPGQFRLLETDHRMVVPVESPTRILVSAEDVLHSWAVPSLGIKMDAVPGRLN
Dape   YMVPTQDLLPGQFRLLETDHRMVVPVESPTRILVSAEDVLHSWAVPSLGIKMDAVPGRLN
Glse   YMIPTQDLNPGQFRLLEADHRMVVPVESPIRILVSAEDVLHSWAVPSLGVKMDAVPGRLN
Naar   YMIPTQDLTPGQFRLLETDHRMVVPVESPIRILVSAEDVLHSWAVPSLGVKMDAVPGRLN
Lioc   YMIPTQDLAPGQFRLLEADHRMVVPVDSPIRVLVTAEDVLHSWAVPALGVKMDAVPGRLN
Opso   YMIPTQDLNPGQFRLLETDHRMVVPVESPIRILVSAEDVLHSWAVPSLGVKMDAVPGRLN
Alte   YMIPTQDLTPGQFRLLETDHRMVIPMESPIRVLVSAEDVLHSWTVPALGVKMDAVPGRLN
Plap   YMVPTQDLTPGQFRLLETDHRMVVPMESPIRILVSAEDVLHSWAVPALGVKMDAVPGRLN
```

To be continued on page 16.

[3/4 of aligned sequences]

To be continued on page 17.

Plal	YMIPTQDLIPGQFRLLEADHRMVVPVESPIRVLVSAEDVLHSWAVPALGVKMDAVPGRLN
Sami	YMIPTQDLAPGQFRLLEADHRMVVPVESPIRVLVSAEDVLHSWAVPALGVKMDAVPGRLN
Rere	YMIPTQDLTPGQFRLLEADHRMVVPVESPIRVLVSAEDVLHSWAVPALGVKMDAVPGRLN
Gama	YMIPTQDLAPGQFRLLEADHRMVIPVESPIRVLVSAEDVLHSWAVPALGVKMDAVPGRLN
Onmy	YMVPTQDLVPGQFRLLETDHRMVVPVESPIRVLVSAEDVLHSWAVPSLGVKMDAVPGRLN
Sasa	YMVPTQDLTPGQFRLLETDHRMVVPVESPIRVLVSAEDVLHSWAVPSLGVKMDAVPGRLN
Cola	YMIPTQDLIPGQFRLLEADHRMVVPVESPIRVLVSAEDVLHSWAVPSLGVKMDAVPGRLN
Dita	YMVPTQDLAPGQFRLLEADHRMVVPAESPIRVLVSAEDVLHSWAVPALGVKMDAVPGRLN
Gogr	YMVPPQDLPLGQFRLLEADHRVVVPTESPIRVLITAEDVLHSWAVPALGVKMDAIPGRLN
Chsl	YMIPTQDLAPGHFRLLEADHRLVVPISVPTRVLITAEDVLHSWAVPALGVKMDAVPGRLN
Atja	YMIPTQDLAPGQFRLLETDHRMVVPAESPIRVLVSAEDVLHSWAVPALGVKMDAVPGRLN
Iido	YMIPTQDLVPGQFRLLETDHRMVVPAESPIRVLVSAEDVLHSWAVPALGVKMDAVPGRLN
Auja	YMIPTQDLLPGQFRLLEADHRMVIPVESPIRILVSAEDVLHSWAVPALGVKMDAVPGRLN
Chag	YMIPTQDLAPGQFRLLEADHRMVIPVESPIRVLVSAEDVLHSWAVPALGVKMDAVPGRLN
Hami	YMVPTQDLTPGQFRLLETDHRMVVPVESPIRILVSAEDVLHSWAVPALGVKMDAVPGRLN
Saun	YMVPTQDLTPGQFRLLETDHRMVVPVESPIRILVSAEDVLHSWAVPALGVKMDAVPGRLN
Nema	YMIPTQDLLPGQFRLLEADHRMVIPIESPIRVLVSAEDVLHSWAVPALGVKMDAVPGRLN
Disp	YMVPTQDLLPGQFRLLETDHRVVVPVESPIRVMVSAEDVLHSWAVPALGVKLDAVPGRLN
Myaf	YMVPTQDLIPGQFRLLETDHRVVVPVESPIRVMVTAEDVLHSWAVPALGVKLDAVPGRLN
Lagu	YMIPTQDLTPGQFRLLEADHRMVVPVESPIRMLVSAEDVLHAWAVPALGIKMDAVPGRLN
Trtr	YMIPTQDLTPGQFRLLEADLRMVVPVESPIRILVSAEDVLHSWAVPALGVKMDAVPGRLN
Zucr	YMIPTQDLSPGQFRLLEADLRMVVPVESPIRILVSAEDVLHSWAVPALGVKMDAVPGRLN
Pxja	YMIPTQDLLPGQFRLLEADHRMVVPVESPIRILVSAEDVLHSWAVPALGVKMDAVPGRLN
Pxlo	YMIPTQDLLPGQFRLLEADHRMVVPVESPIRILVSAEDVLHSWAVPALGVKMDAVPGRLN
Pctr	YMVHTQDLAPGQFRLLEADHRMVVPVESPVRILVSAEDVLHSWTVPALGMKMDAVPGRLN
Apsa	YMIPTQDLSPGQFRLLETDHRMVIPMESPIRILVSAEDVLHSWAVPTLGVKMDAVPGRLN
Cabe	YMVPTQDLAPGHFRLLEADHRMVIPMESPIRVLVSAEDVLHSWAVPTLGTKMDAVPGRLN
Bzze	YMLPTQDLAPGQFRLLETDHRMVIPVESPIRVLVSAEDVLHSWAVPALGVKMDAVPGRLN
Siim	YMINTTDLAPGEFRLLDVDNRVVIPMDSPIRVLVTADDVLHSWAVPTLGIKTDAVPGRLN
Ctru	YMIPTQDLAPGQFRLLEADHRMVIPVESPIRVLVSAEDVLHSWAVPALGVKMDAVPGRLN
Dpbr	YMIPTQDLNPGQFRLLEADHRMVIPIESPIRVLVSAEDVLHSWAVPALGVKMDAVPGRLN
Caki	YMIPTQDLLPGEFRLLETDYRVVLPTNSPLRILTSAEDVLHSWAVPALGVKMDAVPGRLN
Phja	YMIPTQELEAGHFRLLDTDHRMVIPLGTPIRLLISAEDVLHSWAVPALAIKMDAVPGRLN
Brsp	YMIPTSDIFSGHFRLLEADNRMIVPAYSFIRMLITADDVIHAWTVPALGAKVDGIPGRLN
Gamo	YMIPTQDLAPGQFRLLEADHRMVVPVESPIRILISAEDVLHSWAVPALGIKMDAVPGRLN
Lolo	YMIPTQDLAPGQFRLLEADHRMVVPVESPIRILISAEDVLHSWAVPALGIKMDAVPGRLN
Batr	YMVPTESMAGGHFRLLDVDHRMVLPSKTAIRLLVTAEDVIHSWTVQTLGVKLDAVPGRLN
Prmy	YMIPTKDLIPGEYRLLDVDNRLVMPTETPARLLITADDVIHSWAVQTLGMKMDAVPGRLN
Lose	YMTPTQDLLPGQFRLLETDHRMVVPVASPVRMLITAEDVLHSWAVPSLGVKMDAVPGRLN
Loam	YMTPAQDLLPGQFRLLEADHRMVIPVASPIRVLVSAEDVLHSWAVPALGVKMDAVPGRLN
Chab	YMIPTQELTPGQFRLLEADHRMVVPVESPVRVLVTAEDVLHSWAVPALGVKMDAVPGRLN
Chto	YMIPTQELTPGQFRLLEADHRMVVPVESPVRVLVTAEDVLHSWAVPALGVKMDAVPGRLN
Majo	YMIPTQDLVPGQFRLLEVDHRMVVPVETPVRVLVSAEDVLHSWTVPALGVKMDAVPGRLN
Hlst	YMVPTQDLLPGQFRLLEVDHRMVVPVETPVRVLVSAADVLHSWTVPALGVKMDAIPGRLN
Clpe	YMIPTQELAPGQFRLLEADHRMVVPVESPIRVLVSAEDVLHSWAVPALGVKMDAVPGRLN
Mlmr	YMIPTQDLAPGQFRLLEADHRMVVPVESPVRVLVTAEDVLHSWAVPALGVKMDAVPGRLN
Crcr	YMIPTQDLTPGQFRLLEADHRMVIPVESPIRVLVTAEDVLHSWAVPSLGVKMDAVPGRLN
Muce	YMIPTQDLTPGQFRLLEADHRMVIPVESPIRVLVSAEDVLHSWAVPSLGVKMDAVPGRLN
Bege	YMIPTQDLTPGQFRLLEADHRMVIPVESPIRVLVSAEDVLHSWAVPSLGVKMDAVPGRLN
Mela	YMIPTQDLAPGQFRLLETDHRMVIPAESPIRVLVSAEDVLHSWAVPSLGVKMDAVPGRLN
Hats	YMVPTQDLTPGQFRLLEADHRMVVPVESPIRILVSAEDVLHSWAVPSLGVKMDAVPGRLN
Orla	YMIPTQDLTPGQFRLLETDHRMVIPVESPIRVLVSAEDVLHSWAVPSLGVKMDAVPGRLN

[3/4 of aligned sequences]

```
Cosa  YMIPTQDLSPGQFRLLEADHRMVIPIESPIRVLVSAEDVLHSWAVPSLGVKMDAVPGRLN
Exsp  YMIPTQDLTPGQFRLLEADHRMVIPVESPIRVLVSAEDVLHSWAVPSLGVKMDAVPGRLN
Depa  YMIPTQDLTPGQFRLLEADHRMVIPVESPVRVLVSAEDVLHSWAVPSLGVKMDAVPGRLN
Rima  YMVPTQDLTPGQFRLLDVDHRMVIPTESPVRVLITADDVLHSWAVPSLGIKMDAVPGRLN
Fuol  YMVPTQDLSPGQFRLLEADHRMVIPVDSPVRVLVSADDVLHSWAVPSLGIKMDAVPGRLN
Gmaf  YMVPTQELTPGQFRLLETDHRMVVPLESPIRVLISADDVLHSWAVPSLGIKMDAVPGRLN
Xeei  YMIPTQDLTPGQFRLLETDHRMVIPVESPIRVLVSADDVLHSWAVPSLGIKMDAVPGRLN
Pros  YMIPTQDLTPGQFRLLEADHRMVVPVESPIRVLVSAEDVLHSWAVPALGVKMDAVPGRLN
Scmi  YMTPTQDLAPGQFRLLEVDHRMVVPNDAPIRMLISAEDVLHSWAVPALGVKLDAVPGRLN
Rolo  YMIPTQDLAPGQFRLLEADHRMVIPVESPIRILVSAEDVLHSWAVPALGVKMDAVPGRLN
Cere  YMIPTNDLSPGQFRLLEADNRMVVPVESPARILITAEDVLHSWTVPSLGVKMDAVPGRLN
Daga  YMIPTQDLAPGQFRLLETDCRMVVPVESPIRVLVSAEDVLHSWAVPALGVKMDAVPGRLN
Anco  YMVPTQDLPSGQFRLLEADHRMVVPTESPIRVLVSAEDVLHSWAVPALGVKMDAVPGRLN
Dmve  YMIPTNDLSEGQYRLLEADNRMVVPTKAQVRMLITSDDVIHAWAVPALGLKVDAVPGRLN
Dmar  YMIPTNDLSEGQYRLLEADNRMVVPTKAQVRMLITSDDVIHAWAVPALGLKVDAVPGRLN
Anka  YMIPTQDLPSGQFRLLEADHRMVVPTESPIRVLVSAEDVLHSWAVPALGVKMDAVPGRLN
Moja  YMIPTQDLPSGQFRLLEADHRMVVPTESPIRVLVSAEDVLHSWAVPALGVKMDAVPGRLN
Hoja  YMVPTQDLPSGQFRLLEADHRMVVPTESPIRVLVSAEDVLHSWAVPALGVKMDAVPGRLN
Bede  YMIPTQDLTPGQFRLLEADHRMVIPVESPIRILVSAEDVLHSWAVPALGVKMDAVPGRLN
Besp  YMIPTQDLAPGQFRLLEADHRMVIPVESPIRILVSAEDVLHSWAVPAMGVKMDAVPGRLN
Mysp  YMIPTQDLTPGQFRLLEADHRMVVPTESPIRVLVSAEDVLHSWAVPALGVKMDAVPGRLN
Osja  YMIPTQDLTPGQFRLLEADHRMVIPVESPIRVLVSAEDVLHSWAVPALGVKMDAVPGRLN
Sgro  YMIPTQDLTPGQFRLLEADHRMVIPIESPIRVLVSAEDVLHSWAVPALGVKMDAVPGRLN
Pzpa  YMIPTQELMPGQFRLLEADHRMVVPVESPIRVLVSSEDVLHSWAVPALGLKMDAVPGRLN
Zeja  YMIPTTDLVPGQFRLLEADHRMMVPVESPIRVLVSAEDVLHSWAVPALGVKMDAVPGRLN
Znne  YMIPTATLEPGQFRLLEADHRMVVPVESPIRILVSAEDVLHSWAVPALGVKMDAVPGRLN
Zefa  YMIPTQSLEPGQFRLLEADHRMVVPVESPIRVLVSAEDVLHSWAVPALGVKMDAVPGRLN
Acni  YMIPTQDLVPGQFRLLEADHRMVVPVESPIRILVSAEDVLHSWAVPALGVKMDAVPGRLN
Ncrh  YMIPTQDLVPGQFRLLEADHRMVVPVESPIRILVSAEDVLHSWAVPALGVKMDAVPGRLN
Agca  YMIPTQDLTPGQFRLLEADHRMVVPSDSPIRVLVSAEDVLHSWAVPALGVKMDAVPGRLN
Hydy  YMIPTQDLTPGQFRLMEADHRMVIPAESPIRVLVSADDVLHSWAVPSLGIKMDAVPGRLN
Gsac  YMIPTQDLAPGQFRLMETDHRMVIPAESPIRVLVSADDVLHSWAVPSLGIKMDAVPGRLN
Pevo  YMVPTQDLAPGQFRLLEADHRVVIPTESPIRVLVSAEDVLHSWAIPSIGVKVDAVPGRLN
Hiku  YMIPTQDLTPGQFRLLEADHRMVIPVESPIRVLVSAEDVLHSWAVPSLGVKMDAVPGRLN
Inpa  YMLPTQDLAPGQFRLLETDHRMIIPAETPIRVMITAEDVIHSWALPALGVKMDAVPGRLN
Auch  YMIQTPDLGPGQLRLLETDHRMVVPSTSPIRILTSAEDVLHSWAVPSLGVKMDAVPGRLN
Fico  YMIPTQDLTPGQFRLLEADHRMVIPVESPIRILVSAEDVLHSWAVPALGVKMDAVPGRLN
Macs  YMIPTQDLAPGQFRLLEADHRMVVPVESPIRVLVSAEDVLHSWAVPSLGVKMDAVPGRLN
Moal  YMIPTQDLIPGQFRLLETDHRMVVPTDSPVRMLITADDVIHSWAVPTLGVKMDAIPGRLN
Syma  YMIPTQDLTPGQFRLLETDHRMVVPTESPIRMLVTADDVLHSWAIPAFGVKMDAVPGRLN
Mafr  YMIPTQDLSPGQFRLLETDHRMVVPVESPIRILISAEDVLHSWAVPALGVKLDAVPGRLN
Dcpe  YMVPTQDLAPGQFRLLEADHRMVVPVESPIRILVTAEDVLHSWAVPSLGIKMDAVPGRLN
Dcti  YMVPTQDLTPGQFRLLEADHRMVVPVESPIRILVTAEDVLHSWAVPSLGIKMDAVPGRLN
Hehi  YMIPTQDLTPGQFRLLEADHRMVIPVESPIRVLVSADDVLHSWAVPALGVKMDAVPGRLN
Stam  YMIPTQDLTPGQFRLLEADHRMVIPVESPIRVLVSADDVLHSWAVPALGVKMDAVPGRLN
Hogi  YMIPTQDLTPGQFRLLEADHRMVIPVESPIRVLVSADDVLHSWAVPALGVKMDAVPGRLN
Erzo  YMIPTQDLLPGQFRLLEADHRMVIPVEAPIRVLVSADDVLHSWAVPSLGIKMDAVPGRLN
Hxot  YMIPTQDLNPGQFRLMEADHRMVIPVESPIRVLVSADDVLHSWAVPALGIKMDAVPGRLN
Core  YMIPTQDLTPGQFRLLEADHRMVIPVESPIRVLVSADDVLHSWAVPALGIKMDAVPGRLN
Apve  YMIPTQDLNPGQFRLLEADHRMVIPVESPIRVLVSADDVLHSWAVPALGIKMDAVPGRLN
Latj  YMIPTQDLAPGQFRLLEADHRMVIPMESPIRVLISAEDVLHSWAVPSLGVKVDAVPGRLN
Laja  YMIPTQDLTPGQFRLLEADHRMVIPVESPIRMLISAEDVLHSWAVPSLGMKMDAVPGRLN
```

To be continued on page 18.

[3/4 of aligned sequences]

Syja	YMVPTQDLAPGQFRLLETDHRMVIPVESPIRVLISAEDVLHSWAVPALGVKMDAVPGRLN
Epme	YMIPTQDLTPGQFRLLEADHRMVVPLDSPVRVLVSAEDVLHSWTVPALGVKMDAVPGRLN
Grse	YMIPTQDLSPGQFRLLEADHRMVVPVDSPIRVLVSAEDVLHSWAVPALGVKMDAVPGRLN
Clja	YMIPTQDLSPGQFRLLEADHRMVVPVDSPIRVLVSAEDVLHSWAVPALGVKMDAVPGRLN
Ogcy	YMIPTNDLTPGQFRLLEADHRMVIPVESPIRILVTAEDVLHSWTVPTLGVKMDAVPGRLN
Plna	YMIPTQDLTPGQFRLLEVDHRMVVPVEAPVRVLVSAEDVLHSWAVPSLGVKMDAVPGRLN
Lema	YMIPTQDLTPGQFRLLEADHRMVVPVESPIRVLVSAEDVLHSWAVPALGVKMDAVPGRLN
Etzo	YMIPTQDLTPGQFRLLEADHRMVIPVESPIRVLVSAEDVLHSWAVPALGVKMDAVPGRLN
Apse	YMVPTQDLTPGQFRLLEADHRMVVPVESPVRVLVSAEDVLHSWAVPALGVKMDAVPGRLN
Epde	YMIPTQDLAPGQFRLLEADHRMVIPVESPIRILISAEDVLHSWAVPALGVKMDAVPGRLN
Slja	YMIPTQDLTPGQFRLLETDHRMVVPTESPIRVLVSAEDVLHSWAVPALGVKMDAVPGRLN
Bsja	YMIPTQDLTPGQFRLLDTDHRMVVPVESPIRVLVTAEDVLHSWAVPALGVKMDAVPGRLN
Ecna	YMIPTQDLTPGQFRLLEADHRMVIPMDSPVRVLISAEDVLHSWAVPSLGVKVDAVPGRLN
Cohi	YMIPTQELTNGQFRLLEVDHRMVVPESSPLRILISAEDVLHSWAVPALGVKADAVPGRLN
Caar	YMVPTQDLTPGQFRLLEADHRMVIPVDSPIRVLISAEDVLHSWAVPALGVKVDAVPGRLN
Came	YMIPTQDLTPGQFRLLEADHRMVIPMNSPVRVLISAEDVLHSWAVPALGVKVDAVPGRLN
Mema	YMIPTQDLAPGQFRLLEADHRMVIPAESPIRVLISAEDVLHSWAVPALGVKVDAVPGRLN
Lenu	YMIPTQDLLPGQFRLLETDHRMVVPMNAPVRVLVTAEDVLHSWAVPALGVKMDAVPGRLN
Brja	YMIPTQDLTPGQFRLLEADHRMVIPVESPIRILISAEDVLHSWAVPSLGVKMDAVPGRLN
Plma	YMIPTQDLVPGQFRLLEADHRMVVPVESPIRILISAEDVLHSWAVPSLGVKMDAVPGRLN
Emst	YMIPTQDLTPGQFRLLEADHRMVIPVESPIRVLVSAEDVLHSWAVPALGVKMDAVPGRLN
Ptti	YMIPTQDLTPGQFRLLEADHRMVIPVESPIRVLVSAEDVLHSWAVPALGVKMDAVPGRLN
Losu	YMVPTHDLSPGQFRLLETDHRMVIPAESPIRILISAEDVLHSWAVPALGMKMDAVPGRLN
Geoy	YMIPTQDLTPGQFRLLEADHRMVIPVESPIRVLVSAEDVLHSWAVPSLGIKMDAVPGRLN
Dipi	YMIPTQDLTPGQFRLLEADHRMVIPVESPIRVLITAEDVLHSWAVPALGVKMDAVPGRLN
Pama	YMLPTQDLAPGQFRLLEADHRMVVPVESPIRILVSAEDVLHSWAVPALGVKMDAVPGRLN
Leob	YMIPTQDLTPGQFRLLETDHRVVVPSESPIRVLVTAEDVLHSWAVPALGVKMDAVPGRLN
Neba	YMVPTQDLIPGQYRLLETDHRMVVPSDSPVRVLVTAEDVLHSWAVPALGVKMDAVPGRLN
Pdpl	YMVPTQDLMPGQFRLLEADHRMVVPVDSPIRVLISAEDVLHSWAVPSLGIKMDAVPGRLI
Nimi	YMIPTQDLTPGQFRLLEADHRMVIPVESPIRVLISAEDVLHSWAVPTLGIKMDAVPGRLN
Uptr	YMVPTQDLTPGQFRLLEADHRMVIPVESPIRVLVTAEDVLHSWAVPSLGVKMDAVPGRLN
Pesc	YMIPTQDLLPGHFRLLEADHRMVVPTQTPIRLLISAEDVLHSWAVPSLGVKMDAVPGRLN
Baar	YMIPTQDLTSGQFRLLEADHRMVVPVEAPIRILISAEDVLHSWAVPALGVKMDAVPGRLN
Moar	YMIPTQDLTPGQFRLLEADHRMVIPVESPIRVLVSAEDVLHSWAVPALGVKMDAVPGRLN
Toja	YMVPTQDLTPGQFRLLEADHRMVIPVESPVRVLISAEDVLHSWAVPSLGVKVDAVPGRLN
Chau	YMIPTQDLTPGQFRLLEVDHRMIVPTEAPVRVLVSAEDVLHSWAVPALGVKMDAVPGRLN
Chse	YMIPTQDLMPGQFRLLETDHRMVVPVESPIRVLVSAEDVLHSWAVPTLGVKMDAVPGRLN
Enar	YMIPTQDLTPGQFRLLEADHRMVIPVNSPIRVLVSAEDVLHSWAVPALAVKMDAVPGRLN
Hpty	YMIPTQDLTPGQFRLLEADHRMVIPVESPTRMLISAEDVLHSWAVPALGVKMDAVPGRLN
Nana	YMTPTSDLNPGDFRLLETDTRMILPMESPVRVIISADDVIHSWAIPSLGVKMDAVPGRLN
Mcst	YMIPTQDLAPGQFRLLEADHRMVIPVESPIRVLVSAEDVLHSWAVPALGVKMDAVPGRLN
Rhox	YMVPTQDLTPGQFRLLETDHRMVIPVESPIRVLVSAEDVLHSWAVPSLGVKMDAVPGRLN
Opfa	YMIPTQDLAPGQFRLLEADHRMVVPVESPIRMLVSAEDVLHSWAVPALGVKMDAIPGRLN
Paar	YMIPTHDLSPGQFRLLEADHRMVVPVESPVRVLVSAEDVLHSWAVPALGVKMDAVPGRLN
Gozo	YMVPTQDLTPGQFRLLEVDHRMVIPVESPIRVLVSAEDVLHSWAVPSLGVKMDAVPGRLN
Ackr	YMIPTQDLTPGQYRLLEADHRMVVPMESPARILISAEDVLHSWAVPALGVKMDAVPGRLN
Elev	YMVPTQELSPGQFRLLEVDHRMVVPVESPIRVLVSAEDVLHSWALPALGVKMDAVPGRLN
Trdu	YMIPTQDLAPGQFRLLEADHRMVVPVESPIRVLISAEDVLHSWAVPSLGVKMDAVPGRLN
Amoc	YMIPTQDLASGQFRLLDTDHRMVVPIESPIRMLISAEDVLHSWAVPSLGVKMDAVPGRLN
Hame	YMIPTQDLAPGQFRLLETDHRMVVPMESPVRVLVTAEDVLHSWAVPALGVKMDAVPGRLN
Chso	YMIPTQDLAPGQFRLLEADHRMVVPVESPIRVLVSAEDVLHSWAVPALGVKMDAVPGRLN
Lyto	YMIPTQDLTPGQFRLLEADHRMVVPVESPIRVLVSADDVLHSWSVPALGIKMDAVPGRLN

To be continued on page 19.

[3/4 of aligned sequences]

```
Encr   YMIPTQDLTPGQFRLLEADHRMVIPVESPIRVLVSADDVLHSWAVPALGVKMDAVPGRLN
Bvar   YMIPTQDLTPGQFRLLEADHRMVIPTESPIRVLVSAEDVLHSWAVPALGVKMDAVPGRLN
Noco   YMIPTQDLTPGQFRLLEADHRMIAPVESPVRVLVSGEDVLHSWAVPSLGVKMDAVPGRLN
Chsp   YMAQQDNLLNGTFRFLDCDNRMVVPVGCPIRMLITSEDVIHSWAVPALGVKMDAVPGRLN
Arja   YMIPTQDLNPGQFRLLEADHRMVIPVESPIRVLVSADDVLHSWAVPALGIKMDAVPGRLN
Pase   YMIPTNELTEGQYRLLETDNRVVAPIQATIRMIVSSDDVLHSWAVPTLGVKMDAVPGRLN
Trel   YMIPTQDLTPGQFRLLETDHRMVVPVESPIRILVSAEDVLHSWAVPSLGVKMDAVPGRLN
Lifa   YMVPTNDLPSAHFRLLETDHHMVTPMESPIRMLTSAEDVLHSWAVPTLGVKTDATPGRLN
Acur   YMIPTQDLTPGQFRLLEADHRMVVPMESPVRMLVSADDVLHSWAVPALGVKMDAVPGRLN
Ampe   YMIPTNDLTPGQFRLLEADHRMVIPVESPIRVLVSAEDVLHSWAVPALGVKMDAVPGRLN
Urja   YMTPTQDLNPGEFRLLEADHRMIIPVDSPVRLLVSAEDVLHSWAVPSLAVKMDAVPGRLN
Enet   YMIPTQDLAPGQFRLLEADHRMVIPVESPIRVLVSAEDVLHSWAVPSLGIKMDAVPGRLN
Ptbr   YMVPTQDLNPGEFRLLETDHRMVIPVESPIRVLVTAEDVLHSWAVPSLGIKMDAVPGRLN
Safa   YMIPTQDLTPGQFRLLETDHRMVIPVESPIRILVTAEDVLHSWAVPSLGVKMDAVPGRLN
Icae   YMIPTQDLAPGQFRLLEADHRMVIPVESPIRILISAEDVLHSWAVPSLGVKMDAVPGRLN
Asmi   YMIPTQDLNPGQFRLLETDLRMVVPTEAPIRMLVSAEDVLHSWAVPSLGVKMDAVPGRLN
Foal   YMTPTQDLTPGQFRLLEVDHRMVIPTESPIRILVSAEDVLHSWAVPSLGVKMDAVPGRLN
Drze   YMIPTQELTPGGFRLLETDHRVVVPSESPIRILISADDVLHSWAVPAFGVKLDAVPGRLN
Rhas   YMVPTQDLSPGQFRLLEADHRMVVPVESPIRVLVSAEDVLHSWAVPALGVKMDAVPGRLN
Elac   YMVPTQDLAPGQFRLLEADHRMVVPMESPVRVLVSAEDVLHSWAVPSLGVKMDAVPGRLN
Kugu   YMVPTQDLTPGQFRLLETDHRMVLPIESPIRVVITAEDVLHSWAVPALGIKMDAVPGRLN
Plor   YMVPTQDLTPGQFRLLETDHRMVIPVESPIRILISAEDVLHSWAVPSLGVKMDAAPGRLN
Sgun   YMIPTQDLTPGQFRLLEADHRMVIPVESPIRVLVSAEDVLHSWAVPALGVKMDAVPGRLN
Zaco   YMIPTQDLAPGQFRLLEADHRMVIPVESPIRVLVSAEDVLHSWAVPALGVKMDAVPGRLN
Zbfl   YMIPTQDLTPGQFRLLEADHRMVIPVESPIRVLVSAEDVLHSWAVPALGVKMDAVPGRLN
Spba   YMIPTQDLTPGQFRLLEVDHRMVVPVDSPVRTLVSAEDVLHSWALPSLGIKTDAVPGRLN
Game   YMIPTQDLAPGQFRLLEADHRMVIPVESPIRILISADDVLHSWAVPSLGVKMDAVPGRLN
Thth   YMIPTQDLAPGQFRLLEADHRMVIPVESPIRILISADDVLHSWAVPSLGVKMDAVPGRLN
Xigl   YMIPTQDLTPGQFRLLEADHRMVIPVESPIRVLISAEDVLHSWAIPALGVKVDAVPGRLN
Hyja   YMIPTQDLTPGQFRLLEADHRMVIPVESPIRILISAEDVLHSWAVPSLGVKMDAVPGRLN
Psan   YMIATEDLIPGQFRLLEADHRMVIPVESPIRLLISAEDVLHSWAVPSLGVKMDAIPGRLN
Cupa   YMLPTQDLATGQFRLLEADHRMVVPIESPIRVLISAEDVLHSWAVPALGVKMDAVPGRLN
Mpch   YMIPTQDLAPGQFRLLETDHRMVVPFNSPIRVMVTADDVLHSWAVPSLGIKLDAVPGRLN
Char   YMIPTQDLVPGQFRLLETDHRMVIPVESPVRLLVSADDVLHSWAVPSLGVKLDAVPGRLN
Pser   YMLPTQDITSGQYRLLEADHRVILPLESPIRVLTCAEDVLHSWAVPAFGIKMDAVPGRLN
Prol   YMTPTQDLTPGQFRLLEADHRMVTPVESPIRVLISAEDVLHSWAIPALGVKVDAVPGRLN
Plbi   YMVPTQDLTPGQFRLLEADHRMVIPVESPIRVLISAEDVLHSWAVPSLGVKVDAVPGRLN
Calu   YMIQTQDLTPGQFRLLEADHRVVVPSRCPVRVLVTAEDVLHSWALPALGVKADAVPGRLN
Papa   YMVPTQELEPGQFRLLEADHRMVVPVEAPIRVLISAEDVLHSWTVPALGVKADAVPGRLN
Sufr   YMIPTQDLAPGQFRLLEADHRMVVPTESPVRVLVSAEDVLHSWAVPALGVKMDAVPGRLN
Stci   YMAPTQDLTPGQFRLLEVDHRVVLPTESPVRVLVSSEDVIHSWAVPALGVKMDAVPGRLN
Taru   YMVPTQDLAPGQFRLLETDHRMVVPVDSPIRILVSAEDVLHSWAVPSLGVKMDAVPGRLN
Rala   YMIPTQDLAPGQFRLLEADHRMVIPVESPIRVLVSAEDVLHSWAVPALGVKMDAVPGRLN
       *:     :  .  *::: * ::: *     * :  . **:*:*:: ::. * *.  ****
```

To be continued on page 20.

[4/4 of aligned sequences]

```
                   196 200 204
Scca    QTAFIISRPGVYYGQQSEICGANHSFMPIVVEAVPLEHFETWSSLMLEEA---
Muma    QTAFIISRPGVYYGQQSEICGANHSFMPIVVEAIPLEHFEAWSSSMLEEA---
Erca    QATFIATRPGIFFGQQSEICGANHSFMPIAIESTPIKHFESWSSSMLAES---
Pose    QATFIATRPGIFFGQQSEICGANHSFMPIAIESAPVKYFESWSSSMLAES---
Actr    QTAFITSRPGVYYGQQSEICGANHSFMPIVVEAVPLEHFENWSSLMLEES*--
Scal    QTAFITSRPGVYYGQQSEICGANHSFMPIVVEAVPLEHFENWSSLMLEES---
Posp    QTAFITSRPGVYYGQQSEICGANHSFMPIVVEAVPLEHFENWSSLMLEES---
Atsp    QTTFIASRPGVYYGQQSEICGANHSFMPIVVEAVPLQHFENWSSLMLEEA---
Leoc    QTTFIASRPGVYYGQQSEICGANHSFMPIVVEAVPLQHFENWSSLMLEEA---
Amca    QATFMTSRPGVFYGQQSEICGANHSFMPIVVESVPLQHFEDWSLSMIDA----
Osbi    QTTFVTSRPGIYYGQQSEICGANHSFMPIAVEAVPLTHFENWSTSMLETT---
Pabu    QAAFIASRPGVYYGQQSEICGANHSFMPIVVEAVPLKHFEDWSTAMLENT---
Hial    QTAFIASRPGVYYGQQSEICGANHSFMPIVVEAVPLEHFENWSSSMLEDA---
Elha    QTAFIASRPGLYYGQQSEICGANHSFMPIVVEAVPLEHFENWSSLMLEDA---
Mlcy    QTAFIASRPGVFYGQQSEICGANHSFMPIVVEAVPLEHFENWSSLMLEDA---
Algl    QTTFIASRPGVYYGQQSEICGANHSFMPIVVEAVPLEHFEDWSSLMLD*----
Ptgi    QTAFIASRPGIYYGQQSEICGANHSFMPIVVEAVPLEHFENWSSLMLEDA---
Alaf    QTAFIASRPGVYYGQQSEICGANHSFMPIVVEAVPLQHFENWSSLMMEDA---
Nock    QSAFIVSRPGVYYGQQSEICGANHSFMPIVVEAVPLQHFENWSSLMMEDA---
Anja    QTAFIAARPGVYYGQQSEICGANHSFMPIVVEAVPLQHFENWSSMMLEDA---
Gyki    QTAFIAARPGVYYGQQSEICGANHSFMPIVVETVPLHHFETWSSTMLEDA---
Syka    QTAFIAARPGVYYGQQSEICGANHSFMPIVIEAVPLQHFENWSSMMLEDA---
Opma    QTAFVATRPGVYYGQQSEICGANHSFMPIVVEAVPLQHFENWSSMMLKDA---
Comy    QMAFIAARPGVFFGQQSEICGANHSFMPIVVEAVPLQQFEKWSSMMLQEA---
Sasp    QTTFLASRPGVYYGQQSEICGANHSFMPIVVEAVPLEHFENWADMTLQNM---
Eupe    QMTLTAARPGVYYGQQSEICGANHSFMPIVVEAVPLEQFQNWSSTMLENA---
Enja    QTAFITSRPGVFYGQQSEICGANHSFMPIVVEAVPLEHFENWSSLMIEDA---
Same    QTAFIASRPGVFYGQQSEICGANHSFMPIVVESVPLEHFENWSSLMLEDA---
Chch    QTAFIASRPGVFYGQQSEICGANHSFMPIVVEAVPLEHFENWSSLMLEDA---
Grgr    QTAFIASRSGLFYGQQSEICGANHSFMPIVVEAVPLEHFENWSWLMLEDA---
Caau    QTAFIASRPGVFYGQQSEICGANHSFMPIVVEAVPLEHFENWSSLMLEDA---
Cyca    QAAFIASRPGVFYGQQSEICGANHSFMPIVVEAVPLEHFENWSSLMLEDA---
Dare    QTAFIVSRPGVFYGQQSEICGANHSFMPIVVEAVPLEFFENWSSAMLEDA---
Cost    QTAFIASRPGVFYGQQSEICGANHSFMPIVVEAVPLEHFERWSSLMMEDA---
Leec    QTAFIASRPGVFYGQQSEICGANHSFMPIVVEAVPLEHFENWSSLMLEDA---
Fola    QTAFIASRPGVFYGQQSEICGANHSFMPIVVEAVPLSHFENWSTLMLKDA---
Clmc    QTAFIAARPGVFYGQQSEICGANHSFMPIVVEAVPLEHFENWSSLMLEDA---
Phin    QTAFIASRPGVFYGQQSEICGANHSFMPIVVEAVPLEHFENWSSLMLEDT---
Icpu    QTSFITSRPGVFYGQQSEICGANHSFMPIVVEAVPLEHFENWSSLMLEDA---
Psto    QTSFITSRPGVFYGQQSEICGANHSFMPIVVEAVPLEHFENWSSLMLEDA---
Cora    QTAFIASRPGIFYGQQSEICGANHSFMPIVVEAVPLEHFENWSSLMLEDA---
Eisp    QTAFIASRPGVFYGQQSEICGANHSFMPIVVETVPLEHFENWSSLMLEDA---
Apal    QMTLMITRPGLYYGHQSEICGAYHSFMPIAIEAVSLEAFESWAASM*------
Eslu    QTAFITSRPGLFYGQQSEICGANHSFMPIVVEAVPLKHFEEWSTLLLQDA---
Dape    QTAFITSRPGLFYGQQSEICGANHSFMPIVVEAVPLKHFEDWSTLLLQDA---
Glse    QTAFIASRPGVFYGQQSEICGANHSFMPIVVEAVPLKHFENWSSLMLEDA---
Naar    QTAFIASRPGVFYGQQSEICGANHSFMPIVVEAVPLVHFENWSSLMLEDA---
Lioc    QTAFITSRPGVFYGQQSEICGANHSFMPIVVEAVPLDHFENWSSLMLEDA---
Opso    QTTFITSRPGVFYGQQSEICGANHSFMPIVVEAVPLKCFENWSSLMLEDA---
Alte    QTAFIASRPGVFYGQQSEICGANHSFMPITVEAVPLEHFENWSSLMLEDA---
Plap    QTAFIASRPGVFYGQQSEICGANHSFMPIVVEAVPLEHFENWSSLMLEDA---
```

[4/4 of aligned sequences]

```
Plal  QTAFIASRPGVFYGQCSEICGANHSFMPIVVEAVPLKHFENWSSMMLEDA---
Sami  QTAFIASRPGVFYGQCSEICGANHSFMPIVVEAVPLKHFENWSSMMLEDA---
Rere  QTAFIASRPGVFYGQCSEICGANHSFMPIVVEAVPLGYFESWSSFLLQDA---
Gama  QTAFIASRPGVFYGQCSEICGANHSFMPIVVEAVPLKHFENWSSLMLEDA---
Onmy  QTAFIASRPGVFYGQCSEICGANHSFMPIVVEAVPLEHFEKWSTMMLEDA---
Sasa  QTAFIASRPGVFYGQCSEICGANHSFMPIVVEAVPLEHFEKWSTMMLEDA---
Cola  QTAFIASRPGVFYGQCSEICGANHSFMPIVVEAVPLEHFEKWSTMMLEDA---
Dita  QTAFIASRPGVFYGQCSEICGANHSFMPIVVEAVPLEHFENWSSLMLEDA---
Gogr  QTTFITSRPGIFYGQCSEICGANHSFMPIVVEAVPLEHFENWSTLMTEDT---
Chsl  QVSLFTHRPGVYYGQCSEICGANHSFMPVVVEAVPFERFEHWSSLMLEDA---
Atja  QTAFIASRPGVFYGQCSEICGANHSFMPIVVEAVPLEHFENWSTIMLEDA---
Iido  QTAFIASRPGVFYGQCSEICGANHSFMPIVVEAVPLEHFENWSTIMLEDA---
Auja  QTAFITSRSGVFYGQCSEICGANHSFMPIVVEAVPLEHFENWSSLMLEDA---
Chag  QTAFITSRPGVFYGQCSEICGANHSFMPIVVEAVPLEHFEHWSSLMLEDA---
Hami  QTAFITSRPGVFYGQCSEICGANHSFMPIVVEAVPLEHFENWSSLMLEDA---
Saun  QTAFITSRPGVFYGQCSEICGANHSFMPIVVEAVPLEHFENWSSLMLEDA---
Nema  QTAFITSRPGVFYGQCSEICGANHSFMPIVVEAVPLEHFEDWSSLMLEDA---
Disp  QTAFLTSRPGVFYGQCSEICGANHSFMPIVVEAVPLEHFENWSSLMLEDA---
Myaf  QTAFLASRPGVFFGQCSEICGANHSFMPIVVEAVPLEHFENWSSLMLEDA---
Lagu  QTAFIASHPGVFYGQCSEICGANHSFMPIVVEAVPLAHFETWSSRMLEDA---
Trtr  QTAFITSRPGVFYGQCSEICGANHSFMPIVVEAVPLKHFESWSSLMLEDA---
Zucr  QTAFITSRPGVFYGQCSEICGANHSFMPIVVEAVPLKHFESWSSLMLEDA---
Pxja  QTAFIASRPGVFYGQCSEICGANHSFMPIVVEAVPLEHFENWSSLMLEDA---
Pxlo  QTAFIASRPGVFYGQCSEICGANHSFMPIVVEAVPLEHFENWSSLMLEDA---
Pctr  QTTFIASRPGVFYGQCSEICGANHSFMPIVVEAVPLEHFENWSSLVLEDA---
Apsa  QTTFISSRPGVYYGQCSEICGANHSFMPIVIESVPLEHFENWSSLILEDT---
Cabe  QTAFISSRPGVFYGQCSEICGANHSFMPVVVEAVPLQCFEDWSLLMLQDS---
Bzze  QTAFISSRPGVFYGQCSEICGANHSFMPIVVEAVPLEHFESWSSLMLQDA---
Siim  QTTFLSPRTGVSYGQCSEICGANHSFMPIVVETVPLEDFEKWLVLMPTDL---
Ctru  QTAFIASRPGVFYGQCSEICGANHSFMPIVVEAVPLEHFENWSSLMLQDT---
Dpbr  QTAFITSRPGVFYGQCSEICGANHSFMPIVVEAVPLEHFENWSSLMLQDA---
Caki  QTTFINARPGVFYGQCSEICGANHSFMPIVIEVVPLKEFIDWSVLMEA-----
Phja  QATFVTSLPGVYYGQCSEICGANHSFMPIVVESTPLDHFEHWTAAMLESS---
Brsp  QTSFIATRPGLYFGQCSEICGANHSFMPIVVEAVPLNHVEEWLTLQHEEI---
Gamo  QTAFITSRPGVFYGQCSEICGANHSFMPIVVEAVPLEHFESWSSLMLEDA---
Lolo  QTAFITSRPGVFYGQCSEICGANHSFMPIVVEAVPLEHFESWSSLMLEDA---
Batr  QTALMASNPGVFYGQCSEICGANHSFMPIVVEALPTTHYENWLALQNE*----
Prmy  QMALLISRPGVFYGQCSEICGANHSFMPIVVEGIPLHNYKTWISLNMNNS*--
Lose  QTAFIALRPGVFYGQCSEICGANHSFMPIVVEAVPLKHFEDWSTMMID*----
Loam  QTAFITLRPGVYYGQCSEICGANHSFMPIVVEAVPLKHFENWSALMVEDA---
Chab  QTAFITSRPGVFYGQCSEICGANHSFMPIVVEAVPLQHFENWSSLMLEDT---
Chto  QTAFITSRPGVFYGQCSEICGANHSFMPIVVEAVPLQHFENWSSLMLEDA---
Majo  QTAFITSRPGVYYGQCSEICGANHSFMPIVVEAVPLEHFENWSSLMLEDA---
Hlst  QTAFITSRPGVFYGQCSEICGANHSFMPIVVEAVPLEHFENWSSLMLEDA---
Clpe  QTAFITSRPGVYYGQCSEICGANHSFMPIVVEAVPLEHFENWSYSMLEDS---
Mlmr  QAAFIASRPGVFYGQCSEICGANHSFMPIVVEAVPLQHFENWSSLMLQDA---
Crcr  QVAFVASRPGVFYGQCSEICGANHSFMPIVVEAVPLSHFENWSSLMLKDA---
Muce  QVAFVASRPGVFYGQCSEICGANHSFMPIVVEAVPLSHFENWSSLMLKDA---
Bege  QTAFIASHPGVFYGQCSEICGANHSFMPIVVEAVPLEHFENWSSLMLEDA---
Mela  QTAFITSRPGVFYGQCSEICGANHSFMPIVIEAVPLKHFENWSSLMLQDS---
Hats  QTAFIASRPGVFYGQCSEICGANHSFMPIVVEAVPLEHFENWSSLMLEDA---
Orla  QTAFITSRPGVFYGQCSEICGANHSFMPIVVEAVPLEHFENWSFIMLQDA---
```

[4/4 of aligned sequences]

Cosa	QTAFIASRPGVFYGQCSEICGANHSFMPIVVEAVPLEHFEKWSSLMLEDA---
Exsp	QTAFIASRPGVFYGQCSEICGANHSFMPIVVEAVPLEHFENWSSLMLEDA---
Depa	QTAFIASRPGVFYGQCSEICGANHSFMPIVVEAVPLEHFENWSSLMLEDA---
Rima	QTAFIVSRPGIYYGQCSEICGANHSFMPIVVEAIPLKHFENWSTLMLENS---
Fuol	QTAFITSRPGVFYGQCSEICGANHSFMPIVVEAVPLEHFENWSSLMLEDA---
Gmaf	QTAFIISRPGVFYGQCSEICGANHSFMPIVVEAVPLEHFENWSSTMLQDA---
Xeei	QTAFMTSRPGIFYGQCSEICGANHSFMPIVVEAVPLEHFENWSSLMLEDA---
Pros	QTAFIASRPGVFYGQCSEICGANHSFMPIVVESVPLEHFEDWSLLMLQDS---
Scmi	QTFFLTSRPGVFYGQCSEICGANHSFMPIVMESVPLEHFENWSLLMLQDS---
Rolo	QTAFIASRPGVFYGQCSEICGANHSFMPIVVEAVPLEHFENWSSLMLEDA---
Cere	QTAFITSRPGVYYGQCSEICGANHSFMPIVVEALPMKYFEDWTSLMLEDA---
Daga	QTNFIASRPGVFYGQCSEICGANHSFMPIVVEAVPLEHFESWSSSSLQDS---
Anco	QTAFIASRPGVFYGQCSEICGANHSFMPIVVEAVPLEHFETWSSLMLEDA---
Dmve	QTAFIIGRPGVFYGQCSEICGANHSFMPIVVETIDTEDFVFWISTMLES----
Dmar	QTAFIIGRPGVFYGQCSEICGANHSFMPIVVETIDTEDFVFWISTMLES----
Anka	QTAFIASRPGVFYGQCSEICGANHSFMPIVVEAVPLEHFENWSSLMLEDA---
Moja	QTAFIASRPGVFYGQCSEICGANHSFMPIVVEAVPLEHFESWSSLMLEDA---
Hoja	QTAFIASRPGVFYGQCSEICGANHSFMPIVVEAVPLEHFETWSSLMLEDA---
Bede	QTAFIASRPGVFYGQCSEICGANHSFMPIVVEAVPLEHFEDWSLLMLQDA---
Besp	QTAFIASRPGVFYGQCSEICGANHSFMPIVVEAVPLEHFEDWSLLMLQDA---
Mysp	QTAFIASRPGVFYGQCSEICGANHSFMPIVVEAVPLEHFENWSSTMLEDA---
Osja	QTAFIASRPGVFYGQCSEICGANHSFMPIVVEAVPLEHFENWSSMMLEDA---
Sgro	QTAFITSRPGVFYGQCSEICGANHSFMPIVVEAVPLEHFENWSSMMLEDA---
Pzpa	QTAFITSRSGVYYGQCSEICGANHSFMPIVVEAVPLEHFEDWTSFMLEDA---
Zeja	QTAFIASRPGVFYGQCSEICGANHSFMPIVVEVVPLAYFEDWSALMSEDS---
Znne	QTAFIASHPGVFYGQCSEICGANHSFMPIVVEAVPLEHFENWSSLMLEDA---
Zefa	QTAFIVSHPGVFYGQCSEICGANHSFMPIVVEAVPLEHFENWSSLMLEDA---
Acni	QTAFIASRPGVFYGQCSEICGANHSFMPIVVEAVPLEHFENWSSLMLEDA---
Ncrh	QTAFIASRPGVFYGQCSEICGANHSFMPIVVEAVPLEHFENWSSLMLEDA---
Agca	QTAFIASRPGLYYGQCSEICGANHSFMPIVVEAVPLEHFENWSTLMLQDA---
Hydy	QTAFIASRPGVFYGQCSEICGANHSFMPIVVEAVPMTNFEGWSSRMLEDA---
Gsac	QTAFIASRPGIFYGQCSEICGANHSFMPIVVEAVPMTYFEDWSSRMLEDA---
Pevo	QTAFITSHPGVFYGQCSEICGANHSFMPAVIESISLSTFEEWCLTMLLDL---
Hiku	QTAFITSRPGVFYGQCSEICGANHSFMPIVVEAVPLQQFENWSSLMLEDA---
Inpa	QTAFIVTRPGVYYGQCSEICGANHSFMPIVVEAVPLKHFENWTSLALKDA---
Auch	QTAFLTTRPGLFYGQCSEICGANHSFMPIVVEAVPLKFFEDWSMLMLEDS---
Fico	QTAFIASRPGVFYGQCSEICGANHSFMPIVVEAVPLEHFENWSSLMLEDA---
Macs	QTAFITSRPGVFYGQCSEICGANHSFMPIVVEAVPLEHFENWSSLMLEDA---
Moal	QTTFVVSHPGVFYGQCSEICGANHSFMPISVEAIPLIHFEDWSTSMLAKI---
Syma	QTTFVVSRPGVFYGQCSEICGANHSFMPITVEAIPLKHFENWSSMLMDA----
Mafr	QTAFITSRPGIFYGQCSEICGANHSFMPIVVEAVPLEHFENWSSLMLEDA---
Dcpe	QTAFITSRPGVFYGQCSEICGANHSFMPIVVEAVPLEHFENWSTLLLEDA---
Dcti	QTAFITSRPGIFYGQCSEICGANHSFMPIVVEAVPLEHFENWSTLLLEDA---
Hehi	QTAFIASRPGVFYGQCSEICGANHSFMPIVVEAVPLEHFENWSSRMLEDA---
Stam	QTAFIASRPGVFYGQCSEICGANHSFMPIVVEAVPLEHFENWSSRMLEDA---
Hogi	QTAFIASRPGVYYGQCSEICGANHSFMPIVVEAVPLEHFEGWSSRMLEDA---
Erzo	QTAFIATRPGIFYGQCSEICGANHSFMPIVVEAVPLEHFENWSSRMLEDA---
Hxot	QTAFIASRPGIFYGQCSEICGANHSFMPIVVEAVPLEHFENWSSRMLEDA---
Core	QTAFIASRPGVFYGQCSEICGANHSFMPIVVEAVPLEHFENWSSRMLEDA---
Apve	QTAFIASRPGIFYGQCSEICGANHSFMPIVVEAVPLEHFENWSSRMLEDA---
Latj	QTTFIVNRPGVFYGQCSEICGANHSFMPIVIEAVPLAHFENWTSLMMEDA---
Laja	QTAFIASRSGIFYGQCSEICGANHSFMPIVVEAVPLEHFENWSSFMLEDA---

[4/4 of aligned sequences]

```
Syja    QTTFTTARPGVFYGQCSEICGANHSFMPIVIEAVPLEHFEHWSTLMLEDS---
Epme    QTAFITSRPGVFYGQCSEICGANHSFMPIVVEVVPLEHFENWSSFMLQDA---
Grse    QTAFVASRPGIFYGQCSEICGANHSFMPIVVEAVPLEHFENWSSLMLEDA---
Clja    QTAFIASRPGVYYGQCSEICGANHSFMPIVVEAVPLEHFENWSSLMLQDN---
Ogcy    QVAFVASRVGIFYGQCSEICGANHSFMPVVVEAVPLEHFEYWTSAMLEDI---
Plna    QVAFIASRPGVFYGQCSEICGANHSFMPIVVEAVPLEHFENWTLSTLQDA---
Lema    QTAFIASRPGVFYGQCSEICGANHSFMPIVVEAVPLEHFENWSSLMLEDA---
Etzo    QTAFIASRPGVFYGQCSEICGANHSFMPIVVEAVPLEHFENWSSLMLEDA---
Apse    QTAFLTSRPGVYYGQCSEICGANHSFMPIVVEAVPLNFFEQWSSQMLEDA---
Epde    QTAFIASRPGVFYGQCSEICGANHSFMPIVVEAVPLEHFENWSSLMLEDA---
Slja    QTAFITSRPGVFYGQCSEICGANHSFMPIVVEAVPLEHFENWSSLMLEDA---
Bsja    QTAFIASRPGVFYGQCSEICGANHSFMPIVVEAVPLQHFESWSSMLLEDA---
Ecna    QATFIVNRPGVFYGQCSEICGANHSFMPIVVEAVPLEHFENWTSLLIEDA---
Cohi    QTTFIVARPGVFYGQCSEICGANHSFMPIVVEAVPLQYFQDWTSFLLEEA---
Caar    QTTFIVNRPGVYYGQCSEICGANHSFMPIVVEAVPLEHFENWTSSMIEDA---
Came    QTTFIVNRPGVYYGQCSEICGANHSFMPIVVEAVPLEHFENWTSSMIEDA---
Mema    QTTFIINRPGVFYGQCSEICGANHSFMPIVVETVLLEHFENWTWLMIQDA---
Lenu    QTAFATSRPGVFYGQCSEICGANHSFMPIVVEAVPLNHFENWSTLMLEES---
Brja    QTAFITSRPGIFYGQCSEICGANHSFMPIVVEAVPLEHFENWSSLMLEDA---
Plma    QTAFIASRPGVFYGQCSEICGANHSFMPIVVEAVPLEHLENWSSLMLEDA---
Emst    QTAFIASRPGVFYGQCSEICGANHSFMPIVVEAVPLEHFENWSSLMLEDA---
Ptti    QTAFIASRPGVFYGQCSEICGANHSFMPIVVEAVPLEHFENWSSLMLEDA---
Losu    QSAFIPSHPGVFYGQCSEICGANHSFMPIVVEAVPLEHFENWSSLMLEDA---
Geoy    QTAFIASRAGVFYGQCSEICGANHSFMPIVVEAVPLEHFENWSSLLLEDA---
Dipi    QTAFITSRPGVYYGQCSEICGANHSFMPIVVEAVPLEHFENWSSLMLEDA---
Pama    QTAFITSRPGVFYGQCSEICGANHSFMPIVVEAVPLEFFEKWSSLMLEDA---
Leob    QTAFITSRPGVFYGQCSEICGANHSFMPIVVEAVPLEHFENWSTLMLEDA---
Neba    QTAFISSRPGLFYGQCSEICGANHSFMPIVVESVPLGHFEDWSTLLLQES---
Pdpl    QTTFTSSRPGVFYGQCSEICGANHSFMPIVVEAVPLEHFQNWAQMKAQED---
Nimi    QTAFIAARPGVFYGQCSEICGANHSFMPIVIEAVPLNHFESWTALMLEEA---
Uptr    QTAFIASRPGVFYGQCSEICGANHSFMPIVVEAVPLEHFENWSSVMLEDA---
Pesc    QASLAIARPGVYYGQCSEICGANHSFMPIVVEAVPLKHFESWSYNMLQDT---
Baar    QATFTVARPGIFYGQCSEICGANHSFMPIVVEAVPLNHFENWSSFMLQDA---
Moar    QTAFIASRPGVFYGQCSEICGANHSFMPIVVEAVPLEHFENWSSLMLEDA---
Toja    QTTFIVNRPGVFYGQCSEICGANHSFMPIVIEAVPLEHFENWTSLMIEDA---
Chau    QTAFIASRPGVFYGQCSEICGANHSFMPIVVEAVPLAHFEDWSTKALQDV---
Chse    QTAFISSRPGVFYGQCSEICGANHSFMPIVVEAVLLECFEDWLSFVLQDS---
Enar    QTAFMVSRPGVFYGQCSEICGANHSFMPIVVEAVPLEHFENWSSLMLEDA---
Hpty    QTAFIASRPGVFYGQCSEICGANHSFMPIVVEAVPLEHFENWSSFMLEDA---
Nana    QTALMLARPGVFYGQCSEICGANHSFMPIVIEAVPMEHFVNWTYLTFEAA---
Mcst    QTAFITSRPGIFYGQCSEICGANHSFMPIVVEAVPLQHFESWSSLMLEDA---
Rhox    QTAFITSRPGVFYGQCSEICGANHSFMPIVVEAVPLEHFENWSSLMLEDA---
Opfa    QTAFISSRPGVYYGQCSEICGANHSFMPIVVEAVPLGYFESWSSLMLEDA---
Paar    QTAFIASRPGVFYGQCSEICGANHSFMPIVVEAVPLEHFENWSSLMLQDA---
Gozo    QTAFIASRPGVFYGQCSEICGANHSFMPIVVEAIPLEHFESWSSLMLEDA---
Ackr    QTAFITTRPGIFYGQCSEICGANHSFMPIVVESVPLKQFEHWSYLMLENA---
Elev    QTAFITSRPGVFYGQCSEICGANHSFMPIVVEAVPLEHFENWSATMLEDI---
Trdu    QTAFIASRPGIFYGQCSEICGANHSFMPIVVEAVPLEHFENWSSLMLEDA---
Amoc    QAAFIASRPGVFYGQCSEICGANHSFMPIVVEAVPLEHFEKWSSLMLEDA---
Hame    QTAFIASRPGLYYGQCSEICGANHSFMPIVVEAVPLKHFEEWSSLLLQDA---
Chso    QTAFIASRPGVFYGQCSEICGANHSFMPIVVEAVPLEHFENWSSLMLQDS---
Lyto    QTAFIASRPGVFYGQCSEICGANHSFMPIVVEAVPLEHFENWSSRMLEDA---
```

[4/4 of aligned sequences]

```
Encr       QTAFIASRPGVFYGQCSEICGANHSFMPIVVEAVPLEHFENWSSRMLEDA---
Bvar       QTAFITSRPGVFYGQCSEICGANHSFMPIVVEAVPLEHFEKWSSLMLENA---
Noco       QTAFIASRPGVFYGQCSEICGANHSFMPVVVEAVPLQDFEAWSTLMIQDA---
Chsp       QTTFNALRPGLYFGQCSEICGANHSFMPITVEVVSVETFNKWTQKSLN-----
Arja       QTAFIASRPGIFYGQCSEICGANHSFMPIVVEAVPLEHFENWSSRMLEDA---
Pase       QTTFTGLRPGVYYGQCSEICGANHSFMPIVMELYPLDAFHEWAVLKVENA---
Trel       QTAFITSRPGVFYGQCSEICGANHSFMPIVVEAIPLEHFENWSLLMLQDA---
Lifa       QTTFITSRPGLFYGQCSEICGANHSFMPVVVEAVPLALFESWTLNMIDNA---
Acur       QTAFISSRPGVFYGQCSEICGANHSFMPIVVESVPLEHFEAWTTFLMEDA---
Ampe       QTAFIASRPGVFYGQCSEICGANHSFMPVVVEAVPLEHFENWSLLMLQD*---
Urja       QTTFMTSRPGVFYGQCSEICGANHSFMPIVVEAAPLGHFENWSSKVLEDA---
Enet       QVAFITSRPGVFYGQCSEICGANHSFMPIVVEAVPLEHFEDWSSFMLQDA---
Ptbr       QTAFITSRPGVFYGQCSEICGANHSFMPIVVESIPLNHFENWSSLMLQDA---
Safa       QVAFIASRPGVYYGQCSEICGANHSFMPIVVEAVPLKHFENWSSLMLQDASL*
Icae       QTAFIASRPGVFYGQCSEICGANHSFMPIVVEAVPLEHFENWSSLMLEDA---
Asmi       QIAFITSRPGVFYGQCSEICGANHSFMPIVVEAIQLKHFEDWTSSMLEA----
Foal       QTALMASHPGIFYGQCSEICGANHSFMPIVLEAVPLVHFEHWALSMLDS*---
Drze       QTAFMTSHPGVFYGQCSEICGANHSFMPIVVESVPLKHFENWTSIMIKDV---
Rhas       QTAFIASRPGVFYGQCSEICGANHSFMPIVVEAVPLKHFEDWSSLMLEDA---
Elac       QTAFIASRPGVFYGQCSEICGANHSFMPIVVEAVPLTHFEHWSSLMLEDA---
Kugu       QTAFMIARPGVFYGQCSEICGANHSFMPIVIEAVPLNHFENWSYLMLEDA---
Plor       QTAFIASRPGVFYGQCSEICGANHSFMPIVVEAVPLEHFENWSSLMLEDA---
Sgun       QTAFIASRPGVFYGQCSEICGANHSFMPIVVEAVPLEHFENWSSLMLEDA---
Zaco       QTAFIASRPGVFYGQCSEICGANHSFMPIVVEAVPLEHFENWSSLMLEDA---
Zbfl       QTAFIASRPGVFYGQCSEICGANHSFMPIVVEAVPLEHFENWSSLMLEDA---
Spba       QTTFIASRPGVYYGQCSEICGANHSFMPIVVEAVPLEHFENWATYMLEDA---
Game       QTAFIASRPGVFYGQCSEICGANHSFMPIVVEAVPLEHFENWSSLMLEDA---
Thth       QTAFIASRPGVFYGQCSEICGANHSFMPIVVEAVPLEHFENWSSLMLEDA---
Xigl       QTTFIVNRPGVFYGQCSEICGANHSFMPIVVEAVPLEHFENWTSLMIEDA---
Hyja       QVTFIAARSGVFYGQCSEICGANHSFMPIVVEAVPLAHFESWSSMMLEDT---
Psan       QTAFMTSHSGVLYGQCSEICGANHSFMPIVVEAVPLKHFEYWSSLILEDT---
Cupa       QVAFISSRPGVFYGQCSEICGANHSFMPIVVEAVPLEHFENWSSLMLEDA---
Mpch       QTAFVASRPGIFYGQCSEICGANHSFMPIVVEAVPLKHFENWSYLMIKDA---
Char       QTAFIISRPGVYYGQCSEICGANHSFMPIVIEAVPLEHFENWSSLMLEDA---
Pser       QTAFITSRPGVFYGQCSEICGANHTFMPIVIESVPLDHFELWIMSMIQDA---
Prol       QTTFIISRPGVFFGQCSEICGANHSFMPIVVEAVPLQHFENWSSLMIEEA---
Plbi       QATFIVSRPGVFYGQCSEICGANHSFMPVVVEAVPLDHFENWSSLMIEDA---
Calu       QTSFVIQRPGIFYGQCSEICGANHSFMPIVVESTPLQHFEEWSVLMLDEA---
Papa       QTTFITNRPGVFYGQCSEICGANHSFMPIAVETVPLQNFENWASLMASDES--
Sufr       QTAFITSRPGVFYGQCSEICGANHSFMPIVVEAVPLEHFENWSSTMLEDA---
Stci       QTAFTVSRTGLFFGQCSEICGANHSFMPIVVEAIPLKDFESWAMTLLEDL---
Taru       QTAFILSRPGVFYGQCSEICGANHSFMPIVVEAVPLEHFENWSSLMLEDA---
Rala       QTAFIASRPGVFYGQCSEICGANHSFMPIVVEAVPLEHFENWSSLMLEDA---
           *  :     *: :*:******* *:***  :*         *
```
