# Supplementary material for: Structure and variation of the mitochondrial genome of fishes
Source: BMC Genomics. 2016 Sep 7;17(1):719. doi: 10.1186/s12864-016-3054-y (PMC5015259; doi:10.1186/s12864-016-3054-y)
Supplement: Additional file 6: Figure S1-a. — Aligned amino acid sequences of the ATP8 gene in mt genomes of 250 fishes. Figure S1-b. Aligned amino acid sequences of the ATP6 gene in mt genomes of 250 fishes. Figure S1-c. Aligned amino acid sequences of the COI gene in mt genomes of 250 fishes. Figure S1-d. Aligned amino acid sequences of the COII gene in mt genomes of 250 fishes. Figure S1-e. Aligned amino acid sequences of the COIII gene in mt genomes of 250 fishes. Figure S1-f. Aligned amino acid sequences of the Cyt b gene in mt genomes of 250 fishes. Figure S1-g. Aligned amino acid sequences of the ND1 gene in mt genomes of 249 fishes. Figure S1-h. Aligned amino acid sequences of the ND2 gene in mt genomes of 250 fishes. Figure S1-i. Aligned amino acid sequences of the ND3 gene in mt genomes of 250 fishes. Figure S1-j. Aligned amino acid sequences of the ND4L gene in mt genomes of 250 fishes. Figure S1-k. Aligned amino acid sequences of the ND4 gene in mt genomes of 250 fishes. Figure S1-l. Aligned amino acid sequences of the ND5 gene in mt genomes of 250 fishes. Figure S1-m. Aligned amino acid sequences of the ND6 gene in mt genomes of 249 fishes. (ZIP 3250 kb) [file 12864_2016_3054_MOESM6_ESM.zip › Additional file 6 prot align/AF6e-COIII.pdf]

Additional file 6: Figure S1–e. Aligned amino acid sequences of the COIII gene in mt genomes of 250 fishes.

Species name abbreviation followed by aligned amino acid sequences shown by one letter abbreviation. See Additional file 1 for abbreviation of species name. Amino acids shown by magenta letter denote hydrophobic residues. A-G in bold types with yellow background indicate putative transmembrane regions. Asterisk '*' indicates a fully conserved residue. Colon ':' and period '.' indicate 'strong' and 'weak' groups in the level of conservativeness, respectively, in the Gonnet Pam250 matrix, in which the strong and weak groups are defined as strong score >0.5 and weak score =<0.5, respectively (Thompson et al., 1997).

COIII

[1/5 of aligned sequences]

```
                             A                    B
Scca  -MAHQAHPYHMVDPSPWPLTGATAALLMTSGLAIWFHFHSLLLLYLGLTLLLLTMIQWWR
Muma  -MAHQAHAYHMVDPSPWPLTGATAALLMTSGLAIWFHFHSLLLLYLGFTLLLLTMIQWWR
Erca  -MAHQAHAYHMVDPSPWPLTGAVAALLLTSGLAVWFHFKSMTLLVMGLLLMILTMIQWWR
Pose  -MAHQAHAYHMVDPSPWPLTGAVAALLLTSGLAVWFHFKSMTLLAMGLLLMILTMIQWWR
Actr  -MAHQAHAYHMVDPSPWPLTGAVAALLMTSGLAVWFHFNSTVLMTMGLTLLLLTMYQWWR
Scal  -MARQAHAYHMVDPSPWPLTGAVAALLMTSGLAVWFHFNSTVLMTMGLILLLLTMYQWWR
Posp  -MARQAHAYHMVDPSPWPLTGAVAALLMTSGLAVWFHFNSTVLMTMGLALLLLTMYQWWR
Atsp  -MTHQTHAYHMVDPSPWPLTGATAALLMTSGLAIWFHFGSTSLMALGSVLLPLTMYQWWR
Leoc  -MAHQTHAYHMVDPSPWPLTGAIAALLMTSGLAIWFHFGSTSLMTLGSMLLPLTMYQWWR
Amca  -MAHQAHAYHMVDPSPWPLTGAVAALLMTSGLAIWFHFNSTILLIIGQTLLLLTMYQWWR
Osbi  -MAHQAHAYHMVDPSPWPLTGATAALLLTSGLAVWFHYHSTVLMLLGLTLMLLTMYQWWR
Pabu  -MARQAHAYHMVDPSPWPLTGAIAALLMTSGLAIWFHYHSTTLLIIGLALLLLTMYQWWR
Hial  -MARQAHAYHMVDPSPWPLTGAIAALLMTSGLAIWFHFHSTTLMMLGFTLTLLTMYQWWR
Elha  -MAHQAHAYHMVDPSPWPLTGAVAALLMTSGLAIWFHFHSVTLMTLGLVLLLLTMYQWWR
Mlcy  -MAHQAHAYHMVDPSPWPLTGAVAALLMTSGLAIWFHFHSTTLMTLGLILLLLTMYQWWR
Algl  MAHAQAHAFHMVNPSPWPLTGAVSALLVTSGTAIWFHFHSVVPMTLGLILMTLTMYQWWR
Ptgi  -MAHQAHAFHMVDPSPWPLTGAVAALLVTSGTAIWFHFHSTTLMSLGLVLMLLTMYQWWR
Alaf  -MAHQAHAYHMVDPSPWPLTGAIAALLVTSGTAMWFHFQSTTLVSLGMVLMLLTMYQWWR
Nock  -MAHQAHAYHMVDPSPWPLTGAVSALLITSGTAIWFHFQSMTLVSLGMILMLLTMYQWWR
Anja  -MAHQAHAYHMVDPSPWPLTGAVAALLVTSGTAMWFHFQSTTLMTLGMILLLLTMYQWWR
Gyki  -MAHQAHAFHMVDPSPWPLTGAVAALLVTSGTAMWFHFQSLTLITLGMLLLLLTMYQWWR
Syka  -MAHQAHAYHMVDPSPWPLTGAIAALLMTSGTAMWFHHQTTSLMTMGLILLLLTMYQWWR
Opma  -MAHQAHAYHMVDPSPWPLTGAAAALLITSGTAMWFHFQNTILMSLGLILMLLTMYQWWR
Comy  -MAHQAHAFHMVDPSPWPLTGAIAALLVTSGTAMWFHFQNTILMTVGMILMLMTMYQWWR
Sasp  -MPSQMHPYHMVNPSPWPLTGAVAALLMTSGAAMWFHFQSSTLLALGLVLLLLTMLQWWR
Eupe  -MTHQTHPYHMVNPSPWPLSGAIAALLLTSGTAMWFHFQMMTLVILGFMLLLLTMYQWWR
Enja  -MAHQAHAFHMVDPSPWPLTGAVGALLLTSGTAIWFHFHSTTLATLGFVLTILTMYQWWR
Same  -MAHQAHAFHMVDPSPWPLTGAVGALLLTSGTAIWFHFHSTILMTLGLILTLLTMYQWWR
Chch  -MAHQAHAYHMVDPSPWPLTGAVAALLMTSGLAIWFHFHSVTLMTLGTILLLLTMYQWWR
Grgr  -MTHQAHAYHMVDPSPWPLTGAVAALLMTSGLAIWFHFHSTVLMTLGTMLLLLTMYQWWR
Caau  -MAHQAHAYHMVDPSPWPLTGAIAALLMTSGLAIWFHFHSTTLMTLGLILLLLTMYQWWR
Cyca  -MAHQAHAYHMVDPSPWPLTGAIAALLMTSGLAIWFHFHSTTLMTLGLILLLLTMYQWWR
Dare  -MAHQAHAYHMVDPSPWPLTGAVAALLMSSGLAIWFHLHSMTLIVLGMILLILTMIQWWR
Cost  -MAHQAHAYHMVDPSPWPLTGAVGALLMTSGLAIWFHFHSTTLMTLGLILLLLTMYQWWR
Leec  -MAHQAHAYHMVDPSPWPLTGAIGALLMTSGLAIWFHFHSTTLMVLGMILLLLTMLQWWR
Fola  -MAHQAHAYHMVDPSPWPLTGAIGALFLTSGLAIWFHFQSVTLLTLGLILLLLTMYQWWR
Clmc  -MAHQAHAYHMVDPSPWPLTGAVAALLMTSGLAIWFHFHSTTLMTLGLILLLLTMYQWWR
Phin  -MAHQAHAYHMVNPSPWPLTGAIAALLMTSGLAIWFHFHSTTLMTLGLILLLLTMYQWWR
Icpu  -MAHQAHAYHMVDPSPWPLTGAVAALLMTSGLAIWFHFHSTVLMTLGLILLLLTMYQWWR
Psto  -MAHQAHAYHMVDPSPWPLTGAVAALLMTSGLAIWFHFHSTTLMTLGLVLLLLTMYQWWR
Cora  -MTHQAHAYHMVDPSPWPLTGAIAALLMTSGLAIWFHFNTTTLLALGLVLLLLTMYQWWR
Eisp  -MAHQAHAYHMVDPSPWPLTGAVAALLTTSGLAIWFHFHTMTLLSTGLILMLLTMYQWWR
Apal  -MAHQAHAYHMVDPSPWPLTGAVAAFLTTSGLGIWFHYHSTILLSLGLVLMLLTMYQWWR
Eslu  -MAHQAHAYHMVDPSPWPLTGAIAALLLTSGTAIWFHFHSLVLVTAGMILLILTMIQWWR
Dape  -MAHQAHAYHMVDPSPWPLTGAIAALLLTSGTAIWFHFHSLTLLTAGLMLLLLTMIQWWR
                           1
```

To be continued on page 6.

[1/5 of aligned sequences]

```
Glse        -MAHQAHAYHMVDPSPWPLTGAIAALLMTSGLAIWFHFHSVTLMSLGMILLLLTMYQWWR
Naar        -MAHQAHAYHMVDPSPWPLTGAIAALLMTSGLAVWFHFHSTTLMTLGMVLLLLTMYQWWH
Lioc        -MAHQAHAYHMVDPSPWPLSGAIAALLMTSGTAVWFHFHSTTLMTLGLVLLLLTMYQWWR
Opso        -MAHQAHAYHMVDPSPWPLSGATAALLLTSGLAVWFHFHSLTLMYLGLILLLLTMYQWWR
Alte        -MAHQAHAYHMVDPSPWPLTGAVAALLLTSGLAIWFHFHSMTLLSLGLVLTLLTMCQWWR
Plap        -MAHQAHAYHMVDPSPWPLTGAVAALLLTSGLAVWFHFHSMTLLSLGLTLTLLTMLQWWR
Plal        -MAHQAHAFHMVDPSPWPLTGAVAALLMTSGLAIWFHFHSVVLMVAGTTLLLLTMYQWWR
Sami        -MAHQAHAFHMVDPSPWPLTGAVGALLMTSGTAMWFHFHSTTLMTAGTALLLLTMYQWWR
Rere        -MAHQAHAFHMVDPSPWPLTGAVAALLLTSGTAIWFHFHSTTLMGAGTLLLLLTMYQWWR
Gama        -MAHQAHAYHMVDPSPWPLTGAIAALLLTSGTAIWFHFHSTTLMTLGTVLLLLTMYQWWR
Onmy        -MAHQAHAYHMVDPSPWPLTGAIAALLLTSGTAVWFHFHSLTLLTLGNILLLLTMYQWWR
Sasa        -MAHQAHAYHMVDPSPWPLTGAIAALLLTSGTAVWFHFHSLTLLTMGNILLLLTMYQWWR
Cola        -MAHQAHAYHMVDPSPWPLTGAIAALLLTSGTAVWFHFHSLTLLAMGNILMLLTMYQWWR
Dita        -MAHQTHAYHMVDPSPWPLTGAIAALLMTSGLAAWFHFHSYSLLFLGTMLLLLTMYQWWR
Gogr        -MTHQAHAFHMVDPSPWPLTGAIAALLTTSGLAVWFHTHSTTLLALGLALTLLTMYQWWR
Chsl        -MAHQAHAFHMVDPSPWPLTGAVAALLLTSGMAIWFHFHSFVLSAIGLILLLLTMIQWWR
Atja        -MAHQAHAYHMVDPSPWPLTGAIAALLLTSGLAIWFHYNSLITMVLGMTLLLLTMYQWWR
Iido        -MAHQAHAYHMVDPSPWPLTGAVAALLLTSGLAIWFHYNSLITMTLGMTLLLLTMYQWWR
Auja        -MAHQAHAYHMVDPSPWPLTGAVAALLMTSGLAIWFHYHSTTLMVLGTTLLLLTMYQWWR
Chag        -MAHQAHAYHMVDPSPWPLTGAVAALLMTSGLAIWFHYQSTTLMVLGTVLLVLTMYQWWR
Hami        -MAHQAHAFHMVDPSPWPLTGAVAALLMTSGLAIWFHYHSTTLMTLGTTLLLLTMYQWWR
Saun        -MAHQAHAFHMVDPSPWPLTGAVAALLMTSGLAIWFHYHSTTLMTLGTILLLLTMYQWWR
Nema        -MAHQAHAYHMVDPSPWPLTGAVAALLMTSGLAIWFHFHSTTLMTLGTMLLLLTMYQWWR
Disp        -MAHQVHAYHMVDPSPWPLTGAVAALLLTSGLAIWFHFNSTIVMTLGLLLTTLTMLQWWR
Myaf        -MTHQVHAYHMVDPSPWPLIGAVAALLLTSGLAIWFHFNSMILMSLGLFLTTLTMYQWWR
Lagu        -MAHQAHAYHMVDPSPWPLTGATAALLVTSGLAIWFHFHSTTLLSLGLALLLLTMYQWWR
Trtr        -MAHQAHAFHMVDPSPWPLTGAVAALLMTSGLAIWFHFHSTILMTLGLVLMILTSIQWWR
Zucr        -MAHQAHAFHMVDPSPWPLTGAVAALLMTSGLAIWFHFHSTILMTLGLILMLLTSIQWWR
Pxja        -MAHQAHAYHMVDPSPWPLTGAVAALLTTSGLAIWFHFHSTTLMTLGLILMILTMIQWWR
Pxlo        -MAHQAHAYHMVDPSPWPLTGAVAALLTTSGLAIWFHFHSTTLMTLGLILMILTMVQWWR
Pctr        -MTHQAHAFHMVDPSPWPLTGAIAALLMTSGLAIWFHSHSSTLMTLGLILLLLTMLQWWR
Apsa        -MAHQAHAYHMVDPSPWPLTGATAALLMTSGLAIWFHFHSTLLMSIGLILLLLTMLQWWR
Cabe        -MTHQAHAFHMVDPSPWPLTGAIAALLTTSGLATWFHFRSTTLLALGMALLLLTMYQWWR
Bzze        -MAHQAHAYHMVDPSPWPLTGAVAALLMTSGLAVWFHFHSTTLLTLGLVLLLLTMYQWWR
Siim        -MAHQAHAYHMVNPSPWPLTGAIAALSMTCGLAVWFHFNTWGLLALGLILLILTMYQWWR
Ctru        -MAHQAHAYHMVDPSPWPLSGAIAALLMTSGLAIWFHFHSSTLLTLGLILLLLTMYQWWR
Dpbr        -MAHQAHAYHMVDPSPWPLSGAIAALLMTSGLAIWFHFHSTTLMTLGLILLLLTMYQWWR
Caki        -MTHQAHAYHMVDPSPWPLTGAVAALLMTSGLAEWFHFYSTTLMTLGFPLLLLTMYQWWR
Phja        -MAHQAHAYHMVDPSPWPLTGATAALLLTSGLAEWFHFNSMTSMTLGFIVLLLTMFQWWR
Brsp        -MLHHAHPYHLVDASPWPLTGAIAALLMTSGLAMWFHFNVTNLMTLGFILTLLTMYQWWR
Gamo        -MTHQAHAYHMVDPSPWPLTGAVAALLMTSGLAVWFHFHSTTLMALGTVLLLLTMYQWWR
Lolo        -MTHQAHAYHMVDPSPWPLTGAVAALLMTSGLAVWFHFHSTVLMTLGTVLVLLTMYQWWR
Batr        -MTHQTHPYHMVAPSPWPLTGAGSAMTLTIGLVLWFHYKIFSLLALGLLLLLLTSYQWWR
Prmy        -MTHQAHAFHMVDPSPWPLTGASSALFLATGLIMWFHYSSINLLILGLTLLALTMLQWWR
Lose        -MSLRAHPFHMVDPSPWPLTGAVAALLLTSGLAVWFHSDSLTLLIMGIVLLFLTMLQWWR
Loam        -MTHQAHAYHMVDPSPWPLTGAVAALLMTSGLAIWFHFNSMVLIPVGTTLLLLTMLQWWR
Chab        -MAHQAHAYHMVDPSPWPLTGAIAALLMTSGLAIWFHFNSMILMALGLALMLLTMYQWWR
Chto        -MAHQAHAYHMVDPSPWPLTGAIAALLMTSGLAIWFHFNSMILMALGLALLLLTMYQWWR
Majo        -MAHQAHAYHMVDPSPWPLTGAVAALLMTSGLAVWFHFNTTALMGAGIILLLLTMYQWWR
Hlst        -MAHQAHAYHMVDPSPWPLTGAVAALLMTSGLAVWFHFNSTALMNLGLILLLLTMYQWWR
Clpe        -MAHTAHPYHMVCPSPWPLTGAVAALMLTSGLAMWFHYNSKTLMVLGLILLLLTMYQWWR
Mlmr        -MSFHQHPYHMVDPSPWPLTGAVAALLLTSGLAIWFHFHSLVLLTLGLILLLLTMYQWWR
```

To be continued on page 7.

2

[1/5 of aligned sequences]

```
Crcr        -MAHQAHAYHMVDPSPWPFTGAIAALLMTSGLAIWFHYHSTTLMTVGLILLLLTMFQWWR
Muce        -MAHQAHAYHMVDPSPWPFTGAIAALLMTSGLAIWFHYHSTTLMTVGLILLLLTMFQWWR
Bege        -MAHQAHAYHMVDPSPWPLSGAVAALLMTSGLAVWMHFHTTVLMTLGLILLLLTMFQWWR
Mela        -MAHQAHAYHMVDPSPWPLTGAVAALLMTSGLAIWMHFHSTTLMTLGLILLLLTMYQWWR
Hats        -MAHQAHAYHMVDPSPWPLTGAAAAFLTTSGLAIWMHFHSTILMTLGLILLLLTMFQWWR
Orla        -MAHQAHAYHMVDPSPWPLTGAVAALLLTSGTAIWMHFNSLVLMTLGLVLLLLTMYQWWR
Cosa        -MAHQAHAYHMVDPSPWPLTGAVAALLMTSGLAIWMHFHSTILMSLGLVLLLLTMYQWWR
Exsp        -MAHQAHAYHMVDPSPWPLTGAVAALLMTSGLAIWMHFHTMTLMTLGLILLLLTMYQWWR
Depa        -MAHQAHAYHMVNPSPWPLTGAVAALLMTSGLAIWMHFHSVTLMLLGLILLLLTMYQWWR
Rima        -MAHQAHAYHMVDPSPWPLTGAIAALLLTSGLAIWMHTHSAILLTLGLILLLLTMYQWWR
Fuol        -MAHQAHAYHMVDPSPWPLTGAVAALLMTSGLAIWMHFHSTTLMTLGLILLLLTMYQWWR
Gmaf        -MAHQAHAYHMVDPSPWPLTGAVAALLMTSGLAIWMHFHSTTLMTLGLILLLLTMYQWWR
Xeei        -MAHQAHAYHMVDPSPWPLTGAVAALLMTSGLAIWMHFHSTILMSLGLILLLLTMYQWWR
Pros        -MAHQAHAYHMVDPSPWPLTGAIAALLLTSGLAIWFHFHSTILMTLGLVLTLLTMYQWWR
Scmi        -MAHQAHAYHMVDPSPWPLTGAIAALMLTSGLAIWFHFHSTILMTFGLVLTLLTMYQWWR
Rolo        -MAHQAHAYHMVDPSPWPLTGAVAALLMTSGLAIWFHFHSTTLMTLGLMLLLLTMYQWWR
Cere        -MAHQAHAYHMVDPSPWPLTGAVAALLITSGLAVWFHFQSTILMTLGLALLFLTMYQWWR
Daga        -MAHQAHAYHMVDPSPWPLTGAVAALLLTSGLAMWFHFNSMILMSLGLLLLSLTMYQWWR
Anco        -MAHQAHAYHMVDPSPWPLTGAVSALLMTSGLAIWFHYHSTTLMSLGLVLLLLTMYQWWR
Dmve        -MAHQAHAYHMVDPSPWPLTGAVAALMLTSGLAIWFHFNKMTLMALGLVLLLATMYQWWR
Dmar        -MAHQAHAYHMVDPSPWPLTGAVAALMLTSGLAIWFHFNKMSLMALGLVLLLATMYQWWR
Anka        -MAHQAHAYHMVDPSPWPLTGAVAALLMTSGLAIWFHYHSTTLMSMGLILLLLTMYQWWR
Moja        -MAHQAHAYHMVDPSPWPLTGAVAALLMTSGLAVWFHYHTTTLMTMGLILLLLTMFQWWR
Hoja        -MAHQAHAYHMVDPSPWPLTGAVAALLMTSGLAVWFHYHSTTLMTLGLILLLLTMYQWWR
Bede        -MAHQAHAYHMVDPSPWPLTGAVAALLMTSGLAIWFHFHSTTLMTLGLMLLLLTMYQWWR
Besp        -MAHQAHAYHMVDPSPWPLTGAIAALLMTSGLAIWFHFHSTTLMTLGLVLLLLTMYQWWR
Mysp        -MAHQAHAYHMVDPSPWPLTGAVAALLMTSGLAIWFHYHSTTLMVLGTILLLLTMYQWWR
Osja        -MAHQAHAYHMVDPSPWPLTGAVAALLMTSGLAIWFHFHSTTLMILGTMLLLLTMYQWWR
Sgro        -MAHQAHAYHMVDPSPWPLTGAVAALLMTSGLAIWFHFHSTTLMVLGTMLLLLTMYQWWR
Pzpa        -MAHQAHAYHMVDPSPWPLTGAVAALLMTSGLAIWFHFHSTTLMTLGTILLLLTMFQWWR
Zeja        -MTHQAHAYHMVDPSPWPLTGAIAALLMTSGLAVWFHFHSTTLMTLGLILLLLTMYQWWR
Znne        -MAHQAHAYHMVDPSPWPLTGAVAALLMTSGLATWFHFNSTVLMTLGLVLLLLTMYQWWR
Zefa        -MAHQAHAYHMVDPSPWPLTGAVAALLMTSGLAIWFHFNSTTLMTLGLILLLLTMYQWWR
Acni        -MAHQAHAYHMVDPSPWPLTGAVAALLMTSGLAIWFHFHSTTLMTLGLILLLLTMYQWWR
Ncrh        -MAHQAHAYHMVDPSPWPLTGAVAALLMTSGLAIWFHFHSTTLMTLGLILLLLTMYQWWR
Agca        -MAHQAHAYHMVDPSPWPLTGAVAALLMTSGLAIWFHYNSTTLMALGTILLLLTMYQWWR
Hydy        -MTHQAHPFHMVDPSPWPLTGAIAALLMTSGLATWFHFQSTTLMGLGTTLLLLTMYQWWR
Gsac        -MTHQAHPYHMVDPSPWPLTGAIAALLMTSGLATWFHFQSTTLMSLGMALLLLTMYQWWR
Pevo        -MAHQAHAYHMVDPSPWPLTGAIAALSMTSGLAIWFHFHTTSLMVLGTALLLLTMFQWWR
Hiku        -MAHQTHAYHMVDPSPWPLTGAIAALLMTSGLAIWFHFNSTVLMTIGLILLLLTMIQWWR
Inpa        -MAHQAHAYHMVDPSPWPLTGATAAFLLTSGLAMWFHYHTLILLTAGLILLLLTMCQWWR
Auch        -MAHQTHPYHMVDPSPWPLTGAAGAFLMTSGLAAWFHSQSAILLTTGTAILLLTMYQWWR
Fico        -MAHQAHAYHMVDPSPWPLTGAVAALLMTSGLAIWFHYNSTVLMTLGTALLLLTMYQWWR
Macs        -MAHQAHAYHMVDPSPWPLTGAVAALLMTSGLAIWFHFHSTTLMTLGTMLLLLTMFQWWR
Moal        -MAHQAHAYHMVDPSPWPLTGAISALLMTSGLATWFHYKTTTLLFLGTTLLLLTMFQWWR
Syma        -MAHQAHAYHMVDPSPWPLTGATAALLMTSGLAMWFHHDCLILLMTGLLLLLLTMIQWWR
Mafr        -MAHQAHAYHMVDPSPWPLTGAVAALLMTSGLAVWFHFHSTTLILLGTILLLLTMFQWWR
Dcpe        -MAHQAHAFHMVDPSPWPLTGAVAALLMTSGLAIWFHFHSTTLMTLGLALLLLTMLQWWR
Dcti        -MAHQAHAYHMVDPSPWPLTGAIAALLMTSGLAIWFHFHSTTLMTLGLILLLLTMLQWWR
Hehi        -MAHQAHPYHMVDPSPWPLTGAIAALLMTSGLATWFHFRSTTLMTLGTALLLLTMYQWWR
Stam        -MAHQAHPYHMVDPSPWPLTGAIAALLMTSGLATWFHFRSTTLMALGTVLLLLTMYQWWR
Hogi        -MTHQAHPYHMVDPSPWPLTGAIAALLMTSGLATWFHFQSTTLMTLGTILLLLTMYQWWR
```

To be continued on page 8.

[1/5 of aligned sequences]

```
Erzo        -MAHQAHPYHMVDPSPWPLTGAIAALLMTSGLATWFHFQSTILMTLGTALLLLTMYQWWR
Hxot        -MAHQAHAYHMVDPSPWPLTGAIAALLMTSGLATWFHFQSTTLMTLGTALTLLTMFQWWR
Core        -MAHQAHAYHMVDPSPWPLTGAIAALLMTSGLATWFHFQSTTLMTLGTALLLLTMFQWWR
Apve        -MAHQAHPYHMVNPSPWPLTGAIAALLMTSGLATWFHFQSTTLMTLGLVLLLLTMFQWWR
Latj        -MAHHSHPYHMVDPSPWPLTGAIAALLMTSGLAIWFHFHSTSLMLLGTILLTLTVYQWWR
Laja        -MAHQAHAYHMVDPSPWPLTGAIAALLMTSGLAIWFHFHSTTLMSVGLALLLLTMYQWWR
Syja        -MAHQAHAYHMVDPSPWPLTGAIAALLMTSGLATWFHFHALLLMHLGLLLLLLTMYQWWR
Epme        -MAHQAHAYHMVDPSPWPLTGAVAALLMTSGLAIWFHFHSTTLIVLGTILLLLTMYQWWR
Grse        -MAHQAHAYHMVDPSPWPLTGAVAALLMTSGLAIWFHFHSTTLMTLGTVLLLLTMYQWWR
Clja        -MAHQAHAYHMVDPSPWPLTGAVAALLMTSGLAIWFHFNNNTLMILGTVLLLLTMYQWWR
Ogcy        -MAHQAHAYHMVDPSPWPFTGAAAALLTTSGLAIWFHYHSVILMTLGTALLLLTMYQWWR
Plna        -MAHQTHPYHMVDPSPWPLTGAVAALLMTSGLAIWFHFNSISLMTLGTILLLLTMYQWWR
Lema        -MAHQAHAYHMVDPSPWPLTGAIAALLMTSGLAIWFHFHSTTLLTLGLILLLLTMYQWWR
Etzo        -MAHQAHAYHMVDPSPWPLTGAVAALLMTSGLAIWFHFHSTTLMGLGTALLLLTMYQWWR
Apse        -MAHQAHAYHMVDPSPWPLTGAVAALLMTSGLAIWFHYNTMTLMYIGTILLLLTMYQWWR
Epde        -MAHQAHAYHMVDPSPWPLTGAIAALLMTSGLAIWFHFHSTTLMTLGLVLLLLTMYQWWR
Slja        -MAHQAHAYHMVDPSPWPLTGAVAALLVTSGTAIWFHFNSLILMSLGMVLLLLTMYQWWR
Bsja        -MAHQAHAYHMVDPSPWPLTGAVAALLMTSGLAIWFHFHSMNLIMLGTVLLLLTMYQWWR
Ecna        -MAHQAHPYHMVDPSPWPLTGAVAALLMTSGLAIWFHFHSTILMTLGTVLLVLTICQWWR
Cohi        -MTHQMHPYHMVDPSPWPLTGAAAAMLMTSGLAIWFHFHNIILMTLGLVILILTVYQWWR
Caar        -MAHQAHPYHMVDPSPWPLTGAIGALLMTSGLAIWFHFHSTLLMTLGLILVTLTTAQWWR
Came        -MAHQAHPYHMVDPSPWPLTGAVGALLMTSGLAIWFHFHSSLLMTLGLILVTLTTAQWWR
Mema        -MAHQAHAFHMVDPSPWPLTGAVAALLMTSGLAIWFHFHSLPLMTMGLILLIITAGQWWR
Lenu        -MAHQMHAYHMVDPSPWPLTGAIAALLMTSGLAIWFHYHSTTLMTIGTILLLLTMYQWWR
Brja        -MAHQAHAYHMVDPSPWPLTGATAALLMTSGLAIWFHFNSMTLMNIGLVLLLLTMYQWWR
Plma        -MAHQAHAYHMVDPSPWPLTGAIAALLMTSGLAIWFHFHSTTLMTIGLTLLLLTMYQWWR
Emst        -MAHQAHAYHMVDPSPWPLTGAVAALLMTSGLAIWFHFHSTTLMTLGLALLLLTMYQWWR
Ptti        -MAHQAHAYHMVDPSPWPLTGAVAALLMTSGLAIWFHFHSTTLMSLGLVLLLLTMYQWWR
Losu        -MAHQTHAFHMVDPSPWPLTGAIAALLMTSGLAIWFHFHTTTLMLIGSALLLLTMYQWWR
Geoy        -MAHQAHAYHMVDPSPWPLTGAVAALLMTSGLAIWFHFHSTILMTLGTMLLLLTMYQWWR
Dipi        -MAHQAHAYHMVDPSPWPLTGAISALLVTSGTAIWFHFNTTTLMSLGMVLLLLTMYQWWR
Pama        -MAHQAHAYHMVDPSPWPLTGAVAALLITSGTAMWFHFNSMILMTLGTVLLLLTMYQWWR
Leob        -MAHQAHAYHMVDPSPWPLTGAVAALLMTSGLAMWFHFNSTTLMTLGTVLLLLTMYQWWR
Neba        -MAHQAHAYHMVDPSPWPLTGAVGALLMTSGTAMWFHFNSVTLMVLGTILVLLTMYQWWR
Pdpl        -MAHHLHPYHMVDPSPWPLTGAVAALLMTSGLVVWFHHDSILLLSLGLVLLLLTMYQWWR
Nimi        -MAHQAHAYHMVDPSPWPLTGAVAALLLTSGLAIWFHFHSTILLTLGLVLLLLTMYQWWR
Uptr        -MAHQAHAYHMVDPSPWPLTGAVAALLMTSGLAIWFHFHSTTLMVMGTALLLLTMYQWWR
Pesc        -MTHQAHAFHMVDPSPWPLTGAVGALLMTSGLAMWFHYNTMILMTLGVIILLLTMYQWWR
Baar        -MAHQAHAYHMVDPSPWPLTGAVAALLLTAGLVLWFHFDSMTLLCLGLVLLLLTMYQWWR
Moar        -MAHQAHAYHMVDPSPWPLTGAVAALLMTSGLAIWFHFNSTTLMSLGLALLLLTMYQWWR
Toja        -MAHHSHPYHMVDPSPWPLTGAIGALLMTSGLAIWFHYHSTPLMVVGLAVLILTMCQWWR
Chau        -MARQAHAYHMVDPSPWPLTGAVGALLMTSGLAIWFHYHSMTLIYLGTIILLLTMYQWWR
Chse        -MAHQAHAYHMVDPSPWPLTGAVAALLMTSGLAVWFHYHTVTLMLLGMILLLLTMYQWWR
Enar        -MAHQAHAYHMVDPSPWPLTGAVAALLMTSGLAIWFHFHSTTLMVLGTMLLLLTMYQWWR
Hpty        -MAHQAHAYHMVDPSPWPLTGAVAALLMTSGLAIWFHFHSTTLMSLGMALLLLTMYQWWR
Nana        -MAHQAHAYHMVDPSPWPLTGAMAALMMTSGLAIWFHFHTMTLMALGMLLFLLTSYQWWR
Mcst        -MAHQAHAYHMVDPSPWPLTGAVAALLMTSGLAIWFHFHSTTLMMLGTALLLLTMYQWWR
Rhox        -MAHQAHAYHMVDPSPWPLTGAVAALLMTSGLAIWFHFHSTTLMTLGLALLLLTMYQWWR
Opfa        -MAHQAHAYHMVDPSPWPLTGAVAALLMTSGLAIWFHFHSTTLMVLGTALLLLTMYQWWR
Paar        -MTAQAHAYHMVDPSPWPLTGAVAALLMTSGLAIWFHFHTTTLMALGTMLLLLTMFQWWR
Gozo        -MAHQAHAYHMVDPSPWPLTGAVAALLMTSGLAIWFHFHSTTLMTLGLALLLLTMYQWWR
Ackr        -MSHQAHAYHMVDPSPWPLTGAVAALLMTSGLAIWFHFNSMTLMILGMVLLLLTMYQWWR
```

To be continued on page 9.

[1/5 of aligned sequences]

```
Elev       -MAHQAHAYHMVDPSPWPLTGAIAALLMTSGLAIWFHYHSTTLMTLGLILLLLTMYQWWR
Trdu       -MAHQVHAYHMVDPSPWPLTGAVGALLLTSGLAIWMHFNNMTLLTLGLILLLLTMYQWWR
Amoc       -MAHQAHAYHMVDPSPWPLTGAVAALLMTSGLAIWMHFHTMTLMTLGMILLLLTMYQWWR
Hame       -MAHQAHAYHMVDPSPWPLTGAVAALLMTSGLAIWFHYNSTVLMTLGTILLLLTMYQWWR
Chso       -MAHQAHAYHMVDPSPWPLTGAVAALLMTSGLAIWFHFHSTTLMIMGTVLLLLTMYQWWR
Lyto       -MAHQAHPYHMVDPSPWPLTGAIAALLMTSGLATWFHFQSTTLMTLGTALLLLTMYQWWR
Encr       -MAHQAHPYHMVDPSPWPLTGAIAALLMTSGLATWFHFQSTTLMTLGTVLLLLTMYQWWR
Bvar       -MAHQAHAYHMVDPSPWPLTGAVAAMLITSGLAFWFHFHSVILLVLGLILMLLTMYQWWR
Noco       -MAHQAHAYHMVDPSPWPLTGAIAALLMTSGLAVWFHQHSTTLMTLGTILLLLTMYQWWR
Chsp       -MSHQTHAYHMVDPSPWPLTGAAAALFLTSGLTLWFHFHYTTLLYLSLVLMLLTMYQWWR
Arja       -MAHQAHPYHMVDPSPWPLTGAIAALLMTSGLATWFHFQSTILMTLGTILLLLTMFQWWR
Pase       -MTHQAHAFHMVDPSPWPLTGAVAALLLTSGLVCWFHHNSLSVLGLGLLLLALTMYQWWR
Trel       -MSHQTHAYHMVDPSPWPLTGAMAALLMTSGLAVWFHFNSTTLMTMGSALLILTMYQWWR
Lifa       -MTHQAHAYHMVDPSPWPLTGAMGALLMTSGTAVWFHFNSTLLMTLGTMVLLLTMYQWWR
Acur       -MNKQAHAYHMVDPSPWPLTGAIGALMMTSGTAMWFHFNSMKLMALGTIVLLLTMYQWWR
Ampe       -MAHQAHAYHMVDPSPWPLTGAVAALLMTSGLAIWFHFNSTVLMTLGTALLLLTMYQWWR
Urja       -MIHQTHAYHMVDPSPWPLTGAIAAMLMTSGLAMWFQSNSATLLTLGLLLLILTMYQWWR
Enet       -MAHQAHAYHMVDPSPWPLTGAVAALLMTSGLAIWMHYNSTILMLLGTLLLLLTMYQWWR
Ptbr       -MTRQAHAYHMVDPSPWPLTGAIAALLLTSGTAIWMHFQSTTPMVMGLILLLLTMYQWWR
Safa       -MAHQAHAYHMVDPSPWPLTGAVAALLLTSGTAIWMHFHSTTLMTLGLALLLLTMYQWWR
Icae       -MAHQAHAYHMVDPSPWPLTGAIAALLMTSGLAIWFHFHSTTLMTVGVALLLLTMYQWWR
Asmi       -MTRQAHAYHMVDPSPWPLTGAVAALLLTSGLTTWMHFNKLYLMSMGLILLLVTMYQWWR
Foal       -MTHQTHAYHMVNPSPWPLTGATAALFLTAGLALWFHYSLVISLYLGFILLLLTMYQWWR
Drze       -MAHQAHAFHMVDPSPWPLTGAVAALLLTSGLAIWFHLHSSTLMTLGLMLTLLTMYQWWR
Rhas       -MAHQAHAYHMVDPSPWPLTGAVAALLMTSGLAIWFHFNSTTLMAIGTILLILTMYQWWR
Elac       -MAHQAHAYHMVDPSPWPLTGAVAALLMTSGLAIWFHFHSTTLMTIGLILLLLTMYQWWR
Kugu       -MAHQAHAYHMVDPSPWPITGAVAALLMTSGLAIWFHFNSTTLMTLGMVLVILTMIQWWR
Plor       -MAHQAHAYHMVDPSPWPLTGAVAALLMTSGLAIWFHFHSTTLIVLGTALLLLPMLQWWR
Sgun       -MAHQAHAYHMVDPSPWPLTGAVAALLMTSGLAIWFHFNSTTLMGLGTALLLLTMYQWWR
Zaco       -MAHQAHAYHMVDPSPWPLTGAVAALLMTSGLAIWFHFHSVTLMYLGTALLLLTMYQWWR
Zbfl       -MAHQAHAYHMVDPSPWPLTGAVAALLMTSGLAIWFHFHSTTLMALGTALLLLTMYQWWR
Spba       -MAHQAHPYHMVDPSPWPLTGAIAALLMTSGMAMWFHTRSMIMISLGTILLIMTTCQWWR
Game       -MAHQAHAYHMVDPSPWPLTGAVAALLMTSGLAIWFHFHSTTLMTVGTALLLLTMYQWWR
Thth       -MAHQAHAYHMVDPSPWPLTGAVAALLMTSGLAIWFHFHSTTLMTVGTALLLLTMYQWWR
Xigl       -MAHQSHPYHMVDPSPWPLTGAVAALLMTSGLAIWFHFHSVPLMALGTALLVLTVCQWWR
Hyja       -MAHQAHAYHMVDPSPWPLTGAIAALLMTSGLAMWFHFHSTTLMTLGTALLLLTMYQWWR
Psan       -MARQMHPYHIVNPSPWPLTGAIAALLLTSGLVVRFYFQSITLVILGLILLLLTMYQWWR
Cupa       -MAHQAHAYHMVDPSPWPLTGAIAALLMTSGLAIWFHFNSTTLMSVGLILLLLTMYQWWR
Mpch       -MAHQAHAYHMVDPSPWPLTGAVAALLMTSGLAIWFHFHSTILMTLGLVLLLLTMLQWWR
Char       -MAHQAHAYHMVDPSPWPLTGAVAALLMTSGLAIWFHFNSSILMSLGLVLLLLTMYQWWR
Pser       -MAHQAHPYHMVDPSPWPLTGAVAALLMTSGTAIWFHFHSTLLVSLGTVLLVLTICQWWR
Prol       -MAHQAHPYHMVDPSPWPLTGAIAALLMTSGLAIWFHFHSTTLMTLGTILLILTIFQWWR
Plbi       -MAHQAHPYHMVDPSPWPLTGAIAALLMTSGLAIWFHFHSTTLMTIGTVLLILTVFQWWR
Calu       -MAPQAHPYHMVDPSPWPLTGAVGALVLTSGLAVWFHFHSTLPLSLGVALLVLTMYQWWR
Papa       -MAHHIHPYHMVDPSPWPLTGAAAALLMTSGLAIWFHFHSYTLLVIGTMLMIFTIVQWWR
Sufr       -MAHQAHAYHMVDPSPWPLTGAVAALLMTSGLAIWFHYNSTTLMVLGTILLLLTMYQWWR
Stci       -MTHQAHAYHMVDPSPWPLTGAAAALLMTSGLAIWFHYNSTTLMTLGTILLLLTMYQWWR
Taru       -MAHQAHPYHMVDPSPWPLTGAVAALLLTSGLAIWFHFNSTILMTLGLVLLLLTMLQWWR
Rala       -MAHQAHAYHMVDPSPWPLTGAVAALLMTSGLAIWFHFNSTTLMALGTTLLLLTMYQWWR
                 * :*:*  ****: ** .*:  : *    :        .  :       ***:
```

To be continued on page 10.

[2/5 of aligned sequences]

C

```
Scca   DIIREGTFQGHHTPPVQKGLRYGMILFIVSEVFFFLGFFWAFYHSSLAPTPELGGCWPPT
Muma   DVIREGTFQGHHTPPVQKGLRYGMILFITSEVFFFLGFFWAFYHSSLAPTPELGGCWPPT
Erca   DIIREGTFQGHHTPPVQKGLRYGMILFITSEVFFFLGFFWAFYHSSLAPTPELGGVWPPT
Pose   DIIREGTFQGHHTPPVQKGLRYGMILFITSEVFFFLGFFWAFYHSSLAPTPELGGIWPPT
Actr   DIIREGTFQGHHTPPVQKGLRYGMILFITSEVFFFLGFFWAFYHASLAPTPELGGCWPPT
Scal   DIIREGTFQGHHTPPVQKGLRYGMILFITSEVFFFLGFFWAFYHASLAPTPELGGCWPPT
Posp   DIIREGTFQGHHTPPVQKGLRYGMILFITSEVFFFLGFFWAFYHSSLAPTPELGGCWPPT
Atsp   DIVREGTFQGHHTPPVQKGLRYGMILFITSEVFFFLGFFWAFYHSSLAPTPELGGCWPPA
Leoc   DIVREGTFQGHHTPPVQKGLRYGMILFITSEVFFFLGFFWAFYHSSLAPTPELGGCWPPT
Amca   DIIREGTFQGHHTAPVQKGLRYGMILFITSEVFFFLGFFWAFYHSSLAPTPELGGCWPPT
Osbi   DVIRESTYMGHHTPPVQKGLRYGMILFITSEVFFFLGFFWAFFHSSLAPVPELGGCWPPT
Pabu   DIVRESTFLGHHTPPVQKGLRYGMILFITSEIFFFLGFFWAFFHSSLAPTPELGSCWPPT
Hial   DIIREGTFQGHHTPPVQKGLRYGMILFITSEVFFFLGFFWAFFHSSLAPTPELGGCWPPT
Elha   DIVREGTFQGHHTPPVQKGLRYGMILFITSEVFFFLGFFWAFYHSSLAPTPELGGCWPPT
Mlcy   DIIREGTFQGHHTPPVQKGLRYGMILFITSEVFFFAGFFWAFYHSSLAPTPELGGCWPPT
Algl   DVVREGTFQGHHTPPVQKGLRYGMILFITSEVFFFAGFFWAFYHSSLAPTSELGGCWPPT
Ptgi   DIVREGTFQGHHTPPVQKGLRYGMILFITSEVFFFAGFFWAFYHASLAPTPELGGCWPPT
Alaf   DIVREGTFLGHHTPPVQKGLRYGMILFITSEVFFFLGFFWAFYHSSLAPTPELGGCWPPT
Nock   DIVREGTFLGHHTPPVQKGLRYGMILFITSEVFFFLGFFWAFYHSSLAPTPELGGCWPPT
Anja   DIVREGTFQGHHTPPVQKGLRYGMILFITSEVFFFLGFFWAFYHSSLAPTPELGGCWPPT
Gyki   DIVREGTFQGHHTPPVQKGLRYGMILFITSEVFFFLGFFWAFYHSSLAPTPELGACWPPT
Syka   DIVREGTFQGHHTPPVQKGLRYGMILFITSEVFFFLGFFWAFYHASLAPTPELGGCWPPT
Opma   DIVREGTFLGHHTPPVQKGLRYGMILFITSEVFFFLGFFWAFYHSSLAPTPELGGCWPPT
Comy   DIIREGTYQGHHTPPVQKGLRYGMILFITSEVFFFLGFFWAFYHSSLAPTPELGGCWPPT
Sasp   DIVREGTFLGHHSPPVQKGLRYGMILFIVSEVFFFLGFFWAFYHSSLAPTPELGGCWPPS
Eupe   DIIREGTFQGHHSPPVQKGLRYGMILFIVSEVFFFLGFFWAFYHSSLAPTPELGGCWPPT
Enja   DIVREGTFQGHHTPPVQKGLRYGMILFITSEVFFFAGFFWAFYHSSLAPTPELGGCWPPT
Same   DIVREGTFQGHHTPPVQKGLRYGMILFITSEVFFFAGFFWAFYHSSLAPTPELGGCWPPA
Chch   DIVREGTFQGHHTPPVQKGLRYGMILFITSEVFFFLGFFWAFYHSSLAPTPELGGCWPPT
Grgr   DVVREGTYQGHHTPPVQKGLRYGMILFITSEIFFFLGFFWAFHHSSLAPTPELGGCWPPS
Caau   DIIREGTFQGHHTPPVQKGLRYGMILFITSEVFFFLGFFWAFYHSSLAPTPELGGCWPPT
Cyca   DIIREGTFQGHHTPPVQKGLRYGMILFITSEVFFFLGFFWAFYHSSLAPTPELGGCWPPT
Dare   DIIREGTFQGHHTPPVQKGLRYGMILFITSEVFFFLGFFWAFYHSSLAPTPELGGCWPPT
Cost   DIIREGTFQGHHTPPVQKGLRYGMILFITSEVFFFLGFFWAFYHSSLAPTPELGGCWPPT
Leec   DIIREGTFQGHHTPPVQKGLRYGMILFITSEVFFFLGFFWAFYHSSLAPTPELGGCWPPT
Fola   DIIREGTFQGHHTPPVQKGLRYGMILFITSEVFFFLGFFWAFYHSSLAPTPELGGCWPPT
Clmc   DIVREGTFQGHHTPPVQKGLRYGMILFITSEVFFFLGFFWAFYHSSLAPTPELGGCWPPT
Phin   DIIRESTFQGHHTPPVQKGLRYGMILFITSEVFFFLGFFWAFYHSSLAPTPELGGCWPPT
Icpu   DIIREGTFQGHHTPPVQKGLRYGMILFITSEVFFFLGFFWAFYHSSLAPTPELGGCWPPT
Psto   DIVREGTFQGHHTPPVQKGLRYGMILFITSEVFFFLGFFWAFYHSSLAPTPELGGCWPPT
Cora   DIVREGTFQGHHTPPVQKGLRYGMILFITSEVFFFLGFFWAFYHSSLAPTPELGGCWPPT
Eisp   DVIREGTFQGHHTLPVQKGLRYGMILFITSEVLFFAGFFWAFYHSSLAPTPELGGCWPPT
Apal   DVIREGTFQGHHTSPVQKGLRFGMILFITSEVLFFAGFFWAFYHSSLAPTPELGTHWPPT
Eslu   DIIREGTFQGHHTPPVQKGLRYGMILFITSEVFFFLGFFWAFYHSSLAPTPELGGCWPPT
Dape   DIIREGTFQGHHTPPVQKGLRYGMILFITSEVFFFLGFFWAFYHSSLAPTPELGGCWPPT
Glse   DIVREGTFQGHHTPPVQKGLRYGMILFITSEVFFFLGFFWAFYHASLAPTPELGGCWPPT
Naar   DIIREGAFEGDHTPPVQKGLRYGMILFITSEVFFFLGLFWAFYHASLAPTPELGGCWPPT
Lioc   DIIREGTFQGHHTPPVQKGLRYGMILFITSEVFFFLGFFWAFYHASLAPTPELGGCWPPT
Opso   DIIREGTFQGHHTPPVQKGLRYGMILFITSEVFFFLGFFWAFYHASLAPTPELGGYWPPA
Alte   DVIREGTFQGHHTPPVQKGLRYGMILFITSEVFFFLGFFWAFYHSSLAPTPELGGCWPPT
Plap   DVIREGTFQGHHTPPVQKSLRYGMILFITSEVFFFLGFFWAFYHSSLAPTPELGGCWPPT
```

6

To be continued on page 11.

[2/5 of aligned sequences]

```
Plal  DIVREGTFQGHHTPPVQKGLRYGMILFITSEVFFFLGFFWAFYHASLAPTPELGGCWPPT
Sami  DIAREGTFQGHHTPPVQKGLRYGMILFITSDVFFFLGFFWAFYHASLAPTPELGGCWPPT
Rere  DIVREGTFQGHHTPPVQKGLRYGMILFITSEVFFFIGFFWAFYHSSLAPTPELGGCWPPA
Gama  DVIREGTFQGHHTPPVQKGLRYGMILFITSEVFFFLGFFWAFYHSSLAPTPELGGCWPPA
Onmy  DIIREGTFQGHHTPPVQKGLRYGMILFITSEVFFFLGFFWAFYHASLAPTPELGGCWPPA
Sasa  DIIREGTFQGHHTPPVQKGLRYGMILFITSEVFFFLGFFWAFYHSSLSPTPELGGCWPPT
Cola  DIIREGTFQGHHTPPVQKGLRYGMVLFITSEVFFFLGFFWAFYHSSLAPTPELGGCWPPT
Dita  DIIREGTFQGHHTPPVQKGLRYGMILFITSEVFFFLGFFWAFYHSSLAPTPELGGCWPPA
Gogr  DVVREGTFQGHHTPPVQKGLRYGMVLFITSEVFFFLGFFWAFYHSSLAPTPELGGYWPPA
Chsl  DIIREGTFQGHHTPPVQKGLRFGMILFITSEVFFFLGFFWAFYHSSLAPTPELGGFWPPA
Atja  DIVREGTFQGHHTPPVQKGLRYGMVLFITSEVFFFLGFFWAFYHSSLAPTPELGGCWPPT
Iido  DIIREGTFQGHHTPPVQKGLRYGMVLFITSEVFFFLGFFWAFYHSSLAPTPELGGCWPPT
Auja  DIVREGTFQGHHTPPVQKGLRYGMVLFITSEVFFFLGFFWAFYHSSLAPTPELGGCWPPT
Chag  DIVREGTFQGHHTPPVQKGLRYGMVLFITSEVFFFVGFFWAFYHSSLAPTPELGGCWPPT
Hami  DIVRESTFQGHHTPPVQKGLRYGMVLFITSEVFFFIGFFWAFYHSSLAPTPELGGCWPPT
Saun  DIVRESTFQGHHTPPVQKGLRYGMVLFITSEVFFFIGFFWAFYHSSLAPTPELGGCWPPT
Nema  DIIREGTFQGHHTPPVQKGLRYGMVLFITSEVFFFAGFFWAFYHSSLAPTPELGGCWPPT
Disp  DVVREGTFQGHHTPPVQKGLRYGMVLFITSEVFFFAGFFWAFYHSSIVPAPDLGGIWPPS
Myaf  DIIREGTFQGHHTPPVQKGLRYGMILFITSEVFFFAGFFWAFYHSSLNPTPELGGCWPPS
Lagu  DIIREGTFQGHHTPPVQKGLRYGMILFITSEVFFFLGFFWAFFHSSLAPTPELGGCWPPT
Trtr  DIVREGTFQGHHTPPVQKGLRYGMILFITSEVFFFLGFFWAFYHASLAPTPELGGCWPPT
Zucr  DIVREGTFQGHHTPPVQKGLRYGMILFITSEVFFFLGFFWAFYHSSLAPTPELGGCWPPT
Pxja  DIIREGTFQGHHTPPVQKGLRYGMVLFITSEVFFFLGFFWAFYHASLAPTPELGGCWPPT
Pxlo  DIIREGTFQGHHTPPVQKGLRYGMVLFITSEVFFFLGFFWAFYHSSLAPTPELGGCWPPT
Pctr  DIVREGTFQGHHTPPVQKGLRFGMILFITSEVFFFLGFFWAFYHSSLAPAPELGGCWPPT
Apsa  DIIRESTFQGHHTPPVQKGLRLGMILFITSEVFFFLGFFWAFYHSSLAPTPELGGCWPPT
Cabe  DIVREGTFQGHHTPPVQKGLRYGMILFITSEVFFFLGFFWAFYHASLAPTPELGGCWPPA
Bzze  DVIREGTFQGHHTPPVQKGLRYGMILFITSEVFFFLGFFWAFYHASLAPTPELGGCWPPT
Siim  DIVREGTFQGHHTPPVQKGLRYGMILFITSEVFFFLGFFWAFYHASLAPTPELGGHWPPS
Ctru  DIIREGTFQGHHTPPVQKGLRYGMILFITSEVFFFLGFFWAFYHASLAPTPELGACWPPA
Dpbr  DIIREGTFQGHHTPPVQKGLRYGMILFITSEVFFFLGFFWAFYHSSLAPTPELGGCWPPA
Caki  DIIREGTFQGHHTPPVQKGLRYGMILFITSEVFFFLGFFWAFYHASLAPTPELGGCWPPT
Phja  DIIRESTFQGHHTPPVQKGLRYGMILFITSEVFFFIGFFWAFYHASLAPTPELGGCWPPT
Brsp  DIIRESTFQGHHTPPVQKGLRYGMILFITSEVFFFLGFFWAFFHSSLAPTPELGACWPPT
Gamo  DIIREGTFQGHHTPPVQKGLRYGMILFITSEVFFFLGFFWAFYHASLAPTPELGGCWPPT
Lolo  DIVREGTFQGHHTPPVQKGLRYGMILFITSEVFFFLGFFWAFYHSSLAPTPELGGCWPPT
Batr  DVVREATFMGHHTPPVQKGLRYGMALFITSEVCSSLAF-LSMLHSSLAPTHQTGGQWPPS
Prmy  DVVREGTYLGHHTPPVQKSLRYGMILFIASEVFFFLGFFWAFFHSSLAPTPEIGGHWPPT
Lose  DVVREATLLGHHTPPVQKGLRYGMILFIVSEVFFFLGFFWAFYHASLAPTPELGGCWPPA
Loam  DIVREGTFQGHHTPPVQKGLRYGMILFITSEVFFFLGFFWAFYHASLAPTPELGGFWPPA
Chab  DIIREGTFQGHHTPPVQKGLRYGMILFITSEVFFFLGFFWAFYHSSLAPTPELGGCWPPT
Chto  DIIREGTFQGHHTPPVQKGLRYGMILFITSEVFFFLGFFWAFYHSSLAPTPELGGCWPPA
Majo  DIIRESTFQGHHTPPVQKGLRYGMMLFITSEVFFFLGFFWAFYHSSLAPTPELGGCWPPT
Hlst  DIIRESTFQGHHTPPVQKGLRYGMLLFITSEVFFFLGFFWAFYHSSLAPTPELGGFWPPT
Clpe  DVVRESTFQGLHTPPVQKGLRYGMILFITSEVLFFAGFFWAFYHSSLAPTPELGGCWPPS
Mlmr  DITREGTFQGHHTPPVQKGLRYGIILFITSEVFFFLGFFWAFYHSSLAPTPELGGCWPPT
Crcr  DIIREGTFQGHHTPPVQKGLRYGMILFITSEVFFFLGFFWAFYHSSLAPTPELGGCWPPT
Muce  DIIREGTFQGHHTPPVQKGLRYGMILFITSEVFFFLGFFWAFYHSSLAPTPELGGCWPPT
Bege  DIIREGTFQGHHTPPVQKGLRYGMILFITSEVFFFLGFFWAFYHSSLAPTPELGGCWPPT
Mela  DIVREGTFQGHHTPPVQKGLRYGMILFITSEVFFFLGFFWAFYHSSLAPTPELGACWPPT
Hats  DIIREGTFLGHHTPPVQKGLRYGMILFITSEVFFFLGFFWAFYHASLAPTPELGACWPPS
Orla  DIIREGTFQGHHTPPVQKGLRYGMILFITSEVFFFLGFFWAFYHSSLAPTPELGGCWPPT
```

7

To be continued on page 12.

[2/5 of aligned sequences]

To be continued on page 13.

Cosa	DIIREGTFQGHHTPPVQKGLRYGMILFITSEVFFFLGFFWAFYHSSLAPTPELGGCWPPT
Exsp	DIVREGTFQGHHTPPVQKGLRYGMILFITSEVFFFVGFFWAFYHSSLAPTPELGGCWPPT
Depa	DIIREGTFQGHHTPPVQKGLRYGMILFITSEVFFFLGFFWAFYHSSLAPTPELGGCWPPT
Rima	DIIRESTFLGHHTPPVQKGLRYGMILFITSEVFFFLGFFWAFYHSSLAPTPELGGCWPPT
Fuol	DIIREGTFQGHHTPPVQKGLRYGMILFITSEVFFFLGFFWAFYHSSLAPTPELGGCWPPT
Gmaf	DIVREGTFQGHHTPPVQKGLRYGMILFITSEVFFFLGFFWAFYHSSLAPTPELGGCWPPA
Xeei	DIIREGTFQGHHTPPVQKGLRYGMILFITSEIFFFLGFFWAFYHSSLAPTPELGGCWPPT
Pros	DIIREGTFQGHHTPPVQKGLRYGMVLFITSEVFFFLGFFWAFYHSSLAPTPELGGCWPPT
Scmi	DIIREGTFQGHHTPPVQKGLRYGMVLFITSEVFFFLGFFWAFYHASLAPSPELGICWPPT
Rolo	DIIREGTFQGHHTPPVQKGLRYGMVLFITSEVFFFLGFFWAFYHSSLAPTPELGGCWPPT
Cere	DIIREGTFQGHHTPPVQKGLRYGMVLFITSEVFFFLGFFWAFYHSSLAPTPELGGCWPPT
Daga	DIVREGTFQGHHTPPVQKGLRYGMVLFITSEVFFFLGFFWAFYHSSLAPTPELGGCWPPT
Anco	DIVREGTFQGHHTPPVQKGLRYGMVLFITSEVFFFLGFFWAFYHSSLAPTPELGGCWPPT
Dmve	DIIREGTFQGHHTPPVQKGLRYGMVLFITSEVFFFLGFFWAFYHSSLAPTPELGACWPPA
Dmar	DIIREGTFQGHHTPPVQKGLRYGMVLFITSEVFFFLGFFWAFYHSSLAPTPELGACWPPA
Anka	DIVREGTFQGHHTPPVQKGLRYGMVLFITSEVFFFLGFFWAFYHSSLAPTPELGGCWPPT
Moja	DIVREGTFQGHHTPPVQKGLRYGMVLFITSEVFFFLGFFWAFYHASLAPTPELGGCWPPT
Hoja	DIVREGTFQGHHTPPVQKGLRYGMVLFITSEVFFFLGFFWAFYHSSLAPTPELGGCWPPA
Bede	DIIREGTFQGHHTPPVQKGLRYGMVLFITSEVFFFLGFFWAFYHSSLAPTPELGGCWPPT
Besp	DIIREGTFQGHHTPPVQKGLRYGMVLFITSEVFFFLGFFWAFYHASLAPTPELGGCWPPT
Mysp	DIVREGTFQGHHTPPVQKGLRYGMVLFITSEVFFFLGFFWAFYHASLAPTPELGGCWPPT
Osja	DIVREGTFQGHHTPPVQKGLRYGMVLFITSEVFFFLGFFWAFYHSSLAPTPELGGCWPPT
Sgro	DIVREGTFQGHHTPPVQKGLRYGMVLFITSEVFFFLGFFWAFYHSSLAPTPELGGCWPPT
Pzpa	DIIREGTFQGHHTPPVQKGLRYGMILFITSEVFFFLGFFWAFYHSSLAPTPELGGCWPPT
Zeja	DIIREGTFQGHHTPPVQKGLRYGMILFITSEVFFFLGFFWAFYHSSLAPTPELGGCWPPT
Znne	DIIREGTFQGHHTPPVQKGLRYGMVLFITSEVFFFLGFFWAFYHSSLAPTPELGGCWPPT
Zefa	DIVREGTFQGHHTPPVQKGLRYGMVLFITSEVFFFIGFFWAFYHASLAPTPELGGCWPPT
Acni	DIVREGTFQGHHTPPVQKGLRYGMILFITSEVFFFLGFFWAFYHSSLAPTPELGGCWPPT
Ncrh	DIVREGTFQGHHTPPVQKGLRYGMILFITSEVFFFLGFFWAFYHSSLAPTPELGGCWPPT
Agca	DIIREGTFQGHHTPPVQQGLRYGMILFITSEVFFFLGFFWAFYHASLAPTPELGGCWPPA
Hydy	DIVREGTFQGHHTPPVQKGLRYGMVLFITSEVFFFLGFFWAFYHASLAPTPELGGCWPPT
Gsac	DIVREGTFQGHHTPPVQKGLRYGMILFITSEVFFFLGFFWAFYHASLAPTPELGGCWPPT
Pevo	DIVREGTFQGHHTPPVQKGLRYGMILFITSEVFFFLGFFWAFYHASLAPTPELGGCWPPS
Hiku	DIIREGTFQGHHTPPVQKGLRYGMILFITSEVFFFLGFFWAFYHSSLAPTPELGGCWPPS
Inpa	DVVREGTFQGHHTLPVQKSLRYGMILFITSEVFFFLGFFWAFYHSSLTPALELGGSWPPT
Auch	DIIREGTYLGHHTPPVQKGLRYGMILFITSEVFFFLGFFWAFYHSSLAPTPELGGAWPPT
Fico	DIIREGTFQGHHTPPVQKGLRYGMILFITSEVFFFLGFFWAFYHSSLAPTPELGGCWPPT
Macs	DIVREGTFQGHHTPPVQKGLRYGMILFITSEVFFFLGFFWAFYHSSLAPTPELGGCWPPS
Moal	DIIRESTFQGHHTTPVQKGLRYGMVLFITSEVFFFIGFFWAFYHSSLAPAIELGGLWPPT
Syma	DIVRESTFQGHHTQPVQKGLRYGMVLFITSEVFFFIGFFWAFYHSSLAPTPELGGHWPPT
Mafr	DIVREGTFQGHHTPPVQKGLRYGMILFITSEVLFFLGFFWAFYHSSLAPTPELGGSWPPT
Dcpe	DIIREGTFQGHHTPPVQKGLRYGMILFITSEVFFFLGFFWAFYHSSLAPTPELGGCWPPS
Dcti	DIIREGTFQGHHTPPVQKGLRYGMILFITSEVFFFLGFFWAFYHSSLAPTPELGGCWPPS
Hehi	DIVREGTFQGHHTPPVQKGLRYGMILFITSEVFFFLGFFWAFYHASLAPTPELGGCWPPT
Stam	DIVREGTFQGHHTPPVQKGLRYGMILFITSEVFFFLGFFWAFYHSSLAPTPELGGCWPPT
Hogi	DIVREGTFQGHHTPPVQKGLRYGMILFITSEVFFFLGFFWAFYHASLAPTPELGGCWPPT
Erzo	DIIREGTFQGHHTPPVQKGLRFGMILFITSEVFFFLGFFWAFYHASLAPTPELGGCWPPT
Hxot	DVVREGTFQGHHTPPVQKGLRYGMILFITSEVFFFLGFFWAFYHASLAPTPELGGCWPPA
Core	DIVREGTFQGHHTPPVQKGLRYGMILFITSEVFFFLGFFWAFYHASLAPTPELGGCWPPT
Apve	DIIREGTFQGHHTPPVQKGLRYGMILFITSEVFFFLGFFWAFYHASLAPTPELGGCWPPA
Latj	DVVREGTFQGHHTPPVQKGLRYGMILFITSEVLFFLGFFWAFYHSSLAPTPELGGNWPPA
Laja	DIVREGTFQGHHTPPVQKGLRYGMILFITSEVFFFLGFFWAFYHASLAPTPELGGCWPPT

8

[2/5 of aligned sequences]

To be continued on page 14.

```
Syja   DIVREGTFQGHHTPPVQKGLRFGMILFITSEVFFFLGFFWAFYHASLAPTPELGSCWPPT
Epme   DIVREGTFQGHHTPPVQKGLRYGMILFITSEVFFFLGFFWAFYHSSLAPTPELGGCWPPT
Grse   DIVREGTFQGHHTPPVQKGLRYGMILFITSEVFFFLGFFWAFYHASLAPTPELGGCWPPT
Clja   DIVREGTFQGHHTPPVQKGLRYGMILFITSEVFFFLGFFWAFYHASLAPTPELGGCWPPT
Ogcy   DIVREGTFQGHHTPPVQKGLRYGMILFITSEVFFFLGFFWAFYHASLAPTPELGGYWPPS
Plna   DIVRESTFQGHHTPPVQKGLRYGMILFITSEVFFFLGFFWAFYHSSLAPTPELGGCWPPT
Lema   DIIREGTFQGHHTPPVQKGLRYGMILFITSEVFFFLGFFWAFYHASLAPTPELGGCWPPT
Etzo   DIIREGTFQGHHTPPVQKGLRYGMILFITSEVFFFLGFFWAFYHSSLAPTPELGGCWPPT
Apse   DIIREGTYQGHHTPPVQKGLRYGMILFITSEVFFFVGFFWAFYHSSLAPTPEIGGCWPPT
Epde   DIIREGTFQGHHTPPVQKGLRYGMILFITSEVFFFLGFFWAFYHASLAPTPELGGCWPPT
Slja   DIVREGTYQGHHTPPVQKGLRYGMVLFITSEVFFFLGFFWAFYHSSLAPTPELGGCWPPT
Bsja   DIVREGTYQGHHTPPVQKGLRYGMILFITSEVFFFLGFFWAFYHSSLAPTPELGGCWPPT
Ecna   DVVREGTFQGHHTPPVQKGLRYGMILFITSEVLFFAGFFWAFYHSSLAPTPELGGFWPPA
Cohi   DVVREGTFQGHHTFPVQKGLRYGMILFITSEVLFFLGFFWAFYHSSLAPTPELGGQWPPT
Caar   DVVREGTFQGHHTPPVQKGLRYGMILFITSEVLFFLGFFWAFYHSSLAPTPELGGCWPPT
Came   DVVREGTFQGHHTPPVQKGLRYGMILFITSEVLFFLGFFWAFYHSSLAPTPELGGCWPPT
Mema   DVVREGTFQGHHTPPVQKGLRYGMILFIASEVLFFLGFFWAFYHSSLAPTPELGGCWPPT
Lenu   DIIREGTFQGHHTPPVQKGLRYGMILFITSEVFFFLGFFWAFYHASLAPTPELGGCWPPT
Brja   DIIREGTFQGHHTPPVQKGLRYGMILFITSEVFFFLGFFWAFYHASLAPTPELGGCWPPA
Plma   DIIREGTFQGHHTPPVQKGLRYGMILFITSEVFFFLGFFWAFYHASLAPTPELGGCWPPS
Emst   DIVREGTFQGHHTPPVQKGLRYGMILFITSEVFFFLGFFWAFYHASLAPTPELGGCWPPT
Ptti   DIVREGTFQGHHTPPVQKGLRYGMILFITSEVFFFLGFFWAFYHASLAPTPELGGCWPPT
Losu   DIVREGTYQGHHTPPVQKGLRFGMILFITSEVFFFLGFFWAFFHSSLAPTPELGGSWPPT
Geoy   DIIREGTFQGHHTPPVQKGLRYGMILFITSEVFFFLGFFWAFYHASLAPTPELGGFWPPA
Dipi   DIVREGTFQGHHTPPVQKGLRYGMILFITSEVFFFLGFFWAFYHSSLAPTPELGGYWPPA
Pama   DIIREGTFQGHHTPPVQKGLRYGMILFITSEVFFFLGFFWAFYHSSLAPTPDLGGCWPPT
Leob   DIVREGTFQGHHTPPVQKGLRYGMILFITSEVFFFLGFFWAFYHSSLAPTPELGGCWPPT
Neba   DVVREATYQGHHTPPVQKGLRYGMILFITSEVFFFLGFFWAFYHASLAPTPELGGCWPPT
Pdpl   DVVREATYQGHHTLPVQVGLRLGMGLFITSEVLFFTGFFWAFFHSSLAPSPELGSIWPPS
Nimi   DIVREGTFQGHHTPPVQKGLRFGMILFITSEVFFFLGFFWAFYHASLAPTPELGGCWPPT
Uptr   DIVREGTFQGHHTPPVQKGLRYGMVLFITSEVFFFLGFFWAFYHASLAPTPELGGCWPPT
Pesc   DIVREGTFQGHHTPPVQKGLRYGMILFITSEVFFFLGFFWAFYHSSLSPTPELGGCWPPT
Baar   DIVREGTFLGHHTPPVQKGLRYGMVLFITSEVFFFLGFFWAFYHSSLAPTPELGGCWPPT
Moar   DIVREGTFQGHHTPPVQKGLRYGMILFITSEVFFFLGFFWAFYHSSLAPTPELGGCWPPT
Toja   DVIREGTFQGHHTLAVQKGLRYGMILFIISEVFFFLGFFWAFYHSSLAPTPELGGCWPPA
Chau   DIVREATFQGHHTPPVQKGLRYGMILFITSEVFFFLGFFWAFYHSSLAPTPELGGCWPPT
Chse   DIVREGTFQGHHTPPVQKGLRYGMILFITSEVFFFLGFFWAFYHSSLAPTPELGGCWPPT
Enar   DIVREGTFQGHHTPPVQKGLRFGMILFITSEVFFFLGFFWAFYHASLAPTPELGGCWPPT
Hpty   DIVREGTFQGHHTPPVQKGLRYGMILFITSEVFFFLGFFWAFYHSSLAPTPELGGCWPPT
Nana   DVVREGTFQGHHTPPVQKGLRYGMILFITSEVFFIGFFWAFYHSSLAPTPELGGYWPPT
Mcst   DIVREGTFQGHHTPPVQKGLRYGMILFITSEVFFFLGFFWAFYHASLAPTPELGGCWPPT
Rhox   DIVREGTFQGHHTPPVQKGLRYGMILFITSEVFFFLGFFWAFYHASLAPTPELGGCWPPT
Opfa   DIVREGTFQGHHTPPVQKGLRYGMILFITSEVFFFLGFFWAFYHSSLAPTPELGGCWPPA
Paar   DIVREGTFQGHHTPPVQKGLRYGMILFITSEIFFFLGFFWAFYHASLAPTPELGGCWPPT
Gozo   DITREGTFQGHHTPPVQKGLRYGMILFITSEVFFFLGFFWAFYHASLAPTPELGGCWPPT
Ackr   DIIREGTFQGHHTTPVQKGLRYGMILFITSEVFFFLGFFWAFYHSSLAPTHELGGCWPPA
Elev   DIIREGTFQGHHTPPVQKGLRYGMILFITSEVFFFLGFFWAFYHSSLAPTPELGGCWPPT
Trdu   DIVREGTFQGHHTPPVQKGLRYGMILFITSEVFFFLGFFWAFYHASLAPTPELGGCWPPT
Amoc   DIIREGTFQGHHTPPVQKGLRYGMILFITSEVFFFLGFFWAFYHSSLAPTPELGGCWPPT
Hame   DIVREGTYQGHHTPPVQKGLRYGMILFITSEVFFFLGFFWAFYHSSLAPTPELGGCWPPT
Chso   DIVREGTFQGHHTPPVQKGLRYGMILFITSEVFFFLGFFWAFYHSSLAPTPELGGCWPPT
Lyto   DIVREGTFQGHHTPPVQKGLRYGMVLFITSEVFFFLGFFWAFYHASLAPTPELGGCWPPT
```

[2/5 of aligned sequences]

```
Encr    DIVREGTFQGHHTPPVQKGLRYGMILFITSEVFFFLGFFWAFYHASLAPTPELGGCWPPT
Bvar    DIVRESTFLGHHTPPVQKGLRYGMILFITSEVFFFLGFFWAFYHASLAPTPELGGCWPPT
Noco    DIVREGTFQGHHTPPVQKGLRYGMILFITSEVFFFLGFFWAFYHASLAPTPELGGCWPPT
Chsp    DIVREATYLGHHTPPVQKGLRYGMILFITSEVFFFLGFFWAFYHSSLAPTPELGGHWPPT
Arja    DIVREGTFQGHHTPPVQKGLRYGMILFITSEVFFFLGFFWAFYHASLAPTPELGGCWPPA
Pase    DIVREGTYQGHHTPPVQKGLRYGMILFITSEVFFFLGFFWAFFHSSLAPTPELGGCWPPA
Trel    DIIREGTFQGHHTPPVQKGLRYGMILFITSEIFFFLGFFWAFYHSSLAPTPELGGCWPPT
Lifa    DIVREGTFQGHHTPPVQKGLRYGMILFITSEVFFFLGFFWAFYHASLAPTPELGGCWPPT
Acur    DIVREGTFQGHHTPPVQKGLRYGMVLFITSEVFFFLGFFWAFYHSSLAPTPELGGCWPPA
Ampe    DIVREGTFQGHHTPPVQKGLRYGMILFITSEVFFFLGFFWAFYHSSLAPTPELGGCWPPT
Urja    DIVRESTFQGHHTPPVQKGLRYGMILFITSEVFFFLGFFWAFYHASLAPTPELGGCWPPT
Enet    DIVREGTFQGHHTPPVQKGLRYGMILFITSEVFFFLGFFWAFYHSSLAPTPELGGCWPPA
Ptbr    DIVREGTFQGHHTPPVQKGLRYGMILFISSEVFFFLGFFWAFYHSSLAPTPELGGCWPPT
Safa    DIVREGTFQGHHTPPVQKGLRYGMILFITSEVFFFIGFFWAFYHSSLAPTPELGGCWPPT
Icae    DIIREGTYQGHHTPPVQKGLRYGMILFITSEVFFFLGFFWAFYHASLAPTPELGGCWPPT
Asmi    DIIRESTFQGHHTLPVQKGLRYGMILFIASEVFFFLGFFWAFYHSSLAPTPELGGCWPPT
Foal    DIVREGTFQGHHTPPVQKGLRCGMVLFITSEVFFFLGFFWAFYHSSLAPTPDLGGCWPPT
Drze    DIMREATFQGHHTTPVQKGLRCGMVLFITSEIFFFLGFFWAFYHSSLAPTLELGACWPPQ
Rhas    DIVREGTFQGHHTPPVQKGLRYGMILFITSEVFFFLGFFWAFYHASLAPTPELGGCWPPA
Elac    DIIREGTFQGHHTPPVQKGLRYGMILFITSEVFFFLGFFWAFYHASLAPTPELGGCWPPT
Kugu    DIVREGTYQGHHTPPVQKGLRYGMILFITSEVFFFLGFFWAFYHASLAPTPELGSSWPPT
Plor    DIVREGHSKDTHTPPVQKGLRYGMILFITSEVFFFLGFFWAFYHSSLAPTPELGGCWPPM
Sgun    DIVREGTFQGHHTPPVQKGLRYGMILFITSEVFFFLGFFWAFYHSSLAPTPELGGCWPPT
Zaco    DIVREGTFQGHHTPPVQKGLRYGMILFITSEVFFFLGFFWAFYHSSLAPTPELGGCWPPT
Zbfl    DIVREGTFQGHHTPPVQKGLRYGMILFITSEVFFFLGFFWAFYHSSLAPTPELGGCWPPT
Spba    DVVREGTFQGHHTPPVQKGLRFGMILFIASEVLFFAGFFWAFYHSSLAPTPELGGNWPPA
Game    DIIREGTYQGHHTPPVQKGLRYGMILFITSEVFFFLGFFWAFYHASLAPTPELGGCWPPA
Thth    DIIREGTYQGHHTPPVQKGLRYGMILFITSEVFFFVGFFWAFYHSSLAPTPELGGCWPPT
Xigl    DVVREGTFQGHHTPPVQKGLRYGMILFITSEVLFFLGFFWAFYHSSLAPTPELGGHWPPT
Hyja    DIIREGTYQGHHTPPVQKGLRFGMILFITSEVFFFLGFFWAFYHASLAPTPELGGCWPPT
Psan    DIVREATFQGHHTPPVQKGIRYGMILFITSEAFFFLGFFWAFYHASLAPSPELGGCWPPT
Cupa    DIIREGTYQGHHTPPVQKGLRYGMILFITSEVFFFLGFFWAFYHSSLAPTPELGGCWPPT
Mpch    DIVREGTYQGHHTPPVQKGLRFGMILFITSEVFFFLGFFWAFYHSSLAPTPELGGSWPPT
Char    DIIREGTFQGHHTPPVQKGLRYGMILFITSEVFFFLGFFWAFYHSSLAPTPELGGHWPPT
Pser    DVVREGTFQGHHTPPVQKGLRYGMILFITSEVLFFLGFFWAFYHSSLAPTPELGGYWPPS
Prol    DVVREATFQGHHTPPVQKGLRYGMILFITSEVLFFLGFFWAFYHASLAPTPELGGFWPPA
Plbi    DVVREGTFQGHHTPPVQKGLRYGMILFITSEVLFFLGFFWAFYHSSLAPTPDLGGFWPPA
Calu    DIVREGTFQGHHTPPVQKGLRYGMILFITSEVFFFLGFFWAFYHSSLAPTPELGALWPPA
Papa    DVIREATFQGHHTPPVQKSLRYGMILFITSEVLFFLGFFWAFYHSSLAPTPELGGTWPPT
Sufr    DIVREGTFQGHHTPPVQKGLRYGMILFITSEVFFFLGFFWAFYHSSLAPTPELGGCWPPT
Stci    DIIREGTYQGHHTMPVQKGLRYGMILFITSEVFFFLGFFWAFYHASLAPTPELGACWPPT
Taru    DIVREGTFQGHHTPPVQKGLRYGMILFITSEVFFFLGFFWAFYHASLAPTPELGGCWPPT
Rala    DIIREGTFQGHHTPPVQKGLRYGMILFIASEVFFFLGFFWAFYHSSLAPTPELGGCWPPA
        *: **.     *:  **  .:* *: *** *:       .:  :: *:*: *  : *  ***
```

To be continued on page 15.

[3/5 of aligned sequences]

```
                         D                                    E
Scca  GINPLDPFEVPLLNTAVLLASGVTVTWAHHGLMEGNRKEAIQALTLTIILGVYFTALQAM
Muma  GINPLDPFEVPLLNTAVLLASGVTVTWAHHSLMEGNRKEAIQALTLTMILGVYFTSLQAM
Erca  GITPLNPFEVPLLNTAVLLASGVTVTWAHHSLMEGKRVEATQALTLTIILGFYFTALQAM
Pose  GITPLDPFEVPLLNTAVLLASGVTVTWAHHSLMEGKRTEATQALTLTILLGLYFTALQAM
Actr  GIITLDPFEVPLLNTAVLLASGVTVTWAHHSIMERERKQTIQALTLTILLGFYFTALQAM
Scal  GIITLDPFEVPLLNTAVLLASGVTVTWAHHSIMERERKQTIQALTLTILLGFYFTALQAM
Posp  GIIPLDPFEVPLLNTAVLLASGVTVTWAHHSIMERERKQTIQALTLTILLGFYFTALQAM
Atsp  GITPLDPFEVPLLNTAVLLASGVTVTWAHHSLMEGTRKQMIQALTLTIILGFYFTALQTM
Leoc  GITPLDPFEVPLLNTAVLLASGVTVTWAHHSLMEGARKQMIQALTLTIILGFYFTMLQAM
Amca  GINALDPFEVPLLNTAVLLASGVTVTWAHHSLMEGKRNQAIQALTLTILLGLYFTALQAM
Osbi  GITPLDPFEVPLLNTAVLLASGVTVTWAHHSLMEGQRKEAIQSLFLTILLGCYFTMLQAM
Pabu  GIIPLDPFEVPLLNTAVLLASGVTVTWAHHSIMEGGRKETIQSLALTIILGMYFTALQAM
Hial  GIIPLDPFEVPLLNTAVLLASGVTVTWAHHSLMEGERKQAIQSLILTIILGLYFTTLQAM
Elha  GITTLDPFEVPLLNTAVLLASGVTVTWAHHSLMEGERKQAIQSLALTILLGFYFTLLQAM
Mlcy  GITTLDPFEVPLLNTAVLLASGVTVTWAHHSLMEGERKQAIQSLTLTIALGFYFTLLQAM
Algl  GITTLDPFEVPLLNTAVLLASGVTVTWAHHSLMEGERKEAIQSLALTILLGFYFTFLQAM
Ptgi  GIITLDPFEVPLLNTAVLLASGVTVTWAHHSMMEGGRKQAIQSLGLTILLGFYFTFLQAM
Alaf  GITTLDPFEVPLLNTAVLLASGVTVTWAHHSLMEGERKQAIHSLFITIILGFYFTFLQAM
Nock  GIIPLDPFEVPLLNTAVLLASGVTVTWTHHSLMEGERKQAIHSLFLTIILGFYFTFLQAL
Anja  GIITLDPFEVPLLNTAVLLASGVTVTWAHHSIMEGERKQAIQSLTLTIILGFYFTLLQAM
Gyki  GIITLDPFEVPLLNTAVLLASGVTVTWAHHSIMEGERKQAIQSLTLTILLGFYFTFLQAM
Syka  GITALDPFEVPLLNTAVLLASGVTVTWTHHSIMEGERKQAIQSLTITILLGFYFTFLQAM
Opma  GITTLDPFEVPLLNTAVLLASGVTVTWAHHSIMEGERKQAIQSLTLTVILGLYFTLLQAM
Comy  GVTALDPFEVPLLNTAVLLASGVTVTWSHHSIMEGHRKQAIHSLTLTIILGFYFTFLQAM
Sasp  GITPLDPFEVPLLNTAVLLCSGVTVTWAHHCITGNERKQAIQSLALTILLGFYFTLLQAL
Eupe  GISALDPFEVPLLNTAVLLASGVTVTWAHHSLMEKERKQTIHALFLTILLGFYFTLLQAM
Enja  GITTLDPFEVPLLNTAVLLASGVTVTWAHHALMEGERKQAIQSLTLTILLGFYFTFLQGM
Same  GITPLDPFEVPLLNTAVLLASGVTVTWAHHSLMEGERKQAIQSLTLTILLGFYFTFLQGL
Chch  GITTLDPFEVPLLNTAVLLASGVTVTWAHHSLMEGERKQAIQSLTLTILLGFYFTLLQAM
Grgr  GVSTLDPFEVPLLNTAVLLASGVTVTWAHHSIMESDRKQAIHSLFLTILLGFYFTFLQGL
Caau  GITPLDPFEVPLLNTAVLLASGVTVTWAHHSIMEGERKQAIQSLALTILLGFYFTALQAM
Cyca  GITPLDPFEVPLLNTAVLLASGVTVTWAHHSIMEGERKQAIQSLALTILLGFYFTALQAM
Dare  GLTTLDPFEVPLLNTAVLLASGVTVTWAHHSLMEGERKQAIQSLALTILLGLYFTALQAM
Cost  GITPLDPFEVPLLNTAVLLASGVTVTWAHHSLMEGERKQAIQSLALTILLGFYFTALQAM
Leec  GIITLDPFEVPLLNTAVLLASGVTVTWAHHSLMEGERKQAIQSLTLTILLGLYFTALQAM
Fola  GITPLDPFEVPLLNTAVLLASGVTVTWAHHSLMEGARKQAIQALALTIILGVYFTALQAM
Clmc  GITPLDPFEVPLLNTAVLLASGVTVTWAHHSLMEGKRKEAIQSLTLTILLGFYFTALQAM
Phin  GITTLDPFEVPLLNTAVLLASGVTVTWAHHSLMEGGRKQAIQALSLTIILGFYFTALQAM
Icpu  GITPLDPFEVPLLNTAVLLASGVTVTWSHHSLMEGERKQAVQSLTLTILLGFYFAALQAM
Psto  GITTLDPFEVPLLNTAVLLASGVTVTWAHHSLMEGERKQAIQSLALTILLGLYFTVLQAM
Cora  GITTLDPFEVPLLNTAVLLASGVTVTWAHHSIMEGHRKQAIQSLTLTILLGFYFTALQAM
Eisp  GITPLNPMEVPLLNTAVLLASGVTVTWAHHSLMEGERKQAIQALILTILLGFYFTALQAM
Apal  GITPLDPFEVPLLNTAVLLASGVTVTWCHHSLMEGERKQAIQSLTLTIILGLYFTALQAM
Eslu  GIITLDPFEVPLLNTAVLLASGVTVTWAHHSIMEGERKQAIQSLTLTILLGFYFTFLQAM
Dape  GIIPLDPFEVPLLNTAVLLASGVTVTWAHHSIMEGSRKQTTQALTLTILLGFYFTFLQAM
Glse  GITTLDPFEVPLLNTAVLLASGVTVTWAHHSIMEGARKQTIQSLTLTIILGFYFTFLQGM
Naar  GITPLDPFEVPLLNTAVLLASGVTVTWAHHSIMEGARKQAIQSLTLTIILGFYFTFLQGM
Lioc  GITALDPFEVPLLNTAVLLASGVTVTWAHHSIMEGARKQAIQSLTLTIILGFYFTFLQGM
Opso  GITALDPFEVPLLNTAVLLASGVTVTWAHHSIMERTRKQTIQSLTLTIILGFYFTLLQAM
Alte  GITPLDPFEVPLLNTAVLLASGVTVTWTHHSLMEGNRKEAIQSLGLTIILGFYFTLLQAM
Plap  GITPLDPFEVPLLNTAVLLASGVTVTWTHHSLMEGNRKEAIQALTLTILLGFYFTLLQAM
```

To be continued on page 16.

[3/5 of aligned sequences]

To be continued on page 17.

```
Plal  GITTLDPFEVPLLNTAVLLASGVTVTWAHHSIMEGERKQTIHSLTLTILLGFYFTFLQGL
Sami  GITTLDPFEVPLLNTAVLLASGVTVTWAHHSIMEGERKETIQSLTLTILLGFYFTFLQGL
Rere  GITALDPFEVPLLNTAVLLASGVTVTWAHHSIMEGERKQTIHSLALTILLGFYFSFLQGL
Gama  GITTLDPFEVPLLNTAVLLASGVTVTWAHHSIMEGERKQAIQSLALTIILGFYFTFLQGL
Onmy  GITTLDPFEVPLLNTAVLLASGVTVTWAHHSIMEGERKQTIQALTLTILLGFYFTFLQGM
Sasa  GIITLDPFEVPLLNTAVLLASGVTVTWAHHSIMEGERKQTIQALTLTILLGFYFTFLQGM
Cola  GIITLDPFEVPLLNTAVLLASGVTVTWAHHSIMEGERKQAIQSLTLTILLGFYFTFLQGM
Dita  GITALDPFEVPLLNTAVLLASGVTVTWAHHSIMAGERKQAIQSLALTILLGFYFTFLQAL
Gogr  GISALNPFEVPLLNTTTLLASGVTVTWAHHSITTGQQKQATQSLGMTIALGLYFTALQAL
Chsl  GIQALDPFEVPLLNTAVLLASGVTVTWAHHSIMAGDRKQAIHSLTLTILLGGFFTLLQAL
Atja  GITTLDPFEVPLLNTAVLLASGVTVTWAHHSIMEGERKQAIHSLTLTILLGFYFTFLQAM
Iido  GITTLDPFEVPLLNTAVLLASGVTVTWAHHSIMEGERKQTIHSLTLTILLGFYFTFLQAM
Auja  GITTLDPFEVPLLNTAVLLASGVTVTWAHHSIMEGQRKQAIQSLTLTILLGFYFTFLQGM
Chag  GITTLDPFEVPLLNTAVLLASGVTVTWAHHSIMEGERKQAIQSLSLTILLGFYFTFLQGM
Hami  GISTLDPFEVPLLNTAVLLASGVTVTWAHHSIMEGHRDQAIQSLTLTILLGFYFTFLQGM
Saun  GISTLDPFEVPLLNTAVLLASGVTVTWAHHSIMEGQRDQAIQSLTLTILLGFYFTFLQGM
Nema  GITTLDPFEVPLLNTAVLLASGVTVTWAHHSIMEGERKQAIQSLTLTILLGFYFTFLQGM
Disp  GITTLDPFEVPLLNTAVLLASGVTVTWAHHSIMEGERKQATQSLALTILLGFYFTFLQAL
Myaf  GITPLDPFEVPLLNTAVLLASGVTVTWAHHSIMEGERKQAIHSLTLTILLGFYFTFLQGL
Lagu  GVLALNPFEVPLLNTAVLLASGVTVTWAHHSIMEGARKEAIQALALTILLGFYFTFLQAV
Trtr  GITPLNPFEVPLLNTAVLLASGVTVTWAHHSIMEGQRKQTIQSLALTILLGLYFSFLQGL
Zucr  GITTLNPFEVPLLNTAVLLASGVTVTWAHHSIMEGQRKQAIQSLALTILLGFYFTFLQGL
Pxja  GIITLDPFEVPLLNTAVLLASGVTVTWAHHSIMEGERKQAIQSLTLTIILGFYFTFLQAM
Pxlo  GIITLDPFEVPLLNTAVLLASGVTVTWAHHSIMEGERKQAIQSLTLTIILGFYFTFLQAM
Pctr  GITPLDPFEVPLLNTAVLLASGVTVTWAHHSIMAGERKQAIQSLALTILLGFYFTLLQAL
Apsa  GITPLDPFEVPLLNTAVLLASGVTVTWAHHSIMENKRKEAIQSLTLTILLGFYFTLLQAL
Cabe  GITTLDPFEVPLLNTAVLLASGITATWAHHSLMGGERKQAIHSLALTILLGFYFTILQAL
Bzze  GITALDPFEVPLLNTAVLLASGVTVTWAHHSLMEGDRKQAIQSLALTILLGFYFTFLQAM
Siim  GITTLDPFEVPLLNTAVLLASGVTVTWAHHSLMEKKRKEAIQALALTVALGFYFTLLQAM
Ctru  GITPLDPFEVPLLNTAVLLASGVTVTWAHHSLMEGNRKQAIQSLALTILLGFYFTFLQAL
Dpbr  GITALDPFEVPLLNTAVLLASGVTVTWAHHSLMEGERKQAIQSLALTIILGFYFTFLQAM
Caki  GISTLDPFEVPLLNTAVLLASGVTVTWAHHSIMLGERKQAIHSLTLTVLLGLYFTLLQAM
Phja  GIHTLDPFEVPLLNTAVLLASGATVTWAHHSIVAGNRKEAIQSLFLTILLGFYFTFLQAM
Brsp  GISTLNPFEVPLLNTAVLLASGATVTWAHHSILAKERSQAIQSLTLTILLGFYFTALQAL
Gamo  GITTLDPFEVPLLNTAVLLASGVTVTWAHHSIMEGERKQAIHSLTLTILLGFYFTFLQGL
Lolo  GIITLDPFEVPLLNTAVLLASGVTVTWAHHSIMEGERKQAIHSLTLTILLGFYFTFLQAM
Batr  DIFPLNPMSVPLLNTAILLASGVSVTWAHHSIIQSNRRQAIQSLSITALLGIYFTALQAM
Prmy  GITPLNPFEVPLLNTAVLLASGVTVTWAHHSLTQGNRKEAIQSLTLTILLGFYFTILQAV
Lose  GIIPLDPFGVPLVNTAVLLASGITVTWAHQSIMEGDRTEAIMSLFLTILLGLYFTVLQAL
Loam  GITPLDPFEVPLLNTAVLLASGVTVTWAHHSIMEGERKQALQSLLLTILLGFYFTFLQGL
Chab  GIITLDPFEVPLLNTAVLLASGVTVTWAHHSIMEGERKQAIQSLFLTILLGFYFTFLQAM
Chto  GVITLDPFEVPLLNTAVLLASGVTVTWAHHSIMEGERKQAIQSLFLTILLGFYFTFLQAM
Majo  GVITLDPFEVPLLNTAVLLASGVTVTWAHHSIMAGERKQTMQALLLTILLGFYFTFLQAL
Hlst  GITTLDPFEVPLLNTAVLLASGVTVTWAHHSIMFGERKQAIQALTLTILLGFYFTFLQAL
Clpe  GVTALDPFEVPLLNTAVLLASGVTVTWAHHSIMAGQRRQAIHSLLLTVLLGFYFTLLQAM
Mlmr  GITPLDPFEVPLLNTAVLLASGVTVTWAHHSIMEGHRKQAIQSLLLTILLGFYFTFLQAM
Crcr  GITTLNPFEVPLLNTAVLLASGVTVTWAHHSIMEGNRKEAIQSLALTILLGFYFTLLQAM
Muce  GITTLNPFEVPLLNTAVLLASGVTVTWAHHSIMEGNRKEAIQSLALTILLGFYFTLLQAM
Bege  GITTLDPFEVPLLNTAVLLASGVTVTWAHHSIMEGHRKQAIQSLTLTILLGFYFTFLQAM
Mela  GITTLNPFEVPLLNTAVLLASGVTVTWAHHSIMEGNRKQAIQSLALTILLGFYFTFLQAM
Hats  GITTLDPFEVPLLNTAVLLASGVTVTWTHHSIMEGERKQAIHSLALTILLGFYFTFLQGM
Orla  GITTLDPFEVPLLNTAVLLASGVTVTWAHHSIMEGQRKQAIQSLTLTILLGFYFTFLQAM
```

[3/5 of aligned sequences]

To be continued on page 18.

```
Cosa  GITTLDPFEVPLLNTAVLLASGVSVTWAHHSLMEGQRKQAIQSLTLTILLGFYFTFLQAL
Exsp  GITTLDPFEVPLLNTAVLLASGVTVTWAHHSIMEGQRKQTIQSLTLTILLGFYFTFLQAM
Depa  GIMTLDPFEVPLLNTAVLLASGVTVTWAHHSIMEGQRKQAIQSLILTVLLGLYFTFLQAM
Rima  GITTLDPFEVPLLNTAVLLASGVTVTWAHHSIMEGHRKQAIQSLVLTILLGFYFTALQAM
Fuol  GITTLDPFEVPLLNTAVLLASGVTVTWAHHSIMEGHRKQAIQSLALTILLGFYFTALQAL
Gmaf  GINTLNPFEVPLLNTAVLLASGVTVTWAHHSIMEGQRKQALQSLTLTILLGFYFTALQAM
Xeei  GITTLDPLEVPLLNTAVLLASGVTVTWAHHSIMEGLRKQAIQALTLTIILGFYFTYLQAM
Pros  GISPLDPFEVPLLNTAVLLASGVTVTWAHHSIMEGERKQAIHSLGLTILLGFYFTFLQAM
Scmi  GISPLDPFEVPILNTVVLLTSGMTVTWAHHSIMEGERKQTIHALGLTIILGFYFTFLQAM
Rolo  GILPLDPFEVPLLNTAVLLASGVTVTWAHHSIMEGERKQAIQSLALTIILGFYFTFLQAM
Cere  GVITLDPFEVPLLNTAVLLASGVTVTWAHHSLMEGQRKQAIQSLTLTIALGFYFTCLQAM
Daga  GITTLDPFEVPLLNTAVLLASGVTVTWSHHSIMEGKRKQAIQSLTLTILLGFYFTFLQAA
Anco  GITPLDPFEVPLLNTAVLLASGVTVTWAHHSIMEGERKQAIQSLALTILLGFYFTFLQAM
Dmve  GITPLDPFEVPLLNTAVLLASGVTVTWAHHSIMEGERKQAIQSLTLTILLGFYFTFLQAL
Dmar  GVTPLDPFEVPLLNTAVLLASGVTVTWAHHSIMEGERKQAIQSLTLTILLGFYFTFLQAL
Anka  GITTLDPFEVPLLNTAVLLASGVTVTWAHHSIMEGERKQALQSLTLTILLGFYFTFLQAM
Moja  GITTLDPFEVPLLNTAVLLASGVTVTWAHHSIMEGERKQALQSLTLTILLGFYFTLLQAM
Hoja  GITPLDPFEVPLLNTAVLLASGVTVTWAHHSIMEGERKQALQSLTLTILLGFYFTFLQAL
Bede  GITTLDPFEVPLLNTAVLLASGVTVTWAHHSIMEGERKQAIHSLGLTILLGFYFTLLQAM
Besp  GIITLDPFEVPLLNTAVLLASGVTVTWAHHSIMEGERKQAIHSLGLTILLGFYFTFLQAM
Mysp  GITTLDPFEVPLLNTAVLLASGVTVTWAHHSIMEGERKQAIQSLTLTILLGFYFTFLQGM
Osja  GITTLDPFEVPLLNTAVLLASGVTVTWAHHSIMEGERKQAIQALALTILLGFYFTFLQAL
Sgro  GITTLDPFEVPLLNTAVLLASGVTVTWAHHSIMEGERKQAIQSLTLTILLGFYFTFLQAM
Pzpa  GIITLDPFEVPLLNTAVLLASGVTVTWAHHSIMEGERQQAIQSLTLTILLGFYFTYLQAM
Zeja  GITTLDPFEVPLLNTAVLLASGVTVTWAHHSILEGERKQAIQSLTLTILLGFYFTFLQGM
Znne  GITPLDPFEVPLLNTAVLLASGVTVTWAHHSIMEGERKQAIQSLTLTILLGFYFTFLQAM
Zefa  GITPLDPFEVPLLNTAVLLASGVTVTWAHHSIMEGERKQAIHSLTLTILLGFYFTFLQAM
Acni  GITTLDPFEVPLLNTAVLLASGVTVTWAHHSIMEGDRKQTIQSLTLTILLGFYFTFLQAM
Ncrh  GITALDPFEVPLLNTAVLLASGVTVTWAHHSIMEGERKQAIQSLTLTILLGFYFTFLQAM
Agca  GITPLDPFEVPLLNTAVLLASGVTVTWAHHSIMEGERAQAIQSLLLTILLGFYFTFLQAL
Hydy  GITTLDPFEVPLLNTAVLLASGVTVTWAHHSIMEGERKQAIQSLALTILLGFYFTFLQGM
Gsac  GITTLDPFEVPLLNTAVLLASGVTVTWAHHSIMEGERKQAIQSLALTILLGFYFTFLQGM
Pevo  GITTLDPFEVPLLNTAVLLASGVTVTWAHHSIMEGERKQAIQSLALTILLGFYFTFLQGM
Hiku  GVITLDPFEVPLLNTAVLLASGVTVTWAHHSIMEGERKQAIHSLTLTILLGFYFTFLQAM
Inpa  GISPLDPFEVPLLNTAVLLASGVTVTWAHHSIMGQQRKQAIQALALTVLLGLYFTALQAM
Auch  GISPLDPMEVPLLNTAVLLASGVTVTWAHHSLIEQDRRQAIHALALTILLGFYFTFLQAM
Fico  GISTLDPFEVPLLNTAVLLASGVTVTWAHHSIMEGERKQAIQSLTLTILLGFYFTFLQGM
Macs  GISPLDPFEVPLLNTAVLLASGVTVTWAHHSIMEGERKQAIQSLLLTILLGFYFTFLQGL
Moal  GITPLDPFEVPLLNTAVLLASGVTVTWAHHSIMEGARKPALQSLTLTVLLGLYFTALQAM
Syma  GITTLDPFEVPLLNTAVLLASGVTVTWAHHSIMEGKREQAIQSLLLTVLLGLYFTLLQAM
Mafr  GITPLNPFEVPLLNTAVLLASGVTVTWAHHSIMERERKQAIQALTLTILLGFYFTLLQAM
Dcpe  GILPLDPFEVPLLNTAVLLASGVTVTWAHHSIMEGERKQAIHSLTLTILLGFYFTCLQAM
Dcti  GVSPLDPFEVPLLNTAVLLASGVTVTWAHHSIMEGERKQAIHSLTLTILLGFYFTCLQAM
Hehi  GITPLDPFEVPLLNTAVLLASGVTVTWAHHSIMEGERKQAIQSLTLTILLGFYFTFLQAM
Stam  GITPLDPFEVPLLNTAVLLASGVTVTWAHHSIMEGERKQAIQSLALTILLGFYFTFLQGM
Hogi  GITTLDPFEVPLLNTAVLLASGVTVTWAHHSIMEGERKETLQSLLLTIILGFYFTFLQGM
Erzo  GVSTLDPFEVPLLNTAVLLASGVTVTWAHHSIMEGERKQAVHSLGLTILLGFYFTFLQGL
Hxot  GITTLDPFEVPLLNTAVLLASGVTVTWAHHSIMEGERKQAVQSLALTILLGFYFTFLQGL
Core  GITTLDPFEVPLLNTAVLLASGVTVTWAHHSIMEGERKQAVQSLALTILLGFYFTFLQAL
Apve  GITPLDPFEVPLLNTAVLLASGVTVTWAHHSIMEGERKQTIQSLTLTILLGFYFTFLQTL
Latj  GITPLDPFEVPLLNTAVLLASGVTVTWAHHAIMEGKRKQAVQALALTILLGTYFTFLQAL
Laja  GITTLDPFEVPLLNTAVLLASGVTVTWAHHSIMEGERKQAIQSLALTILLGFYFTFLQAL
```

[3/5 of aligned sequences]

To be continued on page 19.

Syja	GITPLDPFEVPLLNTAVLLASGVTVTWAHHSIMEGERKQAIHSLTLTILLGFYFTFLQAM
Epme	GITTLDPFEVPLLNTAVLLASGVTVTWAHHSIMEGERKQAIQSLTLTILLGFYFTFLQAM
Grse	GITTLDPFEVPLLNTAVLLASGVTVTWAHHSIMEGERKQAIQSLTLTILLGFYFTLLQAM
Clja	GITTLDPFEVPLLNTAVLLASGVTVTWAHHSIMEGKRKQTIHSLTLTILLGFYFTFLQGM
Ogcy	GITPLDPFEVPLLNTAVLLASGVTVTWAHHSIMEGHQKQAVQSLTLTIILGFYFTCLQAM
Plna	GITTLDPFEVPLLNTAVLLASGVTVTWAHHSIMEGQRKQALQSLTLTILLGFYFTFLQGM
Lema	GISVLDPFEVPLLNTAVLLASGVTVTWAHHSIMEGERKQAIHSLTLTILLGFYFTFLQAM
Etzo	GITPLDPFEVPLLNTAVLLASGVTVTWAHHSIMEGERKQAIQSLALTILLGFYFTFLQAM
Apse	GITTLDPFEVPLLNTAVLLASGVTVTWAHHSMMEGERAQAIQSLTLTIILGFYFTVLQAL
Epde	GITTLDPFEVPLLNTAVLLASGVTVTWAHHSIMEGNRKQTIQSLTLTILLGFYFTFLQAM
Slja	GIVTLDPFEVPLLNTAVLLASGVTVTWAHHSIMEGERKQAIQSLALTILLGFYFTFLQGM
Bsja	GITTLDPFEVPLLNTAVLLASGVTVTWAHHSIMEGLRAQTTQSLLLTILLGFYFTFLQGL
Ecna	GITPLDPFEVPLLNTAVLLASGVTVTWAHHSIMEGDRKQAIHSLTLTILLGGYFTFLQAL
Cohi	GITALDPFEVPLLNTAILLASGVTVTWAHHSIMETKRKQAIQSLTLTIILGLYFTLLQAL
Caar	GITALDPFEVPLLNTAVLLASGVTVTWAHHSIMEGKRKQAIQSLALTILLGGYFTCLQAM
Came	GITALDPFEVPLLNTAVLLASGVTVTWAHHSIMEGKRKQAIQSLALTILLGGYFTCLQAM
Mema	GITPLDPFEVPLLNTAVLLASGVTVTWAHHSIMEGKRKQAVHSLALTILLGGYFTFLQAM
Lenu	GITTLNPFEVPLLNTAVLLASGVTVTWAHHSIMEGERKQAIHSLTLTILLGFYFTFLQGM
Brja	GLIPLDPFEVPLLNTAVLLASGVTVTWAHHSIMEGTRKQAIQSLVLTILLGFYFTFLQAM
Plma	GITTLDPFEVPLLNTAVLLASGVTVTWAHHSIMEGARKQAVQSLALTILLGFYFTFLQAM
Emst	GITPLDPFEVPLLNTAVLLASGVTVTWAHHSIMEGERKQAIQSLFLTILLGFYFTFLQGM
Ptti	GITPLDPFEVPLLNTAVLLASGVTVTWAHHSIMEGERKQAIQSLLLTILLGFYFTFLQGM
Losu	GITPLDPFEVPLLNTAVLLASGVTVTWAHHSIMEGKRKQAIQSLMLTILLGFYFTLLQAM
Geoy	GVTTLDPFEVPLLNTAVLLASGVTVTWAHHSIMEGERKQAIHSLTLTILLGFYFTFLQGM
Dipi	GITTLDPFEVPLLNTAVLLASGVTVTWAHHSIMEGERKQAIQSLALTILLGFYFTFLQAM
Pama	GISPLDPFEVPLLNTAVLLASGVTVTWAHHSIMEGERKQAIQSLTLTILLGFYFTFLQGL
Leob	GITPLDPFEVPLLNTAVLLASGVTVTWAHHSIMEGERKQAIQSLALTILLGFYFTFLQAL
Neba	GITTLDPFEVPLLNTAVLLASGVTVTWAHHSIMEGERKQAIHSLTLTILLGFYFTFLQGM
Pdpl	GITTLDPFGVPLLNTAVLLASGITVTWAHHSIIEGYRQEAIKALALTVTLGYFFTILQIM
Nimi	GITTLDPFEVPLLNTAVLLASGVTVTWAHHSIMEGERKQAIHSLTLTILLGFYFTFLQAM
Uptr	GITTLDPFQVPLLNTAVLLASGVTVTWAHHSIMEGERAQAIQSLGLTILLGFYFTFLQGM
Pesc	GITTLDPFGVPLLNTAVLLASGVTVTWAHHSITEGDRKQAIHALGLTILLGLYFTLLQAM
Baar	GITPLDPFEVPLLNTAVLLASGVTVTWAHHSIMEGERKQAIHSLTLTILLGFYFTFLQAM
Moar	GITTLDPFEVPLLNTAVLLASGVTVTWAHHSIMEGERKQAIQSLALTILLGFYFTFLQAM
Toja	GITPLDPFEVPLLNTAVLLASGVTVTWAHHSIMETKRKEAIQSLALTILLGAYFTYLQAT
Chau	GITTLDPFEVPLLNTAVLLASGVTVTWAHHAIMEGSRKDAIQGLLVTILLGFYFTALQAM
Chse	GITTLDPFEVPLLNTAVLLASGVTVTWAHHSIMEGQRSQAIQSLLLTILLGFYFTFLQAM
Enar	GILTLDPFEVPLLNTAVLLASGVTVTWAHHSIMEGKRKQAIHSLTLTILLGFYFTFLQAL
Hpty	GITPLDPFEVPLLNTAVLLASGVTVTWAHHSIMEGERKQAIQSLTLTILLGFYFTFLQAM
Nana	GVTPLDPFEVPLLNTAVLLASGVTVTWAHHSIMEGKRNQVIQALTLTILLGLYFTALQAM
Mcst	GITTLDPFEVPLLNTAVLLASGVTVTWAHHSIMEGERKQAIQSLTLTILLGFYFTFLQAM
Rhox	GIVTLDPFEVPLLNTAVLLASGVTVTWAHHSIMEGERKQAIHSLTLTILLGFYFTFLQAL
Opfa	GITTLDPFEVPLLNTAVLLASGVTVTWAHHSIMEGERKQAIQSLTLTILLGFYFTFLQAM
Paar	GITTLDPFEVPLLNTAVLLASGVTVTWAHHSIMEGRRKEAIQSLGLTILLGFYFTFLQAM
Gozo	GITPLDPFEVPLLNTAVLLASGVTVTWAHHSIMEGNRKQAIQSLALTILLGFYFTFLQGM
Ackr	GITPLDPFEVPLLNTAVLLASGVTVTWAHHSIMEGEREQANQALLLTILLGFYFTFLQAM
Elev	GIITLDPFEVPLLNTAVLLASGVTVTWAHHSIMEAQRKQAIQSLTLTILLGFYFTALQAM
Trdu	GVTPLDPFEVPLLNTAVLLASGVTVTWAHHSIMEGHRKEAIQSLTLTILLGFYFTFLQAM
Amoc	GITTLDPFEVPLLNTAVLLASGVTVTWAHHSIMEGLRKQAIQSLALTILLGFYFTFLQGM
Hame	GITTLDPFEVPLLNTAVLLASGVTVTWAHHSIMEGERKQAIQSLTLTILLGFYFTFLQGM
Chso	GLITLDPFEVPLLNTAVLLASGVTVTWAHHSIMEGERKQAIQSLTLTILLGFYFTFLQGM
Lyto	GITALDPFEVPLLNTAVLLASGVTVTWAHHSIMEGARKQAIQSLTLTILLGFYFTFLQAM

[3/5 of aligned sequences]

```
Encr   GITTLDPPFEVPLLNTAVLLASGVTVTWAHHSIMEGERKQAIQSLTLTILLGFYFTFLQGM
Bvar   GITTLDPFEVPLLNTAVLLASGVTVTWAHHSIMEGERKQAIHSLTLTILLGFYFTFLQAL
Noco   GIFTLDPLEVPLLNTAVLLASGVTVTWAHHSIMEGDRKQAIHSLTMTIILGFYFTILQAL
Chsp   GIFPLDPFEVPLLNTAVLLASGVTVTWAHHSILENKRKQTIQALALTILLGFYFTFLQAM
Arja   GITTLDPFEVPLLNTAVLLASGVTVTWCHHSIMAGERKQTVQSLALTILLGFYFTFLQGL
Pase   GITPLDPFEVPLLNTAVLLASGVTVTWAHHSIMEGNRKESIESLTLTILLGFYFTFLQGM
Trel   GVTALDPFEVPLLNTAVLLASGVTVTWAHHSIMEGNRSGAIQALTLTILLGFYFTFLQGM
Lifa   GITTLDPFEVPLLNTAVLLASGVTVTWAHHSIMEGERKQAIHSLTLTILLGFYFTLLQAL
Acur   GITTLDPFEVPLLNTAVLLASGVTVTWAHHSIMEGHRKEAIQSLSLTILLGFYFTFLQGM
Ampe   GITTLDPFEVPLLNTAVLLASGVTVTWAHHSIMEGERKQAIQSLTLTILLGFYFTFLQGL
Urja   GVTPLDPFEVPLLNTAVLLASGVTVTWAHHSIMEGKRKEATQSLFLTIALGLYFTSLQAM
Enet   GITTLNPFEVPLLNTAVLLASGVTVTWAHHSIMEGNRKQAIQSLTLTILLGFYFTFLQGL
Ptbr   GITTLDPFEVPLLNTAVLLASGVTVTWAHHSIMEGNRQQAIQSLFLTILLGFYFTGLQGL
Safa   GITALDPFEVPLLNTAVLLASGVTVTWAHHSIMEGNRKQAIQSLLLTILLGFYFTALQGL
Icae   GITTLDPFEVPLLNTAVLLASGVTVTWAHHSIMEGTRKQAVQSLALTILLGFYFTFLQAL
Asmi   GIMTLNPFEVPLLNTAVLLASGVTVTWAHHSIMEGNRRQTIQALTLTILLGFYFTFLQAL
Foal   GVTPLDPFGVPLLNTAVLLASGATITWSHHSIIEGNRPPAIQALAITIFLGFYFTFLQAV
Drze   GVNVLDPFGVPLLNTAVLLASGATITWCHHSIMEGERTSAIQSLTLTILLGFYFTLLQAI
Rhas   GITTLDPFSVPLLNTAVLLASGVTVTWAHHSIMEGERKQAIHSLGLTILLGFYFTFLQGM
Elac   GITPLDPFEVPLLNTAVLLASGVTVTWAHHSIMEGERKQAIHSLTLTILLGFYFTFLQAM
Kugu   GITTLDPFEVPLLNTAVLLASGVTVTWAHHSIMEGERKQAIQSLILTILLGFYFTFLQAM
Plor   GVTTLDPFEVPLLNTAVLLASGVTVTWAHHSIMEGERKQAIHSLTLTILLGFYFTILQAM
Sgun   GITTLDPFEVPLLNTAVLLASGVTVTWAHHSIMEGERKQAIQSLFLTILLGFYFTFLQGM
Zaco   GITTLDPFEVPLLNTAVLLASGVTVTWAHHSIMEGERKQAIQSLLLTILLGFYFTFLQGM
Zbfl   GITTLDPFEVPLLNTAVLLASGVTVTWAHHSIMEGERKQAIQSLLLTILLGFYFTFLQAM
Spba   GIVPLDPFEVPLLNTAVLLASGVTVTWAHHSIMETKRKQAIHSLALTILLGGYFTMLQAL
Game   GITTLDPFEVPLLNTAVLLASGVTVTWAHHSIMEGNRKETVQSLALTILLGFYFTFLQAL
Thth   GLTTLDPFEVPLLNTAVLLASGVTVTWAHHSIMEGNRKEAIQSLALTILLGFYFTFLQAL
Xigl   GITALDPFEVPLLNTAVLLASGVTVTWAHHSIMEAKRKQAIQSLALTILLGAYFTFLQAM
Hyja   GITPLDPFEVPLLNTAVLLASGVTVTWAHHSIMEGARKQTIQSLTLTILLGFYFTFLQAM
Psan   GITTLDPFEVPLLNTAVLLASGVTVTWAHHSIMEGSRQQAAQSLTLTILLGFYFTVLQAM
Cupa   GITTLDPFEVPLLNTAVLLASGVTVTWAHHSIMEGNRKEAIQSLALTILLGFYFTFLQAM
Mpch   GILPLDPFEVPLLNTAVLLASGVTVTWAHHSIMEGERKQAIQSLTLTILLGMYFTFLQAM
Char   GITPLDPFEVPLLNTAVLLASGVTVTWAHHSIMAGERKQAIQSLALTILLGFYFTLLQAM
Pser   GITALDPFEVPLLNTAVLLASGVTVTWAHHSIMEGKRKQAIQSLALTILLGGYFTFLQGM
Prol   GITPLDPFEVPLLNTAVLLASGVTVTWAHHSIMEGKRKQAIHSLFLTILLGGYFTFLQAL
Plbi   GITPLDPFEVPLLNTAVLLASGVTVTWAHHSIMEGKRKQAIQSLALTILLGGYFTFLQGL
Calu   GINALNPFEVPLLNTAVLLASGVTVTWAHHSIMQGKREQAVQSLALTIFLGVYFSFLQGL
Papa   GVTPLDPFEVPLLNTAVLLASGVTVTWAHHSIMEGRRKQAIQALALTILLGLYFTFLQAM
Sufr   GITPLDPFEVPLLNTAVLLASGVTVTWAHHSIMEGGRNQAIPSLLLTILLGFYFTFLQAM
Stci   GITPLNPFEVPLLNTAVLLASGVTVTWAHHSIMEGERKQAIQSLLLTILLGFYFTFLQGL
Taru   GIIPLNPFEVPLLNTAVLLASGVTVTWAHHSIMEGERKQAIQSLTLTILLGFYFTFLQAM
Rala   GITPLNPFEVPLLNTAVLLASGVTVTWAHHSIMEGERKQAIQSLFLTILLGFYFTFLQGM
         :   *:*: **::**. ** ** : ** *: :    :     .* :*  ** :*: **
```

To be continued on page 20.

[4/5 of aligned sequences]

F

Scca	EYYEAPFTIADGVYGTTFFVATGFHGLHVIIGSTFLAVCLLRQVLYHFTSEHHFGFEAAA
Muma	EYYEAPFTIADGVYGTTFYVATGFHGLHVIIGSTFLAVCLLRQVQYHFTSEHHFGFEAAA
Erca	EYYEAPFTIADGVYGTTFFVATGFHGLHVIIGSTFLVVCLLRQIMYHFTSSHHFGFEAAA
Pose	EYYEAPFTIADGVYGTTFFVATGFHGLHVIIGSTFLMVCLLRQILYHFTSSHHFGFEAAA
Actr	EYYEAPFTIADGVYGSTFFVATGFHGLHVIIGSTFLAICLLRQIQYHFTSEHHFGFEAAA
Scal	EYYEAPFTIADGVYGSTFFVATGFHGLHVIIGSTFLAVCLLRQIQYHFTSEHHFGFEAAA
Posp	EYYEAPFTIADGVYGSTFFVATGFHGLHVIIGSTFLAVCLLRQIQYHFTSEHHFGFEAAA
Atsp	EYYEAPFTIADGVYGSTFFVATGFHGLHVIIGSLFLTVCLLRQIQYHFTPEHHFGFEAAA
Leoc	EYYEAPFTIADGVYGSTFFVATGFHGLHVIIGSLFLLVCLLRQIQYHFTPEHHFGFEAAA
Amca	EYYEAPFTIADGVYGATFFVATGFHGLHVIIGTTFLAVCLLRQIKFHFTSNHHFGFEAAA
Osbi	EYYEAPFTIADGVYGSTFFVATGFHGLHVIIGTTFLAICLLRQIKYHFTSQHHFGFEAAA
Pabu	EYYEAPFTIADGVYGSTFFVATGFHGLHVIIGTSFLAICLMRQIKYHFTSEHHFGFEAAA
Hial	EYYEAPFTIADGVYGSTFFVATGFHGLHVIIGTTFLAVCLLRQIQYHFTSEHHFGFEAAA
Elha	EYYEAPFTIADGVYGSTFFVATGFHGLHVIVGSTFLAVCLLRQIQYHFTSQHHFGFEAAA
Mlcy	EYYEAPFTIADGVYGSTFFVATGFHGLHVIIGSTFLAICLLRQVQYHFTSQHHFGFEAAA
Algl	EYYEAPFTIADGVYGSTFFVATGFHGLHVIIGSTFLAVCFLRQIQHHFTSEHHFGFEAAA
Ptgi	EYYEAPFTIADGVYGSTFFVATGFHGLHVIIGSTFLAVCFLRQVQYHFTSEHHFGFEAAA
Alaf	EYYEAPFTIADGVYGATFFVATGFHGLHVIIGSTFLAVCLLRQVKYHFTSEHHFGFEAAA
Nock	EYYEAPFTIADGVYGSTFFVATGFHGLHVIIGSTFLAVCLLRQVKYHFTSEHHFGFEAAA
Anja	EYYEAPFTIADGVYGSTFFVATGFHGLHVIIGSTFLAVCLLRQIKYHFTSEHHFGFEAAA
Gyki	EYYEAPFTIADGVYGSTFFVATGFHGLHVIIGSTFLAVCLLRQVKYHFTSEHHFGFEAAA
Syka	EYYEAPFTIADGVYGSTFFVATGFHGLHVIIGSTFLAVCLLRQVKYHFTSQHHFGFEAAA
Opma	EYYEAPFTIADGVYGSTFFVATGFHGLHVIIGSTFLAVCLLRQVKFHFTSQHHFGFEAAA
Comy	EYYEAPFTIADGVYGSTFFVATGFHGLHVIIGSTFLAVCLMRQVKYHFTSEHHFGFEAAA
Sasp	EYYEAPFTIADGVYGSTFFVATGFHGLHVIIGSVFLTVCLLRQIKYHFTSQHHFGFEAAA
Eupe	EYYEAPFTIADGVYGATFFVATGFHGLHVIIGSTFLAVCFLRQIKYHFTSQHHFGFEAAA
Enja	EYYEAPFTIADGVYGSTFFVATGFHGLHVIIGSTFLAVCLLRQVLYHFTSNHHFGFEAAA
Same	EYYEAPFTIADGVYGSTFFVATGFHGLHVIIGSTFLAICLLRQVLYHFTSNHHFGFEAAA
Chch	EYYEAPFTIADGVYGSTFFVATGFHGLHVIIGSTFLAICLIRQVQYHFTSEHHFGFEAAA
Grgr	EYYEAPFTIADGVYGSTFFVATGFHGLHVIIGSTFLGICLLRQIRYHFTSEHHFGFEAAA
Caau	EYYEAPFTIADGVYGSTFFVATGFHGLHVIIGSTFLAVCLLRQIQYHFTSEHHFGFEAAA
Cyca	EYYEAPFTIADGVYGSTFFVATGFHGLHVIIGSTFLAVCLLRQIQYHFTSEHHFGFEAAA
Dare	EYYEAPFTIADGVYGSTFFVATGFHGLHVIIGSTFLAVCLLRQVLFHFTSDHHFGFEAAA
Cost	EYYEAPFTIADGVYGSTFFVATGFHGLHVIIGSSFLAVCLLRQIQYHFTSEHHFGFEAAA
Leec	EYYEAPFTIADGVYGSTFFVATGFHGLHVIIGSSFLAVCLLRQIQYHFTSEHHFGFEAAA
Fola	EYYEAPFTIADGVYGSTFFVATGFHGLHVIIGSSFLAVCLLRQIQYHFTSEHHFGFEAAA
Clmc	EYYEAPFTIADGVYGSTFFVATGFHGLHVIIGSTFLAICLLRQIQYHFTSEHHFGFEAAA
Phin	EYYEAPFTIADGVYGSTFFVATGFHGLHVIIGSTFLAICLLRQIQYHFTSQHHFGFEAAA
Icpu	EYYEAPFTIADGVYGSTFFVATGFHGLHVIIGSTFLAICLLRQIQYHFTSEHHFGFEAAA
Psto	EYYEAPFTIADGVYGSTFFVATGFHGLHVIIGTTFLAIGLLRQIQYHFTSEHHFGFEAAA
Cora	EYYEAPFTIADGVYGSTFFVATGFHGLHVIIGSTFLAICLLRQLQYHFTSEHHFGFEAAA
Eisp	EYYEAPFTIADGIYGSTFFVATGFHGLHVIIGSSFLAICLLRQIQYHFTSEHHFGFEAAA
Apal	EYYEAPFTIADGAYGSTFFVSTGFHGLHVIIGSSFLAVCLLRQIKHHFTSEHHFGFEAAA
Eslu	EYYEAPFTIADGVYGSTFFVATGFHGLHVIIGSTFLAVCLLRQIQYHFTSQHHFGFEAAA
Dape	EYYEAPFTIADGVYGSTFFVATGFHGLHVIIGSTFLAVCLLRQIQYHFTSQHHFGFEAAA
Glse	EYYEAPFTIADGVYGSTFFVATGFHGLHVIIGSTFLAVCLLRQIQYHFTSEHHFGFEAAA
Naar	EYYEAPFTIADGVYGATFFVATGFHGLHVIIGSTFLAVCLLRQAQYHFTSEHHFGFEAAA
Lioc	EYYEAPFTIADGVYGSTFFVATGFHGLHVIIGSTFLAVCLLRQAQYHFTSEHHFGFEAAA
Opso	EYYEAPFTIADGVYGSTFFVATGFHGLHVIIGSTFLAICLLRQIQYHFTSQHHFGFEAAA
Alte	EYYEAPFTIADGVYGSTFFVATGFHGLHVIIGSTFLTVCLLRQIQYHFTSEHHFGFEAAA
Plap	EYYEAPFTIADGVYGSTFFVATGFHGLHVIIGSTFLAVCLLRQIQYHFTSEHHFGFEAAA

16

To be continued on page 21.

[4/5 of aligned sequences]

```
Plal  EYYEAPFTIADGVYGSTFFVATGFHGLHVIIGSTFLAICLLRQIQYHFTSEHHFGFEAAA
Sami  EYYEAPFTIADGVYGSTFFVATGFHGLHVLIGSTFLAVCLLRQIHYHFTSEHHFGFEAAA
Rere  EYYEAPFTIADGVYGSTFFVATGFHGLHVFIGSTFLAVCLLRQVQYHFTSGHHFGFEAAA
Gama  EYYDAPFTIADGVYGSTFFVATGFHGLHVIIGSTFLAVCLLRQIQYHFTSEHHFGFEAAA
Onmy  EYYEAPFTIADGVYGSTFFVATGFHGLHVIIGSTFLAVCLLRQVQYHFTSEHHFGFEAAA
Sasa  EYYEAPFTIADGVYGSTFFVATGFHGLHVIIGSTFLAICLLRQIQYHFTSEHHFGFEAAA
Cola  EYYEAPFTIADGVYGSTFFVATGFHGLHVIIGSTFLAVCLLRQIQYHFTSEHHFGFEAAA
Dita  EYYEAPFTIADGVYGSTFFVATGFHGLHVIIGSTFLAVCLLRQVQFHFTSGHHFGFKAAA
Gogr  EYDEAPFTMADSVYGATFFVATGFHGLHVIIGSLFLAVCLLRLTHFHFTPKHHFGFEAAA
Chsl  EYYEAPFSIADGVYGATFFVATGFHGLHVIIGSTFLAVCLLRQIQFHFTAEHHFGFEAAA
Atja  EYYEAPFTIADGVYGSTFFVATGFHGLHVIIGSTFLAVCLLRQIHYHFTSEHHFGFEAAA
Iido  EYYEAPFTIADGVYGSTFFVATGFHGLHVIIGSTFLAVCLLRQIHYHFTSEHHFGFEAAA
Auja  EYYEAPFTIADGVYGSTFFVATGFHGLHVIIGSTFLAICLLRQVQYHFTSKHHFGFEAAA
Chag  EYYEAPFTIADGVYGSTFFVATGFHGLHVIIGSTFLAICLLRQIQYHFTSQHHFGFEAAA
Hami  EYYEAPFTIADGVYGSTFFVATGFHGLHVIVGTTFLAVCLLRQVFYHFTSKHHFGFEAAA
Saun  EYYEAPFTIADGVYGSTFFVATGFHGLHVIIGSTFLAVCLLRQIFYHFTSKHHFGFEAAA
Nema  EYYEAPFTIADGVYGSTFFVATGFHGLHVIIGSTFLAICLLRQIQYHFTSEHHFGFEAAA
Disp  EYYEAPFTIADGVYGSTFFVATGFHGLHVIIGSTFLAVCLLRQIQYHFTSEHHFGFEAAA
Myaf  EYYEAPFTIADGVYGSTFFVATGFHGLHVIIGSTFLAVCLLRLIQHHFTSQHHFGFEAAA
Lagu  EYYEAPFTIADGVYGSTFFVATGFHGLHVIIGTSFLAICLLRQVQYHFTSDHHFGFEAAA
Trtr  EYYEAPFTIADGVYGATFFVATGFHGLHVIIGTTFLAVCLLRQIKYHFTSQHHFGFEAAA
Zucr  EYYEAPFTIADGVYGATFFVATGFHGLHVIIGTTFLGVCLIRQIKYHFTSQHHFGFEAAA
Pxja  EYYEAPFTIADGVYGSTFFVATGFHGLHVIIGSTFLAVCLIRQAQYHFTSEHHFGFEAAA
Pxlo  EYYEAPFTIADGVYGSTFFVATGFHGLHVIIGSTFLAVCLMRQAQYHFTSEHHFGFEAAA
Pctr  EYYEAPFTIADGVYGSTFFVATGFHGLHVIIGSTFLAICLLRQIKYHFTSQHHFGFEAAA
Apsa  EYYEAPFTIADGVYGSTFFVATGFHGLHVIIGSTFLLVCLLRQIKYHFTSEHHFGFEAAA
Cabe  EYYEAPFTIADGVYGSTFFVATGFHGLHVIIGSTFLAVCLIRQVMHHFTSEHHFGFEAAA
Bzze  EYYEAPFTIADGVYGSTFFVATGFHGLHVIIGSIFLAVCLLRQIQYHFTSEHHFGFEAAA
Siim  EYYEAPFTIAESVYGSTFFVATGFHGLHVIIGSAFLTVCILRQINHHFTSEHHFGFEAAA
Ctru  EYYEAPFTIADGVYGATFFVATGFHGLHVIIGSTFLAACLLRQIQYHFTAEHHFGFEAAA
Dpbr  EYYEAPFTIADGVYGATFFVATGFHGLHVIIGSTFLAACLLRQIQYHFTAEHHFGFEAAA
Caki  EYYDAPFTIADSVYGSTFFVATGFHGLHVIIGSTFLIVCLLRQINYQFTSEHHFGFEAAA
Phja  EYYEAPFTIADSVYGSAFFVATGFHGLHVIIGSTFLAVCFLRHIFHQFTSQHHFGFEAAA
Brsp  EYIEAPFTIADGIYGSTFFVATGFHGLHVIIGSTFLLVCLIRQVQFHFTADHHFGFEAAA
Gamo  EYYDAPFTIADGVYGSTFFVATGFHGLHVIIGSTFLAVCLLRQIRYHFTSEHHFGFEAAA
Lolo  EYYEAPFTIADGVYGSTFFVATGFHGLHVIIGSIFLAVCLLRQIRYHFTSEHHFGFEAAA
Batr  EYYLAPFTIADSIYGSTFFVATGFHGLHVIIGTAFLIVCLLRQFHFHFTTTHHFGFEAAA
Prmy  EYYEAPFTIADGVYGSTFFVATGFHGLHVIIGSTFLLVCLLRLVKYHFTTTHHFGFEAAA
Lose  EYYEAPFTLADSVYGSTFFIATGFHGLHVIIGTTFLAVTLLRQVFHHFTSSHHFGFEAAA
Loam  EYYEAPFTLADGAYGSTFFVATGFHGLHVIIGSIFLTVCLIRQVRHHFTLEHHFGFEAAA
Chab  EYYEAPFTIADGVYGATFFVATGFHGLHVIIGSTFLAVCLLRQIQFHFTSEHHFGFEAAA
Chto  EYYEAPFTIADGVYGATFFVATGFHGLHVIIGSTFLAVCLLRQIQFHFTSEHHFGFEAAA
Majo  EYYEAPFTIADGVYGATFFVATGFHGLHVIIGSTFLAVCLLRQAQHHFTSEHHFGFEAAA
Hlst  EYYEAPFTIADGVYGATFFVATGFHGLHVIIGSTFLAVCLMRQVQYHFTSDHHFGFEAAA
Clpe  EYYEAPFTIADGVYGSTFFIATGFHGLHVIIGTLFLAICLLRQIQHHFTSGHHFGFEAAA
Mlmr  EYYEAPFTIADGVYGSTFFVATGFHGLHVIIGSTFLAVCLLRLIQHHFTSEHHFGFEAAA
Crcr  EYYEAPFTIADGVYGSTFFVATGFHGLHVIIGSTFLAVCLFRQIRYHFTSQHHFGFEAAA
Muce  EYYEAPFTIADGVYGSTFFVATGFHGLHVIIGSTFLAVCLFRQIRYHFTSQHHFGFEAAA
Bege  EYYEAPFTIADGVYGSTFFVATGFHGLHVIIGSTFLAVCLLRQVQYHFTSEHHFGFEAAA
Mela  EYYEAPFTIADGVYGSTFFVATGFHGLHVIIGSTFLAVCLCRQIQYHFTSQHHFGFEAAA
Hats  EYYEAPFTIADGVYGSTFFVATGFHGLHVIIGSSFLAICLLRQIQYHFTSEHHFGFEAAA
Orla  EYFEAPFTIADGVYGSTFFVATGFHGLHVIIGSTFLAVCLLRQVQFHFTSEHHFGFEAAA
```

To be continued on page 22.

[4/5 of aligned sequences]

To be continued on page 23.

Cosa EYYEAPFTIADGVYGSTFFVATGFHGLHVIIGSTFLAICLLRQALYHFTSEHHFGFEAAA
Exsp EYYEAPFTIADGVYGATFFVATGFHGLHVIIGSTFLAVCLLRQIQYHFTSEHHFGFEAAA
Depa EYYEAPFTIADGVYGSTFFVATGFHGLHVIIGSTFLAICLFRQIEFHFTSEHHFGFEAAA
Rima EYYEAPFTIADGVYGSTFFVATGFHGLHVIIGSTFLAVCLLRQINFHFTSNHHFGFEAAA
Fuol EYYEAPFTIADGVYGSTFFVATGFHGLHVIIGSSFLAVCLLRQIQHHFTSQHHFGFEAAA
Gmaf EYYEAPFTIADGVYGSTFFVATGFHGLHVIIGSTFLAICLLRQALHHFTSNHHFGFEAAA
Xeei EYYEAPFTIADGVYGSTFFVATGFHGLHVIIGSTFLAICLLRQAQYHFTSEHHFGFEAAA
Pros EYYEAPFTIADGVYGATFFVATGFHGLHVIIGSTFLAVCLLRQIQYHFTSEHHFGFEAAA
Scmi EYYEAPFTIADGVYGATFFVATGFHGLHVIIGSTFLLVCLFRQIQYHFTSEHHFGFEAAA
Rolo EYYEAPFTIADGVYGSTFFVATGFHGLHVIIGSMFLAVCLLRQIQYHFTSEHHFGFEAAA
Cere EYYEAPFTIADGVYGSTFFVATGFHGLHVIIGSMFLAVCLLRQIQYHFTSQHHFGFEAAA
Daga EYYEAPFTIADGVYGSTFFVATGFHGLHVIIGSTFLAVCLIRQVKYHFTSNHHFGFEAAA
Anco EYYEAPFTIADGVYGSTFFVATGFHGLHVIIGSLFLAVCLIRQIFHHFTSEHHFGFEAAA
Dmve EYYEAPFTIADSVYGATFFVATGFHGLHVIIGSTFLAICLLRQVHYHFTSEHHFGFEAAA
Dmar EYYEAPFTIADSVYGATFFVATGFHGLHVIIGSTFLAVCLLRQVHYHFTSEHHFGFEAAA
Anka EYYEAPFTIADGVYGSTFFVATGFHGLHVIIGSTFLAVCLLRQMYHHFTSEHHFGFEAAA
Moja EYYEAPFTIADGVYGSTFFVATGFHGLHVIIGSTFLAVCLLRQIYHHFTSEHHFGFEAAA
Hoja EYYEAPFTIADGVYGSTFFVATGFHGLHVIIGSTFLAVCLLRQVYHHFTSEHHFGFEAAA
Bede EYYEAPFTIADGVYGSTFFVATGFHGLHVIVGSTFLAVCLIRQIQYHFTSEHHFGFEAAA
Besp EYYEAPFTIADGVYGSTFFVATGFHGLHVIIGSTFLAVCLIRQIQYHFTSEHHFGFEAAA
Mysp EYYEAPFTIADGVYGSTFFVATGFHGLHVIIGSTFLAVCLLRQIQYHFTSEHHFGFEAAA
Osja EYYEAPFTIADGVYGSTFFVATGFHGLHVIIGSTFLAICLLRQIQYHFTSEHHFGFEAAA
Sgro EYYEAPFTIADGVYGSTFFVATGFHGLHVIIGSTFLAVCLLRQVQYHFTSEHHFGFEAAA
Pzpa EYYEAPFTIADGVYGSTFFVATGFHGLHVIIGSTFLAVCLLRQILYHFTSDHHFGFEAAA
Zeja EYYEAPFTIADGVYGTTFFVATGFHGLHVIIGSTFLAVCLLRQAQFHFTSDHHFGFEAAA
Znne EYYEAPFTIADGVYGSTFFVATGFHGLHVIIGSLFLAVCLLRQVLHHFTSDHHFGFEAAA
Zefa EYYEAPFTIADGVYGSTFFVATGFHGLHVIIGSTFLAICLIRQVQYHFTSDHHFGFEAAA
Acni EYYEAPFTIADGVYGATFFVATGFHGLHVIIGSTFLAVCLLRQVQFHFTSDHHFGFEAAA
Ncrh EYYEAPFTIADGVYGATFFVATGFHGLHVIIGSTFLAVCLLRQIQFHFTSDHHFGFEAAA
Agca EYYEAPFTIADGVYGSTFFVATGFHGLHVIIGSTFLAVCLLRQIHYHFTSGHHFGFEAAA
Hydy EYHEAPFTIADGVYGSSFFVATGFHGLHVIIGSSFLAVCLLRQVRHHFTAEHHFGFEAAA
Gsac EYYEAPFTIADGVYGSSFFVATGFHGLHVIIGSSFLAVCFLRQIRHHFTAEHHFGFEAAA
Pevo EYYEAPFSIADGVYGSTFFVATGFHGLHVIIGSTFLAVCLIRQIYHHFTSDHHFGFEAAA
Hiku EYYEAPFTIADGIYGSTFFVATGFHGLHVIIGSTFLAICLIRQIQYHFTSQHHFGFEAAA
Inpa EYYEAPFTIADGIYGSTFFVATGFHGLHVIIGSTFLIVCLLRQVHHHFTSQHHFGFEAAA
Auch EYHEAPFTIADGVYGSTFFVATGFHGLHVIIGSTFLAVCLYRQIKYHFTSQHHFGFEAAA
Fico EYYEAPFTIADGVYGSTFFVATGFHGLHVIIGSTFLAVCLLRQIHYHFTSEHHFGFEAAA
Macs EYYEAPFTIADGVYGATFFVATGFHGLHVIIGSTFLAICLLRQIRYHFTSEHHFGFEAAA
Moal EYYEAPFTLADGVYGCTFFVATGFHGLHVIIGTTFLMVCLLRHTHYHFTSNHHFGFEAAA
Syma EYYEAPFTIADGIYGSTFFVATGFHGLHVIIGSTFLTVCLLRQIQHHFTSEHHFGFEAAA
Mafr EYYEAPFTIADGVYGSTFFVATGFHGLHVIIGSTFLTVCLLRQIRYHFTSEHHFGFEAAA
Dcpe EYYEAPFTIADGVYGSTFFVATGFHGLHVIIGSAFLAVCLLRQILFHFTSEHHFGFEAAA
Dcti EYYEAPFTIADGVYGSTFFVATGFHGLHVIIGSAFLAVCLLRQILFHFTSEHHFGFEAAA
Hehi EYYEAPFTIADGVYGSTFFVATGFHGLHVIIGSTFLAVCLLRQIQYHFTSEHHFGFEAAA
Stam EYYEAPFTIADGVYGATFFVATGFHGLHVIIGSTFLAVCLLRQIQYHFTSEHHFGFEAAA
Hogi EYYEAPFTIADGVYGSTFFVATGFHGLHVIIGSTFLAVCLLRQALYHFTSEHHFGFEAAA
Erzo EYYEAPFTIADGVYGSTFFVATGFHGLHVIIGSTFLAVCLYRQIRYHFTSEHHFGFEAAA
Hxot EYYEAPFTIADGVYGSTFFVATGFHGLHVIMGSTFLAVCLIRQILHHFTSEHHFGFEAAA
Core EYYEAPFTIADGVYGSTFFVATGFHGLHVIIGSTFLAVCLIRQILHHFTSEHHFGFEAAA
Apve EYYEAPFTIADGVYGATFFVATGFHGLHVIIGSTFLTICLIRQILHHFTSEHHFGFEAAA
Latj EYNEAPFTIADGVYGCTFFVATGFHGLHVLIGSTFLAVCLIRQILHHFTSEHHFGFEAAA
Laja EYYEAPFTIADGVYGSTFFVATGFHGLHVIIGSTFLAICLLRQIQYHFTSEHHFGFEAAA

[4/5 of aligned sequences]

To be continued on page 24.

```
Syja  EYYEAPFTIADGVYGSTFFVATGFHGLHVIIGSTFLAVCLLRQIQYHFTSDHHFGFEAAA
Epme  EYYEAPFTIADGVYGSTFFVATGFHGLHVIIGSTFLAVCLLRQIQYHFTSEHHFGFEAAA
Grse  EYYEAPFTIADGVYGSTFFVATGFHGLHVIIGSTFLAICLLRQVQYHFTSEHHFGFEAAA
Clja  EYYEAPFTIADGVYGSTFFVATGFHGLHVIIGSSFLAVCLLRQAKFHFTSEHHFGFEAAA
Ogcy  EYFEAPFTIADGVYGSSFFVATGFHGLHVIIGSTFLAVCLMRQTQYHFTSEHHFGFEAAA
Plna  EYYEAPFTIADGVYGSTFFVATGFHGLHVIIGSTFLAVCLIRQAKYHFTSKHHFGFEAAA
Lema  EYYEAPFTIADGVYGSTFFVATGFHGLHVIIGSTFLGICLLRQIKYHFTSEHHFGFEAAA
Etzo  EYYEAPFTIADGVYGATFFVATGFHGLHVIIGSTFLAICLLRQVQYHFTSEHHFGFEAAA
Apse  EYYEAPFTIADGVYGSTFFVATGFHGLHVIIGSTFLAVCLLRQVKYHFTTQHHFGFEAAA
Epde  EYYEAPFTIADGVYGSTFFVATGFHGLHVIIGSTFLAVCLLRQIQYHFTSEHHFGFEAAA
Slja  EYYEAPFTIADGVYGSTFFVATGFHGLHVIIGSTFLAICLLRQINYHFTSEHHFGFEAAA
Bsja  EYYEAPFTIADGVYGSTFFVATGFHGLHVIIGSTFLAVCFVRHLKYHFTTGHHFGFEAAA
Ecna  EYYEAPFTIADGVYGATFFVATGFHGLHVIIGSTFLAVCLHRQIRHHFTSTHHFGFEAAA
Cohi  EYNEAPFTIADGVYGATFFVATGFHGLHVIIGTTFLIICLLRQINYHFTSKHHFGFEAAA
Caar  EYYEAPFTIADGVYGSTFFVATGFHGLHVIIGSTFLAVCFLRQVRHHFTSDHHFGFEAAA
Came  EYYEAPFTIADGVYGSTFFVATGFHGLHVIIGSTFLAVCFLRQLRHHFTSDHHFGFEAAA
Mema  EYNEAPFTIADGVYGATFFVATGFHGLHVLIGSTFLAVCLLRQAQHHFTSDHHFGFEAAA
Lenu  EYYEAPFTIADGVYGSTFFVATGFHGLHVIIGSTFLAVCLLRQVNYHFTQQHHFGFEAAA
Brja  EYYEAPFTIADGVYGSTFFVATGFHGLHVIIGSTFLAVCLLRQIRYHFTSDHHFGFEAAA
Plma  EYYEAPFTIADGVYGSTFFVATGFHGLHVIIGSTFLAVCLLRQIRYHFTSDHHFGFEAAA
Emst  EYYEAPFTIADGVYGSTFFVATGFHGLHVIIGSTFLAICLLRQVQYHFTSEHHFGFEAAA
Ptti  EYYEAPFTLADGVYGSTFFVATGFHGLHVIIGSSFLAVCLLRQVQYHFTSEHHFGFEAAA
Losu  EYHEAPFTIADGVYGSTFFVATGFHGLHVIIGSSFLAICLMRQIAHHFTSGHHFGFEAAA
Geoy  EYYEAPFTIADGVYGSSFFVATGFHGLHVIIGSTFLAVCLLRQIKYHFTSEHHFGFEAAA
Dipi  EYYEAPFTIADGVYGATFFVATGFHGLHVIIGSTFLAVCLLRQIQYHFTSDHHFGFEAAA
Pama  EYFEAPFTIADGVYGSTFFVATGFHGLHVIIGSTFLAVCLLRQIQYHFTSEHHFGFEAAA
Leob  EYYEAPFTIADGVYGSTFFVATGFHGLHVIIGSTFLAVCLLRQIQYHFTSEHHFGFEAAA
Neba  EYYEAPFTIADGVYGSTFFVATGFHGLHVIIGSTFLAVCLLRQIKYHFTSSHHFGFEAAA
Pdpl  EYIEAPFTLADSVYGSTFFVATGFHGLHVIIGSAFLTVCLLRQVWFHFTTQHHFGFEAAA
Nimi  EYYEAPFTIADGVYGATFFVATGFHGLHVIIGSTFLAVCLLRQIQYHFTSEHHFGFEAAA
Uptr  EYYEAPFTIADGVYGSTFFVATGFHGLHVIIGSTFLAICLMRQILFHFTSEHHFGFEAAA
Pesc  EYYEAPFTIADGVYGSTFFVATGFHGLHVIIGTTFLAVCLLRQVQYHFTSEHHFGFEAAA
Baar  EYYEAPFTIADGVYGATFFVATGFHGLHVIIGSTFLAVCLLRQALYHFTSEHHFGFEAAA
Moar  EYYEAPFTIADGVYGSTFFVATGFHGLHVIIGSTFLAICLLRQVQYHFTSEHHFGFEAAA
Toja  EYQEASFTIADGVYGSTFFLATGFHGLHVLIGSTFLAVCLLRQIQYHFTSTHHFGFEAAA
Chau  EYYEAPFTIADGVYGSTFFVATGFHGLHVIIGSTFLAVCLLRQIQYHFTSQHHFGFEAAA
Chse  EYYEAPFTIADGVYGSTFFVATGFHGLHVIIGSTFLAVCLLRQVHYHFTSEHHFGFEAAA
Enar  EYYEAPFTIADGVYGSTFFVATGFHGLHVIIGSTFLAVCLLRQVEYHFTSEHHFGFEAAA
Hpty  EYYEAPFTIADGVYGSTFFVATGFHGLHVIIGSTFLAICLLRQIQYHFTSEHHFGFEAAA
Nana  EYYEAPFTIADAVYGSTFFVATGFHGLHVIIGSSFLIVCLLRQISYHFTSDHHFGFEAAA
Mcst  EYYEAPFTIADGVYGSTFFVATGFHGLHVIIGSTFLAICLLRQIQYHFTSEHHFGFEAAA
Rhox  EYYEAPFTIADGVYGSTFFVATGFHGLHVIIGSTFLAVCLLRQVKYHFTSDHHFGFEAAA
Opfa  EYYEAPFTIADGVYGSTFFVATGFHGLHVIIGSTFLAICLLRQVQYHFTSEHHFGFEAAA
Paar  EYYEAPFTIADGVYGSTFFVATGFHGLHVIIGSTFLAVCLLRQVLYHFTSQHHFGFEAAA
Gozo  EYYEAPFTIADGVYGSTFFVATGFHGLHVIIGSTFLAVCLLRQIQYHFTSDHHFGFEAAA
Ackr  EYYEAPFTIADSVYGATFFVATGFHGLHVIIGSTFLAVCLLRQIHFHFTSEHHFGFEAAA
Elev  EYYEAPFTIADGVYGSTFFVATGFHGLHVIIGSTFLAVCLLRQIKFHFTSDHHFGFEAAA
Trdu  EYYEAPFTIADGVYGSTFFVATGFHGLHVIIGSTFLAICLLRQVLYHFTSEHHFGFEAAA
Amoc  EYYEAPFTIADGVYGSTFFVATGFHGLHVIIGSTFLAVCLLRQVQYHFTSEHHFGFEAAA
Hame  EYYEAPFTIADGVYGSTFFVATGFHGLHVIIGSTFLAVCLLRQVNYHFTSTHHFGFEAAA
Chso  EYYEAPFTIADGVYGSTFFVATGFHGLHVIIGSTFLAVCLLRQVHYHFTSEHHFGFEAAA
Lyto  EYYEAPFTIADGVYGSTFFVATGFHGLHVIIGSSFLAVCLLRQIRYHFTAEHHFGFEAAA
```

[4/5 of aligned sequences]

```
Encr   EYYEAPFTIADGVYGSTFFVATGFHGLHVIIGSSFLAVCLLRQIRYHFTSEHHFGFEAAA
Bvar   EYYEAPFTIADGVYGATFFVATGFHGLHVIIGSTFLAVCLLRQVKYHFTAQHHFGFEAAA
Noco   EYVDAPFTIGDGVYGATFFVATGFHGLHVIIGSTFLAVCLLRQIKHHFTSGHHFGFEAAA
Chsp   EYYEAPFTIADSVYGATFFVATGFHGLHVIIGSTFLAVCFLRQIKFHFTSDHHFGFEAAA
Arja   EYYEAPFTIADGVYGSTFFVATGFHGLHVIIGSTFLAVCLVRQILHHFTSDHHFGFEAAA
Pase   EYYEAPFTLADGVYGSTFFVATGFHGLHVIIGSTFLAVCLLRQLQHHFTSGHHFGFEAAA
Trel   EYKETPFTIADGVYGSTFFMATGFHGLHVIIGSLFLAVCLVRQALFHFTSDHHFGFEAAA
Lifa   EYYEAPFTIADGVYGSTFFVATGFHGLHVIIGSTFLAVCLLRQVQHHFTAEHHFGFEAAA
Acur   EYYEAPFTIADGVYGSTFFVATGFHGLHVIIGSTFLAVCLIRQIQHHFTSEHHFGFEAAA
Ampe   EYYEAPFTIADGVYGSTFFVATGFHGLHVIIGSTFLAVCLLRQVHYHFTSEHHFGFEAAA
Urja   EYYEAPFTIADGVYGSTFFVATGFHGLHVIIGFTFLSICLLRQIRHHFTSDHHFGFEAAA
Enet   EYYEAPFTIADGVYGSTFFVATGFHGLHVIIGSTFLAVCLLRQIQYHFTSEHHFGFEAAA
Ptbr   EYYEAPFTIADGVYGSTFFVATGFHGLHVIIGSSFLAVCLIRQLQFHFTSEHHFGFEAAA
Safa   EYYEAPFTIADGVYGSTFFVATGFHGLHVIIGSTFLAVCLLRQIQYHFTSEHHFGFEAAA
Icae   EYYEAPFTIADGVYGSTFFVATGFHGLHVIIGSTFLAVCLLRQIRYHFTSDHHFGFEAAA
Asmi   EYYEAPFTIADGVYGSTFFVATGFHGLHVIIGSTFLAVCLIRQTFYHFTSKHHFGFEAAA
Foal   EYYEAPFTIADGVYGSTFFVTTGFHGLHVIIGSVFLTVSLVRLCMYHFTTEHHFGLEAAA
Drze   EYYEAPFTIADGVYGSTFFVTTGFHGLHVIIGSTFLAICLLRLIQHHFTSDHHLGMEAAA
Rhas   EYYEAPFTIADGVYGSTFFVATGFHGLHVIIGSTFLAVCLLRQIQYHFTSEHHFGFEAAA
Elac   EYYEAPFTIADGVYGSTFFVATGFHGLHVIIGSTFLAVCLLRQVQYHFTSEHHFGFEAAA
Kugu   EYYEAPFTIADGVYGSTFFVATGFHGLHVIIGSTFLAVCLLRQTYYHFTSDHHFGFEAAA
Plor   EYYEAPFTIADGVYGSTFFVATGFHGLHVIIGSTFLAVCLLRQIRYHFTSEHHFGFEAAA
Sgun   EYYEAPFTIADGVYGSTFFVATGFHGLHVIIGSTFLAVCLLRQIQYHFTSEHHFGFEAAA
Zaco   EYYEAPFTIADGVYGSTFFVATGFHGLHVIIGSTFLAVCLLRQVQYHFTSEHHFGFEAAA
Zbfl   EYYEAPFTIADGVYGSTFFVATGFHGLHVIIGSTFLAVCLLRQIQYHFTSEHHFGFEAAA
Spba   EYMEAPFTIADGVYGATFFVATGFHGLHVIIGSTFLAVCLLRQVRHHFTSTHHFGFEAAA
Game   EYYEAPFTIADGVYGSTFFVATGFHGLHVIIGSTFLAICLLRQIRYHFTSDHHFGFEAAA
Thth   EYYEAPFTIADGVYGSTFFVATGFHGLHVIIGSTFLAVCLLRQIRYHFTSDHHFGFEAAA
Xigl   EYNEAPFTIADGVYGATFFVATGFHGLHVLIGSTFLAVCLLRQVRHHFTSNHHFGFEAAA
Hyja   EYYEAPFTIADGVYGSTFFVATGFHGLHVIIGSTFLAVCLLRQMQYHFTSDHHFGFEAAA
Psan   EYHEAPFTIADGVYGSTFFVATGFHGLHVIIGSTFLAVCLLRQLYYHFTSKHHFGFEAAA
Cupa   EYYEAPFTIADGVYGSTFFVATGFHGLHVIIGSTFLAVCLLRQIQYHFTSDHHFGFEAAA
Mpch   EYYEAPFTIADGVYGSTFFVATGFHGLHVIIGSSFLAVCLLRQVQYHFTSNHHFGFEAAA
Char   EYYEAPFTIADGVYGSTFFVATGFHGLHVIIGSTFLAVCLLRQIRYHFTSEHHFGFEAAA
Pser   EYHEASFTIADGVYGATFFVATGFHGLHVLIGSSFLAVCLLRQIRHHFTSDHHFGFEAAA
Prol   EYHEAPFTIADGVYGATFFVATGFHGLHVLIGSTFLAVCLLRQILHHFTANHHFGFEAAA
Plbi   EYHEAPFTIADGVYGATFFVATGFHGLHVLVGSSFLAVCLLRQILHHFTSDHHFGFEAAA
Calu   EYHEAPFTIADGVYGSTFFVATGFHGLHVLMGTTFLAVCLLRQAQYHFTTLHHFGFEAAA
Papa   EYYEAPFAISDGAYGATFFVATGFHGLHVIIGSTFLAVSLLRQVQHHFTSKHHFGFEAAA
Sufr   EYYEAPFTIADGVYGSTFFVATGFHGLHVIIGSTFLAVCLIRQIQYHFTSEHHFGFEAAA
Stci   EYYEAPFTIADGVYGSTFFVATGFHGLHVIIGSTFLAACLLRQTWYHFTSEHHFGFEAAA
Taru   EYYEAPFTIADGVYGSTFFVATGFHGLHVIIGSTFLAVCLLRQIRFHFTSEHHFGFEAAA
Rala   EYYEAPFTIADGVYGSTFFVATGFHGLHVIIGSTFLAVCLLRQVQYHFTSEHHFGFEAAA
       **  : *::.:. ** :*:::********::*  **   : *   .:**  **:*::***
```

To be continued on page 25.

[5/5 of aligned sequences]

```
                G
Scca     WYWHFVDVVWLFLYVSIYWWGS*
Muma     WYWHFVDVVWLFLYVSIYWWGS-
Erca     WYWHFVDVVWLLLYVSIYWWGS-
Pose     WYWHFVDVVWLFLYVSIYWWGS-
Actr     WYWHFVDVVWLFLYVSIYWWGS*
Scal     WYWHFVDVVWLFLYVSIYWWGS-
Posp     WYWHFVDVVWLFLYVSIYWWGS-
Atsp     WYWHFVDVVWLFLYVSIYWWGS-
Leoc     WYWHFVDVVWLFLYVSIYWWGS-
Amca     WYWHFVDVVWLFLYVSIYWWGS-
Osbi     WYWHFVDVVWLFLYVSIYWWGS-
Pabu     WYWHFVDVVWLFLYVSIYWWGS-
Hial     WYWHFVDVVWLFLYVSIYWWGS-
Elha     WYWHFVDVVWLFLYVSIYWWGS-
Mlcy     WYWHFVDVVWLFLYVSIYWWGS-
Algl     WYWHFVDVVWLFLYVSIYWWGS-
Ptgi     WYWHFVDVVWLFLYVSIYWWGS-
Alaf     WYWHFVDVVWLFLYVSIYWWGS-
Nock     WYWHFVDVVWLFLYVSIYWWGS-
Anja     WYWHFVDVVWLFLYVSIYWWGS-
Gyki     WYWHFVDVVWLFLYISIYWWGS-
Syka     WYWHFVDVVWLFLYVSIYWWGS-
Opma     WYWHFVDVVWLFLYVSIYWWGS-
Comy     WYWHFVDVVWLFLYVSIYWWGS-
Sasp     WYWHFVDVVWLFLYVSIYWWGS-
Eupe     WYWHFVDVVWLFLYISIYWWGS-
Enja     WYWHFVDVVWLFLYVSIYWWGS-
Same     WYWHFVDVVWLFLYVSIYWWGS-
Chch     WYWHFVDVVWLFLYVSIYWWGS-
Grgr     WYWHFVDVVWLFLYVSIYWWGS-
Caau     WYWHFVDVVWLFLYVSIYWWGS-
Cyca     WYWHFVDVVWLFLYVSIYWWGS-
Dare     WYWHFVDVVWLFLYVSIYWWGS-
Cost     WYWHFVDVVWLFLYVSIYWWGS-
Leec     WYWHFVDVVWLFLYVSIYWWGS-
Fola     WYWHFVDVVWLFLYVSIYWWGS-
Clmc     WYWHFVDVVWLFLYVSIYWWGS-
Phin     WYWHFVDVVWLFLYVSIYWWGS-
Icpu     WYWHFVDVVWLFLYVSIYWWGS-
Psto     WYWHFGECGWLFLYVSIYWWGS-
Cora     WYWHFVDVVWLFLYVSIYWWGS-
Eisp     WYWHFVDVVWLFLYVSIYWWGS*
Apal     WYWHFVDVVWLFLFVSIYWWGS-
Eslu     WYWHFVDVVWLFLYVSIYWWGS-
Dape     WYWHFVDVVWLFLYVSIYWWGS-
Glse     WYWHFVDVVWLFLYVSIYWWGS-
Naar     WYWHFVDVVWLFLYVSIYWWGS-
Lioc     WYWHFVDVVWLFLYVSIYWWGS-
Opso     WYWHFVDVVWLFLYVSIYWWGS-
Alte     WYWHFVDVVWLFLYVSIYWWGS-
Plap     WYWHFVDVVWLFLYVSIYWWGS-
```

[5/5 of aligned sequences]

```
Plal    WYWHFVDVVWLFLYVSIYWWGS-
Sami    WYWHFVDVVWLFLYVSIYWWGS-
Rere    WYWHFVDVVWLFLYVSIYWWGS-
Gama    WYWHFVDVVWLFLYVSIYWWGS-
Onmy    WYWHFVDVVWLFLYVSIYWWGS-
Sasa    WYWHFVDVVWLFLYVSIYWWGS-
Cola    WYWHFVDVVWLFLYVSIYWWGS-
Dita    WYWHFVDVVWLFLYVSIYWWGS-
Gogr    WYWHFVDVVWLFLYISIYWWGS-
Chsl    WYWHFVDVVWLFLYVSIYWWGS-
Atja    WYWHFVDVVWLFLYVSIYWWGS*
Iido    WYWHFVDVVWLFLYVSIYWWGS-
Auja    WYWHFVDVVWLFLYISIYWWGS-
Chag    WYWHFVDVVWLFLYISIYWWGS-
Hami    WYWHFVDVVWLFLYISIYWWGS-
Saun    WYWHFVDVVWLFLYISIYWWGS-
Nema    WYWHFVDVVWLFLYISIYWWGS-
Disp    WYWHFVDVVWLFLYISIYWWGS-
Myaf    WYWHFVDVVWLFLYISIYWWGS-
Lagu    WYWHFVDVVWLFLYISIYWWGS-
Trtr    WYWHFVDVVWLFLYISIYWWGS-
Zucr    WYWHFVDVVWLFLYISIYWWGS-
Pxja    WYWHFVDVVWLFLYISIYWWGS-
Pxlo    WYWHFVDVVWLFLYISIYWWGS-
Pctr    WYWHFVDVVWLFLYISIYWWGS-
Apsa    WYWHFVDVVWLFLYISIYWWGS-
Cabe    WYWHFVDVVWLFLYISIYWWGS-
Bzze    WYWHFVDVVWLFLYISIYWWGS-
Siim    WYWHFVDVVWLFLYASIYWWGS-
Ctru    WYWHFVDVVWLFLYISIYWWGS-
Dpbr    WYWHFVDVVWLFLYISIYWWGS-
Caki    WYWHFVDVVWLFLYISIYWWGS-
Phja    WYWHFVDVVWLFLYISIYWWGS-
Brsp    WYWHFVDVVWLFLYVSIYWWGS-
Gamo    WYWHFVDVVWLFLYISIYWWGS-
Lolo    WYWHFVDVVWLFLYISIYWWGS-
Batr    WYWHFVDVVWLFLFTSIYWWGS*
Prmy    WYWHFVDVVWLFLFVSIYWWGS*
Lose    WYWHFVDVVWLFLYISIYWWGS-
Loam    WYWHFVDVVWLFLYISIYWWGS-
Chab    WYWHFVDVVWLFLYISIYWWGS-
Chto    WYWHFVDVVWLFLYISIYWWGS-
Majo    WYWHFVDVVWLFLYISIYWWGS-
Hlst    WYWHFVDVVWLFLYISIYWWGS-
Clpe    WYWHFVDVVWLFLYVSIYWWGS-
Mlmr    WYWHFVDVVWLFLYISIYWWGS-
Crcr    WYWHFVDVVWLFLYISIYWWGS-
Muce    WYWHFVDVVWLFLYISIYWWGS-
Bege    WYWHFVDVVWLFLYISIYWWGS-
Mela    WYWHFVDVVWLFLYISIYWWGS-
Hats    WYWHFVDVVWLFLYISIYWWGS-
Orla    WYWHFVDVVWLFLYISIYWWGS-
```

[5/5 of aligned sequences]

```
Cosa    WYWHFVDVVWLFLYISIYWWGS-
Exsp    WYWHFVDVVWLFLYISIYWWGS-
Depa    WYWHFVDVVWLFLYISIYWWGS-
Rima    WYWHFVDVVWLFLYISIYWWGS-
Fuol    WYWHFVDVVWLFLYISIYWWGS-
Gmaf    WYWHFVDVVWLFLYISIYWWGS-
Xeei    WYWHFVDVVWLFLYISIYWWGS-
Pros    WYWHFVDVVWLFLYISIYWWGS-
Scmi    WYWHFVDVVWLFLYLSIYWWGS*
Rolo    WYWHFVDVVWLFLYISIYWWGS-
Cere    WYWHFVDVVWLFLYISIYWWGS-
Daga    WYWHFVDVVWLFLYISIYWWGS-
Anco    WYWHFVDVVWLFLYISIYWWGS-
Dmve    WYWHFVDVVWLFLYISIYWWGS-
Dmar    WYWHFVDVVWLFLYISIYWWGS-
Anka    WYWHFVDVVWLFLYISIYWWGS-
Moja    WYWHFVDVVWLFLYISIYWWGS-
Hoja    WYWHFVDVVWLFLYISIYWWGS-
Bede    WYWHFVDVVWLFLYISIYWWGS-
Besp    WYWHFVDVVWLFLYISIYWWGS-
Mysp    WYWHFVDVVWLFLYISIYWWGS-
Osja    WYWHFVDVVWLFLYISIYWWGS-
Sgro    WYWHFVDVVWLFLYISIYWWGS-
Pzpa    WYWHFVDVVWLFLYISIYWWGS-
Zeja    WYWHFVDVVWLFLYISIYWWGS-
Znne    WYWHFVDVVWLFLYISIYWWGS-
Zefa    WYWHFVDVVWLFLYISIYWWGS-
Acni    WYWHFVDVVWLFLYISIYWWGS-
Ncrh    WYWHFVDVVWLFLYISIYWWGS-
Agca    WYWHFVDVVWLFLYISIYWWGS-
Hydy    WYWHFVDVVWLFLYISIYWWGS-
Gsac    WYWHFVDVVWLFLYISIYWWGS-
Pevo    WYWHFVDVVWLFLYVSIYWWGS-
Hiku    WYWHFVDVVWLFLYVSIYWWGS-
Inpa    WYWHFVDVVWLFLYISIYWWGS-
Auch    WYWHFVDVVWLFLYVSIYWWGS*
Fico    WYWHFVDVVWLFLYVSIYWWGS-
Macs    WYWHFVDVVWLFLYVSIYWWGS-
Moal    WYWHFVDVVWLFLYISIYWWGS-
Syma    WYWHFVDVVWLFLYISIYWWGS-
Mafr    WYWHFVDVVWLFLYISIYWWGS-
Dcpe    WYWHFVDVVWLFLYVSIYWWGS-
Dcti    WYWHFVDVVWLFLYVSIYWWGS-
Hehi    WYWHFVDVVWLFLYISIYWWGS-
Stam    WYWHFVDVVWLFLYISIYWWGS-
Hogi    WYWHFVDVVWLFLYISIYWWGS-
Erzo    WYWHFVDVVWLFLYISIYWWGS-
Hxot    WYWHFVDVVWLFLYISIYWWGS-
Core    WYWHFVDVVWLFLYISIYWWGS-
Apve    WYWHFVDVVWLFLYISIYWWGS-
Latj    WYWHFVDVVWLFLYISIYWWGS-
Laja    WYWHFVDVVWLFLYISIYWWGS-
```

[5/5 of aligned sequences]

Syja	WYWHFVDVVWLFLYISIYWWGS-
Epme	WYWHFVDVVWLFLYISIYWWGS-
Grse	WYWHFVDVVWLFLYISIYWWGS-
Clja	WYWHFVDVVWLFLYISIYWWGS-
Ogcy	WYWHFVDVVWLFLYISIYWWGS-
Plna	WYWHFVDVVWLFLYISIYWWGS-
Lema	WYWHFVDVVWLFLYISIYWWGS-
Etzo	WYWHFVDVVWLFLYISIYWWGS-
Apse	WYWHFVDVVWLFLYISIYWWGS*
Epde	WYWHFVDVVWLFLYISIYWWGS-
Slja	WYWHFVDVVWLFLYISIYWWGS-
Bsja	WYWHFVDVVWLFLYISIYWWGS-
Ecna	WYWHFVDVVWLFLYISIYWWGS-
Cohi	WYWHFVDVVWLFLYVSIYWWGS-
Caar	WYWHFVDVVWLFLYVSIYWWGS*
Came	WYWHFVDVVWLFLYVSIYWWGS-
Mema	WYWHFVDVVWLFLYVSIYWWGS-
Lenu	WYWHFVDVVWLFLYISIYWWGS-
Brja	WYWHFVDVVWLFLYVSIYWWGS-
Plma	WYWHFVDVVWLFLYVSIYWWGS-
Emst	WYWHFVDVVWLFLYISIYWWGS-
Ptti	WYWHFVDVVWLFLYISIYWWGS-
Losu	WYWHFVDVVWLFLYVSIYWWGS-
Geoy	WYWHFVDVVWLFLYVSIYWWGS-
Dipi	WYWHFVDVVWLFLYISIYWWGS-
Pama	WYWHFVDVVWLFLYISIYWWGS-
Leob	WYWHFVDVVWLFLYISIYWWGC-
Neba	WYWHFVDVVWLFLYVSIYWWGS-
Pdpl	WYWHFVDVVWLFLYAFIYWWGS*
Nimi	WYWHFVDVVWLFLYISIYWWGS-
Uptr	WYWHFVDVVWLFLYVSIYWWGS-
Pesc	WYWHFVDVVWLFLYISIYWWGS-
Baar	WYWHFVDVVWLFLYISIYWWGS-
Moar	WYWHFVDVVWLFLYISIYWWGS-
Toja	WYWHFVDVVWLFLYISIYWWGS-
Chau	WYWHFVDVVWLFLYISIYWWGS-
Chse	WYWHFVDVVWLFLYISIYWWGS-
Enar	WYWHFVDVVWLFLYISIYWWGS-
Hpty	WYWHFVDVVWLFLYISIYWWGS-
Nana	WYWHFVDVVWLFLYISIYWWGS-
Mcst	WYWHFVDVVWLFLYISIYWWGS-
Rhox	WYWHFVDVVWLFLYVSIYWWGS-
Opfa	WYWHFVDVVWLFLYISIYWWGS-
Paar	WYWHFVDVVWLFLYISIYWWGS*
Gozo	WYWHFVDVVWLFLYISIYWWGS-
Ackr	WYWHFVDVVWLFLYVSIYWWGS-
Elev	WYWHFVDVVWLFLYISIYWWGS-
Trdu	WYWHFVDVVWLFLYISIYWWGS-
Amoc	WYWHFVDVVWLFLYISIYWWGS-
Hame	WYWHFVDVVWLFLYISIYWWGS-
Chso	WYWHFVDVVWLFLYISIYWWGS-
Lyto	WYWHFVDVVWLFLYISIYWWGS-

[5/5 of aligned sequences]

```
Encr    WYWHFVDVVWLFLYISIYWWGS-
Bvar    WYWHFVDVVWLFLYVSIYWWGS-
Noco    WYWHFVDVVWLFLYVSIYWWGS*
Chsp    WYWHFVDVVWLFLYISIYWWGS-
Arja    WYWHFVDVVWLFLYISIYWWGS-
Pase    WYWHFVDVVWLFLYISIYWWGS-
Trel    WYWHFVDVVWLFLYASIYWWGS-
Lifa    WYWHFVDVVWLFLYMSIYWWGS-
Acur    WYWHFVDVVWLFLYISIYWWGS-
Ampe    WYWHFVDVVWLFLYISIYWWGS-
Urja    WYWHFVDVVWLFLYISIYWWGS-
Enet    WYWHFVDVVWLFLYISIYWWGS-
Ptbr    WYWHFVDVVWLFLYISIYWWGS-
Safa    WYWHFVDVVWLFLYISIYWWGS*
Icae    WYWHFVDVVWLFLYVSIYWWGS-
Asmi    WYWHFVDVVWLFLYISIYWWGS-
Foal    WYWHFVDVVWLLLYVSLYWWGS-
Drze    WYWHFVDVVWLLLYVSLYWWGS-
Rhas    WYWHFVDVVWLFLYISIYWWGS-
Elac    WYWHFVDVVWLFLYISIYWWGS-
Kugu    WYWHFVDVVWLFLYVSIYWWGS-
Plor    WYWHFVDVVWLFLYISIYWWGS-
Sgun    WYWHFVDVVWLFLYISIYWWGS-
Zaco    WYWHFVDVVWLFLYISIYWWGS-
Zbfl    WYWHFVDVVWLFLYISIYWWGS-
Spba    WYWHFVDVVWLFLYVSIYWWGS-
Game    WYWHFVDVVWLFLYVSIYWWGS-
Thth    WYWHFVDVVWLFLYVSIYWWGS-
Xigl    WYWHFVDVVWLFLYISIYWWGS-
Hyja    WYWHFVDVVWLFLYVSIYWWGS-
Psan    WYWHFVDVVWLFLYISVYWWGS*
Cupa    WYWHFVDVVWLFLYVSIYWWGS-
Mpch    WYWHFVDVVWLFLYISIYWWGS-
Char    WYWHFVDVVWLFLYISIYWWGS-
Pser    WYWHFVDVVWLFLYVSIYWWGS-
Prol    WYWHFVDVVWLFLYISIYWWGS-
Plbi    WYWHFVDVVWLFLYISIYWWGS-
Calu    WYWHFVDVVWLFLYVSIYWWGS-
Papa    WYWHFVDVVWLFLYISIYWWGS-
Sufr    WYWHFVDVVWLFLYISIYWWGS-
Stci    WYWHFVDVVWLFLYISIYWWGS-
Taru    WYWHFVDVVWLFLYISIYWWGS-
Rala    WYWHFVDVVWLFLYISIYWWGS-
        ***** :  **:*:  :****.
```
