# Supplementary material for: Structure and variation of the mitochondrial genome of fishes
Source: BMC Genomics. 2016 Sep 7;17(1):719. doi: 10.1186/s12864-016-3054-y (PMC5015259; doi:10.1186/s12864-016-3054-y)
Supplement: Additional file 6: Figure S1-a. — Aligned amino acid sequences of the ATP8 gene in mt genomes of 250 fishes. Figure S1-b. Aligned amino acid sequences of the ATP6 gene in mt genomes of 250 fishes. Figure S1-c. Aligned amino acid sequences of the COI gene in mt genomes of 250 fishes. Figure S1-d. Aligned amino acid sequences of the COII gene in mt genomes of 250 fishes. Figure S1-e. Aligned amino acid sequences of the COIII gene in mt genomes of 250 fishes. Figure S1-f. Aligned amino acid sequences of the Cyt b gene in mt genomes of 250 fishes. Figure S1-g. Aligned amino acid sequences of the ND1 gene in mt genomes of 249 fishes. Figure S1-h. Aligned amino acid sequences of the ND2 gene in mt genomes of 250 fishes. Figure S1-i. Aligned amino acid sequences of the ND3 gene in mt genomes of 250 fishes. Figure S1-j. Aligned amino acid sequences of the ND4L gene in mt genomes of 250 fishes. Figure S1-k. Aligned amino acid sequences of the ND4 gene in mt genomes of 250 fishes. Figure S1-l. Aligned amino acid sequences of the ND5 gene in mt genomes of 250 fishes. Figure S1-m. Aligned amino acid sequences of the ND6 gene in mt genomes of 249 fishes. (ZIP 3250 kb) [file 12864_2016_3054_MOESM6_ESM.zip › Additional file 6 prot align/AF6f-Cyb.pdf]

Additional file 6: Figure S1–f. Aligned amino acid sequences of the Cyt b gene in mt genomes of 250 fishes.

Species name abbreviation followed by aligned amino acid sequences shown by one letter abbreviation. See Additional file 1 for abbreviation of species name. Amino acids shown by magenta letter denote hydrophobic residues. A-I in bold types with yellow background indicate putative transmembrane regions. Highlighted 'G' and 'H' letters indicate metal binding sites. Numerals on the amino acid sequences correspond to position number of amino acid residues in the human sequence. Asterisk '*' indicates a fully conserved residue. Colon ':' and period '.' indicate 'strong' and 'weak' groups in the level of conservativeness, respectively, in the Gonnet Pam250 matrix, in which the strong and weak groups are defined as strong score >0.5 and weak score =<0.5, respectively (Thompson et al., 1997).

Cyt b

[1/7 of aligned sequences]

```
                                                    34        A    48
Scca  ---MATNIRKTHPLLKIVNHALIDLPAPSNISVWWNFGSLLGLCLIMQIITGLFLAMHYT
Muma  ---MATNIRKTHPLLKIMNHALVDLPAPSNISLWWNFGSLMGLCLLIQILTGLFLAMHYT
Erca  ----MAIMRKTHPLAKIINSAFIDLPAPSNISSWWNMGSLLGLCLIVQIITGLFLAMHYI
Pose  ----MAITRKTHPLAKIINSAFIDLPAPSNISSWWNMGSLLGMCLMSQIITGLFLAMHYV
Actr  ----MANIRKTHPLLKIINGAFIDLPTPSNISVWWNFGSLLGLCLITQILTGLFLAMHYT
Scal  ----MANIRKTHPLLKIINGALIDLPTPSNISVWWNFGSLLGLCLITQILTGLFLAMHYT
Posp  ----MANIRKTHPLLKIINGAFIDLPAPSNISVWWNSGSLLGLCLITQILTGLFLAMHYT
Atsp  ----MANIRKTHPLLKIINGAVIDLPTPSNISAWWNFGSLLGLCLITQVLTGLFLAMHYT
Leoc  ----MANIRKTHPLLKIINGAVIDLPTPSNISAWWNFGSLLGLCLITQTLTGLFLAMHYT
Amca  ----MATIRKTHPLISIINGAFIDLPAPVNISVWWNFGSLLGLCLITQIVTGLFLAMHFT
Osbi  ----MASLRKTHPLAKIVNDALIDLPAPSNISAWWNFGSLLGLCLIIQILTGLFLAMHYT
Pabu  ----MASLRKTHPLAMIANNALVDLPAPSNISAWWNFGSLLGLCLATQILTGLFLAMHYT
Hial  ----MANFRKTHPLIKIANDALVDLPAPSNISVWWNFGSLLGLCLATQILTGLFLAMHYT
Elha  ----MAILRKTHPLLKIANSALVDLPAPSNISAWWNFGSLLGLCLASQIVTGLFLAMHYT
Mlcy  ----MANLRKTHPLLKIANDALVDLPAPSNISAWWNFGSLLGLCLATQILTGLFLAMHYT
Algl  ---------VTNPLLAIANDTFVNLPVPANISAWWNFGSLLGLCLITQILTGLFLAMHYT
Ptgi  ----MANLRKTHPLLKIANDALVDLPAPSNISVWWNFGSLLGLCLITQILTGLFLAMHYT
Alaf  ----MANLRKTHPILKIVNDALVDLPSPSNISAWWNFGSLLGLCLIMQILTGLFLAMHYT
Nock  ----MANLRKTHPILKISNEALVDLPTPSNISAWWNFGSLLGLCLITQILTGLFLAMHYT
Anja  ----MANLRKTHPLLKIANDALVDLPTPSNISAWWNFGSLLGLCLISQILTGLFLAMHYT
Gyki  ----MANLRKTHPILKIANDALVDLPTPSNISAMWNFGSLLGLCLITQILTGLFLAMHYT
Syka  ----MASLRKTHPLLKIANDALVDLPTPSNISAWWNFGSLLGLCLIAQILTGLFLAMHYT
Opma  ----MASLRKDVILVNMVNDSLADLPAPSNISAWWNFGSLLFLCLITQILTGLFLAMHYT
Comy  ----MANLRKNHPLLKIVNDALVDLPAPSNISAWWNFGSLLMLCLITQILTGLFLAMHYT
Sasp  ----MLSPRKAPPLMKIANDALVDLPAPPNLSYLWNFGSLLGLCLIAQILTGLFLAMHYT
Eupe  ----MSNLRKTHPLIKIANNSFVDLPAPSNLSTWWNFGSLLGLCLILQVLTGLFLAMHYT
Enja  ----MASLRKTHPLLKIANDAVVDLPAPSNISVWWNFGSLLGLCLATQILTGLFLAMHYT
Same  ----MASLRKTHPLMKIANDALVDLPAPSNISVWWNFGSLLGLCLAAQILTGLFLAMHYT
Chch  ----MASLRKTHPLIKIANDALVDLPAPSNISVWWNFGSLLGLCLASQILTGLFLAMHYT
Grgr  ----MANLRKTHPLFKIANDALVDLPAPSNISVWWNFGSLLGLCLATQILTGLFLAMHYT
Caau  ----MASLRKTHPLIKIANDALVDLPTPSNISAWWNFGSLLGLCLITQILTGLFLAMHYT
Cyca  ----MASLRKTHPLIKIANDALVDLPTPSNISVWWNFGSLLGLCLITQILTGLFLAMHYT
Dare  ----MTSLRKTHPVLKIANDALVDLPTPLNISAWWNFGSLLGLCLITQILTGLFLAMHYT
Cost  ----MASLRKTHPLFKIANDALVDLPAPSNISVWWNFGSLLGLCLITQILTGLFLAMHYT
Leec  ----MASLRKTHPLMKIANDALVDLPAPSNISVWWNFGSLLGLCLIIQIITGLFLAMHYT
Fola  ----MASLRKTHPLIKIANDALVDLPAPSNISVWWNFGSLLGLCLITQILTGLFLAMHYT
Clmc  ----MANLRKSHPLLKIANSALIDLPAPSNISAWWNFGSLLLLCLIMQILTGLFLAMHYT
Phin  ----MASLRKTHSLLKIANNALIDLPAPSNISAWWNFGSLLLLCLLTQILTGLFLAMHYT
Icpu  -----MITRKTHPLFKIANNALIDLPAPSNISAWWNFGSLLLLCLMAQILTGLFLAMHYT
Psto  -----MVIRKTHPLFKIVNDALIDLPAPSNISVWWNFGSLLLLCLMTQILTGLFLAMHYT
Cora  -----MVTRKTHPLLKIVNDSLIDLPAPSNISAWWNFGSLLLLCLVVQIVTGLFLAMHYT
Eisp  ----MASLRKTHPLLKIVNDALIDLPAPSNISAWWNFGSLLLLCLMAQIITGLFLAMHYT
Apal  ----MASPRKTHPLLKIANGALIDLPAPSNISYWWNFGSLLLLCLITQIATGLFLAMHYT
Eslu  ----MTSLRKTHPVLKIVNDALIDLPAPANISIWWNFGSLLGLCLITQILTGLFLAMHYT
                                                   1
```

To be continued on page 6.

[1/7 of aligned sequences]

```
Dape    ----MTNLRKSHPILKIVNDALIDLPAPANISVWWNFGSLLGLCLATQILTGLFLAMHYT
Glse    ----MASLRKSHPLLKIINDTIIDLPSPSNISAWWNFGSLLGLCLITQILTGLFLAMHYT
Naar    ----MARLRKTHPLLKIANDAFIDLPAPSNISAWWNFGSLLILCLGTQIVTGLFLAMHYT
Lioc    ----MARLRKAHPLLSIVNHSLIDLPAPSNISAWWNFGSLLVMCLIAQILTGLFLAMHYT
Opso    ----MASLRKTHPLLKIINDTFIDLPTPSNISIWWNYGSLLGLCLITQILTGLFLAMHYT
Alte    ----MASLRKTHPLLKIANHALVDLPTPSNISVWWNFGSLLGLCLIAQILTGLFLAMHYT
Plap    ----MASLRKTHPLLKIVNDALVDLPTPSSISVWWNFGSLLGLCLIAQILTGLFLAMHYT
Plal    ----MANLRKTHPLLKISNDALVDLPAPSNISIWWNFGSLLGLCLIIQILTGLFLAMHYT
Sami    ----MANLRKTHPLLKITNDALVDLPAPSSISIWWNFGSLLGLCLIIQILTGLFLAMHYT
Rere    ----MASLRKSHPLLRIANDALVDLPAPSNISVWWNFGSLLSLCLATQILTGLFLAMHYT
Gama    ----MANLRKTHPLLKIANGALVDLPAPSNISVWWNFGSLLGLCLASQILTGLFLAMHYT
Onmy    ----MANLRKTHPLLKIANDALVDLPAPSNISVWWNFGSLLGLCLATQILTGLFLAMHYT
Sasa    ----MANLRKTHPLLKIANDALVDLPAPSNISVWWNFGSLLGLCLATQILTGLFLAMHYT
Cola    ----MANLRKTHPLLKIANDALVDLPAPSNISVWWNFGSLLGLCLATQILTGLFLAMHYT
Dita    ----MASLRKTHPLLKIANDALVDLPAPSNISVWWNFGSLLGLCLVSQILTGLFLAMHYT
Gogr    ----MTSLRKTHPLLKIANSALIDLPTPSNISAWWNFGSLLGLCLASQIVTGLFLAMHYT
Chsl    ----MASLRKTHPLLKIANHALVDLPAPLNISAWWNFGSLLGLCLASQIVTGLFLAMHYT
Atja    ----MASLRKTHPLLKIANDALVDLPTPANISAWWNFGSLLGLCLASQILTGLFLAMHYT
Iido    ----MASLRKTHPLLKIANDALVDLPTPANISAWWNFGSLLGLCLASQILTGLFLAMHYT
Auja    ----MANLRKTHPLLKIANDALVDLPAPSNISVWWNFGSLLGLCLATQILTGLFLAMHYT
Chag    ----MA-LRKTHPLLKIANDALVDLPAPSNISAWWNFGSLLGLCLATQILTGLFLAMHYT
Hami    ----MAILRKTHPLMKIANDALVDLPAPSNISALWNFGSLLGLCLITQIVTGLFLAMHYT
Saun    ----MAILRKTHPLMKIANDALVDLPAPSNISALWNFGSLLGLCLIAQIVTGLFLAMHYT
Nema    ----MANLRKTHPLLKIANDALVDLPSPSNISVWWNFGSLLGLCLIIQIATGLFLAMHYT
Disp    ----MTSLRKTHPLLKIANDAPADLPAPSNISVWWNFGSLLGLCLIIQIATGLFLAMHYT
Myaf    --MTHPTLRKTHPLLKIANDALVDLPAPSNISVWWNFGSLLGLCLIIQIATGLFLAMHYT
Lagu    ----MASLRKIHPLMKITNDMVIDLPAPSNISVWWNFGSLLGLCLFSQILTGLFLAMHYT
Trtr    ----MASLRKTHPLLSIANNALVDLPAPSNISVWWNFGSLLGLCLITQLITGLFLAMHYT
Zucr    ----MASLRKTHPLLSIVNNALVDLPAPSNISVWWNFGSLLGLCLITQLITGLFLAMHYT
Pxja    ----MASLRKTHPLLKIANDALVDLPAPSNISIWWNYGSLLGLCLITQILTGLFLAMHYT
Pxlo    ----MASLRKTHPLLKIANDALVDLPAPSNISIWWNYGSLLGLCLITQILTGLFLAMHYT
Pctr    ----MASLRKSHPLLKIANHILVDLPAPSNISAWWNFGSLLGLCLATQILTGLFLAMHYT
Apsa    ----MTNLRKTHPLLKIINDALVDLPAPSNISAWWNFGSLLALCLITQILTGLFLAMHYT
Cabe    ----MTSLRKTHPLMSIMNGMLVDLPVPSNISAMWNLGSLLFICLATQILTGLFLAMHYT
Bzze    ----MANLRKTHPLLKIVNGALVDLPAPSNISTWWNFGSLLFLCLAAQILTGLFLAMHYT
Siim    ----MANIRKTHIVLKIASLTMVDLPAPINISAWWNFGSLLSLCLITQITTGLILSMHYS
Ctru    ----MANLRKTHPILKIANDALVDLPAPANISVWWNFGSLMFLCLAAQIVTGLFLAMHYT
Dpbr    ----MANLRKTHPILKIVNDALVDLPAPSNISAWWNFGSLLFMCLIAQILTGLFLAMHYT
Caki    ----MTSLRKTHPLLKIANDALVDLPSPSNISIWWNFGSLLGLCLITQLLTGLFLAMHYT
Phja    ----MTNLRKTHPLLKIVNDALIDLPAPANISAMWNFGSLLGLCLVTQILTGLFLAMHYT
Brsp    ----MNHPRHLSPVHAI-GKDAFNLPTPSNISAMWNFGSLLGLCLIIQLVTGLFLAMHYT
Gamo    ----MASLRKTHPILKIANSALVDLPAPSNISVWWNFGSLLGLCLITQLLTGLFLAMHYT
Lolo    ----MASLRKTHPILKIANDALVDLPAPSNISVWWNFGSLLGLCLITQILTGLFLAMHYT
Batr    ----MTHLRKTHPLLKIVNSTIIDLPSPSNISYWWNFGSLLGLCLTIQVITGLFLAMHYA
Prmy    ----MLLFRKRNPLLDMATKSLMDLPSPINLSIWWNTGSLLGLCLVTQMVTGVFIAMHYI
Lose    ----MTSLRKTHPVLKIANDALVDLPAPSNISIWWNFGSLLGLCLIIQILTGLFLAMHYT
Loam    ----MASLRKTHPLLKIANDALVDLPAPSNISAWWNFGSLLALCLIAQVLTGLFLAMHYT
Chab    ----MASLRKTHPLLKIANNALVDLPAPSNISVWWNFGSLLGLCLMAQIITGLFLAMHYT
Chto    ----MASLRKTHPLLKIANNALVDLPAPSNISVWWNFGSLLGLCLMAQILTGLFLAMHYT
Majo    ----MTNLRKTHPLLKITNDTLVDLPAPSNISVWWNFGSLLGLCLIIQLLTGLFLAMHYT
Hlst    ----MTNLRKSHPLLKITNSALIDLPAPSNISVWWNFGSLLGLCLATQLVTGLFLAMHYT
Clpe    ----MASLRKTHPLLKIANNALVDLPAPSNISVWWNFGSLLGLCLIAQILTGLFLSMHYS
```

To be continued on page 7.

[1/7 of aligned sequences]

```
Mlmr      ----MASLRKTHPLLKIANNALVDLPAPSNISVWWNFGSLLGLCLAAQILTGLFLAMHYT
Crcr      ----MTTIRKNHPLFKAANSALIDLPAPASLSSLWNFGSLLGLCLISQIATGLFLAMHYA
Muce      ----MTTIRKNHPLFKAANSALIDLPAPASLSSLWNFGSLLGLCLISQIATGLFLAMHYA
Bege      ----MANLRKTHPLLKIANDALVDLPTPINISVWWNFGSLLGLCLVAQILTGLFLAMHYT
Mela      ----MTNLRKTHPLLKIANDALVDLPTPANISAWWNFGSLLGLCLGAQILTGLFLTMHYC
Hats      ----MANLRKTHPLLKIANDAVVDLPTPANISVWWNFGSLLGLCLIAQILTGLFLAMHYT
Orla      ----MANLRKTHPLLKIANDALVDLPAPSNISVWWNFGSLLGLCLAAQIITGLFLAMHYT
Cosa      ----MANLRKSHPLLKIANDAVIDLPTPANISAWWNFGSLLGLCLITQILTGLFLAMHYT
Exsp      ----MANLRKTHPLFKIANDAVIDLPTPANISAWWNFGSLLGLCLIAQILTGLFLAMHYT
Depa      ----MANLRKTHPLLKIANNALVDLPTPTNISAWWNFGSLLGLCLIAQILTGLFLAMHYT
Rima      ----MTSIRKSHLTLKALNESMIDLPAPKNISAWWNFGSLLSLCLALQILTGLFLAMHYT
Fuol      ----MANLRKTHPLLKIANDALVDLPAPVNISVWWNFGSLLGLCLIAQILTGLFLAMHYT
Gmaf      ----MANLRKTHPLLKVANDALVDLPAPVNISAWWNFGSLLGLCLITQILTGLFLAMHYT
Xeei      ----MANLRKTHPLLKIANNALVDLPAPVNISAWWNFGSLLALCLAAQILTGLFLAMHYT
Pros      ----MANLRKTHPLLKIVNDMIIDLPTPANISTMWNYGSLLGLCLVTQILTGLFLAMHYT
Scmi      MANQQHTLRKAHPLLKIVNDAVIDLPAPSNLTTLWNYGSLMGLCLASQILTGLFLAMHYT
Rolo      ----MANLRKTHPLLKIANDALVDLPTPSNISVWWNFGSLLGLCLATQILTGLFLAMHYT
Cere      ----MARLRKTHPLLKIANDALIDLPAPSNISVWWNFGSLLGLCLVTQILTGLFLTMHYT
Daga      ----MASLRKTHPLLKIANNALVDLPAPSNISVWWNFGSLLGLCLATQILTGLFLAMHYT
Anco      ----MASLRKTHPILKIVNDALIDLPAPSNISFWWNYGSLLFLCLIIQIATGLFLAMHYT
Dmve      ----MASLRKTHPLLKIVNDVLVDLPAPVNISVWWNFGSLLGLCLATQILTGLFLAMHYT
Dmar      ----MASLRKTHPLLKIANDALVDLPAPVNISVWWNFGSLLGLCLATQILTGLFLAMHYT
Anka      ----MASLRKTHPILKIANDALVDLPAPSNISVWWNFGSLLGLCLIIQIATGLFLAMHYT
Moja      ----MASLRKTHPILKIANNALIDLPAPSNISVWWNFGSLLGLCLAIQIATGLFLAMHYT
Hoja      ----MASLRKTHPILKIANDALVDLPAPSNISVWWNFGSLLGLCLIIQIATGLFLAMHYT
Bede      ----MANLRKTHPLLKIANDALIDLPTPSNISTMWNFGSLLGLCLISQILTGLFLAMHYT
Besp      ----MANLRKTHPLLKIANDALIDLPTPSNISTMWNFGSLLGLCLVSQILTGLFLAMHYT
Mysp      ----MASLRKTHPLLKIANDALVDLPAPSNISVWWNFGSLLGLCLITQILTGLFLAMHYT
Osja      ----MASLRKTHPLLKIANDALVDLPAPSNISVWWNFGSLLGLCLVTQILTGLFLAMHYT
Sgro      ----MASLRKTHPLLKTANDALVDLPAPSNISVWWNFGSLLGLCLAAQILTGLFLAMHYT
Pzpa      ----MTHLRKTHPLFKIANDTLIDLPAPINISTWWNFGSLLSLCLMTQILTGLFLAMHYT
Zeja      ----MTNIRKAHPLLKIGSDSLVDLPAPINISTWWNFGSLLGLCLVSQILTGLFLAMHYT
Znne      ----MASLRKTHPLLKIVNDALIDLPAPSNISAWWNFGSLLGLCLVTQILTGLFLAMHYT
Zefa      ----MASLRKTHPLLKIVNDALIDLPAPANISVWWNFGSLLGLCLLTQILTGLFLAMHYT
Acni      ----MASLRKTHPLLKIANDALVDLPTPINISVWWNFGSLLGLCLIAQILTGLFLAMHYT
Ncrh      ----MASLRKTHPLLKIVNDALVDLPTPINISTWWNFGSLLGLCLIAQILTGLFLAMHYT
Agca      ----MASLRKTHPLLKIANDALVDLPTPSNISVWWNFGSLLGLCLITQLVTGLFLAMHYT
Hydy      ----MASLRKTHPLLKIANNALVDLPAPSNISVWWNFGSLLGLCLIVQILTGLFLAMHYT
Gsac      ----MASLRKTHPLLKIANNALVDLPAPSNISVWWNFGSLLGLCLIIQILTGLFLAMHYT
Pevo      ----MASLRKTHPVLKIANNALVDLPAPSNISVWWNFGSLLGLCLITQIATGLFLAMHYT
Hiku      ----MANLRKTHPILKIANNALVDLPAPSNISVWWNFGSLLGLCLIAQILTGLFLAMHYT
Inpa      ----MASLRKTHPLLKIVNDNSIDLPTPQNISAWWNFGSLLGLCLVAQILTGLFLAMHYT
Auch      ----MASLRKTHPLLKVGNDALVDLPTPATLSVWWNFGSLLGLCLIIQIATGLFLAMHYT
Fico      ----MTSLRKTHPIIKLANDALVDLPAPSNISVWWNFGSLLALCLIAQIATGLFLAMHYT
Macs      ----MANLRKTHPLLKIVNDALVDLPAPSNISVWWNFGSLLGLCLAAQILTGLFLAMHYT
Moal      ----MA-LRKRHPLLKIINGALIDLPSPSNISAWWNFGSLLGLCLTLQILTGILLTMHYS
Syma      ----MTSMRKTHPLLKITNDSFIDLPTPSNISALWNFGSLLGLCLMIQILTGFFLAMHYS
Mafr      ----MASLRKTHPLLKIANNALVDLPAPSNISAWWNFGSLLGLCLITQILTGLFLAMHYT
Dcpe      ----MTNLRKTHPLLKIANDALVDLPAPANISVWWNFGSLLGLCLITQIATGLFLAMHYT
Dcti      ----MTSLRKTHPLLKIANDALVDLPAPANISVWWNFGSLLGLCLITQIATGLFLAMHYT
Hehi      ----MASLRKTHPLLKIANHALVDLPAPSNISVWWNFGSLLGLCLIIQILTGLFLAMHYT
Stam      ----MASLRKTHPLLKIANGALVDLPAPSNISVWWNFGSLLGLCLITQLLTGLFLAMHYT
```

To be continued on page 8.

[1/7 of aligned sequences]

```
Hogi    ----MTSLRKTHPLLKIANHALVDLPAPSNISVWWNFGSLLGLCLIIQILTGLFLAMHYT
Erzo    ----MASLRKSHPLLKIANGALVDLPTPSNISVWWNFGSLLGLCLVTQILTGLFLAMHYT
Hxot    ----MASLRKTHPLLKIANSAVVDLPAPSNISVWWNFGSLLGLCLIIQILTGLFLAMHYT
Core    ----MASLRKTHPLLKIANNALVDLPAPSNISVWWNFGSLLGLCLIIQILTGLFLAMHYT
Apve    ----MASLRKTHPLLKIVNSALIDLPAPSNISAWWNFGSLLGLCLIIQILTGLFLAMHFT
Latj    ----MANLRKSHPLLKIANDALVDLPTPVNISAWWNFGSLLGLCLISQILTGLFLAMHYT
Laja    ----MASLRKTHPLLKIANDALVDLPAPSNISVWWNFGSLLGLCLITQILTGLFLAMHYT
Syja    ----MAPLRKSHPLAKIANDALVDLPAPSNISAWWNFGSLLGLCLITQLLTGLFLAMHYT
Epme    ----MANLRKTHPLLKIANDAVVDLPAPANISVWWNFGSLLGLCLGAQILTGLFLAMHYT
Grse    ----MASLRKTHPLLKIANNALVDLPAPSNISIWWNFGSLLGLCLAAQILTGLFLAMHYT
Clja    ----MANLRKTHPLLKIANDALVDLPAPSNISVWWNFGSLLGLCLAAQILTGLFLAMHYT
Ogcy    ----MASLRKTHPLLKIANDAVIDLPTPVNISAWWNFGSLLGLCLITQIITGLFLAMHYT
Plna    ----MTSLRKTHPLLKIVNDAVIDLPAPTNISAWWNFGSLLGLCLATQILTGLFLAMHYT
Lema    ----MASLRKTHPLLKIANDALVDLPTPSNISVWWNFGSLLGLCLATQILTGLFLAMHYT
Etzo    ----MASLRKTHPLLKIANHALVDLPAPSNISVWWNFGSLLGLCLITQILTGLFLAMHYT
Apse    ----MANLRKTHPLLKIANDALVDLPAPSNISIWWNFGSLLGLCLAIQLLTGLFLAMHYT
Epde    ----MAALRKTHPLLKIANDALVDLPAPSNISAWWNFGSLLGLCLITQILTGLFLAMHYT
Slja    ----MASLRKTHPLLKIANDALVDLPAPSNISVWWNFGSLLGLCLITQILTGLFLAMHYT
Bsja    ----MASLRKTHPLLKIANDALIDLPTPSNISVWWNFGSLLGLCLISQIATGLFLAMHYT
Ecna    ----MTSLRKTHPALKIVNDMVIDLPTPSNISAWWNFGSLLGLCLIVQIVTGLFLAMHYT
Cohi    ----MTSLRKSHPLLKIANDALIDLPTPSNISAWWNFGSLLGICLLTQILTGLFLAMHYT
Caar    ----MANLRKTHPLLKIVNDSLIDLPAPSNISAWWNFGSLLALCLATQILTGLFLAMHYT
Came    ----MANLRKTHPLLKIVNDSLIDLPAPSNISAWWNFGSLLALCLATQILTGLFLAMHYT
Mema    ----MANLRKTHPLLKIANDALVDLPAPSNISVWWNFGSLLGLCLIAQILTGLFLAMHYT
Lenu    ----MASLRKTHPILKIANDAVVDLPSPMNISAWWNFGSLLGLCLIAQIVTGLFLAMHYT
Brja    ----MASLRKTHPLLKIVNDALVDLPTPANIFVMWNFGSLLGLCLASQILTGLFLAMHYT
Plma    ----MASLRKTHPLLKIANHALVDLPSPSNISVWWNFGSLLGLCLIAQILTGLFLAMHYT
Emst    ----MASLRKTHPLLKIANNALVDLPAPSNISVWWNFGSLLGLCLIIQILTGLFLAMHYT
Ptti    ----MASLRKTHPLLKIANDALVDLPAPSNISVWWNFGSLLGLCLIAQLLTGLFLAMHYT
Losu    ----MSSLRKTHPLLKIANNALVDLPAPSNISIWWNFGSLLGLCLITQILTGLFLAMHYT
Geoy    ----MASLRKTHPLLKIANDALVDLPAPSNISVWWNFGSLLGLCLISQIVTGLFLAMHYT
Dipi    ----MASLRKTHPLLKIANDALVDLPAPSNISAWWNFGSLLGLCLISQIVTGLFLAMHYT
Pama    ----MASLRKTHPLLKIANHALVDLPAPSNISVWWNFGSLLGLCLISQILTGLFLAMHYT
Leob    ----MASLRKTHPLLKIANDAVVDLPAPSNISVWWNFGSLLGLCLISQILTGLFLAMHYT
Neba    ----MASLRKTHPLLKIANDALIDLPAPSNISAWWNFGSLLGLCLAAQILTGLFLAMHYT
Pdpl    ----MTSLRKTHPLLKIANNALVDLPAPSNISAWWNFGSLLGLCLGIQILTGLFLAMHYT
Nimi    ----MTSLRKTHPLLKIANDALVDLPAPSNISAWWNFGSLLGLCLAAQILTGLFLAMHYT
Uptr    ----MASLRKTHPLIKIANDALVDLPAPSNISVWWNFGSLLGLCLITQIVTGLFLAMHYT
Pesc    ----MAHLRKTHPILKIVNNALVDLPTPASISVWWNFGSLLGLCLVLQILTGLFLAMHYT
Baar    ----MTSLRKTHPLLKIANSALVDLPAPANISAWWNFGSLLGLCLITQILTGLFLAMHYT
Moar    ----MASLRKTHPLLKIANDALVDLPAPSNISAWWNFGSLLALCLITQILTGLFLAMHYT
Toja    ----MASLRKTHPLLKIANNALVDLPAPSNISVWWNFGSLLGLCLAAQILTGLFLAMHYT
Chau    ----MASLRKTHPLLKIANNALVDLPAPSNISVWWNFGSLLGLCLITQILTGLFLAMHYT
Chse    ----MTSMRKTHPLLKIANDALVDLPAPSNISVWWNFGSLLGLCLISQIATGLFLAMHYT
Enar    ----MASLRKTHPLLKIANDALVDLPAPSNISVWWNFGSLLGLCLITQILTGLFLAMHYT
Hpty    ----MASLRKTHPLLKIANDALVDLPAPSNISAWWNFGSLLGLCLITQILTGLFLAMHYT
Nana    ----MASLRKTHPLLKIANDALVDLPAPVNISTWWNFGSLLGLCLIIQILTGLFLAMHYT
Mcst    ----MASLRKTHPLLKIMNHALVDLPTPSNISAWWNFGSLLGLCLITQILTGLFLAMHYT
Rhox    ----MASLRKTHPLLSIANNALVDLPAPANISVWWNFGSLLGLCLATQILTGLFLAMHYT
Opfa    ----MTSLRKTHPLLKIANDALVDLPAPSNISVWWNFGSLLGLCLITQILTGLFLAMHYT
Paar    ----MASLRKTHPLLKIANDALVDLPAPSNISVWWNFGSLLGLCLISQILTGLFLAMHYT
Gozo    ----MASLRKTHPLLKIANDAVVDLPTPSNISVWWNFGSLLGLCLITQLLTGLFLAMHYT
```

To be continued
on page 9.

[1/7 of aligned sequences]

```
Ackr     ----MTSLRKSHPLLKIANHALVDLPAPSNISAWWNFGSLLGLCLMLQIVTGLFLAMHYT
Elev     ----MASLRKTHPLLKIANHALVDLPTPSNISAWWNFGSLLGLCLITQILTGLFLAMHYT
Trdu     ----MANLRKTHPLLKIANNALVDLPAPSNISVWWNFGSLLGLCLAAQILTGLFLAMHYT
Amoc     ----MANLRKTHPLLKIANDALVDLPTPANISVWWNFGSLLGLCLVAQILTGLFLAMHYT
Hame     ----MASLRKTHPLLKIANDAVVDLPAPSNISAWWNFGSLLGLCLATQLLTGLFLAMHYT
Chso     ----MASLRKTHPLLKIANDALVDLPAPSNISVWWNFGSLLGLCLASQILTGLFLAMHYT
Lyto     ----MASLRKTHPLLKIANNALVDLPAPSNISVWWNFGSLLGLCLIIQILTGLFLAMHYT
Encr     ----MASLRKTHPLLKIANDALVDLPAPSNISVWWNFGSLLGLCLIIQILTGLFLAMHYT
Bvar     ----MASFRKSHPLLKIVNNALVDLPAPSSISAWWNFGSLLGLCLLAQILTGLFLAMHYT
Noco     ----MASLRKTHPLLKIANDALVDLPAPSNISVWWNFGSLLGLCLIAQILTGLFLAMHYT
Chsp     ----MTSLRKTDPLIMTANNAVIDLPTPSNISAWWNFGSLLGLCLASQLVTGLFLAMHYT
Arja     ----MASLRKTHPLLKIANSALVDLPAPSNISVWWNFGSLLGLCLIIQILTGLFLAMHYT
Pase     ----MTSLRKTHPLLKIANDALVDLPAPSNISAWWNFGSLLGLCLIIQILTGLFLAMHYT
Trel     ----MANLRKTHPLMKIANDALVDLPAPSNISVWWNFGSLLGLCLATQILTGLFLAMHYT
Lifa     ----MASLRKTHPLFKIANDALVDLPAPSNISVWWNLGSLLGLCLISQILTGLFLAMHYT
Acur     ----MTSLRKSHPLLKMANDALVDLPAPSNISVWWNFGSLLGLCLISQLLTGLFLAMHYS
Ampe     ----MASLRKTHPLLKIANDALVDLPTPSNISVWWNFGSLLGLCLIAQILTGLFLAMHYT
Urja     ----MTSLRKTHPLLKIFNNALIDLPAPSNISAWWNFGSLLGLCLATQILTGLFLAMHYT
Enet     ----MASLRKTHPLLKIANGAVVDLPTPANISVWWNFGSLLGLCLGAQILTGLFLAMHYT
Ptbr     ----MTQLRKSHSLFKIANDALVDMPAPANISAWWNFGSLLGLCLVTQLLTGLFLAMHYT
Safa     ----MANLRKSHPLLKIANDAVVDLPAPANISIWWNFGSLLGLCLIAQLLTGLFLAMHYT
Icae     ----MASLRKTHPLLKIVNHVVIDLPSPSNISAWWNFGSLLGLCLVSQILTGLFLAMHYT
Asmi     ----MA-HRKTHPLLKVANHALVDLPAPANISIWWNFGSLLGLCLATQILTGLFLAMHYT
Foal     ----MTSLRKQHPLLKMANSAVIDLPAPINISAWWNFGSLLGLCLIAQLATGLFLAMHYT
Drze     ----MASLRKTHPLLKIINGTLIDLPTPSNISVWWNFGSLLGLCLIIQILTGLFLAMHYT
Rhas     ----MANLRKTHPLLKIANDALVDLPAPSNISAWWNFGSLLGLCLGAQILTGLFLAMHYT
Elac     ----MAPLRKTHPLLKIVNHALVDLPAPSNISAWWNFGSLLGLCLAAQLLTGIFLAMHYT
Kugu     ----MANLRKTHPLLKIANDALIDLPAPSNISAWWNFGSLLGLCLIIQILTGLFLAMHYT
Plor     ----MASLRKTHPILKIANDAVVDLPTPSNISAWWNFGSLLGLCLISQILTGLFLAMHYT
Sgun     ----MASLRKTHPLLKIANDALVDLPAPSNISAWWNFGSLLGLCLIIQILTGLFLAMHYT
Zaco     ----MASLRKTHPLLKIANDALVDLPAPSNISVWWNFGSLLGLCLVAQLLTGLFLAMHYT
Zbfl     ----MASLRKTHPLLKIANDALVDLPAPSNISVWWNFGSLLGLCLIAQILTGLFLAMHYT
Spba     ----MANLRKTHPLLKIANDALVDLPTPSSISAWWNFGSLLGLCLITQVLTGLFLAMHYT
Game     ----MASLRKTHPLLKIANDALVDLPTPSNISVWWNFGSLLGLCLISQILTGLFLAMHYT
Thth     ----MASLRKTHPLLKIANDALVDLPTPSNISAWWNFGSLLGLCLISQILTGLFLAMHYT
Xigl     ----MANLRKTHPLLKIANDALVDLPTPSNISVWWNFGSLLGLCLAAQVLTGLFLAMHYT
Hyja     ----MANLRKTHPLLKIANDALVDLPSPANISVWWNFGSLLGLCLIVQILTGLFLAMHYT
Psan     ----MASLRKTHPLLKIMNDALIDLPTPASISVWWNFGSLLGLCLMTQILTGLFLAMHYT
Cupa     ----MASLRKTHPLLKIVNDAVIDLPSPSNISVWWNFGSLLGLCLITQILTGLFLAMHYT
Mpch     ----MANLRKTHPLLKIANDALVDLPTPANISAWWNFGSLLGLCLIIQIITGLFLAMHYT
Char     ----MANLRKTHPLLKIANDALVDLPTPSSISAWWNFGSLLGLCLMAQIITGLFLAMHYT
Pser     ----MAKLQKSHPLLKIANDALVDLPTPVNISAWWNFGSLLGLCLITQILTGLFLAMHYT
Prol     ----MASLRKSHPLLKIANDALVDLPAPSNISVWWNFGSLLGLCLITQILTGLFLAMHYT
Plbi     ----MANLRKSHPLLKIANDALVDLPAPSNISVWWNFGSLLGLCLVTQIATGLFLAMHYT
Calu     ----MASLRKTHPLLKVANDALVDLPAPANISVWWNFGSLLGLCLIAQILTGLFLAMHYI
Papa     ----MTSLRKKHPLIKIANSAIIDLPTPSNISGWWNFGSLLGLCLIIQITTGLFLAMHYT
Sufr     ----MASLRKTHPLLKIANGAVVDLPTPSNISAWWNFGSLLGMCLIIQILTGLFLAMHYT
Stci     ----MASLRKTHPLLKIANDAVVDLPAPSNISVWWNFGSLLGMCLILQIVTGLFLAMHYT
Taru     ----MASLRKTHPLLKIVNDMVIDLPTPSNISAWWNFGSLLGLCLITQIITGLFLAMHYT
Rala     ----MASLRKTHPLLKIANDALVDLPTPSNISAWWNFGSLLGLCLIIQILTGLFLAMHYT
                                 ::* *  .:    ** ***: :**  *  **.:::**:
```

To be continued on page 10.

[2/7 of aligned sequences]

```
                                                83    B          97                  116
Scca  ADISMAFSSVIHISRDVNYGWLMRNIHAYGASFFFICIYLHIARGLYYGSYLNKEAWNIG
Muma  ADISMAFSSVVHICRDVNYGWLIRNIHANGASLFFICIYLHIARGLYYGSYLNKETWDIG
Erca  SDINLAFSSVAHICRDVNYGWLIRNIHANSASLFFICIYLHIARGLYYGSYLYMETWNVG
Pose  SDISSAFSSVAHICRDVNYGWLIRNFHANGASLFFICIYLHIARGLYYGSYLYMETWNIG
Actr  ADISTAFSSVAHICRDVNYGWLIRNIHANGASFFFICLYLHVARGMYYGSYLQKETWNIG
Scal  ADISTAFSSVAHICRDVNYGWLIRNVHANGASFFFICLYLHVARGMYYGSYLQKETWNIG
Posp  ADISTAFSSVAHICRDVNYGWLIRNIHANGASFFFICLYLHVARGMYYGSYLYKETWNIG
Atsp  ADITLAFSSVAHICRDVNYGWLLRNIHANGASFFFICIYLHIARGLYYGSYLYKETWNIG
Leoc  ADITLAFSSVAHICRDVNYGWLLRNIHANGASFFFICIYLHIARGLYYGSYLYKETWNIG
Amca  SDISLAFSSVAHICRDVNYGWFLRNIHANGASLFFICLYLHIARGLYYGSYLYKETWNVG
Osbi  SDISTAFSSVAHICRDVNYGWLIRNLHANGASFFFICIYLHVARGLYYGSYLYKETWNIG
Pabu  SDISTAFSSVMHICRDVNYGWLIRNIHANGASFFFICIYFHIARGLYYGSYLYKETWNTG
Hial  SDISTAFSSVTHICRDVSYGWLIRNMHANGASFFFICIYLHVARGLYYGSYLYKETWNIG
Elha  SDISTAFSSVAHICRDVNFGWLIRNLHANGASFFFVCIYLHIGRGLYYGSYLYKETWNIG
Mlcy  SDISTAFSSVTHICRDVNYGWLIRNIHANGASFFFICIYLHIGRGLYYGSYLYKETWNIG
Algl  SDISTAFSSVAHICRDVNYGWLIRSVHATGASFFFICIYIHVARGLYYGSYLYMETWNTG
Ptgi  SDISTAFSSVTHICRDVSYGWLIRNMHANGASFFFICIYMHIARGLYYGSYLYKETWNVG
Alaf  ADISTAFSSVTHICRDVNYGWLIRNIHANGASFFFICIYMHIARGLYYGSYLQKETWFVG
Nock  SDISTAFSSVTHICRDVSYGWLIRNIHANGASFFFICIYMHIARGLYYGSYLQKQTWYVG
Anja  SDISTAFSSVAHICRDVNYGWFIRNLHANGASFFFICLYLHIARGLYYGSYLYKETWNIG
Gyki  SDISTAFSSVAHICRDVNYGWLIRNLHANGASFFFICLYMHIARGLYYGSYLYKETWNIG
Syka  ADISTAFSSVAHICRDVNYGWFIRNLHANGASFFFICLYLHIARGLYYGSYLYMETWNVG
Opma  SDISTAFSSVVHICRDVNYGWLVRNLHANGASFFFICLYLHIARGLYYGSYMYKSTWNIG
Comy  SDISTAFSSVAHICRDVNYGWLIRNLHANGASFFFICLYMHIARGLYYGSYLYKETWNVG
Sasp  PDITMAFSSVAHICRNVNFGWLIRNLHANGASLFFICLYLHVARGLYYGSYVYKKTWNIG
Eupe  SDISTAFSSVAHICRDVNYGWLIRNLHANGASLFFICLYLHIARGLYYGSYNFKMTWNIG
Enja  SDIATAFSSVAHICRDVNYGWLIRNMHANGASFFFICIYAHIARGLYYGSYLYMETWNIG
Same  SDIATAFSSVAHICRDVNYGWLIRSMHANGASFFFICIYAHIGRGLYYGSYLYKETWNIG
Chch  SDISTAFSSVTHICRDVSYGWLIRNMHANGASFFFICIYMHTARGLYYGSYLYKETWNIG
Grgr  SDVSTAFSSVAHICRDVTYGWLIRNIHANGASFFFICVYMHIGRGLYYGSYLYKETWNIG
Caau  SDISTAFSSVTHICRDVNYGWLIRNIHANGASFFFICIYMHIARGLYYGSYLYKETWNIG
Cyca  SDISTAFSSVTHICRDVNYGWLIRNVHANGASFFFICIYMHIARGLYYGSYLYKETWNIG
Dare  SDISTAFSSVVHICRDVNFGWLIRSIHANGASFFFICLYIHIARGLYYGSYLYNETWNIG
Cost  SDITTAFSSVAHICRDVNYGWLIRNIHANGASFFFICIYIHIARGLYYGSYLYKETWNIG
Leec  SDISTAFSSVAHICRDVNYGWLIRSIHANGASFFFICIYMHIARGLYYGSYLYKETWNIG
Fola  SDISTAFSSVAHICRDVNYGWLIRNIHANGASFFFICLYLHIARGLYYGSYLYKETWNIG
Clmc  SDISTAFSSVVHICRDVNHGWLIRNMHANGASFFFICIYLHIGRGLYYGSYLYKETWNIG
Phin  SDISTAFSSVIHICRDVNYGWIIRNIHANGASFFFICIYFHIGRGLYYGSYLYKETWNIG
Icpu  SDISTAFSSVAHICRDVNYGWLIRNLHANGASFFFICIYLHIGRGLYYGSYLYKETWNIG
Psto  SDISTAFSSVAHICRDVNYGWIIRNLHANGASFFFLCIYLHIGRGLYYGSYLYKETWNIG
Cora  SDITSAFSSVAHICRDVNYGWAIRNIHANGASFFFICIYLHIGRGLYYGSYLYKETWNIG
Eisp  SDISTAFSSVAHICRDVNYGWFIRNLHANGASFFFICIYLHIGRGLYYGSFLYKETWNIG
Apal  PDICTAFSSVAHICRDVNYGWLIRNAHANGASLFFVCVYLHIGRGLYYGSYLYKETWNIG
Eslu  SDISTAFSSVCHICRDVNYGWLIRNIHANGASLFFICIYMHIARGLYYGSYLYKETWNIG
Dape  SDISTAFSSVCHICRDVNYGWLVRNIHANGASFFFICIYIHIARGLYYGSYLYKETWNIG
Glse  SDISTAFSSVAHICRDVTYGWLIRNMHANGASFFFICIYMHIGRGLYYGSYLNKETWNIG
Naar  ADISTAFSSVAHICRDVTYGWLIRNIHANGASFFFICIYLHIGRGLYYGSYLMKETWNIG
Lioc  SDISTAFSSVAHICRDVTYGWLIRNMHANGASFFFICLYLHIGRGLYYGSYLLKETWNIG
Opso  SDVSMAFSSVAHICRDVTYGWLIRNIHANGASFFFICIYMHIARGLYYGSYLYKETWNIG
Alte  ADISTAYSSVVHICRDVTYGWLIRSMHANGASFFFICLYIHIARGLYYGSYLYKETWNIG
Plap  ADITTAYSSVTHICRDVTYGWLIRSMHANGASFFFICLYIHIARGLYYGSHLYQETWNIG
                                                   6
```

To be continued on page 11.

[2/7 of aligned sequences]

To be continued on page 12.

```
Plal  AETATAFSSVVHLCRDVNYGWLIRNMHANGASFFFICIYLHIGRGLYYGSFLYKETWNIG
Sami  AETSTAFSSVVHLCRDVNYGWLIRNMHANGASFFFICIYLHIGRGLYYGSFLYKETWNVG
Rere  SDISTAFSSVAHICRDVNYGWLIRNMHANGASFFFICIYLHVGRGLYYGSFLYKETWVLG
Gama  SDISTAFSSVTHICRDVSYGWLIRNMHANGASFFFICIYMHIGRGLYYGSYLYKETWTIG
Onmy  SDISTAFSSVCHICRDVSYGWLIRNIHANGASFFFICIYMHIARGLYYGSYLYKETWNIG
Sasa  SDISTAFSSVCHICRDVSYGWLIRNIHANGASFFFICIYMHIARGLYYGSYLYKETWNIG
Cola  SDISTAFSSVCHICRDVSYGWLIRNIHANGASFFFICIYMHIARGLYYGSYLYKETWNIG
Dita  SDISTAFSSVTHICRDVSYGWLIRNMHANGASFFFICIYLHIGRGLYYGSYLFKETWNIG
Gogr  SDISTAFSSVTHICRDVNYGWLVRNMHANGASFFFICLYTHIGRGLYYGSYLYKETWTVG
Chsl  SDISTAFSSVTHICRDVNYGWMIRNMHANGASFFFICIYLHIGRGLYYGSYLYKETWNIG
Atja  SDISTAFSSVAHICRDVNYGWLIRNLHANGASFFFICIYMHIGRGLYYGSYLYKETWNTG
Iido  SDISTAFSSVTHICRDVNYGWLIRNLHANGASFFFICIYMHIGRGLYYGSYLYKETWNTG
Auja  SDIATAFSSVTHICRDVNYGWLIRNMHANGASFFFICIYMHIARGLYYGSYLYKETWNVG
Chag  SDIATAFSSVTHICRDVNYGWLIRNMHANGASFFFICIYMHIARGLYYGSYLYKETWNVG
Hami  SDVATAFSSVAHICRDVNYGWMIRNLHANGASFFFICIYIHIARGLYYGSYLYMETWNIG
Saun  SDVATAFSSVAHICRDVNYGWMIRNLHANGASFFFICIYIHIARGLYYGSYLYMETWNIG
Nema  SDIATAFSSVTHICRDVNYGWLIRNMHANGASFFFICIYMHIARGLYYGSYLYKETWNTG
Disp  SDIDMAFSSVTHICRDVNYGWLIRNMHANGASFFFICIYLHIGRGLYYGSYLYKETWNVG
Myaf  SSIDTAFSSVAHICRDVNYGWLIRNMHANGASFFFICLYMHIGRGLYYGSYLYMETWNVG
Lagu  SDISTAFSSVVHTCRDVNYGWFIRSLHANGASFFFICIYLHIARGLYYGSYLYKETWTIG
Trtr  SDISMAFSSVVHICRDVNFGWLIRSIHANGASFFFICIYMHIARGLYYGSYLYKETWNVG
Zucr  SDISMAFSSVVHICRDVNFGWLIRSIHANGASFFFICIYMHIARGLYYGSYLYKETWNVG
Pxja  SDISTAFSSVTHICRDVNYGWLIRNMHANGASFFFICIYLHIGRGLYYGSYLYKETWNIG
Pxlo  SDISTAFSSVTHICRDVNYGWLIRNMHANGASFFFICIYLHIGRGLYYGSYLYKETWNIG
Pctr  SDIATAFSSVMHICRDVNYGWLIRNMHANGASFFFICIYLHIGRGLYYGSYLYKETWNVG
Apsa  SDITMAFSSIAHICRDVNYGWLIRNMHANGASFFFICMYLHIGRGLYYGSYLYKETWNIG
Cabe  PDTASAFSSLIHICRDVNYGWLIRNVHANGASVFFICIYIHVARGLYYGSYLYKETWTVG
Bzze  SDIATAFSSVAHICRDVNYGWLIRNMHANGASFFFICIYMHIARGLYYGSYLYKETWNVG
Siim  PDLDTAFQSVVHICRDVNYGWMFRSLHANGASLFFICIYIHIARGLYYGSYMYVHVWNIG
Ctru  SDIATAFSSIAHICRDVNYGWLIRNMHANGASFFFICIYMHIARGLYYGSYLYKETWNVG
Dpbr  SDIATAFSSVAHICRDVNYGWLIRNMHANGASFFFICIYMHIARGLYYGSYLYKETWNVG
Caki  SDIQTAFTSVVHICRDVNYGWLVRNIHANGASFFFICLYLHIARGLYYGSYLFMETWNIG
Phja  ADIETAFSSVVHICRDVNYGWLIRNVHANGASFFFICLYLHIARGLYYGSYLLLETWNIG
Brsp  ANIDFAFSSVVHICRDVNYGWLIRNLHANGASMFFICLYLHIARGLYYESYLFMETWNVG
Gamo  SDIETAFSSVVHICRDVNYGWLIRNMHANGASFFFICLYMHIARGLYYGSYLFVETWNIG
Lolo  SDIETAFSSVVHICRDVNYGWLIRNMHANGASFFFICLYLHIARGLYYGSYLFIETWNIG
Batr  PDTLTAFNSISHITRDVNYGWLIRNLHANGASFFFICLYLHIARGLYYGSYMSKETWNIG
Prmy  PDATLAFSSVAHLTRDVNYGWLLRNLHANGASIFFICMYIHIARGLYYASYMSKNLWNVG
Lose  SDIATAFSSVAHICRDVNYGWLIRNLHANGASFFFICIYMHIGRGLYYGSYLYKETWNIG
Loam  SDIATAFSSVAHICRDVNYGWLIRNLHANGASFFFICIYMHIGRGLYYGSYLYKETWNIG
Chab  SDIATAFSSVGHICRDVNYGWLIRNLHANGASFFFICIYLHIGRGLYYGSYLYKETWNIG
Chto  SDIATAFSSVGHICRDVNYGWLIRNLHANGASFFFICIYLHIGRGLYYGSYLYKETWNIG
Majo  SDISTAFSSVAHICRDVNYGWLIRNMHANGASFFFICIYLHIGRGLYYGSYLYKETWNVG
Hlst  SDISTAFSSVAHICRDVNYGWLIRNIHANGASFFFICIYLHIGRGLYYGSYLYKETWNVG
Clpe  SDIATAFSSVSHICRDVNYGWLMRNMHANGASFFFICIYLHIGRGLYYGSYLYKETWTVG
Mlmr  SDIATAFSSVAHICRDVNYGWLIRNMHANGASFFFICIYLHIGRGLYYGSYLYKETWNIG
Crcr  PETSSAFASVAHICRDVNYGWLIRNMHANGASFFFVCIYIHIGRGLYYGSYLYKETWNIG
Muce  PETSSAFASVAHICRDVNYGWLIRNMHANGASFFFVCIYIHIGRGLYYGSYLYKETWNIG
Bege  SDIATAFSSVAHICRDVNYGWLIRNMHANGASFFFICIYLHIGRGLYYGSYLYKETWNVG
Mela  SDITAAFSSVAHICRDVNYGWLIRNMHANGASFFFICIYMHIGRGLYYGSYLYKNTWNVG
Hats  SDIATAFSSVAHICRDVNYGWLIRNMHANGASFFFICIYLHIGRGLYYGSYLHKETWNVG
Orla  SDIATAFSSVAHICRDVNYGWLIRNMHANGASFFFICIYLHIGRGLYYGSYLYKETWNVG
```

[2/7 of aligned sequences]

To be continued on page 13.

Cosa	SDIATAFSSVAHICRDVNYGWLIRNMHANGASFFFICIYMHIGRGLYYGSFLNKETWNVG
Exsp	ADISTAFSSVAHICRDVNYGWMIRNMHANGASFFFVCIYLHIGRGLYYGSHLNKETWNVG
Depa	SDIATAFSSIAHISRDVNYGWLIRNMHANGASFFFICIYLHIGRGLYYGSFLNKETWNVG
Rima	SDISTAFSSVVHICRDVNYGWLIRSIHANGASFFFICIYLHIGRGLYYGSYLNKEAWSAG
Fuol	SDISTAFSSVAHICRDVNYGWLIRNMHANGASFFFICIYLHIGRGLYYGSYLYKETWNVG
Gmaf	SDISTAFSSVAHICRDVNYGWLIRNMHANGASFFFICIYLHIGRGLYYGSYLFKETWNTG
Xeei	SDISMAFSSVAHICRDVNYGWLIRNMHANGASLFFVCIYFHIGRGLYYGSYLYKETWNIG
Pros	ADITSAFSSVAHICRDVNYGWLIRNIHANGASLFFMCIYMHIGRGLYYGSYLYKEVWNTG
Scmi	ADITMAFSSVAHICRDVNYGWLIRNLHANGASLFFMCIYMHIGRGLYYGSYLYKETWNTG
Rolo	SDIATAFSSVTHICRDVNYGWLIRNMHANGASFFFICIYMHIGRGLYYGSYLYKETWNIG
Cere	SDIATAFSSVTHICRDVNYGWLIRSIHANGASFFFICIYLHIGRGLYYGSYLYKETWNVG
Daga	SDISTAFSSVTHICRDVNFGWLIRNIHANGASFFFICIYLHIGRGLYYGSYLYKETWSIG
Anco	SDITTAFSSVAHICRDVNYGWLIRNMHANGASFFFICLYLHIGRGLYYGSYLNKETWNVG
Dmve	SDITTAFSSVAHICRDVNYGWLIRNLHANGASFFFICIYMHIGRGLYYGSYLYKETWNIG
Dmar	SDITTAFSSVAHICRDVNYGWLIRNLHANGASFFFICIYMHIGRGLYYGSYLYKETWNIG
Anka	SDIATAFSSVTHICRDVNYGWLIRNLHANGASFFFICIYMHVGRGLYYGSYLYKETWNVG
Moja	SDIATAFSSVTHICRDVNYGWLIRNLHANGASFFFICIYMHVGRGLYYGSYLYKETWNVG
Hoja	SDIATAFSSVTHICRDVNYGWLIRNLHANGASFFFICIYMHIGRGLYYGSYLYKETWNVG
Bede	SDIATAFSSVAHICRDVNYGWLIRNLHANGASVFFICIYMHIGRGLYYGSYLYKETWNTG
Besp	SDIATAFSSVAHICRDVNYGWLIRNLHANGASVFFICIYMHIGRGLYYGSYLYKETWNTG
Mysp	SDIATAFSSVTHICRDVNYGWLIRNMHANGASFFFICIYMHIARGLYYGSYLYKEAWNIG
Osja	SDIATAFSSVTHICRDVNYGWLIRNMHANGASFFFICIYMHIARGLYYGSYLYKEAWNVG
Sgro	SDIATAFSSVTHICRDVNYGWLIRNMHANGASFFFICIYMHIARGLYYGSYLYKETWNVG
Pzpa	SDITTAFSSVAHICRDVNHGWLIRNLHANGASFFFVCIYLHIGRGLYYGSYLNQATWNIG
Zeja	SDVSSAFSSVAHICRDVNYGWLIRNLHANGASFFFICIYLHVGRGLYYGSYLNKEAWNIG
Znne	SDVATAFSSVAHICRDVNYGWLIRNIHANGASFFFICLYLHIGRGLYYGSYLYKETWNIG
Zefa	SDIATAFSSVAHICRDVNYGWLIRNFHANGASFFFICLYLHIGRGLYYGSYLYKETWNVG
Acni	ADIATAFSSVAHICRDVNYGWLIRNLHANGASFFFICLYLHVGRGLYYGSYLYKETWNIG
Ncrh	ADIATAFSSVAHICRDVNYGWLIRNFHANGASFFFICLYLHVGRGLYYGSYLYKETWNIG
Agca	ADVTTAFSSVAHICRDVNYGWLIRNLHANGASFFFICIYLHIGRGLYYGSYLYKETWTIG
Hydy	SDIATAFSSVGHICRDVNYGWLIRNLHANGASFFFICIYMHIGRGLYYGSYLYKETWNIG
Gsac	SDIATAFSSVGHICRDVNYGWLIRNLHANGASFFFICIYMHIGRGLYYGSYLYKETWNIG
Pevo	ADISTAFSSVAHICRDVNYGWLLRNMHANGASFFFICIYMHIARGLYYGSYLYKETWNIG
Hiku	SDIATAFSSVTHICRDVNYGWLIRNMHANGASFFFICIYLHIARGLYYGSYLYKETWNVG
Inpa	PDINAAFSSVAHICRDVNHGWLIRNLHANGASFFFLCLYLHIGRGLYYGSYLNKGTWNTG
Auch	PDIDSAFSSVAHICRDVNYGWLIRNMHANGASFFFICIYAHIARGLYYGSYLYKATWNLG
Fico	SDIATAFSSVAHICRDVNYGWLIRNMHANGASFFFICIYMHIARGLYYGSYLYKETWNVG
Macs	SDIATAFSSVAHICRDVNYGWLIRNMHANGASFFFICLYMHIGRGLYYGSYLYKETWNVG
Moal	ADISTAFASVAHICRDVNNGWLIRALHANGASFFFICIYLHIGRGLYYGSYLYKETWLSG
Syma	AEMSFSFASVTHITRDVGYGWFLRNTHANGASILFICMYLHMGRGLYYGSHLYMETWNIG
Mafr	SDISMAFSSVAHICRDVNYGWLIRNIHANGASMFFICIYLHIGRGLYYGSYLYKETWNIG
Dcpe	SDIATAFSSVAHICRDVNYGWLIRNLHANGASFFFICIYLHIGRGLYYGSYLYKETWNVG
Dcti	SDIATAFSSVAHICRDVNYGWLIRNMHANGASFFFICIYLHIGRGLYYGSYLYKETWNVG
Hehi	SDIATAFSSVAHICRDVNYGWLIRNLHANGASFFFVCIYAHIGRGLYYGSYLYKETWNVG
Stam	SDIATAFSSVAHICRDVNYGWLIRNLHANGASFFFVCLYMHIGRGLYYGSYLYKETWNIG
Hogi	SDIATAFSSVAHICRDVNYGWLIRNLHANGASFFFICIYLHIGRGLYYGSYLYMATWNVG
Erzo	SDIATAFSSVGHICRDVNYGWLIRNIHANGASFFFICLYTHIGRGLYYGSYLNKETWNVG
Hxot	SDIATAFSSVGHICRDVNYGWLIRNLHANGASFFFICIYAHIGRGLYYGSYLYKETWTIG
Core	SDIATAFSSVGHICRDVNYGWLIRNLHANGASFFFICIYMHIGRGLYYGSYLYKETWNIG
Apve	SSIETAFSSVGHICRDVNYGWFIRNLHANGASFFFICIYAHIGRGLYYGSFLYKSTWTIG
Latj	SDIATAFTSVTHICRDVNYGWLIRNMHANGASFFFICIYLHIGRGLYYGSYLYKETWNIG
Laja	SDVATAFSSVAHICRDVNYGWLIRNIHANGASFFFICIYMHIGRGLYYGSYLYKETWNIG

[2/7 of aligned sequences]

To be continued on page 14.

```
Syja SDIATAFSSVAHICRDVNYGWLIRNLHANGASFFFICIYLHIGRGLYYGSYLYKETWNIG
Epme SDIATAFSSVAHICRDVNYGWLIRNMHANGASFFFICIYMHIGRGLYYGSYLYKETWNIG
Grse SDIATAFSSVAHICRDVNYGWLIRNMHANGASFFFICIYAHIGRGLYYGSYLYKETWNIG
Clja SDIATAFSSVAHICRDVNYGWLIRNMHANGASFFFICIYLHIGRGLYYGSYLYKETWNIG
Ogcy ADITMAFSSVAHICRDVNYGWFIRNMHANGASMFFVCIYLHIGRGLYYGSYLNKETWNVG
Plna ADIATAFSSVAHICRDVNYGWLIRNMHANGASFFFICIYLHIGRGLYYGSYLYKETWNVG
Lema SDIATAFSSVAHICRDVNYGWLIRNIHANGASFFFICIYLHIGRGLYYGSYLYKETWNIG
Etzo ADIATAFSSVAHICRDVNYGWLIRNIHANGASFFFICIYMHIGRGLYYGSYLYKETWNIG
Apse ADIATAFSSVAHICRDVNFGWLIRNMHANGASFFFICIYLHIGRGLYYGSYMFKETWNVG
Epde SDIATAFSSVAHICRDVNYGWLIRNIHANGASFFFICIYMHIGRGLYYGSYLYKETWNIG
Slja SDIATAFSSVAHICRDVNFGWLIRNVHANGASFFFICIYFHIGRGLYYGSYLYKETWNVG
Bsja SDIATAFSSVTHICRDVNYGWLIRNLHANGASFFFMCIYAHIGRGLYYGSYLYKETWNIG
Ecna ADISAAFSSVAHICRDVNYGWLIRNMHANGASMFFVCIYMHIGRGLYYGSYLNKETWNTG
Cohi ADIATAFPSVAHICRDVNYGWLIRNLHANGASFFFICVYFHIGRGLYYGSYLYKETWNIG
Caar SDIATAFTSVAHICRDVNYGWLIRNMHANGASFFFICIYLHIGRGLYYGSYLYKETWNTG
Came SDIATAFTSVAHICRDVNYGWLIRNMHANGASFFFICIYLHIGRGLYYGSYLYKETWNTG
Mema SDIATAFTSVTHICRDVNYGWLIRNMHANGASFFFICIYLHIGRGLYYGSYLYKETWNIG
Lenu ADINAAFASVTHICRDVNYGWLIRNLHANGASMFFVCIYLHIGRGLYYGSYLYKATWNVG
Brja PDVESAFASVAHICRDVNFGWLIRNLHANGASFFFVCLYLHIGRGLYYGSYLYKETWNIG
Plma PNVESAFDSVAHICRDVNFGWLIRNLHANGASFFFICIYSHIGRGLYYGSYLYKETWNTG
Emst SDIATAFSSVAHICRDVNYGWLIRNMHANGASFFFICIYLHIGRGLYYGSYLYKETWNIG
Ptti SDISMAFSSVAHICRDVNYGWLIRNLHANGASFFFICIYLHIGRGLYYGSYLYKETWNIG
Losu SDITTAFSSVAHICRDVNYGWLIRNMHANGASFFFICIYLHIGRGLYYGSYLYKETWNIG
Geoy ADIDTAFSSVAHICRDVNYGWLIRNLHANGASFFFICIYMHIARGLYYGSYLYKETWNIG
Dipi SDIATAFSSVAHICRDVNYGWLIRNLHANGASFFFICIYLHIGRGLYYGSYLYKETWNIG
Pama SDIATAFSSVAHICRDVNYGWLIRNLHANGASFFFICIYLHIGRGLYYGSYLYKETWNIG
Leob SDIATAFSSVAHICRDVNYGWLIRNLHANGASFFFICIYLHIGRGLYYGSYLYKETWNIG
Neba SDIATAFSSVAHICRDVNYGWLIRNLHANGASFFFICIYLHIGRGLYYGSYLYMETWNIG
Pdpl PNVTSAFDSVAHICRDVNFGWMIRNMHANGASFFFVCIYFHIGRGLYYGSYLYKETWNIG
Nimi SDISMAFSSVAHICRDANYGWLIRNLHANGASFFFICLYLHIGRGLYYGSYLYKETWNIG
Uptr SDIATAFSSVAHICRDVNYGWLIRNMHANGASFFFICIYMHIARGLYYGSYLYKETWNVG
Pesc PDVSSAFSSVVHICRDVNFGWLIRNLHANGASFFFICLYAHIGRGIYYGSYLYKGTWSVG
Baar SDIATAFSSVAHICRDVNYGWLIRNLHANGASFFFICIYLHIGRGLYYGSYLYKETWNIG
Moar SDIATAFSSVAHICRDVNYGWLIRNIHANGASFFFICIYLHIGRGLYYGSYLYKETWNVG
Toja SDIATAFTSVAHICRDVNYGWLIRNMHANGASFFFICIYLHIGRGLYYGSYLYKETWNIG
Chau ADVATAFSSVAHICRDVNYGWLIRNLHANGASMFFICIYLHIGRGLYYGSYLYKETWNIG
Chse SDISTAFSSVAHICRDVNYGWLIRNLHANGASFFFICIYFHIGRGLYYGSYLYKETWNVG
Enar SDIATAFSSVAHICRDVNYGWLIRNIHANGASFFFICIYMHIGRGLYYGSYLYKETWNVG
Hpty SDIATAFSSVAHICRDVNYGWLIRNIHANGASFFFICIYLHIGRGLYYGSYLYKETWNIG
Nana ADTSTAFSSVAHICRDVNYGWLIRNMHANGASFFFICIYLHIGRGLYYGSYLYKETWNIG
Mcst SDIATAFSSVAHICRDVNYGWLIRSIHANGASFFFICIYMHVARGLYYGSYLFKETWNVG
Rhox SDIATAFSSVAHICRDVNYGWLIRNIHANGASFFFICIYMHIARGLYYGSYLYKETWNVG
Opfa SDIATAFSSVAHICRDVNYGWLIRNIHANGASFFFICIYMHIARGLYYGSYLYKETWNVG
Paar SDIATAFSSVTHICRDVNYGWLIRNLHANGASFFFICIYMHIGRGLYYGSYLYKETWNVG
Gozo SDIATAFSSVAHICRDVNYGWLIRNIHANGASFFFICIYMHIGRGLYYGSYLQKETWNIG
Ackr ADVSMAFSSVAHICRDVNFGWLIRNLHANGASFFFVCIYLHIGRGLYYGSYAFKETWNIG
Elev SDIATAFSSVAHICRDVNYGWLIRNIHANGASFFFICIYLHIGRGLYYGSYLYKETWNIG
Trdu SDIATAFSSIAHICRDVNYGWLIRNMHANGASFFFICIYLHIGRGLYYGSYLYKETWNIG
Amoc SDIATAFSSVAHICRDVNYGWLIRNMHANGASFFFICIYLHIGRGLYYGSYLHKETWNVG
Hame SDIATAFSSVAHICRDVNYGWLIRNMHANGASFFFICIYLHVGRGLYYGSYLYKETWNIG
Chso SDIATAFSSVAHICRDVNYGWLIRNMHANGASFFFICIYLHIGRGLYYGSYLYKETWNTG
Lyto PDIATAFSSVGHICRDVNYGWFIRNLHANGASFFFICIYMHVGRGLYYGSYLYKETWNIG
```

[2/7 of aligned sequences]

```
Encr   SDIATAFSSVGHICRDVNYGWLIRNLHANGASFFFICLYMHIGRGLYYGSYLYKETWNVG
Bvar   SDINTAFASVAHITRDVNYGWLLRDMHANGASFFFVCLYLHIGRGLYYGSYLYKETWNVG
Noco   ADINTAFSSVAHITRDVNYGWLIRDMHANGASFFFICIYMHIGRGLYYGSYLYKETWNVG
Chsp   ADINTAFSSVAHICRDVNYGWLIRNLHANGASLFFMCIYFHVGRGLYYGSYLYKKTWNVG
Arja   SDIATAFSSVGHICRDVNYGWLIRNLHANGASFFFICIYAHIGRGLYYGSYLYKETWTVG
Pase   SSVDTAFSSVAHICRDVNYGWLIRNMHANGASFFFICIYMHIGRGLYYGSYLNQATWNVG
Trel   SDIDTAFSSVAHICRDVNFGWMIRNMHANGASFFFVCIYFHIGRGLYYGSYLYKETWNIG
Lifa   SDMATAFSSVAHITRDVNYGWLIRNLHANGASFFFICIYLHIGRGLYYGSYLYKETWNVG
Acur   SDIDTAFSSVVHICRDVNYGWLIRNLHANGASFFFICIYLHIGRGLYYGSFLYKETWNIG
Ampe   SDINTAFSSVAHICRDVNYGWLIRNLHANGASFFFICLYMHIGRGLYYGSYLNKETWTVG
Urja   PDINTAFSSVAHICRDVNYGWFIRNIHANGASLFFICIYMHIGRGLYYGSYLYKETWNVG
Enet   SDIETAFSSVAHICRDVNYGWLIRNMHANGASFFFICIYLHIGRGLYYGSYLYKETWNVG
Ptbr   SDITTAFSSVTHICRDVNYGWLIRNMHANGASFFFICIYLHIGRGLYYGSYLYKETWNVG
Safa   SDITTAFSSVAHICRDVNYGWLIRNMHANGASFFFICIYLHIGRGLYYGSYLYKETWNIG
Icae   PDVESAFASVAHICRDVNFGWLIRNLHANGASFFFICIYSHIGRGLYYGSYLNKETWNTG
Asmi   SDILTAFSSVAHICRDVNYGWLIRNMHANGASFFFICIYLHIGRGLYYGSYLYKETWNVG
Foal   ADIATAFSSVAHICRDVNYGWLIRNMHANGASFFFICIYMHIGRGLYYGSYLYKMTWNVG
Drze   SDVTSAFASIAHICRDVNHGWLIRNLHMNGASFFFICIYLHVGRGLYYGSYLYKEVWNMG
Rhas   SDIATAFSSVAHICRDVNFGWLIRNMHANGASFFFICIYLHIGRGLYYGSYLYKETWNIG
Elac   ADVSMAFSSVAHICRDVNYGWLLRNLHANGASFFFICIYLHIGRGLYYGSYLFKETWNIG
Kugu   SDIATAFSSVAHICRDVNFGWLIRNMHANGASFFFICIYLHIGRGLYYGSYLYMETWNTG
Plor   SDIATAFSSVAHICRDVNYGWLIRNLHANGASFFFICIYLHIGRGLYYGSYLYKETWNIG
Sgun   SDIATAFSSVAHICRDVNYGWLIRNLHANGASFFFICLYLHIGRGLYYGSYLYKETWNIG
Zaco   SDIATAFSSVAHICRDVNYGWLIRNLHANGASFFFICIYLHIGRGLYYGSYLYKETWNVG
Zbfl   SDIATAFSSVAHICRDVNYGWLIRNLHANGASFFFICIYLHIGRGLYYGSYLYKETWNIG
Spba   SDIATAFTSVAHICRDVNFGWLIRNMHANGASFFFICIYLHIGRGLYYGSYLYKETWNIG
Game   PDVESAFASVAHICRDVNFGWLIRNLHANGASFFFVCIYSHIGRGLYYGSYLYKETWNIG
Thth   PDVESAFASVAHICRDVNFGWLIRNLHANGASFFFICIYFHIGRGLYYGSYLYKETWNIG
Xigl   SDIATAFTSVAHICRDVNYGWLIRNMHANGASFFFICIYLHIGRGLYYGSYLYKETWNIG
Hyja   PNVESAFASVAHICRDVNFGWLIRNLHANGASFFFICLYLHIGRGLYYGSYLYKETWNIG
Psan   PNVELAFDSVAHICRDVNFGWLVRNLHANGASFFFICLYFHIGRGLYYGSYLYKKTWSIG
Cupa   PDVESAFASVAHICRDVNFGWLIRNLHANGASFFFICIYMHIGRGLYYGSYLYKETWNIG
Mpch   SDISMAFSSVAHICRDVNYGWLIRNMHANGASFFFICIYLHIGRGLYYGSYMYKETWNIG
Char   SDITTAFSSVAHTCRDVNYGWLIRNLHANGASFFFICIYLHIGRGLYYGSYLYKETWNVG
Pser   SDIATAFSSVAHICRDVNYGWFLRNMHANGASFFFICIYLHIGRGLYYGSYLFKETWNIG
Prol   SDIATAFTSVAHICRDVNYGWLIRNIHANGASFFFICIYLHIGRGLYYGSFLYKETWNVG
Plbi   SDIATAFTSVAHICRDVNYGWLIRSIHANGASFFFICIYLHIGRGLYYGSYLYKETWTIG
Calu   SDINYAFESVTHITRDVHFGWLIRNLHANGASFFFICIYAHIARGLYYGSYLYKATWNLG
Papa   SSIDTAFASVAHISRDVNYGWLIRNMHANGASFFFMCIYLHIGRGLYYGSYLNKATWNTG
Sufr   SDIATAFSSVAHICRDVNYGWLIRSLHANGASFFFICIYLHIGRGLYYGSYLNKETWNIG
Stci   SDIATAFSSVAHICRDVNYGWLIRNLHANGASFFFVCIYFHIGRGLYYGSYTYKATWNVG
Taru   SDISTAFSSVAHICRDVNYGWLIRNLHANGASFFFICLYSHIGRGLYYGSYLSKETWNVG
Rala   SDIATAFSSVAHICRDVNYGWLIRNLHANGASFFFICLYMHIGRGLYYGSYLYKETWNIG
         .   :: *: *  *:.  ** .*  *   .** .:*:*:* *  .**:** *.     *  *
```

To be continued on page 15.

```
            C     130                 D
Scca  VVLLFLLMATAFVGYVLPWGQMSFWGATVITNLLSAFPYIGNLLVQWIWGGFSVDNATLT
Muma  VILLFLLMATAFVGYVLPWGQMSFWGATVITNLLSAFPYIGNMLVQWIWGGFSVDNATLT
Erca  VILLLLTMMTAFVGYVLPWGQMSFWGATVITNLLSAVPYIGDTLVQWIWGGFSVDKPTLT
Pose  VILLLLTMMTAFVGYVLPWGQMSFWGATVITNLLSAIPYIGDTLVQWIWGGFSVDKPTLT
Actr  VILLLLTMMTAFVGYVLPWGQMSFWGATVITNLLSAFPYIGDTLVQWIWGGFSVDNATLT
Scal  VVLLLLTMMTAFVGYVLPWGQMSFWGATVITNLLSAFPYIGDTLVQWIWGGFSVDNATLT
Posp  VVLLLLTMMTAFVGYVLPWGQMSFWGATVITNLLSAFPYIGDTLVQWIWGGFSVDNATLT
Atsp  VLLLLLVMMTAFVGYVLPWGQMSFWGATVITNLLSAFPYIGDTLVQWIWGGFSVDNATLT
Leoc  VLLLLLVMMTAFVGYVLPWGQMSFWGATVITNLLSAFPYIGDTLVQWIWGGFSVDNATLT
Amca  VVLFLLVMMTAFVGYVLPWGQMSFWGATVITNLLSAFPYIGDTLVQWIWGGFSVDNATLT
Osbi  VVLLLLVMMTAFVGYVLPWGQMSFWGATVITNLLSAVPYLGNSLVQWIWGGFSVDNATLT
Pabu  VILLLLVMMTAFVGYVLPWGQMSFWGATVITNLLSAVPYMGDMLVQWIWGGFSVDNATLT
Hial  VVLLLLTMMTAFVGYVLPWGQMSFWGATVITNLMSAVPYVGNDLVQWIWGGFSVDNATLT
Elha  VVLLLLVMMTAFVGYVLPWGQMSFWGATVITNLLSAVPYVGDTLVQWIWGGFSVDKATLT
Mlcy  VILLLLVMMTAFVGYVLPWGQMSFWGATVITNLLSAVPYVGDTLVQWIWGGFSVDNATLT
Algl  VVLLLLVMMTAFVGYVLPWGQMSFWGATVITNLLSAVPYVGESLVQWIWGGFSVDKATLT
Ptgi  VVLLLLVMMTAFVGYVLPWGQMSFWGATVITNLLSAVPYVGDALVQWIWGGFSVDNATLT
Alaf  VILLFLTMMTAFVGYVLPWGQMSFWGATVITNLLSAVPYVGNALVQWIWGGFSVDNATLT
Nock  VILLLLTMVTAFVGYVLPWGQMSFWGATVITNLMSAVPYVGNELVQWIWGGFSVDNATLT
Anja  VVLFLLVMMTAFVGYVLPWGQMSFWGATVITNLLSAVPYVGDSLVQWIWGGFSVDNATLT
Gyki  VILFLLVMMTAFVGYVLPWGQMSFWGATVITNLLSAVPYVGNSLVQWIWGGFSVDNATLT
Syka  VVLFLLVMMTAFVGYVLPWGQMSFWGATVITNLLSAVPYIGNDLVQWIWGGFSVDNATLT
Opma  VVLFLLVMMTAFVGYVLPWGQMSFWGATVITNLLSAVPYVGDMLVQWIWGGFSVDNATLT
Comy  VILFLLVMMTAFVGYVLPWGQMSFWGATVITNLLSAVPYIGNILVQWIWGGFSVDNATLT
Sasp  VILLLLVMMTAFVGYVLPWGQMSFWGATVITNLLSAIPYVGNALVQWIWGGFSIDNATLS
Eupe  VILFLLVMMTAFVGYVLPWGQMSFWGATVITNLLSAVPYVGEMLVQWIWGGFSVDNATLN
Enja  VVLLLLVMMTAFVGYVLPWGQMSFWGATVITNLMSAVPYVGTELVQWIWGGFSVDNATLT
Same  VVLLLLVMMTAFVGYVLPWGQMSFWGATVITNLLSAVPYVGGALVEWIWGGFSVDNATLT
Chch  VVLLLLVMMTAFVGYVLPWGQMSFWGATVITNLLSAVPYVGDALVQWIWGGFSVDNATLT
Grgr  VILLLLVMMTAFVGYVLPWGQMSFWGATVITNLLSAVPYVGDLLVQWIWGGFSVDNATLT
Caau  VVLLLLVMMTAFVGYVLPWGQMSFWGATVITNLLSAVPYMGDMLVQWIWGGFSVDNATLT
Cyca  VVLLLLVMMTAFVGYVLPWGQMSFWGATVITNLLSAVPYMGDMLVQWIWGGFSVDNATLT
Dare  VVLFLLVMMTAFVGYVLPWGQMSFWGATVITNLLSAVPYVGDTLVQWIWGGFSVDNATLT
Cost  VVLLLLIMMTAFVGYVLPWGQMSFWGATVITNLLSAVPYVGNALVQWIWGGFSVDNATLT
Leec  VILLLLVMMTAFVGYVLPWGQMSFWGATVITNLLSAVPYMGNALVQWIWGGFSVDNATLT
Fola  VVLFLLVMMTAFVGYVLPWGQMSFWGATVITNLLSAVPYVGDMLVQWIWGGFSVDNATLT
Clmc  VVLLLLLMMTAFVGYVLPWGQMSFWGATVITNLLSAVPYVGDMLVQWIWGGFSVDNATLT
Phin  VILLLLVMMTAFVGYVLPWGQMSFWGATVITNLLSAVPYIGDALVQWIWGGFSVDNATLT
Icpu  VVLLLLVMMTAFVGYVLPWGQMSFWGATVITNLLSAVPYMGDALVQWIWGGFSVDNATLT
Psto  VVLLLLTMATAFVGYVLPWGQMSFWGATVITNLLSAVPYMGDMLVQWIWGGFSVDNATLT
Cora  VVLLLLVMMTAFVGYVLPWGQMSFWGATVITNLLSAIPYIGNDLVQWIWGGFSVDNATLT
Eisp  VVLFFLVMMTAFVGYVLPWGQMSFWGATVITNLLSAVPYIGNDLVQWLWGGFSVDNATLT
Apal  VILFLLMMVTAFVGYVLPWGQMSFWGATVITNLLSATPYAGTSLVRWVWGGFSVDNATLT
Eslu  VILLLLTMMTAFVGYVLPWGQMSFWGATVITNLLSAVPYIGNDLVQWIWGGFSVDYATLT
Dape  VVLFLLTMMTAFVGYVLPWGQMSFWGATVITNLLSAVPYIGNELVQWIWGGFSVDNATLT
Glse  VVLLLLVMKTAFVGYVLPWGQMSFWGATVITNLLSAVPYVGEALVQWIWGGFSVDNATLT
Naar  VVLLLLVMMTAFVGYVLPWGQMSFWGATVITNLLSAVPYVGEALVQWIWGGFSVDNATLT
Lioc  VVLFLLVMMTAFVGYVLPWGQMSFWGATVITNLLSAVPYVGEALVQWIWGGFSVDNATLT
Opso  VILLLLVMMTAFVGYVLPWGQMSFWGATVITNLLSAIPYVGNTLVQWIWGGFSVDNATLT
Alte  VILFLLTMVTAFVGYVLPWGQMSFWGATVITNLMSAVPYVGNELVQWIWGGFSVDSATLT
Plap  VVLFLLTMMTAFVGYVLPWGQMSFWGATVITNLLSAAPYIGNELVQWIWGGFSVDSATLT
```

To be continued on page 16.

[3/7 of aligned sequences]

Plal	VVLLLLVMMTAFVGYVLPWGQMSFWGATVITNLLSAVPYMGLDLVLWLWGGFSVDSATLT
Sami	VVLLLLVMMTAFVGYVLPWGQMSFWGATVITNLLSAVPYMGLDLVLWLWGGFSVDNATLT
Rere	VVLLLLVMMTAFVGYVLPWGQMSFWGATVITNLLSAVPYVGQALVQWIWGGFSVDNATLT
Gama	VILLLLVMMTAFVGYVLPWGQMSFWGATVITNLLSAVPYVGNDLVQWIWGGFSVDNATLT
Onmy	VVLLLLTMMTAFVGYVLPWGQMSFWGATVITNLLSAVPYVGGALVQWIWGGFSVDNATLT
Sasa	VVLLLLTMMTAFVGYVLPWGQMSFWGATVITNLLSAVPYVGGALVQWIWGGFSVDNATLT
Cola	VVLLLLTMMTAFVGYVLPWGQMSFWGATVITNLLSAVPYVGGALVQWIWGGFSVDNATLT
Dita	VILLLLTMMTAFVGYVLPWGQMSFWGATVITNLLSAVPYVGEALVQWIWGGFSVDNATLT
Gogr	VILLLLTMMTAFVGYVLPWGQMSFWGATVITNLLSAIPYAGQTLVQWIWGGFSVDNATLT
Chsl	VILFLLTMMTAFVGYVLPWGQMSFWGATVITNLLSAVPYAGEALVQWIWGGFSVDNATLT
Atja	VVLLLLVMMTAFVGYVLPWGQMSFWGATVITNLFSAVPYVGDMLVQWIWGGFSVDNATLT
Iido	VVLLLLVMMTAFVGYVLPWGQMSFWGATVITNLFSAVPYVGNMLVQWIWGGFSVDNATLT
Auja	VILLLLVMMTAFVGYVLPWGQMSFWGATVITNLLSAVPYVGNTLVQWIWGGFSVDNATLT
Chag	VILLLLVMMTAFVGYVLPWGQMSFWGATVITNLLSAVPYVGNALVQWIWGGFSVDNATLT
Hami	VILLLLVMMTAFVGYVLPWGQMSFWGATVITNLLSAVPYVGGTLVEWIWGGFSVDKATLT
Saun	VILLLLVMMTAFVGYVLPWGQMSFWGATVITNLLSAVPYVGSTLVEWIWGGFSVDKATLT
Nema	VILLLLVMMTAFVGYVLPWGQMSFWGATVITNLLSAVPYVGGTLVQWIWGGFSVDNATLT
Disp	VVLLLLVMMTSFVGYVLPWGQMSFWGATVITNLLSAVPYVGETLVQWIWGGFSVDNATLS
Myaf	VVLFLLTMMTAFVGYVLPWGQMSFWGATVITNLLSAVPYVGGTLVQWIWGGFSVDNATLT
Lagu	VVLLGLVMATAFVGYVLPWGQMSFWGATVITNLLSAIPYVGGTLVQWIWGGFSVDNATLT
Trtr	VILLFLVMATAFVGYVLPWGQMSFWGATVITNLLSAVPYVGGSLVQWIWGGFSVDNATLT
Zucr	VVLLLLVMATAFVGYVLPWGQMSFWGATVITNLLSAVPYIGNALVQWIWGGFSVDNATLT
Pxja	VVLLLLVMATAFVGYVLPWGQMSFWGATVITNLMSAVPYVGGELVQWIWGGFSVDNATLT
Pxlo	VVLLLLVMATAFVGYVLPWGQMSFWGATVITNLMSAVPYVGGELVQWIWGGFSVDNATLT
Pctr	VALFLLTMMTAFVGYVLPWGQMSFWGATVITNLMSAVPYVGNDLVQWIWGGFSVDNATLT
Apsa	VVLFLVTMMTAFVGYVLPWGQMSFWGATVITNLMSAVPYIGNELVQWIWGGFSVDNATLT
Cabe	VILMILVMATAFVGYVLPWGQMSFWGATVITNLLSAIPYVGDNVVQWIWGGFSVDNATLS
Bzze	VILLLLVMATAFVGYVLPWGQMSFWGATVITNLMSAVPYVGNNLVQWIWGGFSVDNATLT
Siim	IALLMLLMATAFVGYVLPWGQMSYWGATVITNLLSVIPYFGTPIVEWLWGGFSVDNPTLK
Ctru	VVLLLLVMMTAFVGYVLPWGQMSFWGATVITNLLSAIPYVGNALVQWIWGGFSVDNATLT
Dpbr	VILLLLVMMTAFVGYVLPWGQMSFWGATVITNLLSAVPYVGNALVQWIWGGFSVDNATLT
Caki	VILYLLVMMTAFVGYVLPWGQMSFWGATVITNLMSAVPYIGNTLVQWIWGGFSVDNATLT
Phja	VVLFLLVMVTAFVGYVLPWGQMSFWGATVITNLLSAVPNVGNSLVQWVWGGFSVDNATLT
Brsp	VILFLLVMMTAFVGYVLPWGQMSFWGATVITNLVSAVPYIGTSLVQWIWGGFSVDNATLT
Gamo	VVLFLLVMMTSFVGYVLPWGQMSFWGATVITNLMSTVPYVGDALVQWIWGGFSVDNATLT
Lolo	VILFLLVMVTSFVGYVLPWGQMSFWGATVITNLMSTVPYVGNTLVQWIWGGFSVDNATLT
Batr	IILLLLTMATAFMGYVLPWGQMSFWGATVITNLLSAIPYTGQTTVQWLWGGFSIDNPTLI
Prmy	VTIFLVTMLTAFMGYVLPWGQMSFWAATVITNLLSAVPYLGQPLVEWLWGGFSVDAPTLT
Lose	VILLLLVMATAFVGYVLPWGQMSFWGATVITNLMSAVPYIGQTLVQWVWGGFSVDNATLT
Loam	VVLLLLVMMTAFVGYVLPWGQMSFWGATVITNLLSAVPYVGNSLVQWIWGGFSVDNATLT
Chab	VILLLLTMMTAFVGYVLPWGQMSFWGATVITNLLSAVPYVGNTLVQWIWGGFSVDNATLT
Chto	VILLLLTMMTAFVGYVLPWGQMSFWGATVITNLLSAVPYVGGTLVQWIWGGFSVDNATLT
Majo	VVLLLLVMMTAFVGYVLPWGQMSFWGATVITNLLSAVPYMGNTLVQWIWGGFSVDNATLT
Hlst	VVLLLLVMMTAFVGYVLPWGQMSFWGATVITNLLSAVPYVGNTLVQWIWGGFSVDNATLT
Clpe	VVLLLLVMVTAFVGYVLPWGQMSFWGATVITNLLSAVPYIGDSLVQWIWGGFSVDNATLT
Mlmr	VVLLLLVMMTAFVGYVLPWGQMSFWGATVITNLLSAVPYVGNTLVQWIWGGFSVDNATLT
Crcr	VVLLLLVMMTAFVGYVLPWGQMSFWGATVITNLLSAVPYIGESLVQWLWGGFAVDNATLT
Muce	VVLLLLVMMTAFVGYVLPWGQMSFWGATVITNLLSAVPYIGESLVQWLWGGFAVDNATLT
Bege	VILLLLVMMTAFVGYVLPWGQMSFWGATVITNLLSAVPYIGNSLVQWIWGGFSVDNATLT
Mela	VVLLLLVMMTAFVGYVLPWGQMSFWGATVITNLLSAVPYIGNSLVQWIWGGFSVDNATLT
Hats	VVLLLLVMMTAFVGYVLPWGQMSFWGATVITNLLSAVPYIGNSLVQWIWGGFSVDNATLT
Orla	VILLLLVMMTAFVGYVLPWGQMSFWGATVITNLLSAVPYVGNALVQWIWGGFSVDNATLT

To be continued on page 17.

[3/7 of aligned sequences]

To be continued on page 18.

```
Cosa VVLLLLVMMTAFVGYVLPWGQMSFWGATVITNLLSAIPYIGGSLVQWIWGGFSVDNATLT
Exsp VVLLLLVMMTAFVGYVLPWGQMSFWGATVITNLLSAVPYIGNSLVQWIWGGFSVDNATLT
Depa VVLLLLVMMTAFVGYVLPWGQMSFWGATVITNLLSAFPYIGNSLVQWIWGGFSVDNATLT
Rima VILLLLVMMTAFVGYVLPWGQMSFWGATVITNLLSAIPYVGENIVQWLWGGFSVDSATLT
Fuol VILLLLVMMTAFVGYVLPWGQMSFWGATVITNLLSAVPYVGDALVQWIWGGFSVDNATLT
Gmaf VILLLLVMMTAFVGYVLPWGQMSFWGATVITNLLSAVPYMGDTLVQWIWGGFSVDNATLT
Xeei VILFLLAMMTAFVGYVLPWGQMSFWGATVITNLLSAVPYAGDALVQWIWGGFSVDNATLT
Pros VVLLLLTMMTAFVGYVLPWGQMSFWGATVITNLLSAVPYVGDALVQWIWGGFSVDNATLT
Scmi VILFLLTMMTAFVGYVLPWGQMSFWGATVITNLLSAAPYVGDILVQWIWGGFSVDNATLT
Rolo VVLLLLVMMTAFVGYVLPWGQMSFWGATVITNLLSAVPYVGNTLVQWIWGGFSVDNATLT
Cere VVLLLLVMMTAFVGYVLPWGQMSFWGATVITNLLSAVPYVGNTLVQWIWGGFSVDNATLT
Daga VILLLLVMMTAFVGYVLPWGQMSFWGATVITNLLSAVPYVGGTLVQWIWGGFSVDNATLT
Anco VILFLLVMMTAFVGYVLPWGQMSFWGATVITNLLSAVPYVGNALVQWIWGGFSVDNATLT
Dmve VVLLLLVMMTAFVGYVLPWGQMSFWGATVITNLLSAAPYVGGDLVKWIWGGFSVDNATLT
Dmar VVLLLLVMMTAFVGYVLPWGQMSFWGATVITNLLSAAPYVGGDLVKWIWGGFSVDNATLT
Anka VILLLLVMMTAFVGYVLPWGQMSFWGATVITNLLSAVPYVGNALVQWIWGGFSVDNATLT
Moja VILLLLVMMTAFVGYVLPWGQMSFWGATVITNLLSAVPYIGNALVQWIWGGFSVDNATLT
Hoja VILLLLVMMTAFVGYVLPWGQMSFWGATVITNLLSAVPYVGNALVQWIWGGFSVDNATLT
Bede VVLLLLVMMTAFVGYVLPWGQMSFWGATVITNLLSAVPYVGNTLVQWIWGGFSVDNATLT
Besp VVLLLLVMMTAFVGYVLPWGQMSFWGATVITNLLSAVPYVGNTLVQWIWGGFSVDNATLT
Mysp VVLLLLVMMTAFVGYVLPWGQMSFWGATVITNLLSAVPYVGNTLVQWIWGGFSVDNATLT
Osja VVLLLLVMMTAFVGYVLPWGQMSFWGATVITNLLSAVPYVGNTLVQWIWGGFSVDNATLT
Sgro VVLLLLVMMTAFVGYVLPWGQMSFWGATVITNLLSAVPYVGNTLVQWIWGGFSVDNATLT
Pzpa VILLLLVMMTAFVGYVLPWGQMSFWGATVITNLLSAIPYVGNSLVQWIWGGFSIDNATLT
Zeja VVLLLLVMMTAFVGYVLPWGQMSFWGATVITNLLSAIPYVGGTLVQWIWGGFSVDNATLA
Znne VVLFLLVMMTAFVGYVLPWGQMSFWGATVITNLLSAVPYVGDTLVQWIWGGFSVDNATLT
Zefa VVLFLLVMMTAFVGYVLPWGQMSFWGATVITNLLSAVPYVGDILVQWIWGGFSVDNATLT
Acni VVLLLLVMMTAFVGYVLPWGQMSFWGATVITNLLSAVPYVGNTLVQWIWGGFSVDNATLT
Ncrh VVLLLLVMMTAFVGYVLPWGQMSFWGATVITNLLSAVPYVGNTLVQWIWGGFSVDNATLT
Agca VVLLLLVMMTAFVGYVLPWGQMSFWGATVITNLLSAVPYVGNTLVQWIWGGFSVDNATLT
Hydy VVLLLLVMMTAFVGYVLPWGQMSFWGATVITNLLSAVPYIGGSLVQWIWGGFSVDNATLT
Gsac VVLLLLVMMTAFVGYVLPWGQMSFWGATVITNLLSAVPYVGNSLVQWIWGGFSVDNATLT
Pevo VVLLLLVMATAFVGYVLPWGQMSFWGATVITNLFSAIPYVGDSLVQWIWGGFSVDNATLT
Hiku VVLLLLVMATAFVGYVLPWGQMSFWGATVITNLMSAVPYIGNDLVQWVWGGFSVDNATLT
Inpa ILLLLLIMVTAFVGYVLPWGQMSFWGATVITSLLSALPYVGTDLVQWLWGGFSVDNATLT
Auch VVLLLLVMMTAFMGYVLPWGQMSFWGATVITNLLSAFPYVGDTLVQWIWGGFSVDNATLT
Fico VVLLLLVMMTAFVGYVLPWGQMSFWGATVITNLLSAVPYVGNTLVQWIWGGFSVDNATLT
Macs VVLLLLVMMTAFVGYVLPWGQMSFWGATVITNLLSAVPYVGNSLVQWIWGGFSVDNATLT
Moal VALLLLLMATAFVGYVLPWGQMSYWGATVITNLLSAAPYTGPQMVNWVWGGYSVDHATLT
Syma VIMFFLMMTTAFVGYVLPWGQMSLWGATVITNLLSAAPYSGKNLVQWVWGGFSVDAATLT
Mafr VILLLLTMMTAFVGYVLPWGQMSFWGATVITNLLSAVPYIGNTLVQWIWGGFSVDNATLT
Dcpe VILLLLVMMTAFVGYVLPWGQMSFWGATVITNLLSAVPYVGNSLVQWIWGGFSVDNATLT
Dcti VVLLLLVMMTAFVGYVLPWGQMSFWGATVITNLLSAVPYVGNSLVQWIWGGFSVDNATLT
Hehi VVLLLLVMMTAFVGYVLPWGQMSFWGATVITNLLSAVPYVGDALVQWIWGGFSVDNATLT
Stam VVLLLLVMATAFVGYVLPWGQMSFWGATVITNLLSAVPYVGNTLVQWIWGGFSVDNATLT
Hogi VVLLLLVMMTAFVGYVLPWGQMSFWGATVITNLLSAVPYVGNTLVQWIWGGFSVDNATLT
Erzo VVLLLLVMMTAFVGYVLPWGQMSFWGATVITNLLSAVPYVGNSLVQWIWGGFSVDNATLT
Hxot VVLLLLVMMTAFVGYVLPWGQMSFWGATVITNLLSAVPYIGNSLVQWIWGGFSVDNATLT
Core VVLLLLVMMTAFVGYVLPWGQMSFWGATVITNLLSAVPYIGNALVQWIWGGFSVDNATLT
Apve VMLLLLVMMTAFVGYVLPWGQMSFWGATVITNLLSAVPYIGNTLVQWIWGGFSVDNATLN
Latj VILLLLVMMTAFVGYVLPWGQMSFWGATVITNLLSAVPYVGNSLVQWIWGGFSVDNATLT
Laja VILLLLVMMTAFVGYVLPWGQMSFWGATVITNLLSAVPYVGNTLVQWIWGGFSVDNATLT
```

[3/7 of aligned sequences]

To be continued on page 19.

```
Syja  VVLLLLVMMTAFVGYVLPWGQMSFWGATVITNLLSAVPYVGNTLVQWIWGGFSVDNATLT
Epme  VVLLLLVMMTAFVGYVLPWGQMSFWGATVITNLLSAVPYIGNNLVQWIWGGFSVDNATLT
Grse  VVLLLLVMMTAFVGYVLPWGQMSFWGATVITNLLSAIPYVGGTLVQWIWGGFSVDNATLT
Clja  VILLLLVMMTAFVGYVLPWGQMSFWGATVITNLLSAVPYMGDALVQWIWGGFSVDNATLT
Ogcy  VILLLLTMMTAFVGYVLPWGQMSFWGATVITNLLSAFPYIGEILVQWIWGGFSVDNATLT
Plna  VILLLLVMMTAFVGYVLPWGQMSFWGATVITNLLSAFPYIGSALVQWIWGGFSVDNATLT
Lema  VVLLLLVMMTAFVGYVLPWGQMSFWGATVITNLLSAVPYIGNTLVQWIWGGFSVDNATLT
Etzo  VVLLLLVMMTAFVGYVLPWGQMSFWGATVITNLLSAVPYVGNTLVQWIWGGFSVDNATLT
Apse  VVLLLLVMMTAFVGYVLPWGQMSFWGATVITNLLSAVPYVGNTLVQWIWGGFSVDNATLT
Epde  VVLLLLVMMTAFVGYVLPWGQMSFWGATVITNLLSAVPYVGNTLVQWIWGGFSVDNATLT
Slja  VVLLLLVMMTAFVGYVLPWGQMSFWGATVITNLLSAVPYVGNTLVQWIWGGFSVDNATLT
Bsja  VILLLLVMMTAFVGYVLPWGQMSFWGATVITNLLSAVPYVGNMLVQWIWGGFSVDNATLT
Ecna  VVLLLLLMATAFVGYVLPWGQMSFWGATVITNLLSAVPYVGDSLVQWIWGGFSVDNATLS
Cohi  VVLLLLLMGTAFVGYVLPWGQMSFWGATVITNLMSAVPYIGDTLVQWIWGGFSVDNATLT
Caar  VVLLLLLMGTAFVGYVLPWGQMSFWGATVITNLLSAVPYVGNTLVQWIWGGFSVDNATLT
Came  VVLLLLLMGTAFVGYVLPWGQMSFWGATVITNLLSAVPYVGNTLVQWIWGGFSVDNATLT
Mema  VVLLLLVMMTAFVGYVLPWGQMSFWGATVITNLLSAIPYVGNTLVQWIWGGFSVDNATLT
Lenu  VVLLLLVMMTAFVGYVLPWGQMSFWGATVITNLLSAVPYVGNTLVQWIWGGFSVDNATLT
Brja  VILFLLVMMTAFVGYVLPWGQMSFWGATVITNLLSAVPYVGTMLVEWIWGGFSVDNATLT
Plma  VVLLLLVMMTAFVGYVLPWGQMSFWGATVITNLLSAFPYVGTMLVEWIWGGFSVDNATLT
Emst  VVLLLLVMMTAFVGYVLPWGQMSFWGATVITNLLSAVPYVGNTLVQWIWGGFSVDNATLT
Ptti  VVLLLLVMATAFVGYVLPWGQMSFWGATVITNLLSAIPYVGNTLVQWIWGGFSVDNATLT
Losu  VILLLLVMMTAFVGYVLPWGQMSFWGATVITNLLSAVPYVGSTLVQWIWGGFSVDNATLT
Geoy  VVLLLLVMMTAFVGYVLPWGQMSFWGATVITNLLSAVPYVGNTLVQWIWGGFSVDNATLT
Dipi  VILLLLVMMTAFVGYVLPWGQMSFWGATVITNLLSAVPYVGNTLVQWIWGGFSVDNATLT
Pama  VVLLLLVMATAFVGYVLPWGQMSFWGATVITNLLSAVPYVGGTLVQWIWGGFSVDNATLT
Leob  VVLLLLVMMTAFVGYVLPWGQMSFWGATVITNLLSAVPYVGNTLVQWIWGGFSVDNATLT
Neba  VILLLLVMMTAFVGYVLPWGQMSFWGAPVITNLLSAVPYVGNTLVQWIWGGFSVDNATLT
Pdpl  VILLLLTMMTAFVGYVLPWGQMSFWGATVITNLLSAVPYVGDSLVQWIWGGFSVDNATLN
Nimi  VVLFLLVMMTAFVGYVLPWGQMSFWGATVITNLLSAVPYVGNTLVQWIWGGFSVDNATLT
Uptr  VVLLLLVMMTAFVGYVLPWGQMSFWGATVITNLLSAVPYVGNTLVQWIWGGFSVDNATLT
Pesc  VILLLLVMMTAFVGYVLPWGQMSFWGATVITNLLSAIPYVGNTLVQWIWGGFSVDNATLN
Baar  VVLLLLVMMTAFVGYVLPWGQMSFWGATVITNLLSAVPYVGNDLVQWIWGGFSVDNATLT
Moar  VVILLLVMMTAFVGYVLPWGQMSFWGATVITNLLSAVPYVGNTLVQWIWGGFSVDNATLT
Toja  VVLLLLVMMTAFVGYVLPWGQMSFWGATVITNLLSAIPYVGNTLVQWIWGGFSVDNATLT
Chau  VVLLLLVMATAFVGYVLPWGQMSFWGATVITNLLSAIPYIGNTLVQWIWGGFSVDNATLT
Chse  VILLLLVMMTAFVGYVLPWGQMSFWGATVITNLLSAVPYIGSTLVQWIWGGFSVDNATLT
Enar  VVLLLLVMMTAFVGYVLPWGQMSFWGATVITNLLSAVPYVGNTLVQWIWGGFSVDNATLT
Hpty  VVLLLLVMMTAFVGYVLPWGQMSFWGATVITNLLSAVPYIGNTLVQWIWGGFSVDNATLT
Nana  VILLLLVMMTAFVGYVLPWGQMSFWGATVITNLLSAIPYVGGTLVQWIWGGFSVDNATLT
Mcst  VVLLLLVMMTAFVGYVLPWGQMSFWGATVITNLLSAVPYVGNTLVQWIWGGFSVDNATLT
Rhox  VVLLLLVMMTAFVGYVLPWGQMSFWGATVITNLLSAFPYVGNTLVQWIWGGFSVDNATLT
Opfa  VVLLLLVMMTAFVGYVLPWGQMSFWGATVITNLLSAVPYVGNTLVQWIWGGFSVDNATLT
Paar  VVLLLLVMMTAFVGYVLPWGQMSFWGATVITNLLSAVPYVGNTLVQWIWGGFSVDNATLT
Gozo  VILLLLVMMTAFVGYVLPWGQMSFWGATVITNLLSAVPYVGNTLVQWIWGGFSVDNATLT
Ackr  VILLFLTMATAFVGYVLPWGQMSFWGATVITNLLSAIPYVGNTLVQWIWGGFSVDNATLS
Elev  VVLLLLVMMTAFVGYVLPWGQMSFWGATVITNLLSAIPYVGNVLVQWIWGGFSVDNATLT
Trdu  VILLLLTMMTAFVGYVLPWGQMSFWGATVITNLLSAVPYIGNSLVQWIWGGFSVDNATLT
Amoc  VILLLLVMMTAFVGYVLPWGQMSFWGATVITNLLSAIPYVGSSLVQWIWGGFSVDNATLT
Hame  VVLLLLVMMTAFVGYVLPWGQMSFWGATVITNLLSAVPYVGDALVQWIWGGFSVDNATLT
Chso  VILLLLVMMTAFVGYVLPWGQMSFWGATVITNLLSAVPYVGNTLVQWIWGGFSVDNATLT
Lyto  VILLLLVMMTAFVGYVLPWGQMSFWGATVITNLLSAVPYVGNALVQWIWGGFSVDNATLT
```

[3/7 of aligned sequences]

```
Encr    VILFLLVMMTAFVGYVLPWGQMSFWGATVITNLLSAVPYIGSSLVQWIWGGFSVDNATLT
Bvar    VVLLLLVMMTAFVGYVLPWGQMSFWGATVITNLMSAVPYIGSDLVQWIWGGFSVDNATLT
Noco    VVLLLLVMMTAFVGYVLPWGQMSFWGATVITNLLSAVPYVGNALVQWIWGGFSVDNATLT
Chsp    VILLLLTMMTAFVGYVLPWGQMSFWGATVITNLLSAVPYVGNTLVQWIWGGFSVDNATLN
Arja    VVLLLLVMMTAFVGYVLPWGQMSFWGATVITNLLSAVPYIGNALVQWIWGGFSVDNATLT
Pase    VLLLGLLMATAFVGYVLPWGQMSFWGATVITNLLSAVPYVGNTLVQWIWGGFSVDNATLT
Trel    VILLLLVMATAFVGYVLPWGQMSFWGATVITNLLSAVPYVGGTLVQWIWGGFSVDNATLT
Lifa    VILLLLVMMTAFVGYVLPWGQMSFWGATVITNLLSAVPYVGQTLVQWIWGGFSVDNATLT
Acur    VILLLLVMMTAFVGYVLPWGQMSFWGATVITNLLSAIPYVGNDLVQWIWGGFSVDNATLT
Ampe    VVLFLLVMMTAFVGYVLPWGQMSFWGATVITNLLSAVPYVGGTLVQWIWGGFSVDNATLT
Urja    VMLLLLVMITAFVGYVLPWGQMSFWGATVITNLLSAVPYIGNTLVQWIWGGFSVDNATLN
Enet    VVLLLLVMMTAFVGYVLPWGQMSFWGATVITNLLSAVPYVGTSLVQWIWGGFSVDNATLT
Ptbr    VVLLLLVMMTAFVGYVLPWGQMSFWGATVITNLLSAFPYVGDALVQWIWGGFSIDNATLT
Safa    VILLLLVMMTAFVGYVLPWGQMSFWGATVITNLLSAFPYIGDTLVQWIWGGFSIDNATLT
Icae    VILLLLVMMTAFVGYVLPWGQMSFWGATVITNLLSAVPYVGTMLVEWIWGGFSVDNATLT
Asmi    VILLLLVMMTAFVGYVLPWGQMSFWGATVITNLLSAFPYIGNSLVQWIWGGFSVDNATLT
Foal    VVLLLLTMATAFVGYVLPWGQMSFWGATVITNLLSAIPYVGGSVVQWIWGGFSVDNATLT
Drze    VILILLVMMTAFVGYVLPWGQMSFWGATVITNLLSAIPYIGNTLVQWIWGGFSVDNATLN
Rhas    VVLLLLVMMTAFVGYVLPWGQMSFWGATVITNLLSAVPYVGNTLVQWIWGGFSVDNATLT
Elac    VVLLLLVMMTAFVGYVLPWGQMSFWGATVITNLLSAVPYVGTTLVQWIWGGFSVDNATLT
Kugu    VILFLLVMMTAFVGYVLPWGQMSFWGATVITNLLSAVPYIGNTLVQWIWGGFSVDNATLT
Plor    VILLLLVMMTAFVGYVLPWGQMSFWGATVITNLLSAIPYVGNTLVQWIWGGFSVDNATLT
Sgun    VILLLLVMGTAFVGYVLPWGQMSFWGATVITNLLSAVPYVGNTLVQWIWGGFSVDNATLT
Zaco    VVLLLLVMMTAFVGYVLPWGQMSFWGATVITNLLSAVPYVGNTLVQWIWGGFSVDNATLT
Zbfl    VVLLLLVMMTAFVGYVLPWGQMSFWGATVITNLLSAVPYVGNTLVQWIWGGFSVDNATLT
Spba    VVLLLLVMMTAFVGYVLPWGQMSFWGATVITNLLSAIPYVGNTLVQWIWGGFSVDNATLT
Game    VVLLLLVMMTAFVGYVLPWGQMSFWGATVITNLLSAVPYVGTTLVEWIWGGFSVDNATLT
Thth    VVLLLLVMMTAFVGYVLPWGQMSFWGATVITNLLSAVPYVGTTLVEWIWGGFSVDNATLT
Xigl    VVLLLLVMMTAFVGYVLPWGQMSFWGATVITNLLSAVPYVGNALVQWIWGGFSVDNATLT
Hyja    VVLFLLVMMTAFVGYVLPWGQMSFWGATVITNLLSAVPYVGTTLVEWIWGGFSVDNATLT
Psan    VILFLLVMMTAFVGYVLPWGQMSFWGATVITNLLSAVPYVGTTLVEWIWGGFSVDNATLT
Cupa    VVLLLLVMMTAFVGYVLPWGQMSFWGATVITNLLSAVPYVGTMLVEWIWGGFSVDNATLT
Mpch    VVLFLLTMMTAFVGYVLPWGQMSFWGATVITNLLSAVPYVGDMLVQWIWGGFSVDNATLT
Char    VILLLLVMMTAFVGYVLPWGQMSFWGATVITNLLSAVPYVGNMLVEWIWGGFSVDNATLT
Pser    VVLLLLVMMTAFVGYVLPWGQMSFWGATVITNLLSAVPYIGNSLVQWIWGGFSVDNATLT
Prol    VILLLLVMMTAFVGYVLPWGQMSFWGATVITNLLSAVPYVGNTLVQWIWGGFSVDNATLT
Plbi    VVLLLLVMMTAFVGYVLPWGQMSFWGATVITNLLSAVPYVGGTLVQWIWGGFSVDNATLT
Calu    VILLLLVMMTAFVGYVLPWGQMSFWGATVITNLLSAVPYVGDALVEWIWGGFSVDNATLN
Papa    VLLLLLVMATAFVGYVLPWGQMSFWGATVITNLLSAVPYIGNTLVQWIWGGFSVDNATLT
Sufr    VVLLLLVMMTAFVGYVLPWGQMSFWGATVITNLLSAVPYIGNNLVQWIWGGFSVDNATLT
Stci    VVLLLLVMMTAFVGYVLPWGQMSFWGATVITNLLSAVPYIGNDLVQWIWGGFSVDNATLT
Taru    VVLLLLVMATAFVGYVLPWGQMSFWGATVITNLLSAVPYVGNTLVQWVWGGFSVDSATLT
Rala    VVLLLLVMATAFVGYVLPWGQMSFWGATVITNLLSAVPYVGNTLVQWIWGGFSVDNATLT
        : :  : * *:*:********** *.* ***.*.*.  *  *   * *:***:.:*  **
```

To be continued on page 20.

[4/7 of aligned sequences]

```
                182    E           196
Scca    RFFAFHFLLPFLILALSVIHILFLHETGANNPMGINSNTDKISFHPYFSYKDLFGFLIVI
Muma    RFFAFHFLLPFLIMALSIIHLLFLHESGSNNPLGINSDADKVSFHPYFSYKDLLGFFVMI
Erca    RFFAFHFILPFAIAGASLVHILFLHETGSNNPLGINSNADKIPFHPYYTYKDLLGFIILL
Pose    RFFAFHFILPFAIAAASLVHIVFLHETGSNNPMGINSNADKIPFHPYYTFKDLLGFIILL
Actr    RFFAFHFLLPFVIAGASMIHLLFLHQTGSNNPTGLNSDADKVTFHPYFSYKDLFGFILML
Scal    RFFAFHFLLPFVIAGASMIHLLFLHQTGSNNPTGLNSDADKVTFHPYFSYKDLLGFILML
Posp    RFFAFHFLLPFVIAGASMIHLLFLHQTGSNNPTGLNSDADKVPFHPYFSYKDLLGFILML
Atsp    RFFTFHFLLPFIIMGATMLHLLFLHETGSNNPTGLNSDADKVTFHPYFSYKDLLGFTILL
Leoc    RFFTFHFLLPFIIMGTTMLHLLFLHETGSNNPTGLDSDADKVTFHPYFSYKDLLGFTILL
Amca    RFFTFHFLFPFVIAGASMIHLLFLHETGSNNPLGLNSNVDKITFHPYFSYKDLLGFIILL
Osbi    RFFAFHFLLPFMIAGATIIHLLFLHETGSNNPTGLNSNADKVPFHPYFSFKDVLGFMIML
Pabu    RFFAFHFILPFIIAALVIIHLLFLHETGSNNPIGINPNADKIAFHPYFSYKDLMGFATML
Hial    RFFAFHFLFPFAIAGATVLHLLFLHETGSNNPVGLNSDADKITFHPYFSYKDLLGFVALL
Elha    RFFAFHFLLPFVVAAATMLHLLFLHETGSNNPIGINSNADKIPFHPYYTYKDLLGFAILI
Mlcy    RFFAFHFLFPFVIAAVTVLHLLFLHETGSNNPAGLNSDADKISFHPYFSYKDLLGFVILL
Algl    RFFAFHFLLPFVIAGAVMLHLLFLHETGSNNPTGLNSDTDKVPFHPYFSYKDLLGFVILL
Ptgi    RFFAFHFLFPFVILGATVLHLLFLHETGSNNPAGINSDADKISFHPYFSYKDLLGFVALL
Alaf    RFFAFHFLFPFAIVGATVIHLLFLHETGSNNPTGLNSDADKISFHPYFSYKDLLGFAILI
Nock    RFFAFHFLFPFVIAGATLIHLVFLHETGSNNPAGVKLHADKIPFHPYFSYKDLLGFAIML
Anja    RFFAFHFLFPFVVAGATMIHLLFLHETGSNNPVGLNSDADKIPFHPYFSYKDLLGFIIML
Gyki    RFFAFHFLLPFVVLAATVLHLLFLHETGSNNPAGLNSDADKIPFHPYFSYKDLLGFIIML
Syka    RFFAFHFLLPFVVLGATLLHLLFLHETGSNNPVGLNSNADKIPFHPYFSYKDLLGFIILL
Opma    RFFAFHFLFPFLVVAATLLHVVFLHETGSNNPMGLISDADKIPMHPYFTYKDVLGFSIFM
Comy    RFFAFHFLFPFVVVGASLLHIIFLHETGSNNPMGLNSDADKIPFHPYFTYKDFLGFIILI
Sasp    RFFAFHFLLPFVVLGLTLIHLLFLHESGSNNPLGIHSNLDKIPFHPFYTTKDLLGFMLLL
Eupe    RFFAFHFLLPFVVLGITVIHLASLHVTGSVNPVGIISNSDKVPFHPYYTFKDLAGFLILF
Enja    RFFAFHFLFPFVIAGVTILHLLFLHETGSNNPAGLNSDADKIAFHPYFSYKDLLGFAVML
Same    RFFAFHFLFPFVIAGATVLHLLFLHETGSNNPAGLNSDADKISFHPYFSYKDLLGFAVML
Chch    RFFAFHFLFPFVIAATVVLHLLFLHETGSNNPAGLNSDADKISFHPYFSYKDILGFAVLL
Grgr    RFFAFHFLLPFAIIGATLLHLLFLHETGSNNPVGLNSDPDKISFHPYFSYKDLLGFVVLL
Caau    RFFAFHFLLPFIIAAATVIHLLFLHETGSNNPIGLNSDADKISFHPYFSYKDLLGFVIML
Cyca    RFFAFHFLLPFVIAAVTIIHLLFLHETGSNNPIGLNSDADKISFHPYFSYKDLLGFVIML
Dare    RFFAFHFLLPFIIIAMVILHLLFLHETGSNNPLGLNPNMDKIPFHPYFSNKDLLGFVIML
Cost    RFFAFHFLLPFIIAAATILHLLFLHETGSNNPMGLNSDADKVSFHPYFSYKDLLGFAVVL
Leec    RFFAFHFLFPFIIAAATIIHLLFLHETGSNNPAGLNSDMDKISFHPYFSYKDLFGFVVML
Fola    RFFAFHFLFPFIVAAVTILHLLFLHETGSNNPAGLNSDADKISFHPYFSYKDLLGFVVML
Clmc    RFFAFHFLLPFAIVAATGSHALFLHETGSNNPVGLNSDADKISFHPYFSYKDILGFVILL
Phin    RFFAFHFLLPFAIVAATALHALFLHETGSNNPLGLNSDADKIPFHPYFSYKDILGFIILL
Icpu    RFFAFHFLLPFAIIAATLLHALFLHETGSNNPIGLNSDADKISFHPYFSYKDLLGFVFLI
Psto    RFFAFHFLLPFAVVAATLLHALFLHETGSNNPLGLNSDADKISFHPYFSYKDILGFIVLL
Cora    RFFTFHFLLPFIVVAATLIHAMFLHETGSNNPIGVNSDADKISFHPYFSFKDLLGFIILM
Eisp    RFFAFHFLLPFAVVAATILHALFLHETGSNNPTGLNSDSDKIPFHPYFSYKDLLGFIIML
Apal    RFFAFHFLLPFLLIAMTALHALFLHETGSNNPAGLNSDSDKIPFHPYFSYKDLLGFSILL
Eslu    RFFAFHFLFPFIIAAATILHLLFLHETGSNNPVGINSDADKIPFHPYFSYKDLLGFVFML
Dape    RFFAFHFLFPFIIAAATILHLLFLHETGSNNPMGINSDTDKIPFHPYFSYKDLLGFVVML
Glse    RFFAFHFLFPFVIVAATILHLLFLHETGSNNPTGINSNADKISFHPYFSYKDLLGFAILL
Naar    RFFAFHFLFPFVIIAASILHFLFLHETGSNNPVGINSDADKISFHPYFIYKDILGFAITL
Lioc    RFFAFHFLFPFVVIGATILHFLFLHETGSNNPVGINSDADKIPFHPYFTYKDILGFTIVL
Opso    RFFAFHFLFPFLIAAAAILHLLFLHETGSNNPTGISSDTDKIPFHPYYSYKDLLGFLILL
Alte    RFFAFHFLFPFVIVAATVIHLLFLHETGSNNPVGLNSDTDKIPFHPYFTYKDLLGFAVLL
Plap    RFFAFHFLFPFVIIGAVIIHLLFLHETGSGNPAGLNSDVDKVPFHPYFTYKDLLGFTILL
```

To be continued on page 21.

[4/7 of aligned sequences]

To be continued on page 22.

```
Plal  RFFAFHFILPFIIAAATVLHLLFLHETGSNNPVGLNSDADKIPFHSYFIVKDLVGFMVLF
Sami  RFFAFHFILPFIIAAATVVHLLFLHQTGSNNPVGLNSDADKIPFHSYFIIKDLVGFVVLF
Rere  RFFAFHFLFPFVIAAVTVLHLLFLHETGSNNPAGLNSDADKISFHPYFSYKDLLGFAVLL
Gama  RFFAFHFLFPFVIAGATVLHLLFLHETGSNNPAGLNSDADKISFHPYFSYKDLLGFVALL
Onmy  RFFAFHFLFPFVIAAATVLHLLFLHETGSNNPAGINSDADKISFHPYFSYKDLLGFVAML
Sasa  RFFAFHFLFPFVIAAATVLHLLFLHETGSNNPAGINSDADKISFHPYFSYKDLLGFVAML
Cola  RFFAFHFLFPFVIAAATVIHLLFLHETGSNNPAGINSDADKISFHPYFSYKDLLGFVAML
Dita  RFFAFHFLFPFIIFAATVLHLLFLHETGSNNPAGLNSDADKISFHPYFSYKDLLGFVALL
Gogr  RFFAFHFLLPFVIAAFTAIHLLFLHETGSNNPTGLNSDADKIPFHPYFSLKDLLGFTILI
Chsl  RFFAFHFLLPFVVLAATLLHLLFLHETGSNNPAGLNSDADKVPFHPYFSYKDLLGFAILL
Atja  RFFAFHFLFPFVIAAMVIIHLLFLHETGSNNPAGINSDSDKIPFHPYFSYKDLLGFMVLL
Iido  RFFAFHFLFPFVIAAMVVIHLLFLHETGSNNPAGINSDSDKIPFHPYFSYKDLLGFMVLL
Auja  RFFAFHFLFPFVIVAMTVIHLLFLHETGSNNPAGINSDADKISFHPYFSYKDLLGFIALM
Chag  RFFAFHFLFPFVIAAVTVIHLLFLHETGSNNPAGINSDADKISFHPYFSYKDLLGFIIMM
Hami  RFFAFHFLFPFVIIAVTAIHLLFLHETGSNNPTGINSDADKIAFHPYFIYKDLLGFVIML
Saun  RFFAFHFLLPFVIIAVTAIHLLFLHETGSNNPTGINSDVDKIAFHPYFIYKDLLGFVIML
Nema  RFFAFHFLLPFIIAAMALIHLIFLHETGSNNPAGLNSDADKISFHPYFSYKDLLGFVALL
Disp  RFFAFHFLLPFIVAAMTLIHLIFLHETGSNNPAGINSDADKISFHPYFSYKDLLGFVALL
Myaf  RFFAFHFLLPFIVAAMTMIHLIFLHETGSNNPTGINSDSDKISFHPYFSYKDLLGFVALL
Lagu  RFFTFHFLLPFIIAAATLVHLLFLHETGSNNPTGLNSDADKVSFHPYFSYKDLLGFLALL
Trtr  RFFAFHFLLPFVIAAATMIHLLFLHETGSNNPAGLNSDADKISFHPYFSYKDLLGFIILL
Zucr  RFFAFHFLLPFVIAAATMIHLLFLHETGSNNPAGLNSDADKISFHPYFSYKDLLGFIILL
Pxja  RFFAFHFLLPFIVAAATVIHLLFLHETGSNNPAGINSDADKISFHPYFSYKDLLGFVVLL
Pxlo  RFFAFHFLLPFIVAAATVIHLLFLHETGSNNPAGINSDADKISFHPYFSYKDLMGFVVLL
Pctr  RFFAFHFLFPFVIAALTAIHLLFLHETGSNNPAGLNSDADKVTFHPYFSYKDLLGFATLM
Apsa  RFFTFHFLFPFIIAALSALHLLFLHETGSNNPLGLNSNPDKIPFHPYFSYKDLLGFIILL
Cabe  RFFAFHFLLPFLIVGMSAVHILFLHETGSNNPVGLISNSDKVPFHPYFSLKDILGIGALL
Bzze  RFFAFHFLLPFIVAAFSVIHILFLHETGSNNPAGLNSDADKIPFHPYFSYKDVLGFAILL
Siim  RFFSFHFTLPFVMLALSTLHILFLHETGSNNPIGLTNHTDKIPFHPYFVYKDLLGFVIML
Ctru  RFFAFHFLFPFVIAAASVIHILFLHETGSNNPTGLNSNADKISFHPYFVYKDLLGFALLL
Dpbr  RFFAFHFLFPFIITAMAVIHILFLHETGSNNPAGLNSDADKISFHPYFSYKDLLGATIFL
Caki  RFFTFHFLLPFVVMAVTMIHLLFLHETGSTNPTGLNSDMDKIPFHPYFSYKDLLGFIMLL
Phja  RFFAFHFLLPFVVIAATILHFLFLHETGSNNPIGLNSDADKIPFHPYFIYKDFLGFIVLF
Brsp  RFFTFHFLLPFVVAAMTMLHLLFLHETGSNNPTGMNSDLDKIPFHPYFTYKDLLGFMILV
Gamo  RFFAFHFLFPFVVAAFTMLHLLFLHETGSNNPTGINSNADKIPFHPYFTYKDLLGFAVML
Lolo  RFFAFHFLFPFIVAAVTMLHLLFLHETGSNNPTGINSNADKIPFHPYFTYKDLLGFAVML
Batr  RFFTLHFLMPFIIMALAMVHILFLHNTGSNNPMGLNSNLDKVSFHPLYTLKDMLGFLLVL
Prmy  RFFSLHFIFPFIILALVLVHLLFLHEKGSSNPLGLTPNMDKIPFHPYYTVKDVLGFLLLL
Lose  RFFAFHFLFPFLIAAVTAVHLLFLHKTGSNNPLGLNSDSDKISFHPYFSYKDLAGFTALL
Loam  RFFAFHFLFPFIILAMTVIHLLFLHETGSNNPLGLNSDADKISFHPYYSYKDLVGFAIVL
Chab  RFFAFHFLFPFIVAAMTMLHLLFLHETGSNNPLGLNSDADKISFHPYFSYKDLLGFAAVL
Chto  RFFAFHFLFPFIVAAMTMLHLLFLHETGSNNPLGLNSDADKISFHPYFSYKDLLGFAAVL
Majo  RFFAFHFLFPFIVAAMTLIHLLFLHETGSNNPLGLSSNTDKISFHPYFSYKDLLGFMAVL
Hlst  RFFAFHFLLPFVIAAMTLVHVLFLHETGSNNPLGLNSNTDKVSFHPYYSYKDLLGFAVVL
Clpe  RFFTFHFLFPFVIAAMTVIHLLFLHETGSNNPLGLNSDADKIPFHPYFSYKDLLGFVIML
Mlmr  RFFAFHFLFPFIIAALTLIHLLFLHETGSNNPLGLNSDTDKIPFHPYFSYKDLLGFAAML
Crcr  RFFAFHFLLPFVILALTLIHLIFLHETGSNNPLGLPSNSDKIPFHPYYTIKDIFGFLVLL
Muce  RFFAFHFLLPFVILALTLIHLIFLHETGSNNPLGLPSNSDKIPFHPYYTIKDIFGFLVLL
Bege  RFFAFHFLFPFIIAAATMVHLIFLHETGSNNPTGINSDADKISFHPYFSYKDLLGFAILL
Mela  RFLAFHFLLPFVIVAMTMVHLIFLHETGSNNPTGLNSDADKISFHPYFSYKDLLGFTILL
Hats  RFFAFHFLLPFIIAAATLVHLIFLHETGSNNPTGLNSDADKISFHPYFSYKDVLGFAALL
Orla  RFFAFHFLLPFVIAAATVVHLIFLHETGSNNPTGLNSDSDKVSFHPYFSYKDLLGFAALL
```

[4/7 of aligned sequences]

To be continued on page 23.

Cosa	RFFAFHFLFPFIIAAATLIHLIFLHETGSNNPTGINSDADKISFHPYFSYKDILGFAALF
Exsp	RFFTFHFLLPFVIAAMSMIHLIFLHESGSNNPTGINSDADKISFHPYFSYKDILGFAALL
Depa	RFFAFHFLLPFIIAAASMVHLIFLHETGSNNPTGINSDSDKISFHPYFSYKDVLGFAALL
Rima	RFFAFHFLFPFGIIAMTLVHLLFLHEKGSSNPVGINSNADKIYFHPYFIYKDLIGFAWFA
Fuol	RFFAFHFLLPFVVAAATMVHLIFLHETGSNNPTGLNSDADKISFHPYFSYKDILGFALLL
Gmaf	RFFAFHFLFPFIVAAATMVHLIFLHETGSNNPIGLNSDADKISFHPYFSYKDLLGFVFLL
Xeei	RFFAFHFLFPFVIAAATMVHLIFLHETGSNNPTGLNSDADKISFHPYFSYKDMLGFAILL
Pros	RFFAFHFLFPFVIAAMVIIHLLFLHETGSNNPIGINSDADKMPFHPFSIYKDALGFTILL
Scmi	RFFAFHFLFPFIITAMVILHLLFLHETGSNNPIGINSDADKVPFHPYFIYKDALGFTLLL
Rolo	RFFAFHFLFPFVIAAMAVIHLLFLHETGSSNPAGLNSDADKISFHPYFSYKDLVGFVALL
Cere	RFFAFHFLFPFIITAMTIVHFLFLHETGSNNPTGLNSDADKISFHPYFSYKDLLGFVALF
Daga	RFFAFHFLLPFIIAAMTIIHLLFLHETGSNNPTGLNSDADKISFHPYFSYKDLLGFVALF
Anco	RFFAFHFLFPFVIAAAAVIHLLFLHETGSNNPAGVNSNADKISFHPYFSYKDLLGFALML
Dmve	RFFAFHFLFPFIIAAVTAIHILFLHETGSNNPAGLNSDSDKIPFHPYFSYKDLLGFAAML
Dmar	RFFAFHFLFPFLIAAATVIHLLFLHETGSNNPAGLNSDADKIPFHPYFSYKDLLGFAVML
Anka	RFFAFHFLFPFVIAAATMIHLLFLHETGSNNPAGINSDADKISFHPYFSYKDLLGFVVLL
Moja	RFFAFHFLFPFVIAAATMIHLLFLHETGSNNPAGINSDADKISFHPYFSYKDLLGFVVLL
Hoja	RFFAFHFLFPFVIAAATMIHLLFLHETGSNNPAGINSNADKISFHPYFSYKDLLGFVVLL
Bede	RFFAFHFLFPFVIAAMAMIHLLFLHETGSNNPVGLNSDADKISFHPYCTYKDALGFTALL
Besp	RFFAFHFLFPFVIAAMAMIHLLFLHETGSNNPVGLNSDADKISFHPYCTYKDALGFTVLL
Mysp	RFFAFHFLLPFIIAAMTLIHLLFIHETGSNNPAGLNSDADKISFHPYFSYKDLLGFVALL
Osja	RFFAFHFLLPFIIAAMTLIHLLFIHETGSNNPAGLNSDADKISFHPYFSYKDLLGFVALL
Sgro	RFFAFHFLLPFIIAAMTIIHLLFLHETGSNNPAGLNSDADKISFHPYFSYKDLLGFVALL
Pzpa	RFFAFHFLFPFIIAAMTVLHILFLHETGSNNPTGINSNVDKIPFHPYYSYKDLLGFVILL
Zeja	RFFAFHFILPFIVAAMVILHLLFIHETGAFNPVGINSDSDKISFHPYFSYKDLLGFFILL
Znne	RFFAFHFLFPFIIVALTVLHFFFLHETGSNNPTGINSDADKVPFHPYFSYKDLFGLVLLL
Zefa	RFFAFHFLFPFIIAAMTILHFLFLHETGSNNPAGLNSDADKVSFHPYFSYKDLLGFVVML
Acni	RFFAFHFLFPFIIAAMVLLHLLFLHETGSSNPTGINSGADKIPFHPYFTYKDLFGFVILL
Ncrh	RFFAFHFLFPFIIAAMVILHLLFLHETGSSNPTGISSNADKIPFHPYFTYKDLFGFVILL
Agca	RFFAFHFLFPFIIAAMTIIHLLFLHETGSNNPLGLNSDSDKISFHPYFSYKDLLGFAVIL
Hydy	RFFAFHFLFPFVIAGATLVHLLFLHQTGSNNPLGLNSDADKISFHPYFSYKDLLGFVALL
Gsac	RFFAFHFLFPFVIAGATLVHLLFLHQTGSNNPLGLNSDADKISFHPYFSYKDLLGFAALL
Pevo	RFFTFHFLFPFIIVGATAIHLLFLHETGSNNPMGLNSDADKVSFHPYFSYKDLLGFAVMM
Hiku	RFFAFHFLLPFIVAAATMIHLLFLHETGSNNPAGINSDADKISFHPYFSYKDLLGFAALL
Inpa	RFFAFHFFIPFIATAVVALHFLFLHETGSNNPTGLSSSADKVPLHPYFLYKDLLGFLLLL
Auch	RFFTLHFLMPFVIVAATVLHLLFLHETGSNNPLGLTRNADKVSFHPYFSYKDILGFLALL
Fico	RFFAFHFLLPFIIAAASVIHILFLHETGSNNPTGLNSDADKISFHPYFSYKDLLGFGALL
Macs	RFFAFHFLFPFIIAAMTILHFLFLHETGSNNPTGLNSDSDKISFHPYFSYKDLLGFAALL
Moal	RFFSFHFFLPFLIVAMSFIHLIFLHETGSNNPAGLNSNMDKIPFHPYYSFKDLFGYLLFF
Syma	RFFAIHFTLPFITVGLTMLHLLFLHETGSNNPTGLKTEPDKIPFHPYFSYKDVLGFLLLL
Mafr	RFFAFHFLFPFIIAAATMVHLLFLHETGSNNPMGLNSDTDKIPFHPYYSYKDLMGFTLLL
Dcpe	RFFAFHFLLPFIIAAATVIHLLFLHETGSNNPTGLNSDADKISFHPYFSYKDLLGFAALL
Dcti	RFFAFHFLLPFIIAAATVIHLLFLHETGSNNPTGLNSDADKISFHPYFSYKDLLGFAALL
Hehi	RFFAFHFLFPFVIAGATLVHLLFLHQTGSTNPLGLNSDADKMSFHPYFSYKDLLGFVVLI
Stam	RFFAFHFLLPFVIAGATLIHLLFLHETGSNNPLGLNSDADKISFHPYFSYKDLLGFAALL
Hogi	RFFAFHFLFPFVIAGVTFIHLIFLHETGSNNPMGLNSNVDKISFHPYYSYKDLLGFVVLL
Erzo	RFFAFHFLFPFVIAGATLLHLLFLHETGSNNPLGLNSDADKISFHPYFSYKDLLGFAALF
Hxot	RFFAFHFLFPFVIAGATMVHLLFLHQTGSNNPLGLNSDADKISFHPYFSYKDLLGFSALL
Core	RFFAFHFLFPFVIAGATLIHLLFLHETGSNNPLGLNSDADKISFHPYFSYKDLLGFAALL
Apve	RFFAFHFLFPFIIAGLTLIHLLFLHETGSSNPLGLNSNTDKISFHPYFVYKDLLGFATLI
Latj	RFFAFHFLLPFVILAATIIHLLFLHETGSNNPMGLNSDADKISFHPYFSYKDLLGFAALL
Laja	RFFAFHFLFPFVIAGATLIHLLFLHETGSNNPLGLNSDADKIPFHPYFSYKDLLGFAVLL

```
Syja    RFFSFHFLFPFVIAAATLVHLLFLHETGSNNPLGLTSDPDKIPFHPYFSYKDLLGFAVLL
Epme    RFFAFHFLFPFVIAAVTMIHLIFLHETGSNNPTGLNSDADKISFHPYFSYKDLLGFAALL
Grse    RFFAFHFLLPFIIAAITLLHLLFLHETGSNNPLGLNSDADKISFHPYFSYKDLIGFAALL
Clja    RFFAFHFLFPFVILAVTLIHLLFLHQTGSNNPLGLSSDMDKVSFHPYFTYKDLLGFVMLL
Ogcy    RFFAFHYLFPFIIAAMTLIHLIFLHETGSNNPIGLNSDADKISFHPYFSYKDLLGFAILI
Plna    RFFAFHFLLPFIIAAMTMVHLIFLHETGSNNPTGLNSDMDKISFHPYFSYKDLLGFAILL
Lema    RFFAFHFLFPFIIAAATVIHLLFLHETGSNNPLGLNSDADKILFHPYFSYKDLLGFVALL
Etzo    RFFAFHFLFPFVIAGATLIHLLFLHETGSNNPLGLNSDADKVSFHPYFSYKDLLGFAVLL
Apse    RFFAFHFLLPFVIAAATLLHLLFLHETGSNNPVGLNPNADKIPFHPYFTYKDLLGFIVLF
Epde    RFFAFHFLFPFVIAGATIIHLIFLHETGSNNPLGLNSDADKISFHPYFSYKDLLGFAVLL
Slja    RFFAFHFLLPFIIAAFTIVHLLFLHETGSNNPLGLNSDADKISFHPYFSYKDLLGFAAVL
Bsja    RFFAFHFLFPFVILAATLVHLLFLHETGSNNPLGLNSNVDKISFHPYFSYKDLLGFMVLL
Ecna    RFFAFHFILPFIIAAVAVIHLIFLHETGSNNPMGLSSNVDKISFHPYFSYKDLLGFAALI
Cohi    RFFAFHFLLPFVILATTLVHLLFLHETGSNNPTGLNSDSDKIPFHPYFSYKDILGFTVLF
Caar    RFFAFHFLLPFIIAAVFILHVLFLHETGSNNPTGLNSDADKISFHPYFSYKDLLGFAALL
Came    RFFAFHFLLPFIIAAVFILHVLFLHETGSNNPTGLNSDADKISFHPYFSYKDLLGFAALL
Mema    RFFAFHFLFPFVILAATVIHLLFLHETGSNNPTGLNSDADKIPFHPYFSYKDLLGFAVLL
Lenu    RFFTFHFLLPFIIVAVTMIHLFFLHETGSNNPLGLSSNTDKIPFHPYFSYKDLLGFALML
Brja    RFFAFHFLFPFIIAAMTILHLLFLHETGSTNPIGLNSNADKISFHPYFSYKDLLGFAVLL
Plma    RFFAFHFLFPFIIAAMAILHLLFLHETGSNNPIGLNSNTDKISFHPYFSYKDLLGFAVLL
Emst    RFFAFHFLFPFVIAAATMIHLLFLHETGSNNPLGLNSDVDKISFHPYFSYKDLLGFAAVL
Ptti    RFFAFHFLLPFIIAAVTVLHLLFLHETGSNNPLGLNSDVDKISFHPYFSYKDLLGFVVVL
Losu    RFFAFHFLLPFIIAAMTLVHLLFLHQTGSNNPLGLNSDTDKVSFHPYFSYKDLLGFALII
Geoy    RFFAFHFLLPFVILGLTVIHLLFLHETGSNNPLGLNSDADKISFHPYFSYKDLLGFAVLI
Dipi    RFFAFHFLFPFVIVAATLIHLLFLHETGSNNPLGLNSDVDKISFHPYFSYKDLLGFAILL
Pama    RFFAFHFLLPFIVAAMTMLHLLFLHETGSNNPLGLNSDTDKISFHPYFSYKDLLGFAAVI
Leob    RFFAFHFLFPFVIAAATMLHLLFLHETGSNNPLGLNSDSDKISFHPYFSYKDLLGFAAVL
Neba    RFFAFHFLFPFVIAAFTLLHLLFLHETGSNNPLGLTSDTDKISFHPYFSYKDLLGFAAVI
Pdpl    RFFAFHFLFPFVIAAVTMLHVLFLHQTGSNNPIGINSNADKIPFHPYFSYKDLLGFVILL
Nimi    RFFAFHFLLPFIVAAATLLHLLFLHETGSNNPLGLNSDADKIPFHPYFTYKDLLGFAILI
Uptr    RFFAFHFLLPFIIAAMTMIHLIFLHETGSNNPTGLNSDADKISFHPYFSYKDLLGFAALL
Pesc    RFFAFHFLLPFIIAAFVMIHLLFLHETGSNNPIGLNPNAEKISFHPYYTIKDLLGFVVLL
Baar    RFFAFHFLFPFVIAAATLVHLLFLHETGSNNPLGLNSDADKVPFHPYFSYKDLLGFAVLL
Moar    RFFAFHFLLPFIIAAATVIHLLFLHETGSNNPLGLNSDVDKISFHPYFSYKDLLGFAAVL
Toja    RFFAFHFLFPFVIAAVTLIHLLFLHETGSNNPTGLNSDADKISFHPYFSYKDLLGFIILL
Chau    RFFAFHFLLPFIIAAVSMIHLMFLHQTGSNNPLGLNSDMDKISFHPYFSYKDLLGFAVTL
Chse    RFFAFHFLFPFVIAAATMVHLIFLHETGSNNPLGLNSDSDKIPFHPYFSYKDLLGFAIVL
Enar    RFFAFHFLFPFVIVAATVIHLLFLHESGSNNPLGLNSDADKVSFHPYFSYKDLLGFAALL
Hpty    RFFAFHFLFPFIILAATVIHLLFLHETGSNNPLGLNSDADKVSFHPYFSYKDLLGLAVLL
Nana    RFFAFHFLFPFIIAAMTIIHLLFLHETGSNNPIGLNSNADKIPFHPYYSYKDLVGFVLLL
Mcst    RFFAFHFLFPFIIAATAVIHLLFLHETGSTNPLGLNSDTDKISFHPYFSYKDLLGFTALL
Rhox    RFFAFHFLLPFVIAALTMIHLLFLHETGSNNPLGLNSNADKISFHPYFSYKDLLGFVALL
Opfa    RFFAFHFLFPFVIAAATFIHLIFLHETGSNNPLGLNSDADKISFHPYFSYKDLLGFTALL
Paar    RFFAFHFLFPFVIAAATVIHLLFLHETGSNNPLGLNSDADKISFHPYFSYKDLLGFAALL
Gozo    RFFAFHFLFPFVIAAATVIHLLFLHETGSNNPLGLNSDADKISFHPYFSYKDLLGFAALL
Ackr    RFFTFHFLLPFVIVGVTLVHLLFLHETGSNNPLGLNSNADKVPFHPYFTYKDLLGFVIFL
Elev    RFFAFHFLFPFIIAAATMIHLLFLHETGSNNPLGLNSDADKISFHPYFSYKDLLGFIVLL
Trdu    RFFAFHFLLPFITAAATMVHLIFLHETGSNNPTGLNSDADKISFHPYFSYKDLLGFAILL
Amoc    RFFAFHFLFPFVIVAMTLVHLIFLHETGSNNPTGLNSDADKISFHPYFSYKDLLGFAALL
Hame    RFFAFHFLFPFVIAAATVVHLLFLHETGSNNPLGLNSDTDKISFHPYFSYKDLLGFAALI
Chso    RFFAFHFLFPFVIAAAVVLHLLFLHETGSNNPLGLNSDADKISFHPYFSYKDLLGFAALL
Lyto    RFFAFHFLFPFIIAGATLVHLLFLHQTGSTNPLGLNSDADKVSFHPYFSYKDLLGFAALV
```

To be continued
on page 24.

[4/7 of aligned sequences]

```
Encr  RFFAFHFLFPFVVAGATFVHLIFLHQTGSNNPLGLNSDADKISFHPYFSYKDLLGFAALL
Bvar  RFFAFHFLFPFIIVAATMVHLLFLHQTGSNNPLGLNSSTDKISFHPYFTYKDLLGFVTLL
Noco  RFFAFHFLFPFVIAGATMVHLLFLHQTGSNNPLGLNSTSDKIPFHPYFSYKDLLGFAALL
Chsp  RFFTLHFLLPFVIAAFTLVHLLFLHETGSNNPLGLTSNTDKIPFHPYFTYKDLIGFTVLM
Arja  RFFAFHFLFPFVIAGATLIHLLFLHETGSNNPLGLNSDADKISFHPYFSYKDLLGFAAML
Pase  RFFAFHFLLPFIIAAATLIHLLFLHETGSNNPLGLNSNSDKISFHPYFSYKDLLGFAIML
Trel  RFFAFHFLLPFVIAAATVLHLLFLHETGSNNPVDLGSDSDKIPFHPYFSYKDVLGFAGML
Lifa  RFFAFHFLFPFIIAGATLIHLLFLHETGSNNPLGLNSDSDKISFHPYFSWKDLLGFLTLL
Acur  RFFAFHFLFPFVIAAATLIHLLFLHETGSNNPLGLTSNTDKISFHPYFSYKDLGGFGLLL
Ampe  RFFAFHFLFPFVIAGATIIHLLFLHETGSNNPLGLNSDADKISFHPYFSYKDLLGFSALL
Urja  RFFAFHFLLPFITTALVMIHFLFLHETGSTNPLGLNSNTDKISFHPYFSFKDVLGFAALL
Enet  RFFAFHFLFPFVIVAATMVHLFFLHETGSNNPTGLNSDSDKISFHPYFSYKDLLGFAALL
Ptbr  RFFTFHFLFPFVIAAATMVHLIFLHESGSNNPTGLSSDADKISFHPYFSYKDLLGFAALL
Safa  RFFAFHFLFPFVIAAVTMVHLIFLHETGSNNPTGLNSDADKISFHPYFSYKDLLGFAALL
Icae  RFFAFHFLFPFVIAAMAILHLLFLHETGSNNPIGLNSNADKISFHPYFSYKDLLGFALLL
Asmi  RFFAFHFLFPFVIVAGTLIHLVFLHETGSNNPMGLNSNSDKISFHPYFSYKDLLGFAALL
Foal  RFFAFHFLLPFIIAAVTMIHLLFLHETGSNNPVGLNSNVDKITFHPYFSYKDLIGFVVML
Drze  RFFAFHFLFPFIIAAITVIHIFFLHMTGSTNPLGINSDTDKIPFHPYFSYKDLLGFLVML
Rhas  RFFAFHFLLPFIIAAVTVLHLLFLHETGSNNPAGLNSDADKVPFHPYFSYKDLLGFAAML
Elac  RFFTFHFLLPFIITAVTMIHLLFLHETGSNNPTGLNSDADKIPFHPYFTYKDLLGFGIMI
Kugu  RFFAFHFLFPFVIAAVAMLHLLFLHETGSNNPTGLNSNTDKIPFYPYFVYKDLLGFAILL
Plor  RFFAFHFLFPFGILAATVIHLLFLHETGSNNPLGLSSDSDKISFHPYFSYKDLLGFAIVL
Sgun  RFFAFHFLLPFIIAAVTLIHLIFLHETGSNNPLGLNSDMDKISFHPYFSYKDLLGFAALL
Zaco  RFFAFHFLFPFVIAAMTVIHLLFLHETGSNNPLGLNSDVDKISFHPYFSYKDLLGFAAVI
Zbfl  RFFAFHFLLPFIILAATIIHLLFLHETGSNNPLGLNSDVDKISFHPYFSYKDLLGFAVVI
Spba  RFFAFHFLLPFVIMAATMLHLIFLHETGSNNPVGLNSNADKISFHPYFAYKDLLGFVILL
Game  RFFAFHFLFPFVILAMTILHLLFLHETGSNNPIGLNSNADKISFHPYFSYKDLLGFAVLL
Thth  RFFAFHFLFPFVIAAMTILHLLFLHETGSNNPIGLNSNADKISFHPYFSYKDLLGFVILL
Xigl  RFFAFHFLLPFVIMAATIIHLLFLHETGSNNPTGLNSDADKISFHPYFSYKDLLGFAALL
Hyja  RFFAFHFLLPFIVTAMAILHLLFLHETGSNNPLGLNSNTDKISFHPYFSYKDLLGFAVML
Psan  RFFTFHFLLPFIILAMTILHLLFLHETGSNNPIGLNSNTYKVSFHPYFSYKDLLGFAVLL
Cupa  RFFAFHFLFPFVIAAFSVLHLLFLHETGSNNPIGLNSNADKISFHPYFSYKDLLGFAVLL
Mpch  RFFAFHFLFPFIIAAVTIIHLLFLHETGSNNPLGLDSNVDKITFHPYFSYKDLLGFAILM
Char  RFFAFHFLFPFLIAAFTIIHLLFLHETGSTNPVGLNSDADKIPFHPYFSYKDLLGFAILL
Pser  RFFAFHFLLPFVILAATLIHLLFLHETGSNNPTGLNSDADKVPFHPYFSYKDLLGFAVLL
Prol  RFFAFHFLFPFVIAAATVIHLIFLHETGSNNPTGLNSDSDKVPFHPYFSYKDLLGFAALL
Plbi  RFFAFHFLFPFIIAAATVIHLLFLHETGSNNPTGLNSDSDKVPFHPYFTYKDLLGFAVLL
Calu  RFFAFHFVLPFVVLAATVLHLLFLHESGSNNPLGLVSGTDKISFHPYFSVKDIVGFAALV
Papa  RFFSFHFLLPFIIVAVIMLHLLFLHETGSNNPMGLNSNVDKISFHPYFIYKDLLGFVALI
Sufr  RFFAFHFLFPFIIAAATLLHLLFLHESGSNNPLGMNSDVDKIPFHPYFSYKDLLGFAVAL
Stci  RFFAFHFLFPFAIAAVTMIHLIFLHETGSNNPLGLNSNADKIPFHPYFSYKDVLGFAVAL
Taru  RFFAFHFLLPFIVAAAAIVHLIFLHETGSNNPLGLNSNADKIPFHPYFSYKDLLGFTIML
Rala  RFFAFHFLLPFIVAAVTLIHLIFLHETGSNNPLGLSSDMDKISFHPYFSYKDLLGFTIML
      **:::*: :**    .    *   :* .*: **  :     *: ::     **  *
```

To be continued on page 25.

```
                   F                                                   G
Scca   TLLATLALFMPNLLGDAENFIPANPLVTPLHIQPEWYFLFAYAILRSIPNKLGGVLALLF
Muma   FLLALLALFLPNLLGDAENFIPANPLVTPPHIKPEWYFLFAYAILRSIPNKLGGVLALLF
Erca   LIILMLALLSPNLLNDPENFTPANPLVTPPHIKPEWYFLFAYAILRSIPNKLGGVLALLF
Pose   LIIISLALLSPNLLNDPENFTPANPLVTPPHIKPEWYFLFAYAILRSIPNKLGGVLALLF
Actr   VGLTSVALFSPNLLGDPDNFTPANPLVTPPHIKPEWYFLFAYAILRSIPNKLGGVLALLF
Scal   VGLTSVALFSPNLLGDPDNFTPANPLVTPPHIKPEWYFLFAYAILRSIPNKLGGVLALLF
Posp   IGLTAIALFSPNLLGDPDNFTPANPLVTPPHIKPEWYFLFAYAILRSIPNKLGGVLALLF
Atsp   AILSALALLNPNLLGDPENFTPANPLVTPPHIKPEWYFLFAYAILRSIPNKLGGVLALLL
Leoc   ATLSALALLNPNLLGDPENFTPANPLVTPPHIKPEWYFLFAYAILRSIPNKLGGVLALLF
Amca   AGLMFLALFSPNLLGDPENFTPANPLVTPPHIKPEWYFLFAYAILRSIPNKLGGVLALLF
Osbi   LMLASLALFLPNLLGDPENFTPANPLVTPPHIKPEWYFLFAYAILRSIPNKLGGVLALLF
Pabu   LALTTLALFSPNLLGDPENFTPANPLVTPPHIKPEWYFLFAYAILRSIPNKLGGVLALLF
Hial   LALTSIALFSPNLLGDPDNFTPANPLVTPPHIKPEWYFLFAYAILRSIPNKLGGVLALLF
Elha   TALAALSLFSPNLLGDPENFTPANPLVTPPHIKPEWYFLFAYAILRSIPNKLGGVLALLF
Mlcy   MSLTSLALFSPNLLGDPENFTPANPLVTPPHIKPEWYFLFAYAILRSIPNKLGGVLALLF
Algl   VALTSIALFSPNLLGDPDNFTPANPLVTPPHIKPEWYFLFAYAILRSIPNKLGGVLALFF
Ptgi   IALTSIALFTPNLLGDPDNFIPANPLVTPPHIKPEWYFLFAYAILRSIPNKLGGVLALLS
Alaf   IALTSIALFTPNLLGDPDNFTPANPLVTPPHIKPEWYFLFAYAILRSIPNKLGGVLALLA
Nock   MGLTLIALFTPNLLGDPDNFTPANPLVTPPHIKPEWYFLFAYAILRSIPNKLGGVLALLA
Anja   TALTMLALFSPNLLGDPDNFTPANPMVTPPHIKPEWYFLFAYAILRSIPNKLGGVLALLS
Gyki   TALTSLALFYPNALGDPDNFTPANPMVTPPHIKPEWYFLFAYAILRSIPNKLGGVLALLF
Syka   TALASLALFSPNLLGDPDNFTPANPMVTPPHIKPEWYFLFAYAILRSIPNKLGGVLALLS
Opma   PALLMLALFQPNLLGDPDNFTPANPMVTPPHIKPEWYFLFAYAILRSIPNKLGGVLALLS
Comy   ATLTSLALFNPNLLGDPDNFTPANPMVTPPHIKPEWYFLFAYAILRSIPNKLGGVLALLF
Sasp   IALIMLSLFFPYLVGDPDNFSNANPMVTPPHIKPEWTFLFAYAILRSIPNKLGGVLALLA
Eupe   IQLIMLTLFFPTVLGDPDNFTPANPMVTPPHIKPEWYFLFAYAILRSIPNKLGGVMALLF
Enja   LALTSLALFSPNLLGDPDNFTPANPLVTPPHIKPEWYFLFAYAILRSIPNKLGGVLALLF
Same   LVLTSLALFSPNLLGDPDNFVPANPMVTPPHIKPEWYFLFAYAILRSIPNKLGGVLALLF
Chch   LALTSLALFSPNLLGDPENFTPANPLVTPPHIKPEWYFLFAYAILRSIPNKLGGVLALLF
Grgr   LGLASLALFSPNLLGDPENFTPANPLVTPPHIKPEWYFLFAYAILRSIPNKLGGVLALLF
Caau   LALTLLALFSPNLLGDPENFTPANPLVTPPHIKPEWYFLFAYAILRSIPNKLGGVLALLF
Cyca   LALTLLALFSPNLLGDPENFTPANPLVTPPHIKPEWYFLFAYAILRSIPNKLGGVLALLF
Dare   FSLSLLALFSPNLLGDPENFTPANPLVTPPHIKPEWYFLFAYAILRSIPNKLGGVLALLF
Cost   LALTSLSLFSPNLLGDPDNFTPANPLVTPPHIKPEWYFLFAYAILRSIPNKLGGVLALLF
Leec   LALTSLALFSPNLLGDPDNFTPANPLVTPPHIKPEWYFLFAYAILRSIPNKLGGVLALLF
Fola   LGLTTLALFSPNLLGDPENFTPANPLVTPPHIKPEWYFLFAYAILRSIPNKLGGVLALLF
Clmc   MALVSLALFSPNLLGDPENFTPANPLVTPPHIKPEWYFLFAYAILRSIPNKLGGVLALLF
Phin   IALTSLALFSPNLLGDPENFTPANPLVTPPHIKPEWYFLFAYAILRSIPNKLGGVLALLF
Icpu   TALASLALFSPNLLGDPENFTPANPLVTPPHIKPEWYFLFAYAILRSIPNKLGGVLALLL
Psto   TALASLALFSPNLLGDPENFTPANPLVTPPHIKPEWYFLFAYAILRSIPNKLGGVLALLL
Cora   TLLLSLALFSPNLLGDPDNFTPANPLVTPTHIKPEWYFLFAYAILRSIPNKLGGVLALLF
Eisp   TGLISLALFSPNLLGDPENFTPANPLSTPPHIKPEWYFLFAYAILRSIPNKLGGVLALVF
Apal   TTLASLALFSPNLLGDPENFSAANPMITPTHIKPEWYFLFAYAILRSIPNKLGGVLALVF
Eslu   FGLASLALFSPNLLGDPDNFIPANPLVTPPHIKPEWYFLFAYAILRSIPNKLGGVLALLF
Dape   LGLIFLALFLPNLLGDPDNFIPANPLVTPPHIKPEWYFLFAYAILRSIPNKLGGVLALLF
Glse   IALSSLALFSPNLLGDPDNFTPANPLVTPPHIKPEWYFLFAYAILRSIPNKLGGVLALLS
Naar   IALSSLALFAPNLLGDPDNFTPANPLVTPPHIKPEWYFLFAYAILRSIPNKLGGVLALLF
Lioc   LALSSLALFSPNLLGDPDNFTPANPLVTPPHIKPEWYFLFAYAILRSIPNKLGGVLALLF
Opso   FALTSLALFSPNLLGDPDNFTPANPLVTPPHIKPEWYFLFAYAILRSIPNKLGGVLALLF
Alte   AALTALSLFWPNLLGDPDNFTPANPLVTPPHIKPEWYFLFAYAILRSIPNKLGGVLALLA
Plap   MALMTLSLFWPNLLGDPDNFTPANPLVTPPHIKPEWYFLFAYAILRSIPNKLGGVLALLA
```

To be continued
on page 26.

[5/7 of aligned sequences]

To be continued on page 27.

```
Plal  LALVSLALFSPNLLGDPDNFTAANPLVTPPHIKPEWYFLFAYAILRSIPNKLGGVLALLF
Sami  LALVSLALFSPNLLGDPDNFTAANPLVTPPHIKPEWYFLFAYAILRSIPNKLGGVLALLF
Rere  LSLVALALFSPNLLGDPDNFTPANPLVTPPHIKPEWYFLFAYAILRSIPNKLGGVLALLS
Gama  LALTSLALFSPNLLGDSENFIPANPLVTPPHIQPEWYFLFAYAILRSIPNKLGGVLALLF
Onmy  LGLTSLALFAPNLLGDPDNFTPANPLVTPPHIKPEWYFLFAYAILRSIPNKLGGVLALLF
Sasa  LGLTSLALFAPNLLGDPDNFTPANPLVTPPHIKPEWYFLFAYAILRSIPNKLGGVLALLF
Cola  LGLTSLALFAPNLLGDPDNFTPANPLVTPPHIKPEWYFLFAYAILRSIPNKLGGVLALLF
Dita  LALTSFALFSPNLLGDPDNFMPANPLVTPPHIKPEWYFLFAYAILRSIPNKLGGVLALLF
Gogr  LTLTSVALLTPNLLGDPDNFTPANPLVTPPHIKPEWYFLFAYAILRSIPNKLGGVLALLA
Chsl  LALTSFALFAPNLLGDPDNFIPANPLVTPPHIKPEWYFLFAYAILRSIPNKLGGVLALLF
Atja  IALTAIALFSPNLLGDPDNFTPANPLVTPPHIQPEWYFLFAYAILRSIPNKLGGVLALLS
Iido  IALTAIALFSPNLLGDPDNFTPANPLVTPPHIKPEWYFLFAYAILRSIPNKLGGVLALLS
Auja  IALTSLALFSPNLLGDPDNFTPANPLVTPPHIKPEWYFLFAYAILRSIPNKLGGVLALLA
Chag  VALTSLALFSPNLLGDPDNFTPANPLVTPPHIKPEWYFLFAYAILRSIPNKLGGVLALLA
Hami  AGLSALAFFYPNLLGDPDNFTPANPLVTPPHIKPEWYFLFAYAILRSIPNKLGGVLALLA
Saun  AGLSALAFFYPNLLGDPDNFTPANPLVTPPHIKPEWYFLFAYAILRSIPNKLGGVLALLA
Nema  MALASLALFSPNLLGDPDNFTPANPLVTPPHIKPEWYFLFAYAILRSIPNKLGGVLALLF
Disp  MALTCLALFTPNLLGDPDNFTPANPLVTPPHIKPEWYFLFAYAILRSIPNKLGGVLALLF
Myaf  IGLTCLALFTPNLLGDPDNFTPANPLVTPPHIKPEWYFLFAYAILRSIPNKLGGVLALLF
Lagu  SALTSLALFSPNLLGDPDNFIPANPLVTPPHIKPEWYFLFAYAILRSIPNKLGGVLALLF
Trtr  MALTSLALFSPNLLGDPDNFTPANPLVTPPHIKPEWYFLFAYAILRSIPNKLGGVLALLS
Zucr  MALTSLALFSPNLLGDPDNFTPANPLVTPPHIKPEWYFLFAYAILRSIPNKLGGVLALLF
Pxja  LSLTSLALFAPNLLGDPDNFTPANPLVTPPHIKPEWYFLFAYAILRSIPNKLGGVLALLS
Pxlo  LALTSLALFAPNLLGDPDNFTPANPLVTPPHIKPEWYFLFAYAILRSIPNKLGGVLALLS
Pctr  VTLTSLALFSPNLLGDPDNFTPANPLVTPPHIKPEWYFLFAYAILRSIPNKLGGVLALLS
Apsa  TSLTTLALFSPNLLGDPDNFTPANPLVTPPHIKPEWYFLFAYAILRSIPNKLGGVLALLL
Cabe  TGLVALALFVPNLFADPDNFSPADPLTTPPHIKPEWYFLFAYAILRSIPNKLGGVLALAS
Bzze  FALTSLALFSPNLLGDPDNFIPANPLVTPPHIKPEWYFLFAYAILRSIPNKLGGVLALLF
Siim  TLLLCTALFYPNILGDPDNFAPADPFKTPKHIKPEWYFLFAYAILRSFPNKLGGVIALLL
Ctru  VGLTALALFSPNLLGDPDNFIPANPLVTPPHIKPEWYFLFAYAILRSIPNKLGGVLALLF
Dpbr  VGLTSLALFTPNLLGDPDNFIPANPLVTPPHIKPEWYFLFAYAILRSIPNKLGGVLALLA
Caki  IPLASITLFWPNILGDPDNFIPANPLVTPPHIKPEWYFLFAYAILRSIPNKLGGVLALLL
Phja  LALGSLALFAPNLLGDPDNFTPANPLVTPPHIKPEWYFLFAYAILRSIPNKLGGVLALLC
Brsp  LILASLALFSPNLLGDPDNFTPANPLVTPPHIKPEWYFLFAYAILRSIPNKLGGVLALLF
Gamo  LGLTALALFAPNLLGDPDNFTPANPIVTPPHVKPEWYFLFAYAILRSIPNKLGGVLALLF
Lolo  LGLTALALFSPNLLGDPDNFTPANPIVTPPHVKPEWYFLFAYAILRSIPNKLGGVLALLF
Batr  IALSLIAFLSPYMLGDPDNFTPANPLMTPTHIKPEWYFLFAYTILRSIPNKLGGVIALLS
Prmy  FFLIFMALFYPNIFTDPENFTPANPMVTPTHIKPEWYFLFAYAILRAFPDKLGGVIALVM
Lose  IALTALALFSPNLLGDPDNFTPANPLVTPPHIKPEWYFLFAYAILRSIPNKLGGVLALLA
Loam  ITLTALALFAPNLLGDPDNFTPANPLVTPPHIKPEWYFLFAYAILRSIPNKLGGVLALLA
Chab  FALISLALFSPNLLGDPDNFTPANPLVTPPHIKPEWYFLFAYAILRSIPNKLGGVLALLA
Chto  FALISLALFSPNLLGDPDNFTPANPLVTPPHIKPEWYFLFAYAILRSIPNKLGGVLALLA
Majo  LALTSLALFSPNLLGDPDNFTPANPLVTPPHIKPEWYFLFAYAILRSIPNKLGGVLALLA
Hlst  LALSSLALFSPNLLGDPDNFTPANPLVTPPHIKPEWYFLFAYAILRSIPNKLGGVLALLA
Clpe  VALSSLTLFSPNLLGDPDNFTPANPLVTPPHIKPEWYFLFAYAILRSIPNKLGGVLALLS
Mlmr  IALTSLALFSPNLLGDPDNFIPANPLVTPPHIKPEWYFLFAYAILRSIPNKLGGVLALLA
Crcr  FALISLALFAPNLLGDPDNFTPANSMVTPAHIKPEWYFLFAYAILRSIPNKLGGVIALLC
Muce  FTLISLALFAPNLLGDPDNFTPANSMVTPAHIKPEWYFLFAYAILRSIPNKLGGVIALLC
Bege  LALISLALFSPNLLGDPDNFTPANPLVTPPHIKPEWYFLFAYAILRSIPNKLGGVLALLS
Mela  LGLTSLALFLPNLLGDPDNFTPANPLMTPPHIKPEWYFLFAYAILRSIPNKLGGVLALLF
Hats  LALISLALFSPNLLGDPDNYTPANPLITPPHIKPEWYFLFAYAILRSIPNKLGGVLALLS
Orla  VALISLALFSPNLLGDPDNFTPANPLVTPPHIKPEWYFLFAYAILRSIPNKLGGVLALLA
```

[5/7 of aligned sequences]

```
Cosa  VALISLALFSPNLLGDPDNFTPANPLVTPPHIKPEWYFLFAYAILRSIPNKLGGVLALLS
Exsp  IALTSLALFSPNLLGDPDNFTPANPLVTPPHIKPEWYFLFAYAILRSIPNKLGGVLALLS
Depa  ILLVSLALFSPNLLGDPDNFTPANPLVTPPHIKPEWYFLFAYAILRSIPNKLGGVLALLS
Rima  LFLITLVLFIPNLLGDPENFTPANPLVTPPHIKPEWYFLFAYAILRSIPNKLGGVFALLA
Fuol  ATLISLALFSPNLLGDPENFTPANPLVTPPHIKPEWYFLFAYAILRSIPNKLGGVLALLA
Gmaf  TTLVTLSLFSPNLLGDPENFTPANPLITPPHIKPEWYFLFAYAILRSIPNKLGGVLALLA
Xeei  IILTSLALFSPNLLGDPENFTPANPLVTPPHIKPEWYFLFAYAILRSIPNKLGGVLALLA
Pros  TILASLALFSPNLLGDPDNFTPANPLVTPPHIKPEWYFLFAYAILRSIPNKLGGVLALLA
Scmi  MILSSLALFAPNLLGDPDNFTPANPLVTPPHIKPEWYFLFAYAILRSIPNKLGGVLALLA
Rolo  IALTSLALFSPNLLGDPDNFIPANPLVTPPHIKPEWYFLFAYAILRSIPNKLGGVLALLS
Cere  VALVSLALFSPNLLGDPDNFIPANPLVTPPHIKPEWYFLFAYAILRSIPNKLGGVLALLS
Daga  IALVLLALFSPNLLGDPDNFIPANPLVTPPHIKPEWYFLFAYAILRSIPNKLGGVLALLS
Anco  LALTSLALFTPNLLGDPDNFIPANPLVTPPHIKPEWYFLFAYAILRSIPNKLGGVLALLF
Dmve  IALTALALFSPNLLGDPDNFTPANPLVTPPHIKPEWYFLFAYAILRSIPDKLGGVLALLF
Dmar  IALTTLALFSPNLLGDPDNFTPANPLVTPPHIKPEWYFLFAYAILRSIPDKLGGVLALLF
Anka  IALTSLALFAPNLLGDPDNFTPANPLVTPPHIKPEWYFLFAYAILRSIPNKLGGVLALLS
Moja  IALTSLALFAPNLLGDPDNFTPANPLVTPPHIKPEWYFLFAYAILRSIPNKLGGVLALLS
Hoja  LALTSLALFAPNLLGDPDNFTPANPLVTPPHIKPEWYFLFAYAILRSIPNKLGGVLALLS
Bede  VGLSSLALFSPNLLGDPDNFTPANPLVTPPHIKPEWYFLFAYAILRSIPNKLGGVLALLA
Besp  VGLSSLALFSPNLLGDPDNFTPANPLVTPPHIKPEWYFLFAYAILRSIPNKLGGVLALLA
Mysp  MALTSLALFSPNLLGDPDNFTPANPLVTPPHIKPEWYFLFAYAILRSIPNKLGGVLALLS
Osja  MALTSLALFSPNLLGDPDNFTPANPLVTPPHIKPEWYFLFAYAILRSIPNKLGGVLALLS
Sgro  MALISLALFSPNLLGDPDNFTPANPLVTPPHIKPEWYFLFAYAILRSIPNKLGGVLALLS
Pzpa  LALSTLALFSPNLLGDPDNFIPANPLVTPPHIKPEWYFLFAYAILRSIPNKLGGVLALLA
Zeja  TALFALSLFSPNLLGDPDNFTPANPLVTPPHIKPEWYFLFAYAILRSIPNKLGGVLALLA
Znne  LALSSLSFFSPNLLGDPDNFIPANPLVTPPHIKPEWYFLFAYAILRSIPNKLGGVLALLA
Zefa  LALSTLSLFSPNLLGDPDNFIPANPLVTPPHIKPEWYFLFAYAILRSIPNKLGGVLALLS
Acni  LALSILTLFSPNLLGDPDNFIPANPLVTPPHIKPEWYFLFAYAILRSIPNKLGGVLALLA
Ncrh  LALSVLALFSPNLLGDPDNFIPANPLVTPPHIKPEWYFLFAYAILRSIPNKLGGVLALLA
Agca  IPLTSLALFSPNLLGDPDNFTPANPLVTPPHIKPEWYFLFAYAILRSIPNKLGGVLALLA
Hydy  VGLTSLALFAPNLLGDPDNFTPANPLVTPPHIKPEWYFLFAYAILRSIPNKLGGVLALLA
Gsac  IALTSLALFAPNLLGDPDNFTPANPLVTPPHIKPEWYFLFAYAILRSIPNKLGGVLALLA
Pevo  IALTSLALFMPNLLGDPDNFSPANPLVTPPHIKPEWYFLFAYAILRSVPNKLGGVLALLF
Hiku  IALTSIALFTPNILGDPDNFTPANPLVTPPHIKPEWYFLFAYAILRSIPNKLGGVLALLF
Inpa  APLTALAVFSPNLFGDPDNFLRANPMVTPTHIKPEWYFLFAYAILRAIPNKLGGVLALLA
Auch  ALLATLAMFTPNLLGDPDNFTPANPLVTPPHIKPEWYFLFAYAILRSIPNKLGGVLALLA
Fico  LALTSLALFSPNLLGDPDNFTPANPLVTPPHIKPEWYFLFAYAILRSIPNKLGGVLALLA
Macs  IALTSLALFSPNLLGDPDNFTPANPLVTPPHIKPEWYFLFAYAILRSIPNKLGGVLALLF
Moal  LLLTLMSLFSPNLLSDPDNFTQANSLITPPHIKPEWYFLFAYAILRSVPSKLGGVIALAA
Syma  AALTALALFAPNILSDPDNFNSANPLITPTHIKPEWYFLYAYAILRSVPNKLGGVAALAL
Mafr  ITLTSLALFSPNLLGDPDNFTPANPLVTPPHIKPEWYFLFAYAILRSIPNKLGGVLALLA
Dcpe  IALTSLALFSPNLLGDPDNFTPANPLVTPPHIKPEWYFLFAYAILRSIPNKLGGVLALLF
Dcti  IALTSLALFSPNLLGDPDNFTPANPLVTPPHIKPEWYFLFAYAILRSIPNKLGGVLALLF
Hehi  IALTCLALFSPNLLGDPDNFTPANPLVTPPHIKPEWYFLFAYAILRSIPNKLGGVLALLA
Stam  IALTSLALFSPNLLGDPDNFTPANPLVTPPHIKPEWYFLFAYAILRSIPNKLGGVLALLA
Hogi  VALTSLALFSPNLLGDPDNFTPANPLVTPPHIKPEWYFLFAYAILRSIPNKLGGVLALLA
Erzo  AALTSLALFAPNLLGDPDNFIPANPLVTPPHIKPEWYFLFAYAILRSIPNKLGGVLALLA
Hxot  IALTALALFSPNLLGDPDNFTPANPLVTPPHIKPEWYFLFAYAILRSIPNKLGGVLALLA
Core  IALTALALFSPNLLGDPDNFTPANPLVTPPHIKPEWYFLFAYAILRSIPNKLGGVLALLA
Apve  LALSTLAVFSPNLLADPDNFTPANPLITPPHIKPEWYFLFAYAILRSIPNKLGGVLALLA
Latj  LALTCLALFTPNLLGDPDNFTPANPLVTPPHIKPEWYFLFAYAILRSIPNKLGGVLALLA
Laja  TALASLALFSPNLLGDPDNFTPANPLVTPPHIKPEWYFLFAYAILRSIPNKLGGVLALLF
```

To be continued on page 28.

[5/7 of aligned sequences]

To be continued on page 29.

Syja	ISLSSLALFAPNLLGDPDNFTPANPLVTPPHIKPEWYFLFAYAILRSIPNKLGGVLALLF
Epme	IALISLALFSPNLLGDPDNFTPANPLVTPAHIKPEWYFLFAYAILRSIPNKLGGVLALLF
Grse	IALTSLALFSPNLLGDPDNFTPANPLVTPPHIKPEWYFLFAYAILRSIPNKLGGVLALLA
Clja	IALISLALFSPNLLGDPDNFTPANPFITPPHIKPEWYFLFAYAILRSIPNKLGGVLALLA
Ogcy	IALTSLALFAPNLLGDPDNFTPANPLVTPPHIKPEWYFLFAYAILRSIPNKLGGVLALLA
Plna	IALISLALFSPNLLGDPDNFTPANPLVTPPHIKPEWYFLFAYAILRSIPNKLGGVLALLF
Lema	IALTSLALFTPNLLGDPDNFTPANPLVTPPHIKPEWYFLFAYAILRSIPNKLGGVLALLA
Etzo	VALTALALFSPNLLGDPDNFTPANPLVTPPHIKPEWYFLFAYAILRSIPNKLGGVLALLA
Apse	LALASLALFSPNYLGDPDNFTPANPLVTPPHIKPEWYFLFAYAILRSIPNKLGGVLALLA
Epde	IALACLALFAPNLLGDPDNFTPANPLVTPPHIKPEWYFLFAYAILRSIPNKLGGVLALLF
Slja	VALTSLALFSPNMLGDPDNFTPANPLVTPPHIKPEWYFLFAYAILRSIPNKLGGVLALLA
Bsja	TALTSLSLFSPNLLGDPDNFTPANPLVTPPHIKPEWYFLFAYAILRSIPNKLGGVLALLA
Ecna	LLLTTLSLFSPNLLGDPDNFTPANPLVTPPHIKPEWYFLFAYAILRSIPNKLGGVLALLA
Cohi	VFLSSIALFAPNVLGDPDNFTPANPLVTPPHIKPEWYFLFAYAILRSIPNKLGGVLALLF
Caar	TALASLALFSPNLLGDPDNFTPANPLVTPPHIKPEWYFLFAYAILRSIPNKLGGVLALLF
Came	TALASLALFSPNLLGDPDNFTPANPLVTPPHIKPEWYFLFAYAILRSIPNKLGGVLALLF
Mema	IALTSLALFSPNLLGDPDNFTPANPLVTPPHIKPEWYFLFAYAILRSIPNKLGGVLALLF
Lenu	LILASLALFSPNMLGDPDNFTPANPMVTPPHIKPEWYFLFAYAILRSIPNKLGGVLALLA
Brja	MALATLALFSPNLLGDPDNFTPANPMVTPPHIKPEWYFLFAYAILRSIPNKLGGVLALLA
Plma	LALTSLALFSPNLLGDPDNFTPANPMVTPPHIKPEWYFLFAYAILRSIPNKLGGVLALFA
Emst	IALTSLALFSPNLLGDPDNFTPANPLVTPPHIKPEWYFLFAYAILRSIPNKLGGVLALLA
Ptti	IALTSLALFAPNLLGDPDNFTPANPLVTPPHIKPEWYFLFAYAILRSIPNKLGGVLALLA
Losu	AALTSLALFSPNLLGDPDNFTPANPLVTPPHIKPEWYFLFAYAILRSIPNKLGGVLALLA
Geoy	IALTSLALFTPNALGDPDNFTPANPLVTPPHIKPEWYFLFAYAILRSIPNKLGGVLALLA
Dipi	IALTSLALFSPNLLGDPDNFTPANPLVTPPHIKPEWYFLFAYAILRSIPNKLGGVLALLA
Pama	ILLTCLALFTPNLLGDPDNFTPANPLVTPPHIKPEWYFLFAYAILRSIPNKLGGVLALLA
Leob	IALTSLALFSPNLLGDPDNFTPANPLVTPPHIKPEWYFLFAYAILRSIPNKLGGVLALLA
Neba	IFLTCLALFSPNLLGDPDNFTPANPLVTPPHIKPEWYFLFAYAILRSIPNKLGGVLALLA
Pdpl	IILSSMALFSPNELGDPDNFTPANPLVTPPHIKPEWYFLFAYAILRSIPNKLGGVLALLF
Nimi	ICLTSLALFSPNLLGDPDNFTPANPLVTPPHIKPEWYFLFAYAILRSIPNKLGGVLALLA
Uptr	IALVSLALFSPNLLGDPDNFTPANPLVTPPHIKPEWYFLFAYAILRSIPNKLGGVLALLF
Pesc	LVLSYWAFFSPNLLGDPDNFTPANPMVTPPHIKPEWYFLFAYAILRSIPNKLGGVLALLA
Baar	AALTSLALFSPNLLGDPDNFTPANPLVTPPHIKPEWYFLFAYAILRSIPNKLGGVLALLF
Moar	TALTALALFSPNLLGDPDNFTPANPLVTPPHIKPEWYFLFAYAILRSIPNKLGGVLALLA
Toja	IALTSLALFSPNLLGDPDNFTPANPLVTPPHIKPEWYFLFAYAILRSIPNKLGGVLALLA
Chau	VALSSLALFTPNLLGDPDNFIPANPLVTPPHIKPEWYFLFAYAILRSIPNKLGGVLALLA
Chse	LALTSLALFSPNLLGDPDNFTPANPLVTPPHIKPEWYFLFAYAILRSIPNKLGGVLALLA
Enar	TALTSLALFSPNLLGDPDNFTPANPLVTPPHIKPEWYFLFAYAILRSIPNKLGGVLALLS
Hpty	SALTSLALFSPNLLGDPDNFTPANPLVTPPHIKPEWYFLFAYAILRSIPNKLGGVLALLF
Nana	FTLTCLALFAPNLLGDPDNFTPANPLVTPPHIKPEWYFLFAYAILRSIPNKLGGVMALLA
Mcst	TALTSLALFAPNLLGDPDNFTPANPLVTPPHIKPEWYFLFAYAILRSIPNKLGGVLALLS
Rhox	IALTSLALFSPNLLGDPDNFTPANPLVTPPHIKPEWYFLFAYAILRSIPNKLGGVLALLA
Opfa	IALASLALFSPNLLGDPDNFTPANPLVTPPHIKPEWYFLFAYAILRSIPNKLGGVLALLS
Paar	IALTSLALFSPNLLGDPDNFTPANPLVTPPHIKPEWYFLFAYAILRSIPNKLGGVLALLS
Gozo	IALTSLALFAPNLLGDPDNFTPANPLVTPPHIKPEWYFLFAYAILRSIPNKLGGVLALLS
Ackr	SLLAALALFSPNLLGDPDNFTPANPLVTPPHIKPEWYFLFAYAILRSIPNKLGGVLALLA
Elev	TALTTLSLFSPNLLGDPDNFTPANPLVTPPHIKPEWYFLFAYAILRSIPNKLGGVLALLF
Trdu	IALISLALFSPNLLGDPDNFTPANPLVTPPHIKPEWYFLFAYAILRSIPNKLGGVLALLF
Amoc	IALISLALFSPNLLGDPDNFTPANPMVTPPHIKPEWYFLFAYAILRSIPNKLGGVLALLA
Hame	LSLTCLALFAPNLLGDPDNFTPANPLVTPPHIKPEWYFLFAYAILRSIPNKLGGVLALLS
Chso	IALTSLALFAPNVLGDPDNFTPANPLVTPPHIKPEWYFLFAYAILRSIPNKLGGVLALLS
Lyto	IGLTALALFSPNLLGDPDNFTPANPLVTPPHIKPEWYFLFAYAILRSIPNKLGGVLALLA

[5/7 of aligned sequences]

To be continued on page 30.

```
Encr    IGLTALALFSPNLLGDPDNFTPANPLVTPPHIKPEWYFLFAYAILRSIPNKLGGVLALLA
Bvar    TALVMIALFSPNLLGDPDNFTPANPLITPPHIKPEWYFLFAYAILRSIPSKLGGVLALLA
Noco    VALATVALFTPNLLGDPDNFTPANPLVTPPHIKPEWYFLFAYAILRSIPDKLGGVLALLA
Chsp    TLLVSLAMFSPNLLADPDNFTPANPLVTPPHIKPEWYFLFAYAILRSIPNKLGGVLALLS
Arja    LALTALALFSPNLLGDPDNFTPANPLVTPPHIKPEWYFLFAYAILRSIPNKLGGVLALLA
Pase    IALTSLALFSPNLLGDPDNFTPANPLVTPPHIKPEWYFLFAYAILRSIPNKLGGVLALLS
Trel    LTLTVIALFSPNYLGDPDNFSPANPLVTPPHIKPEWYFLFAYAILRSIPDKLGGVLALLC
Lifa    IMLMALALFNPNLLGDPDNFTPANPLVTPPHIKPEWYFLFAYAILRSIPNKLGGVLALLF
Acur    IFLTSLALFSPNLLGDPDNFTPANPLVTPPHIKPEWYFLFAYAILRSIPNKLGGVLALLF
Ampe    VALSSLALFSPNLLGDPDNFIPANPLVTPPHIKPEWYFLFAYAILRSIPNKLGGVLALLF
Urja    LLLTCLALFFPSLLGDPDNFSPANPLVTPPHIKPEWYFLFAYAILRSIPNKLGGVLALLS
Enet    IALISLALFSPNLLGDPDNFTPANPLVTPAHIKPEWYFLFAYAILRSIPNKLGGVLALLF
Ptbr    IALISLALFSPNLLGDPENFTPANPLVTPPHIKPEWYFLFAYAILRSIPNKLGGVLALLF
Safa    IALVSLALFSPNLLGDPDNFTPANPLVTPAHIKPEWYFLFAYAILRSIPNKLGGVLALLF
Icae    VALASLALFSPNLLGDPDNFTPANPMVTPPHIKPEWYFLFAYAILRSIPNKLGGVLALLA
Asmi    FTLTALALFSPNLLGDPDNFIPANPLVTPAHIKPEWYFLFAYAILRSIPNKLGGVLALLF
Foal    VALTTLALFLPNLLGDPDNFTPANPLVTPPHIKPEWYFLFAYAILRSIPNKLGGVLALLF
Drze    IALTSFAMFIPNILGDPDNFIPANPLVTPPHIKPEWYFLFAYAILRSIPNKLGGVLALLF
Rhas    LALTSLALFSPNYLGDPDNFTPANPLVTPPHIKPEWYFLFAYAILRSIPNKLGGVLALLA
Elac    SALYGVALFAPNYLGDPDNFIPANPLVTPPHIKPEWYFLFAYAILRSIPNKLGGVLALLA
Kugu    AALSSLAMFTPNLLGDPDNFTPANPLVTPPHIKPEWYFLFAYAILRSIPNKLGGVLALLF
Plor    LALTSLALFSPNLLGDPDNFTPANPLVTPPHIKPEWYFLFAYAILRSIPNKLGGVLALLA
Sgun    IALTSLALFSPNLLGDPDNFTPANPLVTPPHIKPEWYFLFAYAILRSIPNKLGGVLALLA
Zaco    IALTSLALFSPNLLGDPDNFTPANPLVTPPHIKPEWYFLFAYAILRSIPNKLGGVLALLA
Zbfl    IALTSLALFSPNLLGDPDNFTPANPLVTPPHIKPEWYFLFAYAILRSIPNKLGGVLALLA
Spba    AVLTSLALFSPNLLGDPDNFTPANPLVTPPHIKPEWYFLFAYAILRSIPNKLGGVLALLF
Game    VALASLALFSPNLLGDPDNFTPANPMVTPPHIKPEWYFLFAYAILRSIPNKLGGVLALLA
Thth    VALASLALFSPNLLGDPDNFTPANPMVTPPHIKPEWYFLFAYAILRSIPNKLGGVLALLA
Xigl    VALTSLALFSPNLLGDPDNFTPANPLVTPPHIKPEWYFLFAYAILRSIPNKLGGVLALLA
Hyja    TLLASLALFSPNLLGDPDNFTPANPMVTPPHIKPEWYFLFAYAILRSIPNKLGGVLALLA
Psan    VLLASLALFYPNLLGDPDNFTPANPMVTPPHIKPEWYFLFAYAILRSIPNKLGGVLALLA
Cupa    IALASLALFSPNLLGDPDNFTPANPMVTPPHIKPEWYFLFAYAILRSIPNKLGGVLALLA
Mpch    IALISLSLFSPNLLGDPDNFTPANPLVTPPHIKPEWYFLFAYAILRSIPNKLGGVLALLA
Char    ISLISLSLFAPNLLGDPDNFTPANPLVTPPHIKPEWYFLFAYAILRSIPNKLGGVLALLA
Pser    IALTSLALFSPNLLGDPDNFTPANPLVTPPHIKPEWYFLFAYAILRSIPNKLGGVLALLF
Prol    IALAALALFSPNLLGDPDNFTPANPLVTPPHIKPEWYFLFAYAILRSIPNKLGGVLALLF
Plbi    TALASLALFSPNLLGDPDNFTPANPLVTPPHIKPEWYFLFAYAILRSIPNKLGGVLALLF
Calu    LFLVLIALFTPNALGDPDNFTPANPLVTPPHIKPEWYFLFAYAILRSIPNKLGGVVALLL
Papa    AMLAALALFSPNSLGDPDNFTPANPLVTPPHIKPEWYFLFAYAILRSIPNKLGGVLALLS
Sufr    LALSSLALFSPNLLGDPDNFTPANPLVTPPHIKPEWYFLFAYAILRSIPNKLGGVLALLG
Stci    LVVASLALFSPNLLGDPDNFTPANPLVTPPHIKPEWYFLFAYAILRSIPNKLGGVLALLG
Taru    SALATLALFSPNYLGDPDNFTPANPLVTPAHIKPEWYFLFAYAILRSIPNKLGGVLALLA
Rala    IALTTLALFSPNLLGDPDNFTPANPLVTPPHIKPEWYFLFAYAILRSIPNKLGGVLALLA
          :    .: *   .  * :*:  *: :  ** *::*** **:**:***:.*.***** **
```

[6/7 of aligned sequences]

```
             G                                 H
Scca   SIFILLLVPLLHTSKLRSNIFRPLTQIFFWSLVTNAIILTWIGGQPVEQPF----IMVGQ
Muma   SIFILLLVPLLHTSKQRSIIFRPLTQIFFWVLVANSIILTWIGGQPVEQPF----IMVGQ
Erca   SIIVLMFVPFLHTAKIRTSTFRPLFKITLWILAADVMILTWIGGQPVEDPY----IMIGQ
Pose   SIIILMLVPFLHTSKIRSATFRPLFKITLWILAADVLILTWIGGQPVEDPY----ITIGQ
Actr   SILVLMLVPMLHTSKQRGNTFRPLSQILFWALVADMLVLTWIGGQPVEHPF----VLIGQ
Scal   SILVLMLVPMLHTSKQRGNTFRPLSQILFWTLVADMLVLTWIGGQPVEHPF----VLIGQ
Posp   SILVLMLVPILHTSKQRGNTFRPLSQILFWTLVADMLVLTWIGGQPVEHPF----VLIGQ
Atsp   SILILVVVPVLHTSKQRSNTFRPPSQTLFWILVANMLVLTWIGGQPVEHPF----IIIGQ
Leoc   SILILVVVPTLHTSKQRSNTFRPSSQTLFWILVANMLVLTWIGGQPVEHPF----IIIGQ
Amca   SILILMIVPITHTSKQRSSTFRPLTQILFWTLVADMFILTWIGGQPVEHPF----IIIGQ
Osbi   SILILMTVPFLHTSKMKGITFRPISQFLFWMLVADMTILTWIGGMPVEPPF----ILIGQ
Pabu   SILVLMLVPMLHTSKMRSMTFRPLSQFLFWTLVADMAILTWIGGMPVEHPY----IIIGQ
Hial   SILVLLVVPILHTSKQRGLAFRPLTQFLFWVLVADMLVLTWIGGMPVEHPF----ITIGQ
Elha   SILVLLLVPFLHTSKLRSMTFRPISQFLLWTLVADVAVLTWIGGMPVEHPY----IIIGQ
Mlcy   SILVLMLVPILHTSKQRGLTFRPITQFLFWTLVADVVVLTWIGGMPVEHPF----IIIGQ
Algl   SILVLALVPILHTSKQRGSAFRPTTQLLFWALVADMFALTWIGGMPVEHPF----VIIGQ
Ptgi   SILVLMLVPILHTSKQRGLTFRPVTQILFWTLVADMAVLTWIGGMPVEHPF----VIIGQ
Alaf   SILVLMVVPILHTSKQRGLTFRPLTQFLFWTLVADMVVLTWIGGMPVEHPF----IIIGQ
Nock   SILVLFLVPTLHTSKQRGLTFRPITQFLFWTLVADMIVLTWIGGMPVEDPY----IMIGQ
Anja   SILVLMVVPILHTSKQRGLTFRPASQLLFWILVADMLVLTWIGGMPVEHPY----IIIGQ
Gyki   SILILMLVPFLHTSKQRALTFRPASQILFWLLVADMLVLTWIGGMPVEHPF----IIIGQ
Syka   SILVLLVVPILHTSKQRGLTFRPASQLMFWVLVADMAVLTWIGGMPVEHPF----IIIGQ
Opma   SILVLMVVPMFHTSKHRALTFRPASQLLFWVLVADMFVLTWIGGMPVEDPY----VMIGQ
Comy   SILVLMLVPALHTSKLRGLTFRPLSQLLFWALVADMLVLTWIGGMPVEHPF----IIIGQ
Sasp   SILILLIVPLLHLSKQRGLMFRPVSQLLFWLLISDMFILTWIGGMPVEHPF----IIIGQ
Eupe   SILILLVIPLLHTSKQRNLTYRPTSQTMFWLFATNMLILTWIGGMPMDQPY----TIIGQ
Enja   SILVLMVVPILHTSKQRGITFRPITQFLFWTLVADVIILTWIGGMPVEHPF----IIIGQ
Same   SILVLMVVPILHTSKQRGLTFRPITQFLFWTLVADVVILTWIGGMPVEHPY----VIIGQ
Chch   SILVLMVVPILHTSKQRGLTFRPVTQFLFWALVADVIVLTWIGGMPVEHPF----IIIGQ
Grgr   SILVLMVVPILHTSKQRGLTFRPFTQFLFWTLVADVFILTWIGGMPVEHPF----VIIGQ
Caau   SILVLMVVPLLHTSKQRGLTFRPITQFLFWTLVADMIILTWIGGMPVEHPF----IIIGQ
Cyca   SILVLMVVPLLHTSKQRGLTFRPITQFLFWTLVADMIILTWIGGMPVEHPF----IIIGQ
Dare   SILVLMVVPILHTSKQRGMAFRPVTQFLFWTLVADMLVLTWIGGMPVEHPY----IIIGQ
Cost   SILVLMVVPILHTSKQRGLAFRPITQFLFWALVADMLILTWIGGMPVEHPF----IIIGQ
Leec   SILVLMVVPILHTSKQRGLTFRPMTQILFWALVADMAILTWIGGMPVEHPF----IIIGQ
Fola   SILVLMVVPVLHTSKQRGLTFRPATQFLFWTLVADMIILTWIGGMPVEHPY----IIIGQ
Clmc   SILILMLVPMLHTSKQKALTFRPLSQLLFWALVADVAILTWIGGMPVEHPF----IIIGQ
Phin   SILVLMLIPLLHTSKQQGLTFRPLSQLLFWALIADVIILTWIGGMPVEHPF----IIIGQ
Icpu   SILVLMVVPLLHTSKQQGLTFRPLSQLLFWTLVADVAILTWIGGMPVEHPF----IIIGQ
Psto   SILVLMVVPLLHTSKQQGLTFRPLAQFMFWLLVADVMILTWIGGMPVEHPF----VIIGQ
Cora   SILVLMLIPMLHTSKQRGLTFRPLTQFLFWTLVADVFILTWIGGMPVEHPF----VIIGQ
Eisp   SILILLLVPLLHTSKQQALTFRPLTQLLFWALVADVAVLTWIGAMPVEHPF----IIIGQ
Apal   SVLILMLVPLLHTSKQQGLTFRPLSQLLFWTLIADVVILTWIGGMPVEYPF----TVIGQ
Eslu   SILILMLVPILHTSKQRGITFRPLTQLLFWLLVADMLILTWIGGMPVEHPF----IIIGQ
Dape   SILILMFVPILHTSKQRGLTFRPLAQIIFWLLVATMLILTWIGGMPVEHPF----IIIGQ
Glse   SILVLAAVPFLHTSNQRGLTFRPLTQVLFWTLIADVIILTWIGGMPVEHPF----IIIGQ
Naar   SILVLALVPFLHTSNQRGLTFRPLTQILFWTLVADVLILTWIGGMPVEHPF----VIIGQ
Lioc   SILVLAVVPFLHTSNQRGLTFRPLTQLLFWALVADVMILTWIGGMPVEHPF----IIIGQ
Opso   SILVLMTVPLLHTSNQRSLTFRPLTQILFWALIADVAVLTWIGGMPVEHPY----IIIGQ
Alte   SILVLFVVPILHTSKQRALTFRPITQLLFWTLAADMLILTWIGGMPVEHPY----VLIGQ
Plap   SILVLLVVPILHTSKQRGLTFRPATQLLFWVLVADMLILTWIGGMPVEHPY----VLIGQ
```

To be continued on page 31.

[6/7 of aligned sequences]

```
Plal  SILVLMLVPILHTSKQRGLTFRPFTQFLFWALVADVIILTWIGGMPVEHPF----IEIGQ
Sami  SILVLMLVPILHTSKQRGLTFRPFTQFLFWALVADVIILTWIGGMPVEHPF----VEIGQ
Rere  SILVLLLVPILHTSKQRGLTFRPVTQFLFWALVADVMILTWIGGMPVEHPF----IEIGQ
Gama  SILVLMVVPILHTSKQRGLTFRPLTQFLFWALVADVLVLTWIGGMPVEHPY----IIIGQ
Onmy  SILVLMVVPILHTSKQRGLTFRPLTQFLFWALVADMLILTWIGGMPVEHPF----IIIGQ
Sasa  SILVLMVVPILHTSKQRGLTFRPLTQFLFWTLVADMLILTWIGGMPVEHPF----IIIGQ
Cola  SILVLMVVPILHTSKQRGLTFRPLTQFLFWTLVADMLILTWIGGMPVEHPF----IIIGQ
Dita  SILVLMIVPILHTSKQRGLTFRPLTQFLFWTLVADVMVLTWIGGMPVEHPY----IIIGQ
Gogr  SVLILATVPFLQTSKQQALTFRPLTQLVFWTLIANIAILTWIGGMPVEYPF----VSIGQ
Chsl  SILVLMLVPLLHTSKMRGSTYRPLTQLLFWALVADVLILTWIGGMPVEAPY----IVIGQ
Atja  SILVLMLVPFLHTSKQRGLTFRPLTQTLFWTLVADVVILTWIGGMPVEHPF----IIIGQ
Iido  SILVLMLVPFLHTSKQRGLTFRPLTQILFWALVADVIILTWIGGMPVEHPF----IIIGQ
Auja  SILVLMLVPILHTSKQRGLTFRPLTQLLFWTFVANVIILTWIGGMPVEHPF----IIIGQ
Chag  SILVLMLVPFLHTSKQRGLTFRPLTQILFWTFVANVIILTWIGGMPVEHPF----IIIGQ
Hami  SILVLMLVPILHTSKQRGLMFRPLTQLLFWTFVADVVILTWIGGMPVEHPF----IIIGQ
Saun  SILVLMLVPILHTSKQRGLMFRPLTQLLFWTFVADVVILTWIGGMPVEHPF----IIIGQ
Nema  SILVLMLVPILHTSKQRGITFRPLTQILFWTLVADVIILTWIGGMPVEHPFVIPFVIIGQ
Disp  SILVLMLVPILHTSKQRGITFRPVTQFLFWLLVAEVMILTWIGGMPVEHPF----VIIGQ
Myaf  SILVLMLVPFLHTSKQRANTFRPFTQFLFWLLVADVLILTWIGGMPVEDPY----VIIGQ
Lagu  SILVLVLVPILHTSKQRGLMFRPLTQTLFWTLVADMLILTWIGGMPVEYPF----VIIGQ
Trtr  SILILMLVPILHVSKQRGLTFRPLTQFLFWMLVADVLVLTWIGGMPVEHPF----VIIGQ
Zucr  SILVLMLVPILHTSKQRGLTFRPLTQLLFWALVADVLVLTWIGGMPVEHPF----VIIGQ
Pxja  SILVLMVVPILHTSKQRGLTFRPLTQFLFWTLVADVAILTWIGGMPVEHPF----IIIGQ
Pxlo  SILVLMVVPILHTSKQRGLTFRPLTQFLFWTLVADVAILTWIGGMPVEHPF----IIIGQ
Pctr  SILILTAVPALHASKQRSTTFRPITQLLFWTLVAAVLVLTWIGGMPVEHPF----IIIGQ
Apsa  SILILLAVPILHTSKQRSLTFRPLTQTLFWTLVANVLILTWIGGMPVEHPF----IIIGQ
Cabe  SLLVLILMPYLHTSKQRALTFRPTTQALFWLFVANILILTWIGGVAVEHPF----SLIGQ
Bzze  SILVLMLVPLLHTSKQRGLTFRPLTQILFWTFIANVLILTWIGGMPVEHPF----IIIGQ
Siim  SILVLGLMPFLHTSVHRGMTFRPLGQILFWTFIADVLFLTWVGGMQSVPPF----LVLAR
Ctru  SILVLMLIPLLHTSKQRGLAYRPLTQIIFWTFIADVIILTWIGGMPVEHPF----IIIGQ
Dpbr  SILVLMLIPILHTSKQRGLTYRPLTQLLFWTFIADVVILTWIGGMPVEHPF----IIIGQ
Caki  SILVLMVVPFLHTSKQRGLTFRPLSQLLFWTLVADMLILTWIGGMPVEHPF----TLIGQ
Phja  SVLILMAVPFLHTSKQRSLTFRPYSQVVFWILVADMFILTWIGGMPVEHPF----IVIGQ
Brsp  SILVLMVIPFLRTSKQRGLMFRPMTQILFWFLVADMLILTWIGGMPVEDPF----IIIGQ
Gamo  SILVLMVVPFLHTSKQRGLTFRPLTQMLFWVLVADMLVLTWIGGVPVEHPF----IIIGQ
Lolo  SILILMVVPFLHTSKQRGLTFRPLTQVLFWVLVADMLVLTWIGGVPVEHPF----IIIGQ
Batr  SILVLFLVPLLHTSKELAMTFRPISQILFWTFISNMVILTWVGGLPVEDPY----ITLGQ
Prmy  SILILYLAPALHMSNRAPMTFRPASQLLFWLFMANFVILTWLGGLPVEEPY----ITMGR
Lose  SILVLMLVPVLHTSKQRSLTFRPMAQLLFWTLVADVIILTWIGGMPVEAPF----VVIGQ
Loam  SILILMVVPILHTSKQRSLTFRPITQFLFWTLVANVAILTWIGGMPVEHPF----VIIGQ
Chab  SILVLMLVPALHTSKHRGLTFRPLAQFLFWVLIADVAILTWIGGMPVEHPY----VVIGQ
Chto  SILVLMLVPALHTSKHRGLTFRPLAQFLFWALIADVAILTWIGGMPVEHPY----VVIGQ
Majo  SILILMLVPALHTSKQRGLTFRPLTQLLFWTLIANVAILTWIGGMPVENPF----IEIGQ
Hlst  SILVLMLAPALHTSKQRGLMFRPLTQLLFWTFIANVMILTWIGGQPVENPF----IEIGQ
Clpe  SILVLLLLPALHTSKQRSLTFRPLSQVLFWTLVADVAILTWIGGMPVEHPF----VIIGQ
Mlmr  SILVLLLVPVLHTSKQRGLTFRPLTQLLFWALVADVAVLTWIGGMPVEHPF----IIIGQ
Crcr  SILVLLVVPILHTSKHRSLTFRPITQFLFWLLIADVLVLTWIGGMPVEAPY----IITGQ
Muce  SILVLLVVPILHTSKHRSLTFRPVTQFLFWLLIADVLVLTWIGGMPVEAPY----IITGQ
Bege  SILVLMVIPILHTSKQRSLTFRPFTQFLFWLLVADVAILTWIGGMPVEHPY----IIIGQ
Mela  SILVLMVVPILHTSKQQSLTFRPLAQLLFWLLIADVIILTWIGGMPVEHPF----VIIGQ
Hats  SILVLMVVPILHTSKQRSLTFRPLTQFLFWLLVADVAILTWIGGMPVEHPF----IIIGQ
Orla  SILVLFLVPILHTSKQRSLTFRPFTQFLFWLLVADVMVLTWIGGMPVEHPF----IIIGQ
```

To be continued on page 32.

[6/7 of aligned sequences]

To be continued on page 33.

```
Cosa  SILVLFLVPILHTSKQRSLTFRPLTQLLFWILVADVIILTWIGGMPVEHPF----IIIGQ
Exsp  SILVLFLVPILQTSKQRSLTFRPLSQILFWTLVADVVILTWIGGMPVEHPY----IIVGQ
Depa  SILVLFLVPILHTSKQRSLTFRPLTQLLFWILIADVMILTWIGGMPVEHPF----IIIGQ
Rima  SILILLIVPILHTSKWQNFAFRPLAQIFMGLLVVDVAILTWIGGMPVEPPF----IIIGQ
Fuol  SILILMVVPILHTSKQRSLTFRPLTQFLFWLLVADVAILTWIGGMPVEHPF----VIIGQ
Gmaf  SILVLMVVPLLHTSKQRSLTFRPLTQILFWLLIADVAILTWIGGMPVEHPY----IIIGQ
Xeei  SILILVTVPILHTSKQRSLTFRPITRFLFWLLIADVIILTWIGGMPVEHPY----IIIGQ
Pros  SILVLMVVPFLHTSKQRGLTFRPLTRILFWTLVADVAILTWIGGMPVETPF----IVIGQ
Scmi  SILVLMVVPLLHSSKQRGLTFRPLTRILFWTLVANVMILTWIGGMPVEDPY----IIIGQ
Rolo  SILVLMVVPILHTSKQRGLTFRPLTQFLFWTLIADVIILTWIGGMPVEDPF----VIIGQ
Cere  SILILMTVPFLHTSKQRGLTFRPLTQFLFWTLVADVIVLTWIGGMPVEHPF----ITIGQ
Daga  SILVLMVVPMLHTSKQRSMTFRPITQFLFWTLVADVMVLTWIGGMPVEHPF----IIIGQ
Anco  SILVLMVVPILHTSVQRGLTFRPLSQMLFWTLVADVLILTWIGGMPVEHPF----IIIGQ
Dmve  SILVLMAVPLLHTSKQRGLTFRPLTQMLFWALVADVFILTWIGGMPVEHPF----IIIGQ
Dmar  SILVLMVVPLLHTSKQRGLTFRPLTQTLFWALVADVFILTWIGGMPVEHPF----IIIGQ
Anka  SILVLMVVPILHTSAQRGLTFRPLSQVLFWALVADVLILTWIGGMPVEHPF----IIIGQ
Moja  SILVLMVVPILHTSMQRGLTFRPISQLLFWALVADVLILTWIGGMPVEHPF----IIIGQ
Hoja  SILVLMVVPILHTSMQRGLTFRPLSQVLFWALDADVLILTWIGGMPVEHPF----IIIGQ
Bede  SILVLMVVPFLHTSKQRGLTFRPLTRILFWTLVANVAILTWIGAMPVESPF----IIIGQ
Besp  SILVLMVIPFLHTSKQRGLTFRPLTRILFWTLVANVAILTWIGAMPVESPF----IIIGQ
Mysp  SILVLMVVPILHTSKQRGLTFRPLTQFLFWTLVADVIILTWIGGMPVEHPF----IIIGQ
Osja  SILVLMVVPILHTSKQRGLTFRPLTQFLFWTLVADVIILTWIGGMPVEHPF----IIIGQ
Sgro  SILVLMVVPILHTSKQRGLTFRPLTQFLFWMLVADVLILTWIGGMPVEHPF----IIIGQ
Pzpa  SILILMLVPILHTSKQRGLMFRPMTQTLFWALVADVAILTWIGGMPVEHPF----IIVGQ
Zeja  SILILMLVPILHTSKQQGLTFRPLTQALFWTLVADIAILTWIGGMPVEHPF----ITVGQ
Znne  SILILMLVPILHTSKQRGLMFRPLTQLLFWILVADVMILTWIGGMPVEHPF----IAVGQ
Zefa  SILILMLVPILHTSKQRGLTFRPLTQILFWVLVADVAILTWIGGMPVEHPF----IIVGQ
Acni  SILILMVVPLLHTSKQRGLMFRPLTQILFWTLVADVAILTWIGGMPVEHPF----ITVGQ
Ncrh  SILILMVVPLLHTSKQRGLMFRPLTQILFWTLVADVAILTWIGGMPVEHPF----ITVGQ
Agca  SILVLIVVPILHTSKQQGLAFRPLTQFLFWTLVADVMILTWIGGMPVEHPF----IIIGQ
Hydy  SILVLMVVPILHTSKQRGLTFRPLTQFLFWTLIADVTILTWIGGMPVEDPF----IIIGQ
Gsac  SILVLMVVPILHTSKQRGLTFRPITQFLFWTLIADVAILTWIGGMPVEHPF----IIIGQ
Pevo  SILVLMLVPLLHTSKQRGLTFRPLTQLLFWTLLADVIILTWIGGMPVEHPF----VVIGQ
Hiku  SILVLMLVPILHTSKQRGLTFRPFGQLMFWMLVADMMILTWIGGMPVEPPY----ILIGQ
Inpa  SILVLAVVPFLHTSKQRSLALRPWSQLCLFTLVVTVLILTWIGGMPLEQPL----TTVGQ
Auch  SILILMAMPFLHTSKFRGLNFRPASQWVFWIFLGNVLLLTWIGGMPVEQPY----TLVGQ
Fico  SILVLMLVPILHTSKQRALTFRPLTQILFWAFVADVIILTWIGGMPVEHPF----IIIGQ
Macs  SILILMLVPILHTSKQRGLTFRPLTQFLFWVLIADVLILTWIGGMPVEHPY----IIIGQ
Moal  SIFVLMLVPFLHMSKQQSMTFRPISQLVFWTLIATVAILTWIGGMPVEYPF----ITIGR
Syma  SILALMVLPFIHTSKMRSLTFRPLSQLVFWLFVANIAILTWIGGMPVEPPF----IIIGR
Mafr  SILVLMLVPILHTSKQRSLTFRPISQLLFWTLIADVFILTWIGGMPVEHPF----IIIGQ
Dcpe  SILVLMLVPILHTSKQRGLTFRPITQFLFWTLVADVIILTWIGGMPVEHPF----IIIGQ
Dcti  SILVLMLVPILHTSKQRGLTFRPITQFLFWTLVADVIILTWIGGMPVEHPF----IIIGQ
Hehi  SILVLMLVPFLHTSKQRSLTFRPLTQFLFWTLIADVLILTWIGGMPVSHPF----VIIGQ
Stam  SILVLMVVPILHTSKQRGLTFRPLTQFLFWTLIADVAILTWIGGMPVEHPF----IIIGQ
Hogi  SILVLMVVPFLHTSKQRGLTFRPVTQFLFWTLVADVAILTWIGGMPVEHPY----VIIGQ
Erzo  SILVLMVVPVLHTSKQRGLTFRPLTQFLFWTLIADVVILTWIGGMPVEHPY----IIIGQ
Hxot  SILVLLVVPILHTSKQRGLTFRPITQFLFWTLIADVAILTWIGGMPVEHPF----IIIGQ
Core  SILVLMVVPILHTSKQRGLTFRPMTQFLFWTLIADVAILTWIGGMPVEHPF----IIIGQ
Apve  SILVLLVVPLLHTSKQRTMTFRPLTQTLFWILIADLFILTWIGGMPVENPF----IIIGQ
Latj  SILVLMVVPILHTSKQRSLTFRPLTQFLFWILVADVFILTWIGGMPVEHPF----IVIGQ
Laja  SILVLMLVPILHTSKQRALTFRPITQFLFWTLIADVAILTWIGGMPVEHPF----IIIGQ
```

[6/7 of aligned sequences]

To be continued on page 34.

```
Syja  SILVLMLVPLLHTSKHRALTFRPLTQILFWILIANVLVLTWIGGMPVEDPY----ILIGQ
Epme  SILVLMVVPILHTSKQRGLTFRPITQFLFWLLIADVIILTWIGGMPVEHPF----IIIGQ
Grse  SILVLMLVPILHTSKQRALTFRPLAQFLFWALIADVAILTWIGGMPVEHPF----IIIGQ
Clja  SILVLMIIPTLHTSKQRALTFRPITQFLFWTLIANVAILTWIGGMPVEHPF----IIIGQ
Ogcy  SILVLMVVPFLHTSKQRSLTFRPISQILFWILVADVIILTWIGGMPVEHPF----IIIGQ
Plna  SILVLMVVPILHISKQRGLMFRPLTQFLFWVLVADVIILTWIGGMPVEHPF----IIIGQ
Lema  SILVLMVVPILHTSKQRSLTFRPLTQLLFWALVADVAILTWIGGMPVEHPF----IMIGQ
Etzo  SILVLMVVPILHTSKQRGITFRPISQFLFWTLIADVAILTWIGGMPVEHPF----IIIGQ
Apse  SILILMLVPILHTSKHRSLTFRPFSQIIFWTLVADVTILTWIGGMPVEDPY----IIIGQ
Epde  SILVLMLVPILHTSKQRGLTFRPLTQFLFWTLIATVAILTWIGGMPVEHPF----VIIGQ
Slja  SILVLMLVPILHTSKQRSLTFRPISQFLFWLLIADVIILTWIGGMPVEHPF----IIIGQ
Bsja  SILVLMLVPFLHTSKQRSLTFRPFSQLLFWTLIANVLILTWIGGMPVEDPY----IIIGQ
Ecna  SILVLLLVPILHPSKHRSLTFRPLSQVCFWTLIANVLILTWIGGMPVEHPY----IIVGQ
Cohi  SILILMIVPFLHTSNHRGLTFRPPTQILFWALIADVLVLTWIGGLPVEAPF----VFIGQ
Caar  SILVLMVVPILHTSKQRSLTFRPITQFLFWTLVADVMILTWIGGMPVEHPF----IIIGQ
Came  SILVLMVVPILHTSKQRGLTFRPITQFLFWTLVADVMILTWIGGMPVEHPF----IIIGQ
Mema  SILVLMLVPILHTSKQRGLTFRPISQFLFWTLVADVMILTWIGGMPVEHPF----IIIGQ
Lenu  SILVLALVPFLHTSKHRSLTFRPLSQILFWTLIADVLILTWIGGMPVEHPY----IIIGQ
Brja  SILVLMVVPFLHTSKQRALTFRPVSQLLFWTLIADVVILTWIGGMPAEQPF----IIIGQ
Plma  SILVLMVVPFLHTSKQRALTFRPISQFLFWTLIADVGILTWIGGMPVEYPF----IIIGQ
Emst  SILVLMVVPILHTSKQRGLTFRPVTQFLFWTLIANVAILTWIGGMPVEHPF----IIIGQ
Ptti  SILVLMVVPILHTSKQRGLTFRPVTQFLFWTLIANVAILTWIGGMPVEHPF----IIIGQ
Losu  SILVLMVVPILHTSKQRSLIFRPFTQFLFWALVADVAILTWIGGMPVEPPF----IIIGQ
Geoy  SILVLMVIPILHTSKQRSLTFRPMSQFLFWTLVADVIILTWIGGMPVEHPY----IIIGQ
Dipi  SILVLMVVPILHTSKQRGLTFRPLTQFLFWTLIANVAILTWIGGMPVEHPY----IIIGQ
Pama  SILVLMVVPILHTSKQRSLTFRPVTQFLFWALIANVAILTWIGGMPVEDPY----IIIGQ
Leob  SILVLMVVPILHTSKQRSLTFRPLTQFLFWTLIANVAILTWIGGMPVEHPF----IIIGQ
Neba  SILVLMLVPLLHTSKQRSLTFRPVSQFFFWVLIADVAILTWIGGMPVEDPY----IIIGQ
Pdpl  SILVLMLVPFLHTCKYRSLMFRPLGQIVFWTLVADVLILTWIGGMPVEHPY----VIIGQ
Nimi  SILVLMVVPILHTSKQRGLTFRPLTQFLFWTLIADVLILTWIGGMPVEHPF----IIIGQ
Uptr  SILVLMLVPILHTSKQRGLTFRPLTQFLFWTLVADVAILTWIGGMPVEHPY----IIIGQ
Pesc  SILVLFLLPFLHTSKQRTMTFRPAGQFLFWTLIADVLILTWIGGMPVEQPY----VIIGQ
Baar  SILVLMLVPILHTSKQQGLTFRPLTQLLFWSLVANVAILTWIGGMPVEDPY----IIIGQ
Moar  SILVLMVVPILHTSKQRGLTFRPFTQFLFWMLIANVAILTWIGGMPVEHPF----IIIGQ
Toja  SILVLMVVPILHTSKQRSLTFRPLTQFLFWALVADVVILTWIGGMPVEHPF----IIIGQ
Chau  SILVLMVVPMLHTSKQRGLTFRPLTQFLFWSLIATVMILTWIGGMPVEEPY----IIIGQ
Chse  SILVLMVVPLLHTSKQRSLTFRPLTQFLFWLLIADVAILTWIGGMPVEHPY----IIIGQ
Enar  SILVLMLVPILHTSKQRSLTFRPLTQFLFWTLVADVIILTWIGGMPVEHPF----IIIGQ
Hpty  SILVLMLVPVLHTSKQRSLTFRPLTQLLFWTLIANVAILTWIGGMPVEDPY----IIIGQ
Nana  SILVLMLVPLIHTSKQRSLTFRPITQILFWTLVADVLILTWIGGMPVEHPY----IIIGQ
Mcst  SILVLTVVPILHTSKQRSLTFRPFTQFLFWALIADIIILTWIGGMPVEHPF----IIIGQ
Rhox  SILVLMVVPILHTSKQRSLTFRPLTQALFWLLVADVIILTWIGGMPVEQPF----MIIGQ
Opfa  SILVLMVVPILHTSKQRSLTFRPLTQFLFWTLVADVIILTWIGGMPVEQPF----IIIGQ
Paar  SILVLMVVPFLHTSKQRSLTFRPISQFLFWTLVADVMILTWIGGMPVEHPF----VIIGQ
Gozo  SILVLMIVPILHTSKQRSLTFRPLTQFLFWALVADVVILTWIGGMPVEHPF----IIIGQ
Ackr  SILILMLVPYLHTSKQRSLTFRPASQFLFWTLVADVIVLTWIGGMPVEDPY----IIIGQ
Elev  SILILMLIPLLHASKQRGLTFRPLTQFLFWTLVADVMILTWIGGMPVEHPF----IIIGQ
Trdu  SILVLMVVPILHTSKQRGLTFRPITQFLFWLLVADVAILTWIGGMPVEHPF----VIIGQ
Amoc  SILVLMLVPILHTSKQRSLTFRPLTQFLFWLLIADVIILTWIGGMPVEHPF----IIIGQ
Hame  SILVLMVVPILHTSKQRGLTFRPATQFLFWALVADVVVLTWIGGMPVEHPY----IIIGQ
Chso  SILVLMVVPILHTSKQRSITFRPVTQFLFWALVADVAILTWIGGMPVEHPF----IIIGQ
Lyto  SILILVVVPALHTSKQRGLTFRPVTQFLFWTLIANVAILTWIGGMPVEHPY----IIIGQ
```

[6/7 of aligned sequences]

```
Encr  SILVLMVVPILHTSKQRGLTFRPVTQFLFWTLIADVAILTWIGGMPVEHPY----IIIGQ
Bvar  SILVLMVVPFLHTCKLRSLSMRPLSQILFWALIVNVAVLTWIGGMPVEDPY----IAIGQ
Noco  SILVLLVVPFLHTCKLRSLTFRPLSQLLFWSLIADVAILTWIGGMPVEDPY----IIIGQ
Chsp  SILVLMLMPFLHTSKQRTLTFRPLGQVVFWLLIADIIILTWIGGMPVENPY----ILIGQ
Arja  SILVLLVVPILHTSKQRGLTFRPLTQFLFWTLIADVAILTWIGGMPVEHPF----IIIGQ
Pase  SILVLLTVPLLHTSKQRALTFRPLSQFLFWTLIADVLILTWIGGMPVEDPY----IVIGQ
Trel  SILVLMLVPLLHTSKLRSLTFRPASQFVFWALVADVAVLTWIGGMPVEDPY----IIIGQ
Lifa  SILVLMLVPLLHTSKQRGLTFRPITQFLFWLLIANVAILTWIGGMPVEDPY----IIIGQ
Acur  SILVLMLVPLLHTSKRRSLTFRPLSQLLFWSLVANVGVLTWIGGMPVEDPY----IIIGQ
Ampe  SILVLLVVPILHTSKQRGLTFRPVTQFLFWTLVADVAILTWIGGMPVEDPY----IVIGQ
Urja  SILVLFLIPALHTSKQRSMTFRPIGQFLFWLLITDVIILTWIGGMPVEHPF----TIIGQ
Enet  SILVLMVVPMLHTSKQRGLTFRPLTQCLFWLLIADVMILTWIGGMPVEHPF----VIIGQ
Ptbr  SILVLMLVPILHTSKQRGLTFRPLTQFLFWVLIADVLVLTWIGGMPVEHPF----IIIGQ
Safa  SILVLMLVPILHTSKQRSLTFRPLTQFLFWVLIADVLILTWIGGMPVEHPF----IIIGQ
Icae  SILVLMVVPFLHTSKQRGLTFRPASQFLFWTLIADVVILTWIGGMPAEQPF----IIIGQ
Asmi  SILVLMVVPFLHTSKQRALTFRPLAQATFWLLAADVIILTWIGGMPVEDPY----IAIGQ
Foal  SILVLMLVPMLHTSKQRGNTFRPLTQLLFWTLIADVLILTWIGGMPVEDPY----ILIGQ
Drze  SILILLLVPALHTSKQRSLTFRPLTQLLFWSLVADVIILTWIGGMPVEEPF----IIVGQ
Rhas  SILVLMLVPLLHTSKQRSLTFRPASQFIFWTLVADVMILTWIGGMPVEHPY----IIIGQ
Elac  SILVLLLVPFLHTSKQRALTFRPISQVIFWTLVADVMILTWIGGMPVEHPY----IIIGQ
Kugu  SIMVLMLVPMLHTSKMRSLTFRPISQFLFWTLVADVLILTWIGGMPVEDPY----IIIGQ
Plor  SILVLMIVPLLHTSKQQALTFRPATQFLFWALVANVMILTWIGGMPVEHPF----IIIGQ
Sgun  SILVLMVVPILHTSKQRGLTFRPLTQFLFWTLVANVFILTWIGGMPVEHPY----IIIGQ
Zaco  SILVLMIVPILHTSKQRSLTFRPFTQFLFWTLIANVGILTWIGGMPVEHPF----IIIGQ
Zbfl  SILVLMLVPILHTSKQRSLTFRPFTQFLFWTLIANVAILTWIGGMPVEHPF----IIIGQ
Spba  SILVLMLVPILHTSKQRGLTFRPLSQFLFWTLVADVAILTWIGGMPVEHPF----VIIGQ
Game  SILVLMVVPFLHTSKQRTLTFRPVSQFLFWTLIADVAILTWIGGMPAEQPF----IIIGQ
Thth  SILVLMVVPFLHTSKQRTLTFRPVSQFLFWTLIADVAILTWIGGMPAEQPF----IIIGQ
Xigl  SILVLMVVPILHTSKQRGLTFRPLTQFLFWTLVADVAILTWIGGMPVEHPF----IIIGQ
Hyja  SILILMVVPFLHTSKQRTLTFRPLSQLLFWTLVADVAILTWIGGMPAEQPF----IIIGQ
Psan  SILILLVVPFLHTSKQRAMTFRPFSQVLFWTLVMDVLILTWIGGMPAEQPF----IIIGQ
Cupa  SILVLMVVPFLHTSKQRALTFRPVSQFLFWTLVADVVILTWIGGMPAEQPF----IIIGQ
Mpch  SILILMLVPFLHTSKQRSLTFRPISQFMFWTLIADVLILTWIGGMPVEDPY----IIIGQ
Char  SILVLILVPILHTSKQRSLTFRPLTQLLFWLLVADVIILTWIGGLPVEHPY----VAIGQ
Pser  SILVLMVVPILHTSKQRGLTFRPVTQFLFWALVMDVVILTWIGGMPVEHPF----VIIGQ
Prol  SILILMLVPILHTSKQRSLMFRPFSQFLFWSLVADVMILTWIGGMPVEHPF----VIIGQ
Plbi  SILVLMLVPFLHTSKQRSLMFRPVTQFLFWSLVADVMILTWIGGMPVEHPF----VIIGQ
Calu  SILVLFLVPMLHTSKQRGLTFRPLSQFIFWVLVADVLVLTWIGGMPVETPY----VVVGQ
Papa  SILVLLLVPILHTSKLRTMTFRPISQLLFWTLVADVLILTWIGGMPVEQPY----IIIGQ
Sufr  SILVLMIVPILHTSKQRGLTFRPFTQFLFWTLIADIAILTWIGGMPVEDPY----IIIGQ
Stci  SILILMLVPFLHTSKQRSLVFRPLTQFLFWTLIADIIILTWIGGMPVEHPY----IIIGQ
Taru  SILILMVVPFLHTSKQRSLTFRPLSQFLFWTLIADVVILTWIGGMPVEHPY----IIIGQ
Rala  SILVLMLVPLLHTSKQRGLTFRPLTQFLFWTLIANIVILTWIGGMPVEHPF----VIIGQ
      *:: *   *  : .       **  :  :  :      ***:*.     *        .:
```

To be continued on page 35.

```
Scca   IASVAYFSLFLFVIPITSWCENKFLSLN------------------
Muma   IASISYFALFLIIMPFISWCENKILSLN------------------
Erca   VASVLYFTIFLVFMPASGWIENKMMNRS------------------
Pose   VASILYFLIFLVFMPMSGWIENKMLNRN------------------
Actr   VASTVYFALFLIALPLTGWLENKALNWN*-----------------
Scal   VASTAYFALFLIALPLAGLLENKALNWN------------------
Posp   VASTIYFALFLVALPLTGWLENKILNWN------------------
Atsp   IASVLYFMLFLILIPLTGWLENKILDWA------------------
Leoc   VASVLYFMLFLFFIPLSGWLENKILDWA------------------
Amca   IASIIYFALFLVFAPLAGWVENKMLSW*------------------
Osbi   VASILYFMLFLIFLPLAGLAENKMLQLK------------------
Pabu   IASIFYFALFLILFPLAGLMENKILQLN------------------
Hial   VASVIYFALFLVLSPLAGWVENKALEWN------------------
Elha   VASALYFALFLILMPLAGVVENKMLKLS------------------
Mlcy   VASVLYFMLFLILTPLAGLVENKMLKWN------------------
Algl   VASVIYFAIFLVLTPLTGWLENKVLGEV------------------
Ptgi   GASVLYFSLFLVLNPLVGWLENKALKWA------------------
Alaf   VASTVYFMLFLMFIPLAGWVENKALESS------------------
Nock   VASALYFMLFLVFIPMAGWVENKMIESN------------------
Anja   VASVLYFSLFLVLNPLAGWLENKMMNW*------------------
Gyki   TASVLYFSLFLILNPLVAWLENKMLDW*------------------
Syka   VASVLYFTLFLVLNPLVGWLENKMINW*------------------
Opma   GASLLYFSIFLVLNPVIGWMENKMLEL*------------------
Comy   VASLLYFTLFLVLNPLVGWIENKMLDWQ------------------
Sasp   IASVLYFSLFLFLNPLAAHLENHMIKPKQ-------XXAQHKYMY*
Eupe   VASTWHFSLILLINPLTAWLENKHLIPKK-------QRM*------
Enja   VASLLYFSIFLVLAPVAGWLENKALNWN------------------
Same   VASVLYFSVFLVLAPLAGWAENKVLEWK------------------
Chch   VASAIYFALFLVFIPLAGWFENKALEWA------------------
Grgr   VGSVLYFTLFLVLAPLAGWFENKMLGWA------------------
Caau   IASVLYFALFLVLFPLAGWLENKALKWA------------------
Cyca   IASVLYFALFLIFMPLAGWLENKALKWA------------------
Dare   MASILYFSLFLVLFPITGILENKALQWS------------------
Cost   LASILYFTLFLVLIPLAGWLENKALEWA------------------
Leec   IASILYFALFLVLIPLTGWLENKALEWA------------------
Fola   IASILYFALFLILIPLAGWLENKALEWV------------------
Clmc   IASVLYFAFFLILNPLAGWLENKSLNWS------------------
Phin   VASILYFMLFLIINPLAAWYENKWLNWS------------------
Icpu   VASVLYFSLFLIFSPLMGWLENKTINFK------------------
Psto   IASVLYFSLFLILNPLTGWLENKLLNLH------------------
Cora   VASVLYFALLLILMPATGWLENKLLNWN------------------
Eisp   IASALYFALFLIFFPLAGWCENKMLN*-------------------
Apal   IASALYFALFLILFPLAGWYENKLLKWS------------------
Eslu   VTSVIYFAIFLLLAPLAGWLENKTLE*-------------------
Dape   VSSVIYFMIFLILSPLAGLLENKAIQ*-------------------
Glse   IASLIYFSLFLILVPLAGWAENKILK*-------------------
Naar   IASVLYFSLFLFIMPLVGWVENKALE*-------------------
Lioc   VASLIYFSLFLVIMPVIGWVENKALE*-------------------
Opso   IASLIYFSLFLIMMPLGGWMENKILN*-------------------
Alte   AASVLYFSLFLIFTPIAGWAENKALEWN------------------
Plap   IASVLYFSLFLIFTPLVGWAENKALEWN------------------
```

[7/7 of aligned sequences]

```
Plal    VASVIYFSIFLVLSPLAGWAENKSLKWA------------------
Sami    VASVIYFSIFLILSPLAGLVENKSLKWA------------------
Rere    VASLLYFTIFLVFSPLAGWAENKTLKWA------------------
Gama    VASAIYFAIFLILVPTVGWAENKALEWA------------------
Onmy    VASVIYFTIFLVLSPLAGWAEIKALQWA------------------
Sasa    IASVIYFTIFLVLAPLAGWAENKALEWT------------------
Cola    VASVIYFTIFLVLAPLAGWAENKALEWA------------------
Dita    IASLVYFSIFLVLAPAAGWIENKALEWS------------------
Gogr    LASLAYFSIFLIIIPTTGWLEDKTLKWSRT*---------------
Chsl    LASLIYFSIFLAFLPLAGLLENQALK*-------------------
Atja    VASFLYFMLFLTLMPLAGWIENKTLNWI------------------
Iido    VASFLYFLLFLTLMPLAGWLENKTLNWA------------------
Auja    VASFLYFFLLLILVPLAGWVENKALEWA------------------
Chag    VASFLYFLLLLVLSPLAGWVENKALSWA------------------
Hami    VASFLYFFLLLVLVPTAGWLENKVLEWA------------------
Saun    VASFLYFFLLLVLVPAAGWLENKVLEWA------------------
Nema    IASFIYFSLFLVLAPLAGWVENKALEWN------------------
Disp    VASAVYFSIFLVFAPVAGWAENKALEWA------------------
Myaf    VASALYFSIFLILMPLVGWLENKALAWA------------------
Lagu    VASTLYFLLFLVLAPTAGWLENKSLKWL------------------
Trtr    IASALYFLLFLVLSPAAGWLENKTLEWS------------------
Zucr    VASALYFLLFLVLSPAASWLENKTLEWS------------------
Pxja    VASFLYFFLFLVLTPIAGFMENKALEWN------------------
Pxlo    VASFLYFFLFLVLTPIAGFVENKALEWN------------------
Pctr    IASLLYFMLFLTLIPAAALAENKALEWN------------------
Apsa    MASTLYFLLFLMLIPIAGWTENKILKWN------------------
Cabe    ASSAIYFALLLVIMPITGVLENKTMFSQ------------------
Bzze    VASTTYFLLILVLLPLAGLVENKALEWT------------------
Siim    VASFLYFLILLVLLPLAGLIENKLLGFK------------------
Ctru    VASLLYFVLILILSPFAGWVENKALEWT------------------
Dpbr    VASLLYFILVLILSPFAGWVENKALEWA------------------
Caki    VASVLYFLLFLILFPIAGLLENKALKL*------------------
Phja    LASILYFSLFLVFFPLAGMLENKALKM*------------------
Brsp    IASILYFSLFLLIIPIIGLVENKLLEWG------------------
Gamo    VASVLYFSLFLVLFPLAGMTENKALEWN------------------
Lolo    VASVLYFSLFLVLFPLAGMTENKALEWN------------------
Batr    VSSITHFLLLIIIFPLAGVLENSLTHPKT-------FKN*------
Prmy    ISTAAYFSMVLLIMPLAATMENHLLKKFSSPTPAQECPKN*-----
Lose    VASLLYFFLFLVAAPLVGLAENKYLTWNPQRQ--------------
Loam    AASLLYFSLFLIAMPLIGWVENKFLDWTPKL*--------------
Chab    VASVLYFSLFLVLMPLGGWLENKTLKLT------------------
Chto    VASVLYFSLFLILMPLGGWLENKTLKLT------------------
Majo    AASLLYFSLFLIMMPAASLLENKFV*--------------------
Hlst    AASLLYFSLFLVLMPAAGYLENKLF*--------------------
Clpe    VASLLYFSLFLVLMPLAGWLENKMLGLS------------------
Mlmr    VASLLYFALFLIFMPLTGWLENKTLELL------------------
Crcr    IASALYFSLFLILMPMASWLENKMLTW-------------------
Muce    IASALYFSLFLILMPMASWVENKMLTW-------------------
Bege    IASILYFSLFLIFIPAVGWVENKLLEWQ------------------
Mela    AASVLYFMIFLILMPAASWVENKLINW*------------------
Hats    VASFLYFFLFLILTPTISWVENKVLKWQ------------------
Orla    IASFLYFSLFLIMAPAAGWLENKVLKWQ------------------
```

[7/7 of aligned sequences]

```
Cosa  IASVLYFSLFLILYPTVSWVENKALKWN------------------
Exsp  IASVIYFSIFLVLVPVAGLVENKIIEWQ------------------
Depa  IASILYFSLFLILYPTVSWIENKTLKWN------------------
Rima  IASFLYFFLFLVFFPLSGWLENKMLES-------------------
Fuol  VASFLYFSIFLFLSPTAAWLENKVLGW*------------------
Gmaf  IASVLYFSLFLFFAPAAAWVENKILGW*------------------
Xeei  IASFLYFSLFLFLSPVAAWLENKILELK------------------
Pros  VASLLYFLLILVISPFAAWLENKALQWA------------------
Scmi  IASLLYFMLLLIISPLAARVENKTLQWT------------------
Rolo  VASFVYFFLFLVLAPLAGWVENKALEWT------------------
Cere  VASFLYFFLFLVLLPLAGWVENKTLEWK------------------
Daga  VASFLYFLLFLVLVPLAGWLENKTLGWN------------------
Anco  VASVLYFMLFLVLMPLVSWLENKALRKPIELA--------------
Dmve  MASFLYFFLFLVLLPLAGWAENKILE*-------------------
Dmar  LASFLYFFLFLVLLPLTGWVENKILE*-------------------
Anka  VASLLYFMLFLVFTPLVGWLENKALDKPIEQE--------------
Moja  AASFLYFLLFLVLTPLAGWLENKALDKPLEQE--------------
Hoja  VASLLYFLLFLVVAPVVGLLENKALDKPFEQE--------------
Bede  VASFLYFLLILVLSPLAAWAENKALRWA------------------
Besp  VASFLYFLLILVLSPLAAWAENKALRWT------------------
Mysp  VASFIYFFLFLVLSPIAGWLENKALEWA------------------
Osja  VASFLYFFLFLVLSPLAGWLENKALEWT------------------
Sgro  VASFLYFFLFLVLAPLAGWLENKALEWT------------------
Pzpa  TASILYFSLFLILIPTTGWAENKVLKWK------------------
Zeja  VASILYFSLFLVLIPATGWMENKALEWN------------------
Znne  IASVLYFTLFLILIPTTGWLENKILEWN------------------
Zefa  VASVLYFALFLVIMPTTGWMENKALKWN------------------
Acni  VASVLYFALFLILIPATGWAENKALKWN------------------
Ncrh  VASVLYFALFLIFIPATGWAENKALKWN------------------
Agca  IASVLYFSLFLIIMPLVGWLENKALQWS------------------
Hydy  VASLLYFSLFLVLYPAAGMVENKMLEWS------------------
Gsac  VASVLYFSLFLVLYPGAAVMENKMLEWT------------------
Pevo  IASLIYFALFLIFIPVVGVLENKAIEWA------------------
Hiku  LASVVYFLIFLTALPVSGWMENKILKWN------------------
Inpa  IASLLYFTIILFLMPMLGLIENKMLN*-------------------
Auch  AASFLYFFAIIVLFPLVGWAENQLLVEDE-------CNK*------
Fico  VASFLYFLLLLILSPLAGWVENKVLKWA------------------
Macs  VASILYFLLFLVLAPVAGLVENKILKWS------------------
Moal  VASVLYFSLFLFFFPLASWYENKTLKLN------------------
Syma  IASVSYFTLILILMPLTGWFENKMLNLN------------------
Mafr  IASLIYFLLFLILIPATGWAENKILKK-------------------
Dcpe  IASFLYFFLFLVLTPLAGWLENKALEWS------------------
Dcti  IASFLYFFLFLVLTPVAGWLENKALKWS------------------
Hehi  VASFLYFFLFLVLTPLAGYAEDKALEWA------------------
Stam  VASFLYFLLFLVLTPLAGWMENKALGWS------------------
Hogi  VASILYFSLFLFLFPLAGWVENKALKWN------------------
Erzo  IASFLYFSLFLVLSPLAGWLENKVLEWS------------------
Hxot  IASFLYFFLFLALFPMAGYVENKALGWA------------------
Core  IASLLYFFLFLALFPLAGWVENKALGWA------------------
Apve  IASILYFLLFLTLLPLAGLMENKILDWS------------------
Latj  IASLLYFLLFLVLIPLAGWIENKALGWS------------------
Laja  IASLLYFLIFLVLFPVAGWLENKALGWT------------------
```

[7/7 of aligned sequences]

```
Syja    VASFLYFLLFLVLFPLAGWVESRLLI*-------------------
Epme    VASFLYFFLFLVLTPAVGWAENKMLEWQ------------------
Grse    VASVLYFSIFLFLMPLAGWLENKAFTWS------------------
Clja    IASLLYFSLFLFIFPLTAWLENTAFNWT------------------
Ogcy    LASLLYFLIFLVLFPLAGWLENKALKWS------------------
Plna    IASFLYFLTFLVLFPMASWAENKALQWQ------------------
Lema    VASFLYFFLFLVLTPLTGWLENKVLEWS------------------
Etzo    IASFLYFFLFLFLAPLAGWIENKALGWA------------------
Apse    IASALYFLIFLVFFPLSGWLENKLLGLS------------------
Epde    VASLLYFFLFLVLFPLAGHLENKVLGWK------------------
Slja    VASALYFSLFLLLMPLAGWLENKALGWN------------------
Bsja    IASFTYFFLFLVLMPLTGWVENKIMGWT------------------
Ecna    IASVLYFSIFLIFFPLAGWLENKFLGLN------------------
Cohi    VASVIYFALFLVFIPLAGWLENMAFKQV------------------
Caar    IASVLYFLLFLVLTPLAGWVENKMLDWT------------------
Came    IASVLYFLLFLVFTPLAGWVENKMLGWA------------------
Mema    VASLLYFSLFLVLSPLAGWLENKALEWS------------------
Lenu    IASVLYFSIFLILNPLAGWLENKLLGWN------------------
Brja    VPSLLYFSLFLVFLPLTGWMENKVLGWS------------------
Plma    VASFLYFFLFLVLFPLAGWAENKILGWS------------------
Emst    IASLLYFFLFLVITPLAGWLENKALGWL------------------
Ptti    VASVLYFLLFLVLAPLAGWLENKALGWA------------------
Losu    SASLVYFALFLVIIPMTSLAENKALQWH------------------
Geoy    IASLLYFSIFLILMPLAGWMENKALGWM------------------
Dipi    IASFLYFFLFLVLTPLAGWMENKALGWH------------------
Pama    IASLTYFALFLLIMPMAALVENKVLGWQ------------------
Leob    IASLLYFSLFLIIAPAAGWFENKSLGWR------------------
Neba    IASVLYFAIFLVLMPIAGMVENKVMGWA------------------
Pdpl    VASALYFTIFLVLFPLTGWLENKMFGWS*-----------------
Nimi    VASILYFSLFLILFPLAGWLENKTLEWR------------------
Uptr    AASFLYFFLFLFLVPLAGWVENKALQWA------------------
Pesc    IASALYFSIFLVLLPVAGWLENKLIGW*------------------
Baar    VASLCYFFLFLVLFPLTGLLENKALGWT------------------
Moar    VASFLYFFLFLILTPLAGWLENKALGWL------------------
Toja    IASFLYFLLFLVLTPMAGWLENKALGLL------------------
Chau    VASVLYFCLFLIAMPITGWLENKFLEWS------------------
Chse    LASLLYFLLFLVFMPLAGWLENKSLEWS------------------
Enar    IASFLYFFLFLALIPTAGWLENKALKWS------------------
Hpty    VASLSYFLLFLVLFPMAGWLENKALEWT------------------
Nana    IASILYFLLFLVLMPLSARLENKILKW-------------------
Mcst    VASLLYFLLFLIFTPATGWLENKILEWY------------------
Rhox    IASFLYFFIFLVLTPLAGLVENVVLKWY------------------
Opfa    VASFLYFFLFLVLAPLAGWMENKVLQWH------------------
Paar    VASFLYFSLFLFFMPLAGWLENKALGWA------------------
Gozo    VASFLYFFLFLVLTPLAGWLENKALEWS------------------
Ackr    IASVLYFSLFLILMPMAAWLENKFLGLS------------------
Elev    AASLIYFALFLIIMPLASHLENKALEWT------------------
Trdu    IASFLYFFLFLVLAPLSGWLENKIFEWY------------------
Amoc    IASVLYFSLFLVLVPTVGWVENKFLKWR------------------
Hame    IASVVYFTLFLVFSPIAGWAENKALRWT------------------
Chso    VASFLYFFMFLILAPLAGWLENKALNWS------------------
Lyto    IASVLYFSLFLILSPLAGWAENKALGWS------------------
```

[7/7 of aligned sequences]

```
Encr        VASVLYFSLFLILSPLAGWAENKALGWS------------------
Bvar        VASALYFSIFLILFPLAGWLENKMFSL*------------------
Noco        VASVLYFSIFLIFFPLAGWLENKILGLA------------------
Chsp        LASALYFILFLIMWPLAGWLENKYFKWT------------------
Arja        VASVLYFLLFLTLFPLAGWVENKALGWS------------------
Pase        VASVLYFLLFLVLFPLAGWTENKAFGWT------------------
Trel        VASVYYFASFLIFFPLAGMAENNMMVLK------------------
Lifa        IASFLYFLLFLVLFPLAGLVENKIMGWT------------------
Acur        IASLTYFSLFLILFPLAGLLENEMFNWT------------------
Ampe        IASVLYFSLFLVLFPLVGLAENKVFGWT------------------
Urja        IASLMYFLLFLAFFPLAGILENKVFGWA------------------
Enet        VASFIYFFLFLVLTPLVGWAENKMLEWQ------------------
Ptbr        VASFLYFSLFLFLSPLAGLLENKMLEWR------------------
Safa        VASFLYFFIFLILSPVAALVENKMLEWQ------------------
Icae        VASFLYFSLFLVFLPLAGWAENKILGWS------------------
Asmi        IASFLYFFVFLAIFPSLGWLENKPLY*-------------------
Foal        LASVLYFALFLALMPLVGLVENAILK*-------------------
Drze        IASLIYFLLFMTLIPIAGWVENKTLKW*------------------
Rhas        IASLLYFSIFLVLFPAAGWLENKLLELN------------------
Elac        IASALYFAIFLLVLPMAGWLENKLLALD------------------
Kugu        IASTLYFLIFLVLMPLAGLMENKMFEFS------------------
Plor        VASFLYFFLFLVLAPMTAWMENKALDWS------------------
Sgun        IASVLYFALFLIVTPFVGWLENKALGWV------------------
Zaco        IASVLYFFLFLILTPLTGWLENKALGWL------------------
Zbfl        VASVLYFFLFLILTPLAGWLENKALGWLCISSSASERRSCKPDA*-
Spba        VASLLYFSIFLVFMPLAGLLENKTLGWS------------------
Game        VASVLYFSLFLAFFPLAGWAENKILGWA------------------
Thth        VASVLYFSLFLVFFPLAGWAENKILGWS------------------
Xigl        IASFLYFFLFLVLAPLAGWLENKALGWS------------------
Hyja        IASLLYFSLFLILFPLAGWAENKILGW*------------------
Psan        IASLLYFSLFLILFPLVGSLENKLFDWS------------------
Cupa        VASVLYFSLFLVFFPAAGWVENKILGWA------------------
Mpch        IASLLYFLLFLVFMPLAGIIEDKILLKENNNQ--------------
Char        VASFLYFSLFLVLIPLSGWLENKALKWS------------------
Pser        IASFLYFFLFLILMPATSWVENKALNWG------------------
Prol        VASFLYFFLFLVMIPVTGWLENKILGWQ------------------
Plbi        VASLIYFSLFLVLIPTAGLMENKILGWK------------------
Calu        AASFLYFSIFLCLMPVAGTLENKLLIN*------------------
Papa        IASLLYFLLFLVFMPLAGWVENKMLGLS------------------
Sufr        AASVIYFSLFLIIMPLVSILENKFMGWA------------------
Stci        IASVLYFSLFLFFMPLASLLENKALGLN------------------
Taru        IASVLYFSLFLILMPMAGWLENKMLN*-------------------
Rala        IASVLYFSLFLIFVPLTGWLENKALEWS------------------
              :  :*  .:   *  .  *
```
