# Supplementary material for: Structure and variation of the mitochondrial genome of fishes
Source: BMC Genomics. 2016 Sep 7;17(1):719. doi: 10.1186/s12864-016-3054-y (PMC5015259; doi:10.1186/s12864-016-3054-y)
Supplement: Additional file 6: Figure S1-a. — Aligned amino acid sequences of the ATP8 gene in mt genomes of 250 fishes. Figure S1-b. Aligned amino acid sequences of the ATP6 gene in mt genomes of 250 fishes. Figure S1-c. Aligned amino acid sequences of the COI gene in mt genomes of 250 fishes. Figure S1-d. Aligned amino acid sequences of the COII gene in mt genomes of 250 fishes. Figure S1-e. Aligned amino acid sequences of the COIII gene in mt genomes of 250 fishes. Figure S1-f. Aligned amino acid sequences of the Cyt b gene in mt genomes of 250 fishes. Figure S1-g. Aligned amino acid sequences of the ND1 gene in mt genomes of 249 fishes. Figure S1-h. Aligned amino acid sequences of the ND2 gene in mt genomes of 250 fishes. Figure S1-i. Aligned amino acid sequences of the ND3 gene in mt genomes of 250 fishes. Figure S1-j. Aligned amino acid sequences of the ND4L gene in mt genomes of 250 fishes. Figure S1-k. Aligned amino acid sequences of the ND4 gene in mt genomes of 250 fishes. Figure S1-l. Aligned amino acid sequences of the ND5 gene in mt genomes of 250 fishes. Figure S1-m. Aligned amino acid sequences of the ND6 gene in mt genomes of 249 fishes. (ZIP 3250 kb) [file 12864_2016_3054_MOESM6_ESM.zip › Additional file 6 prot align/AF6g-ND1.pdf]

Additional file 6: Figure S1–g. Aligned amino acid sequences of the ND1 gene in mt genomes of 249*1 fishes.

Species name abbreviation followed by aligned amino acid sequences shown by one letter abbreviation. See Additional file 1 for abbreviation of species name. Amino acids shown by magenta letter denote hydrophobic residues. A-H in bold types with yellow background indicate putative transmembrane regions. Asterisk '*' indicates a fully conserved residue. Colon ':' and period '.' indicate 'strong' and 'weak' groups in the level of conservativeness, respectively, in the Gonnet Pam250 matrix, in which the strong and weak groups are defined as strong score >0.5 and weak score =<0.5, respectively (Thompson et al., 1997). *1 Data of 1 species were not obtained (See text.).

ND1

[1/6 of aligned sequences]

A

```
Scca    -----MLQTTLLYLINPLAYIIPILLATAFLTLIERKILGYMQFRKGPNIVGPYGLLQPI
Muma    -----MLQTILLYLINPLAYIIPILLATAFLTLIERKILGYMQFRKGPNVVGPYGLLQPI
Erca    -------MTIFTLIINPLMYIIPILLAMAFLTLVERKILGYMQLRKGPNIVGPYGLLQPI
Pose    -------MTLITLIINPLMYIVPVLLAMAFLTLVERKILGYMQLRKGPNIVGPYGLLQPI
Actr    -----MTSHLLTYLINPLAYIVPILLAVAFLTLIERKVLGYMQLRKGPNIVGPYGLLQPI
Scal    -----MTSHLLTYLINPLAYIVPVLLAVAFLTLIERKVLGYMQLRKGPNIVGPYGLLQPI
Posp    -----MTSHLLTYLINPLAYIIPILLAVAFLTLIERKVLGYMQLRKGPNIVGPYGLLQPI
Atsp    -----MTKYLMTYIINPLLFIIPILLAVAFLTLIERKVLGYMQLRKGPNIVGPYGLLQPI
Leoc    -----MTKYFMTYIINPLLFIIPILLAVAFLTLIERKVLGYMQLRKGPNIVGPYGLLQPI
Amca    -----MLNVLLTHIISPLAYIIPVLLAVAFLTLIERKVLGYMQLRKGPNIVGPYGLLQPI
Osbi    ------MHEWIILISNPLLCIVPVLLAVAFLTLLERKILGYMQLRKGPNVVGPYGLLQPI
Pabu    ------LTEEGITLINPLSYIIPILVAVAFLTLLERKILGYMQLRKGPNIVGPYGLLQPI
Hial    -----MMKLLIIYIINPLAFIVPVLLAVAFLTLLERKVLGYMQLRKGPNVVGPFGLLQPI
Elha    -----MIELLTTYVINPLAYILPVLLAVAFLTLLERKVLGYMQLRKGPNVVGPYGLLQPI
Mlcy    -----MISTLISHIINPLAYIIPILLAVAFLTLLERKVLGYMQLRKGPNVVGPYGLLQPI
Algl    -------MIIVTHVINPLTYIVPVLLAVAFLTLLERKTLGYMQLRKGPNIVGPTGLLQPI
Ptgi    ------MNIIITHIINPLAYIVPVLLAVAFLTLLERKVLGYMQLRKGPNVVGPYGLLQPI
Alaf    ------MTTLITHIINPLTYIVPVLLAVAFLTLLERKVLGYMQLRKGPNVVGPYGLLQPI
Nock    ------MTTLITHILNPLAYIVPVLLAVAFLTLLERKVLGYMQLRKGPNVVGPYGLLQPI
Anja    ------MNTMLTHIINPLAYIVPVLLAVAFLTLLERKVLGYMQLRKGPNIVGPYGLLQPI
Gyki    ------MNSIMTHIINPLAYIVPVLLAVAFLTLLERKVLGYMQLRKGPNVVGPYGLLQPI
Syka    ------MNTVLTHIINPLAYIVPVLLAVAFLTLLERKVLGYMQLRKGPNVVGPYGLLQPI
Opma    ------MTSILTHIINPLAYIVPVLLAVAFLTLLERKVLGYMQLRKGPNVVGPYGLLQPI
Comy    ------MNLILTHIANPLVYIIPILLAVAFLTLLERKVLGYMQLRKGPNIVGPYGLLQPI
Sasp    -------MNVVIYALLFLSYAVPILLAVAFITLLERTMLGHAQERKGPNIVGPFGLLQSF
Eupe    ------MLMAIIHVISPLMYIIPVLLAVAFLTLLERNILGRMQLRKGPNIVGPLGLLQPF
Enja    -----MLSTIITHIVNPLAYIVPVLLAVAFLTLVERKVLGYMQLRKGPNVVGPYGLLQPI
Same    -----MLNIIMTHIVNPLAYIVPVLLAVAFLTLIERKVLGYMQLRKGPNVVWPYGLLQPI
Chch    -----MLSLLLTYLINPLAYIVPILLAVAFLTLIERKVLGYMQLRKGPNVVGPFGLLQPI
Grgr    -----MLTVLTTHLVSPLTYIVPVLLAVAFLTLVERKVLGYMQLRKGPNIVGPFGLLQPI
Caau    -----MLNTLMTHLINPLAYIVPVLLAVAFLTLIERKVLGYMQLRKGPNVVGPYGLLQPI
Cyca    -----MLNILMTHLINPLAYIVPVLLAVAFLTLIERKVLGYMQLRKGPNVVGPYGLLQPI
Dare    -----MLDILTSHLINPLAYAVPVLIAVAFLTLVERKVLGYMQLRKGPNVMGPRGLLQSV
Cost    -----MLNTLLINLINPLAYIVPVLLAVAFLTLVERKVLGYMQLRKGPNIVGPYGLLQPI
Leec    -----MLNTLLTHLINPLAYIIPVLLAVAFFTLIERKVLGYMQLRKGPNIVGPYGLLQPI
Fola    -----MLNTLLTHLINPLAYIVPVLLAVAFLTLLERKVLGYMQLRKGPNIVGPYGLLQPI
Clmc    -----MLTLLITHLLNPLAYIVPVLLAVAFLTLIERKVLGYMQLRKGPNVVGPYGLLQPI
Phin    -----MPTLLFTHLINPLALIIPVLLAVAFLTLIERKVLGYMQLRKGPNVVGPYGLLQPI
Icpu    -----MMTPLITHIINPLAYIVPVLLAVAFLTLIERKVLGYMQLRKGPNVVGPYGLLQPI
Psto    -----MMTLLITYLINPLAYIVPVLLAVAFLTLVERKVLGYMQLRKGPNVVGPYGLLQPI
Cora    ------MMTLLTHIINPLAYIVPVLLAVAFLTLIERKVLGYMQLRKGPNVVGPFGLLQPI
Eisp    -----MLTLLLTHLINPLAYIVPVLLAVAFLTLIERKVLSYMQLRKGPNVVGPYGLLQPV
Apal    ------ITTLITHLINPLAYIVPILLAVAFLTLIERKILSYMQLRKGPNVVGPYGLLQPI
Eslu    -----MITTLITHIINPLTYIVPVLLAVAFLTLLERKVLGYMQLRKGPNIVGPYGLLQPI
Dape    -----MITLLITHVMNPLTHIVPVLLAVAFFTLLERKVLGYMQLRKGPNIVGPYGLLQPF
                                       1
```

To be continued on page 6.

[1/6 of aligned sequences]

```
Glse      -----MIATLMTFIINPLAFIVPVLLAVAFLTLLERKVLGYMQLRKGPNIVGPYGLLQPI
Naar      -----MIATLVIFIINPLAYIVPVLLAVAFLTLLERKVLGYMQFRKGPNIVGPYGLLQPI
Lioc      -----MITTLMTFIINPLAYIVPVLLAVAFLTLLERKVLGYMQFRKGPNIVGPYGLLQPI
Opso      -----MITPLVTYIINPLAYIIPVLLAVAFLTLLERKVLGYMQLRKGPNIVGPYGLLQPI
Alte      -----MLTILLIYLINPLAYIIPVLLAVAFLTLLERKVLGYMQHRKGPNIVGPYGLLQPI
Plap      -----MLTILITYLINPLAYIIPVLLAVAFLTLLERKVLGYMQLRKGPNIVGPYGLLQPI
Plal      -----MITTILTHVINPLAYIVPVLLAVAFLTLLERKVLGYMQLRKGPNIVGPYGLLQPI
Sami      -----MITSILTYIVNPLAYIVPVLLAVAFLTLLERKVLGYMQLRKGPNIVGPYGLLQPI
Rere      -----MISVLLTHVLNPLAYIVPVLLAVAFLTLLERKVLGYMQLRKGPNIVGPYGLLQPI
Gama      -----MIAALFSYIINPLTYIVPVLLAVAFFTLVERKVLGYMQLRKGPAIVGPYGLLQPI
Onmy      ------MITLITHVINPLAYIVPVLLAVAFLTLLERKVLGYMQLRKGPNIVGPYGLLQPI
Sasa      -----MTTTLITHIINPLAYIVPVLLAVAFLTLLERKVLGYMQLRKGPNIVGPYGLLQPI
Cola      -----MITTLITHVINPLAYIVPVLLAVAFLTLLERKVLGYMQLRKGPNIVGPYGLLQPI
Dita      -----MISTIMTHIINPLAFIVPILLAVAFLTLLERKVLGYMQLRKGPNVVGPYGLLQPI
Gogr      --------MSPTLLVNPLVLAVPVLLAVAFLTLLERKILGYMQLRKGPNTIGPYGLLQPV
Chsl      -----MTHFLFVYIINPLAFIVPVLLAVAFFTLLERKVLGYMQLRKGPNIVGPYGLLQPI
Atja      -----MISTITIYILNPLAFIVPVLLAVAFLTLIERKVLGYMQLRKGPNIVGPYGLLQPI
Iido      -----MISAITIYILNPLAFIVPVLLAVAFLTLIERKVLGYMQLRKGPNIVGPYGLLQPI
Auja      -----MILTLITHILNPLAYIVPVLLAVAFLTLLERKVLGYMQLRKGPNIVGPYGLLQPI
Chag      -----MILALITHIINPLAYIVPVLLAVAFLTLLERKVLGYMQLRKGPNIVGPYGLLQPI
Hami      -----MITTLVLYIINPLAFIVPVLLAVAFLTLLERKVLGYMQLRKGPNIVGPYGLLQPI
Saun      -----MITTLILFIINPLAFIVPVLLAVAFLTLLERKVLGYMQLRKGPNIVGPYGLLQPI
Nema      -----MIHTLMTFIVNPLAYIIPVLLAVAFLTLIERKVLGYMQLRKGPNIVGPYGLLQPI
Disp      -----MINTLVTHILNPLAFIVPVLLAVAFLTLLERKVLSYMQLRKGPNIVGPYGLLQPI
Myaf      -----MIDILITHILNPLTYIVPILLAVAFLTLLERKVLGYMQLRKGPNIVGPYGLLQPI
Lagu      ------MSLIMTHILNPLAYIVPILLAVAFLTLLERKVLGYMQHRKGPNAVGPYGLLQPI
Trtr      -------MIFITHLLNPLAYIVPILLAVAFLTLVERKVLGYMQLRKGPNVVGPYGLLQPI
Zucr      -------MMIVTHLLNPLAYIIPILLAVAFLTLIERKVLGYMQLRKGPNVVGPYGLLQPI
Pxja      -----MISTLTIYILNPLAYIVPVLLAVAFLTLLERKVLGYMQLRKGPNIVGPYGLLQPI
Pxlo      -----MISTLTIYILNPLAYIVPVLLAVAFLTLLERKVLGYMQLRKGPNIVGPYGLLQPI
Pctr      -----VMPVLMSHLIGPLTYIVPVLLAVAFLTLLERKVLGYMQLRKGPNIVGPYGLLQPI
Apsa      -----MISPLFSHIISPLTYIIPVLLAVAFLTLLERKVLGYMQLRKGPNIVGPYGLFQPI
Cabe      -----VYMAALINIGQSFALIVPVLLAVAFLTLIERKVLGYMQLRKGPNVVGPYGLLQPM
Bzze      -----MAHTLITYVINPLALIVPVLLAVAFLTLIERKVLGYMQLRKGPNVVGPYGLLQPI
Siim      ------MRHIFIYVINPLAMIIPVLLAVAFLTLIERKVLGYMQLRKGPNVVGPYGLLQPM
Ctru      -----MMSALTTHIINPLAYIVPVLLAVAFLTLLERKVLGYMQLRKGPNVVGPYGLLQPI
Dpbr      -----MIYTLTTHILNPLAYIVPVLLAVAFLTLIERKVLGYMQLRKGPNVVGPYGLLQPI
Caki      -----MLTSIIKLLVIPITFMIPILLAVAFLTLIERKVLGYIQLRKGPTIVGPYGLLQPF
Phja      ------MTNVTNYLIHPLIYMVPILLAVSFLTLLERKVLGYMQLRKGPNIVGPYGLLQPI
Brsp      ------MITLLNYVIMPLTFMVPILLAVAFFTLIERKVLGYMQLRKGPNVVGPYGVLQPF
Gamo      -----MTNIVVSYILNPLIYMVPVLLAVAFLTLIERKVLGYMQLRKGPNIVGPYGLLQPI
Lolo      -----MAHVVISYILNPLIYMVPVLLAVAFLTLIERKVLGYMQLRKGPNIVGPYGLLQPI
Batr      ----------MINTLSTAMLIIPVLLAVAFLTLLERKVMGHVQARKGPNIVGPSGLLQPF
Prmy      ----------MVTLINLLALIIPVLLTVAFLTLAERKVLGYMQTRKGPNIVGPYGVLQPF
Lose      -----MLPPFMTHLVNPMTFIVPVLLAVAFLTLLERKVLGYMQARKGPNIVGQYGLLQPL
Loam      -----MISTTMIYVINPLAFIVPVLLAVAFLTLLERKVLGYMQLRKGPNIVGPYGLLQPI
Chab      -----MITTLIIYIINPLTFIIPVLLAVAFLTLIERKVLGYMQLRKGPNIVGPYGLLQPI
Chto      -----MITTLIIYIVNPLTFIIPVLLAVAFLTLIERKVLGYMQLRKGPNIVGPYGLLQPI
Majo      -----MTQLLMTYLINPLTFVIFVLLAVAFLTLLERKVLGFMQLRKGPNIVGPHGLLQPL
Hlst      -----MLQLCTSFIINPLIMIVFILLAVAFLTLLERKVLGFMQLRKGPNIVGPHGLLQPI
Clpe      -----MTSPLTTHILSPLTFIVPVLLAVAFLTLIERKVLGYMQLRKGPNVVGPYGLLQPI
Mlmr      -----MTSTLLIYIINPLTFIVPVLLAVAFLTLLERKVLGYMQLRKGPNIVGPYGLLQPI
```

2

To be continued on page 7.

[1/6 of aligned sequences]

```
Crcr      -----MLPAILSTLLNPLILIVFVLLAVALLTLIERKVLGYMQLRKGPNIVGPYGLLQPI
Muce      -----ILPAILSTLLNPLILIVFVLLAVALLTLIERKVLGYMQLRKGPNIVGPYGLLQPV
Bege      -----MMSTLMLFIINPLILIVFVLLAMALLTLVERKVLGYMQLRKGPNIVGPYGLLQPI
Mela      -----MMMTLVLPIINSLILIVFILLAVAILTLVERKVLGYMQLRKGPNVVGPYGLLQPV
Hats      -----MLSKLVLSLINPLILIAFILLAVALLTLVERKVLGYMQLRKGPNVVGPYGLLQPI
Orla      -----MLSKILSYLINPLVIMIFVLLAVALLTLVERKVLSYMQLRKGPNVVGPYGLLQPF
Cosa      -----MISTIINFIINPLIMIIFILLAVALLTLVERKVLGYMQLRKGPNIVGPYGLLQPI
Exsp      -----MLHMILSLIVNPLILTVFVLLAVALLTLVERKVLGYMQLRKGPNVVGPYGLLQPI
Depa      -----MISTITNFIINPLILIIFILLAVALLTLVERKVLGYMQLRKGPNIVGPYGLLQPI
Rima      -----MLPKLLTYLINPLIFMIFILLALAFLTLVERKVIGSMQLRKGPNIAHSYGLFQPF
Fuol      -----MSLTLVFSIINPLVLIVFVMLAVALLTLVERKVLGYMQLRKGPNVVGPYGVLQPF
Gmaf      -----MLPVLLNTIINPLMLFIFIMLAVALLTLLERKVLGYMQLRKGPNVVGPYGLLQPF
Xeei      -----MISTLLYTLINPLALIIFILLAVALLTLVERKVLGYMQLRKGPNVVGPYGLLQPF
Pros      -----MISMLITHILNPLAFIVPVLLAVAFLTLLERKVLGYMQLRKGPNVVGPYGLLQPI
Scmi      -----MTPMLIAHMLNPLAFIIPILLAVAFLTLLERKVLGYMQLRKGPNVVGPYGLLQPI
Rolo      -----MISTLTTHVLNPLAFIVPILLAVAFLTLLERKVLGYMQLRKGPNIVGPYGLLQPI
Cere      -----MTLALIINILNPLSFIIPVLLAVAFLTLIERKVLGYMQLRKGPNIVGPYGLLQPI
Daga      -----MANASIVYLLNPLMLIVPVLLAVAFLTLIERKVLGYMQLRKGPNIVGPYGLLQPI
Anco      -----MISTLITHILSPLAYIVPVLLAVAFLTLLERKVLGYMQLRKGPNIVGPYGLLQPI
Dmve      -----MISMLTTHILNPLAYIVPVLLAVAFLTLIERKVLGYMQLRKGPNIVGPYGLLQPI
Dmar      -----MLSTLTTHILNPLAYIVPILLAVAFLTLLERKVLGYMQLRKGPNIVGPYGLLQPI
Anka      -----MISALITHVLNPLAYIVPVLLAVAFLTLLERKVLGYMQLRKGPNLVGPYGLLQPI
Moja      -----MISALITHIINPLAYIVPVLLAVAFLTLLERKVLGYMQLRKGPNIVGPYGLLQPI
Hoja      -----MISTLVTHILNPLAYIVPVLLAVAFLTLLERKVLGYMQLRKGPNIVGPYGLLQPI
Bede      -----MISILTTHVLNPLAFIVPVLLAVAFLTLLERKVLGYMQLRKGPNVVGPYGLLQPI
Besp      -----MISTLTTHILNPLAFIVPVLLAVAFLTLLERKVLGYMQLRKGPNVVGPYGLLQPI
Mysp      -----MITNLITHLLNPLAFIVPVLLAVAFLTLLERKVLGYMQLRKGPNVVGPYGLLQPI
Osja      -----MITPLITHLLNPLAFIVPVLLAVAFLTLLERKVLGYMQLRKGPNVVGPYGLLQPI
Sgro      -----MITTLITHLLNPLAFIVPVLLAVAFLTLLERKVLGYMQLRKGPNIVGPYGLLQPI
Pzpa      -----MMSFMLTFILSPLAYMVPVLLAVAFLTLIERKVLGYMQLRKGPNVVGPYGLLQPI
Zeja      -----MLTTITNYILNPLAYMVPVLLAVAFLTLIERKVLGYMQLRKGPNIVGPYGLLQPV
Znne      -----MWYLLVAYLINPLTYMVPILLAVAFLTLIERKVLGYMQLRKGPNIVGPYGLLQPI
Zefa      -----MMSTIVTHILNPLAYMVPVLLAVAFLTLIERKVLGYMQLRKGPNIVGPYGLLQPI
Acni      -----MLTTIITYILNPLAYMVPILLAVAFLTLIERKVLGYMQLRKGPNIVGPYGLLQPI
Ncrh      -----MLTTVITYILNPLAYMVPILLAVAFLTLIERKVLGYMQLRKGPNIVGPYGLLQPI
Agca      -----MISTFVTHIINPLAFIVPVLLAVAFLTLIERKVLGYMQLRKGPNVVGPYGLLQPI
Hydy      -----MISYIITHIVNPLTFIIPVLLAVAFLTLLERKVLGYMQLRKGPNVVGPYGLLQPI
Gsac      -----MMSTIITHIINPLTFIVPVLLAVAFLTLLERKVLGYMQLRKGPNIVGPYGLLQPI
Pevo      -----MIHSIITLVLNPLCFIVPVLLAVAFFTLIERKVLGYMQLRKGPNVVGPYGLLQPI
Hiku      -----MTTMILTQIINPLMYIIPVLLAVAFLTLLERKVLGYMQHRKGPNIVGPFGLLQPI
Inpa      ---------MILPFTNLLLIIVPVLLAVAFFTLLERKVLAYMQLRKGPNVVGPYGLLQPI
Auch      LEVKPLSLGVLTHLFNPLAIIIPVILAVAFLTLLERKILGYMQLRKGTNIVGPYGLLQPF
Fico      -----MISLLLTHVINPLAYIVPVLLAVAFFTLIERKVLGYMQLRKGPNVVGPYGLLQPI
Macs      -----MISLIITHLINPLAFIVPVLLAVAFLTLIERKVLGYMQLRKGPNIVGPYGLLQPI
Moal      -----MLPTIMNYIINPLALIIPILLAVALLTLLERKIIGYMQLRKGPNIVGPYGLLQPI
Mafr      ------MTFTLNNIINPLLLIIPILLAVAFLTLLERKVLGYMQLRKGPNIVGPYGLLQPI
Dcpe      -----MMTVLMTHIINPLAYIVPVLLAVAFLTLIERKVLGYMQHRKGPNIVGPYGLLQPI
Dcti      -----MVTLLITHIINPLAYIVPVLLAVAFLTLIERKVLGYMQHRKGPNIVGPYGLLQPI
Hehi      -----MISVLITHILNPLAFIVPVLLAVAFLTLLERKVLGYMQLRKGPNIVGPYGLLQPI
Stam      -----MISTLITHIVNPLTFIVPVLLAVAFLTLLERKVLGYMQLRKGPNIVGPYGLLQPI
Hogi      -----MISALITHVINPLTLIVPVLLAVAFLTLLERKVLGYMQARKGPNIVGPYGLLQPI
Erzo      -----MMSALITHLVNPLAFIVPVLLAVAFLTLLERKVLGYMQLRKGPNIVGPYGLLQPI
```

To be continued
on page 8.

[1/6 of aligned sequences]

```
Hxot     -----MMSTLITHIINPLTFIVPVLLAVAFLTLLERKVLGYMQLRKGPNVVGPYGLLQPI
Core     -----MMHTLVTHIVNPLTFIVPVLLAVAFLTLLERKVLGYMQLRKGPNVVGPYGLLQPI
Apve     -----MLPALMTNIVSPLALMAPVLLAVAFLTLVERKLLGYMQLRKGPNVVGPYGLLQPF
Latj     -----MISTLTTCIVNPLALIVPVLLAVAFLTLIERKVLGYMQLRKGPNIVGPYGLLQPI
Laja     -----MTSTLITHIVNPLAFIVPVLLAVAFLTLLERKVLGYMQLRKGPNVVGPYGVLQPI
Syja     -----MLSLFFTHIINPLALIVPVLLAVAFLTLLERKVLGYMQLRKGPNVVGPYGLLQPI
Epme     -----MISTLFTHIINPLAYIVPVLLAVAFLTLVERKVLGYMQFRKGPNIVGPYGLLQPI
Grse     -----MIHGLFTHLLNPLAYIVPVLLAVAFLTLVERKVLGYMQLRKGPNIVGPYGLLQPI
Clja     -----MTTTLILYIINPLSFIVPVLLAVAFLTLLERKVLGYMQLRKGPNVVGPYGLFQPI
Ogcy     ---------MIPAVINSLTLIIFILLAVALLTLVERKVLGYMQLRKGPNVVGPYGLLQPI
Plna     -----MTSTFLSFILSPLILTLFVLLAVALLTLVERKVLGYMQLRKGPNVVGPYGILQPI
Lema     -----MISALITHIVNPLAFIVPVLLAVAFLTLFERKVLGYMQLRKGPNIVGPYGLLQPI
Etzo     -----MISVLITHIINPLAFIVPVLLAVAFLTLLERKVLGYMQLRKGPNIVGPYGLLQPI
Apse     -----MISIILVHVINPLAYIVPVLLAVAFFTLIERKVLGYMQLRKGPNVVGPYGLLQPI
Epde     -----MISTLITYIINPLAFIVPVLLAVAFLTLIERKVLGYMQLRKGPNVVGPYGLLQPI
Slja     -----MLSMLFTHIINPLAFIVPVLLAVAFLTLLERKVLGYMQLRKGPNIIGPYGLLQPI
Bsja     -----MMSMLITHIINPLIFIVPVLLAVAFLTLVERKVLGYMQLRKGPNVVGPYGLLQPI
Ecna     -----MISTLLTHIINPLTFIVPVLLAVAFLTLLERKVLGYMQHRKGPNIVGPYGLLQPI
Cohi     -----MWTDILTHILNPAAIILPLLLAVAFMTLLERKLLSYMQLRKGPNVVGPYGALQPF
Caar     -----MITALITHLLNPLAFIVPVLLAVAFLTLIERKVLGYMQLRKGPNVVGPYGLLQPI
Came     -----MITALITHLLNPLAFIVPVLLAVAFLTLIERKVLGYMQLRKGPNVVGPYGLLQPI
Mema     -----MISALITHIINPLAFIVPVLLAVAFLTLIERKVLGYMQLRKGPNVVGPYGLLQPI
Lenu     -----MTSALISHIITPLSVIVPVLLAVAFLTLVERKVLGYMQLRKGPNVVGPYGLLQPI
Brja     -----MISTLMTHILSPLTFIVPVLLAVAFLTLIERKVLGYMQLRKGPNIVGPYGLLQPI
Plma     -----MITTLMNHILNPLAFIVPVLLAVAFLTLIERKVLGYMQLRKGPNIVGPYGLLQPI
Emst     -----MISTLITHIINPLTFIVPVLLAVAFLTLLERKVLGYMQLRKGPNIVGPYGLLQPI
Ptti     -----MISTLITHIINPLAFIVPVLLAVAFLTLIERKVLGYMQLRKGPNIVGPYGLLQPI
Losu     -----ISLMLMSYIINPLSLIIPVLLAVAFLTLLERKVLSYMQLRKGPNVVGPYGLLQPI
Geoy     -----MISVLITHILNPLAFIVPVLLAVAFLTLIERKVLGYMQLRKGPNVVGPYGLLQPI
Dipi     -----MISTLITHIINPLTFIVPVLLAVAFLTLIERKVLGYMQLRKGPNIVGPYGLLQPI
Pama     -----MLCTLFTHLINPLAFIVPVLLAVAFLTLLERKVLGYMQLRKGPNIVGPYGLLQPI
Leob     ------MTTLITHIINPLAYIVPVLLAVAFLTLLERKVLGYMQHRKGPNIVGPYGLLQPL
Neba     -----MMVMFTTHIINPFIFIVPVLLAVAFLTLLERKVLGYMQLRKGPNVVGPYGLLQPI
Pdpl     -----MFSHLLTHLLNPLAYIIPVLLAVAFLTLIERKVLGYMQLRKGPNIVGPYGLLQPI
Nimi     -----MIHTMMTHIISPLAFIVPVLLAVAFLTLLERKVLGYMQLRKGPNIVGPYGLLQPI
Uptr     -----MISTLVIHIINPLAFIVPVLLAVAFLTLLERKVLGYMQLRKGPNVVGPYGLLQPI
Pesc     -----MLASLISNVFNPLALIVPVLLAVAFLTLIERKVLGYMQMRKGPNIVGPYGLLQPI
Baar     ---LNALTPIFTHILSPLSYIVAVLLAVAFLTLIERKVLGYMQLRKGPNVVGPYGLLQPL
Moar     -----MITTLITHIINPLAFIVPVLLAVAFLTLLERKVLGYMQLRKGPNIVGPYGLLQPI
Toja     -----MTSALMTHIMNPLAFIVPVLLAVAFLTLIERKVLGYMQLRKGPNIVGPYGLLQPI
Chau     -----MPSTLIIYLINPLTFIIPVLLAVAFLTLLERKVLGYMQLRKGPNVVGPYGLLQPI
Chse     -----MASTLITHIINPLTYIVPVLLAVAFLTLVERKVLGYMQLRKGPNIVGPYGLLQPI
Enar     -----MISTLITHILNPLAFIVPVLLAVAFLTLIERKVLGYMQLRKGPNIVGPYGLLQPI
Hpty     -----MISTLITHIINPLAFIVPVLLAVAFLTLIERKVLGYMQLRKGPNIVGPYGLLQPI
Nana     -----MTTALITHLINPLAFIIPVLLAVAFFTLVERKVLGYMQLRKGPNIVGPYGLLQPI
Mcst     -----MIQTLITHIIHPLTFIIPVLLAVAFLTLLERKVLGYMQLRKGPNIVGPYGLLQPI
Rhox     -----MISALMTQILNPLIFIVPVLLAVAFFTLLERKVLGYMQLRKGPNIVGPYGLLQPI
Opfa     -----MISTLITHIINPLAFIVPVLLAVAFLTLLERKVLGYMQLRKGPNIVGPYGLLQPI
Paar     ------MVTIMMHVVNPLAFIVPVLLAVAFLTLIERKVLGYMQLRKGPNIVGPFGLLQPI
Gozo     -----MISTLITHIINPLAFIVPVLLAVAFLTLVERKVLGYMQLRKGPNIVGPYGLLQPI
Ackr     -----MIKALLAHIIHPLTYIVPILLAVAFLTLIERKVLGYMQLRKGPNVVGPYGLLQPI
Elev     -----MLLTLMTHLINPLAFIIPILLAVAFLTLLERKVLGYMQLRKGPNIVGPYGLLQPV
```

To be continued on page 9.

[1/6 of aligned sequences]

```
Trdu      -----MLSTLISSILNPLILIVFVLLAVALLTLVERKVLGYMQLRKGPNIVGPYGLLQPI
Amoc      -----MLSALITLILNPLILIIFILLAVALLTLVERKVLGYMQLRKGPNVVGPYGLLQPI
Hame      -----MISMLVTHILNPLAYIVPVLLAVAFLTLIERKVLGYMQLRKGPNIVGPYGLLQPI
Chso      ------MYAFALYLINALALIVPVLLAVAFLTLVERKVLGYMQLRKGPNVVGPYGLLQPI
Lyto      -----MMTTIMTHIINPLTFIVPVLLAVAFLTLLERKVLGYMQLRKGPNIVGPYGLLQPI
Encr      -----MMSLIITHIINPLTFIVPVLLAVAFLTLLERKVLGYMQLRKGPNIVGPYGLLQPI
Bvar      -----MPPMLMTHLLNPLTLIVPVLLAVAFLTLIERKVLGYMQLRKGPNIVGPYGLLQPI
Noco      -----MLLTLLTQIVNPLAYIVPVLLAVAFLTLLERKVLGYMQLRKGPNIVGPYGLLHPI
Chsp      -------MVIMTHIINPLTMIVPLLLAVAFLTLFERKILGYMQLRKGPNIVGPSGLLQPI
Arja      -----MASTIIINIINPLALIAPVLLSVAFFTLLERKILGYMQLRKGPNVVGPVGLLQPF
Pase      -----MLTTLITHVINPLAYIVPVLLAVAFLTLLERKVLGYMQLRKGPNVVGPYGLLQPI
Trel      -------MTPLTDLLNPLLIIVPVLLAVAFLTLIERKVLGYMQLRKGPNIVGPYGLLQPV
Lifa      -----VLFSYIMDIINLLARIVPVLLAVAFLTLIERKVLGYMQLRKGPNIVGPYGILQPI
Acur      -----MLATLLTHILNPLAIIVPILLAVAFFTLIERKVLGYMQLRKGPNVVGPYGLLQPI
Ampe      -----MISALMTHIVNPLAYIVPILLAVAFLTLLERKVLGYMQLRKGPNIVGPYGLLQPI
Urja      -----MTSNLLTHLVNPLGYIVPILLAVAFLTLLERKVLGYMQLRKGPNIVGPFGLLQPM
Enet      -----MLPILASYLINPLVLIVFVLLAVALLTLVERKVLGYMQLRKGPNVVGPYGLLQPI
Ptbr      -----LTSALLLYVIQPLILVVPLLLAVALLTLVERKILGHMQARKGPNVVGPTGLLQPF
Safa      -----MMTTILSSIIHPLILIVFVLLAVALLTLVERKVLGYMQLRKGPNVVGPYGLLQPI
Icae      -----MISALMTHILNPLAFIVPVLLAVAFLTLIERKVLGYMQLRKGPNIVGPYGLLQPI
Asmi      -----MIAPILTHLVCPLALIVFILLSVALLTLLERKVLGYMQFRKGPNIVGPYGLLQPI
Foal      -----MLSNFMVYLINPLTFIVPVLLAVAFLTLIERKVLGYMQLRKGPNVVGPYGLLQPV
Drze      -----MISAVMTYIIHPLTLIIPILLAVAFLTLLERKVLGYMQLRKGPNIVGPYGLLQPI
Rhas      -----MIPTILIHILNPLAYIVPVLLAVAFLTLLERKVLGYMQLRKGPNVVGPYGLLQPI
Elac      -----MISLILTHILNPLAYIVPVLLAVAFLTLLERKVLGYMQLRKGPNVVGPYGLLQPI
Kugu      -----MTSIILTHILNPLTLIIPVLLAVAFLTLIERKVLGYMQLRKGPNIVGPYGLLQPV
Plor      -----MISLLITHILNPLAIIVPVLLAVAFLTLLERKVLGYMQLRKGPNIVGPYGLLQPI
Sgun      -----MISTLITHIINPLAYIVPVLLAVAFLTLIERKVLGYMQLRKGPNIVGPYGLLQPI
Zaco      -----MISTLITHIINPLAFIVPVLLAVAFLTLIERKVLGYMQLRKGPNIVGPYGLLQPI
Zbfl      -----MTSTLAAHIINALSFMVPVLLAVAFLTLLERKVLGYMQLRKGPNVVGPYGALQPV
Spba      -----MMSLLITHVINPLAFIVPVLLAVAFLTLIERKVLGYMQLRKGPNIVGPYGLLQPI
Game      -----MITALVTHILNPLAFIVPVLLAVAFLTLIERKVLGYMQLRKGPNIVGPYGLLQPI
Thth      -----MITALMTHILNPLAFIVPVLLAVAFLTLIERKVLGYMQLRKGPNIVGPYGLLQPI
Xigl      -----MMSALITHIINPLAFIVPVLLAVAFLTLIERKVLGYMQLRKGPNIVGPYGLLQPI
Hyja      -----MIHLLMTHILNPLTFIVPVLLAVAFLTLLERKVLGYMQLRKGPNIVGPYGLLQPI
Psan      -----MLKHLATHLIHPLSYIVPTLVAVAYLTLVERKVLSYMQARKGPNVVGPYALLQPL
Cupa      -----MITALMTHLLNPLAFIVPVLLAVAFLTLIERKVLGYMQLRKGPNIVGPYGLLQPI
Mpch      -----MISILLTHIINPLIYIVPVLLAVAFFTLVERKVLGYMQLRKGPNVVGPFGLLQPI
Char      -----MISLLTTHIINPLAFIVPILLAVAFLTLLERKVLGYMQLRKGPNIVGPYGLLQPV
Pser      -----MISSLISYIINPLALIVPVLLAVAFLTLIERKVLGYMQLRKGPNIVGPYGLLQPI
Prol      -----MISTLITHIINPLALIVPVLLAVAFLTLLERKVLGYMQLRKGPNIVGPYGLLQPI
Plbi      -----MISALITHIINPLAFIVPVLLAVAFLTLLERKVLGYMQLRKGPNIVGPYGLLQPI
Calu      ---VFDMSILLTHLINPLVIIALVLLAVAFLTLIERKILGYMQLRKGPNIVGPYGVFQPF
Papa      -----MISGTMLYVINPLALIVPVLLAVAFLTLVERKILGYMQLRKGPNVVGPCGLLQPI
Sufr      -----MISTLITHVISPLAFIVPILLAVAFLTLVERKVLGYMQLRKGPNVVGPYGLLQPI
Stci      -----MISTLITHVINPLTYIVPVLLAVAFLTLIERKVLGYMQLRKGPNIVGPYGLLQPI
Taru      -----MTTMLITHLIHPLTIIVPVLLAVAFLTLIERKVLGYMQLRKGPNIVGPYGLLQPI
Rala      -----MISTLITHIINPLAFIVPVLLAVAFLTLIERKVLGYMQLRKGPNIVGPYGLLQPI
                                 :::  : :** **. :.  * ***       . :: .
```

To be continued
on page 10.

[2/6 of aligned sequences]

	B / C
Scca	ADGLKLFIKEPIRPSASSRSYFLATPTVALALALLMWMPLPLPHSIINLNLGLLFILAIS
Muma	ADGLKLFIKEPVRPSASSPFLFLATPTAALTLALLMWMPLPLPHPIINLNLGLLFILAIS
Erca	ADGVKLFIKEPVKPSTSSPTLFLLTPILALTLALMLWVPLPMPFALTDLNLTILFILAVS
Pose	ADGVKLFIKEPVKPSTASPTLFLLTPTLALTLALILWIPLPMPLALTDLNLTILFILAVS
Actr	ADGIKLFIKEPVRPTTASPFLFLAAPIMALTLALTLWMPLPMPYPVADLNLGILFILALS
Scal	ADGIKLFIKEPVRPTTASPFLFLATPIMALTLALTLWMPLPMPYPIADLNLGILFILALS
Posp	ADGIKLFIKEPVPPTTASPLLFLATPILALTLALTLWMPLPMPYSIADLNLGILFILALS
Atsp	ADGMKLFIKEPIRPSTSSPLLFLATPILALTLALTLWAPMPFPYPLIDLNLGILFILALS
Leoc	ADGMKLFIKEPIRPSTSSPLLFLVTPMLALTLALTLWAPMPFPYPLIDLNLGILFILALS
Amca	ADGLKLFIKEPVRPSTASPLLFLTAPILALTLALALWAPLPLPYPIIDLNLGMLFILALS
Osbi	ADGVKLFIKEPVRPYASSPILFLITPMLALTLALTMWIPMPIPYPVIDINLGVLFILAIS
Pabu	ADGVKLFIKEPVRPSTSSPLLFLITPTLALTLALTLWAPMPMPYPVLDMNLGILFILAIS
Hial	ADGVKLFIKEPVRPSTSSPFLFLATPMLALTLALTLWAPMPIPYPVVDMNLGVLFVLALS
Elha	ADGVKLFIKEPVRPSTSSPFLFLITPMMALTLALTLWAPMPIPYPVIDLNLGILFVLALS
Mlcy	ADGVKLFIKEPVRPSTSSPFLFLATPTLALTLALTLWAPMPMPHPVTDLNLGILFILALS
Algl	ADGVKLFIKEPVRPSTSSPFLFLATPMLALALALALWAPMPIPYPVMDLNLGVLFMLALS
Ptgi	ADGVKLFIKEPVRPSTSSPFLFLATPMLALTLALTLWAPMPIPYPVVDLNLGILFVLALS
Alaf	ADGVKLFIKEPVRPSTASPVLFLATPMLALTLALTLWAPMPIPYPVVDLNLGILFVLALS
Nock	ADGVKLFIKEPVRPSTSSPFLFLMTPMMALILALTLWAPMPIPYPVVDLNLGILFVLALS
Anja	ADGVKLFIKEPVRPSTASPFLFLATPTLALTLALTLWAPMPMPYPVVELNLGILFVLALS
Gyki	ADGVKLFIKEPIRPSTSSPFLFLATPTLALTLALMMWAPIPMPYPVITLNLGMLFVLALS
Syka	ADGVKLFIKEPIRPSTASPFLFLATPTLALTLALTLWAPMPMPYPVVELNLGILFILALS
Opma	ADGVKLFIKEPVRPSNASPILFIIAPTMALALALTLWAPMPMPYPVVEMNLGILFILALS
Comy	ADGVKLFIKEPVRPSTSSPILFLIAPTMALTIALTMWAPMPMPYPVVELSLGVLFVLALS
Sasp	ADGVKLLTKEPVRPSISAPFLFLSTPTLALMLALVMWAPIPMPFPVLELNLSVVFVLAMS
Eupe	ADGMKLFTKEPVRPSTSAPLLFLITPILALTLALTMWAVMPMPFPMVEMNLSMMFILALS
Enja	ADGVKLFIKEPVRPSTSAPFLFLATPTLALTLALTLWAPLPMPHPVTDLNLSMLFILALS
Same	ADGVKLFLKEPVRPSTSSPLLFLVTPTLALTLALTLWAPLPMPFPVVDLNLSMLFILALS
Chch	ADGVKLFIKEPVRPSTSSPFLFLATPMLALTLALMLWAPMPIPYPVIDLNLGILFILALS
Grgr	ADGVKLFIKEPVRPSVASPFLFLLAPVLALALALTLWAPMPLPHPIVDLNLGILFMLALS
Caau	ADGVKLFIKEPVRPSTSSPFLFLAAPVLALTLAMTLWAPMPMPHPVTDLNLGILFILALS
Cyca	ADGVKLFIKEPVRPSTSSPFLFLAAPVLALTLAMTLWAPMPMPHPVTDLNLGILFILALS
Dare	ADGVKLFIKEPIRPSMASPILFLTAPVLALILAMMLWAPMPMPYPVLDLNLGILFIMAIS
Cost	ADGVKLFIKEPIRPSTSSPFLFLATPMLALTLAMTLWAPIPMPHPVTDLNLGVLFILALS
Leec	ADGIKLFIKEPIRPSTSSPFLFLATPMLALTLAMTLWAPMPMPHPVTDLNLGILFLLALS
Fola	ADGVKLFIKEPIRPSTASPTLFLVTPMLALTLAMTLWAPMPMPHPIIDLNLGVLFILALS
Clmc	ADGVKLFIKEPVRPSTSSPFLFLITPMLALTLALTLWAPMPIPYPVTDLHLGILFVLALS
Phin	ADGIKLFIKEPVRPSTSSPFLFLAAPTLALTLALMLWIPMPTPYPIADLNLGILFILALS
Icpu	ADGVKLFIKEPIRPSTSSPFLFLTAPMLALTLALMLWAPMPLPHPVTDLNLGMLFILALS
Psto	ADGVKLFIKEPVRPSTSSPFLFLATPMLALTLALTLWAPMPMPHSMTDLNLGMLFILALS
Cora	ADGVKLFIKEPVRPSTSSPFLFLATPMLAFTLAMTLWAPMPIPYPVMDLNLGILFILALS
Eisp	ADGLKLFIKEPVRPSVASPLLFLITPMLALTLALTLWAPMPLPHPMIDLNLGILFILALS
Apal	ADGVKLFIKEPIRPLASSPVLFLLTPIMALSLALIMWTPMPIPYPMTDLNLGMLFILAIS
Eslu	ADGVKLFIKEPIRPSTSSPLLFLFTPMLALTLALTLWAPMPIPYPVADLNLGILFILALS
Dape	ADGMKLFIKEPVRPSTASPFLFLLTPMLALTLALTLWAPMPLPYPLIDMNLGVLFMLALS
Glse	ADGVKLFIKEPVRPSTSSPFLFLATPMLALTLALTLWAPMPIPYPVTDLNLGVLFILALS
Naar	ADGVKLFIKEPVRPSTSSPFLFLATPVLALTLALALWAPMPIPYPVTDLNLGILFVLALS
Lioc	ADGVKLFIKEPVRPSTSSPFLFLATPILALTLALALWAPMPIPYPVTDLNLGILFVLALS
Opso	ADGLKLFIKEPVRPSTSSPFLFLTTPILALTLALILWAPMPLPYPVMDLNLGVLFILALS
Alte	ADGLKLFIKEPVRPSTSSPFLFLVTPMLALTLALTLWAPMPIPYPVADLNLGILFVLALS
Plap	ADGVKLFIKEPVRPSTSSPFLFLATPMLALTLALTLWAPMPIPYPVTDLNLGILFVLALS

To be continued on page 11.

[2/6 of aligned sequences]

```
Plal    ADGVKLFIKEPIRPSTSSPFLFLATPMLALTLALTLWAPMPIPYPVADLNLGILFVLALS
Sami    ADGVKLFIKEPIRPSTSSPFLFLATPMLALTLALTLWAPMPIPYPVADLNLGVLFVLALS
Rere    ADGVKLFIKEPVRPSTASPFLFLATPMLALTLALTLWAPMPIPYPITDLNLGVLFVLALS
Gama    ADGVKLFIKEPVRPSTSSPFLFLATPMLALTLALTLWAPMPIPYPVADFNLGILFVLALS
Onmy    ADGLKLFIKETVRPSTSSPFLFLATPMLALTLALTLWAPMPIPYPVTDLNLGVLFVLALS
Sasa    ADGLKLFIKEPVRPSTSSPFLFLATPMLALTLALTLWAPMPIPYPITDLNLGVLFVLALS
Cola    ADGVKLFIKEPIRPSTSSPFLFLATPMLALTLALTLWAPMPIPYPVTDLGLGVLFVLALS
Dita    ADGIKLFIKEPIRPSAASPFLFLAAPMLALTLALTLWAPMPMPYPVTDLNLGMLFILALS
Gogr    ADGVKLFTKEPIRPSTSSPLMFLATPILALTLALLLWTPLPAPYPAADLNLGLLFILALS
Chsl    ADGVKLFIKEPVRPSTSFSALFLGTPTLALTLALLLWTPLPMPHPVVDLNLGLLFILAVS
Atja    ADGVKLFIKEPVRPSTSSPILFLSTPMLALTLALVLWAPMPMPHPITNMNLGVLFMLAMS
Iido    ADGVKLFIKEPVRPSTSSPILFLSTPMLALTLALVLWTPMPMPHPVTDMNLGVLFMLAMS
Auja    ADGVKLFIKEPVRPSTSSPFLFLVTPMLALTLALTLWAPMPMPYPVTDLNLGILFILALS
Chag    ADGVKLFIKEPIRPSTSSPFLFLATPMLALTLALTLWSPMPIPYPVVDLNLGVLFVLALS
Hami    ADGVKLFIKEPIRPSTSSPFLFLATPMLALTLALTLWAPMPLPYPVTDLNLGILFILALS
Saun    ADGVKLFIKEPIRPSTSSPFLFLATPMLALTLALTLWAPMPLPYPVTDLNLGILFILALS
Nema    ADGVKLFIKEPIRPSTSSPFLFLATPILALTLALTLWAPMPIPYPVTDLNLGILFVLALS
Disp    ADGVKLFIKEPIRPSTSSPFLFLATPILALTLALTLWAPMPIPYPITDMNLGVLFVLALS
Myaf    ADGVKLFIKEPVRPSTSSPFLFLATPVLALTLALTLWAPMPIPFPVTDLSLGILFVLALS
Lagu    ADGLKLFLKEPVHPSTSAPTLFLITPLLALTLVLTPWVVVPIPYPVADLNLSILFILAIS
Trtr    ADGVKLFLKEPIRPATSSPILFLTTPVLALTLALLLWVTIPIPYPLADLNLGLLFILAIS
Zucr    ADGVKLFLKEPIRPATSSPVLFLVTPVLALTLALLLWVTIPIPYPLADLNLGLLFILAIS
Pxja    ADGVKLFIKEPIRPSTSSPLLFLATPMLALTLALTLWAPMPMPYPVVDLNLGILFMLALS
Pxlo    ADGVKLFIKEPIRPSTSSPLLFLATPMLALTLALTLWAPMPMPYPVVDLNLGILFMLALS
Pctr    ADGVKLFIKEPIRPSTSSPILFLATPVLALTLALTLWAPLPMPHPTLDLNLSILFMLALS
Apsa    ADGIKLFTKEPVRPSTASPLLFMITPIMALTLALALWAPIPMPHAILDLNLGILFMLALS
Cabe    ADGVKLFIKEPVRPSAASPILFTAAPALALALALTLWTPVPMPSPMVTMNLSILFIMALS
Bzze    ADGVKLFIKEPVRPSTASPVLFLLAPMLALTLALTLWVPMSLPHPLADLNLSILFILALS
Siim    ADGLKLFIKEPVRPSTSSPVLFLLAPMLALTLALTLWTPMSMPHPLLDLNLSILFILALS
Ctru    ADGLKLFIKEPVRPSTSSPVLFLLTPMLALTLALTLWAPMPLPYPVLDLNLGILFILALS
Dpbr    ADGVKLFIKEPVRPSTASPVLFLLTPMLALTLALTLWAPMPLPHPVLDLNLGILFILALS
Caki    ADGLKLFIKEPIRPSTSSPLLFIITPILALTFALILWTPMPMPIPAADLNLSILFILALS
Phja    ADGVKLFIKEPIQPSTSSPLLFIFAPVLALTLALILWAPLPMPFSMVDLNLTILFILALS
Brsp    ADGIKLFIKEPVRPSTSSPFLFLISPVLALTIALSLWTLMPMPIPSTDLNFSLLFILALS
Gamo    ADGVKLFIKEPIRPSTSSPSLFVL-PVLALTLALTLWAPMPMPFPVADLNLSILFVLALS
Lolo    ADGVKLFIKEPIRPSTSSPLLFILAPVLALTLALTLWAPMPMPFPVADLNLSILFVLALS
Batr    ADGINFFIKEPMKPLISTHTLFLLMPIMALMLALTLWTIPTIPISLLDINLTILFFLAIS
Prmy    ADGIKLFIKEPIKPLFSTFMIFLLSPILALTLALLLWTPLPLPSSTIDLNLPILFFLALS
Lose    ADGLKLFIKEPVRPSTSSPMLFMTAPVLALALALTMWAPMPLPHPTLDLHLGVLFILAIS
Loam    ADGVKLFIKEPVRPSASSPILFLTAPILALTLALTLWAPMPLPYPVLDLNLGILFILALS
Chab    ADGLKLFIKEPVRPSTSSPLLFLATPMLALTLALTLWSPLPLPYPVIDLNLGILFILALS
Chto    ADGLKLFIKEPVRPSTSSPLLFLATPMLALTLALTLWSPLPLPYPVIDLNLGILFILALS
Majo    ADGVKLFIKEPLRPSPSSPALFLFAPILALSLALILWAPLPLPHPFVNLNLGMLFILAVS
Hlst    ADGVKLFIKEFLRPSPSSPLLFTFAPILALSLALILWSPLPLPFPFINLNLGLLFVLAVS
Clpe    ADGLKLFIKEPVRPSTSSPLLFLAAPILALTLALTLWAPMPLPYPIIDLNLGVLFILALS
Mlmr    ADGVKLFIKEPVRPSTSSPLLFLATPMLALTLALTLWAPMPLPYPVIDLNLGILFVLALS
Crcr    ADGLKLFMKEPIRPLTSSPLLFLATPMLALTLALTLWTPLPLPYSLADLNLGILFILALS
Muce    ADGLKLFMKEPIRPLTSSPLLFLTTPMLALALALTLWTPLPLPCPLADLNLGILFILALS
Bege    ADGLKLFLKEPIRPSTSSPILFLFAPMLALTLALTLWAPMPLPFPITDMTLGILFILALS
Mela    ADGLKLFMKEPVRPSTASSLMFLLAPILALTLALTLWAPMPLPSPMADMTLGVLFILAIS
Hats    ADGLKLFMKEPVRPSTSSPILFLLAPMLALTLALTLWAPMPLPFPMVDMTLGILFVLALS
Orla    ADGLKLFTKEPVRPSTSSPALFLITPIMALTLALTLWAPLPLPFPIADLNLGVLFILALS
```

To be continued on page 12.

[2/6 of aligned sequences]

```
Cosa  ADGLKLFMKEPIRPSTSSPVLFLLAPMMALTLALTLWTPMPLPLPLADLNLGILFILALS
Exsp  ADGLKLFMKEPVRPSTSSPILFLLTPMLALTLAITLWAPMPMPFSVADLNLGILFILALS
Depa  ADGLKLFMKEPVRPSTSSPILFLLAPIMALTLALTLWTPLPLPFPLADMNLGILFILALS
Rima  ADGLKLFIKEPVRPLTTSPLLFTFTPLLAFLLALMLWMPLPIPFPLVDLNLGLLFILALS
Fuol  ADGLKLLTKEPISPSTASPFLFIFTPILAFTLALTLWTPIPLPFPIADLNLGILFILALS
Gmaf  ADGVKLLTKEPIRPSTASPLLFLFTPILAFTLALTLWAPLPLPSPLADLNLGILFILALS
Xeei  ADGLKLLIKEPIRPSTASPLLFIMTPILALTLALTLWTPMPLPFPMADLNLGILFILAIS
Pros  ADGVKLFIKEPVRPSTSSPVLFLATPMLALTLALTLWAPMPLPHPVIDLNLGILFLLALS
Scmi  ADGVKLFIKEPVRPSTSSPILFLATPMLALTLALTLWAPMPLPHPVLDLNLGVLFILALS
Rolo  ADGIKLFIKEPVRPSTSSPVLFLATPILALTLALTLWAPMPIPHPVIDLNLGILFVLALS
Cere  ADGVKLFIKEPVRPSAASPILFLVTPIMALTIALTLWAPLSIPYPVADLNLGILYIIALS
Daga  ADGVKLFIKEPIRPSSASPTLFVAAPVMALAIALVLWSPLSIPYPMTDLNLGILFIIALS
Anco  ADGVKLFIKEPVRPSTSSPVLFLATPILALTLALTLWAPMPFPYPILDLNLGLLFILALS
Dmve  ADGIKLFIKEPVRPSTSSPALFLAAPVLSLTLALTLWAPLPIPHPVMDLNLGILVILTLS
Dmar  ADGIKLFIKEPVGPSTSSSALFLAAPVLSLTLALTLWAPLPIPHSVMDLNLGILVILTLS
Anka  ADGVKLFIKEPVRPSTSSPVLFLATPMLALTLALTLWAPMPIPYPVLDLNLGILFILALS
Moja  ADGVKLFIKEPVRPSTSSPVLFLATPMLALTLALTLWAPMPIPYPVLDLNLGILFILALS
Hoja  ADGVKLFIKEPVRPSTSSPVLFLATPMLALTLALTLWAPMPTPYPVLDLNLGILFVLALS
Bede  ADGVKLFIKEPVRPSTSSPVLFLITPMLALTLALTLWAPMPLPYPVVDLNLGILFILALS
Besp  ADGVKLFIKEPVRPSTSSPVLFLVTPMLALTLALTLWAPMPLPYPVIDLNLGILFILALS
Mysp  ADGVKLFIKEPVRPSTSSPVLFLATPMLALTLALTLWAPMPIPYPVIDLNLGILFVLALS
Osja  ADGVKLFIKEPVRPSTSSPILFLVTPMLALTLALTLWAPMPLPYPVVDLNLGILFVLALS
Sgro  ADGVKLFIKEPVRPSTSSPVLFLATPMLALTLALTLWAPMPIPYPVIDLNLGILFVLALS
Pzpa  ADGVKLFIKEPVRPSTASPTLFIISPALALTLALTLWAPLPLPFPVTDLNLGILFMLALS
Zeja  ADGVKLFIKEPIRPSTSTPALFIFSPMLALTLALVLWAPLPLPFSIIDLNLAILFMLALS
Znne  ADGLKLFIKEPVRPSTSSPSLFIFSPMLALTLALILWAPLPLPFSVLDLNLGVLFLLALS
Zefa  ADGVKLFIKEPIRPSTSAPVLFIFSPMLALTLALMLWAPLPLPFPVIDLNLGVLFMLALS
Acni  ADGVKLFIKEPIRPSTSSPALFIFSPMLALTLALVLWAPLPLPFPVIDLNLGILFMLALS
Ncrh  ADGAKLFIKEPIRPSTSSPALFIFSPMLALTLALVLWAPLPLPFPVIDLNLGILFMLALS
Agca  ADGVKLFIKEPVRPSVSSPVLFLLTPMLALTLALTLWAPMPMPYPVVDLNLGVLFLLALS
Hydy  ADGLKLFIKEPIRPSTASPVLFLLAPILALTLALTLWAPMPLPYPIIDLNLGVLFILALS
Gsac  ADGLKLFIKEPIRPSTASPLLFLLAPILALTLALALWAPMPIPHPVIDLNLGIMFILALS
Pevo  ADGVKLFIKEPVRPSAASPLLFILTPTLALTLALILWAPIPAPYPMADLNLGILFILALS
Hiku  ADGVKLFIKEPVKPSTSSPILFLATPMLALTLALMLWAPLPMPHPVVNVNLGMLLILALS
Inpa  ADGVKLFIKEPLRPSTASPALFLLAPTLALTLALALWAPLPLPHPLIDLNLTILFILALS
Auch  ADGMSLFTKEPVRPSTASPFLFILAPILALTLALTLWAPLPLPYPLMDLNLGIMFILALS
Fico  ADGVKLFIKEPVRPSTSSPILFLLAPMLALTLALTLWAPIPMPCPVADLNLGILFILAIS
Macs  ADGVKLFIKEPVRPSTSSPILFLLAPMLALTLALTLWAPMPLPYPVTDLNLGVLFILALS
Moal  ADGLKLFVKEPILPSTSSQILFILSPMLALTLALTLWAPLPLPSPAADLNLNILFILALS
Mafr  ADGVKLFIKEPIRPSTSSQFLFLLAPMLALTLSLTLWAPIPLPYPLIDLNLTILFILALS
Dcpe  ADGLKLFIKEPVRPSTSSPVLFLLTPMLALTLALMLWAPMPLPYPVTDLNLGILFILAIS
Dcti  ADGLKLFIKEPVRPSTSSPVLFLLTPMLALTLALMLWAPMPLPYPVTDLNLGILFILAIS
Hehi  ADGVKLFIKEPVRPSTSSPVLFLLAPILALTLALTLWAPMPLPYPVIDLNLGILFILALS
Stam  ADGVKLFIKEPVRPSTASPVLFLLAPMLALTLALTLWAPMPMPYPVIDLNLGILFILALS
Hogi  ADGVKLFIKEPVRPSTASPVLFLFAPMLALTLALSLWAPMPLPYPMIDLNLGMLFILALS
Erzo  ADGLKLFIKEPIRPSTASPLLFLLTPMLALTLALTLWAPMPMPYPVVDLNLGILFILALS
Hxot  ADGLKLFIKEPVRPSTSSPVLFLLAPMLALTLALTLWAPMPLPYPVIDLNLGILFLLALS
Core  ADGLKLFIKEPVRPSTSSPVLFLLAPILALTLALTLWAPMPLPYPVFDLNLGILFILALS
Apve  ADGLKLFTKEPIRPLSSAPTMFILTPVLALALALTMWAPLPLPYPIIDLNLGILFILALS
Latj  ADGVKLFIKEPIRPSTSSPLLFLMTPMLALTLALTLWAPMPLPYPVADLNLGVLFILALS
Laja  ADGLKLFIKEPVRPSTSSPVLFLLTPMLALTLALTLWAPMPLPYPTADLNLSILFILALS
Syja  ADGVKLFIKEPVRPSTASPVLFLLAPMLALTLALILWAPMPMPHSMTDLNLSILFILALS
```

To be continued on page 13.

[2/6 of aligned sequences]

To be continued on page 14.

Epme	ADGVKLFTKEPVRPSTASPILFLLAPMLALTLALTLWAPLPMPYPVLDLNLGILFILALS
Grse	ADGVKLFIKEPVRPSTASPVLFLLAPILALTLALTLWAPMPLPYPLLDLNLGVLFILALS
Clja	ADGMKLFIKEPVRPSTSSPLLFITTPMLALTLALTLWAPMPMPSPVINLNMGILFIMALS
Ogcy	ADGLKLFLKEPIRPSASSPILFMIAPMMALTLALTLWTPMPMPYPLADLNLGVLFILALS
Plna	ADGLKLFMKEPVRPSTSSPVLFLLSPMLALALALTLWAPMPLPFPLTDLNLSILFILALS
Lema	ADGVKLFIKEPVRPSTSSPALFILTPMLALTLALTLWAPMPLPYPVTDLNLGILFILALS
Etzo	ADGVKLFIKEPVRPSTASPLLFLLTPILALTLALTLWAPMPMPYPVVDLNLGILFLLALS
Apse	ADGVKLFIKEPIRPSTSSPVLFLLAPMLTLTLALILWAPMPMPYPVIDINLGILFILALS
Epde	ADGVKLFIKEPVRPSTSSPVLFLLAPMLALTLALTLWAPLPMPYPVADLNLGVLFILALS
Slja	ADGVKLFIKEPIRPSTSSPVLFLAAPMLALTLALTLWAPMPLPYPVIDLNLGIMFILALS
Bsja	ADGVKLFIKEPVKPSTSSPILFLLAPMLALTLALTLWAPMPLPYPVIDLNLGILFVLALS
Ecna	ADGVKLFIKEPVRPSASSPTLFILAPILALTLALTLWAPLPLPYPVIDLNLGILFILALS
Cohi	ADGIKLFLKELIRPSSSSPLTFFTGPAMALMLALLMWAPAPIPFSLVSINLSTLYILALS
Caar	ADGVKLFIKEPVRPSTSSPVLFLLAPMLALTLALTLWAPMPLPYPVADLNLGILFILALS
Came	ADGVKLFIKEPVRPSTSSPILFLLAPMLALTLALTLWAPMPLPYPVADLNLGILFILALS
Mema	ADGVKLFIKEPVRPSTSSPILFLLTPMLALTLALTLWAPMPMPYPVVDLNLGILFILALS
Lenu	ADGVKLFIKEPIRPSAASPVLFLLAPILALTLALTLWAPMPMPYPMTNLNLSILFILALS
Brja	ADGVKLFIKEPVRPSTSSPVLFLLAPMLALTLALTLWAPMPLPYPVIDLNLGILFILALS
Plma	ADGVKLFIKEPIRPSTSSPVLFLLAPMLALTLALTLWAPMPLPYPVADLNLSILFILALS
Emst	ADGVKLFIKEPVRPSTSSPILFLLTPMLALTLALTLWAPMPLPYPVIDLNLGILFVLALS
Ptti	ADGVKLFIKEPVRPSTSSPLLFLLTPMLALTLALTLWAPMPLPYPVIDLNLGILFILALS
Losu	ADGIKLFIKEPIRPSTASPLLFSISPVLALTIALMLWMPMPIPYPVLDLNLAILFILALS
Geoy	ADGVKLFIKEPVRPSTSSPILFLFTPMLALTLALSLWAPMPMPFPIADLNLGILFILALS
Dipi	ADGVKLFIKEPVRPSTSSPLLFLLTPMLALTLALTLWAPMPLPYPVVDLNLGILFILALS
Pama	ADGVKLFIKEPVRPSTSSPVLFLLAPMLALTLALTLWAPMPMPYSVIDLNLGILFILALS
Leob	ADGVKLFIKEPVRPSTSSPILFLLTPMLALTLALTLWAPMPMPYPVIDLNLGILFILALS
Neba	ADGLKLFIKEPVRPYTSSPLLFMLTPMLALTLALTLWAPMPMPYPVVDFNLGIMFLLAVS
Pdpl	ADGVKLFIKEPVRPSTSSPVLFLIAPMLALTLALVLWAPMPMPHPVIDINLGVLFILALS
Nimi	ADGIKLFIKEPIRPSTSSPILFIATPMLALTLALTLWAPMPLPYPVIDLNLGILFILALS
Uptr	ADGVKLFIKEPIRPSTASPVLFLLAPMLALTLALTLWAPMPLPYPVTDLNLGILFILALS
Pesc	ADGVKLFLKEPVRPSTSSPILFLFTPVMALAIALTLWTPMPMPFPVVDFGLGILLILALS
Baar	ADGVKLFIKEPVRPSTSSPVLFLLTPMLALTLALTLWAPLPLPYPVTDLNLSILFILALS
Moar	ADGVKLFIKEPVRPSTSSPLLFLLTPMLALTLALTLWSPMPLPYPVIDLNLGILFILALS
Toja	ADGVKLFIKEPIRPSTASPVLFLLTPTMALTIALTLWAPMPIPYPVTDLNLGILFILALS
Chau	ADGLKLFIKEPVRPSTSSPLLFLLTPALALTLALTLWAPMPLPYPVIDLNLGILFILALS
Chse	ADGVKLFIKEPIRPSTSSPLLFLISPIMALTLALALWAPMPLPHPVIDLNLGILFILALS
Enar	ADGVKLFIKEPVRPSTSSPVLFLLTPMLALTLALTLWAPMPLPYPVTDLNLGILFILALS
Hpty	ADGVKLFIKEPVRPSTSSPILFLLTPMLALTLALTLWAPMPIPYPVTDLNLSILFVLALS
Nana	ADGVKLFLKEPVRPSTSSPILFILTPMLALTLALIMWAPLPLPHPIIDLNLGILFIMALS
Mcst	ADGLKLFIKEPVRPSTSSPILFLLAPMLALTLALTLWAPMPLPYPVVDLNLGILFILALS
Rhox	ADGVKLFIKEPVRPSTSSPVLFLLTPMLALTLALALWTPLPLPYPMADLNLGILFILALS
Opfa	ADGVKLFIKEPVRPSTSSPVLFLLTPMLALTLALTLWAPMPLPYPVVDLNLGILFILALS
Paar	ADGVKLFIKEPVRPSTSSPVLFLLTPILALTLALTLWAPMPMPYPVADLNLGILFILALS
Gozo	ADGVKLFIKEPVRPSTSSPVLFLLTPMLALTLALTLWAPMPMPYPVIDLNLGILFILALS
Ackr	ADGIKLFIKEPIRPSTSSPLLFIFSPMLALSLALILWAPMPMPYSFADINLTLLFILALS
Elev	ADGLKLFTKEPVRPSTSSPLLFIFTPMFALILALMLWSPMPLPYPVTDLNLSILFILALS
Trdu	ADGLKLFMKEPVRPSTSSPMLFLFTPVLALTLALTLWTPMPLPFPMADLNLGILFILALS
Amoc	ADGLKLFMKEPVRPSTSSPILFLFMPMLALTLALTLWTPMPLPFPMADLNLGILFILAIS
Hame	ADGVKLFIKEPVRPSTSSPVLFLLAPMLALTLALLLWTPLPLPYPVADLNLGILFILAIS
Chso	ADGVKLFIKEPVRPSTSSPILFIASPTLALTLALTLWAPMPLPYPLADLNLGVLFLLAIS
Lyto	ADGLKLFIKEPVRPSTASPILFLLAPMLALTLALTLWAPLPLPYPVVDLNLGILFILALS
Encr	ADGLKLFIKEPVRPSTASPVLFLLAPMLALTLALTLWAPMPLPYPVIDLNLGILFILALS

[2/6 of aligned sequences]

```
Bvar    ADGLKLFTKEPVRPSTASPILFLLTPMLALTLALTLWTPMPSPYPMADINLGILFILALS
Noco    ADGLKLFTKEPIRPSTSSPLLFLLAPILALTLALALWAPLPLPHPVVDLNLGVLFVLALS
Chsp    ADGLKLFTKEPVRPSTSSPVLFLLAPVLALTLALMLWFPMPLPHPIMEINLNLLLILAIS
Arja    ADGIKLFTKEGLRPLSSSPTMFLLTPVLALTLALGLWSPVPMPKPLVDMNLGILFILALS
Pase    ADGVKLFIKEPVRPSTASPLLFILAPTLALTLALILWSPMPIPHPVMDLNLGILFVLAIS
Trel    ADGVKLFTKEPVRPSTSSPLLFLFAPLLALAISLLLWTPIPLPYPLVDLDLTILFILALS
Lifa    ADGVKLFIKEPIRPSASSPVLFAIAPILALTLALALWTPMPLPYPLADLNLAIMFLLALS
Acur    ADGVKLFIKEPVRPSASSPLLFVLAPMLALILAMVLWTPMPLPYPLVEFNLSIMLVLALS
Ampe    ADGLKLFIKEPVRPSTASPVLFLLAPMLALTLALTLWAPIPMPYPVADLNLGILFILALS
Urja    ADGLKLFIKEPVRPTASYPILFLFSPLLALTVALILWAPMPMPHPVVDLNFGILFILALS
Enet    ADGLKLFMKEPVRPSTSSPVLFLLAPMLALTLALTLWTPIPLPFPLADLNLGILFVLSIS
Ptbr    ADGLKLFIKEPSLPSTSSPILFTLTPMLAFTLALTLWALIPMPSPVADINLNILLILAIS
Safa    ADGLKLFMKEPVRPSTSSPILFLLAPMMALTLALTLWTPIPLPFPMADLNLGLLFILAVS
Icae    ADGVKLFIKEPIRPSTSSPVLFLLAPMLALTLALTLWAPMPLPYPVIDLNLGILFILALS
Asmi    ADGVKLFMKEPIRPSTASPTLFLLAPLMALTLALLLWALMPLPHPIANLNLTMLCVLTIS
Foal    ADGLKLFTKEPILPLTSNGVLFLAAPMLSLALALTLWAPMPLPHPVADISLSILFILALS
Drze    ADGIKLFIKEPVRPSTSSPNLFLFAPILALTLSLAMWMPMPLPYPLMDLNLGVLFILALS
Rhas    ADGVKLFIKEPVRPSTSSPILFLLAPMLALTLAMLLWAPMPLPHPVTDLNLSILFILALS
Elac    ADGVKLFIKEPIRPSTSSPLLFILAPMLALTLALILWAPLPLPHPVTDLNLGILFILALS
Kugu    ADGVKLFIKEPIRPSTSSPLLFILMPMLALTLALSLWTPMPLPYSMADLNLSILFILALS
Plor    ADGVKLFIKEPVRPSTSSPFLFLLTPMLALTLALTLWTPIPLPHPMADLNLSILFILALS
Sgun    ADGVKLFIKEPVRPSTSSPVLFLLTPMLALTLALTLWAPMPLPYPILDLNLGILFILALS
Zaco    ADGVKLFIKEPVRPSTSSPLLFLLTPMLALTLALTLWAPMPLPYPVIDLNLGILFILALS
Zbfl    ADGAKLFIKEPVRPSTSSQLLFLSTPMLALTLALTLWAPMPLPYPVADLNLGILFILALS
Spba    ADGVKLFIKEPVRPSTSSPILFLLAPMLALTLALTLWAPMPLPYPVIDLNLGILFILALS
Game    ADGVKLFIKEPVRPSTSSPVLFLLAPMLALTLALTLWAPMPLPYPVTDLNLGILFILALS
Thth    ADGVKLFIKEPVRPSTSSPVLFLLAPMLALTLALTLWAPMPLPYPVTDLNLGILFILALS
Xigl    VDGVKLFIKEPVRPSTASPVLFLLTPMLALTLALTLWAPMPLPYPVADLNLGILFILALS
Hyja    ADGVKLFIKEPIRPSTSSPVLFLLAPMLALTLALTLWAPLPLPYPVTDMNLGILFILALS
Psan    ADGVKLFTKESLRPYNASTILFTLAPMLSLITALIMWAPMPIPYPVVDINLGLIFILAVS
Cupa    ADGVKLFIKEPVRPSTSSPILFLLAPMLALTLALTLWAPMPLPYPVVDLNLGILFILALS
Mpch    ADGVKLFIKEPVRPSMSSPILFVMAPILALTLALTLWAPIPMPFPVLDLNLGILFILAIS
Char    ADGVKLFIKEPVRPSSSSPALFLVAPVLALTLALSLWIPLPLPHPMADLNLGILFVLALS
Pser    ADGVKLFIKEPVRPSTASPVLFLLTPMLALTLALSLWTPMPLPYPMVDLNLGILFILALS
Prol    ADGIKLFIKEPVRPSTASPVLFLLAPMLALTLALTLWAPMPLPYSTVDLNLGILFVLALS
Plbi    ADGLKLFIKEPVRPSTASPVLFLVAPMLALTLALTLWAPMPFPYPVIDLNLGILFILALS
Calu    ADGIKLFLKEPVRPHASSQALFMLAPMLALTLALLIWAPLPLPHPLANLNLSLLFILAIS
Papa    ADGLKLFIKEPIIPLTSSPILFTLCPVLALTLAMSLWMPMPLPFPIAELNLGILFIMALS
Sufr    ADGVKLFIKEPVRPSTSSPLLFLLTPMLALTLAMTLWAPMPMPYPVIDLNLGILFILALS
Stci    ADGLKLFIKEPIRPSTSSPFLFLLTPMLALTLAMALWAPIPMPYPVVDLNLTILFLLALS
Taru    ADGVKLFIKEPVRPSTSAPILFIIAPTLALTLAMMMWTPMPLPYPILDLNLAILFVLAIS
Rala    ADGVKLFIKEPVRPSTSSPVLFLLTPMLALTLAMTLWAPMPMPYPVIDLNLGILFILALS
        .** .:: **  *  :    *   *  ::    :  *     *    . :   : .:::*
```

To be continued on page 15.

[3/6 of aligned sequences]

```
                C                                                 D
Scca   SLTVYTILGSGWASNSKYALMGALRAVAQTISYEVSLGLILLSMIIFAGGFTLHTFNLAQ
Muma   SLTVYTILGSGWASNSKYALMGALRAVAQTISYEVSLGLILLSMIVFAGGFTLHTFNTTQ
Erca   SLSVYSILGSGWASNSKYALIGALRAVAQTISYEVTLGLIVLSLILFTGAFTLNMFNSAQ
Pose   SLSVYSILGSGWASNSKYALIGALRAVAQTISYEVTLGLIILSLIMFTGAFTLITFNTTQ
Actr   SLAVYSILGSGWASNSKYALIGALRAVAQTISYEVSLGLILLCMIIFTGNFTLHTFNITQ
Scal   SLAVYSILGSGWASNSKYALIGALRAVAQTISYEVSLGLILLSMIIFTGNFTLHTFNVTQ
Posp   SLAVYSILGSGWASTSKYALIGALRAVAQTISYEVSLGLILLCMIVFTGNFTLHTFNTAQ
Atsp   SLAVYSILGSGWASNSKYALIGALRAVAQTISYEVSLGLILLSMIIFSGGFTLHTFNVTQ
Leoc   SLAVYSILGSGWASNSKYALIGALRAVAQTISYEVSLGLILLSMIIFSGGFTLHTFNVTQ
Amca   SLAVYSILGSGWASNSKYALIGALRAVAQTISYEVSLGLILLSMIIFVGGFTLHTFNTTQ
Osbi   SLAVYSILGSGWASNSKYALIGALRAVAQTISYEVSLGLILLSAIILAGGFTLHTFNVAQ
Pabu   SLAVYSILGSGWASNSKYALIGALRAVAQTISYEVSLGLILLSVIIFTGGFTMYTFNITQ
Hial   SLAVYSILGSGWASNSKYALIGALRAVAQTISYEVSLGLILLATIIFAGGFTLHTFNVAQ
Elha   SLAVYSILGSGWASNSKYALIGALRAVAQTISYEVSLGLILLSVIIFSGNFTLHTFGVTQ
Mlcy   SLAVYSILGSGWASNSKYALIGALRAVAQTISYEVSLGLILLATIILAGNFTLYTFGIAQ
Algl   SLAVYSILGSGWASNSKYALIGALRAVAQTISYEVSLGLILLSVIVISGGFTLYTFSVAQ
Ptgi   SLAVYSILGSGWASNSKYALIGALRAVAQTISYEVSLGLILLSIIIITGGFTLHTFNVAQ
Alaf   SLAVYSILGSGWASNSKYALIGALRAVAQTISYEVSLGLILLSAIIITGGFTLHTFNVAQ
Nock   SLAVYSILGSGWASNSKYALIGALRAVAQTISYEVSLGLILLSIIIITGGFTLQTFNVAQ
Anja   SLAVYSILGSGWASNSKYALIGALRAVAQTISYEVSLGLILLSIIIIVGGFNLKTFNIAQ
Gyki   SLAVYSILGSGWASNSKYALIGALRAVAQTISYEVSLGLILLSVVIMVGGFNLTMFNTAQ
Syka   SLAVYSILGSGWASNSKYALIGALRAVAQTISYEVSLGLILLSLIMIAGGFNLKTFNMAQ
Opma   SLAVYSILGSGWASNSKYALLGALRAVAQTISYEVSLGLILLSLIILAGGFDLKTFNLAQ
Comy   SLAVYSILGSGWASNSKYALIGALRAVAQTISYEVSLGLILLSIIIIVGGFDLKTFNTAQ
Sasp   SLAVYSILASGWASNSKFALVGALRAVAQTISYEVSLGLILISVIITTGTFNLKMFNSAQ
Eupe   SLAVYSILGSGWSSNSKYALIGALRAVAQTISYEVTLGLILLSAIIIVGTFNLKLFNIAQ
Enja   SLAVYSILGSGWASNSKYALIGALRAVAQTISYEVALGLILLSTIVFTGGFTLTMFSVTQ
Same   SLAVYSILGSGWASNSKYALIGALRAVAQTVSYEVALGLILLCTIVLTGGFTLSMFSIAQ
Chch   SLAVYSILGSGWASNSKYALIGALRAVAQTISYEVSLGLILLATIVFTGGFTLQMFNTTQ
Grgr   SLAVYSILGSGWASNSKYALIGALRAVAQTISYEVSLGLILLSIIILSGGFTLQTLNVTQ
Caau   SLAVYSILGSGWASNSKYALIGALRAVAQTISYEVSLGLILLSVIIFSGGYTLQTFNTTQ
Cyca   SLAVYSILGSGWASNSKYALIGALRAVAQTISYEVSLGLILLSVIIFSGGYTLQTFNTTQ
Dare   SLAVYSILGSGWASNSKYALIGALRAVAQTISYEVSLGLILLSAVIFSGGYTLQTFNTTQ
Cost   SLAVYSILGSGWASNSKYALIGALRAVAQTISYEVSLGLILLSVIIFTGGYTLQMFSITQ
Leec   SLAVYSILGSGWASNSKYALIGALRAVAQTISYEVSLGLILLSVIIFSGGYTLQMFNVTQ
Fola   SLAVYSILGSGWASNSKYALIGALRAVAQTISYEVSLGLILRSIIIFWGGYTVQTFNTTQ
Clmc   SLAVYSILASGWASNSKYALIGALRAVAQTISYEVSLGLILLSIIVFTGGFTLQTFNVAQ
Phin   SLAVYSILGSGWASNSKYALIGALRAVAQTISYEVSLGLILLSIIIFSGGFTLQMFNTSQ
Icpu   SLAVYSILGSGWASNSKYALIGALRAVAQTISYEVSLGLILLSIIIFTGGFTLQMFNMTQ
Psto   SLAVYSILGSGWASNSKYALIGALRAVAQTISYEVSLGLILLSVIIFTGGFTLQMFNTTQ
Cora   SLAVYSILGSGWASNSKYALIGALRAVAQTISYEVSLGLILLSIMVFSGGFTLQMFNTTQ
Eisp   SLTVYSILGSGWASNSKYALIGAVRAVAQTISYEVSLGLILLSIIIFSGGFTLQTFNVTQ
Apal   SLAVYSILGSGWASNSKYALIGALRAVAQTISYEVSLGLILLSIIIFAGGFTLQTFSASQ
Eslu   SLAVYSILGSGWASNSKYALIGALRAVAQTISYEVSLGLILLTVILFVGGFTLQTFNIAQ
Dape   SLAVYSILGSGWASNSKYALIGALRAVAQTISYEVTLGLILLSSALFVGGFTLQMFNTTQ
Glse   SLAVYSILGSGWASNSKYALIGALRAVAQTISYEVSLGLILLSIIIFTGNFTLQTFNTTQ
Naar   SLAVYSILGSGWASNSKYALIGALRAVAQTISYEVSLGLILLSIIIFTGGFTLQTFNVAQ
Lioc   SLAVYSILGSGWASNSKYALIGALRAVAQTISYEVSLGLILLSIIIFTGGFTLQTFNVAQ
Opso   SLTVYSILGSGWASNSKYALIGALRAVAQTISYEVSLGLILLSIIIFTGGFTLQTFNTAQ
Alte   SLAVYSILGSGWASNSKFALIGTLRAVAQTISYEVSLGLILLSIIIFAGGFTLQTFNTAQ
Plap   SLAVYSILGSGWASNSKYALIGALRAVAQTISYEVSLGLILLSIIIFAGGFTLQTFNVAQ
```

To be continued on page 16.

[3/6 of aligned sequences]

Plal	SLAVYSILGSGWASNSKYALIGALRAVAQTISYEVSLGLILLSIIIFSGGFTLQTFNVTQ
Sami	SLAVYSILGSGWASNSKYALIGALRAVAQTISYEVSLGLILLSVIIFSGGFTLQTFNVAQ
Rere	SLTVYSILGSGWASNSKYALIGALRAVAQTISYEVSLGLILLSIIIFSGGFTLQTFNTTQ
Gama	SLAVYSILGSGWASNSKYALIGALRAVAQTISYEVSLGLILLSVIIFTGGFTLQTFNVAQ
Onmy	SLAVYSILGSGWASNSKYALIGELRAVAQTISYEVSLGLILLSVIIITGGFTLQTFNVAQ
Sasa	SLAVYSILGSGWASNSKYALIGALRAVAQTISYEVSLGLILLSVIIFTGGFTLQTFNVAQ
Cola	SLAVYSILGSGWASNSKYALIGALRAVAQTISYEVSLGLILLSVIIFTGGFTLQTFNVAQ
Dita	SLAVYSILASGWASNSKYALIGALRAVAQTISYEVSLGLILLSIIIFTGSFTLQTFNITQ
Gogr	SLAVYSILGSGWASNSKYALIGALRAIAQTISYEVSLGLILLCTITFAGNFTLQTLNAAQ
Chsl	SLAVYSILGSGWASNSKYALVGALRAVAQTISYEVSLGLILLSMIFFTGGFTLRTFNVAQ
Atja	SLAVYSILGSGWASNSKYALIGALRAVAQTISYEVSLGLILLSVIIFSGGFTLQTFNTTQ
Iido	SLAVYSILGSGWASNSKYALIGALRAVAQTISYEVSLGLILLSVIIFSGGFTLQTFNTTQ
Auja	SLAVYSILGSGWASNSKYALIGALRAVAQTISYEVSLGLILLSIIILTGGFTLQTFNIAQ
Chag	SLAVYSILGSGWASNSKYALIGALRAVAQTISYEVSLGLILLSVIILAGGFTLQTFNVAQ
Hami	SLAVYSILGSGWASNSKYALIGALRAVAQTISYEVSLGLILLSIIIVSGGFTLQTFNVTQ
Saun	SLAVYSILGSGWASNSKYALIGALRAVAQTISYEVSLGLILLSIIIISGGFTLQTFSTTQ
Nema	SLAVYSILGSGWASNSKYALVGALRAVAQTISYEVSLGLILLSVIIFVGGFSLQTFNTAQ
Disp	SLAVYSILGSGWASNSKYALVGALRAVAQTISYEVSLGLILLSVIIFVGGFSLQAFNAAQ
Myaf	SLAVYSILGSGWASNSKYALVGALRAVAQTISYEVSLGLILLSVIIFVGGFSLQTFNSAQ
Lagu	SLSVYSTLGSGWASNSKYALMGALRAVAQTISYEVSLGLILLSVIIFAGSFSLQTFMTAQ
Trtr	SLAVYSTLGSGWASNSKYALMGALRAVAQTISYEVSLGLILLSTVILAGGFTLQTFNIAQ
Zucr	SLAVYSTLGSGWASNSKYALMGALRAVAQTVSYEVSLGLILLSTVILAGGFTLQTFNIAQ
Pxja	SLAVYSILGSGWASNSKYALVGALRAVAQTISYEVSLGLILLNVIIFSGGFTLQTFNVTQ
Pxlo	SLAVYSILGSGWASNSKYALVGALRAVAQTISYEVSLGLILLNVIIFSGGFTLQTFNVTQ
Pctr	SLAVYSILGSGWASNSKYALIGALRAVAQTISYEVSLGLILLCVIVLSGGFTLQTFNTAQ
Apsa	SLAVYSILGSGWASNSKYALIGALRAVAQTISYEVSLGLIILCIIVLSGGFTLQTFNTTQ
Cabe	SLAVYPILAAGWASNSKYALIGALRAVAQTISYEVSLGLILLGSILLAGQFTLQAFGAAQ
Bzze	SLAVYSILGSGWASNSKYALIGALRAVAQTISYEVSLGLILLAVIILTGGFTLYAFNVAQ
Siim	SLAVYSILGSGWASNSKYALIGALRAVAQTISYEVSLGLILLSTIVLTGGFTLHAFSMAQ
Ctru	SLAVYSILGSGWASNSKYALIGALRAVAQTISYEVSLGLILLNTIIFTGGFTLHTFSVAQ
Dpbr	SLAVYSILGSGWASNSKYALIGALRAVAQTISYEVSLGLILLNAIIFTGGFTLQTFSTAQ
Caki	SMAVYSILGSGWASNSKYALMGALRAVAQTISYEVNLGLILLSVIIFSGGFSLQTFSITQ
Phja	SLAVYSILGSGWASNSKYALVGALRAVAQTISYEVSLGLILLSVIIFSGGFTLQMFSVTQ
Brsp	SLAVYSILGSGWASNSKYALVGALRAVAQTISYEVSLGLILLSIIIFSGGFTLQNFHVTQ
Gamo	SLAVYSILGSGWASNSKYALVGALRAVAQTISYEVSLGLILLSVIIFSGGFTLQTFSTTQ
Lolo	SLAVYSILGSGWASNSKYALVGALRAVAQTISYEVSLGLILLSIIIFSGGFTLQTFSMTQ
Batr	SLAVYPLLGSGWASNSKYALLGALRAVAQTISYEVSLALIVITTTILAGGFSLMYFNTTQ
Prmy	SLAVFSILGSGWASNSKYALMGALRAVAQTISYEVVMALILLSLLITGNFSLTSFNTTQ
Lose	SLAVYSTLASGWASNSKYALMGALRAIAQTISYEVSLGLILLSIIMFSGSFTLGNFSVTQ
Loam	SLAVYSILGSGWASNSKYALIGALRAVAQTISYEVSLGLILLSTIIFTGGFTLQTFNTAQ
Chab	SLAVYSILGAGWASNSKYALIGALRAVAQTISYEVSLGLILLSAIIFTGGFTLQTFSTAQ
Chto	SLAVYSILGSGWASNSKYALIGALRAVAQTISYEVSLGLILLSAIIFTGGFTLQTFSTAQ
Majo	SLAVYSILGSGWASNSKYALIGALRAVAQTISYEVSLGLILLAAIIFAGGFTLQTFSTAQ
Hlst	SLAVYSILASGWASNSKYALIGALRAVAQTISYEVSLGLILLTIIIFAGGYTLQTFSTSQ
Clpe	SLAVYSTLGSGWASNSKYALIGALRAVAQTISYEVSLGLILLGAIIFTGGFTLQTFSTAQ
Mlmr	SLAVYSILGSGWASNSKYALIGALRAVAQTISYEVSLGLILLSTIIFAGGFTLQTFNTAQ
Crcr	SLTVYSILGSGWASNSKYALIGALRAVAQTISYEVSMGLILLNAVIFTGGFTLQTFSIAQ
Muce	SLTVYSILGSGWASNSKYALIGALRAVAQTISYEVSIGLILLNAVIFTGGFTLQTFSIAQ
Bege	SLAVYSILGSGWASNSKYALIGALRAVAQTISYEVSLGLILLSTIIFTGGFTLQTFSTAQ
Mela	SLTVYTILGSGWASNSKYALIGALRAVAQTISYEVSLGLILLSTIIFAGGFTLQTFNIAQ
Hats	SLAVYSILGSGWASNSKYALIGALRAVAQTISYEVSLGLILLNAIIFTGGFTLQTFSVAQ
Orla	SLAVYSILGSGWASNSKYALIGALRAVAQTISYEVSLGLILLNAIVFTGGFTLQTFSTAQ

To be continued on page 17.

[3/6 of aligned sequences]

To be continued on page 18.

```
Cosa  SLAVYSILASGWASNSKYALIGALRAVAQTISYEVSLGLILLNAIIFTGGFTLQTFSTAQ
Exsp  SLAVYSILGSGWASNSKYALIGAIRAAAQTISYEVSLGLILLNAVVFTGNFTLQTFSTAQ
Depa  SLAVYSILASGWASNSKYALIGALRAVAQTISYEVSLGLILLNVVIFTGGFTLQTFATAQ
Rima  SLAVYSILGAGWASNSLYALIGAMRAVAQTISYEVSLGLILLSTVIFSGAFTIQTFNVAQ
Fuol  SLAVYSILGSGWASNSKYALIGALRAVAQTISYEVSLGLILLCTIIFTGGFTLQTFSTAQ
Gmaf  SLTVYSILGSGWASNSKYALIGALRAVAQTISYEVSLGLILLSTVLLAGGFTLQTFNTAQ
Xeei  SLAVYSILGSGWASNSKYALIGALRAVAQTISYEVSLGLILLNTIIFTGGFTLQTFSTAQ
Pros  SLAVYSILGSGWASNSKYALVGALRAVAQTISYEVSLGLILLNIIIFTGGFTLQTFNVAQ
Scmi  SLAVYSILGSGWASNSKYALVGALRAVAQTISYEVSLGLILLSIIIFTGGFTLQTFNIVQ
Rolo  SLAVYSILGSGWASNSKYALMGALRAVAQTISYEVSLGLILLNIIIFAGGFTLQTFNITQ
Cere  SLSVYSILGSGWASNSKYALIGALRAVAQTISYEVSLGLILLNAVIFTGGFTLQNFNVAQ
Daga  SLSVYSVLGSGWASNSKYALIGALRAVAQTISYEVSLGLILLNTIIFTGGFALQNFSTTQ
Anco  SLAVYSILGSGWASNSKYALMGALRAVAQTISYEVSLGLILLNIVIFTGGFTLQTFNVAQ
Dmve  SLAVYSLLGSGWASNSKYALMGALRAVAQTISYEVSLGLILLNVVVFTGAFTLQTFNVTQ
Dmar  SLAVYSLLGSGWASNSKYALMGALRAVAQTISYEVSLGLILLNVVVFTGAFTLQTFNVTQ
Anka  SLAVYSILGSGWASNSKYALIGALRAVAQTISYEVSLGLILLNIIIFTGAFTLQTFNVAQ
Moja  SLAVYSILGSGWASNSKYALMGALRAVAQTISYEVSLGLILLNIVIFTGGFTLQTFNITQ
Hoja  SLAVYSILGSGWASNSKYALMGALRAVAQTISYEVSLGLILLNIVIFTGGFTLQTFNVAQ
Bede  SLAVYSILGSGWASNSKYALMGALRAVAQTISYEVSLGLILLNIIIFTGGFTLQTFNIAQ
Besp  SLAVYSILGSGWASNSKYALMGALRAVAQTISYEVSLGLILLNIIIFTGGFTLQTFNVAQ
Mysp  SLAVYSILGSGWASNSKYALMGALRAVAQTISYEVSLGLILLSTIIFTGGFTLQTFNTAQ
Osja  SLAVYSILGSGWASNSKYALMGALRAVAQTISYEVSLGLILLNTIIFAGGFTLQTFNTAQ
Sgro  SLAVYSILGSGWASNSKYALMGALRAVAQTISYEVSLGLILLNIIIFAGGFTLQTFNTAQ
Pzpa  SLAVYSTLGAGWASNSKYALMGALRAVAQTISYEVSLGLILLSVILLSGGFTLQTFNITQ
Zeja  SLTVYSILGSGWASNSKYALVGALRAVAQTISYEVSLGLILLSTILLSGGFTLQTFNITQ
Znne  SLAVYSILGSGWASNSKYALVGALRAVAQTISYEVSLGLILLSAVLLSGGFTLQTFNITQ
Zefa  SLAVYSILGSGWASNSKYALVGALRAVAQTISYEVSLGLILLSVILLSGGFTLQTFSITQ
Acni  SLAVYSILGSGWASNSKYALVGALRAVAQTISYEVSLGLILLSVILLSGGFTLQTFNVTQ
Ncrh  SLAVYSILGSGWASNSKYALVGALRAVAQTISYEVSLGLILLSVILLSGGFTLQTFNETQ
Agca  SLAVYAILGSGWASNSKYALIGALRAVAQTISYEVSLGLILLGLIIFAGSFTLQTFNTAQ
Hydy  SLAVYSILGSGWASNSKYALIGALRAVAQTISYEVSLGLILLSIIIFAGGFTLQTFNIAQ
Gsac  SLAVYSVLGSGWASNSKYALIGALRAVAQTISYEVSLGLILLNIIIFTGGFTLQTFNVAQ
Pevo  SLAVYSILGSGWASNSKYALIGALRAVAQTISYEVSLGLILLSIIIFTGGFTLQMFNYTQ
Hiku  SLAVYSILGSGWASNSKYALIGALRAVAQTISYEVSLGLILLALIIFTGNFTLQSFGITQ
Inpa  SLTVYTILGAGWASNSKYSLIGALRAVAQTISYEVCLGLIILSLIILTGNFALQTFNTTQ
Auch  SLAVYSILGSGWASNSKYALIGALRAVAQTISYEVSLGLIILNAVILTGGFTLQTFNISQ
Fico  SLAVYSILGSGWASNSKYALIGALRAVAQTISYEVSLGLILLCIIIFTGGFTLQAFATTQ
Macs  SLAVYSILGSGWASNSKYALIGALRAVAQTISYEVSLGLILLSTIIFTGGFTLQTFNVTQ
Moal  SLTVYSLLGSGWASNSKYALIGALRAVAQTISYEVTLGLLLLCIIILSGGFNLETFNVTQ
Mafr  SLAVYSILGSGWASNSKYALIGALRAVAQTISYEVSLGLILLCTIIFTGGFTLQTFNTAQ
Dcpe  SLAVYSILGSGWASNSKYALIGALRAVAQTISYEVSLGLILLSIIIFTGGFTLQTFNIAQ
Dcti  SLAVYSILGSGWASNSKYALIGALRAVAQTISYEVSLGLILLSIIIFTGGFTLQTFNTSQ
Hehi  SLAVYSILGSGWASNSKYALIGALRAVAQTISYEVSLGLILLSTIIFTGGFTLQTFNIAQ
Stam  SLAVYSILGSGWASNSKYALIGALRAVAQTISYEVSLGLILLSIIIFTGGFTLQTFNIAQ
Hogi  SLTVYSILGSGWASNSKYALIGALRAVAQTISYEVSLGLILLSIIIFTGGFTLQTFNITQ
Erzo  SLAVYSILGSGWASNSKYALIGALRAVAQTISYEVSLGLILLSIIIFTGGFTLQTFNIAQ
Hxot  SLAVYSILGSGWASNSKYALIGALRAVAQTISYEVSLGLILLNIIIFTGGFTLQTFNIAQ
Core  SLAVYSILGSGWASNSKYALIGALRAVAQTISYEVSLGLILLNIIIFTGGFTLQTFNVAQ
Apve  SFAVYSILGSGWASNSKYALIGALRAVAQTISYEVSLGLILLSIVIFTGGYTLQTFNVAQ
Latj  SLAVYSILGSGWASNSKYALIGALRAVAQTISYEVSLGLILLCAIIFTGGFTLQTFNTAQ
Laja  SLAVYSILGSGWASNSKYALIGALRAVAQTISYEVSLGLILLSVIIFTGGFSLQTFNVAQ
Syja  SLAVYSILGSGWASNSKYALIGALRAVAQTISYEVSLGLILLNIIIFTGGFSLQIFNVAQ
```

[3/6 of aligned sequences]

To be continued on page 19.

```
Epme  SLAVYSILGSGWASNSKYALIGALRAVAQTISYEVSLGLILLNAIIFTGGFTLQTFNTAQ
Grse  SLAVYSILGSGWASNSKYALIGALRAVAQTISYEVSLGLILLNIIIFTGGFTLQTFNIAQ
Clja  SLAVYSILGSGWASNSKYALIGAIRAVAQTISYEVSLGLILLATIIFVGGYTLQTFNVTQ
Ogcy  SLAVYSILGSGWASNSKYALIGALRAVAQTISYEVSLGLILLNAIIFTGGFTLQIFSVAQ
Plna  SLAVYSILGSGWASNSKYALIGALRAVAQTISYEVSLGLILLSIIILAGNFTLQTFSTAQ
Lema  SLAVYSILGSGWASNSKYALIGALRAVAQTISYEVSLGLILLNAIIFTGGFTLQTFSVAQ
Etzo  SLAVYSILGSGWASNSKYALIGALRAVAQTISYEVSLGLILLNIIIFTGGFTLQTFNVAQ
Apse  SLAVYSILGAGWASNSKYALIGALRAVAQTISYEVSLGLILLSTIVLAGNFTLQTFGVAQ
Epde  SLAVYSILGSGWASNSKYALIGALRAVAQTISYEVSLGLILLNIIIFTGGFTLQTFNVAQ
Slja  SLAVYSILGSGWASNSKYALIGALRAVAQTISYEVSLGLILLNIIIFAGGFSLQTFNVAQ
Bsja  SLAVYSILGSGWASNSKYALIGALRAVAQTISYEVSLGLILLNTIIFTGGFTLQIFNVAQ
Ecna  SLAVYSILGSGWASNSKYALVGALRAVAQTISYEVSLGLILLNIIIFTGGFTLQTFNTAQ
Cohi  SLAVYPILCSGWASNSKYALVGSLRAVAQTISYEVVIGLLLLSIIIFAGGFNLQTLTTAQ
Caar  SLAVYSILGSGWASNSKYALVGALRAVAQTISYEVSLGLILLNIIIFTGGFTLQTFNTAQ
Came  SLAVYSILGSGWASNSKYALVGALRAVAQTISYEVSLGLILLNIIIFTGGFTLQTFNTAQ
Mema  SLAVYAILGSGWASNSKYALIGALRAVAQTISYEVSLGLILLSMIIFTGGFTLQTFNTAQ
Lenu  SLAVYSILGSGWASNSKYALVGALRAVAQTVSYEVSLGLILLNAIIFSGGFTLQVFSVTQ
Brja  SLAVYSILGSGWASNSKYALIGALRAVAQTISYEVSLGLILLNTIIFTGGFTLQTFNTAQ
Plma  SLAVYSILGSGWASNSKYALIGALRAVAQTISYEVSLGLILLNAIIFTGGFTLQTFNIAQ
Emst  SLAVYSILGSGWASNSKYALIGALRAVAQTISYEVSLGLILLNAIIFTGGFTLQTFNVAQ
Ptti  SLAVYSILGSGWASNSKYALIGALRAVAQTISYEVSLGLILLNAIIFTGGFTLQTFNVAQ
Losu  SLAVYSILGSGWASNSKYALMGALRAVAQTISYEVSLGLILLSAIMFSGGFTLQVFNTAQ
Geoy  SLAVYSILGSGWASNSKYALIGALRAVAQTVSYEVSLGLILLCIVVFSGGFTLQVFNVTQ
Dipi  SLAVYSILGSGWASNSKYALIGALRAVAQTISYEVSLGLILLNIIIFTGGFTLQTFNVAQ
Pama  SLAVYSILGSGWASNSKYALIGALRAVAQTISYEVSLGLILLCTIIFTGGFTLQIFNVAQ
Leob  SLAVYSILGSGWASNSKYALIGALRAVAQTISYEVSLGLILLSAIIFTGGFTLQMFNVAQ
Neba  SLAVYSILGSGWASNSKYALIGALRAVAQTISYEVSLGLIILCIVILSGNFTLQVLNTTQ
Pdpl  SLAVYSILGSGWASNSKYALIGALRAVAQTISYEVSLGLILLNVVVFAGGFTLQTFNIAQ
Nimi  SLAVYSILGSGWASNSKYALIGALRAVAQTISYEVSLGLILLSAIIFTGGFTLQTFNTAQ
Uptr  SLAVYSILGSGWASNSKYALIGALRAVAQTISYEVSLGLILLSIIIFTGGFTLQMFNVVQ
Pesc  SLAVYSILGSGWASNSKYALIGALRAVAQTVSYEVSLGLILLAAIMFVGNFTLQNFSVTQ
Baar  SLAVYSILGSGWASNSKYALIGALRAVAQTISYEVSLGLILLNIIIFTGGFSLQTFNVTQ
Moar  SLAVYSILGSGWASNSKYALIGALRAVAQTISYEVSLGLILLNAIIFTGGFTLQTFNVAQ
Toja  SLAVYSILGSGWASNSKYALIGALRAVAQTISYEVSLGLILLNAIIFTGGFTLQTFNTAQ
Chau  SLAVYSILGSGWASNSKYALIGALRAVAQTISYEVSLGLILLNIIIFTGGFTLHTFNVAQ
Chse  SLAVYSILGSGWASNSKYALIGALRAVAQTISYEVSLGLILLNTIIFTGGFTLQTFSTAQ
Enar  SLAVYSILGSGWASNSKYALIGALRAVAQTISYEVSLGLILLNAIIFTGGFTLQTFNVAQ
Hpty  SLAVYSILGSGWASNSKYALIGALRAVAQTISYEVSLGLILLNAIIFTGGFSLQTFNVAQ
Nana  SLAVYSILGSGWASNSKYALIGALRAVAQTISYEVSLGLILLSLAIFAGGFTLQTFNISQ
Mcst  SLAVYSILGSGWASNSKYALIGALRAVAQTISYEVSLGLILLNTIIFTGGFTLQTFNIAQ
Rhox  SLAVYSILGSGWASNSKYALIGALRAVAQTISYEVSMGLILLCVILFTGGFTLQTFNITQ
Opfa  SLAVYSILGSGWASNSKYALIGALRAVAQTISYEVSLGLILLNAIIFTGGFTLQTFNIAQ
Paar  SLAVYSILGSGWASNSKYALIGALRAVAQTISYEVSLGLILLSIIIITGGFTLQTFNTAQ
Gozo  SLAVYSILGSGWASNSKYALIGALRAVAQTISYEVSLGLILLNAIIFTGGFTLQTFNIAQ
Ackr  SLTVYSILGSGWASNSKYALMGALRAVAQTISYEVSLGLIVLCVVLLAGGYTLQTFSIAQ
Elev  SLAVYSILGSGWASNSKYALIGALRAVAQTISYEVSLGLILLSSIILTGGFTLQTFNSAQ
Trdu  SLAVYSILGSGWASNSKYALIGALRAVAQTISYEVSLGLILLNAIIFTGGFTLQTFSVAQ
Amoc  SLAVYSILGSGWASNSKYALIGALRAVAQTISYEVSLGLILLNAIIFTGGFTLQTFSEAQ
Hame  SLAVYSILGSGWASNSKYALIGALRAVAQTISYEVSLGLILLNAIIFTGGFTLHAFSVAQ
Chso  SLAVYSILGSGWASNSKYALIGALRAVAQTISYEVSLGLILLNIVIFAGGYTLHTFNIVQ
Lyto  SLAVYSILGSGWASNSKYALIGALRAVAQTISYEVSLGLILLNIIIFTGGFTLQTFNVAQ
Encr  SLAVYSILGSGWASNSKYALIGALRAVAQTISYEVSLGLILLNIIIFTGGFTLQTFNVAQ
```

[3/6 of aligned sequences]

```
Bvar    SLAVYSILGSGWASNSKYALIGALRAVAQTISYEVSLGLILLSIIMLTGGYTLNTFLLAQ
Noco    SLAVYSILGSGWASNSKYALVGALRAVAQTISYEVSLGLILLSVIIFAGGFTLQTFNIAQ
Chsp    SLAVYSILGSGWASNSKYALMGALRAVAQTISYEVSLGLILLCAAMYIGGFSMQAFGHAQ
Arja    SLSVYSILGSGWASNSKYALFGALRAVAQTISYEVSLGLILLCLIFLAGGYTLQMFSTAQ
Pase    SLTVYSILGSGWASNSKYALIGALRAVAQTVSYEVSLGLILLSIVIFTGGFTLQTFNTAQ
Trel    SLAVYSILGSGWASNSKYALIGAIRAVAQTISYEVSLGLLLLSVIILAGGFSLHALNLAQ
Lifa    SLAVYSILGSGWASNSKYALIGALRAVAQTISYEVSLGLILLCTIIFTGGFSLHTFNTTQ
Acur    SLAVYSILGSGWASNSKYALIGALRAVAQTISYEVSLGLILLSLIILSGGFSLQTFNVTQ
Ampe    SLAVYSILGSGWASNSKYALIGALRAVAQTISYEVSLGLILLNVIIFTGGFTLHTFNVAQ
Urja    SLGVYSILGSGWASNSKYALIGALRAVAQTISYEVSLGLILLNIIILAGNFTLQSFNTAQ
Enet    SLAVYSILASGWASNSKYALIGALRAVAQTISYEVTLGLILLSVILFTGGFTLQTFTTAQ
Ptbr    SLAVYSILGSGWASNSKYALIGALRAVAQTVSYEVSLGLILLSTIVLAGGFTLQLFNVVQ
Safa    SLAVYSILGSGWASNSKYALIGALRAVAQTISYEVSLGLILLNAIIFTGGFTLQTFNTAQ
Icae    SLAVYSILGSGWASNSKYALIGALRAVAQTISYEVSLGLILLNTIIFTGGFTLQTFNIAQ
Asmi    SLSVYAILSSGWASNSKYALIGALRAVAQTISYEVSLGLILLAVVIFSGNFSLNAFGALQ
Foal    SLAVYCILGSGWASNSKYALMGALRAIAQTISYEVSLGLIILGGVVFTGAFTLQVFSVTQ
Drze    SLAVYSILASGWASNSKYALMGALRAVAQTISYEVSLGLILLCLIMFTGGFTLQTFNSTQ
Rhas    SLAVYSILGSGWASNSKYALIGALRAVAQTISYEVSLGLILLSAIIFTGGFTLQTFSITQ
Elac    SLAVYSILGSGWASNSKYALIGALRAVAQTISYEVSLGLILLSAIIFTGGFTLKTFSIAQ
Kugu    SLAVYSILGSGWASNSKYALIGALRAVAQTISYEVSLGLILLSTIIFTGGFTLQTFNTTQ
Plor    SLTVYSILGSGWASNSKYALIGALRAVAQTISYEVSLGLILLNIIIFTGGFTLQTFNITQ
Sgun    SLAVYSILGSGWASNSKYALIGALRAVAQTVSYEVSLGLILLSTIIFTGGFTLQTFNVAQ
Zaco    SLAVYSILGSGWASNSKYALIGALRAVAQTISYEVSLGLILLNAIIFTGGFTLQTFNTAQ
Zbfl    SLTVYSILGSGWASNSKYALIGALRAVAQTISYEVSLGLILLSMIIFSGGFTLQTFNVSQ
Spba    SLAVYSILGSGWASNSKYALIGALRAVAQTISYEVSLGLILLSVIIFTGGFTLQTFNITQ
Game    SLAVYSILGSGWASNSKYALIGALRAVAQTISYEVSLGLILLNAIIFTGGFTLQTFNIAQ
Thth    SLAVYSILGSGWASNSKYALIGALRAVAQTISYEVSLGLILLNAIIFTGGFTLQTFNIAQ
Xigl    SLAVYSILGSGWASNSKYALIGALRAVAQTISYEVSLGLILLNIIIFTGGFTLQTFNTAQ
Hyja    SLAVYSILGSGWASNSKYALIGALRAVAQTISYEVSLGLILLNAIIFTGGFTLQTFNTAQ
Psan    SLSVYSILASGWASNSKYALMGALRAVSQTISYEVSLGLILLATVLLTGGFTLQTFSTTQ
Cupa    SLAVYSILGSGWASNSKYALIGALRAVAQTISYEVSLGLILLNAIIFTGGFTLQTFNVAQ
Mpch    SLAVYSILGSGWASNSKYALIGALRAVAQTISYEVSLGLILLNAIIITGGFTLQTFSTAQ
Char    SLAVYSILGSGWASNSKYALIGALRAVAQTISYEVSLGLILLTIIIFSGGFTLQTFNITQ
Pser    SLAVYSILGSGWASNSKYALIGALRAVAQTISYEVSLGLILLAVIILTGGFTLQTFNTTQ
Prol    SLAVYSILGSGWASNSKYALVGALRAVAQTISYEVSLGLILLNIIILTGGFTLQTFNTAQ
Plbi    SLAVYSILGSGWASNSKYALVGALRAVAQTISYEVSLGLILLNIIIFTGGFTLQTFNTAQ
Calu    SLAVYSILGSGWASNSKYALVGALRAVAQTISYEVSLGLILLCVIMFAGAFSLQVFATAQ
Papa    SLAVYSILGSGWASNSKYALIGALRAVAQTVSYEVSLGLILLNVIIFSGGFTLQTFSMTQ
Sufr    SLAVYSILGSGWASNSKYALIGALRAVAQTISYEVSLGLILLCMIIFAGGFTLQTFSTTQ
Stci    SLAVYSILGSGWASNSKYALIGALRAVAQTISYEVSLGLILLSLIIIAGGFTLHTFNVAQ
Taru    SLAVYSILGSGWASNSKYALMGSLRAVAQMISYEVSLGLILLSLIIFTGNFTLQTFNVTQ
Rala    SLAVYSILGSGWASNSKYALIGALRAVAQTISYEVSLGLILLSIIIFTGGFTLQIFSVAQ
        *: *:  * :**:*.* ::*.* :** :* :**** ::*::        * : :  :   *
```

To be continued on page 20.

[4/6 of aligned sequences]

E

```
Scca    ETIWLLIPGWPLAFMWYISTLAETNRAPFDLTEGESELVSGFNIEYAAGPFALFFLAEYT
Muma    ETIWLLIPGWPLALMWYISTLAETNRAPFDLTEGESELVSGFNIEYAGGPFALFFLAEYT
Erca    ETIWLILPAWPMAAMWFISTLAETNRTPFDLTEGESELVSGFNVEYAGGPFALFFLAEYT
Pose    EAVWLIMPAWPLAAMWFISTLAETNRAPFDLTEGESELVSGFNVEYAGGPFALFFLAEYT
Actr    EAIWLLAPGWPLAAMWYISTLAETNRAPFDLTEGESELVSGFNVEYAGGPFALFFLAEYA
Scal    EAIWLLAPGWPLAAMWYISTLAETNRAPFDLTEGESELVSGFNVEYAGGPFALFFLAEYA
Posp    EAIWLLAPGWPLAAMWYISTLAETNRAPFDLTEGESELVSGFNVEYAGGPFALFFLAEYA
Atsp    ESIWLIIPGWPLATMWYISTLAETNRAPFDLTEGESELVSGFNVEYAGGPFALFFLAEYA
Leoc    ESIWLIIPGWPLAAMWYISTLAETNRAPFDLTEGESELVSGFNVEYAGGPFALFFLAEYA
Amca    EAIWLLIPGWPLAAMWYISTLAETNRAPFDLTEGESELVSGFNVEYAGGPFALFFLAEYA
Osbi    ESIWLLAPIWPLATMWYISTLAETNRAPFDLTEGESELVSGFNVEYAGGPFALFFLAEYA
Pabu    KTIWLIMPAWPLAAMWYISTLAETNRAPFDLTEGESELVSGFNVEYAGGPFTLFFLAEYA
Hial    EAVWLVAPAWPLAAMWYISTLAETNRAPFDLTEGESELVSGFNVEYAGGPFALFFLAEYA
Elha    EAIWLLAPAWPLAAMWYISTLAETNRAPFDLTEGESELVSGFNVEYAGGPFALFFLAEYS
Mlcy    EAIWLLAPNWPLAAMWYVSTLAETNRAPFDLTEGESELVSGFNVEYAGGPFALFFLAEYA
Algl    ETMWLVAPAWPLAVMWYISTLAETNRAPFDLTEGESELVSGFNVEYAGGPFALFFLAEYA
Ptgi    EATWLIAPAWPLAAMWYISTLAETNRAPFDLTEGESELVSGFNVEYAGGPFALFFLAEYA
Alaf    ETTWLVAPAWPLAAMWYVSTLAETNRAPFDLTEGESELVSGFNVEYAGGPFALFFLAEYS
Nock    ESTWLVIPAWPLAAMWYVSTLAETNRAPFDLTEGESELVSGFNVEYAGGPFALFFLAEYS
Anja    EATWLLAPAWPLAAMWYISTLAETNRAPFDLTEGESELVSGFNVEYAGGPFALFFLAEYS
Gyki    EAIWLLAPGWPLAAMWYVSTLAETNRAPFDLTEGESELVSGFNVEYAGGPFALFFLAEYS
Syka    ETTWLFAPAWPLAAMWYISTLAETNRAPFDLTEGESELVSGFNVEYAGGPFALFFLAEYS
Opma    ETTWLLAPAWPLAAMWYVSTLAETNRAPFDLTEGESELVSGFNVEYAGGPFALFFLAEYS
Comy    ETTWLIAPTWPLAAMWYVSTLAETNRAPFDLTEGESELVSGFNVEYAGGPFALFFLAEYS
Sasp    ETMWLMIPLWPVMAMWFISTIAETNRAPFDLSEGESELVSGFNVEYTGGPFTLFFIAEYI
Eupe    ETTWLLLPIWPMATMWYISTLAETNRAPFDLTEGESELVSGFNVEYAGGPFALFFLAEYS
Enja    ESIWLLAPAWPLAAMWFVSTLAETNRAPFDLTEGESELVSGFNVEYAGGPFALFFLAEYA
Same    EGIWLLVPAWPLAAMWYISTLAETNRAPFDLTEGESELVSGFNVEYAGGPFALFFLAEYA
Chch    ESIWLIIPAWPLAAMWYISTLAETNRAPFDLTEGESELVSGFNVEYAGGPFALFFLAEYA
Grgr    EATWLLLPAWPLAAMWYISTLAETNRAPFDLTEGESELVSGFNVEYAGGPFALFFLAEYS
Caau    ESIWLLIPAWPLAAMWYISTLAETNRAPFDLTEGESELVSGFNVEYAGGPFALFFLAEYA
Cyca    ESIWLLIPAWPLAAMWYISTLAETNRAPFDLTEGESELVSGFNVEYAGGPFALFFLAEYA
Dare    EDTWLLLPLWPLAIMWFISTLAETNRAPFDLTEGESELVSGFNVEYAAGPFALFFLAEYS
Cost    EGIWLLIPAWPLAAMWYISTLAETNRAPFDLTEGESELVSGFNVEYAGGPFALFFLAEYA
Leec    EAIWLLIPAWPLAAMWYISTLAETNRAPFDLTEGESELVSGFNVEYAGGPFALFFLAEYA
Fola    EALWLLLPACPLAAMWYISTLAETNRAPFDLTEGESELAPGFNVEYAGGPFALLFLAEYA
Clmc    EATWLLLPAWPLAAMWYISTLAETNRAPFDLTEGESELVSGFNVEYAGGPFALFFLAEYS
Phin    EPIWLLFPTWPLAAMWYISTLAETNRAPFDLTEGESELVSGFNVEYAGGPFALFFLAEYA
Icpu    EAIWLLIPAWPLAAMWYISTLAETNRAPFDLTEGESELVSGFNVEYAGGPFALFFLAEYA
Psto    EAVWLLIPAWPLAAMWYISTLAETNRAPFDLTEGESELVSGFNVEYAGGPFALFFLAEYA
Cora    EAMWLMVPAWPLAAMWYISTLAETNRAPFDLTEGESELVSGFNVEYAGGPFALFFLAEYA
Eisp    ETTWLLLPAWPLAAMWYISTLAETNRAPFDLTEGESELVSGFNVEYAGGPFALFFLAEYA
Apal    ETTWLLLPAWPLAAMWYISTLAETNRAPFDLTEGESELVSGFNVEYASGPFALFFLAEYA
Eslu    EGMWLLAPAWPLAAMWYISTLAETNRAPFDLTEGESELVSGFNVEYAGGPFALFFLAEYA
Dape    ESVWLLAPAWPLAAMWYTSTLAETNRAPFDLTEGESELVSGFNVEYAGGPFALFFLAEYA
Glse    ESIWLIIPAWPLAAMWYISTLAETNRAPFDLTEGESELVSGFNVEYAGGPFALFFLAEYA
Naar    ENIWLIAPAWPLAAMWYISTLAETNRAPFDLTEGESELVSGFNVEYAGGPFALFFLAEYA
Lioc    ENIWLIAPAWPLAAMWYISTLAETNRAPFDLTEGESELVSGFNVEYAGGPFALFFLAEYA
Opso    ESIWLIIPGWPLAAMWYISTLAETNRAPFDLTEGESELVSGFNVEYAGGPFALFFLAEYA
Alte    ESIWLLAPAWPLAAMWYISTLAETNRAPFDLTEGESELVSGFNVEYAGGPFALFFLAEYA
Plap    ESIWLLAPAWPLAAMWYISTLAETNRAPFDLTEGESELVSGFNVEYAGGPFALFFLAEYA
```

To be continued on page 21.

[4/6 of aligned sequences]

To be continued on page 22.

```
Plal  ESIWLLVPAWPLAAMWYISTLAETNRAPFDLTEGESELVSGFNVEYAGGPFALFFLAEYA
Sami  ESIWLLIPAWPLAAMWYISTLAETNRAPFDLTEGESELVSGFNVEYAGGPFALFFLAEYA
Rere  ESIWLLVPAWPLAAMWYISTLAETNRAPFDLTEGESELVSGFNVEYAGGPFALFFLAEYA
Gama  EAVWLAAPAWPLAAMWYISTLAETNRAPFDLTEGESELVSGFNVEYAGGPFALFFLAEYS
Onmy  ESIWLLVPAWPLAAMWYISTLAETNRAPFDLTEGESELVSGFNVEYAGGPFALFFLAEYA
Sasa  ESIWLLVPAWPLAAMWYISTLAETNRAPFDLTEGESELVSGFNVEYAGGPFALFFLAEYA
Cola  ESIWLLVPAWPLAAMWYISTLAETNRAPFDLTEGESELVSGFNVEYAGGPFALFFLAEYA
Dita  ESVWLVLPAWPLAAMWFVSTLAETNRAPFDLTEGESELVSGFNVEYAAGPFALFFLAEYA
Gogr  ENMWLILPAWPAAAMWLVSTLAETNRTPFDLAEGESELVSGFNVEYAAGPFALFFLAEYA
Chsl  ESAWLIFSSWPLAAMWFISTLAETNRAPFDLTEGESELVSGFNVEYAAGPFALFFLAEYA
Atja  ESIWLILPAWPLAAMWYISTLAETNRAPFDLTEGESELVSGFNVEYAGGPFALFFLAEYA
Iido  ESIWLVLPAWPLAAMWYISTLAETNRAPFDLTEGESELVSGFNVEYAGGPFALFFLAEYA
Auja  ETIWLIAPAWPLAAMWYISTLAETNRAPFDLTEGESELVSGFNVEYAGGPFALFFLAEYA
Chag  ESIWLLFPAWPLAAMWYISTLAETNRAPFDLTEGESELVSGFNVEYAGGPFALFFLAEYA
Hami  EAIWLIFPTWPLAAMWYISTLAETNRAPFDLTEGESELVSGFNVEYAGGPFALFFLAEYA
Saun  EAIWLILPAWPMAAMWYISTLAETNRAPFDLTEGESELVSGFNVEYAGGPFALFFLAEYA
Nema  ESIWLAVPAWPLAAMWYISTLAETNRAPFDLTEGESELVSGFNVEYAGGPFALFFLAEYA
Disp  ETIWLAVPAWPLAAMWYISTLAETNRAPFDLTEGESELVSGFNVEYAGGPFALFFLAEYA
Myaf  ESIWLAIPAWPLAAMWYISTLAETNRAPFDLTEGESELVSGFNVEYAGGPFALFFLAEYA
Lagu  ESTWMLLATWPLAAMWFISTLAETNRAPFDLTEGESELVSGFNVEYSGGPFALFFLAEYA
Trtr  ETVWLVFPAWPLAAMWYISTLAETNRAPFDLTEGESELVSGFNVEYAGGPFALFFLAEYA
Zucr  ETVWLIFPAWPLAAMWYISTLAETNRAPFDLTEGESELVSGFNVEYAGGPFALFFLAEYA
Pxja  EATWLILPAWPLAAMWYISTLAETNRAPFDLTEGESELVSGFNVEYAGGPFALFFLAEYA
Pxlo  EATWLILPAWPLAAMWYISTLAETNRAPFDLTEGESELVSGFNVEYAGGPFALFFLAEYA
Pctr  EATWLLLPAWPLAAMWFISTLAETNRAPFDLTEGESELVSGFNVEYAGGPFALFFLAEYS
Apsa  EAVWLLIPAWPLAIMWYISTLAETNRAPFDLTEGESELVSGFNVEYAGGPFALFFLAEYS
Cabe  EAMWLALPAAPLAAMWFVSTLAETNRAPFDLTEGESELVSGFNVEYASGPFAFFFLAEYS
Bzze  EAVWLLLPAWPLAAMWFISTLAETNRAPFDLTEGESELVSGFNVEYAGGPFALFFLAEYA
Siim  EATWLLLPAWPLAAMWFISTLAETNRAPFDLTEGESELVSGFNVEYAGGPFALFFLAEYA
Ctru  ESIWLILPAWPLAAMWYISTLAETNRAPFDLTEGESELVSGFNVEYAGGPFALFFLAEYA
Dpbr  ESIWLIVPAWPLAAMWYISTLAETNRAPFDLTEGESELVSGFNVEYAGGPFALFFLAEYA
Caki  EAIWLLLPAWPLAAMWYTSTLAETNRAPFDLTEGESELVSGFNVEYAGGPFALFFLAEYA
Phja  EATWLIFPAWPLAAMWYISTLAETNRAPFDLTEGESELVSGFNVEYAGGPFAMFFLAEYA
Brsp  EAIYLFIPAWPIAIMWYVSTLAETNRAPFDLTEGESELVSGFNVEYAGGPFALFFLAEYA
Gamo  EATWLALPAWPLAAMWYISTLEETNRAPFDLTEGESELVSGFNVEYAGGPFALFFLAEYA
Lolo  EATWLALPAWPLAAMWYISTLAETNRAPFDLTEGESELVSGFNVEYAGGPFALFFLAEYA
Batr  EEVWLILPVWPLAMMWYVSTLAETNRSPFDLAEGESELVSGFNVEYAGGTFALFFLAEYS
Prmy  EVLPLFLPCWPLAVMWFTSIMAETNRAPFDLTEGESELVSGFNVEYAGGPFALFFLAEYS
Lose  EATWLILPTWPLAAMWYISTLAETNRAPFDLTEGESELVSGFNVEYAGGPFALFFLAEYA
Loam  ETIWLILPAWPLAAMWYISTLAETNRAPFDLTEGESELVSGFNVEYAGGPFALFFLAEYA
Chab  EAVWLIMPTWPLAAMWYISTLAETNRAPFDLTEGESELVSGFNVEYAGGPFALFFLAEYA
Chto  EAVWLIMPAWPLAAMWYISTLAETNRAPFDLTEGESELVSGFNVEYAGGPFALFFLAEYA
Majo  EAIWFIIPAWPLAAMWYVSTLAETNRAPFDLTEGESELVSGFNVEYGGGPFALFFLAEYA
Hlst  EAIWFIVPAWPLAAMWYVSSLAETNRAPFDLTEGESELVSGFNVEYSGGPFALFFLAEYS
Clpe  EAIWLIFPAWPLAAMWFTSTLAETNRAPFDLTEGESELVSGFNVEYAGGPFALFFLAEYA
Mlmr  EAIWLVLPAWPLAAMWYISTLAETNRAPFDLTEGESELVSGFNVEYAGGPFALFFLAEYA
Crcr  ESIWLIIPAWPLAAMWYISTLAETNRAPFDLTEGESELVSGFNVEYAGGPFALFFLAEYS
Muce  ESIWLIIPAWPLAAMWYISTLAETNRAPFDLTEGESELVSGFNVEYAGGPFALFFLAEYS
Bege  ESTWLLLPAWPLAAMWYISTLAETNRAPFDLTEGESELVSGFNVEYAGGPFALFFLAEYA
Mela  ETIWLIVPAWPLAIMWYISTLAETNRAPFDLTEGESELVSGFNVEYAGGPFALFFLAEYA
Hats  ESTWLILPAWPLAAMWYISTLAETNRAPFDLTEGESELVSGFNVEYAGGPFALFFLAEYA
Orla  EATWLLLPAWPLAAMWYISTLAETNRAPFDLTEGESELVSGFNVEYAGGPFALFFLAEYG
```

[4/6 of aligned sequences]

```
Cosa    ETTWLIFPAWPLAAMWYISTLAETNRAPFDLTEGESELVSGFNVEYAGGPFALFFLAEYA
Exsp    EATWLILPAWPLAAMWYISTLAETNRAPFDLTEGESELVSGFNVEYAGGPFALFFLAEYA
Depa    EATWLILPTWPLAAMWYISTLAETNRAPFDLTEGESELVSGFNVEYAGGPFALFFLAEYA
Rima    ENIWFIFPAWPLAFMWYTSTLAETNRAPFDLTEGESELVSGFNVEYAAGHFALFFLAEYA
Fuol    ETAWLILPAWPLSAMWFVSTLAETNRAPFDLTEGESELVSGFNVEYAGGPFALFFLAEYA
Gmaf    EQVWLIFPALPLATMWYVSTLAETNRAPFDLTEGESELVSGFNVEYAGGPFALFFLAEYA
Xeei    ENIWLIFPAWPLAAMWYVSTLAETNRSPFDLTEGESELVSGFNVEYAGGPFALFFLAEYA
Pros    ESIWLILPAWPLAAMWYISTLAETNRAPFDLTEGESELVSGFNVEYAGGPFALFFLAEYS
Scmi    ESIWLIIPACPLATMWYISTLAETNRAPFDLTEGESELVSGFNVEYAGGPFALFFLAEYS
Rolo    ESIWLILPAWPLAAMWYISTLAETNRAPFDLTEGESELVSGFNVEYAGGPFALFFLAEYA
Cere    ESVWLVIPMWPLAAMWYISTLAETNRAPFDLTEGESELVSGFNVEYAGGPFALFFLAEYA
Daga    EGVWLVFPMWPLAAMWYISTLAETNRAPFDLTEGESELVSGFNVEYAGGPFALFFLAEYA
Anco    ESIWLILPAWPLAAMWYISTLAETNRAPFDLTEGESELVSGFNVEYAGGPFALFFLAEYA
Dmve    ESIWLIIPTWPLAAMWYISTLAETNRAPFDLTEGESELVSGFNVEYAGGPFALFFLAEYA
Dmar    ESIWLLAPTWPLAAMWYISTLAETNRAPFDLTEGESELVSGFNVEYAGGPFALFFLAEYA
Anka    ESIWLILPAWPLAAMWYISTLAETNRAPFDLTEGESELVSGFNVEYAGGPFALFFLAEYA
Moja    ESIWLILPAWPLAAMWYISTLAETNRAPFDLTEGESELVSGFNVEYAGGPFALFFLAEYA
Hoja    ESIWLILPAWPLAAMWYISTLAETNRAPFDLTEGESELVSGFNVEYAGGPFALFFLAEYA
Bede    ESIWLILPAWPLAAMWYISTLAETNRAPFDLTEGESELVSGFNVEYAGGPFALFFLAEYA
Besp    ESIWLILPAWPLAAMWYISTLAETNRAPFDLTEGESELVSGFNVEYAGGPFALFFLAEYA
Mysp    ESIWLIFPAWPLAAMWYISTLAETNRAPFDLTEGESELVSGFNVEYAGGPFALFFLAEYA
Osja    ESIWLILPAWPLAAMWYISTLAETNRAPFDLTEGESELVSGFNVEYAGGPFALFFLAEYA
Sgro    ESIWLILPAWPLAAMWYISTLAETNRAPFDLTEGESELVSGFNVEYAGGPFALFFLAEYA
Pzpa    ESIWLALPAWPLAAMWYISTLAETNRSPFDLTEGESELVSGFNVEYAGGPFALLFLAEYA
Zeja    EAIWLVLPAWPLAAMWYISTLAETNRAPFDLTEGESELVSGFNVEYAGGPFALLFLAEYA
Znne    EATWLALPAWPLAAMWYTSTLAETNRAPFDLTEGESELVSGFNVEYAGGPFALLFLAEYA
Zefa    EATWLTLPAWPLAAMWYISTLAETNRAPFDLTEGESELVSGFNVEYAGGPFALLFLAEYA
Acni    EATWLTLPAWPLTAMWYISTLAETNRAPFDLTEGESELVSGFNVEYAGGPFALLFLAEYA
Ncrh    EATWLTLPAWPLTAMWYISTLAETNRAPFDLTEGESELVSGFNVEYAGGPFALLFLAEYA
Agca    ESVWLILPAWPLAAMWYVSTLAETNRAPFDLTEGESELVSGFNVEYAGGPFALFFLAEYA
Hydy    ESVWLIIPAWPLAAMWYISTLAETNRAPFDLTEGESELVSGFNVEYAGGPFALFFLAEYA
Gsac    ESVWLIIPAWPLAAMWYISTLAETNRAPFDLTEGESELVSGFNVEYAGGPFALFFLAEYA
Pevo    ESIWLIAPAWPLAAMWFISTLAETNRAPFDLTEGESELVSGFNVEYAAGPFALFFLAEYA
Hiku    ESLWLIVPTWPLAAMWYISTLAETNRAPFDLTEGESELVSGFNVEYAGGPFALFFLAEYA
Inpa    EGTWLILPACPAAIMWFISTLAETNRAPFDLTEGESELVSGFNVEYAGGPFALFFLAEYA
Auch    EAVWLLIPIWPLAAMWYISTLAETNRAPFDLTEGESELVSGFNVEYAGGPFALFFLAEYT
Fico    ESIWLLAPAWPLAAMWYISTLAETNRAPFDLTEGESELVSGFNVEYAGGPFALFFLAEYS
Macs    ESIWLLLPAWPLAAMWYISTLAETNRAPFDLTEGESELVSGFNVEYAGGPFALFFLAEYA
Moal    ENMWLILPSWPLAAMWYVSTLAETNRSPFDLTEGESELVSGFNVEYAGGPFALFFLAEYA
Mafr    ESIWLIIPAWPLAAMWYISTLAETNRAPFDLTEGESELVSGFNVEYAGGPFALFFLAEYA
Dcpe    EAIWLLAPAWPLAAMWYISTLAETNRAPFDLTEGESELVSGFNVEYAGGPFALFFLAEYA
Dcti    EAIWLLVPAWPLAAMWYISTLAETNRAPFDLTEGESELVSGFNVEYAGGPFALFFLAEYA
Hehi    ESVWMLLPAWPLAAMWYISTLAETNRAPFDLTEGESELVSGFNVEYAGGPFALFFLAEYG
Stam    ESVWLVLPAWPLAAMWYISTLAETNRAPFDLTEGESELVSGFNVEYAGGPFALFFLAEYA
Hogi    EAIWLVIPAWPLAGMWYVSTLAETNRAPFDLTEGESELVSGFNVEYAGGPFALFFLAEYA
Erzo    ESVWLILPAWPLAAMWYISTLAETNRAPFDLTEGESELVSGFNVEYAGGPFALFFLAEYA
Hxot    ESVWLILPAWPLAAMWYISTLAETNRAPFDLTEGESELVSGFNVEYAGGPFALFFLAEYA
Core    ESVWLILPAWPLAAMWYISTLAETNRAPFDLTEGESELVSGFNVEYAGGPFALFFLAEYA
Apve    ENMWLILPAWPLATMWFISTLAETNRAPFDLTEGESELVSGFNVEYAGGQFALFFLAEYA
Latj    ESIWLILPAMPLAAMWYISTLAETNRAPFDLTEGESELVSGFNVEYAGGPFALFFLAEYA
Laja    ETIWLIVPAWPLAAMWYISTLAETNRAPFDLTEGESELVSGFNVEYAGGPFALFFLAEYS
Syja    ESIWLILPAWPLAAMWYISTLAETNRSPFDLTEGESELVSGFNVEYAGGPFALFFLAEYA
```

To be continued on page 23.

[4/6 of aligned sequences]

Epme	EATWLLLPAWPLAAMWYISTLAETNRAPFDLTEGESELVSGFNVEYAGGPFALFFLAEYA
Grse	ESIWLILPTWPLAAMWYVSTLAETNRAPFDLTEGESELVSGFNVEYAGGPFALFFLAEYA
Clja	EAIWLILPAWPLAAMWYISTLAETNRAPFDLTEGESELVSGFNVEYAGGPFALFFLAEYA
Ogcy	ESIWLIIPAWPLAAMWYISTLAETNRAPFDLTEGESELVSGFNVEYAGGPFALFFLAEYA
Plna	EAIWLILPAWPLAAMWYISTLAETNRAPFDLTEGESELVSGFNVEYAGGPFALFFLAEYA
Lema	ESTWLILPAWPLAAMWYISTLAETNRAPFDLTEGESELVSGFNVEYAGGPFALFFLAEYA
Etzo	ESIWLIMPAWPLAAMWYISTLAETNRAPFDLTEGESELVSGFNVEYAGGPFALFFLAEYA
Apse	EGIWLVAPAWPLAAMWYISTLAETNRAPFDLTEGESELVSGFNVEYAAGPFALFFLAEYA
Epde	ESIWLVLPAWPLAAMWYISTLAETNRAPFDLTEGESELVSGFNVEYAGGPFALFFLAEYA
Slja	EAVWFILPAWPLAAMWYISTLAETNRAPFDLTEGESELVSGFNVEYAGGPFALFFLAEYA
Bsja	ESVWLILPAWPLAAMWYISTLAETNRAPFDLTEGESELVSGFNVEYAGGPFALFFLAEYA
Ecna	ESVWLLLPAWPLAAMWYISTLAETNRAPFDLTEGESELVSGFNVEYAGGPFALFFLAEYA
Cohi	ENIWFIFPAWPLAAMWFIATLAETKRAPFDLTEGESELVSGYNVEYAGGQFALFFLAEYT
Caar	EGIWLVLPAWPLAAMWYISTLAETNRAPFDLTEGESELVSGFNVEYAGGPFALFFLAEYA
Came	ESIWLVMPAWPLAAMWYISTLAETNRAPFDLTEGESELVSGFNVEYAGGPFALFFLAEYA
Mema	ESIWLALPAWPLAAMWYISTLAETNRAPFDLTEGESELVSGFNVEYAGGPFALFFLAEYA
Lenu	EATWLIFPIWPLAGMWFVSTLAETNRAPFDLTEGESELVSGFNVEYAGGPFALFFLAEYA
Brja	EAIWLAVPAWPLAAMWYISTLAETNRAPFDLTEGESELVSGFNVEYAGGPFALFFLAEYA
Plma	ESIWLIIPAWPLAAMWYISTLAETNRAPFDLTEGESELVSGFNVEYAGGPFALFFLAEYA
Emst	ESIWLILPAWPLAAMWYISTLAETNRAPFDLTEGESELVSGFNVEYAGGPFALFFLAEYA
Ptti	ESIWLILPAWPLAAMWYISTLAETNRAPFDLTEGESELVSGFNVEYAGGPFALFFLAEYA
Losu	ESTWLILPAWPLAAMWYISTLAETNRAPFDLTEGESELVSGFNVEYAGGPFALLFLAEYS
Geoy	ESIWLIVPAWPLAAMWYISTLAETNRAPFDLTEGESELVSGFNVEYAGGPFALFFLAEYA
Dipi	ESVWLVLPAWPLAAMWYISTLAETNRAPFDLTEGESELVSGFNVEYAGGPFALFFLAEYA
Pama	ESIWLIIPTWPLAAMWYISTLAETNRAPFDLTEGESELVSGFNVEYAGGPFALFFLAEYA
Leob	EATWLILPAWPLAAMWYISTLAETNRAPFDLTEGESELVSGFNVEYAGGPFALFFLAEYA
Neba	ESIWLIIPAWPLAAMWYISTLAETNRAPFDLTEGESELVSGFNVEYAGGPFALFFLAEYA
Pdpl	ESIWLILPAWPLAAMWYISTLAETNRAPFDLTEGESELVSGFNVEYAGGPFALFFLAEYA
Nimi	ESIWLIFPAWPLAAMWYISTLAETNRAPFDLTEGESELVSGFNVEYAGGPFALFFLAEYA
Uptr	ESVWLILPAWPLAAMWYISTLAETNRAPFDLTEGESELVSGFNVEYAGGPFALFFLAEYA
Pesc	EGIWLIFPAWPLAAMWYVSTLAETNRAPFDLTEGESELVSGFNVEYAGGPFALFFLAEYA
Baar	ESIWLILPAWPLAAMWYISTLAETNRAPFDLTEGESELVSGFNVEYAGGPFALFFLAEYA
Moar	ESIWLILPAWPLAAMWYISTLAETNRAPFDLTEGESELVSGFNVEYAGGPFALFFLAEYA
Toja	ESVWLILPAWPLAAMWYISTLAETNRAPFDLTEGESELVSGFNVEYAGGPFALFFLAEYS
Chau	ESTWLILPAWPLAAMWYISTLAETNRAPFDLTEGESELVSGFNVEYAGGPFALFFLAEYA
Chse	ESIWLIFPAWPLAAMWYISSLAETNRAPFDLTEGESELVSGFNVEYAAGPFALFFLAEYS
Enar	ESIWLVLPAWPLAAMWYISTLAETNRAPFDLTEGESELVSGFNVEYAGGPFALFFLAEYA
Hpty	ESIWLILPAWPLAAMWYISTLAETNRAPFDLTEGESELVSGFNVEYAGGPFALFFLAEYA
Nana	ESIWLLLPAWPLAAMWYISTLAETNRAPFDLTEGESELVSGFNVEYAGGPFALFFLAEYA
Mcst	ESVWLILPAWPLAAMWYISTLAETNRAPFDLTEGESELVSGFNVEYAGGPFALFFLAEYG
Rhox	ESIWLILPAWPLAAMWYISTLAETNRAPFDLTEGESELVSGFNVEYAGGPFALFFLAEYA
Opfa	ESIWLILPAWPLAAMWYISTLAETNRAPFDLTEGESELVSGFNVEYAGGPFALFFLAEYA
Paar	ESIWLILPAWPLAAMWYISTLAETNRAPFDLTEGESELVSGFNVEYAGGPFALFFLAEYA
Gozo	ESVWLILPAWPLAAMWYISTLAETNRAPFDLTEGESELVSGFNVEYAGGPFALFFLAEYS
Ackr	ESVWLILPAWPLAAMWYISTLAETNRAPFDLTEGESELVSGFNVEYAGGPFALFFLAEYS
Elev	EAIWLIVPMWPLTAMWYISTLAETNRAPFDLTEGESELVSGFNVEYAGGPFALFFLAEYA
Trdu	ESVWLILPAWPLAAMWYISTLAETNRAPFDLTEGESELVSGFNVEYAGGPFALFFLAEYA
Amoc	ESTWLILPAWPLAAMWYISTLAETNRAPFDLTEGESELVSGFNVEYAGGPFALFFLAEYA
Hame	ESVWLILPAWPLAAMWYISTLAETNRAPFDLTEGESELVSGFNVEYAGGPFALFFLAEYA
Chso	EGAWLILPAWPLAAMWYISTLAETNRAPFDLTEGESELVSGFNVEYAGGPFAMFFLAEYA
Lyto	ESVWLILPAWPLAAMWYISTLAETNRAPFDLTEGESELVSGFNVEYAGGPFALFFLAEYA
Encr	ESVWLILPAWPLAAMWYISTLAETNRAPFDLTEGESELVSGFNVEYAGGPFALFFLAEYA

To be continued
on page 24.

```
Bvar    ETTWLILPAWPLAAMWYISTLAETNRAPFDLTEGESELVSGFNVEYAGGPFALFFLAEYA
Noco    ESIWLVLPAWPLAAMWYISTLAETNRAPFDLTEGESELVSGFNVEYAGGPFALFFLAEYS
Chsp    ENMWLLLPAWPLAAMWYISTLAETNRAPFDLTEGESELVSGFNVEYAAGPFALFFLAEYA
Arja    ESVWLILPAWPLATMWYISLLAETNRAPFDLTEGESELVSGFNVEYTGGQFALFFLAEYA
Pase    ESIWLIMPAWPLAVMWYISTLAETNRAPFDLTEGESELVSGFNVEYAGGPFALFFLAEYA
Trel    ESTWLIIPTWPLAAMWYVSTLAETNRAPFDLTEGESELVSGFNVEYAGGPFAMFFLAEYA
Lifa    ESIWLILPAWPLAAMWYISTLAETNRAPFDLTEGESELVSGFNVEYAGGPFALFFLAEYA
Acur    EAIWLILPAWPLAAMWFISTLAETNRAPFDLTEGESELVSGFNVEYAGGPFALFFLAEYS
Ampe    ECVWLILPTWPLAAMWYISTLAETNRAPFDLTEGESELVSGFNVEYAGGPFALFFLAEYA
Urja    ESIWLIIPAWPLAAMWYVSTLAETNRAPFDLTEGESELVSGFNVEYAAGPFALFFLAEYM
Enet    EATWLLLPAWPLAAMWFISTLAETNRAPFDLTEGESELVSGFNVEYAGGPFALFFLAEYA
Ptbr    ETTWLLLPSWPLAAMWFVSSLAETNRTPFDLTEGESELVSGFNVEYSGGLFALFFLAEYA
Safa    EATWLILPAWPLAAMWFISTLAETNRAPFDLTEGESELVSGFNVEYAGGPFALFFLAEYA
Icae    EAIWLIIPAWPLAAMWYISTLAETNRAPFDLTEGESELVSGFNVEYAGGPFALFFLAEYA
Asmi    ENIWLILPAWPLASMWFISTLAETNRAPFDLTEGESELVSGFNVEYAGGPFALFFLAEYA
Foal    EAVWLILPAWPLAMMWYVSTLAETNRSPFDLTEGESELVSGFNVEYAGGPFALFFLAEYA
Drze    ETIWLIVPAWPLAAMWYVSTLAETNRAPFDLTEGESELVSGFNVEYAGGPFALFFLAEYA
Rhas    ESVWLVMPAWPLAAMWYISTLAETNRAPFDLTEGESELVSGFNVEYAGGPFALFFLAEYA
Elac    ESIWLLLPAWPLAAMWYISTLAETNRAPFDLTEGESELVSGFNVEYAGGPFALFFLAEYA
Kugu    ESIWLILPAWPLAAMWYISTLAETNRSPFDLTEGESELVSGFNVEYAGGPFALFFLAEYA
Plor    ESIWLILPAWPLAAMWYISTLAETNRAPFDLTEGESELVSGFNVEYAGGPFALFFLAEYS
Sgun    ESIWLILPAWPLAAMWYISTLAETNRAPFDLTEGESELVSGFNVEYAGGPFALFFLANTP
Zaco    ESVWLIVPAWPLAAMWYISTLAETNRAPFDLTEGESELVSGFNVEYAGGPFALFFLAEYA
Zbfl    ESIWLILPAWPLATMWYISTLAETNRSPFDLTEGESELVSGFNVEYAGGPFALFFLAEYA
Spba    ESTWLILPAWPLAAMWYISTLAETNRAPFDLTEGESELVSGFNVEYAGGPFALFFLAEYA
Game    EAIWLVIPAWPLAAMWYISTLAETNRAPFDLTEGESELVSGFNVEYAGGPFALFFLAEYA
Thth    EAIWLIVPAWPLAAMWYISTLAETNRAPFDLTEGESELVSGFNVEYAGGPFALFFLAEYA
Xigl    ESVWLVLPAWPLAAMWYISTLAETNRAPFDLTEGESELVSGFNVEYAGGPFALFFLAEYA
Hyja    EAIWLAVPAWPLAAMWYISTLAETNRAPFDLTEGESELVSGFNIEYAGGPFALFFLAEYA
Psan    EGIWLIFPAFPLAAMWYVSTLAETGRAPFDLIEGESELVSGFNVEYSAGPFALFFLAEYS
Cupa    EAIWLIIPAWPLAAMWYISTLAETNRAPFDLTEGESELVSGFNVEYAGGPFALFFLAEYA
Mpch    ESIWLIFPAWPLAAMWYISTLAETNRAPFDLTEGESELVSGFNVEYAGGPFALFFLAEYA
Char    ESVWLLLPAWPLAAMWFTSTLAETNRTPFDLTEGESELVSGFNVEYAAGPFALFYLAEYA
Pser    ESIWLLLPAWPLAAMWYISTLAETNRAPFDLTEGESELVSGFNVEYAGGPFALFFLAEYA
Prol    EAVWLVLPAWPLAAMWYISTLAETNRAPFDLTEGESELVSGFNVEYAGGPFALFFLAEYS
Plbi    EAIWLVVPAWPLAAMWYISTLAETNRAPFDLTEGESELVSGFNVEYAGGPFALFFLAEYS
Calu    ENMWLVFPAWPLATMWFVSTLAETNRSPFDLAEGESELVSGFNVEYAGGPFALFFLAEYA
Papa    ESVWLILPGWPLAAMWFVSTLAETNRSPFDLAEGESELVSGFNVEYSGGPFALFFLAEYA
Sufr    ESIWLIFPAWPMAAMWFISTLAETNRAPFDLTEGESELVSGFNVEYAGGPFALFFLAEYA
Stci    ENIWLILPAWPMAAMWYISTLAETNRAPFDLTEGESELVSGFNVEYAGGPFALFFLAEYA
Taru    ESIWLIIPTWPLAAMWYISTLAETNRAPFDLTEGESELVSGFNVEYAGGPFALFFLAEYA
Rala    ESVWLILPAWPLAAMWYISTLAETNRAPFDLTEGESELVSGFNVEYAGGPFALFFLAEYA
        :   :     *   **  : : ** *:**** ******. *:*:** .* *:::::*:
```

To be continued on page 25.

[5/6 of aligned sequences]

```
                      F                              G
Scca NILLMNTLSVILFMGTSYNPLMPQISSLSLMMKASMLTVLFLWIRASYPRFRYDQLMHLV
Muma NILLMNTLSVILFMGISYNPLFPQLSTFNLMMKATLLTFIFLWIRASYPRFRYDQLMHLV
Erca NILLMNALSAILFLGPSFNTVNI---NLNWAIKTTILAIMFLWVRASYPRFRYDQLMHLV
Pose NILLMNALSAILFLGPSFNILTI---NLNWAIKTTLLASLFLWVRASYPRFRYDQLMHLV
Actr NILLMNTLSTILFLGAYHNPMLPEMTALNLMIKASMLSMLFLWVRASYPRFRYDQLMHLV
Scal NILLMNTLSTILFLGAYHNPMLPEMTALNLMIKASMLSMLFLWVRASYPRFRYDQLMHLV
Posp NILLMNTLSTILFLGAYHNPMFPEMTTLNLMIKASMLSMLFLWVRASYPRFRYDQLMHLV
Atsp NILLMNTLSVILFLGAHHNFTTPELTTFNLMLKSSILAMLFLWVRASYPRFRYDQLMHLV
Leoc NILLMNTLSVILFLGAHHNLTTPELTTFNLMLKTSILAMLFLWVRASYPRFRYDQLMHLV
Amca NILLMNTLSAILFLGASHIPTLPTFTTFNLMIKASLLSMVFLWVRASYPRFRYDQLMHLV
Osbi NILLMNTLSTILFLGALHSPLLPEVTTLNLMTKASLLSILFLWIRASYPRFRYDQLMHLM
Pabu NILLMNTLSTIMFLGAMHNPILPELTTFNLMTKASILSILFLWVRASYPRFRYDQLMHLV
Hial NILLMNTLSVVLFLGAFHTPLIPELTAFNLMTKASLLSIMFLWVRASYPRFRYDQLMHLI
Elha NILLMNTLSTILFLGAFHSPTLPELTAFNLMTKAALLSILFLWVRASYPRFRYDQLMHLV
Mlcy NILLMNTLSTILFLGATHNPLLPEMTALNLMTKAATLSIIFLWVRASYPRFRYDQLMHLI
Algl NILLMNTLSVVLFLGALHTPLVPELTALNLMTKAALLSIVFLWVRASYPRFRYDQLMHLV
Ptgi NILLMNTLSTILFLGAFHTPLIPELTAFNLMTKAAMLSIVFLWVRASYPRFRYDQLMHLV
Alaf NILLMNTLSTILFLGAFHIPLYPELTALNLMTKAALLSVVFLWVRASYPRFRYDQLMHLV
Nock NILLMNTLSTILFLGALHIPLFPELTAVNLMTKAALLSVLFLWVRASYPRFRYDQLMHLV
Anja NILLMNTLSTILFLGAMHTPLIPELTTMNLMMKATMLSIMFLWVRASYPRFRYDQLMHLM
Gyki NILLMNTLSTILFLGAMHNPLLPELTTVNLMLKAALLSVMFLWVRASYPRFRYDQLMHLV
Syka NILLMNTLSAILFLGAMHNPLFPQLTTINLMLKAALLSVLFLWVRASYPRFRYDQLMHLV
Opma NILLMNTLSAVLFLGALHNPTMPELTTMNLMLKAALLSVLFLWVRASYPRFRYDQLMHLI
Comy NILLMNTLSTILFIGAMHNPLFPELTTINLMMKATLLSILFLWVRASYPRFRYDQLMHLI
Sasp NIMLMNTLTTIMFWGTPPNTYTPELTTTIMMTKVTTLTAIFLLVRAAYPRLRYDQLMALT
Eupe NILLMNTLSTVLFLGTTQLPNMPELTTISLMLKTTMLSMLFLWVRASYPRFRYDQLMYLT
Enja NILFMNTLSAILFLGATNLPMLPELTTINIMIKAALLSALFLWVRASYPRFRYDQLMHLV
Same NILFMNTLSAVLFMGAAHFPSFPEFTTMNIMFKAALLSGVFLWVRASYPRFRYDQLMHLV
Chch NILLMNTLSTILFLGASHIPMIPELTAINLMTKAALLSIVFLWVRASYPRFRYDQLMHLV
Grgr NILLMNTLSAVLFLGASHLPAFPELTSINLMGKAALLSILFLWVRASYPRFRYDQLMHLI
Caau NILLMNTLSAVLFLGASHIPNMPELTTINLMTKAALLSILFLWVRASYPRFRYDQLMHLV
Cyca NILLMNTLSAVLFLGASHIPSMPELTTINLMTKAALLSIMFLWVRASYPRFRYDQLMHLV
Dare NILLMNTHSTVLFLGASFTPDAPELMTISIATKTAMLSILFLWMRASYPRFRYDQLMHLI
Cost NILLMNTLSAVLFLGASHLPAFPELTTMNLMTKAALLSMVFLWIRASYPRFRYDQLMHLV
Leec NILLMNTLSAILFLGASHTPSIPELTTMNLMAKATLLSMVFLWTRASYPRFRYDQLMHLV
Fola NILLMNTLSAILFLGASHMPAIPELTTINLMTKAALLSVVFLWVRASYPRFRYDQLMHLV
Clmc NILLMNTLSTILFLGASHIPSLPELTSINLMTKAALLSLLFLWVRASYPRFRYDQLMHLV
Phin NILLMNTLSTILFLGASHIPSIPELTSINLMTKAALLSMLFLWVRASYPRFRYDQLMHLV
Icpu NILLMNTLSAILFLGAMHFPHAPELASMNIMMKAALLSMLFLWVRASYPRFRYDQLMHLV
Psto NILLMNTLSTILFLGATHNIIMPEITSINIMTKAALLSMLFLWVRASYPRFRYDQLMHLV
Cora NILLMNTLSAILFLGASHIPTLPQLTSINIMIKAAALSMLFLWVRASYPRFRYDQLMHLV
Eisp NILLMNTLSAVLFLGASHIPHLPELTAINIMTKAALLSILFLWVRASYPRFRYDQLMHLV
Apal NIFLMNTLSVILFLGAS-YATIPELAATNIMTKASLLSLLFLWIRASYPRFRYDQLMHLV
Eslu NILLMNTLSTILFLGATHIPSFPLATTTNLMIKAALLSVLFLWVRASYPRFRYDQLMHLV
Dape NILLMNTLSTILFLGAMHLPALPSFTVMNLMLKAALLSTLFLWVRASYPRFRYDQLMHLV
Glse NILLMNTLSTILFLGASHIPSLPQLTTLNLMTKAACLSVLFLWVRASYPRFRYDQLMHLV
Naar NILLMNTLSAILFLGAYHIPSLPELTSINLMTKAALLSVLFLWVRASYPRFRYDQLMHLV
Lioc NILLMNTLSAILFLGAYHLPAFPELTSVNLMTKAACLSVLFLWVRASYPRFRYDQLMHLV
Opso NILLMNTLSTILFLGASHMPSLPQLTAINLMIKAACLSILFLWVRASYPRFRYDQLMHLV
Alte NILLMNTLSTILFLGASHLPSLPELTAANLMTKAALLSVVFLWVRASYPRFRYDQLMHLV
Plap NILLMNTLSAILFLGASHFPALPELTAANLMTKAALLSVVFLWVRASYPRFRYDQLMHLV
```

To be continued on page 26.

[5/6 of aligned sequences]

```
Plal  NILLMNTLSAVLFLGASHIPSLPELTACNLMTKAALLSVVFLWVRASYPRFRYDQLMHLV
Sami  NILLMNTLSAVLFLGASHLPSLPELTACNLMAKAALLSVVFLWVRASYPRFRYDQLMHLV
Rere  NILLMNTLSAVLFLGASHIPALPELTACNLMTKAALLSVVFLWVRASYPRFRYDQLMHLV
Gama  NILLMNTLSTVLFLGASHFPLVPELTAINLMAKASLLSIMFLWVRASYPRFRYDQLMHLV
Onmy  NILLMNTLSAVLFLGASHIPAFPELTALNLMTKAALLSVVFLWVRASYPRFRYDQLMHLV
Sasa  NILLMNTLSAILFLGASHIPAFPELTAVNLMTKAALLSVVFLWVRASYPRFRYDQLMHLV
Cola  NILLMNTLSTILFLGASHIPAFPELTAMNLMTKAALLSVVFLWVRASYPRFRYDQLMHLV
Dita  NILLMNTLSTILFLGAAHVPLVPELTTANLMTKAALLSLVFLWVRASYPRFRYDQLMHLI
Gogr  NILFMNALSTTLFLGPSLNPHTPELSTLNLMLKTALLSISFLWVRATLPRFRYDQLMHLI
Chsl  NILLMNTLSATLFLGASNIPTLSELSTLSLMTKASLLAVAFLWVRASYPRFRYDQLMHLV
Atja  NILLMNTLSTILFLGASHLPHLPELTTMNLMTKAALLSIVFLWVRASYPRFRYDQLMHLI
Iido  NILLMNTLSTILFLGASHLPHLPELTTMNLMAKAALLSMVFLWVRASYPRFRYDQLMHLI
Auja  NILLMNTLSTVLFLGASHIPTIPELTAINLMTKAALLSVIFLWVRASYPRFRYDQLMHLV
Chag  NILLMNTLSTILFLGASHNPTLPELTAVNLMTKAALLSIVFLWVRASYPRFRYDQLMHLV
Hami  NILLMNTLSTILFLGASHIPTLPELTSMNLMTKAALLSTMFLWVRASYPRFRYDQLMHLV
Saun  NILLMNTLSAILFLGASHIPLFPELTTANLMTKAALLSTMFLWVRASYPRFRYDQLMHLA
Nema  NILLMNTLSTILFLGASHMPALPELTAVNLMTKAAFLSIVFLWVRASYPRFRYDQLMHLV
Disp  NILLMNTLSAILFLGAYHLPALPELTAGNLMTKAALLSVVFLWVRASYPRFRYDQLMHLV
Myaf  NILLMNTLSTILFLGAYHLPAFPELTAANLMTKAAFLPVVFLWVRASYPRFRYDQLMHLV
Lagu  NILFMNTLSAIMFIGGAYT---PELAPATLMTKAALLSLAFIWVRASYPRFRYDQLMHLV
Trtr  NILFMNTLSTVLFLGASHIPTFPELTTINLMIKASLLSLLFLWVRASYPRFRYDQLMHLV
Zucr  NILFMNTLSTVLFLGASHIPTIPELTTINLMVKAALLSLLFLWVRASYPRFRYDQLMHLV
Pxja  NILLMNTLSAVLFLGALHIPLFPELTAMNLMTKASLLSIVFLWVRASYPRFRYDQLMHLV
Pxlo  NILLMNTLSAVLFLGALHIPLFPELTAMNLMTKASLLSIVFLWVRASYPRFRYDQLMHLV
Pctr  NILLMNALTAVLFLGTTHTPTLPELTTTGLMTKAALLATTFLWVRASYPRFRYDQLMHLI
Apsa  NILLMNVLSTILFLGTSYTHMAPELTATNLMTKTALLATLFLWIRASYPRFRYDQLMHLI
Cabe  NILLMNTLSACIFFGTTYFIPHPELTTAGMMTKAALLSMVFLWTRASYPRFRYDQLMHLV
Bzze  NILLMNTLSACLFLGATYTLALPGIATISLMVKASLLSAVFLWTRASYPRFRYDQLMHLV
Siim  NILLMNTLSACLFLGSTHVMTHPELTTTTLMLKAALLSAVFLWTRASYPRFRYDQLMHLV
Ctru  NILLMNTLSATLFLGASHIPSFPELTATNLMTKAALLSVVFLWVRASYPRFRYDQLMHLV
Dpbr  NILLMNTLSATLFLGASHIPSFPELTAMNLMTKAALLSVLFLWVRASYPRFRYDQLMHLV
Caki  NILLMNTLSAILFIGSSFS-LFPEMTTVTLMIKATLLSTLFLWVRASYPRFRYDQLMHLV
Phja  NILLMNTLSAILFIGESYSFMHPELSSLSIMNKAALLSMVFLWVRACYPRFRYDQLMHLV
Brsp  NILMMNTISAILFLNSTL-ASFPEMTSLTLMIKATLLSLAFLWVRGSYPRFRYDQLMHLV
Gamo  NILLMNTLSAVLFLGSSYSTTMPEFTSLTLMTKAALLSMVFLWVRASYPRFRYDQLMHLV
Lolo  NILLMNTLSTILFLGSSYSSTMPELTSLTLMTKAALLSMVFLWVRASYPRFRYDQLMHLV
Batr  NILLMNILTVILFLGPSL-------TAPTISLKLVILVLSFLWVRSSYPRLRYDQLMHLM
Prmy  NILLMSTLTAILFFGTTHLLFMPDISTYLTMAKMVLVTTLFLWVRASYPRFRYDQLMHLT
Lose  NILLMNTLSTILFLGTTFSQAFPEISTANLTLKTILLSILFLWVRASYPRFRYDHLMHLT
Loam  NILLMNTLSATLFLGAAHIPSFPELTAANLMTKAALLSIVFLWVRASYPRFRYDQLMHLI
Chab  NILLMNTLSTALFLGAYHVPTTPELTTINLMIKAALLSVVFLWVRASYPRFRYDQLMHLI
Chto  NILLMNTLSATLFLGAYHLPTTPELTTINLMIKAALLSVVFLWVRASYPRFRYDQLMHLI
Majo  NILLMNTLSATLFLGAHHLPALPSQGATSLMIKVALLAIAFLWVRASYPRFRYDQLMHLV
Hlst  NILLMNTLSAILFLGAHHIPAIAMKGAAGLMAKVMLLATAFLWVRASYPRFRYDQLMHLV
Clpe  NILLMNTLSAVLFLGATHIPAIPALTTLSLMTKTTLLSLAFLWVRASYPRFRYDQLMHLI
Mlmr  NILLMNTLSATLFLGASHMPTIPELTAINLMSKAALLSTMFLWVRASYPRFRYDQLMHLI
Crcr  NILLMNTLSAVLFLGTPLY--YPALTASIIMIKATFLSIVFLWVRASFPRFRYDQLMHLT
Muce  NILLMNTLSAVLFLGTPLY--YPPLTASIIMIKAAFLSIVFLWVRASFPRFRYDQLMHLT
Bege  NILLMNTLSAVLFLGTSILHQAPELTSTILMTKAALLSVIFLWVRASYPRFRYDQLMHLI
Mela  NILLMNTLSAILFLGTSIIYYAPEISTLLLMIKATLLSTVFLWVRASYPRFRYDQLMHLT
Hats  NILLMNTLSAVLFLGSSLLHHTPELTSSTLMTKAALLSVVFLWVRASYPRFRYDQLMHLI
Orla  NILLMNTLSAVLFLGTSTYHNLPELSATLLMLKATLLSVVFLWVRASYPRFRYDQLMHLI
```

To be continued on page 27.

[5/6 of aligned sequences]

Cosa	NILLMNTLSATLFLGTSVYFSSPELTTTSLMIKATLLSVLFLWVRASYPRFRYDQLMHLI
Exsp	NILLMNTLSATLFLGTTIYHFSPELTSMGLMIKAAMLSTLFLWVRASYPRFRYDQLMHLI
Depa	NILLMNTLSATLFLGTSLFHTFPELTSMNLMLKATLLSVLFLWIRASYPRFRYDQLMHLI
Rima	NILLMNTLSAILFFGTSTYHAHPELSSMILMLKATLLSIVFLWVRASYPRFRYDQLMHLI
Fuol	NILLMNTLSTILFLGSSLSHLLPEMTSATLMIKATMLSVVFLWVRASYPRFRYDQLMHLI
Gmaf	NIILMNTLSASIFLGTPFNHSLPELTSLAIMVKTALLVAIFLWVRASYPRFRYDQLMHLT
Xeei	NILLMNTLSAILFLGSSLFHLTPQITSIVLMTKAALLSILFVWVRASYPRFRYDQLMHLI
Pros	NILLMNTLSATLFLGAAHIPTLPELTAMNLMTKAALLSVLFLWVRASYPRFRYDQLMHLI
Scmi	NILLMNTLSATLFLGASHIPAFPELTAMNLMTKAALLSIVFLWVRASYPRFRYDQLMHLI
Rolo	NILLMNTLSAILFLGASHIPALPELTAMNLMTKAALLSIVFLWVRASYPRFRYDQLMHLV
Cere	NILLMNTLSAILFLGASHIPTMPEITSINLMTKAALLSIVFLWVRASYPRFRYDQLMHLV
Daga	NILLMNTLSAVLFLGASHIPTLPELTSVNLMTKAAFLSILFLWIRASYPRFRYDQLMHLV
Anco	NILLMNTLSTILFLGASHIPTFPELTAVNLMTKAALLSVVFLWVRASYPRFRYDQLMHLV
Dmve	NILLMNTLSTILFMGASHIPTLPELTAVNLMTKAAFLSIIFLWVRASYPRFRYDQLMHLV
Dmar	NILLMNTLSTILFLGASHIPTFPELTTINLMTKAALLSVIFLWVRASYPRFRYDQLMHLV
Anka	NILLMNTLSTILFLGASHIPTLPELTAANLMTKAALLSVVFLWVRASYPRFRYDQLMHLV
Moja	NILLMNTLSTILFLGASHIPTLPELTTINLMTKAALLSVVFLWVRASYPRFRYDQLMHLV
Hoja	NILLMNTLSTILFLGASHIPTLPELTAVNLMTKAALLSVVFLWVRASYPRFRYDQLMHLV
Bede	NILLMNTLSATLFLGASHIPTIPELTAMNLMTKAALLSVVFLWVRASYPRFRYDQLMHLI
Besp	NILLMNTLSATLFLGASHIPTIPELTAMNLMTKAALLSVVFLWVRASYPRFRYDQLMHLI
Mysp	NILLMNTLSTILFLGASHTPTLPELTAINIMTKAALLSIVFLWVRASYPRFRYDQLMHLV
Osja	NILLMNTLSTILFLGASHTPTLPELTAMNIMTKAALLSIVFLWVRASYPRFRYDQLMHLV
Sgro	NILLMNTLSTILFLGASHAPTLPELTALNLMTKAAFLSIVFLWVRASYPRFRYDQLMHLV
Pzpa	NILLMNTLSAILFFGASTHTNLPELTSINIMIKAAILSMVFLWVRASYPRFRYDQLMHLV
Zeja	NILLMNTLSAILFFGSSTMPGIPELTSVNIMVKTAILSMLFLWVRASYPRFRYDQLMHLV
Znne	NILLMNTLSAILFFGASVMPTMPELASINIMIKAALLSMVFLWVRASYPRFRYDQLMHLV
Zefa	NILLMNTLSAILFFGASTLPYLPELTSANIMVKAALLSMVFLWVRASYPRFRYDQLMHLV
Acni	NILLMNTLSAILFFGASTIPSFPELTSTNIMTKAAILSMVFLWVRASYPRFRYDQLMHLV
Ncrh	NILLMNTLSAILFFGASTIPSFPELTSTNIMTKAAILSMVFLWVRASYPRFRYDQLMHLV
Agca	NILLMNTLSATLFLGASHIPTIPELTAINLMTKAALLSIVFLWVRASYPRFRYDQLMHLI
Hydy	NILLMNTLSATLFLGASHIPSFPELTAVNLMTKAAFLSVLFLWVRASYPRFRYDQLMHLI
Gsac	NILLMNTLSATLFLGAYHIPSFPELTAVNLMTKAALLSVVFLWVRASYPRFRSDQLMHLI
Pevo	NILLMNTLSTTLFLGAAHTMMSPELSSANLMIKAALMSFCFLWVRASYPRFRYDQLMHLI
Hiku	NILLMNTLSTILFLGASHIPPLPEITTINLMMKATLLSVVFLWVRASYPRFRYDQLMHLI
Inpa	NILFMNALSALLFLGPLLPHAHPELATTVLMIKATLLAVGFLWVRASYPRFRYDQLMHLT
Auch	NILMMNTMSTVLFLGALHLPTFPTLTTLNLMIKATILSMVFLWVRASYPRFRYDQLMHLT
Fico	NILLMNTLSAILFMGASHIPLFPEFTAANLMTKAALLSIVFLWVRASYPRFRYDQLMHLI
Macs	NILLMNTLSATLFLGASHIPTLPELTAVNLMTKAALLSILFLWVRASYPRFRYDQLMHLI
Moal	NILLMNTLSVILFLGSSNTLFSPELMTTNLMTKTALLSILFLWVRASYPRFRYDQLMHLT
Mafr	NILLMNTLSTILFLGAFHIPTLPELTSSNLMLKAASLSLLFLWIRASYPRFRYDQLMHLI
Dcpe	NILLMNTLSAVLFLGASHIPSLPELTAANLMTKAALLSMVFLWVRASYPRFRYDQLMHLI
Dcti	NILLMNTLSAVLFLGASHVPSFPELTAVNLMTKAALLSMVFLWVRASYPRFRYDQLMHLI
Hehi	NILLMNTLSATLFLGASHFPMLPELTAINLMTKAALLSVLFLWVRASYPRFRYDQLMHLI
Stam	NILLMNTLSATLFLGASHIPTLPELTSFNLMTKAALLSVVFLWVRASYPRFRYDQLMHLI
Hogi	NILLMNTLSAALFLGAAHIPSLPELTTINLMLKATLLSVIFLWVRASYPRFRYDQLMHLI
Erzo	NILLMNTLSATLFLGASHIPAFPELTAVNLMTKAALLSVVFLWVRASYPRFRYDQLMHLI
Hxot	NILLMNTLSATLFLGASHIPSIPELTAVNLMTKAALLSVLFLWVRASYPRFRYDQLMHLI
Core	NILLMNTLSATLFLGASHIPSIPELTAVNLMTKAALLSVLFLWVRASYPRFRYDQLMHLI
Apve	NILLMNTLSAMLFLGASHFPAIPELTTLNLMTKATILSLMFLWIRASYPRLRYDQLMHLL
Latj	NILLMNTLSATLFLGAAHLPTTPELTTINLMTKAALLSVLFLWVRASYPRFRYDQLMHLI
Laja	NILLMNTLSATLFLGALHLPTIPELTAINLMTKAALLSVMFLWVRASYPRFRYDQLMHLI
Syja	NILLMNTLSAALFLGASHIPTMPALTTMNLMIKAALLAVVFLWVRASYPRFRYDQLMHLV

To be continued on page 28.

[5/6 of aligned sequences]

Epme	NILLMNTLSATLFLGASHIPATPEMTAVNLMTKAALLSLLFLWVRASYPRFRYDQLMHLI
Grse	NILLMNTLSAILFLGASHIPTIPELTAINLMTKAALLSLVFLWVRASYPRFRYDQLMHLI
Clja	NILLMNTLSTVLFLGAFHIPSLPWMTTINLMIKTASLSAVFLWVRASYPRFRYGQLMHLI
Ogcy	NILLMNTLSAILFLGSTLMHSSPETTTILTMTKAALLSVVFLWVRASYPRFRYDQLMHLI
Plna	NILLMNTLSATLFLGTSLLHHNPDLTSTNLMTKAAFLSVLFLWVRASYPRFRYDQLMHLI
Lema	NILLMNTLSATLFLGASHIPAIPELTTMNLMTKAALLSMVFLWVRASYPRFRYDQLMHLI
Etzo	NILLMNTLSATLFLGASHIPAIPELTAFNLMTKAAILSVVFLWVRASYPRFRYDQLMHLI
Apse	NILLMNTLSATLFLGATHFPALPELTTVNLMTKAALLSIVFLWVRASYPRFRYDQLMHLV
Epde	NILLMNTLSATLFLGASHIPMIPELTAFNLMTKAALLSIVFLWVRASYPRFRYDQLMHLI
Slja	NILLMNTLSATLFLGALFIPAIPELTSVNLMFKAALLSVLFLWVRASYPRFRYDQLMHLI
Bsja	NILLMNTLSAILFLGAFHLPTLPTLTAINLMTKAALLSVVFLWVRASYPRFRYDQLMHLI
Ecna	NILLMNTLSATLFLGASHIPTLPELTATNLMIKAALLSVLFLWVRASYPRFRYDQLMHLI
Cohi	SILLMNLFTVVLFLGGAYTLSIPELTATTCGLKVVTLALFFLWIRASFPRLRYDQLMHLT
Caar	NILLMNTLSATLFLGASHIPTLPELTAVNLMTKAALLSIVFLWVRASYPRFRYDQLMHLI
Came	NILLMNTLSATLFLGASHIPTLPELTAINLMTKAALLSIVFLWVRASYPRFRYDQLMHLI
Mema	NILLMNTLSATLFLGASHFPTIPELTAINLMTKAALLSIVFLWVRASYPRFRYDQLMHLI
Lenu	NILLMNTLSATLFLGASHIPTLPQLTTISLMTKAAFLSVLFLWVRASYPRFRYDQLMHLI
Brja	NILLMNTLSATLFLGASHIPTIPELTATNLMTKAALLSMLFLWVRASYPRFRYDQLMHLI
Plma	NILLMNTLSATLFLGASHIPTMPELTATNLMIKAALLSMVFLWVRASYPRFRYDQLMHLI
Emst	NILLMNTLSATLFLGASHIPTIPELTAVNLMTKAALLSIVFLWVRASYPRFRYDQLMHLI
Ptti	NILLMNTLSATLFLGASHIPTIPELTAMNLMTKAAFLSIVFLWVRASYPRFRYDQLMHLI
Losu	NILMMNTLSAALFLGATHIPMAPEMMTLNLMMKAALLSTIFLWVRASYPRFRYDQLMHLI
Geoy	NILLMNTLSATLFVGATHIPSLPELTAVNLMTKAALLAILFLWVRASYPRFRYDQLMHLI
Dipi	NILLMNTLSATLFLGASHIPTFPELTAMNLMTKAALLSIVFLWVRASYPRFRYDQLMHLI
Pama	NILLMNTLSATLFLGASHIPMFPELTAMNLMTKAALLSVVFLWVRASYPRFRYDQLMHLI
Leob	NILLMNTLSATLFLGASHVPMFPELTAANLMTKAALLSVLFLWVRASYPRFRYDQLMHLI
Neba	NILLMNTLSATLFLGASHFPSMPELTAINLMFKAALLSIVFLWVRASYPRFRYDQLMHLV
Pdpl	NILFMNTLSAMLFLGAYYTPALPELTTVNLMAKAALLSIVFLWVRASYPRFRYDQANALN
Nimi	NILLMNTLSATLFLGALHAPTIPELTAINLMTKAALLSVLFLWVRASYPRFRYDQLMHLI
Uptr	NILLMNTLSATLFLGASHIPSLPELTAANLMTKAALLSVVFLWVRASYPRFRYDQLMHLI
Pesc	NILLMNTLSATLFFGASYIPAMPELTTINLMLKAAILTLVFLWVRASYPRFRYDQLMHLI
Baar	NILLMNTLSATLFLGASHFPTAPELTTANLMTKASLLSIVFLWVRASYPRFRYDQLMHLI
Moar	NILLMNTLSATLFLGASHIPTLPELTAINLMTKAALLSIVFLWVRASYPRFRYDQLMHLI
Toja	NILLMNTLSATLFLGASHIPTIPELTAMNLMTKAAFLSILFLWVRASYPRFRYDQLMHLV
Chau	NILLMNTLSATLFLGACHIPLFPMLTTINMMTKAALLSILFLWVRASYPRFRYDQLMHLI
Chse	NILLMNTLSAILFLGATYLPYLSLLTTTNLMIKATLLSVVFLWVRASYPRFRYDQLMHLI
Enar	NILLMNTLSATLFLGASHIPTIPELTAVNLMTKAALLSIVFLWVRASYPRFRYDQLMHLI
Hpty	NILLMNTLSATLFLGASHIPTIPELTAINLMTKAALLSIVFLWVRASYPRFRYDQLMHLI
Nana	NILLMNTLSIILFMGASHMPLIPELTAMNLMTKAAILSMIFLWVRASYPRFRYDQLMHLI
Mcst	NILLMNTLSATLFLGASHIPALPELTALNLMTKAALLSVLFLWVRASYPRFRYDQLMHLI
Rhox	NILLMNTLSATLFLGASHIPLVPEFTAINLMTKAALLSVLFLWVRASYPRFRYDQLMHLI
Opfa	NILLMNTLSATLFLGASHIPTFPELTAMNLMIKAALLSVVFLWVRASYPRFRYDQLMHLI
Paar	NILLMNTLSATLFLGAYHIPTIPGLTTINLMVKAALLSIVFLWVRASYPRFRYDQLMHLI
Gozo	NILLMNTLSATLFLGASHIPAIPELTAVNLMTKAALLSIVFLWVRASYPRFRYDQLMHLI
Ackr	NILFMNTLSAALFLGASHIPLFPQLTTLNLMTKAAFLSVVFLWVRASYPRFRYDQLMHLI
Elev	NILMMNTLSTVLFLGASHFPNCPELTTLNMMTKAAILSLVFLWVRASYPRFRYDQLMHLI
Trdu	NILLMNTLSATLFLGASIFHTTPEITTTNLMIKATLLSVLFLWVRASYPRFRYDQLMHLI
Amoc	NILLMNTLSATLFLGASLLHFLPELTSINLMTKAALLSVLFLWVRASYPRFRYDQLMHLI
Hame	NILLMNTLSAALFLGAFHTPMFPELTTANLMTKAAILSLLFLWVRASYPRFRYDQLMHLI
Chso	NILLMNTLSAVLFLGALYIPTLPELTSVNLMTKAALLSVLFLWVRASYPRFRYDQLMHLV
Lyto	NILLMNTLSATLFLGASHIPAVPELTAINLMTKAALLSVLFLWVRASYPRFRYDQLMHLI
Encr	NILLMNTLSATLFLGASHIPSLPELTAINLMTKAALLSVVFLWVRASYPRFRYDQLMHLI

To be continued on page 29.

```
Bvar  NILLMNTLSAVLFLGASHIPLLPELTAINIMTKAALLSVLFLWVRASYPRFRYDQLMHLI
Noco  NILLMNTLSTILFLGASHIPSFPELTTTNLMVKAALLSVVFLWVRASYPRFRYDQLMHLI
Chsp  NILLMNTLSAVLFLGTPYLLFMPELTSMSLAIKTTILSTLFLWVRASYPRFRYDQLMHLI
Arja  NILLMNILSVMLFLGSSYIASMPELTTSNMLVKMALLSTVFIWVRASYPRLRYDQLMHLV
Pase  NILLMNTLSTILFLGAAHLPMFPELTTVNLMTKAALLSVLFLWVRASYPRFRYDQLMHLI
Trel  NILLMNTLSAAIFLGAFHFPALPEMTSANIMTKAALLSMVFVWVRASYPRFRYDQLMHLT
Lifa  NILLMNTLSAVLFLGAYHLPALPELTSLNLMTKAALLSLVFLWVRASYPRFRYDQLMHLI
Acur  NILLMNTLSTILFLGAFHVPLIPELTSINLMTKAALLSMVFLWVRASYPRFRYDQLMHLI
Ampe  NILLMNTLSATLFLGASHLPTFPELTAVNLMTKAALLSVLFLWVRASYPRFRYDQLMHLI
Urja  NILLMNTLSATLFLGASHAPHTPGLTTINLMFKAALLSVLFLWVRASYPRFRYDQLMHLT
Enet  NILLMNTLSAVLFLGSTFSLDSPYTTTFTLMVKATLLSVVFLWVRASYPRFRYDQLMHLT
Ptbr  NILFMSTLSSVLFLGSTTWQFFPSLTTISLMTKASFLAVLFIWVRASYPRYRYDQLMHLI
Safa  NILFMNTLSATLFLGTTLLISMPYITTIILMMKATFLAVLFLWVRASYPRFRYDQLMHLI
Icae  NILLMNTLSATLFLGASHIPTLPELTTTNLMIKAALLSMVFLWVRASYPRFRYDQLMHLI
Asmi  NILLMNTLTAVLFLGSTTTLSDSSLMSVTLMIKVALMSVVFLWVRASYPRFRYDQLMHLI
Foal  NILLMNTMSAVMFLGALHMPSMPAMTSLNLMLKASFLALLFLWVRASYPRFRYDQLMHLI
Drze  NILLMNTLSVVLFLGASHTITFPELTTMNLMTKTALLSIVFLWVRASYPRFRYDQLMHLI
Rhas  NILLMNTLSATLFLGASHLPHMSELTTINLMAKAALLSVVFLWVRASYPRFRYDQLMHLI
Elac  NILLMNTLSAILFLGTSHFPHAPQITTMSLMFKAALLSVLFLWVRASYPRFRYDQLMHLI
Kugu  NILLMNTLSATLFLGAYHLPSMPELTTINLMTKAAMLSLVFLWVRASYPRFRYDQLMHLI
Plor  NILLMNTLSATLFLGASHIPALPELTAINLATKAALLSVLFLWVRASYPRFRYDQLMHLI
Sgun  TFFMMNTLSATLFLGASHIPTIPELTAINLMTKAALLSMVFLWVRASYPRFRYDQLMHLI
Zaco  NILLMNTLSATLFLGASHIPTVPEFTTVNLMTKAALLSIMFLWVRASYPRFRYDQLMHLI
Zbfl  NVLLMNTLSATLFLGASHIPLAPELTTISVMIKAALLSSVFLWVRASYPRFRYDQLMHLI
Spba  NILLMNTLSATLFLGATHLPMIPELTAMNLMTKAALLSIMFLWVRASYPRFRYDQLMHLV
Game  NILLMNTLSATLFLGASHIPTLPELTATNLMFKAALLSMVFLWVRASYPRFRYDQLMHLI
Thth  NILLMNTLSATLFLGASHIPTIPELTATNLMIKAALLSMVFLWVRASYPRFRYDQLMHLI
Xigl  NILLMNTLSATLFLGASHIPTIPELTAANLMTKAALLSMVFLWVRASYPRFRYDQLMHLI
Hyja  NILLMNTLSATLFLGASHLPTMPGLTTTNLMIKAALLSMVFLWVRASYPRFRYDQLMHLI
Psan  NILLMNTLSALMFLGPSYIWQMPELGTIILMIKASFLSLAFIWARASFPRFRYDQLMHLI
Cupa  NILLMNTLSATLFLGASHIPTMPELTATNLMIKAALLSMVFLWVRASYPRFRYDQLMHLI
Mpch  NILLMNTLSAILFLGASYIPSIPELTTVNLMTKAALLSLVFLWVRASYPRFRYDQLMHLI
Char  NILLMNTLSVALFLGASHIPSFPELTTVNLMVKASLLSILFLWVRASYPRFRYDQLMHLI
Pser  NILLMNTLSATLFLGASHIPTLPELTAINLMTKAALLSIVFLWVRASYPRFRYDQLMHLI
Prol  NILLMNTLSAVLFLGASHIPTIATLTAINLMTKAALLSVVFLWVRASYPRFRYDQLMHLI
Plbi  NILLMNALSATLFLGASHIPSIPELTSINIMTKAALLSVVFLWVRASYPRFRYDQLMHLI
Calu  NILFMNALTATLFLGTSTHHSTPVSTSTKIMLKLLLLALVFLWIRAAFPRFRYDQLMHLI
Papa  NILLMNTLSTILFLGACHDHLSPQLTTINLMTKAVLLSVLFLWIRASYPRYRYDQLMHLI
Sufr  NILLMNTLSAVLFLGASHIPSFPGLTSANLMTKAAFLSIVFLWVRASYPRFRYDQLMHLI
Stci  NILLMNTLSALLFLGASHIPSIPELTSMNLMTKAALLSMLFLWVRASYPRFRYDQLMHLV
Taru  NILLMNTLSTILFLGALHMPALPELTSINLMSKTAILSLIFLWARASYPRFRYDQLMHLT
Rala  NILLMNTLSATLFLGAYHLPSFPELTAMNLMTKAALLSMVFLWVRASYPRFRYDQLMHLI
      ..::*.  :  :*                   *   :   *:  *.  ** * :   *
```

To be continued on page 30.

[6/6 of aligned sequences]

```
                    H
Scca  WKNFLPLTLAIILWHMALPLATTSLPPLT*--
Muma  WKNFLPLTLAIILWHVALPLAMTSLPPIT*--
Erca  WKNFLPLTLAMIAWHISLPISMAGSPPQL---
Pose  WKNFLPLTLALIIWHISLPISLAGSPPQL---
Actr  WKNFLPITLALVLWHVSLPIASAGLPPQI*--
Scal  WKNFLPITLALVLWHISLPIASAGLPPQV*--
Posp  WKNFLPITLALVLWHISLPIASAGLPPQI*--
Atsp  WKNFLPLTLALVLWHTALPIALASLPPQT*--
Leoc  WKNFLPLTLALVLWHTALPIALASLPPQT*--
Amca  WKNFLPITLAIILWHTALPIAMAGLPPQT*--
Osbi  WKNFLPITLALILWHTALPIAMAGIPPQI*--
Pabu  WKNFLPLTLALILWHISIPLSTASLPPQM---
Hial  WKNFLPLTLALVLWHLALPIALAGLPPQI*--
Elha  WKNFLPLTLALVLWHAALPIALAGLPPQI*--
Mlcy  WKNFLPLTLALVLWHTALPIAIAGLPPQT*--
Algl  WKNFLPLTLALMIWHVALPVSLAGLPPQG*--
Ptgi  WKNFLPLTLALVVWHIALPIALAGLPPQN*--
Alaf  WKNFLPITLALVIWHMALPIALAGLPPQT*--
Nock  WKNFLPVTLALVIWHMALPIAMAGLPPQT*--
Anja  WKNFLPLTLALLIWNLALPITMAGLPPNN*--
Gyki  WKNFLPLALALLMWNMALPVALAGLPPQL*--
Syka  WKSFLPLALALVIWNLALPIATAGLPPN*---
Opma  WKSFLPLALALLIWNLALPIALASLPPH*---
Comy  WKNFLPLSLALVIWNLALSTALASLPPS*---
Sasp  WKNFLPLTLGLLICNLSLITALAGLPPNN*--
Eupe  WKNFLPLTLALLTWNLALAIALTGLPPQ*---
Enja  WKNFLPLTLALILWHTAVPLATAGLPPQL*--
Same  WKNFLPVTLALILWHISLPVGTAGLPPQF*--
Chch  WKSFLPMTLALVLWHSALPIALAGLPPQL*--
Grgr  WKGFLPLTLALTLWHLAFPLALAALPPQG*--
Caau  WKNFLPLTLAFVLWHTALPIALAGLPPQL*--
Cyca  WKNFLPLTLAFVLWHTALPIALAGLPPQL*--
Dare  WKNFLPITLVLVLWHIALPIALAGLPPQT*--
Cost  WKNFLPLTLALVLWHTALPIAFAGLPPQL*--
Leec  WKNFLPLTLALVLWHTALPIALAGIPPQL*--
Fola  WKNFLPLTLALVLWHTALPIAFAGLPPQL*--
Clmc  WKNFLPITLALILWHTALPIAFAGLPPQL*--
Phin  WKNFLPLTLAMILWHMALLTAFASLPPQ*---
Icpu  WKNFLPMTLALILWHAALPVALTGLPPQL*--
Psto  WKNFLPLTLALILWHIALPIALTGLPPQL*--
Cora  WKNFLPLTLALILWHIALPIAFTGLPPQL*--
Eisp  WKSFLPMTIALILWHTALPIAFAGLPPQL*--
Apal  WKNFLPTTLALIIWHTALLIAFAGLPPQL*--
Eslu  WKNFLPLTLAFVLWHLAIPTAFMGLPPQL*--
Dape  WKTFLPLTLALVLWHLALPTALIGLPPHL*--
Glse  WKNFLPLTLALVLWHLALPISFTGLPPQP*--
Naar  WKSFLPLTLALVLWHLALPTAFAGLPPQL*--
Lioc  WKNFLPITLALVLWHLALPTALAGLPPQL*--
Opso  WKSFLPLTLALVLWHLALPIAFAGLPPQL*--
Alte  WKNFLPLTLALVLWHIALPIAFAGLPPQL*--
Plap  WKNFLPMTLALVLWHIALPIAFAGLPPQL*--
```

[6/6 of aligned sequences]

```
Plal  WKNFLPLTLALVLWHLALPIAFAGLPPQL*--
Sami  WKSFLPLTLALVLWHLALPLAFAGLPPQL*--
Rere  WKSFLPLTLALVLWHLALPIAFAGLPPQL*--
Gama  WKNFLPLTLAMVIWHLALPIAFAGLPPQL*--
Onmy  WKSFLPLTLALVLWHLALPIALAGLPPQL*--
Sasa  WKSFLPLTLALVLWHLALPTAMAGLPPQL*--
Cola  WKSFLPMTLALVLWHLALPIALAGLPPQL*--
Dita  WKNFLPLTLALLLWHLALPVAFAGLPPQA*--
Gogr  WKNFLPITLALVLWHLALPIALMGLPPQL*--
Chsl  WKNFLPVTLALVLWHLALPVALAGLPPQL*--
Atja  WKSFLPLTLALVLWHLSLPIALASIPPQT*--
Iido  WKNFLPLTLAMVLWHLSLPTALASIPPQT*--
Auja  WKNFLPLTLALVIWHLALPIAFASLPPQL*--
Chag  WKNFLPVTLALVIWHLALPIAFASLPPQL*--
Hami  WKNFLPLTLALVIWSLAIPIAFAGLPPQL*--
Saun  WKNFLPLTLALIIWSLALPIAFAGLPPQL*--
Nema  WKNFLPLTLALVIWHLALPIALASIPPQF*--
Disp  WKNFLPVTLALVIWHLALPIALASVPPQL*--
Myaf  WKNFLPITLALVIWHLALPIALASIPPQI*--
Lagu  WKNFLPTTLALVVFHLALTTALASTPPQL*--
Trtr  WKNFLPLTLALVILHLSLTVSFASAPPQL*--
Zucr  WKNFLPLTLALVIFHLSLTIALAGAPPQL*--
Pxja  WKNFLPLTLALIIWHLALPIATASLPPHL*--
Pxlo  WKNFLPLTLALIIWHLALPIATASLPPHL*--
Pctr  WKNFLPVTLALVVWHLALPTALAGLPPHL*--
Apsa  WKNFLPLTLALIIWHIALPTSLAGLPPHLLS-
Cabe  WKNFLPLSLAFIIWHLALGVALTGLPPAY*--
Bzze  WKNFLPLSLALIIWHLALPTALAGLPPQL*--
Siim  WKNFLPLSLALIILHLSLPTSLASLPPML*--
Ctru  WKNFLPLTLALVVWHLAVPIAFAGLPPQPW--
Dpbr  WKNFLPLTLALVIWHLALPTAFAGLPPQPW--
Caki  WKNFLPLTLAMVIWHLSLSTAFAGLPPQT*--
Phja  WKNFLPLTLALLIWHLSLSISFAGLPPQM*--
Brsp  WKNFLPLTLGFMIWHLSISISLSGLPPQI*--
Gamo  WKNFLPLTLALVIWHLSLSTACAGLPPHA*--
Lolo  WKNFLPLTLALVIWHLSLSTACAGLPPHA*--
Batr  WKGFLPLTLSLMLFNLALINTMTGIPPM*---
Prmy  WKAFLPLTLALLLFNNSLIYALVGTPPM*---
Lose  WKNFLPLTLALVIWHTSTPITLAGLPPQM*--
Loam  WKNFLPLTLALIIWHLSLPIALAGLPPQM*--
Chab  WKNFLPLTLALVIWHLSLPVTLAGLPPQL*--
Chto  WKNFLPLTLALIIWHLSLPITLAGLPPQL*--
Majo  WKNFLPLTLALIIWHLSLPTALAGIPPQL*--
Hlst  WKSFLPLTLALIVLHLAMPTATAGIPPQM*--
Clpe  WKGFLPLTLAFIIWHLSLPIALAGLPPHP*--
Mlmr  WKNFLPLTLALIIWHLSLPIALTGVPPQP*--
Crcr  WKNFLPLTLALVIWHLALPIALSGLPPQL*--
Muce  WKNFLPLTLALVIWHLALPIALSGLPPQL*--
Bege  WKNFLPLTLALVIWHLALPIAFTGLPPQL*--
Mela  WKNFLPLTLAMVIWHFALSLALAGLPPHL*--
Hats  WKNFLPLTLALVIWHLALPIAFTGLPPQL*--
Orla  WKNFLPLTLALVIWHLSLPISFNGLPPQL*--
```

[6/6 of aligned sequences]

```
Cosa    WKSFLPLTLALVIWHLALPISLLGLPPQM*--
Exsp    WKNFLPLTLALVIWHLAVPISFSGLPPHL*--
Depa    WKNFLPLTLALVIWHLALPISFSGLPPQF*--
Rima    WKNFLPLTLAFVIWHLSLPIAFLGLPPQM*--
Fuol    WKNFLPITLALVIWHLSLPISFSGLPPQL*--
Gmaf    WKNFLPTTLALVIWHLSLPLAFSSFPPQI*--
Xeei    WKNFLPLTLALVIWHLSLPIAFFGLPPQL*--
Pros    WKNFLPLTLALVIWHLALPIAFAGLPPQL*--
Scmi    WKNFLPLTLALVIWHLALPVTFAGLPPQL*--
Rolo    WKNFLPLTLALVIWHLALPIAFVGLPPQL*--
Cere    WKNFLPLTLALVIWHLATPIAFAGLPPYL*--
Daga    WKSFLPMTLALLIWHLSTPIAFAGLPPQP*--
Anco    WKSFLPLTLALVIWHLALPIAFAGLPPQL*--
Dmve    WKNFLPLTLALMLWNLALPVAFTGLPPQL*--
Dmar    WKNFLPLTLALMLWNLALPVAFTGLPPQL*--
Anka    WKNFLPLTLALVVWHLALPIAFAGLPPQL*--
Moja    WKSFLPLTLALVVWHLALPIAFAGLPPQF*--
Hoja    WKNFLPLTLALVVWHLALPIAFAGLPPQL*--
Bede    WKNFLPLTLALVIWHLALPIAFAGLPPQL*--
Besp    WKNFLPLTLALVIWHLALPIAFAGLPPQL*--
Mysp    WKNFLPLTLALVIWHLALPIAFAGLPPQL*--
Osja    WKNFLPLTLALVIWHLALPIAFAGLPPQL*--
Sgro    WKNFLPLTLALVVWHLALPIAFAGLPPQL*--
Pzpa    WKNFLPLTLAMVIWHLALPLSLGGIPPQT*--
Zeja    WKNFLPITLAMVIWHLALPLALAGIPPQI*--
Znne    WKNFLPITLAMMVWNLALPLSLAGIPPQI*--
Zefa    WKNFLPITLAMVVWHLALPLSLAGIPPQI*--
Acni    WKNFLPITLAMVIWHLALPLSLASIPPQI*--
Ncrh    WKNFLPITLAMVIWHLALPLSLASIPPQI*--
Agca    WKNFLPLTLALVIWHLALPIAFAGLPPQI*--
Hydy    WKNFLPLTLALVIWHLSLPIAFAGLPPQA*--
Gsac    WKNFLPLTLALVIWHLSLPIAFAGLPPQV*--
Pevo    WKNFLPITLALLIWHLALPTAFAGLPPQN---
Hiku    WKNFLPLTLALIIWHLSTPLALAGLPPHM*--
Inpa    WKNFLPLSLALVLWHLSLTTSLTGLPPLH*--
Auch    WKNFLPLALAFLIFQLALPIASTGLPSVL*--
Fico    WKNFLPLTLAMVIWHLALPIALAGLPPQL*--
Macs    WKNFLPLTLAMVIWHLALPVAFAGLPPQL*--
Moal    WKSFLPLTLAMVIWHLALPIALTGLPPQL*--
Mafr    WKNFLPITLALVIWHLALPITFAGLPPQT*--
Dcpe    WKNFLPLTLALVMWHLSLPIAFAGLPPQL*--
Dcti    WKNFLPLTLALVMWHLSLPIAFAGLPPQPVV*
Hehi    WKNFLPLTLALVIWHLALPIAFAGLPPQL*--
Stam    WKNFLPLTLALVIWHLALPIAFAGLPPQL*--
Hogi    WKNFLPLTLALIIWHLALPIAFAGLPPQL*--
Erzo    WKNFLPLTLALVIWHLALPIAFAGLPPQL*--
Hxot    WKNFLPLTLALVIWHLALPIAFAGLPPQL*--
Core    WKNFLPLTLALVVWHLALPVAFAGLPPQL*--
Apve    WKNFLPLTLAMVVWHLALPIAFAGLPPQV*--
Latj    WKNFLPLTLALVIWHLALPTAFAGIPPQL*--
Laja    WKNFLPLTLALVIWHLSLPITFAGLPPHL*--
Syja    WKNFLPLTLALVIWHLALPIALAGLPPLL*--
```

[6/6 of aligned sequences]

```
Epme    WKNFLPLTLALVIWHLALPIALAGLPPQT*--
Grse    WKNFLPLTLALIIWHLALPIALAGLPPQT*--
Clja    WKNFLPLTLAFCVLHLSLPITFAGLPSQF*--
Ogcy    WKSFLPLTLALVIWHLALPMALAGLPPQL*--
Plna    WKNFLPLTLALIIWHLSLPITFNGLPPQL*--
Lema    WKNFLPLTLALVIWHLALPIAFAGLPPML*--
Etzo    WKNFLPLTLALVIWHLALPIAFAGLPPQL*--
Apse    WKNFLPLTLSLVIWHLSLPIAFAGLPPQF*--
Epde    WKNFLPLTLALVIWHLALPIALAGLPPQL*--
Slja    WKNFLPLTLALLIWHLALPIAFAGLPPQA*--
Bsja    WKNFLPLTLALVIWHLALPIALAGLPPVL*--
Ecna    WKNFLPLTLALVIWHLALPLAFAGLPPQF*--
Cohi    WKAFLPMSLALIIWHLSLATTFAGLPPM*---
Caar    WKNFLPLTLALVIWHLALPIAFAGLPPQL*--
Came    WKNFLPLTLALVIWHLALPIAFAGLPPQL*--
Mema    WKNFLPLTLALIIWHLALPIAFAGLPPQL*--
Lenu    WKSFLPLTLGLIIWHLALPIAFNGLPPLP*--
Brja    WKNFLPLTLALVIWHLALPTALVGLPPQL*--
Plma    WKNFLPLTLALVIWHLALPIAFAGLPPQL*--
Emst    WKNFLPLTLALVIWHLALPIAFAGLPPQL*--
Ptti    WKNFLPLTLALVIWHLALPIAFAGLPPQL*--
Losu    WKNFLPLTLALVIWHLASPIAFIGLPPQL*--
Geoy    WKNFLPLTLALVIWHLALLVAFAGLPPLI*--
Dipi    WKNFLPLTLALVIWHLALPIAFAGLPPQL*--
Pama    WKNFLPLTLALVVWHLAIPIAFAGLPPHL*--
Leob    WKNFLPLTLAFIIWHLSLPIALAGLPPQL*--
Neba    WKNFLPLTLALVIWHLALPIACAGLPPQL*--
Pdpl    LKNFLPITLALVIWHLALPIAFAGLPPQM*--
Nimi    WKNFLPLTLALIIWHLALPIALAGLPPQL*--
Uptr    WKNFLPLTLALVIWHLSLPIAFAGLPPHL*--
Pesc    WKNFLPLSLALFLWHLALPIAVMGIPPQS*--
Baar    WKNFLPLTLALVIWHLSLPIALAGLPPQL*--
Moar    WKNFLPLTLALVIWHLALPIAFAGLPPQL*--
Toja    WKNFLPITLALVIWHLALPIAFAGLPPQL*--
Chau    WKNFLPLTLALLIWHLALPIAFAGLPPQ*---
Chse    WKSFLPLTLALVIWHLSIPIAFAGLPPQT*--
Enar    WKNFLPLTLALVIWHLALPIAFAGLPPQL*--
Hpty    WKNFLPLTLALVIWHLALPIAFAGLPPQL*--
Nana    WKNFLPLTLAMVIWHLALPIAFAGLPPQL*--
Mcst    WKNFLPLTLALVIWHLALPIAFAGLPPHL*--
Rhox    WKNFLPLTLALIIWHLALPIAFAGLPPQL*--
Opfa    WKNFLPLTLALVIWHLALPIAFAGLPPHL*--
Paar    WKNFLPLTLALIIWHLALPIAFAGLPPQL*--
Gozo    WKNFLPLTLALVIWHLALPIAFAGLPPQL*--
Ackr    WKNFLPLTLALVIWHLALPLAFTGHPPYL*--
Elev    WKNFLPLALALILWNLALPIALASLPPMF*--
Trdu    WKNFLPLTLALVVWHLSLPIALTGLPPQL*--
Amoc    WKNFLPLTLALVIWHLSLPIAFTGLPPQL*--
Hame    WKNFLPITLALVIWHLALPTAFAGLPPQL*--
Chso    WKNFLPLTLAFMIWHLALLIAFAGLPPHL*--
Lyto    WKSFLPLTLALVIWHLALPVALAGLPPQV*--
Encr    WKNFLPLTLALVIWHLALPIAFAGLPPQV*--
```

[6/6 of aligned sequences]

```
Bvar      WKNFLPLTLALVIWHLALPTALAGLPPQL*--
Noco      WKNFLPLTLALVIWHLALPIAFTGLPPQF*--
Chsp      WKNFLPVTLALIVWHMSTPTSFAGHPPQY*--
Arja      WKNFLPMALAMVIWYLAIPVAFAGLPPQV*--
Pase      WKNFLPLTLALIIWHLALPVAFIGLPPQL*--
Trel      WKNFLPLALALIMWHLSLPTAFMGIPPNL*--
Lifa      WKNFLPVTLALIIWHMATPISAAGLPPHL*--
Acur      WKNFLPLALALIIWHLALPIALAGLPPQF*--
Ampe      WKNFLPLTLALVIWHLALPIAFAGLPPQL*--
Urja      WKNYLPLSLGLIILNLGLPLAFEGLPPF*---
Enet      WKNFLPVTLALVVWHLALPTGFAGLPPAS*--
Ptbr      WKNFLPLALALLIWNMSLLMSFAGLPPQ*---
Safa      WKNFLPLTLALVIWHLALPIATAGLPPQY*--
Icae      WKNFLPLTLALVIWHLALPIALAGLPPQL*--
Asmi      WKNFLPLTLALTIWHLSLPISFTGLPTQF*--
Foal      WKNFLPLTLAFITWHLAAPITFNGLPPQ*---
Drze      WKNFLPLTLAMVIWHLALPISFTGLPPQF*--
Rhas      WKNFLPLTLALVIWHLALPIALAGLPPQL*--
Elac      WKNFLPLTLALVIWHLSLPIALAGLPPQL*--
Kugu      WKNFLPLTLALMVWHLALPIAFSGLPPQM*--
Plor      WKNFLPLTLAMVIWHLSLPIAFAGLPPHL*--
Sgun      WKNFLPLTLALIIWHLALPIAFAGLPPQL*--
Zaco      WKSFLPLTLALVIWHLALPIALAGLPPQL*--
Zbfl      WKSFLPVTLALIIWHLALPIAFAGLPPHL*--
Spba      WKNFLPLTLALVIWHLALPTSFAGLPPQL*--
Game      WKNFLPLTLALVIWHLALPIAFAGLPPQL*--
Thth      WKNFLPLTLALVIWHLALPIAFAGLPPQL*--
Xigl      WKNFLPLTLALVIWHLALPIAFAGLPPQL*--
Hyja      WKNFLPLTLALVIWHLALPIAFAGLPPQL*--
Psan      WKNYLPMTLALLMFHLALLIAFAGLPPHL*--
Cupa      WKNFLPLTLALVIWHLALPIAFAGLPPQL*--
Mpch      WKSFLPLTLALVIWHLSLPIAFAGLPPQF*--
Char      WKNFLPLTLALVLWHLALPTAFAGLPPQL*--
Pser      WKNFLPLTLALVIWHLALPIAFAGLPPAI*--
Prol      WKNFLPLTLALIIWHLALPTALAGLPPQL*--
Plbi      WKNFLPLTLALVIWHLALPIAFAGLPPQL*--
Calu      WKSFLPLALALLLWHLALPLSLASLPPQA*--
Papa      WKNFLPLMLAFTIWHMALPIAFAGLPPQP*--
Sufr      WKNFLPLTLALIVWHLALPVAFAGLPPQL*--
Stci      WKNFLPLTLSLIIWHLALPISLGGLPPQL*--
Taru      WKNFLPLTLAFIIWHLALPTTMAGLPPQM*--
Rala      WKNFLPLTLALVIWHLALPIAFSGLPPQL*--
           * :**  : :      .      . *
```
