# Supplementary material for: Structure and variation of the mitochondrial genome of fishes
Source: BMC Genomics. 2016 Sep 7;17(1):719. doi: 10.1186/s12864-016-3054-y (PMC5015259; doi:10.1186/s12864-016-3054-y)
Supplement: Additional file 6: Figure S1-a. — Aligned amino acid sequences of the ATP8 gene in mt genomes of 250 fishes. Figure S1-b. Aligned amino acid sequences of the ATP6 gene in mt genomes of 250 fishes. Figure S1-c. Aligned amino acid sequences of the COI gene in mt genomes of 250 fishes. Figure S1-d. Aligned amino acid sequences of the COII gene in mt genomes of 250 fishes. Figure S1-e. Aligned amino acid sequences of the COIII gene in mt genomes of 250 fishes. Figure S1-f. Aligned amino acid sequences of the Cyt b gene in mt genomes of 250 fishes. Figure S1-g. Aligned amino acid sequences of the ND1 gene in mt genomes of 249 fishes. Figure S1-h. Aligned amino acid sequences of the ND2 gene in mt genomes of 250 fishes. Figure S1-i. Aligned amino acid sequences of the ND3 gene in mt genomes of 250 fishes. Figure S1-j. Aligned amino acid sequences of the ND4L gene in mt genomes of 250 fishes. Figure S1-k. Aligned amino acid sequences of the ND4 gene in mt genomes of 250 fishes. Figure S1-l. Aligned amino acid sequences of the ND5 gene in mt genomes of 250 fishes. Figure S1-m. Aligned amino acid sequences of the ND6 gene in mt genomes of 249 fishes. (ZIP 3250 kb) [file 12864_2016_3054_MOESM6_ESM.zip › Additional file 6 prot align/AF6h-ND2.pdf]

Additional file 6: Figure S1–h. Aligned amino acid sequences of the ND2 gene in mt genomes of 250 fishes.

Species name abbreviation followed by aligned amino acid sequences shown by one letter abbreviation. See Additional file 1 for abbreviation of species name. Amino acids shown by magenta letter denote hydrophobic residues. A-J in bold types with yellow background indicate putative transmembrane regions. Asterisk '*' indicates a fully conserved residue. Colon ':' and period '.' indicate 'strong' and 'weak' groups in the level of conservativeness, respectively, in the Gonnet Pam250 matrix, in which the strong and weak groups are defined as strong score >0.5 and weak score =<0.5, respectively (Thompson et al., 1997).

ND2

[1/6 of aligned sequences]

```
                                      A
Scca     ---MNPTVLTIIISSMGLGTTLTFIGSHWLLVWMGLEINTLAIIPLMIR--QHHPRAVEA
Muma     ---MNPTVLTILISSLGLGTTLTFIGSHWLLVWMGLEINTLAIIPLMIR--QHHPRAVEA
Erca     ---MSPYILFVMLSSLGLGTTLTFTSSHWLLAWMGLEINTLAIIPLMTN--KHHPRAVEA
Pose     ---MSPYILFIMLMSLGLGTTLTFASSNWFLAWMGLEINTLAIIPLMAR--HHHPRAVEA
Actr     ---MNPYVLAILLSSLGIGTTLTFASSHWLLAWMGLEISTLAIIPLMAQ--QHHPRAVEA
Scal     ---MNPYVLAVLLSSLGIGTTLTFVSSHWLLAWMGLEISTLAIIPLMAQ--QHHPRAVEA
Posp     ---MNPYVFAILLSSLGIGTTLTFASSHWLLAWMGLEINTLAIIPLMAQ--QHHPRAVEA
Atsp     ---MNPYVLTILISSLGVGTTLTFMSSHWLLAWMGLEINTMAIIPLMAQ--QHHPRAVEA
Leoc     ---MNPYVLMILISSLGIGTTLTFMSSHWLLAWMGLEINTLAIIPLMAQ--QHHPRAVEA
Amca     ---MNPYPMMILYASLGIGTTLTLTSSHWFMAWMGLEINTLAIIPLMAQ--HHHPRAVEA
Osbi     ---MNPYVYMILLSSLGIGTTLTFASSHWLLAWMGLEINTMAIIPLMAQ--QHHPRATEA
Pabu     ---MNPYIFTILISSLGLGTTITFASSNWLLAWMGLEINTLAIIPLMTQ--QHHPRAIEA
Hial     ---MNPYVLTILVSSLGIGTTLTFASSHWLLAWMGLEINTLAIIPLMAQ--QHHPRAVEA
Elha     ---MNPYVAIILISSLGLGTTLTFASSHWLLAWMGLEINTMAILPLMAR--QHHPRAVEA
Mlcy     ---MNPLIPMILLSSLGLGTTLTFASSHWLLAWMGLEINTLAIIPMMSR--HHHPRAVEA
Algl     ---MNPYVNGFLILSLGLGTTLTMMSSHWFVAWMGLEINTLAIIPLMAR--RGHPRAVEA
Ptgi     ---MNPYVLTVLLTSLGLGTTLTFASSHWLMAWMGLEINTLAIIPLMAQ--HHHPRAVEA
Alaf     ---MALYVLSILIFSMGLGTMLTFASSHWFLAWMGLEINTLAIIPLMAK--SHHPRATEA
Nock     ---MALYVLSILMFSMGLGTMLTFASSHWFLAWMGLEINTLAIIPLMAK--SHHPRATEA
Anja     ---MNPYVTFIMLTSLGLGTTITFASSHWLLAWMGLEINTLAIIPLMAQ--HHHPRAVEA
Gyki     ---MNPWVIFILIASLGLGTTITFASSHWLLAWMGLEINTLAIIPLMTQ--HHHPRAVEA
Syka     ---MNPYVLTIMIMSLGLGTTITFASSHWLLAWMGLEISTLAIIPLMAQ--HHHPRAVEA
Opma     ---MSPYVIYIFLMSLGLGTTITFASSHWLLAWMGLEINTLAILPLMAQ--NHHPRAVEA
Comy     ---MSPYVLFIIIMSLGLGTTTTFASSHWLLAWMGLEISTLAILPLMSQ--YHHPRAVEA
Sasp     ---MNPFVLYIMLLSLGLGTTLTFASSHWLMAWMGLEINTLAIIPLMIQ--QYHPRAVEA
Eupe     ---MNPYVMFLMFTSLGLGTTITFASSHWLMAWMGLEINTLAIIPLMAQ--HRHPRAVEA
Enja     ---MNPYVLTLLLMSLGLGTTLTFMSSHWLLAWMGLEINTLAIIPLMAQ--KHHPRAVEA
Same     ---MNPYVFTILLSTLGLGTTMTFASSHWLMAWMGLEINTLAIVPLMAY--QHHPRAVEA
Chch     ---MNPYVLAILIASLGMGTTITFASSHWLLAWMGLEINTLAIIPLMSK--QHHPRAVEA
Grgr     ---VNPYVLVVLLSSLALGTTITFASSHWLLAWMGLELNTLAILPLMTQ--QHHPRAVEA
Caau     ---MNPYVLMILLSSLGLGTTLTFASSHWLLAWMGLEINTLAITPLMAQ--HHHPRAVEA
Cyca     ---MNPYVLATLLSSLGLGTTLTFASSHWLLAWMGLEINTLAITPLMAQ--HHHPRAVEA
Dare     ---MNPYVLMILMSSLGLGTTLTFSSSHWILAWMGLEINTLAIVPLMAQ--QHHPRAVEA
Cost     ---MNPYVLMTLLSSLGLGTALTFASSHWLLAWMGLEINTLAIIPLMAQ--QHHPRAVEA
Leec     ---MNPYVLAILLSSLGLGTTLTFASSHWLLAWMGLEINTLAITPLMAQ--HHHPRAVEA
Fola     ---MNPYVLMILISSLGLGTTLTFASSHWLLAWMGLEINTLAILRLMAQ--QHHPRAVEA
Clmc     ---MNPYVLAMFLSSLGLGTTITFASSHWLLAWMGLEINTLAILPLMAQ--HHHPRAVEA
Phin     ---MNPYVLTIFLFSLGLGTTLTFMSTHWFLAWMGLEINTLAILPLMAQ--HHHPRAVEA
Icpu     ---MSPYVITILLSSLGLGTALTFMSSHWLLAWMGLEINTLAILPLMAQ--HHHPRAVEA
Psto     ---MSPYIIMILLSSLGLGTALTFMSSHWLLAWMGLEINTLAILPLMAQ--HHHPRAVEA
Cora     ---MNPYVLTIFLLSLGLGTTLTFSSSHWLLAWMGLEINTLAILPLMAQ--HHHPRAVEA
Eisp     ---MNPYVLVILLSSLGLGTTLTFASSHWLLAWMGLEINTLAILPLMAQ--HHHPRAVEA
Apal     ---MSPLLLFIFLSALGLGTTITFASSHWFMAWMGLEVNTLAIIPLMTF--QPHPRAVEA
Eslu     ---MSPYVFTLLIFNLGLGTTMTFASSHWLLAWMGLEINTLAIIPLMTR--QHHPRAVEA
Dape     ---MSPYVLTILVFNLGLGTTLTFASSHWLLAWMGLEINTLAIIPLMAQ--QHHPRAVEA
                              1
```

To be continued on page 6.

[1/6 of aligned sequences]

```text
Glse      ---MNPLVLTLFLSSLGLGTTLTFMSSHWLLAWMGLEINTLAIIPLMAR--HHHPRAVEA
Naar      ---MNPFVLAILLFSLGLGTTLTFTSSHWLLAWMGLEINTLAILPLMAR--HHHPRAVEA
Lioc      ---MNPFVLAILLFSLGLGTMLTLTSSHWLLAWMGLEINTLAILPLMAR--HHHPRAVEA
Opso      ---MNPFILALLLSSLGLGTMLTFASSHWLLAWMGLEINTLAIIPLMAR--HHHPRAVEA
Alte      ---MNPYVLTVLLSSLGLGTTLTFASSHWLLAWMGLEINTLAIIPLMAQ--HHHPRAVEA
Plap      ---MNPYVLTVLLSSLGLGTALTFASSHWLLAWMGLEVNTLAIIPLMAQ--QHHPRAVEA
Plal      ---MNPYVTSLLISALGLGTALTFSSSHWLLAWMGLEINTLAIIPLMTQ--KHSPRSVEA
Sami      ---MNPYVTSFLISALGLGTALTFSSSHWLLAWMGLEINTLAIIPLMTQ--KHSPRSVEA
Rere      ---MNPYVLSLLISALGLGTALTFSSSHWLLAWMGLEVNTLAIIPLMTQ--KHSPRSVEA
Gama      ---MNPMVFAILLSSLGLGTALTFASSHWLLAWMGLEINTLAILPLMTQ--HHHPRAIEA
Onmy      ---MNPYVLTILLSSLGLGTVLTFASSHWLLAWMGLEINTLAIIPIMAQ--QHHPRAIEA
Sasa      ---MNPYVLTILLSSLGLGTILTFASSHWLLAWMGLEINTLAIIPIMAQ--QSHPRAIEA
Cola      ---MNPYVLTILLSSLGLGTVLTFASSHWLLAWMGLEINTLAIIPLMAR--QYHPRAVEA
Dita      ---MHPFVLTCLLSALGLGTVLTFSSTHWLMAWMGLEINTLAILPVMAQ--KHHPRGIEA
Gogr      ---MHPLTTTYLLSSLGLGTTLTLSSSHWLLAWMGLEMNTLAILPLMTH--KHHPRAVEA
Chsl      ---MHPLITFCFLSTLGLGTVLAFSSSHWFLAWMGLELNTLAILPLMAQ--KHHPRAVEA
Atja      ---MNPYVLTVLLFSLGLGTTTTFASSHWLMAWMGLEINTLAIIPLMTQ--HHHPRSVEA
Iido      ---MNPYVLTVLLFSLGLGTTLTFASSHWLLAWMGLEINTLAIIPLMAQ--NHHPRSVEA
Auja      ---MSPYVLTALLLSLGLGTTITFASSHWLLAWMGLEINTLAILPLMAQ--HHHPRAVEA
Chag      ---MSPYILCTLLFSLGLGTAVTFASSHWFLXWMGLEINTLAIIPLMAQ--HHHPRAVEA
Hami      ---MSPFVLTALLFSLSLGTTLTFASSHWLLAWMGLELNTMAILPLMAQ--NHHPRAIEA
Saun      ---MSPFILAALLFSLCLGTTLTFASSHWLLAWMGLELNTMAILPLMAQ--SHHPRAIEA
Nema      ---MNPHILSVLLISLGLGTTITFASSHWLLAWMGLEINTLAIIPLMAQ--YHHPRAVEA
Disp      ---MNPHVLSVLLIALGLGTTITFASSHWLLAWMGLEINTLAIIPLMTQ--HHHPRAVEA
Myaf      ---MNPFILPILLMSLGLGTTLTFASSHWLLAWMGLEINTLAIIPLMAQ--HHHPRAVEA
Lagu      ---MSPPILSLLLISLGLGTTITFASSHWLLAWIGLEINTLAITPLMMQ--HPHPRAVEA
Trtr      ---MSPMILTLFLLSLGLGTTITFASSHWLLAWMGLEINTLAILPLMAQ--HPHPRAVEA
Zucr      ---MSPFILSLFLLSLGLGTTITFSSSHWLLAWMGLEINTLAIMPLMAQ--RPHPRAVEA
Pxja      ---MNPYVLTTLLFSLGLGTTITFASSHWLLAWMGLEINTLAIIPLMAQ--HHHPRAVEA
Pxlo      ---MNPYVLTTLLFSLGLGTTITFASSHWLLAWMGLEINTLAIIPLMAQ--HHHPRAVEA
Pctr      ---MNPHIAATLIFTLGLGTTITFASSHWLLAWMGLEINTLAIIPLMSQ--HHHPRATEA
Apsa      ---MNPYILSIMIFTLGLGTTMTFASSHWLLAWMGLEINTMAIIPLMAQ--QHHPRSVEA
Cabe      ---MSPYIMTALLMSLGLGTTITFASSHWLFAWIGLEINTLAIIPLMAS--HHHPRATEA
Bzze      ---MNPYILATLLFSLGLGTTITFASSHWLLAWMGLEINTLAIIPLMGQ--HHHPRAVEA
Siim      ---VNVQMMFILLSSLMLGTTITCASSHWLYAWLGLEMNTLAMLPLIVR--SQHPRAVEA
Ctru      ---MNPYILSTLLFGLGLGTTITFASTHWLLAWMGLEINTLAIIPLMAQ--HHHPRAVEA
Dpbr      ---MNPYILSTLLFGLGLGTTITFASSHWLLAWMGLEINTLAIIPLMSQ--HHHPRAVEA
Caki      ---MAPPLLSSLLLALGLGTSLTFSSSHWLLAWMGLEINTLAILPLMVS--THQSRGTEA
Phja      ---MSPLILYIFLLALGLGPTLTFASSHWLLAWMGLEISTLAILPLMNQ--HHHPRAVEA
Brsp      ---MPAISILFFITSLILGTAMTLSSNHWFMAWMGLEISTLAIIPLMTM--HHHPRAIEA
Gamo      ---MNPFILSILLLSLGLGTTLTFASSHWLLAWMGLEISTLAIIPLMSQ--HHHPRAVEA
Lolo      ---MNPFVLSILLLSLGLGTTLTFASSHWLLAWMGLEISTLAIIPLMAQ--HHHPRAVEA
Batr      ---MNPLVFFLLSFTVIMGTLMTLMSNHWLTAWMGLELNTLAIIPLMTK--NHHPRTTEA
Prmy      ---MSPFVLFLFLFNLGLGTTITFASSHWLLAWMGLELNTLAIIPLMIQ--HHHPRATEA
Lose      ---MNPTTLFILMSGLALGTTATFASSHWFLAWMGLELNTLAIIPLMSQ--HHHPRAVEA
Loam      ---MNPFILFVLLSGLGLGTTITFASSHWLAAWMGLEISTLAILPLMAQ--HHHPRAVEA
Chab      ---MNPYILFVLLFGLGLGTTITFASSHWLLAWMGIEMNTLAIIPLMAQ--HHHPRGVEA
Chto      ---MNPYILFVLLFGLGLGTTITFASSHWLLAWMGIEMNTLAIIPLMAQ--HHHPRGVEA
Majo      ---MSPYILFALMFGLGLGTTLTFASSHWLLAWMGIEMNTLAFIPLMAQ--RHHPRAVEA
Hlst      ---MSAYILSTLMFGMGLGTTLTFASSHWLLAWMGIEMNTLAFIPLMAR--QHHPRAIEA
Clpe      ---MSPYILSTFLLSLGLGTTITFASSHWLLAWMGVEVSTLAILPLMAQ--HHHPRAVEA
Mlmr      ---MNPYILPTLFLGLGLGTAITFASSHWLLAWMGIEMNTLAILPLMAQ--HHHPRAVEA
                                       2
```

To be continued on page 7.

[1/6 of aligned sequences]

```
Crcr    ---MNPYILTILLFGLGLGTTITFASSHWLLAWMGLEINTLAIIPLMAQ--HHHPRAVEA
Muce    ---MSPLILTILLFGLGLGTTITFASSHWLLAWMGLEINTLAIIPLMAQ--HHHPRAVEA
Bege    ---MNPYILAILLFGLGLGTTITFASSHWLLAWMGLEMNTLAILPLMAQ--YHHPRAVEA
Mela    ---MNPYILMTLAFGLGLGTLITLTSSHWLLAWMGLEMNTLAIIPLLAQ--HHHPRATEA
Hats    ---MNPYILAILLFGLGLGTTITFASSHWFLAWMGLEMNTLAIIPLMAQ--HHHPRAVEA
Orla    ---MNPYVLSILLMGLGLGTTVTFASSHWLLAWMGLEMNTLAILPLMAQ--HHHPRAVEA
Cosa    ---MNPYILTIMLIGLGMGTSITLTSTHWLLAWMGLEMNTMAIIPLMAQ--HHHPRAVEA
Exsp    ---MNPYILATMIFGLGLGTTVTFTSSHWFLAWMGLEINTLAIIPLMAQ--THHPRAVEA
Depa    ---MNPYILTTLLLGLGMGTTITLMSSHWLLAWMGLEMNTLAIIPIMAQ--HHHPRAVEA
Rima    ---MSPYMLIILIFTLGLGTLITLSSSHWLIAWMGLELNTFAILPLMAQ--QHSPRAMEA
Fuol    ---MNPYILTVFLFGLGLGTTITFASSHWLLAWMGLEINTLAILPLMCQ--SHHPRAVEA
Gmaf    ---MTPLIYSTLIISLGIGTTLTFASTHWYLAWMGIEINTLAIIPLMTQ--SHSPRATEA
Xeei    ---MNPYILFILLFALGLGTTTTFASSHWLLAWMGLEINTLAIIPLMTQ--LHHPRAVEA
Pros    ---MNPYILTTLLFGLGLGTTITFASSHWLLAWMGLEMNTLAILPLMAQ--HHHPRAVEA
Scmi    ---MNPYILASLLFGLGLGTTITFASSHWLLAWMGLEMNTLAILPLMTQ--HHHPRAVEA
Rolo    ---MNPYILATLLFGLGLGTTITFASSHWLLAWMGLEMNTLAIIPLMAQ--HHHPRAVEA
Cere    ---MNPYILFTLLFGLGLGTTITFMSTHWLLAWMGLEMNTLAIIPLMSQ--HHHPRSVEA
Daga    ---MNPFVLATLLFGLGLGTTTTFLSSHWLLAWMGLEINTLAIIPLMSK--LHHPRAVEA
Anco    ---MNPLVLAILLLGLGLGTTLTFSSSHWLLAWMGLEINTLAIIPLMAQ--HHHPRAVEA
Dmve    ---MNPYVLAILIFGLGLGTTLTLSSSHWLLAWVGLEISTLSIIPLMAQ--HHHPRAVEA
Dmar    ---MNPYVLAILIFGLGLGTTLTLSSFHWFLAWVGLEISTLSIIPLMAQ--HHHPRAVEA
Anka    ---MNPFVLATLLFGLGLGTTITFSSSHWLLAWMGLEINTLAIIPLMAQ--HHHPRAVEA
Moja    ---MNPLVLATLLLGLGLGTTITFTSSHWLLAWMGLEINTLAIIPLMAQ--HHHPRAVEA
Hoja    ---MNPFVLAILLLGLGLGTTLTFSSSHWLLAWMGLEINTLAIIPLMAQ--HHHPRAVEA
Bede    ---MNPYILATLLFGLGLGTTITFASSHWLLAWMGLEMNTLAIIPLMAQ--HHHPRAVEA
Besp    ---MNPYILATLLFGLGLGTTITFASSHWLLAWMGLEMNTLAIIPLMAQ--HHHPRAVEA
Mysp    ---MNPYILSILLFSLGLGTTITFASSHWLLAWMGLEINTLAIIPIMAQ--QHHPRAIEA
Osja    ---MNPYILTTLLFSLGLGTMITFSSSHWLLAWMGLEMNTLAIIPLMAQ--QHHPRAIEA
Sgro    ---MNPYILTILLFSLGLGTTITFASSHWLLAWMGLEMNTLAIIPLMAQ--QHHPRAIEA
Pzpa    ---MNPLVLSLLMLGLGLGTTITFASSHWLLAWMGLEINTLAIIPLMTQ--HHHPRAVEA
Zeja    ---MNPLVLSLLILSLGLGTTITFASSHWLLAWMGLEINTLAIIPLMAQ--HHHPRAVEA
Znne    ---MNPLVLSLLILSLGLGTTLTFASSHWLLAWMGLEINTLAIIPLMAQ--HQHPRAIEA
Zefa    ---MNPLVLSLLILSLGLGTTLTFASSHWLLAWMGLEINTLAIIPLMAQ--HHHPRAVEA
Acni    ---MNPLVLSLLILGLGLGTTVTFASSHWLLAWMGLEINTLAIIPLMAQ--HHHPRAVEA
Ncrh    ---MNPLVLSLLILGLGLGTTVTFASSHWLLAWMGLEINTLAIIPLMAQ--HHHPRAVEA
Agca    ---MNPHILAILLFGLGLGTTITLTSSHWLLAWMGLEINTLAIIPLMAQ--HHHPRAVEA
Hydy    ---MNPFILSTLLFGLGLGTTITFASSHWLLAWMGLEMNTLAILPLMAQ--HHHPRAVEA
Gsac    ---MNPFILATLLFGLGLGTTITFASSHWLLAWMGLEMNTLAIIPLMAQ--HHHPRAVEA
Pevo    ---MNPYTLIIFFTALGLGTTLTFLSSHWLLAWMGLEMSTLAIIPLMAQ--HHHPRATEA
Hiku    ---MNPYIMSIFLLALGLGTTITFMSSHWLMAWMGLEINTLAIIPLMAQ--HHHPRSVEA
Inpa    ---MSPFIPIILITGLILGTSITLTSSHWLLAWMGLEINTLAIIPLMTK--HHHPRATEA
Auch    ---MNTLATLTLGSSLLLGTLITVSSSHWFYAWLGLEINTLAILPLLAG--PQHARAIEA
Fico    ---MNPYITTILLMGLGLGTTITFASSHWLLAWMGLEMNTLAIIPLMAQ--HHHPRAVEA
Macs    ---MNPYILATLLFGLGLGTTITLSSSHWLLAWMGLEMNTLAIIPLMAQ--HHHPRAVEA
Moal    MQNMNPYLLIVLLTSLGLGTALTLSSTHWLLAWLGLEVGTLAILPLMAQ--PHHPRATEA
Syma    ---MSPYMTTILMVGLGLGTTITLSSTHWLLAWMGLELNTLAIIPLMAM--NHHPRAIEA
Mafr    ---MSPFILSILLLGLGLGTTITFASSHWLLAWMGLEINTLAIIPLMAQ--HHHPRATEA
Dcpe    ---MSPYILMTLIFGLGLGTFVTLTSSHWLLAWMGLEMSTLAVIPLMAQ--HHHPRAVEA
Dcti    ---MSPYILMTLVFGLGLGTFVTLTSSHWLLAWMGLEMSTLAIIPLMAQ--HHHPRAVEA
Hehi    ---MNPYILTTLLFGIGLGTTTTFASSHWLLAWMGLEMNTLAIIPLMAQ--HHHLRAVEA
Stam    ---MNPYILATLLFGLGLGTTITFMSSHWLLAWMGLEINTLAILPLMAQ--HHHPRAVEA
Hogi    ---MNPYILAILLFSLGLGTTLTFASSHWLLAWMGLEINTLAIIPLMAQ--HHHPRAVEA
```

To be continued on page 8.

[1/6 of aligned sequences]

```
Erzo    ---MNPYILATLIFGLGLGTTLTFASSHWLLAWMGLEMNTLAIIPLMAQ--HHHPRAVEA
Hxot    ---MNPYILATLLFGLGLGTTVTFASSHWLLAWMGLEMNTLAIIPLMAQ--HHHPRAVEA
Core    ---MNPYILSTLLFGLGLGTTITFASSHWLLAWMGLEMNTLAIIPLMAQ--HHHPRAVEA
Apve    ---MNPYILATFLFSLGLGTTVTFASSHWFLAWMGLEMNTLAILPLMAQ--HHHPRAVEA
Latj    ---MNPYVMITLLFGLGLGTTITFTSSHWLLAWMGLEINTLAIIPLMAQ--HHHPRAVEA
Laja    ---MNPYILTTLLFGLGLGTTITFASSHWLLAWMGLEINTLAIIPLMAQ--HHHPRAVEA
Syja    ---VHPTLFLLFATGLALGTITAFSSSHWFLAWMGLEIGTLAILPLMAQ--RHHPRGVEA
Epme    ---MNPYISATLLFSLGLGTTITFASSHWLLAWMGLEINTLAILPLMAQ--QHHPRAVEA
Grse    ---MNPHVLATLLLGLGLGTTITFTSSHWLLAWMGLEMNTLAIIPLMAQ--QHHPRAVEA
Clja    ---MSPYMTMIFLFSLGLGTAVTFSSSHWMLAWMGLEMNTLAIVPLMAR--HRHPRAVEA
Ogcy    ---MTPHILTILLMGLGLGTTITFASSHWLLAWMGLEINTLAILPLMAN--RHHPRAVEA
Plna    ---LSPALCSIFLFSLGLGTMIVFTNSHWFLAWIGLEINTLAIIPIMVQLHQHHPRAVEA
Lema    ---MNPYILATLLFGLGLGTTITFASSHWLLAWMGLEMNTLAILPLMAQ--HHHPRAVEA
Etzo    ---MNPYILTALLFGLGLGTTVTVASSHWLLAWMGLEMSTLAIIPLMAQ--HHHPRAVEA
Apse    ---MSPFVLATLVSALGIGTAITLSSSHWLIAWVGLELNTLAILPLMTR--QPHPRAVEA
Epde    ---MNPYILATLLFGLGLGTTITFASSHWLLAWMGLEINTLAIIPLMTQ--HHHPRAIEA
Slja    ---MNPYILFTLICALGLGTTVTFASSHWLLAWMGLEMNTLAILPLMAR--HHHPRAVEA
Bsja    ---MNPYVSAVLLSGLGLGTTLTFASSHWLLAWMGLEINTLAIIPLMAQ--HSHPRAVEA
Ecna    ---MNPLILTILLFALGLGTTITFMSSHWLFAWMGLEINTLAIIPLMTQ--HHHPRSTEA
Cohi    ---MNPYVLAILLFSLGLGTTITFASSHWLLAWMGLEINTLAIIPLMTQ--HHHPRATEA
Caar    ---MNPYILAVLLFGLGLGTTITFASSHWLLAWMGLEINTLAIIPLMAQ--HHHPRAVEA
Came    ---MNPYILAILLFGLGLGTTITFASSHWLLAWMGLEINTLAIIPLMAQ--HHHPRAVEA
Mema    ---MNPYILATFLFGLGLGTTITFASSHWLLAWMGLEINTLAIIPLMAQ--HHHPRAVEA
Lenu    ---MNPYISTILFSGLALGTTITLTSSHWLMAWMGLEINTLAIIPLMSQ--HHHPRATEA
Brja    ---MNPYILTTLLFALGLGTTVTFASSHWLLAWMGLEMNTLAIIPLMAQ--SHHPRAVEA
Plma    ---MNPYILATLLLGMGLGTTITFASSHWLLAWMGLEMNTLAIIPLMAQ--NHHPRAVEA
Emst    ---MNPYILATLLFGLGLGTTITFASSHWLLAWMGLEMNTLAIIPLMAQ--HHHPRAVEA
Ptti    ---MNPYILATLLFGLGLGTTITFASSHWLLAWMGLEMNTLAIIPLMAQ--HHHPRAVEA
Losu    -MSLNPYIFSLLLMSLGLGTTLTFLSSHWLMAWMGLEISTLSIIPLLAK--QNHPRAVEA
Geoy    ---MNPYILATLLFTLGLGTTVTFASTHWLLAWMGLEINTLAIIPMMAQ--HHHPRAVEA
Dipi    ---MNPLILATLLLGLGLGTTITFASSHWLLAWMGLEMNTLAIIPLMAQ--HHHPRAVEA
Pama    ---MSPFILVTLLFALGLGTTIAFASSHWLLAWMGLEINTLAILPLMAQ--HHHPRAVEA
Leob    ---MNPLILATLLFGLGLGTTLTFASSHWLLAWMGLEINTLAIIPLMAQ--LHHPRAVEA
Neba    ---MSPYILVTLLLGLGLGTTITLTSSHWLVAWVGLEINTLAIIPIMAQ--HYHPRAVEA
Pdpl    --MVHPLVIYSLLFLVGLGTAIVFTSSNLLLAWMGLELNMLAMLPLMMQ--HRHPRAVEA
Nimi    ---MSPMLSSTLLFALGIGTTITFASSHWFLAWMGLEINTLAILPLMAQ--YHHPRAVEA
Uptr    --MINPLVISILLITMGLGTIVTFASSHWLLAWMGIELNTLAILPILAG--RHHSRATEA
Pesc    ---MHPALMVIMALGLLLGTTLALSTSHWLIAWMGLEINTIAILPLLAN--KFHPRSIEA
Baar    ---MNPYILATLLFGLGLGTTITFASSHWLLAWMGLEMNTLAIIPLMAQ--HHHPRAVEA
Moar    ---MNPYILATLLFGLGLGTSITFASSHWLLAWMGLEMNTLAIIPLMAQ--HHHPRAVEA
Toja    ---MNPTILPILLLGLVLGTTITFMSSHWLVAWMGLEINTLAIIPLMAQ--HHHPRAVEA
Chau    ---MNPYILTALLFGLGLGTTITLASSHWLLAWMGLEINTLAIIPLMAQ--HHHPRAVEA
Chse    ---MTVTALTTLIFALGIGTTLTFVSSHWLLAWMGLEISTLAILPLMAQ--QHHPRAVEA
Enar    ---MNPYILATLLFGLGMGTTITFASSHWLLAWMGLEMNTLAIIPLMAQ--HHHPRAVEA
Hpty    ---MNPYILATLLFGLGLGTTITFASSHWLLAWMGLEMNTLAIIPLMAQ--HHHPRAVEA
Nana    ---MSPLILTTLLLGLGLGTTITFASSHWLLAWMGLEINTLAILPLMAQ--HHHPRAIEA
Mcst    ---MNPYILATLLFGLGLGTTITFTSSHWLLAWMGLEMNTLAIIPLMAQ--HHHPRAVEA
Rhox    ---MSPYILTVLLFGLGLGTTITFASSHWLLAWMGLEINTLAILPLMAR--HHHPRAVEA
Opfa    ---MNPYLLATLLFGLGLGTTITFTSSHWLLAWMGLEINTLAIIPLMVQ--HHHPRAVEA
Paar    ---MNPYILTILLFSLGLGTTITFMSSHWFLAWMGLEINTLAIIPLMAQ--QHHPRAVEA
Gozo    ---MNPYILATLLFGLGLGTTITFASSHWLLAWMGLEMNTLAIIPLMAQ--HHHPRAVEA
Ackr    ---MLPPIMALFLFGLGLGTLITFSSSHWLLAWIGLEINTFSILPIMAA--SHHPRAVEA
```

To be continued on page 9.

[1/6 of aligned sequences]

```
Elev      ---MNPYVLATLLFSLGLGTTITFASSHWLIAWMGLEVNTLAIVPLMAQ--HHHPRAVEA
Trdu      ---MNPYILAILLFGLGLGTTITFASSHWLLAWMGLEMNTLAIIPLMAQ--HHHPRAVEA
Amoc      ---MNPYILSILLFGLGLGTMTTFASSHWLLAWMGLEINTLAIIPLMAQ--HHHPRAVEA
Hame      ---MNPYILSTLLIGLGLGTTITFASSHWLLAWMGLEMNTLAIIPLMAQ--HHHPRAVEA
Chso      ---MSPYILSIFLFGLGLGTTITFASTHWLTAWMGLEINTLSILPLMAR--QHHPRAVEA
Lyto      ---MNPYILATLLFGLGLGTTITFASSHWLLAWMGLEMNTLAIIPLMAQ--HHHPRAVEA
Encr      ---MNPYILATLLFGLGLGTTITFASSHWLLAWMGLEMNTLAIIPLMAQ--HHHPRAVEA
Bvar      ---MTPYNYALFLFSISLGTTTTFASSNWLLAWTGLEINSLAILPIMAQ--NAHPRAVEA
Noco      ---MSPYVLALLLFSLGLGTTMTFASSHWLLAWVGLEINTLAILPLMAQ--NHHPRAVEA
Chsp      ---MPPMVLILMMLTIGLGTLITVTSSHWLLAWMGLELNTLAIIPIMAH--RHHPRAAEA
Arja      ---MNPYILPVLLFSLGLGTTVTFASSHWFLAWMGLEMNTLAIMPLMAQ--HHHPRAVEA
Pase      ---MNPPLMIIIVFSLGAGTMTTFMSSHWLLAWMGLEINTLAFVPLMAK--QHHPRAVEA
Trel      ---MQVYIKAAFISSLGLGTMTTFAGSHWMFAWAGLEINMLAVLPLMTV--KFHPRASEA
Lifa      ---MSPYVLAAFLISLGLGTTITFASSHWLFAWMGLEINTLAIIPIMAQ--HRHPRSIEA
Acur      ---MSTTVLLSMIFSMTLGTTLVLCSTHLLTVWMGLEMNTLAILIMLTL--NYHVRAVEA
Ampe      ---MNPYILATLLFGLGLGTTITFASSHWLLAWMGLEMNTLAIIPLMAQ--HHHPRAVEA
Urja      ---MNPFILISLLLTLGLGTMITFMSSHWLLAWMGLEINTLAIIPIMAR--YHHPRATEA
Enet      ---MSPFTQMFLFIALGIGTTVTLTSSHWLLAWMGLEVSTLAIAPLMAR--KHHPRAIEA
Ptbr      ---VSPIALTISLTSMFIGTLLTITSSHWLTAWMGLEINTIAILPLMAS--RHLPRSVEA
Safa      ---MSPYIYPIMLMGMLLGTTATMISSHWLLAWMGLELSTIAIIPIMAH--LHHPRAVEA
Icae      ---MNPYILATLLFGLGLGTTITFASSHWLLAWMGLEMNTLAIIPLMAQ--NHHPRAVEA
Asmi      ---MSPFILSIFLMTLGLGTTITFISSHWLLAWMGLELNTLAIIPLMAQ--RHHPRSTEA
Foal      ---MSPLMLPVVVFSLVVGTTITLTSSNWLLAWMGIEINTLAIIPLMIH--NHHPRAAEA
Drze      ---MNPYVLATLLMSLSLGTTITFASSHWLLAWMGLEINTLAILPIMAQ--NHHPRATEA
Rhas      ---MNPYITSVLLFGLGLGTTITFASSHWLLAWMGLEINTLAIIPLMAQ--HHHPRAIEA
Elac      ---MNPYIMAILLFGLGLGTTLTFASSHWLLAWMGLEINTLAIIPLMAQ--HHHPRAIEA
Kugu      ---MNPLILALLLFSLGLGTILTLSSSHWLLAWMGLEINTLAIMPLMAQ--HHHPRATEA
Plor      ---MNPIVLSALLMGLGLGTTITFASSHWLLAWMGLEVNTLAILPLMAR--HHHPRAVEA
Sgun      ---MNPYVLAILLFGLGLGTTITFASSHWLLAWMGLEINTLAIIPLMAQ--HHHPRAVEA
Zaco      ---MAPFVLTILLFGLGLGTTVVFASSHWLLAWMGIEINTLAILPLMAQ--RHHPRGVEA
Zbfl      ---MSPYILATFLFGLGLGTTITFASSHWLLAWMGLEMNTLAIIPLMAQ--HHHPRAVEA
Spba      --MLPPSLTPLFLSMLGIGTLTTLMSSHWLLAWMGLEINSLAILPLMIQ--NQHPRAVEA
Game      ---MNPYILATLLFGLGLGTTITFASSHWLLAWMGLEMNTLAIIPLMAQ--NHHPRAVEA
Thth      ---MNPYILATLLFGLGLGTTITFASSHWLLAWMGLEMNTLAIIPLMAQ--NHHPRAVEA
Xigl      ---MNPYILATLLFGLGLGTTITFASSHWLLAWMGLEINTLAIIPLMAQ--HHHPRAVEA
Hyja      ---MNPYILATLLFGLALGTTITFASSHWLLAWMGLEMNTLAIIPLMAQ--NHHPRAVEA
Psan      ---MSFYVAAIVIPSLGLGTLITLISSHWLLAWMGLEISTLSMIPLMTK--SRHPRAIEA
Cupa      ---MSPYILATLLFGLGLGTTITFASSHWLLAWMGLEMNTLAIIPLMAQ--NHHPRAVEA
Mpch      ---MNPHILSIMLFGLGLGTTITFASSHWLLAWMGLEINTLAILPLMTQ--RHHPRAVEA
Char      ---MNPYILATLLFGLGLGTTITFASSHWLLAWMGLEINTLAILPLMAR--HHHPRAVEA
Pser      ---MNPYILATLIFGLGLGTATTFMSSHWLLAWMGLEINTLAIIPLMAQ--HHHPRAVEA
Prol      ---MNPFILTTLLLGLGLGTTITFASSHWLLAWMGLEINTLAIIPLMAQ--HHHPRAVEA
Plbi      ---MNPYILTTLLFGLGLGTTLTFASSHWLLAWMGLEINTLAIIPLMAQ--HHHPRAVEA
Calu      ---VNPYILSTFLMALGLGTTITFASSHWLLAWLGLEINTLAILPLMAR--HHHPRSVEA
Papa      ---MGPYLTTIMLMSLGLGTTIVFSSHHWMLAWMGLEINTLAMIPLMAQ--KPHPRPVEA
Sufr      ---MSPYVLATLMFSLGLGTTVTFMSSHWLLAWMGLEINTLAIIPLMAH--QYHPRAVEA
Stci      ---MNPYILTTLLFSLGLGTTLAFMSSHWLLAWMGLEINTLAIIPLMAQ--CHHPRAVEA
Taru      ---MNPYITASLLFGLLLGTTITTTSTHWLIAWMGLEINTLAIIPLMAQ--QHHPRAIEA
Rala      ---MNPYILATLLFGLGLGTTITFISSHWLLAWMGLEINTLAIIPLMAQ--HHHPRAVEA
             :           :  *   .    .    * *:*:  ::.  ::        *  **
```

To be continued on page 10.

[2/6 of aligned sequences]

```
              B                                         C
Scca  TTKYFITQATASALLLFASVTNAWTSGEWSLIEMLNPTSATLATAALALKIGLAPLHFWL
Muma  TTKYFITQATASTLLLFASVTNAWTSGEWSLIEMLNPTSATLATVALALKIGLAPLHFWL
Erca  ATKYFLTQAAAAAILLFASIYNAWYSGQWFIQDMSSPVS-TLMTLAIAIKLGIAPMHFWL
Pose  ATKYFITQAAAAALLLFAGLMNAWQSGQWIIQDMSMPTS-ALMTLAIAIKLGVAPMHFWL
Actr  TTKYFLTQATAAAMILFASTTNAWTTGEWNIQEMSNPTALVLITMALALKIGLAPVHYWL
Scal  TTKYFLTQATAAAMILFASTTNAWMTGEWNIQEMSNPTALVLITLALALKIGLAPVHYWL
Posp  TTKYFLTQATAAAMILFASTTNAWLTGEWNIQEMSNPTALTLITMALALKIGLAPIHYWL
Atsp  TTKYFLTQATAAAMILFASTTNAWITGEWNIQQLSHPPSTTLITFALALKIGLAPMHFWM
Leoc  TTKYFLTQATAAAMILFASTTNAWMTGEWNIQQLSHSPSTTLVTFALALKIGLAPMHFWM
Amca  TTKYFLTQATAAAMILFAALSNAWLTGEWAILQTLHPLPSTMLTLGLALKLGLAPMHFWM
Osbi  ATKYFLTQATAAAMILFASTTNAWITGDWNIPQTSHPAATSLLTLALALKIGLAPMHFWL
Pabu  ATKYFLTQATAAALILFAGTMNAWMLGEWNINHMSDPLSTSLLTTALALKIGLAPMHFWL
Hial  TTKYFLTQATAAALILFASTTNAWVMGDWNIQQISHPLATTMIMMALALKIGLAPVHFWM
Elha  TTKYFLTQATAAALILFASLMNAWTTGEWNIQQLSDPLATTLITLGLALKIGLAPVHFWL
Mlcy  ATKYFLIQATAAALILFASLTNAWLTGTWNVQQLTDPTATTLAMMGLALKIGLAPAHFWL
Algl  ATKYFLTQATAAMLVMFAVVINAWMTGEWNIQQLPHSLTSNVVMIALALKVGLAPVHFWY
Ptgi  TTKYFLTQATAAALILFATTTNAWLVGEWDIQHLSHPLAATTVMLALALKVGLAPVHSWM
Alaf  TTKYFIIQAVAAALILFASATNAWMTGEWDIKQLTHPLATNTVMLALALKLGLAPLHLWM
Nock  TTKYFIIQAVAAALILFASATNAWITGEWDIKQLTHPLAANTVMLALALKLGLAPLHLWM
Anja  TTKYFLTQATAAALMLFTTTSNAWITGQWEIQQLSHPMITTITILALGLKVGLAPMHFWL
Gyki  TTKYFLAQAAAAAVLLFATTSNAWVMGEWHIQQLTHPVANTVAMISLGMKIGLAPTHLWL
Syka  TTKYFLTQATAAALMLFAATSNAWLTGQWEIQQLSHPTAAAVTMLALGLKMGMAPMHFWL
Opma  TTKYFLTQATAAAVILFATTMNAWFTGSWEIQQTFHPTIMAITMLSLGLKVGLAPMHFWL
Comy  TTKYFLTQAMATALALFSAALNAWVTGSWEIKQPFHPAIIIIMMLALGLKTGLAPMHFWL
Sasp  SIKYFLTQAMAATLMLFLTTSNAWVTGQWDIQHLSHPALVTMTALALGLKMGLAPLHFWL
Eupe  TVKYFLVQATAATLMLFMATSNAWLEGQWEIEQLSNLTPLMITTLALGLKMGLAPMHFWL
Enja  TTKYFLIQATAAAMILFAASTNAWIMGQWDINHMSHPLTSAMAMTALALKVGLAPLHLWL
Same  TTKYFLVQATAAAMLLFASTTNAWITGQWEITQLSHPIASALAVAALALKVGLAPMHFWL
Chch  TTKYFLTQATAAAMILFASTTNAWVTGTWNIAQLSDPTAVALISIALALKMGLAPAHFWL
Grgr  TTKYFLTQATGAAMILFASTANAWVTGEWNITHLSHPAATALVTVALAMKVGLAPMHFWL
Caau  TTKYFLTQATAAAMILFASTTNAWMTGEWSITDLSDPLANTMFMTALALKIGLAPMHFWM
Cyca  TTKYFLTQATAAAMILFASTTNAWMTGEWSINDLSNPIASTMFMAALALKIGLAPMHFWM
Dare  TTKYFLIQAAAAAMILFTSTTNAWISGQWDVTGMPGPATSTAMMFALALKIGLAPMHFWL
Cost  TTKYFLTQATAAAMILFAATMNAWIMGEWDINSLSHPLASTLVITALALKIGLAPVHFWL
Leec  TTKYFLTQATAAAMILFAATTNAWMTGEWSINELSHPFALAMTITALALKIGLAPMHFWL
Fola  TTKYFLTQATAAAMILFAATTNAWATGEWDINNLTHPLASSLTMMALALKVGLAPMHFWM
Clmc  TTKYFLTQATAAATILFASTINAWATGEWSITHLTYAPATTIATMALALKVGLAPLHFWL
Phin  TTKYFLTQATAAATMLFATTTNAWLTGEWNITLMLHPLSSALIMTALALKVGLAPLHLWL
Icpu  TTKYFLAQAAAAATILFASTINAWTTGEWNIYCLSHPAATILITMALALKVGLAPVHFWM
Psto  TTKYFLAQAAAAATILFASTINAWATGEWNINCLTHPIATTLVTMALALKVGLAPMHFWM
Cora  TTKYFLAQATAAATILFASSMNAWVTGEWNINYSSHQPSTMLIMTALALKLGLAPMHFWL
Eisp  STKYFLTQATAAATILFASTTNAWIMGEWNINDLSHPLPIALITLALALKIGLAPLHFWL
Apal  ATKYFLIQATAAAVLLMATTTNAWYSGEWNINCLTHTPSIILATLALTMKIGIAPLHLWL
Eslu  TTKYFLTQATAAALILFASTSNAWLTGEWAINQMSHPLATSTIMLALALKLGLAPVHFWM
Dape  TTKYFLTQATAAAMVLFAATSNAWLSGEWAIHQTTHPLVTTTLMLALTLKLGLAPAHFWM
Glse  TTKYFLTQATAAATILFASTTNAWLTGQWDILQLSHPLTATTTMLALALKIGLAPLHFWL
Naar  TTKYFLTQATAAATILFASTTNAWLVGEWDILQLSHPIATTITMLALALKIGLAPLHFWL
Lioc  ATKYFLTQATGAAVLLFATTTNAWLLGEWDIYQLSHPIATTMIMLALALKVGLAPLHFWL
Opso  TTKYFLTQATAAATILFASTTNAWLVGEWDILQLSHPLATTATMLALALKIGLAPFHFWL
Alte  TTKYFLTQATAAAMILFASTTNAWMLGEWDITQLSHPAAATMVMIALALKLGLAPMHFWL
Plap  TTKYFLTQATAAAMILFASTTNAWMLGEWDITQLSHPVAATTAMIALALKMGLAPVHFWL
```

To be continued on page 11.

[2/6 of aligned sequences]

```
Plal AAKYFITQAAAAATIMFASTTNAWLTGSWDIQHLSHPAAATIAFMALALKVGLAPVHFWL
Sami AAKYFITQAAAAATIMFASATNAWLTGSWDILHLSHPVSATMAFMALALKVGLAPVHFWL
Rere AAKYFLTQAAAAATIMFASATNAWLTGTWDILHLSHPFCATVAFMALALKLGLAPVHFWL
Gama TTKYFLTQATAAAMILFASTTNAWMSGEWDIHQLSHPVAAAVALMALALKIGLAPVHFWM
Onmy TTKYFLTQATAAAMILFASTTNAWLVGEWEIHQLSHPLATTTVMLALALKLGLAPVHFWL
Sasa TTKYFLTQATAAAMILFASTTNAWLVGEWEIHQLSHPLATTTVMLALALKLGLAPVHFWL
Cola TTKYFLTQATAAAMILFASTTNAWLVGEWDIQQLTHPIAVTTAMLALALKVGLAPVHFWL
Dita ATKYFLTQATAAAVILFAGLTNAWLTGEWSISQLSHPLAANLMFLALALKVGLAPMHFWL
Gogr TTKYFLIQAAAAAMILFASLTNAWFTGEWVIFQLSDPLPASVTALALALKMGLAPMHLWL
Chsl TTKYFLIQATAAAVVLFAALTNAWLTGEWLILQLSHPAPATLTLLALSLKVGLAPVHFWL
Atja TTKYFLTQATAATMILFASTTNAWVTGEWNIQEMSNPLTMTVVTMALALKLGLAPMHFWL
Iido TTKYFLTQATAATVILFASTTNAWITGGWNLQEISSPLTMTMITMALALKLGLAPMHFWL
Auja TTKYFLTQATAAAMILFASTTNAWITGEWDIKQLSHPLATTTTILALALKIGLAPVHFWL
Chag TTKYFLTQAXAAALILFASTTNAWVTGEWNIQQLSHPLASTMLVLALALKIGLAPVHFWL
Hami TTKYFLTQATAAAMILFASITNAWILGEWNIQLTSHPLATTMIMLALSLKVGLAPMHFWL
Saun TTKYFLTQATAAAMILFASTTNAWVLGEWNIQLTSHPLATTMVVLALSLKIGLAPMHFWL
Nema TTKYFLTQATAAAMILFSSTTNAWITGEWDILQLSHPIPATATTIALALKVGLAPLHFWL
Disp ATKYFLTQATAAALILFASTTNAWITGEWSILQLSHPLPATAVTIALALKVGLAPTHFWL
Myaf TTKYFLTQATAAAMILFASTTNAWLTGEWNILQLSHPLPATAMTMALALKLGLAPTHFWL
Lagu STKYFITQATAAAMILFASTTNAWITGQWNITDMTSPLAITIITMALSLKLGLAPTHFWL
Trtr TTKYFLTQATAAAMILFASATNAWYTGEWCITDMSSPLSMTMVIMALALKLGLAPVHFWL
Zucr TTKYFLTQATAAAMILFASATNAWVTGEWNIMNMSSPLSATMAIMALSLKLGLAPVHFWL
Pxja TTKYFLTQATAAAMILFASATNAWLTGEWEIQHISHPIPALATTMALALKVGLAPVHFWL
Pxlo TTKYFLTQATAAAMILFASATNAWLTGEWEIQHISHPIPALATTMALALKVGLAPVHFWL
Pctr TTKYFLIQATAAATILFASATNAWLTGEWEIQHMKHPLPATATTMALALKLGLAPMHFWL
Apsa TTKYFLTQATAAAMILFAGTTNAWMSGEWEIQNMNYPVPTTMMTMALALKIGLAPTHFWL
Cabe ATKYFLVQATASATILFAAISSAWQVGHWVIFDSMDPLSNTLLLAALALKLGLAPLHGWL
Bzze TTKYFLTQATAAAMILFASTMNAWHTGQWDIQQMTDPIPATIVMLALALKVGLAPMHTWL
Siim TVKYFITQITASAMIMMAVLTHVVLSGQWDMKDPHLTVSPVIILLALAMKVGIPPMHAWL
Ctru TTKYFLTQATAAAMILFASTTNAWLTGQWEIQQMSHPLPLTVITLALALKLGLAPLHSWL
Dpbr TTKYFLTQATAAAMILFASTTNAWLTGQWEIQQMTHPLPLTIITLALALKIGLAPLHSWL
Caki SVKYLLIQATGAAIILWGSMLNAQATGHWNIINEKEDMAMLIIITGLALKLGIAPIHAWF
Phja TTKYFITQAGAAALILFATTSNAWITGQWDINFAIHHFPATLLIFALALKMGAAPMHFWL
Brsp ATKYFITQAAAAAIILFASLINALMTSQWDIYFDLRPFPAALLILALALKLGIAPVHVWF
Gamo TTKYFITQAAAAALILFASTTNAWITGQWDINFDLHFFPASMLTMALALKMGLAPVHFWL
Lolo TTKYFITQAAAAALILFASTTNAWITGQWDINFNLHAFPASLLTMALALKMGLAPVHFWL
Batr ATKYFLIQAIASATILLASIFNTWTTGQWNIYYMTHPTASVMVYSALCLKLGLAPLHTWL
Prmy SAKYFMVQATATAILLFSTTSSALMLGQWNIYELDQPLFNALFMLALSLKLGLAPLHAWL
Lose ATKYFLTQATAAAVLLFATVTNAWLTGQWDIQQMTHPLPSTMIVMALALKMGLAPLHTWL
Loam TTKYFLAQATAAAVLLFAGTTNAWLTGQWDIQQALHPLPCTMITLALALKVGLAPLHTWL
Chab TTKYFLTQATAAAVLLFASTTNAWLTGQWDIQQMSHPLPAMMVTTALALKIGLAPLHSWL
Chto TTKYFLTQATAAAVLLFASTTNAWLTGQWDIQQMSHPLPAMMVTTALALKIGLAPLHSWL
Majo TTKYFLAQAAAAAVLLFAALTNAWLTGQWDIQQTLSPTPTAMLTCALALKVGLAPFHTWL
Hlst TTKYFLAQAAAAAMLLFAALTNAWFTTQWDIQNDLNPIPATMIMCALALKVGLAPFHTWL
Clpe ATKYFLTQATAAAVLLFASATNAWITGQWDIQQMSHPLPTTMATIALALKVGLAPLHTWL
Mlmr TTKYFLAQAAAAAMLLFASTTNAWLTGQWDIQQMSHPLPTTMIIIALALKIGLAPLHSWL
Crcr TTKYFLTQATAAAMLLFASTTNAWLTGQWEIQQMSHPLPVTLATLALALKIGLAPFHAWL
Muce TTKYFLTQATAAAMLLFASTTNAWLTGQWEIQQMYHPLSVTLATLALALKIGLAPFHAWL
Bege TTKYFLTQATAAAMLLFASTTNAWLTGQWEIQQMTHPLPTTIIILALALKIGLAPLHAWL
Mela TTKYFLTQATAAAMLLFASTTNAWLSGQWEIQQMTHPLPTTIITLALALKIGLAPLHAWL
Hats TTKYFLTQATAAAMLLFASVTNAWLSGQWDIQQMTHPLPTTIITLALALKIGLAPLHAWL
Orla ATKYFLIQSAAAATILFASSTNAWLSGQWDIQHITHPLPTVMITIALSLKLGLAPLHAWL
```

To be continued on page 12.

[2/6 of aligned sequences]

```
Cosa ATKYFLTQAAAAAMLLFATTTNAWITGQWDLYQLAHPFPTTMITVALALKIGLAPLHAWL
Exsp TTKYFLTQATAATTILFACTTNAWLTGQWDIYQLTHPFPAMMIYLALALKIGLAPLHAWL
Depa TTKYFLTQAAAAAVLLFATATNAWITGQWDIYQLTHPFPVTMITFALTLKIGLAPLHAWL
Rima TIKYFIIQTTAATMLLFASVSNAWLSGQWEIQQMSHPTPLALITIALALKLGLAPMHAWF
Fuol TTKYFLTQATAAAMLLFASITNAWITGQWGLELMNEPLPTTLITMALALKIGLAPLHAWM
Gmaf TTKYFFAQATASATLLFAATSNAFFTGSWDILQTNNPLTYTLMTLALAMKIGLAPLHSWM
Xeei ATKYFLIQATAAAMLLFASISNAWLTGQWNINYMHHPFPSTLIILALALKIGLAPLHGWM
Pros TTKYFLTQATAAAMILFASTTNAWLTGQWDIQQMSHPIPITIITLALALKIGLAPVHSWL
Scmi TTKYFLTQATAAAMILFASTTNAWLTGQWEIQQMSHPLPIACVTMALALKIGLAPVHMWL
Rolo TTKYFLTQATAAAMILFASTTNAWLTGQWDIQQMSHPLPTTIITLALALKLGLAPVHSWL
Cere TTKYFLTQATAAAMILFASTTNAWMTGQWDIQHMSHPLATTMVILALALKLGLAPLHSWV
Daga TTKYFLTQATAAAMILFASTTNAWMTGQWEIQQMSHPLVTTMIIMALALKLGLAPLHSWM
Anco TTKYFLTQATAAALILFASTTNAWMTGQWDIQQLTHPLSTTAITLALALKLGLAPLHFWL
Dmve TTKYFLIQATAAAMILFAATSNAWFTGQWDILQLTHPISANITMLALALKLGLAPLHLWL
Dmar TTKYFLIQATAAAMILFAATSNAWLTGQWDILQLTHPLSADITILALSLKLGLAPLHLWL
Anka TTKYFLTQATAAAMILFASTTNAWMTGQWDIQQLTHPLSTTTVILALALKLGLAPVHFWL
Moja TTKYFLTQATAAAMILFASTTNAWITGQWDIQQLTHPLSTTAITLALALKLGLAPVHFWL
Hoja TTKYFLTQATAAAMILFASTTNAWMTGQWDIQQLTHPLSTTAITLALALKLGLAPMHFWL
Bede TTKYFLTQATAAAMILFASTTNAWLTGQWDIQQMSHPLPITIITMALALKIGLAPVHSWL
Besp TTKYFLTQATAAAMILFASTTNAWLTGQWDIQQMSHPLPITIITLALALKIGLAPVHSWL
Mysp TTKYFLTQATAAAMILFASTTNAWLTGQWDIQQMTHPIPTTILTMALALKIGLAPVHAWL
Osja TTKYFLTQATAAAMILFASTTNAWLTGQWDIQQMTHPVPATILTMALALKIGLAPVHAWL
Sgro TTKYFLTQATAAAMILFASTTNAWLTGQWEIQQVAHPLPATILTMALALKIGLAPVHAWL
Pzpa TTKYFLVQATAAAIILFASTTNAWITGQWDIQQMSHTVPTAMITLALMLKLGLAPAHFWL
Zeja TTKYFLTQATAAALILFASMTNAWITGQWEIQQMTHTIPTTMLTLALMLKLGLAPAHFWL
Znne TTKYFLTQATAAALILFASMTNAWITGQWEIQQMTQMIPTTMLTLALMLKLGLAPAHFWL
Zefa TTKYFLTQATAAALILFASMTNAWITGQWGIQQMDQTIPTTMLTIALMLKLGLAPAHFWL
Acni TTKYFLTQATAAALILFASLTNAWITGQWEIQQMTQTVPTTMLTLALMLKLGLAPAHFWL
Ncrh TTKYFLTQATAAALILFASLTNAWITGQWEIQQMTQTVPTAMLTLALMLKLGLAPAHFWL
Agca TTKYFLTQATAAAMLLFASTTNAWLTGQWEIQQMLHPLPATMITLALALKIGLAPLHSWL
Hydy TTKYFLTQATAAAMLLFASSTNAWMTGQWGIQQLTHPLPITLITLALALKIGLAPLHSWL
Gsac TTKYFLTQATGAAMLLFASTTNAWMTGQWEIQQMTHPLPMTLITLALALKIGLAPLHSWL
Pevo ATKYFITQAAAAAMLLFAAISNTWMTGQWSITQIMHPLPNSLLILALALKLGLAPTHIWL
Hiku STKYFLTQATAAAMILFASSTNAWITGQWSITELSHPAPSTIMMLGLALKIGMAPLHTWL
Inpa ATKYFLTQATAATTLLFAATTNAWISGQWEIQQMTHPLPVTLATLSLALKMGLAPLHAWL
Auch TTKYFLSQATASATILFAAITNAWLTGQWDILQMTHPVPATMLTAALALKIGLAPLHTWV
Fico TTKYFLTQATAAAMLLFASTTNAWLTGQWDIAQMTHPLPTTMITIALALKIGLAPLHGWL
Macs TTKYFLTQATAAAMLLFASLTNAWLTGQWDIGQMTHPLPTTIITLALALKIGLAPVHAWL
Moal ATKYFIVQATAAATLLFAAINNAWLQGHWDIQHMSHPLFTSMITLALLLKLGLAPMHTWM
Syma SIKYFIAQATAATLLLFASTTNAWLSGQWDIQQMTHPLPTTIITLALMLKLGLAPMHAWL
Mafr TTKYFLTQATAAAMLLFASTTNAWISGQWHIMQMTHPIPTTLITLALALKIGLAPMHAWL
Dcpe TTKYFLTQATAAAVLLFASTTNAWLTGQWEITQMTHPLPNIMITMALALKMGLAPLHTWL
Dcti TTKYFLTQATAAAVLLFASTTNAWLTGQWEISQMTHPLPTVMITMALALKIGLAPLHTWL
Hehi ATKYFLIQAAGAAMLLFASTTNAWLTGQWDLLQIAHPFPTALVTLALALKVGLAPVHSWL
Stam TTKYFLTQATGAAILLFASTTNAWLTGQWDIQHMSHPFPVTLITLALALKLGLAPIHAWL
Hogi TTKYFLTQATAAAMLLFASTTNAWLTGQWDIQQMTHPLPTTLIMMALSLKIGLAPMHTWL
Erzo STKYFLTQATGAAMLLFASTTNAWLTGQWDIQQMTHPLPITLITLALALKVGLAPVHSWL
Hxot TTKYFLTQATGAAMLLFASTTNAWLTGQWDIQQMSHPLPVTLITLALALKVGLAPLHSWL
Core TTKYFLTQATGAAMLLFASTTNAWLTGQWDIQQMAHPLPVTMITLALALKMGLAPLHSWL
Apve TTKYFLMQATGAATLLFASITNTWLTGQWDIQQMSHPIPVTLITLALALKVGLAPLHSWL
Latj TTKYFLTQATAAAMLLFASTTNAWLTGQWEIQQMTHPFPTTMIILALALKVGLAPMHSWL
Laja TTKYFLTQATAAAMLLFASTTNAWLTGQWDILQMCHPLPTTMITLALALKIGLAPTHSWL
```

To be continued on page 13.

[2/6 of aligned sequences]

Syja	ATKYFIVQAIGAAAILFATITNGWDMGFWNLDHLTDPLASAIFTLGIALKMGLAPLHTWV
Epme	TTKYFLTQATGAATLLFASTTNAWLTGQWDILQMSHPLTTTMATLALALKVGLAPLHTWL
Grse	TTKYFLTQATAAATLLFASTTNAWLTGQWDIQQMSHPIPTTLTILALALKIGLAPLHLWL
Clja	TTKYFLLQATAAATILFASTINAWMTGEWNIQQTSHIFTTTLITLALALKIGLAPFHMWL
Ogcy	TTKYFLTQATAAATVLFASITNAWISGQWTIEQMNHPLPASIMIVALALKLGLAPLHAWL
Plna	ANKYFFTQIAGAAMLLIAATVNAWNTGQWAISQPCSQSMASLTVLALGLKLGLAPLHLWM
Lema	TTKYFLTQATAAAMLLFASTTNAWLTGQWDIQQMSHPFPTTVIILALSLKIGLAPVHSWL
Etzo	TTKYFLTQATAAAMLLFASTTNAWLTGQWDIQQMTHPLPITLITLALALKIGLAPVHSWL
Apse	TTKYFLIQAAAAATLLFAATTNAWITGQWDIHQMTHPAPATIATLALAMKVGLAPTHFWL
Epde	TIKYFLTQATAAAMLLFASTTNAWITGQWDIQQMSHPLPITMITLALALKIGLAPAHTWL
Slja	TTKYFLMQATAAAMLLFSATTNAWLTGQWDILQMAHPFPVTLVTLALALKIGLAPLHSWL
Bsja	ATKYFLAQATAAAMLLFASTTNAWLTGQWEIQQMSHPLPITMISAALALKIGLAPFHSWM
Ecna	GTKYFLAQATAAAMLLFASVTNAWLTGQWDILQMSHPLPTTMITLALALKIGLAPLHSWL
Cohi	ATKYFLTQATAAAMILFAAISNAWLTGQWEIQQLTNEISIAMFTLAMALKIGLAPVHTWL
Caar	TTKYFLTQATAAAVLLFASTTNAWLTGQWDILQMSHPLPTTMITLALALKIGLAPMHSWL
Came	TTKYFLTQATAAAMLLFASTTNAWLTGQWDIQQMTHPIPTTMITLALALKIGLAPMHSWL
Mema	TTKYFLTQATAAALLFFASTTNAWLTGQWNIQQMAHPLPVTMITLALALKIGLAPTHSWL
Lenu	ATKYFLTQATAAAIMLFSATLNAWLTGLWHIQELTHPMTTTMMLTALALKLGLAPIHSWM
Brja	TTKYFLTQATAAAMLLFASTSNAWLTGQWDITQMSHPLPTTLITLALALKIGLAPVHAWL
Plma	TTKYFLTQATAAAMLLFASTTNAWLTGQWNIEQMTHPLPTTMIILALALKIGLAPLHAWL
Emst	TTKYFLTQATAAAMLLFASTTNAWLTGQWDIQQMSHPLPITMITIALALKIGLAPLHSWL
Ptti	TTKYFLTQATAAAMLLFASTTNAWLTGQWDIQQMSHPLPVTMITIALALKIGLAPTHSWL
Losu	TTKYFLIQAAATATLLFATLSNAWFTGQWNITELTHPLLTSIVILSLALKLGLAPLHGWL
Geoy	ATKYFLTQATAAAMILFASTTNAWLSGQWEIQGMTHPLPITILTVALALKIGLAPLHSWL
Dipi	TTKYFLTQATAAAMLLFASTTNAWLTGQWDIQHMTHPLPLTMITIALALKIGLAPLHSWL
Pama	TTKYFIAQATAAAMLLFASATNAWLVGQWDIQQMAHPLPVTLITLALALKIGLAPLHSWL
Leob	ATKYFLTQATAAAMLLFASTTNAWLTGQWDIQQMSHPLPITMIIIALALKIGLAPLHSWL
Neba	ATKYFLTQATAAAMILFASTTNAWITGQWDIYQMSHPLAITLITLALALKIGLAPLHSWL
Pdpl	TTKYFIIQATAAAVFLFAALVNIWYSYQWNSFHFLHPFTSTLCFCALAMKIGLAPFHAWV
Nimi	TLKYFLTQATAASTLLFATTTNAWLTGQWDIQHMTHPLPATLFTLALALKVGLAPLHIWL
Uptr	SVKYFLIQVTAAAMLLFATCSNAWYTGEWNIQEMAHPLSVTMAIVALALKLGLVPLHTWM
Pesc	TVKYFLVQAAAASVLLFAVITNAWLTGEWSVELTPHPIASNLMILAFAVKLGLAPLHLWM
Baar	TTKYFLTQATAAAMLLFASTTNAWLTGQWDIQQMSHPLPITLITLALALKIGLAPAHSWL
Moar	TTKYFLTQATAAAMLLFASTTNAWLTGQWDILQMSHPLPITMITIALALKIGLAPLHSWL
Toja	TTKYFLTQATAAAMLLFASLTNAWLTGQWDIQQMTHPLPTTMITLALALKIGLAPMHSWL
Chau	TTKYFLTQATAAATLLFAGTTNAWLSGQWDIQQVSHPFPATLLTLALALKIGLAPFHTWL
Chse	TTKYFLIQATAAATLLFASTTNAWISGHWDIQQTSHPLPLTMVTLALALKIGLAPLHSWQ
Enar	TTKYFLTQATAAAMLLFASTTNAWLTGQWEIQQMSHPLPVTMITLALALKIGLAPVHSWL
Hpty	TTKYFLTQATAAAMLLFASTTNAWLTGQWDIQQMSHPLPVTMITLALALKIGLAPVHSWL
Nana	TTKYFLTQATAAAMLLFASTTNAWLTGQWNIYQMTHPFSTTLVLLALALKIGLAPMHSWL
Mcst	TTKYFLTQATAAAMLLFASTTNAWLTGQWDIYQMSHPFPITMIILALALKIGLAPVHSWL
Rhox	TTKYFLTQATAAAVLLFASTTNAWLTGQWDIYQMTHPLPITLITFALALKVGLAPAHFWL
Opfa	TTKYFLVQATAAAMLLFASMTNAWLTGQWDIQQMSHPLPITMITLALALKTGLAPVHAWL
Paar	TTKYFLTQATAAAMLLFASATNAWLTGQWDIQQMSHPLPTMMITLALALKLGLAPVHSWL
Gozo	TTKYFLTQATAAAMLLFASTTNAWMTGQWDIQQMSHPVSTTMIILALALKIGLAPVHAWL
Ackr	TTKYFLIQAMAAALLLAAGVTNAWSSGQWTFSLMEDPLSLLLTTMALSLKLGLAPFHLWV
Elev	TTKYFLTQATAAAMLLFAATTNAWLTGEWQLQEMSHPIPITLITLALALKIGLAPTHAWL
Trdu	TTKYFLTQAAAAATLLFASVTNAWLTGQWEIQQITHPLPSTMITLALALKIGLAPLHAWL
Amoc	ATKYFLTQAAAAAMLLFASTTNAWLTGQWDIQQMTHPLPITMITLALALKVGLAPLHAWL
Hame	ATKYFLTQAAAAAMLLFASTTNAWLTGQWELQQITHPLSLAIVTMALALKIGLAPVHSWL
Chso	TAKYFLAQATAATALLFASTTNAWFSGVWEINQLSHPIPITMMTIAIALKVGLTPLHTWL
Lyto	ATKYFLTQATGAAMLLFASTTNAWMTGQWDIQQMSHPLPITLITLALALKVGLAPVHAWL

To be continued on page 14.

[2/6 of aligned sequences]

```
Encr    TTKYFLTQATGAAMLLFASTTNAWMTGQWDIQQMSHPLPITLITLALALKVGLAPVHAWL
Bvar    TTKYFLVQTGGAATLLFATTLNAWQTGQWNILETADPFATSLITLALMFKLGLAPLHLWL
Noco    ATKYFLIQAGGAAVLLFAGTTNAYLTGQWDIQQAAHPLPIAIITLALALKLGLAPLHSWM
Chsp    ATKYFLAQATAAAMLLFSTIINAWMTGQWCIGQQMSALPHTMIILALALKIGLAPLHSWL
Arja    TTKYFLTQATAAATLLFASTSNAWLTGQWDILQMSHPLPVTLVTLALALKLGLAPIHSWL
Pase    TTKYFLTQATAAAMLLFASASNAWATGQWNIQQMTHPLSSTLAVIALALKIGLAPVHSWL
Trel    ATKYFLVQATASAAILFAALVNSFMFGAWDLFNMIHPLPGSIMILALTLKIGIAPLHMWL
Lifa    TTKYFLTQATAAALLLFAALTNAWTTGQWSIDQMMHPLSITLLTVALMLKLGLAPFHMWL
Acur    ATKYFIIQAAGAAVLLCAGCLNAYLTGTWDINYTWHPLPGMMATLALALKLGLAPVHSWF
Ampe    TTKYFLTQATAAAMLLFASTTNAWLTGQWEIQQMTHPLPTMMITIALALKIGLAPVHSWL
Urja    ATKYFLAQASAAATLLFAATTNAWLTGQWDIQQIMSPIPNALMIIALAMKIGLAPIHAWL
Enet    TTKYFLAQATAAAVLLFASVSNAWLSGQWEISQMTHPLPTSLIILALTLKLGLAPFHGWL
Ptbr    CTKYFLMQAPASALFLFAATSNAWSQGQWETLIMTDSFSTICMLIALMTKMGLAPMYAWF
Safa    TTKYFLVQAAAAALLLFASVTNAWLCGEWFIQQMTHPLPTTMIILALAMKLGLAPLHAWL
Icae    TTKYFLTQATAAAMLMFASTTNAWLTGQWNIEQMTHPIPATMITLALALKIGLAPVHAWL
Asmi    TTKYFLIQATAAATMLFAGTTNAWLTGQWDITTTTHPFPLTLLTLALSLKLGLAPLHTWL
Foal    TTKYFIVQAAAAAMILFSAVIKAWTTGEWVINPFSEPLSTSLLVLALALKLGLAPAHSWL
Drze    TTKYFLIQSTAAALLLFASNINAWMTGQWEINYMIHPLPVTLATLALALKIGLAPVHAWL
Rhas    TTKYFLTQATAAATLLFASMTNAWLTGQWDIQQMVHPVPATMITLALSLKIGLAPLHTWL
Elac    TTKYFLTQATAAATLLFASMTNAWLTGQWDIQQMTHPVALTMVTLALALKIGLAPLHTWL
Kugu    TTKYFLTQATAAATLLFAATTNAWFTGQWEIQQMTHPFPITIITLALALKIGLAPMHPWL
Plor    TTKYFLTQATAAATLLFASITNAWLTGQWDILQMNHPLPTTMMIFALALKVGLAPLHSWL
Sgun    TTKYFLTQATAAAMLLFASTTNAWLTGQWDIQQMSHPLPITMISIALALKIGLAPLHSWL
Zaco    ATKYFLIQATGGAMLLFAATTNAWLTGQWDIQQMSHPFPVIMITVALSLKIGLAPLHSWL
Zbfl    TIKYFLAQATAAAMLLFASATNAWLTGQWDIQQMSHPVPIAMITIALALKIGLAPMHSWL
Spba    TIKYFLTQATAAAMLLFASATNAWLTGQWDIKEMTDPLIIVAITLALALKMGLAPMHSWL
Game    TTKYFLTQATAAAMLLFASTTNAWLTGQWNIEQMTHPIPTTMITLALALKIGLAPVHAWL
Thth    TTKYFLTQATAAAMLLFASTTNAWLTGQWNIEQMTHPIPTTMIMLALALKIGLAPVHAWL
Xigl    TTKYFLTQATAAAMLLFASTTNAWLTGQWDIQQMSHPFPLTLITLALALKIGLAPMHTWL
Hyja    TTKYFLTQATAAAMLLFAGTTNAWLTGQWSIEQMTHPLPTTMMILALALKIGLAPVHTWL
Psan    AAKYFLTQASAGATLLFGAATNAWITGQWAIEQTPHPIAAIIMTIALAMKLGLAPLHTWL
Cupa    TTKYFLTQATAAAMLLFASTTNAWLTGQWSIEQMTHPLPTTMIIIALALKIGLAPVHAWL
Mpch    TTKYFLTQATAAATLLFASTTNAWITGEWNIQQMSNPFSSTLIILALSLKIGLAPMHAWM
Char    TTKYFLTQATAAATILFASTTNAWLTGQWDILQMTHPIPTNMVLLSLALKIGLAPMHSWL
Pser    TTKYFLTQATAAAMLLFASTSNAWLSGQWDIQQMTHPLPTTMIILALALKIGLAPMHSWL
Prol    TTKYFLTQATAAATLLFASMTNAWLTGQWDIQQMTHPLATTMIIIALALKIGLAPMHSWL
Plbi    TTKYFLAQATAAATLLFASTTNAWLTGQWDIQQMTHPLPTTMVVIALALKIGLAPMHSWL
Calu    STKYFLAQATAAATLLFASTTNAWLTGHWGICDTTHPLPTTLVVLALCLKMGLAPMHAWL
Papa    ATKYFLTQAAAALVILFAAILNSWSTGQWFTSFMVQPLPLLILSLGLAMKIGLAPLHFWL
Sufr    ATKYFLTQATAAATLMFASTTNAWLTGQWDIQQTSHPLPITMITIALALKLGLAPLHSWL
Stci    TTKYFLTQATAAATLMFAAVTNAWIEGQWDITQMSHPLPTTIITIALALKIGLAPLHSWM
Taru    TTKYFLTQAAAAATLLLAATTNAWMTGQWELQQATHPVPTTMITIALALKIGLAPLHTWL
Rala    TTKYFLTQATAAAMLLFASTTNAWLTGQWDIQQMTHPLPVTMITIALALKIGLAPLHSWL
          **:: *  .    :          *              .:  * *  * : *
```

To be continued on page 15.

[3/6 of aligned sequences]

```
                  D                                E
Scca  PEVLQGLDLTTGLILATWQKLAPFAILLQLSPLLNSNLLLLFGVTSTIVGGWGGLNQTQL
Muma  PEVLQGLDLTTGLILSTWQKLAPFAILLQLYPLLNPNLLLTLGILSTIIGGWGGLNQTQL
Erca  PEVMQGISISTGLILVTWQKLAPLALLMQISHNIKPELLIILGLMSTIIGGWGGLNQTQI
Pose  PEVMQGITLNTGLILATWQKLAPLALLYQISNNLMPELMIILGLTSTFIGGWGGLNQTQI
Actr  PEVLQGLDLTTGLILSTWQKLAPFALIYQISPMMNPSLVIMLGLASTIIGGWGGLNQTQL
Scal  PEVLQGLDLTTGLILSTWQKLAPFALIYQISQVMNPTLMITLGLSSAVIGGWGGLNQTQL
Posp  PEVLQGLDLTTGLILSTWQKLAPFALIYQISPMMNPTLMITLGLASTIVGGWGGLNQTQL
Atsp  PEVLQGLDLTTGLITATWQKLAPFAIIYQISSTTNPTLLTILGILSTLIGGWGGLNQTQL
Leoc  PEVMQGLDLTTGLIMATWQKLAPFAIIYQISSTTNPTLLTTLGILSTLVGGWGGLNQTQL
Amca  PEVMQGLDLFTGLIMSTWQKLAPFSLIYQLAYAMDSTLLITMGILSTLIGGWGGLNQTQL
Osbi  PEVLQGLNLTTGLILSTWQKLAPFILIYQIAPNTNPTILTSMGILSTLIGGWGGLNQTQL
Pabu  PEVLQGLNLTTGLILSTWQKLAPLALMYQISFNMNTTLLIIFGLTSTLIGGWGGLNQTQL
Hial  PEVLQGLDLTTGLIMSTWQKLAPFALIYQIAPTVSPTVLMSLGVMSAVVGGWGGLNQTQL
Elha  PEVLQGLDLTTGLILSTWQKLAPFALIYQIAPATQPTLLFTLGVLSAMVGGWGGLNQTQL
Mlcy  PEVLQGLDLTMGLILSTWQKLAPFALIYQIAPTVQPTLLITLGIMSALIGGWGGLNQTQL
Algl  PEVLQGLDLTTGLILSTWQKLAPFALIYQVAPGASPVLLACLGVSSALVGGWGGLNQTQL
Ptgi  PEVLQGLDLTTGLIMSTWQKLAPLALIYQVAPTVNPTLLIALGVTSALVGGWGGLNQTQL
Alaf  PEVLQGVTLTTGLIVSTWQKLAPMALLYQIAPAVNPTLLLTLGITSALVGGWGGIDQTQL
Nock  PEVLQGMTLNTGLVIATWQKLAPMALVYQIAPTVSPTLLVALGITSALVGGWGGIDQTQL
Anja  PEVLQGLDLTTGLILSTWQKLAPMALIYQLSPGVEQPLMIMLGVMSALVGGWGGLNQTQL
Gyki  PEVLQGLSLTTGLILSTWQKLAPMILIYQLAPNVNPTVLLLMGLTSAFIGGWGGLNQTQL
Syka  PEVLQGLDLTTGLILSTWQKLAPMILIYQLAPNVNQSLITTMAIASALVGGWGGLNQTQL
Opma  PEVLQGLDLSTGLILSTWQKLAPMALIYQISLETNQSMMVALGLASALIGGWGGLNQTQL
Comy  PEVLQGLDLNIGLILSTWQKLAPMVLIYQLSMEINQTTMIIIGLTSAIIGGWGGLNQTQL
Sasp  PEVMQGLNLYTGMLLITWQKLAPMMLIYQLAPQIKPPLIMALAVLSALIGGWGGLNQTQL
Eupe  PEVLQGLSLTTGLILLTWQKLAPMVLMYQISSQIHPLLILTLATMSVLVGGWGGLNQTQL
Enja  PEVLQGITITTGLILSTWQKLAPLALIMQTANFTHPLLLTTLALSSTLIGGWGGLNQTQL
Same  PEVLQGITLTTGLILSTWQKLAPFALILQIASNAHPYLLTALAVSSTLVGGWGGLNQTQL
Chch  PEVLQGLDLTTGLILSTWQKLAPFALIIQISPNMHPAILTALGLCSTLVGGWGGLNQTQL
Grgr  PEVLQGLTLTTGLILSTWQKLAPFALLLQIAPTVHPLLLTALGLTSTLIGGWGGLNQTQL
Caau  PEVLQGLDLLTGLILSTWQKLAPFALIIQTAQNIDPLLLTLLGVTSTLVGGWGGLNQTQL
Cyca  PEVLQGLDLLTGLILSTWQKLAPFALIIQTAQTIDPLLLTLLGISSTLVGGWGGLNQTQL
Dare  PEVLQGLDLLTGLILSTWQKLAPMALIIQTTQTTDPLILTSLGIASSLIGGWSGLNQTQL
Cost  PEVLQGLDLSTGLILSTWQKLAPFALIIQVAPTIDPYLLTFLGLLSTLVGGWGGLNQTQL
Leec  PEVLQGLDLLTGLILSTWQKLAPFALIIQIAPAIDPMILTSLGLTSTLIGGWGGLNQTQI
Fola  PEVLQGLDLTMGLILSTWQKLAPFALITQMAPNTNPMLLTTLGLLSTLIGGWGGLNQTQL
Clmc  PEVLQGLDLTTGLIMSTWQKLAPFALLVQMTHSTNPTTLIFLGVVSTLVGGWGGLNQTQL
Phin  PEVLQGLNLTTGLILATWQKLAPFALLVQTSHTMHPALLTALGILSTLIGGWGGLNQTQL
Icpu  PPVMQGLTLTTGLIMATWQKLAPFALIIQMAPFTHPLLLTTLGLLSVFIGGWGGLNQTQL
Psto  PPVMQGLDLTTGLIMATWQKLAPFALIIQMAPFTHPQLLTALGLLSVFIGGWGGLNQTQL
Cora  PSVLQGLDLTTGLIMSTWQKLAPFALIVQIAPFIDPTLLMMLGLISTMVGGWGGLNQTQL
Eisp  PEVLQGLDLTTGLILSTWQKLAPLALLIQTAPITQPTLLTTLGLLSTLVGGWGGLNQTQL
Apal  PEVMQGLNLFAALILATWQKLAPIILLMQISPKPCTA-LMTLGLLSVLVGGWGGLNQTQI
Eslu  PEVIQGLDLTTGLVLSTWQKLAPFALLLQVSSTVNPLILTLIGLLSTLIGGWGGLNQTQL
Dape  PEVIQGSTLTTGLILSTWQKLAPFILLLQISSFISPLLLMTIGLLSALVGGWGGLNQTQL
Glse  PEVLQGLDLTTGLILSTWQKLGPFALITQIAPHINSTLLISLGLMSTLVGGWGGLNQTQL
Naar  PEVLQGLDLTTGLILSTWQKLGPFALILQVAPTVNPSLLIFMGLLSTLVGGWGGLNQTQL
Lioc  PEVLQGLDLTTGLILSTWQKLGPFALIIQVAPTVNPSLLVSMGVLSTLIGGWGGLNQTLL
Opso  PEVLQGLDLTTGLILSTWQKLGPFALILQMAPASNSTLLILIGLLSTLVGGWGGLNQTQL
Alte  PEVLQGLDLTTGLILSTWQKLAPFALIIQIAPTMHPMVLTTLGISSAMVGGWGGLNQTQL
Plap  PEVLQGLDLTTGLILSTWQKLAPFALVIQIAPNMHPTMLTTLGVCSALVGGWGGLNQTQL
```

To be continued on page 16.

[3/6 of aligned sequences]

```
Plal   PEVIQGLTLTTGLILSTWQKLAPFALIIQITPALNSSLVLCMGLASTLIGGWGGMNQTQI
Sami   PEVIQGLSLTTGLILSTWQKLAPFALIIQVTPAIDSSLILGMGVASTLVGGWGGMNQTQV
Rere   PEVIQGVTLTTGLILSTWQKLAPFALILQVAPSVHHALVVSVGLASTFIGGWGGINQTQV
Gama   PEVIQGLDLTTGLLLSTWQKLAPFALVVQVAPFAHPAMLTTLGVLSTLIGGWGGLNQVQL
Onmy   PEVLQGLELTTGLILSTWQKLAPFALMIQVAPTINSSLLVTIGLLSTLVGGWGGLNQTQL
Sasa   PEVLQGLELTTGLILSTWQKLAPFALMIQVAPTINSSLLIAMGLLSTLVGGWGGLNQTQL
Cola   PEVLQGLDLTTGLILSTWQKLAPFALMLQVAPTVNSSLIVTLGLLSTLVGGWGGLNQTQL
Dita   PEVLQGLDLTTGLILSTWQKLAPFALLVQVAPSVDPSLLVALSLLSILIGGWGGLNQTQL
Gogr   PEVIQGLDLTTGLVTSTWQKLAPFALMAQTAPSTDHRLLMGVGLLSILIGGWGGLNQTQL
Chsl   PEVLQGLDLTTGLILSTWQKLAPFALIVQTAPSTDPRLLITLGLASTIIGGWGGLNQTQL
Atja   PEVLQGLDLTTGVILSTWQKLAPFALMMQLN-DTNPTLLVALGLTSMIVGGWGGLNQTQT
Iido   PEVLQGLDLTTGVILSTWQKLAPFALMLQLN-NTNPTLMVTLGLMSTIVGGWGGLNQTQT
Auja   PEVLQGLNLTTGLILSTWQKLAPFALIIQIS-SANPTLLVSLGLISTLVGGWGGLNQTQL
Chag   PEVLQGLDLTTGLILSTWQKLAPFALILQLT-SINPTLLVLLGLLSTLVGGWGGLNQTQL
Hami   PEVLQGLDLTTGLILSTWQKLAPFALILQLS-SANSSLLIFLGLTSTLVGGWGGLNQTQT
Saun   PEVLQGLDLTTGLILSTWQKLAPFALILQLS-TINPKLLVFLGLTSTLVGGWGGLNQTQT
Nema   PEVLQGLDLTTGLILSTWQKLAPFALITQLT-STTPTLLILLGLTSTLVGGWGGLNQTQI
Disp   PEVLQGLDLTTGLILSTWQKLAPFALITQLA-STTPALLIFLGLASTLVGGWGGLNQTQL
Myaf   PEVLQGLDLTTGLILSTWQKLAPFALITQLT-STTPALLVLLGLSSTIIGGWGGINQTQL
Lagu   PEVLQGLDLTTGLILSTWQKLAPFALILQLPHS-NYALITTLGLLSILIGGWGGLNQPQI
Trtr   PEVLQGLNLTTGLVLSTWQKLAPFILLIQIPLP-NHTLMTLLALLSVLVGGWGGLNQVQT
Zucr   PEVLQGLDLTTGLILSTWQKLAPFILLVQMSPP-NNDLLIILALLSILVGGWGGLNQLQI
Pxja   PEVLQGLDLTTGLILSTWQKLAPFALILQTP-STNSTLLIILGITSTLVGGWGGLNQTQL
Pxlo   PEVLQGLDLTTGLILSTWQKLAPFALILQTP-STNSTLLIILGITSTLVGGWGGLNQTQL
Pctr   PEVLQGLDLMTGLILSTWQKLAPFALLMQTP-STNSELMILLGLASTVVGGWGGLNQTQL
Apsa   PEVLQGLDLTTGLILSTWQKLAPFALLLQMP--TNQNLLIILGLLSTLVGGWGGLNQTQL
Cabe   PEVLQGLNLTTGLILSTWQKLAPFSILLQTHPH-SPATLVVLGMLSTLVGGWGGLNQTQL
Bzze   PEVLQGLSLTTGLILSTWQKLAPFALILQVQ-NAPSTLLVGLGITSTLVGGWGGLNQTQL
Siim   PEVMQASDYKTAMLVMTWQKIAPVALMMQNRAT-PDNMYTALAMLSVLSAGWAGLNQTHL
Ctru   PEVLQGLDLTTGLILSTWQKLAPFALILQIP-TANATILITLGLMSMLVGGWGGLNQTQL
Dpbr   PEVLQGLDLTTGLILSTWQKLAPFALILQIQ-PTNTTILVILGLTSTLVGGWGGLNQTQL
Caki   PEILQGTNMMTSMILATWQKMAPFILMCEVATS-YQWLTLTLGLCSILIGGLAGINMIHM
Phja   PEVLQGLSLTTGLILSTWQKLAPFILMCQVMPT-NHTLITMLGISSIVVGGLGGINQTQL
Brsp   PEVLQGLNLTTGLILATWQKLAPFILLLQIMPS-SPLLMILLGLMSILVGGWGGINQTQL
Gamo   PEVLQGLDLTTGLILSTWQKLAPFILMCQIM-PVNSSLITFLGVTSTLVGGWGGLNQTQL
Lolo   PEVLQGLDLTTGLILSTWQKLAPFILMCQIM-PVNSSLITFLGIASTLVGGWGGLNQTQL
Batr   PEILQGLNLMTGMILSTWQKLAPMALIVQIPNN-HPNILLTVGMVSMLVGGWGGMNQTQT
Prmy   PEVLQGLDLMTGLILSTWQKLAPLILLLQIPPT-NINILLLLGLTSMLVGGWGGLNQTQL
Lose   PEVLQGIDLTTALLLSTWQKLAPFILLLQIQQP-HPTILVTLGLLSVVVGGWGGLNQTQL
Loam   PEVLQGLDLTTGVILSTWQKLAPFALLLQLQ-QTSPHLLMALGLISTLVGGWGGLNQTQL
Chab   PEVLQGLDLTTGLIMATWQKLAPFALILQLQ-PLNSTMMILLGLTSTLVGGWGGLNQTQL
Chto   PEVLQGLDLTTGLIMATWQKLAPFALILQLQ-PLNSTMLILLGLTSTLVGGWGGLNQTQL
Majo   PEVLQGLDLTTGLILSTWQKLAPFALLFQIH-PLHPTLITTLGLTSALVGGWGGLNQTQL
Hlst   PEVLQGLDLTTGLILSTWQKLAPFALLFQIHPL-HPHFATFIGVTSVVVGGWGGLNQTQL
Clpe   PEVLQGLDLTTGLVLSTWQKLAPFALIVQLH-PANSTIMVLLGLTSVMVGGWGGLNQTHL
Mlmr   PEVLQGLDLTTGLILSTWQKLAPFALILQLQ-PTNPTILMLLGLTSTLVGGWGGLNHTQL
Crcr   PEVLQGLNLTTGLILSTWQKLAPFILLLQIQ-PTSPAMLTILGLTSTLIGGWGGLNQTQL
Muce   PEVLQGLSLTTGLILSTWQKLAPFILLLQIQ-PTNPIMLVILGITSTLIGGWGGLNQTQL
Bege   PEVLQGLDLTTGLVLSTWQKLAPFALLLQIN-PVNPTILIVLGLASTLIGGWGGLNQTQL
Mela   PEVLQGLDLTAGLILSTWQKIAPFALLLQIN-APTPLMLISLSLLSILVGGWGGLNQNQL
Hats   PEVLQGLDLTTGLILSTWQKLAPFALILQLN-LTDSTALILLGLTSTLVGGWGGLNQTQL
Orla   PEVVQGLDLTTGLVLSTWQKLAPFALLLQISPE-IPLLITSLGLISMLAGGWGGLNHTQL
```

To be continued on page 17.

[3/6 of aligned sequences]

To be continued on page 18.

```
Cosa PEVLQGLDLTTGLILSTWQKIAPFALLLQIQ-PTNHTILILLGLTSMLIGGWGGLNQTQL
Exsp PEVLQGLDLTTGLILSTWQKLAPFALLLQIQ-PTDSAIPIILGLTSTLVGGWGGLNQTQL
Depa PEVLQGLDLTTGLIMSTWQKIAPFSLLLQIQ-PTNTNTLIILGILSTLIGGWGGLNQTQL
Rima PEVLQGLNFSTGLILSTWQKLAPFSLLLQIPPT-SSFLLITLGFISVLMGGWGGLNQTQL
Fuol PEVLQGLDLTTGLILSTWQKLAPFCLLLQIN-NPNTALLLTLGLASTLVGGWGGLNQNQL
Gmaf PEVMQGLTLLTGLILSTWQKLAPFCLIYQIQPH-SPNLFMALGLLSIVAGGWGGFNQVQL
Xeei PEILQGLDLTTGLILSTWQKLAPFSLMLQIQ-PANPTILIFLGLMSTLIGGWGGLNQTQL
Pros PEVLQGLDLTTGLILSTWQKLAPFALIMQIQ-PYNPTTMIALGLMSTLVGGWGGLNQTQL
Scmi PEVLQGLDLTTGLILSTWQKLAPFALLLQIQ-PYNPTTILTLGLLSTLIGGWGGLNQTQL
Rolo PEVLQGLDLTTGLILSTWQKLAPFALILQIQ-SINPTIMITLGLMSTLVGGWGGLNQTQL
Cere PEVMQGLNLTPGLILLTWQKLAPFALLVQIQ-TGDLVVITGLEVLSIFMGGWGGLNQTQL
Daga PEVFQGLDLTTGLILSTWQKLAPFALLIQIQ-FTNPPLVLTLGLLSIFIGGWGGLNQTQL
Anco PEVLQGLDLVTGLILSTWQKLAPFALMVQIQ-STNQEILIMLGLASMLVGGWGGLNQTQL
Dmve PEVLQGLDLITGLILSTWQKLAPFALLVQIQ-ETNQSLMILLGLSSMLIGGWGGLNQTQL
Dmar PEVLQGLDLTTGLILSTWQKLAPFALLVQIP-GTTQNLILLLGLLSMLFGGWGGLNQTQL
Anka PEVLQGLDLVTGLILSTWQKLAPFALMVQIQ-STNQEMLIMLGLTSMLIGGWGGLNQTQL
Moja PEVLQGLDLVTGLILSTWQKLAPFALMIQIQ-STNQEILIALGLMSMLVGGWGGLNQTQL
Hoja PEVLQGLDLVTGLILSTWQKLAPFALMVQIQ-STNQEVLIILGLTSMLVGGWGGLNQTQL
Bede PEVLQGLDLTTGLILSTWQKLAPFALILQIQ-PANPTTMIALGLMSTLVGGWGGLNQTQL
Besp PEVLQGLDLTTGLILSTWQKLAPFALLLQIQ-PANPTTMIALGLMSTLVGGWGGLNQTQL
Mysp PEVLQGLDLTTGLILSTWQKLAPFALILQIQ-PNNPTTLILLGLTSTLVGGWGGLNQTQL
Osja PEVLQGLDLTTGLILSTWQKLAPFALILQMQ-PNSTTTLVLLGLTSTLVGGWGGLNQTQL
Sgro PEVLQGLDLTTGLILSTWQKLAPFALILQLQ-LNNADFLIFLGLTSTLVGGWGGLNQTQL
Pzpa PEVLQGLDLTTGLILSTWQKLAPMSLLLQVP-TLNPTLMMIVGLLSTMVGGWGGLNQTQL
Zeja PEVLQGLDLTTGLIMSTWQKLAPMSLLLQIP-SLNSNLLIAIGLTSTMIGGWGGLNQTQL
Znne PEVLQGLDLTTGLILSTWQKLAPLSLLLQIP-NLNSNLLIIIGLTSTMVGGWGGLNQTQL
Zefa PEVLQGLDLTTGLIMSTWQKLAPLALLVQIP-NLNSNLLIFIGLISTMVGGWGGLNQTQL
Acni PEVLQGLDLTTGLIISTWQKLAPLSLLLQIP-SLNPNLLITIGLISTMVGGWGGLNQTQL
Ncrh PEVLQGLDLTTGLIISTWQKLAPLSLLLQIP-SLNSNLLITIGLISTMVGGWGGLNQTQL
Agca PEVLQGLDLTTGLFLATWQKLAPFALLLQLH-PPNSAILIILGISSTLVGGWGGLNQTQL
Hydy PEVLQGLDLTTGLILSTWQKLAPFVLLLQIC-PPSSSLLVVLGLASTLVGGWGGLNQTQL
Gsac PEVLQGLDLTTGLIMSTWQKLAPFALLLHLD-PSNSTLLVVLGLTSTLVGGWGGLNQTQL
Pevo PEVLQGLDLTTGMILSTWQKLAPFALLLQIHES-NPHLLLILGLTSILIGGWGGLNQTQL
Hiku PEVLQGLDLTTGLIMSTWQKLAPFCLLMQINLD-NPV-LITMAIMSTMVGGWGGLNQAQL
Inpa PELLQGLDLLTGLVLSTWQKLAPFALLTQILPT-TPTLFIALGVASTLVGGWGGLNQTQL
Auch PEVLQGLDLNTGLIVATWQKLAPFTLILQTHQS-NSPLLISLGLISILVGGWAGLNQTQL
Fico PEVLQGLDLTTGLILSTWQKLAPFSLLLQMH-DTNSTPLLLLGLTSTLIGGWGGLNQTQL
Macs PEVLQGLDLTTGLILSTWQKLAPFALLLQIH-PTNSTILITLGIASTLVGGWGGLNQTQL
Moal PEVIQGVSLNTGLIVSTWQKIAPFILLLQIKQI-HPPLLTIIGLASILIGGWGGLNQTQL
Syma PEVLQGLTLTMGLILTTWQKLAPFALLLQIQQT-NPLLLTFVALTSIVVGGWAGLNQTQL
Mafr PEVLQGLNLTTGLILSTWQKLAPFALLLQIQ-QTNPTLTIMLGISSMLIGGWAGLNQTQL
Dcpe PEVLQGLNLTTALILSTWQKLAPFALLLQIS-PSNPTVMIMLGLASTIIGGWGGLNQTQL
Dcti PEVLQGLNLTTALILSTWQKLAPFTLLLQIS-PSNPTILIMMGLASTIIGGWGGLNQTQL
Hehi PEVLQGLDLTTGLILSTWQKLAPFALLVQTP-CTNTTLLVILGLTSTVVGGWGGLNQTQL
Stam PEVLQGLDLTTGLVLSTWQKLAPFALLIQIQ-PTNSSILIVLGITSTLVGGWGGLNQTQL
Hogi PEVLQGLDLTTGLILATWQKLAPFALLLQIQ-PSNSLILIILGLASTLIGGWGGLNQTQL
Erzo PEVLQGLDLTTGLILSTWQKLAPFALLVQIQ-PHSPTLLIIILGLTSTLVGGWGGLNQTQL
Hxot PEVLQGLDLTTGLILSTWQKLAPFALLLQIQ-PANSTLLIALGLASTLVGGWGGLNQTQL
Core PEVIQGLDLTTGLILSTWQKLAPFALLLQVQ-PTNSTLLITLGLASTLVGGWGGLNQTQL
Apve PEILQGLDLTTGLILSTWQKLAPFTLLLQIH-PPNSTLLIMLGLTSTLIGGWGGLNQTQL
Latj PEVLQGLHLTTGLILSTWQKLAPFALLLQIQ-PADPLILIALGLTSTLVGGWGGLNQTQL
Laja PEVLQGLDLTTGLILSTWQKLAPLALLLQIQ-PTNSTLLIIILGLMSTLVGGWGGLNQTQL
```

[3/6 of aligned sequences]

```
Syja PEVLQGLDLTTGLILSTWQKLAPFALLLQLPSF-DPFIMLPLCLLSALVGGWGGMNQTQL
Epme PEVLQGLDLTTGLILSTWQKLAPFALLLQIQ-PATPTILIILGVTSTLVGGWGGLNQTQL
Grse PEVLQGLDLSTGLILATWQKLAPFALLLQIQ-PNNPTILIILGLASTLVGGWGGLNQTQL
Clja PEVLQGLDLVTGLLLSTWQKLAPFVLILQIQP-TNSSTLISLGLISTLVGGWGGLNQTQL
Ogcy PEVLQGLDLTTGLIVSTWQKLAPFILLIQMQPA-DPSLPIILGLSSTLIGGWGGLNQTQL
Plna PEVLQGVSFKTGLIISTWQKIAPLILILQMEFN-APHLLIIMGVLSIIIGGLGALNHNHL
Lema PEVLQGFDLTTGLILSTWQKLAPFALLLQMQ-PANPTLLVILGLTSTLVGGWGGLNQTQL
Etzo PEVLQGLDLTTGLILSTWQKLAPFALLLQIQ-PADSTILIVLGLTSTLVGGWGGLNQTQL
Apse PEVLQGLDLTTGLILSTWQKLAPFALLLQMP-HQNPTLLIILGLSSTLVGGWGGLNQTQL
Epde PEVLQGLDLTTGLILSTWQKLAPFALLLQIQ-PSNSTLLIMLGLASTLVGGWGGLNQTQL
Slja PEVLQGLDLTTGLLLSTWQKLAPFALLVQVQ-PTNAPMLIFLGLASTVIGGWGGLNQTQL
Bsja PDVLQGLDLTTGLIMTTWQKLAPFALLLQMQ-PVNSTTLLILGLTSTLIGGWGGLNQTQL
Ecna PEVLQGLDLTTGLILSTWQKLAPFALLLQIN-PTNPTVLIMLGITSTLVGGWGGLNQTQL
Cohi PEVLQGLDLTTGLILSTWQKLAPMALLVQVNMT-NPLILILLAVASTLVGGWGGLNQTQL
Caar PEVLQGLDLTTGLILSTWQKLAPFALFLQLQ-PNNPTLLIILGLSSTLIGGWGGLNQTQL
Came PEVLQGLDLTTGLILSTWQKLAPFALFLQLQ-PNNPTLLILLGISSTLIGGWGGLNQTQL
Mema PEVLQGLDLTTGLILSTWQKLAPFALLLQLH-PTNPTILITLGLLSTLVGGWGGLNQTQL
Lenu PDVMQGLDLLTGLVMSTWQKLAPFILLIQIQPN-APQTITALGLLSTLIGGWGGLNQTQL
Brja PEVLQGLDLTTGLILSTWQKLAPFALLLQIH-PSDSPILIALGLTSTLVGGWGGLNQTQL
Plma PEVLQGLDLTTGLILSTWQKLAPFALLLQIH-PSNSTILIMLGLLSTLVGGWGGLNQTQL
Emst PEVLQGLDLTTGLILSTWQKLAPFALLLQIQ-PTNSTILIALGLASTLVGGWGGLNQTQL
Ptti PEVLQGLDLTTGLILSTWQKLAPFALLLQIQ-PANSSLLILLGLMSTLVGGWGGLNQTQL
Losu PEVLQGLDLSTGLIISTWQKLAPFALLVQISLI-DSFLIIFIGLVSILVGGWGGLNQTQL
Geoy PEVLQGLDLTTGLVLSTWQKLAPFALLLQIY-PSNPTLLIILGITSTLVGGWGGLNQTQL
Dipi PEVLQGLDLTTGLILSTWQKLAPFALLLQIH-PANSTILLSLGIISTLVGGWGGLNQTQL
Pama PEVIQGVDLTTGLILSTWQKLAPFALLLQLQ-PTNTSLLLALGLTSTLIGGWGGLNQTQL
Leob PEVLQGLDLTTGLILSTWQKLAPFALLTQIQ-PTNPTLLIILGITSTLVGGWAGLNQTQL
Neba PEVLQGLDLTTGLIMSTWQKLAPFALLLQLQ-PANPTMMVILGLSSTLIGGWGGLNQTQL
Pdpl PEVLQALELPTAMILATWQKLAPFAVLIQLLPH-NPNLLVLLGLISMLVGGWGGMTQTQL
Nimi PEVLQGLDLGTGLIMSTWQKLAPFALLIQIY-PANSTLLIILGLASTLVGGWGGLNQTQL
Uptr PEVLQGLDLIVGLIVATWQKLGPLVLLFQITPR-DSQLLLVLGAISVLVAGWAGLNQVQV
Pesc PEVLQALDLTTGLILATWQKLAPFALLLYLAPS-KSGLLIGLGLASALIGGCGGLNQTQL
Baar PEVLQGLDLTTGLILSTWQKLAPFALLLQIQ-PTNSPLLIMLGLASTLVGGWGGLNQTQL
Moar PEVLQGLDLTTGLILSTWQKLAPFALLLQIQ-PTNSTILIVLGLTSTLVGGWGGLNQTQL
Toja PEVLQGLDLMTGLILSTWQKLAPFALLLQIQ-QTTAPTLIMLGLASTLIGGWGGLNQTQL
Chau PEVLQGLDLTTGLILSTWQKLAPFALLIQIQ-LVNPSLVMLLGLLSILVGGWGGLNQTQL
Chse PEVLQGLELNTGIIMATWQKLAPFAILTQIQPPDSPL-LLILGITSIFVGGWGGLNQTQL
Enar PEVLQGLDLTTGLILSTWQKLAPLALLLQIQ-PANSTILIFLGLMSTLVGGWGGLNQTQL
Hpty PEVLQGLDLTTGLILSTWQKLAPFALLLQIQ-PTNSTLLVMLGLASTLVGGWGGLNQTQL
Nana PEVLQGLNLTTGLILSTWQKLAPFALILQIQ-PDTP-LLVILGLTSTLVGGWGGLNQTQL
Mcst PEVLQGLDYTTGLLLSTWQKLAPFALLLQIQ-PTNPDLLILLGLSSTLVGGWGGLNQTQL
Rhox PEVLQGLDLTTGLLLSTWQKLAPFALLLQIC-PSNSPLLIALGLASTLIGGWGGLNQTQL
Opfa PEVLQGLDLTTGLLLSTWQKLAPFALLLQIQ-PTNSTLLILLGLSSTLVGGWGGLNQTQL
Paar PEVLQGLDLTTGLILSTWQKLAPFALLLQIQ-PTDSAMLIFLGISSTLIGGWGGLNQTQL
Gozo PEVLQGLDLTTGLILSTWQKLAPFALLLQIQ-AANPTMLIALGLMSTLVGGWGGLNQTQL
Ackr PDVLQGLEFSTGMILSTWQKLAPLALFLQIQPV-DPRITISIGLASIIIGGWGGLDQTQT
Elev PDVLQGLDLTTGLILSTWQKLAPFALLYQIH-NLNPTLLITLGVLSTLVGGWGGLNQTQL
Trdu PEVLQGLDLTTGLILSTWQKLAPFALILQLQ-PSNSTLLIILGLTSTLIGGWGGLNQTQL
Amoc PEVLQGLDLSTGLILSTWQKLAPFSLLLQMQ-TSNPTMLILLGLTSTLVGGWGGLNQTQL
Hame PEVLQGLNLNTGLILSTWQKLAPFALLLQLH-PSNSVMLVALGVTSTLVGGWGGLNQTQL
Chso PEVLQGLDLTTGLILSTWQKLAPIILLMQVQ-PHNGTTFILLGIASTLVGGWGGLNQTQL
Lyto PEVLQGLDLTTGLILSTWQKLAPFALLMQTQ-PLNSSLLIALGLASTLVGGWGGLNQTQL
```

To be continued on page 19.

[3/6 of aligned sequences]

```
Encr    PEVLQGLDLTTGLILSTWQKLAPFALLMQIQ-PANSTLLIILGLASTLIGGWGGLNQTQL
Bvar    PEVIQGCDLPTTLILSTIQKLAPFTVLMQIPFT-IPGILITMSALSIVLGAWGALNQTQL
Noco    PEVFQGLSLTTGLVLATWQKLAPLTLLLQIQPV-NSPLLIFLGLASTLIGGWGGLNQTQL
Chsp    PEVLQGLDLSTGLLLSTWQKLAPFSLLLQLQPQ-DHPLFLIIGLISVLVGGWGGLNQIQL
Arja    PEVLQGLDLTTGLILSTWQKLAPFALLMQIQ-PANSTLLIVLGLASTLVGGWGGLNQTQL
Pase    PEVLQGLDLTTGLIMSTWQKLAPFALLLQIN-PSNSTLLIILGITSTLVGGWGGLNQTQL
Trel    PEVLQGLNLDAAFILATWQKLAPFIMLVQLPSP-HPDLLLALGLLSIMAGGWGGMNQTQT
Lifa    PDVLQGVDLTTGLILSTWQKIAPFIILLQINFQ-NPATLIIIGLTSILVGGWGGFNQTQL
Acur    PEVMQGVDLLVGLVLSTWQKAAPLIVLAQLQLN-NITVLTIVGLTSMIVGGLGTLNQTQL
Ampe    PEVLQGLDLTTGLILSTWQKLAPFALLIQIQ-PTNSTILIALGVTSTLVGGWGGLNQTQL
Urja    PEVLQGLDLATGLLLSTWQKLAPLALLFQIHPL-NPTLTITLGLTSSLVGGWGGLNQTQL
Enet    PEVLQGLDLTMGLVLSTWQKLAPFALLYQISPS-TPALLTTLALLSMMVGGWGGLNQTQL
Ptbr    PEVTQGLNMTTALILCTWQKIAPLILLSQIQLT-DPTPFLVVGALSALIGGWGGLNQTQL
Safa    PEVLQGLDLMTGMILSTWQKLAPMALLIQIQPQ-DPTIFIVLGLASTLIGGWGGLNQTQL
Icae    PEVLQGLDLTTGLILSTWQKLAPFALLLQIH-PTNSTILITLGLASTLVGGWGGLNQTQL
Asmi    PEVLQGLDLITGLILATWQKLAPFAILLQLQPAPS-QLMIALGITSTLVGGWGGLNQTQL
Foal    PDVLQGLNLTTGMILSTWQKIAPLTLMLQASKP-EASLMLTLGVLSIVVGGLGGLSQTQT
Drze    PEVIQGVDLTTGLILSTWQKLPPFTLLLQIYPS-NSPLLIILGITSTFIGGWGGMNQTQL
Rhas    PEVLQGLDLTTGLILSTWQKLAPFALLLQLP-VHNQTTLILLALASTLVGGWGGLNQTQL
Elac    PEVLQGLDLTTGLILSTWQKLAPFALLLQIS-KSDQTVFIILALASTLVGGWGGLNQTQL
Kugu    PEVLQGLDLTTGLILSTWQKLAPFAILMQIN-PTNSNTLIILGLLSTLVGGWGGLNQTQL
Plor    PEVLQGLDLTTGLILSTWQKLAPFALLLQTQ-PTNSTILITLGLASTLIGGWGGLNQTQL
Sgun    PEVLQGLDLTTGLILSTWQKLAPFAILLQIQ-PTNSTLLIMLGLTSTLVGGWGGLNQTQL
Zaco    PGVLQGLDLTTGLILSTWQKLAPFILLLQVP-SPNSTLLVVLGLTSTLIGGWGGLNQTQL
Zbfl    PEVLQGLDLTTGLILSTWQKLAPFALLLQIQ-PANSMILVILGLTSTLVGGWGGLNQTQL
Spba    PEVLQGLDLITGLIISTWQKIAPFTLLIQIQGT-YSPTLIILGILSTLVGGWGGLNQTQL
Game    PEVLQGLDLTTGLILSTWQKLAPFALLLQIH-PTNPAILITLGLASTLVGGWGGLNQTQL
Thth    PEVLQGLDLTTGLILSTWQKLAPFALILQIH-PTNPTLLIVLGVASTLVGGWGGLNQTQL
Xigl    PEVLQGLDLTTGLVLSTWQKLAPFALLLQIQ-NVNPTILVTLGLASTLVGGWGGLNQTQL
Hyja    PEVLQGLDLTTGLILSTWQKLAPFALLMQIY-PENSTLLIMLGLTSTLVGGWGGLNQTQL
Psan    PEVLQGLDFDTGLILVTWQKLAPLGLLAQIPQD-TPALLTTLGLLSILVGGLGGMNQTLV
Cupa    PEVLQGLDLTTGLILSTWQKLAPFALLLQIH-PTNPTILIMLGVSSTLVGGWGGLNQTQL
Mpch    PEVLQGLDLTTGLVMATWQKLAPFALLLQIQ-PNNPTLPVIIGLFSVLIGGWGGLNQTQL
Char    PEVLQGLDLTTGLILSTWQKLAPFALLLQIQ-PNTST-LVLLGLTSTLVGGWGGLNQTQL
Pser    PEVLQGLDLTTGLILSTWQKLAPFALLLQIH-PSNPTTITILGILSTLVGGWAGLNQTQL
Prol    PEVLQGLDLTTGLILSTWQKLAPFALLMQIQ-LDNPTPLIIILGLTSTLVGGWGGLNQTQL
Plbi    PEVLQGLDLTTGLILSTWQKLAPFALLLQIQ-TTNPTPLIIIGLLSALVGGWGGLNQTQL
Calu    PEVLQGIDLYTGLVLSTWQKLAPFALMLQVQTPSTSTPLLLVGALSILVGGWGGLNQTQL
Papa    PEVMQGLTLTTGLILATWQKLAPFVLLIQITPN-DPKIMVMLGLLSILAGGWGGLNQTQL
Sufr    PEVLQGLDLTTGLILSTWQKLAPFALLLQVQ-PTNSNLLMFLGLMSTMVGGWGGLNQTQL
Stci    PEVLQGLTLSTGLLMSTWQKLAPFILLLQIQ-PTNPNLLIFLGIASTFIGGWGGLNQTQL
Taru    PEVMQGLDLTTGLILATWQKIAPFSLLLQIQPN-NQTLLILLAILSMLVGGWGGLNQTQV
Rala    PEVLQGLDLTTGLILSTWQKLAPFALLLQLQ-PSNATLLISLGLLSTLVGGWGGLNQTQL
        * : *.       ..  * **  *. :.                .   *  . .. .  :
```

To be continued on page 20.

[4/6 of aligned sequences]

F G

Scca RKILAYSSIANLGWMITILHYSPSLTLLNLILYMFMTLTTFLLFKTFNSTKINSISSSTL
Muma RKILAYSSIANLGWMITILHYAPNLTLLNLILYIIMTLTTFLLFNIFNSTKINSIASSST
Erca RKIMAYSSIAHLGWIISILHFMPALAMINLFIYLIMTITMFMTFNLLNSPTINTLAMNWS
Pose RKIMAYSSIAHLGWIISIMHFMPTLAIINLIIYIIMTTTMFMTFNVTNSTSISTLAMNWS
Actr RKILAYSSIAHLGWMMIVMQYSPNLAILNLILYITMTSAAFLTFKNVASTKLSTLTLTWS
Scal RKILAYSSIAHLGWMMIVMQYSPNLAILNLILYITMTTATFLTFKNVASTKLSTLALTWS
Posp RKILAYSSIAHLGWMMVIMQYSPSLAMLNLTLYIIMTTATFLTFKNVTSTKLNALALTWS
Atsp RKILAYSSIAHMGWMIIILQFMPKLTLLNLTIYIIMTSAMFMMFKYNNATKINYLTLTWS
Leoc RKILAYSSIAHMGWMIIILQFMPKLTLLNLMIYIIMTSAMFMMFKYNNATKINYLTLTWS
Amca RKILAYSSIAHLGWMIIVLQFLPHLALLALAMYIIMTAATFLALKNLASTKINTLALKWT
Osbi RKILAYSSIAHLGWMITILHFMPALTLLNLTIYIIMTSTMFLSLKSLSATKINSLAITWP
Pabu RKILAYSSIAHLGWMIVILNIMPNLTMLNLFIYIIMTSTMFLAFKNISTTKINTLTMAWS
Hial RKILAYSSIAHLGWMIIVVQLMPNLTLLNLAMYILMTSAIFLTLKNTSATKINTLALTWP
Elha RKILAYSSIAHLGWMIIVLQMMPNLTLLALATYILMTAAMFMTLKTTTSTKINTLALAWP
Mlcy RKILAYSSIAHLGWMIIILQLMPPLTILNLAIYITMTTTMFLSLKTSTTTKINTMATSWA
Algl RKLLAYSSIAHMGWMIIVVKFVPNLTILGLGVYIVMTSATFMALKTVSATKISTLALKWA
Ptgi RKLLAYSSIAHLGWMMIVLKFMPSLMLLNLGIYILMTATAFMALKMNSATKINTLALGWP
Alaf RKLLAYSSITHMGWMIIVLKFMPGLMLLNLVIYVLMTSTVFMALKEVSATKINTLALTWP
Nock RKLLAYSSITHMGWMIIVLKFMPSLMLLNLVIYILMTSTVFMALKEISTTKINTLALAWS
Anja RKILAYSSIAHMGWMMIVMKYLPNLMIMNLMIYIIMTSSAFMALKMTTATKINTLATGWT
Gyki RKILAYSSIAHMGWMMIVLKYFPELMLLNLVIYIIMTSATFMALEAVSATSINALSTMWS
Syka RKLLAYSSIAHMGWMMMVLKYMPNLMLLNLTIYIIMTSATFMALKMAASTNINTLAMGWT
Opma RKILAYSSIAHMGWMMVVLNYAPNLMLLNLTIYIIMTYTAFMTLKTASATKINVLATSWT
Comy RKILAYSSIAHMGWMMIVLTYSPKLMLLNLMVYILMTSTVFMALKTVSATKMNTLGTNWA
Sasp RKIMAYSSIAHMGWMMAVIKYAPDLMMLNLIIYIVLTCSIFMTINMISATKINNLAVGWS
Eupe RKLLAYSSIAHMGWMMAAMKYMPDLTILALMTYIMMTSSIFLMMKLLSATKINELATAWT
Enja RKILAYSSIAHLGWMILMSQMAPQMTLIALITYIVMTSAAFLTLNNINCTKTITLASAWT
Same RKILAYSSIAHLGWMILVAQMAPQMTLMALVAYIAMTAAAFLTLNNVNSTKTITLASAWT
Chch RKILAYSSIAHLGWMIIVLQFAPQLTLLTLATYIVMTSAAFLTLKASSSTKINTLATAWT
Grgr RKILAYSSIAHLGWMVVILQYAPHLTLLTLLAYITMTSATFLTFKASSSTSVNVLALAWT
Caau RKILAYSSIAHMGWMIIVIQYAPQLTLLALGTYIIMTSAAFLTLKMSLTTKVSTLATTWS
Cyca RKILAYSSIAHMGWMIIVIQYAPQLTLIALGTYIIMTSAAFLTLKMSLTTKISTLATTWS
Dare RKILAYSSIAHMGWMIIVIQYAPQLTLIALGTYIFMTSAAFLTLKVLSATKINTLTTTWP
Cost RKILAYSSIAHMGWMLIVLQYTPQLTLLALGTYIVMTTTAFLSFKMSLATKINTLSTAWA
Leec RKILAYSSIAHMGWMMIILQYAPQLTLLALGMYIFMTSTLFLSLKTASATKITTLTTMWS
Fola RKILAYSSIAHMGWMIIILQYTPQLTLIALGLYIFMTSAAFLSLKMASATKMNTLTAAWS
Clmc RKILAYSSIAHLGWMIIILQFMPQLTILALGTYIIMTSATFLTFKMTTSTKINTLATAWS
Phin RKILAYSSIAHLGWMTIIIQFTPQLTIFTLLVYIITTSTAFLTFKFMSTTKINTLTLSWS
Icpu RKILAYSSIAHLGWMIIVTQYKPQLTVLVLITYIIMTSATFLTFKLMATTKINTLAMSWA
Psto RKILAYSSIAHLGWMTVIVQLKPQLTILTLCLYIIMTMATFLTFKLMSATKINTLATSWA
Cora RKMLAYSSIAHLGWMIIILQLQPKLTILTLTIYIIMTSTAFLTFKFLMTTKINTLALAWS
Eisp RKILAYSSIAHLGWMIIILQFAPNLTLLALGTYILMTSTAFLTFKMVSSTKINTLALSWS
Apal RKLMAYSSIAHMGWILSIMSFSPHLALLALLTYILMTMTALLSFKLSKSVKINILALTWS
Eslu RKILAYSSIAHLGWMVLIIQFSPPLATLSLLIYIIMTTSAFLTFKLTSSTTMNALATSWT
Dape RKILAYSSIAHLGWMVMIIQFSPPLALLSLLIYIVMTSSAFLALKTVSSTTMNMLAASWT
Glse RKILAYSSIAHLGWMIIILKFTPSLTLLNLALYLMMTSATFLSFKMSAATNINTLALSWT
Naar RKILAYSSIAHLGWMVLILQFAPSLTLLSLALYLVMTSAAFLSFKSSTATNINVMALSWT
Lioc RKILAYSSIAHLGWMVLILQFAPSLTLLSLTLYLVMTSAAFLSFKSYTATNINFMALSWT
Opso RKILAYSSIAHLGWMILIIQFAPSLALLNFVLYLIMTSATFLSFKTSTATNINMLALSWT
Alte RKILAYSSIAHLGWMIIVLQFAPHLTLLALGTYIAMTSAAFLTLKSTSSTKINTLALAWA
Plap RKILAYSSIAHLGWMIIVLQFAPHLTLLALGTYIAMTSAAFLTLKSTSSTKINTLALAWT

To be continued on page 21.

```
Plal  RKILAYSSIAHLGWMIIVSQVSPSLALLGLTLYIVMTTSAFLTMKATAASSLNTLATAWA
Sami  RKILAYSSIAHLGWMVIVSQISPSLALLGLALYIMMTTSAFLTMKASAASSLNTLATAWT
Rere  RKLLAYSSVAHLGWMIIISQFSSSLSLLALSLYIVMTTSAFLTMKAGAASSLNTLAVSWS
Gama  RKVLAYSSIAHLGWMVLVLQFAPSLTLLALVVYILMTASAFLTFNVVQSTSVSVLATAWA
Onmy  RKILAYLPIAHLGWMVLILQFAPSLTLLSLSLYIVMTSSAFLTLKTNNSLTINTLATSWT
Sasa  RKILAYSSIAHLGWMVLILQYAPSLTLLSLFLYIIMTSSAFLTLKTNNSLTINTLATSWT
Cola  RKILAYSSIAHLGWMVLIMQFAPSLTLLSLIMYIIMTSSAFMTLKTNNSLTINALAISWT
Dita  RKILAFSSIAHLGWMTLVMRFAPSLSILALLLYIVMTSATFLSMKTYGCTTLNGLATSWA
Gogr  RKILAYSSIAHLGWTLLVIQLAPSIALLSLITYIFMTAMTFLSLKTFNCTNLNSLTTSWS
Chsl  RKVLAYSSIAHLGWMALILQFAPPIALLNLSLYITMTSMAFLALKAFNCTNLNNLATSWA
Atja  RKILAYSSIAHLGWMILVLQFSPSLTLLALTLYLVMTSATFLTFKLTSSLNINSLACSWA
Iido  RKILAYSSIAHLGWMILVLQFSPSLTMLALALYLIMTSAMFLTLKLTSSLNINSLACSWT
Auja  RKIMAYSSIAHLGWMILILEFAPPLTVLALSLYILMTTSVFLMLKINNATTINTLASTWT
Chag  RKILAYSSIAHLGWMILILQFAPSLTLLSLILYIVMTSATFLTFKLNGATSINTLATSWA
Hami  RKILAYSSIAHLGWMILIIKFTPHLTLLALLLYVVMTSSVFLTLKLTNGLTINTLATSWT
Saun  RKILAYSSIAHLGWMILILKFTPHLTLLALLLYIIMTSSVFLTLKLNNSMTINALATSWT
Nema  RKILAYSSIAHLGWMVLVLQFAPTLTILALITYIIMTSSAFLTFKISGSTNINTLATSWA
Disp  RKILAYSSIAHLGWMVLVLQFNPSLTILALFTYIIMTSSAFLTFKTAASTNINILATAWA
Myaf  RKILAYSSIAHLGWMILILQFNPSMTIFALTTYIIMTSSAFLTFKTAEAFSINTLATSWA
Lagu  RKILAYSSIAHLGWMVLITQFSPSLTLLALTTYILMTSSAFLLVKFNAATTINALATTWT
Trtr  RKILAYSSIAHLGWMLLISQFSTPLALLALFTYILMTTPAFLILKFTHATTINSLASSWT
Zucr  RKILAYSSIAHLGWMLLVCQFSPPLSLLALFTYILMTTPAFLLLKFTSSLTINSLASAWT
Pxja  RKILAYSSIAHLGWMILVLQFNPQLTLMALLTYFIMTFSTFAVFKINSSTNINTLATSWA
Pxlo  RKILAYSSIAHLGWMILVLQFSPQLTLLALLTYFIMTFSTFAVFKINSSTNINTLATSWA
Pctr  RKLLAYSSIAHLGWMVLVLQFNPQLTTLALLTYLVMTFSAFVVFKINSSTNINTMATSWA
Apsa  RKLLAYSSIAHLGWMILVMQFSPQLTTLALLIYMTMTFSAFMLMKMNNTTNIRSMSMAWA
Cabe  RKILAYSSIAHLGWMVIVMAFAPGLSMLAMIMYIVMTSTIFLVLIASRAFTINSLATAWS
Bzze  RKILAYSSVAHLGWMVLVLQFAPSLTLLALLTYLVMTTSTFLIFKYNEAANINALATSWA
Siim  RKVLAYSSITHLGWTIVILRHSEYLALMNLYVYFIPTIPLLFIFMLMKSDRMGKVGISRH
Ctru  RKVLAYSSIAHLGWMILALQYSPTLTLLALLTYLAMTMATFLLLKLNKATNMNTLATTWT
Dpbr  RKILAYSSIAHLGWMVLVLQYKPSLTLLALLTYFAMTMSIFLLFKLNKATNVNSLATAWT
Caki  RKIIAYSSVAHFGWMMLVVSSNVHLALIALMIYSLATMVIFSALNNSSSSTIQNITFSWT
Phja  RKTLAYSSIAHLGWMIMVMQYSQQLALLTFAIYFVTTMSAFLTINALSSTTINALATSWT
Brsp  RKVLAYSSIAHVGWIILVIQLNQTLSLLALMLYIPMTMTVFLMLMYNSTHSYNSLAISWS
Gamo  RKILAYSSIAHLGWMILVMQFNQQLALLALIIYIPMTFSTFMIFKTNSSTTVNTLAASWA
Lolo  RKILAYSSIAHLGWMVLVMQFNQQLALLALVIYIPMTFSTFMIFKTNSSTTMNTLAASWA
Batr  RKLLAYSSIAHMGWIIVALSLLPSLSHLALLLYIIMTTSTFLGLAHIQATNISHLSLSWI
Prmy  RKILAYSSIAHLGWMVVVMKIAPSISLLALLAYILMTFSLFSMFLLNKTLNINSLSLSWT
Lose  RKILAYSSIAHLGWLIIIMPYSPSLSLLTLALYFVLTFSTFITFKLSNSLNINSLAASWT
Loam  RKILAYSSIAHLGWMILIMQFSPSLTLLALAMYFIMTFSTFTLFKLHDSTNINSLATSWA
Chab  RKILAYSSIAHLGWMMLVLQFSPSLTLLTLFTYLTMTFAAFLLFKLNNSTNINTLASSWA
Chto  RKILAYSSIAHLGWMVLVLQFSPSLTLLTLFTYLTMTFAAFLLFKLNNSTNINTLASSWA
Majo  RKILAYSSIAHLGWMVLVVQFSPPLAKLTLILYITMTAAIFLVLKLNDSKNINTLASSWS
Hlst  RKILAYSSIAHLGWMMIILQFSVPLTKLTLALYIMMTTAMFLMVKMTDSKTINSLSSAWT
Clpe  RKILAYSSIAHLGWMVLVLQYSPSLTMLALLTYITMTVSTFLVFKLAGSTTLNSLATSWA
Mlmr  RKILAYSSIAHLGWMILVLQFSPSLTLLTLLTYMAMTSSLFLVFKLTSSTNINTLATSWA
Crcr  RKILAYSSIAHLGWMILILQLTPSLTLLTLLTYLIMTLSMFLTFKLNKTTTINMLATSWT
Muce  RKILAYSSIAHLGWMILVLQLAPSLTLLTLLTYLIMTLSVFLIFKLNKTTTINMLATSWT
Bege  RKILAYSSIAHLGWMILILQFSPSLTLLALLTYLIMTFSMFLVFKLNNSTTLNTLAISST
Mela  RKILAYSSIAHLGWMVLVLQFSPSLTLLTLSLYIIMTSSLFLSFKTNKTTTINSLATSWA
Hats  RKVLAYSSIAHLGWMILILQFSPSLTFLTLLTYFVMTFSTFLVFKLNKSTTINALATSWA
Orla  RKVLAYSSVAHLGWMMVVMQFSTSLTALALLMYVIMTTSAFLTFKLLKSTDMNSLAMSWA
```

To be continued on page 22.

[4/6 of aligned sequences]

Cosa	RKILAYSSIAHLGWMILVLQFSPTITLLTLLAYITMTLSTFLLFKTNKSTNINSLATSWT
Exsp	RKILAYSSIAHLGWMLLIIQFSPSLTFLTLITYILMTSSTFLIFKANKSTNINSLATSWT
Depa	RKILAYSSIAHLGWMILIMQFSPSLTLLTLIIYITMTLSTFLLFKINNSTNINSLATSWA
Rima	RKILGYSSIAHLGWMILIMQYAPPLTLLALTVYIVLTSSMFLTFLYNQATNINSLAISWT
Fuol	RKILAYSSIAHLGWMILILQFLPPLTLLTLLLYFIMTFSLFISFKVNNASSINSLASSWS
Gmaf	RKILAYSSIAHLGWMVLILSFSPPLTLVAFSTYLMMTFSLFYSFMMTKTTHINSLSTSWA
Xeei	RKILAYSSIAHLGWMALVLQFSPSLTLMTLLIYIIMTFSLFISFKMNHSTNINTLTTSWT
Pros	RKILAYSSIAHLGWMVLIIQFSPSLTLLALLTYFIMTFSTFLAFKLNNSTNINTLATSWA
Scmi	RKILAYSSIAHLGWMTLIIQFSPSLTLLALLTYFIMTFSTFLMFKLNNSTNINSLATSST
Rolo	RKILAYSSIAHLGWMILIAQFAPPLTLLALITYIIMTSSTFLVFKLNNSTNINTLATSWT
Cere	RKIMAYSSITHLGWMALIIQFDTTLTLLPLLTYIIITSSTFLVFKTNNSTNVNSLAISWT
Daga	RKIMAYSSIAHLGWMTLVIQFSSPLALLALFTYMIMTSSLFITFKINSSTTINSLAISWT
Anco	RKLLAYSSIAHLGWMVIVIQFLPSLTLLALFTYFIMTFSTFIVFKLNNSTNINSLATSWA
Dmve	RKILAYSSIAHLGWMVVVVQFSPPLTLLALLLYLVMTFSTFIVFKLNTATSINALATSWA
Dmar	RKILAYSSIAHLGWMVIVVQFSPPLTLLALFTYFVMTFSTFLVFKLNNATSVNALATSWA
Anka	RKLLAYSSIAHLGWMIIVVQFLPSLTLLALFTYFIMTFSTFIVFKLNNSTSINTLATSWA
Moja	RKLLAYSSIAHLGWMVVVVQFLPSLTLLALFTYFIMTFSAFIMFKLNNATSINTLATSWA
Hoja	RKLLAYSSIAHLGWMVIVIQFLPSLTLLALLTYFIMTFSTFIVFKLNNSTSINTLATSWA
Bede	RKILAYSSIAHLGWMILVIQFNPALTLLALLTYFIMTFSTFLAFKLNNSTNINTLATSWA
Besp	RKILAYSSIAHLGWMILIIQFNPSLTLLALLTYFIMTFSTFLVFKLNNSTNINTLATSWA
Mysp	RKILAYSSIAHLGWMMLITQFVPSLALLTLLTYFIMTFSTFLVFKMNNATNINTLATSWA
Osja	RKILAYSSIAHLGWMVLITQFMPSLALLALLTYFIMTFSTFLVFKMNSATNINTLATSWS
Sgro	RKILAYSSIAHLGWMVLVTQFMPSLALLTLLTYFVMTFSTFLVFKLNNSTNINTLATSWA
Pzpa	RKIMAYSSIAHLGWMMLIITFNPKLTLMALVMYITMTLSMFLVFKMNHSLNMNALSTSWS
Zeja	RKILAYSSIAHLGWMVLIISFNPKLTLMALIMYIIMTLSMFLVFKMNHSLNMNTLSTHQT
Znne	RKIMAYSSIAHLGWMILIVSFNPKLTTVALAMYIIMTTSMFLTLKINNSLDVNTLATSWA
Zefa	RKVMAYSSIAHLGWMILIVSFNPKLTLLALSMYILMTTSMFLTLKINNSLDMNTLATSWA
Acni	RKIMAYSSIAHLGWMVLIITFNPKLTLMALIMYIIMTMAMFLVFKTNHTLNMNTLSTSWA
Ncrh	RKIMAYSSIAHLGWMVLIITFNPKLTLMALIMYIIMTMSMFLVFKTNHTLNMNTLSTSWA
Agca	RKILAYSSIAHLGWMILVLQFSPSLTLLTLLTYIVMTFATFLVFKYSKATNINALATSWA
Hydy	RKILAYSSIAHLGWMVLILHFSPPLTTLALMMYLIMTFPAFLVFKLNASTTINSLATSWT
Gsac	RKILAYSSIAHLGWMTLVMHFSPSLTLLTLIMYFIMTFPAFLVFKLNNSTTINSLATSWA
Pevo	RKILAYSSIAHLGWMVMIMQFSPSMALMALLTYIAMTSSTFMIFNFTKSTTTNALATSWT
Hiku	RKILAYSSIAHIGWMVLVLQFSPSLTLLTLVIYILMTFSIFSLFKLNKSTSINTLATSWS
Inpa	RKILAYSSIAHLGWMILILQYSPPLAYLSLLLYFVMTFAPFMTFKLNKSLNINSLATSST
Auch	RKILAYSSVAHLGWAILIVQFSPRLATIAFITYLAMTFSTFLLFKQANSTSINSLATSWT
Fico	RKVLAYSSIAHLGWMVLIMQFAPSLTILTLLTYITMTFSTFLTFKLAKALNVNTLASSWA
Macs	RKILAYSSIAHLGWMILVLQYSPTLTLLTLLMYITMTFSTFLVFKLNNSTNINMLSASWT
Moal	RKILAYSSIAHLGWMVLILQHSTALATFALLIYIFMTSATFITLKKCKATTINMLTTTWT
Syma	RKILAYSSIAHLGWMVMIIQFSPQLTLLTLLIYIIMTFATFITFKTSKATNINTLSTTWT
Mafr	RKILAYSSIAHLGWMLVVIQFSPPLTMLTLFLYFIMTSSAFLSFKLSNSTNINSLAISWA
Dcpe	RKLLAYSSIAHLGWMILIMQFSPSLSLLTLMTYFVMTFSAFLTFKLSDSTNINSLTISWS
Dcti	RKLLAYSSIAHLGWMVLIMQFSPSLSFLTLMLYFVMTFSAFLTFKLNDSTNVNSLAISWA
Hehi	RKILAYSSIAHLGWMVIVLQFSPSLTILTLLTYFIMTFSAFLMFKLNEATSINTLATSWA
Stam	RKILAYSSIAHLGWMILVLQFSPSLTLLTLVLYVLMTTSTFLVFKLNGSTNVNTLATSWA
Hogi	RKILAYSSIAHLGWMIMVMQFSPSLTLLTLATYFVMTISTFLVFKLSKATNINMLALSWA
Erzo	RKILAYSSIAHLGWMVLVLQFSFSLTLLALITYFIVTFSTFLVFKLNGSTNINALATSWA
Hxot	RKILAYSSIAHLGWMILVVQFSPSLTLLTLATYLIMTFSTFLVFKLNSATNINALATSWA
Core	RKILAYSSIAHLGWMVLVVQYSPSLTLLTLLTYLIMTMSTFLVFKLNDATNINALATSWA
Apve	RKILGYSSIAHLGWMVLVLQFSPSLSLLTLFIYIIMTLSTFLIFKLNNSTNINTLATSWA
Latj	RKILAYSSIAHLGWMVLILQFSPTITLITLTTYLLMTTSTFLVFKLNKATNINMLAISWT
Laja	RKILAYSSIAHLGWMILILQFSPSLTLLTLLTYFVMTLSTFLVFKLNKSTSVNQLASSWA

To be continued on page 23.

[4/6 of aligned sequences]

```
Syja  RKVLAYSSIAHLGWITLVLQYSTSFALLALLTYFATTFATFLTFKLNDSTTINQLSYSWA
Epme  RKIMAYSSTAHLGWMILVLQFSPPLTLLTLLTYLVMTSSTFLVFKLNKATNINMLATSWA
Grse  RKILAYSSIAHLGWMILVLQFSPPLTLLTLLTYITMTLSTFLVFKLNKATSINMLATSWA
Clja  RKLLAYSSIAHLGWMILVIQYSPSLTLLTLILYIGMTISLFLTLNLNTTTSINAMATSWT
Ogcy  RKTMAYSSIAHLGWMFLVIQFSPSLTMLTLSTYLIMTTSLFLTLKLNEATTLNMLASSWA
Plna  RKILAYSSTAHLGWMIIVMQFSKSLTLLALVTYIIMTSGAFLLFDSMKAKSINTIMMYLP
Lema  RKILAYSSIAHLGWMILILQFSPALTLLALLTYFIMTSSTFLVFKLNKSTNINTLAISWT
Etzo  RKILAYSSIAHLGWMILVLQFSPSLTLLTLLTYFVMTFSTFLVFKLNKSTTINMLATSWA
Apse  RKILAYSSIAHLGWMVLVLQFSPSLTFLTLILYFAMTFSTFVVFKINKSTNVNSLATSWA
Epde  RKILAYSSIAHLGWMILILQFSPSLTLLALLTYLIMTFSTFLVFKLNKSTNINALATSWA
Slja  RKILAYSSIAHLGWMILVLQFSPSLTLMALVTYLVMTFSMFMIFKLNQSTNINSLAASWA
Bsja  RKILAYSSIAHLGWMALILQFMPSLTLLALLTYFIMTSSMFLAFKLTNSATINAIATSST
Ecna  RKILAYSSIAHLGWMALILPFYPSLTLLALLTYLIMTLSTFLVFKLNKATNINTLAISWS
Cohi  RKVLAYSSISHLGWMILVLHFSPSLSMMTLVIYIIMTSSLFFIFKLNKTTTINSLATSWV
Caar  RKILAYSSIAHLGWMTLIIQFSPSLTLLTLLTYFIMTFSTFLVFKMNKATNVNSLAVSWA
Came  RKILAYSSIAHLGWMTLIIQFSPSLTLLTLLTYFVMTFSTFLVFKINKATNVNSLAISWT
Mema  RKILAYSSIAHLGWMILVLQFYPSLTLMALLTYFIMTFSAFLLFKLNKATNINTLAISWT
Lenu  RKILAYSSIAHLGWMALIMQFSSTLALLTLLTYFIMSIATFMLFKTNKTTTINTLALSST
Brja  RKILAYSSIAHLGWMILVLQFSPSLTLLTLMTYLIMTFSTFLVFKLNKATNINALATSWT
Plma  RKILAYSSIAHLGWMVLVLQFSPSLTLLTLLTYFIMTFSTFLVFKLNKATNINTLATSWT
Emst  RKILAYSSIAHLGWMVLVLQFSPSLTFLTLLTYFIMTFSTFLVFKLNKSTNINALAASWT
Ptti  RKILAYSSIAHLGWMILVLQFSPSLTLLTLLTYLVMTFSTFLVFKLNKATNINTLAISWA
Losu  RKVLAYSSIGHLGWMAIVVQFSTALTLLMLAVYLVMTTALFMVFNMNKTVTTKMLASSAS
Geoy  RKILAYSSIAHLGWMMLVLQFSPSLTLLALLTYFVMTASAFLIFKLNKSANVNTLATSWA
Dipi  RKILAYSSIAHLGWMILVLQYAPSLTFLTLITYFIMTFSTFLVFKLNKSTNINSLATSWA
Pama  RKILAYSSIAHLGWMTLVLQFSPSLALLTLIIYFTMTFSAFLTFKLNNATNINSLAMSWA
Leob  RKILAYSSIAHLGWMILVLQFSPSLTLLALMTYIIMTFSTFLVFKLNKAMNINTLAMSWA
Neba  RKILAYSSIAHLGWMILVLQYAPSLTLLTLVIYLIMTTSAFLTFKMNNVTNINSLALSWT
Pdpl  RKLLAYSSIGHLGWMILALNYFPLLTLAVLLIYISVAVPVFLLLMINKSTSINMLSTSWS
Nimi  RKILAYSSVAHLGWMVLVLQFSPALTLLTLLMYLIMTFSTFLAFKLTNATNINALATAWT
Uptr  RKILAYSSIAHMGWMVVALHFSTALAILTLLIYLVMTAAAYFIFNVNKTNTLYSFHRTP-
Pesc  RKLLAYSSIAHFGWMVLIMQFSPALSMMTFFLYLLMTAAAFLTFKMARATTINQLALSSS
Baar  RKILAYSSIAHLGWMILVLQFSPSLTLLTLLTYFIMTFSTFLVFKLNKSTNINELASSWA
Moar  RKILAYSSIAHLGWMVLILQFSPSLTFMTLITYFIMTFTTFLVFKLNKSTNINSLATSWA
Toja  RKILAYSSIAHLGWMILVLQFSPALTLLTLLVYLIMTTSTFLAFKLNKVTNINSLATSWT
Chau  RKILAYSSIAHLGWMILVLQFSPSLALLTLMVYFIMSSATFLVFKLNNSTNINALATSWA
Chse  RKILGYSSIAHLGWMILVLQYSTLLTLLALLIYIIMTYATFTLFMLKKATSVKTLFALGP
Enar  RKILAYSSIAHLGWMILVLQFSPSLMLLTLLTYIIMTFSTFLVFKLNDATNINTLATSWT
Hpty  RKILAYSSIAHLGWMILILQFSPSLTLLTLLTYFIMTFSTFLVFKLNKSTNINELASSWT
Nana  RKVLAYSSIAHLGWMMVVLQFSPSLTLLTLLIYLIMTSATFLTFKLNKTTNINTLATSWS
Mcst  RKVLAYSSIAHLGWMILILQFSPSLTLLALMTYLVMTSSTFLIFKMNKSTNINMLATSWA
Rhox  RKILAYSSIAHLGWMILVLQFVPSLTLLTLLTYFVMTFSTFLVFKLNNAKSINSLAVSWA
Opfa  RKILAYSSIAHLGWMILILQFSPSLTLLTLLMYFMMTSSTFLTFKLNNSTNINTLATSWA
Paar  RKILAYSSVGHLGWMVLVLQFSPPLTLLTLLTYFIMTSSAFLMFKLNKVTNINALATSWA
Gozo  RKILAYSSIAHLGWMILVMQFSPSLTLLTLFTYLIMTFSTFLVFKLNKSTTINSLSTSWA
Ackr  RKILAYSSIAHMGWIVLILHFSTSLACFTFFLYVFMTLSAFTILDFNKAINMNILAISQK
Elev  RKILAYSSIAHLGWMILILKFSPSLTILALLVYMTMTTATFLTFKLNKATNINTLATCWA
Trdu  RKILAYSSIAHLGWMILILQFSPSITLLTLLTYLIMTFSTFLVFKLNKSTNINTLATSWA
Amoc  RKILAYSSIAHLGWMILVMQFSPSLSLLALMTYFIMTFSAFLVFKLNKATNINSLASSWA
Hame  RKILAYSSIAHLGWMIIVLQFAPSLTLLTLLTYFVMTFSTFIVFKLNNATTINTLSTSWT
Chso  RKILAYSSIAHLGWLTIVLQFSPALSFLTLMTYFVMTFSTFLTFKLNNATSLNALATSWS
Lyto  RKILAYSSIAHLGWMLLVMQFSPSLTLLSLLTYFLMTFSTFLVFKLNNSTTLNALSTSWA
```

To be continued on page 24.

[4/6 of aligned sequences]

```
Encr    RKILAYSSIAHLGWMILVMQFSPSLTLLALLTYFIMTFSTFLVFKLNASTTMNTLATSWA
Bvar    RKIIAYSSIAHLGWVAITLYYSPPLSFLNLLIYFMLTSALILLFILNKATHINALSTTWV
Noco    RKILAYSSTAQLGWMVLVLQFAPSLTTLAILVYIAAATATFLTLKLAKATSINMLAISWA
Chsp    RKILAYSSITHMGWMIMVMQFSPPLAMLTLLTYIIMTTSAFLLFKLKKTYSINQLASSWT
Arja    RKILAYSSIAHLGWMLLVLQFSPSLTFITLLTYILMTSSTFLVFKLTNSTSINALATSWA
Pase    RKILAYSSIAHLGWMLLVLQFSPSLTFLTLILYFVMTFSAFTLFKYNKATTINALSIAWT
Trel    RKIMAYSSMAHMGWIMAIYNFSPTLALLTLALYLIMTSAAFLTLKVLNAFSVKGLARAMF
Lifa    RKILAYSSIAHLGWMIIALQFSPSITLLTLLTYFLMTSAAFLTLKLNNTTSINRLTTAWT
Acur    RKLMAYSSILHLGWIVIISLYSLQLMLLAYIVYTVIMAAMLLVLSHIKSKSLSELAQACT
Ampe    RKILAYSSIAHLGWMILILQFSPSLTLLALLTYLIMTFSTFLVFKLNKATNINALATSWA
Urja    RKILAYSSIAHLGWMTLILHFSPPLFILTLLVYLIISSSTFLVFKLNNSTNINMLAISWA
Enet    RKILAYSSIAHLGWMALVLQYSPSLTILALAIYLIMTATMFMALKLLDSTSMNKLAISWT
Ptbr    RKMLAYSSISHSGWIMVIMQLSPNLALFNFLVYCMITTAAFLTFKTFNTTNFFSLSIKWS
Safa    RKILAYSSIAHLGWMILIIQFAPSLTLLSLLMYFIMTISSFTIFKHLSSTSIPSLATSWS
Icae    RKILAYSSIAHLGWMMLVLQFSPSLTLLTLLTYFIMTFSTFLVFKLNEATNINALATSWT
Asmi    RKILAYSSISHLGWMILVAQFSPALTLLTLIIYVAMTSTVFLTFHLTSAKSISTLIISST
Foal    RKIMAYSSIAHLGWMAVALPLAPHLTLLALTLYIATTYCMFSLLLMAKGLTINSLAISWT
Drze    RKILAYSSIAHLGWMTLVLQFSPSLSFLSMTLYIIMTFSLFMILKLTNSTTINSLAISWT
Rhas    RKILAYSSIAHLGWMVLILQFAPSLTLLALLTYFIMTFSAFLTFKLNNSTTINALATSWA
Elac    RKILAYSSIAHLGWMMLIIQFAPSLTLLALMLYFVMTFSAFLTFKINNATTINSLATSWS
Kugu    RKILAYSSIAHLGWMILVLQFSPQLTLLALMTYLIMTLSTFLIFKLNKATNINSLAISWA
Plor    RKILAYSSIAHLGWMVLVLQFSPHLTLLTLIVYIILTSSTFLVFKLTNTTTINSLASAWA
Sgun    RKILAYSSIAHLGWMILILQFSPSLTFLTLLAYFIMTFSTFLVFKLNKSTNINTLATSWA
Zaco    RKILAYSSIAHLGWMILVLQFSPSLALITLLTYIIMTSSAFLLFKLSQATSINMLATSWT
Zbfl    RKILAYSSVAHLGWMILVLQFSPSLTFLALLTYFVMTLSTFLVFKLNKATNINTLAISWA
Spba    RKILAYSSIAHLGWMTLVMQFSTYLAFMTLLIYLVMTFSMFLTCLLNSATTINMLATSWS
Game    RKILAYSSIAHLGWMMLVLQFSPSLTLLTLLTYFLMTFSTFLVFKLNKATNINSLATSWT
Thth    RKILAYSSIAHLGWMILILQFSPSLTLLTLLTYFIMTFSAFLVFKLNKATNINTLATSWA
Xigl    RKVLAYSSIAHLGWMILILQYSPSLTLLALLTYFVMTFSTFLVFKLNKSTSINALAISWT
Hyja    RKILAYSSIAHLGWMILVLQFSPSLTLLTLLTYLIMTFSTFLVFKLNKATSINALATAWT
Psan    RKILGYSSISNIGWMALAMHYSHPLVLLTLVTYIIMTCTIFMLFKEIEAKSIKCLANAWA
Cupa    RKILAYSSIAHLGWMILILQFSPSLTLLTLLTYFIMTFSTFLVFKLNKATNINALATSWT
Mpch    RKILAYSSIAHLGWMMLILSFLPSLTLLTLLTYFLMTFSTFLVFKTNKATTINMLAISWT
Char    RKILAYSSIAHLGWMLLILQFAPSLTFLALLMYIVMTSSLFLIFKLNKATNINTLATSWA
Pser    RKILAYSSIAHLGWMILVLQFSPSLTLLALLTYFIMTLSAFLVFKLNKATNINMLSTSWT
Prol    RKILAYSSIAHLGWMMLILQFSPLLTLLALITYLIMTSSVFLIFKMNKATTINALAISWT
Plbi    RKVLAYSSIAHLGWMMLILQFSPLLSLLALLTYFTMTFSAFLIFKVNKATTVNALAISWA
Calu    RKLLAYSSIAHLGWMVLIMQFSSGLSLIGLVLYLTMTFPLFIAFKTNNATSINQLAVSWT
Papa    RKILAYSSIAHMGWMAIIVSFSPALATLTILVYFITTTPAFLLLNLTKTTNIGALATSWS
Sufr    RKILAYSSIAHLGWMILIIQFSPSLTLLALITYFIMTTTAFLIFKLNISTNINALATSWA
Stci    RKILAYSSIAHLGWMLLVLQFCPSLSLLALFTYFVMTAAAFTTFKMNNATSISSLTMMWA
Taru    RKILAYSSIAHLGWMVIILQFSPTLAVMTLALYIIMTSSTFLAFMMNKTTTIGTLTISWA
Rala    RKILAYSSIAHLGWMVVVIQFSPSLTFLTLLTYFVMTFSAFLTFKLNNSTTINSLATSWA
        ** :.:    . **           :       *                     .
```

To be continued
on page 25.

```
                                      H                                 I
Scca   KSPLMSVIALMTLLSLGGLPPLSGFMPKWLILQELTKQSLIIPATIMALMALLSLFFYLR
Muma   KSPLLSIIALLTLLSLGGLPPLSGFMPKWLILQELTKQSLFIPATIMALMALLSLFFYLR
Erca   KSPILAATSALGLLSLGGLPPVSGFMPKWLIIQELTNQELAPTATLLALSALLSLFFYLR
Pose   KFPTLSAITMLALLSLGGLPPLSGFLPKWLILQELTNQGLMPTATMIALSALLSLYFYLR
Actr   KTPMMTTMAMMTLLSLGGLPPLTGFMPKWLILQELTKQNLPLTASIMALAALLSLFFYLR
Scal   KTPMMTTMAMMTLLSLGGLPPLSGFMPKWLILQELTKQDLPLTASIMALAALLSLFFYLR
Posp   KTPMMTTMAMMTLLSLGGLPPLTGFMPKWLILQELTKQNLPLTASIMAMAALLSLFFYLR
Atsp   KSPALAALTMPILLSLGGLPPLTGFAPKWLILQELTKQNLAITASIMALATLLSLFFYLR
Leoc   KSPVLATLTMPMLLSLGGLPPLTGFMPKWLILQELTKQNLAITASIMALATLLSLFFYLR
Amca   KAPILVTLTMLILLSLGGLPPLTGFMPKWLILQELTKQDLPLTASIMALAALLSLFFYLR
Osbi   KTPILTVMTMLSLLSLGGLPPLTGFMPKWLILQELTKQNLPILATIMAMSALLSLFFYLR
Pabu   KTPTLTTMTMLSLLSLGGLPPLTGFMPKWLILQELTKQNLPLIATLMAMSALLSLFFYLR
Hial   KTPTLVTLAMLALLSLGGLPPLTGFMPKWLILQELTKQDLPLTATIMALAALLSLFFYLR
Elha   KTPVLTTLTMLTLLSLGGLPPLTGFMPKWLIIQELAKQDLAIAATTMAMTALLSLFFYLR
Mlcy   KTPALATMTTLALLSLGGLPPLTGFMPKWLIIQELTKQNLNPTAMVMAMAALLSLFFYLR
Algl   KAPGLVTLTMLTLLSLGGLPPLSGFLPKWLILHELAKQNLPLIATLMALASLLNLFFYMR
Ptgi   KAPTLTALTMLALLSLGGLPPLTGFMPKWLILQELTKQDLPLVATLMAMAALLSLFFYMR
Alaf   KAPTLTALLMLSTLSLAGLPPLTGFMPKWLILQELTKQELPLIATLMAMTALLSLFFYTR
Nock   KAPTLTALLMLSILSLAGLPPLTGFMPKWLILQELTKQELPLIATLMAMTALLSLFFYTR
Anja   KAPILTTLTMATMLSLGGLPPLTGFMPKWMILQELTKQDLPLIATLMAMTALLSLFFYLR
Gyki   KAPTMITIAMLTLLSLGGLPPLTGFMPKWMILQELTKQHLPVTATILALSALLSLFFYLR
Syka   KAPILTTLIMMTMLSLGGLPPLTGFMPKWLILQELTKQGMPMTATLMAMAALLSLFFYLR
Opma   KAPILTTINLMTMLSLAGLPPLTGFMPKWLILQELTKQGLPLIATLMAMTALLSLFFYLR
Comy   KTPIITVIIMTTMLSLGGLPPMTGFMPKWLILQELTKQELPLTATIMAMSALLSLFFYMR
Sasp   KTPTFVMLMIVILLSLGGLPPLTGFMPKWLIMQELTKQMMPMMATLMAMAALISLFFYLR
Eupe   KAPTLAAINMVTILSLGGLPPLTGFMPKWFIIQELTKQTLPVTPTLLALATLLSLFFYLR
Enja   KAPTMAALTCLILLSLGGLPPLTGFLPKWLIIQELASQGLALTATVIALTALISLFFYLR
Same   KAPTLTALACLILLSLGGLPPLTGFMPKWLILQEVTNQGFPLTATVMAMTALLSLYFYLR
Chch   KAPSLAALATLSLLSLGGLPPLTGFMPKWLILQELTKQSLPLTATIMALSALLSLYFYLR
Grgr   KFPALAALTVLTLLSLGGLPPLTGFMPKWLILQELAKQDLPFLATLAALSALLSLYFYLR
Caau   KSPILTATTALVLLSLGGLPPLTGFMPKWLILQELTKQDLPIIATTMALAALISLYFYLR
Cyca   KSPILTSTTALVLLSLGGLPPLTGFMPKWLILQELTKQDLPIIATAMALTALISLYFYLR
Dare   KSPILAAIATLVMLSLGGLPPLTGFMPKWLILQELTKQDLPATATIMALTALLSLFFYLR
Cost   KNPTLMATTALALLSLGGLPPLTGFMPKWLILQELAKQELPLTATIMALAALLSLYFYLR
Leec   KNPILASTTALALLSLGGLPPLTGFMPKWLILQEMTKQELPLTATIMALAALLSLYFYLR
Fola   KSPVLVSTTALTLLSLGGLPPLTGFMPKWLILQELTKQDLPATATIMALAALLSLYFYLR
Clmc   KTPTAAAATALVLLSLGGLPPLTGFMPKWLILQELTKQELPLIATMAALSALLSLYFYLR
Phin   KEPAIVASAALLLLSLGGLPPLTGFLPKWLILQELTKQNLPLTATIMALTALLSLYFYLR
Icpu   KVPTITAMAALALLSLGGLPPLTGFMPKWLILQELTMQGLPLTATMMTLSALLSLYFYLR
Psto   KAPILTATTALALLSLGGLPPLTGFMPKWLILQELTTQGLPLTATMMALSALLSLYFYLR
Cora   KTPMITMTTMLTLLSLGGLPPLTGFMPKWLILQELTKQQLPLTATIIALSALLSLYFYLR
Eisp   KTPTLVTIATLSLLSLGGLPPLTGFAPKWLILQELTKQELPALATIMAPSALLSLSFYLR
Apal   KNPTLALIIMLVLLSLGGLPPLTGFMPKLLILTELIYQDLPLFATLMALTALLSLFYYLR
Eslu   KSPMMTSLATLILLSLAGLPPLTGFMPKWLILQELVKQDLPMLAFFAAMTALLSLYFYLR
Dape   KSPIIASFTTLVLLSLAGLPPLTGFMPKWLILQELVKQDLPLLAFFAAMSALLSLFFYLR
Glse   KAPPLAALTALVLLSLGGLPPLSGFMPKWLILQELTKQELPLTATLAALSALLSLYFYLR
Naar   KSPALAALTALVLLSLGGLPPFTGFMPKWLILQELTKQGRPLTATLTAMSALLSLYLYLR
Lioc   KSPALAALTALILLSLGGLPPLTGFMPKWLILQELTKQGLPLTATVAAMSALLSLYFYLR
Opso   KTPILATLTTLALLSLGGLPPFTGFMPKWLILQELTKQELPLTATLLALSALLSLYFYLR
Alte   KTPNLAALAALLLLSLGGLPPLTGFMPKWLILQELTKQGLPLTATLMALTALLSLYFYLR
Plap   KTPSLAALTALTLLSLGGLPPLTGFMPKWLILQELTKQGLPLTATLMALTALLSLYFYLR
```

To be continued on page 26.

[5/6 of aligned sequences]

To be continued on page 27.

Plal	KTPALIALASLALLSLGGLPPLSGFMPKWLILQEMTKQGLPLLATLAALTASLSLFFYLR
Sami	KAPALAALASLTLLSLGGLPPLSGFMPKWLILQEMTKQGLPLLATFAALTALLSQFFYLR
Rere	KTPTLIALASLTLLSLAGLPPLSGFMPKWLILQEMTKQGLPVLATLAALSALLSLFFYLR
Gama	KAPALVALTSLVLLSLGGLPPLTGFMPKWLILQELTKQGIPLVATLAALTALLSLYFYLR
Onmy	KSPTLAALTALVLLSLGGLPPLSGFMPKWLILQELTKQGLPLSATLAAMTALLSLYFYLR
Sasa	KSPTLAALTALVLLSLGGLPPLSGFMPKWLILQELTKQGLPLSATLAAMTALLSLYFYLR
Cola	KAPTLAALAILVLLSLGGLPPLSGFMPKWLILQELTKQGLPMSATLAAMTALLSLYFYLR
Dita	KYPVLGALLTPTLLSLGGLPPLSGFLPKWLIIDEMVKQGLQLPATLAALSALLSLYFYLR
Gogr	KAPALAAMLPPTLLSLAGLPPLSGFMPKWLMMYEMVKQGLQLPATVATLSSLLSLYFYLR
Chsl	KTPILAATLTPVLLSLGGLPPLSGFMPKWLVINEMVKQDLPLPATIAALSALLSLYFYLR
Atja	KAPALTALTPLILLSLGGLPPLSGFLPKWMILTELTKQNLPLTATIAAMTALLSLYFYLR
Iido	NTPALTALTPLILLSLGGLPPLSGFLSKWMILTELAKQNLPLTATIAAMTALLSLYFYLR
Auja	KAPALTALAPLALLSLAGLPPMTGFMPKWLILQELTKQQLPLTATLAALSALLSLYFYLR
Chag	KTPALTALVPLSLLSLGSLPPLTGFMPKWLILQELTKQGLPLTATIAALSALLSLYFYLR
Hami	KAPAISTIAPLALLSLGGLPPLSGFMPKWLILQELAKQHLPLTATLAALSALLSLFFYLR
Saun	KAPAISTIAPLTLLSLGGLPPLSGFMPKWLILQELTKQQLPLTATIAAMSALLSLFFYLR
Nema	KTPALTGLAPLVLLSLGGLPPLTGFAPKWLILQELTKQDLPLTATAAALTALLSLYFYLR
Disp	KAPALAGLAPLVLLSLGGLPPLTGFAPKWLILQELTKQDLPLTATISALSALLSLYFYLR
Myaf	KSPALTALSPLILLSLGGLPPLTGFAPKWLILQELTKQDLPLTATIAALTALLSLYFYLR
Lagu	KSPALTALAPLILLSLGGLPPLLGFAPKWMILLELTKQQMILTATAAAMMALLSLYFYLR
Trtr	KSVVLTALTPLVLFSLGGLPPLSGFAPKWLILQELTKQGLPIVATLAAMMALLSLYFYLR
Zucr	KSTVLVATAPFILFSLGGLPPLSGFAPKWLILQELTKQDMPIIATMAAMMALLSLYFYLR
Pxja	KTPALTALTPLILLSLGGLPPLTGFAPKWLILQELTKQDLPLTATLAAMTALLSLYFYLR
Pxlo	KTPALTALTPLVLLSLGGLPPLTGFAPKWLILQELTKQDLPLTATFAAMTALLSLYFYLR
Pctr	KAPALATLTPLIMLSLGGLPPLTGFAPKWLILQELTKQELPLVATLAALAALLSLFFYLR
Apsa	KNPALTMLAPLIMLSLGGLPPLTGFSPKWLILQELTKQELPLTATLAALTALLSLYFYLR
Cabe	AGPLMAALTSLCLLSLGGLPPLSGFMPKLLIIHELTKQELAIPATITALSALLSLYFYLR
Bzze	NNPALTALAPLVLLSLGGLPPLTGFAPKWLILQELTKQGLGLTAALAAITALLSLYFYLR
Siim	YGPIMTALSPLLLLALAGLPPFPIFYMKLYIIGEMIYQDIGVTATLIALASLLGLYFYAR
Ctru	KAPALTALLPLLLLSLGGLPPLTGFAPKWLILQELTKQDLAPTATFAALTALLSLYFYLR
Dpbr	KSPALTALLPLLLLSLGGLPPLTGFAPKWLILQELTKQDLAPTATFAALTALLSLYFYLR
Caki	KYPALMALFPLILLSLGGLPPLSGFLPKWLIIQELTNQGLILIIFIAAISALLSLFFYIR
Phja	KAPALTSMVPLVLLSLAGLPPLSGFLPKWLILEELTKQATSGTALLIAWSALLSLWFYVR
Brsp	KSSMILVMAPFAFLSLAGLPPLSGFLPKWLIMEELTKQDIPMIALVAAISSLLSLYFYIH
Gamo	KTPALTAITPMILLSLGGLPPLSGFMPKWMILQELTKQDIPLTASIAALSALLSLYFYLR
Lolo	KTPALTAITPLVLLSLGGLPPLSGFMPKWMILQELTKQDIPLTASVAALSALFSLYFYLR
Batr	KTPILTCLILLALITLGGLPPLSGFMPKWLIISEMTKQGMLPLTLLAAMSSLLSLFFYTR
Prmy	KSPFMMVSVPALLLTLAGLPPLTGFMPKWLILQELANQNLVLLAVLATMSSLLSLFFYLR
Lose	KLPLLAALIPLIMLSLGGLPPLTGFMPKWLILSELTKQGMSLTATLIALSALLSLYFYLR
Loam	KAPALTALAPLLMLSLGGLPPLSGFMPKWLILLELTKQDLAPAATFAALSALLSLYFYLR
Chab	KTPVSQPSPPWSYYPSGACHHSLAFMPKWLILQELTKQDLPLIATVAALSALLSLYFYLR
Chto	KAPALTALAPLILLSLGGLPPLTGFMPKWLILQELTKQDLPLIATVAALSALLSLYFYLR
Majo	KAPTIAALAPLILLSLGGLPPLTGFMPKWLILHELTKQELSLLATIMAIGALLSLYFYLR
Hlst	KAPAIAALTPLILLSLGGLPPLTGFLPKWFILHELTKQSLTPLATTLAISALLSLYFYLR
Clpe	KVPMIAAITPLILLSLAGLPPLTGFLPKWLILHELTKQDLPLPATLAALGALLSLYFYLR
Mlmr	KAPALTSITPLALLSLGGLPPLTGFMPKWLILQELTKQGLALTATIAAMSALLSLYFYLR
Crcr	KAPTLTSMSPMILLSLGGLPPLTGFMPKWLILQELTKHDLALIATMAALTALLSLYFYLR
Muce	KTPTLTSMSPMILLSLGGLPPLTGFMPKWLILQELTKHDLALIATTAALTALLSLYFYLR
Bege	KMPVITSLAPLILLSLGGLPPLTGFMPKWMILQELTKQDLASIATIAALSALLSLYFYLR
Mela	KTPTLTLLIPPILLSLGGLPPLTGFMPKWLILQELTKQDLALAATVAALSALLSLYFYLR
Hats	KTPIMTSLTPLILLSLGGLPPLTGFMPKWLILQELTKQDLGLTATVAALSALLSLYFYLR
Orla	KTPSVTALAPMMLLSLGGLPPLSGFLPKWLIIQELTKQELGLVATVAALSALLSLFFYLR

[5/6 of aligned sequences]

To be continued on page 28.

```
Cosa KAPVLTSITPLILLSLGGLPPLTGFVPKWMILQELSKQDLAPVATLAALSALLSLYFYLR
Exsp KSPALTSLTPLILLSLGGLPPLTGFMPKWLILQELTKQDLGLTATLAALSALLSLYFYLR
Depa KTPTLTSLTPLVLLSLGGLPPLTGFMPKWLILQELSKQHLAPIATLAALSALLSLYFYLR
Rima KTPMLTALTPLVLLSLGGLPPLTGFLPKWAILQELTKQGLTPVAALTALSALLSLYFYLR
Fuol KTPVISSVLPLVLLSLGGLPPLSGFMPKWLILQELAKQELAPLATLAALSALLSLYFYLR
Gmaf KIPTLTALTPLILLSLGGLPPLTGFLPKWMILQELTKQDLALVAVLAALFSLFSLYFYLR
Xeei KAPALTSIIPLTLLSLGGLPPLTGFMPKWLILQELTKQDLAPAATLAALSALLSLYFYLR
Pros KSPALTSLVPLVLLSLGGLPPLTGFAPKWLILQELTKQELAPAATIAALTALLSLYFYLR
Scmi KTPALTALVPLILLSLGGLPPLTGFAPKWLILQELTKQELAPIATLAALTALLSLYFYLR
Rolo KSPALTALVPLILLSLGGLPPLTGFAPKWLILQELTKQGLAPTATVAALSALLSLYFYLR
Cere KVPTLAALTPLLLLSLGGLPPLTGFMPKWLILQELTKQQLSPTATFAALAALLSLYFYLR
Daga KTPVLAAMTPLLLLSLGGLPPMTGFMPKWMIIQELTKQDLAPTGTLAALAALLSLYFYLR
Anco KAPALTAIVPLALLSLGGLPPLTGFAPKWLILQELTKQELPLTATLAALSALLSLYFYLR
Dmve KSPVLTAITPLALLSLGGLPPMTGFMPKWLILQELTKQELPLTATLAALSALLSLYFYLR
Dmar KSPVLTTLAPLALLALGGLPPMTGFMPKWLILQELTKQELPLTATLAALSALLSLYFYLR
Anka KSPALTAIVPLALLSLGGLPPMTGFAPKWLILQELTKQELPLTATLAALSALLSLYFYLR
Moja KAPALTAIIPLALLSLGGLPPMTGFAPKWLILQELTKQGLPLTATLAALSALLSLYFYLR
Hoja KAPALTAIVPLALLSLGGLPPLTGFAPKWLILQELTKQELPLTATLAALSALLSLYFYLR
Bede KAPALTALMPLVLLSLGGLPPLSGFAPKWLILQELTKQGLAPTATLAALTALLSLYFYLR
Besp KSPALTALMPLVLLSLGGLPPMTGFAPKWLILQELAKQGLAPTATLAALTALLSLYFYLR
Mysp KTPILTTLTPLMLLSLGGLPPLTGFAPKWLILQELTKQELATTATLAAMTALLSLYFYLR
Osja KTPILTTLTPLMLLSLGGLPPLTGFAPKWLILQELTKQELATTATLAALTALLSLYFYLR
Sgro KTPVLTALTPLMLLSLGGLPPLTGFAPKWLILQELTKQQLATTATIAAMTALISLYFYLR
Pzpa KTPILTMLFPLILLSLGGLPPLSGFMPKWLILQELTKQELPLTATIAALSALLSLYFYLR
Zeja KMPILTALSPLILLSLGGLPPLSGFMPKWLILQELTKQELPLTTTIAALSALLSLYFYLR
Znne KTPVLTALTPLMLLSLGGLPPLSGFMPKWLILQELTKQELPLTATIAALSALLSLYFYLR
Zefa KTPILTALTPLILLSLGGLPPLSGFMPKWLILQELTKQELPLTATIAALSALLSLYFYLR
Acni KTPILTALTPLILLSLGGLPPLSGFMPKWLILQELTKQELPLTATIAALSALLSLYFYLR
Ncrh KTPILTALTPLILLSLGGLPPLSGFMPKWLILQELTKQELPLTATIAALSALLSLYFYLR
Agca KAPALTALMPLVLLSLGGLPPLPGFMPKWLILQELTKQDLAPTATLAALTALLSLYFYLR
Hydy KAPALTSLAPLILLSLGGLPPLAGFMPKWLILQELSKQCLAPAATLAAFAALLSLYFYLR
Gsac KAPALTSLTPFILLSLGGLPPLVGFMPKWLILQELTKQELAAVATLAAFAALLSLYFYLR
Pevo TAPVVTALAPLALLSLGGLPPLTGFMPKWLIIQELTKQQLAPVATAAALAALLSLYFYLR
Hiku KSPTITALAPLILLSLGGLPPLTGFGPKWMILSELTKQELNTIATITALSALLSLYFYLR
Inpa KNPVLTALLLLSLLSLGGLPPLSGFMPKLLILQELTQQNLPLIATLAALSSLLSLYFYLR
Auch TSPMLAAIAPLILLSLAGLPPLTGFMPKWLILQELIKQDLGFTATLAAMAALLSLYFYLR
Fico KLPALTALVPLVLLSLGGLPPLTGFMPKWLILQELTKQDLAPLATLAALSALLSLYFYLR
Macs KAPALTALTPLVLLSLGGLPPLSGFMPKWLILQELSKQDLAPIATLAALTALLSLYFYLR
Moal KAPTITLVAPLMLLSLGGLPPLSGFAPKWFILQELTQQNFIPHASIAALAALLSLYYYLR
Syma KAPLLTLLTPLILLSLGGLPPLTGFMPKWLILQELTKQSITFTTLIMVMSTLLSLYFYLR
Mafr KTPTLTSLAPLILLSLAGLPPLTGFMPKWLILQELTKQDLAPTATLAALSALLSLYFYLR
Dcpe KAPAMTALTPLVLLSLAGLPPLTGFMPKWLILQELTKQSLAPVATLAALSALLSLYFYLR
Dcti KSPAMTALTPLVLLSLAGLPPLSGFMPKWLILQELTKQSLAPVATLAALSALLSLYFYLR
Hehi KAPALTALAPLLLLSLGGLPPLTGFMPKWLILQELAKQDLAPAATLAAMTALLSLYFYLR
Stam KSPTLTALAPLVLLSLGGLPPLTGFMPKWLILQELSKQDLAPMATLAAMTALLSLYFYLR
Hogi KAPALMTMTPLILLSLGGLPPLTGFMPKWLILQELTKQELNLTATLAALSALLSLYFYLR
Erzo KAPALTALAPLILLSLGGLPPLTGFMPKWLILQELANQDLAATATLAALTALLSLYFYLR
Hxot KAPALTSLTPLVLLSLGGLPPLTGFMPKWLILQELTKQDLAATATLAALTALLSLYFYLR
Core KAPALTCLTPLVLLSLGGLPPLTGFMPKWLILQELTKQDLATTATFAALTALLSLYFYLR
Apve KTPALMALTPLMLLSLGGLPPLTGFMPKWLILQELTKQDLATTATLAALTALLSLYFYLR
Latj KTPALTSLTPLILLSLGGLPPLTGFMPKWLVLQELTKQDLSALATLTALTALLSLFFYLR
Laja KTPAITSLTPLILLSLGGLPPLTGFMPKWLILQELTKQDLAITATFAALTALLSLYFYLR
```

[5/6 of aligned sequences]

To be continued on page 29.

Syja KTPVLTSCLPLIMFSLAGLPPLFGFTPKWLILQTLVNHDFSLVATLMALTALLSLFFYMR

Epme KAPALTTLTPLILLSLGGLPPLTGFMPKWLILQELTKQDMAPIATLAALTALLSLYFYLR

Grse KAPALTTLTPLILLSLGGLPPLTGFMPKWLILQELTKQELAPTATLAALSALLSLYFYLR

Clja KAPLLAAMAPLLLLSLGGLPPLSGFMPKWLILQELTKQNVPFTATMAAMTALLSLYFYLR

Ogcy KTPILASITPLILLSLAGLPPLTGFLPKWFILQELSNQGLPLIATLAALSALLSAYFYLR

Plna TNPSFFIVTFAILMSLSGFPPLTGFITKWIILDELIKQQLLALAMVAALSALLSAFFYFR

Lema KSPAITCLAPLILLSLGGLPPLTGFMPKWLILQELTKQELPCTATLAALTALLSLYFYLR

Etzo KAPALTALTPLVLLSLGGLPPLTGFMPKWLILQELTKQDLAPTATLAAMSALLSLYFYLR

Apse KTPALTAITPLILLSLGGLPPLSGFMPKWLILQELTKQGLALTATITALSALLSLYFYLR

Epde KAPALTSLAPLLLLSLGGLPPLTGFMPKWLILQELTKQDLAPTATLAALTALLSLYFYLR

Slja KTPALATLAPLVLLSLGGLPPLTGFMPKWLILQELSKQHLPAIATVAALSALLSLYFYLR

Bsja KTPALTALMPLVLLSLAGLPPLTGFMPKWLILHELTKQDLIPIATFAALTALLSLYFYLR

Ecna KTPIITTLTPLLLLSLGGLPPLTGFMPKWFILQELTKQNLPLLATFTALTALLSLYFYLR

Cohi KNPTLTYLMPLVLFSLGGLPPLTGFIPKWMILNELTSNNLPALAMLTALTALLSLYFYVR

Caar KTPVITSLAPLILLSLGGLPPLTGFMPKWLILQELTKQDLPVLATMAALTALLSLYFYLR

Came KTPIITSLAPLVLLSLGGLPPLTGFMPKWLILQELTKQDLPLLATMAALTALLSLYFYLR

Mema KAPALTSLAPLILLSLGGLPPLTGFMPKWLILQELTKQDLAPLATLAALTALLSLYFYLR

Lenu KLPMMTAIAPLLLLSLGGLPPLTGFMPKWMIIQELTKQDLAPLATIAAISALLSLFFYLR

Brja KAPALTALAPLILLSLGGLPPLTGFMPKWLILQELSKQDLAPVATLAALTALLSLYFYLR

Plma KSPALTSLTPLILLSLGGLPPLTGFMPKWLILQELSKQDLAPMATMAALSALLSLYFYLR

Emst KAPVLTALAPLVLLSLGGLPPLTGFMPKWLILQELAKQDLAPAATLAALTALLSLYFYLR

Ptti KTPALTSLAPLVLLSLGGLPPLTGFMPKWLILQELTKQDLAPTATLAALTALLSLYFYLR

Losu NPVIFTIATPLLFLSLAGLPPLMGFMPKLLILKELMQQEFILLAIMIAMSALLAIYFYLR

Geoy KAPALTSLAPLILLSLGGLPPLTGFMPKWLILQELTKQDLAPTATFAALTALLSLYFYLR

Dipi KAPALTSLTPLLLLSLGGLPPLTGFMPKWLILQELTKQDLAPIATLAALTALLSLYFYLR

Pama KAPMITALAPLILLSLGGLPPLTGFMSKWLILQELTKQELLPLATLAALSALLSLYFYLR

Leob KAPALTAFTPLILLSLGGLPPLTGFMPKWLILQELAKQDLAPTATLAALTALLSLYFYLR

Neba KTPVLTALTPLVLLSLGGLPPLSGFLPKWLILQELTKQNLEPTATFAALTALLSLYFYLR

Pdpl RTSFTISIAAILFLSLAGLPPLTGFVPKLLVVSELVQQGYLTPALLALLVTLLSLTYYLR

Nimi KAPALTALMPLVLLSLGGLPPLSGFMPKWLILQELSKQDLALTATLAALTALLSLYFYLR

Uptr SNPATITVAILVLLSLGGLPPLTGFITKWLIIQELTVQSHGALVTLLVFSALLSLYYYLR

Pesc KYPILTALTPLTLLSLAGLPPLTGFAPKWLIVQELVKQGLPVVATLAIMSALFSLYFYLR

Baar KAPALTSLVPLTLLSLGGLPPMTGFMPKWLILQELTKQELAPTATLAALTALLSLYFYLR

Moar KAPALTSLTPLVLLSLGGLPPLTGFMPKWLILAELTKQDLAPTATLAALTALLSLYFYLR

Toja KAPVIASLTPLILLSLGGLPPLTGFMPKWLILQELTKQDLGTLATLTALTALLSLYFYLR

Chau KAPIITSLTPLVLLSLGGLPPLTGFMPKWLILQELTKQSLTPMATLAALAALLSLYFYLR

Chse KNPILTMLLPLLLMSLAGLPPLTGFLTKLLILKELAKQGLAPTAALAILGTLLSAFFYIR

Enar KAPALTSLAPLVLLSLGGLPPLTGFMPKWLILQELAKQDLAPTATLAALTALLSLYFYLR

Hpty KAPALTSLTPLVLLSLGGLPPLTGFMPKWLILQELTKQDLAPTATLAALTALLSLYFYLR

Nana KNPTLTTLTPLVLLSLGGLPPLTGFMPKWLILQELTKQNLALVATMIALSALLSLYFYLR

Mcst KTPALTSLAPLILLSLGGLPPLTGFMPKWLILQELTKQDLAPTAMLAALTALLSLYFYLR

Rhox KTPVLTALTPLILLSLGGLPPLTGFMPKWLILQELTKQGLAPTATLAALSALLSLFFYLR

Opfa KAPVITTLAPLILLSLGGLPPLTGFVPKWLILQELTKQDLAPTATLVALTALLGLYFYLR

Paar KAPVLTSLAPLVLLSLGGLPPLTGFMPKWLILQELTKQMLIPTATLAALSALLSLYFYLR

Gozo KAPVLTSLTPLILLSLGGLPPMTGFMPKWLILQELTKQDLAPTATLAALTALLSLYFYLR

Ackr NSPVISATAVLVLLSMAGLPPLTGFAPKAMILYELSINNLPLIALVAALSALLNMFFYLR

Elev KAPTLTALTPLVLLSLAGLPPLTGFMPKWLILQELTKQSLPVTATIAALSALLSLYFYLR

Trdu KAPALTALTPLILLSLGGLPPLTGFMPKWLILQELTKQGLTPTATLAALSALLSLYFYLR

Amoc KMPVITSLTPLILLSLGGLPPLTGFLPKWLILQELAKQALPLTATLAALTALLSLYFYLR

Hame KTPAITCLTPLMLLSLGGLPPLTGFMPKWLILQELSKQGLAPTATLAALSALLSLYFYLR

Chso KAPMLTALTPLVLLSLGGLPPLTGFMPKWLIIQELTKQDLPLLAALTAMTALLSLYFYLR

Lyto KAPALTALAPLILLSLGGLPPLTGFMPKWLILQELAKQDLALTATLAALAALLSLYFYLR

[5/6 of aligned sequences]

To be continued on page 30.

```
Encr    KAPALTALAPLILLSLGGLPPLTGFMPKWLILQELAKQDLAVTATLAALAALLSLYFYLR
Bvar    KAPALTALTPLVLLSLGGLPPLTGFMTKWLVIQELTGQGLALVATLTALGALLSLYFYLR
Noco    KAPALVALTPLGLLSLAGLPPMTGFMPKWLILQELSKQELAPTGTLAALTALLSLYYYLR
Chsp    MFPALTAMTPLVLLSLGGLPPLTGFMPKWLILQELTTQALTPLATLMAMSALFSLYFYLR
Arja    KAPAFTSLTPLVLLSLGGLPPLTGFMPKWLILQELTKQDLGATATLAALTALLSLYFYLR
Pase    NAPALTALSPLLLLSLGGLPPLAGFMPKWLILQELAGQGLGLIATFAALTALLSLYFYLR
Trel    NLPMLTALTPMTLLSLGGLPPLSGFAPKWLITNEIVVNKLPLMATIMTLVTLFSLYFYLR
Lifa    YAPAITALVPLIMLSLGGLPPLTGFLPKWLIIQELTKQALPLTATLAALSALFSLYFYLR
Acur    KAPALTATTPLILLSLAGLPPLSGFLPKLMILSELAKNRLIIPATVMTLIALLSTFAYMR
Ampe    KTPALTALTPLLLLSLGGLPPLTGFMPKWLILQELTKQGLAPTATLAALTALLSLYFYLR
Urja    KMPALSALAPPLLLSLGGLPPLTGFMPKLLIIQELTKQNQALTATVIALSSLLSLFFYLR
Enet    KAPLVSLLLPLVLLSLAGLPPLTGFAPKWMILQELSTQGLPVVATIAALTALLSLFFYLR
Ptbr    KAPILALCTATTLMSLGGIPPLLGFAPKLLILKMLLTENLALLACMMAFFSLPSLFFYVR
Safa    KAPALTALAPFTLLSLGGLPPLSGFAPKWMILQELTKQQLPLIATVAALSALLSLYFYLR
Icae    KAPALTALTPLILLSLGGLPPLTGFMPKWLILQELSKQDLAPVATLAALTALLSLYFYLR
Asmi    KTPAIACLVPLTLLSLGGLPPMTGFAPKLLILQELTKQTLPLTATITALSALLSLYFYLR
Foal    KNNALLISTPIILFSLGGLPPTLGFTPKWFILMELTSHNMMIVALMIALSSLLSLYFYVR
Drze    KTPVLTTITPLILLSLGGLPPLSGFMPKWLILHELTKQGINFIATLAAFSALLSLNFYIR
Rhas    KTPPITAMAPLILLSLGGLPPLTGFAPKWLILQELTKQSLPLTATLAAMTALLSLYFYLR
Elac    KAPILTSTAPMILLSLGGLPPLTGFMPKWLILQELAKQGLPATATIAALTALLSLYFYLR
Kugu    KTPALMSITPLILLSLGGLPPLTGFMPKWLIIQELTKQNLPITATLAALTALLSLYFYLR
Plor    KNPIITALMPLLLLSLAGLPPLTGFAPKWLILQELTNQDLALTATLAALTALLSLYFYLR
Sgun    KAPTLTALTPLILLSLGGLPPLTGFMPKWLILQELAKQDLGLTATLAALTALLSLYFYLR
Zaco    KAPALTALTPLILLSLGGLPPLTGFMPKWLILQELTKQSLAPTATIAALTALLSLYFYLR
Zbfl    KTPVLTSLTPLVLLSLGGLPPLTGFMPKWLILQELTKHDLAPAATIAALTALLSLYFYLR
Spba    KNPVLTVSTPLVLLSLGGLPPLTGFAPKWLILQELPKHDLGLLATTTALTALLSLFFYLR
Game    KAPALTSLTPLVLLSLGGLPPLTGFMPKWLILQELSKQDLAPMATLAALTALLSLYFYLR
Thth    KAPALTSLTPLVLLSLGGLPPLTGFMPKWLILQELSKQDLAPVATLAALSALLSLYFYLR
Xigl    KAPILTSLTPLVLLSLGGLPPLTGFMPKWLILQELAKQDLAPLATLAALSALLSLYFYLR
Hyja    KAPALTALTPLVLLSLGGLPPLTGFMPKWLILQELSKQDLAPVATLAALTALLSLYFYLR
Psan    RNPVFTACIPLVLLSLAGLPPLTGFMPKWLIILKLCEHNLAFLATVAALSALLSLRFYLQ
Cupa    KAPALTSLTPLVLLSLGGLPPLTGFMPKWLILQELSKQDLAPVATLAALSALLSLYFYLR
Mpch    KTPVLTALTPLILLSLGGLPPLTGFMPKWLIIQELTKQELGLTATLAALSALLSLYFYLR
Char    KSPMLTSVTPLLLLSLGGLPPLSGFMPKWLILQELTKQDLPLAATLAALTALLSLYFYLR
Pser    KAPIITALAPLTLLSLGGLPPLTGFMPKWLILQELTKQELAPLATLAALTALLSLYFYLR
Prol    KTPILTALIPLVLLSLGGLPPLTGFMPKWFILQELTKQDLATLATLAALTALLSLYFYLR
Plbi    KTPALTALAPLVLLSLGGLPPLTGFMPKWFILQELTKQDLPALATIAALTALLSLYFYLR
Calu    KSPSLTALTPLLLLSLGGLPPLAGFMPKWLILTELTKQGLVAIATLAAFSALLSLYFYLR
Papa    KTPILTMITPLLMLSLAGLPPLSGFMPKLLIITELTKQQLGGLATLAAMFTLFTVYFYLR
Sufr    KTPVLASLTPLILLSLGGLPPLTGFMPKWLILQELTKQDLALPATLAALTALLSLYFYLR
Stci    KTPVLTAMVPLILLSLGGLPPLTGFMPKWMIIVELSKQGLPLIATLAALSALLSLYFYLR
Taru    KTPIISSLMPLILLSLGGLPPLTGFMPKWLILQELTKQDLGMTATLAALSALLSLYFYLR
Rala    KAPALTSLAPLVLLSLGGLPPLTGFMPKWLILQELTKQDLAPTATLAALSALLSLYFYLR
                        ..      *   *  :   :  .              :   * :
```

[6/6 of aligned sequences]

```
                                                      J
Scca     LCYATTLTKAPGPINMASTWRTKSHQPT---LIL-LTSASISIFMLPMTPLILMLMT----------
Muma     LCYATTLTMNPNPTNMLSSWRTKPNHFT---LIL-LTSATLSILLLPLTPAIFTLTS----------
Erca     LSYSLSLTIMPNTHPQTLNWNMKTT-TP---LPL-SLMMMMTLTMLPITPTIISLF-----------
Pose     LSYSLTTTVTPNTHAQTLNWGTKTK-TS---FIL-PVVMVMTITMLPISPSIISLF-----------
Actr     MCYAMTLTIAPNTNTNTSTWRQKSSQTT---MTL-SITTTLALILLPITPAIMALTT----------
Scal     VCYAMTLTIAPNTNNNASTWRQKSSQTT---MTL-SITTTLALALLPITPAILALTT----------
Posp     MCYAMTLTISPNTNNNTSTWRQKSSQTT---MTL-SITTLLALTLLPTTPTIMALMT----------
Atsp     LCYTMTLTLAPNTTNTPSTWRIKSPQTT---MPL-AMATTLTILLLPITPTILSLIM----------
Leoc     LCYAMTLTLSPNTTNTSSTWRIKSPQMT---MPL-ATISTLTIMLLPITPTIFSLIM----------
Amca     LCYTMTLTISPAPNNSQLPWRYNPTVSS---TTL-PLYMVLAMMLLPMTPATMSMVM----------
Osbi     LCYSSTLTTFPNTNNTTTSWHKKTTQMT---MPL-TIMAILTLMLLPTTPIITTMMI----------
Pabu     MCYAMTLTISPNIYNTTTPWRFMHQTTT---LL--PTLMITTLALLPITPTIITMMT----------
Hial     LCYAMTLTISPNITNTMSPWRLKSNQTT---MPL-SLSMTAALMLLPITPAMLALAS----------
Elha     LCYAMTMTISPNTNNTATSWRLNSLQTN---LPL-AIATAGATMLLPMTPTILNLIH----------
Mlcy     LCYTTALTISPNTNNTTSHWRTPPTQTN---PTL-PLTMTGTIMLLPITPAILSLTS----------
Algl     VSYVMCLTLFPGTSNTTALWRLYSTQAI---LPL-SMALVAALMLLPVTPLIEAMTL----------
Ptgi     LCYTMTLTISPNTNNTTSPWRLNSTRTT---LPL-STTIAATLMLLPLTPAVMAMTL----------
Alaf     MCYAMTLTISPGTNNNYTTWRLHKMRKH---LPL-AISTTGTLMLLPLTPAMTTML-----------
Nock     MCYTMTLTISPGTNNNYTTWRLYKMRKY---LPL-AITTTGTLMLLPLTPTMTTML-----------
Anja     LCYAMTLTISPNTNNAKTPWRLKSKQMT---MPL-STTMILTAMMLPVAPAVMAMTT----------
Gyki     LCYSMTLTSTPNTNNNKAPWRLRTT-IK---TPL-SLMTIASVAMLPITPTAMALLT----------
Syka     LCYAMTLTISPSTNNTKTPWRLKSTQTT---MPL-SIMIIMTMLLLPMTPTAMAMMT----------
Opma     ICYTMSLTISPNTNNTKTPWRMKSKQMT---MPL-SIATAMTMMLLPMTPTTVILITTMTL------
Comy     ICYATTLTISPNTNNAKTPWQLKSSLMP---TPL-PGMTAMAIMLLPVTPMFMAMMM----------
Sasp     VCYGSSLTTSPNTNNALTPWRLKSMIPS---TLI-SSTSVATTMLLPLTPTILMIVSW*--------
Eupe     MCYSLSLTSAPNTNNSKTAWRFKPSMMM---FPL-STTVIISSMMLPLAPTMLAMAVTTTM*-----
Enja     LTYAVTLTLSPQTSYATTPWRIQAKQST---LAL-SLATISATCLLPLTPTVFTLLT----------
Same     LSYAMTLTLSPYTITSTAPWRAATKQSS---LPL-ATAIVLSTCLLPLTPTALALLA----------
Chch     LCYAMALTIAPNVNNSSAPWQLLSTQST---LPL-ALTTVAALALLPATPAVLAMLT----------
Grgr     LSYALALTISPGTTNGTTSWRLPAQPSM---LPL-TTFTIFTLGMLPLTPTLLALMT----------
Caau     LCYAMTLTISPNTTNSTTPWRTQTTQAS---MPL-ALFTMATLGLLPMTPAILTLTT----------
Cyca     LCYAMTLTISPNMINSTTPWRTQTTQTS---LPL-ALFTTAALGLLPMAPAILMLTT----------
Dare     LCHAMTLTTSPNTINSAPHWRVQTTQNS---LPL-TISVTVTMGLLPLTPAILMLTT----------
Cost     LCYAMALTISPVTTNSMLPWRTSNSQST---LTL-ALTTTIALGLLPITPALLALTT----------
Leec     LCYAMTLTISPNTVSGKTPWRTQTTQPV---MTL-ALMTTMALGLLPITPAILMLVT----------
Fola     LSYAMALTIYPNTTNALTPWRTNTTQST---LAL-ALMMIGALGLLPLTPTIMALFF----------
Clmc     LCYAMTLTISPNTNNSVAPWRLTSTQMT---LPL-AISMTLTLMLLPLTPATMSLMY----------
Phin     LCYSMILTISPNTSNSFTPWRLNNFQMS---LPL-STLMTATLLLLPISPALMTLLF----------
Icpu     LCYAMTLTISPNTNNSSAPWRLQNTQAT---APL-ATLMIMTLLLLPLTPLAQTLTN----------
Psto     LCYSMTLTMAPNTNNALTPWRLQNTQTK---NLL-VITMTLTLMLLPLTPLAQTLIN----------
Cora     LTYAMTLTISPNTNNATMPWRLTNNQMT---LPL-TIFSTMTLMLLPITPTIQALIH----------
Eisp     LCYTMAQTITPNTINATPTWHLTHTQKT---LLT-ATAASTTTMLLPLTPLLFSLTL----------
Apal     LCYIILLTTSPNTNQAKAPWRLIPNTKS---MTI-ALTSALSTALLPFTPLLLVLTMTH*-------
Eslu     LCYTVALTSSPNTITMLTPWRLNPTQTS---PLN-PLTTMLTLALLPLTPAILALVIY*--------
Dape     ICYTMTLTSAPAPSSYSTPWRLNPLRPS---YFM-PLTITLSLMLLPLTPPILTLVLP*--------
Glse     LCYAMTLTTSPNTLPSSSPWRLYFTQTT---LPL-STLTLGTLTLLPLTPAIETLLSP---------
Naar     LCYAMAFTASPNTLTSAAPWRLSFTQPT---LPL-SLTTMGALTLLPLTPAIAAFLTI---------
Lioc     LCYALSFMASPSTLNSSSTWRLSFTQPT---LPL-STATMGALALLPLTPAIAAFLTI---------
Opso     LCYSMTLTLSPATLNTTSPWRLSSTQPA---LPL-SLTMIGTLTLLPLTPAIKALLAL---------
Alte     LCYAMTLTISPNTNNSTTPWRLPNTQNT---LPL-SLMMVAALALLPLTPATMALVT----------
Plap     LCYAMTLTISPNTNNSTTPWRLPSTQNT---LPL-SLTTTTALALLPLTPAAMALVT----------
```

[6/6 of aligned sequences]

```
Plal  ICYAMTLTISPNTLGASPSWRFSSNQSS---LLL-ALSTVGTLGLLPITPFFLSAFSWA--------
Sami  ICYTMALTFSPNTSGSSPSWRFEGGQTS---FLL-ALSTVSALGLLPITPLFLAAFAWS--------
Rere  ISYAMALTVSPNTVGASLSWRSSGNHST---LLL-ASSTVMALGLLPLTPAFLSLYSLG--------
Gama  LCYAMSLTTSPNTTLATAPWRVCTLRPS---LPL-SATSVAALALLPVTPAFATFFGL*--------
Onmy  LCYALTLTIYPNTLTATAPWRLNFTMIT---LPL-SITTIMALGLLPLTPAVTAMLAL*--------
Sasa  LCYAMTLTIYPNTLTATAPWRLNFTLIT---LPL-SIITILALGLLPLTPAVTTLLTL*--------
Cola  LCYAMTLTIWPNTLAATTPWRLDFTHTT---LPL-SLVTMLALGLLPLTPAVAALLNL*--------
Dita  LCYAATLTSAPNVLTKVTPWRLVSPGGH---LLL-ASLSTVALGTLPLAPTILAWIA*---------
Gogr  LCYAVILTSPPNVYTKTTPWRLTPFLNP---LPL-TVLSSVTLGILPLTPSMLTLLN*---------
Chsl  VCYAMTLTSPPNTFIKVTPWRLLHPKIH---FPL-ALASIAALGLLPVTPAILAWFT*---------
Atja  LSYAMTLTTSPTTLPHTATWRLPSKQLT---LML-AMATTLALGLLPLSPMVLTVMMH*--------
Iido  LSYAMALTTSPTTLSHTAPWRLPSKQLT---LIL-AFTTTLTLGLLPLSPMILAVMSF*--------
Auja  ICYAMTLTISPNTLTACSPWRLPTTQST---LLL-STSLVFSLALLPTTPTICTLILY*--------
Chag  LCYAMTLTISPNTLTSPAPWRLPALQQT---LLL-SALMVMALMLLPLTPTALTLLSP*--------
Hami  VSYSMTLTMSPNTVSASAPWRLQNHQFT---LLL-AFMTTLTILLLPITPTILSILPS---------
Saun  ISYSMALTMSPNTVSASTPWRLNNPQST---LLL-STATALALLLLPITPTIISTLPM---------
Nema  ICYAMTLTISPNTSSGLAPWRHKSPSPT---LLL-SSSAAAALLLLPLTPTALAILSP---------
Disp  ICHAMTLTVSPNTSTSLSPWRHSPATST---LLL-SVSTAGALLLLPLTPTALTLLPL---------
Myaf  VCYAMTLTVSSNTTASLATWRHKTYRPA---LLM-STSTCATILLLPVTTAALALIPN---------
Lagu  LSYAISLTAAPNTLPMSSTWRLNPAQLP---LVL-PPMLIITLTALPLTPTALSLLTP---------
Trtr  LSYSLALTMAPNTPPASAPWRSIPQQSS---SLL-AITTMMSILMLPIMPSTLSLFYL---------
Zucr  VSYAAALTMAPNTPPASAPWRPSPTQSS---LVL-SLTTISSLFMLPLMPTALTLFCL---------
Pxja  LCYAMTLTISPNTTIGVTPWRLTSTQLT---LPL-AIATMATLSLLPLTPTALALLTP*--------
Pxlo  LCYAMTLTISPNTTIGVTPWRLTSTQLT---LPL-AIATMATLSLLPLTPTALALLTP*--------
Pctr  ICYAAALTGPPNTLTNTAPWRLLTHQLT---LPL-AISASASLLLLPFTPTALALLAP---------
Apsa  ISYALTLTISPNTQMNTSPWRLTTNQLS---TPL-SISSTMTMMMLPMTPTMLSMLSP---------
Cabe  LAYAYTLTSSPNTPASCAQWRLQPISQA---ALM-PITIVAALLLLPMTPTALALMAQ---------
Bzze  LTYAMTLTMSPNTATGPIQWRLKAARNT---LPL-AVATVSALMLLPATPAAVALLTH---------
Siim  LAYTMSLTSWPNLSTAMANWRLRTKKVT---IVL-AVTAAAAILMLPLLPTQISFMMWYWSMR----
Ctru  LSYAMTLTSSPNNLMATTPWRLLPTQIT---SPL-TLSTMATISLLPLTPALIALLTP---------
Dpbr  LSYAMTLTTSPNNLVATTPWRLLPSQST---VPL-ALSTMASISLLPLTPTLAALLTP---------
Caki  VVYTIMAITAPNFFGYTPSKFFRTSFKT---SPL-SIPSVMALMLLPLLPALWAFFSL*--------
Phja  ICYSTTLTSFPSNTLAKLPWRLHATKPLYI-----SSAIMVSFSILPLAPALTSCFC----------
Brsp  LLYTLSVMMAPNLAHGGMAWRLYPLKSP---LPI-AIAFVLSTLAIPFSPLIYSLLPI*--------
Gamo  VSYAMTLTISPNNLNATTPWRLQTTAST---LPL-AISATISAMLLPLAPATLALLSL---------
Lolo  VSYAMTLTISPNNLNATTPWRLQTTSST---LPL-AISTTISAMLLPLAPATLALLSP---------
Batr  LAFITTLTISPNNTPLSISWRTTHTNFN---MLL-SPLLASSIFLIPLTPLLLYAFMP*--------
Prmy  LSYSIMLISSPNNTTHTAAWRQNHKTLY---FPL-SITTILSTLSLPLTPLLLAVMM*---------
Lose  LSYAVALTIPPANMFSSTPWRLYTNQLK---MPL-AVSISIATLLLPLAPTTAALLQL*--------
Loam  LSYAMTLTLSPSSLTASTPWRLLNTQLT---LPL-AITTMLALLLLPLTPGTTALLAL---------
Chab  LSYAMTLTMAPNNLMGTSPWRLHSPQPT---MPA-TISTMMTLSLLPLTPAMLTVITL---------
Chto  LSYAMTLTMAPNNLMGTSPWRLHSPQPT---MPA-TISTMMTLSLLPLTPAMLTVITL---------
Majo  LSYAMALTISPNNISGTPPWRFTSPSLK---LIL-AILTTCSIALLPLAPALASLSPL---------
Hlst  LSYAMALTINPNSTATTPPWRFPTPQMN---LPL-AIATAMAISLLPMAPALKTLSSF---------
Clpe  LSYAMTLTMSPNTGVSTAPWRLHSPHQT---MPL-ALSAALTVLLLPLAPALTALFLI---------
Mlmr  LSYAMTLTMSPNTLTSTTSWRFPPPRST---MPL-TLSATVATLLLPLTPALTSALTL---------
Crcr  LSYAITLTMSPNNLTGTLPWRLHPSQLT---LLL-TFSTSTTILLLPLTPTILTSLTSLCC------
Muce  LSYTITLTISPSNLTGTLPWRLHPSQLT---LPL-TFSTSATILLLPLTPTILTTLTSLTSLTSLRC
Bege  LSYAMTLTISPNNLTGTAPWRLSSTQST---TPL-AIAISMTLCLLPLTPAISTLLIF---------
Mela  LCYAMTLTMSPNTIINTMPWRLPSTQLS---LPL-SITASLTICLLPLTPTILTMTTL---------
Hats  LSYAMTLTISPNNLTGTTPWRLPSSQLT---LPL-AISTAMTTCLLPLTPAVMALLAP---------
Orla  VCYCLTFTSSPNNLAGVAPWRLESKQIS---LPL-ATSTSLSILLLPISPAILSLTLPLF-------
```

[6/6 of aligned sequences]

```
Cosa    ISHAMTLTTSPNCLPGITPWRLPLTQSL---LPL-ATSASATMCLLPIAPTLVTLFNP---------
Exsp    LSHAMTLTISPNNLAGTTPWRLTPNHQT---MPL-ATVTSATICLLPLTPALTTILTI---------
Depa    ISYAMTLTTSPNNTPGMTPWRLHTNQTS---LLL-AMSTSMTICLLPLTPALLTLFTP---------
Rima    LSYATALTTPPNNLPATLPWRIIFKQPT---TFL-AVLITMSLSLLPLSPSVMAFFTF---------
Fuol    ISYAMTLTMTPNILINTLPWRLSSNQHT---LPL-AFTTSITICLLPLTPATIALLTF---------
Gmaf    LSYAMTLTMPPNNPAGTLPWRLNPSHNT---LPP-ALTTTMTIFLLPITPAFMTLFFL---------
Xeei    LSYAMTLTISPNNIMSTLPWRSPTKQLT---LPL-AIFTTATISFLPLTPVMLALFTF---------
Pros    LSYAMTLTMSPNNLTNTMPWRLPTLQNT---LPL-ATSTMATITLLPLTPAAIALLTP---------
Scmi    LSYAMALTMSPNNLTNTTSWRLPTLQHT---LPL-ALSTMATIALLPLTPAAIALLTP*--------
Rolo    LSYASTLTMYPNNTAGTTPWRLPLLQPT---MPL-AISAMATVSLLPLTPAAVALLTP---------
Cere    LSYAAALTMSPNNLNGSTPWRLQPLQTF---SPL-AILATAAISFLPMAPAVVALLTP*--------
Daga    LSYTTALTLSPGSASGTAPWRLQPVQSF---MPI-AILGMATISLLPLAPAALALLT*---------
Anco    LSYAISLTISPNHLMGTTPWRLNTLQLT---LPL-AVSTMATLALLPLAPAATALLAP---------
Dmve    LSYAMTLTTAPNNLMTTTPWRLHSPQLT---LPL-AISMLTTISLLPMTPTAMTLLTP*--------
Dmar    LSYAMTLTASPNNLMTTTPWRLPSLQPT---LPL-AVSILATVSLLPLAPTILTLLTP*--------
Anka    LSYAVSLTILPNHLTGTTPWRLHSSQLT---LPL-AISTLATISLLPLTPAAIALLAP*--------
Moja    LSYAISLTMLPNHLTGTAPWRLCPTQLT---LPL-AISTLATISLLPLTPAAIALLTP---------
Hoja    LSYAISLTILPNHLTGTTPWRLHTSQLT---LPL-AISTLATISLLPLTPAATALLTS*--------
Bede    LSYAMTLTMSPNNLTNTMPWRLPPLQHT---MPL-ALSTMATISLLPLTPAATALLTP---------
Besp    LSYAMTLTMSPNNLTNTMPWRLPLLQHT---MPL-ALSTMATISLLPLTPAATALLTP---------
Mysp    LSYAMTLTISPNNLSSTTPWRFQSSQAT---MPL-AITMMGTIALLPLTPTAMALLTP---------
Osja    LSYAMTLTISPNNLSNTTPWRFQPLQAT---MPL-AITMMGTISLLPLTPTAMALLTP---------
Sgro    LSYAMTLTMSPNTLSGTTPWRLQPLQTT---LPL-AITMMGTISLLPLTPTAMALLIP*--------
Pzpa    LSYAMTLTTSPNTIMNITPWRLKSLQLT---TPL-SISIALTTLLLPLTPALMTL------------
Zeja    LSYAMTLTTSPNMTTNTTPWQLKSPQLT---LPL-SMSMALALLLLPMTPTLLTL------------
Znne    LSYAMTLTMSPNTTVNTTLWRLKSNQSS---APL-SVSIALATLLLPLTPALLIL------------
Zefa    LSYAMALTTSPNTTINTAPWRLKTNQNS---IPL-STSIALTAMLLPMTPTILAI------------
Acni    LSYAMTLTTSPNTTLNTAPWRLKSAQST---VPL-SISIALAMLLLPMTPTLLAL------------
Ncrh    LSYAMTLTTSPNTTMNTAPWRLKSAQST---APL-SISIALTMLLLPMTPTLLAL------------
Agca    LTYAMALTISPNNLVGITPWRLPSLQLT---LPL-TISITATTLLLPLTPAVKALLTL---------
Hydy    LSYAMALTMSPNNTSATSPWRFSSPQLT---LPL-AISATATLLLLPLSPAVVALLTF---------
Gsac    LSYAMALTMSPNSTTATTPWRYSSSQAT---LPL-AISTTATLLLLPLAPAAIALFTI---------
Pevo    LTYSMAMTISPNNTPATAPWRLTSKNSV---MPL-SLTLIMALMLLPATPTIMALAMT---------
Hiku    LSYAMALTLSPNTTPMTNQWRFNTKQLT---IHL-SISTIMSLMLLPLMPSLMTIIN----------
Inpa    LSHAMALTTFPNSPLGTTPWRLLPTHKT---LPL-TLAISTTIILFPHTPTTLALLGL---------
Auch    LSYAMTLTISPNTLQSIAPWRLRSLTLS---LPL-AISTCAVVLLLPLTPSLLALLPI---------
Fico    LSYAMALTMSPNTLPGSTPWRFTSTQQT---LPL-AVSTSASLLLLPLTPAICALTLP---------
Macs    LSYALALTMSPNNIAGSTPWRLNSTQLT---LPL-ATSTMATLLLLPLTPAVSALLL----------
Moal    LSYSITLSLSPNNLPECLQWRVNYPISTPLSSAL-PAALALTLFLLPLTPSALTLII----------
Syma    LAYSTTLTISPNNLNGTTQWRFPQSQKT---LPL-MLSITTTILLLPSTPLILPLLI----------
Mafr    LTYAMALTISPNNLTGTTPWRLSSNQLT---LPL-TISITATIFLLPLSPTILTLLTP---------
Dcpe    LAYAMTLTTSPNNISGTTPWRLTSNQTT---LPL-AFSTTGTLMLLPLMPATSALFTL---------
Dcti    LAYAMTLTTSPNNLTGTTPWRLDSNQST---LPL-AISTTGALMLLPLTPATSALFAL---------
Hehi    LSYAVTLTISPNNLTAISPWRLPTLQLT---LPL-ATSAMATLLLLPLTPAAMALMTL---------
Stam    LSYAMTLTMSPNISTGSTPWRFQSSQLA---FPV-AISTMATLLLLPTAPMMVALMTL---------
Hogi    LSYAMTLTISPNTLTGTMPWRLPTHQIN---FPL-AITSTATILLLPITPAIVASLTL---------
Erzo    LSYAMALTMSPNNTTGTTPWRLQSSQSS---LPV-AISTTATLLLLPLAPAAITLLAP---------
Hxot    LSYAMTLTMSPNNTAGTTPWRLPSSQLT---LPL-AISTTATLLLLPLTPAAIALLAL---------
Core    LSYAMALTMSPNNTAGTTPWRFPSLQNT---LPL-AISTTTTLLLLPLAPAATALLTL---------
Apve    LSYAMALTMPPNNTTGTTPWRLPSLQLT---MPL-AISTTTTLLLLPLTPATTAFLTL---------
Latj    LSYAMTLTLSPNNSTGTTPWRFPPSQPN---LPL-AISTTTSILLLPLTPAAMALLTP---------
Laja    LSYAMTLTMSPNNLIGVTPWRLLTPQLT---MPL-AFFTTSTLLLLPLTPALKALLTL---------
```

[6/6 of aligned sequences]

```
Syja    LAFAMTLTMSHNDTLGVSPWRLSPTQDM---LIL-ATTITISLLSLPLTPVIVFLLSR---------
Epme    LTYAMTLTMSPNNTTGTAPWRIPTSQMT---LPL-AILTLATLSLLPLTPTMLTLLTL---------
Grse    LTYAMTLTMSPNNPTGTTPWRLQSSQPT---LPL-AASTTATLSLLPLTPAAIALLFP---------
Clja    LSYSMTLTMSPNNLTGSTPWRLHSSQPT---LML-SAASTITVLLLPLTPTAIMMLLL---------
Ogcy    ISYTMILTLSPNNIVGTATWRLLSTKAT---LPL-ALSTTLTILLLPLAPTLMALFMF---------
Plna    LAYVMALTLPPNTTYTQTSWRATSLSNL--AMPL-AVTTSLTMTLLPLMPALAALMSI---------
Lema    LSYAMTLTMAPNNLTGTTPWRVYPQQLT---LPL-ASSTMMALLLLPLTPSMLALLSP---------
Etzo    LSYAMALTMSPNNLAGTTPWRLQHSQFT---LPL-ALTTTGTLLMLPLAPAVVALLSL---------
Apse    LSYAMTLTTPPNTPMGLSPWRLHTSQGT---LPL-AISTTATLALLPLTPLVAALFSP*--------
Epde    LSYAMTLTMSPNNLAGTTPWRLPSTQLT---LSL-AISTISALTLLPLAPAAMALLTP---------
Slja    LTYAMTLTMSPNNLPGTAPWRLTPTQLS---LPL-ATSTMVTIALLPLTPATSALLTGLWP------
Bsja    LSYAMTLTMSPNNLTGTSFWRLQAPQPT---LPL-ALTTTSTIMLLPLTPTAIALLSP---------
Ecna    LSYSMTLTMFPNNLMGTTPWRFFTQSTS---LPL-ALSISMAIALLPLAPAMMALLTP---------
Cohi    LSYAMTLTSFPNTITSTAYWRFLPNQAS---FPL-SMFMSGTILLLPVTPAIVALFYL---------
Caar    LSYAMTLTMFPNNLMGTAPWRFHTPQLN---LPL-AISTSATILLLPLTPAITALLTP---------
Came    LSYAMTLTMFPNNLAGTTPWRFHTPQLN---LPL-AISTSATILLLPLTPAITALLTL---------
Mema    LSYAMTLTMFPNNLTGTTPWRFNSPQLT---LPL-AISTTATLSLLPLTPLATALLTP---------
Lenu    LSYNMTLTMFPNIITATTPWRLPLSKPS---LPL-AISATASLLLLPLTPTIIAMLTP---------
Brja    LSYAMTLTMSPNNLPGTTSWRLPSLQTT---LPL-SLSLMATLSLLPLTPAVTTVLAL---------
Plma    LSYAMTLTMSPNNLTGTPSWRLPSLQLT---LPL-TTSLMATLCLLPLTPAAMAMLTL---------
Emst    LSYAMTLTMSPNNLAGTTPWRLPSSQLT---LPV-AVATMAALSLLPLTPATVALLTL---------
Ptti    LTYAMTLTISPNSLSGTTPWRLPTTQLT---LPL-AVSTMATLSLLPLTPAVMALLTL---------
Losu    LIYVMAMTTFPLPASAPAIWRKISTKWS---VII-SFLISSTIMAMPLFPLIYAVISYFTG*-----
Geoy    LTYAMTLTLSPNNQTGITPWRLPSTQMK---LPL-TLTTMGAIMLLPLTPAFMALLVL---------
Dipi    LSYAMTLTMSPNNLIGTTPWRLQSSQLT---LPL-AISTTATLLLLPLTPAMIALLTI*--------
Pama    LAYALTLTVSPNTLIGTTPWRYPTPHLT---LLL-STTTIATILLLPLTPAIVMLLTL---------
Leob    LSYALTLTLAPNNLTGTTPWRLPNRQFT---LPL-ATSTVATVSLLPLTPAIIAVLTF---------
Neba    LSYSMTMTMAPNNLSGTTPWRFSLLQPT---LFP-TLAITGAIGLLPIAPLIWTTLTV---------
Pdpl    LLYAMASTISPNNVASLLPWRIHPTRLS---LPL-AVTSVIAISAIPLLPAAYMLTQ*---------
Nimi    LSYAMALTMFPNTPTGTPPWRFQSSQIT---FPL-AISVTATLMLLPLTPTITALLTS---------
Uptr    LSHALTLTMPPRHVMVTALWRLDPPNPTPL---M-PAFIAMAIALLPIMPIIASLTLK*--------
Pesc    LAYAMAMTLPPNTMAGIPHWRLLPPNAS---LPL-AAFTAATLFLLPLTPGLLALMTP---------
Baar    LSYAMTLTSSPNNLTGATPWRLSSTQTT---LPL-ATSTMATILLLPLTPAATALLSL---------
Moar    LSYALTLTISPNNLVGTTPWRLPSTQLT---LPL-AISTMATISLLPLTPAMIALLTL---------
Toja    LSYAMTLTVSPSNLTGTTSWRFYPSQPT---MPL-AIATMASMTLLPLTPATVALLTP---------
Chau    LSYAMTLTASPNTLTGTTPWRLTSSQLT---LPL-AISTVATISLLPLTPAMVALLTL---------
Chse    LSVATTLTLSPNILTGAAPWRLSSSRLT---LPL-AISSMLSIALLPLTPTLIALLTH---------
Enar    LSYAMALTISPNNLLATTPWRLPSSQLT---LPL-AVSTVATLSLLPLTPAALALLTL---------
Hpty    LSYAMTLTMAPNNLTGTAPWRLPSSQLT---LPL-STSTTATLLLLPLAPAATALLAL---------
Nana    LSYAMTLTMSPNNLTGITPWRLPSPQLT---LHL-SLSISTTILLLPLTPLISTIMTP---------
Mcst    LSYAMTLTMFPNNLTGTTPWRLPSSQLT---LPL-TLSTMAALSLLPLTPATTALLTL---------
Rhox    LSYAATLTISPNNLASIPTWRLPSTQST---LPL-AFSTMATITLLPLTPAAATLLAL---------
Opfa    LSYAMTLTMSPNNLAGTTPWRLRPSQFT---MPI-ALSTMTTLSLLPLTPAMIALLTQ---------
Paar    LSYTMTLTLSPNNLLASTPWRLMTSQTT---LPT-AIFTTTSLLLLPLTPTALILLTH---------
Gozo    LTYAMTLTMAPNNLAGTTPWRLPSSQLT---LPL-AISTMASLSLLPLMPAAVAFLAL---------
Ackr    LTYIVALSLSPSTILACAPWRLPSSKFK---TYS-AMACIITLAFLPLTPDIMAIFLP---------
Elev    LTYAITLTISPNTSNGLAPWRFTMMKNT---TLL-SSAVMGTLLLLPLSPSATSFLYF---------
Trdu    LSYAMTLTISPNNLTGLTPWRLPSTQLT---YPL-ATSTAMTICLLPLTPAISALLIS---------
Amoc    LCYAMTLTMSPNNLVGTAPWRLPSFQLT---LPL-ATTTTMTILLLPLAPAATTLMTI---------
Hame    LSYALALTMAPNVISGITPWRLPTSQAT---LPL-ATSATATMLLLPLTPAVVALVVL---------
Chso    LSYAMALTISPNNLAGTTPWRLPSLQST---LPL-ALATVGTLLLLPLTPTITSLLSL---------
Lyto    LSYAMTLTMSPNNATGTTPWRFPPSQLT---LPA-AVSATATLLLLPLTPATMALLTI---------
```

[6/6 of aligned sequences]

```
Encr   LSYAMALTMSPNNITGVTPWRFSCSQLT---LPV-ATSTTAALLLLPLTPAAVALLTI---------
Bvar   ITYAMALTISPNTLTTTMPWRLPPSHLT---LPL-AAATATSLLLLPLTPAAITLLAP---------
Noco   LTYAMTLTMFPNNLAGTLPWRLPSHQLT---LPL-ACLAIATISLLPLTPAALALVAL---------
Chsp   LSYSMTLTMFPHNMLAAAPWRLNPPNNN---SLITAYMTMTTALMLPMAPTTMTLLTY---------
Arja   LSYAMTLTMSPNNTTGTAPWRFPYLHPT---MLL-AVSTAATLLLLPLTPAAVTFLTL---------
Pase   LSYAMALTVSPNNVSSIPTWRLPSLQST---LPL-ATATVATLLLLPLMPAATALLAL---------
Trel   LTNSLSLTMSPNSNVGLLPLFLSKSPIAPM---L-PLVISGSMMLIPLIPVITAAMPACK-------
Lifa   LTYNSALVISPNNLPPTAMWRLSPAKGT---LLL-ATLASAALMLLPLSPLATTLTDL---------
Acur   MATVMALIGAPGSPLLLPQWRNMSLPSN---LPM-ALVITLTLLLLPFSSLILTLIPS---------
Ampe   LSYAMTLTLSPNNLSGVTPWRLPSSQLT---LPL-AVSTTAALCLLPLTPAAMALLTL---------
Urja   LTYVMTLTISPNNLAGTTPWRLQPHKMK---LPL-ATLISFSLLLLPLTSTSMALLCT---------
Enet   VSYAVVLTISPATTPTIARWRLDSSFPT---FFL-AALLIGTLSLLPLAPLLLALTST---------
Ptbr   TVVVLTLTTISKPLSSSSPWRILTLKSS---ALL-AWAIMASALFLPISPLILSLLVF---------
Safa   LTYSMSLTISPNTTLNSAPWRLLRQHNT---LHI-ALPTTMTILLLPLTPAALTLLS----------
Icae   LSYAMTLTMSPNNLAGTTPWRLPSIQPT---LPL-ATSLVATLSLLPLTPAATAMLTL---------
Asmi   LTYASSLTLSPNITSTPTMWRLPLFSST---MPL-AFFSTLSILLFPLLPALATPNYF---------
Foal   LSYALALTKAPNTISGAAPWRLHLTSKD---MFM-ASFVTMIILFLPASPLILTLFPF---------
Drze   LSYAVALTMAPNNLSGTAHWRSTSNQPT---LPI-LITATATLMLLPLTPTIKTLILT---------
Rhas   LSYAITLTMSPNNLTGTTPWRLPSLQLS---LPL-SAATMMALLLLPLTPAITALLSNP--------
Elac   LSYAMALTMSPNNLSGTTPWRLCSSQIS---LPL-STSTLMACALLPLTPAVTALLTF*--------
Kugu   LSYAMTLTMSPNNLTGTSTWRLPSLQQT---LLL-ATSAVATIALLPLTPAAIALLT*---------
Plor   LSYAMTLTMFPNNQTGSTPWRLLSQQHT---LPL-ALSLTASITLLPLTPTLTALPTL---------
Sgun   LSYAMTLTISPNNLIGTTPWRLPSSQLT---LPL-TISTMATISLLPLTPATMALLSL---------
Zaco   LSYAMTLTMSPNNPIGIAPWRQPSSQSS---TLL-AIAMMATLLLLPLTPAAVALIAH*--------
Zbfl   LSYAMTLTMSPNNLAGTTPWRLPSPQLA---LPL-AISTTATLSLLPLTPATITLLTL---------
Spba   VTYAMAVTLFPLHLTSTAPWRFSNNQLT---LPL-ATSTVATISLLPLTPAITALLTP*--------
Game   LSYAMALTMSPNNLPGTASWRLPSLQLT---LPL-ALSIVATLTLLPLTPAVTAMLTL---------
Thth   LSYAMTLTMSPNNLSGTASWRLPSLQPT---LPV-ATSLVATLALLPLTPAITAILTL---------
Xigl   ISYAMTLTMSPNNLTGVTPWRFSSPQLT---LPL-AIATTATISLLPLAPAAIALLTP---------
Hyja   LSYAMTLTMSPNNLTGVTPWRLPSLQLT---LPL-ALSLMATLSLLPLAPAAATILTL---------
Psan   LIYAMMTIMPSSATLGSKPWRLRNRMGS---MTM-GLGVSASLFLTPLIPILLVFLSP---------
Cupa   LSYAMTLTMSPNNLTGITSWRLPSLQPT---LPL-ALSLVATLTLLPLTPAATALLTL---------
Mpch   LSYAMTLTMSPNILTGSAPWRLTSNFNT---LPL-AIATISTISLLPLMPAMVTLLQL---------
Char   LSYTMALTLAPNSLTGITPWRLPHLQTT---MPT-SIFTTLSLFLLPLTPAAITLLIL*--------
Pser   LSYAMTLTASPNNSLGSMPWRFQSNQPT---MPL-AISTTAAISLLPLAPSAIALLTP---------
Prol   LSYAMTLTMAPNNLTGTTPWRFSSPQLT---LPL-AISSTTATLLLPLAPATLALLTT---------
Plbi   LSYAMTLTMFPNNLVGVTPWRFYSPQFT---LPL-AISTAATTLLLPLAPAAVALLIT---------
Calu   LSYAVALTIPPNNTSGTLGWRLFLPCTP---LPL-AFSAALSLTLLPLTPGITALLIT---------
Papa   VAYSLSLTLSPNNLAGTTPWRFTTKQNT---LPL-ATIATLSMMLLPLAPAMLTLTKP*--------
Sufr   ITYASTLTISPNTPTGTTPWRLPSTQST---LLL-STATTATICLLPLTPALAALLTT---------
Stci   IAYASTLTVSPNTTMGTPLWRTNTTQAT---LPL-ALTLTMTAVLLPLAPGALAMLSF---------
Taru   LSYAMTLTISPNNLTGTLPWRTQTNKKT---LPT-AILLSSSILLLPLTPEVLTLFNT---------
Rala   LTYAMTLTISPNNLPGTIPWRFASAQLT---LPL-AITTTATLTLLPLTPSILMLFTL---------
                                                     *
```
