# Supplementary material for: Structure and variation of the mitochondrial genome of fishes
Source: BMC Genomics. 2016 Sep 7;17(1):719. doi: 10.1186/s12864-016-3054-y (PMC5015259; doi:10.1186/s12864-016-3054-y)
Supplement: Additional file 6: Figure S1-a. — Aligned amino acid sequences of the ATP8 gene in mt genomes of 250 fishes. Figure S1-b. Aligned amino acid sequences of the ATP6 gene in mt genomes of 250 fishes. Figure S1-c. Aligned amino acid sequences of the COI gene in mt genomes of 250 fishes. Figure S1-d. Aligned amino acid sequences of the COII gene in mt genomes of 250 fishes. Figure S1-e. Aligned amino acid sequences of the COIII gene in mt genomes of 250 fishes. Figure S1-f. Aligned amino acid sequences of the Cyt b gene in mt genomes of 250 fishes. Figure S1-g. Aligned amino acid sequences of the ND1 gene in mt genomes of 249 fishes. Figure S1-h. Aligned amino acid sequences of the ND2 gene in mt genomes of 250 fishes. Figure S1-i. Aligned amino acid sequences of the ND3 gene in mt genomes of 250 fishes. Figure S1-j. Aligned amino acid sequences of the ND4L gene in mt genomes of 250 fishes. Figure S1-k. Aligned amino acid sequences of the ND4 gene in mt genomes of 250 fishes. Figure S1-l. Aligned amino acid sequences of the ND5 gene in mt genomes of 250 fishes. Figure S1-m. Aligned amino acid sequences of the ND6 gene in mt genomes of 249 fishes. (ZIP 3250 kb) [file 12864_2016_3054_MOESM6_ESM.zip › Additional file 6 prot align/AF6i-ND3.pdf]

Additional file 6: Figure S1–i. Aligned amino acid sequences of the ND3 gene in mt genomes of 250 fishes.

Species name abbreviation followed by aligned amino acid sequences shown by one letter abbreviation. See Additional file 1 for abbreviation of species name. Amino acids shown by magenta letter denote hydrophobic residues. A-C in bold types with yellow background indicate putative transmembrane regions. Asterisk '*' indicates a fully conserved residue. Colon ':' and period '.' indicate 'strong' and 'weak' groups in the level of conservativeness, respectively, in the Gonnet Pam250 matrix, in which the strong and weak groups are defined as strong score >0.5 and weak score =<0.5, respectively (Thompson et al., 1997).

ND3

[1/2 of aligned sequences] A

To be continued on page 6.

```
Scca        -MSLIMSSVVATALVSLILAFIAFWLPSLKPDNEKLSPYECGFDPLGSARLPFSMRFFLI
Muma        -MNLIMSSVATTALVSLILALIAFWLPSLNPDNEKLSPYECGFDPLGSARLPFSLRFFLV
Erca        -MNLILTMILISSLISITLAFVAFWLPQMNPDMEKLSPYECGFDPLGSARLPFSMRFFLI
Pose        -MNLILTMILISSSISTILAIVAFWLPQMNPDSEKLSPYECGFDPLGSARLPFSIRFFLV
Actr        -MNLIMAVLAIAITLSCILAVVAFWLPQMNPDSEKLSPYECGFDPLGSARLPFSLRFFLV
Scal        -MNLITAVLAIAITLSCVLAVIAFWLPQMNPDSEKLSPYECGFDPLGSARLPFSLRFFLV
Posp        -MNLAMTILAIAIALSCILATVAFWLPQMNPDSEKLSPYECGFDPLGSARLPFSLRFFLV
Atsp        -MNLIAMILIITAILSCILAMVAFWLPQMTPDSEKLSPYECGFDPLGSARLPFSLRFFLI
Leoc        -MNLIAMILIITTILSCILAMVAFWLPQMVPDSEKLSPYECGFDPLGSARLPFSLRFFLI
Amca        -MNLTMTIFTITAALSCILAIVAFWLPQMSPDSEKLSPYECGFDPLGSARLPFSMRFFLV
Osbi        -MNLITSTLSIAILLTILLAMVSFWIPQMSPDTEKLSPYECGFDPLGSARLPFSLRFFLV
Pabu        -MNLTIT-LATATILSLILAVVSFWLPHMNPDTEKLSPYECGFDPLGSARLPFSIRFFLI
Hial        -MNLIMVILTIATILCLVLATVAFWLPQMNPDAEKLSPYECGFDPLGSARLPFSLRFFLV
Elha        -MNLLTTILAITTILSLVLIIVSFWLPQMSPDSEKLSPYECGFDPLGSARLPFSLRFFLV
Mlcy        -MNLITTILTITSILSMVLLIVSFWLPQMNPDSEKLSPYECGFDPLGSARLPFSLRFFLV
Algl        -MNLILTVILIVSALSCILAIVSFWLPQMNPDAEKLSPYECGFDPLGSARLPFSMRFFLV
Ptgi        -MNLITTIMAITTILSCILAIVSFWLPQMSPDAEKLSPYECGFDPLGSARLPFSLRFFLV
Alaf        -MNLITTILTITTLLTCVLAIVSFWLPQMNPDAEKLSPYECGFDPLGSARLPFSLRFFLV
Nock        -MNLITAILMITTILSCVLALVSFWLPQMNPDAEKLSPYECGFDPMGSARLPFSLRFFLV
Anja        -MNPIISILTITTTLSCVLITVSFWLPQMNPDSEKLSPYECGFDPLGSARLPFSMRFFLV
Gyki        -MNPIISIFIITTALSCVLIFVSFWLPQMSPDSEKLSPYECGFDPLGSARLPFSMRFFLV
Syka        -MNPIISVFMITIALSCILITISFWLPQMNPDSEKLSPYECGFDPLGSARLPFSMRFFLV
Opma        -MNPIISTIMITTALSCILITVSFWLPQMNPDSEKLSPYECGFDPIGSARLPFSMRFFLV
Comy        -MNPIISIMIITSILSSVLIIVSFWLPQMNPDSEKLSPYECGFDPLGSARLPFSIRFFLV
Sasp        -MYQVILILIISAALAFLIISASLWMPQMHPATEKLSPYECGFDPMGSARLPFSIHFFVV
Eupe        -MNPIISTLIVAAVLSLVLITASFWLPLFNLSSEKLSPYECGFDPLGSARLPFSLHFFLV
Enja        -MNLITTILAIATALSLVLMTVSFWLPQMAPDAEKLSPYECGFDPLGSARLPFSMRFFLV
Same        -MSLIMTIMAISSALSIILIIVSFWLPQMNPDAEKLSPYECGFDPLGSARLPFSMRFFLV
Chch        -VNLITTIILITMILSMVLAIVAFWLPQMNPDAEKLSPYECGFDPLGSARLPFSLRFFLV
Grgr        -INLITATIIITALLSFVLIIVGFWFPQANPDAEKLSPYECGFDPLGSARLPFSLRFFLV
Caau        -MNLIMTILTITAALSLILATVSFWLPQMNPDAEKLSPYECGFDPLGSARLPFSLRFFLV
Cyca        -MNLIMTILTITVALSLILATVSFWLPQMNPDAEKLSPYECGFDPLGSARLPFSLRFFLV
Dare        -MNLFATILIIMTTLSLVLALVSFWLPQMNSDTEKLSPYECGFDPLGSARLPFSLRFFLV
Cost        -MNLITTILIITMTLSLILAIVSFWLPQMNPDAEKLSPYECGFDPLGSARLPFSIRFFLI
Leec        -MNLIVMILTITTALSMILAIVSFWLPQMNPDAEKLSPYECGFDPLGSARLPFSLRFFLV
Fola        -MNLVISILAITIILSSILAVVSFWLPQMNPDAEKLSPYECGFDPLGSARLPFSIRFFLV
Clmc        -MNLITTILLITTALSVLLALVSFWLPQMNPDAEKLSPYECGFDPLGSARLPFSLRFFLV
Phin        -MNLITTTLFICSLLSLLLALLSFWLPQMSPDMEKLSPYECGFDPLGSARLPFSLRFFLV
Icpu        -MNLIMVVTLITLILSTILATVSFWLPQMTPDAEKLSPYECGFDPLGSARLPFSLRFFLV
Psto        -MNLIMIMMAISTALSTILALVSFWLPQMTPDTEKLSPYECGFDPLGSARLPFSLRFFLI
Cora        -MNLMATIFTISIILSLVLALVSFWLPQMNPDTEKLSPYECGFDPLGSARLPFSLRFFLV
Eisp        -MNLIMTILLISTGLSLLLALISFWLPQMNQDAEKLSPYECGFDPLGSARLPFSLRFFLV
Apal        -INLLLLMMVISASLSLILTIVAFWLPQTNPDMEKLSPYECGFDPLGSARLPFSMRFFLI
Eslu        -MNLISTIFVITITLSAILATLSFWLPQMSPDTEKLSPYECGFDPLGTARLPFSLRFFLV
Dape        -MNLITTMIIITTTLSLILTTVSFWLPQMSPDTEKLSPYECGFDPLGSARLPFSLRFFLV
```

[1/2 of aligned sequences]

```
Glse    -MNLITAVLIITILLSTVLATLSFWLPQLNPDAEKLSPYECGFDPLGSARLPFSLRFFLV
Naar    -MNLIMTVFTITISLSLILATVSFWLPQITPDAEKLSPYECGFDPLGSARLPFSLRFFLV
Lioc    -MNLVMTVFTITIALSLVLAAVSFWLPQLNPDAEKLSPYECGFDPLGSARLPFSLRFFLV
Opso    -MNLVMVVIIITTSLSLFLATVSFWLPQIAPDAEKLSPYECGFDPRGSARLPFSLRFFLI
Alte    -MNLITTILLITITLSTLLAIVSFWLPQMTPDAEKLSPYECGFDPLGSARLPFSLRFFLI
Plap    -MNLISTVLLITIALSTILALVAFWLPQMTPDAEKLSPYECGFDPLGSARLPFSLRFFLI
Plal    -MNLVSSVIFITILLSTVLAIVSFWLPQLNPDAEKLSPYECGFDPLGSARLPFSLRFFLI
Sami    -MNLVTSVVSITILLSTVLAIVSFWLPQLNPDAEKLSPYECGFDPLGSARLPFSLRFFLI
Rere    -MNLITTVILITVLLSTVLAIVSFWLPQLNPDAEKLSPYECGFDPLGSARLPFSLRFFLI
Gama    -MNLIVTVIAITTALSVVLALVSFWLPQISPDAEKLSPYECGFDPLGSARLPFSMRFFLV
Onmy    -MNLITTIITITITLSAVLATISFWLPQISPDAEKLSPYECGFDPLGSARLPFSLRFFLI
Sasa    -MNLITTIIAITITLSAVLATISFWLPQMTPDAEKLSPYECGFDPLGSARLPFSLRFFLI
Cola    -MNLITTIVTITIALSMVLATVSFWLPQITPDAEKLSPYECGFDPLGSARLPFSLRFFLI
Dita    -MNVIAAVMMITLALSALLAVVSFWLPQINPNAEKLSPYECGFDPLGSARLPFSLRFFLV
Gogr    -MNMVSLAITTASSLSIILTTISFWLPQTAPTAEKLSPYECGFDPLGSARLPFSLRFFLV
Chsl    -MNVVTAVIVITSALSLILVSLSFWAAQLTPGNEKASPYECGFDPLGSARLPFSMRFFLV
Atja    -MNLVSTVISIAVILSMVLAMVSFWLPQLNPDAEKLSPYECGFDPLGSARLPFSLRFFLV
Iido    -MNLISTVISIAIILSTILALVSFWLPQLNPDAEKLSPYECGFDPLGSARLPFSLRFFLV
Auja    -MNLIATVLLIATALSSVLALVSFWLPQMNPDTEKLSPYECGFDPLGSARLPFSMRFFLV
Chag    -MNLVSTVLMIALALSSLLAFVSFWLPQLNPDTEKLSPYECGFDPLGSARLPFSLRFFLV
Hami    -MNLLTTILLITSALSAILALVSFWLPQMTPDTEKLSPYECGFDPLGSARLPFSLRFFLV
Saun    -MNLLATILLITTALSAVLALVSFWLPQMTPDTEKLSPYECGFDPLGSARLPFSLRFFLV
Nema    -MNLISTIILIAMLLSLVLTAVSFWLPQMTPDSEKLSPYECGFDPLGSARLPFSLRFFLV
Disp    -MNLISTILLIAVVLSFILTTVSFWLPQMTPDSEKLSPYECGFDPLGSARLPFSLRFFLV
Myaf    -MNLISTILLIAVLLSLVLTIVSFWLPQMNPDSEKLSPYECGFDPLGSARLPFSLRFFLV
Lagu    -MSLVMTALTIAAALSIVLITVSFWLPQLIPDYEKLSPYECGFDPLGSARLPFSLRFFLI
Trtr    -MNLVVTSLLIALTLSTVLIFISFWIPQLSPDHEKLSPYECGFDPLGSARLPFSLRFFLV
Zucr    -MNLVMTSLVIALLLSVVLIFISFWIPQLSPDHEKLSPYECGFDPLGSARLPFSLRFFLV
Pxja    -MNLISTIMIIAILLSTILTIVSFWLPQLNPDYEKLSPYECGFDPLGSARLPFSLRFFLV
Pxlo    -MNLISTIMIIAILLSTILTIVSFWLPQLNPDYEKLSPYECGFDPLGSARLPFSLRFFLV
Pctr    -MNLMLTVVTITAALSALLATISFWLPLTNPDYEKLSPYECGFDPLGSARLPFSLRFFLV
Apsa    -MNLILTILIITSTLSAILATISFWLPLMKPDYEKLSPYECGFDPLGSARLPFSLRFFLV
Cabe    -INPLVMFISTAAALSIVLMLVAFWLPVATPDSEKLSPYECGFDPLGSARLPFSLRFFLV
Bzze    -MNLVTTFITIAVALSIILAIVSFWLPQMTPDCEKLSPYECGFDPLGSARLPFSLRFFLV
Siim    -MNMVTVFAALAIIISAVLTTISFWMPQMSPDYEKLSPYECGFDPLGSARLPFSLRFFLV
Ctru    -MNLTTTVLIITALLSAILATVSFWLPQITPDHEKLSPYECGFDPVGSARLPFSLRFFLV
Dpbr    -MNLTTTVLTITILLSTILATVSFWLPQITPDHEKLSPYECGFDPVGSARLPFSLRFFLV
Caki    -MNTISATVLIASIMSLVLMLVSFWLPQLNPDYEKLSPYECGFDPLGSARLPFSLRFFLV
Phja    -MNLVVMVMMIASVLSLLIILISFWLPQLNPDYEKLSPYECGFDPLGTARLPFSMRFFLI
Brsp    -VNLLLTTISIALLLSLILMLVSFWLPQLVPDYEKLSPYECGFNPLGSARLPFSLRFFLI
Gamo    -MNLISTVILIASALSLILILVSFWLPQLSPDYEKLSPYECGFDPLGSARLPFSLRFFLI
Lolo    -MNLISTVILIASALSLVLILVSFWLPQLNPDYEKLSPYECGFDPLGSARLPFSLRFFLI
Batr    -MNLLTSFI-LILFLSALLGILSLWLPQTSPSLNKLSPYECGFDPLKSARLPFSLRFFLI
Prmy    MINPLIV-TLTISALSLIMIMVSFWLPMISPNNEKLSPYECGFDPLGSARLPFSLRFFLV
Lose    -MNLTTSTFVITSTLSMALMMVAFSLPQMVPDQEKLSPFECGFDPLGSARLPFSLRFFLV
Loam    -MNLVTTIVCITGTLSLILATVAFWLPQMTPDHEKLSPYECGFDPLGSARLPFSLRFFLV
Chab    -MNLVMTILAITMTLSVLLAIVSFWLPQMTPDHEKLSPYECGFDPLGSARLPFSLRFFLV
Chto    -MNLVMTILTITITLSVLLAIVSFWLPQMTPDHEKLSPYECGFDPLGSARLPFSLRFFLV
Majo    -MNLMLTVIAIAIALSTILALVSFFLPQMTPDHEKLSPYECGFDPLGSARLPFSLRFFLV
Hlst    -MSLMMTIITITIGLSMILALVSFFLPQMTPDHEKLSPYECGFDPLGSARLPFSLRFFLV
Clpe    -MNLVLTVIAIALTLSTLLIVVSFWLPLVAPDHEKLSPYECGFDPVGSARLPFSIRFFLI
Mlmr    -MNLIITIIIIAVSLTAVLAIVSFWLPLMFPDHEKLSPYECGFDPVGSARLPFSLRFFLV
```

To be continued on page 7.

[1/2 of aligned sequences]

```
Crcr    -VNLITSMLMITTLLSVLLCIVSFWLPQMTPDFEKLSPYECGFDPLGSARLPFSLRFFLV
Muce    -MNLITSMLTIATLLSVLLCIVSFWLPQMTPDFEKLSPYECGFDPLGSARLPFSLRFFLV
Bege    -MNLVMTIILISLVLSMLLTIISFWLPQMTPDYEKLSPYECGFDPLGSARLPFSLRFFLV
Mela    -MNLATTLLLTSATLSVILAIVSFWLPLMTPDYEKLSPYECGFDPLGSARLPFSLRFFLI
Hats    -MNLVTTIILIAITLSTVLAIVSFWLPQMTPDYEKLSPYECGFDPLGSARLPFSLRFFLV
Orla    -MNLVMVTLLISLVLATVLAIASFFLPQMTPDYEKLSPYECGFDPVGSARLPFSIRFFLV
Cosa    -MNLMMTIILISTLLSVILAIVSFWLPQMNPDYEKLSPYECGFDPLGSARLPFSLRFFLV
Exsp    -MNLLLTVILISTALSIILAIVSFWLPQMTPDYEKLSPYECGFDPLGSARLPFSLRFFLV
Depa    -MNLVMTIILVSTLLSTILAIISFWLPQMNPDYEKLSPYECGFDPLGSARLPFSLRFFLV
Rima    -MNMIIASTTIAMALSTLLILVSFWLPASNPDYEKLSPYECGFNPFASTRLPFSLRFFLV
Fuol    -INLIMIMMFIASILSLVLAVVAFWLPQMNPDYEKLSPYECGFDPLGSARLPFSLRFFLV
Gmaf    -MNLVMTTLILSLLLSLLLAIVAFWLPLMMPDYEKLSPYECGFDPLGSARLPFSIRFFLV
Xeei    -MNLVTIMIFISLTLSTLLAITAFWLPLMNMNYEKLSPYECGFDPLGSARLPFSLRFFLV
Pros    -MSLVLTIIVITALLSSILALVSFWLPQMTPDSEKLSPYECGFDPLGSARLPFSIRFFLI
Scmi    -MSLILTIVIITALLSSILALISFWLPQMSPDSEKLSPYECGFDPLGSARLPFSLRFFLV
Rolo    -MNLVLTIIIIAISLSSILATVSFWLPQISPDYEKLSPYECGFDPLGTARLPFSLRFFLI
Cere    -MNLVLTIMILTIALSTILAIVSFWLPQISSDYEKLSPYECGFDPLGTARLPFSLRFFLV
Daga    -MSLLQVVIAASVLISSFLALLSFWLPQMSSDYEKLSPYECGFDPLGTARLPFSLRFFLV
Anco    -MNLVMTVIMIAAVLSLILATISFWLPQLSPDYEKLSPYECGFDPLGTARLPFSLRFFLV
Dmve    -MNLATTVATIAIALSLLLATISFWLPQLSPDYEKLSPYECGFDPVGTARLPFSLRFFLV
Dmar    -MNLTTTVATLAILLSLLLATISFWLPQLNPDYEKLSPYECGFDPVGTARLPFSLRFFLV
Anka    -MNLVTTVMVIAVVLSSILAIVSFWLPQLSPDYEKLSPYECGFDPLGTARLPFSLRFFLV
Moja    -MNLLATVMIIAVTLSFILAIVSFWLPQLSPDYEKLSPYECGFDPLGTARLPFSLRFFLI
Hoja    -MNLVATVMAIAVILSAVLATISFWLPQLSPDYEKLSPYECGFDPLGSARLPFSLRFFLV
Bede    -MNLLLTIITIAVLLSSVLALVSFWLPQMTPDYEKLSPYECGFDPLGSARLPFSLRFFLI
Besp    -MNLVLTIITITALLSSVLALVSFWLPQMTPDYEKLSPYECGFDPLGSARLPFSLRFFLI
Mysp    -MNLLSMIIAITAALSVILAIVSFWLPQMNPDYEKLSPYECGFDPLGTARLPFSLRFFLV
Osja    -MNLLSTIMIITAALSIILAIVSFWLPQMNPDYEKLSPYECGFDPLGSARLPFSLRFFLV
Sgro    -MNLVLMIITIAAILSIVLTIVSFWLPQMNPDYEKLSPYECGFDPLGTARLPFSLRFFLV
Pzpa    -MNLISTIMIISALISLVLSIISFWLPLLNPDSEKLSPYECGFDPLGSARLPFSLRFFLV
Zeja    -MNLASTILFISALIPLVLAVVSFWLPLLNPDSEKLSPYECGFDPLGSARLPFSLRFFLV
Znne    -MNMVSTIMMISALISLVLAVVSFWVPQLTPDTEKLSPYECGFDPLGSARLPFSLRFFLV
Zefa    -MNMISTLMLISALISLVLATVSFWLPQLNPDSEKLSPYECGFDPLGSARLPFSLRFFLV
Acni    -MNLVSTIMLISALISLVLATISFWLPQLSPDSEKLSPYECGFDPLGSARLPFSLRFFLV
Ncrh    -MNLVSTIMLISALISLVLATVSFWLPQLSPDSEKLSPYECGFDPLGSARLPFSLRFFLV
Agca    -MNLVTTIIAISITLSTILAFVSFWLPQMMPDHEKLSPYECGFDPLGTARLPFSLRFFLV
Hydy    -MNLITTVIAITATLSLVLTLVSFWLPQTTPDHEKLSPYECGFDPLGSARLPFSLRFFLV
Gsac    -MNLVTTVVSITAALSLVLALVSFWLPQMTPDHEKLSPYECGFDPLGSARLPFSLRFFLV
Pevo    -MNLVTTTILIATVLSLILALVSFWMPQMTPDPEKLSPYECGFDPLGSARLPFSIRFFLI
Hiku    -MNLLITILLITTTLSILLALISFWLPQMNPDTEKLSPYECGFDPLGSARLPFSLRFFLV
Inpa    -MNLTTTTLLTTALLSTALLILAFWLPQTKPDYEKLSPYECGFDPLGSARLPFSMRFFLV
Auch    -MNLMVAVPAISLAISVILTVVSFWLPLVTPDSEKLSPYECGFDPLGSARLPFSLRFFLV
Fico    -MNLITTVLAITTLLSTVLALVSFWLPLMTPDSEKLSPYECGFDPLGSARLPFSMRFFLV
Macs    -MNLITTIVAIAVALSMVLALVSFWLPQITPDPEKLSPYECGFDPLGSARLPFSLRFFLV
Moal    -MNLIIAFMLMTTLIPMALMLISFWLPMMTPDYEKLSPYECGFDPLGSARLPFSIRFFLV
Syma    -MNLMMTFMSIALVLSLILSVIAFWLPLTKPDHEKLSPYECGFDPLGTARLPFSIRFFLI
Mafr    -MK-LMTIVIITSTLSLILTMVAFWLPQLSPDYEKLSPYECGFDPLGTARLPFSIRFFLV
Dcpe    -MNLITTIMMISALLTTVLAFVSFWLPQMNPDPEKLSPYECGFDPLGTARLPFSLRFFLV
Dcti    -MNLITTIMLISALLTTVLAFVSFWLPQMNPDPEKLSPYECGFDPLGTARLPFSLRFFLV
Hehi    -MNLAMAVITITIMLSLVLAIVSFWLPQMTPDHEKLSPYECGFDPLGSARLPFSLRFFLV
Stam    -MNLVTTVITIALLLSIVLAIVSFWLPQMTPDHEKLSPYECGFDPLGSARLPFSLRFFLV
Hogi    -MNLTMAIITITALLSTILALVSFWLPQMTPDHEKLSPYECGFDPLGSARLPFSLRFFLV
```

To be continued on page 8.

[1/2 of aligned sequences]

```
Erzo   -MNLVTTVIAITTTLSIILALVSFWLPQMTPDHEKLSPYECGFDPLGSARLPFSLRFFLV
Hxot   -MNLVTTVITIASLLSVILAIVSFWLPQMTPDHEKLSPYECGFDPIGSARLPFSLRFFLV
Core   -MNLVTTVIAITTLLSIILAIVSFWLPQMTPDHEKLSPYECGFDPVGSARLPFSLRFFLV
Apve   -MNLIAVVVTIATLLAMILAIVSFWIPQMAPDYVKLSPYECGFDPSNSARLPFSLRFFLV
Latj   -MSLILTVVIITASISAILIIVSFWLPQMNPDHEKLSPYECGFDPLGTARLPFSLRFFLV
Laja   -MNLIMTIIAITVALSTLLAIVSFWLPQMTPDHEKLSPYECGFDPLGSARLPFSLRFFLI
Syja   -MNLITTVITIAIALSTILAVVAFWLPHMAPDHEKLSPYECGFDPLGSARLPFSLRFFLV
Epme   -MNLVSTVMTIALALSMALAILSFWLPSMSPDHEKLSPYECGFDPLGTARLPFSLRFFLV
Grse   -MNLISTTIAITTALSLVLAIISFWLPQISPDYEKLSPYECGFDPLGTARLPFSLRFFLI
Clja   -MNLLLSIAFITVALSIILALVSFWLPSINPDYEKLSPYECGFDPLGSARLPFSVRFFLV
Ogcy   -MNLIMTMLLISIILSTALIIISFWLPQITPDQEKLSPYECGFDPLGTARLPFSMRFFLV
Plna   -MNLITAVITISITLSTILIIVSFWLPQMTPDYEKLSPYECGFDPLGTARLPFSLRFFLV
Lema   -MNLVTTIISIATALCVVLAIVSFWLPQMTPDHEKLSPYECGFDPLGSARLPFSLRFFLV
Etzo   -MNLISTIIVITTILPIILALVSFWLPQMTPDHEKLSPYECGFDPLGSARLPFSLRFFLV
Apse   -MNLLTTVMIITAALSTILAIVSFWLPQMNPDQEKLSPYECGFDPLGSARLPFSLRFFLV
Epde   -MNLITTVVTIAIALSTVLALVSFWLPQMSPDHEKLSPYECGFDPLGSARLPFSLRFFLV
Slja   -MNLVLTIIIITGLLSAVLAIVSFWLPQMTPDHEKLSPYECGFDPLGSARLPFSLRFFLV
Bsja   -MNLVTTIITIALLLSIALLLISFWLPQMNPDHEKLSPYECGFDPLGSARMPFSLRFFLV
Ecna   -MNIVMMILLIAIALSTVLAIVSFWLPQMNPDSEKLSPFECGFDPLGSARLPFSLQFFLV
Cohi   -MNSLTTFLAIIVILLLAMLFISFWIPQMNPDPEKLSPFECGFDPLGTARLPFSLRFFLV
Caar   -MNLITTVIAIATLLSIILAIVSFWLPQMSPDHEKLSPYECGFDPLGSARLPFSLRFFLV
Came   -MNLITTVIAIATLLSIILAIVSFWLPQMNPDHEKLSPYECGFDPLGSARLPFSLRFFLV
Mema   -MNLVTTIIAIAIALSTILAIVSFWLPQITPDHEKLSPYECGFDPLGSARLPFSLRFFLV
Lenu   -MNLLMTIIFISSALSVILAMVSFWLPQMNPDYEKLSPYECGFDPLGSARLPFSLRFFLI
Brja   -MNLITTIILIATALSTLLAIVSFWLPQMTPDHEKLSPYECGFDPLGSARLPFSLRFFLV
Plma   -MSLITTVIAIATALSTILAIVSFWLPQMTPDHEKLSPYECGFDPLGSARLPFSLRFFLV
Emst   -MNLVTTIITIAIVLSIILAIVSFWLPQMTPDHEKLSPYECGFDPLGTARLPFSLRFFLV
Ptti   -MNLVTTIIIIAIALSTILAIVSFWLPQMTPDHEKLSPYECGFDPLGSARLPFSLRFFLV
Losu   -VNLTLMTILITAGLSTALIVVSFWLPQVKPDYEKLSPYECGFDPLGSARLPFSIRFFLV
Geoy   -MNLITAVILIATALSVVLAIVSFWLPQMTPDHEKLSPYECGFDPLGSARLPFSLRFFLV
Dipi   -MNLVTVIMVIAIALSTILAIVSFWLPQMTPDHEKLSPYECGFDPLGSARLPFSLRFFLV
Pama   -MNLVTTILIITAILSIILAIVSFWLPQMSPDYEKLSPYECGFDPLGSARLPFSLRFFLV
Leob   -MNLMTTIMLISTALSIVLALVSFWLPQMTPDHEKLSPYECGFDPLGSARLPFSLRFFLV
Neba   -MNLIVLITTITAMLSVILAAVSFCLPQLTPDQEKLSPYECGFDPLGSARLPFSLRFFLV
Pdpl   -MNLLLTVVLICLLLATILATVSFWLPQMLPDHEKLSPYECGFDPLGTARLPFSLRFFLV
Nimi   -MNLITTIFIITTLLSAILATISFWLPQMTPDHEKLSPYECGFDPLGTARLPFSLRFFLV
Uptr   -MNLMTTIVAITVLLSTILAIVSFWLPLMNPDPEKLSPYECGFDPLGSARLPFSLRFFLV
Pesc   -MNLLTAMLSVTTVLSALLMFISFWLPQLSPDYEKLSPYECGFDPVGSARLPFSMRFFLV
Baar   -MNLITTILVIAIALSTLLAIVSFWLPQMSPDHEKLSPYECGFDPLGSARLPFSLRFFLV
Moar   -MNLITTIFIIAIALATILAIVSFWLPQMTPDHEKLSPYECGFDPLGTARLPFSLRFFLV
Toja   -MYLILTVIAITALLSTILITVSFWLPLLTPDHDKLSPYECGFDPLGTARLPFSLRFFLV
Chau   -MNLITAIFGISVLLSLILIMVAFWLPLMGPDHEKLSPYECGFDPLGTARLPFSLRFFLV
Chse   -MNLVMTILLITLTLSAILATVSFWLPQMTPDQEKLSPYECGFDPLGSARLPFSIRFFLV
Enar   -MNLITTIITIAILLSTVLAIVSFWLPQITPDYEKLSPYECGFDPLGSARLPFSLRFFLV
Hpty   -MNLTTTIIGIAVALSTILAIVSFWLPQMSPDHEKLSPYECGFDPLGSARLPFSLRFFLV
Nana   -MNLITTIISISATLTLALAIISFWLPMIKPDHEKLSPYECGFDPLGTARLPFSLRFFLV
Mcst   -MSLITTIIFITLAISVVLTIVSFWLPQMNPDHEKLSPYECGFDPLGTARLPFSLRFFLV
Rhox   -MNLIPTIIVIATVLSTVLALVSFWLPQMSPDHEKLSPYECGFDPLGTARLPFSLRFFLV
Opfa   -MNLVTTIITIAIALSTILAIVSFWLPQMSPDHEKLSPYECGFDPLGTARLPFSLRFFLV
Paar   -MNIITTVITITILLSSVLAIVSFWLPLMSPDHEKLSPYECGFDPVGSARLPFSLRFFLV
Gozo   -MNLVTTIITITIILSTILAIVSFWLPQMTPDHEKLSPYECGFDPLGSARLPFSLRFFLI
Ackr   -MPMLMTMIIFATALSALLMFISFWLPQMNPDQEKLSPYECGFDPLGTARLPFSLRFFLV
```

To be continued on page 9.

[1/2 of aligned sequences]

```
Elev      -MNLIMTIIMLTTALCALLAVVSFWLPLMNPDHEKLSPYECGFDPLGSARLPFSLRFFLI
Trdu      -MNLITTMLIISITLSTILAIISFWLPQMTPDHEKLSPYECGFDPLGSARLPFSLRFFLI
Amoc      -MNLVTTVILIAITLSMILAIVSFWLPQMTPDHEKLSPYECGFDPLGSARLPFSMRFFLV
Hame      MTSLITTCIMIALALSIVLALVSFWLPQMAPDYEKLSPYECGFDPLGSARLPFSLRFFLV
Chso      -MNIITTYIFVTILLSCIVAIISFWLPQMTPDYEKLSPYECGFDPLGSARLPFSLRFFLV
Lyto      -MNLITTVVTITAALSVLLALVSFWLPQMTPDHEKLSPYECGFDPLGTARLPFSLRFFLV
Encr      -MNLVTTVITITAALSVILAIVSFWLPQMTPDHEKLSPYECGFDPLGSARLPFSLRFFLV
Bvar      -MNLVTTMLLITAALSSLLALVSFWLPQMSPDNEKLSPYECGFDPLGSARLPFSLRFFLV
Noco      -MNLVMTIIAIASLLSTILAIVSFCLPQMSPDYEKLSPYECGFDPLGSARLPFSLRFFLI
Chsp      -MSLTTTIVMICFLLSTLLATISFWLPQMRPDHEKLSPYECGFDPLGSARLPFSIRFFLI
Arja      -MNTTVAMISIGALLSLVLALVAFWLPMKSVTLEKLSPFECGFDPGLSARLPFSLRFFLV
Pase      -MSLMVMIINITCILGALLMLVSFWLPQMMPDHEKLSPYECGFDPLGTARLPFSLRFFLV
Trel      -MNLLSAVLFVAAMLSALIAMVSFWLPQLTPDQEKLSPYECGFDPLGSARLPFSMRFFLV
Lifa      -MNMVIVTLAISILLSMVLAMVSFWLPLMTPDHEKLSPYECGFDPLGSARLPFSLRFFLV
Acur      -MNLFTTTILIILLITLALIIVSFWLPQMTPDYEKLSPYECGFDPLGSARLPFSLRFFLV
Ampe      -MNLIMTVITITIVLSTVLAIVSFWLPQMTPDHEKLSPYECGFDPLGSARLPFSLRFFLV
Urja      -MNLLMTIIITATTLSSILLLISFWLPQMSPDQEKLSPYECGFDPFNSARLPFSLRFFLV
Enet      -MNLITAVISISCLLSAILAIVSFWLPQITPDYEKLSPYECGFDPLGSARLPFSMRVFSG
Ptbr      -MNTLLAIIIIAILLSLLLSLITYWLPQITPDSEKLSPYECGFDPLGSARLPFSMRFFLV
Safa      -MNLIMIMITISAVLSTILALVSFWLPQMSPDHEKLSPYECGFDPLGSARLPFSMRFFLV
Icae      -MNLITTIIAITATLSIVLAIVSFWLPQMTPDHEKLSPYECGFDPLGSARLPFSLRFFLV
Asmi      --ITMMTMALVAMTLSTLLMIVSFWLPLMSPDYEKLSPYECGFDPLGSARLPFSMRFFLI
Foal      -MNTIVAVLFISTALALILAFISFYLPQMNPDPEKLSPYECGFDPLGSARLPFSLRFFLI
Drze      -MNLLLIILLISLTLSFIMAVVAFSFPPMTPDPEKLSPYECGFDPQGSARLPFSLRFFLV
Rhas      -MNLITTVIIISVALSTILAIVSFWLPLMSPDQEKLSPYECGFDPLGSARLPFSMRFFLV
Elac      -MNLVTTVIIIAVILSSVLAIVSFWLPMMSPDQEKLSPYECGFDPLGSARLPFSLRFFLV
Kugu      -MNLITTMILITLALSAILATISFWLPQMNPDQEKLSPYECGFDPLGSARLPFSLRFFLV
Plor      -MNLVMTVITITVALSTILAIVAFWLPQMSPDYEKLSPYECGFDPLGSARLPFSLRFFLV
Sgun      -MNLVTTIITIAAVLSAVLAVVSFWLPQMTPDHEKLSPYECGFDPLGSARLPFSLRFFLV
Zaco      -MNLITTIITITIVLSTVLAIVSFWLPQMSPDHEKLSPYECGFDPLGSARLPFSLRFFLV
Zbfl      -MNLIATIMLITVTLSIVLATVSFWLPQMTPDHEKLSPYECGFDPLGTARLPFSLRFFLI
Spba      -MSLITTIILITLILSVILATVSFWLPQMNPDYEKLSPYECGFDPLGSARLPFSLRFFLI
Game      -MSLITTIILIAAALSAILALVSFWLPQMTPDHEKLSPYECGFDPLGSARLPFSLRFFLV
Thth      -MSLITTIITIAAALSTVLAVVSFWLPQMTPDHEKLSPYECGFDPLGSARLPFSLRFFLV
Xigl      -MNLVTTVITIAVALSTVLAIVSFWLPQMTPDHEKLSPYECGFDPLGTARLPFSLRFFLV
Hyja      -MSLVPTIITIAIALSTVLALVSFWLPQMTPDHEKLSPYECGFDPLGSARLPFSLRFFLI
Psan      -MSLLTAIVMITTMLAIVLIFLAMWLPTLNPDYEKLSPFECGFDPMASARNPFSLHFFLV
Cupa      -MSLVTTIIAIAAVLSTILAIVSFWLPQITPDHEKLSPYECGFDPLGSARLPFSLRFFLV
Mpch      -MNLFTTIITLTIGLSIILAIVSFWLPQMNPDYEKLSPYECGFDPLGTARLPFSLRFFLV
Char      -MSLIITSIALALLLTLILALVSFWLPHMNSDSEKLSPYECGFDPLGSARLPFSLRFFLV
Pser      -MNLVTSAITIAIILSLVLALVSFWLPQMTPDHEKLSPYECGFDPLGTARLPFSLRFFLV
Prol      -MSLLMTIITITALLSTILAIVSFWLPQISPDHEKLSPYECGFDPMGSARLPFSLRFFLI
Plbi      -MSLLLTIITITALLSTVLAIVSFWLPQITPDHEKLSPYECGFDPMGSARLPFSLRFFLI
Calu      -MSLLMTIIGIASILCWVLVIVSFWLPIMNPNHEKLSPYECGFDPVGSARLPFSVRFFLV
Papa      -MN-VMLMVIIATTLSAILITISYLLPQTNPYYEKLTPYECGFDPIGSARLPFSMHFFLV
Sufr      -MNLIATIILLTTLLSAILAIVSFWLPQMTPDHEKLSPYECGFDPLGSARLPFSLRFFLI
Stci      -MNLITAIVLIPIVLSSVLALVSFWLPLMTPDHEKLSPYECGFDPLGSARLPFSMRFFLV
Taru      -MNLLTTMLIITTALSLILMTVSFWLPALTPDYQKLSPYECGFDPLGSARLPFSLRFFLV
Rala      -MNLVTTIITIAVALSTVLALVSFWLPQMTPDHEKLSPYECGFDPLGTARLPFSLRFFLV
                                                 * :*:****:*  ::* ***::. *
                         :   :
```

To be continued on page 10.

[2/2 of aligned sequences]

```
                 B                                     C
Scca  AILFLLFDLEIALLLPLPWGNQLFSPFSTLLWTTTILVLLTLGLIYEWFQGGLEWAE-
Muma  AILFLLFDLEIALLLPLPWGNQLLTPLYTLLWAAAILILLTLGLIYEWLQGGLEWAE-
Erca  AILFLLFDLEIALLLPLPWSIHL-NPLYVLMWAFMIIMLLTIGLIYEWVQGGLEWAE-
Pose  AILFLLFDLEIALLLPLPWGIHL-DPMLMLTWAFVIIMLLTVGLIYEWLQGGLEWAE-
Actr  AILFLLFDLEIALLLPLPWGDQLASPTIALLWTTTILSLLTLGLIYEWTQGGLEWAE*
Scal  AILFLLFDLEIALLLPLPWGDQLASPTITLFWATTILILLALGLVYEWTQGGLEWAE-
Posp  AILFLLFDLEIALLLPLPWGDQLASPTTALLWAMAILVLLTLGLIYEWTQGGLEWAE-
Atsp  AILFLLFDLEIALLLPLPWSDQLASPVTTLIWATMILTLLTLGLIYEWLQGGLEWAE-
Leoc  AILFLLFDLEIALLLPLPWSDQLTSPTTTLIWATVILMLLTLGLIYEWLQGGLEWAE-
Amca  AILFLLFDLEIALLLPLPWGDQLISPSLTLFWATSILILLTLGLIYEWLQGGLEWAE-
Osbi  AILFLLFDLEIALLLPLPWGDQLTSPTQTLTWASIILVLLTLGLIYEWIQGGLEWAE-
Pabu  AILFLLFDLEIALLLPLPWGDQLYMPIHTFFWAAAILMLLTLGLIYEWTQGGLEWAE-
Hial  AILFLLFDLEIALLLPLPWGNQLSDPTETFFWATAVLILLTLGLVYEWIQGGLEWAE-
Elha  AILFLLFDLEIALLLPLPWGDQLLSPTETLFWATAILILLTLGLIYEWTQGGLEWAE-
Mlcy  AILFLLFDLEIALLLPLPWGDQLLSPTETFFWATTILTLLTLGLAYEWTQGGLEWAE-
Algl  AILFLLFDLEIALLLPLPWGNQLPVPTETFYWATILLVLLTLGLAYEWAQGGLEWAE-
Ptgi  AILFLLFDLEIALLLPLPWGDQLLVPMQTFLWATAVLILLTLGLIYEWTQGGLEWAE-
Alaf  AILFLLFDLEIALLLPLPWGNQLLIPMQTFFWASAILILLTLGLIYEWLQGGLEWAE-
Nock  AILFLLFDLEIALLLPLPWGNQLLIPMQTFFWASSILVLLTLGLIYEWLQGGLEWAE-
Anja  AILFLLFDLEIALLLPLPWGDQLPNTTHTFFWAMSIIILLTLGLVYEWIQGGLEWAE-
Gyki  AILFLLFDLEIALLLPLPWGNQLVTTTQTLFWATLIIILLTIGLAYEWAQGGLEWAE-
Syka  AILFLLFDLEIALLLPLPWGDQLPDPAQTFFWATSILVLLTLGLAYEWAQGGLEWAE-
Opma  AILFLLFDLEIALLLPLPWGDQLPDAIQTLFWAMFILILLTAGLIYEWIQGGLEWAE-
Comy  AILFILFDLEIALLLPLPWSDQLPNVIQTFFWAMSILFLLTVGLIYEWVQGGLEWAE-
Sasp  AILFLLFDLEIALLLPLPWMAELPDTLNSFFWATLIVILVTLGFAYEWVKGGLEWAE*
Eupe  AILFLLFDLEIALLLPLPWGTQLPNVLNTLSWAISIIILLTLGLAYEWTQGGLEWAE*
Enja  AILFLLFDLEIALLLPLPWGNQLLEPKTTLIWVIAVLGLLTLGLVYEWLQGGLEWAE-
Same  AILFLLFDLEIALLLPLPWGTQLLEPLTTVLWATAILVILTLGLVYEWIQGGLEWAE-
Chch  AILFLLFDLEIALLLPLPWGNQLLLPSTTFFWAAAVLILLTLGLIYEWTQGGLEWAE-
Grgr  AILFLLFDLEIALLLPLPWGDQLPLPSNTLFWAAAVLVLLTLGLIYEWAQGGLEWAE*
Caau  AILFLLFDLEIALLLPLPWGDQLNNPTGTFFWATTVLILLTLGLIYEWTQGGLEWAE-
Cyca  AILFLLFDLEIALLLPLPWGDQLHNPTGTFFWATTVLILLTLGLIYEWTQGGLEWAE-
Dare  AVLFPLFDLEIALLLPLPWGDQLNNPMETLFWAMTVLILLTLGLAYEWAQGGLEWAE-
Cost  AILFLLFDLEIALLLPLPWGDQLFNPAGTLLWASAVLILLTLGLIYEWTQGGLEWAE-
Leec  AILFLLFDLEIALLLPLPWGDQLINPTGTFFWATAVLILLTLGLVYEWVQGGLEWAE-
Fola  AILFLLFDLEIALLLALPWGDQLYSATGTFFWATAVLILLTLGLIYEWTQGGLEWAE-
Clmc  AILFLLFDLEIALLLPLPWGNQLSNPTLTFFWAAAILTLLTLGLIYEWMQGGLEWAE-
Phin  AILFLLFDLEIALLLPLPWGNQL-HPAQTLFWATTILILLTLGLVYEWLQGGLEWAE-
Icpu  AILFLLFDLEIALLLPLPWGNQLLTPAYTLLWAATILILLTLGLIYEWVQGGLEWAE-
Psto  AILFLLFDLEIALLLPLPWGNQLPDPSYTLLWAATVLILLTLGLIYEWVQGGLEWAE-
Cora  AILFLLFDLEIALLLPLPWANQLPEPTITFLWASIILILLTAGLIYEWIQGGLEWAE-
Eisp  AILFLLFDLEIALLLPLPWSNQLHTPTTTFLWATAILALLTLGLIYEWIQGGLEWAE-
Apal  AILFLLFDLEIALLLPLPWANQLHAPTTTFIWATLILTLLTLGLIYEWIQGGLEWAE*
Eslu  AILFLLFDLEIALLLPLPWADQLSSPTLTFLWATTILTLLTLGLIYEWIQGGLEWAE-
Dape  AILFLLFDLEIALLLPLPWANQLPAPSFTLFWTATVLSLLTLGLIYEWVQGGLEWAE-
Glse  AILFLLFDLEIALLLPLPWGDQLLTPLLTFSWTTSILILLTLGLAYEWVQGGLEWAE*
Naar  AILVLLFDLEIALLLPLPWGDQLLTPTTTFLWVTAVLTLLTLGLAYEWAQGGLEWAE*
Lioc  AILFLLFDLEIALLLPLPWGDQLPTPTITFLWVTAVLTLLTLGLAYEWAQGGLEWAE-
Opso  AILFLLFDLEIALLLPLPWGDQLLTPFMTLLWATTVLALLTLGLAYEWVQGGLEWAE*
Alte  AILFLLFDLEIALLLPLPWGDQLLTPINTFLWATAVLALLTLGLIYEWTQGGLEWAE-
Plap  AILFLLFDLEIALLLPLPWGDQLLAPVNTFLWATVVLALLTLGLVYEWTQGGLEWAE-
```

[2/2 of aligned sequences]

Plal	AILFLLFDLEIALLLPLPWGDQLAAPAITFTWAVAVLALLTLGLIYEWVQGGLEWAE*
Sami	AILFLLFDLEIALLLPLPWGDQLDAPATTFTWAVAVLTLLTLGLIYEWIQGGLEWAE-
Rere	AILFLLFDLEIALLLPLPWGDQLVSPTLTFTWAVAVLALLTLGLIYEWVQGGLEWAE-
Gama	AILFLLFDLEIALLLPLPWGDQLATPSATFAWAVAVLTLLTFGLIYEWVQGGLEWAE-
Onmy	AILFLLFDLEIALLLPLPWGDQLHTPTLTLIWSTAVLALLTLGLIYEWTQGGLEWAE-
Sasa	AILFLLFDLEIALLLPLPWGDQLTTPALTLAWSAAVLALLTLGLIYEWTQGGLEWAE-
Cola	AILFLLFDLEIALLLPLPWGDQLDTPTLTLAWSAAVLTLLTLGLIYEWTQGGLEWAE-
Dita	AILFLLFDLEIALLLPLPWGDQLSTPSTTLFWTTTVLALLTIGLAYEWSQGGLEWAE-
Gogr	AILFLLFDLEIALLLPLPWASQLSSPSITLAWTFVILILLTLGLAYEWTQGGLEWAE-
Chsl	AILFLLFDLEIALLLPLPWGDQMTAPFITFAWAAVILTLLTLGLAYEWAQGGLEWAE*
Atja	AILFLLFDLEIALLLPLPWADQMPTPTQTVLWATTVLILLTLGLIYEWTQGGLEWAE-
Iido	AILFLLFDLEIALLLPLPWADQMPAPTETLLWATTVLILLTLGLIYEWTQGGLEWAE-
Auja	AILFLLFDLEIALLLPLPWGDQLTTPISTFCWTTIVLSLLTLGLAYEWTQGGLEWAE-
Chag	AILFLLFDLEIALLLPLPWGDQLTTPTHTFYWATAVLALLTLGLAYEWTQGGLEWAE-
Hami	AILFLLFDLEIALLLPLPWGDQLDSPLLTLSWATTILILLTLGLVYEWIQGGLEWAE-
Saun	AILFLLFDLEIALLLPLPWGDQLATPTLTLSWTTAILILLTLGLVYEWIQGGLEWAE-
Nema	AILFLLFDLEIALLLPLPWGDQMSSPLHTFMWATAVLWLLILGLVYEWTQGGLEWAE-
Disp	AILFLLFDLEIALLLPLPWGDQLANPLFTFLWATSVLWLLILGLIYEWTQGGLEWAE-
Myaf	AILFLLFDLEIALLLPLPWGDQLTNPLSTFLWATSVLWLLILGLIYEWTQGGLEWAE*
Lagu	AILFLLFDLEIALLLPLPWGDQLPFPSTTFTWAASVLFLLTLGLIYEWLQGGLEWAE-
Trtr	AILFLLFDLEIALLLPLPWGNHLASPLETFTWASLVLILLTLGLIYEWYQGGLEWAE-
Zucr	AILFLLFDLEIALLLPLPWGNHLACPQETFIWATLVLILLTLGLIYEWSQGGLEWAE-
Pxja	AILFLLFDLEIALLLPLPWGDQLANPLFTFSWATAVLVLLTLGLVYEWTQGGLEWAE-
Pxlo	AILFLLFDLEIALLLPLPWGDQLANPLFTFSWATAVLVLLTLGLVYEWTQGGLEWAE-
Pctr	AILFLLFDLEIALLLPLPWADQLVSPTNTLAWATLVLLLLTLGLIYEWTQGGLEWAE-
Apsa	AILFLLFDLEIALLLPIPWANQLTSPTHTLMWATAVLLLLTAGLIYEWKQGGLEWAE-
Cabe	AILFLLFDLEIALLLPLPWADQAPSPLMTLTWTNIVLALLTLGLAYEWVQGGLEWAE-
Bzze	AILFLLFDLEIALLLPLPWGDQLVSPLTTFIWATTVLALLTLGLIYEWMQGGLEWAE-
Siim	AILFLLFDLEIALLLPLPWGDHMPSPMVTFLWAASLIILLTLGLIYEWLQGGLEWAE-
Ctru	AILFLLFDLEIALLLPLPWGDQLASPVLTFVWATTVLLLLTLGLIYEWMQGGLEWAE-
Dpbr	AILFLLFDLEIALLLPLPWGDQLSSPVLTFIWAAIVLFLLTLGLIYEWIQGGLEWAE-
Caki	AILFLLFDLEIALLLPIPWADQLDNPSLTFLWTSCVLLLLTLGLIYEWLQGGLEWAE-
Phja	AILFLLFDLEIALLLPLPWGDQLNNPSLTLLWAMSVIILLTLGLIYEWLQGGLEWAE-
Brsp	AILFLLFDLEIALLLPLPWGNQLLNPLSSFFWATTVLILLTLGLIYEWIQGGMEWAE-
Gamo	AILFLLFDLEIALLLPLPWGDQLSNPTLTFMWATSVLALLTLGLIYEWLQGGLEWAE-
Lolo	AILFLLFDLEIALLLPLPWGDQLSNPALTFMWATSVLALLTLGLIYEWLQGGLEWAE-
Batr	AILFLLFDLEIALLLPLPWGSHLENPTITASLASMILILLTLGLIYEWFHGALEWAE*
Prmy	AILFLLFDLEIALLLPLPWSSHLCSPMVT-TWASSILLILTVGLAYEWSQGGLEWAE*
Lose	AILFLLFDLEIALLLPLPWGDQLISPLTTFQWAATIIFLLTLGLVYEWKQGGLEWAE-
Loam	AILFLLFDLEIALLLPLPWGDQLASPLMTFLWATAVLVLLTLGLIYEWLQGGLEWAE-
Chab	AILFLLFDLEIALLLPLPWGNQLASPLLTVTWASIVLILLTLGLIYEWLQGGLEWAE-
Chto	AILFLLFDLEIALLLPLPWGNQLTSPLLTVAWASIVLILLTLGLIYEWLQGGLEWAE-
Majo	AILFLLFDLEIALLLPLPWGNQLSSPTLTFTWATLVLTLLTLGLIYEWIQGGLEWAE-
Hlst	AILFLLFDLEIALLLPIPWGNQLESPLLTFLWATLVLVILALGLLYEWLQGGLEWAE-
Clpe	AILFLLFDLEIALLLPLPWGVQLEAPHLTFYWATLVLALLTVGLIYEWLQGGLEWAE-
Mlmr	AILFLLFDLEIALLLPLPWGSQLEFPLLTFFWASLVLVLLTLGLVYEWLQGGLEWAE-
Crcr	AILFLLFDLEIALLLPLPWGDQLPSPLTTLVWALLILTLLTLGLIYEWTQGGLEWAE-
Muce	AILFLLFDLEIALLLPLPWGDQLPSPLTTLVWALLILTLLTLGLIYEWTQGGLEWAE-
Bege	AILFLLFDLEIALLLPLPWGDQLPTPLTTFAWASAVLILLTLGLIYEWAQGGLEWAE-
Mela	AILFLLFDLEIALLLPLPWGDQLLSPLMAFTWAATVLLLLTLGLIYEWVQGGLEWAE-
Hats	AILFLLFDLEIALLLPLPWGDQLSTPLTTFTWAAAVLVLLTLGLIYEWLQGGLEWAE-
Orla	AILFLLFDLEIALLLPLPWGDQLSSPLMTFFWASAILMLLTLGLIYEWLQGGLEWAE-

[2/2 of aligned sequences]

```
Cosa    AILFLLFDLEIALLLPLPWGDQLPSPVMTFSWASIVLVLLTLGLIYEWTQGGLEWAE-
Exsp    AILFLLFDLEIALLLPLPWGDQLSSPLTTFIWASAILGLLTLGLIYEWIQGGLEWAE-
Depa    AILFLLFDLEIALLLPLPWGDQLSFPMTTFSWASTILILLTLGLIYEWTQGGLEWAE-
Rima    AILFLLFDLEIALLLPLPWGDQFQTPLITFMWASTVLIFLTLGFAYEWAQGGLEWAE-
Fuol    AILFLLFDLEIALLLPLPWGDQLPSPMTTFSWASLVLILLTLGLIYEWIQGGLEWAE-
Gmaf    AILFLLFDLEIALLLPLPWGDQLNNPHLTFTWATILLILLTLGLIYEWIQGGLEWAE-
Xeei    AILFLLFDLEIALLLPLPWGNQLSTPLLTFGWASTILTLLTLGLIYEWIQGGLEWAE-
Pros    AILFLLFDLEIALLLPLPWGDQLPSPLHTFTWATAVLVLLTLGLIYEWLQGGLEWAE-
Scmi    AILFLLFDLEIALLLPLPWGDQLPDPRETLTWATAVLLLLTLGLIYEWMQGGLEWAE-
Rolo    AILFLLFDLEIALLLPLPWGDQLTTPLLTFVWATAVLALLTLGLIYEWTQGGLEWAE-
Cere    AILFLLFDLEIALLLPLPWGVQLISPLLTFTWATAVLVLLTLGLIYEWAQGGLEWAE-
Daga    AILFLLFDLEIALLLPLPWGNQLLSPLLTFFWAVVILTLLTLGLIYEWIQGGLEWAE-
Anco    AILFLLFDLEIALLLPLPWGDQLATPLLTFTWATAILVLLTLGLIYEWMQGGLEWAE-
Dmve    AILFLLFDLELALLLPLPWGNQLTTPFYTFAWAAAILGLLTLGLIYEWIQGGLEWAE-
Dmar    AILFLLFDLELALLLPLPWGNQLITPFYTFAWAAAILGLLTLGLIYEWIQGGLEWAE-
Anka    AILFLLFDLEIALLLPLPWGDQLTTPLLTFTWATAVLVLLTLGLIYEWIQGGLEWAE-
Moja    AILFLLFDLEIALLLPLPWGDQLTTPLLTFIWATAVLVLLTLGLVYEWTQGGLEWAE-
Hoja    AILFLLFDLEIALLLPLPWGDQLATPLLTFTWATAVLALLTLGLIYEWIQGGLEWAE-
Bede    AILFLLFDLEIALLLPLPWGDQLVSPLYTFIWATAVLMLLTLGLIYEWTQGGLEWAE-
Besp    AILFLLFDLEIALLLPLPWGDQLVSPLYTFIWATAVLMLLTLGLIYEWTQGGLEWAE-
Mysp    AILFLLFDLEIALLLPLPWGDQLASPLLTFTWAITILVLLTLGLIYEWTQGGLEWAE-
Osja    AILFLLFDLEIALLLPLPWGDQLSSPLLTFTWAITILVLLTLGLIYEWTQGGLEWAE-
Sgro    AILFLLFDLEIALLLPLPWGDQLSSPLLTFTWAITILLLLTLGLIYEWTQGGLEWAE-
Pzpa    AILFLLFDLEIALLLPLPWGDQLHNPTMTFLWALIVLMLLTLGLVYEWLQGGLEWAE-
Zeja    AILFILFDLEIALLLPLPWGDQLSSPTQTLIWATTVLTLLTLGLIYEWIQGGLEWAE-
Znne    AILFILFDLEIALLLPLPWGDQLTSPTLTFIWATTVLALLTLGLVYEWLQGGLEWAE-
Zefa    AILFLLFDLEIALLLPLPWGDQLTNPTLTFIWAATVLVLLTLGLIYEWLQGGLEWAE-
Acni    AILFLLFDLEIALLLPLPWGDQLNNPTLTFIWATAVLTLLTLGLIYEWLQGGLEWAE-
Ncrh    AILFLLFDLEIALLLPLPWGDQLNDPTLTFIWATAVLMLLTLGLIYEWLQGGLEWAE-
Agca    AILFLLFDLEIALLLPLPWGDQLSSPLLTFLWAAAVLTLLTLGLIYEWLQGGLEWAE-
Hydy    AILFLLFDLEIALLLPLPWGDQLTSPLSTFLWATAVLILLTLGLIYEWAQGGLEWAE-
Gsac    AILFLLFDLEIALLLPLPWGDQLASPLFTFLWATAVLTLLTLGLIYEWMQGGLEWAE-
Pevo    AILFLLFDLEIALLLPLPWGDQMPVPLMTFFWASAVLILLTLGLVYEWIQGGLEWAE-
Hiku    AILFLLFDLEIALLLPLPWGMQLMSPFYTFMWASSVVILLTLGLIYEWIQGGLEWAE-
Inpa    AILFLLFDLEIALLLPLPWANQLTHPPLTLFLASLVLVLLTLGLIYEWAQGGLEWAE-
Auch    AILFLLFDLEIALLLPTPWGDQLPAPLTTFFWVSAILILLILGFIYEWAEGGLEWAE-
Fico    AILFLLFDLEIALLLPLPWGDQLPFPLTTFVWASSVLLLLTLGLIYEWLQGGLEWAE-
Macs    AILFLLFDLEIALLLPLPWGDQLSSPLVTFLWAASVLTLLTIGLIYEWVQGGLEWAE-
Moal    AILFLLFDLEIALLLPLPWSSQLPLPNHTFFWASTILILLTLGLIYEWQQGGLEWAE-
Syma    AILFLLFDLEIALLLPLPWGSQLTNPLLTLFWASTVLLLLTIGLIYEWLQGGLEWAE-
Mafr    AILFLLFDLEIALLLPLPWGDQLSSPLLTFTWASTILTLLTLGLIYEWMQGGLEWAE-
Dcpe    AILFLLFDLEIALLLPLPWGDQLTSPVTTFLWASSVLILLTLGLIYEWLQGGLEWAE-
Dcti    AILFLLFDLEIALLLPLPWGDQLASPLTTFLWASSVLILLTLGLIYEWLQGGLEWAE-
Hehi    AILFLLFDLEIALLLPLPWGDQLTSPLLTLFWAVAVLILLTLGLIYEWIQGGLEWAE-
Stam    AILFLLFDLEIALLLPLPWGDQLTSPLLTFLWAAAVLALLTLGLIYEWTQGGLEWAE-
Hogi    AILFLLFDLEIALLLPLPWGDQLSSPLLTFFWASAVLILLTLGLIYEWLQGGLEWAE-
Erzo    AILFLLFDLEIALLLPLPWGDQLTSPLLTFAWATAVLALLTLGLIYEWLQGGLEWAE-
Hxot    AILFLLFDLEIALLLPLPWGDQLTSPLLTFLWAAAVLALLTLGLIYEWLQGGLEWAE-
Core    AILFLLFDLEIALLLPLPWGDQLTAPLTTFLWATAVLMLLTLGMIYEWLQGGLEWAE-
Apve    AILFLLFDLEIALLLPLPWGDQLASPMLTLLWATTILTLLTLGLIHEWLQGGLEWAE-
Latj    AILFLLFDLEIALLLPLPWGDQLESPLLTFLWASAILSLLTLGLIYEWMQGGLEWAE-
Laja    AILFLLFDLEIALLLPLPWGDQLSSPLLTFFWASVVLILLTLGLIYEWTQGGLEWAE-
```

[2/2 of aligned sequences]

```
Syja    AILFLLFDLEIALLLPLPWGDQLTTPLLTFIWASAVLILLTLGLIYEWTQGGLEWAE-
Epme    AILFLLFDLEIALLLPLPWGDQLASPMLTFLWASVVLALLTLGLIYEWLQGGLDWAE-
Grse    AILFLLFDLEIALLLPLPWGDQLPSPLLTFLWASAVLLLLTLGLIYEWIQGGLDWAE-
Clja    AILFLLFDLEIALLLPLPWGTQTPHPLFTLISASTFLLLLAVGLIYEWLQGGLEWAE-
Ogcy    AILFLLFDLEIALLLPLPWADQLPSPLTTFTWASTVLLLLTLGLIYEWLQGGLEWAE-
Plna    AILFLLFDLEIALLLPLPWGDQLPTPTMTFSWASSILVLLTLGLIYEWIQGGLEWAE-
Lema    AILFLLFDLEIALLLPLPWGDQLTSPTTTFLWASAVLALLTLGLIYEWIQGGLEWAE-
Etzo    AILFLLFDLEIALLLPLPWGDQLAIPLLTFLWASAVLALLTLGLIYEWLQGGLEWAE-
Apse    AILFLLFDLEIVLLLPLPWSNQLLSPLLTFSWAAAILILLTLGLVYEWMQGGLEWAE-
Epde    AILFLLFDLEIALLLPLPWGDQLSSPLLTFTWATAVLALLTLGLIYEWMQGGLEWAE-
Slja    AILFLLFDLEIALLLPLPWGDQLPSPLLTFIWASSVLGLLTLGLVYEWLQGGLEWAE-
Bsja    AILFLLFDLEIALLLPLPWGDQLSSPLLTFFWASAVLIILTLGLIYEWLQGGLEWAE-
Ecna    AILFLLFDLEIALLLPLPWGDQLDNPLLTFTWASLVLLLLTLGLVYEWIQGGLEWAE-
Cohi    AILFLLFDLEIALLLPLPWGIQLESPLLTFVWISTVLVLLTIGLIYEWSQGGLEWAE-
Caar    AILFLLFDLEIALLLPLPWGDQLDTPVVTFLWASLVLALLTLGLIYEWIQGGLEWAE-
Came    AILFLLFDLEIALLLPLPWGDQLDTPILTFLWASLVLALLTLGLIYEWIQGGLEWAE-
Mema    AILFLLFDLEIALLLPLPWGDQLSSPLLTLSWASIVLILLTLGLVYEWMQGGLEWAE-
Lenu    AILFLLFDLEIALLLPLPWGNQLLSPLTTLIWASAVLAILTLGLIYEWVQGGLEWAE-
Brja    AILFLLFDLEIALLLPLPWGDQLPSPLTTFFWASSVLILLTLGLIYEWLQGGLEWAE-
Plma    AILFLLFDLEIALLLPLPWGDQLPLPLTSFLWASAVLILLTLGLIYEWLQGGLEWAE-
Emst    AILFLLFDLEIALLLPLPWGDQLSSPLITFLWASAVLVLLTLGLIYEWLQGGLEWAE-
Ptti    AILFLLFDLEIALLLPLPWGDQLSSPLLTFFWASAVLILLTLGLIYEWLQGGLEWAE-
Losu    AILFLLFDLEIALLLPLPWSDQLPSPTLTFFWTSAILTLLTTGLIYEWTQGGLEWAE-
Geoy    AILFLLFDLEIALLLPLPWGDQLSSPLLTFSWASAVLVLLTLGLIYEWLQGGLEWAE-
Dipi    AILFLLFDLEIALLLPLPWGDQLSSPLLTFLWATAVLTLLTLGLIYEWLQGGLEWAE-
Pama    AILFLLFDLEIALLLPLPWGDQLPSPLLTLLWAFAVLVLLTLGLIYEWLQGGLEWAE-
Leob    AILFLLFDLEIALLLPLPWGDQLPSPLLTFTWASAVLVLLTLGLIYEWVQGGLEWAE-
Neba    AILFLLFDLEIALLLPLPWGNQASTPEMTFFWASMVLIILTLGLIYEWIQGGLEWAE-
Pdpl    AILFLLFDLEIALLLPLPWGVQLSSPTLTFFWASALLILLTLGLVYEWLQGGLEWAE-
Nimi    AILFLLFDLEIALLLPLPWGDQLLSPLLTFLWATAVLALLTLGLIYEWLQGGLEWAE-
Uptr    AILFLLFDLEIALLLPLPWGDQLSDPLTTFFWATAVLVLLTLGLIYEWFQGGLEWAE-
Pesc    AILFLLFDLEIALLLPLPWCVQLPSPLATFTWATLILVLLTVGLVYEWLQGGLEWAE-
Baar    AILFLLFDLEIALLLPLPWGDQLASPLLTFLWASIVLALLTLGLIYEWTQGGLEWAE-
Moar    AILFLLFDLEIALLLPLPWGDQLSSPLLTFLWASAVLILLTLGLIYEWLQGGLEWAE-
Toja    AILFLLFDLEIALLLPLPWGDQLVSPLLTFFWATAVLVLLILGLIYEWTQGGLEWAE-
Chau    AILFLLFDLEIALLLPLPWGDQLTSPLLTFLWAAAVLTLLTLGLVYEWLQGGLEWAE-
Chse    AILFLLFDLEIALLLPLPWGDQLTSPLMTFLWASAVLALLTLGLAYEWLQGGLEWAE-
Enar    AILFLLFDLEIALLLPLPWGDQLSSPLLTFLWASAVLILLTLGLIYEWMQGGLEWAE-
Hpty    AILFLLFDLEIALLLPLPWGDQLPSPLLTFLWASTVLVLLTLGLIYEWTQGGLEWAE-
Nana    AILFLLFDLEIALLLPLPWGDQLPSPLLTLTWALVVLILLTLGLVYEWTQGGLEWAE-
Mcst    AILFLLFDLEIALLLPLPWGDQLSSPLTTFMWASTVLLLLTLGLIYEWLQGGLEWAE-
Rhox    AILFLLFDLEIALLLPLPWGDQLSSPLLTFLWATAVLVLLTLGLIYEWLQGGLEWAE-
Opfa    AILFLLFDLEIALLLPLPWGDQLSSPLTTFLWATTVLALLTLGLIYEWLQGGLEWAE-
Paar    AILFLLFDLEIALLLPLPWGDQLLYPLITFSWATSILILLTLGLIYEWMQGGLEWAE-
Gozo    AILFLLFDLEIALLLPLPWGDQLSCPLTTFLWASAVLGLLTLGLVYEWVQGGLDWAE-
Ackr    AILFLLFDLEIALLLPLPWGNQLPSPMLTFLWASAILMILTLGLVYEWLQGGLEWAE-
Elev    AILFLLFDLEIALLLPLPWSNQLISPTTTFLWASAVLTLLTLGLIYEWIQGGLEWAE-
Trdu    AILFLLFDLEIALLLPLPWGDQLSSPLMTFTWAFAVLVLLTLGLIYEWIQGGLEWAE-
Amoc    AILFLLFDLEIALLLPLPWGDQLSSPLLTFTWAFAILVLLTLGLIYEWLQGGLEWAE-
Hame    AILFLLFDLEIALLLPLPWGDQLTSPLLTFFWASAVLMLLTIGLIYEWIQGGLEWAE-
Chso    AILFLLFDLEIALLLPLPWGNQLSSPLTTFIWATAILVLLTLGLIYEWIQGGLEWAE-
Lyto    AILFLLFDLEIALLLPLPWGDQLASPLLTFLWATAVLTLLTLGLIYEWLQGGLEWAE-
```

[2/2 of aligned sequences]

```
Encr   AILFLLFDLEIALLLPLPWGDQLASPLFTFLWATTVLVLLTLGLIYEWLQGGLEWAE-
Bvar   AILFLLFDLEIALLLPLPWGDQLASPLLTFSWASVVLSLLTLGLIYEWAQDGLEWAE-
Noco   AILFLLFDLEIALLLPLPWGDQLASPLVSFSWATALLTLLTLGLIYEWAQDGLEWAE-
Chsp   AILFLLFDLEIALLLPLPWGNQLPIPSLTFMWATTIVILLTLGLIYEWLKGGLEWAE-
Arja   AILFLLFDLEIALLLPLPWGDQQACPLNAVILTAAVLVILTLGLVYEWLQGGLEWAE-
Pase   AILFLLFDLEIALLLPLPWGDQLASPLITFLWAATVLAILTLGLIYEWVQGGLEWAE-
Trel   AILFLLFDLEIALLLPLPWADQLPSPQTTVTWTAVVLILLTLALVYEWLQGGLEWAE-
Lifa   AILFLLFDLEIALLLPIPWGNQLSCPTMTFLWASAVLTLLILGLVYEWLQGGLEWAE-
Acur   AILFILFDLEIALLIPLPWGDQLPSPLTTVTWASTIIVLLTLGLVYEWVQGGLEGAE-
Ampe   AILFLLFDLEIALLLPLPWGDQLPSPLITFIWASAVLALLTLGLIYEWIQGGLEWAE-
Urja   AILFLLFDLEIALLLPLPWANQFSPPILTFMWATIILALLTIGLAYEWLQGGLEWAE-
Enet   AILFLLFDLEIALYTPFPWGDQLFSPLTTFTWAAALFILLTAGLIYEWLQGGLEWAE-
Ptbr   AILFLLFDLEIALLLPLPWSNQLISPLTTFLWASLLLALLTLGLIYEWLQGGLEWAE-
Safa   AILFLLFDLEIALLLPLPWGDQLSSPLTTFLWAALVLALLTLGLIYEWIQGGLEWAE-
Icae   AILFLLFDLEIALLLPLPWGDQLPSPITTFLWASAVLVLLTLGLIYEWLQGGLEWAE-
Asmi   AILFLLFDLEIALLLPLPWADQLSSPLFTFLWTTVLLTLLTLGLVYEWLQGGLEWAE-
Foal   AILFLLFDLEIALLLPLPWADHLPSPVTTFAWAALILLMLTAGLIYEWSQGGLEWAE-
Drze   AILFLLFDLEIALLLPLPWGCQLPSPLLTFTLASSIIIILTLGLIYEWLQGGLEWAE-
Rhas   AILFLLFDLEIALLLPLPWGDQLPTPLLTFSWASLVLLLLTLGLIYEWIQGGLEWAE*
Elac   AILFLLFDLEIALLLPLPWGDQLPAPTITFFWAALVLLLLTLGLIYEWAQGGLEWAE-
Kugu   AILFLLFDLEIALLLPLPWGDQLPSPLLTFTWATIVLLLLTLGLIYEWTQGGLEWAE-
Plor   AILFLLFDLEIALLLPLPWGDQLPSPLLTFLWAAAVLILLTLGLIYEWLQGGLEWAE-
Sgun   AILFLLFDLEIALLLPIPWGDQLSSPLLTFLWASAVLVILTLGLIYEWLQGGLEWAE-
Zaco   AILFLLFDLEIALLLPLPWGDQLSSPLFTFLWASSVLALLTLGLIYEWLQGGLEWAE-
Zbfl   AILFLLFDLEIALLLPLPWGDQLPSPLLTFLWASAVLALLTLGLIYEWLQGGLEWAE-
Spba   AILFLLFDLEIALLLPLPWGDQLASPLLTFLWASAVLILLTLGLIYEWLQGGLEWAE-
Game   AILFLLFDLEIALLLPLPWGDQLPSPLLTFLWASAVLALLTLGLIYEWLQGGLEWAE-
Thth   AILFLLFDLEIALLLPLPWGDQLPSPLSTFLWASTVLVLLTLGLIYEWLQGGLEWAE-
Xigl   AILFLLFDLEIALLLPLPWGDQLASPLLTFFWASAVLILLTLGLIYEWMQGGLEWAE-
Hyja   AILFLLFDLEIALLLPLPWGDQLPSPLTTFFWASAILILLTLGLIYEWLQGGLEWAE-
Psan   AILFLLFDLEIALLLPLPWADQLSSPLTTFFLTFVVLALLTSGLLYESVQGGLEWAQ-
Cupa   AILFLLFDLEIALLLPLPWGDQLTSPLTTFLWAAAVLTLLTLGLIYEWLQGGLEWAE-
Mpch   AILFLLFDLEIALLLPLPWGDQLMSPLITFMWASAILILLTLGLIYEWIQGGLEWAE-
Char   AILFLLFDLEIALLLPLPWGDQLTAPFLTFVWATIVLSLLTLGLIYEWLQGGLEWAE-
Pser   AILFLLFDLEIALLLPLPWGDQLSFPLHTFLWASTILILLTAGLIYEWIQGGLEWAE-
Prol   AILFLLFDLEIALLLPLPWGDQLPTPLLTFTWATAVLFLLTLGLIYEWIQGGLEWAE-
Plbi   AILFLLFDLEIALLLPLPWGDQLASPLLTFTWATAVLSLLTLGLIYEWMQGGLEWAE-
Calu   AILFLLFDLEIALLLPLPWGHQLVTPTSTFTWTAAVLLLLTFGLIYEWLQGGLEWAE-
Papa   AILFLLFDLEIALLLPLPWGDQLENPLMTFTWASAVLILLTLGLIYEWAQGGLEWAQ-
Sufr   AILFLLFDLEIALLLPLPWGNQLDSPLLTFLWASIVLFIATLGLIYEWVQEGLEWAE-
Stci   AILFLLFDLEIALLLPLPWGDQLSSPLLTFFWASAVLILLTAGLIYEWLQGGLEWAE-
Taru   AILFLLFDLEIALLLPLPWGDQLPSPTLTLMWTSALLILLTIGLAYEWLQGGLEWAE-
Rala   AILFLLFDLEIALLLPLPWGDQLSSPLLTFFWASLVLALLTLGLIYEWLQGGLEWAE-
       *:*. *****:.*     **  .             .. :   .: :*  . .:: *:
```
