# Supplementary material for: Structure and variation of the mitochondrial genome of fishes
Source: BMC Genomics. 2016 Sep 7;17(1):719. doi: 10.1186/s12864-016-3054-y (PMC5015259; doi:10.1186/s12864-016-3054-y)
Supplement: Additional file 6: Figure S1-a. — Aligned amino acid sequences of the ATP8 gene in mt genomes of 250 fishes. Figure S1-b. Aligned amino acid sequences of the ATP6 gene in mt genomes of 250 fishes. Figure S1-c. Aligned amino acid sequences of the COI gene in mt genomes of 250 fishes. Figure S1-d. Aligned amino acid sequences of the COII gene in mt genomes of 250 fishes. Figure S1-e. Aligned amino acid sequences of the COIII gene in mt genomes of 250 fishes. Figure S1-f. Aligned amino acid sequences of the Cyt b gene in mt genomes of 250 fishes. Figure S1-g. Aligned amino acid sequences of the ND1 gene in mt genomes of 249 fishes. Figure S1-h. Aligned amino acid sequences of the ND2 gene in mt genomes of 250 fishes. Figure S1-i. Aligned amino acid sequences of the ND3 gene in mt genomes of 250 fishes. Figure S1-j. Aligned amino acid sequences of the ND4L gene in mt genomes of 250 fishes. Figure S1-k. Aligned amino acid sequences of the ND4 gene in mt genomes of 250 fishes. Figure S1-l. Aligned amino acid sequences of the ND5 gene in mt genomes of 250 fishes. Figure S1-m. Aligned amino acid sequences of the ND6 gene in mt genomes of 249 fishes. (ZIP 3250 kb) [file 12864_2016_3054_MOESM6_ESM.zip › Additional file 6 prot align/AF6j-N4L.pdf]

Additional file 6: Figure S1–j. Aligned amino acid sequences of the ND4L gene in mt genomes of 250 fishes.

Species name abbreviation followed by aligned amino acid sequences shown by one letter abbreviation. See Additional file 1 for abbreviation of species name. Amino acids shown by magenta letter denote hydrophobic residues. A-C in bold types with yellow background indicate putative transmembrane regions. Asterisk '*' indicates a fully conserved residue. Colon ':' and period '.' indicate 'strong' and 'weak' groups in the level of conservativeness, respectively, in the Gonnet Pam250 matrix, in which the strong and weak groups are defined as strong score >0.5 and weak score =<0.5, respectively (Thompson et al., 1997).

ND4L

[1/2 of aligned sequences] **A** **B**

Scca	MSPMYFSFSSAFMLGLMGLAFNRSHLLSALLCLEGMMLTLFVATATWSLMLNSTSSSILP
Muma	MSPMYFSFTSAFILGLMGLAFNRSHLLSALLCLEGMMLTLFIATAIWSMMLNSTSSSIIP
Erca	MTHILFTISTAFMLGLTGLTFHRTHLLSALLCLEGMMLSLFIALAMWSTQNDMMIFSSSP
Pose	MTHILFTISTAFMLGLSGLTFNRTHLLSALLCLEGMMLSLFIALAMWCSQNETMMFSSTP
Actr	MTPVHFSFSSAFMLGLMGLTFHRTHLLSALLCLEGMMLSLFIALSLWSLQLESTTYATAP
Scal	MTPVHFSFSSAFMLGLMGLTFHRTHLLSALLCLEGMMLSLFIALSLWSLQLESTAYAAAP
Posp	MTPVHFSFSSAFMLGLMGLTFHRTHLLSALLCLEGMMLSLFIALSLWSLQLESTTHATAP
Atsp	MTPVHFSFSAAFILGLMGLAFHRTHLLSALLCLEGMMLSLFIALSMWSLQLESMSYATTP
Leoc	MTPVHFSFSAAFILGLMGLAFHRTHLLSALLCLEGMMLSLFIALSMWSLQLESMSYATTP
Amca	MTPVHFSFSSAFILALMGLAFHRTHFLSALLCLEGMMLSLFIALSLWSLQLEATTHAATP
Osbi	MTSVHFSFSSAFTLGLMGLAFHRTHLLSALLCLEGMMLSLFIALSLWALQLQTIMFSAAP
Pabu	MTPMHFSFSSAFILSLMGLAFHRTHLLSALLCLEGMMLSLFIALSIWSMQFDLTTYTSTP
Hial	MTPVHFSFSSAFILGLMGLAFHRTHLLSALLCLEGMMLSLFIALSLWTLQLESTAYSAAP
Elha	MTPVHFSFSSAFILGLTGLALHRTHLLSALLCLEGMMLSLFIALSLWALRLESVIFSTAP
Mlcy	MTPVHFSFSSAFMLGLMGLAFHRTHLLSALLCLEGMMLSLFIALSLWALQSDSTVSSTAP
Algl	MTPVHFGFASAFILGLMGVASHRTHLLSTLLCLEGMMLSLYIALALWGLQLESVAYSVAP
Ptgi	MTPVHFSFTSAFILGLMGLAFHRTHLLSALLCLEGMMLSLYIALALWALQLESITYSAAP
Alaf	MTPVHFSFTSAFILGFMGLAFNRMHLLSALLCLEGMMLSLYIALALWALKLDSMTFSAAP
Nock	MTPVHFSFTSAFVLGFMGLAFNRMHLLSALLCLEGMMLSLYIMMALWALKLDSMTFSAVP
Anja	MTPVHFSFTLAFTLGFSGLAFHRKHLLSALLCLEAMMLSLYIAMALWSFQTESTTFSSAP
Gyki	MTPIHFSFTTAFILGLSGLAFHRKHLLSALLCLEAMMLSLYVAMALWSIQMDSSVFSPTP
Syka	MTPAHFSFTLAFVLGISGLALHRKHLLSALLCLEAMMLSLYLAIALWAHQMGVTGFSSAP
Opma	MTPLHFSFSTAFILGFAGLAFHRKHLLSALLCLEAMMLSLYVATAMWSFQTGATIFSSAP
Comy	MGPTHLSFMLTFVLGFLGLAFNRKYLLSALLCLEMMMLSIYMNMTMWSYQTGSNIFSSTP
Sasp	MSSHQFSLSLSFALALLGFMYNQKHLLLALLCLEAMMLTLYILMAIWPSQTEHINPSPLP
Eupe	MSHLYFSLSMMFTLGFSGLALNRKHLLLALLCLETMMLLLYISTALWASQTGDIATTAIP
Enja	MTPTHFSFTTAFILGLSGVAFHRTHLLSALLCLEGMMLSLFIALSLWSLQMGATNFTPAP
Same	MTPVHLTFSLGFVLGLTGLAFHRTHLLSALLCLEGMMLSLFLALSLWVLQTEVASFSAAP
Chch	MTPVHFSFSSAFILGLMGLAFHRTHLLSALLCLEGMMLSLFIALSLWSLQLEATGYSAAP
Grgr	MTPVQFSFTTAFLLGLMGLAFHRTHLLSALLCLEGMMLSLFIALSLWALQLGVPVYTAAP
Caau	MTPVHFSFSSAFILGLMGLAFHRTHLLSALLCLEGMMLSLFIALALWALQFESTGFSTAP
Cyca	MTPVHFSFSSAFILGLMGLAFHRTHLLSALLCLEGMMLSLFIALALWALQFESTGFSTAP
Dare	MTPTHFSLNAAFMLGLAGLTFHRVHLLSALLCLEGMMLSLFISMALWTLKTESMSLSTAP
Cost	MTPVHFSFSSAFILGLMGLTFHRTHLLSALLCLEGMMLSLFIALALWALQFEMTGFSAAP
Leec	MTPVHFSFSSAFALGLMGLAFHRTHLLSALLCLEGMMLSLFIALALWTLQFESTMFSVAP
Fola	MTPVHFSFTSAFILGLMGLAFYRTHLLSALLCLEGMMLSLFIALALWALQFESTGFSTAP
Clmc	MTPVHFSFTSAFVLGLMGLAFHRTHLLSALLCLEGMMLSLFIALALWALQLETTAFSTAP
Phin	MTPVHFTFSSAFTLGLMGLTFHRTHLLSALLCLEGMMLSLYMALALWALQLEAATFSSAP
Icpu	MTPVYFSFTSAFTLGLTGLAFHRTHLLSALLCLEGMMLSLFIALALWMLQLESTAFSAAP
Psto	MTPVYFTFTSAFTLGLTGLAFHRNHLMSALLCLEGMMLSLFLALALWMLQLEATAFSAAP
Cora	MTPVHFSFTSAFLLGLTGLAFHRTHLLSALLCLEGMMLSLFIALGLWALQLESTAFSAAP
Eisp	MTPVHFSFTSAFALGLMGLAFHRTHLLSALLCLEGMMLSLFLAMALWALQLESVTFSAAP
Apal	TSLTHFSFTSAFSLGLMGLAFHRTHLLSALLCLEGMMLSLFVALSLWALQLEATASSTIP
Eslu	MTPVHFSFTAAFTLGLMGLAFHRTHLLSALICLEGMMLSLFIALSLWTLQLEATAFSTAP
Dape	MTPVHFSFTMAFILGLMGLAFHRAHLLSALLCLEGMMLSLFIAFSLWALQLETTTFSTAP

To be continued on page 6.

[1/2 of aligned sequences]

```
Glse  MTPVHFSFTSAFVLGLTGLAFHRTHLLSALLCLEGMMLSFIALSLWTLQLETIGFSTAP
Naar  MTPVHFSFTSAFALGLMGLAFHRTHLLSALLCLEGMMLSFIALSLWALQLEATSYSTAP
Lioc  MTPVHFSFTSAFALGLMGLAFHRTHLLSALLCLEGMMLSFIALSLWALQLETTSYSSAP
Opso  MTPVHFSFTSAFVLGLMGLAFHRTHLLSALLCLEGMMLSFIALSLWTLQFESTHYSTAP
Alte  MTPAHFSFSSAFILGLMGLAFHRTHLLSALLCLEGMMLSFIALSLWALQLETTGYSSAP
Plap  MTPVHFSFSSAFILGLMGLAFHRTHLLSALLCLEGMMLSFIALSLWALQLEATGYSSAP
Plal  MTPTHFSFTSAFILGLMGLAFHRTHLLSALLCLEGMMLSYIALSIWALQAEATGFSSAP
Sami  MTPTHFSFTSAFILGLMGLAFHRTHLLSALLCLEGMMLSYIALSVWALQAEVTGFSCAP
Rere  MTPVHFSFSSAFILGLMGLAFHRTHLLSALLCLEGMMLSYIALSVWALQLEASGFSCAP
Gama  MTPVHFTFTSAFVLGLMGLAFHRTHLLSALLCLEGMMLSFIALSLWSLQLDSTGYSSAP
Onmy  MTPVHFSFTSAFILGLMGLAFHRTHLLSALLCLEGMMLSFIALSLWALQMEATGYSVAP
Sasa  MTPVHFSFTSAFILGLMGLAFHRTHLLSALLCLEGMMLSFIALSLWALQMEAIGYSVAP
Cola  MTPVHFSFTSAFVLGLMGLAFHRTHLLSALLCLEGMMLSFIALSLWALQMEATGYSVAP
Dita  MTPSHFSFTAAFALGSMGLAFHRTHLLSALLCLEGMMLSYIALSLWALQLETTGFSATP
Gogr  MTPTYFTLSSTFTLGFMGLAFHRTHLLSALLCLEGMMLSLYTSLALWTLQLEVSNLSTTP
Chsl  MTPMHFSFTSAFILGFMGLAFHRTHLLSALLCLEGMMLSFIALSMWALQLEATEFSTAP
Atja  MTPTQFTFTSAFVLGLMGLTFHRTHLLSALLCLEGMMLALFIALSLWTLQLDSTNCSSAP
Iido  MTPTQFTFTSAFVLGLTGLTFHRTHLLSALLCLEGMMLALFIALSLWTLQLDSTNYSSAP
Auja  MTPTHFAFSSAFTLGLMGLAFHRTHLLSALLCLEGMMLSFIALSLWALQLEATAYSTAP
Chag  MTPAHFAFSSAFMLGLMGLAFHRVHLLSALLCLESMMLSFIALSLWTLQLGATGYSAAP
Hami  MTPTHFAFSTAFTLGLMGLAFHRTHLLSALLCLEGMMLSFVALSLWTLQLESIGYTATP
Saun  MTPTHFAFSTAFILSLMGLAFHRTHLLSALLCLEGMMLSFIALSLWTLQLETIGYSATP
Nema  MTPTHFAFSSAFLLGLMGLAFHRTHLLSALLCLEGMMLSFIALSIWALQLGATGYSAAP
Disp  MTPTHFTFSSAFILGLMGLAFHRTHLLSALLCLEGMMLSFIALSIWALQLGTTGYSAAP
Myaf  MTPAHFAFSSAFILGLMGLAFHRTHLLSALLCLEGMMLSFIALSIWTLQLGTTGYSAAP
Lagu  MSPTQFSFSSMFVLGLVGLAFHRTHLLSALLCLEGMMLSFIALAIWALQFNVVGYSTTP
Trtr  MTPVHFTFSTMFILGLMGLAFHRTHLLSALLCLEGMMLSFLALATWAMQLGVSGFSAAP
Zucr  MTPVHFTFSAMFILGLMGLAFHRTHLLSALLCLEGMMLSFLALAIWAMQFGVSGFSAAP
Pxja  MTPVHFSFSSAFILGLMGLAFHRTHLLSALLCLEGMMLSFIALSLWALQLDATGYSTSP
Pxlo  MTPVHFSFSSAFILGLMGLAFHRTHLLSALLCLEGMMLSFIALSLWALQLDATGYSTSP
Pctr  MTPAHFTFSSAFMLGLMGLAFHRTHLLSALLCLEGMMLALFLALSLWALQLGATGPTTSP
Apsa  MTPTHFTFSSAFLLGLMGLAFHRTHLLSALLCLEGMMLSFIALSIWSLQLDSTNYTTSP
Cabe  MTPVRVTFMSAFALGLLGLALHRVHLISALLCLEAMMLTLYVTLSVWALEHTSASHITCP
Bzze  MTPTHFAFSSAFILGLSGLAFHRTHLLSALLCLEGMMLSFIALSLWTLQLDATSYSASP
Siim  MTPAYFALTSAFILGLSGLIFHRTHLLSALLCLEGVMLALFAYLSLLTLQFGMADHASTP
Ctru  MTPAHFAFSSAFALGLTGLAFHRTHLLSALLCLEGMMLSFIALSLWTLQLDATEYSTSP
Dpbr  MTPTHFAFSSAFTLGLTGLAFHRTHLLSALLCLEGMMLSFIALSLWTLQLDSTEYSTSP
Caki  MTPIAFTTSSAFFLSLMGLTFHRTHLLSALICLEGMMLSLFTSFSLWALQSDTASFSTVP
Phja  MSPTHYLISSAFLLSFLGLSFHRTHLLSLLLCLEAMMLTLFIMLSLWTLELDSSIQSSTP
Brsp  MSSIHFILTTSFLAGLSGLALHRTHLISVLLCLETMMLTLFIALSLWSLEMETSSYASAP
Gamo  MTPTHFTISSAFLLGMMGLAFHRTHLLSALLCLEAMMLALFIALSLWSLQLDATGCSTAP
Lolo  MTPTHFTISSAFLLGLMGLAFHRTHLLSALLCLEAMMLALFIALSLWSLQLDATGYSTAP
Batr  MTWIHLVTMTAFLLTMMGLTVHLTHLLSTLLCLEALMLILFMALAAWTLDLHLGSSFVLP
Prmy  VLLQQLIFSVIFYLALVGLTVHRTHFLSMLLCLEMIMLILFITISIWASNQNSLMMAPMP
Lose  MRLATMTFSTAFMLGMVGLTFNRDHLLSALLCLEGMMLALFIALSLWTLQLGSTNFSSSP
Loam  MTPTNFTFSAAFMLGLAGLTFHRTHLLSALLCLEGMMLALFLALSLWSLQLGVTGFSATP
Chab  MTPIQFTFSSSFMLGLAGLAFHRTHLLSALLCLEGMMLSFIALSLWTMQLTATNFSVSP
Chto  MTPIQFTFSSGFMLGLAGLAFHRTHLLSALLCLEGMMLSFIALSLWTMQLTATNFSVSP
Majo  MSQAQFAIFSAFSLGLTGLALHRTHLLSALLCLEGMMLSFIALSLWAVQLSATNFSAAP
Hlst  MSQVQFTITSSFMLGLTGLVLHRTHLLSALLCLEGMMLSFIALSLWTMQLTTINFASAP
Clpe  MTPAHYALSSTFVLGLAGLTFHRTHLLSALLCLEGMMLSFMALSLWAMQLATTSFSASP
Mlmr  MTPTHFALSSTFMLGLTGLACHRTHLLSALLCLEGMMLSFIALSLWTMQLSTTNFSASA
```

To be continued on page 7.

[1/2 of aligned sequences]

Crcr	MTPTHFAFSSAFLLGLAGLAFNRTHLLSALLCLEGMMLSLFIALSVWTLQLSSMNFSVAP
Muce	MTPTHFAFSSAFLLGLAGLAFNRTHLLSALLCLEGMMLSLFIALSIWTLQLSSMNFSVAP
Bege	MTPVHFAFSSTFILGLAGLAFNRIHLLSALLCLEGMMLSLYIALALWTLQLDSTSFSTSP
Mela	MSPIHFTFSATFILGLAGLAFHRSHLLSALLCLESMMLSLFLALSLWTLQLNSTSFSTSP
Hats	MTPTHFAFSSTFILGLAGLAFHRTHLLSALLCLEGMMLSLFIALSLWTLQMDSTVFSTSP
Orla	MTLTHYAFSSSYFMSFLGLIFYRKHLLSALLCLEAMMLILFISLCLWGLILASTVFSAGP
Cosa	MTPTHFAFSSMFFLGLAGLAFHRTHLLSALLCLEGMMLSLFIALSLWVLQLDSTSFSPSP
Exsp	MTPTHFTFSSLFFLGLAGLAFHRTHFLSALLCLEGMMLSLFLALSLWALQLDSTNFAASP
Depa	MSPIHYTFSLMFFLGLAGLTFHRTHLLSALLCLETMMLSLFMVLSLWVLQLDSTSFSPSP
Rima	MSPLHFTFTAMFTLSLLGLTFYRTHLLSALLCLESIMLSLFLAFSLCALKFDSSAFSVAP
Fuol	MTPIHFAFSSTFMLGLAGLAFHRTHLLSALLCLEGMMLSLFIALSLWTLELNSTSFASSP
Gmaf	MTPTHFAFSSAFMLGLAGLAFHRTHFLSALLCLEGLMLSLFIALSLWTLQFNTMNSASLP
Xeei	MTPVHFVFSSSFMLGLAGLAFHRTHLLSALLCLEGMMLSLFIALSTWTLQFNSTNFAPSP
Pros	MTPIHFSFSSAFMLGLVGLAFHRTHLLSALLCLEGMMLSLYIALSLWALQLDATGYSASP
Scmi	MTPIHFSFSSAFILGLVGLTFHRTHLLSALLCLEGMMLSLFIALSLWTLQLDATGYSASP
Rolo	MTPVHFAFSSAFILGLVGLAFHRTHLLSALLCLEGMMLSLFIALSLWALQLDAAGYSASP
Cere	MTPTQFTFSLAFVLGLAGLVFHRTHLLSALLCLEGMMLSLFIALSLWALQLDATGYSASP
Daga	MTPVHFALSSAFILGLMGLTFHRTHLLSALLCLEGMMLSLFSALSLWALQLDSTGFSTSP
Anco	MTPTHFTFSSAFILGLMGLAFHRTHLLSALLCLEGMMLSLFIALSLWALQLDATGYSASP
Dmve	MTSTHLAFSSAFLLALVGLTLHRTRLLSALICLEGMMLSLFIGLTLWALQLGATTHLSAP
Dmar	MTPTHLAFSSAFLLALVGLTLHRTRLLSALICLEGMMLSLFIGLTLWALQLDATAHLSSP
Anka	MTPVHFTFSSAFILGLMGLAFHRTHLLSALLCLEGMMLSLFIALSLWTLQLDSTGYSASP
Moja	MTPVHFAFSSAFILGLMGLAFHRTHLLSALLCLEGMMLSLFIALSLWALQLDSTGYSASP
Hoja	MTPVHFAFSSAFILGLMGLAFHRTHLLSALLCLEGMMLSLFIALSLWALQLDSTGYSASP
Bede	MTPVHFSFSSAFILGLMGLAFHRTHLLSALLCLEGMMLSLYIALSLWALQLDATGYSASP
Besp	MTPVHFSFSSAFILGLVGLAFHRTHLLSALLCLEGMMLSLYIALSLWALQLDATGYSASP
Mysp	MTPTHFAFSSAFILGLVGLTFHRTHLLSALLCLEGMMLSLFIALSLWALQLDTTIYSASP
Osja	MTPTHFTFSSAFILGLVGLTFHRTHLLSALLCLEGMMLSLFIALSLWALQLDTTVYSASP
Sgro	MTPVHFTFSSAFILGLIGLTFHRTHLLSALLCLEGMMLSLFIALSLWALQLDSTAYSASP
Pzpa	MTPVHFSVSSAFILGFTGLAFHRTHLLSALLCLEGMMLSLFTALSLWALQLDATGYATSP
Zeja	MTLAHFSVSSAFLLGLMGLAFHRTHLLSALLCLEGMMLSLFIALSLWALQLDAVGYVTSP
Znne	MTPIHFSISSAFILGLMGLAFHRTHLLSALLCLEGMMLSLFIALSLWALQLDATGYASSP
Zefa	MTPTHFSVSSAFILGLMGLAFHRTHLLSALLCLEGMMLSLFIALSLWSLQLDAAGYSSSP
Acni	MTPIHFSVSSAFILGFMGLAFHRTHLLSALLCLEGMMLSLFIALSLWALQLDATGYATSP
Ncrh	MTPIHFSVSSAFILGFMGLAFHRTHLLSALLCLEGMMLSLFIALSLWALQLDATGYATSP
Agca	MTPVHFSFSSAFMLGLTGLAFHRTHLLSALLCLEGMMLSLFVGLSLWALQLDSTSFSTAP
Hydy	MTPVHFAFSSAFTLGLTGLAFHRTHLLSALLCLEGMMLSLFIAFSLWTLQLDSTNFSGAP
Gsac	MTPVHFAFSSAFMLGLTGLAFHRTHLLSALLCLEGMMLSLFIALSLWTLQLDSTNFSGAP
Pevo	MTPTQFAFSSAFIMGTAGLTFHRTHLLSALLCLEGMMLSLLIALAFWSLELDSAGPMASP
Hiku	MTPVHFAFSTTFMLGLMGLTFHRNHLLSALLCLEGMMLSLYVALSLWTLQLNSINFSPTP
Inpa	MTPLHFSFSSAFFLGLSGLAFHRTHLLSALLCLEGMMVALFIALSLWTLQLNSTSLSSAP
Auch	MTPVHFTFSTAFALGLLGLAFHRIHLLSVLLCLEGVMLSLFLAISLWSLQLNSTTTTPLP
Fico	MTPAHFAFSSSFVLGLMGLAFHRTHLLSALLCLEGMMLSLFIALSLWTLQLDSTNFSSSA
Macs	MTPVHFTFSSAFILGLMGLAFHRTHLLSALLCLEGMMLSLFIALSLWTLQLDSTSFTTSP
Moal	MTPIQMTFSTMFLLGLMGLTFHRTHLLSALLCLESMMLSLFLATSLWALQLSTINIATVP
Syma	MTPIHFTFSAMFSLGLMGLTFHRTHLLSTLLCLESMMLSLFIALSMWTLQFNATNLSLSP
Mafr	MTPTHFTFSSAFTLGLLGLAFHRTHLLSALLCLEGMMLTLFIALSLWTLQLNSTGFSTSP
Dcpe	MSPTHFAFSTAFALALTGLAFHRTHLLSALLCLEGMMLSLFIALSLWALQLDSTNLSSAP
Dcti	MSPTHFAFSTAFALALTGLAFHRTHLLSALLCLEGMMLSLFIALSLWALQLDSTNLSSAP
Hehi	MTPAHFAFSSAFTLGLTGLAFHRTHLLSALLCLEGMMLSLFIGLSIWTLQLGSTSFSAAP
Stam	MTPVHFAFSSAFLLGLSGLAFHRTHLLSALLCLEGMMLSLFIALSLWTLQLDSTSFSAAP
Hogi	MTPAHFAFSSTFLLGLMGLAFHRTHLLSALLCLEGMMLSLFIALSIWTLQLDTTSFSCAP

To be continued on page 8.

[1/2 of aligned sequences]

Erzo	MTPVHFAFSSAFMLGLAGLAFHRTHLLSALLCLEGMMLSLFIALSLWTLQLGSTSFSAAP
Hxot	MTPVHFAFSSAFMLGLTGLAFHRTHLLSALLCLEGMMLSLFIALSLWTLQLGSTSFSAAP
Core	MTPVHFAFSSAFLLGLTGLAFHRTHLLSALLCLEGMMLSLFVALSLWTLQLGATSFSTAP
Apve	MTPVHFAFSSAFMLGLTGLAFHRTHLLSALLCLEGMMLSLFIALSLWTLQLEATNLSTTP
Latj	MTPTHFAVTSAFMLGLTGLTLHRYHLLSALLCLEGMMLSLFIALSLWTLQLDATNFSVSP
Laja	MTPVHFAFSSAFMLGLTGLAFHRTHLLSALLCLEGMMLSLFLALSLWTLQLDSTSFSTSP
Syja	MTPVRFAFSAAFTLGLTGLAFHRTHLLSALLCLEGMMLSLFLALSLWTLQLNSTNFAASP
Epme	MTPVHFTFSSAFILGLTGLAFHRTHLLSALLCLEGMMLSLFIAFSLWTVQLSSTSFCTAP
Grse	MTPTHFACSSAFLLGLAGLAFHRTHLLSALLCLEGMMLSLFIAFSLWTLQLGSTSFSSAP
Clja	MSPLCFIFSSAFVTALLGLTMNRKHLLSALICLEGMMLSLFMALTSWTFQLGAMNFSTAP
Ogcy	MTPAHFAFSSAFSLGLAGLAFHRTHLLSALLCLEGMMLSLYIALSMWTLDLNTTSFSASP
Plna	-MLEIFVFSSTFTLALVGLVFHRTHLLSALLCLEAMMLSLFLALSLWAQQLNSTGSSTSP
Lema	MTPTHFAFSSTFLLGLTGLAFHRTHLLSALLCLEGMMLSLFIALSLWTLQLDSTNFSAAP
Etzo	MTPVHFAFSSAFILGLTGLAFHRTHLLSALLCLEGMMLSLFIALSLWTLQLDSTNFSAAP
Apse	MAVTHSAFTIAFLTGLFGLAFNRIHLLSALLCLEAMMLSLFLGLSLWMLQLNSTTFAATP
Epde	MTPVHFAFSSAFMLGLSGLAFHRTHLLSALLCLEGMMLSLFIALSLWTLQLDSTNFSASP
Slja	MTPVHFTFSATFLLGLTGLAFYRTHLLSALLCLEGMMLSLFIALSLWTLELNSTSFSTAP
Bsja	MTPVHFAFSSAFTLGLMGLVFHRTHLLSALLCLEGMMLSLFIALSVWTLQLNATNFAISP
Ecna	MTPTHFAFSSAFMLGLMGLAFHRQHLLSALLCLEGMMLSLFIALSLWTLQLDSTSFSASP
Cohi	MSPAQFTFSIAFFLGIVGLALHRHHLLSTLLCLEGMMLSLFIGMSLWSLQLDSSNFSSLP
Caar	MTPVHFAFSSTFMLGLAGLAFHRYHLLSALLCLEGMMLSLFVALSLWTLQLDSTSFSASP
Came	MTPVHFAFSSTFMLGLAGLAFHRYHLLSALLCLEGMMLSLFVALSLWTLQLDSTSFSASP
Mema	MTPTHFAFSSSFVLGLTGLTFHRTHLLSALLCLEGMMLSLFIALSLWTLQLDSTNFSSSA
Lenu	MTPIQFSFSSAFILGLTGLAFNRAHLLSALLCLEGMMLSLFLALSLWTLHLNATSFTTAP
Brja	MTPTHFAFSSTFMLGLMGLALHRTHLLSALLCLEAMMLSLFIALSIWTLQLDSTNFSASP
Plma	MTPVHFAFSSTFMLGLAGLAFHRTHLLSALLCLEAMMLSLFIALSIWTLQLDSTNFSASP
Emst	MTPVHFTFSSAFMLGLTGLAFHRTHLLSALLCLEGMMLSLFIALSLWTLQLNTTSFSASP
Ptti	MTPVHFTFSSAFMLGLTGLAFHRTHLLSALLCLEGMMLSLFIALSLWALQLSTTHFSASP
Losu	VTPTYFAISSAFTLGLSGLAFNRSHLLSALLCLEGMMLSLYIAFSLWSLQLNSTNSSSLP
Geoy	MSPAQFAFSSMFLLGLTGLAFHRTHLLSALLCLEGMMLSLYMALSLWTLQLEATNFSASS
Dipi	MTPGHFTFSAAFMLGLTGLAFHRTHLLSALLWLEGMMLSLFIALSLWTLQLNATNFSAAP
Pama	MTPVHFTFSSAFMLGLTGLALHRTHLLSALLCLEGMMLSLFIALSVWALQLSTVSFSASP
Leob	MTPVHFTFSSAFVLGLMGLAFHRTHLLSALLCLEAMMLSLYVALSLWSLQLDATVFSASP
Neba	MRAIQFAFSAAFFLGLSGLSFHRTHLLSALLCLEGMMLSLFIGLSVWTLQLSAANFSPAP
Pdpl	MTPTHFALSSAFLLGLSGLAFNRTHLLSALLCLEGMMLSLFVALSIWTLQSSTTSATATP
Nimi	MTPVHYAFSSAFVLGLTGLAFHRTHLLSALLCLEGMMLSLFIALSLWTLQLNSTNFSTAP
Uptr	MTPVHFAFSSTFILGLMGLAFNRVHLLSALLCLEGMMLSLFIAFSLWTLQLDSSNFSTSP
Pesc	MTPIHLAASSTFILAMVGLAVHREHLLSALLCLEGMMLSLFITLSLWTLHMDASYLSVGP
Baar	MTPVHFAFSSAFMLGLSGLAFHRTHLLSALLCLEGMMLSLFIALSLWTLQLNSTSFSASP
Moar	MTPVHFTFSATFMLGLTGLAFHRTHLLSALLCLEGMMLSLFIALSLWTLQLNATNFSASP
Toja	MTPTHFAFSSAFLLGLTGLAFNRFHLLSALLCLEGMMLSLFVALSLWTLQLDTTNFSASP
Chau	MTPTHFAFSSAFMLGLSGLALHRTHLLSALLCLEGMMLSLFLALSLWALQLNVSIFSASP
Chse	MTPVHFTFSSAFILGLMGLAFHRTHLLSALLCLEGMMLSLFIAMSLWTLQLDATSFSPSP
Enar	MTPVHFAFSSTFILGLAGLAFHRTHLLSALLCLEGMMLSLFIALSLWTLQLDSTNFSASP
Hpty	MTPVHFAFSSAFMLGLTGLAFHRTHLLSALLCLEGMMLSLFIALSLWTLQLDSTNFSASP
Nana	MTPTHFAFSSAFVLGLTGLAFHRTHLLSALLCLEGMMLSLFIALSLWTLQLNSTNFSASP
Mcst	MTPVQFAFSSTFLLGLTGLAFHRTHLLSALLCLEGMMLSLFIALSLWTLQLDSTNFSPAP
Rhox	MTPTHFAFSTTFILGLMGLASHRTHLLSALLCLEGMMLSLFIAFSLWTLQLGSSSFSASP
Opfa	MTPVHFAFSSTFLLGLTGLAFHRTHLLSALLCLEGMMLSLFIALSLWTLQLDSTSFSTSP
Paar	VTPVHFAFSSAFLLGLSGLAFHRTHLLSALLCLEGMMLSLFIALSLWALQLNSTNFSASP
Gozo	MTPVHFAFSSSFLLGLTGLAFHRTHLLSALLCLEGMMLSLFIALSLWALQLDSTNFSASP
Ackr	MTPVHFFLSMAFILGLAGLAFNRGHLLSALLCLEGIMLTLYLALALSAIQLGTINFLSAP

To be continued on page 9.

[1/2 of aligned sequences]

```
Elev      MTPIHFAFSSAFLLGLSGLAFHRTHLLSALLCLEGMMLSLFIALSLWSLQLDSSSFLTSP
Trdu      MTPTHFAFSSTFLLGLAGLAFHRTHLLSALLCLEGMMLSLFIALSMWTLQLNSTNFSASP
Amoc      MTPTHFAFSSAFTLGLAGLAFHRTHLLSALLCLEGMMLSLFIALSLWTLHLDSTNFSASP
Hame      MTPTHFAFSAAFTLGLAGLTFHRTHLLSALLCLEGMMLSLFVALSLWSLQLNSTSFSIAP
Chso      MMPIHFAFTTAFALGLTGLAFQRTHLLSALLCLEGMMLSLYVALSLWSLQLDSSGFSPAP
Lyto      MTPVHFAFSSAFMLGLTGLAFHRTHLLSALLCLEGMMLSLFIALSLWTLQLGSTSLSAAP
Encr      MTPVHFAFSSAFMLGLTGLAFHRTHLLSALLCLEGMMLSLFIALSLWTLQLGVTSFSAAP
Bvar      MTPVHFAFSSAFVLGLTGLAFNRTHLLSALLCLEGMMLSLFIALSVWALQLDSTNFSVAP
Noco      MTSIQFTFSSAFILGLTGLAFNRTHLLSALLCLEGMMLSLYIALSIWTLETGSTSSSALP
Chsp      MTTSHLALSSAFILGLTGLTFYRTHLLSALLCLESMMLSLFIILSLWSLQLNSANFSASP
Arja      MTPVHFAFSSAFMLGLTGLAFHRTHLLSALLCLEGMMLSLFIALSLWALQLGSANFSAVP
Pase      MAVTMHTLMATFMLSLTGLAFHRTHLLSALLCLEGMMLALFLAFSFWALQFTSATLSPAP
Trel      MSPTHFSISATFLIAMTGLVLCRTHLLSALLCLEMMMLSLYIGLSMWALQIHSTTPSLSP
Lifa      MSPLHFTFSSTFLLSLTGLVFHRTHFLSALLCLESMMLSLFLALSLWALQLGSTNFSASP
Acur      MTPVLYTLMSSFLLGLVGLTFHRIHLLSALLCLEGMMLTLFIALSLWTLEFNTSNHAMTP
Ampe      MTPVHFAFSSAFILGLTGLAFHRTHLLSALLCLEGMMLSLFIALSLWTLQLDSTSFSASP
Urja      MPSAQFAYMCSFMLALIGLALHRMHLLSALLCLEGMMLALFVALSLWTFQLGAPNFSTSP
Enet      MTPAQFTFSLAFVLGLTGLTFHRTHLLSALLCLEGMMLSLFIALSLWVLQLDSTALSASP
Ptbr      VSPAQFTFTSAFIIGLAGLAFTRTHLLSALLCLEGMMLSLFIGLTVWTLTLDSLSSSSSP
Safa      MMPAQFAFSSAFIVGLAGLAFHRTHLLSALLCLEGMMLSLFIALSLWMLSTDSTTFSASP
Icae      MTPVHFAFSSTFMLGLAGLAFHRTHLLSALLCLEAMMLSLFIALSIWTLQLDSTSFSASP
Asmi      -MFYHFILTSAFTISLTGLAFQRTHLMSALLCLEGMMLSLYTFLSLWSLQLSSINFTPSP
Foal      MTLTNFSFLSAFLLGLTGLSFNRTHLLSALLCLEGMMLSLYLAFSLWALTLDATNFSSAP
Drze      MQPMHFAITMTFVLGLSGLAFHRYHLLSALLCLEGMMISLFIALSVWTLQFGVTNFSIAP
Rhas      MTPVHFTFSTAFLLGLAGLAFHRTHLLSALLCLEGMMLSLFLALSLWTLDLNSTNFSASP
Elac      MTPVHFTFSTAFLLGLAGLAFHRMHLLSALLCLEGMMLSLFLALSLWTLELNTTSFSTSP
Kugu      MTPVQFAFSSSFILGLTGLAFNRNHLLSALLCLEGMMLSLFLALSLWSLQMNTTSFSSAP
Plor      MTPVHFTFSSVFLLGLTGLAFHRTHLLSALLCLEGMMLSLFIALSLWTLQLNATNFSASP
Sgun      MTPVHFTFSSAFVLGLMGLAFHRTHLLSALLCLEGMMLSLFIALSLWTLQLGATNFSASP
Zaco      MTPVHFTFSSAFMLGLTGLAFHRTHLLSALLCLEGMMLSLFLALSLWTLQLNTTNFSASP
Zbfl      MTPVHFTFSSAFVLGLTGLTYHRTHLLSALLCLEGMMLSLYIALSLWTLQLDSTNFSATP
Spba      MTPIHFAFSSTFILGLTGLAFHRHHLLSALLCLEGMMLSLFIALSLWALQLNSTSLSASP
Game      MTPVHFAFSTTFMLGLTGLAFHRTHLLSALLCLEAMMLSLFIALSIWTLQLDSTSFSASP
Thth      MTPVHFAFSTTFMLGLTGLAFHRTHLLSALLCLEAMMLSLFIALSIWTLQLDSTNFSASP
Xigl      MTPVHFAFSSTFMLGLTGLAFHRYHLLSALLCLEGMMLSLFIALSLWTLQLDSTNFSASP
Hyja      MTPVHFTFTTTFLLGLAGLAFHRTHLLSALLCLEAMMLSLFIALSIWTLQLGSTNFSASP
Psan      MSAIHLALSMSFVMGATGLVFNRTHLLSALLCLEAMMLSLYLLLAVWSLQTDSANFSAAP
Cupa      MTPVHFAFSSTFMLGLTGLAFHRTHLLSALLCLEAMMLSLFIALSIWTLQLDSTNFSASP
Mpch      MTPIHFTISTAFMLSLSGLAFHRTHLLSALLCLEGMMLSLFIAISLWSLQLNSMNFSMAP
Char      MTPVHFTFSSAFILGLAGLAFHRTHLLSALLCLEGMMLSLFVALALWSLQFNVTNFSAAP
Pser      MTPVHFAFSSTFLLGLTGLAFHRFHLLSALLCLEGMMLSLFIALSLWTLQLDSTNFSASP
Prol      MTPTHFAFSSAFLLGLTGLAFHRFHLLSALLCLEGMMLSLFIALSLWTLQLDSTNFSASP
Plbi      MTPTHFAFSSAFFLGLTGLAFHRFHLLSALLCLEGMMLSLFVALSLWTLQLDSTNFSASP
Calu      MTPSHFAFSMAFALGLTGVALHRHHLLSALLCLEAMMLSLFVALSLWSLNLSTASFSPAP
Papa      MTPVHFTLSSTFVLGLAGFTLHRFHLLSALLCLEGMMLSLFATISLWLLQMNFTSLSTTP
Sufr      MTPVHFSFSTAFILGLAGLTFHRTHLLSALLCLEGMMLSLFIALSLWSMQFSSASFSVLP
Stci      MTPVHFSFSAAFMLGLSGLAFHRTHLLSALLCLEAMMLSLFIALSLWTMNFASMSFSTPP
Taru      MTLIQLSFTSVFFLGLFGLAFYRVHLLSALLCLESMMLALFLALSTWSLQMSSTSFSAAP
Rala      MTPVHFTFSSAFMLGLTGLAFHRTHLLSALLCLEGMMLSLFIALSLWTLQLNSTSFSAAP
                     :      *.     ::  *: ** :*: :
```

To be continued on page 10.

[2/2 of aligned sequences]

C

```
Scca    MILLTFSACEASAGLAILVATSRSHGSDNLQNLNLLQC*
Muma    MIMLTFSACEASAGLAILVAASRSHGSDNLQNLNLLQC*
Erca    LLLLALSACEAGLGLSLLVATSRTHGSDHLQNLNLLQC*
Pose    LLLLALSACEAGLGLSLLVATSRTHGSDHLKNLNLLQC*
Actr    MLLLAFSACEAGAGLALLVATTRTHGTDHLQNLNLLQC*
Scal    MLLLAFSACEAGAGLALLVAATRTHGTDHLQNLNLLQC*
Posp    MLLLAFSACEAGAGLALLVAATRTHGTDHLQNLNLLQC*
Atsp    ILLLAFSACEASAGLALLVAATRTHGTSHLQNLNLLQC*
Leoc    VLLLAFSACEASAGLALLVAATRTHGTSHLQNLNLLQC*
Amca    MLLLAFSACEASAGLALLVATARTHGTDRLQSLNLLQC*
Osbi    MLLLAFSACEASAGLALLVATARTHGTDHLQNLNLLQC*
Pabu    MFLIAFSACEASAGLALLVATARTHGTDHLQNLNLLQC*
Hial    MLLLAFSACEASAGLALLVATARTHGTDRLQNLNLLQC*
Elha    MLLLAFSACEASAGLALLVATSRTHGTDRLQNLSLLQC*
Mlcy    MLLLAFSACEASAGLALLVATSRTHGTDRLQNLNLLQC*
Algl    MFLLAFSACEAGAGLALLVATSRTHGTSHLQNLNLLKC*
Ptgi    MLLLAFSACEASAGLALLVATSRTHGTDHLKNLNLLQC*
Alaf    ILLLAFSACEASAGLALLVATSRTHGTDHLRALNLLRC*
Nock    ILLLAFSACEASAGLALLVATSRTHGTDHLRALNLLRC*
Anja    MMLLAFSACEASAGLALLVATSRTHGTDHLMNLNLLQC*
Gyki    MMLLAFSACEASAGLALLVATSRTHGTDHLKALNLLQC*
Syka    MLLLALSACEASAGLALLVATSRTHGTDHLKALNLLQC*
Opma    MMLLAFSACEASAGLALLVAASRTHGTDLLKNLNLLQC*
Comy    MILLAFSACEASAGLALLVATTRTHGTVYLNNLNILKC*
Sasp    ILMLAFSACEAGAGLALLVSISRTHTTEHLENMTILQC*
Eupe    VYVLAFSACEAGTGLALLVATSRTHATDHLENLNILQC*
Enja    MMLLAFSACEASAGLALLVATARTHGTDRLQNLNLLQC*
Same    MMLLAFSACEASTGLALLVATARTHGSDRMQSLNLLQC*
Chch    MLLLAFSACEASAGLALLVATARTHGTDRLQNLNLLQC*
Grgr    MLLLAFSACEASAGLALLVATARTHGTDRLQNLNLLQC*
Caau    MLLLAFSACEASTGLALLVATARTHGTDRLQNLNLLQC*
Cyca    MLLLAFSACEASTGLALLVATARTHGTDRLQNLNLLQC*
Dare    MLLLAFSACEASAGLALLVATARTHGSDHMKNLNLLQC*
Cost    MLLLAFSACEASAGLALLVATARTHGTDRLQNLNLLQC*
Leec    MLLLAFSACEASAGLALLVATARTHGTDGLQNLNLLQC*
Fola    MLLLAFSACEASAGPGLLVATARTHGTDRLQNLNLLQC*
Clmc    MLLLAFSACEASAGLALLVATARTHGTDRLQNLNLLQC*
Phin    VLLLAFSACEAGAGLALLVATARTHGTDHLQNLNLLQC*
Icpu    ILLLAFSACEAGAGLALLVATARTHGTDRLQSLNLLQC*
Psto    ILLLAFSACEASAGLALLVATARTHGTDRLQALNLLQC*
Cora    LLLLTFSACEASAGLALLVATARTHGTDRLQSLNLLQC*
Eisp    LLLLAFSACEASAGLALLVATARTHGTDRLQNLNLLQC*
Apal    MLLLAFSACEASTGLALLVATARTHGTDRLQNLNLLQC*
Eslu    MLLLAFSACEASAGLALLVATARTHGTDRLQNLNLLQC*
Dape    MLLLAFSACEASAGLALLVASTRTHGSDRLQNLSLLQC*
Glse    MLLLAFSACEAGAGLALLVATARTHGTDRLQSLNLLQC*
Naar    MLLLAFSACEASAGLALLVATARTHGTDRLQSLNLLQC*
Lioc    MLLLAFSACEASAGLALLVATARTHGTDRLQSLNLLQC*
Opso    MLLLAFSACEASTGLALLVATARTHGTDRLLSLNLLQC*
Alte    MLLLAFSACEASAGLALLVATARTHGTDRLQSLNLLQC*
Plap    MLLLAFSACEASAGLALLVATARTHGTDRLQSLNLLQC*
```

[2/2 of aligned sequences]

```
Plal  MLLLAFSACEASAGLAILVATARTHGTDRLQSLNLLRC*
Sami  MLLLAFSACEASAGLAILVATARTHGTDHLQSLNLLRC*
Rere  MLLLAFSACEASAGLAILVAAARTHGTDRLQSLNLLRC*
Gama  MLLLAFSACEAAAGLALLVATARTHGTDHLQSLNLLQC*
Onmy  MLLLAFSACEASAGLALLVATARTHGTDRLQSLNLLQC*
Sasa  MLLLAFSACEASAGLALLVATARTHGTDRLQSLNLLQC*
Cola  MLLLAFSACEASAGLALLVATARTHGTDRLQSLNLLQC*
Dita  MFLLAFSACEASAGLALLVAASRAHGSDHLQSLNLLRC*
Gogr  LLMLAFSACEASTGLALLVATSRTHGTDHLHNLNLLQC*
Chsl  ILLLAFSACEASVGLALLVATSRAYGADHLQSLSLLRS*
Atja  MLFLAFSACEAGAGLALLVATARTHGTDHLQSLNLLQC*
Iido  MLFLAFSACEAGAGLALLVATARTHGTDHLQSLNLLQC*
Auja  MLLLAFSACEAGAGLALLVATARTHGTDHLQTLNLLQC*
Chag  ILLLAFSACEASAGLALLVATARTHGTDRLQSLNLLQC*
Hami  LLLLAFSACEASAGLALLVATARTHGTDHLQNLNLLQC*
Saun  LLLLAFSACEASAGLALLVATARTHGTDHLQNLNLLQC*
Nema  MLLLAFSACEASAGLALLVATARTHGTDRLQSLNLLQC*
Disp  MLLLAFSACEASAGLALLVATARTHGTDHLQSLNLLQC*
Myaf  MLLLAFSACEASAGLALLVATARTHGTDRLQSLNLLQC*
Lagu  MFLLSLSACEASVGLALLVATARTHGTDRLQSLNLLQC*
Trtr  LLLLAFSACEASAGLALLVATARTHGTDRLQSLNLLQC*
Zucr  LLLLAFSACEASAGLALLVATARSHGTDRLQSLNLLQC*
Pxja  MLLLAFSACEASAGLALLVATARTHGTDRLQSLNLLQC*
Pxlo  MLLLAFSACEASAGLALLVATARTHGTDRLQSLNLLQC*
Pctr  LLLLAFSACEASAGLALLVATARTHGTDHLQSLNLLQC*
Apsa  LLLLAFSACEASAGLALLVATARTHGTDRLQSLNLLQC*
Cabe  LILLAFSACEASAGLALLVAMNRTHGSDSLQSLSLLQC*
Bzze  LLLLAFSACEASAGLALLVATARTHGSDHLQSLNLLQC*
Siim  LLLLSFSACDAGVGLALLVATARTHGSDRMQSLNLLQC*
Ctru  MLLLAFSACEASAGLALLVATARTHGTDRLQSLNLLQC-
Dpbr  MLLLAFSACEASAGLALLVATARTHGTDRLQSLNLLQC*
Caki  LCILAFSACEASAGLALLVATARTHGTDHLKKLNLLKC*
Phja  LLMLAFSACEASAGLALLVATARTHGTDHLKNLSLLQC*
Brsp  LLMLAFSACEAGTGLALLVATTRTHGTDHLMNMNLLKC*
Gamo  MLMLAFSACEASAGLALLVATARTHGTDHMQALNLLQC*
Lolo  MLMLAFSACEASAGLALLVATARTHGTDHLQNLNLLQC*
Batr  MLLLTLSACEASTGLALLTATTRTHTTTQLSSMNMLQC*
Prmy  LLLLTFSACEMATGLALLVATARTHATDHLTSLNILQC*
Lose  LLLLAFSACEASAGLALLVATARTHGSDRPQLLTLLQC*
Loam  LLLLAFSACEASAGLALLVATTRTHGSDRLQTLNLLQC*
Chab  MLLLAFSACEASTGLALLVATARTHGTDRLQSLNLLQC*
Chto  MLLLAFSACEASTGLALLVATARTHGTDRLQSLNLLQC*
Majo  ILLLAFSACEASVGLALLVAMARTHGTDRLQNLNLLQC*
Hlst  ILLLAFSACEASMGLALLVAMTRTHGSDHLRSLSLLQC*
Clpe  MILLAFSACEASAGLALLVATARTHGTDHMHSLNLLQC*
Mlmr  MLLLAFSACEASAGLGLLVATARTHGTDRLQNLNLLQC*
Crcr  MILLAFSACEASAGLGLLVATARTHGTDRLKNLSLLQC*
Muce  MILLAFSACEASAGLGLLVATARTHGTDRLKNLSLLQC*
Bege  MILLAFSACEASAGLALLVATARTHGTDHLQSLNLLQC*
Mela  LLLLAFSACEASVGLALLVATARTHGTDRLQNLNLLQC*
Hats  MILLAFSACEASAGLALLVATARTHGTDRLQSLNLLQC*
Orla  MILLAFSACEASAGLALLVAMARTHGTDRLKNLSLLRC*
```

[2/2 of aligned sequences]

Cosa	MILLAFSACEASAGLALLVATARTHGTDHLKNLNLLQC*
Exsp	VLLLAFSACEASAGLALLVATARTHGTDRLQSLNLLQC*
Depa	MILLAFSACEASAGLALLVATARTHGTDHLQSLNLLQC*
Rima	MILLTFSACEASIGLALLVATVRTHGTDQLQNFNVLQC*
Fuol	MILLAFSACEAGAGLALLVATARTHGTDNLQSLNLLQC*
Gmaf	MILLAFSACEAGAGLALLVATTRTHSNSRLQSLNLLQC*
Xeei	MILLAFSACEASAGLALLVATARTHGTDRLQSLNLLQC*
Pros	MLLLAFSACEASAGLALLVATARTHGTDRLQSLNLLQC*
Scmi	MLLLAFSACEASAGLALLVATARTHGTDRLQSLNLLQC*
Rolo	MLLLAFSACEASAGLALLVATARTHGTDRLQSLNLLQC*
Cere	MLLLAFSACEASAGLALLVATARTHGTDRLQNLSLLQC*
Daga	MILLAFSACEASAGLALLVATARTHGTDRLQNLNLLQC*
Anco	MLLLAFSACEASAGLALLVATARTHGTDRLQSLNLLQC*
Dmve	MFLLALSACEASTGLALLVATARTHGSDHLQSLNLLQC*
Dmar	MFLLALSACEASTGLALLVATARTHGSDRLQSLNLLQC*
Anka	MLLLAFSACEASAGLALLVATARTHGTDRLQNLNLLQC*
Moja	MLLLAFSACEASAGLALLVATARTHGTDRLQSLNLLQC*
Hoja	MLLLAFSACEASAGLALLVATARTHGTDRLQSLNLLQC*
Bede	MLLLAFSACEASAGLALLVATARTHGTDRLQSLNLLQC*
Besp	MLLLAFSACEASAGLALLVATARTHGTDRLQSLNLLQC*
Mysp	MLLLAFSACEAGAGLALLVATARTHGTDHLQSLNLLQC*
Osja	MLLLAFSACEASAGLALLVATARTHGTDHLQSLNLLQC*
Sgro	MLLLAFSACEASAGLALLVATARTHGTDRLQSLNLLQC*
Pzpa	MLMLTFSACEASAGLALLVATARTHGTDQLQSLNLLQC*
Zeja	MLMLAFSACEASAGLALLVATARTHSTDHLQSLNLLQC*
Znne	MLMLVFSACEASAGLALLVATARTHGTDRLQSLNLLQC*
Zefa	MLMLAFSACEASAGLALLVATARTHGTDRLQSLNLLQC*
Acni	MLMLAFSACEASAGLALLVATARTHGTDRLQSLNLLQC*
Ncrh	MLMLAFSACEASAGLALLVATARTHGTDRLQSLNLLQC*
Agca	MLLLAFSACEASAGLGLLVATARTHGSDRLQSLNLLQC*
Hydy	LLLLAFSACEASAGLALLVAAARTHGTDHLQNLNLLQC*
Gsac	MLLLAFSACEASAGLALLVAAARTHGTDHLQNLNLLQC*
Pevo	MILLAFSACEASAGLALLVATSRTHGGDRLKNLNLLQC*
Hiku	MLMLAFSACEASAGLALLVATSRTHGTDRLQALNILQC*
Inpa	ILLLAFSACEASTGLALLVATARTHGSDRMQNLNLLQC*
Auch	MLLLALAACGASAGLALLVATARTHGNDRMQTLSLLKC*
Fico	MLLLAFSACEASAGLALLVATTRTHGSDRLQNLNLLQC*
Macs	MLLLAFSACEASAGLGLLVATARTHGSDRLQSLNLLQC*
Moal	MLLLAFSACEASSGLALLVATARTHGSDHMQNLNLLQC*
Syma	MLLLAFSACEASCGLALLIATARTHGSDHMQNMNILKC*
Mafr	MLLLAFSACEASTGLALLVATARTHGTDRLQSLNLLQC*
Dcpe	MLLLAFSACEASTGLAILVATARTHGSDRLQSLNLLQC*
Dcti	MLLLAFSACEASTGLAILVATARTHGSDRLQSLNLLQC*
Hehi	MLLLAFSACEASAGLALLVATARTHGSDRLQTLNLLQC*
Stam	MLLLAFSACEASAGLALLVATARTHGTDRLQSLNLLQC*
Hogi	MLLLAFSACEAGAGLALLVATARTHGSDRLKNLNLLQC*
Erzo	MLLLAFSACEASAGLALLVATARTHGTDRLQSLNLLQC*
Hxot	MLLLAFSACEASAGLALLVATARTHGTDRLQSLNLLQC*
Core	MLLLAFSACEASAGLALLVATARTHGTDRLQSLNLLQC*
Apve	VLLLAFSACEASAGLALLVATTRTHGTSRLQSLNLLQC*
Latj	MLLLAFSACEASAGLALLVATARTHGTDRLQSLNLLQC*
Laja	MLLLAFSACEASAGLALLVATARTHGSDHLQTLNLLQC*

[2/2 of aligned sequences]

Syja	MLLLAFSACEASAGLALLVATARTHGSDRLQNLNLLQC*
Epme	MLLLAFSACEASAGLALLVATARTHGTDHLKSLNLLQC*
Grse	MLLLAFSACEASAGLALLVATARTHGTDHLQNLNLLQC*
Clja	MLLLAFSACEAGVGLALLVAAVRTHGSDELHNLNLLRC*
Ogcy	MILLAFSACEASAGLSLLVATARTHGTDRLQNLNLLQC*
Plna	MFLLAFSACEASTGLALLVAATRTHGNDHLQNFNLLKC*
Lema	MLLLAFSACEASAGLALLVATARTHGSDRLQNLNLLQC*
Etzo	MLLLAFSACEASAGLALLVATARTHGTDRLQSLNLLQC*
Apse	ILLLALSVCEASAGLALLVATTRTHGSDRLQNLNLLQC*
Epde	MLLLAFSACEASAGLALLVATARTHGTDRMHGLNLLQC*
Slja	MLLLAFSACEASAGLALLVATARTHGTDRLQNLNLLQC*
Bsja	MLLLAFSACEASAGLALLVATARTHGSDRLQTFNLLQC*
Ecna	MLLLAFSACEASAGLALLVATTRTHGSDQLQNLNLLQC*
Cohi	MFLLAFSACEASAGLALLVATSRTHGTDRLQSLSLLQC*
Caar	MLLLAFSACEASAGLALLVATARTHGTDRLQSLNLLQC*
Came	MLLLAFSACEASAGLALLVATARTHGTDRLQSLNLLQC*
Mema	MLLLAFSACEASAGLALLVATARTHGTDRLQSLNLLQC*
Lenu	MILLAFSACEASAGLALLVATARTHGTDQLKNLNILQC*
Brja	MLLLAFSACEASAGLALLVATSRTHGSDRLQGLSLLQC*
Plma	MLLLAFSACEASAGLALLVATSRTHGSDRLQSLNLLQC*
Emst	MLLLAFSACEASAGLALLVATARTHGTDRLQSLNLLQC*
Ptti	MLLLAFSACEASAGLALLVATARTHGTDRLQSLNLLQC*
Losu	MLILAFSACEAGVGLALLVATARTHGTNQLQNLNLLQC*
Geoy	LLLLAFSACEASAGLALLVATARTHGSDRLQTLNLLQC*
Dipi	MLLLAFSAWEASAGLALLVATARTHGTDRLQSLNLLQC*
Pama	LLLLAFSACEASAGLALLVATARTHGSDHLQSLNLLQC*
Leob	MLLLAFSACEASAGLALLVATARTHGTDRLQSLNLLQC*
Neba	LILLAFSACEASAGLALLVATARSHGSAHLKTLNLLRC*
Pdpl	MFLLAFSACEASAGLALLVATARTHGTDRLQSLNLLQC*
Nimi	MLLLAFSACEASAGLALLVATSRTHGSDRLQSLSLLQC*
Uptr	MLLLAFSACEASAGLALLVATSRTHGTDRLQTLNLLQC*
Pesc	MLLLTFSACEASAGLALLVATTRTHGTSRLQNLNLLKC*
Baar	MLLLAFSACEASAGLALLVATTRTHGTDRLQGLNLLQC*
Moar	MLLLAFSACEASAGLALLVATARTHGTDRLQSLNLLQC*
Toja	MLLLAFSACEASAGLALLVATARTHGTDRLQSLNLLKC*
Chau	MLLLAFSACEASAGLALLVATTRTHGSDRMQNLNLLQC*
Chse	MLLLAFSACEASAGLALLVATARTHGTDRLQSLNLLQC*
Enar	MLLLAFSACEASAGLALLVATARTHGSDRLQTLNLLQC*
Hpty	MLLLAFSACEASAGLALLVATARTHGTDRLQNLNLLQC*
Nana	MLLLAFSACEASAGLALLVATARTHGSDRLQSLNLLQC*
Mcst	MLLLAFSACEASAGLALLVATARTHGSDRLQNLNLLQC*
Rhox	MILLALSACEASAGLALLVATARTHGSDHLQTLNLLQC*
Opfa	MLLLAFSACEASAGLALLVATARTHGSDRLQNLNLLQC*
Paar	MLLLAFSACEASAGLALLVATARTHGSDRLQNLNLLQC*
Gozo	MLLLAFSACEASAGLALLVATSRTHGSDRLQSLNLLQC*
Ackr	MILLAFSACEAGLGLALLVATARTHGTDRLQSLNLLQC*
Elev	MLLLAFSACEAGAGLALLVATARTHGSDHLQSLSLLQC*
Trdu	MLLLTFSACEASAGLALLVATARTHGTDRLQNLNLLQC*
Amoc	MILLAFSACEASAGLALLVATARTHGTDRLQNLNLLQC*
Hame	MLLLAFSACEAGAGLALLVATARTHGTDRLQSLNLLQC*
Chso	MILLAFSACEASAGLALLVATSRTHGSDRLKNLNLLQC*
Lyto	MLLLAFSACEASAGLALLVATSRTHGTDHLQGLNLLQC*

[2/2 of aligned sequences]

```
Encr    MLLLAFSACEASAGLALLVATARTHGTDHLQTLNLLQC*
Bvar    MFLLTFAACEAGAGLALLVATARTHGTDRLQNLNLLQC*
Noco    LFLLAISACEASAGLALLVATARTHGTDRLQSLNLLQC*
Chsp    LLLLVFSACEASVGLALLVATTRTHGSDHINNLNLLQC*
Arja    LVLLAFSACEASAGLSLLVAAARTHGTTRLKSLNLLQC*
Pase    MLLLAFSACEASAGLALLVATARTHGSDHLQTLNLLQC*
Trel    MVLLSFSACEASAGLALLVATARTHGSDRLQSFNLLRC*
Lifa    MLLLAFSACEASAGLALLVATARTHGSDHLQSFTLLRC*
Acur    MVLLAFSACEASAGLALLVATARTHGTDRLQSLNLLQC*
Ampe    MLLLAFSACEASAGLGLLVATARTHGSDRLQTLNLLQC*
Urja    LFLLAFSACEAGTGLALMVATARTHSCDHVKMLSLLKC*
Enet    MILLAFSACEASTGLALLVATARTHGTDCMQNLNLLQC*
Ptbr    LLLLAFSVCEASTGLALLVATVRTHGSDRLQALNLLQC*
Safa    ILLLAFSACEASTGLALLVATSRTHGSDRLHALNLLQC*
Icae    MLLLAFSACEASAGLALLVATTRTHGSDRLQTLNLLRC*
Asmi    LLLLAFSACEAGAGLSLLVATAHTYETDHLHNLTLLQC*
Foal    MFLLTFSACEAGTGLALLVATARTHGSDHLKTLSLLQC*
Drze    MVLLAFSACEASAGLALLVATSRTHGSDLLKTLNLLQC*
Rhas    MLLLAFSACEASAGLALLVATARTHGTDRLQSLNLLQC*
Elac    MLLLAFSACEASAGLALLVATSRTHGTDRLQSLNLLQC*
Kugu    MLLLAFSACEASAGLALLVATARTHGTDRLQNLNLLQC*
Plor    MLLLAFSACEASAGLALLVATARTHGTDHLQNLNLLQC*
Sgun    MILLAFSACEASAGLALLVATARTHGTDHLQSLNLLQC*
Zaco    MLLLAFSACEASAGLALLVATARTHGTDRLQSLNLLQC*
Zbfl    MLLLAFSACEASAGLALLVATARTHGTDHLQNLNLLQC*
Spba    ILLLAFSACEASAGLALLVATARTHGTDRLQNLNLLQC*
Game    MLLLAFSACEASAGLALLVATSRTHGSDRLQSLNLLQC*
Thth    MLLLAFSACEASAGLALLVATSRTHGSDRLQSLNLLQC*
Xigl    MLLLAFSACEASAGLALLVATARTHGTDRLQSLNLLQC*
Hyja    MLLLAFSACEASAGLALLVATTRTHGSDRLQSLNLLQC*
Psan    MFLLAFSACEASTGLALMVALTRTHGCDHVKNFNLLRC*
Cupa    MLLLAFSACEASAGLALLVATSRTHGSDHLQSLNLLQC*
Mpch    MILLAFSACEASAGLALLIATARTHGTDRLQNLNLLQC*
Char    MLLLAFSACEAGAGLALLVATARTHGTDRLQSLNLLQC*
Pser    MLLLAFSACEASAGLALLVATARTHGTDRLQSLNLLQC*
Prol    MLLLAFSACEASAGLALLVATARTHGTDRLQSLNLLQC*
Plbi    MLLLAFSACEASAGLALLVATARTHGTDRLQSLNLLQC*
Calu    MILLAFSACEAGTGLSLLVATARTHGTDRLKNLSLLQC*
Papa    LLLLAFSACEASAGLALLVATARTHGTNRLKSLNLLQC*
Sufr    VFLLAFSACEASTGLALLVATARTHGSDRLQALNLLQC*
Stci    ILLLAFSACEASTGLALLVATARTHGTDRLQSLNLLQC*
Taru    LLLLAFSACEAGVGLALMVATARTHGSDHLQNLNLLQC*
Rala    MLLLAFSACEASAGLALLVATARTHGTDRLQSLNLLQC*
        : .: ::.   . * .:: :  :::       :.:*:.
```
