# Supplementary material for: Structure and variation of the mitochondrial genome of fishes
Source: BMC Genomics. 2016 Sep 7;17(1):719. doi: 10.1186/s12864-016-3054-y (PMC5015259; doi:10.1186/s12864-016-3054-y)
Supplement: Additional file 6: Figure S1-a. — Aligned amino acid sequences of the ATP8 gene in mt genomes of 250 fishes. Figure S1-b. Aligned amino acid sequences of the ATP6 gene in mt genomes of 250 fishes. Figure S1-c. Aligned amino acid sequences of the COI gene in mt genomes of 250 fishes. Figure S1-d. Aligned amino acid sequences of the COII gene in mt genomes of 250 fishes. Figure S1-e. Aligned amino acid sequences of the COIII gene in mt genomes of 250 fishes. Figure S1-f. Aligned amino acid sequences of the Cyt b gene in mt genomes of 250 fishes. Figure S1-g. Aligned amino acid sequences of the ND1 gene in mt genomes of 249 fishes. Figure S1-h. Aligned amino acid sequences of the ND2 gene in mt genomes of 250 fishes. Figure S1-i. Aligned amino acid sequences of the ND3 gene in mt genomes of 250 fishes. Figure S1-j. Aligned amino acid sequences of the ND4L gene in mt genomes of 250 fishes. Figure S1-k. Aligned amino acid sequences of the ND4 gene in mt genomes of 250 fishes. Figure S1-l. Aligned amino acid sequences of the ND5 gene in mt genomes of 250 fishes. Figure S1-m. Aligned amino acid sequences of the ND6 gene in mt genomes of 249 fishes. (ZIP 3250 kb) [file 12864_2016_3054_MOESM6_ESM.zip › Additional file 6 prot align/AF6k-ND4.pdf]

Additional file 6: Figure S1–k. Aligned amino acid sequences of the ND4 gene in mt genomes of 250 fishes.

Species name abbreviation followed by aligned amino acid sequences shown by one letter abbreviation. See Additional file 1 for abbreviation of species name. Amino acids shown by magenta letter denote hydrophobic residues. A-K in bold types with yellow background indicate putative transmembrane regions. Asterisk '*' indicates a fully conserved residue. Colon ':' and period '.' indicate 'strong' and 'weak' groups in the level of conservativeness, respectively, in the Gonnet Pam250 matrix, in which the strong and weak groups are defined as strong score >0.5 and weak score =<0.5, respectively (Thompson et al., 1997).

ND4

[1/8 of aligned sequences]

```
                                   A
Scca    MLKILIPTIMLFPTTWFMNKKWLWSSITTHSLLISLLSLSWFKWNMDIGWDFSNQYLAID
Muma    MLKILIPTIMLFPTAWFSHKKWLWTTTSAHSLLIATMSLLWFKWNMDIGWDFSNQYLAVD
Erca    MLKLLIPTVMMFPMIWVLKPKWLWSMTTTHSLLIASYSLSLFKYHSTAQWSNMSFTMATD
Pose    MLKLLIPTIMLFPTIWVLSPKWLWSITTAHSLMIATLSLTLFKYYSTTPWSNLNYMMATD
Actr    MLKIMIPTLMLFPTTWLVTPKWLWTTTTAQALVIATASLTLLNWNSETGWTSSNPYLGTD
Scal    MLKVLIPTLMLFPTTWLVTPKWLWTTTTAQALVIATTSLTLLNWNSETGWTSLNPYLGTD
Posp    MLKILIPTLMLFPTTWLATPKWLWATTTAQALIIATASLTLLNWSSETGWTSTNPYLGTD
Atsp    MLKILIPTLMLLPTTWLVSPKWLWTTTTAQSLLVACLSFVWLKRSTEPGWTTLNSYLAID
Leoc    MLKILIPTLMLLPTTWLSSPKWLWTTTTAQSLMIASLSLTWLKRSAEPGWLTLNSYLAID
Amca    MLKILIPTLMMLPTTWLTPNKWVWTVTTTQSLIIATLSLSWLKWDAETGWTSSNLHMATD
Osbi    MLKILIPTLMLIPTTWLTPKSWLWATTTTQSLIIAFLSLSWLKWSTETGWYTLNQYLATD
Pabu    MLKVLIPTIMLIPTTWFTNKSWLWTTTTLQSLLIALFSLHWLKWDTEVGWTTSNSYLATD
Hial    MLKILIPTLMLYPTIWLAPQKWLWTTTTAQSMVIALLSLSWLKWNSETGWSTSNLYLATD
Elha    MLKILVPTLMLFPTTWLAPKKWLWTSTTAQSLLIALFSLTWLKWTAETGWTTSNFYLATD
Mlcy    MLKILLPTLMLFPTIWLTPKKWLWTTTTAQSLMIAFSSLFWLKWASETGWSTSNTYMATD
Algl    MLKILVPTIMLIPTTWLVNKKWLWATTTSQSLVIALFSLLWVKWDFEAGWGASNSYLGID
Ptgi    MLKILIPTLMLYPTTWLVNKKWLWATTTAQSLVIALISLTWLKWDSEAGWSTSNHYLATD
Alaf    MLKILIPTIMLYPTTWLVNKKWLWATATAQSLIIALMSLNWLKWDSESGWATSNSYMATD
Nock    MLKILIPTIMLYPTTWLVNKKWLWVTTTAHSLVIALMSLSWLKLDSETGWYSSNPYMATD
Anja    MLKVLIPTIMLIPTTWLVDKKWLWITTTYQSFIIASISLVWFKWDSEMGWSTTNTYLATD
Gyki    MLKVLIPTIMMIPTTWMVNKKWLWITTSSQGLMIAIVSLTWMKWESETGWSTSNLYLATD
Syka    MLKVLIPTIMLIPTTWLINKKWLWTTTTYQSLMIAAVSLTWLKWDTETGWSTSNQYLATD
Opma    MLKVLIPTIMLIPTTWLINKKWLWTTTTYQSFTIAAISLTWLKWDSESGWLTSNPYLATD
Comy    MLKVLIPTIMLIPTAWTVNKKWMWTTITYQSFFISAMSLIWLKWDSETGWSALNPYLGTD
Sasp    MLKVLIPTLTMIPTTWFMDKKWLWSLTTSQSLLIAGFGLTWLKCSPDTEWSTTNTYLSTD
Eupe    MLKILIPTFLLIPTTWLVNKKWLWTTTTSQALIIAAISLLWFKWSPEMTWSTSGAHLATD
Enja    MLKVLIPTLMLFPTIWLSPQKWLWTATTAHSLVIALLSFSWLSWPSETGWSASNTYMAID
Same    MLKVLIPTLMLFPTIWLTPKKWLWTSTVSHSLVIALLSLTWLNWTAETGWTLPSNYMAID
Chch    MLKILIPTLMLFPTIWLAPTKWLWTCTTAQSLFIATISLTWLKWDSEIGWGTTNSYLATD
Grgr    MLKILIPTLMMFPLIWLAPAKWLWASATAQALFIALASLTWLKWNSETGWAAPDALLATD
Caau    MLKVLIPTIMLFPTIWLTSPKWLWTTTTAHSLLIASISLMWFKWTSETGWTSSNTYLATD
Cyca    MLKVLIPTIMLFPTIWLTSPKWLWTTTTAHSLLIASISLTWLKWTSETGWTSSNMYLATD
Dare    MLKVLIPTIMLFPTIWLSSSKWLWTTTTMNSFLIAFISLTWLKWTSDTGWNASNSYMAAD
Cost    MLKVLMPTIMLFPTIWLSSPKWLWSTTIAHSLLIALTSMSWLSWSSETGWSSSNLYLATD
Leec    MLKVLFPTLMLFPTIWLSTPKWLWTTTTAQSLFIAFISLYWLKWTSEIGWSTSNTYLATD
Fola    MLKVLIPTIMLFPTIWLTPPKWLWSTTTAHGLLIALISLTWLKWSSEVGSATSSLYLASD
Clmc    MLKILIPTIMLFPTIWLSSPKWLWTTTTAQSLIIALTSLTWLKLNSETGWMTSNLYMGTD
Phin    MLKILIPTAMLFPTIWLTPAKWLWTTTTAQSLLIATISLSWLKWNTETGWTASNTYMAVD
Icpu    MLKILIPTIMLFPTIWLSPSKWVWTTTTLQSLIIALVSLSWINWSSETGWASSNLYMGTD
Psto    MLKILIPTIMLFPTIWMTSPKWLWTTTTFQSFIIALISLTWLKYPLETGWASSNHYMGTD
Cora    MLKILIPTIMLFPTIWLVSPKWLWSTSTMQSLLIALISLSWLKWPSETGWSALNTYLGTD
Eisp    MLKILIPTLMLFPTIWLTSPKWLWTTMTAQSLLIAALSLAWFGFYQETGWAASNQYMAID
Apal    MLKILMPTMMLIPTIWLSPQKWLWTATTTQSLLIATVSLAWFGLYSETGWTTPNPYMATD
Eslu    MLKILIPTFMLFPTIWLVPGKWLWTATTAHSLVVASASLSWFKWCSETGWVAPNLYLALD
Dape    MLKILIPTLMLFPTTWLAPNKWLWTTITAQSLMVALMSLMWFNWTMETGWTAPNMYIALD
                               1
```

To be continued on page 6.

[1/8 of aligned sequences]

```
Glse      MLKILIPTLMLFPTIWLSPTKWLWPISAGHSLIIALISLTWLKWTSETGWSASNLYLATD
Naar      MLKILIPTLMLFPTIWLTPKKWLWSTLTTQSLIIALASLAWLKWTSETGWSTSNLYLATD
Lioc      MLKILIPTLMLFPTIWLTPKKWLWSTLTAHSLLIALASLTWLKWSSETGWSTSNLYLATD
Opso      MLKILIPTLMLFPTIWLTPTKWLWPTSTAHALAIALASLTWLKWSSETGWTSSSYYLATD
Alte      MLKILIPTLMLFPTIWLGPTKWLWTTTTAQSLLIALTSLSWLKWNSEIGWSSSNSYLATD
Plap      MLKILIPTLMLFPTIWLTPSKWLWTTTTAQSLFIALISLSWLKWDSEIGWSSSNLYMATD
Plal      MLKILIPTIMLFPTIWLTPAKWLWPTSVAQSLVIALASMTWLSWTSETGWATSNLYLATD
Sami      MLKILVPTIMLFPTIWLAPAKWLWPTTVAQSLMIALASLSWLKWTSEAGWTTSNLYLATD
Rere      MLKILIPTIMLFPTIWLSPPKWLWPTSVAQSLVIALASLSWFKCSSETGWSNPSLYLATD
Gama      MLKILIPTIMLFPTIWLAPTKWLWPASIAQSLVIALMSLSWFKYSSETGWSTTNLYIATD
Onmy      MLKILIPTLMLFPTIWLSPAKWLWTTSIAQSLIIALASLSWLKWSSETGWSSSNLYLATD
Sasa      MLKILIPTLMLFPTIWFSPAKWLWTTSIAQSLVIALASLSWLKWSSETGWSSSNLYLATD
Cola      MLKILIPTLMLFPTIWLSPAKWLWATSTAQSLLIALASLSWLKWSSETGWTSSNLYLATD
Dita      MLKILLPTIMLFPTIWLTPKKWLWSTSLAQSLIIALASLCWLNWSSETGWSAPGYYLAFD
Gogr      MLMVLIPTIMLLPTIWLTPHKWLWPTLLMQSLIIALMSLSWLKWPSETGWMSPSHYLAPD
Chsl      MLKVLIPTIMLFPAIWLSPPKWLWPSSLIYSLAIAAVSLSWLKWPAETGWHFSNFYLASD
Atja      MLKILIPTLMLFPMIWLTPHKWLWPASLAHSLTIALASLSWFINTSETGWSTLNPYMATD
Iido      MLKILIPTLMLFPMIWLTSHKWLWPTSLAHSLAIAFTSLFWLVNTSETGWSTLNSYMATD
Auja      MLKVLLPTLMLFPTIWFSPKKWLWTISLVQSTLIALVSLSWFKWTSETGWTTLNLYLGTD
Chag      MLKILLPTLMLFPTIWFAHKKWLWTISLTQSLLIALISLTWLKNPSETGWSTLNPYLGTD
Hami      MLKILVPTLMLFPTIWLAPKKWLWPVSLVQSLLIALASLLWLKTSAGVGWGTLNLYLGTD
Saun      MLKILMPTLMLFPTVWLAPKKWLWTSVLTQSLLIALASLLWLKNTSETGWLTLNPYLGTD
Nema      MLKILVPTLMLFPTVWMTPFKWLWPSSLAHSLIISLISLHWLKNSSETGWSSMNPLMATD
Disp      MLKILLPTLMLFPTIWLTPTKWLWPSSLAHGLIIALVSLHWLAHPSETGWSFVSPLMGTD
Myaf      MLKILLPTLMMLPLIWLTQPKWVWPASLAHSLVIALASFHWLMNTSETGWSLMNPLMATD
Lagu      MLKIFTMTIMLLPTIWLAPPKWLWAFSLTQSFLIATLSLHWLNLWSETGWNMTNLLLALD
Trtr      MLKILLPSIMLLPTIWLTPFKWVWTITLGQTMLIALASFMWFNLPSETGWTMANPYLAVD
Zucr      MLKVLFASVMLLPSIWLTSPKWLWTFTLAQSLLIAFLSFTWLNLFCETGWMMTSMYLAVD
Pxja      MLKILVPTIMLFPTAWLTPAKWLWPTSLGQSLIIALLSLQWLINPSETGWLTLNTLMATD
Pxlo      MLKILVPTLMLFPTAWLTPAKWLWPTSLGQSLIIALLSLQWLINPSETGWLTLNTLMATD
Pctr      MLTVLVSTLMLFPTTLLSSHKLLWSTTLGHSLIIALLSLTWLKSATGTGWSALGPLMATD
Apsa      MLTILIPTLMLFPTTLLTPPKLLWPTSLGHSTIIALISLTWFKNTTETGWSFLNPLLGTD
Cabe      MLMVLIPTLMLVPVTWLSPPKWVWAATLMHSLLIAVISLKWLYTGHETGWHELTPSMGLD
Bzze      MLKILLPTIMLVPTTWFTPAKWLWPTTLAHSLIIALASLTWLKNTSETGWYTLNPYMALD
Siim      MLKVLIPTIMLLPTTWLTPQKWLWSTTLTHSLLVALSSLMWLKNTSEVGWWALGPYMAND
Ctru      MLKILLPTLMLVPTTWLAPTKWLWPTTLTHSLIIALVSLTWLKSPAEAGWSTLNLFMATD
Dpbr      MLKILIPTLMLVPTTWLTPSKWLWPTTLTHSLVIALMSLTWLKNPAETGWSTLNPWMATD
Caki      MLKILIPTLLLIPTACFVHSKWLWTTTLIQSFTIALVSFVWLNNTSETGWNTLNFFIATD
Phja      MLKILIATLFLLPTTWLSPMKWLYSTTMLQSMLIALGSLYWFNYSSETGRTALNLYIGTD
Brsp      MLKILIPTFFLLPSTWLTPSKWLWSTLLAQSTFISLMSLTWLNFSSGTKWSTLNLLMASD
Gamo      MLKILIPTLFLLPTTWLTSSKWLWPTALTQSMLIALGSITWLNNTTDTGWTALNSYIGTD
Lolo      MLKILIPTLFLLPTTWLTSGKWLWPTALTQSMLIALASLTWLNNSTDVGWAALNSYIATD
Batr      MLKILLPTLSLLTSTLLTNKKWLWSTSLFHSLLISTVALTLFFNFSITEHSTTNPLTITN
Prmy      MLKLILPNILFSLILWVFPQKWFWPMSLFYMLLLAILNTPILYTPPMTPWALTNLYLSTD
Lose      MLKILIPTIMLIPAALIVNGKWLWPSTLLYSTLIALASLTWLKNLNETGWSLINPYMATD
Loam      MLKVLIPTLMLMPTIMAAKAKWLWPTTLLHSLLIALISLTWLKNLGETGWSHLNPLMATD
Chab      MLKILLPTLMLLPTTWVGPIKWLWPTTLFHSLLIATLSLTWFKNPSETAWTSLSPWMATD
Chto      MLKILLPTLMLLPTTWLGPIKWLWPTTLFHSLLIATLSLTWFKNPSETAWTSLSPWMATD
Majo      MLKILMPTLALIPTLWLLSPAWLWTAALIYGLLLATLALPLVGNVGEMGWSTLNLYLTVD
Hlst      MLKIILPTLMLIPSVWLSSKTWLWNSTLMFSLIIASLALPLVNSLGETGWSALNPHLAVD
Clpe      MLKILIPALMLIPTAWLAPAKWLWPTALFNSIMIAAVSLPLLKNLSETGWSSLNLYMATD
Mlmr      VLKILIPTLMLMPTAWLATPKWLWPTTLFHSLTIAVISLTWLKNLSDTGWSCLNLYMATD
                                   2
```

To be continued on page 7.

[1/8 of aligned sequences]

Crcr	MLKILIPSIFLIFTAWTAPKKQLWSTTLAYSLIIALISLSWLVNPAETGWSFLGLHMATD
Muce	MLKILIPSIFLVLTAWTTPKKQLWSTTLAYSLIIALISLSWLVNPTETGWSFLGPHMATD
Bege	MLKILIPTIMLLPTTWIIPFPRLWATTLSYSLIIAIVSLTWLKPPAETGWTSLNSYMATD
Mela	MLKILVPTVMLLPMAWTIPFHQLWPTALLYSLTIAFMSLTWLKTTAEAGWAFLSPYMATD
Hats	MLKILIPTIMLIPTTWLTPARQLWSTVLLYSLVIAIASLTWLKTPAETGWTTLSSYMATD
Orla	MLMVLAPTMMLLPMIWLCSSAHLWTSALAHSMAIALISLSWLCLPMEAGWSSLNTFVATD
Cosa	MLKVLFPTIMLLPLTWLCPQKKLWSLTLLYSLMISFLSLLWLKMPLQTNWTHLNLYMATD
Exsp	MLKVLLPTIMLLPVTWLIPHKKLWATTLMYSLIIALISLSWLKMPAETGWTCLSLYMATD
Depa	MLKILIPTIMLLPTTWLTPHKNLWSTTLLYSLLIAFLGLSWLKMPSETGWVHLTPYMATD
Rima	MLMILMPTILLFVAAWLTPSRHLWTTTASYSFLIAVMSLKWLNMPADTGWMFLTLNLATD
Fuol	MLKILIPSVLMLATTWTSPYKQLWSTTLIYSLLVSTLSFTWLAMPTDTGWYYINNYMASD
Gmaf	MLKILLPSILLLPSTWLMPKKHLWSTTLSYSLMIAGTSLAWLAIPSENGWSFLNCFMASD
Xeei	MLKILTPTIFLLLTTWVTPSKHLWSTSLLHSLVIATVSLIWLISSAETGSSFLGTYTATD
Pros	MLKVLIPTLMLFPTAWLAPTKWLWPTTLGQSLMIALISLTWLNRLSETGWSALNTFMATD
Scmi	MLKVLIPTLMLFPTTWLVPAKWLWPTTLGQSLMIALAALTWLGCLSEVGWSALNSFMATD
Rolo	MLKILIPTLMLVPTAWLTPAKWLWTMTLAQSLIIALASLTWLIHFSETGWSTMNTYMATD
Cere	MLKVLIPTLMLAMMVWLIPAKWLWAAALGHSLVIALISLNWLGCLSETGWSQLNMFMATD
Daga	MLKILIPISMLTLTVWMSPAKWMWTLALAHSLMIALGSLPWLAWLSETGWSNMNLYMAAD
Anco	MLKVLLPTLMLIPTAWLVPAKWLWTTTLGQSMVIALVSLTWLNNMTEAGWTTLTPYMATD
Dmve	MLKILVPTLMLIPTTWLTPTKWLWTTALGQSLMIALMSLAWFNNMSETGWSTLNPYMATD
Dmar	MLKILIPTLMLVPTIWLAPTKWLWTATLGQSLLIALMSLAWFNNMSETGWSTLNPYMATD
Anka	MLKILIPTLMLIPTTWLVPTKWLWTTTLGQSMIIALASLTWLNNTTEAGWTTLSPYMATD
Moja	MLKILIPTLMLIPTAWLVPAKWLWTTTLGQSMMIALVSLIWLNSTAETGWSTLNPYMATD
Hoja	MLKILFPTLMLVPMAWLVPAKWLWTSTLGQSMMIALISLTWLSNTTETGWSTLNSYMATD
Bede	MLKILIPTLMLVPTAWLTPTKWLWPTALGQSLVIALISLTWLGHLSETGWSTLNTFMATD
Besp	MLKILIPTLMLVPTTWLTPAKWLWPTTLGQSLVIALISLTWLGHLSETGWSTLNTFMATD
Mysp	MLKILIPTIMLIPTIWLTPAKWLWTTALNQSLIIALLSLTWFKNMSETGWSCLNPYMATD
Osja	MLKILIPTIMLIPTIWLSPTKWLWTTALNQSLIIALLSLTWFKNMSETGWSSLNPYMATD
Sgro	MLKILIPTIMLIPTVWLTPAKWLWPTALNQSLIIALASLTWLKNSSETGWSTLNPYMATD
Pzpa	MLKILIPTLMLFPTTWLTPSKWLWPTSFAHSMVIAFMSLSWLKNSTESGWTTLNTLMGTD
Zeja	MLKILIPTLMLLPTTWLLPSKWLWPASLAHSTVIAFTSLHWLKNSSESGWTMLNPLMGTD
Znne	MLKILIPTLMLMPTTWLVPNKWLWPTSLTHSMIIALISLYLLKNSSEQGWTALNTLMGTD
Zefa	MLKILIPTLMLLPLPWLTPKKWLWPTSLMHSMIIALISLYWLKNSSEEGWTTLNPMMGTD
Acni	MLKILIPTLMLFPTAWLTPSKWLWPTTLAHSTIIALISLHWLKNSSETGWTTLNTLMGTD
Ncrh	MLKILIPTLMLFPTAWLTPSKWLWPTTLAHSTIIALISLHWLKNSSETGWTTLNTLMGTD
Agca	MLKILIPSLMLVPTAWMAPTKWLWPTSLLHSLLIALASLMWLKNSSETGWSCLNPYMATD
Hydy	MLKVLIPTLMLIPTTWLTKPKQLWPTTLAHSLFIALLSLTWFINMSETGWTSLGSYLATD
Gsac	MLKILIPTLMLIPTTWLTKPNWLWPSTLAHSLLIAVFSLTWFANMSETGWTSLNYYLATD
Pevo	MLKILLPTIMLLPLSWLTPMKWLWTSTLAHSLLIALISMTWLKNLSETGWSTINMFLATD
Hiku	MLKILIPTIMLIPTTWLTPKKWLWPSTLTHSLIIALFALTWLIWPSETAWSNLSGSMATD
Inpa	MLKILLPTIMLIPTICLTPPKWLWPASLAHSLIIALISLTWFTSLTNMTWTSINLYMMCD
Auch	VLKILMPTTMLLPMAWLSPASWLWPNVLCYSLAIAILSLPWLKTCSETGWIVLNKLLASD
Fico	MLKILVPTIMLLPTIWLSAPKWLWPTSLLHSITIAIASLVWLNETSETGWSSLNTMLATD
Macs	MLKILLPTLMLVPTTWLVPSKWLWPSTLAHSLIIAFASLLWLSSAMDTGWSSLSSYLATD
Moal	VLKILIPTLLLIPTSWLAKAKWLWPTTLLHSFIITLFSLAWLKNTSETNWSTLNPYMATD
Syma	MLKIIMPSLMLIPTALLAKTKWLWPMTLSYSLIIALFSLTWFKNLSETNWHSLNIYSASD
Mafr	MLKILIPTLMLVPTTWTSPIKWLWPTTLTHSLIIALVSFTWLKNPSETGWHSINMCMALD
Dcpe	MLKILVPSIMLVPMVWTVREKWLWLVATAHSFTIAVASLSWLKTTTETGWTYINSFLGTD
Dcti	MLKILIPSIMLVPMVWSVREKWLWLVAIAHSFTIAVASLSWLKNTAETGWTYINSFLGTD
Hehi	MLKILIPTLMLLPTAWLTPAKWLWPTTLSHSLVIALASLTWLKNTSETGWSCLTPFMATD
Stam	MLKILIPTLMLIPTAWLAPAKWLWPTTLAHSLVIATFSLSWLKNLSETGWSALNSYMATD
Hogi	MLKILIPTIMLIPTTWLTSPKWVWPTALSHSLIIALGSLLWLKNTSETGWSTLNPLMATD

To be continued on page 8.

[1/8 of aligned sequences]

Erzo	MLKILIPTLMLIPTAWATKPKWLWPTTLAHSLVIAVLSLSWLVNMSETGWSNLNPYLATD
Hxot	MLKILIPTLMLIPTAWMAPPKWLWPTTLMHSLLIALFSLSWLTNMAETGWSTLNPYMATD
Core	MLKILIPTLMLVPTTWMAPSKWLWPTTLMHSLLIALASLSWLSSTAETGWSCLNFYMATD
Apve	MLKILIPTLMLIPTTWMAPPKWLWPVTLGHSLTIALFSLSWLTNTSESGWTSLNPFMATD
Latj	MLKIIIPTIMLIPTTWLTPAKWLWPAILAHSLSIALVSTTWLMRLTETGWSFLGSYMATD
Laja	MLKVLIPTLMLIPTIWLAPTKWLWPTTLLHSLIIALTSLSWLKNLSETGWSHLNLYMATD
Syja	MLKILLPTLMLVPTIWATPNKWLWPATLAHSLIIALASLSWLKTPSETGWTSLNSYMATD
Epme	MLKILLPTLMLVPATWLTPAKWLWPTALGHSMLIALISLSWLKHAMETGWSTLSLFMATD
Grse	MLKILIPTLMLVPTTWMAPAKWLWPATLMHSLLIALASLTWLKNIMETGWSSINPYMATD
Clja	MLKILIPTVMLVPTIWLTPPKWLWPATLAHSLLITLISFTWFKFTSESGWYFINSHMATD
Ogcy	MLKMLIPTMMLMPLIWTTPKKKLWHLTLAYSLTIALSSLTWLNTWSEAGWAFMNTYLATD
Plna	MLKVLFSSIMLVPFIFLISPKQLWISTLTYSLAIAMMSLPWLKTFSTHSWTFLTSNLGVD
Lema	MLKILIPTLMLVPTIWLAPAKWLWPTTLLHSLIIALTSLTWLKNTLETGWSSLNVYMATD
Etzo	MLKILIPTLMLIPTAWGVPAKWLWPTTLAHSLIIALASLIWLKNTAEVGWSNLGLYMATD
Apse	MLKILIPTLMLVFTTWNVRAKWLWPTVLAHSLLISVLSLTWFANTSETGWKSLNSYLATD
Epde	MLKILIPTLMLIPTAWMVSPKWLWPTALLHSLIIALASLTWLKNLAETGWASLSLYMATD
Slja	MLKVLIPTLMLIPTIWLTPNKWLWSTTLMHSLIIALVSLTWLKNLAETGWTTLNLYMATD
Bsja	MLVILIPTLMLIPTAWLIRPNWLWPVTLAYSLMIALTSFFWFKNLTETGMSYLTPYTATD
Ecna	MLKILLPTLMLVPTIWVTPAKWLWPTTLAHSFLIALTSFSWLKNFSETGWMSLNKYMATD
Cohi	MLKILIPTLLLIPSAWFTPAKWLWTSSLAHSFIIVLVSLPLLKDTSFSQWTMLSSYLGVD
Caar	MLKILIPTLMLVPTTWLTPAKWLWPTTLAHSFIIALASLSWLKNLSETGWSCLNNYMATD
Came	MLKILIPTLMLVPTTWLTPAKWLWPTTLAHSFVIALASLTWLKNLSETGWSCLNNYMATD
Mema	MLKILIPTLMLVPTTWLTPAKWLWPTTLAHSFIIALFSLTWLKNLSETGWTSLSPYMATD
Lenu	MLKILIPTLMLLPTAWLAPTKWLWTTSLLHSLMIALASLTWLHNTSETGWTNLNQLMATD
Brja	MLKILIPTLMLVPTTWFTPPKWLWPTTLAHSLIIALISLTWLDNLTEAGWTSLTPYLATD
Plma	MLKILIPTLMLVPTTWLTPPKWLWPTALAHSLIIALASLSWLSNLSETGWTSLSQFMATD
Emst	MLKILIPTLMLVPTAWMAPAKWLWPTTLMHSLVIALASLTWLKNLSETGWSALNPYMATD
Ptti	MLKILIPTLMLVPTTWLVSAKWLWPTALLHSLVIALASLTWLKNLSETGWSCLNPYMATD
Losu	MLKILIPTLMLTPMTCLTPLKWLWPTTLLHSLLISLASLIWLKNSTDTGWSFLTPIMATD
Geoy	MLKILFPTLMLIPTIWLAPHKWLWPTTLAQSLLVALASLTWLKNFSEAGWTSLNSFMATD
Dipi	MLKILIPTIMLVPTAWMAPAKWLWPTTLLHSLIIALASLTWLKNLSETGWSFLSPYMATD
Pama	VLKILLPTLMLVPTIWLAPAKWLWPTTLLHSFIIALISLTWLINLSEVGWSSLGPYLATD
Leob	MLKILIPTILLVPTTWLTPAKWLWPTALLHSLIIALLSLSWLKNLAETGWAFLSPYMATD
Neba	MLMILTATLMLFPTVWLVRYSWLWSITLFYSMTIAVVGVTMPKNLAHTGWYEINPYMAVD
Pdpl	VLKIIIPTFMLLPTTWLVPLKGLWSSTLSYSFLIALISLTWLKCPSETGWTSLTSFLAVD
Nimi	VLKILVPTLMLVPTTWLVPSKWLWPTTLTHSLLIALVSINWFKNPSETGWTFLNSYMATD
Uptr	MLKILIPTFMLMPTTWLAPAKWLWPTALAHSFIIAVASLAWFKYVPETGWSELSLYMATD
Pesc	MLKVLLPTIMLIPTIWLAPFKWVWTSTLLHSLIIALMSFSWLKNLAETGWTSLNPYMGLD
Baar	MLKILIPTLMLVPTTWMAPAKWLWPTTLLHSLAIALVSLTWLKNLSETGWTSLSLYMATD
Moar	MLKILIPTLMLIPTAWLTPSKWLWPTSLLHSLIIALASLTWLKNLSETGWSSLNTYMATD
Toja	MLKILIPTLMLVPTAWMSSPKWLWPTALTQSFIIALLSLSWLKNLSETGWSSLGPYMATD
Chau	MLIILVPTFLLVLTTWLTPAKWLWPTTLMHSLIIALASLHWLENMSEVGWSHLSTYMATD
Chse	MLKILVPTLMLIPMIWLASPKWLWSTALTHSLIIALASFSWLKNLSETGWSTLNTFMATD
Enar	MLKILIPTLMLVPTIWMVPGKWLWPTTLLQSLLIALASFSWLKNFSETGWSSLNPYMATD
Hpty	MLKILIPTLMLVPTTWMVPAKWLWPTTLLHSLIIALTSLLWLKNLSETGWTSLNLYLATD
Nana	MLKILIPTLMLIPTTWFTNTKWLWPTTLAHSLIIAFLSLTWLKNMSETSWTSLNPYMATD
Mcst	VLTILIPTLMLIPTTWLTPSKWLWPTTLFHSLIVALVSLTWLKQMSETGWSCLNPYMATD
Rhox	MLKVLIPTLMLIPTTWLTPQKWLWPTTLFHSLAVALVSLTWLENLTEAGWSCLNQFMATD
Opfa	MLKVLIPTLMLIPTTWLAPSKWLWPTTLLHSLLVALISLTWLKNLSETGWSCLNLYMATD
Paar	MLKILIPTLMLIPTAWFTSGPLLWPTVLLHSLTISVASFTWFKNPAETGWSFMNANMAAD
Gozo	MLKILIPTLMLVPTIWLTPAKWLWPTTLLHSLLIALVSLTWLKNLSETGWSSLNLYMATD
Ackr	MLKILIPTMMLIPTTWLSSPKWLWPTTLLHALLIATASLTWFKNLAETGWSSLNLYMATD

To be continued
on page 9.

[1/8 of aligned sequences]

```
Elev  MLKILVPTIMLIPTAWLTPAKWLWSTTMLHSLLIAMVSFTWLSSVLETGWNSINLFMATD
Trdu  MLKILLPTIMLVPTIWAIPAKHLWSTTLSYSLIISLTSLTWLKSPAESGWSFLSPYMATD
Amoc  MLKILLPTIMLIPTTWLSPAKQLWTTTLAYSLVIALASLAWFNSPTETGWSCMNSYMATD
Hame  MLKILIPTLMLIPAIWLSPTKWFWQMATSNSLLIATISLLWMKKPWDTGWSYLTSMTGID
Chso  MLKILIPTLALIPLSWACSKKWLWHTTTANSLLIATLSLPWLKTSLDAGWTHLNPLMGTD
Lyto  MLKILIPTLMLVPTAWAVKPKWLWPTTLTQSLIIALLSLSWLVNMSETGWSGLNSYMATD
Encr  MLKILIPTLMLIPTTWMVKPKWLWPTTLTQSLVIALLSLSWLVNMSETGWSSLNGYMATD
Bvar  VLKVLVPTFMLIPTTLLTPTKWLWPTTLAHSLMISLISMSWLKNMTEAGWTSLSPYMATD
Noco  MLKILIPTLMLIPTIWLVPAKWLWPSTLVHSLVIAVASFTWFKNTSETGWTTLNSYMATD
Chsp  MLKILIPTIMLIPTVWMVTPKWLWTTALFHSLIIALTSLQWINFMTETGWNFLNSYLAND
Arja  MLKILVPTLMLIPTAWMASPKWLWPTTLMHSLLIALFSLSWLINMAETGWSSLNPFLATD
Pase  MLKLLLPTIMLIPTAWFLPAAHLWHGMVLQSLVVAASSLHTLKFLAHTGQSNISPFFSID
Trel  MLKILVPTIMLIPTTWLSPPKWLWSSALTHSMLITLASFYWLENLTETGWSSLNSFLATD
Lifa  LLKVLIPTLAMFPIVWLSPKKWLWPTSLLHSFTIALISLSWLKYHGETGWTSLNQFMATD
Acur  MLKIILPTILLVPMVWLVSSRWLWPWMQLHGLVIALISLSWCKPSFFLGWIFLGPFTGAD
Ampe  MLKILIPTIMLIPTTWLVPHKWLWSSTLLNSLVIAACSLTWMKKLAETGAASINNYLAVD
Urja  MLKLFAPTLMLSPLASFSPFKWLWPSSFLGSILIATLSFSWLKNISETGWTSLNLYLASD
Enet  MLKVLIPTLMLAPTVALVPQNKIWTTTLAYSFLIACMSLTFFRNMQETAWALMNPYMATD
Ptbr  MLKILLPTIMLFPTIWFAPAKKIWSLPLMSSLIIAFSSLLFFYSPEETGWMMLNSSMGSD
Safa  MLKILAPTIMLIPTIWLLPSKKLWSISLSYSLIIAIYSLTLLNNPAEVGWTMLGQYLATD
Icae  MLKILIPTLMLIPTTWLAPPKWLWPTALTHSLIIALASLTWLDSLAETGWTSLNLYMATD
Asmi  MLKILTATVLLIPMVWTTPIAKPWSLSLLYSLTLAFLSLLWFNLPASTNWSFLNSYMATD
Foal  MLKILIPTLMMIPTTWLTPRKWLWPTALYQSLFLAFLSLSWLKNTSQPGWYFINSYMATD
Drze  MLKILTPLIMMTLSICLIPKKWLWSSTLGHSFMVAFISLFLLKESTIARWFYLSPTLGAD
Rhas  MLKILIPTIMLVPTTWAVNPKWLWPTTLAQSLVIAIISLTWLKAPAETGWSCLNPLMATD
Elac  MLKILIPTIMLIPTAWLTPAKWLWPTTLAQSLTIALASLTWLINTSETGWSALNYFMATD
Kugu  MLKILIPTLMLIPTIWLTPTKWLWPTALAHSLLIATASLSWLKNLTETGWSSLNPYMATD
Plor  MLKLLIPTLMLVPTTWMASPKWLWPTTLLHSLLIALASLTCLKNTSETGWYFLGPHMATD
Sgun  MLKILIPTLMLIPTTWLTPAKWLWPTTLLHSLIIAVASLTWLKNLSETGWSNLSPYMATD
Zaco  MLKILIPTIMLVPTAWMIPTKWLWPTTLAHSMLIAFASLAWLKNLSETGWSCLNPYMATD
Zbfl  MLKILIPTIMLIPTTWAVPAKWLWPTTLMHSLVIALASLSWLKNLSETGWSSLNPYMATD
Spba  MLKILIPTFMLIPTTWLLPPKWLWPTTLAHSFIIATLSLTWLQTLSETGWSSLNLTMATD
Game  MLKILIPTLMLVPTAWLTPPKWLWPTALTHSLIIALASLTWLESLSETGWASLNLYMATD
Thth  MLKILIPTLMLVPTTWLTPPKWLWPTTLAHSLIIALASLTWLESLSETGWTSLNLYMATD
Xigl  MLKILIPTLMLAPTTWLSSPKWLWPSTLAHSFVIALFSLTWLKNLSETGWSSLNLYMATD
Hyja  MLKILIPTLMLIPTTWLVLPKWLWTTTLAHSLIIALASLTWLENLSETGWTSLNSYMAVD
Psan  MLKILVPMIMMIPTTLFVPSASIWSTAGCQSLMIAMASLTWLIKLSETGWTHVNTYLAID
Cupa  MLKILIPTLMLVPTTWMVSPKWLWPTTLGHSLIIALASLTWLESLSETGWTSLNQYMATD
Mpch  MLKVLIPTLMLIPTAGFAPTKWLWSVTLAHSLIISLISLSWLKNISETGWTMLNPYMATD
Char  MLKLLIPTLMLIPTTWAAPKKWLWPTTLAHSLTIALISLSWLKNMSETGWSCLGPYMATD
Pser  MLKILIPTLMLAPTAWLTPAKWLWPTTLAHSFLIALASFSWLPNLSETGWHSLNPYMATD
Prol  MLKILIPTLMLIPTAWLVKPNWLWPTTLTHSFCISLASLSWLKNLSETGWSSLNLCMATD
Plbi  MLKILIPTFMLIPTAWLLKPNWLWPVTLLYSFCISLISLSWLKNLSETGWSSLNLFMATD
Calu  MLKVLLPTLMLIPTIWLSKASWLWSSVLSHSFCIAMISLTWFCTPSETGSVFLSQYVATD
Papa  MLKILLPTLMLIPTACLAHPNWLWPTSLTHAFLVAMLSLTWLKNPSETGWSTLNTYMATD
Sufr  MLKILIPTLLMVPTTWLVPPKWLWPSSLFHSLLIAAVSLIWLKNISETGWASLNFYLATD
Stci  MLKILIPTLALVPVTWMVPKKWLWPTVLLHSLLIATASLSWFKYITETGWTTTNNYLATD
Taru  MLKILIPTTMLILATWLTPPKWLWPSSLLNSLLIALTSLLWLKNASETGWTFLNPYLATD
Rala  MLKILLPTLMLMPVTWLTPAKWLWPSTLLHSLLIAFISLTWLKNLSETGWSFLSPYLAAD
      :* ::    :            :          :                         :
```

To be continued on page 10.

[2/8 of aligned sequences]

```
                          B                                      C
Scca  PLSAPLLILTCWLLPLMILASQNHITPEPLTRQRIYISLLISLQVFLIMAFSATEMILFY
Muma  PLSTPLLILTCWLLPLMILASQNHISPEPLTRQRTYISLLIFLQFFLIMAFSATEMILFY
Erca  MISTPLIILTCWLLPLMILASQNHITTEPINRQRTYITLLTSLQLLLIMAFSATEIILFY
Pose  MISTPLIVLTCWLLPLMILASQNHISPEPINRQRSYITLLVSLQTLLIMAFSATEIILFY
Actr  PLSTPLLVLTCWLLPLMILASQNHISPEPISRQRTYITLLVSLQLFLIMAFGATEIILFY
Scal  PLSTPLLVLTCWLLPLMILASQNHISPEPIGRQRTYITLLVSLQLFLIMAFGATEIILFY
Posp  PLSTPLLVLTCWLLPLMILASQNHISPEPVSRQRTYITLLVSLQLFLIMAFGATEIILFY
Atsp  PLSTPLLVLTCWLLPLMIMASQNHIFPEPINRQRMYISLLVSLQAFLIMAFGATEIILFY
Leoc  PLSTPLLVLTCWLLPLMIMASQNHISPEPINRQRMYISLLISLQVFLIMAFGATEIILFY
Amca  PLSTPLLILTCWLLPLMLLASQNHLHLEPLNRQRTYITLLVSLQVFLIMAFGATEIILFY
Osbi  PLSTPLLVLTCWLLPLMILASQNHTLQEPINRQRSYITLLVFLQISLIMAFGATEIIMFY
Pabu  PLSTPLLVLTCWLLPLMILASQNHIRSEPMNRQRSYIMLLILLQSFLIMAFGATEMIMFY
Hial  PLSTPLLVLTCWLLPLMILASQNHVSPEPVNRQRTYITLLVSLQTFLIMAFGATEIIMFY
Elha  PLSTPLLVLTCWLLPLMILASQNHISPEPVNRQRTYITLLISLQALLIMAFGATEIIMFY
Mlcy  PLSTPLLVLTCWLLPLMILASQNHTMPEPTNRQRTYLTLLASLQALLIMAFSATEIIMFY
Algl  PLSTPLLVLTCWLLPLTILATQNHAMSEPEYRQRTYITLLISLQAFLIMAFGATEVMMFY
Ptgi  PLSTPLLVLTCWLLPLMMLASQNHISPEPENRQRVYITLLVSLQTFLIMAFGATEIIMFY
Alaf  PLSTPLLVLTCWLLPLMILASQNHISPEPENRQRTYITLLISLQVFLIMAFGATEIIMFY
Nock  PLSTPLLVLTCWLLPLMILASQNHISPEPENRQRTYITLLISLQVFLIMAFGATEIIMFY
Anja  PLSTPLLVLSCWLLPLMILASQNHMRPEPINRQRSYITLLISLQMFLIMAFGATEIIMFY
Gyki  PLSIPLLILSCWLLPLMIIASQNHMSTEPISRQRTFIMLLTFLQIFLILAFSSTEVMMFY
Syka  PLSTPLLVLSCWLLPLMILASQNHMTYEPVSRQRSYITLLISLQIFLTMAFGATEIIMFY
Opma  PLSTPLLVLSCWLLPLMILASQNHMSLEPTNRQRSYITLLISLQILLTLAFGATEIIMFY
Comy  QLSTPLLILSCWLLPLTIMASQNHMMKEPETRQRTYITLLISLQMLLTLAFGATEIIMFY
Sasp  LLSAPLLMLSCWLLPLMILASQNHMRPEPLTRQQSYITLMILLQLALIMAFGATEIALFY
Eupe  PLSTPLLVLSCWLLPLMILASQNHMNPEPLTRQRSYITLLILLQLALILAFGATEIIMFY
Enja  PLSSPLLVLTCWLLPLMILASQNHTQTEPISRQRMYITLLTSLQAFLILAFGATEVIMFY
Same  PLSAPLLVLTCWLLPLMILASQNHTQPEPAARQRMYLSLLASLQVFLIMAFGATEIIMFY
Chch  PLSTPLLVLTCWLLPLMILASQNHLAPEPINRQRIYISLLTSLQAFLIMAFGATEIILFY
Grgr  PLSTPLLILTCWLLPLMILASQNHIAPEPLSRQRTYISLLVSLQAFLIMAFGATEIIMFY
Caau  PLSTPLLVLTCWLLPLMILASQNHINPEPISRQRLYITLLASLQTFLIMAFGATEIIMFY
Cyca  PLSTPLLVLTCWLLPLMILASQNHINPEPISRQRLYITLLASLQTFLIMAFGATEIIMFY
Dare  PLSTPLLVLTCWLLPLMILASQNHINSEPVNRQRMYITLLASLQTFMIMAFGATKIIMFY
Cost  PLSTPLLVLTCWLLPLMILASQNHISPEPVNRQRLYITLLVFLQTFLIMAFGATEIIMFY
Leec  PLSAPLLILTCWLLPLMILASQNHINPEPISRQRLYITLLAFLQTFLIMAFGATEIIMFY
Fola  PLSTPLLGLTCWLLPLMVLASQNHITPEPIIRQRLYITLLASLQTFLIMAFGATEIIMFY
Clmc  PLSTPLLVLTCWLLPLMILASQNHINPEPLSRQRMYITLLASLQTFLIMAFGATEIIMFY
Phin  PLSAPLLILTCWLLPLMMIASQNHIGPEPMNRQRIFISLLTSLQMFLIMAFGATELILFY
Icpu  PLSTPLLVLTCWLLPLMILASQNHIKAEPIARQRNYITLLASLQTFLIMAFGATEIIMFY
Psto  PLSTPLLVLTCWLLPLMILASQNHIKTEPINRQRTYITLLTSLQTFLIMAFGATEIIMFY
Cora  PLSTPLLVLTCWLLPLMILASQNHIKNEPLSRQRTYITLLTSLQMFLIMAFGATEIMMFY
Eisp  PLSTPLLILTCWLLPLMIIASQNHIQPEPISRQRTYITLLASLQMFLIMAFGATEIIMFY
Apal  PLSTPLLILTCWLLPMMILASQNHTRSEPLNRQRTFLTLLTSLQIFLIIAFSATEILLFY
Eslu  PLSFPLAVLTCWLVPLMIIASQNHVAHEPLTRQRTYISLLVSLQVFLILAFSAMDILTFY
Dape  PLSAPLLILTCWLLPLMIIASQKHIVPEPLTRQRTYISLLISLQVFLILAFGATEILMFY
Glse  PLSTPLLVLSCWLLPLMILASQNHMALEPKNRQRTYISLLTSLQMFLIMAFGATEIILFF
Naar  PLSTPLLILSCWLLPLMILASQNHMASEPENRQRTYISLLASLQMFLIMAFGATEIILFY
Lioc  PLSTPLLVLSCWLLPLMILASQNHTASEPTNRQRTYLSLLASLQMFLIMAFGATEIILFY
Opso  PLSTPLLVLSCWLLPLMIIASQSHMTPEPKSRQRTYISLLTSLQMFLIMAFGATEIIMFY
Alte  PLSTPLLELTCWLLPLMILASQNHISPEPISRQRTYISLLTSLQMFLIMAFGATEIIMFY
Plap  PLSTPLLVLTCWLLPLMILASQSHISPEPISRQRMYVSLLASLQTFLIMAFGATEIIMFY
```

To be continued on page 11.

[2/8 of aligned sequences]

To be continued on page 12.

```
Plal PLSTPLLVLSCWLLPLMILASQNHISPEPVNRQRTYISLLASLQMFLILAFGATEIIMFY
Sami PLSTPLLVLSCWLLPLMILASQNHISPEPVNRQRTYIALLASLQMFLILAFGATEIIMFY
Rere PLSTPLLVLSCWLLPLMILASQNHICPEPIGRQRTYISLLASLQMFLILAFGATEIIMFY
Gama PLSTPLLVLSCWLLPLMILASQGHAATEPINRQRTLISLLASLQVFLILAFGATEVIMFY
Onmy PLSTPLLVLTCWLLPLMILASQSHLSPEPLNRQRAYISLLVSLQTFLVLAFGATEIIMFY
Sasa PLSTPLLVLTCWLLPLMVLASQNHISPEPLNRQRTYISLLVSLQMFLILAFGATEIIMFY
Cola PLSTPLLVLTCWLLPLMILASQNHINPEPLNRQRTYISLLVSLQMFLILAFGATEIIMFY
Dita PLSAPLLVLSCWLLPLMILASQNHVCPEPTGRQRVYISLLVSLQALLILAFGATETIMFY
Gogr LLSTPLLILTCWLLPLMILASQNHISLEPTGRQRSFMTLLVLLQTSLILAFGSTEITMFF
Chsl PLSTPLLILSCWLLPLMILASQNHITPEPLARQRTYLSLLTSLQMFLILAFGATEIIMFY
Atja SLSTPLLILTCWLLPLMIMASQKHTYNEPMNRQRAYLSLLVSLQVFLILAFGATEIIMFY
Iido PLSTPLLVLTCWLLPLMVMASQNHTYNEPVNRQRTYLSLLVSLQTFLIMAFGATEIIMFY
Auja PLSTPLLVLTCWLLPLMILASQNHLFVEPANRQRTYISLLVTLQTCLILAFSATEILMFY
Chag PLSTPLLVLTCWLLPLMILASRNHISPEPLNRQRTYLSLLTSLQVFLIMAFGATEIIMFY
Hami PLSTPLLVLTCWLLPLMILASQNHISPEPDTRQRIYLSLLTSLQVFLIMAFGATEIILFY
Saun PLFTPLLILTCWLLPLMVLASQNHISPEPATRQRIFITLLVSLQMFLILAFGATEIILFY
Nema PLSTPLLILSCWLLPLMILASQNHMAHEPQNRQRTYISLLTSLQFFLVLAFGATEIFMFY
Disp PLSTPLLILSCWLLPLMILASQNHMTHEPISRQRTYISLLTSLQFFLILAFGATEVFMFY
Myaf PLSTPLLVLSCWLLPLMILASQNHMSHEPMNRQRTYITLLTSLQFFLILAFGATEVFMFY
Lagu PLSSPLLILTCWLLPLMIIASQSHLKAEPINRQRSFLSLLTLLQAFLIAAFGATETILFY
Trtr ALSSPLLILTCWLLPLMVLASQNHLKSEPAVRQRTFLSLLVSLQILLIMAFSATELILFY
Zucr PLSSPLLVLTCWLLPLMVIASQNHLKTEPISRQRTFLSLLTVLQILLILAFSATELVLFY
Pxja PLSTPLLVLTCWLLPLMIIASQNHISPEPISRQRTYIMLLTSLQLFLILAFGATEITMFY
Pxlo PLSTPLLVLTCWLLPLMIIASQNHISPEPISRQRTYIMLLTSLQLFLILAFGATEITMFY
Pctr PLSAPLLVLSCWLLPLMLLASQKHMALEPPARQRTYVLLLASLQLFLIVAFGATEILLFY
Apsa PLSAPLLVLSCWLLPLMILASQNHMSLEPPARQRTYIMLLTSLQLFLVLAFGATEIILFY
Cabe PLSTPLLVLTCWLLPLMILASQGHMQIEPANRQRTFITLLISLQTFLIVAFGATEMIMFY
Bzze PLSTPLLVLTCWLLPLMILASQNHTRSEPVNRQRTYITLLASLQFFLILAFSATEILMFY
Siim PLSSPLLVLTCWLLPLMILASQNHTQLEPANHQRIYITLLTSLQFFLIMAFSATEMIMFF
Ctru PLSTPLLVLTCWLLPLMILASQNHTAQKPANRQRVFITLLISLQFFLILAFSATEVIMFY
Dpbr PLSTPLLVLTCWLLPLMILASQNHTAQEPDNRQRMFITLLISLQFFLILAFSATEVIMFY
Caki PLSTPLLILSCWLLPLMILASQNHLASEPLFRQRSFIVVITTLQSFLIMAFGATEFIMFY
Phja PLSTPLLALSCWLLPLMIMASQHHLLTEPVNRQRTFISILTILQIFLILAFSATEMIMFF
Brsp SLSTPLLVLSCWLLPLMIIASQNHMTSEPMVRQRIYISLLVTLQVFLIVTFSTTEMVMFF
Gamo PLSTPLLVLSCWLLPLMLLASQNHLSSEPMNRQRMYITLLATLQLFLILAFGATEMIMFY
Lolo PLSTPLLVLSCWLLPLMILASQNHLSSEPVNRQRMYITLLATLQLFLILAFGATEMIMFY
Batr SISTPLIVLSCWLLPLTIMASQHHMTPEPKNHQRLYIFLLTTLQLFLVLAFSSAELIMFF
Prmy PLSSPLLVLTLWLMPLMILASQNHLKTETFPRQRSFLFILTLLHISLILAFSALNALLFY
Lose PLSTPLLVLTCWLLPLAILASQAHMASEPMTRQRTYIILLTSLQIFLTMAFGATEIIMFY
Loam PLSTPLLVLTCWLLPLMILASQAHTASEPVGRQRLYIILLTSLQAFLIMAFSATEMILFY
Chab PLSTPLLILTCWLLPLMILASQNHMRPEPAGRQRTYIMLLTSLQIFLIMAFGATEIIMFY
Chto PLSTPLLVLTCWLLPLMILASQNHMRPEPAGRQRTYIMLLTSLQIFLIMAFGATEIIMFY
Majo PVSAPLIVLTCWLLPLMILASQNHMSTEPINRQRTFLTLLVLLQFLLTLAFSAAEFILFY
Hlst PLSAPLIMLTCWLLPLMILASQNHMASEPISRQRMFLTLLILLQFLLILAFSATELISFY
Clpe PLSSPLLVLTCWIQPLAIIASQNHMAAEPLVRQRMYLALLASLQLFLILAFSATEVLMFY
Mlmr PLSTPLLVLTCWLLPLMILASQNHMTPEPLNRQRVYITLLTSLQLFLIMAFGATEIIMFY
Crcr PLSTPLLVLTCWLLPLMILASQNHISPEPENRQRMYITLLTSLQIFLIMAFGATEMFMFY
Muce PLSTPLLVLTCWLLPLMILASQNHISPEPENRQRMYITLLTSLQIFLIMAFGATEMFMFY
Bege PLSTPLLVLTCWLLPLMILASQNHTSTEPQNRQRMYISLLTSLQIFLILAFSATEVIMFY
Mela PLSTPLLVLTCWLLPLMILASQNHMSAEPQNRQRTYISLLTSLQIFLIMAFGATEMIMFY
Hats PLSTPLLVLTCWLLPLMILASQNHTSAEPENRQRMYISLLTSLQIFLIMAFGATEVIMFY
Orla PLSTPLLVLTCWLLPLMILASQNHMAKEPINRQRTYISLLVSLQIFLVLAFGATEMIMFY
```

[2/8 of aligned sequences]

Cosa	HLSTPLLVLTCWLLPLMIIASQNHMSQEPINRQRTFILLLTSLQIFLILAFSATEMIMFY
Exsp	PLSTPLLVLTCWLLPLMILASQNHMAMEPENRQRTYILLLTSLQIFLILAFGATELIMFY
Depa	PLSTPLLILTCWLLPLMILASQNHMATEPENRQRTYILLLTSLQAFLILAFSATEVIMFY
Rima	PLSTPLLVLTTWLLPMMVLASQNHMSSEPEPRQRAFIALLTSLQIFLILAFSATELIMFY
Fuol	PLSTPLLVLTCWLLPLMIIASQNHLKAEPENRQRTYISLLISLQIFLILAFSATEMIMFY
Gmaf	PISTPLLVLTCWLLPLMILASQNHLMLEPTNRQRTFISLLISLQIFLILAFSATELILFY
Xeei	PLSTPLLVLTCWLLPLMIIASQNHLMMEPMNRQRLYISLLISLQMFLILAFSATEVIMFY
Pros	PLSTPLLVLTCWLLPLMILASQNHMTLEPQNRQRMYITLLTSLQVFLILAFGATEIIMFY
Scmi	PLSTPLLVLTCWLLPLMILASQNHMTPEPQNRQRMYISLLTSLQMFLILAFSATEIIMFY
Rolo	PLSTPLLVLTCWLLPLMILASQNHMAPEPHNRQRVYISLLTSLQVFLILAFGATELIMFY
Cere	PLSTPLLILTCWLLPLMILASQNHMFVEPLNRQRMYISLLALLQVFLIMAFSATELIMFY
Daga	PLSAPLLVLTCWLLPLAILASQNHMMNEPLNRQRTYISLLTLLTLFLILAFTATELIMFY
Anco	PLSTPLLVLTCWLLPLMILASQNHMGVEPINRQRTYLSLLTSLQVFLIMAFGATEIIMFY
Dmve	PLSTPLLTLTCWLLPLMILASQNHMALEPVSRQRTYITLLTSLQLFLILAFGATEIIMFY
Dmar	PLSTPLLTLTCWLLPLMILASQSHMSLEPMNRQRTYITLLSSLQLFLILAFGATEIIMFY
Anka	PLSTPLLVLTCWLLPLMILASQNHMNQEPNNRQRTYISLLTSLQIFLILAFGATEIIMFY
Moja	PLSTPLLVLTCWLLPLMILASQNHMSPEPKNRQRTYISLLTSLQVFLILAFGATEIIMFY
Hoja	PLSTPLLVLTCWLLPLMILASQNHMSSEPTNRQRTYISLLTSLQIFLILAFGATEIIMFY
Bede	PLSTPLLVLTCWLLPLMILASQNHMTPEPQNRQRMYISLLTSLQVFLILAFGATEIIMFY
Besp	PLSTPLLVLTCWLLPLMILASQNHMTPEPQNRQRMYISLLTSLQVFLILAFGATEIIMFY
Mysp	PLSTPLLVLTCWLLPLMILASQNHLSPEPLNRQRMYISLLTTLQIFLIMAFSATEIILFY
Osja	PLSTPLLVLTCWLLPLMILASQNHLSPEPLNRQRMYISLLTTLQIFLIMAFSATEIILFY
Sgro	PLSTPLLVLTCWLLPLMILASQNHTKAEPHNRQRTYISLLTTLQIFLIMAFGATEIIMFY
Pzpa	PLSTPLLILTCWLLPLMILASQHHMATEPLSRQRTYIMLLSSLQFFLILAFSATEILMFY
Zeja	PLSTPLLILTCWLLPLMILASQHHMTQEPLSLQRTYITLLSSLQLFLILAFSATEILMFY
Znne	PLSTPLLILTCWLLPLMILASQNHMAQEPLSRQRTYITLLASLQFFLILAFSSTEIIMFY
Zefa	PLSTPLLILTCWLLPLMILASQNHMAPEPLSRQRTYISLLSSLQVFLILAFSATEILMFY
Acni	PLSTPLLILTCWLLPLMILASQHHMAQEPLSRQRVYITLLSSLQFFLILAFGATEILMFY
Ncrh	PLSTPLLILTCWLLPLMILASQHHMAQEPLNRQRVYITLLSSLQFFLILAFGATEILMFY
Agca	PLSTPLLVLTCWLLPLMILASQNHTAHEPANRQRMYISLLASLQIFLIMAFGATEVIMFY
Hydy	SLSTPLLVLTCWLLPLMIIASQAHTAAEPCNRQRTYITLLTSLQIFLILAFGATEIILFY
Gsac	SLSTPLLVLTCWLLPLMIIASQNHTAAEPCNRQRVYITLLTSLQIFLILAFSATEIIMFY
Pevo	CLSTPLLVLSCWLLPLMILASQNHTAQEPENRQRMYISLLVSLQMFLILAFGATEMIMFY
Hiku	PLSTPLLILTCWLLPLMILASQNHTTNDPINRQRLYISLLASLQMFLILAFSATELIMFY
Inpa	PLSTPLLALTCWLLPLMILASQNHMQAEPTTRQRTYLLALTALQTFLILAFSATELLLFY
Auch	PLSAPLLVLTCWLLPLMILASQNHTKQEPTNHQRTFICLLAFLQIFLVAAFGATEMIMFY
Fico	SLSTPLLVLTCWLLPLMILASQNHTAQEPLNRQRTYISLLTSLQVFLIMAFGATEVILFY
Macs	PLSTPLLVLTCWLLPLMILASQNHTRQEPINRQRAYITLLASLQIFLIMAFGATEIIMFY
Moal	PLSSPLLTLTCWLLPLMILASQNHLAQDPLVRQRIYITLLVTLQLSLIMAFAATELTMFY
Syma	PLSTPLLILTCWLLPLTILASQNHISQEPINRQRTYIILLTTLQLSLILAFSATELIMFY
Mafr	PLSTPLLILTCWLTPLMILASQNHMSTEPASRQRIYITLLISLQFFLILAFSATEIIMFY
Dcpe	PLSTPLLILTCWLLPLMLLASQKHVNSEPINRQRTFISLLISLQVFLIMAFGATEIILFY
Dcti	PLSTPLLILTCWLLPLMLLASQKHVNDQPINRQRTFISLLISLQIFLIMAFGATEIILFY
Hehi	PLSTPLLVLTCWLLPLMILASQGHTALEPINRQRMYITLLTSLQVFLIMAFSATELLMFY
Stam	TLSTPLLVLTCWLLPLMILASQNHTALEPINRQRMYITLLTSLQVFLIMAFGATEIIMFY
Hogi	GLSTPLLILTCWLLPLMILASQNHTALEPLNRQRMYISLLTSLQIFLIMAFSSTEIILFY
Erzo	ALSTPLLVLTCWLLPLMILASQNHTATEPVNRQRTYITLLTSLQIFLILAFGATEIIMFY
Hxot	PLSTPLLVLTCWLLPLMILASQNHTTSEPLNRQRTFITLLTSLQIFLIMAFGATEIIMFY
Core	ALSTPLLVLTCWLLPLMILASQNHTASEPLSRQRTYLTLLTSLQFFLIMAFGATEIIMFY
Apve	PLSTPLLVLTCWLLPLTILASQNHTSHEPLNRQRAYITLLTSLQIFLILAFSATEIIMFY
Latj	PISTPLLMLTCWLLPLMILASQNHLALEPVNRQRTYISLLISLQFFLIIAFSATEMAMFY
Laja	SLSTPLLVLTCWLLPLMILASQNHTALEPSNRQRMFLTLLTSLQIFLIMAFSATEIILFY

To be continued on page 13.

[2/8 of aligned sequences]

To be continued on page 14.

```
Syja PLSTPLLVLTCWLLPLMILASQNHTAQEPINRQRTYITLLTSLQIFLIMAFSATEIILFY
Epme TLSTPLLVLTCWLLPLMILASQNHTATEPINRQRMYITLLTSLQIFLILAFGATELIMFY
Grse PLSTPLLVLTCWLLPLMILASQNHTSAEPINRQRMYITLLTSLQIFLIMAFGATEVIMFY
Clja PVSSPLLILSCWLLPLMILASQNHLAHEPMNRQRLYLTLLTSLQFFLVMAFSATEILLFY
Ogcy PLSSPLLVLTCWLLPLMILASQNHMTLEPENRQRMYITLLTSLQMFLILAFGATEIIMFY
Plna SLSAPLLVLTCWLLPLMILASQHHLKTEPLIRQRTYLALLTSLQILLILAFGATELLLFF
Lema PLSTPLLVLTCWLLPLMILASQNHTASEPINRQRVFITLLTSLQAFLIMAFSATEIIMFY
Etzo SLSTPLLILTCWLLPLMILASQNHTASEPLNRQRMYITLLTSLQFFLILAFGATEVIMFY
Apse LLSTPLLVLSCWLLPIMILASQNHLTPEPINRQRTYISLLISLQAFLIMAFGSVEIVLFF
Epde PLSTPLLVLTCWLLPLMILASQSHTALEPLNRQRMYITLLTSLQAFLIMAFGATEIIMFY
Slja PLSTPLLVLSCWLLPLMILASQNHTALEPINRQRMYISLLTSLQFFLILAFSTTEIIMFY
Bsja AISAPLLVLTTWLLPLMILASQAHLHHEPLSRQRMYVSLLASLQFFLILAFSATEIIMFY
Ecna ALSTPLLVLTCWLLPLMILASQNHTSAEPINRQRMYITLLTSLQFFLILAFSATEIIMFY
Cohi SFSAPLLVLTCWMLPLMILASQNHMSYEPITRQRVYISLLTALQFFLILAFGATELIMFY
Caar ALSTPLLVLTCWLLPLMILASQNHTSSEPINRQRMYITLLTSLQFFLILAFGATEIIMFY
Came ALSTPLLVLTCWLLPLMILASQNHTSSEPINRQRMYITLLTSLQFFLILAFGATEVIMFY
Mema PLSTPLLVLTCWLLPLTILASQNHMNSEPTNRQRMYITLLTSLQFFLILAFSATEIIMFY
Lenu PLSTPLLVLTCWLLPLMILASQHHMTPEPLPRQRAYITLLTSLQALLILAFSATEIMMFY
Brja PLSTPLLVLTCWLLPLMILASQNHTALEPLGRQRTYITLLTSLQIFLILAFSATEIIMFY
Plma PLSTPLLVLTCWLLPLMILASQNHTALEPVGRQRMYITLLTSLQIFLIMAFSATEVIMFY
Emst PLSTPLLVLTCWLLPLMILASQNHTNLEPINRQRMYITLLTSLQIFLIMAFGATEIIMFY
Ptti PLSTPLLVLTCWLLPLMILASQNHTAQEPINRQRMYITLLTSLQIFLIMAFGATEIIMFY
Losu ALSAPLLSLTCWLLPLMILASQNHMSSEPGGRQRAFIVLLALLQTFLILAFSATEMILFY
Geoy TLSTPLLVLSCWLLPLMILASQNHTALEPVNRQRMYLTLLTSLQMFLIMAFGATEIIMFY
Dipi PLSTPLLVLTCWLLPLMILASQNHTASEPINRQRTYIMLLTSLQIFLIMAFGATEIIMFY
Pama PLSSPLLVLTCWLLPLMILASQNHTSMDPINRQRMYITLLTSLQFFLILAFGATEVIMFY
Leob PLSTPLLVLTCWLLPLMILASQNHTSSEPTNRQRMYITLLTSLQIFLILAFSATEVIMFY
Neba ALSAPLICLTCWLLPLSILASQKHVQHDPENRQRLFLSVLVVLQLAIIMAFAVTDMVMFY
Pdpl QLSAPLLVLSCWLLPLMILASQNHLTLEPANRQRMYVTILIILQLFLILAFSATDLMLFF
Nimi PLSTPLLVLTCWLLPLMILASQNHTASEPTNRQRMYITLLTSLQIFLILAFSATEVIMFY
Uptr PLSTPLLVLSCWLLPLMILASQNHLSSEPVNRQRMYITLLTSLQAFLIMAFGATEILMFY
Pesc PLSTPLLVLTCWLLPLMVIASQNHLASEPHNRQRTYLMVMTSLQIFLILAFSATELILFY
Baar PLSTPLLILTCWLLPLMILASQNHTALEPINRQRTYIMLLTSLQIFLIMAFSATEVIMFY
Moar PLSTPLLVLTCWLLPLMILASQNHTASEPVNRQRMYISLLTSLQVFLIMAFSATEIIMFY
Toja PLSTPLLVLTCWLLPLMILASQNHMTPEPINRQRMYITLLTSLQFFLILAFSATEIIMFY
Chau PLSTPLLILTCWLLPLMILASQNHTKHEPVSRQRMYITLLTSLQILLIMAFGATELMMFY
Chse PLSTPLLVLTCWLLPLMILASQNHLISEPISRQRMYITLLTTLQIFLILAFGATEIIMFY
Enar ALSTPLLVLTCWLLPLMILASQNHTALEPLNRQRMYITLLTSLQAFLIMAFSATEVILFY
Hpty PLSTPLLVLTCWLLPLMILASQNHTALEPINRQRMYITLLTSLQAFLIMAFSATEIIMFY
Nana PLSAPLLVLTCWLLPLMILASQSHISPEPANRQRMYITLLTSLQFFLILAFSATEIIMFY
Mcst PLSTPLLVLTCWLLPLMILASQNHMAPEPLSRQRMFITLLTSLQLFLIMAFSATEIIMFY
Rhox PLSTPLLVLTCWLLPLMILASQNHTSSEPINRQRMYISLLTSLQIFLIMAFGATEIIMFY
Opfa PLSTPLLVLTCWLLPLMILASQNHTALEPINRQRMYITLLTSLQLFLIMAFSATEIIMFY
Paar PISTPLLILSCWLLPLMILASQHHVASEPLARQRMYITLLTLLQFFLILAFSATELIMFY
Gozo PLSTPLLVLTCWLLPLMILASQNHTAHEPENRQRMFITLLTSLQAFLIMAFSATEVIMFY
Ackr PLSTPLLVLSCWLLPLMILASQKHTAHEPLPRQRLYISLMVSLQVFLIMAFSATEMIMFY
Elev PISTPLMILTCWLLPLTIIASQNHTSSEPINRQRMYITLLTSLQFFLILAFSATELIMFY
Trdu PLSTPLLALTCWLLPLMILASQNHTASEPSSRQRTYITLLTSLQIFLIMAFGATEVIMFY
Amoc PLSTPLLALTCWLLPLMILASQNHTSSEPTGRQRMYITLLTSLQIFLILAFSATEVIMFY
Hame PLSSPLLVLSCWLLPLMIIASQSHMASEPVQRQRLYITLMVLLQSSLILAFTATEVLLFY
Chso PLSGPLLVLSCWLLPLMILASQNHLASEPISRQRTYISLMIALQAFLILAFSATELIMFY
Lyto PLSTPLLVLTCWLLPLMIMASQNHTALEPINRQRTYITLLTSLQIFLIMAFGATEIIMFY
```

[2/8 of aligned sequences]

```
Encr   PLSTPLLVLTCWLLPLMIMASQNHTTLEPLNRQRTYITLLTSLQIFLIMAFGATEIIMFY
Bvar   TLSTPLLVLTCWLLPLMIIASQTHMAPEPPNRQRAYISLLISLQFFLVLAFSATELLMFY
Noco   LLSTPLLVLTCWLLPLMILASQHHMGNEPKNRQRTYITLLTSLQFFLILAFSATELIMFY
Chsp   PLSTPLLVLTCWLLPLMILASQNHMSTEPLNRQRMYLMHLTSLQMFLIMAFSATELIMFY
Arja   TLSTPLLVLTCWLLPLMILASQKHTASEPLNRQRTYLILLTSLQIFLILAFGATEIIMFY
Pase   NISGPLICLACWLLPLMIMASQNHMESQPINNQRAYLMSLATLQVLLILAFATNELMSFY
Trel   PLSTPLLVLTCWLLPLTILASQSHASKEPLSRQRLYISLLVTLQVFLIMAFGATEFMMFY
Lifa   PLSTPLLVLTCWLLPLMILASQGHLKHEPLNRQRLYITLLISLQTFLILAFSATELIMFY
Acur   PVSAPLLILSCWLFPLMLLASQYHLSVEPDFRQRMFICLTSLLQLFIVLAFAATEFLMFF
Ampe   SLSAPLMALTCWLLPLMILASQHHMSHEPANRQRMYLTLLTSLQAFLIMAFSATEIILFY
Urja   PLSSPFLALSCWLLPLMILASQNHMLIEPKNRQRNYIFLLATLQAFLILAFSATEIIMFY
Enet   PLSTPLLVLTCWLLPLMILASQSHMSSEPVARQRIYIALMISLQFFLILAFSATEIIMFY
Ptbr   PVSSPLIILTCWLLPLMILASQNHISPEPLNYQRTYISLLASLQLFLILAFSATELLMFY
Safa   PLSSPLTVLTCWLLPLMILASQNHTAHDPLNRQRTYITLLTSLQMFLILAFNATELIMFY
Icae   PLSTPLLVLTCWLLPLMILASQSHTALEPVGRQRMYITLLTSLQIFLIMAFSATEIIMFY
Asmi   ALSIPLLTLTCWLLPLMIMASQNHTAHEPFNRKRLFITLLISLQVFLIMAFSSTEMMMFY
Foal   NLSTPLLILSCWLLPLMVLASQNHMSTSPMNQQRTYITLLVLLQFFLLLAFSATDFILFF
Drze   SISMPLLVLTCWMFPIMILASQNNIRFEPINRQRSFIALMVSLHIFLILTFAATDFILFY
Rhas   ALSTPLLVLTCWLLPLMILASQNHTALEPINRQRMYITLLTSLQIFLIMAFSATEVLLFY
Elac   PLSTPLLVLTCWLLPLMILASQNHTMPEPINRQRMYITLLISLQMFLIMAFSATEVILFY
Kugu   PLSTPLLILTCWLLPLMILASQNHTSTEPINRQRSYISLLTSLQIFLILAFSATEIIMFY
Plor   PLSTPLLILTCWLLPLMILASQNHTALEPINRQRTYITLLTSLQIFLIMAFTATELIMFY
Sgun   PLSTPLLVLTCWLLPLMILASQNHTTLEPVNRQRTYITLLTSLQIFLIMAFGATEVIMFY
Zaco   PLSTPLLILTCWLLPLMILASQNHTASEPANRQRTFITLLISLQIFLILAFSATEIIMFY
Zbfl   SLSTPLLVLTCWLLPLMILASQNHTTLEPINRQRMYITLLTSLQIFLIMAFSATEIIMFY
Spba   PLSTPLLVLTCWLLPLMILASQNHTSTEPINRQRMYITLLISLQFFLILAFSATEVIMFY
Game   PLSTPLLVLTCWLLPLMILASQNHTALEPIGRQRMYITLLTSLQIFLILAFSATEIIMFY
Thth   PLSTPLLVLTCWLLPLMILASQNHTAQEPINRQRMYITLLTSLQFFLILAFSATEIIMFY
Xigl   PLSTPLLVLTCWLLPLMILASQNHMSSEPINRQRMYITLLTSLQFFLILAFSATEVIMFY
Hyja   ALSTPLLVLTCWLLPLMILASQNHTSLEPLNRQRMYITLLTSLQIFLIMAFGATEVIMFY
Psan   PLSAPLMVLSCWLLPLMILACQKTTMKEPVNRQRMHVIILTSLSIFLVLAFSSTEIVMFF
Cupa   PLSTPLLVLTCWLLPLMILASQNHTAQEPLNRQRMYITLLTSLQIFLILAFSATEIIMFY
Mpch   PLSSPLLVLTCWLLPLMILASQNHTASEPMNRQRTFIMLLTSLQFFLILAFSATEMIMFY
Char   PLSTPLLVLTCWLLPLMILASQNHTSHEPINRQRTYITLLISLQFFLILTFTATEILMFY
Pser   PLSTPLLILTCWLLPLMILASQNHTNAEPLSRQRLYITLLTTLQFFLILAFSATEMIMFY
Prol   ALSTPLLVLTCWLLPLMILASQNHTASEPINRQRMYITLLTSLQFFLILAFGATEIIMFY
Plbi   ALSTPLLVLTCWLLPLMILASQKHTASEPLGRQRMYITLLASLQFFLILAFSATELIMFY
Calu   PLSTPLLVLSCWLLPLMLIASQNHTSAEPLPRQRLYMTLLVTLQLFLILAFGATELILFY
Papa   PLSTPLLVLSCWLLPLMILASQSHMAKEPINRQRIFISLLISLQFFLILAFSATELIMFY
Sufr   GLSTPLLVLTCWLLPLMILASQNHLTPEPLSRQRMYISLLTSLQFFLILAFGATEVLMFY
Stci   TLSTPLLVLSCWLLPLMILASQNHVSHEPLARQRLYISVLTSLQIFLILAFGATEAMMFY
Taru   PLSTPLLILSCWLLPLMILASQNHTSHEPINRQRMYITLLASLQFFLILAFSATEMIMFY
Rala   TLSTPLLVLTCWLLPLMILASQNHMAHEPVSRQRTYIMLLTSLQFFLILAFGATEVIMFY
        .  *:   *:  *:  *:  ::* :     .    ::  :     *    :  :*    .    *:
```

To be continued on page 15.

D

```
Scca  IMFEATLIPTLIIITRWGNQTERLNAGTYFLFYTLIGSLPLLIALLFMQNDLNTLSMFII
Muma  IMFEATLIPTLIIITRWGNQTERLNAGTYFLFYTLIGSLPLLIALLFMQNNLGSLSMFII
Erca  IMFEATLIPTLIIITRWGNQTERLNAGTYFLFYTLAGSLPLLVALLSLYKSTGSLSMISM
Pose  IMFEATLIPTLIIITRWGNQAERLNAGTYFLFYTLAGSLPLLVALLYLYNTAGSLSIISM
Actr  IMFEATLIPTLIIITRWGNQTERLNAGTYFLFYTLAGSLPLLVALLTLQKELGSLSMLII
Scal  IMFEATLIPTLIIITRWGNQTERLNAGTYFLFYTLAGSLPLLVALLILQKELGSLSMLII
Posp  IMFETTLIPTLIIITRWGNQTERLNAGTYFLFYTLAGSLPLLVALLILQKEMGSLSMLII
Atsp  IMFEATLIPTLAIITRWGNQAERLNAGTYFLFYTLAGSLPLLVALLTLQKDTGTLSMLLI
Leoc  VMFEATLIPTLAIITRWGNQAERLNAGTYFLFYTLVGSLPLLIALLILQKDVGTLSMLLI
Amca  IMFEATLIPTLVIITRWGNQTERLNAGVYFLFYTLAGSLPLLVALLTLQKDIGTLSMLIM
Osbi  VMFETTLIPTLIIITRWGNQTERLNAGTYFLFYTLAGSLPLLVAILIMQKDTGTLSMLII
Pabu  IMFEATLIPTLIIITRWGNQAERLNAGTYFLFYTLAGSLPLLVALLILHKNTGTLSVLTL
Hial  IMFEATLIPTLIIITRWGNQTERLNAGTYFLFYTLAGSLPLLVALLILQKDVGTLSMITI
Elha  VMFEATLIPTLIIITRWGNQTERLNAGTYFLFYTLAGSLPLLVALLTLQKDVGTLSMLTI
Mlcy  VMFEATLIPTLIIITRWGNQTERLNAGTYFLFYTLAGSLPLLIALLILQKDTGTLSMLLT
Algl  VMFEATLIPTLVIITRWGNQMERLDAGVYFMFYTLAGSLPLLVALLLLHKDLGTLSMLTM
Ptgi  VMFEATLIPTLIIITRWGNQTERLNAGIYFLFYTLAGSLPLLVALLMMQKDLGTLSMLTI
Alaf  VMFEATLIPTLIIITRWGNQTERLNAGIYFLFYTLAGSLPLLVALLIMQKDLGTLSMLTI
Nock  VMFEATLIPTLIIITRWGNQTERLNAGIYFLFYTLAGSLPLLVALLILQKDLGTLSMLIM
Anja  VMFEATLIPTLIIITRWGNQTERLNAGTYFLFYTLAGSLPLLVALLALQKDLGTLSMLTI
Gyki  IMFEATLIPTLIVITRWGNQAERLNAGTYFLFYTLAGSLPLLVALLALQKDLGTLSMFII
Syka  IMFEATLIPTLIIITRWGNQTERLNAGTYFLFYTLAGSLPLLVALLAMQKDLGTLSLLSI
Opma  IMFEATLIPTLIIITRWGNQTERLNAGTYFLFYTLAGSLPLLVALLSLHKDLGTLSMLTM
Comy  IMFEATLIPTLIIITRWGNQMERLNAGTYFLFYTLTGSLPLLVALLILQKELGTLSMLII
Sasp  VMFEATLIPTMIIITRWGSQTERLNAGTYFMFYTLMGSLPLLVALLMMHKDLGSLSLLLT
Eupe  VMFEATLIPTLIIITRWGNQTERLNAGIYFLFYTLAGSLPLLVALLMMYKDQGTLSMWML
Enja  IMFEATLVPTLIIITRWGNQAERLNAGTYFLFYTLAGSLPLLVALLALQASTGSLSMITL
Same  IMFEATLVPTLIIITRWGNQAERLNAGTYFLFYTLAGSLPLLVALLSLQSSTGSLSMVTL
Chch  IMFEATLVPTLMIITRWGNQTERLSAGTYFLFYTLAGSLPLLVALLLLQNDTGTLSMLTL
Grgr  VMFEATLVPTLVIITRWGNQAERLNAGTYFLFYTLAGSLPLLVALLLLHKTTGTLSVHAL
Caau  IMFEATLIPTLIIITRWGNQTERLNAGTYFLFYTLAGSLPLLVALLLLQQSTGTLSMLVL
Cyca  IMFEATLIPTLIIITRWGNQTERLNAGTYFLFYTLAGSLPLLVALLLLQQSTGTLSMLVL
Dare  IMFEATLIPTLIIITRWGNQAERLNAGTYFLFYTLAGSLPLLVALLLLQQSTGTLSMLVL
Cost  VMFEATLIPTLIIITRWGNQTERLNAGTYFLFYTLAGSLPLLVALLLLQHSTGTLSMLIL
Leec  IMFEATLIPTLIIITRWGNQTERLNAGTYFLFYTLAGSLPLLVALLMLQQTTGTLSMLIL
Fola  IMFEATLIPTLIIITRWGNQTERLNAGTYFLFYTLAGSLPLLVALLLLQQTNGTLSLLIL
Clmc  IMFEATLIPTLIIITRWGNQTERLNAGTYFLFYTLAGSLPLLVALLLLQQSTGTLSMLIL
Phin  VMFEATLIPTLIIITRWGNQAERLNAGTYFLFYTLAGSLPLLVALLLLQQNMGTLSLMIL
Icpu  IMFEATLIPTLIIITRWGNQAVRLSAGTYFLFYTLAGSLPLLVALLLLHQNTGSLSMLII
Psto  IMFEATLIPTLIIITRWGNQAERLSAGTYFLFYTLTGSLPLLVALLLLHTDVGTLSMTTI
Cora  IMFEATLIPTLIIITRWGNQTERLNAGTYFLFYTLAGSLPLLVALLLLHQNMGTLSMITL
Eisp  VMFEATLIPTLIIITRWGNQTERLNAGTYFLFYTLAGSLPLLVALLLLQQSTGTLSMLIL
Apal  IAFEATLIPTLIIITRWGNQTERLDAGTYFLFYTLAGSLPLLVALLILQKTTGTLSLPIL
Eslu  VMFEATLIPTLIIITRWGSQTERLSAGTYFMFYTLLGSLPLLVALLYLLNDTGTLSMHTL
Dape  VMFEATLIPTLIIITRWGNQTERLNAGTYFLFYTLAGSLPLLVALLFLYKDTGTLSMFTL
Glse  IMFEATLVPTLIIITRWGNQTERLNAGTYFLFYTLAGSLPLLVSLLLLQNHTGTLSMLIL
Naar  VMFEATLVPTLIIITRWGNQAERLNAGTYFLFYTLAGSLPLLVALLLLQNHAGTLSMLTL
Lioc  VMFEATLVPTLIIITRWGNQAERLNAGTYFLFYTLAGSLPLLVALLLLQNHTGTLSLLTL
Opso  IMFEATLIPTLIIITRWGNQTERLNAGTYFLFYTLAGSLPLLIALLLLQTHTGTLSLFTL
Alte  VMFEATLLPTLIIITRWGNQTERLNAGTYFLFYTLAGSLPLLVALLLLQNSTGTLSMLTL
Plap  VMFEATLLPTLIIITRWGNQTERLNAGTYFLFYTLAGSLPLLVALLLLQNSTGTLSMLTL
```

To be continued on page 16.

[3/8 of aligned sequences]

Plal	VMFEATLVPTLIIITRWGNQAERLNAGTYFLFYTLAGSLPLLVALLLLQNETGTLSLITL
Sami	VMFEATLVPTLIIITRWGNQAERLNAGTYFLFYTLAGSLPLLVALLLLQNETGTLSLLTL
Rere	VMFEATLVPTLIIITRWGNQAERLNAGTYFLFYTLAGSLPLLVALLLLQADTGTLSLITI
Gama	VMFEATLLPTLVIITRWGNQTERLNAGTYFLFYTLAGSLPLLVALLLLQADAGSLSMLTL
Onmy	VMFEATLLPTLIIITRWGNQTERLNAGTYFLFYTLAGSLPLLVALLLMQNDNGTLSMFTL
Sasa	IMFEATLLPTLIIITRWGNQTERLNAGTYFLFYTLAGSLPLLVALLLLQNDSGTLSMFTL
Cola	IMFEATLLPTLIIITRWGNQTERLSAGTYFLFYTLAGSLPLLVALLLLQNDNGTLSMLTL
Dita	VMFEATLIPTLFIITRWGNQAERLNAGVYFLFYTLAGSLPLLVSLLLLQKDTGTLSLLTL
Gogr	IMFEATLIPTLFIITRWGNQTERLNAGTYFLFYTLTGSLPLLIALLLLQKDTTTLSLMTL
Chsl	IMFEATLIPTLFLITRWGNQTERLNAGTYFLFYTLAGSLPLLVALLLLQKDTGTLSLLTL
Atja	IMFEATLIPTLIIITRWGNQAERLNAGTYFLFYTLAGSLPLLVSLLMLQNNLGSLSMLTL
Iido	IMFEATLVPTLIIITRWGNQTERLNAGTYFLFYTLAGSLPLLVALLMLQNNLGSLSMLTL
Auja	VMFEATLVPTLFIITRWGNQTERLNAGTYFLFYTLAGSLPLLVALLMLQNDTGTLSMLTL
Chag	VMFEATLVPTLFIITRWGNQTERLNAGTYFLFYTLAGSLPLLVALLLLQNDTGTLSMLTL
Hami	VMFEATLIPTLFIITRWGNQTERLNAGTYFLFYTLAGSLPLLVALLILQNETGTLSMLTL
Saun	VMFEATLIPTLFIITRWGNQTERLNAGTYFLFYTLAGSLPLLVALLMLQNETGTLSMLTL
Nema	IMFEATLIPTLMIITRWGNQAERLNAGTYFLFYTLAGSLPLLVALLLLQNEAGTLSLLTL
Disp	VMFEATLIPTLILITRWGNQAERLNAGTYFLFYTLAGSLPLLVALLLLQNETGTLSLLTL
Myaf	IMFEATLIPTLILITRWGNQAERLNAGTYFLFYTLAGSLPLLVALLLLQNETGTLSLLTL
Lagu	LMFEATLIPTLFIITRWGNQTERLGAGTYFLFYTLAGSLPLLVALLFLQNNTGSLSMLTL
Trtr	IMFEATLIPTLLIITRWGNQAERLNAGTYFLFYTLAGSLPLLVALLLLQNDTGTLSMLTL
Zucr	IMFEATLIPTLLVITRWGNQAERLNAGTYFLFYTLAGSLPLLVALLLLQNDTGTLSMLIL
Pxja	IMFEATLVPTLIIITRWGNQTERLNAGTYFLFYTLAGSLPLLVALLMLQNDAGTLSMLTL
Pxlo	IMFEATLVPTLIIITRWGNQTERLNAGTYFLFYTLAGSLPLLVALLMLQNDAGTLSMLTL
Pctr	VMFEATLIPTLILITRWGNQTERLNAGTYFLFYTLAGSLPLLVALLLLQNDTGTLSMLTL
Apsa	IMFEATLIPTLMIITRWGNQAERLNAGTYFLFYTLAGSLPLLVALLLLQNCTGTLSLLTL
Cabe	VMFEATLIPTLVLIARWGNQPARLNASTYFLLYTLTGSLPLLVALLALYNEMGTLSLLVM
Bzze	VMFEATLIPTLIIITRWGNQTERLNAGTYFLFYTLAGSLPLLVSLLLLQNDTGTLSLLTL
Siim	TMFEATLIPTLIIITRWGNQTERLNAGTYFLFYTMAGSLPLLVALLSLQKDVGTLSMLTL
Ctru	VMFEATLIPTLIIITRWGNQTERLNAGTYFLFYTLAGSLPLLVALLLLQNDTGTLSLLTL
Dpbr	VMFEATLIPTLIIITRWGNQTERLNAGTYFLFYTLAGSLPLLVALLLLQNDTGTLSLLTL
Caki	IMFEATLIPTLLVITRWGNQMERLNAGTYFLFYTLAGSLPLLIALMMIQNSMETLSLLML
Phja	IMFEATLIPTLSLITRWGNQTERLNAGTYFLFYTLAGSLPLLVALLMLQNDMGTLSMLIV
Brsp	IMFEATLIPTLLIITRWGNQAQRLNAGTYFLFFTLAGSLPLLVALLTLQNNLGSLSFLII
Gamo	VMFEATLIPTLLVITRWGNHTERLNAGTYFLFYTLAGSLPLLVALLMLQNNTGTLSLLII
Lolo	VMFEATLIPTLLVITRWGNQTERLNAGTYFLFYTLAGSLPLLVALLMLQNNAGTLSLLIL
Batr	ILFEATLIPTIMIITRWGIQPERLSATTYFLFYTMASSMPLLIALLYLQEKIGSLSIFFT
Prmy	IMFEATLIPTLMIITRWGNQTERISAGTYFLFYTIISSMPLLIVLLVAQKDLGSLSFPLI
Lose	VMFEATLIPTLILITRWGNQTERLNAGTYFLFYTLAGSLPLLVALLWLQNNTGSLSLLTL
Loam	VMFEATLIPTLILITRWGNQTERLNAGTYFLFYTLAGSLPLLVALLWLQNNAGTLSLLTL
Chab	IMFEATLIPTLILITRWGNQTERLNAGTYFLFYTLAGSLPLLVALLILQNNTGTLSLLTL
Chto	IMFEATLIPTLILITRWGNQTERLNAGTYFLFYTLAGSLPLLVALLILQNNTGTLSLLAL
Majo	VMFEATLIPTLILITRWGTQKERLNAGSYFLLYTLVTSLPLLIALLLLQNITGSLSLLTL
Hlst	VTFEATLIPTLALITRWGAQKERLNAGTYFLMYTLITSLPLLVALLILQNTTGSLSPLTL
Clpe	VMFEATLIPTLIIITRWGNQAERLNAGTYFLFYTLAGSLPLLVALLLLQNSTGTLSLLTL
Mlmr	IMFEATLIPTLILITRWGNQTQRLNAGTYFLFYTLAGSLPLLVALLLLQNTTGTLSLLTF
Crcr	VMFEATLIPTLIIITRWGNQAERLNAGTYFLFYTLAGSLPLLVALLLLQNSTGTLSLLTL
Muce	VMFEATLIPTLIIITRWGNQAERLNAGTYFLFYTLAGSLPLLVALLLLQNSTGTLSLLTL
Bege	IMFEATLIPTLIIITRWGNQTERLNAGTYFLFYTLAGSLPLLVALLLLQNTTGTLSLLTL
Mela	LMFEATLIPTLIIITRWGNQAERLNAGTYFLFYTLAGSLPLLIALLLLQNSAGTLSLLTL
Hats	IMFEATLIPTLIIITRWGNQTERLNAGTYFLFYTLAGSLPLLVALLLLQNTTGTLSLLTL
Orla	IMFEATLIPTLIIITRWGNQTERLNAGIYFLFYTLAGSLPLLVALLLLQNSTGSLSFLTT

To be continued on page 17.

[3/8 of aligned sequences]

To be continued on page 18.

```
Cosa    VMFEATLIPTLFIITRWGNQTERLNAGTYFLFYTLAGSLPLLVALLLLQNSVGTLSLLTL
Exsp    VMFEATLIPTLFIITRWGNQTERLNAGTYFLFYTLAGSLPLLVALLLLQNSTGSLXLLTL
Depa    VMFEATLIPTLFIITRWGNQTERLNAGTYFLFYTLAGSLPLLIALLLLQNSTGTLSLLTL
Rima    VMFEATLIPTLIIITRWGNQMERLNAGTYFLFYTLIGSMPLLVALLMFYTKTGSLSFLIL
Fuol    IMFEATLIPTLIIITRWGNQMERLNAGTYFLFYTLAGSLPLLVALLVYQSSMGSLSFLSL
Gmaf    IMFEATLIPTLLIITRWGNQTERLNAGTYFLFYTLAGSLPLLIALLMMQNSTGTLSFLTL
Xeei    VMFEATLIPTLIIITRWGNQMERLNAGTYFLFYTLAGSLPLLVALLVFQSSTGTLSFLTT
Pros    VMFEATLIPTLIIITRWGNQTERLNAGTYFLFYTLAGSLPLLVALLLLQNDTGTLSLLTL
Scmi    IMFEATLIPTLIIITRWGNQTERLNAGTYFLFYTLAGSLPLLVALLLLQNDTGTLSLLTL
Rolo    VMFEATLIPTLIIITRWGNQTERLNAGTYFLFYTLAGSLPLLVALLLLQGDAGTLSMLTL
Cere    IMFEATLIPTLMIITRWGNQAERLNAGTYFLFYTLAGSLPLLVALLLLQNDAGTLSMLTL
Daga    VMFEATLIPTLFIITRWGNQTQRLNAGTYFLFYTLAGSLPLLVALLLLQNDTGGLSMLTL
Anco    VMFEATLIPTLIIITRWGNQTERLNAGTYFLFYTLAGSLPLLVALLLLQNETGTLSLLTL
Dmve    VMFEATLIPTLIIITRWGNQTERLNAGTYFLFYTLAGSLPLLMALLLLQNEAGTLSMITL
Dmar    VMFEATLIPTLIIITRWGNQTERLNAGTYFLFYTLAGSLPLLVALLLLQNEAGTLSMVTL
Anka    VMFEATLIPTLFIITRWGNQTERLNAGTYFLFYTLAGSLPLLVALLLLQNETGTLSMITL
Moja    VMFEATLIPTLIIITRWGNQTERLNAGTYFLFYTLAGSLPLLIALLLLQNETGTLSMITL
Hoja    VMFEATLIPTLIIITRWGNQTERLNAGTYFLFYTLAGSLPLLVALLLLQNETGTLSLLTL
Bede    VMFEATLIPTLIIITRWGNQTERLNAGTYFLFYTLAGSLPLLVALLLLQNDTGTLSMLTL
Besp    VMFEATLIPTLIIITRWGNQTERLNAGTYFLFYTLAGSLPLLVALLLLQNDTGTLSMLTL
Mysp    VMFEATLIPTLIIITRWGNQTERLNAGTYFLFYTLMGSLPLLVALLLLQNSTGTLSMLAL
Osja    VMFEATLIPTLILITRWGNQTERLNAGTYFLFYTLMGSLPLLVALLLLQNNTGTLSMLTL
Sgro    VMFEATLIPTLIIITRWGNQTERLNAGTYFLFYTLMGSLPLLIALLLLQNSTGTLSMLTL
Pzpa    IMFEATLIPTLIIITRWGNQKERLNAGTYFLFYTLAGSLPLLVALLLLQNSTGTLSMLTM
Zeja    VMFEATLIPTLIIITRWGNQKERLNAGTYFLFYTLAGSLPLLVALLLLQNSTGTLSMLTT
Znne    VMFEATLIPTLLIITRWGNQKERLNAGTYFLFYTLAGSLPLLVALLLLQNSTGTLSMLIT
Zefa    IMFEATLIPTLIIITRWGNQKERLNAGTYFLFYTLAGSLPLLVALLLLQNSTGTLSMLIT
Acni    VMFEATLIPTLIIITRWGNQKERLNAGTYFLFYTLAGSLPLLVALLFLQNSTGTLSMLTT
Ncrh    VMFEATLIPTLIIITRWGNQKERLNAGTYFLFYTLAGSLPLLVALLLLQNSTGTLSMLTT
Agca    VMFEATLIPTLMLITRWGNQTERLNAGVYFLFYTLAGSLPLLVALLLLQNSTGTLSLLIL
Hydy    IMFEATLIPTLIIITRWGNQTERLNAGVYFLFYTLAGSLPLLVALLLLQNSAGTLSLLTI
Gsac    VMFEATLIPTLVIITRWGNQTERLNAGVYFLFYTLAGSLPLLVALLLLQNSTGTLSLLTI
Pevo    VMFESTLIPTLVLITRWGNQTERLKAGTYFLFYTLAGSLPLLVALLLLQNSTGTLCLLTL
Hiku    VMFEATLIPTLLIITRWGNQTERLNAGTYFLFYTLAGSLPLLVALLMLQNKTGSLSMLTL
Inpa    LMFEATLIPTLILITRWGNQAERLLAGNYFLFYTLAGSLPLLIALLALQNNTGTLSLLIL
Auch    VMFEATLLPTLVLITRWGNQAERLNAGTYFLFYTLAGSLPLLVALLFLHSYMGSLSMLTL
Fico    VMFEATLIPTLIIITRWGNQTERLNAGTYFLFYTLAGSLPLLVALLLLQNTTGTLSMLTL
Macs    VMFEATLIPTLVIITRWGNQAERLNAGTYFLFYTLAGSLPLLVALLLLQNNTGTLSILTL
Moal    VMFETTLIPTLILITRWGNQMERLNAGTYFLFYTLASSLPLLITLLALQNKTGTLSLLII
Syma    VMFETTLIPTLIIITRWGNQTERLNAGTYFLFYTLASSLPLLLALLSMHNSLGSLSLLTM
Mafr    IMFEATLIPTLIIITRWGNQTERLNAGTYFLFYTLAGSLPLLVALLILQNNTGSLSLLIL
Dcpe    VMFEATIIPTLFIITRWGSQKERLNAGTYFLFYTLVGSLPLLVALLVLQNNIGSLSLLTV
Dcti    VMFEATIIPTLLIITRWGSQKERLNAGTYFLFYTLMGSLPLLVALLVLQNNIGSLSLLTV
Hehi    VMFEATLIPTLILITRWGNQAERLNAGVYFLFYTLAGSLPLLVALLLLQKDTGSLSLLTI
Stam    VMFEATLIPTLIIITRWGNQTERLNAGIYFLFYTLAGSLPLLVALLLLQNSTGTLSLLTL
Hogi    VMFEATLIPTLIIITRWGNQTERLNAGVYFLFYTLAGSLPLLVALLILQNTTGTLSLLTL
Erzo    VMFEATLIPTLIIITRWGNQTERLNAGVYFLFYTLAGSLPLLVALLLLQGNVGTLSILTL
Hxot    VMFEATLIPTLILITRWGNQTERLNAGIYFLFYTLAGSLPLLVALLLLQNSAGTLSLLTL
Core    VMFEATLIPTLILITRWGNQTERLNAGIYFLFYTLAGSLPLLVALLLLQNSTGTLSLLTL
Apve    VMFEATLIPTLILITRWGNQAERLNAGVYFLFYTLAGSLPLLIALLLLQNSAGTLSLLTL
Latj    IAFEATLIPTLVIITRWGNQAERLSAGIYFLFYTLTASLPLLIALLVLQHHTGSLSILTA
Laja    IMFEATLIPTLILITRWGNQTERLNAGTYFLFYTLAGSLPLLVALLLLQNTTGTLSLLTL
```

[3/8 of aligned sequences]

To be continued on page 19.

```
Syja  IMFEATLIPTLILITRWGNQKERLNAGTYFLFYTLAGSLPLLVALLLLQNNLGTLSLLII
Epme  VMFEATLIPTLILITRWGNQTERLNAGVYFLFYTLAGSLPLLVALLLLQNHTGTLSLLTL
Grse  VMFEATLIPTLIIITRWGNQAERLNAGIYFLFYTLAGSLPLLVALLLLQNTSGTLSLLTL
Clja  IMFEATLIPTLILITRWGNQKERLNAGNYFLFYTLAASLPLLIALLLNKQATGTGSLLAL
Ogcy  VMFEATLIPTLVIITRWGNQAERLNAGTYFLFYTLAGSLPLLIALLLLQNNTGTLSMLTL
Plna  VAFEATLIPTVIIITRWGSQKERIYAGIYLIFYTLTGSLPLLVALLALKAELGSLSFITL
Lema  VMFEATLIPTLFLITRWGNQTERLNAGTYFLFYTLAGSLPLLVALLLLQNSTGTLSLLTL
Etzo  VMFEATLIPTLIIITRWGNQTERLNAGVYFLFYTLAGSLPLLVALLLLQNTSGTLSLITL
Apse  LMFEATLIPTLIIITRWGSQAERLNAGTYFLFYTLAGSLPLLVSILYLQNCTGTTSLLIM
Epde  VMFEATLIPTLIIITRWGNQTERLNAGTYFLFYTLAGSLPLLVALLLLQNSTGTLSLLTL
Slja  VMFEATLIPTLMLITRWGNQAERLNAGTYFLFYTLAGSLPLLVALILLQDSVGTLSLLTL
Bsja  IMFEATLIPTLFLITRWGNQTERLNAGTYFLFYTLAGSLPLLVALLLLQNTTGTLSLLTL
Ecna  VMFEATLIPTLIIITRWGNQTERLNAGTYFLFYTLAGSLPLLVALLLLQNNTGTLSLLTI
Cohi  VMFEATLIPTLMVITRWGSQKERLNAGTYFLMYTLLASLPLLVTLLKIQATSGTLSLLLT
Caar  VMFEATLIPTLIIITRWGNQTERLNAGTYFLFYTLAGSLPLLVALLLLQNNTGSLSLLTL
Came  VMFEATLIPTLIIITRWGNQTERLNAGTYFLFYTLAGSLPLLVALLLLQNNTGSLSLLTL
Mema  IMFEATLIPTLIIITRWGNQTERLNAGTYFLFYTLAGSLPLLVALLLLQNTTGTLSLLTL
Lenu  ITFEATLIPTLIIITRWGNQAERLNAGTYFLFYTLLSSLPLLVALLVLQNTTGTLSFLTL
Brja  IMFEATLIPTLVIITRWGNQTERLNAGTYFLFYTLAGSLPLLVALLLLQNTTGTLSLLTL
Plma  IMFEATLIPTLVIITRWGNQTERLNAGTYFLFYTLAGSLPLLVALLLLQNNTGTLSLLTL
Emst  VMFEATLIPTLILITRWGNQTERLNAGTYFLFYTLAGSLPLLVALLLLQNSTGTLSLLTL
Ptti  IMFEATLIPTLFLITRWGNQTERLNAGTYFLFYTLAGSLPLLVALLLLQNSTGTLSLLTL
Losu  VMFEATLIPTLLLITRWGSQAERLNAGTYFLFYTLASSLPLLIALIFLQHSSGTLSLLTL
Geoy  VMFEATLIPTLIIITRWGNQTERLNAGTYFLFYTLAGSLPLLVALLLLQNTTGSLSLLTL
Dipi  VMFEATLIPTLILITRWGNQTERLNAGTYFLFYTLAGSLPLLVALLLLQNSTGTLSLLTL
Pama  IMFEATLIPTLILITRWGNQTERLNAGTYFLFYTLAGSLPLLVALLLLQNTAGSLSFLTL
Leob  VMFEATLIPTLMLITRWGNQTERLNAGTYFLFYTLAGSLPLLVALLLLQNSTGTLSLLTL
Neba  IMFEATLIPTLIAITRWGSQWQRLNAGAYFLFFTLAASLPLLIGILYLCKIAGSASFMMI
Pdpl  LAFEATLIPTLIIITRWGNQAKRLDAGTYFLFYTLVGSMPLLVTLLFLQKAPATHSTLIL
Nimi  VMFEATLIPTLFLITRWGNQAERLNAGTYFLFYTLAGSLPLLVALLLLQNSAGTLSLLTL
Uptr  VMFEATLIPTLVIITRWGNQMERLNAGTYFLFYTLAGSLPLLIALLALQNHTGTLSLLTL
Pesc  IMFEATLIPTLIIITRWGNQMERLNAGIYFLFYTLAGSLPLLVALLLLQDSTGSLSLLTL
Baar  IMFEATLIPTLILITRWGNQAERLNAGTYFLFYTLAGSLPLLVALLLLQNSTGTLSLLTL
Moar  VMFEATLIPTLFLITRWGNQTERLNAGTYFLFYTLAGSLPLLVALLLLQNSTGTLSLLTL
Toja  VMFEATLIPTLIIITRWGNQTERLNAGTYFLFYTLAGSLPLLVALLLLQNSTGTLSLITL
Chau  VMFEATLIPTLIIITRWGNQKERLNAGMYFLFYTLASSLPLLVALLMLQKNVGTLSLLPA
Chse  VMFEATLLPTLVLITRWGNQMERLSAGFYFLFYTLAASLPLLIALLLLQNSTGTLSLLTL
Enar  VMFEATLIPTLILITRWGNQAERLNAGTYFLFYTLAGSLPLLVALLLLQNNTGTLSLLTL
Hpty  VMFEATLIPTLILITRWGNQTERLNAGTYFLFYTLAGSLPLLVALLLLQNSTGTLSLLTL
Nana  VMFEATLIPTLILITRWGNQTARLSAGTYFLFYTLAGSLPLLVALLLLQNNTGTLSLLTM
Mcst  IMFEATLIPTLILITRWGNQTERLNAGTYFLFYTLAGSLPLLIALLVLQNNTGTLSLLTL
Rhox  VMFEATLIPTLILITRWGNQTERLNAGTYFLFYTLAGSLPLLVALLLLHNDTGTLSLLVL
Opfa  IMFETTLIPTLILITRWGNQTERLNAGTYFLFYTLAGSLPLLVALLLLQNTTGTLSLLTI
Paar  IMFEATLIPTIIIITRWGNQAQRLTAGIYFLFYTLAGSLPLLIALLYLQKMSGSLSLMTL
Gozo  VMFEATLIPTLILITRWGNQTERLNAGTYFLFYTLAGSLPLLVALLLLQNNTGTLSLLTL
Ackr  IMFEATLIPTLFLITRWGNQAERFNAGTYFLFYTLAGSLPLLIALLTLQHTVGTLSLLTL
Elev  VMFEATLIPTLILITRWGNQTERLNAGTYFLFYTLVGSLPLLVALLILQNSTGSLSLLTL
Trdu  IMFEATLIPTLVIITRWGNQTERLNAGTYFLFYTLAGSLPLLVALLLLQNSTGTLSLLTL
Amoc  IMFEATLIPTLIIITRWGNQTERLNAGTYFLFYTLAGSLPLLVALLLLQNSTGTLSLLTL
Hame  VMFEATLIPTLFLITRWGNQTERLNAGTYFLFYTLAGSLPLLVALLILQNTTGSLSMLTL
Chso  VMFEATLIPTLFLITRWGNQAERLNAGTYFLFYTLAGSLPLLVALLVMHNTTGSLSTLTL
Lyto  IMFEATLIPTLIIITRWGNQTERLSAGVYFLFYTLAGSLPLLVALLLLQNSAGTLSLLTL
```

[3/8 of aligned sequences]

```
Encr  VMFEATLIPTLIIITRWGNQTERLNAGIYFLFYTLAGSLPLLVALLLLQNSAGTLSLLTL
Bvar  VMFEATLLPTLMIITRWGNQKERLNAGVYFLFYTLAGSLPLLVALLTLQNNAGTLSLLTM
Noco  LMFEATLLPTLMIITRWGNQAERLNAGMYFLFYTLASSLPLLVALLILQNTSGTLSLLTL
Chsp  IMFEATLIPTLFLITRWGKQMERLNAGTYFLFYTLMSSLPLLVALLLLQKDTGTLSLITL
Arja  VMFEATLIPTLILITRWGNQTERLNAGVYFLFYTLAGSLPLLVALLLLQNSAGTLSLFTI
Pase  VMFEATLIPTLFIITRWGTQKARLSAGTYFLFYTLAGSLPLLVALLFQQLTSGTLSLVIL
Trel  LMFEATLIPTLIIITRWGNQMERLNAGVYFLFYTLASSLPLLVALLLLKKSMGGLSMVIL
Lifa  VMFEATLIPTLILITRWGNQAERLNAGTYFLFYTLAGSLPLLVALLLLQNSVGSLSIMTA
Acur  IMFEATLIPTLFLIMRWGNQPERVYAGMYFLFYTLAGSLPLLIALLYMQDYMGSMSLLIT
Ampe  IMFEATLIPTLIIITRWGNQTERLNAGTYFLFYTLAGSLPLIVALLLLQNSTGTLSILTL
Urja  IMFEATLLPTLIIITRWGNQTERLNAGTYFLFYTLAGSLPLLVGLLALNSNSGTLSLLVL
Enet  VMFEATLIPTLIIITRWGNQTQRLNAGIYFLFYTLAGSLPLLVSLLQLQNNIGTLSLLTL
Ptbr  IMFEATLIPTFFIITRWGNQPERLDAGYYFLFYTLVGSLPLLLALFYIQNLTGTLSTLTL
Safa  IMFEATLIPTLMIITRWGNQTERLNAGTYFLFYTLAGSLPLLVALLHLHNVTGTLSTLTL
Icae  IMFEATLIPTLVIITRWGNQTQRLNAGTYFLFYTLAGSLPLLVALLLLQNNTGTLSLLTL
Asmi  VMFEATLIPTLILITRWGNQAQRLNAGTYFLFFTLAGSLPLLVALLTLQSYSGSLSLLTL
Foal  VSFEATLLPTLAIITRWGNQKDRLNAGTYFLFYTLAGSLLSLIALLALHGDTGTLSLIAL
Drze  TMFEATLVPTLFLITRWGSQEKRLMAGSYFLFYTLTGSLPLLIALLVMYKHLGSLSLILW
Rhas  VMFEATLIPTLILITRWGNQTERLNAGTYFLFYTLAGSLPLLVALLLLQNTAGTLSLLTL
Elac  IMFEATLVPTLIIITRWGNQTERLNAGTYFLFYTLAGSLPLLVALLLLQNTAGTLSLLTL
Kugu  VMFEATLIPTLIIITRWGNQTERLNAGTYFLFYTLAGSLPLLVALLLLQNNTGSLSLLIL
Plor  VMFEATLIPTLFLITRWGNQAERLNAGIYFLFYTLAGSLPLLVALLLLQNNAGTLSLLTI
Sgun  VMFEATLIPTLFLITRWGNQTERLNAGTYFLFYTLAGSLPLLVALLLLQNSTGTLSLLTL
Zaco  VMFEATLIPTLILITRWGNQTERLNAGTYFLFYTLAGSLPLLVALLLLQNDTGTLSLLTL
Zbfl  IMFEATLVPTLILITRWGNQTERLNAGTYFLFYTLAGSLPLLVALLLLQNNTGTLSLLTL
Spba  IMFEATLIPTLIIITRWGNQAERLNAGTYFLFYTLAGSLPLLVALLLLQNNTGSLSLLTL
Game  IMFEATLIPTLVIITRWGNQTERLNAGTYFLFYTLAGSLPLLVALLLLQNNAGTLSLLTL
Thth  IMFEATLIPTLVIITRWGNQTERLNAGTYFLFYTLAGSLPLLVALLLLQNSTGTLSLLTL
Xigl  VMFEATLIPTLIIITRWGNQTERLNAGTYFLFYTLAGSLPLLVALLLLQNSTGTLSLLTL
Hyja  IMFEATLIPTLVIITRWGNQTERLNAGTYFLFYTLAGSLPLLVALLLLHNNTGTLSLLTL
Psan  IMFEATLMPTLVIITRWGAQQERLIAGIYFIFYTLAGSIPLLVSILMLENTTGTLSLLTI
Cupa  IMFEATLIPTLVIITRWGNQTERLNAGTYFLFYTLAGSLPLLVALLLLQNSTGTLSLLTL
Mpch  VMFEATLIPTLIIITRWGNQTERLNAGTYFLFYTLAGSLPLLVALLLLSDSTGSLSLLTL
Char  IMFEATLIPTLIIITRWGNQTERLNAGTYFLFYTLAGSLPLLIALLLLQNSAGTLSLLTL
Pser  VMFEATLIPTLIIITRWGNQTERLNAGTYFLFYTLAGSLPLLVALLLLQNNTGTLSLLAL
Prol  VMFEATLIPTLIIITRWGNQTERLNAGTYFLFYTLAGSLPLLVALLLLQNSAGTLSLLTL
Plbi  VMFEATLIPTLIIITRWGNQTERLNAGTYFLFYTLAGSLPLLVALLLLQNTSGTLSLLTL
Calu  VMFEATLVPTLLIITRWGNQPDRINAGTYFLFYTLAGSMPLLVALLVLANKTGTLSILTI
Papa  VMFEATLIPTLILITRWGNQAERLNAGTYFLFYTLAGSLPLLVALLSLQNSSGTLSLIIV
Sufr  VMFEATLIPTLILITRWGNQTERLNAGTYFLFYTLAGSLPLLVALLLLQNTAGTLSLLTL
Stci  VMFEATLIPTLILITRWGNQAERLNAGTYFLFYTLAGSLPLLVALSLLYSSTGSLSFFII
Taru  VMFEATLIPTLILITRWGNQAERLNAGTYFLFYTLAGSLPLLIALLLLQNSNGSLSLLML
Rala  VMFEATLIPTLILITRWGNQTERLNAGTYFLFYTLAGSLPLLVALLLLQNSTGTLSLLAL
        **:*::**.  * *** :  *. *  *::::*:  *:  :: :
```

To be continued on page 20.

[4/8 of aligned sequences]

E

```
Scca    QYSHL----PN--PSSWANKFWWTACLIAFLVKMPLYGVHLWLPKAHVEAPIAGSMILAA
Muma    QHSQY----TN--LYSWADKFWWTACIIAFLVKMPLYGVHLWLPKAHVEAPIAGSMILAA
Erca    FMNNN----IS--SDTWTNSLLWAACLMAFLVKMPLYGVHLWLPKAHVEAPIAGSMILAA
Pose    NL-IS----IH--PDTWANTFLWVACLIAFLVKMPLYGVHLWLPKAHVEAPIAGSMILAA
Actr    QYMQP----AP--LCTWADKIWWAACLIAFLVKMPLYGAHLWLPKAHVEAPVAGSMVLAA
Scal    QYMQP----TP--LCTWADKIWWAACLIAFLVKMPLYGAHLWLPKAHVEAPIAGSMVLAA
Posp    QYMQP----AP--LCTWADKIWWAACLIAFLVKMPLYGVHLWLPKAHVEAPVAGSMVLAA
Atsp    QYSQP----AL--TPSWTNKIWWAACLLAFLVKMPLYGIHLWLPKAHVEAPIAGSMVLAA
Leoc    QYSPP----TL--LPSWTNKIWWAACLLAFLVKMPLYGIHLWLPKAHVEAPIAGSMVLAA
Amca    QHAPT----LS--LTSSGSKIWWAACLVAFLVKMPLYGAHLWLPKAHVEAPIAGSMILAA
Osbi    QYTKS----AE--PLSWANKIWWTGCLLAFLVKMPLYGVHLWLPKAHVEAPIAGSMVLAA
Pabu    QYSPN----LN--LHTWANNIWWASCLMAFLVKMPLYGIHLWLPKAHVEAPVAGSMILAA
Hial    QYSQP----LN--LSSWGDKIWWAGCLIAFLVKMPLYGVHLWLPKAHVEAPIAGSMVLAA
Elha    QYTQP----IT--SNSWGSKIWWAACLVAFLVKMPLYGVHLWLPKAHVEAPIAGSMVLAA
Mlcy    QYTNT----TM--PNSWGSKMWWAGCLMAFLVKMPLYGVHLWLPKAHVEAPIAGSMVLAA
Algl    QYVQP----LV--LSSWGDKIWWAGCLVAFLVKMPLYGVHLWLPKAHVEAPVAGSMILAA
Ptgi    QYSQP----MS--LSSWGDKIWWVGCLLAFLVKMPLYGVHLWLPKAHVEAPVAGSMVLAA
Alaf    QYTQP----MS--MTSWGDKIWWAGCLIAFLVKMPLYGVHLWLPKAHVEAPVAGSMVLAA
Nock    HYTQP----MS--LNSWGDKIWWAGCLLAFLVKMPLYGVHLWLPKAHVEAPVAGSMVLAA
Anja    QYTKP----LI--LSTWGDKLWWAGCLMAFLVKMPLYGVHLWLPKAHVEAPVAGSMVLAA
Gyki    QHA-D----IH--ISSWGVKFWWAACLVAFLVKMPLYGVHLWLPKAHVEAPIAGSMVLAA
Syka    QYTSP----LT--LYSWGDKIWWVGCLLAFLVKMPLYGVHLWLPKAHVEAPVAGSMVLAA
Opma    QYTDP----LP--LLSWGDKVWWAGCLMAFLVKMPLYGVHLWLPKAHVEAPVAGSMVLAA
Comy    QYNKP----FN--LISWGDKMWWVACLVAFLVKMPLYGVHLWLPKAHVEAPVAGSMILAA
Sasp    QHEMP----YP--NTSWGDKIWWLGCLMAFLVKMPLYGVHLWLPKAHVEAPVAGSMVLAA
Eupe    QHTKP----LI--LSSWGNKLWWAGCLMAFLVKLPLYGVHLWLPKAHVEAPVAGSMVLAA
Enja    NFNEL----PS--LTHQADKLWWAACLLAFLVKMPLYGVHLWLPKAHVEAPIAGSMVLAA
Same    NFCQP----LT--LMSWGDKIWWAGCLVAFLVKMPLYGVHLWLPKAHVEAPIAGSMVLAA
Chch    QFSNP----LP--LSSWGDKIWWAGCLIAFLAKMPLYGIHLWLPKAHVEAPIAGSMVLAA
Grgr    GLLPQ----LT--LTSYGDKIWWAGCLIAFLVKMPLYGVHLWLPKAHVEAPIAGSMVLAA
Caau    QYSQP----LQ--LNSWGHMIWWAACLIAFLVKMPLYGVHLWLPKAHVEAPVAGSMVLAA
Cyca    QYSQP----LQ--LNSWGHMFWWAGCLIAFLVKMPLYGVHLWLPKAHVEAPVAGSMVLAA
Dare    QYSDP----LL--LNSWGHKIWWAGCLIAFLVKMPLYGMHLWLPKAHVEAPVAGSMILAA
Cost    QYSQP----LT--LNAWGHKIWWAGCLIAFLVKMPLYGVHLWLPKAHVEAPVAGSMVLAA
Leec    QYSQP----LT--LNSWGHSIWWAGCLIAFLVKMPLYGVHLWLPKAHVEAPVAGSMVLAA
Fola    QHSQP----LA--LTSWGHKIWWAGCLIAFLVKMPLYGVHLWLPKAHVEAPVAGSMVLAA
Clmc    QYSQP----IA--LHSWGNKIWWAGCLIAFLVKMPLYGVHLWLPKAHVEAPVAGSMVLAA
Phin    QHSQP----AL--SSSWGDKIWWMSCLIAFLVKMPLYGVHLWLPKAHVEAPVAGSMVLAA
Icpu    QYSQP----MA--LSSWGDKIWWAGCLIAFLVKMPLYGVHLWLPKAHVEAPVAGSMVLAT
Psto    QYSQP----LN--LHTWGDKIWWAGCLIAFLVKMPLYGIHLWLPKAHVEAPVAGSMVLAA
Cora    QYSQP----MT--LLSWSDKIWWLGCLIAFLVKMPLYGVHLWLPKAHVEAPVAGSMVLAA
Eisp    QYSHP----LA--LLSWGDKIWWAGCLIAFLVKMPLYGVHLWLPKAHVEAPVAGSMVLAA
Apal    QLSNL----ST--LNTEGDKIWWIACLFAFLVKMPLYGVHLWLPKAHVEAPVAGSMVLAA
Eslu    QYIQM----TN--PDTLGTMLWWVACLLAFLVKMPLYGVHLWLPKAHVEAPVAGSMVLAA
Dape    QYTKT----LN--LTSCGQKLWWAACLLAFLVKMPLYGVHLWLPKAHVEAPIAGSMVLAA
Glse    PYSQP----LH--LSSYGDKLWWAGCLLAFLVKMPLYGVHLWLPKAHVEAPVAGSMVLAA
Naar    PYTQP----LH--LSSFGDKLWWAGCLLAFLVKMPLYGIHLWLPKAHVEAPVAGSMVLAA
Lioc    PYAQP----LT--LSSFGDKLWWAGCLLAFLVKMPLYGIHLWLPKAHVEAPVAGSMVLAA
Opso    PYAQP----LC--LSSYADKMWWAGCLLAFLVKMPLYGVHLWLPKAHVEAPIAGSMVLAA
Alte    QYSQP----LS--LFSWGDKIWWAGCLIAFLVKMPLYGVHLWLPKAHVEAPIAGSMVLAA
Plap    QYAQP----LS--LFSWGDKIWWAGCLIAFLVKMPLYGVHLWLPKAHVEAPIAGSMVLAA
```

To be continued on page 21.

[4/8 of aligned sequences]

```
Plal  QYSQP----LH--LSAWGDKLWWAGCLIAFLVQMPLYGVHLWLPKAHVEAPIAGSMVLAA
Sami  QYSQP----LH--LSTWGDKLWWAGCLLAFLVKMPLYGVHLWLPKAHVEAPIAGSMVLAA
Rere  QYAQP----LG--LSSWADKLWWMGCLMAFLVKMPLYGVHLWLPKAHVEAPIAGSMVLAA
Gama  QYSAP----LT--LMTWADKMWWAACLVAFLVKMPLYGVHLWLPKAHVEAPIAGSMVLAA
Onmy  QYTQP----LH--LLTWGDKLWWAACLLAFLVKMPLYGVHLWLPKAHVEAPIAGSMILAA
Sasa  QYTQP----MH--LLTWGNKLWWAACLLAFLVKMPVYGVHLWLPKAHVEAPIAGSMILAA
Cola  QYTQP----LH--LLTWGDKLWWAACLLAFLVKMPLYGVHLWLPKAHVEAPIAGSMVLAA
Dita  QYSPP----LQ--LSTWGSKLWWAGCLLAFLVKMPLYGTHLWLPKAHVEAPIAGSMVLAA
Gogr  QFTKP----LQ--PSSYGDKLWWAGCLAAFLVKMPLYGVHLWLPKAHVEAPIAGSMVLAA
Chsl  QYSRP----LQ--LLSWGDKLWWAGCLIAFLVKMPLYGTHLWLPKAHVEAPIAGSMVLAA
Atja  AYSQP----LT--LISWGDKIWWAGCLLAFLVKMPLYGVHLWLPKAHVEAPIAGSMVLAA
Iido  TYSEP----LT--LASWGDKIWWAGCLLAFLVKMPLYGVHLWLPKAHVEAPIAGSMVLAA
Auja  QFSDP----LS--LLTPGGKFWWLACLLAFLVKMPLYGVHLWLPKAHVEAPIAGSMVLAA
Chag  QYSNP----LN--LSTWGDSLWWAACLLAFLVKMPLYGVHLWLPKAHVEAPVAGSMVLAA
Hami  HYSKP----IY--LSPWGGKLWWAACLLAFLVKMPLYGIHLWLPKAHVEAPVAGSMVLAA
Saun  HYTKP----LS--LSPWGGKLWWAACLLAFLVKMPLYGIHLWLPKAHVEAPVAGSMVLAA
Nema  QYGKP----LL--LSTWSNKMWWAGCLIAFLVKMPLYGVHLWLPKAHVEAPIAGSMILAA
Disp  QFSKP----LL--MTTWSNKIWWAGCLIAFLVKMPLYGAHLWLPKAHVEAPIAGSMVLAA
Myaf  QYGAP----LL--LTTWSNKIWWAGCLIAFLVKMPLYGVHLWLPKAHVEAPIAGSMVLAA
Lagu  QYMEP----LA--PSPAAGGLWWAACLLAFLVKMPLYGVHLWLPKAHVEAPIAGSMILAA
Trtr  PYSKP----MA--FTPLGGSIWWTACLLAFLVKMPLYGVHLWLPKAHVEAPIAGSMVLAA
Zucr  LYSKP----LV--STPLGGTLWWAACLLAFLVKMPLYGIHLWLPKAHVEAPIAGSMILAA
Pxja  PYTKP----MY--LLTYGDKIWWAGCLLAFLVKMPLYGVHLWLPKAHVEAPVAGSMVLAA
Pxlo  PYTKP----LH--LLTYGDKIWWAGCLLAFLVKMPLYGVHLWLPKAHVEAPVAGSMVLAA
Pctr  QYLGP----LK--LETFGDKIWWAACLLAFLVKMPLYGVHLWLPKAHVEAPVAGSMVLAA
Apsa  QYYQP----LN--MLTIGDKMWWAGCLIAFLVKMPLYGVHLWLPKAHVEAPIAGSMILAA
Cabe  QYPQA----MV--LVSHADMIWWTGCVIAFLVKMPLYGIHLWLPKAHVEAPIAGSMVLAA
Bzze  QFSET----AQ--LSTYADKLWWAGCLVAFLVKMPLYGVHLWLPKAHVEAPIAGSMVLAA
Siim  HFSHT----TQ--LPTLTNNLWWAGCLIAFLVKMPLYGIHLWLPKAHVEAPIAGSMVLAA
Ctru  PYSET----IQ--LSSYADKLWWAGCLLAFLVKMPLYGVHLWLPKAHVEAPIAGSMILAA
Dpbr  QYSET----MQ--LSSYADKLWWAGCLLAFLVKMPLYGVHLWLPKAHVEAPIAGSMVLAA
Caki  QYSKP----LS--VLSLADKFWWAACMIAFLVKMPLYGVHLWLPKAHVEAPIAGSMVLAA
Phja  NYMDP----QF--SPSMTNKIWWAACMIAFFVKMPLYGMHLWLPKAHVEAPIAGSMILAA
Brsp  EYLEP----TH--FPPIAAKLLWIACILAFLVKLPLYGMHLWLPKAHVEAPVAGSMVLAA
Gamo  PYAKP----LL--LMPFGSKIWWAACMIAFLVKMPLYGMHLWLPKAHVEAPVAGSMVLAA
Lolo  PYTKP----LL--LTPFGSNIWWAACMIAFLVKMPLYGMHLWLPKAHVEAPVAGSMVLAA
Batr  QFSTT--------MNPSNSILWLGCTLAFFVKMPLYGIHLWLPKAHVEAPIAGSMILAA
Prmy  QQLSN----LS--SPTFSNNLWWLACMMAFLVKMPLYGIHLWLPKAHVEAPIAGSMVLAA
Lose  QFSDP----LE--LKNITHKMWWAGCILAFLVKMPLYGMHLWLPKAHVEAPIAGSMILAA
Loam  LYSDP----LQ--LGVYAHKLWWAGCVLAFLVKMPLYGMHLWLPKAHVEAPVAGSMILAA
Chab  QYSTP----IA--LTTFASKLWWAGCLLAFLVKMPLYGVHLWLPKAHVEAPVAGSMILAA
Chto  QYSAP----IA--LTTFASKLWWAGCLLAFLVKMPLYGVHLWLPKAHVEAPVAGSMILAA
Majo  TYSPS----LP--LTTYGDKLWWVGSLLAFLVKMPMYGTHLWLPKAHVEAPVAGSMILAA
Hlst  SYSTP----LT--MTTWGDKLWWAGFLLAFLVKLPMYGTHLWLPKAHVEAPVAGSMILAA
Clpe  QYSNP----AT--PDAYATKLWWVGCLLAFLVKMPLYGVHLWLPKAHVEAPVAGSMVLAA
Mlmr  QYSSP----AL--LTTYADKLWWVGCLLAFLVKMPLYGMHLWLPKAHVEAPVAGSMVLAA
Crcr  QFCPP----AP--LSSYADKMWWAGCLIAFLVKMPLYGAHLWLPKAHVXAPIAGSMVRAX
Muce  QFCPP----FP--LSSYADKMWWAGCLIAFLVKMPLYGAHLWLPKAHVEAPIAGSMVLAA
Bege  QYTLP----AQ--LSTYADKLWWAGCLLAFLVKMPLYGAHLWLPKAHVEAPIAGSMILAA
Mela  QYKPP----MQ--LFTYADKLWWASCLLAFLVKTPLYGIHLWLPKAHVEAPIAGSMILAA
Hats  QYTTP----LQ--LSTSADKFWWAGCLLAFLVKMPLYGAHLWLPKAHVEAPIAGSMILAA
Orla  QFFPP----LQ--LHTEASKFWWAGCLLAFLVKMPLYGAHLWLPKAHVEAPIAGSMVLAA
```

To be continued on page 22.

[4/8 of aligned sequences]

```
Cosa    QFTNP----LP--LSSYADKIWWTGCLLAFLVKMPLYGTHLWLPKAHVEAPIAGSMILAA
Exsp    QHATI----SP--LSTYADKIWWAGCLIAFLVKMPLYGAHLWLPKAHVEAPIAGSMVLAA
Depa    QLTNP----SP--LTTYADKIWWTGCLLAFLVKMPLYGAHLWLPKAHVEAPIAGSMILAA
Rima    SYNPP----HL--IFSFSNKIWWAACLLAFLVKMPLYGMHLWLPKAHVEAPIAGSMVLAA
Fuol    TNLPM----TC--TGLYADKMWWVACLLAFLVKMPLYGVHLWLPKAHVEAPIAGSMVLAA
Gmaf    PYLSL----IP--AHDWAHKFWWTACLLAFLVKMPLYGAHLWLPKAHVEAPIAGSMVLAA
Xeei    SFLPS----TP--LDTWASKTWWVACLLAFLVKMPLYGAHLWLPKAHVEAPIAGSMVLAA
Pros    QYTKP----LQ--LTSYADKLWWAGCLVAFLVKMPLYGVHLWLPKAHVEAPIAGSMILAA
Scmi    QYTKP----LQ--LSSYADKLWWAGCLLAFLVKMPLYGVHLWLPKAHVEAPIAGSMILAA
Rolo    QYTKP----LQ--LLSYADKLWWAGCLLAFLVKMPLYGVHLWLPKAHVEAPIAGSMVLAA
Cere    QYSKP----LQ--FSSYTNKIWWVGCLLAFLVKMPLYGVHLWLPKAHVEAPIAGSMVLAA
Daga    PFSNT----QQ--LISYADKAWWLGCLMAFLVKMPLYGMHLWLPKAHVEAPIAGSMILAA
Anco    QYSKP----LE--LLTYGDKLWWAGCLVAFLVKMPLYGVHLWLPKAHVEAPIAGSMVLAA
Dmve    QYTKP----LQ--LLTYSDKIWWAGCLLAFLVKMPLYGMHLWLPKAHVEAPIAGSMVLAA
Dmar    QYVKP----LQ--LLTYSDKVWWAGCLLAFLVKMPLYGMHLWLPKAHVEAPIAGSMVLAA
Anka    QYSKP----LQ--LITYGDKLWWAGCLLAFLVKMPLYGVHLWLPKAHVEAPIAGSMVLAA
Moja    QYSKP----FQ--LLTFGDKLWWAGCLVAFLVKMPLYGIHLWLPKAHVEAPIAGSMVLAA
Hoja    QYSKP----FQ--LLTYGDKLWWAGCLVAFLVKMPLYGVHLWLPKAHVEAPIAGSMVLAA
Bede    QYAKP----LQ--LVSYADKLWWAGCLLAFLVKMPLYGVHLWLPKAHVEAPIAGSMVLAA
Besp    QYAKP----LQ--LTSYADKLWWAGCLLAFLVKMPLYGVHLWLPKAHVEAPIAGSMVLAA
Mysp    QYVKP----LQ--LLSLGDKLWWAACLIAFLVKMPLYGVHLWLPKAHVEAPVAGSMVLAA
Osja    QYVKP----LQ--LLSLGDKLWWAACLIAFLVKMPLYGVHLWLPKAHVEAPVAGSMVLAA
Sgro    QYAKP----FQ--LLSLGDKLWWAACLIAFLVKMPLYGVHLWLPKAHVEAPVAGSMVLAA
Pzpa    QYNDM----PL--TESWSQNLWWAGCMIAFLVKMPLYGMHLWLPKAHVEAPVAGSMILAA
Zeja    QYNDT----LL--MTSWGNKIWWAGCMIAFLVKMPLYGVHLWLPKAHVEAPIAGSMILAA
Znne    QYNDL----AL--TQSWSHKIWWAGCMIAFLVKMPLYGVHLWLPKAHVEAPIAGSMILAA
Zefa    PYNDL----TL--TTSWSHKLWWAGCMIAFLVKMPLYGVHLWLPKAHVEAPIAGSMILAA
Acni    QYNDL----SL--MTSWGHKLWWAVCMIAFLVKMPLPGVHLWLPKAHVEAPIAGSMILAA
Ncrh    QYNDL----NL--MTSWGHKLWWAGCMIAFLVKMPLYGVHLWLPKAHVEAPIAGSMILAA
Agca    QYSAP----TQ--LITYADKLWWAGCMLAFLVKMPLYGVHLWLPKAHVEAPVAGSMILAA
Hydy    PYSDP----AH--LISFADKLWWASCILAFLVKMPLYGMHLWLPKAHVEAPVAGSMILAA
Gsac    QYSDP----FH--LSTFADKLWWASCLLAFLVKMPLYGVHLWLPKAHVEAPVAGSMILAA
Pevo    QYSPS----TY--DLSYANKLWWLACLLAFLVKMPLYGVHLWLPKAHVEAPIAGSMVLAA
Hiku    QFSNP----LN--LIHLGDKLWWTGCLLAFLVKMPLYGIHLWLPKAHVEAPIAGSMILAA
Inpa    QYTAN----NL--STSPTNLVWWLGCTVAFLVKMPLYGVHLWLPKAHVEAPIAGSMVLAA
Auch    QFCNI----SL--LPTNASKLWWLACLLAFLVKMPLYGVHLWLPKAHVEAPIAGSMVLAA
Fico    HYSSC----PE--LSSYADKLWWAGCLVAFLVKMPLYGVHLWLPKAHVEAPIAGSMVLAA
Macs    QYANP----IA--LSSYGDKLWWAGCLLAFLVKMPLYGVHLWLPKAHVEAPIAGSMILAA
Moal    QLLGP----IP--ISPYVNIIWWTTSLLAFLVKMPLYGVHLWLPKAHVEAPIAGSMILAA
Syma    QLSNP----SL--TPSYTNLLWWLGCNLAFLVKMPLYGVHLWLPKAHVEAPIAGSMILAA
Mafr    QYSDP----LN--LTPFASKLWWAGCLLAFLVKMPLYGVHLWLPKAHVEAPIAGSMVLAA
Dcpe    QYSPL----DS--HHLSTNKLWWAACLMAFLIKLPLYGLHLWLPKAHVEAPIAGSMILAA
Dcti    QYSPL----DA--SHLSTNKLWWTACLMAFVIKLPLYGLHLWLPKAHV*APIAGSMILAA
Hehi    QYANT----TP--LSSYADKLWWAGCLIAFLVKMPLYGAHLWLPKAHVEAPVAGSMVLAA
Stam    QYSDP----FQ--LTTYADKLWWAGCLLAFLVKMPLYGVHLWLPKAHVEAPVAGSMVLAA
Hogi    QFANP----IQ--LYSYSDKLWWAGCLIAFLVKMPLYGVHLWLPKAHVEAPVAGSMILAA
Erzo    QYTDP----LQ--LVSYGDKLWWAGCLLAFLVKMPLYGVHLWLPKAHVEAPVAGSMILAA
Hxot    PYSDP----VE--MTSYAHKLWWAGCLLAFLVKMPLYGVHLWLPKAHVEAPVAGSMILAA
Core    QYSDP----IE--LSSYAHKLWWAGCLLAFLVKMPLYGVHLWLPKAHVEAPVAGSMILAA
Apve    QYSNP----TE--LTSYAHKLWWAGCLLAFLVKMPLYGMHLWLPKAHVEAPIAGSMILAA
Latj    QYPHP----IH--HMSYTNKLWWSGFMLAFLVKLPLYGVHLWLPKAHVEAPVAGSMILAA
Laja    QYSNP----LH--LTTYGDKLWWAGCLLAFLVKMPLYGVHLWLPKAHVEAPVAGSMILAA
```

To be continued on page 23.

[4/8 of aligned sequences]

```
Syja   QYSEP----LH--LSTYADKLWWAGCLLAFLVKMPLYGVHLWLPKAHVEAPVAGSMVLAA
Epme   PLSHP----LN--LSSYTDKLWWAGCLLAFLVKMPLYGVHLWLPKAHVEAPVAGSMVLAA
Grse   QYSNP----LQ--LASYADKLWWAGCLLAFLVKMPLYGVHLWLPKAHVEAPIAGSMILAA
Clja   CYSEP----TS--LMYLKDKLWWTACIFAFLVKLPLYGFHLWLPKAHVEAPIAGSMILAA
Ogcy   QYSPA----LQ--LLSYGDKLWWAGCLLAFLVKMPLYGAHLWLPKAHVEAPIAGSMVLAA
Plna   QHIFW----PQ--PSSFGTHFLVMVSMMAFLIKMPLYGVHLWLPKAHVEAPIAGSMVLAA
Lema   QYMNP----FP--LLTYADKLWWAACLLAFLVKMPLYGVHLWLPKAHVEAPIAGSMILAA
Etzo   QFSDP----FQ--LTSFADKLWWAGCLMAFLVKMPLYGVHLWLPKAHVEAPVAGSMILAA
Apse   HYTPA----FP--MSSFADKFLWAGCLLAFLVKMPLYGIHLWLPKAHVEAPIAGSMVLAA
Epde   QYSNP----VP--LTTYADKLWWAGCLLAFLVKMPLYGVHLWLPKAHVEAPVAGSMILAA
Slja   QYSNP----LP--LLSAADKLWWAGCLLAFLVKMPLYGVHLWLPKAHVEAPVAGSMVLAA
Bsja   QYSEL----AM--SGSYSSKFWWVALMLAFLVKMPLYGAHLWLPKAHVEAPVAGSMILAA
Ecna   QFSDP----IL--LSTYADKIWWAGCLIAFLVKMPLYGVHLWLPKAHVEAPIAGSMILAA
Cohi   PYSAT----TP--VISFADKLWWLGCMLAFFVKLPLYGVHLWLPKAHVEAPVAGSMILAA
Caar   QYSNP----IP--LATYADKLWWAGCLLAFLVKMPLYGVHLWLPKAHVEAPIAGSMVLAA
Came   QYSNP----IP--LSTYADKLWWAGCLLAFLVKMPLYGVHLWLPKAHVEAPIAGSMVLAA
Mema   QYSSP----IN--LHSYADKLWWAGCLLAFLVKMPLYGVHLWLPKAHVEAPIAGSMILAA
Lenu   QFNSQ----MP--LTSFTDKLWWAGCLLAFLVKMPLYGVHLWLPKAHVEAPVAGSMVLAA
Brja   QYATP----FP--LESFGDKLWWAGCLLAFLVKMPLYGVHLWLPKAHVEAPIAGSMILAA
Plma   QYSTP----LQ--LVSYADKLWWAGCLLAFLVKMPLYGVHLWLPKAHVEAPIAGSMILAA
Emst   QYSNP----AH--LVTYADKLWWAACLLAFLVKMPLYGVHLWLPKAHVEAPVAGSMILAA
Ptti   QFSNP----LQ--LTTYADKLWWAGCLLAFLVKMPLYGVHLWLPKAHVEAPIAGSMVLAA
Losu   QYLKP----LSLELSNHAHKLLWASCLVAFLVKMPLYGVHLWLPKAHVEAPIAGSMILAA
Geoy   QYSHI----SL--SPSFADKLWWAGCLVAFLVKMPLYGAHLWLPKAHVEAPVAGSMVLAA
Dipi   QYSNP----LP--LITYADKLWWAGCLLAFLVKMPLYGVHLWLPKAHVEAPIAGSMVLAA
Pama   SYSTP----FQ--LLSYGDKLWWAACLLAFLVKMPLYGVHLWLPKAHVEAPVAGSMILAA
Leob   QFSDP----LQ--LISYGDKLWWAGCLLAFLVKMPLYGVHLWLPKAHVEAPVAGSMVLAA
Neba   PFMGL----TH--VSSIAQLMWWLGCVVAFMVKMPLYGMHLWLPKAHVEAPIAGSMVLAA
Pdpl   QYTDP----LF--LNTFQTKLWWTGCLLAFLVKMPLYGIHLWLPKAHVEAPVAGSMVLAA
Nimi   QFSNP----VP--LTTVADKIWWMGCLLAFLVKMPLYGVHLWLPKAHVEAPIAGSMVLAA
Uptr   QYAHA----MP--LTTYADKLWWAACLLAFLVKMPLYGVHLWLPKAHVEAPVAGSMVLAA
Pesc   QFASP----MS--LSTHTSKLWWAGCLLAFLVKMPLYGLHLWLPKAHVEAPIAGSMILAA
Baar   QYSPH----VQ--LTTYADKLWWAGCLLAFLVKMPLYGVHLWLPKAHVEAPVAGSMILAA
Moar   QYSNP----LL--LTTYADKLWWAGCLLAFLVKMPLYGVHLWLPKAHVEAPVAGSMVLAA
Toja   QYTNA----IQ--LSSYADKLWWAGCLLAFLVKMPLYGVHLWLPKAHVEAPIAGSMVLAA
Chau   PLPIT----GM--PMTSSTKLWWVACLLAFLVKMPLYGAHLWLPKAHVEAPIAGSMILAA
Chse   QYAPP----LQ--LSSYADKIWWASCLLAFLVKMPLYGVHLWLPKAHVEAPVAGSMVLAA
Enar   QYTNP----LL--LSTYADKLWWAGCLLAFLVKMPLYGVHLWLPKAHVEAPVAGSMILAA
Hpty   QYSDP----IQ--LSTYADKLWWAGCLLAFLVKMPLYGVHLWLPKAHVEAPVAGSMILAA
Nana   QYSNP----IQ--LASYTDKLWWAGCLLAFLVKMPLYGMHLWLPKAHVEAPIAGSMVLAA
Mcst   QYSYP----FL--PNTYADKLWWAACLLAFLVKMPLYGTHLWLPKAHVEAPVAGSMILAA
Rhox   QYSNP----IP--LTTYADKLWWAACLLAFLVKMPLYGAHLWLPKAHVEAPVAGSMVLAA
Opfa   QYSSP----LL--LTTYADKLWWAGCLLAFLVKMPLYGAHLWLPKAHVEAPVAGSMILAA
Paar   HYYYS----LP--SCLSGMKLWWVACLLAFLVKMPLYGLHLWLPKAHVEAPIAGSMVLAA
Gozo   QYSSP----IL--LTTYAHKLWWAGCLLAFLVKMPLYGVHLWLPKAHVEAPVAGSMILAA
Ackr   PYSPS----LQ--LASYTHKLWWVGCLLAFHVKMPLYGAHLWLPKAHVEAPIAGSMILAA
Elev   QYNNP----IH--LMSYADKFWWAACLMAFLVKMPLYGVHLWLPKAHVEAPIAGSMILAA
Trdu   QYAPS----LQ--LSSFADKLWWAGCLLAFLVKMPLYGAHLWLPKAHVEAPIAGSMVLAA
Amoc   QYTPA----ME--LSSYADKFWWAGCLLAFLVKMPLYGAHLWLAKAHVEAPIAGSMILAA
Hame   LYNQP----LS--LVNWGDNFWWAACLLAFLVKMPLYGVHLWLPKAHVEAPIAGSMILAA
Chso   QFTPT----LT--TTSYANKACWAACLMAFLVKMPLYGVHLWLPKAHVEAPVAGSMVLAA
Lyto   QYSDP----VQ--LASYADKLWWAGCLLAFLVKMPLYGVHLWLPKAHVEAPVAGSMILAA
```

To be continued on page 24.

[4/8 of aligned sequences]

```
Encr      QYSDP----VQ--LTSYGDKLWWAGCLLAFLVKMPLYGVHLWLPKAHVEAPVAGSMVLAA
Bvar      QYLDS----SP--LASYADKMWWAGCLLAFLVKMPLYGVHLWLPKAHVEAPVAGSMILAA
Noco      QYMGP----LS--LTTHADKLWWAGCLLAFLVKMPLYGVHLWLPKAHVEAPVAGSMILAA
Chsp      QYSKP----SM--HLMTSNKLWWLGCMMAFLVKMPLYGFHLWLPKAHVEAPIAGSMVLAA
Arja      QYSDP----VE--LTSYAHKLWWAGCLLAFLVKMPLYGVHLWLPKAHVEAPVAGSMILAA
Pase      THLEMWPGMTG--PTMHTSIFWWAACLLAFLVKMPLYGMHLWLPKAHVEAPIAGSMILAA
Trel      DFSSP----FP--LSSVGDMIWWTACLLAFFTKMPLYGLHLWLPKAHVEAPIAGSMILAA
Lifa      PLLDL----PL--LDLYSHKMWWAGCLIAFLVKMPLYGAHLWLPKAHVEAPVAGSMILAA
Acur      PFSKA----MM--FTPIASKFWWGACMLAFLVKLPLYGVHLWLPKAHVEAPVAGSMVLAA
Ampe      PYTNP----LP--LATYADKLWWAGCLMAFLVKMPLYGVHLWLPKAHVEAPVAGSMILAA
Urja      PLINP----LS--PNNFSNKLWWAACLLAFLVKMPLYGIHLWLPKAHVEAPVAGSMILAA
Enet      TYAPQ----MS--LSTYADKIWWAGCLLAFLVKMPLYGGHLWLPKAHVEAPIAGSMILAA
Ptbr      PLSDL----HL--DHSYAHKLLWVSCLLAFLIKTPLYGVHLWLPKAHVEAPVAGSMVLAA
Safa      PLIQP----LH--LTSYADKFLWAGCLLAFLVKMPLYGVHLWLPKAHVEAPVAGSMVLAA
Icae      QYSAP----LQ--LVSYADKLWWAGCLLAFLVKMPLYGVHLWLPKAHVEAPIAGSMVLAA
Asmi      ISTPD----LP--LTTLGDKIWWLGCLAAFLVKAPLYGLHLWLPKAHVEAPIAGSMILAA
Foal      HMTPT----PN--TFSYTNKLWWLACVLAFLVKLPLYGVHLWLPKAHVEAPIAGSMVLAA
Drze      FYTAQ----AP--MTAYATELWWLSCLLAFVIKLPLYGLHLWLPKAHVEAPVAGSIVLAA
Rhas      QYTPT----LP--MVTIADKMWWAGCLLAFLVKMPLYGVHLWLPKAHVEAPIAGSMVLAA
Elac      QYTPA----PS--MISVADKLWWAGCLLAFLVKMPLYGVHLWLPKAHVEAPIAGSMILAA
Kugu      QYTPH----TP--LTNYADKLWWAGCLLAFLVKMPLYGAHLWLPKAHVEAPIAGSMVLAA
Plor      QYSNP----FP--LLTFADKIWWAGCLLAFLVKMPLYGLHLWLPKAHVEAPVAGSMVLAA
Sgun      QFSNP----IP--LTTYADKLWWMACLLAFLVKMPLYGVHLWLPKAHVEAPVAGSMVLAA
Zaco      QYASC----IQ--LTTYADKLWWAACLIAFLVKMPLYGAHLWLPKAHVEAPVAGSMVLAA
Zbfl      QYSNY----IH--LTTYSDKLWWAACLLAFLVKMPLYGVHLWLPKAHVEAPVAGSMILAA
Spba      QYSNF----MH--LTSYAHKLWWAGCLLAFLVKMPLYGVHLWLPKAHVEAPIAGSMVLAA
Game      QYSAP----LQ--LVSYADKLWWAGCLLAFLVKMPLYGVHLWLPKAHVEAPIAGSMILAA
Thth      QYAPP----LQ--LMSYADKLWWAGCLLAFLVKMPLYGVHLWLPKAHVEAPIAGSMVLAA
Xigl      QYSDP----LY--LNSYADKLWWAGCLLAFLVKMPLYGVHLWLPKAHVEAPIAGSMVLAA
Hyja      QFSSP----LQ--LISYADKLWWAGCLLAFLVKMPLYGVHLWLPKAHVEAPIAGSMILAA
Psan      LYCSP----TE--LDLQTDKLWWVGCLMAFMTKIPLYGVHLWLPKAHVEAPIAGSMILAA
Cupa      QYAAP----LQ--LVSYADKLWWAGCLLAFLVKMPLYGVHLWLPKAHVEAPIAGSMVLAA
Mpch      QYSNP----IP--LLTYADKLWWAGCLIAFLVKMPLYGVHLWLPKAHVEAPIAGSMILAA
Char      QYTAH----PP--LLTYADKLWWAGCLLAFMVKMPLYGVHLWLPKAHVEAPIAGSMVLAA
Pser      HYSNP----TP--LTSYAHSFWWAGCLLAFLVKMPLYGLHLWLPKAHVEAPIAGSMILAA
Prol      HYTDP----TH--MTSYGDKLWWAGCLLAFLVKMPLYGVHLWLPKAHVEAPIAGSMILAA
Plbi      HYGDP----LT--LSSYADKMWWAGCLLAFLVKMPLYGVHLWLPKAHVEAPIAGSMILAA
Calu      HFSTN----MA--STTMGDKLWWAACMLAFLVKMPLYGAHLWLPKAHVEAPVAGSMVLAA
Papa      QYSSP----NC--PNLIMNKIWWAACLLAFLVKMPLYGAHLWLPKAHVEAPVAGSMILAA
Sufr      QHTTF----LC--PDTWANKFWWIGCMLAFLVKMPLYGAHLWLPKAHVEAPVAGSMVLAA
Stci      QYLGP----LQ--LSTLLDKLWWAGCMLAFLVKMPLYGMHLWLPKAHVEAPVAGSMILAA
Taru      PHLGQ----LE--LTSYADKIWWAGCILAFLVKMPLYGVHLWLPKAHVEAPIAGSMVLAA
Rala      PYSDP----LS--LTTYADKLWWAGCLVAFLVKMPLYGVHLWLPKAHVEAPVAGSMILAA
                                    **  :  *:  * **** **** **:***:: *
```

To be continued on page 25.

```
          F                        G
Scca  VLLKLGGYGMMRII-IMLNPITKEMAYPFIILAIWGIVMTSSICLRQTDLKSMIAYSSVS
Muma  VLLKLGGYGMMRII-IMLNPLTKEMAYPFLILAIWGVVMTSSICLRQTDLKSLIAYSSVS
Erca  VLLKLGGYGMIRMT-LILEPATKSLAYPFIILALWGIIMTGSICMRQSDMKSLIAYSSVS
Pose  VLLKLGGYGMIRMT-IILEPTTKSLAYPFIILALWGIIMTGSICMRQSDMKSLIAYSSVS
Actr  VLLKLGGYGMMRMI-IMLEPASKNLAYPFIILALWGIIMTGSICLRQTDLKSLIAYSSVS
Scal  VLLKLGGYGMMRMI-VMLEPTSKNFAYPFIILALWGIIMTGSICLRQTDLKSLIAYSSVS
Posp  VLLKLGGYGMMRMI-IMLEPASKNLAYPFIILALWGIIMTGSICLRQTDLKSLIAYSSVS
Atsp  VLLKLGGYGMMRVV-VLLDPLTKELAYPFIILALWGAIMTGTTCLRQTDLKALIAYSSVS
Leoc  VLLKLGGYGMMRMV-ILLDPLTKELAYPFIILALWGAIMTGSTCLRQTDLKALIAYSSVS
Amca  VLLKLGGYGMMRIM-VMLSPLTEQLAYPFIILALWGIIMAGSICLRQTDLKSLIAYSSVS
Osbi  VLLKLGGYGMMRIT-ILLDPLTKELAYPFIVLALWGVIMTGSICLRQTDLKSLIAYSSVS
Pabu  VLLKLGGYGMMRIM-VVLTPMTKELAYPFIILALWGIIMTGSICLRQTDLKSLIAYSSVS
Hial  VLLKLGGYGMMRMM-IMLNPLTKEMAYPFIILALWGIIMTGSICLRQTDLKSLIAYSSVS
Elha  VLLKLGGYGMMRMM-IMLTPMTKELAYPFIILALWGIIMTGSICLRQTDLKSMIAYSSVS
Mlcy  VLLKLGGYGMMRMT-AMLTPMTKELAYPFIALATWGIIMTGSICLRQTDLKSLIAYSSVS
Algl  VLLKLGGYGMMRMM-IVLEPLTKELAYPFITLALWGIVMTGAICLRQADLKSLIAYSSVS
Ptgi  VLLKLGGYGMMRMM-VMLDPLTKELAYPFIILALWGIIMTGSICLRQTDLKSLIAYSSVS
Alaf  VLLKLGGYGMMRMM-VMLNPLTKEMAYPFIILALWGIIMTGSICLRQTDLKSMIAYSSVS
Nock  VLLKLGGYGMMRMM-VMLNPLTKEMAYPFIVLALWGIIMTGSICLRQTDLKSMIAYSSVS
Anja  VLLKLGGYGMMRMI-IILTPLTKELAYPFIILALWGIIMTGSICLRQTDLKSMIAYSSVS
Gyki  VLLKLGGYGMMRVM-TVLTPMTKELAYPFIVLALWGIIMTGSICMRQTDLKSMIAYSSVS
Syka  VLLKLGGYGLMRMM-VVLDPLTKELAYPFIILALWGIIMTGSICLRQTDLKSMIAYSSVS
Opma  VLLKLGGYGMMRMM-IMLTPLTKELAYPFIVLALWGIIMTGSICLRQTDLKSMIAYSSVS
Comy  VLLKLGGYGMMRIM-VLLDSQMKELSYPFIVLALWGIIMTGSICLRQTDLKSMIAYSSVS
Sasp  VLLKLGGYGIMRMA-AILTSLTKMLSYPFMVLSLWGIIMTGANCLRQSDLKALVAYSSVS
Eupe  VLLKLGGYGMMRIF-MILTPPTKEMSYPVIILALWSIIMTGIICLRQIDLKSMIAYSSVG
Enja  VLLKLGGYGMIRIT-PILDPLTKEMAYPFIVLALWGIVMTGTICLRQTDLKSLIAYSSVS
Same  VLLKLGGYGMIRMT-TVLDPLTKEMAYPFIVLALWGIIMTGSICLRQTDLKSLIAYSSVS
Chch  ILLKLGGYGMMRII-IILDPLTKEMAYPFIVLALWGIVMTGSICLRQTDLKSLIAYSSVS
Grgr  VLLKLGGYGMIRVM-VMLDPLTKEMAYPFIILALWGIIMTGSICLRQTDLKSLIAYSSVS
Caau  VLLKLGGYGMMRMM-VMLDPLSKELAYPFIILALWGIIMTGSICLRQTDLKSLIAYSSVS
Cyca  VLLKLGGYGMMRMM-VMLDPLSKELAYPFIILALWGIIMTGSICLRQTDLKSLIAYSSVS
Dare  VLLKLGGYGMMRMM-VMLDPLSKQLAYPFIILALWGVIMTGLVCLRQTDLKSLIAYSSVG
Cost  VLLKLGGYGMMRMM-VMLDPLSKELAYPFIILALWGIIMTGSICLRQTDLKSLIAYSSVS
Leec  VLLKLGGYGMMRMM-VMLDPLSKELAYPFIILALWGIIMTGSICLRQTDLKSLIAYSSVS
Fola  VLLKLGGYGMMRMM-VVLDPLSKELAYPFIILALWGIIMTGSICLRQTDLKSLIAYSSVS
Clmc  ILLKLGGYGMMRMM-VILDPLTKDMAYPFIILALWGIIMTGSICLRQTDLKSLIAYSSVS
Phin  ILLKLGGYGMMRMM-IILMPITKEMAYPFMILALWGIIMTGSICLRQTDLKSLIAYSSVS
Icpu  ILLKLGGYGMMRMM-LMLDPLSKDMVYPVIALALWGVLMTGSICLRQTDLKSLIAYSSVS
Psto  ILLKLGGYGMMRMM-IILDPLSKDLIYPIIALALWGVIMTGSICLRQTDLKSLIAYSSVS
Cora  ILLKLGGYGMMRMM-IMLDPLSKEMAYPFIILALWGIIMTGSICLRQTDLKSLIAYSSVS
Eisp  ILLKLGGYGMMRIT-VMLDPLTKDLAYPFIILALWGIIMTGSICLRQTDLKSLIAYSSVS
Apal  VLLKLGGYGMMRIT-IVLDPLTKEMAYPFIILALWGIIMTGSICLRQTDLKSLIAYSSVS
Eslu  VLLKLGGYGMMRMM-VVLGPLTKEMAYPFIILALWGIIMTGSICLRQTDLKSLIAYSSVS
Dape  VLLKLGGYGMMRMV-VILGPPTKEMAYPFIILALWGVIMTGSICLRQTDLKSLIAYSSVS
Glse  VLLKLGGYGMMRIM-VLLDPLTKELAYPFIILALWGVIMTGSICLRQTDLKSLIAYSSVS
Naar  VLLKLGGYGMMRMM-VMLDPLTKELAYPFIALALWGVIMTGSICLRQTDLKSLIAYSSVS
Lioc  VLLKLGGYGMMRMM-VMLDPLTKELAYPFIALALWGVIMTGSICLRQTDLKSLIAYSSVS
Opso  VLLKLGGYGMMRMM-VMLDPLTKELAYPFIILALWGVVMTGSICLRQTDLKALIAYSSVS
Alte  VLLKLGGYGMMRMM-VMLDPLTKEMAYPFIVLALWGIIMTGSICLRQTDLKSLIAYSSVS
Plap  VLLKLGGYGMMRMM-VMLDPLTKEMAYPFIVLALWGIIMTGSICLRQTDLKSLIAYSSVS
```

To be continued on page 26.

[5/8 of aligned sequences]

To be continued on page 27.

```
Plal VLLKLGGYGMMRMM-LMLDPLSKELAYPFIVLALWGVIMTGSICLRQTDLKSLIAYSSVS
Sami VLLKLGGYGMMRMM-LMLDPLSKELAYPFIVLALWGVIMTGSICLRQTDLKSLIAYSSVS
Rere VLLKLGGYGMMRMM-LMLDPLSKELAYPFIVLALWGVIMTGSICLRQTDLKSLIAYSSVS
Gama VLLKLGGYGMMRMV-LVLDPLTKDLAYPFIVLALWGIIMTGSICLRQTDLKSLIAYSSVS
Onmy VLLKLGGYGMMRMM-VMLDPLTKELAYPFIVLALWGIIMTGSICLRQTDLKSLIAYSSVG
Sasa VLLKLGGYGMMRMM-VMLDPLTKELAYPFIVLALWGIIMTGSICLRQTDLKSLIAYSSVG
Cola VLLKLGGYGMMRMM-IMLDPLTKELAYPFIVLALWGIIMTGSICLRQTDLKSLIAYSSVG
Dita VLLKLGGYGMMRMM-PMLDPLTKNLAYPFIALALWGVIMTGSICLRQTDLKSLIAYSSVS
Gogr VLLKLGGYGMIRMT-LVLNPLTKHLAYPIIALALWGVVVMGSACLRQTDLKSLIAYSSVS
Chsl VLLKLGGYGMIRMV-IILNPLTELLALPFITLALWGIIMTGSICLRQTDLKSLIAYSSVS
Atja VLLKLGGYGMIRMM-IMLDPLSKELAYPFIILALWGIIMTGSICLRQTDLKSLIAYSSVS
Iido VLLKLGGYGMIRMM-AVLDPLSKELAYPFIILALWGIIMTGSICLRQTDLKSLIAYSSVS
Auja VLLKLGGYGMIRIM-LILDPLSKELAYPFIILALWGVIMTGSICLRQTDLKSLIAYSSVS
Chag VLLKLGGYGMMRMM-LILDPLSKELAYPFIVLALWGIIMTGSICLRQTDLKSLIAYSSVS
Hami VLLKLGGYGMIRIM-IILEPLSSDLAYPFISLALWGIIMTGSICLRQTDLKSLIAYSSVS
Saun VLLKLGGYGMIRIM-VILEPQSTDLAYPFIALALWGIIMTGSICLRQTDLKSLIAYSSVS
Nema VLLKLGGYGMMRMM-ALLDPLSKELAYPFIILALWGVIMTGSICLRQTDLKSLIAYSSVS
Disp VLLKLGGYGMMRMM-LLLDPLSKELAYPFIVLALWGVIMTGSICLRQTDLKSLIAYSSVS
Myaf VLLKLGGYGMMRMM-LLLDPLSKELAYPFIALALWGVIMTGSICLRQTDLKSLIAYSSVS
Lagu VLLKLGGYGMVRIM-VVLAPLSKELSYPFIILALWGVVMTASVCLRQTDLKSLIAYSSVG
Trtr VLLKLGGYGMIRIM-VILEPVSKNLVYPFIIFALWGVVMTGSICLRQTDLKSLIAYSSVG
Zucr VLLKLGGYGMIRMM-VILEPLSKNLIYPFIIFALWGVVMTGSICLRQTDLKSLIAYSSVG
Pxja VLLKLGGYGMMRIM-IMLDPLSKEMAYPFIILALWGIIMTGSICLRQTDLKSLIAYSSVS
Pxlo VLLKLGGYGMMRIM-IMLDPLSKEMAYPFIILALWGIIMTGSICLRQTDLKSLIAYSSVS
Pctr VLLKLGGYGMMRVM-TVLEPLSKEMVYPFIALALWGVIMTGSICLRQTDLKSLIAYSSVS
Apsa VLLKLGGYGMMRMM-MMLTPLSKEMAYPFIILALWGVVMTGTICLRQTDLKALIAYSSVS
Cabe VLLKLGGYGMMRMM-TLLEPLTLDMCFPFIALALWGMAMTSSICMRQNDLKSLIAYSSVS
Bzze VLLKLGGYGMMRMM-IVLEPLTKELSYPFIIFALWGIIMTGSICLRQTDLKSLIAYSSVS
Siim VLLKLGGYGMMRIM-TIMQPLTAQLNYPFMILALWGVLMTSSICLRQADLKSLIAYSSVS
Ctru VLLKLGGYGMMRML-IVLEPLTKELSYPFIIFALWGVIMTGSICLRQTDLKSLIAYSSVS
Dpbr VLLKLGGYGMMRMM-VMLEPLTKELSYPFIIFALWGVIMTGSICLRQTDLKSLIAYSSVS
Caki VLLKLGGYGMMRLM-VILGPLSKEVIYPFMILALWGVIMTGSACLRQTDLKALIAYSSVS
Phja VLLKLGGYGIMRIM-VLLTPISKEMIYPFIILALWGVVMAGSICLRQTDLKALIAYSSVS
Brsp VLLKLGGYGMIRLM-LIISSIPEKTVYPFIVLALWGVIMXSSICLRQTDLKSLIAYSSVG
Gamo VLLKLGGYGMMRLM-IVLDPLSKEMVYPFIVLALWGVIITGSICLRQTDLKSLIAYSSVS
Lolo VLLKLGGYGMMRLM-VMLEPLSKEMVYPFIVLALWGVIMTGSICLRQTDLKSLIAYSSVS
Batr VLLKLGGYGLIRIT-PMLKPMTSHMIYPFIILSIWGLVMTCTICLREPDLKALIAYSSVS
Prmy ILLKLGGYGMIRIL-PLIKPLPCYMAYPFIILAIWGIIMTSSICLCQMDLKALIAYSSVS
Lose VLLKLGGYGMMRIL-LLLQPLTKEMCYPFIVLALWGVIMTGCVCLRQTDLKSLIAYSSVA
Loam VLLKLGGYGMMRML-MMLEPLTKELSYPFIILALWGVIMTGSICLRQTDLKSLIAYSSVS
Chab VLLKLGGYGMMRML-VILNPLTKELSYPFIILALWGVIMTGSICLRQTDLKSLIAYSSVS
Chto VLLKLGGYGMMRML-VILNPLTKELSYPFIILALWGVIMTGSICLRQTDLKSLIAYSSVS
Majo VLLKLGGYGLIRIL-VVLEPLTQDLCYPFIILSVWGIIMTSAACLRQTDLKSLIAYSSVS
Hlst VLLKLGGYGLIRIL-TVLEPLTQELSYPFIILSVWGIVMTSAACLRQTDLKSLIAYSSVS
Clpe ILLKLGGYGMIRVL-IVLDPLTKELCYPFVVLALWGVLMASSVCLRQTDLKSLIAYSSVS
Mlmr VLLKLGGYGMMRML-VTLEPLTKELSYPFIIFALWGVVMTSSICLRQTDLKSLIAYSSVS
Crcr VLLKLGGYGMMRMM-ILLEPLTKELSYPFIIFALWGIIMTGSICLRQTDLKSLIAYSSVS
Muce VLLKLGGYGMMRMM-MLLEPLTKELSYPFIIFALWGIIMTGSICLRQTDLKSLIAYSSVS
Bege VLLKLGGYGMIRMM-TMLEPLTKELSYPFIIFALWGVIMTGSICLRQTDLKSLIAYSSVS
Mela VLLKLGGYGMIRIM-SLLEPLSKELCYPFLIFALWGVIMTASTCLRQPDLKSLIAYSSVS
Hats VLLKLGGYGMIRMM-TLLEPLTKELSYPFIVLALWGVIMTGSICLRQTDLKSLIAYSSVS
Orla VLLKLGGYGMMRII-IILDPLTKQLSYPFIILALWGVVMTGSICLRQTDLKSLIAYSSVS
```

[5/8 of aligned sequences]

To be continued on page 28.

Cosa	VLLKLGGYGMMRIM-LMLEPLTKELSYPFIILALWGVIMTSSICLRQTDLKSLIAYSSVS
Exsp	VLLKLGGYGMMRMM-TSLEPLTKELSYPFIVLALWGVLMTGSICLRQTDLKSLIAYSSVS
Depa	VLLKLGGYGMMRIM-PVLEPLTKELSYPFIILALWGVVMTGSICLRQTDLKSLIAYSSVS
Rima	VLLKLGGYGMMRVT-NILEPLTKDMCYPFIILALWGVVMTGLICLRQTDLKSLIAYSSVG
Fuol	VLLKLGGYGMMRVM-TILTPLTEELSYPFIILALWGLIMTGSICLRQTDLKSLIAYSSVS
Gmaf	VLLKLGGYGMIRIL-IIFAPSTKELSYPFIILALWGVVMTGSICLRQTDLKALIAYSSVS
Xeei	VLLKLGGYGMMRIL-IILDPLTKELSYPFIILALWGVLMTGSICLRQTDLKSLIAYSSVS
Pros	VLLKLGGYGMMRMV-MMLDPLTKELSYPFIIFALWGIIMTGSICLRQTDLKSLIAYSSVS
Scmi	VLLKLGGYGMMRMM-TMLDPLTKELSYPFIIFALWGIIMTGAICLRQTDLKSLIAYSSVS
Rolo	VLLKLGGYGMMRMM-IMLEPLTKELCYPFIIFALWGIIMTGSICLRQTDLKSLIAYSSVS
Cere	VLLKLGGYGMMRMM-IMLEPLTMELCYPFIIFALWGIIMTGSICLRQTDLKSLMLLLC-E
Daga	VLLKLGGYGMIRVV-VMLEPLTKELCYPFIVFALWGIVMTSSICLRQTDLKSLIAYSSVS
Anco	VLLKLGGYGMMRMM-VMLDPLTKELSYPFIIFALWGIIMTGSICLRQTDLKALIAYSSVS
Dmve	VLLKLGGYGMMRMM-TMLEPLTKEMSYPFIILALWGIIMTGSICLRQTDLKALIAYSSVS
Dmar	VLLKLGGYGMMRMM-IMLEPLTKEMSYPFIILALWGIIMTGSICLRQTDLKALIAYSSVS
Anka	VLLKLGGYGMMRMM-IMLDPLTKELSYPFIIFALWGIIMTGSICLRQTDLKALIAYSSVS
Moja	VLLKLGGYGMMRMM-IMLDPLTKELSYPFIIFALWGIIMTGSICLRQTDLKALIAYSSVS
Hoja	VLLKLGGYGMMRMM-VMLDPLTKELSYPFIILALWGIIMTGSICLRQTDLKALIAYSSVS
Bede	VLLKLGGYGMMRMM-VMLDPLTKELSYPFIIFALWGIIMTGSICLRQTDLKSLIAYSSVS
Besp	VLLKLGGYGMMRMM-IMLDPLTKELSYPFIIFALWGIIMTGSICLRQTDLKSLIAYSSVS
Mysp	VLLKLGGYGMMRML-VILDPLTKELSYPFIILALWGIIMTGSICLRQTDLKSLIAYSSVS
Osja	VLLKLGGYGMMRML-VVLDPLTKELSYPFIILALWGIIMTGSICLRQTDLKSLIAYSSVS
Sgro	VLLKLGGYGMMRIL-VVLDPLTKELSYPFIILALWGVIMTGSICLRQTDLKSLIAYSSVS
Pzpa	VLLKLGGYGMMRIM-VTLNPLSKEMVYPFIILALWGVIMTGSICLRQTDLKSLIAYSSVS
Zeja	VLLKLGGYGMMRIM-VTLNPLSKEMIFPFIVLALWGVVMAGSICLRQADLKSLIAYSSVS
Znne	VLLKLGGYGMMRMM-VTLNPLSKEMIFPFIILALWGVIMTGSICLRQTDLKSLIAYSSVS
Zefa	VLLKLGGYGMMRIM-VTLDPLSKEMLFPFIILALWGVIMTGSICLRQTDLKSLIAYSSVS
Acni	VLLKLGGYGMMRIM-VTLNPLSKEMIFPFIILALWGVIMTGSICLRQTDLKSLIAYSSVS
Ncrh	VLLKLGGYGMMRIM-VTLNPLSKEMIFPFIILALWGVIMTGSICLRQTDLKSLIAYSSVS
Agca	VLLKLGGYGMMRML-VVLEPLTKELCYPFIIFALWGVIMTGSICLRQTDLKSLIAYSSVS
Hydy	VLLKLGGYGMMRMV-VILDPLTKDLSYPFIIFALWGVVMTGSICLRQTDLKSLIAYSSVS
Gsac	VLLKLGGYGMIRMI-PILDPLTKELGYPFIIFALWGVVMTGSICLRQTDLKSLIAYSSVS
Pevo	VLLKLGGYGMMRIM-IMLEPMTKELSYPFIVFALWGVIMTGSICLRQTDLKSLIAYSSVS
Hiku	VLLKLGGYGMMRLM-NILEPLSKQLSYPFMILALWGIIMTGLICLRQNDLKSLIAYSSVS
Inpa	VLLKLGGYGLLRMV-PLLAPLTKEMSYPFMVLSLWGVIMTSSICLRQTDLKSLIAYSSVS
Auch	ILLKLGGYGMIRIS-PALVPLTKQLTYPLIALALWGVIMTSSICLRQTDLKSLIAYSSVS
Fico	VLLKLGGYGMMRIM-VMLDPLTKELSYPFIILALWGIIMTGSICLRQTDVKSLIAYSSVS
Macs	VLLKLGGYGMMRMM-IVLEPLTKELSYPFIVFALWGVIMTGSICLRQTDMKSLIAYSSVS
Moal	VLLKLGGYGMMRMM-LILSPLAKELCYPFIILALWGVLMTSSICLRQTDLKSLIAYSSVS
Syma	ILLKLGGYGLIRMM-MVTGPLAKELSYPFIILALWGIIMTGSICVRQTDLKSLIAYSSVG
Mafr	VLLKLGGYGMMRIT-IMLDPLTKELSYPFIILALWGVVMTASICLRQTDLKSLIAYSSVS
Dcpe	VLLKLGGYGMMRMT-ALLDPLTKELSYPFIILALWGLVMTGAICLRQTDLKSLIAYSSVS
Dcti	VLLKLGGYGMMRMM-VLLDPLSKELSYPFIILALWGLIMTGCICLRQTDLKSLIAYSSVS
Hehi	VLLKLGGYGMIRMM-VMLDPLTKELSYPFIILALWGVIMTGSTCLRQTDLKSLIAYSSVS
Stam	VLLKLGGYGMMRIV-VMLDPLTKELSYPFIIFALWGVIMTGSICLRQTDLKSLIAYSSVS
Hogi	VLLKLGGYGMMRVV-VMLEPLTQELSYPFIIFALWGVIMTGSICLRQTDLKSLIAYSSVS
Erzo	VLLKLGGYGMMRMV-VVLDPLTKELSYPFIVFALWGVIMTGSICLRQTDLKSLIAYSSVS
Hxot	VLLKLGGYGMMRMV-VMLEPLTKELSYPFIVFALWGVIMTGSICLRQTDLKSLIAYSSVS
Core	VLLKLGGYGMMRMV-VMLEPLTKELSYPFIVFALWGVVMTGSICLRQTDLKSLIAYSSVS
Apve	VLLKLGGYGMMRMI-VMLEPLTKELSYPFIIFALWGVIMTGSICLRQTDLKSLIAYSSVS
Latj	VLLKLGGYGMMRMV-AILDPLTKELSYPFIIFALWGLIMTGSTCLRQADLKSMIAYSSVA
Laja	VLLKLGGYGMMRML-IILDPLTKELSYPFIVFALWGVIMTGSICLRQTDLKSLIAYSSVS

```
Syja  VLLKLGGYGMMRIL-VVLEPLTKELSYPFIIFALWGVIMTGSICLRQTDLKSLIAYSSVS
Epme  VLLKLGGYGMMRMI-VMLDPLTKELSYPFLIFALWGVIMTSSICLRQTDLKSLIAYSSVS
Grse  VLLKLGGYGMMRMI-IILEPLTKELSYPFIILALWGVIMTGSICLRQTDLKSLIAYSSVS
Clja  VLLKLGGYGMLRMT-SMFGTSTTTLYYPFLILGLWGVIMTGSVCLRQPDLKAIIAYSSVS
Ogcy  VLLKLGGYGMMRIL-TILDPLTSKLCYPFIILALWGVVMTGSICLNQTDLKALIAYSSVS
Plna  VLLKLGGYGMIRML-AVVQHEANMMAYPFITLSLWGMIMTGLICLRHTDLKAVIAYSSVG
Lema  VLLKLGGYGMMRMM-ILLEPLTKELCYPFIIFALWGVVMTGSICLRQTDLKSLIAYSSVG
Etzo  VLLKLGGYGMMRIV-VVLEPLTKDLSYPFIIFALWGVIMTGSICLRQTDLKSLIAYSSVS
Apse  VLLKLGGYGMMRIM-VLLEPLTKELSYPFIILALWGVVMTGSICLRQTDLKSLIAYSSVG
Epde  VLLKLGGYGMMRML-VMLEPLTKELSYPFIIFALWGVVMTGSICLRQTDLKSLIAYSSVS
Slja  VLLKLGGYGMMRML-IILEPLTKELSYPFIALALWGVIMTGSICLRQTDLKSLIAYSSVS
Bsja  VLLKLGGYGMIRVL-MMLEPVTKQLCYPFIILALWGVIMTGSTCLRQTDLKSLIAYSSVS
Ecna  VLLKLGGYGMLRMM-TVLEPLTKELSYPFIILALWGIIMTGSICLRQTDLKSLIAYSSVS
Cohi  VLLKLGGYGMIRMT-CLLEPMTPQMSYPFIILALWGTIMTGLICLRQTDLKLLIAYSSVS
Caar  VLLKLGGYGMMRMM-TMLEPLTKELSYPFIIFALWGVIMTGSICLRQTDLKSLIAYSSVS
Came  VLLKLGGYGMMRMM-IMLEPLTKELSYPFIIFALWGVIMTGSICLRQTDLKSLIAYSSVS
Mema  VLLKLGGYGMMRMM-TMLEPLTKELSYPFILLALWGVIMTGSICLRQTDLKSLIAYSSVS
Lenu  VLLKLGGYGMMRIL-PLLEPLTKQLSYPFILLALWGMIMTGSICLRQSDLKSLIAYSSVS
Brja  VLLKLGGYGMMRMM-TLLEPLTKELSYPFIIFALWGVIMTGSICLRQTDLKSLIAYSSVS
Plma  VLLKLGGYGMMRMM-IMLEPLTKELSYPFIIFALWGVIMTGSICLRQTDLKSLIAYSSVS
Emst  VLLKLGGYGMMRML-VMLEPLTKELSYPFIIFALWGVIMTGSICLRQTDLKSLIAYSSVS
Ptti  VLLKLGGYGMMRML-VVLEPLTKELSYPFLILALWGVIMTGSICLRQTDLKSLIAYSSVS
Losu  VLLKLGGYGMMRVL-TILEPLTKELNYPFIILALWGVLMTSSICLRQTDLKSLIAYSSVS
Geoy  VLLKLGGYGMMRIL-VTLDPLTEELCYPFIILALWGVIMTGSICLRQTDLKSLIAYSSVS
Dipi  VLLKLGGYGMMRML-VMLEPLTKELSYPFIIFALWGVIMTGSICLRQTDLKSLIAYSSVS
Pama  VLLKLGGYGMMRML-TIFEPATEKMSYPFIIFALWGVIMTGSICLRQTDLKSLIAYSSVS
Leob  VLLKLGGYGMMRML-IVLEPLTKELSYPFIIFALWGVIMTGSICLRQTDLKSLIAYSSVS
Neba  VLLKLGGYGLMRVS-PVLAPLTMKACYPFMVMALWGVIMTGSICLRQTDLKSLIAYSSVS
Pdpl  VLLKLGGYGMIRMIMLLAEPLDKELSYPFIILALWGIIMTGSICLRQTDLKSLIAYSSVS
Nimi  VLLKLGGYGMMRVL-PVLEPLTKELSYPFIIFALWGVVMTGLICLRQTDLKSLIAYSSVG
Uptr  VLLKLGGYGMMRMM-MILEPLTKELSYPFIIFALWGVIMTGSICLRQTDLKSLIAYSSVS
Pesc  VLLKLGGYGMLRIL-SVLDPLTKELSYPFIILALWGVVMTGSICLRQVDLKSLIAYSSVS
Baar  VLLKLGGYGMMRML-VMLDPLTKELSYPFIILALWGVIMTGSICLRQTDLKSLIAYSSVS
Moar  VLLKLGGYGMMRML-VVLEPLTKELSYPFIIFALWGVIMTGSICLRQTDLKSLIAYSSVS
Toja  VLLKLGGYGMMRMM-IALEALTKELSYPFIILALWGVIMTGSICLRQTDLKSLIAYSSVS
Chau  VLLKLGGYGMMRML-PMLEPLTKELSYPFILLALWGVVMTGSICMRQTDLKSLIAYSSVS
Chse  VLLKLGGYGMMRML-VVLEPLTKELSYPFIALALWGVVMTGSICLRQTDLKSLIAYSSVS
Enar  VLLKLGGYGMMRIM-VVLEPLTKELSYPFIIFALWGVIMTGSICLRQTDLKSLIAYSSVS
Hpty  VLLKLGGYGMMRML-VMLEPLTKELSYPFIIFALWGVVMTGSICLRQTDLKSLIAYSSVS
Nana  VLLKLGGYGMMRMM-TMLEPLTKQMSYPFIILALWGVVMTSSICLRQTDLKSLIAYSSVS
Mcst  VLLKLGGYGMMRMM-TMLEPLTKELSYPFIIFALWGVIMTSSICLRQTDLKSMIAYSSVS
Rhox  VLLKLGGYGMMRMM-IMLEPLTKQLSYPFIIFALWGIVMTGSICLRQTDLKSLIAYSSVS
Opfa  VLLKLGGYGMMRMM-TMLEPLTKELSYPFIVFALWGVIMTGSICLRQTDLKSLIAYSSVS
Paar  VLLKLGGYGMIRIM-TTLEPYTKELSYPFIILALWGMIMTGSICLRQTDLKSLIAYSSVS
Gozo  VLLKLGGYGMMRMM-VMLEPLTKELSYPFIIFALWGVIMTGSICLRQTDLKSLIAYSSVS
Ackr  VLLKLGGYGMIRIM-IILEPLTEQLSYPFIILALWGIIMTSAICLRQTDLKSLIAYSSVS
Elev  VLLKLGGYGMIRML-TVLEPLSKELSYPFIILALWGVIMTGSICLRQTDLKSLIAYSSVS
Trdu  VLLKLGGYGMMRMM-IMLEPLTKELSYPFIIFALWGVIMTGSICLRQTDLKSLIAYSSVS
Amoc  VLLKLGGYGMMRMM-TMLEPLTKELSYPFIIFALWGVIMTGSICLRQTDLKSLIAYSSVS
Hame  VLLKLGGYGMMRVL-IVLNPLTKDLAYPFIVLALWGVVMTGTICLRQTDLKSLIAYSSVG
Chso  VLLKLGGYGIIRVL-TILEPLTKEMAYPFIILALWGIIMTGSICLRQTDLKALIAYSSVS
Lyto  VLLKLGGYGMMRMV-VMLDPLTQELSYPFIVFALWGVIMTGSICVRQTDLKSLIAYSSVS
```

To be continued on page 29.

[5/8 of aligned sequences]

```
Encr     VLLKLGGYGMIRMV-VMLDPLTQELSYPFIVFALWGVIMTGSICLRQTDLKSLIAYSSVS
Bvar     VLLKLGGYGMMRMM-TNLEPLSKDLTYPFIIFALWGVLMTSSICLRQTDLKSLIAYSSVS
Noco     VLLKLGGYGMMRMM-LVLEPLTKEMSYPFMVFALWGIMMAGSICLRQTDLKSLIAYSSVS
Chsp     VLLKLGGYGMMRML-MILSPLTKEIAYPFIILALWGIIMTGAICLRQLDLKSLIAYSSVS
Arja     VLLKLGGYGMMRMV-VMLEPLTKELSYPFIVFALWGVIMTGSICLRQTDLKALIAYSSVS
Pase     VLLKLGGYGMMRVM-ILLEPLTKELSYPFIILALWGVLMTSTICLRQTDLKSLIAYSSVS
Trel     VLLKLGGYGMIRIM-PMLSPLTNIMSLPFLILALWGVVMTGTICLRQTDLKSLIAYSSVG
Lifa     ILLKLGGYGIIRML-PLLEPLTKELSYPFILLALWGIIMTGSICLRQTDLKSLIAYSSVS
Acur     ILLKLGGYGMIRMM-SLLQPLTHELMYPFIALALWGVLMASSVCLRHTDLKALIAYSSVS
Ampe     VLLKLGGYGMMRLM-IMLDPLTKELSYPFIIFALWGVVMTSSICLRQTDLKSLIAYSSVS
Urja     VLLKLGGYGMMRMA-IILGPLTKELYYPLISLALWGVIMTASICLTQVDLKSLIAYSSVS
Enet     VLLKLGGYGMMRII-VILDPLTDLMAYPFIALALWGVVMTGTICLRQTDLKALIAYSSVS
Ptbr     VLLKLGGYGMIRII-LILDPLTDIMAYPFLVMALWGMIMAGSICLRQTDLKSLIAYSSVS
Safa     VLLKLGGYGMLRII-LLLDPLTKEMSYPFMVLAMWGLVMAGSVCMRQTDLKSLIAYSSVS
Icae     VLLKLGGYGMMRMM-IMLEPLTKELSYPFIIFALWGVIMTGSICLRQTDLKSLIAYSSVS
Asmi     ILLKLGGYGMMRIT-MALGPISTKLSYPLIMLALWGVVMTSSICLRQTDLKSLIAYSSVS
Foal     ILLKLGGYGLMRIV-PLLTPLTPTLSYPFMILALWGGVMTASICTRQTDLKSLIAYSSVG
Drze     VLLKLGGYGMLRMM-PFVEPVTKDLSYPFIVLAVWGTIMTGSMCLRQLDLKALIAYSSVG
Rhas     VLLKLGGYGMMRMM-ILLEPLTKELSYPFIILALWGVIMTGSICLRQTDLKSLIAYSSVG
Elac     VLLKLGGYGMMRMM-VMLEPLTKELSYPFIILALWGVIMTGSICLRQTDLKSLIAYSSVG
Kugu     VLLKLGGYGMMRMM-IMLEPLTKELSYPFIILALWGVIMTGTICLRQTDLKSLIAYSSVG
Plor     VLLKLGGYGMIRML-TILEPATKELSYPFIIFALWGVVMTGSICLRQTDLKSLIAYSSVS
Sgun     VLLKLGGYGMMRIL-VVLEPLTKELSYPFIIFALWGIIMTGSICLRQTDLKSLIAYSSVS
Zaco     VLLKLGGYGMMRML-VILEPLTKELSYPFIILALWGVIMTGSICLRQTDLKSLIAYSSVS
Zbfl     VLLKLGGYGMMRML-VILEPLTKELSYPFIIFALWGVIMAGTICLRQTDLKSLIAYSSVS
Spba     VLLKLGGYGMIRMM-TVLEPLTKELGYVFIIFALWGIIMTGSICLRQTDLKSLIAYSSVS
Game     VLLKLGGYGMMRMM-VMLEPLTKELSYPFIVFALWGVIMTGSICLRQTDLKSLIAYSSVS
Thth     VLLKLGGYGMMRMM-IMLEPLTKELSYPFIVFALWGVIMTGSICLRQTDLKSLIAYSSVS
Xigl     VLLKLGGYGMMRMM-VMLEPLTKDLSYPFIVFALWGVIMTGSICLRQTDLKSLIAYSSVS
Hyja     VLLKLGGYGMMRMM-TMLEPLTKELSYPFIIFALWGVIMTGSICLRQTDLKSLIAYSSVS
Psan     VLLKLGGYGMIRLM-GILEPVTHELCYPFMVFTLWGVIMTGTICLRQSDLKAMIAYSSVS
Cupa     VLLKLGGYGMMRMM-IMLEPLTKELSYPFIIFALWGVIMTGSICLRQTDLKSLIAYSSVS
Mpch     VLLKLGGYGMMRMM-IMLEPLTKELSYPFIVLALWGVIMTSSICLRQTDLKSLIAYSSVS
Char     VLLKLGGYGMMRMM-PMLEPLTKELSYPFIILALWGVIMTGSICLRQADLKSLIAYSSVS
Pser     VLLKLGGYGMMRMM-TMLEPLTKELSYPFIIFALWGVVMTGSICLRQTDLKSLIAYSSVG
Prol     VLLKLGGYGMIRMM-TMLEPLTKELSYPFIIFALWGVVMTGSICLRQTDLKSLIAYSSVS
Plbi     VLLKLGGYGMIRMM-TMLEPLTKELSYPFIIFALWGVIMTGSICLRQTDLKSLIAYSSVS
Calu     VLLKLGGYGMIRVL-PMLEPLTEQLSYPFILLALWGVIMTGVICLRQTDLKSLIAYSSVS
Papa     VLLKLGGYGMMRVM-TVLEPMTKELSYPFITLAVWGIIMTGTICLRQTDLKSLIAYSSVS
Sufr     VLLKLGGYGMMRML-TVLGPLTKELCYPFIILALWGVIMTGSICLRQTDLKSLIAYSSVS
Stci     VLLKLGGYGMIRML-VILDPLTKELSYPFIILALWGIIMTGSICLRQTDLKSLIAYSSVS
Taru     VLLKLGGYGMMRIL-VTLDPLTKELSYPFIVLALWGVIMTGSICMRQTDLKSLIAYSSVS
Rala     VLLKLGGYGMMRML-VMLEPLTKQLSYPFIILALWGVIMTGSICLRQTDLKSLIAYSSVS
         :********::*:              .: :   *.  :    *  . *:* ::   .
```

To be continued on page 30.

```
     H                                 I
Scca HMGLVAGAILIQTPWSFAGAITLMIAHGLVSSALFCLANTNYERIHSRTLLLARGVQVIL
Muma HMGLVAAAILIQTPWSFAGATMLMIAHGLISSALFCLANTNYERIHSRTLLLARGIQIIL
Erca HMGLVASGILIQTTWGFTGAIILMIAHGLTSSALFCLANTTYERTNSRTLLLARGMQIIL
Pose HMGLVASGILIQTPWGFTGAIILMIAHGLTSSALFCLANTTYERTHSRTLLLARGMQIIM
Actr HMGLVVAGILIQTPWGFTGAIILMIAHGLASSALFCLANTNYERLHSRTLLLARGMQAVL
Scal HMGLVVAGILIQTPWGFTGAIILMIAHGLASSALFCLANTNYERLHSRTLLLARGMQTIL
Posp HMGLVVAGILIQTPWGFTGAIILMIAHGLASSALFCLANTNYERLHSRTLLLARGMQAIL
Atsp HMGLVTGGILIQTPWGFTGAIILMIAHGLVSSALFCLANTVYERTNTRTLLITRGMQTFL
Leoc HMGLVVGGILIQTPWGFTGAIILMIAHGLVSSALFCLANTVYERTNTRTLLITRGMQTLL
Amca HMGLVAAGILIQTPWGFTGAIVLMIAHGLVSSALFCLANTNYERTHSRTLLLARGLQILL
Osbi HMGLVASGILIQTPWSFTGAIILMIAHGLVSSALFCLANTNYERTHSRTLLLTRGLQTIL
Pabu HMGLVAAGILIQTPWGFTGAIILMIAHGLVSSALFCLANTNYERMHSRTLLLARGMQTIL
Hial HMGLVAGGILIQTPWGFTGAIILMIAHGLVSSALFCLANTNYERIHSRTLLLARGLQMIL
Elha HMGLVAGGILIQTPWGFTGAIILMIAHGLVSSALFCLANTNYERVHSRTLLLARGLQMIL
Mlcy HMGLVAGGILIQTPWGFTGAIILMIAHGLVSSALFCLANTNYERIHSRTLLLARGLQMIL
Algl HMGLVVGGILIQTPWGFTGALILMISHGLVSSALFCLANTNYERIHSRTLLLARGLQMVL
Ptgi HMGLVAGGILIQTPWGFTGAIILMIAHGLVSSALFCLANTNYERIHSRTLLLARGLQMIL
Alaf HMGLVAGGILIQTPWGFTGAIILMIAHGLVSSALFCLANTNYERIHSRTLLLARGLQMIL
Nock HMGLVAGGILIQTPWGFTGAIILMVAHGLVSSALFCLANTNYERIHSRTLLLARGLQMIL
Anja HMGLVAGGILIQTPWGFTGAIILMIAHGLVSSALFCLANTNYERTHSRTLLLARGLQMIL
Gyki HMGLVAAGIMIQTPWGFTGAIILMIAHGLVSSALFCMANTNYERTHSRTLLLARGLQTLL
Syka HMGLVAGGILIQTPWGFTGAIILMIAHGLVSSALFCLANTNYERTHSRTLLLARGLQMIL
Opma HMGLVAGGILIQTPWGFTGAIILMIAHGLVSSALFCLANTNYERTHSRTLLLARGLQMML
Comy HMGLVAAGIFIQTPWGFTGAIILMIAHGLVSSALFCLANTNYERTHSRTLLLARGLQTIL
Sasp HMGLVVAGVMTQTPWGFTGSLILMISHGLTSSLLFCLTNTNYERTHSRTLLLTRGMCMIM
Eupe HMGLVTGGIMIQTPWSFAGATILMISHGLASSTLFCLANTNYERIHSRMLLLARGLQMIL
Enja HMGLVAGGILTQTPWGLTGAIILMIAHGLTSSALFCLANTSYERTHSRTMALARGMQTLF
Same HMGLVAGGILIQTPWGLTGAIILMIAHGLVSSALFCLANTSYERTHSRTMVLARGLQMLF
Chch HMGLVAGGILVQTPWGFTGALILMIAHGLTSSALFCLANTSYERTHSRTMALTRGLQVIF
Grgr HMGLVAGGILVQTPWGLTGAIILMIAHGLASSALFCLANTSYERTHSRTMVLARGLQVVL
Caau HMGLVAGGILIQTPWGFSGAIILMIAHGLVSSALFCLANTAYERTHSRTMILARGLQIIF
Cyca HMGLVAGGILIQTPWGFSGAIILMIAHGLVSSALFCLANTAYERTHSRTMILARGLQVIF
Dare HMGLVAGGILIQTPWGFTGAIILMIAHGLTSSALFCLANTSYERTHSRTMILARGLQMVL
Cost HMGLVAGGILIQTPWGFTGAIILMIAHGLVSSALFCLANTAYERTHSRTMILARGLQMIF
Leec HMGLVAGGILIQTPWGFTGAIILMIAHGLVSSALFCLANTTYERTHSRTMLLARGLQMIL
Fola HMGLVAGGILIQTPWGFTGAIILMIAHGLVSSALFCLANTAYERTHSRTMILARGLQMIF
Clmc HMGLVAAGILIQTPWGFTGAIILMIAHGLVSSALFCLANTAYERTHSRTMILARGLQMIF
Phin HMGLVASGVLTQTPWGFTGAIILMIAHGLTSSALFCLANTTYERTHSRTMVLARGMQLIF
Icpu HMGLVAGGILIQTPWGFTGALVLMIAHGLVSSALFCLANTTYERTHSRTMILARGMQIIF
Psto HMGLVAGGILIQTPWGFTGALVLMIAHGLVSSALFCLANTTYERTHSRTMVLARGLQIIF
Cora HMGLVAGGILIQTPWGFTGAIILMIAHGLVSSALFCLANTTYERTHSRTMVLARGMQLLL
Eisp HMGLVAAGILIQTPWGFTGAIILMIAHGLVSSALFCLANTAYERTHSRTMILARGLQMIF
Apal HMGLVAGGILIQTPWGFTGAIILMIAHGLASSALFCLANTTYERTHSRTMILARGMQMIF
Eslu HMGLVASGILIQTPWGFTGAIILMVAHGLASSALFCLANTSYERTHSRTMLLTRGLQMIL
Dape HMGLVASAILIQTPWSFTGAIVLMIAHGLASSALFCLANTGYERTHSRTMLLTRGLQMIF
Glse HMGLVASGILIQTPWGFTGAIILMIAHGLASSALFCLANTSYERTHSRTMLLARGLQMIF
Naar HMGLVAGGILIQTPWGFTGAIILMIAHGLASSALFCLANITYERTHSRTMLLARGLQMIF
Lioc HMGLVAGGILIQTPWGFTGAIILMIAHGLASSALFCLANITYERTHSRTMLLARGLQMIF
Opso HMGLVASGILIQTPWGFTGAIILMIAHGLASSALFCLANTTYERTHSRTMLLTRGLQMIF
Alte HMGLVAGGILIQTPWGFTGAIILMIAHGLASSALFCLANTSYERTHSRTMVLARGLQMLF
Plap HMGLVAGGILIQTPWGFTGAIILMIAHGLASSALFCLANTNYERTHSRTMVLARGLQMLF
```

To be continued on page 31.

[6/8 of aligned sequences]

```
Plal HMGLVAGGILIQTPWGFTGAIILMIAHGLASSALFCLANTAYERTHSRTMLLARGLQMAL
Sami HMGLVAGGILIQTPWGFTGAIILMIAHGLASSALFCLANTAYERTHSRTMLLARGLQMVL
Rere HMGLVAGGILIQTPWGFTGVIILMIAHGLASSALFCLANTTYERTHSRTMLLARGLQMIL
Gama HMGLVAGGILIQTPWGFTGAIILMIAHGLTSSALFCLANTSYERTHSRTMLLARGMQMVL
Onmy HMGLVAGGILIQTPWGFTGAIILMIAHGLASSALFCLANTSYERTHSRTMLLARGMQMIL
Sasa HMGLVAGGILIQTPWGFTGAIILMIAHGLASSALFCLANTSYERTHSRTMLLARGMQMIL
Cola HMGLVAGGILVQTPWGFTGAIILMIAHGLASSALFCLANTSYERTHSRTMLLARGMQMIL
Dita HMGLVAGGILIQTPWGISGAIILMIAHGLASSALFCLANTNYERTHSRTMLLMRGMQAVL
Gogr HMALVAGGILIQTPWGFYGATMLMIAHGLTSSALFCLANTYYERVNTRTMLIIRGMQIIS
Chsl HMGLVVGGILIQTPWGVAGATILMIAHGLASSALFCLANTSYERTHSRVMFLMRGTQTIL
Atja HMGLVAGGILIQTPWGFTGAIILMIAHGLASSALFCLANTNYERTHSRTMLLARGLQTVL
Iido HMGLVAGGILIQTPWGFTGAIILMIAHGLASSALFCLANTNYERTHSRTMLLARGLQTVL
Auja HMGLVAGGILIQTPWGLTGALILMIAHGLASSALFCLANTNYERTHSRTMLLARGLQMVL
Chag HMGLVAGGILIQTPWGFTGALVLMIAHGLASSALFCLANTNYERTHSRTMLLARGLQAVL
Hami HMGLVAAGILIQTPWGFTGALTLMIAHGLASSALFCLANTNYERTHSRTMLLARGLQAAL
Saun HMGLVAAGILIQTPWGFTGALILMIAHGLASSALFCLANTNYERTHSRTMLLARGLQAVL
Nema HMGLVAGGILIQTPWGFTGALILMIAHGLASSALFCLANTNYERTHSRTMILARGMQTVL
Disp HMGLVAGGILIQTPWGFTGALVLMIAHGLASSALFCLANTNYERTHSRTMVLARGMQTML
Myaf HMGLVAGGILIQTPWGFTGALVLMIAHGLASSALFCLANTTYERTHSRTMILARGMQTIL
Lagu HMGLVASGILTQTYWGLTGAIILMVAHGLTSSALFCLANTNYERTHTRTMLLARGLQMVL
Trtr HMGLVVAGILTQTPWGLTGAVILMIAHGLTSSALFCLANTNYERTHSRTMLLARGLQSVF
Zucr HMGLVAAGILIQTPWGLAGAIILMIAHGLTSSALFCLANTNYERTHTRTMLLARGLQSIF
Pxja HMGLVAGGILIQTPWGFTGALILMIAHGLASSALFCLANTNYERTHSRTMVLARGLQMIL
Pxlo HMGLVAGGILIQTPWGFTGALILMIAHGLASSALFCLANTNYERTHSRTMVLARGLQMIL
Pctr HMGLVAGGILIQTPWGFTGALTLMIAHGLASSALFCLANTTYERTHSRTMVLARGLQMLL
Apsa HMGLVAAGILIQTPWGFTGALTLMIAHGLASSALFCLANTNYERTHSRTMILARGLQGLL
Cabe HMGAVAAGILVQTSWGFTGALALMIAHGLTSSALFCLANTNYERIHSRTIVLARGLQLVL
Bzze HMGLVAGGILIQTAWGFTGALILMIAHGLTSSALFCLANTNYERTHSRTMILARGLQMVL
Siim HMGLVIGGILSQTPWGFTGALVLMIAHGLTSSALFCLANVNYERTHSRTMLLSRGLQILL
Ctru HMGLVAAAILIQTPWGFTGALILMIAHGLTSSALFCLANTNYERTHSRTMVLARGLQMVL
Dpbr HMGLVAGAILIQTPWGFTGALILMIAHGLTSSALFCLANTNYERTHSRTMILARGLQMVL
Caki HMGLVAGGILIQTPWGFTGALTLMIAHGLTSSALFCLANTNYERTHSRTMLLARGLQIAL
Phja HMGLVVAGLLIQTPWGFKGALVLMIAHGLTSSALFCLANINYERIHSRTMLLARGLQIAL
Brsp HMGLVAAAILIQTPWGLTGAIILMIAHGLASSALFCLANTNYERTHTRTMLLTRGLQSAL
Gamo HMGLVAGGILIQTPWGFTGALILMIAHGLASSALFCLANTNYERTHSRTMLLARGLQIAL
Lolo HMGLVAGGILIQTPWGFTGALILMIAHGLASSALFCLANTNYERTHSRTMLLARGLQIAL
Batr HMAMVISAILIQTQWSISGASIMMIAHGLTSSALFCLANMNYERLHSRTLLMMRGLQMLL
Prmy HMGLVIASILIQTQWSLTGATILMIAHGFTSSALFCLANTNYERTHSRTMLLTRGLQVLL
Lose HMGLVIAGILIQTPWGFTGALILMIAHGLTSSALFCLANTNYERLHSRTLMLARGLQMAL
Loam HMGLVVGGILIQTPWGFTGALILMIAHGLTSSALFCLANTNYERTHSRTMVLARGLQMAL
Chab HMGLVVGGILIQTPWGFTGALTLMIAHGLTSSALFCLANTNYERTHSRTMILARGLQVLL
Chto HMGLVVGGILIQTPWGFTGALTLMIAHGLTSSALFCLANTNYERTHSRTMILARGLQILL
Majo HMGLVVGGIFSQTTWGLAGATVLMIAHGLTSSALFCLANIQYERTHNRTMVLARGLQSTM
Hlst HMGLVMGGVFSQSSWGLAGATILMIAHGLTSSALFCLANIQYERTHNRTLVLARGLQSAM
Clpe HMGLVAAGILTQTPWGFTGALVLMIAHGLTSSALFCLANTNYERTHSRTMILARGMQVLM
Mlmr HMGLVVGGILIQTPWGFSGAMILMIAHGLTSSALFCLANTQYERTHSRTMILARGMQMLL
Crcr HMGLVAAGILIQTPWGFSGALILMIAHGLTSSALFCLANTNYERTHSRTMVLARGLQMAL
Muce HMGLVAAGILIQTPWGFSGALILMIAHGLTSSALFCLANTNYERTHSRTMVLARGLQMAL
Bege HMGLVAAGILIQTPWGLTGALILMIAHGLTSSALFCLANTNYERTHSRTMVLARGLQMVL
Mela HMGLVTAGILIQTPWGLTGALILMIAHGLTSSALFCLANTNYERTHSRTMILARGLQTAI
Hats HMGLVAAGILIQTPWGLTGALILMIAHGLTSSALFCLANTNYERTHSRTMVLARGLQMVL
Orla HMGLVAAGILIQTPWGLTGALILMIAHGLTSSALFCLANTNYERTHSRTMLLARGLQMIL
```

To be continued on page 32.

[6/8 of aligned sequences]

```
Cosa    HMGLVAAAILIQTPWSFTGALILMIAHGLTSSALFCLANTNYERTHSRTMVLARGLQMVL
Exsp    HMGLVAAGILIQTPWGFTGALILMIAHGLTSSALFCLANTNYERTHSRTMVLARGMQMAL
Depa    HMGLVAAGILIQTPWSFTGALILMIAHGLTSSALFCLANTNYERTHSRTMILARGLQMVL
Rima    HMGLVTAGILIQTPWSFTGALILMIAHAFTSSALFCLANTNYERTHSRTMILARGLQTIF
Fuol    HMGLVAAGILIQTPWGFTGALILMIAHGLTSSALFCLANTNYERTHSRTIVLARGLQMIM
Gmaf    HMGLVAAAILIQTPWAFTGALVLMIAHGLTSSALFCLANTNYERSHSRTMLLVRGLQTLL
Xeei    HMGLVAAGILIQTPWGFTGALVLMIAHGLTSSALFCLANTNYERTHSRTMLLARGLQMAL
Pros    HMGLVAGGILIQTPWGFTGSLILMIAHGLASSALFCLANTNYERTHSRTMVLARGLQMVL
Scmi    HMGLVAGGILIQTPWGFTGALILMIAHGLASSALFCLANTNYERTHSRTMILARGLQMVL
Rolo    HMGLVAGGILIQTPWGFTGALILMIAHGLASSALFCLANTNYERTHSRTMILARGLQMVL
Cere    PYGPVVEESWFKPREASQGAMTLMIAHGLASSALFCLANTNYERTHSRTMLLTRGLQMVF
Daga    HMGLVASGILIQTPWGFTGALTLMIAHGFTSSALFCLANTNYERTHSRTMILARGLQVLL
Anco    HMGLVAGGILIQTPWGFTGALVLMIAHGLASSALFCLANTNYERTHSRTMVLARGLQMVL
Dmve    HMGLVVGGILIQTPWGFTGALILMVAHGLASSALFCLANTNYERTHSRTMILARGLQMVL
Dmar    HMGLVVGGILIQTPWGFTGALVLMIAHGLASSALFCLANTNYERTHSRTMVLARGLQMVL
Anka    HMGLVAGGILIQTPWGFTGALILMIAHGLASSALFCLANTNYERTHSRTMVLARGMQMVL
Moja    HMGLVAGGILIQTPWGFTGALVLMIAHGLASSALFCLANTNYERTHSRTMILARGLQMVL
Hoja    HMGLVAGGILIQTPWGFTGALILMIAHGLASSALFCLANTNYERTHSRTMVLARGLQMVL
Bede    HMGLVAGGILIQTPWGFTGALILMIAHGLASSALFCLANTNYERTHSRTMVLARGLQMVL
Besp    HMGLVAGGILIQTPWGFTGALILMIAHGLASSALFCLANTNYERTHSRTMVLARGLQMVL
Mysp    HMGLVVGGILIQTPWGLTGALLLMIAHGLASSALFCLANTNYERTHSRTMLLARGLQMVL
Osja    HMGLVVGGILIQTPWGLTGALLLMIAHGLASSALFCLANTNYERTHSRTMLLARGLQMVL
Sgro    HMGLVVGGILIQTPWGFTGALLLMIAHGLASSALFCLANTNYERTHSRTMLLARGLQMIL
Pzpa    HMGLVAGGIMIQTPWGFTGALILMIAHGLASSALFCLANTNYERMHSRTMLLARGMQMVM
Zeja    HMGLVAGGILIQTPWGFTGALILMIAHGLASSALFCLANTNYERTHSRTMLLTRGLQMVL
Znne    HMGLVAGGILIQTPWGFTGALILMIAHGLASSALFCLANTNYERTHSRTMLLARGLQMLM
Zefa    HMGLVAGGILIQTPWGFTGALILMIAHGLASSALFCLANTNYERTHSRTMLLARGLQMIM
Acni    HMGLVAGGILIQTPWGFTGALILMIAHGLASSALFCLANTNYERTHSRTMLLARGLQMVM
Ncrh    HMGLVAGGILIQTPWGFTGALILMIAHGLASSALFCLANTNYERTHSRTMLLARGLQMVM
Agca    HMGLVVGGILIQTPWGFTGALILMIAHGLTSSALFCLANTNYERTHSRTMVLARGLQMAL
Hydy    HMGLVVAAILIQTPWSFSGALILMIAHGLASSALFCLANTNYERTHSRTLVLARGLQMAL
Gsac    HMGLVVAAILIQTPWSLSGALILMIAHGLASSALFCLANTNYERTHSRTLLLGRGLQMAL
Pevo    HMGLVAAGILIQTPWGTTGALVLMIAHGLTSSALFCLANTNYERTHSRTMVLARGLQAIL
Hiku    HMGLVTTGILIQTPWGFTGALVLMIAHGLTSSALFCLANTNYERTHSRTMLLARGLQMVL
Inpa    HMGLVTAGILTQTTWGAAGALALMIAHGLTSSALFCLANTNYERTHTRTLSLTRGLQGLL
Auch    HMSLVAAGILTQTPWGISGALILMIAHGLTSSALFCLANTSYERTHTRAMLLTRGLQALF
Fico    HMGLVAGGILIQTPWGFTGALILMIAHGLTSSALFCLANTNYERTHSRTMVLARGLQMLL
Macs    HMGLVAGGILIQTPWGFTGALILMIAHGLTSSALFCLANTSYERTHSRTMVLARGMQMIL
Moal    HMGLVAAAILIQTPWGLTGALILMIAHGLTSSALFCLANTNYERTHSRTMILTRGSQMIL
Syma    HMSLVAAAILIQTPWSIAGALILMIAHGLTSSALFCLANTNYERTHTRTMTLTRGMQVVL
Mafr    HMGLVIAAILTQTPWGFTGALILMIAHGLTSSALFCLANTNYERTHSRTMILTRGLQIIL
Dcpe    HMGLVAGGILIQTPWGFSGAMILMISHGLTSSALFCLANTNYERTHSRTMILARGLQMAL
Dcti    HMGLVAGGILIQTPWGFSGAMVLMITHGLTSSALFCLANTNYERTHSRTMVLARGLQMAL
Hehi    HMGLVVGGILIQTPWGFAGAVILMIAHGLTSSALFCLANTNYERLHSRTMLLARGLQMVL
Stam    HMGLVVGGILIQTPWGFAGALILMIAHGLTSSALFCLANTNYERTHSRTMLLARGLQMVL
Hogi    HMGLVVGGILIQTPWGFTGALILMIAHGLTSSALFCLANTNYERTHTRTMVLARGLQMAL
Erzo    HMGLVVGGILTQTPWGFTGALILMIAHGLTSSALFCLANTNYERTHSRTMLLARGLQMVL
Hxot    HMGLVVGGILIQTPWGFSGALILMIAHGLASSALFCLANTSYERTHSRTMILARGLQMVL
Core    HMGLVVGGILIQTPWGFSGALVLMIAHGLASSALFCLANTSYERTHSRTMLLARGLQMVL
Apve    HMGLVVGGILTQTPWGFTGALILMIAHGLASSALFCLANTNYERTHSRTMLLARGLQMAL
Latj    HMGLVVGGILIQTQWSFTGALILMVAHGLTSSALFCLANTNYERTHSRVMLLTRGLQMAL
Laja    HMGLVVAGILIQTPWGFTGALILMVAHGLTSSALFCLANTNYERTHSRTLVLARGLQVAL
```

To be continued on page 33.

[6/8 of aligned sequences]

To be continued on page 34.

```
Syja  HMGLVVGGILIQTPWGFTGALILMIAHGLTSSALFCLANTNYERTHSRTMVLARGLQMAL
Epme  HMGLVVGGILIQTPWGFTGALILMIAHGLTSSALFCLANTNYERTHSRTMLLARGLQIML
Grse  HMGLVIGGILIQTPMGFAGALILMIAHGLTSSALFCLANTNYERTHSRTMILARGLQMIL
Clja  HMGLVIAGILIQTSWGLSGAMVLMIAHGLTSSAYFCLANSNYERTHTRTLLLARGLQMIL
Ogcy  HMGLVAAGILTQTPWGFTGALILMIAHGLTSSALFCLANTNYERTHSRTMILARGLQTIL
Plna  HMGMAAAGIFTQTQWGLTGAMLIMVSHGLTSSALFCLANTNYERYHSRTMLLARGLQLIT
Lema  HMGLVVAGILIQTPWGFSGALILMIAHGLTSSALFCLANTNYERTHTRTMVLARGLQVVL
Etzo  HMGLVVGGILIQTPWGFSGALILMIAHGLTSSALFCLANTNYERTHSRTMLLARGLQMVL
Apse  HMGLVAAAILIQTPWSFTGALILMIAHGLTSSALFCLANTNYERTHSRTMLLARGLQTVF
Epde  HMGLVVGGILIQTPWGFTGAIILMIAHGLTSSALFCLANTNYERTHSRTLVLTRGLQMAL
Slja  HMGLVVGGILIQTPWGFTGSLILMIAHGLTSSALFCLANMNYERTHSRTMVLARGLQMAL
Bsja  HMGLVIAGILTQSPWGITGSLILMISHGLTSSALFCLANTNYERTHSRTMLLARGLQAAL
Ecna  HMGLVAGGILIQTPWGFTGALILMIAHGLTSSALFCLANTNYERTHSRTMILARGMQMVL
Cohi  HMGLVAGGILTQSVWGFSGALALMIAHGLTSSALFCLANTIYERTHTRTMILTRGMQVMF
Caar  HMGLVAGGILIQTPWGFTGALILMIAHGLTSSALFCLANTNYERTHSRTMVLARGLQMVL
Came  HMGLVAGGILIQTPWGFTGALILMIAHGLTSSALFCLANTNYERTHSRTMILARGLQMVL
Mema  HMGLVAGGILIQTPWGFTGALILMIAHGLTSSALFCLANTNYERTHTRTMVLARGLQMIL
Lenu  HMGLVVAGALLQTPWSFSGALILMIAHGLTSSALFCLANTNYERTHNRTMLLTRGLQTAF
Brja  HMGLVAGGILIQTPWGFSGALILMIAHGLTSSALFCLANTNYERTHSRTMVLARGLQMVL
Plma  HMGLVAGGILIQTPWGFTGALILMIAHGLTSSALFCLANTNYERTHSRTMVLARGLQMIL
Emst  HMGLVVGGILIQTPWGFTGALILMIAHGLTSSALFCLANTNYERTHSRTMVLARGLQMAL
Ptti  HMGLVVGGILIQTPWGFTGALILMIAHGLTSSALFCLANTNYERTHSRTMVLARGLQMAL
Losu  HMALVASAILIQTPWGATGALVLMIAHGLTSSALFCLANTNYERTHSRTMILARGLQITL
Geoy  HMGLVVGGILIQTPWGFTGALILMIAHGLTSSALFCLANTNYERTHSRTMVLARGLQMVL
Dipi  HMGLVVGGILIQTPWGFTGALILMIAHGLTSSALFCLANTNYERTHSRTMVLARGLQMVL
Pama  HMGLVIGGILIQTPWSFTGALILMIAHGLTSSALFCLANTNYERTHSRTMVLARGLQMAL
Leob  HMGLVVGGILIQTPWGFTGALILMIAHGLTSSALFCLANTNYERTHSRTMVLARGLQMAL
Neba  HMGLVAAGILTQTDWGFTGALVLMIAHGLTSSALFCLANTNYERTHSRTMLLVRGMQMVL
Pdpl  HMGLVAAGILIQTPWGLTGAIILMIAHGLTSSALFCLANTNYERTHSRTMLLARGLQMVL
Nimi  HMGLVVGGILVQTPWGFTGALILMIAHGLTSSALFCLANTNYERTHSRTMILARGLQVAL
Uptr  HMGLVAGGILIQTPWGFTGALVLMIAHGLASSALFCLANTNYERTHSRTMLLARGLQMVL
Pesc  HMGLVIAGILIQTPWGVAGGIIMMIAHGLTSSALFCLANTNYERTFTRTMLLTRGLQAIL
Baar  HMGLVVGSILIQTPWGFTGALILMIAHGLTSSALFCLANTNYERTHSRTMVLARGLQMIL
Moar  HMGLVVGGILIQTPWGFTGALILMIAHGLTSSALFCLANTNYERTHSRTMVLARGLQMAL
Toja  HMGLVAGGILIQTPWGFTGALILMIAHGLTSSALFCLANTNYERTHSRTMILARGLQMVL
Chau  HMGLVIAAILIQSPWALSGAMILMIAHGLTSSAMFCLANTAYERTHSRTMLLTRGMQMVL
Chse  HMGLVVGGILIQTPWGFTGALILMIAHGLTSSALFCLANTNYERTHSRTMVLARGLQMAL
Enar  HMGLVVGGILIQTPWGFTGALILMIAHGLTSSALFCLANTNYERTHSRTMVLARGLQMAL
Hpty  HMGLVVGGILIQTPWGFTGALILMIAHGLTSSALFCLANTNYERTHSRTMILARGLQMAL
Nana  HMGLVVGGILTQTPWGFSGALILMIAHGLTSSALFCLANTNYERTHSRTMVLTRGLQTIL
Mcst  HMGLVIGGILTQTPWGFTGALILMIAHGLTSSALFCLANTNYERTHSRTMVLARGLQIAL
Rhox  HMGLVVGGILIQTPWGFTGALILMIAHGLTSSALFCLANTNYERTHSRTMVLARGLQIAL
Opfa  HMGLVVGGILIQTPWGFTGALILMIAHGLTSSALFCLANTNYERTHSRTLVLARGLQMAL
Paar  HMGLVVGGILIQTPWGLTGALILMIAHGLTSSALFCLANTNYERTHSRTLVLARGLQLAL
Gozo  HMGLVVGGILIQTPWGFTGALILMIAHGLTSSALFCLANTNYERTHSRTMVLARGLQMAL
Ackr  HMGLVIAAILVQTPWGLTGALTLMIAHGLTSSALFCLANTNYERTHTRTMILARGLQVAL
Elev  HMGLVVAGILTQTSWGITGAMILMIAHGLTSSALFCLANTNYERTHSRTMILARGLQMAL
Trdu  HMGLVAAGILIQTPWGFTGALILMIAHGLTSSALFCLANTNYERTHSRTMVLARGLQMVL
Amoc  HMGLVAAGILIQTPWGFTGALILMIAHGLTSSALFCLANTNYERTHSRTMILARGLQMAL
Hame  HMGLVVAGILAQTSWGFTGALVLMIAHGLTSSALFCLANTNYERTHTRTMVLARGLQTAL
Chso  HMGLVVAGILSQTPWGLTGALILMIAHGLTSSALFCLANTNYERTHSRTMVLARGLQMAL
Lyto  HMGLVVGGILIQTPWGFTGALILMIAHGLASSALFCLANTNYERTHSRTMLLARGLQMVL
```

[6/8 of aligned sequences]

```
Encr  HMGLVVGGILIQTPWGFSGALILMIAHGLASSALFCLANTNYERTHSRTMLLARGLQMVL
Bvar  HMGLVISGILIQTPWGFTGALILMIAHGLTSSALFCLANTNYERTHSRTMLLARGLQIAL
Noco  HMGLVVAGILVQTPWGFSGALILMIAHGLTSSALFCLANTNYERIHSRSILLARGLQMVL
Chsp  HMGLVVGGILIQTPWGLTGALIIMIAHGLTSSALFCLANMNYERTHTRVMMLTRGLQMSI
Arja  HMGLVVGGILIQTPWGFTGALVLMIAHGLASSALFCLANTSYERTHSRTMLLARGLQMVL
Pase  HMGLVAAAILIQTPWSFTGALILMIAHGLTSSALFCLANINYERTHSRTMILARGMQMAL
Trel  HMGLVAAALLTHTQWALAGSVILMVAHGLTSSALFCLANANYERTQSRTMMLARGMQMAL
Lifa  HMGLVVGGILTQTPWGLSGAIILMVAHGLTSSALFCLANTNYERTHSRVMVLARGLQTVL
Acur  HMGLVIAAILTQTPWGLTGAMILMIAHGLTSSALFCLANTNYERTHSRVLFLARGLHMAL
Ampe  HMGLVAAAILIQTPWSFTGALILMIAHGLTSSALFCLANMNYERTHSRTMVLARGLQVAL
Urja  HMGMVAAGILIQTPWGFTGALILMIAHGLTSSALFCLANMNYERTHSRIMVLSRGLQMAL
Enet  HMGLVAAGILIQTPWGLTGSLVLMIAHGLTSSALFCLANTNYERTHSRTMILARGLQMVL
Ptbr  HMGLVAAGILIQTPWGLAGSIVLMIAHGLTSSALFCLANMNYERTHSRSIILSRGLQVIF
Safa  HMGLVAAGILIQTPWGLAGAIALMIAHGLTSSALFCLANTNYERTHSRTMLLARGLQMTL
Icae  HMGLVAGGILIQTPWGFTGALILMIAHGLTSSALFCLANTNYERTHSRTMVLARGLQMVL
Asmi  HMGLVTAAILIQTPLAYSGALILMIAHGLTSSALFCLANTNYERTHSRTLLLTRGLQTAL
Foal  HMGLAAAGILTQSSCGYSGAMMLMIAHGLTSSALFCLANVNYERTHSRTLTLARGLQTLL
Drze  HMGLVAAGILIQTPWGHSGALTIMIAHGLTSSALFCLANTNYERTNSRTMMLTRGLHMML
Rhas  HMGLVAGGILIQTPWGFTGALILMIAHGLTSSALFCLANTNYERTHSRTMVLARGMQMVL
Elac  HMGLVAGGILIQTPWGFTGALILMIAHGLTSSALFCLANTNYERTHSRTMMLARGLQVVL
Kugu  HMGLVAGGILIQTPWGFTGALILMIAHGLTSSALFCLANTNYERTHSRTMMLARGLQMIL
Plor  HMGLVVGGILIQTPWGLTGALILMIAHGLTSSALFCLANTNYERTHSRTMVLARGLQMAL
Sgun  HMGLVVGGILIQTPWGFTGAIILMIAHGLTSSALFCLANTNYERTHSRTMVLARGLQMAL
Zaco  HMGLVVGGILIQTPWGFTGALILMIAHGLTSSALFCLANTNYERTHSRTMLLARGLQVAL
Zbfl  HMGLVVGGILIQTPWGFTGALILMIAHGLTSSALFCLANTNYERAHSRTMVLARGLQMAL
Spba  HMGLVAGGILIQTPWGFSGALILMIAHGLTSSALFCLANTNYERTHSRTMILARGLQMVL
Game  HMGLVAGGILIQTPWGFTGALILMIAHGLTSSALFCLANTNYERTHSRTMVLARGLQMVL
Thth  HMGLVAGGILIQTPWGFTGALILMIAHGLTSSALFCLANTNYERTHSRTMVLARGLQMVL
Xigl  HMGLVAGGILIQTPWGFTGALILMIAHGLTSSALFCLANTNYERTHSRTMLLARGLQMVL
Hyja  HMGLVAGGILIQTPWGFTGALILMIAHGLTSSALFCLANTNYERTHSRTMVLARGLQMAL
Psan  HMGLVTGALLTQSVWGLAGALVLMIAHGLTSSVLFYIAATHYDRTHTRSIILMRGLQKFL
Cupa  HMGLVAAGILIQTPWGFTGALILMIAHGLTSSALFCLANTNYERTHSRTMVLARGLQMVL
Mpch  HMGLVAGGILIQTPWGFTGALVLMIAHGLTSSALFCLANTNYERTHSRTMVLARGMQMIF
Char  HMGLVVGGILIQTPWGFTGALVLMIAHGLTSSALFCLANTNYERTHNRTMILARGLQMVL
Pser  HMGLVAGGILIQTPWGFSGALILMIAHGLTSSALFCLANTNYERTHSRTMLLARGLQMIL
Prol  HMGLVAGGVLIQSPWGLTGSLILMIAHGLTSSALFCLANTNYERTHSRTMVLARGLQMAL
Plbi  HMGLVAGGILIQSPWGLTGALTLMIAHGLTSSALFCLANTNYERTHSRTMVLARGLQVAL
Calu  HMGLVIAAILVQSPWGLTGATILMIAHGLTSSSLFCLANTNYERTHTRTLLLARGLQVVL
Papa  HMGLVAAGIMIQTPWGLTGALILMIAHGLTSSALFCLANTNYERTHSRTMLLARGLQVIL
Sufr  HMGLVVGGILVQTSWGFTGALILMIAHGLTSSALFCLANTNYERTHSRTLLLARGLQVAL
Stci  HMGLVVGGILIQTPWGFTGALILMIAHGLTSSALFCLANTNYERTHSRTMILARGLQTIL
Taru  HMGLVVGGILIQTPWGFTGALILMIAHGLTSSALFCLANTSYERTHSRTMLLARGMQMIL
Rala  HMGLVVGGILVQTPWGFTGALILMIAHGLTSSALFCLANTNYERTHSRTMVLARGLQVAL
           .  .       :   .   *    :*::*.:  **   *  ::    *:*   .* : : **
```

To be continued on page 35.

```
                    J                                          K
Scca   PLMATWWLLANLANLALPPSPNLVGELLIISSLFNWSNWTILLTGIGVLITASYSLYMFL
Muma   PLMATWWFLANLANLALPPSPNLMGELFIITSLFNWSNWTLILTGSGVLITASYSLYMFL
Erca   PLMATWWFIMSLMNMALPPLPNLMGELIIMVSLFNWSSWTILLTGTGTLITATYSLYLYL
Pose   PLMATWWLIMSLANMALPPLPNLMGELMIMISMFNWSAWTILLTGTGTLITAGYSLYLFV
Actr   PLMATWWFIANLANLALPPLPNLMGELVIISSMFNWSSWTIILTGGGTLITASYSLYMYL
Scal   PLMATWWFIANLANLALPPLPNLMGELVIITSMFNWSSWTIVLTGGGTLITASYSLYMYL
Posp   PLMAAWWFIANLANLALPPLPNLMGELIIITSMFNWSSWTIILTGTGTLITASYSLYMYL
Atsp   PLTAVWWFIASLANLALPPFPNLTGEMAIMASLFYWSEWTLILTGLAALITIIYTMYMFM
Leoc   PLTATWWFIASLANLALPPFPNLTGEMAIMASLFYWSEWTLILTGLAALITIIYTMYMFM
Amca   PLMASWWFIANLANLALPPLPNLMGELIIITSMFNWSPWTIVLTGLGTLITASYSLYMFL
Osbi   PLMTTWWFIANLANLALPPLPNLMGELAIITSMFNWSPWTIILTGLGTLITAGYSLYVFL
Pabu   PLMASWWFISNLANLALPPLPNLMGELTIISSAFNWSYWTIVLTGLGTLITAAYSLYMFL
Hial   PLMAAWWFIANLANLALPPLPNLMGELMIITSMFNWSYWTIILTGLGTLITASYSLYMFL
Elha   PLMAAWWFIANLANLALPPLPNLMGELMIITSMFNWSYWTILLTGLGTLITASYSLYMFL
Mlcy   PLMAAWWFIMNLANLALPPLPNLMGELMIITSLMNWSLWTILLTGLGTLITASYSLYMFL
Algl   PLMATWWFFASLANLALPPLPNFVGEVMIIMSLFNWSHWTIIFTGLGTLITATYSLYVFQ
Ptgi   PLMAAWWFIANLANLALPPLPNLMGELMIITSMFNWSYWTILLTGLGTLITASYSLYMFL
Alaf   PLMAAWWFLANLANLALPPLPNLMGELMIITSMFNWSWLTIILTGLGTLITASYSLYMFL
Nock   PLMAAWWFLANLANLALPPLPNLMGELMIITSMFNWSWITIILTGLGTLITASYSLYMFL
Anja   PLMAAWWFIANLANLALPPLPNLMGELMIITSMFNWSYWSIALTGLGTLITAGYSLYMFL
Gyki   PLMATWWFISSLANLALPPLPNLMGELMIITSMFNWSRLTIILTGLGTLITAAYSLYMFL
Syka   PLMTAWWFIANLANLALPPLPNLMGELMIITSMFNWSPWTIIMTGLGTLITASYSLYMFL
Opma   PLMTAWWFISNLANLALPPLPNLMGEIMIITAMFNWSYWSIVLTGLGTLITAGYSLYMFL
Comy   PLMASWWFIMNLANLALPPLPNLMGEIMIITAMFNWSYWSIIITGLGTLITAGYSLYMFL
Sasp   PLMSAWWLIACLANLALPPLPNLMGELMIITSLFNWTYWSIILTGLGTLITAGYSLYMFL
Eupe   PLAAIWWLAASLANLALPPLPNLMGELMIISSIFNWSQWSIILTGLGTLITAGYSLYMFT
Enja   PLTATWWFIANLANLALPPLPNLMGEMMIITTMFNWSPWSIVLTGLGTLITAGYSLYMFL
Same   PLTATWWFIANLANLALPPLPNLMGEIMIITTMFNWSPWTLVLTGLGTLITAGYSLYMFL
Chch   PLTATWWFAANLANLALPPLPNLMGELMIITALFNWSPWTLILTGAGTLITAGYSLYLFL
Grgr   PLTAAWWFGASLANLALPPLPNLMGELMIITTLFAWSPWTLILTGAGTLITAGYSLYLFL
Caau   PLTAVWWFIANLANLALPPLPNLMGELMIITTLFNWSPWTILLTGLGTLITAGYSLYMFL
Cyca   PLTAVWWFIANLANLALPPLPNLMGELMIITTLFNWSPWTILLTGLGTLITAGYSLYMFL
Dare   PLATVWWFIANLANLALPPLPNLMGELMIITALFNWSPWTIIITGMGTLITANYSLYMFL
Cost   PLTAVWWFIANLANLALPPLPNLMGELTIISTLFNWSPWTILLTGTGTLITAGYSLYLFL
Leec   PLAAAWWFIANLANLALPPLPNLMGELMIITTLFNWSPWTIMLTGVGTLITAGYSLYLFL
Fola   PLTAVWWFIANLANLALPPLPNLMGELMIITTLFNWSPLTIILTGTGTLITAGYSLYLFL
Clmc   PLAATWWFIANLANLALPPLPNLMGELLIITAMFNWSPWTLALTGAGTLITAAYSLHLFL
Phin   PLTTTWWFFASLANLALPPLPNLMGELLIITTLFNWSPWTLIMTGIGTLITAAYSLYLFL
Icpu   PLTAVWWFLSNLANLALPPLPNLMGELVIITALFNWSPWTLILTGAGTLITAAYSLYLFL
Psto   PLTATWWFIANLANLALPPLPNLMGELMIITTMFNWSPWTLVLTGAGTLITAAYSLYLFL
Cora   PLTATWWFIANLANLALPPLPNLMGELLIITAMLNWSPWTLIITGTGTLITAAYSLYLFL
Eisp   PLTAAWWFIANLANLALPPLPNLMGELMIISAMFNWSPWTLIMTGAGTLITAAYSLYLFL
Apal   PLATVWWFIANLANLALPPLPNLMGELTIISAMFNWSKWTIILTGAGTLITAAYSLHLFL
Eslu   PLMAAWWFLANLANLALPPLPNLMGELMIIVSVFNWSSWTILLTGLGTLITASYSLHLFL
Dape   PLMATWWFMANLANLALPPLPNLMGELMIIVSLFNWSYWTILLTGLGTLITASYSLYLFL
Glse   PLTATWWFLANLANLALPPLPNLMGELMIITSMFNWSPWTIALTGTGTLITASYSLYLFL
Naar   PLTATWWFIANLANLALPPLPNLMGELMIISSMFNWSPWTLALTGAGSLITASYSLYLFL
Lioc   PLMATWWFIANLANLALPPLPNLMGELMIITSMFNWSPWTLILTGAGTLITASYSLYLFL
Opso   PLMTTWWFAASLANLALPPLPNLMGELMIITSLFNWSLWTLALTGTGTLITASYSLYLFL
Alte   PLTAVWWFTANLANLALPPLPNLMGEIMIISTMFNWSPWTLALTGLGTLITAGYSLYLFL
Plap   PLTTVWWFTANLANLALPPLPNLMGEIMIITTMFNWSPWTLALTGLGTLITAGYSLYLFL
```

To be continued on page 36.

[7/8 of aligned sequences]

```
Plal  PLTATWWFVANLANLALPPLPNLMGELVIITAMFNWSYWTIAITGVGTLITASYSLYLFL
Sami  PLMTTWWFIANLANLALPPLPNLMGELMIITAMFNWSYWTLLITGLGTLITASYSLYLFL
Rere  PLMATWWFLANLANLALPPLPNLMGELMIITSLFNWSQWTLVLTGVGTLITAGYSLYLFL
Gama  PLMATWWFIANLANLALPPLPNLMGELVIITSLFNWSGWTIALTGVGTLITAAYSLYLFL
Onmy  PLMTTWWFVASLANLALPPLPNLMGELMIITSMFNWSYWTLILTGLGTLITASYSLYLFL
Sasa  PLMTTWWFVASLANLALPPLPNLMGELMIITSMFNWSHWTLLLTGLGTLITASYSLYLFL
Cola  PLMTTWWFVASLANLALPPLPNLMGELMIITTMFNWSHWTLLLTGVGTLITASYSLYLFL
Dita  PLMTAWWFIASLANLALPPLPNLMGELLIISSMFNWSYWTLVLTGAGTLITAAYSLHLFL
Gogr  PTITTWWLTASLANLALPPLPNLMGELLIISSMFNWSYWTLLLTGAGTLITATYSLHLYL
Chsl  PLMTTWWFLASLANLALPPLPNLMGELLIISSMFNWSYFTLILTGTGTLITAAYSLHLFL
Atja  PLMTAWWFISSLANLALPPLPNLMGELMIITSLYNWSYWSIILTGMGTLITAAYSLYMFL
Iido  PLMATWWFIASLANLALPPLPNLMGELMIITSLYNWSYWSIILTGMGTLITAAYSLYMFL
Auja  PLMTSWWFLANLANLALPPLPNLMGELMIITSLFNWSYWSIILTGAGTLITASYSLYMFL
Chag  PLMGTWWFFANLANLALPPLPNLMGELVIISSLFNWSSWTLILTGVGTLIAAGYSLYMFL
Hami  PLMTTWWFLANLANLALPPLPNLMGELVIITSLFNWSNWTLILTGLGTLITASYSLYMFL
Saun  PLMTTWWFLANLANLALPPLPNLMGELIIISSLFNWSYWTLILTGLGTLITASYSLYLFL
Nema  PLMATWWFITNLANLAVPPLPNLMGELMIITSLFNWSPWTLALTGLGTMITAGYSLYMFL
Disp  PLMATWWFISNLANLALPPLPNLMGELMIITSLFNWSPWTLILTGAGTLITAGYSLYMFL
Myaf  PLMATWWFVSSLANLALPPLPNLMGELMIITSLFQLIALNLILTGAGTLITAGYSLYMFL
Lagu  PLMAAWWFIMSLANLALPPLPNLMGELMIISSLFNWSPLTLATTGAGVLITAGYSLHMFL
Trtr  PLLATWWFLTTLANLALPPLPNLMGELMIMTSMFNWSPFTLILTGAGAFITAAYSLHMFI
Zucr  PLLAVWWFLASLANLALPPLPNLMGELMIMTSMFNWSPFSLILTGAGALITAAYSLHMFL
Pxja  PLMTTWWFIASLANLALPPLPNLMGELMIITSLFNWSWWTLALTGTGTLITAGYSLYMFL
Pxlo  PLMTTWWFIASLANLALPPLPNLMGELMIITSLFNWSWWTLALTGTGTLITAGYSLYMFL
Pctr  PLAATWWFIASLANLALPPLPNLMGEIMIITSLFNWSPWTLLCTGLGTLITAGYSLYLFL
Apsa  PLMATWWFIASLANLALPPLPNLMGEIMIITSLFNWSLWTLICTGAGVLITASYSLYMFL
Cabe  PLMATWWLLATLANLALPPLPNYIGELVLVTALFKWSNWTLPLTGLAILITAAYSLYMFL
Bzze  PLMTTWWFIASLANLALPPLPNLMGELMIITSLFGWSNWTLLLTGTGTLITAGYSLYMFL
Siim  PLTTTWWFITALANLALPPLPNLMGELMIIVSLFNWSNWTLLLTGAGMLITAAYSLHMFL
Ctru  PLMASWWFISSLANLALPPLPNLMGELMIITSLFNWSPWTLVLTGAGTLITAGYSLYMYL
Dpbr  PLMATWWFISSLANLALPPLPNLMGELMIITSLFNWSPWTLILTGTGTLITASYSLYMFL
Caki  PLMTSWWFFTCLANLALPPLPNLMGELAILTSLLAWSPWTLILTGGGTLITATYSLYVFL
Phja  PLMSSWWLFASFANLALPPLPNLVGEIMIVTALFNWSPWTLALTGTGMLITATYSLYMFL
Brsp  PLMTLWWFFMTLANLALPPLPNLMGELIILTALFNWSPWTLLLTGVGTLITATYSLYMFL
Gamo  PLMTTWWFIASLANLALPPLPNLMGELMILTSLFNWSAWTLILTGIGTLITAAYSLYMFL
Lolo  PLMTTWWFIASLANLALPPLPNLMGELMILTSLFNWSAWTLILTGAGTLITAAYSLYMFL
Batr  PLMMTWWLLMCLSNLALPPTINFMAELTIVASLLQWSPWTFLLLGLGIVITSTYTLTMFI
Prmy  PLMSAWWLLTCLLNLALPPTINLVAELTIISSLFKWSPWTLLFTGGGVLVTAAYSLSMFL
Lose  PLMTGWWFIASLANLALPPLINLMGELMIITALFNWSPWTLILTGVGTLISAIYSLYMFL
Loam  PLMTAWWFIASLANLALPPLPNLMGELMIITSLFSWSWWTLVLTGAGTLITAGYSLYMFL
Chab  PLMATWWFIASLANLALPPLPNLMGELMILTSLFNWSYWTIALTGGGTLITAGYSLYMFI
Chto  PLMATWWFIASLANLALPPLPNLMGELMILTSLFNWSYWTIALTGGGTLITAGYSLYMFI
Majo  PLLTTWWLIATLANLGLPPFPNFLGEIMAITALYEWSPFSLILTGAGTVITAAYSLYMFL
Hlst  PLLTTFWLIATLANLGLPPLPNFLGEILSITALYEWSCYSLILTGAGTVITAAYSIYMFT
Clpe  PLMASWWFIACLANLALPPLPNLMGELLILTSLFSWSPWTIALTGAGTLITASYSLYMFL
Mlmr  PLMTSWWFIASLANLALPPLPNLMGELMILTSLFNWSPWTIALTGAGTLITATYSLYMFL
Crcr  PLMTSWWFIASLANLALPPLPNLMGELMIISSLFNWSWWTLALTGIGTLITAAYSLYMFL
Muce  PLMTSWWFIASLANLALPPLPNLMGELMIISSLFNWSWWTLTLTGIGTLITAAYSLYMFL
Bege  PLMATWWFIASLANLALPPLPNLMGELMIICSLFHWSWWTLMITGLGTLITAGYSLYMFL
Mela  PLMTTWWFIASLANLGLPPLPNLMGELMIITALFSWSKWTLLFTGAGTLITASYSLYMFL
Hats  PLMTTWWFLASLANLALPPLPNLMGELMIISSLFNWSWWTLALTGLGTLITAGYSLYMFL
Orla  PLLATWWFLFTLANLALPPLPNLMGELMIITSLFNWSNWTLLLTGAGTLITASYSLHMFL
```

To be continued on page 37.

To be continued on page 38.

```
Cosa PIMTTWWFLASLANLALPPLPNLMGEIMIVTSLFNWSPWTLLLTGLGMLITAGYSLYMFL
Exsp PLMTSWWFLASLANLALPPLPNLMGELMILSSLFNWSPWTLALTGTGTLITAGYSLYMFL
Depa PIMTTWWFLTSLANLALSPLPNLMGEVMILTSLFHWSPWTLALTGLGTLITAGYSLHMFS
Rima PLMTAWWFIASLANLALPPLPNLLGELMIISSLFSWAPFTLLLTGLGTLITASYTLYMLI
Fuol PLMAAWWFLASLANLALPPLPNLMGEIMIISSLFNWSFFTLILTGAGTLITASYSLYMFL
Gmaf PLTALWWFTATLANLALPPLPNLMGELMIITSLFNWSMWTLILTGLGMTITAGYSLYMFL
Xeei PLMTAWWFIASLANLALPPLPNLMGELMIISSLFNWSMWTLALTGLGMLITAGYSLYMFL
Pros PLMTAWWLIASFANLALPPLPNLMGELMIITSLFNWSWFTLLLTGTGTLITAGYSLYMFL
Scmi PLMTTWWFIASLANLALPPLPNLMGELVIITSLFNWSWWTLLLTGAGTLITASYSLYMFL
Rolo PLMTAWWFTASLANLALPPLPNLMGELMIITSLFNWSWWSLLLTGAGTLITAGYSLYMFL
Cere PLMTTWWFIASLANLALPPLPNLMGELMIISSLFNWSEWSLLITGSGTMITAGYSLYMFL
Daga PLMATWWFIASLANLALPPLPNLMGELMIISSLFGWSPLSLAITGLGVILTASYSLYLFL
Anco PLMTTWWFIASLANLALPPLPNLMGELMIIASLFNWSWWTIFLTGAGTLITAGYSLYMFL
Dmve PLMTSWWFIASLANLALPPLPNLMGELMIITALFNWSWWTLALTGAGTLITASYSLYMFL
Dmar PLMTSWWFIASLANLALPPLPNLMGELMIITSLFNWSWWTLVLTGAGTLITASYSLYMFL
Anka PLMTTWWFIASLANLALPPLPNLMGELMIITSLFSWSWWTIFLTGAGTLITAGYSLYMFL
Moja PLMTTWWFIASLANLALPPLPNLMGELMIITSLFNWSWWTILLTGAGTMITAGYSLYMFL
Hoja PLMTTWWFIASLANLALPPLPNLMGELMIITSLFNWSWWTIILTGAGVIITAGYSLYMFL
Bede PLMTTWWFIASLANLALPPLPNLMGELMIITSLFNWSWWTLLLTGTGTLITAGYSLYMFL
Besp PLMTTWWFIASLANLALPPLPNLMGELMIITSLFNWSWWTLLLTGTGTLITAGYSLYMFL
Mysp PLMTTWWFIASLANLALPPLPNLMGELMIITSLFNWSWWTLLLTGAGTLITAGYSLYMFL
Osja PLMTTWWFIASLANLALPPLPNLMGELMIITSLFNWSWWSLLLTGAGTLITAGYSLYMFL
Sgro PLMTTWWFMASLANLALPPLPNLMGELMIITSLFNWSWWTLLLTGAGTLITAGYSLYMFL
Pzpa PLMATWWFTASLANLALPPLPNLMGEIMIISSLFNWSWLTIILTGTGTLITAGYSLYLFL
Zeja PLMTTWWFTANLANLALPPLPNLMGEIMIISSLFNWSWFTIALTGTGTLITAGYSLYVFL
Znne PLMATWWFTASLANLALPPLPNLMGEIMIITSLFNWSWFTIILTGAGTLITAGYSLYLFL
Zefa PLMASWWFTASLANLALPPLPNLMGEIMIISSLFNWSWFTIILTGAGTLITAGYSLYLFL
Acni PLMTTWWFTASLANLALPPLPNLMGEIMIISSLFNWSWFTIALTGTGTLITAGYSLYLFL
Ncrh PLMATWWFTASLANLALPPLPNLMGEIMIISSLFNWSWFTIALTGTGTLITAGYSLYLFL
Agca PLMTSWWFIASLAYLALPPLPNLMGELMIITSLFNWSWWTLALTGAGTLITAGYSLYMFL
Hydy PLMCTWWFISTLANLGLPPLPNLMGELMIIVSLFSWSWWTLVLTGAGTLITVSYSLYMFI
Gsac PLMTTWWFIASLANLGLPPLPNLMGELMIITSLFSWSWWTLALTGAGTLITVSYSLYMFI
Pevo PLMATWWFIASLANLALPPLPNLMAEIMIISSLFNWSYFTILITGAGTLITAGYSLYMFL
Hiku PLMATWWFTASLANMALPPLPNLMGELMIISSLFNWSPWTLILTGLGTLITAGYSLYLFL
Inpa PLMAAWWLISSLANLALPPLPNLMAELVIITSLFNWSYWSLLLTGTATMLTAWYSLHMAT
Auch PLMTAWWFTASLANLALPPLPNLLGELMIMTSLFDWSWWTIIMTGLGTLVTATYTLYLFL
Fico PLMTAWWFIASLANLALPPLPNLMGELLIISSLFMWTPWTIALTGLGTLITAGYSLYLFL
Macs PLMTAWWFIASLANLALPPLPNLMGELMIITSLYNWSWPTLLLTGAGTLITAGYSLYLFL
Moal PLMATWWLITCLANLALPPLPNLMGELIIISALLGWSWWTIIFTGAGALITATYSLYMYL
Syma PLMVLWWFFICLTNLALPPLPNLMGELMIIISLFNWSFWTIIFTGLGALITAIYSLYMFL
Mafr PLMTMWWFTASLANLALPPLPNLMGELTIITSLFGWSWWTLLLTGAGTLITANYSLYMFL
Dcpe PLMTTWWLIASLANLALPPIPSSMAELMIITALFNWSQWTLVFTGLGTLITAGYSLYLFI
Dcti PLMTTWWLVASLANLALPPIPSSMAELMIITALFNWSQWTLVFTGIGTLITAGYSLYLFI
Hehi PLMTTWWFIASLANLALPPLPNLMGELLIITSLFGWSWWTLVLTGAGTLITASYSLYMFL
Stam PLMTTWWFIASLANLALPPLPNLMGELMIVTSLFNWSWWTLALTGGGMLITASYSLYMFL
Hogi PLMTSWWFIASLANLALPPLPNLMGELMIISSLINWSWWTLVLTGAGVLITASYSLYMFL
Erzo PLMTTWWFIASLANLALPPLPNLMGELMIITSLFNWSWWTLLLTGVGTIITASYSLYMFL
Hxot PLMTAWWFIASLANLALPPLPNLMGELMIITSLFNWSWWTIVLTGAGTLITASYSLYMFL
Core PLMTAWWFIASLANLALPPLPNLMGELMIITSLFSWSWWTIIMTGAGTLITASYSLYMFL
Apve PLMTAWWFITSLANLALPPLPNLMGELMIITALLNWSWWTIILTGTGTLITASYSLYMFL
Latj PLMTAWWFLSTLANLALPPSPNLMAELMLVAALCNWSPRTLVLTGAGILITAAYSLYMFL
Laja PLMTAWWFIASLANLALPPLPNLMGELMIITSLFNWSWWTLALTGTGTLITAGYSLYMFL
```

[7/8 of aligned sequences]

To be continued on page 39.

```
Syja  PLMTSWWFIASLANLALPPLPNLMGELMIITSLFNWSWWTLALTGAGTLITAGYSLYMFL
Epme  PLMSAWWFFASLANLALPPLPNLMGELMIISSLFNWSWWTIVLTGGGTLITASYSLYMFL
Grse  PLMATWWFTASLANLALPPLPNLMGELMIIASLFNWSWWTIVLTGAGTLITATYSLYMFL
Clja  PLMTAWWFISTLANLALPPLPNLMGELLIISALFDWSWWTLALTGTGTLITAAYSLYMFI
Ogcy  PLMATWWFIASLANLALPPLPNLMGELMIITSLFDWSPWTLALTGTGTLITAGYSLYMFL
Plna  PLMASWWFIAILANLAQPPLPNSLAEVMITAALYDWSPWAVPLLGGGALITAAYSLYMFL
Lema  PLMTSWWFIASLANLALPPLPNLMGELMIITSLVNWSWWTLILTGAGTLITAGYSLYMFL
Etzo  PLMTAWWFIASLANLALPPLPNLMGELMIVTSLFNWSWWTLALTGAGMLITASYSLYMFL
Apse  PLMASWWFLASLANLALPPLPNLMGELMIITSLFNWSWFTLLLTGAGTLITAGYSLYMFL
Epde  PLMTTWWFIASLANLALPPLPNLMGELMIITSLFNWSWWTLVLTGAGTLITASYSLYMFL
Slja  PLMASWWFLGSLANLALPPLPNLMGELMIITSLFNWSWWTLALTGAGTLITAGYSLYMFL
Bsja  PLMAMWWFLASLANLALPPLPNLMGELMIITAMFNWSWWTIALTGAGTLITAGYSLYMFL
Ecna  PLMTTWWFIASLANLALPPLPNLMGELMIITSLFNWSWWTIALTGTGTLITAGYSLYMFL
Cohi  PLMTLWWFISSLANLAFPPFPNLMGELLLITALFKWSWWTLLLTGTATLITAGYSLYMFV
Caar  PLMATWWFIASLANLALPPLPNLMGELMIITSLFNWSWWTLALTGTGTLITAGYSLYMFL
Came  PLMATWWFIASLANLALPPLPNLMGELMIITSLFNWSWWTLALTGAGTLITAGYSLYMFL
Mema  PLMTAWWFTASLANLALPPLPNLMGELMIITSLFNWSWWTLALTGTGTLITAGYSLYMFL
Lenu  PLMATWWFLFSLANLALPPLPNLMGELMVISSLFNWSPWTLILTGAGTLITAAYSLYMFL
Brja  PLMATWWFIGSLANLALPPLPNLMGELMIITSLFNWAPWTLALTGAGTLITAGYSLYMFL
Plma  PLMTTWWFIASLANLALPPLPNLMGELMIITSLFNWSNWTLALTGAGTLITAGYSLYMFL
Emst  PLMTTWWFIASLANLALPPLPNLMGELMIITSLFNWSWWTLALTGAGTLITAGYSLYMFL
Ptti  PLMTTWWFIASLANLALPPLPNLMGELMIITSLFNWSWWTLILTGAGTLITAGYSLYMFL
Losu  PLMTTWWFIASLANLALPPLPNLMGELMIITSLFNWSWWTLAMTGTGTLITAGYSLYMFL
Geoy  PLMTAWWFIASLANLALPPLPNLMGELMIITSLISWSWWTLALTGAGTLITAGYSLYMFL
Dipi  PLMTTWWFIASLANLALPPLPNLMGELMIITSLFNWSWWTLALTGAGTLITAGYSLYMFL
Pama  PLMTAWWFISSLANLALPPLPNLMGELMIISSLFNWSWWTLALTGAGTLITAGYSLYMFL
Leob  PLMTAWWFLASLANLALPPLPNLMGELMIISSLFNWSWWTLVLTGAGTLITAGYSLYMFL
Neba  PLMTIWWFIASLANLALPPLPNLMGELMIISSLFSWSWWTIIITGAGTLITVGYSLYMFL
Pdpl  PLMTTWWFISSVANLALPPLPNLMGELLIITSLFNWSWWTLVLTGAGTLITAGYSLFMFL
Nimi  PLMGTWWFFASLANLALPPLPNFMGELMIITSLFNWSGWTLLLTGAGTLITAGYSLYMFI
Uptr  PLMTAWWFIASLANLALPPLPNLMGEIMIITSLFNWSPWTLALTGAGTLITAGYSLYMFL
Pesc  PLMAAWWFIASLANLALPPFPNLMGELMIIVSLFNWSWWTIILTGTGTLITATYSLYMFL
Baar  PLMATWWFIASLANLALPPLPNLMGELMIIASLFNWSWWTLALTGAGTLITAGYSLYMFL
Moar  PLMTTWWFIASLANLALPPLPNLMGELMIITSLFNWSWWTLVLTGAGTLITAGYSLYMFL
Toja  PLAATWWFIASLANLALPPLPNLMGELMIITSLFNWSWWTLALTGAGTLITAGYSLYMFL
Chau  PLMVSWWFAASLANLALPPLPNLMGELAIITSLFNWSPWTIILTGAGTLITAGYTLYMFL
Chse  PLMTTWWFIASLANLALPPLPNLMGELMILSSLFNWSWWTLALTGAGTLITAGYSLYMFL
Enar  PLMTSWWFIASLANLALPPLPNLMGELMIITSLFNWSWWTLALTGAGTLITAGYSLYMFL
Hpty  PLMTTWWFIASLANLALPPLPNLMGELMILTSLFNWSWWTLALTGAGTLITAGYSLYMFL
Nana  PLMTAWWFIASLANLALPPLPNLMGELKIIVSLFNWSWWTIMLTGAGTLITAAYSLYMFL
Mcst  PLTATWWFIASLANLALPPLPNLMGELMIITALFNWSWWTLMLTGTGTLITAAYSLYMFL
Rhox  PLMTTWWFIASLANLALPPLPNLMGELMIIVSLFNWSWWTLLLTGAGTLITASYSLYMFL
Opfa  PLMTTWWFIASLANLALPPLPNLMGELMIISSLFNWSWWTLLLTGAGTLITAGYSLYMFL
Paar  PLMTTWWFIASLANLALPPLPNLMGELMVIASLFGWSWWTLILTGAGALITAGYSLYMFL
Gozo  PLMTTWWFVASLANLALPPLPNLMGELMIITSLFNWSWWTLALTGGGTLITAGYSLYMFL
Ackr  PLMSFWWLFGTLANLALPPLPNLMGELMIIVSLFNWSYFTLLITGLGTLITAAYSLYMFI
Elev  PLMTTWWFFSSLANLALPPLPNLMGELMIITSLFNWSWWTIALTGSGTLITAGYSLYMFL
Trdu  PLMTAWWFITSLANLALPPLPNLMGELMIIASLFHWSWWTIALTGAGTLITAGYSLYMFL
Amoc  PLMTAWWFIASLANLALPPLPNLMGELMIITSLFNWSWWTLALTGAGTLITAGYSLYMFL
Hame  PLMAAWWFIATVANLALPPLPNLMAELTVISGMWKWSWWTIILTGAGTLITAGYSLYMFL
Chso  PLMATSWFIASLANLALPPLPNLMAELMVICTMFDWSPWTLILTGAGTLITAAYSLYMFL
Lyto  PLMTTWWFIASLANLALPPLPNLMGEIMIITSMFNWSWWTLLLTGAGTLITASYSLYMFL
```

```
Encr PLMATWWFVASLANLALPPLPNLMGEIMIITSMFNWSWWTLVLTGAGTLITASYSLYMFL
Bvar PLMTAWWFIASLANLALPPLPNLMGELMIVVSIFNWSKWSLMLTGAGILTTASYSLYMFI
Noco PLMATWWFFASLANLALPPLPNLMGELMIITSLINWSPWTLALTGSGVLITASYSMYMFI
Chsp PLMTTWWFIASLANLALPPFPNLMGELAIITSLFNWSQWTLILTGGGMLITASYSLYMFL
Arja PLMTAWWFIASLANLALPPLPNLMGELMIITSLFNWSWWTIALTGAGTLITASYSLYMFL
Pase PLTATWWFIASLANLALPPLPNLMGELMIILSLFNWAWWTLLLTGAGTLITAAYSLHMFL
Trel PLMSSWWFAGSLANLALPPLPNLMGELMIITALFNWSQWTLLLTGLGTLITASYSLYMYL
Lifa PLMTLWWLFMSLANMALPPLPNLMGELMIIVSLFNWAPWTLIITGVGIVITAGYSLYMYQ
Acur PVMTVWWFISSLANLALPPLPNLMGELVIMVALIKWSKWTLIFTGLGTLITACYSLFMYS
Ampe PLMATWWFVSSLANLALPPLPNLMGELIIITSLFNWSWWTLVFTGAGTLITASYSLYMFL
Urja PLMGTWWFFLSLANIALPPLPNLMGELMILSSLYNWSWPSLIFTGTGIVITASYTLHMFL
Enet PLMMTWWFLATLANLALPPLPNLMGELMIIVSTFNWSWASILLTGTGTLITASYSLYMFS
Ptbr PLLSTWWFLMSLANLALPPSPNLMGELMIITATFGWSWWTLLLTGPATLITAGYSLYMFL
Safa PLMTSWWLLMTLANLALPPSPNLMGELMIIVSAFNWSYWTLILTGTATLITAAYSLYMFL
Icae PLMTTWWFIASLANLALPPLPNLMGELMIITSLFNWSHWTLVLTGAGTLITAGYSLYMFL
Asmi PLMAFWWLFSSVANLALPPLPNLMGELFIITSLCNWSLGSLAFTGTGMLITAAYTLHMFL
Foal PLMTLWWIIAVLANLALPPLPNLMGELLIMTSLFGWSYWTLIFTGLAALITAAYSLFMFL
Drze PLMSTWWLMAALANLALPPLPNLMGELMIILSLFNWTWISIVLMGIAALITAAYSLYLYL
Rhas PLMTTWWFISSLANLALPPLPNLMGELMIITSLFNWSWWTIALTGTGTLITAGYSLYMFL
Elac PLMTAWWFIASLANLALPPLPNLMAELMIIVSLFNWSWWTIALTGGGTLITAGYSLYMFL
Kugu PLMMFWWLLSSLANLALPPLPNLMGELMIITSLFNWSQWTLALTGLGTLITAGYSLYMFL
Plor PLMASWWFIASLANLALPPLPNLMGELMIITSLFNWSWWSIALTGAGTLITASYSLYMFL
Sgun PLMATWWFIGTLANLALPPLPNLMGELMIISSLLNWSWWTLLLTGVGTLITAGYSLYMFL
Zaco PLMTTWWFIGSLANLALPPLPNLMGELMIITSLFNWSWWTLALTGVGTLITAGYSLYMFL
Zbfl PLMATWWFVGSLANLALPPLPNLMGELMIMTSLFNWSWWTLILTGAGTLITAGYSLYMFL
Spba PLMTTWWFIASLANLALPPLPNLMGELMIITSLFNWSWWTLALTGTGTLITAGYSLYMFL
Game PLMTTWWFIASLANLALPPLPNLMGELMIITSLFNWSHWTLVLTGAGTLITAGYSLYMFL
Thth PLMTTWWFIASLANLALPPLPNLMGEIMILTSLFNWSHWTLALTGAGTLITAGYSLYMFL
Xigl PLMATWWFFASLANLALPPLPNLMGELMIITSLFNWSWWTLALTGAGTLITAGYSLYMFL
Hyja PLMTTWWFLASLANLALPPLPNLMGELMIITSLFNWSHWTILLTGAGTLITASYSLYMFL
Psan PLIGMWWFFAILANMAMPPLPNFTGEIMIISSLFKWSNWTIILTGTATMITAGYSLFNYL
Cupa PLMTTWWFIASLANLALPPLPNLMGELMIITSLFNWSYWTLLLTGAGTLITAGYSLYMFL
Mpch PLMASWWFITSLANLALPPLPNLMGELMILTSLFNWSCWTIALTGAGTLITAAYSLFMFL
Char PLATTWWFIASLANLAFPPLPNLMGELVIITSLFNWSWWTLALTGTGTLITAAYSLHMFL
Pser PLMATWWFIASLANLALPPLPNLMGELMIITSLFNWSWWTLILTGAGALITASYSLFMFL
Prol PLMATWWFIASLANLALPPLPNLMGELMIIISLFNWSWWTLALTGTGTLITAGYSLYMFL
Plbi PLMATWWFISSLANLALPPLPNLMGELMIITSLFNWSWWTLALTGSGTLITAGYSLYMFL
Calu PLMATWWFIASLANLALPPLPNLMGELMIITSLFNWAPWTIIITGLGTLITAAYSLYMFL
Papa PLMATWWLVASLANLALPPLPNLMGELAIITSLFNWSQWTLVLTGLGTLITAGYSLYMFL
Sufr PLMATWWFLASLANLALPPLPNLMGELMVITSLFNWSWWTLILTGGGTLITAGYSLYMFL
Stci PLMATWWFIMALANLALPPLPNLMGELMIISSLFNWSWSTIVLTGLGTLITAGYSLYMFL
Taru PLMAAWWFIASLANLALPPLPNLMGELMIITSLFNWSPWTIGLTGLGTLITAGYSLYMFL
Rala PLMTAWWFIASLANLALPPLPNLMGELMIITSMFNWSWWTLALTGTGTLITAGYSLYMFL
     *     *:    .  :. *  .  .*:            .    * .   :  *::
```

To be continued on page 40.

[8/8 of aligned sequences]

```
Scca      MTQRGLTSKHLMNLNPSHTREHLLLTLHVLPVLLLILKPELIWGWTF--
Muma      MTQRGPTSNHLISLNPSHTREHLLLSLHIMPVLLLILKPELIWGWTF--
Erca      SSQRGPAPDNILLIEPTHTREHLLLILHIIPMVMLMIKPEFIWGWCW--
Pose      SSQRGPTSNNS-SMEPSHTREHLLLTLHIIPVLLLMAKPELIWGWCW--
Actr      MTQRGPVSTLIMAVEPSHTREHLLMALHLIPIILLMLKPELMWGWCF*-
Scal      MTQRGPVSTFIMAVEPSHTREHLLMALHLIPIALLMLKPELMWGWCF--
Posp      MTQRGPVTTLITAIEPSHTREHLLMALHLIPIIMLTLKPELMWGWCF--
Atsp      TTQWGPVPPSMSNIEPPNSREHLLISLHLIPVLLLILKPELVWGWYH--
Leoc      TTQWGPVPPSMNNIEPPHSREHLLISLHLIPILLLILKPELVWGWYY--
Amca      MTQRGPTPTHIISIEPSHTREHLLMAMHLIPVIMLITKPELMWGWAF--
Osbi      MTQRGPTPLHTISLSPTHTREHLLMTLHLIPVLLLITKPELMWGMFY--
Pabu      MTQRGPTPTYFYSLDPSYTREHLLIFLHMMPVMLLILKPELMWGWYA--
Hial      MTQRGPTPTHIIALEPSHTREHLLMALHLIPVLLLVLKPELMWGWCF--
Elha      MTQRGPTPNHIIALEPSHTREHLLMALHLIPVILLVLKPELMWGWCF--
Mlcy      MTQRGPTPNHIMALEPSHTREHLLMTLHLIPVLLLMLKPELMWGWCF--
Algl      ATQQGPVPGHINVLEPSHTREHLLITLHLIPVMLLALKPELVWGWCL--
Ptgi      MTQRGPAPNHIIALEPSHTREHLLIAMHLIPVLLLVLKPELMWGWCM--
Alaf      MTQRGPVPNHIIALEPSHTREHLLIAMHLIPVLLLVLKPELMWGWCL--
Nock      MTQRGPVPNHIIALEPSHTREHLLIAMHLVPVMLLVLKPELIWGWCS--
Anja      MTQRGPTPNHIIGLEPSHTREHLLIAMHLIPVLLLVLKPELMWGWCL--
Gyki      MTQRGPTPNHIIGLEPSHTREHLLITLHLSPLILLMLKPELMWGWCM--
Syka      MTQRGPTPNHIMGLEPTHTREHLLIAMHLVPVLLLILKPELMWGWCL--
Opma      MTQRGPIPKHIIGMEPSHTREHLLITMHLAPVLLLVLKPELMWGWCL--
Comy      MTQRGPTPNHITNLEPSHSREHLLISMHLIPVILLTLNPMPIWGAFL--
Sasp      MTQHGTVPPHLIILSPTFTREHLMINMHFIPLLLLVLKPELIWGQHL--
Eupe      VTQRGPTPNHLISLHPTYTREHLTVSLHLLPLVLLVLKPELIWGWCM--
Enja      MTQRGQTPSHITALPPYHTREHLLISLHLIPIILLMVKPELMWGWFY--
Same      MTQRGPVPAHITGLTPYHTREHLLIALHLIPVILLILKPEFMWGWFY--
Chch      MTQRGPVPTHITGLPPFHTREHLLMALHLVPIVLLITKPELMWGWCY--
Grgr      MTQRGPVPAHLLGLEPYHTREHLLMAMHLLPISLLITKPELMWGWHH--
Caau      MSQRGPTPNHITGLQPFHTREHLLMTLHLIPVILLVTKPELMWGWCY--
Cyca      MSQRGPTPNHIMGLQPFHTREHLLMTLHLIPVILLVTKPELMWGWCY--
Dare      TSQRGSIPEHITNLSPSHTREHLLMTLHLIPIILLMLKPELMWGWCN--
Cost      MSQRGPTPTHITGLPPFHTREHLLMALHLIPVILLIAKPELMWGWCY--
Leec      MTQRGTTPNHIMGLPPFHTREHLLMVLHLLPVILLVMKPELMWGWCY--
Fola      MSQRGPTPKHIVGLPPFHTREHLLMALHLIPVLLLMTKPEIMWGWCY--
Clmc      TSQRGPAPAHIMGLQPFHTREHLLMALHLVPVILLVTKPELMWGWCY--
Phin      LTQRGPTPHHIINLPPFHTREHLLLALHLSPIMLLILKPELIWGWCF--
Icpu      MSQRGPLPQHIINLQPFHTREHLLMILHLLPVALLITKPEIMWGWWY--
Psto      MTQRGPLPQHIINLQPFHTREHLLMTLHLLPVILLITKPEIMWGWWY--
Cora      KTQRGTLPTHIINLQPYHTREHLLMILHLLPIIMLITKPELMWGWWY--
Eisp      MSQRGPAPTHIINLQPFHTREHLLMALHLLPVILLITKPELMWGWCY--
Apal      TTQRGPTPPHMLNLQPSHTREHLLMTLHLASIMLLVTKPELMWGWWY--
Eslu      TTQRGPTPQHFISMDPSHTREHLLMALHLIPLLLLVLKPELMWGWCF--
Dape      ATQRGPLPNHILSLDPSHSREHLLMTLHLLPLLLLVLKPELMWGWCF--
Glse      TTQRGPLPTHIMTLAPYHTREHLLLLLHLIPIALLITKPELMWGWCF--
Naar      TTQRGPLPSHIIALEPPHTREHLLLLLHLLPITLLVLKPELMWGWCF--
Lioc      MTQRGPLPSHIIALEPTHTREHLLLLLHLLPITLLVLKPELMWGWCF--
Opso      MTQRGPLPTHMMALEPPHTREHLLLLLHLLPVALLILKPELIWGWCS--
Alte      MTQRGPAPAHMIGLTPYHTREHLLMSLHLIPIILLITKPELMWGWCH--
Plap      MTQRGPAPAHMIGLTPCHTREHLLMTLHLIPILLLITKPELIWGWCH--
```

[8/8 of aligned sequences]

```
Plal      TSQRGPLPAHIIALDPSHTREHLLLTLHLLPILLLVLKPELMWGWCF--
Sami      TSQRGPLPSHIIALEPSHTREHLLLTLHLLPIVLLVLKPELMWGWCF--
Rere      TSQRGPLPSHIVGLEPSHTREHLLLTLHLLPIMLLILKPELMWGWCF--
Gama      VSQRGPVPPHVIALPPSYTREHLLLALHLLPVVLLVAKPELMWGWSF--
Onmy      MTQRGPLPSHIIALEPTHTREHLLIILHLIPIVLLILKPELMWGWCF--
Sasa      MTQRGPLPSHIIALEPTHTREHLLITLHLIPIILLILKPELMWGWCF--
Cola      MTQRGPLPSHIIALEPTHTREHLLITLHLIPIILLILKPELMWGWCF--
Dita      TTQRGPLPRHATSLEPSHTREHLLIALHLLPIALLVLKPEHVWGWCL--
Gogr      VTQQGPSPQHITSLEPPHTREHLLLILHLLPIILLILKPELILG*----
Chsl      TTQRGPVPNSILALEPTHTREHLLLALHLSPIALLVLKPEHMWGWCF--
Atja      MTQRGQLPTHIVTLDPSHTREHLLMVLHLLPLLILILKPELIWGWST--
Iido      MTQRGPLPTHILALDPSHTREHLLMVLHLLPLLILILKPELIWGWCT--
Auja      MTQRGPLPQHMYALEPTHTREHLLIALHLAPLLLLILKPELIWGWCN--
Chag      MTQRGPIPQHMLALDPTHTREHLLIVLHLLPLALLILKPELIWGWCF--
Hami      MTQRGPLPQHMYALEPSHTREHLLISLHLLPLLLLVLKPELIWGWFS--
Saun      MTQRGPLPQHMFALEPSHTREHLLISLHLIPLLLLILKPELIWGWFS--
Nema      MTQRGPLPTHIIALDPSHTREHLLIALHILPLILLLTKPELMWGWCA--
Disp      MTQRGPLPVHIISLDPSHTREHLLMTLHVIPLLLLVLKPELMWGWCA--
Myaf      MTQRGPIPAHIITLDPSHSREHLLITLHLMPLLLLMTKPELMWGWCM--
Lagu      VSQRGALPHHIIYLEPTHTREHALMLLHLVPLLLLILKPELMWGWTA--
Trtr      TTQRGGLPLHLMSLDPTHTREHILIVLHIFPLFMLMVKPELIWGWLY--
Zucr      TTQRGNLPLHLVGLDPTHTREHILMALHILPLLMLMLKPELIWGWLY--
Pxja      MTQRGPLPPHIIAMDPSHTREHLLMALHLIPLLLIILKPELIWGWTA--
Pxlo      MTQRGPLPPHIIAMDPSHTREHLLMALHLIPLLLIILKPELIWGWTA--
Pctr      MTQRGPVPPHIICTPPSHTREHILITLHLAPLMLLILKPELIWGWSA--
Apsa      MTQRGNTPAHMLSMNPSYTREHVLMTLHLIPLLLLIFKPELVWGWCS--
Cabe      LTQRGPTTKHAADLVPTYCREHLLLALHILPLLLIILKPSLISGWFA--
Bzze      MTQRGPLPAHIVALEPSHSREHLLIALHLLPLVLLILKPELIWGWTT--
Siim      TTQRGPLPAHTLITDPAHSREHLLITLHLVPLILLVLKPELIWGWAV--
Ctru      MTQRGPLPDHITALDPTHSREHLLLSLHLLPLLLLILKPELVWGWTA--
Dpbr      MTQRGPLPNHITALAPTHTREHLLLALHLLPLLLLILKPELVWGWTA--
Caki      MSQRGPLPQHMFFLTPSHTREHLLLTLHLAPLILITLKPALIWGWFA--
Phja      INQRGPLPPHITVLSPSHTRENLLIALHLVPLLLLIPKPNLLWGNFS--
Brsp      TTQRGPLPHHIMGLIPTHTREHLLMTLHLIPLILIILKPELIWG*----
Gamo      MSQRGPLPQHMLALPPSYTREHLLMALHLIPLLLIILKPALLWGWFA--
Lolo      MSQRGPLPQHMLALPPSYTREHLLMALHLIPLLLIILKPALLWGWFA--
Batr      ITQRNL-NKNLPQTQPSHSREHLLMLLHLIPLILLLLKPELVSLPGPLI
Prmy      VTQRGPAKQE-APVTPSHSREHLLLALHILPVLLLSLKPSLTWGLFL*-
Lose      MSQRGSLPTHITALEPSHSREHLLLALHLMPLLLLTLKPELIWGWSI--
Loam      MTQRGPLPAHILALEPSHSREHLLMALHLLPLLLLTLKPELIWGWSM--
Chab      MTQRGPAPPHILALEPSHTREHLLMALHLLPLMLLISKPTLVWGWTA--
Chto      MTQRGPAPPHILALEPSHTREHLLMALHLLPLMLLVSQPTLVWGWTA--
Majo      MSQRGPVPNHMMTLEPVTAREHLLMILHLGPLMLLTLKPELICGWAV--
Hlst      MTQRGSIPDHLMALEPVTAREYLLIYLHLAPLILLALKPEMICGWAI--
Clpe      MTQRGPTPTHLLALEPSHTREHLLMALHLAPLTLLVLKPELIWGWAA--
Mlmr      MTQRGPVPSHIFALEPSHTREHLLMALHLLPLALLILKPELIWSWTS--
Crcr      MTQRGPLPKHIIALDPSHSREHLLLALHLLPLLLLILKPEIIWGWTA--
Muce      MTQRGPLPKHIIALDPSHSREHLLLALHLLPLLLLILKPEIIWGWTA--
Bege      MTQRGPLPNHILALDPTHSREHLLITLHLLPLILLILKPELIWGWTA--
Mela      VTQQGPLPPHIIALDPTHSREHLLMALHLIPLLLLITKPELLWGWTA--
Hats      MTQRGPIPAHILALDPTHSREHLLIALHLIPLILLTTKPELIWGWTT--
Orla      TTQRGPVTSPVVAIEPTHTREHLLMVLHLLPLLLLVLKPTLIWGWTL--
```

[8/8 of aligned sequences]

```
Cosa    MTQRGPIPNHIISLNPTHTREHLLITLHLIPLLLLILKPELIWGWTA--
Exsp    MTQRGPLPMHMIAINPTHSREHLLITLHMIPLLLLVMKPELIWGWTA--
Depa    MTQRGPLPSHIIALNPTYTREHLLILLHLAPLFLLILKPELIWGWAT--
Rima    TTQRGPLPQHIATLAPSHTREHLLIFFHLFPLMQLMFSPDLISGYFI--
Fuol    LTQRGPLPHHLLSLQPTHTREHLLLVLHLLPLILLIAKPELIWGWTA--
Gmaf    TSQRGPLPSHLISWDPSHSREHLLLTMHLVPLLLLMLKPELLWGWNAC*
Xeei    MTQRGPLPNHLLSLDPSHSREHLLLTLHLLPLILLIFKPEITWGWMA--
Pros    MTQRGPLPAHILALDPTHTREHLLMALHLIPLVLLIMKPELIWGWTA--
Scmi    MTQRGPLPTHIITLDPTHSREHLLMALHLIPLILLITKPELVWGWTA--
Rolo    MTQRGPLPTHIIALDPSHSREHLLMALHLIPLILLILKPELIWGWTA--
Cere    MTQRGPLPTHTIALYPTHSREHLLMALHLIPLLLLIMKPELIWGWAA--
Daga    MTQRGSLPPHAIALSPSHSREHLLMALHLIPLVLLVAKPELIWGWTA--
Anco    MTQRGPLPSHIIALPPSHSREHLLMALHLIPLMLLILKPDLIWGWVA--
Dmve    MTQRGPLPPHIISLDPTHSREHLLIALHLVPLLLLILKPELIWGWAT--
Dmar    MTQRGPLPPHIISLNPTYSREHLLIALHLVPLLLLILKPELIWGWAT--
Anka    MTQRGPLPSHIIALSPSHSREHLLMALHLIPLMLLILKPELVWGWVA--
Moja    MTQRGPLPSHIIALAPSHSREHLLLALHLLPLMLLILKPELVWGWTA--
Hoja    MTQRGPLPSHIIALPPSHSREHLLMALHLIPLLLIILKPELVWGWVV--
Bede    MTQRGPLPTHIIALDPSHSREHLLMALHLIPLILLIMKPELIWGWTA--
Besp    MTQRGPLPTHIIALDPSHSREHLLMALHLIPLILLIMKPELIWGWTA--
Mysp    MTQRGPLPPHIIALEPSHSREHLLMALHLIPLLLLILKPDLIWGWSA--
Osja    MTQRGPLPPHIISLEPSHSREHLLMALHLIPLLLLILKPELIWGWSA--
Sgro    MTQRGPLPPHIIALEPSHSREHLLMTLHLIPLLLLILKPELIWGWIA--
Pzpa    MTQRGPLPPHIIALPPSHTREHLLLALHLLPLIMIITKPALVSGWLA--
Zeja    ATQRGPLPPHITALPPTHTREHLLMALHLLPLMMIIAKPALISGWLA--
Znne    MSQRGPLPPHIIALAPSHTREHLLMALHLLPLIMIITKPALVSGWFA--
Zefa    MTQRGPLPQHITALSPSHTREHLLMALHLLPLIMIIAKPALVSGWFA--
Acni    MTQRGPLPPHIIALAPSHTREHLLMTLHLLPLIMLITKPALISGWCA--
Ncrh    MTQRGPLPPHIIALAPSHTREHLLMTLHLLPLIMLITKPALISGWCA--
Agca    MTQRGPLPPHMLALEPSHTREHLLMALHLIPLALLILKPELIWGWIA--
Hydy    MTQRGPLPLHMIALDPTHTREHLVMALHLIPLLLLVLKPELVWGWTT--
Gsac    MTQRGPLPAHMIALDPSHTREHLIMALHLIPLLLLVLKPELVWGWAT--
Pevo    MTQRGPTPQHLSAIFPSYSREHLLMALHILPLVLLVLKPELIWGWTT--
Hiku    MTQRGPLTNHLLHMEPSHSREHLIMMLHLTPLILLILKPELLWGWIL--
Inpa    AIQHGPPPTHLLTLSPSHTREHLLMTLHLLPLLLLVLNPSLIWGWAA--
Auch    TTQRGPTPAHIIDLQPTHSREHLLITLHLLPLILLILKPELSGGWAC--
Fico    MTQRGPLPPHVVALEPSHSREHLLMALHLIPLLLLVLKPELVWGWAA--
Macs    MTQRGPLPAHLLALDPTHSREHLLMALHLIPLALLIAKPELIWGWAA--
Moal    MTQRGPVPNHMISMNPSHSREHLLLTLHLLPLLLLVFKPTTIWGSAY--
Syma    NTQRGSLMTHLLSLDPTHSREHLLIALHALPLLLVMLKPELIWGWTF--
Mafr    TTQRGPLPHHITHLDPTHSREHLLMSLHLLPLILLMLKPNLIWGWAA--
Dcpe    MTQRGPLPPHIQTLDPSHTREHLLITLHLIPLILLILDPQLVWGWTG--
Dcti    MTQRGPLPPHIQTLDPSHTREHLLVALHLIPLILLILDPQLVWGWTG--
Hehi    MTQRGPLPAHIISLNPSYTREHLVMALHLLPLLLLVLKPELVWGWTA--
Stam    MTQRGPLPEHILALNPSHTREHLVMALHLLPLMLLILKPELVWGWVA--
Hogi    MTQRGPLPAHIMALDPSHTREHLVVALHLLPLMLLIIKPELIWGWAA--
Erzo    MTQRGPIPAHVLALDPSHTREHLLMALHLLPLLLLVLKPELIWGWTS--
Hxot    MSQRGPLPAHIIALAPSHTREHLLMALHLIPLLLLILKPELIWGWAT--
Core    MSQRGPLPNHLLALDPSHTREHLLMALHLLPLVLLVLKPELIWGWAS--
Apve    MTQRGPLPAHIVALLPSYTREHLLMTLHLVPLLLLILKPELIWGWAT--
Latj    TTQRGRLPPHIKALDPSHSREHLVVTLHLTPILLLILKPELIWTWFT--
Laja    MSQRGPLPTHILALDPSHSREHLLMALHLLPLMLLILKPELIWASVL--
```

[8/8 of aligned sequences]

```
Syja    MTQRGPLPPHLLALPPSHSREHLLMALHLLPLILLILKPELIWGWAA--
Epme    MTQRGPLPTHIIGLDPSHSREHLLMALHLLPLLLLILKPELIWGWTI--
Grse    TTQRGPLPTHIIALDPSHTREHLLMTLHLLPLILLILKPELIWGWTG--
Clja    MTQRGPLPKHIISIDPTHTREHLLIILHLIPLLLLIIKPELIWAWTA--
Ogcy    MTQRGPLPNHIIALDPSHSREHLLITLHILPLMLLILKPELIWGWAA--
Plna    TIQRGPLPSHISARDPSHSREHLLITLHLLPLLLLITNPELAWILF*--
Lema    MTQRGPLPTHIIALDPTHSREHLLMALHLLPLLLLILNPALIWGWTA--
Etzo    MTQRGPIPPHLIALDPSHSREHLLMALHLLPLILLMLKPELIWGWAF--
Apse    MTQRGPLPSHILALEPTHSREHLLLTLHLLPLLLLILKPELIWGWTI--
Epde    MSQRGPLPAHITALDPSYSREHLLMALHLLPLILLILKPELIWGWTA--
Slja    MTQRGPIPAHIIALDPSHSREHLLIALHLLPLLLLVLKPELIWGWTA--
Bsja    MTQRGPLPPHIKNITPSHSREHLLMALHLLPLILLVLKPELIWGWTS--
Ecna    MTQRGPIPLHITAIEPSHSREHLLIFLHLLPLLLLILKPELIWGWTF--
Cohi    LTQRGPAPLHSTNLEPSHTREHLLIFLHLMPLIMLIFKPELILAPIL--
Caar    MTQRGPLPGHIIALDPSHSREHLLILLHLLPLILLILKPELIWGWTA--
Came    MTQRGPLPGHIIALEPSHSREHLLILLHILPLILLILKPELIWGWTA--
Mema    MTQRGPLPPHIISLDPSHTREHLLMALHLLPLLLLTLKPELIWGWTT--
Lenu    TTQRGPLPTHMLNLPPTHSREHLLITLHLLPLILLILKPELIWGWTY--
Brja    MTQRGPLPAHVLALNPSHSREHLLLALHLLPLVLLILKPELIWGWTT--
Plma    MTQRGPLPIHIIALDPSHSREHLIMALHLLPLILLILKPELIWGWTA--
Emst    MTQRGPLPAHIIALDPSHSREHLLMALHLLPLILLILKPELVWGWAT--
Ptti    MTQRGPLPAHIIALDPSHSREHLLMALHLLPLLLLILKPELIWGWTA--
Losu    TTQRGPTPPHMMALDPSHTREHLLMTLHLLPLILLILKPELMWGWAA--
Geoy    MTQRGPLPAHMMGLDPSHTREHLLMTLHILPLLLLILKPDLIWGWAA--
Dipi    MTQRGPLPTHIIALDSSHSREHLLMTLHLLPLMLLILKPELIWGWAA--
Pama    MTQRGQLPMHITALEPSHSREHLLMALHLLPLGLLILKPELIWGWTA--
Leob    MTQRGPLPAHILALSPSHSREHLLMALHLLPLVLLILKPELIWGWAA--
Neba    MTQRGYLPPHILAIEPTHTREHLLMALHLLPLILLVLKPELFWGWSA--
Pdpl    TTQRGPLPAHILALEPSHTREHLLISLHLIPLLLLILKPELICGWAL--
Nimi    TTQRGPLSAHILALEPSHSREHLLVALHLLPLLLLMLKPQLITGWTA--
Uptr    MTQRGPLPPHILALDPSHTREHLLMALHLIPLILLILKPELIWGWTG--
Pesc    MTQRGPLPTHLHGLEPSHSREHLLMALHLLPLLLIICKPELIWGWTA--
Baar    MTQRGPLPTHIIALDPSHSREHLLIALHLLPLLLLILKPKVIWGWAA--
Moar    MTQRGPLPAHIIALDPSHSREHLLMALHLLPLILLILKPELIWGWAA--
Toja    MTQRGPLPMHIIALDPSHSREHLLIILHLLPLILLILKPELIWGWTA--
Chau    SIQRGTLPTHIIALDPSHSREHLLMALHMLPLFLLILKPELIWGLAG--
Chse    MTQRGPLPAHITALDPSHSREHLLIILHLIPLLLLILKPELIWGWTA--
Enar    MTQRGPLPAHVMALDPSHSREHLLMALHLLPLLLLIIKPELVWGWAA--
Hpty    MTQRGPLPAHILALDPSHSREHLLMALHLLPLMLLILKPELVWGWTA--
Nana    MTQRGTTPAHLLALEPTHSREHLLMALHLLPLILLILKPELIWGWTT--
Mcst    MTQRGPLPTHLMALDPTHSREHLLMVLHLAPLALLILKPELIWGWTA--
Rhox    MTQRGPLPAHMTALSPSHSREHLLITLHLLPLLLLILKPELIWGWTF--
Opfa    MTQRGPLPAHIIALDPSHSREHLLMALHLLPLLLLILKPELIWGWTA--
Paar    MTQRGPLPAHIIALDPSHSREHLLITLHLLPLILLILKPGLIWGWLI--
Gozo    MTQRGPLPAHMIALDPSHSREHLLMALHLLPLLLITLKPELIWGWAA--
Ackr    TTQRGPLPAHILGLEPSHTREHLLMALHFIPLFLLILKPQLVWAWII--
Elev    MTQRGPLPPHILTLEPSHSREHLLLTMHTLPLFMLIFKPELIWGWTA--
Trdu    MTQRGPLPAHINSLDPTHSREHLLLALHLLPLILLISKPELIWGWTA--
Amoc    MTQRGPIPAHMMALHPTHSREHLLIILHLLPLILLILKPELVWGWSA--
Hame    MTQRGPLPAHIINLPPSFTREHFLMALHLLPLLLLMLKPSLVWGWF*--
Chso    MSQRGQLPPHMTALPPTYSREHLLIALHLVPLLLLTLKPELIWGWM*--
Lyto    MTQRGPLPTHIAALAPSHTREHLLLALHLLPLLMLVMKPELIWGWAS--
```

[8/8 of aligned sequences]

```
Encr      MTQRGPLPTHIIALDPSHTREHLLMALHLIPLLLLVLKPELIWGWAA--
Bvar      MTQRGPLPSHLTTIEPSYTREHLLMALHLLPLVLLTLKPELIWGWAN--
Noco      TTQRGPLPSHLIALDPSHTREHLIMALHLLPLILLILKPHLIWGWAS--
Chsp      STQRGPLTPHAMVLEPTHTREHLLLTMHLLPLTLLIMKPELMWGWTA*-
Arja      MSQRGPLPAHIIALDPSHTREHLLMALHLIPLLLLILKPELIWGWATC-
Pase      TTQRGQMPPHIIALNPSYSREHLLITLHLLPLLLLMLKPELIWGSLT--
Trel      MTQRGPLTTHLTNLPPSHSREHLLLSLHVVPLVLLVLRPELMLAWIA--
Lifa      MTQLGQLPPHITAMDPSHTREHLLIILHATPLILLILKPELVWGWTS--
Acur      TTQRGPLTTHMISLAPTQSREHLLLALHIVPLLLLMTHPYLIWSLIN--
Ampe      MTQRGPLPAHIIALDPTHSREHLVAVLHLLPLLLLILKPELIWGFTA--
Urja      LTQRGPLPHHIKHIPPSHSREHLTASLHLLPLIWLVTKPETVCGWCT--
Enet      MTQRGRVPSHLTAIDPSHSREHLMLTLHMIPLALLTLKPELIWGWAS--
Ptbr      MTQRGPLPHHITNLPPTHSREHLTVTLHLIPLVLLISKPELILGWIS--
Safa      MTQRGPLPPHIIALQPTYSREHLLITLHLLPLLLIILKPETILGWAA--
Icae      MTQRGPLPTHIIALDPSHSREHLLMALHLLPLALLVLKPELIWGWTT--
Asmi      TTQQGPLPAHILSLSPTHTREHLLIMLHLMPLGLVILKPEIIWGWCF--
Foal      MTQRGQAPLHIKFPDPSFTREHLLMTFHLLPLLLLILTPSLITTL*---
Drze      TTQRGPAPTHISSFEPSHTREHLLLILHLIPLLLLTLKPEYTWGVIS--
Rhas      MTQRGPIPSHIIALDPSHTREHLLVALHLLPLLLLIAKPELIWGWTA--
Elac      MTQRGPLPAHILALPPSHTREHLLMALHLLPLLLLILKPELIWSWSA--
Kugu      MTQRGPIPTHIMAINPSHSREHLTIALHLIPLVLLMLKPELIWGWTA--
Plor      MTQRGPLPAHIIALEPSHSREHLLITLHLLPLILLILKPELIWGWTA--
Sgun      MTQRGPLPAHILALDPSHSREHLLIALHLIPLLLLILKPELIWGWTA--
Zaco      MTQRGPLPAHLIALDPSHSREHLLMALHLVPLILLILKPELVWGWTA--
Zbfl      MTQRGPLPDHLTALEPSHSREHLLMTLHLIPLVLLILKPELIWGWAA--
Spba      MTQRGNLPNHIISLDPPHTREHLLITLHLLPLILLILKPELIWGWTA--
Game      MTQRGPLPAHIIALDPSHSREHLLMALHLLPLALLILKPELIWGWTA--
Thth      MTQRGPLPAHIIALDPSHSREHLLIALHLLPLILLILKPELIWGWTA--
Xigl      MTQRGPLPAHIIALDPSHSREHLLILLHLLPLLLLILKPELIWGWTA--
Hyja      MTQRGPLPTHIIALDPSHSREHLIIILHLLPLALLILKPELIWGWTS--
Psan      KTQRGELPSHIKTLAPSDTREHILIVLHLAPLALIMLKPEVVWGWPYS*
Cupa      MTQRGPLPAHIIALDPTHSREHLLMALHLLPLALLILKPELIWGWTA--
Mpch      MTQRGPTPAHIITLDPTHSREHLLIALHLIPLILLITKPELIWGWTA--
Char      TTQRSTVPAHIIALDPTHSREHLLMALHLLPLVLLTLKPELIWGWTA--
Pser      TTQRGPLPGHMLNLTPSHTREHLLITLHLLPLILLITKPELIWGWTT--
Prol      MTQRGPLPTHILALEPSHTREHLLIALHLLPLILLVLKPELIWGWTA--
Plbi      MTQRGPLPTHVIALEPSHTREHLLIALHLIPLILLVLKPELIWGWTA--
Calu      TTQRGPNSSHVLALEPSHTREHLLITLHLVPLLLLMCKPELIWGLTS--
Papa      MTQRGPLPTHILALDPSHTREHLLMTLHLVPLLTLVVKPELIWGWTA--
Sufr      MTQRGQLPLHILSIQPTHSREHLLIVLHLTPLFLLILKPELVWGWTA--
Stci      MTQRGKVPNHLLAIEPTHSREHLLIALHLLPLLLLILKPELISGWAA--
Taru      MTQRGPAPSHLLALEPSHTREHLLIALHLLPLILIITKPELIWGWTA--
Rala      MTQRGPLPAHILALEPSHSREHLLIALHLLPLILLILKPELIWGWAA--
            *                 **    :*   :  :   *
```
