# Supplementary material for: Structure and variation of the mitochondrial genome of fishes
Source: BMC Genomics. 2016 Sep 7;17(1):719. doi: 10.1186/s12864-016-3054-y (PMC5015259; doi:10.1186/s12864-016-3054-y)
Supplement: Additional file 6: Figure S1-a. — Aligned amino acid sequences of the ATP8 gene in mt genomes of 250 fishes. Figure S1-b. Aligned amino acid sequences of the ATP6 gene in mt genomes of 250 fishes. Figure S1-c. Aligned amino acid sequences of the COI gene in mt genomes of 250 fishes. Figure S1-d. Aligned amino acid sequences of the COII gene in mt genomes of 250 fishes. Figure S1-e. Aligned amino acid sequences of the COIII gene in mt genomes of 250 fishes. Figure S1-f. Aligned amino acid sequences of the Cyt b gene in mt genomes of 250 fishes. Figure S1-g. Aligned amino acid sequences of the ND1 gene in mt genomes of 249 fishes. Figure S1-h. Aligned amino acid sequences of the ND2 gene in mt genomes of 250 fishes. Figure S1-i. Aligned amino acid sequences of the ND3 gene in mt genomes of 250 fishes. Figure S1-j. Aligned amino acid sequences of the ND4L gene in mt genomes of 250 fishes. Figure S1-k. Aligned amino acid sequences of the ND4 gene in mt genomes of 250 fishes. Figure S1-l. Aligned amino acid sequences of the ND5 gene in mt genomes of 250 fishes. Figure S1-m. Aligned amino acid sequences of the ND6 gene in mt genomes of 249 fishes. (ZIP 3250 kb) [file 12864_2016_3054_MOESM6_ESM.zip › Additional file 6 prot align/AF6l-ND5.pdf]

Additional file 6: Figure S1–I. Aligned amino acid sequences of the ND5 gene in mt genomes of 250 fishes.

Species name abbreviation followed by aligned amino acid sequences shown by one letter abbreviation. See Additional file 1 for abbreviation of species name. Amino acids shown by magenta letter denote hydrophobic residues. A-O in bold types with yellow background indicate putative transmembrane regions. Asterisk '*' indicates a fully conserved residue. Colon ':' and period '.' indicate 'strong' and 'weak' groups in the level of conservativeness, respectively, in the Gonnet Pam250 matrix, in which the strong and weak groups are defined as strong score >0.5 and weak score =<0.5, respectively (Thompson et al., 1997).

ND5

[1/12 of aligned sequences]

To be continued on page 6.

```
Scca      -------------MNTIFNSSFLLIFITLMIPLVT-----SLSPKKLNPSPSSFYVKTAV
Muma      -------------MHIIFNSSFLLIFIILTLPLIS-----SLSPKELKPNWSSLYVKTAV
Erca      -------MFI-DQLPQMFLTCLSLTMVLLILPIIL-----SMVTKPL-NNWP-LYVKNSV
Pose      -------MSI-PQLSQMFLTCLSLTIIILALPIIL-----SIMFKPL-NNWP-HYVKNAV
Actr      ---------M-HSPTLIFSSTLLIIFTLLTYPLIV-----SLSPNPLNKKWATTHVKTAV
Scal      ---------M-HSPTLIFSSTLLMIFILLTYPLIV-----STNPSPLNKKWATTHVKTAV
Posp      ---------M-HPPTLIFNSTLLIILFLLTYPLIV-----SLNPSPLNKKWATTHVKTAV
Atsp      -----------MHLATVFNSNLAVILLILAAPLLM-----TLAPTPL-KNWA-LRVKTAI
Leoc      -----------MHLATMFNSNLAVILLILATPLLM-----TLTPSPL-KNWA-IRVKTAI
Amca      ---------M-HPTTLIFNSSLFIIFFLLAFPLFT-----TLSPSPLKKDWATSKVKTAV
Osbi      ---------M-LSTTTIFNTSFLIILAILAFPILT-----ALSPDPTKKNWATTHVKTAI
Pabu      ---------M-HLTMLIFNSSFILIFMLLILPIIM-----TMNPQPLKPTWALSQVKTAI
Hial      ---------M-HSTTLIFNSSLLIIFALLIYPLLT-----SMSPTPAKKDWALSHVKTAV
Elha      ---------M-HPTTLIFSSTLLTIFALLILPLFT-----TLNPTPQKKDWAVTHVKTAV
Mlcy      ---------M-HATTLMFTSSLLTILALLTYPLTT-----TMNPNPSQKTWATAQAKTSV
Algl      ---------M-HLSTLGFNSSSLTVFVLLLFPILT-----TLGSAHTKKDWASSYVLPAI
Ptgi      ---------M-HSTTLIFNSSLLIIFALLIFPIVM-----TLNPTPMKKDWAVTHVKTAV
Alaf      ---------M-HPTTLIFNSSLIIIFALLLFPILT-----TMNPTPLKKDWAATHAKTAV
Nock      ---------M-HSTTMIFNSSLIIIFALLLFPILT-----TLSPTPVKKDWATTHVKTAV
Anja      ---------M-PLTILTLNSSLLIILTLLIYPIMM-----TLNPNPVKKDWAVTHVKTAV
Gyki      ---------M-HNTTLMFTSTLILTLTVLILPIIT-----TLSPTPQKKTWALNQVKTAV
Syka      ---------M-HPTILMFNSTIIIILIILIYPIAM-----TLNPAPMKKDWAATHVKTAV
Opma      ---------M-YSTILTFNSTLFAILILLTYPIIT-----TLSPNQLTKDWALTHVKTAV
Comy      ---------M-HLTTLVLNSSLLMILALLTYPILM-----SLSPTPQKKDWARTHVKTAV
Sasp      ---------M-PSTPMMLNSSLLLIMAL-LSPPTL----MLSTTNMAKTTSALGRTQVIV
Eupe      ---------M-PSTSLIFNSSIFIIIVVLMTPPAL----MSLNSSMTKT-GISPHPVAAI
Enja      -----------MQTTLVMTTSFILIFVMLAYPLMT-----TINPTPVKGQWATTHVKTAV
Same      -----------MQTTLILSSSLTLIFALLAYPILT-----TISPTPKPDGWAVSHVKTAV
Chch      ---------M-QSTAMILSSSLVLVLGLLLYPLFT-----TLSPEPRKEGWAVTHVKTAV
Grgr      ---------V-QPVMIILSSTHLLVLGLLLYPLLT-----TLHPSLRHGNWALTHVKTAV
Caau      -------------MTLIMHSSLLLIFFILMYPLLT-----TLNPNQQGSNMA-GMTKTAV
Cyca      -------------MTLIMHSSLLLIFFILVYPLLT-----TLNPNQQDSNMA-GMTKIAV
Dare      ------------MTDMIMPLTLILIFAVLSYPLLK--------PKSYSKSNNSFQAWNAV
Cost      ---------M-HPTTLILSSSLILVIAILIYPLLT-----TLYPEPKDQKWAIAHVKTAV
Leec      ---------M-HPTTLILSSSLIMIILILIYPLLT-----TLSPTLKNPNWAVTHVKTAV
Fola      ---------M-HPTPLILSSSLLLVIIILIYPVLT-----SLNPNPQNPKWATSHVKTAV
Clmc      ---------M-YTTSLILSSSLILMLSILLYPLLM-----TLNPRPQNPEWAVTHVKTAV
Phin      -----------MSAPLALSSSFMLTLIILMVPLLN-----TLKPNPCPPLWASAYVKTAV
Icpu      -------------MADIMTTTLLLTLAILMWPLMT-----TLSPTPLDQKWALKYVKTAV
Psto      ------------MVNTIMTTTLLLTLTTLIWPLIM-----TLSPKPLKQDWALKYAKTAV
Cora      ------------MTNLVMNSTLILMLTILIYPLLM-----TLSPNPQKPNWATTHVKTAV
Eisp      ---------M-YPTTLILSSSFILMIALLTYPLTT-----TLTPSQQAPSWAATHVKTAV
Apal      -------------IALTLSSSLILTIIILLYPLIT-----TLNPKPQNQNWALTHVKTAT
Eslu      ---------M-HTTTLAMNTSLLMIFALLIFPLLT-----SLSPTPLHKTWAVTHVKTAV
Dape      ---------M-HTSTLAMSSSLLMVLTLLATPLFI-----SLSPLPLQKSWALTHVKTAV
```

[1/12 of aligned sequences]

Glse ---------M-HPSTLVLNSSLLLIFALLIFPLIT-----TLSPNHLPKTWALSHVKTAV
Naar ---------M-HSTSLILSSNLLVIFALLIYPLIT-----TLSPNPLPQSWALTHVKTAV
Lioc ---------M-HSTSLILSSNLLIIFALLIYPLMT-----TLSPNTLPQTWALSHVKTAV
Opso ---------M-CPPTLILSSSLLIIFTLLIFPLVN-----TLCLNPSPPLWPLTLTKTAV
Alte ---------M-QPTTLIFSTSLVLIFGLLLYPLVT-----TLDPNPQKSNWAVTHVKTGV
Plap ---------M-QPTALILSTSLVLIFGLLLYPLAT-----TLNPSPRESNWAVTHVKTGV
Plal ---------M-HLTSTILSSSLLLIFALLLYPLLT-----TLSPSPLHKEWALTHVKNAV
Sami ---------M-HLMSTILSSSLLLIFALLLYPLIA-----TLSPSPVHKGWALTHVKAAV
Rere ---------M-HLTPLVLSSSLLLVFALLLYPLVT-----TLAPSPVHKGWGLTHVKTAV
Gama ---------M-HPTTLVLSSSLLTIFVLLIYPLLN-----TLSPSPRPLDWAETHVKNAV
Onmy ---------M-HPTTLILSSSLLMIFTLLIYPLIT-----TLTPTPQHKNWALTHVKTAI
Sasa ---------M-HPTTLILSSTLLMIFALLLYPLIT-----TLNPTPQQENWALTHVKTAI
Cola ---------M-HPTTLILSSTLLMILALLIYPLLT-----TLDPNQRPGNWALTHVKTSV
Dita ---------M-HPTTLALTSSLLITLTLLSLPLIS-----TLAPRPRPEGWALTDVKTAV
Gogr ---------M-HPTSLILTSNLLLIFTLLILPMAS-----ASNLSPRPKDWTLTHVKTAV
Chsl ---------M-PLSSLVSTSSLFLTIALLILPLLL-----TLSPTPKQSSWAETHVKTAV
Atja ---------M-HSSALIMTSNLLMIFLLLLYPLIT-----TLSPQPLKNSWALTHVKTSV
Iido ---------M-HSSALIMTSNLLAIFLVLVYPLIT-----TLSPKPRKTNWALTHVKTSV
Auja ---------M-HPTAVIMASSLLLIFILLTYPLAL-----TLQPKSYKPSWSLVYVKTAV
Chag ---------M-HPTTLIMSTSLLLVFFLLVYPLMT-----TLSSSPRQNDWALTHVVNAV
Hami ---------M-HPTTLFMNSSLILIFILLIFPLFS-----SLDPAPLKKDWAPSYVTVAV
Saun ---------M-HPTTLLMNSSLILIFVLLLSPLLL-----TLNPSPLKKDWALSHVTMAV
Nema ---------M-HPTTITSSSSLLLVLLLLVIPVLT-----TLSPHPLPPQWATSHVKTAV
Disp ---------M-HLMTITTSSLLLVLALL-TLPVLT-----TLSPNPVPSHWATSQVKTAV
Myaf ------------MMSTITSSSLLLVITLLILPILT-----TLNPTPTPLNWATSQVKTAV
Lagu ---------M-LSLPTLLNTSFLLIFMLLLYPLAS-----SLSPSPKAPHWANSHVKTAV
Trtr ---------M-HTTTLMMNTSLMIIFILLLTPLFS-----TLSPHPKNAQWARTHIKTSV
Zucr ---------M-HPTTLMMNSSLMIIFALLLLPLLS-----ALNPTPNTPQWAHSYVKTSV
Pxja ---------M-HPTTLTISSSLLVIFTLLIFPLVT-----TLSPNPQHSEWALSHVKTAV
Pxlo ---------M-HPTTLTISSSLLVIFTLLIFPLAT-----TLSPNPHHSEWALSHVKTAV
Pctr ---------M-HSTTLMIASGLLTTLALLLSPLLA-----TMHPRPPQATCLAPRVKTAV
Apsa ---------M-NPTTLMISSSILITFSILIFPLLK-----ITSPQPPLNPWATQYVKTAV
Cabe MLNAPSTATMDLITSTATTSGLIIIIIFLLTPVAL-----SFINPSRDPVKACDMAVNAI
Bzze ---------M-HPTPLMMTSSLIIIFIILAYPLLT-----TMKPEPQGPAWALSQVMTAV
Siim ---------M-HTTPLVMTSSLILIFMLLAYPVIT-----TLTPNPKTADWSKTHATLAV
Ctru ---------M-HPTALMMTSSLIIIFTLLVYPLLT-----TLTPQPQDNTWALSHVKTAV
Dpbr ---------M-HPTALMMTSSLIIIFILLMYPLVT-----TLNPSPQNDTWARTHVKTAV
Caki ---------M-HSSSLLYTSSLLIMFTTLMYPLFY-----STTKSSPNDPQLAYHTKTAI
Phja ----------MFVVKNFYGMILMLMFMTLLLPLVV-----DLMAEPLQKDKAALNIKTAI
Brsp ---------M-PSSSMIYSSSLLMMLLVLLWPLIS-----SLLLLPFPSYWYSSYTKMAV
Gamo ---------M-HPTTLMYTSSLLLMFAVLLYPLLV-----TFTSLPLNNDWASSHAKTAV
Lolo ---------M-HPTTLMYTSSLLLMFAILLYPLLV-----TFTSLPLNNDWASSHAKTAV
Batr -----------MYYTLIVTTSFLTMLMLMLSPLII---------STIYKTTNDTVFTKAI
Prmy ----------MCLSHLMMPSCFLMVFIMLIFPLVP---------QILLNKPLHLKIKNSV
Lose ---------M-THSPVIMTSSFLIIFALLIYPVIT-----PPH-HNLSKSWALTHPMTAV
Loam ---------M-HPTSTMMASSLLIIFALLTYPVLT-----TLTPNPARGTWALSQVKTAV
Chab ---------M-HLAPTMMASSLIIIFMLLTYPILT-----TLSPEPKTPDWAITQVKTAV
Chto ---------M-HLAPTMMASSLIIIFMLLTYPILT-----TLSPEPKTPDWAVTQVKTAV
Majo ----------MFSTPAMMASSLLIILTLLILPVLT-----TFSPDNKNENWSPTQAKAAV
Hlst ----------MFTTPAIMASSLLVTLTLLTLPLVT-----TLSPNPKNNTWATMQVKLAV
Clpe ---------M-HYTTIMMASSLTIIFALLAYPLLT-----TLGPQPKQSHWPLSQVTTAV
Mlmr ---------M-HITPLMMSSSLMIIITLLAAPILT-----SMRPEPQAPHWALTQVKTAV

To be continued on page 7.

[1/12 of aligned sequences]

```
Crcr      ---------M-LTSTLFMTSCLVFTFLLLVHPLLT-----Y---QSQKNDWPLLKVKNAV
Muce      ---------M-LTSTLFMTSCLVFTFLLLAHPLLT-----Y---QSQKNDWPLLKVKNAV
Bege      ---------M-HHVPLMMTSSLILIFLLLLSPIVT-----SLSPNPRAPDWALVQVKTAV
Mela      ---------M-YLTSLIMTTSLALIFLLLLYPIIM-----TFVPSPQNTNHSPIQAKTAV
Hats      ---------M-NHTPLMMTSSLVLIFFLLLFPVIT-----TFSLKTQAPDWALTHVKTAV
Orla      ---------M-HLTTMVLSSSLLTIFSILILPVLG-----TLNPSPPGSLWATNSVKTAV
Cosa      ---------M-YYSPLIMTSTLIMIFIILTYPILV-----TLTPNDQDPYQNIEKTKTAV
Exsp      ---------M-PCTPLMMTSSLFFSLILLAVPALS-----TFVNSPPPFPTLSAQIKTAV
Depa      ---------M-HFTPLIMASTFNVILLILILPIVS-----TLNPKIMNQKQFSMDIKTAV
Rima      ---------M-ALTNLITLVTTLTIFMVLLYPLGI----L-PIALFFKQPWPLSHVKTSV
Fuol      ---------M-HLTPLIMSSSLIIVLTILFLPLLS-----TLSPTPLKPQWALQNVKTAV
Gmaf      ---------M-PSTALLLPSCMMIIFLILLFPVLT-----TLSPTPLITGWALTKTKNAV
Xeei      ---------M-HLSPLFMSSCLLIIFFLLLYPIFS-----TLTPKPLSTNWALTKVKTAI
Pros      ---------M-NSVPLIMTSGLLTIFTLLIYPLLT-----TFNPHPLKDTWALSQVKTAV
Scmi      ---------M-HPTTLIMTSSLLTIFTLLLYPLMT-----TFNPTPQKNTWALTQVKTAV
Rolo      ---------M-HPTTLMMASSLMTIFALLIYPLAT-----TFNPQPQGHTWALSQVKTAV
Cere      ---------M-HPSTLLMTSSLMIMFALLIHPLIT-----TLSPEPLNSKWTTLKAKNAV
Daga      ---------M-TPIVVMMTSSLLVTMALLMYPVIN-----TFTPGPLNYNWASMQVKTMV
Anco      ---------M-HPTALLMTSSLMIIFTLLIFPLMT-----TFHPQPLKAAWATTHVKTAV
Dmve      ---------M-HPTALLMTSSLMIIFTLLIYPLAT-----TFSPHPLKATWATSHVKTAV
Dmar      ---------M-HPTALLMTSSLMVIFTLLIYPLAT-----TFSPHPLMASWATSHVKTAV
Anka      ---------M-HPTALLMTSSLMIIFTLLIYPLLT-----TFHPTPLKTAWATTHVKTAV
Moja      ---------M-HPTALLMTSSLMIIFTLLIYPLIT-----TFHPHPLKTTWATTHVKTAV
Hoja      ---------M-HPTALLMTSSLLVVFTLLIFPLVT-----TFSPHPLKAAWATTHVKTAV
Bede      ---------M-HSTTIIMTSSLLTIFTLLLYPLLT-----TLSPNPQENTWALAQVKTAV
Besp      ---------M-HSTTIIMTSSLLTIFTLLLYPLMT-----TLSPNPQENTWALTQVKTAV
Mysp      ---------M-HPTTLTMASSLLIIFALLIYPLFS-----TFTPEPKPSSWALTHVKTAV
Osja      ---------M-HPTTLMMTSSLLIIFTLLIYPLLS-----TLTPNPKAPSWALSHVKTAV
Sgro      ---------M-HPTTLIMTSSLLIIFTLLIYPLLT-----TFNPTPKENSWALSHVKNAV
Pzpa      ---------M-DLAILVYNSCLIVVIVVLLFPILT-----SLYQKHTPKNWAVTHVTTAV
Zeja      ---------M-HPTLLTTSSSLLLVMALLLYPILM-----TFSNKQTPPEWASTHVKTAV
Znne      ---------M-HPIMITTSSCLLIVLALLLFPVLT-----TLSPKPTPPNWASSYVKTAV
Zefa      ---------M-HPTMITTSTCLLVVLVLLLFPILT-----TFSPKPSPHHWASSHVKTAV
Acni      ---------M-HPIMITTSSCLLVVMVLLLFPILT-----ALSPKSTPHNWASTHVKTAV
Ncrh      ---------M-HPIMITTSSCLLVVMVLLLFPILT-----ALSPKSTPHNWASTHVKTAV
Agca      ---------M-HPTPLMMTSSLIIIFALLMFPVLT-----TLNPRPQNANWALSQVKTAV
Hydy      ---------M-WHTPPVIYSGMIIVVTILFFTLLS-----SLMPTTSTPNQTAQHITTAV
Gsac      ---------M-WQTPSVLYSGMIIILVVLSFALLS-----ALVQGNSVLNQTVPNITSAV
Pevo      ---------M-HTTPLFMTSSLMIIFIILLFPVMS-----TLTPQKAQPTWALTHVKTAV
Hiku      ---------M-HSTSLMMSSSLLMIFTLLTTPLIS-----TMTTKSYPSDWSISWVKNSV
Inpa      ----------MHLSPTTFSTTLLLTLTLLTFPLLL-----ALKPNTPHNSSLVAQTKTAV
Auch      ---------M-PPTPLILSSSLVIVIITLLAPVVL-----SVPPYSTKPKWLLPQAKTAV
Fico      ---------M-NPTSLMMTSSLIAIFLLLAFPLLT-----SLAPTPLAPTWALSHVKTAV
Macs      ---------M-HPTSLMMASSLVIIFLLLSYPVLL-----SFSPSPLPSNWALTQVKTAV
Moal      ----------MPSLHIILTTSFIITLVILISPVFT-----SILYKSLNPNWHTHHIKTAV
Syma      ----------MPILATTLTSSLLIILLLLTYPLFT-----TL-SNKLLKNWHTNQTKTAI
Mafr      ---------M-HYAPIFLTSNLLIIFALLIYPVLT-----TLTPAPLKTDWALSKTKTAV
Dcpe      ---------M-TMPSLILSSVLASTLTLLVYPVLK-----IFSTKTKNHASALSLVKTSV
Dcti      ---------M-TVPSLILSSTLAATLTLLVYPVLK-----IFSTKAKDHALALNFVKTSV
Hehi      ---------M-HPSSLIMSSSLVIIFLLLAYPIFT-----TLEPRPRNPGWAVSHVKTAV
Stam      ---------M-HPTSLMMTSSLLMIFLLLLYPVLT-----TLNPHPQDTTWASTHVKTSV
Hogi      ---------M-HPSSLIMSSSLIIIFILLIYPVLT-----TLTPRPQDPGWALTHVKTAI
```

To be continued on page 8.

[1/12 of aligned sequences]

```
Erzo    ---------M-HPPSLMMTTSLIIILALLAYPVLS-----TLTPHPQQTSWATSHVKTGV
Hxot    ---------M-HPTSLMMTSSLIIIFTLLAYPVLS-----TLTPRIQDPSWAVTHVKTAV
Core    ---------M-HPTSLMMTSSLVIIFALLAFPVLT-----TLAPQTKAPDWATTHVKTAV
Apve    ---------M-HPTSLAMTSSLAIVFTLLAFPVLS-----TLTPHIKAPNWAVTHVKTAV
Latj    ---------M-HPTSLMMTSSLIIIFVILTYPVLT-----TLSPRPQDPHWALSHVKTAV
Laja    ---------M-HTTSLIMTSSLIIIFTLLTYPILT-----TLTPQPKGTAWAVTQVKTAV
Syja    ---------M-HPSPLMMTSSLIIIFTLLAYPLLT-----TMTPKPQNPDWALSQVKTAV
Epme    ---------M-HTSSVIMTSSLLIIFSLLIFPVLT-----TFNPLPRKEDWALTHVKLAV
Grse    ---------M-TPTSVTMATSLMLVFALLLLPVIT-----TFSPEPKDKLWPLTHVKTAV
Clja    -----------------MASSLLIIYGLLFYPLIM-----TMSPKPQKEDWALTQVTTAI
Ogcy    -----------MSTSLAITSGMTFVMVLLFCPLLM-----LLSPNTPMKNWA-ANVKTTV
Plna    ---------V-HYSSTTMTSSMLIVFVLLTFPVMS-----TITPSPLESNWALTKAKTAV
Lema    ---------M-HTTSLMMTSSLVIIFLLLLYPVVT-----TLSPKPQDNTWALSHVKTAV
Etzo    ---------M-HPTSLMMTSSLIIIFILLAFPVFT-----TLSPDPRGHDWALSYVKTAV
Apse    ---------M-HPTSIMMTSSLIVIFGLLSYPLLT-----TLTPSPSAKHWALSQVKTAI
Epde    ---------M-HPTPLMMTTSLILIFTLLAYPLLT-----TLSPRPLEDNWALIQVKTAV
Slja    ---------M-HTSSLIMTSSLMIILSLLFLPVLT-----TLSPKPLPITWALLQVKTAV
Bsja    ---------M-HSTSLMMTSSLVLIFMLLAYPIAT-----TLTPAPKNPDWALTHVKTAV
Ecna    ---------M-HPTSLMMTSSLIIIFFILAYPILT-----TLNPNEQHPQWALSHVKSAV
Cohi    ---------M-LSPTNIIPSLLLLTSILLTTPLII-----NLFKLSKNFELTAARVTLAV
Caar    ---------M-HPTSLMMTSSLIIIFTLLAYPILT-----TLNPRPQHPDWALLQVKTAV
Came    ---------M-HPTSLMMTSSLIIIFTLLVYPVLT-----TLNPRPQDPEWALTQVKTAV
Mema    -----------MYLPFALTSSLLCVFLVLISPVFT-----TFSPRPKDPSWALTHVTKAV
Lenu    ---------M-HPTAITMGSSLILIFIILSFPLLL-----SMHPNNQNT---ALHAKTAV
Brja    ---------M-HPTSLMMSTSLVITFFLLAFPILT-----TLSPKPQAPDWALTQVKTAV
Plma    ---------M-HPTSLMMTTSLIIIFSLLTYPVLT-----TLSPHPKAPDWALTQVKTAV
Emst    ---------M-HPTSLMMTSSLIIIFTLLAYPVLT-----TLNPRPREADWALSQVKTAV
Ptti    ---------M-HPTSLMMTSSLIIIFSLLVYPVLT-----TLTPRPQDANWALSQVKTAV
Losu    ---------V-NSSSLVMTSSLALILALLFYPLMM-----TLNPRLKSSDWALMQIKTAV
Geoy    ---------M-YTTPVMMTTSLVTILLVLAYPVLT-----TLSPNPTSKDWALSHVKTAV
Dipi    ---------M-HPTSLMMTSSLIIIFSLLAYPVLT-----TLSPNPQSHDWALTQVKTAV
Pama    ---------M-HPSSLIMASSLITIFLLLSYPVLT-----TLSPNPQKNDWALLKVKTAV
Leob    ---------M-HPTPLMMTSSLIIIFSLLAYPVLT-----TLTPTPQGPNWALTHVKTAV
Neba    ---------M-LPLSVMMTSSLILIFFLLAYPVFT-----TLSPNPRDPNWAITHVKTAV
Pdpl    ---------M-LNITSIFAATSLTILILLASPLFT-----TLDPNPKNAKWAMSHVKSAV
Nimi    ---------M-SMTPLALTSSLISIFFFLAQPLIT-----PSTP-----APATPLVKSTI
Uptr    ---------M-CSTSLMMTSSLVIIFSLLIYPVLT-----TLSPTPSAPDWALAQVKTAV
Pesc    ---------M-TVSVAITTTLLLGVLAALIFPVLLFILAFALAQQTFMPGWSDKFIKTSV
Baar    ---------M-HPPSLMMASSLLLIFSLLTYPLLT-----TLTPQPLAPNWALTQTKTAV
Moar    ---------M-HPTSLMMTSSLIIIFVLLAYPVLT-----TLNPTPRKADWALSHVKTAV
Toja    ---------M-HPTPLMTTSSLMIIFTLLAYPIFT-----TLSPFPQKHDWALSHVKTAV
Chau    ---------M-HPTPLMMSSTLILIFITLLYPLLT-----TPTSHPLKETWALYQVKTAV
Chse    ---------M-HPTMIVMTSSLILIFTLLSLPVIT-----SLSPKLKKPDWASTHVKTAV
Enar    ---------M-HTSSLILTSSLITIFLLLIYPVLT-----TLSPQPQKADWALSQVKTAV
Hpty    ---------M-HPTSLMMTSSLIIIFTLLTYPVLT-----TFTPRSHGTNWALTQVKTAV
Nana    ---------M-CSTSLIMTSTLISMLTLLMYPLLT-----TFSNSSSDTNWPITHVKTAV
Mcst    ---------M-HSTSLMMTSSLIIMFMLLMYPVLT-----TLTPRPQESDWALVQVKTAV
Rhox    ---------M-TSTSLTMASSLVIIFALLAYPVLT-----TLTPQPRPTDWALSHVKTAV
Opfa    ---------M-QPTSLMMTSSLIIIFTLLAYPVLT-----TLTPQPQPSNWALIQVKTAV
Paar    ---------M-HTPSVLMTTSLATIFLLLALPVFS-----TFSPIPRPTNWALSEVKAAV
Gozo    ---------M-HPTSLMMTSSLILIFLLLLYPVLT-----SFSPQPQENDWALTQVKTAV
Ackr    MTMAPVDLTTIKAIAFSLNSTLLTIFLILLYPIAM-----SLSPYPAPMGWARTHVKTAV
```

To be continued on page 9.

[1/12 of aligned sequences]

```
Elev     ----------MYTSPLIMASSLIITFLILLYPVMM-----SWTTSSLPSNWSMLQVKTAV
Trdu     ---------M-HLTSTMMTTSLILIFLLLTSPILS-----SFSPTPLPPNWALTQVKTAV
Amoc     MLNKAIEMNT-LTSPLHLTSCMILVFFILSYPILT-----SLSPKPLKTDWAISHVKKAV
Hame     ----------MPVPIYPPTSSILLVLALLMYPILD-----RLFPNNTSTKHLANYIKTAV
Chso     ---------M-HSSTLILASSLVLVLVLLTLPILT-----TLSPKPKTPEWATSQVKTTV
Lyto     ---------M-HPTSLMMTSSLIIIFALLAYPVLS-----TLSPRIQAPAWPVSHVKTAV
Encr     ---------M-HPTSLMMTSSLILIFALLASPVLS-----TLSPRTQAPSWAVSHVKSAV
Bvar     ---------M-HSTPLVMTTSLMIIFLTLLYPLLT-----ALSPTPLKPTWTISHVATAV
Noco     ----------MFFPASVLTSSLMITLFLLGSPLLL-----SLFSAPAKPSWATTHVKPAV
Chsp     ----------MHLPSLIMTTTLMMIFVLLLYPLLT-----TLSPTIKNPPWASLKVETAV
Arja     ---------M-HPTSLTMTSSLIIIFTLLACPVLS-----TLSPHVKAPSWAITHVKTAV
Pase     MFCVSTSLEDIINIPAVLASMLLIVLLLLAAPVIL-----TILPKAHQPLWLPAAIKMSV
Trel     ---------M-HFPPAYFSSSLMMVLMLLCNPILT-----SLDPYKPKPKWPLSQVKTAV
Lifa     -------MAQ-QTQQHLMASSLMLILLLLTLAILW-------LPTRRPNPTRSQEAQVLV
Acur     ---------M-PLMIVVNTFCFLLVFAILAYPLVL-----SLSRYPKPPEWHSTYIKPCV
Ampe     ---------M-HPTSLMMTSSLIIIFTLLIYPVLT-----TLSPEPRDPNWAVSQVKTAV
Urja     ---------M-PCLPSLLATNTLCILALLMFPLMT-----TLGAKTYNARRFTTLTQTNV
Enet     ---------M-PQTPLILSSSLLLILFLLTLPVLV-----TFLRPSPDPAWPLTQAKSAV
Ptbr     ----------MQLSALMMTSSMLGIFTVLTLPVIS-----TLSPSPKHSPWPLEKVMTSV
Safa     ---------M-HLTPLIMSSSMILIFVILVYPTLT-----TLSPKPTSPSWPLSMAKNSV
Icae     ---------M-HPTALMMTTSLVIIFSLLTYPVIT-----TLSPRPQAPDWAVTQVKTAV
Asmi     --------------MTNMTPIFLSIFIVLLVPLFK-----------SKPLFFPNDSVLAV
Foal     ---------M-PILET-LISSLLVVLVMLLTPLVW---------NTPFK-PYPLKVKTTI
Drze     ---------M-HPIPYVMTSSLITIFFILVYPIIT-----TLTPKLLHMKWALLQAKTAE
Rhas     ---------M-HPTLITMASSLIIIFVLLLYPLIT-----TLSPRPKEPSWAVIQVKTAV
Elac     ---------M-HPTPVTMTTSLIVIFALLLYPVLT-----TFSPTPKNETWALLQVKTAV
Kugu     ---------M-HPLSIMMTSSLIIIFILLLYPVLT-----TLPLYPLKTNWALTQVKTAV
Plor     ---------M-HPTSLMMTSSLITIFILLLYPVLT-----TLNPNPQKEGWALSQVKTAV
Sgun     ---------M-HPTSLMMTSSLIVIFTLLVLPAIS-----TLTPRPQASDWALTQVKTAV
Zaco     ---------M-HPTSLTMTSSLIIIFTLLAYPLLT-----TLNPRPREDTWALTQVKTAV
Zbfl     ---------M-HPTSLMMASSLLIIFTLLIYPVLT-----TLNPSPKEADWALTHVKTAV
Spba     ---------M-HPTPLIMTSSLIMIFILLAYPLIT-----TLHPQPSAPNWALSHVKTSV
Game     ---------M-HPTSLMMTTSLIIIFSLLAYPVFT-----TLSPRPRAHDWALTQVKTAV
Thth     ---------M-HPTSLMMTTSLIIIFSLLAYPVFT-----TLSPRPQAPNWALTQVKTAV
Xigl     ---------M-HPTSLMMTSSLIIIFALLAYPLLT-----TLSPQPREPHWALSHIKTAV
Hyja     ---------M-HPSSFMMTTSLITIFLLLVSPVLT-----TLSPSPRAADWALTQVKTAV
Psan     ----------MYLPSLVMSTSLAVILLVLLYPVFA-----TLFPKPENTDSVSMQPKNAV
Cupa     ---------M-HPTSLMMTTSLIIIFSLLVYPVLT-----TLSPQPQTPDWALTQVKTAV
Mpch     -----------MFYSLIFSSSLILILTTLSYPVFL-----TMIPTWNSNIPLSHHVKTAV
Char     ---------M-HPTSIMMTTCFLIIFATLAFPVIS-----ALYFKPHKPEWALSHVKTAV
Pser     ---------M-HPALLMMTSSLIIIFVLLTFPLIS-----TLSPNPKTPDWALTHVKTAV
Prol     ---------M-SPNMLIMSSSLAIVFTLLTFPLAT-----SLLPSPRKLQWANSHVTMAV
Plbi     ---------M-HPTSLMISSSLVTIFALLAYPLVT-----TIRPTPRGPQWATSYVKTAV
Calu     ---------M-LNNMYIATSALLLIFTLLLYPVAT-----TLVPTRNTPTEGPSQIKTAV
Papa     ----------MHSTSTMMTTTLIMIFTILVTPILM-----TLLPHTKKPKWTAVEMKTAI
Sufr     ---------M-HPSALMMTSSLIIIFIFLLYPLLT-----TLTPNPQHPDWALAKVKTAV
Stci     ---------M-PPCALMMTSCLMTIFALLLYPVMT-----SIHPSPLQQNWALTHPKTSV
Taru     ---------M-HSTPLIMTSTLIIIFALLIYPVLT-----TFSPKPQNPNWALIQVKTAV
Rala     ---------M-HPTSLMMTSSLLVIFTLLVYPILT-----TLSPHPKQGDWALTHVKTAV
```

To be continued on page 10.

[2/12 of aligned sequences]

```
              A                                                   B
Scca  KISFFISLIPLFIFLDQGLESIVTNWNWMNMGPFNINMSFKFDLYSIMFTPVALYVTWSI
Muma  KISFFISLIPLFIFLDQGLESIVTNWNWMNMGPFDINMSFKFDLYSIIFTPVALYVTWSI
Erca  KMSFFTSLIPSIIYLNTNMQSCVIYYRWMLVSPIEINISLQFDQYSMIFMSIALYVTWSI
Pose  KLSFLVSLIPSLMCLNLNLQSFTIYYRWLLIPPTEINISFQFDQYSMIFMTIALYVTWSI
Actr  QTAFYASLLPLAVFFDQGMEVITTNWHWMNIATFDINISFKFDQYSIIFTPVALYVTWSI
Scal  QTAFYVSLLPLAMFFDQGMEVITTNWHWMNIATFDINISFKFDQYSIIFTPVALYVTWSI
Posp  QTAFYISLLPLAMFFDQGMETITTNWHWMNIATFNINVSFKFDQYSIIFTPVALYVTWSI
Atsp  QTAFFASLFQLFLFLTQGLQTTTTSWYWMKISTFNITISFKFDFYSIIFIPVALYVSWSI
Leoc  QTAFFASLFQLSLFLTQGLHTTTTSWHWMKISTFNISLSFKFDFYSIIFIPVALYVSWSI
Amca  QMAFFTSLIPLFIFLDQGMEVITTNWHWMNIMSFDINTSFKFDQYSIIFTPVALYVTWSI
Osbi  QWAFFISLIPMVIFLDQGIETITTNFHWMNTMTFNINTSFKFDFYSIIFVPVALYVTWSI
Pabu  HMAFLISLLPMFIFMDQGMETITTNWHWMNTTTFDINLSLKFDSYSIMFTPIALYVTWSI
Hial  QMAFFVSLIPLFIFMDQGVEVITTNWQWMNTLTFDINTSFKFDQYSIIFTPIALYVTWSI
Elha  QMAFFISLLPLFIFLDQGMETITTNWQWMNTATFDINTSFKFDQYSIIFTPVALYVTWSI
Mlcy  QAAFYISLLPLFIFTDQGMETITTSWQWMNTMTFDINISFKFDQYSIIFMPVALYVTWSI
Algl  TTALFVSLLPLFLLMDQGLEATALNWQWMNTMIFNINSSFKFDYYSVLFTSIALFVTWSI
Ptgi  QMAFFVSLLPLFIFLDQGMEAITTNWQWMNTMTFDINTSFKFDQYSIIFTPIALYVTWSI
Alaf  QLAFFVSLLPLFIFLDQGMETITTNWQWMNTMTFDINTSFKFDQYSIIFTPIALYVTWSI
Nock  QMSFFVSLLPLFIFLDQGMETITTNWQWMNTLTFDINTSFKFDQYSIIFTPIALYVTWSI
Anja  QTAFFVSLIPLFLFLDQGMETVLTNWQWANTMTFDLNTSFKFDHYSIIFTPVALYVTWSI
Gyki  QVAFFISLAPLCVFLDQGLETITTNWSWINTMTFDLNMSFKFDQFSIIFTPIALYVTWSI
Syka  QMAFFVSLIPLFIFLDQGMEAITTNWQWMNTMTFDVNTSFKFDQYSIIFTPIALYVTWSI
Opma  QMAFFVSLIPLCIFLDQGMEVISTNWQWTNTMTFDLNTSFKFDQYSLIFTPVALYVTWSI
Comy  QMAFFISLIPLCIFLDQGVEVISTNWQWTNTMTFDLNISFKFDQYSIIFTPIALYVTWSI
Sasp  KTAFFVSLLPLILFLDQGMEATMTNWQWSNTMAYNLVMSFKFDVYAIIFTPVALYVTWSI
Eupe  RTAFLISLIPLLIFLDQGMETVVTNWQWSNTMTFDLNMSFKFDHYSIVFTPIALYVTWSI
Enja  STAFAVSLLPLFIFLDQGLEAVVTTWHWMNTSTFSISVSLKLDFYSIIFTPIALFVTWSI
Same  SAAFVVSLLPLFIFLDQGVETIVTTWHWMNTSTFNISVSLKFDAYSIIFTPIALYVTWSI
Chch  STAFMISLLPLLIFLDQGTESIVTNWHWMNTTTFDVNISFKFDHYSLIFTPVALYVTWSI
Grgr  STAFMVSLLPLFVFLDQGAEVIVTNWHWMNTTTFDINISFKFDHYSLIFTPIALYVTWSI
Caau  SSAFFISLLPLMIFLNLKTEGIITNWQWMNTQTFDVNISFKFDHYSLIFVPIALYVTWSI
Cyca  SSAFFVSLLPLMIFLNLKTEGIITNWQWMNTQTFDVNISFKFDHYSLIFVPIALYVTWSI
Dare  HVSFLISLIPLTMMLYKESDHVVMCWSWMNTQAFNVDLSFKFDYYSVTFTSIALFITWSI
Cost  STAFLVSLLPLMIFLDQGAETIVTNWHWMNTAPFDINVSFKFDNYSIIFTPIALYVTWSI
Leec  STAFFVSLLPLMLFLDQGAEIIITNWQWMNTTLFDVNVSFKFDHYSLIFTPIALYVTWSI
Fola  SSAFLISLLPLAVFLDQGTETIVTNWHWMNTTMFDINISFKFDYYSLVFTPIALYVTWSI
Clmc  STAFFISLIPLMIFLDQGTESIVTNWHWMNITNFDINISFKFDHYSLIFTPIALFVTWSI
Phin  KAAFYSSLIPLMIFLDQGTESIVTNWHWANIMTFDINTSFKLDHYSLIFTSVALFVTWSI
Icpu  STAFFINTIPLIIFLDQGTESIITTWNWMNIMNFDINISFKFDHYSLIFTPIALYVTWSI
Psto  STAFFINIIPLIIFLDQGTETIITTWNWMNISSFDINVSFKFDHYSLIFTPVALYVTWSI
Cora  STAFFISLIPLAIFLDQGTENIISSWHWMNIATFDINISFKFDHYSLIFTPIALFVTWSI
Eisp  STAFFVSLLPLMIFLDQGTETIVTNWHWMNTMNFDINISFKFDHYSLIFTPIALFVTWSI
Apal  STAFYISLIPLMIFLDQGTETITTNWHWMNITNFDINISFKFDHYSIIFTPIALFVTWSI
Eslu  KMAFLVSLLPLFIFLNEGAETIISTWHWMNTLVFDINLSFKFDHYSIIFTPIALYVTWSI
Dape  KLAFLVSLFPLFIFLNEGAEIIITNWHWMNTLIFDINISFKFDHYSIIFTPVALYVTWAI
Glse  KSAFIVSLLPLFIFLNEGAETIITNWQWMNTNTFDINLSFKFDHYSIIFTPIALYVTWSI
Naar  KTAFLVSLLPLFIFLNHGTETIVTNWQWMNTSTFDISLSFKFDHYSIIFTPIALYVTWSI
Lioc  KAAFLVSLLPLFIFLNHGTETIVTNWQWMNTGTFDISLSFKFDHYSIIFTPIALYVTWSI
Opso  KMAFFTSLLPLFIFLDQGAETIITSWHWMNINTFDMNLSFKFDHYSLVFVPVALYVTWSI
Alte  STAFAVSLFPLFIFLDQGVETIVTNWHWMNTATFDINISLKFDHYSIIFTPIALYVTWSI
Plap  STAFAVSLLPLFIFLDQGVETIVTNWHWMNTATFDVNISLKFDHYSIIFTPIALYVTWSI
```

To be continued on page 11.

```
Plal  KAAFLVSLLPLFIFLNSGAETIVTAWQWMNILSFDINISFKFDHYSIIFTPVALYVTWSI
Sami  KAAFLVSLLPLFIFLNSGAETIVTAWQWMNTLSFDINISFKFDHYSIIFTPVALYVTWSI
Rere  KTAFLVSLLPLSIFLDSGAETVVTAWQWMNTLSFDINLSFKFDHYSVIFTPIALYVTWSI
Gama  KISFFVSLVPLFLFLDEGAETIVTNWRWMNILAFDVNLSFKFDHYSLIFTPIALYVTWSI
Onmy  KMAFLVSLLPLFVFLDQGTETIVTNWQWMNTTTFDINLSFKFDHYSIIFTPIALYVTWSI
Sasa  KMAFLVSLLPLFIFLDQGTETIVTNWQWMNTTTFDINLSFKFDHYSIIFTPIALYVTWSI
Cola  KMAFLVSLLPLFIFLDQGTETIVTNWQWMNTSTFDVNLSFKFDHYSIIFTPIALYVTWSI
Dita  KAAFFVSLIALFLFLDEGVETIATAWQWMNTSTFDVNLSFKFDYYSAVFVPVALYVTWSI
Gogr  KASFFFSLLPLFILLDSGTETILTSWQWMDMMTFDTNLSFKFDHFSSTFIPVALFVTWSI
Chsl  KMAFFTSLLPLLIFLDEGTQTIMTNWQWMNVYTFDINLSFKFDCYSTVFVPVALYVTWSI
Atja  KMAFFISLVSLFLFLNEGTETVTTTWLWMNNLTFDINMSFKFDHYSIIFTPIALYVTWSI
Iido  KMAFFISLISLFLFLNEGTETVTTTWAWMNNMTFDINMSFKFDHYSVIFTPIALYVTWSI
Auja  KMAFFVSLLPLCLFLNEGSETIITNWSWMNTLSFDINMSLKFDHYSIIFIPIALYVTWSI
Chag  KMAFFISLLPLFIFLNEGAETIVTNWSWMNTLTFDINLSFKFDHYSIIFTPIALYVTWSI
Hami  KTAFLVSLFPLFIYLNEGSEAIVTNWNWMNIHTFDINLSFKFDHYSVIFTPIALYVTWAI
Saun  KTAFLVSLFPLFIFLNEGSETIITSWNWMNTLTFDINLSLKFDHYSIVFTPIALYVTWSI
Nema  KMAFFVSLIPLFMFLNEGTETIITNWAWMNTLSFDINLSFKFDHYSIIFTPIALYVTWSI
Disp  KLAFFVSLAPLFVFLNEGTETIVTSWTWMNTLTFDINLSFKFDHYSIIFTPIALYVTWSI
Myaf  KLAFFVSLIPLFIFLNEGTETIVTNWTWMNTLAFDINLSFKFDFYSIIFTPIALYVTWSI
Lagu  KLAFLTSLLPLTLFLNEGVEASTASWTWMNTNSFNILVSLKYDYYSVIFTPIALYVTWSI
Trtr  KMAFFVSLAPLFIFLDTGTESINSTWTWVSTASFDFSVSFKFDFYSVMFIPIALYVTWSI
Zucr  KLAFFVSLVPLILFLDSGLQSLTSSWTWMNTSSFDISISLKFDFYSIIFTPVALYVTWSI
Pxja  KTAFLVSLIPLCLFLNEGVETIVTNWTWMNTNTFDINLSFKFDHYSIIFIPIALYVTWSI
Pxlo  KTAFLVSLIPLCLFLNEGVETIVTNWTWMNTNTFDINLSFKFDHYSIIFIPIALYVTWSI
Pctr  KTAFIISLAPLCLFLNDGLEAVVTSWSWINTNTFDINLSFKFDNYTLIFLPIALYVTWSI
Apsa  KTAFLTSLLPLALYTNEGLETIVTNWTWMNTHSFNINTSFKFDCYSLTFIPIALYVTWSI
Cabe  FMSFLISLLPLTLLLFQGADNVTTSWSWLSISSFTLSLSFKFDFYSATFIPVALFVSWSI
Bzze  KMAFLVSLFPLFLFLNEGAETIITSWTWMNTITFDINISFKFDSYSIVFIPVALYVTWSI
Siim  KTSFFISLLPLFLFFSEGTETVTTTWAWMNTNTFDISISLKFDIYSIAFIPIALYVTWSI
Ctru  KMAFLVSLLPLFLFLNEGAETIITSWTWMNTNTFDINISLKFDHYSIIFIPVALYVTWSI
Dpbr  KTAFFISLLPLFLFLNEGAETIITSWSWMNTNTFDINISLKFDHYSIIFIPVALYVTWSI
Caki  KTAFMISLVPLFTFINLGMETVTSTWTWMAFATLDISVSFKFDHYSVIFIPIALYVTWSI
Phja  KYAFLISLPATLLFYIDNQQTTFATLDWEMTSSLSTNFSFYFDRFSIPFVSAALFVSWAI
Brsp  KTAFFISLIPLLLFLDSGMQSVTTTWTWMLTTSLDISLSFKFDEYSAIFIPIALYVTWSI
Gamo  KSAFLISLAPLSLFLSTGMEAVTSSWTWMVTTTLDITLSFKFDHYSIIFIPIALYVTWSI
Lolo  KTAFLISLAPLSLFLSSGMETVTSSWTWMVTTTLDITLSFKFDYYSIIFIPIALYVTWSI
Batr  KAAFLISLLPLCIFIDQGIETTTSSLVWMDSLTHSTPISFKLDLLSLLFCPVALFVSWAI
Prmy  KLAFLTSLLPLCLFINEGLETTTACFTWMNFLTLPVFISFKFDLYSIMFMPVALYVTWAI
Lose  KLAFFTSLLPLFLFLKNGEEVIMVTWDWVTTHAFIINISFKFDMYSIIFTPVALYVTWSI
Loam  KLAFFTSLLPLFLFINEGAEAIITSWTWTNTHTFDINISLKFDIYSIIFTPVALYVTWSI
Chab  KLAFFVSLLPMLAFTNEGTETIITTWSWMNTLAFDINISLKFDIYSIIFTPVALYVTWSI
Chto  KLAFFVSLLPMLAFTNEGTETIITTWSWMNTLAFDINISLKFDIYSIIFTPVALYVTWSI
Majo  KLAFFISLLPLFLFLNEGAETTITVWDWVKIFTLDINVTLKFDSYSIIFMSVALYVTWSI
Hlst  MLAFFTSLFPLFIFLNEGAETTITIWHWANIFTLDMNITLKFDAYSIVFIPVALYVTWSI
Clpe  KLAFFVSLLPLFLFLNGGTETIITTCGWLNTLTFDVNITLKFDIYSVIFTPVALYVTWSI
Mlmr  KLAFLVSLLPLLLFVSEGTETIVTTWAWMNTLTFDINISLKFDIYSTIFTPVALYVTWSI
Crcr  MLAFFSSLPPLFMYLYTGAETVSSEWFWYHIQSLNGYISFKFDFYAIVFVPIALYVTWSI
Muce  MLAFFSSLPPLFMYLYTGAETVSSEWFWYHIQSLNGYISFKFDFYAIVFVPIALYVTWSI
Bege  KLAFFISLLPLFLFLNQGAETIVTTWAWTNTLMFDINISLKFDFYSIIFTPIALYVTWSI
Mela  KLAFFVSLIPLFLFINEGAEAITTTWSWANTLTFDINISLSLDLYAIIFVPIALYVTWSI
Hats  KMAFFVSLLPLFLFLNEGAETIITTWAWTNTLTFDINISLKFDFYSLIFIPIALYVTWSI
Orla  KVAFFVSLLPLFIFLNEGVEAIMTNWEWMNTLMFNINISFKFDLYSIVFTPVALYVTWSI
```

To be continued
on page 12.

[2/12 of aligned sequences]

```
Cosa    KMAFFVSLIPLSLFLNEGTEIISTNWSWMNTLLFDINMSLKFDFYSIIFTPIALYVTWSI
Exsp    KMAFFVSLLPLFLFLNEGAETVTTNLVWMNTLLFDVNISLKFDLYSTVFLPIALYVTWSI
Depa    KVTFFISLIPLFLFFNNGTEIIIISWTWTNTLMFDINISFKFDFYSIIFIPIALYVTWAI
Rima    KVAFFLSLIPLALSFDQGTVTTPTESAWITKLTLDLNFNLNLDFYALMFSSVAFYVTWSI
Fuol    KLSFLFSLLPLFLFLDQGTETVVSSWNWMNTQAFNIIISFKFDLYSCVFVPVALYVTWSI
Gmaf    KLAFFISLFPLFLFFDQGAETIITAWSWMNTLTFDINISFKFDTYSCIFIPVALYVTWSI
Xeei    KLAFFVSLLPLFLFLDQGTEMITTAWSWTNTLTFDINISLKFDFYSCIFTPVALYVTWSI
Pros    KMAFLVSLIPLFLFLNEGAEVIITNWNWMNTATFDINISFKFDHYSIIFIPIALYVTWSI
Scmi    KLAFLVSLIPLFLFLNEGAEMILTHWNWMNTTTFDINISLKFDHYSTIFIPIALYVTWSI
Rolo    KMAFFVSLAPLFLFLNEGAETIITSWSWMNTTTFDINISLKFDHYSIIFIPIALYVTWSI
Cere    KMAFFVSLIPLTLFMNEGVETVTTNWNWINTNTFDINISFKFDYYSVIFIPIALYVTWSI
Daga    KTAFFVSLIPLSLFLNEGVEVISTSWNWINTTAFDINISLKFDYYSVVFIPIALYVTWAI
Anco    KTAFFISLAPLFLYLDEGAETIITNWNWMNTNTFDINLSFKFDHYSIIFIPIALYVTWSI
Dmve    KTAFLISLAPLFIYINEGAETIITNWSWMNTNTFDINLSFKFDHYSIIFLPIALYVTWSI
Dmar    KTAFLISLAPLFIYLNEGAETIITNWSWMNTNTFDINLSFKFDHYSIIFLPIALYVTWSI
Anka    KTAFFISLAPLFLYMNEGAETIITNWNWMNTNTFDINLSFKFDHYSIIFIPIALYVTWSI
Moja    KTAFFVSLAPLFLYLNEGAETIITNLTWMNTNTFDINISFKFDHYSIIFIPIALYVTWSI
Hoja    KAAFFVSLAPLFLYLNEGAETIITNWNWMNTHTFDINLSFKFDHYSIIFIPIALYVTWSI
Bede    KMAFLVSLIPLFLFLNEGAEVIITNWSWMNTFTFDINISLKFDHYSIIFIPIALYVTWSI
Besp    KMAFLVSLIPLFLFLNEGAEVIITNWNWMNTATFDVNISLKFDHYSIIFIPIALYVTWSI
Mysp    KAAFFVSLIPLFLFLNEGAETIVTNWNWMNTNTFDINLSFKFDFYSIIFIPIALYVTWSI
Osja    KTAFFVSLIPLFLFLNEGAETIITNWNWMNTATFDINLSFKFDYYSIIFIPIALYVTWSI
Sgro    KTAFFVSLIPLFLFLNEGAETIITNWTWMNTATFDINISFKFDHYSIIFIPIALYVTWSI
Pzpa    KTAFFISLPPLFLFLSIGTEATISTWTWMSTNTFDINLSFKFDHYTVIFTPVALYVTWAI
Zeja    KTAFFVSLIPLLLFLNEGTETIITNWSWMNTNTFNITMSFKFDHYSVIFIPIALYVTWSI
Znne    KTAFFVSLVPLFLFLNEGTETIVTNWSWINTNTFDINMSFKFDHYSIIFTPIALYVTWAI
Zefa    KTAFFVSLAPLFLFLNEGTETIITNWSWMNINTFDINMSFKFDHYSIIFIPIALYVTWAI
Acni    KTAFFVSLAPLFLFLNEGAETIITNWAWMNTNTFDINMSFKFDHYSIVFIPIALYVTWAI
Ncrh    KTAFFVSLAPLFLFLNEGTETIITNWAWMNTNTFDINMSFKFDHYSIVFIPIALYVTWAI
Agca    KLAFFVSLLPLCLFLNEGAEAIITNWTWMNTHTFDINISLKFDHYSIIFTPIALYVTWSI
Hydy    KLSFFVSLMPLFTFFNEGAETILTSWVWMNTTCFEINLSFKIDQYSVVFTTVALYVTWSI
Gsac    KLSFFISLLPLFMFFNEGAETIVSSWTWMNTTCFEINLSFKFDQYSVIFTTVALYVTWSI
Pevo    MVAFAISLLPLALFLNEGIETIASTWTWMNTLMFNINISLKFDLYSIVFVPIALYVTWSI
Hiku    KMCFMISLLPLMLFINEGLETIITTWSWMNTMTFDINISFKFDLYSVIFTPVALYVTWSI
Inpa    KLAFFTSLLPLFMFLHDGLDTLATSWSWMNTLTHNFNISFKFDYYSVLFVPLALYVTWSI
Auch    KSAFMVSLLPLFLFLAQGTETVMTSWTWTNASMFNITISFKFDLYSIIFLPVALYVTWAI
Fico    KMAFFVSLLPLFLFLNEGAETIVTTWSWMNTHTFDVNISLKFDHYSVIFTPIALYVTWSI
Macs    KMAFLVSLLPLSLFLSEGAEAIVTTWSWMNTLTFDVNISLKFDHYSIIFTPIALYVTWSI
Moal    KMAFFTSLLPLFLHLHTGTELTITNLHWMNIMTFDINTSFKLDHLSITFVPIALYVTWAI
Syma    KLAFFVSLLPLSLYLCNGTETTITNWHWTNTLTFDINISFKFDHYSIIFTPIALYVTWSI
Mafr    KLAFLVSLLPLFLLLSQGTESIITNWTWMNTHTFDINLSFKFDFYSTMFVPIALYVTWAI
Dcpe    MMAFWLSLASLFMFYAHGTQTILTSSTWFHTETFNITMNMKFDTLSITFIPVALYVTWSI
Dcti    TVAFWFSLASLFLFYSYGTQTILTSSTWFHTETFNITINMKFDTLSIIFIPIALYVTWSI
Hehi    ALAFFVSLIPLFLFLNEGAEAIITSWNWMNTLTFDVNISFKFDHYSVIFVPIALYVTWSI
Stam    KLAFFVSLLPLFLFFNEGAEAIITSWTWMNTLTFDINLSFKFDYYSVIFTPIALYVTWSI
Hogi    KLAFLISLLPLFLFLNEGAETIITSWTWMNTLAFDVTISLKFDSYSIIFTPVALYVTWSI
Erzo    KLAFFVSLFPLFLFFNEGAETIVTSWTWMNTYCFDVNISFKFDYYSIIFTPIALYVTWSI
Hxot    KLAFFVSLLPLFLFFNEGAEAIVTSWTWMNTTCFDISISFKFDHYSIIFTPIALYVTWSI
Core    KLAFFVSLLPLFLFFNEGAEAVVTSWTWMNTTCFDINISFKFDHYSIIFTPIALYVTWSI
Apve    KLSFFVSLLPLFLFFNEGTETIITSWTWMNTTCFDINLSFKFDHYSIIFTPIALYVTWSI
Latj    KLAFFVSLLPLFLFLNEGLETIITNWNWMNTATFDINISLKFDHYSVIFTPIALYVTWSI
Laja    KLSFFVSLLPLFLFLNEGAEMVITNWNWMNTMTFDVNISLKFDHYSIIFTPIALYVTWSI
```

To be continued on page 13.

To be continued on page 14.

Syja KLAFFVSLLPLCLFFNEGLETIITTWNWTNTTTFDVNVSFKFDHYSIIFTPVALYVTWSI
Epme KLAFFVSLLPLFLFLNEGAETIVSSSNWMNTATFDVNLSLKFDLYSIIFTPIALYVTWSI
Grse KLAFLISLLPLLLHLNHGSETIISTWSLLSNSAFDVTLSFKFDRYAIIFTPVALYVTYSI
Clja KLALLISLIPLLTFLNEGAETIVTTLTWMNTSSFDVSISFKFDHYSIIFIPIALYVTWSI
Ogcy MLAFFFSLFPLFIFLSKGAETSSHSLHLMTILTLDIKIGIQVDAYSLIFTPVALFVTWSI
Plna KLAFFVSLLPLFLFLHEGAEAITTSWTWMSISTFDINISFKFDLYSTIFTPIALYVTWSI
Lema KLAFFTSLLPLSLFLNEGAETIITSWTWMNTLTFDVSISLKFDSYSIIFTPVALYVTWSI
Etzo KLAFLVSLLPLFLFINEGAETIVTSWSWLNTLAFDINISFKFDHYSIIFTPIALYVTWSI
Apse KMAFFVSLFPLFLFLSEGAEAIITSWSWMNTTTFDVNISLKFDHYSIIFTPVALYVTWSI
Epde KLAFFISLLPLSLFLNEGAEMIITSWNWMNTTMFDVNISFKFDHYSIIFTPIALYVTWSI
Slja KWAFLVSLLPLALFLNEGAETIITNWTWMNTLAFDINISFKFDHYSIIFTPIALYVTWSI
Bsja KTAFFVSLLPLFLFLNEGAETIITNWNWANTQTFDINISLKFDHYSIIFTPVALYVTWSI
Ecna KTAFIVSLLPLCLYLTQGTETIITTWNWMNTQSFDVNISLNFDCYSIIFTPIALYVTWSI
Cohi KLSFFSSLAALLIFLAQGMEMVITSWTWIETNPFTVFLSFKFDNYSLTFLPVALFVTWSI
Caar KLAFFVSLLPLFIYLNQGLETIITNWNWMNTLTFDINISLKFDHYSIIFTPIALYVTWSI
Came KLAFFVSLLPLFIYLNQGLETIITNWNWMNTLTFDINISLKFDHYSIIFTPIALYVTWSI
Mema KLAFLLSLIALFLFLNEGLVVIVTSWTWFNTLIFDVNISLKFDYYSMIFVPIALYVTWSI
Lenu KLAFFTSLLPLFIMLNQGTETIISTWSWTNTMTFDINISFKFDHYSVIFMPIALYVTWSI
Brja KLAFFVSLLPLCLFMNEGAETVITTWNWMNTYTFDINISLKFDHYSIIFTPIALYVTWSI
Plma KLAFLVSLLPLFLFMNEGAETIITSWNWMNTLTFDINISLKFDHYSIIFTPIALYVTWSI
Emst KLAFFVSLLPLFLFLNEGAETIITNWNWMNTMTFDVNISFKFDHYSIIFTPIALYVTWSI
Ptti KLAFFVSLLPLFLFLNEGAETIITNWNWMNTLTFDVNISFKFDHYSIIFTPIALYVTWSI
Losu KLAFFISLLPLFLFLNEGLELIVMSWTWTNTLTFDVNLGFKFDFYAIIFTPIALYVTWSI
Geoy KLAFFISLLPLALFLNEGAETIITTWEWMNTLTFNISVSFKFDFYSIIFTPIALYVTWSI
Dipi KFAFFVSLLPLFLFLNEGAETIITNWTWMNTLTFDINISFKFDHYSIIFTPVALYVTWSI
Pama KLAFFVSLLPLFLFLNEGAEAIITNWTWMNTLTFDINISFKFDHYSIIFTPIALYVTWSI
Leob KLAFFVSLLPLCLFLNEGAETIVTNWTWMNTLTFDINISFKFDHYSIIFTPIALYVTWSI
Neba KYAFLVSLFPLFLFLNEGTETIISNWTWMNTTAFDIKISLKFDFYSIIFTPVALYVTWSI
Pdpl LVAFLTSLVPLLIFLNDGTEVVIADWLWFNVLTFHAKLSFKFDFYSVFFTPIALYVTWSI
Nimi KTAFFVSLLPLFLFLNEGTEAVVTGLTCLETPFFNINISLKFDHYSLIFIPIALFVTWSI
Uptr KFAFLISLLPLFLFFNEGAEAVITNWTWMNTLTFDINISLKFDHYSIIFVPIALYVTWSI
Pesc QLAFFLSLFPLFFHLFTGVEPFLTNYTWMTPLAFNISISFKFDYYAIVFTPVALYVSWAI
Baar KLAFFISLLPLSLFLNEGAEAIITNWNWMNTTTFDVNISFKFDHYSIIFTPVALYVTWSI
Moar KLAFFISLLPLFLFLNEGAETIITNWNWMNTNTFDVNISLKFDHYSIIFTPVALYVTWSI
Toja KLAFFISLLPLFLFLNEGLETIITNWNWMNTITFDINISLKFDHYSIIFVPIALYVTWSI
Chau KLAFFISLLPMFLFFNEGAEAIITNWNWMNTLTFDINISFKFDHYAIIFTPVALYVTWSI
Chse KLAFFVSLLPLSLFLDQGAEMVITTWKWMNTLTFDISISFKFDFYSLIFTPIALYVTWSI
Enar KLAFLVSLIPLFLFLNEGAETIVTNWNWMNTVTFDINISFKFDHYSIIFTPIALYVTWSI
Hpty KLAFFVSLLPLFLFLNEGAEMIITNWNWMNTTTFDVNISFKFDHYSIIFTPIALYVTWSI
Nana KLAFLTSILPLCFFLNEGAEAIITSWSWTNTLTFDINISFKFDSYSIIFISVALYVTWSI
Mcst KLAFFISLLPLFLFLNEGTETIITNWNWMNTLTFDVNISFKFDHYSIIFTPIALYVTWSI
Rhox KLAFFVSLLPLCLFLNEGAETIVTSWSWMNTGTFDINISLKFDHYSIIFTPIALYVTWSI
Opfa KLAFFVSLLPLFLFLNEGAEMIITSWNWMNTLTFDVNISFKFDHYSIIFTPIALYVTWSI
Paar KLSFFVSLLPLLIFFNEGAETIITNWNWMNTLTFDINISLKFDHYSIIFCPIALYVTWSI
Gozo KLAFFVSLLPLFLFLNEGAEMIITNWSWMNTQSFDVNISFKFDHYSIIFTPIALYVTWSI
Ackr KIAFFSSLLPLFIFLHTGMQAVLSDLTWTSLMTFDLRTTLMIDNYTIVFVPILLYVSWSI
Elev KLAFFISLIPLFLFLNEGAEVVVTAWTWTNTLAFDINISFKFDYYSTLFTPIALYVTWAI
Trdu KWAFFTSILPLCLFLNEGAETIITNWTWMNTHTFDINISLKFDIYSIIFTPVALYVTWSI
Amoc KLAFFTSLLPLFLFLNQGTETIMTDWKWMGTDTFNLCISLKFDFFSTIFTPIALFVTWSI
Hame KLSFFISLMPLAYLLFAKPESTVNSWTLIKTESFSMTFSCNFDLYALVFIPIALYVTWAI
Chso KTAFFVSLLPLFIFLNEGTEMIITNWNWMNTSTFDINLSFKFDHYSIIFVPIALYVTWSI
Lyto KLAFFVSLLPLFLFFNEGTETIITSWSWMNTTCFDVNISLKFDHYSIIFTPIALYVTWSI

[2/12 of aligned sequences]

```
Encr  KLAFFISLLPLFLFFNEGAETIVTSWSWMNTTCFDVNISLKFDHYSIIFTPVALYVTWSI
Bvar  KLAFFTSLLPLFLFLNEGAEAVITTWNWMSTNTFDVSLSFKFDHYSLIFTPVALYVTWAI
Noco  KGAFFVSLIPLILFINFGKQQVISTWTWTSTLTFDMSVSFKFDHYSLIFTPVALYVTWSI
Chsp  KLAFFLSLLPLALFINEGAQVIITNWIWMTTETFNISISFKFDFYSIIFTPIALYVTWAI
Arja  KLSFFISLLPLFLFFNEGAETIVTSWTWMNTLCFDISLSFKFDHYSIIFTPIALYVTWSI
Pase  LLAFLASLAPLAFYLFAGVEVVVYSREWMNLGSFDLNISLMFDLYSLVFLPVALYVTWSI
Trel  KWAFLGSLFPLFFFISDGLETVTSSWQWLSNTPFNINISLKFDLYSVIFIPVALYVSWSI
Lifa  RTTFFCSLIPLVLFMYSGWQSTTSSYCWFQTNSFMLSLNFKFDLYTVIFLPILFFVTWSI
Acur  MLAFFISLIPLLMYMNYGTESVATSCIWMDMSPFDINISFKFDQYSIIFTSVALYVTWSI
Ampe  KLAFFVSLLPLCLFLNEGAETIITNWNWMNTGTFDVNISFKFDHYSLIFTPIALYVTWSI
Urja  MLALLMSLPPLLILLSEGLDTVICTSNWINATTLNINVSLKYDLYSAIFTPIALYVTWSI
Enet  KTTFFISLLPLCIFLSQGTESIITSWTWMTTETFNVVVSLKFDIYTIVFIPTALFVTWSI
Ptbr  KLAFFISLIPLSLFLNEGAQIMTTTWSWFPLLNFPISVSFNFDIYSVVFIPIALYVTWSI
Safa  MISFFVSLVPLVLFMNEGAEAIITNFSWINIHSFNISLSFNFDLYSTVFVPIALYVTWSI
Icae  KLAFFVSLLPLFLFMNEGVEAVITSWSWMNTHTFDVNISLKFDHYSITFTPIALYVTWSI
Asmi  KHAFLISLIPLLFFLSTGFQTVVSSGTWFNCQSFHFTASLNLDTYSIIFTPLALLVTWSI
Foal  KLAFFVSLIPLALFLNTGLEALTYTWSWTKFSYLPLSVSFKFDLYTIIFTPIALYVTWSI
Drze  KLSFLVSLLPLFLFFTENAEVIITNWTWMITLTFNINISFKFDFYSIIFTPVALYVTWSI
Rhas  KLAFFVSLLPLFLFLSEGAETILTTWSWMNTYTFDINISLKFDFYSVIFTPIALYVTWSI
Elac  KLAFFTSLLPLFLFLSEGAETVITNWNWTNTHTFDINISLKFDHYSLIFTPVALYVTWSI
Kugu  KLAFLTSLFPLFIFLSEGVETIITNWNWMNTTAFNVNISFKFDHYSIIFIPIALYVTWSI
Plor  KLAFFISLLPLSLFLNEGTETIITNWSWMNTTTFDVNISFKFDHYSIIFTPVALYVTWSI
Sgun  KLAFLVSLLPLSLFLNEGAEAIITNWTWMNTLTFDVNISFKFDHYSIIFTPIALYVTWSI
Zaco  KLAFFISLLPLFLFLNEGAEAVITNWTWMNTLSFDVNISFKFDHYSIIFTPIALYVTWSI
Zbfl  KLAFFISLLPLFLFLNEGAEAIVTSWNWMNTLTFDVNISFKFDHYSIIFTPIALYVTWSI
Spba  KLAFFVSLLPLFMYLDEGTETIITTWTWMNTQTFDVNISLKFDHYSITFTPIALYVTWSI
Game  KLAFFVSLLPLFLFMNEGAETIITSWSWMNTHTFDINISLKFDHYSIIFTPIALYVTWSI
Thth  KLAFFVSLLPLFLFMNEGAEAIITNWTWMNTLTFDINISLKFDHYSIIFTPIALYVTWSI
Xigl  KLAFFVSLLPLFLFLNEGLETIVTNWNWMNTLTFDINISLKFDHYSIIFTPIALYVTWSI
Hyja  KLAFFVSLLPLFLFMNEGTEAIITNWSWMNTLTFDVNLSLKFDHYSIIFVPIALYVTWSI
Psan  KLAFLVSLLPLFLFMNEGAETVITSWNWMNTHTFDVNISLKFDHYSIIFIPIALYVTWSI
Cupa  KLAFLVSLLPLFLFMNEGAETIITSWNWMNTHTFDVNISLKFDHYSIIFTPVALYVTWSI
Mpch  KTAFFISLLPLSLHLSLGVVTILTTWSWMNIHTFDINISFKFDLYSLIFIPIALYVTWSI
Char  KLAFLVSLLPLFLFLTEGAETITTAWNWANIFTFDLNISLKFDHYSIIFTPIALYVTWSI
Pser  KLAFFISLLPLCLFLNEGLETITTNWTWMNTHTFDINISLKFDHYSIIFTPIALYVTWSI
Prol  KMAFFVSLLPLALYLHEGSETIITIWTWMNTNSFSINIGLKLDFYSIIFVPIALYVTWSI
Plbi  KMAFFVSLLPLALFLNEGAETIVTNWTWMNTNTFDVNISLKFDFYSIIFTPIALYVTWSI
Calu  KLAFFVSLIPLFLFLNHGTEQIVFMHPWMGMASFDINISFKFDFYSLIFVPIALYVSWSI
Papa  KLAFLTSLVPLFLFLSEGLELITTNWSWMNINTFDINISLKFDAYSVVFTPIALYVTWSI
Sufr  KLAFLVSLLPLALFLNEGTQIMTTTWSWMNTLMFDINISLYFDHYSIIFTPVALYVTWSI
Stci  KMAFFVSLIPLTLFLNQGAEIITSSWSWMSTQTFDIHISFYFDSYSIIFTPVALFVTWSI
Taru  KYAFLVSLLPLCLHLNEGTESIITNLNWMNTLTFDINISLKFDSYSIIFTPVALYVTWSI
Rala  KLAFFVSILPLFLFLNEGAETIITNWTWMNTHTFDVNISFKFDHYSIIFTPVALYVTWSI
         :  .                                   *  :  *    : ::::*
```

To be continued on page 15.

[3/12 of aligned sequences]

C D

```
Scca  LEFALWYMHLDPNINRFFKYLLLFLISMIILVTANNMFQLFIGWEGVGIMSFLLIGWWYS
Muma  LEFALWYMHSDPNMNRFFKYLLLFLISMIILVTANNMFQLFIGWEGVGIMSFLLIGWWYS
Erca  LEFAMYYMHTDILINRFFKYLLTFLISMMILVTANNMFQLFIGWEGVGIMSFLLIGWWYG
Pose  LEFAIYYMQTDILINRFFKYLLTFLIAMLILVTANNMFQLFIGWEGVGIMSFLLIGWWHG
Actr  LEFASWYMHSDPNMNRFFKYLLLFLIAMITLVTSNNMFQLFIGWEGVGIMSFLLIGWWYG
Scal  LEFASWYMHSDPNMNRFFKYLLLFLIAMITLVTANNMFQLFIGWEGVGIMSFLLIGWWYG
Posp  LEFASWYMHSDPNMNRFFKYLLLFLIAMITLVTANNMFQLFIGWEGVGIMSFLLIGWWYG
Atsp  LEFALWYMHADPRMDQFFKYLLIFLITMIILVTANNLYQLFIGWEGVSIMSFLLISWWYG
Leoc  LEFALWYMHADPRMDQFFKYLLVFLITMIILVTANNLYQLFIGWEGVSIMSFLLISWWYG
Amca  LEFASWYMHSDPNMNRFFKYLLMFLIAMIVLVTANNMFQLFIGWEGVGIMSFLLIGWWYG
Osbi  MEFASWYMHSDPNMNQFFKYLLLFLIAMLILVTANNMFQLFIGWEGVGIMSFLLIGWWYG
Pabu  LEFASWYMHSDPHMNKFFKYLLLFLIAMLTLVTANNLFQLFIGWEGVGIMSFLLIGWWYG
Hial  LEFASWYMHADPNMNRFFKYLLMFLVAMIILVTANNMFQLFIGWEGVGIMSFLLIGWWYG
Elha  LEFASWYMHADPNMNRFFKYLLLFLVAMIILVTANNMFQLFIGWEGVGIMSFLLIGWWYG
Mlcy  LEFASWYMHTDPNMNRFFKYLLMFLVAMMILVTANNMFQIFIGWEGVGIMSFLLIGWWYA
Algl  LEFATWYMHLDPNMNRFFKYLMIFLISMIILVTANNMFQLIIGWEGVGIMSFLLIGWWHA
Ptgi  LEFASWYMHADPNMNRFFKYLLMFLIAMVILVTANNMFQLFIGWEGVGIMSFLLIGWWYG
Alaf  LEFATWYMHADPNINRFFKYLLMFLIAMIILVTANNMFQLFIGWEGVGIMSFLLIGWWYG
Nock  LEFASWYMHADPNINRFFKYLMMFLIAMIILVTANNMFQLFIGWEGVGIMSFLLIGWWYG
Anja  LEFASWYMHADPNMNRFFKYLLMFLVAMIILVTANNLFQLFIGWEGVGIMSFLLIGWWYG
Gyki  LEFASWYMSTDPYMNRFFKYLLMFLIAMVTLVTANNMFQLFIGWEGVGIMSFLLIGWWYG
Syka  LEFATWYMHADPNMNRFFKYLLTFLVAMIILVTANNMFQLFIGWEGVGIMSFLLIGWWYG
Opma  LEFALWYMHADPDINRFFKYLLMFLVAMIILVTANNMFQLFIGWEGVGIMSFLLIGWWYG
Comy  LEFALWYMHADPNMNRFFKNLLMFLVAMILLVTANNMFQLFIGWEGVGIMSFLLIGWWHG
Sasp  IGFTMWYMEADPDISRFLKYLLIFLTTMIILVTANNLLQLFIGWEGVGIMSFLLIGWWYG
Eupe  MEFTSWYMQTDPNLNRFFKYLLIFLIAMIILVTANNLFQLFIGWEGVGIMSFLLIGWWYG
Enja  LEFAAWYMHEDPNMNRFFKYLLTFLIAMVILVTANNMFQLFIGWEGVGIMSFLLIGWWYG
Same  LEFASWYMHADPFMNRFFKYLLMFLIAMIILVTANNMFQLFIGWEGVGIMSFLLIGWWYG
Chch  LEFASWYMHADPNMNRFFKYLLLFLVAMIILVTANNMFQLFIGWEGVGIMSFLLIGWWHG
Grgr  LEFASWYMHADPLMNRFFKYLLLFLVAMIVLVTANNMFQLFIGWEGVGIMSFLLIGWWHG
Caau  LEFALWYMHSDPYIDRFFKYLLTFLVAMIILVTANNMFQLFIGWEGVGIMSFLLIGWWHG
Cyca  LEFALWYMHSDPNIDRFFKYLLTFLVAMIILVTANNMFQLFIGWEGVGIMSFLLIGWWHG
Dare  LEFASWYMASYPQKELFYKYLLLFLMSMIILVTANNLFQLFIGWEGVGIMSFLLIGWWFG
Cost  LEFASWYMHSDPLMNRFFKYLLLFLVAMILLVTANNMFQLFIGWEGVGIMSFLLIGWWYG
Leec  LEFASWYMHSDPYMNRFFKYLLLFLVAMIILVTANNMFQLFIGWEGVGIMSFLLIGWWYG
Fola  LEFASWYMHADPYVNRFFKYLLTFLIAMITLVTANNMFQLFIGWEGVGIMSFLLIGWWYG
Clmc  LEFASWYMHSDPYMNRFFKYLLLFLVAMIILVSANNMFQLFIGWEGVGIMSFLLIGWWYG
Phin  LEFATWYMSSDPHMNRFFKYLLLFLMAMMILVSANNMFQLFIGWEGVGIMSFLLIGWWYG
Icpu  LEFASWYMHSDPYLNRFFKYLLLFLVAMVILVTANNMFQLFIGWEGVGIMSFLLIGWWHG
Psto  LEFASWYMHSDPYLNRFFKYLLLFLVAMITLVTANNMFQLFIGWEGVGIMSFLLIGWWHG
Cora  LDFASWYMSADPYMNRFFKYLLLFLVAMVILVTANNMFQLFIGWEGVGIMSFLLIGWWYG
Eisp  LEFATWYMHADPNMNRFFKYLLLFLVAMIILVTANNMFQLFIGWEGVGIMSFLLIGWWYG
Apal  LEFASWYMHADPYMNRFFKYLLLFLVAMIILVTANNMFQLFIGWEGVGIMSFLLIGWWYG
Eslu  LEFAFWYMHADPNINRFFKYLLLFLIAMITLVTANNMFQLFIGWEGVGIMSFLLIGWWYG
Dape  LEFAFWYMHTDPNINRFFKYLLLFLIAMIMLVTANNMFQLFIGWEGVGIMSFLLIGWWYG
Glse  LEFASWYMHADPNMNRFFKFLLLFLVAMIVLVTANNLFQFFIGWEGVGIMSFLLIGWWYG
Naar  LEFASWYMHADPNMNRFFKFLLLFLVTMIVLVTANNMFQLFIGWEGVGIMSFLLIGWWHG
Lioc  LEFASWYMHADPNMNRFFKFLLLFLVAMIILVTANNMFQFFIGWEGVGIMSFLLIGWWYG
Opso  LEFASWYMHTDPNTDQFFKYLLLFLVAMIVLVTANNMFQLFIGWEGVGIMSFLLIGWWHG
Alte  LEFASWYMHADPNINRFFKYLLLFLVAMIILVTANNMFQLFIGWEGVGIMSFLLIGWWYG
Plap  LEFASWYMHADPNMNQFFKYLLLFLVAMIILVTANNMFQLFIGWEGVGIMSFLLIGWWYG
```

To be continued on page 16.

[3/12 of aligned sequences]

To be continued on page 17.

```
Plal  LEFASWYMHADPNMNRFFKYLLLFLVAMVVLVTANNMFQFFIGWEGVGIMSFLLIGWWYG
Sami  LEFASWYMHADPNMNRFFKYLLLFLVAMVVLVTANNMFQFFIGWEGVGIMSFLLIGWWYG
Rere  LEFASWYMHADPNVNRFFKYLLLFLVAMIVLVTANNMFQLFIGWEGVGIMSFLLIGWWYG
Gama  LEFASWYMHSDPCMNRFFKYLLLFLVAMIVLVTANNMFQLFIGWEGVGIMSFLLIGWWYG
Onmy  LEFASWYMHADPNMNRFFKYLLLFLIAMIILVTANNMFQLFIGWEGVGIMSFLLIGWWHG
Sasa  LEFASWYMHADPNMNRFFKYLLLFLIAMIILVTANNMFQLFIGWEGVGIMSFLLIGWWYG
Cola  LEFASWYMHADPNMNRFFKYLLLFLVAMIVLVTANNMFQLFIGWEGVGIMSFLLIGWWYG
Dita  LEFASWYMHSDPNMNRFFKYLLLFLVAMITLVTANNMFQLFIGWEGVGIMSFLLIGWWGG
Gogr  LEFAHWYMHTDPNMDKFFMYLLLFLMAMITLVTANDMFQLFIGWEGVGIMSFLLISWWSG
Chsl  LEFASWYMHADPNMNRFFKYLLLFLVAMIALVTVNNLFQLFIGWEGVGIMSFLLIGWWYG
Atja  LEFATWYMHNDPNMNRFFKYLLMFLVAMIILVTANNMFQLFIGWEGVGIMSFLLIGWWYG
Iido  LEFATWYMHNDPNMNRFFKYLLMFLVAMIILVTANNMFQLFIGWEGVGIMSFLLIGWWYG
Auja  LEFAAWYMHSDPNMNRFFKYLLLFLVAMIILVSANNLFQLFIGWEGVGIMSFLLIGWWYG
Chag  LEFASWYMHADPNMNRFFKYLLLFLVAMIILVTANNMFQIFIGWEGVGIMSFLLIGWWYG
Hami  LEFASWYMHADPNMNRFFKYLLLFLVAMIILVTANNMFQLFIGWEGVGIMSFLLIGWWHA
Saun  LEFASWYMHADPNMNRFFKYLLLFLVAMIILVTANNMFQLFIGWEGVGIMSFLLIGWWHA
Nema  LEFASWYMHSDPQMNRFFKYLLIFLIAMVILVSANNMFQIFIGWEGVGIMSFLLIGWWHG
Disp  LEFASWYMHADPQMNRFFKFLLIFLIAMVVLVSANNMFQIFIGWEGVGIMSFLLIGWWYG
Myaf  LEFASWYMHADPQMNRFFKFLLIFLVAMVILVSANNMFQFFIGWEGVGIMSFLLIGWWYG
Lagu  LEFALWYMHADPNISRFFKYLLMFLVAMVILVTANNMFQLFIGWEGVGIMSFLLIGWWYG
Trtr  LEFALWYMHSDPNVNRFFKYLLVFLIAMLALVTANNMFQLFIGWEGVGIMSFLLIGWWYG
Zucr  LEFALWYMHSDPNINRFFKYLLIFLIAMITLVTANNMFQLFIGWEGVGIMSFLLIGWWYG
Pxja  LEFASWYMHADPNMNRFFKYLLTFLVAMIILVSANNMFQLFIGWEGVGIMSFLLIGWWYG
Pxlo  LEFASWYMHADPNMNRFFKYLLTFLVAMIILVSANNMFQLFIGWEGVGIMSFLLIGWWYG
Pctr  LEFATWYMHTDPHIDRFFKYLLVFLVAMLILVSANNMFQLFVGWEGVGIMSFLLIGWWHG
Apsa  LEFASWYMHTDPNMNRFFKYLLIFLLAMILLVSANNLFQLFIGWEGVGIMSFLLIGWWYG
Cabe  LQFTTWYMHSDPQVARFTKYLLIFLTSMLLLVSANNMFQLFIGWEGVGIMSFLLIGWWYG
Bzze  LEFASWYMHSDPCMNRFFKYLLVFLIAMVILVSANNMFQLFIGWEGVGIMSFLLIGWWYG
Siim  LEFALYYMHSDPYMNRFFKYLLIFLTSMIVLVSANNMFQLFIGWEGVGIMSFLLIGWWYG
Ctru  LEFAAWYMHTDPYINRFFKYLLTFLIAMITLVSANNMFQLFIGWEGVGIMSFLLIGWWYG
Dpbr  LEFASWYMHTDPYINRFFKYLLIFLIAMITLVSANNMFQLFIGWEGVGIMSFLLIGWWYG
Caki  LEFALWYMQSDPFISRFFKYLLIFLIAMLILISANNLFQFFIGWEGVGIMSFLLIGWWHG
Phja  LEFALWYMHADPLVNRFFKYMLIFLLMMLVFLTANNLFQLFIGWEGMGIMSFLLIGWWHG
Brsp  LEFATWYMHSDPQINRFFKYLLTFLVAMLILVSANNLFQLFIGWEGVGIMSFLLIGWWYG
Gamo  LEFATWYMHSDPFMNRFFKYLLTFLVAMLILVSANNLFQLFIGWEGVGIMSFLLIGWWHG
Lolo  LEFATWYMHADPLINRFFKYLLTFLVAMLILVSANNLFQLFIGWEGVGIMSFLLIGWWHG
Batr  LEYTLWYMHQDPNINTFMKYLLTFLVTMIILVTANNVFQLFIGWEGVGIMSFLLIGWWHG
Prmy  LEFAIWYMASDPFIDTFFKFLLMFLLTMLILVTANNLLQLFVGWEGVGIMSFLLIGWWFG
Lose  LEFASWYMHADLHKDRFFKFLLIFLIAMITLVTANNLFQLFIGWEGVGIMSFLLIGWWHG
Loam  LEFASWYMHADPYMNRFFKYLLIFLIAMIVLVTANNMFQIFIGWEGVGIMSFLLIGWWYG
Chab  LEFASWYMHSDPLMNRFFKYLLIFLITMIILVTANNMFQLFIGWEGVGIMSFLLIGWWYG
Chto  LEFASWYMHSDPLMNRFFKYLLIFLITMIILVTANNMFQLFIGWEGVGIMSFLLIGWWYG
Majo  LEFALWYMNSDQHIDRFFKYLLIFLMAMLTLVTADSMFPLFVGWEGVGIMSFLLIGWWYG
Hlst  LEFASWYMSTDPQMNRFFKYLLIFLIAMITLTTADSMLPLFVGWEGVGIMSFLLIGWWYG
Clpe  MEFASWYMHTDPHINQFFKYLLVFLITMLILVTAGNMFQLFIGWEGVGIMSFLLIGWWFG
Mlmr  MEFASWYMHSDPYMNRFFKYLLVFLIAMIVLVTANNMFQLFIGWEGVGIMSFLLIGWWYG
Crcr  LEFASWYMHTDPLMGRFFKYLLIFLIAMIILVTANNLFQFFIGWEGVGIMSFLLIGWWHG
Muce  LEFASWYMHTDPLMGRFFKYLLIFLIAMIILVTANNLFQFFIGWEGVGIMSFLLIGWWHG
Bege  LEFASWYMHSDPLMNRFFKYLLIFLIAMIILVTANNMFQLFIGWEGVGIMSFLLIGWWYG
Mela  LEFASWYMHADPLVDRFLKYLLIFIIAMIILVTANNLFQLFIGWEGVGIMSFLLIGWWYG
Hats  LEFASWYMHADPFMNRFFKYLLVFLVAMIILVTANNMFQLFIGWEGVGIMSFLLIGWWYG
Orla  LEFASWYMHDDPNMNRFFKYLLIFLIAMIVLVTANNMFQLFIGWEGVGIMSFLLIGWWFG
```

[3/12 of aligned sequences]

To be continued on page 18.

```
Cosa   LEFASWYMHSDPYKDRFFKYLLIFLIAMIILVTANNLFQLFIGWEGVGIMSFLLIGWWYG
Exsp   LEFASWYMHADPNKDRFFKYLLIFLIAMMILVTANNMFQLFIGWEGVGIMSFLLIGWWYG
Depa   LEFASWYMHSDPYKDRFSKYLLVFLIAMIILVTANNMFQLFIGWEGVGIMSFLLISWWYA
Rima   LEFALWYMHTDPLINRFFMHLLAFLIAMVVLVTANNMLQLFVGWEGVGIMSFLLIGWWYG
Fuol   LEFASWYMHSDPNMNRFFKYLLIFLIAMIILVTANNLFQLFIGWEGVGIMSFLLIGWWYG
Gmaf   LEFASWYMHSDPKMNQFFKYLLVFLIAMIILVTANNMFQLFIGWEGVGIMSFLLIGWWYG
Xeei   LEFASWYMHADPNMNRFFKYLLIFLIAMIILVTANNMFQLFIGWEGVGIMSFLLIGWWYG
Pros   LEFASWYMHSDPNMNRFFKYLLIFLVAMIILVSANNMFQLFIGWEGVGIMSFLLIGWWYG
Scmi   LEFALWYMHADPNMNQFFKYLLTFLIAMIILVSANNMFQLFIGWEGVGIMSFLLIGWWSG
Rolo   LEFASWYMHADPNMNQFFKYLLIFLVAMIILVSANNMFQLFIGWEGVGIMSFLLIGWWYG
Cere   LEFASWYMHSDPNVNQFFKYLLVFLMAMLLLVSANNMFQLFIGWEGVGIMSFLLIGWWYG
Daga   LEFALWYMNADPEMNRFFKYLLIFLVAMIVLVSANNMFQLFIGWEGVGIMSFLLIGWWYG
Anco   LEFASWYMHADPYMNRFFKYLLIFLVAMLILVSANNMFQLFIGWEGVGIMSFLLIGWWYG
Dmve   LEFASWYMHADPYMNRFFKYLLFFLVAMLILVTANNMFQLFIGWEGVGIMSFLLIGWWYG
Dmar   LEFASWYMHTDPYMNRFFKYLLFFLVAMIILVTANNMFQLFIGWEGVGIMSFLLIGWWYG
Anka   LEFASWYMHADPYMNRFFKYLLTFLVAMIILVSANNMFQLFIGWEGVGIMSFLLIGWWYG
Moja   LEFASWYMHSDPYMNRFFKYLLTFLVAMIILVSANNMFQLFIGWEGVGIMSFLLIGWWYG
Hoja   LEFASWYMHADPYMNRFFKYLLIFLVAMIILVSANNMFQLFIGWEGVGIMSFLLIGWWYG
Bede   LEFASWYMHADPNMNRFFKYLLTFLVAMIILVSANNMFQLFIGWEGVGIMSFLLIGWWYG
Besp   LEFASWYMHADPNMNQFFKYLLTFLVAMIILVSANNMFQLFIGWEGVGIMSFLLIGWWYG
Mysp   LEFASWYMHADPNMNRFFKYLLTFLVAMIILVSANNMFQLFIGWEGVGIMSFLLIGWWYG
Osja   LEFASWYMHSDPNMNRFFKYLLTFLVAMIILVSANNMFQLFIGWEGVGIMSFLLIGWWYG
Sgro   LEFASWYMHADPNMNQFFKYLLTFLVAMIILVSANNMFQLFIGWEGVGIMSFLLIGWWYG
Pzpa   LEFATWYMHSDPQMNRFFKYLLIFLVAMVTLVSANNMFQLFIGWEGVGILSFLLIGWWYG
Zeja   LEFATWYMHADPYMNRFFKYLLIFLVAMVILVSANNLFQLFIGWEGVGIMSFLLIGWWYG
Znne   LEFATWYMHSDPQMNRFFKYLLIFLVAMVVLVSANNMFQLFIGWEGVGIMSFLLIGWWSG
Zefa   LEFATWYMHTDPQMNRFFKYLLIFLVAMIILVSANNMFQLFIGWEGVGIMSFLLIGWWYG
Acni   LEFALWYMHADPQINRFFKYLLIFLVAMIILVSANNMFQLFIGWEGVGIMSFLLIGWWYG
Ncrh   LEFALWYMHADPQINRFFKYLLIFLVAMIILVSANNMFQLFIGWEGVGIMSFLLIGWWYG
Agca   LEFASWYMHADPYMNRFFKYLLIFLIAMIILVTANNMFQLFIGWEGVGIMSFLLIGWWYG
Hydy   LEFASWYMHSDPNMGRFFKFLLIFLMAMLTLVTANNMFQLFIGWEGVGIMSFLLIGWWGG
Gsac   LEFASWYMHSDPNISRFFKYLLIFLMAMLTLVTANNMFQLFIGWEGVGIMSFLLIGWWGG
Pevo   LEFASWYMHADPQMNQFFKYLLIFLIAMIILVTANNLFQLFIGWEGVGIMSFLLIGWWYG
Hiku   LEFASWYMHSDPVMNRFFKYLLMFLIAMLILVTSNNMFQLFIGWEGVGIMSFLLIGWWYG
Inpa   LEFASWYMHTDPQINRFFKYLLIFLISMLILTTANNMFQLFIGWEGVGIMSFLLIGWWRG
Auch   LEFATWYMHSDPNIERFVKFLLIFLVAMLILVTANNMFQLFIGWEGVGIMSFLLIGWWHG
Fico   LEFASWYMHADPYMNRFFKYLLIFLIAMIVLVTANNMFQLFIGWEGVGIMSFLLIGWWYG
Macs   LEFASWYMHADPFMNRFFKYLLIFLIAMIVLVTANNMFQLFIGWEGVGIMSFLLIGWWYG
Moal   LEFASWYMKSDPNINQFFKFLLIFLIAMVTLVTANNMFQLFIGWEGVGIMSFLLIGWWWA
Syma   LEFASWYMHTDPNINQFFKYLLIFLIAMIILVTANNLFQLFIGWEGVGIMSFLLIGWWWS
Mafr   LEFASWYMSSDPNINKFFKYLLIFLIAMIILVTSNNMFQLFIGWEGVGIMSFLLIGWWFS
Dcpe   LEFATWYMHDDPEVTRFFSHLLLFLALMIFFVLADNMFQLFVGWEGIGIMSFLLIGWWCG
Dcti   LEFAAWYMHDDPEITQFFSHLLLFLALMISFVLADNMFQLFVGWEGVGIMSFLLISWWCG
Hehi   LEFASWYMHADPYMNRFFKYLLIFLIAMIILVTANNLFQLFIGWEGVGIMSFLLIGWWYG
Stam   LEFATWYMHADPYMNRFFKYLLIFLIAMIILVTANNLFQLFIGWEGVGIMSFLLIGWWYG
Hogi   LEFASWYMHSDPFINRFFKYLLIFLIAMIILVTANNLFQLFIGWEGVGIMSFLLIGWWYG
Erzo   LEFAAWYMHADPNMNRFFKYLLIFLIAMIVLVTANNLFQLFIGWEGVGIMSFLLIGWWYG
Hxot   LEFASWYMHADPNMNRFFKYLLIFLIAMIILVTANNMFQLFIGWEGVGIMSFLLIGWWYG
Core   LEFASWYMHADPYMNRFFKYLLIFLIAMIILVTANNMFQLFIGWEGVGIMSFLLIGWWYC
Apve   LEFASWYMHTDPNINRFFKYLLIFLISMIILITANNMFQLFIGWEGVGIMSFLLIGWWHG
Latj   LEFASWYMHADPDMNRFFKYLLIFLITMIILVTANNMFQLFIGWEGVGIMSFLLIGWWHG
Laja   LEFAAWYMHADPYMNRFFKYLLVFLIAMIILVTANNMFQIFIGWEGVGIMSFLLIGWWYG
```

[3/12 of aligned sequences]

```
Syja LEFASWYMHADPYMNRFFKYLLVFLIAMIILVTANNMFQIFIGWEGVGIMSFLLIGWWYG
Epme LEFASWYMHADPNMNRFFKYLLVFLIAMIILVTANNLFQLFIGWEGVGIMSFLLIGWWHG
Grse LEFASWYMASDPDMNQFFKYLLIFLVAMIILVTANNLFQLFIGWEGVGIMSFLLIGWWFA
Clja LEFASWYMHTDPFMNRFFKYLLTFLIAMILLVTANNMFQLFIGWEGVGIMSFLLIGWWYG
Ogcy LEFSLSYMSQEPQKNRFFKYMLSFLICMIILVTANNMFQFFIGWEGVGILSFMLIGWWHG
Plna LEFAYWYMHADPNIDRFFKYLLIFLITMIILVTANNMLQLFIGWEGVGIMSFLLIGWWYG
Lema LEFAAWYMHADPYMNRFFKYLLIFLIAMIMLVTANNMFQFFVGWEGVGIMSFLLIGWWYG
Etzo LEFASWYMHADPFVNRFFKYLLIFLIAMIVLVTANNLFQLFIGWEGVGIMSFLLIGWWYG
Apse LEFASWYMHSDPNMNRFFKYLLIFLIAMIVLVTANNMFQLFIGWEGVGIMSFLLIGWWYA
Epde LEFASWYMHADPYMNRFFKYLLVFLIAMVILVTANNMFQIFIGWEGVGIMSFLLIGWWYG
Slja LEFASWYMHADPYMNRFFKYLLIFLIAMVVLVTANNMFQLFIGWEGVGIMSFLLIGWWYG
Bsja LEFASWYMHSDPYMNRFFKYLLTFLIAMIILVTANNMFQFFIGWEGVGIMSFLLIGWWYG
Ecna LEFASWYMHADPYMNRFFKYLLIFLIAMIILVTANNMFQLFIGWEGVGIMSFLLIGWWYG
Cohi LEFASWYMHADPLKDTFFKYLLTFLLAMIILTTANNMFQLFIGWEGVGIMSFLLISWWHA
Caar LEFASWYMHSDPYMNRFFKYLLIFLIAMIILVTANNMFQIFIGWEGVGIMSFLLIGWWYG
Came LEFASWYMHSDPYMNRFFKYLLIFLIAMIILVTANNMFQIFIGWEGVGIMSFLLIGWWYG
Mema LEFASWYMHADPYMNRFFKYLLVFLIAMLILVTANNMFQLFIGWEGVGIMSFLLIGWWYG
Lenu LEFAMWYMSTDPFMNRFSKYLLTFLITMIILVTANNMFQLFIGWEGVGIMSFLLIGWWFG
Brja LEFASWYMHADPQMNRFFKYLLIFLIAMIILVTANNMFQLFIGWEGVGIMSFLLIGWWYG
Plma LEFASWYMHADPYMNRFFKYLLVFLIAMIILVTANNMFQLFIGWEGVGIMSFLLIGWWYG
Emst LEFASWYMHADPFMNRFFKYLLVFLIAMIILVTANNMFQIFIGWEGVGIMSFLLIGWWYG
Ptti LEFASWYMHADPFMNRFFKYLLVFLIAMIILVTANNMFQIFIGWEGVGIMSFLLIGWWYG
Losu LEFASWYMHTDPNVGRFFKYLLIFLIAMITLVTASNMFQLFIGWEGVGIMSFLLIGWWHG
Geoy LEFASWYMHADPFMNRFFKYLLVFLIAMLVLVTANNMFQLFIGWEGVGIMSFLLIGWWYG
Dipi LEFASWYMHADPYMNRFFKYLLIFLIAMIILVTANNMFQIFIGWEGVGIMSFLLIGWWYG
Pama LEFASWYMHADPYMNRFFKYLLIFLIAMITLVTANNMFQLFIGWEGVGIMSFLLIGWWYG
Leob LEFASWYMHADPFMNRFFKYLLIFLIAMIILVTANNMFQIFIGWEGVGIMSFLLIGWWYG
Neba LEFASWYMHSDPFMNRFFKYLLIFLITMIILVTANNMFQLFIGWEGVGIMSFLLIGWWYG
Pdpl LDFASWYMHKDPYMNRFFKYLLIFLITMVVLVTANNLLQLFIGWEGVGIMSFLLIGWWYS
Nimi LEFTFWYMYSSPLLDLFYDYLLTFLIAMIILVTAGNLFQLFIGWEGVGIMSFLLIGWWHG
Uptr LEFASWYMHSDPYMNRFFKYLLVFLIAMIILVTANNMFQIFIGWEGVGIMSFLLIGWWYG
Pesc LEFASWYMHQDPLKHRFFKFLLVFLIAMIVLVTANNMFQLFIGWEGVGIMSFLLIGWWHG
Baar LEFASWYMHADPYMNRFFKYLLIFLITMIILVTANNMFQFFIGWEGVGIMSFLLIGWWYG
Moar LEFASWYMHADPFMNRFFKYLLIFLIAMIILVTANNMFQLFIGWEGVGIMSFLLIGWWYG
Toja LEFASWYMHADPYMNRFFKYLLIFLIAMIVLVTANNMFQLFIGWEGVGIMSFLLIGWWYG
Chau LEFAAWYMHADPQMDRFFKYLLTFLIAMIILVTANNMFQLFIGWEGVGIMSFLLIGWWYG
Chse LEFASWYMHSDPFMNRFFKYLLIFLIAMIILVTANNMFQLFIGWEGVGIMSFLLIGWWFG
Enar LEFASWYMHADPFMNRFFKYLLVFLIAMIVLVTANNMFQLFIGWEGVGIMSFLLIGWWYG
Hpty LEFASWYMHADPYMNRFFKYLLVFLVAMIILVTANNMFQLFIGWEGVGIMSFLLIGWWYG
Nana LEFASWYMHADPNVNRFFKYLLMFLIAMIILVSANNMFQLFIGWEGVGMMSFLLIGWWYG
Mcst LEFAAWYMHADPCMGRFFKYLLIFLIAMIILVTANNMFQLFIGWEGVGIMSFLLIGWWYG
Rhox LEFASWYMHSDPYMNRFFKYLLIFLIAMIVLVTANNMFQLFIGWEGVGIMSFLLIGWWYG
Opfa LEFASWYMHADPYMNRFFKYLLVFLIAMIILVTANNMFQLFIGWEGVGIMSFLLIGWWYG
Paar LEFASWYMHSDPNMNRFFKYLLIFLIAMIILVTANNMFQLFIGWEGVGIMSFLLIGWWHG
Gozo LEFASWYMHADPYMNRFFKYLLVFLIAMVILVTANNMFQIFIGWEGVGIMSFLLIGWWYG
Ackr LEFAEWYMHNDPDMNRFFKYLLIFLIMMMILVTANNLFQLFIGWEGVGIMSFMLIGWWAG
Elev LEFASWYMHADPYMNRFFKYLLIFLVAMITLVSANNMFQFFIGWEGVGIMSFLLIGWWYG
Trdu LEFASWYMHTDPNMNRFFKYLLIFLIAMIILVTANNMFQLFIGWEGVGIMSFLLIGWWYG
Amoc LEFASWYMHTDPNMNRFFKYLLIFLIAMIILVTANNMFQLFIGWEGVGIMSFLLIGWWYG
Hame LEFAIWYMSDDMNIGRFFLYLLVFLMAMMILVTANNLYQLFIGWEGVGMMSFLLIGWWHA
Chso LEFASWYMHSDPYMNRFFKYLLIFLIAMLVLVTANNMFQLFIGWEGVGIMSFLLIGWWYG
Lyto LEFASWYMHADPNMGRFFKYLLVFLIAMVILVTANNMFQLFIGWEGVGIMSFLLIGWWYG
```

To be continued on page 19.

[3/12 of aligned sequences]

```
Encr  LEFASWYMHADPNMNRFFKYLLVFLIAMVVLVTANNMFQLFIGWEGVGIMSFLLIGWWYG
Bvar  LEFASWYMHSDPYMTRFFKYLLIFLIAMIILVTANNLFQLFIGWEGVGIMSFLLIGWWFG
Noco  LEFATWYMQDDPDMNRFFKYLLIFLIAMIVLVTANNLFQLFIGWEGVGIMSFLLIGWWSG
Chsp  LEFATWYMHSDPKMNQFFKYLLLFLIAMITLVTANNMMQIFIGWEGVGIMSFLLIGWWYG
Arja  LEFASWYMHADPNMNRFFKYLLIFLISMITLVTANNMFQLFIGWEGVGIMSFLLIGWWHG
Pase  LSFATWYMASDPEASRFFKYLLIFLISMLLLVTANNLFQLFIGWEGVGIMSFLLIGWWHG
Trel  LEFARWYMHQDPLVNRFFKYLLIFLIAMIVLVTANNMFQLFIGWEGVGIMSFLLIGWWWG
Lifa  IQFATSYMNEDMYQGRFFKYLYIFVIAMVILVTADNWFQLLIGWEGVGVMSFKLIGWWYG
Acur  LEFATWYMHADPLVGRFFKYLLIFLIAMLVLVTANNLFQIFIGWEGVGIMSFLLIGWWHA
Ampe  LEFASWYMHADPQMNRFFKYLLVFLIAMIILVTANNLFQIFIGWEGVGIMSFLLIGWWYG
Urja  IQFASWYMNEDPQVDRFFKYLLIFLIAMIVLVTANNMFQLFIGWEGVGIMSFLLIGWWHG
Enet  LEFALWYMHADPYSPRFFKYLLVFLIAMLLLVSANNMFQIFIGWEGVGIMSFLLISWWGA
Ptbr  LQFAYWYMHSDPNICRFFKYLLMFLIAMLILVSANNMFQLFIGWEGVGIMSFLLIGWWYG
Safa  LEFASWYMHSDPNISRFFKYLLIFLTAMLVLVTANNMFQLFIGWEGVGIMSFLLIGWWYG
Icae  LEFASWYMHADPFMNRFFKYLLVFLIAMIILVTANNMFQLFVGWEGVGIMSFLLIGWWYG
Asmi  LTFALWYMSDEPNMALFFKYMLMFLMAMLVLVTANNIFQLFIGWEGVGIMSFLLIGWWRG
Foal  LEFTSWYMASDPLIDRFAKFLLMFLVAMLMLITANNVFQLFIGWEGVGIMSFVLIGWWGG
Drze  LEFASWYMHTDPYMNRFFKYLLIFLIAMIILVTANNMFQLFIGWEGVGIMSFLLIGWWYG
Rhas  LEFASWYMHADPYMNRFFKYLLTFLIAMIILVTANNMFQLFIGWEGVGIMSFLLIGWWYG
Elac  LEFASWYMHSDPYMNRFFKYLLTFLIAMIVLVTSNNMFQLFIGWEGVGIMSFLLIGWWYG
Kugu  LEFASWYMHSDPHMNRFFKYLLIFLIAMIVLVTANNMFQLFIGWEGVGIMSFMLIGWWYG
Plor  LEFASWYMHADPFMNRFFKYLLIFLVAMITLVTANNMFQLFIGWEGVGIMSFLLIGWWYG
Sgun  LEFASWYMHADPQMNRFFKYLLVFLIAMIILVTANNMFQLFIGWEGVGIMSFLLIGWWYG
Zaco  LEFASWYMHADPYMNRFFKYLLTFLIAMVILVTANNMFQLFIGWEGVGIMSFLLIGWWYG
Zbfl  LEFASWYMHADPFMNRFFKYLLIFLIAMIILVTANNMFQLFIGWEGVGIMSFLLIGWWYG
Spba  LEFASWYMHADPFMNRFFKYLLTFLIAMIILVTANNMFQLFIGWEGVGIMSFLLIGWWYG
Game  LEFASWYMHADPFMNRFFKYLLIFLIAMIVLVTANNMFQLFIGWEGVGIMSFLLIGWWYG
Thth  LEFASWYMHADPFMNRFFKYLLVFLIAMIILVTANNMFQLFIGWEGVGIMSFLLIGWWYG
Xigl  LEFASWYMHSDPYMNRFFKYLLIFLIAMIILVTANNMFQLFIGWEGVGIMSFLLIGWWYG
Hyja  LEFAAWYMHADPFMNRFFKYLLIFLIAMIILVTANNMFQLFIGWEGVGIMSFLLIGWWYG
Psan  MEYAAWYMHSDPFVNRFFKYLLIFLIAMIILVTANNMFQFFIGWEGVGIMSFLLIGWWYG
Cupa  LEFASWYMHADPFMNRFFKYLLVFLIAMIILVTANNMFQIFIGWEGVGIMSFLLIGWWYG
Mpch  LEFAVWYMHSDPLINRFFKYLLIFLISMVILVTANNMFQIFIGWEGVGIMSFLLIGWWYG
Char  LEFASWYMHSDPYMNRFFKYLLIFLIAMIILVTANNMFQLFIGWEGVGIMSFLLIGWWYG
Pser  LEFASWYMHADPNMNRFFKYLLTFLIAMIILVTANNMFQLFIGWEGVGIMSFLLIGWWYG
Prol  LEFASWYMHADPQMNRFFKYLLTFLIAMIILVTANNMFQLFIGWEGVGIMSFLLIGWWYG
Plbi  LEFASWYMHADPHMNRFFKYLLTFLIAMIILVTANNMFQLFIGWEGVGIMSFLLIGWWYG
Calu  LEFASWYMSSDPLIGRFFKYLLIFLIAMLVLVTANNMFQLFIGWEGVGILSFLLIGWWHA
Papa  LEFASWYMNKDPHIDRFFKYLLIFLITMIILVTANNMFQLFIGWEGVGIMSFLLIGWWHA
Sufr  LEFASWYMHSDPNMNRFFKYLLTFLIAMIILVTANNMFQLFIGWEGVGIMSFLLIGWWYS
Stci  LEFAAWYMHADPNMNRFFKYLLVFLIAMIILVTANNMFQLFIGWEGVGIMSFLLIGWWYS
Taru  LEFASWYMHSDPFMNRFFKYLLVFLIAMIILVTANNMFQLFIGWEGVGIMSFLLIGWWYA
Rala  LEFASWYMHADPYMNRFFKYLLTFLVAMIILVTANNMFQLFIGWEGVGIMSFLLIGWWYG
      : ::  **       *  :  *:  *: :    .   :::****:.::** **.**
```

To be continued on page 20.

[4/12 of aligned sequences]

To be continued on page 21.

```
                                 E
Scca  RTDANTAALQAVIYNRIGDIGLILSMAWLAMNLNSWEIQQIFILSK--DKDLTLPLLGLV
Muma  RADANTAALQAVIYNRIGDVGLILSMAWLATNLNSWEIHQLFILSK--NKDLTLPLLGLV
Erca  RTDANMAAMQAVIYNRVGDIGLMLAMAWLLINMNSWDIQQLFIMTK--NMDTTIPAMGLL
Pose  RADANMAALQAVIYNRVGDIGLMLSMSWLLINTNSWDIQQLFILTK--NMDMTLPAAGLL
Actr  RADANTAALQAVIYNRVGDIGLILSMAWFAMNMNTWEIQQMFAFSQ--DNQATLPLMGLI
Scal  RTDANTAALQAVIYNRVGDIGLILSMAWFAMNMNTWEIQQMFASSQ--DNQTTLPLMGLI
Posp  RADANTAALQAVIYNRVGDIGLILSMAWFAMNMNTWEIQQMFASSQ--DNPATLPLMGLI
Atsp  RADANTAALQAVIYNRVGDIGLVLSMTWFVMNLNTWEIEQTFSSIH--GVPATLPLMGLI
Leoc  RADANTAALQAVIYNRVGDIGLILSMTWFVMNLNTWEIEQTFSFLN--DTPTTLPLLGLI
Amca  RTDANTAALQAVIYNRVGDIGLILSMAWFAMNLNTWEIQQMFISSH--DTQMTLPLMGLI
Osbi  RADANTAALQAVIYNRVGDIGLILAMAWLAMNVNSWEMQQMFITTK--EIDLTLPLLGLV
Pabu  RADANTAALQAVIYNRVGDIGLIMAMAWTATNFNSWEMQQMFILSN--NTDLTLPLMGLI
Hial  RADANTAALQAVIYNRVGDIGLILSMAWLAMNLNSWEIQQIFASSK--SMDLTLPLMGLI
Elha  RADANTAALQAVIYNRVGDIGLILSMAWFAMNLNSWEIQQMFITSK--NMDLTLPLMGLI
Mlcy  RADANTAALQAVIYNRVGDIGLIMSMAWLATSLNSWEMQQMFISSQ--DMDLTLPAMGLI
Algl  RADANVSALQAILYNRTGDIGLILAMAWIATKLNSWEFSQVFSASK--GMDLTLPLLALI
Ptgi  RADANTAALQAVIYNRVGDIGLILSMAWIAMNTNSWEIQQVFAASK--EMDLTLPLVGLI
Alaf  RADANTAALQAVVYNRVGDIGLILSMAWLAMNTNSWEIQQLFAVSK--EMDLTMPLMGLI
Nock  RADANTAALQAVVYNRVGDIGLILSMAWLAMNTNSWEIQQLFAVSK--EMDLTIPLMGLI
Anja  RADANTAALQAVIYNRVGDIGLILAMAWMAMNLNSWEIQQVFIISK--EMDLTLPLMGLV
Gyki  RADANTAALQAVLYNRVGDIGLIMSMAWLAMNMNSWELQQIFAMAQ--DMDLTAPLMGLI
Syka  RAAANTAALQAVIYNRVGDIGLIFSMAWIAMNLNSWEIQQVFIASK--EMDLTLPLIGLI
Opma  RADANTAALQAVIYNRVGDIGFILSMAWLAMNLNTWEIQQMFITSK--EMDLTLPLIGLI
Comy  RADANTAALQAVIYNRVGDIGLVLSMAWIAMNLNSWEIQQVFITSK--EMNLTLPLAGLI
Sasp  RADANTAALQAVIYNRIGDIGLILTMAWMAMNFNTWELQQLFILSK--DVDLTLPLMGLV
Eupe  RADANTAALQAVIYNRVGDIGLILTMAWMAMNFNAWEIQQIIVLSK--EMDLTLPLMGLV
Enja  RADANTAALQAVIYNRVGDIGFILTMAWFATKLNSWEIQQIFSLSK--DFNTTLPALGLV
Same  RADANTAALQAVIYNRVGDIGLIMTMAWFAMNLNSWEMQQIFALSH--NMDMTLPLLGLI
Chch  RADANTAALQAVIYNRVGDVGLIMSMAWFAMNLNSWEMQQIFSSSK--DMNLTLPLLGLV
Grgr  RADANTAALQAVIYNRVGDVGLIMSMAWFATNLNSWEMQQMFSSSQ--AFDLTLPLMGLI
Caau  RADANTAALQAVIYNRVGDIGLIMTMAWFAMNLNSWEIQQIFVLSK--NFDMTIPLMGLA
Cyca  RADANTAALQAVIYNRVGDIGLIMTMAWLAMNLNSWEIQQIFALSK--NFDMTIPLMGLA
Dare  RTEANTASLQAVIYNRMGDIGFILTLAWMAMYLNSWDIQQIFILSK--DFDMTIPQIGLI
Cost  RADANTAALQAVLYNRVGDIGLIMTMAWLAMNFNSWEIQQIFFLSK--NFDMTLPLLGLI
Leec  RADANTAALQAVLYNRVGDIGLIMSMAWIAMNLNSWEIQQIFYLSK--THDMTLPLIGLI
Fola  RADANTAALQAWIYNRVGDIGLIMSMAWLAMNLNSWEIQQIFFLSK--DFDMTLPLMGLI
Clmc  RADANTAALQAVLYNRVGDIGLILSMAWIAMNMNSWEIQQIFMLSK--DYDMTLPLLGLI
Phin  RADANTAALQAVLYNRVGDIGLILSMAWIATNLNSWEIQQILLTSK--DMNMTLPLLGLI
Icpu  RADANTAALQAVIYNRVGDVGLILTIAWIAMNLNSWEIPQIFLLSK--DFDMTLPLMGLI
Psto  RADANTAAMQAVIYNRVGDVGLILAMAWIAMNFNSWEIPQIFLLSK--DFDMTLPLMGII
Cora  RADANTAAMQAVLYNRVGDIGLILSIAWIAMNMNSWEISQIFLVSK--YFDLTLPLLGLI
Eisp  RAEANTAALQAVIYNRVGDIGLMLAMAWIATNLNSWEIQQIFFSAK--DFDMTLPLLGLI
Apal  RADANTAALQAVIYNRVGDIGLMLSMAWIVSNLNSWEMQQIFFSAK--DMDMTLPLIGLI
Eslu  RADANTAALQAVIYNRVGDIGLILSMAWFMMNTNSWEIHQMFINSQ--NLDLTLPLMGLI
Dape  RTEANTAALQAVIYNRVGDVGLILSMAWFATNLNSWEIHQMFANPQ--NLDFTLPLLGLI
Glse  RADANTAALQAVLYNRVGDIGLILSMAWFATNLGSWEIQQMFFISK--NLDLTILFLGLI
Naar  RADANTAALQAVLYNRVGDIGLILSMAWFATNLGSWEIQQMFSTSK--DFDLTLPLLGLI
Lioc  RADANTAALQAVLYNRVGDIGLILAMAWLATNLGSWEIQQMFASSK--DFDLTLPLLGLI
Opso  RADANTAALQAVVYNRVGDVGLILSMAWFATNLGSWEIQQMFFSSK--NLDLTLPLSGLI
Alte  RADANTAALQAVLYNRVGDIGLIMAMAWFAMNLNSWEIQQMFFASK--EFNLTLPLIGLV
Plap  RADANTAALQAVLYNRVGDIGLIMAMAWFAMNLNSWEIQQMFFVSK--EFDLTLPLVGLI
```

[4/12 of aligned sequences]

```
Plal  RADANTAALQAVIYNRVGDIGLILSMAWFAMKLNSWEMQQMFSSAQ--GFDLTLPLLGLI
Sami  RADANTAALQAVIYNRVGDIGLILSMAWFAMQLNSWEMQQMFASSQ--GLDLTLPLMGLI
Rere  RADANTAALQAVVYNRVGDIGLILSMAWFAVHLNSWEMQQMFTSAK--AFDLTLPLLGLV
Gama  RADANTAALQAVLYNRVGDIGLILSMAWLATNYNSWELQQIFSTTR--GDDLTLPLLGLI
Onmy  RADANTAAMQAVIYNRVGDIGLILSMAWFATNLNSWEIQQMFASSK--GLDLTLPLMGLI
Sasa  RADANTAAMQAVIYNRVGDIGLILSMAWFATNLNSWEIQQMFASSK--ELDLTLPLMGLI
Cola  RADANTAAMQAVVYNRVGDIGLILSMAWFATNLNSWEIQQMFASSK--DLDLTLPLMGLI
Dita  RADANTAALQAVLYNRVGDIGLILSMAWFATNLNSWEMQQIFASSQ--DLDLTLPLLGLI
Gogr  RTAANTAALQAILYNRVGDIGLILAMVWLATNFNSWEIQQMLPLSK--NHDLTLPLLGLI
Chsl  RADANTAALQAVLYNRVGDIGLILSMAWLAMNFNSWEIQQIFASAK--ELDLTLPLLGLI
Atja  RADANTAALQAVIYNRVGDIGLILSMAWMATNLNSWEMQQMFASSQ--KMDLTLPLMGLI
Iido  RADANTAALQAVVYNRVGDIGLIISMAWMATNLNSWEMQQMFASSQ--NLDLTLPLMGLI
Auja  RADANTAALQAVIYNRVGDIGLILSMAWLATNLNSWEMQQLFISSK--SHDLTLPLMGLI
Chag  RADANTAALQAVIYNRVGDIGLILSMAWLAANLNTWEMQQLFAASK--QQDLTLPLMGLI
Hami  RADANTAALQAVIYNRVGDIGLILSMAWLATNLNSWEMQQMFAASK--NHDLTLPLMGLI
Saun  RADANTAALQAVIYNRVGDIGLILSMAWLATNLNSWEMQQMFASSK--DHDLTLPLMGLI
Nema  RADANTAALQAVMYNRVGDIGLILAMAWLATNLNSWEIQQMFASSK--DLDLTLPLMGLI
Disp  RADANTAALQAVIYNRVGDIGLILAMAWLAMNLNSWETQQMFAASK--DMNLTLPLLGLI
Myaf  RADANTAALQAVIYNRVGDIGLILAMAWLATNLNSWEIQHMFSASK--NMDLTLPLMGLI
Lagu  RADANTAALQAVLYNRVGDIGLILAMAWIAAHLNSWELSQVFFISQ--DFNLTLPLLGLI
Trtr  RADANTAALQAVLYNRVGDVGLILSMAWLAINLNSWELQQIFLASN--HLDMTLPLMGII
Zucr  RADANTAALQAVLYNRVGDIGLILAMAWLATNLNSWELEQIFSASK--HLDLSLPLMGVI
Pxja  RADANTAALQAVLYNRVGDIGLILSMAWLATNLNSWEMQQMFSMSK--DFDLTLPLLGLI
Pxlo  RADANTAALQAVLYNRVGDIGLILSMAWLATNLNSWEMQQMFSMSK--DFDLTLPLLGLI
Pctr  RADANTAALQAVLYNRIGDIGLILSMAWLATNLNSWEIQQLFSLSK--DFNMTLPLLGLI
Apsa  RADANTAALQAVVYNRIGDIGLIMSMAWLAMNLNSWEIQQIFHTAK--DFDMTLPLMGLI
Cabe  RADANTAALQAMLYNRAGDIGMVLAMAWFSITFNSWETQQAFVTTR--DMDTTLPLLGLI
Bzze  RTDANTAALQAVVYNRVGDIGLILAMAWFATNLNSWEMQQMFAVAR--DFDLTLPLLGLI
Siim  RADANTAALQAVVYNRVGDIGLVMAMAWFATNLNSWEIQQMLTTAH--NYDLTLPLMGII
Ctru  RADANTAALQAVIYNRVGDVGLLLAMAWFATNLNSWEMQQMFVTAS--NTDLTLPLLGLI
Dpbr  RTDANTAALQAVIYNRVGDIGLILAMAWFATNLNSWEMQQMFATAN--KLDLTLPLLGLI
Caki  RTDANTAALQAVLYNRIGDIGMIFGLAWIAINIGSWDLQQIMITNS--SVNPTMPLLALI
Phja  RADANTAAMQAVAYNRVGDVGLIMCMAWLATNIDSWDIQHIVILTK--NLDLTFPMLGII
Brsp  RADANTAALQAVIFNRVGDIGLILSMAWLATQLNTWDIQQLFMINK--NLDLTIPLLGLI
Gamo  RADANTAALQAVLYNRVGDIGLILGMAWLATNVNSWDIQQMFILSK--NLDMTLPLLGLI
Lolo  RADANTAALQAVLYNRVGDIGLILGMAWLATNVNSWDIQQMFILSK--NLDLTLPLLGLI
Batr  RADANTAAMQAIIYNCIGDLGLIFMLAWTAIFLNTWDFTQLFYMSQ--NHDMTTPLLALV
Prmy  RAEANAAALQAVIYNRVGDVGLILAMAWAATHLNTWEFQQLFFLGH--SLSLTTPLLGLI
Lose  RTDANTSALQAISYNRVGDIGLVLMMAWMATTFNSWELPQIYLNPR--NADFTLPLMGVI
Loam  RADANTAALQAVLYNRVGDIGLILAMAWMATNLNSWELQQVFSCTK--SVDLTLPLLGLI
Chab  RADANTAALQAVLYNRIGDIGLIFAMAWLATNLNSWEMHQVFASSK--NTALTLPLLGLI
Chto  RADANTAALQAVLYNRIGDIGLIFAMAWLATNLNSWEMHQVFASSE--NTALTLPLLGLI
Majo  RADANTAALQAIVYNRIGDVGLILSMAWFATHLYSWDFSQIFASTK--DMNVTLPLLGLI
Hlst  RADANTAALQAIIYNRVGDVGLILAMVWFATHLYTWDMTQIFMLTK--NLNLNIPLLGLI
Clpe  RADASTAALQAVLYNRVGDIGLILAMAWMATNMNSWEMTQLFASTE--GTNLTLPLLGLI
Mlmr  RADANTAALQAVVYNRVGDIGLIFTMAWLAMNVNSWEMHQMFSGTN--NTDLTLPLLGLI
Crcr  RSDANTAALQAVIYNRVGDIGLLFAMAWFATTLNTWDWQQIFAIAKYYNPDLTFPLMGLI
Muce  RSDANTAALQAVIYNRVGDIGLLFAMAWFATTLNTWDWQQIFAIAKYYNPDLTFPLMGLI
Bege  RTDANTAALQAVLYNRVGDIGLILAMAWIATNLNSWEMQQMFATAK--DFNLTLPLLGLI
Mela  RADANTAALQAVIYNRVGDIGLILAMAWIATNLNSWEIQQIFITAK--NFDLTLPLLGLI
Hats  RADANTAALQAVLYNRVGDIGLILAMAWIAMNLNSWEMQQMFATAK--GFDLTFPLLGLI
Orla  RADANTAALQAVVYNRVGDIGLILAMAWMAVNLNSWDMQQLFSMTT--NQDMTLPLLGLV
```

To be continued on page 22.

```
Cosa    RADANTAALQAVLYNRVGDIGLILAMAWIAMNLNSWELQQVFSSTN--NMDLTLPLLGLI
Exsp    RADANTAALQAVLYNRVGDIGLILAMAWIAMTTNSWELQQIFTLTK--DMNLTLPLIGLI
Depa    RADANTAALQAVLYNRIGDIGLILAMAWMATNLNSWELQQMLATTN--KTNLTLPLIGLI
Rima    RADANTAALQAVLYNRLGDIGIILVMAWMAMNLHSWDMNQLFTAPQ--DLDLTLPLLGLI
Fuol    RADANTAALQAVLYNRVGDIGLILTMAWMATNLHSWEINQIFVLAK--DFDMTLPLIGLI
Gmaf    RTDANTAALQAVLYNRIGDVGLILTMVWTATRLNTWDLEQLFSLSN--EHDLTLPLFGLI
Xeei    RADANTAALQAVLYNRVGDIGLILAMAWMATNLNSWEIQQVFSSSK--NLDLTFPLLGLI
Pros    RADANTAALQAVVYNRVGDIGLILAMAWMATTLNSWEMQQMFILAQ--QYDLTLPLLGLI
Scmi    RADANTAALQAVVYNRVGDIGLILAMAWVATNLSSWEMQQMFATSK--EHNLTLPLLGLI
Rolo    RADANTAALQAVVYNRVGDIGLILAMAWMATNLNSWEMQQMFAASK--DFDLTLPLLGLI
Cere    RADANTAALQAVVYNRVGDIGLILAMAWMAMNLNSWEMQQMFYISK--DLDLTLPLLGLI
Daga    RADANTSALQAVVYNRVGDVGLVLAMAWMAMNLNSWEMQQVFYAST--SSNLTLPLLGLI
Anco    RADANTAALQAVVYNRVGDIGLILAMAWMAMNLNSWEMQQMFFISK--ELDLTLPLLGLI
Dmve    RADANTAALQAVVYNRVGDIGLILAMAWMAMNLNSWEMQQMFSTSK--GLDLTLPLMGLI
Dmar    RADANTAALQAVVYNRVGDIGLILAMAWMAMNLNSWEMQQMFSTSK--EFDLTLPLMGLI
Anka    RADANTAALQAVVYNRIGDIGLILAMAWMATNLNSWEMQQMFFTSK--EFDLTLPLLGLI
Moja    RADANTAALQAVMYNRIGDIGLILAMAWMAMNLNSWEMQQMFSTSK--ELNLTLPLLGLI
Hoja    RADANTAALQAVLYNRIGDVGLILAMAWMAMNLNSWEMQQMFYTSK--EFDLTLPLLGLI
Bede    RADANTAALQAVVYNRVGDIGLILAMAWMATNLNSWEMQQMFAASK--DFDLTLPLLGLI
Besp    RADANTAALQAVVYNRVGDIGLILAMAWMATNLNSWEMQQMFAASK--DFDLTLPLLGLI
Mysp    RADANTAALQAVLYNRVGDIGLILAMAWMATNLNSWEMQQMFATSK--NLDLTVPLLGLI
Osja    RADANTAALQAVLYNRVGDIGLILAMAWMATNLNSWEMQQMFAASK--DLDLTLPLLGLI
Sgro    RADANTAALQAVMYNRVGDIGLILAMAWMATNLNSWEMQQMFASSK--DMDLTIPLLGLI
Pzpa    RADANTAALQAVLYNRIGDIGLILSMAWMATNLNSWEIQQLTTLSK--TMDMTIPLLGLI
Zeja    RASANTAALQAVMYNRVGDIGLILSMAWLATTFNSWEIQQLIMLSK--NSDMTIPLLGLI
Znne    RADANTAALQAVLYNRIGDIGLILSMAWLAKTLNSWEIQQMIILSK--DLDMTIPLLGLI
Zefa    RADANTAALQAVLYNRIGDIGLILSMAWLAKTLNSWEIQQMIFLSK--DLDMTVPLLGLI
Acni    RADANTAALQAVLYNRVGDIGLILSMAWLATTLNSWEIQQLFTLSK--DLDMTIPLLGLI
Ncrh    RADANTAALQAVLYNRVGDIGLILSMAWLATTLNSWEIQQLFTLSK--DLDMTIPLLGLI
Agca    RADANTAALQAVLYNRVGDIGLIFAMAWMATNLNSWEMQQMFTTAK--DFDLTPPLIGLI
Hydy    RADANTAALQAVVYNRIGDIGLIFTMAWMATNLNSWEMQQIFISSK--DLDLTFPLLGLI
Gsac    RADANTAALQAVVYNRIGDIGLIFAMAWMATKLNSWEIQQIFVASK--DFDLTFPLLGLI
Pevo    RADANTAALQAVLYNRVGDVGLIMAMAWIATKMNSWELHQLFTLGT--KQDMTLPLLGLI
Hiku    RADANVAAIQAVVYNRVGDIGLILFMAWIAMNLNSWEINQMFTLAE--NKNLTLPLLGLV
Inpa    RTDATTAAIQAVVYNRIGDIGLILAMAWMAMNLNSWDFQQIFHLAQ--HQDLTLPLLGFI
Auch    RADANTAALQAVLYNRIGDIGLILAMVWMATTLNSWEMHQIMATAN--NFDLTLPLLGLI
Fico    RADANTAALQAVLYNRVGDIGLIMAMAWMAAKMNSWEMHQMFSISE--NYDLTFPLLGLI
Macs    RADANTAALQAVLYNRVGDVGLILAMAWMATNLNSWEMPHIFAVSK--DMDMTLPLLGLI
Moal    RADANTAALQAVLYNRVGDVGLIFALGWMATNLNSWEMQQMFITAK--SLDMNAPLIGLI
Syma    RTDANTAALQAVIYNRIGDIGLILAMAWLASNLNSWEMQQMLINTK--NLDLTLPLLGFI
Mafr    RTDANTAALQAVIYNRVGDIGLIFSMAWLATNLGSWEMQQMFLNAK--NLDLTFPLLGLI
Dcpe    RADANTAALQAVLYNRIGDIGLILSMAWMAMNLNAWQLDQIFALAQ--DHDLTLPLVGFI
Dcti    RADANTAALQAVLYNRIGDIGLILSMAWMAMNLNAWQLDQLFILAQ--THDLTLPLVGFI
Hehi    RADANTAALQAVVYNRVGDIGLLFTMAWMATNANSWELQQIFVATK--NMDLTLPLLGLI
Stam    RADANTAALQAVIYNRVGDVGLIFAMAWLATTLNSWEMQQIFAAAK--DFDLTLPLLGLI
Hogi    RADANTAALQAVVYNRVGDIGLIFALAWMATNLNSWEFQQMFMISK--NYDLTYPLVGLI
Erzo    RADANTAALQAVVYNRVGDVGLILAMAWMAMNFNSWEMQQVFSASK--DFDLTLPLLGLI
Hxot    RADANTAALQAVVYNRVGDVGLIFAMAWMAMNLNSWEMQQIFAASK--DFDLTYPLLGLI
Core    RTDANTAALQAVVYNRVGDIGLIFAMAWMAMNLNSWEMQQVFIASK--DFDLTFPLLGLI
Apve    RTDANTAALQAVVYNRIGDVGLIFAMAWMAVNLNSWEMQQIFVTSK--DFDLTFPLLGLI
Latj    RADANTAALQAVLYNRIGDIGLIFFMAWVAMNLNSWEMQQMFAVAK--DMDLTYPLLGLI
Laja    RADANTAALQAVLYNRIGDIGLIFAMAWMATNLNSWEMQQMFAAST--DFDLTFPLLGLI
```

To be continued on page 23.

To be continued on page 24.

```
Syja  RTDANTAALQAVLYNRVGDIGLIFAMAWMAMNLNSWEMQQMFVISK--NQDLTFPLLGLI
Epme  RADANTAALQAVVYNRVGDVGLIFAMAWMVSHLNSWELQQIFATAK--EFDLTYPLLGLI
Grse  RANANTAALQAVLYNRAGDVGLIIAMVWMLTNLNTWQTDQIILIAK--NFNLTLPLMGLI
Clja  RADANTAALQAVLYNRVGDIGLIFAMAWMAMNLNTWEFHQIFTASK--ELDLTAPLVGLI
Ogcy  RADANTSALQAVLYNRVGDVGLFLAMAWFAANTSSWDYQQIFASMK--DLNPALPALGLI
Plna  RADANSAALQAVLYNRVGDIGLILAMAWIATNLNSWEMQQMFFTAK--SYDMTLPLLGLI
Lema  RADANTAALQAVLYNRVGDIGLIFAMAWTATNLNSWEMQQMFITAK--DFDMTFPLLGLI
Etzo  RADANTAALQAVVYNRVGDVGLIFAMAWMATNLNSWEMQQLFVTAK--EFDLTFPLLGLI
Apse  RADANTAALQAVLYNRVGDIGLIFTMAWMATNLNSWEMQQIFSVAS--SYDLTYPLIGLI
Epde  RADANTAALQAVLYNRVGDIGLIFTLAWMAVNLNSWEMQQIFAAAK--DLDMTFPLIGLI
Slja  RANANTAALQAVLYNRVGDVGLIFAMAWMATNLNSWEFSQIFSAAK--GLDLTYPLIGLI
Bsja  RTDANTAALQAVLYNRVGDIGLIFAMAWMATNLNSWEMQQMFVTAK--NLDLTFPLLGLI
Ecna  RADANTAALQAVLYNRVGDIGLIFTMAWMATNLNSWELQQVFISAK--SFDLTYPLIGLI
Cohi  RADANTAALQAVIYNRIGDIGLIFSLAWMAINLNSWEFQQIFTLQT--DTDFTLPLIGFI
Caar  RADANTAALQAVLYNRVGDIGLIFAMAWMATNLNSWEMQQMFATAK--DLDLTCPLLGLI
Came  RADANTAALQAVLYNRVGDIGLIFAMAWMATNFNSWEMQQMFAAAK--DFDLTYPLLGLI
Mema  RADANTAALQAVLYNRVGDIGLIFAMAWMATNLNSWEMPQMFAASK--DFDLTYPLLGLI
Lenu  RADANSAALQAVLYNRIGDIGLILTMAWAAMNLNSWEMQQIFSTSN--LHDLTLPLLGLV
Brja  RADANTAALQAVLYNRVGDIGLILAMAWMATNLNSWEMQQMFVTAK--NLDMTLPLLGLI
Plma  RADANTAALQAVVYNRVGDIGLILTMAWMATNLNSWEMQQMFITAK--NFDLTLPLLGLI
Emst  RADANTAALQAVLYNRVGDIGLIFAMAWMATNLNSWEMQQMFAAAK--DFDLTVPVLGLI
Ptti  RADANTAALQAVLYNRVGDIGLIFAMAWMATNLNSWEMQQMFAAAK--DFDLTFPLLGLI
Losu  RACANTAALQAVLYNRIGDIGLIFAMAWMATNLNSWEMQQVTMAAH--NMDLTLPLLGLI
Geoy  RADANTAALQAVLYNRVGDIGLIFAMAWMATNLNSWEMQQLFVAAK--GLDLTYPLLGLI
Dipi  RADANTAALQAVLYNRVGDIGLIFAMAWMATNLNSWEMQQMFAASK--NMDLTFPLLGLI
Pama  RADANTAALQAVLYNRVGDIGLIFAMAWMAMNLNSWEMQQMFATAK--DLDLTFPLLGLI
Leob  RADANTAALQAVLYNRVGDIGLIFAMAWMAMNLNSWEMQQIFSAAK--GMDLTFPLLGLI
Neba  RADANTAALQAVLYNRVGDIGLILAMAWMATNLNSWEFQQMFSATE--NMNITLPLLGLI
Pdpl  RADANTAALQAVLYNRVGDIGILFVLAWTATNLHTWDLHHIATAAKTLNLNITLPLMALI
Nimi  RADANTAALQAVILNRIGDIGLFLALASIAMKFNSLEMSELFVLAQ--NEDLTLPLLGLI
Uptr  RADANTAALQAVIYNRVGDIGLILAMAWMATNLNSWEMHQIFASTK--DYDLTLPLLGLI
Pesc  RADANTAALQAVIYNRVGDLGLIFAMAWMASNFNSWDLQQVYAMPL--SADVTFPLLGLI
Baar  RADANTAALQAVLYNRVGDIGLIFAMAWLAMNLNSWEMQQMFATTK--NLDLTFPLLGLI
Moar  RADANTAALQAVLYNRVGDIGLIFAMAWMATNLNSWEMQQMFAAAK--NLDLTFPLLGLI
Toja  RADANTAALQAVLYNRVGDIGLIFAMAWMATNLNSWEMQQMFAAAK--NFDLTFPLLGLI
Chau  RADANTAALQAVLYNRVGDIGLILSMAWIAMNLNSWEMQQIFAAAK--NMDLTLPLLGLI
Chse  RADANTSALQAVLYNRVGDVGLILAMAWFAMNLNSWEMQQMYAAAK--GLDLTLPLMGLI
Enar  RADANTAALQAVLYNRVGDVGLIFAMAWMATNLNSWEMQQLFAAAK--DMDLTFPLLGLI
Hpty  RADANTAALQAVLYNRVGDIGLIFAMAWMATNLNSWELQQMFATAK--DLDLTFPLLGLI
Nana  RADANTAALQAVVYNRVGDIGLIFAMAWFATNLNSWEMQQMFITSK--NLDLTPPLLGLI
Mcst  RTDANTAALQAVVYNRVGDIGLIFAMAWMATSLNSWEMQQMLAASK--DFDLTFPLLGLI
Rhox  RADANTAALQAVVYNRVGDIGLILAMAWMAMNLNSWEMQQMFAASK--GLDLTLPLLGLI
Opfa  RADANTAALQAVIYNRVGDIGLIFAMAWMAMNLNSWEMQQMFAAAK--NFDLTFPLLGLI
Paar  RADANTAALQAVLYNRLGDIGLILAMAWMAVNLNSWEMQQIFASAK--NLDLTFPLLGLI
Gozo  RADANSAALQAVLYNRVGDIGLIFAMAWMATNFNSWEMQQMFASAK--NFDLTFPLLGLI
Ackr  RVNANVAALQAVIYNRVGDIGLILAMAWLMANAKSWDMQLIHLNTK--DLDLTLPLCGLI
Elev  RTDANTAALQAVLYNRVGDIGLIMAMAWFATNINCWEMQQMFILAK--ELDLTLPLLGLV
Trdu  RADANTAALQAVIYNRVGDIGLIFAMAWTATSLNSWEMQQMFTLSK--DFDLTYPLIGLI
Amoc  RTDANTAALQAVLYNRVGDIGLIFTMAWFATNLNSWDLQQVFTASK--DMDLTFPLLGLI
Hame  RADANTASFQAVAYNRVGDVGFLILMAWALNGFNTWQIEEINQLAL--GKDATPGLIGAI
Chso  RSDANTAALQAVLYNRVGDIGLILAMAWMAMNLNSWELHHLFICAK--HYDLTLPLLGLV
Lyto  RADANTAALQAVVYNRVGDIGLIFAMAWMAVTLNSWEMQQIFAASK--DFDLTYPLLGLI
```

[4/12 of aligned sequences]

To be continued on page 25.

```
Encr  RADANTAALQAVVYNRVGDIGLIFAMAWMAMNLNSWEMQQIFATSK--DLDLTFPLLGLI
Bvar  RTDANTAALQAVVYNRVGDIGLIFAMSWMALNLNSWEMDQMFLAPK--NFDLTLPLLGLI
Noco  RADANTAALQAVVYNRVGDIGLIFAMAWMATNMNSWELQQIFIALK--GFNVTPPVLGLI
Chsp  RADANTAALQAVIYNRVGDIGLIMAMAWIAMNFNSWELQQMFSVKE--NFDMTLPLLGLI
Arja  RADANTAALQAVVYNRVGDVGLIFAMAWMAMNLNSWEMQQIFVASK--DFDLTFPLLGLI
Pase  RADANTAALQAVIYNRVGDIGLICALAWLAMNSNSWELQQIFAASK--DPNMILPLVGLI
Trel  RTDANTAALQAVLYNRVGDIGLILAMAWAATNLGSWELQNLFSSSS--SWDLTLPAMGLI
Lifa  RANANTASLQAILYNRVGDIGLLLVMGWLILNTGSWDSLCL-PLSEFTNCDLTIPLLGLI
Acur  RADANTAALQAVLYNRVGDIGLIFAMAWIAINLNSWELTQIYSTAK--ELDLTLPLVGMI
Ampe  RADANTAALQAVVYNRVGDIGLIFAMAWMAMNLNSWEMQQMFATAQ--SFDLTFPLLGLI
Urja  RADANSAALQAVIYNRIGDIGLIFAMAWAAMNLNSWELQQMLSSAK--DLDLTLPLLGLI
Enet  RADANTAALQAVIYNRIGDIGLILAMAWMATNLNSWDIQQILLASK--NMDTTLPLMGLI
Ptbr  RSDANTAALQAVLYNRVGDIGLILAMAWLATTLDSWDLQQIFLLSK--DKDLTLPLIGVI
Safa  RADANTAALQAVLYNRVGDIGLILAMAWLATTLNSWELQQVFMASK--DMDLTLPLIGLI
Icae  RADANTAALQAVVYNRVGDIGLILAMAWMATNLNSWEMQQMFVTAK--SFDLTLPLLGLI
Asmi  RADANTAALQAVLYNRIGDVGFILLLAMAILVFDSWELMNIQELAK--NNTPTPLLIGVI
Foal  RADANTAALQAVIYNRVGDIGLILVLAWTAIKFNSWELDQIFILSE--NSNTLLPALGLI
Drze  RADANTAALQAVLYNRIGDIGMILAMAWMATNMNSWNMQQIFISSH--NFDLTLPLIGLI
Rhas  RADANTAALQAVLYNRVGDIGLIFAMAWMATNLNSWEMQQIFSTSK--TYDLTFPLLGLI
Elac  RADANTAALQAVIYNRVGDIGLIFAMAWMATNLNSWEMQQIFSTTK--DIDLTFPLLGLI
Kugu  RTDANTAALQAVLYNRIGDIGLILAMAWIATNLNSWELQQLFCTSK--NYDLTLPLLGLI
Plor  RADANTAALQAVVYNRVGDIGLIFTMAWIATNLNSWEMQQVFTAAK--NLDLTLPLLGLI
Sgun  RADANTAALQAVLYNRVGDIGLIFAMAWMAMNLNSWEMQQMFAAAK--DMDLTYPLIGLI
Zaco  RADANTAALQAVLYNRVGDIGLIFAMAWIATNLNSWEMQQVFSAAK--DFDLTFPLLGLI
Zbfl  RADANTAALQAVLYNRVGDIGLIFAMAWTATNLNSWEMQQMFAAAK--DLDLTFPLLGLI
Spba  RADANTAALQAVLYNRVGDIGLIFAMAWMATNLNSWEMQQMFAAAK--DLDLTLPLLGLI
Game  RADANTAALQAVVYNRVGDIGLILAMAWMATNLNSWEMQQMFIAAK--DFDLTLPLLGLI
Thth  RADANTAALQAVVYNRVGDIGLILAMAWMATNLNSWEMQQMFVTAK--NFDLTLPLLGLI
Xigl  RSDANTAALQAVLYNRVGDIGLIFAMAWMATNLNSWEMQQMFATAK--DFDLTPPLLGLI
Hyja  RADANTAALQAVVYNRVGDIGLILAMAWMATNLNSWEMQQMFAAAK--DFDLTLPLLGLI
Psan  RADASTAALQAVMYNRLADIGLILTMAWMMAKLNSWEMQQLFMNAK--NFDLNLPLLGLI
Cupa  RADANTAALQAVVYNRVGDIGLILAMAWMATNLNSWEMQQMFVTAK--NFDMTLPLLGLI
Mpch  RADANTAAMQAVVYNRVGDIGLIFAMAWFATNLNSWEMQQMFSITK--NLNITPPLMGLI
Char  RADANTAALQAVLYNRVGDIGLVFFMAWLATNLNSWEMQQMFAASQ--SFDLTLPLLGLI
Pser  RADANTAALQAVVYNRVGDIGLILTMAWMATNLNSWEMQQMFSTSK--DLDLTLPLIGLI
Prol  RADANAAALQAVIYNRVGDIGLIFAMAWMALNLNSWDLEQLFAASK--NMDLSFPLLGLI
Plbi  RADANTAALQAVLYNRVGDIGLIFAMAWMATNLNSWEMQQIFATSK--DMDLTYPLLGLI
Calu  RADANTAALQAVLYNRVGDIGLIFTMAWMATNLGTWEIHQITLASK--GLDLTYPLMGVV
Papa  RADANTAALQATLYNRVGDIGLMCAMAWMATNLNSWEMQQIFLASK--DLDLTIPLLGLI
Sufr  RADANTAALQAVLYNRVGDIGLIFAMAWMATNLNSWEIQQIFSNAN--SMDLTFPLLGLI
Stci  RADANTAALQAVLYNRVGDIGLILAMAWMMANLNSWEMQQIFSTSQ--NMDLTLPLLGLI
Taru  RADANTAALQAVIYNRVGDIGLIIAMAWMATHLNSWELQQIFFSTK--NMDMTLPLVALI
Rala  RADANTAALQAVLYNRVGDIGLIFAMAWMAMNLNSWEMQQMFSAAK--DMDLTFPLIGLI
      *  *. :::**    *   .*:*:.  :          :                    .
```

```
          F                     G
Scca LAAAGKSAQFGLHPWLPSAMEGPTPVSALLHSSTMVVAGIFLLIRLHPLIQDNQFILTTC
Muma LAAAGKSAQFGLHPWLPSAMEGPTPVSALLHSSTMVVAGIFLLIRLHPLIQDNKLILTVC
Erca LAAMGKSAQFGLHPWLPAAMEGPTPVSALLHSSTMVVAGIFLLIRLHPFLENNNKVLTAA
Pose LAATGKSAQFGLHPWLPAAMEGPTPVSALLHSSTMVVAGIFLLIRLHPLIENNNNILTAA
Actr LAATGKSAQFGLHPWLPSAMEGPTPVSALLHSSTMVVAGIFLLIRLHPLMEHNQVALTTC
Scal LAATGKSAQFGLHPWLPSAMEGPTPVSALLHSSTMVVAGIFLLIRLHPLMEHNQVALTTC
Posp LAATGKSAQFGLHPWLPSAMEGPTPVSALLHSSTMVVAGIFLLIRLHPLMEHNQLALTTC
Atsp LAATGKSAQFGLHPWLTSAMEGPTPVSALLHSSTMVVAGIFLLIRFHPLIGQNPTALTIC
Leoc LAATGKSAQFGLHPWLTAAMEGPTPVSALLHSSTMVVAGIFLLIRFHPLIAQNPTALTIC
Amca LAATGKSAQFGLHPWLPSAMEGPTPVSALLHSSTMVVAGIFLLIRLHPLMEHNQLALTTC
Osbi LAATGKSAQFGLHPWLPSAMEGPTPVSALLHSSTMVVAGIFLLIRLHPLMETNQTVLTTC
Pabu LAATGKSAQFGLHPWLPSAMEGPTPVSALLHSSTMVVAGIFLLIRLHPLMEHNQTALTTC
Hial LAATGKSAQFGLHPWLPSAMEGPTPVSALLHSSTMVVAGIFLLIRLHPLMENNQTALTTC
Elha LAATGKSAQFGLHPWLPSAMEGPTPVSALLHSSTMVVAGIFLLIRLHPLMENNQTALTTC
Mlcy LAATGKSAQFGLHPWLPSAMEGPTPVSALLHSSTMVVAGIFLLIRLHPLMEDNQLALTTC
Algl LAATGKSAQFGLHPWLPSAMEGPTPVSALLHSSTMVVAGVFLLVRLHPLMENNQMALTIC
Ptgi LAATGKSAQFGLHPWLPSAMEGPTPVSALLHSSTMVVAGIFLLIRLHPLMENNQTALTIC
Alaf LAATGKSAQLGLHPWLPSAMEGPTPVSALLHSSTMVVAGIFLLIRLHPLMENNQTALTIC
Nock LAATGKSAQLGLHPWLPSAMEGPTPVSALLHSSTMVVAGIFLLIRLHPLMENNQTALTVC
Anja IAATGKSAQFGLHPWLPSAMEGPTPVSALLHSSTMVVAGIFLLIRLHPMMENNQTVLSTC
Gyki LAATGKSAQFGLHPWLPAAMEGPTPVSALLHSSTMVVAGIFLLIRLHPIMQNNQTALTTC
Syka LAATGKSAQFGLHPWLPSAMEGPTPVSALLHSSTMVVAGIFLLIRLHPLMENNQTALTTC
Opma LAATGKSAQFGLHPWLPSAMEGPTPVSALLHSSTMVVAGIFLLIRLHPMMENNQVALTTC
Comy LAATGKSAQFGLHPWLPSAMEGPTPVSALLHSSTMVVAGIFLLIRLHPLMENNQTALTTC
Sasp LAAMGKSAQFGLHPWLPSAMEGPTPVSALLHSSTMVVAGIFLLIRLHPLMENNPMIQSTC
Eupe MAATGKSAQFGLHPWLPSAMEGPTPVSALLHSSTMVVAGIFLLVRLHPMMECNQTIQSIC
Enja IAATGKSAQFGLHPWLPSAMEGPTPVSALLHSSTMVVAGIFLLIRLHPFMASNETIMTIC
Same IAATGKSAQFGLHPWLPSAMEGPTPVSALLHSSTMVVAGIFLLIRLHPLTQSNPTALTIC
Chch LAATGKSAQFGLHPWLPSAMEGPTPVSALLHSSTMVVAGIFLLIRLHPLMSNNQTVLTTC
Grgr LAATGKSAQFGLHPWLPSAMEGPTPVSALLHSSTMVVAGIFLLIRMYPLLEGNQTALTLS
Caau LAATGKSAQFGLHPWLPSAMEGPTPVSALLHSSTMVVAGIFLLIRLHPLMENNQLALTTC
Cyca LAATGKSAQFGLHPWLPSAMEGPTPVSALLHSSTMVVAGIFLLIRLHPLMENNQLALTTC
Dare IAATGKSAQFTLHPWLPSAMEGPTPVSALLHSSTMVVAGIFLLIRLHPIMETNKLAPTIC
Cost LAATGKSAQFGLHPWLPSAMEGPTPVSALLHSSTMVVAGIFLLIRLHPLMEDNNLALTTC
Leec LAATGKSAQFGLHPWLPSAMEGPTPVSALLHSSTMVVAGIFLLIRLHPLMENNDLALTIC
Fola LAATGKSAQFGLHPWLPAAMEGPTPVSALLHSSTMVVAGIFLLIRLHAIMENNQLALTTC
Clmc LAATGKSAQFGLHPWLPSAMEGPTPVSALLHSSTMVVAGIFLLIRLHPLMENNPLALTTC
Phin LAATGKSAQFGLHPWLPSAMEGPTPVSALLHSSTMVVAGIFLLIRLHPLMESNQLVLTIC
Icpu LAATGKSAQFGLHPWLPSAMEGPTPVSALLHSSTMVVAGIFLLIRLHPLMQDNQLALTVC
Psto LAATGKSAQFGLHPWLPSAMEGPTPVSALLHSSTMVVAGIFLLIRLHPLMENNQLALTTC
Cora IAATGKSAQFGLHPWLPSAMEGPTPVSALLHSSTMVVAGIFLLIRLHPLMENNNLALTIC
Eisp LAATGKSAQFGLHPWLPSAMEGPTPVSALLHSSTMVVAGIFLLIRLHPLMEDNPLALTTC
Apal LAATGKSAQFGLHPWLPSAMEGPTPVSALLHSSTMVVAGIFLLIRLHPLMENNPTALTIC
Eslu LAATGKSAQFGLHPWLPSAMEGPTPVSALLHSSTMVVAGIFLLIRLHPLMENNQMALTTC
Dape LAATGKSAQFGLHPWLPAAMEGPTPVSALLHSSTMVVAGIFLLIRLHPLMENNQTALSTC
Glse LAATGKSAQFGLHPWLPSAMEGPTPVSALLHSSTMVVAGIFLLIRLHPMMENNPFALTLC
Naar LAATGKSAQFGLHPWLPSAMEGPTPVSALLHSSTMLVAGIFLLIRLHPLMENNPFALTLC
Lioc LAATGKSAQFGLHPWLPSAMEGPTPVSALLHSSTMVVAGIFLLIRLHPLMENNPFALTLC
Opso LAAAGKSAQFGLHPWLPAAMEGPTPVSALLHSSTMVVAGIFLLIRLHPLMEYNPFALTLC
Alte VAATGKSAQFGLHPWLPSAMEGPTPVSALLHSSTMVVAGIFLLIRLHPLMQDNQTALTIT
Plap LAATGKSAQFGLHPWLPSAMEGPTPVSALLHSSTMVVAGIFLLIRLHPLMQDNQMALTIC
```

To be continued on page 26.

[5/12 of aligned sequences]

```
Plal  LAATGKSAQFGLHPWLPSAMEGPTPVSALLHSSTMVVAGIFLLIRTSPLMENNQTALTTC
Sami  LAATGKSAQFGLHPWLPSAMEGPTPVSALLHSSTMVVAGIFLLIRTSPLMENNPTALTTC
Rere  LAATGKSAQFGLHPWLPSAMEGPTPVSALLHSSTMVVAGIFLLIRLSPLMENNPLVLTTC
Gama  VAATGKSAQFGLHPWLPSAMEGPTPVSALLHSSTMVVAGIFLLIRLSPLMAGNPVALTTC
Onmy  LAATGKSAQFGLHPWLPSAMEGPTPVSALLHSSTMVVAGIFLLIRLHPLMEDNQTALTVC
Sasa  LAATGKSAQFGLHPWLPSAMEGPTPVSALLHSSTMVVAGIFLLIRLHPLMENNQTALTTC
Cola  LAATGKSAQFGLHPWLPSAMEGPTPVSALLHSSTMVVAGIFLLIRLHPLMENNQTALTTC
Dita  LAAAGKSAQFGLHPWLPSAMEGPTPVSALLHSSTMVVAGIFLLVRLAPLLETNQTALTTC
Gogr  LAATGKSAQFGLHPWLPSAMEGPTPVSALLHSSTMVVAGIFLLIRFSPLMEINQVALTSC
Chsl  LAATGKSAQFGLHPWLPSAMEGPTPVSALLHSSTMVVAGIFLLIRLSPLMQDNQTALTTC
Atja  LAATGKSAQFGLHPWLPSAMEGPTPVSALLHSSTMVVAGIFLLIRLSPLMEDNQLALTTC
Iido  LAATGKSAQFGLHPWLPSAMEGPTPVSALLHSSTMVVAGIFLLIRLSPLMENNQLALTTC
Auja  LAATGKSAQFGLHPWLPSAMEGPTPVSALLHSSTMVVAGIFLLIRLSPLMENNPTALTTC
Chag  LAATGKSAQFGLHPWLPSAMEGPTPVSALLHSSTMVVAGIFLLIRLNPLMENNQAALTTC
Hami  LAATGKSAQFGLHPWLPSAMEGPTPVSALLHSSTMVVAGIFLLIRLSPLMEGNQTALTTC
Saun  LAATGKSAQFGLHPWLPSAMEGPTPVSALLHSSTMVVAGIFLLIRLSPLMEDNQVALTTC
Nema  LAATGKSAQFGLHPWLPSAMEGPTPVSALLHSSTMVVAGIFLLIRLSPLIENNQAALTTC
Disp  LAATGKSAQFGLHPWLPSAMEGPTPVSALLHSSTMVVAGIFLLIRLSPLIENNQTALTTC
Myaf  LAATGKSAQFGLHPWLPSAMEGPTPVSALLHSSTMVVAGIFLLIRLSPLMENNETALTVC
Lagu  LAATGKSAQFGLHPWLPAAMEGPTPVSALLHSSTMVVAGIFLLIRLSPMMEHNQVALTTC
Trtr  LAATGKSAQFGLHPWLPSAMEGPTPVSALLHSSTMVVAGIFLLIRLSPVMEHNQVALSTC
Zucr  LAAAGKSAQFGLHPWLPSAMEGPTPVSALLHSSTMVVAGIFLLIRLSPLLEHNQLALSVC
Pxja  LAATGKSAQFGLHPWLPSAMEGPTPVSALLHSSTMVVAGIFLLIRLSPLMENNQTALTAC
Pxlo  LAATGKSAQFGLHPWLPSAMEGPTPVSALLHSSTMVVAGIFLLIRLSPLMENSQTALTTC
Pctr  IAATGKSAQFGLHPWLPAAMEGPTPVSALLHSSTMVVAGIFLLIRLSPLMEGNQTALTTC
Apsa  LAATGKSAQFGLHPWLPAAMEGPTPVSALLHSSTMVVAGIFLLVRLSPLMENNQTALTTC
Cabe  LGATGKSAQFGLHPWLPAAMEGPTPVSALLHSSTMVVAGIFLLIRLAPLMQFNNTALTIC
Bzze  LAATGKSAQFGLHPWLPSAMEGPTPVSALLHSSTMVVAGIFLLIRLSPLMENNPLALTTC
Siim  LAATGKSAQFGLHPWLPSAMEGPTPVSALLHSSTMVVAGIFLLIRLSPLVATNDTALTTC
Ctru  LAATGKSAQFGLHPWLPSAMEGPTPVSALLHSSTMVVAGIFLLIRLSPLIQDNPTALTTC
Dpbr  LAAAGKSAQFGLHPWLPSAMEGPTPVSALLHSSTMVVAGIFLLIRLSPLIQDNPTALTTC
Caki  LAATGKSAQFGLHPWLPAAMEGPTPVSALLHSSTMVVAGIFLLVRFSPMMENNPLPLSLC
Phja  IAATGKSAQFGLHPWLPAAMEGPTPVSALLHSSTMVVAGVFLLIRLNPLITNSPQALTLC
Brsp  LAATGKSAQFGLHPWLPAAMEGPTPVSALLHSSTMVVAGIFLLIRLNPLMENNPAALSTC
Gamo  LAATGKSAQFGLHPWLPAAMEGPTPVSALLHSSTMVVAGIFLLIRMSPLMENNQTALTLC
Lolo  LAATGKSAQFGLHPWLPAAMEGPTPVSALLHSSTMVVAGIFLLIRLSPLMENNQTALSLC
Batr  LAASGKSAQFGLHPWLPAAMEGPTPVSALLHSSTMVVAGVFLMIRTSPIMTNHPMVLSTC
Prmy  LAAAGKSAQFGLHPWLPSAMEGPTPVSALLHSSTMVVAGIFLLIRVSPLLSNNPLALTLC
Lose  LAAAGKSAQFGLHPWLPAAMEGPTPVSALLHSSTMVVAGVFILIRMSPLMEDNPVALTTC
Loam  LAATGKSAQFGLHPWLPAAMEGPTPVSALLHSSTMVVAGIFLLIRMSPLMENNQTALTAC
Chab  IAATGKSAQFGLHPWLPSAMEGPTPVSALLHSSTMVVAGIFLLIRMSPLMEHNPTALTTC
Chto  IAATGKSAQFGLHPWLPSAMEGPTPVSALLHSSTMVVAGIFLLIRMSPLMEHNPTALTTC
Majo  LAATGKSAQFGLHPWLPSAMEGPTPVSALLHSSTMVVAGIILMIRFSPLMENNQLALTIT
Hlst  IAATGKSAQFGLHPWLPAAMEGPTPVSALLHSSTMVVAGVILMIRFNPLMTDNPLCLSIV
Clpe  VAATGKSAQFGLHPWLPSAMEGPTPVSALLHSSTMVVAGIFLLIRMSPLMQHNHTALTTC
Mlmr  IAATGKSAQFGLHPWLPSAMEGPTPVSALLHSSTMVVAGIFLLIRMSPLLGDNQTALTAC
Crcr  IAATGKSAQFGLHPWLPSAMEGPTPVSALLHSSTMVVAGIFLLIRVSPLLEQNQTALTTC
Muce  IAATGKSAQFGLHPWLPSAMEGPTPVSALLHSSTMVVAGIFLLIRVSPLLEQNQTALTTC
Bege  LAATGKSAQFGLHPWLPSAMEGPTPVSALLHSSTMVVAGIFLLIRISPLMENNPTALTTC
Mela  LAAAGKSAQFGLHPWLPSAMEGPTPVSALLHSSTMVVAGIFLLIRLFPLMEGNSTASTTC
Hats  LAATGKSAQFGLHPWLPSAMEGPTPVSALLHSSTMVVAGIFLLIRLSPMMESNPTALTTC
Orla  LAATGKSAQFGLHPWLPSAMEGPTPVSALLHSSTMVVAGIFLLIRLNPLMDNNPLVLSTC
```

To be continued on page 27.

[5/12 of aligned sequences]

```
Cosa   LAATGKSAQFGLHPWLPSAMEGPTPVSALLHSSTMVVAGIFLLIRISPIMENNQTALTTC
Exsp   VAATGKSAQFGLHPWLPSAMEGPTPVSALLHSSTMVVAGIFLLIRLSPLLENDTTASTIC
Depa   LAAAGKSAQFGLHPWLPSAMEGPTPVSALLHSSTMVVAGIFLLIRLSPLIEDDRMASTIC
Rima   LAAMGKSAQFGLHPWLPAAMEGPTPVSALLHSSTMVVAGIFLLVRFSPLFENNQMALTAC
Fuol   LAATGKSAQFGLHPWLPSAMEGPTPVSALLHSSTMVVAGIFLLIRLSPIMENNTTALTIC
Gmaf   LAAMGKSAQFGLHPWLPAAMEGPTPVSALLHSSTMVVAGIFLLIRLNPLLENNKLALTTC
Xeei   LAATGKSAQFGLHPWLPSAMEGPTPVSALLHSSTMVVAGIFLLIRLSPILENNQIALTTC
Pros   LAATGKSAQFGLHPWLPSAMEGPTPVSALLHSSTMVVAGIFLLIRLSPLMENNQTALTIC
Scmi   LAATGKSAQFGLHPWLPSAMEGPTPVSALLHSSTMVVAGIFLLIRLSPLMENNQTALTLC
Rolo   LAATGKSAQFGLHPWLPSAMEGPTPVSALLHSSTMVVAGIFLLIRLSPLLENNQTALSTC
Cere   LAAAGKSAQFGLHPWLPSAMEGPTPVSALLHSSTMVVAGIFLLIRLNPLMESNKLALTTC
Daga   LAAAGKSAQFGLHPWLPAAMEGPTPVSALLHSSTMVVAGIFLLIRLSPLMQNNQAALTIC
Anco   LAATGKSAQFGLHPWLPSAMEGPTPVSALLHSSTMVVAGIFLLIRLSPLMENNQTALTTC
Dmve   LAATGKSAQFGLHPWLPSAMEGPTPVSALLHSSTMVVAGIFLLVRLSPLMENNPMALSTC
Dmar   LAATGKSAQFGLHPWLPSAMEGPTPVSALLHSSTMVVAGIFLLVRLSPLMENNQAALSTC
Anka   VAATGKSAQFGLHPWLPSAMEGPTPVSALLHSSTMVVAGIFLLIRLSPLMEDNQTALSTC
Moja   LAATGKSAQFGLHPWLPSAMEGPTPVSALLHSSTMVVAGIFLLIRLSPLMENNQTALSTC
Hoja   LAATGKSAQFGLHPWLPSAMEGPTPVSALLHSSTMVVAGIFLLIRLSPMMENNQTALSTC
Bede   LAATGKSAQFGLHPWLPSAMEGPTPVSALLHSSTMVVAGIFLLIRLSPLLENNQTALSIC
Besp   LAATGKSAQFGLHPWLPSAMEGPTPVSALLHSSTMVVAGIFLLIRLSPLLENNQTALTTC
Mysp   LAATGKSAQFGLHPWLPSAMEGPTPVSALLHSSTMVVAGIFLLIRLSPLMENNQTALSIC
Osja   LAATGKSAQFGLHPWLPSAMEGPTPVSALLHSSTMVVAGIFLLIRLSPLMENNQTALSIC
Sgro   LAATGKSAQFGLHPWLPSAMEGPTPVSALLHSSTMVVAGIFLLIRLSPLMENNQTALSIC
Pzpa   LAATGKSAQFGLHPWLPSAMEGPTPVSALLHSSTMVVAGIFLLIRLNPLMENNQIVLSTC
Zeja   LAATGKSAQLGLHPWLPSAMEGPTPVSALLHSSTMVVAGIFLLIRLAPLMENNQTALSTC
Znne   LAATGKSAQFGLHPWLPAAMEGPTPVSALLHSSTMVVAGIFLLIRLSPLMENNQTALSTC
Zefa   LAATGKSAQFGLHPWLPSAMEGPTPVSALLHSSTMVVAGIFLLIRLSPLMENNQTALSVC
Acni   LAATGKSAQFGLHPWLPSAMEGPTPVSALLHSSTMVVAGIFLLIRLSPLMENNQTALSTC
Ncrh   LAATGKSAQFGLHPWLPSAMEGPTPVSALLHSSTMVVAGIFLLIRLSPMMENNQTALSTC
Agca   IAATGKSAQFGLHPWLPSAMEGPTPVSALLHSSTMVVAGIFLLVRMSPLMENNQTALTTC
Hydy   VAAAGKSAQFGLHPWLPSAMEGPTPVSALLHSSTMVVAGIFLLVRLSPLLEDNQTALTIC
Gsac   VAAAGKSAQFGLHPWLPSAMEGPTPVSALLHSSTMVVAGIFLLVRLSPLLEGNQTALTIC
Pevo   LAATGKSAQFGLHPWLPSAMEGPTPVSALLHSSTMVVAGIFLLIRVSPLIEQNTLASTLC
Hiku   LAATGKSAQFGLHPWLPSAMEGPTPVSALLHSSTMVVAGIFLLIRMSPLMENNSTILTTC
Inpa   LAATGKSAQFGLHPWLPSAMEGPTPVSALLHSSTMVVAGIFLLIRISPVMQYHQTALTIC
Auch   LAATGKSAQFGLHPWLPSAMEGPTPVSALLHSSTMVVAGIFLLIRTSPLMQNNPSALTLC
Fico   LAATGKSAQFGLHPWLPSAMEGPNPVSAVLHYSTMVVAGIFLLIRMSPLMQGNSTALTLC
Macs   LAATGKSAQFGLHPWLPSAMEGPTPVSALLHSSTMVVAGIFLLIRMSPLLETNQTALTLC
Moal   LAATGKSAQFGLHPWLPSAMEGPTPVSALLHSSTMVVAGIFLLVRLSPLMQDNPFALTTC
Syma   LAAAGKSAQFSLHPWLPSAMEGPTPVSALLHSSTMVVAGIFLLVRMGPLMQNNQTALTIC
Mafr   LAATGKSAQFGLHPWLPSAMEGPTPVSALLHSSTMVVAGIFLLIRMAPLMHNNQTVLTTC
Dcpe   IAATGKSAQFGLHPWLPSAMEGPTPVSALLHSSTMVVAGIFLLIRISPLMENNQVALTLC
Dcti   IAAAGKSAQFGLHPWLPSAMEGPTPVSALLHSSTMVVAGVFLLIRISPLMENNPTALTLC
Hehi   IAATGKSAQFGLHPWLPSAMEGPTPVSALLHSSTMVVAGIFLLVRTSPLLENNQTALTTC
Stam   IAATGKSAQFGLHPWLPSAMEGPTPVSALLHSSTMVVAGIFLLVRMSPLMETNETASTIC
Hogi   IAATGKSAQFGLHPWLPSAMEGPTPVSALLHSSTMVVAGIFLLIRMSPLLEQNETALTIC
Erzo   LAATGKSAQFGLHPWLPSAMEGPTPVSALLHSSTMVVAGIFLLIRMSPLLEGNQTALTTC
Hxot   LAATGKSAQFGLHPWLPSAMEGPTPVSALLHSSTMVVAGIFLLVRMSPLLENNQTALTTC
Core   LAATGKSAQFGLHPWLPSAMEGPTPVSALLHSSTMVVAGIFLLVRMSPLLENNQTALTTC
Apve   LAATGKSAQFGLHPWLPSAMEGPTPVSALLHSSTMVVAGIFLLVRMSPLLENNQTALTTC
Latj   IAATGKSAQFGLHPWLPSAMEGPTPVSALLHSSTMVVAGIFLLIRMSPLMENNQTALTTC
Laja   IAATGKSAQFGLHPWLPAAMEGPTPVSALLHSSTMVVAGIFLLIRMSPLMENNQTALTTC
```

To be continued on page 28.

[5/12 of aligned sequences]

Syja	LAATGKSAQFGLHPWLPSAMEGPTPVSALLHSSTMVVAGIFLLVRLSPLMENNQTALTTC
Epme	VAATGKSAQFGLHPWLPAAMEGPTPVSALLHSSTMVVAGIFLLVRMSPLLENNTTALTTC
Grse	LAASGKSAQFGLHPWLPSAMEGPTPVSALLHSSTMVVAGIFLLVRVSPFLEMSEAALTLC
Clja	VAATGKSAQFGLHPWLPSAMEGPTPVSALLHSSTMVVAGIFLLIRMSPLMEQNPTAQTTC
Ogcy	LAATGKSAQFGLHPWLPSAMEGPTPVSALLHSSTMVVAGIFLLIRFNSIMETSENALTIC
Plna	LAATGKSAQFGLHPWLPSAMEGPTPVSALLHSSTMVVAGIFLLIRMSPLMETNSTALTTC
Lema	IAATGKSAQFGLHPWLPSAMEGPTPVSALLHSSTMVVAGIFLLVRLSPLMEHNQTALTTC
Etzo	VAATGKSAQFGLHPWLPSAMEGPTPVSALLHSSTMVVAGIFLLIRMSPLLADNSTALTVC
Apse	VAATGKSAQFGLHPWLPSAMEGPTPVSALLHSSTMVVAGIFLLIRMSPLLAGNQTALTIC
Epde	IAATGKSAQFGLHPWLPSAMEGPTPVSALLHSSTMVVAGIFLLVRMSPLMENNQTALTTC
Slja	VAATGKSAQFGLHPWLPEAMEGPTPVSALLHSSTMVVAGIFLLIRMSPLLESNKTALTIC
Bsja	LAATGKSAQFGLHPWLPSAMEGPTPVSALLHSSTMVVAGIFLLIRVSPLMENNQTALTTC
Ecna	IAATGKSAQFGLHPWLPSAMEGPTPVSALLHSSTMVVAGIFLLIRTSPLMQNNQLALTTC
Cohi	VAATGKSAQFGLHPWLPAAMEGPTPVSALLHSSTMVVAGIFLLIRVSPLMESNQTALTIC
Caar	IAATGKSAQFGLHPWLPSAMEGPTPVSALLHSSTMVVAGIFLLVRMSPLMENNQTALTAC
Came	VAATGKSAQFGLHPWLPSAMEGPTPVSALLHSSTMVVAGIFLLVRMSPLMENNQTALTTC
Mema	IAATGKSAQFGLHPWLPSAMEGPTPVSALLHSSTMVVAGIFLLVRMSPLMENNQTALTIC
Lenu	LAATGKSAQFGLHPWLPSAMEGPTPVSALLHSSTMVVAGIYLLVQMSPLLSNNKTALTTC
Brja	LAATGKSAQFGLHPWLPSAMEGPTPVSALLHSSTMVVAGIFLLIRLSPLMENNQTALSLC
Plma	VAATGKSAQFGLHPWLPSAMEGPTPVSALLHSSTMVVAGIFLLIRMSPLLENNQTALTLC
Emst	VAATGKSEQFGMHPWLPCAMEGPTPVYAMLHSSTMDVAGIFLLVRMSPLMENNQTALTTC
Ptti	LAATGKSAQFGLHPWLPSAMEGPTPVSALLHSSTMVVAGIFLLVRMSPLMENNQTALTTC
Losu	LAATGKSAQFGLHPWLPSAMEGPTPVSALLHSSTMVVAGIFLLVRMSPLMENNQTALTTC
Geoy	IAATGKSAQFGLHPWLPSAMEGPTPVSALLHSSTMVVAGIFLLVRMSPLMEDNPLALTIC
Dipi	LAATGKSAQFGLHPWLPSAMEGPTPVSALLHSSTMVVAGIFLLIRMSPLLENNQTALTTC
Pama	IAATGKSAQFGLHPWLPSAMEGPTPVSALLHSSTMVVAGIFLLIRMSPLMENNQTALTTC
Leob	LAATGKSAQFGLHPWLPSAMEGPTPVSALLHSSTMVVAGIFLLIRTSPLMENNPTALTTC
Neba	LAATGKSAQFGLHPWLPSAMEGPTPVSALLHSSTMVVAGIFLLIRVSPMLENNHIALTTC
Pdpl	VAAAGKSAQFGLHPWLPSAMEGPTPVSALLHSSTMVVAGIFLLIRMAPLLEQSPAALCTC
Nimi	LAATGKSAQFGLHTWLPAAMEGPTPVSALLHSSTMVVAGIFLLIRLSPLLANNQMALTIC
Uptr	VAATGKSAQFGLHPWLPSAMEGPTPVSALLHSSTMVVAGIFLLIRMSPMLENNQTALTLC
Pesc	LAATGKSAQFGLHPWLPAAMEGPTPVSALLHSSTMVVAGIFLLIRMAPLMENNQTALTAC
Baar	LAATGKSAQFGLHPWLPSAMEGPTPVSALLHSSTMVVAGIFLLIRMSPLMQENQTALTTC
Moar	VAATGKSAQFGLHPWLPSAMEGPTPVSALLHSSTMVVAGIFLLIRLSPLMENNQTALTTC
Toja	LAATGKSAQFGLHPWLPSAMEGPTPVSALLHSSTMVVAGIFLLIRMSPLMENNQTALTVC
Chau	LAATGKSAQFGLHPWLPAAMEGPTPVSALLHSSTMVVAGIFLLIRMSPLLENNQVALTTC
Chse	LAATGKSAQFGLHPWLPAAMEGPTPVSALLHSSTMVVAGIFLLVRMSPLMENNPTALTTC
Enar	IAATGKSAQFGLHPWLPSAMEGPTPVSALLHSSTMVVAGIFLLIRMSPLMENNQTALTVC
Hpty	IAATGKSAQFGLHPWLPSAMEGPTPVSALLHSSTMVVAGIFLLVRMSPLMENNQTALTTC
Nana	LAAAGKSAQFGLHPWLPSAMEGPTPVSALLHSSTMVVAGIFLLIRLSPMLENNPPALTTC
Mcst	IAATGKSAQFGLHPWLPSAMEGPTPVSALLHSSTMVVAGIFLLIRMSPLMENNPIALTTC
Rhox	LAATGKSAQFGLHPWLPSAMEGPTPVSALLHSSTMVVAGIFLLIRMSPLLETNPAALTIC
Opfa	IAATGKSAQFGLHPWLPSAMEGPTPVSALLHSSTMVVAGIFLLVRMSPLLENNQTALTIC
Paar	IAATGKSAQFGLHPWLPSAMEGPTPVSALLHSSTMVVAGIFLLIRMSPLLENNQTALTIC
Gozo	IAATGKSAQFGLHPWLPSAMEGPTPVSALLHSSTMVVAGIFLLIRMSPLMENNPTALTVC
Ackr	LAATGKSAQFGLHPWLPAAMEGPTPVSALLHSSTMVVAGIFLLIRFYPLMENNQAAHSLC
Elev	LAATGKSAQFGLHPWLPAAMEGPTPVSALLHSSTMVVAGIFLLIRMSPLLDTSPMVATIC
Trdu	IAATGKSAQFGLHPWLPSAMEGPTPVSALLHSSTMVVAGIFLLIRMSPMLENNQTALTIC
Amoc	IAATGKSAQFGLHPWLPSAMEGPTPVSALLHSSTMVVAGIFLLVRMSPLLENNPTALTTC
Hame	LAATGKSAQFGLHPWLPAAMEGPTPVSSLLHSSTMVVAGIFLLIRFSDLLATSQLASTIC
Chso	LAATGKSAQFGLHPWLPSAMEGPTPVSALLHSSTMVVAGIFLLVRTSPMMEHNQTALTTC
Lyto	LAATGKSAQFGLHPWLPSAMEGPTPVSALLHSSTMVVAGIFLLVRMSPLLEGNQTALTTC

To be continued on page 29.

[5/12 of aligned sequences]

To be continued on page 30.

```
Encr    VAATGKSAQFGLHPWLPSAMEGPTPVSALLHSSTMVVAGVFLLVRMSPLLEGNQTALTTC
Bvar    IAAAGKSAQFGLHPWLPSAMEGPTPVSALLHSSTMVVAGIFLLIRMHPLFENNNNALTIC
Noco    IAAAGKSAQFGLHPWLPSAMEGPTPVSALLHSSTMVVAGIFLLIRMNPMIEKSPAALTLC
Chsp    IAAAGKSAQFGLHPWLPAAMEGPTPVSALLHSSTMVVAGIFLLVRMSPMMENNQAALTTC
Arja    LAATGKSAQFGLHPWLPSAMEGPTPVSALLHSSTMVVAGIFLLVRMSPLLEGNQIALTTC
Pase    IAATGKSAQFGLHPWLPAAMEGPTPVSALLHSSTMVVAGIFLLVRLSPLMENNQAALTIC
Trel    LAATGKSAQFGLHPWLPSAMEGPTPVSALLHSSTMVVAGIFLLIRMSPFLQNNQTALTAC
Lifa    LAAAGKSALFGLHPWLPAAMEGPTPVSALLHSSTMVVAGVFLSIRLSPMMENNQIILSTC
Acur    IAASGKSAQFGLHPWLPSAMEGPTPVSALLHSSTMVVAGIFLLIRMAPLLEVNKTAQTIC
Ampe    IAATGKSAQFGLHPWLPSAMEGPTPVSALLHSSTMVVAGIFLLIRMSPLMETNQTALTTC
Urja    LAAAGKSAQFGLHPWLPSAMEGPTPVSALLHSSTMVVAGIFLLVRMSPFMETNQTALTLC
Enet    LAATGKSAQFGLHPWLPSAMEGPTPVSALLHSSTMVVAGIFLLIRMSPLITDNPIALTTC
Ptbr    LAATGKSAQFGLHPWLPAAMEGPTPVSALLHSSTMVVAGVFLLIRMSPLLDLNASAQTTC
Safa    LAATGKSAQFGLHPWLPSAMEGPTPVSALLHSSTMVVAGIFLLIRLSPLMDNNYTAQTTC
Icae    VAATGKSAQFGLHPWLPSAMEGPTPVSALLHSSTMVVAGIFLLVRMSPLMENNQAALTLC
Asmi    LAAMSKSAQFGMHPWLPSAMEGPTPVSALLHSSTMVIAGVFLLVRFYPVIKTSPLALTTC
Foal    LAATGKSAQFGLHPWLPAAMEGPTPVSALLHSSTMVVAGIFLLIRMNPLIAQNPHALTTC
Drze    IAAAGKSAQFGLHPWLPSAMEGPTPVSALLHSSTMVVAGIFLLIRLNPLMENNQMALTLC
Rhas    VAATGKSAQFGLHPWLPSAMEGPTPVSALLHSSTMVVAGIFLLVRMSPLMENNPTSLTIC
Elac    VAATGKSAQFGLHPWLPSAMEGPTPVSALLHSSTMVVAGIFLLIRMSPLMEHNTTALTIC
Kugu    LAATGKSAQFGLHPWLPSAMEGPTPVSALLHSSTMVVAGIFLLIRMSPLLENNHTALTIC
Plor    IAATGKSAQFGLHPWLPSAMEGPTPVSALLHSSTMVVAGIFLLIRISPLMENNQTALTIC
Sgun    VAATGKSAQFGLHPWLPSAMEGPTPVSALLHSSTMVVAGIFLLVRMSPLMENNQTALTIC
Zaco    IAATGKSAQFGLHPWLPSAMEGPTPVSALLHSSTMVVAGIFLLIRMSPLMENNQTALTTC
Zbfl    LAATGKSAQFGLHPWLPSAMEGPTPVSALLHSSTMVVAGIFLLIRMSPLMENNQTALTIC
Spba    IAATGKSAQFGLHPWLPSAMEGPTPVSALLHSSTMVVAGIFLLVRMSPLMENNHTALTIC
Game    VAATGKSAQFGLHPWLPSAMEGPTPVSALLHSSTMVVAGIFLLVRMSPLMENNQTALTLC
Thth    VAATGKSAQFGLHPWLPSAMEGPTPVSALLHSSTMVVAGIFLLVRMSPLMENNQTALTLC
Xigl    IAATGKSAQFGLHPWLPSAMEGPTPVSALLHSSTMVVAGIFLLVRMSPLMENNQTALTIC
Hyja    VAATGKSAQFGLHPWLPSAMEGPTPVSALLHSSTMVVAGIFLLIRMSPLMENNPTALTLC
Psan    MAAAGKSAQFGFHPWLPSAMEGPTPVSALLHSSTMVVAGVFLLIRMSPLMEDNPMALTLC
Cupa    LAATGKSAQFGLHPWLPSAMEGPTPVSALLHSSTMVVAGIFLLIRMSPLMENNQTALTLC
Mpch    VAATGKSAQFGLHPWLPSAMEGPTPVSALLHSSTMVVAGIFLLIRMAPLMENNPFALTTC
Char    LAATGKSAQFGLHPWLPSAMEGPTPVSALLHSSTMVVAGIFLLVRMSPLLENNQTALTTC
Pser    IAATGKSAQFGLHPWLPAAMEGPTPVSALLHSSTMVVAGIFLLIRLNPLMENNQTALTIC
Prol    VAATGKSAQFGLHPWLPAAMEGPTPVSALLHSSTMVVAGIFLLVRMSPLLENNPMALTTC
Plbi    VAATGKSAQFGLHPWLPSAMEGPTPVSALLHSSTMVVAGIFLLVRMSPLLENNPTALTTC
Calu    LAATGKSAQFGLHPWLPSAMEGPTPVSALLHSSTMVVAGIFLLVRMGPLLEHNPLVLTTC
Papa    IAATGKSAQFGLHPWLPSAMEGPTPVSALLHSSTMVVAGIFLLIRLNPLIENNQMALTTC
Sufr    LAATGKSAQFGLHPWLPSAMEGPTPVSALLHSSTMVVAGIFLLIRLSPLMEHNQTALTTC
Stci    LAATGKSAQFGLHPWLPAAMEGPTPVSALLHSSTMVVAGIFLLIRLSPLLNNNPTALTTC
Taru    LAATGKSAQFGLHPWLPSAMEGPTPVSALLHSSTMVVAGIFLMIRISPLLETNPTALTLC
Rala    VAATGKSAQFGLHPWLPSAMEGPTPVSALLHSSTMVVAGIFLLIRLSPLMEHNPTALTIC
        :.* .**  : :* **  *****.** ::** *** :**: : ::    .
```

[6/12 of aligned sequences]

```
                 H                   I                  J
Scca  LCLGAITTLFTATCALTQNDIKKIIAFSTSSQLGLMMVTIGLNQPQLAFLHICTHAFFKA
Muma  LCLGALTTLFTATCALTQNDIKKIVAFSTSSQLGLMMVTIGLNQPQLAFLHICTHAFFKA
Erca  LCLGAITTFFTAACALTQNDIKKIVAFSTSSQLGLMMVAIGLNQPQLAFMHICTHAFFKA
Pose  LCLGAITTLFTATCALTQNDIKKIVAFSTSSQLGLMMVAIGLNQPQLAFLHICTHAFFKA
Actr  LCLGATTTLFTAACALTQNDIKKIVAFSTSSQLGLMMVTIGLNQPQLAFLHICTHAFFKA
Scal  LCLGAATTLFTATCALTQNDIKKIVAFSTSSQLGLMMVTIGLNQPQLAFLHICTHAFFKA
Posp  LCLGATTTLFTAACALTQNDIKKIVAFSTSSQLGLMMVTIGLNQPQLAFLHICTHAFFKA
Atsp  LCLGALSTMFAATCALTQNDLKKIVAFSTSSQLGLMMVTIGLNQPQLAFLHICTHAFFKA
Leoc  LCLGALSTMFAATCALTQNDLKKIVAFSTSSQLGLMMVTIGLNQPQLAFLHICTHAFFKA
Amca  LCLGALTTLFTATCALTQNDIKKIVAFSTSSQLGLMMVTIGLNQPQLAFLHICTHAFFKA
Osbi  LCLGALTTLFTATCALTQNDIKKIIAFSTSSQLGLMMVTIGLNQPQLAFLHICTHAFFKA
Pabu  LCLGALTTLFTATCALTQNDIKKIIAFSTSSQLGLMMVTIGLNQPQLAFLHICTHAFFKA
Hial  LCLGALTTLFTATCALTQNDIKKIVAFSTSSQLGLMMVTIGLNQPQLAFLHICTHAFFKA
Elha  LCLGALTTLFTATCALTQNDIKKIVAFSTSSQLGLMMVTIGLNQPQLAFLHICTHAFFKA
Mlcy  LCLGALTTLFTATCALTQNDIKKIVAFSTSSQLGLMMVTIGLNQPQLAFLHICTHAFFKA
Algl  LCLGALTSLFAATCALTQNDIKKIVAFSTSSQLGLMMVAIGLNQPQLAFLHICTHAFFKA
Ptgi  LCLGALTTLFTATCALTQNDIKKIVAFSTSSQLGLMMVTIGLNQPQLAFLHICTHAFFKA
Alaf  LCLGALTTLFTATCALTQNDIKKIVAFSTSSQLGLMMVTIGLNQPQLAFLHICTHAFFKA
Nock  LCLGALTTLFTATCALTQNDIKKIVAFSTSSQLGLMMVTIGLNQPQLAFLHICTHAFFKA
Anja  LCLGALTTLFTATCALTQNDIKKIVAFSTSSQLGLMMVTIGLNQPQLAFMHICTHAFFKA
Gyki  LCLGALTTLFTAACALTQNDIKKIVAFSTSSQLGLMMVTIGLNQPQLAFMHICTHAFFKA
Syka  LCLGALTTLFTATCALTQNDIKKIVAFSTSSQLGLMMVTIGLNQPQLAFMHICTHAFFKA
Opma  LCLGALTTLFTATCALTQNDIKKIVAFSTSSQLGLMMVTIGLNQPQLAFLHICTHAFFKA
Comy  LCLGALTTLFTATCALTQNDIKKIVAFSTSSQLGLMMVTIGLNQPQLAFLHICTHAFFKA
Sasp  LCLGALTTLFTATCALTQNDIKKIIAFSTSSQLGLMMVTIGLNQPQLAFMHICTHAFFKA
Eupe  LCLGALTTLFTATCALTQNDIKKIIAFSTSSQLGLMMVTIGLNQPQLAFIHICTHAFFKA
Enja  LCLGALTTLFTATCALTQNDIKKIVAFSTSSQLGLMMVTIGLNQPQLAFLHICTHAFFKA
Same  LCLGALTTLFTATCALTQNDIKKIVAFSTSSQLGLMMVTIGLNQPQLAFFHICTHAFFKA
Chch  LCLGALTTLFTATCALTQNDIKKIVAFSTSSQLGLMMVTIGLNQPQLAFLHICTHAFFKA
Grgr  LCLGALTTVFTATCALTQNDIKKIVAFSTSSQLGLMMVTIGLGQPQLAFLHICTHAFFKA
Caau  LCLGALTSLFTATCALTQNDIKKIVAFSTSSQLGLMMVTIGLNQPQLAFLHICTHAFFKA
Cyca  LCLGALTSLFTATCALTQNDIKKIVAFSTSSQLGLMMVTIGLNQPQLAFLHICTHAFFKA
Dare  LCLGALTTLFAATCALTQNDIKKIVAFSTSSQLGLMMVAIGLNQPHLAFFHICTHAFFKA
Cost  LCLGALTTLFTAACALTQNDIKKIVAFSTSSQLGLMMVTIGLNQPQLAFLHICTHAFFKA
Leec  LCLGALTTLFTAACALTQNDIKKIVAFSTSSQLGLMMVTIGLNQPQLAFLHICTHAFFKA
Fola  LCLGALTSLFTAACALTQNDIKKIVAFSTSSQLGLMMVTIGLNQPQLAFLHICTHAFFKA
Clmc  LCLGALTTLFTATCALTQNDIKKIVAFSTSSQLGLMMVTIGLNQPQLAFLHICTHAFFKA
Phin  LCLGALTTLFTATCALTQNDIKKIIAFSTSSQLGLMMVTIGLNQPQLAFLHICTHAFFKA
Icpu  LCLGALTTLFTATCALTQNDIKKIVAFSTSSRLGLMMVTIGLNQPQLAFLHICTHAFFKA
Psto  LCLGALTTLFTATCALTQNDIKKIVAFSTSSQLGLMMVTIGLNQPQLAFLHICTHAFFKA
Cora  LCLGALTTLFTATCALTQNDIKKIVAFSTSSQLGLMMVTIGLNQPQLAFLHICTHAFFKA
Eisp  LCLGALTTLFTATCALTQNDIKKIVAFSTSSQLGLMMVTIGLNQPQLAFLHICTHAFFKA
Apal  LCLGALTTLFTATCALTQNDIKKIVAFSTSSQLGLMMVTIGLNHPQLAFLHICTHAFFKA
Eslu  LCLGALTTLFTATCALTQNDIKKIVAFSTSSQLGLMMVTIGLNQPQLAFLHICTHAFFKA
Dape  LCLGALTTLFTATCALTQNDIKKIIAFSTSSQLGLMMVTIGLNQPQLAFLHICTHAFFKA
Glse  LCLGALTTLFTATCALTQNDIKKIIAFSTSSQLGLMMVTIGLNQPQLAFLHICTHAFFKA
Naar  LCLGALTTLFTATCALTQNDIKKIIAFSTSSQLGLMMVTIGLGQPQLAFLHICTHAFFKA
Lioc  LCLGALTTLFTATCALTQNDIKKIIAFSTSSQLGLMMVTIGLNQPQLAFLHICTHAFFKA
Opso  LCLGALTTLFTATCALTQNDIKKIIAFSTSSQLGLMMVTIGLNQPQLAFLHICTHAFFKA
Alte  LCLGALTTLFTATCALTQNDIKKIVAFSTSSQLGLMMVTIGLNQPQLAFLHICTHAFFKA
Plap  LCLGALTTLFTATCALTQNDIKKIVAFSTSSQLGLMMVTIGLNQPQLAFLHICTHAFFKA
```

To be continued on page 31.

[6/12 of aligned sequences]

```
Plal    LCLGALTTLFTATCALTQNDIKKIVAFSTSSQLGLMMVTIGLDQPQLAFLHICTHAFFKA
Sami    LCLGALTTLFTATCALTQNDIKKIVAFSTSSQLGLMMVTIGLNQPQLAFLHICTHAFFKA
Rere    LCLGALTTLFTATCALTQNDIKKIVAFSTSSQLGLMMVTIGLNQPQLAFLHICTHAFFKA
Gama    LCLGALTTLFTATCALTQNDIKKIVAFSTSSQLGLMMVTIGLGQPQLAFLHICTHAFFKA
Onmy    LCLGALTTLFTATCALTQNDIKKIVAFSTSSQLGLMMVTIGLNQPQLAFLHICTHAFFKA
Sasa    LCLGALTTLFTATCALTQNDIKKIVAFSTSSQLGLMMVTIGLNQPQLAFLHICTHAFFKA
Cola    LCLGALTTLFTATCALTQNDIKKIVAFSTSSQLGLMMVTIGLNQPQLAFLHICTHAFFKA
Dita    LCLGALTTFFTATCALTQNDIKKIVAFSTSSQLGLMMVTLGLNQPHLAFLHICTHAFFKA
Gogr    LCLGALTSLFTAACALTQNDIKKIVAFSTSSQLGLMMVTLGLNQPQLAFLHICTHAFFKA
Chsl    LCLGALTTLFTATCALTQNDIKKIVAFSTSSQLGLMMVTIGLNQPQLAFLHICTHAFFKA
Atja    LCLGALTTLFTAACALTQNDIKKIVAFSTSSQLGLMMVTIGLNQPQLAFLHICTHAFFKA
Iido    LCLGALTTLFTAACALTQNDIKKIVAFSTSSQLGLMMVTIGLNQPQLAFLHICTHAFFKA
Auja    LCLGALTTLFTATCALTQNDIKKIVAFSTSSQLGLMMVTIGLNQPQLAFMHICTHAFFKA
Chag    LCLGALTTLFTATCALTQNDIKKIVAFSTSSQLGLMMVTIGLNQPQLAFLHICTHAFFKA
Hami    LCLGALTTLFTATCALTQNDIKKIVAFSTSSQLGLMMVTIGLNQPQLAFLHICTHAFFKA
Saun    LCLGALTTLFTATCALTQNDIKKIVAFSTSSQLGLMMVTIGLNQPQLAFLHICTHAFFKA
Nema    LCLGALTTLFTATCALTQNDIKKIVAFSTSSQLGLMMVTIGLNQPQLAFLHICTHAFFKA
Disp    LCLGALTTLFTATCALTQNDIKKIVAFSTSSQLGLMMVTIGLNQPQLAFLHICTHAFFKA
Myaf    LCLGALTTLFTATCALTQNDIKKIVAFSTSSQLGLMMVTIGLNQPQLAFLHICTHAFFKA
Lagu    LCLGALTTLFTAICALTQNDIKKIVAFSTSSQLGLMMVTIGLNQPQLAFLHICTHAFFKA
Trtr    LCLGALTTVFTAICALTQNDIKKIVAFSTSSQLGLMMVTIGLNLPQLAFLHICTHAFFKA
Zucr    LCLGAFTTLFTAICALTQNDIKKIVAFSTSSQLGLMMVTIGLNLPQLAFLHICTHAFFKA
Pxja    LCLGALTTLFTAACALTQNDIKKIVAFSTSSQLGLMMVTIGLNQPQLAFLHICTHAFFKA
Pxlo    LCLGALTTLFTAACALTQNDIKKIVAFSTSSQLGLMMVTIGLNQPQLAFLHICTHAFFKA
Pctr    LCLGALTTLFTATCALTQNDIKKIVAFSTSSQLGLMMVTIGLGHPQLAFLHICTHAFFKA
Apsa    LCLGALTTLFTATCALTQNDIKKIVAFSTSSQLGLMMVTIGLNQPQLAFLHICTHAFFKA
Cabe    LCLGSLTALVNAFFALVQNDIKKIVAFSTSSQLGLMMVTIGLNQPQLAFLHICTHAFFKA
Bzze    LCLGALTTLFTATCALTQNDIKKIVAFSTSSQLGLMMVTIGLNQPQLAFLHICTHAFFKA
Siim    LCLGALTTSFTATCALTQNDIKKIVAFSTSSQLGLMMVTIGLNQPQLAFLHICTHAFFKA
Ctru    LCLGALTTLFTATCALTQNDIKKIVAFSTSSQLGLMMVTIGLNQPQLAFLHICTHAFFKA
Dpbr    LCLGALTTLFTATCALTQNDIKKIVAFSTSSQLGLMMVTIGLNQPQLAFLHICTHAFFKA
Caki    LCLGAMTTTFTAICALTQNDIKKIIAFSTSSQLGLMMVTIGLNQPQLTFFHICTHAFFKA
Phja    LCLGALTALFSAICALTQNDIKKIIAFSTSSQLGLMMVSIGLNQPELAFLHIFTHAFFKA
Brsp    LCLGAITTMFTATCALTQNDIKKIVAFSTSSQLGLMMVTIGLNQPQLAFLHICTHAFFKA
Gamo    LCLGALTTMFTATCALTQNDIKKIVAFSTSSQLGLMMVTIGLNQPQLAFLHICTHAFFKA
Lolo    LCLGALTTMFTATCALTQNDIKKIVAFSTSSQLGLMMVTIGLNQPQLAFLHICTHAFFKA
Batr    LCLGAMTTMFTAACALTQNDIKKIVAFSTSSQLGLMMVAIGLNMPHLAFFHICTHAFFKA
Prmy    LCLGAITTVFTATCALTQNDIKKIIAFSTSSQLGFMLVTVGLNMPQLAFFHICTHAFFKA
Lose    LCLGALTTLFTATCALTQHDIKKIIAFSTSSQLGLMMVTIGLNQPQLAFLHICTHAFFKA
Loam    LCLGALTTLFTATCALTQNDIKKIVAFSTSSQLGLMMVTIGLNQPQLAFLHICTHAFFKA
Chab    LCLGALTTLFTATCALTQNDIKKIIAFSTSSQLGLMMVTIGLNQPQLAFFHICTHAFFKA
Chto    LCLGALTTLFTATCALTQNDIKKIIAFSTSSQLGLMMVTIGLNQPQLAFFHICTHAFFKA
Majo    LCLGALTSFFAAVCALTQNDIKKIIAFSTSSQLGLMMVSIGLNQPQLAFLHICTHAFFKA
Hlst    MCLGALTSFFAAVCALTQNDIKKIVAFSTSSQLGLMMVAVGLNQPQLAFLHVCTHAFFKA
Clpe    LCLGALTTLFTALCALTQNDIKKIVAFSTSSQLGLMMVTIGLNQPQLAFLHICTHAFFKA
Mlmr    LCLGALTTLFTATCALTQNDIKKIVAFSTSSQLGLMMVTIGLNQPQLAFLHICTHAFFKA
Crcr    LCLGALTTFFTAACALTQNDIKKIVAFSTSSQLGLMMVTIGLNQPELAFLHICTHAFFKA
Muce    LCLGALTTFFTAACALTQNDIKKIVAFSTSSQLGLMMVTIGLNQPELAFLHICTHAFFKA
Bege    LCLGALTTLFTATCALTQNDIKKIVAFSTSSQLGLMMVTIGLNQPQLAFLHICTHAFFKA
Mela    LCLGALTTLFTATCALTQNDIKKIVAFSTSSQLGLMMVTIGLNQPQLAFLHICTHAFFKA
Hats    LCLGAMTTLFTATCALTQNDIKKIVAFSTSSQLGLMMVTIGLNQPQLAFLHICTHAFFKA
Orla    LCLGALTTVFTATCALTQNDIKKIIAFSTSSQLGLMMVTIGLNQPQLAFLHICTHAFFKA
```

To be continued on page 32.

[6/12 of aligned sequences]

To be continued on page 33.

```
Cosa LCLGALTTLFAATCALTQNDIKKIVAFSTSSQLGLMMVAIGLNQPQLAFLHICTHAFFKA
Exsp LCLGALTTLFTATCALTQNDIKKIVAFSTSSQLGLMMVTIGLNQPQLAFFHICTHAFFKA
Depa LCLGALTTLFTATCALTQNDIKKIIAFSTSSQLGLMMVSIGLNQPQLAFLHICTHAFFKA
Rima LCVGALTTLFTATCALTQNDIKKIVAFSTSSQLGLMMVAIGLNQPHLAFLHICTHAFFKA
Fuol LCLGALTTLFTATCALTQNDIKKIVAFSTSSQLGLMMVTIGLNQPQLAFLHICTHAFFKA
Gmaf LCLGALTTLFTAICALTQNDIKKIVAFSTSSQLGLMMVTIGLNQPQLAFLHICTHAFFKA
Xeei LCLGALTTLFTATCALTQNDIKKIVAFSTSSQLGLMMVTIGLNQPQLAFLHICTHAFFKA
Pros LCLGALTTLFTATCALTQNDIKKIVAFSTSSQLGLMMVTVGLNQPQLAFLHICTHAFFKA
Scmi LCLGALTTLFTATCALTQNDIKKIVAFSTSSQLGLMMVTIGLNQPQLAFLHICTHAFFKA
Rolo LCLGALTTLFTATCALTQNDIKKIIAFSTSSQLGLMMVTIGLNQPQLAFLHICTHAFFKA
Cere LCLGALTTLFTATCALTQNDIKKIIAFSTSSQLGLMMVAVGLNQPQLAFLHICTHAFFKA
Daga LCLGALTTLFTATCALTQNDIKKIIAFSTSSQLGLMMVAIGLGQPNLAFVHICTHAFFKA
Anco LCLGALTTLFTATCALTQNDIKKIVAFSTSSQLGLMMVTIGLNQPQLAFLHICTHAFFKA
Dmve LCLGALTTLFTATCALTQNDIKKIIAFSTSSQLGLMMVTIGLNQPQLAFLHICTHAFFKA
Dmar LCLGALTTLFTATCALTQNDIKKIIAFSTSSQLGLMMVTIGLNQPQLAFLHICTHAFFKA
Anka LCLGALTTLFTATCALTQNDIKKIVAFSTSSQLGLMMVTIGLNQPQLAFLHICTHAFFKA
Moja LCLGALTTVFTATCALTQNDIKKIVAFSTSSQLGLMMVTIGLNQPQLAFLHICTHAFFKA
Hoja LCLGALTTLFTATCALTQNDIKKIVAFSTSSQLGLMMVTIGLNQPQLAFLHICTHAFFKA
Bede LCLGALTTLFTATCALTQNDIKKIVAFSTSSQLGLMMVTIGLNQPQLAFLHICTHAFFKA
Besp LCLGALTTLFTATCALTQNDIKKIVAFSTSSQLGLMMVTIGLNQPQLAFLHICTHAFFKA
Mysp LCLGALTTLFTATCALTQNDIKKIVAFSTSSQLGLMMVAIGLNQPHLAFLHICTHAFFKA
Osja LCLGALTTLFTATCALTQNDIKKIVAFSTSSQLGLMMVAIGLNQPHLAFLHICTHAFFKA
Sgro LCLGALTTLFTATCALTQNDIKKIVAFSTSSQLGLMMVAIGLNQPHLAFLHICTHAFFKA
Pzpa LCLGALTTLFTATCALTQTDIKKIIAFSTSSQLGLMMVSIGLNQPQLAFLHICTHAFFKA
Zeja LCLGALTTLFTAICALTQNDIKKIIAFSTSSQLGLMMVTIGLNQPQLAFLHICTHAFFKA
Znne LCLGALTTLFTATCALTQNDIKKIVAFSTSSQLGLMMVTIGLNQPQLAFLHICTHAFFKA
Zefa LCLGALTTLFTATCALTQNDIKKIVAFSTSSQLGLMMVTIGLNQPQLAFLHICTHAFFKA
Acni LCLGALTTLFTATCALTQNDIKKIVAFSTSSQLGLMMVTIGLNQPQLAFLHICTHAFFKA
Ncrh LCLGALTTLFTATCALTQNDIKKIVAFSTSSQLGLMMVTIGLNQPQLAFLHICTHAFFKA
Agca LCLGALTTLFTATCALTQNDIKKIVAFSTSSQLGLMMVTIGLNQPQLAFLHICTHAFFKA
Hydy LCLGALTTLFAATCALTQNDIKKIVAFSTSSQLGLMMVAIGLNQPHLAFLHICTHAFFKA
Gsac LCLGALTTLFTATCALTQNDIKKIVAFSTSSQLGLMMVAIGLNQPHLAFLHICTHAFFKA
Pevo LCLGALTTVFTAICALTQNDIKKIVAFSTSSQLGLMMVTIGLNQPQLAFLHICTHAFFKA
Hiku LCLGALTTLFTAVCALTQNDIKKIVAFSTSSQLGLMMVTIGLNQPQLAFLHICTHAFFKA
Inpa LCLGALTTVFTAICALTQNDIKKIIAFSTSSQLGLMMVTIGLNQPQLAFLHICTHAFFKA
Auch LCLGALTTFFTATCALTQNDIKKIIAFSTSSQLGLMMVAIGLNQPHLAFLHICTHAFFKA
Fico LCLGALTTLFTATCALTQNDIKKIVAFSTSSQLGLMMVTIGLNQPQLAFLHICTHAFFKA
Macs LCLGALTTLFTATCALTQNDIKKIVAFSTSSQLGLMMVTIGLNQPQLAFLHICTHAFFKA
Moal LILGALTTLFTTICALTQNDIKKIIAFSTSSQLGLMMVTIGLNQPYLAFLHICTHAFFKA
Syma LTLGAITTLFTAICALTQNDIKKIIAFSTSSQLGLMMVTIGLNQPSLAFLHICTHAFFKA
Mafr LCLGALTTLFTATCALTQNDIKKIVAFSTSSQLGLMMVAIGLNQPQLAFLHISTHAFFKA
Dcpe LCLGALTTLFTATCALTQNDIKKIVAFSTSSQLGLMMVTLGLNQPHLAFLHICTHAFFKA
Dcti LCLGALTTLFTATCALTQNDIKKIVAFSTSSQLGLMMVTIGLNQPQLAFLHICTHAFFKA
Hehi LCLGALTTLFTATCALTQNDIKKIVAFSTSSQLGLMMVTIGLNQPQLAFLHICTHAFFKA
Stam LCLGALTTLFTATCALTQNDIKKIVAFSTSSQLGLMMVTIGLNQPQLAFLHICTHAFFKA
Hogi LCLGALTTLFTATCALTQNDIKKIVAFSTSSQLGLMMVTIGLNQPQLAFLHICTHAFFKA
Erzo LCLGALTTLFTATCALTQNDIKKIVAFSTSSQLGLMMVTIGLNQPQLAFLHICTHAFFKA
Hxot LCLGALTTLFTATCALTQNDIKKIVAFSTSSQLGLMMVTIGLNQPQLAFLHICTHAFFKA
Core LCLGALTTLFTATCALTQNDIKKIVAFSTSSQLGLMMVTIGLNQPQLAFLHICTHAFFKA
Apve LCLGALTTLFTATCALTQNDIKKIIAFSTSSQLGLMMVTIGLNQPQLTFLHICTHAFFKA
Latj LCLGALTTLFTATCALTQNDIKKIVAFSTSSQLGLMMVTIGLNQPQLAFLHICTHAFFKA
Laja LCLGALTTLFTATCALTQNDIKKIVAFSTSSQLGLMMVTIGLNQPQLAFLHICTHAFFKA
```

[6/12 of aligned sequences]

To be continued on page 34.

Syja	LCLGALTTLFTATCALTQNDIKKIVAFSTSSQLGLMMVTIGLNQPQLAFLHICTHAFFKA
Epme	LCLGALTTLFTATCALTQNDIKKIVAFSTSSQLGLMMVTIGLNQPQLAFLHICTHAFFKA
Grse	LCLGAVTTFFTATCALTQNDIKKIIAFSTSSQLGLMMVAIGLNQPELAFFHICTHAFFKA
Clja	LCLGALTTMFTATCALTQNDIKKIVAFSTSSQLGLMMVTIGLNQPQLAFLHICTHAFFKA
Ogcy	LCLGALTTMFTAACAVTQNDIKKIVAFSTSSQLGLMMVAIGLNQPQLAFLHICTHAFFKA
Plna	LCLGALTTLFTATCALTQNDIKKIVAFSTSSQLGLMMVTIGLNQPQLAFFHICTHAFFKA
Lema	LCLGALTTFFTATCALTQNDIKKIVAFSTSSQLGLMMVTIGLNQPQLAFLHICTHAFFKA
Etzo	LCLGALTTLFTATCALTQNDIKKIVAFSTSSQLGLMMVTLGLNQPQLAFLHICTHAFFKA
Apse	LCLGALTTLFTATCALTQNDIKKIVAFSTSSQLGLMMVTIGLSQPQLAFLHICTHAFFKA
Epde	LCLGALTTLFTATCALTQNDIKKIVAFSTSSQLGLMMVTIGLNQPQLAFLHICTHAFFKA
Slja	LCLGALTTLFTATCALTQNDIKKIVAFSTSSQLGLMMVTIGLNQPQLAFLHICTHAFFKA
Bsja	LCLGALTTLFTATCALTQNDIKKIVAFSTSSQLGLMMVTIGLNQPHLAFLHICTHAFFKA
Ecna	LCLGALTTLFTATCALTQNDIKKIVAFSTSSQLGLMMVTIGLGQPQLAFLHICTHAFFKA
Cohi	LCLGALTTLFTATCALTQNDIKKIVAFSTSSQLGLMMVTIGLNQPQLAFLHICTHAFFKA
Caar	LCLGALTTFFTATCALTQNDIKKIVAFSTSSQLGLMMVTIGLNQPQLAFLHICTHAFFKA
Came	LCLGALTTFFTATCALTQNDIKKIVAFSTSSQLGLMMVTIGLNQPQLAFLHICTHAFFKA
Mema	LCLGALTTMFTATCALTQNDIKKIVAFSTSSQLGLMMVTIGLNQPHLAFLHICTHAFFKA
Lenu	LCLGALTTLFTATCALTQNDIKKIIAFSTSSQLGLMMVTIGLNQPQLAFLHICTHAFFKA
Brja	LCLGALTTLFTATCALTQNDIKKIVAFSTSSQLGLMMVTIGLNQPQLAFLHICTHAFFKA
Plma	LCLGALTTLFTATCALTQNDIKKIVAFSTSSQLGLMMVTIGLNQPQLAFLHICTHAFFKA
Emst	LCLGALTTLFTATCALTQNDIKKIVAFSTSSQLGLMMVTIGLNQPQLAFLHICTHAFFKA
Ptti	LCLGALTTLFTATCALTQNDIKKIVAFSTSSQLGLMMVTIGLNQPQLAFLHICTHAFFKA
Losu	LCLGALTTLFTATCALTQNDIKKIVAFSTSSQLGLMMVTIGLNQPQLAFLHICTHAFFKA
Geoy	LCLGALTTLFTATCALTQNDIKKIVAFSTSSQLGLMMVTIGLNQPQLAFLHICTHAFFKA
Dipi	LCLGALTTLFTATCALTQNDIKKIVAFSTSSQLGLMMVTIGLNQPQLAFLHICTHAFFKA
Pama	LCLGALTTVFTATCALTQNDIKKIVAFSTSSQLGLMMVTIGLNQPQLAFLHICTHAFFKA
Leob	LCLGALTTLFTATCALTQNDIKKIVAFSTSSQLGLMMVTIGLNQPQLAFLHICTHAFFKA
Neba	LCLGALTTMFTATCALTQNDIKKIVAFSTSSQLGLMMVTIGLNQPQLAFLHICTHAFFKA
Pdpl	LCLGAFTTLFTATCALTQNDIKKIVAFSTSSQLGLMMVAVGLNQPQLAFLHICTHAFFKA
Nimi	LGLGSLTTLLTATCALTQNDIKKIIAFSTSSQLGLMMVAIGLNQPQLAFLHICTHAFFKA
Uptr	LCLGALTTLFTATCALTQNDIKKIVAFSTSSQLGLMMVTIGLNQPQLAFLHICTHAFFKA
Pesc	LCLGALTTFFTGACALTQNDIKKIVAFSTSSQLGLMMVAIGLNQPHLAFLHICTHAFFKA
Baar	LCLGALTTLFTATCALTQNDIKKIVAFSTSSQLGLMMVTIGLNQPQLAFLHICTHAFFKA
Moar	LCLGALTTLFTATCALTQNDIKKIVAFSTSSQLGLMMVTIGLNQPQLAFLHICTHAFFKA
Toja	LCLGALTTLFTATCALTQNDIKKIVAFSTSSQLGLMMVTIGLNQPQLAFLHICTHAFFKA
Chau	LCLGALTTLFTATCALTQNDIKKIVAFSTSSQLGLMMVTIGLNQPQLAFLHICTHAFFKA
Chse	LCLGALTTLFTATCALTQNDIKKIVAFSTSSQLGLMMVTIGLNQPQLAFLHICTHAFFKA
Enar	LCLGALTTLFTATCALTQNDIKKIVAFSTSSQLGLMMVTIGLNQPQLAFLHICTHAFFKA
Hpty	LCLGALTTLFTATCALTQNDIKKIVAFSTSSQLGLMMVTIGLNQPQLAFLHICTHAFFKA
Nana	LCLGALTTLFTAICALTQNDIKKIVAFSTSSQLGLMMVTIGLNQPQLAFLHICTHAFFKA
Mcst	LCLGALTTLFTATCALTQNDIKKIVAFSTSSQLGLMMVTIGLNQPQLAFLHICTHAFFKA
Rhox	LCLGALTTLFTATCALTQNDIKKIVAFSTSSQLGLMMVTIGLNQPQLAFLHICTHAFFKA
Opfa	LCLGALTTLFTATCALTQNDIKKIVAFSTSSQLGLMMVTIGLNQPQLAFLHICTHAFFKA
Paar	LCLGALTTLFTATCALTQNDIKKIVAFSTSSQLGLMMVTIGLNQPQLAFLHICTHAFFKA
Gozo	LCLGALTTLFTATCALTQNDIKKIVAFSTSSQLGLMMVTIGLNQPQLAFLHICTHAFFKA
Ackr	LCLGALTTFFTAACALTQNDLKKIIAFSTSSQLGLMMVTIGLGQPHLAFLHICTHAFFKA
Elev	LCLGALTTLFTATCALTQNDLKKIIAFSTSSQLGLMMVAIGLNQPELAFLHICTHAFFKA
Trdu	LCLGALTTLFTATCALTQNDIKKIVAFSTSSQLGLMMVTIGLNQPQLAFLHICTHAFFKA
Amoc	LCLGALTTLFTATCALTQNDIKKIVAFSTSSQLGLMMVTIGLNQPQLAFLHICTHAFFKA
Hame	LWLGAMTTFVTAICALTQSDIKKIIAFSTSSQLGLMMVTVGLNQPQLAFLHICTHAFFKA
Chso	LCLGALTTVFTATCALTQNDIKKIVAFSTSSQLGLMMVTIGLNQPQLAFLHICTHAFFKA
Lyto	LCLGALTTLFTATCALTQNDIKKVIAFSTSSQLGLMMVTIGLNQPQLAFLHICTHAFFKA

[6/12 of aligned sequences]

```
Encr    LCLGALTTLFTATCALTQNDIKKIVAFSTSSQLGLMMVTIGLNQPQLAFLHICTHAFFKA
Bvar    LCLGALTTFFTAACALTQNDIKKIIAFSTSSQLGLMMVTIGLNQPQLAFLHICTHAFFKA
Noco    LCLGGLTSMFTAFCALTHNDLKKVIAFSTASQLGLMMVTIGLNQPQLAFLHICMHAFFKA
Chsp    LCLGALTTLFTATCALTQNDIKKIIAFSTSSQLGLMMVTIGLNQPQLAFLHICTHAFFKA
Arja    LCLGALTTLFTATCALTQNDIKKIVAFSTSSQLGLMMVTIGLNQPQLAFLHICTHAFFKA
Pase    LCLGALTTLFTATCALTQNDIKKVIAFSTSSQLGLMMVTIGLNQPQLAFLHICTHAFFKA
Trel    MCLGAMTTMFTATCALTQNDIKKIVAFSTSSQLGLMMVTIGLNQPQLAFLHICTHAFFKA
Lifa    LCLGALTCLFASMCALAQNDVKKIVAFSTSSQLGLMMVSIGLGQPHLAFFHVCTHAFFKS
Acur    LCLGALTTLFTAACALTQNDIKKIVAFSTSSQLGLMMVTIGLNQPQLAFFHICTHAFFKA
Ampe    LCLGALTTLFTATCALTQNDIKKIVAFSTSSQLGLMMVTIGLNQPQLAFLHICTHAFFKA
Urja    LCLGALTTLFTATCALLQNDIKKIIAFSTSSQLGLMMVAIGLNQPQLAFFHISTHAFFKA
Enet    LCLGALTTLFTAICALTQNDIKKIVAFSTSSQLGLMMVTIGLGQPQLAFFHICTHAFFKA
Ptbr    LCLGALTTLFTAISAITQNDIKKIVAFSTSSQLGLMMVTIGLNQPQLAFLHICTHAFFKA
Safa    LCLGALTTLFTATCALTQNDIKKIVAFSTSSQLGLMMVTIGLNQPQLAFLHICTHAFFKA
Icae    LCLGALTTLFTATCALTQNDIKKIVAFSTSSQLGLMMVTIGLNQPQLAFLHICTHAFFKA
Asmi    LCLGALTSFFAALCAISQNDIKKIIAFSTSSQLGLMMVALGLGYPSLAFMHICMHAFFKA
Foal    LWLGALTTFFTALCALTQNDIKKIVAFSTSSQLGLMMVTIGLNQPQLAFLHICTHAFFKA
Drze    LCLGALTTFFTAICALTQNDIKKIVAFSTSSQLGLMMVTIGLNQPQLAFLHICTHAFFKA
Rhas    LCLGALTTLFTATCALTQNDIKKIVAFSTSSQLGLMMVTIGLGQPQLAFLHICTHAFFKA
Elac    LCLGALTTLFTATCALTQNDIKKIVAFSTSSQLGLMMVTIGLGQPQLAFLHICTHAFFKA
Kugu    LCLGALTTLFTATCALTQNDIKKIVAFSTSSQLGLMMVTIGLNQPQLAFLHICTHAFFKA
Plor    LCLGALTTLFTATCALTQNDIKKIIAFSTSSQLGLMMVTIGLNQPQLAFLHICTHAFFKA
Sgun    LCLGALTTLFTATCALTQNDIKKIVAFSTSSQLGLMMVTIGLNQPQLAFLHICTHAFFKA
Zaco    LCLGALTTLFTATCALTQNDIKKIVAFSTSSQLGLMMVTIGLNQPQLAFFHICTHAFFKA
Zbfl    LCLGALTTLFTATCALTQNDIKKIVAFSTSSQLGLMMVTIGLNQPQLAFLHICTHAFFKA
Spba    LCLGALTTLFTATCALTQNDIKKIVAFSTSSQLGLMMVTIGLNQPQLAFLHICTHAFFKA
Game    LCLGALTTLFTATCALTQNDIKKIVAFSTSSQLGLMMVTIGLNQPQLAFLHICTHAFFKA
Thth    LCLGALTTLFTATCALTQNDIKKIVAFSTSSQLGLMMVTIGLNQPQLAFLHICTHAFFKA
Xigl    LCLGALTTLFTATCALTQNDIKKIVAFSTSSQLGLMMVTIGLNQPQLAFLHICTHAFFKA
Hyja    LCLGALTTLFTATCALTQNDIKKIVAFSTSSQLGLMMVTIGLNQPQLAFLHICTHAFFKA
Psan    LCLGALTTLYAATCALTQNDIKKIIAYSTTSQLGLMMVTIGLKQPQLAFLHICTHAFSKA
Cupa    LCLGALTTLFTATCALTQNDIKKIVAFSTSSQLGLMMVTIGLNQPQLAFLHICTHAFFKA
Mpch    LCLGALTTFFTATCALTQNDIKKIVAFSTSSQLGLMMVAIGLNQPQLAFLHICTHAFFKA
Char    LCLGAMTTLFTATCALTQNDIKKIVAFSTSSQLGLMMVTIGLNQPQLAFLHICTHAFFKA
Pser    LCLGAITTLFTAACALTQNDIKKIVAFSTSSQLGLMMVTIGLNQPQLAFLHICTHAFFKA
Prol    LCLGALTTLFTATCALTQNDIKKIIAFSTSSQLGLMMVTIGLNQPQLAFLHICTHAFFKA
Plbi    LCLGALTTLFTATCALTQNDIKKIVAFSTSSQLGLMMVTIGLNQPQLAFLHICTHAFFKA
Calu    LCLGALTTVFTALCALTQNDIKKIIAFSTSSQLGLMMVAIGLNQPQLAFLHICTHAFFKA
Papa    LCLGALTTFFTATCALTQNDIKKIIAFSTSSQLGLMMVTIGLNQPQLAFLHICTHAFFKA
Sufr    LCLGALTTLFTATCALTQNDIKKIVAFSTSSQLGLMMVTIGLNQPQLAFLHICTHAFFKA
Stci    LCLGALTTVFTATCALTQNDIKKIVAFSTSSQLGLMMVTLGLNQPQLAFLHICTHAFFKA
Taru    LCLGALTTLFTATCALTQNDIKKIVAFSTSSQLGLMMVTIGLNQPQLAFLHICTHAFFKA
Rala    LCLGALTTLFTATCALTQNDIKKIVAFSTSSQLGLMMVTIGLNQPQLAFLHICTHAFFKA
        : :*. :       *: : *:**::*:**:*:**:*:*::**  * *:*.*:  *** *:
```

To be continued on page 35.

[7/12 of aligned sequences]

```
                                                       K
Scca   MLFLCSGSIIHSLNDEQDIRKMGGLHKLLPFTSSSLTVGSLALTGMPFLSGFFSKDAIIE
Muma   MLFLCSGSIIHSLNDEQDIRKMGGLHKLLPFTSTSLTIGSLALTGMPFLSGFFSKDAIIE
Erca   MMFLCSGSIIHSLNDEQDIRKMGGIHKIMPMTSTCLMIGSLALMGTPFLAGFFSKDAIIE
Pose   MLFLCSGSIIHSLNDEQDIRKMGGIHKTLPLTSSCLTIGSLALMGTPFLAGFFSKDAIIE
Actr   MLFLCSGSIIHSLNDEQDIRKMGGLHTMLPLTSTCLTIGSLALTGMPFLSGFFSKDAIIE
Scal   MLFLCSGSIIHSLNDEQDIRKMGGLHTMLPLTSSCLTIGSLALTGMPFLSGFFSKDAIIE
Posp   MLFLCSGSIIHSLNDEQDIRKMGGLHTMLPFTSTCLTIGSLALTGMPFLSGFFSKDAIIE
Atsp   MLFLCSGSIIHDFKDEQDIRKLGNIHALLPLTSACMVVGSLALTGMPFLSGFFSKDAIIE
Leoc   MLFLCSGSIIHDFKDEQDIRKLGHVHALLPFTSACMVVGSLALTGMPFLSGFFSKDAIIE
Amca   MLFLCSGSIIHSLNDEQDIRKMGGLHTILPFTSSCLTVGSLALTGMPFLAGFFSKDAIIE
Osbi   MLFLCSGSIIHSLNNEQDIRKMGGLHTLLPFTSSSLTLGSLALTGTPFLAGFFSKDAIIE
Pabu   MLFLCSGSIIHSLNNEQDIRKMGGLHKLMPLTSSCLTIGSLALTGTPFLAGFFSKDTIIE
Hial   MLFLCSGSIIHSLNDEQDIRKMGGLHNLLPFTSSCITIGSLALTGTPFLAGFFSKDAIIE
Elha   MLFLCSGSVIHSLNDEQDIRKMGGLHNFLPFTSSCLTIGSLALTGTPFLAGFFSKDAIIE
Mlcy   MLFLCSGSIIHSLNDEQDIRKMGGMHNLMPFTTSCMTIGSLALTGTPFLAGFFSKDAIIE
Algl   MLFLCSGAIIHSLNDEQDLRKMGGLHNLLPLTSSCMMVGSLALTGVPFLAGFFSKDSIIE
Ptgi   MLFLCSGSVIHSLNDEQDIRKMGGLHNVLPFTSSCMTIGSLALTGTPFLAGFFSKDAIIE
Alaf   MLFLCSGSVIHSLNDEQDIRKMGGLHNMLPFTSSCMTIGSLALTGTPFLAGFFSKDAIIE
Nock   MLFLCSGSIIHSLNDEQDIRKMGGLHNMLPFTSSCMTVGSLALTGTPFLAGFFSKDAIIE
Anja   MLFLCSGSIIHSLNDEQDIRKMGGLHKLLPFTSSCMTIGSLALTGTPFLAGFFSKDAIIE
Gyki   MLFLCSGSVIHSLNNEQDIRKMGGLHKTMPFTSSCMTLGSLALTGTPFLAGFFSKDAIIE
Syka   MLFLCSGSIIHSLNDEQDIRKMGGLHKMLPLTSSCMTIGSLALTGTPFLAGFFSKDAIIE
Opma   MLFLCSGSIIHSLNDEQDIRKMGGLHKTLPFTSSCMMIGSMALTGTPFLAGFFSKDAIIE
Comy   MLFLCSGSIIHSLNDEQDIRKMGGLHKTLPLTSSCMTIGSLALTGMPFLAGFFSKDAIIE
Sasp   MLFLCSGSIIHSLGDEQDIRKMGGLYKTLPLTMSCVTIGNLALTGFPFLSGFFSKDAIIE
Eupe   MLFLCSGSVIHSLGDEQDIRKMGGLYQTLPFTTTCMTIGNLALTGTPFLSGFFSKDTIIE
Enja   MLFLCSGSIIHSLNDEQDIRKMGGLHHLAPFTSTCTTIGSLALTGTPFLAGFFSKDAIIE
Same   MLFLCSGSVIHSLNDEQDIRKMGGLHNLAPFTSTCLTIGSLALTGTPFLAGFFSKDAIIE
Chch   MLFLCSGSIIHSLNDEQDIRKMGGLHNLTPFTSSCLTIGSLALTGTPFLAGFFSKDAIIE
Grgr   MLFLCSGSIIHSLNDEQDIRKMGGMHNLVPFTSTCMTIGSLALTGTPFLAGFFSKDAIIE
Caau   MLFLCSGSIIHSLNDEQDIRKMGGLFSIMPATSTYFTIGSLALTGTPFLAGFFSKDAIIE
Cyca   MLFLCSGSIIHSLNDEQDIRKMGGLFNIMPATSTYFTIGSLALTGTPFLAGFFSKDAIIE
Dare   MLFLCSGAIIHSLNNEQDIRKMGGTYHTLPMTTNYLTIGKMALMGTPFLAGFFSKDAILE
Cost   MLFLCSGSIIHSLNDEQDIRKMGGLQNLMPLTSTCLTIGSLALTGTPFLAGFFSKDAIIE
Leec   MLFLCSGSIIHSLNDEQDIRKMGGLQNLLPFTSTCLTIGSLALAGTPFLAGFFSKDAIIE
Fola   MLFLCSGSIIHSLNDEQDIRKMGGLQNLLPFTSTCLTIGSLALTGTPFLAGFFSKDAIIE
Clmc   MLFLCSGSIIHSLNDEQDIRKMGGLHKLMPFTSSCLTIGSLALTGTPFLAGFFSKDAIIE
Phin   MLFLCSGSIIHNLNNEQDIRKMGGLHKPLPLTSSCLTIGSLALTGTPFLAGFFSKDAILE
Icpu   MLFLCSGSIIHSLNDEQDIRKMGGLHKLMPFTSSCLIIGSLALTGMPFLTGFFSKDAIIE
Psto   MLFLCSGSIIHSLNDEQDIRKMGGLHKLMPFTSSCLIIGSLALTGMPFLAGFFSKDAIIE
Cora   MLFLCSGSIIHSLNDEQDIRKMGGLFKLLPFTSTCLTIGSLALTGTPFLTGFFSKDAIIE
Eisp   MLFLCSGAIIHSLNDEQDIRKMGGLHKLMPFTSSCLTIGSLALTGTPFLAGFFSKDAIIE
Apal   MLFLCSGMIIHSLNDEQDIRKMGGLHKLLPTTSSCMTIGSLALTGTPFLAGFFSKDAIIE
Eslu   MLFLCSGSIIHSLNNEQDIRKMGGMHNLAPLTSSCLTIGSLALTGTPFLAGFFSKDAIIE
Dape   MLFLCSGSIIHNLNNEQDIRKMGGMHSLTPYTSSCLTIGSLALTGTPFLAGFFSKDAIIE
Glse   MLFLCSGAVIHSLNDEQDIRKMGGLHYLTPFTSSCMTIGSLALTGTPFLAGFFSKDAIIE
Naar   MLFLCSGAVIHSLNDEQDIRKMGGLHHLTPFTSSCMTIGSLALTGTPFLAGFFSKDAIIE
Lioc   MLFLCSGAVIHSLNDEQDIRKMGGLHHLTPFTSSCMTIGSLALTGTPFLAGFFSKDAIIE
Opso   MLFLCSGVMIHSLNNEQDIRKMGGLHHLTPFTSSCMVVGSLALTGTPFLAGFFSKDAIIE
Alte   MLFLCSGSIIHSLNDEQDIRKMGGLHNLMPFTSSCLTIGSLALTGTPFLAGFFSKDAIIE
Plap   MLFLCSGSIIHSLNDEQDIRKMGGLHNLLPLTSSCLTVGSLALTGTPFLAGFFSKDAIIE
```

To be continued on page 36.

[7/12 of aligned sequences]

To be continued on page 37.

```
Plal MLFLCSGSIIHSLNDEQDIRKMGGLHNLTPFTSSCMTIGSLALTGTPFLAGFFSKDAIIE
Sami MLFLCSGSIIHSLNDEQDIRKMGGLHNLTPFTSSCLTIGSLALTGTPFLAGFFSKDAIIE
Rere MLFLCSGSIIHSLNDEQDIRKMGGLHTLTPFTSSCMAIGSLALTGTPFLAGFFSKDAIIE
Gama MLFLCSGSVIHSLNDEQDIRKMGGMHRLTPFTSTALTIGSLALTGTPFLAGFFSKDAIIE
Onmy MLFLCSGSIIHSLNDEQDIRKMGGMHNLTPSTSSCLTIGSLALTGTPFLAGFFSKDAIIE
Sasa MLFLCSGSIIHSLNDEQDIRKMGGMHNLTPFTSSCLTIGSLALTGTPFLAGFFSKDAIIE
Cola MLFLCSGSIIHSLNDEQDIRKMGGMHNLTPFTSSCLTIGSLALTGTPFLAGFFSKDAIIE
Dita MLFLCSGSLIHSLNNEQDIRKMGGLYNLAPFTSSSITIGSLALTGTPFLAGFFSKDAIIE
Gogr MLFLCSGSLIHNLNNEQDIRKMGGLMNLAPFTSSCITIGSLALTGTPFLAGFFSKDAIIE
Chsl MLFLCSGSLIHSLNNEQDIRKMGGLFNLAPFTSSCMTLGSLALTGTPFLAGFFSKDAIIE
Atja MLFLCSGSIIHSLNDEQDIRKMGGMSNLAPLTSSCLTIGSLALTGTPFLAGFFSKDAIIE
Iido MLFLCSGSIIHSLNDEQDIRKMGGMNNLAPLTSSCLTIGSLALTGTPFLAGFFSKDAIIE
Auja MLFLCSGSIIHSLNDEQDIRKMGGLHYLTPFTSSCLTIGSLALTGTPFLAGFFSKDAIIE
Chag MLFLCSGSIIHSLNDEQDIRKMGGLHHLTPFTSTCFTIGSLALTGTPFLAGFFSKDAIIE
Hami MLFLCSGSIIHSLNDEQDIRKMGGLHHLTPLTSSCLTIGSLALTGTPFLAGFFSKDAIIE
Saun MLFLCSGSIIHSLNDEQDIRKMGGLQHLTPFTSSCLTIGSLALTGTPFLAGFFSKDAIIE
Nema MLFLCSGSIIHSLNDEQDIRKMGGLHHLAPFTSSCLTIGSLALTGTPFLAGFFSKDAIIE
Disp MLFLCSGSIIHSLNDEQDIRKMGGMHHLAPFTSSCLTIGSLALTGTPFLAGFFSKDAIIE
Myaf MLFLCSGSIIHSLNDEQDIRKMGGMHHLAPFTSTCLTIGSLALTGTPFLAGFFSKDAIIE
Lagu MLFLCSGSIIHSLNDEQDIRKMGGLHHLAPFTSSCLTLGSLALAGTPFLAGFFSKDAIIE
Trtr MLFLCSGSIIHSLNDEQDIRKMGGLHSLTPLTSSCLTIGSFALTGTPFLAGFFSKDAIIE
Zucr MLFLCSGSIIHSLNDEQDIRKMGGMHNLTPFTSSCLTVGSLALTGTPFLAGFFSKDAIIE
Pxja MLFLCSGSIIHSLNDEQDIRKMGGMHHLTPFTSSCLTIGSLALTGTPFLAGFFSKDAIIE
Pxlo MLFLCSGSIIHSLNDEQDIRKMGGLHHLTPFTSSCLTIGSLALTGTPFLAGFFSKDAIIE
Pctr MLFLCSGTIIHSLNDEQDIRKMGGMHHLAPFTSTCMIVGSLALTGTPFLAGFFSKDAIIE
Apsa MLFLGSGAIIHSLNNEQDIRKMGGMHHLAPLTSSCMTIGSLALTGTPFLAGFFSKDAIIE
Cabe LLFLCSGSIIHSLDNEQDIRKMGGVQHHMPVTTSALAVASLALTGTPFLTGFFSKDAIIE
Bzze MLFLCSGAIIHSLNDEQDIRKMGGMHHLTPFTSTSLTIGSLALTGTPFLAGFFSKDAIIE
Siim MLFLCCGSIIHSLNDEQDIRKMGGMHNLAPMTSSCLTIGSLALTGTPFLAGFFSKDAIIE
Ctru MLFLCSGSIIHSLNDEQDIRKMGGMHRLTPFTSSCLTVGSLALTGTPFLAGFFSKDAIIE
Dpbr MLFLCSGSIIHSLNDEQDIRKMGGMHRLTPFTSSSLTIGSLALTGTPFLAGFFSKDAIIE
Caki MLFLCSGSVIHALNNEQDIRKMGGLHHLAPLTSSCLTIGSLALTGTPFLAGFFSKDAIIE
Phja MLFMCAGSFIHMLNDEQDIRKMGGMMYLAPDTSAYFTIGCLALSGMPFLSGFFSKDAIIE
Brsp MLFLCSGSIIHSLNDEQDIRKMGGMHTLTPFTSSCLTVGSLALTGTPFLTGFFSKDTIIE
Gamo MLFLCSGSVIHSLNDEQDIRKMGGLHHLAPFTSSCLTVGSLALTGTPFLAGFFSKDAIIE
Lolo MLFLCSGSVIHSLNDEQDIRKMGGLHHLAPFTSSCLTVGSLALTGTPFLAGFFSKDAIIE
Batr MLFLCSGSIIHCLNNEQDIRKMGGMFKTAPLTSSCLTLGTLALAGFPFLAGFYSKDPILE
Prmy MLFMCSGSIIHSLDNEQDIRKMGGLHNLLPTTSTCLTIGSLALTGFPFLAGFYSKDLIIE
Lose MLFLCSGSVIHSLNDEQDIRKMGGMHFCLPLTSSSITLGSLALTGTPFLSGFFSKDAIIE
Loam MLFLCSGSIIHSLNDEQDIRKMGGMHRLAPFTSSSLTMGSLALTGTPFLAGFFSKDAIIE
Chab MLFLCSGSIIHSLNDEQDIRKMGGMHPLMPLTSSCLTIGSLALTGTPFLAGFFSKDAIIE
Chto MLFLCSGSIIHSLNDEQDIRKMGGMHPLMPLTSSCLTIGSLALTGTPFLAGFFSKDAIIE
Majo MLFLCSGSIIHSLHDEQDIRKMGGMHNVTPLTSSCLSIGSLALAGTPFLAGFFSKDAIIE
Hlst MLFLCSGSIIHSLNDEQDIRKMGGMHNISPVTSSCLTIGSLALTGTPFLAGFFSKDAIIE
Clpe MLFLCSGSIIHSLNDEQDIRKMGGMHHLAPFTSSSLTIGSLALTGTPFLAGFFSKDAIIE
Mlmr MLFLCSGSIIHSLNDEQDIRKMGGMHNLAPVTSSCLTIGSLALTGTPFLAGFFSKDAIIE
Crcr MLFLCSGAIIHSLNDEQDIRKMGGMHRLAPVTSSCLIIGSLALTGTPFLAGFYSKDAIIE
Muce MLFLCSGAIIHSLNDEQDIRKMGGMHRLAPVTSSCLIIGSLALTGTPFLAGFYSKDAIIE
Bege MLFLCSGSIIHSLNDEQDIRKMGGMHYLTPFTSSCLTIGSLALTGTPFLAGFFSKDAIIE
Mela MLFLCSGSIIHSLNDEQDIRKMGGMHTLTPFTSSCMTLGSLALTGTPFLAGFFSKDAIIE
Hats MLFLCSGSIIHSLNDEQDIRKMGGMHNLAPFTSSCLTLGSLALAGTPFLAGFFSKDAIIE
Orla MLFLCSGSIIHSLNDEQDIRKMGGLHHLLPFTSSCLTIGSLALTGTPFLAGFFSKDAIIE
```

[7/12 of aligned sequences]

To be continued on page 38.

Cosa	MLFLCSGSIIHSLNDEQDIRKMGGMHHLTPLTSSCLTIGSLALTGTPFLAGFFSKDAIIE
Exsp	MLFLCSGSIIHSLNDEQDIRKMGGMQHLAPFTSSCLTIGSLALTGTPFLAGFFSKDAIIE
Depa	MLFLCSGSIIHNLNDEQDIRKMGGMHHLTPFTSSCLTIGSLALMGTPFLAGFFSKDAIIE
Rima	MLFLCSGSLIHALDNEQDIRKMGSMLQLAPFTSSCMTLGSLALTGMPFLAGFFSKDAIIE
Fuol	MLFLCSGSIIHSLNDEQDIRKMGGMHHLAPFTSSCLIIGSLALTGTPFLAGFFSKDAIIE
Gmaf	MLFICSGSIIHSLNDEQDIRKMGGMHHLTPLTSSCFTIGSLALTGTPFLAGFFSKDAIIE
Xeei	MLFLCSGSIIHSLNDEQDIRKMGGLHHLTPFTSSCLTLGSLALTGTPFLAGFFSKDAIIE
Pros	MLFLCSGSIIHSLNDEQDIRKMGGLHHLVPTTTTCLTIGSLALTGTPFLAGFFSKDAIIE
Scmi	MLFLCSGSIIHSLNDEQDIRKMGGLHHLLPTTTASLTIGSLALTGTPFLAGFFSKDAIIE
Rolo	MLFLCSGSIIHSLNDEQDIRKMGGLHHLTPTTSSCLTIGSLALTGTPFLAGFFSKDAIIE
Cere	MLFLCSGSIIHSLNNEQDIRKMGGLHHLAPTTSSCLTIGSLALTGVPFLAGFFSKDAIIE
Daga	MLFLCSGSIIHSLNDEQDIRKMGGLHRLMPTTSSCLTIGSLALTGMPFLAGFFSKDAIIE
Anco	MLFLCSGSIIHSLNDEQDIRKMGGLQNLAPTTSSCLTIGSLALTGTPFLAGFFSKDAIIE
Dmve	MMFLASGSIIHALNDEQDIRKMGGIRYLLPETTACLTIGSLALTGTPFLAGFYSKDAIIE
Dmar	MMFLASGSIIHALNDEQDIRKMGGIRYLLPKTTACLTIGSLALTGTPFLAGFYSKDAIIE
Anka	MLFLCSGSIIHSLNDEQDIRKMGGLHHLVPTTTTCLTIGSLALTGTPFLAGFFSKDAIIE
Moja	MLFLCSGSIIHSLNDEQDIRKMGGLQHLTPTTTTCLTIGSLALTGTPFLAGFFSKDAIIE
Hoja	MLFLCSGSIIHSLNDEQDIRKMGGLQHLAPTTSSCLTIGSLALTGTPFLAGFFSKDAIIE
Bede	MLFLCSGSIIHSLNDEQDIRKMGGMHHLAPTTSSCLTIGSLALTGTPFLAGFFSKDAIIE
Besp	MLFLCSGSIIHSLNDEQDIRKMGGMHHLTPTTSSCLTIGSLALTGTPFLAGFFSKDAIIE
Mysp	MLFLCSGSIIHSLNDEQDIRKMGGMHQLAPVTSSCLTIGSLALTGTPFLAGFFSKDAIIE
Osja	MLFLCSGSIIHSLNDEQDIRKMGGMQQLAPITSSCLTIGSLALTGTPFLAGFFSKDAIIE
Sgro	MLFLCSGSIIHALNDEQDIRKMGGMHQLVPITSSCLTIGSLALTGTPFLAGFFSKDAIIE
Pzpa	MLFLCSGSIIHALNDEQDIRKMGGMHKLMPFTSSCLIVGSLALTGTPFLAGFFSKDAIIE
Zeja	MLFLCSGSIIHSLNDDQDIRKMGGMQNLTPFTSSCLTIGSLALTGTPFLAGFFSKDAIIE
Znne	MLFLCSGSIIHALNDEQDIRKMGGMQNLTPLTSSCLTIGSLALVGTPFLAGFFSKDAIIE
Zefa	MLFLCSGSIIHALNDEQDIRKMGGMQNLAPLTSTCLTIGSLALTGTPFLAGFFSKDAIIE
Acni	MLFLCSGSIIHALNDEQDIRKMGGMQSLTPFTSSCLTIGSLALTGTPFLAGFFSKDAIIE
Ncrh	MLFLCSGSIIHALNDEQDIRKMGGMQNLTPFTSSCLTIGSLALTGTPFLAGFFSKDAIIE
Agca	MLFLCSGSIIHSLNDEQDIRKMGGMHHLTPFTSSCLTLGSLALTGTPFLAGFFSKDAIIE
Hydy	MLFLCSGSVIHSLSDEQDIRKMGGMHHLTPLTSSCLTIGSLALAGTPFLAGFFSKDAILE
Gsac	MLFLCSGSVIHSLNDEQDIRKMGGMHFLTPFTSSCLTIGSLALAGTPFLAGFFSKDAILE
Pevo	MLFLCSGSIIHSLNDEQDIRKMGGMHNLTPFTSSCLTIGSLALTGTPFLAGFFSKDAIIE
Hiku	MLFLCSGSLIHSLNDEQDIRKMGGMHFLTPFTSSSLTLGSLALTGTPFLAGFFSKDAIIE
Inpa	MLFLCSGSIIHCLNNEQDIRKMGGMHHLLPTTTSCLTIGSLALTGTPFLAGFYSKDAIIE
Auch	MLFLCSGSIIHSLNNEQDIRKMGGLHHLAPVTSSCLILGSLALTGTPFLAGFFSKDAIIE
Fico	MLFLCSGSIIHSLNDEQDIRKMGGMQELTPFTSSCLTLGSLALTGTPFLAGFFSKDAIIE
Macs	MLFLCSGSIIHSLNDEQDIRKMGGMHHLTPFTSSCFTLGSLALTGTPFLAGFFSKDAIIE
Moal	MLFLCSGVIIHALNNEQDIRKMGGLHYLIPTTTSCLTIGSLALMGTPFLAGFFSKDAIIE
Syma	MLFICSGVIIHSLNNEQDIRKMGGLHHVIPMTSSCLTIGSLALMGMPFLAGFFSKDTIIE
Mafr	MLFLCSGSIIHSLNDEQDIRKMGGTHHLLPITSSCLTIGSLALTGTPFLAGFFSKDAIIE
Dcpe	MLFLCSGSIIHSLNDEQDIRKMGGMHHLAPFTSSCLTLGSLALAGTPFLAGFFSKDAIIE
Dcti	MLFLCSGSIIHSLNDEQDIRKMGGMHNLTPFTASCLTLGSLALTGTPFLAGFFSKDAIIE
Hehi	MLFLCSGSIIHSLNDEQDIRKMGGMHHLAPFTSSCLTIGSLALTGTPFLAGFFSKDAIIE
Stam	MLFLCSGSIIHSLNDEQDIRKMGGMHHLAPFTSSCLTIGSLALTGTPFLAGFFSKDAIIE
Hogi	MLFLCSGSIIHSLNDEQDIRKMGGMHQLTPFTSSCLTIGSLALTGTPFLAGFFSKDAIIE
Erzo	MLFLCSGSVIHSLNDEQDIRKMGGMHHLTPFTSSCLTIGSLALTGTPFLAGFFSKDAIIE
Hxot	MLFLCSGSVIHSLNDEQDIRKMGGMHHLTPFTSSCLTIGSLALTGTPFLAGFFSKDAIIE
Core	MLFLCSGSVIHSLNDEQDIRKMGGMHHLTPFTSSCLTIGSLALTGTPFLAGFFSKDAIIE
Apve	MLFLCSGSVIHSLNDEQDIRKMGGMHHLTPFTSSCLTIGSLALTGTPFLTGFFSKDAIIE
Latj	MLFLCSGSIIHSLNDEQDIRKMGGMHRLTPFTSSCLTIGSLALTGTPFLAGFFSKDAIIE
Laja	MLFLCSGSIIHSLNDEQDIRKMGGMHHLTPFTSSCLSIGSLALTGTPFLAGFFSKDAIIE

[7/12 of aligned sequences]

To be continued on page 39.

```
Syja  MLFLCSGSIIHSLNDEQDIRKMGGMHHLTPFTSSCLSIGSLALTGTPFLAGFFSKDAIIE
Epme  MLFLCSGSIIHSLNDEQDIRKMGGMHHLTPFTSTCLTIGSLALTGTPFLAGFFSKDAIIE
Grse  MLFLCSGSIIHSLNDEQDIRKMGGMHRLTPFTSACMTIGSLALMGTPFLAGFYSKDAIIE
Clja  MLFLCSGTIIHSLNDEQDIRKMGGMHHLTPFTSSCMTIGSLALTGTPFLAGFFSKDAIIE
Ogcy  MLFLCSGMIIHCTNSEQDIRKMGGMSTLAPLTSSCFTVGSLALMGTPYLAGFYSKDAIIE
Plna  MLFLCSGSIIHSLSDEQDIRKMGGMHHLAPFTSSCLTIGSLALTGTPFLAGFFSKDAIIE
Lema  MLFLCSGSIIHSLNDEQDIRKMGGMHHLAPFTSSCLTIGSLALTGTPFLAGFFSKDAIIE
Etzo  MLFLCSGSIIHSLNDEQDIRKMGGMHHLTPFTSSCLTIGSLALTGTPFLAGFFSKDAIIE
Apse  MLFLCSGSIIHCLNDEQDIRKMGGMHHLTPFTSSALTIGSLALTGTPFLAGFFSKDAIIE
Epde  MLFLCSGSIIHSLNDEQDIRKMGGMHHLTPFTSSCLTIGSLALTGTPFLAGFFSKDAIIE
Slja  MLFLCSGSIIHSLNDEQDIRKMGGMQHLTPFTSSCLTLGSLALTGTPFLAGFFSKDAIIE
Bsja  MLFLCSGAIIHSLNDEQDIRKMGGMHHLAPFTSSCMTLGSLALTGTPFLAGFFSKDAIIE
Ecna  MLFLCSGSIIHSLNDEQDIRKMGGMHNLTPFTSSCLTIGSLALTGTPFLAGFFSKDAIIE
Cohi  MLFMCSGSILHSLNDEQDIRKMGGMHKALPFTSSCLIIGNLALTGTPFLAGFFSKDAIIE
Caar  MLFLCSGSIIHSLNDEQDIRKMGGMHNLTPFTSSCLTIGSLALTGTPFLAGFFSKDAIIE
Came  MLFLCSGSIIHSLNDEQDIRKMGGMHHLTPFTSSCLTIGSLALTGTPFLAGFFSKDAIIE
Mema  MLFLCSGSTAKAANDEQDIRKMGGMHHLTPFTSSCFTLGSLALTGTPFLAGFFSKDAIIE
Lenu  MLFLCAGSIIHSLNNEQDIRKMGGMHHLAPFTSTCMTLGSLALTGTPFLAGFFSKDAIIE
Brja  MLFLCSGSIIHSLNDEQDIRKMGGMHHLTPFTSSCLTIGSLALTGTPFLAGFFSKDAIIE
Plma  MLFLCSGSIIHSLNDEQDIRKMGGMHHLTPFTSSCLTIGSLALTGTPFLAGFFSKDAIIE
Emst  MLFLCSGSIIHSLNDEQDIRKMGGMHHLTPFTSSCFTIGSLALTGTPFLAGFFSKDAIIE
Ptti  MLFLCSGSIIHSLNDEQDIRKMGGMHHLTPFTSSCLTLGSLALPRTPFLAGFFSKDAIIE
Losu  MLFLCSGSIIHSLDNEQDIRKMGGMHHLVPLTSSCLTLGNLALTGTPFLAGFFSKDAIIE
Geoy  MLFLCSGSIIHSLNDEQDIRKMGGMHHLTPFTSSCLTLGSLALTGTPFLAGFFSKDAIIE
Dipi  MLFLCSGSIIHSLNDEQDIRKMGGMHHLTPFTSSCLTIGSLALTGTPFLAGFFSKDAIIE
Pama  MLFLCSGSIIHSLNDEQDIRKMGGMHHLTPFTSSCLTIGSLALTGTPFLAGFFSKDAIIE
Leob  MLFLCSGSIIHSLNDEQDIRKMGGMHHLTPFTSSCLTIGSLALTGTPFLAGFFSKDAIIE
Neba  MLFLCSGSIIHSLDNEQDIRKMGGMHHLLPFTSSCMTLGSLALAGTPFLAGFFSKDAIIE
Pdpl  MLFLCSGSIIHSLNDEQDIRKMGGLHYMLPITSSCLILGNLALAGSPFLAGFFSKDAIIE
Nimi  MLFLCAGSFIHCLNDEQDIRKMGGMLHLAPVTSTCFTIGSLALAGTPFLAGFFSKDAIIE
Uptr  MLFLCSGSIIHSLNDEQDIRKMGGMHHLTPHTSSCLTLGSLALTGTPFLAGFFSKDAIIE
Pesc  MLFLCSGSTIHSLNDEQDIRKMGGIQVLTPLTSSCLMLGSFALMGFPFLAGFYSKDAIIE
Baar  MLFLCSGSIIHSLNDEQDIRKMGGMQHLTPFTSSCLSVGSLALTGTPFLAGFFSKDAIIE
Moar  MLFLCSGSIIHSLNDEQDIRKMGGMHHLTPFTSSCLTIGSLALTGTPFLAGFFSKDAIIE
Toja  MLFLCSGSIIHSLNDEQDIRKMGGMHHLTPFTSSCLTIGSLALTGTPFLAGFFSKDAIIE
Chau  MLFLCSGSIIHSLNDEQDIRKMGGMHHLAPFTSSCMTIGSLALTGTPFLAGFFSKDAIIE
Chse  MLFLCSGAIIHSLNDEQDIRKMGGMHRLTPFTSSCLTIGSLALTGTPFLAGFFSKDAIIE
Enar  MLFLCSGSIIHSLNDEQDIRKMGGMHHLTPFTSSCLTIGSLALTGTPFLAGFFSKDAIIE
Hpty  MLFLCSGSIIHSLNDEQDIRKMGGMHHLTPFTSSCLTIGSLALTGTPFLAGFFSKDAIIE
Nana  MLFLCSGSIIHSLNDEQDIRKMGGMHHLTPLTSSCITIGSLALTGTPFLAGFFSKDAIIE
Mcst  MLFLCSGSIIHSLNDEQDIRKMGGMHHLTPFTSSCLTIGSLALTGTPFLAGFFSKDAIIE
Rhox  MLFLCSGSIIHSLNDEQDIRKMGGMHHLTPFTSSCLTIGSLALTGTPFLAGFFSKDAIIE
Opfa  MLFLCSGSIIHSLNDEQDIRKMGGMHHLTPFTSSCLTVGSLALTGTPFLAGFFSKDAIIE
Paar  MLFLCSGSIIHSLNDEQDIRKMGAMHNLTPFTSSCLTVGSLALTGFPFLAGFFSKDAIIE
Gozo  MLFLCSGSIIHSLNDEQDIRKMGGMHHLTPFTSSCLTIGSLALTGTPFLAGFFSKDAIIE
Ackr  MLFLCAGSFIHCLNGEQDIRKMGGMFYWAPFTSTCFTIGSLALAGTPFLSGFYSKDAIME
Elev  MLFLCSGSIIHSLNDEQDIRKMGGLYNLTPITSSCLTIGSLALTGTPFLAGFFSKDAIIE
Trdu  MLFLCSGSIIHSLNDEQDIRKMGGMHYLTPFTSSCLTIGSLALTGTPFLAGFFSKDAIIE
Amoc  MLFLCSGSIIHSLNDEQDIRKMGGMNHLVPFTSSCLTLGSLALTGTPFLAGFFSKDAIIE
Hame  MLFICAGSIIHCLNNEQDIRKMGGVYRLAPFTCACLTLGSLALTGTPFLAGFFSKDAIIE
Chso  MLFLCSGAIIHSLNDEQDIRKMGGMHHLTPFTSSCLTLGSLALTGTPFLAGFFSKDAIIE
Lyto  MLFLCSGSVIHSLNDEQDIRKMGGMHHLTPFTSSCLTIGNLALTGTPFLAGFFSKDAIIE
```

[7/12 of aligned sequences]

```
Encr    MLFLCSGSVIHSLNDEQDIRKMGGMHHLTPFTSSCLTIGSLALTGTPFLAGFFSKDAIIE
Bvar    MLFLCSGSLIHSLNDEQDIRKMGGMHHLTPFTSSCLLVGSLALTGTPFLAGFFSKDSIIE
Noco    MLFLCSGSIIHSLGGEQDIRKMGALHRQTPWTSSCFIIGTLALTGMPFLSGFFSKDAIIE
Chsp    MLFLCSGSIIHALNDEQDIRKMGGKQKLTPITSSCLTIGSLALTGTPFLTGFYSKDAIIE
Arja    MLFLCSGSIIHSLNDEQDIRKMGGMHHLTPFTSSCLTIGSLALTGTPFLAGFFSKDAIIE
Pase    MLFLCAGSLIHSLNDEQDLRKMGGLNYLTPFTSACMTVGSLALTGTPFLAGFFSKDAIIE
Trel    MLFLCSGSIIHSLNDEQDIRKMGGLHRLLPFTSSCLTVGSLALAGTPFLTGFFSKDAIIE
Lifa    MLFLCSGSIIHTLVGEQDIRKMGGLSRVIPLTSTCMVLGSLALMGTPYLAGFYSKDTIIE
Acur    MLFLCSGSIIHSLNDEQDIRKMGGMHTLTPFTSSCLTLGSLALTGTPFLAGFFSKDAIIE
Ampe    MLFLCSGSIIHSLNDEQDIRKMGGMQHLTPFTSTCLTIGSLALTGTPFLAGFFSKDAIIE
Urja    MLFLCAGSIIHNLNNQQDIRKMGHIHQLMPVTTSCLTLGSLALAGTPFLAGFFSKDAIIE
Enet    MLFLCSGSIIHSLNDEQDIRKMGALHNLTPFTSTALTIGSLALTGTPFLAGFFSKDAIIE
Ptbr    MLFLCSGSIIHCLNDEQDIRKMGGMQNITPVTTSALTIGSLALTGTPFLAGFFSKDAIIE
Safa    MLFLCSGSIIHCLNDEQDIRKMGGMNNLAPTTASALTIGSLALTGTPFLAGFFSKDAIIE
Icae    MLFLCSGSIIHSLNDEQDIRKMGGMHHLAPFTSSCLTIGSLALTGTPFLAGFFSKDAIIE
Asmi    MLFLTAGSFIHYLDNEQDIRKMGHMYYAAPTTTAVLSTGCLALAGTPFLAGFYSKDAIIE
Foal    MLFLCSGAVIHSLNDEQDIRKMGGMYYLTPFTASCINVGSLALAGFPFLAGFFSKDAIIE
Drze    MLFLCSGSIIHSLNDEQDIRKMGGMHHLAPFTSSCLTLGSLALTGTPFLAGFFSKDAIIE
Rhas    MLFLCSGSIIHSLNDEQDIRKMGGMHHLAPFTSSCLTVGSLALTGTPFLAGFFSKDAIIE
Elac    MLFLCSGSIIHSLNDEQDIRKMGGMHHLTPFTSSALTIGSLALTGTPFLAGFFSKDAIIE
Kugu    MLFLCSGTIIHSLNDEQDIRKMGGMHHLTPFTSTCLTIGSLALTGTPFLAGFFSKDAIIE
Plor    MLFLCSGSIIHSLNDEQDIRKMGGMHYLTPFTSSCLTIGSLALTGTPFLAGFFSKDAIIE
Sgun    MLFLCSGSIIHSLNDEQDIRKMGGMHHLTPFTSSCLTLGSLALTGTPFLAGFFSKDAIIE
Zaco    MLFLCSGSIIHSLNDEQDIRKMGGMHHLTPFTSSCLTIGSLALTGTPFLAGFFSKDAIIE
Zbfl    MLFLCSGSIIHSLNDEQDIRKMGGMHHLTPFTSSCLTIGSLALTGTPFLAGFFSKDAIIE
Spba    MLFLCSGSIIHSLNDEQDIRKMGGMHHLTPFTSSCLTIGSLALTGTPFLAGFYSKDAIIE
Game    MLFLCSGSIIHSLNDEQDIRKMGGMHHLTPFTSSCFTLGSLALTGTPFLAGFFSKDAIIE
Thth    MLFLCSGSIIHSLNDEQDIRKMGGMHHLTPFTSSCLTLGSLALTGTPFLAGFFSKDAIIE
Xigl    MLFLCSGSIIHSLNDEQDIRKMGGMHHLTPFTSSCLTIGSLALTGTPFLAGFFSKDAIIE
Hyja    MLFLCSGSIIHSLNDEQDIRKMGGMHYLTPFTSSCLTIGSLALTGTPFLAGFFSKDPIIE
Psan    MLFLCSGSIIHSLNDEQDIRKMGGMHRLVPVTSSCIIIGSLALTGFPFLAGFFSKDPIIE
Cupa    MLFLCSGSIIHSLNDEQDIRKMGGMHHLTPFTSSCLTIGSLALTGTPFLAGFFSKDAIIE
Mpch    MLFLCSGSIIHSLNDEQDIRKMGGMHHLTPFTSSCFSLGSLALTGTPFLAGFFSKDAIIE
Char    MLFLCSGSIIHSLNDEQDIRKMGGMHHLAPFTSSCLTIGSLALTGTPFLAGFFSKDAIIE
Pser    MLFLCSGSVIHSLNDEQDIRKMGGTHHLTPLTSSCLTIGSLALTGTPFLAGFFSKDAIIE
Prol    MLFLCSGSIIHSLNDEQDIRKMGGMHHLTPFTSSCLTIGSLALTGTPFLAGFFSKDAIIE
Plbi    MLFLCSGSIIHSLNDEQDIRKMGGMHRLAPFTSSCLTIGSLALTGTPFLAGFFSKDAIIE
Calu    MLFLCSGSAIHSLDNEQDIRKMGTMLHLTPLTASCLTIGSLALAGTPFLAGFFSKDAIIE
Papa    MLFICSGSSIHSLKDEQDIRKMGAMHHLTPFTSSCFILGSLALTGVPFLAGFFSKDMIIE
Sufr    MLFLCSGSIIHSLNDEQDIRKMGGMQHLTPFTSSCLTLGSLALTGTPFLAGFFSKDAILE
Stci    MLFLCSGSIIHSLNDEQDIRKMGGMQHLTPFTSSCLTLGSLALTGTPFLAGFFSKDAIIE
Taru    MLFLCSGSIIHSLNDEQDIRKMGGMHHLTPVTSSCLTIGSLALTGTPFLAGFFSKDAIIE
Rala    MLFLCSGSIIHSLNDEQDIRKMGGMHHLTPFTSSCLTIGSLALTGTPFLAGFFSKDAIIE
        ::*: .*   :    :**:**:*     * *       . :**   *:*:**:*** *:*
```

To be continued on page 40.

[8/12 of aligned sequences]

```
                                      L
Scca   AMNTSHLNAWALILTLVATSFTAIYSLRLIFFTLMKFPRFNSFSPINENNPMVINPLKRL
Muma   SMNTSHLNAWALILTLVATSFTAIYSLRLIFFALMNYPRFNTLSPINENNPLVINPIKRL
Erca   ALNTSHLNAWALMLTLIATSFTAVYSLRIIYFVSMSTTRMLPLSPVNENNPLIINPIKRL
Pose   ALNTSNLNAWALMLTLIATSFTAVYSLRIIYFVLMNNPRTLPLSPINENNPLIANPIKRL
Actr   ALNTSHLNAWALTLTLIATSFTAVYSFRVIFFASMGSPRFLPLSPLNENNPTVINPIKRL
Scal   ALNTSHLNAWALTLTLIATSFTAVYSLRIIFFASMGSPRFLPLSPLNENNPMVINPIKRL
Posp   ALNTSHLNAWALTLTLIATSFTAVYSFRVIFFASMGSPRFLPLSPLNENNPTVINPIKRL
Atsp   AMNTSHLNAWALTLTLLATSFTAVYNFRITFYALMNFPRCIFLPPPKEDFSPVRNPIKRL
Leoc   AMNTSYLNAWALTLTLLATSFTAVYNFRITFYALMNFPRCIFLPPPKEDYSPVRNPIKRL
Amca   ALNTSHLNAWALVLTLIATSFPAVYSFRVIFFASMGFPRFLPMLPINENNPTILNPIKRL
Osbi   ALNTSYLNAWALTLTLLATSFTAVYSFRVVFFALMNHPRFLTLSPINENTPTVINPIKRL
Pabu   ALNTSHLNAWALATTLIATSFTAIYSFRLILFSSMGYPRFFPLSPINENNPTVINPIKRL
Hial   ALNTSYLNAWALALTLIATSFTAIYSFRVVFFASMGHPRFSSLSPINENDPAIINPIKRL
Elha   ALNTSYLNAWALTLTLVATSFTAVYSFRVIFFASMKSPRFLPLSPINENNPSVINPIKRL
Mlcy   ALNTSHLNAWALTLTLIATSFTAIYSFRIIFFASMGTPRFLPMSPINENNPSVINPIKRL
Algl   ALNTSHLNAWALALTLVSTAFTAVYSFRMAFLALMGHPRFLPLSPINENTPSASNPIKRL
Ptgi   ALNTSYLNAWALTLTLIATSFTAVYSFRVIFFASMGHPRSLPLSPINENNPAVMNPIKRL
Alaf   ALNTSYLNAWALTLTLIATAFTAVYSFRVIFFASMGHPRFLPLSPINENNPAVINPIKRL
Nock   ALNTSYLNAWALVLTLIATAFTAVYSFRVIFFASMGHPRFLPLSPINENNPTVINPIKRL
Anja   AMNTSYLNAWALTLTLIATSFTAVYSFRIIFFASMGQPRFLPLSPINENNPAVLNPIKRL
Gyki   ALNTSLLNAWALVLTLLATSFTAIYSFRIIFFASMGQPRFLPLSPINENATTLLGPIKRL
Syka   AMNTSYLNAWALTLTLIATSFTAIYSFRVIFFASMGQPRFLPLSPINENNPAVLNPLKRL
Opma   AMNTSYLNTWALTLTLIATSFTAIYSLRIVFFASMGQPRHSSLPPINENNPLVINPIKRL
Comy   AMNTSHLNAWALILTLIATSFTAVYSLRIVYFALMGQPRSTPMVPTNENNQMVMNPIKRL
Sasp   AMNTSHLNSWALILTLMATSFTAVYSLRMTLFTMMDHPRLTPLITTNENNPTAMNPIKRL
Eupe   AMNTSYLNSWALILTLVATSFTAAYSLRITFFTLMGQPRFLPLPSINENNPMVLNPIKRL
Enja   ALNTSHLNAWALILTLVATSFTAAYSLRVIFFVSMGTPRFLPLSPINENDPQVINPIKRL
Same   ALNTSYLNAWALVLTLIATSFTAVYSFRVVFFVTMGTPRFLPLSPINENDPAVINPIKRL
Chch   ALNTSHLNAWALVLTLIATSFTAIYSFRVIFFVSMGTPRFLPLSPINENNPSVINPIKRL
Grgr   ALNTSYLCSWALVLTLLATSFTAVYSLRVVFFVSMGTPRFLPLSPIDENNPSVINPIKRL
Caau   ALNTSHLNAWALTLTLIATSFTAVYSFRLVYFVVMGTPRFLALSPINENNPLVINSIKRL
Cyca   ALNTSYLNAWALTLTLIATSFTAVYSFRLVYFVIMGTPRFLPLSPINENNPLVINSIKRL
Dare   AMTTSHLNAWALTLTLIATSFTAVYSFRMIYLVCLGSPRHKTYETIDENHI-PTNTIQRL
Cost   ALNTSHLNAWALILTLIATSFTAVYSFRVVYFVTMGTPRFLALSPINENNPSVINPIKRL
Leec   ALNTSNLNAWALTLTLIATSFTAIYSFRVIFFVTMGTPRFLPLSPINENNLLVINPIKRL
Fola   AMNTSYLNAWALTLTLIATSFTAVYSFRVVFFVSMGTPRFLPLSPINENNPAVINPIKRL
Clmc   ALNTSYLNAWALALTLIATSFTAVYSFRVVFFVTMGTPRFLPLSPINENDPAVINPIKRL
Phin   ALNTSSLNAWALALTLIATSFTAAYSLRMIFLVTMGPPRFLPLSPLNENNPAIMNPLKRL
Icpu   ALNTSHLNAWALALTLIATSFTAVYSLRVIFFVVMGSPRFSTLSPINENHPAVIASIERL
Psto   ALNTSHLNAWALALTLIATSFTAVYSLRVIFFVTMGSPRFPALSPINENHQTLTAPIERL
Cora   ALNTSYLNAWALTLTLIATSFAAVYSFRVIYFVTMGTPRFSSMTPINENNPAVMNPIKPL
Eisp   ALNTSYLNAWALTLTLIATSFTAIYSFRVVFFVTMNTPRFLPLSPINENNPALINSIKRL
Apal   ALNTSYLNAWALTLTLIATSFTAIYSFRVIYFVSMKTPRFLPMSPINENSPTAINPIKRL
Eslu   AMNTSHLNAWALVLTLLATSFTAVYSLRVVFFVSMGFPRFNTSSPINENNPLIINPLKRL
Dape   ALNTSHLNAWALILTLLATSFTAIYSLRIIFFVSMGFPRFNSISPINENNPSIINPLKRL
Glse   ALNTSHLNAWALILTLLATSFTAIYSLRLTFFVSMGYPRFSAFSPINENNPSLINPLKRL
Naar   ALNTSYLNAWALVLTLLATSFTAIYSLRLIYFVSMGYPRFSSLSPINENNPSVINPLKRL
Lioc   ALNTSYLNAWALSLTLLATSFTAVYSLRLIYFVSMGYPRFSTLSPINENNPSVINPLKRL
Opso   ALNTSYLNAWALVLTLLATSFTAIYSLRLIYFVLIDHPRFTALSPVNENNPSVINPLKRL
Alte   ALNTSYLNAWALALTLIATSFTAVYSFRVVFFVSMGTPRFLALSPINENNPSIINPLKRL
Plap   ALNTSYLNAWALALTLIATSFTAVYSFRVVFFVSMGTPRFLALSPINENNPSVINPIKRL
                                                 36
```

To be continued on page 41.

Plal	ALNTSYLNAWALALTLLATSFTAIYSLRVVYFVAMGHPRFPALSPINENNPSVINPIKRL
Sami	ALNTSYLNAWALALTLLATSFTAIYSLRVVYFVAMGHPRFPALSPINENNPSVINPIKRL
Rere	ALNTSYLNAWALALTLLATSFTAVYSLRVVFFVSMGHPRFPALSPINENNPSVINPIKRL
Gama	ALNTSYLNAWALVLTLLATSFTAVYSLRVVFFVSMGHPRFPSLSPINENNPSVINPIKRL
Onmy	ALNTSHLNAWALTLTLLATSFTAIYSLRVIFFVSMGHPRFTATAPVNENNPSVINPIKRL
Sasa	ALNTSHLNAWALTLTLLATSFTAVYSLRVVFFVSMGHPRFTATAPINENNPSVINPIKRL
Cola	ALNTSHLNAWALTLTLLATSFTAVYSLRVVYFVSMGHPRFTAISPINENNPSVINPIKRL
Dita	ALNTSHLNAWALALTLIATSFTAVYSTRMVFLVVMGNPRFPSHSPINENNPTIINPIKRL
Gogr	ALNTSYLNAWALTLTLIATSFTATYSTRLTLLVAVGHPRFLSLPPLNENNPLVINPLKRL
Chsl	ALNTSYLNAWALTLTILATSFTAIYSIRMIFLVSMGAPRFLTLSPVNENNSRIFNPIKRL
Atja	ALNTSYLNAWALTLTLLATSFTAIYSLRVIFFVSMGHPRFNTTSPINENNPSVINPIKRL
Iido	ALNTSYLNAWALALTLLATSFTAVYSLRVIFFVSMGHPRFNTNSPINENNPAVINPIKRL
Auja	ALNTSYLNAWALALTLLATAFTAVYSMRVVFFVSMGYPRFSPLSPINENNPAVINPIKRL
Chag	AVNTSHLNAWALVLTLLATSFTAVYSLRVVFFVTMGHPRFLPLSPINENNPMVINPIKRL
Hami	ALNTSYLNAWALTLTLLAASFTAIYSMRVVFFVSLRHPRFPPLSPINENNPAVLNPIKRL
Saun	ALNTSYLNAWALTLTLLATSFTAIYSLRVVYFVSLHHPRFTPLPPINENNPAVINPIKRL
Nema	ALNTSHLNAWALTLTLIATAFTAVYSLRVVFFVSMGHPRFSPLSPINENNPAVINPLKRL
Disp	AMNTSHLNAWALTLTLLATSFTAVYSLRVVFFVSMGHPRFLALSPINENNPAVINPIKRL
Myaf	AMNTSHLNAWALTLTLLATSFTAVYSLRVVYFVSMGHPRFLPLSPINENNPSVINPIKRL
Lagu	ALNTSHINAWALATTLIATALTAAYSLRVIFFVSMGFPRFSTYAPINENNPQVINPIKRL
Trtr	ALNTSHINAWALVLTLLATSFTAVYSLRVIFYVSMGTPRFSSLPPINENNPFVLNPIKRL
Zucr	ALNTSHINAWALILTLLATSFTAVYSLRVVFFVSMGYPRFATLSPINENNPSVINPIKRL
Pxja	ALNTSHINAWALVLTLLATSFTAVYSLRVVFFVSMGFPRFPAMSPINENNPAVINPIKRL
Pxlo	ALNTSHINAWALVLTLLATSFTAVYSLRVVFFVSMGFPRFSAMSPINENNPAVINPIKRL
Pctr	AMNTSYINAWALALTLLATSFTAAYSLRMIFFVSMGHPRFPALSPINENNSTVINPIKRL
Apsa	ALNTSNINAWALTLTLLATSFTAIYSLRIIFFVVMGYPRFSALAPINENNPAVINPLKRL
Cabe	ALNTSYLNACALVITLIATSFTAVYSMRIVTFVVIGSPRNKAFPPINENSPNLIQPISRL
Bzze	ALNTSHLNAWALLLTLLATSFTAIYSLRIVFFVSMGHPRFNSLSPIKEDVSAVINPIKRL
Siim	ALNTSHLNAWALSLTLIATSFTAVYSLRLVFFVSMGNPRFNAFSPINENTPKVINPIKRL
Ctru	ALNTSHLNAWALTLTLLATSFTAIYSLRVIFFVSMGHPRFTALAPINENNPAVINPIKRL
Dpbr	ALNTSHLNAWALTLTLLATSFTAIYSLRVIFFVSMGHPRFPPLSPINENNPTVINPIKRL
Caki	ALNTSTLNAWALTLTLFATSLTAVYSLRVIFFVSMGSPRFSCHSPINENNPSLINPLKRL
Phja	ALNTSHINATALVFTLIATAFTAVYSLRLIFSVVMGQPRYPALSFSNEDNPLVMNPLTRL
Brsp	ALTNSYINAWALILTLIATSFTAAYSLRLIFYVTMGTPRFLPFSPINENNATVLNPLKRL
Gamo	ALNTSHVNAWALTLTLIATSFTAIYSLRVIFFVTMGTPRFLPLSPINENNPAVINPLKRL
Lolo	ALNTSQVNAWALTLTLIATSFTAIYSLRVIFFVTMGTPRFLPSSPINENNSAVINPLKRL
Batr	TMNNSHINSWAILLTLVATTFTAIYSLRMLVYVNLTFPRHTPLPPLNENFPNLMNPLKRL
Prmy	AMNTSYLNGWALTMTLVATSFTAIYSTRLIYLVVLGRPRFLPLAPLNENSPALINPLKRL
Lose	AANTSYLNAWALTLTLLATSFTAIYSLRIMLFVSVGTPRFHPLSALNENNPNIINPIKRL
Loam	ALNTSHLNAWALALTLLATSFTAIYSLRIVYFVSMGHPRFNPLPPINENSPAVINPIKRL
Chab	ALNTSYLNAWALTLTLLATSFTAIYSFRVIFFVCMGHPRFNTLPPINENNPAVINPIKRL
Chto	ALNTSYLNAWALTLTLLATSFTAIYSFRVIFFVCMGHPRFNTLPPINENNPAVINPIKRL
Majo	ALNTSYLNAWALILTLLATTFTAIYSLRLIFFVSMGHPRFNSFTPINENSPTVMNPIMRL
Hlst	ALNTSHLNAWALALTLLATTLTAIYSLRLIFFVSMGHPRFNSYSPINENDPAVINPLKRL
Clpe	ALNTSYLNAWALTLTLLATSLTAVYSLRLIFFVSMGHPRFAPLAPINENNPAVINPIKRL
Mlmr	ALNTSYLNAWALTLTLLATSFTAIYSLRLIFFVSMGHPRFNSLPPINENNPAVINPIKRL
Crcr	ALNSSHLNAWALVLTLLATSFTAVYSLRLIFYVSLGHPRFNTFSPINENNPAVINPLTRL
Muce	ALNSSHLNAWALVLTLLATSFTAVYSLRLIFYVSLGHPRFNTFSPINENNPAVINPLTRL
Bege	ALNTSYLNAWALVLTLLATSFTAIYSLRVVFFVSMGYPRFNSLPPINENNPTVINPIKRL
Mela	AMNTSYLNAWALVLTLLATSFTAVYSLRMIFFVSMGSPRFNPLSPINENNPAVINPIKRL
Hats	ALNTSYLNAWALVLTLLATSFTAVYSLRVVFFVSMGHPRFNPLSPINENNPAVINPIKRL
Orla	ALNTSYLNAWALVLTLVATSFTAVYSLRVVFFVSMGHPRFNPISPINENNLTVMNPIKRL

To be continued on page 42.

[8/12 of aligned sequences]

```
Cosa  ALNTSYLNAWALALTLLATSFTAVYSLRVVFFVSMGHPRFNSLSPINENNPAVINPIKRL
Exsp  ALNTSYLNAWALTLTLIATSFTAVYSLRVIFFVSMGNPRFNPLSPINENSPEVINPIKRL
Depa  ALNTSYLNAWALALTLLATSFTAIYSLRIVFFVSMGYPRFNPLPPINENNPTMMNPIKRL
Rima  ALNTSYLNAWALSLTLIATMFTAVYSFRMIYFVSITYPRFNTFLPINENNPTLVKPLKRL
Fuol  ALNSSYLNAWALILTLLATSFTAIYSLRVIYFVSIHHPRFNAFSPINENNTAVINPIKRL
Gmaf  ALNTSYLNAWALTLTLIATSFTAVYSLRIIYFVLMEYPRFNPLMPINENNPSITNPLKRL
Xeei  ALNTSYLNAWALVLTLIATSFTAIYSLRMVFFVSMGNPRFNSFSPINENNPSVINPIKRL
Pros  ALNTSYLNAWALALTLLATSFTAIYSLRVVFFVSMGHPRFNTLSPINENNPTVINPIKRL
Scmi  ALNTSHLNAWALALTLLATSFTAIYSLRVIFFVSMGHPRFNSLTPINENNPTVVNPIKRL
Rolo  ALNTSHLNAWALVLTLIATAFTAVYSLRLVFFVSMGHPRFITLPPINENNPAVINSIKRL
Cere  ALNTSHLNAWALILTLLATSFTAIYSLRVVFFVSMGSPRFPTLTPINENNPAVISPIKRL
Daga  ALNTSHLNAWALTLTLVATSFTAIYSLRLVFFVSMGTPRFISMAPINENSMQVLAPLKRL
Anco  AMNTSHLNAWALALTLLATSFTAIYSLRVVYFVSMGHPRFIPLAPINENNPAVMNPIKRL
Dmve  AMNTSYINAWALVLTLLATSFTAIYSLRIFYLVSAGFPRFATLRPPADTSPAAINPIKRL
Dmar  AMNTSYINAWALVLTLLATSFTAIYSLRIFYLVSAGYPRFATLLPPSDPNPASIAPIKRL
Anka  AMNTSHLNAWALTLTLLATSFTAVYSLRVVFFVSMGHPRFITLPPINENNPAVLNPIKRL
Moja  AMNTSHLNAWALALTLLATSFTAIYSLRVVYFVSMGYPRFITLPPINENNPAVMNPIKRL
Hoja  AMNTSHLNAWALILTLLATSFTAVYSLRVVYFVSMGHPRFIPLPPINENNPAVMNPIKRL
Bede  ALNTSHLNAWALALTLLATSFTAIYSLRVVFFVSMGHPRFNALSPINENNPTVINPIKRL
Besp  ALNTSHLNAWALALTLLATSFTAVYSLRVVFFVSMGHPRFNPLSPINENNPTVINPIKRL
Mysp  ALNTSYLNAWALTLTLLATSFTAIYSLRVVFFVSMGHPRFTPLSPINENDPTTINPIKRL
Osja  ALNTSYLNAWALTLTLLATSFTAIYSLRVVFFVSMGHPRFIPLSPINENDPTTINPIKRL
Sgro  ALNTSYLNAWALTLTLLATSFTAIYSLRVVFFVSMGHPRFIPLAPINENDPTTINPIKRL
Pzpa  ALNTSYINAWALALTLLATSFTAVYSLRLIYFVSMGHPRFATLPPINENNPAVINPLKRL
Zeja  ALNTSHINAWALTLTLLATSFTAVYSLRVIYFVSMGHPRFAPLSPINENNPSVINPLKRL
Znne  ALNTSHINAWALMLTLLATSFTAIYSLRIVYFVSMGFPRFTPITPINENNPSVINPLKRL
Zefa  AMNTSHINAWALILTLLATSFTAIYSLRVVYFVSMGHPRFITLPPINENNPSVINPLKRL
Acni  ALNTSHINAWALALTLLATSFTAIYSLRIVYFVSMGHPRFTPLAPINENNPSVINPLKRL
Ncrh  ALNTSHINAWALALTLLATSFTAIYSLRVVYFVSMGHPRFTPLAPINENNPAVINPLKRL
Agca  ALNTSHLNAWALTLTLLATSFTAIYSLRVVFFVSMGHPRFNSLSPINENNPAVINPIKRL
Hydy  ALNTSHLNAWALALTLLATSFTAIYSLRVIFFVTMGHPRFNSVSPIDENSPFVVNPIKRL
Gsac  ALNTSHLNAWALVLTLLATSFTAIYSLRVIFFVSMGYPRFNSLSPINENNPAVINPIKRL
Pevo  ALNTSHLNAWALVLTLIATSFTAVYSLRVVFFVSMGYPRFNALSPINENNPSVINPVKRL
Hiku  SMNTSYLNAWALLLTLIATSFTAVYSLRIIYYVNMGYPRFNPLSPINENSKPVINPIKRL
Inpa  ALTTSNLNAWALTLTLLATAMTAIYSLRIVFLVNMGHPRFPSLSPLNENYKANTQPIKRL
Auch  ALNTSNLNAWALFLTLLATSFTAAYSLRIVFFVSIAHPRLPTTTPIDENNPAIIKPLARL
Fico  AMNTSHLNAWALTLTLLATSFTAIYSLRVVFFVSMGFPRFNSLSPINENNPSVINPIKRL
Macs  ALNTSHLNAWALTLTLLATSFTAIYSLRVIFFVAMGHPRFNSLSPINENNPAVINPIKRL
Moal  ALNNSYLNAWALALTLLATSLTAAYSLRMIFFVNMNFPRFPALSPINENIKSITNPLKRL
Syma  SMNTSYLNAWALTLTLLATALTAAYSMRLIFLVNMGSPRFNSLPPINENTPTVINPIKRL
Mafr  ALNTSHLNAWALTLTLLATSFTAIYSLRIIYFVNMNHPRFNAFSPINENNPTLTNPLKRL
Dcpe  ALNTSHLNAWALVLTLLATSFTAVYSLRVIFFVSMGQPRFNPLSPINENNPAVINPIKRL
Dcti  ALNTSHLNAWALVLTLLATSFTAVYSLRVIFFVCMGQPRFNSLSPINENNPAVINPIKRL
Hehi  ALNTSHLNAWALVLTLLATSFTAIYSLRVVFFVSMGHPRFNSISPINENNPAVINPLKRL
Stam  ALNTSHLNAWALTLTLLATSFTAIYSLRVVFFVTMGHPRFNSLSPINENNSAVINPIKRL
Hogi  ALNTSHLNAWALALTILATSFTAIYSLRVVFFVSMGHPRFNALSPINENNPAVINPIKRL
Erzo  ALNTSHLNAWALTLTLLATSFTAIYSLRVVFFVSMGHPRFNSFSPINENNPAVINPIKRL
Hxot  ALNTSHLNAWALTLTLLATSFTAIYSLRVVYFVSMGHPRFNSLSPINENNTAVINPIKRL
Core  ALNTSYLNAWALTLTLLATSFTAIYSLRVVYFVSMGHPRFNPLSPLNENNPSVINPIKRL
Apve  ALNTSHLNAWALTLTLLATSFTAIYSLRMVFFVTMGCPRFNSLSPINENNSALMNPIKRL
Latj  ALNTSYLNAWALALTLLATSFTAVYSLRVIYFVSMGHPRFNPLSPINENNPTVINPIKRL
Laja  ALNTSHLNAWALTLTLLATSFTAVYSLRVVFFVSMGHPRFNPLSPINENNPAVINPIKRL
```

To be continued on page 43.

[8/12 of aligned sequences]

```
Syja  ALNTSHLNAWALTLTLLATSFTAIYSLRVVFFVSMGHPRFSPLSPINENNPAVINPIKRL
Epme  ALNTSYLNAWALTLTLLATSFTAIYSLRVVYFVSMGRPRFNALSPINENNPAVLNPIKRL
Grse  ALNTSHLNAWALTMTLLATSFTAVYSIRLISYVSLGMPRFNPFSPINENNSMVINPIKRL
Clja  SLTTSYINAWALFLTLLATAFTAIYSLRVIFFVPMNFPRFNPCSPINENHPNVLNPIKRL
Ogcy  AMTTSHLNAWALFLTILATSFTGVYSLRLIFLVTMGNPRFTPLSPINENDPQLINPIKRL
Plna  ALNNSYLNAWALLLTLLATSFTAAYSLRIIFFVSLGHPRFTTLPPINENNPALMNAIKRL
Lema  ALNTSHLNAWALILTLLATSFTAIYSLRVVFFVSMGHPRFSPLSPINENNPAVINPIKRL
Etzo  ALNTSHLNAWALVLTLLATSFTAIYSLRVVYFVSMGHPRFNALSPINENNPAVVNPIKRL
Apse  SLNTSHLNAWALTLTLLATSFTAIYSLRVVYFVSMGQPRFPSLSPINENNLTVINPIKRL
Epde  ALNTSYLNAWALTLTLLATSFTAIYSLRVVFYVSMGHPRFNSLSPINENNPAVIKPIKRL
Slja  ALNTSHINAWALILTLLATSFTAIYSLRVVFFVSMGHPRFNSFSPINENNPAVINPIKRL
Bsja  ALNNSYLNAWALALTLLATSFTAVYSLRLVFFVVMGHPRFNPLSPINENTPSVINPLKRL
Ecna  ALNTSHLNAWALILTLLATSFTAIYSLRVIFFVSMGTPRFNSLSPINENNPTVINPIKRL
Cohi  ALNTSYLNAWALSITLIATSFTAVYSLRLVYFVSLGNPRSGSFMPINETGALLTGPLGRL
Caar  ALNTSHLNAWALILTLLATSFTAIYSMRIVFFVVMGYPRFNALSPINENNPAVINPIKRL
Came  ALNTSHLNAWALVLTLLATSFTAIYSMRIVFFVVMGHPRFNALSPINENNPAVINPIKRL
Mema  ALNTSHLNAWALTLTLIATSFTAVYSLRVVYFVVMGHPRFASLSPINENDPAVINPIKRL
Lenu  AMNTSYTNAWALTLTLIATSFTAIYSLRIIFLVSMGHPRFNPISPINENNPNLINPIKRL
Brja  ALNTSHLNAWALTLTLLATSFTAIYSLRVVFFVSMGHPRFNTLSPINENNPTVINPIKRL
Plma  ALNTSHLNAWALTLTLLATSFTAIYSLRVVFFVSMGHPRFNALSPVNENNPTVINPIKRL
Emst  ALNTSHLNAWALTLTLLATSFTAIYSLRVIFFVAMGHPRFNSLSPINENNPAVINPIKRL
Ptti  ALNTSHLNAWALTLTLLATSFTAIYSLRVVFFVAMGHPRFNSLSPINENNPAVINPIKRL
Losu  SMNTSHLNAWALTLTLIATTFTAIYSLRLTFFVSMGHPRFKPLSPINENNYAVVNPIKRL
Geoy  ALNNSFLNAWALVLTLLATSFTAIYSLRVVFFVSMGHPRFAPLSPINENNPAVINPIKRL
Dipi  ALNTSHLNAWALTLTLLATSFTAIYSLRVVFFVSMGHPRFNSLSPINENNPTVINPIKRL
Pama  ALNNSYLNAWALAMTLLATSFTAIYSLRVVFFVSMGHPRFSSLSPINENNPSVINPIKRL
Leob  ALNTSYLNAWALALTLVATSFTAIYSLRVVFFVSMGHPRFNTLSPINENNPAVINPIKRL
Neba  ALTTSYLNAWALTLTIIATSFTAVYSLRVIFFVSMGHPRFITLAPINENNPTVINPIKRL
Pdpl  ALNTSYLNAWALALTLLATSFTAIYSFRMIFYVCLGHPRFNPLSPINENNPTMTHAITRL
Nimi  ALASSHLNAWALALTLIATSFTAVYSLRLIFFVVIGYPRFSGYIPINENNPAVINPLKRL
Uptr  ALNTSHLNAWALALTLLATAFTAVYSLRVVYFVSMGHPRFNALSPINENNPAVINPIKRL
Pesc  ALNTSYLNAAALALTLMALTFTAVYSIRLMLHAFSAHPRLHPLVPIREESPAQRNPIKRL
Baar  ALNTSHLNAWALTLTLLATSFTAIYSLRIVFFVSMGHPRFNSLSPINENNPAVINPIKRL
Moar  ALNTSHLNAWALTLTLLATSFTAIYSLRVVFFVSMGHPRFNSLSPINENNTAVINPIKRL
Toja  ALNTSYLNAWALTLTLLATSFTAIYSLRVVYFVAMGFPRFNTLSPINENNPTVINPIKRL
Chau  ALNTSYLNAWALALTLVATSFTAIYSLRVIFFVSMGHPRFNPLSPINENDPKVINPIKRL
Chse  ALNTSHLNAWALVLTLLATSFTAVYSLRVIYFVSMGHPRFNPLSPINENNPSVINPIKRL
Enar  ALNTSHLNAWALTLTLLATSFTAIYSLRVVFFVSMGHPRFNSLSPINENNPAVINPIKRL
Hpty  ALNTSHLNAWALTLTLLATSFTAIYSLRAVFFVAMGHPRFNSLSPINENNPAVLQPIKRL
Nana  ALNTSHLNAWALTLTLLATSLTAAYSLRVIFFVNMGHPRFNPLSPINENNHTVINPIKRL
Mcst  ALNTSHINAWALTLTLLATSFTAIYSLRVIFFVSMGHPRFNTLSPINENNPMVINPIKRL
Rhox  ALNTSHLNAWALTLTLIATSFTAIYSLRVVFFVSMGYPRFNSLSPINENNPAVINPIKRL
Opfa  ALNTSHLNAWALALTLLATSFTAIYSLRVVFFVSMGHPRFNPLSPINENNPAVINPIKRL
Paar  ALNTSHLNAWALALTLLATSFTAIYSLRIVFFVTMGHPRFNALSPINENFPTVVNPIKRL
Gozo  ALNTSHLNAWALTLTLIATSFTAIYSLRVVYFVSMGHPRFNPLSPINENNPAVINPIKRL
Ackr  ALLTSPVNAWALALTVIAVMFTVVYSLRAIFYLALFNPRFAPLSPINENNPAVINPLKRL
Elev  ALNTSSINAWALTLTIIATSFTAAYSLRIVFFVTMGHPRFKPLSPINENNPSLINPLKRL
Trdu  ALNTSYLNAWALFLTLLATSFTAIYSLRVIFFVSMGHPRFNPLSPINENNSTVINPIKRL
Amoc  ALNTSYLNAWALVLTLLATSFTAVYSLRIVFFVSMGNPRFTPLSPINENNPTVINPIKRL
Hame  AATTSTSNACALLITLVATGMTAAYSLRIVFYVVMGRPRINPLSPANENTPHVINPLKRL
Chso  ALNTSHLNAWALTLTLIATSFTAIYSLRVVFFVSMGHPRFNPLSPINENNTAVINPIKRL
Lyto  ALNTSHLNAWALVLTLLATSFTAIYSLRVVFFVSMGYPRFNPLSPLNENNPAVINPLKRL
```

To be continued on page 44.

```
Encr  ALNTSHLNAWALVLTLLATSFTAIYSLRVVFFVSMGHPRFNSLSPINENNPAVINPIKRL
Bvar  ALNTSHLNAWALTLTLLATSFTATYSLRLIYFVIMGNPQFNVHPPINENNPLVINPLKRL
Noco  AMTTSYLNTWALLLTLFATAFTAIYSVRLIYFVLMPNPRSMTITPIGEANPQVLNPLKRL
Chsp  ALNTSHINAWALILTLIATSFTAVYSLRIVYMVLMKNPRFNPLSPINENIPAVTKPLKRL
Arja  ALNTSHLNAWALTLTLLATSFTAIYSLRVVFFVSMGHPRFNSFSPINENTPAVINPIKRL
Pase  ALNTSVLNAWALALTLLATSFTAVYSLRIIFFAVMNYPRYQALTPIDENSRAVINPIKRL
Trel  ALNTSHLNAWALILTLIATSFTAVYSLRVVFFVCMGHPRFNAISPLNENDPQLIKPIKRL
Lifa  AALNSPVNAWALGLTAIATSFTAVYSLRIMYYFSILSPRYPALITLHQDDHNTNAPILRL
Acur  SLTTSHLNAWALVLTLLATSFTAVYSLRLTFFVVMGTPRFNCLSPINENDPLVIKPIKRL
Ampe  ALNTSHLNAWALVLTLLATSFTAIYSLRVVYFVSMGHPRFNSLSPINENNPAVINPIKRL
Urja  AMTSSHLNTWALFLTLVATSFTAVYSLRLIFSVSLGHPRFDPLSPINENFSAVTSPIKRL
Enet  ALNTSYLNAWALTLTLLATSFTAVYSLRLAYFSVLGHPRFNPLSPINENTPLVINPLKRL
Ptbr  ALNTSYLNAWALTLTLLATSLTAAYSLRVVYFVCMGNPRFNVLSPINENNQLVLKPIKRL
Safa  ALNTSHLNAWALILTLLATSFTAVYSLRVVFFVCMGNPRFNAYTPINENDPHTVNPIKRL
Icae  AMNTSHLNAWALTLTLLATSFTAIYSLRVVFFVSMGHPRFNAFSPINENNPLVINPIKRL
Asmi  ALNTSNVNIVALSLTLAATSLTAVYSLRLIYHVAAGTPRFTSFLPIDETHPLINKAFKYL
Foal  AMNTSSLNTFALLLTLIATSFTAAYSLRLIFLVSLNTTRMAPLLPIDESPAEVINPIKRL
Drze  ALNTSHLNAWALILTLMATSFTAVYSLRIVYYVSMGYPRFNVLPPINENDPSVINPIKRL
Rhas  ALNTSYLNAWALTLTLLATAFTAVYSLRVVFFVSMGHPRFNPLSPINENNPAVINPIKRL
Elac  ALNTSYLNAWALILTLLATSFTAVYSLRVVFFVSMGHPRFNTLSPINENNPAVLNPIKRL
Kugu  ALNTSHLNAWALTLTLLATSFTAIYSLRVVFFVSMGHPRFNTLPPINENNPTVMNPIKRL
Plor  ALNTSHLNAWALTLTLLATSLTAIYSLRVVFFVSMGYPRFNPLSPINENNPAVINPIKRL
Sgun  ALNTSHLNAWALTLTLLATSFTAIYSLRVIFFVSMGHPRFNSFSPINENNQAVINPIKRL
Zaco  ALNTSHLNAWALALTLLATSFTAIYSLRVVFFVSMGHPRFNPLSPINENNPSVINPIKRL
Zbfl  ALNTSYLNAWALTLTLLATSFTAIYSLRVVFFVSMGHPRFNPLSPINENNSAVINPIKRL
Spba  ALNTSHLNAWALTLTLLATSFTAIYSLRVVFFVTMGYPRFNPLSPINENNPAVMNPIKRL
Game  ALNTSHLNAWALTLTLLATSFTAIYSLRVVFFVSMGHPRFNSLSPINENNPAVINPIKRL
Thth  ALNTSHLNAWALTLTLIATSFTAIYSLRVVFFVSMGHPRFNSLSPINENNPAVINPIKRL
Xigl  ALNTSHLNAWALTLTLLATSFTAIYSLRVVFFVSMGHPRFNPLSPINENNPTVINPIKRL
Hyja  ALNTSHLNAWALTLTLLATSFTAIYSLRVVFFVSMGRPRFNAFSPINENNPMVINPIKRL
Psan  ALNTSYLNAWALTLTLLATSFTAIYSLRVVYYVSMGYPRFNTLCPINENNPMLINPLKRL
Cupa  ALNTSHLNAWALTLTLLATSFTAIYSLRVVFFVSMGHPRFNALSPINENNPAVINPIKRL
Mpch  ALNTSYLNAWALTLTLLATSFTAIYSLRLIFFVSMGYPRFNTLSPINENNPSVINPLKRL
Char  ALNTSHLNAWALVLTLIATSFTAIYSLRVVFFVSMGHPRFNTLSPINENNLAVINPIKRL
Pser  ALNTSHLNAWALTLTLLATSFTAIYSLRIISFVPINFPRFNSLSPINENHPAVTNSIKRL
Prol  AMNTSHLNAWALALTLLATSFTAAYSFRLVFYVSMGQPRFTPITPINENNPALINPIKRL
Plbi  ALNTSHLNAWALTLTLLATSFTAIYSFRVIFFVPMGYPRFSPLSPINENNPAVINPLKRL
Calu  ALNTSELNAWALTLTLLATSFTAVYSFRLIYYVVMGYPRFPSLSPINENSATLINPLKRL
Papa  ALNTSYLNAWALALTLLATSLTAAYSLRIIFFVFMHHPRFLPLPPINENSTALLNATKRL
Sufr  ALNNSYLNAWALALTLIATSFTAVYSLRVVFFVSMGFPRFNPLSPINENNPAVINPIKRL
Stci  ALNTSHLNAWALALTLIATSFTAIYSLRIIFLVSMGRPRFNSLSPINENNPAVINPIKRL
Taru  SLTTSQLNAWALCLTLLATSFTAIYSLRVVFYVSMGHPRFNSLSPINENNPSVINPIKRL
Rala  ALNTSHLNAWALVLTLLATSFTAIYSLRVVFFVSMGHPRFNSLSPINENNPAVINPIKRL
      :  .*     *:  *   :  :   *. *           :       :          *
```

To be continued on page 45.

```
                      M                                N
Scca  AYGSIIAGLIITSNLPPAKTQIMTMSPLLKLSALLVTILGLLLALELTNLTHS-------
Muma  AYGSIIAGLIITLNLTPTKTQIMTMSPLLKLSALLVTIMGLLLALELTNLTNS-------
Erca  AWGSIIAGFILYNFILPNKTQMMTMPMSLKLTALIVSLLGLIIAFELALLTNK-------
Pose  AWGSIVAGLILCQYILPNKTQMLTMPPTLKLTGLIVSLLGLITALELASMTNK-------
Actr  AWGSILAGLFITSNFLPAKTPIMTMPTTLKLSALLVTALGLLVALELTNLTNK-------
Scal  AWGSILAGLLITSNFLPAKTPIMTMPTTLKLSALLVTALGLLMALELTSLTNK-------
Posp  AWGSILAGLFITSNFLPTKTPIMTMPTTLKLSALLVTTLGLLMALELTNLTNK-------
Atsp  AWGSILAGLLITLNCPKTETPVLTMPTTLKLGALLVTILGLLSAMWLKTLMNKLPNEPLH
Leoc  AWGSILAGFLITLNCPKTETPILTMPMTLKLSALLVTILGLLSAMWLKTLMNKLPNKSPA
Amca  AWGSILAGLLITSNFLPTKTPIMTMPLSLKMAALLVTTIGLLIAIELTSLTNK-------
Osbi  AWGSIIAGFLITSNLTPTKTQIMTMPSLLKLSALIVSIAGLLTAIELASMTNK-------
Pabu  AWGSIIAGLLISSNLLPTKTPVMTMPTMLKLAALLVSILGLMIALELASLTNK-------
Hial  AWGSIIAGLIITSNYLPTKTPVMTMPPALKLAALLVTIIGLLTAMELANLTNK-------
Elha  AWGSIVAGLIITSNFLPTKTPIMTMPTALKMSALLVTITGLLVAMELANLTNK-------
Mlcy  ALGSIIAGLIITSNLPSTKTPIMTMPTTLKMSALMVTITGLLMAIELSSLTNK-------
Algl  AWGSIIAGLVISSNFLPMKTPVMTMPVLLKLSALLVTIAGASVAAQLASLTTK-------
Ptgi  AWGSIVAGLIITSNFLPTKTPVMTMPPLLKLSALLVTILGLLTALELTNLTNK-------
Alaf  AWGSIVAGLIITANFLPTKTPVMTMPPLLKLAALLVTIMGLLTAMELANLTNK-------
Nock  AWGSIVAGLIITANFLPTKTPVMTMPPLLKLAALLVTIMGLLTAMELANLTNK-------
Anja  AWGSIIAGLIITSNFLPMKTPVMTMAPTLKLSALMVTAIGLLTAMELANLTNK-------
Gyki  AWGSIVAGLILTSNFMPNKTQIMTMPSPLKLGALIVTIMGLIMAMELANLTNK-------
Syka  AWGSIIAGLIITSNFLPTKTPIMTMPHMLKLSALLVTILGLIIAMDLTNLTNK-------
Opma  AWGSIIAGLIITSNFTPKNTPVMTMPETLKLSALIVTILGLIIAMDLANLTNK-------
Comy  AWGSIIAGVIITSNFLPTKTPILTMPTSLKLSALLVTIVGLITAMELANLTNK-------
Sasp  AWGSIIMGLVMSSHLLPTKSATLTMPPLLKTAAVLVTIVGLYAALELANLASK-------
Eupe  GWGSIIAGLMVMSNLLPMKTPTMTMPLTLKLSALLVTVFGLLAALELANLTNT-------
Enja  AWGSIVAGLILTSNILPLNTPIMTMPPVLKLAALLVTIIGLLTALELANLTSK-------
Same  AWGSIVAGLILISNTLPTKTPVMTMPPMLKLAALIVTIIGLLTAMELATLTSK-------
Chch  AWGSIIAGLIITSNFLPHKTPVMTMPMILKLAALGVTILGLLTAMELASMTNK-------
Grgr  AWGSIVAGLLLISNILPLKTPVMTMPAILKLAALAVTIIGLLAALEMASFTTK-------
Caau  AWGSIVAGLIITQNFLPMKTPIMTMPTTLKMAALLVTIAGLLVAMELANMTSK-------
Cyca  AWGSIIAGLIITQNFPPMKTPIMTMSITLKMAALMVTIAGLLVAMELANLTSK-------
Dare  AWGSIIAGLIISYTMIPLKTPILTMPIYLKLAAILVTLLGIILGLEISTLANK-------
Cost  AWGSIIAGLIITSNFLPSKTPVMSMPLALKLAALLVTIIGLLVALELSALTNK-------
Leec  AWGSIIAGLIITSNFLPYKTPIMTMPTLLKMAALMVTIVGLLVAMELTTLTNK-------
Fola  AWGSIIAGLIITSNLLPSKSPVMTMPPTLKMAALMVTAIGLFTAMELATLTSK-------
Clmc  AWGSIIAGLIITSNFLPSKTPIMTMPPILKLAALTVTIMGLLAAMELASMTSK-------
Phin  AWGSITAGLIISLNFLPLKTPTMTMPLALKMAALTVTLTGLLISLELASLTSK-------
Icpu  AWGSIIAGLIITSNFLPSKAPVMTMPLSLKIAAMAVTILGVIIALTLATTTSK-------
Psto  AWGSIIAGLILTLNFLPSKTPTMTMPPSLKLAALLVTVVGLIIALALATATTK-------
Cora  AWGSIIAGLIITSNFLPSKVPVMTMPTSLKLAAIMVTITGVLIAMALTTMTAK-------
Eisp  AWGSIIAGLIITSNLLPLKPSLMTMPPALKLAALIVTLLGLTIAMELASLTSK-------
Apal  AWGSMIAGLIITSNLLPLKTPVMTMPPALKLAALTVSVAGLLIAMELASLTSK-------
Eslu  AWGSIIAGLLITLNLLPSKTPIMTMPPLLKLSALLVSILGLLLALELASLTSK-------
Dape  AWGSIVAGLIITLNFLPSKTPIMSMPLLLKISALLVSLLGLLIALELASLTSK-------
Glse  AWGSVIAGLLITSNFLPSKTPIMTMPPALKLSALLVTILGLLIALELASLTNK-------
Naar  AWGSVIAGLLITANFLPSKTPIMTMPTPLKLAALLVTILGLLIALELASLTNK-------
Lioc  AWGSIVAGLLITSNFLPSKTPIMTMPSSLKLAALFVTILGLLIALELASLTNK-------
Opso  AWGSLIAGLLISANLVPLKTPAMTMPLPLKLSALLVTLMGLLVALELASLTNK-------
Alte  AWGSIVAGLIITSNFIPSKTPIMTMPPLLKLAALAVTITGLLMAMELASLTSK-------
Plap  AWGSIVAGLILTSNLLPSKTPVMTMPPLLKLAALLVTIAGLLTAMELASLTSK-------
```

To be continued on page 46.

[9/12 of aligned sequences]

```
Plal AWGSIVAGLIITSNFLPTKTPVMTMPPLLKLSALIVTIVGLLTALELASLTSK-------
Sami AWGSIVAGLIITSNFLPSKTPVMTMPPLLKLSALLVTILGLLTALELASLTSK-------
Rere AWGSIVAGLIITSNFLPSKTPIMTMPPLLKLSAIIVTILGLLVALELASLTAK-------
Gama AWGSIVAGLILTSSFLPSKTPIMTMAPLLKISALLVSIFGLIIALELASLTGK-------
Onmy AWGSIIAGLLITSNFLPTNTPVMTMPTHLKLAALLVTISGLLIALELASLTNK-------
Sasa AWGSIIAGLLITSNFLPSKTPIMTMPLPLKLAALLVTISGLLIALELASLTNK-------
Cola AWGSIVAGLLITSNFLPSKTPVMTMPPALKLAALLVTILGLLVALELASLTSK-------
Dita AWGSVIAGLLITSNILPPKTPVMTMPPLLKTSALAVSILGLLVALELAALTNK-------
Gogr AWGSMIAGLMITSFLPPSKTPIMTMPTPLKLGALIVTILGLLTALELASLATK-------
Chsl AWGSIVAGLLITSHIIPLKTPTMTMPPLLKLAALTVSILGLLIALELASLTNK-------
Atja AWGSIIAGSIITSNILPLKTPVMTMPPALKLSALIVTVLGLIIALEMASLTNK-------
Iido AWGSIIAGSIITSNILPMKTPVMTMPLALKLSALAVTALGLITALEMASLTNK-------
Auja AWGSIVAGLILTSNFPLSNTPVMTMPPLLKLAALAVTITGLLIALELASLTSK-------
Chag AWGSILAGLLLTSNFLPLKTPVMTMPPLLKLSALIVTIIGLLTALELASLTSK-------
Hami AWGSILAGLIITLNFLPNKTPVMTMPPLLKLAALVVTILGLLIALELATLTSK-------
Saun AWGSIFAGLIITLNLTPNKTPIMTMAPPLKLAALIVTITGFLVALELATLTSK-------
Nema AWGSIIAGLLITSNITPLKTPIMTMHPTLKMGALLVTLLGLITAMELASLTSK-------
Disp AWGSIIAGLLITSNITPLKTPVMSMHPSLKLSALIVTILGLLTALELASLTSK-------
Myaf AWGSIIAGLLITSNITPLKTPIMTMHPSLKLSALIVTILGLMTALELASMTSK-------
Lagu AWGSIAAGLVITLNINALKTPTMTMPAPLKLGALAVSILGLLVALELAMATSK-------
Trtr AWGSLVAGLLISSNINFVKTPVMTMPPLLKTSALIVSILGLLIAFELAQMTSK-------
Zucr AWGSIAAGLLISYNINFVKTPVMTMPFTLKASALIVTIMGLVIALELAHMTTK-------
Pxja AWGSIVAGLLITSNIIPLKTPVMTMPTTLKLAALAVTAIGLFTALELASLTNK-------
Pxlo AWGSILAGLLITSNIIPLKTPVMTMPTTLKLAALAVTAIGLFTALELASLTNK-------
Pctr AWGSILAGLLITSSMLPMKTPIMTMPPTLKIAALAVTALGALTALELATMTKN-------
Apsa VWGSITAGLLITSNLTPTNTPIMTMPITLKIAALMVTILGLLTATELSALTNK-------
Cabe VWGSIFAGFFLSAYILPAKTPVLTMPFYAKLGALGVTILGLLIALELGRLAGD-------
Bzze AWGSIVAGLVITYNLLPLKTPVMSMPPLLKLAALLVTIIGLITALEIQSFTTK-------
Siim AWGSIIAGLILTSNLLPQKTPVMSMPLMLKLAALLVTVLGVLTALELASLASK-------
Ctru AWGSIVAGFILTSNITPLKTPIMSMPAPLKTAALAVTILGLLIALELATMTTK-------
Dpbr AWGSIIAGLIITSNILPLKTPVMSMPLTLKLAALTVTILGLLIALELATMTTK-------
Caki AWGSIFGGLMIILNTNLIKTPVLTMPFNLKLAALLVSLLGLTIALDLALLTTK-------
Phja SVASILVGTFIAVSSFSWEPPVMTMPLPLKLAALLVSLLALMIALQLSTYSKN-------
Brsp AWGSIIGGFLIIYNIHLFKTPVLTMPIELKLAALLVTIMGLISALQLSMLTTL-------
Gamo AWGGIFGGLLVMLNINLFKTPVLTMPAELKLAALAVSILGLLTALELATLTSK-------
Lolo AWGSIFGGLLIMLNTNLFKTPVLTMPTELKLAALAVSILGLLIALELATLTTK-------
Batr AWGSVIAGFVIFSHILPTKPPILTMPTTIKLTTLFLLTIGLLSALQMSSLFNK-------
Prmy AYGSLAAGLLILSSSTPLKTIPMTMPTSIKLAAIMVTLLGFFTALELSFFTNK-------
Lose AWGSLAAGSLLISMITPLKTPVMSMPPTLKLSALLVSIVGLLVALEIARLDIN-------
Loam AWGSILAGLLITSNITPLKTPVMSMPLLLKVAALAVTITGLLIALELARLTNK-------
Chab AWGSIVAGLLITSNITPMKTPVMTMPLLMKTAALSVTILGLLLALELALLTNK-------
Chto AWGSIVAGLLITSNIAPMKTPVMTMPLLMKTAALSVTILGLLLALELALLTNK-------
Majo AWGSIVAGLLITTNLAPVKSPIMTMPPLMKLAAILVTITGLAFAVELASLTSK-------
Hlst AWGSIIAGLLITASLLPMKTPVLTMPPLMKLTAIIITALGLALALELASLTNK-------
Clpe AWGSILAGLLITSNMSPLKTPIMTMPPLMKLAALLVSLLGLILALELASLTGK-------
Mlmr AWGSILAGLLITSNITPLKTPIMSMPPLLKLAALLVTILGLLTALELASLTNK-------
Crcr AVGSIMAGLLITSYLTPQKTPIMTMTPLMKLTALIVTIAGLLIALELSELARK-------
Muce AVGSIMAGLLITSYLTPQKTPIMTMTPLMKLTALIVTIAGLLIALELSELARK-------
Bege AWGSIVAGFLITSNISPLKTPVMSMPPSLKLAALIVTILGLLVAFELASLTSK-------
Mela AYGTIIAGLLITSNMLPIKTPVMTMPPLLKLAALTVSILGALTALELASLTTK-------
Hats AWGSIIAGLLITSNIIPLKTPIMSMPPVLKLAALLVTILGVLTALELASLTSK-------
Orla AWGSILAGLLITANISPLKTPLMTMPFVLKMAALTVTIVGLLMALELASLASQ-------
```

To be continued on page 47.

[9/12 of aligned sequences]

```
Cosa  AWGSIFAGLLIMTNITPLKTPVMSMPFTLKMAAIIVTVIGLLTALELASMTTK-------
Exsp  AWGSILAGLLITSNILPLKTPVMTMHPTLKMAAILVTIIGVLTALELASLTTK-------
Depa  ALGSIIAGLLITANMFPLKTPVMSMPPILKMAALIVTIIGLLTALELASMTTK-------
Rima  AWGSIVAGFLIIMHTLPAKSSVMTMPFTLKVSALALSLTGLIMALKLASMATKF------
Fuol  AWGSLIAGLIITSNLIPLNTPVMTMHPLLKLAALLVTILGFLTALELACLTSK-------
Gmaf  AWGSIIAGFLITSNTLPLKTPLMTMPTPLKLTALAVTLLGLLTALEIAKLSSK-------
Xeei  AWGSITAGLLITSNILPHNTPILVMPSSLKLAALMVSVLGLLAALELASMTSK-------
Pros  AWGSIVAGLLLTSNITPLKTPVMTMPPLLKLSALIVTILGLIIALELASLTSK-------
Scmi  AWGSLIAGLLITSNIVPLKTPVMTMPPLLKLSALIVTILGLTIALELASLTTK-------
Rolo  AWGSIVAGLLITSNIMPLKTPVMSMPPLLKLAALTVTVLGLLTALELASLTNK-------
Cere  AWGSIIAGLFISLNTPPLKVPIMTMSPPLKVAALVVTMLGLLLALELATLTSK-------
Daga  AWGSLLAGFLITLNAPPLKTPVMTMHPLIKLSALAVTVLALLMALELSALTSK-------
Anco  AWGSIVAGLLITSNILPLKTPVMTMPPTLKLSALIVTALGLLTALELASLTNK-------
Dmve  AWGSIIAGLLITSNMLPLKTPTMTMHPTLKLSALAVTLAGLLIALELASLTNK-------
Dmar  AWGSIIAGLLITSNMLPLKTPVMTMPPTLKLSALAVTLTGLLIALELASLTNK-------
Anka  AWGSIIAGLLITSNTLPLKTPVMTMPPLLKLSALIVTILGLLTALELASLTNK-------
Moja  AWGSLVAGLLITSNTLPLKTPVMTMPPLLKLSALIVTILGLLTALELASMTNK-------
Hoja  AWGSIVAGLLITSNILPLKTPIMTMPPLLKLSALIVTILGLLTALELASLTNK-------
Bede  AWGSIIAGLLITSNITPLKTPVMTMPPLLKLSALIVTILGLIIALELASLTSK-------
Besp  AWGSIVAGLLITSNITPLKTPVMTMPPLLKLSALIVTILGLIIALELASLTSK-------
Mysp  AWGSIIAGLLITSNTLPLKTPIMTMHPLLKLSALIVTIAGLLTALELAQMTNK-------
Osja  AWGSILAGLLITSNTLPIKTPIMTMHPVLKLSALIVTIMGLLIALELALMTSK-------
Sgro  AWGSIFAGLLITSNTLPIKTPIMTMHPILKLSALIVTILGLLIALELAMLTSK-------
Pzpa  ALGSIFTGLLIILNYLPIKTPVLTMSPTLKIAALAVTILGFLIALDLAKQSSS-------
Zeja  ALGSIIAGLLITLNYSPLKTPVLTMPLSLKIAALIVTILGLLTALELAQLTTT-------
Znne  ALGSIIAGFLITLNYLPLKTPVLTMPLTLKLAALAVTILGLVTAFELAQMTTT-------
Zefa  ALGSIIAGFLITLNYLPLKTPILTMPPSLKLAALAVTIMGLLMAFELAQMTSA-------
Acni  ALGSIIAGLLITLNYSPLKTPVLTMPLPLKLAALAVTILGLLTALELAQLTTS-------
Ncrh  ALGSIIAGLLITLNYSPLKTPVLTMPLPLKLAALAVTILGLLTALELAQLTTS-------
Agca  AWGSIVAGLLITSNIIPLKTPVMSMPPLLKLAALTVTVLGLLLALELASLTSK-------
Hydy  AWGSIIAGLLITSAIVPLKTPIISMPLPLKLAALIVSITGLLLAIELASLTSK-------
Gsac  AWGSIIAGFLITSTIIPMKTPIITMPPVLKLAALIVSVLGLLIALELASLTAK-------
Pevo  AWGSIVAGLIITTNLLPLKTPVMSMPAYMKLAAIAVTIAGLTTALDLAKMTNK-------
Hiku  AWGSIIAGLIITSNMTPMKTPIMTMPYYTKMAALIVTAIGLITALEMAQNTSK-------
Inpa  AWGSIAAGLLITSALPPTHTEILSMPLHLKLAALTITILGLLIALELANLAQK-------
Auch  AVGSIIAGLLIISCILPSKTPTLTMDLPLKLAALTVTAFGLLGALDLASLTTK-------
Fico  AWGSIVAGLLITSNFLPLKTPIMTMPPLLKLAALAVTALGLITALELASLTSK-------
Macs  AWGSIFAGLLLTSNILPLKTPVMSMPTSLKLAALIVSILGLLIALELASLTSK-------
Moal  AWGTILAGLLMTCAVLPPKPPMLSMPPYIKLTALVITILGLTIALELALFTNK-------
Syma  AWGSILAGLLMTSYTLPLKTPTLTMPLTLKTAALLITLLGLLTALNLANLTST-------
Mafr  AWGSIIAGLLITSYTIPLKTPTMTMHPSLKLAALIVSAVGLLIALDLASLTNK-------
Dcpe  AYGSIAAGLLITMNIVPTKTPVMTMAPALKLAALLVSVIGLLIALDLASLTHK-------
Dcti  AYGSIAAGLLITINIVPMKTPVMTMPPTLKLAALLVSILGLLIALDLASLTHK-------
Hehi  AWGSIVAGLLIISSITPLKTPVMSMPPLLKLAALTVTIMGLLIALELATLTNK-------
Stam  AWGSIIAGLLITSNILPLKTPVMTMPPLLKLAALAVTITGLLLALELASLTTK-------
Hogi  AWGSIIAGLLITSNLLPLKTPIMSMPLILKLAALTVTIIGLILALELASLTNK-------
Erzo  AWGSIVAGLLITSNILPLKTPIMTMPPLLKLAALVVTITGLLLALELASLTTK-------
Hxot  AWGSIVAGLLITSSITPLKTPIMTMHPLLKLAALAVTVIGLLLALELASLTSK-------
Core  AWGSIIAGLLITSSITPLKTPIMTMPPLLKLAALLVTIAGLILALELASLTTK-------
Apve  AWGSIIAGLLITSNLTPLKTPIMTMAPMLKLTALIVTVAGLLLALELTSLTNK-------
Latj  AWGSIIAGLLITSNIIPIKTPIMSMPAVLKLAALTVTILGLLLALELATLTNK-------
Laja  AWGSIIAGLLITSNITPLKTPIMSMPLLLKMAALVVTILGLIVALELATLTNK-------
```

To be continued
on page 48.

```
Syja  AWGSIIAGLLITSHILPLKTPVMSMPPLLKLAALTVTILGLLTALELATLTSS-------
Epme  AWGSIVAGLLITSNLIPLKTPVMSMPPLLKLAALTVTILGLMVALELASLTSK-------
Grse  AWGSIIAGFLYSSTLIPLKTPTLTMPLSLKLAALAVTIFGLLYGMELAWLTSL-------
Clja  ALGSIIAGLFITQNIIPIKTPTMSMPPLLKLAALTVTIIGLLLALELASLTSK-------
Ogcy  VLGSIIAGLLISQNILPTKPSILTMPFELKILAMAVSILGFLIAADLAYSAHK-------
Plna  AWGSIVAGLLIAYNMTPSKTPIMTMPLFLKLQALIVSILGLLIALDLASITSQ-------
Lema  AWGSIIAGLLITSNITPLKTPVMSMPPLLKLAALLVTIMGLLLALELATLTNK-------
Etzo  AWGSIIAGLLITSNIVPLKTPVMTMPPLLKLAALIVTIGGLLLALELASLTNK-------
Apse  AWGSIVAGLIITSSIIPLKTPVMSMPPLLKLAALIVTILGLLTALELASLTNK-------
Epde  AWGSIIAGLLITSNILPLKTPIMTMPPSLKLAALIVTILGLLIALDLASLTNK-------
Slja  AWGSILAGLVLTSNIIPLKTPVMSMPPLLKLAALIVSVLGLLLALELASLTSK-------
Bsja  AWGSIIAGLLITSNITPLKTPIMSMPPLLKLAALFVTILGLALALELASLTSK-------
Ecna  AWGSIIAGLLITSNFIPTKTPIMSMPPLLKLAALLVTIIGLLTALELASLTNK-------
Cohi  AWGSIIAGFLITSTMIPVKTAVLTMPLVVKLTALIISIIGLLIGLEISSHANK-------
Caar  AWGSIIAGLLITSNILPLKTPIMTMPPLLKLAALAVTILGVLIALELASLTNK-------
Came  AWGSIIAGLLITSNILPLKTPVMTMPPILKLAALAVTILGVLIALELASLTSK-------
Mema  AYGSIIAGLLITSNTLPLKTPIMSMPAYLKLAALAVTILGLLTALELASLTNK-------
Lenu  AWGSITAGLIITSNILPLKTPIMTLPPLSKLAALLVSVTGLILAVELASLTNK-------
Brja  AWGSILAGLLITSNISPLKTPIMSMPPLLKLAALIVSILGLLIALELASLTNK-------
Plma  AWGSIIAGLLITSNITPLKTPIMTMPPLLKLAALIVTILGLIIALELASLTNK-------
Emst  AWGSIIAGLLITSNIIPLKTPVMSMPPLLKLAALTVTILGLLLALELASLTSK-------
Ptti  AWGSIVAGLLITSNILPLKTPVMSMPPLLKLAALTVTILGLLLALELASLTSK-------
Losu  AWGSVVAGLLITSNILPMKTPVLSMPPLLKLAALIVTIAGLILALELASLTSK-------
Geoy  AWGSIIAGLLITSNILPLKTPVMTMPPLLKLAALLVTILGLLIALELASLTNK-------
Dipi  AWGSIIAGLLITSNILPLKTPVMSMPPLLKLAALIVTILGLILALELATLTSK-------
Pama  AWGSIIAGLLITSNIVPLKTPVMTMPPLLKLAALVVTILGLLLALELASLTNK-------
Leob  AWGSIIAGLLITSNILPLKTPVMSMPPLLKLAALIVTILGALLALELASLTSK-------
Neba  AWGSIIAGLLITSNITPAKTPIMTMPPALKLAALVVSVLGLVMALELATMTTK-------
Pdpl  AAGSIVAGFLLSRSMTPLKTSVMTMPLSLKMAAILITLVGFLIAMELSSLSEK-------
Nimi  AWGSIIAGLLITLTMTPLKTPVLTMPDHLKLSALAVSLLGFVVALDLASITNK-------
Uptr  AWGSIVAGFLITLNTLPLKTPVMTMPTSLKLAALIVTILGLLTALELASLTSK-------
Pesc  ALGSIIAGFFLIYSLIPLDTPVLTMPLPMKLMAIGVTLMGVSLAAELSNLAPR-------
Baar  AWGSIIAGLLIISNITPLKTPVMSMPPLLKLAALIVTILGLLTALELASLTSK-------
Moar  AWGSIIAGLLITANIMPLKTPVMSMPPLLKLAALTVTILGLLLALELASLTSK-------
Toja  AWGSIIAGLLITSNITPLKTPIMSMPPLLKLAALTVTILGFLTALELASLTNK-------
Chau  AWGSIIAGLLITSHIVPMKTPIMTMPPLLKLAALLVTITGLLLALELASLTSK-------
Chse  AWGSIIAGLLITTNITPMKTPVMTMPPLLKLAALIVTILGLIVALELASLTNK-------
Enar  AWGSILAGLLITSNITPLKTPIMTMPPLLKLAALAVTILGLLIALELASLTTK-------
Hpty  AWGSIIAGLLITSNIIPLKTPVMSMPPLLKLAALTVTILGLLLALELASLTSK-------
Nana  AWGSIIAGLLITSNMLPTKTPVMSMSPLMKTTALIVTAIGLLASLELALLTNK-------
Mcst  AWGSIVAGLLITSSITPLKTPVMSMPPMLKLAALIVTISGLLVALELASLTSK-------
Rhox  AWGSIIAGLLITSHISPLKTPVMTMPPLLKLAALAVTISGLLIALELASLTSM-------
Opfa  AWGSIVAGLLITSNITLSKTPVMSMPPLLKLAALTVTILGLLIALELASLTSK-------
Paar  AWGSIVAGLLITSNITPLKTPVMSMPPLLKLAALTVTIAGLLAALELASLTTK-------
Gozo  AWGSIIAGLLITSNILPLKTPVMTMHPLLKLAALSVTILGLLIALELASLTNK-------
Ackr  AWGSIVAGYIITHNLLPLKTPVLTMPLQLKLITLMIMILAFTLAYQLLLSY-K-------
Elev  AWGSILSGLLLTSNINTLNLPIMTMAPLLKLSALIVSVLGLFIALDLTSFTNN-------
Trdu  AWGSVIAGLLITSNIAPLKTPVMSMPFLLKAAALMVTIIGLLTALELASLTNK-------
Amoc  AWGSIIAGLLITSNVLPMKTPVMSMPPLLKLAALTVTLLGVLTAIELASLTNK-------
Hame  AWGSIASGLLLFSYLMPEKTPLMTMHPVLKTAALMVTLAGLLLAFELAALAAK-------
Chso  AWGSIIAGLLITSNILPLKTPVMTMPPLLKLAALVVTIGGLLIALELASLTTK-------
Lyto  AWGSIVAGLLITSSIVPLKTPIMTMPPLLKLAALLVTVAGLLLALELASLTNK-------
```

To be continued on page 49.

[9/12 of aligned sequences]

```
Encr   AWGSIVAGLLITSSIVPLKTPIMTMPPLLKLAALLVTITGLILALELASLTSK-------
Bvar   AGGSIVAGLLITANTHPLTTPVMSMPFLLKVAALIVTLVGFLLAASLASLMTG-------
Noco   VWGSIFAGLWLTLSINPLKIPVMSMPPTIKLTALLVTISGLLIALWFITPSGA-------
Chsp   AWGSIMAGLLITNNIYPLKIPAMTMTPALKLAALAVTAMGLLTALELASLTNK-------
Arja   AWGSIVAGLLITSSITPLKTPIMTMPPLLKLAALIVTILGLLLALELASLTTK-------
Pase   ALGSIIAGLLIIWNITPLKTPIMTMSPLLKLAALIVTILGLVTALPLTSLTNT-------
Trel   ALGSIAAGLLISSSILPLKTPVMTMPFTLKLAALTVSVLGLLAALELASATTM-------
Lifa   ALASIIAGFLVIMCFMPSKTPVHTMPLMLKTLALLVSVMGFVYATQLSRLTTS-------
Acur   AWGSICAGLLVTSNTLPTKTPVMSMPLPLKMAALTVTILGLIIALDLASLTSK-------
Ampe   AWGSIVAGLLITSNIIPMKTPVMSMPPLLKLAALVVTILGLLTALELASLTSK-------
Urja   AWGSVIAGLLIFHNMMPVKTPVLSFPPLLKLAALLVTIFGLIIAHSLASRTAM-------
Enet   AWGSIVAGLLITSNILPLNTPIMSMPPLLKLAALLVTIAGFLVALELASLTTQ-------
Ptbr   AWGSIIAGLLITSNLTPIKTPVMSMPMVLKLSALLVTILGLLIALDLALFTNK-------
Safa   AWGSILAGLIITSNILPMKTPVMTMPPLLKLAALAVSILGLLTALELASLTNK-------
Icae   AWGSIIAGLLITSNITPLKTPVMSMPPLLKLAALAVTILGLVIALELASLTSK-------
Asmi   ILGTLIAGALYSSYVITPTTPVLTMPFLTKTAALSVSLGAFLLGLTLTQHATT-------
Foal   AWGSILAGLLITLNMSPIKTPVMVMTPLMKLSALIVTIIGLLAALHLVLNNKQH------
Drze   AWGSIIAGLLITSNIVPMKTPVMSMPPTLKMAALLVTTTGLLLALDLSWLTNK-------
Rhas   AWGSIIAGLLITSNLLPPKTPIMTMPPLLKLAALLVTVIGLLTALELASLTSK-------
Elac   AWGSIIAGLLITSNILPSNTPIMTMPPILKLAALLVTIIGLVTALELASLTNK-------
Kugu   AWGSIVAGLLITSNITPLKTPIMTMPLTLKLAAIIVTILGLLTALELASLTTK-------
Plor   AWGSIIAGLLITSNIVPLKTPVMTMPPTLKLAALLVTITGLLLALELASLTNK-------
Sgun   AWGSIIAGLLITSNILPLKTPVMSMPPLLKLAALIVTILGLLMAFELASLTSK-------
Zaco   AWGSIIAGLLITTNALPMKTPVMSMPPLLKLAALTVSVLGLLIALELASLTSK-------
Zbfl   VWGSIIAGLLITSNMLPLKTPVMSMPPLLKLGALTVSILGLLLALDLASLTTK-------
Spba   AWGSIIAGLLITSNLTPLKTPVMTMPTSLKLAALTVTIMGLLIALELASLTNK-------
Game   AWGSIVAGLLLTSNITPLKTPIMSMPPLLKLAALAVTILGLIIALELASLTSK-------
Thth   AWGSIIAGLLITSNITPLKTPVMSMPPLLKLAALAVTILGLIIALELASLTSK-------
Xigl   AWGSIIAGLLITSNMLPLKTPVMSMPLLLKLAALAVTITGLLIALELASLTNK-------
Hyja   AVGSIVAGLLITSNILPLKTPVMSMPPLLKLAALIVTILGLITALELASLTNK-------
Psan   AFGSIIAGTLITSNIIPLKVPIMTLPAMYKLTAILVSTLGLLAALQLASLANK-------
Cupa   AWGSIIAGLLITSNILPLKTPVMSMPPLLKLAALIVTILGLIIALELATLTSK-------
Mpch   AWGSIIAGLLITSHMTPLKTPILSMPLPIKLAALLVTIFGLMIALDLASLTTK-------
Char   AWGSIIAGLLITSNLLPTKTPVMSMPLPLKLTALVVTTIGLLIALELASLTNK-------
Pser   AWGSIIAGLLITSNIIPLKTPVMSMPPILKLAAITVTAIGLLVALELASLTSN-------
Prol   AWGSIIAGLLIFSNISPLKTPVMSMPPALKLAALAVTISGLLVAMAIVAIVNK-------
Plbi   AWGSIIAGLLITSNITPLKTPVMSMPPLLKLAALAVTILGLLIAMELAMLTNK-------
Calu   AWGSIAAGLLIIQNMVPNKTPIMSMPLSLKLGALSVTILGLVTALELVSLTSK-------
Papa   AWGSILAGFLITSNITPLKTPVMTMPPALKTAALAVTVLGLITAIQLAALTKT-------
Sufr   AWGSIVAGLLITSNIIPLKTQVMTMPAPLKMAALTVTVLGLLLALELASLTNK-------
Stci   AWGSILAGFLITSNLLPEKTPVMTMPTSLKLAALLVTALGLLIALELASLTNK-------
Taru   AWGSIIAGLLITTNLLPTKTPVMSMPMVVKLTALIVTILGLLIALELASLTSK-------
Rala   AWGSIIAGLVITSNITPLKTPIMSMPPLLKLAALAVTVTGLLLALELASLTNK-------
          . :  *               :      *    : :   .    .    :
```

To be continued on page 50.

[10/12 of aligned sequences]

To be continued on page 51.

```
Scca   -------QFKIYPTFNanaPYHHFSNMLGYFPPIIHRLLPKINLNWAQHISTHLIDQTWN
Muma   -------HFKTNPT----LHYHHFSNLLGYFPSIIHRLLPKTSLNWAQYISTHLIDQTWN
Erca   -------QIKIHPT----KILHNFSNMLGFYPHTMHRLMSKLPLMFGQINATQTTDQSWT
Pose   -------QIKISAT----KLTHNFSNMLGFYPHITHRLMSKLPLTLGQVFATQTSDQLWM
Actr   -------QLKITPT----IPLHNFSNMLGYFPSIIHRLTPKIKLSLGQTMATHLIDQTWL
Scal   -------QLKITPT----IPLHNFSNMLGYFPSTIHRLAPKIKLNLGQTMATHLIDQTWL
Posp   -------QLKITPT----LPLHNFSNMLGYFPSIIHRLAPKIKLNLGQTMATHLIDQTWL
Atsp   TQSMTPLQKSKIPMNNPIKVIFNFSNMTGYIQTIMHRLAPKTSLSMGQLMATQLMDQTWL
Leoc   SNT--MTQKSKIPMDNPAKIIFNFSNLTGYIPTIMHRMAPKANLTMGQLMATQLMDQTWL
Amca   -------QMKTTPI----LQTHNFSNMLGYFPSIMHRLAPKTNLLMGQTMATHLVDQMWF
Osbi   -------QFKIMPT----NTPHNFSNMLGYFPTTIHRLVPKMNLILGQTMASQLTDQTWF
Pabu   -------QFKATPS----LFTHNFSNMLGYFPTFIHRTLPKINLHIGQTIAFHLVDQTWF
Hial   -------QFKTTPT----IWAHNFSNMLGYFPNIIHRMTPKMNLILGQKMATQLVDQTWF
Elha   -------QFKTNPT----MPAHNFSNMLGYFPAVIHRMTPKINLMLGQTVASQLVDQTWF
Mlcy   -------QFKTTPT----MPTHNFSNMLGFFPMVIHRMTPKINLTMGQAVASQLIDQTWF
Algl   -------QVKSSLN----TAPYNFSVMLGYYQTVVHRHVSKVNLMLGQKTMTQLVDQTWL
Ptgi   -------QFKVSPN----LPMHNFSNMLGYFPAVMHRMTPKLNLVLGQTAATQMVDQTWF
Alaf   -------QFKITPL----IIPHNFSNMLGYYPAIIHRLLPKLNLTLGQTMATQVVDQTWF
Nock   -------QFKVTPN----IIAHNFSNMLGYFPTIVHRMAPKVNLMLGQTMATQLVDQTWF
Anja   -------QYKTKPY----TKTHNFSNMLGYFPAVIHRMAPKLTLVLGQKVATQLVDQTWF
Gyki   -------QLKMTPE----IKTHNFSNMLGYYPSIIHRAFPKAMLVLGQTAATQAVDQSWF
Syka   -------QFKVNPN----MPTHNFSNMLGYFPDIIHRAVPKLNLVLGQTMATQLVDQTWF
Opma   -------QFKIKPN----LPTHNFSNMLAFFPAIFHRTLPKLTLTLGQTAATQLVDQTWF
Comy   -------QFKIYPN----MTTHNFSNSLAFFPTIIHRMAPKLNLVLGQTAATQMVDQTWF
Sasp   -------QHKALPH----LEMYRFSNMLGYFPSLIHRSAPKYTLLIGQRTASQLIDQTWL
Eupe   -------QYKIFPQ----KQMNYFSNMLGFFPTLIHRLIPHLVLSTGQKMATQLIDQGWF
Enja   -------QVKITPT----IKVHNFSNALGYFPATVHRLVPKVTLIMGQTMANQMADQTWL
Same   -------QFKPTPM----IKLHNFSNMLGYFPATVHRLAPKLNLVLGQTMANQLVDQSWF
Chch   -------QFKTTPI----ILTHNFSNMLGYFPAAIHRLLPKINLTLGQTIASQTVDQTWF
Grgr   -------QLKVFPN----TPLHNFSNMLGYFPALMHRLAPTTNLALGQTVANQLVDQNWF
Caau   -------QMKITPI----IPLHHFSNMLGFFPTIVHRLLPKLKLTLGQSAATQ-LDKTWL
Cyca   -------QVKITPM----ISTHHFSNMLGFYPMIIHRLIPKLKLTLGQSAATQ-LDKTWL
Dare   -------INKNTTP----GIPFHFSISLIFFP-ILHRLIPMRKLFMGESAAT-KIEKSWS
Cost   -------QFKITPV----MPLHHFSNMLGFFPMIIHRITPKLNLTLGQSIATQLVDQTWF
Leec   -------QFKINPT----TSIHHFSNMLGYFPMIIHRLIPKLNLTLGQSIATQLVDVTWL
Fola   -------QYKTNPI----TQTHHFSNMLGFFPSVVHRLMKKLNLTLGQLAATQMVDQTWF
Clmc   -------QFKPTPV----LPLHNFSNMLGYFPATIHRLMPKLNLTLGQLIATQLVDQTWL
Phin   -------QFKISPT----RPTYQFSNMLAYFPTIIHRLMPKLNLTLGQLIATQLVDQTWT
Icpu   -------QIKITSL----LTPHNFSNMLGFFPSIIHRSLPKLNLVLGKQA--TKLDRQWM
Psto   -------QIKISPS----LLLHNFSNMLGFFPPIIHRMMPKLNLSLGKQA--TKFDRQWL
Cora   -------QYKVTPT----LNLHNFSNMLGYFTSIIHRLVPQLTLTLGKKA--TKFDRQWM
Eisp   -------QLKPTPT----LLPHNFSNMLGYFPTTVHRLIPKLNLVLGQSIAAQ-FDQTWL
Apal   -------QHKTSPI----LITHNFSNMLGYFPTTIHRLIPKLNLTLGQSIAT-LFDQTWL
Eslu   -------TFKALPN----LPLHSFSNMLGFYPSIIHRLTPKLNLILGQKIATQMVDQTWF
Dape   -------TFKSHPN----MALYTFSNLLGFFPTIIHRMAPNLNLVLGQKIATQLVDQTWF
Glse   -------QFKITPN----LSTHHFSNLLGFFPAIIHRVTPKLSLSLGQTIATQLIDQTWL
Naar   -------QFKTTPN----LPAHHFSNLLGFFPAIFHRITPKLNLVLGQTIATQLVDQTWL
Lioc   -------QLKTTPN----LPAHHFSNMLGFFPAIIHRITPKLNLILGQTIATQLVDQTWL
Opso   -------QFKTTPV----LPTYHFSNSLGFFPAIFHRFTPKMNLVLGQTIATQLVDQTWL
Alte   -------QFKTTPT----IPLHNFSNMLGYFPAAVHRLIPKLNLTLGQTIATQLVDQTWF
Plap   -------QLKATPT----ISPHNFSNMLGYFPATIHRLIPKLNLTLGQTFATQLVDQTWF
```

[10/12 of aligned sequences]

To be continued on page 52.

```
Plal  -------QFKITPS----LTLHNFSNMLGFFPATVHRSLPYLNLSLGQAIASQMVDQTWF
Sami  -------QFKITPS----LTLHNFSNMLGFFPATVHRSMPYLNLSLGQAIASQMVDQTWF
Rere  -------QFKTSPS----LSAHNFSNMLGFFPATVHRLLPFLNLSLGQSIAGQMVDQTWF
Gama  -------QFKPTPS----LSSHNFSNMLGYFPTLVHRLAPKLNLILGQSIASQGVDQTWF
Onmy  -------QFKLHPT----LTLHNFSNMLGFFPAIIHRLTPKLNLTLGQTIASQMVDHTWF
Sasa  -------QFKTTPN----LITHNFSNMLGFFPAIIHRLAPKLNLTLGQTIASQMVDQTWF
Cola  -------QFKTTPN----LVTHNFSNMLGFFPAIVHRLAPKLNLTLGQAIASQMVDQTWF
Dita  -------QYKPSPN----LPAHNFSNMLGFFPTLVHRVTPFVSLNLGQTIASQTLDLTWL
Gogr  -------QHKTAPN----PLTHNFSILLGFFPTIIHRSTPQLSLSFGQTFANQALDLAWL
Chsl  -------QLKPTPN----LLAHNFSNMLAFFPAIIHRLTPKLSLAMGQSIATQVLDLSWM
Atja  -------QFKPIPQ----KTTHHFSVMLGYFPTVLHRLMPKVNLILGQTIANQTIDQTWL
Iido  -------QFKTTPQ----QTTHHFSNMLGYFPSVLHRLLPKVNLILGQTIANQTIDQTWL
Auja  -------QFKTTPQ----IALHNFSNMLGYFPHIIHRLAPKITLTLGQTIANQTIDQTWL
Chag  -------QFKPTPH----LPLHNFSNMLGFFPAIIHRFTPKINLSLGQAIASQMLDQTWL
Hami  -------QHKPLPY----LVPHNFSNMLGYFPNIIHRTLPKTFMILGQTIATQMVDQTWL
Saun  -------QFKPLPR----LSPHNFSNMLGYFPNIIHRTLPKTFMVLGQSIATQMIDQTWL
Nema  -------QLKVNPT----PTPHHFSNMLGYFPTIVHRAAPKIALVLGQTVASQTIDQTWL
Disp  -------QVKVTPT----LATHHFSNMLGYFPAITHRATPKIGLVLGQSVASQTVDQTWL
Myaf  -------QIKINPT----LTSHHFSNMLGYFPAVTHRAAPKVGLILGQSLASQTIDQAWL
Lagu  -------QLKPSPL----PIPHHFSNMLGFFPTVIHRLMPKVNLTLGQKMATQAMDQTWL
Trtr  -------QLKPLPL----LPAHHFSNMLGFFPSITHRLLPKLALILGQKVASQMIDQTWF
Zucr  -------QLKSLPI----LPIHHFSNMLGFFPSIIHRLAPKLGLLLGQKLASQMMDQTWL
Pxja  -------QFKTTPQ----LPLHHFSNMLGYFPAVVHRLAPKLSLLMGQLVASQMVDQTWL
Pxlo  -------QFKTTPQ----LPLHHFSNMLGYFPAIIHRLAPKLSLLMGQLVASQMVDQTWL
Pctr  -------QFKPTPN----PAPHHFSNMLGFFPAIAHRITPKMSLVLGQAMATQTVDQTWL
Apsa  -------QYKILPS----LTTHHFSNMLGFFPAVMHRITPKITLNLGQTAANQMIDQTWL
Cabe  -------HFQPTSK----PTFYSFSSLTGFVPAVVHRLVPNLSLVMAQAVASQTVDQAWL
Bzze  -------QFKPTPT----LSTHHFSNMLGFFPSVVHRMVPKLGLVLGQAIAAQMVDQTWL
Siim  -------QLKPTPT----LAPYRFSNMLGFFPAVLHHFMPKLTLVLGQTVAAQTMDQTWM
Ctru  -------QFKPHSK----LTPHHFSNMLGFYPATIHRMTPKLNLMLGQTIASQMVDQTWL
Dpbr  -------QFKAQPK----LAPHHFSNMLGFYPTTVHRLAPKLNLILGQTIASQMVDQTWL
Caki  -------QIKITPM----KTPHHFSNLLGFFPLITHRLNSKISLQLGQKIATQMVDQTWL
Phja  --------FEFSPK----NKYYQFSNSLGYFPTINHRKVPEIGLVLGQKIASQMVDQAWL
Brsp  -------QLKMKPD----LNSYHFSTSLGFFPSLIHRYIPSLKLLLGQKIASQTIDQTWL
Gamo  -------QFKVTPL----RTPHHFSTSLGFVPAIIHRQIPQLSLLLGQKIASQMVDQTWL
Lolo  -------QLKVTPL----QVSHHFSNSLGFIPAIIHRQAPQLSLILGQKIASQMVDQTWL
Batr  -------KTPFLHS----PSNWDFFTLLGFFPQTFHRMISKTTLRLGHLSASLMLDLTWT
Prmy  -------QLTSTLA----SPSFNFSTSLGFFPSITHRLLPKYTLFLGHFIASQTLDLYAM
Lose  -------PLRTSPS----NTPYRFSNMLGFFNSIIHTKIATLFLTFGQAIAGQTTDQTWY
Loam  -------PLKPTPK----IGTHHFSNMLGFFPAIIHHHAPKINLTLGQTIASQMIDQTWL
Chab  -------QFKPTPK----LTPHHFSNMLGFFPMIIHRLTPKLSLLLGQTIATQLIDQTWL
Chto  -------QFKPTPK----LTPHHFSNMLGFFPMIIHRLTPKLSLLLGQTIATQLIDQTWL
Majo  -------QFSTTPK----LLPHHFSNMTGFFPTVVHRLAPKVTLLLGQDIATNTLDQTWL
Hlst  -------QLNITPK----LPPHHFSNMTGYFPTVVHRLLPKTTLLLGQEIAINIMDQTWL
Clpe  -------QFSPTPN----LALHRFSNMLGFFPMIAHRLVPKLNLTLGQSVATQMIDQTWL
Mlmr  -------QFNTTPK----LYPHHFSNMLGFFPTIAHRLPPKLNLLLGQTIATQMIDQAWL
Crcr  -------QHKSTPR----SALHYFSNLLGFFPSIIHRLAPKTNLTLGQKIANQSVDQTWL
Muce  -------QHKSTPR----SALHYFSNLLGFFPSIIHRLAPKTNLTLGQKIANQSVDQTWL
Bege  -------QFKPTPY----LALHHFSNMLGFFPAIMHRISPKIALLLGQTIATQMIDQAWL
Mela  -------QFKSAPN----LTLHHFSNMLGFFPAITHRSLPKLSLLLGQSIASQTIDQAWL
Hats  -------QFKPTPN----LPLHHFSNMLGFFPQIIHRMSPKINLLLGQTIAGQMVDQTWL
Orla  -------QFKIKPA----SAPHHFSNMLGFYPSVVHRLAPKTNLVLGQLIANQTIDQTWL
```

[10/12 of aligned sequences]

```
Cosa    -------QIKTNPN----PTTHHFSNMLGFFPAITHRLAPKLNLTLGQFIASQTIDQTWL
Exsp    -------QFKTLPN----LNLHHFSNMLGFFPAVVHRMMPKLNLILGQTIAAQMVDQTWM
Depa    -------QVKTKPN----LTTHHFSNMLGFFPPIIHRMAPKLSLMLGQTIASQTIDQTWL
Rima    --------NNPNKS----FVAYHFSIMLGFFPTIIHRIIPKLALTLGQKIANQMIDQAWI
Fuol    -------QFKIFPQ----HTPHHFSNMLGFYPHIVHRFLPKINLLFGQAIATQAIDQIWL
Gmaf    -------QFKKTNN----SPSHPFSIMLGFYPHVAHRSVPLTALVFGQAIATHLVDQTWL
Xeei    -------QFKSIPN----LKTHHFSNMLGFFPHIIHYSFPKINLILGQTIANQTIDQAWL
Pros    -------QFKPAPH----LPLHHFSNMLGFFPLIIHRLTPKMNLVLGQTMANQMIDQTWL
Scmi    -------QFKPLPH----QPLHHFSNMLGFFPLLIHRLAPKINLILGQTVANQMIDQTWL
Rolo    -------QHKPTPH----LVPHHFSNMLGFFPAIIHRLAPKLNLVLGQTMASQMVDQTWL
Cere    -------QYNPTPS----LNPHHFSNMLGFFPTVVHRLAPKMSLILGQTMANQTIDQAWL
Daga    -------QHKTTPY----LTPHRFSNMLGFFPMVTHRLTPKLFMALGQTLANQTIDQTWL
Anco    -------QFKPTPH----LQPHHFSNMLGFFPAIIHRLTPKLNLLLGQSISSQMVDQTWL
Dmve    -------QFKPVPQ----VGPHHFSNMLGFFPAIIHRYTPKINLLLGQTMASQTVDQAWL
Dmar    -------QFKPSPL----AGPHHFSNMLGFFPAIVHRYTPKVNLLLGQTMASQTVDQAWL
Anka    -------QFKPTPR----LPLHHFSNMLGFFPATMHRLTPKLSLLLGQTIASQMVDQAWL
Moja    -------QFKPTPH----LQLHHFSNMLGFFPTTMHRLTPKLSLLMGQTIASQMVDQTWL
Hoja    -------QFKPTPH----LHPHHFSNMLGFFPAVMHRLTPKVNLLLGQTIASQMVDQTWF
Bede    -------QFKPTPH----LPLHHFSNMLGFFPTIIHRLAPKMNLILGQTMANQMIDQTWL
Besp    -------QFKPAPH----LPLHHFSNMLGFFPMIIHRLAPKMNLILGQTMANQMIDQTWL
Mysp    -------QFKPVPH----TAPHHFSNMLGFFPAIIHRMAPKINLILGQSIASQMVDQTWL
Osja    -------QFKPLPY----STPHHFSNMLGFFPAVIHRLMPKLNLTLGQSIASQMVDQTWL
Sgro    -------QFKTTPH----LTLHHFSNMLGFFPAIIHRFAPKMNLILGQSIASQMVDQTWL
Pzpa    -------QMKITPK----LTFYKFSSMLGFFPNFTHRLIQKMALALGQTVANQLVDQTWL
Zeja    -------QLKTAPK----LLAHNFSNMLGFFPSLIHRLLPEMSLVLGQNISSQMVDQTWL
Znne    -------QLKPSPK----LAPHNFSNMLGFFPSLIHRLIPKMSLTLGQMMANQMVDQTWL
Zefa    -------QFKPLPK----LTPHNFSNMLGFFPILIHRLLPKISLTLGQAVATQTVDQTWL
Acni    -------QLKTTPK----LNPHNFSNMLGFFPSLVHRLLPKMSLTLGQTVATQMVDQTWL
Ncrh    -------QLKAAPK----LKPHNFSNMLGFFPSLVHRLLPKMSLTLGQTVATQMVDQTWL
Agca    -------QYNPTPR----LNVHHFSNMLGFFPMIIHRLAPKLTLTLGQTIASQLIDQTWL
Hydy    -------QYKPTPY----LSPHHFSNMLGYFPSIIHRLTPKLGLSVGQAVATQMLDQTWM
Gsac    -------QYHPTPR----LTPHHFSNMLGFFPSVIHRLTPKFGLTLGQTIASQMLDQTWI
Pevo    -------QLKPTPS----KTPHHFSNMLGFFPAITHRLLPKMNLVLGQTLASQMVDQTWL
Hiku    -------QLNIIPK----QTPHSFSNMLGFYPSIIHRLPMKLSLQLGQNIASQTIDTTWL
Inpa    -------QHKAQPR----KPSHNFSNMLGFFPLITHRLSPKLSLVLGQTLATQMVDQLWL
Auch    -------QYKITPN----LPLHHFSSMLGYYPSIIHRLFPKLALLFGQKIATQAIDQTWL
Fico    -------QFKPTPT----LTTHHFSNMLGFFPAVIHRFLPKINLVLGQTISSQTVDQTWL
Macs    -------QFKALPL----LIPHHFSNMLGFFPAIIHRIPPKLNLALGQTIANQMVDQTWL
Moal    -------QRPYMPK----Q-YFYFSTMLGYYPTIMHRFFPYTFLKLSQTIATMTIDQTWL
Syma    -------QTKLNPK----IVPHHFSNMLGYFPTVVHRLTPSLTLNLGQTIASQMIDQTWL
Mafr    -------QYKITPQ----TTPHHFSNMLGFFPTIMHRLTPKLNLIFGQTLANQMIDQTWL
Dcpe    -------QHKVLPR----LDTLRFSNMLAFFPALLHRSFPKLSLVLGFSIAVQTID-TWL
Dcti    -------QYKVLPS----LGAHLFSSMLGFFPTVLHRLTPKLVLILGQLIATQTVDQTWL
Hehi    -------QYKITPN----LVTHHFSNMLGFFPSIIHRFTPKLNLVLGQTLASQLIDQTWL
Stam    -------QFKVLPN----LTTHHFSNMLGYFPTLIHRLPPKLNLVLGQTIASQMVDQTWL
Hogi    -------QFKTTPN----LVPHHFSNMLGFFPSVVHRFAPKLNLALGQTIASQMVDQTWL
Erzo    -------QHRATPL----LTTHHFSNMLGFFPPIIHRLTPKLGLILGQTVASQMVDQTWL
Hxot    -------QYQTSPN----LATHHFSNMLGFFPTIIHRLTPKINLVLGQTIASQMVDQTWL
Core    -------QYQTTPN----LAPHHFSNMLGFFPTIIHRLTPKANLVLGQAIASQMVDQTWL
Apve    -------QYQTKPN----LTAHNFSNMLGFFPTIVHRLSPKINLTLGQTIANQMVDQTWL
Latj    -------QFKPTPT----LTPHHFSNMLGFFPTIIHRLTPKLNLFLGQAIASQTIDQTWL
Laja    -------QHKPIPQ----LVAHRFSNMLGFFPTIVHRFTPKLNLTLGQTIASQMVDQTWL
```

To be continued on page 53.

[10/12 of aligned sequences]

To be continued on page 54.

```
Syja  -------QFKPAPL----QTPHHFSNMLGFFPAVIHRLTPKLNLVLGQSIATQMVDQTWL
Epme  -------QFKPIPH----LPSFRFSNMLGFFPTIMHRFPPAMGLGMGQSIASQMIDQTWL
Grse  -------QPMPIPK----QTPFRFSNLLGYYPIVVHRLAPKLGLVTGYTIASQMVDHSWL
Clja  -------QIKTTPQ----LNLHHFSNMLGFFPMVTHRLAPKINLILGQAIATQTIDQTWM
Ogcy  -------QLKSVPI----KALHDPSNMLGFYPTVIHRAISKMFLLLGQKIADQLIDKIWM
Plna  -------QFKTTPQ----TTTHHFSNMLGFFPLIIHRLVPKSNLSLGQTIASQVIDQTWL
Lema  -------QYKPIPQ----LTPHHFSNMLGFFPTTLHRLAPKLNLMLGQSIASQMVDQTWL
Etzo  -------QFKPTPY----LATHHFSNMLGFFPIIVHRISPKLNLVLGQAVASQMVDQTWL
Apse  -------QFKPTPE----LTPHHFSNMLGFFPTIIHRLPPKLNLVLGQVIASQTIDQTWL
Epde  -------QYKTTPS----LTPHHFSNMLGFFPSAIHRFTPKLSLALGQTIASQMVDQAWL
Slja  -------QFKPSPM----LPTHHFSNMLGFFPMIMHRLIPKLTLSLGQSIASQMVDQTWL
Bsja  -------QYNPTPN----LLTHHFSNMLGFFPTIIHRLTPKLNLSLGQMIASQMMDQTWL
Ecna  -------QFKPLPH----LSAHHFSNMLGFFPTIIHRLPPKMNLTLGQMIASQMVDQTWL
Cohi  -------QLKPSPK----FTLYPFATLLGFFPSIIHRLVPLFNLRFGQKIASQMIDFTWF
Caar  -------QYSPTPT----LPTHHFSNMLGFFPAVIHRLTPKLNLVLGQFIASQLVDQTWL
Came  -------QYSPTPT----LPAHHFSNMLGFFPAIIHRFTPKLNLTLGQLIASQLVDQTWL
Mema  -------QFKPTPI----LPTHHFSNMLGFFPPIIHRLTPKLSLILGQTIASQMIDQTWL
Lenu  -------QFKIKPL----TTPHLFSNMLGFYPTLIHRFIPKTGLFLGQTIASQTMDQAWL
Brja  -------QFKPLPK----LSPHHFSNMLGFFPAIVHRFTPKLNLVLGQTIAGQMIDQSWL
Plma  -------QFKPIPM----LTTHHFSNMLGFYPSIIHRFTPKLNLILGQTIASQMIDQTWL
Emst  -------QYKPTPQ----LASHHFSNMLGFFPAVIHRLTPKLNLTLGQMIASQMIDQTWL
Ptti  -------QFKPTPQ----LISHHFSNMLGFFPAVIHRLTPKLNLTLGQAIASQMIDQTWL
Losu  -------QFKTSPN----LPPHHFSNMLGFFPPITHRILPKMSLTLGQTIANQLIDQTWL
Geoy  -------QFKPSPQ----LDLHHFSNMLGFFPAVVHRLLPKLNLTLGQAIASQMVDQTWL
Dipi  -------QYNPTPR----LATHHFSNMLGFFPMIIHRLTPKLNLTLGQTIASQTIDQTWL
Pama  -------QFKSIPQ----LTPHHFSNMLGFFPAVIHRLTPKLNLVLGQAIASQMIDQTWF
Leob  -------QYKPAPQ----LIPHHFSNMLGFFPAVIHRLTPKLNLTLGQAIASQMVDQTWL
Neba  -------QFKVAPI----LNTHHFSNMLGFFPPVIHRLIPKTSLVLGQTIASQMLDQTWL
Pdpl  -------QQKPKPN----SRAYLFSCSLGYFTTIIHRFAPKLNLTLGQKLANQIVDLIWL
Nimi  -------QFKPTPE----STPHHFSNMLGFFPSIVHRTMSKLALLLGQKIASQMIDLTWL
Uptr  -------QFKPTPA----LTAHHFSNMLGFFPAVVHRLAPKMNLVLGQAVASQMMDQTWL
Pesc  -------QNYPFDD----QPTHQFSNMLGFYPEVVHRLLPKITLTVAQKIATHTIDLTWL
Baar  -------QYKSTPQ----LVPHHFSNMLGFFPSIIHRFTPKLNLALGQTIASQMVDQTWL
Moar  -------QFKPTPQ----LVTHHFSNMLGFFPAIIHRLTPKLNLVLGQTIASQMVDQTWL
Toja  -------QFNPTPK----LTTHHFSNMLGFFPAIIHRLSPKLNLLLGQTAASQMVDQTWL
Chau  -------QINVKPQ----SAPHHFSNMLGFFPIIIHRLMPKLNLTLGQTIANQTLDQSWL
Chse  -------QFKATPK----LPHHRFSNMLGFFPLIMHRAPTKFSLALGQSIASQMVDQTWL
Enar  -------QYKSTPS----LNPHHFSNMLGFFPSIIHRFTPKLNLALGQAIASQMVDQTWL
Hpty  -------QYKTTPQ----LTAHHFSNMLGFFPAIIHRLTPKLNLILGQTIASQMVDQTWL
Nana  -------QFKICPT----SATHHFSNMLGYFPSIVHRLVPKLNLVLGQNIANQTIDQAWL
Mcst  -------QYKPVPK----LTTHHFSNMLGFFPTIIHRFTPKLNLMLGQTIASQMIDQTWL
Rhox  -------QHKPTPH----FTPHHFSNMLGFFPALIHRLTPKLNLVLGQALASQTIDQTWL
Opfa  -------QYKATPQ----LATHHFSNMLGFFPAIIHRLTPKLNLTLGQAIASQMIDQTWL
Paar  -------QFKPIPH----LSPHHFSNMLGFFPTIVHRLVPKLNLTLGQTIASQMVDQAWL
Gozo  -------QFKTTPQ----LTSHHFSNMLGFFPAIIHRLTPKLGLALGQTIASQTIDQTWL
Ackr  -------QLKPSFK----SALYHFSNMLGFYPTILHRVLPYYFLLLAQKIANQMLDLAWL
Elev  -------QNQPVSK----ANSFMFSNMLGFFPTIIHRFSPLVSLTAGQTIATRSIDQTWL
Trdu  -------QYKPMPD----LSPHHFSNMLGFFPMVIHRLIPKLSLILGQAIASQTVDQTWL
Amoc  -------QFKTTPN----LTAHHFSNMLGFFPAIIHRLTPKLNLVLGQTIAAQTVDQTWL
Hame  -------HYKPRPK----REPYLFSLLLSYFPTLIHRSLPMMTLSMGQKFASQSVDQTWL
Chso  -------QFKPTPS----LSSHHFSNMLGFFPSVIHRLAPKINLTLGQLIASQMVDQTWL
Lyto  -------QFQKTPN----LPLHHFSNMLGFFPPVVHRLAPKLNLILGQTIASQMLDQSWI
```

[10/12 of aligned sequences]

To be continued on page 55.

```
Encr      -------QFQKTPY----LAPHHFSNMLGFFPSIIHRAAPKLNLVLGQTVASQMLDQTWI
Bvar      -------QKKISP------TPHHFSLLLGFFPTIIHQLFPKFALTLGQTAATQMVDQTWM
Noco      -------RIQTTLF----PTPHHFSSMLGYYPTLVHRMTPKLSLMWGQTIADQTIDQNWL
Chsp      -------QLMIMPA----HTPHHFSNMLGFFSAVMHRLLPKITLKFGQTIATQMIDQAWM
Arja      -------QHQTTPN----KATHHFSNMLGFFPTIIHRLTPKLNLVLGQTIASQMVDQTWL
Pase      -------QFKAMPA----QHPHHFSVMLGFFPTLLHRFMPHTTLSLGQVIASQTIDQTWL
Trel      -------QVKVTPK----MTPHRFSNMLGFFTTLIHRKPSKLGLVLGQFLACQTVDQTWL
Lifa      -------SDEFHAK----IFVHAFLTLLGYYHFVAMRVLADFVLRQANTLAKKMLDIYWL
Acur      -------QLKTTPL----TTPHHFTNMLGFFPSLVHRVSPKVSLLVGQTIASQMIDQTWL
Ampe      -------QFKPTPQ----LNLHHFSNMLGFFPMIVHRFTPKLNLALGQTIATQMIDQAWL
Urja      -------QGKSTPPLT--PPFSAFATLLGYFTLILHPLLPKLAFLLGQKIANQLVDQLWL
Enet      -------QFKVAPY----LPAHNFSNMLGFFPALVHRLPPSLALSFGQVIANQAVDQTWL
Ptbr      -------QLSPIPK----RTPYLFSNMFGFYPSLIHRLAPKITLILGQHVATQMIDLIWF
Safa      -------QFKPTPF----ITTHHFSNMLGYFPTLVHRLNPKISLSLGQFVATQMVDQTWM
Icae      -------QLKPTPL----LTPHHFSNMLGFFPPIIHRFVPKLNLVLGQTIANQMVDQTWL
Asmi      -------RQTMSPK----PPIYYFSTLLGYFSHYFHRTAPKTILTFGQFLALLLSDQTWL
Foal      -------HLGPNPK----INPYRFSTMLGFFPSITHRLLPYTNLAFAHKIATQILDLSWT
Drze      -------QLKTTPN----LNMHHFSNMLGFYPTVTHRLIPKMSLTLGQKMAGQMVDQNWL
Rhas      -------QFKPLPQ----TQPHHFSNMLGFFPAIIHRLPPKLNLLLGQAIASQMVDQTWF
Elac      -------QFKPTPL----LAPHHFSNMLGFFPSIIHRLPPKINLLLGQYIASQMVDQTWF
Kugu      -------QFKQTPK----LTTHHFSNMLGFFPSIMHRAPNKIGLILGQKMASQIIDLAWL
Plor      -------QFKPTPK----LIPHHFSNMLGYFPMIIHRFTPKLNLVLGQTIATQMIDQTWL
Sgun      -------QLKPSPK----LSTHHFSNMLGFFPHIIHRLTPKLNLILGQAIASQMIDQTSL
Zaco      -------QLKPSPL----HTPHHFSNMLGFFPPIIHRLTPKLNLLLGQSIASQMVDQTWL
Zbfl      -------QFKTTPR----LISHHFSNMLGFFPAIVHRLTPKLNLSLGQMIASQMIDQSWL
Spba      -------QFKPTPM----LTTHHFSNMLGFFPAIIHRLTPKLNLILGQTIASQMIDQTWF
Game      -------QFKPTPT----LTPHHFSNMLGFFPHIIHRFTPKLNLVLGQTIASQMVDQTWL
Thth      -------QFKPTPM----LTTHHFSNMLGFFPHIIHRFTPKLNLVLGQAIASQMVDQTWL
Xigl      -------QFKPTPT----LTTHHFSNMLGFFPAIVHRLTPKLNLTLGQTVASQMVDQTWL
Hyja      -------QLKPTPT----LTAHHFSNMLGFFPPIIHRFTPKLNLVLGQTIANQLIDQTWL
Psan      -------QIKAYPV----LKPHNLSNMTAFFSSIINPAMSKLGLSLGQKGANQSADQTWM
Cupa      -------QLKPTPM----LTPHHFSNMLGFFPHIIHRFTPKLNLVLGQTVASQMIDQTWL
Mpch      -------QFKLKPN----HTLHHFSNMLGFFPTIIHRLMPKMNLILGQKIASQTIDLTWL
Char      -------QFKTTPY----QPAHHFSNMLGFFPSIMHRLAPKLNLILGQTAASQTIDQTWL
Pser      -------QPKTAST----PINPHFFNQLGFFPAISHRFAPKTTMILGSAIASQAIDLTWL
Prol      -------PSQPAPY----RNPHHFSLMLGFYPHILHRLAPKLGLLLGQSIASQMVDQTWL
Plbi      -------QFKPTPI----LNVHNFSNMLGFFPAIVHRLTPKLGLVLGQDIANQMVDQTWL
Calu      -------QLKATPK----LAPYTFSNMLGYFPTIVHRNAPKIGLILGQTVANQLLDQIWL
Papa      -------RAKVTPF----IPTHHFSNMLGFFPMIIHRTTPLLALVLGQMISTQTIDQTWL
Sufr      -------QFKPHPT----LTPHHFSNALGFFPSIIHRVTPKLNLALGQTLASQTIDQTWL
Stci      -------QFKQSPV----LPTHHFSNMLGFFPTVIHRAAPKITLTLGQTIASQTVDQTWL
Taru      -------QLKPTPH----LSPHHFSNMLGFFPTIVHRASPKINLILGQTIATQIIDLTWL
Rala      -------QFKPTPM----LAPHHFSNMLGFFPAVVHRLMPKINLTLGQTIASQMVDQTWL
                                       :             :  .        :
```

```
                                                  O
Scca    EKIGPKSTLIQQIPLIKLSTHPQQGYIKTYLTLLFLTLTLIILVVFI-*-----------
Muma    EKIGPKSNLIQQTSLIKLSTQPQQGLIKTYLTLLFLTLTLVILITLT-*-----------
Erca    EMMGPKGIANLQLTLAQKITHMQKGLIKTYLSITMLSLLVITAMIIY-F*----------
Pose    EKLGPKGIAHAQLLTTQKITHAHKGLIKTYLSITVLSLFVIMLLIMF-I*----------
Actr    EKVGPKGITTSQIPLIKATSNIQQGLIKTYLTIFFLTTTLSILLITL-I*----------
Scal    EKVGPKGITTSQIPLIKATNSIQQGLIKTYLTIFFLTTTLSILLITL-I*----------
Posp    EKMGPKGVTTNQIPMIKATNNIQQGLIKTYLTIFFLTTALSILLITL-I*----------
Atsp    EITLPKGISSSQLPMAKLTSDIPQGMIMTYLTTFIFTTLLSTLLLIL-TQPDLPI-LA--
Leoc    EISLPKGITSSQLPLTKLTSDIPRGMVMTYLTTFIFTTLLSTLLLIL-TQPDLPI-LT--
Amca    EKAGPKGITSIQLPMIKITSNTQRGLIKTYLMLFLLTVVLAVTLINI-I*----------
Osbi    EKAGPKGVATKQIPMIEITNNIQQGLIKTYLTMFFLTVALTISLSLL-LT*---------
Pabu    EKVGPKGTSLNQKPMILSTNDIQQGMIKTYLMLFLLTSALTTIIVTT-RHA*--------
Hial    EKIGPKGISSNQIPMIKITNNIQQGMIKTYLTIFFLTTTLAILTTMA-I*----------
Elha    EKAGPKGVASNQLPMVKLVNDAQRGMIKTYLSIFLLTTALATAAMTS-H*----------
Mlcy    EKVGPKGAASCQIPMIKLTNDLQRGMIKTYLSIFLLTTALITISFTL-H*----------
Algl    EKAGPKGVGNSFIPVSNTVNNAQQGVIKAYLAVFFITITLTALYTLS-V*----------
Ptgi    EKAGPKGIATNQLPMINITNNTQQGMIKTYLTIFFLTTALAVTLTML-I*----------
Alaf    EKAGPKGIAASQIPMIKTVNSAQQGIIKAYLAVSFLTVLFTALLMTY-T*----------
Nock    EKAGPKGIAASQIPIIKMVNSPQQGIIKAYLAISFLTVLFTTLLVGL-S*----------
Anja    EKLGPKGIVNIQLPMIKIINNPQQGLIKVYLATFFLTITLIIIMMMV-F*----------
Gyki    EKAGPKGTAATQISMIKVANNPQQGLIKTYLSAFLLTSVIATLIVLL-T*----------
Syka    EKTGPKGAANAQIPMIKIINDPQQGLIKAYLAMFLLTNALVIIMMLY-*-----------
Opma    EKAGPKGVATAQIPMIKMVNDPQQGLIMSYLAMFFLTVALAVWMSW*-------------
Comy    EMTGPKGITKAQIPMIKIVNNPQQGLIKNYLSMFFLTNILVIATMTL-Y*----------
Sasp    EKLGPKGVTSMQLPAIKLVTKTQQGLIKIYLLSSVLTILVGLFMFSF-SDHS*-------
Eupe    EKLGPQGVVTSQLLMVKLINKPQHGLIKTYLTTFFLTVVLVSLMTIL-N*----------
Enja    EASGPKGLSSVQLKMSSITSDMQQGMIKTYLTTFLITLALATLMVLI-*-----------
Same    EAAGPKGLASTQTKMSTLISDAQRGIIKTYLVIFLITTGLATLLASA-*-----------
Chch    EAAGPKGLSALQVKMSAITSDTQRGMIKTYLTIFLITTSLAVLTAAI-*-----------
Grgr    EAVGPKGVARAQAKVSTYASDAQQGLIKSYLTIFAITTGLALLVASY-*-----------
Caau    EAMGPKGLALTQMTMAKVTNDVSRGMIKTYLTIFLLTLILAILPVLL-*-----------
Cyca    ETVGPKGLALTQMAMAKITNDITRGMIKTYLTIFLLTLILATIPVLL-*-----------
Dare    ELFGPCGIAFTLMTVATFVKDHRMASIKSYLAVFLTSIILKMMMLKLYT*----------
Cost    EASGPKGASSLQVKMAKTVSDMQQGMIKTYLTIFLLTTTIAILLAMN-*-----------
Leec    EAVGPKGVSSLQTKMSKTINDTQRGLIKTYLTIFLLSTTLAILLATI-*-----------
Fola    EAAGPKGASSTQIKMAKITSDTQRGMIKTYLTIFLLTTALATRLATI-*-----------
Clmc    ENAGPKGLSSTQIKMATTTSNMQRGMIKTYLTMFLLTSTLAVLFIII-*-----------
Phin    EKTGPKGLSSTQIKLATLTGNIQQGMIKTYLTMFLLTNIMAILYITI-*-----------
Icpu    E-MGPKGLHPLHFTLSSMFDNINTNIIKVYLTFYLISTALIIVLLST-I*----------
Psto    E-IGPKGLHPAHHYISALFDKTNTNIIKVYLTSYLISTALAITLLFT-L*----------
Cora    E-LGPKGMVPIHMSISAKFDNMDSNMIKIYLTIYLLTSIMAIILLTT-I*----------
Eisp    EASGPKGLSHTQMKMATITSNTQRGMIKTYLTIFLLTNVLATILILT-*-----------
Apal    ESSGPKGLANAQIKMASTTSDSQRGMIMTYLTIFLLTATLTLFIFF*-------------
Eslu    EKIGPKGVSSINLPMIITTNDVQKGMIKTYLTLFFLTLILALFISII-*-----------
Dape    EKGGPKGVTSLNLPLITLTSDTQKGLIKTYLTLFFLTFTLALFFSAS-*-----------
Glse    EKIGPKALISSNLPLVTSTSNAQQGMIKTYLSLFFLTTSLVLLLSSL-NSS*--------
Naar    EKVGPKTVVSSHLPLITSTSNAQQGMIKTYLSLFFLTTILALLLTSL-YSS*--------
Lioc    EKVGPKAATSAHLPMITSTSNAQQGMIKTYLSLFFLTMTLALLLTSL-YSA*--------
Opso    EKVGPKAIPLSHLPFITSTSDAQRGLIKTYLTLFFLTMALALLLTN*-------------
Alte    EMAGPKGLASAQMKMITTTSNAQRGMIKTYLTIFLLTTTLATLLATL-*-----------
Plap    EMAGPKGLASAQLKMITTTSNAQRGMIKTYLTIFLLTTTLATLLAIL-*-----------
```

To be continued on page 56.

[11/12 of aligned sequences]

```
Plal   EKAGPKAMASLHLPASAATTDLQQGMIKTYLALFFLTISLAVLAALI-*-----------
Sami   EKVGPKAMSAIHLPAASTTTDLQQGMIKKYLSLFFLTIALAVIMALV-*-----------
Rere   EKVGPKAVASGQLPLVTSTTEVQQGMIKTYLAMFFLSLTLAIVFALV-*-----------
Gama   EKVGPKAIVSANLPLVSGVTGVQRGMVKTYLTLFFLSTTLAVFLASV-KRLVGFLGLSLV
Onmy   EKVGPKELFQLTCLMVTTTSNIQQGMIKTYLTLFFLSTTLAVLLTLT-*-----------
Sasa   EKIGPKGVVSTHLPMVTTTSNIQQGMIKTYLTLFFLSTALAVLLTLT-*-----------
Cola   EKIGPKGVVSTHLPMITTTSNMQQGMIKTYLTLFFLSTTLAVLLTST-*-----------
Dita   EKTGPKAISSLNLPVIIKANEFQQGLIKVYLGLFFLTVTIFILVTAF-*-----------
Gogr   EKTGPKSASSSHIPAINLTNEIQQGLIKTYLSLFFLTLALTVLL-------ISPN-CP*-
Chsl   EKVGPKAASASHLPAIIMATDIQQGLIKTYLRLSLLTLALATLIFV*------------
Atja   EKTGPKTVTLSHLPLISATSNAQQGMIKTYLTIFLMSSALAILLMIA-*-----------
Iido   EKTGPKAATSSHLPLISAMSNAQQGMIKTYLTIFLMSSALAILLMLA-*-----------
Auja   EKTGPKAIASMNLPIITTASNAQQGLIKTYLTLFLLTLSLSLLLFSL-*-----------
Chag   EKSGPKTAASVHIPLASTTSNMQQGLIKTYLTLFLLTLTLTILITFY-*-----------
Hami   EKTGPKAIVSAHLPLSTLTSNIQRGLIKTYLTIFLLTLSLSTLILLI-*-----------
Saun   EKTGPKAITSASLPLATMTSNIQRGLIKTYLTIFLLTLALTSLILMF-*-----------
Nema   EKAGPKTATSAQLPLISATSNAQQGAIKAYLTLFFITLALATLLVSY-*-----------
Disp   EKAGPKMITSAQLPLISATSNAQQGLIKTYMSLFFMTLCLTTLFSAN-L*----------
Myaf   EKTGPKAITSAQLPLVSSTSNMQQGYIKTYLSLFFMTLCLATLLTTN-L*----------
Lagu   EKSGPKAATTPQLPLISATSNIQRGLVKTYFTLLMMTLTLSVLLST*------------
Trtr   EKIGPKATTLMQIPMISTTSNAQQGLIKTYISMFTLSLLFAILITLL-PA*---------
Zucr   EKIGPKAITQNQIPLISTTSNAQQGAIKTYLTMFALSLMLALLIT*-------------
Pxja   EKVGPKAVITTNIPMVTTTSNIQQGMIKTYLTLFFLTLVLMTLLMIP-*-----------
Pxlo   EKVGPKAVIATNTPMVTTTSNIQQGMIKTYLTLFFLTLVLMTLLMIP-*-----------
Pctr   EKCGPKMVTSSQTLMAQNINNLQQGLIKTYLTLFFLTLVLMMFLASP-*-----------
Apsa   EKIGPKMITSSQIPLIHTTSNAQKGLIKTYLTLFFLTLTLTTVLLIT-*-----------
Cabe   EKVGPKAIETSTKRLATTASNVQRGLIKIYLVSFVLTLAIALLLVHA-*-----------
Bzze   EKTGPKAIPNYNLPLVTTTSNAQQGMIKTYLTLFVLTITLASLLVAL-*-----------
Siim   EKTGPKAIPQLNLPMISWASNMQRGMIMAAPTSLVISLILTFVFLTI-*-----------
Ctru   EKAGPKAIASSNLPLITTTSNTQQGLIKTYLTLFLLTLVLTTLLVAL------------
Dpbr   EKTGPKTIASTNLPLITTTSNTQQGMIKTYLTLFLLTLALTVFLVVT-*-----------
Caki   EKFGPKTISDTILPLSASASNMQRGLIKPYLLMYLTTLLMTLMVFIV-KT*---------
Phja   EKTGPKAISQLTLQFLYFASNTQQKYTKTHLILFLTCMMTLITTMD*------------
Brsp   EKVGPKMAQLLSTSMSHSISNIQQGSIKTYLILFIMTLALLTPIMVF-N*----------
Gamo   EKTGPKAIANATTPLASATSNMQQGLIKTYLTLFLMTLVLVTLISAT-*-----------
Lolo   EKTGPKAIANATTPLAAATSNIQQGLIKTYLTLFLITLILVTLISVA-*-----------
Batr   EKLGPQHIAVLNKNSSNYTSNLQRGYFKTSSLMFLLTILLAITSQVVQN*---------
Prmy   EKAGPHSLATTNRTTSIYISGAQKGKVKTYLVSTLLTFSLFALMIFF-PLM*--------
Lose   EKLGPKMFVKINKPLAANVSDAQRGTIKTFLTLYLLTLAILIFISHL-*-----------
Loam   EKAGPKAAASLNIPLITSTSNTQQGMIKTYLTLFLLTLTLAVVILTI-*-----------
Chab   EKVGPKTMASVNTPMITTTSNIQQGAIKTYLTLFLLTLALAALMLAI-*-----------
Chto   EKTGPKTMASMNTPMITTTSNIQQGAIKTYLTLFLLTLALAALMLAI-*-----------
Majo   EKTGPKAVTTINTPLITTTSNLQQGMTKTYLAFFLLTLVFAVTALML-*-----------
Hlst   EKTGPKALSTLNLPLIKITSDLQQGMTKTYLGFFLFTLVFMVTTLVF-*-----------
Clpe   EASGPKAIVSLNNPLIKTTSDAQRGAIKTYLTLFLLTMTLSPLALLI-*-----------
Mlmr   EKSGPKAIVSMNIPLVTTTSNTQQGAIKTYLTLFLLTLTLATLTLMI-*-----------
Crcr   EKAGPKFLALLNTRMTILASNLQQGLIKTYFLLFLLTLSIALILLLL-PH*---------
Muce   EKAGPKFLALLNTRMTILASNLQRGQIKTYFLLFLLTLSIALILLLL-PH*---------
Bege   EKTGPKAITTLNLPLATTASNIQQGMIKTYLSLFFITTALSMLMLLN-*-----------
Mela   EKTGPKSLVDLNTPVIATTSNIQQGMVKTYLSLFFFTTILATLLLVY-*-----------
Hats   EKSGPKAITALNTPLVTTTNNIQRGMIKTYLSLFFLTTALATLLLLH-*-----------
Orla   EKTGPKMTASVNLPLISSTSNLQQGVIKTYFLMFFFTMILAVLILVI-*-----------
```

To be continued on page 57.

```
Cosa    EKSGPKAVASTSIPIILTTDNIQRGMIKTYLSLFFFTMTLAILLLY*-------------
Exsp    EKVGPKTVSSMNKPLATTMSNIQRGMIKTYLSLFFFTIAMTALLLFF-*-----------
Depa    EKSGPKAITSLNTPLASTTNNIQRGMIKTYLSLFLLTMTLAMFLLLI-*-----------
Rima    EKLGPKMISLLNKIFAITTDNLQRGMIKIFLCLFLLTSTIALLALFN-LF*---------
Fuol    EKTGPKTICSLNLPLASSVSNIQQGSVKIYLSLFFFTTLLSLTILLA-*-----------
Gmaf    EKSGPKATFNLSLRPITMTSNIQQGMIKTYLSLFFFTSLLTLLFC*--------------
Xeei    EKSGPKAAYSMNLPLITTSSNVQQGIVKTYLSLFFFTTLLAMAFMLP-*-----------
Pros    EKTGPKTLISANLPLITTTSNLQQGLVKTYLTLFLLTLTLTLLLSSL-*-----------
Scmi    EKAGPKTLIATNLPLITTTSNIQRGMIKTHLTFFLFTLTFIALLSSP-*-----------
Rolo    EKAGPKALVSMHLPLVTTTSNTQQGMIKTYLTLFLLTLTLTILLFNS-*-----------
Cere    EKLGPKTITSAQLPLITTTSNAQRGLIKTYLTLFFLTLTLVVILSHP-*-----------
Daga    EKAAPKAIAFGQLKFITTTSNIQQGLVKIYLTLFLLTLTLSIALLNP-*-----------
Anco    EKTGPKMLASAHLPLVTSTSNAQQGMIKTYLTLFLFTLTLMILLFNT-*-----------
Dmve    EKTGPKMLTTLNMPIITSASNAQQGMVKTYLTIFLFTLALMALLFNT-*-----------
Dmar    EKTGPKMLATVNMPIITSASNAQQGMIKTYLTIFLFTLALMTLLFNT-*-----------
Anka    EKTGPKMLTSAHLPLVTSTSNAQQGMIKTYLTLFLFTLTLMIMLFNT-*-----------
Moja    EKAGPKTLTSAHLPLVTSTSNAQQGMIKTYLTLFLFTLTLMALMFNT-*-----------
Hoja    EKAGPKMLTSSQLPLVTSTSNAQQGMIKTYLTLFLFTLTLMVLLFNT-*-----------
Bede    EKTGPKTLITANLPLITTTSNTQQGMIKTYLTLFLLTLTLAALLFTP-*-----------
Besp    EKTGPKTLITANLPLITTTSNTQQGMIKTYLTLFLLTLTLAALLFTP-*-----------
Mysp    EKTGPKALVHMHLPLVTTTSNAQQGMIKTYLTLFLLTLTLTVLLIIP-*-----------
Osja    EKTGPKALISTHLPLITTTSNTQQGMIKTYLTLFLLTLALAILLIIP-*-----------
Sgro    EKSGPKALVSLHLPLVTTTSNAQQGMIKTYLTLFLLTLTLAILLTLP-*-----------
Pzpa    EKAGPKSMTLSQLPMINMTNNVLKGFIKTYLTLFFLTFTLVLWKILT-*-----------
Zeja    ENTGPKYTTSAQPPMIALTNNVQQGIIKTYLTLFFLTFSLVLLITFA-*-----------
Znne    EKVGPKYMTLAQTPMISLTTNVQQGLIKTYLTLFFFTFSLVMMITLT-*-----------
Zefa    EKTGPKSMTSAQVPMISLTTNIQQGLIKTYLTLFFLTFSLAMMISLS-*-----------
Acni    EKVGPKSMTLAQIPMITLTSNIQQGIIKTYLTLFFLTFSLVLLILLP-*-----------
Ncrh    EKIGPKSMTLAQIPMITLTSNVQQGIIKTYLTLFFLTFSLVLLILLP-*-----------
Agca    EKSGPKAVASLNLPLITTTSNTQRGMIKTYLTLFLLTLTLAILILSF-*-----------
Hydy    EKIGPKAVISSRSPLITTTSNTQRGLVKTYLTLFLLTLALALFLNFF-*-----------
Gsac    EKIGPKAIVYHTTPLITTTSNTQRGLVKTYLALFLLTLALAMLLASF-*-----------
Pevo    EKTGPQATVTANTPLSSYTSNAQQGAIKTYLMIFLLTLALACIILWT-*-----------
Hiku    EKIGPKATATLNLPLITTTSNTQKGVIKTYLMLFILTFTLAMALLI*--------------
Inpa    EKTGPKSTISNNIPLVTTTSNIQQGLIKTYLTLFLATTLLTALLTTY-*-----------
Auch    EALGPKAASSLSTPLITLTSDAQQGTPKTFLTLFLLTLVLALAS-------MIP*-----
Fico    EKVGPKAVTSASIPLITNTSNAQKGAIKTYLTFFLLTLALMTITLIF-*-----------
Macs    EKVGPKSLISANLPLVTTTSNTQHGAIKSYLTFFLLTLALATLTLIY-*-----------
Moal    EKTGPKTITSLNLSLATTTSNIQKGMIKTFLSLFFTTLMIIILLNYY-*-----------
Syma    EKSGPKATASLNMPLITTISNTQKGMIKTYLTLFFITSIIMILLITN-*-----------
Mafr    EKTGPKATTHLNLPLITTSSNIQQGAIKTYFMLFILTLTLSILTLY*-------------
Dcpe    EKTGPKGIVSLNHQPITSTSNAQRGIIKTYLGLFFLTMALSTLLVAL-L*----------
Dcti    EKTGPKAITSSNLPLVSTTSNAQRGIIKTYLTLFFLTIAFATLVIAR-*-----------
Hehi    EKVGPKAISSSNMPLITTTSNTQQGMIKTYLTLFLLTLTLAALLFTR-*-----------
Stam    EKVGPKAVATSTLPLVTTTSNTQQGLIKTYLTLFLLTLALSTLIFLL-*-----------
Hogi    EKVGPKAIALSNSPLITTTSNTQQGLIKTYLTLFVLTLTLVTLLATY-*-----------
Erzo    EKTGPKAVASSNIPLITTTSNTQQGLIKTYLSLFLLTLSLSVLLTTY-*-----------
Hxot    EKTGPKAIASSSLPLITTTSNTQQGLIKTYLALFLLTLTLTILVTIY-*-----------
Core    EKAGPKAIASISLPLITTTSNTQQGLIKTYLALFLLTLTLTVLITAY-*-----------
Apve    EKTGPKAIITSNLPLITTTSNTQQGLIKTYLAFFLLTLTLAILLTMN-*-----------
Latj    EKSGPKALTNLNLPMITTTSNIQQGLIKTYLSLFLLTLTLALLISY*-------------
Laja    EKTGPKAVTSLNIPLITTTSNAQQGMIKTYLTLFLLTLLFATLLFSN-*-----------
```

To be continued on page 58.

[11/12 of aligned sequences]

```
Syja   EKTGPKAIASLNMPLITTTSNTQQGMIKTYLTLFLLTLALLTLTFVY-*-----------
Epme   EKTGPKAAANLNSPLITSTSNTQRGLVKTYLIFFLITMVFTLLIFFT-*-----------
Grse   EKLGFQAAPKFKYIFITGLTDAQQGNIKPYLSLFLLTLTLAIMAFVF-*-----------
Clja   EKVGPGAMKKFTASSIKTVNMYQRGLVKTYIILFMMTITLCSALTVV-KD*---------
Ogcy   EKAGPKSMSILNMALAHHMNKIQMGKIKTYLALFFLTLALSTLPLLF-F*----------
Plna   EKAGPKGIASLTMPLISTTSNMQQGMIKTFLSFFLLTLVLAMFLLLI-*-----------
Lema   EKTGPKAVSSLNLPLISTTSNIQQGMIKTYLALFLLSLALATLIFAT-*-----------
Etzo   EKSGPKALATLNTPLITSTSNAQRGMIKTYLTLFLLTLALTTLVFIN-*-----------
Apse   EKTGPKALTSFNLPLVTTTSNTQHGKIATYLVFFIMALELAVALTFI-*-----------
Epde   EKTGPKAVASHNLPLITTTSNVQQGLIKTYLTLFLLTLTLTTLIFIL-*-----------
Slja   EKTGPKAAASLNIPLITTTSNAQKGKIKAYLAQFLLTVAIAVLLLIL-*-----------
Bsja   EKSGPKAVASLNTPLITTASNIQQGMIKTYLTLFLLTLAIALLVVIF-*-----------
Ecna   EKTGPKAITSLNLPLISTASNIQRGMVKTYLSLFLLTLLLIIPIISF-*-----------
Cohi   KEIGPNAISTFNTTLASVTSNMQRGSVKTHLTTFLHTLLFLIFLIVI-*-----------
Caar   EKVGPKTITSTNLPLISTASNIQQGMVKTYLSIFFLTLTLALLFILS-*-----------
Came   EKTGPKAITSLNLPLISTASNIQQGMVKTYLSIFFLTLIIMLLLILP-*-----------
Mema   EKTGPKAITSSNLPIIKTTSNIQQGLVKTYLSFLFITLALAILLFS*-------------
Lenu   EKTGPKATITLNMKPIKLTSDIQQSLIKNYLTILLLTLTLATAFLLI-*-----------
Brja   EKIGPKALTSSNMPLITTTSNAQQGMIKTYLTLFLLTLTLTSLLVAY-*-----------
Plma   EKTGPKTLASSNIPLITTTSNTQQGMIKTYLALFLLTLTLATLLVSY-*-----------
Emst   EKTGPKAVASSNIPLITTASNAQQGMIKTYLTFFLLTLALATLVFVL-*-----------
Ptti   EKSGPKATASLNLPLITTTSNTQQGMIKTYLTLFLLTFTLATLIFIL-*-----------
Losu   EKTGPKAISALNMPLITTTSNIQQGMIKTYLMLFFLTLSLTTLIFLI-*-----------
Geoy   EKVGPKASASLNMPMASATSNIQRGMVKTYLTLFLLTFTLAVLMLMA-*-----------
Dipi   EKSGPKAVASLNTPLITTTSNTQQGMIKTYLTLFLLTLVLATLIFTL-*-----------
Pama   EKTGPKALASLNSPLITTTSNAQHGMIKTYLALFLLTLTLATLLLLI-*-----------
Leob   EKIGPKAVASASLPMITTASNTQQGMIKTYLILFALTLTIAILALFL-*-----------
Neba   EKVGPKATISINKPMATSTSDIQRGMIKTYLALFMLTTTVAVLMLAP-*-----------
Pdpl   EKTGPKTVSSVNIALSSTTNDFQQGMIKTYLATFFITLILATLTTAL-*-----------
Nimi   EKLIPKALARLNKPLVTWTSNTQRGKIKTFLTFFLITLALVVLVVIF-*-----------
Uptr   EKVGPKSVVTHSLPLITLTSNAQRGAIKMYLITFLLTLALTGLSLVF-*-----------
Pesc   EKIGPKFLTSRIIPLITTTSDAQQGKVMSYIALFLLTSALAMLTFYF-SF*---------
Baar   EKLGPKALASYNLPLITTTSNTQQGMIKTYLTLFLLTLILMTLIFTY-*-----------
Moar   EKSGPKAAASLNIPLVTTTSNTQQGMIKTYLTLFLLTLTLATLVFIL-*-----------
Toja   EKVGPKATISMNLPLITSTSNIQQGMIKTYLALFLLTLALVVLLFTH-*-----------
Chau   EKTGPKAIITPNVLLATTTSNAQQGTIKAYLTLFLLTTSFASLIYSL-*-----------
Chse   EKTGPKAVTSSNTPLASTVSNIQRGLIKTYLITFLLTLALAALALMF-*-----------
Enar   EKVGPKALTSSMKPLITTTSNTQQGMIKTYLTLFFLTLTLAILTFFY-*-----------
Hpty   EKTGPKAVASSNIPLITTTSNTQQGMIKTYLTLFLLTLVLTTLIFTY-*-----------
Nana   EKTGPKAALSLNLPLITTTSNMQQGMIKTYLTMFFLTLSLMILILVI-*-----------
Mcst   EKTGPKAVTSSNLPLITTTSNAQQGMIKTYLTLFLITLALSALMFAL-*-----------
Rhox   EKTGPKAMFTSNLPLITTTSNTQQGFIKTYLTLFLMSLIIVALAFTN-*-----------
Opfa   EKTGPKAIASSNLPLITTTSNAQQGMIKTYLALFLITLALSTLMFIY-*-----------
Paar   EKVGPKAISSLNIPLITTASNVQQGMIKTYLTLFLLTLSLATLTLTL-*-----------
Gozo   EKTGPKALASLNLPLITTTSNTQQGVIKTYLTLFLLTLALTTLLFIF-*-----------
Ackr   EKILPKAIRPVFLPMIKMVSNAQKGVVSSYITFSIISFFLLITFFVLLYSPESPSTYPPG
Elev   EEAGPKALVHMNTPIASSISNIQQGIIKTYLTLYILTLFLFMVVLIT-*-----------
Trdu   EKIGPKATATLNLPLITTTNNIQQGMIKTYLSLFFFTFGLALLLLLY-*-----------
Amoc   EKTGPKAIVSLNEPLIKTTNNIQQGMIKTYLSLFFFTLAMALLLLLT-*-----------
Hame   EKIGPQAVNSSNRPMISTTSNLQRGLLMTHLAMLALTTTLAIILGCQ*------------
Chso   EKSGPKAVSAVNLPLITSTSNAQQGMIKTYLIMFVLTLALAVLVTSL-*-----------
Lyto   EKIGPKAVASSNMPLVTTISNTQQGLIKTYLALFLLSLALSLLAITY-*-----------
```

To be continued on page 59.

```
Encr   EKIGPKAVLSSNIPLVTTTSNTQQGLIKTYLALFLLSLTLALLMATY-*-----------
Bvar   EKVGPKALIATNMPLVTLASNAQQGTIKVYLMLFVLTLATVSLMFFF-*-----------
Noco   EEIGPKATATLSKPLIIFTSEMQQGRMKTHLLFFFLGLTLMTSMIIY-*-----------
Chsp   EKTIPKAVVAATLPMATFTSNIQRGLIKTHMSFLVITLITSFIMLNS-*-----------
Arja   EKTGPKAIATSSLPLITTTSDTQQGLIKTYLALFLLTLVFTILLTTG-*-----------
Pase   EKTGPQALALSNRPLITTTSNIQRGLVMTYLAFFLITVMLAALVLLA-*-----------
Trel   EKVGPKATSSLILPLIKKTAELQRGNPMASLGPLLVSLVDYLLTFNL-TTRMAPR-SPPR
Lifa   KEVTTMMIYRSLSMVSPFVSNAQKGLFKDAMMTTMFSLFVLLCLGCAYVSVI*-------
Acur   EKSGPKLMSSQLTPLISAVSNVQQGLIKTHLMLFLLTLVVITLPFIL-NST*--------
Ampe   EKTGPKALISSNLPLVTTTSNVQQGMIKTYLALFLLTLTLATLASLY-*-----------
Urja   EKMGPKAASLASTYATKTISEIPQGHAKIFLGLFALSVTLAPLSFMI-*-----------
Enet   EKIGPKSTASLITPLVSSTDALQQGLVKTFLSLFFATTALALLF-------LAY------
Ptbr   EKTGPKTTIALNKPGAQVVSNIQQGNIKVYLLTLFLTLSIATLLIIA-I*----------
Safa   EKTGPKAISEINNPIISTVSNIQQGMIKTYLSLFFFTLAISTVAFAL-*-----------
Icae   EKAGPKALVSSNIPLITTTSNAQQGMIKTYLALFLLTLTLTTLLVSY-*-----------
Asmi   EKVAPKLANWANMPLIHTTDNLQRGKIKLHMYLFILTLTIVLLALCFSNYTDAPLFLASH
Foal   ETLGPKGLSYQSLQAASKVTDTQKGLIKSYMLLFMLTIISVFLISLI-Y*----------
Drze   EKIGPKAIVSLNIPLITLTSNTQKGMIKTYLILFMLTLMLAIILLPI-K*----------
Rhas   EKTGPKAVSSLNLPLITTTSNAQRGMVKTYLALFFLTFMLALLLISL-DRTQSPTTLPT*
Elac   EKSGPKAIYNTNLPLITTASNAQRGMVKTFLTLFLLTFLLSLLLMTY-*-----------
Kugu   EKIGPKTLTSLNTPLITTTSNTQQGMIKTYLTLFLLTLMLMTLLLVL-*-----------
Plor   EKSGPKAVASSNIPLITTTSNIQQGMIKTYLTLFLLTLALATVLLTF-*-----------
Sgun   EKTGPKAIASLNMPLITTTSNTQQGMIKTYLSIFFLTLALTTVIFTL-*-----------
Zaco   EKSGPKAVSSLNMPLITSTSDIQQGMIKTYLTLFLLTLALATLVFLL-*-----------
Zbfl   EKSGPKATASLNTPLITTTSNIQQGLIKTYLTFFFLTLAFALIITLA-MTSLL*------
Spba   EKVGPKALASLNLPVVTITSNIQRGMIKTYLTLFVLTLAFTALLTTY-*-----------
Game   EKTGPKALASSNIPLITTTSNTQQGMIKAYLALFLLTLTLATLLVSY-*-----------
Thth   EKSGPKALATSNLPLITTTSNAQQGMIKTYLALFLLTLTFATLLVSY-*-----------
Xigl   EKTGPKAITSYNLPLVTTTSNIQQGMIKTYLSLFLLTLTLMTLLLAY-*-----------
Hyja   EKTGPKALSSYNIPLITTTSNAQQGMIKIYLALFLLTLTFAALLISY-*-----------
Psan   EKVGPKAVASYNIRMATTTSDTQRGSIKTYLTMFFMTLGFATPVAYF-*-----------
Cupa   EKTGPKALASSNIPLITTTSNAQQGMIKTYLSLFLLTLALATLLIIY-*-----------
Mpch   EKTGPKAVQALNMPLVTTTSNMQRGMIKAYITMFLLSILLAIFIIY*-------------
Char   EKTGPKAVSNTLLPLITTTSNIQQGMIKTYLTIFFLTLTLALLLVFF-*-----------
Pser   EKTGPKAITSSNLPLVSTTSNIQRGMIKTYLMLTLTTLFLATLAIIP-*-----------
Prol   EKTGPKAISSSNIPLASATSNIQKGMIKTFLLSFVLSLALMILIFTI-*-----------
Plbi   EKTGPKAIASSNLPLVSSTSNIQRGMIKTYLTLFLLTLALMIPTLIP-*-----------
Calu   EKAGPKAVSAINTPLSSTISNLQRGAIKSFLAMFLLSVPAMFSVMWY-LN*---------
Papa   EKVGPKAVSTSNIPLISSISNIQQGKIKTYLTMFSLTLATATLTLLS-*-----------
Sufr   EKVAPKALISINAPLITTTSNTQRGMIKTYLTLFLFTLAMATMLLAL-*-----------
Stci   EKLAPKALVSLNKPLVTSTSNIQKGMVKTYLTLFLLTLSLAIILISL-*-----------
Taru   EKVGPKTISSINTPLISTISNIQQGSIKTYLVLFLTTLALSTLVLLT-*-----------
Rala   EKTGPKALASINSPLITTTSNLQRGMIKTYLTLFLLTLALAVLILAL-*-----------
       :
```

To be continued on page 60.

[12/12 of aligned sequences]

```
Scca  ------------------------
Muma  ------------------------
Erca  ------------------------
Pose  ------------------------
Actr  ------------------------
Scal  ------------------------
Posp  ------------------------
Atsp  -*----------------------
Leoc  -*----------------------
Amca  ------------------------
Osbi  ------------------------
Pabu  ------------------------
Hial  ------------------------
Elha  ------------------------
Mlcy  ------------------------
Algl  ------------------------
Ptgi  ------------------------
Alaf  ------------------------
Nock  ------------------------
Anja  ------------------------
Gyki  ------------------------
Syka  ------------------------
Opma  ------------------------
Comy  ------------------------
Sasp  ------------------------
Eupe  ------------------------
Enja  ------------------------
Same  ------------------------
Chch  ------------------------
Grgr  ------------------------
Caau  ------------------------
Cyca  ------------------------
Dare  ------------------------
Cost  ------------------------
Leec  ------------------------
Fola  ------------------------
Clmc  ------------------------
Phin  ------------------------
Icpu  ------------------------
Psto  ------------------------
Cora  ------------------------
Eisp  ------------------------
Apal  ------------------------
Eslu  ------------------------
Dape  ------------------------
Glse  ------------------------
Naar  ------------------------
Lioc  ------------------------
Opso  ------------------------
Alte  ------------------------
Plap  ------------------------
```

[12/12 of aligned sequences]

```
Plal   ------------------------
Sami   ------------------------
Rere   ------------------------
Gama   LTPELRTVWVATPTRKPQASPHQ*
Onmy   ------------------------
Sasa   ------------------------
Cola   ------------------------
Dita   ------------------------
Gogr   ------------------------
Chsl   ------------------------
Atja   ------------------------
Iido   ------------------------
Auja   ------------------------
Chag   ------------------------
Hami   ------------------------
Saun   ------------------------
Nema   ------------------------
Disp   ------------------------
Myaf   ------------------------
Lagu   ------------------------
Trtr   ------------------------
Zucr   ------------------------
Pxja   ------------------------
Pxlo   ------------------------
Pctr   ------------------------
Apsa   ------------------------
Cabe   ------------------------
Bzze   ------------------------
Siim   ------------------------
Ctru   ------------------------
Dpbr   ------------------------
Caki   ------------------------
Phja   ------------------------
Brsp   ------------------------
Gamo   ------------------------
Lolo   ------------------------
Batr   ------------------------
Prmy   ------------------------
Lose   ------------------------
Loam   ------------------------
Chab   ------------------------
Chto   ------------------------
Majo   ------------------------
Hlst   ------------------------
Clpe   ------------------------
Mlmr   ------------------------
Crcr   ------------------------
Muce   ------------------------
Bege   ------------------------
Mela   ------------------------
Hats   ------------------------
Orla   ------------------------
```

[12/12 of aligned sequences]

```
Cosa    ------------------------
Exsp    ------------------------
Depa    ------------------------
Rima    ------------------------
Fuol    ------------------------
Gmaf    ------------------------
Xeei    ------------------------
Pros    ------------------------
Scmi    ------------------------
Rolo    ------------------------
Cere    ------------------------
Daga    ------------------------
Anco    ------------------------
Dmve    ------------------------
Dmar    ------------------------
Anka    ------------------------
Moja    ------------------------
Hoja    ------------------------
Bede    ------------------------
Besp    ------------------------
Mysp    ------------------------
Osja    ------------------------
Sgro    ------------------------
Pzpa    ------------------------
Zeja    ------------------------
Znne    ------------------------
Zefa    ------------------------
Acni    ------------------------
Ncrh    ------------------------
Agca    ------------------------
Hydy    ------------------------
Gsac    ------------------------
Pevo    ------------------------
Hiku    ------------------------
Inpa    ------------------------
Auch    ------------------------
Fico    ------------------------
Macs    ------------------------
Moal    ------------------------
Syma    ------------------------
Mafr    ------------------------
Dcpe    ------------------------
Dcti    ------------------------
Hehi    ------------------------
Stam    ------------------------
Hogi    ------------------------
Erzo    ------------------------
Hxot    ------------------------
Core    ------------------------
Apve    ------------------------
Latj    ------------------------
Laja    ------------------------
```

[12/12 of aligned sequences]

```
Syja ------------------------
Epme ------------------------
Grse ------------------------
Clja ------------------------
Ogcy ------------------------
Plna ------------------------
Lema ------------------------
Etzo ------------------------
Apse ------------------------
Epde ------------------------
Slja ------------------------
Bsja ------------------------
Ecna ------------------------
Cohi ------------------------
Caar ------------------------
Came ------------------------
Mema ------------------------
Lenu ------------------------
Brja ------------------------
Plma ------------------------
Emst ------------------------
Ptti ------------------------
Losu ------------------------
Geoy ------------------------
Dipi ------------------------
Pama ------------------------
Leob ------------------------
Neba ------------------------
Pdpl ------------------------
Nimi ------------------------
Uptr ------------------------
Pesc ------------------------
Baar ------------------------
Moar ------------------------
Toja ------------------------
Chau ------------------------
Chse ------------------------
Enar ------------------------
Hpty ------------------------
Nana ------------------------
Mcst ------------------------
Rhox ------------------------
Opfa ------------------------
Paar ------------------------
Gozo ------------------------
Ackr *-----------------------
Elev ------------------------
Trdu ------------------------
Amoc ------------------------
Hame ------------------------
Chso ------------------------
Lyto ------------------------
```

[12/12 of aligned sequences]

```
Encr  ------------------------
Bvar  ------------------------
Noco  ------------------------
Chsp  ------------------------
Arja  ------------------------
Pase  ------------------------
Trel  VNS*--------------------
Lifa  ------------------------
Acur  ------------------------
Ampe  ------------------------
Urja  ------------------------
Enet  ------------------------
Ptbr  ------------------------
Safa  ------------------------
Icae  ------------------------
Asmi  QN*---------------------
Foal  ------------------------
Drze  ------------------------
Rhas  ------------------------
Elac  ------------------------
Kugu  ------------------------
Plor  ------------------------
Sgun  ------------------------
Zaco  ------------------------
Zbfl  ------------------------
Spba  ------------------------
Game  ------------------------
Thth  ------------------------
Xigl  ------------------------
Hyja  ------------------------
Psan  ------------------------
Cupa  ------------------------
Mpch  ------------------------
Char  ------------------------
Pser  ------------------------
Prol  ------------------------
Plbi  ------------------------
Calu  ------------------------
Papa  ------------------------
Sufr  ------------------------
Stci  ------------------------
Taru  ------------------------
Rala  ------------------------
```
