# Supplementary material for: Structure and variation of the mitochondrial genome of fishes
Source: BMC Genomics. 2016 Sep 7;17(1):719. doi: 10.1186/s12864-016-3054-y (PMC5015259; doi:10.1186/s12864-016-3054-y)
Supplement: Additional file 6: Figure S1-a. — Aligned amino acid sequences of the ATP8 gene in mt genomes of 250 fishes. Figure S1-b. Aligned amino acid sequences of the ATP6 gene in mt genomes of 250 fishes. Figure S1-c. Aligned amino acid sequences of the COI gene in mt genomes of 250 fishes. Figure S1-d. Aligned amino acid sequences of the COII gene in mt genomes of 250 fishes. Figure S1-e. Aligned amino acid sequences of the COIII gene in mt genomes of 250 fishes. Figure S1-f. Aligned amino acid sequences of the Cyt b gene in mt genomes of 250 fishes. Figure S1-g. Aligned amino acid sequences of the ND1 gene in mt genomes of 249 fishes. Figure S1-h. Aligned amino acid sequences of the ND2 gene in mt genomes of 250 fishes. Figure S1-i. Aligned amino acid sequences of the ND3 gene in mt genomes of 250 fishes. Figure S1-j. Aligned amino acid sequences of the ND4L gene in mt genomes of 250 fishes. Figure S1-k. Aligned amino acid sequences of the ND4 gene in mt genomes of 250 fishes. Figure S1-l. Aligned amino acid sequences of the ND5 gene in mt genomes of 250 fishes. Figure S1-m. Aligned amino acid sequences of the ND6 gene in mt genomes of 249 fishes. (ZIP 3250 kb) [file 12864_2016_3054_MOESM6_ESM.zip › Additional file 6 prot align/AF6m-ND6.pdf]

Additional file 6: Figure S1–m. Aligned amino acid sequences of the ND6 gene in mt genomes of 249*[1] fishes.

Species name abbreviation followed by aligned amino acid sequences shown by one letter abbreviation. See Additional file 1 for abbreviation of species name. Amino acids shown by magenta letter denote hydrophobic residues. A-F in bold types with yellow background indicate putative transmembrane regions. Asterisk '*' indicates a fully conserved residue. Colon ':' and period '.' indicate 'strong' and 'weak' groups in the level of conservativeness, respectively, in the Gonnet Pam250 matrix, in which the strong and weak groups are defined as strong score >0.5 and weak score =<0.5, respectively (Thompson et al., 1997). *1 Data of 1 species were not obtained (See text.).

ND6

[1/4 of aligned sequences] **A** **B**

```
Scca   MVYF---VSMMMIGLILGLMGVASNPSPYYAALGLVTAAGVGCGLLVSY-GGSFMS----L
Muma   MMYF---MFVMMIGLILGLMGVASNPSPYYAALGLVTAAGVGCGLLVGH-GGSFMS----L
Erca   -MVM---MVVFSVMFLISLIAVASNPSPYFAALGLMIGAGVGCGMLTQF-GMTFLS----M
Pose   -MVL---MVVFSVIFLVSLIAVASNPSPYFAAFGLMVGAGVGCGMLMQC-GMTFLS----V
Actr   MFYF---TFLVLIGLVLGLVGVASNPAPYFAALGLVVAAAVGCGILVGH-GGSFLS----L
Scal   MFYF---TFLVLIGLVLGLVGVASNPAPYFAALGLVVAAAVGCGILVGH-GGSFLS----L
Posp   MFYF---TFLVLVGLVLGLVGVASNPAPYFAALGLVVAAAVGCGILVGH-GGSFLS----L
Atsp   MVYL---VFLFLFGLVLGLVAVASNPAPFFGALGLVAAAAFGCGVLVGY-GGSFLS----L
Leoc   MVYL---VFLFLLGLVLGLIAVASNPAPFFGAFGLVVAAAFGCGILVVY-GGSFLS----L
Amca   MTYF---VVLFLIGFVLGVVAVASNPAPYFAALGLVFAAAGGCGVLVGH-GGSFLS----L
Osbi   MSYV---MFLLMVFLVLGLVAVASNPAPYYAALGLVLAAAAGCCILVSY-GGSFLS----L
Pabu   MTYV---VMIFMVGFVLGLVAVASNPAPYFAALGLVVAAAVGCGLLIEC-GGSFLS----L
Hial   MTYF---VFLFLIGLVLGLVAVASNPAPYFAALGLVVAAAVGCGVLVGH-GGSFLS----L
Elha   MTYL---IFLFLIGLVLGLVAVASNPAPYFAALGLVVAAAVGCGVLVGH-GGSFLS----L
Mlcy   MAYF---TFLFLVGLVLGFVAVASNPAPYFAALGLVVVAAIGCGMLASH-GGSFLS----L
Algl   MAYP---FCLSLIGLVLGLVAVASNPAPYFGALGLVVAATAGCSILVGH-GGSFLS----L
Ptgi   MTYL---IFLFLIGLVLGLVAVASNPAPYFAALGLVLAAAAGCGVLVGH-GGSFLS----L
Alaf   MTYF---IFLFLVGLVLGLVAVASNPAPYFAALGLVVAAAAGCGVLASH-GGSFLS----L
Nock   VTYF---IFLFLIGLVLGLVAVASNPAPYFAALGLVVAAAAGCGVLASH-GGSFLS----L
Anja   MSYF---LFLFLVMLVLGFLGVASNPAPYFAALGLVLAAAGGCGVLAGY-GGSFIS----L
Gyki   MEYF---SFLFMMLLVLGVLGVASNPAPYFAALGLVLGAGGGCGMLAGC-GGSFVS----L
Syka   MSFF---IFLFLVTMVLGFLGVASNPAPYFAALGLVVAAAGGCGVLVGY-GGSFVS----L
Opma   MTYF---ISLFLVLLVLGVVVVASNPAPYFAALGLVVAAAGGCGVLASY-GGSFIS----L
Comy   MMLF---IVLFLVLMTFGAVCVSCNPSPFYSIFGLVLSTLAGCMMLASY-GGTFVS----L
Sasp   MNLL---NYSLLAVLLLGVLGVSSNPAVYYAALSLVLAAAGGCGIVGIL-GGSFMS----L
Eupe   MNFF---IFFSLMLLVLGVLGVASNPSPYFAALGLVLAAAGGCGVVAGH-GGSFIS----L
Enja   MSYL---FCGWLLLLILGLVGVASNPAPYFAALGLVVVAGAGCAILAGC-GASFLA----L
Same   ----MSFQYIWLAGFVIGVIGIMTNPSPYFAALGLVMCSAATCCILVFG-GSTFLA----V
Cech   MTYL---VSLFLFGLVVGLVAVASNPAPYFAALGLVVAAGVGCGVLIGH-GGLFLS----L
Grgr   ITYL---MCVLLCGVVVGLIAVASNPAPYYAALGLVLASGVGCGVLAGH-GGTFLC----L
Caau   MTYF---MFLLLMALVVGLVAVASNPTPYFAALGLVVAAGVGCGVLVGH-GGSFLS----L
Cyca   MTYF---MFLLLMALVVGLVAVASNPTPYFAALGLVVAAGVGCGVLVGH-GGSFLS----L
Dare   MAFY---LSFLMAALVGGMIAIASNPAPYFAAFGLVVVAGVGCGILVSY-GGSFLS----L
Cost   MTYL---VSLLLMALVVGLVAVASNPAPYFAALGLVVAAGVGCGVLISH-GGSFLS----L
Leec   MTYL---VSLLLMALIMGLVAVASNPAPYFAAFGLVVAAGVGCGVLIGH-GGSFLS----L
Fola   MTYL---VSLLLMALIVGLIAVASNPAPYFAALGLVVAAGVGCGVLVSH-GGSFLS----L
Clmc   MNYI---FCLFLLGLVVGLVAVASNPAPYFAALGLVVAAGVGCGILVGH-GGSFLS----L
Phin   MNYV---FSLLLLGFVVGLVAVASNPAPYFAALGLVVVAGVGCGVLVGH-GGSFLS----L
Icpu   MTYF---LDLFLLSLVVGLVGVASNPAPYFAALGLAVAGGVGCGVLVGH-GGSLVG----L
Psto   MMYF---LDLLLLSLVVGLVGVASNPAPYFAALGLAMAAGVGCVVLVGH-GGSLIG----L
Cora   MEYL---LDLFLLGLVVGLIGVASNPAPYFAALGLVIAAGVGCGILVGH-GGSLLS----L
Eisp   MTYL---VSLFLLGLVVGLVAVASNPAPYFAALGLVVAAGVGCGVLVSH-GGSFLS----L
Apal   MTYT---VFLFLMGLVMGLVGVASNPAPYFSALGLVVAAGGGCGVLVWH-GGSFLS----L
Eslu   MTYL---VSLFLLGLIFGLVAVASNPAPYFAALGLVIAAGAGCGVLVGH-GGSFLS----L
Dape   MTYL---VSLFLLGLVLGLVAVASNPAPYFAALGLIIAAGTGCGLLASQ-GGSFLS----L
```

To be continued on page 6.

[1/4 of aligned sequences]

Glse	MTYF---VSLFLFGLVLGLVAVASNPAPYFAALGLVVAAGVGCGVLVGH-GGSFLS----L
Naar	MTYF---MSLFLFGLVLGLVAVASNPAPYFAALGLVVAAGVGCGVLVGH-GGVFLS----L
Lioc	MTYF---MSLFLFGLVLGLVAVASNPAPYFAALGLVIAAGVGCGVLVGH-GGVFLS----L
Opso	MTYL---VSLLLFGLILGLVAVASNPAPYFAALGLVLAAGAGCGVLVGH-GGAFLS----L
Alte	MTYL---VSLFLLGLVVGLVAVASNPAPYFAAFGLVVAAGVGCGVLVGH-GGAFLS----L
Plap	MTYL---VSLFLLGLILGLVAVASNPAPYFAALGLVVAAGVGCGVLVGH-GGAFLS----L
Plal	MAYL---VSLFLVGLVLGLIAVASNPAPYFAALGLVVAAGAGCGVLVGY-GGSFLS----L
Sami	MAYT---MCLFLIGLVLGLVAVASNPAPYFAALGLVMAAGAGCGVLVGH-GGSFLS----L
Rere	MTYL---VSLFLFALVLGLVAVASNPAPYFAALGLVVAAGVGCGVLVGH-GGSFLS----L
Gama	MTYL---VCLFLLGFVLGFVAVAYNPAPYFAALGLVVAAGVGCGVLVGH-GGSFLS----L
Onmy	MTYL---VSLFLLGLVLGLVAVASNPAPYFAALGLVVAAGVGCGVLVGH-GGSFLS----L
Sasa	MTYI---VSLFLLGLVLGLVAVASNPAPYFAALGLVVAAGVGCGVLVGY-GGSFLS----L
Cola	MTYL---VSLFLLGLVLGLVAVASNPAPYFAALGLVVAAGVGCGVLVGH-GGSFLS----L
Dita	MTYL---VSLFLLCLVLGLVGVASNPAPYFAALGVVVAAGAGCGVLVGH-GGSFLS----L
Gogr	MTYL---VFLFLLVLVVGLVGVASNPTPYFAALGLVVAAGAGCGVLVGC-GGSFLS----L
Chsl	MTYF---MSLFLITFVAGLVGVASSPAPYFAALGLVVAAGAGCGVLVGH-GGSFLS----L
Atja	MTYL---MFLFLFGLIVGLVGVASNPAPYFAALGLVLAAGCGCGILVGF-GGSFLS----L
Iido	MTYL---MFLFLLGLIVGLVGVASNPAPYFAALGLVLAAGCGCGILVGF-GGSFLS----L
Auja	MSYL---MSLFLFGLILGLVGVASNPAPYFAALGLVLAAGLGCGVLVGH-GGSFLS----L
Chag	MTYF---LSLLMLGFVVGVIAVASSPAPFFAALGVVVAAAFSCGMLSAH-GGCFLS----L
Hami	MAYL---MSLFLFGFVIGMVGVASNPAPYFAALGLVLSAGFGCGVMLSC-GGSFLS----L
Saun	MAYL---MSLFLFGFVVGMVGVASNPAPYFAALGLVLSAGFGCGVMLSC-GGSFLS----L
Nema	---M---MSLFMLGLVVGLVAVASNPSPYFAALGLVVVAGLGCGVLIGH-GGSFLS----L
Disp	MTYL---MSLLMLGLVFGLVAVASNPSPYFAALGLVVVAGLGCGILVGH-GGSFLS----L
Myaf	VTYL---MSLLVLGLVFGLVAVASNPSPYFAALGLVVVAGLGCGVLVGY-GGSFLS----L
Lagu	MTYL---MFMMLLGLVLGLVAVASNPSPYFAALGLVGVAGFGCGVLVEF-GGTFLS----L
Trtr	MTYI---MMLLLLGLVLGMVGVASNPSPYFAALGLVAVAGLGCGVLMQY-GGSFLS----L
Zucr	MTYI---MVMLLLGLVLGMVGVASNPSPYFAALGLVVVAGLGCGVLMHY-GGSFLS----L
Pxja	MTYL---MSLFLFGLVLGLVAVASNPSPYFAALGLVGVAGVGCGVLVGY-GGSFLS----L
Pxlo	MTYL---MSLFLFGLVLGLVAVASNPSPYFAALGLVGVAGVGCGVLVGY-GGSFLS----L
Pctr	MSYF---MSFLLVGVVLGLVAVASNPSPYYAALGLVAVAVLGCGVVSGY-GGSFLC----L
Apsa	MSYF---MSLMLIGVVIGLIAVASNPSPYYAALGLVGVAIFGCGVLSGY-GGSFLC----L
Cabe	MLLA---MKVGICWMIVGLVGVASNPSPYFGALGLVMAASGGCVTLACY-GGPFLS----L
Bzze	MTYV---MSLFLFGLVLGLVAVASNPSPYFAALGLVVVAGMGCGVLVGH-GGPFLS----L
Siim	MKFM---MWVFLYGLVIGMAGVASNPSPYFAAFGLVVVAGMSSGLLVSY-GGSFFS----L
Ctru	MTYI---MSLFLLGMVLGLVGVASNPSPYFAALGLVVVAGMGCGVLIGF-GGPFLS----L
Dpbr	MTYI---MSLFLMGLVLGLVGVASNPSPYFAALGLVVVAGMGCGVLVGF-GGPFLS----L
Caki	MFYM---MMMMMVGFVLGAAALASNPSPVYAVLGLVLMAGMACMILINS-GGPFLS----L
Phja	----MMYLLVVLAVMNLGVIFVASNPTPNFASLGLVFAAGLGCVVVLGS-GGSFLA----L
Brsp	MSYV---LILILIGMILGVVSVVSSPSPYFAALGLVVMAAAGCSVLVVS-GGSFLS----L
Gamo	MAYI---MLTLLIGMVLGVISVASNPSPYFAALGLVLVAGVGCVVLMGH-GGSFLS----L
Lolo	MAYI---MLTLLIGMVLGVISVASNPSPYFAALGLVLVAGVGCGVLMGH-GGSFLS----L
Batr	MMIK---DMILSVGLIFGLVVVVANPFPFYGALGLVLASGLGCLVLLGL-EGSFLS----L
Prmy	MVYM---TVLVLGALFVGAITVASNPSPYFGALGLVVVAGMGCILLMEA-GESFLS----L
Lose	MTFV---VPLFLVSLIMGMVVVASNRSPYFAALGLVVVAGSSCGILVGC-GGSFLP----L
Loam	MTYV---VYLFLLGLVVGMVVVASNPSPYFAALGLVIVAGLGCGILVGH-GGSFLS----L
Chab	MTYL---MCLLLIGLVLGLVAVASNPSPYFAALGLVVVAGLGCGVLVGH-GGCFLS----L
Chto	MTYL---MCLLLIGLVLGLVAVASNPSPYFAALGLVVVAGLGCGVLVGH-GGCFLS----L
Majo	MSYL---MFLFLFGLVVGLVVVASNPSPYFAAFGLVAVAGAGCGVLTGV-GAGFLS----L
Hlst	MSYY---MSFLLFGLMGGLMVVSSNPSPYFAALGLVVVAGFGCGLLTGT-GAVFLS----L
Clpe	MSYF---MFMLLLGLVGGLVAVASNPSPYFAALGLVVVAGVGCGILLGF-GGSFLS----L
Mlmr	MTYF---MSLLLFGLVLGLVAVASNPSPYFAALGLVVVAGLGCGVLVGY-GASFLS----L

To be continued on page 7.

[1/4 of aligned sequences]

```
Crcr  MTFM---LVVFLVGLVLGMAAVASNPSPYFAALGLVAVAGMGCGILLGH-GGSFLS----L
Muce  MTFT---LVVFLVGLVLGMAAVASNPSPYFAALGLVVVAGMGCGILLGH-GGSFLS----L
Bege  MAYI---MYLLLFGLVLGLVAVASNPSPYFGALGLVMVAGMGCGVLVGH-GGSFLS----L
Mela  MVFF---MFSLLFGLVLGLAAVASNPSPYFAALGLVVVAGTGFGVLVGH-GGSFLS----L
Hats  MAYI---MYLLLFGLVVGLAAVASNPSPYFAALGLVVVAGMGCGVLVGH-GGSFLS----L
Orla  MVYM---TYLLLIGLVLGLAAVASNPSPYFAALGLVTVAGMSCGILVGY-GGSFLS----L
Cosa  MAYV---MYFLLFGLVVGLAAVASNPSPYFAALGLVVVSGVGCGVLVSY-GGSFLS----L
Exsp  ML---ALWYTCLLSMILGMASVASNPIPHIAALFLVLVAGVGCGSLAIH-GGTYFA----L
Depa  MVYI---MYLFLFGLILGLSAVASNPSPYFAALGLVVVAGVGCGVLVEH-GGSFLS----L
Rima  MKYL---LFLLLFLLILGVVGVASNPSPGFAALSLVMVAGSGCSLLAGQ-GGYFLA----L
Fuol  MIYV---MYFLLFSLVLGLAAVASNPSPYFAALGLVVVAGVGCGVLIGH-GGGFLS----L
Gmaf  MSYL---MCVLLLGLVLGLVAVASNPSPYFAAMGLVVVAGVGCGVLVGT-GGCFLS----L
Xeei  MVYM---MYFLLFGLVLGLAGVACNPSPYFAALGLVMVAGVGCGILLGH-GGSFLS----L
Pros  MNFI---LSLLLFGLVLGVIAVASNPSPYFAALGLVLVSGIGCGILFGM-GGPFLS----L
Scmi  -------MSLFLLGLVLGLIAVASNPSPYFAALGLVVVAGLGCGLLVGH-GGSFLS----L
Rolo  MTYF---MSLFLFGLVLGLVAVASNPSPYFAALGLVVVAGLGCGVLVGH-GGPFLS----L
Cere  -------MSLLLFGFVLGLIAVASNPSPYFAALGLVVVAGLGCGLLIGY-GGPFLS----L
Daga  MTYL---MSFLLFGFVVGLIAVASNPSPFYAALGLVVVAGLGCALLVGH-GGPFLS----L
Anco  MTYV---MSLFLFGLVMGLVAVASNPSPYFAALGLVVVAGLGCGVLVGH-GGSFLS----L
Dmve  MPIL---ETFFVSGILFGMLMVVCTSSTYYASLGLIFVCVLGSGAVCCS-GGVFLC----L
Dmar  MPML---EIFFVSGVLFGMLMVVCTSSTYYASLGLIFVCVLGSGAVCCS-GGVFLC----L
Anka  MTYV---MSLFLFGLVLGLVAVASNPSPYFAALGLVVVAGLGCGVLVGH-GGSFLS----L
Moja  MTYV---MSLFLFGLVLGLVAVASNPSPYFAALGLVVVAGLGCGVLVGH-GGSFLS----L
Hoja  MTYV---MSLFLFGLVLGLVAVASNPSPYFAALGLVVVAGLGCGVLVGH-GGSFLS----L
Bede  MTYF---MSLFLFGLVLGLIAVASNPSPYFAALGLVVVAGLGCGVLVGH-GGPFLS----L
Besp  MTYF---MSLFLFGLVLGLIAVASNPSPYFAALGLVVVAGLGCGVLVGH-GGPFLS----L
Mysp  -------MSLFLFGLVLGLVAVASNPSPYFAALGLVAVAGLGCGVLVGY-GGPFLS----L
Osja  MTYI---MSLFLFGLVLGLVAVASNPSPYFAALGLVGVAGLGCGVLVGY-GGPFLS----L
Sgro  MTYF---MSLFLFGLILGLVAVASNPSPYFAALGLVGVAGLGCGVLVGY-GGPFLS----L
Pzpa  MTYI---TLVLLLGFVVGLMGVASNPSPYYAALGLVVVAGAGCGMLMMY-GGGFLS----L
Zeja  MTYI---MYVLLLGLVLGLVGVASNPSPYYAALGLVMVAGAGCGVLVVC-GGGFLS----L
Znne  -------MSVLLLGFVLGLVGVASNPSPYYAALGLVLVSGAGCGVLVAY-GGGFLS----L
Zefa  MTYA---MSVLLLGFVLGLVGVASNPSPYYAALGLVLVAGAGCGVLVAC-GGGFLS----L
Acni  VTYL---MSVLLLGLVLGLVGVASNPSPYYAALGLVLVAGAGCGVLVVC-GGGFLS----L
Ncrh  MTYL---MSVLLLGLVLGLVGVASNPSPYYAALGLVLVAGAGCGILVVC-GGGFLS----L
Agca  MTYI---MSLLLCGLIFGLIAVASNPSPYFAALGLVIVAGLGCGVLVGH-GGSFLS----L
Hydy  MTFV---MCLFLLGLVLGLVAVASNPSPYFAALGLVAVAGMGCGVLVSH-GGPFLS----L
Gsac  MTYV---MSLFLLGLVLGLVAVSSNPSPYFAALGLVMVAGMGCGVLVSH-GGPFLS----L
Pevo  MTYI---MFVLLLALVLGLIAVASNPSPYFAALGLVVVAGAGCGILVGS-GGSFLS----L
Hiku  MTYL---MFLLLMGLIFGLIAVASNPSPYFAALGLVVVAGMGCGVMVSF-GGPFLS----L
Inpa  MVYV---TGLFMIGFVAGLVAVASNPSPYYGAFGLVVAGGMGCGILVSH-GAAFLA----L
Auch  MSYT---LHMLLFGLVAGMVAVASNPSPYFAALGLVVVAGAGSGVLAGH-GGSFLS----L
Fico  MTYF---MCLFLLGLVLGLVGVASNPSPYFAALGLVVVAGMGCGVLVAY-GGAFLS----L
Macs  MTYV---MSLFLFGLVLGLIAVASNPSPYFAALGLVIVAGVGCGILVWH-GGPFLS----L
Moal  MSYL---VCVLLFGLMVGSVGVASNPSPYFAAMGLVMVSGVGCGVLICF-GGSFLP----L
Syma  MAYV---MFLFLFGLMLGLVAIASNPSPYFAALGLVVVSGMACGVLVSH-GGPFLS----L
Mafr  MAYI---MYLFLFGLVVGLAAVASNPSPYFAALGLVMVAGVGCGVLVGF-GGSFLS----L
Dcpe  MTYL---MLLFLFGLVLGLIAVASNPSPYFAALGLVIVAGMGCGVLVGH-GGAFLS----L
Dcti  MTYI---MSLFLFGLVLGLIAVASNPSPYFAALGLVIVAGMGCGVLVGH-GGAFLS----L
Hehi  MTYI---MSLFLIGLVLGLVAVASNPSPYFAALGLVVVAGMGCGVLVGY-GGPFLS----L
Stam  MTYI---MSLFLLGLVLGLVAVASNPSPYFAALGLVVVAGMGCGVLVGH-GGPFLS----L
Hogi  MTFI---MSLFLIGLVLGLVAVASNPSPYFAALGLVGVAGMGCGVLLGH-GGPFLC----M
```

To be continue
on page 8.

[1/4 of aligned sequences]

Erzo	MTYI---MSLFLLGLVLGLVAVASNPSPYFAALGLVVVAGMGCGVLIGH-GGPFLS----L
Hxot	MTYI---MSLFLLGLVLGLVAVASNPSPYFAALGLVVVAGMGCGVLVGH-GGPFLS----L
Core	MTYV---MCLFLLGLVLGLVAVASNPSPYFAALGLVVVAGMGCGVLVGH-GGPFLS----L
Apve	MTYV---MFLSLLSLVLGLVAVASNPSPYFAALGLVVVAGVGCGVLAVH-GGPFLC----L
Latj	MSFI---MYLILFCFVLGLVAVASNPSPYFAALGLVVVAGMGCGVLVGH-GGPFLS----L
Laja	MTFI---MFLFLFGLVLGLIAVASNPSPYFAALGLVMVAGMGCGVLVGH-GGSFLS----L
Syja	MIYV---MMFFLVGLVLGLAAVASNPSPYFAALGLVVVAGMGCGVLVGY-GGSFLS----L
Epme	MCFT---ILCFLICWIVGAIAVASNPSPFFGALGLVAVAGVGCGVLVEF-GSPFLA----L
Grse	MSYL---MLLFLLGLVLGLVAVASNPSPYFAALGLVVVAGMGCGVLTSH-GGSFLS----L
Clja	--------------MPLLNLTLRRTFSRLYHALALFCSHWACCHIISSR-WPHITNWKRYI
Ogcy	MAYM---MSMLLFGLVFGLAAVASNPSPYFGALGLVVVSGLGCGVLVGY-GGSFLS----L
Plna	MIYL---MSVFLIGLVIGLAGVASNPSPYFAALGLVVAAGCGCGVLVGH-GGPFLS----L
Lema	MTYM---MSLFLVGLVLGLVAVASNPSPYFAALGLVVVAGMGCGVLIGH-GGPFLS----L
Etzo	MTYI---MCLFLLGLVLGLVAVASNPSPYFAALGLVVVAGMGCGVLINY-GGPFLS----L
Apse	MLTS---VLLFLFGWVVGSIVVASNPSPYFAALGLVFVAGMGGSVLLLH-GASYLC----F
Epde	MTYI---MALFLFGLVLGLVAVASNPSPYFAALGLVVVAGMGCGVLVGH-GGPFLS----L
Slja	MVYI---LYLLLIVMIFGLAAVASNPSPYFAAFGLVLVAAVGCGVLAGY-GGSFLS----L
Bsja	MTLT---MSMLLFGLVLGLVAVACNPSPYFAALGLVGVAGLGCGVLVCH-GGSFLS----L
Ecna	MTFI---MYLLLFLLILGLVAVASNPSPYFAALGLVLVAGMGCGVLVSH-GGPFLS----L
Cohi	MTFV---VFSMMVVFMTGMAVVALTPSPYFAALGLVMVSGMGCGILLWY-GAPFLS----L
Caar	MTFI---MYLVLFSFVLGLVAVASNPSPYFAALGLVVVAGMGCGVLIGH-GGPFLS----L
Came	MTFI---MYLFLFCFVFGLIAVASNPSPYFAALGLVVVAGMGCGVLVGH-GGPFLS----L
Mema	MTFI---MCFVLCSLVLGLVAVASNPSPYFAALGLVVVAGMGCGVLVGH-GGPFLS----L
Lenu	MSYI---VSFFLLGLVLALVAVASNPSPYFGALGLVVAAGLGCCILAGH-GGSFLS----L
Brja	MTYM---MCLLLFGLVSGLVAVASNPSPYFAALGLVVVAGMGCGVLVGH-GGSFLS----L
Plma	MTYL---MCLFLVGLVLGLVAVASNPSPYFAALGLVVVAGMGCGVLVGH-GGSFLS----L
Emst	MTYI---MSLFLFGLVLGLVAVASNPSPYFAALGLVVVAGLGCGVLVGH-GGSFLS----L
Ptti	MTYI---MSLFLFGLVLGLVAVASNPSPYFAALGLVVVAGLGCGVLVGH-GGSFLS----L
Losu	-MLV---MNLLLISLVSALAAVASHPSPHFGALGLVVAAAVGCWVLVLN-GGLFLS----L
Geoy	MSLL---AFVFLMGALVGLLAVAANPSPFFAALGLVAAAGMGCGVLVLY-GGSFLS----L
Dipi	MTYI---MSLFLFGLVLGLVAVASNPSPYFAALGLVVVAGLGCGVLVGH-GGSFLS----L
Pama	MSFF---MSLSLSGLVLGLVVVASNPSPYFAALGLVAAAGVGCGILVWH-GGSFLC----F
Leob	MTVI---MNILLLGLLLGLIAVASNPSPYFAALGLVVVSGFGCGVLLGH-GGCFLS----L
Neba	MAWV---LFLLLLGLIFGLIVVACNPSPYFAALGLVVVSAVGCGILVGH-GGVFLP----L
Pdpl	MTFI---MNLFLFCFVFGLIAVASNPSPYFAALGLVVVSGVGCGLLLGY-SGPFLS----L
Nimi	MTYI---MCLLLFGLVVGVVAVASNPSPYFAALGLVVVAGLGCGVLVWH-GGHYLS----L
Uptr	MVYT---MSLFLFCLVLGLVAVASNPSPYFGALGLVVVAGMGCGILMGH-GGPFLS----L
Pesc	MTYM---TYLLMGGLVLGSIGVASNPSPYFAAFGLVLVSGMGCGVLAEQ-GGSFLS----L
Baar	MTFI---MSLFLFGLVLGLVAVASNPSPYFAALGLVVVAGMGCGALVSH-GAPFLS----L
Moar	MTYI---MSLFLLGLVLGLVAVASNPSPYFAALGLVVVAGLGCGVLVGH-GGSFLS----L
Toja	MTYA---MYLILFLFVLGLVAVASNPSPYFAALGLVVVAVMGCWVLVGH-GGSFLS----L
Chau	MSYI---VVFFLFGLVFGLVGVAANPSPFFAAFGLVVVAIFGCGALAQE-GGVFLS----L
Chse	MTYI---MSLFSVGLVLGVVAVASNPSPYFAALGLVVVAGFGCGVLAGH-GGSFLS----L
Enar	MTYI---MYLFLLGLVLGLAAVASNPSPYFAALGLVVVAGMGCGVLVGH-GGPFLS----L
Hpty	MTYI---MSLFLFGLVLGLVAVASNPSPYFAALGLVVVAGMGCGALVGH-GGPFLS----L
Nana	MTYF---MFMLLVGLVLGLIAVASNPSPYFAALGLVVVAGVGCGVLVGH-GGPFLS----L
Mcst	-------MFLFLFGFILGLVAVASNPSPYFAALGLVVVAGMGCGVLMGH-GGPFLS----L
Rhox	MTLL---MYLSVFSWILALIAVASNPSPYFAALGLVVVAGSGCGVLIAH-GGPFLS----L
Opfa	MTFV---MFLFLFGLILGLVAVASNPSPYFAALGLVVVAGMGCGVLVGH-GGPFLS----L
Paar	MTFI---MFLFLLGLVLGLVGVASNPSPYFAALGLVAAAGMGCGVLVGH-GGPFLS----L
Gozo	MTYI---MSLLLLGLVLGLVAVASNPSPYFAALGLVVVAGMGCGVLVGH-GGPFLS----L
Ackr	MTYI---MTISCWGLVGGMIGVASNPSPHFAALGLVVAAGFGCGVLAGS-GAVFLS----L

To be continued on page 9.

[1/4 of aligned sequences]

```
Elev    MMYV---MFLLMVGLVVGLVGVASNPSPYFAALGLVLVAGVGCGLLAGH-GGAFLS----L
Trdu    MVYI---MFMFLLGLVLGLAAVASNPSPYFAALGLVAVAGMGCGVLVGH-GGSFLS----L
Amoc    MVYI---FFLFLFCLVLGLAAVASNPSPYFAALGLVMVAGAGCGVLVGH-GGSFLS----L
Hame    MDLTTIFFLIGLGGLVVGLVAVASNPSPYFGALGLVGVAGLGCVILTSH-GGSFLA----V
Chso    MSFL---VVLFTVGLVLGLIGVASNPSPYFAALALVVVAWAGCGLLTSY-GGSFLA----L
Lyto    MTYI---MSLFLLGLVLGLVAVASNPSPYFAALGLVVVAGMGCGVLVGH-GGPFLS----L
Encr    MTYI---MSLFLLGLVLGLVAVASNPSPYFAALGLVVVAGMGCGVLVGH-GGPFLS----L
Bvar    MTYF---VSFFLLGLVLGLVAVASNPSPYFAALGLVVVAGMGCGVITGH-GGSFLS----L
Noco    MLYC---GYLLMFGVVVGMAAVAANPSPFYAALGLVMVAAMGCGFIMCL-GGTFSC----L
Chsp    MAVF---TYLLLFGFVFGVVLTVSNPAPHLAAFGLVFSACVGCVVLMYY-GSSFLC----L
Arja    MTYV---MFLFLLGLVLGLVAVASNPSPYFAALGLVVVAGMGCGMLLYH-GGLFLS----L
Pase    MSFM---AFVFMGGLTIGMVGVASNPSPYYGALGLVLVAGMGCGGLVVS-GGSFLS----L
Trel    MDLF---VMIFLLGLVLGAAAVASNPSPYFGALGLMVAAGMGGGLLMVV-GGSFLS----L
Acur    MSYS---MIFLLVGVLFGVVAIASNPAPYYGAFGLVVMAGMGCGLILSF-GGPLLA----L
Ampe    MTYI---MSLFLFGLVLGLVAVASNPSPYFAALGLVVVAGMGCGVLVGH-GGPFLS----L
Urja    MTYI---ITLFLLGLVLGLIAVASNPSPYYGALGLVAAAGMGCGVLLGY-GGCFMS----L
Enet    MAYV---MVMFLFGLVLGMGAVASNPSPYFAALGLVVVAGMGCGVLAGH-GGPFLS----L
Ptbr    -VYYT--MYILFVGVLVGAASVAANPSPYYAAFGLVVMAVSGCGVVGLL-GGTLLS----L
Safa    MSYL---IILFLFGLIVGLAAVGSNPSPFFAALGLVMVAGIGCGILVGW-GGSFLS----L
Icae    MTYM---MCLFLFGLVLGLVAVASNPSPYFAALGLVVVAGMGCGVLVGY-GGSFLS----L
Asmi    MNYI---FLVPIGALVIGLVGVASVPSPYFGALGLVLAAGGSCGVLLSQ-GGTFLS----L
Foal    MSYI---SSSCLLVVVLGLVLVACNPSPYFAALGLVLVAGAGCGVLMIE-GGSFLA----L
Drze    MTYL---MCSFLIGLVFGLIAVASNPAPYFAALGLVLVAAAGCGILVIY-GGAFLS----L
Rhas    MTYI---MCVFMFGLIMGLVAVASNPSPYFAALGLVVVSGMGCGVLVGH-GAPFLS----L
Elac    MTYV---MCIFMVGLVMGLVVVASNPSPYFGALGLVVVSGMGCGMLVGH-GAPFLS----L
Kugu    MTYL---MCMFLLGLVLGLVVVASNPSPYFAALGLVVVAGMGCGVLVGH-GGPFLS----L
Plor    MTYF---MFLFLFGLVLGLVAVASNPSPYFAALGLVVVAGLGCGVLVGH-GGSFLS----L
Sgun    MTYV---MSLFLFGLVLGLVAVASNPSPYFAALGLVVVAGLGCGVLVGH-GGSFLS----L
Zaco    MTYI---MCLFLLGLVLGLVAVASNPSPYFAALGLVVVAGLGCGVLVGH-GGSFLS----L
Zbfl    MTFS---MSLFLCGLILGLVAVASNPSPYFAALGLVTVAGLGCGVLAGH-GGSFLS----L
Spba    MTFV---LYLILFCFMMGLVAVASNPSPYFGALGLVVVAGMGCGILVGH-GGPFLS----L
Game    MTYM---MCLLLFGLVLGLVAVASNPSPYFAALGLVVVAGLGCGVLVGH-GGSFLS----L
Thth    MTYM---MCLLLFGLVLGLVAVASNPSPYFAALGLVVVAGMGCGVLVGH-GGSFLS----L
Xigl    MTFI---MYLLLFSFVMGLVAVASNPSPYFAALGLVVVAGVGCGVLVGH-GGPFLS----L
Hyja    MTYM---MCLFLFGLVLGLVAVASNPSPYFAALGLVVVAGMGCGVLVGY-GGSFLS----L
Psan    MTFM---MCLFLFGLVMGTTAVGSSPSPYYGGLGLVVAAGMGCGILVCC-GGSFLS----L
Cupa    MTYM---MCLLLFGLVLGLVAVASNPSPYFAALGLVVVSGMGCGVLIGH-GGSFLS----L
Mpch    MTYV---MFLLLFGLVLGLISVASNPSPYFAALGLVMVAGMGCGILVYY-GGPFLS----L
Char    MTYI---VFLFLVGLVLGLIAVASNPSPYFAALGLVVVAGMGCGVLVGH-GGSFLS----L
Pser    MAIF---MCLILFLLVLGLVAVASNPSPYFAALGLVLVAGVGCAVLVGH-GGPFLS----L
Prol    MTFV---MCLVLFCLVLGLVAVASNPSPYFAALGLVVVAGMGCGVLIGH-GGPFLS----L
Plbi    MTFV---MYLVLFSLVLGLVAVASNPSPYFAALGLVVVAGMGCGVLVGH-GGPFLS----L
Calu    VTFI---MWLVLFCFVVALVAVASNPSPYFAALGLVAAASVGCALLTGY-GGSFLS----L
Papa    MKFF---MCLILFLFILGLVGVASNPSPFFAALGLVMVAGAGCVMLVGH-GGSFLS----L
Sufr    MVYI---MSLFTLGLVLGLLAVASNPSPYFAAFGLVIVSGVGCGVIAGC-GGPLLS----L
Stci    MTYV---MSLLLFGLVLGLLAVASNPSPYFAALGLVVVAGLGCGVLLGH-GGSFLP----L
Taru    MFYA---LVFLLLGMLVVMIVLSTNPAPFYGVFNLVLVALLCCGASVLH-GGTFLS----L
Rala    MTYI---MFLFLFGLVLGLVAVASNPSPYFAALGLVVVAGLGCGVLVGH-GGSFLS----L
                                         : :                    .
```

To be continued on page 10.

[2/4 of aligned sequences]

```
                C                                  D
Scca  IL-FLIYLG-GMLVVFAYTAALAAEPYPEAWGDWSVLMYVGIYLTG-LVIA-GKYFL---
Muma  IL-FLIYLG-GMLVVFAYTAALAAEPYPESWGDWSVLLYVGLYLVG-LFVS-GKYFM---
Erca  IL-FLIYLG-GMLVVFAYSAALAAEPYPSAWGSLEVFSYVVGYLML-VVVM-WMLLV---
Pose  IL-FLIYLG-GMLVVFAYSAALAAEPYPPAWGSWEVFSYVLGYMVL-VIMV-WAVFV---
Actr  VL-FLIYLG-GMLVVFAYSAALAAEPYPETWGDWSVLFYVVVYITG-VLVV-GGLVG---
Scal  VL-FLIYLG-GMLVVFAYSAALAAEPYPETWGDWSVLLYVVAYIAG-VLIL-GGLVG---
Posp  IL-FLIYLG-GMLVVFAYSAALAAEPYPETWGDWSVLFYVVVYVVG-VLIV-GTLVS---
Atsp  IL-FLIYLG-GMMVVFAYSAALAAEPYPETWGDWSVLVCVVISAVG-VLGA-GYWFV---
Leoc  IL-FLIYLG-GMMVVFAYSAALAAEPYPETWGAWSVLVYVIVSVIG-VLGA-GYWFV---
Amca  VL-FLIYLG-GMLVVFAYTAALAAEPYPEAWGDWSVLGYVLLYMVG-LLVI-GGWLS---
Osbi  VL-FMIYLG-GMLVVFAYSAALAAEPYPESWFDLSVFSYVVVYILG-LMVV-GLCFF---
Pabu  VL-FLIYLG-GMMVVFAYSAALAAEPYPEAWGDWSVLGYVVVYVVG-VVLV-GWYFW---
Hial  IL-FLIYLG-GMLVVFAYSAALAAEPYPESWGDWSVLVYVVMYLLG-VMVV-GGWLS---
Elha  IL-FLIYLG-GMLVVFAYSAALAAEPYPESWGDWSVFSYVVIYVLG-VALV-GGWLS---
Mlcy  IL-FLIYLG-GMLVVFVYSAALAAEPYPESWGDWSVLGYVVVYLFS-VGLV-GGWLS---
Algl  VL-LVIYLG-GMMVVFAYSAALAAEPYPETWGDWSVLVYVVIYTIA-VLIG-ACFFM---
Ptgi  VL-FLIYLG-GMLVVFAYSAALAAEPYPESWGDWSVLGYVVIYTLG-VVMA-AGVLS---
Alaf  IL-FLIYLG-GMLVVFAYSAALAAEPYPEAWGSWSVLGYVVFYISV-VVLG-VFALS---
Nock  IL-FLIYLG-GMLVVFAYSAALAAEPYPEAWGSWSVLGYVLFYASV-IALG-VCYSY---
Anja  VL-FLIYLG-GMLVVFAYSAALAAEPYPESWGDWSVLGYVVGYVFL-CVLG-IGMFY---
Gyki  VL-LLIYLG-GMLVVFAYSAALAAEPYPESWGDWSVAGYVVGYSLL-CVVA-GIMCI---
Syka  VL-FLIYLG-GMLVVFAYSAALAAEPYPESWGDWSVLGYVVGYILL-SVLG-VVTFC---
Opma  VL-FLIYLG-GMLVVFAYSAALAADPYPEGLGDWSVMMRAAGCFSI-IFVG-VLMGG---
Comy  IL-FLIYLG-GMLVAFVFCVAVASEAYLEGLESASVLVRFVLCLLI-SFIF-C-MIS---
Sasp  VL-LLIYLG-GMLVVFVYSVALTADPHPESWVGWDVAGRVGGYIFV-SILG-VAVFC---
Eupe  VL-FLIYLG-GMLVVFAYSVALAAEPFPEAWGGWSVMGRMMTYTFL-CGVF-FLMFY---
Enja  VL-FLIYLG-GMLVVFAYSAALAAEPHPEGWWEASVLEYVVCYLVA-VVVAAGY-FS---
Same  VL-FLIYLG-GMLVVFAFTAALAADPFPEGTLEAAPYEFLILVVLG-ISLLVAQ-SW---
Cech  VL-FLIYLG-GMLVVFAYSAALAAEPFPESWVDRSVIWYVLVYLIG-VVAV-AGLFW---
Grgr  VL-FLIYLG-GMLVVFAYSAALAAEPFPETWGDKSVMSLVVVYSSG-VLAG-GVWSW---
Caau  VL-FLIYLG-GMLVVFAYSAALAAEPYPEAWGSRSVLGYVLVYLLG-VGLV-AGFFW---
Cyca  VL-FLIYLG-GMLVVFAYSAALAAEPFPEAWGSRSVLGYVLVYLLG-VGLV-AGVFW---
Dare  IL-FLIYLG-GMLVVFAYSAALAAEPFPEAWGDRVVFWRVMVYGLV-VIVA-AGFLL---
Cost  VL-FLIYLG-GMLVVFAYSAALAAEPFPESWGDRSVLGYVLVYLIG-VGLL-VNFFC---
Leec  VL-FLIYLG-GMLVVFAYSAALAAEPFPEAWGDRSVMGYVLIYVLG-VGLM-VVLFW---
Fola  VL-FLIYLG-GMLVVFAYSAALAAEPFPEAWGDRSVVGYVMIYLLG-VGLM-VGVFW---
Clmc  VL-FLIYLG-GMLVVFAYSAALAAEPFPESWGDRSVIGYVLAYLLV-VGVA-GWWAQ---
Phin  VL-FLIYLG-GMLVVFAYSAALAAEPFPESWGDRSVAGYVLMYVLV-VVFA-GVWVL---
Icpu  ML-FLIYLG-GMLVVFAYSAALAAEPFPETWGAGSVKVYVLMYLFG-VGVA-VWWFW---
Psto  ML-FLIYLG-GMLVVFAYSAALASEPFPETWGAGSVKTYVLMYLFV-VGVA-GWWFW---
Cora  LL-FLVYLG-GMLVVFAYSAALAAEPFPETWGVRSVKIYVIVYMLS-VGVA-VWWYC---
Eisp  VL-FLIYLG-GMLVVFAYSAALAAEPFPESWGDRSVMGYVLVYLLG-LGFV-GGWLC---
Apal  VL-FLIYLG-GMLVVFAYSAALAAEPYPESWGDRSVVGYVLLYVVG-VVLV-GWWFY---
Eslu  VL-FLIYLG-GMLVVFAYSAALAAEPFPETWGDRSVLVYVLFYILG-VMAV-VGWFW---
Dape  VL-FLIYLG-GMLVVFAYSAALAAEPFPETWGNWSVLSYILIYFLG-VVGL-VVWFW---
Glse  VL-FLIYLG-GMLVVFAYSAALAAEPFPESWGESSVLGYVVGYLGV-VGFT-GVWLC---
Naar  VL-FLIYLG-GMLVVFAYSAALAAEPFPESWGDTSVLGYVIGYLGL-VGLA-GGWFW---
Lioc  VL-FLIYLG-GMLVVFAYSAALAAEPFPESWGDSSVLGYVAGYLVV-LGLV-AGWAW---
Opso  VL-FLIYLG-GMLVVFAYSAALAAEPFPETWGDSSVVGYVGGYLGV-VGLG-VAWLG---
Alte  VL-FLIYLG-GMLVVFAYSAALAAEPFPETWGDRSVIGYVVAYLVG-VVLA-ASWFW---
Plap  VL-FLIYLG-GMLVVFAYSAALAAEPFPETWGDRSVAGYVVAYLVG-VVLA-AGWFW---
```

To be continued on page 11.

[2/4 of aligned sequences]

```
Plal  VL-FLIYLG-GMLVVFAYSAALAAEPYPETWGDRSVLGYVVGYSIV-VAVT-AGWLS---
Sami  IL-FLIYLG-GMLVVFAYSAALAAEPYPETWGDRSVLGYVAGYFLA-VSAM-AGWLS---
Rere  VL-FLIYLG-GMLVVFAYSAALAAEPFPETWGDRSVLGYVVAYVVV-VAVT-AGWFS---
Gama  VL-FLIYLG-GMLVVFAYSAALAAEPFPEAWGDRSVLGYVGAYLAG-AAGF-AGVFW---
Onmy  VL-FLIYLG-GMLVVFAYSAALAAEPFPESWGDRSVLGYVVVYTVG-VVLV-AGLFW---
Sasa  VL-FLIYLG-GMLVVFAYSAALAAEPFPESWGDRSVLGYVVVYTVG-VMLV-AGWFW---
Cola  VL-FLIYLG-GMLVVFAYSAALAAEPFPESWGDRSVLGYVVAYVVG-VALV-AGWFW---
Dita  VL-FLIYLG-GMLVVFAYSAALAAEPYPESWGDRSVLGYVGGYLAG-VGAM-GGWFW---
Gogr  VL-LLVYLG-GMLVVFAYSAALAAEPHPESWGDWSVVGYVGGYMTG-VVAG-GGWLW---
Chsl  VL-FLIYLG-GMLVVFAYSAALAAEPYPESWVDRSVLGHALVYLLG-VTVS-VWYLG---
Atja  VL-FLIYLG-GMLVVFAYSAALAAEPFPETWGDRSVLGYVAIYLLG-VLFF-STWFW---
Iido  VL-FLIYLG-GMLVVFAYSAALAAEPFPETWGDRSVLGYVAIYLMG-LLFF-SVWFW---
Auja  VL-FLIYLG-GMLVVFAYSAALAAEPFPVSWGDRSVMGYVLMYLTG-VVLI-GGLVG---
Chag  VL-FLIYLG-GMLVVVAYSAALTAEPFPATWGDRSVMRYVVFYTLG-VLIS-MMWFW---
Hami  VL-FLVYLG-GMLVVFAYSAALAAEPYPTSWGDRSVVGYVVVYLSG-VVLV-GVSFM---
Saun  VL-FLVYLG-GMLVVFAYSAALAAEPYPTSWGDRSVAGYVVVYLGG-VVMA-GFGLL---
Nema  VL-FLIYLG-GMLVVFAYSAALAAEPFPESWGDRSVMVYVVGYLLV-VALM-GSWFW---
Disp  VL-FLIYLG-GMLVVFAYSAALAAEPFPESWGDRSVVVYVVGYLLV-VGLV-AGWFW---
Myaf  VL-FLIYLG-GMLVVFAYSAALAAEPFPEAWGDRSVVVYVFGYLAA-VGAV-GGWFW---
Lagu  VL-FLIYLG-GMLVVFAYSAALAAELHPEAWGGWSVMGLGLTYMAG-VVGG-YLYLG---
Trtr  VL-FLIYLG-GMLVVFAYSAALAAEPYPEAWGGQAVLGYVLLYLGG-VLVA-GLYLW---
Zucr  VL-FLIYLG-GMLVVFAYSAALAAEPYPEAWGGWAVLGYVLLYLGG-VLVA-GLYCW---
Pxja  VL-FLIYLG-GMLVVFAYSAALAAEPYPESWGDRSVLGYVAGYLVV-VGIF-CGWFW---
Pxlo  VL-FLIYLG-GMLVVFAYSAALAAEPYPESWGDRSVLGYVAGYLVV-VGVF-CGWFW---
Pctr  VF-FFIYLG-GMLVVFAYSAALAAEPFPISWGDSSVFGSVLAYLLL-VGAL-FVGLS---
Apsa  VF-FFIYLG-GMLVVFAYSAALAAEPFPVAWGDQSVIMSVLGYLVF-ISML-LWYFS---
Cabe  VL-FLIYLG-GMLVVFAYSAALAAEPYPEAWGARPVAMSAVVYVVV-LAVS-LALSM---
Bzze  VL-FLIYLG-GMLVVFAYSAALAAEPYPESWGSRSVMVYVGGYLLG-LGLV-SALFW---
Siim  VL-FLIYLG-GMLVVFAYCAALAAEPYPESWGSRSVLGYVMAYLGG-VLLA-VKVWG---
Ctru  VL-FLIYLG-GMLVVFAYSAALAAEPYPESWGSRSVMSYVGVYLLG-VLLV-SGPFW---
Dpbr  VL-FLIYLG-GMLVVFAYSAALAAEPYPESWGSRPVMFYVGVYLLG-VLLV-SGPFW---
Caki  LF-FLVYLG-GMLVVFAHCVALAAERYPKAWGDVIFLGALVGVVVG-YSVA-VIVGW---
Phja  VL-FLIYLG-GMLVVFAYCVALAAEEYPEGLGAWSVSSVIAGYVAL-LAGGTVF-FM---
Brsp  VL-FLIYLG-GMLVVFAYSAALAAEPYPEAWGEWSVLGAVGGYLVV-IMGAVIWFYN---
Gamo  VL-FLIYLG-GMLVVFAYCAALAAEPYPEAWGEWSVLGSVLGYLLL-VVGA-GSWFW---
Lolo  VL-FLIYLG-GMLVVFAYCAALAAEPYPEAWGEWSVLGSVLGYLLL-VFGG-VSWFW---
Batr  VL-FLIYLG-GMLVVFAYTTALAAEVYPRGLGTWEISRNVTVNFVL-VMVV-FKLVY---
Prmy  VL-FLIYLG-GMMVVFVYTTALAADSYPEVYYAENSLGGMMINIAF-IVFLVGLVWW---
Lose  VL-FLIYLG-GMLVVFAYSTALAAEPYPDGYLGGSPLVSMIMCLGA-VGVLLVY-LF---
Loam  VL-FLIYLG-GMLVVFAYSAALAAEPFPEGLGSRPIALSVVFYLGG-AGLV-ASSFL---
Chab  VL-FLIYLG-GMLVVFAYSAALAAEPYPESLGSRPIAGSMVIYSLG-VVLV-SSVFW---
Chto  VL-FLIYLG-GMLVVFAYSAALAAEPYPESLGSRPIAGSMVIYSLG-VVLV-SSVFW---
Majo  VL-FLVYLG-GMLVVFAYSAALAAEPYPEGVGSRPVASSALMYLGV-VGLV-SSMFW---
Hlst  VL-FLIYLG-GMMVVFAYSAALAAEPYPEGVGSRPVALLIVMYLFV-ITIA-GVLFG---
Clpe  VL-FLIYLG-GMLVVFAYSAALAAEPYPESFGSSLVAPTVGAYLLG-VGFI-SSFFW---
Mlmr  VL-FLIYLG-GMLVVFAYSAALAAEPYPEGLGSRPVAALMVIYLLG-VGLV-SSPFW---
Crcr  VL-VLIYLG-GMLVVFAYSAALAAEPYPESWGDWPVMGMLSLYIIC-VLLL-VNMFS---
Muce  VL-VLIYLG-GMLVVFAYSAALAAEPYPEGWGDWPVMGMLSLYIAC-VLLS-VNMFS---
Bege  IL-FLIYLG-GMLVVFAYSAALAAEPYPEGWGSWPVLGMAVLYLMG-VVMG-FGLFW---
Mela  IL-FLIYLG-GMLVVFAYSAALAAEPYPEGWGSWPVLGMMLVYLLG-VGVV-AGGFW---
Hats  IL-FLIYLG-GMLVVFAYSAALAAEPYPEGWGSWPVMGMMLVYLVG-VLIS-GALFL---
Orla  IL-FLIYLG-GMLVVFAYSAALASEPYPEGWGSWPVSTLMLAYVLL-VVCV-SVFFS---
```

To be continued
on page 12.

[2/4 of aligned sequences]

```
Cosa   IL-FLIYLG-GMLVVFAYSAALAAEPYPESWGSWSVLGVLLVYILG-LVIV-GSLFF---
Exsp   IL-LIIYLG-GMLIVFVYSMALASDPYSEEWWVWSAAGVMMTFIFF-VAVLAGL-FW---
Depa   IL-FLIYLG-GMLVVFAFASAMAAEPHPKGWAHWPVLALMAFYLVW-VIIT-LLMFS---
Rima   IL-FLIYLG-GMLVVFAYSVAFTGSDYVEGWGTRRISLAAAGYLLL-VWGG-GVILS---
Fuol   IL-FLIYLG-GMLVVFAYSAALAAEPYPEGWGSWPVLGLVLGYLLG-VGSV-GLLLG---
Gmaf   VL-FLIYLG-GMLVVFAYSAAMAAEPFPEGWGSWPTLRLVGGYLVG-VAVA-RGLLG---
Xeei   IL-FLIYLG-GMLVVFAYSAALAAEPYPEGWGSWPVLGLMIVCILG-VLAV-GAFME---
Pros   VL-FLIYLG-GMLVVFAYSAALAAEAYPESWGSWSVMGYVLAYCGI-VLLF-SGYFW---
Scmi   VL-FLIYLG-GMLVVFAYSAALAAEPHPEGWGSRSVVGYVMAYCVG-VLLV-SGCFW---
Rolo   VL-FLIYLG-GMLVVFAYSAALAAEPYPESWGSWSVLGYVMAYMIG-ATLA-SGFFW---
Cere   VL-FLIYLG-GMLVVFAYSAALAAEPFPESWCNWSVVGYAMTYLIL-VVSI-SSYFW---
Daga   VL-FLIYLG-GMLVVFAYSAALAAEPFPESWGNWSVLGQALVYFLG-VALA-FFHFY---
Anco   VL-FLIYLG-GMLVVFAYSAALAAEPFPESWGSRSVMGYVAAYLVG-VSLV-AGLSF---
Dmve   TL-SLIYLA-GMLVVFIFCTALSAEAYPEDEGSSWNV-YVVV-TVG-VFWV-VTCLF---
Dmar   AL-ALIYLA-GMLVVFIFCTALSPEAYPEDEGSSWNM-NLVA-MVG-IFWV-VTCFF---
Anka   VL-FLIYLG-GMLVVFAYSAALVAEPFPESWGSRSVVGYVVAYLVG-VSLV-AGLSY---
Moja   VL-FLIYLG-GMLVVFAYSAALAAEPFPESWGSRSVVGYVVVYLVG-VCLM-AGFSF---
Hoja   VL-FLIYLG-GMLVVFAYSAALAAEPFPESWGSRSVMGYVAAYLVG-LSVV-AGLSF---
Bede   VL-FLIYLG-GMLVVFAYSAALAAEPYPESWGSRSVVGYVVAYSIG-VFLV-SGCFW---
Besp   VL-FLIYLG-GMLVVFAYSAALAAEPYPESWGSRSVMGYVVAYSVG-VLLV-SGCFW---
Mysp   VL-FLIYLG-GMLVVFAYSAALAAEPHPESWGSRSVAGYFLLYLIG-VFAA-SSYFW---
Osja   VL-FLIYLG-GMLVVFAYSAALAAEPYPESWGSRSVVGYVLAYLVG-VLVA-SSYFW---
Sgro   VL-FLIYLG-GMLVVFAYSAALAAEPYPESWGSRSVVGYVVAYLIG-VLLV-SSYFW---
Pzpa   VL-FLIYLG-GMLVVFAYSAALAAEPYPEAWGGRAVLGYVIAYVVL-VLGA-LWCVS---
Zeja   VL-FLIYLG-GMLVVFAYSAALAAEPYPEAWGGEAVFESVVVYGML-VLGA-FLWAG---
Znne   VL-FLIYLG-GMLVVFAYSAALAAEPYPEAWGGWAVFGYVITYAVL-VWSA-ALWMG---
Zefa   VL-FLIYLG-GMLVVFAYSAALAAEPYPEAWGGRAVFGYVVIYIGL-VLGA-ALWAG---
Acni   VL-FLIYLG-GMLVVFAYSAALAAEPYPEAWGGRAVFGYVLIYIML-VLGA-SWWAG---
Ncrh   VL-FLIYLG-GMLVVFAYSAALAAEPYPEAWGGRAVFGYVLIYIML-VLGA-SWWAG---
Agca   VL-FLIYLG-GMLVVFAYSAALAAEPYPESWGSWPVAMSMLIYLAG-VVLV-SGLFW---
Hydy   VL-FLIYLG-GMLVVFAYSAALAAEPFPEGWGSRQVGAYAGLYVGG-VAGL-SSVFR---
Gsac   VL-FLIYLG-GMLVVFAYSAALAAEPFPEGWGSRQVGVYMVLYGAG-VGLV-SNMFW---
Pevo   IL-FLIYLG-GMLVVFAYSSALAAEPYPETLGSRSVAIYVGSYLLA-VVGG-SVSLV---
Hiku   VL-FLIYLG-GMLVVFAYSAALAAEPYPESWGSWSVAIYGVLYFMG-GVLV-LSYLY---
Inpa   IL-FLVYLG-GMLVVFAYAAALAAEPYPEGLLSGEVAIYVGVYVLV-LFLG-ASMFL---
Auch   TL-FLIYLG-GMLVVFAYSAALAAEPYPESWGSGYVAMYLGLYVAG-VVLG-GGVLC---
Fico   VL-FLIYLG-GMLVVFGYSAALAAEPYPESWGSRSVVVYTLVYILG-VVGV-SGLFW---
Macs   IL-FLIYLG-GMLVVFAYSAALAAEPYPESWGSRSVAVSMVGYMVG-VTLI-SGMFW---
Moal   VF-FLIYLG-GMMVVFAFSAALSAEPYPYGWGNRSVKMYVAAYVLG-VGCAWKV-LL---
Syma   VL-FLVYLG-GMLVVFAYSAALAAEPYPESWGSWSVFVYMMLYVGG-VILA-VLCFW---
Mafr   VL-FLIYLG-GMLVVFAYSAALAAEPYPEGWGSGSVMVYVVVYVLG-VVLI-SGMFW---
Dcpe   IL-FLIYLG-GMLVVFAYSAALAAEPYPESWGSRSVVVYMGAYVVA-VATA-SILFW---
Dcti   IL-FLIYLG-GMLVVFAYSAALAAEPYPESWGSRSVVVYMGVYLVA-VVVA-SVLFW---
Hehi   VL-FLIYLG-GMLVVFAYSAALAAEPYPESWVSGPVVGDMVMYLVG-VGVA-SSVFW---
Stam   VL-FLIYLG-GMLVVFAYSAALAAEPFPETWGSRPVIAYMVMYVAG-VCLV-SSLFW---
Hogi   VL-FLIYLG-GMLVVFAYSAALAAEPYPESWGSRSVGVYAMVYLVG-VVVA-SSMFW---
Erzo   VL-FLIYLG-GMLVVFAYSAALAAEPFPEGWGSWPVAVYMLMYMLG-VGLV-SSTLW---
Hxot   VL-FLIYLG-GMLVVFAYSAALAAEPFPESFGSRPVLLYMVMYVLG-VVVV-SSTVW---
Core   VL-FLIYLG-GMLVVFAYSSALAAEPFPESWGSRPVLLYMAMYAVG-VVMV-SSVVW---
Apve   VL-FLIYLG-GMLVVFAYTAALAAEPFPETWGSRTVLLYMVAYIVV-VVGV-SSTVW---
Latj   IL-FLIYLG-GMLVVFAYSAALAAEPYPEAWGSWPVVVHMAVYMAG-VVLA-FVVFG---
Laja   VL-FLIYLG-GMLVVFAYSAALAAEPYPESWGSRAIVTYMAVYMVG-GGLV-TVLFW---
```

To be continued
on page 13.

[2/4 of aligned sequences]

```
Syja IL-FLIYLG-GMLVVFAYSAALAAEPYPESWGSWEVAGYMVVYVVG-VIMV-SGLFW---
Epme IL-FLIYLG-GMLVVFAYSAALAAEPYPESWVDSTVLIYMACYTVG-VVVV-SSKFW---
Grse VL-FLIYLG-GMLVVFAYSAALAAEPYPESWGDRSVAGYMVGYMGA-VLLA-SAMFW---
Clja ILHFCYYLPFWNVVVFSIAAAASSDEKPKLLEPTPFVLTVLVGISFFSFNALNLYYVKHF
Ogcy VL-FLIYLG-GMLVVFAYSAALAAEPYPETWGSLPVLGLMVFYLFG-VVVF-SLGFV---
Plna VL-FLIYLG-GMLVVFAYSAALAAEPYPENWGDLPVLVTVLFYALG-LVVA-AIWFW---
Lema VL-FLIYLG-GMLVVFAYSAALAAEPFPETWGSWPVLGYMLVYVSG-VVGM-SGIFW---
Etzo VL-FLIYLG-GMLVVFAYSAALAAEPYPESWGSRPVVGYMAVYVVG-VLFI-SSKFW---
Apse VL-LLVYLG-GMLVVFAYTSAMAADPYPETLGSLGVLKGLVVYLSA-LFVM-CVFLW---
Epde VL-FLIYLG-GMLVVFAYSAALAAEPYPESWGSRFVAVDTVMYVLG-MTLI-SGLCW---
Slja VL-FLIYLG-GMLVVFAYSAALAAEPYPETWGSRPVLMYMVVYLAG-VGVI-AAWFW---
Bsja VL-FLIYLG-GMLVVFAYTAALAAEPFPEGWGSGRVMVSGVGYVGG-VFWG-AYYAF---
Ecna IL-FLIYLG-GMLVVFAYSAALAAEPYPETWGSRPVIMSVMIYVLG-VLLV-GGIFW---
Cohi VL-FLIYLG-GMLVVFAYSTALAAEPFPELWGGGSVMSSMFLCMSV-VVFL-SSFFW---
Caar VL-FLIYLG-GMLVVFAYSAALAAEPYPETWGSRPVTMYMVAYVLA-VAVV-SGLFW---
Came VL-FLIYLG-GMLVVFAYSAALAAEPYPETWGSRPVTMYMIAYVLI-VVVV-SGLFW---
Mema IL-FLIYLG-GMLVVFAYSAALAAEPYPETWGSGPVLVYMVMYLVG-VVLI-SWSLW---
Lenu IL-FLIYLG-GMLVVFAYSAALAAEPYPETWGSWPVMVWGVIYLVG-TFLG-SCFFW---
Brja VL-FLIYLG-GMLVVFAYSAALAAEPYPESWGSPAVLLYMVVYGAG-VVGV-CGLFW---
Plma VL-FLIYLG-GMLVVFAYSAALAAEPYPESWGSPAVVLYMAIYMVG-VISA-CVLLW---
Emst VL-FLIYLG-GMLVVFAYSAALAAEPYPESLGSRPVAVYMLMYVVG-VALV-SGLFW---
Ptti VL-FLIYLG-GMLVVFAYSAALAAEPYPESWGSRPVAAYMVMYVAG-VALV-SGLFW---
Losu VL-FLIYLG-GMLVVFVYSTALAAEPYPKSLGHRSVLANVLSYIAV-ACLI-CVSLY---
Geoy IL-FLIYLG-GMLVVFAYSAALAAEPYPETWGSLPVVLYMVGYLVV-TGIM-AGLLW---
Dipi VL-FLIYLG-GMLVVFAYSAALAAEPYPESLGSRPVAASMLVYLVG-VGLV-SGLYW---
Pama IL-FLIYLG-GMLVVFAYCAALAADSHWEGLGSRSVALSILIYGVA-MLVF-VGLFY---
Leob VL-FLIYLG-GMLVVFAYSAALAAEPYPQTWGDRSVLVSVMFYLVS-VAVF-AGVFS---
Neba IL-FLIYLG-GMLVVFAYSAALAAEPFPESLGSRRVIMVMLIYCLA-AFLV-GGYFY---
Pdpl VL-FLIYLG-GMLVVFAYSAALAAEPYPETWGSRSVMMYMTMYGVG-VVMS-AGYFW---
Nimi VL-FLIYLG-GMLVVFAYSAALAAEPYPEGWGSGAVVAYIFVYLVG-VVAV-SGLFW---
Uptr IL-FLIYLG-GMLVVFAYSAALAAEPYPETWGNRSVLAYMAMYVGG-LLMA-AAISG---
Pesc VL-FLIYLG-GMLVVFAYSAALAAEPYPESWGEWKVLSRVLGYVIG-VVSI-GSLFW---
Baar VL-FLIYLG-GMLVVFAYSAALAAEPYPESWGSGGVAVYMLVYVVG-VVLV-SGLFW---
Moar IL-FLIYLG-GMLVVFAYSAALAAEPYPEGLGSRPVAASMVVYLVG-VALV-SGLFW---
Toja IL-LLIYLG-GMLVVFAYSAALAAEPYPESWGSWPVLVNMMIYLVG-VILV-SALFW---
Chau IL-FLIYLG-GMLVVFAYSAALAADPHPESLGDSSVMGYMAFYLAV-VVVA-SGLFW---
Chse IL-FLIYLG-GMLVVFAYSAALAAEPYPETWGSRSVAVYMSVYVLG-VVLA-STLFQ---
Enar VL-FLIYLG-GMLVVFAYSAALAAEPYPETWGSGPVIMYMAMYVVA-VVLV-SSFFW---
Hpty VL-FLIYLG-GMLVVFAYSAALAAEPYPESWGSWSVAAYMVMYALG-VALA-SGLFW---
Nana VL-FLIYLG-GMLVVFAYSAALAAEPYPESWGSRSVVVYMFIYLAV-VALA-FMIFW---
Mcst VL-FLIYLG-GMLVVFAYSAALAAEPYPESWGSRSVVVYMVIYIVI-VIMV-SGLFW---
Rhox VL-FLIYLG-GMLVVFAYSAALAAEPYPETWGSEPVLVYAVVYILS-VAGA-AALVG---
Opfa VL-FLIYLG-GMLVVFAYSAALAAEPYPESWGSRSVATYMVVYVVV-VVLV-SGLFW---
Paar VL-FLIYLG-GMLVVFAYSAALAAEPYPESWVNWSVGMYILVYLAG-VVLA-SCLFW---
Gozo VL-FLIYLG-GMLVVFAYSAALAAEPYPESWGSRPVAMYMLLYVVA-VVLV-SGLFW---
Ackr VL-LLIYLG-GMLVVFAYSVALAAEPYPESWGNKSVRFLGAGYIGG-VCVM-AGCFW---
Elev VL-FLIYLG-GMLVVFAYSAALAAEPYPETWGSRSVVVYMGAYMVL-VLLC-SVIFW---
Trdu IL-FLIYLG-GMLVVFAYSAALAAEPYPEGWGSWPVLGVMVAYVLG-VGVA-AALFW---
Amoc VL-FLIYLG-GMLVVFAYSAALAAEPYPEGLGSWPVLGVMLTYALG-AVMA-SVFFW---
Hame IL-FLIYLG-GMLVVFAYAAALAAEAYPEGWLTGSVALYTGLYLIV-VMFATIS-VR---
Chso VL-FLIYLG-GMLVVFAYSSALAAEIYPEAWLRAGPGVAMYLYAFA-VAGATLI-YF---
Lyto VL-FLIYLG-GMLVVFAYSAALAAEPFPEGWGSRPVVAYMVLYTLG-VCLA-SSAVW---
```

To be continued on page 14.

[2/4 of aligned sequences]

```
Encr      VL-FLIYLG-GMLVVFAYSAALAAEPFPEGWGSRPVVAYMVLYVLG-VCLA-SSAVC---
Bvar      VL-LLIYLG-GMLVVFAYSAALAAEPFPESWGSGSVMLYMFLYLMG-TGVV-ASMFF---
Noco      VL-FLIYLG-GMLVVFAYSVALCSDALPTGLAEGSVLKSMVGYLGL-TGVV-ALFQQ---
Chsp      IL-FLIYLG-GMLVVFAYCVSLAADPYPEGWGNLSVTANIIVYMLG-LVFV-GLWYY---
Arja      VL-FLIYLG-GMLVVFAYSAALAAEPFPKTWGSRSVMLYATLYVVV-VGVI-ASTLW---
Pase      VL-FLIYLG-GMLVVFAYSAALAAEPFPESWWSLEVGMTIVFYFLG-IFMV-FGLVW---
Trel      VL-FLIYLG-GMLVVFAYTAALSSEEYPQTLGSWPVGIRMFTYLAA-VGWS-VLIVS---
Acur      VL-FLIYLG-GMLVVFAFSTALAAEPHPESWVNREVADTSVKYVLG-IIIF-AMMYV---
Ampe      VL-FLIYLG-GMLVVFAYSAALAAEPYPETWGSRPVAVYMGLYLAG-VVLV-SGMFW---
Urja      VL-FMIYLG-GMLVVFAYSAALSAEPFPESWGSREVKGYVGGFAVA-AVTS-YALLG---
Enet      VL-FLIYLG-GMLVVFAYSAALAAEPYPESWGELACMWVMGAYLGG-SLLG-ACLLW---
Ptbr      VL-VLIYLG-GMLVVFAYSAAMAAEPYPEAWLSLPVFLSMLVYMLG-AAGAGVI-LM---
Safa      IL-FLIYLG-GMLVVFAYSAALAAEPYPESWMSWPVLATMVCYLVG-VGVV-GVLV----
Icae      VL-FLIYLG-GMLVVFAYSAALAAEPYPESWGSPAVVLYMVIYTVG-VISA-CVLLW---
Asmi      IL-FLIYLG-GMLVVFAYTAAIAADPYPEAWGSLSVCCLGGGYMMG-IWVG-FMVFK---
Foal      VL-FLIYLG-GMLVVFAYSVALAAEPYPEAWMSRSVFSSMCAYLSL-VYAFFLF-FD---
Drze      VL-FLIYLG-GMLVVFAYTAALAAEPYPEGWGTRAVAFYTVVYMGF-ALGL-CYFLQ---
Rhas      VL-FLIYLG-GMLVVFAYSAALAAEPYPETLGSRAVALNVGMYLAG-VLVA-ASFFW---
Elac      IL-FLIYLG-GMLVVFAYSAALAAEPYPETLGSRSVALHVLMYSXA-VSLG-GAYFW---
Kugu      VL-FLIYLG-GMLVVFAYFIALAAEPYPETLGSWPVMMQMVGYVGV-LLVA-CSFFW---
Plor      IL-FLIYLG-GMLVVFAYSAALAAEPYPESWGDWSVGGSMLAYAVV-VALV-SGLFL---
Sgun      VL-FLIYLG-GMLVVFAYSAALAAEPFPETLGSGPVVFSMVVYMVG-VVLA-SGLFW---
Zaco      VL-FLIYLG-GMLVVFAYSAALAAEPYPESWGSRPIAASMFVYVVG-VVLA-SGLLW---
Zbfl      VL-FLIYLG-GMLVVFVYSVALAAEPFPESWGSQPVAMSAMVYVLW-VMLA-AGLCW---
Spba      VL-FLIYLG-GMLVVFAYSAALAAEPYPETWGSRPVVAYTVIYVVG-LGLV-SMLFW---
Game      VL-FLIYLG-GMLVVFAYSAALAAEPYPESWGSPAVVLYMVVYAVG-VALA-CALLW---
Thth      VL-FLIYLG-GMLVVFAYSAALAAEPYPETWGSPAVVLYMVIYSVG-VVLA-CMALW---
Xigl      VL-FLIYLG-GMLVVFAYSAALAAEPYPESWGSRPVVMYMGAYLVG-VVVV-SGLFW---
Hyja      VL-FLIYLG-GMLVVFAYSAALAAEPYPESWGSPGVVMYMVVYLAG-VVLV-CGVMS---
Psan      VL-FLIYLG-GMLVVMAYSSFLAAERFPYSWERKEVSMYMALFAAV-NSQV-FLLWP---
Cupa      VL-FLIYLG-GMLVVFAYSAALAAEPYPESWGSPAVVLYMVIYAVG-VILA-CALFW---
Mpch      IL-FLIYLG-GMLVVFAYSAALAAEPFPEGWGSRSVIFLMFMYGGV-VVCM-FWMFS---
Char      VL-FLIYLG-GMLVVFAYSAALAAEPYPETWGSRPVVFYMMMYLLG-VVLV-GGVFW---
Pser      VL-FLIYLG-GMLVVFAYSAALAAEPYPEAWGSWSVVVNMAGYVVV-VLLV-SAFFW---
Prol      VL-FLIYLG-GMLVVFAYSAALAAEPYPETWGSRPVMVYSASYMVG-VVLV-SVLFW---
Plbi      VL-FLIYLG-GMLVVFAYSAALAAEPYPESWGSWSVMVYGVVYVAG-VAMV-SGLFW---
Calu      VL-LLIYLG-GMLVVFAYSVALAAEPYPEAWGSLPVLTNTLLLMVA-AVGM-SLVLW---
Papa      IL-FLIYLG-GMLVGFDYSAALAAEPYPEGWWSRTVLGYVVIYLAG-VVLM-SSFFW---
Sufr      IL-FLIYLG-GMLVVFAYTAALAAEPYPESLGSGPVFESMLGYGVT-VALA-SCFFW---
Stci      VL-FLIYLG-GMLVVFAYSAALAAEPYPESWGSGPVLASMLSYAFA-VGVV-SALFW---
Taru      VL-LMIYMG-GMLVVFIYSSALSADKDPKAPTGWQVVLFFFGYFFF-VFGILYT-NM---
Rala      VL-FLIYLG-GMLVVFAYSAALAAEPYPESWGSRPVVMLMLVYTVI-VAAV-SGMFW---
           :    *:    :: .       .
```

To be continued on page 15.

[3/4 of aligned sequences]

```
                E                                                F
Scca  VGKWGDS-WAGVEELSSFEVVRGDFGGVALLYSLGGWMLVLSGWVLLVTLFVVLEVTRGL
Muma  VKGWGLH-WTGVEELSNLDMVRGDFLGVALLYSDGGWMLVLGGWVLLLTLFVVLELTRGL
Erca  ND------FGAFDEIEGCVMSVRRYVGVSEMYNAGGYMLLVAGWVLLVALLVVLELTRGF
Pose  GD------FGVLDDIEGHFGAVRRYVGVAEVYNVGGYMLLVAGWVLLMALLVVLELTRGY
Actr  GDWYSCS-WVVVDEFKDLSLLRGDFSGVALMYSLGGAMLVVSGWVLLLTLFVVLELTRGL
Scal  GDWYSYS-WVVVDEFKDLSLLRGDFSGVALMYSLGGVMLVVSGWVLLLTLFVVLELTRGL
Posp  NDWYSCS-WVVVDEFKDLSLLRGDFSGVAFMYSLGGFMLMVSGWVLLLTLFVVLELTRGL
Atsp  DGWDKFL-WV-VDEFEGFVVLRGDFSGVALVYDFGGGMLIICGWVLLLTLFVVLELIRGM
Leoc  GEWDKFM-WVA-EEFMELGVLRGDFSGVALVYDLGGGMLIVCGWVLLLTLFVVLELIRGM
Amca  VDWYINS-WVVIDDFGTFSLLRGDFSGVSVIYSFGGGMLIVSGWVLLLSLFVVLELTRGL
Osbi  EGWYDGF-WV-VDELKEFSLLRGDFGGVALMYSLGGWVLVVCGWVLLLTLFVVLELTRGL
Pabu  GGWFDYC-WV-VDEFKDFSFQRADFSGVALMYSFGGGMLMICGWVLLLALFVVLELTRGL
Hial  GGWYKYS-WVVVDEFKEFSVLRGDSGGVALMYSSGGAMLIICGWVLLLTLFVVLELTRGL
Elha  VGWYSNS-WFVVDEFKDFSVLRGDFSGVALMYSLGGGMLIVCGWILLVTLFVVLELTRGL
Mlcy  FGWYGYS-WVVADELEGFSVLRGDYSGVSLMYSLGGGMLVVCGWVLLLALFVVLELTRGL
Algl  GSWYDFG-WAVVDEFKDFSVLRGDFSGAALMYSLGGEMLFICGWVLLLTLFVVLELTRGV
Ptgi  TGWYNYT-WLVVDEFKDFSVVRGDLSGVALMYSLGGGMLIVCGWVLLLTLFVVLELTRGL
Alaf  GGWYDFS-WVVADEYEHFSVVRGDCSGVAYIYSSGGWMLLICGWALFLTLFVVLELTRGL
Nock  GGWYNYN-LTVADEYDYYSLVRGDCSGVAFIYSSGGGMLLICGWALFLTLFVVLELTRGL
Anja  VEWFDCY-CGIVDEYRDFSVLRGDFSGVSFIYYLGGGMLIICGWALLLTLFVVLELTRGR
Gyki  GKEGRGS-FMM-DEFHGFSTLRGDFGGVSYMYCLGGEMLMVCGWGLLLTLFVVLELSRGR
Syka  GGWLNYH-CGLVDEYRDFSALRGDFSGVSFMYNLGGEMLIVCGWALLLALFVVLELTRGR
Opma  GRSDYYC-GVV-DEFRDFSALRGDFSGVSYIYYYGGGMLAVSGWALLLTLFVVLELTRGR
Comy  MPLKHFC-GVV-DEHQDLSPLRGDFAGVSMIYGPGGGMLMACGWALFLSLFVVLELTRGR
Sasp  ATTPLGY-NGVVGESHGLLFLSGGFCGVSLIYCWG-LLLFICGWGLLLTLFVVYELIRGK
Eupe  TGNFHYY-GIA-DEYQYFSVLCGDLIGVSWMYYWG-WLLIICGWGLLLTLFVILELVRSC
Enja  GDWYNYL-LPSVSTFKEFFVLSEDTGGVGVMYSMGGGLLVICAAVLLLSLFVVLEIVRGV
Same  RTCYNFA-VGGFNNLKEFLMAPTDTSGVSMLYSFGGVMVVVCALALLVTLFVVLELTRGS
Cech  GGWYESS-WVVVDGLKGFFVLRGDVSGVALMYSFGGGMLVVCAWVLLLTLFVVLELTRGL
Grgr  ETWYGGS-WVMGDEFKELSVVRGDSGGAARMYSEGAPMLVLCAWVLLVTLLVVLELTRGM
Caau  GGWYEGS-WVVVDGLKEFSVLRGDVSGVAVMYSSGGGMLVICAWVLLLTLLVVLELTRGL
Cyca  GGWYEGS-WVVVDGLKEFSVLRGDISGVAVMYSSGGGMLVICAWVLLLTLLVVLELTRGL
Dare  TGDTGLL-MSV-DAFKEFSVIRADVSGVAMMYSSGGKMLVICAWVLLLTLFVVLEVTRGL
Cost  EGWYEGS-WMVIDSFKEFSMLRGDTSGVAMMYSSGGGMLVICAWVLLLTLFVVLELTRGL
Leec  ENWYEGS-WVVIDSLKEFSVLRGDTSGVAMMYSSGGTMLVICAWVLLLTLFVVLELTRGL
Fola  EGWYEGS-WVVIDGLKEFSVLRGDTSGVAEMYSYGGGMLVICAWVLLLTLFVVLELTRGL
Clmc  GGWYEGS-WFVVDS-KDFFVLRGDTSGVALLYSLGGWMLIVSGWVLLLTLFVVLELTRGL
Phin  GGWYEGS-WVIMDV-KEFFMVRGDVSGVSMIYSVGGGMLVVCGWVLLLTLFVVLELTRGL
Icpu  GGH--GG-YWVIDEFKEFFMVRGDIGGISLMYSVGGGMLVVCAWVLLLCLFVILELTRGL
Psto  SGY--GG-WVVVDEFKEFFMVRGDVGGVSLMYSVGGGMLVVCAWVLLLCLFVVLELTRGL
Cora  GGWY-GS-YLVVDEFKEFFTLRGDFNGVALMYSFGG-VLVICAWVLLLSLFVVLEVTRGL
Eisp  GGWFEGS-WVVGEGVKEFFVLRGDAVGVAMMYSWGGGMLIVCGWVLLLTLFVVLELTRGL
Apal  GGWYEGS-WVVGDSMKGFGLLRGDIGGVVEMYFLGGGMLIVCGWVLLLTLFVVLELTRGF
Eslu  GGWHEAC-WVAIDELKELVVLRGDVSGVALMYSSGGGLLMLCAWVLLLTLFVVLELTRGL
Dape  GGWYEAC-WVVLDELKELAVLRGDTGGAALLYDSGGGLLILCAWVLLLTLFVVLELTRGL
Glse  GGWHGVT-WVVVDEFKEFSVLRGDTSGVAIMYSSGGPMLIICAGVLLLTLFVVLELTRGL
Naar  GGWFGAS-WAVVDEFKEFSVLRGDTSGVALMYSSGGGALVICAGVLLLTLFVVLEMTRGL
Lioc  TGWFGVD-WAVVDEFKEFSVLRGDSSGVALMYSSGGGVLVVCAGVLLLTLFVVLEMTRGL
Opso  GGWLGVS-WVVVDELKEFSVLRGDSSGVAMMYSEGGGMLVICAGVLLLTLFVVLELTRGL
Alte  GGWYEGS-WVVVDEFKEFFVLRGDSSGVALMYSFGGGMLVVCAWVLLLTLFVVLELTRGL
Plap  GGWYESS-WVVVDEFKEFFVLRGDSSGVALMYSFGGGMLVVCAWVLLLTLFVVLELTRGL
```

To be continued on page 16.

[3/4 of aligned sequences]

```
Plal  NGWFETS-WVVVDEFKELGVVRGDSGGVALMFSSGGGVLVVCAGVLLLTLFVVLELTRGL
Sami  GEWHESS-WVVVDEFKEFAVVRGDSGGVALMFSSGGGVLVACAGVLLLTLFVVLELTRGL
Rere  SGWFEAS-WVVVDEFKELSFSRADTSGVALMFSSGGGLLVVSACVLLLTLFVVLELTRGL
Gama  GGWYEGS-WTVVDEFKDLSVFRGDTSGVAVMYSSGGGMLVACAWVLLLTLFVVLELTRGL
Onmy  GGWYETS-WVAVDEFKEFSVLRGDTSGVALMYSHGGGMLIACAWVLLLTLFVVLELTRGL
Sasa  SGWYETS-WVVVDEFKEFSVLRGDTSGVALMYSYGGGMLIVCAWVLLLTLFVVLELTRGL
Cola  GGWYETS-WVAVDEFKEFSVVRGDTSGVALMYSSGGGLLVVCAWVLLLTLFVVLELTRGL
Dita  GGWYGGP-WAV-EEYGEYGVLRADVGGVAVMYSSGGEMLVTCAWVLLLTLFVVLELSRGA
Gogr  GGWYESC-WMMGDEFSESCVLRGDSSGVALMYFTGGEMLVVCVWVLLMTLFVVLELTRGV
Chsl  AGWHEGS-WETAEGLKEFSILRGDTSGVAQVYSLGGEMLAVCALALLLTLLGVLELTRGL
Atja  GSWYGGS-WVVVEEMKDFVVVRGDVGGVSLMYSYGGLMLVLGAWVLLLTLFVVLELTRGL
Iido  GAWYGGS-WFVVEEMKDFVVVRGDVGGVSLMYSYGGPMLVLGAWVLLLTLFVVLELTRGL
Auja  EGWYEIS-WVVVDELAEFAVLRGDVSGVALMYSFGGGMLVVCAWVLLLALFVVLELTRGL
Chag  GGWYGVG-VMGVSEVHNMVMLREDVSGVAWIYSFGGGLLVGCAWALMLVLIVVLELTRGL
Hami  GGWYDIT-WITVDEMKEFMMLRGDVAGVALMYSLGGEMLVVCAWALLLALFVVLELTRGL
Saun  GGWYDIT-WITVDEMKEFMVLRGDVAGVALMYSLGGEMLVVCAWALLLALFVVLELTRGL
Nema  GGWYEAS-WGTVDELQSFAVIRADSGGVAVMYSSGGLMLVVCAWVLLLTLFVVLELTRGL
Disp  YGWHEAS-WGIVDELQSFAMVAADTAGVAMMYSSGGPMLLACAWVLLLTLFVVLELTRGL
Myaf  SGWYEAS-WGVVDELQGFSVVVADTAGVAVMYSWGAPILVICAWVLLLTLFVVLELTRGL
Lagu  GKWSEYS-WVPVGELNGLSLFSGDTSGVALMYSSGGGLLVLVAWALLLTLGVVLELTRGF
Trtr  GGWFESS-WVPEVELKEFTVMRGDTNGVALMYSLGGGVLVLAAWVLLLTLGVVLELTRGL
Zucr  GGWFEST-WVPEVELKGFTVTRGDTSGVALMYSSGGGVLVLAAWVLLLTLGVVLELTRGL
Pxja  GGWYEAS-WVPVDELKEFSALSGDTGGVALMYSFGGSMLLISAWVLLLTLFVVLELTRGL
Pxlo  GGWYESS-WVPVDELKEFSALCGDTGGVALMYSFGGSMLLISAWVLLLTLFVVLELTRGL
Pctr  GGWYEGS-WFPVNELKEFAVFMGDASGVAVMYSLGGGMLLVGAWVLLLTLFVVLELTRGL
Apsa  GCWYVDS-WASVNEFKDFTMYMGDISGVSLMYSFGGGMLVVGAWILLVTLFVVLELTRGL
Cabe  KDGGGSL-WAPYGAVACPWTVVAEMEGVAMLYQEGATMLVVTAGVLLLALFVVLELARGR
Bzze  GGWYETS-WVPADEQGDFSMFRGDIGGVALVYSSGGGMLVISAWVLLLTLFVVLELTRGL
Siim  GAWYPGD-WYLVEEADQLSLTRVDTGGVAAMYSFGGSVLLCSAWALLVALFVVLELTRGM
Ctru  GGWYEVS-WVPADELEGLSMFRGDIGGVALMYSSGGALLVIGAWVLLLTLFVVLELTRGL
Dpbr  GGWYEMS-WVPADELVGFSMFRGDIGGVALMYSSGGGLLVIGAWVLLLTLFVVLELTRGL
Caki  VNWFGVG-SESGYDFDDYSFICPEGEGLGLLYDKGGWLLLFCVIILLVVLVVVLELTRGR
Phja  EEWYQYI-WVPESKSSDYGLIIPDVGGAGVMYHQGAGMLLIGAFVLLLTLFVVLEICRGL
Brsp  SLSEGEW-LTSEDT-DGKGVFGLDSEGVALMYSSGGGLLILSAWVLLLTLFVVLSVTWGR
Gamo  GGWYEGM-WVPVDELIEFSVVAADSGGVALMYSLGGGLLVVSAWVLLLTLLVVLELTRGL
Lolo  GGWYEGA-WVPVDELIEFSVVAADSGGVALMYSLGGGLLVVSAWVLLLTLLVVLELTRGL
Batr  EGLSGLS-WVVGPSEG--FKYIMVELGVVELYQIGYLMLFGGVVGLLVTLFSVLSLTWGF
Prmy  WE-AGGF-WAASMS--NELSSLLGGWGVGEVYGGGGKMLVVGGWVLFLTLFVVLELTRGL
Lose  GGRPHPL-GRSVEEFGEFFIIRADVGGVAVVYSSGGEMLFLSGWVLLLTLFVVLELTRGL
Loam  GGWFESS-WQSVEEFGELLVSRGDIGGVALMYSSGGGMLLISAWVLLLTLFVVLELTRGL
Chab  GGWYEGS-WLCVEELGEFCAVRGDVAGVAMMYSLGGGMLVISAWVLLLTLFVVLELTRGL
Chto  GGWYEGS-WLCVEELGEFCAVRGDVAGVAMMYSLGGGMLVISAWVLLLTLFVVLELTRGL
Majo  GGWYSGS-WLCAEELGEFGVSRADVAGLSFMYSTGGGMLVIGAWVLLLTLFVVLELTRGL
Hlst  EGWFRDS-CLCLEELEEFSLNRGDVVGVSLMYEGGGGVLCLAAFVLFVTLLGVLEVSRGL
Clpe  EGWYEAS-WVPVGELFEFCIQRADTSGVAMMYCWGGEMLVASAWVLLLTLFVVLELTRGL
Mlmr  GGWYEVS-WLSVEELGEFCVLRGDVGGVAMMYSFGGGMLVICAWGLLLTLFVVLELTRGL
Crcr  EGWYWGG-GCTGEESGGMMMIRGDIGGVALLYSMGGGMLIVGGWVLLLTLFVVLELTRGL
Muce  GGWYWGG-GCTGEELGGMAMIRGDIGGVALLYSMGGGMLIVGGWVLLLTLFVVLELTRGL
Bege  GGWYEES-WVCVDELGEFSVLRGDVGGVALMYSSGGGLLIVGAWVLLLTLFVVLELTRGL
Mela  GGWYEGS-WISADELSEVSVFRGDIGGIALMYLAGGKLLIMGAWVLLLTLFVVLELTRGL
Hats  GGWYEES-WVSVDELSELSVFRGDVGGVALMYSAGGGLLILAAWVLLLTLFVVLELTRGL
Orla  GGWFEGS-WVTVDEFSEFALFRGDSGGIALMYSSGGGILMLGGWVLLLTLFVVLELTRGL
```

To be continued on page 17.

[3/4 of aligned sequences]

To be continued on page 18.

```
Cosa  GGWYEGS-WMTADEFSELSMFRGDMGGVALMYSAGGGLLVLGAWVLLLTLFVVLELTRGL
Exsp  GGWYKGA-LYLTGDYVGMSVHCGDVWGMVNIYSTGGGFLILGGLALLLALFVVLELVRGL
Depa  GGWYEVS-PETVDEFNELFVFRGDMGGVALMYSTGGGLLILGAWTLLLTLFVVLELTRGL
Rima  TNLNEEF-WTVGASFGEFMSLEYSLVGVAHLYMGGGWLLVAGAWALLLVLLAVLELVRGA
Fuol  SKWYMES-WVACDEFGEFYIGRGDVGGVGILYSSGGFMLTMGAWVLLLTLFVILELTRGM
Gmaf  KNWYEED-WSGFDSILGLTVLRGDVGGVSLMYSSGGWLLMGGGWVLLLTLFVVLELTRGL
Xeei  GGWYFES-WGVLDELNEGSVLRGDIEGVAMMYSSGGFLLILGGWVLLLTLFVVLELTRGM
Pros  GGWYEVS-WVPADEIKEFSVFRGDVGGVALMYSLGGGMLIISAWVLLLTLFVVLELTRGL
Scmi  GGWYEVS-WVPVDDVKEFSVFRGDMGGVALMYSLGGGMLIVSAWVLLLTLFAVLELTRGL
Rolo  GGWYEVS-WVPADELKEFAVFRGDVGGVALMYSLGGGMLVISAWVLLLTLFVVLELTRGL
Cere  GDWYEAS-WVPADELKVFSVFRGDAGGVALIYSSGGEMLLLSAWVLLLTLFVVLELTRGT
Daga  GGWSEAS-WVPVDELKTFSMFRGDMGGVPLIYSSGGGLLLISAWVLLLALFTVLELTRGM
Anco  GGWYEFS-WVPVDELKDFVVLRGDSGGVALMFSVGGGLLMISAWVLLLTLFVVLELTRGL
Dmve  WNWVPSY-KDAGVGVDDQSHCGVDQGAVALMYSSGGGLMMLGAWALLLALFVVLELTRGL
Dmar  WSWVPSY-KDLGVGVEDQSHCGVDQGAVALMYSSGGGLMMLGAWALLLALFVVLELTRGL
Anka  GGWYEYS-WVPVDELKDFVVLRGDLSGVALMFSLGGGMLLISAWALLLTLFVVLELTRGL
Moja  EGWYEFS-WVPVDELKDLVVHRGDLGGVALMFSLGGGMLMISVWVLLLTLFVVLELTRGL
Hoja  GGWYEFS-WVPVDELKDLVVLRGDSGGVALMFSLGGGMLMISAWVLLLTLFVVLELTRGL
Bede  GGWYEVS-WVPADELKEFSVFRGDIGGVALMYSLGGGMLVISAWVLLLTLFVVLELTRGL
Besp  GGWYEVS-WVPADELKEFSMFRGDIGGVALMYSLGGGMLVISAWVLLLTLFVVLELTRGL
Mysp  GGWYEAS-WVPVDELKEFSMFRGDTSGVALMYSLGGGMLVICAWVLLLALFVVLELTRGL
Osja  GGWYEAS-WVPADELKEFSMFRGDIGGVALVYSLGGGMLVISAWVLLLTLFVVLELTRGL
Sgro  GGWYEVS-WVPADELKEFSMFRGDVGGVALVYSLGGGMLVISAWVLLLTLFVVLELTRGL
Pzpa  GEWYVGS-WVPVYELKALGVVSGDQGGVALMFSFGGPVLVLGAWALLLTLFVVLELIRGL
Zeja  GDWYEGS-WVPVYELKAFSVVPGDLGGVALMFSFGGVMLNLGAWVLLLTLFVVLELIRGL
Znne  SGWYEES-WVPVYELKAFSILPGDFGGVALLYSLGGYMLILGAGTLLLSLFVVLELIRGV
Zefa  SGWYEGS-WVPIYELKAFSVLPGDFGGVALMFSFGGFLLILGAGALLLTLFVVLELIRGV
Acni  GGWYEGS-WVPVYELKSFSVVPGDFGGVALMFSFGGVMLILGAWVLLLTLFVVLELIRGL
Ncrh  GGWYEGS-WVPVYELKSFSVVPGDFGGVALMFSFGGVMLILGAWVLLLTLFVVLELIRGL
Agca  GGWYEFS-WTTVEELGEFSVVRGDMGGVALMYSLGGGMLLISAWVLLLTLFVVLELTRGL
Hydy  EGWYEES-WVPADEPGSFNTLRGDIGGVAMMYSSGGQMLVLSAWALLLTLFVVLELTRGL
Gsac  EGWYEVS-WVPADELDGFTMFRGDIGGVAMMYSLGGGMLVLSAWALLLTLFVVLELTRGL
Pevo  GEWYEGS-WVPCGEMSTGGVIHSDMGGVGMIYSEGGWMLVVSAWMLLLCLFVVLELTRGV
Hiku  GGWNESS-WVPVDESSGLSLFRGDMSGVALMYSSGGWVLVISAWVLLLTLFVVLELTRGL
Inpa  GNWSESE----AGEFEGFLVWGGDMSGVSAVYAEGWAILMLGAWVLLMTLFVVLEVIRGH
Auch  GGWYEGS-WIPVEEQMDFSVFQGDAGGVALMYSFGGGGLVIGGWALLVALFVALELTRGL
Fico  GGWYEGS-WVPVDELTEFSVLRGDMGGVALVYSSGGAMLVVCAWVLLMTLFVVLELTRGS
Macs  GGWHEGS-WVPVDELVELCMFRGDMGGVAFMYSSGGGMLVISAWVLLLTLFVVLELTRGM
Moal  GKWSGFW-GGILDESGELLVLRGDMMGVAAMHDVGGWMLVLGGLGLLLTLFVVLELVRGV
Syma  GGWFGFW-GSVSDEVLEVGVLRGDFGGVAMVYCLGGKMLGVGVWVLLLSLFVVLELVRGL
Mafr  GEWYAVF-WVPVDEVSEFSMIRGDVGGVALMYSFGGGMLLVGGWVLLLTLFVVLELVRGL
Dcpe  GGWYEGS-LVPSDDLVMFSSFRGDMAGVALVYSFGGGMLILSVWVLLLTLLVVLELTRGL
Dcti  GGWYEGS-WVPSDDLAMFSSLRGDMAGVALVYSSGGGMLILSVWVLLLTLLVVLELTRGL
Hehi  GGWYESS-WVPADELSELSVLRGDIGGVALMYSLGGGMLVLSAWVLLLTLFVVLELTRGL
Stam  GGWFESS-WVSADEFGDFSVFRGDMGGVSLMYSWGGGMLVISAWVLLLTLFVVLELTRGL
Hogi  GGWYEFS-WTPVDDHGGFSVLRGDVGGVALMYSFGGGMLIISAWVLLLTLFVVLEVTRGL
Erzo  SGWYESS-WVPADELGDFSVFRGDIGGVALMYSSGGGMLVISAWVLLLTLFVVLELTRGL
Hxot  GGWYESS-WVPADELGDFSVFRGDMGGVALMYSSGGGMLVMSAWVLLLTLFVVLELTRGL
Core  GGWHEVS-WVPADELGEFSVFRGDIGGVALMYSSGGGMLVLSAWVLLLTLFVVLELTRGL
Apve  GGWYESS-WVPTDELESFSVLRRDMSGLALMYSEGGGVLVMSAWVLLLTLFVVLELTRGL
Latj  GAWYESS-WVSVDELGEFSMLRGDVGGVALMYSSGGWMLIIGGWVLLLTLFVVLELTRGL
Laja  GGWYESS-WVPVDEFGEFSVFRGDIGGVALMYSLGGGVLVISAWVLLLTLFVVLELTRGL
```

[3/4 of aligned sequences]

To be continued on page 19.

```
Syja GGWYEAS-WVPADELGGFCMFRGDIGGVALMYSLGGFMLVISAWVLLLTLFVVLELTRGL
Epme HEWVGGL-SGSADEWGPFTVMRADHDGVAVMYSEGGWMLIVGAGVLLLTLFVVLELTRGL
Grse GGWYEFS-WVPVEECVEFSMVRGDLEGVAMMYFLGGGMLIIGAWVLLLTLFVVLELTRGL
Clja AGGVTNF-MKIMNSFYESDFVRSELEALSYLYLSSSPLLFFVGVLLLLTLVCILALIKGG
Ogcy GGWYEGS-WFSSDEYGEMLMFRGDVGGVAVMYSLGAGFLIVGGGVLLLTLFVVLELTRGV
Plna KEGFESF-FVSVEVCSDRCVVQADTVGIAWMYSSGGGCLIVSAGVLLLTLFGVMELTRGL
Lema GGWYESS-WMTADELGDFLMVRGDVGGVALMYSLGGGMLVISAWVLLLTLFVVLELTRGL
Etzo GEWYEFS-WVPGDELDELSVFRGDIGGVALMYSAGGGMLVVSAWVLLLTLFVVLELTRGL
Apse RGWAAES-GDFAFEGDALGMSRGDIEGVAYMYSAGGWTLMMCGWGLLLTLFVVMELIRGP
Epde AEWYEGS-WMSADELGEFSVLRGDTGGVALMYSLGGGMLVTSAWVLLLTLFVVLELTRGL
Slja TGWYEYS-WVTPDGLEEASMFRADMGGVARMYLEGGGMLMISAWVLLLTLFVVLELTRGL
Bsja GQWHEAS-WLSSEEGSDFYMFRGDVGGVAMMYSGGGGMLIISAWALLLTLFVVLELTRGL
Ecna GGWYESS-WMSVDELMEFSILRGDMAGVAMMYSSGGLMLIISAWVLLLTLFVVLELTRGL
Cohi EGWSGVF-WGGVNELEDFLLLRGDAGGIGEMYSLGGGLLIISVWVLLLALFAVLEMTRGL
Caar GGWYESS-WGSVDELGEFSVFRGDMAGVALMYSLGGWMLIISAWVLLLTLFVVLELTRGL
Came GGWYESS-WGSADELAEFSVFRGDMAGVALMYSLGGWMLIISAWVLLLTLFVVLELTRGL
Mema GGWYESS-WVSADELGGLSVFRGDIGGVALVYSLGGWMLVISAWVLLLTLFVVLELTRGL
Lenu GGWYSPF-WVVADEFGEFSVLRADTGGVGLLYSVGGGMLVIAVFVLLLALFVVLELTRGL
Brja GGWYEAS-WASADGVGEFFVFRGDMGGVALMYSLGGGVLVISAWVLLLTLFVVLELTRGI
Plma EGWYESS-WVPVDDMGEFSVFRGDVGGVALMYSSGGGMLVISAWVLLLTLLVVLELTRGV
Emst GGWYESS-WVSVDELGEFSVLRGDTGGVALMYSLGGGMLVISAWVLLLTLFVVLELTRGL
Ptti GGWYESS-WVSVDELGDFSTVRGDIGGVALMYSLGGGMLVISAWVLLLTLFVVLELTRGL
Losu ETWFSDS-WLVADEFDELAAARGDIEGVAMLYSFGGEMLLVGGYALFLALLAALELTRGV
Geoy SGWYEAS-WAPLEDRGDFFLFRGDFGGVGMIYGLGGGMLVIGAWVLLLTLFVVLELTRGL
Dipi GGWYEFS-WASVDELGEFSVLRADIGGVALMYSLGGGMLVISAWVLLLTLFVVLELTRGL
Pama GGWYESS-WVTAEEFHELAVHRGDAVGVALMYLSGGKMLMIAAWTLLLTLFVVMELVRGM
Leob MDWYVTS-WGSVEEMEAFSVIRADAGGVASMYNWGGGMLFVTAGVLLLALFVVLELTRGL
Neba DEWSFSS-WVTAEEGSEFSMSRGDLEGIGMLYSHGGLLLLIAAGVLLLTLFAVLELSRGA
Pdpl GGWSEVS-WIPADELNEFSLFRADIGGVGEMYGLGGGMLAISGLVLLSTLFVVLELTRGL
Nimi GGWYETS-WVPVDELGEMSVLRGDSGGVALMYGGGGGMLVVGAWVLLLTLFVVLELTRGL
Uptr GVWYEGS-WIPADELGEFSVFRGDAGGVALMYSLGGGMLVLSAWVLLLTLFVVLELTRGL
Pesc GGWHEFS-GSPGEQSGNFSVLGWDTTGVGEMYHVGGPLLVVSAWVLLLTLFVVLELVRGF
Baar EGWHEAS-WVPGDELGEFAMFRGDIGGVALMYSLGGGMLVISAWVLLLTLFVVLELTRGL
Moar GGWYESS-WVSADELSEFSVFRGDMGGVALVYSFGGGMLVISAWVLLLTLFVVLELTRGL
Toja GGWYGFS-WVSADELGGFSVFRGDVGGVALMYSLGGLMLIIGAWVLLLTLFVVLELTRGL
Chau EEWHEYS-WGL-SDFTDFSVSRGDTGGAAFMYSLGGGMLIICGWVLFLTLFVVLELTRGL
Chse GGWYEFS-WTSVDELGEFSVLRGDIGGVALMYSLGGGLLVVGAWVLLLTLFVVLELTRGL
Enar GGWFETS-WVPVDELEEFSGLRGDMGGVALMYSWGGGMLVISAWVLLLTLFVVLELTRGL
Hpty GGWYEFS-WVPVDELGEFSVFRGDVGGVALMYSLGGGMLVISAWVLLLTLFVVLELTRGL
Nana GGWYESS-WVSADELCEVSVFRGDIGGVALMYAPGGGMLVIGAWVLLLTLFVVLELTRGL
Mcst SGWHEFS-WVPVDELGEFFILRGDAEGVALMYSFGGGMLVISAWVLLLTLFVVLELTRGM
Rhox GGWYESS-WVPADELSEFSVFRGDMGGVAFMYSLGGGMLVIGAWVLLLTLFVVLELTRGL
Opfa SGWYEAS-WVPVDELGEFSVFRGDAGGVAWMYSFGGGMLVIGAWVLLLTLFVVLELTRGL
Paar GGWYEFS-WVPVDELGVFFTLRGDTGGVALLYSFGGWMLIFSAWVLLLALFVVLELTRGL
Gozo SGWYESS-WVSVDELGELSVFRGDTGGVALMYSLGGGMLVISAWVLLLTLFVVLELTRGL
Ackr EWWAGYL-NIPVEGGVDMYFNRADTLGASLLYKDAGGLLLISGIVLLLVLFVVLELTRGM
Elev KGWFDFS-WVPACDTSDFVSMHACMGGVAVIYSLGGLPLVISAWILLLTLLVVLELTRGL
Trdu GGWYEGA-WVSADEFVEFSVFRGDVGGVALMYSMGGGVLVMGAWVLLLTLFVVLELTRGL
Amoc GGWYEGS-WTSADEFVEFSLFRGDIAGVALMYSMGGGILIMGAWVLLLTLFVVLELTRGL
Hame QGWYDPS-LASVDDMCDFLMVSGDVSGVALMYSYGGGMLVFSAWMLLLTLFVVLELTRGL
Chso YHWEGGD-LALDGVVDWLSPFAGDVEGVALMYESGGGMLILSVWVLLLTLFVVLELVRGL
Lyto GGWYESS-WVPAEEFGEFSTFRGDIGGVAMMYSLGGGMLVISAWVLLLTLFVVLELTRGL
```

[3/4 of aligned sequences]

```
Encr   GGWYESS-WVPADELGDFSMSRGDIGGVALMYSLGGGMLVISAWVLLLTLFVVLELTRGL
Bvar   GGWYEFS-WGVVDEFCEFSLFRGDTGGVAVMYSWGGGMLVLSAWVLFLTLFVVLELTRGL
Noco   SSEAPLF-WWFVGQESEMSVMQGETEGVSEAYGSGGILLLTCGWALLVALYVALEVSRGC
Chsp   GECFGGS-WVPIYEREMCAVISPDPVGVSVLYDSGGGMMVLGAWALLTTLFVVFQLSRGG
Arja   GGWYEAS-WVPAQELGDFSVFRGDMGGVALMYSSGGGILIMTAWALLLTLLVVLELTRGL
Pase   EGWYEVS-WLTAYEQVEFCLVPGDVEGVALMYAGGGGLLVVAVWVLLLALFVVLELTRGL
Trel   GG--CGS-YFKGGGPSDVAVARVDTSGVAQLYWSGGWILVLTAWVLVLALLVVLELTRGG
Acur   GDWQEGM-FVPATDLVYNSTLRVDSGGVGALYGWGGGMLILAGWVLFLTLVVVLELTRGL
Ampe   GGWYEGS-WISVDELAGFSVLRGDTGGVALMYSQGGGMLVISAWVLLLTLFVVLELTRGL
Urja   GGRSNGV-WACMDEGPSFSISRGDAVGAASMYSSGGGLLFISTGCTFLTLFVVLELTRGR
Enet   GGWQEGA-WAPVGGGQEFFVMRGDMAGVAVMYSMGGGMLVLAGGVLLLALLVILELTRGV
Ptbr   SGWMGGG-LVLEGEAGGLGLEYGNIAGVSFLYSTGGGLLLAAGWGLLLTLFAVLELVRGL
Safa   VDWGGGASVEPVVGGLELGVLRGDISGVALMYSMGGGMLVGGAWVLLLTLFVVLELTRGA
Icae   GGWYEVS-WVPADDMGELSVFRGDVGGVALMYSAGGGMLVISAWVLLLTLFVVLELTRGM
Asmi   EQWGHAW-WTILGESGNLFMSCGDTVGTVPLYSSGGAVLATAGYVLLLTLTVILVLVRGQ
Foal   GKFSGCW-GGHDWG-AGFELVRGDMMGVALMYSGGGMMLVISAWVLLLTLFVVLELTRGL
Drze   GGWCDSS-WVTVDELEDFSLFQGDIGGVAMMYHFGGYMLIISAWALLLTLFAVLELTRGL
Rhas   GGWFNGS-WVTVDELVDFSVFRGDMAGVALMYSMGGWMLVVSAWVLLLTLFVVLEVSRGQ
Elac   GGWYADS-WATADELGEFYVSRGDTMGVAMMYSSGGWMLVVGAWVLLLTLFVVLELSRGL
Kugu   GGWYEGS-WVVVDELACYSLFRGDMGGVALMYSLGGWMLVISAWVLLLTLFVVLELTRGL
Plor   GGWYEVS-WVPADELGEFSVSRGDVGGVAVMYSLGGGMLAVSAWVLLLTLFVVLELTRGL
Sgun   GGWYESS-WMSVEELGEFMVLRGDVAGVALMYSVGGGMLIIGAWVLLLTLFVVLELTRGL
Zaco   GGWYESS-WVSVDELGEFSMLRGDIGGVAMMYSSGGGMLVIGAWVLLLTLFVVLELTRGL
Zbfl   GGWYGPS-WLPADELGEFLVFRGDVGGVALMYSLGGEMLVVSAWVLLLTLFVVLELTRGL
Spba   GGWYESS-WMSSDEVGEFSVFRGDVGGVALMYSAGGWLLVVSAWVLLLTLFVVLELTRGL
Game   GGWYEVS-WVPADDMGEFSVFRGDVGGVALMYSLGGGMLVISAWVLLLTLFVVLELTRGM
Thth   GGWYEVS-WVPADDMEEFAVFRGDVGGVALMYSLGGGMLVISAWVLLLTLFVVLELTRGL
Xigl   GGWYESS-WVSVDELGEFSVFRGDVGGVALMYSLGGWMLIISAWVLLLTLFVVLELTRGL
Hyja   GGWYEAS-WVPADDMAEFSVFRGDMGGVALMYSLGGGMLVISAWVLLLTLFVVLELTRGM
Psan   VDWYQTS-WAPMDDMEEFIVYRSDMEGVAMMYSSGGGMLVITVWALLLTLLVVLEVARIG
Cupa   GGWYEAS-WVPADDMEEFSVFRGDVGGVALMYSLGGGMLVISAWVLLLTLFVVLELTRGM
Mpch   EGWHEFS-WVPADELVEFSLFRGDVEGIALMYSFGGGFLIIGVWVLLLTLFVVLELTXGL
Char   GGWYEGS-WVASDEMFEFSVVRGDIGGVAVMYSSGGGLLVIGAWVLLLTLFVVLELTRGL
Pser   GGWYESS-WGCADECGELSVFRGDMGGVALMYSEGGGMLVISAWVLLLTLFVVLELTRGL
Prol   GGWYESS-WVLVDELGEFSVFRGDIGGVALMYSSGGWMLLISAWVLLLTLFVVLELTRGM
Plbi   GGWYESS-WVLVDELAEFSVFRGDIGGVALMYSSGGWLLIISAWVLLLTLFVVLELTRGL
Calu   GGWYEAS-WMGADDTEEFPATRGDMDGVALMYSSAAGLLVAGAAALFLTLLVVLELTRGL
Papa   KMWHGFP-WGVMGGYCDFSSFRGDTGGVALVYCSGGWMLLISTWALLLALFVVLELTRGL
Sufr   GGWYEFS-WLSVDELGEMLTVRGDVSGVALMYSLGGGLLAVSAWVLVLTLFIVLELTRGF
Stci   GGWYDFS-WAVQDEGEAFSSIRGDTSGVALMFSEGGGLLVISAWVLLLTLFVVLELTRGL
Taru   VLDEEVW-WGVSGEMDWDAVFRGDMDGVSLMYSSGGGVLLLGAWVLLLTLLVVLELVRGL
Rala   GGWYEFS-WLSADELGEFLVSRGDIGGVALMYSLGGGMLVIGAWVLLLTLFVVLELTRGL
                                .    . .   :        .  *     :
```

To be continued on page 20.

[4/4 of aligned sequences]

```
Scca    SWGTLRAV*--
Muma    SWGTLRMV*--
Erca    GRGCLRAV*--
Pose    SRGCLRAV*--
Actr    SRGALRAV*--
Scal    GRGALRAV*--
Posp    SRGALRAV*--
Atsp    SRGCLRAI*--
Leoc    GRGCLRAI*--
Amca    SRGCLRAV*--
Osbi    IRGALRAV*--
Pabu    SRGALRAV*--
Hial    SRGALRAV*--
Elha    SRGALRAV*--
Mlcy    SRGALRAV*--
Algl    GRGTLRTI*--
Ptgi    SRGALRAV*--
Alaf    SRGTLRAV*--
Nock    SRGTLRAV*--
Anja    SRGALRAI*--
Gyki    ELGALRAI*--
Syka    SRGALRAI*--
Opma    SRGALRAI*--
Comy    SLGALRSI*--
Sasp    GRGTLRMIGE*
Eupe    GSGSLRTI*--
Enja    DRGTLRAV*--
Same    ARGALRAP*--
Cech    SRGALRAV*--
Grgr    NRGVLRAV*--
Caau    SRGALRAV*--
Cyca    SRGALRAV*--
Dare    SYGVLRAI*--
Cost    SRGSLRAV*--
Leec    ERGTLRAV*--
Fola    SRGTIRAV*--
Clmc    SRGALRAV*--
Phin    SRGALRAV*--
Icpu    SRGALRAV*--
Psto    SRGALRAV*--
Cora    SRGALRAV*--
Eisp    GRGALRAV*--
Apal    SRGALRAV*--
Eslu    NRGTVRAI*--
Dape    SRGTVRAV*--
Glse    ARGALRAV*--
Naar    NRGALRAV*--
Lioc    NRGALRAV*--
Opso    NRGALRAV*--
Alte    SRGTLRAV*--
Plap    SRGTLRAV*--
```

[4/4 of aligned sequences]

```
Plal    SRGTLRAV*--
Sami    SRGALRAV*--
Rere    SRGVLRAV*--
Gama    SRGSLRAV*--
Onmy    SRGALRAV*--
Sasa    SRGALRAV*--
Cola    SRGALRAV*--
Dita    GRGALRAV*--
Gogr    GRGSLRAV*--
Chsl    SRGTLRAV*--
Atja    SRGTLRAV*--
Iido    SRGTLRAV*--
Auja    GRGALRAV*--
Chag    QRGPLRSV*--
Hami    SRGTLRAV*--
Saun    SRGALRAV*--
Nema    SRGALRAV*--
Disp    SRGALRAV*--
Myaf    SRGALRAV*--
Lagu    SRGTLRAV*--
Trtr    ARGTLRAV*--
Zucr    SRGALRAV*--
Pxja    SRGALRAV*--
Pxlo    SRGALRAV*--
Pctr    GRGALRAV*--
Apsa    GRGALRAV*--
Cabe    TWGVLRAI*--
Bzze    SRGVLRAV*--
Siim    SRGALRAV*--
Ctru    SRGTLRAV---
Dpbr    GRGTLRAV*--
Caki    SQGALRAI*--
Phja    SRGTLRSV*--
Brsp    AEGSLRVV*--
Gamo    ARGALRAV*--
Lolo    ARGALRAV*--
Batr    GWGALR*----
Prmy    SRGSLREV*--
Lose    GRGVLRAVY*-
Loam    GRGALRAV*--
Chab    SRGTLRAV*--
Chto    SRGTLRAV*--
Majo    SRGALRVA*--
Hlst    SRGSLRVV*--
Clpe    ARGAVRAV*--
Mlmr    GRGTLRAV*--
Crcr    SRGAIRAV*--
Muce    SRGAIRAV*--
Bege    SRGALRAI*--
Mela    SRGTLRAV*--
Hats    NRGALRAV*--
Orla    SRGTLRAV*--
```

[4/4 of aligned sequences]

```
Cosa      SRGALRMV*--
Exsp      SRGALRPV*--
Depa      SRGALRAV*--
Rima      SRGAVRVV*--
Fuol      GRGTLRAV*--
Gmaf      GRGALRAV*--
Xeei      VRGALRAV*--
Pros      SRGTLRAV*--
Scmi      SRGALRAV*--
Rolo      SRGTLRAV*--
Cere      SRGALRAV*--
Daga      SRGALRAI*--
Anco      GRGTLRAV*--
Dmve      SRGTLRAV*--
Dmar      SRGTLRAV*--
Anka      ARGTLRAV*--
Moja      GRGALRAV*--
Hoja      GRGALRAV*--
Bede      SRGTLRAV*--
Besp      SRGTLRAV*--
Mysp      SRGALRAV*--
Osja      SRGALRAV*--
Sgro      SRGALRAV*--
Pzpa      SRGTLRAV*--
Zeja      SRGTLCAV*--
Znne      SRGALRAV*--
Zefa      SRGALRAV*--
Acni      SRGALRAV*--
Ncrh      SRGALRAV*--
Agca      SRGALRAV*--
Hydy      SRGSVRAV*--
Gsac      SRGSVRAV*--
Pevo      SRGTLRVV*--
Hiku      ARGTLRVV*--
Inpa      ARGALRAV*--
Auch      SRGAVRMV*--
Fico      SRGALRVV*--
Macs      SRGALRMV*--
Moal      SRGGLRTV*--
Syma      SRGSIRAVY*-
Mafr      SRGTLRAV*--
Dcpe      SRGSLRTP*--
Dcti      SRGSLRAV*--
Hehi      SRGALRAV*--
Stam      SRGMLRAV*--
Hogi      SRGALRAV*--
Erzo      SRGALRAV*--
Hxot      SRGMLRAV*--
Core      SRGTLRAV*--
Apve      SRVALRAV*--
Latj      SRGALRAV*--
Laja      GRGAVRAV*--
```

[4/4 of aligned sequences]

```
Syja    ARGTLRAV*--
Epme    SRGTLRAV*--
Grse    NRGALRAV*--
Clja    GKGPNRPL*--
Ogcy    SRGALRAV*--
Plna    SRGTIRAV*--
Lema    SRGALRAV*--
Etzo    SRGTLRAV*--
Apse    SRGALRAV*--
Epde    SRGALRAV*--
Slja    SRGALRAV*--
Bsja    GRGSLRAV*--
Ecna    NRGALRAV*--
Cohi    SRGALRAV*--
Caar    SRGALRAV*--
Came    SRGALRAV*--
Mema    SRGALRAV*--
Lenu    GRGVLRAV*--
Brja    GRGTVRAV*--
Plma    DRGAVRAV*--
Emst    SRGTLRAV*--
Ptti    SRGTLRAV*--
Losu    SRGAVRAL*--
Geoy    GRGTLRAV*--
Dipi    SRGTLRAV*--
Pama    SRGALRSI*--
Leob    NRGALRAV*--
Neba    NRGALRVV*--
Pdpl    SRGAIRAV*--
Nimi    SRGSLRAV*--
Uptr    SRGALRTV*--
Pesc    SRGALRAV*--
Baar    SRGALRAV*--
Moar    SRGALRAV*--
Toja    SRGALRAV*--
Chau    SRGTMRVV*--
Chse    SRGTLRAV*--
Enar    SRGTLRAV*--
Hpty    ARGTLRAV*--
Nana    SRGAVRAV*--
Mcst    GRGTVRAV*--
Rhox    SRGTLRGV*--
Opfa    SRGTLRAV*--
Paar    SRGTLRAV*--
Gozo    SRGALRAV*--
Ackr    SRGALRAV*--
Elev    ARGTLRAV*--
Trdu    SRGALRAV*--
Amoc    SRGALRAV*--
Hame    SRGAVRAV*--
Chso    ARGTIRAV*--
Lyto    SRGALRAV*--
```

[4/4 of aligned sequences]

Encr	SRGTLRAV*--
Bvar	SRGALRAV*--
Noco	SRGPVRPIK*-
Chsp	WQGALRAV*--
Arja	SRGSLRAV*--
Pase	SRGAIRAV*--
Trel	ERGAIRVV*--
Acur	SRGTLRAV*--
Ampe	SRGALRAV*--
Urja	GRGPLRAV*--
Enet	SRGALRAV*--
Ptbr	SRGAIRIK*--
Safa	SRGALRYV*--
Icae	DRGTVRAV*--
Asmi	EKGGIRMI*--
Foal	GRGSLRAV*--
Drze	GRGALRAI*--
Rhas	SRGTLRAV*--
Elac	NRGALRAV*--
Kugu	SRGALRAV*--
Plor	GRGTLRAV*--
Sgun	SRGTLRAV*--
Zaco	NRGTLRAV*--
Zbfl	ARGTLRAV*--
Spba	GRGALRAV*--
Game	GRGAVRAV*--
Thth	SRGTVRAV*--
Xigl	SRGALRAV*--
Hyja	ERGTVRAV*--
Psan	DRGAIKAV*--
Cupa	DRGTVRAV*--
Mpch	SRGTLRAV*--
Char	SRGALRAV*--
Pser	SRGALRAV*--
Prol	SRGALRAV*--
Plbi	SRGALRAV*--
Calu	SRGALRTI---
Papa	SRGALRAI*--
Sufr	SRGVLRSV*--
Stci	NRGALRAV*--
Taru	GRGALRAV*--
Rala	SRGALRAV*--
