# Supplementary material for: Structure and variation of the mitochondrial genome of fishes
Source: BMC Genomics. 2016 Sep 7;17(1):719. doi: 10.1186/s12864-016-3054-y (PMC5015259; doi:10.1186/s12864-016-3054-y)
Supplement: Additional file 10: Figure S2. — Aligned nucleotide sequences of the 12S rRNA gene in the mt genomes of 249 fishes. (PDF 537 kb) [file 12864_2016_3054_MOESM10_ESM.pdf]

Additional file 10: Figure S2. Aligned nucleotide sequences of the 12S rRNA gene in mt genomes of 249[*1] fishes.

Species name abbreviation followed by aligned nucleotide sequences. See Additional file 1 for abbreviation of species name. Highlighted letters with red background indicate stem regions, which are denoted by 1 and 1', 2 and 2' and so forth. Unhighlighted red letters indicate bulge structures. Highlighted green and blue letters show regions losing stem structure and tertiary interaction sites, respectively. 'HVR' indicates hyper variable region. See the legend of Additional file 5 for details. *1 Data for one species were not obtained (See text.).

```
             1          2        1'         3        4     5            6
Scca   TGTT-AGGTTTGGTCCTGA-CCTTAGTGTTAATTGCA-ACTAAAATT-ATAC-ATGCAA
Muma   TACA-AGGTTTGGTCCTGG-CCTTAGTGTTAATTGTA-ACTAGAATT-ATAC-ATGCAA
Erca   -CAA-AGATTTGGTCCTAG-TCTTACTATCATTTTCG-ACTAAACTT-ACAC-ATGCAA
Pose   -CAA-AGATTTGGTCCTGG-TCTTACTATCATTTTTCAACTAAACTT-ACAC-ATGCAA
Actr   -CAA-AGGCTTGGTCCTGG-CCTTACTATCAATTTTA-ACCCAATTT-ACAC-ATGCAA
Scal   -CAA-AGGCTTGGTCCTGA-CCTTACTATCAATTTTA-ACCCAATTT-ATAC-ATGCAA
Posp   -CAA-AGGCTTGGTCCTGG-CCTTACTATCAATTTTA-ACCCAATTT-ACAC-ATGCAA
Atsp   -CAA-AGGCTTGGTCCTGA-CCTTGCTATCAGTTTTA-GCCGAACTT-ACAC-ATGCAA
Leoc   -CAA-AGGCTTGGTCCTGA-CCTTGCTATCAGTTTTA-GCCGAACTT-ACAC-ATGCAA
Amca   -CAA-AGGCTTGGTCCTGA-CTTTACTATCATTTCTA-ACTGAACTT-ACAC-ATGCAA
Osbi   -CAA-AAGCATGGTCCCGA-CTTTTATGTCAGCTACA-ACTAAAATT-ACAC-ATGCAA
Pabu   CTAC-AGGCCTGGTCCCGG-CCTTAATATCAACTTTT-GCTAAACTT-ACAC-ATGCAA
Hial   -CAA-AGGCCTGGTCCTGG-CTTTACTGTCGGCTTTA-ACCAAAATT-ACAC-ATGCAA
Elha   -CAA-AGGCCTGGTCCTGG-CTTTACCATCAACCCTA-ACCGAGCTT-ACAC-ATGCAA
Mlcy   -CAA-AGGCCTGGTCCCGG-CTTTACTGTCAATTCCA-ACAGAACTT-ACAC-ATGCAA
Algl   -CAA-AGGCTTGGTCCTGA-CTTTGACATCAATTTTG-GTCTGATTT-ACAC-ATGCAA
Ptgi   -CAA-GAGTCTGGTCCTGA-CTTTAACATCAGTTTTG-GTCGAACTT-ACAC-ATGCAA
Alaf   -CAA-AGGCCTGGTCCTGA-CTTTAGCGTCAGTTTTG-ACTAAAATT-ACAC-ATGCAA
Nock   -CAA-AGGCCTGGTCCTGA-CTTTACCATCAGTTTTG-ACTAAACTT-ACAC-ATGCAA
Anja   -CAA-AAGCCTGGTCCTGA-CTTTAACATCAGTTCTG-GCCTGACTT-ACAC-ATGCAA
Gyki   -AAA-AAGCCTGGTCCTGA-CTTTAACGTCAGTTGTA-ACCCAACTT-ACAC-ATGCAA
Syka   -CAA-AAGCCTGGTCCTGA-CTTTAACGTCAGTTGTG-GCCCAACTT-ACAC-ATGCAA
Opma   -CAA-AGGCCTGGTCCTGA-CTTTAATATCAGTTTTA-ACCCAAATT-ATAC-ATGCAA
Comy   -CAA-AAGCTTGGTCCTGA-CTTTAATATCAATTATG-GCCTAAATT-ACAC-ATGCAA
Sasp   -TAA-AGGTCTAGTCCTGG-CTTTAACATCAGCTACT-ACCCAATTT-ACAC-ATGCAA
Eupe   -TAA-AGGTCTGGTCCTGG-CTTTAACATCACTTATA-ATCTAATTT-ACAC-ATGCAA
Enja   -CAA-AGGTTTGGTCCTAG-CCTTACTATCAGCTTTA-GCTCAAATT-ACAC-ATGCAA
Same   -CAA-AGGTTTGGTCCTGA-CTTTACTATCAGCTTTA-ACCCGACTT-ACAC-ATGCAA
Chch   -CAA-AAGCTTGGTCCTGA-CTTTACCGTCAGCTTTA-ACCTGATTT-ACAC-ATGCAA
Grgr   -CAA-AGGCTTGGTCCTGG-CTTTACTGTCAGCTTTA-ACCCGGATT-ATAC-ATGCAA
Caau   -CAA-AGGCATGGTCCCGA-CCTTATTATCAGCTTTA-ACTCAACTT-ACAC-ATGCAA
Cyca   -CAA-AGGCATGGTCCCGA-CCTTATTATCAGCCCTA-ACTCAACTT-ACAC-ATGCAA
Dare   -CAA-AGGCATGGTCCCGA-CCTTTTGATCAGCTTTT-ACCTAATTT-ACAC-ATGCAA
Cost   -CAA-AGGCTTGGTCCTGA-CTTTACCATCAGCTTTA-GCCCGATTT-ACAC-ATGCAA
Leec   -CAA-AGGCTTGGTCCTGA-CTTTTCTATCAGCTTTA-GCACGACTT-ACAC-ATGCAA
Crla   -CAA-AGGCTTGGTCCTGA-CTTTACTATCAGCCGTA-GCACAACTT-ACAC-ATGCAA
Clmc   -CAA-AGGCCTGGTCCCGA-CTTTACTATCGGCTTTA-GCCCAAATT-ATAC-ATGCAA
Phin   -TAA-AGGCCTGGTCCTGA-CTTTACTGTCAGCCTTA-ATCCAATTC-ATAC-ATGCAA
Icpu   -CAA-AGGCTTGGTCCTGG-CTTTACTATCAGCTTTA-GCCCAATTT-ATAC-ATGCAA
Psto   -CAA-AGGCTTGGTCCTGA-CTTTACTATCAGCTTTA-ACTCAATTT-ATAC-ATGCAA
Cora   -CAA-AGGTTTGGTCCTGA-CTTTACTGTCAGCTTTA-ACCTAATTC-ATAC-ATGCAA
Eisp   -CAA-AGGCTTGGTCCTGA-CTTTACTATCAGCTGTA-GCCCAATTT-ATAC-ATGCAA
Apal   -TAA-AAGCTTGGTCCTGA-CTTTACTATCAGCTATA-GCCCAATTT-ATAC-ATGCAA
```

Eslu	-CAA-AGGCTTGGTCCTGA-CCTTACTATCAGCTTTA-ACTAAATTT-ACAC-ATGCAA
Dape	-CAA-AGGCTTGGTCCTGA-CTTTATTATCAGCTTTA-ATTAAACTT-ACAC-ATGCAA
Glse	-CAA-AGGCTTGGTCCTGA-CTTTACTATCAGCTTTT-ACCAAAACT-ACAC-ATGCAA
Naar	-CAA-AGGCTTGGTCCTGA-CTTTACCATCAGCTTTT-GCCAGATCT-ACAC-ATGCAA
Baoc	-CAA-AGGCTTGGTCCTGA-CTTTACTATCAGCTTTT-ACCAAATTT-ACAC-ATGCAA
Opso	TTAA-AGGCTTGGTCCTGG-CTTTACTATCAGCTTTATACTAAACCT-ATAC-ATGCAA
Alte	-CAA-AGGCTTGGTCCTGA-CTTTACTATCAGCTTTA-GCCTGGTTT-ACAC-ATGCAG
Plap	-CAA-AGGCTTGGTCCTGA-CTTTACTATCAGCTTTA-GCCTAGTTT-ACAC-ATGCAA
Plal	-CAA-AGGCTTGGTCCTGA-CTTTACTTTCAGCTCAA-ACCAAATTT-ATAC-ATGCAA
Sami	-CAA-AGGCTTGGTCCTGA-CTTTACCTTCAGCTTCA-GCCAAATTT-ATAC-ATGCAA
Rere	-CAA-GGGCTTGGTCCTGA-CTTTACTTTCAGCTCAA-ACTACATTT-ATAC-ATGCGA
Gama	-CAA-AGGCTTGGTCCTGA-CTTTACTATCAGCTTTA-ACCAGATTT-ACAC-ATGCAA
Onmy	-CAA-AGGCTTGGTCCTGA-CTTTACTATCAGCTCTA-ACTGAACTT-ACAC-ATGCAA
Sasa	-CAA-AGGCTTGGTCCTGA-CTTTACTATCAGCTCTA-ACTGAACTT-ACAC-ATGCAA
Cola	-CAA-AGGCTTGGTCCTGA-CTTTATTATCAGCTTTA-ACTGAACTT-ACAC-ATGCAA
Dita	-CAA-AGGCTTGGTCCTGA-CTTTACCGTCAGCTTTA-ACCGGATTT-ACAC-ATGCAA
Gogr	-AAA-AGATTTAATCCTAA-CCTTTCTATCAGCTCTA-ACTCAAACT-ACAC-ATGCAA
Chsl	-CAA-AGGCCTGGTCCTGG-CTTTATTGTCAGCTAGA-ACTATATTT-ATAC-ATGCAA
Atja	-CAA-AGGTTTGGTCCTGA-CTTTACTATCAGCTTTG-ACTATACTT-ACAC-ATGCAA
Iido	-CAA-AGGTTTGGTCCTGA-CTTTACTATCAGCTTTG-ACTATACTT-ACAC-ATGCAA
Auja	-CAA-AAGCTTGGTCCTGG-CTTTTATGTCAGCTCTT-ACTAAACTT-ACAC-ATGCAA
Chag	-CAA-AAGCTTGGTCCTGG-CTTTACTGTCATGTTTA-ACCAGATTT-ATAC-ATGCAA
Hami	-TAA-AAGCTTGGTCCTGA-CTTTATTATCAGCTCCG-ACTAAATTT-ACACCATGCAA
Saun	-TAA-AAGCTTGGTCCTGA-GTTTATTGTCAGCTATT-ACTAAATTT-ACAC-ATGCAA
Nema	-CAA-AGGCTTGGTCCTGA-CTTTACTATCAGCCCTA-ACCGAACTT-ACAC-ATGCAA
Disp	-CAACAAGCCTGGTCCTGA-CTTTGTCATCCGCCTTA-ACCAAATTT-ATAC-ATGCAA
Myaf	-CAA-AAGCCTGGTCCTGA-CTTTATTGTCTGCCCTA-ACCAAGATT-ATAC-ATGCAA
Lagu	-CAA-AGATTTGGTCCTAA-TCTTTCCATCGGCCCAG-GTCAGAATT-ACAC-ATGCAA
Trtr	-CAA-AGGCTTGGTCCTGA-CTTTACTATTGGCTTTG-ATTACACTT-ATAC-ATGCAA
Zucr	-CAA-AGGCTTGGTCCTGA-CTTTACTATTGGCCTCG-ATTATACTT-ATAC-ATGCAA
Pxja	-CAA-AGGCTTGGTCCTGA-CTTTACTATCAGCTCTA-GCTAAACTT-ACAC-ATGCAA
Pxlo	-CAA-AGGCTTGGTCCTGA-CTTTACTATCAGCTCTA-GCTAAACTT-ACAC-ATGCAA
Pctr	-CAA-AGGCTTGGTCCTGA-CTTTACTATCAGCTTTA-ACCAAACTT-ATAC-ATGCAA
Apsa	-TAA-AGGCTTGGTCCTAA-CTTTACTGTCAGCTTTA-GCTAAACTT-ACAC-ATGCAA
Cabe	-CAA-AAACTTGGTCCCGA-TTTTATTGTCAGCTATA-GCCAAATTT-ACAC-ATGCAA
Bzze	-CAA-AGGCTTGGTCCTGA-CTTTACTATCAACTTTA-GCTAAATTT-ACAC-ATGCAA
Siim	-CAA-AGGCTTGGTCCTGA-CCTTACTGTCAACTATA-ACTGAACTT-ACAC-ATGCAA
Ctru	-CAA-AGGCTTGGTCCTGA-CTTTACTATCAACTTTA-GCCAAATTT-ACAC-ATGCAA
Dpbr	-CAA-AGGCTTGGTCCTGA-CTTTACTATCAACTTTA-GCCAAATTT-ACAC-ATGCAA
Caki	-CAA-AGGTTTGGTCCTAG-CTTTATTATCAGCTTTA-ACCTATTTT-ACAC-ATGCAA
Phja	-TAA-AAGTTTGATCCTGA-CTTTACTATCAGTTTTA-ACCTAATTT-ACAC-ATGCAA
Brsp	-CAA-AAGCTTGGTCCCGG-CTTTACTATCAGCTGTA-ATCTAAATT-ACAC-ATGCAA
Gamo	-CAA-AGGTTTGGTCCTGA-CTTTACTATCAATTGTA-CCCTAATTT-ACAC-ATGCAA
Lolo	-CAA-AGGTTTGGTCCTGA-CTTTACTATCAATTCTA-CCCCAATTT-ACAC-ATGCAA
Batr	-TAA-AAACTTAGTCTTGG-TTTTATTATTTTCTACC-ACTTAATTC-ATAC-ATGCAA
Prmy	-TAA-AAATTTGGTCTTAA-TTTTATTATCTGTTTTT-ACTAGAATT-ATAC-ATGCAA
Loli	TTAA-AAGTTTGGTCCTGA-CTTTACTGTCAACTTTA-GCTAGGTTT-ATAC-ATGCAA
Loam	-TAA-AAGTTTGGTCCTGA-CTTTATTATCAACTATA-GCTAAACTT-ACAC-ATGCAA
Chab	-CAA-AGGCTTGGTCCTGA-CTTTGCTATCAGTTTTA-GCCTAATTT-ACAC-ATGCAA
Chto	-CAA-AGGCTTGGTCCTGA-CTTTGCTATCAGTTTTA-GCCTAATTT-ACAC-ATGCAA
Majo	ACAA-AGGCTTGGTCCTGA-CTTTACTATCAGCTCTA-GCTAGATTT-ACAC-ATGCAA
Hlst	-TAA-AAGTTTGGTCCTGG-CTTTAATGTCAGTTTTA-ACTCAAATT-ACAC-ATGCAA

Clpe	-CAA-AAGTTTGGTCCTGA-CTTTACTATCAATTTGA-CCCTGGCTT-ATAC-ATGCAA
Mlmr	-CAA-AGGTTTGGTCCTGA-CTTTACTATCAATTTTA-GCCCAACTT-ATAC-ATGCAA
Crcr	-CAA-AGGCTTGGTCCTAG-CTTTTCTATCATCTCTA-GCCCAGATT-ACAC-ATGCAA
Muce	-CAA-AGGCTTGGTCCTAG-CTTTTCTATCATCTCTA-GCCCAGATT-ACAC-ATGCAA
Bege	-CAA-AGGCTTGGTCCTGA-CTTTCCTGTCAGCTTAA-ACTAAATTT-ACAC-ATGCAA
Mela	-CAA-AGGCTTGGTCCTGG-CTTTTCTGTCAACTCTA-GCTAGACTT-ACAC-ATGCAA
Hats	-CAA-AGGCTTGGTCCTGA-CTTTTCTGTCAGCTTTA-GCTAGATTT-ACAC-ATGCAA
Orla	-CAA-AAGTTTGGTCCTGA-CTTTTCTATCAACTCTA-GCTAAACTT-ACAC-ATGCAA
Cosa	-CAA-GAGTTTGGTCCTGA-CTTTTCTATCAGCTCTA-GCTAAACTT-ACAC-ATGCAA
Exsp	-CAA-AGGTTTGGTCCTGT-CTTTACTGTCAGCTTTA-GCTAAACTT-ACAC-ATGCGA
Depa	-CAA-AAGTTTGGTCCTGA-CTTTACTATCAATTGTA-GCTAAACTT-ACAC-ATGCAA
Rima	-TAA-AAGTTTGGTCCTGA-CTTTACCATCAGCTCTG-GCTAAATTT-ACAC-ATGCAA
Fuol	-CAA-AGGCTTGGTCCTGA-CTTTACTATCAGCTTTA-GCAGTATTT-ATAC-ATGCAA
Gmaf	-CAA-AAGCTTGGTCCTGA-CTTTACTATCAGCTTTA-GCAACACTT-ACAC-ATGCAA
Xeei	-TAA-AAGCTTGGTCCTGA-CTTTTCTATCAGCTCTA-GCAAAACTT-ACAC-ATGCAA
Pros	-CAA-AGGCTTGGTCCTGA-CTTTCCTATCAACTTTA-ACTAGATTT-ATAC-ATGCAA
Scmi	-CAA-AGGCTTAGTCCTGA-CTTTACTATCAACTCTA-ACTAGACTT-ACAC-ATGCAA
Rolo	-CAA-AGGCTTGGTCCTGA-CTTTGCTGTCGACTTTA-ACCGGACTT-ACAC-ATGCAA
Cere	-CAA-AAGCTTGGTCCTGG-CTTTACTATCAGCTCTA-ACCAAACTT-ACAC-ATGCAA
Daga	-CGA-AGGCTTGGTCCTGA-CTTCACTATCAACTTTA-ACTAAACTT-ATAC-ATGCAA
Anco	-CAA-AGGCTTGGTCCTGG-CTTTACTATCAACTTTT-ACTAAACTT-ACAC-ATGCAA
Dmve	-CAA-AGGATTAGTCCTAA-CTTTAGTATCACCTTTT-ACTGGATTT-ACAC-ATGCAA
Dmar	-CAA-AGGATTAGTCCTAA-CTTTAGTATCACCTTTT-ACTGGATTT-ACAC-ATGCAA
Anka	-CAA-AGGCTTGGTCCTGG-CTTTACTATCAACTCTT-ACCAAACTT-ACAC-ATGCAA
Moja	-CAA-AGGCTTGGTCCTGG-CTTTACTATCAACTTTT-ACCAAACTT-ACAC-ATGCAA
Hoja	-CAA-AGGCTTGGTCCTGG-CTTTACTATCAACTTTT-ACCAAACTT-ACAC-ATGCAA
Bede	-CAA-AGGCTTGGTCCTGA-CTTTACTATCGACTTTA-ACTAGATTT-ACAC-ATGCAA
Besp	-CAA-AGGCTTGGTCCTGA-CTTTACTATCGACTTTA-ACTAGATTT-ACAC-ATGCAA
Mysp	-CAA-AGGCTTGGTCCTGA-CTTTACTATCAGCTTTA-GCTAAACTT-ACAC-ATGCAA
Osja	-CAA-AGGCTTGGTCCTGA-CTTTACTATCAACTTTTAGCCAAACTT-ACAC-ATGCAA
Sgro	-CAA-AGGCTTGGTCCTGA-CTTTACTATCAGCTTTA-GCCAAACTT-ACAC-ATGCAA
Pzpa	-CAA-AGGCTTGGTCCTGA-CTTTTCTATCAGCTCTA-GCCAAATTT-ACAC-ATGCAA
Zeja	-CAA-AGGCTTGGTCCTGA-CTTTTCTATCAGCTTTA-ACCAAATTT-ATAC-ATGCAA
Znne	-CAA-AGGCTTGGTCCTGG-CTTTTCTGTCAGCTCTA-ACCAAATTC-ATAC-ATGCAA
Zefa	-CAA-AGGCTTGGTCCCGG-CTTTTCTGTCAGCTCTA-ACTAAATTT-ATAC-ATGCAA
Acni	-CAA-AGGCTTGGTCCTGA-CTTTTCTATCAGCTTTA-ACCAAATTT-ACAC-ATGCAA
Ncrh	-CAA-AGGCTTGGTCCTGA-CTTTTCTATCAGCTTTA-ACCAAATTT-ACAC-ATGCAA
Agca	-CAA-AGGCTTGGTCCTGA-CTTTACTATCAATTATA-GCTAAACTT-ACAC-ATGCAA
Hydy	-CAA-AGGCTTGGTCCTGA-CTTTGCTGTCAACTTTA-GCTCAACTT-ACAC-ATGCAA
Gsac	-CAA-AGGCTTGGTCCTGA-CTTTGACATCAACTTTA-GCCAAACTT-ACAC-ATGCAA
Pevo	-CAA-AGGCTTGGTCCTGA-CTTTTCTGTCAGCTTTG-GCTAGACTT-ACAC-ATGCGA
Hiku	-CAA-AGGCTTGGTCCTAG-CTTTACTATTATTTATA-ACCAAACTT-ACAC-ATGCAA
Inpa	-CAC-AGGTTTGGTCCTGG-CTTTTCTATCAATTCTA-GCCGAGCTT-ATAC-ATGCAA
Auch	-TAA-AGGCTAGGTCCTGG-CCTTGCAATCAGCCTAG-GCTACACTT-ACAC-ATGCAA
Fico	-CAA-AGGCTTGGTCCTGA-CTTTACTATCAACTCTG-ACTAAACTT-ACAC-ATGCAA
Macs	-CAA-AGGCTTGGTCCTGA-CTTTGCTGTCAACTCTA-GCTGTACTT-ACAC-ATGCAA
Moal	-CAA-AGGTTTGGTCCTGG-CTTTATCATCAACTATA-ACTAAACTT-ACAC-ATGCAA
Syma	-CAA-AGGTTTGGTCCTAG-CTTTGTTATCAGCTATA-CCTGGACTT-ATAC-ATGCAA
Mafr	-CAA-AGGTTTGGTCCTGA-CTTTTCTGTCAACTCTA-ACTAAACTT-ACAC-ATGCAA
Dcpe	-CAT-AAGCTTAGTCCCAA-CTTTGCTATCGATTCTG-GCTAGATTT-ACAC-ATGCAA
Dcti	-CAT-AAGCTTAGTCCCAA-CTTTGCTATCGATTTTG-GCTAAATTT-ACAC-ATGCAA
Hehi	-CAA-AGGCTTGGTCCTGA-CTTTACTATCAACTTTA-GCCAAATTT-ACAC-ATGCAA

```
Stam   -CAA-AGGCTTGGTCCTGA-CTTTACTGTCAACTTTA-GCTAAATTT-ACAC-ATGCAA
Hogi   -CAA-AAGCTTGGTCCTGA-CTTTACTATCAGCTTTA-GCCAAATTT-ACAC-ATGCAA
Erzo   -CAA-AGGCTTGGTCCTGA-CTTTACTATCAACTTTA-GCCAAACTT-ACAC-ATGCAA
Hxot   -CAA-AGGCTTGGTCCTGA-CTTTACTATCAACTTTA-GCCAAACTT-ACAC-ATGCAA
Core   -CAA-AGGCTTGGTCCTGA-CTTTATTATCAACTTTA-GCCAAACTT-ACAC-ATGCAA
Apve   -CAA-AGGCTTGGTCCTGA-CTTTACTATCAACTTTA-GACAAATTT-ACAC-ATGCAA
Latj   -CAA-AGGCTTGGTCCTAA-CTTTACTGTCAGCTCTA-GCTAGATTT-ACAC-ATGCAA
Laja   -CAA-AAGCTTGGTCCTGA-CTTTACTATCAACTCTA-GCTAAACTT-ACAC-ATGCAA
Syja   -CAA-AAGTTTGGTCCTGA-CTTTAGTATCAACTTTA-GCTAGACTT-ACAC-ATGCAA
Epme   -CAA-AGGCTTGGTCCTGA-CTTTCCTATCAGCTTTA-ACCAAATTT-ACAC-ATGCGA
Grse   -CAA-AGGCTTGGTCCTGA-CTTTTCTATCAACTCTA-ACTAGACTT-ACAC-ATGCAA
Clja   -CAG-AAGCTTGGTCCTAG-CTTTATTAACAGCTCTA-GCTAAACTT-ACAC-ATGCAA
Ogcy   -TAA-AAATTTGGTCCTGA-TTTTACTGTCGGCTTTA-GCCAGATTT-ACAC-ATGCAA
Plna   -CAA-AAGCTTGGTCCTGG-CTTTTCTATTAGCTCTA-GCTAAACTT-ACAC-ATGCAA
Lema   -CAA-AGGCTTGGTCCTGA-CTTTATTATCAACTTTA-GCCAAACTT-ACAC-ATGCAA
Etzo   -CAA-AGGCTTGGTCCTGA-CTTTACTATCAACTCTA-GCTAAACTT-ACAC-ATGCAA
Apse   -CAA-AGGCTTGGTCCTGA-CTTTACTGTCAACTGTA-GCTAAACTT-ACAC-ATGCAA
Epde   -CAA-AGGCTTGGTCCTGA-CTTTACTATCAACTTTA-GCTAAACTT-ACAC-ATGCAA
Slja   -CAA-AGGCTTGGTCCTGG-CTTTACTATCAGCTCTA-GCTAAACTT-ACAC-ATGCAA
Bsja   -TAA-AGGCTTGGTCCTGA-CTTTACTAACAGCTTTG-TCCTAACCT-ACAC-ATGCAA
Ecna   -CAA-AGGCTTGGTCCTGA-CTTTACTGTCTGCTTTA-GCCATATTT-ACAC-ATGCAA
Cohi   -CAA-AGGTTTGGTCCTGG-CTTTACTGTCAGCTTTA-GTTAAACTTTACAC-ATGCAA
Caar   -CAA-AGGTTTGGTCCTGA-CTTTACTGTCAGCTTTA-GCTAGATTT-ACAC-ATGCAA
Came   -CAA-AGGTTTGGTCCTGA-CTTTACTGTCAGCTTTA-GCTAGATTT-ACAC-ATGCAA
Mema   -CAA-AGGCTTGGTCCTGA-CTTTACTATCAGCTTTA-GCCAAACTT-ACAC-ATGCAA
Lenu   -CAA-AGGTTTGGTCCTGA-CCTTACTGTCAATTCTA-GCTAAAACT-ATAC-ATGCAA
Plma   -TAA-AGGCTTGGTCCTGA-CTTTACTATCAACTCTA-GCTAAACTT-ACAC-ATGCAA
Emst   -CAA-AGGTTTGGTCCTGA-CTTTACCATCAACTTTA-GCTAAACTT-ACAC-ATGCAA
Ptti   -CAA-AGGTTTGGTCCTGA-CTTTACTATCAACTTTA-GCTAAACTT-ACAC-ATGCAA
Losu   -CAA-AGGTTTGGTCCTGG-CCTTACTAACAGCTCTA-ACCAAATTT-ACAC-ATGCAA
Geoy   -CAA-AAGCTTGGTCCTGG-CTTTGTTGTCAGCTTTA-ACTTAATTT-ACAC-ATGCAA
Dipi   -CAA-AGGTTTGGTCCTGA-CTTTATTATCAACTTTA-GCTAAACTT-ACAC-ATGCAA
Pama   -CAA-AGGTTTGGTCCTGA-CTTTTCTACCAGCTTTA-GCTAGACTT-ACAC-ATGCAA
Leob   -CAA-AGGTTTGGTCCTGA-CTTTCCTGTCAGCTTTA-ACTCAACTT-ACAC-ATGCAA
Neba   -CAA-AAGTTTGGTCCTGA-CTTTATTGTCAGCTTTA-GCTAGATTT-ATAC-ATGCAA
Pdpl   -CAA-AGGCTTGGTCCTGA-CTTTACTGTCAGCTTTA-GCTTGACTT-ACAC-ATGCAA
Nimi   -CAA-AAGCTTGGTCCTGA-CTTTACTATCAACTTTA-GCTATATTT-ACAC-ATGCAA
Uptr   -CAA-AGGCTTGGTCCTGA-CTTTACTATCAGCTCTA-GCTAGATTT-ACAC-ATGCAA
Pesc   -CAA-AAGCCTGGTCCTGA-CTTTATTGTCAGCTATT-ACTAAACTT-ACAC-ATGCAA
Baar   -TAA-AAGTTTAGTCCTGG-CTTTACTGTCAGCTTTA-GCCGAAACT-ACAC-ATGCAA
Moar   -CAA-AGGTTTGGTCCTGA-CTTTACTATCAACTTTA-GCTAAACTT-ACAC-ATGCAA
Toja   -CAA-AGGTTTAGTCCTGA-CTTTACTATCAGCCCTA-GCCTAATTT-ACAC-ATGCAA
Chau   -CAA-AGGTTTGGTCCTGA-CTTTACTGTCAGCTATA-GCTAAATTT-ACAC-ATGCAA
Chse   -CAA-AGGTTTGGTCCTGG-CCTTCCTAACAACTATA-GCTAAACTT-ACAC-ATGCAA
Enar   -CAA-AAGCTTGGTCCTGA-CTTTACTATCAACTTTA-GCTAGACTT-ACAC-ATGCAA
Hpty   -CAA-AAGCTTGGTCCTGA-CTTTACTATCAACTTTA-GCTAAACTT-ACAC-ATGCAA
Nana   -CAA-AAGCTTGGTCCTGG-CTTTGACCTCAGCTCTA-GCCAAATTT-ACAC-ATGCAA
Mcst   -CAA-AGGCTTGGTCCTGA-CTTTACTATCAGCTCTA-ACCAAACTT-ACAC-ATGCAA
Rhox   -CAA-AGGCTTGGTCCTGA-CTTTACTATCAACTCTA-GCTAAACTT-ACAC-ATGCAA
Opfa   -CAA-AGGCTTGGTCCTGA-CTTTACTATCAGCTCTA-GCCAAACTT-ACAC-ATGCAA
Paar   -CAA-AGGTTTGGTCCTGG-CCTTGCTCTCAACTTTT-ACTAAACTT-ACAC-ATGCAA
Gozo   -CAA-AGGCTTGGTCCTGA-CTTTATTATCAGCTTTA-GCTAGACTT-ACAC-ATGCAA
```

```
Ackr    -CAA-AGGCTTGGTCCTGA-CCTTGCTATCAACTTTA-GCTATGCTT-ACAC-ATGCAA
Elev    -CAA-AAGCTTGGTCCTGA-CTTTACTATCAACTTTA-GCTAAAATT-ACAC-ATGCAA
Trdu    -CAA-AGGCTTGGTCCTGA-CTTTACTGTCAGCTTTG-GCTAAACTT-ACAC-ATGCAA
Amoc    -TAA-AGGCTTGGTCCTGA-CTTTACTGTCAGCTCTG-GCTAAACTT-ACAC-ATGCAA
Hame    -CAA-AGGCTTGGTCCTGA-CTTTGCCATCAACTTTA-ACTAGATTT-ACAC-ATGCAA
Chso    -CAA-AGGTTTGGTCCCGGCCTT-ATTCTCAACTGTA-GCTGAACTT-ACAC-ATGCAA
Lyto    -CAA-AGGCTTGGTCCTGA-CTTTACTATCAACTTTA-GCTAAACTT-ACAC-ATGCAA
Encr    -CAA-AGGCTTGGTCCTGA-CTTTACTATCACCTTTA-GCTAAACTT-ACAC-ATGCAA
Bvar    -CAA-AGGCCTGGTCCTGA-CTTTATTATCAGCTTCA-GCCAAACTT-ACAC-ATGCAA
Noco    -CAA-AGGCTTGGTCCTGA-CTTTATCATCAGCTTCA-GCCGAACTT-ACAC-ATGCAA
Chsp    -CAA-AAGTTTGGTCCCGG-CTTTATAGTCACCTTTG-GCCCAACTT-ACAC-ATGCAA
Arja    -CAA-AGGCTTGGTCCTGA-CTTTATTATCAACTTTA-GCCAAATTT-ACAC-ATGCAA
Pase    -CAA-AAGTTTGGTCCTGG-CTTTCTTATCTGCTGTA-ACTAGACAT-ACAC-ATGCAA
Trel    -CAA-AGGCTTGGTCCTGA-CTTTTCTCTCAGCTTTG-GCTAAGTTT-ACAC-ATGCAA
Lifa    -TAA-AAGCTTAGTCCTGG-CTTTACTGTCAGCTTTA-GTCCAACTT-ACAC-ATGCAA
Acur    -CAA-AAGCTTGGTCCTCG-CTTTCCTATCAGCTGCG-ACTAATATT-ACAC-ATGCAA
Ampe    -CAA-AGGCTTGGTCCTGA-CTTTACTATCAACTTTA-GCTAAACTT-ACAC-ATGCAA
Urja    -CAA-AGGCTTGGTCCTGA-CCTTGCTATCATCTTTA-GCCAGACTT-ACAC-ATGCAA
Enet    -CAT-AGGCTTGGTCCTGA-CTTTCCTATCAGCTTTA-GCTTAATTT-ACAC-ATGCAA
Ptbr    -CAA-AGGCTTGGTCCTGA-CCTTGTTGTCAACTTTA-GCTTAATTT-ACAC-ATGCAA
Safa    -CAA-AGGCTTGGTCCTGA-CTTTACTGTCAACTCTG-GCTAGATTT-ACAC-ATGCAA
Icae    -CAA-AGGCTTGGTCCTGA-CTTTACTGTCAACTCTA-ACTAAACTT-ACAC-ATGCAA
Asmi    -AAG-AAACTTGGTCCTGA-TTTTATTGTCTTCTCTAGCTTCAACTT-ACAC-ATGCAA
Foal    -CAA-AGGCTTGGTCCTGA-CTTTATTATCAGTATTA-GCTGAGTTT-ATAC-ATGCAA
Drze    -CAA-AGGTTTGGTCCTAG-CTTTAATATCAGTTTTA-ACTAAACTT-ACAC-ATGCAA
Rhas    -CAA-AAGCTTGGTCCTGA-CTTTACTATCAGCTCTG-GCCAGACTT-ACAC-ATGCAA
Elac    -CAA-AAGCTTGGTCCTGA-CTTTACTATCAGCTTTG-GCCAAATTT-ACAC-ATGCAA
Kugu    -CAA-AGGCTTGGTCCTGA-CTTTACTATCAGCTTTA-GCCAAACTT-ACAC-ATGCAA
Plor    -CAA-AAGCTTGGTCCTGA-CTTTACTATCAACTCTA-GCCAAACTT-ACAC-ATGCAA
Sgun    -CAA-AAGTTTGGTCCTGA-CTTTGCTATCAGCTTTA-GCTAAGCTT-ACAC-ATGCAA
Zaco    -CAA-AAGTTTGGTCCTGA-CTTTACTAACAACTCCA-GCTAGATTT-ACAC-ATGCAA
Zbfl    -CAA-AGGTTTGGTCCTGA-CTTTACTAACAACTTTA-TCTAAACTT-ACAC-ATGCAA
Spba    -CAA-AGGCTTGGTCCTGA-CTTTACTGTCAGCTTTG-ACTAGATTT-ACAC-ATGCAA
Game    -CAA-AGGCTTGGTCCTGA-CTTTACTGTCAACTCTA-ACTAGACTT-ACAC-ATGCAA
Thth    -CAA-AGGCTTGGTCCTGA-CTTTACTGTCAACTCTA-GCTAAACTT-ACAC-ATGCAA
Xigl    -CAA-AGGCTTGGTCCTGA-CTTTACTGTCAGCTTTA-GCTATACTT-ACAC-ATGCAA
Hyja    -CAA-AGGCTTGGTCCTGA-CTTTTCTGTCAGCTTTA-GCTAGATTT-ATAC-ATGCAA
Psan    -GAA-AGGCTTGGTCCTGA-CTTTTCCGTCAGCTTTA-GCTAGATTT-ATAC-ATGCAA
Cupa    -CAA-AGGCTTGGTCCTGA-CTTTACTGTCAACTCTA-GCTAAACTT-ACAC-ATGCAA
Mpch    -CAA-AGGCTTGGTCCTGA-CTTTATTATCAGCTACA-ACTAAACTT-ACAC-ATGCAA
Char    -CAA-AGGTTTGGTCCTGA-CTTTACTATCAGCTGTA-GCCACACTT-ACAC-ATGCAA
Pser    -CAA-AAGTTTGGTCCCGG-CTTTACTGTCAACTGTA-GCTAAACTT-ACAC-ATGCAA
Prol    -CAA-AGGCTTGGTCCTGA-CTTTACTGTCGACTCTA-ACTAGACTT-ACAC-ATGCAA
Plbi    -CAA-AGGCTTGGTCCTGA-CTTTACTGTCGACTTTA-ACTAAACTT-ACAC-ATGCAA
Calu    -CAA-AGGCTTGGTCCTGG-CCTTACTGTCGGCTGTA-ACTCAAATT-ACAC-ATGCAA
Papa    -CAA-AAGTTTAGTCCTGA-CTTTACTATCAGCTCTA-GCAGTGCTT-ACAC-ATGCAA
Sufr    -CAA-AGGCTTGGTCCTGA-CTTTTCTGTCAACTCTA-ACCTAACTT-ACAC-ATGCAA
Stci    -CAA-AAGCTTGGTCCTGA-CTTTTCTATCAACTCTA-ACCTATCTT-ACAC-ATGCAA
Taru    -CAA-AAGCTTGGTCCTGA-CTTTACTAACAACTCTG-ATCAAACTT-ACAC-ATGCAA
Rala    -CAA-AGGCTTGGTCCTGA-CTTTACTAACAACTCTA-GCTTAACTT-ACAC-ATGCAA
                     **       *                         *  * ****
```

```
               7     7'      8          9 ¦ HVR  ¦10 ¦  HVR   ¦10'
Scca    GTTTCAGCCCCCCTGTGAG---AATGCCCTAAGT-----ATTCTATTA-----AATAATT
Muma    GTTTCAGCCCTCCTGTGAG---AATGCCCTAATT-----AATCTATTA-----AATAATT
Erca    GTATCCGCACTCCGGTGAA---AATGCCCTTAATC----TTCCTAGAA-----GAAGACA
Pose    GTATCCGCGCTCCGGTGAA---AATGCCCTTAATC----TTCCTAGTA-----GAAGACG
Actr    GTCTCCGCACCCCTGTGAG---AATGCCCTTAATC----CCCCTGCCACATA-GGGGAAA
Scal    GTCTCCGCACCCCTGTGAG---AATGCCCTTAATC----CCCCAACCACATA-GGGGAAA
Posp    GTCTCCGCACCCCTGTGAG---AATGCCCTTAATC----CCCCACCACATA--GGGGAAA
Atsp    GTATCCGCACCCCCGTGAG---AATGCCCTTTAAC----CCCAACCA------GGAGATA
Leoc    GTATCCGCACCCCTGTGAG---AATGCCCTTCAAAC---CCTAAGCA------GGAGATA
Amca    GTATCCGCCCCCCTGTGAG---GATGCCCTTAACCT---CCCCCCAC------GGAGATA
Osbi    GTGTCCGCGCCCCCGTGAG---AATGCCCTCACT-----GCCTAGC-------GGTTTAG
Pabu    GTATCCGCACCCCAGTGAG---AATGCCCTCAAAAC---CTAAA---------GGACTAG
Hial    GTATCCGCACCCCCGTGAG---GATGCCCTCGATC----CCCCCTCC------GGAGACG
Elha    GTCTCCGCACCCCTGTGAG---GATGCCCTCGATC----CTCTAAT-------GAGGACG
Mlcy    GTCTCAGCAAACCCGTGAG---AATGCCCTCAATC----CCCTACT-------GGAGACG
Algl    GTTTCCGCGTCCCCGTGAG---AATGCCCTTGGTC----CCCCTCAG------GGGGACG
Ptgi    GTCTCCGCACACCCGTGAG---GATGCCCTTGATCC---CCCAATT-------GGGGACG
Alaf    GTATCCGCACCCCGGTGAG---AATGCCCTTGAT-----CTTCCTCC------GAGGACA
Nock    GCCTCCACGCCCCAGTGAG---AATGCCCTTGTT-----CCCCTTAT------GGGGACA
Anja    GTACCCGCGCACCCGTGAG---AATGCCCTATATC----CCCTCCC-------GGGGAAA
Gyki    GTACCCGCACCCCTGTGAG---AATGCCCTTTCCC----CCCGGCA-------GGAAAAA
Syka    GTATCCGCACCCTCGTGAG---AATGCCCTTCATC----CCCCTCCC------GGGGAGA
Opma    GTACCCGCACCCCCGTGAG---AATGCCCCTCATCC---CCCACGCC------GGGGAAA
Comy    GTACCCGCACCCCTGTGAG---AATGCCCTCTTTC----CTCTAAT-------GAAGAAG
Sasp    GTACCCGCACACCCGTGAG---AATGCCCTGAACC----CTCTCTAT------GAGGAAC
Eupe    GTATCTGCACACCCGTGAG---AATGCCCTACGCC----CCTTACTTAT----AGGGATT
Enja    GTCTCCGCAGTCCCGTGAG---GATGCCCCCAACT----TCCCACCC------GGAAATA
Same    GCCTCCGCACCCCTGTGAG---GATGCCCTCAATC----CCCCGTCC------GGGGACG
Chch    GTATCCGCACCCCTGTGAG---AATGCCCTCATTC----CCCCGCCC------GGGGACG
Grgr    GTATCCGCACCCCTGTGAG---GATGCCCTCGGTG----CTCCATCC------GAGAACG
Caau    GTCTCCGCACCCCAGTGAA---TATGCCCTCAATCC---CCCTACCC------GGGGACG
Cyca    GTCTCCGCAACCCAGTGAA---TATGCCCTCAATCC---CCCCACCC------GGGGACG
Dare    GTCTCCGCACCCCTGTGAA---TACGCCCTCAATCC---CCAAACCT------GGGGACG
Cost    GTCTCCGCAAACCCGTGAG---AATGCCCTCAATCC---CCCGCCC-------GGGGACG
Leec    GTATCCGCAGCCCTGTGAG---GATGCCCTTAATC----CCCAGCCC------GAGGACG
Crla    GTCTCCGCAGCCCCGTGAG---GATGCCCTTAAT-----CCCTGCCC------GGGGACG
Clmc    GTATCCGCACCCCTGTGAG---AATGCCCTCAATC----CTCCACCC------GAGAACG
Phin    GTATCCGCACCCCCGTGAG---AATGCCCCCAGC-----CTCCCCCCA-----GAGC-CG
Icpu    GTCTCCGCACCCCTGTGAG---AATGCCCTCAATC----CCCTGCCC------GGGGACG
Psto    GTATCCGCATCCCTGTGAG---AATGCCCTCAATC----CTCTGCCC------GAGAACG
Cora    GTATCCGCACCCCTGTGAG---AATGCCCTCAATCC---CCCTCCC-------GGGGACG
Eisp    GTATCAGCACCCCAGTGAG---AATGCCCTCAATC----CCCCGTCC------GAGGATG
Apal    GTATCCGCACCCCCGTGAG---AATGCCCTCAATT----CCCCATCC------GGGAGCG
Eslu    GTCTCCGCACCCCTGTGAG---GATGCCCTTAATC----CCCTGCCC------GGGGCTG
Dape    GTCTCCGCACTCCTGTGAG---AATGCCCTTAATC----CCCTGCCC------GGGGCTG
Glse    GTCTCCGCACCCCTGTGAG---AATGCCCTTAATC----CCCCGCCC------GGGGACG
Naar    GTCTCCGCCCCCCTGTGAG---GATGCCCTTAATC----CCCTGCCC------GGGGACG
Baoc    GTCTCCGCCCCCCTGTGAG---GATGCCCTTAATC----CCCCGCCC------GAGGACG
Opso    GTCTCCGCATCCCTGTGAG---AATGCCCTTAATC----CCCCGCCC------GGGGGTG
Alte    GTCTCCGCACCCCTGTGAG---AATGCCCTTAATC----CCCTGTCC------GGGGACG
Plap    GTCTCCGCACCCCTGTGAG---AATGCCCTTAATC----CCCCACCC------GGGGACG
```

Plal	GTCTCCGCACCCCCGTGAG---GATGCCCTTTACC----CCCTGCCC------GGGGGCA
Sami	GTCTCCGCGTCCCTGTGAG---GATGCCCTTTACC----CCCCGCCC------GGGGGCA
Rere	GTCTCCGCAGGGGTGTGAG---GATGCCCTTTGCCC---CCCCCCCC------GGGGGCA
Gama	GTCTCCGCACCCCTGTGAG---GATGCCCTTAATC----CCCCGGCC------GGGGATG
Onmy	GTCTCCGCATTCCTGTGAG---GATGCCCTTAAT-----CCCTGCCC------GGGGACG
Sasa	GTCTCCGCATTCCTGTGAG---GATGCCCTTAAT-----CCCTGCCC------GGGGACG
Cola	GTCTCCGCACTCCTGTGAG---GATGCCCTTAATC----CCCTGCCC------GGGGACG
Dita	GTTTCCGCACCCCCGTGAG---GATGCCCTCAATT----TCCCACCC------GGAAACG
Gogr	GCCTCCGCCCCCCAGTGAG---AATGCCCTTAATC----CCCTAACCT-----GGGATC-
Chsl	GTCTCCGCGACCCTGTGCG---AATGCCCTTTACC----CTCCCTTG------GAGGA-G
Atja	GTATCCGCACCCCTATGAG---AATGCCCTTAACT----TCCAGGACT-----AGAAGAA
Iido	GTATCCGCACCCCTATGAG---AATGCCCTTAACT----TCCAGGACT-----GGAAGAA
Auja	GCCTCTACAACCCCGTGAG---AATGCCCTCAATC----TCCCGCCCG-----GAGA-CG
Chag	GTTTCCGCACCCCTGTGAG---GATGCCCTCAAACT---TTCCCAGCC-----GAAAACG
Hami	GCATCCACCCCCCTGTGAG---AATGCCCTCGATC----CCCTATTT------GGAAACG
Saun	GCATCTACCCCCCTGTGAG---AATGCCCTCGATCC---CCTAATT-------GGAAACG
Nema	GTATCCGCACCCCCGTGAG---AATGCCCTCAAAACC--CCGCCC--------GGGAATG
Disp	GTATCCGCACCCCCGTGAG---AATGCCCCTAAAA----TCCTACCC------GGAAACG
Myaf	GTATCCGCACCCCCGTGAG---AATGCCCTCAAAA----TCCCGCCC------AGAAACG
Lagu	GCGTCGGCGCCCCCGTGAG---AATGCCCTCAACT----TCCTACCC------GAAGAAA
Trtr	GTATCCGCGCCCCTGTGAG---AATGCCCTCAAC-----CCCTACCC------GGGGCTA
Zucr	GTATCTGCACCCCTGTGAG---AATGCCCTCAAC-----CCCCGACC------GGGGCTA
Pxja	GTATCCGCGCCCCTGTGAG---AATGCCCTCAGTT----ACCTGCCC------GGAAACA
Pxlo	GTATCCGCACCCCTGTGAG---AATGCCCTCAGTT----ACCTGCCC------GGAAACA
Pctr	GTATCCGCGCCCCCGTGAG---AATGCCCTCAGCT----TCCTGCCC------GGAAGCA
Apsa	GTATCCGCGCCCCCGTGAG---AATGCCCTCAACT----TCCCAGCC------GGAAACA
Cabe	GTATCCGCGCCCCTGTGAG---AATGCCCTCATCCT---CCCGCCC-------GGAAGAG
Bzze	GTCTCCGCCTCCCCGTGAG---AATGCCCTCATCT----TCCCGCAC------GGAACAG
Siim	GTCTCCGCACCCCTGTGAG---AATGCCCTCATAT----TCCTGCCC------GGAAGAG
Ctru	GTATCCGCCACCCCGTGAG---AATGCCCTCACCT----CCCCGTCC------GGGAAAG
Dpbr	GTATCCACGCCCCAGTGAG---AATGCCCTCACCT----CCCCCTCC------GGGAAAG
Caki	GTCTCCGCACCCCTGTGAG---AATGCCCTTATTGT---CCTGCCCA------GGAACTA
Phja	GTCTCTGCATTCCAGTGAA---AATGCCCTCGGTTA---CCCTAATCC-----GGGACTG
Brsp	GTCTCCGCACCCCTGTGAA---GAAGCCCTCAGCCC---CCCAACCG------GAGGCCC
Gamo	GTCTCCGCCTCCCCGTGAG---AATGCCCTTAATG----TCCTGCCC------GGAATTA
Lolo	GTCTCCGCATCCCCGTGAG---AATGCCCTTAATG----TCCTGCCC------GGAATTA
Batr	GACTCATCACCCCAGTGAG---GCAGGCCTATGACCC--TTTGCTTA------AAAGGCA
Prmy	GCCTCCACAACCCTGTGAG---AATGCCCATTAC-----TCTATTTC------GGACAAA
Loli	GTCTCCGCACCCCTGTGAATC--ATGCCTATGCAT----CTCCTCCTA-----GAGAGCA
Loam	GTCTCCACGCCCCTGTGAAGT--ACGCCCTATGTGT---CTCCCCCCA-----GAGAACA
Chab	GTATCCGCCCCCCCGTGAG---AATGCCCTTTAGTC---CCCTGCCC------GGGGAAA
Chto	GTATCCGCCCCCCCGTGAG---AATGCCCTTTAGTC---CCCTGCCC------GGGGAAA
Majo	GCATCCGCCCCCCTGTGAG---AATGCCCTCTTCGTC--CTCCCTTTC-----GAGAACA
Hlst	GCATCCACATTCCGGTGAA---AACGCCCTACCGTA---CCCCACCCC-----GGGTACA
Clpe	GTATCCGCACCCCTGTGAG---AATGCCCCACAGTT---CCCCGCCT------GGGAACA
Mlmr	GTATCCGCGCCCCGGTGAG---AATGCCCTACAATT---CCCTACTA------GGGAACA
Crcr	GTCTCCGCTCCCCCGTGAG---AATGCCCTGCAGTTC--CCCTACCC------GGGAACA
Muce	GTCTCCGCTCCCCCGTGAG---AATGCCCTGCAGTTC--CCCTACCC------GGGAACA
Bege	GTATCCGCCCGCCCGTGAG---AATGCCCCACAGTT---TCCCGCCC------GGAAACA
Mela	GTATCCGCATACCCGTGAG---GATGCCCTACAGTC---CCCCGCCC------GGAGACA
Hats	GTATCCGCCTACCCGTGAG---AATGCCCCACAGTT---TCCCGCCC------GGAAACA
Orla	GTATCCGCAGTCCCGTGAG---AATGCCCTACAGTT---TCCTTAAT------GGAAACA

Cosa	GTATCCGCACCCCCGTGAG---AATGCCCTTTAGTT---CCCCACCC------GGGAACA
Exsp	GTATCCGCACCCCTGTGAG---AATGCCCTAAAGTT---CCCTA-CC------GGAAACA
Depa	GTATCAGCACCCCCGTGAG---AATGCCCTGCAGTT---TCCTTTTA------GGAAACA
Rima	GTATCCGCACCCCCGTGAG---AATGCCCTGCTT-----TTCTTCAT------GAAAAAA
Fuol	GTATCCGCACTCCTGTGAG---AATGCCCTCAGTC----TCCTCTTCA-----GGAAACA
Gmaf	GTATCCGCACCCCAGTGAA---AATGCCCCCCGCC----TTCTTAAA------GAAGACA
Xeei	GTATCCGCACCCCCGTGAA---TATGCCCTACAATC---TTCTTCTAAG----GAAATTA
Pros	GTATCCGCACCCCTGTGAG---AATGTCCTCACCGC---CCCGTCC-------GGGGCTG
Scmi	GTATCCGCACCCCTGTGAG---AATGCCCTCCCCTC---CCTGCCCA------GGGAACG
Rolo	GTCTCCGCACCCCTGTGAG---AATGCCCTCATCC----CCCTGCCC------GGAACTG
Cere	GTCTCCGCACCCCCGTGAG---AATGCCCTCATCC----CCCCGCTC------GGGATCA
Daga	GTCTCCGCAATCCCGTGAA---AATGCCCTTATCC----CCCCGCTC------GGAAATA
Anco	GTCTCCGCACCCCTGTGAG---AATGCCCTCAGTC----CCCCGCCC------GGGAACA
Dmve	GTCTCCGCACCCCCGTGAG---AATGCCCTTGAGT----CCTAAAACT-----AGGGACA
Dmar	GTCTCCGCACCCCTGTGAG---GATGCCCTTGAGTC---CTAGAACT------AGGGACA
Anka	GTCTCCGCACCCCCGTGAG---AATGCCCTCAGTC----CCCCACCC------GGGAACA
Moja	GTCTCCGCACCCCCGTGAG---AATGCCCTCAGTC----CCCCACCC------GGGAACA
Hoja	GTATCCGCACCCCCGTGAG---AATGCCCTCAGTC----CCCCTCCC------GGGAACA
Bede	GTATCCGCACCCCTGTGAG---AATGCCCTCATCTC---CCTGCCC-------GGGGCTG
Besp	GTATCCGCACCCCTGTGAG---AATGCCCTCATCT----CCCTGCCC------GGGGCTG
Mysp	GTCTCAGCACCCCCGTGAG---AATGCCCTCAGCC----CTCTGCCC------GAGGACA
Osja	GTCTCAGCACCCCCGTGAG---AATGCCCTCAGTC----CCCCGCCC------GGGGACA
Sgro	GTCTCCGCACCCCCGTGAG---AATGCCCTCAGTC----CCCTGCCC------GGGGACA
Pzpa	GTCTCCGCACCCCCGTGAG---AATGCCCTAAGTA----TCCCCC--------GGAACTA
Zeja	GTCTCCGCGCCCCCGTGAG---AATGCCCTAAATA----CCCTGCCC------GGAATTA
Znne	GTTTCCGCACCCCTGTGAG---AATGCCCTAGATA----ACCTGCCCG-----GAATT-T
Zefa	GTTTCCGCACCCCTGTGAG---AATGCCCTAGATA----ACCTGCCC------GGAATTT
Acni	GTCTCCGCACCCCCGTGAG---AATGCCCTAAATA----CCCTGCCC------GGAATTA
Ncrh	GTCTCCGCACCCCCGTGAG---AATGCCCTAAATA----CCCTGCCC------GGAATTA
Agca	GTATCCGCACCCCTGTGAG---AATGCCCTACAGTT---CCCCGCCC------GGGAACA
Hydy	GTATCCGCACCCCTGTGAG---AATGCCCTACAGCC---CCCCGCCC------GGGGGCA
Gsac	GTATCCGCACCCCTGTGAG---GATGCCCTACAGTT---CCCCGCCC------GGGAACA
Pevo	GTATCCGCAACCCCGTGAA---AATGCCCTCCAAT----CTCTTGC-------GAGAACG
Hiku	GCATCCGCACTCCCGTGAG---AATGCCCTTAACC----CTCTTAT-------GAGATCA
Inpa	GTCTCCGCCCCCCAGTGAG---AATGCCCCCCCCGTC--TTCCCCGCC-----GAAGACA
Auch	GTATCCGCCCACCCGTGAG---GATGCCCAAGTT-----CTCTATTT------GAGACCT
Fico	GTATCCGCACCCCTGTGAG---GATGCCCTACAGCT---CTCCATCC------GAGAGCA
Macs	GTATCCGCACCCCTGTGAG---GATGCCCTACAGTT---CTCCCGC-------GAGAACA
Moal	GTATCCGCACCCCCGTGAG---AATGCCCTTAAC-----CCTCGTTC------AGGGTAG
Syma	GTCTCCGCCCACCCGTGAG---AATGCCCTTAGCC----CTCCGCCC------GAGGGAA
Mafr	GTATCCGCACCCCCGTGAG---AATGCCCCACAGTT---TTCTTCCC------GAAAACA
Dcpe	GTATCCGCACCCCTGTGAG---GATGCCCTTTATT----CTTCTCCCA-----AAGATTT
Dcti	GTATCCGCACCCCTGTGAG---GATGCCCTACATT----CTTCTCCCA-----AAGAGCT
Hehi	GTATCCGCCCCCCTGTGAG---AATGCCCTACAGCT---CCCTGCCC------GGGAGCA
Stam	GTATCCGCACCCCTGTGAG---AATGCCCTACAGTT---CCCCGCCC------GGGAACA
Hogi	GTCTCCGCCCCCCTGTGAG---GATGCCCTACATT----CCCCGCCC------TGGAATA
Erzo	GTATCCGCCCCCCTGTGAG---AATGCCCTACAGTT---CCCCGCCC------GGGAACA
Hxot	GTATCCGCACCCCTGTGAG---AATGCCCTACAGTC---CCCCGCCC------GGGAACA
Core	GTATCCGCACCCCTGTGAG---AATGCCCTACAGTT---CCCCGCCC------GGGAACA
Apve	GTATCCGCACTCCTGTGAG---AATGCCCTACAGTC---CCCCGTCC------GGGAACA
Latj	GTATCCGCACCCCCGTGAG---AATGCCCATGATT----CCCTGTTT------GGGAACA
Laja	GTATCCGCGCCCCTGTGAG---AATGCCCTATCGT----CCCCGCCC------GGGAACA

Syja	GTATCCGCAACCCCGTGAG---AATGCCCTTACGTC---CTCCACCAC-----GAAGACA
Epme	GTATCCGCACCCCTGTGAG---AATGCCCTACAGTT---CCCCGCCC------GGGAACA
Grse	GTATCCGCATCCCCGTGAG---AATGCCCCACAGTTC--CCCATCC-------GGGAACA
Clja	GTATCCGCCCCCCCGTGAA---AATGCCCAAATAAAAC-CCCTAACG------GGGAAAA
Ogcy	GTATCCGCACCCCCGTGAG---AATGCCCCACAACT---CTCCACCC------GAGAACG
Plna	GCCTCCACGCCCCCGTGAG---AATGCCCTATAGTT---CTCTGCCC------GAGAACA
Lema	GTATCCGCGCACCCGTGAG---AATGCCCTTACAGTT--TCCAGCAT------GGAAACA
Etzo	GTATCCGCATCCCCGTGAG---AATGCCCTACAGTT---CCCTGCCC------GGGAACA
Apse	GTATCCGCACCCCCGTGAG---AATGCCCCACAGCT---CCCTGCCC------GGGAGCA
Epde	GTATCCGCACTCCTGTGAG---AATGCCCTACAGTT---CCCTGCCC------GGGAACA
Slja	GTATCCGCGCCCCCGTGAG---GATGCCCTATAGATT--CCCTACTA------GGAAGCT
Bsja	GTATCCGCACCCCGGTGAG---AATGCCCCCACAGTC--CCCCGCCC------GGAGACA
Ecna	GTATCCGCACCCCTGTGAG---AATGCCCATATTC----TCCTGTAT------GGAGATA
Cohi	GTATCCGCCCCCCGGTGAG---GATGCCCTTACTC----TTCTTGCACT----GAAGATA
Caar	GTATCCGCCCCCCTGTGAG---AATGCCCTTAGTG----CCCTTATT------GAGCACA
Came	GTATCCGCCCCCCTGTGAG---GATGCCCTTAGTA----CCCTTCTC------GGGCACA
Mema	GTATCCGCACCCCCGTGAG---AATGCCCACAGTC----CCCTGCTT------GGGAACA
Lenu	GTATCCGCATCCCTGTGAG---AATGCCCCAACAGTC--TCCTTACT------GGAGACA
Plma	GTATCCGCACCCCCGTGAG---AATGCCCCACAGTT---TTCTGCTC------GAAAACA
Emst	GTATCCGCACCCCTGTGAG---AATGCCCTACAGTT---CCCTGTCT------GGGAACA
Ptti	GTATCCGCGCCCCTGTGAG---AATGCCCCACAGTT---CCCTGTTT------GGGAACA
Losu	GTATCCGCACTCCCGTGAG---AATGCCCTAACAGTC--CCCTGAAT------GAGGACA
Geoy	GTATCCGCGCCCCTGTGAG---GATGCCCTACAGTT---CCCTGACT------GGGAACA
Dipi	GTATCCGCACTCCTGTGAG---AATGCCCTAGTT-----TCCTGCTT------GGTAACA
Pama	GTATCCGCGCCCCCGTGAG---AATGCCCTATAGTC---TCCTGCCC------GGAAACA
Leob	GTATCCGCACCCCCGTGAG---AATGCCCTGCAGCC---TCCTGCCC------GGAGACA
Neba	GTATCCGCGAACCCGTGAG---AATGCCCTAAGTGC---TCCTCACCC-----GGAGCCA
Pdpl	GTCTCCGCACCCCCGTGAG---GATTGCCCATTGGCC--TCCCGACC------GGAGGCC
Nimi	GTATCCGCACCCCTGTGAG---AATGCCCTAATAGCT--CCCCGCCC------GGGAACA
Uptr	GCATCCGCCCCCCTGTGAG---AATGCCCTCTTCGTC--CTCCTTTTC-----GAGAACA
Pesc	GTATCCGCGTCCCTGTGAA---AATGCCCTATGCG----CCCTAGCC------GGGCGCA
Baar	GTATCCGCACCCCTGTGAG---AATGCCCTTGAACC---CCCCGCCAC-----GGGGACA
Moar	GTATCCGCACCCCTGTGAG---AATGCCCCACAGTT---CCCCGCCC------GGGAACA
Toja	GTATCCGCCCCCCTGTGAG---AATGCCCACAGTT----CCCCTCTC------GGGAACA
Chau	GTATCCGCGCCCCTGTGAG---AATGCCCTGTAAC----CTCTGGAT------GAGATCA
Chse	GTATCCGCACTCCCGTGAG---AATGCCCTTCAGTT---CCCTGCCC------GGGAACA
Enar	GTATCCGCACTCCCGTGAG---AATGCCCCACAGTT---TCCTGCTC------GGAAACA
Hpty	GTATCCGCACTCCTGTGAG---AATGCCCTACAGTC---CCCCGCCC------GGGAACA
Nana	GTATCCGCATCCCCGTGAG---AATGCCCAACAGTT---CCCCGCCCC-----GGGAACA
Mcst	GTATCCGCGCCCCAGTGAG---GATGCCCTACAGTT---CCCCGCCC------GGGAACA
Rhox	GTATCCGCACCCCCGTGAG---AATGCCCTAACAGTT--CCCTGCCC------GGGAACA
Opfa	GTATCCGCACCCCCGTGAG---AATGCCCCAACAGTT--CCCTGCCC------GGGAACA
Paar	GTATCCGCGCCCCCGTGAG---AATGCCCTACAGTT---CCCCGTCC------GGGAACA
Gozo	GTATCCGCACCCCCGTGAG---AATGCCCTACAGTT---TCCCGTCC------GGAAACA
Ackr	GTATCCGCACCCCCGTGAG---AATGCCCTATGGT----TCCTATTT------GGAAACA
Elev	GTATCCGCACTCCCGTGAG---AATGCCCCACAGTT---CTCTTAA-------GAGAACA
Trdu	GTCTCCGCCCCCCTGTGAG---AATGCCCACAGTT----TTCTGCCC------GAAAACA
Amoc	GTATCCGCACCCCTGTGAG---AATGCCCTCCAGTC---TCCCGCCC------GGAAACA
Hame	GTATCCGCACCCCCGTGAG---AATGCCCCATAACC---TCCTGAATT-----GGAGATG
Chso	GTATCCGCACCCCCGTGAA---AATGCCCCCCGCCC---CCCACC-C------GGGGGCA
Lyto	GTATCCGCACTCCTGTGAG---AATGCCCCACAGTT---CCCCGCCC------GGGAACA
Encr	GTATCCGCACTCCTGTGAG---AATGCCCTAATAGTT--CCCCGTCC------GGGAACA

```
Bvar    GTATCCGCCCCCCCGTGAG---GATGCCCTTCGCT----CCCCGCCC------GGGACCA
Noco    GTATCCGCACCCCTGTGAG---AATGCCCTCAGTT----CCCTGCCT------GGGAACA
Chsp    GTATCCGCCCCCCAGTGAA---AACGCCCAAGACCC---CCCTACTC------GGGACCA
Arja    GTATCCGCGCCCCTGTGAG---AATGCCCTACAGTT---CCCCGCCC------GGGAACA
Pase    GTATCCGCACCCCTGTGAG---AACGCCCGAACAGAC--CTCTGCCC------GAGATCA
Trel    GTCTCCGCACCCCCGTGAG---AAGGCCCTAAGCTTC--CCCTACC-------GGGAGCA
Lifa    GTATCCGCACCCCCGTGAG---GATGCCCCACAGCC---CCCAGCC-------GGAGCCA
Acur    GTATCCGCACACCAGTGAG---AATGCCCTAACAGTC--AACTGAAT------GAAGACA
Ampe    GTATCCGCACCCCTGTGAG---AATGCCCTACAGTT---CCCTGCCC------GGGAACA
Urja    GTATCCGCCCCCCGGTGAAT-TAATGCCCACATAGTCCCCCCACCCC------GGGAACA
Enet    GTCTCCACGAGCCCGTGAG---GATGCCCGTAGTC----TCTTACCC------AGAAACA
Ptbr    GTCTCCGCATCCCTGTGAG---AATGCCCTAATAC----TCCTGAATA-----GGAGAAA
Safa    GTCTCCGCACCCCCGTGAG---AATGCCCTACAGTT---TCCCTTCC------TGAAACA
Icae    GTATCCGCAACCCTGTGAG---AATGCCCCACAGTT---TTCTGCCC------GAAAACA
Asmi    GTCTCCGCACCCCCGTGAA---AATGCCCTTCTTTC---TTTCTTCAAGA---AATAGGA
Foal    GTCTCCGCACCCCTGTGAG---GATGCCCACAACT----TTCTAAT-------GAGAACT
Drze    GTATCCGCATCCCTGTGAG---AATGCCCCTACAGCT--CTCTTAC-------GAGAACA
Rhas    GTATCCGCGTTCCCGTGAG---AATGCCCTACGATT---CCCCTACA------GGAATCA
Elac    GTATCCGCACCCCTGTGAG---AATGCCCTACCGTT---CCCCCTTTACTCCCGGGAACA
Kugu    GTATCCGCGCCCCTGTGAG---AATGCCCTCAGTC----CCCCAACC------GGGAACA
Plor    GTATCTGCACCCCGGTGAG---AATGCCCTTACAGTT--CCCCGCCC------GGGAACA
Sgun    GTATCCGCACCCCTGTGAG---AATGCCCTCAATC----CCCCGCCC------GGGAATA
Zaco    GTATCCGCACCCCTGTGAG---AATGCCCTACAGTC---CCCTGCTT------GGGAACA
Zbfl    GTATCCGCACCCCTGTGAG---AATGCCCCACAGTT---CCCTGCCC------GGGAACA
Spba    GTATCCGCCCTCCTGTGAG---GATGCCCATTTCT----CCCTGTTC------GGGAACA
Game    GTATCCGCGACCCTGTGAG---AATGCCCCACAGTT---TTCCGCCC------GAAAACA
Thth    GTATCCGCGACCCTGTGAG---AATGCCCCACAGTT---TTCCGCCC------GAAAACA
Xigl    GTATCCGCCCCCCTGTGAG---AATGCCCATAATT----CCCTGCTT------GGGAACA
Hyja    GTATCCGCGTCCCCGTGAG---AATGCCCTTGTT-----TTCCGCCCA-----GAAACCA
Psan    GTATCCGCACCCCCGTGAG---AATGCCCTTGTT-----GTCCGCTTA-----GAAACCA
Cupa    GTATCCGCGACCCTGTGAG---AATGCCCATAGTT----TTCTGCCC------GAAAACA
Mpch    GTATCCGCACCCCTGTGAG---AATGCCCTACAGTC---TCCCTTACTAC---GGAAACA
Char    GTATCCGCACCCCTGTGAA---AATGCCCCACAGCT---CTCCGCCC------GAGAACA
Pser    GTATCCGCGCCCCCGTGAG---AATGCCCTTGATC----CCCCGCCT------GGGGACA
Prol    GTATCCGCCCCCCTGTGAG---AATGCCCATAACG----CCCTGCTC------GGGAACA
Plbi    GTATCCGCCCCCCTGTGAG---AATGCCCACAACT----CCCTGCTT------GGGAACT
Calu    GTATCCGCACCCCCGTGAG---AATACCCATAGCC----CCCTTTAT------GAGGGCA
Papa    GTATCCGCACCCCCGTGAG---AATGCCCTTATGATA--CCTTGT--------AGGTACT
Sufr    GTCTCCGCACCCCCGTGAA---GATGCCCTATAGTT---CCCCGCTC------GGGAACA
Stci    GTCTCCGCGCCCCCGTGAG---AATGCCCTGCAACT---TCCGCCCTC-----GGAAATA
Taru    GTATCCGCATCCCAGTGAA---AATGCCCCCCGCC----CCCCGTCC------GGAAATA
Rala    GTATCCGCACCCCTGTGAG---AATGCCCCACAGTT---TCCTGCCC------GGAAACA
        *   *  *       **           *
```

```
           9'    11     12     ¦                 HVR               ¦
Scca    AG-GGGCGGGCATCAGGC-ACACATTTTACA----------------------------
Muma    AG-GAGCAGGTATCAGGC-ACACGCA---------------------------------
Erca    AG-GAGCAGGCATCAGGC-ACGTATTC--------------------------------
Pose    AG-GAGCCGGCATCAGGC-TCGTGTT---------------------------------
Actr    AG-GAGCAGGTATCAGGC-ACGCACCC--------------------------------
Scal    AG-GAGCAGGTATCAGGC-ACGCACCC--------------------------------
Posp    AG-GAGCAGGTATCAGGC-ACGCAACC--------------------------------
Atsp    AG-GAGCAGGCATCAGGC-ACACAACCC-------------------------------
Leoc    AG-GAGCAGGCATCAGGC-ACACGACC--------------------------------
Amca    AG-GAGCCGGCATCAGGC-ACACACACAT------------------------------
Osbi    AG-GAGCCGGTATCAGGC-ACACAAA---------------------------------
Pabu    AG-GAGCAGGTATCAGGC-ACGCATACA-------------------------------
Hial    AG-GAGCAGGTATCAGGC-ACGCACCAC-------------------------------
Elha    AG-GAGCGGGCATCAGGC-ACGCAACA--------------------------------
Mlcy    AG-GAGCCGGCATCAGGC-ACGCATAC--------------------------------
Algl    AG-GAGCCGGCATCAGGC-ACACTTAAT-------------------------------
Ptgi    AG-GGGCCGGCATCAGGC-ACGCAATTAA------------------------------
Alaf    AG-GAGCCGGCATCAGGC-ACGCAATTC-------------------------------
Nock    AG-GAGCCGGCATCAGGC-ACACTAAAAC------------------------------
Anja    AG-GAGCCGGCATCAGGC-ACACCCGT--------------------------------
Gyki    AG-GAGCAGGCATCAGGC-ACACATT---------------------------------
Syka    AG-GCGCCGGCATCAGGC-ACACTAAATT------------------------------
Opma    GG-GAGCTGGCATCAGGC-ACACCCCCG-------------------------------
Comy    AG-GAGCCGGTATCAGGC-ACACCAATCC------------------------------
Sasp    AG-GAGCTGGCATCAGGC-TCACAAAC--------------------------------
Eupe    AG-GAGTTGGCATCAGGC-TCACCTAA--------------------------------
Enja    GG-GAGCCGGCATCAGGC-ACAATTAT--------------------------------
Same    AG-GAGCTGGTATCAGGC-ACACGATTT-------------------------------
Chch    AG-GAGCTGGCATCAGGC-ACGCTCA---------------------------------
Grgr    AG-GGGCTGGTATCAGGC-ACGCACC---------------------------------
Caau    AG-GAGCGGGCATCAGGC-ACAAATA---------------------------------
Cyca    AG-GAGCGGGCATCAGGC-ACAAACA---------------------------------
Dare    AG-GAGCAGGTATCAGGC-ACAAAATT--------------------------------
Cost    AG-GAGCAGGCATCAGGC-ACAAAT----------------------------------
Leec    AG-GAGCGGGCATCAGGC-ACTAATATC-------------------------------
Crla    AG-GAGCGGGCATCAGGC-TCAATTAT--------------------------------
Clmc    AG-GAGCCGGCATCAGGC-ACGAACT---------------------------------
Phin    GG-GAGCTGACATCAGGC-ACAACC----------------------------------
Icpu    AG-GAGCAGGCATCAGGC-ACACTTTCTACC----------------------------
Psto    AG-GAGCAGGCATCAGGC-ACAACTAACC------------------------------
Cora    AG-GAGCAGGCATCAGGC-ACAATCAT--------------------------------
Eisp    AG-GAGCAGGTATCAGGC-ACATAGAT--------------------------------
Apal    AG-GAGCAGGTATCAGGC-ACACTAC---------------------------------
Eslu    AG-GAGCTGGCATCAGGC-ACACAT----------------------------------
Dape    AG-GAGCTGGCATCAGGC-ACGCCCC---------------------------------
Glse    AG-GAGCGGGCATCAGGC-ACGCTTA---------------------------------
Naar    AG-GATCTGGCAGCAGGC-ACGCTAC---------------------------------
Baoc    AG-GAGCCGGTATCAGGC-ACGCCACC--------------------------------
Opso    AG-GAGCTGGTATCAGGC-ACGCCTCC--------------------------------
Alte    AG-GAGCCGGCATCAGGC-ACACTTTG--------------------------------
Plap    AG-GAGCCGGCATCAGGC-ACGCTCTCC-------------------------------
```

```
Plal    AG-GAGCTGGTATCAGGG-ACGCCCACC-------------------------------
Sami    AG-GAGCCGGTATCAGGG-GCGCGCAAA-------------------------------
Rere    AG-GAGCGGGCATCAGGG-ACGCGCACTTCA----------------------------
Gama    AG-GAGCCGGCATCAGGG-TCACCCC---------------------------------
Onmy    AG-GAGCCGGCATCAGGG-ACGCCCA---------------------------------
Sasa    AG-GAGCCGGCATCAGGG-ACGCCCAA--------------------------------
Cola    AG-GAGCTGGCATCAGGG-ACGCCCC---------------------------------
Dita    AG-GAGCCGGCATCAGGG-GCACCCT---------------------------------
Gogr    AG-GAGCCGGTATCAGGG-ACACCCCCAT------------------------------
Chsl    AG-GAGCTGGTATCAGGG-GCGCAACAC-------------------------------
Atja    AG-GAGCCGGCATCAGGG-ACATCAAACTAAAA--------------------------
Iido    AG-GAGCTGGCATCAGGG-ACATCAAACTAAA---------------------------
Auja    AG-GAGCAGGCATCAGGG-ACGTTTTAC-------------------------------
Chag    AG-GAGCAGGTATCAGGG-ACGCACGCAC------------------------------
Hami    AG-GAGCGGGCATCAGGG-TCATTGTAAG------------------------------
Saun    AG-GAGCGGGCATCAGGG-TCGCCATATC------------------------------
Nema    AG-GGGCAGACATCAGGG-ACGCCCC---------------------------------
Disp    AG-GAGCGGACATCAGGG-ACGCGCC---------------------------------
Myaf    AG-GAGCAGGCATCAGGG-ACACTTCC--------------------------------
Lagu    AG-GAGCCGGCATCAGGG-ACAGACCCCCTCC---------------------------
Trtr    GG-GAGCCGGCATCAGGG-ACAACCT---------------------------------
Zucr    GG-GAGCTGGTATCAGGG-ACAAAC----------------------------------
Pxja    AG-GAGCTGGCATCAGGG-ACGCTTTCACAC----------------------------
Pxlo    AG-GAGCTGGCATCAGGG-ACGCTTACAC------------------------------
Pctr    AG-GAGCTGGTATCAGGG-ACAATCCGT-------------------------------
Apsa    AG-GAGCCGGTATCAGGG-ACACACTACCC-----------------------------
Cabe    AG-GAGCCGGTATCAGGG-ACAGCCCT--------------------------------
Bzze    AG-GAGCCGGCATCAGGG-ACAACCCG--------------------------------
Siim    AG-GAGATGGTATCAGGG-ACAATACGT-------------------------------
Ctru    AG-GAGCAGGTATCAGGG-ACTTCCC---------------------------------
Dpbr    AG-GAGCCGGTATCAGGG-ACGACCCGC-------------------------------
Caki    AG-GAGCAGGTATCAGGG-ACGAGTAA--------------------------------
Phja    AG-GAGCAGGCATCAGGG-ACATCCCTT-------------------------------
Brsp    AG-GAGCAGGTATCAGGG-CCCAATA---------------------------------
Gamo    AG-GAGCAGGTATCAGGG-ACATCTAATAGTTTA-------------------------
Lolo    AG-GAGCAGGCATCAGGG-ACGTCAAATC------------------------------
Batr    TA-GAGCTGGTATCAGGG-ACATAAAT--------------------------------
Prmy    CC-GGGCAGATATCAGGG-ACGCCTTT--------------------------------
Loli    AA-GAGCAGGCATCAGGG-ACAACTT---------------------------------
Loam    AG-GAGCAGGCATCAGGG-ACAAGCACA-------------------------------
Chab    AG-GAGCCGGTATCAGGG-ACTACCAACT------------------------------
Chto    AG-GAGCCGGTATCAGGG-ACTACCAACT------------------------------
Majo    AG-GAGCTGGTATCAGGG-ACACAT----------------------------------
Hlst    AG-GAGTAGGTATCAGGG-ACGACATTTTT-----------------------------
Clpe    AG-GAGTTGGTATCAGGG-ACAAACA---------------------------------
Mlmr    AG-GAGCCGGTATCAGGG-ACAAGAAACCAA----------------------------
Crcr    AG-GAGCTGGTATCAGGG-ACAGCCTAATAAAGCT------------------------
Muce    AG-GAGCTGGTATCAGGG-ACAGCCTAATAAAGCT------------------------
Bege    AG-GAGCCGGTATCAGGG-TCAATCCTCCC-----------------------------
Mela    AG-GAGCTGGTATAAGGG-ACAATTAT--------------------------------
Hats    AG-GAACTGGTATCAGGG-TCACTAAATACT----------------------------
Orla    AG-GAGCTGGTATCAGGG-ACAATAT---------------------------------
```

```
Cosa    AG-GAGCTGGTATCAGGA-ACAAAAA-------------------------------
Exsp    AG-GAGCTGGTATCAGGG-TCAAAAT-------------------------------
Depa    AG-GAGCCGGTATCAGGG-ACAAACTA------------------------------
Rima    AG-GAGCTGGTATCAGGG-ACAACTTC------------------------------
Fuol    AG-GAGCTGGTATCAGGG-ACAATAAAC-----------------------------
Gmaf    AG-GAGCTGGTATCAGGG-ACGCACA-------------------------------
Xeei    AG-GAGCTGGTATCAGGG-ACACACAG------------------------------
Pros    AG-GAGCCGGTATCAGGG-ACGCCCT-------------------------------
Scmi    AG-GAGCCGGTATCAGGG-ACGCCCC-------------------------------
Rolo    AG-GAGCTGGTATCAGGG-ACGCTACC------------------------------
Cere    AG-GAGCTGGTATCAGGG-ACGCGTTAT-----------------------------
Daga    AG-GAGCTGGCATCAGGG-ACGCACA-------------------------------
Anco    AG-GAGCTGGTATCAGGG-ACGCCCATAT----------------------------
Dmve    AG-GAGCAGGTATCAGGG-ACACCCC-------------------------------
Dmar    AG-GAGCAGGTATCAGGG-ACGCCCCCC-----------------------------
Anka    AG-GAGCGGGTATCAGGG-ACGCCCC-------------------------------
Moja    AG-GAGCTGGTATCAGGG-ACGCCCC-------------------------------
Hoja    AG-GAGCTGGTATCAGGG-GCGCCCC-------------------------------
Bede    AG-GAGCCGGCATCAGGG-TCGCCCC-------------------------------
Besp    AG-GAGCCGGCATCAGGG-GCACTCC-------------------------------
Mysp    AG-GAGCAGGCATCAGGG-ACGCTCTC------------------------------
Osja    AG-GAGCAGGCATCAGGG-ACGCCTTC------------------------------
Sgro    AG-GAGCAGGTATCAGGG-ACGCCCCT------------------------------
Pzpa    AG-GAGCGGGCATCAGGG-ACGCACA-------------------------------
Zeja    AG-GAGCTGGCATCAGGG-ACGCTAAC------------------------------
Znne    AG-GAGCTGGTATCAGGG-ACCCAACTTT----------------------------
Zefa    AG-GAGCTGGTATCAGGG-ACCCTATCT-----------------------------
Acni    AG-GAGCTGGCATCAGGG-ACGCTTT-------------------------------
Ncrh    AG-GAGCTGGCATCAGGG-ACGCTTT-------------------------------
Agca    AG-GAGCCGGCATCAGGG--ACACTCCTCCCT-------------------------
Hydy    AG-GAGCTGGTATCAGGG-ACATTT--------------------------------
Gsac    AG-GAGCTGGTATCAGGG-ACATCTTTT-----------------------------
Pevo    AG-GAGTGGGTATCAGGG-ACACCAAA------------------------------
Hiku    AG-GAGCTGGTATCAGGT-ACAAATA-------------------------------
Inpa    AG-GAGCCGGCATCAGGG-ACAACCACCC----------------------------
Auch    CG-GAGCAGGTATCAGGG-GCGAGCCCCC----------------------------
Fico    AG-GAGCTGGTATCAGGG-ACAACTAC------------------------------
Macs    AG-GAGCTGGTATCAGGG--ACACCCAT-----------------------------
Moal    AG-GAGCTGGTATCAGGG-ACACTCCCT-----------------------------
Syma    AG-GAGCTGGTATCAGGG-ACACTCC-------------------------------
Mafr    AG-GAGCTGGTATCAGGG-ACACAACCCT----------------------------
Dcpe    AG-GAGCGGGCATCAGGG-ACACAAT-------------------------------
Dcti    AG-GAGCAGGTATCAGGG-ACACAAT-------------------------------
Hehi    AG-GAGCTGGTATCAGGG-ACACACC-------------------------------
Stam    AG-GAGCTGGTATCAGGG--ACATTTTAT----------------------------
Hogi    AG-GAGCCGGTATCAGGG-ACAAACACTG----------------------------
Erzo    AG-GAGCTGGTATCAGGG--ACATCCCAC----------------------------
Hxot    AG-GAGCTGGTATCAGGG--ACACCCCAT----------------------------
Core    AG-GAGCTGGTATCAGGG--ACATCCTAT----------------------------
Apve    AG-GAGCTGGTATCAGGG--ACACTCAT-----------------------------
Latj    AG-GAGCCGGTATCAGGG--GCACCTAAC----------------------------
Laja    AG-GAGCTGGTATCAGGT-ACAACTAT------------------------------
```

```
Syja    AG-GAGCTGGTATCAGGG-ACATCCCTACATT---------------------------
Epme    AG-GAGCAGGTATCAGGG-ACATATCCC-------------------------------
Grse    AG-GAGCTGGTATCAGGG-ACATCGTACACAT---------------------------
Clja    AG-GAGCTGGTATCAGGG-ACACATACT-------------------------------
Ogcy    AG-GAGCTGATATCAGGG-ACATCCACGA------------------------------
Plna    AG-GAGCCGGTATCAGGG-ACAATATTC-------------------------------
Lema    AG-GAGCTGGTATCAGGG-ACATCAATT-------------------------------
Etzo    AG-GAGCTGGTATCAGGG-ACGCCACTG-------------------------------
Apse    AG-GAGCTGGTATCAGGG-TCTTTTCTTAAG----------------------------
Epde    AG-GAGCTGGTATCAGGG--ACATTATAT------------------------------
Slja    GG-GAGCAGGTATCAGGG-ACAGATTCT-------------------------------
Bsja    AG-GAGCTGGTATCAGGG-ACACTATAACT-----------------------------
Ecna    AG-GAGCTGGTATCAGGG-ACACAACTCT------------------------------
Cohi    AG-GAGCTGGTATCAGGG--ACACAAGC-------------------------------
Caar    AG-GAGCCGGTATCAGGG-ACACAACATACG----------------------------
Came    AG-GAGCCGGTATCAGGG-ACACAACACA------------------------------
Mema    AG-GAGCTGGTATCAGGG-ACCCCCCCCCC-----------------------------
Lenu    AG-GAGCTGGTATCAGGG-ACCCCCC---------------------------------
Plma    AG-GAGCTGGTATCAGGG-TCACCCCATTT-----------------------------
Emst    AG-GAGCCGGTATCAGGG--ACACTTAATTT----------------------------
Ptti    AG-GAGCTGGTATCAGGG-ACAACTAACC------------------------------
Losu    AG-GAGCCGGTATCAGGG-ACATATATT-------------------------------
Geoy    AG-GAGCTGGTATCAGGG-ACATCTTTCGATCGTTGCGCCCTCCATCCTACTCTTCCCC
Dipi    AG-GAGCTGGTATCAGGG-ACAACCCACTCT----------------------------
Pama    AG-GAGCTGGTATCAGGG-ACATACAA--------------------------------
Leob    AG-GAGCCGGTATCAGGG-ACAATACCCG------------------------------
Neba    AG-GAGCTGGTATCAGGG-TCACCCTATCCATCTCATAATATTTTATGTTATGT-----
Pdpl    GG-GAGCTGGTATCAGGG-ACAATATT--------------------------------
Nimi    AG-GAGCTGGTATCAGGG-ACAACCCGCC------------------------------
Uptr    AG-GAGCTGGTATCAGGG-ACACAT----------------------------------
Pesc    AG-GAGCTGGCATCAGGG-ACATCTTAATT-----------------------------
Baar    AG-GAACCGGTATCAGGG-ACAACCCCCGT-----------------------------
Moar    AG-GAGCTGGTATCAGGG-ACAACTCACC------------------------------
Toja    AG-GAGCTGGTATCAGGG-TCACCTTTCCTT----------------------------
Chau    AG-GAGCTGGTATCAGGG-ACACTTAAAT------------------------------
Chse    AG-GAGCTGGTATCAGGG--ACACCCTAAT-----------------------------
Enar    AG-GAGCCGGTATCAGGG-ACATACTA--------------------------------
Hpty    AG-GAGCCGGTATCAGGG-ACAACTAC--------------------------------
Nana    AG-GAGCTGGTATCAGGG-ACATCCTTAT------------------------------
Mcst    AG-GAACTGGTATCAGGG-ACATATTAT-------------------------------
Rhox    AG-GAGCTGGCATCAGGG-ACATTCCCTT------------------------------
Opfa    AG-GAGCTGGTATCAGGG-ACACCCCATT------------------------------
Paar    AG-GAGCTGGTATCAGGG-TCGACACCT-------------------------------
Gozo    AG-GAGCTGGTATTAGGG-ACATCCATA-------------------------------
Ackr    AG-GAGCTGGTATCAGGG-CCACCTACC-------------------------------
Elev    AG-GAGCTGGTATCAGGG-TCAAAC----------------------------------
Trdu    AG-GAGCTGGTATCAGGG-ACACTTTCC-------------------------------
Amoc    AG-GAGCCGGTATCAGGG-ACGGTAGAC-------------------------------
Hame    AG-GAGCTGGTATCAGGG-ACAATTATT-------------------------------
Chso    AG-GAGCTGGTATCAGGG-ACAAATCCAT------------------------------
Lyto    AG-GAGCAGGTATCAGGG--ACACCTCTAGC----------------------------
Encr    AG-GAGCTGGTATCAGGG-ACAACCCTA-------------------------------
```

```
Bvar    AG-GAGCCGGTATCAGGG-ACACCCTA--------------------------------
Noco    AG-GAGCTGGTATCAGGG-ACAATT----------------------------------
Chsp    AAGGAGCCGGTATCAGGG-ACATAATATG------------------------------
Arja    AG-GAACTGGTATCAGGG-ACATACTGT-------------------------------
Pase    AG-GAGCCGGCATCAGGG-ACCCCCTTAT------------------------------
Trel    AG-GAGCAGGTATCAGGG-ACTGACCCTAC-----------------------------
Lifa    AG-GAGCCGGTATCAGGG-GCATAAA---------------------------------
Acur    AG-GAGCAGGCATCAGGG-ACATAAGTTATTACTTA-----------------------
Ampe    AG-GAGCTGGTATCAGGG-ACACTAGT--------------------------------
Urja    AG-GAGCCGGCATCAGGG-CCAACTCTAC------------------------------
Enet    AG-GAGCCGATATCAGGG-ACACACCCGC------------------------------
Ptbr    AG-GAGCGGGTATCAGGG-ACGCTCTACGA-----------------------------
Safa    AG-GAGCAGGTATCAGGG-ACACTAC---------------------------------
Icae    AG-GAGCCGGTATCAGGG--ACACCCAATT-----------------------------
Asmi    GG-GAGTGGATATCAGGG-ACATCTCA--------------------------------
Foal    AG-GAGCTGGTATCAGGG-TCATAA---------------------------------
Drze    AG-GAGCAAGCATCAGGG-ACAAATATAAA-----------------------------
Rhas    AG-GAGCCGGTATCAGGG-ACAACTTCAGAC----------------------------
Elac    AG-GAGCTGGTATCAGGG-ACAACCTATTGT----------------------------
Kugu    AG-GAGCTGGTATCAGGG-ACAAACTTTAAAAC--------------------------
Plor    AG-GAGCTGGTATCAGGG-ACCTAAAACT------------------------------
Sgun    AG-GAGTTGGTATCAGGG-ACATACTA--------------------------------
Zaco    AG-GAGCCGGTATCAGGG-ACAATTACT-------------------------------
Zbfl    AG-GAGCTGGTATCAGGG-ACAATCTCCTTGT---------------------------
Spba    TG-GAGCCGGTATCAGGG-ACACTCCTT-------------------------------
Game    AG-GAGCTGGTATCAGGG-ACCCCCTACC------------------------------
Thth    AG-GAGCTGGTATCAGGG-ACACCCAATT------------------------------
Xigl    AG-GAGCCGGTATCAGGG-ACACTCTACTA-----------------------------
Hyja    AG-GAGCTGGTATCAGGG--ACATCCCTTAT----------------------------
Psan    AG-GAGCTGGTATCAGGG-ACAACCCTAAC-----------------------------
Cupa    AG-GAGCTGGTATCAGGG-ACACCCAATCT-----------------------------
Mpch    AG-GAGCTGGTATCAGGG-ACATAAAATC------------------------------
Char    AG-GAGCCGGTATCAGGG-TCGATACAC-------------------------------
Pser    AG-GAGCCGGTATCAGGG-ACAAGCCATAT-----------------------------
Prol    AG-GAGCTGGCATCAGGG-ACAGACATA-------------------------------
Plbi    AG-GAGCCGGTATCAGGG-ACAAGCCCAG------------------------------
Calu    TG-GAGTTGGTATCAGGG-GCACGACTAGATCTAAT-----------------------
Papa    AG-GAGCTGACATCAGGG--ACACAAACG------------------------------
Sufr    AG-GAGCTGGTATCAGGG-ACAATCCAAA------------------------------
Stci    AG-GAGCTGGCATCAGGG-ACAATAACA-------------------------------
Taru    GG-GAGTTGGTATCAGGG-ACACAAATT-------------------------------
Rala    AG-GAGCTGGTATCAGGG--ATCTCAACT------------------------------
           *       *   ***
```

```
                          12'          11'  13         13' 8'  14       14'¦ HVR ¦
Scca   ---------TGT-GGCCCAAGACGCCTTGCTCA---GC-CACACCCT---AAGGGTT-----
Muma   ---------CGT-AGCCCAAGACACCTTGCT--A-AGC-CACACCCC--CAAGGGATTTC--
Erca   ----------AC-GGCCCAAAACGCCTTGCT--T-TGC-CACACCCC--CACGGGAT-TT--
Pose   ---------CAC-GGCCCAAAACGCCTTGCTTT---GC-CACACCCC--CACGGGAT-----
Actr   ----------GC-AGCCCAAGACGCCTTGCTA---AGC-CACACCCC--CAAGGGAA-----
Scal   ----------GC-AGCCCAAGACGCCTTGCTA---AGC-CACACCCC--CAAGGGAA-----
Posp   ----------GC-AGCCCAAGACGCCTTGCTA---AGC-CACACCCC--CAAGGGAACTC--
Atsp   ----------GT-AGCCCAAGACGCCTTACTTA---GT-CACACCCC--CAAGGGAACTC--
Leoc   ---------CGT-AGCCCAAGACGCCTTACTTA---GT-CACACCCC--CAAGGGAACTC--
Amca   ---------TGT-AGCCCAAAACGCCTTGCTTA---GC-CACCCCCC--CAAGGGAATTC--
Osbi   ---------TGT-AGCCTAAAACGCCTTGCTTA---GC-CACACCCC--CAAGGGCACCC--
Pabu   ---------TGC-AGCCAAAAACACCTTGTTAA---AC-CACACCCC--CAAGGGTACTC--
Hial   ----------GC-AGCCCAAGACGCCTTGTTTA---GC-CACACCCC--CAAGGGAA-----
Elha   ----------GC-AGCCCAAGACGCCTTGTTC---AGC-CACACCCC--CAAGGGAA-----
Mlcy   ----------GC-AGCCCAAGACGCCTTGTT--C-AGC-CACACCCC--CAAGGGAA-CT--
Algl   ----------GT-AGCCCAAGACGCCTTGCTTA---GC-CACACCCC--CAAGGGGGCCCGA
Ptgi   ---------TGC-AGCCCAAGACGCCTTGCTT---AGC-CACACCCC--CAAGGGAA-----
Alaf   ----------GC-AGCCCAAGACGCCTTGCTCA---GC-CACACCCC--CAAGGGAATCC--
Nock   ----------GT-AGCCCAAGACGCCTTGCT--T-AGC-CACACCCC--CAAGGGAA-TT--
Anja   ----------GT-AGCCCAAAACGCCTTGCTC---AGC-CACGCCCA--CAAGGGAATTC--
Gyki   ---------TGT-AGCCCAAAACGCCTTGCTT---AGC-CACACCCC--CAAGGGAACGC--
Syka   ----------GT-AGCCCAAGACGCCTTGCCTA---GC-CACACCCC--CAAGGGGACCC--
Opma   ---------GGT-AGCCCAAAACGCCTTGCT--A-AGC-CACACCCC--CAAGGGAATTC--
Comy   ---------GGT-AGCCCAAAACGCCTTGCTTA---GC-CACACCCC--CAAGGGAATTC--
Sasp   ----------GT-AGCCCAAAACGCCTTGCT--A-AGC-CACACCCC--CAAGGGAAATT--
Eupe   ----------GT-AGCCCAAAACGCCTTGTTAA---AC-CACACCCC--CAAGGGAA-CC--
Enja   ----------GT-AGCCCAAAACGCCTTGCTCA---GC-CACACCCC--CAAGGGAA-----
Same   ----------TA-AGCCCAAGACGCCTTGCTT---AGC-CACACCCC--CAAGGGAACT---
Chch   ---------AGC-TGCCCAAGACGCCTTGCT--T-TGC-CACACCCC--CAAGGGAACTC--
Grgr   ----------GC-AGCCCATGACGCCTTGCTAC---GC-CACCCCCC--CAAGGGAATTC--
Caau   ----------TT-AGCCCAAGACGCCTAGCC--G-AGC-CACACCCC--CAAGGGAATTC--
Cyca   ----------TT-AGCCCAAGACGCCTAGCCA---AGC-CACACCCC--CAAGGGAA-----
Dare   ---------TTT-AGCCCAAGACGCCTAGCCAA---GC-CACACCCC--CAAGGGGATCC--
Cost   ---------TTT-AGCCCAAGACGCCTTGCCAT---GC-CACACCCC--CAAGGGAACTC--
Leec   ----------TT-AGCCCAAGACGCCTTGCCAA---GC-CACACCCC--CAAGGGAA-----
Crla   ----------TT-AGCCCAAGACGCCTTGCC--A-CGC-CACACCCC--CAAGGGAA-CT--
Clmc   ----------AC-AGCCCAAGACGCCTTGCTTA--CGC-CACACCCC--CAAGGGAACTC--
Phin   ----------CC-CGCCCAAGACGTCTTGCTTT---GC-CACACCCC--CAAGGGAACTC--
Icpu   ----------CC-CGCCCAAGACGCCTTGCTAC---GC-CACACCCC--CAAGGGAACTC--
Psto   ----------TC-TGCCCAAGACGCCTTGCTAA---GC-CACACCCC--CAAGGGACTTC--
Cora   ----------TT-GGCCCAAGACGCCTTGCTTT---GC-CACACCCC--CAAGGGAATTC--
Eisp   ----------CT-AGCCCAAGACGCCTTGCTAC---GC-CACGCCCC--CAAGGGAA-----
Apal   ----------TT-AGCCCAAGACGCCTTGCTAT---GC-CACACCCT--CAAGGG-A-----
Eslu   ---------TGT-AGCCCAAGACGCCTTGCTAA---GC-CACACCCC--TACGGGTA-----
Dape   ----------GC-AGCCCAAGACGCCTTGCT--A-AGC-CACACCCC--CACGGGCAATC--
Glse   ---------AGC-AGCCCAAAACGCCTTGCT--A-TGC-CACACCCC--TAAGGGAACTC--
Naar   ---------AGC-AGCCCAATACGCCTTGCTA---ATC-CACACCCC--CAAGGGAACTC--
Baoc   ----------GC-AGCCCAAGACGCCTTGCTAA---GC-CACACCCC--CAAGGGAA-----
Opso   ----------GC-AGCCCAAAACGCCTTGCTA---AGC-CACACCCC--TAAGGGTATTC--
Alte   ----------TT-AGCCCAAGACGCCTTGCTAC---GC-CACACCCC--CAAGGGAACTC--
Plap   ----------AC-AGCCCAAGACGCCTTGCTAC---GC-CACACCCC--CAAGGGAG-----
```

```
Plal    ----------GC-AGCCCAAGACGCCTTGTT--T-AGC-CACACCCC--CAAGGGTATT---
Sami    ----------GC-AGCCCAGGACGCCTTGTTCA---GC-CACACCCC--CAAGGGTACTC--
Rere    ----------GC-AGCCCAAGACGCCTTGTTTA---GC-CACACCCC--CAAGGGTA-----
Gama    ----------AC-AGCCCAAGACGCCTTGCTAA---GC-CACACCCC--CAAGGGAACTC--
Onmy    ---------GGC-AGCCCACGACGCCTTGCTA---AGC-CACACCCC--CAAGGAAACTC--
Sasa    ----------GC-AGCCCAAGACGCCTTGCTAA---GC-CACACCCC--CAAGGAAACCC--
Cola    ---------GGC-AGCCCAAGACGCCTTGCT--A-AGC-CACACCCC--CAAGGAAACT---
Dita    ----------GT-AGCCCAAGACGCCTTGCT--A-AGC-CACACCCC--CAAGGGAATTC--
Gogr    ----------AA-AGCCCAAGACGCCTTGCTTA---GC-CACACCCC--CAAGGGAA-----
Chsl    ----------GC-AGCCCACAACGCCTTGCT--T-AGC-CACACCCC--CAAGGGTACTC--
Atja    ---------GAT-AGCCCAAAACGCCTTGCTTA---GC-CATACCCC--CAAGGGAACTC--
Iido    ---------GAC-AGCCCAAAACGCCTTGCTTA---GC-CATACCCC--CAAGGGAA-----
Auja    ----------TT-AGCCCAAGACGCCTTGCTT---AGC-CACACCCC--TAAGGGAATTC--
Chag    ----------GC-AGCCCAAGACGCCTTGCTC---AGC-CACACCCC--TAAGGGAC-----
Hami    ----------CC-AGCCCAAGACGCCTTGCTAC---GC-CACACCCC--TAAGGGTACTC--
Saun    ----------CC-TGCCCAAAACGTCTTGCTCC---GC-CACACCCC--CAAGGGAA-----
Nema    ----------GC-AGCCCAAGACGTCTTGCTTA---GC-CACACCCC--CAAGGGAACTC--
Disp    ---------CGT-AGCCCACGACGTCTTGCACA---GC-CACACCCC--TAAGGGATCTC--
Myaf    ----------GT-AGCCCAAGACGCCTTGCTT---AGC-CACACCCC--CAAGGGAACTC--
Lagu    ----------CT-AGCCCAAGACGCCTTGCTCA---GC-CACACCCT--CAAGGGAA-----
Trtr    ----------TT-AGCCCATGACGCCTTGCTTC---GC-CACACCCT--TAAGGGTACTC--
Zucr    ---------TTT-AGCCCATGACGCCTTGCTTA---GC-CACACCCC--TAAGGGTA-----
Pxja    ----------GC-AGCCCAAGACGCCTTGCT--T-AGC-CACACCCT--CAAGGGAA-CT--
Pxlo    ----------GC-AGCCCAAGACGCCTTGCTTA---GC-CACACCCT--CAAGGGAA-----
Pctr    ----------GT-GGCCCAAGACGCCTTGCTCA---GC-CACACCCC--CAAGGGAA-----
Apsa    ----------GT-AGCCCAAGACACCTTGCTAA---AC-CACACCCA--CACGGGTCTTC--
Cabe    ----------GT-TGCCCACGACGCCTTGCTCA---GC-CACACCCC--CACGGGAATTC--
Bzze    ----------AT-AGCCCACGACGCCTTGCTTA---GC-CACACCCT--CAAGGGAATTC--
Siim    ---------ATT-AGCCGACGACACCTTGCACA---GC-CACGCCCC--CAAGGGTAATC--
Ctru    ----------TC-CGCCCACTACGCCTTGCT--A-AGC-CACACCCC--CAAGGGAC-TT--
Dpbr    ----------TC-AGCCCATGACACCTTGCTA---AGC-CACACCCC--CAAGGGACTTC--
Caki    ----------TC-AGCCCAATACACCTTGTTT---TGC-CACACCCC--CACGGGTAATC--
Phja    ----------TT-AGCCCAAGACGCCTTGCTCA---GC-CACGCCCC--CACGGGTATTC--
Brsp    ----------TG-AGCCCATGACGCCTAGCTAA---GC-CACTCCCC--CACGGGAACTC--
Gamo    ----------CT-AGCCCATAACGCCTTGCTC---AGC-CACACCCC--TACGGGTATTC--
Lolo    ----------TC-AGCCCATAACGCCTTGCT--C-AGC-CACACCCC--TACGGGTATTC--
Batr    ---------TAT-AGCCCACAACACCACGCTT---AGC-CAC--CCT--TAA----ACAC--
Prmy    ---------GGC-AGCCCATGACATCTTGCT--T-AGC-CACACCTC--CAAAGGA--CA--
Loli    ---------GTA-AGCCCATGACGCCTCGTCA---AGC-CACTCCCC--CACGGGTACTC--
Loam    ---------CTT-GGCCCATGACGCCTTGCTAA---GC-CACACCCC--CACGGGAACTC--
Chab    ----------GA-AGCCCACGACACCTTGCCCAC-AGC-CACACCCT--CAAGGGAATTC--
Chto    ----------GA-AGCCCACGACACCTTGCCCAC-AGC-CACACCCT--CAAGGGAATTC--
Majo    ---------TGT-AGCCCACGACACCTTGCTTT---GC-CACACCCC--CAAGGGAACTC--
Hlst    ----------TC-AGCCCACGACGCCTCGCTC---ATG-GCCACTATA-CAAGTCAAATT--
Clpe    ----------CA-CGCCCACGACACCTTGCTCAT-AGC-CACACCCC--CAAGGGGT-----
Mlmr    ----------AA-AGCCCACGACACCTTGCTCAT-AGC-CACAACCC--CAAGGGGCCCCC-
Crcr    ----------AC-CGCCCACGACACCTTGCTTA---GC-CACACCCC--CAAGGGATTTC--
Muce    ----------AC-CGCCCACGACACCTTGCTTA---GC-CACACCCC--TAAGGGATTTC--
Bege    ----------CC-CGCCCATGACACCTTGCTT---AGC-CACACCCC--CAAGGGAATTC--
Mela    ----------TT-AGCCCACGACACCTTGCTTA---GC-CACACCCC--CAAGGGAATTC--
Hats    ----------TT-AGCCCACGACACCTTGCTC---AGC-CACACCCC--CAAGGGAATTC--
Orla    ----------AA-TGCCCATAACACCTTGCTT---AGC-CACACCCC--CAAGGGAA-----
```

```
Cosa    ----------TT-AGCCCATAACACCTTGCT--C-AGC-CACACCCC--CAAGGGAATTC--
Exsp    ----------CT-AGCCCATGACACCTTGCT--A-AGC-CACACCCC--CAAGGGAT-TT--
Depa    ----------TT-AGCCCACAACACCTTGCTT---TGC-CACACCCT--CAAGGGAATTC--
Rima    ----------TT-AGCCCATAACGCCTTGCTTA---GC-CACACCCC--CAAGGGAA-----
Fuol    ----------TT-GGCCCATGACACCTTGCTT---AGC-CACACCCC--TAAGGGAATTC--
Gmaf    ----------GC-AGCCCATGACACCTTGCT--A-AGC-CACACCCC--CAAGGGAACT---
Xeei    ----------CT-AGCCCACGACACCTTGCT--T-AGC-CACACCCC--CAAGGGAATTC--
Pros    ----------GC-AGCCCAAGACGCCTTGTTCA---GC-CACACCCC--CAAGGGACCTC--
Scmi    ----------GC-AGCCCAAGACGCCTTGTTCA---GC-CACACCCC--TAAGGGAA-----
Rolo    ----------GC-AGCCCAAGACGCCTTGCTTA---GC-CACACCCC--CAAGGGAACTC--
Cere    ---------CGC-AGCCCAAGACACCTTGCTCA---GC-CACACCCT--TAAGGGAGCTC--
Daga    ---------TGC-AGCCCAAGACGCCTTGCTC---AGC-CACACCCC--CAAGGGAA-----
Anco    ----------GC-AGCCCAAGACGCCTTGCTAA---GC-CACACCCC--CAAGGGAACTC--
Dmve    ---------GGT-AGCCCATGACGCCTTGTTTA---GC-CACACCCC--CAAGGGAACTC--
Dmar    ----------GC-AGCCCATGACATCTTGTT--T-AGC-CACACCCC--CAAGGGAG-CT--
Anka    ---------GGC-AGCCCAAGACGCCTTGCT--A-AGC-CACACCCT--CAAGGGAACTC--
Moja    ---------GGC-AGCCCAAGACGCCTTGCT--A-AGC-CACACCCC--CAAGGGAA-CT--
Hoja    ---------GGC-AGCCCAAGACGCCTTGCTAA---GC-CACACCCT--CAAGGGAACTC--
Bede    ----------GC-AGCCCAAGACGCCTTGTTCA---GC-CACACCCC--CAAGGGAGCTC--
Besp    ----------GC-AGCCCAAGACGCCTTGTTCA---GC-CACACCCC--CAAGGGAACTC--
Mysp    ----------GC-AGCCCAAAACGCCTTGCT--T-AGC-CACACCCC--CAAGGGAACTC--
Osja    ----------GC-AGCCCAAAACGCCTTGCTT---AGC-CACACCCC--CAAGGGAA-----
Sgro    ----------GC-AGCCCAAAACGCCTTGCTCA---GC-CACACCCC--CAAGGGAACTC--
Pzpa    ---------TGC-AGCCCATAACGCCTTGCTTA---GC-CACACCCC--CAAGGGACCTC--
Zeja    ----------GC-AGCCCAAGACGCCTTGCTTA---GC-CACACCCT--CAAGGGCCTTC--
Znne    ----------TT-AGCCCAAGACGCCTTGCT--T-AGC-CACACCCC--CAAGGGAC-CT--
Zefa    ----------TT-AGCCCAAGACGCCTTGCTTA---GC-CACACCCC--TAAGGGAG-----
Acni    ---------AGC-AGCCCAAGACGCCTTGCTT---AGC-CACACCCC--CAAGGGAGCTC--
Ncrh    ---------AGC-AGCCCAAGACGCCTTGCTTA---GC-CACACCCC--CAAGGGAACTC--
Agca    ----------TC-AGCCCACGACGCCTTGCTTA---GC-CACACCCC--CAAGGGAA-----
Hydy    ----------TT-AGCCCACGACGCCTTGCTTA---GC-CACACCCT--CAAGGGAA-----
Gsac    ----------TG-AGCCCACGACACCTTGCTT---AGC-CACACCCC--CAAGGGAA-----
Pevo    ----------GT-TGCCCACGACACCTTGCTCA---GC-CACACCCC--CAAGGGAAA-C--
Hiku    ----------TT-TGCCCATAACACCTTGCTT---AGC-CACACCCC--CAAGGGAATTC--
Inpa    ----------TT-AGCCCACGACGCCTTGCT--T-AGC-CACACCCC--CAAGGGGCTCC--
Auch    ---------CTC-AGCCCATAACACCTTGTTCA---GC-CACACCCC--CAAGGGCA-----
Fico    ----------CT-AGCCCACAACACCTTGCTCA---GC-CACACCCC--CAAGGGAA-----
Macs    ----------TC-AGCCTACGACACCTTGCTT---AGC-CACACCCC--CAAGGGAA-----
Moal    ----------GT-AGCCCAAGACACCTTGTTTA---GC-CACATCCC--CAAGGAAA-----
Syma    ----------GT-AGCCCACAACACTTTGCTC---AGC-CACACCCC--CAAGGGTA-----
Mafr    ----------AA-AGCCCACGACACCTTGCACA---GC-CACACCCC--CAAGGGAA-----
Dcpe    ---------TGTTAGCCGATGACGCCTTGTTTA---GC-CACACCCC--TAAGGGAACTC--
Dcti    ---------TGTTTGCCCACAACGCCTTGTTTA---GC-CACACCCC--CAAGGGAACTC--
Hehi    ---------TGTAAGCCCATGACACCTTGCT--T-AGC-CACACCCT--CAAGGGAA-CT--
Stam    ----------TA-AGCCCATGACACCTTGCTTA---GC-CACACCCT--CAAGGGAACTC--
Hogi    ----------TT-AGCCCACGACACCTTGCTT---AGC-CACACCCC--TAAGGGAA-----
Erzo    ----------TG-AGCCCATGACGCCTTGCT--T-AGC-CACACCCT--CAAGGGAA-CT--
Hxot    ----------TA-AGCCCATGACACCTTGCT--T-AGC-CACACCCT--CAAGGGAA-CT--
Core    ----------TG-AGCCCACGACACCTTGCTTA---GC-CACACCCT--CAAGGGAACTC--
Apve    ----------CG-AGCCCATAACACCTTGCTTA---GC-CACACCCT--CAAGGGAA-----
Latj    ----------TA-AGCCCACAACACCTTGCTTT---GC-CACACCCT--CAAGGGAACCC--
Laja    ---------GTT-AGCCCACGACACCTTGCTTA---GC-CACACCCC--CAAGGGAACTC--
```

```
Syja    ----------AT-AGCCCAAGACACCTTGCTT---AGC-CACCCCCT--CAAGGGAATCC--
Epme    ---------TAT-AGCCCACAACACCTTGCT--T-AGC-CACACCCT--CAAGGGAATCC--
Grse    ----------CT-TGCCCATGACACCTTGCTTA---GC-CACACCCC--CAAGGGAACGC--
Clja    ----------TT-AGCCCACGACACCTTGCA--T-AGC-CACACCCC--CAAGGGTAATC--
Ogcy    ---------GATAAGCCCACAACATCTTGCTC---AGC-CACACCCC--CAAGGGAATTC--
Plna    ---------ATT-AGCCCATGACGCCTTGCTTA---GC-CACACCCC--CAAGGGAATTC--
Lema    ----------AA-AGCCCACGACGCCTTGCTC---AGC-CACACCCC--CAAGGGAACTC--
Etzo    ----------CT-AGCCCACGACACCTTGCT--T-AGC-CACACCCC--CATGGGAACT---
Apse    ---------AAATAGCCCACGACACCTTGCTTA---GC-CACACCCT--CAAGGGATCTC--
Epde    ----------TC-AGCCCATGACACCTTGCTTA---GC-CACACCCT--CAAGGGAA-----
Slja    ---------TCT-AGCCCACAACACCTTGTTCA---GC-CACACCCC--CAAGGGTA-----
Bsja    ----------GA-AGCCCACGACACCTTGCTCA---GC-CACACCTT--CAAGGGTAATC--
Ecna    ---------TGT-AGCCCATAACACCTTGCTTA---GC-CACACCCC--CAAGGGAACCC--
Cohi    ----------TC-AGCCCACAACACCTTGCTA---AGC-CACACCCC--CAAGGGAA-----
Caar    ----------TT-AGCCCACGACACCTTGCTTA---GC-CACACCCC--CAAGGGAATCC--
Came    ---------TGT-AGCCCACAACACCTTGCT--T-AGC-CACACCCC--CAAGGGAATCC--
Mema    ----------AC-AGCCCACGACACCTTGCTT---AGC-CACACCCT--CAAGGGAA-----
Lenu    ----------CC-CGCCCACGACACCTTGCTTA---GC-CACACCCC--CAAGGGAACTC--
Plma    ----------CA-AGCCCACGACGCCTTGCT--T-AGC-CACACCCT--CAAGGGAACTC--
Emst    ----------TA-AGCCCATGACGCCTTGCTTA---GC-CACACCCT--CAAGGGAACTC--
Ptti    ----------CT-AGCCCATGACACCTTGCTTA---GC-CACACCCT--CAAGGGAACTC--
Losu    ---------TAT-AGCCCATAACACCTTGCT--C-AGC-CACACCCC--CAAGGGAAATT--
Geoy    CCCCCCCCGCGC-CGCCCAAAACACCTTGCT--T-TGC-CACACCCT--CAAGGGAACTC--
Dipi    ----------CA-AGCCCATGACACCTTGCTTA---GC-CACACCCT--CAAGGGAATTC--
Pama    ---------TAT--GCCCACGACACCTTGCT--C-AGC-CACACCCC--CAAGGGTACTC--
Leob    ----------TT-AGCCCACAACACCTTGCT--C-AGC-CACACCCT--CAAGGGAACTT--
Neba    ----------TT-TGCCCATGACACCTTGCTTA---GC-CACACCCC--CAAGGGTATTC--
Pdpl    ----------TT-AGCCCACGACGCCTTGCTT---AGC-CACACCCC--CAAGGGTA-----
Nimi    ----------GT-AGCCCACGACACCTTGCTTT---GC-CACACCCT--CAAGGGAA-----
Uptr    ---------TGT-AGCCCACGACACCTTGCT--T-TGC-CACACCCC--CAAGGGAACTC--
Pesc    ----------AT-TGCCCATAACGCCCTGCT--T-AGC-CACACCCC--CACGGGAA-TC--
Baar    ----------TT-AGCCCAAGACACCTTGCTTTA-TGC-CACACCCTATTAATGGAGCCC--
Moar    ----------TA-AGCCCATGACACCTTGCTTA---GC-CACACCC--TCAAGGGAA-CT--
Toja    ----------CA-AGCCCACGACACCTTGCT--T-AGC-CACACCCT--CAAGGGAA-TT--
Chau    ----------GT-AGCCCACGACGCCTTGCTTA---GC-CACACCCC--CAAGGGAG-----
Chse    ----------TA-AGCCCACGACACCTTGCTAA---GC-CACACCCC--CAAGGGAATTC--
Enar    ----------CT-AGCCCATGACACCTTGCTT---AGC-CACACCCT--CAAGGGAACTC--
Hpty    ----------CC-AGCCCATAACACCTTGCTT---AGC-CACACCCT--CAAGGGAA-----
Nana    ----------TT-AGCCCACGACACCTTGCTT---AGC-CACACCCC--CAAGGGAACC---
Mcst    ---------TAT-AGCCCACGACGCCTTGCTTA---GC-CACACCCC--CAAGGGAA-----
Rhox    ----------AC-AGCCCACGACGCCTTGCTT---AGC-CACACCCT--CAAGGGAA-----
Opfa    ----------TA-AGCCCACGACACCTTGCTTA---GC-CACACCCT--CAAGGGAATTC--
Paar    ----------TC-AGCCCACAACACCTTGCTCA---GC-CACACCCC--CAAGGGATTGC--
Gozo    ----------TT-AGCCCACGACACCTTGCTTA---GC-CACACCCT--CAAGGGAACTC--
Ackr    ----------GT-AGCCCACGACACCTTGCT--C-AGC-CACACCCC--CAAGGGAATTC--
Elev    ---------TTT-AGCCCATGACACCTTGCTTA---GC-CACACCCC--CAAGGGAATTC--
Trdu    ----------GC-AGCCCATGACACCTTGCTTA---GC-CACACCCT--CAAGGGAACTC--
Amoc    ---------ACC-TGCCCATGACACCTTGCTT---AGC-CACACCCT--CAAGGGACATC--
Hame    ----------TT-AGCCCACGACGCCTTGCTT---GGC-CACACCCC--CAAGGGAATTC--
Chso    ----------TT-AGCCAACGACACCTAGCCTA---GC-CACACCCC--CAAGGGACTAC--
Lyto    ----------TG-AGCCCATGACGCCTTGCTT---AGC-CACACCCT--CAAGGGAA-----
Encr    ---------GTG-AGCCCACGACGCCTTGCTTA---GC-CACACCCT--CAAGGGAACTC--
```

```
Bvar    ----------GT-AGCCCACGACACCTTGTTTA---GC-CACCCCCC--CAAGGGTA-----
Noco    ----------TT-AGCCCATGACACCTTGTTT---AGC-CACACCCT--TAAGGGAACTC--
Chsp    ----------CC-CGCCCACAACACCTGGTTTT--AGC-CACCCCCT--CACGGGTA-----
Arja    ----------AG-AGCCCACGACATCTTGCT--A-AGC-CACACCCT--CAAGGGAACTC--
Pase    ----------GG-AGCCCATGACGCCTAGCT--T-AGC-CACACCCC--CAAGGGAATG---
Trel    ----------CT-AGCCCAAGACGCCTTGCCCA---GC-CACACCCC--TAAGGGAACTC--
Lifa    ----------TT-AGCCCATAACACCTTGCTC---AGC-CACACCCC--CAAGGGTACTC--
Acur    ----------CT-AGCCCACGACACCTTGCTAAT--GC-CACACCCC--CACGGGTATAC--
Ampe    ----------GT-AGCCCACGACACCTTGCTTA---GC-CACACCCC--CAAGGGAA-----
Urja    ----------TC-CGCCCATGACGCCTTGCT--T-AGC-CACACCCC--CAAGGGAACCC--
Enet    ----------AC-TGCCCACGACATCTTGCTTT---GC-CACGCCCC--CAAGGGAA-----
Ptbr    ---------AGC-AGCCCATGACGCCTTGTTTA---AC-CACACCCC--CAAGGGCT-----
Safa    ---------AGT-AGCCCACTACACCTTGCTT---AGC-CACACCCC--CAAGGGATCTC--
Icae    ----------TA-AGCCCACGACGCCTTGCTT---AGC-CACACCCT--CAAGGGAA-----
Asmi    ---------GAT-GGCCTAAAACATCTAGCTA---AGC-CACACCCC--CAAGGGTT-----
Foal    ----------TA-CGCCCACGACGCCTTGCTC---AGC-CACACCCC--CAAGGGTCTAC--
Drze    ---------TTT-AGCCCATAACGCTTTGCT--T-AGC-CACACCCC--CAAGGGATTTC--
Rhas    ---------GTT-AGCCTACAACACCTTGCTCA---GC-CACACCCC--CAAGGGAACTC--
Elac    ----------TT-AGCCCACGACACCTTGCTC---AGC-CACACCCC--CAAGGGAA-----
Kugu    ---------TTT-AGCCTACAACACCTTGCTTA---GC-CACACCCA--CAAGGGAACTC--
Plor    ---------TAA-AGCCCACGACACCTTGCTTA---GC-CACACCCC--CAAGGGAA-----
Sgun    ----------CT-AGCCCATGACACCTTGCTTA---GC-CACACCCT--CAAGGGAACTC--
Zaco    ----------GA-AGCCCATGACACCTTGCTTA---GC-CACACCCT--CAAGGGAATTC--
Zbfl    ----------TT-AGCCCATGACACCTTGCTT---AGC-CACACCCT--CAAGGGAACTC--
Spba    ---------AGT-AGCCCACGACACCTTGCTAA---GC-CACGCCCT--CAAGGGAATTC--
Game    ----------CG-AGCCCATGACGCCTTGCT--T-AGC-CACACCCA--CAAGGGATCTC--
Thth    ----------AA-AGCCCATGACGCCTTGCTTA---GC-CACACCCT--CAAGGGAACTC--
Xigl    ----------TT-AGCCCATGACGCCTTGCTTA---GC-CACACCCT--CAAGGGAA-----
Hyja    ----------TG-AGCCCACGACGCCTTGTTC---AGC-CACACCCC--CAAGGGAA-----
Psan    ----------TG-AGCCCATGACGCCTTGTT--T-AGC-CACACCCC--CAAGGGAA-TT--
Cupa    ----------CA-AGCCCATGACGCCTTGCTTA---GC-CACACCCT--CAAGGGAACTC--
Mpch    ----------AT-AGCCCATGACGCCTTGCTTA---GC-CACACCCT--CAAGGGAACTC--
Char    ----------CC-AGCCCACGACGCCTTGCTC---AGC-CACACCCC--CAAGGGAA-----
Pser    ---------CTT-GGCCCATGACACCTTGCCTC--AGC-CACACCCC--CAAGGGAATCC--
Prol    ---------TCT-GGCCCACGACGCCTTGCT--T-AGC-CACACCCT--CAAGGGAACTC--
Plbi    ----------CT-AGCCCACGACGCCTTGCTTA---GC-CACACCCT--CAAGGGAC-----
Calu    ----------GT-AGCCCAGAACACCTTGCTTA---GC-CACACCCC--CAAGGGTACTC--
Papa    ----------TA-AGCCCAAGACGTCTTGCTTTTTTGC-CACACCCC--CAAGGGAAATC--
Sufr    ---------ATT-AGCCCAAGACACCTTGCTTA---GC-CACGCCCT--CAAGGGAA-----
Stci    ---------ATT-AGCCCAAGACGCCTTGCT--T-AGC-CACACCCT--CAAGGGAA-CT--
Taru    ---------TGT-AGCCCATGACACCTAGCTTT---GC-CACGCCCC--CAAGGGAA-----
Rala    ----------GA-AGCCCAAGACACCTTGCT--T-AGC-CACACCCT--CAAGGGAC-CT--
                       ** *  **
```

```
          ¦ HVR ¦ 6'                 15                  15'     5'     4'
Scca -----TTCAGCAGTAATAAACATTGATTACATAAGCGT-AAGCTTGAATCAGTTA-AAGT
Muma --------AGCAGTAATAAACATTAATTCA-TGAGCGA-AAGCTTGAATTAGTTA-AAGT
Erca -------CAGCAGTGATAAACATTAGGCAA-TCAGCGA-AAGCTAGACCTAGTTA-TTGT
Pose -----TTCAGCAGTGATAAACATTAGGCA-ATCAGCGA-AAGCTAGACCTAGTTA-TGGT
Actr -----CTCAGCAGTGATAAACATTGAGC-TATGAGCGC-AAGCTCGACTCAGCCA-GAGT
Scal -----CTCAGCAGTGATAGACATTGAGC-CATGAGCGT-AAGCTCGACTCAGCCA-GAGT
Posp --------AGCAGTGATAAACATTGAGCCA-TGAGCGC-AAGCTCGACTCAGCCA-GAGT
Atsp --------AGCAGTGATAGACATTAAGC-AATAAGCGA-AAGCTTGACTTAGTTA-AAGC
Leoc --------AGCAGTGATAAACATTAAGC-AATAAGCGA-AAGCTTGACTTAGTTA-AAGC
Amca --------AGCAGTGATAAATTTTAAGC-AATAAGCGA-AAGCTTGACTTAGTTA-ATGT
Osbi --------AGCAGTGATTAACATTAAAT--ATAAGCGA-AAGCTTGATTTAGTTA-TAGT
Pabu --------AGCAGTGATTAATATTAAAA-CATGAACGA-AAGCTCAATTAGTTA-AAGC
Hial -----TTCAGCAGTGATAAACATTGAGC-CATAAGCGA-AAGCTTGACTCAGTTA-AAGT
Elha -----CTCAGCAGTGATAGACATTAAGC-CATAAGCGA-AAGCTTGACTTAGCTA-ACGT
Mlcy -------CAGCAGTGATAAACATTAGGCAA-TTAGTGA-AAACTAGACCTAGCTA-AAGT
Algl AGGGGTTCCGCAGTGATAGACATTAAGC-TATGAGTGA-AAGCTCGACTTAGTTA-GGGC
Ptgi -----CCCAGCAGTGATAAACATTAAGC-CATAAGCGA-AAGCTCGACTTAGTTA-AAGC
Alaf --------AGCAGTGATAAACATTAAGC-CATAAGCGA-AAGCTAGACTTAGTTA-AAGC
Nock -------CAGCAGTGATAAACATTAAGCTA-TAAGCGA-AAGCTAGACTTAGTCA-AAGT
Anja --------AGCAGTGATAGATATTAAGCAA-TAAGCGA-AAGCTTGACTTAGTCA-AGGC
Gyki --------AGCAGTGACAAACATTAAGC-CATGAGCGA-AAGCTTGACTTAGTTA-GGGT
Syka --------AGCAGTGATAAATATTAAGC-CATAAGCGA-AAGCTTGACTTAATTA-AGGC
Opma --------AGCAGTAATTAATATTAGGC-CATAAGCGA-AAGCTTGACTTAATTA-GAGT
Comy --------AGCAGTGATTAATATTATAA-TATGAGCGA-AAGCTCGACCTAATTA-AGAC
Sasp -------CAGCAGTGATAGACATTAAGCTA-TAAGAGA-AAACTCGACTTAATTA-GGAG
Eupe --------AGCAGTGATAAACATTAAGC-CATGAGGGA-AAGCTCGACTTAATTA-AGAT
Enja -----TTCAGCAGTGATAAACATTAAGC-CATAAGCGA-AAGCTTGACTTAGTCC-GAGC
Same ----CTTCAGCAGTGATAAACATTAAGC-CATAAGTGA-AAACTTGACTTAGTTA-GGGT
Chch --------AGCAGTGATAAACATTAGGCATATAAGTGC-AAACTTGACCTAGTCA-AGGT
Grgr --------AGCAGTGATTAACATTGAGC-TATAAGTGA-AAACTTGACTTAGCTA-GGGT
Caau --------AGCAGTGATAAACATTAAGCCA-TAAGTGA-AAACTTGACTCAGTTA-GTGT
Cyca -----TTCAGCAGTGATAAACATTAAGC-CATAAGTGA-AAACTTGACTCAGTTA-GCGT
Dare --------AGCAGTGACAAACATTAAGC-TATAAGTGA-AAACTTGACTCAGTTA-AGGT
Cost --------AGCAGTGATAAATATTAAGC-CATAAGTGA-AAACTTGACTTAGTTA-GAGC
Leec -----TTCAGCAGTGATAAATATTAAGC-CATAAGTGA-AAACTTGACTTAGTTA-GTGC
Crla -------CAGCAGTGATAAAAATTAAGCCA-TGAGCACTAAGCTTGACTTAGTTA-GTGT
Clmc --------AGCAGTAATAAATATTAAGC-CATAAGTGA-AAACCTGACTTAGTTA-GGGC
Phin --------AGCAGTAGTAAACATTAAGC-CATAAGTGA-AAACTTGACTTAGTTA-GGAT
Icpu --------AGCAGTAATAGACATTAAGC-CATAAGTGT-AAACTTGACTTAGTTA-GGGC
Psto --------AGCAGTAATAAATATTAAGC-CATAAGTGA-AAACTTGACTTAGTTA-GAGT
Cora --------AGCAGTAGTAAATATTAAGC-CATAAGTGA-AAACTTGACTTAGTTA-AGGT
Eisp -----CTCAGCAGTAATAAACATTAAGC-AATAAGTGA-AAACTTGACTTAATTA-GGGT
Apal -----TCCAGCAGTAATTAACATTAAGC-AATAAGTGA-AAACTTGACTTAGTTA-GGGC
Eslu -----CTCAGCAGTGATAAATATTAAGT-GATAAGCGA-AAGCTTGACTTAGTTA-TTGT
Dape --------AGCAGTGATAAATCTTAAAA-AATAAGCGA-AAGCTTGACTTAGTTA-TTAT
Glse --------AGCAGTGATAAACATTAAGCCA-TAAGCGT-AAGCTTGACTTAGTTA-AGGA
Naar --------AGTACTGATAAACATTAAGC-CATGAGCGA-AAGCTTGACTTAGTTA-AGGT
Baoc -----CTCAGCAGTGATAAACATTAAGCC-ATAAGCGT-AAGCTTGACTTAGTTA-AGGT
Opso --------AGCAGTAACAAACATTAAGCCA-TGAGCGA-AAGCTTGACTTAGTTA-AAGT
Alte --------AGCAGTGGTAAACATTAAGC-CATAAGTGA-AAACTTGACTTAGTTA-AGGC
Plap -----CTCAGCAGTGGTAAACATTAAGCCCATAAGTGA-AAACTTGACTTAGTTA-GGGC
```

```
Plal   -------CAGCAGTGATAGACATTAAGCAA-TAAGCGA-AAGCTTGACTAGGTTA-CGGT
Sami   --------AGCAGTGATAGACATTAAGC-AATAAGCGA-AAGCTTGACTAGGTTA-CGGT
Rere   -----TTCAGCAGTAATAGACATTAAGC-CATGAGCGA-AAGCTCGACTAGGTTA-AGGT
Gama   --------AGCAGTGATAGACATTAAGC-CATGAGCGG-AAGCTCGACTTAGTTA-AGGT
Onmy   --------AGCAGTGATAAATATTAAGC-CATAAGCGA-AAGCTTGACTTAGTTA-AGGT
Sasa   --------AGCAGTGATAAATATTAAGC-CATAAGCGA-AAGCTTGACTTAGTTA-AAGT
Cola   -------CAGCAGTGATAGATATTAAGCTA-TAAGCGA-AAGCTTGACTTAGTTA-AGGT
Dita   --------AGCAGTGATAAATATTAAGCCA-TGGGCGG-AAGCCCGACTTAGTTA-AGGT
Gogr   -----CTCAGCAGTGAAAAACTTTAAGC-AATAAGCGA-AAGCTAGACTCAATCA-GGGT
Chsl   --------AGCAGTGATAAATTTTAAGCCA-TAAGCGA-AAGCTTGACTTAGTTC-CGGT
Atja   --------AGCAGTGATAAACATTAAGC-TATTAGTGA-AAACTTGACTTAGTTA-AAGA
Iido   -----CTCAGCAGTGATAAACATTAAGC-CATTAGTGA-AAACTTGACTTAGTTA-AAGA
Auja   --------AGCAGTGATAAACATTAAGCCA-TAAGTGA-AAACTTGACTTAGTTA-AAGT
Chag   -----CTCAGCAGTGATAGACATTAAGC-AATGAGTGA-AAACTCGACTTAGTTA-CGGT
Hami   --------AGCAGTGATAAACATTAAGC-CATGAGCGA-AAGCTCGACTTAATGA-AAGT
Saun   -----CTCAGCAGTGATAGACATTAAGC-TATGAGTGA-AAACTCGACTTAGTGA-AGGT
Nema   --------AGCAGTGATAAACATTAGGC-AATAAGTGA-AAACTTGACCTAGTTA-TGGT
Disp   --------AGCAGTAATAAACATTAGGC-AATAAGTGT-AAACTTGACCTAGTTA-CGGT
Myaf   --------AGCAGTAATTAACATTAGGC-AATAAGTGT-AAACTTGACCTAACTA-CGGT
Lagu   -----TTCAGCAGTGATAAATATTGAGA-TATAAACGA-AAGCTTGACTCAGTTA-AGAC
Trtr   --------AGCAGTAATAAACATTAAGC-CATAAGTGC-CAACTTGACTTAATTA-AGAT
Zucr   -----CTCAGCAGTAATAAACATTAAGC-CATAAGCGT-CAGCTTGACTTAATTA-AGAT
Pxja   -------CAGCAGTGATAAACATTAAGCCA-TAAGTGC-AAACTTGACTTAGTCA-AAGC
Pxlo   -----CTCAGCAGTGATAAACATTAAGC-CATAAGTGC-AAACTTGACTTAGTCA-AAGC
Pctr   -----CTCAGCAGTAGTAGACATTAAAC-CATAAGCGC-AAGCTTGACTTAGTTA-GGGC
Apsa   --------AGCAGTGATAAACATTAAGC-CATAAGTGC-AAACTTGACTTAGTTA-CAGC
Cabe   --------AGCAGTGATAAACATTAGGC-AATTAGCGA-AAGCTTGACCTAATTA-TGGT
Bzze   --------AGCAGTGATAAACATTAAGC-CATAAGTGA-AAACTTGACTTAGTTA-AGGC
Siim   --------AGCAGTGATAAACATTAAGC-TATAAGTGA-AAACTTGACTTAGTTA-AAGT
Ctru   -------CAGCAGTGATAAACATTAAGTA--TAAGTGA-AAACTTGACTTAGTCA-AGGC
Dpbr   --------AGCAGTGATAAACATTAAGCCA-TAAGTGA-AAACTCGACTTAGTTA-AGGC
Caki   --------AGCAGTGATAAACTTTTAGC-TATAAGTGA-AAACTTGACTCAGTTA-AGGT
Phja   --------AGCAGTGAGAAAATTTAAGC-CATAAGTGA-AAGCTTGACTTAGTTA-AGGT
Brsp   --------AGCAGTGATAAAAATTAAG--TATTTGAGC-AAACTTGACTTAGTTA-AGAT
Gamo   --------AGCAGTGATAAAATTTAAGC-CATAAGTGA-AAGCTTGACTTAGTTA-AGGG
Lolo   --------AGCAGTGATAAAATTTAAGC-CATAAGTGA-AAGCTTGACTTAGTTA-GGGT
Batr   --------CGCAGTAGTTAATATTAGGCAA-TGAGCGA-AAGCTTGACCTAGTCA-AAGT
Prmy   -------CAGCAGTAGTTAATATTAGGCAA-TGGGTGA-AAACCTGACCTAGTTA-TAGT
Loli   --------AGCAGTAATAAATATTAAGCCA-TGAGCGA-AAGCTCGACTTAGCCA-CAAG
Loam   --------AGCAGTGATAAACATTAAGC-CATAAGCGA-AAGCTTGACTTAGTTA-AAGT
Chab   --------AGCAGTGATAAATATTAAGCAA-TAAGTGA-AAACTTGACTTAGATA-GGGC
Chto   --------AGCAGTGATAAATATTAAGCAA-TAAGTGA-AAACTTGACTTAGATA-GGGC
Majo   --------AGCAGTGACAGACATTAAGC-CATTAGCGA-AAGCTTGACTTAGTTA-AAGC
Hlst   --------AGCAGTGATAGACATTGAGC-CATAAGTGA-AAGCTTGACTCAGTCA-GGGT
Clpe   -----CCCAGCAGTAATAAATATTAAGC-AATAAGTGA-AAGCTTGACTTAGCTA-AGGT
Mlmr   -----TCCAGCAGTAACAAACATTAAGC-AATAAGTGA-AAACTTGACTTAGCCA-AGGC
Crcr   --------AGCAGTGATTAACCTTAAGC-CATAAGTGA-AAACTTGACTTAGCCA-CGGC
Muce   --------AGCAGTGATTAACCTTAAGC-CATAAGTGA-AAACTTGACTTAGCCA-CGGC
Bege   --------AGCAGTGATAAATATTAAGCCA-TAAGTGA-AAACTTGACTTAGTTA-GAGT
Mela   --------AGCAGTGATAAACATTAAGC-TATAAGTGA-AAACTTGACTTAGTTA-AAGC
Hats   --------AGCAGTGATAAACATTAAGCCA-TAAGTGA-AAACTTGACTTAGTTA-AAGC
Orla   -----CTCAGCAGTGATAGACATTAAGC-AATAAGTGA-AAACTTGACTTAATTA-AGGC
```

```
Cosa    --------AGCAGTGATAAATATTAAGCAA-TAAGTGA-AAACTTGACTTAGTTA-AAGC
Exsp    -------CAGCAGTGATAAACATTAAGCTA-TAAGTGA-AAACTTGACTTAGTTA-AAGC
Depa    --------AGCAGTGATAAATATTAAGCAA-TAAGTGA-AAACTTGACTTAGTTA-AAGC
Rima    -----CGCAGCAGTGATAAACCTTAAGC-AATAAGTGT-AAGCTTGACTTAATGA-AAGC
Fuol    --------AGCAGTAATAAATATTAAGCCA-TAAGTGA-AAACTTGACTTAGTTA-AAGC
Gmaf    -------CAGCAGTGATAAACATTAAGCAA-TAAGTGA-AAACTTGACTTAGTAA-AAGC
Xeei    --------AGCAGTGATAAACATTAAGCCA-TAAGTGT-AAACTTGACTTAGTTA-AAGC
Pros    --------AGCAGTAATAAACATTGAGC-TATAAGTGA-AAACTTGACTCAGTCA-AAGT
Scmi    -----CTCAGCAGTGATAAACATTGAAC-CATAAGTGA-AAACTTGAATCAGTTA-AAGT
Rolo    --------AGCAGTGATAAACATTAAGC-CATAAGTGA-AAACTTGACTTAGTTA-AAGT
Cere    --------AGCAGTGATAAACATTAAGC-CATAAGTGA-AAACTTGACTTAGTTA-AAGT
Daga    -----CTCAGCAGTAATAAACATTAAGC-CATAAGCGA-AAGCTCGACTTAGTTA-AGGT
Anco    --------AGCAGTGATAAACATTAAGC-CATAAGTGA-AAACTTGACTTAGTTA-CAGT
Dmve    --------AGCAGTGATAGACATTAAGC-CATAAGTGA-AAACTTGACTTAGTTA-CAGT
Dmar    -------CAGCAGTGATAGACCTTAAGCTA-TAAGTGA-AAACTTGACTTAGTTA-CAGT
Anka    --------AGCAGTGATAAACATTAAGCCA-TAAGTGA-AAACTTGACTTAGTTA-CGGT
Moja    -------CAGCAGTGATAAACATTAAGCCA-TGAGTGA-AAACTCGACTTAGTTA-CGGT
Hoja    --------AGCAGTGATAAACATTAAGC-CATAAGTGA-AAACTTGACTTAGTTA-CGGT
Bede    --------AGCAGTGATAAACATTAAGC-CATAAGTGA-AAACTTGACTTAGTCA-AAGT
Besp    --------AGCAGTGATAAACATTGAGC-TATAAGTGA-AAACTTGACTTAGTCA-AAGT
Mysp    --------AGCAGTGATAAACATTAAGC-AATAAGTGA-AAACTTGACTTAGTCA-AAGC
Osja    -----CTCAGCAGTGATAAATATTAAGC-AATAAGTGA-AAACTTGACTTAGTTA-AAGC
Sgro    --------AGCAGTGATAAATATTAAGC-AATAAGTGA-AAACTTGACTTAGTTA-AAGC
Pzpa    --------AGCAGTGGTAAACATTAAGC-CATAAGTGA-AAACTTGACTTAGTTA-AAGC
Zeja    --------AGCAGTAATAAAAATTGAGC-CATAAGTGA-AAACTTGACTTAGTTA-AAGT
Znne    -------CAGCAGTAGTAAATATTAAGCCA-TAAGTGA-AAACTTGACTTAGTTA-AGGT
Zefa    -----TTCAGCAGTAATAAATATTAAGC-CATAAGTGA-AAGCTTGACTTAGTTA-AGGT
Acni    --------AGCAGTGATAAAAATTAAGCTA-TAAGTGA-AAACTTGACTTAGTTA-AAGT
Ncrh    --------AGCAGTGATAAAAATTAAGC-TATAAGTGA-AAACTTGACTTAGTTA-AAGT
Agca    -----CTCAGCAGTGACAGACATTAAGC-CATAAGTGA-AAACTTGACTTAGTTA-AAGC
Hydy    -----TTCAGCAGTGACAGACATTAAGC-CATAAGTGA-AAACTTGACTTAGTCA-GAGC
Gsac    -----TTCAGCAGTGACAAACATTAAGC-TATAAGTGA-AAACTTGACTTAGTTA-AAGC
Pevo    --------AGCAGTGATAGACATTAAGC-AATAAGTGA-AAACTTGACTTAGTTA-AAGC
Hiku    --------AGCAGTGATAAACATTAAGC-CATAAGTGT-AAACTTGACTTAGTTA-AGGT
Inpa    --------AGCAGTAACAAACATTAGGCCA-TAAGTGA-AAACTTGACCTAGCCA-CGGT
Auch    -----CTCAGCAGTGATTAACCTTAGGC-AATAAGCGA-AAGCTTGACCTAGTGG-TAGC
Fico    -----TTCAGCAGTGATAAACATTAAGC-AATGAGCGA-AAGCTCGACTTAGTTA-TAAG
Macs    -----CTCAGCAGTGATAGACATTAAGC-TATAAGTGA-AAACTCGACTTAGTTA-AAGC
Moal    -----ACCAGCAGTGATAAACATTAAGC-CATTAGTGA-AAACTTGACTCAGTTA-AAGT
Syma    -----ATCAGCAGTAATAAACCTTAAGC-CATAAGTGA-AAACTTGACTTAGTCA-AAGC
Mafr    -----CTCAGCAGTGATAAACATTAAGCC-ATAAGTGA-AAACTTGACCTAGCTA-TGGT
Dcpe    --------AGCAGTGATAAACCTTAAGC-TATAAGTGA-AAACTTGACTTATTTA-GTGC
Dcti    --------AGCAGTGATAAACCTTAAGC-TATAAGTGA-AAACTTGACTTAGTTA-GTGC
Hehi    -------CAGCAGTGATAAACATTAAGCCA-TAAGTGA-AAACTTGACTTAGTTA-AAGC
Stam    --------AGCAGTGATAAACATTAAGC-CATAAGTGA-AAACTTGACTTAGTTA-AAGC
Hogi    -----CTCAGCAGTGATAAATATTAAAC-CATAAGTGA-AAACTTGATTTAGTTA-AAGC
Erzo    -------CAGCAGTGATAAACATTAAGCCA-TAAGTGA-AAACTTGACTTAGTCA-AAGC
Hxot    -------CAGCAGTGATAAACATTAAGCCA-TAAGTGA-AAACTTGACTTAGTTA-AAGC
Core    --------AGCAGTGATAAACATTAAGC-CATAAGTGA-AAACTTGACTTAGTTA-AAGC
Apve    -----CTCAGCAGTGACAAACATTAAGC-CATAAGTGA-AAACTTGACTTAATTA-AAAC
Latj    --------AGCAGTGATAGACTTTAAGC-TATAAGTGA-AAACTTGACTTAGTTA-AAGC
Laja    --------AGCAGTGATAGACATTAAGC-CATAAGTGA-AAACTTGACTTAGTCA-AAGC
```

```
Syja    --------AGCAGTGATAAACATTAAGC-CATAAGTGA-AAACTTGACTTAGTTA-A-GC
Epme    --------AGCAGTGATAGACATTAAGCCAATGAGTGC-AAACTTGACTTAGTTA-TGGT
Grse    --------AGCAGTGATAAACATTAAGC-CATGAGTGA-AAACTTGACTTAGTTA-AAGC
Clja    --------AGCAGTGACAAATATTAAGCCA-TAAGTGT-AAACTTGACTTAGTTA-CACG
Ogcy    --------AGCAGTGATAAACATTAAGC-TATAAGTGA-AAACTTGACTTAGTCA-AGGC
Plna    --------AGCAGTGATAAATATTAAGC-CATAAGTGA-AAACTTGACTTAGTTA-ATGC
Lema    --------AGCAGTGATAAACATTAAGC-CATAAGTGA-AAACTTGACTTAGTTA-AAGC
Etzo    -------CAGCAGTGATAAACATTAAGCCA-TAAGTGA-AAACTTGACTTAGTTA-AAGC
Apse    --------AGCAGTGATAAACATTAAGC-CATAAGCGA-AAGCTTGACTTAGTTA-AAGC
Epde    -----CTCAGCAGTGATAGACCTTAAGC-CATGAGTGA-AAACTCGACTTAGTCA-AAGC
Slja    -----CTCAGCAGTGATAGACATTAAGC-TATAAGTGA-AAACTTGACTTAGTTA-AAGC
Bsja    --------AGCAGTGGTAAATATTAAGC-CATAAGTGA-AAACTTGACTTAATTA-AGGC
Ecna    --------AGCAGTGATAAATATTAAGC-CATAAGTGA-AAGCTTGACTTAGTTA-AAGC
Cohi    -----CCCAGCAGTGATAAATTTTAAGC-CATAAGTGA-AAACTTGACTTAGTTA-AGGC
Caar    --------AGCAGTGATAAATATTAAGC-CATAAGTGA-AAACTTGACTTAGTTA-AAGC
Came    --------AGCAGTGATAAATATTAAGCCA-TAAGTGA-AAACTTGACTTAGTTA-AAGC
Mema    -----CTCAGCAGTGACAGACATTAAGC-CATAAGTGA-AAACTTGACTTAGTCA-AAGC
Lenu    --------AGCAGTAACAAACATTAAGC-AATAAGCGA-AAGCTTGACTTAATTA-TAGC
Plma    --------AGCAGTGATAAACCTTAAGCTA-TAAGTGA-AAACTTGACTTAGTTA-AAGC
Emst    --------AGCAGTGATAGACATTAAGC-CATAAGTGA-AAACTTGACTTAGTCA-AAGC
Ptti    --------AGCAGTGATAAACATTAAGC-CATAAGTGA-AAACTTGACTTAGTTA-AAGC
Losu    -------CAGCAGTGATAAACATTAAGCAA-TAAGTGA-AAGCTTGACTTAGTTA-TGGT
Geoy    --------AGCAGTGATAGATATTAAGCCA-TAAGTGA-AAACTTGACTTAGTTA-AGGT
Dipi    --------AGCAGTGATAAACATTAAGC-CATAAGTGA-AAACTTGACTTAGTCA-AAGC
Pama    --------AGCAGTGATAAACATTGACA-TATAAGTGA-AAACTTGACTCAGTCA-AAGC
Leob    -------CAGCAGTGATTAATATTTAGCCA-TAAGTGA-AAACTTGACTCAGTTA-GAGC
Neba    --------AGCAGTAGTAAACATTGAGC-TATAAGCGA-AAGCTTGACTCAGTTA-GAGC
Pdpl    -----GTCAGCAGTGATAAACATTAAGC-CATGAGTGA-AAACTTGACTTAGTTA-AAGC
Nimi    -----TTCAGCAGTGATAAACCTTAAGCC-ATAAGTGA-AAACTTGACTTAGTAA-AAGC
Uptr    --------AGCAGTGACAGACATTAAGCCA-TAAGTGA-AAGCTTGACTTAGTTA-AAGC
Pesc    -------CAGCAGTGATAAACATTAAGCAA-TAAGCGA-AAGCTTGACTTAGTTA-ATGA
Baar    --------AGCAGTGATAGACATTAAGC-CATAAGTGA-AAACTTGACTTAGTCTTAAGG
Moar    -------CAGCAGTGATAGACATTAAG-CCATAAGTGA-AAACTTGACTTAGTCA-AAGC
Toja    -------CAGCAGTGATAGACATTAAGCCA-TAAGTGA-AAACTTGACTTAGCCA-AGGC
Chau    -----TCCAGCAGTGATAGATTTTAAGCC-ATAAGTGA-AAACTTGACTTAGTTA-ATGC
Chse    --------AGCAGTGATAAACATTAAGC-CATAAGTGA-AAACTTGACTTAGTTA-AAGC
Enar    --------AGCAGTGATAGACATTAAGCCA-TAAGTGA-AAACTTGACTTAGTCA-AAGC
Hpty    -----CTCAGCAGTGATAAACATTAAGC-CATAAGTGA-AAACTTGACTTAGTCA-AAGC
Nana    --------AGCAGTGATAAACATTAAGCTA-TAAGTGA-AAACTTGACTTAGTTA-AAGC
Mcst    -----TCCAGCAGTGATAGACATTAAGC-TATGAGTGA-AAACTTGACTTAGTCA-AGGT
Rhox    -----TTCAGCAGTGATAAACATTAAGC-AATAAGTGA-AAACTTGACTTAGTTA-AGGC
Opfa    --------AGCAGTGATAGACATTAAGC-TATAAGTGA-AAACTTGACTTAGTCA-AGGC
Paar    --------AGCAGTGATAAACATTAAGC-TATGAGTGA-AAACTTGACTTAGTTA-AAGA
Gozo    --------AGCAGTGATAAACATTAAGC-CATAAGTGA-AAACTTGACTTAGTTA-AAGC
Ackr    --------AGCAGTGATAAATATTAAGC-CATGAGTGA-AAACTTGACTTAGTTA-AAGC
Elev    --------AGCAGTGATAAATATTAAGC-CATAAGTGA-AAACTTGACTTAGTTA-AAGC
Trdu    --------AGCAGTGATAGACATTAAGC-CATAAGTGA-AAACTTGACTTAGTTA-AAGC
Amoc    --------AGCAGTGATAGATATTAAGCCA-TAAGTGA-AAACTTGACTTAGTTA-AGGC
Hame    --------AGCAGTGACAAACATTAAGC-CATGAGTGA-AAACTTGACTTAGTTA-GTGT
Chso    --------AGCAGTGATAGACATTAAGC-AATGAGTGA-AAACTCGACTTAGTTA-AAGC
Lyto    -----CTCAGCAGTGATAAACATTAAGC-CATAAGTGA-AAACTTGACTTAGTTA-AAGC
Encr    --------AGCAGTGATAAACATTAAGC-CCTAAGTGA-AAACTTGACTTAGTCA-AAGC
```

```
Bvar  -----TTCAGCAGTGATAGATTTTAAGC-CATAAGTGA-AAGCTTGACTTAGTAG-AAAG
Noco  --------AGCAGTGATAGATATTAAGCCA-TAAGTGA-AAACTTGACTTAGTAA-AAGC
Chsp  -----TTCAGCAGTGATAAAAATTAAGA-TATAAGTGA-AAACTTGACTTAATCA-GGAC
Arja  --------AGCAGTGATAAACATTAAGC-CATGAGTGA-AAACTTGACTTAGTTA-AAGC
Pase  -------CAGCAGTGATAAACATTAAGCAA-TAAGCGA-AAGCTTGACTTAGTTA-TAGT
Trel  --------AGCAGTGATTGACATTAAGT--ATAACCGA-AAGTTTGACTTAGCAA-CAGC
Lifa  --------AGCAGTGACAGACATTAAAT-AATAAGTGA-AAACTTGAATTAGTTA-GGGA
Acur  --------AGCAGTGATTTATTTTAAGC-TATGAGTGT-AAACTTGACTTAGATA-AAGC
Ampe  -----CTCAGCAGTGATAGACATTAAGCC-ATAAGTGA-AAACTTGACTTAGTCA-AAGC
Urja  --------AGCAGTGATAAATCTTAAGCAA-TAAGTGA-AAACTTGACTTAGTTA-GGGC
Enet  -----CTCAGCAGTGATAAACATTAAGC-GATGAGCGA-AAGCTCGACTTAATTA-GGGC
Ptbr  -----CGCAGCAGTGATAAATATTAAGC-AATAAGTGA-AAACTTGACTTAATTA-GAGC
Safa  --------AGCAGTGATAAATATTAAGCAA-TAAGTGA-AAACTTGACTTAGTTATAAGC
Icae  -----CTCAGCAGTGATAAACCTTAAGC-TATAAGTGA-AAACTTGACTTAGTTA-AAGC
Asmi  -----TTCAGCAGTGATAAACATTAAGC-TATTAGTGA-AAACTTGACTTAATTA-AGGC
Foal  --------AGCAGTGATAAATATTAAGC-CATAAGTGA-AAACTTGACTTAGCAG-CAGC
Drze  --------AGCAGTGTCAGACATTAAGCTA-TGAGTGT-AAACTCGACTTAATTA-TGGT
Rhas  --------AGCAGTGATAGACATTAAGC-CCTAAGTGT-AAACTTGACTTAGTTA-AAGC
Elac  -----CTCAGCAGTGATAGACATTAAGC-CATTAGTGA-AAACTTGACTTAGTTA-AGGC
Kugu  --------AGCAGTGACAGACATTAAGC-CATAAGTGA-AAACTTGACTTAGTTA-AAGC
Plor  -----TTCAGCAGTGATAAACATTAAGC-CATAAGTGA-AAACTCGACTCAGTCA-AGGC
Sgun  --------AGCAGTGATAAACATTAAGC-CATAAGTGA-AAACTTGACTTAGTCA-AAGC
Zaco  --------AGCAGTGACAAACATTAAGC-CATAAGTGA-AAGCTTGACTTAGTTA-AAGC
Zbfl  --------AGCAGTGATAAACATTAAGCCA-TAAGTGA-AAACTTGACTTAGTCA-AAGC
Spba  --------AGCAGTGATAAACATTAAGC-CATGAGCGA-AAGCTTGACTTAGTTA-AAGC
Game  --------AGCAGTGATAAACCTTAAGC-TATGAGTGC-AAACTTGACTTAGTTA-AAGC
Thth  --------AGCAGTGATAAACCTTAAGC-TATAAGTGA-AAACTTGACTTAGTTA-AAGC
Xigl  -----CTCAGCAGTGATAGATATTAAGC-TATAAGTGA-AAACTTGACTTAGTTA-AAGC
Hyja  -----TTCAGCAGTGATAAACCTTAAGC-TATAAGTGA-AAACTTGACTTAGTTA-AAGC
Psan  -------CAGCAGTAATAAACCTTAAGCTA-TGAGTGA-AAACTTGTCAAAGTCA-AAGC
Cupa  --------AGCAGTGATAAACCTTAAGC-TATAAGTGA-AAACTTGACTTAGTTA-AAGC
Mpch  --------AGCAGTGATAAATATTAAGC-CATGAGTGA-AAACTTGACTTAGTTA-AAGT
Char  -----CTCAGCAGTGATAAACATTAAGC-CATAAGTGA-AAGCTTGACTTAGTCA-AGGC
Pser  --------AGCAGTGAGTAACATTAAAC-TATAAGTGC-AAACTTGACTTAGTTA-AAGC
Prol  --------AGCAGTGATAAATATTAAGC-CATAAGTGC-AAACTTGACTTAGTTA-AGGT
Plbi  -----CTCAGCAGTGATAAATATTAAGC-CATAAGTGA-AAACTTGACTTAGTTA-AGGT
Calu  --------AGCAGTGATTAACATTAAGC-CATGAGCGA-GAGCTCTACTCAGTTA-GAGT
Papa  --------TGCAGTGATCAACATTAAGC-AATGAGTGA-AAACTCGACTTAGCTA-AAGC
Sufr  -----CTCAGCAGTGACAAACATTAAGC-CATGAGTGA-AAACTTGACTTAGTTA-GGGT
Stci  -------CAGCAGTGATAAACATTAAGCCA-TAAGTGA-AAGCTTGACTTAGTCA-AGGT
Taru  -----TTCAGCAGTGATAAACATTAAGC-CATAAGTGA-AAACTTGACTTAGTCA-TGAT
Rala  -------CAGCAGTGATAAACATTAAGCCA-TAAGTGA-AAACTTGACTTAGTCA-CAGC
               * * *     *  **       *        *
```

```
      4'¦  HVR    ¦16   A        17       B  A' B'      17'         16'
Scca  CGACA------GAGTTGGTAAA-TCTCGTGCCAGCCACC-GCGGTTATACGAGT----GACTC
Muma  CGACA------GAGTTGGTTAA-TCTCGTGCCAGCCACC-GCGGTTATACGAGT----AACTC
Erca  TAATATA----GAGTCGGTAAA-ACTCGTGCCAGCCACC-GCGGTTATACGAGA----GACTC
Pose  TAAATA-----GAGCCGGTAAA-ACTCGTGCCAGCCACC-GCGGTTATACGAGA----GGCTC
Actr  TAAGA------GGGCCGGTAAA-ACTCGTGCCAGCCACC-GCGGTTATACGAGA----GGCCC
Scal  TAAGA------GGGCCGGTAAA-ACTCGTGCCAGCCACC-GCGGTTATACGAGA----GGCCC
Posp  TAAGA------GGGCCGGTAAA-ACTCGTGCCAGCCACC-GCGGTTATACGAGA----GGCCC
Atsp  TAAGA------GAGTCGGTCAA-ACTCGTGCCAGCCACC-GCGGTTATACGAGA----GACTC
Leoc  TAAGA------GAGCCGGTAAA-ACTCGTGCCAGCCACC-GCGGTTATACGAGA----GGCTC
Amca  TAAGA------GGGCCGGTAAA-ACTCGTGCCAGCCACC-GCGGTTATACGAGA----GGCCC
Osbi  TAAGA------GGGTCGGTTAA-ACTCGTGCCAGCCGCC-GCGGTTATACGAGA----GACCC
Pabu  AAAACA-----GAGCCGGTAAA-ACTCGTGCCAGCCACC-GCGGTTATACGAGA----GGCTC
Hial  TAAGA------GGGCCGGTAAA-ACTCGTGCCAGCCACC-GCGGTTATACGAGA----GGCCC
Elha  CAAGA------GGGCCGGTAAA-TCTCGTGCCAGCCACC-GCGGTTATACGAGA----GGCCC
Mlcy  TACAA------GGACTGGTAAA-TCTCGTGCCAGCCACC-GCGGTTATACGAGA----GGTCC
Algl  CAAGA------GAGCCGGTAAA-ACTCGTGCCAGCCACC-GCGGTTATACGAGA----GGCTC
Ptgi  CAAGA------GAGCCGGTAAA-ACTCGTGCCAGCCACC-GCGGTTATACGAGA----GGCTC
Alaf  CAAGA------GAGCCGGTAAA-ACTCGTGCCAGCCACC-GCGGTTATACGAGA----GGCTC
Nock  CAAGA------GAGCCGGTAAA-ACTCGTGCCAGCCACC-GCGGTTATACGAGA----GGCTC
Anja  CAAAA------GAGCCGGTAAA-ACTCGTGCCAGCCACC-GCGGTTATACGAGG----GGCTC
Gyki  TGACA------GGGCCGGTAAA-ACTCGTGCCAGCAACC-GCGGTTACACGAGA----GGCCC
Syka  CAAATTA----GAGCCGGTAAA-ACTCGTGCCAGCCACC-GCGGTTATACGAGG----GGCTC
Opma  TAATAAA----GAGCCGGTAAA-ACTCGTGCCAGCCACC-GCGGTTATACGAGA----GGCTC
Comy  CAAACA-----GAGCCGGTAAA-ATTCGTGCCAGCCACC-GCGGTTATACGTAT----GACTC
Sasp  TAATTA-----GGGCCGGTAAA-ACTCGTGCCAGCCACC-GCGGTTATACGAGA----GGCTC
Eupe  TAACCT-----AAGCCGGTAAA-ATTCGTGCCAGCCACC-GCGGTTATACGAAA----GGCTT
Enja  TAAGA------GGGCCGGTAAA-ACTCGTGCCAGCCACC-GCGGTTATACGAGA----GACCC
Same  TAAGA------GGGCCGGTAAA-ACTCGTGCCAGCCACC-GCGGTTATACGAGG----GACCC
Chch  TAATA------GAGCCGGTTAA-ACTCGTGCCAGCCACC-GCGGTTATACGAGA----GGCTC
Grgr  TAGTTCCATA-GGGCCGGTAAA-ACTCGTGCCAGCCACC-GCGGTTATACGAGA----GGCTC
Caau  TAAGA------GGGCCGGTAAA-ACTCGTGCCAGCCACC-GCGGTTAGACGAGA----GGCCC
Cyca  TAAGA------GGGCCGGTAAA-ACTCGTGCCAGCCACC-GCGGTTAGACGAGA----GGCCC
Dare  AAAGA------GAGCCGGTAAA-ACTCGTGCCAGCAACC-GCGGTTAAACGAGA----GGCTC
Cost  TAAGA------GGGCCGGTAAA-ACTCGTGCCAGCCACC-GCGGTTATACGAGA----GGCCC
Leec  TAAGA------GGGCCGGTAAA-ACTCGTGCCAGCCACC-GCGGTTATACGAGA----GGCCC
Crla  TAAGA------GGGCCGGTAAA-ACTCGTGCCAGCCACC-GCGGTTATACGAGA----GGCCC
Clmc  TAAGA------GGGTCGGTAAA-ACTCGTGCCAGCCACC-GCGGTTATACGAGA----GACCC
Phin  TATAAA-----GGGTCGGTAAA-ACTCGTGCCAGCCACC-GCGGTTATACGAGA----GACCC
Icpu  TATTA------GGGCCGGTAAA-ATTCGTGCCAGCCACC-GCGGTTATACGAAA----GACCC
Psto  TAAAA------GGGCCGGTAAA-ATTCGTGCCAGCCACC-GCGGTTATACGAAA----GACCC
Cora  TAAAA------GAGTCGGTAAA-ATTCGTGCCAGCCACC-GCGGTTATACGAAA----GACTC
Eisp  TAAGA------GGGCCGGTAAA-ACTCGTGCCAGCCACC-GCGGTTATACGAGA----GGCCC
Apal  TAACA------GGGCCGGTAAA-TCTCGTGCCAGCCACC-GCGGTTATACGAAA----GACCC
Eslu  TAAAA------GGGCCGGTAAA-ACTCGTGCCAGCCACC-GCGGTTATACGAGA----GGCCC
Dape  TTAGA------GGGCCGGTAAA-ACTCGTGCCAGCCACC-GCGGTTATACGAGA----GGCCC
Glse  AAACA------GGGTCGGTAAA-ACTCGTGCCAGCCACC-GCGGTTATACGAGA----GACCC
Naar  AAACA------GGGCCGGTAAA-ACTCGTGCCAGCCACC-GCGGTTATACGAGA----GGCCC
Baoc  AAGCA------GGGCCGGTAAA-ACTCGTGCCAGCCACC-GCGGTTATACGAGA----GGCCC
Opso  AAATA------GAGTCGGTAAA-ACTCGTGCCAGCCACC-GCGGTTATACGAGA----GACTC
Alte  TAAGA------GGGCCGGTAAA-ACTCGTGCCAGCCACC-GCGGTTATACGAGA----GGCCC
Plap  TAAGA------GGGCCGGTAAA-ACTCGTGCCAGCCACC-GCGGTTATACGAGA----GGCCC
```

```
Plal  TTTTA------GGGCCGGTTAA-TCTCGTGCCAGCCACC-GCGGTTATACGAGT----GGCCC
Sami  TTTTA------GGGCCGGTTAA-TCTCGTGCCAGCCACC-GCGGTTATACGAGT----GGCCC
Rere  TTTTA------GGGCCGGTTAA-TCTCGTGCCAGCCACC-GCGGTTATACGAGT----GGCCC
Gama  TAAGA------GGCCCGGTAAA-ACTCGTGCCAGCCACC-GCGGTTATACGAGA----GGGCC
Onmy  TAAGA------GGGCCGGTAAA-ACTCGTGCCAGCCACC-GCGGTTATACGAGA----GACCC
Sasa  TAAGA------GGGCCGGTAAA-ACTCGTGCCAGCCACC-GCGGTTATACGAGA----GGCCC
Cola  TAAGA------GGGCCGGTAAA-ACTCGTGCCAGCCACC-GCGGTTATACGAGA----GGCCC
Dita  TAAACA-----GGGCCGGTAAA-ACTCGTGCCAGCCACC-GCGGTTATACGAGA----GGCCC
Gogr  TCCTAA-----GGGCCGGTTAA-GCTCGTGCCAGCCGCC-GCGGTTATACGAAA----GACCC
Chsl  TTTTA------GGGTCGGTAAA-ACTCGTGCCAGCCACC-GCGGTTATACGAGA----GACTC
Atja  CAACA------GGGCCGGTAAA-ACTCGTGCCAGCCACC-GCGGTTATACGAGA----GGCCC
Iido  CAACA------GGGCCGGTAAA-ACTCGTGCCAGCCACC-GCGGTTATACGAGA----GGCCC
Auja  AAATA------GGGCCGGTAAA-ACTCGTGCCAGCCACC-GCGGTTATACGAGA----GACCC
Chag  TTATACA----GGGCCGGTTAA-TCTCGTGCCAGCCACC-GCGGTTATACGGGCA---GGCCC
Hami  GTTTA------GGGCCGGTTAA-ACTCGTGCCAGCCACC-GCGGTTATACGAGA----GGCCC
Saun  AATAAA-----GGGCCGGTAAA-ACTCGTGCCAGCCACC-GCGGTTATACGAGA----GGCCC
Nema  TATCTTTTTGAGGGCCGGTAAA-ACTCGTGCCAGCCACC-GCGGTTATACGAGA----GGCCC
Disp  TATAAGA----GGGCCGGTAAACACTCGTGCCAGCCACC-GCGGTCAAACGAGGCAG-CGCCC
Myaf  TAAAA------GGGCCGGTAAA-ACTCGTGCCAGCCACC-GCGGTCATACGAGTGAT-AGCCC
Lagu  CCCCA------GGGCCGGTAAA-ACTCGTGCCAGCCACC-GCGGTTATACGAGA----GGCCC
Trtr  CAATA------GGGCCGGTAAA-ACTCGTGCCAGCCACC-GCGGTTATACGAGA----GGCCC
Zucr  CACTA------GGACCGGTAAA-ACTCGTGCCAGCCACC-GCGGTTATACGAGA----GGTCC
Pxja  TTAGA------GGGCCGGTAAA-ACTCGTGCCAGCCACC-GCGGTTATACGAGA----GGCCC
Pxlo  TTAGA------GGGCCGGTAAA-ACTCGTGCCAGCCACC-GCGGTTATACGAGA----GGCCC
Pctr  TAACA------GGGCCGGTAAA-ACTCGTGCCAGCCACC-GCGGTTATACGAGC----GGCTC
Apsa  AACCA------GAGCCGGTAAA-ACTCGTGCCAGCCACC-GCGGTTATACGAGC----GACTC
Cabe  TAAGA------GGGCCGGTAAA-TCTCGTGCCAGCCACC-GCGGTTATACGGGAA---GGCCC
Bzze  TAACA------GGGCCGGTAAA-ACTCGTGCCAGCCACC-GCGGTTATACGAGTA---GGCCC
Siim  TATTA------GGGCCGGTAAA-TCTCGTGCCAGCTACC-GCGGTTACACGGGGG---AGCCC
Ctru  TAACA------GGGCCGGTAAA-ACTCGTGCCAGCCACC-GCGGTTATACGAGA----GACCC
Dpbr  TAACA------GGGCCGGTAAA-ACTCGTGCCAGCCACC-GCGGTTATACGAGA----GACCC
Caki  TAAAA------GGGTCGGTAAA-CCTCGTGCCAGCCACC-GCGGTTAAACGGGA----GACCC
Phja  TAAGA------GAGCCGGTAAATACTCGTGCCAGCCACC-GCGGTTAAACGAGAA---GGCTC
Brsp  AAACA------GAACCGGTTAA-ACTCGTGCCAGCCACC-GCGGTTATACGAGG----GGTTC
Gamo  AAAGA------GGGCCGGTAAA-ACTCGTGCCAGCCACC-GCGGTTATACGAGA----GGCCC
Lolo  TAAGA------GGGCCGGTAAA-ACTCGTGCCAGCCACC-GCGGTTATACGAGA----GGCCC
Batr  AAAAAA-----TGTGGGCCAAT-GCTCGTGCCAGCCACC-GCGGTTATACGGG-----CCACC
Prmy  AATTTA-----TAGTTGGTCAA-ATTCGTGCCAGCCACC-GCGGTTACACGAAT----AACTA
Loli  CCAAGA-----GGGCCGGTAAA-CCTCGTGCCAGCCACC-GCGGTTATACGAGG----GGCCC
Loam  TAAGA------GGGCCGGTAAA-ACTCGTGCCAGCCACC-GCGGTTATACGAGA----GGCCC
Chab  TAATTGA----GGGCCGGTAAA-CCTCGTGCCAGCCACC-GCGGTTATACGAAG----GGCCC
Chto  TAATTAA----GGGCCGGTAAA-CCTCGTGCCAGCCACC-GCGGTTATACGAAG----GGCCC
Majo  TAAGA------GGGCCGGTAAA-ACTCGTGCCAGCCACC-GCGGTTATACGAGA----GGCCC
Hlst  TAAGA------GGGTCGGTAAA-ACTCGTGCCAGCTACC-GCGGTTATACGAGA----GACCC
Clpe  TCAGA------GGGCAGGTAAAAACTTGTGCCAGCCACC-GCGGTTACACTTGT----TGCCC
Mlmr  TAAGA------GGGCCGGTAAA-ACTCGTGCCAGCCACC-GCGGTTATACGGGAT---GGCCC
Crcr  TAATA------GGGCCGGTAAA-TCTCGTGCCAGCCACC-GCGGTTATACGAAA----GACCC
Muce  TAATA------GGGCCGGTAAA-TCTCGTGCCAGCCACC-GCGGTTATACGAAA----GACCC
Bege  TTAAA------GGGCCGGTAAA-ACTCGTGCCAGCCACC-GCGGTTATACGAGA----GGCCC
Mela  TAAGA------GAGCCGGTAAA-ACTCGTGCCAGCAACC-GCGGTTATACGAGA----GGCCC
Hats  TTAGA------GGGCCGGTAAA-ACTCGTGCCAGCCACC-GCGGTTATACGAGA----GGCCC
Orla  TAAGA------GAACCGGTTAA-ACTCGTGCCAGCCGCC-GCGGTTATACGAGG----GGTTC
```

```
Cosa    TAAGCAA----AGGCCGGTAAA-ACTCGTGCCAGCCACC-GCGGTTAGACGAGA----GGCCT
Exsp    TAAGA------AGGCCGGTAAA-ACTCGTGCCAGCCACC-GCGGTTATACGAGA----GGCCT
Depa    TAAAT-A----AAGCCGGTAAA-ACTCGTGCCAGCCACC-GCGGTTAGACGAGA----GGCTT
Rima    CAAATA-----GAGCCGGTTAA-TTTCGTGCCAGCCACC-GCGGTCATACGAAT----GGCTC
Fuol    TAACA------GAGCCGGTTAAAACTCGTGCCAGCCACC-GCGGTTAAACGAGA----GGCTC
Gmaf    TAATAA-----GGGCCGGTTAA-ACTCGTGCCAGCCACC-GCGGTTATACGAG-A---GGCCC
Xeei    TAAAA------GAGCCGGTTAA-ACTCGTGCCAGCCACC-GCGGTTATACGAGA----GACTC
Pros    TATAGA-----GGGCCGGTAAA-ACTCGTGCCAGCCACC-GCGGTTATACGAGC----GGCCC
Scmi    TAATA------GGGCTGGTAAA-ACTCGTGCCAGCCACC-GCGGTTATACGAGC----AGCCC
Rolo    AAATTA-----GGGCCGGTAAA-ACTCGTGCCAGCCACC-GCGGTTATACGAGA----GGCCC
Cere    AAAC--AA---GGGCCGGTAAA-ACTCGTGCCAGCTACC-GCGGTTATACGAG-A---GGCCC
Daga    AACA--A----GAGCCGGTAAA-ACTCGTGCCAGCTACC-GCGGTTATACGA-GA---GGCTC
Anco    AAAT--A----GGGCCGGTAAA-ACTCGTGCCAGCCACC-GCGGTTATACGAG-A---GACCC
Dmve    AATTCTA----GGGCCGGTAAA-ACTCGTGCCAGCCACC-GCGGTTAGACGAGA----GGCTC
Dmar    AACTCTA----GGGCCGGTAAA-ACTCGTGCCAGCCACC-GCGGTTAGACGAG-A---GGCTC
Anka    AAAC--AA---GGGCCGGTAAA-ACTCGTGCCAGCCACC-GCGGTTATACGAGA----GACCC
Moja    AAAC-AA----GGGCCGGTAAA-ACTCGTGCCAGCCACC-GCGGTTATACGAG-A---GACCC
Hoja    AAAC--AA---GGGCCGGTAAA-ACTCGTGCCAGCCACC-GCGGTTATACGAG-A---GACCC
Bede    TAA--G-A---GGGCCGGTAAA-ACTCGTGCCAGCCACC-GCGGTTATACGAGC----GACCC
Besp    TAA--G-A---GGGCCGGTAAA-ACTCGTGCCAGCCACC-GCGGTTATACGAGC----GACCC
Mysp    AAA---GA---GGACCGGTAAA-ACTCGTGCCAGCCACC-GCGGTTATACGAGA----GGTCC
Osja    AAA--GA----GGACCGGTAAA-ACTCGTGCCAGCCACC-GCGGTTATACGAGA----GGTCC
Sgro    AAAG-A-----GGACCGGTAAA-ACTCGTGCCAGCCACC-GCGGTTATACGAGA----GGTCC
Pzpa    TTAG-A-----GGGCCGGTAAA-ACTCGTGCCAGCCACC-GCGGTTATACGAGA----GGCCC
Zeja    TAGTAA-----GGGCCGGTAAA-ACTCGTGCCAGCCACC-GCGGTTATACGAGC----GACCC
Znne    TTTT-A-----GGGCCGGTAAA-ACTCGTGCCAGCCACC-GCGGTTATACGAG-A---GACCC
Zefa    TTA--AA----GGGCCGGTAAA-ACTCGTGCCAGCCACC-GCGGTTATACGAGA----GGCCC
Acni    TTAAA------GGGCCGGTAAA-ACTCGTGCCAGCCACC-GCGGTTATACGAGA----GGCCC
Ncrh    TTAAA------GGGCCGGTAAA-ACTCGTGCCAGCCACC-GCGGTTATACGAGA----GGCCC
Agca    TAA-GA-----GGGCCGGTAAA-ACTCGTGCCAGCCACC-GCGGTTATACGAGA----GGCCC
Hydy    TAA--GA----GGGCCGGTAAA-ACTCGTGCCAGCCACC-GCGGTTATACGAGA----GGCCC
Gsac    TAAG--A----GGGCCGGTAAA-ACTCGTGCCAGCCACC-GCGGTTATACGAGA----GGCCC
Pevo    CAAGA------GGGTCGGTAAA-ACTCGTGCCAGCAACC-GCGGTTATACGAGA----GACCC
Hiku    TTTTA------GGGCCGGTAAA-ACTCGTGCCAGCCACC-GCGGTTATACGAG-A---GGCTC
Inpa    TATTC------GGGCCGGTAAA-ACTCGTGCCAGCCACC-GCGGTTATACGAGA----GACCC
Auch    CAACA------GAGCCGGTAAA-CCTCGTGCCAGCCACC-GCGGTGATACGGAG----GGCTC
Fico    CCTA-GA----GGGCCGGTAAA-ACTCGTGCCAGCCACC-GCGGTTATACGAGA----GGCCC
Macs    TAAG--A----GGGCCGGTAAA-ACTCGTGCCAGCCACC-GCGGTTATACGAGA----GGCCC
Moal    TA-A-AA----GGGTCGGTCAA-ACTCGTGCCAGCCACC-GCGGTTATACAAGA----GACTC
Syma    TAAA--A----GAGTCGGTAAA-TCTCGTGCCAGCCACC-GCGGTTATACAAGA----GACTC
Mafr    TAA--TA----GAGCCGGTAAA-ACTCGTGCCAGCCACC-GCGGTTATACGAGA----GGCTC
Dcpe    CAAGA------GAGCCGGTAAA-TTTCGTGCCAGCCACC-GCGGTTATACGGAA----GGCTC
Dcti    CA-AGA-----GAGCCGGTTAA-TTTCGTGCCAGCCACC-GCGGTTATACGGAA----GGCTC
Hehi    TAAG-A-----GGGCCGGTAAA-ACTCGTGCCAGCCACC-GCGGCTATACGAG-A---GGCCC
Stam    TAGCA------GGGCCGGTAAA-ACTCGTGCCAGCCACC-GCGGTTATACGAGA----GGCCC
Hogi    TTAG--A----GGGCCGGTAAA-ACTCGTGCCAGCAACC-GCGGTTATACGAGA----GGCCC
Erzo    TAAGA------GGGCCGGTAAA-ACTCGTGCCAGCCACC-GCGGTTATACGAG-A---GGCCC
Hxot    TAAGA------GGGCCGGTAAA-ACTCGTGCCAGCCACC-GCGGTTATACGAG-A---GGCCC
Core    TAAG-A-----GGGCCGGTAAA-ACTCGTGCCAGCCACC-GCGGTTATACGAGA----GGCCC
Apve    TAAA-A-----GGGCCGGTAAA-ACTCGTGCCAGCCACC-GCGGTTATACGAGA----GGCCC
Latj    TAAGAA-----GGGCCGGTTAA-ACTCGTGCCAGCCACC-GCGGTTATACGAGA----GGCCC
Laja    TA-AGA-----GGGCCGGTAAA-ACTCGTGCCAGCCACC-GCGGTTATACGAGA----GGCCC
```

```
Syja    TAGACT-----GGGCCGGTAAA-TCTCGTGCCAGCCACC-GCGGTTATACGAGA----GACCC
Epme    TAA---AA---GGGCCGGTAAA-ACTCGTGCCAGCTACC-GCGGTTATACGAGA----GGCCC
Grse    TAA--GA----GGGCCGGTAAA-ACTCGTGCCAGCTACC-GCGGTTATACGAGA----GGCTC
Clja    CTAA--GA---GGGCCGGTAAA-TCTCGTGCCAGCCACC-GCGGTTATACGAGA----GGCCC
Ogcy    TAATTGTTA--GGGCCGGTAAA-ACTCGTGCCAGCCACC-GCGGTTATACGAGTA---GACCC
Plna    TTAA-A-----GGGCCGGTAAA-AGTCGTGCCAGCCACC-GCGGTTATACGACA----GGCCC
Lema    TAAAA------GAGCCGGTAAA-ACTCGTGCCAGCCACC-GCGGTTATACGAGA----GGCTC
Etzo    CAA---GA---GGGCCGGTAAA-ACTCGTGCCAGCCACC-GCGGTTATACGAG-A---GGCCC
Apse    TTAG-A-----GGGCCGGTAAA-ACTCGTGCCAGCCACC-GCGGTTATACGAGA----GGCCC
Epde    TA-A-GA----GGGCCGGTAAA-ACTCGTGCCAGCCACC-GCGGTTATACGAGA----GACCC
Slja    TAA--GA----GGGCCGGTAAA-ACTCGTGCCAGCCACC-GCGGTTATACGAGTT---GGCTC
Bsja    CAAG--A----GGGTCGGTAAA-ACTCGTGCCAGCCACC-GCGGTTATACGAG-A---GACCC
Ecna    TAAG-A-----GGGCCGGTAAA-ACTCGTGCCAGCCACC-GCGGTTATACGAGA----GGCCC
Cohi    TAATT-A----GGGTTGGTAAA-TTTCGTGCCAGCCACC-GCGGTTAGACGAAT----GACCC
Caar    TAAGA------GAGCCGGTAAA-ACTCGTGCCAGCCACC-GCGGTTATACGAGA----GGCTC
Came    TAA---GA---GAGCCGGTAAA-ACTCGTGCCAGCCACC-GCGGTTATACGAGA----GGCTC
Mema    TAAG--A----GGGCCGGTAAA-ACTCGTGCCAGCCACC-GCGGTTATACGAGA----GGCCC
Lenu    TATA-AA----GGGCCGGTAAA-ACTCGTGCCAGCCACC-GCGGTTATACGAGA----GACCC
Plma    TATTT-AA---GGGCCGGTAAA-ACTCGTGCCAGCCACC-GCGGTTATACGAGA----GGCCC
Emst    TAAG-A-----GGGTCGGTAAA-ACTCGTGCCAGCCACC-GCGGTTATACGAGA----GACCC
Ptti    TAAG-A-----GAGTCGGTAAA-ACTCGTGCCAGCCACC-GCGGTTATACGAGA----GACCC
Losu    TAAG---A---GGGCCGGTAAA-ATTCGTGCCAGCCACC-GCGGTTATACGAATA---GGCCC
Geoy    TTA---GA---GGGCCGGTAAA-ATTCGTGCCAGCCACC-GCGGTTATACGAAA----GGCCC
Dipi    TA---AGA---GGGCCGGTAAA-ACTCGTGCCAGCCACC-GCGGTTATACGAGA----GGCCC
Pama    TAA---GA---GGGCCGGTAAA-ACTCGTGCCAGCCACC-GCGGTTATACGGGA----GGCCC
Leob    TAA---GA---GGGCCGGTAAA-ACTCGTGCCAGCCACC-GCGGTTATACGAG-A---GGCCC
Neba    TAAG---A---GAGTCGGTCAA-CCTCGTGCCAGCCACC-GCGGTTATACGAGG----GACTC
Pdpl    TAAT-AA----GGGCCGGTAAA-ACTCGTGCCAGCCACC-GCGGTTATACGAGG----GGCCC
Nimi    TAA--GA----GGGCCGGTCAA-ACTCGTGCCAGCCACC-GCGGTTATACGAGA----GGCCC
Uptr    TAAGA------GGGCCGGTAAA-ACTCGTGCCAGCCACC-GCGGTTATACGAGA----GGCCC
Pesc    TAAA-GA----GGGCCGGTAAA-ACTCGTGCCAGCCACC-GCGGTTATACGAG-G---GGCCC
Baar    CTAATAA----GGGCCGGTAAA-ACTCGTGCCAGCCACC-GCGGTTATACGAGTCA--GGCCC
Moar    TAAG-A-----GGGTCGGTAAA-ACTCGTGCCAGCCACC-GCGGTTATACGAGA----GACCC
Toja    TAAC-A-----GGGCCGGTAAA-ACTCGTGCCAGCCACC-GCGGTTATACGAG-G---GACCC
Chau    TAGT-GA----GAGTCGGTAAA-ACTCGTGCCAGCCACC-GCGGTTATACGAGA----GACTC
Chse    TAAGA------GGGTCGGTAAA-ACTCGTGCCAGCCACC-GCGGTTAGACGAGG----GACTC
Enar    TAAG--A----GGGCCGGTAAA-ACTCGTGCCAGCCACC-GCGGTTATACGAGA----GGCCC
Hpty    TAA--GA----GGGTCGGTAAA-ACTCGTGCCAGCCACC-GCGGTTATACGAGA----GACCC
Nana    TAA--GA----GGGCCGGTAAA-ACTCGTGCCAGCCACC-GCGGTTATACGAGA----GGCCC
Mcst    TAA--GA----GGGCCGGTAAA-ACTCGTGCCAGCCACC-GCGGTTATACGAGA----GACCC
Rhox    TAA--GA----GGGCCGGTAAA-ACTCGTGCCAGCCACC-GCGGTTATACGAGA----GGCTC
Opfa    AA---AGA---GGGCCGGTAAA-ACTCGTGCCAGCCACC-GCGGTTATACGAGA----GGCCC
Paar    TA-ATCCT---GGGCCGGTCAAAACTCGTGCCAGCCACC-GCGGTTAAACGTGATTATGGCCC
Gozo    TAAG---A---GGGCCGGTTAA-ACTCGTGCCAGCCACC-GCGGTTATACGAGA----GGCCC
Ackr    TATT--AA---GGGCCGGTAAA-ACTCGTGCCAGCCACC-GCGGTTATACGAGA----GGCCC
Elev    TAA---TA---GGGCCGGTTAA-ACTCGTGCCAGCTACC-GCGGTTATACGAGG----GGCCC
Trdu    CAA---GA---GGGCCGGTAAA-ACTCGTGCCAGCCACC-GCGGTTATACGAG-A---GGCTC
Amoc    CTAG--A----GGGCCGGTAAA-ACTCGTGCCAGCCACC-GCGGTTATACGAGA----GGCTC
Hame    TAACA------GGGCCGGTAAA-TCTCGTGCCAGCCACC-GCGGTTATACGAGA----GACCC
Chso    TAAG--A----GGGCTGGTAAA-TCTCGTGCCAGCCACC-GCGGTTATACGAAA----GGCCC
Lyto    TAAG--A----GGGCCGGTAAA-ACTCGTGCCAGCCACC-GCGGTTATACGAGA----GGCCC
Encr    TAAG-A-----GGGCCGGTAAA-ACTCGTGCCAGCCACC-GCGGTTATACGAGA----GGCCC
```

```
Bvar    CTAC-CA----GGGCCGGTAAA-CCTCGTGCCAGCCACC-GCGGTTATACGTGG----GACCC
Noco    TAAG--A----GGGCCGGTAAA-ACTCGTGCCAGCCACC-GCGGTTATACGAGA----GGCCC
Chsp    CAAA--AA---GAGCCGGTAAA-ACTCGTGCCAGCCACC-GCGGTTAGACGTAGA---GGCTC
Arja    TAAG--A----GGGCCGGTAAA-ACTCGTGCCAGCCACC-GCGGTTATACGAGA----GGCCC
Pase    TATACATA---GGGCTGGTCAAGACCCGTGCCAGCCACC-GCGGTTAGACGGG-A---AGCCC
Trel    CAA--G-A---GGGCCGGCAAA-ACTCGTGCCAGCAGCC-GCGGTTATACGAGA----GGCTC
Lifa    TAACA------GAGCCGGTAAA-CATTCGTGCCAGCAGC-CGCGGTTACACGGTT---GGCTC
Acur    CAACA------AGGCCGGTCAA-TCTCGTGCCAGCCACC-GCGGTTACACGGAA----GGCCT
Ampe    TAA--GA----GGGCCGGTAAA-ACTCGTGCCAGCCACC-GCGGTTATACGAGA----GGCCC
Urja    TAAA--TA---GAGCCGGTAAA-ACTCGTGCCAGCCACC-GCGGTTATACGAGA----GGCTC
Enet    TAAC-A-----GGGCCGGTAAA-ACTCGTGCCAGCCACC-GCGGTTATACGAGA----GGCCC
Ptbr    TT-A-AA----GGGCCGGTAAA-ACTCGTGCCAGCCACC-GCGGTTATACGAGA----GGCCC
Safa    CTACA------GGGCCGGTTAA-ACTCGTGCCAGCCACC-GCGGTTATACGAGA----GGCCC
Icae    TAA--GA----GGGCCGGTAAA-ACTCGTGCCAGCCACC-GCGGTTATACGAGA----GGCCC
Asmi    TATT-AA----GGGTCGGTAAA-ATTCGTGCCAGCAACC-GCGGTTATACGAAA----GACCC
Foal    TTAC--A----GGGCCGGTAAA-CCTCGTGCCAGCTACC-GCGGTGATACGGAG----GGCCC
Drze    TAATT-A-A--GGGCCGGTAAA-ACTCGTGCCAGCCACC-GCGGTTATACGGGA----GACCC
Rhas    CAAG-A-----GAGCCGGTAAA-ACTCGTGCCAGCCACC-GCGGTTATACGAGA----GGCTC
Elac    CAAC--A----GAGCCAGCAAA-ACTCGTGCCAGCCGCC-GCGGTTATACGAGG----GGCTC
Kugu    TAAA-CTA---GGGCCGGTAAATACACGTGCCAGCCACC-GCGGTTACACGTCCGAA-GGCCC
Plor    TAA--GA----GGGTCGGTAAA-ACTCGTGCCAGCCACC-GCGGTTATACGAGA----GACCC
Sgun    TAA---G-A--GGGTCGGTAAA-ACTCGTGCCAGCCACC-GCGGTTATACGAGA----GACCC
Zaco    TAA---G-A--GGGTCGGTAAA-ACTCGTGCCAGCCACC-GCGGTTATACGAGA----GACCC
Zbfl    TAAG--A----GGGTCGGTAAA-ACTCGTGCCAGCCACC-GCGGTTATACGAGA----GACCC
Spba    CATTCA-----GGGCCGGTAAA-ACTCGTGCCAGCCACC-GCGGTTATACGAGA----GGCCC
Game    TAA---GA---GGGCCGGTAAA-ACTCGTGCCAGCCACC-GCGGTTATACGAGA----GGCCC
Thth    TAAG-A-----GGGCCGGTAAA-ACTCGTGCCAGCCACC-GCGGTTATACGAGA----GGCCC
Xigl    TAA--GA----GGGCCGGTAAA-ACTCGTGCCAGCCACC-GCGGTTATACGAGA----GGCCC
Hyja    TAAG--A----GGGCCGGTAAA-ACTCGTGCCAGCCACC-GCGGTTATACGAGA----GGCCC
Psan    TAAG-A-----GAGCCGGTAAA-ACTCGTGCCAGCTACC-GCGGTTACACGAGA----GGCTC
Cupa    TAAG-A-----GGGCCGGTAAA-ACTCGTGCCAGCCACC-GCGGTTATACGAGA----GGCCC
Mpch    AAAA-A-----GAGCCGGTAAA-ACTCGTGCCAGCCACC-GCGGTTATACGAGA----GGCTC
Char    TAAG--A----GAGTCGGTAAA-ACTCGTGCCAGCCACC-GCGGTTATACGAGG----GACCC
Pser    TAAGA------GGGCCGGTAAA-ACTCGTGCCAGCCACC-GCGGCTATACGATGA---GGCTC
Prol    TAA---GA---GGGCCGGTAAA-ACTCGTGCCAGCCACC-GCGGTTATACGAGA----GGCCC
Plbi    TAA--GA----GGGCCGGTAAA-ACTCGTGCCAGCCACC-GCGGTTATACGAGA----GGCCC
Calu    TAACCA-----GAGCCGGTAAA-ACCCGTGCCAGCCACC-GCGGTTATACGGGA----GGCTC
Papa    TATT--T-A--GAGCCGGTAAAACCTTGTGCCAGCTACC-GCGGTTACACGAG-A---GGCTC
Sufr    TTCA-GA----GGGCCGGTAAA-ACTCGTGCCAGCCACC-GCGGTTATACGAGG----GGCCC
Stci    TAAGA------GGGCCGGTAAA-ACCCGTGCCAGCCACC-GCGGTTATACGGGG----GGCCC
Taru    CTA--AA----GAGTCGGTAAA-ACTCGTGCCAGCCACC-GCGGTTATACGAGA----GACCC
Rala    TAAG-A-----GGGCCGGTAAA-ACTCGTGCCAGCCACC-GCGGTTATACGAGA----GGCCC
                            *          *      *   **
```

```
                    3'     ¦  HVR  ¦18  C      19       20¦       HVR        ¦
Scca      ACATTAACACT--T-CCCGGCGTAAAGTGTGATTTAAGGATGACCTCCAAATAAC---
Muma      ATATTAACACA--C-CCCGGCGTAAAGAGTGGTTTA-AGAATGACCTTTTAATAAC--
Erca      AAAATGATAGT--CCTTCGGCGTAGAGTGTGGTTAA-CGTAAATTAAT--AA--AC--
Pose      AAAATGATAGT--CCTTCGGCGTATGGTGTGGTTA--GCAATTAAATTATAAACT---
Actr      CAACTGATAG---TCCACGGCGTAAAGCGTGATTAAAGGATGCCTACTACAC------
Scal      CAACTGATAG---TTCACGGCGTAAAGCGTGATTAAAGGATACCCACTGCAC------
Posp      CAACTGATAGT--CCAC-GGCGTAAAGCGTGATTAAAGGACGCCCACTACAC------
Atsp      TAACTGACAGC--CCA-CGGCGTAAAGCGTGATTATAGGATGCTACCCTA----AC--
Leoc      CAACTGATAGC--CCAC-GGCGTAAAGCGTGATTATAGGATGCTGCCCCAAC------
Amca      CAATTGATAGA--CATC-GGCGTAAAGCGTGATTACAGGCTGCTGCTATAAAT-----
Osbi      CAGTTGACAC---TAT-CGGCGTAAAGTGTGACTAC-AGAAAAATAT-----AAAAC-
Pabu      AAGCTGATAAT--T-ATCGGCGTAAAGAGTGATA---ATCCTAAT--------ATAC-
Hial      TAGTTGACAG---CTATCGGCGTAAAGCGTGATTAT-AGGACGCTAAAC--AAC----
Elha      GAGTTGATGG---TCACCGGCGTAAAGAGTGATTAT-AGCATGTTAAAATAAC-----
Mlcy      AAATTGACAGC--CA-TCGGCGTAAAGAGTGGTTAT-AGACCCCTACA--CA--AC--
Algl      AAGTTGATGT---CCGCCGGCGTAAAGAGTGATTACAGGAGTGTGTATA--AC-----
Ptgi      AAATTGATGC---TCGTCGGCGTAAAGAGTGATTACAGGAATATAAACAAC-------
Alaf      AAATTGACACT--CCAC-GGCGTAAAGAGTGATTAAAGGAGTACCCC---AA-C----
Nock      AAATTGATGAT--C-AGCGGCGTAAAGAGTGATTAAAGGAGTAAA-A--T--AAC---
Anja      AAATTGATATT--ACAC-GGCGTAAAGCGTGATTAA-AAAACAA----ACAA---AC-
Gyki      GAATTGACACA--TCAC-GGCGTAAAGTGTGATTAG-AGATAAACC---AGAC-----
Syka      AAGTTGACACT--TTTTCGGCGTAAAGCGTGATTAA-GGACTATAAT-----A-AAC-
Opma      AAATTGATATT--C-CACGGCGTAAAGCGTGATTAA-AGAAAAT----AAATAC----
Comy      AAACTGATATC--TCTC-GGCGTAAAGCGTGATTAG-AGAAAAAAGACAAC-------
Sasp      AAGCTGACACC--TC-ACGGCGTAAAGCGTGATTA--GAAATATA-A--TTA-AC---
Eupe      AAACTGATGCA--TCA-CGGCGTAAAGCGTGATTA--GAATATTTAAC-----A-CAA
Enja      TAGTTGATTGA----AGCGGCGTAAAGAGTGGTTAT-GGAATTTTCTAC-CC------
Same      TAGTTGATTT---AATC-GGCGTAAAGAGTGGTTAT-GGAGAATAAGAAAC-------
Chch      TAGTTGACGAA--C-TACGGCGTAAAGCGTGGTCAC-GGAGAGCAAT----CAATAC-
Grgr      TAGTTGACAGT--CGC--GGCGTAAAGAGTGATTAG-GGCGCACCCC-----CGAAC-
Caau      TAGTTGATATT--ACAACGGCGTAAAGGGTGGTTAA-GGATAA---ACAAAAA-----
Cyca      TAGTTGATATT--ATAACGGCGTAAAGGGTGGTTAA-GGATA-AAC-AAAAA------
Dare      TAGTTGATATA--CTA-CGGCGTAAAGGGTGGTTAA-GGAACAATG-----TAAAA--
Cost      TAGTTGATGGG--CA-C-GGCGTAAAGGGTGGTTAA-GGTTTAATT-TAAA-------
Leec      TAGTTGTTAAA--T-ACCGGCGTAAAGGGTGGTTAA-GGAGA-GCAAAGA--------
Crla      TAGTTGATAGG--T-G-CGGCGTAAAGGGTGGTTAA-GGAGAG-C--AAGAAT-----
Clmc      TAGTTGATAGA--TAC--GGCGTAAAGAGTGGTCTA-GGACCCACAAC---AAAA---
Phin      AAGTTGACAAA--C-AC-GGCGTAAAAAGTGGTTAT-GTGTTATACTAAA--------
Icpu      TAGTTGCTAG---CCA-CGGCGTAAAGGGTGGTTAA-GGACAACAATG-------AA-
Psto      TAGTTGATAG---ACA-CGGCGTAAAGGGTGGTTAA-GGAAGACAAATA-----A---
Cora      TAGTTGATAGA--CA--CGGCGTAAAGGGTGGTTA--GGATAACAAC-----AAAA--
Eisp      TAGTTGACAG---C-CACGGCGCAAAGAGTGGTTAA-GGAGTCCTACCA-AA------
Apal      AAGTTGATAG---TCA-CGGCGTAAAGAGTGGTTAAGGGAACTA-ATA----AA----
Eslu      TAGTTGATAA---TTGTCGGCGTAAAGAGTGGTTT--AGAAAATAA-TTT----AA--
Dape      TAGTTGATAAA--C-ATCGGCGTAAAGAGTGGTTA--AGACATAAAA----TAA----
Glse      AAGTTGATAGT--T-TTCGGCGTAAAGAGTGGTTAG-GGAGTC-TTTTAAC-------
Naar      AAGTTGATGGT--TACC-GGCGTAAAGAGTGGTTAC-GGAACTTTT--AAAC------
Baoc      AAGTTGATGAC--CAC-CGGCGTAAAGAGTGGTTAC-GGAAGCACTTCAC--------
Opso      AAGTTGATA-T--TTACCGGCGTAAAGAGTGGTTAC-GGAAC------TTAATAAAC-
Alte      CAGTTGATAG---GTG-CGGCGTAAAGAGTGGTTAT-GGAGTATACCCA-----GC--
Plap      CAGTTGATAGA--T-A-CGGCGTAAAGAGTGGTTAA-GGAATACACCGAAC-------
```

Plal	AAGTTGAAAGT--TA-CCGGCGTAAAGAGTGGTTAG-GGAAACA--AAAAAC------
Sami	AAGTTGAAAGT--CAC-CGGCGTAAAGAGTGGTTAG-GGGGACAA-------GAAAC-
Rere	AAGTTGAAAG---TCGCCGGCGTAAAGAGTGGTTA--GGAAAAGAT-CAAA----C--
Gama	AAGTAGATAGA--CAT-CGGCGTAAAGTGTGGTTAG-GGATTTGT-----CAGC----
Onmy	TAGTTGATAAC--TACC-GGCGTAAAGAGTGGTTAT-GGAAAAT-AT---TTAA----
Sasa	TAGTTGATAAC--TACC-GGCGTAAAGAGTGGTTAC-GGAAAAATATT---TA-A---
Cola	TAGTTGATAAT--CA-CCGGCGTAAAGAGTGGTTA--GGAATTAT-A--TTTAA----
Dita	GAGTTGACAGA--C-GCCGGCGTAAAGAGTGGTTAGAGGAAAT------AGGAAC---
Gogr	AAGTTGATAAT--C-ACCGGCGCAAAGCATGGTTAA-GGACATAATAA-AC-------
Chsl	AAGTTGACACC--C--GCGGCGTAAAGAGTGGTTAG-GGGCAAGACAAC---------
Atja	AAGTTGATAGG--AAT-CGGCACAAAGGGTGGTTA--AGAAAACTAAGCA----G---
Iido	GAGTTGATAG---GAATCGGCACAAAGGGTGGTTA--AGAAAACTAAAT--GG-----
Auja	AAGTTGACT-T--ACACCGGCGTAAAGTGTGGTTAG-GGCCCCA----ACCTC-TAA-
Chag	AAGTTGACAG---AATTCGGCGTAAAGAGTGGTTAC-GGATACAATTTAC--------
Hami	GAGTTGATAAA--CATC-GGCGTAAAGTGTGGTTA--GGACTTTTCCC-ACC------
Saun	GAGTTGATAA---ACATCGGCGTAAAGTGTGGTTA--GGATTTTT-CCCA----C---
Nema	AAGCTGATAGG--A-AACGGCGTAAAGAGTGGTTAG-GGAAATTTTA-----AT-AC-
Disp	AAGCGGATGAC--CAA-CGGCGTAAAGAGTGGTTAG-GGAGACCCCCAA-----C---
Myaf	AAGTGGACAGT--CAAC-GGCGTAAAGCGTGGTTAG-AGAATTACCC---CCAC----
Lagu	AAGTCGACAGC--C-TGCGGCGTAAAGAGTGGTTA--AGCGCCCCC-CCCCCA-AC--
Trtr	AAGTCAATAGC--C-CCCGGCGTAAAGAGTGGTTA--GGATTACCCTA-----A--AC
Zucr	AAGTCAATAG---TACCCGGCGTAAAGAGTGGTTA--GGAGAACTA-AAA----AC--
Pxja	AAGTTGATAGG--T-ACCGGCGTAAAGCGTGGTTAA-GGTTAA-C--ACAAAC-----
Pxlo	AAGTTGATAGG--T-ACCGGCGTAAAGCGTGGTTAA-GGTTAACG-TAAA----C---
Pctr	AAGTTGATTAT--A-CCCGGCGTAAAAGGTGGCTAG-GGTAAC------TAAACA---
Apsa	AAGTTGACAAA--CAC-CGGCGTAAAGCGTGATTAA-GGTTTACATT-----A-AC--
Cabe	AAGTTGACGAA--GCC-CGGCGTAAAGCGTGGTTA--AGAGTACATC------AAAC-
Bzze	AAGTTGATAAA--TGC-CGGCGTAAAGCGTGGTTAA-GGAAAGACTC-----A-AAC-
Siim	AAGTTGATAGC--CTAC-GGCGTAAAGGGTGGTTA--AGAGAACAACAT---AAAC--
Ctru	AAGTTGATAAC--TC-CCGGCGTAAAGTGTGGTTAA-GGAAAACC-T-A--GA--AC-
Dpbr	AAGTTGATGA---CCCCCGGCGTAAAGCGTGGTTAA-GGAATC-----CTT-AAAAC-
Caki	AAGTTGATAGA--CAAC-GGCGTAAAGCGTGGTTAA-AGTATTAATT---AAA-----
Phja	AAGTTGATAGG--AAT-CGGCATAAAGTGTGGTTTA-GGAATAAA-----AATAA---
Brsp	AAGTTGATAGT--GCGC-GGCGTAAAGTGTGGTCT--ATTAATCATATT---AA----
Gamo	AAATTGATGAA--AAAC-GGCGTAAAGCGTGGTTA--AGAAAAAAGAG---AAAA---
Lolo	AAGTTGATGAA--A-AACGGCGTAAAGCGTGGTTA--AGAAAACAAG------AAAAA
Batr	AAGAAAATA-A--ACTC-GGCACAAAGGGTGGTTAA-AGACTGA----CTATC-AAA-
Prmy	AAACTGATAAA----ACCGGCACAAAGGGTGGTTA--ATATAA-C--TCAGAA-----
Loli	GAGCTGACAG---TCCCCGGCGTAAAGCGTGGTTAA-GGTACACCCCTAATACAAAT-
Loam	AAGTTGATAAC--AGT-CGGCGTAAAGCGTGGTTA--GGCCATCAACC-----CCCAC
Chab	AAATTGATAG---CCATCGGCGTAAAGGGTGGTTA--AGATTAT-----AT--TCAAA
Chto	AAATTGATAG---CCATCGGCGTAAAGGGTGGTTA--AGATTAT------ATTCAAA-
Majo	AAGTTGATAA---GCATCGGCGTAAAGGGTGGTTAG-GGAAAACTATAC------AA-
Hlst	AAATTGACAAG--T-TC-GGCGTAAAGCGTGGTTAA-GGATAACTT----AAAG----
Clpe	GAGTTGATAGA--A-GCCGGCGTAAAGGGTGGTTA--GGATCT-TCTATG-AA-----
Mlmr	AAATTGACAGG--T-GTCGGCGTAAAGGGTGGTTA--TGATTTTA----ATAAGA---
Crcr	AAGCTGATAGA--TGC-CGGCGTAAAGAGTGGTTAA-GTATCTTGAT-----AGAAC-
Muce	AAGCTGATAGA--TGC-CGGCGTAAAGAGTGGTTAA-GTATCTTGAT-----AGAAC-
Bege	AAGTTGACAG---TTACCGGCGTAAAGAGTGGTTA--AGAAAAA------TTTTAAAC
Mela	AAGTTGACAGC--CAT-CGGCATAAAGCGTGGTTA--GGAGAATTTT-----AAAC--
Hats	AAGTTGACAG---CCACCGGCGTAAAGAGTGGTTA--AGAGATA-----ATG-AAAAC
Orla	AAGCTGATAG---ATAACGGCGTAAAGAGTGGTTA--ATTTAA-AAGAGAAGC-----

```
Cosa    AAGTTGATAGC--C-ACCGGCGTAAAGAGTGGTTAG-GGATAAAAT-----GAAAAC-
Exsp    AAGTTGACAGA--C-AACGGCGTAAAGAGTGGTTAA-GGAAAAA-T--TTCTAC----
Depa    AAATTGATAAT--TATC-GGCGTAAAGCGTGGTTA--AGAATA-------ATACATAC
Rima    AAGTTGATGG---TCCCCGGCGTAAAGCGTGGTTAA-AGACCCCT-CCCCC--AC---
Fuol    GAGTTGATAGC--CTTC-GGCGTAAAGCGTGGTTAA-GGAAC-------TATCAGAC-
Gmaf    AAGTTGATAAA--AT-ACGGCGTAAAGCGTGGTTAA-AAGCCCCC-A--CTAAAC---
Xeei    AAGTTGATAGT--C-TTCGGCGTAAAGCGTGGTTAA-AAATGA-A-TTGACAA-----
Pros    AAGCTGATAGT--CCT-CGGCACAAAGCGTGGTTAA-GGAATAATAT-----AAAC--
Scmi    AAGCTGATAAA--T-ATCGGCGTAAAGAGTGGTTAA-GGAGT-AAAACAAAA------
Rolo    AAGTTGATAG---ATACCGGCGTAAAGAGTGGTTAG-GGCACAACTTCTA-----AC-
Cere    AAGTTGATAGA--TAT-CGGCGTAAAGAGTGGTTAG-GGCACATTA------A-AAC-
Daga    AAGTTGATCG---ATTACGGCGTAAAGAGTGGTTAA-GGAAG-ACC-TAAAC------
Anco    AAGTTGATAAG--CAA-CGGCGTAAAGAGTGGTTAA-GGAAACATCT-----ATAAC-
Dmve    AAGCTGATGAA--TCA-CGGCGTAAAGAGTGGTTAA-GGAATCCCCAGA----AC---
Dmar    AAGCTGATAAA--C-AACGGCGTAAAGAGTGGTTAA-GGGACAC-T--CACAAC----
Anka    AAGTTGATAAG--C-AACGGCGTAAAGAGTGGTTAA-GGAAACAC-----CCCAAC--
Moja    AAGTTGATAAG--CA-ACGGCGTAAAGAGTGGTTAA-GGAAACCCCC-A--CAA-AC-
Hoja    AAGTTGATAAG--CAA-CGGCGTAAAGAGTGGTTAA-GGAAACACCC-----A-AAC-
Bede    AAGCTGATAGT--C-ACCGGCGTAAAGAGTGGTTAGAGAACAACCC-----AA-AC--
Besp    AAGCTGATAGT--CAC-CGGCGTAAAGAGTGGTTAGAGGACAGCCCAA----AC----
Mysp    AAGTTGATAGA--A-ACCGGCGTAAAGAGTGGTTA--GGATAATCTTA----TTTAAC
Osja    AAGTTGATGG---AAGCCGGCGTAAAGAGTGGTTAG-GGCGCCCACCCTAAAC-----
Sgro    AAGTTGATGAT--ATACCGGCGTAAAGAGTGGTTAG-GGAAAACAAAATAA----AC-
Pzpa    AAGTTGATAGC--CTAACGGCGTAAAGCGTGGTTAG-AGAAATTAAAAT----AC---
Zeja    AAGTTGATAGC--CTA-CGGCGTAAAGCGTGGTTA--AGAAAATAAAAT----AC---
Znne    AAGTTGACAGC--CCAACGGCGTAAAGCGTGGTTA--ACTACCCCCC-C--CCCAAC-
Zefa    AAGTTGACAAC--TCAACGGCGTAAAGCGTGGTTA--ACTACCCCC-CCCCCCCAC--
Acni    AAGTTGATAGC--CCAACGGCGTAAAGCGTGGTTA--GGAAAAA-----CTAAACAAC
Ncrh    AAGTTGATAGC--CCAACGGCGTAAAGCGTGGTTA--GGAAAACTAA-----ACAAC-
Agca    AAGTTGATAG---ACACCGGCGTAAAGGGTGGTTA--AGATATTCTAAA----AC---
Hydy    AAGTTGATGAA--A-AACGGCGTAAAGCGTGGTTA--ACTTAAAATCAAA-AC-----
Gsac    AAGTTGATGA---ATTCCGGCGTAAAGAGTGGTTA--ACCTAAAATTAAAAC------
Pevo    AAGTTGACAGA--TATC-GGCATAAAGCGTGGTTAA-GGAAACATAAAAA--------
Hiku    AAGATAATAGA--AGTC-GGCGTAAAGAGTGGTTA--ACTATAAAATA-----AC---
Inpa    AAACTGATAGA--T-CACGGCGTAAAGCGTGGTTAA-ACCACAA------ACAAAA--
Auch    AAGTTGATCGC--C-GTCGGCGTAAGGTGTGGTT---ACAGTAAAAA-CTAGA---G-
Fico    AAGTTGATAG---CCTACGGCGTAAAGAGTGGTTA--AGATGTCT-AATTT--AT---
Macs    AAGTTGACAG---ATGCCGGCGTAAAGAGTGGTTA--AGATTCCT-TATTC-AAC---
Moal    AAGTTGATGA---CAGACGGCGTAAAGAGTGGTTA--AGAAAATCC-CAA----AC--
Syma    AAACTGATAAC--ACAACGGCGTAAAGAGTGGTTA--ACAAAAACCCAAAC-------
Mafr    AAGTTGACAAA--CTA-CGGCGTAAAGAGTGGTTA--ACAACATTCCAAAA-------
Dcpe    AAGTTGATAGC--C-ATCGGCGTAAAGCGTGGTTAG-GGAGCCCGTA-----AAAAC-
Dcti    AAGTTGATAGC--CCTC-GGCGTAAAGTGTGGTTAA-GGAAGTCCCCTTAAAC-----
Hehi    GAGTTGATACC--AT-TCGGCGTAAAGAGTGGTTAT-GGAAAATAAA-A------AC-
Stam    AAGTTGTCAGT--TAC-CGGCGTAAAGAGTGGTTAA-AGAATAATA-----TAGAC--
Hogi    AAGTTGATAT---GCATCGGCGTAAAGCGTGGTTA--AGACTGTAT-AAAAC------
Erzo    AAGTTGAAAGA--C-ATCGGCGTAAAGAGTGGTTA--ACTAAAA-A--TTAAAC----
Hxot    AAGTTGATAGA--C-ACCGGCGTAAAGAGTGGTTA--ACTTAAAAC-C--TCATAC--
Core    AAGTTGACAAA--CACC-GGCGTAAAGCGTGGTTA--ACTTAAAAATCGTAC------
Apve    AAGTTGACAG---ACACCGGCGTAAAGAGTGGTTA--ACTTAAAAC-TTAA---AC--
Latj    AAGTTGACAAA--CCA-CGGCGTAAAGAGTGGTTAA-GGAAACTTAAA----AC----
Laja    AAGTTGATAGT--CACC-GGCGTAAAGGGTGGTTA--GGATAAAATTAAAGAC-----
```

```
Syja  AAGTTGATGCA--CACC-GGCGTAAAGAGTGGTT---AGTCAAGACTTTT--AAAA--
Epme  AAGTTGACAGG--C-ACCGGCGTAAAGCGTGGTTAA-GGAACAAA------ACACAC-
Grse  AAGTTGATAGA--T-CCCGGCGTAAAGAGTGGTTA--AGATTAGGTCC-----GACAC
Clja  AAGTTGTTAAT--C-ATCGGCGTAAAGGGTGGTTA--AAATAA-A-ATTTTAC-----
Ogcy  AAGTTGACAGA--CTAC-GGCGTAAAGAGTGGTTA--GGAGAAAAATATT-AAAC---
Plna  AAGTTAATAGA--CATC-GGCGCAAAGCGTGGTTA--AGAAAACTTTACAAC------
Lema  AAGTTGATGAA--CCCC-GGCGTAAAGAGTGGTTA--AGAGAGATCA---AAAC----
Etzo  AAGCTGATAGA--CA-CCGGCGTAAAGCGTGGTTA--GGATTTTT-A--TAAAAC---
Apse  AAGCTGACAGC--TACC-GGCGTAAAGAGTGGTTA--ATAACCCCGCCA---TA-C--
Epde  AAGTTGACAGA---CATCGGCGTAAAGCGTGGTTA--AGACAAATT-GAAG---AC--
Slja  AAGTTGATAG--A--CGCGGCGTAAAGCGTGGTTA--GGGGAAT------AACAAC--
Bsja  AAGTTGTTAAA--TCA-CGGCGTAAAGAGTGGTTA--AAATGTATTAA-----A-AAA
Ecna  GAGTTGACAGA--TAAC-GGCGTAAAGCGTGGTTA--AGGGTGTCCTAA-------AC
Cohi  AAGTTGACAG---AATACGGCGTAAAGGGTGGTTAG-GGAAT-ATTAATA-C------
Caar  AAGTTGACAGA--CAA-CGGCGTAAAGAGTGGTTAA-GGGAAATACATA----AC---
Came  AAGTTGACAG---ACAACGGCGTAAAGCGTGGTTAA-GGAAAA-T-ATGTAAC-----
Mema  AAGTTGATAA---ACAGCGGCGTAAAGAGTGGTTAA-GGAACAC-TGACA-AAC----
Lenu  AAATTGACAGT--ACC-CGGCATAAAGTGTGGTTA--AGAAATAA-AC-----ACAA-
Plma  AAGTTGACAGA--C-ACCGGCGTAAAGCGTGGTTAA-GGAAAAAC------TTAAAC-
Emst  AAGTTGTTAGA--TACC-GGCGTAAAGAGTGGTTA--AGATAAGTCAGACAC------
Ptti  AAGTTGATAGA--CACC-GGCGTAAAGAGTGGTTA--AGACTTACTTA---ACAC---
Losu  AAGTTGTTAGA--C-ATCGGCGTAAAGCGTGGTTA--GGTA-ATTAAA-T--ATCCAG
Geoy  AAGTTGACCAC--C-ACCGGCGTAAAGCGTGGTTA--GGTTTATGTAC-A-ATATGAC
Dipi  AAGTTGATAAG--C-ATCGGCGTAAGGAGTGGTTA--AGACAAATCAA-----ATAC-
Pama  AAGTTGTTAAA--A-ATCGGCGTAAAGGGTGGTTA--AGAG-CAAGCT--TAAAAT--
Leob  AAGTTGACAAC--CA-TCGGCGTAAAGAGTGGTTA--AGATTAGC-C--CTCCAT---
Neba  GAGTTGACAAT--CCC-CGGCGTAGAGCGTGGTTA--AACATATTTGC-----AAAC-
Pdpl  AAGTTGACAG---TCCACGGCGTAAAGGGTGGTTAG-GGAAT-T-CTATTAAAC----
Nimi  AAGTCGATAGT--CCA-CGGCGTAAAGAGTGGTTAG-AGAACC-CCAATTAC------
Uptr  AAGTTGATAAG--C-ATCGGCGTAAAGGGTGGTTAG-GGAAAACT-----ATACAA--
Pesc  GAGTTGACAAT--CA-TCGGCGTAAAGAGTGGTTA--GGATGTTTA-C--CAA-AC--
Baar  AAGTTGACAAA--CAC--GGCGTAAAGAGTGTTTA--AGGTAACCAAAGA----AG--
Moar  AAGTTGATAGA--CA-TCGGCGTAAAGAGTGGTTAA-GACAGATC-T---C--GCAC-
Toja  AAGTTGATAGA--CA-GCGGCGTAAAGAGTGGTTAA-GGACAACC---A--AAA-AC-
Chau  AAGTTGACAAT--CAT-CGGCGTAAAGAGTGGTTA--AGATGTA--TAAAAAC-----
Chse  AAGTTGTTAA---GTATCGGCGTAAAGCGTGGTTA--AGACAACTCAAAC----AC--
Enar  AAGTTGATAGA--CCTC-GGCGTAAAGAGTGGTTA--AGATAAGA----TTAA-AAC-
Hpty  AAGTTGATAA---ATATCGGCGTAAAGCGTGGTTA--AGATA----GACTGAAAC---
Nana  AAGTTGAGGAA--CAAC-GGCGTAAAGAGTGGTTA--AAATAA----ATCTCAAAC--
Mcst  AAGTTGATAGA--C-TACGGCGTAAAGAGTGGTTA--AGATAT-ATTTTAAAC-----
Rhox  AAGTTGATAG---ACTACGGCGTAAAGCGTGGTTAA-GGAAA-A-CTATAAAAC----
Opfa  AAGTTGATAGA--CTC-CGGCGTAAAGCGTGGTTA--AGACAAATTTT---AAAC---
Paar  AAGTTGACAGC--CTAC-GGCGTAAAGGGTGGTTAA-GGAAAAAAACA-AAAC-----
Gozo  AAGTTGATAAA--CCC-CGGCGTAAAGAGTGGTTA--AGATAGATTAA-----ACAC-
Ackr  AAGCTGATAGC--C-ATCGGCGTAAAGAGTGGTTA--GGAAAAACAA----GAAC---
Elev  AAGTTGTAAGA--CAT-CGGCGTAAAGGGTGGTTAA-GGA-AAGTTA------AAAC-
Trdu  AAGTTGACAGA--CAT-CGGCGTAAAGAGTGGTTA--GGAAGTTTTT---A-AAC---
Amoc  AAGTTGACAGA--CAAC-GGCGTAAAGAGTGGTTAG-GGAAA-------TTTTAAAC-
Hame  AAGTCGATGGC--CTTC-GGCGTAAAGCGTGGTTA--GGACAAACCTA---AAAC---
Chso  AAGTTGAAAAAC-ATTC-GGCGTAAAGGGTGGCTAA-GGACCTATT-----TCAAAC-
Lyto  AAGTTGAAAG---ACCCCGGCGTAAAGAGTGGTTA--AGTTAG-AAT-TTTAC-----
Encr  AAGTTGACAG---ACATCGGCGTAAAGAGTGGTTA--AGTTAAA---ATTGT----AC
```

```
Bvar    AAGTTGATAGA--A-CCCGGCGTAAAGCGTGGTTAGGGGT-AAC-ACAAAC-AT----
Noco    AAGTTGATTAT--TCAC-GGCGTAAAGAGTGGTTAG-GGAACAA----ATAG--TAC-
Chsp    AAGCTGACTT---TTTCCGGCGTAAAGGGTGGTTA--AATAAAAT-AAAT---AC---
Arja    AAGTTGATAGC--C-GGCGGCGTAAAGAGTGGTTA--ACTTTAAACT------CACAC
Pase    AAGTAGATAAG--TT-ACGGCGTAAAGTGTGGTTAA-GCAAATAT-A--CTAAAA---
Trel    AAGTTGACAGA--C-AGCGGCGTAAAGGGTGGTTA--GAAGAACTT-----CACAC--
Lifa    AAGTTGATGGC--TGTC-GGCGTAAGGTGTGGTTA--GCAGACAATTT---GAAC---
Acur    AAGTTGATAGA--TTT-CGGCGTAAAGAGTGGTTA--ACAATTTTATA-----AAAG-
Ampe    AAGCTGATAGA--CCC-CGGCGTAAAGAGTGGTTAA-GCTAAA-CTTAAAAC------
Urja    AAGCTGATAGC--CCAACGGCGTAAAGAGTGGTTAA-GCGAGT-A-TTTTCAA-----
Enet    AAGTTGATAG---TATTCGGCGTAAAGAGTGGTTA--AACTAGACT-TAAA----C--
Ptbr    AAGCTGACAAC--TCTTCGGCGTAAAGGGTGGTTA--AAATAA-CA-ACTT---AT--
Safa    AAGCTGACAGA--CCCTCGGCGTAAAGTGTGGTTA--AATAGT-----GTA-CCTAC-
Icae    AAGTTGACAG---ATATCGGCGTAAAGCGTGGTTAA-GCTAA-A-CTA--AAAC----
Asmi    AAGCAGACAA---TACCCGGCGTAAAGAGTGGTTT--ATTTA-----AATTTTAC---
Foal    AAATTGATAAA--TAC--GGCGTAAAGGGTGGTTA--GATTTA----ACTTAAAAC--
Drze    AAATTGATAAA--C-AACGGCGTAAAGAGTGGTTA--GCAATTAAA------ATGAAC
Rhas    AAGCTGATAGA--CGCC-GGCGTAAAGGGTGGTTA--ACAAATAACA----AAAC---
Elac    AAGTTGATGG---CCCTCGGCGTAAAGAGTGGTTA--ACAAATATAACAC--------
Kugu    AAGTTGATAGA--TGA-CGGCGTAAAGAGTGGTT---ACATATTAAAC-----AAAAC
Plor    AAGTTGATAAA--C-ACCGGCGTAAAGAGTGGTT---ATGACTAAC-AATTAA-AC--
Sgun    AAGTTGATAGA--CAG-CGGCGTAAAGCGTGGTTA--ACAACAAAACC-----TAAAC
Zaco    AAGTTGTTAGA--TAC-CGGCGTAAAGAGTGGTTA--ACATAAATT------ATAGAC
Zbfl    AAGTTGTTAGA--CACC-GGCGTAAAGAGTGGTTA--GCTACTA----TTACT-TAC-
Spba    AAGTTGACAGA--CCG-CGGCGTAAAGCGTGGTTAA-GCAAAAACAAAA----AC---
Game    AAGTTGACAGA--C-ACCGGCGTAAAGCGTGGTTAA-GCTAAAC----TTAAAC----
Thth    AAGTTGACAGA--CACC-GGCGTAAAGCGTGGTTAA-GCTATACCAAA-AC-------
Xigl    AAGTTGACAGA--C-AACGGCGTAAAGAGTGGTTAA-GCAAACCA-AAAAA---C---
Hyja    AAGTTGACAG---ACGCCGGCGTAAAGTGTGGTTAA-GCAAAAT-T-TTA-AAC----
Psan    AAGTTGACAGA--CA-TCGGCGTAAAGAGTGGTTA--GCGGGGAATTC-T--TAAAC-
Cupa    AAGTTGACAGA--CGCC-GGCGTAAAGCGTGGTTAA-GCTAAACTAC-AAC-------
Mpch    AGGTTGATAAT--TAA-CGGCGTAAAGAGTGGTTA--AATTAACAAC-----AAAAC-
Char    AAGTTGATA----GTCACGGCGTAAAGAGTGGTTA--ACAAAA-ACTATAAAC-----
Pser    AAGCTGACAGA--CAAC-GGCGTAAAGAGTGGTTA--GCGATTACAAA---AAA----
Prol    GAGTTGACAGA--C-AGCGGCGTAAAGGGTGGTTA--GCGGGTTGA----CCAAAC--
Plbi    AAGTTGACAAA--C-AACGGCGTAAAGAGTGGTTA--GCGGATT-TACTAA-AC----
Calu    AAGTTGACAGA--ACCACGGCGTAAAGGGTGGTTAA-GCAGGAATAT---ACAC----
Papa    AAGTTGATAGT--CAA-CGGCACAAAAGGTGGTTA--GCTAGAACACT----A-AAT-
Sufr    AAGCTGACAGA--CTATCGGCGTAAAGAGTGGTTA--ACAGAAACA-CAGCTAAAC--
Stci    AAGCTGATAGA--C-ACCGGCGTAAAGCGTGGTTA--GCAATATTTAA-T--ACAAT-
Taru    AAGTTGTTAGC--C-AACGGCGTAAAGGGTGGTTA--GAACTAAAA-ACAACAAAC--
Rala    AAGTTGTTAGT--CC-TCGGCGTAAAGAGTGGTTA--GCAATAGCCT-A--CAG-AC-
                          ***   *      **
```

```
             21                  22       23              23'¦  HVR  ¦22'¦    HVR    ¦
Scca         TATAGTTA--TGACCT---CAT-CAAGCTGT--TATACGCA--TTC--GTCAACGGAAT-AAT-
Muma         TAAAGTTT--AGACCT---CAT-AAAGCTGTT--ATACGCAC--TC--ATCAGTGGA--ATAAT
Erca         TAAGGATA--AAATAT---CTT-AAAGCTGTC--ATACGC-TAA-A--AAATATATG--AAAAC
Pose         GAGAACAA--AATACC---TTT-A-AGCTGT--CATACGCT-A-AT--AAGTATATGA--ATAT
Actr         TAGAGCCA--AAAGCC---TCC-TAAGCCGT--CATACGCA-CCT---GAAGGCCCGA--AGCC
Scal         TAGAGCCA--AAAGCC---CCC-CAAGCTGT--CATACGCA-CCT---GAGGGCCCGA--AGCC
Posp         TAGAGTCA--AAACCC---CCC-TAAGCTGTC--ATACGCATT-T---GGAGGCTC-G-AAGCC
Atsp         TAAAGTCA--AAATCC---TCC-TAAGCTGTC--ATACGCACT-T-G-GGAGC-ATGA--AGCC
Leoc         TAAAGTCA--AAATCT---CCC-TAAGCCGTC--ATACGCACT---T-GGCAGCATGA--AGCC
Amca         TAAAGCGG--AAATCT---CTC-TCGACCGTT--ATACGTATA--T--GAAAAGATGA--AAAT
Osbi         TAAAGCCA--AAACCT---CTC-AAAGCCGTT--ATACGCATA--TC-GAGACTCGTA--GGTC
Pabu         TAAAGTTA--AATACC---TTC-CAGGCTGTT--ATACGTACT-CC--GAAGACTACA--AAAC
Hial         TAAAGCCA--AAAACC---CTC-CGGGCCGT--CATACGCA--TC-C-GAGGGCGCGA--GGCC
Elha         TAAAGCCA--AAGCCC---TTC-CCGGCTGT--CATACGCA-CCC---GAAGGCATGA--GGCC
Mlcy         TAAAGCCA--AAACTC---CTC-CCAGCTGTC--ATACGCAC-C-C--GAAGACAAG--AGGCC
Algl         TAAAGCCG--AAGACC---TCC-TGGGCTGT--TTTACGCA-TCT---GGGGGCTATTA-AGCC
Ptgi         TAAAGCCG--AATACC---CCT-CAGGCTGT--TATACGCATCT----GGGGAAATGA--AGCC
Alaf         TAAAGCCG--AACCCC---CCT-TAGGCCGTC--ATACGCTTC--T--AGGGACATGA--AGCC
Nock         TAAAGCCG--TACCCC---TCT-TAGGCCGTC--ATACGCTT-CT---AGAGATGTG--AAGCC
Anja         TAAAGCCA--AACACT---TCC-CAAGCTGTC--ATACGCTAC-C---GGATAAAACG-AAGCC
Gyki         TAGAGCCA--AACACC---CCT-TATGCTGTC--ATACGCCAT--G--GGGGTCACGA--AGAT
Syka         TAAAGCCA--AACACC---TCC-CAAGCTGTC--ATACGCAATC----GGAGGCAGGA--GGCC
Opma         TAAAGCCG--AACACC---TCC-TTAACTGTT--ATACGTTTA-AA--GAGAC-ACG--AAGCC
Comy         TAAAGAAG--AACAGC---TTT-CATGCCGTA--TCAAGCTTA---C-AAAGACCTGA--AAAA
Sasp         TAAAGCTA--AACACC---CCC-TAAGCTGTA--ATACGCCCC-C---AAGAGGTAG--AGAAT
Eupe         TAAAGCCA--AACGCA---CCC-CCAACTGTA--ATACGCCTCC----GGGAGACAGA--AAAT
Enja         TAAAGCAG--AAAACC---TCT-CAAACTGT--TATACGCA--C-CC-AGAGGTTGAA--ACCC
Same         TAAAGCCG--AAGACC---TCT-TAGGCCGT--CATACGTA-CCT---AGAGGCTCGA--ATAA
Chch         TAAAGCCA--AACGCC---TCC-CAGGCTGTC--ATACGCATC-C---GGAGGTACG--AAGCC
Grgr         TAAAGCCA--AAGGTC---CCC-TAAGCCGTT--ATACGCATT-TC--GGAGACATTA--GACC
Caau         TAAAGTCA--AATGGC---CCC-TTGGCCGTC--ATACGCTT-CT---AGGCGTCCG--AAGCC
Cyca         TAAAGTCA--AATGGC---CCC-TTGGCCGT--CATACGCT-TCTA--GGAGTCC-GA--AGCC
Dare         TAAAGCCA--AACGGC---CCT-TTAACTGTT--ATACGCTTT-T---AGATGCTAGA--GGCC
Cost         TAAAGCCA--AAAGAC---CTC-TTGGCTGTC--ATACGCCCC-T---GAGTGTCTGA--AGAC
Leec         TAAAGCCA--AAGGGC---CTC-TTGGCCGT--CATACGCT-TACT--GAGTGTCCGA--AGCC
Crla         TAAAGCCA--AGGGAC---CTC-TTGGCCGTC--ATACGCTT--CT--GAGTATCCA--AAGC-
Clmc         TAAAGCCA--AAGACC---TCC-CAAGCTGTC--GCACGCACC-CC--GGAGGCACGA--AGCC
Phin         TAAAGCCA--AAAACT---TCC-CAAGCTGTC--GCACGCACA--AC-GGAAATAAAA--AGCC
Icpu         TAAAGCTA--AAGACC---CCC-TGGGCCGTC--ATACGCATT-TC--GGGGGCACGA--AACC
Psto         TAAAGCTA--AAGACC---CTC-TAAGCTGTC--ATACGCACC-CC--GAGAGCACGA--AACC
Cora         TAAAGCTA--AAGACT---TAC-CAAGCCGTC--ATACGCCCA-T---GGAAGAACGA--AAAC
Eisp         TAAAGTCA--AATGTT---TCC-TAGGCCGT--TATACGTT--TTCT-AGAAACATGA--AGCC
Apal         TAAAGCCA--AACACC---TCC-CAGGCCGT--TGCACGTT-TTCT--GGAAACACGA--AGCC
Eslu         TAAAGCCG--AACACC---TCC-TCAGTTGT--TATACACA-TT-T--GAAGATATGA--AGCC
Dape         TAAAGCCA--AACACC---TTT-TCAGTTGTT--ATACACATT--T--AAAGGCTTG--AAGCC
Glse         TAAAGCCG--AACACC---CCC-TAGGCTGTT--ATACGCAC-CT---GGGAGTATG--AAGCC
Naar         TAAAGCCG--AACACC---CTT-TAAGCTGTC--ATACGCACC-T---GAGGGCACGA--AGCC
Baoc         TAAAGCCG--AACAAC---CTC-TAGGCTGT--CATACGCAC-CT---GAGGCTACGA--AGCT
Opso         TAGAGCCA--AACACC---TCC-TAGGCTGTC--ATACGCACC--T--GAAGACAC-G-AAGCC
Alte         TAAAGCCG--AAGACC---CCC-TAGGCTGTC--ATACGCACCT----GGAGGCACGA--AGCC
Plap         TAAAGCCG--AAGACC---CCC-TAGGCTGT--CACACGCA-C-CT--GGGGGCACGA--AGCC
```

```
Plal    TAAAGCCG--AACACC---CTC-CAGGCCGTT--ATACGCTTC-T---GAGGGCACG--AAGCT
Sami    TAAAGCCG--AACACC---CTC-CAGGCTGTT--ATACGCTC-CC---GAGGGCGGGA--AGCC
Rere    TAAAGTTG--AATAAC---CCC-TAGGCCGT--TGTACGCT--CCT--GGGGTAATGA--AAAT
Gama    TAAAGCCG--AATACC---TCC-AAGGCTGTT--ATACGCACCC----GGAGGTCTGA--AGCC
Onmy    TAAAGCCG--AACACC---CCC-TCAGCCGTC--ATACGCACC--T--GGGAGCACGA--AGAC
Sasa    TAAAGCCG--AACACC---CCC-TCAGCCGTC--ATACGCACC--T--GGGGGCACGA--AGAT
Cola    TAAAGCCG--AACACC---CCC-TTGGCTGTC--ATACGCACC--T--GGGGGCACG--AAGCC
Dita    TAGAGCTG--AACTTC---CCC-CCGGCTGTC--ATACGCAC--CC--GGAGGAACG--GAACC
Gogr    TGAAGCTG--AACCCT---CCC-CCTGCTGT--TATACGCC---CCC-GGAAAAATAT--ACCC
Chsl    TAAAGCCG--AACATC---CCC-CAAGCCGTT--GCACGCTT-AC---GGAGGAATG--AAGCC
Atja    TAAAGCCG--AACCTT---CTC-CCAGCTGTC--ATACGCAAA-C---GAAGAGACGA--AGCC
Iido    TAAAGCCG--AACCTC---TTC-CCAGCTGT--CATACGCA-AAC---GAAGAGACGA--AGCC
Auja    TAAAGTCG--AACACC---TCC-CGAACTGTT--ATACGCACA-C---GGAGACAA-G-AAGCA
Chag    TAAAGTTA--AACACC---CCC-CTTGCTGT--AATACGCG--GCC--GGGGGTAGGA--GACC
Hami    TAAAGTTA--AACACC---CCC-AGAACTGTT--ATACGCTCC--C--GGGAGTAGGA--GGCC
Saun    TAAAGTTA--AACACC---CCC-AGAACTGT--TATACGCT-CCC---GGGGACAGGA--AGCC
Nema    TAAAGCTA--AACGCC---TGC-AAAGCCGTT--ATACGCACC-C---GAAGGCATGA--AAAC
Disp    TAAAGCTG--AATGCC---CAC-ACAGCCGTG--ATACGCATC-C---GATGGCATGA--AACC
Myaf    TAAAGCTG--AAGTAA---CTC-CAGGCTGTC--GCACGCATC--T--GAGAATATTA--AACC
Lagu    TAAAGCCA--AACGCC---CCC-AAAGCCGT--TATACGCA-CTCC--GAAGGGCAGA--GGAT
Trtr    TAGAGCCC--TACGCC---CTC-ATGGCTGTT--ATACGCACC--C--GAGAGGCGGA--TGAA
Zucr    TAGAGCCC--TACATC---CCC-ATGGCTGT--TATACGCA---CC-CGGGGAATGGA--TGAC
Pxja    TAGAGCCA--AACACC---ATC-AAAGCTGTT--ATACGCAC----CCGATAGTATG--AAGAT
Pxlo    TAGAGCCG--AACACC---ATC-AAAGCTGT--TATACGCA---C-CCGATAGTATGA--AGAT
Pctr    TAGGGCCG--AACCCC---CTC-TAAGCCGT--TATACGCA---CAC-GAAGGCATGA--AGCA
Apsa    TAGAGCCA--AACCAC---TTC-TAAGCCGTT--ATACGCAAT----CGAAGAAATGA--AGCC
Cabe    TAAAGCCG--AAGACT---ATC-AAGGCTGTC--GCACGCATG---TCGAGAGCGCGA--AGAA
Bzze    TAAAGTCG--AACACC---CTC-AGAGCTGTT--ATACGCAT----CCGAGGGCATGA--AGAA
Siim    TAAAGTCG--AATGCC---CCC-AAGGCCGTT--GTACGCATA--C--GGGGGTGCGA--AGCA
Ctru    TAAAGCCG--AACACC---CTC-AAAGCTGTT--ATACGCAT-C---CGAGGGCATG--AAGAA
Dpbr    TAAAGCCG--AACACC---CTC-AAAACTGTT--ATACGTATT----CGAGGGCAT-G-AAGAA
Caki    TAGGGTCA--AATAGC---TTC-TAAGCAGTC--AAATACTAT--C--GAAGCCACAA--AGCA
Phja    TAGGATTG--AAGTGC---TTC-AAGGCAGTG--ATAAGC-TT---CCGAAGTCACGA--AACA
Brsp    TAGGATCA--AATAAT---CGC-AACGCTGTG--ATATGTTTA---C-GAAATCATTA--AGAC
Gamo    TATGGCCG--AACAGC---TTC-AAAGCAGTT--ATACGCATC--C--GAAGTCACGA--AGAA
Lolo    TAGGGCCG--AACAGC---CTC-AAAGCAGTT--ATACGCATT---C-GAGGCCACG--AAGCT
Batr    TGAAGTCA--AACACT---AGC-ATAAGTAGT--GAAAAGCTG-----GTTTAGCATG-AAGC-
Prmy    TAAAGTTA--AACATA---CTA-CCAGACGT---ATAAACCTAAA---CACTATATG--AAACC
Loli    TAAAGTCG--AATGCC---CTC-AAAGCTGTT--ATACGCTCT----CGAAGGCAA-G-AAGCC
Loam    TAAAGTCG--AATGCC---CTC-AAAGCTGTT--ATACGCAC----CCGAGGGTTAGA--AGTT
Chab    TAAAGCCA--AATGCC---TTC-AAAGCTGTA--ATAAGCATC----CGAAGATAA-G-AGGCT
Chto    TAAAGCCA--AATGCC---TTC-TAGGCTGTA--ATAAGCATC----CGAAGATAA-G-AGGCT
Majo    TAAAGCCG--AAC-ATC--TTC-AAAACTGTT--ATACGC-CC---TCGAAGATTTGA--AGCC
Hlst    TAAAGCCG--AATATA---CTC-AAGGCTGTT--ATAAGCTCC----CGATTACAAGA--AGCC
Clpe    TAGAGCCA--AATGCC---CTC-AAAGCTGT--CATAAGCA---C-CCGAGGGGAAGA--AGCC
Mlmr    TAAAGACA--AATGCC---TTC-AGAGCTGTA--CTACGCAAT---T-GAAGGTGAGG-AAGCC
Crcr    TAAAGCCG--AACGCC---CTC-AAGACCGTT--ATACGTTTC-C---GAAGGTATGA--AGAC
Muce    TAAAGCCG--AACGCC---CTC-AAGACCGTT--ATACGTTT-CC---GAAGGTATGA--AGCC
Bege    TAAAGTTG--AATACT---CTC-CAAACTGTT--ATACGTACA-C---GAGAACCA-G-AAACC
Mela    TAAAGCCA--AACACT---CTC-AGAACTGTT--ATACGTACC-C---GAGAATTAGA--GGTC
Hats    TAAAGCCG--AACACT---CTC-AGGACTGTT--ATACGTGTC-C---GAGAGCAA-G-AAGCT
Orla    TAAAGTGG--AATGTT---TTC-AAAGCTGT--TATACGCA---CCC-GAAAATAAGA--AGAC
```

```
Cosa    TAAAGCCG--AATATC---CCC-ACAGCTGTT--ATACGCAC----CCGAAGATAAG--AAGCC
Exsp    TAAAGCCG--AACATC---CTC-AAGACTGTC--GTACGTTT----CCGAGGATATG--AAGTC
Depa    TAAAGTCG--AATCAT---TTC-TAAGCTGTT--ATACGCACT----CGAAAGTAG-G-AAGAA
Rima    TAAAGCCG--AACGCT---CTC-ATAGCTGT--TATACGTC---TTA-GAAAGTATGA--AGCC
Fuol    TAAAGTCG--AACTCT---CTC-ATGGCTGTT--ATACGCACC----CGAGAGCAT-G-AAGTC
Gmaf    TAAGACTA--AACCTT---TCC-AAAGCTGTT--ATACGCACC----CGGAAATATG--AAACT
Xeei    TAAAGCCA--AACTCT---CTC-AAGGCTGTT--ATACGCAC----CCGAGAGCATG--AAGAT
Pros    TAAAGCCG--AATGCC---CTC-AGAGCTGTT--ATAAGCATC----CGAGAGTTCGA--AGCC
Scmi    TAAAGCCG--AACGCC---CTC-AAGGCTGT--TATACGCA---TCC-GAAGGTACGA--AGCA
Rolo    TAAAGCCG--AAC-GCC--CTC-AGAGCTGTT--ATACGCACC---C-GAAGGTATGA--AGAA
Cere    TAAAGCAA--AACACC---CCC-AAGGCTGTT--ATACGCAC----CCGGAGGAATGA--GTAA
Daga    TAAAGCGG--AACCCC---ACC-AAGGCTGT--TATACGCA---CCC-GGAGACGTGA--ATAT
Anco    TAAAGCCG--AACACC---CTC-AGAGCTGTT--ATACGCAC----CCGAGAATATGA--AGCA
Dmve    TAAAGCCA--AAC-GCC--CCC-CAAGCCGTT--GTACGCTCC---C-GGGGGTAGGA--AGCC
Dmar    TAAAGCCA--AACACC---CCC-CAAGCCGTT--GTACGCCT----CCGGAGGTAGG--AAGCC
Anka    TAAAGCCG--AACACC---CTC-AAAGCTGTT--ATACGCAC----CCGAGGACATG--AAGCA
Moja    TAAAGCCG--AACACC---CTC-AAAGCTGTT--ATACGCACC----CGAGGGCATG--AAGCA
Hoja    TAAAGCCG--AACATT---CTC-AGAGCTGTT--ATACGCAC----CCGAGAACATGA--AGCA
Bede    TAAAGCCG--AACGCC---TTC-AAGGCTGTT--ATACGCA-T---CCGAAGGTACGA--AGAA
Besp    TAAAGCCG--AACGCC---CTC-AAAGCTGTT--ATACGCATC---C-GAAGGTACGA--AGAA
Mysp    TAAAGCCA--AACACC---TTC-AGAACTGTT--ATACGTACC---C-GAAGGCATG--AAGAA
Osja    TAAAGCCA--AACACC---TTC-AGAACTGT--TATACGTA---CCC-GAAGGCATGA--AGAA
Sgro    TAAAGCCA--AACACC---TTC-AGAACTGTT--ATACGT-AC---CCGAAGGCATGA--AGAA
Pzpa    TGGAATCA--AACACT---CCC-AAAGCTGTT--ATAAGCATA---T-GAGAATACGA--AGCC
Zeja    TAGGGCCG--AACCTC---CAC-ACAGCTGTT--ATACGCATA---C-GAGAATATGA--AGCC
Znne    TAGGGCCA--AACACC---CTC-AAAGCCGTT--ATACGCAC----ATGAGGGCTTG--AAGAT
Zefa    TAGGGCCA--AACACC---CAC-AAAGCTGT--TATACGCA---TGTTGAGGATTTGA--AGCA
Acni    TAGGGCTA--AACATC---CCC-AGAGCTGTT--ATACGCAAA----CGAGGATAT-G-AAAAT
Ncrh    TAGGGCTA--AACATC---CCC-AGAGCTGTT--ATACGCAAA----CGAGGATATGA--AAAT
Agca    TAAAGCCG--AATACC---TTC-AAAGCCGT--TATACGTA---CT-CGAAGGTAAGA--AGCC
Hydy    TAAAGCCG--AACACC---CCC-AAAGCTGT--TATACGCA---C-CCGGAGGTTAGA--AGTC
Gsac    TAAAGCCG--AACGCC---CCC-AAAGCTGT--TATACGCA---TCCGGAGGTGA-GA--AGTT
Pevo    TAAAGCCG--AACGCC---CCC-AGAGCTGTC--ATACGCTCC---C-GAGAGTAGGA--AGAC
Hiku    TAAAGTTA--AACATC---TTC-CAAGCTGTT--ATACGCACC--C--GAAGGTATGA--GATT
Inpa    TAAAGCCA--AACACT---TCC-ACAGTCGTT--ATACACAA----CCGAAAGTTAG--AAGAC
Auch    TAAAACTA--AAAGAA---AAC-GGAGCAGT--GTAACGCCAACCACCGTATGTCGGA--GAAC
Fico    TAAAGCCG--AATGCC---CCC-AAAGCTGT--TATACGCT---TCC-GGAGGTAAGA--AGAA
Macs    TAAAGCCG--AACGCC---TCC-AGAACTGT--TATACGCC---CCC-GGAGGTAAGA--AGTT
Moal    TAAAGCCA--AATTTT---CTC-CTCGCTGT--TATACGCA-CCC---GAGACATAAGA-AGTA
Syma    TAAGGTCG--CACATC---CTC-ATGGTAGT--GATACACA---TCC-GAACATACGA--AGCC
Mafr    TAAAGTCG--AATGCT---TTC-AAAGCTGT--CATACGCA---C-CTGAAAGTAAGA--AGCC
Dcpe    TAAAGTCA--AACGCC---CTC-ACTGCAGTC--ATACGCCCC----CGAGGGTAAGA--AGCC
Dcti    TAAAGTCA--AACGCC---CTC-ACTGCAGTC--ATACGCCCC---C-GAGGGTAAGA--AGCC
Hehi    TAAAGCCG--CACACC---TTC-AAAGCTGTT--ATACGCAT-C---CGAAGGCAAG--AAGAC
Stam    TAAAGCCG--AACACC---TTC-AAGGCAGTT--ATACGCAC----CCGAAGGTTAGA--AGCC
Hogi    TAGAGCCG--AATATC---TTC-AAGACTGT--TATACGTA---CCC-GAAGATGAGA--AGTC
Erzo    TAAAGTCG--AACATC---TTC-AAGGCTGTT--ATACGCAT----CCGAAGACAAG--AAGTT
Hxot    TAAAGCCA--AACATC---TTC-AAGACTGTT--ATACGCAA----CCGAAGACAGG--AAGTT
Core    TAAAGCCA--AACATC---TTC-AAGACTGTT--ATACGTAAC---C-GAAGACAGGA--AGTT
Apve    TAAAGACA--AACATC---TTC-AAGACTGT--TATACGTA---CCC-GAAGACAGGA--AACT
Latj    TAAAGTCG--AACGCT---CCCTAGAGCTGTT--ATACGTGTA---C-GAGAGTACGA--AGCT
Laja    TAAAGCCG--AACACC---TTC-AAGGCTGTT--ATACGCACC---C-GAAAGTAAGA--AGCT
```

```
Syja    TAAAGCCG--AACACC---CTC-AGAGCTGTT--ATAAGCATC---C-GAAGGTACGA--AGTT
Epme    TAAAGCCG--AACGCT---TAC-TAGGCTGTT--ATACGCTT----ACGAAAGTAAG--AAGCA
Grse    TAAAGCCG--AACAAC---CTC-AATGCTGTT--ATACGCA-T---CCGAAGGTCAGA--AGTA
Clja    TAAAGTCG--AACGCC---TAC-AGGACCGTT--ATACGTATT---CCGAAGGTATG--AAGAT
Ogcy    TAAAGTCG--AAAATC---TTC-AAGACTGTA--ATACGTTCC--C--GAAGATATGA--AGCC
Plna    TAAAGCCG--AATGCT---TTC-ACGACTGTC--GTACGTCCC---T-GAAAGTAAGA--AGCT
Lema    TAAAGCCG--AATGCT---TTC-AAAGCTGTT--ATACGCTTC--C--GAAAGTAAGA--AGCC
Etzo    TAAAGCCG--AACACC---CTC-AGAGCTGTT--ATACGCACC----CGAAGGTAAG--AAGAC
Apse    TAAAGCCG--AACATC---TCC-AAAGTTGTA--CAACGCACT--C--GAAGACATGA--AGAC
Epde    TAAAGCCG--AACCCC---CTC-AAAGCTGT--TATACGCA---TC-CGAGGGCAAGA--AGCT
Slja    TAAAGTCG--AATTGC---CTC-CTAGCAGT--TATACGCT---CAC-GAGGGCTTAGA-AGCA
Bsja    TAGAGCCG--AACACT---TAC-AAAGTTGTT--ATAAGCAC----ACGAAATTAAGA--AGCC
Ecna    TAAAGCCG--AATATC---TCC-AGGACTGTT--ATACGT-TT---CCGGAGAAACGA--AGAT
Cohi    TAAAGCCG--AACACC---TTC-CAAGCTGT--TATACGCT---TAT-GAAGAACTGA--AGCA
Caar    TAAAGCGG--AACACC---CTC-ACAGCTGTT--ATACGCTTC---C-GAGGGCATGA--ACCA
Came    TAAAGCGG--AACCTC---CTC-CTAGCTGTT--ATACGCTT----CCGAGGAAGTG--AACCC
Mema    TAAAGCCA--AACACT---TTC-AGAGCTGT--TATACGCA---CCT-GAAAGCATGA--AGCC
Lenu    TAAGGCCA--AACTCT---TTC-AAGGCTGTT--ATACGCAAC----CGAAAGAAAGA--AGAT
Plma    TAAAGCCG--AACACC---TTC-AGGGCAGTT--ATACGTTT----CCGAAGGTACG--AAGCC
Emst    TAAAGTCG--AATACC---TTC-AGAGCTGTT--ATACGCACC---C-GAAGGCAAGA--AGTT
Ptti    TAAAGCCG--AACGCC---TTC-AGAGCTGTT--ATACGCATC--C--GAAGGTAAGA--AGCC
Losu    TAAAGCCG--AACGCC---TTC-TAGGCTGTC--ATACGTGTATTTGCGAAGGTGAG--AAGCC
Geoy    TAAAGTCG--AACTCC---TTC-AAGGCCGTT--TAACGTGT----TCGAAAGGGAG--AAGCA
Dipi    TAAAGCCG--AATACC---TTC-ACAGCTGTC--ATACGCAAC---ACGAAGGCTAGA--AGTT
Pama    TAAAGCCG--AACGCC---TTC-TAGGCTGTT--ATACGCATC---C-GAAGGTAAG--AAGCC
Leob    TAAAGTCG--AATGTC---TTC-AAGGCTGTT--ATACGCACC----CGAAGACTAG--AAGCC
Neba    TAAAGCCG--AAAACA---TTC-ACAGCAGTC--ATACGCAT------GAAAGTAATA--AGCC
Pdpl    TAAAGCCG--AACGCT---TAC-AAAGCTGT--TATACGCA---CAC-GAAAGTAAGA--AGAT
Nimi    TAAAGCCG--AACCCC---TTC-AAGGCTGT--TATACGCA---CACCGAAGAGGAGA--AGCC
Uptr    TAAAGCCG--AACATC---TTC-AAAACTGTT--ATACGCCC----TCGAAGATCTG--AAGCC
Pesc    TAAAGCCG--AACGCC---GTC-CAAGCTGTT--ATACGCGTT----TGATGGTAAG--AAGCA
Baar    TAAAGCCG--AACGCCC--CTC-AAAGCTGTC--ATACGCATC---C-GAGGGTGACGA-AGCC
Moar    TAAAGCCG--AACGCC---CTC-AAAGCTGTT--ATACGCACC---C-GAGGGT-A-AGAAGTT
Toja    TAAAGCCG--AACGCT---CTC-AAAGCTGTT--ATACGCACTA---CGAAAGTATG--AAGCT
Chau    TAGAGCTA--AATACC---CTC-AAAGCTGT--TATACGCT---C-TCGAAGGTAAGA--AGCC
Chse    TAAAGCCG--AAC-ACC--TTC-TCTACTGTT--ATACGTTCC---C-GAAGGTAAGA--AGCC
Enar    TAAAGCCG--AATGTC---TCC-AGAGCTGTT--ATAAGCACC----CGGAGATAA-G-AAGTT
Hpty    TAAAGCCG--AACGCC---CTC-AGAGCCGT--TATACGCA---CCC-GAAGGTAAGA--AGAC
Nana    TAAGGTTG--AATATT---TTC-AGGGCTGTT--ATACGCAAC----CGAAAACAA-G-AAACC
Mcst    TAAAGCCG--AACGCC---CTC-AAAGCTGT--TATACGCC---TTC-GAGGGTAAGA--AGCC
Rhox    TAAAGCCG--AATGCC---CTC-AGAGCTGT--TATACGCT---CAC-GATGGTTAGA--AGCC
Opfa    TAAAGCCG--AACGCC---CTC-AGAGCTGTT--ATACGC-TC---CCGAGGGTAAGA--AGCC
Paar    TAAAGCTA--AATGCC---CCC-CCGGCTGTT--ATACGCTCT---C-GGGGGTAAGA--AAAT
Gozo    TAAGGCCG--AACGCC---CCC-TAGGCTGTT--ATACGCATC----CGGGGGTAAGA--AGTT
Ackr    TAAAGCCG--AACAGA---TTT-GAGGTTGTT--ATACACGTA---C-AAATACCGG--AAGCC
Elev    TAAAGCCG--AATACT---ATC-AAGGCTGTT--ATACGCGC----CCGATAATAAGA--AGAT
Trdu    TAAAGCCG--AACGCC---CTC-AGAACTGTT--ATACGTAC----CCGAGAGCAAGA--AGCC
Amoc    TAAAGCCA--AACGTC---CTC-AAAGCTGTT--TAACGCTC----CCGAGTTAA-G--AAGAT
Hame    TAAAGATA--AACGAT---TTC-AAAGCTGTT--ATACGCTCA--T--GAAAATCTGA--AGAT
Chso    TAGAGCTA--AATTTC---TTC-AAAGCTGTT--ATACGCTCA--T--GAAAACCAGA--AAAT
Lyto    TAAAGCCG--AACATC---CTC-ACGGCTGT--TATACGCA---CCC-GAGGATAAGA--AGCT
Encr    TAAAGCCG--AAC-ATC--CTC-CAGGCTGTT--ATACGC-AT---CCGAAGATAAGA--AGTT
```

```
Bvar    TAAAGCCG--AATACC---TTC-AAAGCTGT--TATACGCA---T-ATGAGGGTGAGA--AGTC
Noco    TAAAGCCG--AATGTT---TTC-AAAGCTGTT--ATACGCATC----CGAAAACAA-G-AAGTT
Chsp    TAAAGCCG--AATGCTT--CTT-AAAGCTGT--CATACGCC-TTT---ATAAGCAAGA--AGCC
Arja    TAAAGCCA--AACATC---TTC-AAGACTGTT--ATACGTACA---C-GAAGGCAGG--AAGTT
Pase    TAAAGCGG--AACACC---CTC-CAGGCTGTT--ATACGTTAC----TGAGGTTAAAT-AAACA
Trel    TAGAGCCG--AACGCC---TCC-AGCGCTGTC--TAAAGCA-C---CCGGAGGTAAGA--AGTG
Lifa    TAGAGCTG--TATGGA---CCC-CGAGCCGTC--TAAGGTTTA---GAACGGGTAAGA--AAGT
Acur    TAAAGTAG--AACCCT---TTC-AAAGCAGTT--ATACGCATT----TGAAT-TGTGA--ACCC
Ampe    TAAAGCCG--AACACC---CTC-ACAGCTGT--TATACGCA--C-CC-GAGAGTAAGA--AGCC
Urja    TAAAGCCG--AGAATC---AAC-AAAGCAGTT--ATACGCAC----CCGAAAAAA-A--AAGAC
Enet    TAAAGCCG--AACTCC---CTC-ATGGCTGT--TATACGCA---CCC-GAGGGTAAGA--AGCC
Ptbr    TAAAGTCA--AACTCC---TCC-CTAACTGT--TGTACGTA---TT-TGGAGGAAAGA--GGTC
Safa    TAAGGTCG--AATGCA---GTC-AAAACTGTT--ATACGTAAT----CGAATGCTA-G-AAGCC
Icae    TAAAGCCG--AACACC---TTC-AGGGCAGT--TATACGCA---TCC-GAAGGCACGA--AGCC
Asmi    TAGAGCCA--AATACC---CTC-AAGACTGT--TATACGTG---TTC-GAGAATATAGA-AGCC
Foal    TAGGGCCG--AACTCT---CCC-CTGACTGTT--GTACGTTAC-----GGAATGAAG--AAAAC
Drze    TAAAGCCA--TACACC---CTC-TTAGTTGTG--ATACACTG----ATGAACGGGGG--AGGAT
Rhas    TAAAGCCG--AACTTC---TCC-AAGGCTGTC--ATACGCACC--C--GGAGAAATGA--AGAA
Elac    TAAAGCCA--AACACC---TTC-ATAGCTGT--CATACGCA---CTT-GAAGACAGGA--AGAC
Kugu    TAAAGCCG--AATATC---TTC-AAGGCTGTG--GTAAGCAC----CCGAAGATTTGA--AGCC
Plor    TAAAGCCG--AACGCT---CTC-AGAGCTGT--TATACGCA---T-CCGAAAGTAAGA--AGCC
Sgun    TAAAGCCG--AACACT---CTC-AAAGCTGTT--ATACGCAC----TCGAGAGTATGA--AGTC
Zaco    TAAAGCCG--AACGCC---CTC-AGAACTGTT--ATACGTTT----CCGAAGGTGAGA--AGTC
Zbfl    TAAAGCCG--AACACC---TTC-AAAGCTGTT--ATACGCACC----TGAAGATAG-G-AAGTT
Spba    TAAAGCCG--AACGAC---CTC-AAGGCTGTT--ATACGCTTC----CGAAGGTACGA--AGCT
Game    TAAAGCCG--AACACC---TTC-AGGGCAGTT--ATACGCATC----CGAAGGCACG--AAGCC
Thth    TAAAGCCG--AACACC---TTC-AGGGCAGTT--ATACGCATC---C-GAAGGCACGA--AGCC
Xigl    TAAAGCCG--AACGCT---CTC-AGAGCTGT--TATACGCA---T-CCGAAGGTATGA--AGCC
Hyja    TAAAGCCG--AACGCC---TTC-AAGGCAGTC-TGAATGCA---TTC-GAAGGTACGA--AGCC
Psan    TAAAGCCA--AACGCC---TTC-AAAGCAGTC-CGAATGCATT----CGAAGGTACG--AAGCC
Cupa    TAAAGCCG--AATACC---TTC-AAGGCAGTT--ATACGCATT--C--GAAGGCACGA--AGCC
Mpch    TAAGGCCG--AAAATT---TTC-ACAGCTGTT--ATACGCACT---C-GAAGATAAGA--AGCT
Char    TAAAGCCG--AACACC---TTC-AAAGCTGT--TATACGCA---TTC-GAAAGTAAGA--AGCC
Pser    TAAAGCCG--AACGCT---CTC-TAAGCTGTT--ATACGCGCA--TGCGAGAGTATGA--AGCC
Prol    TAAGGCCG--AACGCT---CCC-AAAGCTGTT--ATAAGCACC---C-GGGAGTATG--AAACC
Plbi    TAGAGCCG--AACGCT---TTC-AAAGCTGT--TATACGCA---C-CCGAAAGTATGA--AACC
Calu    TAAAGTCG--AAAGAA---TTC-AGAGTTGTG--ATAAGCCCA--C--GAAGACATGA--AGAA
Papa    TAAGGCTG--AACGCTT--TTCTTACAGCTGT-T-ATAAGCTC-C---TAAAAGTATGA--AAAC
Sufr    TAGAGCCG--AATGCT---TTC-AAGGCTGT--TTTACGCA---CC-CGAAAGCAAGA--AGAA
Stci    TAAAGCCG--AATGCT---TTC-AAGGCTGTT--ATACGCAT----CCGAAAGCTAG--AAGTA
Taru    TGAGACCG--AACACC---TTC-AAGGCTGT--TATACGCT---TCC-GAAGCAACGA--AGAA
Rala    TAAAGCCA--AACATC---TTC-AAAGCTGTT--ATACGCTCT----CGAAGACAAG--AAGAC
```

```
                          ¦HVR¦  24        24'  21'  ¦      HVR     ¦20'            19'
Scca    CAACA--AC---GAAAGTGACTCTAAAT-TACCAGGAATCTTGATG-------TCACGACA
Muma    CAACA--AC---GAAAGTGACTTTACATATTAA-GGAGCCTTGATG-------CCACGATA
Erca    CATTA--AC---GAAAGTTACCTTAT--------A--TAAGTGAAC-------CCACGAAA
Pose    CATTA--AC---GAAAGTTATCTCAT-TAAA--------TGTGAAC-------CCACGAAA
Actr    CAACC--AC---GAAGGTAGCTCTACCTAACAAGGACCCCTTGAAC-------CCACGACA
Scal    CAACC--AC---GAAGGTAGCTCTACCCGACAAGGATCCCTTGAAC-------CCACGACA
Posp    CAACC--AC---GAAGGTAGCTCTACCTACAAG-GACCCCTTGAAC-------CCACGACA
Atsp    CCTCC--AC---GAAGGTAACTTTATAACCCACAGCCACCCTGAAC-------TCACGATA
Leoc    CCTCC--AC---GAAGGTAACTTTATAGCCTACAGCCACCCTGAAC-------TCACGATA
Amca    CAACC--AC---GAAAGTAGCTTTATACAAAACAGCCACCTTGAAC-------TCACGACA
Osbi    CCTAA--AC---GTAAGTAGCTTTAACAT------A---TCTGAAC-------TCACGAAA
Pabu    CTATAG-AC---GAAAGTAACTTTAAAATGA----A--TACGAAAC-------TCACGAAA
Hial    CTATT--AC---GAAAGTAGCTTTAACAAAAAG-C--ACCTAGAAC-------TCACGACA
Elha    CCTAC--AC---GAAAGTAGCTTTAACCACAA--GACCCCTAGAAC-------TCACGACA
Mlcy    CAACC--AC---GAAAGTAGCTTTAATCACA---AACCCCTAGAAC-------CCACGACA
Algl    CTTTA--AC---GAAGGTAGCTTTAAAAGATTA--CATCCTCGAGT-------TCACGATA
Ptgi    CCAAA--AC---GAAGGTAGCTTTAATAT----ATTAACCTCGAAT-------TCACGACA
Alaf    CCGAA--AC---GAAAGTAGCTTTAAATAAACA---CCCCTTGAAC-------TCACGACA
Nock    CCAAA--AC---GAAAGTAGCTTTAATTTA-----ACCCCTCGAAT-------TCACGACA
Anja    CCACT--AC---GAAAGTGGCTTTAACAC---------CTTTGAAC-------TCACGACA
Gyki    CAACG--AC---GAAGGTGGTTCTAATAACCTAA----TCTTGAAC-------TCACGACA
Syka    CCCCA--AC---GAAAGTAGCTTTAATAAAC----AAATCTTGAAT-------TCACGACA
Opma    CCATA--AC---GAAAGTAGCTTTAACCCAAA-----ATCTTGAAC-------TCACGAAA
Comy    CAATA--AC---GAAAGTAACTTTAAACAT-------TACTTGAAT-------TCACGACC
Sasp    CTTTG--AC---GAAAGTAGCTTTA----------ACAGTTTGAAT-------TCACGATA
Eupe    CTATA--AC---GAAAGTAGCTTTAACAC-------C--TTTGAAT-------TCACGATA
Enja    CTTAC--AC---GAAAGTGACTTTATTTTCGC----CTACCAGAAG-------CCACGAAA
Same    CAAAC--AC---GAAAGTAGCTTTACCCC---TTCCT-GCCAGAAC-------CCACGAGA
Chch    CAATA--AC---GAAAGTAGCTTTATTACCGCC----AACCTGACC-------CCACGACA
Grgr    CACCC--AC---GAAAGTAGCTTTACCACAA----ACCACCCGACC-------TCACGACA
Caau    CTAAT--AC---GAAAGTAACTTTAATAAA-----CCCACCTGACC-------CCACGAAA
Cyca    CTAAT--AC---GAAAGTAACTTTAATAAAC-----CTACCTGACC-------CCACGAAA
Dare    CAAAC--AC---GAAAGTAGCTTTAAATT-A----ATTACCTGAAC-------CCACGAAA
Cost    CACAC--AC---GAAGGTAGCTTTATTATTA---GTCCACCTGACC-------CCACGAAA
Leec    CAAAA--AC---GAAAGTAGCTTTAATATAGC----CCACCTGACC-------CCACGAAA
Crla    CAAAT--AC---GAAAGTAGCTTTAATAAA----ACCCACCTGACC-------CCACGAAA
Clmc    CAAAC--AC---GAAGGTAGCTTTATTACATTC-----TCCTAACC-------CCACGAAA
Phin    CCAAC--AC---GAAAGTAGCTTTAAA---------TAAACTGACG-------CCACGAAA
Icpu    CTAAC--AC---GAAAGTAGCTTTAAAAACAC------ACCTGACC-------CCACGAAA
Psto    CAAGC--AC---GAAAGTAGCTTTAAACATTATT----ACCTGACC-------CCACGAAA
Cora    CACAT--AC---GAAAGTAACTTTATTA--T----AACATCCGAAC-------CCACGAAA
Eisp    CAATTACAC---GAAAGCAACTTTATGCTAAA----CGACCTGACC-------CCACGAAA
Apal    CAATC--AC---GAAAGTAGCTTTACATAAACC-----ACCTGACC-------CCACGAAA
Eslu    CTGCC--GC---GAAAGCAGCTTTAAGTAT---------CCTGAAC-------CCACGACA
Dape    CCCTC--GC---GAAAGCAGCTTTAATTCA---------TCTGAGC-------CCACGACA
Glse    CCACCCTAC---GAAAGTGGCTTTAC-CCC-----CCCCCCCGAAC-------CCACGATA
Naar    CCCTC--AC---GAAAGTGGCTTTACCCCCCCC-----CCCCGAAC-------CCACGACA
Baoc    CCCCC--AC---GAAAGTGGCTTTACCCC--------TCCCCGAAC-------CCACGACA
Opso    CCCTT--AC---GAAAGTAGCTTTACCCCT-----CCCTCCCGAAC-------CCACGATA
Alte    CAATC--AC---GAAAGTAGCTTTACTCAAGCCC----ACCCGACC-------CCACGACA
Plap    CTGTC--AC---GAAAGTAGCTTTATTCAAGC----CCGCCCGACC-------CCACGACA
```

```
Plal    CCACT--AC---GAAAGTGGCTTTAAC---------ACACCTGAAC-------CCACGACA
Sami    CCACT--AC---GAAAGTGGCTTTAA---------CTCACCTGAAC-------CCACGACA
Rere    CTACC--AC---GAAAGTGACTTTACACCTAC------TTCTGAAC-------CCACGACA
Gama    CCCTT--AC---GAAAGTAGCTTTATTACTA---G---ACCTGAAC-------CCACGACA
Onmy    CTACT--GC---GAAAGCAGCGTTTAACTAT-------GCCTGACC-------CCACGACA
Sasa    CTACT--AC---GAAAGCAGCTTTAATTAT--------ACCTGAAC-------CCACGACA
Cola    CCACT--GC---GAAAGCAGCTTTAATC-------ACCACCTGAAC-------CCACGACA
Dita    CCACCC-AC---GAAAGTGGCTCTACCTTC--------ACCTGACC-------CCACGACA
Gogr    CTATC--AC---GAAAGTAGCTTCATCCCC--------ACCTGAAC-------CCATGACA
Chsl    CCCTT--AC---GAAAGTAGCTTTAC---------GCCACCCGAAC-------CCACGATA
Atja    CACAT--AC---GAAGGTAGCTTTATTAA-TA-------TCTGAAC-------CCACGGCA
Iido    CCTAT--AC---GAAGGTAGCTTTAT--TAAT------ATCTGAAC-------CCACGACA
Auja    CCGCT--AC---GAAAGTGACTTTAAACC---------CCCTGAAC-------CCACGACA
Chag    CCGCT--AC---GAAAGTTGCTTTAATAA---CCC---TCCCGACC-------CCACCACA
Hami    CAACA--AC---GAAAGTGACTTTA----------AATATCCGACC-------CCACGACA
Saun    CAGCA--GC---GAAAGTGACTTTAA----AC------CTCCGACC-------CCACGACA
Nema    CCCCC--AC---GAAAGTGGCTTTAACCCCA----C---CCCGAAC-------CCACGACA
Disp    CCACC--AC---GAAAGTGACTTTA--ATCT-CG-----CCCGAAC-------CCACGAAA
Myaf    CGACC--AC---GAAAGTGGCTTTAACCTA--------ACTCGAAC-------CCACGAAA
Lagu    TCTCC--AC---GAAGGTGGCTTTAAAC---------CAGCTGAAC-------CCACGAAA
Trtr    CTCCC--AC---GAAAGTGGCTTTA--ATTA-------TACTGAAC-------CCACGAGA
Zucr    CTCCT--AC---GAAAGTGGCTTTA-ATAT--------AACTGAAC-------CCACGAAA
Pxja    CCGCC--AC---GAAAGTGACTCTAACCAT------ACCCCTGAAT-------CCACGAAA
Pxlo    CCACC--AC---GAAAGTGACTCTAACCACA------CCCCTGAAT-------CCACGAAA
Pctr    CCAAC--AC---GAAAGTAGCCCTACCC--------CTCCCCGAAC-------CCACGAAA
Apsa    CTATC--AC---GAAAGTAGCTCTACATA---------CCCCGAAC-------CCACGAAA
Cabe    CCTTT--AC---GAAAGTAGCTTTA----------ATCATCTGAAC-------CCACGAAA
Bzze    CCACC--AC---GAAAGTGACTTTACACA---------CCCTGAAC-------CCACGAAA
Siim    CAACT--AC---GAAAGTGACTTTAAATA---------CACTGAGC-------CCACGAAA
Ctru    CCACC--AC---TAAAGTGACTTTAAT-------ACCATCCTGAAC-------CCACGAAA
Dpbr    CCACC--AC---CAAGGTGGCTTTAAAC-------CCCACCTGAAC-------CCACGAAA
Caki    CACTC--AC---GAAAGTAACCCTAGTACC---------CCTGACC-------CCACGAAA
Phja    CAGTCA-AC---GAAAGTAATTCTACC---A------ACCCTGACA-------CCACGAAA
Brsp    TCAT---AC---GAAAGTTATCCTAATATT------CTTAAT-----------CCACGAGA
Gamo    CAATC--AC---GAAAGTTGCCCTAAA--AC-------CTCCGATT-------CCACGAAA
Lolo    CAATC--AC---GAAAGTGGCCCTACAAA--------TCTCTGATT-------CCACGAAA
Batr    CCATT--AT---ACAAGCAACTCCATTC----------ACTTGACC-------CCACGAAA
Prmy    CTCTCC-AC----AATGAAACTTTATC--------CC--CTTGACC-------CCACGTAA
Loli    CACTC--AC---GTAAGTAACTTTATT---------AA-ACTGAAC-------CCACGAAA
Loam    CAAAT--AC---GAAAGTAACTTTA--TA------AC--TCTGAAT-------CCACGAAA
Chab    CAACT--AC---GAAAGTGGCTTTACA---------AGATCTGACC-------CCACGAAA
Chto    CAACT--AC---GAAAGTGGCTTTACA---------AAATCTGACC-------CCACGAAA
Majo    CCACC--AC---GAAAGTGGCTTTAT--ATAAC------CCCGAAC-------CCACGAGA
Hlst    CGACT--AC---GAAAGTGACTTTACAAT---------ACCTGATC-------CCACGAAA
Clpe    CAACT--AC---GAAAGTGGCTCTACGG---------AGTCTGACC-------CCACGAAG
Mlmr    CGACT--AC---GAAAGTGACTTTAAAAAT---------TCCGACC-------CCACGAAA
Crcr    CAACT--AC---GAAAGTAACTTTAAC---T-----ATATCCGACT-------CCACGAAA
Muce    CAACC--AC---GAAAGTAACTTTAATTA--------TATCCGACT-------CCACGAAA
Bege    CCTCA--AC---GAAAGTGACTTTAAC---------CTTTTTGACC-------CCACGAAA
Mela    CCCCC--AC---GAAAGTGGCTTTAACC--C----A--TTCTGACT-------CCACGAAA
Hats    CCCCT--AC---GAAAGTGGCTTTAA---------CCCTTCCGAAC-------CCACGAAA
Orla    CAAGA--AC---GAAAGTAACTTTA----------ACTTTATGAAT-------CCACGAAA
```

```
Cosa    CC-T---AC---GAAAGTAGCTTTAATCCA---------CCCGACT-------CCACGAAA
Exsp    CCCCT--AC---GAAAGTGGCTTTAACT---------CTCCTGACC-------CCACGAAA
Depa    CTATT--AC---GAAAGTAACTTTAATACA---------TCTGACT-------CCACGAAA
Rima    CCCCC--AC---GAAAGTGACTTTAATAC---------CCTTGAAC-------CCACGAAA
Fuol    CAAAC--GC---GAAAGTAACTTTAAATA---------TCCTGACC-------CCACGAAA
Gmaf    CAACT--AC---GAAAGTGGCCTTAATT-------TCCCCTTGACC-------CCACGAAA
Xeei    CAATA--AC---GAAAGTAGCTTTAA-ATA-----T---TTTGACT-------CCACGAAA
Pros    CCATC--AC---GAAAATGGCTTTACCCCCT----CCCCCCTGACC-------CCACGAAA
Scmi    CCCCT--AC---GAAAGTAACTTTAAAC---C----CCGCCTGACC-------CCACGAAA
Rolo    CCACC--AC---GAAAGTGGCTTTACCCCCACC------CCCGAAC-------CCACGAAA
Cere    CCACC--AC---GAAAGTGGCTTTACCCC------CCCCCCTGACC-------CCACAAAA
Daga    CCACC--AC---GAAAGTGGCTTTAATA---------AGCCCGAAT-------CCACGAAA
Anco    CCACC--AC---GAAAGTGGCTTTACTCT---------TCCTGAAC-------CCACGAAA
Dmve    CCACC--AC---GAAAGTGGCTTTAATC-C--------CCCTGACT-------CCACGAAA
Dmar    CCACC--AC---GAAAGTGGCTTTAATT---------CTCCTGACC-------CCACGAAA
Anka    CCACC--AC---GAAAGTGGCTTTACCCA---------TCCTGAAC-------CCACGAAA
Moja    CCACC--AC---GAAAGTGGCTTTAA--------CTTTTCCTGAAC-------CCACGAAA
Hoja    CCACT--AC---GAAAGTGGCTTTACCTT---------TCCTGAAC-------CCACGAAA
Bede    CCATC--AC---GAAAGTGGCTTTACCCCCC----A---CCCGAAC-------CCACGAAA
Besp    CCATC--AC---GAAAGTGGCTTTACCCCCCA-------CCCGAAC-------CCACGAAA
Mysp    CCACT--AC---GAAAGTGGCTTTACTAG---------CCCTGAAC-------CCACGAAA
Osja    CTACC--AC---GAAAGTGGCTTTATTAA---------CCCTGAAC-------CCACGAAA
Sgro    CCACC--AC---GAAAGTGGCTTTAC---CAG------CCCTGAAC-------CCACGAAA
Pzpa    CACCC--AC---TAAAGTGATTCCACCAATC--------CCCGACC-------CCACGAAA
Zeja    CCCCT--AC---AAAAGTGGCCCCAACCTAC--------TCTGACC-------CCACGAAA
Znne    CTCCT--AC---AAAAGTGACCCTAAAC-------CCT-ACTGAAC-------CCACGAAA
Zefa    CCCCC--AC---AAAAGTGGCCCTAAACCC-------CCACTGACC-------CCACGAAA
Acni    CCCCC--AC---AAAAGTGGCCCCAA---------CTAACCCGACC-------CCACGAAA
Ncrh    CCCCC--AC---AAAAGTGGCCCCA--ACTT----A---CCCGACC-------CCACGAAA
Agca    CAACT--AC---GAAAGTGGCTTTACATAT---------TCTGACC-------CCACGAAA
Hydy    CAACT--AC---GAAGGTGGCTTTACCC---------AACCTGAAC-------CCACGAAA
Gsac    CAACC--AC---GAAGGTGGCTTTA--------TTTA-ACCTGAAC-------CCACGAAA
Pevo    CATCC--AC---GAAAGTGACTTTACA---------TTACCTGAAC-------CCACGAAA
Hiku    CATTT--AC---GAAAGTGACTTTA---CAA-------AACTGAAC-------CCACGAAA
Inpa    CAGCT--AC---GAAAGTGGCTTTAAAC-----------ACTTGACC------CCACGAAA
Auch    CAGAA--AC---GAAAGTAGTTTTATACA----------TCTTGATT------CCACGAAG
Fico    CCACC--AC---GAAAGTGGCTTTATGTGA---------TCTGAAC-------CCACGAAA
Macs    CCACC--AC---GAAAGTGGCTTTATT---------TAACCTGAAC-------CCACGAAA
Moal    CTATC--AC---GAAAGTAGCTTTATATAA---------TCTGACC-------CCACGAAA
Syma    CACTC--AC---GAAAGTAACCTTAC----------AAATCTGACT-------CCACGAAA
Mafr    CAATC--AC---GAAGGTAACTTTACATAAT--------TCTGACC-------CCACGAAA
Dcpe    CCACC--AC---GAAAGTGACTTTA--CACA-------ACCTGAAC-------CCACGAAA
Dcti    CCACC--AC---GAAAGTGACTTTATGTA---------GCCTGAAC-------CCACGAAA
Hehi    CAACC--AC---GAAGGTAGCTTTACA-------A--CCCCTGACC-------CCACGAAA
Stam    CAATC--AC---GAAAGTAGCTTTAT---------TTCTCCTGAAT-------CCACGAGA
Hogi    CAATA--AC---GAAAGTAGCCTTAGTA---------TAACTGAAC-------CCACGAAA
Erzo    CAACC--AC---GAAGGTGACTTTATTTAT---------ACTGAAC-------CCACGAAA
Hxot    CAACC--AC---GAAAGTGGCTTTATTT-------GA--TCTGAAC-------CCACGAAA
Core    CTCCC--AC---GAAAGTCGCTTTATCTAG---------TCTGAAC-------CCACGAAA
Apve    CAACC--AC---GAAAGTAACTTTAT--TTA-------ATCTGACC-------CCACGAAA
Latj    CAACC--AC---GAAAGTGACTTTA-TAAC--------CCCTGACT-------CCACGAAA
Laja    CAATC--AC---GAAAGTGGCTTTACT---------CCTTCCGAAT-------CCACGAAA
```

```
Syja   CAATC--AC---GAAAGTAGCTTTAAATC----------ACATGACC------CCACGAAA
Epme   CATCC--AC---GAAGGTGGCTTTAT-TAC--------ACCTGAAC-------CCACGAAA
Grse   CAACT--AC---GAAAGTGGCTTTAACT-------A---CCTGAAT-------CCACGAAA
Clja   CAATT--AC---GAAAGTGGCTTTAT-TAC-----A---TTTGACC-------CCACGAAA
Ogcy   CCACC--AC---GAAAGTGACTTTAAC-CCA-------ATCTGAAT-------CCACGAAA
Plna   CAACT--AC---GAAAGTGGCTTTAATAGT---------TCTGACC-------CCACGAAA
Lema   CAATC--AC---GAAAGTGGCTTTA-CTTT--------ACCTGACC-------CCACGAAA
Etzo   CAACC--AC---GAAAGTGGCTTTA----------CAACGCTGAAC-------CCACGAAA
Apse   CTGCC--AC---GAAAGTGACTTTA-CACTC--------TTTGAAC-------CCACGAAA
Epde   CAATC--AC---GAAAGTGGCTTTACCTC----------CCTGAAT-------CCACGAAA
Slja   CAACC--AC---GAAAGTAGCTTTAACA--------AA-CCTGACT-------CCACGAAA
Bsja   CAATC--AC---GAAAGTGGCTTTATATA---------ATTTGAAC-------CCACGTAA
Ecna   CAACT--AC---GAAAGTGGCTTTAT---AAAA------CCTGAAT-------CCACGAAA
Cohi   CAACT--AC---GAAAGTGGCTTTAAAAC---A------CCTGAAC-------CCACGAAA
Caar   CGACT--AC---GAAAGTGGCTTTAC-AT-CA-------CCTGAAC-------CCACGAAA
Came   CAACT--AC---GAAAGTGGCTTTAT---------CCAACCTGAAC-------CCACGAAA
Mema   CAACC--AC---GAAAGTGGCTTTATC---------ACCCCTGAAC-------CCACGAAA
Lenu   CAACA--AC---GAAAGTGACCTTAGCCCA--------ACCTGAAC-------CCACGAAA
Plma   CCACC--AC---GAAAGTGGCTTTAT-AAT--------CCCTGATC-------CCACGAAA
Emst   CAATC--AC---GAAAGTGACTTTATGCC---------ATCTGAAC-------CCACGAAA
Ptti   CAACT--AC---GAAAGTGGCTTTACAACA---------TCTGAAT-------CCACGAAA
Losu   CAATT--AC---GAAAGTAGCTTTATA-------ATA--ACTGACT-------CCACGAAA
Geoy   CAACT--AC---GAAAGTGACTTTAC-AAA---------CCTGACT-------CCACGAAA
Dipi   CAACT--AC---GAAAGTGGCTTTA--TAAT----A---TCTGAAC-------CCACGAAA
Pama   CAATC--AC---GAAAGTAGCTTTATATTT---------TCTGACC-------CCACGAAA
Leob   CAGCT--AC---GAAAGTGACTTTATC---------TTATCTGACC-------CCACAAAA
Neba   CACTT--AC---GAAGGTAGCTTTACAAT------A--TTTTGAAC-------CCACGAAA
Pdpl   CAACA--AC---GAAAGTGGCTTTATTCA---------CCCTGAAC-------CCACGAAA
Nimi   CGCCC--AC---GAAAGTGGCTTTA--CAA--------TCTTGAAC-------CCACGAAA
Uptr   CCACC--AC---GAAAGTGGCTTTATATAAC--------CCCGAAC-------CCACGAGA
Pesc   CACCC--AC---GAAAGTGGCTTTA---------CATACTCTGAAT-------CCACGAAA
Baar   CAATC--AC---GAAAGTGGCTTTATATT----------CCTGACC-------CCACGAAA
Moar   CAACT--AC---GAAAGTGGCTTTA---------CTCCATCTGAAT-------CCACGAAA
Toja   CGACC--AC---GAAAGTGGCTTTA---------CTCCCCCTGAAC-------CCACGAAA
Chau   CAACT--AC---GAAAGTGGCTCTAT-CATA--------TCTGATT-------CCACGAAA
Chse   CACTC--AC---GAAAGTAGCTTTATTTCA---------TCTGACC-------CCACGAAA
Enar   CAATT--AC---GAAAGTAGCTTTATTTCA---------GCCGAAC-------CCACGAAA
Hpty   CAATC--AC---GAAAGTGGCTTTAAACA---CCCCCCCCTGAAC-------CCACGAAA
Nana   CAACT--AC---GAAAGTGACCTTACCACA---------AATGAAC-------CCACGAAA
Mcst   CAATC--AC---GAAAGTGGCTTTAA-----C----ACTTCCGAAC-------CCACGAAA
Rhox   CAATT--AC---GAAAGTAGCTTTA---------CACAACCTGAAC-------CCACGAAA
Opfa   CAATC--AC---GAAAGTGGCTTTAT---------ACCAACTGAAC-------CCACGAAA
Paar   CAACA--AC---GAAAGTGGCTTTACCCCC------CCCCCCGATT-------CCACGAAA
Gozo   CAACC--AC---GAAAGTGGCCTTATACT---------CCCTGAAC-------CCACGAAA
Ackr   CTATC--AC---GAAAGTAGCTTTACAA---------GATCTGAAC-------CCACGAAA
Elev   CAACC--AC---GAAAGTGGCTTTAT---------ATTCCCTGAAC-------CCACGAAA
Trdu   CCACT--AC---GAAAGTGGCTTTATATC----------CCCGACC-------CCACGAAA
Amoc   CCATT--AC---GAAAGTAGCTTTACCCAT---------CCTGACC-------CCACGAAA
Hame   CAACT--AC---GAAAGTGACTTTAAA--AT--------CCTGACC-------CCACGAAA
Chso   CAACC--AC---GAAAGTGGCTCTAATT--ACT------CCTGACA-------CCACGAAA
Lyto   CAGCC--AC---GAAGGTAGCTTTA-TT---------TGTCTGAAC-------CCACGAAA
Encr   CAACC--AC---GAAGGTGGCTTTAT---TTAG------TCTGAAC-------CCACGAAA
```

```
Bvar    CAAAC--AC---GAAAGTAGCTTTAAAATA---------CCCGAAC-------CCACGAAA
Noco    CAATC--AC---GAAAGTAGCTTTACTAAA--------CCCTGAAC-------CCACGAAA
Chsp    CAACA--AC---GAAAGTAGCTTTATT---------ATAATTGAAG-------CCACGAAA
Arja    CAACC--AC---GAAAGTGGCTTTATTTA--------A-TCTGAAC-------CCACGAAA
Pase    CATCT--AC---GAAAGTGGCTTTAATA----------CTCCCCAT-------CCACGAAA
Trel    CCCCT--AC---GAAAGTAGCTCTAAGAAC------A--TTTTAAC-------CCACGAGA
Lifa    CAATA--AC---GAAAGTAGCTTTATCTA---------GTCTGATG-------CCACGAAA
Acur    CATCA--AC---GAAAGTGGCTTTATAC----------CTCTGATT-------CCACGAAA
Ampe    CAACT--AC---GAAAGTGGCTTTAC-AAC--------CCCTGAAC-------CCACGAAA
Urja    CGATC--AC---GAAAGTAGCTTTAA---------CACCCCTGAGC-------CCACGAAA
Enet    CCTCC--AC---GAAAGTGGCTTTAA---TAA------TTTTGACC-------CCACGAAA
Ptbr    CTACT--AC---GAAAGTGACTTTAAACA----------ATTGAAC-------CCACGAAA
Safa    CCGTC--AC---GAAAGTAACCTTATA----------ATATTGAAC-------CCACGAAA
Icae    CCACC--AC---GAAAGTGGCTTTA---------CAACTCCTGACT-------CCACGAAA
Asmi    CACCC--AC---GAAAGTAGCTTTATAAA----------ATAAATA-------CCACTAAA
Foal    CATAA--AC---GAAAGTAGCCCTAATCT---------ATTTGAAC-------CCACGAAA
Drze    CTAAC--AC---GAAAGTAGCTTTAA--AA---------TACTGAT-------CCACGAAA
Rhas    CCCCT--AC---GAAAGTGGCTTTAAAC----------CTCTGAAC-------CCACGAAA
Elac    CTTCC--AC---GAAAGTGGCTTTA--------AAAA-CTCTGAAC-------CCACGAAA
Kugu    CATTA--AC---GAAGGTAGCTTTA----------TTCAATTCGAC-------CCACGAAA
Plor    CAACC--AC---GAAAGTAGCTTTACA-CC---------CCTGAAC-------CCACGAAA
Sgun    CAATC--AC---GAAAGTGGCTTTAC---------TTATTCTGAAC-------CCACGAAA
Zaco    CGATC--AC---GAAAGTGGCTTTACA--------TT-AACTGAAC-------CCACGAAA
Zbfl    CAACC--AC---GAAAGTGGCTTTATACTT---------ACTGAAC-------CCACGAAA
Spba    CAATC--AC---GAAAGTGGCTTTA--ATCCACA-----CCTGACT-------CCACGAAA
Game    CCACC--AC---GAAAGTGGCTTTATAATT---------CCTGACC-------CCACGAAA
Thth    CCACC--AC---GAAAGTGGCTTTATGAA---------CCCTGACC-------CCACGAAA
Xigl    CAACT--AC---GAAAGTGGCTTTA--CAT-------CACCTGAAC-------CCACGAAA
Hyja    CCACC--AC---GAAAGTGGCTTTATG---------ACTCCTGATT-------CCACGAAA
Psan    CAACC--AC---GAAAGTGGCTTTA---------TGACTCCTGAAT-------CCACGAAA
Cupa    CCACC--AC---GAAAGTGGCTTTAT---------GACCCCTGACT-------CCACGAAA
Mpch    CAACT--AC---GAAAGTGACCTTA-TA--T----G---TTTGAAC-------CCACGAAA
Char    CAACT--AC---GAAAGTGGCTTTACAA---------CACCCGAAC-------CCACGAAA
Pser    CAACT--AC---GAAAGTGGCTTTACT--AC-------ACCTGAAC-------CCACGAAA
Prol    CAATT--AC---GAAAGTAGCCTTATTCAC---------CCTGAAC-------CCACGAAA
Plbi    CAACT--AC---GAAAGTAGCTCTACCTAT---------CCTGAAC-------CCACGAAA
Calu    CACCC--AC---GAAAGTGACTTTAATA---------CCCCTGACT-------CCACGAAA
Papa    CAGCT--AC---GAAAGTGGCCTTA-TAA--------AGTCCGAAC-------CCACGAAA
Sufr    CAACG--AC---GAAAGTGGCCCTATCCA--------ATTCCCAAC-------CCACGAAA
Stci    CAACT--AC---GAAGGTGGCTTTATA---------ACTTCTGAAC-------CCACGAAA
Taru    CAATA--AC---GAAAGTAGCCTCACTA----------ACTCGAAC-------CCACGAAA
Rala    CAACA--AC---GAAAGTGGCTTTA---------AATTTTCCGAAC-------CCACGAAA
                      *                                        *
```

```
             25         26              26'     25'               27
Scca  GTTGGG-CCCCAAACTAGGATTA-GATACCCTACTATGCCCT-ACCATAAACTTAGACAAT
Muma  GTTGAG-ACCCAAACTAGGATTA-GATACCCTACTATGCCCA-ACCATAAACTTAGACAAT
Erca  ACCAAG-ATACAAACTGGGATTAAGATACCCCACTATGCTTG-GAATTAAACTAAGACGGT
Pose  ACCAAG-ACACAAACTGGGATTA-GATACCCCACTATGCTTG-GAACTAAACTAAGGCGGC
Actr  ACTGAG-ACACAAACTGGGATTA-GATACCCCACTATGCTCA-GTCATAAACCTTGGTAAT
Scal  ACTGAG-GCACAAACTGGGATTA-GATACCCCACTATGCTCA-GTCATAAACTTTTGGCCA
Posp  ACCGAG-ACACAAACTGGGATTA-GATACCCCACTATGCTCA-GTCATAAACTTTGGTGAT
Atsp  GCTAAG-ACACAAACTGGGATTA-GATACCCCACTATGCTTAAGCCCTAAACCTAAATAAT
Leoc  GCTAAG-ACACAAACTGGGATTA-GATACCCCACTATGCTTA-GCCCTAAACCTAAATAAT
Amca  GCTAAG-ACACAAACTGGGATTA-GATACCCCACTATGCTTA-GCTATAAACCTTGATAAT
Osbi  GCTGGG-AAACAAACTGGGATTA-GATACCCCACTATGCCCA-GTCATAAACTTTAATGGT
Pabu  GCTGAG-AAACAAACTGGGATTA-GATACCCCACTATGCTCA-GCCGTAAACTTTGATAGT
Hial  GCTGGG-AAACAAACTGGGATTA-GATACCCCACTATGCCCA-GCCATAAACCCTGATAAT
Elha  GCCAAG-ACACAAACTGGGATTA-GATACCCCACTATGCTTG-GCCGTAAACCTTGATGAT
Mlcy  GCCAGG-ACACAAACTGGGATTA-GATACCCCACTATGCCTG-GCCTTAAACCCTGATAGC
Algl  GCTAAG-GCACAAACTGGGATTA-GATACCCCACTATGCTTA-GCCTTAAACCTTGATAAT
Ptgi  ACTGAG-ACACAAACTGGGATTA-GATACCCCACTATGCTCA-GCCTTAAACCTTGATGAT
Alaf  GCTAAG-AAACAAACTGGGATTA-GATACCCCACTATGCTTA-GCCTCAAACTTTGGTAAT
Nock  GCTAAG-GAACAAACTGGGATTA-GATACCCCACTATGCTTA-GCCTCAAACTTTGGTAAT
Anja  GTTGAG-AAACAAACTGGGATTA-GATACCCCACTATGCTCA-ACCTTAAACAACGATGAC
Gyki  GCCAAG-ATACAAACTGGGATTA-GATACCCCACTATGCTTG-GCCTTAAACTAAGGCGAT
Syka  GTTAAG-GAACAAACTGGGATTA-GATACCCCACTATGCTTG-ACCGTAAACAACGATGAC
Opma  GTTAAG-AAACAAACTGGGATTA-GATACCCCACTATGCTTA-ACCGTAAACAACGACGGA
Comy  GTAAAG-AAACAAACTGGGATTT-GATACCCCACTATGCTTA-ACTGTAAACAAAGATGAT
Sasp  GTTAAG-AAACAAACCGGGATTA-GATACCCCACTATGCTTA-ACCGTAAACAATGATGGT
Eupe  GTTAAG-AAACAAACTGGGATTA-GATACCCCACTATGCTTA-ACCTTAAACAACGATGCA
Enja  GCTGGG-ACACAAACTGGGATTA-GATACCCCACTATGCCTA-GCCGTAAACTTTAATGCT
Same  GCTGGG-ATACAAACTGGGATTA-GATACCCCACTATGCCCT-GCCGTAAACTTAGATATT
Chch  ACTGAG-AAACAAACTGGGATTA-GATACCCCACTATGCTCA-GTCGTAAACTTAGGTATT
Grgr  GCCGAG-GAACAAACTGGGATTA-GATACCCCACTATGCTCA-GCCGTAAACTTAGATGCT
Caau  GCTGAG-AAACAAACTGGGATTA-GATACCCCACTATGCTCA-GCCGTAAACTTAGACATC
Cyca  GCTGAG-AAACAAACTGGGATTA-GATACCCCACTATGCTCA-GCCGTAAACTTAGACATC
Dare  GCTAAG-AAACAAACTGGGATTA-GATACCCCACTATGCTTA-GCCATAAACAAAGACATT
Cost  GCTGAG-GAACAAACTGGGATTA-GATACCCCACTATGCTCA-GCCATAAACCTAGACGTT
Leec  ACTGAG-AAACAAACTGGGATTA-GATACCCCACTATGCTCA-GCCGTAAACCTAGACGTT
Crla  GCTGAG-AAACAAACTGGGATTA-GATACCCCACTATGCTCA-GCTATAAACCTAGACGTT
Clmc  GCTAAG-AAACAAACTGGGATTA-GATACCCCACTATGCTTA-GCCCTAAACTTAGATGCT
Phin  GCTAAG-AAACAAACTGGGATTA-GATACCCCACTATGCTTA-GCCTTAAACTCTGATAAA
Icpu  GCTAAG-AAACAAACTGGGATTA-GATACCCCACTATGCTTA-GCCCTAAACCCAGATGTC
Psto  GCTAAG-AAACAAACTGGGATTA-GATACCCCACTATGCTTA-GCTATAAACCTAGATGTC
Cora  GCTAAG-AAACAAACTGGGATTA-GATACCCCACTATGCTTA-GCCTTAAACCAAGATATT
Eisp  GCTAAG-AAACAAACTGGGATTA-GATACCCCACTATGCTTA-GCCCTAAACTTAGATGTC
Apal  GCTAAG-AAACAAACTGGGATTA-GATACCCCACTATGCTTA-GCCCTAAACCTAGATGTA
Eslu  GCTATG-GTACAAACTGGGATTA-GATACCCCACTATGCATA-GCCATAAATTTTGATAAA
Dape  GCTGTG-AAACAAACTGGGATTA-GATACCCCACTATGCACA-GCCATAAATTTTGAGGAG
Glse  GCTATG-ACACAAACTGGGATTA-GATACCCCACTATGCTTA-GCCGTAAACTTTGATTTT
Naar  GCTATG-ATACAAACTGGGATTA-GATACCCCACTATGCTTA-GCTATAAACCTTGATAGA
Baoc  GCTATG-ATACAAACTGGGATTA-GATACCCCACTATGCTTA-GCTATAAACCTTGATAGA
Opso  ACTATG-CCACAAACTGGGATTA-GATACCCCACTATGCTTA-GCCGTAAACTTTGATAAA
Alte  GCTGAG-ATACAAACTGGGATTA-GATACCCCACTATGCTCA-GCCGTAAACTTAGATGTC
Plap  GCTGAG-ACACAAACTGGGATTA-GATACCCCACTATGCTCA-GCCGTAAACTTAGATGTC
```

```
Plal    ACTAAG-ATACAAACTGGGATTA-GATACCCCACTATGCTTA-GCCGTAAACTTTGATATT
Sami    GCTAAG-GTACAAACCGGGATTA-GATACCCCGCTATGCTTA-GCCATAAACTTTGATATT
Rere    ACTAAG-ATACAAACTGGGATTA-GATACCCCACTATGCTTA-GCCGTAAACTTTGATGTT
Gama    GCCACG-AGACAAACTGGGATTA-GATACCCCACTATGCGTG-GCCGTAAAACTTGACAGC
Onmy    GCTAAG-AAACAAACTGGGATTA-GATACCCCACTATGCCTA-GCCGTAAACCTTGATAGA
Sasa    GCTACG-ACACAAACTGGGATTA-GATACCCCACTATGCCTA-GCCGTAAACTTTGATGGA
Cola    GCTATG-ATACAAACTGGGATTA-GATACCCCACTATGCCTA-GCCGTAAACTTTGATGGA
Dita    ACTAGG-GAACAAACTGGGATTA-GATACCCCACTATGCTTA-GCCGTAAACACTGATACG
Gogr    TCTAAG-AGACAAACTGGGATTA-GATACCCCACTATGCTTA-GTCACAAACACTGACACC
Chsl    GCTAGG-AGACAAACTGGGATTA-GTAAACCCACTATGCCTA-GTCGTAAACCTCGACACT
Atja    GCTAAG-ACACAAACTGGGATTA-GAGACCCCACTATGCTTA-GCCGTAAACTTTGATAGA
Iido    GCTAAG-ACACAAACTGGGATTA-GAGACCCCACTATGCATA-GCCGTAAACTTTGATAGA
Auja    GCCTTG-ACACAAACTGGGATTA-GATACCCCACTATGCCTG-GCCGTAAACTTTGATAAT
Chag    GCCAGG-AAACAAACTGGGATTA-GATACCCCACTATGCCTG-GCCATAAACCTCGATTTT
Hami    GCTAAG-GCACAAACTGGGATTA-GATACCCCATTATGCTTAAGCTATAAACTTTGATAGA
Saun    GCTATG-ACACAAACTGGGATTA-GATACCCCATTATGCCTA-GCCATAAACTTTGATAGA
Nema    GCTTTG-ACACAAACTGGGATTA-GATACCCCACTATGCCCA-GCCGTAAACTTTGATAGT
Disp    GCTAGG-ACACAAACTGGGATTA-GATACCCCACTATGCCTA-GCCGTAAACATTGATAGA
Myaf    GCTGAG-GGACAAACCGGGATTA-GATACCCCGCTATGCTCA-GCCGTAAACATCAATAAG
Lagu    GCTATA-GAACAAACTGGGATTA-GATACCCCACTATGTATA-GCTATAAACCTTGATAGG
Trtr    GCTATA-GAACAAACTGGGATTA-GATACCCCACTATGTGTA-GCCGTAAACTCTGATAGC
Zucr    GCTATA-GAACAAACTGGGATTA-GATACCCCACTATGTATA-GCTGTAAACTTCGATAGC
Pxja    GCTATA-AAACAAACTGGGATTA-GATACCCCACTATGTATA-GCCTTAAACCTTGATAGA
Pxlo    GCTATA-AAACAAACTGGGATTA-GATACCCCACTATGTATA-GCCTTAAACCTTGATAGA
Pctr    GCCACA-AAACAAACTGGGATTA-GATACCCCACTATGTGTG-GCCCTAAACTTTGATAGA
Apsa    TCCATA-TAACAAACTGGGATTA-GATACCCCACTATGTATG-GCTATAAACAAAGATGGT
Cabe    GCTAAG-GTACAAACTGGGATTA-GATACCCCACTATGCTTA-GCCGTAAACGCAGGTGTG
Bzze    GCTAAG-ACACAAACTGGGATTA-GATACCCCACTATGCTTA-GCCCTAAATATAGATAGT
Siim    ACTAAG-GCACAAACTGGGATTA-GATACCCCACTATGCTTG-GTTGTAAACAAAGATAGC
Ctru    GCTAAG-ACACAAACTGGGATTA-GATACCCCACTATGCTTA-GTCCTAAACCTTGATAGA
Dpbr    GCTAAG-ACACAAACTGGGATTA-GATACCCCACTATGCTTA-GTCCTAAACATTGATAGA
Caki    GCCATA-ACACAAACTGGGATTA-GATACCCCACTATGTATG-GCTGTTAAAATTGATGGT
Phja    ACCATA-AAACAAACCGGGATTA-GATACCCCATTATGTATG-GTCGTTAACACTGATGGC
Brsp    GCCAAG-ACCTAAACAGGGATTA-GATACCCCTTTATACTTG-GCCCTAAACAATAGCATT
Gamo    GCCATA-AAACAAACTGGGATTA-GATACCCCACTATGTATG-GTCGTTAACATTGATGGT
Lolo    GCCATA-AAACAAACTGGGATTA-GATACCCCACTATGTATG-GTTGTTAACATTGATGGT
Batr    ACTAAA-GAACAAACTGGGATTA-GACACCCCACTATGTCTG-ATCGTAAACTTT------
Prmy    GCTATT-ATACAAACTAGGATTA-GACACCCTACTATGCTTA-GTCGTAAACTTT------
Loli    GCTGCG-GCACAAACTGGGATTA-GAAACCCCACTATGCCCA-GCCCTAAACATTGGTAGC
Loam    GCTACG-GCACAAACTGGGATTA-GAAACCCCACTATGCCTA-GCCCTAAACATTGGCAAC
Chab    GCTAGG-GCACAAACTGGGATTA-GATACCCCACTATGCCTA-GCCGTAAACAATGACAAT
Chto    GCTGGG-GCACAAACTGGGATTA-GATACCCCACTATGCCTA-GCCGTAAACAATGACAAT
Majo    GCCAGG-AAACAAACTGGGATTA-GATACCCCACTATGCCTG-GCTGTAAACATTGATAAA
Hlst    GCTAGG-GTACAAACTGAGATTA-GATACCCCACTATGCCTA-GCCTCAAACAATGATAAT
Clpe    GCTGGG-GCACAAACTGGGATTA-GATACCCCACTATGCCCT-GCCGTAAACCAAGATAAT
Mlmr    GCTGAG-ACCCAAACTGGGATTA-GAGACCCCACTATGCCCA-GCCCTAAACTATGATAAT
Crcr    GCTGTG-AAACAAACTGGGATTA-GATACCCCACTATGCCCA-GCCCTAAACTTTGATAAT
Muce    GCTGTG-AAACAAACTGGGATTA-GATACCCCACTATGCCCA-GCCCTAAACTTTGATAAT
Bege    GCTGTG-AAACAAACTGGGATTA-GAAACCCCACTATGCCCA-GCCCTAAACTTTGATAAC
Mela    GCTGAG-AAACAAACTGGGATTA-GATACCCCACTATGCTCA-GCCCTAAACTTTAATAGT
Hats    GTTGGG-AAACAAACTGGGATTA-GATACCCCACTATGCCCA-ACCCTAAACTTTGATAGA
Orla    ACTATG-AAACAAACTGGGATTA-GATACCCCACTATGCATA-GCTGTAAACTTTGATGAA
```

Cosa	GCTGTG-AAACAAACTGGGATTA-GATACCCCACTATGCACA-GCCCTAAACTCTGATAAA
Exsp	GCTGTG-ACACAAACTGGGATTA-GATACCCCACTATGCTCA-GCCGTAAACATAGATAAA
Depa	GCTGTG-AAACAAACTGGGATTA-GATACCCCACTATGCACA-GCCCTAAACTATGACAGT
Rima	GCTGTG-ACACAAACTGGGATTA-GATACCCCACTATGCACA-GCCGTAAACCTTAGCTAA
Fuol	GCTGTG-AAACAAACTGGGATTA-GATACCCCATTATGCACA-GCCATAAACTTTGATAAA
Gmaf	GCTGCG-AAACAAACTGGGATTA-GATACCCCACTATGCACA-GCCATAAACTTTGATAGA
Xeei	GCTGTG-AAACAAACTGGGATTA-GATACCCCACTATGCACA-GCCGTAAACTTTGGTAGC
Pros	GCTACG-CAACAAACTGGGATTA-GATACCCCACTATGCCTA-GCCCTAAACATTGATGAT
Scmi	GCTACG-AAACAAACTGGGATTA-GATACCCCACTATGCCTA-GCCCTAAACATTGATGGT
Rolo	GCTATG-TCACAAACTGGGATTA-GATACCCCACTATGCCTA-GCCCTAAACATTGATAAC
Cere	GCTACG-GTCAAAACTGGGATTA-GATACCCCACTATGCCTA-GCCCTAAACATTGATAGC
Daga	GCTATG-ATACAAACTGGGATTA-GATACCCCACTATGCCTA-GCCCTAAACATTGATAGC
Anco	GCTACG-ACACAAACTGGGATTA-GATACCCCACTATGCTTA-GCCTTAAACATTGATAGT
Dmve	GCTACG-GCACAAACTGGGATTA-GATACCTCACTATGCCTA-GCCCTAAACAATGACAGC
Dmar	GCTATG-GCACAAACTGGGATTA-GATACCTCACTATGCCTA-GCCCTAAACACTGATAGC
Anka	GCTAAG-ACACAAACTGGGATTA-GATACCCCACTATGCCTA-GCCCTAAACATTGATAGT
Moja	GCTATG-ACACAAACTGGGATTA-GATACCCCACTATGCCTA-GCCCTAAACATTGACAGT
Hoja	GCTATG-ACACAAACTGGGATTA-GATACCCCACTATGCTTA-GCCCTAAACATTGATAGT
Bede	GCTACG-TAACAAACTGGGATTA-GATACCCCACTATGCCTA-GCCCTAAACATAGATAGT
Besp	GCTACG-TAACAAACTGGGATTA-GATACCCCACTATGCCTA-GCCCTAAACATAGATAGT
Mysp	GCTATG-CCACAAACTGGGATTA-GATACCCCACTATGCTTA-GCCATAAACATCGATAGT
Osja	GCTATG-TCACAAACTGGGATTA-GATACCCCACTATGCTTA-GCCATAAACATCGATAGT
Sgro	GCTATG-AAACAAACTGGGATTA-GATACCCCACTATGCTTA-GCCATAAACATAGATAGC
Pzpa	GCTACA-AAACAAACTGGGATTA-GATACCCCACTATGTGTA-GCTGTAAACTTTGATGGT
Zeja	GATGCA-AAACAAACTGAGATTA-GATACCCCACTATGTGCA-TCCGTTAACCTTGATGGT
Znne	GCTACA-AAACAAACTGGGATTA-GATACCCCACTATGTGTA-GCCGTAAACCTTGATGGT
Zefa	GCTACA-AAACAAACTGGGATTA-GATACCCCACTATGTGTA-GCCGTAAACCTTGATGGT
Acni	GCTGCA-AAACAAACTGGGATTA-GATACCCCACTATGTGCA-GCCGTAAACCTTGATGGA
Ncrh	GCTGCA-AAACAAACTGGGATTA-GATACCCCACTATGTGCA-GCCGTAAACCTTGATGGA
Agca	GCTATG-ACACAAACTGGGATTA-GATACCCCACTATGCCTA-GCCATAAACATCGACAAC
Hydy	GCTAAG-TTACAAACTGGGATTA-GATACCCCACTATGCTTA-GCCCTAAACATTGGTAGT
Gsac	GCTACG-GCACAAACTGGGATTA-GATACCCCACTATGCCTA-GCCCTAAACATTGATAGA
Pevo	GCCATGAAAACAAACTGGGATTA-GATACCCCACTATGCTTG-GCCGTAAACAATAATAAG
Hiku	ACTATG-AAACAAACTGGGATTA-GATACCCCACTATGCATA-GTCTTAAACAAAAGTATT
Inpa	GCTAAG-GCACAAACTGGGATTA-GATACCCCACTATGCTTA-GCCCTAAACATAAATAGC
Auch	GCTAAG-AAACAAACTGGGACTA-GATACCCCACTATGCTTA-GCTGTAAACAATGATGGG
Fico	GCTAGG-AAACAAACTGGGATTA-GATACCCCACTATGCCTA-GCCCTAAACATCGACAGG
Macs	GCTATG-TCACAAACTGGGATTA-GATACCCCACTATGCTTA-GCCGTAAACATTGATAGA
Moal	GCTATG-GTACAAACTGAGATTA-GATACCTCACTATGCATA-GCCATAAACAATAATAAA
Syma	GCTATG-ACACAAACTGGGATTA-GATACCCCACTATGCTTA-GCTATAAACCTTAATAGT
Mafr	GCTAGG-CCACAAACTGGGATTA-GATACCCCACTATGCCCA-GCCTTAAACATTAATAGC
Dcpe	GCTAGG-GAACAAACTGGGATTC-GATACCCCACTATGCCTA-GCCGTAAACTAAGATAGG
Dcti	GCTAGG-GAACAAACTGGGATTC-GATACCCCACTATGCCTA-GCCGTAAACAAAGATAGA
Hehi	GCTCTG-GCACAAACTGGGATTA-GATACCCCACTATGCCTA-GCCCTAAACCTTGGCAAT
Stam	GCTATG-GTACAAACTGGGATTA-GATACCCCACTATGCCTA-GCCCTAAACATTGATAGT
Hogi	GCCACG-GTACAAACTGGGATTA-GAGACCCCACTATGCTTG-GCCGTAAACTTTGATAGT
Erzo	GCTAAG-ACACAAACTGGGATTA-GATACCCCACTATGCCTA-GCCCTAAACCTTGATAGT
Hxot	GCTACG-GAACAAACTGGGATTA-GATACCCCACTATGCCTA-GCCGTAAACATTGATAGT
Core	GCTAAG-GAACAAACTGGGATTA-GATACCCCACTATGCCTA-GCCTTAAACATTGATAGT
Apve	GCTACG-GCACAAACTGGGATTA-GATACCCCACTATGCCTA-GCCCTAAACATTGATAGT
Latj	GCTGAG-AAACAAACTGGGATTA-GATACCCCACTATGCTTA-GCCCTAAACATTGACTTC
Laja	ACTAGG-ACACAAACTGGGATTA-GATACCCCACTATGCCTA-GCTGTAAACATTGATAGT

```
Syja    GCTAGG-GCACAAACTGGGATTA-GATACCCCACTATGCCTA-GCTGTAAACATAGATAGC
Epme    GCCAAG-ATACAAACTGGGATTA-GATACCCCACTATGCTTC-GCCCTAAACATTGATAGC
Grse    GCTAAG-GCACAAACTGGGATTA-GATACCCCACTATGCTTT-GCCCTAAACATTGATAGT
Clja    GCTAAG-GTACAAACTGGGATTA-GATACCCCACTATGCTTA-GCCGTAAACACTGGTAGC
Ogcy    GCTGAG-GCACAAACTGGGATTT-GATACCCCACTATGCTCA-GCCATAAACTTTGATAGT
Plna    GTTGGG-AAACAAACTGGGATTA-GATACCCCACTATGCCCA-ACCCTAAACCTTGATAGA
Lema    GCTACG-ACACAAACTGGGATTA-GATACCCCACTATGCCTA-GCCTTAAACATTGGCAAC
Etzo    GCTATG-ATACAAACTGGGATTA-GATACCCCACTATGCCTA-GCCGTAAACATTGGTAAC
Apse    GCTAAG-AAACAAACTGGGATTA-GATACCCCACTATGCCTA-GCCCTAAAATTAGATAGA
Epde    GCTATG-ACACAAACTGGGATTA-GATACCCCACTATGCCTA-GCCCTAAACATTGATAGT
Slja    GCTAAG-GCACAAACTGGGATTA-GATACCCCACTATGCTTA-GCCCTAAACATTGACAAC
Bsja    GCTAGG-ACACAAACTGGGATTA-GATACCCCATTATGCCTA-GCCGTCAACATCGACAGC
Ecna    GCTAAG-AAACAAACTGGGATTA-GATACCCCACTATGCTTA-GCCCTAAACATTGATTGT
Cohi    GCTAAG-AAACAAACTGGGATTA-GATACCCCACTATGCTTA-GCCCTAAACATTGACTGT
Caar    GCTAAG-AAACAAACTGGGATTA-GATACCCCACTATGCTTA-GCCTTAAACATTGATTAT
Came    GCTAAG-AAACAAACTGGGATTA-GATACCCCACTATGCTTA-GCCTTAAACATTGATTGT
Mema    GCTAAG-AAACAAACTGGGATCA-GATACCCCACTATGCTTA-GCCCTAAACATCGATCAC
Lenu    GCTAGG-AAACAAACTGGGATTA-GATACCCCACTATGCCTA-GCCGTAAACTAAAACAGT
Plma    GCTATG-GAACAAACTGGGATTA-GATACCCCACTATGCTTA-GCCGTAAACATTGATAGA
Emst    GCTATG-ACACAAACTGGGATTA-GATACCCCACTATGCCTA-GCCCTAAACATCGACAGT
Ptti    GCTATG-ATACAAACTGGGATTA-GATACCCCACTATGCCTA-GCCATAAACATTGATAGT
Losu    GCCAGG-AAACAAACTGGGATTA-GATACCCCACTATGCCTG-GTCCTAAAAATAGATAAC
Geoy    GCTAAG-GCACAAACTGGGATTA-GATACCCCACTATGCTTA-GCCCTAAACATAGATAGA
Dipi    GCTATG-GTACAAACTGGGATTA-GATACCCCATTATGCCTA-GCCCTAAATATTGATAGA
Pama    GCTAAG-ATACAAACTGGGATTA-GATACCCCACTATGCTTA-GCCGTAAACATTGACAGT
Leob    GCTAGG-GCACAAACTGGGATTA-GATACCCCACTATGCCTA-GCCATAAACATTGACAGT
Neba    ACTGGG-AAACAAACTGGGATTA-GAGACCCCACTATGCCTA-GTCTTAAACACAAACGGT
Pdpl    GCTAAG-GAACAAACTGGGATTA-GATACCCCACTATGCTTA-GCCCTAAACAATGACTGT
Nimi    GCTAAG-GTACAAACTGGGATTA-GATACCCCACTATGCTTA-GCCCTAAACATTGACAAC
Uptr    GCCAGG-GAACAAACTGGGATTA-GATACCCCACTATGCCTG-GCTGTAAACATTGATAAA
Pesc    ACTATG-ATACAAACTGGGATTA-GATACCCCACTATGCGTA-GACGTAAAAATTGATAGT
Baar    GCCATG-AAACAAAATGGGATTA-GATACCCCACTATGCTTG-GCCCTAAAAATTGATAGC
Moar    GCTATG-ACACAAACTGGGATTA-GATACCCCACTATGCCTA-GCCCTAAACATTGACAGT
Toja    GCTGGG-GAACAAACTGGGATTA-GATACCCCACTATGCCCA-GCCCTAAACATTGAACAA
Chau    GCTAGG-GCACAAACTGGGATTA-GATACCCCACTATGCCTA-GCCCTAAACATTGACAGT
Chse    GCTAGG-GCACAAACTGGGATTA-GATACCCCACTATGCCTA-GCCATAAACATAGACAGT
Enar    GCTAAG-GTACAAACTGGGATTA-GATACCCCACTATGCCTA-GCCTTAAACATCGATAGT
Hpty    GCTATG-ATACAAACTGGGATTA-GATACCCCACTATGCCTA-GCCCTAAACATCGATAGC
Nana    GCTAGG-ACACAAACTGGGATTA-GATACCCCACTATGCCTA-GCCATAAACATTGATAGT
Mcst    GCTATG-ACACAAACTGGGATTA-GATACCCCACTATGCCTA-GCCTTAAACCTTGATAGT
Rhox    GCTATG-GCACAAACTGGGATTA-GATACCCCACTATGCTTA-GCCCTAAACATCAATAGT
Opfa    GCTATG-ACACAAACTGGGATTA-GATACCCCACTATGCTTA-GCCATAAACATTGATAGT
Paar    GTTATG-GTACAAACTGGGATTA-AATACCCCATTATGCCTA-ACCCTAAACATTGATAGT
Gozo    GCTAGG-ATACAAACTGGGATTA-GATACCCCACTATGCCTA-GCCCTAAACATCGATAGT
Ackr    GCCAGG-GAACAAACTGGGATTA-GATACCCCACTATGCCTG-GCCTTAAACTTTGACAGC
Elev    GCTATG-ATACAAACTGGGATTA-GATACCCCACTATGCTTA-GCCCTAAACATTGATGGG
Trdu    GCTGCG-AAACAAACTGGGATTA-GATACCCCACTATGCCCA-GCCCTAAACCTTGATAGT
Amoc    GCTGTG-ACACAAACTGGGATTA-GATACCCCACTATGCTCA-GCCCTAAAATTTGATAGG
Hame    GCTATG-GTACAAACTGGGATTA-GATACCCCACTATGCTTA-GCCCTAAACTTAGATATT
Chso    GCTATG-GCACAAACTGGGATTA-GATACCCCACTATGCGTA-GTCGTAAACC-TAACAGC
Lyto    GCTACG-GCACAAACTGGGATTA-GATACCCCACTATGCCTA-GCCCTAAACATTGATAGT
Encr    GCTACG-GCACAAACTGGGATTA-GATACCCCACTATGCCTA-GCCCTAAACATTGATAGT
```

```
Bvar  GCTAGG-AGACAAACTGGGATTA-GATACCCCACTATGCCTA-GCCCTAAACATTTATAGT
Noco  GCTAGG-GAACAAACTGGGATTA-GAGACCCCACTATGCCTA-GCCGTAAACATTGATAGA
Chsp  GCCAAG-AAACAAACTGGGATTA-GATACCCCACTATGCCTA-GCCCTAAACATTGGTGAT
Arja  GCTACG-GTACAAACTGGGATTAAGATACCCCACTATGCCTA-GCCCTAAACATTGATAGT
Pase  GCTTTG-GCACAAACTGGGATTA-GATACCCCACTATGCTTA-GCCTTAAACCTTGATGAC
Trel  GCTAGG-GCACAAACTGGGATTA-GATACCCCACTATGCCTA-GCCATAAACATAGATGAT
Lifa  ACTGGG-AGACAAACTGGGATTA-GATACCCCACTATGCCTA-GTTGTAAAAGTTGATGGG
Acur  GCTAAG-AAACAAACTGGGATTA-GATACCCCACTATGCTTA-GCCTCAAACATTGATAGT
Ampe  GCTATG-ACACAAACTGGGATTA-GATACCCCACTATGCTTA-GCCCTAAACATCGATAGC
Urja  GCTAGG-ACACAAACTGGGATTA-GATACCCCACTATGCCTA-GCCTTAAATAAAGGCAGC
Enet  GCTGAG-AAACAAACTGGGATTA-GATACCCCACTATGCCCA-GCTGTAAACATTGATAGC
Ptbr  GCTATG-GCACAAACTGGGATTA-GATACCCCACTATGCTTA-GCCCTAAACTTTTATACT
Safa  GCTAGG-ACACAAACTGGGATTA-GATACCCCACTATGCCTA-GCACTAAACCTAGATGAA
Icae  GCTATG-TCACAAACTGGGATTA-GATACCCCACTATGCTTA-GCCGTAAACATTGATAGA
Asmi  GCTAAG-ACACAAACTGGGATTA-GATACCCCACTATGCTTA-GCCCTAAACCTAGACAAC
Foal  GCTAGG-AAACAAACTGGGATTA-GATACCCCACTATGCCTA-ACTATAAACCCCGATAAT
Drze  GCTGAG-AAACAAACTGGGATTA-GATACCCCACTATGCTCA-GTCGTAAACTATGGTAGT
Rhas  GCCAGG-ATACAAACTGGGATTA-GATACCCCACTATGCCTG-GCCCTAAACAAAGGCAGC
Elac  GCTAGG-ATACAAACTGGGATTA-GATACCCCACTATGCCTA-GCCATAAACAAAAGTGGC
Kugu  GCTAGG-ACACAAACTGGGATTA-GATACCCCACTATGCCTA-GCCATAAACATAGATAGT
Plor  GCTAAG-ATACAAACTGGGATTA-GATACCCCACTATGCTTA-GCCCTAAACATTGGTAAC
Sgun  GCTAGG-GCACAAACTGGGATTA-GATACCCCACTATGCCTA-GCCCTAAACATTGACAAT
Zaco  GCTAGG-GTACAAACTGGGATTA-GGTACCCCACTATGCCTA-GCCCTAAACATCGGTAGC
Zbfl  GCTAAG-ACACAAACTGGGATTA-GATACCCCACTATGCCTA-GCCTTAAACATTGATGAC
Spba  GCTGAG-AAACAAACTGGGATTA-GATACCCCACTATGCTCA-GTCCTAAACATCGATCGT
Game  GCTATG-ATACAAACTGGGATTA-GATACCCCACTATGCCTA-GCCATAAACATTGATAGA
Thth  GCTATG-ACACAAACTGGGATTA-GATACCCCACTATGCCTA-GCCGTAAACATTGATAGA
Xigl  GCTAAG-AAACAAACTGGGATTA-GATACCCCACTATGCTTA-GCCCTAAACATCGATTAT
Hyja  GCTAAG-GAACAAACTGGGATTA-GATACCCCACTATGCTTA-GCCGTAAACATTGATAGA
Psan  GCTAAG-AAACAAACTGGGATTA-GATACCCCACTATGCTTA-GCCGTAAACATTGATAGT
Cupa  GCTATG-GCACAAACTGGGATTA-GATACCCCACTATGCTTA-GCCGTAAACATTGATAGA
Mpch  GCCAAG-ACACAAACTGGGATTA-GATACCCCACTATGCTTG-GCCCTAAACATTGATAGA
Char  GCTAAG-AAACAAACTGGGATTA-GATACCCCACTATGCTTA-GCCTTAAACATTGATAGC
Pser  GCTAAG-AAACAAACTGGGATTA-GATACCCCACTATGCTTA-GCCTTAAACATTGATTGT
Prol  GCTAAG-GAACAAACTGGGATTA-GATACCCCACTATGCTTA-GCCCTAAACATCGATTGT
Plbi  GCTAAG-GAACAAACTGGGATTA-GATACCCCACTATGCTTA-GCCCTAAACATCGATTGC
Calu  GCTGGG-ACACAAACTGGGATTA-GATACCCCACTATGCTCA-GCCCTAAACATAGACTGA
Papa  GCTAAG-AAACAAACTGGGATTA-GAGACCCCACTATGCTTA-GCCCTAAATTGATTAAAT
Sufr  GCTAAG-GCACAAACTGGGATTA-GATACCCCACTATGCCTA-GCCCTAAACATTGATGGT
Stci  GCTAAG-AAACAAACTGGGATTA-GATACCCCACTATGCTTA-GCCCTAAACATTGATAGG
Taru  GCTAGG-ACACAAACTGGGATTA-GATACCCCACTATGCCTA-CCCCTAAACAC-GATATG
Rala  GCTACG-GTACAAACTGGGATTA-GATACCCCACTATGCCTA-GCCGTAAACATTGATAGT
                 ***     **       * *     ***            **
```

```
        ¦      HVR    C'       ¦ 27'  18' 28          29
Scca    A-C----------CCTACTA---TATTGTCGCCAGAG-TACTACAAGC--GCT-------
Muma    AC-----------TTCACCA---TATTGTTCGCCAGAG-TACTACAAGC--GCT-------
Erca    ATA----------AATACCC---AGCCGTCCGCCCGGT-TACTACGAGC--GTA-------
Pose    A-T---------AAATACTA---AGCCGCCCGCCAGGT-TACTACGAGC--GCA-------
Actr    ---------AAATTACACA----TATTACCCGCCAGGG-TACTACGAGC--GCT-------
Scal    --------TAAATCACACA----TATTGCCCGCCAGGG-TACTACGAGC--GCT-------
Posp    A-A--------ATTACACA----TATCACCCGCCAGGG-TACTACGAGC--GCT-------
Atsp    AAG----------ATACCGAA--TATTATCCGCCAGGG-GACTACAAAG--CGCT------
Leoc    AGA----------AT-ACAAA--TATTATCCGCCAGGG-GACTACAAGC--GCC-------
Amca    AAA----------AT-ACAAA--TATTATCCGCCAGGG-GACTACGAGC--GTT-------
Osbi    AAC-----------ACACTCA--TACCACTCGCCAGGG-AACTACGAGC--GCA-------
Pabu    AAAA-----------TACAAT--TACTACCCGCCAGAG-TACTACAAGCG-AAT-------
Hial    A-A----------GGTACAAA--TATTATCCGCCAGGG-GACTACAAGC--ATT-------
Elha    ----------AAA-ACACAAA--TATCATCCGCCAGGG-GACTACAAGC--GTC-------
Mlcy    AAG----------A-TACAAA--TACTATCCGCCCGGG-AACTACAAGC--GCT-------
Algl    A-T----------GATACAGA--TATTATCCGCCAGGG-GACTACAAGC--GCT-------
Ptgi    -----------AAAGTACAGA--TATCATCCGCCAGGG-GACTACAAGC--GCC-------
Alaf    AAA-----------GTACAAA--TATTACCCGCCAGGG-GACTACAAGC--ACT-------
Nock    AA-----------TATACACA--TATTACCCGCCAGGG-GACTACAAGC--ACT-------
Anja    A-A----------AATACAAA--TATCATCCGCCAGGG-GACTACGAGC--GTT-------
Gyki    AAA----------CCTACAAA--CTTCGCCCGCCAGGG-AACTACGAGC--CCT-------
Syka    ATAA-----------TACAAA--TATCATCCGCCAGGG-GACTACGAGC--GCC-------
Opma    AA-----------TTTACAAA--TACCGCCCGCCAGGG-AATTACGAAC--ACT-------
Comy    AAT----------AC-ACAAA--CATCATCCGCCAGGG-GATTACGAGC--AA--------
Sasp    AT----------ATACACA-C--CACTATCCGCCAGGG-AATAACGAGC--ACCC------
Eupe    ----------------TA------TGCATCCGCCAGGG-TACTACAAGC--TC--------
Enja    A-A----------TGTACAACT--AGCATCCGCCAGGG-AACTACGAGC--ACC-------
Same    T-----------CAATACAATA--GATATCCGCCAGGG-GACTACGAGC--GCT-------
Chch    CC--------TTTACCAC----C-AATACCCGCCTGGG-AACTACGAGC--GCT-------
Grgr    AGC-----------CTACCCT---CGCATCCGCCTGGG-GACTACGAGC--AA--------
Caau    CA-----------ACTACAAT--AGATGTCCGCCAGGG-TACTACGAGC--ATT-------
Cyca    C-----------AGCTACAATA--GATGTCCGCCAGGG-TACTACGAGC--ATT-------
Dare    T---A--------ATCACAAC---AATGTTCGCCCGGA-CACTACAAGC--AT--------
Cost    ACT----------TC-ACAAA---AACGTCCGCCAGGG-TACTACGAGC--ATT-------
Leec    A-T-------TCCACAACA-----AACGTCCGCCAGGG-TACTACGAGC--GTT-------
Crla    TA--------ATCACAAC---A--AACGTCCGCCAGGG-TACTACGAGC--GTC-------
Clmc    TTA----------CATACAAA---CGCATCCGCCTGGG-AACTACGAGC--GCT-------
Phin    AAA----------CT-ACAA-T--TTTATCCGCCAGGG-GACTACGAGC--GCT-------
Icpu    -CT--------CTTACACA-----CACATCCGCCCGGG-TACTACGAGC--A-C-------
Psto    CC-----------CTTACATA---AACATCCGCCCGGG-TACTACGAGC--A-C-------
Cora    ATTT-----------TACAAA---ACTATCCGCCAGGG-TACTACGAGC--A-T-------
Eisp    ----------------TA------AACATTCGCCCGAG-TACTACGAGC--ACC-------
Apal    CTA-------TTCACCACA-----AACATCCGCCCGAT-TACTACGAGC--CCC-------
Eslu    A-A----------TATACAAT---TTTATCCGCCAGGG-AACTACAAGC--ATC-------
Dape    AC-----------TATACAAT---TTCCCCCGCCCGGG-AACTACAAGC--ACT-------
Glse    AA---------ACAATACAATT--TATATCCGCCAGGG-AACTACAAGC--GCC-------
Naar    -AT-------AATACAACT-----AATATCCGCCAGGG-GACTACAAGC--GCC-------
Baoc    A-A-------TATACAACT-----GATATCCGCCAGGG-TACTACAAGC--GCC-------
Opso    AA--------AATACAAC-T----AATATCCGCCAGGG-GACTACAAGC--GCC-------
Alte    AT--------CCCACAATC-----GACATCCGCCAGGG-AACTACGAGC--G-CT------
Plap    C-T-------TCCACAACC-----GACATCCGCCCGGG-GACTACGAGC--ACT-------
```

```
Plal    AA----------CTCACCCC--T-AATATCCGCCAGGG-GACTACAAGC--GTT-------
Sami    AAT----------TTACCCTT---AATGTCCGCCAGGG-GACTACAAGC--GC--------
Rere    A-A-------TATACAACT-----AGCATCCGCCAGGG-AACTACAAGC--GTC-------
Gama    ATAA-------GTACAATT-----TCTGTCCGCCAGGG-GACTACAAGC--ATC-------
Onmy    A-A-------TATACAATT-----GATATCCGCCAGGG-AACTACAAGC--GCC-------
Sasa    AAC--------ATACAACT-----GACATCCGCCAGGG-GACTATAAGC--GCC-------
Cola    AA--------CATACAAC----T-AACATCCGCCAGGG-AACTACAAGC--GCC-------
Dita    AA--------AATACAAC-T----AGTATCCGCCCGGG-AACTACAAGC--GCC-------
Gogr    ----------------CCA-----CCACTCCGCCAGGG-GACTACGAGC--GTA-------
Chsl    CA--------TATACAGC----G-AGCGTCCGCCAGGG-AATTACAAGC--ACC-------
Atja    A-----------AATTGAA-----TCTATCCGCCCGGG-AACTACAAGC--G-CT------
Iido    A-A---------AATTAAA-----TCTATCCGCCTGGG-AACTACAAGC--GCT-------
Auja    A-A--------AGTACATC-T---TCTATCCGCCTGGG-AACTACTAGC--GCA-------
Chag    G-------ATACATACACAC----ATTATCCGCCAGGG-TACTACGAGC--GCT-------
Hami    -AA-------CTTACAACA-----CCTATCCGCCTGGG-AACTACGAGC--GCT-------
Saun    A-T-------CTTACAACC-----TCTATCCGCCTGGG-AACTACGAGC--GCT-------
Nema    ACCC--------CACAATT-----TCTATCCGCCCGGG-AACTACAAGC--ACC-------
Disp    AAC------GCCACAAAT------TCTGTCCGCCCGGG-TACTACAAGC--ATT-------
Myaf    CAT-------CTCACAACA-----CTTATTCGCCTGGG-AACTACAAGC--ATT-------
Lagu    C-C--------CCTACACG-----TCTATCCGCCCGGG-GATTACGAGC--ATC-------
Trtr    TCAT--------TTACATA-----GCTATCCGCCAGGG-TACTACGAGC--GTT-------
Zucr    TCA--------AGTACATTA----ACTATCCGCCCGGG-TACTACGAGC--GTT-------
Pxja    AT--------AACACAAC-C----CCTATCCGCCTGGG-AACTACGAGC--ACC-------
Pxlo    A-T-------AACACAACC-----CCTATCCGCCCGGG-AACTACGAGC--ACC-------
Pctr    A-T--------AACACACCC----CCTATCCGCCAGGG-GACTACAAGC--ACC-------
Apsa    GTAT---------CACACA-----ACCATTCGCCCGGG-AACTACAAGC--ACT-------
Cabe    AGTA---------ATACCC-C---CCTACTCGCCAGGG-AACTACTAGC--GG--------
Bzze    TAT-----------TTACCAA---ACTGTCCGCCAGGG-GACTACGAGC--AC--------
Siim    GTA-----------ATACTA-C--ACTATCCGCCCGGG-TACTACGAGC--ACC-------
Ctru    CCT--------A-CACACC-C---ACTATCCGCCCGGG-TACTACGAGC--ACC-------
Dpbr    CC---------CCCACACC-C---ACTATCCGCCCGGG-TACTACAAGC--ACC-------
Caki    AC---------TTCACTCGA----ACCATCCGCCCGGG-AACTACGAGC--CTC-------
Phja    TTG----------CATACCTA---GTCATCCGCCTGGG-GACTACGAGC--GA--------
Brsp    CAC--------TA-ACACAG----AATGCTCGCCCGGG-TATTACGAGC--TCTTAT----
Gamo    T-T--------TATACCCAA----ACCATCCGCCTGGG-AACTACGAGC--AAT-------
Lolo    TT----------TATACTTA--A-ACCATCCGCCTGGG-TACTACGAGC--ATT-------
Batr    ----------------GTAC----------CGCCAGGA-GACTACGAGC--CAC-------
Prmy    ----------------GACTC---------CGCCTGGA-TACTACGAGT--TAA-------
Loli    -CC--------TGTACACC--C--GCTGCCCGCCCGGA-TACTACGAGC--ATA-------
Loam    ACA---------AAACACCC----GTTGCCCGCCAGGG-CACTACGAGC--AT--------
Chab    -GT---------ACCACCCC-CC-ATTGTCCGCCCGGG-AACTAAAAGC--ACG-------
Chto    -GT---------ACCACCCC-CC-ATTGTCCGCCCGGG-GACTAAAAGC--ACG-------
Majo    -AT--------ACCACAAAT----ATTATCCGCCCGGG-TACTACGAGC--A-CT------
Hlst    -AA--------CTTACACAC----ATTATCCGCCAGGG-GATTACAAGC--A-A-------
Clpe    A-A---------GTTACCAC----ATTATCCGCCCGGG-AACTACGAGC--GCA-------
Mlmr    GA----------ACAACCCC--C-GTTATCCGCCCGGG-AACTACAACC--GCA-------
Crcr    TTAT---------TACACCC----ATTATCCGCCTGGG-AACTACGAGC--TTC-------
Muce    TTA---------TCACACCC----ATTATCCGCCTGGG-AACTACGAGC--TT--------
Bege    -AA--------AATACATT--C--GCTATCCGCCTGGG-AACTACGAGC--ATC-------
Mela    ACTA----------TTAT-TG---ACTATTCGCCTGGG-AACTACGAGC--GCT-------
Hats    TG---------AATACATC-C---CCTATCCGCCCGGG-AACTACGAGC--ACT-------
Orla    ---------CTGTTACAT-T----ATCATTCGCCAGGG-TACTACGAGC--ACC-------
```

```
Cosa     AA---------CCTACATT-T---ATTATCCGCCAGGG-TACTACGAGC--TCT-------
Exsp     AC---------TTTACATA-A---CTTATCCGCCAGGG-TACTACGAGC--ATT-------
Depa     ATT----------TACACCA----ACTGTTCGCCAGGG-TACTACAAGC--TCT-------
Rima     C-C--------CCTACATAA----TTAGCCCGCCAGAA-GATTACAAGC--ATA-------
Fuol     AAA----------TTTACAAT---ATTATCCGCCCGGG-TACTACGAGC--ATA-------
Gmaf     AA-----------ACTACAAA-T-TCTATCCGCCCGGG-AACTACGAGC--ATA-------
Xeei     AC-----------CTAACAAT-T-ACTACCCGCCCGGG-TACTACGAGC--ATT-------
Pros     ----------------AC--C---CCCATCCGCCCGAA-AACTACAAGC--ATT-------
Scmi     A-A---------GCCCACCCC---GCCATCCGCCCGGA-AACT-CGAGC--ACT-------
Rolo     AT-----------TTTACCCC---GTTATCCGCCCGGG-AACTACGAGC--A-TT------
Cere     AGT-----------CCACTGA---GCTGTCCGCCTGGG-AACTACGAGC--AG--------
Daga     --------ATC-CTACACC-----GCTATCCGCCCGGG-AACTACAAGC--ATT-------
Anco     ATT---------ATACACCC----ACTATCCGCCCGGG-AACTACGAGC--AC--------
Dmve     GTC---------ACACGTACC---GCTGTCCGCCCGGG-TACTACGAGC--ACT-------
Dmar     GT---------CGCACATA--CC-GCTATCCGCCCGGG-TACTACGAGC--ACT-------
Anka     AC---------TTTACACC-C---ACTATCCGCCAGGG-AACTACGAGC--ACC-------
Moja     AT---------CCTACACC-C---ACTGTCCGCCCGGG-AACTACGAGC--ACC-------
Hoja     TCA---------ATACACCC----ACTATCCGCCCGGG-AACTACGAGC--AC--------
Bede     AACA-----------TACTCT---ACTATCCGCCCGGG-AACTACGAGC--ATT-------
Besp     A-----------ACATACTCC---ACTATCCGCCCGGG-AACTACGAGC--A-TT------
Mysp     AT----------TATCACCT--C-ACTATCCGCCAGGG-AACTACGAGC--AAT-------
Osja     -----------ACACCACCCC---ACTATCCGCCCGGG-AACTACGAGC--AAT-------
Sgro     AT-----------CTCACCCC---GCTATCCGCCCGGG-AACTACGAGC--A-AT------
Pzpa     TACT-------AATACCTAC----CCCATCCGCCTGGG-AACTACAAGC--GAC-------
Zeja     A----------ATTATACATC---CCCATCCGCCTGGG-AACTACGAGC--ATA-------
Znne     AA---------CCTGTACC--CT-TCCATCCGCCTGGG-AACTACGAGC--GCT-------
Zefa     A-A--------CCCTTACCCCC-CCCCATCCGCCTGGG-AACTACGAGC--ACC-------
Acni     AGC----------AATACCCTT--CCCATCCGCCTGGG-AACTACGAGC--ACC-------
Ncrh     AGCA----------ATACCCTT--CCCATCCGCCTGGG-AACTACGAGC--ACC-------
Agca     A-A--------ACTACACCT----GTTGTCCGCCTGGG-AACTACGAGC--ACT-------
Hydy     T-C-------TTTACAATC-----ACTACCCGCCTGGG-AACTACGAGC--ATC-------
Gsac     A--------TTTTACAACC-----ACTATCCGCCCGGG-GACTACAAGC--ATC-------
Pevo     AGA-------------ACTA----CTTATTCGCCAGGG-TACTACGAGC--ACT-------
Hiku     TTA----------ATTACAT----CATACTCGCCAGGG-TACTACGAGC--TTT-------
Inpa     AC-----------CCCACCTCC--GCTATTCGCCCGGG-AACTACTAGC--CCC-------
Auch     A-C--------CTCACACTCAA--CCCATCCGCCCGGA-TACTACGAGC--GCA-------
Fico     C-T-------CGTACTTCG-----CCTGTCCGCCAGGG-TACTACAAGC--ACC-------
Macs     ----------ACC-CTACCCC---ACCATCCGCCCGGG-AACTACGAGC--ATC-------
Moal     C-C--------CTAATATC-----CCTATTCGCCTGGG-AATTACGAGC--CTC-------
Syma     T---------CTATACAT-A----ACTATTCGCCAGGG-TACTACGAGC--ACT-------
Mafr     A-C--------TCTACATTA----GCTATTCGCCAGGG-AACTACGAGC--CCC-------
Dcpe     ACCA---------TACACA-----TCTATCCGCCTGAA-AACTACGAGC--GCA-------
Dcti     ACC--------CT-ACACA-----TCTATCCGCCCGAA-TACTACGAGC--GCA-------
Hehi     AT---------ATCACATAC----CCTGCCCGCCTGGG-AACTACGAGC--ATC-------
Stam     ACT---------CTACATCC----ACTATCCGCCTGGG-AACTACGAGC--AT--------
Hogi     --------ACTACTACACCC----ACTATCCGCCCGGG-GACTACGAGC--ATT-------
Erzo     GA---------CCTACGCC-C---ACTATCCGCCTGGG-GACTACGAGC--ATC-------
Hxot     AT---------CCTACACC-C---ACTATCCGCCCGGG-AACTACGAGC--AAA-------
Core     ACT--------AT-ACACCC----ACTATCCGCCCGGG-TACTACGAGC--AAT-------
Apve     A-C--------TCTACACCC----ACTATCCGCCAGGG-AACTACGAGC--AAC-------
Latj     G---------CACTACACCCG-A-TATTTCCGCCCGGG-TATTACGAGC--A-CT------
Laja     GTA-------AT-ACGACC-----ACTATCCGCCCGGG-AACTACGAGC--ACT-------
```

```
Syja   CAT---------CGCTACCCT---GCTATCGCCTGGG-GACTACGAGC--GTA-------
Epme   TT--------TACACAAC-C----CCTATCGCCTGGA-AACTACGAGC--AAC-------
Grse   GCAC---------TACACCC----ACTATCGCCCGGG-AACTACGAGC--ACC-------
Clja   TG-----------ACAACAAA-C-ACTGCCGCCTGGA-CACTACGAGC--ACC-------
Ogcy   A-A--------ACTACACCC----ACTATCGCCTGGG-AACTACGAGC--ATC-------
Plna   CAT----------TG-GTT-----TCTATCGCCTGGG-TACTACTGGC--GTT-------
Lema   -AC--------TTTACACCT----GCTGCCGCCAGGA-AACTACGAGC--ATT-------
Etzo   AC---------AATACACC--C--ATTACCGCCTGGG-GACTACAAGC--ATC-------
Apse   ACA-------CTACCTACA-----CCTATCGCCCGGG-TACTACGAGC--ACC-------
Epde   A-C--------ACTACACCC----ACTATCGCCCGGG-TACTACGAGC--ACC-------
Slja   T-T--------TACACACCC----GTTGTCGCCTGGG-AACTACGAGC--AGC-------
Bsja   ACC---------TTACACCC----GCTGTCGCCCGGG-AACTACGAGC--AA--------
Ecna   -TT--------AATACATCA----AACATCGCCCGGG-AATTACGAAC--A-TC------
Cohi   ---------TTATTACATA-----AACATCGCCTGGG-AACTACGAAC--ATT-------
Caar   T---------TACCACATTT----AACATCGCCTGGG-AATTACGAAC--A-CT------
Came   TT---------ACTACATC--A--AACATCGCCTGGG-AATTACGAAC--ATT-------
Mema   --------ACC-CTACACTT----AACATCGCCCGGG-AACTACGAAC--ACC-------
Lenu   TCAT----------TAACTCA---ACTGTTCGCCTGGG-GACTACGAGC--GCT-------
Plma   AT---------AATACACC-T---TCTATCGCCTGGG-TACTACGAGC--ATT-------
Emst   AAA--------CT-ACACCT----GCTGTCGCCTGGG-AACTACGAGC--ATC-------
Ptti   A-A--------CCTACACCT----ACTATCGCCCGGG-TACTACGAGC--ATC-------
Losu   AC---------AGTACATC-T---GTTATCGCCGGGG-TACTACGAGC--ACT-------
Geoy   AT-----------GCCACCC--T-TCTATCGCCCGGG-TACTACGAGC--ATT-------
Dipi   GAAC---------TACACCC----ACTATCGCCCGGG-TACTACGAGC--ATC-------
Pama   TG---------AATACATT---T-TCTGTCGCCCGGG-TACTACGAGC--ATT-------
Leob   GT---------TATACACC---C-ACTGTCGCCTGGG-AACTACGAGC--GCA-------
Neba   TGGC---------GCCACGTC---CCCGTTCGCCTGGT-AACTACGAGCTCACCGAAATTC
Pdpl   --------GGCACTACACCC----CACATCGCCCGGG-TATTACGAAC--GTC-------
Nimi   A-C--------CATACACCT----GTTGTCGCCTGGG-AACTACGAGC--ATT-------
Uptr   AT---------ACCACAAA--T--ATTATCGCCCGGG-TACTACGAGC--ACT-------
Pesc   CC---------ACTACAC--CC--GCTATCGCCTGGG-GACTACGAGC--ACT-------
Baar   A---------CACTACACCC----GCTATCGCCTGGG-AATTACGTGC--AACC------
Moar   A---------ACATACACC--T--ACTGTCGCCTGGG-TACTACGAGC--ATC-------
Toja   AC--------ACCACAAC-T----AACTTCGCCCGGG-AATTACGAAC--ATA-------
Chau   T-A--------ATTACACCC----ACTGTCGCCTGGG-AACTACGAGC--ACC-------
Chse   TCA----------CTTACCCT---ACTGTCGCCAGGG-AATTACGAGC--A-TT------
Enar   A-T----------AATACAACT--ATTATCGCCTGGG-AACTACGAGC--ATC-------
Hpty   ---------GCACTACACCT----GCTATCGCCTGGG-AACTACGAGC--ACC-------
Nana   ACA---------CCACATA-A---ACTATCGCCCGGG-CACTACGAGC--GCT-------
Mcst   A-C--------TGCACACCT----ACTATCGCCTGGG-AACTACGAGC--ACC-------
Rhox   A---------CAATACACCC----ACTATTCGCCCGGG-AACTACGAGC--ACT-------
Opfa   ATA--------CTACAACT-----ACCATCGCCCGGG-AACTACGAGC--AC--------
Paar   AAG--------TC-ACACCC----ACTATCGCCTGGG-TACTACAAGC--ACC-------
Gozo   GCAA---------TACATCC----ACTATCGCCTGGG-TACTACGAGC--GTC-------
Ackr   AA---------CCTACACC--C--GCTGTCGCCCGGG-AATTACGAGC--ACC-------
Elev   -GTA--------CTACCCTC----CCCATCGCCTGGG-AACTACGAGC--AT--------
Trdu   TCC---------CTACACCC----ACTATCGCCCGGG-TACTACGAGC--AC--------
Amoc   C-C--------CCTACGCC--C--CCTATCGCCCGGG-TACTACGAGC--ATC-------
Hame   A-A--------TCTACTTAT----TTTATCGCCTGGG-AACTACGAGC--ATC-------
Chso   T---------CCTTACAT-T---CGCTGTTCGCCCGGG-AACTACGAGC--GTA-------
Lyto   --------ACC-CCACACCC----ACTATCGCCTGGG-GACTACGAGC--AGC-------
Encr   -AT--------TATACACCC----ACTATCGCCTGGG-AACTACGAGC--A-TT------
```

```
Bvar    T-C-------CCTACAACC-----TCTATTCGCCTGGA-AACTACGAGC--GCG-------
Noco    C-C-------AATACAAC-C----CCTATCCGCCCGGG-AACTACGAGC--ACC-------
Chsp    ----------GCT-ATACCTC---ATCATCCGCCCGGG-AATTACAAGC--ATT-------
Arja    GT---------AGTACATC---C-ACTATCCGCCCGGG-AACTACGAGC--AAT-------
Pase    GA---------CTCACAAA--AT-GTCATCCGCCCGGG-TACTACGAGC--GCA-------
Trel    TTTT---------CACAAGT----TTCATCCGCCAGGG-TATTACGAGC--TCC-------
Lifa    TAA--------C-TACACA-----CCCATCCGCCCGGG-TACTACAAGC--GCA-------
Acur    ACAC---------CACATAT----ACTATCCGCCCGGT-TACTACGAGC--ATT-------
Ampe    G-C--------ACTACATCT----GCTATCCGCCTGGG-AACTACGAGC--ATC-------
Urja    C----------TGAACACC-----AAACCCCGCCAGGG-AAGTACGAGC--ACG-------
Enet    ACA--------AATACACAA----GCTATTCGCCCGGG-TACTACGAGC--ACC-------
Ptbr    T-A----------TTCACAAA---AGTATACGCCAGGG-TATTACGAGC--ATA-------
Safa    CAT--------ATTACGTA-----ATCATCCGCCTGGG-AACTACGAGC--ACT-------
Icae    A---------TAATACACTT----TCTATCCGCCTGGG-AACTACGAGC--ATT-------
Asmi    TTTTATCTTC-ACCCGACTTCC--GTTGTCCGCCAGGG-AACTACGAGC--ATA-------
Foal    A-C--------AACACAT------ATTATCCGCCCGGG-TATTACGAGC--ATA-------
Drze    AA--------TCTACAAT---TA-TCTACCCGCCTGGG-AATTACGAGC--GAA-------
Rhas    ATT--------CTCACACCT----GCTGCTCGCCTGGG-AACTACGAGC--ATT-------
Elac    ---------AAGCCCACCTCT---GCCACTCGCCAGGG-AACTACGAGC--ATA-------
Kugu    ATCA--------CTACACCT----ACTATCCGCCTGGG-AACTACGAGC--GC--------
Plor    G-C--------CCCACACCC----GTTACTCGCCCGGA-AACTACGAGC--ATC-------
Sgun    GAA--------CTACAACC-----ATTGTCCGCCTGGG-AACTACGAGC--AC--------
Zaco    A-T-------TCTACAGCT-----GCTACCCGCCTGGG-TACTACGAGC--ATC-------
Zbfl    A-T-------CCTACAAC---T--GTCATCCGCCCGGG-TACTACGAGC--ATC-------
Spba    C-T-------ACCACAACC-----CCGATCCGCCTGGG-AATTACGAAC--ACT-------
Game    AT---------TTTACACC---C-TCTATCCGCCTGGG-TACTACGAGC--ATT-------
Thth    ATT--------TT-ACACCC----TCTATCCGCCTGGG-TACTACGAGC--ATT-------
Xigl    T-A--------ATTACACCC----AATATCCGCCCGGG-AATTACGAGC--ATC-------
Hyja    --------ATA-TCACACAAA---TCTATCCGCCTGGG-TACTACGGGC--ATT-------
Psan    TT---------ATTACATA-AA--TCTATCCGCCTGGG-TACTACGGGC--ATT-------
Cupa    -GT--------CCTACACCC----TCTATCCGCCTGGG-TACTACGAGC--ATT-------
Mpch    CTAT---------TACACTC----CCTATTCGCCAGGG-TACTACGAGC--ATT-------
Char    --------ACC-CTACATCA----GCTATTCGCCAGGG-GACTACGAGC--ATA-------
Pser    A-C--------CCTACACCT----ACAATCCGCCCGGG-AATTACAAAC--ATT-------
Prol    AC---------ATTACACT--C--AACATCCGCCCGGG-AATTACAAAC--GTT-------
Plbi    A-T--------CATACACTC----CATATCCGCCCGGG-AATTATGAAC--GTC-------
Calu    A-A--------TTAACACAT----TCTATCCGCCCGGG-AACTACGGAC--ACT-------
Papa    ATA---------T-ACATA-----CACTTCTGCCTGGA-AATTACGAAC--GC--------
Sufr    C-C--------CTTACATTTA-A-GCCATCCGCCAGGG-GACTACGAGC--ATT-------
Stci    AG---------ATTACACC-----TCTATCCGCCTGGG-GACTACGAGC--TTA-------
Taru    A-A--------ACTACGTA-----CATATCCGCCTGGT-TACTACGAGC--ATT-------
Rala    AA---------ACTACATG--C--GCTATCCGCCTGGG-AACTACGAGC--ATT-------
                                       ***      *
```

```
          29'    28'   2'    30                 31     32
Scca  -AGCTT-AAAACCCAAAGGACTTGGCGGTGCCCCACAC-CCACCTAGAGGAGCCTGTT-CT
Muma  -AGCTT-AAAACCCAAAGGACTTGGCGGTATCCCACAC-CCACCTAGAGGAGCCTGTT-CT
Erca  -AGCTT-AAAAACCAAAGGACTTGGCGGTGCTTCAGAA-CCACCTAGAGGAGCCTGTT-CT
Pose  -AGCTT-AAAACCCAAAGGACTTGGCGGTGCTTCAGAA-CCACCTAGAGGAGCCTGTT-CT
Actr  -AGCTT-AAAACCCAAAGGACTTGGCGGTGCCCCAGAC-CCACCTAGAGGAGCCTGTT-CT
Scal  -AGCTT-AAAACCCAAAGGACTTGGCGGTGCCCCAGAC-CCACCTAGAGGAGCCTGTT-CT
Posp  -AGCTT-AAAACCCAAAGGACTTGGCGGTGCCCCAGAC-CCACCTAGAGGAGCCTGTT-CT
Atsp  -AGCTTTAAAACCCAAAGGACTTGGCGGTGTCCCAAAC-CCACCTAGAGGAGCCTGTT-CT
Leoc  -AGCTT-AAAACCCAAAGGACTTGGCGGTGTCCCAAAC-CCACCTAGAGGAGCCTGTT-CT
Amca  -AGCTT-AAAACCCAAAGGACTTGGCGGTGCCCCAGAC-CCACCTAGAGGAGCCTGTT-CT
Osbi  TCGCTT-AAAACCCAACGGACTTGGCGGTGCCTCACACTCCACCTAGAGGAGCCTGTT-CT
Pabu  -AGCTC-GAAACCCAACGGACTTGGCGGTGCCCCAGAC-CCACCTAGAGGAGCCTGTT-CT
Hial  -AGCTC-GAAACCCAAAGGACTTGGCGGTGCCCCAGAC-CCACCTAGAGGAGCCTGTT-CT
Elha  -AGCTT-AAAACCCAAAGGACTTGGCGGTGCCCCACAC-CCACCTAGAGGAGCCTGTT-CT
Mlcy  -AGCTT-TAAACCCAAAGGACTTGGCGGTGCCCCAAAC-CCACCTAGAGGAGCCTGTT-CT
Algl  -AGCTT-AAAACCCAAAGGACTTGGCGACACCTTAGAT-CCCCCTAGAGGAGCCCGTT-CT
Ptgi  -AGCTT-AAAACCCAAAGGACTTGGCGGTGCCTCAGAC-CCACCTAGAGGAGCCTGTT-CT
Alaf  -AGCTT-AAAACCCAAAGGACTTGGCGGTGCCTCAAAC-CCATCTAGAGGAGCCTGTT-CT
Nock  -AGCTT-AAAACCCAAAGGACTTGGCGGTGCCTCAAAC-CCATCTAGAGGAGCCTGTT-CT
Anja  -AGCTT-AAAACCCAAAGGACTTGGCGGTGCCTCAAAC-CCACCTAGAGGAGCCTGTT-CT
Gyki  -AGCTT-AAAACCCAAGGGACTTGGCGGTGCCTCAAAC-CCACCTAGAGGAGCCTGTC-CT
Syka  -AGCTT-AAAACCCAAAGGACTTGGCGGTGCCTTAAAC-CCACCTAGAGGAGCCTGTT-CT
Opma  -AGTTT-AAAACCCAAAGGACTTGGCGGTGCTTCAAAA-CCCCCTAGAGGAGCCTGTC-CT
Comy  -AGCTT-AAAACCCAAAGGACTTGGCGGTGCCTCAAAC-CCACCTAGAGGAGCCTGTT-CT
Sasp  -AGCTT-AAAACCCAAAGGACTTGGCGGTATCTCAGAC-CCACCTAGAGGAGCCTGTC-CT
Eupe  TAGCTT-AAAACCCAAAGGACTTGGCGGCACCTCAAAC-CCACCTAGAGGAGCCTGTC-CT
Enja  -AGCTT-AAAACCCAAAGGACTTGGCGGTGCCTCAGAC-CCCCCTAGAGGAGCCTGTT-CT
Same  -AGCTT-AAAACCCAAAGGACTTGGCGGTGCTTCAGAC-CCCCCTAGAGGAGCCTGTT-CT
Chch  -AGCTT-AAAACCCAAAGGACTTGGCGGCACCTCAGAC-CCACCTAGAGGAGCCTGTT-CT
Grgr  TAGCTT-GAAACCCAAAGGACTTGGCGGCGCCTCAGAC-CCACCTAGAGGAGCCTGTT-CT
Caau  -AGCTT-AAAACCCAAAGGACCTGACGGTGTCTCAGAT-CCCCCTAGAGGAGCCTGTT-CT
Cyca  -AGCTT-AAAACCCAAAGGACCTGACGGTGTCTCAGAC-CCCCCTAGAGGAGCCTGTT-CT
Dare  AAGCTT-AAAACCCAAAGGACCTGACGGTGCCTTAGAT-CCCCCTAGAGGAGCCTGTT-CT
Cost  -AGCTT-AAAACCCAAAGGACTTGGCGGTGCCTTAGAC-CCCCCTAGAGGAGCCTGTT-CT
Leec  -AGCTT-AAAACCCAAAGGACTTGGCGGTGCCTTAGAC-CCCCCTAGAGGAGCCTGTT-CT
Crla  -AGCTT-AAAACCCAAAGGACTTGGCGGTGCCTTAGAC-CCCCCTAGAGGAGCCTGTT-CT
Clmc  -AGCTT-AAAACCCAAAGGACTTGACGGTGTCTCAGAC-CCACCTAGAGGAGCCTGTT-CT
Phin  -AGCTT-AAAACCCAAAGGACTTGACGGTGTCTCACCT-CCACCTAGAGGAGCCTGTT-CT
Icpu  -AGCTT-AAAACCCAAAGGACTTGGCGGTGTCTCAGAC-CCACCTAGAGGAGCCTGTT-CT
Psto  -AGCTT-AAAACCCAAAGGACTTGGCGGTGTCTCAGAC-CCACCTAGAGGAGCCTGTT-CT
Cora  -AGCTT-AAAACCCAAAGGACTTGGCGGTGTCTCAGAC-CCACCTAGAGGAGCCTGTT-CT
Eisp  -AGCTT-AAAACTCAAAGGACTTGGCGGTGCTCCAGAC-CCCCCTAGAGGAGCCTGTT-CT
Apal  -AGCTT-AAAACTCAAAGGACTTGGCGGCGTCTCC-AC-CCGACTAGAGGAGCCTGTT-CT
Eslu  -AGCTT-AAAACCCAAAGGACTTGGCGGTGCTTCAGAC-CCACCTAGAGGAGCCTGTT-CT
Dape  -AGCTT-AAAACCCAAAGGACTTGGCGGTGCCTCAGAC-CCACCTAGAGGAGCCTGTT-CT
Glse  -AGCTT-AAAACCCAAAGGACTTGGCGGTGCCCCAGAC-CCACCTAGAGGAGCCTGTT-CT
Naar  -AGCTT-AAAACCCAAAGGACTTGGCGGTGCCCCAGAC-CCACCTAGAGGAGCCTGTT-CT
Baoc  -AGCTT-AAAACCCAAAGGACTTGGCGGTGCCCCAGAC-CCACCTAGAGGAGCCTGTT-CT
Opso  -AGCTT-AAAACCCAAAGGACTTGGCGGTGCCCCAGAC-CCACCTAGAGGAGCCTGTT-CT
Alte  -AGCTT-AAAACCCAAAGGACTTGGCGGTGCCTCAGAC-CCACCTAGAGGAGCCTGTT-CT
Plap  -AGCTT-AAAACCCAAAGGACTTGGCGGTGCCTCAGAC-CCACCTAGAGGAGCCTGTT-CT
```

```
Plal    -AGCTT-AAAACCCAAAGGACTTGGCGGTGCCTCATAC-CCACCTAGAGGAGCCTGTT-CT
Sami    TAGCTT-AAAACCCAAAGGACTTGGCGGTGCCTCATAC-CCACCTAGAGGAGCCTGTT-CT
Rere    -AGCTT-AAAACCCAAAGGACTTGGCGGTGCCTCACAC-CCACCTAGAGGAGCCTGTT-CT
Gama    -AGCTT-AAAACCCAAAGGACTTGGCGGTGCCTCAGAC-CCACCTAGAGGAGCCTGTT-CT
Onmy    -AGCTT-AAAACCCAAAGGACTTGGCGGTGCCTCAGAC-CCACCTAGAGGAGCCTGTT-CT
Sasa    -AGCTT-AAAACCCAAAGGACTTGGCGGTGCCTCAGAC-CCACCTAGAGGAGCCTGTT-CT
Cola    -AGCTT-AAAACCCAAAGGACTTGGCGGTGCCTCAGAC-CCACCTAGAGGAGCCTGTT-CT
Dita    -AGCTT-AAAACCCAAAGGACTTGGCGGTGCCTTACAC-CCACCTAGAGGAGCCTGTT-CT
Sigr    -AGCTT-AAAACCCAA-GGATTTGGCGGCACTTCAAAC-CCATCTAGAGGAGCCTGTT-CT
Chsl    -AGCTT-AAAACTCAAAGGACTTGGCGGTACCTCAAAC-CCCCCTAGAGGAGCCTGTT-CT
Atja    -AGCTT-AAAACCCAAAGGACTTGGCGGTGCTTCAGAC-CCACCTAGAGGAGCCTGTT-CT
Iido    -AGCTT-AAAACCCAAAGGACTTGGCGGTGCTTCAGAC-CCACCTAGAGGAGCCTGTT-CT
Auja    -AGCTT-AAAACCCAAAGGACTTGGCGGTGCTTCAGAC-CCCCCTAGAGGAGCCTGTT-CT
Chag    -AGCTT-AAAACCCAAAGGACTTGGCGGTGTTTCAGAT-CCACCTAGAGGAGCCTGTT-CT
Hami    -AGCTT-AAAACCCAAAGGACTTGGCGGTGCTTCAGAC-CCCCCTAGAGGAGCCTGTC-CT
Saun    -AGCTT-AAAACCCAAAGGACTTGGCGGTGCTTCAGAC-CCCCCTAGAGGAGCCTGTC-CT
Nema    -AGCTT-AAAACCCAAAGGACTTGGCGGTGCTTTAGAC-CCACCTAGAGGAGCCTGTT-CT
Disp    -ATCTT-AAAACCCAAAGGACTTGGCGGTGCTTTAGAC-CCACCTAGAGGAGCCTGTC-CT
Myaf    -AGCTT-CAAACCCAAAGGACTTGGCGGTGCTTTAGAC-CCACCTAGAGGAGCCTGTC-CT
Lagu    -AGCTT-AAAACCCAAAGGACTTGGCGGTACCTTAGAC-CCCCCTAGAGGAGCCTGTT-CT
Trtr    -AGCTT-AAAACCCAAAGGACTTGGCGGTGCCTCAGAC-CCCCCTAGAGGAGCCTGTT-CT
Zucr    -AGCTT-GAAACCCAAAGGACTTGGCGGTGCCTCAGAC-CCCCCTAGAGGAGCCTGTT-CT
Pxja    -AGCTT-AAAACCCAAAGGACTTGGCGGTGCTTTAGAC-CCCCCTAGAGGAGCCTGTC-CT
Pxlo    -AGCTT-AAAACCCAAAGGACTTGGCGGTGCTTTAGAC-CCCCCTAGAGGAGCCTGTC-CT
Pctr    -AGCTT-AAAACCCAAAGGACTTGGCGGTGCTTTAGAC-CCCCCTAGAGGAGCCTGTT-CT
Apsa    -AGCTT-AAAACCCAAAGGACTTGGCGGTGCTTTATAC-CCCCCTAGAGGAGCCTGTT-CT
Cabe    CAGCTT-AAAACCCAAAGGACTTGGCGGCGCTTTAGAC-CCACCTAGAGGAGCCTGTT-CT
Bzze    CAGCTT-AAAACCCAAAGGACTTGGCGGTGCTTTAGAC-CCACCTAGAGGAGCCTGTT-CT
Siim    -AGCTT-GAAACCCAAAGGACTTGGCGGTGCTTCAGAC-CCACCTAGAGGAGCCTGTT-CT
Ctru    -AGCTT-AAAACCCAAAGGACTTGGCGGTGCTTTAGAC-CCCCCTAGAGGAGCCTGTT-CT
Dpbr    -AGCTT-AAAACCCAAAGGACTTGGCGGTGCTTTAGAC-CCCCCTAGAGGAGCCTGTT-CT
Caki    -AGCTT-AAAACCCAAAGGACCTGGCGGTGCTTTAGAT-CCCCCTAGAGGAGCCTGTT-CT
Phja    AAGCTT-AAAACCCAAAGGACTTGACGGTGCTTTAG-T-CCCCCTAGAGGAGCCTGTT-CT
Brne    -AGCCT-AAAACCTAAAGGACCTGGCGGTGCTTTACAT-CCATCTAGAGGAGCCTGTC-CT
Gamo    -AGCTT-AAAACCCAAAGGACTTGGCGGTGCTTTAGAC-CCCCCTAGAGGAGCCTGTT-CT
Lolo    -AGCTT-AAAACCCAAAGGACTTGGCGGTGCTTTAGAC-CCCCCTAGAGGAGCCTGTT-CT
Batr    -AGCTT-AAAATCCAAAAGACTTGGCGGCACTTTACAA-CC-CCTAGAGGAGCCTGTT-TC
Prmy    -CGCTT-AAAAACCAAAAGACCTGGCGGTGCTTTAT-T-CCCCCTAGAGGAGCCTGTT-TC
Loli    -AGCTT-AAAATCCAAAGGACTTGGCGGTGCTTTAGAC-CCACCTAGAGGAGCCTGTT-CT
Loam    TAGCTT-AAAACCCAAAGGACTTGGCGGTGCTTTAGAC-CCACCTAGAGGAGCCTGTT-CT
Chab    -AGCTT-GAAACCCAAAGGACTTGGCGGTGCTTAAAAT-CCACCTAGAGGAGCCTGTT-CT
Chto    -AGCTT-GAAACCCAAAGGACTTGGCGGTGCTTAAAAT-CCACCTAGAGGAGCCTGTT-CT
Majo    -AGCTT-AAAACCCAAAGGACTTGGCGGTGCTTTAGAC-CCCCCTAGAGGAGCCTGTTTCT
Hlst    -TGCTT-AAAACCCAAAGGACTTGACGGTGCTTTAGAC-CCACCTAGAGGAGCCTGTT-CT
Clpe    -AGCTT-AAAACCCAAAGGACTTGGCGACGCTTAAGAC-CCACCTAGAGGAGCCTGTT-CT
Mlmr    -AGATT-GAAACCCAAAGGACTTGGCGGTGCTTTAGA--CCCCCTAGAGGAGCCTGTT-CT
Crcr    -AGCTT-AAAACCCAAAGGACTTGGCGGTGCTTAAGAT-CCACCTAGAGGAGCCTGTT-CT
Muce    CAGCTT-AAAACCCAAAGGACTTGGCGGTGCTTAAGAT-CCACCTAGAGGAGCCTGTT-CT
Bege    -AGCTT-AAAACCCAAAGGACTTGGCGGTGCTTTAGAT-CCACCTAGAGGAGCCTGTT-CT
Mela    -AGCTT-AAAACCCAAAGGACTTGGCGGTGCTTTAGAT-CCCCCTAGAGGAGCCTGTT-CT
Hats    -AGCTT-AAAACCCAAAGGACTTGGCGGTGCTTTAGAC-CCACCTAGAGGAGCCTGTT-CT
Orla    -AGCTT-AAAACCCAAAGGACTTGGCGGTGCTTTAGAC-CCACCTAGAGGAGCCTGTT-CT
```

Cosa	-AGCTT-AAAATCCAAAGGACTTGGCGGTGCTTTAGAC-CCACCTAGAGGAGCCTGTT-CT
Exvo	-AGCTT-GAAACCCAAAGGACTTGGCGGTGCTTCAAAC-CCACCTAGAGGAGCCTGTT-CT
Depa	-AGCTT-AAAATCCAAAGGACTTGGCGGTGCTTTAGAT-CCACCTAGAGGAGCCTGTT-CT
Rima	-AGCTC-AAAACTCAAAGGACTTGGCGGCGCTTTAAAC-CCATCTAGAGGAGCCTGTC-CT
Fuol	-AGCTT-AAAACCCAAAGGACTTGGCGGTGCTTTAGAT-CCACCTAGAGGAGCCTGTT-CT
Gmaf	-AGCTT-AAAACCCAAAGGACTTGGCGGTGCTTTAGAC-CCCCCTAGAGGAGCCTGTT-CT
Xeei	-AGCTT-AAAACCCAAAGGACTTGGCGGTGCTTTAGAC-CCCCCTAGAGGAGCCTGTT-CT
Pros	-AGCTT-AAAACCCAAAGGACTTGGCGGTGCTTTAGAC-CCACCTAGAGGAGCCTGTC-CT
Scmi	-AGCTT-AAAACCCAAAGGACTTGGCGGTGCTTCA--C-CCACCTAGAGGAGCCTGTC-CC
Rolo	-AGCTT-AAAACCCAAAGGACTTGGCGGTGCTTTAGAC-CCACCTAGAGGAGCCTGTT-CT
Cere	CAGCTT-AAAACCCAAAGGACTTGGCGGTGCTTTAAAC-CCATCTAGAGGAGCCTGTT-CT
Daga	-AGCTT-AAAACCCAAAGGACTTGGCGGTGCTTTA-AC-CCCCCTAGAGGAGCCTGTT-CT
Anco	CAGCTT-AAAACCCAAAGGACTTGGCGGTGCTTTAGAC-CCACCTAGAGGAGCCTGTT-CT
Dmve	-AGCTT-AAAACCCAAAGGACTTGGCGGTGCTTCATAC-CCACCTAGAGGAGCCTGTT-CT
Dmar	-AGCTT-AAAACCCAAAGGACTTGGCGGTGCTTCACAC-CCACCTAGAGGAGCCTGTT-CT
Anka	-AGCTT-AAAACCCAAAGGACTTGGCGGTGCTTTAGAC-CCACCTAGAGGAGCCTGTT-CT
Moja	-AGCTT-AAAACCCAAAGGACTTGGCGGTGCTTTAGAC-CCACCTAGAGGAGCCTGTT-CT
Hoja	CAGCTT-AAAACCCAAAGGACTTGGCGGTGCTTTAGAC-CCACCTAGAGGAGCCTGTT-CT
Bede	-AGCTT-AAAACCCAAAGGACTTGGCGGTGCTTTAGAC-CCACCTAGAGGAGCCTGTC-CT
Besp	-AGCTT-AAAACCCAAAGGACTTGGCGGTGCTTTAGAC-CCACCTAGAGGAGCCTGTC-CT
Mysp	-AGCTT-AAAACCCAAAGGACTTGGCGGTGCTTTAGAC-CCACTTAGAGGAGCCTGTT-CT
Osja	-AGCTT-AAAACCCAAAGGACTTGGCGGTGCTTTAGAT-CCACTTAGAGGAGCCTGTT-CT
Sgro	-AGCTT-AAAACCCAAAGGACTTGGCGGTGCTTTAGAT-CCACTTAGAGGAGCCTGTT-CT
Pzpa	-AGCTT-TAAACCCAAAGGACTTGGCGGTGCTTTAGAC-CCCCCTAGAGGAGCCTGTT-CT
Zeja	-AGCTT-AAAACCCAAAGGACTTGGCGGTGCTTTAGAC-CCCCCTAGAGGAGCCTGTT-CT
Znne	-AGCTT-AAAACCCAAAGGACTTGGCGGTGCTTTAGAC-CCCCCTAGAGGAGCCTGTT-CT
Zefa	-AGCTT-AAAACCCAAAGGACTTGGCGGTGCTTTAGAC-CCCCCTAGAGGAGCCTGTT-CT
Acni	-AGCTT-AAAACCCAAAGGACTTGGCGGTGCTTTAGAC-CCCCCTAGAGGAGCCTGTT-CT
Ncrh	-AGCTT-AAAACCCAAAGGACTTGGCGGTGCTTTAGAC-CCCCCTAGAGGAGCCTGTT-CT
Agca	-AGCTT-AAAACCCAAAGGACTTGGCGGTGCTTTAGAC-CCCCCTAGAGGAGCCTGTC-CT
Hydy	-AGCTT-AAAACCCAAAGGACTTGGCGGTGCTTTAGAC-CCACCTAGAGGAGCCTGTT-CT
Gsac	-AGCTT-GAAACCCAAAGGACTTGGCGGTGCTTTAGAT-CCACCTAGAGGAGCCTGTT-CT
Pevo	-AGCTT-AAAACCCAAAGGACTTGGCGGTGCTTTAGAC-CCACCTAGAGGAGCCTGTT-CT
Hiku	-AGCTT-AAAACCCAAAGGACTTGGCGGTGCTTTACAC-CCACCTAGAGGAGCCTGTT-CT
Inpa	-AGCTT-TAAACCCAAAGGACTTGGCGGTGCTTAAAAT-CCAACTAGAGGAGCCTGTT-CT
Auch	-AGCTT-AAAACCCAAAGGACTTGGCGGTGCTTTAGAC-CCAACTAGAGGAGCCTGTT-CT
Fico	-AGCTT-AAAACCCAAAGGACTTGGCGGTGCTTTAGAC-CCACCTAGAGGAGCCTGTT-CT
Macs	-AGCTT-GAAACCCAAAGGACTTGGCGGTGCTTTAGAC-CCACCTAGAGGAGCCTGTT-CT
Moal	-AGCTT-AAAACCCAAAGGACTTGGCGGTGCTTAAGAC-CCACCTAGAGGAGCCTGTT-CT
Syma	-AGCTT-AAAACCCAAAGGACTTGGCGGTGCTTAAGAC-CCATCTAGAGGAGCCTGTC-CT
Mafr	-AGCTT-AAAACCCAAAGGACTTGGCGGTGCTTTAAAT-CCACCTAGAGGAGCCTGTT-CT
Dcpe	-AGCTT-AAAACTCAAGGGACTTGGCGGCGCTTTATAA-CCATCTAGAGGAGCCTGTT-CT
Dcti	-AGCTT-AAAACTCAAGGGACTTGGCGGCGCTTTATAA-CCATCTAGAGGAGCCTGTT-CT
Hehi	-AGCTT-AAAACCCAAAGGACTTGGCGGTGCTTTAGAC-CCCCCTAGAGGAGCCTGTT-CT
Stam	CAGCTT-GAAACCCAAAGGACTTGGCGGTGCTTTAGAT-CCACCTAGAGGAGCCTGTT-CT
Hogi	-AGCTT-AAAACCCAAAGGACTTGGCGGTGCTTTAGAC-CCCCCTAGAGGAGCCTGTT-CT
Erzo	-AGCTT-GAAACCCAAAGGACTTGGCGGTGCTTTAGAT-CCACCTAGAGGAGCCTGTT-CT
Hxot	-AGCTT-AAAACCCAAAGGACTTGGCGGTGCTTTAGAT-CCACCTAGAGGAGCCTGTT-CT
Core	-AGCTT-AAAACCCAAAGGACTTGGCGGTGCTTTAGAT-CCACCTAGAGGAGCCTGTT-CT
Apve	-AGCTT-GAAACCCAAAGGACTTGGCGGTGCTTTAGAC-CCACCTAGAGGAGCCTGTT-CT
Latj	-AGCTT-GAAACCCAAAGGACTTGGCGGTGCTTAATAT-CCACCTAGAGGAGCCTGTT-CT
Laja	-AGCTT-GAAACCCAAAGGACTTGGCGGTGCTTTAGAT-CCACCTAGAGGAGCCTGTT-CT

Syja	-AGCTT-AAAACCCAAAGGACTTGGCGGTGCTTTAGAT-CCACCTAGAGGAGCCTGTT-CT
Epme	-AGCTT-AAAACCCAAAGGACTTGGCGGTGCTTTAGAC-CCACCTAGAGGAGCCTGTT-CT
Grse	-AGCTT-AAAACCCAAAGGACTTGGCGGTGCTTTAGAT-CCACCTAGAGGAGCCTGTT-CT
Clja	-AGCTT-AAAATCCAAAGGACTTGGCGGTGCTTCAGAT-CCACCTAGAGGAGCCTGTC-CT
Ogcy	-AGCTT-AAAACCCAAAGGACTTGGCGGTGCTTAAGAT-CCATCTAGAGGAGCCTGTT-CT
Plna	-AGCTT-AAAACCCAAAGGACTTGGCGGTGCTTTAGAC-CCACCTAGAGGAGCCTGTC-CT
Lema	-AGCTT-AAAACCCAAAGGACTTGGCGGTGCTTTAGAC-CCACCTAGAGGAGCCTGTT-CT
Etzo	-AGCTT-GAAACCCAAAGGACTTGGCGGTGCTTTAGAT-CCACCTAGAGGAGCCTGTT-CT
Apse	-AGCTT-GAAACCCAAAGGACTTGGCGGCGCTTCACAC-CCACCTAGAGGAGCCTGTT-CT
Epde	-AGCTT-GAAACCCAAAGGACTTGGCGGTGCTTTAGAT-CCACCTAGAGGAGCCTGTT-CT
Slja	-AGCTT-GAAATCCAAAGGACTTGGCGGTGCTTTAGAT-CCACCTAGAGGAGCCTGTC-CT
Bsja	CAGCTT-AAAACCCAAAGGACTTGGCGGTGCTTCAGAT-CCCCCTAGAGGAGCCTGTT-CT
Ecna	-AGTTT-AAAACCCAAAGGACTTGGCGGTGCTTTACAT-CCACCTAGAGGAGCCTGTT-CT
Cohi	-AGTTT-AAAACCCAAAGGACTTGGCGGTGCTTAATAT-CCACCTAGAGGAGCCTGTC-CT
Caar	-AGTTT-AAAACCCAAAGGACTTGGCGGTGCTTAACAT-CCACCTAGAGGAGCCTGTT-CT
Came	-AGTTT-AAAACCCAAAGGACTTGGCGGTGCTTAACAT-CCACCTAGAGGAGCCTGTT-CT
Mema	-AGTTT-AAAACCCAAAGGACTTGGCGGTGCTTTACAT-CCACCTAGAGGAGCCTGTT-CT
Lenu	-AGCTT-AAAACCCAAAGGACTTGGCGGTGCTTCAAAC-CCACCTAGAGGAGCCTGTT-CT
Plma	-AGCTT-GAAACCCAAAGGACTTGGCGGTACTTTAGAT-CCCCCTAGAGGAGCCTGTT-CT
Emst	-AGCTT-GAAACCCAAAGGACTTGGCGGTGCTTTAGAT-CCACCTAGAGGAGCCTGTT-CT
Ptti	-AGCTT-AAAACCCAAAGGACTTGGCGGTGCTTTAGAT-CCACCTAGAGGAGCCTGTT-CT
Losu	-AGCTT-AAAACTCAAAGGACTTGGCGGTGCTTTAGAT-CCACCTAGAGGAGCCTGTC-CT
Geoy	-AGCTT-AAAACCCAAAGGACTTGGCGGTGCTTTAGAC-CCATCTAGAGGAGCCTGTT-CT
Dipi	-AGCTT-AAAACCCAAAGGACTTGGCGGTGCTTTAGAT-CCACCTAGAGGAGCCTGTT-CT
Pama	-AGCTT-AAAACCCAAAGGACTTGGCGGTGCTTTAGAC-CCACCTAGAGGAGCCTGTT-CT
Leob	-AGCTT-AAAACCCAAAGGACTTGGCGGTGCTTTAGAC-CCCCCTAGAGGAGCCTGTT-CT
Neba	AAGCTT-AAAAACCAAAGGACTTGGCGGTGCTTCAAAC-CCCCCTAGAGGAGCCTGTT-CT
Pdpl	-AGTTT-AAAACCCAAAGGACTTGGCGGTGCTTTATAT-CCCCCTAGAGGAGCCTGTT-CT
Nimi	-AGCTT-AAAACCCAAAGGACTTGGCGGTGCTTTAGAC-CCACCTAGAGGAGCCTGTT-CT
Uptr	-AGCTT-AAAACCCAAAGGACTTGGCGGTGCTTTAGAC-CCCCCTAGAGGAGCCTGTTTCT
Pesc	-AGCTT-AAAACCCAAAGGACTTGGCGGTGCTTCAAAC-CCCCCTAGAGGAGCCTGTT-CT
Baar	-TGCCT-AAAACCCAAAGGACTTGGCGGTGCTTTAGAC-CCCCCTAGAGGAGCCTGTC-CT
Moar	-AGCTT-AAAACCCAAAGGACTTGGCGGTGCTTTAGAT-CCACCTAGAGGAGCCTGTT-CT
Toja	-AGTTT-AAAACCCAAAGGACTTGGCGGTGCTTAACAT-CCACCTAGAGGAGCCTGTC-CT
Chau	-AGCTT-AAAACCCAAAGGACTTGGCGGTGCTTTAGAC-CCACCTAGAGGAGCCTGTT-CT
Chse	-AGCTT-AAAACCCAAAGGACTTGGCGGTGCTTCAAAC-CCACCTAGAGGAGCCTGTT-CT
Enar	-AGCTT-AAAACCCAAAGGACTTGGCGGTGCTTTAGAT-CCACCTAGAGGAGCCTGTT-CT
Hpty	-AGCTT-GAAACCCAAAGGACTTGGCGGTGCTTTAGAT-CCACCTAGAGGAGCCTGTT-CT
Nana	-AGCTT-AAAACCCAAAGGACTTGGCGGTGCTTAAGAT-CCACCTAGAGGAGCCTGTT-CT
Mcst	-AGCTT-AAAACCCAAAGGACTTGGCGGTGCTTTAGAT-CCACCTAGAGGAGCCTGTT-CT
Rhox	-AGCTT-AAAACCCAAAGGACTTGGCGGTGCTTTAGAT-CCACCTAGAGGAGCCTGTT-CT
Opfa	TAGCTT-AAAACCCAAAGGACTTGGCGGTGCTTTAGAT-CCACCTAGAGGAGCCTGTT-CT
Paar	-AGCTT-CAAACCCAAAGGACTTGGCGGTGCTTCAGAT-CCCCCTAGAGGAGCCTGTT-CT
Gozo	-AGCTT-AAAACCCAAAGGACTTGGCGGTGCTTTAGAC-CCACCTAGAGGAGCCTGTT-CT
Ackr	-AGCTT-AAAACCCAAAGGACTTGGCGGTGCTTTAAAC-CCCCCTAGAGGAGCCTGTC-CT
Elev	AAGCTT-AAAACCCAAAGGACTTGGCGGTGCTTTAGAT-CCACCTAGAGGAGCCTGTT-CT
Trdu	TAGCTT-AAAACCCAAAGGACTTGGCGGTGCTTTAGAT-CCACCTAGAGGAGCCTGTT-CT
Amoc	-AGCTT-AAAACCCAAAGGACTTGGCGGTGCTTCACAT-CCGTCTAGAGGAGCCTGTT-CT
Hame	-AGCTT-AAAACCCAAAGGACTTGGCGGTGCTTTAGAT-CCACCTAGAGGAGCCTGTT-CT
Chso	-AGCTT-AAAACCCAAAGGACTTGACGGTGCTTTATAT-CCCCCTAGAGGAGCCTGTC-CT
Lyto	-AGCTT-AAAACCCAAAGGACTTGGCGGTGCTTTAAAC-CCACCTAGAGGAGCCTGTT-CT
Encr	-AGCTT-AAAACCCAAAGGACTTGGCGGTGCTTTAGAT-CCACCTAGAGGAGCCTGTT-CT

```
Bvar      -AGCTT-AAAACCCAAAGGACTTGGCGGTGCTTTACAT-CCTTCTAGAGGGACACGTC-CT
Noco      -AGCTT-AAAACCCAAAGGACTTGGCGGTGCTTTAGAT-CCTACTAGAGGAGCCTGTT-CT
Chsp      -AGCTC-AAAACCCAAAGGACCTGGCGGTGCTTTAAAC-CCCTCTAGAGGAGCCTGTC-CT
Arja      -AGCTT-AAAACCCAAAGGACTTGGCGGTGCTTTAGAT-CCACCTAGAGGAGCCTGTT-CT
Pase      -AGCTT-AAAACCCAAAGGACTTGGCGGTGCTTTATAC-CCCTCTAGAGGAGCCTGTC-CA
Trel      -AGCTT-GAAACCCAAAGGACTTGGCGGTGCTTTAGAC-CCACCTAGAGGAGCCTGTC-CT
Lifa      -AGCTT-GAAACCCAAAGGACTTGGCGGTGCTTTAGAC-CCCCCTAGAGGAGCCTGTT-CT
Acur      -AGCTT-AAAACCCAAAGGATTTGGCGGTGCTTTATAC-CCCCCTAGAGGAGCCTGTC-CT
Ampe      -AGCTT-GAAACCCAAAGGACTTGGCGGTGCTTTAGAT-CCACCTAGAGGAGCCTGTT-CT
Urja      -AGCTT-AAAACCCAAAGGACTTGGCGGTGCTTTAGAT-CCACCTAGAGGAGCCTGTC-CT
Enet      -AGCTT-AGAACCCAAAGGACTTGGCGGTGCTTAAGAC-CCCCCTAGAGGAGCCTGTT-CT
Ptbr      -AGCTT-AAAACCCAAAGGACTTGGCGGTGCTTAATAA-CCCCCTAGAGGAGCCTGTT-CT
Safa      -AGCTC-AAAACCCAAAGGACTTGGCGGTGCTTAACAT-CCCCCTAGAGGAGCCTGTT-CT
Icae      -AGCTT-GAAACCCAAAGGACTTGGCGGTACTTTAGAT-CCCCCTAGAGGAGCCTGTT-CT
Asmi      -AGCTT-GAAACCCAAAGGACTTGGCGGTGCTTTAGAC-CCACCTAGAGGAGCCTGTT-CT
Foal      -AGCTT-AAAACCCAAAGGACTTGGCGGTGCTTTAAAC-CCCCCTAGAGGAGCCTGTT-CT
Drze      -AGTTT-AAAACCCAAAGGACTTGGCGGTGCTTTAGAC-CCCCCTAGAGGAGCCTGTT-CT
Rhas      -AGCTT-AAAACCCAAAGGACTTGGCGGTGCTTTAGAT-CCACCTAGAGGAGCCTGTT-CT
Elac      -AGCTT-AAAACCCAAAGGACTTGGCGGTGCTTTAGAC-CCCCCTAGAGGAGCCTGTT-CT
Kugu      AAGCTT-AAAACCCAAAGGACTTGGCGGTGCTTTAGTC-CCACCTAGAGGAGCCTGTT-CT
Plor      -AGCTT-AAAACCCAAAGGACTTGGCGGTGCTTTAGAT-CCACCTAGAGGAGCCTGTT-CT
Sgun      CAGCTT-AAAACCCAAAGGACTTGGCGGTGCTTTAGAT-CCACCTAGAGGAGCCTGTT-CT
Zaco      -AGCTT-AAAACCCAAAGGACTTGGCGGTGCTTTAGAT-CCACCTAGAGGAGCCTGTT-CT
Zbfl      -AGCTT-AAAACCCAAAGGACTTGGCGGTGCTTTAGAC-CCACCTAGAGGAGCCTGTT-CT
Spba      -AGTCT-GAAACCCAAAGGACTTGGCGGTGCTTTACAT-CCACCTAGAGGAGCCTGTC-CT
Game      -AGCTT-GAAACCCAAAGGACTTGGCGGTACTTTAGAT-CCCCCTAGAGGAGCCTGTT-CT
Thth      -AGCTT-GAAACCCAAAGGACTTGGCGGTACTTTAGAT-CCCCCTAGAGGAGCCTGTT-CT
Xigl      -AGTTT-AAAACCCAAAGGACTTGGCGGTGCTTAACAT-CCACCTAGAGGAGCCTGTT-CT
Hyja      -AGCCT-AAAACCCAAAGGACTTGGCGGTTCTTTAGAT-CCCCCTAGAGGAGCCTGTT-CT
Psan      -AGCCT-AAAACCCAAAGGACTTGGCGCTTCTTTAGAC-CCACCTCGAGGAGCCTGTT-CT
Cupa      -AGCTT-AAAACCCAAAGGACTTGGCGGTACTTTAGAT-CCCCCTAGAGGAGCCTGTT-CT
Mpch      -AGCTT-AAAACCCAAAGGACTTGGCGGTGCTTAAGAT-CCACCTAGAGGAGCCTGTT-CT
Char      -AGCTT-AAAACCCAAAGGACTTGGCGGTGCTTAAGAT-CCACCTAGAGGAGCCTGTT-CT
Pser      -AGTTC-AAAACCCAAAGGACTTGGCGGTGCTTTACAT-CCACCTAGAGGAGCCTGTT-CT
Prol      -AGTTT-AAAACCCAAAGGACTTGGCGGTGCTTAACAT-CCACCTAGAGGAGCCTGTT-CT
Plbi      -AGTTT-AAAACCCAAAGGACTTGGCGGTGCTTAACAT-CCACCTAGAGGAGCCTGTT-CT
Calu      -AGTCT-GAAAGCCAAAGGACCTGGCGGTGCTTCACAT-CCATCTAGAGGAGCCTGTC-CT
Papa      AAGTTT-AAAACCCAAAGGACTTGGCGGTGCTTAGAAT-CCACCTAGAGGAGCCTGTT-AT
Sufr      -AGCTT-AAAACCCAAAGGACTTGGCGGTGCTTTAGAT-CCACCTAGAGGAGCCTGTT-CT
Stci      -AGCTT-AAAACCCAAAGGACTTGGCGGTGCTTTAGAT-CCCCCTAGAGGAGCCTGTT-CT
Taru      -AGCTT-AAAACCCAAAGGACTTGGCGGTGCTTTAAAA-CCATCTAGAGGAGCCTGTT-TT
Rala      -AGCTT-AAAACCCAAAGGACTTGGCGGTGCTTTAGAT-CCACCTAGAGGAGCCTGTT-CT
                   **   **   *  ** **            **   * ****  *  **
```

```
               33          33'           34 ¦      HVR      ¦ 35
Scca  ATAACC-GATAATCCCCG-TTAAACCTCACCACTTCTTGCCA----TTACCGTCTATAT
Muma  ATGACC-GATAATCCCCG-TTCAACCTCACCACTTCTTGCCAT----TACCGTCTATAT
Erca  ATAATC-GATAATCCTCG-TTAAACCTCACCACATCTTGC------ATTCAGCCTATAT
Pose  ATAATC-GATAATCCTCG-TTCAACCTCACCACATCTTGC------ATCCAGCCTATAT
Actr  AGAACC-GATAATCCCCG-TTAAACCTCACCACTTCTTGT--C-A-TTTCCGCCTATAT
Scal  AGAACC-GATAATCCCCG-TT-AACCTCACCACTTCTTGT----CATTTCCGCCTATAT
Posp  AGAACC-GATAATCCCCG-TTAAACCTCACCACTTCTTGT-CATTT---CCGCCTATAT
Atsp  AGACCC-GATACTCCCCGGTTAAACCTCACCACTTCTTGTCA----ATCCCGCCTATAT
Leoc  AGAACC-GATATTCCCCG-TTAAACCTCACCACTTCTTGTC---AAT-CCCGCCTATAT
Amca  AGAACC-GATAATCCTCG-TTAAACCTCACCACTCCTTGTTAA-T---CCCGCCTATAT
Osbi  ATAACT-GAAACTACCCG-TTAAACCTCACCGTTTCTAGCCAT--C----AGTCTATAT
Pabu  ATAACT-GACACCCCCCG-TTAAACCTCACCACTCCTAGCCA---ACCCA-GTTTATAT
Hial  AGAACT-GATAACCCCCG-TTTAACCTCACCACCCCTTGCC----AACCCCGTCTATAT
Elha  AGAACC-GATAACCCCCG-TTAAACCTCACCACCCCTTGT----CAACCCCGCCTATAT
Mlcy  ATAACC-GATAACCCCCG-TTAAACCTCACCGCTTCTAGCC---AACCCC-GCCTATAT
Algl  ATAACC-GATGATCCTCG-TTTAACCTCACCATCCCTTGTA----AACCCAGCCTATAT
Ptgi  ATAACC-GATAATCCCCG-TTAGACCTCACCACCCCTTGT----CAACCCCGCCTATAT
Alaf  ACAACT-GATAATCCTCG-TTAAACCTCACCACCCCTTGTTAA-C---CCCGCCTATAT
Nock  ATAACT-GATAATCCTCG-TTAAACCTCACCACTTCTTGTTAA-TT---CCGCCTATAT
Anja  ATAACC-GATAACCCCCG-TTAAACCTCACCATCTCTTGC-CTAAA---CCGCCTATAT
Gyki  ATAACC-GATGATCCCCG-TTTAACCTCACCTCTCCTTGCCA--AT--CCCGCCTATAT
Syka  ATAACC-GATAATCCCCG-TTAAACCTCACCACCCCTTGTTA---AATCCCGCCTATAT
Opma  ATAACC-GATAATCCCCG-TTTAACCTCACCACTCCTTGCCAA-C---TCAGTTTATAT
Comy  ATAACC-GATAACCCCCG-TTTAACCTCACCACTTCTAGTT---TTA-CCAACCTATAT
Sasp  ATAATC-GATACACCCCG-CTAAACCTCCCCTTTTCTTGCT---AAATCC-GCCTATAT
Eupe  ATAATC-GATAAACCCCG-CTAAACCTTGCCTCCCCTTGCCAA--A--ACCGCCTATAT
Enja  AGAACC-GATAACCCCCG-TTCAACCTCACCACTCCTTGCCC----TTTCCGCCTATAT
Same  AGAACC-GATAACCCCCG-TTCAACCTCACTACTCCTTGC----TTTTCCCGCCTATAT
Chch  AGAACC-GATAATCCCCG-TTAAACCTCACCACTTCTTGTTAT-C---ACAGCCTATAT
Grgr  AGAACC-GATAACCCCCG-TTAAACCTCACCACCCTTTGTTCG--C--CCCGTCTATAT
Caau  AGAACC-GATAACCCCCG-TTCAACCTCACCACTCCTAGCCAA-C---CCAGCCTATAT
Cyca  AGAACC-GATAACCCCCG-TTCAACCTCACCACTTCTAGC----CACCCCAGCCTATAT
Dare  AGAACC-GATTATCCCCG-TTAAACCTCACCATTTCTAGC-AA--A-ATCAGCCTATAT
Cost  AGAACC-GATAACCCCCG-TTAAACCTCACCACTTCTGGTT---AAC-CCCGCCTATAT
Leec  AGAACC-GATAATCCCCG-TCAAACCTCACCACTTCTAGTCA----TTCCCGCCTATAT
Crla  AGAACC-GATAACCCCCG-TTAAACCTCACCACTTCTAGTCAT-C---CCCGCCTATAT
Clmc  AGAACC-GATAATCCCCG-TTAAACCTCACCACCCCTTGTTTT-C---CCCGCCTATAT
Phin  AGAACC-GATATTCCTCG-TTTAACCTCACCATCCCTTGCC---CCC-GCCGCCTATAT
Icpu  ATAACC-GATAACCCCCG-TTAAACCTCACCACTTCTTGTTTA----TCCCGCCTATAT
Psto  AGAACC-GATAACCCCCG-TTAAACCTCACCACTTCTTGTTTT----CCCCGCCTATAT
Cora  AGAACC-GATAACCCCCG-TTAAACCTCACCACTTCTTGTTA---A-TCCCGCCTATAT
Eisp  AGAACC-GATAACCCCCG-TTCAACCTCACCACCTCTTGTTC----ATACCGCCTATAT
Apal  AGAACC-GATAACCCCCG-TTCAACCTCACCACCCCTTGTT----TTCACAGCCTATAT
Eslu  ATAACC-GATAACCCCCG-CTTAACCTCACCAGCCCTTGTT----CACCCCGCCTATAT
Dape  ATAACC-GATAACCCCCG-CTTAACCTCACCGCCCCTTGTTCC-T---CCCGCCTATAT
Glse  AGAACC-GATACCCCCCG-TTCAACCTCACCACCCCTTGTTAA-C---CCCGCCTATAT
Naar  AGAACC-GATACCCCCCG-TTCAACCTCACCACCTCTTGTTA--AC--CCCGCCTATAT
Baoc  AGAACC-GATACCCCCCG-TTCAACCTCACCACCTCTTGTCA----ACCCCGCCTATAT
Opso  AGAACC-GATACCCCCCG-TTCAACCTCACCACCTCTTGTTAATC----CCGCCTATAT
Alte  AGAACC-GATAACCCCCG-TTAAACCTCACCACCCCTTGTTAA----TCCCGCCTATAT
Plap  AGAACC-GATAACCCCCG-TTAAACCTCACCACCCCTTGTTA----ACCCCGCCTATAT
```

```
Plal    TGAATC-GATAATCCCCG-TTCAACCTCACCACCCCTTGC----TCGACCCGCCTATAT
Sami    AGAATC-GATAATCCCCG-TTCAACCTCACCACCCCTTGTTTG--A--CCCGCCTATAT
Rere    AGAATC-GATACTCCCCG-TTCAACCTCACCACCCCTTGTT----CAACCCGCCTATAT
Gama    AGAACC-GATAACCCCCG-TTTAACCTCACCTCCCCTTGTT----ATCACCGCCTATAT
Onmy    AGAACC-GATAACCCCCG-TTCAACCTCACCACCCCTTGTTT--TA--CCCGCCTATAT
Sasa    AGAACC-GATAACCCCCG-TTCAACCTCACCACCTCTTGTTTT-C---CCCGCCTATAT
Cola    AGAACC-GATAACCCCCG-TTCAACCTCACCACCTCTTGT----TTTCCCCGCCTATAT
Dita    AGAACC-GATAACCCCCG-TTCAACCTCACCACCTCTTGTCGT----CCCCGCCTATAT
Gogr    AAAACC-GATTATCCCCG-TTCAACCTTACCACCCCTTGCCA----ACCCCGCCTATAT
Chsl    TAAACC-GATAATCCCCG-TTCAACCTCACCACCTCTCGT-TT-T---CCAGTCTATAT
Atja    AGAACC-GATAATCCACG-TTCAACCTCACCACCTCTTGTTTAA---CACAGCCTATAT
Iido    AGAACC-GATAATCCCCG-TTCAACCTCACCACCCCTTGTT----AATACAGCCTATAT
Auja    AGAACC-GATGATCCCCG-TTCAACCTCACCATTTCTTGC-TTGTC---CCGCCTATAT
Chag    AGAATC-GATAGTCCCCG-CTTAACCTCACCATCCCTGGCTTC-AAACCCCGCCTGTAT
Hami    AGAACC-GATGATCCCCG-TTCAACCTCACCATTTCTTGTTCA-T---TCCGCCTATAT
Saun    AGAACC-GATGATCCCCG-TTCAACCTCACCACTTCTTGTT----TCTCCCGCCTATAT
Nema    AGAACC-GATACCCCCCG-TTCAACCTCACCACTTCTTGTTA---TCCCC-GCCTATAT
Disp    AGAACC-GATACCCCCCG-TTCAACCTCACCACTTCTGGCTCGAT-ATTCCGCCTGTAT
Myaf    AGAACC-GATAACCCCCG-TTCAACCTCACCACTTCTAGCTC--AA--CCCGCCTATAT
Lagu    AGAACC-GATACTCCCCG-TCTAACCTCACCACCCCTAGCCC---CAAGCCGCTTATAT
Trtr    AGAACC-GATACTCCCCG-TTTAACCTTACCCTCTCTTGTTC---CCACT-GCCTATAT
Zucr    AGAACC-GATACCCCCCG-TTAAACCTTTCCCTCTCTAGTT----CCCACTGCCTATAT
Pxja    AGAACC-GATAACCCCCG-TTCAACCTCACCACCCCTTGTTAA-GA---CCGCCTATAT
Pxlo    AGAACC-GATAACCCCCG-TTCAACCTCACCACCCCTTGTTA----AAACCGCCTATAT
Pctr    AAAACC-GATACTCCCCG-TTGAACCTCACCACCTTTTGTTA----ACCCCGCCTATAT
Apsa    AAAACC-GATAACCCCCG-TTTAACCTCACCCCCTTTTGTT----TAACCCGCCTATAT
Cabe    ATAACC-GATAACCCCCG-TTAAACCTCACCTCTCCTAGT-TA--T-CACAGCCTATAT
Bzze    AGAACC-GATAACCCCCG-TTCAACCTCACCCCTCCTTGTTTC--C--CCCGCCTATAT
Siim    AGAACC-GATAATCCCCG-TTCAACCTCACCCCTCCTTGCAAT-T---TCAGCCTATAT
Ctru    AGAACC-GATAACCCCCG-TTTAACCTCACCCCTCCTAGTT---CCTTCC-GCCTATAT
Dpbr    AGAACC-GATAACCCCCG-TTTAACCTCACCCCTCCTAGTTCATC----CCGCCTATAT
Caki    ATAACT-GACAACCCCCG-TTAAACCTCACCACCCCTTGTCA--AA--TCCGCCTATAT
Phja    AGAACT-GATGATCCCCG-TTTAACCTCACCACCTCTTGCTTA--T--TCTGTTTGTAT
Brsp    AGAACT-GATATTCCCCA-TTAAACCTCACCCTCCCTGGCT---TAA-TCCGCCTATAT
Gamo    AGAACT-GATAACCCCCG-TTTAACCTCACCATCCTTTGTTT--TC--CCCGCCTATAT
Lolo    AGAACT-GATAACCCCCG-TTAAACCTCACCATCCTTTGTTTA-A---CCCGCCTATAT
Batr    AAAACC-GATACTCCCCG-CTTCACCTCACCCCTTCTAGC-CTTTT---CAGCCTATAT
Prmy    ATAACC-GATAATCCACG-TTAAACCTTACCCTTTCTTGCATC-C-----AGCCTATAT
Loli    AAAACC-GATACCCCCCG-TTAAACCTCACCCCCCCTTGCACTAT----CCGCCTATAT
Loam    AGAACC-GATACCCCCCG-TTAAACCTCACCCCTTCTTGTCAT--T--ACCGCCTATAT
Chab    AGAACC-GATAACCCCCG-TTCAACCTCACCCTTTCTTGTTATCC----CCGCCTATAT
Chto    AGAACC-GATAACCCCCG-TTCAACCTCACCCTTTCTTGTTATCC----CCGCCTATAT
Majo    AGAACC-GATAATCCCCG-TTCAACCTCACCCCTCCCTGTCAA----TTCTGCCTATAT
Hlst    AGAACC-GACAACCCCCG-TTCAACCTCACCCTCCCTTGTTT--TC--CCCGCCTATAT
Clpe    AGAACC-GATAATCCCCG-TTCAACCCGACCCTTTCTTGTTT--ATACCCCGCCTGTAT
Mlmr    AGAACC-GATGATCCCCG-TTCAACCTCACCCTTTCTTGCTCC-C---CCCGCCTGTAT
Crcr    AGAACC-GATACCCCCCG-TTCAACCTCACCCTCTCTTGTTT---A-TCCCGCCTATAT
Muce    ATAACC-GATACCCCCCG-TTCAACCTCACCCTCTCTTGTTTA--T--CCCGCCTATAT
Bege    AGAACC-GATACTCCCCG-TTAAACCTCACCCTCTCTTGTTCATC----CCGCCTATAT
Mela    AGAACC-GATAACCCCCG-TTTAACCTCACCCCCTCTTGCTC---T-TACCGCTTATAT
Hats    AGAACC-GATAACCCCCG-TTAAACCTCACCCTCCCTTGCTCTTT----CCGCCTATAT
Orla    AGAACC-GATAACCCCCG-TTAAACCTCACCCTCCCTTGT----TCTTTCCGCCTATAT
```

```
Cosa    AGAACC-GACAACCCCCG-TTAAACCTCACCCTCCCTTGTTTA-TTACCC-GCCTATAT
Exsp    AGAACC-GATAATCCCCG-TTAAACCTCACCCTCTCTTGTTCT-TC---CCGCCTATAT
Depa    AGAACC-GATAACCCCCG-TTCAACCTCACCCTTTCTTGT-TTATC---CCGCCTATAT
Rima    ATAACC-GATAATCCCCG-TTAAACCTTACCTTCTCTTGCT------CCCAGCCTATAT
Fuol    AGAACC-GATAACCCCCG-TTAAACCTCACCCCCTCTGGT-TATTT---CCGCCTATAT
Gmaf    AGAACC-GATAACCCCCG-TTAGACCTCACCTTTTCTTGT----CTATCCCGCCTATAT
Xeei    AGAACC-GATAACCCCCG-TCTAACCTCACCTTCCCTTGCCTA-T---CCCGCCTATAT
Pros    AGAACC-GATACCCCCCG-TTCAACCTCACCTCCCCTTGTTT---C-CCCCGTCTATAT
Scmi    AGAACC-GACACCCCCCG-TTCAACCTCACCTCCCCTTGTTT----TTTCTGTCTATAT
Rolo    AGAACC-GATAACCCCCG-TTCAACCTCACCCCCCCTTGTTTT----TACCGCCTATAT
Cere    AGAACC-GATAATCCCCG-TTCAACCTCACCCCCCCTTGTTTT--C--CCCGCCTATAT
Daga    AGAATC-GATAACCCCCG-TTTAACCTCACCCCCCCTTGT----TCATCCCGCCTATAT
Anco    AGAACC-GATAACCCCCG-TTCAACCTCACCCTCTCTTGTTTT--C--CCCGCCTATAT
Dmve    AGAACC-GATAATCCCCG-TTCAACCTCACCCTCTCTTGTCCC---ATTCCGCCTATAT
Dmar    AGAACC-GATAATCCCCG-TTCAACCTCACCCTCTCTTGTCCC-ACT--CCGCCTATAT
Anka    AGAACC-GATAACCCCCG-TTCAACCTCACCCTCTCTTGTCCC-CCCCCCCGCCTATAT
Moja    AGAACC-GATAACCCCCG-TTCAACCTCACCCTCTCTTGTT---TTCCCC-GCCTATAT
Hoja    AGAACC-GATAACCCCCG-TTCAACCTCACCCTCTCTTGTTTT--C--CCCGCCTATAT
Bede    AGAACC-GATAACCCCCG-TTCAACCTCACCCCCCTTTGTTT---TTCCC-GCCTATAT
Besp    AGAACC-GATAACCCCCG-TTCAACCTCACCCCCCTTTGTTTT----TCCCGCCTATAT
Mysp    AGAACC-GATACTCCCCG-TTCAACCTCACCTTTTCTTGTTTA-A---CCCGCCTATAT
Osja    AGAACC-GATAATCCCCG-TTCAACCTCACCTCTCCTTGT----TCAACCCGCCTATAT
Sgro    AGAACC-GATAATCCCCG-TTCAACCTCACCTTTCCTAGTTTA----ACCCGCCTATAT
Pzpa    AGAACT-GATAATCCCCG-TTAAACCTCACCCCCCCTTGTTA----ATACCGCCTATAT
Zeja    ATAACT-GATAACCCTCG-TTTAACCTCATCACCCCTTGTTCA----TACCGCCTATAT
Znne    AGAACC-GATAACCCCCG-TTCAACCTCACCACCCCTTGTTAA-TA---CCGCCTATAT
Zefa    AGAACT-GATAACCCCCG-TTAAACCTCACCAGCCCTTGTTA----ATACCGCCTATAT
Acni    AGAACT-GATAACCCCCG-TTAAACCTCACCGCCCCTTGTTAACA----CCGCCTATAT
Ncrh    AGAACT-GATAACCCCCG-TTAAACCTCACCGCCCCTTGTTA---ACACC-GCCTATAT
Agca    AGAACC-GATAACCCCCG-TTCAACCTTACCCTTCCTTGTT----TTTCCCGCCTATAT
Hydy    AGAACC-GATAACCCCCG-TTCAACCTCACCTCTCCTTGTTT----ATCCCGCCTATAT
Gsac    AGAACC-GATAACCCCCG-TTCAACCTCACCTCCCCTTGT----TAATACCGCCTATAT
Pevo    AGAACC-GATAACCCCCG-TTCAACCTCACCCTTTCTTGTT---ATC-TCCGCCTATAT
Hiku    ATAACT-GATAACCCCCG-TTAAACCTCACCCTATTTTGCCTA-AT---CAATCTATAT
Inpa    ATAATC-GATAACCCCCG-CTCAACCTCACCTTCCCTTGCC------CTCAGTCTATAT
Auch    CCAACC-GACACTTCCCG-TTAAACCTCACCCTTTCTCGTAA--C-TTACAGCCTGTAT
Fico    AGAACC-GATACCCCCCG-TTCAACCTCACCCTCCCTTGTT----TTACCCGCCTATAT
Macs    AGAACC-GATAACCCCCG-TTCAACCTCACCCTCCCTTGT----TTCTCCCGCCTATAT
Moal    ATAACC-GATAATCCCCG-TTAAACCTCACCCTCTCTTGCT----TTTCCCGTCTATAT
Syma    ATAACC-GATACTCCCCG-TTAAACCTTACCCTCCCTTGT----TTAATCCGCCTATAT
Mafr    ATAACC-GATAACCCCCG-TTCAACCTCACCTTCTTTTGTTA----ACCCCGCCTATAT
Dcpe    AGAACC-GATTCCCCCCG-TTCAACCTCACCCTCTCTTGCTC---CTTCCCGTCTATAT
Dcti    AGAACC-GATTCCCCCCG-TTCAACCTCACCCTCTCTTGCTCC-T-T-CCCGTCTATAT
Hehi    AGAACC-GATAACCCCCG-TTCAACCTCACCCTTCCTTGTT---TATCCC-GCCTATAT
Stam    AGAACC-GATAACCCCCG-TTCAACCTCACCTTTTCTTGTTTT--C--CCCGCCTATAT
Hogi    AGAACC-GATGACCCCCG-TTCAACCTCACCTTTTCTTGT----TAAACCCGCCTATAT
Erzo    AGAACC-GATAACCCCCG-TTCAACCTCACCTTTCCTTGTTTT-C---CCCGCCTATAT
Hxot    AGAACC-GATAACCCCCG-TTCAACCTCACCTTTCCTTGTTTT-TC---CCGCCTATAT
Core    AGAACC-GATAACCCCCG-TTCAACCTCACCCTTCCTTGTT---TTA-CCCGCCTATAT
Apve    AGAACC-GATAACCCCCG-TTCAACCTCACCCTTCCTTGTT----TTTCCCGCCTATAT
Latj    ATAACC-GACAATCCCCG-TTTAACCTCACCCCCTCTTGCTTC----TACCGCCTATAT
Laja    AGAACC-GATAATCCCCG-TTCAACCTCACCCTTCCTTGTTC--ATT-CCCGCCTATAT
```

```
Syja    ATAACC-GATAACCCCCG-TCTAACCTCACCCTTCCTTGTTC--TT--CCCGCCTATAT
Epme    ATAACC-GATAACCCCCG-TTTAACCTCACCTTTCCTAGTCAT----TCCCGCCTATAT
Grse    AGAACC-GATAACCCCCG-TTCAACCTCACCTTTCCTTGTTT---TTTCC-GCCTATAT
Clja    AGAACC-GATAGTCCCCG-TTCAACCTCACCTTTCCTTGTTAA-TA--CCCGCCTATAT
Ogcy    AGAACC-GATAATCCCCG-TTAAACCTCACCCCCTTTTGCCCC-TT--TCCGCCTATAT
Plna    AGAACC-GATAATCCCCG-TTAAACCTCACCTTCTCTTGCA---ATT-TCAGTCTATAT
Lema    AGAACC-GATAACCCCCG-TTCAACCTCACCCTTCCTTGTTT--TT--TCCGCCTATAT
Etzo    AGAACC-GATAACCCCCG-TTCAACCTCACCTCCCCTTGT----TCATCCCGCCTATAT
Apse    AGAACC-GATGACCCCCG-TTCAACCTCACCTTCTCTTGTTCT-C---CCCGCCTATAT
Epde    AGAACC-GATAACCCCCG-TTCAACCTCACCCTTCCTTGTT----CTCCCCGCCTATAT
Slja    AGAACC-GATAATCCCCG-TTTAACCTCACCCCCTCTTGCTC--T-GATCCGCCTATAT
Bsja    GGAACC-GATAATCCCCG-TTCAACCTCACCCTTTCTTGTTTT--T--CCCGCCTATAT
Ecna    AGAACC-GATAACCCCCG-TTTAACCTCACCCTCCCTTGTTTT----TTCCGCCTATAT
Cohi    ATAACC-GATAATCCCCG-TTAAACCTCACCTTCTCTCGC----AATATCAGCCTATAT
Caar    AGAACC-GATAACCCCCG-TTAAACCTCACCCTCTCTAGTTTAA---TACCGCCTATAT
Came    AGAACC-GATAATCCCCG-TTTAACCTCACCCCCCCTAGCTTT-T---TCCGCCTATAT
Mema    AGAACC-GATAACCCCCG-TTCAACCTCACCCTCCCTTGC----TTATCCCGCCTATAT
Lenu    GTAACC-GATACCCCCCG-TTTAACCTCACCCTTTCTTGCCC---C-TCCCGCCTATAT
Plma    ATAACC-GATAACCCCCG-TTCAACCTCACCCTCCCTTGTCTC----TCCCGCCTATAT
Emst    AGAACC-GATAACCCCCG-TTCAACCTCACCCTTCCTTGTT---TTT-CCCGCCTATAT
Ptti    AGAACC-GATTACCCCCG-TTCAACCTCACCTTTCCTTGTCCC-T---CCCGCCTATAT
Losu    AGAACC-GATAATCCCCG-TTCAACCTCACCCCTTCTTGCACT-TATATCAGCCTATAT
Geoy    AGAACC-GATACTCCCCG-TTAAACCTCACCCCTCTTTGTCTC-T---TCCGCCTATAT
Dipi    AGAACC-GATAACCCCCG-TTCAACCTCACCCTTCCTTGTTT---ACCCC-GCCTATAT
Pama    AGAACC-GATAATCCCCG-TTCAACCTCACCCTTCCTTGCTTG-T---CCCGCCTATAT
Leob    AGAACC-GATAATCCCCG-TTCAACCTCACCCTTTCTTGT----TAATCCCGCCTATAT
Neba    ATAACC-GATAATCCCCG-TTAAACCTCACCCTTCCCAGCCCA--ATTCCAGCTTATAT
Pdpl    AGAACC-GATAACCCCCG-TTCAACCTCACCCTCTCTTGC--C-CCTTTCCGCCTATAT
Nimi    AGAACC-GATAATCCCCG-TTCAACCTCACCCTTCCTTGTCC----TTTCCGCCTATAT
Uptr    AGAACC-GATAATCCCCG-TTCAACCTCACCCCTCCCTGTCA----ATTCTGCCTATAT
Pesc    AGAACC-GATACTCCCCG-TTCAACCTCACCCCTTCTTGCC---CAATCA-GCCTATAT
Baar    ATAATC-GATGACCCCCG-TTTAACCTCACCCTTCCTTGTTTT----TCCCGCCTATAT
Moar    AGAACC-GATAACCCCCG-TTCAACCTCACCCTTCCTTGTTTT----TCCCGCCTATAT
Toja    ATAACC-GATAACCCCCG-TTTAACCTCACCCTTTCTTGTT---TAAACC-GCCTATAT
Chau    AGAACC-GATAATCCCCG-TTAAACCTCACCCTTCCTTGTCA----TTTCCGCCTATAT
Chse    AGAACC-GATAATCCCCG-TTCAACCTCACCCTTCCTTGTTCT----TACCGCCTATAT
Enar    AGAACC-GATAACCCCCG-TTCAACCTCACCCTTCCTTGT-TTTTC---CCGCCTATAT
Hpty    AGAACC-GATAACCCCCG-TTCAACCTCACCCTTCCTTGT----TTTCCCCGCCTATAT
Nana    AAAACC-GATAACCCCCG-TTAAACCTCACCCCCTCTTGT-TATCC---CAGCCTATAT
Mcst    AGAACC-GATAACCCCCG-TTCAACCTCACCCTTCCTTGTTT----AACCCGCCTATAT
Rhox    AGAACC-GATAACCCCCG-TTCAACCTCACCCTTCCTTGC--T-CTTTTCCGCCTATAT
Opfa    AGAACC-GATAACCCCCG-TTCAACCTCACCCTTCCTTGTCCT--C--CCCGCCTATAT
Paar    AGAACC-GATGATCCCCG-TTAAACCTCACCTCTTCTTGTTCC-TAT-CCCGCCTATAT
Gozo    GGAACC-GATACCCCCCG-TTCAACCTCACCCCTCCTTGTT----TTTCCCGCCTATAT
Ackr    AGAACC-GATTCCCCCCG-TTAAACCTCACCTTTCCTTGCCCA-A---CCCGCCTATAT
Elev    AGAACC-GATAACCCCCG-TTAAACCTCACCCTTCCTAGCCTT--T-TTCCGCCTATAT
Trdu    AGAACC-GATAACCCCCG-TTAAACCTCACCCTCTCTTGTTCT--T--CCCGTCTATAT
Amoc    AGAACC-GATAACCCCCG-TTGAACCTCACCCTCCCTTGT-TAATA---CCGCCTATAT
Hame    AGAACC-GATAATCCCCG-TTAAACCTCACCTTTCCTTGTCTT-TA--TCCGCCTATAT
Chso    AGAACC-GATAATCCCCG-TTTAACCTCACCTTTTCTTGCT----TATTCCGCCTATAT
Lyto    AGAACC-GATAGCCCCCG-TTCAACCTCACCTTTCCTTGT----TTTACCCGCCTATAT
Encr    AGAACC-GATAACCCCCG-TTCAACCTCACCTTTCCTTGTTTT----TCCCGCCTATAT
```

```
Bvar    AGAACC-GACAACCCCCG-TTCAACCTCACCTTTCCTTGTTT----CTCCCGCCTATAT
Noco    AGAACC-GATAACCCCCG-TTCAACCTCACCTTTCCTTGT-TTTTC---CCGCCTATAT
Chsp    ATAACC-GATAACCCCCG-TTAAACCTCACCCTCTTTTGT----TCCAACCGCCTATAT
Arja    AGAACC-GATAACCCCCG-TTCAACCTCACCCTTCCTTGTTTT-T---CCCGCCTATAT
Pase    AGAACC-GATAATCCTCG-TTAAACCTCACCTCTCCTTGCAACGTGACCCAGTCTATAT
Trel    AGAACC-GATAACCCCCG-TTTAACCTCACCCCCTCTTGTTT---AGTCA-GTCTATAT
Lifa    AGAAAC-GACTCTACACG-TTTAACCTCACCCCTCCTAGT-A--AG--CCAGCCTATAT
Acur    AAAATC-GATAACCCTCG-TTTAACCTCACCCCTTCTAGTTCA--TTTTCCGCCTGTAT
Ampe    AGAACC-GATAACCCCCG-TTCAACCTCACCCCTTCTTGTTT----ATCCCGCCTATAT
Urja    AAAACC-GATGACCCCCG-TTCAACCTCACCCTCCCTTGTCTT-T---TCCGCCTATAT
Enet    GGAACC-GATACCCCCCG-TTAAACCTCACCTTCCCTGGCT----AATCCCGTCTATAT
Ptbr    AGAACC-GATACCCCTCG-TTAAACCTCACCCTTTCTTGCC----CAACCCGTCTATAT
Safa    AGAACC-GATAACCCTCG-TTAAACCTCACTTTTTCTTGCCCGCCA---CCGCCTATAT
Icae    GTAACC-GATGACCCCCG-TTCAACCTCACCCTCCCTTG---T-CTCTCCCGCCTATAT
Asmi    ATAACC-GATAACCCCCG-TTAAACCTCACCCTCACTTGT----TTA-TCAGCCTATAT
Foal    ACAACC-GATAATCCCCG-TTAAACCTTACCCTTTTTTGTTCTTTC---CCGCCTGTAT
Drze    AGAACC-GATAATCCCCG-TTCAACCTCACCCTCTCTTGCACT-TTATTCAGCCTATAT
Rhas    AGAACC-GATAACCCCCG-TTCAACCTCACCCTTTCTGGTCTT-T---CCCGCCTATAT
Elac    AGAACC-GATAACCCCCG-TTCAACCTCACCCTCCCTTGT----TGTCCCCGCCTATAT
Kugu    AGAACC-GATAATCCCCG-TTCAACCTCACCCCTTCTTGTTTA--TATCCAGCCTGTAT
Plor    AGAACC-GATACCCCCCG-TTAAACCTCACCCCTCCTTGTTA----CTTCCGTCTATAT
Sgun    AGAACC-GATAACCCCCG-TTAAACCTCACCCTTCCTTGTTTA--T--CCCGCCTATAT
Zaco    AGAACC-GATGCCCCCCG-TTCAACCTCACCCTTTCTTGTTTT----CCCCGCCTATAT
Zbfl    AGAACC-GATAACCCCCG-TTCAACCTCACCCTTCCTTGT-TTTCC---CCGCCTATAT
Spba    ATAACC-GATAAACCCCG-TTCAACCTCACCCTCTCTTGCTT--T-ATTCCGCCTATAT
Game    ATAACC-GATGACCCCCG-TTCAACCTCACCCTCCCTTGTTTT-T---CCCGCCTATAT
Thth    ATAACC-GATGACCCCCG-TTCAACCTCACCCTCCCTTGTT---TCT-CCCGCCTATAT
Xigl    AGAACC-GATAACCCCCG-TTCAACCTCACCCTCCCTTGCTT----TTCCCGCCTATAT
Hyja    GTAACC-GATACTCCCCG-TTAAACCTCACCCTCTCTTGT----TTTTCCCGCCTATAT
Psan    ATAACC-GATAATCCCCG-TTAAACCTCACCCTCTCTTGTT---TTTCCC-GCCTATAT
Cupa    ATAACC-GATGACCCCCG-TTTAACCTCACCCTCCCTTGTTTT-T---CCCGCCTATAT
Mpch    AGAACC-GATAACCCCCG-TTCAACCTCACCCTCTCTTGTTT---T-TCCCGCCTATAT
Char    AGAACC-GATAACCCCCG-TTTAACCTCACCCTCCCTTGT----TTATCCCGCCTATAT
Pser    AGAACC-GATAACCCCCG-TTCAACCTCACCCTCCCTTGTTCC-TT--TCCGCCTATAT
Prol    AGAACC-GATAACCCCCG-TTAAACCTCACCCTCTCTTGCCTC-A---TCCGCCTATAT
Plbi    AGAACC-GATAACCCCCG-TTAAACCTCACCCTCTCTTGTTT----TATCCGCCTATAT
Calu    ATAACC-GATAATCCCCG-TTAAACCTCACCCCCCCTTGCTCC-TGT-CCCGCCTATAT
Papa    GTAATC-GATGATCCCCG-TTCAACCTCACCCTCTCTAGCTCA--A--TCAGCCTGTAT
Sufr    AGAACC-GATAACCCCCG-TTCAACCTCACCTTTCCTTGTT----CAACCCGCCTATAT
Stci    GGAACC-GATAATCCCCG-TTAAACCTCACCTTTTCTTGTTCT-C---CCCGCCTATAT
Taru    AAAACC-GATACTCCCCG-TTCAACCTCACCCCTCCTTGTTT----TAACCGCCTATAT
Rala    AGAACC-GATAACCCCCG-TTAAACCTCACCCTTCCTTGTT---TCTCCC-GCCTATAT
                **       * *    ***          *             * ***
```

```
                35        36              36'
Scca    ACC-GCCGTCGTCAGCTC-ACCCGGT-GAGGGTT----AAAA------AGTAAGCAAAAA
Muma    ACC-GCCGTCGTCAGCTC-ACCCTGT-GAAGG-GT---CAAA------AGTAAGCAAAAA
Erca    ACC-GCCGTCGCCAGCCT-ACCTTCT-GAGAGAC----CTAA------AGTAGGCTAAAT
Pose    ACC-GCCGTCGCCAGCCT-ACCTTTT-GAAAGAC----CCTA------AGTAGGCATAAT
Actr    ACC-GCCGTCGTCAGCTT-ACCCTGT-GAAAGAC----TAAT------AGTAAGCAAAAA
Scal    ACC-GCCGTCGTCAGCTT-ACCCTGT-GAAAGAC-C---AAT------AGTAAGCAAAAA
Posp    ACC-GCCGTCGTCAGCTT-ACCCTGT-GAAAGACC---AA-T------AGTAAGCAAAAA
Atsp    ACC-GCCGTCGCCAGCTT-ACCCTGT-GAAGGAAA----AAC------AGTAAGCAAAGA
Leoc    ACC-GCCGTCACCAGCTT-ACCCTGT-GAAGGAAAA----AC------AGTGAGCAAAAA
Amca    ACC-GCCGTCGTCAGCTT-ACCCTGT-GAAGGGCTA----AT------AGTAAGCAGAAA
Osbi    ACC-GCCGTCGCAAGCTC-ACCCTAT-GAAGGAAA---TAGT------AGCAAGCAAGAA
Pabu    ACC-ACCGTCGCAAGCTC-ACCCTAT-GAAGGTCT----AAT------AGTAAGCAAAAT
Hial    ACC-GCCGTCGTCAGCTT-ACCCTGT-GAAGGAT----TAAT------AGTAAGCAAGAT
Elha    ACC-ACCGTCGCCAGCTT-ACCCTGT-GAAGGA--C--CAAT------AGTAAGCCTAAT
Mlcy    ACC-GCCGTCGCCAGCTT-ACCCTAT-GAAGGTC----CAAC------AGTAAGCCCAAA
Algl    ACC-GCCGTCGTCAGCTT-ACCTTGT-GAGGGAT----TAGT------AGTAAGCTTAAT
Ptgi    ACC-GCCGTCGCCAGCTT-ACCTTAT-GAGGGAC----TAAT------AGTAAGCCTAAT
Alaf    ACC-GCCGTCGCCAGCTC-ACCTTGT-GAAAGATAA----AT------AGTGAGCCTAAT
Nock    ACC-GCCGTCGCCAGCTC-ACCTTGT-GAAAGAC----TAAT------AGTGAGCTTAAT
Anja    ACC-GCCGTCGCCAGCTT-GCCTCTT-GAGAGATT---AA-A------AGCAAGCCTAAT
Gyki    ACC-GCCGTCGCCAGTTT-ACCTTTT-GAAAGAACA----AC------AGTAAGCAAAAC
Syka    ACC-GCCGTCGCCAGCTT-ACCTTAT-GAAAGCAC----AAC------AGTAAGCCAAAT
Opma    ACC-ACCGTCGCCAGCTT-ACCCCCT-GAGGG-TA---TAAC------AGTAAGCTAAAT
Comy    ACC-GCCGTCCCCAGCTT-GCCTTGT-GAAAGACAG----AT------AGCAAGCCAAAT
Sasp    ACC-GCCGTCGCCAGCTT-ACCTTCT-AAGAGAA----TGGT------AGTGAGCCCAAA
Eupe    ACC-ACCGTCGTCAGCTT-ACCTCTC-AAGAGAA----TGAT------AGCGAGCCCAAT
Enja    ACC-ACCGTCGCCAGCTT-ACCCTGT-GAAGGA-------AA------AGTAAGCGAAAT
Same    ACCCACCGTCGCCAGCTTTACCCTGT-GAAGGAA----CTAC------AGTAAGCAGGAT
Chch    ACC-GCCGTCGTCAGCTT-ACCCTGT-GAAGG-AT---GCCT------AGTAAGCAAAAT
Grgr    ACC-ACCGTCGCCAGCTT-ACCCCGT-GAGGGCCC----TAT------AGTAAGCAAAAT
Caau    ACC-GCCGTCGTCAGCTT-ACCCTGT-GAAGGTAA---TAAA------AGTAAGCAAAAT
Cyca    ACC-GCCGTCGTCAGCTT-ACCCTGT-GAAGGTA-A--TAAA------AGTAAGCAAAAT
Dare    ACC-ACCGTCGCCAGCTT-ACCCTGT-GAAGGAAT----AAC------AGTAAGCAAGAT
Cost    ACC-GCCGTCGTCAGCTT-ACCCTGT-GAAGGACTA----AT------AGTAAGCAAAGT
Leec    ACC-GCCGTCGTCAGCTT-ACCCTGT-GAAGGAT----TAAT------AGTAAGCTAAAT
Crla    ACC-GCCGTCGTCAGCTT-ACCCTGT-GAAGGCT----CAAT------AGTAAGCAAAGT
Clmc    ACC-GCCGTCGCAAGCTT-ACCCTGT-GAAGGCCCT----AC------AGTAAGCAAAAT
Phin    ACC-GCCGTCGCAAGCTT-ACCCTAT-GAAGGCCCC----AC------AGTAAGCACAAT
Icpu    ACC-GCCGTCGTCAGCTT-ACCCTGT-GAAGGCCT----AAC------AGTAAGCAAAAT
Psto    ACC-GCCGTCGTCAGCTT-ACCCTGT-GAAGGCTT----AAT------AGTAAGCAAAAT
Cora    ACC-GCCGTCGCCAGCTT-ACCCTAT-GAAGGCAA----AAC------AGTAAGCAAAAT
Eisp    ACC-GCCGTCGTCAGCTT-ACCCTGC-AAAGGCC----AAAC------AGTAAGCAAAAC
Apal    ACC-GCCGTCGTCAGCTT-ACCCTGT-GAAGGTA----AAAT------AGTAAGCAAAAT
Eslu    ACC-ACCGTCGCCAGCTT-ATCCTGT-GAAGGTC----CCAT------AATAAGCAAAAT
Dape    ACC-ACCGTCGCCAGCTT-ATCCCAT-GAGGG-AT---TTAT------AATAAGCAAAAG
Glse    ACC-GCCGTCGTCAGCTT-ACCCTGT-GAAGG-CC---TCAT------AGTAAGCAAAAA
Naar    ACC-GCCGTCGTCAGCTT-ACCCTGT-GAAGGCCTC----AT------AGTAAGCAAAAA
Baoc    ACC-GCCGTCGTCAGCTT-ACCCTGT-GAAGGTC----TCAT------AGTAAGCAAAAA
Opso    ACC-GCCGTCGTCAGCTT-ACCCTGT-GAAGGCCT---CA-T------AGTAAGCAGAAA
Alte    ACC-GCCGTCGTCAGCTT-ACCCTGT-GAAGGCCC----CAT------AGTAAGCAAAAT
Plap    ACC-GCCGTCGTCAGCTT-ACCCTGT-GAAGGCC----CCAT------AGTAAGCAAAAT
```

```
Plal    ACC-GCCGTCGTCAGCTC-ACCCTGT-GAAGGAC----TTAA------AGTGAGCAAAAT
Sami    ACC-GCCGTCGTCAGCTC-ACCCTGT-GAAGGTGT----TGA------AGTGAGCAAAAT
Rere    ACC-GCCGTCGTCAGCTT-ACCCTGT-GAAGGTT----CCAT------AGTAAGCAAAAT
Gama    ACC-GCCGTCGTCAGCAT-ACCCTGT-GAAGGGTT----CAT------AGTATGCAAGGT
Onmy    ACC-ACCGTCGTCAGCTT-ACCCTGT-GAAGGCCCC----AT------AGTAAGCAAAAT
Sasa    ACC-ACCGTCGTCAGCTT-ACCCTGT-GAAGGCCTT----AT------AGTAAGCAAAAT
Cola    ACC-ACCGTCGTCAGCTT-ACCCTGT-GAAGGAT----TTAT------AGTAAGCAAAAT
Dita    ACC-GCCGTCGTCAGCTT-ACCCTGT-AAAGG-AC---CCAT------AGTAAGCAAAAT
Gogr    ACC-GCCGTCGCCAGCTT-ACCCCTC-AAGTGCC----CCCC------AGTAAGCAAAAT
Chsl    ACC-GCCGTCGCAAACTT-ACCCTGT-GAGGG-AA---TCAT------AGTAAGTAAAAA
Atja    ACC-GCCGTCGTCAGCTT-ACCCCGC-AAGGGACT----CAT------AGTAAGCATAAT
Iido    ACC-GCCGTCGTCAGCTT-ACCCCGC-AAGGGCC----TTAT------AGTAAGCACAAT
Auja    ACC-GCCGTCGTCAGCTT-ACCCTTT-GAAGGCCC---CA-T------AGTAAGCAAAAT
Chag    ACC-GCCGTCGTCAGCTT-ACCCCTT-GAAGGAA----TGAT------AGTAAGCGAAAC
Hami    ACC-GCCGTCGCCAGCCT-ACCCTCT-AAGGGACCC----AA------AGTAGGCAAAAT
Saun    ACC-GCCGTCGCCAGCCT-ACCCTCT-AAGGGAC----CCAA------AGTAGGCAAAAT
Nema    ACC-GCCGTCGTCAGCTT-ACCCTGT-GAAGGCCT----AGT------AGTAAGCTAAAT
Disp    ACC-TCCGTCGTCAGCTT-ACCCCGT-GAGGGAAC----CAT------AGTAAGCTAAAT
Myaf    ACC-ACCGTCGTCAGCTT-ACCCTGT-GAAGGATAA----AT------AGTAAGCTAAAT
Lagu    ACC-GCCGCCGCCAGCTT-ACCCCGT-GAGGGAC----TAAA------AGTAAGCAAGAC
Trtr    ACC-GCCGCCGTCAGCTT-ACCCTGT-GAAGGTAA----AAT------AGTAAGCATAAA
Zucr    ACC-GCCGCCGTCAGCTT-ACCCTAT-GAAGGGA----TAAT------AGTAAGCATAAA
Pxja    ACC-GCCGTCGTCAGCTT-ACCCTGT-GAAGGAA----ATAT------AGTAAGCAAAGT
Pxlo    ACC-GCCGTCGTCAGCTT-ACCCTGT-GAAGGGA----GTAT------AGTAAGCAAAAT
Pctr    ACC-ACCGCCGTCAGCTT-ACCCTGT-GAAGGCC----CCAT------AGTAAGCAAAAT
Apsa    ACC-GCCGTCGTCAGCTT-ACCCTGT-GAAGGCCC----CAT------AGTAAGCAAAAT
Cabe    ACC-GCCGTCGTCAGTCT-ACCCCGT-GAGGGATA----AGA------AGTAGACAAAAT
Bzze    ACC-GCCGTCGTCAGCTT-ACCCTGT-GAAGGTC----ACAT------AGTAGGCAGAAT
Siim    ACC-TCCGTCGCCAGCCT-ACCCCCT-GAGAGTTTA----AT------AGTAGGCTCAAT
Ctru    ACC-GCCGTCGTCAGCTT-ACCCTGT-GAAGGCC----CCAT------AGTAAGCAAAAT
Dpbr    ACC-GCCGTCGTCAGCTT-ACCCTGT-GAAGGCCT---CA-T------AGTAAGCAAAAT
Caki    ACC-ACCGTCGTAAGCTT-ACCCTACTGAAGGCTAA----AA------AGTAAGCCCCAA
Phja    ACT-TCCGTCGTCAGCTA-ACCTTGC-AAAAGAAAG--GAAC------AGTTAGCACAAA
Brsp    ACC-GCCGTCGTCAGCTA-ACCTTAT-GAAAGAGAATCCCAA------CGTTAGCTCAAG
Gamo    ACC-ACCGTCGTCAGCTT-ACCCTGT-GAAGGAAA-----AT------AGTAAGCATA-A
Lolo    ACC-ACCGTCGTCAGCTT-ACCCTGT-GAAGG-TA---AAAT------AGTAAGCATAAA
Batr    ACC-CCCGTCTCCAGTTC-ACCTTAA-AAAAGAAC---AA-A------ATTGAACACAAC
Prmy    ACC-GCCGTCTTTAGTCT-ACCTTA--AAAAGTT----AAAT------ATTAGGCAAAAT
Loli    ACC-GCCGCCGTCAGCTT-ACCCTGT-GAAGGTTT---AG-T------AGTAAGCAAAAT
Loam    ACC-CCCGCCTTCAGCTT-ACCCTGT-GAGGGAT----TAGT------ACTAAGCAAAGT
Chab    ACC-GCCGTCGTCAGCTT-ACCCTGT-GAAGGCCT---AA-T------AGTAAGCAAAAT
Chto    ACC-GCCGTCGTCAGCTT-ACCCTGT-GAAGGCCT---AA-T------AGTAAGCAAAAT
Majo    ACC-GCCGTCGCAAGCCT-ACCCTGT-GAAGGAAC----CAT------AGTGGGCTATAC
Hlst    ACC-GCCGTCGTCAGCCT-ACCCCCT-GAGGGTTAA----AT------AGTAAGCGAAAT
Clpe    ACC-TCCGTCGTCAGCTT-ACCCTGT-GAAGGCC----TAAA------AGTAAGCACAAT
Mlmr    ACC-TCCGTCGTCAGCTT-ACCCTGT-AAAGG-CC---CAAC------AGTAAGCAAAAT
Crcr    ACC-GCCGTCGTCAGCCC-ACCCTGT-GAGGGTCC----AAT------AGTGAGCAGGAT
Muce    ACC-GCCGTCGTCAGCCC-ACCCTGT-GAGGGTC----CAAT------AGTGAGCAGGAT
Bege    ACC-GCCGTCGTCAGCTT-ACCCTAT-GAAGGCCC---CA-A------AGTAAGCAAAAT
Mela    ACC-GCCGTCGTCAGCTT-ACCCTGT-GAGGGACT----AAT------AGTGAGCAAAAT
Hats    ACC-GCCGTCGCCAGCTT-ACCCTGT-GAAGGCCT---AA-T------AGTAAGCAAAAT
Orla    ACC-GCCGTCGTCAGCTT-ACCCTGT-GAAGGAC-A---AAT------AGTAAGCAAAAC
```

```
Cosa          ACC-GCCGTCGTCAGCTT-ACCCCAT-GAAGG-AC---TTAT------AGTAAGCAAGAC
Exsp          ACC-GCCGTCGTCAGCCT-ACCCTGT-GAAGGCC----TCAT------AGTGAGCATAAT
Depa          ACC-GCCGTCGTCAGCTT-ACCCTGT-GAAGGATC---TA-T------AGTAAGCAAAAC
Rima          ACC-GCCGTCGTCAGCCC-ACCCTGT-GAAGGCC----CACT------AGTAGGCAAAAC
Fuol          ACC-GCCGTCGTCAGCCC-ACCCTGT-GAAGGTAT---AA-T------AGTGAGCCTAAT
Gmaf          ACC-GCCGTCGTCAGCTT-ACCCTGT-GAAGGAT----TAAT------AGTAAGCAAAAC
Xeei          ACC-GCCGTCGTCAGCAT-ACCCTGT-GAAGG-GA---CAAT------AGTAAGCAAAAT
Pros          ACC-ACCGTCGTCAGCTG-ACCCTGT-GAAGGACT----TAT------AGTAAGCAAAAT
Scmi          ACC-ACCGTCGTCAGCTG-ACCCTAT-GAAGGAC----CCAG------AGTAAGCACAAT
Rolo          ACC-ACCGTCGTCAGCTT-ACCCTGT-GAAGGCCT----TAT------AGTAAGCAAGAT
Cere          ACC-GCCGTCGTCAGCTT-ACCCTGT-GAAGGTA----AAAT------AGTAAGCAGAAT
Daga          ACC-GCCGTCGTCAGCTT-ACCCTAT-GAAGGTA-T---TAT------AGTAAGCAAGGT
Anco          ACC-GCCGTCGTCAGCTT-ACCCTGT-GAAGGTT----TAAT------AGTAAGCAAAAT
Dmve          ACC-ACCGTCGTCAGCTT-ACCCTCT-GAGGGTCT----TAT------AGTAAGCAAGAC
Dmar          ACC-ACCGTCGTCAGCTT-ACCCTTT-GAGGGTC----TCAT------AGTAAGCAAGAC
Anka          ACC-GCCGTCGTCAGCTT-ACCCTGT-GAAGG-CC---CCAT------AGTAAGCAAAAT
Moja          ACC-GCCGTCGTCAGCTT-ACCCTGT-GAAGGTC----TTAT------AGTAAGCAAAAT
Hoja          ACC-GCCGTCGTCAGCTT-ACCCTGT-GAAGGTC----TTAT------AGTAAGCAAAAT
Bede          ACC-ACCGTCGTCAGCTT-ACCCTGT-GAAGGCCC----TAT------AGTAAGCAAAAT
Besp          ACC-ACCGTCGTCAGCTT-ACCCTGT-GAAGGCCC----TAT------AGTAAGCAAAAT
Mysp          ACC-GCCGTCGTCAGCTT-ACCCTGT-AAAGG-CC---CTAT------AGTAAGCAAAAA
Osja          ACC-GCCGTCGTCAGCTT-ACCCTGT-GAAGGCC----CCAT------AGTAAGCAAAAA
Sgro          ACC-GCCGTCGCCAGCTT-ACCCTGT-GAAGGTCT----TAT------AGTAAGCAAAAG
Pzpa          ACC-ACCGTCGTCAGCTT-ACCCTGT-GAAGGAAA----AAT------AGTAAGCAAAAT
Zeja          ACC-GCCGTCGTCAGCTT-ACCCTGT-GAAGGAAA----AAC------AGTAAGCAAAAT
Znne          ACC-GCCGTCGTCAGCTT-ACCCTGT-GAAGGTT----TAAC------AGTAAGCAAGAC
Zefa          ACC-GCCGTCGTCAGCTT-ACCCTGT-GAAGGCA----CAAT------AGTAAGCAAAGT
Acni          ACC-GCCGTCGTCAGCTT-ACCCTGT-GAAGGTAA---AA-C------AGTAAGCAAAAT
Ncrh          ACC-GCCGTCGTCAGCTT-ACCCTGT-GAAGGTAA----AAC------AGTAAGCAAAAT
Agca          ACC-GCCGTCGTCAGCTT-ACCCTGTTGAAGGCC----CCAC------AGTAAGCAAAAT
Hydy          ACC-ACCGTCGTCAGCTT-ACCCTGT-GAGGGAT----TAGT------AGTGAGCTAAAC
Gsac          ACC-ACCGTCGTCAGCTT-ACCCTGT-GAGGGAC----TAAT------AGTAAGCTAAAC
Pevo          ACC-GCCGTCGTCAGCTT-ACCCTGT-AAAGGACCC----AA------AGTAAGCAATAT
Hiku          ACC-GCCGTCGTCAGCTT-ACCTTAT-GAAAGAACA----AC------AGTGAGCAGAAT
Inpa          ACC-GCCGTCGTCAGCTT-ACCCTTT-GAGGA-AC---TTAA------AGTAAGCCTAAT
Auch          ACC-GCCGTCGCCAGCCT-ACCCTAT-GATGGAC----CCGC------AGTGAGCTTGAA
Fico          ACC-GGCGTCGTCAGCTT-ACCCCGT-GAGGGAC----AAAC------AGTAAGCAAAAT
Macs          ACC-GCCGTCGTCAGCTT-ACCCTGT-GAAGGT--T--TAAT------AGTAAGCAAAAT
Moal          ACC-GCCGTCGTCAGCTT-ACCCC-T-AAGGGTT----CAAC------AGTAAGCAAAAT
Syma          ACC-GCCGTCGTCAGCTT-ACCCCTT-AAGAGCC-A---AAC------AGTAAGCAAATC
Mafr          ACC-GCCGTCGTCAGCTT-ACCCCCC-AAGGGAT----AAAC------AGTAAGCATAAT
Dcpe          ACC-GCCGTCGTCAGCTC-ACCCTTT-GAAGGAAA----AAC------AGTGGGCATAAC
Dcti          ACC-GCCGTCGTCAGCTC-ACCCTTT-GAAGGAAAA----AC------AGTGGGCGCAAC
Hehi          ACC-GCCGTCGTCAGCTT-ACCCTGT-GAAGGCC----TAAA------AGTAAGCACAAC
Stam          ACC-GCCGTCGTCAGCTT-ACCCTGT-GAAGGTCT----TAT------AGTAAGCAAAAT
Hogi          ACC-GCCGTCGTCAGCTT-ACCCCGT-AAGGGAC-A---AAC------AGTAAGCAAAAT
Erzo          ACC-GCCGTCGTCAGCTT-ACCCTGT-GAAGGTC----TAAT------AGTAAGCAAAAC
Hxot          ACC-GCCGTCGTCAGCTT-ACCCTCT-GAAGGTC----TAAT------AGTAAGCAAAAC
Core          ACC-GCCGTCGTCAGCTT-ACCCTGT-GAGGGACTA----AT------AGTAAGCAGAAC
Apve          ACC-GCCGTCGTCAGCTT-ACCCTGT-GAAGGAC----GAAT------AGTAAGCAAAAC
Latj          ACC-ACCGTCGTCAGCCT-ACCCTGT-GAAGGCCC----AAT------AGTATGCAGAAC
Laja          ACC-GCCGTCGTCAGCTT-ACCCTGT-GAAGGTCTA----AT------AGTAAGCATAAT
```

```
Syja ACC-GCCGCCGTCAGCTT-ACCCTGT-GAAGGATCC----AT------AATAAGCAAAAT
Epme ACC-GCCGTCGCCAGCTT-ACCCTGT-GAAGG-CC---CCCT------AGTAAGCTTAAT
Grse ACC-GCCGCCGTCAGCCT-ACCCTGT-GAAGGCCC----AGC------AGTGAGCAAAAT
Clja ACC-GCCGTCGCCAGCTT-TCCCCGT-GAGGG-TT---AAAT------AGTTAGCACAAT
Ogcy ACC-GCCGTCGTCAGCTT-ACCCTGT-AAGGGATCA----AT------AGTAGGCTCAAT
Plna ACC-GCCGTCTTCAGTTT-ACCCCGC-GAGGGGCGC----AT------AGTAAGCGCAAT
Lema ACC-ACCGTCGTCAGCTT-ACCCTGT-GAAGGCCTT----AT------AGTAAGCAAAAT
Etzo ACC-GCCGTCGTCAGCTT-ACCCTGT-GAAGGTA----AAAT------AGTAAGCAAAAT
Apse ACC-GCCGTCGTCAGCCC-ACCCTGT-GAAGGCCCA----GT------AGTGAGCAGAAT
Epde ACC-GCCGTCGTCAGCTT-ACCCTGT-GAAGGAC----TCAT------AGTAAGCAAAAT
Slja ACC-GCCGTCGCAAGCTT-ACCCTTT-GAGGGAC----TAAT------AGTAAGCACAAT
Bsja ACC-GCCGTCGCAAGCTC-ACCCTGT-GAAGGAT----AAAC------AGTGAGCCCAAC
Ecna ACC-ACCGTCGTCAGCTT-ACCCTGT-GAAGGCCT----AAT------AGTAAGCAAAAT
Cohi ACC-GCCGTCGCCAGCTT-ACCCTTT-GAAGGAC----TAAC------AGTAAGCACAAT
Caar ACC-ACCGTCGCCAGCTT-ACCCTGT-GAAGGCCT----AAT------AGTAAGCACAAT
Came ACC-ACCGTCGCCAGCTT-ACCCTGT-GAAGG-AC---TAAT------AGTAAGCACAAT
Mema ACC-ACCGTCGTCAGCTT-ACCCTGT-GAAGGC--C--TAAT------AGTAAGCAAAAT
Lenu ACC-GCCGTCGTCAGCTT-ACCCTGT-AAAGGCCC----CAT------AGTAAGCAAAAT
Plma ACC-GCCGTCGTCAGCTT-ACCCTGT-GAGGG-TT---TAAT------AGTAAGCAAAAT
Emst ACC-ACCGTCGTCAGCTT-ACCCTGT-GAAGGCCTT----AT------AGTAAGCAAAAT
Ptti ACC-ACCGTCGTCAGCTT-ACCCTGT-GAAGGACTC----AT------AGTAAGCAAGAT
Losu ACC-ACCGTCGTAAGTTT-ACTCTGT-GAAGAGC----CCGT------AGTAAGCGAAAT
Geoy ACC-GCCGTCGTAAGCTT-ATCCCGT-GAGGG-TC---TAGT------ATTTAGCTTAAT
Dipi ACC-ACCGTCGTCAGCTT-ACCCTGT-GAAGGCCC----AAC------AGTAAGCAGAAT
Pama ACC-ACCGTCGCCAGCTT-ACCCTGT-GAAGG-GT---AAAA------AGTAAGCGAAAT
Leob ACC-ACCGTCGTCAGCTT-ACCCTCT-GAAGGTC----TAAT------AGTAAGCACAAT
Neba ACC-GCCGTCGTCAGCTT-ACCCCGT--AAGGGTC----CACCCAAAAAGTAGGCGCAAT
Pdpl ACC-GCCGTCGTCAGCTT-ACCCTGT-GAAGGAG-A--TAAA------AGTGAGCAAAAT
Nimi ACC-GCCGTCGTCAGCTT-ACCTTGT-GAAAGAC----TAAT------AGTAAGCGAAAT
Uptr ACC-GCCGTCGCCAGCCT-ACCCTGT-GAAGG-AA---CCAT------AGTGGGCCATAC
Pesc ACC-GCCGTCGTCAGCTA-ACCCTGT-GAAGGTC----TAGC------AGTAAGCAAGAT
Baar ACC-GCCGTCGCCAGCTT-ACCCTGT-GAAGGACT----AAT------AGTGAGCAGAAT
Moar ACC-ACCGTCGTCAGCTT-ACCCTAT-GAAGGCCC----TAT------AGTAAGCAAAAT
Toja ACC-ACCGTCGTCAGCTT-ACCCTGT-GAAGGCC----TAGT------AGTGAGCAGGCT
Chau ACC-ACCGTCGCCAGCTT-ACCCTGT-GAAGGCC----CAAT------AGTAAGCACAAT
Chse ACC-ACCGTCGCCAGTTT-ACCCTGT-GAAGGTCT----AAC------AGTAAACACAAT
Enar ACC-GCCGTCGTCAGCTT-ACCCTGT-GAAGGAAC---TA-T------AGTAAGCAAGAT
Hpty ACC-GCCGTCGTCAGCTT-ACCCTGT-GAAGGCC----CAAT------AGTAAGCTAAAT
Nana ACC-GCCGTCGTCAGCTT-ACCCTGT-GAGGGTCT---AA-A------AGTAAGCAGAAT
Mcst ACC-GCCGTCGTCAGCTT-ACCCTGT-GAAGGAC----CCAT------AGTAAGCAAAAT
Rhox ACC-GCCGTCGCCAGCTT-ACCCTAT-GAAGGAC----CCAC------AGTAAGCAAAAT
Opfa ACC-GCCGTCGTCAGCTT-ACCCTGT-GAAGGCCT----AAT------AGTAAGCAAAAT
Paar ATC-TCCGTCGCCAGCTT-ACCCTGT-GAAGGCTCAT---AA------AGTAAGTCAAAC
Gozo ACC-GCCGTCGTCAGCTT-ACCCTGT-GAAGGTTA----TGT------AGTAAGCAAAAT
Ackr ACC-ACCGTCGTCAGCTT-ACCCTGT-GAAGG-CC---TACT------AGTAAGCGAAAT
Elev ACC-ACCGTCGTCAGTTT-ACCCTAT-GAAGGATT----AAC------AGTAAGCAAGAC
Trdu ACC-GCCGTCGTCAGCTT-ACCCTAT-GAAGGAG----CCAC------AGTAAGCAAAAC
Amoc ACC-GCCGTCGTCAGCCT-ACCCTGT-GAAGGACT---AG-A------AGTGAGCAAGAT
Hame ACC-GCCGTCGTCAGCTT-ACTCTGT-GAAGACCCC----AT------AGTAGGCACAAT
Chso ACC-GCCGTCTCCAGTCT-ACCCTGT-AAAGGACCC----AT------AGTAGGCGTAAT
Lyto ACC-GCCGTCGTCAGCTT-ACCCTGT-GAAGGTC-T---AAT------AGTAAGCAAAAC
Encr ACC-GCCGTCGTCAGCTT-ACCCTGT-GAAGGCCA----AAT------AGTAAGCAAAAC
```

```
Bvar    ACC-ACCGTCGTAAGCTG-ACCCTGT-GAAGGAA----TTAT------AGTAAGCTTAAC
Noco    ACC-GCCGTCGTCAGCCC-ACCCTAT-GAAGGTTT---AA-A------AGTAGGCTAAAT
Chsp    ACC-GCCGTCATCAGCCT-ACCCTGT-GAAGGC--C--TAAT------AGTGGGCATAAC
Arja    ACC-GCCGTCGTCAGCTT-ACCCTGT-GAAGG-TC---TAAT------AGTAAGCAAAAC
Pase    ACC-GCCGTCGTAAGCCC-ACCCTGT-GAAGGAC----TAGT------AGTGGGCTCAAC
Trel    ACC-ACCGTCGTCAGCTC-ACACTCT-GAAGGAAC----AAA------AGTGAGCGAAAT
Lifa    ACC-ACCGTCGCCAGCCC-ACCCCGT-GAGGGAATA----AC------AGTAGGCTAGAC
Acur    ACC-TCCGTCGTAAGCTT-ACCCTGT-GAAGGACT----AAT------AGTAAGCTCAAC
Ampe    ACC-GCCGTCGTCAGCTT-ACCCTGT-GAAGGAA----GAAT------AGTAAGCATAAT
Urja    ACC-GCCGTCGTCAGCTT-ACCCTAT-GAAGG-AC---GAAC------AGTGAGCACAAC
Enet    ACC-GCCGTCGTCAGCTT-ACCCCCT-GAGGGGT----CCAC------AGTAAGCAAGAT
Ptbr    ACC-GCCGTCGTCAGCTC-ACCCTGT-GAAGGTC----TTAA------AGTAAGCATAAT
Safa    ACC-GCCGTCGTCAGCTT-ACCCTAT-GAAGGACA---AA-C------AGTAAGCAGAAT
Icae    ACC-GCCGTCGTCAGCTT-ACCCTGT-GAGGGTT----TAAT------AGTAAGCAAAAT
Asmi    ACC-GCCGTCGTCAGTTC-ACCCTAT-GAAGGCT----TAAT------AGTGAGCACAAT
Foal    ACC-TCCGTCTCCAGCTT-ACCCTGT-GAAGGATT---AA-T------AGTAAGCACAAC
Drze    ACC-GCCGTCGCCAGCTT-ACCCTGT-GAGAG-AC---GAAT------AGTAAGCACAAT
Rhas    ACC-ACCGTCGTCAGCTT-ACCCTAT-GAAGGCCTT----AT------AGTAAGCAAAAC
Elac    ACC-GCCGTCGTCAGCCT-ACCCTAT-GAAGGAC----ATAA------AGTAAGCAAAAT
Kugu    ACC-TCCGTCTCCAGCTT-ACCCTGT-GAAGGTCC----AAT------AGTAGGCACAAC
Plor    ACC-GCCGTCGTCAGCCT-ACCCTGT-GAAGGTC----TAAT------AGTAAGCAAAAT
Sgun    ACC-ACCGTCGTCAGCTT-ACCCTGC-GAAGACCC----AAT------AGTAAGCACAAT
Zaco    ACC-ACCGTCGTCAGCTT-ACCCTGT-GAAGGCCT----CAT------AGTAAGCAAGAT
Zbfl    ACC-ACCGTCGTCAGCTT-ACCCTGT-GAAGGTTA---AAAC------AGTAAGCAAAAT
Spba    ACC-GCCGTCGTCAGCTT-ACCCTGT-GAAGGCCC----AAT------AGTAAGCACAAT
Game    ACC-GCCGTCGTCAGCTT-ACCCTGT-GAAGG-TC---TAAT------AGTAAGCAAAAT
Thth    ACC-GCCGTCGTCAGCTT-ACCCTGT-GAAGGTCTA----AT------AGTAAGCAAAAT
Xigl    ACC-ACCGTCGTCAGCTT-ACCCTGT-GAAGGCC----TAAT------AGTAAGCAGAAT
Hyja    ACC-GCCGTCGTAAGCTT-ACCCTGT-GAAGGAAAT--ATAT------AGTAAGCAAAAT
Psan    ACC-GCCGTCGTAAGCTT-ACCCTCT-GAGGGGAAT--TTGT------AGTAAGCACAAT
Cupa    ACC-GCCGTCGTCAGCTT-ACCCTGT-GAGGGTTTA----AC------AGTAAGCAAAAT
Mpch    ACC-GCCGTCGTCAGCTT-ACCCTGT-GAAGGATC----AAC------AGTAAGCAAAAA
Char    ACC-GCCGTCGTCAGCTT-ACCCTAT-GAAGGTC-T---AAT------AGTAAGCAAAAT
Pser    ACC-ACCGTCGTCAGCTC-ACCCTGT-GAAGGATTA----AT------AGTGAGCAAAGT
Prol    ACC-ACCGTCGTCAGCTT-ACCCTGT-GAAGG-CT---TAAC------AGTAAGCAAAGT
Plbi    ACC-ACCGTCGTCAGCTT-ACCCTGT-GAAGGAT----TTAC------AGTAAGCAAAAT
Calu    ACC-GCCGTCGTCAGCTT-ACACTGA-GAAGGGATA----AA------AGTAAGCAAAAC
Papa    ACC-TCCGTCGCAAGCTT-ACCCTAGCAAAGGTT----CAGT------AGTAAGCAATAA
Sufr    ACC-GCCGTCGTCAGCTT-ATCCTGT-GAAGGCT----TCTT------AATGAGCAAAAT
Stci    ACC-GCCGTCGTCAGCTT-ACCCTAT-GAAGGAA----CAAC------AGTTAGCAGAAT
Taru    ACC-ACCGTCGTCAGCCT-ACCCTGT-GAAGGGC----AAAT------AGTAGACAAAAT
Rala    ACC-ACCGCCGTCAGCTT-ACCCTGT-GAAGGCA----CAAT------AGTAAGCAAAAT
        *      *  *   *              *
```

```
      37¦  HVR   ¦37'           35'          34'
Scca  G-AATTAA----ACTCC---TAAACGTCAGGTCGAGGTGTAGCGA-ATGAAGTGGA-A
Muma  G-AATT----AAACTCC---AAAACGTCAGGTCGAGGTGTAGCAA-ATGAAGTGGG-A
Erca  A-AGTA-----AAACTT---AACACGTCAGGTCAAGGTGTAGCTT-ATGATATGGG-A
Pose  A-AGTAA-----AACTT---AATACGTCAGGTCAAGGTGTAGCTT-ATGATGTGGG-A
Actr  T-GGCA----CACCCAA---AA-ACGTCAGGTCGAGGTGTAGCGA-ATGAAGTGGA-A
Scal  T-GGCA-----CACCCA---AAAACGTCAGGTCGAGGTGTAGCGA-ATGAAGTGGA-A
Posp  T-GGCA-----CACCCA---AAAACGTCAGGTCGAGGTGTAGCGA-ATGAAGTGGA-A
Atsp  G-GGAA-----CACCCC---AAAACGTCAGGTCGAGGTGTAGCGT-ACGAAGTGGG-A
Leoc  G-GGGACA-----CCCC---AAAACGTCAGGTCGAGGTGTAGCGT-ATGAAGTGGA-A
Amca  G-AACT-CA----CTTA---AAAACGTCAGGTCGAGGTGTAGCGT-ATGGAGTGGA-A
Osbi  G-GGCTTCG-----CCC---AGAACGTCAGGTCGAGGTGTAGCAA-ATGAAACGGG-A
Pabu  G-ATTAAAA-----CTC---AGCACGTCAGGTCGAGGTGTAGCGA-ATGGAGCGGG-A
Hial  G-GGTA-CA----ACCC---AGAACGTCAGGTCGAGGTGTAGCGT-ATGAGGTGGG-A
Elha  G-AGCA-----CAGCTC---AAAACGTCAGGTCGAGGTGTAGCGA-ATGGGGTGGG-A
Mlcy  G-AGCA-----TAACTC---AAAACGTCAGGTCGAGGTGTAGCGC-ACGAAGCGGG-A
Algl  G-GGTAGA-----GCCC---AGAACGTCAGGTCGAGGTGTAGCTA-ATGGGGTGGG-A
Ptgi  G-GGTA----AAT-CCC---AAAACGTCAGGTCGAGGTGTAGCGA-ATGGGGTGGG-A
Alaf  G-AGAATCA----TCCC---AAAACGTCAGGTCGAGGTGTAGCGA-ATGGGGTGGG-A
Nock  G-GGAA-----CATCCC---AGAACGTCAGGTCGAGGTGTAGCGA-ATGAAGTGGG-A
Anja  G-GGTT-----CTGCCC---AAAACGTCAGGTCGAGGTGTAGCGA-ATGAGATGGA-A
Gyki  G-GGTAT-T----ACCC---AACACGTCAGGTCGAGGTGTAGCGA-ATGGAGGGGG-A
Syka  G-GGTCCCC----CCCA---TAAACGTCAGGTCGAGGTGTAGCGA-ACGGAGTGGG-A
Opma  G-GGCCC----CCACCC---AGCACGTCAGGTCGAGGTGTAGCGA-ACGCAGTGGG-A
Comy  G-GGTTTT-----ACCC---AGAAAGTCAGGTCGAGGTGTAGTTA-ATGAAGTGGG-A
Sasp  T-AATA-----AAATTA---AAAACGTCAGGTCAAGGTGTAGCGG-ATGAAAAGGA-A
Eupe  G-AGTAAAA-----CTC---AAAACGTCAGGTCAAGGTGTAGCGT-ATGAGGAGGA-A
Enja  G-GAGAC-----TCTCC---AAAACGTCAGGTCGAGGTGTAGCAC-ACGGAGTGGG-A
Same  G-AGCA----TTG-CTC---AAAACGTCAGGTCGAGGTGTAGCGT-ACGAAGTAGG-A
Chch  G-AGCAA----G-ACTC---AAAACGTCAGGTCGAGGTGTAGCGC-ATGAAGTGGG-A
Grgr  G-GGCACAG-----CCC---AGAACGTCAGGTCGAGGTGTAGCGT-ACGGGGTGGG-A
Caau  G-GGTA-----CAACCC---AAAACGTCAGGTCGAGGTGTAGCGC-ATGAAGTGGG-A
Cyca  G-GGCA-----CAACCC---AAAACGTCAGGTCGAGGTGTAGCGC-ATGAAGTGGG-A
Dare  G-GGCATA-----GCCC---AGAAAGTCAGGTCGAGGTGTAGCAC-ATGAAATGGG-A
Cost  G-GACACA-----ATCC---AAAACGTCAGGTCGAGGTGTAGCGA-ACGAAGTGGG-A
Leec  G-GAT-AC----ATTCC---AAAACGTCAGGTCGAGGTGTAGCGA-ACGAAGTGGG-A
Crla  G-GGCA-----CAACCC---AAAACGTCAGGTCGAGGTGTAGCGT-ACGAAGTGGG-A
Clmc  G-GGTA-CA----TCCC---AAAACGTCAGGTCGAGGTGTAGCTC-ACGAGGTGGA-A
Phin  G-GGCACA-----GCCC---AAAACGTCAGGTCGAGGTGTAGCTT-ACGAGATGGA-A
Icpu  G-GGC-----CCGCCCA---AAAACGTCAGGTCGAGGTGTAGCGT-ACGAAGTGGG-A
Psto  G-GGC-----CCGCCCA---AAAACGTCAGGTCGAGGTGTAGCGT-ACGAAGTGGG-A
Cora  G-GACAAAA----CCCA----AAACGTCAGGTCGAGGTGTAGCGC-ACGAAGTGGG-A
Eisp  G-GGCAC-----AGCCC---AAAACGTCAGGCCGAGGTGTAGCGT-ACGAGATGGG-A
Apal  G-GGCACA-----ACCC---AAAACGTCAGGCCGAGGTGCAGCAC-ATGGGGTGGG-A
Eslu  G-GGTAAA-----ACCC---AGAACGTCAGGTCGAGGTGTAGCGC-ATGAGCTGGG-A
Dape  G-GGCAA----A-ACCC---AAAACGTCAGGTCGAGGTGTAGCGC-ATGGGGCGGG-A
Glse  G-GATA-----TAACCC---AAAACGTCNGGTCGAGGTGTAGCAN-ATGGGGTGGG-A
Naar  G-GG-TAAA----ACCC---TAAACGTCAGGTCGAGGTGTAGCGT-ATGGGGTGGG-A
Baoc  G-GGCAG-----ACCCC---CGAACGTCAGGTCGAGGTGTAGCGC-ATGAGGTGGG-A
Opso  G-AGTA-----CAACCC---CAAACGTCAGGTCGAGGTGTAGCGC-ATGAGGTGGG-A
Alte  G-GAC-----AAAACCC---AGAACGTCAGGTCGAGGTGTAGCGT-ACGGGGTGGA-A
Plap  G-GGTAA-----AACCC---AGAACGTCAGGTCGAGGTGTAGCGT-ACGGGGTGGG-A
```

```
Plal    G-GGCA-----AAGCCC---AAAACGTCAGGTCAAGGTGCAGCGT-ATGGGGTGGG-A
Sami    G-GGTACA-----ACCC---AAAACGTCAGGTCAAGGTGCAGCGT-ATGAGGTGGG-A
Rere    G-GACATA-----ACCC---AGAACGTCAGGTCAAGGTGCAGCGT-ATGGGGTGGG-A
Gama    G-GGTAGAG-----CCC---AAAACGTCAGGTCGAGGTGTAGCGT-ATGAGGAGGG-A
Onmy    G-GGCAA-A-----ACC---AAAACGTCAGGTCGAGGTGTAGCGC-ATGAGGTGGG-A
Sasa    G-GGCA-AA----ACCC---AAAACGTCAGTCGGAGGTGTAGCGC-ATGGGGTGGG-A
Cola    G-GGCA-----TGACCC---AAAACGTCAGGTCGAGGTGTAGCGC-ATGGGGTGGG-A
Dita    G-GGCA-----CAGCCC---GGAACGTCAGGTCGAGGTGTAGCGT-ATGGGGTGGA-A
Gogr    A-GGCAC-----AACCT---AAAACGTCAGGTCGAGGTGTAGCGC-ACGCAGTGGA-A
Chsl    G-GGCA-----CAGCCC---AGAACGTCAGGTCGAGGTGCAGCAT-ACGAGGTGGG-A
Atja    T-AGCA-----TCGCTC---AATACGTCAGGTCGAGGTGTAGCAT-ATGAGGTGGG-A
Iido    T-AGCATC-----GCTC---AATACGTCAGGTCGAGGTGTAGCAT-ATGAGGTGGG-A
Auja    G-GGCA-----CAGCCC---AAAACGTCAGGTCGAGGTGTAGCGT-ATGAAGTGGG-A
Chag    A-GGTA----CCC-CCT---ATTACGGCAGGTCAAGGTGCAGCGC-ATGAGATGGA-A
Hami    A-AATA-CT----TCTT---AAAACGTCAGGTCGAGGTGTAGCGA-ATGAAATGGG-A
Saun    A-AGAACT-----TCTT---AAAACGTCAGGTCGAGGTGTAGCGA-ATGAAGTGGG-A
Nema    T-GGCACAG----CCCA----AAACGTCAGGTCGAGGTGTAGCGT-ACGAAGTGCGGA
Disp    T-GGCA----CACCCCC---AAAACGTCAGGTCAAGGTGCAGCGC-ACGAAGTGTGGA
Myaf    T-GGCAC-A----GCCC---AAAACGTCAGGTCGAGGTGTAGCGC-ACGAAGTGGC-A
Lagu    T-GATAC-----AACCA---AAAACGTCAGGTCGAGGTGTAGCGC-ATGGGGTGGG-A
Trtr    T-GGTAAAG----CCTA----AAACGTCAGGTCGAGGTGTAGCGC-ATGAGACGGT--
Zucr    T-GGTAAA-----GCCT---AAAACGTCAGGTCGAGGTGTAGCGC-ATGAGAAGGT--
Pxja    T-GGTA-----AAACCC---AAAACGTCAGGTCGAGGTGTAGCGT-ATGAGGTGGG-A
Pxlo    T-GGTAG-----AACCC---AAAACGTCAGGTCGAGGTGTAGCGT-ATGAGGTGGG-A
Pctr    T-GGC-AA----ACCCC---AAAACGTCAGGTCGAGGTGTAGCGT-ATGAGGTGGG-A
Apsa    T-AGCACAG----CCCA---AA-ACGCCAGGTCGAGGTGTAGCGC-ATAAGGGGGG-A
Cabe    T-GGCACC-----GCCC---AAAACGTCAGGTCGAGGTGTAGCCA-ATGGAGAGGG-A
Bzze    T-GGTATAA-----CCC---AAAACGTCAGGTCGAGGTGTAGCGT-ATGGAGAGGG-A
Siim    T-GGCA-CA----GCCC---AGTACGTCAGGTCGAGGTGTAGCCT-ATGTAGGGGG-A
Ctru    T-GGCA-----AAACCC---AAAACGTCAGGTCGAGGTGTAGCGC-ATGAAGGGGG-A
Dpbr    T-GGCA-----AAACCC---AAAACGTCAGGTCGAGGTGTAGCGC-ATGAAGGGGG-A
Caki    T-AGTAA-T----ACTA---AAGACGTCAGGTCGAGGTGTAGCGT-ATGGGATGGG-A
Phja    T-GATATAA---ACTCA---AAAACGTCAGGTCGCAGTGCAGCGA-ATGAGGTGGG-A
Brsp    A-AGCTA---TTTCCTA---AAGACGTCAGGTCGAGGTGTAGCGT-ATGGGAAGGG-A
Gamo    A-TGCAA-A----GCCA---AAAACGTCAGGTCGAGGTGTAGCGT-ATGGGATGGG-A
Lolo    T-GGCAA----A-GCCA---AAAACGTCAGGTCGAGGTGTAGCGA-ATGGGATGGG-A
Batr    ATAGCA-----TAGCCC---AACACGGCAGGTCGAGGTGTAGCCA-ATGAAGGGGG-A
Prmy    TTAATA----CTCCCTT---AAAACGTCAGGTCGAGGCGTAGCCA-ATGAAAGGGC-G
Loli    T-GTTT-----TAACCC---AAAACGCCAGGTCGAGGTGTAGCTA-ATGGGAGGGG-A
Loam    T-GCCACAC-----CCC---ACAACGTGATGTCGAGGTGTGTCCT-ATGAAGGGGG-A
Chab    T-GGCA----CAG-CCC---AAAACGTCAGGTCGAGGTGTAGCGT-ATGAAAGGGG-A
Chto    T-GGCA----CAG-CCC---AAAACGTCAGGTCGAGGTGTAGCGT-ATGAGAGGGG-A
Majo    T-GGT-----AACACCC---CAAACGTCAGGTCGAGGTGTAGCAT-ATGGAGGGGG-A
Hlst    T-GG-CCCA----CCCC---AACACGTCAGGTCGAGGTGTAGCGC-ATGAGAGGGG-A
Clpe    T-GGCAG--ACCCCCCC---AATACGTCAGGTCGAGGTGCAGCTG-ATGGAAGGGA-A
Mlmr    A-GGCAA----CCCCCC---CCCACGTCAGGTCAAGGTGCAGCGA-ATGAAAGGGA--
Crcr    C-GGTAGAA-----CCC---AAAACGCCAGGTCGAGGTGTAGCGT-ATGAGAGGGG-A
Muce    C-GGTAGAA-----CCC---AAAACGCCAGGTCGAGGTGTAGCGT-ATGAGAGGGG-A
Bege    T-AGTA-----TAACCC---AAAACGTCAGGTCGAGGTGTAGCGC-ATGGCAGGGG-A
Mela    C-AGTACAA-----CTC---AAAACGCCAGGTCGAGGTGTAGCAT-ATGAGGGGGG-A
Hats    C-AGTA-----CAACTC---AAAACGTCAGGTCGAGGTGTAGTAC-ATGGGAGGGG-A
Orla    C-AGTA-----ACACTC---AAAACGTCAGGTCGAGGTGTAGCAT-ATGAGAGGGG-A
```

```
Cosa    C-AGTA-----AAACTC---AAAACGCCAGGTCGAGGTGTAGCGC-ATGAGAGGGG-A
Exsp    C-AGTA-----AAACTT---AAAACGCCAGGTCGAGGTGTAGCAT-ATGAGAGGGG-A
Depa    C-AGTA-----AAACTA---AAAACGTCAGGTCGAGGTGTAGCAT-ATGAAAGGGG-A
Rima    T-GGTCCA-----GCCC---AAAACGTCAGGTCGAGGTGTAGCAT-ATGAGAAGGA-A
Fuol    T-GGTA-----AAACCC---AAAACGCCAGGTCAAGGTGTAGCAT-ATGAGAGGGA-A
Gmaf    T-GGTA-----AAACCC---AAAACGCCAGGTCGAGGTGTAGCAT-ACGAAAAGGA-A
Xeei    T-GGTT-----TAACCC---AAAACGTCAGGTCGAGGTGTAGCAT-ATGAGAAGGA-A
Pros    T-GACAAAC-----CCC---AAGACGTCAGGTCGAGGTGTAGCGC-ATGAGGAGGG-A
Scmi    T-GAT-AA----AACCC---AAGACGTCAGGTCGAGGTGTAGCGT-ATGAGGAGGG-A
Rolo    T-GTT-----AAAACCC---AAAACGTCAGGTCGAGGTGTAGCGC-ATGGGGCGGG-A
Cere    T-GATAAAA-----TCC---GAAACGTCAGGTCAAGGTGTAGCAT-ATGGGAGGGA-A
Daga    T-GATA-----AAATCC---AAAACGTCAGGTCAAGGTGTAGCAT-ACGGGGAGGG-A
Anco    T-GGTACAA-----CCC---AAAACGTCAGGTCGAGGTGTAGCGT-ATGAGATGGG-A
Dmve    T-GGCA-----TAACCC---CAAACGTCAGGTCGAGGTGTAGCGC-ATGAGAGGGG-A
Dmar    C-AGCA-----TAGCCC---AACACGTCAGGTCGAGGTGTAGCGT-ATGAGAGGGA-A
Anka    T-GGCA-----CAGCCT---AAAACGTCAGGTCGAGGTGTAGCGTTATGAGATGGA-A
Moja    T-GGCA-----CAGCCC---AAAACGTCAGGTCGAGGTGTAGCGT-ATGAGATGGG-A
Hoja    T-GGCACAG-----CCC---AAAACGTCAGGTCGAGGTGTAGCGT-ATGAGATGGG-A
Bede    T-GGTAAAA-----CCC---AAAACGTCAGGTCGAGGTGTAGCGC-ATGGGAGGGG-A
Besp    T-GGTA-----AAACCC---AAAACGTCAGGTCGAGGTGTAGCGC-ATGGGAGGGG-A
Mysp    T-GATTT----AAATCC---AAAACGTCAGGTCGAGGTGTAGCGC-ATGGAAAGGA-A
Osja    T-GATG----TAAATCC---AAGACGTCAGGTCGAGGTGTAGCGC-ATGAAGAGGA-A
Sgro    T-GATT----AAAATCC---AAGACGTCAGGTCGAGGTGTAGCGC-ATGGAAAGGA-A
Pzpa    C-AGTA-----AAACTC---AAAACGTCAGGTCGAGGTGTAGCGA-ATGGGGGGGG-A
Zeja    T-AGTA-----ACACTC---AAAACGTCAGGTCGAGGTGTAGCGT-ATGGGGTGGA-A
Znne    T-AGTA-----AGACTC---AAAACGTCAGGTCGAGGTGTAGCGA-ATGGGTTGGG-A
Zefa    T-AGTAA-----AACTC---AAAACGTCAGGTCGAGGTGTAGCGA-ATGAGCTGGG-A
Acni    T-AGTA-----AAACTC---AAAACGTCAGGTCGAGGTGTAGCGC-ATGAGGCGGG-A
Ncrh    T-AGTAAAA-----CTC---AAAACGTCAGGTCGAGGTGTAGCGC-ATGAGGCGGG-A
Agca    T-GGTATA-----CCCC---AAAACGTCAGGTCGAGGTGTAGCGT-ATGGAAGGGA-A
Hydy    T-GGTAC-----AACCC---TAAACGTCAGGTCGAGGTGTAGCGT-ATGGAGAGGG-A
Gsac    T-GGTA----TAA-CCC---TAAACGTCAGGTCGAGGTGTAGCGT-ATGTGGAGGG-A
Pevo    T-GGCACA-----ACCC---AAAACGTCAGGTCGAGGTGTAGCGT-ATGCAAGGGG-A
Hiku    T-AGTAACA----ACCC---AAAACGTCAGGTCGAGGTGTAGTTT-ATAATAGGGG-A
Inpa    T-GGCA-----CAGCCC---AAAACGCCAGGTCAAGGTGTAGCAT-ACGAGAAGGA-A
Auch    C-GATAACC-CTACTCC---AGAATGACAGGTCGAGGCGCAGCTT-ATGAAAGGGGCT
Fico    T-GGCACA-----CCCC---AAAACGTCAGGTCGAGGTGTAGCGT-ATGAGAGGGG-A
Macs    T-GGCA-----AAGCCC---AAAACGTCAGGTCGAGGTGTAGCGT-ATGAGAGGGG-A
Moal    T-GGTCCCT----CCCC---AAAACGTCAGGTCGAGGTGTAGCGA-ATGAGGGGGG-A
Syma    C-AGTA-----CAACTC---CAAACGTCAGGTCGAGGTGTAGCGC-ATGGGAGGGA-A
Mafr    T-GGTTC-----ATCCC---CAAACGTCAGGTCGAGGTGTAGCGC-ATGAGAAGGA-A
Dcpe    T-GGTAATG-----CCT---AAAACGTCAGGTCGAGGTGTAACGT-ATGAGAGGGATA
Dcti    T-GGTAA-----TACCT---AAAACGTCAGGTCGAGGTGTAACGT-ATGAGAGGGA-A
Hehi    T-GGCA-----AAACCC---AAAACGTCAGGTCGAGGTGTAGCGC-ATGGAGGGGG-A
Stam    T-GGCACA-----GCCC---AAAACGTCAGGTCGAGGTGTAGCGT-ATGGAAAGGG-A
Hogi    T-GGCA-----TAGCCC---AAAACGTCAGGTCGAGGTGTAGCGT-ATGGAAAGGG-A
Erzo    T-GGCA-----TAGCCC---TAAACGTCAGGTCGAGGTGTAGCGC-ATGGGAAGGG-A
Hxot    T-GGTA-----AAACCT---AAAACGTCAGGTCGAGGTGTAGCGT-ATGGAAGGGG-A
Core    T-GGTACA-----ACCT---AAAACGTCAGGTCGAGGTGTAGCGT-ATGGAGGGGG-A
Apve    T-GGCACA-----ACCT---AAAACGTCAGGTCGAGGTGTAGCGT-ATGGAAGGGG-A
Latj    T-AGCA-----TAGCCC---AGAACGTCAGGTCAAGGTGTAGTAC-ATGAGGGGAGAA
Laja    T-GGCAGA-----GCCC---AGAACGTCAGGTCGAGGTGTAGCGT-ATGGAGGGGG-A
```

Syja	T-GG-CACA----GCCC---AAAACGTCAGGTCGAGGTGTAGCGT-ATGGAAGGGG-A
Epme	T-GGCA-----CAGCCC---AAAACGTCAGGTCGAGGTGTAGCGT-ATGGAAAGGG-A
Grse	C-AGTACAA-----CTC---AAAACGTCAGGTCGAGGTGTAGCGC-ATGGAAAGGG-A
Clja	T-GGTCC----TAGCCC---AGTACGTCAGGTCGAGGTGTAGCTT-ATGGAAAGGG-A
Ogcy	C-AGTAATA-----CTC---AAAACGCCAGGTCGAGGTGTAGCTA-ACAAGGGGGG-A
Plna	C-AGTTTA-----GCTC---AGTACGTCAGGTCGAGGTGCAGCTT-ACGAGAAGGG-A
Lema	T-GGCAC-A----GCCC---AGAACGTCAGGTCGAGGTGTAGCGA-ATGGAAGGGG-A
Etzo	T-GGTA-----CAGCCT---AAAACGTCAGGTCGAGGTGTAGCGC-ATGGGGAGGG-A
Apse	T-GGCA-TA----GCCC---AAAACGTCAGGCCGAGGTGTAGCGT-ATGAGAAGGG-A
Epde	T-GGCA-TA----GCCC---AAAACGTCAGGTCGAGGTGTAGCGT-ATGGAGGGGG-A
Slja	C-GGC-AT----TGCCC---AATACGTCAGGTCGAGGTGTAGCGA-ATGAGGGGGG-A
Bsja	T-GGTACTG-----CCC---AATACGTCAGGTCGAGGTGTAGCGT-ATGAAAGGGG-A
Ecna	T-GGT-----AAAACCC---AGAACGTCAGGTCGAGGTGTAGTGC-ATGAGAGGGG-A
Cohi	T-GGTA----CAA-CCC---AATACGTCAGGTCGAGGTGTAGCCT-ATGAGAAGGG-A
Caar	T-GGCA-----TAGCCC---AAAACGTCAGGTCGAGGTGTAGTGC-ATGGGAGGGG-A
Came	C-GGCA-----CAGCCC---AGAACGTCAGGTCGAGGTGTAGTGA-ATGGGAGGGG-A
Mema	T-GGCA-----TAGCCC---AAAACGTCAGGTCGAGGTGTAGCGT-ATGAGAGGGG-A
Lenu	T-GGCACAG-----CCC---TAAACGTCAGGTCGAGGTGTAGCGA-ACGAAAGGGG-A
Plma	T-GGCA-----CCACCC---AGAACGTCAGGTCGAGGTGTAGCGC-ATGAGAGGGG-A
Emst	T-GGCACA-----GCCC---AAAACGTCAGGTCGAGGTGTAGCGT-ATGGAGGGGG-A
Ptti	T-GGCA-TA----GCCC---AAAACGTCAGGTCGAGGTGTAGCGT-ATGGAAAGGG-A
Losu	T-GGCA-----CAGCCC---AAGACGTCAGGTCGAGGTGTAGCTT-ACGAAGGGGGCT
Geoy	C-GGCA-----CAGCCC---CAAACGTCAGGTCGAGGTGTAGCTA-ATGGAGGGGG-A
Dipi	T-GGCACAC-----CCC---AAAACGTCAGGTCGAGGTGTAGCGC-ATGGAAGGGG-A
Pama	T-GGCAC----T-GCCC---AAAACGTCAGGTCGAGGTGTAGCGA-ATGGAAGGGG-A
Leob	T-GGCA-----TAGCCC---AAAACGTCAGGTCGAGGTGTAGCGC-ATGAAAGGGG-A
Neba	T-GGTTTAC----CCCC---AGCAAGTCAGGTCGAGGTGTAGCGT-ATGGAAGGGG-A
Pdpl	T-GGCA-----CTGCCC---AAAACGTCAGGTCGAGGTGTAGCGA-ATGAGAGGGG-A
Nimi	T-GGTAC-----AGCCC---TAAACGCCAGGTCGAGGTGTAGCGT-ATGGAGGGGG-A
Uptr	T-GGTA-----ATACCC---CAAACGTCAGGTCGAGGTGTAGCAT-ATGGAGGGGG-A
Pesc	C-GGTA-----CAACCC---AAAACGCCAGGTCGAGGTGTAGCAT-ATGAAGGGGG-A
Baar	T-GGCA-----CAGCCC---AAAACGTCAGGTCAAGGTGTAGCGC-ATGGAAGGGG-A
Moar	T-GGCA-----TAGCCC---AAAACGTCAGGTCGAGGTGTAGCGT-ATGGAAGGGG-A
Toja	C-GGCA-----TAGCCC---CAAACGTCAGGTCGAGGTGTAGTGT-ATGAGAGGGA-A
Chau	C-GGCAA-----TGCCC---AAAACGTCAGGTCGAGGTGTAGCGA-ATGGAAGGGG-A
Chse	C-GGC-----ACAGCCC---AGAACGTCAGGTCGAGGTGTAGCGA-ATGGAGGGGG-A
Enar	T-GGCA-----CAGCCT---AAAACGTCAGGTCGAGGTGTAGCGT-ATGGAAGGGG-A
Hpty	T-GGCA----CAA-CCC---AAAACGTCAGGTCGAGGTGTAGCGT-ATGGAGGGGG-A
Nana	T-AGCA-----TAGCCC---AAAACGTCAGGTCGAGGTGTAGCGC-ATGAGGGGGG-A
Mcst	T-GGC-AC----AACCC---AAAACGTCAGGTCGAGGTGTAGCGT-ATGGTAGGGG-A
Rhox	T-GGCA-----CAGCCC---AGAACGTCAGGTCGACGTGTAGCGT-ATGGAAGGGG-A
Opfa	T-GGCATGG-----CCC---AAAACGTCAGGTCGACGTGTAGCGT-ATGGAGGGGG-A
Paar	C-GGTGAAAATTCACCC---CCAACGTCAGGTCGAGATGTAGCTC-ATGAAGAGGG-A
Gozo	T-GGCATAG-----CCC---AAAACGTCAGGTCGAGGTGTAGCGC-ATGGAAGGGG-A
Ackr	T-GGTAA----A-GCCC---AGAACGTCAGGTCGAGGTGTAGCGC-ATGGAAAGGG-A
Elev	T-GGCACCA----GCCT---AAAACGTTAGGTCGAGGTGTAGTGC-ATGGAAGGGG-A
Trdu	T-AGCACAA-----CTC---AAAACGCCAGGTCGAGGTGTAGCAT-ATGAGAGGGG-A
Amoc	T-GGTA-----AGACCC---AAAACGTCAGGTCGAGGTGTAGTGT-ATGAGAGGGG-A
Hame	C-GGCAC-A----GCCC---AAAACGCCAGGTCGAGGTGTAGCGT-ATGGAAAGGG-A
Chso	T-GGTT-----TAGCCC---CAGACGACAGGTCGAGGTGTAGCGT-ATGAAAAGGG-A
Lyto	T-GGCT-----TGGCCC---AGAACGTCAGGTCGAGGTGTAGCGC-ATGGGAGGGG-A
Encr	T-GGT-----AGAACCC---AAAACGTCAGGTCGAGGTGTAGCGT-ATGGGAAGGG-A

```
Bvar            T-GGCAC-----AGCCC---AGAACGTCAGGTCGAGGTGTAGTGT-ATGGAAAGGG-A
Noco            T-GGCA-----TAGCCC---AGCACGTCAGGCCGAGGTGTAGCGT-ATGGAAAGGG-A
Chsp            T-AGCA-----CGCCCC---AAAATGTCAGGCCGAGGTGTAGCGT-ATAAGAGGGG-A
Arja            T-GGTAG----A-ACCT---AAAACGTCAGGTCGAGGTGTAGCGT-ATGGAGGGGG-A
Pase            C-AGCA-----TTGCTT---CAAACGTCAGGTCGAGGTGTAGCCT-ATGGAGAGGG-A
Trel            T-GGTACAA-----CCC---AGAACGTCAGGTCGAGGTGTAGCCT-ACGAGGGGGG-A
Lifa            T-GAACATA----TCCC---CGTATGTCAGGTCGAGGTGTAGCTA-ATGAAGGGGG-A
Acur            T-GGTAACG-----CCC---AAAACGTCAGGTCAAGGTGCAGCGT-ATGAAGGGGG-C
Ampe            T-GGCAC-----AGCCC---AAAACGTCAGGTCGAGGTGTAGCGC-ATGGAGGGGG-A
Urja            C-GGCATT---CAACCC---AAAACGTCAGGTCGAGGTGTAGCGC-ACGGAAGGGG-A
Enet            T-AGTAAA-----ACTC---AAAACGTCAGGTCGAGGTGTAACGT-ATGGGAAGGT-T
Ptbr            C-AGAAATA----CCTT---AAAACGTCAGGTCGAGGTGTAGCAT-ATGAAAGGGC-A
Safa            C-AGTA-----ATACTC---AAAACGTCAGGTCGAGGTGTAGCAA-ATGAAAGAGG-A
Icae            T-GGCA-----CCACCC---AGAACGTCAGGTCGAGGTGTAGCGC-ATGAGAGGGG-A
Asmi            C-AGCA----TAA-CCC---AAAACGTCAGGTCGAGGTGTAGTAT-ATGTGAGGGG-A
Foal            T-GGTT-----TCACCC-CCTAAACGTCAGGTCGAGGTGCAGCGC-ATGAAAGGGT-T
Drze            T-GGTA-----AAACCC---TAAACGTCAGGTCGAGGTGTAGCGA-ATGAGGGGGA-A
Rhas            T-GGCA-AA----GCCT---AAAACGTCAGGTCGAGGTGTAGCGC-ATGAAAGGGG-A
Elac            T-GGCA----CAG-CCC---AAAACGTCAGGTCGAGGTGTAGCGA-ATGAGAGGGG-A
Kugu            T-GGTAAA-----ACCC---AGAACGTCAGGTCAAGGTGCAGCTT-ATGAAAGGGA-A
Plor            T-GGCAC-----AGCCC---AGAACGTCAGGTCGAGGTGTAGCAT-ATGGAAGGGG-A
Sgun            T-GGCACA-----GCCC---AAAACGTCAGGTCGAGGTGTAGCGT-ATGGAGGGGG-A
Zaco            T-GGCACA-----GCCC---AAAACGTCAGGTCGAGGTGTAGCGC-ATGGAAGGGG-A
Zbfl            T-GGCA-----TAGCCC---AAAACGTCAGGTCGAGGTGTAGCGT-ATGGAAGGGG-A
Spba            C-GGCA-----CAGCCC---AGTACGTCAGGTCGAGGTGTAGTGT-ATGAGAGGGG-A
Game            T-GGCA-----CCGCCC---AGAACGTCAGGTCGAGGTGTAGCGC-ATGAGAGGGG-A
Thth            T-GGCACC-----GCCC---AGAACGTCAGGTCGAGGTGTAGCGC-ATGAGAGGGG-A
Xigl            T-GGCAT-----AGCCC---AAAACGTCAGGTCGAGGTGTAGTGC-ATGAGAGGGG-A
Hyja            C-GGCA-----CTGCCC---AGAACGTCAGGTCGAGGTGTCGCGC-ATGAGAGGGG-A
Psan            C-GGCA-----CTGCCC---AGAACGTCAGGTCGAGGTGTCGCGC-ATGAGAGGGG-A
Cupa            T-GGCA-CC----ACCC---AGAACGTCAGGTCGAGGTGTAGCGC-ATGAGAGGGG-A
Mpch            T-GGCAAAG-----CCC---AAAACGTCAGGTCGAGGTGTAGCGT-ATGAGAGGGG-A
Char            T-GGCA-----CAGCCC---AAAACGTCAGGTCGAGGTGTAGCGC-ATGAGAGGGG-A
Pser            T-GGCAC-A----GCCC---AAAACGTCAGGTCGAGGTGTAGCGT-ATGAGAGGGG-A
Prol            T-GGCA-----AAGCCC---AAAACGTCAGGTCGAGGTGTAGTGA-ATGAGGGGGG-A
Plbi            T-GGCAC-----AGCCC---AAAACGTCAGGTCGAGGTGTAGTGA-ATGAGGGGGG-A
Calu            T-AGCA-AA----GCCC---AGAACGTCAGGTCGAGGTGCAGCTC-ATGGGAGGGGCA
Papa            C-GATTTACG--TGCCC---CCAACGTCAGGTCAAGGTGCAGCCC-ACGAGAGGGG-A
Sufr            T-GGCA-AA----GCCT---AGTACGTCAGGTCGAGGTGTAGCGT-ACGGAAAGGG-A
Stci            T-GGCA-----CAGCCC---AAAACGTCAGGTCGAGGTGTAGCGT-ACGGAAAGGG-A
Taru            T-GGC-AC----AGCCA---AAAACGTCAGGTCGAGGTGTAGCGA-ATGGAGGGGG-A
Rala            T-GGCA-----CAGCCC---AAAACGTCAGGTCGAGGTGTAGCGT-ATGGAAGGGG-A
                                         *            *       *
```

```
                    32'        38          38'               39
Scca    AGAAATGGGCTACATTTTTTTA--C--C--AAAAAAT--AC--GGACA---GTAAACTGAAA
Muma    AGAAATGGGCTACATTTTTTAC------C-AAAAACAT-AC--GAATG---GTAAATTGAAA
Erca    AGAAATGGGCTACATTTTCTAG--A--CT-AGAACAT--AC--GAACA---ACACTATGAAA
Pose    AGAAATGGGCTACATTTTCTAG--A--TT-AGAACAC--AC--GGACG---ACACTATGAAA
Actr    AGAAATGGGCTACATTTTCTGACAC-----AGAAAATACAC--GAATA---ACACTGTGAAA
Scal    AGAAATGGGCTACATTTTCTGAC-A---C-AGAAAATACAC--GAATA---ACACTGTGAAA
Posp    AGAAATGGGCTACATTTTCTGACAC-----AGAAAACACAC--GAATA---ACACTGTGAAA
Atsp    AGAAATGGGCTACATTTTCTATAT-----CAGAACA-TAAC--GAACA---ACACCATGAAA
Leoc    AGAAATGGGCTACATTTTCTATG----TC-AGAATAT-TAC--GAACA---ACACCATGAAA
Amca    AGAAATGGGCTACATTTTCTAAC-T--AC-AGAA-TACTAC--GGATG---ATTCTATGAAA
Osbi    AGAAATGGGCTACATTTTCTGAACA---ACAGAA-CAT-AC--GAAC---ATAATTATGAAA
Pabu    AGAAATGGGCTACATTTTCTAACAC-----AGAAAA-C-AC--GGAT---AATTCTATGAAA
Hial    AGAAATGGGCTACATTTTCTGCAAG--AC-AGAATATT-AC--GAACGA--CTACCATGAAA
Elha    AGAAATGGGCTACATTTTCTGAAAC--C--AGAATACT-AC--GGATG---ACATAGTGAAA
Mlcy    AGAAATGGGCTACATTTTCTAACC---CT-AGAACATT-GC--GAACG---ACACAGTGAAA
Algl    AGAAATGGGCTACATTTTCTGGT----TC-AGAATACT-AC--GGAT-A--ATGCTTTGAAA
Ptgi    AGAAATGGGCTACATTTTCTGACCC--AC-AGAATATC-AC--GAATA---TTGCTCTGAAA
Alaf    AGAAATGGGCTACATTTTCTGACCA---TCAGAA-TATTAC--GAAAA---ATGCCATGAAA
Nock    AGAAATGGGCTACATTTTCTGGTT---TC-AGAATATT-AC--GAAAT---ATGTTATGAAA
Anja    TGAAATGGGCTACATTTTCTGA-TA--C--AGAAAAAC-AC--GAAAA---GTGCCATGAAA
Gyki    AGAGATGGGCTACATTTTCTGGC----AACAGAA-CACTAC--GAAGA---GCACAATGAAA
Syka    AGAAATGGGCTACATTTTCTGCCCC-----AGAATACT-AC--GGAA---AGTGTCATGAAA
Opma    AGAGATGGGCTACATTTTCTGAAA---C--AGAACATT-AC--GAAAG---GTGTCATGAAA
Comy    AGAGATGGGCTACATTTTCTAAC----CC-AGAATATT-AC--GAAAA---GTATTATGAAA
Sasp    TGAGATGGGCTACATTTTCTGACC---C--AGAAAAC--AC--GAAAA---TACAAATGAAA
Eupe    TGAGATGGGCTACATTTTCTGAACC-----AGAAAA-CTAC--GAAT---AGTATAATGAAA
Enja    AGAAATGGGCTACATTGCCTGACTT-----AGGCTACCAAC--GGAAA---GTCGCCTGAAA-
Same    AGAAATGGGCTACATTGTCTGAACC-----AGATCATTCAC--GGAAA---GTTGTCTGAAA
Chch    AGAAATGGGCTACATTTTCTACTCC-----AGAATACT-AC--GAAAG---GCACCATGAAA
Grgr    AGAGATGGGCTACATTTTCTACCCC-----AGAATAT--AC--AGAC---AGTGCCCTGAAA
Caau    AGAAATGGGCTACATTTTCTAACAT-----AGAATATC-AC--GAACAT--GCACCATGAAA
Cyca    AGAAATGGGCTACATTTTCTAAT-A--T--AGAATATTA-C--GAAC-AT-GCACCATGAAA
Dare    AGAAATGGGCTACACTTTCTACCCC-----AGAATAT--AC--GAAA--ATGTAACATGAAA
Cost    AGAAATGGGCTACATTTTCTTTT----AC-AGAATATT-AC--GAACA---ACACTATGAAA
Leec    AGAAATGGGCTACATTTTCTAATTT-----AGAATATC-AC--GAACA---GTACCCTGAAA
Crla    AGAGATGGGCTACATTTTCTATAAT-----AGAATAAG-AC--GAATA---GCATCATGAAA
Clmc    AGAAATGGGCTACATTTTCTAT--A--GC-AGAA-TATCAC--GAACG---GCACCGTGAAA
Phin    AGAAATGGGCTACATTTTCTACA----AC-AGAATAT-TAC--GAACT---GCACTATGAAA
Icpu    AGAAATGGGCTACATTTTCTATACC----TAGAATATTAC---GAATG---GCACCATGAAA
Psto    AGAAATGGGCTACATTTTCTATCCA--TATAGAATATTAC---GAATG---ACATACTGAAA
Cora    AGAAATGGGCTACATTTTCTATTGC-----AGAAAATC-AC--GAAT---GGTTTACTGAAA
Eisp    AGAAATGGGCTACATTTTCTACAAT-----AGAATATTCTAC-AGAC---GGCGCCCTGAAA
Apal    AGAAATGGGCTACATTTTCTACAAT-----AGAATATTTTAACGAACG---GTAGCATGAAA
Eslu    AGAAATGGGCTACATTTTCTAATTC-----AGAATATAC----GGAACT--ACACTGTGAAA
Dape    AGAAATGGGCTACATTTTCTAAAC----T-AGAATAT--AC--GAACC---CTGTTGTGAAA
Glse    AGAAATGGGCTACATTCTCTAACC---TT-AGAGTATTAAC--GAATG---ACTCTGTGAAA
Naar    AGAAATGGGCTACATTCTCTAA-----TTTAGAGTA--CAC--GGACA---ACACTGTGAAA
Baoc    AGAAATGGGCTACATTCTCTGACCC--TC-AGAGTAT--AC--GAACG---ATGTTGTGAAA
Opso    AGAAATGGGCTACATTCTCTAA-CC--T--AGAGCAATAC---GAATA---GTACTGTGAAA
Alte    AGAAATGGGCTACATTTTCTGACT-----CAGAATACTAC---GAACG---GCACCATGAAA
Plap    AGAAATGGGCTACATTTTCTGA--C--CC-AGAATACT-AC--GAACG---GCACCCTGAAA
```

```
Plal    AGAAATGGGCTACATTACCTAA-CT---C-AGGTCATT-AC--GGAGG---GAGCTGTGAAA
Sami    AGAAATGGGCTACATTACCTACCAC-----AGGTTATT-AC--GGAG---GGGGCTGTGAAA
Rere    AGAAATGGGCTACATTCCCTAAT----TT-AGGGCACT-AC--GGAT-A--GTGCTGTGAAA
Gama    AGAAATGGGCTACATTCCCTGTTTT-----AGGGAAAA-AC--GGAC---GGGGGCGTGAAA
Onmy    AGAAATGGGCTACATTCTCTAAA-----TTAGAGC-ACTAC--GAACC---ACGCTGTGAAA
Sasa    AGAAATGGGCTACATTCTCTAA--A--TT-AGAGCA-CTAC--GAACC---ACGCTGTGAAA
Cola    AGAAATGGGCTACATTCTCTAAAT---T--AGAGCATT-AC--GAACC---ACGCTGTGAAA
Dita    AGAAATGGGCTACATTTCCTGGA----CC-AGGATATC-AC--GGAAG---GTGTTATGAAA
Gogr    AGAAATGGGCTACATTTCCTATA-A--T--AGGTACCAC----GACAG---ATACTATGAAA
Chsl    AGAAATGGGCTACATTCCCCAAC----CT-AGGGAACT-AC--GAATG---CTGCCATGAAA
Atja    AGAAATGGGCTACACTCTCTAGCA-----CAGAGTA--CAC--GAATG---ATATGATGAAA
Iido    AGAAATGGGCTACACTCTCTAAT----GC-AGAGTAC--AC--GAAT-G--ATGTGATGAAA
Auja    AGAAATGGGCTACATTCTCTAA-AT-----AGAGAAAC-AC--GAAAG---ATGTGATGAAA
Chag    AGAAATGGGCTACATTCTCTTCTTC--AC-AGAGAA-C-AC--GGAAG---GTCCAATGAAA
Hami    AGAGATGGGCTACATTCTCTAGA----CC-AGAG-AATTAC--GAAAG---AGGTTTTGAAA
Saun    AGAGATGGGCTACATTCTCTACTAA--CC-AGAGAACC-AC--GAAA-G--AGATTTTGAAA
Nema    AGAAATGGGCTACATTTTCTGAC-C--C--AGAAAACTTTA-CGGAAG---GAAAAATGAAA
Disp    AGAGATGGGCTACATTTTCTGACC----C-AGAA-AATTAC--GAAAG---GAAAAATGAAA
Myaf    AGAGATGGGCTACATTTTCTGAC-----CCAGAAA-ACTAC--GAAAGA--AGAATATGAA-
Lagu    AGCAATGGGCTACATTCTCTACCCC-----AGAATAT--AC--GAAAA---GGCTGTTGAAA
Trtr    AGTAATGGGCTACATTCTCTAAT-T--A--AGAGTACT-AC--GGAAG---GGGGGTTGAAA
Zucr    AGAAATGGGCTACATTCTCTGATTC-----AGGGTACC-AC--GGAAG---AGGGATTGAAA
Pxja    AGAGATGGGCTACATTCTCTACTAT-----AGAGTAT--AC--GAACG---GTATATTGAAA
Pxlo    AGAGATGGGCTACATTCTCTACTAC-----AGAGTATT-AC--GAACG---GTATATTGAAA
Pctr    AGAAATGGGCTACATTCTTTCAAAC--C--AGAGAAC--AC--GAACA---GTGGGCTGAAA
Apsa    AGAAATGGGCTACATTCTCTCAATG-----AGATTATT-AC--TAAT---GGTAAATTGAAA
Cabe    AGAAATGGGCTACATTTTCTCACG-----GAGAAAACT-AC--GGAA---AACGAACTGAAA
Bzze    AGAAATGGGCTACATTTTCTACACA-----AGAAAATT-AC--GGAG---GGTAAACTGAAA
Siim    AGAAATGGGCTACATTTTCTTATCA-----AGAAAAC--AC--GGACT---GAGGGATGAAA
Ctru    AGAAATGGGCTACATTTTCTTAGTC-----AGAAAAT--AC--GAAAA---GTGTGCTGAAA
Dpbr    AGAAATGGGCTACATTTTCTTA-----CACAGAAAAT--AC--GAAAA---GTGTACTGAAA
Caki    AGAAATGGGCTACATTCCCTAAT-----GAAGGGA--ACAC--GAATG---GTAGTTTGAAA
Phja    TAAAATGGGCTACATTCTCTATTAT-----AGAGAAC--AC--GAAT---GATAACTTGAAA
Brsp    AGAGATGGGCTACATTTTCTATA----CT-AGAAAACAC----GAATG---ATTATCTGAAA
Gamo    AGAAATGGGCTACATTCTCTGTT-----ACAGAGA-A-TAC--GAATT---GTAATTTGAAA
Lolo    AGAAATGGGCTACATTCTCTACTAC-----AGAGA-AT-AC--GAATG---GTATTTTGAAA
Batr    TGAAATGGGCTACATTTTCTTA------A-AGAAAA-A-AC--GAATT---GAACATTGAAA
Prmy    CAAAATGGGCTACATTCCCTTAT-------AGGGAACC-AC--GAATC---AAGGGCTGAAA
Loli    TGAAATGGGCTACATTCCCTAA-----ATAAGAGAAA--AC--GAACGA--CTGTCCTGAAA
Loam    AAAAATGGGCTACATTCACTATTTA-----AGAGAA-T-AC--TAAC---GATGTATTGAAA
Chab    AGAAATGGGCTACATTCCCTAA-----CAAAGCGAAT--AC--GAATA---GTGCATTGAAA
Chto    AGAAATGGGCTACATTCCCTAA-----CAAAGCGAAT--AC--GAATA---GTGCATTGAAA
Majo    AGCAATGGGCTACATTCACTGCATC-----AGTGA--ATAC--GAAGG---ATGCACTGAAA
Hlst    TGAAATGGGCTACATTCCCTAC-----TACAGGGCA--TAC--GAATT---ACATGTTGAAA
Clpe    AGAAATGGGCTACATTCCCTGTATA-----AGCGCAT--AC--GGATA---ATACACTGAAA
Mlmr    AGAAATGGGCTACATTCCCTAAC-A---A-AGTGCA-C-AC--GGATAG--GACGGCTGAAA
Crcr    AGAAATGGGCTACATTTCCTAACAC-----AGGA-AAT-AC--GAAT---AATGCACTGAAA
Muce    AGAAATGGGCTACATTTCCTAACAC-----AGGAAA-T-AC--GAAT---AATGTACTGAAA
Bege    AGAAATGGGCTACATTTTCTGC-----CACAGAAAAC--AC--GGACA---ATGAAATGAAA
Mela    AGAAATGGGCTACATTTTCTGAAAC-----AGAAAAT--AC--GGAT---TATGTAATGAAA
Hats    AGAAATGGGCTACATTCTCTAC-----CTCAGAACAT--AC--GGATA---ATGAAATGAAA
Orla    AGAAATGGGCTACATTCCTTGCT-T--CA-AGGAAAAC-----GGATA---GTATAATGAAA
```

```
Cosa    AGAAATGGGCTACATTCCCTGAC----TT-AGGGTAT--AC--GAAAA---ATACAATGAAA
Exsp    AGAAATGGGCTACATTCCCTAAATC-----CGGGCAT--AC--GGATA---GTATAATGAAA
Depa    AGAAATGGGCTACATTCCCTAAAT------AGGGTAT--AC--GAAAA---ATATGATGAAA
Rima    AGAGATGGGCTACATTTCCTAC--A--C-AGGGAACC--AC--GGAT-A--GTACAATGAAA
Fuol    AGAAATGGGCTACATTTTCTGCTTA--A--GGAAA-T--AC--GGATT---ACACTATGAAA
Gmaf    AGAAATGGGCTACATTTCCTTCCCT--C--AGGAAACA--C--GAATT---GTGCTATGAAA
Xeei    AGAAATGGGCTACATTTCCTCAATG-----AGGAAACC-AC--GGAAT---GTGTCATGAAA
Pros    AGAGATGGGCTACATTCTCTACCCC--C--AGAGAAAC-AC--GAATGTAATAGTACTGAAA
Scmi    GGAGATGGGCTACATTCTCTACCCC-----AGAGAAAT-AC--GAATGTACTAGTATTGAAA
Rolo    AGAAATGGGCTACATTCCCTACTAC-----AGGGAA--TAC--GAACG---GTGTATTGAAA
Cere    AGAAATGGGCTACATTCCCTATCAC-----AGGGAAT--AC--GAAA---GATGTAATGAAA
Daga    AGAAATGGGCTACATTCCCTACC-T--TT-AGGGAATA--C--GGAAG---GTAAAATGAAA
Anco    AGAAATGGGCTACATTCTCTACCCC-----AGAGAACA--C--GAAC---GGTGTAATGAAA
Dmve    AGAAATGGGCTACATTCTCTATCA-----CAGAGAAC--AC--GGACG---GTACAATGAAA
Dmar    AGAAATGGGCTACATTCTCTACCCTCCCCCAGAG--AACAC--GAATA---GTACAATGAAA
Anka    AGAAATGGGCTACATTTTCTCTGCC--AT-AGAGAATT-AC--GGATG---GTGTAATGAAA
Moja    AGAAATGGGCTACATTCTCTGCCA---C--AGAGAAT--AC--GAACG---GTGTAATGAAA
Hoja    AGAAATGGGCTACATTCTCTGCCCC-----AGAGAACA--C--GAACG---GTGTAATGAAA
Bede    AGAGATGGGCTACATTCTCTACTAC-----AGAGAACC-AC--GAATGTATTAGTACTGAAA
Besp    AGAGATGGGCTACATTCTCTACTA-----CAGAGAACTAC---GAATGTATTAGTACTGAAA
Mysp    AGAAATGGGCTACATTCCCTGCT-C--C--AGGGAAC-AC---GAATA---GTACACTGAAA
Osja    AGAAATGGGCTACATTCCCTGCCA----C-AGGGAAT--AC--GAATA---GTACACTGAAA
Sgro    AGAAATGGGCTACATTCCCTAA-CT----CAGGG--AATAC--GAATA---ATACACTGAAA
Pzpa    AGAAATGGGCTACATTCTCTCTTG------AGAG-CAT-AC--GAATG---ATAGATTGAAA
Zeja    AGAAATGGGCTACATTCTCTCCC------GAGAGCA--TAC--GAATG---ATGTTTTGAAA
Znne    AGAAATGGGCTACATTTTCTCTCG------AGAATAT--AC--GAATA---GTGTGTTGAAA
Zefa    AGAAATGGGCTACATTTTCTCTTG------AGAAAAA--AC--GAATG---GTGTGTTGAAA
Acni    AGAAATGGGCTACATTCTCTCT-----TG-AGAGTAT--AC--GAATG---ATGTATTGAAA
Ncrh    AGAAATGGGCTACATTCTCTCTTG------AGAGCAT--AC--GAA---TGATGTATTGAAA
Agca    AGAGATGGGCTACATTCCCTTAATT--A--AGGGCAC--AC--GGAA-G--GTGCACTGAAA
Hydy    AGAAATGGGCTACATTCGCTGCAAC-----AGCGAAC--AC--GAATG---ATGAACTGAAA
Gsac    AGAAATGGGCTACATTCGCTACAA---AT-AGCGAAC--AC--GAATG---ATGTCCTGAAA
Pevo    AGAAATGGGCTACATTCGCTGAC----AC-AGCGTATTAAA-CGAATT--AGCCCACTGAAA
Hiku    AGAAATGGGCTACACTCATTTA------TTAATGA-A-TAC--GGATG---GTATATTGAAA
Inpa    AGAAATGGGCTACATTCTCTAACAC---TAGCAGA-C--AC--GAATT---ATGCGTTGAAA
Auch    AAGAATGGGCTACATTCGCTTACTT--CA-AGCGCAT--AC--GGAAACT-TAACACTGAAA
Fico    AGAAATGGGCTACATTCGTTAGT----CT-AACGAACTTAC--GAAA-A--CCATAATGAAA
Macs    AGAAATGGGCTACATTCATTAAT-T--T--AATGAACC-AC--GGAAG---ACGTACTGAAA
Moal    AGAAATGGGCTACATTCTCTAACCC--T-AGCAGAT---AC--GAAA-A--ATGAACTGAAA
Syma    AGAGATGGGCTACATTCTCTACC-A--CTAGTAGTTC------GAATA---ACGCATTGAAA
Mafr    AGAAATGGGCTACATTCACTACCTA-----AGTGAAC--AC--GAATG---ATAAATTGAAA
Dcpe    AGAAATGGGCTACATTTGCTTACTC--A--AGTGAGCAC----CTAGA-C-CATAACTGAAA
Dcti    AGAAATGGGCTACATTTGCTTA--C--TCAAGTGAGC-AC---CTAGAC--CATAACTGAAA
Hehi    AGAAATGGGCTACATTCCCTACAAT-----AGGGAAC--AC--GAATG---GTGCACTGAAA
Stam    AGAAATGGGCTACATTCTCTATAAT----TAGTGAAT--AC--GGAT---GATGTACTGAAA
Hogi    AGAAATGGGCTACATTCCCTGAA-G--A--AGGGTACA--C--GAACG---ATGAACTGAAA
Erzo    AGAAATGGGCTACATTTGCTATTAT-----AGCAAAT--AC--GGATG---GTGTTCTGAAA
Hxot    AGAAATGGGCTACATTCGCTACTAC-----AGCGAAC--AC--GGAAG---GTGCACTGAAA
Core    AGAAATGGGCTACATTCGCTACC----AC-AGCGAAC--AC--GAATG---ATGCGCTGAAA
Apve    AGAAATGGGCTACATTCGCTACT----GT-AGCGAAC--AC--GAAA-G--ACGGACTGAAA
Latj    AGAAATGGGCTACATTCATTACCCT-CCCTAATGAA--CAC--GAATG---ATGCACTGAAA
Laja    AGAAATGGGCTACATTCCCTGAC----AC-AGGGAA-ACAC--GGAT-G--ACACACTGAAA
```

```
Syja   AGAAATGGGCTACATTCACTGA-----AACAGTGAA--TAC--AGATG---GCATGATGAAA
Epme   AGAAATGGGCTACATTCCCTGTTAC-----AGCGAATC-AC--GGATAA--TAATACTGAAA
Grse   AGAAATGGGCTACATTCCCTCAATA--TC-AGGGAAC--AC--GAA---AAGTGCACTGAAA
Clja   TGAGATGGGCTACATTGTTTTAATG--C--AAACAAAT-AC--GAAAGA--TTACTCTGAAA
Ogcy   AGAAATGGGCTACATTCCCTAAA-----TCAGGGA-A-CAC--GAACA---ATATGATGAAA
Plna   TAAGATGGGCTACATTCCCTGAT----TC-AGGGTAT--AC--GGATA---ATGCATTGAAA
Lema   AGAAATGGGCTACATTCTTTAAT-----AAAAAG--AATAC--GAATG---ACTGACTGAAA
Etzo   AGAAATGGGCTACATTTCCTGCTA---C--AGGAAATA--C--GGACG---ATGCACTGAAA
Apse   AGAAATGGGCTACAATTGCTTA--A--AC-AGCTAAAAC----GAATTG--TAACGCTGAAA
Epde   AGAAATGGGCTACATTCCCTAAT----GC-AGAGAAT--AC--GAATG---ACACATTGAAA
Slja   AGAGATGGGCTACATTCCCTGACTC-----AGGGAATT-AC--GAATGG--TCATGCTGAAA
Bsja   AGAAATGGGCTACATTCTCTAATAC-----AGAGCATA-CG--GAAG---GCTACATTGAAA
Ecna   AGAAATGGGCTACATTCACTAATTA----TAGTG--AACAC--GAATA---AAGTATTGAAA
Cohi   AGAGATGGGCTACATTCACTAACAT-----AGTGAAT--AC--GAATA---GTATATTGAAA
Caar   AGAAATGGGCTACATTCGCTGTCTG--C-CAGCGA-A-CAC--GAATG---ACGCATTGAAA
Came   AGAAATGGGCTACATTCGCTGCCCA--C--AGCGAAC--AC--GAATG---CTACACTGAAA
Mema   AGAAATGGGCTACATTCACTAGCAG--T--AGTGAATTTAC--GAAAA---ATGCATTGAAA
Lenu   AGAAATGGGCTACATTCCCTGTAAC-----AGGGAACA-AC--GAA---AGTCTCTATGAAA
Plma   AGAAATGGGCTACATTCGCTAATAC-----AGCGAAT--AC--GAATG---ATGTACTGAAA
Emst   AGAAATGGGCTACATTCCCTAAT----AC-AGTGAAT--AC--GGACG---ATATACTGAAA
Ptti   AGAAATGGGCTACATTCACTAA--C--AC-AGTG-AACTAC--GAACG---ATACGCTGAAA
Losu   GGAGATGGGCTACATTCTCTATTTC-----AGAGAAT--AC--GGATG---GTGTAATGAAA
Geoy   AGAAATGGGCTACATTTCCTCGCGA--C--AGGAAA-T-AC--GGATA---ACGTACTGAAA
Dipi   AGAAATGGGCTACATTCACTGCATC-----AGTGAA-T-AC--GGA---TAGTGCATTGAAA
Pama   AGAAATGGGCTACATTCCCTTTTTAAACATAGGGT--ACTA-CGAAAG---ATGCACTGAAA
Leob   AGAAATGGGCTACATTCACCCTTTA--C--GGTGTACT-AC--GAAAGA--TGCAATTGAAA
Neba   AGAAATGGGCTACATTCTCTATAGC---AGAGAATA---CA--GAGG---ATAGTCCTGAAA
Pdpl   AGAAATGGGCTACATTCGCTAACA---GTAGCGAA-C--AC--GGATG---TTGCATTGAAA
Nimi   AGAAATGGGCTACATTCTCTAG--C--AT-AGAGAAC--AC--GGATG---ACGTGTTGAAA
Uptr   AGTAATGGGCTACATTCACTGCATC-----AGTGAAT--AC--GAAAG---GTGCGCTGAAA
Pesc   AGCAATGGGCTACATTCTCTCCCA---C--AGAGAAT--AC--GGATG---ATGAATTGAAA
Baar   AGAGATGGGCTACATTCTCTAATG-----CAGAGAA--TAC--GGACG--ATCGCACTGAAA
Moar   AGAAATGGGCTACATTCCCTAATAA----CAGTGAA--TAC--GGACG---ATACACTGAAA
Toja   AGAGATGGGCTACATTCGCTAACAA--C--AGCGAAC--AC--GAATA---ATGCACTGAAA
Chau   AGAAATGGGCTACATTTACTAA--C--AC-AGTAAAT--AC--GGATGA--TTACCCTGCAA
Chse   AGAAATGGGCTACATTCCCTAATAC----TAGGGCA--TAC--GAATG---ATATGCTGAAA
Enar   AGAAATGGGCTACATTCCCTAATTT-----AGTGAAT--AC--GGATA---ATATGTTGAAA
Hpty   AGAAATGGGCTACATTCCCTAACA----C-AGCGAAT--AC--GAACG---ATGCACTGAAA
Nana   AGAAATGGGCTACATTCCCTAATAT-----AGGGAAT--AC--GAAAG---ACAAATTGAAA
Mcst   AGAAATGGGCTACATTCACTAGTAC-----AGTGAAT--AC--GAATGA--TGGTACTGAAA
Rhox   AGAAATGGGCTACATTCCCTAACA----C-AGTGAAT--AC--GAAAG---ATGCACTGAAA
Opfa   AGAAATGGGCTACATTCACTAAGAC-----AGTGAAC--AC--GAAC---GATGTACTGAAA
Paar   AAAAATGGGCTACATTCCCTGG----TTACAGGG---CATA--CAGAT--GACAGACTGAAA
Gozo   AGAAATGGGCTACATTCCCTATTTT-----AGGGAAT--AC--GAAC---GATGCTTTGAAA
Ackr   AGAGATGGGCTACATTCCCTAGCAA--T--AGGGTAC--AC--GGATA---ATACACTGAAA
Elev   AGCAATGGGCTACATTCGCTACTC------AGCGAAAA--C--GAAC---GACAAAATGAAA
Trdu   AGAAATGGGCTACATTCCCTGCCGC-----AGGGAA-T-AC--GAAC---AATGTAATGAAA
Amoc   AGAAATGGGCTACATTTCCTAAATC-----AGGAAACA--C--GAACA---ATGAACTGAAA
Hame   AGAAATGGGCTACATTCAGTAAA-----TTAATGC-A-TAC--GGATG---ACATCCTGAAA
Chso   AGAGATGGGCTACATTCAGTGAT-------AGCACTGAATAC-GGAGA---GTTGATTGAAA
Lyto   AGAAATGGGCTACATTCGCTGCA-A--C--AGCGAAC-A-C--GGACG---ATGCACTGAAA
Encr   AGAAATGGGCTACATTCGCTAG-CA----TAGCG--AATAC--GGACG---ATGCACTGAAA
```

```
Bvar  AGAAACGCGTCCCATTTTCTATTAA-----AGAACCC--CC--GATA----ATGTGCTGAAA
Noco  AGAAATGGGCTACATTCCCTAATTT-----AGGGCAC--AC--GAATG---ATATGCTGAAA
Chsp  AGAGATGGGCTACATTCTCTGAA-C--C--AGAGAACTTAC--GAAAA---GTGTAATGAAA
Arja  AGAAATGGGCTACATTCGCTACTAC-----AGCGAAC--AC--GAATG---GTGTAATGAAA
Pase  TGAGATGGGCTACATTCGCTAGCTA--T--AGCGCATA-AC--GGATGG--CATTCCTGAAA
Trel  AGAGATGGGCTACATTTGCTAACAC-----AGCACATTCAC--GCAGG---ACCAATTGAAA
Lifa  AGAGATGGGCTACATTTTCTATA------CATAGATCAAAC--GAATT---ATGCTCTGAAA
Acur  AGAGATGGGCTACATTTTCTATTAT-----AGAACAT--AC--GAAT---AACTCATTGAAA
Ampe  AGAAATGGGCTACATTCCCTAA--T--GT-AGTGAAT--AC--GAACG---ATGCACTGAAA
Urja  TGAGATGGGCTACATTCCCTAAT-G--A--AGGGAA-T-AC--GAATG---ATAGACTGAAA
Enet  AGAAATGGGCTACATTCCTTGTT----CCCAAGGAAA--AC--GGAT-A--ATGACATGAAA
Ptbr  AGAAATGGGCTACATTTCCTATA----CT-AGGACAT--AC--GGAAT---GTGTGTTGAAA
Safa  AGAAATGGGCTACATTTCCTACGCC--GC-AGGAAAT--AC--GAAAA---GTTTACTGAAA
Icae  AGAAATGGGCTACATTCGCTAGTA----C-AGCGAA-T-AC--GAACG---ATGCACTGAAA
Asmi  TTAAATGGGCTACATTCTCTACTAG--TT-AGAGCAT--AC--GAAAG---GTGTATTGAAA
Foal  AGTAGTGGGCTACATTTACTATTTT-----AGTATAT-ACG--AACAA--AGTG--CTGAAA
Drze  TGAAATGGGCTACATTCACTAATA----CTAGTGAAA--AC--GAATG---TTGTTCTGAAA
Rhas  AGAAATGGGCTACATTCACTGA--T--GC-AGTG-AAC-AC--GAACG---ATAGCCTGAAA
Elac  AGAAATGGGCTACATTTACTGAT----AC-AGTATAC--AC--GAACG---ATGCATTGAAA
Kugu  AGAAATGGGCTACATTTACTGAAAC-----AGTACAT--AC--GAAC---GACCAATTGAAA
Plor  AGAAATGGGCTACATTCTCTAATCT--C--AGAGAACAC----GCACG---ACATAATGAAA
Sgun  AGAAATGGGCTACATTCCCTGCCCC-----AGGGAATC-AC--GAAC---GATACTCTGAAA
Zaco  AGAAATGGGCTACATTCCCTCCCCT--C--AGGGAATT-AC--GGACG---ATGTACTGAAA
Zbfl  AGAAATGGGCTACATTCCCTAATTC-----AGCGCAT--AC--GGACG---ACACACTGAAA
Spba  AGAGATGGGCTACATTCGCTAACA----TTAGCGAA--TAC--GAACG---CTGCATTGAAA
Game  AGAAATGGGCTACATTCGCTAACG----T-AGCGAA-T-AC--GAACG---ATGTACTGAAA
Thth  AGAAATGGGCTACATTCGCTAGT----AT-AGCGAAT--AC--GAACG---ATGCACTGAAA
Xigl  AGAAATGGGCTACATTCGCTAGTAG--T--AGCGAAT--AC--GAATG---TTGCATTGAAA
Hyja  AGAAATGGGCTACATTCCCTAATAT--T--AGCGATTACAC--GAATGA--TTGTAATGAAA
Psan  AGAAATGGGCTACATTCTCTAACAT--T--AGAGAAC--AC--GAATGA--TTGTAATGAAA
Cupa  AGAAATGGGCTACATTCGCTAAC----AC-AGCG-AAT-AC--GAACG---ATGTACTGAAA
Mpch  AGAAATGGGCTACATTTTCTAAATT-----AGAAAACT-AC--GAATA---ATACATTGAAA
Char  AGAAATGGGCTACATTCACTACC-A--T--AGTGAATA--C--GAATG---ATGAACTGAAA
Pser  AGAAATGGGCTACATTCTCTAAA-----TTAGAGA-A-CAC--GAATG---ATGCATTGAAA
Prol  AGAAATGGGCTACATTTGCTAAAGC--T--AGCAAAT--AC--GAATA---ATGCATTGAAA
Plbi  AGAAATGGGCTACATTTGCTAAATA--T--AGCAAACAC----GAATG---TTGCATTGAAA
Calu  AGAGATGGGCTACATTTGCTCG--C-TTAGAGCAAAT-AC---AGATA---CCTCGTTGAAA
Papa  AGAAATGGGCTACATTCTCTGACAC--TC-AGAGAACTTAC--GAATG---GCATGTTGAAA
Sufr  AGCAATGGGCTACATTACCTGCC----CC-AGGTAATC-AC--GAACG---ATATATTGAAA
Stci  AGAAATGGGCTACATTTTCTAGCAT-----AGAAAAT--AC--GGATG---ATATACTGAAA
Taru  CAAAATGGGCTACATTCTCTGCCTA--G--AGAA-C---AC--GAAAG---ATGTGCTGAAA
Rala  AGAAATGGGCTACATTCCCTACCCC-----AGGGAACT-AC--GGACG---ATGTATTGAAA
            * *   ** *                                            *
```

```
                      39'                  31'     40             40'
Scca   --------AATTACCC------AAAGGTGGATTTAGCAGTAAGAGAAGGTCA-GAGTACTTCTCT
Muma   --------ATATACC-T-----AAAGGTGGATTTAGCAGTAAGAGAAGATCAGA-GTACTTCCCT
Erca   -------CCTGTGTTT------GAAGGAGGATTTAGCAGTAAAGGGAGAATAGA-GCGCTCCTAT
Pose   -------CCTGTGTCT------AAAGGAGGATTTAGCAGTAAGCGGGGAGTAGA-GTGCCCCACT
Actr   -------CCAGTGATT------GAAGGTGGATTTAGCAGTAAAAAGAAAA-TAGAAAATTCTTTT
Scal   -------CCAGTGATT------GAAGGTGGATTTAGCAGTAAAAAGAAAATAGAAAA-TTCTTTT
Posp   CC-------AGTGATT------GAAGGTGGATTTAGCAGTAAAAAGAAAATAG-AGAATTCTTTT
Atsp   -------CTGGTATTT------TAAGGTGGATTTAGCAGTAAAAAGAAAATAGA-GAGTTCTTTT
Leoc   T------TG-GTATTT------TAAGGTGGATTTAGCAGTAAAAAGAAAA-TAGAGAGTTCTTTT
Amca   CA----TAG--AATCTTT----GAAGGTGGATTTAGCAGTAAAAAGAAAGCAG-AGAGTTCTTTT
Osbi   -------CACGTTACTT-----GAAGGAGGATTTAGTAGTAAAAAGAAAATAG-AGTGTTCTTTT
Pabu   CT-------AGAATTT------CAAGGCGGATTTAGCAGTAAAAAGAAAA-TAGAGTGTTCTATT
Hial   -T------TGGTGTCT------GAAGGTGGATTTAGCAGTAAAAAGAAAGCA-GAGTGTTCTTTT
Elha   -C------CAATGCCT------GAAGGTGGATTTAGCAGTAAAAAGGGAAC-AGAGAGCCCTTTT
Mlcy   CC-------TGAGTCC------AAAGACGGATTTAGCAGTAAAAAAGAAA-TAGAGAGTCTTTTT
Algl   -------CAGGCATTGT-----GAAGGTGGATTTAGCAGTAAAGAGTGAAGTAGAGTGTCCTCTT
Ptgi   TA------AAGCACCCT-----GAAGGTGGATTTAGCAGTAAAAAGAAA-ACAGAGTGTTCTTTT
Alaf   CA------TGGCGTACA-----GAAGGTGGATTTAGCAGTAAAGAGAAAACAGAGT-GTTCTCTT
Nock   TA------TGACATA-AA----GAAGGTGGATTTAGCAGTAAAGAGAAAGCAGAGT-GTTCTCTT
Anja   TA------AG-CACGACT----GAAGGTGGATTTAGCAGTAAAAAGAAAATAGAGA-GTTCTTTT
Gyki   -------TGTGTGCGGCC----GAAGGTGGATTTAGCAGTAAAAAGAAAG-AAGAGAGTTCTTTT
Syka   AC-------AACACTAAT----GAAGGTGGATTTAGCAGTAAAAAGAAAA-TAGAGTGTTCTTTT
Opma   CA------GAACACAATT----GAAGGTGGATTTAGCAGTAAAAAGAAAATA-GAGAATTCTTTT
Comy   GA------AAACACAA----CTGAAGGTGGATTTAGCAGTAAAAAGATAA-TAGAGAGTTCTTTT
Sasp   T----------GTATAT-T---GAAGGTGGATTTAGTAGTAAAATGAAAATAGAG-TGTTCTTTT
Eupe   -T----AAATACATACA-----GAAGGTGGATTTTACAGTAAAAGAGGAACAGTAGAGCCCCTTT
Enja   A-------GACTATTT------GAAGGTGGATTTAGCAGTAAGAGGGGAATA-GAGTGCCCCTTT
Same   C-------GACCACTC------GAAGGTGGATTTAGCAGTAAAGGGGGAA-TAGAGCGCCCCCTT
Chch   T---------AAGTAC-CC---GAAGGTGGATTTAGCAGTAAAAAGAAAATAGA-GAGTTCTTTT
Grgr   TC------GGACAC-TC-----GAAGGTGGATTTAGCAGTAAAAAGAAAATAG-AGTGTTCTTTT
Caau   -C------A-GTGCTT------GAAGGAGGATTTAGTAGTAAAAGGGAAATAGA-GTGTCCCTTT
Cyca   C-----AA---TGCT-T-----GAAGGAGGATTTAGTAGTAAAAGGGAAATAGA-GTGTCCCTTT
Dare   -C----AA---TACTT------GAAGGAGGATTTAGCAGTAAAAGAGAAGCAG-AGTGTCTCTTT
Cost   ---------CTAGTACTT----AAAGGAGGATTTAGTAGTAAAAAGGAAA-TAGAGTGTCCTTTT
Leec   -A----A--T-TACTC------GAAGGAGGATTTAGTAGTAAAAAGGAAAT-AGAGTGTCCTTTT
Crla   AC----T-TAATGCTT------GAAGGAGGATTTAGTAGTAAAAAGGAAAT-AGAGTGTCCTTTT
Clmc   CT-------AGTGCCC------GAAGGTGGATTTAGTAGTAAAAAACAAA-TAGAGAGTCTTTTT
Phin   CA----A--AGTGCCC------GAAGGTGGATTTAGCAGTAAAAATAAAC-TAGAGTGTTTTTTT
Icpu   ------AT-AATGCCT------GAAGGTGGATTTAGTAGTAAAAAGCAAAT-AGAGTGTCCTTTT
Psto   ------TA-AATGTCT------GAAGGTGGATTTAGCAGTAAAAAACAAAC-AGAGTGTCCTTTT
Cora   -C----A--CATACCT------GAAGGAGGATTTAGTAGTAAAAAGCAAA-TAGAGAGTCCTTTT
Eisp   TC------ATGCGCCC------GAAGGTGGATTTAGCAGTAAAAAGCAAAT-AGAGAGTCCTTTT
Apal   -T------ATGCGCC---C---GAAGGTGGATTTAGTAGTAAAAAGCAAACAG-AGTATCCTTTT
Eslu   -C----C--AGTGCC---C---AAAGGTGGATTTAGCAGTAAACAGAAAATAG-AGTGTTCTCTT
Dape   CC----A---ACAGCC------AAAGGTGGATTTAGCAGTAAACAGAAAATAG-AGTGTTCTATT
Glse   -C----C--AGATTCT------GAAGGCGGATTTAGCAGTAAACAGAAAATAG-AGTGTTCTATT
Naar   -C------CAGTGTCT------TAAGGTGGATTTAGCAGTAAACAGAAAA-CAGAGAGTTCTCTT
Baoc   -C----C--AACATTT------TAAGGTGGATTTAGCAGTAAACAGAAAATAGA-GAGTTCTCTT
Opso   CC----A---GTAGTT------AAAGGTGGATTTAGCAGTAAATAGAAAATAG-AGAATTCTTTT
Alte   ------CG-GGTGCCC------GAAGGTGGATTTAGCAGTAAAAAGAAAATAGA-GAGTTCTTTT
Plap   -C----G--GGTGCCC------GAAGGTGGATTTAGCAGTAAAAAGAAAATAGA-GAGTTCTTTT
```

```
Plal    CC-------AGTTCTT------GAAGGTGGATTTAGCAGTAAGGGAAAATAG-AGAGTTCCTCT
Sami    C-------CGGCCCCT------AAAGGTGGATTTAGCAGTAAGGGTAAATAG-AGAGTTCTCCT
Rere    -C----CA--GCCCC-T-----GAAGGTGGATTTAGCAGTAAGAGGGAAATA-GAGTGTTCCTCT
Gama    -C----G--GCCCCCT------GAAGGTGGATTTAGCAGTAGACTGGAAGCA-GAGAGTCCCGTC
Onmy    -T------CAGCGTCCG-----AAGGTGAAATTTAGCAGTAAACAGAAAA-CAGAGAGTTCTCTT
Sasa    C-------CAGCGTC-----C-AAAGGTGGATTTAGCAGTAAATAGAAAATAGA-GAGTTCTCTT
Cola    CC-------AGCGTCC------GAAGGTGGATTTAGCAGTAAACAGAAAGCAGA-GAGTTCTCTT
Dita    -C------CCACACC-C-----TAAGGTGGATTTAGCAGTAAGAGGGAGCAGA-GCGCCCCCCT
Gogr    -C------AAGTATCC------AAAGCCGGATTTAGCAGTAAGGTAGAAAACAGCGTGTTCTCCT
Chsl    -------TTGGTGTT-CT----GAAGGAGGATTTAGCAGTAAGAAAAGACCAGG-ACACTCTTCT
Atja    ------A-CCACATCT------AAAGGAGGATTTAGCAGTAAGCAGGGAATAG-AAAGCCCTACT
Iido    -G----AT--ACATC-T-----AAAGGAGGATTTAGCAGTAAGCAGGGAATAG-AGAGCCCTACT
Auja    CA-------CACATCC------GAAGGTGGATTTAGCAGTAAGCAGAAAAAAG-TGTGTTCTGCT
Chag    CT-------CACACC--C----TAAGGAGGATTTAGCAGTAAGAAGAAAGAAAGAAAGTTCTTCT
Hami    TA------A-ACCCCA------GAAGGAGGATTTAGCAGTAAGCACAAAAATAGAGAGCTTGGCT
Saun    -C----AA--ACCCCT------GAAGGCGGATTTAGCAGTAAGCACGAAAACAGCGAGTTCGGCT
Nema    A-------ACAAACC----C--GAAGGAGGATTTAGCAGTAAGCAGAAAGTAGA-GCGTTCTGCT
Disp    ------AAC--CCCCT------GAAGGAGGATTTAGCAGTAAGCAAGGAGCAG-AGTGCCCTGCT
Myaf    -A----AA--ACTT------CCGAAGGAGGATTTAGCAGTAATCAGGGAGCAG-AGCGCCCTAAT
Lagu    AC----A--CACCCCT------AAAGGAGGATTTAGCAGTAAGAGGAAAACAGA-GTGTTCCCCT
Trtr    TA----C---CCCCT----C--AAAGGAGGATTTAGCAGTAAGCGAGGAACTAGAGCGTCCCGCT
Zucr    -T----AT--CCCTT-T-----GAAGGAGGATTTAGCAGTAAGTGGAGAAATAGAGTGTCCCGCT
Pxja    CA----T---ATACCC------AAAGGAGGATTTAGCAGTAAGTAAAAAA-TAGAGCGTTTTACT
Pxlo    -T----AT--ACACCT------AAAGGAGGATTTAGCAGTAAGTAAAAAA-TAGAGCGTTTTACT
Pctr    TC----GC--CCGCTT------AAAGGAGGATTTAGCAGTAAGTAAAAACAG--AGTGTTTTACT
Apsa    -T----AT--TAACCT------GAAGGAGGATTTAGCAGTAAGTAGAAAA--AGAGTTTTCCACT
Cabe    AC----C---GCATAC------GAAGGAGGATTTAGCAGTAAGCGGGGAACAT-AGTGCCCCTCT
Bzze    -T----AC--TTACCC------GAAGGAGGATTTAGCAGTAAGCAGAAAATAG-AGCGTTCTGCT
Siim    -------TGCCCTCTC------CAAGGCGGATTTAGCAGTAAGAAGGAAATAG-AGTGTCCTGCT
Ctru    CA-------CACACCT------AAAGGAGGATTTAGCAGTAAGCAGTAAATAG-AGTGTTCTGCT
Dpbr    CG----T---ACACCT------AAAGGAGGATTTAGCAGTAAGCAGAAAATAG-AGTGTTCTGCT
Caki    -A----A--ACTACACT-----GAAGGAGGATTTAGTAGTAAGAAAGGAG-TAGAGTGCCTTACT
Phja    -A----A--GTTTTCT------GAAGGAGGATTTAGTAGTAAGTGAAGAGTAG-AGTGCCTCACT
Brsp    AGAAT-T-CTTAATACT-----GAAGGTGGATTTAGTAGTAGGAGGGAAT-AAGAGAGTCCCCCT
Gamo    ------AAAATTACCT------GAAGGAGGATTTAGCAGTAAGTAGGGAC-TAGAGTGCCCTGCT
Lolo    AG----AA--ACACCT------GAAGGAGGATTTAGCAGTAAGTAAGGAATA-GAGCGCCCTACT
Batr    CA-------CGTTCAT------GAAGGCGGATTTAGTAGTAAACAGAGAACA-GAGTGCTCTATT
Prmy    CG----C---CCTTAA------GAAGGAGGATTTAGAAGTAAGTGAATTTA--GAGCGGTCCACT
Loli    CC----G--CACGTCT------GAAGGAGGATTTAGCAGTAAGCAGAAAATA-GAGTGTTCTGCT
Loam    -C----A--CACGTCC------AAAGGAGGATTTAGCAGTAAGCAAAAAATAG-AGCGTTTTGCT
Chab    AC----T---GTACTT------GAAGGAGGATTTAGTAGTAAGCAAAAAATAGA-GCGTTATGCT
Chto    AC----T---GTACTT------GAAGGAGGATTTAGTAGTAAGCAAAAAATAGA-GCGTTATGCT
Majo    ------AA-AGCATCT------GAAGGTGGATTTAGCAGTAAGAAGGGAGCA-GAGTGCCCCTCT
Hlst    -T----A--CATATCT------GAAGGAGGATTTAGCAGTAAGCAAAAAATAG-AGAGTTAAGCT
Clpe    -------CGCGTATTT------GAAGGAGGATTTAGCAGTAAGCACAAAGTAG-AGCGTTGTGCT
Mlmr    -------CGTGTTCTT------GAAGAAGGATTTAGCAGTAAGCACAAAATAG-AGCGTTGTACT
Crcr    T-------AAGCATATT-----GAAGGAGGATTTAGCAGTAAATGAAAAGCA-GAGCGTTTCATT
Muce    -------TAAGCATATT-----GAAGGAGGATTTAGCAGTAAATGAAAAACAG-AGCGTTTCATT
Bege    CA-------AACATTT------GAAGGAGGATTTAGCAGTAAGCAAGAAATAG-AGTGTCTTGCT
Mela    AC----A----CATTAT-----GAAGGAGGATTTAGTAGTAAGCAAAAAATAG-AGTGTTTTGCT
Hats    A--------AACATTA------GAAGGAGGATTTAGAAGTAAGCAAAAAATA-GAGTGTTTTGCT
Orla    CG-------TGTATTA------GAAGGAGGATTTAGCAGTAAGCAGTAAATA-GAGAGTTCTGCT
```

Cosa	-A----A--TGTGTTA------GAAGGAGGATTTAGCAGTAAGAAGAAAACA-GAGTGTTCTACT
Exsp	A-------GTATATTA------GAAGGAGGATTTAGCAGTAAGCAGTAAATA-GAGTGTCCTGCT
Depa	-A------GCACATTA------GAAGGAGGATTTAGCAGTAAGTAGAAAATA-GAGTGTTCTACT
Rima	-T------GTGTACATT-----GAAGAAGGATTTAGCAGTAAGTAAAAAAT-AGAGTGTTTTACT
Fuol	--------CAGTGTAT------GAAGGAGGATTTAGTAGTAAGTGAAAAATAGA-GTGTTTCACT
Gmaf	--------TAACACAT------GAAGGTGGATTTAGCAGTAAGCAGAAAACAGA-GAGTTCCGCT
Xeei	T------TACTCACAT------GAAGGAGGATTTAGTAGTAAGCAGAAAACAGA-GTGTTCTGCT
Pros	-------CGTACAACT------GAAGGAGGATTTAGAAGTAAGTGGAAAACAGA-GCGTCCCACT
Scmi	-------TATACAACT------GAAGGAGGATTTAGAAGTAAGTGAGAAACAGA-GCGTCCCACT
Rolo	-------TATATACCC------GAAGGAGGATTTAGCAGTAAGTGGAAAATAGA-GCGTTCCACT
Cere	-C------TTACACCT------AAAGGAGGATTTAGCAGTAAGTAGGAAGTAGAG-AGTCCAACT
Daga	T-------TTTTATCC------GAAGGAGGATTTAGCAGTAAGTAGGAAAGCAGAGAGTTCCACT
Anco	-------CATACACCT------GAAGGAGGATTTAGCAGTAAGCAGAAAATAGAG-CGTTCTACT
Dmve	-------TAAATACCTT-----GAAGGAGGATTTAGCAGTAAGTATAAAG-TAGAACATTTTACT
Dmar	-------CAAATACCTT-----GAAGGAGGATTTAGCAGTAAGTGAAAAGTAG-AACATTTTACT
Anka	-------TACACACCT------GAAGGAGGATTTAGCAGTAAGCAGAAAACAG-AGCGTTCTACT
Moja	C-------ATACACCT------GAAGGAGGATTTAGCAGTAAGTAAAAAATAG-AGCGTTCTACT
Hoja	-------CACACACCT------GAAGGAGGATTTAGCAGTAAGTAGAAAATAG-AGCGTCCTACT
Bede	C-------ATACAACT------GAAGGAGGATTTAGCAGTAAGTGGAAAA-TAGAGCGTTCCACT
Besp	-------TATACAACT------GAAGGAGGATTTAGCAGTAAGTGGAAAAT-AGAGCGTTCCACT
Mysp	-------CACGTACTT------GAAGGAGGATTTAACAGTAAGCAGGAAACA-GAGCGTTCTGCT
Osja	-------CACGTACTT------GAAGGAGGATTTAACAGTAAGTAGAAAATA-GAGTGTCCTACT
Sgro	-------CGCGTATTT------GAAGGAGGATTTAATAGTAAGTAGAAAATA-GAGTGTCCTACT
Pzpa	-------TATCTACCT------GAAGGAGGATTTAGCAGTAAGTGGTAAATA-GAGAGTTCCACT
Zeja	-------CATACACCT------GAAGGAGGATTTAGCAGTAAGTGGCAAGCA-GAGAGCCCCACT
Znne	C-------ACACACCT------GAAGGAGGATTTAGCAGTAAGTGGAAAATA-GAGCATCCCACT
Zefa	-------CACCCACCT------AAAGGAGGATTTAGCAGTAAGTGGTAAATA-GAGTGTCCCACT
Acni	C-------ATACACCT------AAAGGAGGATTTAGCAGTAAGTGGCAAATA-GAGCGTCCCACT
Ncrh	CA-------TACACCT------AAAGGAGGATTTAGCAGTAAGTGGCAAA-TAGAGCGTCCCACT
Agca	-------TATACACCT------GAAGGAGGATTTAGCAGTAAGCAGAAAATAG-CGTGTTCTACT
Hydy	-------CATTCATCT------GAAGGAGGATTTAGCAGTAAGTAGAAAAT-AGAGTGTTCCACT
Gsac	T-------GTACATCT------GAAGGAGGATTTAGCAGTAAGTAGAAAA-TAGAGTGTTCTACT
Pevo	-------CGTGGGTAT------GAAGGAGGATTTAGAAGTAAGAGGGAAA-TAGAGCGTCCCCCT
Hiku	-------CATAAACCT------AAAGGAGGATTTAGCAGTAAGAAGAAAA-TAGAGTGTTCATCT
Inpa	-------CCCGCACAT------GAAGCAGGATTTAGAAGTAAGCAGAAAAT-AGAGCGTTCTACT
Auch	-------TACATAGGTT-----GAAGGTGGATCTAGCAGTAAGAGGGGAAT-AGCGTGCCCCTCT
Fico	------CCGTATGGTC------AAAGGAGGATTTAGCAGTAAGCGGAGAGT-AGAGCGCTCCACT
Macs	AC------GTATGTCT------GAAGGAGGATTTAGCAGTAAGTGGAGAAT-AGAGTGCCCCACT
Moal	--------ACACATTT------GAAGACGGATTTAGAAGTAAATAGAAAAT-AGAGTGTTCTATT
Syma	TA-------TGCGTCTT-----GAAGATGGATTTAGAAGTAAATAGAAAAC-AGAGAGTTCCACT
Mafr	-C------ATTTATCT------GAAGGAGGATTTAGCAGTAAGCAGGAAAT-AGAGCGTTCTGCT
Dcpe	TA----G---TTTGGTA-----GAGCAAGGATTTAGCAGTAAGCGTAAGAGTAGAGCGCTACGCT
Dcti	A-------TGTTTGGTA-----GAGCAAGGATTTAGCAGTAAGCGTAAGAGTAGAGCGCTACGCT
Hehi	TA----C---GCACCT------GAAGGAGGATTTAGTAGTAAGCGGGAAATAG-CGTGTTCCGCT
Stam	-------GAGACATCT------GAAGGAGGATTTAGTAGTAAGCAGGAAATAG-AGTGTTCCGCT
Hogi	CA-------ATCATCT------AAAGGAGGATTTAGCAGTAAGTAGGAAGTA-GAGTGTCCTACT
Erzo	------TAT-ACACCT------GAAGGAGGATTTAGCAGTAAGCGGAAAATAGA-GTGTTCCGCT
Hxot	CG----T---ACACCT------GAAGGAGGATTTAGCAGTAAGCGGGAAATAGA-GCGTCCCGCT
Core	-------CACGCATCT------GAAGGAGGATTTAGCAGTAAGCTGGAATTAG-AGCGTCCCGCT
Apve	-C------GTTCGCCT------GAAGGAGGATTTAGCAGTAAGCAGGAACTAG-AGCGTCCCACT
Latj	-G----CCATGCAGCT------GAAGGAGGATTTAGTAGTAAGAGAGAAGCAG-AGCGTCTCCCT
Laja	-------TGTGTGCCT------GAAGGAGGATTTAGTAGTAAGTGGGGAATAGAG-CGCCCCCCT

```
Syja    -------CACATGCCT------GAAGGAGGATTTAGCAGTAAGCAGGAAATAGAG-TGTCCCGCT
Epme    ------CGTGCATCTA------GAAGGAGGATTTAGCAGTAAGCAGAAA-GCAGAGCGTTCCGCT
Grse    -------CGTACATTC------AAAGGAGGATTTAGCAGTAAGAAGAAAA-TAGAGTGTTCTCCT
Clja    -------TGTGTGTCG------GAAGGAGGATTTAGAAGTAAGTAGAAA-ATAGAGCGTTCTACT
Ogcy    -------CGCATATTA------GAAGGAGGATTTAGCAGTAAGCAGAAAA-TAGAGTGTTCTGCT
Plna    -------CATGCATTT------GAAGGCGGATTTAGCAGTAAGCTGAAAA-TAGAGTGTTCAACT
Lema    -------CGTCTTTCC------GAAGGAGGATTTAGCAGTAAGCAGGAAA-TAGAGTGTTCTGCT
Etzo    CG-------TTCATCT------GAAGGAGGATTTAGCAGTAAGCAGGAAAT-AGAGCGTCCCGCT
Apse    -------TGACAGACAT-----GAAGGAGGATTTAGAAGTAAGAGGGCAAACAGAGTGTCCCCCT
Epde    -------CATGTGTCT------GAAGGAGGATTTAGCAGTAAGCAGGAAA-TAGAGTGTTCCGCT
Slja    -------TGCATACACT-----GAAGGAGGATTTAGTAGTAAGCTGAAAG-CAGAGTGTTCAGCT
Bsja    -------CATGTACCT------GAAGGAGGATTTAGTAGTAAGCAGGAAGTAG-AGTGTCCCGCT
Ecna    -------TATACTTCT------GAAGGAGGATTTAGCAGTAAGTGGGAA-ATAGAGAGTCCCTCT
Cohi    -------CATATACTT------GAAGGAGGATTTAGCAGTAAGCAGGAA-ATAGAGTGTTCTGCT
Caar    -------CATGCAGCT------GAAGGAGGATTTAGCAGTAAGCAGAAAA-CAGAGTGTTCCGCT
Came    -------CATGTAGCT------GAAGGAGGATTTAGCAGTAAGCAGAAAGCAG-AGTGTTCCGCT
Mema    -------CATGCAATT------GAAGGAGGATTTAGTAGTAAGCAGGAAGTAG-AGTGTCCCGCT
Lenu    -T----A--ATATTCT------GAAGGAGGATTTAGCAGTAAGTAAAAAACA-GAGAGTTTTACT
Plma    AC------GTTCATCC------GAAGGAGGATTTAGCAGTAAGTGGGAAATA-GAGTGTTCCACT
Emst    -------TGTATATCT------GAAGGAGGATTTAGCAGTAAGCAGGAAA-TAGAGTGTTCCGCT
Ptti    CA-------CGTATCT------GAAGGAGGATTTAGCAGTAAGCAGAAAATAG-AGCGTTCCGCT
Losu    CT----T--ACACACA------GAAGGAGGATTTAGAAGTAAGCGGAAAATAGA-GTGTTCCGCT
Geoy    -------TACGCGTTT------GAAGGTGGATTTAGTAGTAAGCAGTAAACAGA-GTGTGCTGCT
Dipi    -------CATGCACTT------GAAGGAGGATTTAGCAGTAAGTAGAAAG-CAGAGTGTTCCACT
Pama    C------CGTGCCTCT------GAAGGAGGATTTAGCAGTAAGCGGAAAGTAGA-GCGTTCCACT
Leob    C--------ATTCATCA-----GAAGGAGGATTTAGCAGTAAGCAGAAAGCAGA-GCGTTCTGCT
Neba    ------CAAGACATCC------AAAGGTGGATTTAGCAGTAAGGAGGGGAACAGAGTGCCCCCCT
Pdpl    -------CATGCAGCT------GAAGGTGGATTTAGTAGTAAGCGGAAAG-TAGAGTGTTCCGCT
Nimi    -------CACACGCCT------GAAGGAGGATTTAGCAGTAAGCGGGAAATA-GAGCGTCCCGCT
Uptr    -------GACGCATCT------GAAGGTGGATTTAGCAGTAAGAGGGGAGCAGA-GTGCCCTTCT
Pesc    CA-------TTTATCT------GAAGGAGGATTTAGCAGTAAGCAGAAAATAGA-GTGTTCTGCT
Baar    ------TGTGCATACT------AAAGGAGGATTTAGCAGTAAGCAGGGAAGCAGAGTGTCCCGCT
Moar    -------TGTGAATCC------GAAGGAGGATTTAGCAGTAAGCAGGAAATAGA-GTGTTCCGCT
Toja    CA----A--CGCAATT------GAAGGAGGATTTAGCAGTAAGCAGGAAACAG-AGTGTTCAGCT
Chau    -------CGAGTATCT------GAAGGAGGATTTAGCAGTAAGCAAAAAGCA-GAGTGTTTTGCT
Chse    ------CG-CACATCT------GAAGGAGGATTTAGCAGTAAGCAGGAAGTA-GAGTGTCCTGCT
Enar    TA-------CATATCT------GAAGGAGGATTTAGCAGTAAGCAGAAAG-TAGAGTGTCCTGCT
Hpty    T-------GTTCATCC------AAAGGAGGATTTAGCAGTAAGCGGGAAACA-GAGCGTCCCACT
Nana    TA-------TTTACCA------GAAGGAGGATTTAGCAGTAAGCAGAGAACA-GAGTGCTCCGCT
Mcst    A-------CGTATATCT-----AAAGGAGGATTTAGCAGTAAGCAGAAAA-TAGAGTGTTCCGCT
Rhox    -------CGTGCACCAC-----GAAGGAGGATTTAGCAGTAAGCAGGAAG-TAGAGTGTCCCGCT
Opfa    -------CGTATGTCC------GAAGGAGGATTTAGCAGTAAGCAGAAAATAG-AGCGTTCTGCT
Paar    -------TTTCTGTTT------GAAGGTGGATTTAGTAGTAAGCAGGAAA-TAGAGTGTTCTGCT
Gozo    -------CAAGCATCC------GAAGGAGGATTTAGCAGTAAGCAGAAAAT-AGAGAGTTCCGCT
Ackr    CA-------CGTATATT-----GAAGGTGGATTTAGTAGTAAGCAGAAAA-TAGAGTGTTCCGCT
Elev    T-------GTTTGTCT------AAAGGAGGATTTAGAAGTAAGTAAAAAATAG-AGTGTTTTACT
Trdu    -T------ATGCATTA------GAAGGAGGATTTAGCAGTAAGCAGAAAATAG-AGCGTTCCGCT
Amoc    CA-------AGCATTT------GAAGGTGGATTTAGCAGTAAGCAAGAAAACAGAGTGTTCTGCT
Hame    -------AAGATGTCT------GAAGGAGGATTTAGCAGTAAGTAGAGAA-TAGAGTGCTCTACT
Chso    TA--------TTGACTT-----GAAGGTGGATTTAGCAGTAAGCGGGGAG-TAGAGAGCCCCACT
Lyto    C-------GTCCATCT------GAAGGAGGATTTAGCAGTAAGCAGAAAATA-GAGTGTTCCGCT
Encr    -------CGTTCATCT------GAAGGAGGATTTAGCAGTAAGCAGGAAATA-GAGTGTTCCGCT
```

```
Bvar     CG----CATGTTTTAT------GAAGTAGAATTTAGCAGTAAGCAGGAAGCA-GAGTGTCCTGCT
Noco     CG-------CACATCTT-----GAAGGAGGATTTAGCAGTAAGTAGGAAGTA-GAGCGTCCTGCT
Chsp     T------ATTACACCT------AAAGGAGGATTTAGCAGTAAATAATAAAC-AGAGTGTTTTATT
Arja     CG-------TACACCT------GAAGGAGGATTTAGCAGTAAGCAGGAAA-TAGAGCGTCCCGCT
Pase     TA--------AATGCCT-----GAAGGTGGATTTAGTAGTAAGCTGAGAAATAGTGTGTCCAGCT
Trel     T------CACCAGTCCC-----CAAGGAGGATTTAGAAGTAAGTGGGGAGTAGA-GTGCCCCCCT
Lifa     G------CATGCATCT------GAAGGTGGATTTAGCAGTAAGCGGAAATTAGA-ATGCTCCGCT
Acur     -C----AT-GCTGTTT------GAAGGAGGATTTAGCAGTAAGTAAAAA-CTAGAGAGTTTTACT
Ampe     ------AG-TGCATCT------GAAGGAGGATTTAGCAGTAAGCAGGAAAT-AGAGTGTCCCGCT
Urja     -C----A--TCTATACT-----GAAGGAGGATTTAGTAGTAAGCAAGGAA-TAGAATGCCCTGCT
Enet     -T----AT--TCATT-T-----GAAGGAGGATTTAGCAGTAAGTGGAAAG-CAGAGCGTTCCACT
Ptbr     -T------ATACACTAT-----GAAGGAGGATTTAGTAGTAAATAGAAAAT-AGAGAGTTCTATT
Safa     GC----T---AAACTA------GAAGGAGGATTTAGTAGTAAGGGAGAAAT-AGAGTGTCTCCCT
Icae     AC------GTACATC-C-----GAAGGAGGATTTAGCAGTAAGTGGAAAA-TAGAGTGTTCCACT
Asmi     TT----T---ACGTCA------GAAGGAGGATTTAGCAGTAAGTGAGAAACATGAGTGTCTCACT
Foal     CAC------CACTTTC------AAAGGAGGATTTAGCAGTAAGAGGGGAAC-AGAGTGCCCCCCT
Drze     -------TTAACAACT------GAAGGAGGATTTAGCAGTAAGTGGGGGGT-AGAGCACCCCACT
Rhas     TA-------ACTAT-CT-----AAAGGAGGATTTAGCAGTAAGGAGGAAAATAGAGCGTTCCCCT
Elac     TC----A--GACATCT------GAAGGAGGATTTAGCAGTAAGAAGGAAAATAGAGCGTTCCCCT
Kugu     CC------ATTCGCCT------GAAGGAGGATTTAGCAGTAAGAGGAAAATAG-AGCGTTCCACT
Plor     ------ACACATGTCAC-----AAAGGAGGATTTAGTAGTAAGCAGGAAAT-AGAGAGTTCCGCT
Sgun     TA-------AGTATCT------AAAGGAGGATTTAGCAGTAAGCAGAAAATAG-AGCGTTCCGCT
Zaco     TA-------CATATCT------GAAGGAGGATTTAGCAGTAAGCAAAAAATAG-AGTGTTTTGCT
Zbfl     TA-------CGTATCC------GAAGGAGGATTTAGCAGTAAGCAGAAAATAG-AGTGTTCCGCT
Spba     -------TATGCAACT------GAAGGAGGATTTAGTAGTAAGCCGGAAACAG-AGTGTCCCGCT
Game     AC----AT--ACATCT------GAAGGAGGATTTAGCAGTAAGTGGAAAATAG-AGTGTCCCACT
Thth     A------CGTCCATCT------GAAGGAGGATTTAGCAGTAAGTGGAAAATAG-AGTGTTCCACT
Xigl     -T------ATGCGACT------GAAGGAGGATTTAGCAGTAAGCAGAAAGCA-GAGCGTCCCGCT
Hyja     A------CGTACATC-T-----GAAGGAGGATTTAGCAGTAAGGGGAAAATA-GAGTGTTCCCCT
Psan     AA----G--TACATCT------GAAGGAGGACTTAGCAGTAAGTGGAAAATAG-----------
Cupa     TT------GTACATCC------GAAGGAGGATTTAGCAGTAAGTGGAAAATA-GAGTGTTCCACT
Mpch     -C-----A-TGTGTTT------AAAGGAGGATTTAGCAGTAAGCAGAAAATA-GAGCGTTCAGCT
Char     -------TGTTCATCC------GAAGGAGGATTTAGCAGTAAGCGGAGAAT-AGAGTGCTCCACT
Pser     -CATGCATGTATATTATT----GAAGGCGGATTTAGTAGTAAGCAGGAAATAG-AGAGTCCCGCT
Prol     CAT-------GCAATT------GAAGGAGGATTTAGTAGTAAGCAGGGAATA-GAGTGTCCCGCT
Plbi     -CA------TGCAACT------GAAGGAGGATTTAGCAGTAAGCAGGAAGTAG-AGCGTCCCGCT
Calu     CA-------CGAGA--TT----GAAGGAGGATTTAGTAGTAAGCAGGGAACAGAGC-GCCCTCCT
Papa     ACA------TATGC--CT----GAAGGAGGATTTAGCAGTAAGTAGGAAATAG-AGTGTCCTACT
Sufr     -AA---T---ACATCT------GAAGGAGGATTTAGTAGTAAGAGAAGAGTAGA-GAGCTTCTCT
Stci     TAC-------ATATCT------GAAGGAGGATTTAGTAGTAAGAAGAGAATAGA-GTGCCCTTCT
Taru     -TG------CACACCC------GAAGGAGGATTTAGCAGTAAGCAAGAAATAGA-GTGTCATGCT
Rala     CA-------TACATCT------GAAGGAGGATTTAGCAGTAAGTGAAAAATAGAG-CGTTTCACT
                                *      *  *    ****
```

```
                         30'         D   41     42   ¦    HVR    ¦
Scca   GAAACT--GGCTCTGGGGCGCGCACACACCGCCCGTCACTCTCCTCAATAAATACACT--
Muma   GAAATT--GGCTCTGGGATGCGCACACACCGCCCGTCACTCTCCTCAAA-----AACCTA
Erca   GAAGCC-CGGCGCTGAAGCGCGCACACACCGCCCGTCACTCTCCTCG------AAACCAT
Pose   GAAGCTT-GGCGCTGAAGCGCGCACACACCGCCCGTCACTCTCCTCGAAAC---AACT--
Actr   GAAGCC--GGCTATGGGGCGCGCACACACCGCCCGTCACTCTCCTCAAAG---GAACA--
Scal   GAAGCC--GGCTATGGGGCGCGCACACACCGCCCGTCACTCTCCTCAAAGGGACGC----
Posp   GAAGC--CGGCTATGGGGCGCGCACACACCGCCCGTCACTCTCCTCA-------AAGGAA
Atsp   GAAGTT--GGCTCTGGGACGCGCACACACCGCCCGTCACTCTCCTCGA------ACATAA
Leoc   GAA-GT-TGGCTCTGGGACGCGTACACACCGCCCGTCACCCTCCTCG-AATTGAACCTAC
Amca   GAA-GC-CGGCTCTGAGGCGCGCACACACCGCCCGTCACTCTCCTCA--AAT-TGGACAA
Osbi   GAAACT--GGCTCTGAGGCGCGCACACACCGCCCGTCACTCTCCACT--AAACATTTTAT
Pabu   GAAACC--GGCTCTGGAGCGCGCACACACCGCCCGTCACTCTCCACT--ACATCCATTAA
Hial   GAAGCC--GGCTCTGGGACGCGCACACACCGCCCGTCACTCTCCCCTAACATGGTACAAA
Elha   GAAATA--GGCCCTGGGGCGCGCACACACCGCCCGTCACTCTCCTCAAAACACACACC--
Mlcy   GAAACC--GGCCATGGGACGCGCACACACCGCCCGTCACTCTCCTCA------AAACAAC
Algl   GAAGCT--GGCTCTAAGGCACGCACACACCGCCCGTCACTCTCCTCGAATTACATGAGCA
Ptgi   GAAACT--GGCTCTGAGGCGCGCACACACCGCCCGTCACTCTCCTCAAGTCATAAAAT--
Alaf   GAA-AT-CGGCTCTGAGGCGCGTACACACCGCCCGTCACTCTCTTCACCAGTCAAGCAAC
Nock   GAAATT--GGCTCTGAGGCGCGTACACACCGCCCGTCACTCTCTTCACC----AGTCAAG
Anja   GAA-AC-AGGCTCTGAGGCGCGTACACACCGCCCGTCACTCTCCTCG-------AACAAT
Gyki   GAA-AC-CGGCCCTGAGGCGCGTACACACCGCCCGTCACTCTCCTCGAA-------AAA-
Syka   GAAGCC--GGCTCTAAGGCGCGTACACACCGCCCGTCACTCTCCTCG--AA-TAAAT-TC
Opma   GAAACT--GGCCCTGAAGCGCGTACATACCGCCCGTCACCCTCCTTTA-------GCC-A
Comy   GAA-GT-TGGCTCTGAGGCGCGTACACACCGCCCGTCACTCTCCTCA-AGGTAAGTAAAT
Sasp   GAAACC--GGCCCTGAGATACGTACACACCGCCCGTCACCCTCCTCA------ACCGCAC
Eupe   GAAGCT--GGCCCTGAGGCGCGTACACACCGCCCGTCACCCTCCTC---AATCAAACCAA
Enja   GAAGCC--GGCTCTGAGGCGCGCACACACCGCCCGTCACTCTCCCCAACAACCCTAAT--
Same   GAAGCC--GGCTCTGAAGCGCGCACACACCGCCCGTCACTCTCCCCGACAACTACTCA--
Chch   GAATCC--GGCTCTGAGGCGCGCACACACCGCCCGTCACTCTCCCCAACT---CTAGGCC
Grgr   GAATTT--GGCTCTGAGGTGTGCACACACCGCCCGTCACCCTCCCCTCCAGGACCTGTCC
Caau   GAACCC--GGCTCTGAGACGCGTACACACCGCCCGTCACTCTCCCCTGTC---AAAATGC
Cyca   GAACCC--GGCTCTGAGACGCGTACACACCGCCCGTCACTCTCCCCTGTCAAAACGCA--
Dare   GAACCC--GGCTCTAAGGCGCGTACACACCGCCCGTCACTCCCTCCG--GTCA---ATTG
Cost   GAA-CC-CGGCTCTGAGGCGCGTACACACCGCCCGTCACTCTCCCCG---CCAAATGCAA
Leec   GAACTC--GGCTCTGAGGCGCGTACACACCGCCCGTCACTCTCCCCTGTCACC--TAG--
Crla   GAACCC--GGCTCTGAGGCGCGTACACACCGCCCGTCACTCTCCCCT------GTAATAG
Clmc   GAA-CC-AGGCTCTGAGACGCGCACACACCGCCCGTCACTCTCCCCA--TCA-CC-CAAT
Phin   GAA-AC-AGGCTCTGAGACGCGTACACACCGCCCGTCACCCTCCCCA-CCCCCCCCCACC
Icpu   GAATTA--GGCTCTGAGACGCGCACACACCGCCCGTCACTCTCCCCTCT-----GTATAT
Psto   GAATTA--GGCTCTGAGACGCGCACACACCGCCCGTCACTCTCCCCACA-----TTTATA
Cora   GAACCA--GGCTCTGAGACACGCACACACCGCCCGTCACTCTCCCCT--ACACAAAAACC
Eisp   GAATTA--GGCTCTGGAGCGCGCACACACCGCCCGTCACCCTCCCCTCATTA-ATTAT--
Apal   GAA-TA--GGCTCTGAGATGAGCACACACCGCCCGTCACCCCCTCCT---CTCAACTC-A
Eslu   GAAACT--GGCTCTGAAGCGCGCACACACCGCCCGTCACTCTCTCCAAG-TTCAACCCTA
Dape   GAAACT--GGCTCTGAGGCGCGCACACACCGCCCGTCACTCTCTCCAA-------G---T
Glse   GAAACT--GGCTCTGAGGCGCGCACACACCGCCCGTCACTCTCCCCGGGT---TCACTTT
Naar   GAA-AC-TGGCTCTGAGGCGCGCACACACCGCCCGTCACTCTCCCCAAGTCCGCTTTCCC
Baoc   GAAACT--GGCTCTGAGGCGCGCACACACCGCCCGTCACTCTCCCCAAGTCC--GCTT--
Opso   GAAAC--TGGCTCTGAGGCGCGCACACACCGCCCGTCACTCTCCCCA-------AGTCCA
Alte   GAATCC--GGCTCTGAGGCGCGCACACACCGCCCGTCACTCTCCCCGAA-----CCC-CA
Plap   GAATCC--GGCTCTGAGGCGCGCACACACCGCCCGTCACTCTCCCCGCACCC--TCAA--
```

Plal	GAAGCC--GGCTCTGAGGCGCGCACATACCGCCCGTCACTCTCTCCA------GG---TT
Sami	GAAGCC--GGCTCTGAGGCGCGTACATACCGCCCGTCACTCTCTCCG--GG---TTCACT
Rere	GAAGCC--GGCTCTGAGGCGCGCACACACCGCCCGTCACTCTCCCCAAATTCACCAA-TT
Gama	GAAACT--GGCTCTGAGGCGCGCACACACCGCCCGTCACTCTCCCCA--AGTCTATTTTT
Onmy	GAA-AC-TGGCTCTGAGGCGCGCACACACCGCCCGTCACTCTCCCCAAGTTC----AACC
Sasa	GAA-AC-TGGCTCTGAGGCGCGCACACACCGCCCGTCACTCTCCCCA--AGTTCAATTAA
Cola	GAAACT--GGCTCTGAGGCGCGCACACACCGCCCGTCACTCTCCCCA------AG--TTC
Dita	GAAGTT--GGCTCTGAGGCGCGTACACACCGCCCGTCACTCTCCCCGAGT---TCGCCAC
Gogr	GAAACA--GGGCCTGAAGCGCGTACACACCGCCCGTCACTCCCCCCT-CCCTCAACAC--
Chsl	GAAGCA--GGATATGAGGTGCGTACACACCGCCCGTCACCCTCGGCTAGT-------TCA
Atja	GAAACT--GGCTCTGAAGCACGTACACACCGCCCGTCACTCTCCCCAA------GTTCGT
Iido	GAAACT--GGCTCTGAAGCACGTACACACCGCCCGTCACTCTCCCCAAGTCCATTAA--A
Auja	GAA-AC-CGGCTCTGAAGCGCGCACACACCGCCCGTCACTCTCCCCA-------AATCAT
Chag	GAAGCC--GGCTCTGGAACGCGCACACACCGCCCGTCACTCTCCCCAACTTCCGACCT--
Hami	GAA-AC-TGGCTCTGAAGCGCGTACACACCGCCCGTCACTCTCCCCG--AATATTATT--
Saun	GAAACC--GGCTCTGAAGCGCGTACACACCGCCCGTCACTCTCCCCGAATACCCTAT-TC
Nema	GAAACT--GGCCCTTAAGCGCGCACACACCGCCCGTCACTCTCCCCG--AA-TCCAAATT
Disp	GAAATT--GGCCCTTAAGCGCGCACACACCGCCCGTCACTCTCCCCGA------ACCCAA
Myaf	GAA-GC-CGGCCCTTAAGCGCGTACACACCGCCCGTCACTCTCCCCAAGTCT----ACC-
Lagu	GAAGCC--GGCTCTAAGGTGCGCACACACCGCCCGTCACTCTCCCCCGCCCC-CGCAA--
Trtr	GAAGCC--GGCTCTGAGGCGCGCACATACCGCCCGTCGCTCTCCCCC--AATGTTTACCA
Zucr	GAAGCC--GGCTCTGAGGCGCGCACATACCGCCCGTCGCTCTCCCCCAGTGTTTATGCTA
Pxja	GAAACT--GGCCCTGAAGCGCGCACACACCGCCCGTCACTCTCCCCA------AGTTTAT
Pxlo	GAAACT--GGCCCTGAAGCGCGCACACACCGCCCGTCACTCTCCCCAAGTTTATTAA---
Pctr	GAAACC--GGCCCTAAAGCGCGCACACACCGCCCGTCACTCTCTCCAAGCGCAGGAAA--
Apsa	GAAACT--GGCCCTGAAGCGCGCACACACCGCCCGTCACTCTCCCCA--AATAAGCA--C
Cabe	GAAACC--GGCCCTAAAGCGTGCACACACCGGCCGTCACCCTCCCCA--AAGTTGCACAC
Bzze	GAAACT--GGCCCTGAAGCGCGCACACACCGCCCGTCACTCTCCCC---AAAGACTTTTA
Siim	GAA-AC-TGGCTCTGAAGCGCGCACACACCGCCCGTCACTCTCCCCTAGACACCTATAAA
Ctru	GAAACC--GGCCCTGAAGCGCGCACACACCGCCCGTCACTCTCCCCA------AGCCCTC
Dpbr	GAAAC--CGGCCCTGAAGCGCGCACACACCGCCCGTCACTCTCCCCA-------AGCCCC
Caki	GAA-AA-TGGCCCTAAAGCGCGTACAGACCGCCCGTCACTCTCCCCGAA-------A---
Phja	GAATAC--GGACCTAAAGCGCGTACACACCGCCCGTCACCCTCTCC----GATTATTTTT
Brsp	GAA-TT-AGGCCCTAAAGCGCGTACATACCGCCCGTCACCCTCCCCC--------TTCCC
Gamo	GAA-AA-CGGCCCTGAAGCGCGCACACACCGCCCGTCACTCTCTCCAAATA-----AACC
Lolo	GAAAAC--GGCCCTGAAGCGCGCACACACCGCCCGTCACTCTCTCCAAA-----TAGACC
Batr	GAACAC-AAACCTTAAAGTGCGCACAAACTGCCCGTCACCCCCCTCA-------CA----
Prmy	GAAATT--GGCACTAAAGCATGCACATACCGCCCGTCACCCTCCCCA------TCTTTCC
Loli	GAAACATCGGCTCTAAAGCGCGCACACACCGCCCGTCACTCTCCCCG-------AGCTAT
Loam	GAAAAT--GGCCCTGAAGCGCGCACACACCGCCCGTCACTCTCCTC---AAGCTAACGT-
Chab	GAAAC--TGGCCCTTAAGCGCGCACACACCGCCCGTCACTCTCCCCA-------AGCTGA
Chto	GAAAC--TGGCCCTTAAGCGCGCACACACCGCCCGTCACTCTCCCCA-------AGCTGA
Majo	GAAACC--GGCTCTTGAGCGTGCACACATCGCCCGTCACTCTCCCCGA------ACCCGT
Hlst	GAA-AC-TGGCCCTGAAGCGAGCACACACCGCCCGTCACTCTCCCCCGCCTA--AAATCT
Clpe	GAAGCC--GGCCCTAAAGCACGTACACACCGCCCGTCACTCTCCCCGAACA---GTAA--
Mlmr	GAACCT--GGACCTAAGGCACGTACACACCGCCCGTCACTCTCCCCCA-------AA-CA
Crcr	GAAGCC--GGCTCTAAAGCGCGCACACACCGCCCGTCACTCTCCCCA--AAACATAA-TC
Muce	GAAATC--GGCTCTAAAGCGCGCACACACCGCCCGTCACTCTCTCC---AAAACATACT-
Bege	GAAAC--TGGCCCTGAAGCGCGCACACACCGCCCGTCACTCTCCCCA-------AGCCAT
Mela	GAAACC--GGCCCTGAAGCGCGCACACACCGCCCGTCACTCTCCCCG--A-ATACAGGCC
Hats	GAAGC--TGGCCCTGAAGCGCGCACACACCGCCCGTCACTCTCCCCA-------AGCCAC
Orla	GAAACT--GGCCCTGAAGCGCGCACACACCGCCCGTCACTCTCCCCAAATCCAGTT----

Cosa	GAAACT--GGCCCTGAAGCGCGCACACACCGCCCGTCACTCTCCCCAAAA---A-TAAAC
Exsp	GAAACT--GGCCCTGAAGCGCGCACACACCGCCCGTCACCCTCCCCA------AAACCCC
Depa	GAAACT-TGGCTCTGAAGCGCGCACACACCGCCCGTCACTCTCCCCC-------AAAAAC
Rima	GAAACC--GGCCCTAAAGCGAGCACACACCGCCCGTCACTCTCTCCTAGTCTACCGCCCC
Fuol	GAA-TA-CGGCCCTGAAGCGCGCACACACCGCCCGTCACTCTCCCCA-------GGCTTA
Gmaf	GAAACC--GGCCCTGAAGCGCGCACACACCGCCCGTCACTCTCCCCA------AGTTCTT
Xeei	GAACCT--GGCCCTGAAGCGCGCACACACCGCCCGTCACTCTCCCCAAGC---C---AGT
Pros	GAAATT--GGCCCTGAAGCGCGCACACACCGCCCGTCACTCTCTCCC--GGCCTACCCTT
Scmi	GAAACC--GGCCCTGAAGCACGCACATACCGCCCGTCCCCCTCTCCGAGCTT--AGAA--
Rolo	GAAATT--GGCCCTGAAGCGCGCACACACCGCCCGTCACTCTCCCCAAG-----CCC---
Cere	GAAACA--GGCCCTAAAGCGCGCACACACCGCCCGTCACTCTCCCC---AAACCCAC--C
Daga	GAAACT--GGCCCTAAAGCGCGCACACACCGCCCGTCACTCTCCCCAA----AACCC---
Anco	GAAACT--GGCCCTGAAGCGCGCACACACCGCCCGTCACTCTCCCCA--AAAA-TTTTA-
Dmve	GAAGCT--GGCCCTGAAGCGTGCACACATCGCCCGTCACTCTCTCCGA------GCCGAC
Dmar	GAAGCT--GGCCCTGAAGCGTGCACACATCGCCCGTCACTCTCTCCA------AAGCACT
Anka	GAAACT--GGCCCTGAAGCGCGCACACACCGCCCGTCACTCTCCCCAAAA---AACTAAA
Moja	GAAACT--GGCCCTGAAGCGCGCACACACCGCCCGTCACTCTCCCCA------AAAAT-T
Hoja	GAAACT--GGCCCTGAAGCGCGCACACACCGCCCGTCACTCTCCCCA--AAAAACTTTC-
Bede	GAAACC--GGCCCTGAAGCGCGCACACACCGCCCGTCACTCTCTCC---AAGCTTAT--T
Besp	GAAACC--GGCCCTGAAGCGCGCACACACCGCCCGTCACTCTCTCCAA------GCT-TA
Mysp	GAAACT--GGCCCTGAAGCGCGTACACACCGCCCGTCACTCTCCCCAA-------ACAAA
Osja	GAAACT--GGCCCTGAAGCGCGCACACACCGCCCGTCACTCTCCCCAAACCAAACCCA--
Sgro	GAAACC--GGCCCTGAAGCGCGCACACACCGCCCGTCACTCTCCCCAA------ACTCAA
Pzpa	GAAACT--GGCCCTGAAGCGCGTACACACCGCCCGTCACTCTCCCCAA------ATTACC
Zeja	GAAACT--GGCCATGAAGCGCGTACATACCGCCCGTCACTCTCCCCAA------ATTACC
Znne	GAAATT--GGCCCTGAAGCGCGCACACACCGCCCGTCACTCTCCCCG------AAT-TAT
Zefa	GAAACT--GGCCCTGAAGCGCGCACACACCGCCCGTCACTCTCCCCTGTTGGCCCCC---
Acni	GAAAC--TGGCCCTGAAGCGCGCACACACCGCCCGTCACTCTCCCCA-------AAT---
Ncrh	GAAACT--GGCCCTGAAGCGCGCACACACCGCCCGTCACTCTCCCCA--AATTGCCCCTA
Agca	GAAACT--GGCCCTGAAGCGCGCACACACCGCCCGTCACTCTCCCCGAATTATTATAACT
Hydy	GAAACC--GGCCCTGAAGCGCGCACACATCGCCCGTCACTCTCCCCGAGCCT-AATTT--
Gsac	GAAATT--GGCCCTGAAGCGCGCACACATCGCCCGTCACTCTCCCCAA-GCCTAACAA--
Pevo	GAA-AC-AGGCCCTGAAGCGCGCACACACCGCCCGTCACTCTCCCCA-GTGT-AACAA--
Hiku	GAA-AT-TGGCTCTAAAGCGCGCACACACCGCCCGTCACTCTCCCCAATAAT----ATA-
Inpa	GAAACC--GGCCCTTAAGCGCGCACACACCGCCCGTCACTCTCCCCAACC---CACCCAA
Auch	GAAGCA--GGCCAGAAAGCGCGCACACACCGCCCGTCACTCTCCCTTAGCCACATACCAC
Fico	GAAACA--GGCCCTGAAGCGCGCACACACCGCCCGTCACTCTCCCCTATTTCTACACACA
Macs	GAAACT--GGCCCTGAAGCGCGCACACACCGCCCGTCACTCTCCCCAGAC-TACTAAC--
Moal	GAAACC--GGCCCTAAAGCGCGTACACACCGCCCGTCACTCTCCCCAAACAATCACATCA
Syma	GAAACT--GGCCCTAAAGCGCGCACACACCGCCCGTCACTCTCCCCAAACAATCTCAA--
Mafr	GAAACC--GGTTCTAAAGCGCGCACACACCGCCCGTCACTCTCCCCAAACCA--CCAT--
Dcpe	GAAACC--GGCACTGAAGCGAGTACACACCGCCCGTCACTCTCCCCG--ATGGCTCCACC
Dcti	GAA-AA-CGGCACTGAAGCGAGTACATACCGCCCGTCACTCTCCCCG---ATGG-CTCCA
Hehi	GAAATC--GGCCCTGAAGCGCGCACACACCGCCCGTCACTCTCCCCA------AGCTTAC
Stam	GAAATT--GGCCCTGAAGCGCGCACACACCGCCCGTCACTCTCCCCA--AG---CTTACA
Hogi	GAAACT--GGCCCTGAAGCGCGCACACACCGCCCGTCACTCTCCCCAA--AAACCGTA--
Erzo	GAAATT--GGCCCTGAAGCGCGCACACACCGCCCGTCACTCTCCCCA------AGCCTGC
Hxot	GAAATC--GGCCCTGAAGCGCGCACACACCGCCCGTCACTCTCCCCG------AAAACAC
Core	GAA-AT-TGGCCCTGAAGCGCGCACACACCGCCCGTCACTCTCCCCA-----AAAGCCCC
Apve	GAAACT--GGCCCTGAAGCGCGCACACACCGCCCGTCACTCTCCCCAAAACCACAAA--C
Latj	GAAACC--GGCTCTTAAGCGCGCACACACCGCCCGTCACCCTCCCCGA------GCACCC
Laja	GAA-AT-TGGCCCTGAAGCGCGCACACACCGCCCGTCACTCTCCCCA-AAAT-TACAAGT

```
Syja  GAA-AT-CGGCCCTGAAGCGCGTACATACCGCCCGTCACTCTCCCGAACCT--TAATAT
Epme  GAAATT--GGCCCTGAAGCGCGCACACACCGCCCGTCACTCTCTCCAAAC---ATGTCTT
Grse  GAAACA--GGCTCTGAAGCGCGCACACACCGCCCGTCACTCTCCCC---AAGCCCGCC-T
Clja  GAACCC--GGCCCTGAAGCGTGTACACACCGCCCGTCGCTCCCCCCGAGC---TT-TATT
Ogcy  GAA-GC-AGGCCCTGAAGCGCGTACACACCGCCCGTCACTCTCCCCGGAATA----AAT-
Plna  GAA-GC-AGGCACTGAAGCGCGTACATACCGCCCGTCACCCTCGCCG-AGTCTATTAAAC
Lema  GAA-AT-TGGCCCTGAAGCGCGCACACACCGCCCGTCACTCTCCCCGAGCTC----AC--
Etzo  GAAATT--GGCCCTGAAGCGCGCACACACCGCCCGTCACTCTCCCCA------AGCCTAC
Apse  GAA-A--TGGTTCTGAAGCGTGCACACACCGCCCGTCACTCTCCCCA--AGTTAGAATAT
Epde  GAAATC--GGCCCTGAAGCGCGCACACACCGCCCGTCACTCTCCCCAAGCCCCATAAATT
Slja  GAAATC--GGCTCTGAAGCGCGCACACACCGCCCGTCACTCTCTCCGATAATTGGAAA--
Bsja  GAAACT--GGCTCTGGAGCGCGCACACACCGCCCGTCACTCTCCCC---GAG--CTATAG
Ecna  GAAAAT--GGCTCTAAAGCGCGCACACACCGCCCGTCACCCTCCCCAA------GC--AC
Cohi  GAAGCC--GGCTCTTAAGCGCGTACATACCGCCCGTCACCCTCGCCAAGCCCTACCAA--
Caar  GAAACC--GGCTCTTAAGCGCGCACACACCGCCCGTCACCCTCCCCAA------GCACCT
Came  GAAGCC--GGCTCTTAAGCGCGCACACACCGCCCGTCACCCTCCCCAAGC---AACTGGA
Mema  GAAACC--GGCCCTAAAGCATGCACACACCGCCCGTCACCCTCCCCGAACC-CACAAC--
Lenu  GAAGAT--GGCCCCGAAGCGCGCACACACCGCCCGTCACTCTCCCCA--AA--TACCCTA
Plma  GAAATC--GGCTCTGAAGTGCGTACACACCGCCCGTCACTCTCCCCAAGC---TGACCAG
Emst  GAA-AT-CGGCCCTGAAGCGCGCACACACCGCCCGTCACTCTCCCCG-AGCTTACAAACT
Ptti  GAA-AC-CGGCCCTGAAGCGCGCACACACCGCCCGTCACTCTCCCCG--AGC-TT-ACAA
Losu  GAACCC--GGCTCTGAAGCGCGCACATACCGCCCGTCACTCTCTCCA------AGCCCCT
Geoy  GAAACT--GGCTCTGAAGCGCGCACACACCGCCCGTCACTCTCCCCAAGC---AACTTTT
Dipi  GAAAAT--GGCCCTGAAGCGCGCACACACCGCCCGTCACTCTCCCCG--AGCTCCCCAAA
Pama  GAAACC--GGCTCTTAAGCGCGCACACACCGCCCGTCACTCTCCCCGA-------AAC-T
Leob  GAAACT--GGCCCTGAAGCGCGCACACACCGCCCGTCACTCTCCCCG------AGCTTTC
Neba  GAAGAC--GGCCCTGAAGTGCGTACACACCGCCCGTCACTCTCCCCA--AGCACATTCAT
Pdpl  GAAATT--GGCCCTAAAGCGCGTACACACCGCCCGTCGCCCTCTCCGGCCCCCACACA--
Nimi  GAAATT--GGCCCTGAAGCGCGCACACACCGCCCGTCACCCTCCCCGAGACTAATCAA--
Uptr  GAAATC--GGCTCTTGAGCGTGCACACATCGCCCGTCACTCTCCCCGAAC---CCGTCTA
Pesc  GAAGTC--GGCCCTGAAGCGCGCACACACCGCCCGTCACTCTCCCCA------AAAGC-C
Baar  GAAGCC--GGCCCTGAAGCACGTACACACCGCCCGTCACTCTCCCCAA------ACGACC
Moar  GAAACT--GGCCCTGAAGCGCGCACACACCGCCCGTCACTCTCCCCGAGCTT--ACAAG-
Toja  GAAACC--GGCTCTTGAGCGCGCACACACCGCCCGTCACCCTCCCCA------AGTCT-A
Chau  GAAATT--GGCCCTGAAGCGTGCACACACCGCCCGTCACTCTCTCCGAACTAA-AACT--
Chse  GAAGAC--GGCTCTGAAGCGCGCACACACCGCCCGTCACTCTCCCCGAG-----CTA---
Enar  GAA-AC-TGGCCCTGAAGCGCGCACACACCGCCCGTCACTCTCCCCA-------AGCTCC
Hpty  GAAACC--GGCCCTGAAGCGCGCACACACCGCCCGTCACTCTCCCCCAAACCTCTAAA--
Nana  GAAAT--CGGCTCTAAGGCGCGCACACACCGCCCGTCACTCTCCCCG-------AAAAAC
Mcst  GAAATT--GGCTCTGAAGCGCGCACACACCGCCCGTCACTCTCCCTAAATTATAAAAA--
Rhox  GAAATT--GGCCCTGAAGCGCGCACACACCGCCCGTCATTCTCCCCGAGCTTAAATTT--
Opfa  GAAATT--GGCCCTGAAGCGCGCACACACCGCCCGTCACTCTCCCC----GAGCATAGCA
Paar  GAA-AC-CGGCCCTGAAGCGCGCACACACCGCCCGTCACTCTCCCCATGCATAAACTTCC
Gozo  GAAACC--GGCCCTGAAGCGCGCACACACCGCCCGTCACTCTCCCCA--AATCTACAAAA
Ackr  GAAACT--GGCCCTAAAGCACGCACACACCGCCCGTCACTCTCCCCGA---------GCT
Elev  GAAACT--GGCCCTAAAGCACGCACACACCGCCCGTCACTCTCTCCA--AAA----ACCC
Trdu  GAAATC--GGCCCTGAAGCGCGCACACACCGCCCGTCACTCTCCCC---AAGCCAACAAT
Amoc  GAAACA-TGGCAATAAAGCACGCACATATCGCCCGTCACTCTCCCCG-------AGCTAA
Hame  GAA-TC-TGGCCCTGAAGCGCGCACACACCGCCCGTCACTCTCCCCGAGCTA----ATA-
Chso  GAAATC--GGCCCTGAAGCGTGTACACACCGCCCGTCACTCTCCCCGAGCAG--AAAAAC
Lyto  GAAATT--GGCCCTGAAGCGCGCACACACCGCCCGTCACTCTCCCCAA--G---CCCA--
Encr  GAAATT--GGCCCTGAAGCGCGCACACACCGCCCGTCACTCTCCCCAA------GCCTAC
```

```
Bvar        GAAATT--GGCTCTGAAGCGCGCACATACCGCCCGTCACTCTCCCGGAACCT-CTCGA--
Noco        GAA-AT-TGGCTCTGAAGCGCGCACATACCGCCCGTCACTCTCCCGG-------AAAAAT
Chsp        GAAATA--GGCCATAAAGCATGCACACACCGCCCGTCACTCTCTCTAAATGCATTGAA--
Arja        GAAACT--GGCCCTGAAGCGCGCACACACCGCCCGTCACTCTCCCGAA-------GAACA
Pase        GAAAGA--GGCTCTGAAGCGCGTACATACCGCCCGTCACTCTCCCGG------AAAATGA
Trel        GAAGCT--GGCCCTGAAGCACGTACACACCGCCCGTCACCCTCCCG---A-GAATGTT-C
Lifa        GAA-GA-CGGCAATGAAGCGCGTACAGACCGCCCGTCATTCTCCCGGAATCT--ATATTC
Acur        GAAGAC--GGCAATGAAGCGCGCACACACCGCCCGTCATTCCCACCT--TTAAAGTAGAA
Ampe        GAAACT--GGCCCTGAAGCGCGCACACACCGCCCGTCACTCTCCCGAAACCCA-GAAC--
Urja        GAACTT--GGCCCTGAAGCGCGCACACACCGCCCGTCACCCTCCCGAAAA---CGAATTA
Enet        GAAGTT--GGCCCTAAAGCGCGCACACACCGCCCGTCATTCTCCCGAAGCTTATTAAACC
Ptbr        GAAGCT--GGCCCTTAAGCGCGCACACACCGCCCGTCACTCTCCTCTAACCAAACACAAT
Safa        GAAAT--TGGCCCTTAAGCGCGCACACACCGCCCGTCACTCTCCCGG-------AGCTAA
Icae        GAAATC--GGCTCTGAAGTGCGTACACACCGCCCGTCACTCTCCCGAAGCTCACCAAC--
Asmi        GAAACC--GGCTCTAAAGCGCGCACACACCGCCCGTCACTCTCCCGAAAAAAAATTAA--
Foal        GAA-GT-CGGCCATAAAGCGCGTACACACCGCCCGTCACTCTCTCGAAATAATAGC-ATT
Drze        GAAACC--GGCCCTGAAGCGCGCACATACCGCCCGTCACTCTCCACAGTA---AAAAATT
Rhas        GAA-AC-TGGCCCTGAAGCGCGCACACACCGCCCGTCACTCTCCCGA--AAC-CCACTGA
Elac        GAAACT--GGCCCTGAAGCGCGCACACACCGCCCGTCACTCTCCCGGAGCTTAAATTT--
Kugu        GAAACT--GGCTCTAAAGCACGCACACACCGCCCGTCACTCTCCCGG--AAACACCAATT
Plor        GAAATA--GGCCCTTAAGCGCGCACACACCGCCCGTCACTCTCCCGAAGCCTACACAA--
Sgun        GAAACC--GGCCCTGAAGCGCGCACACACCGCCCGTCACTCTCCCGG--AG---CTTAAT
Zaco        GAAATT--GGCCCTGAAGCGCGCACACACCGCCCGTCACTCTCCCGA--AG---CTCACA
Zbfl        GAAAC--CGGCCCTGAAGCGCGCACACACCGCCCGTCACTCTCCCGG-------AGCTTA
Spba        GAAATT--GGCTCTAAAGCGCGTACATACCGCCCGTCACCCTCTCTAG------GCCTAT
Game        GAAACC--GGCTCTGAAGTGCGTACACACCGCCCGTCACTCTCCCGAA-------GCTCA
Thth        GAA-AT-CGGCTCTGAAGTGCGTACACACCGCCCGTCACTCTCCCGA-AGCTTACCAATT
Xigl        GAAACT--GGCCCTAAAGCGCGCACACACCGCCCGTCACCCTCCCGGAGCCC--TAA---
Hyja        GAAGCC--GGCTCTGAAGTGCGCACATACCGCCCGTCACTCTCCCGAAGC-TCACAAG--
Psan        ----CC--GGCTCTGAAGAGCGCACATACCGCCCGTCACTCTCCCGA------AAAAT-A
Cupa        GAA-AT-CGGCTCTGAAGTGCGTACACACCGCCCGTCACTCTCCCGA--AGC-TCATCAA
Mpch        GAAACC--GGCCCTAAAGCGCGCACACACCGCCCGTCACTCTCCCGA--AGCTATAAACT
Char        GAAACC--GGCCCTAAAGCGCGCACACACCGCCCGTCACCCTCCCGCA----GCTTAA--
Pser        GAAACC-TGGCTCTAAAGCGCGCACACACCGCCCGTCACCCTCCCGGAGCTA----ATAT
Prol        GAAATT--GGCCCTAAAGCGCGCACACACCGCCCGTCACCCTCCCGAA-------GCCCT
Plbi        GAAACT--GGCCCTAAAGCGCGCACACACCGCCCGTCACCCTCCCGGAGCCCCCTGAA--
Calu        GAA-AC-CGGCACTGAAGCACGTACACACCGCCCGTCACCCTCACCA--AGC-GC-CTGG
Papa        GAAACC--GGCCCCTAAGCACGTACACACCGCCCGTCAATCTCCCTG--AAAACATTGAA
Sufr        GAAACC--GGCTCTGAAGCGCGCACACACCGCCCGTCACTCTCCCGAAGCTCCAATCAAC
Stci        GAAATT--GGCCCTGAAGCGCGCACACACCGCCCGTCACCCTCCCGT------AGCTCTT
Taru        GAAACC--GGCTATGAAGCGCGCACACACCGCCCGTCACTCTCCCGAAACTCT--TAA--
Rala        GAAATT--GGCCCTGAAGCGCGCACACACCGCCCGTCACTCTCCCGG------AGCTA-C
                                 * *** *  * * ***   * *
```

```
                   ¦                      HVR                      ¦ 42'
Scca    -----TATT----T-TTATAAAAATTTTACCAA---------AA-CA-AGAGGAGGCAAGT
Muma    CTTATTTTTTTATA-AATACATTT-----CTTT---------AACA--AGAGGAGGCAAGT
Erca    TTA-ACAAAATTTA-TAAAAAACTATATATAG----------A----AAGAGGAGGCAAGT
Pose    ------CACACTTT-TAATAA-AACACTACTAA---------CA-AGAAGAGGAGGCAAGT
Actr    -----CCCCAAGTA-TATAAATCTACAACCCAA---------ACA---AGAGGAGGCAAGT
Scal    -----CCTA---A--GTACATAAATCTATAACC---------CAAACAAGAGGAGGCAAGT
Posp    TACC-----CCCAG-TATATAAAAATATAA-CC---------CAAGCAAGAGGAGGCAAGT
Atsp    CC---A-TTTAACTTAATAAGCAACAAACCAA-----------AACA-AGAGGAGGCAAGT
Leoc    TTAACT-TA------ATAAACAGAAAGCCAGAA-------------CAAGAGGAGGCAAGT
Amca    ACCCTA-TA------AATAAAAAAACACCCTTA----------ACAA--GAGGAGGCAAGT
Osbi    CACTGACATTCAACAAAATAACTAATATAAATATGTC--ACCAAC-ACAGCGGAGACAAGT
Pabu    AG-------CTATCACCAGCTAAACTTCATAATTAACCAAAAATACCTAGAGGAGACAAGT
Hial    A-----ATTTAA-----ATAAAACATTACAACC---------GA-ACAAGAGGAGACAAGT
Elha    -----AACAAAGGT-ACATAAACAACTAG--AA---------AAAACAAGAGGAGGCAAGT
Mlcy    -AC-CCCACATATA-CAAAAACAAACAACCAAC---------AC---AAGAGGAGGCAAGT
Algl    A-----TGAATAT----ATAAGCTTAAAGGTAG---------CT-ATAAGAGGAGGTAAGT
Ptgi    -----TAAAGATAC-TTAAGAAACAAGAAGACA---------T----AAGAGGAGGCAAGT
Alaf    AAAATATTT------AATAAAA-CTGCAGACTA----------CCTA--GAGGAGACAAGT
Nock    CA---ACAAAAATA-TATAATAAGCCTGCAGAC---------TAC-CTAGAGGAGACAAGT
Anja    AATAAA---ATAAT-C-CATAAAACAATAA-GA---------ACAAAAAGAGGAGGCAAGT
Gyki    CGAAATGCCC-----ATATATAAAATTTTAGAC---------CAATAGAGAGGAGGCAAGT
Syka    A--------AAACATCTATAAACACCA--AAAC--------ACAAGAAAGAGGAGACAAGT
Opma    CAAACAAAAAATTT-A-TAAAAC--CTCAACTA---------GC-AAAAAAGGAGGCAAGT
Comy    GAATTACTT------TTATAAACACTTAATTTG---------GCCTACAGAGGGGGCAAGT
Sasp    TAG-TATTAACATG-TATAGT---GCATAG-----------------AGAGGAGGCAAGT
Eupe    -----------ATTATTTATAATTACTTGACATGA----TATTC----AGAAGAGGCAAGT
Enja    ------TCACGAGT-AATTTAACCCTACACATG---------TA-AAAAGGGGAGGCAAGT
Same    -----CACCAAAGT-AAGTAACGCAATATTTGT---------CAC--AAGGGGAGGCAAGT
Chch    CAGAAAATT--GTA-AACAGCCAGCAACCCTAG---------CCCCCAAGGGGAGGCAAGT
Grgr    AT---------TGGACTCTAACCCCATCCCGGGTCCG--CTTTTGTATAGGAGAGGCAAGT
Caau    ACCAGAAATATCTA-ATATAATAG----CA--C---------TGAC-AAGGGGAGGCAAGT
Cyca    -----CCAA---AA-ATACATAATACAACAGCA---------CTGACAAGGGGAGGCAAGT
Dare    CA---------CAAGAAATAATTAATACAGAAG-------CACAGACAAGGGGGGAAAAGT
Cost    CAATAGTTA------A-TAACATACAAGCACAA------------ACAAGGGGAGGCAAGT
Leec    -----CAACAAATG-TTTTTAACACCAAAGCGC---------CG-ACAAGGGGAGGCAAGT
Crla    CA---ATCAATGTA-AATAACAA-CAAAGCACC---------AAC--AAGGGGAGGCAAGT
Clmc    AACCAAGTA------AATAACAACAAACCAATG----------AAAG-CGGGGAGGCAAGT
Phin    ACAACAGTA------ACTAACAAAATATTCCCC-----------TAAGCGGGGAGGCAAGT
Icpu    TT---ACCCAACATTCCTAATACAT-TACACAT---------CAACA-CGGGGAGGCAAGT
Psto    AC--TACCCAATATAT-AATAAA-ACACATAAT---------TA--CACGGGGAGGCAAGT
Cora    T--------AACAATAGATAATACATC--AAAA--------AACACATCGGGGAGGCAAGT
Eisp    ------ACAAAGCT-ATATAACACAACCTCTTA---------TA-CTAAGGGGAGGCAAGT
Apal    C-----AACTTAGT-ATTTAACACATTAACATG---------AA-TTC-GGGGGGGCAAGT
Eslu    T-----TATTAAAT-AAAAAATATATCGA-ACA---------AA-----GGAGAGGCAAGT
Dape    TTAACACCCTTTTA-AATAAAAA--ATTAACAG---------AA-CAAAGGAGAGGCAAGT
Glse    CAACATAT--AAAT-AAGAAAATA----CACGA----------ATC-AAGGGGAGGCAAGT
Naar    AAAATTAACT-----AAGAAAACAACTGGACTA---------------A-GGGGAGGCAAGT
Baoc    ------TCCCAAGT-AACTAAGAAAACAACTGG----------A-CTAAGGGGAGGCAAGT
Opso    TCTTTCTCTAAATA-ACTAAGAAAACA---ACT---------AGACTAAGGGGAGGCAAGT
Alte    AC--CTTTAAAAGTA--AATAAC-AAAATTAAC---------TCACCAAGGGGAGGCAAGT
Plap    ------CCAAAAGT-AAATAA-CAAAATTGACT---------CA-CCAAGGGGAGGCAAGT
```

```
Plal      CAC-TTTCTTTGGT-TCTTAACAAGATTTCCGA---------ACA--AAGGGGAGGCAAGT
Sami      CA---------GTGCAGTTCTTAACAAGACAAC-------CGAA-CAAAGGGGAGGCAAGT
Rere      ------CTAGTAA----CTAACAAGAAAACTCG---------AG-TAAAGGGGAGGCAAGT
Gama      ---------TTGGTTCATAAAAGCACT-ACTGG--------A---CAAAGGGGAGGCAAGT
Onmy      TGTCCTTCTA-----ACTAAGAAGTTAAC--CG---------AACAA-AGGGGAGGCAAGT
Sasa      C-CCTTCTA------ACTAAGAAATTAACCGAA----------CAAA--GGGGAGGCAAGT
Cola      AAT-CTACCCTTCT-AACTAAGAAGTTAACCGA---------ACA--AAGGGGAGGCAAGT
Dita      CAACAGT---AATT-AAAAAGTTT-----CTTG---------AACT-AAGGGGAGGCAAGT
Gogr      ------CCCCCCCC-TTCTTAACACAAACCACA---------AC-CTAAGGGGGGAGAAGT
Chsl      CGCGACTCTTGTAT-ATACTAGAC----AGCAA---------GACA-AAGCAGAGGCAAGT
Atja      T----ATAACAATTAAATAAAAAATTACTAGAC---------CC-AA--GGGGAGGCAAGT
Iido      ------TATTTAA----ATAAAAAACTACCAGA---------C--CCAAGGGGAGGCAAGT
Auja      CTACCT---ACATT-T-CATAAA-CAAAAC-TT---------AAAACAAGGGGAGGCAAGT
Chag      -----TCTAAATAC-ATAAAACCCCCGTTCCCC---------G----CAGGGGAGGCAAGT
Hami      -CTAACTTT------ACCTAAAAACTTTTATCC----------TCA--AGGGGAGGCAAGT
Saun      ------ACCCTAC----CTAAAAAATTTTAATC------------TTAAGGGGAGGCAAGT
Nema      TC-------TTATATAATTAATCCGAATATAT---------AGGGCAAAGGGGAGGCAAGT
Disp      CC---C-TCCGATAACATATTATGTCAACTGG-----------GTAA-AGAGGAGGCAAGT
Myaf      TTAACCCCTA-----ACCTATAACGTAACACCT---------GGCAAAAGGGGAGGCAAGT
Lagu      ------GAAGAACT-CCCTAAAAGGTAACGCAC---------GG-CCCAGGGGAGGCAAGT
Trtr      AA-------A-ACATCTTTAAACCCCCAAC-T---------AAAACCAAGAGGAGATAAGT
Zucr      T-----AACTATAT--TTTAATACCCCATAATT---------AA-CTAAGAGGAGATAAGT
Pxja      TA--AAACATATTA-ATTAAAAAATTAGCCAA----------ACC--AAGGGGAGGCAAGT
Pxlo      ------AACATATT-AA-CTAAAAAATTAGCCA---------AA-CCAAGGGGAGGCAAGT
Pctr      -----AAAAAG--T-AAATAAACACAAAACTGA---------CC-CA-AGGAGAGGCAAGT
Apsa      C--------TAAACTAAATAAAACA---ATAAA--------ATTAACAAGGGGAGGCAAGT
Cabe      TA---------ATTACTACGTCAGAAATA-ACA-------GCCATATAAGGGGAGGCAAGT
Bzze      -----------ACTACGTTAACTAAAACATCATATG---TACAA----AGGGGAGGCAAGT
Siim      AACGTACCT------AACAAG--CTAAACATGT----------AGCA--GGGGAGACAAGT
Ctru      CAA-ATTTTATATT-TAAAAACATACAAACG-C---------A----GAGGGGAGGCAAGT
Dpbr      CACGAATTAAATA----CTTAAAAATAAACAAA---------T-GCAAAGGGGAGGCAAGT
Caki      CAATTCTCTT-----ATTCTTAATAATTTAAAT---------AAAACAAGGTGAGATAAGT
Phja      GT---------AAG-ATTTAAATAAAACATTTT------TTAACCGCGAGGAGAGGCAAGT
Brsp      AAAAAA-CC------ATAAGGTT------AGGG-----------TACCCGGAGAGGAAAGT
Gamo      CTAGATATTA-----CCTAAAATGCTTTT--TA---------TAATA-AGGGGAGGCAAGT
Lolo      CTAAATA-T---TA-CCTAAAATGTTTTATATA------------ATAAGGGGAGGCAAGT
Batr      --ATCA---CTCAT-TACGTAAA----TGA-GT---------TCTCTAAGAGGGGGAAAGT
Prmy      CT----CCAAATTA-AGTAAAACGCTATTTAA----------GAC--AAGGGGAGGTAAGT
Loli      -AATACTGAAAAA---TTAATAATAAGACACAA---------CTGCAAAGGGGAGGCAAGT
Loam      -----------CTACATATAATTAAATCATAATAACT--GCAAA-----GGGGAGGCAGGT
Chab      CAGGTCTTTATAC----TCTAAAACCTTATGGA---------CAGCAAAGGGGAGGCAAGT
Chto      CAGGTCTTTATAC---TCTAAAACCTTAT-GGA---------CAGCAAAGGGGAGGCAAGT
Majo      CT---ATGCTA-GTACTTAACAATA-AGGACAT---------CACCA-AGGGGAGGCAAGT
Hlst      GA---TAGAT-----ACCTTAACAAATGTCTTA---------AGGCAATGGGGAGGCAAGT
Clpe      ------CTCTCACT-AACTAAAACCCTATA-AA---------AG-AAGAGGGGAGGCAAGT
Mlmr      ATAG--ACTAAATA-ATTAAACCACCATTAAAA---------GC-TAAAGGGGAGGCAAGT
Crcr      T--------TTTCATTCATAATCCCCT--TAAA--------TCAAATTCGGGGAGAAAAGT
Muce      -----------CTTTTTATCCATAATTCCCTTAAATC--AAATTC----GGGGAGAAAAGT
Bege      CAACACCTAATAA---G-TAAGACCCTAT-CTT---------AGGCAAAGGGGAGGCAAGT
Mela      C--------TGCAATAAATAAAAAGCT--ACTC--------AAAAGAA-GGGGAGGCAAGT
Hats      TTTAACCCTTAAA------TAAAAACGAACCAG---------G-CAAGAGGGGAGGCAAGT
Orla      -----TTAA---AC-ATAAATAAACCCTTCAAG---------GAATAAAGGGGAGGCAAGT
```

```
Cosa    CTTAAAT---AATT-AATCAATT-----AAATT---------AATA-AAGGGGAGGCAAGT
Exsp    TA---AAAAGATTA-AGTAAAACCATAGATC-----------CAATAAAGGGGAGGCAAGT
Depa    AA-AGA-----AAA-TAACTAATACATTAA-CA---------AAATAAAGGGGAGGCAAGT
Rima    A-----ACCCTAC----ATAAAAAGCCTTACCA---------GC-A-AAGGAGAGACAAGT
Fuol    AATATA-----AGA-TTTTTAATGAACTAAATA---------AAGCAAAGGGGAGGCAAGT
Gmaf    ATA-CATTAA--TA-ACTAATACCTCACC-AGA---------ACA--AAGGGGAGGCAAGT
Xeei    AATCTTC-ATCCTT-AATAAACTA----AATAG----------GCA-AAGGGGAGGCAAGT
Pros    C--------TAA-TTAACTAATACCCC--GCCA--------ATTGCAAAGGGGAGGCAAGT
Scmi    -----TCTCAAAAT-ATTTAAACCCCCCCCCCG---------CT-TAAAGGAGAGACAAGT
Rolo    AC--CGCACCAAATA--ATTAATTATTTTATTT---------CCGCAAAGGGGAGGCAAGT
Cere    -----------TAAACGATAATTAATTAGTTATAACC--CTTTA----AGGGGAGGCAAGT
Daga    -----CCAAATCAA-TAACTAATAAATTAATTA---------ACATTAAGGGGAGGCAAGT
Anco    ------------ACCTAATAACTAA---AACCTTAAT--AC-AAA-AAAGGGGAGGCAAGT
Dmve    AC---T-ACTAATTAACTAAAACCCCAATGCAG---------GACAC-AGGAGAGGCAAGT
Dmar    AC---CATCTATTA-ATTAATACCCCCGTACAG---------CATTACAGGAGAGGCAAGT
Anka    AACAAGT---AACT-AAATACCC-----ACCAC---------AATA-AAGGGGAGGCAAGT
Moja    TTT-CATCAA-ATA-ACTAA-ACCCTTAATACA---------ATA--AAGGGGAGGCAAGT
Hoja    ------------ACCAAATAAATAA---ACCTTTAAC--AC-AAT-AAAGGGGAGGCAAGT
Bede    AT-------TCTTTATAACTAAAATATTATTC---------ATTGCAAAGGGGAGGCAAGT
Besp    TT---ATTCTTTATAACTAAAACAGTACCCATT---------GCAAA--GGGGAGGCAAGT
Mysp    CCCA--TTAAACTA-ACATAAAC-AACTACTCA---------TC-CAAAGGGGAGGCAAGT
Osja    -----CTAAATTAA-CCTATACA-ACTACCTAT---------ACA--AAGGGGAGGCAAGT
Sgro    AC--TACTAAACTAAACCTTAAAACATTACCCA---------TGA-AAAGGGGAGGCAAGT
Pzpa    CT---TTAATGCTTTTATAAAAACTTA-TGTAA---------AACA--AGGGGAGGCAAGT
Zeja    CC---CTTAACATTTTATAAAACTTTTTACTAA---------ATGAA--GGGGAGGCAAGT
Znne    CC---CCT-AACTA-TCTATAAAACAATTATTA---------AAA-AGAGGGGAGGCAAGT
Zefa    ------AACCTTTC-A---TAAAACCTTTATTA---------AA-CAAAGGGGAGGCAAGT
Acni    --TGCCCCTAAACA-TTTCATAAAACTTTTATT---------AAATAAAGGGGAGGCAAGT
Ncrh    AA-------C-ATTTCATTAAAACTTTTAT-T---------AAATA-AAGGGGAGGCAAGT
Agca    C-----AAGTAA-----CTAAAACCCTA-AATT---------CC-TAAAGGGGAGGCAAGT
Hydy    -------CATAGTT-AACTAAAGCCTAGTATC----------TG-CAAAGGGGAGGCAAGT
Gsac    -----CACAATTAC-TTAAAAACTAATAATTGC---------A----AAGGGGAGGCAAGT
Pevo    -TAACA-AT------CCATAAACAATAATAAGA-----------CAAAAGGGGAGGCAAGT
Hiku    ATCACAATTA-----ATTAAAACCCTAAA--TA---------AATACAAGGGGAGGCAAGT
Inpa    ATAACAT---ACCT-AATA-CATT----ACTCC---------ACCA-AAGGGGAGGCAAGT
Auch    GGTAACATCATTTT-ATTCCAAAAGGTTCATGA---------TA-ACGAGGGGAGGAAAGT
Fico    -------TAATTT----CTAATAAGATAAAGAG---------CC-A-GAGGGGAGGCAAGT
Macs    -----TTTGA---T-AACTAATACCTTA--AGA---------TCTCTAAGGGGAGGCAAGT
Moal    C-----CCTAA------CTAAAACACTATCAGC---------CA-AAGAGGGGAGACAAGT
Syma    -----CTTA---TA-TAATAATAAACTACCAAC---------CTCAAGAGGGGGGACAAGT
Mafr    ------TACCCCCT-AACTAAACACAAAACAAT---------CA-CAAAGGGGAGGCAAGT
Dcpe    AA-------C-ATAATTAATATGCAATTAT-G---------CCTAT-AAGGGGAGGTAAGT
Dcti    TTAACA-TA------ATTAATACCCAATCAGCC-----------TATGAGGGGAGGCAAGT
Hehi    CAC-TTTTAATAAT-TAAAAACCCAAAAATCGC---------G----GAGGGGAGGCAAGT
Stam    AA-----------TCAATTAATTAAAACCCTAG-------AATTGCAAAGGGGAGGCAAGT
Hogi    -----GTAAAACAC-ATAAATAAAACCCTATTA---------ATATAAAGGGGAGGCAAGT
Erzo    TA---ATTCAATTA-ACTAAACC-CTAAAAAGG---------GCA--AAGGGGAGGCAAGT
Hxot    CC---ACTCAGTTA-ATTAAAACCTAATAATCA---------TA---GAGGGGAGGCAAGT
Core    AATCAGTTA------ACTAAAACCTAATAATCA------------AAAAGGGGAGGCAAGT
Apve    ------CAATTAA----TTAAAACCTAATAACC---------A--ACAAGGGGAGGCAAGT
Latj    GG---CATAACATAA-ATAAAACCAAACGA-AC---------GCGAA--GGAGAGGAAAGT
Laja    CTATTA-TA------ACTAATACCCTATTAATA-----------TGAA-GGGGAGGCAAGT
```

```
Syja    AAACCTAAAT-----ACCTAAACACCTATAATA---------CTCCTA-GGGGAGGCAAGT
Epme    TTTAAATTTAACTT-AAAACTTTC-----CACT---------AACACAAGGGGAGGCAAGT
Grse    AC-------TAACAATAACTAATAC-CCATAA---------ACAGCTAAGGGGAGGCAAGT
Clja    TATCAACTATAATT-AATACACTA----TCTTG---------TGCT-AAGGGGGGATAAGT
Ogcy    ATAACATAAT-----ACTAATGCCTTATG--AA---------AAAAATAGGGGAGGCAAGT
Plna    TTAAACATA------ACTAATAAGTGTATTTGA------------TAAAGGGGAGGCAAGT
Lema    -GAACTTTTA-----ATAAATAAAATCCCGCCA---------CTGCAAAGGGGAGGCAAGT
Etzo    TGA-CTAAAG--TA-ACTAAAACCTTAAA-ATC---------GCG--AAGGGGAGGCAAGT
Apse    TACCTAGTA------ACTAAACACTTAACCCTA----------CAAA--GGGGAGGCAAGT
Epde    T-----AATTAA-----CTAAAAACCTACTATC----------G-CGAAGGGGAGGCAAGT
Slja    -----TGAAACCTT-AACTAAAAAGAGAACAGC---------TA-AAGAGGGGAGGAAAGT
Bsja    -----------AACCCTATAACTAATAACCCATGAC---TGCAA----AGGGGAGGCAAGT
Ecna    CC--AACACCCCTTAA--CTAAACTATACCGAC---------CGCTACAGGAGAGGAAAGT
Cohi    -----TTATTTTTT-CTAAATCACTTATAAAGC---------T----AAGGAGAGGAAAGT
Caar    GG---ACCTAAATTTTCTTAAACCTAAACAACC---------GCGAA--GGAGAGGAAAGT
Came    CCTAAAATTTCTTT-AAACCCTAA----CAA-C---------CGCG-AAGGAGAGGAAAGT
Mema    -----CA-AACCAT-AAATAATAAACTAT--AA---------CCGCAAAGGGGAGGAAAGT
Lenu    A--------CAACTTAACTAAAATACA--AGAC--------ATAACAA-GGGGAGGCAAGT
Plma    CCCTAAT---AACT-AAAATACTA-----AAAC---------CACG-AAGGGGAGGCAAGT
Emst    CAGATAACT------A---AAGCCCTATAATTG------------CAAAGGGGAGGCAAGT
Ptti    ACTCAAATA------CTTAAAACCTTAAAATTG----------CAAA--GGGGAGGCAAGT
Losu    ACCCCATCTTATTA-TCTAAAAAGCTAAATAAT---------GCT--GAGGAGAGACAAGT
Geoy    TAATCTTTTTAAAT-AAAATCCCG----CCTTT----------GCA-AAGGGGAGGCAAGT
Dipi    AC-------CCAACTAACTAAAACCAGCAC-T---------AGCAATTAGGGGAGGCAAGT
Pama    TTACCAAAACATTA-ATTAAAAT--GCCAAAAT---------CA-TAGAGGGGAGGCAAGT
Leob    AAA-TTTTGCCCTA-AATAAAACACTTCCCACC---------GCT--AAGGGGAGGCAAGT
Neba    AA-------TAACCTAAATAAAACGTA-AAAAA--------AG--CATAGGGGAGGCAAGT
Pdpl    -----CTTAATTTA-CATAAACCTACGTCTAAA---------CCCTACAGGAGAGGAAAGT
Nimi    ------ATTCAATT-AAATAA-AACCCCATTAT---------AG-TAAAGAGGAGGCAAGT
Uptr    TTCTCGT---ACTT-AACAATACG----GACAC---------CACT-AAGGGGAGGCAAGT
Pesc    TAT-TACTCACGTA-ATTAATCCCACACCGCTG---------CCC--CAGGGGAGGCAAGT
Baar    CT---AATTTAAATAACTAATTCAACTACCACC---------ACGAACAGGGGAGGCAAGT
Moar    ------TTTGAAT--AACTAAAACCTCATAAT-----------TGCAAAGGGGAGGCAAGT
Toja    CAA-ACTCAACATA-ACTAA-ACCCCTATAACT---------GCA--TAGGGGAGGAAAGT
Chau    ------TTTAACAT-AACTAA-TATGCTATAAC---------TG-TAGAGGGGAGGCAAGT
Chse    AG--ATTTTAAATTA--ACTAAA-ACACTACAA---------CTGTAAAGGGGAGGCAAGT
Enar    AAAAAT---TTTGT-AAC-TAAGACCCTAC-AA---------CTGCAAAGGGGAGGCAAGT
Hpty    -----CTTAAATAA-CTAAAACC--CTACTACC---------ACA--AAGGGGAGGCAAGT
Nana    ---ATTAATCAACT-TAACTAAAACCTAAG-AC---------ACATAGAGGGGAGGCAAGT
Mcst    -----TCTCATATA-ACTAAGACCCTAAAGTCA---------C---AGAGGGGAGGCAAGT
Rhox    -----TCATTTAAC-TAAAACCCTAAGACTGCT---------AA-----GGGGAGGCAAGT
Opfa    GT---------TAC-CTGTAACTAAAACCTCAA---G--ATATTGTAAAGGGGAGGCAAGT
Paar    AAAACA-TA------ATTAATTTGCTAGACTTG-----------CACAAGGGGAGGCAAGT
Gozo    ---------TCAAATAACTAAACCCT--ATAAG--------AC-ACAAAGGGGAGGCAAGT
Ackr    AATAAAATTCAATA-AATAATAAACAATACCAG------------CTAAGGGGAGGCAAGT
Elev    TA---------ACATATATACCTAAAACGTAAA-------ATTAAACAAGGGGAGGCAAGT
Trdu    -----------CACCCTATAAATAATATATT-TTACC--GGTAA----AGGGGAGGCAAGT
Amoc    TACGAA---TATAA-TACCTAAAAAATTAC-AT---------CTGCAAAGGGGAGGCAAGT
Hame    AAAGCACATA-----ATTAATAAACCAGA--CC---------TGCAA-AGGGGAGGCAAGT
Chso    TCTTTACC-TAAAAA-GCCATAAAAATGTAAA----------------GGGGAGGCAAGT
Lyto    -----CCAACTTAA-TTAACTAAACCCTAATAA---------TCGCAAAGGGGAGGCAAGT
Encr    -C--AACTTAATTAA---TTAAACCATAATAAT---------CGC-AAAGGGGAGGCAAGT
```

```
Bvar            ------AGTAATTT-ACTTAAAACCCTAGAACT---------GGCCTTAGGGGAGGCAAGT
Noco            TTCACC---CTAAT-TATATAAAACCTTAG-AG---------CAAAAGAGGGGAGGCAAGT
Chsp            -----CTTTTG-AT-ACCTAACAAACCCT-AAA---------AATTACAAGGGAGAGAAGT
Arja            CCAACTAGT---TA-ATTAAAACCTAATAACAT------------AAAAGGGGAGGCAAGT
Pase            AAA-TAAAACCTTA-CATAAAACATTATCGAAT---------ATA--GAGGGGAGGCAAGT
Trel            AC-------CTATTGTAACTAAAGAGTAATAAC--------ACCCTCCAGGGGAGGCAAGT
Lifa            -----TAAAT-----TCCTAATAAGTTACAATC---------GGTAAA-GGGGAGGCAAGT
Acur            C--------TTAAATAACTAAAAAATTAATACG--------AC-ACCA-GGTGGGGTAAGT
Ampe            ------CTTAA-GT-AAATAA-ATCATTATCAC---------CA-AGAAGGGGAGGCAAGT
Urja            TACAAATTTTAC-T-AAATCAATA----CACAT---------AGTA-AAGGGGAGGCAAGT
Enet            ------CTTATAA----CTAAGAAACCGAGACT---------AG-TAAAGGGGAGGCAAGT
Ptbr            T-----AATTTA-----CTAAAACAATAAAAGG----------G-ACCAGAGGAGATAAGT
Safa            AAAATATCAAT------TCATAATACCTATTAA---------CAGCAAAGGGGAGGCAAGT
Icae            -----CAAAATAAC-TAAAACACTACAATCGCG---------AA-----GGGGAGGCAAGT
Asmi            -----AT--GCTCC-CTAATCGA-AATAA-AAG---------ATA--AAGGGGAGGCAAGT
Foal            --CCACTACT-TAAG-ATGACAAAACCTTTTAT---------TACCC--GGAGAGACAAGT
Drze            ACATGAT---AACT-AATAATTACAGAAAAAAA---------AACA-AAGAGGAGACAAGT
Rhas            ATACAAGTA------AATAACACACAAAAGACA----------TAAA--GGGGAGGCAAGT
Elac            -----TAAACATAA-ATAAACCACAACAACTGT---------A----AAGGGGAGGCAAGT
Kugu            AA---------ACCTTAATTTAAACCACCACAT-------TATC-TAAAGGGGAGGCAAGT
Plor            ------ACCCAAAT-AACCTAAACCCTACTATT---------G--CAAAGGGGAGGCAAGT
Sgun            AA---------ACTCAACTAACTAAAACCCTAT-------AATTGCAAAGGGGAGGCAAGT
Zaco            CCC--TAAAG-----TAAATAAAACCCTATACC----------TGCAAAGGGGAGGCAAGT
Zbfl            CATATA----CAAG-TAAATAAAACCCTAC-AA---------TTGCAAAGGGGAGGCAAGT
Spba            AG---A-ACTACATTTATCTAAAACCCTTCGAC---------TGCAA-AAGGGAGGAAAGT
Game            CTAT--TCAACGTA-ACTAAAACACT-TTAACC---------GC--GAAGGGGAGGCAAGT
Thth            TATGTATCT------A---AAACGCTTTAACTG------------CGAAGGGGAGGCAAGT
Xigl            ------ACTCACTT-AA-CTAAAACCCTATAAT---------TG-CGAAGGGGAGGAAAGT
Hyja            -----TTCCAC-AT-AACTAAAATGCTATTAAT---------ACGCGAAGGGGAGGCAAGT
Psan            CAA-ATTTAACATA-AATAATATGCTTTATTAC---------ATA--AAGGGGAGGCAAGT
Cupa            -CCCACCTA------ACTAAAACAACATAACTG----------CAA--AGGGGAGGCAAGT
Mpch            T--------CTACATAATTAAAACATT--AAA---------ACTGCAAAGGGGAGGCAAGT
Char            -----CTTAAATAA-TAAATAATAACACAAACA---------CTGTTAAGGGGAGGCAAGT
Pser            AAAACAACTT-----ACTTTAAAACAAGATACC---------TGCGATAGGGGAGGAAAGT
Prol            ACAAGACTAAACTA-ACTAAAACCAACAACCCG------------CGAAGGGGAGGAAAGT
Plbi            ------CTA-ATCT-AATTAAAAACCAACAACC---------CG-CGAAGGGGAGGAAAGT
Calu            AAAACCCTA------ACTAAGATGAGCCTAACT----------GCAA-AGGAGAGGAAAGT
Papa            -----------CACCACACTAAATAAAAAAATAAGTC--GATACCTGGAAGGGAGAAAAGT
Sufr            C-----TCATAA-----CTAATTCACCAACTTC---------TG-CTGAGGGGAGGCAAGT
Stci            AA---AAGTAATTT-CATAAAACGCTATA-ACT---------GCA--AAGGGGAGGCAAGT
Taru            -----TTTAAAAAT-AACTAATAAGCCACCAAA---------AG-AAAAGGGGAGGCAAGT
Rala            CAC-AAACCATTTA-ATTTAAGCCATTAAAACT---------GCA--AAGGGGAGGCAAGT
                                                                    * *  * **
```

```
           41'      D'   43          43'
Scca       CGTAACATCGTAAGTGTACTGGAAAGTGCACTTGGAAC-------
Muma       CGTAACATCGTAAGTGTACTGGAAAGTGCACTTGGAAT-------
Erca       CGTAACATCGTAAGCGTACCAGAAGGTGTGCTTGGAATAAT----
Pose       CGTAACATCGTAAGCGTACCGGAAGGTGTGCTTGGAATAT-----
Actr       CGTAACATCGTAAGTGTACCGGAAGGTGCACTTGGAACAAC----
Scal       CGTAACATCGTAAGTGTACCGGAAGGTGCACTTGGAACAAC----
Posp       CGTAACATCGTAAGTGTACCGGAAGGTGCACTTGGAACAAC----
Atsp       CGNAACATCGTAAGTGTACCGGAAGGTGCACTTGGAATAAC----
Leoc       CGTAACACCGTAAGTGTACCGGAAGGTGCACTTGGAATAAC----
Amca       CGTAACATCGTAAGTGTACCGGAAGGTGCACTTGGATCAC-----
Osbi       CGTAACACCGTAAGTGTACCGGAAGGTGCACTTGGATTAAC----
Pabu       CGTAACATCGTAAGTGTACCGGAAGGTGTACTTGGAACAAT----
Hial       CGTAACATCGTAAGTGTACCGGAAGGTGCACTTGGAATAAG----
Elha       CGTAACATCGTAAGTGTACCGGAAGGTGCACTTGGAATAAC----
Mlcy       CGTAACATCGTAAGTGTACCGGAAGGTGCACTTGGAACAAT----
Algl       CGTAACAACGTAAGTGTACCGGAAGGTGCACTTGGAACTAT----
Ptgi       CGTAACATCGTAAGTGTACCGGAAGGTGCACTTGGAATAAC----
Alaf       CGTAACATCGTAAGTGTACCGGAAGGTGCACTTGGAACAAC----
Nock       CGTAACATCGTAAGTGTACCGGAAGGTGCACTTGGAACAAC----
Anja       CGTAACACCGTAAGTGTACCGGAAGGTGCACTTGGATAAAT----
Gyki       CGTAACATCGTAAGTGTACTGGAAGGTGCACTTGGAATAAT----
Syka       CGTAACATCGTAAGTGTACCGGAAGGTGCACTTGGATAAAA----
Opma       CGTAACATCGTAAGTGTACCGGAAGGTGCACTTGGAAGAAT----
Comy       CGTAACATCGTAAGTGTACCGGAAGGTGCACTTGGATAAA-----
Sasp       CGTAACATCGTAAGTGTACCGGAAGGTGTACTTGGAAAAATA---
Eupe       CGTAACATCGTAAGCGTACCGGAAGGTGTGCTTGGATATAT----
Enja       CGTAACATCGTAAGTGTACCGGAAGGTGCACTTGGAATAAC----
Same       CGTAACATCGTAAGTGTACCGGAAGGTGCACTTGGAATAAT----
Chch       CGTAACATCGTAAGTGTACCGGAAGGTGCACTTGGAGTAAC----
Grgr       CGTAACAACGTAAGTGTACCGGAAGGTGCACTTGGACTAAT----
Caau       CGTAACACCGTAAGTGTACCGGAAGGTGCACTTGGATCAAACC--
Cyca       CGTAACACCGTAAGTGTACCGGAAGGTGCACTTGGATCAAACC--
Dare       CGTAACAACGTAAGTGTACCGGAAGGTGCACTTGGACATTATC--
Cost       CGTAACATCGTAAGTGTACCGGAAGGTGCACTTGGATCAAAT---
Leec       CGTAACATCGTAAGTGTACCGGAAGGTGTACTTGGATCAAATA--
Crla       CGTAACATCGTAAGTGTACCGGAAGGTGCACTTGGATCAAACC--
Clmc       CGTAACATCGTAAGTGTACCGGAAGGTGCACTTGGAACAT-----
Phin       CGTAACATCGTAAGTGTACCGGAAGGTGCACTTGGATCAC-----
Icpu       CGTAACATCGTAAGTGTACCGGAAGGTGCACTTGGAACAAT----
Psto       CGTAACATCGTAAGTGTACCGGAAGGTGCACTTGGAACAAT----
Cora       CGTAACATCGTAAGTGTACCGGAAGGTGCACTTGGAACAAT----
Eisp       CGTAACATCGTAAGTGTACCGGAAGGTGCACTTGGAATAAC----
Apal       CGTAACAACGTAAGTGTACCAGAAGGTGTACTTGGAACAAC----
Eslu       CGTAACATCGTAAGTGTACCGGAAGGTGCACTTAGTTTAAT----
Dape       CGTAACATCGTAAGTGTACCGGAAGGTGCACTTAGTTTAAT----
Glse       CGTAACATCGTAAGTGTACCGGAAGGTGCACTTGGAACAAT----
Naar       CGTAACATCGTAAGTGTACCGGAAGGTGCACTTGGAACAAC----
Baoc       CGTAACATCGTAAGTGTACCGGAAGGTGTACTTGGAACAAC----
Opso       CGTAACATCGTAAGTGTACCGGAAGGTGCACTTGGAATAAG----
Alte       CGTAACATCGTAAGTGTACCGGAAGGTGCACTTGGAATAAC----
Plap       CGTAACATCGTAAGTGTACCGGAAGGTGCACTTGGAATAAC----
```

```
Plal    CGTAACATCGTAAGTGTACCGGAAGGTGCACTTGGGACAAC----
Sami    CGTAACATCGTAAGTGTACCGGAAGGTGCACTTGGGACAAC----
Rere    CGTAACATCGTAAGTGTACCGGAAGGTGCACTTGGGACAAT----
Gama    CGTAACATCGTAAGTGTACCGGAAGGTGCACTTGGAAAAAC----
Onmy    CGTAACATCGTAAGTGTACCGGAAGGTGCGCTTGGAATAAC----
Sasa    CGTAACATCGTAAGTGTACCGGAAGGTGCACTTGGAATAAC----
Cola    CGTAACATCGTAAGTGTACCGGAAGGTGCACTTGGAATAAC----
Dita    CGTAACATCGTAAGTGTACTGGAAAGTGCACTTGGAATAAC----
Gogr    CGTAACACCGTAAGTGTACCGGAAGGTGCACTTGGAATAAC----
Chsl    CGTAACATCGTAAGTGTACTGGAAAGTGTACTTGGGATAA-----
Atja    CGTAACATCGTAAGTGTACTGGAAAGTGCACTTGGATAAAC----
Iido    CGTAACATCGTAAGTGTACTGGAAAGTGCACTTGGATAAAC----
Auja    CGTAACATCGTAAGTGTACCGGAAGGTGCACTTGGAACAAAT---
Chag    CGTAACACCGTAAGTGTACCGGAAGGTGCACTTGGATGAAT----
Hami    CGTAACATCGTAAGTGTACCGGAAGGTGCACTTGGACAAA-----
Saun    CGTAACATCGTAAGTGTACCGGAAGGTGCACTTGGAAAAA-----
Nema    CGTAACATCGTAAGTGTACCGGAAGGTGCACTTGGTTAACA----
Disp    CGTAACATCGTAAGTGTACCGGAAGGTGCACTTGGAATATA----
Myaf    CGTAACATCGTAAGTGCACCTGAAGGTGTACTTGGAAAAAT----
Lagu    CGTAACAACGTAAGTTTACCGGAAGGTGAACTTGGAAAAC-----
Trtr    CGTAACATCGTAAGTGTACCGGAAGGTGCACTTGGTGTAAC----
Zucr    CGTAACATCGTAAGTGTACCGGAAGGTGTACTTGGAATAAT----
Pxja    CGTAACATCGTAAGCGTACCGGAAGGTGTGCTTGGAAAAAC----
Pxlo    CGTAACATCGTAAGCGTACCGGAAGGTGTGCTTGGAAAAAC----
Pctr    CGTAACATCGTAAGCGTACCGGAAGGTGCGCTTGGAAAAAC----
Apsa    CGTAACATCGTAAGCATACCGGAAGGTGTGCTTGGATTAAC----
Cabe    CGTAACATCGTAAGTGTACCGGAAGGTGCACTTGGAAAAT-----
Bzze    CGTAACATCGTAAGTGTACCGGAAGGTGCACTTGGAAAAAC----
Siim    CGTAACATCGTAAGTGTACCGGAAGGTGTACTTGGACAAACCC--
Ctru    CGTAACATCGTAAGTGTACCGGAAGGTGTGCTTGGAAAAAT----
Dpbr    CGTAACATCGTAAGTGTACCGGAAGGTGCACTTGGAAAAAC----
Caki    CGTAACATCGTAAGTGTACCGGAAGGTGTACTTGGACAAAT----
Phja    CGTAACATCGTAAGTGTACCGGAAGGTGCCCTTGGACAAT-----
Brsp    CGTAACATCGTAAGTGTACCGGAAGGTGCACTTGAGTAT------
Gamo    CGTAACATCGTAAGCGTACCGGAAGGTGCGCTTGGATGAAC----
Lolo    CGTAACATCGTAAGCGTACCGGAAGGTGCGCTTGGATGAAT----
Batr    CGTAACAACGTAAGTGTACTGGAAAGTGCACTTGAATA-------
Prmy    CGTAACAACGTAAGTGTACTAGAAAGTGTACTTGACAA-------
Loli    CGTAACATCGTAAGTGTACCGGAAAGTGCACTTGGAGAAA-----
Loam    AGTAACATCGTAAGTGTACCGGAAAGTGCACTTGGAAAAAC----
Chab    CGTAACATCGTAAGTGTACCGGAAGGTGCACTTGGAAAAGC----
Chto    CGTAACATCGTAAGTGTACCGGAAGGTGCACTTGGAAAAGC----
Majo    CGTAACATAGTAAGTGTACCGGAAGGTGCACTTGGAACAT-----
Hlst    CGTAACATCGTAAGTGTACCGGAAGGTGCACTTGGAAAAAC----
Clpe    CGTAACATCGTAAGTGTACCGGAAGGTGCACTTGGAAAAGA----
Mlmr    CGTAACATCGTAAGTGTACCGGAAGGTGTACTTGGTAAAG-----
Crcr    CGTAACATCGTAAGTGTACCGGAAGGTGCACTTGGAAAAAC----
Muce    CGTAACATCGTAAGTGTACCGGAAGGTGCACTTGGAAAAAC----
Bege    CGTAACATCGTAAGTGTACCGGAAGGTGCACTTGGATAAAC----
Mela    CGTAACATCGTAAGTGTACCGGAAGGTGCACTTGGAATATAT---
Hats    CGTAACATCGTAAGTGTACCGGAAGGTGCACTTGGACAAAT----
Orla    CGTAACATCGTAAGTGTACCGGAAGGTGCACTTGGTAAAAT----
```

Cosa	CGTAACATGGTAAGTGTACCGGAAGGTGCACTTGGTTAAC-----
Exsp	CGTAACATGGTAAGTGTACCGGAAGGTGCACTTGGTTAAC-----
Depa	CGTAACATGGTAAGTGTACCGGAAGGTGCACTTGGTTTAT-----
Rima	CGTAACATGGTAAGTGTACTGGAAAGTGCACTTGGATAAA-----
Fuol	CGTAACATGGTAAGTGTACCGGAAGGTGCACTTGGAAAAT----
Gmaf	CGTAACATGGTAAGTGTACCGGAAGGTGCGCTTGGAAAAC----
Xeei	CGTAACATGGTAAGTGTACCGGAAGGTGCACTTGGAAAAT----
Pros	CGTAACATGGTAAGCGTACCGGAAGGTGTGCTTGGATAAAC----
Scmi	CGTAACATGGTAAGTGTACCGGAAGGTGCACTTGGAAAAAC----
Rolo	CGTAACATGGTAAGTGTACCGGAAGGTGTACTTGGAAAAAT----
Cere	CGTAACATGGTAAGTGTACCGGAAGGTGTACTTGGAAAAAT----
Daga	CGTAACATGGTAAGTGTACCGGAAGGTGTACTTGGAAGAAT----
Anco	CGTAACATGGTAAGTGTACCGGAAGGTGCACTTGGAAAAAAT---
Dmve	CGTAACACAGTAAGTGTACCGGAAGGTGCACTTGGAATAAG----
Dmar	CGTAACACAGTAAGTGTACCGGAAGGTGCACTTGGAGCAAG----
Anka	CGTAACATGGTAAGTGTACCGGAAGGTGCACTTGGAAAAAT----
Moja	CGTAACATGGTAAGTGTACCGGAAGGTGCACTTGGAAAAAC----
Hoja	CGTAACATGGTAAGTGTACCGGAAGGTGCACTTGGAAAAAT----
Bede	CGTAACATGGTAAGTGTACCGGAAGGTGTACTTGGAAAAAC----
Besp	CGTAACATGGTAAGTGTACCGGAAGGTGTACTTGGAA-AAAT---
Mysp	CGTAACATGGTAAGTGTACCGGAAGGTGCACTTGGAACAAC----
Osja	CGTAACATGGTAAGTGTACCGGAAGGTGCACTTGGAACAAC----
Sgro	CGTAACATGGTAAGTGTACCGGAAGGTGCACTTGGAATTACAT--
Pzpa	CGTAACATGGTAAGCGTACCGGAAGGTGCGCTTGGATCAAC----
Zeja	CGTAACATGGTAAGCGTACCGGAAGGTGCGCTTGGAT-TAAT---
Znne	CGTAACATGGTAAGCGTACCGGAAGGTGCGCTTGGATAAAC----
Zefa	CGTAACATGGTAAGCGTACCGGAAGGTGCGCTTGGATAAAT----
Acni	CGTAACATGGTAAGCGTACCGGAAGGTGCGCTTGGACAAAC----
Ncrh	CGTAACATGGTAAGCGTACCGGAAGGTGCGCTTGGACAAAC----
Agca	CGTAACATGGTAAGTGTACCGGAAGGTGCACTTGGAAAAAT----
Hydy	CGTAACATAGTAAGTGTACCGGAAGGTGCACTTGGCAAAG-----
Gsac	CGTAACATAGTAAGTGTACCGGAAGGTGCACTTGGCAAAG-----
Pevo	CGTAACATGGTAAGTGTACCGGAAGGTGCACTTGGACAAAC----
Hiku	CGTAACATGGTAAGTGTACCGGAAGGTGTGCTTGGAATAC-----
Inpa	CGTAACATGGTAAGTGTACCGGAAGGTGCACTTGGAAAAT-----
Auch	CGAAACATGGTAAGTGTACCGGAAGGTGCACTTGGATAACC----
Fico	CGTAACATGGTAAGTGTACCGGAAGGTGTACTTGGAAAAAC----
Macs	CGTAACATGGTAAGTGTACCGGAAGGTGCACTTGGAAAAAT----
Moal	CGTAACATGGTAAGTGTACCGGAAGGTGCACTTGGATAAT-----
Syma	CGTAACATGGTAAGTGTACCGGAAGGTGCACTTGGGTCAT-----
Mafr	CGTAACATGGTAAGTGTACCGGAAGGTGTACTTGGAAAAAT----
Dcpe	CGTAACATGGTAAGTGTACCGGAAGGTGCACTTGGGCAAACACA-
Dcti	CGTAACATGGTAAGTGTACCGGAAGGTGCACTTGGGTAAACATA-
Hehi	CGTAACATGGTAAGGGTACCGGAAGGTGCACTTGGTAATAT----
Stam	CGTAACATGGTAAGTGTACCGGAAGGTGCACTTGGAAAAAT----
Hogi	CGTAACATGGTAAGTGTACTGGAAAGTGCACTTGGAAAAT-----
Erzo	CGTAACATGGTAAGTGTACCGGAAGGTGCGCTTGGAAAAAT----
Hxot	CGTAACATGGTAAGTGTACCGGAAGGTGCACTTGGAAAAAT----
Core	CGTAACATGGTAAGTGTACCGGAAGGTGCACTTGGTAAAAT----
Apve	CGTAACATGGTAAGTGTACCGGAAGGTGCACTTGGTACAAT----
Latj	CGTAACATGGTAAGCGTACCGGAAGGTGTGCTTGGTTAAAT----
Laja	CGTAACATGGTAAGTGTACCGGAAGGTGTACTTGGAGAAAT----

Syja	CGTAACATGGTAAGTGTACCGGAAGGTGTACTTGGAATAAA----
Epme	CGTAACATGGTAAGTGTACCGGAAGGTGCACTTGGCAAAAC----
Grse	CGTAACATGGTAAGTGTACCGGAAGGTGCACTTGGAAAAACT---
Clja	CGTAACATGGTAAGTGTACCGGAAGGTGCACTTGGCATAAAC---
Ogcy	CGTAACATGGTAAGTGTACCGGAAGGTGCACTTGGATAAAT----
Plna	CGTAACATGGTAAGTGTACCGGAAGGTGCACTTGGAA-AAAT---
Lema	CGTAACATGGTAAGTGTACCGGAAGGTGTACTTGGAAAAAT----
Etzo	CGTAACATGGTAAGTGTACCGGAAGGTGCACTTGGAAAAAT----
Apse	CGTAACATGGTAAGTGTACCGGAAGGTGCACTTGGAAGAAAC---
Epde	CGTAACATGGTAAGTGTACCGGAAGGTGCACTTGGAAAAAT----
Slja	CGTAACATGGTAAGTGTACCGGAAGGTGTACTTGGATGAAT----
Bsja	CGTAACATGGTAAGTGTACCGGAAGGTGTACTTGGACTAAC----
Ecna	CGTAACATGGTAAGCGTACCGGAAGGTGCGCTTGGAATAAC----
Cohi	CGTAACATGGTAAGCGTACCGGAAGGTGTGCTTGGATAATC----
Caar	CGTAACATGGTAAGCGTACCGGAAGGTGCGCTTGGTTAAAC----
Came	CGTAACATGGTAAGCGTACCGGAAGGTGCGCTTGG-TTAAAC---
Mema	CGTAACATGGTAAGTGTACCGGAAGGTGTACTTGGAAAAAC----
Lenu	CGTAACATGGTAAGCGTACCGGAAGGTGCGCTTGGAAAAAC----
Plma	CGTAACATGGTAAGTGTACCGGAAGGTGCACTTGGAAAAAT----
Emst	CGTAACATGGTAAGTGTACCGGAAGGTGCGCTTGGAA-AAAT---
Ptti	CGTAACATGGTAAGTGTACCGGAAGGTGCACTTGGAAAAAT----
Losu	CGTAACATGGTAAGCGTACCGGAAGGTGCGCTTGGAATAAT----
Geoy	CGTAACATGGTAAGTGCACCGGAAGGTGTACTTGGACTAAC----
Dipi	CGTAACATGGTAAGTGTACCGGAAGGTGTACTTGGAATATA----
Pama	CGTAACATGGTAAGTGTACCGGAAGGTGCACTTGGACAAC-----
Leob	CGTAACATGGTAAGTGTACCGGAAGGTGCACTTGGAAAAACT---
Neba	CGTAACATGGTAAGTGTACCAGAAGGTGTACTTGGAAAAT-----
Pdpl	CGTAACATGGTAAGCGTACCGGAAGGTGTGCTTGGAGTAACAAC-
Nimi	CGTAACATGGTAAGTGTACCGGAAGGTGCACTTGGAATAAACAAT
Uptr	CGTAACATAGTAAGTGTACCGGAAGGTGCACTTGGAACAT-----
Pesc	CGTAACATGGTAAGTGTACCGGAAGGTGCACTTGGACAAACAT--
Baar	CGTAACATGGTAAGTGTACCGGAAGGTGTACTTGGATCAAAT---
Moar	CGTAACATGGTAAGTGTACCGGAAGGTGCACTTGGATAAAT----
Toja	CGTAACATGGTAAGTGCACCGGAAGGTGTACTTGGTAGAAT----
Chau	CGTAACATGGTAAGCGTACCGGAAGGTGCGCTTGGAAAAACT---
Chse	CGTAACATGGTAAGCGTACCGGAAGGTGCTCTTGGAATAAACT--
Enar	CGTAACATGGTAAGTGTACCGGAAGGTGCACTTGGA-AAAAT---
Hpty	CGTAACATGGTAAGTGTACCGGAAGGTGCACTTGGAAAAAT----
Bsbs	CGTAACATGGTAAGTGTACCGGAAGGTGCACTTGGAAAAAA----
Mcst	CGTAACATGGTAAGTGTACCGGAAGGTGCACTTGGTAAAAC----
Rhox	CGTAACATGGTAAGTGTACCGGAAGGTGCACTTGGAACAAA----
Opfa	CGTAACATGGTAAGTGTACCGGAAGGTGCACTTGGAAAAAT----
Paar	CGTAACATGGTAAGTGTACCGGAAGGTGCACTTGGCTAATCTG--
Gozo	CGTAACATGGTAAGTGTACCGGAAGGTGCACTTGGATAAAT----
Ackr	CGTAACATGGTAAGTGTACCGGAAGGTGCACTTGGATCAAAT---
Elev	CGTAACATGGTAAGTGTACCGGAAGGTGCACTTGGAAAACAAT--
Trdu	CGTAACATGGTAAGTGTACCGGAAGGTGTACTTGGAAAAAC----
Amoc	CGTAACATGGTAAGCGTACCGGAAGGTGCGCTTGGAACAAT----
Hame	CGTAACATGGTAAGTGTACCGGAAGGTGCACTTGGGAGAACT---
Chso	CGTAACATGGTAAGTGTACCGGAAGGTGCACTTGGACAAAAATAT
Lyto	CGTAACATGGTAAGCGTACCGGAAGGTGCGCTTGGAAAAAT----
Encr	CGTAACATGGTAAGTGTACCGGAAGGTGCACTTGGAAAAAT----

```
Bvar      CGTAACATGGTAAGTGTACCGGAAGGTGCACTTGGAATAACTTAT
Noco      CGTAACATGGTAAGTGTACCGGAAGGTGCACTTGGA-TCAATAAT
Chsp      CGTAACATGGTAAGTGTACCAGAAGGTGCACTTGGTAAAA-----
Arja      CGTAACATGGTAAGTGTACCGGAAGGTGCGCTTGGAAAAAT----
Pase      CGTAACATGGTAAGTGTACCGGAAGGTGTACTTGGCGAAATATA-
Trel      CGTAACAAGGTAAGCGCACCGGAAGGTGCGCTTGGAAAAATATC-
Lifa      CGTAACAAGGTAAGTGTACCGGAAGGTGCGCTTTGCCTATC----
Acur      CGTAACATGGTAAGTGTACCGGAAGGTGCCCTTGGGTAAACAA--
Ampe      CGTAACATGGTAAGTGTACCGGAAGGTGTACTTGGAAAAAT----
Urja      CGTAACATGGTAAGTGTACTGGAAGGTGTACTTGGAATAAC----
Enet      CGTAACATGGTAAGTGTACCGGAAGGTGTACTTGGTAGAAAC---
Ptbr      CGTAACATGGTAAGTGTACCGGAAGGTGCACTTGGAAAAAAT---
Safa      CGTAACATGGTAAGTGTACCGGAAGGTGTACTTGGGTAAAT----
Icae      CGTAACATGGTAAGTGTACCGGAAGGTGCACTTGGAAAAAT----
Asmi      CGTAACATGGTAAGTGTACCGGAAGGTGTACTTGGAAAAAT----
Foal      CGTAACATGGTAAGCGTACCGGAAGGTGCTCTTGGAAAA-T----
Drze      CGTAACATGGTAAGCGTACTGGAAAGTGCGCTTGGTAAAAACA--
Rhas      CGTAACATGGTAAGTGTACCGGAAGGTGCACTTGGAAAAC-----
Elac      CGTAACATGGTAAGTGTACCGGAAGGTGCGCTTGGAAAAAC----
Kugu      CGTAACATGGTAAGTGTACCGGAAGGTGTACTTGGC---------
Plor      CGTAACATGGTAAGCGTACCGGAAGGTGCGCTTGGAAAAAT----
Sgun      CGTAACATGGTAAGTGTACCGGAAGGTGCACTTGGAAAAAT----
Zaco      CGTAACATGGTAAGTGTACCGGAAGGTGCACTTGGCAAAAC----
Zbfl      CGTAACATGGTAAGTGTACCGGAAGGTGCACTTGGAAAAAT----
Spba      CGTAACATGGTAAGCGTACCGGAAGGTGTGCTTGGCAAAAT----
Game      CGTAACATGGTAAGTGTACCGGAAGGTGCACTTGGTAAAAT----
Thth      CGTAACATGGTAAGTGTACCGGAAGGTGCACTTGGAA-AAAT---
Xigl      CGTAACATGGTAAGTGTACCGGAAGGTGCGCTTGGAAAAAT----
Hyja      CGTAACATGGTAAGTGTACCGGAAGGTGCACTTGGATAAATAT--
Psan      CGTAACATGGTAAGTGTACCGGAAGGTGTACTTGGAAAAACAT--
Cupa      CGTAACATGGTAAGTGTACCGGAAGGTGCACTTGGAAAAAT----
Mpch      CGTAACATGGTAAGTGTACCGGAAGGTGTACTTGGAAAAAT----
Char      CGTAACATGGTAAGTGTACCGGAAGGTGCACTTGGAAAAAT----
Pser      CGTAACATGGTAAGCGTACCGGAAGATGTGCTTGGAAAAAC----
Prol      CGTAACATGGTAAGTGTACCGGAAGGTGCACTTGGAAAAAT----
Plbi      CGTAACATGGTAAGTGTACCGGAAGGTGTACTTGGAAAAAT----
Calu      CGTAACATGGTAAGTGTACCGGAAGGTGCACTTGGTCAAGA----
Papa      CGTAACATGGTAAGTGTACCGGAAGGTGTACTTGGAATAC-----
Sufr      CGTAACATGGTAAGTGTACTGGAAAGTGCACTTGGAAAAACT---
Stci      CGTAACATGGTAAGTGTACTGGAAAGTGCACTTGGTTATAT----
Taru      CGTAACATGGTAAGTGTACCGGAAGGTGCACTTGGAAAAAC----
Rala      CGTAACATGGTAAGTGTACCGGAAGGTGCACTTGGAAAAAT----
           * ****  *****   **  ***  **  ***
```
