# Supplementary material for: Structure and variation of the mitochondrial genome of fishes
Source: BMC Genomics. 2016 Sep 7;17(1):719. doi: 10.1186/s12864-016-3054-y (PMC5015259; doi:10.1186/s12864-016-3054-y)
Supplement: Additional file 11: Figure S3. — .Aligned nucleotide sequences of the 16S rRNA gene in the mt genomes of 250 fishes. (PDF 893 kb) [file 12864_2016_3054_MOESM11_ESM.pdf]

Additional file 11: Figure S3. Aligned nucleotide sequences of the 16S rRNA gene in mt genomes of 250 fishes.

See the legend of Additional file 10 for details.

```
Scca          -------------ACCTTAA-AGCTAGCCTA------ACCCACCATT-------AAATAA
Muma          -------------ACCTTAA-AACTAGCCTATATACCAATCTAACCAG------ACCTTA
Erca          -------------GCATTAA-AGCTAGCCTAAC--TATGTAT--AACC--------GTAT
Pose          -------------GCATTAT-AGCTA-----GCCTGACCA--TGCATC---------TAA
Actr          -------------GCTAAAT-AGCTAGCCTCACC---ACACACATCAC------AAATGA
Scal          -------------GCTAAAT-AGCTAGCCTCA---CCACACACGTCAC------AAATGG
Posp          -------------GCTAAAT-AGCTAGCCTCACCACAAACACACAAAT---------A-A
Atsp          -------------GCTACAC-AGCTAGCCAA-ACCAAT--ACTTCAAC----------TT
Leoc          -------------GCCATAC-AGCTAGCCA--AACCAATATCTT-----------AACTT
Amca          ------------AGCTGAAT-ATCTAG-CTTAACCACAAAATACCCAA---------ATA
Osbi          -------------GCTGCCA-AGCTAGCCAA-TCCAACCCCTTTAAAC----------AA
Pabu          -------------GCTACAA-AGCTAGCCGAACTATTTAAACCCTGGA------CCAAAT
Hial          -------------GCTGTAA-AGCTAGCC----TAAAAATGATAACCA------AAGTGA
Elha          -------------GCTAAAT-AGCTAGCCTAACCACCAACACCAAACC-----------A
Mlcy          -------------GCTAAAC-AGCTAGCC-TAACCCCC---CAAACCC------AAACAT
Algl          -------------GCTGAAT-AGCTAGCCGAACA---AATTAAGT--T-------AAGTG
Ptgi          -------------GCTAGAT-AGCTAGCTTA----AACAAA---AACA--------CCAA
Alaf          -------------GCCAAAC-AGCTAGCTGAAAAACTAAAAATAACTG-----------A
Nock          -------------GCTGAAT-AGCTAGCT---TA----AAC--ACTAA------AATAAA
Anja          -------------GCAAAAC-AGCTAGCC---TAA--CCACAATAAAA---------CAA
Gyki          -----------AAGCCAAAT-AGCTAGCC----------TG--AACAC--------AACC
Syka          -------------GCAAAAA-AGCTAGCCTAAACAT----AACAAACA-------AATGA
Opma          -------------GCAAAAA-AGCTAGCTCAA-----ATATACCAAAT------AAACGA
Comy          -------------GCAAAAC-AGCTAGC-CTAAACATTTAA-CAAAA-----------T-
Sasp          -------------GCAGAAC-AACTAGCTCGA----CAAAT---TAAA--------ACAA
Eupe          -------------GCGAAAC-GGCTAGCCCAACTA-----ACATAAAC-----------A
Enja          -------------GCCACAA-AGCTAGCTCTA---ACCATATAGC--A--------A-AA
Same          -------------ACCAAAC-AGCTAGCTCAGACCATAGAACCC--AA------GTTGAT
Chch          -------------GCTATAC-AGCTAGCTTACCCAA-----TGGGTAA------AACTCA
Grgr          -------------GCCGAAT-AGCTAGCTTAAAC-----TAACGGGAC------TAACTT
Caau          -------------GCCAAAC-AGCTAGCTTAACTAC-CTAATAACTA-------------
Cyca          -------------GCCAAAC-AGCTAGCTTAA---CTACTTA---ATA--------ACTA
Dare          -------------GCTAAAT-AGCTAGCT----TAACCATCAAGAT--------CAACTT
Cost          -------------GCTAAAT-AGCTAGCTT-A---ATTACTATACTAA--------ATTA
Leec          -------------GCCAAAC-AGCTAGCTTAAAC----ATT---AAAT------TAATAC
Crla          -------------GCCAAAC-AGCTAGCTTAACAA-----CCTAGAC--------AACTA
Clmc          -------------GCTATAA-AGCTAGCTTAAC-----CACCAAAACC------AACCC-
Phin          -------------GCCAAAA-AGCTAGCTTTCTAACCAAAAATCATAA---------CAC
Icpu          -------------GCTAAAC-AGCTAGCTTAATTA----CCTAAA-TA------CCTAAA
Psto          -------------GCTAAAC-AGCTAGCTTAA------ACACCAAAAA------TTACCC
Cora          -------------GCCATAA-AGCTAGCTTAAACACATCAAC-CAACT-----------A
Eisp          -------------GCTAAAC-AGCTAGCAAAC---AC---CAAGATCA--------TCAA
Apal          -------------GCCAAAA-AGCTAGCTT-----AATTATTAAGACA---------ACC
Eslu          -------------GCTCGCC-GACACACCCGGAT--------------------TAAAAC
Dape          -------------GCTGACT-AGCTCGCCGACACCCC--CGGTT-----------AAATA
Glse          -------------GCTAAAC-AGCTAGCCCCCACA---ATTGGTTAAC----------AC
Naar          -------------GCTGAAA-AGCTAGCC----TAGACAT-TTATT--------TAACAC
```

```
Baoc    -------------GCTGAAA-AACTAGCCTAGATATT--TTTTTAACA-----------T
Opso    -------------GCTGAAC-AGCTAGCCTAA---GCATTTGAATA-A----------CACA
Alte    -------------GCTCAAC-AGCTAGCTTAACCGCCTTAGAATAAAC-----------T
Plap    -------------GCTCAAC-AGCTAGCTTAA---CCGCCTAGA-ATA---------AACC
Plal    -------------GCTAACT-AGCTAGCCAAACAC-CTGG--ACTA
Sami    -------------GCCAAAC-AGCTAGCCGAACACTTGGAT-TAATT-----------T-
Rere    -------------GCCAGTT-AGCTAGCC-AACC-ACT----TGGATT------AA-TAC
Gama    -------------GCTGATC-AGCTAGCTCGCA--CATCTGGTTAATT---------ACCA
Onmy    -------------GCTGACT-AGCTAGCCAACATATTTGGT-CC-----------AACAC
Sasa    -------------GCTGACT-AGCTAGCCAA-CACACTTGGTCTAAC-------------
Cola    -------------GCTGACT-AGCTAGCCCACACATTTGGTCT-AACA-----------C
Dita    -------------GCTGAAT-AGCTAG-CCTTATACACCACGGCCAAC---------ACA
Gogr    -------------GCTAACA-ACCTAG-CACATG------------CC------CCGACA
Chsl    -------------GCTGAAT-AGCTAGCCAACT--CCCAAAGG-CCC-----------CC
Atja    ---------GCTTAAAAGCT-AGCCTACTTAATAGTTCAAAAACCTT-------------
Iido    -------------GCTTAAA-AGCTAGCCTACCT---AAAGTTAA-AA------AAACTT
Auja    -------------GCCCAAC-ACCTAGCCC-CACAGACAAATAAACTA-----------A
Chag    -------------GCTCAAC-AGCTAGCCCAAACAACTAAGC-T--AA------ACCCCC
Hami    -------------GCCCACC-AGCTAGCCCCCACCCCTAATATA----------AAA---
Saun    -------------GCCCACC-AGCTAGCCCCCACC--C-CACAACTAA---------ATA
Nema    ------------TCCTTAAC-AGCTAGCCCCC----CCCCCCG-CTAA----------AA
Disp    ------------TCCTAAAA-AGCTAGCCCAA-----CCCCTGAAAAA------CGACCA
Myaf    ------------ACCTAAAA-AGCTAGCCTAACT-------CAAAAAA-----------A
Lagu    -------------GCTCAAC-AACTAGCTCGCCCC-----C-------------CCCCAA
Trtr    ------------CGCTAAAT-AGCTAGCCC--AAACAATAACT------------AAACT
Zucr    ------------TGCTAAAT-AGCTAGCC-------CAAAT--AACAT------TTAAGT
Pxja    ------------TGCCTAAT-AGCTAGCCCAAATTATCTCA-CC-----------AAAAA
Pxlo    ------------TGCCTAAT-AGCTAGCCCAA---ACTACCCACC-A---------AAAA
Pctr    -------------GCCTAAA-AACTAGCCC-ACCCACCACAA-CAATA---------AGA
Apsa    ------------AACCTAAA-AGCTAGCCCGCC--CATAACATAATAA------------
Cabe    ------------CGCCCAGC-AGCTAGCC--TTAACCCACGCCT-----------AAAAG
Bzze    ------------CGCCTTAA-AGCTAGCAC-GAACCCCAAAAAT-----------AACAA
Siim    ------------AGCCTAAA-AACTAGCTCAACCAT-TAAAAAC-----------AACAA
Ctru    ------------CGCCTAAC-AGCTAGCCTCCCCCACAAAAACAAACC-----------C
Dpbr    ------------CGCCTAAC-AGCTAGCCTCACCCG-----TAAAAAC-------AACCA
Caki    ------------AACCTAAG-AGCTAGCCTAC-----TCAATCTTAAA------TTTATA
Phja    ----------------TAAC-GCCTAA--CAGCTA----GCCTAA-AC------CAA--A
Brsp    ----------TTCACCTAAC-AGCTAGCC---TCA--CCTCTTAAAAA---------TCT
Gamo    -------------GCCTAAC-AGCTAGCCT-------CAAAAATAA-A-----------A
Lolo    ------------TGCCTAAC-AGCTAGCCTCA---ACAATCAAATTTT--------ATTA
Batr    ----------AACACTGAGCTGTCTTACGCCTACA--GCTAGTCATAT---------TTC
Prmy    -----------------------ACTGCCA-------ATTTGCTA-GT------CAAATA
Loli    ------------CACTCAAC-AGCTAGCCAC-CCA--TCTAATCACAA---------CAG
Loam    ------------TACCTAAT-AGCTAGCCACA---CCAACTAAACACA--------ACAA
Chab    ------------CACCACAC-AGCTAGCC-TTACAACCT---AAAGAC------AA-CAA
Chto    ------------CACCACAC-AGCTAGCCTTACAA----CCTAAA-GA------CAACAA
Majo    ------------TGCCTTAC-AGCTAGCCCGT------CCAAATAAAA------GAAACA
Hlst    -----------CAGCCCTGA-AACTAGCCAAAAC---CTTCAATC-A-------CAACAA
Clpe    ------------CACCCCCC-AGCTAGCCCGACAGTTTA-AAACCAAC---------C-A
Mlmr    ------------CACCCTAA-AGCTAGCC----TTTTAATTAAAGA--------CACCAC
Crcr    ------------TCCTAAAC-AGCTAGCCCA-CCAACGCAACTAATAC----------TT
Muce    ------------TCCTAAAC-AGCTAGCCTACC--AACGCA---ACTA--------ATAC
```

```
Bege    ------------CACTCAAC-AGCTAGCCCTACC---ATATA-CT--T------CAACAC
Mela    ------------CGCCCACT-AGCTAGCTCG-CACAAC--ATCATAAC----------AT
Hats    ----------AACGCCTAGC-AGCTAGCCCGCTCAAACAAAAAC-----------AACAC
Orla    ------------ATCTAAAA-AGCTAGCC----CAAAGCC-TTAAT--------TAAAGA
Cosa    --------------CCCACA-ACCCAACACCAATTACCCAT-TAT-------------T-
Exsp    ------------AGCCAAAC-AGCTAG-CCCATCCCATTAAAACCATC---------ATA
Depa    ------------CGCCCAAA-AGCTAGCCTAC----AACCCAAAACCA--------ACAC
Rima    --ATTATTCTGACACCTAAC-AGCTAGCTTACCTA------TTAAACT------ACACCA
Fuol    ------------AGCCCAAC-AGCTCGCCTCC------AACCCTAAAT------TCAACA
Gmaf    ------------AACCCTAT-AGCTAGCCC--CCA-----ACCTAAAA------AAATAT
Xeei    ------------AGCCCAAC-AGCTAGCCTACTTACCCTAAAACCAAC---------AAT
Pros    ------------CGCCCCAC-AGCTAGCCC-ACAA----CATTTA-AT------CAACAA
Scmi    ------------CGCCTCAC-AACTAGCCCAAAGA--TATTTTAAATC------AACAA-
Rolo    ------------CGCCCAAC-AGCTAGCCCGAAACCCCAACCCAACAT------CCCAAC
Cere    ------------CACCAAAC-AGCTAGCCA-CCAAACCATGAGT-----------AATCC
Daga    ------------CGCTAAAA-AGCTAGCC----CAAGATCAGGGGA--------TAATAC
Anco    ------------CGCCCAAC-AGCTAGCCCACACCCACC--CCCCAAC----------AA
Dmve    ------------CGCCCAAC-AGCTAGCCCA-CAA--CCAACCCACAA---------CAA
Dmar    -----------ACGCCCAAC-AGCTAGCCCAAA----AAC-AACC-CA------CAACAA
Anka    ------------CGCCCAAT-AGCTAGCCCACA-----ACCCCACA-C------AACAA-
Moja    ------------TGCCCCAC-AGCTAGCCCACACCCCCCCCCCCCCAACAAACAACCATT
Hoja    ------------AGCCCAGC-AGCTAGCCCACAATA----CCACCCCC-------AACAA
Bede    ------------CGCCCCAC-AGCTAGCCC-A---CAAAATCCAACCA--------ACAA
Besp    ------------CGCCCCAC-AGCTAGCCCACAAAATCCAACCAACAA------------
Mysp    ------------TGCCAAAA-AGCTAG-CCCGCCTACC--AAACTAAC---------AAC
Osja    ------------CGCCGAAC-AGCTAGCCC-----GCCCTCCCCCCTA--------ACAA
Sgro    ------------AACCCAAC-AGCTAGCC-CACCTACC-----AACCT------AA-CAA
Pzpa    -----------ACGCCCAAC--CGTAGCCCAAAA---AACATTTATAA---------ATT
Zeja    ------------CTCCCAGT-TGCTAGCCCAACAAACGTTCCTAAAAA-----------T
Znne    ------------CACCTAAC-TGCTAGCCCCCCA---CC-CATTT--T------TAAACC
Zefa    ------------CGCCCAAC-TGCTAGCTCA-ATAGTATCATTAAATT------------
Acni    ------------CGCCCAAT-TGCTAGCTCAAAA----GAC--ATTTT------TAAAAG
Ncrh    ------------CGCCCAAT-TGCTAGCTCAA------AAGACATTTT------TAAAAG
Agca    ------------CGCCCAAC-AGCTAGCCCTCC-CCCAAAACCTAACA-----------A
Hydy    ------------TGCCCAAA-AGCTAGCCCACCT----AACCAACCTC------AAATAA
Gsac    ------------TGCCTAAC-AGCTAGCCCGCCCAAACAATTTCAACG-----------A
Pevo    ------------GGCCTCAC-AGCTAGCCCCACTA-----A--AAATA------CAACAA
Hiku    ------------TACCCATA-TGCTAGCTCAAACAC-----TAAATTC------AAACAA
Inpa    ------------CACCTAAC-AGCTAGCCCACCCATCC---CCC----------CCACAC
Auch    ------------CGCCGAAA-AGCTAGCCT-A---CTACGCAAACACA--------ACAA
Fico    ------------TGCCCAGC-AGCTAGCCCCAC-CCCAAGAGCAACAA------------
Macs    ------------AGCCTTAC-AGCTAGCCTACAATA--AAAAGC-----------AACAA
Moal    ------------TGCCAAAC-AGCTAGCTTCTA-ACCAAAAC-AACA-----------A
Syma    ------------TACCAAAC-AGCTAGCAGC-CCTAA--AAACACAAC----------AA
Mafr    ------------CACTAAGC-AACTAGCCCCACCCACCAAAAACAATA---------AAC
Dcpe    ------------CGCCCCCC-AGCTAGC-CCACAT-----C-TAAAAG------CAACAA
Dcti    ------------CGCCCCCC-AGCTAGCCCAC-----ACCTAAAAAA-------CAACAA
Hehi    ------------CGCCCAAC-AGCTAGCCCACA--AACACAAC-AACA--------ACCA
Stam    ------------TGCTTAAC-AGCTAGCCCCCTCT--ACCAAGAACAA---------CAA
Hogi    ------------CGTCCAAC-AGCTAGCGCATTA----AACCAATCAC------AATATA
Erzo    ------------CGCCTAAC-AGCTAGCCCACCCACACAACCCCAACA-----------A
Hxot    ------------TGCCTAAC-AGCTAGCCCACCCAAACAATTTCAACA-----------A
```

```
Core    ------------CGCCCAAC-AGCTAGCCCA--AC----TAAGAAATT------TCAACA
Apve    ------------CGCCCAAC-AGCTAGCCCATTAA--AACAATCACAA---------CAA
Latj    ------------CGCCTATT-AGCTAGCCC--CCACTTAAAC-A--AA------CAACAA
Laja    ------------CGCCCAAC-AGCTAGCCCCA-----CCAATCAAGAA------CAACAA
Syja    ----------GACGCCCAAC-AGCTAGCCTCTCC----ATCAAAAAAC------AAC-AA
Epme    ------------CACTGACC-CGCTAGCCCAACCAAACCAA--A--AA------CAACAA
Grse    ------------CGCCGACT-AGCTAGCCCG-CTTATCCAAAAACAAC----------AA
Clja    -----------ACACTAACA-AAATAGCCTGACC-----ACCTAA-AA------CAACAC
Ogcy    -------------GCCTAAC-AGCTAGCTCACCCAA---CATAAACCA------AAAGTT
Plna    ------------CACCTATC-AGCTAGCC--CCTCCAACAACACCAA----------AAT
Lema    ------------AGCCCAAC-AGCTAGCC-CCACACCC----AAAAAC------AA-CAA
Etzo    ------------CGCCCAAC-AGCTAGCCCCCAAA----GCCAGA-AA------CAACAA
Apse    ------------AGCCCATT-AGCTAGCTCGCACC-----CCAAAATG------CAACAA
Epde    ------------AGCCAAAC-AGCTAGCCCCCTCA--ACCAAAAATAA---------CAA
Slja    ------------GGCTCATC-AGCTAGCCCCACCATTAGTGCAAGTACTAA-TAATTATT
Bsja    ------------TGCTAAAA-AGCTAGCCCCTCCCCCAAAAACAACAA------------
Ecna    ------------AGCCAACC-AGCTAGCCCTAAT-ACTAAATTC--AA------CAACCA
Cohi    ----------CAGCTTAATT-AGCTAGCCTA-CCTAAC--AACCCAAT----------CA
Caar    ------------TGCCTATT-AGCTAGCCCCCTCAA----CTAAACTT-------AACAA
Came    ------------TGCCTATT-AGCTAGCCCCACCA--ATCAAACCCAA---------CAA
Mema    ------------CACCCACA-AGCTAGCCC-CACCCACAACAAC-----------AACAA
Lenu    ------------AGCCCAAC-AACTAGCCCCACCCCAC--CCAAACAC-------AACAC
Brja    ------------CGCCAACC-AGCTAGCCCGCCCCAACAAAACTAACA---------CCC
Plma    ------------AGCCAACC-AGCTAGCCCACATC--AGTAAAAACAA---------CAA
Emst    ------------CGCCCAAC-AGCTAGCCCACCC---CAACAAAA-AC------AACAAA
Ptti    ------------CGCCCAAC-AGCTAGCCCCTCTTAACCAAAAGCAAC---------AAA
Losu    ------------AGCCCATT-AGCTAGCTCACCCCCCAAAAACTTAAC---------CTG
Geoy    ------------TGCTGACG-AGCTAGCTTTA---ACCCTCGAACCTA--------ACGT
Dipi    ----------CGTGCCCAAC-AGCTAGCCCAA----TAATTAAAATCA--------ACAA
Pama    ------------CGCCGATC-AGCTAGCCCACCCAACAAAAATC-----------AACAC
Leob    ------------AGCCCAAC-AGCTAGCCCAACTCCCCAAAACAACCA------------
Neba    ------------CGCCCATC-AGCTAGCCTATCAACTTAAAAACAACA-----------A
Pdpl    ------------CGCCAATC-AGCTAGCTCCGCCC-----A-TAACTT------CAACAA
Nimi    ------------CGCCCATT-AGCTAGCCGC-CCCAGCCAAAAACAAC----------AA
Uptr    ------------TGCCTTAC-AGCTAGCCCACCAA-----ATTAAAAG------AAACAT
Pesc    ----------TTAACCAAGC-AGCTAGCCCCCCCCCCCCCCCCCATTT------CAAGTA
Baar    ------------CGCCCACC-AGCTAGCCCA-TCCAACCAAAAACAAC----------AC
Moar    ------------CGCCCAATAGCTAGCCCCCTAAACCA--A----AAACAA----CACA-
Toja    ------------AGCCCATT-AGCTAGCC-CCATCACTTTACAAAAAC------AA-CAA
Chau    ------------AGCTCAAC-AGCTAGTCCACCCAACCAAAAAC-AAA------CTCCCC
Chse    ------------TACCAAAA-AACTAGCCCCCCT----CCCCAACAGC------AACAA-
Enar    ------------AGCCCAAC-AGCTAGCCCCCCT----TTACCAAAAA------CAACAT
Hpty    ------------GGCCCAAC-AGCTAGCCCCTCCAACCAAA--GATAA------CAAATC
Nana    ------------CACTCAAC-AGCTAGCCCCCCCCCCCCAAA-AAGCA------CAACAT
Mcst    ------------AGCCAACT-AGCTAGCCCCATCATTACCGAGACCGAAAA---CAACCA
Rhox    ------------CGCCCATT-AGCTAGCCCCAAACTCAAAACCAAAAAC------AACCA
Opfa    ------------TGCCCACC-AGCTAGCCCCCAGACCAAAAAC------------AACCC
Paar    -----------CTGCCAAAT-AGCTAGCCTAA---ATGAACTTAAC-A--------AAAA
Gozo    ------------TGCCCAAC-AGCTAGCCCC-ACCCCTAAAAAA----------CAACAG
Ackr    ------------CACCCACC-AGCTAGCCT-ACAA----TTAAAA-CA------CAATTA
Elev    -------------GCCCATA-AACTAGCCCCC----CGCCTGA-ATAA--------GTAA
Trdu    ------------TGCCCAAC-AGCTAGCCCATTCA-----TCAACCC-------CAAATC
```

```
Amoc    ------------TGCCTAAC-AGCTAGCCCGCCCTTATAAAATC-----------ATCAA
Hame    ------------AGCCTAAC-AGCTAGCCCA-AACCCCTAAATACAAT------------
Chso    ------------CGCCCAAT-AGCTAGCCCACTCAC-TAACAAACAACA------CACC-
Lyto    ------------AGCTTTAT-AGCTAGCCCACT--CAAACAAC-CTCA--------ACCA
Encr    ------------AGCTTTAT-AGCTAG-CCCGCTCAAACAACTTCAAC---------ACC
Bvar    ------------AGCCAACT-AGCTAGCCCATAGAAACAAATTCAACA-----------C
Noco    ------------TACCGAAC-AGCTAGCCCCCCA---AGACAACT--T------CAACAT
Chsp    ------------AGCCTTAC-AGCTAGCCCACCAC----TAAAAA----------CAACA
Arja    ------------CGCCCAAC-AGCTAGCCCACCCAAGCAAGTTCAAC----------AAA
Pase    ------------CACCCAAC-AGCTAGCCCCCCACCTAAAATC------------AACTA
Trel    ------------AGCCCTAC-AGCTAGCCTGATCACAA---CAAATGC------AAACTG
Lifa    ------------CACCTACC-AGCTAGCCGTAACATTAAAAGCAAAA-----------TA
Acur    ------------TACCCATC-AGCTAGCTCAAAGTC-TAATATC-----------AACAA
Ampe    ------------TGCCCAAC-AGCTAGCCACCACCCCCAAA--AACAA------CAAACC
Urja    -----------TCGCCAAAA-AGCTAGCCCCACC----CCCAAAAATC------AAAACC
Enet    ------------TGCTTAAA-CGCTAGCTTAATTCCACCCC--TAACC-----------T
Ptbr    -------------GCCCACT-AGCTAGCTCTC---ACATTAAAGT--A--------ACAA
Safa    ------------AGCCCAGC-AGCTAGCCTAACCAA-----CAAACCT-------AAAGC
Icae    -----------ACGCCAACC-AGCTAGCC----CAACT-CAACAAAAC------CAACAA
Asmi    ------------CACCAAAT-AGCTAGCC-CACCCAAT----ATAACT------AA-TTA
Foal    ------------GGTTTAAC-AACTAGCACAC--TGACATATTT-TTA------AAATTA
Drze    ------------ATCAAAAC-AACTAGCC----CATAAA--AAAAT--------CAATCA
Rhas    -----------CACCCCAAC-AGCTAGCCCCCCCC----CCTTAA-AA------CAACAA
Elac    -----------ACACCCAAC-AGCTAGCCCCTCC---CTCCAACT--A------CAACAA
Kugu    ------------AGCTCAAC-AGCTAGCCCGACC----ATACAAAAAC------AACCAA
Plor    ------------CGCCCATC-AGCTAGCC-CCTCCCCT----AAAAAC------AA-CAA
Sgun    ------------CGCCCATT-AGCTAGCCCAAC--ACCCAAA--ACCA--------ACAC
Zaco    ------------CGTCCAAC-AGCTAGCCCCCCT--CCCAAA--AAG-------CAACAA
Zbfl    ------------CGCCCAAC-AGCTAGCCCCC-----TATATCAAATA------CAACAA
Spba    ------------CGCCCATT-AGCTAGCCCCACCTATAAACC-ACC-A--------ACAA
Game    ------------AGCCGACC-AGCTAGCCCCCTCCACCAAAAAC-----------AACAA
Thth    ------------TGCTGACC-AGCTAGCCCAC---CCTAACAAAAACA--------ACAA
Xigl    ------------CGCCCA-TCAGCTAGCCCCACCC--ACCAATAACAA---------CAA
Hyja    ------------TGCCGACC-AGCTAGCCCG-CCCAACAAAAAACAAC----------AC
Psan    ------------TGCCAACC-AGCTAGCCCGCCCCCCCCAACAATAAA------CAACAA
Cupa    ------------CGCCAAAC-AGCTAGCCCACCCC-----AATAAAAA------CAACAA
Mpch    ------------TACCCAAC-AACTAGCCT--CA----AACCAAACCC------AA-CAA
Char    ------------AACCAAAC-AGCTAGCCCC-ACCCCATAAAACCAAA----------AA
Pser    ------------CACCCATT-AGCTAG-CCCACCCAACCAAAAACAAC---------AAA
Prol    ------------CGCCCATT-AGCTAGCC----CAACCCCTTCACC--------TAACAA
Plbi    ------------CGCCTATT-AGCTAGCCCCACCCC--TTAATACAAC------AAACCC
Calu    ------------CACCCACA-GGCTAGCCCAACCAACCA--AATCCAC---------CAA
Papa    ------------CACTAGCA-AACTAGCCCAAAC---ACCCCAAA-CA------CAACGA
Sufr    ------------AGCCCAAT-AGCTAGCCTACTT---ACATAAA--AT------CAACAA
Stci    ------------AGCCCACT-AGCTAGCCCAAAC---AAT-TAAA-AC------CAACAA
Taru    ------------CACCTAAC-AGCTAGCCC--CCACCCCACCCACAAC----------AA
Rala    ------------CGCTTAAA-AGCTAGCCCCCCCCC----CCAAAAGC-------AACCC
```

```
                                                                              1      1'
Scca    T--AACACTATTAATTCTA--CTTACATTACA--ACCTT-A--AACTAAAAC-ATT---
Muma    TAGATCTAATTTATACC-----AC------AA--AC---TTTTAACTAAAAC-ATT---
Erca    AAAAATATTAAAAT------A--AATACTCAT---AAAACT-AAAACAAAAC-ATT---
Pose    CCA-A-ATAAATATT---TAAATAAACACACA--AAAA-ATAAAAACAAAAC-ATT---
Actr    ATATTTATATATA-ACCCC-CCATAAGATCAA--AAAAA--ATAAACAAACC-ATT---
Scal    ATACTTATACATAAACCCC--CATAAGATCAA--AAAAA-AT-AAACAAACC-ATT---
Posp    ATATTTATATATAACCCCCCATAAAAACAAAA------AAAATAAACAAACC-ATT---
Atsp    ACAAATATACATAACCCCACATATT--CTTAA---AA-ACATAAACCAAATC-ATT---
Leoc    ACAAATATACATAACCCCA--TAAATTTTTAA--AGACCTA--AACCAAATC-ATT---
Amca    CAC-AAATATAAATACCCTCACATACCATTAA--ACTAAACCAA-CCAAACC-ATT---
Osbi    ACAACAATATATACAGGCCAATAACCCTAAAC---CTTCAAAC---TAAACC-ATT---
Pabu    ACTCAACTACATATAGCCAGACAACATATTAA--AAATACT--AACTAAACC-ATT---
Hial    ACAAACATATATAACCCCA-CATGT---TTTG--AAA-GTACTAAACAAACC-ATT---
Elha    CACAACCATAAATACCCAACCACACACTAACA--GAAACC----ACTAAACC-ATT---
Mlcy    ACAACCATAAATACCACCA-CACAACAAGA-----CTCACC--AACTAAACC-ATT---
Algl    ATCA--ATTATAG-ATAGC-GAGTTGTTTAGT--GGCGT--ATGGCTAAACC-ATT---
Ptgi    ATGAACAACCATAGATATAAA--AGTAGAAAC---AAAACC-GCAACAAACC-ATT---
Alaf    AAACCCATAAAT-ACCCACAA----CCAAAAA--CCCAA----AACCAAAAC-ATT---
Nock    CCATAAACCATACATACTC-ATTCCCCCAACA--ATTG-----AAACAAAAC-ATT---
Anja    ATGACCAAGCATATATAACAA-AATAAACCCA---AATATA--AAATAAAAC-ATT---
Gyki    GTAACCCCAAAATATATAT-ATAAAGAACCTA--TAATTTCAC-ACTAAAAC-ATT---
Syka    ACAACCATATATACCACACCCAAAG----AAA--C----ATTAAACTAAAAC-ATT---
Opma    CCAACCATAAATAAT---------AGACAAAA--AC--A-AT-AACTAAAGC-ATT---
Comy    AATCAACCATAAATTACA----AAACAAA-AA----ATTTAAAA-ATAAAAC-ATT---
Sasp    A---CAACCAAACAAATATATAACAAAACTAAATAATTTAATTAATTAAAAC-ATT---
Eupe    AGTAACCAC-TATAAATCAAAAAA---TAAAA--CTAA------ACTAAAAC-ATT---
Enja    TTAATAATCTAATTACCTACTTAACAATAA----AAAAATAGAAACCAAATC-ATT---
Same    AATAT---AACTAATTCTTCAAG-AAGTTGA---CAACGCA--AACTAAACC-ATT---
Chch    CAACATTTCTCTA-CCCAAAACAAAACCCCAA--AC---ATTAAAACAAACC-ATT---
Grgr    TAGAATATAGATATCCCC---CCACAACTTAA--AATGAAACAAAACAAATC-ATT---
Caau    AACAATATAAATAAAATAAGATAGACCTAACA--CTAAAAAT----TAAATC-ATT---
Cyca    AACAATATAAATAAAACAAAAT-AAGCCTAAC--ACCAA---AAACTAAACC-ATT---
Dare    AAAAACATCAATAACCTAT-AATCAACCTAAA--ACC-ACA--AACTAAACC-ATT---
Cost    ATAA-CATAAATAAAAGTACAA--AACCTAAA-----ATTATTAATCAAATC-ATT---
Leec    AACAACATAAATAAAATAA-AATGAACCTAAA--T---CTACAAACTAAACC-ATT---
Crla    AACAACATAAATAAAACAA-CA-AA-ACCTTA--AAAAATAAAC--TAAACC-ATT---
Clmc    AACAACATTCAACAATTTT-ACTAAACCCAAA--CATCACA--AACTAAAAC-ATT---
Phin    CACCATTCCCACCCACCTTACACA-ACACACA---AATAACTTAATTAAAAC-ATT---
Icpu    ACAAC--ATTACAACATT--TAAAAAACCTAC--AATACTCCAAACTAAAAC-ATT---
Psto    ATCAACATCAAAAATCTTT--AAAAGACCCCA--ACAAC-TCAAATTAAAAC-ATT---
Cora    ACAAC-ATTAATAAGTT-TAGAAAAACTAAAA--TTAAAT---AACCAAAAC-ATT---
Eisp    A-CAACATTACCACATCTTCT--TACACCACC--AATAAACTAAACCAAACC-ATT---
Apal    TAACAACATTATCTTGTCTTACACAACCCCAC--C---CAACTAATCAAACC-ATT---
Eslu    CCCAACATATATA--CCCC-AACAAACCTTTA--AAAAAATCAA--CAAACC-ATT---
Dape    ACCCCTATAGATACCCGAACAAAC-CTTAAAC--TTAAAC----AACAAACC-ATT---
Glse    CACAATATAAATACCCCTACACAAC--CTTAC---CA--AATTAAACAAACC-ATT---
Naar    CACAATATATATTCCTTTG-CATATCCTGCAC--T---AGATTAAACAAACC-ATT---
Baoc    CACAAT-ATAAATTCCTTTGCATA-ACTTGCA--CTTACCT--AAACAAACC-ATT---
Opso    ACAATATACTAT--ACCCCCACATAACCTA----CATTAAATTAAACAAACC-ATT---
Alte    AACAACATAAATAACCCAACAAAA--ACCAAA--AATA-AT--AAACAAATC-ATT---
Plap    GACAACATAAATAACCCAACA--AAACCCAAA--AGTAA---TAAACAAACC-ATT---
```

```
Plal    ATTTTACATTATAAATATTCCCTCACAACCTGACAACTTG--TGAACAAATC-ATT---
Sami    ATTAGTATAAATAACCCCTCATAACCTAG-AA----GCTTGTGA-ACAAATC-ATT---
Rere    CACAACATAAATACCCCCT-CACTACTTAGAG--AGTAAG---AAACAAATC-ATT---
Gama    --CGACATACCTGAACCCGCA--TA-ACAAAC----TACACCCCATCAAACC-ATT---
Onmy    CACAACATACATACCCCAATAAA-ACTTAGAA--TTAAGT---CAACAAACC-ATT---
Sasa    ACCACAACATACATACCCCTATAAAACTTAAA---ACTAAGTCAA-CAAACC-ATT---
Cola    CACAACATATATACCCCCACAAAAC-TTAGAA--TTAAGTC--AA-CAAACC-ATT---
Dita    CTGATTAGCCACAACCCACCAAGACCTGAATA--C----AACGA-ACAAACC-ATT---
Gogr    GCCCCCAACAACAACCCTAATAAAACCCCCAG--CCCACA---AAC-AAACC-ATT---
Chsl    CTCCCAACAACCTGCCTTTAA---AAATTTAT---TTCCAACCCAACAAACC-ATT---
Atja    AAACATAGAAATAAAACTA-AGAAACCCAAAA--GAACAAGA----CAAACC-ATT---
Iido    ATACATAAACATAAAACT--AACAACCCAAAA--AAA----TGAAACAAACC-ATT---
Auja    CTCTCTATTAATT-TTATAAAAAAGATCGAAC--ACAAAAA--TAACAAAAC-ATT---
Chag    ACTAT--TTAATAATTTATAACA-CACTAAA----TAAACT--AAATAAACC-ATTTGT
Hami    ACCCCAAAATATATTCCTTTCTA---GAATAT--ACA---A--AAACAAACC-ATT---
Saun    TTCCATATATATACTTCC--------CCCACC---CCGCAATTAAACAAACC-ATT---
Nema    CAACCC-CCTATAAATATTTACTCATTA-----TTTCTTCCCTAA-CAAACC-ATT---
Disp    CCCTTTA--AAT---------CAAGTCCCTAA--AAAAC-ACTCAACAAACC-ATT---
Myaf    AACAACCCCATTTAAATTAAAACCCCTTAAAA--CAAGC-----AACAAAAC-ATT---
Lagu    C---ATAAATATA-ACCAC-AGCAAACCTCCC--CCCAGA-GTAAACAAACC-ATT---
Trtr    ACTTATACAACCTAACTTAATTATACATTTAA--A-ATGTA--ACTTAAACC-ATT---
Zucr    AGAATATATAACTAATCTA-GTAATACACTTA--AAATATAA--ATTAAATC-ATT---
Pxja    TTAATTATAAATACCCCTTAATTAACTTAAAA--A-ATTTA--AA-CAAACC-ATT---
Pxlo    TTAATTATAAAT--ACCCCTTAATTAACTT----AAAAAATCTAAACAAACC-ATT---
Pctr    TCTTACTAAACCCCCCCACAAGAACTAAA--AA--C---AAC--AAACAAATC-ATT---
Apsa    TCAAATATTACTAACCCAAAAGAAAACTA--A---ACCTATAAA-ATAAACC-ATT---
Cabe    AAACTACTAACCAGGCTAAAAGGACACCAACC--C------CACCCTAAAAC-ATT---
Bzze    CCACCCATCAATAACCTTATAAAACATACACA--TGTAC-----AACAAACC-ATT---
Siim    ACAGACACTAATAACCCATAAAA-ACCTAAAA--ACCAC----AATCAAATC-ATT---
Ctru    AA-ACCATTAATACCCCCGAATGC--CCGACG--CACTCAT--AATTAAACC-ATT---
Dpbr    CAAACCATAAATACCCCCACATACCCTACA-C--ACC--TACAA--TAAACC-ATT---
Caki    AT-AGAATTAAAAACTTTC--AATATACAAA-------------GTAAACC-ATT---
Phja    CTTAAGATTTACATAAAG----TTAAAGCTTA--CAATAACTAAAATAAATC-ATT---
Brsp    AT---------TATGAAATAA-GATCAAATTA---TACATTT-AAACAAAAC-ATT---
Gamo    ATTTT-ACTATTATGGACCTAAAAACTCATAA--TAAACTT--AAACAAATC-ATT---
Lolo    -TTG-TGAAACTAAAAACTCA-------TAAT-----ATAATTAAATAAACC-ATT---
Batr    CCCTTCCCCATCTTTATCATAAAAACAT-AAA---CTCA---CACTCAAGT-ATT---
Prmy    T-AACAACTATAACCCACA-AAATCCCCTTAA--ACCCC---TAAATAAAAT-ATT---
Loli    ATAAGTATAAATAACCCCTAA-GACACCCCCG---TTTTTAT-AAACAAACC-ATT---
Loam    AACTACATAAATACCCCCTAAC-CACCCCACCCCACCCACCTTAAACAAACC-ATT---
Chab    ACAAACATTAATTACCCCC-AACAACCCAAAT--GCAAGTCG-AAACAAAAC-ATT---
Chto    ACAAACATTAATTACCCC---CAACAACCCAA--TGCAAGTTGAAACAAAAC-ATT---
Majo    TATCACATTAATAACCCCT--AATACGCTTAA--CACCA-ACTAAACAAATC-ATT---
Hlst    CACCATATAAATAAACCCA-AATGCAATAAGA--ACTT---TACACCAAACC-ATT---
Clpe    GAGCACATCAATAACCCCAACTGCCCTAACAA------TTTCTTAACAAACC-ATT---
Mlmr    ACAAACATCAATAACCCCT-AAA--ACCCTAC--ATAAACCTTAAACAAACC-ATT---
Crcr    ATT--TATACACACCTCCCAATGTA-CTGAAA---TA--AACAAACCAAACC-ATT---
Muce    TTATTTATATACACCTCCCAA--TGTACTGAA---ATAAAC-AAACCAAACC-ATT---
Bege    -TCAAC-TATTTATACCCT-AAAAAACACTTA--ATACA--TCAACCAAACC-ATT---
Mela    TTAGATATAGATATACAC----ACA-CCAACA---AATAAGC-AAACAAATC-ATT---
Hats    ATAAATACTCATAACCCCTAATACACCCAACA--ACCA-----AACTAAATC-ATT---
Orla    CCAAATAT-TACTAGTAAT-TAAAAACCCAAG--G---ATATTAAATAAATC-ATT---
```

```
Cosa      AATAACCCCTAAAACACG----AAACAAA-AT----------AA-ACAAATC-ATT---
Exsp      AAC-ATATAATTAAACCCTAAGTTACTAACAA--A---AACTAA-CTAAACC-ATT---
Depa      CCACT-ATTAATAACCCCTAAAACACT-------TAAAAAATTAAACAAACC-ATT---
Rima      AACACTATTATTACCCCC---TAACATCTTAA--CAAACAATAAT-TAAAAC-ATT---
Fuol      ATCCTTATCAATAACCCCT--AATTCCTTTAT--TCAAT-T--AATTAAATC-ATT---
Gmaf      AAATATATTTCT---TTCC-AACCCCAC-----------CTCA-TCAAAAAC-ATT---
Xeei      AAAAATATTACTAAACCCAAATTCCCTTAACA--ACCTAATC----TAAACC-ATT---
Pros      CCAAACATAAATAACCCT---AAAAACCCCAA--ACAAAAC--AAACAAACC-ATT---
Scmi      CTAATTATTTATACCCCTT-GAATTCCCCAAA--GAGA--C--AAACAAACC-ATT---
Rolo      ATAAATACCCTTAAATACC--CCTAATTT------AAAT-AC-AAACAAACC-ATT---
Cere      CCCCCCATAATTAACCTAAAATAAACTAAACC------AAGAAAT--AAATC-ATT---
Daga      CTCAATATAATCCTCCCCA-AATAA---TTTC--AAG-CAAGTACGTAAATC-ATT---
Anco      ATAACTATTAATACCCCTTAATAAC--CTCAA---GAAAGACCAAACAAACC-ATT---
Dmve      ACAACCATAAATAACCTTTAATGACCTAAAAC---CACATA--GAACAAACC-ATT---
Dmar      CCAACCATTAATACCCCCC-AATAACCTAAAG--CCAT---CTAAACAAACC-ATT---
Anka      ACAATTATTAATACCCCCT-AATAACCCTAAA--TACAATT--AATCAAACC-ATT---
Moja      AATACCCACTAACAACCTTAAGAC------AA--GA---GTTAAACCAAACC-ATT---
Hoja      ACAACTATTAATACCCCTTAATGACCTTAAGA--TAA--GACAAACCAAACC-ATT---
Bede      TCAAACATAAATACCCCCAAA--TACCCCAAA-----CCAAACAAACAAACC-ATT---
Besp      TCAAACATAAATACCCCCAAACACCCCA---A--ACCTAACAAA-C-AAATC-ATT---
Mysp      CAA-ACATAAATAACCCCCAATACCCCAAACA--AA---ACAAA-ACAAACC-ATT---
Osja      CTAAACATAAAT--AACCCCTAATACCCTA----AACAACATAAAACAAACC-ATT---
Sgro      CTAACTATAAATAAACCCT-AATACCCCAAAC--CTAAAAT--AAACAAACC-ATT---
Pzpa      CTAAATATACTCAACAATTAATTAACCTAAAA---CCTC---TCAACAAACC-ATT---
Zeja      TA-AATATTTTTAACAACAAATAA--ACAAAA---ACCCCC--AAACAAACC-ATT---
Znne      ACCAATATTTTTA-ACAAT-AAATAACATAAA--AACCC--CCAAACAAACC-ATT---
Zefa      ACAAATATTTTTAACGATAAATAACCTAA-AA--ACTTTTTAA---CAAACC-ATT---
Acni      TTTAATACTACTAATAATA-GATAACCTAAAA--CCCCCAC--AA-CAAACC-ATT---
Ncrh      TTTAATACTACTAATAATA--GATAACCTAAA--ACCCC-AT--AACAAACC-ATT---
Agca      ACCCTTATTAATTAACCCCAAATACACCAACA--AACCCTG--CAACAAACC-ATT---
Hydy      ATCAATCTAAATAACCCCG-AAGACACCACTA--TT--TAAATTACCAAACC-ATT---
Gsac      TTCAATCTTGATAACCCCAAAGGACACTTAAC--TTAAATT--AAACAAACC-ATT---
Pevo      ATAATTATTAATA-ACCCC-AAATTTACTAAA--AACACA-C-AAACAAACC-ATT---
Hiku      -AAACTAAAAATAACCCCTAACACCATAAAAA--GT---AAT--ATTAAAAC-ATT---
Inpa      AACCA-CCATATTTAACCGAAAA-TAAACCAC--CTATATA-AAACCAAACC-ATT---
Auch      CCAA-TATTAATACCCCCTAACATACTACAAA-----TCACTAGACCAAATC-ATT---
Fico      TTCCATATAAATAACCCCTCATATCCTAA--A--TGACGACTAA-ACAAACC-ATT---
Macs      ACAACTATGAATACCCCCTCAGACACTAGAAC--CTAAAT---AAACAAACC-ATT---
Moal      ACAATTAATAAT-AACCCCACATTAATTACCC--AAAAAAC--CAATAAATC-ATT---
Syma      ACCACCATAACTAAACCCAAATACA--CTAAC---CA-TAACAAAACAAAAT-ATT---
Mafr      CCACATTCATAACCCCTAAAACACCAAC--AC--C---ACCTCAAACAAACC-ATT---
Dcpe      CCCACCATTAATA-CCCCC-TCATCACCCACA--AATCAA--TAAACAAATC-ATT---
Dcti      CTCAACATTAATACCCCCT--CACACACGACA--AATTA-AC-AAACAAATC-ATT---
Hehi      ACCACTATTTATAACCCCAAA--TACACGAGTG--TTTTAATTAAACAAACC-ATT---
Stam      CTCAATATTAATAACCCCCAATTTACTATTAA---CCTC---TAAACAAACC-ATT---
Hogi      CCCCCTATAAATAACCCCT-AACACACTAAGA--CTTAATT--AAGCAAACC-ATT---
Erzo      ACCATTATTAATACCCCCTAAGTACACTAGAG--TTTATTT--AAACAAACC-ATT---
Hxot      ACCCCTGTTAATAACCCCTAAGTACCCCAGTA--TTTATTTT-AAACAAACC-ATT---
Core      AACCCTGTTAATAACCCCT-AAATACCCCAGT--ATTTAAATTAAACAAACC-ATT---
Apve      ATCCATGTCAATAACCCCTAAATACACCAGTA---TTTATTTTAAACAAACC-ATT---
Latj      ACTATCATAAATAACCCCAAAAT-CACGCAT---TCTCCTA--AAACAAACC-ATT---
Laja      GTCACTGTTAATAACCCCA--AATGCACCAAC--ATCATTATTAAACAAATC-ATT---
```

```
Syja    CCTATTATTAATAACCCCC-AATACCCTATTA--ACATGTAAAAAGTAAACC-ATT---
Epme    AACACCATAACTAACCCCAAAGA-CACGAATG--TCTCCAC--GAACAAACC-ATT---
Grse    ATCACTATCAATACCCCCTAATACAATCCACC---CACAATTTAATCAAATC-ATT---
Clja    ATCACTATTTTTACCCAC----AAATATCCTT--AACCCTATTAAATAAAAC-ATT---
Ogcy    TACCCTATAAATAACCCC---TAAGACATTAC--CCCTAAACAAAATAAACC-ATT---
Plna    CCTTTTATTAATACCCCTAAAT-CATACAACC--CTTAAACAAA--CAAACC-ATT---
Lema    AACATCATTAATACCCCCA-AAAACACTAACG--TTTTTC---AAACAAACC-ATT---
Etzo    ATCACCATTTATACCCCT--TAATACACTAAT--GT-TATGTTTAACAAACC-ATT---
Apse    TCCATTGTAAATAACCCCT-AACCACACCTTA--TACCCCTAAGACCAAACC-ATT---
Epde    ATCACCATTAATATCCCCAAA-TACACCCCCC----CCCAACA-AACAAACC-ATT---
Slja    CA-ATATAAACTAAACCCTAATACCCCTTAAC--CC---ACCAAAACAAACC-ATT---
Bsja    ACCACTATCAATACCCCCAAATACACCACCAG--AC-TTAATAAACCAAACC-ATT---
Ecna    AATAT---TAATAAACCCTAAAT-CACCATA---AAACTAT--AAATAAAAC-ATT---
Cohi    AATACTTCTACTAACCCCTAATACA-CTAAAA---A-T-ATTCAACTAAACC-ATT---
Caar    GTAACCATGAATA--ACCCCAAAAGCACTAAA--CTT--ATTCAAACAAAAC-ATT---
Came    ACAAACATGAATAAACCCAAAAACACTTCAAA---CTCAA--TAAACAAAGC-ATT---
Mema    ACCCCCATTAATAACCCC-TAATGCACAAACT--TCCACTT--AACTAAAAC-ATT---
Lenu    CCCCCTATAAATACCCCCTCACAGTCTTAA-C--CAT--AAAGATCTAAACC-ATT---
Brja    CAATATTAATACCCCCGAATACCCCTTCCCCC--C---CCCCCAAACAAACC-ATT---
Plma    CCCCCTATATATAACCCCTAATACACCTCCTC---CTCTAAATAAACAAACC-ATT---
Emst    C-CCTCATTAATAACCCCA-AACACACTGACA--ACTCAA-CCAAACAAACC-ATT---
Ptti    CCC-CCATTAATACCCCCTAACACCATAGACT--CACCCCTAA--ACAAATC-ATT---
Losu    AAAAATTTATGTAACCATAAATACCACTACTA--C---TATAAGAACAAAAC-ATT---
Geoy    TACCACATAAACTAAACCCCAAATTATCTG----AAACTCCACAAACAAATC-ATT---
Dipi    ATACCCATTAATAACCCCTAAAACACTA------CACCGCGCCAAACAAACC-ATT---
Pama    ACCCCCATTAATAACCCCTAACATACTAACAT--ATAACCA--AA-CAAATC-ATT---
Leob    CCCACTATAACTAACCCCAAAGA-CACGC--A--CCCCCACAAA-ACAAATC-ATT---
Neba    ACCAC-ATTAATTAACCCCTAACACACTGTAA--CCAACTC--AAACAAATC-ATT---
Pdpl    ATAAATTTCACTA-ACCCA-ACATGCATGTAA--ATTCAACTTGACTAAACC-ATT---
Nimi    ATTCCCATCAATACCCCCTAAAACA-CTCAAA---ATTGTACTAAACAAACC-ATT---
Uptr    ATCAT-ATTAATA-ACCCC-TAAGACACTTAA--CACCAA-CTAAACAAATC-ATT---
Pesc    ATAAATATTTATAACCCCA-AACACACCA-TA--TCACAAAC--AACAAATC-ATT---
Baar    CCCACCATTAATACCCCCAAATGCA--CTAAC---ATTAAACTGAATAAACC-ATT---
Moar    -CACCCATTAATAACCCCAAACACA--CTAAC---TAAACACTAAACAAACC-ATT---
Toja    ATCAAAATAAATAAACCCA-AAGACCCAAA-----CAAGTCT-AACCAAACC-ATT---
Chau    AC-CATCTAAATACCCCCCAATA-CACGCAAT--ATATACC--AAACAAAAC-ATT---
Chse    ACCAATATCCATACCCCCA-AACATATTACCT--GTTTTCCT-TAACAAACC-ATT---
Enar    ATCCCTATTTATACCCCCT-AATACACCAACA--TT--CCCCTAAACAAACC-ATT---
Hpty    ACCATTAATT--AACCCCA-AATACACTAATA--TACTATTA--AACAAACC-ATT---
Nana    -CCATATAAAATAACCCCT-AATTAACTACAC--ACCC--ACAAAACAAATC-ATT---
Mcst    T-CTATATTAATAACCCCA-AATTCACGAATG--CCCCCA-CTTAACAAACC-ATT---
Rhox    ATCAACATCCATACCCCCAAAGACACTAA-CC--CTAGAACAAA-ACAAACC-ATT---
Opfa    ATCAATATTTATACCCCCAAACACACGAATAC--TTACGTT--AAACAAACC-ATT---
Paar    GCAAATATTTAT--ACCCCTTCATATATCA---AATTTTTATTAAACAAACC-ATT---
Gozo    ACCATTATTTATAACCCCACATGCACAAATGT--TTTCACA--AAACAAATC-ATT---
Ackr    AACCCTGTTTATACCCCC---ACATCCCTTAG--AACCCCTATAAACAAATC-ATT---
Elev    AAAATA-TTAACTACCACTAATACACT------TAATTTAAACAA-CAAATC-ATT---
Trdu    AACCCCTTATATATATCCC-CA-AACACACCA--TTCTCTCAACAACAAACC-ATT---
Amoc    TCCCCTATCAATACCCCCAAAGATACTCTAAA--CACAA-----AACAAAAC-ATT---
Hame    AGAACCCCTTCAATTAACCCCACACACAG-TA--AATTCACAAA-ACAAACC-ATT---
Chso    ACCAT---AACTAACCCCACATGCA--C--TA--AATAAAGTTAAACAAACC-ATT---
Lyto    CCCCCTGTTAATAACCCCTAAG-TACACTAGT---ATTTACATAAACAAACC-ATT---
```

```
Encr    CCC-TTGTTAATAACCCCTAAGTACCCCAGTA--TTTATATTAA-ACAAACC-ATT---
Bvar    AACACCATTAAT-ACCCCTAAATACCCTAATG--TGCAGTT--AAATAAATC-ATT---
Noco    ACCACCATTAATA-CCCCC-AGATGCACAACT--CAAGA--TTAAACAAATC-ATT---
Chsp    AACA-TATTAATAAACCA--GAATGTATCA-T--ACATAACTAAACTAAACC-ATT---
Arja    CCC-CTGTTAATAACCCCTAACTACCCTAGTA--TTTATATTAA-ACAAACC-ATT---
Pase    ATAGCTATAAATAAACCCTAAGACATCAAAGG--ACTAACT--AAACAAAAC-ATT---
Trel    CAGAACAGGAATAACCCCAAAAACAGAAACAC--CTC--AATTAAACAAACC-ATT---
Lifa    AACAATATTTATAGCCCCACAAATACTTA-CA---TAAATTTAA-ATAAACC-ATT---
Acur    ATCCACGTCAATAACCATTAATA-CCCTTAAA--ACCTCT---AAACAAAGC-ATT---
Ampe    CCCATCAATAACCCCTCAC-ACACGACACACA--AG---TGA--AACAAATC-ATT---
Urja    GCCAATTTACCTAACCCCA-AACATACCACAA--ACTAGA---AACTAAACC-ATT---
Enet    AACAATACCCCTACCCCCTGATGC--TTTATG--CAAT-TA--AACTAAACC-ATT---
Ptbr    TTAATTAT--AAATATCT-TAGAATAACAT----AAAATATTAAATCAAAAC-ATT---
Safa    CCCGACATTAATAAACCCGAAGAATCGCA--C--AAC--AATATATTAAAAC-ATT---
Icae    CCCAATATTTATAACCCCA-AACACACATCTC--CTC-CTATTAAACAAACC-ATT---
Asmi    TAC----TGAATAAAACAT-AATAAACTAATT--CTTATA---AAATAAATC-ATT---
Foal    A--CTATTTTTAA-CCC-TATCATCAATTGAA--TAAGTGTATAAACAAATC-ATT---
Drze    ATAACCATCAATA-CCCAA-AAATAACCTATT--TACTAAA--AACTAAACC-ATT---
Rhas    CCCAACATCAATAACCCC----TAACACCGAA--CCCAACACAAAACAAACC-ATT---
Elac    GTCAACATAGACTAACCCC-AAATACCCTCCC--ATCCA--TTAACCAAACC-ATT---
Kugu    ACAAATTTAAATAACCCCA-AAAACACTGACC--ACAACTTAAAA-CAAACC-ATT---
Plor    ACCCCCATTAATAACCCCA-AACCCACTTTAA--CTAACCT--AAACAAATC-ATT---
Sgun    ACCCCCATTCATACCCCCAAA--TACACTAAT---TTACCTCAAAACAAACC-ATT---
Zaco    AACCCTATCAATACCCCCAAA--TACACCAAC---ACCTCCTAAAACAAACC-ATT---
Zbfl    ACCCATATTAATAACCCCA--TACATTCGTAT--GTTAC-GTTAAACAAACC-ATT---
Spba    TCAACCATTAATT-AGCCCCTAATCCACTATA-AAATACCCTAAGCTAAACC-ATT---
Game    CCCACTATAAATACCCCCAAACGCACGCCTCC--CCGCATT--AAACAAACC-ATT---
Thth    CTCAATATAAATAACCCCAAA--CACACTACT--CCTCCTATAAAC-AAACC-ATT---
Xigl    ATCACTATTAATAACCCCGAA-AACACACTAA---ATTTATT-TACTAAACC-ATT---
Hyja    CCCAATATTAATACCCCCAAAGACC-CCCCCC---CCCCGCTTAATCAAACC-ATT---
Psan    CCCAATATTAATAACCCCT-AATACACCACCT--CCCTAT-TTAACCAAACC-ATT---
Cupa    CCCAATATTCATA-AACCC-AAATATACGCCT--CCCCAA-TTAAACAAACC-ATT---
Mpch    AACACTATTAATAACCCCT-CAAACCCTATAA--AT--ATAA--AACAAACC-ATT---
Char    ATCACCATAAATAACCCCAAATACA-CAACCC---AATACACCAACTAAATC-ATT---
Pser    TCA-ACATAAATAACCCCAAATACACAACCCC--CA---TCCAA-CCAAACC-ATT---
Prol    ACCCATATAAATAACCCCT-AAAGCACGTAAC--ACCCACGT-AGCCAAACC-ATT---
Plbi    CCATTCATA---ACCCCT---AAAGCACGAAA--CACCCACGTAGCTAAACC-ATT---
Calu    ACCCCCTTCAATAAACCCAAACACC--CACCA--A---TACACAAGTAAAAC-ATT---
Papa    GACCTTATAAATAAACCCC-AACACACTCAAC--CAA----CCAAACAAAAC-ATT---
Sufr    CCAAACATAAATA-ACCCC-ATA-AACACTAC--AAAAT--AAAAACAAACC-ATT---
Stci    CTAAACATTAATAAACCCT-TATTTACTCACA--ACA----CTAAACAAACC-ATT---
Taru    ACCACTATAAATACCCCCTAAGATA-CTTAAC---TA--AACAAAACAAATC-ATT---
Rala    ACCCCTATTTATACCCCCTAATAGCACTAACC--CCC--T-TAAAACAAACC-ATT---
                                                       **    ***
```

```
               2            3         3'        2' ¦     HVR     ¦
Scca    CTTCACCCTTAAGTA--TGGGCGACAGAACAAGGACCTC------AGCGC--AATAGCTTA
Muma    -TTAACCTTCTAGTA--TGGGTGACAGAACAATAACTC------AAGAGC--AATAGCTTA
Erca    T-GTTAACTCAAGTA--TAGGAGATAGAAAAAGACTA------ATAGAGC--AATAGTTA-
Pose    -TGTCAACTTTAGTA--TAGGCGATAGAAAAAGATT------TACAGAGC--TATAGCAAT
Actr    TAAT-ACCCCCAGTA--TAGGCGATAGAAAAGGACA-----AAGCAGCGC--AATAGAGAA
Scal    TAAT-CCCCCTAGTA--TAGGCGATAGAAAAGGACA-----AAGCAGCGC--AATAGAAAA
Posp    -TAATTTCCCCAGTA--TAGGCGATAGAAAAGGACAA----AACAGCGC--AATAGAGAA
Atsp    T--CTACCCCCAGTA--TAGGCGATAGAAAAGGAAAT------TTGAGC--AATAGAAAA
Leoc    T-GTAC-CCCCAGTA--TAGGCGATAGAAAAGGAAAA-----TTTTGAGC--AATAGAAAA
Amca    -TTTCCTCCCTAGTA--TAGGCGATAGAAAAGGATC-----CTTTAAAGC--TATAGAAAA
Osbi    T-TTCCTCCCTAGTA--TTGGAGAAAGAAAAGGATACT-----CTTGAGC--AATAG-AAA
Pabu    C-TTCCATCTTAGTA--TAGGAGATAGAAAAGAAAAT-----TTTCGAGC--CACAAAAAT
Hial    T-TCCCACCTTAGTA--TGGGCGACAGAAAAGGAAAT-------TAGAGC--TATAGAGAA
Elha    CTCC-CCCCTTAGTA--TGGGCGACAGAAAAGGAAAAA-----CCAGAGC--AATAGAAAA
Mlcy    T-TCCCCTTCTAGTA--TAGGAGACAGAAAAAAGAG------ACCAGAGC--AATAGAAAA
Algl    TTTC-CTTCTTAGTA--TAGGCGATAGAAAAGGAGAAT------TTGAGC--AATAGAAGA
Ptgi    T-TTCCACCCCAGTA--TAGGCGATAGAAAAGGAAAA-----CTTCAAGC--GATAGAAA-
Alaf    TAAC-CTCCCCAGTA--TAGGCGATAGAAAAGGAAA-----AATTTAAGC--AATAGAAAA
Nock    TTAC-CTCCCCAGTA--TAGGCGATAGAAAAGGAAA------ATTTGAGC--GATAGAAAA
Anja    C-TTCCCCCTAAGTA--TAGGTGATAGAAAAGGACAAA------ACGCGC--AATAGAAAA
Gyki    C-TACCCCCCTAGTA--TAGGTGATAGAAAAGGTC-------CCCAGAGC--AATAGAAAA
Syka    -CTTCCACCTTAGTA--AGGGAGACAGAAAAGGAAATC----TCTAGAGC--CATAGAAAA
Opma    TTTC-CACCTAAGTA--TAGGCGATAGAAAAGGACA---CACCTTGAAGC--TATAGAAAA
Comy    C-TCCCACCTTAGTA--TGGGCGACAGAAAAGGATA-----TTATAGAGC--CATAGAAAA
Sasp    C-TTCCCCCCTAGTA--TGGGCGACAGAAAAGGATCA-------AGGAGC--AATAGAAA-
Eupe    CTCC-CACCTAAGTA--TAGGCGATAGAAAAGGAAC-------TAAGAGC--GATAAAGAA
Enja    T-GACCTTCCCAGTA--TGGGCGACAGAAAAGAATTC-----CCAGAAGC--AATAGAAA-
Same    C-GACCACCCCAGTACCGGGCAGACAGAAAAGGAAAA------ATGACGC--TATAGACAA
Chch    -TTACCGCCTAAGTA--CGGGTGACGGAAAAGGCAACTACACAAATAAGC--AATAGAAAT
Grgr    TTAC-CACCTGAGTA--TGGGCGACAGAAAAGGTC-----ACCAAAAAGC--CATAGAAAA
Caau    C-TTTTACCTGAGTA--TGGGAGACAGAAAAGGTTC------CACAAAGC--AATAGAAAC
Cyca    T-TTTTACCTGAGTA--TGGGAGACAGAAAAGGTTCC------ACAAAGC--GATAGAAA-
Dare    A-TATTATCCTAGTA--TGGGAGACAGAAAAGATA----AAATACTAAGC--AATAGAGAA
Cost    T-TTCCACCTTAGTA--CGGGCGACGAAAAAGGTTCC-------GTGAGC--AATAGAAA-
Leec    TTTC-CACCTTAGTA--CGGGCGACAGAAAAGGTA------AACTTAAGC--AATAAAGAG
Crla    TTTC-CACCTTAGTA--CGGGCGACGGAAAAGGAT-----CCGATTAAGC--GATAGAAAA
Clmc    CTCC-TGCCTTAGTA--CGGGCGACAGAAAAGGCAA---TAAACCAAAGC--AATAGAGAA
Phin    C-TCCCACCTTAGTA--CGGGCGACGAAAAAGGCACA--TCTTCTTAAGC--AATAGTAAA
Icpu    CTAC-CGCCTTAGTA--TGTGTGACAGAAAGGGCA----C--CACAAAGC--AATAATAAA
Psto    TTAC-CGCCCAAGTA--TGTGAGACAGAAAAGGCACA------CTAAAGC--TATAGAAAA
Cora    TTTC-CGTCCAAGTA--TATGAGATAAAAAAGACACA-------AAAAGC--AATAGATAA
Eisp    T-TACTGCCTTAGTA--CGGGCGACGGAAAAGGCACC--ACATTATAAGC--GATAGAAA-
Apal    -TTCCTGCCTTAGTA--TGGGCGACAGAAAAGGCACC--A-CCCAAAAGC--AATAGAAAC
Eslu    TTTC-CTCCTTAGTA--TAGGCGATAGAAAAGGAAATA------CCGAGC--AATAGAAAA
Dape    T-TTCCTCCTTAGTA--TAGGCGATAGAAAAGGATCC------TCTGAGC--AATAGAAAA
Glse    T-TTCCCCCTTAGTA--CGGGCGACAGAAAAGGGAATA-------CGAGC--AATAGAAAA
Naar    T-TTCCCCCTTAGTA--CGGGCGACAGAAAAGGGACC-------CTGAGC--AACAGAGAA
Baoc    TTTC-CCCCTTAGTA--TGGGCGACAGAAAAGGGAC------CC-TGAGC--AACAGAGAA
Opso    T-TTCCCCCTTAGTA--CGGGCGACAGAAAAGGGACC--C-----TGAGC--AACAGAGA-
Alte    TTTC-CACCTTAGTA--TGGGAGACAGAAAAGGAAC------CACAAAGC--AATAGAGAA
Plap    T-TTCCGCCCTAGTA--TGGGAGACAGAAAGGGAACC------ACAAAGC--AATAGAGA-
```

Plal	T-TTCCACCTGAGTA--CGGGCGACGGAAAGGGAGCA-----AATTGAGC--GACAGAGA-
Sami	T-TTCCACCTTAGTA--CGGGCGACGGAAAAGGGCA------ATTTGAGC--CATAGAAAA
Rere	T-TTCCACCTTAGTA--TGGGAGACAGAAAGGGACATT-------TGAGC--AATAGAGAA
Gama	T-TTCCACCTGAGTA--AGGGAGACAGAAAAGGACC---TT----GAAGC--AACAGAGA-
Onmy	T-TTCCACCTTAGTA--GGGGCGACCGAAAAGGAGAT--AA---TTGAGC--AACAGAAAA
Sasa	T-TTCCACCTTAGTA--CGGGCGACGGAAAAGGAAACAAT----TTGAGC--AACAG-AAA
Cola	TTTC-CCCCTTAGTA--TGGGCGACAGAAAAGGGAA----TAAT-TGAGC--AACAGAGAA
Dita	-TTTCCATCCTAGTA--CGGGAGACGGAAAGGAGCA-----CTTTTGAGC--GACAGAGAT
Gogr	TTTC-CACTCTAGTA--TGGGAGACAAAAAGAATT----AG-CCCTGAGC--AACAGAGAG
Chsl	T-TTCCTCCCTAGTA--CGGGCGACAGAAAAGAAGAT--AT----TGAGC--TATAGTAA-
Atja	T-TTCCCCCTAAGTA--TGGGAGACAGAAAAGGAAAA----CCTTAGAGC--AATAGAAAA
Iido	TTTC-CCCCCAAGTA--TGGGAGACAGAAAAGGAA---AACT---AGAGC--AATAGAAAA
Auja	TTTC-CCCCTTAGTA--CGGGCGACAGAAAAGGGAA----CGCACGGCGC--AACAGACAA
Chag	GTTTATTCCCAAGTA--CGGGCGACGGAAAAGGAAAACGA----CGGAGC--AATAGAAAC
Hami	C-TTCCTCCTAAGTA--TGGGCGACAGAAAAGGACA--GT----AGGAGC--TACAGATAA
Saun	C-TTCCTCCCCAGTA--CGGGCGACAGAAAAGGATAGCA------GGAGC--TACAGACAA
Nema	T-TTCCCCCCTAGTAC-TGGGCGATAGAAAAGGGCCT------ACGGAGC--TACAGAGA-
Disp	TTAC-CTCCCCAGTACCCGGGCGACAGAAAAGGAAATCC-----CGGAGC--AATAGAAAA
Myaf	TTAC-CTCCTCAGTA--CGGGCGACAGAATAGGAAA----T-CA-AGAGC--AATAGAGAA
Lagu	TTTC-CCCTTAAGTA--TGGGAGACAGAAATAGGA-------ACAGAAGC--AATAGAGAC
Trtr	C-TCCCACTTTAGTA--TGGGCGATAGAAACAGGTC------ATTGGAGC--CATAGACTA
Zucr	T-TCCCGCCCTAGTA--TAGGTGACAGAAATGGAA-------ACAGGAGC--AATAGAACT
Pxja	T-TTCCACCCTAGTA--TGGGCGACAGAAAAGGAAC-------ACGGAGC--AACAGAAAA
Pxlo	T-TTCCACCCTAGTA--TGGGCGACAGAAAAGGAACA--C-----GGAGC--AACAGAAA-
Pctr	-TTTCCACCCCAGTA--TGGGAGACAGAAAAGGAA-C--A-A---GGAGC--AATAGACAG
Apsa	C-TACCACCACAGTA--TGGGAGACAAAAAAGGAC-------CTCGGAGC--AATAGACAA
Cabe	C-TCCCCCCTAAGTA--TAGGAGATAGAAAAGGACAC--G----ATGAGC--AATAGAGAA
Bzze	T-TTCCGCCCGAGTA--TAGGAGATAGAAAAGGACCA--GA---CGGAGC--AATAGATAA
Siim	T-TTCCCCCCGAGTA--CAGGAGATGGAAAGGGGACA--CA--GAGAAGC--TACAGATAA
Ctru	TTTC-CACCCCAGTA--CGGGCGACAGAACAGGACC-----TGCGAGAGC--GATAGACAA
Dpbr	-CTCCCACCCTAGTA--TGGGCGACAGAAAAGGAT------CTAGAGAGC--AATAGAGAA
Caki	TATA-AACCCCAGTA--TGAGAGACAAAAAAGGAT-----ATT--AGAGC--TATAGACAT
Phja	TTAA-ACCCTTAGTA--TGGGCGACAGAAAAGGAA----A---AAAGAGC--TATAGATAA
Brsp	C-TATTTCCCTAGTA--TGGGCGACAGAAAAGGACCC------CGAGAGC--TATAGACAA
Gamo	TTAC-CCCCTGAGTA--CGGGCGACAGAAAAGGAGA----AA---AGAGC--AACAGACAA
Lolo	T-TACCCCCTAAGTA--TGGGCGACAAAAAAGGAAAA-------AAGAGC--AACAGATA-
Batr	C--CTCAGGATAGTA--TGGCCGATAGAAAAGCTTA-------AATGAAC--TATACAACA
Prmy	TTAC-CGCTCTAGTA--TGGACGATAGAAAAAGGCC-------TATGAAC--TATAGAAAA
Loli	T-TTCCCCCTTAGTA--CGGGCGACAGAAAAGGACCTT-------CGAGC--GATAGACTA
Loam	T-TTCCCCCCTAGTA--TGGGCGACAGAAAAGGACCT--T---AGTGAGC--AATAGAAA-
Chab	T-TTCCCCCCTAGTA--TAGGCGATAGAAAAGGGCC-------TTAGAGC--GATAGATAT
Chto	TTTC-CCCCCTAGTA--TAGGCGATAGAAAAGGGC----C--TTAG-AGC--GATAGATAT
Majo	TTAC-CCCCTTAGTA--TGGGCGACAGAAAAGGAAT-------AAGGAGC--AATAGAGAA
Hlst	TTTC-CGCCCTAGTA--CGGGAGACAGAAAAGGAC----TAAT--TGAGC--AATAGAGAA
Clpe	-TTTCCTCCTTAGTA--TAGGCGATAGAAAAGGATAC-------CGGAGC--GATAGAGAC
Mlmr	C-TCCCCCTTTAGTA--TAGGCGATAGAACAAGACAT------A-AGAGC--AATAGAAGA
Crcr	T-TACCCCCTTAGTA--TGAGCGACAGAAATGAAATCAC-----CGGAGC--AATAG-AAA
Muce	T-TACCCCCTTAGTA--TGAGCGACAGAAATGAAATC--ACC---AGAGC--AATAGAAA-
Bege	TTAC-CGCTTTAGTA--CAGGAGATGAAAAAAGAAC---TA----GGCGC--AATAGAAAA
Mela	T-TTCCACTTTAGTA--TAGGTGATAGAAAAAGACATT------CGACGC--AATAG-AGA
Hats	C-TTCCCCCTAAGTA--TAGGAGATAGAAAAGGAACC-------AGGCGC--GATAGAAAA
Orla	T-TTCCTCCATAGTA--TGGGAGACAGAAAGGGACT-------AAGGAGC--TATAGACAA

```
Cosa    T-TTCCCTTATAGTA--TATGAGATAGAAAATAAGCT-----T-TGGAGT--AACAGAACA
Exsp    -TTTCCCCTCTAGTA--TGGGAGACAGAAAGAGAAC-----AA--GGCGC--AATAGAGAA
Depa    T-TTCCTTTTTAGTA--TGAGAGACAGAAAATATA-------ACAGAGC--TATAGATAC
Rima    CTAC-CCCCTAAGTA--TGGGCGACAGAAAAGGAC-----CT-TAAAAGC--TATAGAAAA
Fuol    TTTC-CTCCCTAGTA--CGTGCGACAGAACAGGAAA---TACGCGAGCGC--TATAGATAA
Gmaf    TTTC-CTCCTTAGTA--AAGGCGATAGAAAAGGAA----AAACACGGAGC--CATAGAAGC
Xeei    T-TTCCTCCTAAGTA--AAAGTGACAGAAAAGGATCA--ACTATTAGAGC--CATAGAAAA
Pros    TTCC-CTTCCTAGTA--CGGGAGACAGAAAAGAGA----A--TCAGGAGC--AATAGAAAC
Scmi    TTCC-CTTCTTAGTA--TGGGAGACAGAAAAGAAAA------TCAGGAGC--AATAGAAAT
Rolo    TTTC-CCTCTTAGTA--CGGGCGACGGAAAAGAGAC------CCAGGAGC--AATAGAAAT
Cere    T-TACCCTCTTAGTA--CGGGCGACCGAACGGAGACC--A----GCGAGC--AATAGAATT
Daga    TT-TCCCTCCTAGTA--CGGGCGACGGAAAAGAGACC-----CAGCGAGC--GATAGATAT
Anco    T-TTCCCCCTTAGTA--TGGGCGACAGAAAAGGATCCA-------GGAGC--AACAGAAAA
Dmve    T-TTCCCCCTTAGTA--TGGGCGACAGAAAAGGACCC-----A--GGAGC--AACAGAGAA
Dmar    TTTC-CCCCTTAGTA--TGGGCGACAGAAAAGGACCC----A---GGAGC--AACAGAAAA
Anka    TTTC-CCCCTTAGTA--TGGGCGACAGAAAAGGATC-------AAGGAGC--AATAGAAAA
Moja    -TTTCCCCCTTAGTA--TGGGCGACAGAAAAGGACCC-------AGGAGC--AATAGAAAA
Hoja    -TTTCCCCCTTAGTA--TGGGCGACAGAAAAGGAC------CTATGGAGC--AACAGAAAA
Bede    T-TCCCCTCTTAGTA--TGGGAGACAGAAAAGAGAA------TTAGGAGC--AATAGAAA-
Besp    TT-TCCCTCTTAGTA--CGGGAGACAGAAAAGAGA------ATTAGGAGC--AATAGAAAC
Mysp    -TTCCCATCCTAGTA--TGAGCGACAGAAAAGATACT------CGCGAGC--AATAGAGAC
Osja    T-TCCCATCCTAGTA--TGAGCGACAGAAAAGAGACA--CG----CGAGC--GATAGAAA-
Sgro    T-TTCCGTCCTAGTA--TGTGCGACAGAAAAGAAAC-----TATGCGAGC--GATAGAGAC
Pzpa    T-TTCCCCCTAAGTAA-CAGGCGATAGAAAAGGGA----CCT---GGAGC--AACAGACGA
Zeja    TTTC-CCCCTAAGTAACTAGGCGATAGAAAAGGGAC------CATTGAGC--AATAGATGA
Znne    TT-TT-ACCCTAGTA--AAGGAGATAGAAAAGGAAA---C--A--GGAGC--AATAGATAA
Zefa    TT-TT-ACCTTAGTA--AAGGCGATAGAAAAGGAC------CCACGGAGC--AACAGATAA
Acni    TTTC-CCCCCAAGTA--AAGGCGATAGAAAAGGGAC-------CAGGAGC--TATAGACAA
Ncrh    TTTC-CCCCTAAGTA--AAGGCGATAGAAAAGGGACC-------AGGAGC--TATAGACAA
Agca    TTTC-CACCCTAGTA--TAGGCGATAGAAAAGGAAG----CCCCAGGAGC--AATAGAGAA
Hydy    TTCC-CTCCTTAGTA--TAGGCGATAGAAAAGGAC-----TCA-CGGCGC--AATAGAGAA
Gsac    TTTC-CACCTTAGTA--TAGGCGATAGAAAAGGA------CATATGGCGC--TATAGAGAA
Pevo    T-TTT-ACCCAAGTA--TCGGCGAGAGAAAGGGAC------CAACAGCGC--TATAGAAAC
Hiku    -TTATAACCCTAGTA--ACGGAGACTGAAAAGGAAA------AATGAAGC--TACAAAACT
Inpa    CTCACACTCTTAGTA--AAGGCGATAGAAAAGAGA----CA---CGGAGC--AATAGACAA
Auch    T-TTCCACCTAAGTA--TGAGCGACAGAAAAGGACAC--ATTTTTAGAGC--AATAGAGA-
Fico    T-TACCCCCCTAGTA--TGGGCGACAGAAAAGGAC-------AAGGGAGC--TATAGAGAA
Macs    T-TTCCCCCCAAGTA--CGGGCGACAGAAAAGGGACC--AA---TTGAGC--GATAGAGAA
Moal    CTAC-CCATTTAGTA--TGTGCGACAGAAAAAGAAA-------TTTAAGC--TATAGAAAA
Syma    T--AACCTCCAAGTA--TGGAAGACAGAAAAGACTATA-------TACGC--AATAG-AGA
Mafr    -TTAT-ACCCTAGTA--TGGGCGACAGAAAAGGAAAT--A-T---GGAGC--AATAGAATA
Dcpe    TTTC-CACCTAAGTA--TGGGCGACAGAAAAGGAA----C--TTCGGAGC--GATAGAGAA
Dcti    TTTC-CACCTAAGTA--TGGGCGACAGAAAAGGA------ACTTTGGAGC--AATAGAGAA
Hehi    T-TTCCCCTTTAGTA--TGGGCGACAGAAAAAGGACC------CAGGAGC--AATAGAGA-
Stam    T-TTCCCCCCAAGTA--TGGGCGACAGAAAAGGGC------CACAGGAGC--GATAGAGAA
Hogi    TTTC-CCCCCTAGTA--TGGGAGACAGAAAAGGAAC------AAAGAAGC--AATAGAAAA
Erzo    TTTT-CCCCTAAGTA--TAGGCGATAGAAAAGGGCT-----TA-CGGCGC--AATAGAGAA
Hxot    TTTC-CCCCTTAGTA--TAGGCGATAGAAAAGGGCC-----CA-CGGCGC--AATAGAGAA
Core    TTTC-CCCCTTAGTA--TAGGCGATAGAAAAGGAC----A--AGAGGCGC--AATAGAGAA
Apve    T-TCCCCCCTTAGTA--TAGGCGATAGAAAAGGG------CCCCCGGCGC--AATAGAAAA
Latj    TTTATCACCTGAGTA--TGGGCGACAGAAAAGGACTC--TG---TGGAGC--GATAGAAAA
Laja    TTTC-CCCCTTAGTA--TGGGCGACAGAAAAGGGA-------CCAGGAGC--AATAGAGAA
```

```
Syja    TTTC-CCCCTTAGTA--TGAACGACAAAAAGGAC-----CC--TAGAGC--AACAGAGAA
Epme    TTT-CCCCTTTAGTA--TGGGCGACAGAAAAAGAA----CA---CGGAGC--GATAGAGAA
Grse    T-TTCCTCCCTAGTA--TGGGCGACAGAAAAGAACATA------CGGAGC--AATAG-ACA
Clja    CTCC-CCCCTTAGTA--TAGGCGATAGAAAAGGAA----A---TAAGAGC--AACAGAGAA
Ogcy    TTTC-CACCCAAGTA--TGGGAGACAGAAAAGGAA-----AA-TTGAAGC--AATAGAAAA
Plna    TT-TCACCTTAAGTA--TAGGCGATAGAAAAAGATC-------TAGGAGC--GATAGAAAA
Lema    TT-TCCACCTAAGTA--TGGGCGACAGAAAAGGAACT-----A-CGGCGC--AATAGATAA
Etzo    TTTC-CCCCTGAGTA--TGTGCGACAGAAAAGGGC----C--CGTGGAGC--AATAGAGAA
Apse    TTTC-CCCCTAAGTA--TGGGCGACAGAAAAGGAT----AAGTC-GGAGC--AACAGAAAC
Epde    T-TTCCCCCCTAGTA--TGGGCGACAGAAAAGGG-----CCCGA-GGAGC--AATAGAGAA
Slja    -TTTCCCCCTTAGTA--TGGGCGACAGAAAAGGAACT-----CGAGGAGC--TATAGAGAA
Bsja    TTCCCCTCCCTAGTA--TAGGTGATAGAAAAGAGAC-----ACAAGGCGC--AACAGAGAA
Ecna    T-TGCACCCTTAGTA--TAGGAGATAGAAAAGGAATA--TA---CGGAGC--AATAGAAAA
Cohi    C-ATCTTCCCTAGTA--TAGGCGATAGAAAAGGAATAA------AAGAGC--AACAGATAT
Caar    -TTTCCTCCCTAGTA--TGGGCGACAGAAAAGGA----C-CTTCGGGCGC--AATAGAACA
Came    T-TTCCCCCTAAGTA--TGGGAGACAGAAAAGGAC----CACAAGGGCGC--AATAGAAAA
Mema    C-TACCCCCTCAGTA--CGGGCGACGGAAAAGGGCCC--CG---TGGAGC--AATAGATAT
Lenu    -CTTCCCCCTTAGTA--TAGGAGATAGAAAAGGA----A--CAGTGGCGC--CATAAAAAA
Brja    -TTTCCCCCTTAGTA--TAGGAGACAGAAAAGGAACA--A-C---AGAGC--AATAGAGAA
Plma    T-TACCCCCTCAGTA--TGGGCGACAGAAAAGGAA----CAGTTG-GAGC--AATAGAGAA
Emst    TTTC-CCCCTAAGTA--TAGGCGATAGAAAAGGGAC---CAC---GGAGC--AATAGAGAA
Ptti    -TTTCCTCCCTAGTA--CAGGCGATAGAAAAGGAAC-----TGT-GGAGC--TACAGAAAA
Losu    -TTTCCCCCCTAGTA--CGGGAGACAGAAAAGGAACA--A-T--TGAAGC--CATAGACAA
Geoy    T-TTCCTCCCAAGTA--CGGGCGACGAAAAAGGAAGC--CT----AAAGC--AATAGAAA-
Dipi    T-TTCCCCCTTAGTA--TAGGCGATAGAAAAGGAACT--CCT----GAGC--AATAGAAA-
Pama    T-TTCCCCCCAAGTA--TGAGCGACAGAAAAGGAACT------ACGGAGC--AACAGAGAA
Leob    T-TTCCCCCTAAGTA--TAGGCGATAGAAAAGGAA-------TTAGGAGC--GATAGAAAA
Neba    TTCC-CTCTCTAGTA--TGTGCGACAGAAAAAGAGC----AA-T-AGCGC--TATAGAAAA
Pdpl    CT-TT-TCCCTAGTA--TAGGCGATAGAAAAGGAA----C-CCAGGGAGC--GATAGAGAA
Nimi    T-TTCCTCCCAAGTA--CGGGCGACAGAAAAGGAACCG------TCGCGC--CATAG-AGA
Uptr    TTAC-CCCCTTAGTA--TGGGCGACAGAAAAGGAA----CAA---GGAGC--AATAGAGAA
Pesc    T-TCCCCCCCTAGTA--TGGGCGACAGAAAAGGAG----ACCAG--GAGC--TATAGAGAA
Baar    T-TTCCCCCTTAGTA--TGGGAGACAGAAAAGGGCCCA------AGGAGC--AATAGAGAA
Moar    TT-TCCCCCTGAGTA--TGGGCGACAGAAAAGGGACT-----GT-GGAGC--AACAGAGAA
Toja    T-TTCCTCCCTAGTA--TAGGCGATAGAAAAGGAC-----TTAAGGGAGC--TATAGAAAA
Chau    T-TTCCCCTTTAGTA--TAGGCGATAGAAAAAGTAC--CC----CGGAAC--GACAGAAAA
Chse    TTTC-CCCTTTAGTA--TAGGCGATAGAAAAAGATC---TCCG---GAGC--AATAGAGAA
Enar    TTTC-CCCCTGAGTA--TGGGCGACAGAAAAGGAA-----CTG-TGGCGC--TATAGAGAA
Hpty    T-TTCCCCCTTAGTA--CGGGCGACAGAAAAGGGC----CCGAG--GAGC--AATAGAGAA
Nana    TTTC-CTCCCTAGTA--TGGGCGACAGAAAAGGAC-------AAAGGAGC--TATAGAGAA
Mcst    TTAC-CCCTCTAGTA--TGGGCGACAGAAAGAGACT---TAG---GGAGC--TATAGAGTA
Rhox    T-TTCCCCCTTAGTA--TGGGCGACAGAAAAGGAA------CCATGGAGC--AATAGAAAA
Opfa    T-TTCCCCCCTAGTA--TGGGCGACAGAAAAGGGACT--ACTAATGGAGC--GATAGAGAA
Paar    T-TTCCACTTTAGTA--TGGGAGACAGAAAAAGAACT--C----CAGCGC--AATAGAGA-
Gozo    T-TTCCCCCTTAGTA--TGGGCGACAGAAAAGGAAC---TC---CGGCGC--AATAGAGAA
Ackr    TTTC-CCCTTTAGTA--CAGGCGATGGAAAAAGAA----C---TTAGAGC--TATAGAAAA
Elev    T-TTCCCCCTTAGTA--TGGGTGACAAAAAAGGATC---ATG----GCGC--AATAGAGA-
Trdu    TTCC-CACCTAAGTA--CGGGCGACGGAAAAGGAC----CTAG---GAGC--AACAGAGAA
Amoc    T-TTCCTCCCTAGTA--CGGGCGACGGAAAGGGAACT--AG-----GAGC--AATAGAAAA
Hame    T-TTCCATCCTAGTA--TAGGAGATAGAAAAGAGC------CTA-GGAGC--AATAGAGAT
Chso    T-TTCCCCCTAAGTA--TGGGCGACAGAAAAGGAAC---ATT---GGAGC--AATAGAGAA
Lyto    T-TTCCCCCTTAGTA--TAGGCGATAGAAACGGACCC--CC----GGCGC--AATAGAAA-
```

```
Encr    -TTTCCCCCTTAGTA--TAGGCGATAGAAACGGGAC-----ACC-GGCGC--AATAGAGAA
Bvar    TTCC-CCCCTTAGTA--TGGGAGACAGAAAAGGGAC----T-TC-GGAGC--AATAGAGAG
Noco    TTAC-CCCCTTAGTA--TGGGCGACAGAAAAGGGAC---TT-A--GGAGC--AATAGAGAA
Chsp    CTCC-CCCCTCAGTAACCAGGCGATAGAAAAGGAC-----C--ACCGAGC--TATACAAAA
Arja    -TTTCCCCCTTAGTA--TAGGCGATAGAAAAGAGA------CTAAGGCGC--AATAGAGAA
Pase    T-TCAACCCTTAGTA--TGGGAGACAGAAAAGGGACC--CC---GGGAGC--TATAGAGAA
Trel    -TTACCCCTTTAGTA--TCGGTGAGAGAAAAACACA-----C-CA-GAGC--AACAGAACA
Lifa    T-TCCACCCCTAGTA--CCGGCGACCAAAAAGGGA------CAACAGAGC--AATAGAGAA
Acur    T-TCCCTTTTTAGTA--TGGGCGACAGAAAAAGAATT--AG-----AAGC--AATAGAAAA
Ampe    T-TTCCCCCCTAGTA--TGGGCGACAGAAAAGGAT----CCCGATTGAGC--AATAGAGAA
Urja    TTTC-CCCCTAAGTA--TCGGAGACAGAAACGGGTC---ACCAAT-GAGC--GATAGAGAA
Enet    CTCC-CTCTTTAGTA--CAGGCGATGGAAAAAGAAC-------TATAAGC--AATAGAAAG
Ptbr    T-TACCCTTTGAGTA--TAGGCGATAAAAACAAGAAA--T-----GAAGC--TATA-ATA-
Safa    -TTATTGCTGAAGTA--TGGGCGACAGAAACACAA-------AATAGAGC--AACAGACAA
Icae    T-TACCCCCTCAGTA--TGGGCGACAGAAAAGGAACT----A-TTGGAGC--GATAGAGAA
Asmi    TCTTAACCTAAAGTA--TAGGAGATTGAATAGGATAA------AAGGAGC--TATAGACAA
Foal    T-TTCCGCCTTAGTA--TGGGAGACAAAAAAGGCC-------TTAGAAGC--GACAGAAAA
Drze    T-TTTTACCTTAGTA--TGGGAGACAGAAAAGGAAAAG----T---GAGC--AATAGAAAA
Rhas    TTTC-CCCCTTAGTA--TGGGCGACAGAAAAGGAA----A---CAGGAGC--AATAGAAAA
Elac    TTTC-CTCCTTAGTA--TGGGCGACAGAAAAGGACC---CA----GGCGC--CATAGAAAA
Kugu    TTCC-CTCCCCAGTA--TAGGCGATAGAAAAGGAC-----ACA-CGAAGC--TATAGAAAA
Plor    T-TATCCCCTGAGTA--TAGGCGAAAGAAAAGGAACC-----ATCGGCGC--AATAGAAAA
Sgun    T-TACCCCCTTAGTA--TAGGCGATAGAAAAGGACAT------AAGGAGC--AACAGAGA-
Zaco    TTTC-CCCCTTAGTA--TGGGCGACAGAAAAGGCACT------ACGGAGC--TATAGATAA
Zbfl    TTTC-CCCCCTAGTA--TAGGTGATAGAAAAGGAA------ATACGGAGC--AACAGAGAA
Spba    T-CTCCCCTTAAGTA--CGAGCGACGGAAAAAGGACC-----CGCGGAGC--GATAGAGA-
Game    T-TACCCCCTCAGTA--TGGGCGACAGAAAAGGAACT--AT---AGGAGC--AATAGAGAA
Thth    T-TACCCCCCTAGTA--TGGGCGACAGAAAAGGAACC--A---TTGGAGC--GATAGAGA-
Xigl    C-TCCCCCCTTAGTA--TGGGCGACAGAAAAGGAACTA-----GCGGAGC--AATAGAGAA
Hyja    T-TACCCCCCCAGTA--TGGGCGACAGAAAAGGAACTAT-----CGGAGC--TATAG-AGA
Psan    TTAC-CCCCTGAGTA--CGGGCGACAGAAAAGGAAC---TACC--GGAGC--GATAGAAAA
Cupa    TTTC-CCCCTTAGTA--TGGGCGACAGAAAAGGAA----TATACAGGAGC--AATAGAGAA
Mpch    TTTC-CCCCCTAGTA--TGGGCGACAGAAAAGGAAC---GTCT-AAGAGC--AATAGATAA
Char    T-TTCCACCCTAGTA--CGGGAGACAGAAAAGGACACA------CGGAGC--TATAG-AAA
Pser    -TCTCCCCCCTAGTA--TAGGCGATAGAAAAGGAA------CCACGGCGC--AATAGAAAA
Prol    C-TTCCACCTGAGTC--CAGGCGATAAAAAAGGAAAT------CCGGAGC--AATAGAAAA
Plbi    CTCC-CCCCTAAGTC--CAGGCGATAAAAAAGGAA-----AT-TTGGAGC--AATAGAAAA
Calu    -CTCCCTCCCTAGTA--TAGGCGACAGAAAAGGAAAA--A-----GGAGC--TATAGCACT
Papa    TTTT--TCCTTAGTA--CGGGAGACGAAAAAGGAC---AC-CC--GGAGC--TATAGAGAA
Sufr    TTTC-CACTCTAGTA--TAGGTGATAGAAAAAGACC---A--T--AGAGC--AATAGAAAA
Stci    TTTC-CTCTTTAGTA--TAGGAGATAGAAAGACAT---A-C-T--TGAGC--GATAGAAAA
Taru    T-TTCCACCCTAGTA--TAGGAGATAGAAAAGGAACTA-------GGAGC--TATAG-ATA
Rala    -TTTCTTCCCCAGTA--TAGGCGATAGAAAAGGGA------CTA-GGAGC--CATAGAGAA
                   ***        **   **
```

```
                   4     4'                                  ¦    HVR    ¦
Scca    -TGTACCGCAAGGGAAA-GCTGAAAAAGAAA-TGAAATAAAT---AATTA-AAGTACTAA
Muma    -TGTACCGCAAGGAAAA-GCTGAAAAAGAAA-TGAAATA-AATCATTA---AAGTACTAA
Erca    TAGTACCGCAAGGGAAT-GCTGAAAGAGAAA-TGAAATAAAT---AG-TTAAAGCGCTAC
Pose    -AGTACCGCAAGGGAAA-GCTGAAAGAGAAA-TGAAATA-AATCGTTA---AAGCATAAC
Actr    -AGTACCGCAAGGGAAA-GCTGAAAGAGAAAATGAAACAACT---TG-TTAAAGCAATAA
Scal    -AGTACCGCAAGGGAAA-GCTGAAAGAGAAAATGAAACAACT---TGTTA-AAGCAATAA
Posp    -AGTACCGCAAGGGAAA-GCTGAAAGAGAAAATGAAACA-ACTTGTTA---AAGCAACAA
Atsp    CAGTACCGCAAGGGAAA-GCTGAAAAAGAAA-TGAAACAACC----CATCAAAGCACCAA
Leoc    CAGTACCGCAAGGGAAA-GCTGAAAAAGAAA-TGAAACAACC----CATCAAAGCACTAA
Amca    -AGTACCGCAAGGGAAC-GATGAAAGAGAAA-TGAAATA-ACCCGTTA---AAGCACCAA
Osbi    AAGTACCGCAAGGGAAC-GCTGAAAAAGAAA-TGAAACAAAC----CATTCAAGCACAAA
Pabu    -AGTACCGCAAGGGAAA-GCTGAAAAAGAAA-TGAAACAACC----CATTCAAGCATAAA
Hial    -AGTACCGTAAGGGAAA-GCTGAAAGAGAAA-TGAAATAACT---C-ATTAAAGCACAAA
Elha    -AGTACCGCAAGGGAAA-GCTGAAAGAGAAG-TGAAACAACCCGTAA----AAGCAAAGA
Mlcy    -AGTACCGCAAGGGAAA-GCTGAAAAAGAAA-TGAAAAAACC---C-ATAAAAGCAAAAA
Algl    -AGTACCGCAAGGGAAA-GCTGAAAGAGAGG-TGAAATAGTT---CG-TAAAAGCATAAA
Ptgi    AAGTACCGCAAGGGAAA-GCTGAAAGAGAAA-TGAAACAACC---CA-TAAAAGCAATAA
Alaf    -AGTACCGCAAGGGAAA-GCTGAAAGAGAAA-TGAAATAACCCGTTA----AAGCACAAC
Nock    -AGTACCGCAAGGGAAA-GCTGAAAGAGAAA-TGAAATAACC---CG-TCAAAGCATAAA
Anja    -AGTACCGCAAGGGAAA-GCTGAAAGAGAAA-TGAAACAATCCAT-AT---AAGCAAAAA
Gyki    -AGTACCGCAAGGGAAA-GCTGAAAAAGAAA-TGAAACAACC---C-GATAAAGCAAAAA
Syka    -AGTACCGCAAGGGAAA-GCTGAAAAAGAAA-TGAAACA-ACGCATAT---AAGCAACAA
Opma    -AGTACCGCAAGGGAAA-GCTGAAAAAGAAA-TGAAAAAACT---CATAA--AGCAATAG
Comy    -AGTACCGCAAGGGAAA-GCTGAAAAAGAAA-TGAAAAA-ACCCATAA---AAGCATAAA
Sasp    AAGTACCACAAGGGAAA-GTTGAAATAGAAA-TGAAATAAAT---AT-CACAGGCAATAT
Eupe    --GTACCGCAAGGGAAA-GTTGAAAAAGAAA-TTAAATAAACACCAA----AAGCAACAT
Enja    AAGTACCGCAAGGGAAA-GCTGAAAGAGAAA-TGAAATAACC---CA-TTAAAGCACAGA
Same    -AGTACCGCAAGGGAAA-GCTGAAAGAGAAG-TGAAATAACG----CACTAAAGTAAATA
Chch    -AGTACCGCAAGGGAAA-GCTGAAAGAGAAA-TGAAACA-ACTCATCA---AAGCGACAA
Grgr    -AGTACCGCAAGGGAAA-GCTGAAAGAGAAA-TGAAACAACC---CATT-TAAGCCTAGG
Caau    -AGTACCGCAAGGGAAA-GCTGAAAGAGAAG-TGAAATA-ACCCATAT---AAGCAGTAA
Cyca    TAGTACCGCAAGGGAAA-GCTGAAAGAGAAA-TGAAATAACC---CA-TATAAGCACTAA
Dare    -AGTACCGCAAGGGAAC-GCTGAAAGAGAAA-TGAAATAACC---C-GTAAAAGCAATAA
Cost    AAGTACCGCAAGGGAAA-GCTGAAAGAGCAA-TGAAACAAGT---CA-TTTAAGCACAAA
Leec    -AGTACCGCAAGGGAAA-GCTGAAAGAGTAA-TGAAATAATC---CA-TATAAGCACAAA
Crla    -AGTACCGCAAGGGAAA-GCTGAAAAAGAAA-TGAAACAACC---CATA-TAAGCACCAC
Clmc    -AGTACCGCAAGGGAAA-GCTGAAAGAGAAA-TGAAACAACC---CA-TTAAAGCCAAAA
Phin    -AGTACCGCAAGGGAAA-GCTGAAAAAGAAA-TGAAACAACTCAT-CA---AAGCACAAC
Icpu    -AGTACCGCAAGGGAAC-GCTGAAAGAGAAG-TGAAACAAAT---AATT-AAAGCACAAC
Psto    -AGTACCGCAAGGGAAC-GCTGAAAGAGAAA-TGAAATAAAT---CATTA-AAGCCTCAC
Cora    -AGTACCGCAAGGGAAA-GCTGAAAAAGAAA-TGAAATAAACCATCA----AAGCACAAC
Eisp    AAGTACCGCAAGGGAAA-GCTGAAAGAGAAA-TGAAACAACC---CA-TATAAGCACAAA
Apal    -AGTACCGCAAGGGAAA-GCTGAAAAAGAAA-TGAAATA-ATTACACA---A-GCACAAT
Eslu    -AGTACCGTAAGGGAAA-GCTGAAAAAGAAG-TGAAACAAGT---CATT-CAAGCCTTGA
Dape    -AGTACCGCAAGGGAAA-GCTGAAAAAGAAA-TGAAACAACT----CATATAAGCCCTAA
Glse    -AGTACCGCAAGGGAAA-GCTGAAAAAGAAA-TGAAACAACC----CATCCAAGCTTAGA
Naar    -AGTACCGCAAGGGAAA-GCTGAAAGAGAAA-TGAAACAACC---C-ATCAAAGCCTAAA
Baoc    -AGTACCGCAAGGGAAA-GCTGAAAGAGAAA-TGAAACAACCCATTA----AAGCCTAAA
Opso    AAGTACCGCAAGGGAAG-GCTGAAAAAGAAA-TGAAATAACC---TA-TTAAAGCCTAAA
Alte    -AGTACCGCAAGGGAAA-GCTGAAAGAGAAA-TGAAACAACCCATTT----AAGCCTAAA
Plap    AAGTACCGCAAGGGAAA-GCTGAAAGAGAAA-TGAAACAACC---CA-TTTAAGCCTAAA
```

```
Plal    AAGTACCGCAAGGGAAA-GCTGAAAAAGAAG-TGAAACAACC---CA-TATAAGCACCAT
Sami    -AGTACCGCAAGGGAAA-GCTGAAAGAGAAG-TGAAATA-ACCTATAC---AAGCACTGA
Rere    -AGTACCGCAAGGGAAA-GCTGAAAGAGAAA-TGAAACAACC---C-ATCTAAGCGCCTA
Gama    AAGTACCGTAAGGGAAA-GTTGAAAGAGTGA-TGAAAAAGTC---CA-TATAAGCACAGT
Onmy    -AGTACCGCAAGGGGAA-GCTGAAAGAGAAT-TGAAATAACC----CATTTAAGCCTAGA
Sasa    AAGTACCGCAAGG-AAA-GCTGAAAGAGAAA-TGAAACAACC----CATTTAAGCCTAGA
Cola    -AGTACCGCAAGGGAGA-GCTGAAAGAGAAC-TGAAACAACCCATTT----AAGCCTAGA
Dita    -AGTACCGCAAGGGAAA-GCTGAAAGAGGAA-TGAAACA-ACTCACTC---AAGCCCCAG
Gogr    -AGTACCGCAAGGGAAA-GCTGAAAGAGAAA-TGAAACAACC---CACT-TAAGCCTAGT
Chsl    A-GTACCGCAAGGGAAG-GCTGAAATAGAAG-TGAAATAACT---TG-TTTAAGCCCGAA
Atja    -AGTACCGCAAGGGAAA-GCTGAAAGAGAAA-TGAAATA-ATTTATTC---AAGCCTAAA
Iido    -AGTACCGCAAGGGAAA-GCTGAAAGAGAAA-TGAAACAAAT---TA-TTCAAGCCTGAA
Auja    -AGTACCGCAAGGGAAA-GTTGAAAAAGAAA-TGAAATAACCCCTTT----AAGCCCCAA
Chag    -AGTACCGCAAGGGAAA-GCTGAAAAAGAAG-TGAAATAAAC----CTCCAAAGCCTAAA
Hami    -AGTACCGCAAGGGAAT-GCTGAAAAAGAAA-TGAAAGAATTAT-CTTTTAAAGCCTTAA
Saun    -AGTACCGCAAGGGAAC-GCTGAAAAAGAAA-TGAAAGAACTATTTATC--AAGCAAAGA
Nema    AAGTACCGCAAGGGAAA-GCTGAAAGAGAAA-TGAAACAACT---AC-CCCAAGCCAAAT
Disp    -AGTACCGCAAGGGAAA-GCTGAAAGAGAAG-TGAAACAACT---ACCCC-AAGCTGTGA
Myaf    -AGTACCGCAAGGGAAA-GCTGAAAGAGAAA-TGAAACAAATACCCC----AAGCCATAA
Lagu    CAGTACCGCAAGGGAAA-GTTGAAAGAGATA-TGAAA-AAGC---CATT-CAAGCAAAGA
Trtr    -AGTACCGCAAGGGAAA-ACTGAAATATTAAATGAAAC--------ACTCAAGCAAACT
Zucr    -AGTACCGCAAGGGAAA-GCTGAAAAATCCAATGAAAAT--------ACTTAAGCGTAGA
Pxja    -AGTACCGCAAGGGAAA-GCTGAAAGAGAAA-TGAAACAACT----TGTTTAAGCCGCAA
Pxlo    AAGTACCGCAAGGGAAA-GCTGAAAGAGAAA-TGAAACAACT---TG-TTTAAGCCACAA
Pctr    -AGTACCGCAAGGGAAA-GTTGAAAGAGAAA-TGAAAAA-ACTCATCT---AAGCCAATG
Apsa    -AGTACCGCAAGGGAAA-GCTGAAAAAGAAG-TGAAACA-ACTCATACA--A-GCATAAA
Cabe    -AGTACCGCAAGGGAAA-GCTGAAAGAGAAG-TGAAACAAAC----CATGCAAGCCCTGA
Bzze    -AGTACCGCAAGGGAAC-GCTGAAAGAGAAA-TGAAATAAAC----CAGGAAAGCCCAGA
Siim    -AGTACCGCGAGGGAAC-GATGAAAGAGAAA-TGAAACAACC----CATAAAAGCCCTAA
Ctru    -AGTACCGCAAGGGAAC-GCTGAAAGAGAAA-TGAAACAAACCAGTA----AAGCCCAGA
Dpbr    -AGTACCGCAAGGGAAC-GCTGAAAGAGAAA-TGAAACA-AATTAGTA---AAGCCCAAA
Caki    -AGTACCGCAAGGGAAA-ACTGAAAAAGAAA-TGAAACAAAT---CATTT-AAGTACTGA
Phja    -AGTACCGTAAGGGAAA-GCTGAAAAAGAAA-TGAAATAAAC---CATT-TAAGCGCAGA
Brsp    -AGTACCGCAAGGGAAA-GCTGAAAAAGAGA-TGAAATAGATCTC-TT---AAGTTTTAT
Gamo    -AGTACCGCAAGGGAAC-GCTGAAAAAGAAA-TGAAATAAACCATTT----AAGCACCAA
Lolo    AAGTACCGCAAGGGAAC-GCTGAAAAAGAAG-TGAAATAAAC---CA-TTTAAGCACCGA
Batr    -AGTACCGCAAGGAAAA-GCTGAAAGACCC--TCAATGAAACAAGTAAT--TAGCACTAA
Prmy    -AGTACCGCAAGGGAAA-GCTGAAAAAGAAT-TGAAAGAA-T---AA-TTTAAGCACAAA
Loli    -AGTACCGCAAGGGAAC-GCTGAAAGAGAAA-TGAAAGAACCCCA-CG---ACGTTTAAA
Loam    AAGTACCGCAAGGGAAA-GCTGAAAGAGATA-TGAAAAAGCC---CA-GTGAAGTTTAGA
Chab    -AGTACCGCAAGGGAAC-ACTGAAAGAGAAA-TGAAATAACT---C-AGCAAAGTAATAA
Chto    -AGTACCGCAAGGGAAC-ACTGAAAGAGAAA-TGAAATAACT---C-AGCAAAGTAATAA
Majo    -AGTACCGCAAGGGAAA-GCTGAAAGAGAAG-TGAAATAAAT---CAGTTTAAGCATAAA
Hlst    -AGTACCGCAAGGGAAA-GATGAAAGAGTAA-TGAAACAACC----A-ACAAAGCAATGA
Clpe    -AGTACCGCAAGGGAAC-GCTGAAAGAGAAA-TGAAATA-ACCCAG-A---AAGTACTAA
Mlmr    -AGTACCGCAAGGGAAC-GCTGAAAGAGAAA-TGAAACAACC---CTGTAGAAGTAACAA
Crcr    AAGTACCGCAAGGGAAC-GCTGAAAGAGAAA-TGAAATAACC----ATCCAAAGTTACAA
Muce    AAGTACCGCAAGGGAAC-GCTGAAAGAGAAA-TGAAATAACC---AT-CCAAAGTTACAA
Bege    -AGTACCGCAAGGGAAC-GCTGAAAAAGAAA-TGAAACAACG---CA-GTAAGGTAACAT
Mela    AAGTACCGTAAGGGAAC-GCTGAAAGAGAAA-TGAAATAACT----CAGTAAAGCACAAC
Hats    -AGTACCGCAAGGGAAC-GCTGAAAGAGAAA-TGAAATAATT----CAGTAAAGCACAAC
Orla    -AGTACCGCAAGGGAAA-GCTGAAAGAGAAA-TGAAATAAAT---C-AGTCAAGAGAAGT
```

```
Cosa    -AGTACCGCAAGGGAAC-GCTGAAAAAGAAA-TGAAAAA---CCAGTA---AAGCAAAGA
Exsp    -AGTACCGCAAGGGAAC-GCTGAAAGAGAAA-TGAAACA-AACCAGTA---AAGCACTAA
Depa    AAGTACCGCAAGGGAAA-GCTGAAAAAGAAA-TGAAATAAAC---CA-TCAAAGCCAAAA
Rima    -AGTACCGCAAGGGAAC-GCTGAAAGAAAAA-TGAAACAAAT---TAGT-AAAGTATAAC
Fuol    -AGTACCGTAAGGGAAT-GCTGAAAGAGAAA-TGAAACAACC---CAGTC-AAGCACAGA
Gmaf    -AGTACCGCAAGGGAAC-GCTGAAAGAGAAA-TGAAAGAACC---CAG-TAAAGTAAAAA
Xeei    -AGTACCGCAAGGGAAC-GCTGAAAGAGAAA-TGAAAAA-ACCCAGTC---AAGCACCAA
Pros    -AGTACCGCAAGGGAAA-GCTGAAAGAGAAA-TGAAACAAAC---CATT-TTAAGCCCAA
Scmi    -AGTACCGCAAGGGAAA-GTTGAAAAAGAAA-TGAAATAAAC---CA-TTTAAGCCCTAA
Rolo    -AGTACCGCAAGGGAAA-GCTGAAAAAGAAA-TGAAATAACT---CATTC-AAGCTACAA
Cere    -AGTACCGCAAGGGAAA-GCTGAAAGAGAAA-TGAAATAAAT----CATTTAAGCCTTAA
Daga    -AGTACCGTAAGGGAAA-GCTGAAAAAGGAA-TGAAAGAGCC---C-ATTTAAGCTTCAA
Anco    -AGTACCGCAAGGGAAA-GCTGAAAAAGAAA-TGAAATAACC----CATTCAAGCCTAAA
Dmve    -AGTACCGCAAGGGAAA-GCTGAAATAGTAA-TGAAATAACCCAT-AT---AAGCCCCAA
Dmar    -AGTACCGCAAGGGAAA-GCTGAAAAAGAAA-TGAAACAACCCATCA-ATTAAGCCCAAA
Anka    -AGTACCGCAAGGGAAA-GCTGAAAAAGAAA-TGAAACAACC---CA-TTTAAGCCCAAA
Moja    -AGTACCGCAAGGGAAA-GCTGAAAAAGAAA-TGAAATA-ACCCATTA---AAGCTTAAA
Hoja    -AGTACCGCAAGGGAAA-GCTGAAAAAGAAA-TGAAACA-ACCCATTC---AAGCCTAAA
Bede    CAGTACCGCAAGGGAAC-GCTGAAAGAGAAA-TGAAACAAAC---CATTTTAAGCCCAAA
Besp    -AGTACCGCAAGGGAAC-GCTGAAAGAGAAA-TGAAACA-AACCATTTT--AAGCCCAAA
Mysp    -AGTACCGCAAGGGAAA-GCTGAAAAAGAAA-TGAAACA-ACCCATTC---AAGCCAAAA
Osja    TAGTACCGCAAGGGAAA-GCTGAAAAAGAAA-TGAAACAACC---CA-TTTAAGCTAAAA
Sgro    -AGTACCGCAAGGGAAA-GCTGAAAAAGAAA-TGAAATAAAC---C-ATTCAAGTCAAAA
Pzpa    -AGTACCGCAAGGGAAA-GCTGAAAGAAAAA-TGAAAGAAATC--CAAT--AAGCCTAAG
Zeja    -AGTACCGCAAGGGAAC-CCTGAAAAAGAAA-TGAAACAACTCAACACTACAAGAACCCA
Znne    -AGTACCGCAAGGGAAC-GCTGAAAGAAAAA-TGAAACAAAT---TA-AATAAGTGAGAC
Zefa    -AGTACCGCAAGGGAAC-GCTGAAAAAGAAA-TGAAATA-AATTAAAT---AAGCATATT
Acni    -AGTACCGCAAGGGAAC-GCTGAAAAAGAAG-TGAAACAAAT---CA-AATAAGCCTAAT
Ncrh    -AGTACCGCAAGGGAAC-GCTGAAAAAGAAG-TGAAACAAAT---CAAAT-AAGCCTAAT
Agca    -AGTACCGCAAGGGAAA-GCTGAAAGAGAAA-TGAAACAACCCAGTT----AAGCATAAA
Hydy    -AGTACCGCAAGGGAAT-GCTGAAAGAGAAG-TGAAACAACT---CA-TAAAAGCTCAGA
Gsac    -AGTACCGCAAGGGAAC-GCTGAAAGAGAAA-TGAAACAACCCAGAC----AAGCTAAGA
Pevo    -AGTACCGCAAGGGAAA-GCTGAAAGAGAAG-TGAAATAAAT---AA--TAAAGCCACAC
Hiku    -AGTACCGCAAGGGAAA-GCTGAAAGAGAAA-TTAAACA-AGTAATTT---AAGTATATA
Inpa    -AGTACCGCAAGGGAAC-GCTGAAAAAGAAA-TGAAAAAACC----CAGAAAAGTGTAAA
Auch    CAGTACCGCAAGGGAAAAGCTGAAAGAAAAA-TGAAATAC-T---CAACTCAAGCACCAA
Fico    -AGTACCGCAAGGGAAA-GCTGAAAGAGAAG-TGAAACA-ACTCACACG--A-GCAAAGA
Macs    -AGTACCGCAAGGGAAA-GCTGAAAGAGAAG-TGAAACAACT----CAGCAAAGCAATAA
Moal    -AGTACCGCAAGGGAAA-GCTGAAAAAGAAA-TGAAACAAATCAGTC----AAGCAAAAA
Syma    AAGTACCGCAAGGGAAC-GCTGAAAAAGAAA-TGAAAAAAAT----CAGTTAAGTTAAAA
Mafr    -AGTACCGCAAGGGAAC-AATGAAAAAGAAA-TGAAACA-ACTCAACA---AAGTACAAA
Dcpe    -AGTACCGCAAGGGAAT-GCTGAAAAAGAAA-TGAAATAACT---CATTACAAGCCCTAA
Dcti    -AGTACCGCAAGGGAAT-GCTGAAAAAGAAG-TGAAACAACT---CATTACAAGCCCTAA
Hehi    AAGTACCGCAAGGGATT-GCTGAAAGAGAAA-TGAAAGAACC---CA-GTAAAGCCAAGT
Stam    -AGTACCGCAAGGGAAC-GCTGAAAGAGAAA-TGAAAAAACCCAGTAA----AGCTTAAA
Hogi    -AGTACCGCAAGGGAAC-GCTGAAAAAGAAA-TGAAATAACC---CA-GTAAAACTTTAA
Erzo    -AGTACCGCAAGGGAAA-GCTGAAAAAGAAG-TGAAAGAAATCAGTA----AAGCCTAGA
Hxot    -AGTACCGCAAGGGAAC-GCTGAAAGAGAAG-TGAAACAACCCAGTA----AAGCCTAAA
Core    -AGTACCGCAAGGGAAT-GCTGAAAGAGAAA-TGAAACAAGC---CAGT-GAAGCCTAAG
Apve    -AGTACCGCAAGGGAAC-GCTGAAAGAGAAA-TGAAACAACCCAG-TA---AAGCCTAAA
Latj    -AGTACCGCAAGGGAAC-GCTGAAATAGAGA-TGAAACAGCC----CAGTAAAGCCCTAA
Laja    -AGTACCGCAAGGGAAC-GCTGAAAAAGTAAATGAAAGAACC---CAGTA-AAGCATAGA
```

```
Syja    -AGTACCGCAAGGGAAC-GCTGAAAGAGAAA-TGAAAAACCC---AG-TTTAAGCCTAAA
Epme    -AGTACCGCAAGGGAAT-GCTGAAAAACTAAATGAAATAAAC----AAGTGAAGCCCAAA
Grse    AAGTACCGCAAGGGAAC-CCTGAAAGAGAAA-TGAAACAAGT----CAGTAAAGCCCCAA
Clja    -AGTACCGCAAGGGAAC-ATTGAAATAAAAA-TGAAACAAAT---ATAG-CAAGTTTTAA
Ogcy    -AGTACCGCAAGGGAAA-GCTGAAACAGAAA-TGAAACAACCCA-TAAC-CAAGCCCAAA
Plna    -AGTACCGCAAGGGAAT-GCTGAAAGAGAAA-TGAAATA-ACCCAGTT---AAGTCCAAA
Lema    -AGTACCGCAAGGGAAC-GCTGAAAGAGAAA-TGAAAGAACC---C-AGCAAAGCTACAA
Etzo    -AGTACCGCAAGGGAAC-GCTGAAAGAGAAA-TGAAATAACC---CAGT-GAAGCCTACA
Apse    -AGTACCGCAAGGGAAC-GCTGAAAGAGAAA-TGAAACAAAT---CAGT-AAAGCACAAA
Epde    -AGTACCGCAAGGGAAA-GCTGAAAGAGAAA-TGAAATAACCCAGATA---AAGCCTAGA
Slja    -AGTACCGCAAGGGAAC-GCTGAAAGAGAAG-TGAAACATACTCAC-----AAGCCCGAA
Bsja    -AGTACCGCAAGGGAAC-GCTGAAAAAGTAA-TGAAACA-ACCCAGTA---AAGCTTTAA
Ecna    -AGTACCGCAAGGGAAA-GCTGAAAGAGAGA-TGAAAAAGCC----CAGTAAAGCCTAAT
Cohi    CAGTACCGCAAGGGAAA-GCTGAAAGAGAGG-TGAAAAAGTC----CAATAAAGGTAAGA
Caar    -AGTACCGCAAGGGAAA-GCTGAAAGAGAAA-TGAAATA-AACCAGTA---AAGCTAAAA
Came    -AGTACCGTAAGGGAAA-GCTGAAAGAGAGA-TGAAACAGACCAGTAA----AGCCATAA
Mema    -AGTACCGCAAGGGAAC-GCTGAAAGAGAGA-TGAAACAGAC----CAGTAAAGCCTAAA
Lenu    -AGTACCGCAAGGGAAA-GCTGAAATAGAAA-TGAAAAA-ACCCAGTCCCGAAGCCCAAG
Brja    -AGTACCGTAAGGGAAC-GCTGAAAGAGAAA-TGAAACA-ACCCAGTA---AAGCCCAAA
Plma    -AGTACCGCAAGGGAAC-GCTGAAAGAGAAA-TGAAATAACCCAGTAT---AAGCCTTAA
Emst    -AGTACCGCAAGGGAAT-GCTGAAAGAGAAG-TGAAACAATC---CA-GTAAAGCCTAAA
Ptti    -AGTACCGCAAGGGAAA-GCTGAAAGAGAAA-TGAAACA-ACTCAGTA---AAGCATATA
Losu    -AGTACCGCAAGGGAAC-GCTGAAAAAGAAA-TGAAATA-ACCCAGTA---AAGCACAAA
Geoy    AAGTACCGCAAGGGAAT-GCTGAAAAAGAAA-TGAAATAAAC---CA-GTAAAGCTTGGA
Dipi    AAGTACCGCAAGGGAAT-GCTGAAAGAGAAA-TGAAACAACC---CA-GTAAAGCTGAAA
Pama    -AGTACCGCAAGGGAAC-GCTGAAAGAGGAG-TGAAACAACC----CAGTAAAGCCTAGA
Leob    -AGTACCGCAAGGGAAC-GCCGAAAGAGAAA-TGAAACA-ACCCAGTTA--AAGCCCAAA
Neba    -AGTACCGCAAGGGAAC-GCTGAAAGAGAAA-TGAAACAACTCAGTA----AAGCAAAAA
Pdpl    -AGTACCGCAAGGGAAT-GCTGAAAGAGAGA-TGAAAGAGAC---CAGT-TAAGCCATAA
Nimi    AAGTACCGCAAGGGAAT-GCTGAAAGAGAAA-TGAAACAAAC----CAGTAAAGCCCCAA
Uptr    -AGTACCGCAAGGGAAA-GCTGAAAGAGAAG-TGAAATAAAT---CAGTCTAAGCATAAT
Pesc    -AGTACCGCAAGGGAAT-GCTGAAAGAGAAG-TGAAAAAACC---C-AGTAAAGCAAATA
Baar    -AGTACCGCAAGGGAAC-GCTGAAAGAGAAA-TGAAAGAACC----CAGTAAAGCCTAGA
Moar    -AGTACCGCAAGGGAAC-GCTGAAAGAGAAA-TGAAACAAAC----CAGTAAAGCCTAGA
Toja    -AGTACCGCAAGGGAAA-GCTGAAAGAGTGA-TGAAACAGCC---C-AGTAAAGCCTAAA
Chau    -AGTACCGCAAGGGAAC-GCTGAAAGACACA-TGAAACAGGA----CAAT-AGGCCTAAA
Chse    -AGTACCGCAAGGGAAC-GTTGAAAGAGAAC-TGAAACAACC---CA-GTTAAGTCTAAA
Enar    -AGTACCGCAAGGGAAC-GCTGAAAGAGAAT-TGAAATAACC---CA-GTTAAGCAAAAA
Hpty    -AGTACCGCAAGGGAAC-GCTGAGAAGAGAAATGAAAAAACC---C-AGTAAAGCCTAGA
Nana    -AGTACCGCAAGGGAAC-GCTGAAAGAGAAA-TGAAATAAACC----AGTAAAGTATAAA
Mcst    -AGTACCGTAAGGGAAC-GCTGAAAGAGTAG-TGAAACAGCT---CA-GTAAAGCTTAGA
Rhox    -AGTACCGCAAGGGAAA-GCTGAAAGAGAGA-TGAAATA-GCCCAATA---AAGCCTAAA
Opfa    -AGTACCGCAAGGGAAC-GCTGAAAGAGCAAATGAAATAACC----CAGTGAAGCCTTAA
Paar    AAGTACCGCGAGGGAAC-GCTGAAAGAGAAA-TGAAATAACC---CA-GTAACGCTTAAC
Gozo    -AGTACCGCAAGGGAAC-GCTGAAAGAGAAA-TGAAACAACT----CAGTAAAGCCTAAG
Ackr    -AGTACCGCAAGGGAAC-GCTGAAAGAGAAA-TGAAACAACC---CAGT-TAAGTAATAT
Elev    AAGTACCGTAAGGGAAA-GTTGAAAGAGAAA-TGAAATAACC---CA-GTAAAGCCTAAA
Trdu    -AGTACCGCAAGGGAAA-GCTGAAAGAGTAA-TGAAATAACC---CAGT-AAAGCACTAA
Amoc    -AGTACCGCAAGGGAAA-GCTGAAAGAGAAA-TGAAACAACC----CAGTAAAGCCCAAA
Hame    -AGTACCGCAAGGGAAG-GCTGAAAGAGAAA-TGAAATA-ATCCAGAA---AAGCCTAAA
Chso    -AGTACCGCAAGGGAAA-GCTGAAAGAGAAA-TGAAACAACC----CAGTTAAGCTTAAA
Lyto    AAGTACCGCAAGGGAAC-GCTGAAAGAGAGA-TGAAAAAGAC---CA-GTGAAGCCTAAA
```

```
Encr      -AGTACCGCAAGGGAAT-GCTGAAAGAGAAA-TGAAACA-ACCCAGTG---AAGCCTAAG
Bvar      -AGTACCGCAAGGGAAA-GCTGAAAAAGGAA-TGAAACAACCCAGTA----AAGCCTTAA
Noco      -AGTACCGCAAGGGAAC-GCTGAAAGAGAAA-TGAAATAACC---CA-GTAAAGCACCAA
Chsp      -AGTACCGCAAGGGAAA-GCTGAAAAAGAAA-TGAAATAACT---CAAT-TAAGCCCTAA
Arja      -AGTACCGCAAGGGAAT-GCTGAAAGAGAAA-TGAAACA-AACCAGTA---AAGCCTAAA
Pase      -AGTACCGCAAGGGAAA-GCTGAAAGAGAAA-TGAAACAATG----CATTAAAGTAATAA
Trel      -AGTACCGCAAGGGAAT-GCTGAAAGAGAGA-TGACAAA-GGTCGTGT---AAGCATTAA
Lifa      -AGTACCGCAAGGGAAA-GCTGAAATAGCAG-TGAAACA-AGTCAGTG---AAGTAAAGA
Acur      -AGTACCGCAAGGGAAC-GCTGAAAGAGAAG-TGAAA-AACC----CAGTAAAGTCAAAA
Ampe      -AGTACCGCAAGGGAAC-GCTGAAAGAGAAG-TGAAATAACC---C-ATTAAAGCATAGA
Urja      -AGTACCGCAAGGGAAA-GCTGAAAAAGAAA-TGAAATAAAC---CAAGTTAAGCCTGAA
Enet      -AGTACCGCAAGGGAAA-GCTGAAAAAGAAA-TGAAACAAACCTCTA----AAGCGCGAG
Ptbr      AAGTACCGCAAGGGAAA-GCTGAAAAAGAAA-TGAAACAAAT---CA-GTAAAGCACATA
Safa      -AGTACCGCAAGGGAAT-GCTGAAATAGAAA-TGAAAAA-AATCAGTT---AAGCACAGA
Icae      -AGTACCGCAAGGGAAC-GCTGAAAGAGAAA-TGAAATAACC---C-AGTGAAGCCTAGA
Asmi      -AGTACCGCAAGGGAAT-GCTGAAATAGAAA-TGAAA-AACC---C-AGTTAAGTATAAT
Foal      -AGTACCGCAAGGGAAA-GTTGAAAAAGAAA-TGAAAAATTA-----AGCAACACCCCAA
Drze      -AGTACCGCAAGGGAAT-GCTGAAAGAATAA-TGAAAAATTT---A---TAAGGATAAAA
Rhas      -AGTACCGCAAGGGAAA-GCTGAAAGAGAAA-TGAAACAACC---AACT-GAAGGAGAAA
Elac      --GTACCGCAAGGGAAA-GCTGAAAGAGAAA-TGAAATAACC---CA-GTAAAGTACAAA
Kugu      -AGTACCGCAAGGGAAT-GCTGAAAGAGAGA-TGAAACAGCC---CA-GTAAAGCATTAA
Plor      -AGTACCGCAAGGGAAC-GCTGAAAGAGAAA-TGAAATAACC---C-AGTTAAGCTACAA
Sgun      AAGTACCGCAAGGGAAA-GCTGAAAGAGAAA-TGAAACAACC---CA-GTAAAGCACAAA
Zaco      -AGTACCGCAAGGGAAC-GCTGAAAGAGAAA-TGAAATAACC---CA-GTTAAACCCAAA
Zbfl      -AGTACCGCAAGGGAAC-GCTGAAAGAGAAA-TGAAACAACC---CAGTA-AAGCCAAAA
Spba      AAGTACCGCAAGGGAAC-GCTGAAAGAAAGA-TGAAACAGAT---TA-GTAAAGCACAAA
Game      -AGTACCGCAAGGGAAC-GCTGAAAGAGAAA-TGAAACAACC----CAGTGAAGCCTAAA
Thth      AAGTACCGCAAGGGAAC-GCTGAAAGAGAAA-TGAAACAACC---CA-GTAAAGCCTAAA
Xigl      -AGTACCGCAAGGGAAC-GCTGAAAGAGAGG-TGAAAAAGCCCAG-TA---AAGCCTAAA
Hyja      AAGTACCGCAAGGGAAT-GCTGAAAAAGAAA-TGAAATAATT----CAGTGAAGCCAAAA
Psan      -AGTACCGAAAGGGAAC-GCTGAAAAAGAAA-TGAAATAACC---CA-GTCAAGCTTAAA
Cupa      -AGTACCGCAAGGGAAC-GCTGAAAGAGAAA-TGAAATAACC---CAGT-AAAGCCTAAA
Mpch      -AGTACCGCAAGGGAAC-GTTGAAAGAGAAA-TGAAACAAAT---CA-GTAAAGTAAGAA
Char      AAGTACCGCAAGGGAAC-GCTGAAAGAGTAA-TGAAACAATC----CAGTAAAGTACAGA
Pser      -AGTACCGCAAGGGAAA-GCTGAAAGAGAGA-TGAAACA-GCCCAGTA---AAGTCTAAA
Prol      -AGTACCGCAAGGGAAA-GCTGAAAAAGAGA-TGAAATAGCC---C-AGTAAAGCTTAAT
Plbi      -AGTACCGCAAGGGAAA-GCTGAAAGAGAGA-TGAAAAAGCC---CAGT-AAAGCTTAAA
Calu      -AGTACCGCAAGGGAAG-GCTGAAAGAGCAA-TGAAACA-ACGC--TA---AAGTAAAGA
Papa      -AGTACCGCAAGGGAAA-GCTGAAAGAGAAA-TGAAAAGA-----CA-ACAAAGTACAAA
Sufr      -AGTACCGCAAGGGAAA-GCTGAAAGAGAAA-TGAAATAACC---CA-GTTAAGCCAGAA
Stci      -AGTACCGCAAGGGAAA-GCTGAAAAAGAAG-TGAAACAACC---CA-GTTAAGCGATAA
Taru      AAGTACCGCAAGGGAAC-GCTGAAAGAGAAA-TGAAATAACC----CAGTAAAGTAAAAA
Rala      -AGTGCCGCAAGGGAAT-GCTGAAAGGGAAG-TGAAACA-ACCCAGTA---AAGCCTTAA
            ** **   ***        ** *
```

```
                      5          5'                      6      HVR
Scca    AAA---GCA-G-AG-ATTAACCCTG-GTACC-TTTGGCATCATGATTTAATTAGAA--AAA
Muma    AAA---GCA-G-AG-ATTACACCTG-GTACC-TTTTGCATCATGATTTAGCTAGAA--AAA
Erca    ACA---GCA-G-AG-ATCAAATCTGGGTACC-TTTTGCATCATGATCTAGTAAGTT-A-TA
Pose    ACA---GCA-G-AG-ATAAAACCTG-GTACC-TTTTGCATCATGGTCTAGCAAGTC-AGAT
Actr    AAA---GCA-A-AG-ATTAAAACTT-GTACC-TTTTGCATCATGATTTAGCCAGTT-CTTA
Scal    AAA---GCA-A-AG-ATTAAAACTT-GTACC-TTTCGCATCATGATTTAGCCAGTT-CTTA
Posp    AAA---GCA-A-AG-ATTAAATCTT-GTACC-TTTTGCATCATGATTTAGCCAGTT-CTTG
Atsp    AAA---GCA-G-AG-ATTAAATCTG-GTACC-TTTTGCATCATGATTTAGCTAGTC-TA-C
Leoc    AAA---GCA-G-AG-ATTAAATCTG-GTACC-TTTTGCATCATGATTTAGCCAGT---CTA
Amca    AAA---GCA-A-AG-ATTAAATCTT-GTACC-TTTTGCATCATGATTTAGCCAGAC-ACAC
Osbi    AAA---GCA-G-AG-ATTAACACTG-GTACC-TTTTGCATCATGATCTAGCTAGCA-TAAA
Pabu    AAA---GCA-G-AG-ATTTAGCCTG-GTACC-TTTTGCATCATGACTTAGCCAGAA--TAC
Hial    GAA---GCA-G-AG-ATTAAACCTG-GTACC-TTTTGCATCATGATTTAGCCAGTA-AAAT
Elha    AAA---GCA-G-AG-ATTAAGTCTG-GTACC-TTTTGCATCATGACTTAGCTAGCA-CCAA
Mlcy    AAA---GCA-G-AG-ACGCAACCTG-GTACC-TTTTGCATCATGATTTAGCTAG-A-TCAA
Algl    AAA---GCA-G-AG-ACTAAGTCTG-GTACC-TTTTGCATCATGATTTAGTTAGTG--TAA
Ptgi    AAA---GCA-G-AG-ACTAAACCTG-GTACC-TTTTGCATCATGATTTAGCTAGTA-AGAT
Alaf    AAA---GCA-G-AG-ACTAAACCTG-GTACC-TTTTGCATCATGATTTAGTAAGTA-CAA-
Nock    AAA---GCA-G-AG-ATTAACACTG-GTACC-TTTTGCATCATGATTTAGCAAGTA-CAA-
Anja    AAA---GCA-G-AG-ACTAAAACTG-GTACC-TTTTGCATCATGGTTTAGCAAGTA-AAAA
Gyki    AAA---GCA-G-AG-ATTAGACCTG-GTACC-TTTTGCATCATGATCTAGCAAGAA-A-GC
Syka    AAA---GCA-G-AG-CTAAACCCTG-GTACC-TTTTGCATCATGATTTAGCAAGCA-CACA
Opma    AAA---GCA-G-AG-ATTAAAACTG-GTACC-TTTTGCATCATGATTTAGCAAGTA-TAAC
Comy    AAA---GCA-G-AG-ATTAAAACTG-GTACC-TTTTGCATCATGATTTAGCAAGCA-AAAC
Sasp    AAT---GCA-A-AG-ACTAAAACTT-GTACC-TTTTGCATCATGATTTAGCAAGTT-GAGT
Eupe    AAA---GCA-G-AG-ATTAAATCTG-GTACC-TTTTGCATCATGATTTAGCAAGTA--CAA
Enja    GAA---GCA-G-AG-ATCACCTCTG-GTACC-TTTTGCATCATGATTCAGCAAGTT-A-AC
Same    AAA---GCA-GTAG-ATTACACCTT-GTACC-TTTTGCATCATGATCTAGCCAGTA-AT-T
Chch    AAA---GCA-G-AG-ATTAAAACTG-GTACC-TTTTGCATCATGATCTAGCCAGCA-TAC-
Grgr    AAA---GCA-G-AG-ACTAGTCCTG-GTACC-TTTTGCATCATGATTTAGCAAGC--ACCC
Caau    AAA---GCA-A-AG-ATTAAACCTT-GTACC-TTTTGCATCATGATTTAGCCAGTA--CAC
Cyca    AAA---GCA-A-AG-ATTAAACCTG-GTACC-TTTTGCATCATGATTTAGCCAGTA-C-AC
Dare    AAA---GCA-A-AG-ATTAAACCTT-GTACC-TTTTGCATCATGATTTAGCCAACA-CC-A
Cost    AAA---GCA-G-AG-ATTAATGCTG-GTACC-TTTTGCATCATGATTTAGCAAGAA-ATCG
Leec    AAA---GCA-G-AG-CTCAAACCTG-GTACC-TTTTGCATCATGATTTAGCCAGTA-ATAA
Crla    AAA---GCA-G-AG-ACACAACCTG-GTACC-TTTTGCATCATGATTTAGCCAGAA--CAC
Clmc    AAA---GCA-G-AG-ACTAACCCTG-GTACC-TTTTGCATCATGATTTAGCCAGCA-GCCG
Phin    AAA---GCA-G-AG-ACTAACTCTG-GTACC-TTTTGCATCATGATTTAGCCAGCA-AA-A
Icpu    AAA---GCA-G-AG-ACTAGACCTG-GTACC-TTTTGCATCATGATTTAGCCAGCC-C--C
Psto    AAA---GCA-G-AG-ACTAAACCTG-GTACC-TTTTGCATCATGATTTAGCTAGC--CCTT
Cora    AAA---GCA-G-AG-ACTAAAACTG-GTACC-TTTTGCATCATGATTTAGCTAGT--TATT
Eisp    AAA---GCA-G-AG-ATACAACCTG-GTACC-TTTTGCATCATGATTTAGCCAGCA-CC-C
Apal    AAA---GCA-G-AG-ACACAACCTG-GTACC-TTTTGCATCATGATTTAGCCAGAA-CTAT
Eslu    AAA---GCA-G-AG-ACAGAACCTG-GTACC-TTTTGCATCATGATTTAGCTAGTA-A-AT
Dape    AAA---GCA-G-AG-ATAAAACCTG-GTACC-TTTTGCATCATGCTTTAGCTAGCA--AAC
Glse    AAA---GCA-G-AG-ATTACCCCTT-GTACC-TTTTGCATCATGATCTAGCCAGCA--AAC
Naar    AAA---GCA-G-AG-ATTAACCCTT-GTACC-TTTTGCATCATGATCTAGCCAGCA-AA-C
Baoc    AAA---GCA-G-AG-ATTAACCCTT-GTACC-TTTTGCATCATGATCTAGCCAGCA-A-AC
Opso    AAA---GCA-A-AG-ATTAACCCTT-GTACC-TTTTGCATCATGATTTAGCCAGCA-AA-C
Alte    AAA---GCA-G-AG-ATAAACCCTG-GTACC-TTTTGCATCATGATTTAGCCAGCA-TTAT
Plap    AAA---GCA-G-AG-ATCAGCCCTG-GTACC-TTTTGCATCATGATTTAGCCAGTA-CCAT
```

Plal	AAA---GCA-A-AG-ATTAAAGCTT-GTACC-TTTTGCATCATGATCTAGCCAGA--ATAC
Sami	AAA---GCA-A-AG-ATTTAAACTT-GTACC-TTTTGCATCATGATCTAGCCAGTA--AAC
Rere	GAA---GCA-A-AG-ATTAGCTCTT-GTACC-TTTTGCATCATGATCTAGCTAGAA--TAC
Gama	GTA---GCA-G-AG-CCTAGCTCTG-GTACC-TTTTGCATCATGATTTAGCCAGTA-TAGC
Onmy	GAA---GCA-G-AG-ATTAAATCTG-GTACC-TTTTGCATCATGATTTAGCCAGCA--CAC
Sasa	AAA---GCA-G-AG-ATTAAATCTG-GTACC-TTTTGCATCATGATTTAGCCAGCA--AAC
Cola	AAA---GCA-G-AG-ATTAAATCTG-GTACC-TTTTGCATCATGATTTAGCCAGCA-AAC-
Dita	GAA---ACA-G-AG-ACACAACCTG-GTACC-TTTTGCATCATGGTTTAGCCAGA-A-CAC
Gogr	ACA---GCA-G-AG-ATTAAACCTG-GTACC-TCTTGCATCATAATTTAGCCAGCA-CA-C
Chsl	AAA---ACA-G-AG-ATAAAGCCTG-GTACC-TTTTGCATCATGAATTAGCCAGAA-TA-C
Atja	AAA---GCA-G-AG-ATTAACACTG-GTACC-TTTTGCATCATGATTTAGCAAGTA-AT-C
Iido	AAA---GCA-G-AG-GCTAAACCTG-GTACC-TTTTGCATCATGATTTAGCAAGTA-AC-C
Auja	AAA---GCA-G-AG-CTAAACCCTG-GTACC-TTTTGCATCATGATTAAGCAAGTA--AAT
Chag	GAA---GCA-G-AG-ATTAACTCTG-GTACC-TTTTGCATCATGATTAAGCCAGTA-TA-C
Hami	AAA---GCA-A-AG-ACTAACACTT-GTTCC-TTTTGCATCATGATTGAGCTAGAA-AACT
Saun	AAA---GCA-A-AG-ATTAATACTT-GTACC-TTTTGCATCATGATTAAGCTAGTA-AACT
Nema	AAA---GCA-G-AG-ACCACCACTG-GTACC-TTTTGCATCATGACTTAGCTAGTA--TAT
Disp	AAA---GCA-G-AG-ATAACCCCTG-GTACC-TTTTGCATCATGATCTAGCCAGTA-T-GC
Myaf	AAA---GCA-G-AG-ATAACCCCTG-GTACC-TTTTGCATCATGATTTAGCAAGAA-CCCC
Lagu	GCA---GCA-G-AG-CTATCCCCTG-GTACC-TCTTGCATCATGATCTAGCCAGTC-ATAC
Trtr	ATA---GCA-G-AG-CTACACCCTG-GTACC-TTTTGCATCATGATCCAGCAAGTC--ATT
Zucr	GAA---GCA-A-AG-ATAACCCCTT-GTACC-TTTTGCATCATGATCAAGCAAGTC-A-TT
Pxja	AAA---GCA-G-AG-ATTAAAACTG-GTACC-TTTTGCATCATGATTTAGCCAGTA-AAAC
Pxlo	AAA---GCA-G-AG-ATTAAAACTG-GTACC-TTTTGCATCATGATTTAGCCAGTA-AAAC
Pctr	AAA---GCA-G-AG-ACTAGAACTG-GTACC-TTTTGCATCATGATTTAGTTAGAA-GCCC
Apsa	AAA---GCA-G-AG-ACTACACCTG-GTACC-TTTTGCATCATGAATTAACAAGAA-AACC
Cabe	AAA---GCA-G-AG-ATTACACCTG-GTACC-TTTTGCATTATGATTTAGCCAGAC-AAGC
Bzze	AAA---GCA-G-AG-ATCAACACTG-GTACC-TTTTGCATCATGTTTTAGCCAGTA-ATAT
Siim	AAA---GCA-G-AG-ATTCAAGCTG-GTACC-TTTTGCATCATGATTTAGCTAGCA-ACGC
Ctru	AAA---GCA-G-AG-CTTAATACTT-GTACC-TTTTGCATCATGATTTAGCTAGCA-ACAC
Dpbr	AAA---GCA-G-AG-ATTAATGCTG-GTACC-TTTTGCATCATGATTTAGCTAGCA-ACAT
Caki	ATA---GCA-G-AG-ATTTCCCCTG-GTACC-TTTTGCATCATGATTTAGCAAGTA--AAA
Phja	GAA---GCA-A-AG-CTTATAGCTT-GTACC-TTTTGCATCATGATTTAGCAAGTA-AAAC
Brsp	ATA---GAA-G-AG-ATTAACCCTG-GTTCC-TCTTGCATCATGATTTAACTAG-A-AACA
Gamo	GCA---GCA-G-AG-TTTTCTACTG-GTACC-TTTTGCATCATGATTTAGCAAGAA-AACT
Lolo	ACA---GCA-G-AG-TTTACCACTG-GTACC-TTTTGCATCATGATTTAGCAAGTA-AACT
Batr	AAA---GCA-A-AG-TTAAGACCTT-GTACC-AAAAGCATAATGGATTAGCTTGCC--ACA
Prmy	AAA---GCA-G-GC-ATTAAACCCG-GTACC-TTTTGCATCATGGATTAGCTAG---TCAA
Loli	AAA---GCA-G-AG-ATTCAA-CTG-GTACC-TTTTGCATCATGTTTTAACCAGCA-ACAC
Loam	AAA---GCA-G-AG-ATTAAA-CTG-GTACC-TTTTGCATCATGTTTTAGCCAGTA-ACAC
Chab	AAA---GCA-G-AG-ATTTTAACTG-GTACC-TTTTGCATCATGGTTTAGCCAGTA-CC-C
Chto	AAA---GCA-G-AG-ATTTTAACTG-GTACC-TTTTGCATCATGGTTTAGCCAGTA-CC-C
Majo	AAA---GCA-G-AG-ATTAAACCTG-GTACC-TTTTGCATCAGGCTTTAGCTAGTA-CCTA
Hlst	AAA---GCA-G-AG-AAAAC-CCTG-GTACC-TTTTGTATCATGATTTAGCTAGTA-AA-C
Clpe	AAA---GCA-G-AG-CTTTCACCTG-GTACC-TTTTGCATCATGATTTAGCCAGAA-CTCT
Mlmr	GAA---GCA-G-AG-ATTTTACCTG-GTACC-TTTTGCATCATGATTTAGCCAGCA-AA-C
Crcr	AAA---GCA-G-AG-CTTATCCCTG-GTACC-TTTTGCATCATGATTTAGCTAGTA-ATAG
Muce	AAA---GCA-G-AG-CTTATCCCTG-GTACC-TTTTGCATCATGATTTAGCTAGTA-ATAG
Bege	AAA---GCA-G-AG-CTATTTCCTG-GTACC-TTTTGCATCATGTTTTAGCCAGAG-CCCC
Mela	AAA---GCA-G-AG-CTCACCCCTG-GTACC-TTTTGCATCATGTTTTAGCCAGAG-CCCC
Hats	AAA---GCA-G-AG-TTTTATCCTG-GTACC-TTTTGCATCATGTTTTAGCTAGAG-CCAC
Orla	AAA---GCA-G-AG-TTTAACCCTT-GTACC-TTTTGCATCATGAATTAGCCAGTT-TA-A

```
Cosa    AAA---GCA-G-AG-ACCAAAACTG-GTACC-TTTTGCATCATGAATTAGCCAGTA-ACCT
Exsp    AAA---GCA-G-AG-ATTAAAGCTG-GTACC-TTTTGCATCATGAATTAGCCAGTA-ATAC
Depa    AAA---GCA-G-AG-CTACAACCTG-GTACC-TTTTGCATCATGAATTAACTAGTA-AAAC
Rima    AAA---GCA-G-AG-CTTAACCCTG-GTACC-TTTTGCATCATGCCTTAGTGAG-TT-AAC
Fuol    AAA---GCA-G-AG-ATTAATTCTG-GTACC-TTTTGCATCATGATTTAGCAAGT--TTAC
Gmaf    GAA---GCA-G-AG-ACCAACCCTG-GTACC-TTTTGCATCATGATTTAGCTAGTC-CTAG
Xeei    AAA---GCA-G-AG-ACTAACCCTG-GTACC-TTTTGCATCATGATTTAGCAAGT--CACT
Pros    AAA--AGCA-G-AG-ATCCAAACTG-GTACC-TTTTGCATCATGACTTAGCCAGTA-CTAT
Scmi    AAA---GCA-G-AG-ATATAGCCTG-GTACC-TTTTGCATCATGACTTAGCCAGCA-CTAT
Rolo    AAA---GCA-G-AG-ATTTAAACTG-GTACC-TTTTGCATCATGATTTAGCTAGTA-TCAC
Cere    AAA---GCA-G-AG-ATTAAAACTG-GTACC-TTTTGCATCATGAATTAGCTAG-C-ACAC
Daga    GAA---GCA-G-AG-ATCTAAACTG-GTACC-TTTTGCATCATGAATTAGCTAGT--CCAC
Anco    AAA---GCA-G-AG-ATTTAACCTG-GTACC-TTTTGCATCATGATTTAGCTAGTA-CC-C
Dmve    AAA---GCA-G-AG-ATTTAAGCTG-GTACC-TTTTGCATCATGATTTAGCCAG-T-ACCC
Dmar    AAA---GCA-G-AG-AATCAAACTG-GTACC-TTTTGCATCATGATTTAGCTAGTA-CCCC
Anka    AAA---GCA-G-AG-ATTCAAACTG-GTACC-TTTTGCATCATGATTTAGCTAGTA-CC-C
Moja    AAA---GCA-G-AG-ATTCAAACTG-GTACC-TTTTGCATCATGATTTAGCTAGTA-CCAC
Hoja    AAA---GCA-G-AG-ATTTAAACTG-GTACC-TTTTGCATCATGATTTAGCTAGTA--CCC
Bede    AAA---GCA-G-AG-ATCTAAACTG-GTACC-TTTTGCATCATGACTTAGCCAGTA-CTAT
Besp    AAA---GCA-G-AG-ATTTAAACTG-GTACC-TTTTGCATCATGACTTAGCCAGTA-CTAT
Mysp    AAA---GTA-G-AG-ATTTAAACTG-GTACC-TTTTGCATCATGTTTTAGCCAGCA-AAAC
Osja    GAA---GTA-A-AG-ATTTAAACTT-GTACC-TTTTGCATCATGTTTTAGCCAGCA-CCCC
Sgro    AAA---GCA-G-AG-ATTAACACTG-GTACC-TTTTGCATCATGTTTTAGCCAGTA-CTAC
Pzpa    ATA---GCA-G-AG-CTTTACCCTG-GTACC-TTTTGCATCATGATTTAGCAAGCA-AAAC
Zeja    ATA---GCA-G-AG-ACTCCC-CTG-GTACC-TTTTGCATCATGATTTAGCAAGTA-AAGC
Znne    ATA---GCA-G-AG-ATTTCCCCTG-GTACC-TTTTGCATCATGATTTAGCAAGTA-AAGC
Zefa    ATA---GCA-G-AG-ACCCACCCTG-GTACC-TTTTGCATCATGATTTAGCAAGTA-AGAC
Acni    ATA---GCA-G-AG-ACCCCAACTG-GTACC-TTTTGCATCATGATTTAGCAAGTA-AAAC
Ncrh    ATA---GCA-G-AG-ACCCCAACTG-GTACC-TTTTGCATCATGATTTAGCAAGTA-AGAC
Agca    AAA---GCA-G-AG-CTTCCACCTG-GTACC-TTTTGCATCATGATTTAGCCAGTA-TCC-
Hydy    AAA---GCA-G-AG-ATTATAACTG-GTACC-TTTTGCATCATGATTTAGCAAGTG-TAAC
Gsac    GAA---GCA-G-AG-ATTTAACCTG-GTACC-TTTTGCATCATGATTTAGCAAGTG-TAAC
Pevo    AAA---GCA-G-AG-ATTCCCCCTG-GTACC-TTTTGCATCATGAATCAGCCAGTA-AAC-
Hiku    ATA---GCA-A-AG-ATTAAATCTT-GTACC-TTTTGCATCATGGTTTAACAAGA--TACC
Inpa    AAA---GCA-G-AG-ACTAAAACTG-GTACC-TTTTGCATCATGATTTAGCAAGCA-C-CT
Auch    AAA---GCA-A-AG-CTTAAAGCTT-GTACC-TTTTGCATCATGGTCTAGCCAGCA-TTAC
Fico    AAA---GCA-G-AG-ATTACCCCTG-GTACC-TTTTGCATCATGATTTAGCCAGAA-CAAC
Macs    AAA---GCA-G-AG-AATAAACCTG-GTACC-TTTTGCATCATGATTTAGCTAGAA-CAAC
Moal    AAA---GCA-G-AG-ACTCATCCTG-GTACC-TTTTGCATCATGAATTAGTTAGAC-ACC-
Syma    AAA---GCA-G-AG-ATTATACCTG-GTACC-TTTTGCATCATGATTTAGCAAGCC-CCC-
Mafr    AAA---GCA-G-AG-ACTTAAACTG-GTACC-TTTTGCATCATGATTTAGCAAGTA--CCC
Dcpe    AAA---GCA-G-AG-ATTATTACTG-GTACC-TTTTGCATCATGATTTAGCCAGTA-CCCC
Dcti    GAA---GCA-G-AG-TTTATTGCTG-GTACC-TTTTGCATCATGATTTAGCCAGTA-CCTC
Hehi    AAA---GCA-G-AG-ATTTACCCTG-GTACC-TTTTGCATCATGATTTAGCTAGCG-TGAC
Stam    AAA---GCA-G-AG-GCTAACCCTG-GTACC-TTTTGCATCATGATTTAGCCAGTG-TTGA
Hogi    AAA---GCA-G-AG-ATTAACCCTG-GTACC-TTTTGCATCATGATTTAGCCAGTG-TAAC
Erzo    GAA---GCA-G-AG-ATTAAAGCTG-GTACC-TTTTGCATCATGATTTAGTGAGTG-TACA
Hxot    AAA---GCA-G-AG-ATCTAAACTG-GTACC-TTTTGCATCATGATTTAGCAAGTG-TAAC
Core    AAA---GCA-G-AG-ATTAAAGCTG-GTACC-TTTTGCATCATGATTTAGCAAGTG-TACC
Apve    AAA---GCA-G-AG-ATAAAACCTG-GTACC-TTTTGCATCATGATTTAGCAAGTG-TACA
Latj    AAA---GCA-G-AG-ATTAGACCTG-GTACC-TTTTGCATCATGACTTAGCTAGTA-CC-C
Laja    AAA---GCA-G-AG-ATTTAAACTG-GTACC-TTTTGCATCATGATTTAGCTAGTA-AACC
```

```
Syja    AAA---GCA-G-AG-ACTTTACCTG-GTACC-TTTTGCATCATGATTTAGCAAGTA-ATCT
Epme    AAA---GCA-G-AG-ACATTACCTG-GTACC-TTTTGCATCATGATTTAGCCAGTG-CAAC
Grse    GAA---GCA-G-AG-ATTTCACCTG-GTACC-TTTTGCATCATGATTTAGCCAGAG-AAAA
Clja    AAA---GCA-G-AG-ACTAAACCTG-GTACC-TTTTGCATCATGATTTAGCAAGT-A-AAA
Ogcy    AAA---GCA-G-AG-ACCCACCCTG-GTACC-TTTTGCATCATGATTTAGCCAGTA-ACCC
Plna    AAA---GCA-G-AG-ACTAACCCTG-GTACC-TTTTGCATCATGCTTTAGCCAGTA-CTAA
Lema    AAA---GCA-G-AG-ATTTTTCCTG-GTACC-TTTTGCATCATGATTTAGCCAGAAATTAC
Etzo    AAA---GCA-G-AG-ATATAACCTG-GTACC-TTTTGCATCATGATTTAGCCAGCG-TAAC
Apse    AAA---GTA-G-AG-TTTAATCCTG-GTACC-TTTTGCATCATGATTTAGCAAGTA-GCAG
Epde    AAA---GCA-G-AG-ATTATAGCTG-GTACC-TTTTGCATCATGATTTAGCCAGTA-AAAT
Slja    ACA---GCA-G-AG-ATTCTCCCTG-GTACC-TTTTGCATCATGATTTAGCTAGTA-AACC
Bsja    AAA---GCA-G-AG-ATTCCAACTT-GTACC-TTTTGCATCATGATTTAGCCAGTA-AACC
Ecna    AAA---GCA-G-AG-ATATACCCTG-GTACC-TTTTGCATCATGAATTAGCCAGTA-AA-A
Cohi    AAA---GCA-G-AG-ATTACCCCTG-GTACC-TTTTGCATCATGATTTAGCTAG-C-AACA
Caar    AAA---GCA-G-AG-ATTTTCCCTG-GTACC-TTTTGCATCATGATTTAGCCAGTA-C-CT
Came    AAA---GCA-G-AG-ATTACTCCTG-GTACC-TTTTGCATCATGATTTAGCCAGCA--CAT
Mema    AAA---GCA-G-AG-ATTAAACCTG-GTACC-TTTTGCATCATGATTTAGCCAGCA--CCC
Lenu    AAA---GCA-A-AG-ATTAATCCTT-GTACC-TTTTGCATCATGATTTAGCTAGCA--TAA
Brja    AAA---GCA-G-AG-ATCCCCCCTG-GTACC-TTTTGCATCATGATTTAGCCAGTA-CCCC
Plma    AAA---GCA-G-AG-ATTTAACCTG-GTACC-TTTTGCATCATGATTTAGCCAGTA-ATAC
Emst    AAA---GCA-G-AG-ATTTTAACTG-GTACC-TTTTGCATCATGATTTAGCCAGAA-AAAT
Ptti    AAA---GCA-G-AG-ATATAGCCTG-GTACC-TTTTGCATCATGACTTAGCCAGTA--ACC
Losu    AAA---GCA-G-AG-ATTAACACTG-GTACC-TTTTGCATCATGATTTAGCCAGTA-AAAC
Geoy    AAA---GCA-G-AG-ACTCACCCTG-GTACC-TTTTGCATCATGATTTAGCCAGTA-ACCA
Dipi    AAA---GCA-G-AG-ATTTTAACTG-GTACC-TTTTGCATCATGATTTAGCAAGTA-CA-C
Pama    AAA---GCA-G-AG-ATCAAAACTG-GTACC-TTTTGCATCATGATTTAGCCAGTA-AC-C
Leob    AAA---GCA-G-AG-ATAATTCCTG-GTACC-TTTTGCATCATGATTTAGCAAGTA-CC-C
Neba    AAA---GCA-G-AG-ATTCGCCCTG-GTACC-TTTTGCATCATGATTTAGCCAGTA-ACAC
Pdpl    AAA---GCA-G-AG-ATAAACCCTT-GTACC-TTTTGCATCATGATTTAGCTAGTA-CAA-
Nimi    AAA---GCA-G-AG-ATTTACACTG-GTACCCTTTTGCATCATGAGTTAGCCAGAA-AATC
Uptr    AAA---GCA-G-AG-ATTAAAACTG-GTACC-TTTTGCATCAGGCTTTAGCTAGTA-CCCA
Pesc    AAA---GCA-G-AG-CTACCCCCTG-GTACC-TTTTGCATCATGAATTAGCCAGTA-CTAC
Baar    AAA---GCA-G-AG-ATTTCAACTG-GTACC-TTTTGCATCATGATTTAGCCAGCA-AAAC
Moar    AAA---GCA-G-AG-ATTTTAACTG-GTACC-TTTTGCATCATGATTTAGCCAGAA-AAAG
Toja    AAA---GCA-G-AG-ATCAAAACTG-GTACC-TTTTGCATCATGATTTAGCCAG-A-ACAT
Chau    AAA---GCA-G-AG-ATTTAAACTG-GTACC-TTTTGCATCATGATACAGCCAGAA-AACT
Chse    AAA---GCA-G-AG-ATTCTAGCTG-GTACC-TTTTGCATCATGAATTAGCCAGTA-AAGT
Enar    AAA---GCA-G-AG-ATTTTTACTG-GTACC-TTTTGCATCATGTTTTAGCTAG-A-CAAC
Hpty    GAA---GCA-G-AG-ATTTTAGCTG-GTACC-TTTTGCATCATGATTTGGCTAGCA-ATAC
Nana    AAA---GCA-G-AG-ATTTGAGCTG-GTACC-TTTTGCATCATGATTTAGCCAGCA-AA-T
Mcst    AAA---GCA-G-AG-ATTCAGGCTG-GTACC-TTTTGCATCATGATTTAGCAAGAA-CTAT
Rhox    AAA---GCA-G-AG-ATTCAACCTG-GTACC-TTTTGCATCATGATTTAGCAAGTA-ACCC
Opfa    AAA---GCA-G-AG-ATTCAACCTG-GTACC-TTTTGCATCATGATTTAGTTAGTA-ATAA
Paar    AAA---GCA-G-AG-ACTTCACCTG-GTACC-TTTTGCATCATGATCTAGCCAGAA-ACCC
Gozo    AAA---GCA-G-AG-ATTCTAACTG-GTACC-TTTTGCATCATGATTTAACTAGAA-TACC
Ackr    AAA---GCA-G-AG-ACCCTC-CTG-GTACC-TTTTGCATCATGATTTAGCCAGTA-AA-C
Elev    AAA---GCA-G-AG-CATAGTGCTG-GTACC-TTTTGCATCATGTTTTAGCTAGAA-ATAT
Trdu    AAA---GCA-G-AG-ATTTCCCCTG-GTACC-TTTTGCATCATGATTTAGCCAGAA-AGCC
Amoc    AAA---GCA-G-AG-ACACAACCTG-GTACC-TTTTGCATCATGATTTAGCTAGTA-TACC
Hame    AAA---GCA-A-AG-ACTAACCCTT-GTACC-TCTTGCATCATGATTTAGCTAGCA-ATAA
Chso    AAA---GCA-G-AG-ATTAAAGCTG-GTACC-TTTTGCATCATGAACTAGCGAGA--ATAA
Lyto    AAA---GCA-G-AG-ATTAAAGCTG-GTACC-TTTTGCATCATGATTTAGCAAGTG-TAAC
```

```
Encr      AAA---GCA-G-AG-ATCAAAACTG-GTACC-TTTTGCATCATGATTTAGCAAGTG-TAAC
Bvar      GAA---GCA-G-AG-ATAAAACCTG-GTCCC-TTTTGCATCATGATCTAGCTAGTG-AAAC
Noco      AAA---GCA-G-AG-TTAAACCCTG-GTACC-TTTTGCATCATGATTTAGCCAGTG-ATTT
Chsp      AAA---GCA-G-GC-ATAACCCCCC-GTACC-TTTTGCATCATGAATTAATGAGAA-CACT
Arja      AAA---GCA-G-AG-ATTATGCCTG-GTACC-TTTTGCATCATGATTTAGCAAGTG-TACC
Pase      AAA---GCA-G-AG-ATACACTCTG-GTACC-TTTTGCATCATGATTCAGCAAGCA-ACCA
Trel      AAA---GCA-G-AG-AGCC--TCTG-GTACC-TTTTGCATCATGATTTAGCCAGAA-CAAG
Lifa      AAA---GCA-G-AG-ATGAAACCTG-GTACC-TTTTGCATCATGATTTAGCTAG-A-ACGA
Acur      GAA---GCA-A-AG-TTTAAGCCTT-GTACC-TTTTGCATCATGGTTTAGCAAGAA-ATAT
Ampe      AAA---GCA-G-AG-ATTTAAACTG-GTACC-TTTTGCATCATGATTTAGCTAGAA-AGCC
Urja      AAA---GCA-A-AG-ACTAACTCTT-GTACC-TTTTGCATCATGATTTAGCCAGCA-CC-C
Enet      AAA---GCA-A-AG-ATCAGCCCTT-GTACC-TTTTGCATCATGAATTAGCCAGAAATTAA
Ptbr      AAA---GCA-A-AG-ATTAAATCTT-GTACC-TTTTGCATCATGGTTTAGCTAGTC-AGAA
Safa      AAA---GCA-G-AG-ACTTACTCTG-GTACC-TTTTGCATCATGATTTAGCGAGCC-ATCT
Icae      AAA---GCA-G-AG-ATTTTACCTG-GTACC-TTTTGCATCATGATTTAGCTAGTA-CTTC
Asmi      AAA---ACA-G-AG-ATTTATCCTG-GTACC-TTTTGCATCATGATTTAGTGAGCA--T-C
Foal      AAA---GCA-G-AG-CTTAGTTCTT-GTACC-TTTTGCATCATGTTTTAGTAAGCG-TAAA
Drze      AAA---GCA-G-AG-ACTAATCCTG-GTACC-TTTTGCATCATGATTTAGCAAGCA-ATAT
Rhas      AAA---GCA-G-AG-ATTAATACTG-GTACC-TTTTGCATCATGACTTAGCCAGCA-AACC
Elac      AAA---GCA-G-AG-CTTAAGCCTG-GTACC-TTTTGCATCATGACCTAGCCAGTA-AGCC
Kugu      AAA---GTA-G-AG-ACCAAACCTG-GTACC-TTTTGCATCATGATTTAGCCAGTA-ACCC
Plor      AAA---GCA-G-AG-ATCACACCTG-GTACC-TTTTGCATCATGATTTAGCTAGAAAAC-C
Sgun      AAA---GCA-G-AG-AT-ACCCCCTCGTACC-TTTTGCATCATGATTTAGCTAGAA-AGCC
Zaco      GAA---GCA-G-AG-ATTAGAACTG-GTACC-TTTTGCATCATGATTTAGCCAGTA-CTCA
Zbfl      AAA---GTA-G-AG-ATTCAACCTG-GTACC-TTTTGCATCATGATTTAGCTAGAA-AAGT
Spba      AAA---GCA-G-AG-ATTTAACCTG-GTACC-TTTTGCATCATGATTTAGCCAGTA--TCC
Game      AAA---GCA-G-AG-ATTACACCTG-GTACC-TTTTGCATCATGATTTAGCTAGTA-TTAC
Thth      AAA---GCA-G-AG-ATTTTACCTG-GTACC-TTTTGCATCATGATTTAGCTAGTA-CTAT
Xigl      AAA---GCA-G-AG-ATTTGACCTG-GTACC-TTTTGCATCATGATTTAGCCAGCA-CC-C
Hyja      AAA---GCA-G-AG-ATTTCACCTG-GTACC-TTTTGCATCATGATTTAGCTAGTA-CCCC
Psan      AAA---GCA-G-AG-ATAATACCTG-GTACC-TTTTGCATCATGATTCAGCTAGTA-ATTC
Cupa      AAA---GCA-G-AG-ATTTTACCTG-GTACC-TTTTGCATCATGATTTAGCTAGTA-TTAC
Mpch      AAA---GCA-G-AG-ACTAATCCTG-GTACC-TTTTGCATCATGATTTAGCTAGAA-AATT
Char      AAA---GCA-G-AG-ATTAACCCTG-GTACC-TTTTGCATCATGATTTAGCCAGTA--AGC
Pser      AAA---GCA-G-AG-ATTACCTCTT-GTACC-TTTTGCATCATGATTTAGCCAG-A-AACC
Prol      AAA---GCA-G-AG-ATTAGAACTG-GTACC-TTTTGCATCATGATTTAGCTAGCA-CT-T
Plbi      GAA---GCA-G-AG-CTTAAAGCTG-GTACC-TTTTGCATCATGATTTAGCTAGC--ACTT
Calu      ACA---GCA-G-AG-CTTACCCCTG-GTACC-TTTTGCATCATGAGTTAGCTAGTA--TAC
Papa      AAA---GCA-G-AG-ACTAACCCTG-GTACC-TTTTGCATCATGATTTAGCAAGTA-TA-A
Sufr      AAA---GCA-G-AG-ATCTACCCTG-GTACC-TTTTGCATCATGATTTAGCCAGTA--CCC
Stci      TAA---GCA-G-AG-ATTACACCTG-GTACC-TTTTGCATCATGATTTAGCTAGCA-CCTC
Taru      AAA---GCA-G-AG-ATTACACCTG-GTACC-TTTTGCATCATGATTTAGCTAGTA--TAA
Rala      AAA---GCA-G-AG-ATGAAACCTG-GTACC-TTTTGCATCATGATTTAGCTAGTA--TTC
                  *                  * **     * ** *
```

```
           7      8      8'     7'        9       10                 11
Scca  CTAGGCAAAGAGACC-TTAAGTCTACCCTCCCGAAACTAA-AC---GAGCTACTCCGAAG-C
Muma  CTAGGCGAAAAGATC-TTAAGTCTATCCTCCCGAAACTAA-AC---GAGCTACTCCGAAG-C
Erca  CCAAGCAAAATGAAT-TGTAGTTTGATACCCCGAAACTAG-AC---GAGCTACTTCGAAG-C
Pose  CCAAGCAAAATGATT-TATAGTTTGACCCCCCGAAACTAG-AC---GAGCTACTTCGAAG-C
Actr  TCAGGCAAAGAGAAC-TTTAGTCTGACCCCCCGAAACTAG-AC---GAGCTACTCCGAGA-C
Scal  TCAGGCAAAGAGAAC-TTTAGTCTGACCCCCCGAAACTAG-AC---GAGCTACTCCGAGA-C
Posp  TCAGGCAAAGAGAAC-TTTAGTCTGACCCCCCGAAACTAG-AC---GAGCTACTCCGAGA-C
Atsp  CCAGGCAAAGAGAAC-TTAAGTCTGCCTTCCCGAAACTAG-AC---GAGCTACTCCGAGA-C
Leoc  CCAGGCAAAGAGAAC-TTAAGTCTGCCTTCCCGAAACTAG-AC---GAGCTACTCCGAGA-C
Amca  CCAGGCAAAAAGAAT-TTTAGTCTGTGCCCCCGAAACTAA-AC---GAGCTACTTCGAGA-C
Osbi  TCAGGCAAAGAGGCC-TTCAGTCTGAGCCCCCGAAACTAG-AT---GAGCTACTTCAAGA-C
Pabu  TTAAGCAAAGAGAAC-TTTAGTTTAATTTCCCGAAACTAA-AC---GAGCTACTCCGAGA-C
Hial  TCAGGCAAAGAGCAC-TTAAGTCTGACACCCCGAAACTAA-AC---GAGCTACTCCGAGA-C
Elha  CCAGGCAAAGAGAAC-TTTAGTCTGGCCCCCCGAAACTAG-AC---GAGCTACTCCGGGA-C
Mlcy  CCAGGCAAAGAGACC-TACAGTCTGACACCCCGAAACTAA-AC---GAGCTACTTCGAGA-C
Algl  TCGAGCAAAGTGGTT-TTTAGTTCGAACTCCCGAAACCGA-GC---GAGCTACTCCGGAG-C
Ptgi  TCAAGCAAAGAGAAC-TTTAGTTTGAAACCCCGAAACTAG-AC---GAGCTACTCCGGGA-C
Alaf  TCAAGCGAAGAGAAC-TTTAGTTTGAAACCCCGAAACTAG-AC---GAGCTACTCCGGGG-C
Nock  TCAAGCAAAGAGAATTTTTAGTTTGAAACCCCGAAACTAG-AC---GAGCTACTCCGGGG-C
Anja  TCAAGCAAAGAGAAC-TTTAGTTTGAAACCCCGAAACTAG-AC---GAGCTACTCCGGGG-C
Gyki  CCAAGCGAAGAGAAC-TTAAGTTTGACCCCCCGAAACCAG-GA---GAGCTACTTCGGAG-C
Syka  TCAAGCAAAGAGAAC-TTCAGTTTGAAACCCCGAAACTAG-AC---GAGCTACTCCGAGG-C
Opma  CCAAGCAGAAAGAAT-CTTAGTTTGGAACCCCGAAACTAG-AC---GAGCTACTCCGGAG-C
Comy  TCAGGCAAGAAGCAT-CATAGTCTGAGACCCCGAAACTAG-AC---GAGCTACTCCGAAG-C
Sasp  TCAGGCGAAGGGAAC-TTTAGTCTGAAGCCCCGAAACTAG-GC---GAGCTACTTCGGGG-C
Eupe  TCAAGCAAAGAGAAC-TGTAGTTTGAAACCCCGAAACTAG-AC---GAGCTACTCCGGGG-C
Enja  TCAAGCAAAGAGCAC-TCTAGTTTGCACCCCCGAAACTTG-AC---GAGCTACTCCGAGA-C
Same  TCAAGCAAAGAGACC-TCTAGTTTGAACCCCCGAAACCGG-AC---GAGCTACTCCGGGG-C
Chch  CCAAGCAAAGAGAAC-TGTAGTTTGACTCCCCGAAACTAA-GT---GAGCTACTCCGAGA-C
Grgr  TTGAGCGAAGGGCAC-TATAGTTCAGGACCCCGAAACCAA-GC---GAGCTACTCCGAGA-C
Caau  CCAAGCAAAGAGACC-TTTAGTTTGAAACCCCGAAACCAG-GT---GAGCTACCCCGAGA-C
Cyca  CCAAGCAAAGAGACC-TTTAGTTTGAAACCCCGAAACCAG-GT---GAGCTACCCCGAGA-C
Dare  CTAGGCAAAGAGACC-TTTAGTTTAAAGCCCCGAATCCAG-GT---GAGCTACCCCGAGA-C
Cost  ACAAGCAAAGAGACC-TTTAGTTTGTTACCCCGAAACCAA-GT---GAGCTACCCCGGGA-C
Leec  ACAAGCAAAGCGACC-TTTAGTTTGTAACCCCGAAACCAA-GT---GAGCTACCCCGGGA-C
Crla  CCAAGCAAAGAGACC-TTTAGTTTGAAACCCCGAAACCAA-GT---GAGCTACCCCGGGA-C
Clmc  TTGAGCAAAGAGAAC-TTTAGTTCAGACCCCCGAAACCAA-GT---GAGCTACCTCGAGA-C
Phin  CTGAGCAAAAAGAAT-TTTAGTTCAAGACCCCGAAACCAA-GC---GAGCTACCTCGGGA-C
Icpu  AAGAGCAAAGCGCAC-TTTAGTTCAAGCCCCCGAAACTAA-GT---GAGCTACCCCGAGA-C
Psto  CTGAGCAAAGCGTAC-TTTAGTTCAAAACCCCGAAACTAC-GT---GAGCTACCCCGAAA-C
Cora  CTGAGCAAAGAGTAC-TTTAGTTCAAAACCCCGAAACTAA-GT---GAGCTACCCCAAGA-C
Eisp  CTGAGCAAAGAGCCC-TTTAGTTCATAACCCCGAAACCAA-GT---GAGCTACCCCGAGA-C
Apal  TTGAGCAAAGAGCAC-TTCAGTTCAAAACCCCGAAACCAA-GC---GAGCTACCTCGAGA-C
Eslu  CCGAGCAAAGAGCAC-TTTAGTCCGGACCCCCGAAACTAG-AC---GAGCTACTCCGGGA-C
Dape  CCGAGCAAAGAGCAC-TTTAGTTCGGGCCCCCGAAACTAG-AC---GAGCTACTCCGGGA-C
Glse  CTTAGCGAAGAGACC-TTTAGTTATGGCCCCCGAAACTAG-AC---GAGCTACTCCGAGA-C
Naar  TTAAGCAAAGAGACC-TTTAGTTTAAGCCCCCGAAACTAA-AC---GAGCTACTCCGAGA-C
Baoc  CTAAGCAAAGAGATC-TTTAGTTTAAGTCCCCGAAACTAG-AC---GAGCTACTCCGAGA-C
Opso  CAAGGCAAAGAGACC-TTTAGTCACGGCCCCCGAAACTAA-AC---GAGCTACTCCGAGA-C
Alte  CCGAGCAAAGAGAAC-TTTAGTTCGAGACCCCGAAACCGA-GT---GAGCTACTCCGAGA-C
Plap  CCGAGCAAAGAGAAC-TTTAGTTCGAGCCCCCGAAACCGA-GT---GAGCTACTCCGAGA-C
```

```
Plal      TCAGGCAAAGAGAAC-TTTAGTTTGAGCCCCCGAAACTGG-AC---GAGCTACTCCGAGA-C
Sami      CCAGGCAAAGAGAGC-TTTAGTCTGGGCCCCCGAAACTGG-AC---GAGCTACTCCGAGA-C
Rere      TCAAGCGAAGAGCAC-TTTAGTTTGAGCCCCCGAAACTGG-AC---GAGCTACTCCGAGA-C
Gama      TCAGGCGAAGTGAAC-TTTAGTCTTAGCCCCCGAAACTGG-AC---GAGCTACTCCGAGG-C
Onmy      CTGAGCAAAGAGAAC-TTTAGTTTAGGCCCCCGAAACTAG-AC---GAGCTACTCCGGGA-C
Sasa      CCGAGCAAAGAGAAC-TTTAGTTCAGGCCCCCGAAACTAG-AC---GAGCTACTCCGGGA-C
Cola      CCGAGCAAAGAGAAC-TTTAGTTCAGGCCCCCGAAACTAG-AC---GAGCTACTCCGGGA-C
Dita      CCAGGCAAGGAGAAC-TTTAGTCCGGACCCCCGAAACTAC-GT---GAGCTACTCCGAGG-C
Gogr      TCAAGCAAAAAGAAC-TTTAGTTTGAACCCCCGAAACTAG-AC---GAGCTACTCCGAAA-C
Chsl      TTAAGCAAAGAGAGC-TTTAGTTTAAGCCCCCGAAACTAG-AC---GAGCTACTCCGAGA-C
Atja      TTAGGCAAAGAGAAC-TTTAGTCTAAGCCCCCGAAACTAG-AC---GAGCTACTCCAAGA-C
Iido      TTAGGCAAAGAGAAC-TTTAGTCTAAGCCCCCGAAACTAG-AC---GAGCTACTCCAGGA-C
Auja      TAAAGCAAAGAGCCC-TTTAGTTTTATTTCCCGAAACTAA-AC---GAGCTACTCCGAGA-C
Chag      TAAAGCGAAGCGCCC-TTTAGTTTTATACCCCGAAACTAG-AC---GAGCTACTCCGAGA-C
Hami      TAAAGCAAAGCGCCC-TTTAGTTTTACTTCCCGAAACCAG-AC---GAGCTACTCCGAGA-C
Saun      TAAAGCAAAGCGCCC-TTTAGTTTTACCCCCCGAAACCAG-AC---GAGCTACTCCGAGAAC
Nema      CTGAGCAAAGAGACC-TTTAGTACAGACCCCCGAAACTAG-AC---GAGCTACTTCAAGA-C
Disp      CTGAGCAAAGTGAAC-TTTAGTTCAGTCCCCCGAAACTAG-AC---GAGCTACTTCAAGA-C
Myaf      CTGAGCAAAGTGAAC-TTTAGTTCAGCCCCCCGAAACTAG-AC---GAGCTACTTCAAGG-C
Lagu      TCAGGCAAAGTGACC-TTTAGTCTGATTTCCCGAAACTAG-AC---GAGCTACTTCAAGA-C
Trtr      GCAGGCAAAGAGGTC-TTTAGTCTGACCCCCCGAACCCAG-GT---GAGCTACTTCGAGG-C
Zucr      CCAGGCACAGAGCAC-TGTAGTCTGACA-CCCGAAACCAA-GT---GAGCTACTCCGAGG-C
Pxja      TCAAGCACAGTGCCC-TTTAGTTTGATACCCCGAAACTGA-GC---GAGCTACTTCAAGA-C
Pxlo      TCAAGCACAGTGCCC-TTTAGTTTGATACCCCGAAACTGA-GC---GAGCTACTTCAAGA-C
Pctr      TCAGGCAAAGTGCCC-TGTAGTCTGGACCCCCGAAACTGAAGC---GAGCTACTCCAAGA-T
Apsa      TCAAGCAAAGCGACC-TAAAGTTTGAGACCCCGAAACTGAAGC---GAGCTACTCCAAGA-C
Cabe      TTAGGCAGAGAGCTC-TGCAGTCTAAGACCCCGAAACCGG-GT---GAGCTACTCCGGAA-C
Bzze      TCAAGCAAAGAGAAC-TATAGTTTGACACCCCGAAACTAG-GT---GAGCTACTCCAAGG-C
Siim      CCAAGCAGAAAGAAT-TATAGTTTGGTTCCCCGAAACTAA-GT---GAGCTACTCCGAGG-C
Ctru      ACAAACAAAGAGCCC-TTTAGTTTGGTCCCCCGAAACTAG-AT---GAGCTACTCCAAGA-C
Dpbr      ATAAACAAAGAGCCC-TTTAGTTTAATGCCCCGAAACTAG-AT---GAGCTACTCCGAGA-C
Caki      CCAAGCAAAGAGCCC-TTTAGTTTGTTACCCCGAAACTGT-GC---GAGCTACTTCAAGA-C
Phja      ACAAGCAAAGAGCCC-TTTAGTTTGTGACCCCGAAACTGA-GC---GAGCTACTTCAAGA-C
Brsp      CCAAGCAAAGTGACC-TTTAGTTTGCTACCCCGAAACTGC-GC---GAGCTACTCCGAGA-C
Gamo      ACAAGCAAAGAGCCC-TTTAGTTTGTAACCCCGAAACTGA-GC---GAGCTACTCCAAGA-C
Lolo      ACAAGCAAAGAGCCC-TTTAGTTTGTAACCCCGAAACTGA-GC---GAGCTACTCCAAGA-C
Batr      ATAAGCAAAAAAAAC-TTTAGTTTATATCCCCGAAACTAG-TT---GAGCTACTTCGTGC-C
Prmy      AAGAGCACAGCCTCTCTTAAGTTCTATCCCCCGAAACTGT-AG---GAGCTACTTCAGGC-C
Loli      CCAAGCAAAGTGCCC-TTTAGTTTGGGCCCCCGAAACTAA-GC---GAGCTACTCCAAGA-C
Loam      CCAAGCAAAGAGCCC-TTTAGTTTGGACCCCCGAAACTAA-GC---GAGCTACTCCAAGA-C
Chab      CCAAGCGAAGAGCCC-TACAGTTTGAACCCCCGAAACTAA-GC---GAGCTACTCCAAGA-C
Chto      CCAAGCGAAGAGCCC-TACAGTTTGAACCCCCGAAACTAA-GC---GAGCTACTCCAAGA-C
Majo      TCAAGCAAAAGGAAT-TTTAGTTTGAGACCCCGAAACCAA-GT---GAGCTACTCCAAGA-C
Hlst      ACAGGCAAAGAGCCC-TTTAGTCTGGAACCCCGAAACTAA-GA---GAGCTACTCCAAGA-C
Clpe      CCAAGCAAAGAGCCC-TTTAGTTTGACCCCCCGAAACTAG-AC---GAGCTACTCCAAGA-C
Mlmr      CCAAGCAAAGAGCCC-TTCAGTTTGAGCCCCCGAAACTAA-GC---GAGCTACTCCAAGA-C
Crcr      TCAAGCAAAAAGCAT-TTTAGTTTGACCCCCCGAAACCAA-GT---GAGCTACTCCAAGA-C
Muce      TCAAGCAAAAAGCAT-TTTAGTTTGACCCCCCGAAACCAA-GT---GAGCTACTCCAAGA-C
Bege      TCAAGCAAAAAGCAT-TTTAGTTTGACTACCCGAAACCAG-AC---GAGCTACTTCAAGA-T
Mela      TCAAGCAAAAAGCAT-TGTAGTTTGACAACCCGAAACTAA-GG---GAGCTACTCCAGGC-C
Hats      TCAAGCAAAAAGCAT-TTTAGTTTGAATACCCGAAACTAG-GC---GAGCTACTCCAAGA-C
Orla      TCAAGCAAAAAGAAC-TGTAGTTTGAAACCCCGAAACTTA-GT---GAGCTACTTCAAGA-C
```

Cosa	TTAAGCAAAATGAAT-TTTAGTTTAAACCCCCGAAACTAA-GT---GAGCTACTCCAAGA-C
Exsp	TTAAGCAAAATGAAT-TTTAGTTTAACTTCCCGAAACTAG-GT---GAGCTACTCCAAGG-C
Depa	CTAAGCAAAAAGATT-TTTAGTTTAGTACCCCGAAACTAA-GT---GAGCTACTTCAAGA-C
Rima	TTAAGCAAAAAGTTT-T-TAGTTTAACCCCCCGAAACTGG-GT---GAGCTACTCCAAGA-C
Fuol	TCAAGCAAAAAGCAT-TTTAGTTTGATATCCCGAAACTAG-AC---GAGCTACTCCAGGA-C
Gmaf	TTAAGCAAAAAGATT-TTTAGTTTGACATCCCGAAACTAG-AC---GAGCTACTCCAAGG-C
Xeei	CCAAGCAAAAAGCCT-TTTAGTTTGACTTCCCGAAACTAG-AC---GAGCTACTCCAAGA-C
Pros	TCGAGCACAGCGTCC-TTTAGTTCGACACCCCGAAACTAG-AC---GAGCTACTCCAAGG-C
Scmi	TCGAGCACAGCGTTC-TTTAGTTCGACACCCCGAAACTAG-AC---GAGCTACTCCAAGA-C
Rolo	CCGAGCAAAGAGCCC-TTTAGTTCGGCACCCCGAAACTGG-AC---GAGCTACTCCAAGA-C
Cere	CCAAGCGAAGAGCCC-TTTAGTTTGGTATCCCGAAACTGGAGC---GAGCTACTTCAAGA-C
Daga	CCAAGCAAAGAGCCC-TTTAGTTTGACACCCCGAAACTGAGGC---GAGCTACTTCAAGA-C
Anco	TTAAGCAAAGAGCCC-TTTAGTTTAACACCCCGAAACTGA-GC---GAGCTACTCCAAGA-C
Dmve	CTAAGCAAAGAGCCC-TTTAGTTTAGCACCCCGAAACTGA-CA---GAGCTACTCCAAGC-C
Dmar	CTAAGCAAAGAGCCC-TTTAGTTTAGCCCCCCGAAACTGA-GC---GAGCTACTCCAAGA-C
Anka	TTAAGCAAAGAGCCC-TTTAGTTTAACACCCCGAAACCGG-GC---GAGCTACTCCAAGA-C
Moja	TTAAGCAAAGAGCCC-TTTAGTTTAATACCCCGAAACTGA-GC---GAGCTACTCCAAGA-C
Hoja	TTAAGCAAAGAGCCC-TTTAGTTTAATACCCCGAAACTGA-GC---GAGCTACTCCAAGA-C
Bede	TCGAGCACAGAGTTC-TTTAGTTCGACACCCCGAAACTAG-AC---GAGCTACTCCAAGA-C
Besp	TCGAGCATAGAGTCC-TTTAGTTCGACACCCCGAAACTAG-AC---GAGCTACTCCAAGG-C
Mysp	TCAAGCAAAGAGCCC-TTTAGTTTGAAACCCCGAAACTGG-AC---GAGCTACTCCAAGG-C
Osja	CCAAGCAAAGAGCCC-TTTAGTTTGACACCCCGAAACTGG-AC---GAGCTACTCCAAGA-C
Sgro	TCAAGCAAAGAGCCC-TTTAGTTTGATACCCCGAAACTGG-AC---GAGCTACTCCAAGA-C
Pzpa	CCAAGCAAAGAGACC-TATAGTTTGAGACCCCGAAACTAA-GC---GAGCTACTCCAAGA-C
Zeja	CCAAGCAAAGAGCCC-TATAGTTTGAGACCCCGAAACTTAAGC---GAGCTACTCCAAGA-C
Znne	TCAAGCAAAGAGACC-TGTAGTTTGAGACCCCGAAACTAA-GC---GAGCTACTCCAAGA-C
Zefa	CCAAGCAAAGCGACC-TGTAGTTTGAGACCCCGAAACTAA-GC---GAGCTACTCCAAGA-C
Acni	CCAAGCAAAGAGACC-TATAGTTTGGAACCCCGAAACTAA-GC---GAGCTACTCCAAGA-C
Ncrh	CCAAGCAAAGAGACC-TATAGTTTGGAACCCCGAAACTAA-GC---GAGCTACTCCAAGA-C
Agca	TCAAGCAAAGAGCAC-TTTAGTTTGACACCCCGAAACTAA-GT---GAGCTACTCCAAGA-C
Hydy	TTAAGCAAAGAGAAC-TTTAGTTTAACATCCCGAAACTAC-GT---GAGCTACTCCAAGA-C
Gsac	TTAAGCAAAGAGACC-TATAGTTTAATGCCCCGAAACTAC-GT---GAGCTACTCCAAGA-C
Pevo	CCTAGCAAAGAGCCC-TTTAGTTAGAAACCCCGAAACCGA-GT---GAGCTACTCCAAGA-C
Hiku	TCAAGCAAAGAGATC-TTTAGTTTGCTACCCCGAAACTAA-GC---GAGCTACTCCGAGA-C
Inpa	CTAAGCAAAGAGCCC-TCTAGTTTAAAACCCCGAAACCAA-GT---GAGCTACTCCAAGA-C
Auch	TTAGGCAAAGCGCAC-TTTAGTCTAAGCCCCCGAAACCAG-GT---GAGCTACTTCAAGA-C
Fico	TCAAGCAAAAAGAAT-TGTAGTTTGATACCCCGAAACTAC-GT---GAGCTACTTCAGGG-C
Macs	CTAAGCAAAGAGCAC-TTTAGTTTAGGACCCCGAAACCAG-GC---GAGCTACTCCAAGA-C
Moal	CCAAGCAAAGAGACC-TATAGTTTGTTTCCCCGAAACTAA-GT---GAGCTACTCCGAGA-C
Syma	TCAAGCAAAACGTCCTTTAAGTTTGACACCCCGAAACTAC-GT---GAGCTACTCCAAGA-C
Mafr	CTAAGCAAAGAGAAC-TTTAGTTTATAACCCCGAAACCAA-GT---GAGCTACTCCAAGA-C
Dcpe	TCTAGCAAAAAGAAC-TTTAGTTAGAGCCCCCGAAACCAA-GT---GAGCTACTCCAAGG-C
Dcti	TCTAGCGTAGAGAAC-TTTAGTTAGAACCCCCGAAACCAA-GT---GAGCTACTCCAAGG-C
Hehi	CCAAGCAAAGAGTGC-TTTAGTTTGACACCCCGAAACTAG-GG---GAGCTACTCCAAGA-C
Stam	CCAAGCAAAATGAAT-TTTAGTTTGACAGCCCGAAACTAA-GT---GAGCTACTCCAAGA-C
Hogi	TCAGGCAAAGAGCAC-TTTAGTCTGTTTACCCGAAACTAT-AT---GAGCTACTCCAAGA-C
Erzo	CCAAGCAAAGAGTGC-TTTAGTTTGACGCCCCGAAACTAA-GT---GAGCTACTCCAAGA-C
Hxot	CCAAGCAAAGCGTAC-TTTAGTTTGACGTCCCGAAACTAG-GT---GAGCTACTCCAAGA-C
Core	TCAAGCAAAGCGAAC-TTTAGTTTGACGTCCCGAAACTAC-GT---GAGCTACTCTAAGA-C
Apve	CCAAGCAAAGAGCAC-TTTAGTTTGACGTCCCGAAACTAC-GT---GAGCTACTCCAAGA-C
Latj	CCAAGCAAAGAGTACCTTTAGTTTGATACCCCGAAACTAACGT---GAGCTACTCCAAGA-C
Laja	TCAAGCAAAGCGTAC-TTTAGTTTGACCCCCCGAAACCAGTGT---GAGCTACTCCAAGA-C

```
Syja    CAAGCAAGTAATGC-TTTAGTTTGATTCCCCGAAACTAG-GT---GAGCTACTCCAAGT-C
Epme    CCAAGCAAAGAGCAC-TTTAGTTTGACAACCCGAAACTTT-GT---GAGCTACTCCAAAG-C
Grse    TCAAGCAAAATGCAT-TTTAGTTTGACAACCCGAAACTAT-GT---GAGCTACTCCGAGA-C
Clja    TCAAGCAAAGTGTAC-TTTAGTTTGACATCCCGAAACTAA-GT---GAGCTACTCCAAGG-C
Ogcy    CCAAGCGAAGAGAAC-TATAGTTTGGCCCCCCGAAACTGA-GT---GAGCTACTCCAAGA-C
Plna    TCAAGCAAAAAGCAT-TTTAGTTTGATTCCCCGAAACTAC-GT---GAGCTACTTCAAGA-C
Lema    TCAGGCAAAGAGAAC-TTTAGTCTGACACCCCGAAACTAA-GT---GAGCTACTCCAAGA-C
Etzo    CCAAGCAAAGTGTGC-TTTAGTTTGATAACCCGAAACTAA-GT---GAGCTACTCCAAGA-C
Apse    TCAAGCGAAGAGCAC-TTTAGTTTGACACCCCGAAACCAA-GT---GAGCTACTCCAAGA-C
Epde    TCAAGCAAAGAGCAC-TTTAGTTTGATTCCCCGAAACTAG-GT---GAGCTACTCCAAGA-C
Slja    TTAAGCAAAGAGAAC-TTTAGTTTAAGACCCCGAAACTATGGT---GAGCTACTCCGAGA-C
Bsja    CCAAGCAAAGAGCAC-TTTAGTTTGACACCCCGAAACTAC-GT---GAGCTACTCCAAGA-C
Ecna    CTAAGCAAAGAGCCC-TTAAGTTTAGTACCCCGAAACTAG-GT---GAGCTACTCCAAGA-C
Cohi    TTAAGCGAAGAGCAC-TTTAGTTTAATACCCCGAAACTAGCGT---GAGCTACTCCAAGA-C
Caar    TCAAGCAAAGAGAGC-TTTAGTTTGAAACCCCGAAACTGC-GT---GAGCTACTCCAAGA-C
Came    TCAAGCAAAGAGAAC-TTTAGTTTGAAATCCCGAAACTGT-GT---GAGCTACTCCAAGA-C
Mema    TCAAGCAAAGAGCAC-TTTAGTTTGACCACCCGAAATTAG-GT---GAGCTACTCCAAGA-C
Lenu    TTGAGCGAAAAGAAT-TTTAGTTCAAGCCCCCGAAACCAG-AC---GAGCTACTCCAGGA-C
Brja    TCAAGCGAAGAGCAT-TTTAGTTTGATCCCCCGAAACTAC-GT---GAGCTACTCCAAGA-C
Plma    CCAAGCAAAGAGAAC-TTTAGTTTAGGACCCCGAAACTAC-GT---GAGCTACTCCAAGA-C
Emst    CCAAGCAAAGAGCAC-TTTAGTTTGGCTCCCCGAAACTAA-GT---GAGCTACTCCAAGG-C
Ptti    CCAAGCAAAGAGCAC-TTTAGTTTAAAGCCCCGAAACCGA-GC---GAGCTACTCCAAGA-C
Losu    CCAAGCAAAGAGAAT-TTTAGTTTGACCCCCCGAAACTAG-AT---GAGCTACTCCAAGG-C
Geoy    TCAAGCAAAGTGCAC-TTTAGTTTGATTTCCCGAACCTAC-GC---GAGCTACTCCAAGA-C
Dipi    CCAAGCAAAGAGAAC-TTTAGTTTGACACCCCGAAACTAA-GT---GAGCTACTCCAAGA-C
Pama    CCAAGCAAAGAGGAC-TTTAGTTTGGCCCCCCGAAACTAA-GT---GAGCTACTCCAGGT-C
Leob    CCAAGCAAAGAGCAC-TTTAGTTTGCTACCCCGAAACTAG-AC---GAGCTACTCCAAGA-C
Neba    CCAAGCAAAGCGCCC-TTTAGTTTGGTTTCCCGAAACTAA-GT---GAGCTACTCCGAGC-C
Pdpl    TTAAGCAAAGAGAAC-TTTAGTTTAAAGCCCCGAAACCGG-GT---GAGCTACTCCAAGA-C
Nimi    TCAAGCAAAGAGCAC-TTTAGTTTGATTCCCCGAAACTAC-GT---GAGCTACTTCAAGA-C
Uptr    TCAAGCAAAGAGAAC-TTTAGTTTGAAACCCCGAAACTAG-GT---GAGCTACTCCAAGA-C
Pesc    TCAAGCAAAGGGCAC-TTTAGTTTGAAACCCCGAAACTAG-AT---GAGCTACTTCAAGC-C
Baar    CCAAGCAAAGAGTAC-TTTAGTTTGAATCCCCGAAACTAGTGT---GAGCTACTCCAAGC-C
Moar    TCAAGCAAAGAGCAC-TTTAGTTTGACTCCCCGAAACTAA-GT---GAGCTACTCCAAGA-C
Toja    CCAAGCAAAGAGCAC-TTTAGTTTGATGCCCCGAAACTAGTGT---GAGCTACTTCAAGA-C
Chau    CCGAGCAAAGAGCAC-TTTAGTTCGGCTCCCCGAAACTAA-GT---GAGCTACTCCAAAA-C
Chse    CCAAGCAAAGCGTAC-TTTAGTTTGCTTTCCCGAAACTAA-GT---GAGCTACTCCAAGA-T
Enar    TCAAGCAAAGAGATC-TTTAGTTTGATTCCCCGAAACTAA-GT---GAGCTACTCCAAGA-C
Hpty    CCAAGCAAAAAGTAT-TTTAGTTTGGTTCCCCGAAACTAA-GT---GAGCTACTCCAAGA-C
Nana    TCAAGCAAAGAGCAC-TTAAGTTTGACACCCCGAAACTAA-GT---GAGCTACTCCAAGA-C
Mcst    CCAAGCAAAGAGCCC-TTTAGTTTGACTTCCCGAAACTGG-GT---GAGCTACTCCAAGA-C
Rhox    CCGAGCAAAGAGACC-TTTAGTTCGGCCCCCCGAAACTAA-GC---GAGCTACTCCAAGA-C
Opfa    TCAAGCAAAGAGTTC-TTTAGTTTGACCCCCCGAAACTAG-GT---GAGCTACTCCAAGA-C
Paar    CCAAGCGAAGAGAAC-TTTAGTTTGATACCCCGAAACTGGCGT---GAGCTACTCCAAGA-C
Gozo    CCAAGCAAAGCGCGC-TTTAGTTTGGTACCCCGAAACTAA-GT---GAGCTACTCCAAGA-C
Ackr    CCAAGCAAAGAGCCC-TTTAGTTTGTCCCCCCGAAACTAA-GT---GAGCTACTTCAAGA-C
Elev    TCAGGCAAAGAGAAC-TTTAGTCTGAACCCCCGAAACTAA-GT---GAGCTACTCCAAGA-C
Trdu    TCAAGCAAAAAGCAT-TATAGTTTGATACCCCGAAACTAA-GC---GAGCTACTCCAAGG-C
Amoc    TCAAGCAAAAAGAAT-TTTAGTTTGACACCCCGAAACTAG-GT---GAGCTACTCCAAGA-C
Hame    TTAAGCAAAGAGCCC-TTTAGTTAAGTACCGCAAGGGAAG-GCTGAAAACCGAAATGAAG-T
Chso    CCAGGCGAAAAGATT-TTTAGTCTGATGTCCCGAAACCAA-GT---GAGCTACTCCAAGA-C
Lyto    CCAAGCAAAGCGTGC-TTTAGTTTGATACCCCGAAACTGG-GT---GAGCTACTCCAAGA-C
```

```
Encr      CCAAGCGAAGCGTGC-TTTAGTTTGGTATCCCGAAACTGG-GT---GAGCTACTCCAAGA-C
Bvar      TCAAGCAAAGCGTTC-TTTAGTTTGAAAACCCGAAGCTAA-GG---GAGCTACTCCAAGA-C
Noco      TCAAGCAAAAAGATT-TTTAGTTTGTTAACCCGAAACTAA-GG---GAGCTACTCCAAGA-C
Chsp      CCTGGCAAAATGAGT-TTTAGTCAGGCCCCCCGAAACTAA-GT---GAGCTACTCCAAGA-T
Arja      CCAAGCAAAGAGTAC-TTTAGTTTGACGTCCCGAAACTAC-GT---GAGCTACTCCAAGA-C
Pase      TTAAGCAAAGAGCCC-TTTAGTTTAATACCCCGAAACTAGCGT---GAGCTACTCCAAGA-C
Trel      CTAAGCACAGAGTAC-TTCAGTTTTAGACCCCGAAACTAG-GT---GAGCTACTCCGAGA-C
Lifa      CGAAGCAAAGTGAAC-TTTAGTTTCCTACCCCGAAACTAG-GT---GAGCTACCCCAGGA-C
Acur      TCAAGCAAAGAGTAC-TTTAGTTTGACTTCCCGAAACTAA-GT---GAGCTACTCCAAGA-C
Ampe      CCGAGCGAAGAGAAC-TTTAGTTTGCTTTCCCGAAACTAA-GT---GAGCTACTCGAGGA-C
Urja      CCAAGCATAAAGAAC-TTTAGTTTGACACCCCGAAACTAG-GT---GAGCTACTTCGAGT-C
Enet      TCAAGCAAAGGGATC-TTTAGTTTGAGACCCCGAAACCTG-GC---GAGCTACCTCAGAG-C
Ptbr      TTAAGCAAAAAGAAT-TTTAGTTTAATACCCCGAAACCAG-GG---GAGCTACCCCAAGA-C
Safa      TCAAGCAAAAAGCCC-TGTAGTTTGAAAACCCGAAACCAA-GT---GAGCTACTCCAAGA-C
Icae      CCAAGCGAAGAGCAC-TTTAGTTTGGAACCCCGAAACTAT-GT---GAGCTACTCCAAGA-C
Asmi      TAAAGCGAAATGTTT-TTTAGCTTACAGCCCCGAAACCAG-GC---GAGCTACTCCAAGA-C
Foal      TTAAGCAAAGCGAGC-TGTAGTTTAATTACCCGAAACCAA-GC---GAGCTACTCCGAGA-C
Drze      TCAAGCAAAGAGCAC-TTTAGTTTGAAATCCCGAAACCTA-GT---GAGCTACTCCAAGA-C
Rhas      ACAAGCAAAGAGCGC-TTTAGTTTGGGACCCCGAAACTAA-GT---GAGCTACTCCAAGA-C
Elac      CTGAGCAAAACGAAT-TTTAGTTTAGACCCCCGAAACTAA-GT---GAGCTACTCCAAGA-C
Kugu      CCAAGCAAAAAGTAT-TTTAGTTTGACACCCCGAAACCAG-GT---GAGCTACTCCAAGA-C
Plor      TCAAGCAAAGAGCAC-TTTAGTTTGATTCCCCGAAACTAG-GT---GAGCTACTCCAAGA-C
Sgun      CCAAGCAAAGAGCCC-TTTAGTTTGACTTCCCGAAACTTA-GT---GAGCTACTCCAAGA-C
Zaco      TCAAGCAAAGAGCAC-TTTAGTTTGACTCCCCGAAACTGT-GC---GAGCTACTCCAAGA-C
Zbfl      CCAAGCAAAGAGAAC-TTTAGTTTGACTCCCCGAAACTAA-GT---GAGCTACTCCAAGA-C
Spba      CCAAGCAAAAAGTTT-TTTAGTTTGATACCCCGAAACTAA-GG---GAGCTACTCCAAGA-C
Game      CCAAGCAAAGAGCCC-TTTAGTTTGGACCCCCGAAACTAC-GT---GAGCTACTCCAAGA-C
Thth      CCAAGCAAAGAGAAC-TTTAGTTTGGACCCCCGAAACTAG-GT---GAGCTACTGCAAGA-C
Xigl      TCAAGCGAAGAGCAC-TTTAGTTTGATACCCCGAAACTAG-GT---GAGCTACTCCAAGA-C
Hyja      CCAAGCAAAGAGCAC-TTTAGTTTGGACCCCCGAAACTAC-GT---GAGCTACTCCAAGA-C
Psan      CCAAGCAAAGAGCAC-TTTAGTTTGGACCCCCGAAACTAC-GC---AAGCTACTCCAAGA-C
Cupa      CCAAGCAAAGAGCAC-TTTAGTTTGGGCCCCCGAAACTAC-GT---GAGCTACTCCAAGA-C
Mpch      TCAAGCAAAGAGCAC-TTTAGTTTGCCTTCCCGAAACTAA-GT---GAGCTACTCCAAGA-C
Char      CCAAGCAAAGAGCAC-TTTAGTTTGACCCCCCGAAACTAA-GT---GAGCTACTCCAAGA-C
Pser      CCAAGCAAAGAGCAC-TTTAGTTTGACTCCCCGAAACTAGCGT---GAGCTACTCCAAGA-C
Prol      TCAAGCAAAGAGAACTTAAAGTTTGTAACCCCGAAACTGA-GT---GAGCTACTCCAAGA-C
Plbi      TCAAGCAAAGAGAACCTAAAGTTTGTAACCCCGAAACTGA-GT---GAGCTACTCCAAGA-C
Calu      CCAAGCAAGGAGCAC-CATAGCTTGATACCCCGAAACTGG-GT---GAGCTACTCCAGGA-C
Papa      CCAAGCGAAGAGCCC-TTTAGTTTACTACCCCGAAACTAG-GT---GAGCTACTCCAAGA-C
Sufr      CTAAGCAAAAAGAAT-TTTAGTTTAAACCCCCGAAACTTA-TT---GAGCTACTCCAGGA-C
Stci      TTGGGCAAAACGATT-TTTAGTCCAATCCCCCGAAACTAA-GT---GAGCTACTCCGAGA-C
Taru      TTAGGCAAAGAGCAC-TTTAGTCTAACACCCCGAAACTGA-AT---GAGCTACTCCAAGA-C
Rala      CTAGGCAAAAAGAAT-TTTAGTCTAGTCCCCCGAAACTAA-GT---GAGCTACTCCGAGA-C
                              *        * * *             * *
```

```
                 12          HVR          12'     13        13'
Scca    AGCA------TT-AT------------AGAGC-TAACCC--GTCTCTGTGGCAAAAGA-G
Muma    AGCATT---A---------------TTAGAGC-TAACCC--GTCTCTGTGGCAAAAGA-G
Erca    AGTTG----TAA----------------GGAC-TAACCC--GTCTCTGTGGCAAAAGG-G
Pose    AGTTAA--------------------AAGGAC-CAACCC--GTCTCTGTGGCAAAAGA-G
Actr    AGCCT-------AA------------TAGGGC-AAACCC--GTCTCTGTGGCAAAAGA-G
Scal    AGCCT----A---AC------------AGGGC-AAACCC--GTCTCTGTGGCAAAAGA-G
Posp    AGCC-------TA------------ACAGGGC-AAACCC--GTCTCTGTGGCAAAAGA-G
Atsp    AGTCTACCA------------------AGGAC-CAATCC--GTCTCTGTGGCAAAAGA-G
Leoc    AGTCTA---CCA----------------GGGC-TAATCC--GTCTCTGTGGCAAAAGA-G
Amca    AGCCTC---CTA----------------GGGC-CAACCC--GTCTCTGTGGCAAAAGA-G
Osbi    AGCCTAC---A---------------CAGGGC-AAACCC--GTCTCTGTGGCAAAAGA-G
Pabu    AGCTTT---AATG------------A--GAGC-CAACCC--GTCTCTGTAGCAAAAGA-G
Hial    AGCCTA---TC---------------CAGGGC-CAACCC--GTCTCTGTGGCAAAAGA-G
Elha    AGCC--------CATAT--------G--GGGC-CAACCC--GTCTCTGTGGCAAAAGA-G
Mlcy    AGCCCC---TTAA---------------GGGC-CAACCC--GTCTCTGTGGCAAAAGA-G
Algl    AGCC-------GTT------------TAGGGC-CAACCC--GTCTCTGTGGCAATAGA-G
Ptgi    AGCCTA---TAA--------------TAGGGC-CAACCC--GTCTCTGTGGCAAAAGA-G
Alaf    AGCCT--------AC-A--------CCAGGGC-CAACCC--GTCTCTGTGGCAAAAGA-G
Nock    AGCCTA---AAC--A-------------GGGC-CAACCC--GTCTCTGTGGCAAAAGA-G
Anja    AGCCTA---ATTA---------------GGGC-CAACCC--GTCTCTGTGGCAAAAGA-G
Gyki    AGCC------CAA-------------TGGGGC-AAACCC--GTCTCTGTGGCAAAAGA-G
Syka    AGTCT------T-------------AAAGGAC-TAACCC--GTCTCTGTGGCAAAAGA-G
Opma    AGTT------TT-GA------------AGAAC-CAACCC--GTCTCTGTGGCAAAAGA-G
Comy    AGTCTA---AA----------------AGGAC-TAACCC--GTCTCTGTGGCAAAAGA-G
Sasp    AGCCCA---AAT----------------GAGC-GAACCC--ATCTCTGTAGCAAAAGA-G
Eupe    AGCC------AATAATT--------G---GGC-GAACCC--GTCTCTGTTGCAAAAGA-G
Enja    AGCCAA---CTAT---------------GGGC-CAACCC--GTCTCTGTGGCAAAAGA-G
Same    AGCCTA---TTGT--------------AGGGC-CAACCC--GTCTCTGTGGCAAAAGA-G
Chch    AGCCTA---A-----GA--------ACTGGGC-CAACCC--GTCTCTGTGGCAAAAGA-G
Grgr    AGCCTG---CAGA--------------AGGGC-CAACCC--GTCTCTGTGGCAAAAGA-G
Caau    AGCCTA---TTG---------------AGGGC-CAACCC--GTCTCTGTGGCAAAAGA-G
Cyca    AGCCTA---TT--------------A-AGGGC-CAACCC--GTCTCTGTGGCAAAAGA-G
Dare    AGCCTA---TTTAACT----------TAGGGC-CAACCC--GTCTCTGTGGCAATAGA-G
Cost    AGCCTA------T------------A-AGGGC-CAACCC--GTCTCTGTGGCAAAAGA-G
Leec    AGCCTA---TC-----------------GGGC-CAACCC--TTCTCTGTGGCAAAAGA-G
Crla    CGCCAA---CAT----------------GGGC-CAACCC--ATCTCTGTGGCAAAAGA-G
Clmc    AGCCTA---TTA--------------CAGGGC-CAACCC--GTCTCTGTGGCAAAAGA-G
Phin    AGCCAA---ATC--------------TAGGGC-AAACCC--GTCTCTGTGGCAAAAGA-G
Icpu    AGCCTA---TTAATT------------AGGGC-CAACCC--GTCTCTGTGGCAAAAGA-G
Psto    AGCCTA---TCAATT------------AGGGC-CAACCC--GTCTCTGTGGCAAAAGA-G
Cora    AGTCT-----ATTATAT--------A--GGAC-CAACCC--GTCTCTGTGGCAAAAGA-G
Eisp    AGCCTA---C---------------ATAGGGC-CAACCC--GTCTCTGTGGCAAAAGA-G
Apal    AGCCTA---TCT--------------CAGGGC-CAACCC--GTCTCTGTAGCAAAAGA-G
Eslu    AGCCTA---ACAT--------------AGGGC-CAACCC--ATCTCTGTGGCAAAAGA-G
Dape    AGCCCA---A-AA-------------GGGGGC-AAACCC--GTCTCTGTGGCAAAAGA-G
Glse    AGCCTATCTA------------------GGGC-CAACCC--GTCTCTGTGGCAAAAGA-G
Naar    AGCCTA---TC---------------CAGGGC-CAACCC--GTCTCTGTGGCAAAAGA-G
Baoc    AGCC--------TATCT--------A--GGGC-CAACCC--GTCTCTGTGGCAAAAGA-G
Opso    AGCCTG---TCCA---------------GGGC-CAACCC--GTCTCTGTAGCAAAAGA-G
Alte    AGCCT--------AT-C--------ATAGGGC-CAACCC--GTCTCTGTGGCAAAAGA-G
Plap    AGCCTA---TC--------------ATAGGGC-CAACCC--GTCTCTGTGGCAAAAGA-G
```

```
Plal    AGCCTA---T-TA-------------TAGGGC-ACACCC--GTCTCTGTTGCAAAAGA-G
Sami    AGCCTA---TC--------------GCAGGGC-CTATCC--GTCTCTGTTGCAAAAGA-G
Rere    AGCCTA---TTAT--------------AGGGC-CAACCC--GTCTCTGTTGCAAAAGA-G
Gama    AGCCTA---TG-G-------------CAGGGC-CAACCC--GTCTCTGTGGCAAAAGA-G
Onmy    AGCCTA---TTG--------------TAGGGC-CAACCC--GTCTCTGTGGCAAAAGA-G
Sasa    AGCCTAT--TA---------------TAGGGC-CAACCC--GTCTCTGTGGCAAAAGA-G
Cola    AGCCT-----ATTA--T--------A--GGGC-CAACCC--GTCTCTGTGGCAAAAGA-G
Dita    AGCCTA---T-A-------------GCAGGGC-CAACCC--ATCTCTGTGGCAAAAGA-G
Gogr    AGCCTA---TCAT---------------GGGC-CAACCC--GTCTCTGTGGCAAAAGA-G
Chsl    AGCCTT---TTA----------------GGGC-CAACCC--CCCTCTGTTGCAAAAGA-G
Atja    AGCCTA---TAAC--------------AGGGC-CAACCC--GTCTCTGTGGCAAAAGA-G
Iido    AGCCTA---TTA--------------TAGGGC-CAACCC--GTCTCTGTGGCAAAAGA-G
Auja    AGCCT--------ATTA---------TAGGGC-CAACCC--GTCTCTGTGGCAAAAGA-G
Chag    AGCCTA---TTGA--------------AGGGC-CAACCC--GTCTCTGTGGCAAAAGA-G
Hami    AGCCTA---TT----------------AGGGC-CAACCC--GTCTCTGTGGCAAAAGA-G
Saun    AGCCTT---TT---------------TAGGGC-TAACCC--GTCTCTGTGGCAAAAGA-G
Nema    AGCCTA---TGT--------------T-GGGC-GAACCC--GTCTCTGTGGCAAAAGA-G
Disp    AGCCTA---C---GT------------TGGGC-AAACCC--GTCTCTGTTGCAAAAGA-G
Myaf    AGCCTAAGGCAATATCC--------GAAGGGC-ACACCC--GTCTCTGTAGCAATAGA-G
Lagu    AGCCTA---ATA-A--------------GGGC-TAACCC--GTCCCTGTGGCAAAAGG-G
Trtr    TGCCTA---ACATT-----------AAAGGGC-TAACCC--GTCTCTGTGGCAAAAGA-G
Zucr    AGCCT-----AGA-------------TAGGGC-TAACCC--GTCTCTGTGGCAAAAGA-G
Pxja    AGCCTA---TTTA------------ATAGGGC-AAACCC--GTCTCTGTGGCAAAAGA-G
Pxlo    AGCCTA---TTTAA------------TAGGGC-AAACCC--GTCTCTGTGGCAAAAGA-G
Pctr    AGCCTG---TAAT------------TTAGGGC-AAACCC--GTCTCTGTGGCAAAAGA-G
Apsa    AGCCT----TAT-------------ATAGGGC-ACACCC--GTCTCTGTGGCAAAAGA-G
Cabe    AGCCTA---TAAA------------GAAGGGC-ACACCC--GTCTCTGTCGCAAAAGA-G
Bzze    AGCCCG---TAAA-------------AGGGGC-ACACCC--GTCTCTGTGGCAAAAGA-G
Siim    AGCTTG---TATA------------AAAGAGC-AAACCC--GTCTCTGTTGCAAAAGA-G
Ctru    AGCCC--------GACA--------AAGGGGC-AAACCC--GTTTCTGTAGCAAAAGA-A
Dpbr    AGCCCG---TTA-------------AAGGGGC-AAACCC--GTTTCTGTGGCAAAAGA-A
Caki    AATCTA---TC--A--------------GGAT-AAACCC--GTCTCTGTGGCAAAAGA-G
Phja    AGCCTA---TAATTAT---------TTAGGGC-AAATCC--GTCTCTGTGGCAATAGA-G
Brsp    AGCCT-----TTT---------------GGGC-CTACCC--GTCTCTGTGGCAAAAGA-G
Gamo    AGCCT-----ATAA-----------A--GGGC-AAACCC--GTCTCTGTGGCAAAAGA-G
Lolo    AGCCTA------C------------ATAGGGC-AAACCC--GTCTCTGTGGCAAAAGA-G
Batr    AGTC-A---CTGA-------------C-GAAC-ACACCC--ATCTCTGTGGCAAAAGA-G
Prmy    -ATCAA---CTAA--------------AGGATTATACCC--CCCTCTGTGGCAAAAGA-G
Loli    AGCCTT---CATA-------------AAGGGC-AAACCC--GTCTCTGTGGCAAAAGA-G
Loam    AGCCTA---TTT-------------AAAGGGC-ACACCC--GTCTCTGTGGCAAAAAGAG
Chab    AGCCTA---T-CA-------------TAGGGC-AAACCC--GTCTCTGTGGCAAAAGA-G
Chto    AGCCTA---TCA--------------TAGGGC-AAACCC--GTCTCTGTGGCAAAAGA-G
Majo    AGCCTG---ACA-AT------------AGGGC-GAACCC--GTCTCTGTGGCAAAAGA-G
Hlst    AGCCTA---TTAAAG-------------GGGC-CAACCC--GTCTCTGTGGCAAAAGA-G
Clpe    AGCCCA-----TA------------ATGGGGC-AAACCC--GTCTCTGTGGCAAAAGA-G
Mlmr    AGCCTA---TAAA-------------TAGGGC-GAACTC--GTCTCTGTGGCAAAAGA-G
Crcr    AACCTAAA-TC---------------TAGGGT-AAACCC--GTCTCTGTGGCAAAAGA-G
Muce    AACCTA---AATC-------------TAGGGT-AAACCC--GTCTCTGTGGCAAAAGA-G
Bege    AACCTA---ATAAA------------TAGGGC-CAACCC--GTCTCTGTGGCAAAAGA-G
Mela    AGCCTAATAAA---------------AAGGGC-CAACCC--GTCTCTGTGGCAATAGA-G
Hats    AGCCTG----ACA------------ACAGGGC-CAACCC--GTCTCTGTGGCAAAAGA-G
Orla    AGCCTG---AATAA------------CAGGGC-TAACCC--GTCTCTGTGGCAAAAGA-G
```

```
Cosa     AGCCTA---AAT-------------ATAGGGC-AAACCC--GTCTCTGTGGCAAAAGA-G
Exsp     AGCCTG---ACA-------------ACAGGGC-AAACCC--TTCTCTGTGGCAAAAGA-G
Depa     AGCCCA---A--A------------AGGGGGC-AAACCC--GTCTCTGTGGCAAAAGA-G
Rima     AGTCTT---TTA-T-------------AGGAC-ACACCC--GTCTCTGTGGCAAAAGA-G
Fuol     AGCCTA---ATA-TT------------AGGGC-ACACCC--GTCTCTGTGGCAAAAGA-G
Gmaf     AGCCTA---ATA-AA------------AGGGC-CAATCC--GTCTCTGTGGCAAAAGA-G
Xeei     AGCCTA---TTA-------------ATAGGGC-AAACCC--GTCTCTGTGGCAAAAGA-G
Pros     AGCCTA---TAA--------------CAGGGC-AACCCC--GTCTCTGTAGCAAAAGA-G
Scmi     AGCCTA---TAATAA-----------TAGGGC-AAACCC--GTCTCTGTCGCAAAAGA-G
Rolo     AGCCT----ATT-AT------------AGGGC-AAACCC--GTCTCTGTGGCAAAAGA-G
Cere     AGCCTA---TTTT--------------AGGGC-AAACCC--GTCTCTGTTGCAAAAGA-G
Daga     AGCCTA---TTG--------------TAGGGC-AAACCC--GTCTCTGTTGCAAAAGG-G
Anco     AGCCTATCC------------------AGGGC-AAACCC--GTCTCTGTGGCAAAAGA-G
Dmve     AGCCTA----TCA-------------TAGGGA-TAACCC--GTCTCTGTGGCAAAAGA-G
Dmar     AGCCTA---TCA--------------TAGGGC-TAACCC--GTCTCTGTGGCAAAAGA-G
Anka     AGCCTA---TA---------------TAGGGC-AAACCC--GTCTCTGTGGCAAAAGA-G
Moja     AGCCTA---T---------------ATAGGGC-AAACCC--GTCTCTGTGGCAAAAGA-G
Hoja     AGCCTA---TA---------------TAGGGC-AAACCC--GTCTCTGTGGCAAAAGA-G
Bede     AGCCTA----T-A------------ACAGGGC-AAACCC--GTCTCTGTGGCAAAAGA-G
Besp     AGCCTA---TA--------------ACAGGGC-AAACCC--GTCTCTGTGGCAAAAGA-G
Mysp     AGCCTA---T-T-------------ATAGGGC-CAACCC--GTCTCTGTGGCAAAAGA-G
Osja     AGCCTA---TT-A-------------TAGGGC-CAACCC--GTCTCTGTGGCAAAAGA-G
Sgro     AGCCCA---T-AA-------------TGGGGC-CAACCC--GTCTCTGTGGCAAAAGA-G
Pzpa     AGCCCA---AT---------------TGGGGC-TAACCC--GTCCCTGTGGCAAAAGG-G
Zeja     AGCCC--------AAC----------TGGGGC-AAACCC--GTCCCTGTGGCAAAAGG-G
Znne     AGCCC------AAT------------TGGGGC-AAACCC--GTCCCTGTGGCAAAAGG-G
Zefa     AGCCCA---AT---------------TGGGGC-AAACCC--GTCCCTGTGGCAAAAGG-G
Acni     AGCCCA---ATT--G-------------GGGC-CAACCC--GTCCCTGTGGCAAAAGG-G
Ncrh     AGCCCA---AT---T------------GGGGC-CAACCC--GTCCCTGTGGCAAAAGG-G
Agca     AGCCT--------GTTA--------ATAGGGC-AAACCC--GTCTCTGTGGCAAAAGA-G
Hydy     AGCCTG---TTA--A-----------TAGGGC-ACACCC--GTCTCTGTTGCAAAAGA-G
Gsac     AGCCT--------ATTA--------ATAGGGC-ACACCC--GTCTCTGTTGCAAAAGA-G
Pevo     AGCCTA---TTA-AT------------AGGGC-CAACCC--GTCTCTGTGGCAAAAGA-G
Hiku     AGTCAA---AA---CAT--------AATGGAC-AAATCC--GTACCTGTGGCAAAAGG-T
Inpa     AGCCT----AATA------------ACTGGGT-TAACCC--GTCCCTGTGGCAAAAGG-G
Auch     AGCCTA---AT-A------------TAAGGGC-GAACCC--GTCCCTGTAGCAAAAGG-G
Fico     AGCTTA---CTT-------------ATAGAGC-AAACCC--GTCTCTGTGGCAAAAGA-G
Macs     AGCCTA---ACAA-------------TAGGGC-CAACCC--GTCTCTGTGGCAAAAGA-G
Moal     AGCCT--------AATA--------ATAGGGC-GAACCC--GTCTCTGTGGCAAAAGA-G
Syma     AGTCTAC--AA---------------CAGGAC-CAACCC--GTCTCTGTGGCAAAAGA-G
Mafr     AGCCCA---TA---------------TGGGGC-CAACCC--GTCTCTGTGGCAAAAGA-G
Dcpe     AGCCTA---GCA-AT------------AGGGC-CAACCC--GTCTCTGTAGCAAAAGA-G
Dcti     AGCCTA---GTA-AT------------AGGGC-CAACCC--GTCTCTGTAGCAAAAGA-G
Hehi     AGCCTA---TTTA-------------TAGGGC-GAACCC--GTCTCTGTGGCAAAAGA-G
Stam     AGCCTA---TTAA-------------TAGGGC-CAACCC--GTCTCTGTGGCAAAAGA-G
Hogi     AGCCTA---TTT--A-----------TAGGGC-AAACCC--GTCCCTGTGGCAAAAGG-G
Erzo     AGCCT-----ATTA-AT--------A--GGGC-ACACCC--GTCTCTGTGGCAAAAGA-G
Hxot     AGCCT-----ATCA-AT--------A--GGGC-GTACCC--GTCTCTGTGGCAAAAGA-G
Core     AGCCTA---TTAAT-------------AGGGC-ATACCC--GTCTCTGTGGCAAAAGG-G
Apve     AGCCTA---TTAA-------------TAGGGC-TTACTC--GTCTCTGTAGCAAAAGA-G
Latj     AGCCTG---TTTA-------------CAGGGC-AAACCC--GTCTCTGTGGCAAAAGA-G
Laja     AGCCTA---AT--AT------------AGGGC-ACACCC--GTCTCTGTGGCAATAGA-G
```

```
Syja    AGCCTA---ATA--C------------AGGGC-CAACCC--GTTTCTGTGGCAAAAGA-A
Epme    AGCCTA---ATCA------------ATAGGGC-CAACCC--GTCTCTGTGGCAAAAGA-G
Grse    AGCCTATT-AA---------------TAGGGC-GAACCC--GTCTCTGTGGCAAAAGA-G
Clja    AGCCTA---AAAAA------------TAGGGC-GAACCC--GTCTCTGTTGCAAGAGA-G
Ogcy    AGCCTA---TAAAT-------------AGGGC-ACACCC--GTCTCTGTGGCAAAAGA-G
Plna    AGCCTA---AAAT------------ATAGGGC-CAACTC--GTCTCTGTGGCAAAAGA-G
Lema    AGCCTA---TCAT-------------TAGGGC-ACACCC--GTCTCTGTGGCAAAAGA-G
Etzo    AGCCTA---TTAAT-------------AGGGC-AAACCC--GTCTCTGTGGCAAAAGA-G
Apse    AGCCT-----CTA--------------AGGGC-GCACCC--GTCTCTGTGGCAAAAGA-G
Epde    AGCCTA---ATA--------------TAGGGC-GAACCC--GTCTCTGTTGCAAAAGA-G
Slja    AGCCTA---TGTAAAAG--------TTAGGGC-GAACCC--GTCTCTGTGGCAAAAGA-G
Bsja    AGCCTA---CACA------------ATAGGGC-GAACCC--GTCTCTGTGGCAATAGA-G
Ecna    AGCCTA---TTAA--------------AGGGC-AAACCC--GTCTCTGTGGCAAAAGA-G
Cohi    AGCCTATT-A----------------TAGGGC-AAACCC--GTCTCTGTGGCAAAAGA-G
Caar    AGCCTA---TTT-------------ATAGGGC-AAACCC--GTCTCTGTGGCAAAAGA-G
Came    AGCCTA---TTTA-------------TAGGGC-AAACCC--GTCTCTGTGGCAAAAGA-G
Mema    AGCCTA---TCCA------------TAAGGGC-AAACCC--GTCTCTGTGGCAAAAGA-G
Lenu    AGCC-A---ACA-------------ATTGGGC-AAACCC--GTCTCTGTGGCAAAAGA-G
Brja    AGCCTA---TCA--------------TAGGGC-AAATCC--GTCTCTGTGGCAAAAGA-G
Plma    AGCCTA---TCAA-------------TAGGGC-GAACCC--GTCTCTGTGGCAAAAGA-G
Emst    AGCCTA---TCAA-------------TAGGGC-AAACCC--GTCTCTGTGGCAAAAGA-G
Ptti    AGCCTA---GTA-------------TAAGGGC-AAACCC--GTCTCTGTGGCAAAAGA-G
Losu    AGCCTG---ACTT------------ACAGGGC-AAACCC--GTCTCTGTGGCAAAAGA-G
Geoy    AGCCTG---TTTA-------------GAGGGC-AAACCC--GTCTCTGTGGCAAAAGA-G
Dipi    AGCCTA---TTCA------------TTAGGGC-AAACCC--GTCTCTGTGGCAAAAGA-G
Pama    AGCCTA---TT--------------GTAGGGC-AAACCC--GTCTCTGTGGCAAAAGA-G
Leob    AGCCTG---TAG-------------CAGAGGC-CAACCC--GTCTCTGTGGCAAAAGA-G
Neba    AGCCT-----ATCA-----------A--GGGC-GAACCC--GTCTCTGTGGCAAAAGA-G
Pdpl    AGCCTA---GTATAT------------AGGGC-AAACCC--GTCTCTGTGGCAAAAGA-G
Nimi    AGCCTAA--AA---------------TAGGGC-GAACCC--ATCTCTGTAGCAAAAGA-G
Uptr    AGCCTG---ACA-AT------------AGGGC-GAACCC--GTCTCTGTGGCAAAAGA-G
Pesc    AGCCT----A-TA-------------TAGGGC-AAACCC--GTCTCTGTGGCAAAAGA-G
Baar    AGCCTATTATAGCCTGGCACTATAAATAGGGC-GAACCC--GTCTCTGTGGCAACAGA-G
Moar    AGCCTAT---CAA-------------TAGGGC-GAACCC--GTCTCTGTGGCAAAAGA-G
Toja    AGCCTG---TTTA-------------CAGGGC-AAACCC--GTCTCTGTGGCAAAAGA-G
Chau    AGCCT----ATCT------------A--GGGC-AAACCC--GTCTCTGTAGCAAAAGA-G
Chse    AGCCTA---GTA--A-----------TAGGGC-GAACCC--GTCTCTGTGGCAAAAGA-G
Enar    AGCCTA---TAA--A-----------TAGGGC-ACACCC--GTCTCTGTGGCAAAAGA-G
Hpty    AGCCT----AATA-------------CAGGGC-AAACCC--GTCTCTGTGGCAAAAGA-G
Nana    AGCCTA---ACTA-------------AAGGGC-CAACCC--GTCTCTGTGGCAAAAGA-G
Mcst    AGCCTA---TTAA-------------TAGGGC-AAACCC--GTCCCTGTGGCAAAAGG-G
Rhox    AGCCTA---AA--------------ATAGGGC-AAACCC--GTCCCTGTGGCAAAAGG-G
Opfa    AGCCTG---TTAGT-----------A--GGGC-AAACCC--GTCCCTGTTGCAAAAGG-G
Paar    AGCCTA---TTAA-------------TAGGGC-ACACCC--GTCTCTGTGGCAAAAGA-G
Gozo    AGCCTA---ATTA------------ATAGGGC-ACACCC--GTCTCTGTTGCAAAAGA-G
Ackr    AGCCTA---TTAAT------------TAGGGC-TAACCC--GTCTCTGTCGCAAAAGA-G
Elev    AGCCTA---TTT--------------TAGGGC-ACACCC--GTCTCTGTGGCAAAAGA-G
Trdu    AGTCTA---ATTTAT------------AGGAC-CACCCC--ATCTCTGTGGCAAAAGA-G
Amoc    AGCCTA---AAAA-------------CAGGGC-ACCCCC--GTCTCTGTGGCAAAAGA-G
Hame    AAACCA---AA---------------CA-CTC-GAACCC--TTCTCTGTGGCAAAAGA-G
Chso    AGCCTA----TAA-------------CAAGGGCAAACCC--GTCTCTGTGGCAAAAGA-G
Lyto    AGCCTA---TTAA-------------TAGGGC-ACACCC--GTCTCTGTGGCAAAAGA-G
```

```
Encr    AGCCTA---TTA-------------ATAGGGC-ACACCC--GTCTCTGTGGCAAAAGA-G
Bvar    AGCCT--------ATTA--------ATAGGGC-AAACCC--GTCTCTGTCGCAAAAGA-G
Noco    AGCCTG---TTAAT------------AGGTGC-GCACCC--GTCTCTGTTGCAAAAGA-G
Chsp    AGCCTT---AT-----------------GGGC-ACACCC--GTCCCTGTAGCAAAAGG-G
Arja    AGCCTA---TTA-------------ATAGGGC-ACACCC--GTCTCTGTGGCAAAAGA-G
Pase    AGCCTA---CCAA------------GCAGGGC-CTACCC--GTATCTGTAGCAAAAGA-T
Trel    AGCC----------TTT--------AAAGGGC-GTACCC--GTCTCTGTGGCAAAAGA-G
Lifa    AGCCTT---TC--------------TTAGGGC-GCACCC--GTCTCTGTTGCAAAAGA-G
Acur    AACCTA---AT-G-------------TAGGGT-GAACCC--GTCTCTGTGGCAAAAGA-G
Ampe    AGCCTA---TAAT-------------TACGGC-CAACCC--GTCTCTGTGGCAAAAGA-G
Urja    AGTCTA---GTA--A-----------TAGGAC-TAACCC--GTCTCTGTGGCAAAAGA-G
Enet    AGCCT--------AAAT--------ACAGGGC-ACACCC--GTTTCTGTGGCAAAAGA-A
Ptbr    AGTCTA---CATTC-----------GTAGGAC-CTACCC--GTCTCTGTGGCAAGAGA-G
Safa    AGTCTA---ATT-------------AATAGGA-CTACTCCCGTCTCTGTGGCAAAAGATG
Icae    AGCCTA---TCAA-------------TAGGGC-AAACCC--GTCTCTGTGGCAAAAGA-G
Asmi    GGTCTA---CTAT-------------TAGGAC-CCACCCCCGGCTCTGTTGCAAAAGA-G
Foal    AACCTT---ATT-------------GAAGGGT-AAACCC--GTCTCTGTGGCAAAAGA-G
Drze    AGCCTA---GAATA------------TAGGGC-AAACCC--GTCTCTGTAGCAAAAGA-G
Rhas    AGCCTA---ACA--------------TAGGGC-AAACCC--GTCTCTGTGGCAAAAGA-G
Elac    AGCCTA----TAA---------------GGGC-GCACCC--GTCTCTGTGGCAAAAGA-G
Kugu    AGCCTA----TA---------------AGGGC-GAACCC--GTCTCTGTCGCAAAAGA-G
Plor    AGCCTA---TCAT-------------TAGGGC-CAACCC--GTCTCTGTGGCAAAAGA-G
Sgun    AGCCTA---TAAA-------------CAGGGC-AAACCC--GTCTCTGTGGCAAAAGA-G
Zaco    AGCCTAC---CAA-------------TAGGGC-AAACCC--GTCTCTGTGGCAAAAGA-G
Zbfl    AGCCTA---TCA-AT------------AGGGC-AAACCC--GTCTCTGTGGCAAAAGA-G
Spba    AGCCTA---CCAA-------------TAGGGC-GAACCC--GTCTCTGTGGCAAAAGA-G
Game    AGCCTA---TCAA-------------TAGGGC-AAACCC--GTCTCTGTGGCAAAAGA-G
Thth    AGCCTA---TC-A------------ATAGGGC-AAACCC--GTCTCTGTGGCAAAAGA-G
Xigl    AGCCTA---TTTAT------------TAGGGC-GAACCC--GTCTCTGTGGCAAAAGA-G
Hyja    AGCCTATC-AA---------------TAGGGC-AAACCC--GTCTCTGTGGCAATAGA-G
Psan    AGCCTA---TAAATA-----------TAGGGC-AAACCC--GTCTCTGTGGCAATAGA-G
Cupa    AGCCTA---TCA-AT------------AGGGC-AAACCC--GTCTCTGTGGCAAAAGA-G
Mpch    AGCCTA---A-A--A-----------TAGGGC-CAACCC--GTCTCTGTGGCAAAAGA-G
Char    AGCCTATT-T----------------TAGGGC-CAACCC--GTCTCTGTGGCAAAAGA-G
Pser    AGCCTA---TCT-------------ATAGGGC-AAACCC--GTCTCTGTGGCAAAAGA-G
Prol    AGCCTA---TTTA-------------TAGGGC-AAACCC--GTCTCTGTGGCAAAAGA-G
Plbi    AGCCTA---TTTAT-------------AGGGC-GAACCC--GTCTCTGTGGCAAAAGA-G
Calu    AGCCTA------T------------ATAGGGC-AAACCC--GTCTCTGTGGCAAAAGA-G
Papa    AGCCTA---CAAAC------------CAGGGC-AAACCC--GTCTCTGTAGCAAAAGA-G
Sufr    AGTCTC-----AAA------------TAGGAC-GCACCC--GTCTCTGTGGCAAAAGA-G
Stci    AGCCTA---TTAA-------------TAGGGC-ACACCC--GTCTCTGTGGCAAAAGA-G
Taru    AGCCTTTA------------------TAGGGC-ACATCC--GTCTCTGTGGCAAAAGA-G
Rala    AGCCTA---TAA-------------ATAGGGC-ACACCC--GTCTCTGTGGCAAAAGA-G
                                              *       **** ****  *
```

```
                13'        11'      14      14'        10'          9'   15
Scca    TGGGAAGACTTCCGAGTAGTGGTGACAAGCCTACCGAGTTTAGTGATAGCTGGTTACCCAA
Muma    TGGGAAGACTTCCGAGTAGCGGTGAAAAGCCTACCGAGTTTAGTGATAGCTGGTTACCCAA
Erca    TGGGAAGACTTCCAAGTAGAGGTGATAAGCCTAACGAGCCTAGTAATAGCTGGTTACTCAA
Pose    TGGGAAGACTTCCAAGTAGAGGTGACAAGCCTAACGAGCCTAGTGATAGCTGGTTACTTGA
Actr    TGGGAAGATCTCCGAGTAGAGGCGACAAACCTAACGAGCCTAGTAATAGCTGGTTGCTCAG
Scal    TGGGAAGATCTCCGAGTAGAGGCGACAAACCTAACGAGCCTAGTAATAGCTGGTTGCTCAG
Posp    TGGGAAGATCTCCGAGTAGAGGCGACAAACCTAACGAGCCTAGTAATAGCTGGTTGCTCAA
Atsp    TGGAAAGATCTCCGAGTAGCGGTGATAGACCTAACGAGCCTAGTGATAGCTGGTTGCTTAG
Leoc    TGGAAAGATCTCCGAGTAGCGGTGACAGACCTAACGAGTCTAGTGATAGCTGGTTGCTTAG
Amca    TGGGAAGATCTCCAAGTAGTGGTGACAGACCTAACGAGTTTAGTTATAGCTGGTTGCTTAG
Osbi    TGGGAAGATCTTCAAGTAGAGGTGACAAACCTAACGAATCTAGTGATAGCTGGTTGCTTGA
Pabu    TGGGATGATCCCTGAGTAGAGGTGACAAACCTAACGAGTTTAGTTATAGCTGGTTGCTTAA
Hial    TGGGAAGATCTCCGAGTAGAGGTGACAGACCTAACGAGTTTAGTTATAGCTGGTTGCTTAG
Elha    TGGGAAGATCCCCGAGTAGAGGTGAAAAATCTAACGAGTCTAGTCATAGCTGGTTGCTTAG
Mlcy    TGGGAAGATCTCCAAGTAGAGGTGACAAACCTAACGAGTTTAGTGATAGCTGGTTGCTTAA
Algl    TGGGAAGACTCCCGAGTAGAGGTGACAGACCTATCGAGCTCGGTTATAGCTGGTTGCTTAG
Ptgi    TGGGACGATCCCCGAGTAGAGGTGACAAACCTATCGAGCCTAGTTATAGCTGGTTGCTTAG
Alaf    TGGGAAGACCCCCGAGTAGAGGTGACAAACCTATCGAGTCTAGTTATAGCTGGTTGCTTAG
Nock    TGGGAAGACCCCCGAGTAGAGGTGATAAACCTATCGAGCCTAGTTATAGCTGGTTGCTTAG
Anja    TGGGAAGACCCCCGAGTAGAGGTGATAAGCCTACCGAGCCTAGTTATAGCTGGTTGCTTAA
Gyki    TGGGGAGACTCCCGAGTAGAAGTGACAAGCCTACCGAACCTGGTGATAGCTGGTTGCTCAG
Syka    TGGGAAGACCCCCGAGTAGAGGTGAAAAGCCTACCGAGCCTAGTTATAGCTGGTTGCTTAA
Opma    TGGGAAGACCCCCGAGTAGGAGTGAAAAACCTAACGAGCCTAGTTATAGCTGGTTGCTTAA
Comy    TGGGAAGACTTCCGAGTAGAGGTGAAAAGCCTACCGAGCCTAGTTATAGCTGGTTGCTTAA
Sasp    TGGGAAGACCCCCAAGTAGAGGTGACAAACCAACCGAGCCCAGTTATAGCTGGTTGCTTAA
Eupe    TGGGAAGACCCCCGAGTAGAGGTGATAGACCTATCGAGCCTAGTGATAGCTGGTTGCTTAA
Enja    TGGGAAGATCTCCGAGTAGAGGCAAAAAACCTACCGAGCCAAGTTATAGCTGGTTGCCCAA
Same    TGGGAAGACCCCCGAGTAGAGGTGAAAGACCTACCGAGTCAGGTTATAGCTGGTTGCCCAA
Chch    TGGGAAGATCTCCGAGTAGAGGTGACAGACCTACCGAACTTAGTGATAGCTGGTTGCCCGA
Grgr    TGGGAAGAGCTCCGAGTAGAAGTGACAGACTTACCGAGCTTGGTGATAGCTGGTTGCCCAG
Caau    TGGGAAGAGCTCCGGGTAGAAGTGACAGACCTACCGAACCTGGTGATAGCTGGTTGCCTAA
Cyca    TGGGAAGAGCTCCGGGTAGAAGTGACAGACCTACCGAACCTGGTGATAGCTGGTTGCCTAA
Dare    TGGGAAGAGCTCCGGGTAGAAGTGACAGACCTATCGAACCCGGCAATAGCTGGTTGTCTGG
Cost    TGGGAAGAGCCCCGGGTAGAGGTGACAGACCTACCGAACTTGGTGATAGCTGGTTGCCTAA
Leec    TGGGAAGAGCCCCGGGTAGAGGTGATAAGCCTACCGAACTTGGTGATAGCTGGTTGCCTAA
Crla    TGGGGAGAGCCCCGGGTAGAGGTGATAGACCTACCGAACTTGGTGATAGCTGGTTGTCTGG
Clmc    TGGGAAGATCTCCAGGTAGAGGTGACAGACCTACCGAACTTGGTGATAGCTGGTTGCCTAG
Phin    TGGGAAGATCCCCGGGTAGAGGTGAAAAGCCTACCGAGCCTGGTGATAGCTGGTTGCCTAA
Icpu    TGGGAAGATCTCCGGGTAGAGGTGACAAACCTACCGAACTTAGTTATAGCTGGTTGCCTAA
Psto    TGGGAAGATTTCCGGGTAGAGGTGACAAACCTACCGAACCTAGTTATAGCTGGTTGCCTAA
Cora    TGGGAAGATCTTTGGGTAGGGGTGACAGACCTACCGAACTTAGTTATAGCTGGTTGCCTAA
Eisp    TGGGAAGATCTCCCGGTAGAGGTGACAGACCTATCGAACTTGGTGATAGCTGGTTGCCTAA
Apal    TGGGAAGATCTCCGGGTAGAAGTGACAAACTTATCGAGCTTGGTGATAGCTGGTTGCCTAA
Eslu    TGGGAAGATCCCCGAGTAGAGGTGACAAGCCTACCGAGTTTAGTTATAGCTGGTTGCTTAA
Dape    TGGGAAGAGCCCTGAGTAGAGGTGATAAACCTATCGAGTTTAGTTATAGCTGGTTGCTTAA
Glse    TGGGAAGAGCTCCGAGTAGAGGTGATAAACCTACCGAGTTTAGTTATAGCTGGTTGCTTAA
Naar    TGGGAAGAGCTCCGAGTAGAGGTGATAAACCTACCGAGTTTAGTTATAGCTGGTTGCTTAA
Baoc    TGGGAAGAGCTCCGAGTAGAGGTGACAAACCTACCGAGTCTAGTTATAGCTGGTTGCTTAA
Opso    TGGGAAGAGCTCCGAGTAGAGGTGATAAGCCTACCGAGTTTAGTTATAGCTGGTTGCTTAA
Alte    TGGGAAGAACTCCGAGTAGAGGTGACAAACCTACCGAACCCGGTTATAGCTGGTTGCCTAA
Plap    TGGGAAGAACTCCGAGTAGAGGTGACAAACCTACCGAACCCGGTTATAGCTGGTTGCCTAA
```

```
Plal    TGGGAAGAGCTCCGAGTAGAAGTGAAAGACCTATCGAGTCTAGTTATAGCTGGTTGTTCGA
Sami    TGGAGAGAGCTCCGAGTAGAAGTGAAATACCTATCGAGTCTAGTTATAGCTGGTTGTTTAG
Rere    TGGGAAGAGCTCCGAGTAGAAGTGAAATACCTATCGAGTCTAGTTATAGCTGGTTGCCCAG
Gama    TGGGAAGAACTCTGAGTAGAGGTGATAAACCTACCGAGTCTAGTTATAGCTGGTTGTCTAA
Onmy    TGGGACGAGCCCCGAGTAGAGGTGATAAACCTATCGAGCCTAGTTATAGCTGGTTGCTTAG
Sasa    TGGGACGAGCCCCGAGTAGAGGTGACAAACCTATCGAGCCTAGTTATAGCTGGTTGCTTAG
Cola    TGGGAAGAGCCCCGAGTAGAGGTGATAAACCTATCGAGCCTAGTTATAGCTGGTTGCTTAG
Dita    TGGGAAGAACTTCGAGTAGAGGTGAAAAGCCTACCGAACCTAGTTATAGCTGGTTGCTTGG
Gogr    TGGGGAGAATTCCGAGTAGAGGTGAAAGACCTACCGAGCCTAGCTATAGCTGGTTGCTTAC
Chsl    TGGGATGATCTCCGAGTAGAGGCAAAAAACCAACCGAGCCTAGTTATAGCTGGTTGCTTAG
Atja    TGGGAAGAGCTTCGAGTAGAGGTGACAAACCTACCGAGCCTAGTAATAGCTGGTTGTCTAA
Iido    TGGGAAGAGCTTTGAGTAGAGGTGACAAACCTACCGAGCCTAGTAATAGCTGGTTGTCTAA
Auja    TGGGAAGAGCTCCGAGTAGAGGTGATAAACCTACCGAGTTTAGTTATAGCTGGTTGCCTAG
Chag    TGGGAACAACTCTGAGTAGAGGTGACAGACCTACCGAGCCTAGTTATAGCTGGTTGCCTAG
Hami    TGGGAAGAACTCCGAGTAGAGGTGATAAGCCTACCGAGCCTGGTTATAGCTGGTTGCCTAA
Saun    TGGGAAGAGCTCCGAGTAGAGGTGATAAGCCTACCGAGCCTGGTTATAGCTGGTTGCCTAA
Nema    TGGAAAGAGCTTCAAGTAGAGGTGACAAACCTATCGAGCCTAGTTATAGCTGGTTGCCTGG
Disp    TGGGACGATCTTCAAGTAGAGGTGACAAACCTATCGAGCCTAGTCATAGCTGGTTGTCTGA
Myaf    TGGGACGAGCTTTAAGTAGAGGTGACAAACCTATCGAGCCTAGTCATAGCTGGTTGTTTAA
Lagu    TGGGAAGAGCTTCAAGTAGAGGTGATAAACCTACCGAGCCTAGTAATAGCTGGTTGCTTAG
Trtr    TGGGGTGAACTCCAAGTAGAGGTGATAAACCTACCGAACCTGGTGATAGCTGGTTATCTAG
Zucr    TGGGATGAACGCTGAGTAGAGGTGATAAACCTACCGAACCTGGTGATAGCTGGTTATCTAG
Pxja    TGGGAAGAGCTTCAAGTAGAGGTGATAAACCTACCGAGCCCAGTTATAGCTGGTTGCCTGA
Pxlo    TGGGAAGAGCTTCAAGTAGAGGTGATAAACCTACCGAGCCCAGTTATAGCTGGTTGCCTGA
Pctr    TGGGAAAATCTTCAAGTAGAGGTGACAAGCCTACCGAGCTCAGTTATAGCTGGTTGCCTGG
Apsa    TGGGAAGATCTTTAAGTAGAGGTGACAAACCTACCGAGCTCAGTTATAGCTGGTTGCCTGA
Cabe    TGGGAAGATTCCTGAGTAGAGGCGATAAACCTACCGAACCCGGTTATAGCTGGTTGTCTGG
Bzze    TGGGAGGACCTTCGAGTAGGGGTGACAGACCTACCGAACCTAGTTATAGCTGGTTGCCTTA
Siim    TGGGAAGACCTTTGAGTAGTGGTGACAGACCTATCGAACTTAGTTATAGCTGGTTGCCTGG
Ctru    TGGGGAGAGCTTCGAGTAGAGGTGATAAACCTACCGAGTCTAGTTATAGCTGGTTGCCCAA
Dpbr    TGGGAAGAGCTTCGAGTAGAGGTGACAAACCTACCGAGTCTAGTTATAGCTGGTTGCCCAA
Caki    TGGGAAGAGCTTTGAGTAGAGGTGATAAACCTATCGAGCACAGTTATAGCTGGTTGCCTAA
Phja    TGGAGAGAGCTTCAAGTAGAGGTGATAAGCCTACCGAGCACAGTTATAGCTGGTTGCCTGA
Brsp    TGGGGAGAGCTCCGAGTAGTGGTGATATACCTATCGAGCCCAGTTATAGCTGGTTGTCTGA
Gamo    TGGGAAGAGCTTTGAGTAGAGGTGACAAACCTACCGAGCCCAGTTATAGCTGGTTGCCTGT
Lolo    TGGGAAGAGCTTTGAGTAGAGGTGATAAACCTACCGAGCACAGTTATAGCTGGTTGCCTGA
Batr    TGGGAAGAGCACCAAGTAGAGACAAAACATCTAACAAAATTAGTTATAGCTGGTTCCTCAA
Prmy    CGGGAAGAGCCTCAAGTAGAGACGAAACATCTACCGTATACAGTTATAGCTGGTTGCCCAA
Loli    TGGGAAGAGCTTTGAGTAGAGGTGACAAGCCTACCGAGCTTAGTTATAGCTGGTTCCCTGG
Loam    TGGGAAGAGCTTTGAGTAGAGGTGACAGACCTACCGAGCTTAGTTATAGCTGGTTCCCTGG
Chab    TGGGGAGAGCTTTGAGTAGAAGTGAAAAGCCTACCGAGCCTAGTCATAGCTGGTTGCCTGG
Chto    TGGGGAGAGCTTTGAGTAGAAGTGAAAAGCCTACCGAGCCTAGTCATAGCTGGTTGCCTGG
Majo    TGGGACGAGCTTCGAGTAGAGGTGACAAACCTACCGAACTTGGTTATAGCTGGTTGCCCGG
Hlst    TGGGAAGATCTTTGAGTAGAGGTAAAAAGCCTACCGAGCTTAGTTATAGCTGGTTGCCCGA
Clpe    TGGGAAGAGCTTTGAGTAGAGGTGACAAACCTACCGAGCCTAGTTATAGCTGGTTGCCTGG
Mlmr    TGAGAAGAGCTTTGAGTAGAGGTAAAAAACCAACCGAGCCTAGTTATAGCTGGTTGCCTGG
Crcr    TGGGAAGAGCTTTGAGTAGGGGTGACAGACCTACCGAACTTGGTTATAGCTGGTTGCTTGA
Muce    TGGGAAGAGCTTTGAGTAGGGGTGACAGACCTACCGAACTTGGTTATAGCTGGTTGCTTGA
Bege    TGGGAAAAGCTTCAAGTAGAGGTGATAAACCTACCGAGCCTGGTTATAGCTGGTTACCTGA
Mela    TGGGAAGAGCTTTGAGTAGAGGTGACAGACCTACCGAACTTAGTTATAGCTGGTTACCTGA
Hats    TGGGAAGAGCTTCGAGTAGAGGTGACAGACCTACCGAGCCTAGTTATAGCTGGTTACCTGA
Orla    TGGGATGATCTTCAAGTAGAGGTGACAGACCTATCGAACTAAGTTATAGCTGGTTGCTCGT
```

Cosa	TGGGAAGAGCTTTGAGTAGAGGTGATAAACCTACCGAACTTAGTAATAGCTGGTTGCCTGA
Exsp	TGGGAAGAACTTTGAGTAGCGGTGAAAAGCCTCCCGAACCTAGTAATAGCTGGTTGCCTGA
Depa	TGGGAAGATCTTTGAGTAGAGGTGACAGACCTACCGAACTTAGTAATAGCTGGTTATCTGA
Rima	TGGGAAGAGCTTTGAGTAGGGGTGACAGACCTACCGAACCTGGTTATAGCTGGTTGCCTAA
Fuol	TGGGAAGATCCTTGAGTAGAGGTGATAAGCCTACCGAGCCTAGTTATAGCTGGTTGCCTGA
Gmaf	TGGAAAGACCTTTGAGTAGAGGTGATAAACCTACCGAGCCTAGTTATAGCTGGTTGCCTGT
Xeei	TGGGGAGAGCTTTGAGTAGAGGTGACAGACCTACCGAGCCTAGTTATAGCTGGTTGCCTGA
Pros	TGGGAAGAACTTTGAGTAGAGGTGACAGACCTATCGAGTCTAGTTATAGCTGGTTGCCTAA
Scmi	TGGGAAGAGCTTTGAGTAGCGGTGACAGACCTATCGAGTCTAGTTATAGCTGGTTGCCCAA
Rolo	TGGGAAGAGCTTTGAGTAGAGGTGACAGACCTACCGAGCCCAGTTATAGCTGGTTACCTAG
Cere	TGGGAAGAGCTTTGAGTAGAGGTGACAGACCTACCGAGCCCAGTAATAGCTGGTTTCCTAA
Daga	TGGGAAGAGCTTTGAGTAGAGGTGATAAACCTAACGAGCCCGGTGATAGCTGGTTTCCTGG
Anco	TGGGAAGATCTTCGAGTAGAGGTGACAGACCTACCGAGCCCAGTAATAGCTGGTTGCCTAA
Dmve	TGGGGAGAGCTTCGAGTAGAGGTGAAAGGCCTACCGAGCCCAGTTATAGCTGGTTGCCTAA
Dmar	TGGGGAGAGCTTTGAGTAGAGGTGAAAAGCCTACCGAGCCCAGTAATAGCTGGTTGCCTAA
Anka	TGGGAAGAACTTTGAGTAGAGGTGACAGACCTACCGAGCCCAGTTATAGCTGGTTGCCTAG
Moja	TGGGAAGAGCTTTGAGTAGAGGTGAAAGACCTACCGAGCCCAGTCATAGCTGGTTGCCTAA
Hoja	TGGGAAGAGCTTTGAGTAGAGGTGACAGACCTACCGAGCCCAGTTATAGCTGGTTGCCTAA
Bede	TGGGAAGAACTTTGAGTAGAGGTGACAGACCTACCGAGCCTAGTTATAGCTGGTTGCCTAA
Besp	TGGGAAGAACTTTGAGTAGAGGTGACAGACCTACCGAGTCTAGTTATAGCTGGTTGCCTAA
Mysp	TGGGAAGAGCTTTGAGTAGAAGTGACAGACCTACCGAGCCCAGTAATAGCTGGTTGCCTGG
Osja	TGGGAAGAGCTTTGAGTAGAAGTGACAGACCTACCGAGCCCAGTTATAGCTGGTTGCCTGA
Sgro	TGGGAAGAGCTTCGAGTAGAAGTGACAGACTTACCGAGCCCAGTTATAGCTGGTTGCCTGA
Pzpa	TGGGAAGAGCTTTGAGTAGAGGTGACAAATCTACCGAGCATAGTTATAGCTGGTTGCCTAA
Zeja	TGGGAAGAGCTTCGAGTAGAGGTGACAGACCTACCGAGCATAGTTATAGCTGGTTGCCTGA
Znne	TGGGAAGATCTTTGAGTAGAGGCGATAAACCTACCGAGCATAGTTATAGCTGGTTGTTTGA
Zefa	TGGGAAGAGCTTTGAGTAGAGGCGATAAACCTACCGAGCATAGTTATAGCTGGTTGTTTGA
Acni	TGGGAAGAGCTTTGAGTAGAGGTGATAAACCTACCGAGCATAGTTATAGCTGGTTGCCTGA
Ncrh	TGGGAAGAGCTTTGAGTAGAGGTGATAAACCTACCGAGCATAGTTATAGCTGGTTGCCTGA
Agca	TGGGATGAGCTTTGAGTAGAGGTGACAGACCTACCGAACTTAGTTATAGCTGGTTGCCTAG
Hydy	TGGGAAGAGCTTTGAGTAGAGGTGATAGACCTACCGAACATAGTTATAGCTGGTTGCCCAG
Gsac	TGGGGCGAGCTTTGAGTAGAGGTGACAGACCTACCGAACCTAGTTATAGCTGGTTGTCCAA
Pevo	TGGGAAGAGCTTTGAGTAGAGGTGACAGACCTACCGAACCCGGTTATAGCTGGTTGCCTGC
Hiku	TGGAATGAGCTCCGAGTAGAGGTGATAAACCAAACGAGCTTGGTTATAGCTGGTTGCCTGA
Inpa	TGGGAAGAGCTTCGAGTAGAGGCGAAAAACCTACCGAGCTTGGTGATAGCTGGTTGCCTGA
Auch	TGGGACGATCTTCAAGTAGAGGTGACATACCTATCGAACCTGGCTATAGCTGGTTGCTTGA
Fico	TGGGAAGACCTTCAAGTAGAGGTGATAAACCTACCGAACCTAGTTATAGCTGGTTGTCCAA
Macs	TGGGAAGAGCTTCGAGTAGAGGTGACAAACCTACCGAGCCCGGTTATAGCTGGTTGCCTGG
Moal	TGGGGAGAGCTCCGAGTAGAGGTGACAGATCTACCGAACTTAGTTATAGCTGGTTGCCTGA
Syma	TGGGAAGAGCTTTGAGTAGAGGTGATAAACCTACCGAACCTAGTTATAGCTGGTTACCTGA
Mafr	TGGGAAGAGCTTCGAGTAGAGGTGACAGACCTATCGAACTTGGTTATAGCTGGTTGCCCGA
Dcpe	TGGGAAGAACTTCGAGTAGAGGTGACAGACCTACCGAACTTGGTTATAGCTGGTTGCCTGA
Dcti	TGGGAAGAACTTCGAGTAGAGGTGACAGACCTACCGAACTTGGTTATAGCTGGTTGCCTGA
Hehi	TGGAACGAGCTTTGAGTAGAGGTGATAAACCTACCGAACCTAGTTATAGCTGGTTGCCCGG
Stam	TGGGAAGAGCTTCGAGTAGAGGTGATAGACCTACCGAACCTAGTTATAGCTGGTTGCCTGA
Hogi	TGGGGAGAGCTTTGAGTAGAGGTGAAAAACCTACCGAATATAGTTATAGCTGGTTGCCCGA
Erzo	TGGGAGGAGCTTTGAGTAGAGGTGACAGACCTACCGAACTTAGTTATAGCTGGTTGTTCAA
Hxot	TGGGGAGAGCTTTGAGTAGAGGTGACAAACCTACCGAACCTAGTTATAGCTGGTTGTCCAA
Core	TGGGGAGAGCTTTGAGTAGAGGTGACAAACCTACCGAACCTAGTTATAGCTGGTTGTCCAA
Apve	TGAGGAGAACTTTGAGTAGAGGTGACAAACCTACCGAACCTAGTTATAGCTGGTTGTCCAA
Latj	TGGGAAGAGCTTTGAGTAGAGGTGACAGACCTACCGAACTTAGTTATAGCTGGTTGCCTGA
Laja	TGGGAAGAGCTTTGAGTAGAGGTGACAGACCTACCGAACCTGGTTATAGCTGGTTGCCTGA

```
Syja   TGGGAAGAACTTCGAGTAGTGGTGACAGACCTATCGAACCTAGTTATAGCTGGTTGCCTGG
Epme   TGGGAAGACTTTCGAGTAGAGGTGACAAACCTACCGAACTTAGTAATAGCTGGTTGCCCAA
Grse   TGGGAAGAGCTCTGAGTAGAGGTGACAGACCTACCGAACTTAGTTATAGCTGGTTGCCCGA
Clja   TGGGAAGACCTTTGAGTAGGGGTGATAGACCTACCGAACTTAGTTATAGCTGGTTATCCGG
Ogcy   TGGGAAGAGCTTCGAGTAGAGGTGACAGACCTACCGAACTCAGCTATAGCTGGTTATCCGA
Plna   TGAGAAGATCTTCAAGTAGAGGTGAAACATCTACCGAACTTAGTTATAGCTGGTTGCTTAG
Lema   TGGGAAGAGCTTTGAGTAGAGGTGACAGACCTACCGAACTAAGTTATAGCTGGTTGTCTGG
Etzo   TGGGAAGAGCTTTGAGTAGAGGTGACAGACCTACCGAACTTAGTTATAGCTGGTTGCCTGA
Apse   TGGGAAGATCTTTGAGTAGAGGTGACAGACCTACCGAACTTGGTTATAGCTGGTTGCCCAG
Epde   TGGGAAGAGCTTTGAGTAGAGGTGACAGACCTACCGAACCTAGTTATAGCTGGTTGCCTGA
Slja   TGGGAAGAGCTCCGAGTAGTGGTGACAGACCTATCGAACCTAGTTATAGCTGGTTGCCTGG
Bsja   TGGGAAGAGCTTCGAGTAGAGGTGACAAGCCTACCGAACCTAGTTATAGCTGGTTGCCTGA
Ecna   TGGGAAGAGCTTCGAGTAGCGGTGACAGACCTACCGAACCTAGTAATAGCTGGTTGCCTGA
Cohi   TGGGAAGAGCTTTGAGTAGAAGTGACAGACCTATCGAACCTAGTTATAGCTGGTTGCCTGT
Caar   TGGGAAGAGCTTCGAGTAGGGGTGACAGACCTATCGAACCCAGTTATAGCTGGTTGCCCGG
Came   TGGGGAGAGCTTCGAGTAGGGGTGACAGACCTATCGAACTCAGTTATAGCTGGTTGCCCGG
Mema   TGGGACGAGCTTCGAGTAGAGGTGACAGACCTACCGAACCTAATTATAGCTGGTTGCCTGG
Lenu   TGGGAAGAGCCTAGAGTAGAGGTGAAAAGCCTATCGAGTCTAGTTATAGCTGGTTGCCTAA
Brja   TGGAAAGAGCTTTGAGTAGAGGTGACAAACCTACCGAACCTAGTTATAGCTGGTTGCCTGA
Plma   TGGGAAGAGCTTTGAGTAGAGGTGACAGACCTACCGAACCTAGTTATAGCTGGTTGCCTGA
Emst   TGGGAAGAACTTTGAGTAGAGGTGACAGACCTACCGAACCTAGTTATAGCTGGTTGCCTGA
Ptti   TGGGAAGAGCTTTGAGTAGAGGTGACAGACCTACCGAGCCCAGTTATAGCTGGTTGCCTGA
Losu   TGGGAAGAACTTCGAGTAGGAGTGACAGACCTATCGAATCTAGTTATAGCTGGTTGCCTGA
Geoy   TGGGGAGATCTTCGAGTAGAGGTGACAGACCTATCGAGCCTAGTTATAGCTGGTTGCCTAA
Dipi   TGGGAAGAGCTTCGAGTAGAGGTGACAGACCTACCGAACTTAGTTATAGCTGGTTGCCTGG
Pama   TGGGAAGAACTTTGAGTAGAGGTGATAAATCTACCGAACTTAGTAATAGCTGGTTGTCTGA
Leob   TGGGAAGAGCTTCGAGTAGAGGTGACAGACCTACCGAGCCTAGTTATAGCTGGTTCCCTGG
Neba   TGGGAAGAGCTCCGAGTAGAGGTGATAGATCTACCGAACTTAGTGATAGCTGGTTGCCTAA
Pdpl   TGGGAAGAGCTTTGAGTAGGGGTGACAGACCTACCGAACCTGGTTATAGCTGGTTGCCTGA
Nimi   TGGGATGAGCTTTGAGTAGCGGTGACAGACCTATCGAACCTAGTTATAGCTGGTTGCCTGA
Uptr   TGGGACGAGCTTCGAGTAGAGGTGACAGACCTACCGAACTTGGTTATAGCTGGTTGCCCGG
Pesc   TGGGACGAGCTTCAAGTAGTGGTGACAGACCTATCGAATCTAGTTATAGCTGGTTGCCTGG
Baar   TGGAAAGAGCTTTGAGTAGAGGTGATATACCTACCGAACCTAGTTATAGCTGGTTGCCTGA
Moar   TGGGAAGAACTTTGAGTAGAGGTGACAGACCTACCGAACTTAGTTATAGCTGGTTGCCTGG
Toja   TGGGAAGAGCTTCAAGTAGCGGTGACAGACCTACCGAACCTAGTTATAGCTGGTTGCCTAA
Chau   TGGGAAGAATTTTGAGTAGCGGTGACAGACCTATCGAACTTAGTTATAGCTGGTTGCCTGA
Chse   TGGGAAAAGCTTTGAGTAGTGGTGACAGACCTATCGAACTTAGTTATAGCTGGTTGCCTGA
Enar   TGGGAAGAGCTTTGAGTAGAGGTGACAGACCTACCGAACTTAGTTATAGCTGGTTGCCTGG
Hpty   TGGGAAGAGCTTCGAGTAGAGGTGACAGACCTACCGAACTTAGTTATAGCTGGTTGCCTGA
Nana   TGGGAAGAGCTTTGAGTAGAGGTGACAGACCTACCGAACTTAGTTATAGCTGGTTGCCTGA
Mcst   TGGGAAGAGCTTCGAGTAGGGGTGACAGACCTATCGAACCCAGTTATAGCTGGTTGCCTGA
Rhox   TGGGATGAGCTTTGAGTAGAGGTGACAGACCTACCGAGCCTAGTTATAGCTGGTTGCCTGG
Opfa   TGGGAAGAGCTTTGAGTAGAGGTGACAGACCTATCGAACCTAGTTATAGCTGGTTGCCTGA
Paar   TGGGACGAGCTTTGAGTAGAGGTGACAGACCTATCGAACCCAGTTATAGCTGGTTGCCCAA
Gozo   TGGGAAGAGCTTTGAGTAGAGGTGACAGACCTACCGAACTTAGTTATAGCTGGTTGCCTGA
Ackr   TGGGGAGAGCTTCGAGTAGCGGTGACAGACCTACCGAACTTAGTTATAGCTGGTTGCCTAC
Elev   TGGGAAGAGCTTTGAGTAGAGGTGATAGACCTACCGAACTTAGTTATAGCTGGTTACCTGA
Trdu   TGGGAAGAACTTCGAGTAGAGGTGATAGACCTACCGAGCCTAGTTATAGCTGGTTGCCTGA
Amoc   TGGGAAGATCTTTGAGTAGAGGTGACAGACCTACCGAACCTAGTGATAGCTGGTTGCCGGG
Hame   TGGGAAGATCTTCGAGTAGCGGTGACAGACCTACCGAACCTAGTTATAGCTGGTTGCTTGG
Chso   TGGGAAGAGCTTTGAGTAGAGGTGACAGACCTATCGAACTTGGTCATAGCTGGTTGCCTGA
```

```
Lyto    TGGGAAGAGCTTTGAGTAGAGGTGATAGACCTACCGAACCTAGTTATAGCTGGTTGTTCAA
Encr    TGGGAGGAGCTTTGAGTAGAGGTGACAGACCTACCGAACCTAGTTATAGCTGGTTGTTCAA
Bvar    TGGGGAGAGCTTTGAGTAGAGGCAACATACCTACCGAACTTAGTTATAGCTGGTTGCCCAC
Noco    TGGGACGAGCTTTGAGTAGAGGTGACAAGCCTACCGAACTTAGTTATAGCTGGTTGCCCAA
Chsp    TGGGAAAAGCTTTGAGTAGAGGTGATAAACCTACCGAACTTAGTGATAGCTGGTTGTCTAA
Arja    TGGGGAGAGCTTTGAGTAGAGGTGACAAACCTACCGAACCTAGTTATAGCTGGTTGTCCAA
Pase    TGGGGAGAGCTTCGAGTAGAGGTGACAAGCCTATCGAACCTAGTTATAGCTGGTTGCCTGG
Trel    TGGGATGAGCTCCGAGTAGAGGTGACAAACCTAACGAACTTAGTTATAGCTGGTTGCCCGG
Lifa    TGGGAAGAGCCTCGGGTAGAGGTGAAAAATCTACCGGACCTAGTTATAGCTGGTTGCCTGG
Acur    TGGGACGAGCTTAGAGTAGAGGTGACAAACCTATCGAACTTAGTGATAGCTGGTTGCCTGA
Ampe    TGGGAAGAGCTTTGAGTAGAGGTGACAGACCTACCGAACTTAGTTATAGCTGGTTGCCTGA
Urja    TGGGAAGAACTTTGAGTAGAGGTGAAAGACCTTTCGAACCTAGTAATAGCTGGTTGCCTGC
Enet    TGGGGAGACTCTCAGGTAGAGGTGATAAGCCTACCGAGCCTGGTTATAGCTGGTTGCCCGT
Ptbr    TGGGGAGAGCTTTGGGTAGAGGTGATAAACCTACCGAACCTGGTGATAGCTGGTTGCTTGA
Safa    TGGGAAGAGCTTTGAGTAGAGGTGAAAAGCCTACCGAACTTGGTGATAGCTGGTTGCCCGA
Icae    TGGGAAGAGCTTTGAGTAGAGGTGACAAACCTACCGAACCTAGTTATAGCTGGTTGCCTGA
Asmi    TGGGGAGAGCTTCGAGTAGAGGTAACAAGCCTACCGAGCCTGATGATAGCTGGTTATCTGA
Foal    TGGGAAGAGCTCCGAGTAGAAGCGAAAAACTTATCGAGCTTGGTTATAGCTGGTTGCCTGA
Drze    TGGGAAGAGCTTTGAGTAGAGATGAAAAGTCTACCGAACAGGGTGATAGCTGGTTGCCTGT
Rhas    TGGGAAGAGCTTTGAGTAGAGGTGACAGACCTACCGAACTTAGTTATAGCTGGTTGCCTAA
Elac    TGGGAAGAGCTTTGAGTAGAGGTGACAGACCTACCGAACTTAGTTATAGCTGGTTGCCTAA
Kugu    TGGGAAGAGCTTTGAGTAGAGGTGACAGACCTACCGAACCTGGTTATAGCTGGTTGCCTGA
Plor    TGGGAAGAGCTTCGAGTAGAGGTGACAGACCTATCGAACCTAGTTATAGCTGGTTACCTGA
Sgun    TGGGAAGAGCTTCGAGTAGAGGTGACAGACCTACCGAACTAAGTTATAGCTGGTTACCTGT
Zaco    TGGGAAGAGCTTAGAGTAGAGGTGACAAACCTACCGAGCCCAGTTATAGCTGGTTGCCTGG
Zbfl    TGGGAAGAGCTTCGAGTAGAGGTGACAGACCTACCGAACTTAGTTATAGCTGGTTGCCCGG
Spba    TGGGAAGATCTTCGAGTAGAGGTGACAGACCTACCGAACTTAGATATAGCTGGTTGCCTGA
Game    TGGGAAGAGCTTTGAGTAGAGGTGACAGACCTACCGAACCTAGTTATAGCTGGTTGCCTGA
Thth    TGGGAAGAGCTTTGAGTAGAGGTGACAGACCTACCGAACCTAGTTATAGCTGGTTGCCTGA
Xigl    TGGGAAGAGCTTTGAGTAGAGGTGACAGACCTACCGAACCTAGTTATAGCTGGTTGCCTGG
Hyja    TGGGAAGATCTTTGAGTAGAGGTGACAGACCTACCGAACCTAGTTATAGCTGGTTGCCTGA
Psan    TGGGAAGATCTTTGAGTAGAGGTGACAAACCTACCGAGCCTAGTTATAGCTGGTTGCCTGG
Cupa    TGGGAAGAGCTTTGAGTAGAGGTGACAGACCTACCGAACCTAGTTATAGCTGGTTGCCTGA
Mpch    TGGGAAGAGCTTAGAGTAGAGGTGACAGACCTACCGAACTTAGTTATAGCTGGTTGCCTGA
Char    TGGGAAGAGCTTTGAGTAGAGGTGACAAACCTACCGAACTTAGTTATAGCTGGTTGCCTAG
Pser    TGGGAAGAGCTTTGAGTAGAGGTGACAGACCTACCGAACCTAGCAATAGCTGGTTGCCTGA
Prol    TGGGAAGAGCTTTGAGTAGAGGTGACAGACCTACCGAACTCAGTTATAGCTGGTTGCCCGT
Plbi    TGGGAAGAGCTTTGAGTAGAGGTGACAAACCTACCGAACTTAGTTATAGCTGGTTGCCTGT
Calu    TGGGAAGATCCTTGAGTAGAAGTGACAGACTTACCGAACTCAGTTATAGCTGGTTGTCTGA
Papa    TGGGAAGATCTTTGAGTAGAGGCGACAAACCTATCGAACCCAGTTATAGCTGGTTGCTTGG
Sufr    TGGGAAGATCTTTGAGTAGAGGTGATAAACCTACCGAACTTAGTTATAGCTGGTTGCCTGA
Stci    TGGGAGGAGCTCTGAGTAGAGGTGATAGACCTACCGAACTTAGTTATAGCTGGTTGCCTGA
Taru    TGGAAAGAGCTTTGAGTAGAGGTGATAAACCTACCGAGTTCAGTTATAGCTGGTTGCCCGA
Rala    TGGGAGGAGCTCTGAGTAGAGGTGACAAACCTACCGAACTTAGTGATAGCTGGTTGCTCGG
         *     *        ****    * *       *          **********
```

```
                16       16'      17      ¦              HVR
Scca   GAAAAGAACTTTAATTCTGCATTAATCCCCT--------------------------TT
Muma   GAAAAGAACTTTAGTTCTGCATTAACTCTTT--------------------------AC
Erca   GAAATGAATAAAAGTTCAGCCTCAAATATTC--------------------------TA
Pose   GAAATGGATAAAAGTTCAGCCTCAAAAAATC--------------------------TA
Actr   GAAATGAATATTAGTTCAGCCTCAAGGCTTC--------------------------TA
Scal   GAAATGAATATTAGTTCAGCCTCAAGGCTTC--------------------------TA
Posp   GAAATGAATATTAGTTCAGCCTCAAGGCTTC--------------------------TA
Atsp   GAAACGAATATTAGTTCAGCCTCAAGACATA--------------------------CT
Leoc   GAAACGAATATTAGTTCAGCCTCAAGACATG--------------------------CT
Amca   GAAATGGATACAAGTTCAGCTTTAAGGATAC--------------------------TC
Osbi   AAAATGAATTTTAGTTCAGCCATATAGCCTG--------------------------CT
Pabu   AAAATGAATATAAGTTCAGCCTTACACTCTC--------------------------CT
Hial   GAAATGGATATTAGTTCAGCCCCATGGCCTT--------------------------CT
Elha   GAAATGAATATTAGTTCAGCCCCACGACCTG--------------------------CT
Mlcy   GAAATGAATATAAGTTCAGCCTCCAGAGCCT--------------------------AC
Algl   GAAATGGATATTAGTTCAGCCTTATGTTATG--------------------------CT
Ptgi   GAAATGAATATTAGTTCAGCCTTACACTATT-------------------------CTC
Alaf   GAAATGAATTTTAGTTCAGCCCCACCCGTTC---------------------------T
Nock   GAAATGAATTTTAGTTCAGCCCTACTCGTTC----------------------------
Anja   GAAATGAATGTTAGTTCAGCCTTATGTAATT------------------------CTAT
Gyki   AAAATGAATATAAGTTCAGCGCGGTGAACTT----------------------------
Syka   GAAATGAATGTTAGTTCAGCCTTATATATTC----------------------------
Opma   GAAATGAATGTTAGTTCAGCCCCACATATTC----------------------------
Comy   GAAAAGAATATGAGTTCAGCCTTATAAGTTT----------------------------
Sasp   GAAATGAATGTTAGTTCAGCCTAATATAATT-----------------------CTTTA
Eupe   AAAATGGATATCAGTTCAGCCTTAATTAATC----------------------------
Enja   TAAATGAATACAAGTTCAGCCCTGCCTAGCC----------------------------
Same   GAAATGAATAGAAGTTCAGCCCCGTTGCGCC--------------------------CT
Chch   GAAATGGATAGAAGTTCAGCCTTATCCCCCT---------------------------C
Grgr   GAAATGGATAAGAGTTCAGCCCCCCAACCAC--------------------------CT
Caau   GAAATGGATAGAAGTTCAGCCTCGTACTCCC----------------------------
Cyca   GAAATGAATAGAAGTTCAGCCTCGTGCACCT-----------------------CAAA-
Dare   GAGATGAATAAAAGTTCAGCTTCGTACACCC--------------------------C-
Cost   GAAATGAATAGAAGTTCAGCCTCGTGCTCCC-----------------------TTTAA
Leec   GAAATGAATAGAAGTTCAGCCTCATACACCC--------------------------TC
Crla   GAAATGAATAGAAGTTCAGCCTCGCACCCCT---------------------------T
Clmc   GAAATGGATAGAAGTTCAGCCTCGTACTCCC---------------------------C
Phin   AAAATGAATAGAAGTTCAGCCTCGTGCTCCT----------------------------
Icpu   GAAATGAATAGAAGTTCAGCCTCGTACTCCT---------------------------C
Psto   GAAATGAATAGAAGTTCAGCCTCACACTCCT--------------------------TA
Cora   GAAGTGGATAGAAGTTCAGCCTCACACCCCT----------------------------
Eisp   AAAATGGATAAAAGTTCAGCCTCATACTCCT-----------------------CACC-
Apal   GAATTGGATAAAAGTTCAGCCTCGTACTCCT----------------------------
Eslu   GAAATGAATAGAAGTTCAGCTCTATGGGCCT-------------------------CTC
Dape   GAAATGAATAGAAGTTCAGCTCTTCGGTCCT--------------------------CT
Glse   AAAATGAATAGAAGTTCAGCCCCCTGGCTCC---------------------------T
Naar   GAAATGAATAGAAGTTCAGCCCTCTCGCTCC--------------------------C-
Baoc   GAAATGAATAGAAGTTCAGCCCTCTCGCTCC----------------------------
Opso   GAAATGAATAGAAGTTCAGCCCCCTGGCTCC-----------------------TCTAG
Alte   GAAGTGGATATAAGTTCAGCCCCTCACCCCT----------------------------
Plap   GAAATGGATAGAAGTTCAGCCCCTCACCCCT-----------------------CACG-
```

```
Plal    GAAATGAATAGGAGTTCAGCCCCCTAGCTTC------------------------TCAA
Sami    GAAATGAATAGGAGTTCAGCCCCCTGGCTTT----------------------------
Rere    GAAATGAATAGGAGTTCAGCCCCCCAACTTT----------------------------
Gama    GAAGCGAATAGAAGTTCAGCCCTTCAAGTCA-----------------------CCCAT
Onmy    GAAATGAATAGAAGTTCAGCCCCCCGGCTTT----------------------------
Sasa    GAAATGAATAGAAGTTCAGCCCCCTGGCCTT--------------------------CT
Cola    GAAATGAATAGAAGTTCAGCCCCCTGGCTTT----------------------------
Dita    AAAATGAATAGAAGTTCAGCCTTCTGAGTTC--------------------------TT
Gogr    AAAATGAGTAGAAGCTCAGCCTCCTGGGTTA--------------------------TT
Chsl    GAAATGAATAGAAGTTCAGCCTCCCGTATTC-----------------------TCCAG
Atja    GAAATGGATAGAAGTTCAGCCTTCAAGACTT--------------------------CC
Iido    GAAATGAATAGAAGTTCAGCCCTCAAGACTA--------------------------TT
Auja    GAAATGGATAGAAGTTCAGCCCTTTAGCTTC---------------------------C
Chag    GAAGTGGATAGAAGTTCAGCCCTCCGACCCT--------------------------TC
Hami    GAAATGAATAGAAGTTCAGCCCCCAAGATTC----------------------------
Saun    GAAATGGATAGAAGTTCAGCCCCTGACCTTC--------------------------CC
Nema    GAAATGGATAGAAGTTCAGCCTCCCGATAAC------------------------CCTC
Disp    AAAGTGTATATGAGTTCAGCCTTCCGGCTCC-------------------------CCC
Myaf    GAAGTGAATTTAAGTTCAGCCTCCTGACTCC----------------------------
Lagu    AAAGTGAATATTAGTTCAGCCCCCTGCCTGC-------------------------CCC
Trtr    GAAGCGAATATAAGTTCAGCCCCCATCTTCC---------------------------C
Zucr    AAAATGAATATAAGTTCAGCCCCCGCCCCTC----------------------------
Pxja    GAAATGAATAGAAGTTCAGCCTTTTAGCTTC---------------------------T
Pxlo    GAAATGAATAGAAGTTCAGCCTTTTAGCTTC-----------------------TTAAC
Pctr    AAAATGAATATAAGTTCAGCCCCTCGGCCCC--------------------------CC
Apsa    GAAATGAATAAAAGTTCAGCCCTTTAATCTC----------------------------
Cabe    GATATGAATAGGAGTTCAGCCCCCTGGACCC--------------------------AC
Bzze    GAAATGGATAGAAGTTCAGCCCCATGCCTTC--------------------------TC
Siim    GAAACGGATATAAGTTCAGCCCTGCGGTTTC--------------------------TC
Ctru    GAAACGAATAGAAGTTCAGCCTCTCAGGTTC---------------------------T
Dpbr    GAAACGAATAGAAGTTCAGCCTCCCGGATTC--------------------------TC
Caki    GAAATGGATAGAAGTTCAGCCCTTAAGCCAT-------------------------TCC
Phja    GAAATGAATAGGAGTTCAGCCCTTTAAAATT-------------------------CCC
Brsp    GAAATGAATAGAAGTTCAGCCCTCATAAATT-----------------------TTCCT
Gamo    GAAATGAATAGGAGTTCAGCCCTTTAAGTCT-------------------------TTC
Lolo    GAAATGAATAGAAGTTCAGCCCTCTAAGTCT-----------------------TTC--
Batr    GAATAAAGTATAAGCTTGGCCCTTTGTAACA----------------------------
Prmy    GAAATGAATAGAAGTTCAGCCCCGTAGCTCT--------------------------TT
Loli    GAAATGGATAGGAGTCCAACCCCCTAGGTTC-------------------------TTT
Loam    GAAATGGATAGGAGTTCAGCCTTCTAAGTTC-----------------------TTCA-
Chab    AAATTGGATAAAAGTTCAGCCTCCAGGCTTT-------------------------TCC
Chto    AAATTGGATAAAAGTTCAGCCTCCAGGCTTT-------------------------TCC
Majo    GAAATGAATAGCAGTTCAGCCCTACGGCTTC-------------------------TCC
Hlst    GAAATGGATAGAAGTTCAGCCTTCAAGGTTC--------------------------T-
Clpe    GATTTGGATAGAAGTTCAGCCTCCCCGCTTC--------------------------TT
Mlmr    GACTTGGATAGAAGTTCAGCCTCCCGGCCCT--------------------------T-
Crcr    AAAATGAATAGGAGTTCCGCTTCTTAAATTC--------------------------TT
Muce    AAAATGAATAGGAGTTCCGCTTCTTAAATTC-----------------------TTACC
Bege    AAATTGAATAGAAGTTCAGCCCCTTGAATTC--------------------------TC
Mela    AAATTGGATAGAAGTTCAGCCTCTTAAGTTC--------------------------TC
Hats    AAATTGAATAGGAGTTCAGCCTCTTGAATTC--------------------------TT
Orla    GAAATGGATAGAAGTTCAGCCTTTTGCTTTC--------------------------C-
```

```
Cosa    TAAATGAATAGAAGTTCAGCCTATTAACTTT----------------------------
Exsp    GAAATGAATAGAAGTTCAGCCTCCTCCCTTC--------------------------TT
Depa    GAAATGAATACAAGTTCAGCCTATTGCTTTT-----------------------TTAAA
Rima    AAATTGAATAAAAGTTCAGCCTTTATTATTC--------------------------TT
Fuol    GAACTAAATATAAGTTTAGCCTCTTATCTTC-------------------------TTA
Gmaf    GAAATAAATTTAAGTTTAGCCTTTAATTATC-------------------------TCA
Xeei    GAACTAAATAAAAGTTTAGCCTCTTATTTTC--------------------------TC
Pros    GAAATGGATATGAGTTCAGCCCCCTGGACTC-------------------------CCC
Scmi    GAAATGGATATAAGTTCAGCCCCTTAGATTT-----------------------TCCCG
Rolo    GAAATGAATAGTAGTTCAGCCCCCCGACTTC--------------------------CC
Cere    GAAGTGGATAGAAGTTCAGCCCCTTAACTTA--------------------------CC
Daga    GAAATGGATAGAAGTTCAGCCCCCCAACTTA--------------------------T-
Anco    GAAATGAATAGAAGTTCAGCCTCCGGGATTC--------------------------CC
Dmve    GAAATGAATATAAGTTCAGCCTCCGAGCTTC--------------------------CC
Dmar    GAAATGAATATAAGTTCAGCCTCCAAGCCTT--------------------------CC
Anka    AAAATGAATAGAAGTTCAGCCTCCAAATATT------------------------CCTA
Moja    GAAATGAATAGAAGTTCAGCCTCCGAGAATT--------------------------CC
Hoja    GAAATGAATAGAAGTTCAGCCTCCGAGATTC--------------------------CT
Bede    GAAATGGATAGAAGTTCAGCCCCCCGTATTT-----------------------CCCAA
Besp    GAAATGGATAAAAGTTCAGCCCCCCGTATTT----------------------------
Mysp    AAAATGAATAGAAGTTCAGCCTCCAAGCTTC--------------------------CC
Osja    GAAATGAATAGAAGTTCAGCCTCCAAGCTTC-----------------------CCACA
Sgro    GAAATGAATTTAAGTTCAGCCCTCTAGATTT-------------------------CCA
Pzpa    GAAATGAATAAAAGTTCAGCCCTCCGGCTTC--------------------------CT
Zeja    GAAATGAATAGAAGTTCAGCCCCCCGGCTTC---------------------------C
Znne    GAAATGAATAGAAGTTCAGCTCTCCGGCTTC--------------------------CC
Zefa    GAAATGAATAGAAGTTCAGCTCTCCGGCTTC----------------------------
Acni    GAAATGAATAGAAGTTCAGCCCCCCGGCTTA--------------------------CT
Ncrh    GAAATGAATAGAAGTTCAGCCCCCCGGCTTA-------------------------CTG
Agca    GAAACGGATAGAAGTTCAGCCTCCCGGCTTC---------------------------T
Hydy    GAAATGAATAGAAGTTCAGCCTTCCGGCTTC--------------------------TC
Gsac    GAAATGAATAGAAGTTCAGCCTCCCGGCTTC---------------------------T
Pevo    GAAATGAATAGAAGTTCAGCCCCACCCCTAC-------------------------TTT
Hiku    AAAATGAATAAAAGTTCAGCCTATATTATAC--------------------------CC
Inpa    GAAATGAATAGAAGTTCAGCCTCTTAACTTC--------------------------TT
Auch    AAAATGGATAGAAGTTCAGCCTTGCCCTTTC-----------------------CTCAG
Fico    GAAATGAATAGAAGTTCAGCCCCATACTTTA----------------------------
Macs    GAAATGAATAGAAGTTCAGCCTCACGGCTTC---------------------------T
Moal    GAACTGAATATAAGTTCAGCCTCCCAGCTTC---------------------------T
Syma    AAATTGAATTTAAGTTCAGCCTCCCAAATTC--------------------------TC
Mafr    GAACTGGATAGAAGTTCAGCCTCCAGGCTTC--------------------------TT
Dcpe    GAAATGAATAGAAGTTCAGCCCCATAGCCAC-------------------------CCT
Dcti    GAAATGAATAGAAGTTCAGCCCCATAACCAC-------------------------CCT
Hehi    GAACTGGATAGAAGTTCAGCCCCCCAGATTC-----------------------TTTAT
Stam    GAATTGAATAGAAGTTCAGCCTCCCGGGTTC--------------------------TT
Hogi    GAATTGAATAGAAGTTCAGCCCCTCCGTTCC--------------------------TC
Erzo    GAAATGAATAGAAGTTCAGCCTTATGGCTTC----------------------------
Hxot    GAAATGAATAGAAGTTCAGCCTTTCAGCTTC----------------------------
Core    GAAATGAATAGAAGTTCAGCCTCTTGGCTTC--------------------------TT
Apve    GAAATGAATAAAAGTTCAGCCTCTAGGCTTC--------------------------TC
Latj    GAACTGGATAGAAGTTCAGCCTCCCGGCTTC--------------------------TC
Laja    GAAATGGATAGGAGTTCAGCCTCCCGGCTTC-------------------------TCT
```

```
Syja    GAACTGGATAGAAGTTCAGCCTCACGGCTTC--------------------------TC
Epme    TAACTGGATAGAAGTTCAGCCTCCCCGCTTC--------------------------TT
Grse    GAATTGAATAGAAGTTCAGCCTCCCGGCTTC--------------------------TC
Clja    GAAATGGATATGAGTTCAGCCTTCCGGGTTC--------------------------TC
Ogcy    AAACTGAATAGAAGTTCAGCCTTTATGAATT-------------------------CTC
Plna    GAACTGGATAGAAGTTCAGCCTCTTAGCTTC--------------------------TT
Lema    GAAATGGATAGGAGTTCAGCCTCTCGGATTC----------------------------
Etzo    GAATTGAATAGAAGTTCAGCCTCCCCGATTC--------------------------TT
Apse    GAAATGAATAGGAGTTCAGCCTTTTGGCTTC--------------------------TT
Epde    GAATTGGATAGAAGTTCAGCCTCTCGGCTTC--------------------------TC
Slja    AAGATGGATAGAAGTCCAGCCTCATGGCTTC--------------------------TC
Bsja    GAAATGGATAGAAGTTCAGCCTCCCGGCTTC----------------------------
Ecna    GAAGTGGATATAAGTTCAGCCCTGTAATTTT--------------------------CT
Cohi    GAAATGAATAGAAGTTCAGCCTCCCAGCTTC--------------------------T-
Caar    GAACTGGATAGAAGTTCAGCCTTATAGCTTC--------------------------TC
Came    GAATTGGATAAAAGTTCAGCCTCATGGCTTC--------------------------TC
Mema    GAACTGGATAGAAGTTCAGCCCTACGGCTTC--------------------------TC
Lenu    GAAATGGATTTAAGTTCAGCCTCCTGGCTTC--------------------------TA
Brja    GAATTGGATATGAGTTCAGCCTCTAGGCTTC--------------------------TT
Plma    GAATTGGATAGGAGTTCAGCCTCCAGGCTTC--------------------------TC
Emst    GAATTGGATAGAAGTTCAGCCTCCCGGCTTC--------------------------TT
Ptti    GAACTGAATAGAAGTTCAGCCTCCCGGCTTC----------------------------
Losu    AAAATGGATAGGAGTTCAGCCTTTGGGCTTC--------------------------TT
Geoy    GACCTGAATAGGAGTTCAGCCTCTTGGCTTC-----------------------TTAAT
Dipi    GAATTGGATAAAAGTTCAGCCCCTTGGCTTC-----------------------TTCCC
Pama    GAAATGGATAGAAGTTCAGCCTCACGGCTTC---------------------------T
Leob    GAATTGGATAGAAGTTCAGCCTCTTGGCTTC----------------------------
Neba    GAAATGGATATAAGTTCAGCCTACAGACTTCCTTACTCCAGCCGTAACTTTGACGTTGC
Pdpl    GAGCCGGATAAAAGTTCAGCCTTAAAAGATT-------------------------CTT
Nimi    GAAATGAATAGAAGTTCAGCCTTTCGAATTC-------------------------TC-
Uptr    GAAATGAATAGCAGTTCAGCCCTACGGCTTC-------------------------TCC
Pesc    GAACTGGATACGAGTTCAGCCCCCAGGCTTC-------------------------T--
Baar    GAATTGGATAT-AGTTCAGCCTCCCGGCTTC----TCTCCTCAACACGTCGAAATTTTT
Moar    GAAATGGATAGAAGTTCAGCCTCCCAGCTTC-----------------------TTTTT
Toja    GAACTGGATAGAAGTTCAGCCTCCCGGCTTC-------------------------TCC
Chau    GAAATGGATAGGAGTTCAGCCTATTGGCTTC--------------------------TT
Chse    GAACTGGATAGAAGTTCAGCCTCCCGGCTTC--------------------------TC
Enar    GAATTGGATAGAAGTTCAGCCTCCCGGATTC--------------------------TT
Hpty    GAATTGGATAGAAGTTCAGCCTCCCGGCTTC--------------------------T-
Nana    GAATTGAATAGGAGTTCAGCTTCTAGGCTTC------------------------TTAA
Mcst    GAAATGGATAGAAGTTCAGCCTCTTAGCTTC------------------------TC--
Rhox    GAATTGGATACAAGTTCAGCCTCTCGGCTTC------------------------TC--
Opfa    GAATTGGATAGAAGTTCAGCCTCCCGGTTTC------------------------T---
Paar    GAAATGGATAGAAGTTCAGCCTCTCGGCTTC-----------------------TTTAT
Gozo    GAACTGGATAGAAGTTCAGCCTCCCGGCTTC------------------------TT--
Ackr    GAATTGGATAGAAGTTCAGCCCCTTGGCTTC------------------------TT--
Elev    GAAATGGATAGAAGTTCAGCCTTTTGGCTTC------------------------TCTA
Trdu    AAACTGAATAAAAGTTCAGCCCTTTAAATTC------------------------TC--
Amoc    GAAATGAATAGAAGTTCAGCCTCACAAATTC------------------------TT--
Hame    AAAATGGATAGAAGTTCAGCCTTTTAAATTC----------------------------
Chso    GAAATGGATAGAAGTTCAGCCTTTTTAATTC-------------------------TTT
Lyto    GAAATGAATAGAAGTTCAGCCTCCCGGCTTC-----------------------TCTTT
```

```
Encr    GAAATGAATAGAAGTTCAGCCTCCTGGCTTC----------------------------
Bvar    GAAATGGATAAAAGTTCAGCCTTACGGATTC--------------------------T-
Noco    GAAATGAATAGTAGTTCAGCCTCCCGGCTTC--------------------------TC
Chsp    AAAATGAATAGAAGTTCAGCCTCCCAAATTC--------------------------TT
Arja    GAAGTGAATAGAAGTTCAGCCTCTCGGCTTC--------------------------TC
Pase    GAAATGAATAGAAGTTCAGCCCTTTGGCTTA--------------------------TT
Trel    GAAATGAGTAAAAGCTCAACCTGCCGGATTC--------------------------TC
Lifa    GAAATGGAT-CTAGTTCAGCCTCTTGGTTTC----------------------------
Acur    GAACTGAATAGAAGTTCAGCCTCTAGTTTTT--------------------------CT
Ampe    GAAATGAATAGAAGTTCAGCCTCCCGGCTTC--------------------------T-
Urja    CAATTGAATAGAAGTTCAGCCTCTTGACTTC--------------------------TT
Enet    GAACTGGATAGGAGTTCAGCCCTTTTAATTC---------------------------T
Ptbr    GAAATGAATAGAAGTTCAGCTTTTAAACTTC-----------------------TC---
Safa    GAAATGAATAGAAGTTCAGCCCTTAATGCTC--------------------------TT
Icae    GAATTGGATAGGAGTTCAGCCTCCAGGCTTC--------------------------T-
Asmi    AAAATGGATATGAGTTCAGCCCTTAATATTT--------------------------C-
Foal    GAAATGGATAACAGTTCAGCACTATTATTTC------------------------CTC-
Drze    AAAATGAATAGAAGTTCAGCCTTTTATATTC--------------------------T-
Rhas    GAACTGAATAGAAGTTCAGCCTTTTGATTTC--------------------------TT
Elac    GAACTGAATAGAAGTTCAGCCTTTTAGCTTC--------------------------TT
Kugu    GAAACGAATAGAAGTTCTGCCTCCTGCGTTA--------------------------TT
Plor    GAATTGGATAGAAGTTCAGCCTCTCGGCTTC----------------------------
Sgun    GAAATGGATAGAAGTTCAGCCTCCCGGCTTC-----------------------TTTAA
Zaco    GAATTGGATAGAAGTTCAGCCTCTCGGCTTC--------------------------TT
Zbfl    GAATTGGATAGAAGTTCAGCCTCTAGGCTTC-----------------------TTT--
Spba    GAAATGAATAGGAGTTCAGCCTCCCGGCTTC-----------------------TCTCT
Game    GAATTGGATAGGAGTTCAGCCTCCAGGCTTC-----------------------T----
Thth    GAATTGGATAGGAGTTCAGCCTCCAGGCTTC-----------------------TCT--
Xigl    GAATTGGATAGAAGTTCAGCCTTGCGGCTTC-----------------------TC---
Hyja    GAATTGGATAAGAGTTCAGCCTCCAGGCTTT-----------------------CT---
Psan    AAATTGGATAAGAGTTCAGCCTCTAGGATTC-----------------------TT---
Cupa    GAATTGGATAGGAGTTCAGCCTCCCGGCTTC-----------------------TCC--
Mpch    GAAATGAATAGAAGTTCAGCTTTTTGGTTTC-----------------------TC---
Char    AAATTGAATATAAGTTCAGCCCCCTGCCTTC-----------------------T----
Pser    AAACTGGATATAAGTTCAGCCTCCCGGCTTC-----------------------TC---
Prol    GAATTGGATAGAAGTTCAGCCCTCTGGGTTC-----------------------C----
Plbi    GAATTGAATAGAAGTTCAGCCCGCTGGGTTC-----------------------TC---
Calu    GAGATGGATATTAGTTCAGCCCTCCTCCTTT-----------------------TT---
Papa    GAATTGAATTTAAGTTCAGCCTCTAAGCTTC-----------------------TT---
Sufr    GAAATGGATAGAAGTTCAGCCCCCTAGACCC-----------------------TT---
Stci    GAATTGGATAGAAGTTCAGCCCTTTTTACTT----------------------------
Taru    GAACTGAGTATAAGCTCAGCCTTTTGGCTTC-----------------------TT---
Rala    GAATTGGATAGAAGTTCAGCTTCCTGGTTTC-----------------------TC---
         *          *
```

```
                                    HVR                                   ¦ 17'
Scca   CTACTAAAT-------AAGAATCTTCTTATTAAAGTTAAACA-----TAGAGATTAATAG
Muma   TATCT-AGACAAGAA----------TTTCTCGTCAAAGAAAA--CTATAAGAGTTAATAG
Erca   AGTATAC-TAAAGTACCACCTAA----AAAAATTT---TAA------GAACATTTGAGAG
Pose   AAAAATAT---------ATAAGTACTACCTAAA------AATTTTAAGAAAGTTTGAGAG
Actr   CTGTCACCCAGGTCATTACCAACAAAGAC-ACCAA-----------GAAAACCTTAAGAG
Scal   CTGTCACCC-------AAGTTATTACCAACCAAGACACCAA-----GAAAACCTTAAGAG
Posp   CTGTCAACCAG----------GTCATCACCAACAAAGACACCA--AGAAAACCTTAAGAG
Atsp   CTTAAC--CACCCCAAGTG-----CCTACTCCA--CCAAGGTGCCCGACAATCTTGAGAG
Leoc   CCTAACCGCCCCAA-GTACTTA-----CTTCACCAAGGC--ACTC-GATAATCTTGAGAG
Amca   AAACCAAC---------TACAGTGTTACCACCAA---AGACATAAAGTTAGCCTTAAAAG
Osbi   CTAA----CCAAACTT------------ACCCTAAAAAGAAAACGA-GTAGCTATAAAAG
Pabu   AAATCAAAA----------------------ACAAAGAATTAA-AGAGCCTACGTAAGAG
Hial   -TAGATCAAATGATAAGATATTTACCAAATAAGAAC----CAAAG--ATAACCATGAGAG
Elha   TAAATCAAAAAAGTTAATTCTACCAAAGAATT-----------AGAGACAGTCCTGGGAG
Mlcy   TTAAATCAAACACCA----CACCAAAGACTAAAAG------------ACAGCCCGGAGAG
Algl   TTGGTCAATGGGTGGGTCGCCTATTAAGG--ATGA---------GAGTCAGAGATAAGAG
Ptgi   AGATCAGGAAACCACCACTGAT----AAAGAAC--C-------AGAGTAATATGTAAGAG
Alaf   ---TTAAATCA-------AAAATACCCAAAAA------GAATAAAAGTTACCCGTAGGAG
Nock   TTAAATCAAGTTT---TACTTAACAAGAATAAA------------AGTTACCCGTAGGAG
Anja   AACCAAAACTT----------------ATTAACAAAAAGAACAAAAGCAATACATAAGAG
Gyki   ----CTAACCC--TATATAAAAAGAGGAAA----------------GCAAGACACAGCAG
Syka   TAAAACCCAAAACC---------C----AAACAATAAGG-GCCCAAGTAATACATAAGAG
Opma   CTTAAACC--------AATACAACACACAACAAGGAAC-----AAAGTGATATGAGGGAG
Comy   ---CT--------CAA--AAATCAAAATAAAAG---AAT-AA---AGTAACACATAAGAG
Sasp   ACTA-CACACCC-TAAAA-AGA-----CAA--------------AAGCAATATATATGAG
Eupe   -CTATAATTACATCATATACAC--ATAAAATAAT-----------AGAGAAACTTAAGAG
Enja   -CGACTCACACCAGTTTTACGAAA---AAAGACAAA-------GAGCTCACTCGCAGGAG
Same   CACCCACA-ACAGTT--TTAC------TTA-AACAAGGC-ATGA-GGACACCCCCGGGAG
Chch   CAGTCACCGATGTGCC--A------CTACCTACTAGACACCCAAGAGAGACCGATAAGAG
Grgr   TAGGCCACCTTAAGGTCACTCACATGCACCTGC-CACAGGCCTCAAGAAAGATGGGGGAG
Caau   ---CAAAT--------CAAGTAAACATTAACAAGACAACAAGA---GAAATACACGAGAG
Cyca   TCACACAAACAAAATAA------------GACT--------A-AGAGAAACACATGAGAG
Dare   -AAATTAAAAAATTAAATTTAATTTAACACAAAATTAAGAG------AAAAATACGAAAG
Cost   TCATA--CAAGTAATATCTAACTTAGAA---AA-TA-------AG--AAAAGCATGAGAG
Leec   TAATCAATTAAGA---AACATCTAAATTAAGTAAA-----AAG----CAAAATATGAGAG
Crla   GA----GTTAAATAAGAAACACTCTAACATAACACAAAA--AG----AGAAACGCGAGAG
Clmc   AGCTCACACAAGCAC---CAGCACGCCATGAGACC----GA-----GAAACACACGAGAG
Phin   CACCTC--CTA----------------GACAACTATGAA--ACAGAGAAAAACACGAGAG
Icpu   AT----CTCACAAATACTTTTAT-----ATTAACGAGCCCGAG----AAACCTACGAAAG
Psto   ACTCAAAAA-------TAAAT--TTAAAATACACGAGATAAAG----AAATATGTGAGAG
Cora   CAAGCCAATATTCAACAGTATTATGACAA--------------CGAGCAAGATATGAGAG
Eisp   TCACACCACATCTTTATGTC---CCACGA-G-CCCGA---------GAAACACATGAGAG
Apal   CCGCCCAAA-----------CATCACCCACAAC---ACGAACCAGAGAAACTTACGAGAG
Eslu   AA----TACCCTTAA-GGTAAAACTA--ATTTTGTTTAC--AA----GAGCCCAAAGAAG
Dape   TCCTCC----CCAAAAGTAAC------ACTAAATTTG-TTTAC-A-AGAGACCAAAGAAG
Glse   CCAACCTTTTAAG---------TTTATCTT---ACTCCGGCCCTGAGGAAACCAGTGGAG
Naar   -CAAGCCCCGA---AAGTTTTTCTAACG----CCGGCCC----CGGTGACACCAGAGGAG
Baoc   -CTAAGCCCCGAAAGTTTATCTTACCCCGGCCTC----------AGTGAAACCAGAGGAG
Opso   CCCTTAAAGTACT---ACTAAG-----ACCGGCC-CTG-------AAGAAACCACTGGAG
Alte   CAGGTCAC----------AACAGTAACACGAATACTAGAC-CACGAGAAAAGAAAGGGAG
Plap   TCACAAAAGTAACACAAATA------CTAGA-------CCAC--GAGAGAAGAAAGGGAG
```

```
Plal   ATCCCTAAGATCCTT----CTT-----CTAG-------TGATTAAGAGAAACCGGGGGAG
Sami   ---TCAAG---CCCATGAGAACCTTCTTCTTAA----GGCGAA--GAGAAACCGGGGGAG
Rere   -TCAAGTCATTAAGACCCTACTTTTGTTGATTCC-----------GAGAAGCTGAGGGAG
Gama   CCTCCCAAGTGAAACCTGCTTT----GGTG-CC---------G---GAGAGTTGAAGGAG
Onmy   CTTAG---GACCTTAAGGTA------AAACTAATATT-GTCCC-AAAGAAACCAGGAGAG
Sasa   TAGGAC-CTCAAGGTA------------AAACTAACCTTGTCCCAAAGAAACCAAGAGAG
Cola   CTTAGGACCCTAAGGTAAAACTAACCTCGTCC-----------CATAGAAACCAAGGGAG
Dita   AAACCCCC----------CCGGCGCA----CGACA-CCTGGGCTGCAGAAGTTAGAAGAG
Gogr   TT----AGCCCCTAAGTTCTTCT-----AATGC----TAAACTTAAAGGAACCAGAAGAG
Chsl   CCTCCCCCATGACCCCACTAAC----TTCG-GCC----------CCAGAATACGAGAGAG
Atja   TCCCCCGC--------CACTAGTATAA---CGAACAGTTGGCCCCAGGAAACTAGAAGAG
Iido   CCTCCCCCACCACTCGTA-ACACAACAA-----T----TGGCCCCAGGAAACTAGAGGAG
Auja   CCTCCCAA----------TTCACCC---CCTATATTGGT---CACAGGAAACTAAAGGAG
Chag   CCGCCCCG-ACTGCTCCAAGC------AC---GCTCGGCCCCG-GGATCAAGCGGAGGCG
Hami   -CCGAGCCC---------------------CCCACTACGACTT-AAGGAATCTTAGGGAG
Saun   AACCC---------CTCCCT-------CAGGCC----------CAGGGAAGCTCAAGGAG
Nema   AGTCCCGTAAATACTATTTAAC-----CCA-------GACCACAGGATCACTCGAGAGAG
Disp   CATCCAAGA-------CATTTATGTCCCCCTGGACC--AACTG----CGTACCGGAAGAG
Myaf   -TTACATCAACGGCGCCCACGCCACCATAGATT----------GACGGCAGTCAAGAGAG
Lagu   CG----CTAAACTAGGCCAC--ACCCCC-CTAGTGCC----AG----GTAACCAGAGGAG
Trtr   ACTAGCCCCCATAATGTATGTT-----AAACCTTTGGCTAGGG-TGTTAAGTCTGGGGAG
Zucr   -CCCTAGCCCC-TATAATAGACG-TAAAATCATTGGCTAT-GGT--ATTAAAGCTGGGAG
Pxja   --TAACCCAAACCA-GAATTAC-----CCAGACCTGGGCTC----AAGAAACTAAAAGAG
Pxlo   CCAAACCAGACTT---ACCCAG-----ACCTGGG--------CTCAAGAAACTAAAAGAG
Pctr   ACCCCAAGCGGG-----------AATAACCCC-GCCAG-GCCCCGGAGGAGCCAAGGGAG
Apsa   ---TTA--ACCCCTAGACGATAATCCCCAA-CAG-GCAA-T---GAAAGACTTAAAAGAG
Cabe   ----AGTCCAAC--------AA-----ATACACAAGGGCCCC--CACAGGCCCGGGGGAG
Bzze   ----CCTTCCCAAGCGTT--AA-----CTCAGCTTGACCA----AAAGAAGACATCGGAG
Siim   ----CACTCCA----AATTAAC-----ATAAGCTAGGACAAAA-AGAACCGCCGCAGAAG
Ctru   CCCCTCAA----------ACCGCACCAACTGTGTC-CGACA--CTAAGAAACTGGGAGAG
Dpbr   CCCTCGAACCACCCT-------------TACTGACCCCGACACTAAGAACGCCGCGAGAG
Caki   CCGC------------CCACAAAACACCACGGTA----------AAGGAAAATTAAGGAG
Phja   TC----ATCCCATAAGTTATTACACAA-TT---------TGATAAAGGAAATTAAAGGAG
Brsp   AATTCAAACTTCAATTTACCTTGT--AATTTTCAGAAAAGACTTAAGGAAGTATGAGGAG
Gamo   CCCCCTCACCCAT--GCTTA---CGCTAAAATT-------GATTAAGGAAACTAAAGGCG
Lolo   CCCCA--TCACCCATGCTTACGCTAAAATTGAT-T---------AAGGAAACTAGAGGAG
Batr   ------AGAAAACCC-------CAAAATAG--GC---------CCCCCTGTAACAAAAAG
Prmy   AATTCTACTAA----------------TAGAACCT----------AAAGAACTACTGGAG
Loli   CACCCA---TT-----------------TTGATACTCAAGGCCCAAAGATACTAGGTGAG
Loam   CTCCAACCCTTAAATTA------------AGGTGA---------AAAGAAGTCAGAAGAG
Chab   GATCACATTTCTTTT-TTCTATCTGATCTCGGTTGTC------AACAAAAACCTGGGGAG
Chto   GA----TCACATT---TCTTTTTTCTATCTGATCTCCGTTGTCAACAAAAACCTGGGGAG
Majo   TCTCAACTC-------TGTAGTTCTTTTACCGACAGTTTAAGA----ATAACCGTAGGAG
Hlst   -----CATACCAAAACCCTAG----ACCCAGGACT---------AGAGAAACTAGAAGAG
Clpe   TCTTTCGCACC----------TTCACATGACCCCATTTGATCAAGAAGACCACGGGAGAG
Mlmr   -TCCACCACACTTTCAAATGACCCCCTTCTGG--CCAAGAA------GAAGCCGAGAGAG
Crcr   ACCCCCCTCGATTTTAT--CCCCTATAACAGACAGTAGAATATCAAGAAAATTAAGAAAG
Muce   CCCCTCGATTTTATCCCCTAAT----AACAGACAGCAGAATATCAAGAAAATTAAGAAAG
Bege   AAGCC---CAAACTGGCTACCCTCAGCTAAGACTA-----AAA----GAAATCTAGAGAG
Mela   CCCTCACCTTGATATC------------TTTCTAAGTGAGACACCAAGAAATTAAGAGAG
Hats   ----TCTTCCTTAATAGTTGTT-----CTTAAT--TAAGACACAA-AGAAATCTAGAGAG
Orla   -TAAATTTAAGTCTAGCATTACTTAATACAAATGA---------CTAGGAATCAAAAGAG
```

```
Cosa    ---TTATCT--CCCAACTGGCTCACCCTAAAAGCT-AAATATC--AAGAAGTTAATAGAG
Exsp    GCCCCTAT---------TATGGAAACACCTTA-ATCC-AGAGAAAAAGAAGAGAAGAGAG
Depa    CCAACCATAATCATT----TAT--------------TTA-GGCACCTAAAATCAATAGAG
Rima    TA----ATCAAAAC-TAATTTAATTTTAACTAGAC------AACAAGAAATAATAAAGAG
Fuol    CACCAAAAG-------TGGACACCCTAATTT----TAGGT-TCTGAGAAGACACTGAGAG
Gmaf    TA----CCCCCAACGGCCTA--ACCAACAGCCCCGGCCC-----AAAGATTATTAAAGAG
Xeei    CCCCCAAC--------CTCTTGGTCCTCCTTAGACCAGGTCCAGAGAACTCATAAGAGAG
Pros    GC----CCCAACCCAGTCTTCACTCAAGCTGGATTCAGGTCTT----CAAGCCAGGGGTG
Scmi    CCCCCGCCCAGTCTT---CACTAAAACTGGACTAC--------AAGGCAAGCTAAGGGTG
Rolo    ATCCCACCC-------TAGCCTCACTCAACCTGGATG----CC-GGTTAAACCGAGGGAG
Cere    ----ACACCAACCCTATCTTAG-----TTCTATTCGGGCTAC---AGTAAATTAAGGGAG
Daga    -TACGCCA----CCCCGGCAT--ACTAAGCCCGGG------CTTTAATAAGTTAGAGGAG
Anco    AAC----CCAAACCAG-----TCTCACCCG-----ACTTGGCCCCAGGAAGCCCAGAGAG
Dmve    ACCCCAAGCAAGT--------------CTCACCAACATTGGCCCTAGGAAGCCCAGAGAG
Dmar    CACCCCAAGCAAGTCTCA-CCCACATTGGCCCT------------AGGAAGCCCAGAGAG
Anka    CCCCAAATCAGTTTT----ACTAAACTTGGCT--C----------AAGGAAACTTGGAGAG
Moja    CACCCCAAACTAGTC----------TTACAAGCCTTGGCCC---CAGGAAACCCGAAGAG
Hoja    AATCCAAACTAGTCT-------------CACCAAATTTGGC-CCCAGGAAGCCCAGAGAG
Bede    CC-----CAACCCAGTCTTTACTTAAGCTGGAC-------ATCAGGCCTAGCCGAGGGAG
Besp    ---CCCAAC--CCAACCCAGTCGTTTACTTAAGCTGGATATTTAGGCTTAGCCGAGGGAG
Mysp    ACACTCAA----------ACAACCCCTA--CCATTAAC-GAAAACAGGAAACTCAGAGAG
Osja    CTCAAGCAACACTAATTTACCATGA--------------GTAT--AGGAAACCCAGAGAG
Sgro    AATCAAACCGAATC------ATCTATGCACG--TACC------CG-GAAAACTAGAAGAG
Pzpa    AATCCAC---CTAGTACCAC-------TAATATTG--GCTACA--GGAATACCAGATGAG
Zeja    TACCCCAC----------CTTAGTA--CTACTAAATTACGGCTACAGGAAACCAGAGGAG
Znne    AACCCCCCTTAGTATA--AC-TAATATTG---GCCC--------CAGGAAACTAGAGAAG
Zefa    ----CCAAC--CCCCCTTAGTACCAC--T-AAAATTGGCC---CCAGGAAACCAGAGAAG
Acni    GCCCCACCTTAGTAC---TACTAAAATTG-GG---TACA-------GGAAACCAGAGGAG
Ncrh    CCCCACCT---------TAGTACTACTAAAATTG-GA----CACAGGAAACCAGAGGAG
Agca    ----TTACTCATA----TACGTCGCAACTCCTTTATGACATGTTAAGTAAACCGAGAGAG
Hydy    TTTTCACCCCGGCCT--AACACCAATTGATGATAT----------AAGAAACCGGAAGAG
Gsac    CTTTTCAC----------ATTAGTCTAACACCTATTGATGTATTTAAGAAACCATGAGAG
Pevo    AA----CCGACCAAG-CATC--GCCTGAATTGCTTAC-TA-AG----ATAGCCGTGGGAG
Hiku    TAATTCACCTAAAATT--T------AAATGAATA-GATTCAGCTAAATGATAATATAGAG
Inpa    TTTCCCGCCTCTTTTCCC---------TTTTACAAATTGGCCA-CAAGAAGTTAAGAGAG
Auch    CCACCCTCACTTTAGTCTCCACTCATAG---------GAATCTAACGAATAGCGCAAGAG
Fico    ---CCTCG--TC-CCACAGT-TTCACTTCTT--TAGTCA----CAGGGCAAGTATCGGAG
Macs    CCACTC----ACCTCAGTACT------ACTTAGTCTGACAC-T-ACAGAACCTTTGAGAG
Moal    ----CTAC----------ACACTATCACACCCAGACAGTTACTAAAGAACACCGAGAGCG
Syma    CATACAACACA-----------------CCACC-TTAGTTAACAAAGAACTTTGAGAGAG
Mafr    TATTC---CCCG-----------AC--ACCCA-ACGA-TAAC---AAGAAACCAGGAGAG
Dcpe    GT----CTCACTTCAGCTT---ACTCATATTGACACAAG--AA----GAAACTATGGGAG
Dcti    GTCTCACCC-------CAGTTAACTCCGATCGACAC-AA-----GGAGGGGCTATGGGAG
Hehi    TCAAATCAGTATTACCCCACCT----GA-------TA-ACA---CAAGAAGCTGTGAGAG
Stam    TTTTCACTACGGAATAACCC-------CTCCCTGAT-AACCCTTTAAGAAACCGCGAGAG
Hogi    TCTTCATCTTCGTTT---ACCCTAACTGATATCAA-----------AGGAACTGAGGGAG
Erzo    TCACTTCACCTTAGTTTTACCCCTATCGATGCA----------TAAAGAAACCATAAGAG
Hxot    TCTTTTCACCTTAGTCTGACCCCTATTGATGCAT---------TAAAGAAACCGAAAGAG
Core    TG----TTCACACTAGTCTAACCCCTAAATTGATGCG--TAAA----GAAACCAACAGAG
Apve    TTTTCACCTTGGCCT------------AACCTCTACAGATGCATTAAGAAACCAGAAGAG
Latj    CATTCATACATTATTCTAAAT------GATTACCCCTAGATGA-AAAGAAACCGCGAGAG
Laja    ATTCAAAGCCTATTAACCGCCAAACAAAACGATAGATGCAAGA----GAAACCGAGAGAG
```

```
Syja   CCTTCCTCC---------CCCTCCCCTCAGATACC-----A----AAGAAACCATGAGAG
Epme   CCTTCTC---TAATTCCTTAC------TTTTAACAG-ATACTG-TGAGAAACCGGGAGAG
Grse   CACTCCACCCCTGGTT------------CCACCGCCAATGATCCAAAGAAACCGCGAGAG
Clja   ------ACCGCACACCTCCA-ACCATA-CC---GCCATCTAAA----GAAACCAGAAGAG
Ogcy   CA----ATCACAGCCTAACTTATTCAAGTCTACTCCCCCGAATAAAAGATTCAGAAAGAG
Plna   TATTC--C--------CCCCAATATTTTATACAACTAAGATAATTAAGAAGCTAAAAGAG
Lema   -TTACCTCACTCTCGTCTAAACACCCAACATTGACCCGAAA------GAAACCGCGAGAG
Etzo   TA----TTCACCTCAGTATTACC-----CCTTCTGATACCTAA----GAAGCGAAGAGAG
Apse   TC----CACACATGCCGGTTACACCG--AAAGCATTTA---AG----CAGGTCAAAAGAG
Epde   TCTTCACCCCCCCCCCATATCT-----TGGTCTTACCGATATTTAGAGAAACCGTGAGAG
Slja   TCCTCAACACCCGGAG--GCACTGCCTACATCATAAGGATAATTTCAGAAACCATGAGAG
Bsja   ---TCCTC--TCACCCCAAATACTT--------CAGACACCA-A--AGAAACCGAGAGCG
Ecna   TTCTTCAT-ATATTTACTTCC------TTA-AA---TGAT--CACAAGAAATTATAGGAG
Cohi   CTCTTCACCTATTGTA-------CACACAAA--ACAATGATTTAG-AGAAACTGTGAGAG
Caar   CTTTCATACTAATAC--------T----AAATACCGAACGATAAAAAGAAACTGTAAGAG
Came   CCTTCAT---ACAATAATAA-------CAACCCAAT-GATTAA--AAGAAACCGTGAGAG
Mema   ----CTTCCCAACCCGTTCCAA-----CACACCCCTGGCACTTTAGAGAAACCGTACGAG
Lenu   CATTCAAACCATACA-------------CATCAAGAACC-----CAAGAAACCAGGGGAG
Brja   TCCTCCCCCGGA-----------CCTTACCAAC-ACCGACACCTAAAGAAACCAAGAGAG
Plma   TCTTCACT--ACGGTTCTTT-------CACCCCTACCGACACACAAAGAAGCCTAGAGAG
Emst   TCTTCACCCTCCGTCTAAACCCCATTTTGACACCT---------AAAGAAACCGAGAGAG
Ptti   TTTCCTCC-------CCCTCCGTCTTACCCCCTTT--T-GACACAAAGAAACCGAGAGAG
Losu   TACTCACTAAGCATCTTACAATTCACGTACTATGAAGCGGACAACAAGAAACCCATAGAG
Geoy   TCCCACTACCTATTTCCTTCCTCAATCCCA-------CGATATTAAAGAAACCCTGAGAG
Dipi   TCACCCCTTGTCAAT----CCC-----ACA-------TGACCCCTGAGAAACCAAGAGAG
Pama   --TACTTCAAATTTCGTATAAA-----CCTTAATTAGATTTCT-AAAGAAACCGAGAGAG
Leob   ---TCCCC--TCACTTTAGTATTTACTCCTTA-CAGA-CAACC-AAAGAAACCTTGAGAG
Neba   TGCTCTAACCCAGCAGCATAATTTATTGAAACCTTTAAGAGATAAAAGAAATCTGTAGAG
Pdpl   CA----CTCGAAACAACCTCAAAATTGTATAGACATACTTCAA----GAAACTTTAAGAG
Nimi   CCCTCAACCCCGTCTT------------CACCCCCG-GACAACCAAAGAAATCGAAAGAG
Uptr   TC----TCAACTCTG-TAGT--ACTTTTACCGACAGCCTA-AG----AAAACCGTAGGAG
Pesc   -CCGCTCATCC--CCCAGCCATTATCCCGATTA-----------AGAGAAACCAGGGGAG
Baar   AACACTATCGTTAAAG-----CCCCCTTCTGACACCCCCCCCCCCAGAAACCGTGAGAG
Moar   TCACTTTT---CGTTTCAACCCCT------TTTTGATAACTT---AAGAAACTGAGAGAG
Toja   ACTCATCTCCGTTATACGCTATCCCCTCCCGACACT--CA-----GAGAAGCCGTGAGAG
Chau   TACTCTGCCCCCTTCCCCCCCCCCTTTTTTACCCGGACGCCA-AGAGAAACCAATAGAG
Chse   CACTCACACCTTATC---TTCTAACCCATTAGACACTTT-----AAAGAAACCGAGAGAG
Enar   TACTCGCTTCTGTCTTAACCCTTTTCTGACACTAT----------AAGAAGCCGAGAGAG
Hpty   -TTCTTCACCT--TGTCTCAACCCTCCCATTGACACCT------AAAGAAACCGCGAGAG
Nana   CTCATATCGG-TAT--CACCACCC-----CAGA--CTTT------AAGAAACCAAGAAAG
Mcst   ATTTCAACTCCGCCTGG---CCCGTCCCGACACCT---------AGAGAAACTAAGAGAG
Rhox   CCGTTAATCTGTTCGACTCACCCTCCTTCCCCCCTGAA-CGCCAAAAGAAACCAAGAGAG
Opfa   -CTAGTCACCTTCGTCCAACCC-----ACTCAGACACC-TA-A-AGAAAACCCGCGAGAG
Paar   TCACACTTGTTCT--TACTTAA-----ACAGATA--------TAAGAGAAACCGAAAGAG
Gozo   TCCTCACCCTTCGTCTTTA---------CCCCTACTGACGCCC-AAAGAAACCGCGAGAG
Ackr   ----------ACC----CTTCCCCTAACCTTCCCCGGATTATA----GAAGACATGGGAG
Elev   GTCATAGTAGATATTA--------------GACCCCAA---------GAAACCAAAAGAG
Trdu   CA----TTCCCATTGGCCTAAGGCCTTCACACCGAACAA--AA----GAAGTTAAAGGAG
Amoc   CCCCTC---AACTGTCTTTTA------AGGCGGTTAGATTAAG-AAAGAAGTTAAGAGAG
Hame   ---TACCC--TCAACTAGGAACACC--TCAAACCCGC-CAATTA-TAGAAGCTAAAAGAG
Chso   ATTC-----ATAA-------CCAAACAGATAATAA-----------GATACTAAAGAGAG
Lyto   TCACCCTAGGTTAACCCCTATC----GATGTACT----------TAAGAAACCGGGGGAG
```

```
Encr        TCTTTTCA--------CTTTAGTCTAACCC-CTATTGATGTGCTTAAGAAACCGAGAGAG
Bvar        CCCTTCATTCATAGGGGCAGACCCCCTACCACCCCTGATAGCCTTAAGAAACCATAAGAG
Noco        CGTTCACCTCAGTGTAC-ACACATTCCTGATGTTT---------CAAGAAACCGTGAGAG
Chsp        AC----ATCCCATAAACAATACTTTAAAAATAAAAGAGTTAAA----GAATAAGAGAGAG
Arja        TTTTCACC----------TTAGTCTGACCCCTATTGAT-GCATTAAAGAAACTAAGAGAG
Pase        ----TTTTTGAA----TACCCC-----ATAAATTAAACATAA--AAAGAAACTAAAGGAG
Trel        CCGTCCCTTTTCGATT--G------TGTCGACCCA---TGACCAAGAGAACCTGACAGAG
Lifa        ---TTAAC---TCCCACCCTCTTATTAAGGGCTGAAGACG-ACAGAGAATACCATTAGAG
Acur        TGGTTCTATAATCAACACTAC------GATAACATAGATTTTT-TAAGAAAACAGGAGAG
Ampe        -TTCCTGACCCC-TGTCCTAACCCCTA--------GAGACACCCAAAGAAACCGAGAGAG
Urja        CATTGACTCAATCCC---TAC--AACCAT--GACA-----------AGAAATTAAGAGAG
Enet        CGATACCC----------CCTTGGTATATCCTTACAACGTCTAAGGAGAAGTAAAAGGAG
Ptbr        -TAAACACCTAATAATTTTC-------ATCCG---------TTAAGAGAAGTCTAAAGAG
Safa        CA-----ATCACGCG-------------CATCGTCGATA----AAAGAGCCGTTAAGGAG
Icae        -CTCTTCA----CCACGGTCTTACCCATACCGA------CACCTAAAGAAGCCTAGAGAG
Asmi        -TTAAAACCCCTAAGGCCCTGTCTTTAAAGTAGTA----------AAGAAACTTAAGGAG
Foal        T-CCC-TTTTA-CCCCTTTTACACTAATT--ACAAACAGGCATAGAAGAAGTAATAGCAG
Drze        -TAATTTATAAACATAA--TAAAACACCATAATA--T--TA----AGATAATTATTAGAG
Rhas        TA----GCCAAACAAGGCGACGCCCTA----CCCAGACCACGAGAA-TTAATCAAAAGAG
Elac        CAACC---CGACCTGATTACATCTCCCC---CGGTCCA------AAGAAAGCTAAAAGAG
Kugu        TTCTCCCCGATGGTT--ACTCCCACCCC---TGACAAA------AAGTAAACCAAGAGAG
Plor        -TT--CATCTCCTGGCCCCTACGCCCCCTCT------ACACCAAAGAACCACCGAGAGAG
Sgun        TCACCCCTGTCTTACTCCGTTC-----------TGACACCC---TAAGAAACCGCGAGAG
Zaco        TCTTCCCC-----TCACGTCCTAGACCCA---CTTC-GACACTTTAAGAAACCGACAGAG
Zbfl        ACTCCTCC--------TATTCTAATCCCTTAGAAAC--CTAAA----GAAACCAAGAGAG
Spba        TCACACTTGCACCAATGCTTTA-----ATAG-------ATAACTAGAGAAACCGTTAGAG
Game        CTCTTC----CTCCCGGTCTT------ACCCCTACCGATCCAC-AGAGAAACCTAGAGAG
Thth        ATTCACCGCGGTCTTACCCC---TACCGATG-CAC--------TAAAGAAGCCTAGAGAG
Xigl        CATTCACCTCCGTTTCAACT----------TCCTCCGGATAATCCGAGAAACTGCGAGAG
Hyja        CCCTTCAACCCCGGTA-------CTTCTCCACTACCGATATCTAAAAGAAGCCTAGAGAG
Psan        CCTTCCACCCCGGTACCCACCCACTACCGATACCT---------AAAGAAGCCTACAGAG
Cupa        C-----TTCACCAAGGTCTC--ACCCCTACCGACACCT----A--AAGAAGCCGAGAGAG
Mpch        CTTTCAACTTTATTT--ATT-TAAACTGAT--TCA----------AAAGAACCAAAGAAG
Char        TCATTCA--CACAACT-------CCTAATGCCCCCGGTTACCCG--AGAAGACAGGGGAG
Pser        -CATTCAC----------CCCACCCCAA------CAGATAC-TAAAGAACACCGCGAGAG
Prol        -CCACTCATGCACTG---TTATTACCCATCAGATG--CA----ACGAGAAACTAGAGGTG
Plbi        CA------CTCATG--CACTTTGTTTAACCCTTCAGAC--GCAGCGAGAAACCAGGGGTG
Calu        ACCTCATGGGC--------------CACAGTGCTCTTATGATAAAAAGAAGGGGGAGGAG
Papa        AAGCCACC-TCGTGCCTA-TTATTATAGGAAA-------------AAGAAACTAAGAGAG
Sufr        TTTT----CAAATTCGTACCACTTAACCCGACCAA---------GAGAGCCCTAAGGGAG
Stci        CTCAAATCAAAGCCA-TCAAGCCCGATTAA---------------AAGAATATTAAGGAG
Taru        AACTCCATAACTATT--------TATATTAACCCG-----ACTTTAAGAAACCAAAAGAG
Rala        TCTTCACCCTTGTTT--------T----CATCCCACCCGATGCCCGAGAAACCAAGAAAG
                                                                       *
```

```
                     18                18'       19      19'           20
Scca    TTATTTAGAAGAGGAACA-GCCCTTCTAAATT-AAGATACAACTTTTT---AAGGTGGTT
Muma    TTATTTAGAAGAGGTACA-GCCCTTCTAAACT-AAGATACATCTTTTA---AAGATGGGA
Erca    TTATTCATAAGAGGTACA-GCTCTTATGAACA-GGGAAACAACCCCCA---AAGGAGGAA
Pose    TTATTCACAGGAGGTACA-GCTCCTGTGAACT-GGGAAACAACCCAAT---AAGGAGGAA
Actr    TTATTCAAGAGAGGTACA-GCTCCCTTGAAAA-AGAACACAACCTTAA---CAGGCGGAT
Scal    TTATTCAAGAGAGGTACA-GCTCCCTTGAAAA-AGAACACAACCTTAA---CAGGTGGAT
Posp    TTATTCAAGAGAGGTACA-GCTCCCTTGAAAA-AGAACACAACCTTAA---CAGGCGGAT
Atsp    TTACTCAAAGGAGGTACA-GCTCCTTTGAGAA-AGAATACAACCTTAA---CGGATGGAT
Leoc    TTACTCAAAGGAGGTACA-GCTCCTCTGAGAA-AGAACACAACCTTAA---TAGATGGAT
Amca    TTATTCAAAGGAGGTACA-GCTCCCTTGAAAA-AGAACACAACCTTAA---AATGTGGAT
Osbi    TTAGTCAATGGAGGTACA-GCTCCCTTGAAAA-AGAACACAACCTTTA---CAGAAGGGT
Pabu    TTAATTGAAGGAGGTACA-GCTCCTTCAAAAA-AGAAAACAACCTTCA---CAGATGGGC
Hial    TTAGTCAAAAGAGGTACA-GCTCCTTTGACAA-AGAATACAACCTTAA---AAGATGGAT
Elha    TTAGTCAGAGGAGGTACA-GCTCCCCTGAAAA-AGAACACAACCTTCA---CAGGAGGAT
Mlcy    TTAGTCAGAGGGGGTACA-GCCCCTCTGAAAA-AGAACACAACCTTACA--CAGGAGGAT
Algl    TTAGTCAAAGGAGGTACA-GCTCTTTTGAAAA-AGGATACAATCTTCT---CAGGTGGAT
Ptgi    TTAGTCAGAGGAGGTACA-GCTCCCCTGAAAA-AGAACACAACCTTAA---CAGGAGGAT
Alaf    TTAGTCAAAGGAGGTACA-GCTCCTTTGAAAA-AGAACACAACCTTTC---CAGGAGGAT
Nock    TTAGTCAAAGGAGGTACA-GCTCCTTTGAAAA-AGAACACAACCTTAA---CAGGAGGAT
Anja    TTAGTCAAAGGGGGTACA-GCCCCTTTGAAAC-AGAACACAACCTTATT--CAGGAGGAC
Gyki    TTAGCCAAAAGGGGTACA-GCCCTTTTGGAAA-AGGACACAACCTTATTT-AGGGAGGGA
Syka    TTAGTCAAAGGGGGTACA-GCCCCTTTGAAAC-AGAACACAACCTTTT--ACAGGAGGG-
Opma    TTAGTCAAAGGGGGTACA-GCTCCTTTGAAAC-AGGACACAACCTTAT---CAGGCGGAC
Comy    TTAGTCGAAGGGGGTACA-GCTCCTTCGAAAA-AGAACACAACCTTAC--CCAGGTAAA-
Sasp    TCAGTCAGTGGGGGTACA-GCCCCCCCGACTA-AGGACACAACCTTACA--TTGATGGCC
Eupe    TTAGTCAAGGGGGGTACA-GCCCCCTTGAAAA-AGAATACAACCTTTA---TAGGAGGAC
Enja    TTAATCAGAGGAGGTACA-GCTCCCCTGATAC-AGGATACAACCTTTT---TAGGAGACA
Same    TTAGTCAAGGGAGGTACA-GCTCCCTTAACAA-AGGACACAACCTTAGA--CAGGAGGCT
Chch    TTATTCAGAAGGGGTACA-GCTCTTCTGAACC-AGGACACAACCTTAC--ACAGGAGGT-
Grgr    TTAGTCAGAGGAGGTACA-GCTCTTCTGAAGC-AGGACACAACCT-TT--ATAGGAGGCT
Caau    TTAGTTAAAGGGGGTACA-GCCCCTTTGACAA-AGGATACAACCTTTC---TAGGAGGAT
Cyca    TTAGTTAAAGGGGGTACA-GCCCCTTTAACAA-AGGACACAACCTTTC---CAGGAGGAT
Dare    TTAGTTAAAGAGGGTACA-GCCCCTTTAACAA-AGGATACAACCTTTT---CAGGAGAAT
Cost    TTAGTTAAAGGGGGTACA-GCCCCTTTAATTA-AGGATACAACTTTAT---CAGGCGGAT
Leec    TTAGTTGAAGGGGGTACA-GCCCCTTTAACCA-AGGACACAACCTTAAA--CAGGAGGAT
Crla    TTAGTCAAAGGGGGTACA-GCCCCTCTGATAA-AGAATACAACCTTAG--CCAGGAGGAT
Clmc    TTAGTCAAAGAGGGTACA-GCCCCTTTGATCA-AGGACACAACCTTACT-ACAGGAGGTT
Phin    TTAATCAAAAGAGGTACA-ACTCTTTTGACC--AGGACACAACCTTACA--CAGGAGATT
Icpu    TTAGTCAAAGGGGGTACA-GCCCCTTTGAAAC-AGGACACAACCT-CA--CCAGGAGGTT
Psto    TTAGTCAAAGGGGGTACA-GCCCCTTTGAAAC-AGGACACAGCCTCA---CCAGGAGGTT
Cora    TTAGTCAAAGGGGGTACA-GCCCCTTTGAAAA-AGGAAACAACCTACA---CAGGAGGTT
Eisp    TTAATCAAAGGGGGTACA-GCCCCTATGAAAC-AGGACACAACCTTAA---CAGGAGGTT
Apal    TTAGTCAAAGGGGGTACA-GCCCCTATGACAC-AGGACACAACCTTCT---CAGGTGGTT
Eslu    TTAATCAAAAGAGGTACA-GCTCTTTTGAACA-AGGACACAACCTTA---ACAGGTGTTT
Dape    TTAGTCAAAGGAGGTACA-GCTCCTTTGAATA-AGGACACAACCTTGA---CAGGTGGTT
Glse    TTAGTCAAAAGAGGTACA-GCTCTTTTGAACA-AGGACACAACCTTAC---TAGGTGGTT
Naar    TTAGTCAAAAGAGGTACA-GCTCTTTTGAACA-AGGACACAACCTTAT---CAGGCGGTT
Baoc    TTAGTCAAAAGAGGTACA-GCTCTTTTGAACA-AGGACACAACCTTAC---CAGGCGGTT
Opso    TTAGTCAAAAGAGGTACA-GCTCTTTTGAACA-AGGACACAACCTTAC---CAGGCGGTT
Alte    TTAGTCAGAGGAGGTACA-GCTCCTCTGAACC-AGGACACAACCTTAC---CAGGAGGCC
Plap    TTAGTCAGAGGAGGTACA-GCTCCCCTGAACC-AGGACACAACCTTAT---CAGGAGGCC
```

Plal	TTATTCGAAGGAGGTACA-GCTCCTTCGAACA-AGGACACAACCTT-TG--CAGGCGGTC
Sami	TTATTCAAGGGAGGTACA-GCTCCTTTGAACC-AGGACACAACCTTAA---CAGGCGGTC
Rere	TTAGTCGAAAGAGGTACA-GCTCCTTCGAACC-AGGACACAACCTTAC---CAGGCGGTT
Gama	TAAGTCAAAAGAGGTACA-GCTCTTTTGATAG-AGGACACAACCTTAC---CAGGTGGTT
Onmy	TTAGTCAAAGGAGGTACA-GCTCCTTTGAACA-AGGACACAACCTTAA---CAGGCGGCT
Sasa	TTAATCAAAGGAGGTACA-GCTCCTTTGAACA-AGGACACAACCTTAA---CAGGCGGCT
Cola	TTAGTCAAAGGAGGTACA-GCTCCTTTGAACA-AGGACACAACCTTAA---CAGGCGGCT
Dita	TTAGTCAAGAGAGGTACA-GCTCTCTTGAAAT-AGGACACAACCTTTT---CAGGCGGTT
Gogr	TAAGTCAAGGGAGGTACA-GCTCCTTTGAACC-GGGATACAACCC-CC--TCAGGCGGTA
Chsl	TTACTCAAGTGAGGAACA-GCTCCCTTGACCA-AGGACACAACCTTCA---CAGGAGGTT
Atja	TAAATCAAAGGAGGTACA-GCTCCTTTGAGTA-AAGATACAACTTTAA---AAGAAGGTT
Iido	TAAATCAAAGGAGGTACA-GCTCCTTTGAATA-AAGATACAACTTTAA---AAGAAGGTT
Auja	TTAGTTAAAGAAGGTTCA-GCTTCTTTAAAA--AAGATACAACTTTTT---TAGGAGATT
Chag	TTAGTCAAAGAAGGTCCA-GCTTCTTTGAACA-AAGATACAACTTTTT---TAGGAGGCT
Hami	TTAGTTAAAGAAGGTCCA-GCTTCTTTAAAAC-AAGACACAACTTTTT---TAGGAGACT
Saun	TTAGTTAAAGAAGGTCCA-GCTTCTTTAAACA-AAGACACAACTTT-TT--CAGGAGACA
Nema	TTAGTCGAAGAGGGTACA-GCCCCTTCGAAAA-AGGACACAACCTC-CC--AGGGAGATA
Disp	TTAGTCAAAGAGGGTACA-GCCCCTTTGAAAC-AGGACACAACCTCC---CCGGGAGACT
Myaf	TTAGTCAAAGAGGGAACA-GCCCCTTTGAAAA-AGGACACAACCTCCA---CGGGAGATA
Lagu	TTAGTCAAGAAAGGTTCA-GCTTTCTTGAAA--AGGACACAACCTTC---CTGGGAGGGT
Trtr	TAATTCAAGGGAGGTTCA-GCTCCCTTGAAAG-AGGATACAACCTTACG--CAGGTGGAT
Zucr	TTATTCAAGGGAGGTTCA-GCTCTCTTGAAGG-AGGATACAACCTTATTAACGGGAGGAT
Pxja	TTAGCCAACGGAGGTACA-GCTCCTTTGGAA--AAGATACAACTTTAT---TAGGAGGTG
Pxlo	TTAGCCAACGGAGGTACA-GCTCCTTTGGAA--AAGATACAACTTTAT---CAGGAGGTG
Pctr	TTAGTCAAAAGAGGTACA-GCTCTTTTGAAA--AAGATACAACTTAAT---CAGGAGGCC
Apsa	TTAGTCAGAAGAGGTACA-GCTCTTCTGAAA--AAGATACAACTTATT---CAAGAGGTT
Cabe	TTATTCAGAGGGGGTACA-GCTCCTCTGATAACAAGATACAACTTTAA---AAGGTGAGT
Bzze	TTAGTCAAAGGAGGTACA-GCTCCTTTGAAA--AAGATACAACTTTGC---GAGGAGGGT
Siim	TTAATCAAAGGAGGTACA-GCTCCTTTGAAA--AAGACACAACTTTAC---TAGGAGAGT
Ctru	TTATTCGAAGGGGGTACA-GCCCCTTCGACCC-AAGACACAACTTTTA---TAGGAGGCT
Dpbr	TAATTCGAAGGGGGTACA-GCCCCTTCGACCC-AAGATACAACTTTTC---TGGGGGGCT
Caki	TTAGACAGAAGGGGTACA-GCTCTTCTGAATA-AAGAAACAACCTTAG---CAGGTGAAC
Phja	TTATTCAAAAGGGGCACA-GCTCTTTTGATAA-AAGAAACAACTTTTA---CAGGTGGCC
Brsp	TTAGTCAAAAGAGGTTCA-GCTCGTTTGACTT-TGGAAACAGCCTC-TT--TAAGTGATT
Gamo	TTAATCAAAAGGGGTACA-GCCCTTTTGATAG-AAGAAACAACTTTAA---CAGGTGACC
Lolo	TTAATCAAAAGGGGTACA-GCCCTTTTGATAA-AAGAAACAACTTTAA---CAGGTGGCC
Batr	GTCGTCAAAGGGGGTACA-GCCCATTTGACAT-AAGAAACAACTTC-TC--ATAAAGGTT
Prmy	TTAATTAAAGGGGGGACA-GCTCCTTTAATAC-CAGAAACAACTGCCT---AATGCGGAT
Loli	CAAGTGGAAAGGGGAACA-GCCCTTTCTACCA-AAGACACAACTTT-TA--TAGGAGGAT
Loam	TTAATCAAGGGGGGTACA-GCCCCCTTGAAAA-AAGATACAACTTTAG---CAGAAGGAT
Chab	TTAATCAAAGGGGGTACA-GCCCCTTTGATAC-AAGATACAACTTTTT---TAAGAGGAT
Chto	TTAATCAAAGGGGGTACA-GCCCCTTTGATAC-AAGATACAACTTTTT---TAAGAGGAT
Majo	TTAGTCAAAGGGGGTACA-GCCCCTTTGACAC-AGGACACAACCTTCC---CAGGAGGGT
Hlst	TTATTCAAAGGAGGTACA-GCCCCTTTGATAA-AGGACACAACCTTTTA--CAGGTGGAT
Clpe	TTAGTCAAAGGGGGTACA-GCCCCTTTGACAC-AGGACACAACTTTTT---CAAGTGGAT
Mlmr	TTAATCAAAGGGGGTACA-GCCCCTTTGATAG-AAGATACAACTTTTT---CAAGAGGAT
Crcr	TTAATCAAAGGGGGTACA-GCCCCTTTGAAAC-AAGGTACAACTTTTT---TAGGAGGGT
Muce	TTAATCAAAGGGGGTACA-GCCCCTTTGAAAC-AAGGTACAACTTTTT---TAGGAGGGT
Bege	TTATTCAAAGGGGGTACA-GCCCCTTTGAAAC-AAGACACAACTTTTA---CAGGCGGAT
Mela	TTAGTCAAAGGGGGTCCA-GCCCCTTTGAATA-AAGAAACAACTTTTC---TGGGTGGAT
Hats	TTAGTCAAAGGGGGTACA-GCCCCTTTGAAAC-AAGACACAACTTTACC--CAGGAGGCT
Orla	TTAATCAAAGAGGGTACA-GCCTATTTGATGG-AAGATACAACTTTAC---TAGGAGGCT

Cosa	TTAATTAAAGGGGGTACA-GCCCCTTTAAAAA-AAGACACAACTTTAA---CAGAAGGTA
Exsp	TTATTCAAAGAAGGGACA-GCTTCTTTGAAAA-AAGACACAACTTTTT---TAGGAGGGT
Depa	TTAGTCAAAAGAGGTACA-GCTCTTTTGAAA--AAGATACAACTTT-TA--TAGGAGGAA
Rima	TTAGTCAAAGGAGGAACA-GCCCCTTTGAAAC-AAGAAACAACTT-TT--CCAGAAGGAT
Fuol	TTAGTCAAAAGGGGGACA-GCCCTTTTGAACC-AAGATACAACTTTT---TTAGCTGGAT
Gmaf	TTATTCAAAGGGGGTACA-GCCCCTTTGTACA-AAGACACAACTTTA---ATGAGAGGAT
Xeei	TTAGTCAAAGGGGGTACA-GCCCCTTTGAATC-AAGATACAACTTTCC---TGGGTGGAT
Pros	TTAGTCAAAGGAGGTACA-GCTCCTTTGAAAC-AAGACACAACTTTAC---CAGGAGGAT
Scmi	TTAGTCAAAGGAGGTACA-GCTCCTTTGAAAC-AAGACACAACTTTAT---CAGGAGGGT
Rolo	TTATTCAAAGAAGGTACA-GCTCCTTTGAAAT-AAGATACAACTTTA---ACAGGAGGCT
Cere	TTAATCAAAGGAGGTACA-GCTCCTTTGAAAT-AAGACACAACTTTAA---CAGGAGGCT
Daga	TTAGTCAAAGGAGGTACA-GCTCCTTTGAGA--AAGATACAACTTTTC---CAGGTGGGT
Anco	TTAGTCAAAAGAGGTACA-GCTCTTTTGAAAC-AAGACACAACTTTAC---CAGGCGGAT
Dmve	TTAGTTAAAGGAGGTACA-GCTCCTTTAAAA--AAGACACAACTTT-CC--CAGGTGGAT
Dmar	TTAGTTAAAGGAGGTACA-GCTCCTTTAAAA--AAGATACAACTTTTC---CAGGCGGAT
Anka	TTAGTCAAAAGAGGTACA-GCTCTTTTGAAAC-AAGATACAACTTTGT---CAGGAGGAT
Moja	TTAGTCAAAAGAGGTACA-GCTCTTTTGAAAC-AAGATACAACTTTAA--TTAGGCGGAT
Hoja	TTAGTCAAAAGAGGTACA-GCTCTTTTGAAAC-AAGATACAACTTTAC---CAGGAGGAT
Bede	TTATTCGAAGGAGGTACA-GCTCCTTTGAAAC-AAGACACAACTTTAC---CAGGAGGAT
Besp	TTATTCGAAGGAGGTACA-GCTCCTTTGAAAC-AAGACACAACTTTAC---CAGGAGGAT
Mysp	TTATTCAAAGAAGGTACA-GCTCCTTTGAAAA-AAGAAACAACTTAAC---CAGGAGGAT
Osja	TTATTCAAAGAAGGTACA-GCTCCTTTGAAAA-AAGAAACAACTTTTC---CAGGTGGGT
Sgro	TTATTCAAAGAAGGTACA-GCTCCTTTGAAAA-AAGAAACAACTTTGA---TAGGTGGGT
Pzpa	TTAATTAAAGGAGGTACA-GCTCCTTTATTA--TAGAAACACCTTT-TA--CAGGTGGCT
Zeja	TTAATTAAAGGGGGTACA-GCTCCTTTTTAAT-AGAA-ACACCTTTCA---CAGATGGCC
Znne	TTAGTTAAAGGAGGTACA-GCTCCTTTTTAAT-AGAA-ACACCTTTTA---CAGATGGTC
Zefa	TTAATTAAAGGAGGTACA-GCTCCTTTTTAAT-AGAA-ACACCTTTTA---CAGATGGCC
Acni	TTAGTTAAAGGAGGTACA-GCTCCTTTTTAAT-AGAA-ACACCTTTTA---CAGATGGCC
Ncrh	TTAGTTAAAGGAGGTACA-GCTCCTTTTTAAT-AGAA-ACACCTTTT---ACAGATGGCC
Agca	TTAGTCAAAGGGGGGACA-GCCCCTTTGACAC-AAGACACAACTTTAA---CAAGAGGAT
Hydy	TTAGTCAAAGGGGGTACA-GCCCCTTTGAACC-AAGGTACAACTTTGT---CAGGAGGGT
Gsac	TTAGTCAAAGGGGGTACA-GCCCCTTTGAACA-AAGACACAACTTTAT---CAGGAGGGT
Pevo	TTAATCAAAAGGGGTACA-GCCCTTTTGATAA-AGAATACAACCTTA---AGCACAGGGT
Hiku	TTAGTCAAAAGGGGTACA-GCTCTTTTGACAT-AGGACACAACCTTA---TAATGAGGTT
Inpa	TTACCCAAAGGGGGGACA-GCCCCTTTGACCC-AAGAGACAACTTTCA---CGGGAGGAT
Auch	TTAGTCACAGGAGGTACA-GCTCCTCTGATAT-AGGACACAGCCTTACA--CAGGAGGGT
Fico	TTAGTCAAAGGGGGTACA-GCCCCTTTGACAA-AGGATACAACTTTCC---CAGGAGGAT
Macs	TTAATCAAAGGAGGTACA-GCTCCTTTGATAA-AAAACACAACTTTTT---CAGGTGGGT
Moal	TAAGTTAAAGGGGGAACA-GCTCCTTTAACAT-AAGATACAACTTTAT---CAGAAGGAT
Syma	TTACTTAAAGGGGGAACA-GCTCCTTTAAGAC-AAGATACAACTTTTAC--CAGAAGGAT
Mafr	TTAGTCAAAGGGGGGACA-GCCCCTTTGAAC--AGAACACAATTTTAT---CAGAAGGTT
Dcpe	TTACTCAGAGGGGGTACA-GCTCCTCTGACAA-AAGACACAACTTTC---CCGGGAGGGT
Dcti	TTATTCAGAGGGGGTACA-GCTCCTCTGACAA-AAGACACAACTTTC---CCGGGAGGGT
Hehi	TTATTCAAAGGGGGTACA-GCCCCTTTGAAAC-AAGATACAACTTTCC---CGGGAGGAA
Stam	TTAGTCGAAGGGGGTACA-GCCCCTTTGAACC-AAGACACAACTTT-TA--TAGGAGGTT
Hogi	TTAGTCAAAGGGGGTACA-GCCCCTTTGAATT-AAGACACAACTTTTT---AAGGAGGAT
Erzo	TTAGTCAAAGGGGGTACA-GCCCCTTTGAACC-AAGACACAACTTTCC---CAGGAGGGT
Hxot	TTAGTCAAAGGGGGTACA-GCCCCTTTGAATC-AAGACACAACTTTAC---CAGGAGGGT
Core	TTAGTCAAAGGGGGTACA-GCCCCTTTGAATC-AAGACACAACTT-TA--CTAGGAGGGT
Apve	TTAGTCAAAGGGGGTACA-GCCCCTTTGAATC-AAGACACAACTTTAC---CAGGAGGGT
Latj	TTACTCAAAGGAGGTACA-GCTCCTTTGAACC-AAGATACAACTTTCT---AAGGAGGGT
Laja	TTAGTCAAAAGAGGTACA-GCTCTTTTGAAAC-AAGAAACAACTTTC---CCAGGAGGGT

```
Syja  TTATTCAAAGGGGGAACA-GCCCCTTTGAAAC-AAGAAACAACTTTCC---CAGGAGGGT
Epme  TTAGTCAAAGGGGGTACA-GCCCCTTAGAAAC-AAGATACAACTTTTT---TAGGAGGAT
Grse  TTAGTCAAAGGGGGTACA-GCCCCTTTGAAAC--AGACACAACTTT-AT--CAGAAGGAT
Clja  TTAATCAAAGGGGGTACA-GCTCCTTTGATAA-AAAATACAACTTCTC---CAGGTGGGT
Ogcy  TTATTCGAAAGGGGTACA-GCCCTTTCGAAAT-AAGAAACAACTT-TA--ACAGAAGGTA
Plna  TTAGTCAAAAGAGGTTCA-GCTCCTTTGAAAC-AAGACACAACTTTTC---CAGGAGGGA
Lema  TTAGCCAAAGGGGGTACA-GCCCCTTTGAAAC-AAGATACAACTTTTC---CAGGAGGGT
Etzo  TTAGTCAAAGGGGGTACA-GCCCCTTTGAATC-AAGATACAACTT-TT--CCAGGAGGGT
Apse  TTAGTCAAAGGGGGTACA-GCCCCTTTGAAA--AAGATACAACTTTA---CCAGAAGGGT
Epde  TTAGTCAAAGGGGGTGCA-GCCCCTTTGAAAC-AAGACGCAACTTT-CC--CAGGAGGGT
Slja  TTAATCAGAGGAGGCACA-GCTCCTCTGATAA-AAGATACAACTTTAG---CAGGAGGGT
Bsja  TTAATCAAAGGAGGGACA-GCCCCTTTGATAC-AAGACACAACTTTTA---CAGAAGACT
Ecna  TTAGTCAAAAGGGGTACA-GCCCTTTTGAACC-AAGATACAACTTTAT---AAGGAGGGA
Cohi  TTAGTCAAAGGAGGAACA-GCTCCTTTGAAAC-AAGATACAACTTTTT---TAATTGGGT
Caar  TTAGTCAAAGGGGGTACA-GCCCCTTTGAATA-AAGACACAACTTTTC---TAGGAGGAT
Came  TTAGTCAAAGGGGGTACA-GCCCCTTTGAATA-AAGATACAACTTT-TT--TAGAAGGAT
Mema  TTAGCCAAAGGAGGTACA-GCCCCTTTGAACC-AAGAAACAACTTTAC---CAGGAGGGT
Lenu  TTAGTCAAAGGGGGTTCA-GCCCCTTTGACAA-AGGACACAACCTTAC---CAGTAGGAA
Brja  TTAATCAAAGGGGGTACA-GCCCCTTTGATAT-AAGACACAACTTTTA---CAGGAGGGT
Plma  TTAATCAAAGGGGGTACA-GCCCCTTTGAAAC-AAGACACAACTTT-CC--CAGGAGGGT
Emst  TTAATCAAAGGGGGTACA-GCCCCTTTGATAC-AAGACACAACTTTCC---CAGGAGGGT
Ptti  TTAGTCAAAGGGGGTACA-GCCCCTTTGATAC-AAGACACAACTTTTT---CAGGAGGGT
Losu  TTAGTCAAAGGAGGTACA-GCTCCTTTGACCT-AAGACACAACTTTTC---AGACTGGGT
Geoy  TTATTCAAAGGGGGTACA-GCCCCTTTGAAAC-AAGATACAACTTTTT---GAAAAGGCT
Dipi  TTAATTAAAGGGGGGACA-GCCCCTTTAATCA-AAGACACAACTTT-TA--CAGGAGGAT
Pama  TTAATCTTAGGGGGTACA-GCCCCTAAGATCA-AGGACACAACCTTCA---CAGGTGGGT
Leob  TTAGTCAAAGGGGGCACA-GCCCCTTTGACAT-AAGACACAACTTTTT---TAGTAGGCT
Neba  TTAATCAAAAGGGGGACA-GCCCTTTTGATAA-AAGAAACAACTTTAC---TAGGAGGAT
Pdpl  TTAGTCAAAGGGGGTACA-GCTCCTTTGACCA-GAGACACAACTCTTT---CAGGAGGGT
Nimi  TTAGTTAAAGGGGGTACA-GCCCCTTTAACAC-AAGACACAACTTTCC---CAGGAGGGT
Uptr  TTAGTCAAAGGGGGTACA-GCCCCTTTGATAC-AGGACACAACCTTC---CCAGGAGGGT
Pesc  TTAATCAAAGGGGGTACA-GCCCCTTTGACCC-AAGACACAACTTTTT---CAGGCGGGT
Baar  TCATTCAAAGGAGGTACA-GCTCCTTTGAAAC-AAGACACAACTTTTC---CAGGAGGGT
Moar  TTAATCAAAGGGGGTACA-GCCCCTTTGATAC-AAGACACAACTTTTC---CAGGAGGAT
Toja  TTAGTCAAAGGGGGTACA-GCCCCTTTGAATC-AAGATACAACTTTTC---CAGGAGGGT
Chau  TTAATCAAAGGGGGTACA-GCCCCTTTGATAT-AAGACACAACTTTTA---TAGGAGGAT
Chse  TTAGTTAAAGGAGGAACA-GCTCCTTTAATAC-AAGACACAGCTTTTT---CAGGAGGGT
Enar  TTAGTCAAAGGGGGTACA-GCCCCTTTGAATC-AAGACACAACTTTTT---CAGGAGGAT
Hpty  TTAGTCAAAGGGGGTACA-GCCCCTTTGAAAC--AGATACAACTTTTC---CAGGAGGGT
Nana  TTAGTCAAGGGGGGCACA-GCCCCCTTGAAAA-AGGACACAACCTTAC---CAGATGGGT
Mcst  TTAGCCAAAGGGGGAACA-GCTCCTTTGCAAT-AAGATACAACTTTAC---CAGGAGGGT
Rhox  TTAGTCAAAGGGGGCACA-GCCCCTTTGAAAC-AAGACACAACTTTAC---CAGAAGGGT
Opfa  TTAGTCAAAGGGGGAACA-GCCCCTTTGAAAC-AAGACACAACTTTAC---CAGGAGGGT
Paar  TTAGTCAAAGGAGGTACA-GCTCCTTTGAAAA-AGGACACAACCTTAT---CAGGAGGAT
Gozo  TTAGTCAAAGGGGGTACA-GCCCCTTTGAAAT-AAGATACAACTTTAC---CAGGTGGGT
Ackr  TTAATCGAAGGGGGTACA-GCTCCTTTGATAT-AAGACACAACTTTAA--ACAGGAGGGT
Elev  TTAGTCAAAGGAGGTACA-GCTCCTCTGAATA-AAGAAACAACTTT-TA--TAGATGGAT
Trdu  TTAGTCAAAGGGGGTACA-GCCCCTTTGAAAC-AAGATACAACTTTC---CAAGGAGGGT
Amoc  TTAGTCAAAAGGGGTACA-GCCCTTTTGAAAT-AAGATACAACTTTTC---CAGGAGGAT
Hame  TTAGTCAAGGGGGGTACA-GCCCCTTTGAACA-AGGACACAACCTTAA---CAGGAGGTT
Chso  TTACCCAAAGGGGGTACA-GCCCCTTTAGGAC-AAGACACAACTTCCC---CAGGAGGAT
Lyto  TTAGTCAAAGGGGGTACA-GCCCCTTTGAACC-AAGATACAACTTTAC---CAGGAGGGT
```

```
Encr TTAGTCAAAGGGGGTACA-GCCCCTTTGAACC-AAGACACAACTTTAT---CAGGAGGGT
Bvar TTAATCAAAGGGGGCCCA-GCCCCTTTGAAAC-AAGACACAACTTTTT---CAGAAGGAT
Noco TTATTCAAAGGGGGTACA-GCCCCTTTGAAAC-AAGACACAACTTTTC---CAGGAGGGT
Chsp TTAGTCAAAGGAGGAACA-GCCCCTTTGAAAC--AGATACAACTTATA--ACAGAAGGAC
Arja TTAGTCAAAGGGGGTACA-GCCCCTTTGAATC-AAGACACAACTTTAT---TAGGCGGGT
Pase TTATTCAAAGGAGGCACA-GCTCCTTTGAAAC-AAGATACAACTTTAT---TAGGTGAAT
Trel TAATTCAAAGGGGGTTCA-GCCCCTTTGAGTG-AGGATACAACCTTAT---AAGCAGGCC
Lifa TTAGTCGAAGGAGGTACA-GCCCCTTCGACCA-AAGATACAACTTTTT---AAGACGGCA
Acur TCAGTCCTAAGAGGTACA-GCTCTTAGGACAA-AGAGTACAGCCTT-CC--AAGGAGGAA
Ampe TTATTCAAAGGGGGTACA-GCCCCTTTGAAAT-AAGATACAACTTTAT---CAGGAGGAT
Urja TTAATTAAAGGGGGGACA-GCCCCTTTGAAAT-AAGACACAACTTTTA---CAGGTGGCT
Enet TTACTCAAGAGGGGGACA-GCTCTCTTGAGAT-AAGACACAACTTTTT---TGATTGGGT
Ptbr TTAGTTTAAAGGGGTCCA-GCCCTTTAAACAA-AAGAAACAACTTTAT---TAGATGGAT
Safa TTAGTCATAGGGGGTCCA-GCCCCTATGACGA-AAGAAACAACTTTAT--AAGGACGGCT
Icae TTAATCAAAGGGGGTACA-GCCCCTTTGAGAC-AAGACACAACTTTCC---CAGGAGGGT
Asmi TTAGTCAAAGGAGGAACA-GCTCCTTTGACAA-AAGAATCAACTTTTC---TAAGAGGTT
Foal TTATTTTAAAGGGGTTCA-GCCCTTTAAAAAC-AGAATACAGCCCCCT---TAGAAGGGT
Drze TTAGTCAAAAGAGGAACA-GCCCTTTTGACTA-AGAAAACAATCTTAC---AAGGAGGTT
Rhas TTAGTCGAGGGGGGTACA-GCCCCCTCGAAAA-AAGATACAACTTTAC--CATGGAGGAA
Elac TTAGTCAAGAGGGGTACA-GCCCTCTTGAAA--GAGATACAACTCTAA--CAAGCAGGTA
Kugu TTAGTCAAAAGGGGTACA-GCCCCTTTGAAA--AAGATACAACTTTAAT--AGGAAGGGT
Plor TTAATCAAAGGGGGTACA-GCCCCTTTGATAC-AAGAAACAACTTTAC---CAGGAGGAT
Sgun TTAATCAAAGGGGGTACA-GCCCCTTTGATAA-AAGACACAACTTTTC---CAGGAGGGT
Zaco TTAATCAAAGGAGGTACA-GCCCCTTTGATAC-AAGACACAACTTTCC---TAGGAGGTT
Zbfl TTAGTCAAAGGGGGTACA-GCCCCTTTGACAT-AAGATACAACTTTA---CCAGGAGGTA
Spba TTAGTCAAAGGGGGTCCA-GCCCCTTTGAAAC-AAGACACAACTTTTA---TAGGAGGGT
Game TTAATCAAAGGGGGTACA-GCCCCTTTGAGAC-AAGATACAACTTTCC---CAGGAGGGT
Thth TTAATCAAAGGGGGTACATGCCCCTTTGAGAC-AAGATACAACTTTTC---CAGGAGGGT
Xigl TTAGTCAAAGGGGGTACA-GCCCCTTTGAACC-AAGACACAACTTT-TC--CAGGAGGGT
Hyja TTAATCAAAGGGGGAACA-GCTCCTTTGACCC-AAAACACAACTTTCC---CAGGAGGGT
Psan TTAGTCAAAGGAGGAACA-GCTCCTTTGACAT-AAGACACAACTTTCC---CAGGAGGGT
Cupa TTAATCAAAGGGGGTACA-GCCCCTTTGAGAC-AAGACACAACTTTC---CCAGGAGGGT
Mpch TTAGTTAAAGGAGGTACA-GCTCCTTTAAATA-AAGATACAACTTTTT---CAGATGGAT
Char TTAGTCAAACGGGGTACA-GCCCATTTGACCC-AAGATACAACTTTTT---TAGGAGGGT
Pser TTAGTCAAAGGGGGTACA-GCCCCTTTGAACC-AAGACACAACTTTAC---CAGGTGGGT
Prol TTAGTCAAAGGGGGTACA-GCCCCTTTGAAAC-AAGACACAACTTTTA---CAGCAGGAT
Plbi TTAGTCAAAGGGGGTACA-GCCCCTTTGAAAC-AAGACACAACTT-TT--CCAGCAGGAT
Calu TTAGTCGAAGGGGGTTCA-GCCCCTTTGAAAA-AAGACACAACTTTTT---AAGGAGGAT
Papa TTAATTAAAGGTGGTACA-GCCCCTTTAAAA--CAGACACAACTTTTA---CAGAAGGGT
Sufr TTAGTTACAGGAGGTACA-GCCCCTGTGACAC-AAGAAACAACTTTAC---CAGGAGGAT
Stci TTAGCTAAAGGAGGTACA-GCTCCTTTATTTA-AAGACACAACTTTAC---CCGGAGGAT
Taru TTAATCAAAGGGGGTACA-GCCCCTTTGATAC-AAGAAACAACTTTTAA--CAGGAGGAT
Rala TTAATCGAAGGAGGTACA-GCTCCTTCGACAC-AAGAAACAACTTTTT---CAGGAGGAT
                   **  **  *                     *
```

```
                          ¦          HVR         ¦      21   22          22'   21'
Scca    AATG-ATCATA-ATTA--TTAAGGTTTTT-----TCCTCAGTGGGCCTAAAAGCAGCCACCTG
Muma    AATG-ATCATAATTATT-----AAGGTT--TC-CACCCCAGTGGGCCTAAAAGCAGCCACCTG
Erca    AAAG-ATCATAATATA----TAAGGA--CAA--TATTTAAGTGGGCCTGAAAGCAGCCACCTT
Pose    AA-AGATCATAATAATACA--AGGATAAG---ATCC--AAGTGGGCCTGAAAGCAGCCACCTT
Actr    AAA-GATCACATTAAATT--AAAGGAACTTT---GTTTCAGTGGGCCTAAAAGCAGCCACCTG
Scal    AAAG-ATCATA-TTAAATCAAAGGAACTT----TGTTTCAGTGGGCCTAAAAGCAGCCACCTG
Posp    AA-AGATCATATTCAAACC--AAAGGAAATTTGTTT--CAGTGGGCCTAAAAGCAGCCACCTG
Atsp    AAAG-ATCACAACA---CAACAGGACCTTCT---GCTCCAGTGGGCCTAAAAGCAGCCACCTG
Leoc    AAAG-ATCATAACATAAC--AGG-ACTTCCC---GCTCCAGTAGGCCTAAAAGCAGCCACCTG
Amca    AA-AGATCATAATGATAAA--AGGACCCC--TACTT--CAGTGGGCCTGAAAGCAGCCATCTG
Osbi    AAAG-ATCATATTTC----TTAAGGTACTCT---GTTACAGTGGGCCTAAAAGCAGCCATCTA
Pabu    AAGG-ATTATA-TTA-ATT-AAGGCACTCT----ACTACAGTGGGCTTGAAAGCAGCCACCTG
Hial    AAGG-ATCATAATGACCA--A-GGTAATC-T---GCTTCAGTGGGCTTAAAAGCAGCCATCTG
Elha    AAGG-ATCATAATC----AAAAAGGCACCCT---GCTCCAGTGGGCCTAAAAGCAGCCACCTG
Mlcy    AAGG-ATCACAATAAAA----AAGACACCATCAAGCCACAGTGGGCCTAAAAGCAGCCACCAA
Algl    AAG-GATCATATTATTT---TAAGGTAAATT---GCTTTAGTGGGCCTAAGAGCAGCCATCTG
Ptgi    AAGG-ATCATATT--AAT--TAAGGTACCC---TGCTTCAGTGGGCCTAAGAGCAGCCACCTG
Alaf    AAAG-ATCATAATATT--C--AAGGTAAACT---GCTTCAGTGGGCCTAAAAGCAGCCAACTG
Nock    AAA-GATCATA-ACAAATCAAGGTAAACTGC-----CTCAGTGGGCCTAAAAGCAGCCAACTG
Anja    AAGG-ATCATACCATA----AAAGGACAAAATTA-CCTCAGTGGGCCCAAAAGCAGCCACCTG
Gyki    AAGG-ATCA----AAACC--CAAGGCATCTT----CCCCAGTGGGCCTAAAAGCAGCCACCTG
Syka    CAAGGATCATAATAATTAA---GGTATATTAGCCGCCTCAGTGGGCCTAAAAGCAGCCACCTG
Opma    AAGG-ATCAAAGATTA--CTAAGGATTAGAA--TCCCTCAGTGGGCCAAAAGCAGCCACCTG
Comy    TA-AGGATTATACTAAAA---AAGGACATGACTTTCAACAGTGGGCCGAAAGCAGCCACCTC
Sasp    AAGG-ACCATACCAAAA----A-ATAAACTT--T-TCTCAGTGGGCCTAAAAGCAGCCACCTA
Eupe    AAAG-ATCATAA------ATTAAAAAATATA---CACCCAGTGGGCCAAAAGCAGCCACCTA
Enja    AAAG-AATATTGC-CAA-A-CAAGGC-CTTG--GGTCTCAGTGGGCCTGAAAGCAGCCATCTG
Same    AAGG-AATATATT---AAC-CAAGGCCACAG---GTTTCAGTGGGCCTAAAAGCAGCCACCTC
Chch    TAAGGACCATAGTCAAC-----AAGGAC--ATCCGTTTCAGTGGGCCTAAAAGCAGCCACCTG
Grgr    AAGG-GTCACACT--TTTT-CAAGGCAAAAC---GTTCCAGTGGGCCTGAAAGCAGCCATCTG
Caau    AA-AGATCATAATACATAA--AACATA----CT-GTTCTAGTGGGCCTAAAAGCAGCCACCTA
Cyca    AAAG-ATCATAATACAT-----AAAACATAC--TGTTCTAGTGGGCCTAAAAGCAGCCATCTA
Dare    AAAG-ATCATAATTTACA--AAACACGT--T---GTCGTAGTGGGCCTGGAAGCAGCCACCTA
Cost    AGGG-ATTATAATTAATA---AAATTTATCG----TTTTAGTGGGCCTAAAAGCAGCCATCTA
Leec    AAG-GGTCAAATTTTC-CAAAACCAGTC------GCCTCAGTGGGCCTAAAAGCAGCCATCTG
Crla    AAGG-ATCATACT-TAAC--AAAACA-C-GTC--GTCTCAGTGGGCCAAAAGCAGCCATCTG
Clmc    AAG-GATCATATTAAATAAGATTAC-----C---GCTTCAGTGGGCCTAAAAGCAGCCACCTG
Phin    AAAG-ACTATACTTAC----TAAGAT-TA--C-TGCTTCAGTGGGCCTAAAAGCAGCCACCTG
Icpu    AAAG-ATTATACT-AAATA---AG-ATACGCC--GCTCCAGTGGGCCTAAAAGCAGCCATCTG
Psto    AAAG-ATTACA-TTAAAT-AAGATAAAAC----CGCTCTAGTGGGCCTAAAAGCAGCCATCCA
Cora    AAGG-ATTATATTTA----C-AAAGATTACC---GCTTCAGTGGGCCTAAAAGCAGCCATCTG
Eisp    AAGG-ACCACACTCATTT---AGACTCC-----TGCTCCGGTGGGCCTAAAAGCAGCCATCCG
Apal    AA-AGATCATA--TACCCT--AAA-ACA--AC-TGTTTCAGTGGGCCAAAAGCAGCCACCCG
Eslu    AAGG-ATCATAA----TTACTAAGGC-ACC-T--GTTACAGTGGGCCTAAGAGCAGCCACCTA
Dape    AAGG-ATCATAATTA--TT-AAGGCTC--CT---GTTATAGTGGGCCTAAAAGCAGCCACCTA
Glse    AAGG-ATCATATTAA--ACAAGGTGT--TCT---GCTTCAGTGGGCCTAAAAGCAGCCATCTA
Naar    AAAG-ATCATAAT-AACT--AAGGTGAAC-T---GTTCCAGTGGGCCTAAAAGCAGCCACCTG
Baoc    AAAG-ATCACAATA----ATTAAGGTCACCT---GTTCCAGTGGGCCTAAAAGCAGCCATCTG
Opso    AAGG-ATCATAATAATTAA----GGTGC-T---TGCCCCAGTGGGCTTAAAAGCAGCCACCTG
Alte    AAGG-ATCATACTCAA--C-AAGGTCAACC----GTTTCAGTGGGCCTAAAGGCAGCCACCTG
Plap    AAGG-ATCACCCTCAAC-----AAGGTCGGC--CGTTTCAGTGGGCCTAAAAGCAGCCACCTG
```

```
Plal    AAGG-ATCAAAATTCTT----A-AGGAAAAC--TGTTTCAGTGGGCCTAAGGGCAGCCACCTG
Sami    AA-GGATCATAATT--TT---GAAGGCATACTGTTC--CAGTGGGCTTAAGGGCAGCCATCTG
Rere    AAGA-ATCAAAGT--AAC--TTAGGTTAGCT---GCTTCAGTGGGCTTAAGGGCAGCCATCTG
Gama    AATG-ATCATATA-TAC---CAAGGG-CCTG--TGCTCCAGTGGGCCTAAAAGCAGCCACCTG
Onmy    AAGG-ATCATA---GTTCC-AAGGTAA-CCT---GTTACAGTGGGCCTAAGAGCAGCCACCTG
Sasa    AAGG-ATCATAATTA----CTAAGGCAACCT---GTTACAGTGGGCCTAAGAGCAGCCACCTG
Cola    AAGG-ATCATAATTA----CTAAGGTAACCT---GTTACAGTGGGCCTAAGAGCAGCCACCTG
Dita    GA-GGATCATAAT-ACTTA--AGGTGAAC--TATCT--TAGTGGGCCTGAAAGCAGCCACCTG
Gogr    AAAG-ATCATAA--ACCAC---AGGC-CTCAC--GTTATAGTAGGCCCAAAAGCAGCCACCTA
Chsl    AAGA-AGCACAA--CAAG--AAAGGT-CACC--TGTTTTAGTGGGCCTAAAAGCAGCCACCTA
Atja    AA-AGATCATAATTAACTA--AGGAAATT-GTTC----TAGTGGGCCTAAAAGCAGCCACCTA
Iido    AAA-GATCATA---ATTAGCAAGGAAATTGT-----TCTAGTGGGCCTAAAGGCAGCCACCTA
Auja    AAAG-ATCATAAAAGC--ACAAAGGAAACTT---GTTCTAGTGGGCCTAAAAGCAGCCACCTA
Chag    AAAG-ACCAGAGTTA-AAC-AAGGGGCAAAA---ATTTTAGTGGGCTTAAAAGCAGCCACCTA
Hami    AAAG-ATCATAATTA-ACC-AAAGGAGCCTT---ATTTTAGTGGGCCTAAAAGCAGCCACCTA
Saun    AAAG-ATCATAATTAAA---CCCAAG-GAGTCTTATTTTAGTGGGCCTAAAAGCAGCCACCTA
Nema    AAAG-ATCATACTTCACTT--AAAGGAAGCC--TGTTCAAGTGGGCCTAAAAGCAGCCACCTA
Disp    AAGG-ATCATA-ATCAACCCAAGGAAACT----TGTTTTAGTGGGCCTAAAAGCAGCCACCTA
Myaf    AAAG-ATCAAATTCC---ACTAAGGAAACCT---ATTCTAGTAGGCCTAAAAGCAGCCACCTA
Lagu    AAT--ATATTAGC-TACC-CAAGGTA-A-ACT--ATCCAGGTAGGCCTAAAAGCAGCCACCCT
Trtr    AAAA-ATCATT-TAATCA--AGGTATTTGTG---GCCCAAGTAGGCCTAAAAGCAGCCACCAC
Zucr    AAAA-ATCATTTTACT-T--AAGGTCACTACA--GCCTTTGTAGGCCTGAAAGCAGCCACCAC
Pxja    AAAG-ATCAAAATTATTTA-CTAGGTATATT---GTTTTAGTGGGCCTAAAAGCAGCCATCTT
Pxlo    AAAG-ATCAAAATTATTTA-CTAGGTATAT---TGTTTTAGTGGGCCTAAAAGCAGCCATCTT
Pctr    AA-AGATCATA--TAAAAC--A--AGGAAAAT-TATCCCAGTGGGCCTGAAAGCAGCCACCTA
Apsa    AA-AGATCATATATTA-----G--ACAGGGACTTAACCAGGTGGGCCTAAAAGCAGCCACCCC
Cabe    AAGG-ATCATAACTTATTA-TAAGGCCTCGT---GCTGTAGTAGGCCTGAAAGCAGCCATCTA
Bzze    GAAG-ATCACA--TTAACT-CAAGGTAGCAT---ACCCCGGTGGGCCTAAAAGCAGCCACCCG
Siim    AAAG-ATCATA--ATTTTA-AAAAGCAACGT---ACTAACGTGGGCCTAAGAGCAGCCACCTA
Ctru    AAAG-ATCACATCCGC--TTAAGGTAAAAT----ATTTTGGTGGGCCTAAAAGCAGCCACCCA
Dpbr    AAAG-ATCATG-CCCGCCC---AAGGTA--CTATATTTTGGTGGGCCTAAAAGCAGCCACCCA
Caki    CAGG-ATCATA-TCAATCAAGGACTAA--AA----TTTAAGTGGGCCTAAAAGCAGCCACCTT
Phja    CAAG-ATCATACTATCAAA----GGA-TTTTA--ACCTAAGTGGGCCTAAAAGCAGCCATCTA
Brsp    TAAG-ATCATAATGTT----TAAGG-ATA--CTAATTTTAGTAGGCCTAGAAGCAGCCACCTA
Gamo    CAAG-ATCATATTACC---CAAG-GATTTCAA---ATTAAGTGGGCCTAAAAGCAGCCATCTT
Lolo    CAAG-ATCATATTACCCA---AGGATTCCAA----ATTAAGTGGGCCTAAAAGCAGCCATCTT
Batr    ATGG-TTCACA---------CTAAACAAAACATTTTATACGTAGGCCTAAAACCAGCCACCAA
Prmy    AAA-GTTCATATTAAA----AATCACCC-AC---TTTAGAGTAGGCTTAATACCAGCCACCCC
Loli    AAAG-ATCATAATATT----TTAAGGAAAA-GTACTGTTGGTGGGCCTAAGAGCAGCCACCCC
Loam    AAAG-ATCATACGAAATTT--AAAGGAAAGT--CCTCTTGGTGGGCCTAAAAGCAGCCACCCC
Chab    AAAG-ATCCTACCAAGACATTAAGGCATCAT---ACTTAGGTAGGCCTAAAAGCAGCCAACCT
Chto    AAAG-ATCCTACCAAAACATTAA-GGCATCAT--ACTTAGGTAGGCCTAAAAGCAGCCACCCT
Majo    AAAG-ATCATA-TTTTTCATAAGGTTCTA----TGTCTTGGTGGGCTTGAGAGCAGCCACCCT
Hlst    AAA-GATCACTTTAAAC---AAGGATGT-AC---ATCAAGGTGGGCTTAATAGCAGCCACCCT
Clpe    AA-AGATCGTG--CACACT--TACG--------TACTTGGGTGGGCCTAAAAGCAGCCACCCC
Mlmr    AAAG-ATCATA---TATT--AAAC-CG-TA-T---ACTAGGTGGGCCTAAAAGCAGCCATCCT
Crcr    ACAG-ATCATATTTC-ACTTAAGGAACTTAT---GCCTTGGTGGGCCTAAAAGCAGCCATCCA
Muce    ACAG-ATCATATTTTACT--TAAGGAACTTA--TGCCTTGGTGGGCCTAAAAGCAGCCATCCA
Bege    AAG-GATCAAATTTAC----CAAGGTTAAAT---ACCCAGGTGGGCCTAAAAGCAGCCATCCT
Mela    AAAG-ATCAAATTCATA----AAGGTAAGTC---ATTGAGGTGGGCTTAAAAGCAGCCATCCT
Hats    AAGG-ATCAAG--CTCAAC-CAAGGCACAAT---ATTTTGGTGGGCCTAATAGCAGCCACCCC
Orla    AAAA-ATCACAAT-ATTA--AAGGTCAA--T---GCCCAGGTGGGCCTAAAAGCAGCCACCCT
```

```
Cosa    AA-AGATTAAAATTAACT-----TAAGGCATTATGTTTTAGTGGGCCTAAAAGCAGCCACCCT
Exsp    AA-AAATCAAACTAAGTTA----AGGCCTTA--TGTTCAGGTGGGCCTAAAAGCAGCCACCCT
Depa    AAAG-ACTATATTAA---C--AAAGGCATTA--CGTTTAAGTGGGCCTAAAAGCAGCCAACCC
Rima    AAAATATTATA---------CAAGG-GC-CAT--TCCTCAGTGGGCCTAAAAGCAGCCACCTC
Fuol    AATA-ATTATA-AATCAT-AAAGTTCA------TGCTTAGGTGGGCCTAAAAGCAGCCACCCT
Gmaf    AATA-AACATAAA-AAAT-T--AGGTA-AGT---GCCAAAGTGGGCCTAAAAGCAGCCACCCT
Xeei    TA-TAATCAAAATTAATTA--AAGCTAAT----GTTTTATGTGGGCCTAAAAGCAGCCATCAA
Pros    AAAG-ATCACA----AGTACTAAGGAAGAA-T--GCTCCAGTGGGCCTAAAAGCAGCCACCTG
Scmi    AAA-GATCATAAGTACCAAGGAAAATATTGT---GCCCCAGTGGGCCTAAAAGCAGCCATCCG
Rolo    AAAG-ATCATA-ATTA--CCAAGGAAAAG----TGTTTCAGTGGGCCTAAAAGCAGCCATCTT
Cere    AAGG-ATCATA---TAT-A-TAAAGGTACGT---ACCTCAGTGGGCCTAAAAGCAGCCACCTT
Daga    AAGG-ATCATACTTATTA--A-GGCAGG--T---GCCTGAGTGGGCCTAAAAGCAGCCACCTC
Anco    AAAG-ATCAAAATAA--TAAAGGAAA--AAT---GTTTCGGTGGGCCTAAAAGCAGCCATCCT
Dmve    ATAG-ATCAAAATTAA----ACAAGGCA---CATGTCTTAGTGGGCTTGGAAGCAGCCACCCC
Dmar    ATA-GATCAAA--ATTAAGCAAGGTAT--GT---GTTTTAGTGGGCTTGGAAGCAGCCACCAT
Anka    AAA-GATCAAAATTGCAAAGGGAAA----AT---GTTTAGGTGGGCCTAAAAGCAGCCACCCC
Moja    AAAG-ATCAAAATTACA-----AAGGAA--AAATGTTTCGGTGGGCCTAAAAGCAGCCACCCC
Hoja    AAAG-ATCAAAATTAA-TA---AAGGAA--AAATGTTTCGGTGGGCCTAAAAGCAGCCATCCC
Bede    AAAG-ATCATAATTATTA---AGGAAACAAG--TGCTCCAGTGGGCCTAAAAGCAGCCACCTC
Besp    AA-AGATCATAATTACC------AAGGAATCAGTGCTCCAGTGGGCCTAAAAGCAGCCACCTC
Mysp    AA-AGATCATAAT-TATCA----AGGACAAA--TGTTACAGTGGGCCTAAAAGCAGCCATCAC
Osja    AAAG-ATCATAATTA---C-CAAGGA-CGGA--TGTTCCAGTGGGCCTAAAAGCAGCCACCAC
Sgro    AAAG-ATCATAATAAA----CAAGGGTAGAT---GTTTTAGTGGGCCTAAAAGCAGCCACCAC
Pzpa    AAAG-ATCAAATTA------A-TTAAGGAGCCTTATTTCAGTGGGCCTATAAGCAGCCACCTA
Zeja    AAAA-ATCAAACTAGA--TCAAGGAACCTC----ATTTTAGTGGGCCTAAAAGCAGCCACCTA
Znne    AAA-GATCAAAATAAAT---TAAGGATTATT---ATTTTAGTGGGCCTAAAAGCAGCCACCTA
Zefa    AA-AGATCAAAATAAATT----AAGG--AGTCCTATTTTAGTGGGCCTAAAAGCAGCCACCTA
Acni    AAA-GATCAAA-ATAGATTAAGGAACCTTAT-----TTTAGTGGGCCTAAAAGCAGCCACCTA
Ncrh    AAAG-ATCAAA-ATAGAT-TAAGGAACCT----TATTTTAGTGGGCCTAAAAGCAGCCACCTA
Agca    AAAG-ATCAAAATTAC--CTAAAGGTAAAAT---GTTTTGGTGGGCCTAAAAGCAGCCATCCC
Hydy    AAA-GATCATACTAAGCAAGGAA--AGT------ACTCCCGTGGGCTTGAAAGCAGCCATCCT
Gsac    AAAG-ATCATAATAAA--ACAAAGGGAAGT----ACTCTGGTGGGCTTGAAAGCAGCCATCCT
Pevo    AAAG-ATTATAAT-TTTC-A--AGGCAT-GAT--ACTCAAGTGGGCCTAAGAGCAGCCATCTA
Hiku    -AAAGATCAAAAATAATA----AAGTTC--ACATGCCTTAGTGGGCCTGAAAGCAGCCACCAA
Inpa    AAAA-ATACAA--CA-CCC-AAGATACAAC----ACCACAGTGGGCCTAAAAGCAGCCATCTG
Auch    AAAG-ATCATTTTACCTC---AAGGCCTTAC----CCGCAGTAGGCTTGAAAGCAGCCACCTT
Fico    AA-GGATCATACACAT-----AAAGG--CACGATGCACCGGTGGGCCTAAAAGCAGCCATCCT
Macs    AAAG-ATCATAATCACGAC-AAGGCAA-AAT---GTTCGGGTGGGCCTAAAAGCAGCCACCCC
Moal    AAAG-ATCATAATAAT--C--AAGGTACCAA---CTTTTCGTAGGCCTAAAAGCAGCCATCCT
Syma    ACGG-CTCATTATATC---TAAAGGCATTTT---ATACCTGTGGGCTTAAAAGCAGCCATCCT
Mafr    AA-AGATTATAA-TACCTC--AAGGA--AATA-TGTCTAAGTAGGCCTAAAAGCAGCCACCAC
Dcpe    AAAG-ATCATA---ATTC-TCAAGG--TAAA--GTATTACGTAGGCTTAAAAGCAGCCATCCA
Dcti    AAAG-ATCATA-ATTT--TTAAGGTAAAG-----TATTATGTAGGCTCAAAAGCAGCCATCTA
Hehi    AAAG-ATCATAAT-TAAA--TAAAGG-TAAG--TATTTGGGTGGGCCTAAAAGCAGCCATCCC
Stam    AAAG-ATCATACCAAA----TTAAAGG-CATAATGTTTTGGTGGGCCTAAAAGCAGCCATCCC
Hogi    AAG-GATCATA-TTAAAAATAAGGACATTAT---ACTCTGGTGGGCCTAAAAGCAGCCATCCA
Erzo    AAAG-ATCATAATAAA---CCAAAGGCAAAT---ATTTGGGTGGGCCTAAAAGCAGCCATCCC
Hxot    AAAG-ATCATAATAA----TTAAAGCTAAAT---GTTCTGGTGGGCCTAAAAGCAGCCATCCC
Core    AAAG-ATCATAATAAC-TA---AAG-CTAGAT--GTTCCGGTGGGCCTAAAAGCAGCCATCCC
Apve    AAAG-ATCATAATAAC----TAAAG-CTA--AATATTCTGGTGGGCCTAAAAGCAGCCATCCC
Latj    AAAG-ATCATAATCT-AAC-AAGGCA-AAAT---GTTCCGGTGGGCCTAAGAGCAGCCACCCC
Laja    AAAG-ATCATA-ATAAGAATAAGGTAAAA----TGTTTTGGTGGGCCTAAAAGCAGCCATCCC
```

```
Syja    AAA-GATCATAATATATTTTAAGGTAATCGT---GTTCTGGTGGGCTAAAAGCAGCCATCCC
Epme    AAAG-ATCATAATTA-AAT-AAGGCA-AAAC---ATCAGGTGGGTTGAAAGCAGCCATCCC
Grse    TAAG-ATCATAATAA----TCAAGGCAATGT---GTCCCAGTTGGCCTAAAAGCAGCCATCCC
Clja    CTGG-ATCATATTAGTTTATAA-AGGTAAATT--ATATAAGTGGGTTAGAAGCAGCCATCAA
Ogcy    -AAAGATCACAAT--TAACCCA-AGGCATTAA--ACCCAGGTTGGCTTAAAAGCAGCCACCCC
Plna    AA-AGATCACAGCACCATA--AAGGC--AAA-GTACATCAGTAGGCCTAAAAGCAGCCACCCC
Lema    AAAG-GTCATAAT-AAAC--AAAAGTAGGAT---GTTCTGGTGGGCTAAAAGCAGCCATCCC
Etzo    AAAG-ATCATAATTACAGA---AG-GTATAAT--ATTTAGGTGGGCTAAAAGCAGCCATCCC
Apse    TAAG-ATCACAGC-ACTTATTAA-GGGAACGT--GTTCTAGTTGGCCTAAGAGCAGCCACCCA
Epde    AAAG-ATCATAATTAA----TTAA-GGTAA-AATGTTCTGGTGGGCTAAAAGCAGCCACCCC
Slja    AAAG-ATCATATTTATATT---AAGGCT--AAGTGTCCGAGTGGGCTAAGAGCAGCCACCCC
Bsja    AA-AGATCATATTCACATTTA--AAGGTAAGCATGTTCTGGTTGGCTTAAAAGCAGCCCTCCC
Ecna    ATAG-ATCATAAT---ACT-AAAGGTAAAAT---GTTTTAGTGGGCTAAAAGCAGCCACCTA
Cohi    AAAG-ATCATATTA--TTTAAAGTA-----AAAAATCTCAGTGGGCTGAAAGCAGCCACCCA
Caar    AAAG-ATCATAATTTTCAA---GGCA----AAACGTTTTGGTGGGCTAAAAGCAGCCATCCC
Came    AAAG-ATCATAATT------TT-TAAGGAAGAATGTTCCGGTGGGCTAAAAGCAGCCATCCC
Mema    ATCA-ATCATA--TTA-TT-CAAGGCAAAAT---GCCAAGGTGGGCTAAAAGCAGCCATCCT
Lenu    AAAG-ATCATA-TTCCCTA---AGGAAA--CCCTGCTTAAGTGGGCTAAAAGCAGCCATCTT
Brja    AA-AGATCATA--ATCACC--AAGG--ACATA-TGCCCTAGTGGGCTAAAAGCAGCCACCTA
Plma    AAAG-ATCATAATTA-----CTA-AAGGTAATATGCCCTGGTGGGCTAAAAGCAGCCACCCC
Emst    AAA-GATCATAATAAATTTAAAGGTAAA-AT---GTTTTGGTGGGCTAAAAGCAGCCATCCC
Ptti    AA-AGATCATATTTAAATT--AAA--GGTAGTATGTTTTAGTGGGCTAAAAGCAGCCATCCC
Losu    AA-AGATCATAACAACCCT--AAA--GGTAGGGTGTTTTTGTGGGCTAAAAGCAGCCACCCC
Geoy    AAAG-ATCACACCCCACCC-TAAGGT-TAGA--TGTTCGAGTGGGCTAAAAGCAGCCACCTC
Dipi    AAAG-ATCATAATCAAATC--AAAGGTAAAA--TGTCCAGGTGGGCTAAAAGCAGCCATCCT
Pama    AAAG-ATCATAATTAACC--CAAGGTAAAGC---ACCCCGGTGGGCTAAAAGCAGCCACCCA
Leob    AA-AGATCATAAACCCTC---GAAGG--ACGGTTATTTAGGTGGGTTAAAAGCAGCCATCCT
Neba    AAAG-ATCATAATTTCA-ACAAAGGTAGAAT---ATTCTGGTGGGTTAAAAGCAGCCATCCA
Pdpl    AAAG-ATCATAGT-ATTC-TAA-AGGATCTCC--GTCTTAGTTGGCTTGAAAGCAGCCACCAC
Nimi    ATAG-ATCACATTCAAATTCCAAGGTAAAAT---GTTTTGGTGGGCTAAGAGCAGCCACCCC
Uptr    AAAG-ATCATATT-TTAT-ATAA-GGTGCTAT--GTCTTGGTGGGTTAAGAGCAGCCACCCT
Pesc    GTGG-ATCAAAATATGAC--AA-GGTA-ACC---CCCTGGGTGGGCTGAAAGCAGCCATCCC
Baar    AAAG-ATCATAACAA--CCAAAGGTACAAAT---GTCCTGGTGGGCTAGGAGCAGCCACCCC
Moar    AAAG-ATCATAATTAAACTA-AAGGCAAAAT---GTTCTGGTGGGCTAAAAGCAGCCATCCC
Toja    AAAG-ATCATATTAAACC---AAGGCAGAAT---GTTTAGGTGGGCTAAAAGCAGCCATCCT
Chau    AAAG-ATCATAATTA-AAC-AAGGCAAAGA----GTCTAAGTGGGCTAAGAGCAGCCACCCC
Chse    AAA-GATCACAATTTAAACAAGGAATAA-----TATCTAGGTGGGCTAAAAGCAGCCATCCA
Enar    AAA-GATCATATTTAAACAAGGTAAAAT------GTTCTGGTGGGCTAAAAGCAGCCATCCC
Hpty    AAAG-ATCATAATAAACT--AAAGGTAGAAT---GTTCTGGTGGGCTAAAAGCAGCCATCCT
Nana    ATCG-ATCATAATATAA---CAA-GGCAACAT--ACCCTAGTGGGCTAAAAGCAGCCATCTT
Mcst    AAA-GATCATAATAAGCC--AAGGCAAA-AT---GTTCTAGTGGGCTAAAAGCAGCCACCTA
Rhox    AA-AGATCATAATAAATT---TAAG--GTAAGATGTTCTGGTGGGCTAAAAGCAGCCATCCC
Opfa    AAAG-ATCATAATAAACT--AAAGGTAAAAT---GTTCTGGTGGGCTAAAAGCAGCCACCCC
Paar    AAAG-ATCATA-TTCTCAA-CAAAGTATTT---TATTCTTGTGGGCTAATAGCAGCCATCTA
Gozo    AAAG-ATCATAGTAA-AAT-AAGGTAGAAT----GTTCCGGTGGGCTAAAAGCAGCCATCCT
Ackr    AAAG-ATCATATC----GTCTAAGATAAA-AA--TCCCTCGTGGGTTAAAAGCAGCCACCAT
Elev    AAAG-ATCACAAACAAC-----AAGGATGAG--TGTTCAGGTGGGTTAAAACCAGCCATCCA
Trdu    AAAA-ATCACAAC-AAACTTAAAGGAT-AGAT--GTCCTAGTGGGCTAAAAGCAGCCACCTA
Amoc    AAAG-ATCATACCCATTTT-AAGGAAA-AAC---GCTTTGGTGGGCTAAAAGCAGCCATCCA
Hame    AA-AGATCAAAGTTAAC-----AAGG--ACCAGTCTTTAAGTGGGCTAAAAGCAGCCACCTT
Chso    AAAG-ATCAAAAT-AACTAT--AGGAGA-GCAT-GTTTTAGTGGGTTAAGAGCAGCCATCCC
Lyto    AAAG-ATCATAA--TACC--TAAAGG-AGAA--TATTTGGGTGGGCTAAAAGCAGCCATCCC
```

```
Encr        AA-AGATCATAAT-AAACC--AAAGG--TAA-ATATTTGGCTGGGCCTAAAAGCAGCCATCCC
Bvar        AAAG-ATCATAATAAC--T--AAGGTAA-CA---CCTCCCCTGGGCTCGAAAGCAGCCACCCC
Noco        AAA-GATCATAATTACA---CAAGGTAAGTA---ACTCTCCTGGGCCCGAAAGCAGCCACCCC
Chsp        TAGG-ACCATAAACCCATA----AGACAACAT--ACTTTCCTGGGCTTAAAAGCAGCCATCAG
Arja        AA-AGATCATAAT-AATTA--A--AGCTGAA--TACTCTGGTGGGCCTAAAAGCAGCCATCCC
Pase        ATAG-ATCATAATACAA-G-CAAGGCTCAAT---GTTCCGGTGGGCTTAAGAGCAGCCACCCG
Trel        AAAG-ATCGTATTTACGT----AAGGAC--TTGTGACAATCTGGGCCTAGAAGCAGCCATCAT
Lifa        AA-AGATCATAGTCA-TA---CA--AGG-AGACTGCTCAGGTGGGCCTAAAAGCAGCCACCCT
Acur        GAAG------ATA-TTTAA-AAGGTAT-AAA---TGTCCTCTGGGCTTGAAAGCAGCCATCAC
Ampe        TAAG-ATCATATTAAAAT--AAAGGCAAAAT---GTTCTGGTGGGCCTAAAAGCAGCCATCCT
Urja        AAA-GATCATA-CAAACTCAAGGCACAGT-----GTTTTGGTGGGCCTAAAAGCAGCCACCCA
Enet        AAAG-ACCATAATAGC--T-AAGGTAAAAC----ACATTAGTAGGCCTAAAAGCAGCCATCTT
Ptbr        AAAG-CTTATAATACAT-T-TTAGGT-TAAA--TACACTCGTGGGCTTGAAAGCAGCCATCTA
Safa        AAGG-ATTAAC-CTAATAA---AGGCAC--CCGTACACTAGTGGGCTTAAAAGCAGCCATCTT
Icae        AAAG-ATCATAATTACTA--AAGGTAACAAT---GCCCTGGTGGGCCTAAGAGCAGCCACCCT
Asmi        AAAG-GTCCTATT--CAA--AAAGGTTAAAA---ACTAGGCTGGGCTTAGAAGCAGCCAGCCC
Foal        AAGG-ATCATAAA-CAACCTTAAGGTATTAT---ATTTAAGTGGGCTTAGAAGCAGCCACCCT
Drze        AAGG-ATCAAAA--TATT--AAAGGTATT-T---ACTTGGGTGGGCTTAGAAGCAGCCAACCC
Rhas        AAAG-ATCAAAGCACCTCA----GGC-ATTAT--GTTTTAGTGGGCCTAAAAGCAGCCACCCT
Elac        AAAG-ATCAAAAGATA----AAAGGCATTAC---ATTCAAGTGGGCCTAAAAGCAGCCACCCT
Kugu        AAA-GATCCTAGTACAAAACTAAGTAAC--T---GTTACTCTGGGCCCAAAAGCAGCCATCAA
Plor        TAAG-ATCATAATTAAAC--CAAGGCATAAT---GTTCTGGTGGGCCTAAAAGCAGCCACCCC
Sgun        AAAG-ATCACACTAAACTATTAAGGTTCCTG--TGTTTAGGTGGGCCTAAAAGCAGCCATCCC
Zaco        AAA-GATCATAATTAGACCA--A-GGCAAAA--TGTTTTAGTGGGCTTAAAAGCAGCCACCTC
Zbfl        AAAG-ATCATA-ACTA--TTAAGGCGTAA----TGTTTTGGTGGGCCTAAAAGCAGCCATCCC
Spba        AAAG-ACCATA-CTAT-AT-CAAAGAATTAA--TGTTTTCGTGGGCCTAAAAGCAGCCACCCT
Game        AAAG-ATCATAATTA--AC-AAGGTAATAAT---GCCCTGGTGGGCCTAAAAGCAGCCATCCT
Thth        AAAG-ATCATATTTACCC---AAGGTAACAA--TGCCCAGGTGGGCCTAAAAGCAGCCATCCT
Xigl        AAAG-ATCATAAT-AA----TCAAGGA-AC-AATGTTCTGGTGGGCCTAAAAGCAGCCATCCC
Hyja        AAAG-ATCATAATT--ACTAAAGGGA---CAT--GCCCTGGTGGGCTTAAGAGCAGCCACCCA
Psan        TAA-GATCATAATTAAT---TAAGGGAT-GT---GCCCTGGTGGGCTTAAGAGCAGCCACCCA
Cupa        AAAG-ATCATAAT-TACT-AAAGGCAAT-AAT--GCCCTGGTGGGCCTAAAAGCAGCCACCCT
Mpch        AAA-GATCATAATAACTAAAGGATAAGT------GTTTTAGTGGGCCTAAAAGCAGCCATCTA
Char        AAAG-ATCATATTA--CCAAAGGGAA--A---ATGTTTTAGTGGGCCTAAAAGCAGCCACCTA
Pser        AA-AGATCAAAATATACCA--AGGAA--ATA--TGTTCTGGTGGGCCTAAGAGCAGCCATCCA
Prol        AAAG-ATCATACTCAATC--AAGGACAAA-T---GTTTTAGTGGGCCTAAAAGCAGCCACCTT
Plbi        AAAG-ATCATATT--CAAA-TAAGG-ACAGAT--GTTTTAGTGGGCCTAAAAGCAGCCACCTT
Calu        AA-AGATAGCA-----ACC--AA--GGGCAG--CGCTTCAGTGGGCCTGAAAGCAGCCAACTT
Papa        AAG-GATCACACTGATCACAAAGGAGTATGT---A-CCCTCTGGGCTTTAAAGCAGCCACCTT
Sufr        TAA-GATCAAATTAAATT--AAAGGCATTAT---GTTTAGGTGGGCCTAAAGGCAGCCATCCC
Stci        AAA-GATTATA--AT-AGTTAAGGGACTAAT---ATTTTGGTGGGCCTGAAAGCAGCCATCCT
Taru        AAGG-ATCATAAAA--AATCAAGGCACC-GC---GCTTAAGTAGGCTTAGAAGCAGCCACCAC
Rala        AAAG-ATCATATTTTACTA---AAGGTC--TGATGTTAAGGTGGGCCTAAAAGCAGCCATCCT
                                                      ** ***         ***** *
```

```
                 23         23'      ¦            HVR            ¦
Scca   --TTAAGTAA-GCGTCACAGCTCTAGTTTTTTAAA--A-----ACCCA--TAATTTA
Muma   --TTAAGTAA-GCGTCGCAGCTCCAGTCTAACAC--TAA----A--CCTATAATTTA
Erca   T---TAGATA-GCGTTATAGCTCAAATA---ATACTCCA-----CCCAAA-TATCCG
Pose   --TAAAGAAA-GCGTTATAGCTTAAATACT--C---AAA----AACCCGAATATCCT
Actr   C--ACAGAAA-GCGTTAAAGCTCAGACAAAA-CCC--------CACCCTATTATCCC
Scal   --CACAGAAA-GCGTTAAAGCTCAGACAAAACCTC--A-----TCCCA--TTATCCC
Posp   --CACAGAAA-GCGTTAAAGCTCAGACA--AAAC--TCC----ACCCTA-TTATCCC
Atsp   C--ATAGAAA-GCGTTAAAGCTCAGGCAGAAATCC-CCC------AATT---ATAAC
Leoc   C--ACAGAAA-GCGTTAAAGCTCAGGCAGAAG---CCA-------TCCAATTATAAC
Amca   --AAAAGAAA-GCGTTAAAGCTCAAGCAGG--A---C-A----TACCCAATTATACC
Osbi   A--TTAAAAA-GCGTTAAAGCTTAAACA---GAAAACAA------ACCTATCATCCC
Pabu   T--AAAGAAA-GCGTTAAAGCTCAAGTAAA-----ACATA----AGCCTATTATTAC
Hial   --AGTAGAAA-GCGTTAAAGCTCAGGCA--GAATCAAA---ACCTATT----ATCCT
Elha   A--ACAGAAA-GCGTCAAAGCTCAGGCAGGACCACA-TT-----AACCTATTATTCT
Mlcy   --TACAGAAA-GCGTCAAAGCTCAAGCAAACAAACA-------AGTCCATTATTAC-
Algl   G--ATAGAAA-GCGTTAAAGCTC-AGGTAAA-ATTAAA--------CCTTTAATACT
Ptgi   T--ATAGAAA-GCGTTAAAGCTCAAGCA----GAATTAA-----ACCCGT-AATACC
Alaf   TCAATAGAAA-GCGTTAAAGCTCCAGCAGAAGAAAACCC-----CT-----AATTCC
Nock   TTAACAGAAA-GCGTTAAAGCTCAGGCA------GGAAA----GATCCTATAATTCC
Anja   T--AAAGAAA-GCGTTAAAGCTCCGGTA-AA--TACAGA------CCA-ATAATAAA
Gyki   --TAAAGATA-GCGTTAAAGCTCAAGCA---TACTTTA---ACCAAGAA---TGTAA
Syka   --AACAGAAA-GCGTTAAAGCTCCGGCA---GAA--AAC----TCACCAATAATAAA
Opma   --GAAAGAAA-GCGTTATAGCTCAAGGA-AGAACC--C-----ACCAA--CAATAAC
Comy   T-AATAGAAA-GCGTTAAAGCTCAAGAACAGCC----A-----CA-CCAAAAATAAA
Sasp   T--AAAGAAA-GCGTCAAAGCTCAGACAC--AAGACAAA-------TAAATAATAAT
Eupe   T--AAAGAAA-GCGTCAAAGCTCCGTATAGAACAAA-T--------TGC--AATCAC
Enja   A--TCTGAAA-GCGTTAAAGCTCCGGC----CAAAAGAA-----AGCCTTTTATTTA
Same   A--ACAGAAA-GCGTTAAAGCTCAGACCAA------TCCA----AGCCTATTATAAC
Chch   --AACAGAAA-GCGTTAAAGCTCAGACGGAA-AA--TAT----AATCCTATTATCCC
Grgr   --TTGAGAAA-GCGTTAAAGCTCGGACTG--AACTCAA-----TCCT--ATTATGCC
Caau   --AACAGAAA-GCGTTAAAGCTCAGACAGAC-A--GAAG----T--TTATTA-TCCT
Cyca   A--ACAGAAA-GCGTTAAAGCTCAGACA---G-AAAAAA-----G-TTTATTATCCC
Dare   --AAAAGAAA-GCGTTAAAGCTCAGGTAAGAAGAAATT---CATA-------ATTCT
Cost   A--GCAGAAA-GCGTTAAAGCTCAAAC----GATGTAAA-----ATTTAT-TATTTC
Leec   --AACAGAAA-GCGTTAAAGCTCAAGCG----ACATAAA----GTT--TATTATTCT
Crla   --ATCAGAAA-GCGTTAAAGCTCAAACGAC----ACAA-----AGTTTA-TTATTCT
Clmc   --GTCAGAAA-GCGTTAAAGCTCAGGCA-----GATTAC----AAATCTATTATCCC
Phin   T-CTAAGAAA-GCGTTAAAGCTCAAACAGAAA----AAA------ATCTATTATCCC
Icpu   --AACAGAAA-GCGTTAAAGCTCCGGCAGACTACA--A-----TCTA---TTATTTA
Psto   --AACAGAAA-GCGTTAAAGCTCTGGCGGGATAA--AA-----ATCCA--TTATACA
Cora   A--ACAGAAA-GCGTTAAAGCTCAAGCAGACAA-----A-----AATCACTTATACC
Eisp   C--ATAGAAA-GCGTTAAAGCTCAGGCA---G-ACAAAA-----GTCTATA-ATTCA
Apal   --TATAGAAA-GCGTTACAGCTCAAACAGATTAA--ATC----T----AACAATCCA
Eslu   --TGTAGAAA-GCGTTAAAGCTCAGACAGA----TACC-----AGCTC--TTATTCT
Dape   T--AAAGAAA-GCGTTAAAGCTCAAACAGATG------CC----CGCCTCTTATCCT
Glse   T--GCAGAAA-GCGTTAAAGCTCAAACAGACTACAAACC------TTTT---ATTCT
Naar   --CACAGAAA-GCGTTAAAGCTCAGACAGACAAACAAA---CCTTTT-----ATCCC
Baoc   C--ACAGAAA-GCGTTAAAGCTCAGACAGA--CAAA-CA-----AACTTTTTATCCT
Opso   A--ACAGAAA-GCGTTACAGCTCAGACAAACCCCTTTTA-----AACCTTTTATTCT
Alte   C--ACAGAAA-GCGTTAAAGCTCAGACG----GACCAAA-----GACCCTTTATTCT
Plap   C--ACAGAAA-GCGTTAAAGCTCAGACG---G-ACCAAA-----GACCCCTTATCCT
```

```
Plal    A--TTAGAAA-GCGTTAAAGCTCAGACA----GTATAGT-------CCTCTTATCCT
Sami    --ACTAGAAA-GCGTTAAAGCTCAGGCAGCAC-----A-----CA-CCTCTTATCCT
Rere    --AATCGAAA-GCGTTAAAGCTCAGGCA-GCGACCG--------C-CCACTTATTCT
Gama    C--TCAGAAA-GCGTTAAAGCTCAGACAC--CCCTCAGC-----CCCTTT-TATTTT
Onmy    C--ACAGAAA-GCGTTAAAGCTCAGACAGAT---ACAAA-----C-CTCTTA-TCCT
Sasa    C--ATAGAAA-GCGTTAAAGCTCAGACA---GATA-TAA------GCCTCTTATCCT
Cola    C--ATAGAAA-GCGTTAAAGCTCAGACAGACAC-----G-----AACCTCTTATTTT
Dita    --GACAGAAA-GCGTTAAAGCTCAAACAGATCAGCCTTT----AAACCTCTAATTTG
Gogr    --TTCAGAAG-GCGTTACAGCCCAAGCAAATCACA--C-----CCCCC--CCATCTC
Chsl    A--ATAGAAA-GCGTTAAACCTCAAACAG--GCCCT--------CACCTCTAATTAC
Atja    --TGAAGAAA-GCGTTAAAGCTCATACAACCTA--TAAA----T--CCGATTATACT
Iido    --TGAAGAAA-GCGTTAAAGCTCATACAAT---TTATTA----AT-CCAATTATTCT
Auja    A--GTAGAAA-GCGTTAAAGCTCAGACAAC-CTCCTCTA-----A-CCTATTATCCC
Chag    A--GCAAAAA-GCGTTAAAGCTTATATGGACATCATTCAG----CCCCCCTAATTAT
Hami    C--ACAAAAA-GCGTTAAAGCTTAAATAAG-----ACCTA----C-CCTATTATCCC
Saun    C--AGAAATA-GCGTTAAAGCTTAAA--TA--AAAACCC------CCCCATTATCCC
Nema    A--AAAGAAA-GCGTTAAAGCTCAGACAC--TCCATACA-----C-CCCTTAATACC
Disp    --TAAAGAAA-GCGTTAAAGCTCATACAAATTT---CG-----ACCCT--TAATACC
Myaf    A--GAAGAAA-GCGTTACAGCTCC-TATAAACCCAG-C--------CCCCTAATACC
Lagu    ---CCCGAAA-GCGTTAAAGCTCAAGTAGT----CCTA-----AACCAC-TAATTCC
Trtr    A--TCAGACA-GCGTTAAAGCTCAAGC-CACA---TCA------ACCCTATTATTAG
Zucr    --TCCTGATA-GCGTTAAAGCTCAAG----CTGTATCT---ACCCTATT---ATTCT
Pxja    G--TTAGAAA-GCGTTAAAGCTCAAACACCTA---TTATT----AACCTTTTATTAT
Pxlo    G--TTAGAAA-GCGTTAAAGCTCAAAC--ACCTATTATT-----AACCTTTTATTAT
Pctr    --AGCAGAAA-GCGTTAAAGCTCAATTA---ATG--ATC----CAACCAGTTATACT
Apsa    --AACAGAAA-GCGTTATAGCTCAACTAAAATC---GCC----CCATTT---ATAAC
Cabe    T--GTAGATA-GCGTTAAAGCTCAAGCACAT-----TTA-----ACCCACCCATGCC
Bzze    A--AGAGAAA-GCGTTAAAGCTCAAGTATGAA---CTAA-----ACCTATT-ATTCT
Siim    --GAGAGAAA-GCGTTAAAGCTCGAGTAC-TA---ACTA-----GCCAATT-ATACT
Ctru    C--GTAGAAA-GCGTTAAAGCTCAAATA----TAAACAA-----AACCCATTATCCT
Dpbr    --TGAAAAAA-GCGTTAAAGCTTAAATATAATCA--A-------ACCCATTA-TCCC
Caki    --TAAAGAAA-GCGTTAAAGCTCTAACTATCTAGC-AC-----ACCCT--TTATACT
Phja    ---ATAGAAA-GCGTTAAAGCTCAAATTAA--ATATCA-----TCTTG--ATATCCC
Brsp    C-CCTCGAAA-GCGTTAAAGCTCAAATT-AA--ATCAAT------CCTCACATCCCG
Gamo    A--TCAGAAA-GCGTTAAAGCTCAAATTAGCCTAT---A-----TCCTCATA--TAC
Lolo    A--CTAGAAA-GCGTTAAAGCTCAAAT----TAGACATC-----ATCCTCATATACT
Batr    C--CATGAAA-ACGTCATAGTTC-----TA-AAAATCCT------GCTACCAATCTC
Prmy    --AAAAGAAA-GCGTTAAAGCTCAAA-------TACCCC----ACAATTAA-ATTCC
Loli    T--ATAGAAA-GCGTTAAAGCTCAAGTATAG--TACTAC------CCATACATCCCG
Loam    G--ACAGAAA-GCGTTAAAGCTCAAATA---C-GGAGCA-----CCCTATATATTCT
Chab    --TACAGAAA-GCGTTAAAGCTCGAGT--ATAACTA-------ACCCTATAAATACT
Chto    --TACAGAAAAGCGTTAAAGCTCGAGTATA-ACTAA-C-----CCTAT--AAATACT
Majo    --ATTAGAAA-GCGTTAAACCTCGGACATACCTAA-AC-----ACCTA--TTATACG
Hlst    --AGTAGAAA-GCGTTAAAGCTCAA--ATG--TAAAAAA----ATCTAATT-ATTAC
Clpe    --CTCAGAAA-GCGTTACAGCTCGAGTA--CAGC--CCC----AGCCT---CATCCC
Mlmr    --CATGGAAA-GCGTCTCAGCTCAAGTATAACCCCCCC---CCCCCCCCCCAATACT
Crcr    A--CCAGAAA-GCGTTAAAGCTCTAACAT----TCCTTC------CTCCCTAATACT
Muce    A--CCAGAAA-GCGTCAAAGCTCTAACA----TTCCTTC-----CTCCCT-AATACT
Bege    A--GAGAAAA-GCGTTATAGCTCAAGTATAG-ACA--------CAACTAATAATTCT
Mela    A--ATAAAAA-GCGTTAAAGCTTGAACATT-GCACTCAA------ACCCCTAATTCC
Hats    A-AACAGAAA-GCGTTAAAGCTCAAATATACC---ACAA-----GCCCCTA-ATCCT
Orla    --AATAAATA-GCGTTAAAGCTTAAGCATAAAAATCA----CCTACA-----ATTCT
```

```
Cosa        --AGAAGAAA-GCGTTACAGCTCAAACATAACT----C-----CAACCAATAATACT
Exsp        --AAAAGAAA-GCGTTATAGCTCGAACATT--A---TTA----CTTCCTTTAATACT
Depa        G--AGAGAAA-GCGTCAAAGCTCAAACAT--AAAGAAAG-----CCCTTAATAA--T
Rima        --AAAAGAAA-GCGTTAAAGCTCAAAATCTCAAACCCC-----TT-----AAATCCC
Fuol        --GAGAGATA-GCGTTAAAGCTCAAACATAACTT--AA-----ACTAA--TTATATC
Gmaf        ---TAAGAAA-GCGTTAAAGCTCAGACACACCCCTTCC-----CACCTC-AAATCCC
Xeei        --AAAAAATA-GCGTTAAAGCTTAAACATACCC--CTCC----CCGCCTATTATTTT
Pros        --AACAGAAA-GCGTTAAAGCTCAAGCATCCGCCCA-C-----CCT-C--AAATCCC
Scmi        --CACAGAAA-GCGTTAAAGCTCAGACAC---TCCTCCC----CCACCTTAAATCCC
Rolo        --AATAGAAA-GCGTTAAAGCTCAGACACAACCTC-CA-----ACCCC--CGATACT
Cere        A--AAAGATA-GCGTTAAAGCTCAAGCACATG---CCCG-----ACCTAAT-ATACT
Daga        --TGAAGAAA-GCGTTAAAGCTGAAGCAC-GTATTACA---GCCCCCT----ATAAT
Anco        A--GCAGAAA-GCGTTAAAGCTCAAACATACTTATATCC------CCTT---ATCCT
Dmve        C--ATAGAAA-GCGTTAAAGCTCAAACATACC-TGCCCC------CCCCCCAATTCT
Dmar        --AGCAGAAA-GCGTTAAAGCTCAGACATA--CTCACAC----CTCCCCCAA-TCCC
Anka        --AACCGAAA-GCGTTAAAGCTCAAACA----TAATGAC----TCACCCCCAATTCT
Moja        --AATAGAAA-GCGTTAAAGCTCAAACATAATCA--CCT----A--CCCCTTATACC
Hoja        --AACAGAAA-GCGTTAAAGCTCAAACATACTTA--AT------AACCCCTTATACT
Bede        A--ACAGAAA-GCGTTAAAGCTCAGGCA---CATCTACC-----AACCCCATATTCT
Besp        --AACAGAAA-GCGTTAAAGCTCAGACACAC----CTAC----CAACCCCATATTCT
Mysp        --AATAGAAA-GCGTTAAAGCTCGGACATATCT--ACCA----CAACCTTATATACT
Osja        A--ACAGAAA-GCGTTAAAGCTCAGAC----ATAGCCCT-----TAAATTCAACCTT
Sgro        --AATAGAAA-GCGTTAAAGCTCAGAC--ATAGCTCTTACTTCAACCCCCAGATATT
Pzpa        A--ACAGAAA-GCGTCAAAGCTAAAA--TAAATAAATCA------TCCCTATCTAAC
Zeja        A--AGAAAAA-GCGTTAAAGCTGAAATA----AAAACTA-----CTCCTTTTATTAC
Znne        A--TTATAAA-GCGTTAAAGCTAAAGTAAA--CTAAAA------TTCCTTTTATACT
Zefa        --GTCATAAA-GCGTTAAAGCTAGAATAAAATA---AAC----ATTCCTTTTATAAT
Acni        --AATAGAAA-GCGTTAAAGCTAAAATAAA--TCTAACT----ACTCCTTTTATAAT
Ncrh        --AATAGAAA-GCGTTAAAGCTAAAATAAACCTATTAC-----TCCTT--TTATAAT
Agca        C--ATAGAAA-GCGTTAAAGCTCAAACATCCCTACCGTC-----CCCTCCCTATCCC
Hydy        --TGAAGAAA-GCGTCACAGCTCAAGTACT--CTACTTA----AACCCTCTTATTCT
Gsac        C--TAAGAAA-GCGTCAAAGCTCAAGTACC-CTACTTAA-----ATCCTCTTATCCT
Pevo        --CGAAGAAA-GCGTTAAAGCTCGTGCAT---CTAAC------TGCCAT-CAATACT
Hiku        --GATAGAAA-GCGTTAAAGCTCAAGCAC---TA--TTT----AAACTTTTAATTCA
Inpa        C--CCAGACA-GCGTTACAGCTCAAGT--GTT---ATCAC----AACCCCCGATCCC
Auch        T--AAAGAAA-GCGTTGTAGCTCAGATC---GGCAAATA-----TGCCTAAAATACG
Fico        --AGTAGAAA-GCGTTAAAGCTCAAGCATTCCC---TTC----GCCTCT--AATCAA
Macs        T--ATAGAAA-GCGTTAAAGCTCAGACATAAA-----CCC----ATCCTTTTATTCC
Moal        T--ATAGAAA-GCGTCAAAGCTCAAATACCCCT----TC-----CACCCCTTATTAC
Syma        A--ACAGAAA-GCGTCAAAGCTCGTATAA--CACAATCA------ACCTATTATCCT
Mafr        --AATAGAAA-GCGTTAAAGCTCAGACA---TAA--CTT----TCTCCTCTTATCCT
Dcpe        --TAGTAAAA-GCGTTCAAGCTTAAACTCA---CCCTT-----CGCTTA-TAATCCG
Dcti        --TAGTAAAA-GCGTCCAAGCTTAAACTCATCCCT--A-----ACTTA--TAATAAG
Hehi        A--ATAGAAA-GCGTTATAGCTCAAATAC--ATCACTAC-----CCCTCTCTATCCT
Stam        T--ACAGAAA-GCGTTAAAGCTCAGACATATAGTAAAAC------CCTACTTATAAT
Hogi        --AATAGAAA-GCGTTACAGCTCGAGTA-----TTAATT----ACTCCTTTTATACT
Erzo        T--ATAGAAA-GCGTTAAAGCTCAGATATAAACCCT--A-----TCCTTCTTATCCT
Hxot        C--TTAGAAA-GCGTTAAAGCTCAGACATGCGACC---A-----TACTTCTTATCCT
Core        --CTTAGAAA-GCGTTAAAGCTCGAACATATACCCGCC-----CCTTC--TTATTCT
Apve        --CTTAGAAA-GCGTTAAAGCTCTAGCATATTCCCCAAC------CCTTCTTATTCT
Latj        C--ATAGAAA-GCGTTAAAGCTCAGACATA----CCCTTT----CGCCCCATATTCC
Laja        --TCTAGAAA-GCGTTAAAGCTCAAACATACCCCC-TA-----TCCTA--TTATCCC
```

```
Syja    --TTTAGAAA-GCGTTAAAGCTCGAAC-----ACACTCT----TTTCCTCTTATCCC
Epme    A--TCAAAAA-GCGTTAAAGCTCAGACACCTAATCCCATA----AGCCCCAAGTCCC
Grse    C--ATAGAAA-GCGTTACAGCTCAAACA---CTCTTCTA------GCCCTAAATCCC
Clja    --TAAAGAAA-GCGTTAAAGCTCAAATTAATAAAACTA-----CCCCT--CTATAAC
Ogcy    --TAAGGAAA-GCGTTAAAGCTCAGACGACCAAACCCC-----CACCCCAAAAATTC
Plna    --CTAAGAAA-GCGTTAAAGCTCGAATATATAC--CACC-------CCTTAAATCCC
Lema    --CTTAGAAA-GCGTTAAAGCTCAGACATCACACTA--------AGCTCTCTATTCT
Etzo    --CGTAGAAA-GCGTTAAAGCTCAAATATTCAACT--A-----CCCCC--TTATTTG
Apse    --AGCAGAAA-GCGTTAAAGCTCAAACACC----TCCC-----CGCCC--CTATTAT
Epde    T--ATAGAAA-GCGTTAAAGCTCAGACATATC-ACTTAC------CCTTTCA-TCCT
Slja    --TACGAAAA-GCGTTAAAGCTTAAACATGATAG--CCC-------CCTAAAATAAC
Bsja    --TACAGAAA-GCGTTAAAGCTCGGGCATACTAC-CCGC----CTCCTCCCTATATT
Ecna    A--ATAGAAA-GCGTTATAGCTCAGACATAAA--ATTCTA----AACCTTAAATCAA
Cohi    A--A-AGAAA-GCGTTAAAGCTCAGATAC---ACAAAAA------ACTATTTATTTA
Caar    --AACAGAAA-GCGTTAAAGCTCAAACGTCCCCC--AAC----CTCCCTATA-TCCT
Came    A--ACAGAAA-GCGTTAAAGCTCAGACATA-ATACTCCC------TCCCTATATCCT
Mema    T--ATAGAAA-GCGTTACAGCTCAAGCATTTC---CCAC-----GCCCATT-ATACC
Lenu    --AAAAGAAA-GCGTTATAGCTCAAC-ACCCCTT--T-------CCCCACAAATTAC
Brja    --CATAGAAA-GCGTTAAAGCTCAAGCA--TTAA--ACC----TGCCCACCTATTCA
Plma    C--ATAGAAA-GCGTTAAAGCTCAAG--CATTAAACCTC------CCCAAATATTTA
Emst    --TATAGAAA-GCGTTAAAGCTCAGACATA--TCACCTA----CCCCTCCCCATCCT
Ptti    --TATAGAAA-GCGTTAAAGCTCAAACATAACC---CCA----CCCCTCCCCATTCT
Losu    --TATAAAAA-GCGTTAAAGCTTAAACA--TCAA--CCC----AACCTAATTATTCC
Geoy    C---TAGAAA-GCGTTATAGCTCAGAC----ATCCTTCT-----TACCTATAATCCC
Dipi    C--CCAGAAA-GCGTTAAAGCTCAAACAT--ACAACCAC-----CCCTCCCTATCCT
Pama    T--TCAGAAA-GCGTTAAAGCTCAGGTGCACC---CAACC----TTCCGTCGATCCC
Leob    --GTTAGAAA-GCGTTAAAGCTCCAATATATTC---TCA----ACTCCTTAGATCCC
Neba    T--AATGATA-GCGTTACAGCTCAAATATGCCCCT---C-----CCCTTAAAATTAG
Pdpl    --CACAGAAA-GCGTTTAAGCTCGTACAAA---CCCCA-----CTCCTT-TAATTCC
Nimi    T--ATAGAAT-GCGTTAAAGCTCAAATAT---AATTACC------ACCCCTAATCCT
Uptr    --ATTAGAAA-GCGTTAAACCTCGGACATA--CCGACA-----CACCTA-TTATCCG
Pesc    --CACAGAAA-GCGTTAAAGCTCAGACATCCTAC-CAA---CCCCTATT---ATCCC
Baar    A--ACAGAAA-GCGTTAAAGCTCAGACATACCCCTACCC------TCTC---ATCCC
Moar    T--ACAGAAA-GCGTTAAAGCTCAGACATACTATTACCC----CTCCTT---ATCCC
Toja    --TAAAGAAA-GCGTTAAAGCTCGAAC--ATAACTC-------CCTCCCCCAATACT
Chau    C--ATAGAAA-GCGTTAAAGCTCAGACTAATT---ACGCC----GCCTCATAATTCC
Chse    --TATAGAAA-GCGTTAAAGCTCAGACATA----CCTCT----TCTCCCCCTATTCC
Enar    --CATAGAAA-GCGTTAAAGCTCAGAC-----ATATCAA----ATCCCTCTTATCCT
Hpty    --TATAGAAA-GCGTTAAAGCTCAGACATACCGC-TA----CCCCTCTC---ATCCT
Nana    A--ATAGAAA-GCGTTAAAGCTCAAGTACTAATT-TA----CAAGCCTATT-ATCCT
Mcst    ---ATAGAAA-GCGTTAAAGCTCAGACATA--CTACCCT----ACCCTATT-ATCCC
Rhox    --AATAGAAA-GCGTTAAAGCTCAGACATACCC----CC----TATCCTTTTATCCT
Opfa    G--ACAGAAA-GCGTTAAAGCTCGGACATACC---ACAC-----ACCCTCTAATCCT
Paar    A--ATAGAAA-GCGTTAAAGCTCAAAT--TTTCCCCCTC-----ACTTCTTATT-CT
Gozo    C--ATAGAAA-GCGTCAAAGCTCCAACATA-----CCCCC----TACCTCACATCCC
Ackr    ---CAAGAAA-GCGTTACAGCTCAGATACCATTTTT-A-----ATCAC--ATATCCC
Elev    T--ATAGAAA-GCGTTACAGCTCCTACAC--AGTCCTAA-----T-CCTACGATCCT
Trdu    --ACCAGAAA-GCGTTAAAGCTCGAGCATT----ACCT-----AGCCAA-CC-TTCT
Amoc    T--ACGGAAA-GCGTTTAAGCTCAAGACTTAC---AACAC----C-CCCAAAATACC
Hame    --AGTAAAAA-GCGTTACAGCTTAAATTTCAAA---ATT----TGACCTTAGATACC
Chso    T--AATGAAA-GCGTTACAGCTCAAACACCCTCCACCAC-----CCGCAGAT--CCC
Lyto    C--TCAGAAA-GCGTTAAAGCTCAAATAT--AATACGGA-----CCCTTCTTATTCT
```

```
Encr    --CTTAGAAA-GCGTTAAAGCTCGGATATACCA---TCA----ACCCTTCTTATTCT
Bvar    -CTAAAGAAA-GCGTCTTAGCTC-AGAATAACCTTGCCC-----CGCTCTAAATGCC
Noco    C-TAAAGAAA-GCGTCTAAGCTCGAAACAAT-CTTGCCC----CTCCCCCCAATCCT
Chsp    --AACAAACA-GCGTTAAAGCTTAAATATAAATG---T-----CCCCC--CAATCCT
Arja    --CGTAGAAA-GCGTTAAAGCTCAGGTATGCTA--CTTA----AACCTTCTTATTTT
Pase    T-GTAAGAAA-GCGTTAAACCTCAAACATGCC---TACC-----CTCCCCCAATCTC
Trel    T-AAGAGAAA-GCGTTACAGCTCAGTTACACCTG--CCC----CTTCCCCCTATTAG
Lifa    --AAATGAAA-GCGTCAAAGCTCAAGCATAAGA----A-----TAGCCTCTAATTCC
Acur    C--CAAGAAA-GCGTTTAAGCTCGTTCATTAT---ACCTC----AACTCGTAATTTC
Ampe    --TACAGAAA-GCGTTAAAGCTCAGATATACTAAATAA---CCCTCCCT---ATCCT
Urja    --CCCAGAGG-GCGTTAAAGCTCAAACAC-----CTCCC----CCGCCCCATATTCT
Enet    A--TCAGAAA-GCGTTAAAGCTCAAGTG----TTCCTAG-----CGCCTTGGATACT
Ptbr    T--AAAAAAA-GCGTTAAAGCTTAAGT----ATTAATTC-----AACCTATAATTCC
Safa    --AACAAAAA-GCGTTAAAGCTTGAGTACCCCTA--A-------CGCCAATAATTTT
Icae    --TATAGAAA-GCGTTAAAGCTCAAGCATTAAACCTCA---CCCATAT----ATTCA
Asmi    --CTTCAAAA-GCGTTAAAGCTCAAGCA-AATATTA--------CACCTATAATTTA
Foal    ----TAGAAA-GCGTTACAGCTCTTATATAATTTTAAA-----CCCTTA---ATACT
Drze    --TAAAGAAA-GCGTCATAGCTCGCATACATAGAATTA---AGCCCTTT---ATACC
Rhas    --AATAGAAA-GCGTTAAAGCTCAAACATATCCCTCTG-----CCCCT---TATCCC
Elac    T--AAAGAAA-GCGTTAAAGCTTAAATGTCT-GCCACCC----CCCCCCCCCATCAC
Kugu    --AAAAGAAA-GCGTTAAAGCTCAAAC-----AGATACA----CCACTATTTCTCCC
Plor    --CAAAGAAA-GCGTTAAAGCTCAGACATACCTCC----------GCCCGATATCCC
Sgun    T--GTAGAAA-GCGTTAAAGCTCAAACAC--ATACCTC------CCCTAT-TATCCT
Zaco    T--ACAGAAA-GCGTTAAAGCTCAGACATA-CCCTACC------CCCT-TAAATCCT
Zbfl    --CACAGAAA-GCGTTAAAGCTCAGACATAGCCATACC-----ACCTA--TAATTCT
Spba    A--ATAGAAA-GCGTTAAAGCTCAAAC--ATACAATTAA-----ATTCTATAATACC
Game    A--ATAGAAA-GCGTTAAAGCTCAAGCATTAA---ACCTC----ACCCATATATTTA
Thth    T--ACAGAAA-GCGTTAAAGCTCAAGCA---TTACACCT-----TCCCACATATTCA
Xigl    C--GCAGAAA-GCGTTAAAGCTCGGACATACC-TTACCT------CCCTATA-TACT
Hyja    A--ACAGAAA-GCGTTCAAGCTCAAGCAT-TTAACCTTA------CCCATATATCTC
Psan    --AACAGAAA-GCGTTCAAGCTCAAGCATC--TAACCTT----ACCCATAT-ATTCC
Cupa    --TACAGAAA-GCGTTAAAGCTCAAGCATTA-AACCTT-----ACCCAT-ATATTCA
Mpch    ---CAAGAAA-GCGTTAAAGCTCAGACACA--CCCCCTC----A-TCCATTAATTCA
Char    A--ACAGAAA-GCGTTAAAGCTCAGACATATTAATAACC------CCTATA-ATCCG
Pser    --AATAGAAA-GCGTTCAAGCTCAGACATCCCC--TTCC----CCA---AAAATCCC
Prol    --AACAGAAA-GCGTTAAAGCTCAGACATAGACACCTC---CATAT------ATACC
Plbi    --AACAGAAA-GCGTTAAAGCTCAGACAT--GAAGCCC-----TCCC--GTTATACC
Calu    --AGCAGAAA-GCGTTAAAGCTCAAGCG--CAA---CAT----AACCCATAAATCTC
Papa    C-TTACAAAA-GCGTAGTAGCTCAAACAACAACCTACCA----AAACCCATAATTAA
Sufr    A--ACTGAAA-GCGTTAAAGCTCGAACATTT-TAC--------TTCCCC-CAATCCC
Stci    --TAAAGAAA-GCGTTAAAGCTCAAATAT-----TGCTC----TTCCCATAAATTCC
Taru    ----AAGAAA-GCGTTAAAGCTCTAGCAC----ATCCCT------GCCACAAATACC
Rala    --AATAGAAA-GCGTTAAAGCTCAAACATTATTA--A-------ACCTAATTATCCC
                    ***   *
```

```
                                       20'                          15'      24
Scca    --GATATT------TATTCAAAAACCCCCTTAAC---C--CTATTGGGTTATTTTATATA
Muma    --GATATT------TA-CTCATAACCCCCTTT--ATCC---TATTGGGTTATTTTATA-A
Erca    --GATAAA------ACCT-CTAAATCCCCTATA--CA----TATTGAGTTACTCTAT-TA
Pose    --GATAAA------CCC-TCTAAATCCCCTATAAA------TATCAAGTTACTCTAT--T
Actr    --GATAAA------ACAATCACAATCCCCTA-AACC-----TACAGAGCCCCTCTATAT-
Scal    --GATAAA------ACGATCACAATCCCCTAA---A-C--CTACAGAGCCGCTCTATACA
Posp    --GATAAA------ACAATCACAATCCCCTAAC---T----TACTGAGCTACTCTATACA
Atsp    --GACAAA------CACATCTGACTCCTCCTAA--TAG---TACTAAGCTACTCTATTT-
Leoc    --GACAAA------TACATCTGACTCCTCCTAAT--AC---TACTAAGCTACTCTATT--
Amca    --GAT-AA------CCAATCTCACTCCCCTCTAAC------TACTAAGCTGTTCTATA--
Osbi    --GACAAT------TA-ATCTCACCCCTCCAACAT------TATCAAGCTATTCTATACT
Pabu    --AATAAA------AATCTCCAACCCCACTAAA---AA---TATTAAGCCGCCCTATG-T
Hial    --GATAAT------AA-ATCCTACTCCTCTAAAA--G----TACTAAGCCACTCTATAC-
Elha    --GATACC------CGGTCCA-ACTCCCCTAAG---AA---TACTAAGCCATTCTATGC-
Mlcy    --GATAAA-------ACATCCCACTCCCCTAACA------ACACTAAGCCATTCTATGC-
Algl    --GATTAT------TTT-TCCTAGTCCCCTAAA---CA---TACTAAGCTATTCTATGT-
Ptgi    --GATAAC------CCAT-CCTACTCCCCTAAAA--A----TACTAAGCCATTCTATGCC
Alaf    --GATAAA------A-AATCATATTCCCCTTAA---TA---TACTAAGCCGTTCTATGCT
Nock    --GACAAT------AAA-TCCCAATCCCCTAATT--T----TACTAAGCCATTCTATGC-
Anja    --GACAAT------ACACTCCCCACCCCCTAAAA--A----TATTAAGCTATCCTATGCA
Gyki    --AATAAC------TAAGCCACACCCCCCAAAA--A----ATACTGAGCCCCCCCATGA-
Syka    --GATAAA------TTTCTCCCCACCCCCTA-AAAA-----TATTAAGCCATCCTATG-C
Opma    --GATAAC------ATAATCCTAATCCCCTATA---A---ATATTAAGCCACTCTATGTA
Comy    --AATAAC------C-ACTCCTAATTTCCTAATAA------TATTAAGCTACCCTATTT-
Sasp    --GATTTT-----TAACCTCCAAACCCCCAAGTAA------TATTAGGCCTCACTATACT
Eupe    --GATAAA------TTCTCTTTA-TCCCCAAAAAA------TATTAAGCTACTCCATTT-
Enja    --ATCTTA------AT--ATCTGCTCCCCTAAACCT-----TATTGGGCCTCTCCATGCA
Same    --ATTAAA------A-TCTCCAAAGCCCCTAA---TAT---TATTGGGCCGCCCTATG-C
Chch    --GCTATA------CCACCCCCCACCCCCTA---ATTT---TACCGGGCCATTTTATGCC
Grgr    --GATA-C------CACCCCCCACCCCCCTAGTT--T----TACTGGGCCGCCCCATGCC
Caau    --GAT-AA------TATATCTCACTCCCCTA--AATAC---TATTAGGCCAACCCATGCC
Cyca    --GATAAA------A-AATCTTACTCCCCTAAGT--AC---TATTAGGCCAACCCATGCC
Dare    --GACAAT------AATATCTTACTTCCCTAAAA------ATACCAGACTAATCCATAC-
Cost    --AATAAA------ATA-TCCCACTCCCCTAA-A--CG---TATTAGGCCACTCCATGAT
Leec    --GATAA-------ACAACCCCACTCCCCTTA-T--AT---TATTAGGCCATTCCATGC-
Crla    --GATAAA-------CAATCCCACTCCCCTAAAA--T----TACCAGACTATTCCATGCC
Clmc    --GATACT------CCCATCCTAAACCCCTCCAA--AA---TACTAGGCCACTCCATGC-
Phin    --AG-CAT------TACCTCTAATATCCCTACTT--A----TATTAGGCCTCTCCATGAA
Icpu    --A-ATAT------TTTATCTTAAACCCCTAATC--A----TATTAGGCCAATCCATGCC
Psto    --AATACA------TT-ATCTAAAACCCCTA-AC---C--TTATTAGGCCACTCCATGCC
Cora    --AATACC------CTAATCCAAAACCCCTAAT---AT---TATTAGGCCGCTCCATGCC
Eisp    --GATAAC------AA-ATCCAAAACCCCTAA-C--CA---CATTAGGCCTCTCCATGCC
Apal    --GATATT------A-TATCTTAAACCCCTA--ACCC----TACTAGGCCCTCCTATGCT
Eslu    --GATAGA---------CATCCCAACCCCTAATAT------TATTAAGCCCTTCCATTC-
Dape    --GATAAA-------CTAATCCTAACCCCTAAAAA------TATTAAGCCCTTCCACC-C
Glse    --GATAA-------A--CTCTCCAACCCCCTTTTTT-----TATTAAGCCACCCCATGCC
Naar    --GATACT------T--ATTCTTACCCCCTAATC------TTACTAAGCCGCCCCATGCC
Baoc    --GATATT-------AATT-CTTACCCCCTAATCT------TACTAAGCCGCCCCATGCC
Opso    --GATAA-------ATG-CTCCCACCCCCTAACTT------TACTAAGCCACCCCATGCC
Alte    --GATAAC-------CAACCCCAACCCCCTAATAG------TATTAGGCCGCCCTATGCC
Plap    --GATAGC------C-ACCCCCAACCCCCTAA-TAT-----TACTAGGCCGCCCTATGCC
```

```
Plal   --GAT--A-----ACCTTTCCTCCACCCCTAGTTT------TACCGAACCATCCCATGCC
Sami   --GATAAC------C-TCTCCCCACCCCTAATTT------TACTAAACCGCCCCATGCC
Rere   --GATAGA--------GTTTTCTACCCCCTACTAT------TACTGAGCCATCCCACGC-
Gama   --GATAAA--------TAGTCTTACCCCCTAAGTT------TAATAGACCGTCCCACATT
Onmy   --GATAAG-------AAATCCCACCCCCCTAACCG------TACTAAGCCGTTCCATG-C
Sasa   --GATAAA-------AAATCCTACCCCCCTAACCG------TACTAAGCCGTTCCATGCT
Cola   --GATAAG--------AAATCCTACCCCCTAACCG------TACTAAGCCGTTCCATGCC
Dita   --GATAAA--------A-AGTCCCACCCCCTTCCAC-----CACCAAGCCCCCCTATGCA
Gogr   --G-TTGT-------AGCCCCCCATCCCCTAACCC-CCCCCACTAAGCCATTTTATGCC
Chsl   --GCCCAG--------TAATTCTAACCCCTAACCAC-----TACTAAGCCACCCCATGTT
Atja   --GAT-AA------AACATCTTAAACCCCTATA---AC---TATTAGACCACCCCATGCA
Iido   --GATAAA-------ACATCTTAAACCCCTATAAC------TATTAGACCATCCCATGCA
Auja   --GA-CAA------AAAATCTTACACCCCTAACTAG-----TACTAGGCCACTTCATGCC
Chag   --GGTATA------ATTTTCTTATTCCCCTGCATTC-----TACTAGGCCATTTCATG-C
Hami   --GATAAA------TTA--TCTTTTCCCCTTAAAT------TATTAGGCCATTCCATG-C
Saun   --GATAAT--------TAATCTTTTCCCCTTGCAT------TAATAGGCTATCCCATGCC
Nema   --GA-ACC-----AAACTCTAAACTCCCCACCCCC------TACCAGGCCGTTCCATGCA
Disp   --GA-ACC-----AAGCTCCACACTCCCCACCC--A----CTATCAGACCCTCCCATAAG
Myaf   --GAACAC------AACTCTTCAGTCCCCTTTCAC------TATTAAACCCTCCCATCA-
Lagu   --AATCAA------CCAGTCAAGCCCCCCTCCTAA------TACTAAGCTATTCTACCCA
Trtr   --GAACAC------CCG-TTTTATTCCCCTAATCC------TACTAGATCACTCTATT--
Zucr   --GATTAT-------ATGTTTTACTCCCCCTAAC------TTACTAGATCACTCTATTT-
Pxja   --GTTATC-------ATA-TCTTCTCCCCCTAAATA-----TATCAGGCCGCCTCATG--
Pxlo   --GTTATC-------ATA-TCTTCTCCCCCTAAATA-----TATCAGGCCGCCTCATGCA
Pctr   --GATAGA------A-AATCTTTATCCCCTA--CAAC----TACCCGGCCTCCCCACGCA
Apsa   --GACA-A------CCAATCTTCAACCCTTAACAA------TATCAGGCCGCCCTATGCC
Cabe   --GATAGC------TCAATCCCAACCCCCTAACCC------TACCAGACCATTATGTA-C
Bzze   --GATAAT------ACCATCTAAACCCCCTACCTA------TACAAGGCCTTCTCATA-C
Siim   --GATAAC------CCAATCTTACCCCCCTGCCCCC-----TACCAGGCCTTCTCAT--T
Ctru   --GACAAC------CTCTTCTTAAGCCCCTTCACCC-----TATTGAGCCCCTCCACATC
Dpbr   --GACAAC------TTAATCTTAAGCCCCCA-ACCCCC---TATTGGGCCCCTCCATA-C
Caki   --GATATA-----TATCTTTCCCCCGCCCTGCTACT-----TAATAGGCTATCTTATGCC
Phja   --GATAAA------AAATCTCCCTCCCTGTTA-ATC-----TACCAGGCTGTCTTATGCT
Brsp   --AACCTC------CCTCTCTTTTATCCTTAAAAA------TATCAGACTAATTTATTT-
Gamo   --TGATAT------TACATCTCCCTCCCTGCCCCT------TACCAGGCTGTCTTATGCC
Lolo   --GATATT------ATA-TCTCCCTCCCTGCT-CTC-----TACCAGGCTGTCTTATGCC
Batr   --GATATC------ACACACCAAAACCTAAACCCC------CATTGAGGCCCATCACACT
Prmy   --GATCCA-------TTAACTTAGTCCCACAACT-------TATTGGGCCCTACTATCT-
Loli   --AA-AAC------TTAATCTGAACCCCCTAAAGAC-----TACCAGGGAGCCCTATTCA
Loam   --GACAAC------ATAATCTTAATCCTCTAA-AAC-----TACCAGGAAGCCCTATGCG
Chab   --AACAAT------TATTTCTTATTCCCTTG-TAC-----ATACCAGGCCCTCCCATGC-
Chto   --AACAAT------TATTTCTTATTCCCTTGTACA------TACCAGGCCCTCCCATGCA
Majo   --GATAAT------TACATCCCATCCCCCTAAT--T----CTACCGGGCCGCCCCATACC
Hlst   --GATAAT------C-AAACTTACTCCCCTAACCT------TACCGGGCCTCTCCATGC-
Clpe   --CCTAAT------TTGATCTGATTCCCCTC--AACA----TACCAGGCCTTCCCATGCT
Mlmr   --GACAAT------TTAATCTTATTCCCTTAAAT------ATACCAGGCCCTTCCATGC-
Crcr   --GATAAC------AAG-TCTTAGCCCCCTAA-CCA-----TATCAAGCCGCTCCATCAA
Muce   --GATAAC------AAGT-CTTAGCCCCCTAACCA------TATCAAGCCGCTCCATCGA
Bege   --GAT-AT------TCTATCCTACTCCCCTAACCC------TATCAGGTCCTCCCATAT-
Mela   --GATTTT------AGCCTCCTACTCCCCCTTCTCC-----TATCAGGTTTTCCCATATC
Hats   --GATTAT------TTT-TCCAACGCCCCTAACCC------TATCAGGTCTTCCTATAAT
Orla   --GATAAC------TTAATTTGAAGCCCCTTATA------TTAACGAGCTATTTCATAT-
```

```
Cosa    --AATAAC------TTACTCTTACACCTCTTTAAAACC---TACCAGGCCTTTCTATAAA
Exsp    --GATCAA------CCAATTTTACACCCCTA--ATTC----TAACAGGCCTTTCTATTTT
Depa    --AGTAAA-----CAAATCTCAAACCCCTTAAT--T-----TATCAGAATTTTCCACTAA
Rima    --GCCTAT-----AAGATTTTATTCCCCTTGATT-------TATTAGGCTATTTTATTAA
Fuol    --AACAAT------AA-ATTATAAGCCGCTAGAA-T----TTACCAGGCCCTCCTAT-TT
Gmaf    --GATAAC------ATAATAATATTCCCTCGACAA------TACCAGGCCTTTCTATCTT
Xeei    --GAT-AA------CAAATTATATTCCCCTT--AAACC---TACCAGGCCATTCTATTTT
Pros    --GACTGA------CATATCTCAGCCCCCTAATCCT------ACTAGGCCCTCCCATGCC
Scmi    --GACAAA------TATATCTTAACCCCCTAATCC------TATTGGGCCCTCCCATGC-
Rolo    --GATAAA-------TAATCTAAACCCCCTA-AA-C----CTACTAGGTCCTCTCATGCA
Cere    --GATTAT------TTA-TCCCACCCCCCTAACCC------CACTAGGACCTCCTATG-C
Daga    --GATTAT-------ATTTCCAACCCCCCTGCCCCC----ACACCAGGACCTCTCATGC-
Anco    --GATAAC------CA-ATCTTAAACCCCTGACCCAG----TATTAGGCCTCCTCATGCA
Dmve    --GATAA-------ACAATCTAAATCCCCGACCCGA-----TATTAGGCCCCCTCATGCA
Dmar    --GATAAA------AA-ATCTAAATCCCCAACCTGA-----TATTAGGCCCCCTCATGC-
Anka    --GATAAC------TA-ATCTAAAACCCCTACCACA-----TACTAGGCCTCCTCATGC-
Moja    --GATAAA------TA-ATCTAAACCCCCTACCCACAA---TATTAGGCCTCCTCATG-C
Hoja    --GATAAA------TAAT-CTAAACCCCCTA-ACCGAA---TATTAGGCCTCCTCATG-C
Bede    --GATTAA------TCA-TCTAAACCCCCTAA-TCC-----TATTAGGCCCTCCCATGCA
Besp    --GATTAA------TCA-TCTAAACCCCCTAATCC------TACTAGGCCCTCCCATGCA
Mysp    --GATAAA------CAAATCTTAATCCCCTC--TCCT----TACCAGGCCCATCCATGCA
Osja    --ATATAATGATACAAAATCTTAACCCCCTACCAA------TACCAGGCCCATCCATGCA
Sgro    --GATACA------TAAATCTTACACCCCTAACCC-----GTATCAGGCCTACCCATGC-
Pzpa    --GTTAAT------A-AATCTTACACCCCTAATAC------TATTAGGCCATCTCATGCC
Zeja    --GTTAAT------A-AATTTTTTACCTCTGATGTC-----TATCAGGCCTTCTCATGCT
Znne    --G-TTAT------TAAATCTCTCACCTCTATATT------TATCAAACCATCTCATGC-
Zefa    --GTTACT------AAA-TCTCCCACCTCTGTAGT------TATCAAACCATCTCATGCC
Acni    --GTTAAT------GA-ATCTCCCACCTCTGATATT-----TATCAGACCATCTCATGC-
Ncrh    --GTTAAT------GA-ATCTCCCACCTCTGATA-T----TTATCAGGCCATCTCATGCC
Agca    --GATAAT------AAAATCTTATTCCCCTAATTT------TACTAGGCCATCCCATGAT
Hydy    --GATCAC------CTAATCTTATCCCCCTAA-TTA-----TACTGGGCCTTCCCATGC-
Gsac    --GATCAC------CAAATCTTATCCCCCTATATA------TACTGGACCATCCCATGCC
Pevo    --AATAAA-------ACATCTCAACCCGTGCAAAAACT----AGCAGGCTGTTCTATTTA
Hiku    --GATA-A------ATATTCTAAAACCCTTA---AAAT---CATCAGGCCTTTTCAT--A
Inpa    --AACATC------TATACTTTAACCCCCAACTC-------TACCAGGCCCTTCCATG-C
Auch    --GATAAT------TCAATCTTAACCCCCTAT-CAC-----TATCAAGCCGTTCAATACA
Fico    --GATA-C------AAAATCCTACTCCCCTAGATA------TATTGGACCATCCCATTCC
Macs    --GATATA------ATAATCTCAACCCCCTAAATA------TACCAGGCCGTTCCACA-C
Moal    --GATAGC------CAA-TCTAACTCCTCTAATAC------TACCAGGCTTTTTCATGCC
Syma    --GATAAT------TATACCCCAATCCTCTAACCC------TACCAGGTCTTCTCATGCA
Mafr    --AATAAC------ACAATCTCAATCCCCTAATAT------TACCGGGCTGCTCCATGCA
Dcpe    --GATAAA------TATATCTCAACCCCCCAACATT------ACCGGGCCGCCCCATACA
Dcti    --GATAAT------AATATCTTAACCCCCCAGC-A-----TTACCGGGCCGCCCCATACA
Hehi    --GATCAT------TAATTCTTACTCCCCCCTTTCC-----TACCGGGCCATCCCATGCA
Stam    --GATCAT------AAAATCTTATACCCCTAGTAC------TACCAGGCCGTTCCATGCC
Hogi    --GATCAC------TTAGTCTTAATCCCCTACATT------TACTGGGCCGTCCCATGC-
Erzo    --GATCAA------ATAATCTTATCCCCCTATACA------TACTGAACCGCCCCATGCC
Hxot    --GATCAC------TTAATCTTACCCCCCTAACCG------TACTGGACCATCCCATGCC
Core    --GATCCC------CTTATCTTACCCCCCTAAACGT------ACTGGACCATCCCATGCC
Apve    --GATCAA------CAAATCTTACCCCCCTAATCG------TACTGGACTATCCCATGCC
Latj    --GATAAC------CCAATCTTAGCCCCCTA-ATCC-----TATCAGGCCGCCCCATG-C
Laja    --GATAAC------CCAATCTTAGCCCCCTAGT--C----TTACCAGGCCGCTCCATACA
```

```
Syja    --GATAAT------TTAATCTAAACCCCCTAACACT-----TACCAGGCCCTCCCACAC-
Epme    --GACTAT------CTTTTCTTACTCCCCTT-ATCC-----TACTGGGCCGTCCCATG-C
Grse    --GACCAC------CCAATCTTACTCCCCTAACCC------TACCGGGCCGTTCCATGCA
Clja    --GATAAC------CCCTTCCTAACCCCCTAGCTTT-----TACCGGATTTTTCCATGCA
Ogcy    --AGATGA------AAATTCTCAAACCTCTAATCTT------ATCGGACTACTCTATTCC
Plna    --GAC-A------TAAACTCTTGCCCCCCTAATCC------TACTAAGCCGCCCCATGCC
Lema    --GATAAT------T-TACCTCACCCCCCTAACTC------TACCAGACCGACCCATGC-
Etzo    --GATCAT------AAAATCTTATCCCCCTAATTTT------ACCAGGCCGTCCCATGCA
Apse    --CCGGAT--CACATTAATCTTACCCCCCTAAACAT------ACTGAGCCTTCCCATACA
Epde    --GATAAT------TTAATCTTAACCCCCTAATCC------TACCAGGCCGTCCCCTGCA
Slja    --GATAAT------TTTATCTTAACCCCCTA--GACCC---TACCAGGCCGCTCCATA-C
Bsja    --GATAAT------TAAATCTTAAGCCCCTACTCC------TATCAGGCCGCCCCATGCC
Ecna    --GATAAC------A-ATTCTCAACCCCCTA-ACCA-----TAACAGGCCCTCCCATG-C
Cohi    --GATAAG------CCAATCTTAACCCCTTAGCCTT-----TAACAGGCCGTTCCATACT
Caar    --GATATC------CTAATCTTAATCCCCTA-ATAT-----TACCGGGCCTCCCCATG-C
Came    --GATACC------ACAATCTTAATCCCCTAACA--T----TACCGAGCCTCCCCATGCA
Mema    --GATAAC------CCAATTCCAACCCCCTAACC--T----TACCAGGCCATCCTATG-C
Lenu    --GATAAT------ACCATCTTTATCCCCTA-ACAA-----TAAAAGGCCATTCCACC-C
Brja    --GATAAC------A-CCTCCCAACCCCCTA--ACAC----TACCAGGCCTTCTCATGCA
Plma    --GATAAC------CAAATCCCAGCCCCCTAACAT------TATCAGGCCGTCTCATGCA
Emst    --GATAAT------TTTATCTTAGCCCCCTCATCAT------ACCAGGCCGTCCTATGC-
Ptti    --GATAAT------TTAATCTTAACCCCCTT--CAATC---TACCAGGCCATCCCATGCA
Losu    --GACATT------ATAATCTTAACCCCTCC--GCAC----TATCAGGCCTTCCCATGCA
Geoy    --ACCAAA------ATAATCTTAAGCCTTTAAATT------TATTAGGCCTTCCCATGCC
Dipi    --GATAAT------CTTATCTTAACCCCCTAGCC-C-----TACCAGGCCGTTCCATGCA
Pama    --GATAAT------GCAATCTCATCCCCCTCTCAA------TATCAGACTATCCACTG--
Leob    --GACAAT------CCTATCTTACCCCACTAAACC------TACCAGGGCGCTCTATACA
Neba    --GATAAA------AATATCTTAAACCCCTTTAAT------TATTAGGCCACTCTATTAT
Pdpl    --GATAAT------TTTATCTTATCCCCCTAACTTT------ATCAGGCCGCCCCATGCC
Nimi    --GACAAC------CCCATCTTAACCCCCTAACCT------TACCAGGCCGTCCCATGCA
Uptr    --GATAAT------TATATCCCATCCCCCTAATTCT------ACCGGGCCGCCCCATACC
Pesc    --GATAAA------CTCCTCCCAACCCCCTAATT------TTACCAGGCCGTCCCATAC-
Baar    --GACAAT------TTAATCTTAACCCCCTAGTAT------TACCAGGCCGTTCCATACA
Moar    --GATAAG------CCAATCTTATTCCCCTAATCC------TACTAGGCCGCCCCATGCA
Toja    --GATAAC------CAAATCTCAACCCCCTAGTC------TTATTAGGCCTTCCTATGC-
Chau    --GATAAT------TTAATCTTAATCCCCTGACTT------TACCAGGCCTCCCCATG-C
Chse    --GATAAC------CCAGTCTTATCCCCCTGAATT------TATCAGGCCGCCCCATGC-
Enar    --GATAAT------AAAATCTTATTCCCCTAACCT------TACCAGGCCTTCCCATGC-
Hpty    --GATAAT------TTAATCTCAACCCCCTAATC------CTACCAGGCCATCCCATGC-
Nana    --GATAAA------ATATTCCCAACCCTCTAACCC-----CTACCAGGCCATCCCATGC-
Mcst    --GATAAT------TAAATCTTAACCCCCTCATCCT------ACCAGGCCGTCCCATGC-
Rhox    --GATAAC------CTAATCTTAACCCCCTAGCCC------TACCAGGCCGCCCCATGCC
Opfa    --GACAAT------TAAATCCTAGCCCCCTAATCC------TACCAGGCCGCCCCATG--
Paar    --GATATC------ATAATCTTAATCCCCTGAAAT------TATTGGGCCGTTTCATGCA
Gozo    --GATGAC------CCAATCTTAACCCCCTA-ACCC-----TACCAGGCCGCCCCATG-C
Ackr    --GACACC------CCTATCTTACCCCTCTAACCCT------ACTAGGCCGTCCCATACC
Elev    --GATAAT------ATAATCTTAATCCCCTACCCCC-----TAACAGGTTATTCTATGCC
Trdu    --AATAAAGACAACACAATTTCACCCCCCTAAGCCT------ACCAGGCCGTTCCATAAT
Amoc    --GACAAT------TAA-TCTTAAACCCCTAAACC------CACCCGGCCGCCCCATG-C
Hame    --GACAAT------TAA-TTCTTAACCCCTAACCC------TACCAAGCCATCCTATGCA
Chso    --GATAAT------CCC-ATCTTAACCCCTAACAA------TACCAGGCCGCCCCATGCC
Lyto    --GATCAC------CACATCTTATCCCCCTAAACA------TAATGAACCACCCCATGCA
```

```
Encr    --GATCAC------ATAATCTTATCCCCCTA--AACA----TACTGAACCATCCCATGCC
Bvar    --GATCAT------CTAATCTTAGTCCTCTACCTT------TACTGGGTCGTCCTATGCA
Noco    --GATCAT------TTAATCTTATCCCCCTAAACT------TATTGAGCCGTCCCATGC-
Chsp    --GACCAC------TCAACCCTAACCCTCCTACCAT------ACTAGACTGTTCCCTGAC
Arja    --GATCAC------CTAATCTTACCCCCCTA--AACA----TACTGGACCGTCCCATGCC
Pase    --GATATA------TACCTCTCAATTCCCTTTAAT------TACCAGGCCTATCCATA-C
Trel    --GATT-A------TATTTCTTCAGCCCCCA---ATTC---TACCGGGCCCCTCCATTCA
Lifa    --GATAAC------TCACTCTTAACCCGCTCCTAA------TACCACGCCAATCTATATC
Acur    --TGTATA------GGATTCTTATTCCCCTTAAAAT-----TATCAGGCCCTCCTATA-C
Ampe    --GATAAC------CAAATCTTACTCCCCTAACC------CTACCGGGCCGCCCCATGC-
Urja    --GATGAT------TTTATCTTAGACCCCTAAATT------TACCGGGCCAGCCTATGC-
Enet    --GGTAAA------TATTTCTTAACCCTTCAAGCCT-----TATCGGGCCATTCCATTTT
Ptbr    --AATATA------CT-CACTTATTCCCCTTAATAT-----TATCAAGCTGCCTTATGTC
Safa    --ATTACC------CCCCTCCGAACCCTCCG-AGAA-----TATCGTGCTACTTTATG-C
Icae    --GATAAT------CACATCCTAACCCCCTAATACT------ATCAGGCCATCTCATGC-
Asmi    --GATATT------AC-CCCTAAAACCCTTATATTCC---CTACCAGATTCTCCTATGC-
Foal    --GAAAAC------AGTTCTCAACCCTCTTCAAC-------TATCAGGCTATACTATTT-
Drze    --AATATA------AATATCCCAATCCCCTAAAAC-----TTAACAGGCCTTTCCATAT-
Rhas    --GACAAG------CACATCTTAAACCCCTTA-TTT-----TATTAGGCCTTTCCATGCC
Elac    --GACGAA------ACCATCTTAAACCTCTTAACC------TATTAGGCCTCCCTATGC-
Kugu    --GATAAA------CACATCTTATCCCTCCAA-ACC-----TATCAGGCCATTCTATGC-
Plor    --GATAAC------TATTTCTTAATCCCCTAACTC------TATCAGGCCGCCCCATGC-
Sgun    --GATAAT------ACCTTCTTAACCCCCTAACCA------TAACAGGTTATCCCATGCC
Zaco    --GATAAT------CCTATCTCACCCCCCTAACAC------TACCAGGCCATCCTATGCA
Zbfl    --GATAAC------CCACTCTTAATCCCCTAACC------TTACCGAGCCATCCCATGCC
Spba    --GATAAC------CCAATCTTAACCCCCTAACCCC-----TATCAGGCCGTCCTATGCC
Game    --GATAAC------CAAATCCCAACCCCCTAACAT------TATCAGGCCATCTCATG-C
Thth    --GATAAC------CATATCCCAACCCCCTAA-TAT-----TATCAGGCCATCTCATGCA
Xigl    --GATAAC------CAAATCTTAACCCCCTAACTC------TACCAGGCCGCCCCATGCA
Hyja    --GATAAC------CAAATCCCAACCCCCTAAACAC-----TATCAGGCCATCTCATGCA
Psan    --GATAAC------CAAATCTCAACCCCCTTAATAT-----TACCAGGCCATCTCATGC-
Cupa    --GATAAA------CATATCCTAACCCCCTAACACT------ATCAGGCCATCTCATGCA
Mpch    --GATAAA------ATA-TCTTATTCCCCTAG-TAA-----TATCAGGCCGCTCCATAT-
Char    --GACAAC------CCACTCTTAACCCCCTACTTCC-----CACTAGGCTGTTTCATGCA
Pser    --GATAAC------CCAATCTTATCCCCCTA--AACT----TATCAGGCCGCCCCATGCA
Prol    --GATAAC------CTTATCTTAATCCCCTAAAT------TTAACGAGCCCCCCTATGCC
Plbi    --GATAAC------CTTATCTTAATCCCCCACATTT------AACAGGCCCTCCTATGCA
Calu    --ATTAAA------ACTTTCTAAAGCCCCATAATT------TATCAGACCTTTCCACCC-
Papa    --GATAAT-------ACACCCCACCCCCCTGCAACAC----TACCAAGCCACCCCATA--
Sufr    --GATAAC-----ACAAATCTAAATCCCCTAAATT----TATATCAGGCCCTCCTATGC-
Stci    --AATAAA------TTTTCTGATTCCCCCACTAT-------TAACAGGCCTCCCTATGC-
Taru    --AATAAA------ACACTCCTAACCCCTTCCCC-------TACCGGGCTTTTCTATGCT
Rala    --GATAAT------CCTTTCTTAATCCCCTATAAC------TACCGAGCCGTCCTATG-C
```

```
                  24'              6'   A        25
Scca    AAA---TTATAAAAGAACTTA-TGT-TAAAATGAGTA-A-TAAGAGGAT--------AA----
Muma    ----AAATATAAAAGAACTTA-TGC-TAAAATGAGTA-A-TAAAGAGAA--------CA-AAT
Erca    T------ATTAGAAGAAATTA-TGC-TAGAACTAGTA-A-TAAGAAAAA------------GA
Pose    ----TAA-ATAGAAGAAACAA-TGC-TAGAACTAGTA-A-TAAGAAAAA---------TGATT
Actr    ----AACTATAGAAGCAATAA-TGC-TAAAATTAGTA-A-CAAGAAGGTA-------C-GACC
Scal    GC-----TATAGAAGCAATAA-TGC-TAAAATTAGTA-A-CAAGAAGGC--------AC--GA
Posp    ----ACT-ATAGAAGCAATAA-TGC-TAAAAT-AGTA-A-CANGAAGGC--------N-CGAC
Atsp    -TT-----ATAGAAGAAATAA-TGC-TAAAATTAGTA-A-TAAGAAGAC---------ATGAT
Leoc    T---T--TATAGAAGAAATAA-TGC-TAAAATCAGTA-A-TAAGAAGGCA-C---------GA
Amca    ----AAT-ATAGAAGTAATAA-TGC-TAAAATTAGTA-A-TAAGAAGGC--------ACGAC-
Osbi    TAT-----ATAGAAGAAACAA-TGC-TAGAATAAGTA-A-CAAGAAGAT-AT--------AAT
Pabu    ----AACCATAGAAAAGACAA-TGC-TAAGATTAGTA-A-CAGGAAGAAA----------TGA
Hial    ----AACTATAGAAGCGATAA-TGC-TAAAATAAGTA-A-TAAGAAGGTATG--------ACC
Elha    --A--CCCATAGAAGAGACAA-TGC-TAAGATCAGTA-A-TAAGAAGGCA----------CAA
Mlcy    ----AAACATAGAAAAAACAA-TGC-TAAAATCAGTA-A-TAAGAAGACAC-------GA---
Algl    ----TGACATAGAAGCAATAA-TGC-TAAAATTAGTA-A-CAAGAAGAAA-------TTGTTC
Ptgi    CCC-----ATAGAAGAGATAA-TGC-TAAAATCAGTA-A-TAAGAAGGAAC----------AA
Alaf    AA-----CATAGAAGAGATAA-TGC-TAAAATGAGTA-A-TAAGAAGAAAC----------GA
Nock    ----TAACATAGAAGAGATAA-TGC-TAAAATGAGTA-A-TAAGAAGGGA----------TGA
Anja    CA-----CATAGGAGAAACAA-TGC-TAAAATCAGTA-A-TAAGAGGGCCC---------AAG
Gyki    ----GCATATGGAGAAGATAA-TGC-TAGAACTAGTA-A-TAAGAGGAGCCAC----G--CC-
Syka    ----AGACATAGGAAAGATTA-TGC-TAAAATGAGTA-A-TAAGAAGGAGT------ATTAGA
Opma    CA-----CATAGAAGAAATGA-TGC-TAAAATGAGTA-A-TAAGAGAGCACT--------CAG
Comy    -------TATAGGAGAAATTA-TGC-TAAAATGAGTA-A-TAAGAGAAACTAG-------ACC
Sasp    AT--T---ATAGAAGAGATTA-TGC-TAAAATTAGTA-A-TAAGAGAGGCCC---------GC
Eupe    -----CACATGGAAAAAATAA-TGC-TAAAATTAGTA-ACTAGAAGGCCAT----------GA
Enja    CCC-----ATGGAAGAGACTA-TGC-TGGAACGAGTA-A-CAAGAAGAAT-----------GA
Same    ----CATCATAGGGGAGACCA-TGC-TAGAACGAGTA-A-TAAGAAGGATA----------GA
Chch    ----AAACATAGAAGAGACTA-TGC-TAGAATGAGTA-A-CAAGAAGAACA--------GCCC
Grgr    CC-----CATGGGAACGACCA-TGC-TAGAATGAGTA-A-CAAGAGGAGA-------AGATC-
Caau    ----CAC-ATGGAAGAGATTA-TGC-TAAAATGAGTA-A-CAAGAAGGCCCGC--------C-
Cyca    CCC-----ATGGAAGAGATTA-TGC-TAAAATGAGTA-A-CAAGAAGGCCC----------GC
Dare    ----AAATATGGAAGAAATTA-TGC-TAAAATGAGTA-A-CAAGAAGAACCT-------GTAC
Cost    TAC-----ATGGAAGAGACCA-TGC-TAAAATGAGTA-A-CAAGAGAC------------AAC
Leec    ----CCACATGGAAGAGACTA-TGC-TAAAATGAGTA-A-CAAGAGGG----------CACGA
Crla    CC-----CATGGAAGAAACTA-TGC-TAAAATGAGTA-A-CAAGGGGG---------TCATGG
Clmc    ----CAACATGGAGGAGACCC-TGC-TAAAATGAGTA-A-CAAGAAGG----A----AACCTC
Phin    AT-----CATGGAGGAGACCC-TGC-TAAAACGAGTA-A-TAAGAAGA-----------AACC
Icpu    CG-----CATGGATGAGATAC-TGC-TAAAATGAGTA-A-TAAGAAGG---AA----CCC--C
Psto    TA-----CATGGAAGAAATAC-TGC-TAAAATGAGTA-A-TAAGAAAG---------AACCC-
Cora    -----AACATGGAAACGACAC-TGC-TAAAATGAGTA-A-TAAGAAGGTC-TA----CCAAGC
Eisp    AAC-----ATGGAGGAGATTC-TGC-TAAAATGAGTA-A-CAAGAAAGACAC--------CCC
Apal    ----GAC-ATAGAAGAGACCC-TGC-TAAAATGAGTA-A-CAAGAAGAT---------ACCCC
Eslu    -C-----CATGGAAAAGATTA-TGC-TAAAATGAGTA-A-TAAGAGGG---------AACAAC
Dape    -------TGTGGAAAAGATTC-TGC-TAAAATGAGTA-A-TAAGAGGAA----------ACAA
Glse    CCC--CCCCTGGGAGCGACTA-TGC-TAGAATGAGTA-A-TAAGAGGGA---------ACGAC
Naar    CCCCCCCCCTGGGGGCGATTA-TGT-TAGAATGAGTA-A-TAAGAGGGGACA---------AC
Baoc    CCA--CCCTTGGGGGCGATTA-TGC-TAGAATGAGTA-A-TAAGAGGG----------TGCAA
Opso    CCC-TCCCCTGGGAGAGACTA-TGC-TAGAATGAGTA-A-TAAGAGGG---GA-------CAA
Alte    C-----CCATAGGAGAGACAA-TGC-TAAAATGAGTA-A-TAAGAAGG-----------ACGC
Plap    CCC-----ATAGGAGAGATAA-TGC-TAAAATGAGTA-A-TAAGAAGGAC-----------GC
```

```
Plal   CC--C---ATGGGAGTGACCC-TGC-TAGAATGAGTA-A-TAAGAGGGGCTA---------CC
Sami   -----CCCATGGGAGCGACCC-TGC-TAGAATGAGTA-A-TAAGAGGGGC--A----CACACC
Rere   ----CTCCGTGGGAATGACCA-TGC-TAGAATGAGTA-A-TAAGAGGGGTTA------CC---
Gama   TAT-----GTGGAAGAGACCA-TGC-TATAATGAGTA-A-CAAGGGGGT-AA---------GA
Onmy   ----CCCCATGGAAGAGATTA-TGC-TAGAATGAGTA-A-TAAGAGAGT--------AC--AA
Sasa   CAC-----ATGGAAGCGATTA-TGC-TAGAATGAGTA-A-TAAGAGAG-GAC--------AAC
Cola   -----CCCATGGAAGAGATTA-TGC-TAGAATGAGTA-A-TAAGAGGG---GA----C--AA-
Dita   ----CCC-ATAGGAGAGCTTA-TGC-TAAAATGAGTA-A-TAGGAGGGC---------ACGC
Gogr   CC-----CATAAAAGACCCCC-TGC-TAAAATTAGTA-A-TAAGAGAC---CC----CAC---
Chsl   TCC-----ATGGGGGAGCCCC-TGC-TAATTATGAGTA-A-TAAGGGAGGCTAGCCTTCGCTGC
Atja   -----TACATGGGAAAGATTA-TGC-TAAAATGAGTA-A-TAAGGGACT--------ATAAG-
Iido   ----CAACATGGAAAAGATTA-TGC-TAAAATGAGTA-A-TAAGGGATTA----------TGA
Auja   T-----CCATGAAAGAGATTA-TGC-TTATATGAGTA-A-TAAGAGGA----------AATGA
Chag   ----CCCCATGAAAGAGACCA-TGC-TTATATGAGTA-A-CAAGAGGGC--------CAGCCC
Hami   ----CCCCATGGAAGAAATTA-TGC-TTATATGAGTA-A-TAAGAGGT---------ACATGA
Saun   CC-----CATGGGAGAAACGA-TGC-TTATATGAGTA-A-TAAGAGGCACAC---------GA
Nema   AG--C---ATGGAAAAGACCC-TGC-TAAGATGAGTA-A-TAAGAGGGA-CA---------GC
Disp   CC------CTGGGAAAGACCC-TGC-TAGAATGAGTA-A-TAAGAGGA----G----CCTAC-
Myaf   -----ACCCTGGGAGAGACCC-TGC-TAAAATGAGTA-A-TAAGAGAG----------TACGC
Lagu   --------GTAGAAGAAACCA-TGC-TAGAATGAGTA-A-CAAGAGGC---------CCAAGC
Trtr   T---ATATATAGAAAAGACTA-TGC-TAGAACGAGTA-A-TAAGGGGCCT-AT-------ACA
Zucr   ----GGAAATAGAAGAGATTA-TGC-TAGAACGAGTA-A-TAAGGGGTACTAT----T--TAA
Pxja   C---AACCATGAGAGTGACCA-TGC-TAAAATGAGTA-A-TAAGAGGGTA-C--------GA-
Pxlo   ACC-----ATGAGAGTGACCA-TGC-TAAAATGAGTA-A-TAAGAGGG---TA-------CGA
Pctr   ----CCC-GTGGGGGAGATAA-TGC-TAAAATGAGTA-A-TAAGAGGGC---------AA-GC
Apsa   TC--AGCCATAGGAGAGACTA-TGT-TAATATGAGTA-A-CAAGAGGG----------CCTGC
Cabe   T----CTTACATAAGTGATTC-TGC-TAATATGAGTA-A-TAAGGGGCTC-C-----AAACGG
Bzze   A----ATTATGTGAGTGACTA-TGC-TAATATGAGTA-A-TAAGAGAACA-T-------CCGG
Siim   C---AACCATGTGAGTGATTA-TGC-TAATATGAGTA-A-CAAGAGTGAA-G--------GAA
Ctru   A-----ATGTGGAAGAGACCA-TGC-TAATATGAGTA-A-TAAGAGGG----------AGACC
Dpbr   ----TAATATGGAAGAGACCA-TGC-TAATATGAGTA-A-TAAGAGGGAA--A----AC----
Caki   CC-----CATAAGAATAATTA-TGC-TAAAATGAGTA-A-TAAGAAGA----A----A--AT-
Phja   TT-----CATAAGAACAATTA-TGC-TAAAATGAGTA-A-TAAGAAGA---AT----CTAATT
Brsp   -------TATAAAAGAAATTA-TGT-TAAAATGAGTA-A-TAAGAATT--------------A
Gamo   -----CCCATAAGAACAATTA-TGC-TAAAATGAGTA-A-TAAGAAGA----A----TTTAAT
Lolo   CCC-----ATAAGAACAATTA-TGC-TAAAATGAGTA-A-TAAGAAGA-ATAT-------AAT
Batr   CT-----GATGTAGATAA----TGC-TAATATGAGTA-A-TATGGAAC---------------
Prmy   ----A---ATAGTAGAGACAA-TGC-TAGTATGAGTA-A-TAAGAGGCC--------TCA---
Loli   -------TATAGGAGCTATAC-TGT-TAATATGAGTA-A-TAAGAAAGTACCCT---ACAAGG
Loam   ATC-----ATAGGAATTATAC-TGC-TAATATGAGTA-A-TAAGAGAACATA--------AGG
Chab   ----AAACATGGAAGAGATAA-TGC-TAATATGAGTA-A-TAAGAGGGCTACA----CCCC--
Chto   AA-----CATGGAAGAGATAA-TGC-TAATATGAGTA-A-TAAGAGGG---CT----CCACCC
Majo   CG-----TATGGGAGTGACCA-TGC-TAATATGAGTA-A-TAAGAGAG----T----CAAGG-
Hlst   ----AACCATGGAAAAGATAA-TGC-TAATATGAGTA-A-TAAGAAAGA--CT----AACCCT
Clpe   ----AAC-ATGGGAAAGATTA-TGC-TAATATGAGTA-A-CAAGAGGGC--------CACCCC
Mlmr   ----AAGCATGGAAAAGATTA-TGC-TAATATGAGTA-A-TAAGAGGGCT---------ACCC
Crcr   AGC-----ATGGAAGTGATTA-TGC-TAAAATGAGTA-A-TAAGAGAAT---------ACGTC
Muce   AGC-----ATGGAAGTGATTA-TGC-TAAAATGAGTA-A-TAAGAGAATAC----------GT
Bege   ----CCTTATGGGAGTGATCA-TGC-TAATATGAGTA-A-TAAGAAAG---AA----C--ACT
Mela   AT------ATGGGAATAATAA-TGC-TAATATGAGTA-A-TAAGAGAGCAA---------GAC
Hats   T---ACATATAGGAGCGACTA-TGC-TAGTATGAGTA-A-CAAGAGAGCC-C--------TGA
Orla   -----TTTATGAAAGAAATTA-TGC-TAGTATGAGTA-A-TAAGAAGTTCTG---------AA
```

Cosa	-----A-TATAGAAGCGATTA-TGC-TAATATGAGTA-A-TAAGAGGC---------TACAAC
Exsp	----GTA-ATAGAAGAGATAA-TGC-TAATATGAGTA-A-TAAGAGATG--------AAGAT-
Depa	--G-T---GTGGAAACAATTA-TGT-TAATATGAGTA-A-TAAGAGGCAC-----------AA
Rima	C-------ATAAAAGCAACCA-TAC-TAACATGAGTA-A-TAAGAGACCC----------GAA
Fuol	TC-----TATAGGAAAGATTA-TGC-TAATATGAGTA-A-TAAGAGAA---------CTTGT-
Gmaf	AA------ATAGAAGAGATTA-TGC-TAAAATGAGTA-A-TAAGAGAA---------T-ACAC
Xeei	----CCTAATAGAAAAGATTA-TGC-TAAAATGAGTA-A-TAAGAGAAT--------ACGCT-
Pros	CC-----CATGGAAGAGATCC-TGC-TAATATGAGTA-A-TAAGAAAA---TC----CCAGAT
Scmi	----CCCCATGGAAAAAATAC-TGC-TAACATGAGTA-A-CAAGAAAA----C----CCCAGA
Rolo	GC-----CATGAAAGAGACCA-TGC-TAATATGAGTA-A-TAAGAAGG----T----AGCAGA
Cere	A----ACCATAGGAGAGACTA-TGC-TAATATGAGTA-A-TAAGAAGGAA-A-----ATTAAA
Daga	----GACCATGAAAGAGACCA-TGC-TAATATGAGTA-A-CAAGGGGGCACTC----ATAGAC
Anco	ACC-----ATGAGAGAGTTAA-TGC-TAAAATGAGTA-A-TAAGAGGGA---------A--GC
Dmve	AC-----CATGAGAGAGTTTA-TGC-TAAAATGAGTA-A-TAAGAGGGCAC----------GC
Dmar	----CACCATGAGAGAGTTTA-TGC-TAAAATGAGTA-A-CAAGAGGG----------CACGC
Anka	----AACCATGAGAGAGTTAA-TGC-TAAAATGAGTA-A-TAAGAGGTTA-----------GC
Moja	----AGCCATGAGAGAGTTAA-TGC-TAAAATGAGTA-A-TAAGAGGG----T----AG-CCC
Hoja	----ACCCATGAGAGAGTTTA-TGC-TAAAATGAGTA-A-TAAGAGGGTA-----------GC
Bede	GCC-----ATGGGAGAGACCC-TGC-TAATATGAGTA-A-CAAGAAAATTTCT-------AGA
Besp	-----ACCATGGGAGAGACCC-TGC-TAATATGAGTA-A-CAAGAAAA----T----TCTAGA
Mysp	----ACCCATGTAAGAGATCC-TGC-TAATATGAGTA-A-TAAGGAAGC--------ATAAGA
Osja	ACC-----ATGTATGAGATCC-TGC-TAATATGAGTA-A-TAAGAATGTCCTT-------AGA
Sgro	----AACCATGTGAGAGATCC-TGC-TAATATGAGTA-A-TAAGAACG-TACC----AGA---
Pzpa	CC-----CATGAGAGTGATAA-TGC-TAAAATGAGTA-A-TGAGAAAATAAAA--------CT
Zeja	T-----TCATGAGAGTGATAA-TGC-TAAAATGAGTA-A-TAAGAAGC----------ATATG
Znne	----CCCCATGAGAGTGATAA-TGC-TAAAATGAGTA-A-TAAGAAGTT--------ATAAC-
Zefa	-----TCCATGAGAGTGATAA-TGC-TAAAATGAGTA-A-TAAGAAGT----A----ATAA--
Acni	----CCCCATGAGAGTGATAA-TGC-TAAAATGAGTA-A-TAAGAAGTTA----------TAA
Ncrh	CC-----CATGAGAGTGATAA-TGC-TAAAATGAGTA-A-TAAGAAGT---------TATAA-
Agca	TAA--ACCATGGGAGAGATTA-TGC-TAATATGAGTA-A-TAAGAGAGC--------CC---T
Hydy	----TTTCATGGGAGTGACGA-TGC-TAATATGAGTA-A-TAAGGGGG---C-----CTACGC
Gsac	T-----TCATGGGAGTGATTA-TGC-TAATATAAGTA-A-TAAGGGAGC--------CAATGC
Pevo	AC-----TATAGAAACAATTA-TGC-TGATATGAGTA-A-TAAGAGAA---------T-AAGA
Hiku	----ATACATGAAAATGATAA-TGG-TAATATGAGTA-A-TAAGAGAAGT--T----AAGATT
Inpa	----AAACATGGAAGTGACAA-TGC-TAATATGAGTA-A-TAAGAAAGC-----------AAG
Auch	ACT-----ATTGAAGCGAAAA-TGC-TAGAATGAGTA-A-CAAGAGACCCC---------AGA
Fico	-T--AA--ATGGGAGTAATTA-TGC-TAATATGAGTA-A-TAAGAGAA---------CCAAGC
Macs	----AACTGTGGAAGTGACCA-TGC-TAATATGAGTA-A-TAAGAAAT---------TAAGAA
Moal	CA-----CATGAAAGTAATTA-TGC-TAATATGAGTA-A-TAAGAGAGC--------TC--GC
Syma	TAC-----ATGAGAGCGATTC-TGC-TAATATGAGTA-A-TAAGAGAGCTAC--------GCC
Mafr	----ACC-ATGGAAGAAATTA-TGC-TAATATGAGTA-A-TAAGAGAAC---------CACAC
Dcpe	AA-----TATGGGAGTGATCC-TGC-TAATATGAGTA-A-TAAGAGAA---------ATAACA
Dcti	GA-----TATGGGAGTGATCC-TGC-TAATATGAGTA-A-TAAGAGAA----A----TAACA-
Hehi	AAC-----ATGGGAGAGACCC-TGC-TAATATGAGTA-A-TAAGAGAGCCAA---------GC
Stam	CC-----CATGGAAGCGACTA-TGC-TAATATGAGTA-A-TAAGAGAGCTCCG--------CC
Hogi	----AACCATGGGAGCGACCA-TGC-TAATATGAGTA-A-TAAGAGACCC----------CGC
Erzo	-----TACATGGGGGTGACTA-TGC-TAATATGAGTA-A-CAAGAGAG---CC----TCGGA-
Hxot	-----AACATGGGAGTGACTA-TGC-TGAAATGAGTA-A-TAAGAGAG-C-TA----CCGG--
Core	CC-----CATGGGAGTGACTA-TGC-TAATATGAGTA-A-TAAGAGGG---CC----AAAGGC
Apve	TG-----CATGGGAGTAACTA-TGC-TAATATGAGTA-A-TAAGAGAGCCT--------ACGC
Latj	----AAACATGGGAGCGACCA-TGC-TAACATGAGTA-A-TAAGAGGATG-GC----CTACGC
Laja	AA-----TATGGAAGTGACCA-TGC-TAATATGAGTA-A-TAAGAGAG----C----CTCGC-

```
Syja    ----AACTGTGGGAGTGACTA-TGC-TAATATGAGTA-A-CAAGAGAG---C-----CCCGGC
Epme    ----ACACATGGGAGCGATCC-TGC-TAAAATTAGTATA-TAAGAGAAC---C----TAACGA
Grse    AAC-----ATGGAAGCGACTA-TGC-TAATATGAGTA-A-TAAGAGAGCTACC-------GCC
Clja    AA-----CATGGAAGTAATAA-TGC-TAATATGAGTA-A-TAAGAGAG---CA----CTCGCC
Ogcy    CC-----AATAGAAGAAACAA-TGC-TAAAATGAGTA-A-TAAGAGGA---AA----CCCGCC
Plna    ----TCC-ATGGGAGTGACCC-TGC-TAAAATGAGTA-A-TAAGAGACT--------CCAAA-
Lema    ----AAACATGGGCGTGACTA-TGC-TAAAATGAGTA-A-TAAGAGAGTTAA------GAC--
Etzo    AA-----CATGGGAGTGACCC-TGC-TAAAATGAGTA-A-TAAGAGAG---CC----CACGCC
Apse    CC-----TATGGGAGAGATTA-TGC-TAATATGAGTA-A-TAAGAGAT---------CCCCGC
Epde    AA-----CAGGGGAGCGACCA-TGC-TAATATGAGTA-A-TAAGAGAACATCCG---CT----
Slja    ----CTATATGGAAGTGATAA-TGC-TAGAATGAGTA-A-TAAGAGAGC--------CAGACT
Bsja    -----AGCATGGGGACGACCA-TGC-TAATATGAGTA-A-TAAGAGAAC--------ATAAGA
Ecna    ----TAACATGGGAATGATAA-TGC-TAATATGAGTA-A-TAAGAGAGA--------TACCGA
Cohi    ATT-----ATGGAAGTGATTA-TGC-TAAAATGAGTA-A-TAAGAGAGC--TT-----AAGGC
Caar    ----ACCCATGGGGGCGACCC-TGC-TAAAATGAGTA-A-TAAGAGAGCA--T----ACC---
Came    CT-----CATGGGGGCGACCC-TGC-TAAAATGAGTA-A-TAAGAGAGCTACA--------CC
Mema    A----CACATAGGAGTGACCA-TGC-TAATATGAGTA-A-TAAGAGGACA----------CGC
Lenu    ----CGTGGAAGAA---ACCA-TGC-TAATATGAGTA-A-TAAGAGACTA--C----CAA-GG
Brja    ----AAC-ATGAGAGTGCCCA-TGC-TAATATGAGTA-A-TAAGAAGGC---------CCCGG
Plma    AA-----CATGAGAGTGCACA-TGC-TAATATGAGTA-A-TAAGAGAACACCC--------GT
Emst    ----AAACATAGGAGTGACCA-TGC-TAATATGAGTA-A-TCAGAGAGTA-------TTA--C
Ptti    ----AAC-ATGGGAGTGATAA-TGC-TAACATGAGTA-A-TAAGAGAGC--------ACAAC-
Losu    ----ACC-ATGGGAGAGACCA-TGC-TAATATGAGTA-A-TAAGAGGGC--------TTCCGC
Geoy    CCC-----ATGGGAACGACCA-TGC-TAAAATGAGTA-A-TAGGAAGGGCCAA-------CGC
Dipi    CGA-C---ATGGAAGCGAACA-TGC-TAATATGAGTA-A-TAAGAGAGCCTC---------GC
Pama    C---AAACAGTGGAGAAATAA-TGC-TAATATGAGTA-A-TAAGAGAGAA-C--------AAA
Leob    AC--CT--ATAGAAGCGATTA-TGC-TAATATGAGTA-A-CAAGAGGGGG--T----CATAGC
Neba    -----GTTATAGAAGTGATAA-TGC-TAATATGAGTA-A-TATGAGACC--------------
Pdpl    CC-----CATGGGTGTGATTA-TGC-TAATATGAGTA-A-CAAGATGGGCCAA----CTACGC
Nimi    ACC-----ATGGGAGCGACCA-TGC-TAATATGAGTA-A-TAAGAGGGCAAAT-----ACGCC
Uptr    CT-----TATGGGAGTGACCA-TGC-TAATATGAGTA-A-CAAGAGAGTCAA---------GA
Pesc    ----TACTATGGGAGCGATCA-TGC-TAATATGAGTA-A-CAAGAATGGCTTC----C--GCC
Baar    ACT-----ATGGAAGCGACCC-TGC-TAAAATGAGTA-A-TAAGAGAGC---------CTCGC
Moar    G-----ACATGGGGGTGTCCA-TGC-TAATATGAGTA-A-TAAGAGAGTAA-------ACCAA
Toja    ----CCACATAGGAGTGATAA-TGC-TAAAATGAGTA-A-TAAGAGAGTACC-----CAACCC
Chau    ----AGACATGGGAGAGATTA-TGC-TGAAATGAGTA-A-TAAGAGAGT---G----ACACGA
Chse    ----AACCATGGGGGCGATTA-TGC-TAATATGAGTA-A-TAAGAGAGTA-------CTTAGA
Enar    ----AAACATGGGAGTGACCA-TGC-TAATATGAGTA-A-TAAGAGAGT--------CCTCGA
Hpty    ----CTACATGGGAGCGACCA-TGC-CAATATGAGTA-A-TAAGAGAGCACCA----C--CCC
Nana    ----AAACATGGAAGAGACCA-TGC-TAATATGAGTA-A-TAAGAGAACA-------C--TCC
Mcst    ----AAACATGGGAGTGACCA-TGC-TAATATGAGTA-A-TAAGAGAGCC-------CTAAGC
Rhox    -----CCCATGGGAGCGATTA-TGC-TAGAATGAGTA-A-TAAGAGAGC--------CCTGAC
Opfa    C---AAACATGGGAGTGACCA-TGC-TAATATGAGTA-A-TAAGAGAGGC-CC-------CGC
Paar    TAC-----ATGAAAGCGACCA-TGC-TAGAATGAGTA-A-TAAGAGAAAT-CC-------AGC
Gozo    ----ACACATGGGGGTGACCA-TGT-TAATATGAGTA-A-TAAGAGAGC--------ACCCGC
Ackr    CT-----TATGGGAGTGATTA-TGC-TAAAATGAGTA-A-TAAGAAGGCC-------TGGC--
Elev    TC--C---ATAGAAGAGATAA-TGC-TAATATGAGTA-A-TAAGAGACA-CA---------GA
Trdu    AC-----TATGGAAGCGTTTA-TGC-CAATATGAGTA-A-TAAGAGAACCC------------
Amoc    ----CTCCATGGGAGCAATTA-TGC-TAAAATGAGTA-A-TAAGAGGGC--------ACTCAA
Hame    -----TCCATAGGAGTGACCC-TGC-TAATATGAGTA-A-TAAGAGCGC--------TCAGAC
Chso    --C---CCATGGGAGTGACCA-TGC-TAGTATGAGTA-A-TAAGAGAA---------TAAGAC
Lyto    CAC-----ATGGGGGTGACTA-TGC-TAATATGAGTA-A-TAAGAGAGCCT--------CTGG
```

```
Encr      ----TGC-ATGGGAGTGATTA-TGC-TAATATGAGTA-A-TAAGAGAGC--------CTTTGG
Bvar      AG-----CATAGGAGTGACCA-TGC-TAGTATGAGTA-A-TAAGAGAGC--------CCTCAC
Noco      ----CAACATGGGAGTGACCA-TGC-TAATATAAGTA-A-TAAGAAAGCC-------CTGGAC
Chsp      T------ACTGGGAAGCATTCCTAT-TAATATGAGTA-A-TAAGTAAAGC-------AAGCCT
Arja      ----CGC-ATGGGAGTGATTA-TGC-TAATATGAGTA-A-TAAGAGAGC--------TAAAGA
Pase      T---TATTATGGAAGCGATTC-TGC-TAAAATGAGTA-A-CAAGAGGGGG-CT----AGACAC
Trel      ----ATTAATGGAAGAGACCA-TGC-TAAAATTAGTA-A-TAAGAGGGC--------AAGACC
Lifa      -------AATAGAAGAGATTA-TGC-TAATATGAGTA-A-TATGAAAAC--------TACAAC
Acur      G---CACTATAGGAGTAACAC-TGC-TAGAATGAGTA-A-CTGGAAAACT-------AAAAGA
Ampe      ----AAACATGGGAGCGACCA-TGC-TAATATGAGTA-A-TAAGAGAGCTTAA----G--CC-
Urja      ----AACCATAGGCGTGACCA-TGC-TAATATGAGTA-A-TTGGGGGCCTG-----------A
Enet      TAA---CTATGGAAGAGATAA-TGC-TAGTATGAGTA-A-CGTGAGGTTG----------AGC
Ptbr      CCC-----ATAAAGGCATTTC-TGC-TAGAATGAGTA-A-TAAGAGAACT-----------GA
Safa      ----CACCATAAAAATATTAA-TGC-TAGAATGAGTA-A-CAAGAGCCAA--C----GAA---
Icae      ----AGACATGAGAGTGCACA-TGC-TAATATGAGTA-A-CAAGAGAGCCCC--------GTC
Asmi      ----AAACATAGGAAAAAATA-TGC-TAATATGAGTA-A-CTAGAGAAA---------GAC--
Foal      -----AATATAGTAAAAATAA-TGC-TAGTATGAGTA-A-TAAGGGGAA----------GCC-
Drze      ----AATTATGGAAGTGATAA-TGC-TAAAATGAGTA-A-CAAGAGACCTTAA----C-AT-C
Rhas      CA-----CATGGAAGAGACCA-TGC-TAACATGAGTA-A-TAAGAGGGA--------CTAGTC
Elac      ----GAACATAGAAGAGAATA-TGC-TAGAATGAGTA-A-TAAGAGGGCC-------C-GACC
Kugu      ----ACCCATAGAAGTGACTA-TGC-TAATATGAGTA-A-TAAGAGAAA--------ACTAGA
Plor      ----AGTCATGGGAGCGATCA-TGC-TAAAATGAGTA-A-TAAGAGGGCTAC------GCC--
Sgun      CAC-----ATGGGAGAAATTA-TGC-TAATATGAGTA-A-TAAGAGAACAC----------GC
Zaco      ----ACC-ATAGGAGCGATCA-TGC-TAATATGAGTA-A-TAAGAGAGTT--T-----AACGC
Zbfl      CA-----CATGGGAGTGATCC-TGC-TAATATGAGTA-A-TAAGAGAAC--------CTACG-
Spba      AAC-----ATAGGAGCGACTA-TGC-TAATATGAGTA-A-TAAGAGACC--CC-------CGA
Game      ----AAACATGAGAGTGCACA-TGC-TAATATGAGTA-A-CAAGAGAGC--------TCTGCC
Thth      TAC-----ATGAGAGTGCACA-TGC-TAATATGAGTA-A-CAAGAGAGCCTC--------CGC
Xigl      GA-----CATGGGCGTGACTA-TGC-TAATATGAGTA-A-CAAGAGAGTACCTA---CCC--C
Hyja      TAC-----ATGAGAGTGCACA-TGC-TAATATGAGTA-A-TAAGAGAGT--AC-----CCCGC
Psan      ----ATACATGAGAGTGTATA-TGC-TGATATGAGTA-A-TAAGAGCGCA-------ACCCCG
Cupa      GA-----CATGAGAGTGCACA-TGC-TAATATGAGTA-A-TAAGAGAGG--------ACTCGC
Mpch      ----CATTATGGAGGTGATAA-TGC-TAATATGAGTA-A-TAAGAGAGTA----------AGA
Char      AAC-----ATGAAAGCACCAA-TGC-TAAAATGAGTA-A-TAAGAGAGC--AC-----CGACC
Pser      ----ACC-ATGGAGGTGTCCA-TGC-TGATATGAGTA-A-TAAGAGAGT--------CACACC
Prol      ----AAACATAGGGATGACCA-TGC-TAATATGAGTA-A-TAAGAGAATACTA----A-GTAC
Plbi      TCA----CATAGGAACGACTA-TGC-TAATATGAGTA-A-TAAGAAAGTA-------TAACCA
Calu      ------T-GTGGAAGAGAACA-TGC-TAATATGAGTA-A-CAAGAAGACA-------CTACGA
Papa      -----AATATGGGAGAGACCA-TGC-TAATATGAGTA-A-TAAGAGAAG--------TAATCC
Sufr      ----AACCATAGGACTGATTA-TGC-TAATATGAGTA-A-TAAGAAAGTA-------CTGACT
Stci      ----CCCCATAGGAAAGATTA-TGC-TAAAATGAGTA-A-TAAGAAAGAC---------AAGA
Taru      TCC-----ATAGAAGAAATTA-TGC-TAAAATGAGTA-A-TAAGGGGCC-----------GAC
Rala      ----CTGCATAGGAGCGACTA-TGC-TAGAACGAGTA-A-TAAGAGAATT--A----AT--GA
                                *       *  **** *
```

```
                26    27    B   28           28'
Scca      ACCTCT-CCCGACACAAGTGTAT-GTCA-GAAAGAATTAAATCACTGATAA--TTA-AA
Muma      --CTCT-CCCGACATAAGTGTAC-GTCA-GAAAGAATTAAATCACTGACAA--TTA-AT
Erca      TTTTCT-CCCAGCACAAGTGTAA-GTTA-GACAGGACAAA-CCACTAACAA--TTA-AA
Pose      --TTCT-CCTCGCACAAGTGTAA-GTTA-GAACGGACAAA-CCACTAACAG--TTA-AA
Actr      --TTCT-CCAAGCACACGTGTAA-GTCA-GACCGGACCCG-CCACTGACAA--ATA--A
Scal      CCTTCT-CCAAGCACACGTGTAA-GTCA-GATCGGACCCG-CCACTGACAA--ATA--A
Posp      CTTCTT-CCCAGCACATGTGTAA-GTCAAGATCGGACTCA-CCGCTGACAA--ATA-AC
Atsp      C-TTCT-CCAAGCATACGTGTAA-GTCA-GATCGGACACA-CCACTGACAA--GTA-A-
Leoc      CCTTCT-CCAAGCATACGTGTAA-GTCA-GATCGGACACA-CCACTGACAA--ATA--A
Amca      -CTTCT-CCAAGCATCCCTGCAA-GTCA-GACCGGACTCC-CCACTGACAA--ATA--A
Osbi      C-TTCT-CTCAGCACATGTGTAA-GTCA-GATCGGACCAC-CCACTGACAA--TTAGAA
Pabu      TCTTCT-CCAAGCGCACGTGTTC-ATCA-GACCGGACAAC-CCACTGATAA--ATA-AA
Hial      --TTCT-CCTAGCACATGTGTAA-GTCA-GATCGGACACC-CCACTGACAA--TTA-A-
Elha      CCTTCT-CCTAGCACATGTGTAA-GTCA-GATCGGACCAA-CCACTGACAA--ACA-A-
Mlcy      TCTTCT-CCAAGCACATGTGTAC-GTTA-GACCGAACTAA-TCACTAACAA--ATC-AA
Algl      --TTCT-CCTGGCATGTGTGTAG-GTCA-GATCGGACAAG-CCGCTGACAA--TTA--G
Ptgi      CCTTCT-CCCGGCACATGTGTAA-GTCA-GATCGGACCCC-CCACTGAC--AAATA-A-
Alaf      TCTTCT-CCCTGCACACCTGCAA-GTCA-GATTGGACCAC-CCACTGACAA--CTA--A
Nock      CCTTCT-CCTCGCACACCTGTAA-GTCA-GATTGGACCAT-CCACTGATAA--ATA--A
Anja      CCCTCT-CCTAGCACATGTGTAA-GTCA-GAACGGACCGA-CCACTGACAA--TCA--A
Gyki      -CCTCT-CCCCGCACGATTGTGA-GTCA-GGCAGGACCAA-CCACTGATAA--CTA--A
Syka      CCTTCT-CCCGGCACATGTGTAA-GTCA-GCTCGGACAGA-CCACTGACAA--TTA--A
Opma      CCCTCT-CCAAGCACAAGTGTAA-GTCA-GAACGGACAAA-CCACTGACAA--TTA--A
Comy      --CTCT-CCGGGCACACGTGTAA-ATTA-GAGCGGACAAA-CCACTGACAA--CTA--A
Sasp      CTCTCTCCT-GACACATGTGTAA-ACCA-GAACGGACTAA-CCACTGGC--AATTA--A
Eupe      CCCTCTCC-CAGCACTTGTGTAA-GTCA-GAACGGAACAA-CCACTGACAA--TTA--A
Enja      ACTTCTCCA-CGCACAAGTGTAT-GTCA-AATTGGACCAA-CCATCGAC--AATCA--A
Same      CCTTCTCC-CAGCACACGTGTAA-GTCG-AATCGGACGAC-CCATCGAC-A--ATT-AA
Chch      --TTCT-CCCAGCACGTGTGTAA-ACCA-GATCGGACACC-CCTCTGGAAA--TTA-A-
Grgr      --CTCTCC-CTGCACACGTGTAG-GCCG-GGACGAACCAA-GCACCGGC-A--AAT-AA
Caau      -CTTCTCC-AAGCACAAGTGTAA-GCCA-AATCGGACTAG-CCATTGGCAA--CTA--A
Cyca      CCTTCTCCA-AGCACAAGTGTAA-GCCA-AACCGGACCAA-CCATTGGC--AACTA-A-
Dare      --TTCT-CCAAGCACAAGTGTAA-GCCA-AGTTGGACAAA-CCATTGGCAA--TTA-A-
Cost      ATCTCTCCA-GGCATAAGTGTAA-GCCA-GCCCGGATAAG-CCACCGGC--AATTA-A-
Leec      CCCTCTCC-TAGCACAAGTGTAA-TTTA-GACCGGACTAA-CCACTGAAAA--TTA-A-
Crla      CCCCCTCC-CGGCACAAGTGTAA-GCCA-GACCGGACCCA-CCACTGGCAA--TTA-A-
Clmc      -CTTCTCCTCGGCCTACGTGTAA-GTTA-GATCGGACAAC-CCACTAACAA--TTA-A-
Phin      CCTTCTCC-TGACCCATGTGTAA-GTCA-GACCGGACCAA-CCACTGATAA--TTA-A-
Icpu      -CTTCTCC-TAGCCTACGTGTAC-GCTA-AATTGGACCTA-CCACTAGC-A--ATT-AA
Psto      CTTTCTCC-TAGCCTACGTGTAC-ACTA-GATCGGACCCA-CCACTAGTAA--TTA-C-
Cora      CCTTCTCC-TAGCCTAAGTGTAA-ATTA-GATTGGACCAA-CCACTAAAACATTTA-A-
Eisp      CTTTCTCCT-CACCCAAGTGTAA-GTCA-GACCGGACCCG-CCACTGAC--AATTA-A-
Apal      C-TTCTCCTAAGCCCAAGTGTAA-ACTA-GATCGGACCCT-CCACTAGCAA--TTA-A-
Eslu      -CCTCTCC-TGGCACCTGTGTAA-GTCG-GGCCGGACCCC-CCACCGATTC--TTA-A-
Dape      TCCTCTCC-CCGCACTAGTGTAT-ATCG-GGCCGGATATA-CCACCGAT-C--CTT-AA
Glse      C-CTCTC-CCAGCACATGTGTAA-GTCG-GATCGGACCCC-CCGCCGATTA--ATA-A-
Naar      -CCTCTCC-CAACACATGTGTAA-GTCG-GACCGGACCCC-CCACCGACAA--ATA-A-
Baoc      CCCTCTCC-CAACACATGTGTAA-GTCG-GACCGGACTCC-CCGCCGACAA--GTA-A-
Opso      CCCTCTCCC-GGCACATGTGTAA-GTTG-GACCGGACCCC-CCACCAAC--AAGTA-A-
Alte      CCTTCTCC-CCGCACATGTGTAA-GTTA-GTCCGGACCTT-CCACTAACAA--ATA--A
Plap      CCTTCTCCC-AGCACACGTGTTA-GTTA-GCCCGGACTCC-CCACTAAC--AAATA--A
```

```
Plal    CCCTCTCCC-CGCACATGTGTAA-GTTG-GACCGGACCCC-CCGCCGAC--AAATA--A
Sami    -CCTCT-CCTTGCACATGTGTAA-GTCG-GACCGGACCCC-CCACCGACAA--ATA--A
Rere    CCCTCT-CCCCGTACACGTGTAT-GTCG-GACCGGACCCC-CCACCGATGA---TT-AA
Gama    CCCCCTCCC-CGCACCCGTGTAA-ATCG-GACCGGACACC-CCGCCGAC--TATTA-A-
Onmy    CTCTCTCC-CAGCACATGTGTAA-GTCG-GACCGGACCCC-CCACCGAC-A--AAT-AA
Sasa    T-CTCTC-CCAGCACATGTGTAA-GTCG-GACCGGACCCG-CCACCGACAA--ATA--A
Cola    CCCTCTCC-CAGCACATGTGTAA-GTCG-GACCGGACCCC-CCACCGACAA--ATA--A
Dita    CCCTCTCC-CCGCGCAAGTGTAA-GTCA-GACCGGACCCC-CCCCTGACAG--ATA--A
Gogr    --CCCTCC-AAGCACATGTGAAA-GTCA-AACCGGACCCA-CCATCGAC-A--AAT-AA
Chsl    CTCCCTCCC-GGCACAAGTGTAA-GTCG-CACTGGACCCC-CCGGCGAC--AAATA-A-
Atja    -TCCCTCC-CAGCACAGGTGTAC-ATCG-GAACGGACAAG-CCACCGAAAG--TTA-A-
Iido    ATCCCTCC-CCGCACATGTGTAT-ATCG-GAACGGACAAA-CCACCGAAAA--TTA-A-
Auja    CCCTCTCC-CTGCACACGTGTAA-TTCG-GAACGGACTCA-CCACCGACAA--TTA-A-
Chag    --CTCTCCGTAGCACATGTGTAA-GTCG-GATTGGACACA-CCACCGACA---TTT-AG
Hami    CCCTCTCC-GTGCACACGTGTAA-GTCG-GAACGGACCCC-CCACCGACA---ATT-AA
Saun    CCCTCTCC-CAGCACACGTGTAA-GTCG-GAACGGACCCC-CCGCCGACAG--TTA-A-
Nema    CCCTCTCCAATGCACAAGTGTAC-CTCG-GAACGGACCCC-CCACCGAC--AGTCA-A-
Disp    TCCTCTCC-CTGCACACGTGTAA-ATCG-GAACGGACCCA-CCGCCGACAA--CTA--A
Myaf    CCCTCTCCGTCACACATGTGTAA-GTCG-GAGCGGACCCG-CCCCCGATAA--CTA-A-
Lagu    GCCTCTCC-TTGCACAAGTGTAC-CCCG-GCCTGGACCCC-CCACCGGAAA--CTA-CC
Trtr    ACCCCTCC-CTGCATAAGTGTAC-CTCG-GACCGGACCAC-CCCCCGAACA--TTA-TA
Zucr    -CCCCTCC-TTGCATAAGTGTAA-CTCG-GACCGGATTCC-CCACCGAAAT--TTA-TC
Pxja    CCCTCTCC-CTGCACATGTGTAA-GTCG-GAACGGACCTA-CCACCGACAA--ATA-AC
Pxlo    CCCTCTCCC-TGCACATGTGTAA-GTCG-GAACGGACCTC-CCACCGAC--AAATA-AA
Pctr    CCCTCTCC-CAGCACCCGTGTAA-GTCG-GAGCGGACCCG-CCACCGACCA--TTA-AC
Apsa    CCCTCTCC-AAACACCAGTGTAT-ATCG-GAACGGACACC-CCACCGACCA--TTA--A
Cabe    CCCCCTCC-TGGCACACGTGTAT-AGCG-AAACGAACCC--TCATCGCCAA--CTA-A-
Bzze    TTCTCTCC-CAACACAAGTGTAA-ATCG-GATCGGACCCC-CCGCCGACTA--TTA-A-
Siim    --CTCTCCTACACACAAGTGTAA-GTCG-AAACGGACCAC-CCATCGACCA--TTA-A-
Ctru    CCCTCTCC-TTGCACAAGTGTAA-ATCG-GAACGGACCCA-CCACCGACCC--CTA-TC
Dpbr    -CCTCTCC-TTGCACAAGTGTAA-ATCG-GAACGGACCCA-CCACCGACCC--CGA-CC
Caki    TCTTCTCC-CAGCATAAATGTAA-GTCA-GACCGGACCAC-CCACCGATTA--CTA-CA
Phja    C-TTCTCC-TGACACGTGTGTAT-GTTG-GAACGGACCCC-CCACCAACAG--TTA-TT
Brsp    TATTCTCC-TTGCACATGTGTAC-CTCG-GAACGGACCAG-CCACCGAATA--TTA-AT
Gamo    TCTTCTCC-TAGCACATGTGTAA-GTCG-GAACGGACCTC-CCACCGACTA--TTA-AT
Lolo    TCTTCTCCC-AGCACATGTGTAA-GTCG-GAACGGACCTC-CCACCGAC--TATTA-AT
Batr    -CACCTCC-TAGCACAACTATAA-GTCG-TAAT----------ATTGATCT--TTA-TT
Prmy    -CCTCTCC-TTACATACCTGTTT-ATCA-GCCCGGAACCA-CGATTGAAAA--TTA-CT
Loli    CTTTCTCC-TAGCACCCGTGTAC-ATCG-GAACGAACCC--CCACCGAAAA--TCA-A-
Loam    TTCTCTCCT-AGCACAAGTGTAA-ATCG-GAACGAACCC--CCACCGAA--AATTG-A-
Chab    -CCTCTCC-CTGCACAAGTGTAA-ATTG-GAACGGACCCC-CCACCGAACT---TT-AA
Chto    CCCTCTCC-CTGCACAAGTGTAA-ATTG-GAACGGACCAC-CCACCGAACT--TT-A-A
Majo    CTCTCTCC-TTGCACAAGTGTAA-GTCA-GAACGAACCCG-CCACTGAACC--TT-A-A
Hlst    --TTCTCCCACACACAAGTGTAC-ATCG-GCCCGGACCAC-CCACCGAAAC--TCA-A-
Clpe    -CCTCTCCCCCGCACAACTGTAT-ATCG-GATCGGACACC-CCGCCGAACT--TTA-A-
Mlmr    -CCTCTCC-CCGCACAAGTGTAA-GTCG-GAACGGACCCC-CCACCGAGCT--TTA-A-
Crcr    T-CTCTC-CCCGCACATGTGTAA-ATCG-GAGCGGACTCC-CCACCGAAAC--TTA--A
Muce    CTCTCTCCC-CGCACATGTGTAA-ATCG-GAGCGGACTCC-CCACCGAA--ACTTA-A-
Bege    --TTCTCC-CCGCTCAAGTGTAA-CTCG-GAACGGACCCC-CCACCGAAAA--TTA-A-
Mela    T-CTCTC-CACGCCCAAGTGTAA-CCCG-GAACGGACCCC-CCGCCGGCCA--TTA--A
Hats    CTCTCTCC-GTACACAAGTGTAA-TTCG-GAACGGACCCC-CCACCGAGCA--TTA-A-
Orla    -CTTCTCC-TTGCACACGTGTAA-ATCG-GAACGGACAAA-CCACCGAATT--TTA-A-
```

Cosa	--CTCTCCC-CGCACAAGTGTAA-ATCA-TAACAGACAAA-CTACTGAACA--TTA-A-
Exsp	-TCTCTCC-AAGCATAAGTGTAT-ATCA-GAACAGACATC-CTGCCGAGAA--TTA-A-
Depa	ACCTCTCCA-AGCACAAGTGTAA-ATCA-ATACAGATTAA-CTATTGAA--TATTA-A-
Rima	T-CTCTCT-AAGAGTATCTGTAA-CTCG-GAACGGACCCA-CCGCCGAA-A--ATT-AA
Fuol	TTCTCTCC-ATGCATAAGTGTAA-ATCG-GAACGGACAGA-CCACCGAACA--TTA-A-
Gmaf	TTCTCTCC-CCGCACATGTGTAA-GCCG-GCTCGGACCAC-CCACCGGCAA--TTA-A-
Xeei	-TCTCTCC-GAACACATGTGTAC-ATCG-GAACGGACCAC-CCTCCGAAAT--TTA-A-
Pros	-TTTCTCC-TTGCACAAGTGTAA-GTCG-GAACGGACCTT-CCACCGACCC--CTA-AC
Scmi	TTTTCTCC-TTGCACACATGTAA-GTCG-GATCGGACTCC-CCACCGACCC--CTA-AC
Rolo	CCTTCTCC-CCGCACACGCGTAT-ATCG-GAACGGACTCC-CCACCGACCC--ACA-AC
Cere	CCTTCTCC-TAGCACACGCGTAC-ATCA-GAACGGACCCC-CCACCGACCA--TTA-AT
Daga	-CCCCTCC-TAGCACGCGTGTAC-ATCG-GAACGGACCAA-CCTCCGACTA--TTA-AA
Anco	CCCTCTC-CTTGCACACGTGTAC-ATCG-GAACGGACTAC-CCACCGATAA--ATA-AA
Dmve	CCCTCTCC-TTGCACTAGTGTAC-ATCG-ACATGAACCCA-CCACCGACCA--TTA-AA
Dmar	CCCTCTCC-TTGCACAAGTGTAC-ATCG-ACATGGACCCA-CCACCGACCA--TTA-AA
Anka	CCCTCTCC-CCGCACACGTGTAA-GTCG-GAACGGACAAC-CCACCGACCA--TTA-AA
Moja	--CTCTCCC-CGCACACGTGTAT-GTCG-GAACGGACCCA-CCACCGACCA--TTA-AA
Hoja	CCCTCTCC-TAGCACACGTGTAT-GTCG-GAACGGACCTT-CCACCGACCA--TTA-AA
Bede	TTTTCTCCT-TGCACAAGTGTAC-ATCG-GAACGGACCCC-CCACCGAC--CCTTA-AC
Besp	TTTTCTCC-TTGCACAAGTGTAC-ATCG-GAACGGACCCC-CCACCGACCC--TTA-AC
Mysp	CTTCCTCC-CCGCACAAGTGTAC-ATCA-GAACGGACCCTTCCACTGACCA--ATT-AC
Osja	CCTTCTCCC-TGCACACGTGTAC-ATCA-GAACGGACTCC-CCCACTGACCAATTA-AC
Sgro	CGTTCTCC-ACGCACACGTGTAC-ATCA-GAACGGACTAACCCACTGATCC--ATT-AC
Pzpa	--TCTTCC-TGACACATGTGTAC-CACG-GAGCGGATAAA-CCACCGTCCA--CTA-AT
Zeja	ACTTCTCC-TAACACATGTGTAA-GTCG-GAACGGAAAAA-CCACCGACCA--CTA-AC
Znne	--TTCTCC-TAACACATGTGTAC-CTCG-GAACGGAAAAT-CCACCGACTA--ATA-GC
Zefa	-CTTCTCC-TAATACATGTGTAT-CTCG-GAACGGAAAAC-CCACCGATCA--TTA-GC
Acni	-CTTCTCCCAAACACATGTGTAC-GCCG-GAACGGATAAG-CCACCGACCA--CTA-AC
Ncrh	-CTTCTCC-CAACACATGTGTAC-ACCG-GAACGGATAAG-CCACCGACCA--CTA-AC
Agca	CTCTCTCC-CCGCACAAGTGTAT-CTCG-GAACGGACCCC-CCACCGAACC--TTA-C-
Hydy	CCCCCTCC-CCGCACACGTGTAC-ACCG-GAACGGACCAC-CCGCCGACTC--TTA-A-
Gsac	CTCCCTCC-CCGCACACGTGTAC-ATCG-GAACGGACAAC-CCGCCGACCT--TTA-A-
Pevo	CTCTCTCC-TAGCACAAGTGTAA-CTCG-GAACGAACT-C-ACACCGAAAA--CCA-A-
Hiku	CTCTTCTT-TAGCAAAAGTGTGC-CTCG-GATCGAAC-TA-ACACCGAAAT--TTA-A-
Inpa	CTTTCTCC-TAGCACCAGTGTAA-ATCG-GCACGGAACAA-CCACCGAACA--TTA-A-
Auch	CTCTCTCCC-AGCACAAGCGTAC-CTCG-GAACGAACCC--GCACCGAA--ATTCA-A-
Fico	CTCTCTCC-ACGCACATGTGTAC-CTCG-GAACGAACCT--GCACCGAATT--TTA-A-
Macs	-TTTCTCC-CTGCACAAGTGTAA-ATCG-GAACGAACCC--CCACCGAAT---CCT-AA
Moal	CTCTCTCC-CCACACAAGTGTAA-ATCG-AAACAAACCC--CAACCGAAAA--TTA-A-
Syma	T-CTCTC-CAGACACTAACGTAC-ATCA-AAACGAACCC--ACATCGAAAA--TTA--A
Mafr	CTCTCTCC-CAGCATCTGTGTAC-ATCG-GAACGGACACC-CCACCGAATA--TTA-A-
Dcpe	CTCTCTCC-TAGCACATGTGTAA-ATCG-GAACGAACTC--ACACCGAACT--TTA-A-
Dcti	CTCTCTCC-CGGCACATGTGTAA-ATCG-GAACGAACCC--ACACCGAAC---TTT-AA
Hehi	CTCTCTCCC-TGCACACATGTAA-TTCG-GAACGAAC-CC-GCACCGAG--CATTA-A-
Stam	-TCTCTCC-CTGCACACGTGTAA-GTCG-GAACGGACTCC-CCGCCGACCC--TTA-A-
Hogi	ATCTCTCC-TAGCACATGTGTAA-ATCG-GAACGGACTCC-CCTCCGAACA--TTA-A-
Erzo	CTCTCTCC-TTGCACATGTGTAC-GTCG-GAACGGACACC-CCGCCGACTC--TTA-A-
Hxot	CTCTCTCC-CTGCACACGTGTAT-ATCG-GAACGGACAAC-CCACCGAACC--TTA-A-
Core	-TCTCTCC-CCGCACACGTGTAC-ATCG-GAACGGACACT-CCACCGAC-A--ATT-AA
Apve	CTCTCTCC-CTGCACACGTGTAC-CTCG-GAACGGACAAC-CCGCCGACTC--TTA-A-
Latj	CCCTCTCC-TAGCACACGTGTAC-ATCG-AAACGGACCCG-CCACCGAAA---CTT-AA
Laja	CTCTCTCC-TTGCACAAGTGTAA-ATCG-GACTGGACTCC-CCGCCGAAAA--TTA--A

```
Syja    -TCTCTCC-CTGCACACGCGTAA-ATCG-GAGTGGACCTC-CCACCGAACC--TTA-A-
Epme    CTCTCTCC-CTGCATGCGTGTAA-ATCGGAAACGGACAAC-CCACCGACC---CTT-AA
Grse    T-CTCTCTCCAACACACGTGTAA-CTCG-AAACGGACAAA-CCATCGAATA--CTA--A
Clja    -TCTCTCC-CCGCACAAGTGTAA-ATCG-GAGCGGACCAC-CCACCGAATA--ATA-AC
Ogcy    C-CTCTCC-TGACACCCGCGTAG-ATCG-GATCGAACCAA-GCTCCGAA-A--ATT-AA
Plna    -TCTCTCC-TTGTACAAGTGTAA-ATCG-AAACGGACCCC-CCACCGAAAC--TTA-A-
Lema    -TCTCTCC-CCGCACATCCGTAA-CTCG-GAACGGACTA-ACCACCGAACT---TT-AA
Etzo    -TCTCTCC-TTGCACGTGTGTAA-ATCG-GAACGGACACC-CCACCGAA-C--CTT-AA
Apse    ATCTCTCC-CTGCACAAGTATAT-ATCG-GAACGAACTA--ACACCGACAA--TTA-A-
Epde    -TCTCTCC-TTGCACACGTGTAA-ATCG-GAATGGACTTC-CCACCGAATA--TTA-A-
Slja    --CTCTCCT-CGCACAAGTGTAA-TTCG-ATACGGACCTA-CCATCGAAAA--TTA-A-
Bsja    CTCTCTCC-TCGCACACGTGTAA-ATCG-GAACGGACCCC-CCACCGAACT--TTA-A-
Ecna    CCCTCTCC-CTGCCCATGTGTAT-ATCG-GAACGGACAAA-CCACCGACC---ATT-AA
Cohi    T-CTCTC-CTGGCACAAGTGTAA-ACCG-GAAAGGACTTC-CCGCCGGAAA--TTA--A
Caar    CTCTCTCC-TAGCACATGTGTAA-GTCG-GAACGGACCAA-CCACCGAATA--ATA-A-
Came    CTCTCTCC-TAGCACATGTGTAA-ATCG-GAACGGACCCC-CCACCGAACA--ATA-A-
Mema    CCCTCTCC-CTGCACACGTGTAC-ATCG-GAACGGACCAA-CCACCGAACA--TTA-AA
Lenu    CTCTCTCC-CGGCACAAGCGTGA-ATCG-AAACGAACC-C-CCATCGATCA--TTA-A-
Brja    CTTTCTCC-TGACACACGTGTAA-ATCA-GAACGAACCC--CCACTGAAAC--TTA-C-
Plma    CTCTCTCC-TCGCACACGTGTAA-CTCG-GAACGAACC-C-CCACCGAAAC--TTA-A-
Emst    CTCTCTCC-CTGCACACGCATAA-ATCG-GAACGGACCCC-CCACCGAACC--TTA-A-
Ptti    CTCTCTCC-TCGCACACGTGTAA-ATCG-GAACGGACCTC-CCACCGAACC--TTA-AC
Losu    CCCTCTCC-CCGCACACGTGTAA-ATCG-GTACGGACCAA-CCACCGAAAC--TTA-A-
Geoy    CTTTCTCCC-CGCACAAGTGTAC-ATCG-GAACGAACAAA-GCCCCGAACC--TTA-A-
Dipi    CTCTCTCCT-CGCACAAGTGTAA-ATCG-GAACGGACATT-CCACCGAA--ATTTA-A-
Pama    CTCTCTCC-CTGCACAAGTGTAC-CTCG-GAACGGACCAA-CCACCGAATC--TTA-A-
Leob    CCCTCTCC-TTGCACAAGTGTAC-ATCA-GAACGGACCCC-CCACCGCAAA--TTA-A-
Neba    -TCTCTCC-TTACGCAAGTGTAC-ATCG-GAACGGAGCCA-CCACCGAAAC--TTA-A-
Pdpl    CCCTCTCC-CAGCACACGTGTAC-ATCG-GAACGAACCCC-CCACCGAAAA--TTA-AC
Nimi    T-CTCTC-CACGCACAAGTGTAA-CTCG-GATCGGACTCC-CCGCCGAACC--TTA--A
Uptr    CTCTCTCC-TTGCACAAGTGTAA-GTCA-GAACGAACC-C-GCACTGAACC--TTA-A-
Pesc    -TTTCTCC-TAGCACACATGTAC-CTCG-GAGCAGACTA-ACTACCGAACC--TTA-A-
Baar    CTCTCTC-CTTGCACACGTGTAA-ATCG-GAACGGACCCC-CCACCGAAAT--CTA-A-
Moar    CTCTCTC-CCTGCACACCTGTAC-ATCG-GAACGGACCAC-CCACCGAATA--TTA-A-
Toja    CTCTCTCC-CCGCACACGTGTAA-ATCG-GAACGGACCC-CCCACCGAAAC--TTA-AA
Chau    CTCTCTCC-TATCACAAGTGTAA-ATCG-AAATGGACCTA-CCATCGAAT---ATT-AA
Chse    CTCTCTCC-CCGCACAAGTGTAA-ATTG-GAACGGACTAC-CCACCAAACC--TTA-A-
Enar    CTCTCTCC-CTGCACACCTGTAA-ATCG-GAATGGACCTT-CCACCGAATC--TTA-A-
Hpty    -TCTCTCC-CTGCACACGTGTAA-ATCG-GAACGGACCC-CCCGCCGAACC--TTA-A-
Nana    TTCTCTCC-GCGCACACATGTAA-ATCG-GAACGGACCC-CCCACCGAATA--TTA-AC
Mcst    CTCTCTCC-TTGCACACGTGTAA-ATCG-CAATGGACCAA-CCACCGAACT--TTA-A-
Rhox    -TCTCTCCC-CGCACAAGTGTAA-CTCG-GAATGGACCTA-CCACCGAACC--TTA-A-
Opfa    CTCTCTCC-TTGCACACGTGTAA-ATCG-AAATGGACCCG-CCACCGAATC--TTA--A
Paar    CTCTCTCCT-CGCACAAGTGTAA-ATCG-GAATGGACACC-CCGCCGAA--CATTA-AT
Gozo    CTCTCTCC-TTGCACACGTGTAA-ATCG-GAACGGACCCC-CCACCGAAT---CTT-AA
Ackr    -CTTCTCC-CCCCACAAGTGTAA-ATCG-TAATGGACCAA-CCACCGAAAA--TTA--C
Elev    CTCTCTCCA-CGCACAAGTGTAC-ATCG-GCATGGACCAT-CCACCGAA--CCTTA-A-
Trdu    GCCTCTCC-CCGCACAAGTGTAA-CTCG-GAACGAACCCT-TCACCGACCA--TTA-A-
Amoc    CCCTCTCC-CCGCACATGTGTAT-ATCG-GAACGGACCCC-CCACCGAAC---TTT-AA
Hame    -TCTCTCCCTGACACAAGTATAA-ATCG-GAACGGACCCC-CCACCGAAAC--TTA-A-
Chso    -TCTCTCC-GTGCACACGTGTAC-GTCG-GAACGAACCC--CCGCCGATCC--TTA-A-
Lyto    CTCTCTCCC-TGCACACATGTAC-GTCG-GAACGGACATC-CCGCCGAC--CATTA-A-
```

```
Encr    CTCTCTCC-CTGCACACGTGTAC-GTCG-GAACGGACACC-CCACCGACCA--TTA-A-
Bvar    CTCTCTCC-TTGCATGCGTGTAC-ATCG-GTACGGATCAA-CCACCGAATA--TTA-A-
Noco    TATTCTCC-CCGCACGCGTGTAA-ATCG-GAATGGACCCC-CCACCGAATA--TTA-A-
Chsp    -TATCTCCTCCGCATACCTGTAA-ACCG-AACCGAACTCT-TCGCCGGA-A--ATT-AA
Arja    CTCTCTCC-CCGCACACGTGTAC-GTCG-GAACGGACAAA-CCGCCGACTC--TTA-A-
Pase    CCCTCTCC-TTTCACAAGTGTAAGATCG-ATATGGACCCG-CCATCGTCAA--TTA-A-
Trel    --CTCTCCG-GGCATAAGTGTAC-GTCG-GAACGGAC-CC-TCACCGACAA--TTA-A-
Lifa    -TTTCTCCCGCGCACAAGTGTAC-ATCA-GATCGGATAAA-CCACTGATAA--TTA-A-
Acur    TTTTCTCC-TGGTATTTGTGTAA-ATCG-GGTTGGACATC-CCGCCGAG-A--ATT-AA
Ampe    -TCTCTCC-TTGCACGAGTGTAA-ATCA-AAACGGACCT-CCCATCGAATC--TTA-A-
Urja    CCCCCTCC-CTGCACACGTGTAA-ATCG-GAACGGACCCC-CCACCGAACT--TTA--A
Enet    ACCTCTCC-TGGCACAAGCATAC-CTCG-GAACGGACTAA-CCCCCGAACT--TTA--A
Ptbr    ATCTCTCCT-GCCATAAGTGTAA-ATCA-GATTAGACTTA-CTACTGAA--AATTA-TT
Safa    -GCTCTCC-TAACACCTGTGTAA-ATCA-GAACGGACCCC-CCACTGAAAA--TTA-A-
Icae    -TCTCTCC-TTGCACACGTGTAA-ATCG-GAACGAAC-C-CCCACCGAAAC--TTA-A-
Asmi    -TCTCTCC-AAGCACAAGTGTAC-ATCG-GAACGGACAC-TCCACCGAAAT---TT-AA
Foal    -CCCCTCC-TTGCACAAGTGTAA-GTCG-GAGCGAACAC-CCGCCGACTCT--TAA---
Drze    -TCTCTCT-ATACATAAGTGTAA-ATTA-GAACGAACCC--ACACTAAACA--TTA-A-
Rhas    -CCTCTCC-CAGCACCTGTGTAC-ATCA-GAACGAACC-C-CCACTGAA-A--ATT-AA
Elac    --CTCTCC-TAGCACCCGTGTAT-ATCA-GAACGAACC-C-CCACTGAAAA--TTA--A
Kugu    ATCTCTCC-CCGCACACATGTCA-ATCA-GAACGAACC-C-GCACTGAAAA--TTA-A-
Plor    -CCTCTCC-TTGCACACGTGTAA-ATCG-GAACGAACCC--CCACCGAACC---TT-AA
Sgun    CTCTCTCCT-AGCACACGTGTAA-ATCG-GAACGAACCC--CCACCGAG--TATTA-A-
Zaco    CTCTCTCCT-TGCACACACGTAT-ATCG-GAGCGGACCT--CCCACCGAA-TCTTA-A-
Zbfl    CCCTCTCC-CCGCACACGTGTAA-ATCG-AAACGGACCAT-CCATCGAAAC--TTA--A
Spba    CTCTCTCCC-TGCATGCGTGTAA-ATCG-GAACGGACCCC-CCACCGAA--CATTA-A-
Game    -TCTCTACCTTGCACACGTGTAA-ATCG-GAACGAACCC--CCACCGAAA---CTT-AA
Thth    CTCTCTCCT-TGCACACGTGTAA-ATCG-GAACGAACCC--CCACCGAA--ACTTA-A-
Xigl    CTCTCTCC-TTGCACACGTGTAA-ATCG-GAACGGACCTA-CCACCGAATC--TTA-A-
Hyja    -TCTCTC-CCTGCACACGTGTAT-ATCG-GCTACGAACCC-CCACCGAAAC--TTA--A
Psan    TCCTCTCC-CTGCACATGTGTAT-ATCG-AGTACGAACCC-CCACCGAAAC--TTA-A-
Cupa    CTCTCTCC-TTGCACACGTGTAT-ATCG-GAACGAACCC--CCACCGAAA---CTT-AA
Mpch    CTCTCTCC-ACGCACACGTGTAT-ATCG-AAACGGACACT-CCACCGAAAA--TTA--C
Char    T-CTCTC-CTAGCACGCACATAC-ATCG-GAACGGACCCC-CCACCGAACA--TTA--C
Pser    CTCTCTCC-CCGCATAAGTGTAA-ATCG-AAACGGACCCC-CCATCGATAT--TTA-AA
Prol    -TCTCTCC-TCGCACATGTGTAA-ATCG-GAACGGACCC-CCCACCGACTC--TTA-A-
Plbi    CTTTCTCC-TTGCACATGTGTAA-ATCG-GAACGGACCCC-CCACCGAA-T--CTT-AA
Calu    GCTTCTCC-CCGCACACCCATAA-ATCG-GAACGGACAAC-CCACCGAATA--TTA-A-
Papa    CCCTCTCC-CAGTACAAGTGTAA-GTTG-GCACACGACCA-AAGCCAACTA--TTA--A
Sufr    --TTCTCC-AAGCACAAGTGTAC-ACCG-GAACGAACCC--GCACCGAACA--TTA--A
Stci    CTTTCTCC-AGGCACAAGTGTAT-ATCG-GAACGAACCC--GCACCGAACA--TTA--A
Taru    C-CCCTC-CAAGCACAAGTGTAC-ATCA-GAACGAAC-CC-CCACCGAAAT--TCA--A
Rala    TTCTCTCC-CAGCATAAGTGTAC-ATCG-GAACGAACCC--CAACCGAATC--CTA-A-
```

```
          29
Scca      CGATCCCAGAC-T-GAGGCCATC--ATAACT-TCATTTC-TTGA---CTAGAAA---ACCC
Muma      CGACCCCAGACT--GAGGTCATT--ATACTA-TT--A--AATCATTAACTAGAAAACCTTA
Erca      CGAACCCCATAA--GAGGGAAGA--ATAATGGACATAT---ATAAATCAAGGAAAGCCTAT
Pose      CGAACCCACCGG--AGGAGCAAA--TAATA--CACAT-AACAAA-A-ACAAGAAAACC--C
Actr      CGGACCCAACCAA-A-GAGGGAA--ATACAG-AATAATAATAG-A-AATCAAGAAAACCCT
Scal      CGGACCCAACC-A-AAGAGGGAA--ATACAG-TATAATA-ATAGA-AGTCAAGAAAACCCT
Posp      GGACCCA-CCAA---AGAGGGAA--ATNCRG-AATACCAATG-AAACTCAAGAAAA-CCTG
Atsp      CGAACCCAACCAA--AGAGGGCA--GTA--TTCACACAAACCCCAA-ACAAGAAGA--ACT
Leoc      CGAACCCAAC-CA-AAGAGGGCA--AT-ATTTATAT-ATCCTAAA---CAAGAAAAGCTTA
Amca      CGAACTCACACA--AAGAGAGAA--TTACAC-AAATA-GCACGACATACTAGAAGACCCTG
Osbi      TGAACCCAACCAA--AGAGGGCA--TTA--CAGACCAAAAA-CTAT-CCTAGAAAATCCTG
Pabu      AGAACCCAATCAA--AGAGGGAA--TCACAAT-ATAAAACATAAACA---AGAAAAACATG
Hial      CGAACCCAACA-A-AAGAGGGAA--GCACAA-G--ACAC-AACATAACCAAGAAAAATCTG
Elha      CGGACCCAATAC--AAGAGGGAA--ATGAAA-CAAAAACCAAACCCAAGAAGAA---GCTA
Mlcy      CGGACCCAAA-CC-AAGAGGGAA--ATGCA--ACAAACA-CCAATAAACCAAGAAAAAACT
Algl      CGAACCCAAACAG-A-GGGGCGT--GTAGAA-CAAACGATAAC-----CTGGAAAATCTTA
Ptgi      CGAACCCAGATA--AAGAGGGAA--ACGCAGAAAACAA---ACAACACCAAGAAATACCTA
Alaf      CGAACCCTAAAC--AAGAGGGCA--TCATCTTCAATAAACACAA--ATCAGGAAAACCCC-
Nock      CGAACCCTATGATA-A-GAGGGAA--TCATTTTATGATAATTGAG-ATCAAGAAAACCCAA
Anja      CGGACCCAAACAG--AGAGAAAAA--GAACAAACTACAAAAA-ACAAGAAAAAT--CTATT
Gyki      CGGACCTAATA-----GAAGGCA--AAGCAA-AG-GAAA-AATAACATACAAGAATTCCAT
Syka      CGAACCCAAA-AAT-GAGGGGACA--ACAACA-ACCTA-CAA-TAAAACAAGAAAACCCTG
Opma      CGGACCCAGACC-C-TGAGGGAAA--AACAAT-TGAACAAATAAA---CAAGAAGATCTTG
Comy      CGGACCCAAA-A--AAGAGGGTA--AAACAA-C-AAA-ACTCAATAAACAAGAAAAACCTA
Sasp      CGAACCCAGTAAT--ACTTGAGGG--CCAAGTAGAAGGT-ATTATTGTATCTAGAAAATTC
Eupe      CAGC-CCCAGAA--GAGAGTAAA--ACAACA-AACAC-CATAACGCAAGAAAATACTGTCA
Enja      CGAACCCAACAA--AAGAGGGCC--CTACACACCCGCC-ACCTCTGACCAAGAAAACTATG
Same      CGAACCCAATAAA--AGAGGGTA--TTACATC-ACTGCCATCCCAGGCCAAGAAAACCATG
Chch      CGAACTCAATCA--AAGAGAGCA--GTGCGA-AGTTA--AAAAGAGACCAGGAAGGCCCCG
Grgr      CGAACTCAACT-C-TAGAGAGCA--CTGTGG-TTCCCAACAAAAACAGG----AAAAACCC
Caau      CGAACTCAACCC--AAGAGAGCA--ATGTGG-TATTA-CAAAAAAA-CCTAGAAAAAC--C
Cyca      CGAACTCAATCA--AAGAGAGCA--ATGTGAATTACAA-AA---AAACCAAGAAAAATCCA
Dare      CGAGCCCAAAC-A-AAGAGGGCA--ATGTGA----GTTA-AAAACTGA---GAAAAATCCA
Cost      CGAGCCCAATAA--AAGAGGGTA--ATATAACTAA----TATAAATTACAAGAAAATCATA
Leec      CGAACCCAGA-AAA-A-GAGGGCA--GTGT-T-GACAA-TAAAAAAATCAAGAAAACCCCA
Crla      CGAACCCAAAA--A-AAGAGGGAT--GCATG-----GAAGACAAAAAACCAAGAAAACCCC
Clmc      CGAACCCACCCCA-A-GAGGGAA--AAATGA-TATATAATAAAAC-CAAG-AAAAACCCAC
Phin      CGAACTCACCCAA---GAGAGTAATGT-TCCCAAAACCATAA-ACAAGAAAAC----CCCA
Icpu      CGAACCCAATT-A-AAGAGGGCA--TTATG--GTTATA--TTAAATATC--AAGAAATCTC
Psto      CGAACCCAATC-A-AAGAGGGAA--ATGTGA-ACACCTA-AAAC--ACCAAGAAAACCTCA
Cora      CGAACCCAAATAC-AAGAGGGAA--ATGTGA-GCCAAAT-AAACAA--CAAGAAAACTTCA
Eisp      CGAACCTAATCA--AAGAAGGCC--ACGTGGCCTAATA-CA-ATTAACCAAGAAAACCCCA
Apal      CGAACC-CAACCA--AAGAGGGCA--TTGTGA-CCTC--AACAAATAACAAGAAAACCCCA
Eslu      CGAACCCAAACC-A-AGAGTGTAT--TGTAA-------AC-CCAA--ACAAGAAAAATCTA
Dape      CGAACCCAAAACA--AGAGGGCC--TTGTAAAT----------AATCACAAGAAAAATTTA
Glse      CGAACCCAAAAAA--AGAGGGTA--ATA--CAAA-CTAAGTAAT---ACTAGAAAAACACG
Naar      CGAACCCAACA-C-CAGAGGGAA--ATACGA-ATTAAAA-AATAATG-CTAGAAAAACACG
Baoc      CGAACCCAGAAA--AAGAGGGTA--ATATGA-GCCAAATAAAATGCTAGAAAAA---CACA
Opso      CGAACCCAATAT--AAGAGGGTA--ATACA---AACCA-GAAAAACTGCTAGAAAAACACG
Alte      CGAACCCAACCC--AAGAGAGCA--CTGTGG--ATTTA-AACACA-ACCTAGAAAACCCCA
Plap      CGAACCCAACCC--AAGAGAGCA--CTGCGGATTTAAA-TA---TAGCCTAGAAAGTCCCG
```

```
Plal    CGAACCCAACCCT--AGAGGGGAA--TTTGGACTGAATT--ACAA----CCAAGAAAACTC
Sami    CGAACCCAATGC--AAGAGGGAA--CTTTGG---ACT-TTAGCGCAACCAAGAAGCGCCCA
Rere    CGAACCCAACTGT-TAGAGGGAA--ATTCA--AGCTTAA-CTA-GAAACGAGAAGAGCCTG
Gama    CGAACCCAATTG--AAGAGGGAA--AAACGGACTAAGC---TGACCA-CCGGAAGGTCCCG
Onmy    CGAACCCAAACCA--AGAGGGAA--CTGTAGGCC-AGA--ACAAACACCAAGAAAAACCTA
Sasa    CGAACCCAAGCCA--AGAGGGAA--CTG--TAGCCCAGAAC-AAATACCGAGAAAAACCTA
Cola    CGAACCTAAACC--AAGAGGGAA--ATGCAG-GCCAGAAGAGAAAC--CGAGAAAAGCCTA
Dita    CGAACCCACCCC--TAGAGAGAA--CTTCGG-ACCAA-ATCAGTTAACCAGGAAAA-ACCC
Gogr    CGAACCCAAAC-C-TAGAGGGAA--TCATAA-ACTATA--C---ACAACCAAGAAAATCCT
Chsl    CGAACTCAAACT--CAGAGAGCAGTCTGACCAGGGTTT---CACACAAGAAGA----CCCC
Atja    CGACCCCAAACA--AAGAGGGAA--ATGAAT-AAAAT-AAATGTAA-ACAAGAAATACACT
Iido    CGACCCCAAAACAACAGAGGGAA--ATGAAC-AAGACTAAATATA-AACAAGAAAAACACC
Auja    TGTATCTA--CA--AAGAAGCAA--CTGTGG--CCTAAACCTATA-AACAAGAAAACCCCA
Chag    CGACCCCAACCCC--AGAGGGAA--TTGAGCTTAGCCACAGTTTAA-ACTAGAAGAACACT
Hami    TGGACCCAGA-----AGAGGGTA--CTATGAA-ATAAA---AAATAAACTAGATAAAACCA
Saun    TGGTCCCAAAAGA--GGGCACTG-TGGAAAAAACTAATA-AACTAGATAA------AACCA
Nema    CGACCCCAACCC--AAGAGGGCA--GTGTAAGACCAC---ACAAATACCTAGAAAACCCAA
Disp    CGGTCCCCATC-C-GAGGGCCA----TGTAT-TACTACA-CGAAT-ACCTAGAAACCTCAA
Myaf    CGACGCCCCAAA--GAGGCTACT--GTTA-A-ACCACACAAAAACCAAGAAAAT-CTAACA
Lagu    CGACCCCACCC-C-CAGAGGGCA--CTGAAC-CC-CAA--CCAAACAACCAGAAGATCGTT
Trtr    CGACCTCAAC-AT-CTGAGATGA--ATGGAATGAAT-ATAAAGTA---CTAGAAAAGCATC
Zucr    CGACCTCAAACC-GCAGAGGTG--ATTGGG-AA-GTAA--CTTGATTTCTAGAAAAGCCCC
Pxja    CGGCCCCAAA-TA-AAGAGGGAA--ATGTATAATAA-ATAAACTA---CTAGAAAAATATA
Pxlo    CGGCCCCAAATA--AAGAGGGAA--ATGTA---TAATA-AATAAACCACTAGAAAAATATA
Pctr    CGGCCTCAAACA--AAGAGAGCC--CTGCAC-AAAA--CAC-AGAATACAAGAAAAACGTG
Apsa    CGACCTCACAAC--CAGAGAGTA--CTACAA-AAT-------ACTAAACAAGAAAAATTTG
Cabe    CGACGCCAAT-AC-AAGAGGCAA--CTGGGCCACGA-ATTAAACAC--TTAAAAGACCACC
Bzze    CGACCCCAAC-CC-AAGAGGGCA--TTGAGTAATAA-ATTAAACAA--CTAGAAGAACACT
Siim    CAACCCCCAC-CA-AAGAGGGCA--CTGGGTAACAA-ACCAAACAA--CAAGAGAACTACC
Ctru    CGGCCCCAACCC--CAGAGGGTA--ATGAAT--GAAAATAGGAC--AACAAGAAAACCATT
Dpbr    CGGCCCCAACCC--CAGAGGGTA--TTGAAT-AA-GA--ATAATACAACAAGAAAACCATT
Caki    CGACCCCAAAC-C-TAGAGGG-A--CT-------TAAGT-TAAAA-AACAAGAAAACCACT
Phja    CGACCCCAAAC-C-AAGAGGGCC--ATAAAC-T-------ATCTA-AACAAGAAAACCATT
Brsp    CAGCTCAAT----------AGAG--GAACTTAAGGCAATCA-ACTAGAAAA-----CCCCT
Gamo    CGACCCCAAACC--CAGAGGGTA--ATAGGT-CAAAAAAAC--------AAGAAAAACACC
Lolo    CGACCCCAAACC--CAGAGGGAA--ATAAGAAACA----TTTAA----CAAGAAAAACACT
Batr    CGATCCTATAAT--AGAAGGCACCAGCCTAAAAATAAACTACAAGCTC-------CCCCCC
Prmy    CGGCCCCAA-CA----GAGGGTA--ATGAAT-ACTACAT-AAACA--ACTAGAAAAACAAA
Loli    CGGTCCCAACCCA--AAGAGGGCACTGAATAAAAACCAGAAAGACCAGAAAAA----TATT
Loam    CGGCCCCAAAAG--AAGAGGGAA--CTGAGCAAAAAAT-AA---AAAACTAGAAAAACACC
Chab    CGGTCCCAAA-CA-AAGAGGGTA--GTGAG--AAAGACA-CCAA-AAACTGGAAAACCCCC
Chto    CGGCCCCAAAC-A-AAGAGGGTA--GTGAGA-AAGACA--CCA-AAAACTGGAAAACCCCC
Majo    CGGCCCCAACA-A-AAGAGGGAA--GTGAGA-AACAAAC-AGAT--AACAAGAAAATTACT
Hlst    CGGTCCCAA-GAA-TAGAGGGCA--ATTAAA-AATAATTAAAAAT--ACTAGAAAAATTTT
Clpe    CGGCCCCAACCAA---AGAGGGCA--CTGAGA-AGGG--AATAAAGGACAAGAAAAACCCT
Mlmr    CGGCCCCAACC-A-AAGAGGGCA--ATGTCC-AA-GGAA-CCAAATAACAGGAAACCCCCC
Crcr    CGGGCCCAAACCA--AGAGGGCA--TTG---AATAGAA-GACCTCACACTAGAAAACTATC
Muce    CGGGCCCAAACC--AAGAGGGCA--TTGAATAGAAGAC---CTCACACTAGAAAAC-TATC
Bege    CGGCCCCAATAAA-A-GAGGGAA--TTAGGC-TATAAAACAAA-G-AACTAGAAAACCACC
Mela    CGGTCCCATA-AC--TGAGGGTA--ATA---GGCCAAAAATA-GAGAACTAGAAGACCACC
Hats    CGGCCCCAAA-AA-AAGAGGGAA--CCGGACCATAA-AC-AAAAAA--CTAGAAACCCACC
Orla    CGGCCCCAATT-A-AAGAGGGGA--GGCCGG-AT-GGAA-AAGAGAA-CAAGAAAATTCCG
```

```
Cosa    CGACTCCAAATA--AAGAGGATA--TTA-GA-T-TAT-ATATAAAGAACTAGAAAACTCTC
Exsp    CGGCCCCAAGCA--AAGAGGGCA--TTGGAA-AATTA-AATAGACA-ACTAGAAAACCTTC
Depa    CGGCCCCAAATA--AAGAGGGTA--TTACAAAAAAAAT--ATAA--AACAAGAAAACCTTG
Rima    CGGCCCTAGCT-G-AAGGGCTTA--TACTAC-ACAAAATCAACTAG------AAACCCCTA
Fuol    CGGCCCCAACC-A-AAGAGGGAA--TTAGAG-CTAAAAC-AGAA--AACAGGAAATATCTC
Gmaf    CGGCCCCAAAT-A-A-GAGGTTA--ATAAGA-CC-CAA--AAAGATAACAAGAAACCCCCT
Xeei    CGGCCCTAATAA--AAGAAGGAA--ATAGAG-TAAAA-ATAAATA--ACAAGAAAAACCTC
Pros    CGGCCCCAATC-A-AAGAGGGGA--CTGAAT-AATAAA--TAA-AATCCCAGAAAAATATT
Scmi    CGGACCCAATCAA-A-GAGGGTA--TGAAAG-AGACGAGTAGCCC-CCAA-GAAAACCATT
Rolo    CGGCCCCAACC-A-AAGAGGGAA--TTGAAT-AATAA-A-CTAGA-GTACTAGAAAGACAC
Cere    CGGCCCCAATTAG-TAGAGGGTA--GTGAACACTAA-ACAAAACAC--T-AGAAGAACGTT
Daga    CGGCCCCAACA-G-CAGAGGGAA--ATGAAC-A--ACAA-ATTATAAACTGGAAGTGCGTT
Anco    CGGCCCCAAACAA--AGAGGGAA--ATG--AACA-ATAAAACATAA-ACTAGAAAAACACT
Dmve    CGGCCCCAACCAA--AGAGGGAATTGAAAGATAAAAGTAATAAACCAGAAAAT----CACT
Dmar    CGGCCCCAA-TCA-CAGAGGGGA--GTGAAC-AGTAAACAACAAA---CTAGAAGACCACT
Anka    CGACTCCAACCAA-A-GAGGCTA--ATGAAA-AATAAAATCATAA-ACCA-GAAAACCATT
Moja    CGGCCCCAATCA--AAGAGGGAA--ATGAAA-TTCAA--AACTATAAACCAGAAGACCATT
Hoja    CGGCCCCAACCA--AAGAGGGAA--ATGAAA-AACAA--AACCACAAACCAGAAAAATATT
Bede    CGGCCCCAATCA--AAGAGGGTA--TTGAATAATAAAC-CACAAA--ACTAGAAAAACATT
Besp    CGGCCCCAATAG--AAGAGGGTA--TTGAAT-A-ATA-AACCATAAAACTAGAAAAACATT
Mysp    CGGACCCAAAAA--AAGAGGGAA--TTGAGC-AACAA-ACCT-GTAAACAAGAAAAACACT
Osja    CGGACCCAACCA--AAGAGGGAA--CTGA--ACAATAA-ACCAGCCAACCAGAAAAACACT
Sgro    CGGACCCAAA-AA-AAGAGGGAA--CTGAGC-AAACAAA-GCAATAAACCAGAAGAACACT
Pzpa    CGGCCCCAAGAAA--AGAGGGAAGTAAAAACTAGCTAAATTAAACCAGAAGA----CCTTT
Zeja    CGACCCCAAACA--AAGAGGGAA--GTGTAG--ACTAACCAGGCCAAACTAGAAGCCCCTA
Znne    CGACCCCAAAAAA-A-GAGGGAA--GTGAAA-TCTATGCCCAA----ACTAGAAAACCTTT
Zefa    CGACCCCAACAA--AAGAGGGAA--GTGAA----ATA-TGTGCCCAAACTAGAAAACCTTT
Acni    CGACCCCAAAAAA-A-GAGGGAA--GTGAAA-ATTAATAGACCAA-ACTA-GAAAACCTTT
Ncrh    CGACCCCAAAA-T-AAGAGGGAA--GTGA-A-AATTAAT-AGACCAAACTAGAAAACCTTT
Agca    CGCCCCCAAACA--AAGAGGGTA--TTGAAC--TACAAATAAAAC-AACTGGAAGACCCCC
Hydy    CGAACCCAAC-AAA-A-GAGGGAA--TTGGAT-AACAA-ACCAAACAACTAGAAAACTATC
Gsac    CGAACCCAACCA--AAGAGGGAC--CTGGGT--AATAAACTAAAT-AACTAGAAAACTACC
Pevo    CGGACTCAAAC-A-AAGAGAGCC--CTGGGC-TC-TATC-ACAAAAAACAAGAAAAACACC
Hiku    TGGTCCCACTAC--CAGAGGGTA--ATGTTG-TAAAA--GCAAAGGAACAAGAAAAAGCAA
Inpa    CGGCCCCAAACCC--AGAGGGCA--TTAAACC-CCCAA--ACAGACAACTAGAAACCCAAC
Auch    CGGCCCCAAAAC--CAGAGGAGA--CTGATCATTAACT-CTCAACCCACAAGAAAACCCCT
Fico    CGGCCCCAACAA--AAGAGGGAA--TTGGGC-AATCA-GCACAATCGCCAAGAAAACCCCC
Macs    CGGCCCCAATCAC--AGAGGGAA--CTGAACAAT-AAA--CAAAACAACTAGAAAAACACC
Moal    TGGCCCCAAAAA--AAGAGGGGA--TTGAAT--AACTAATAGATT-A-CTAGAAAAACAAC
Syma    TGGCCCCAAACAA--AGAGAGCC--CTGA-TAAACCTAAAC-AGATAACTAGAAAACTAAT
Mafr    CGGCCCCAATTA--CAGAGGGTA--ATGAAC-ACTA--AACCACTACACCAGAAAACCACC
Dcpe    CGGCCCCAAAC-A-CAGAGGGCA--ATGAAC-AA-AAAA-C-AGAAAACCAGAAATTCACC
Dcti    CGGCCCCAATC-G-TAGAGGGTA--ATGAAC-AAAAAGC-AGAAA-ACCAGAAATTCACCC
Hehi    CGACCCCAAACG--AAGAGGGCC--CTGAACAACAACC---CAAACAACCAGAAAAAAATT
Stam    CGGCCCCAAATAA--AGAGGGTAATGAATTATGGTATAG--ACAACTAGAAAAACCTCCAA
Hogi    CGGCCCCAGACAA-A-GAGGGAA--CTGAGC-AACAACCTAAAGA-ACTA-GAAAAACACT
Erzo    CGACCCCAAGCA--AAGAGGGCC--CTGAAT-AATAGACCAAACAA--CTAGAAAAACATT
Hxot    CGGCCCCAAATA--AAGAGGGCA--GTGGAT-GTTAGACCAAAAAA--CTAGAAAAATATC
Core    CGGCCCCAAAC-A-AAGAGGGCA--TTGGAC-AACAAA--CCAAACAACTAGAAAAGCCTT
Apve    CGACCCCAAATAA--AGAGGGCACCGGATCACAAACCAAAAA-ACTAGAAAAT----TATC
Latj    CGGCCCCAAACAA--AGAGGGAA--TTGAAAA-ACAAACACAACAT-ACTAGAAAACCACC
Laja    CGATCCCAAAC-A-AAGAGGGTA--CTGAAC-GATAAAC-CGGAT-AACGAGAAAATCATT
```

```
Syja    CGGCCCCAAAACAA-A-GAGGGAA--CTGAAA-AACTAAACCAGAG-ACCAGAAAAACATT
Epme    CGGACCCAAATAA-TAGAGGGAA--CTGAACC-ACAGACGAAACA--ACTAGAAAACCACC
Grse    CGGCCCCAACCTATCAGAGGGAA--TTGA-TGGACATAAACCAAACAACTAGAAAAACACC
Clja    CGGCCCCAAGC-C-AAGAGGGTA--CTGAAC-C-AAGTACATAGATAACTGGAAAACCGTT
Ogcy    CGGCCCCAAAC-A-AAGAGGGTA--TTGAAC-AATAAACCAAACAACCA---GAAAAATTC
Plna    CGAACCCAAACA--AAGAGGGCA--CCGAAC-AACAA-ATAAACC--CCTAGAAAACCATT
Lema    CGGACCCAAA-CA-AAGAGGGCA--ATAGA--TGGCAGA-CTAAACAACTGGAAAAACAT-
Etzo    CGGCCCCAATC-T-AAGAGGGAA--CTGAGC-AACAGA--CCAAACAACCAGAAAAACATT
Apse    CGGCCCCAAGA--A-AGAGGGAAC--TGAAAT-ATTATGGCCAGGCCACTAGAAGACCATC
Epde    CGGCCCCAAACAA--AGAGGGCACTGAACAACAAACCAGATAA-CCAGAAAAA----CATC
Slja    CGGCCCCAATAA--AAGAGGGAA--ATGAAT-AACCA--TACAAACAACTAGAAAAATTAT
Bsja    CGGCCCCAAACA--AAGAGGGAA--TTGGAC-ATACA-AACCAAACAACCAGAAAAACGTC
Ecna    CGCCCCCAAACAA--AGAGGGCA--TTAAA-C-ATCATCTTAAAACCACTAGAAAACCGTT
Cohi    CGTCCCCAAAAAA--AGAGGGAA--TTG---AGTACTTTAGAAAACTACAAGAAGACTACT
Caar    CGGCCCCAAACAAA-GAGGGAACT-GGACAA-ACGCA--CAAATTACACTAGAAAATCGTC
Came    CGGCCCCAAACAA--AGAGGGTATTGGACAAACGTACAAATACAAAACTAGAAAATCGTCC
Mema    CGACCTCAAA-CA-AAGAGAGCA--CTGAAAAACAA-ACCAAACAAA-CTAGAAAACCATC
Lenu    CGACCCCAAACTAA-AAGAGGGTA-ATGAAC-AA-AA--CCCAAAACCCTAGAAAAAAGTT
Brja    CGGCCCCAAACA--AAGAGGGCA--ATGGAC-AAGA--AATAAAGA-GCCAGAAAAACGTC
Plma    CGGCCCCAAACGA--AGAGGGTAATGAACAACAATAAAACAACCAGGAAA------CCATC
Emst    CGGCCCCAA-ACA-AAGAGGGTA--CTGAAC-AACAGACCAAACA--ACCAGAAAAACATC
Ptti    CGGCCCCAAACA--AAGAGGGTA--CTGAAC-AACAG-ACCAAACA-ACCAGAAAACCACC
Losu    CGACCCCAATCAA-CAGAGGGCA--CTAGAT-AACTTTAACCTAATAACAAGAAGAACATC
Geoy    CGAACCCAATCA--AAGAGAAGA--ATGA--AAGTCCC-GAAAAGAAGCAAGAAAATTATT
Dipi    CGGGCCCAAACA--AAGAGGGCA--TTAGGCAACTAAC--AAAAC-AACTAGAAAAACACC
Pama    CGCCCCCAAG-CA-AAGAGGGAA--ATGGATTTCAA-ATAAATTAA--CTAGAAAAACATC
Leob    CGGCCTCAAACA--AAGAGGGCA--CTGAAG-AATGA-AC-TAGACAACTAGAAAAACATC
Neba    CGACCCCAAACA--CAGAGGGAA--TTGAAC-TACTACTCCTACCTTTCAAGAAAATACTT
Pdpl    CGGCCCCAAAC-A-AAGAGGGAA--GTTGGT-AT-GAAT-TTAAGAAACTAGAAAACCACC
Nimi    CGGACCCAAACAA--AGAGGGCC--TTG---AACAACAAACCCGCCAACTAGAAAAACACT
Uptr    CGGCCCCAACA-A-AAGAGGGAA--GTGAGA-AA-TAA--ACAGATAACAAGAAAATTACT
Pesc    CGACCCCAAACCA-A-GAGGGTA--CTGAAC-AA-TACT-CAAAATTACAAGAAACCCCCC
Baar    CCGCCCCAAACAA--AGAGGGCA--CTG--AATG-ACATACCACAATACCAGAAAACCATT
Moar    CGGCCCCAAACAAAGA--GGGTAATGAACAACAAACCAAAC----AACCA-GAAAATCATC
Toja    CGGCCCCAAA-CA-AAGAGGGTA--CTGAA--TAACAAT-ATAACCCACTAGAAAATCATT
Chau    CGGCCCCAAGCAA--AGAGGGCA--TTGGGCT-ACAAC-CTTAAACGTCTAGAAAAGCCCC
Chse    CGGCCCCAAACAA-A-GAGGGAA--CTGAAC-AGCAAATAAAACA-ACTA-GAAAACCGTC
Enar    CGGCCCCAAA-TAA-A-GAGGGTA--ATGAACAACAA-ATTAAAC-AACTAGAAGAACATC
Hpty    CGGCCCCAAAATA-AAGAGGGTA--CTGAAC-AA-CAAA-CCAAATAACCAGAAAAACATC
Nana    CGGCCCCAAGACA-AAGAGGGAA--CTGAAA-AA-CAAA-ATAAAATACAAGAAAAACCCC
Mcst    CGGCCCCAA-AAA-AAGAGGGAA--CTGAAC-AACAGACCAAACA--ACCAGAAAACCACT
Rhox    CGGCCCCAAACA--AAGAGGGTA--ATGAAT-A-CCA-AACTAAACAACCAGAAAATCACT
Opfa    CGGCCCCAAA-CA-AAGAGGGAA--TTGAACAGCAA-ACCAAACAA--CCAGAAAACCACC
Paar    CGCCCCCAAACA--AAGAGGGCA--TTGAA-CAAACAA-ATCAAACAACTAGAAAAACAAT
Gozo    CGGCCCCAAACAA--AGAGGGTA--TTGAACA-ACAAA---CACACAACTAGA-AAAGCGT
Ackr    CGGCCCCAAAA-A-AAGAGAGCA--ATGAGC-CTTTCA--CTATAGAACAAGAAATACCCT
Elev    CGGCCCCAAACA--AAGAGGGTA--TTAAATCATAGC---ACAG-------GAAAACTATT
Trdu    CGGCCCCAAAAC-A-AAGAGGGCC--CTAGGC-A-AAAA-ACAAACAACTAGAAAACCCCC
Amoc    CGGCCCCAAGCAA--AGAGGGTA--CTGTGTAAA-AAA--TTAAATAACAAGAAAACCCCA
Hame    CGGACCC-AACAA--AAGAGGGCA--CTGGAC-CGTA-CACATCAAAACCAGAAAATACTC
Chso    CGGACCCAATACA--AAGAGGAA--ATAGGGC-CACACG-ATAGAGAACAAGAAAA-ACAC
Lyto    CGGCCCCAAACA--AAGAGGGCA--CTGAATAACAGAT---CAAACAACAAGAAAAGCATT
```

```
Encr    CGGCCCCAATCA--AAGAGGGCA--CTGAAT-AATAG-ATTAAACA-ACAAGAAAAGCGTT
Bvar    CGGCCCCAAACA--AAGAGGGCA--TTGGAT--AATAAACCAAAC-AACCAGAAAATTACC
Noco    CGGCCCCAAATAC-A-GAGGGCA--ATGGAT-TATAACCCAAA-C-AACAAGAAAATCCTC
Chsp    CGGCCCCACCA-A-ACGAGGGCC--CTGTAC-AACAT---CTTAATTACAAGAAAACACCC
Arja    CGGCCCCAAATA--AAGAGGGTA--CTGGAT-CATAG-ACCAAACAACTAGAAAAATATCC
Pase    CGGCCCCAAATTAAAAGAGGGCA--ATGGGCACTTATACTAAACAA--CAAGAAATACCCC
Trel    CGGCCTCAAACT--GAGAGAGTA--ATGAGC-TACAT--TGTGTTTATCTAGAAAGCCACT
Lifa    CGGCCCCAAGCA--AAGAGGGCA--TTAAAC-T-AAA-AAGAGAATAACTAGAACTGCATC
Acur    CGGCCCCCCTCAC--AGAGGGCC--ATGAAAAATTAAC--CTTAACCACTTGTTAACCTTT
Ampe    CGGCCCCAACCA--AAGAGGGAC--ATGGAC-AA-CAAA-CCAAACAACCAGAAAACCATC
Urja    CGGCCCCAAACAA-A-GCGGGAA--CTGAAT-ATTAAACCTAACA-ACCA-GAAAACCATT
Enet    CGG-CTCCACTA--TTGAGGACA--CTGCAC--ATCTA-AAACTATAACAAGAAAACCCTT
Ptbr    CGGCCCCA-AA--AAGAGGGTA--ATG----ATTTATT-AATAAAAAACAAGAAAAATA-A
Safa    CGGCCC----CAAA-AAGAGGGTA--ATGGCCTA-GA--AAAAGAGAACTAGAAAAACCCC
Icae    CGGCCCCAAAC-A-AAGAGGGTA--ATGAAC-A--ATAA-GTAAGCAACCAGAAAACCATC
Asmi    TGGCCCCAAACCA-CAGAGTGAA---CTGA--AATATAA-CTACAAAACAAGAAAAATCTC
Foal    CGGCTCCAAACAA-AGAGGGCCT---TGGGC--CTATCATAAACAA--CTAGAAAACCGCC
Drze    CGGCCCCAAGCTA-AAGAGGGTA--ATGTGC---CAAAA-ATAACCAACCAGAAATATACA
Rhas    CGGCCCCAATC-A-AAGAGGGCC--TTGAGC-CGAAGCAGACTAA-AACTAGAAGATCGCT
Elac    CGGCCCCAATAAA-AAGAGGGTA--ATGAAC-CAAAAAGCCCGTG-AACCAGAAGCCCCTT
Kugu    CGGCCCCAAACAA-A-GAGGGAA--CAGAGT-AATAACACTGAAA-TACCAGAAAAACACC
Plor    CGGCCCCAAAATA-AAGAGGGTA--TTGAA--CAACAAA-CCTGCTAACTAGAAAACTATT
Sgun    CGGCCCCAAACA--AAGAGGGAA--ATGAACAACATACCAAACGACTAGAAAACCACTCAA
Zaco    CGGCCCCAAACA--AAGAGGGCA--CTGAACAACAGACCAAAC---AACAAGAAAAACGTC
Zbfl    CGGCCCCAAAC-A-AAGAGGGAA--CTGAAC-AACAAAT-TAAAG-AACAAGCAAACCATC
Spba    CGGCCCCAAGCA--AAGAGGGCA--TTGAA--GAATTA-ATCAAACAGACCGGAAAACCCT
Game    CGGCCCCAAACAA--AGAGGGCA--ATGAACATA-TAA--GTAAGCAACCAGAAAATCATC
Thth    CGGTCCCAAACA--AAGAGGGCA--ATG-AACAACAAG-TA-AGCAACCA-GAAAACCATC
Xigl    CGGCCCCAAACAA--AGAGGGCACTGAAAAATGACCCAAGCAAACCAGAAAAC----CATC
Hyja    CGGTCCCAAACAA--AGAGGGCC--CTG---AACAACA-AGTAACCAACTAGAAAAACATC
Psan    CGGCCCCAA-ACA-AAGAGGGAA--ATGAAC-AGCAAGTAAGTA---ACCAGAAAACCATC
Cupa    CGGCCCCAAAC-A-AAGAGGGTA--ATGAAC-AA-CAAG-TTAGACAACCAGAAAACCATC
Mpch    CGGCCCCAAA-CAA-A-GAGGGAA--TTGAATGATAA-ATTAAAC-AACAAGAAGACCATT
Char    CGGCCCCAAACAA--AGAGGGCA--ATG---CACAACC-AACAAACAACCAGAAAACCCCC
Pser    CGGCCCCACACC--AAGAGGGCA--TTAGGA-AGCAA-ACCAAGTGAACAAGAAAATCGCC
Prol    CGGCCCCAATC-A-AAGAGGGAA--CTGGAA-AATGTCA-TCGATTGACTAGGAAAACATC
Plbi    CGGCCCCAATC-A-AAGAGGGTA--TTGGAA-ATCACCACAAATTTAGGCCAGAAAAACAT
Calu    CGGACCCAAAACA--TAGAGGGCC--CTGGGA-AAC--AGCAAATGAACTGGAAGACCACC
Papa    CGGCCCCAGA-CAA-AAGAGGGCA--CTGAA---AACAAAATAAAGAACAAGAAGAACTTT
Sufr    CGGCCCCAACCAA-A-GAGGGTA--GTG-AT-AATAAAAAAGA-T-AACAAGAAAAACGCT
Stci    AGGCCCCAA-CTA-CAGAGGGAA--ATGAA--AATGTTTTACAAA-TACAAGAAAAACAAT
Taru    CGGACCCAACCAA--AGAGGGAA--ATA---AATATTA-AACTCACAACAAGAAAAACATT
Rala    CGGCCCCTACAC--AAGAGGGTA--TTGAAC-TACAA--ATTAAACAACTAGAAAACCCCC
```

```
                   29' B' 30        30' 27'          26'     31
Scca    TATCTTACTAT--TCGTTAACCCA-ACACAGGAGTGTCT------TAAGGAAAG-ATTAA
Muma    TTCTTCT---AT-TCGTTAATCCC-ACACAGGAATGTCAC-----C-AGGAAAG-ATTAA
Erca    TAT---A-TTTACTCGTTAACCTT-ACACAAGAGTGCACA------AAGGAAAG-ACTAA
Pose    TATTAACCTCA--TCGTTAATCTT-ACACAAGAGTGC-CCA-----AAGGAAAG-ACTAA
Actr    GTAAAACACA-A-CCGTTAACCCA-ACACAGGAGTGCACCG-C-C-AAGGAAAG-ACTAA
Scal    GTAAAACACAA--CCGTTAACCCA-ACACAGGAGTGCGCCA-C--CAAGGAAAG-ACTAA
Posp    TAAAACACAA---CCGTTAATCCC-ACACAGGAGTGCTCAC----CAAGGAAAG-ACTAA
Atsp    TATTGAATATAAATCGTTAATCTT-ACACAAGAATGCCC-------ATGGAAAG-ACTAA
Leoc    TTAAATATT--AATCGTTAACCTT-ACACAAGAATGCAC-------ATGGAAAG-ACTAA
Amca    TAATAA--CTA--TCGTTACCCCT-ACACAGGAGTGCACCC-----AAGGAAAG-ACTAA
Osbi    TATTAC-----AATCATTAATCCT-ACACCGGTGTGCTATTCA---AAAGAAAG-ACTAA
Pabu    AGCAAAATT--A-TCGTCAACCCA-ACACAGGAGCGCAC----TACAAGGAATG-ACTAA
Hial    TGACAA---ATA-TCGTTAACCCA-ACACAGGAGTGCAAA------AAGGAAAG-ACTCA
Elha    CAACACAATA---CCGTTAGCCCT-ACACAGGAGTGCCC-C-----AAGGAAAG-ACTAA
Mlcy    GCTACC--CACTACCGTTAACCCA-ACACAGGAGTGCAGCC-TCAGAAGGAAAG-ACTAA
Algl    CAGTGAGATC-A-TCGTTAGCCCG-ACACAGGAGTGCCTAT-G---ATGGAAAG-ATTGA
Ptgi    CGA---AAAATGATCGTTAACCCA-ACACAGGTGTGCCA------CAGGGAAAG-ACTAA
Alaf    ATGAAGCAAA---TCGTTAACCCA-ACACAGGAATGCACAA------AGGAAAG-ACACA
Nock    TGAAGAAA---A-TCGTTGACCCA-ACACAGGGGTGCACA------AAGGAAAG-ACATA
Anja    TAATACCACA-AACCGTTAACCCA-ACACAGGAGTGCCTA-----A-AGGAAAG-ACTA-
Gyki    GCCAAA---ACAACCGTCACCCCA-ACACAGGAGTGCGCC------CGGGAAAG-ACTAA
Syka    TATTACCTACAA-TCGTTAGCCCC-ACACAGGAGTGCCCATA---AAAGGAAAG-ACTAA
Opma    TATGAGAACAA--CCGTTAACCCA-ACACAGGTGTGCAAT---A--AAGGAAAG-ACTAA
Comy    TTAAAAC-A---ACCGTTAACCCA-ACACAGGAGTGCCACT----TAAGGAAAG-ACTAA
Sasp    TAC--TATCACATTCGTTAACCCA-ACACAGGAGTGCCTT-----AATGGAAAG-ACTAA
Eupe    AAACCACAAA---CTGTTAACCCG-ACACAGGAGTGCATCT-----AAGGAAAG-ACAAA
Enja    TTC--CCCAGA-ATCGTTACCCCG-ACACAGGAGTGCCAGA---ACAAGGAAAG-ACTTA
Same    TAG-TGAAA--CATCGTTACCCCA-ACACAGGAGTGCTCTA--GCCAGGGAAAG-ACTTA
Chch    CATTCAAATAAA-TCGTTACCCCC-ACACAGGTGTGCTAAAC---TAGGGAAAG-ACAAA
Grgr    GC-CACCTGAA--TCGTTGACCCT-ACACAGGGGTGCCCC------TGGGAAAG-ACTAA
Caau    CACAACCTAAGTATCGTTACCCCC-ACACTGGAGTGCAACA-----AAGGAAAG-ACTAA
Cyca    CAA--CTAAACTATCGTTACCCCC-ACACTGGAGTGCCCT-----AAAGGAAAG-ACTAA
Dare    CAACA----GACATCGTTACCCCC-ACACTGGAGTGCATAT-----AAGGAAAG-ACTAA
Cost    TA---AAATAATATCGTTAATCCC-ACACTGGAATGCCCC------AAGGAAAG-ACTAA
Leec    CAACACCTG--AATCGTAAAACCC-ACACTGGAGTGCTAT-----CCAGGAAAG-ACTAA
Crla    AT-GTTATAACAATCGCTAACCCC-ACACTGGAGTGCCC-----C-CAGGAAAG-ACTAA
Clmc    CTA--ACC---CATCGTTAACCCC-ACACCGGAAGGCCCC-C-AACAAGGAAAG-ACTAA
Phin    CAC-ACCC---CTTCGTTAACCCC-ACACTGGCGCGCCACC----ACAGGAAAG-ACTA-
Icpu    CA-TACACCTGCATCGTTAACCCC-ACACCGGAAGGCAGA-----ATAGGAAAG-ACTAA
Psto    CATAAAA-TAA--TCGTTAAACCC-ACACCGGAAGGCCTA----CATAGGAAAG-ACTAA
Cora    CACGACTTAA---TCGTTAACCCC-ACACCGGAAGGCTAC------AAGGAAAG-ACTAA
Eisp    CGC--ACACACAATCGTTAATCCT-ACACTGGAGGGATAC-------AGGAAAG-ACTAA
Apal    CACCAC-CAA---TCGTTAACCTT-ACACCAGAGCGCAACT-----AAGGAAAG-ACTAA
Eslu    CA-TAAAC--CAATCGTTAACTCC-ACACTGATGTGCACC--C---AAGGAAAG-ACCTA
Dape    CA-TAAAAT--AATCGTTAACTCC-ACACTGAAGTGCCT------CCGGGAAAG-ACCTA
Glse    TACATT---TAAATCGTTAACCCC-ACACAGGAGTGCCCCC-----AGGGAAAG-ACCTT
Naar    TATACT---ACTATCGTTAACCCC-ACACAGGAGTGTTCCC-----CGGGAAAG-ACCTA
Baoc    TATTCCGCAA---TCGTTAACCCC-ACACAGGAGTGTTCTC-----TGGGAAAG-ACCTT
Opso    TAT--AA-AATAATCGTTATTCCT-ACACAGGGGTGCCC------TAAGGAAAG-ACTAA
Alte    CACGCACACA---TCGTTACCCCT-ACACAGGAGTGCCTAT---AAGAGGAAAG-ACCCA
Plap    CAC--ACGCAC-ATCGTTAGCCCC-ACACAGGAGTGCCCCC---AAGAGGAAAG-ACCCA
```

```
Plal    CAA--AGTGACCATCGTTAATCCC-ACACAGGAGTGCCTA------GAGGAAAG-ACTAA
Sami    ATGAAAC-G---ATCGTTAGCCCC-ACACAGGAGTGCACCA------AGGAAAG-ACTAA
Rere    ATC-CA--CAACATCGTTGACCCT-ACACAGGAGTGCTTG-------AGGAAAG-ACCTA
Gama    TTG---TAATGAATCGTTGACCCC-ACACTGGAGTGCA-T-----AAGGGAAAG-GCCTA
Onmy    CA-CCAACA--AATCGTTACCCCC-ACACAGGAGTGCCC-----CAAGGGAAAG-ACCCA
Sasa    CATCAA---CAAATCGTTAACCCC-ACACAGGAGTGCTCAC-----AGGGAAAG-ACCTA
Cola    CAAAACTA-A---TCGTTAAACCC-ACACAGGAGTGCCCAC-----AAGGAAAG-ACCCA
Dita    GAATGTAACA---TCGTTAACCCT-ACACAGGAGCGCCCCA-----AGGGAAAG-ACCTA
Gogr    AA-TAATCCTATATCGTTAACCCC-ACACAGGACTGCATA-----TAAGGAAAG-ACCTA
Chsl    AGA---TGCTTCATCGTTAACCCA-ACACAGGAGTGCCC------CGAGGAAAG-ACTAA
Atja    CAATATATTTCGATCGTTAAACCT-ACACAGGAGTGCCACC-----CGGGAAAG-ACTAA
Iido    CAACAATTTT-AATCGTTAACCCC-ACACAGGAGTGCCCCC------AGGAAAG-ACTAA
Auja    CACCTATAAA---CCATTAACCCC-ACACAGGAGTGCTTAC-----CAGGAAAG-ACCAA
Chag    CAATAAAAC--AATCGTTATCCCC-ACACAGGAGTGCCC----CACCCGGAAAA-ACGAA
Hami    TAATCAACT--A-CCATTAGACCC-ACACAGGAGTGCAA-----CTAAGGAAAG-ACTAA
Saun    CAAGATATA---ACCATTAAACCC-ACACAGGTCTGCCAAC-----AGGGAAAG-ACTA-
Nema    CAC--GAACCGAATCGTTAGCCCT-ACACAGGTGTGCAGC-----CCAGGAAAG-ACTAA
Disp    CAT-AAC-TAA--CCGTTAACCCA-ACACAGGTGTGCACG-----CCCGGAAAG-ACTTA
Myaf    TA--TAATAG---TCGTTAACCCA-ACACAGGTGTGTGCCC------CGGAAAG-ACTAA
Lagu    CA-AATAATA---TCGTTAACCTT-ACACCAGAGTGCAAC--C---AAGGAAAG-ACTAA
Trtr    CACCCTCCCC-CATCGTTAACCTA-ACACAAGAATGCAC-----C-AGGGAAAG-ACTAA
Zucr    CACATT---AA-ACCGTTGACCTA-ACACAAGAATGCACC------AAGGAAAG-ACTAA
Pxja    CACCTTAAT--AATCGTTAACCCT-ACACAGGAGTGCAT-----ACAAGGAAAG-ACTAA
Pxlo    CAC--CTTAATAATCGTTAACCCC-ACACAGGAGTGCATA-----CAAGGAAAG-ACTAA
Pctr    CACCCCCAAA---TCGTTACCCCC-ACACAGGAGTGCAACA----AGTGGAAAG-ACTAA
Apsa    TACCTAAAC---ACCGTTAACCCT-ACACCGGAGTGT-AAT-----AAGGAAAG-ACTAA
Cabe    CAGTACACA---CACGTTGACCCC-ACACCGGAGTGCAC------CAAGGAAAG-ACTTA
Bzze    CAACAACCG----ACGTTAACCCC-ACACTGGTGTGCCT-----GCAAGGAAAG-ACTGA
Siim    CAACAACAA----ATGTTAATCCA-ACACCGGCGTGCTA-----CCCAGGAAAG-ACTAA
Ctru    CAACACAAAG---CCGTTAGCCCC-ACACTGGAGTGCCCAC-----AAGGACAG-ACTAA
Dpbr    CACATCTATAG--CCGTTAACCCC-ACACTGGTGTGCCCACA-----AGGACAG-ACTAA
Caki    TAACTAA-T-A--TCGTTGACCCT-ACACAGGAATGCACC------AAGGAAAG-ACTAA
Phja    TA-G-AATTTA--TCGTTAACCCC-ACACAGGTGTGCCCC------AAGGAAAG-ACTAA
Brsp    TAAACCTA----ACTGTTAACCCT-ACACAGGAGTGCACC-----A-AGGAAAG-ACTTT
Gamo    TATTTTAT-A---TCGTTAACCCC-ACACAGGTGTGCCTAA------AGGAAAG-ACTAA
Lolo    TA---TT-TTTTATCGTTAACCCCCACACAGGTGTGCCCA------AAGGAAAG-ACTAA
Batr    CAACACAA----ACCGTTAACCCG-ACACAGGTGTGCTGCA-----AAGGAAAG-ACAA-
Prmy    C--TCATTAA-A-CCGTAGACCCC-ACACAGGCATGTGCCC-----TAGGAAAG-ATTAA
Loli    CAATGCAT----ACCGTTAACCTC-ACACTAGAGTGCCCCC----A-AGGAAAG-ACTA-
Loam    CAG---TAACTAACCGTTAACCTC-ACACTAGAGTGCTGCC-----AAGGAAAG-ACTAA
Chab    CAAAA----CCCACCGTTAACCCC-ACACTGGCGTGCTTCA-----AGGGAAAG-ACTAA
Chto    CA-AACCCCA---CCGTTAACCCC-ACACTGGCGTGCTTC----CAAGGGAAAG-ACTAA
Majo    CAGCTCACCCA--CCGTTAACCCC-ACACTGGTGTGCCTA----C-AAGGAAAG-ACTAA
Hlst    AAACATTTTA-AACCGTTACCCCT-ACACTGGAGTGTTACA-----AAGGAAAG-ACTAA
Clpe    CAGCAACCAA---CCGTTGACCCC-ACACTGGTGTGCAT-C----AAGGGAAAG-ACAGA
Mlmr    CAATAA---GTTACCGTTACCCCC-ACACTGGTAGTCCCCC-----AAGGGAAA-GCTGA
Crcr    CAAAAC---CCCACCGTTAACCCC-ACACTGGCGTGCCCTC-----AGGGAAAG-GTTAA
Muce    CAA---AACCCCACCGTTAATCCC-ACACTGGCGTGCCCT-----CAGGGAAAG-GTTAA
Bege    CATAAA--TT-A-CCGTTAACCCC-ACACTGGCGCGCCCCA------TGGAAAG-ACTAA
Mela    CAAATA---TCC-CCGTTAACCCT-ACACTGGAGCGCCTCC-----GTGGAAAG-ACTAA
Hats    CAAAAGATA----CCGTTAACCCC-ACACTGGTGTGTCC-----CCACGGAAAG-ACTAA
Orla    -ACATA---ATTACCGTTACTCCT-ACACCGGTGTGCTCTT-----TAGGAAAG-ACAAA
```

```
Cosa    TAATAACTA---ACCGTTAACCCC-ACACTGGTGTGCCCCC------CGGAAAG-ACTAA
Exsp    CAACAA--AT--ACCGTTGACCCT-ACACTGGAATGCTAAC-----CTGGAAAG-ACCAA
Depa    TAC--AA--ATAACCGTTAACCCC-ACACTGGTGTGCCTT------CCGGAAAG-ACTAA
Rima    TA-AACACTAA-ACCGTTAACCCC-ACACAGGCACTCCCC-----TGAAGAAAG-ACTAA
Fuol    TATTTAT-TAA--CCGTTAACCCC-ACACTGGTGTACAAA----C-TTGGAAAG-ACTAA
Gmaf    TA--A-ACAAATACCGTTACCCCT-ACACAGGAGTGCCCC-----T-GGGAAAG-ACTAA
Xeei    TAATAAAAT--AACCGTTAACCCC-ACACTGGCGTGCAAGC-----CCGGAAAG-ACTAA
Pros    CA-ACCATCAA--CCGTTAGCCCC-ACACTGGAGTGCCCA-----CAAGGAAAG-ACTAA
Scmi    TGATTACC---CACCGTTAACCCC-ACACTGGCGTGCCC-----ACAAGGAAAG-ACTAA
Rolo    TCAATTATAAA--CCGTTAGCCCC-ACACCGGAGTGCACA-----CAAGGAAAG-ACTAA
Cere    CAACACTTT--GGCCGTTACCCCC-ACACCGGAGTGCTT-----ACAAGGAAAG-ACTAA
Daga    CATATT---ATG-CCGTTGATCCT-ACACTGGGGTGCTTAA-----AAGGAAAG-ACTAA
Anco    CAACAAAA----ACCGTTAACCCC-ACACTGGTGTGCAAC------AAGGAAAG-ACTAA
Dmve    CAACAACC----ACCGTTAACCCA-ACACTGGCGTGCACCC-----CAGGGAAG-ACTA-
Dmar    CAAC-AGC---CACCGTTAGCCCC-ACACTGGCGTGCAATT-----AAGGAAAG-ACTAA
Anka    CAATTAAC---AATCGTTAACCCC-ACACTGGTGTGCT------AAAAGGAAAG-ACTAA
Moja    CAACAAC---AA-CCGTTAACCCC-ACACTGGTGTGCAAC------AAGGAAAG-ACTAA
Hoja    CAATAA-CAG---CCGTTAACCCC-ACACTGGTGTGCAACA------AGGAAAG-ACTAA
Bede    CA---AATACTGGCCGTTAACCCC-ACACTGGAGTGCCCAC-----AAGGAAAG-ACTAA
Besp    CAAATACTA---GCCGTTGACCCC-ACACTGGAGTGCCCAC-----AAGGAAAG-ACTAA
Mysp    CAAAACAACACCACCGTTAACCCT-ACACTGGAGTGCCTGC-----AAGGGAAG-ACTAA
Osja    CAA--CCAAACCACCGTTAGCCCT-ACACTGGAGTGC-CT----ACAAGGAAAG-ACTAA
Sgro    CAACAAA-TTTAACCGTTAACCCC-ACACTGGAGTGCAAAC-----AAGGAAAG-ACTAA
Pzpa    TACTTAAC----ACCGTTAACCCT-ACACAGGAGTGTTATC-----AAGGAAAG-ACCA-
Zeja    CACTTATTTA---TCGTTAGCCCT-ACACAGGAGTGTTTTC-----AAGGAAAG-ACAAA
Znne    CACTAA-ACA-A-TCGTTAACCCT-ACACAGGAGTGTAATT-----AAGGAAAG-ACCAA
Zefa    CACTAA-AA---ATCGTTAACCCA-ACACAGGAGTATAAAT-----AAGGAAAG-ACAAA
Acni    CACCTATA---A-TCGTTAACCCA-ACACAGGAGTGTATT-----TAAGGAAAG-ACAAA
Ncrh    CACCTA--TAA--TCGTTAACCCA-ACACAGGAGTGTATT--T---AAGGAAAG-ACAAA
Agca    CAACAATCAA---CCGTTAACCCC-ACACTGGTGTGCCCAT-----AAGGAAAG-ACTAA
Hydy    CAAAAACT---AGCCGTTAACCCC-ACACAGGTGTGCCCC------CGGGAAAG-ACTAA
Gsac    CAACATTTGA---CCGTTAACCCC-ACACAGGTGTGCCC-C-----CGGGAAAG-ACTAA
Pevo    CA--CCAT-CACACCGTTAACCTC-ACACAAGCGTGCTCC--C---AAGGAAAG-ACTAA
Hiku    CAAT--AATAAA-CCATCAACCCC-ACACCGGATTGCTAA-----CCAGGATAA-ACTAA
Inpa    TAACCAAAC--A-CCGTTGAACCA-ACACCGGAGTGCCC-----CATAGGAAAG-ACCTA
Auch    CAGT-AAACCCTCCCGTTAACCCT-ACACAGGAGTGCACCC-----CGGGAAAG-ACTTA
Fico    CAAAAACTT---ACCGTTAACCCT-ACACTGGTGTGCCCAC-----TAGGAAAG-ACTAA
Macs    CA-GACAAA--AACCGTTAATTCC-ACACTGATGTGCCC-----CCAAGGAAAG-ACTAA
Moal    CAACAATCAT---CCATTAATCTT-ACACCAGCGTGTGATA-----AAGGAAAG-ACTAA
Syma    CAACAT---ACA-CCATTAACTCT-ACACTTA-GTGTAATA-----AAGGAAAG-ACTAA
Mafr    CACCAACCA---ACCGTTAACCTC-ACACCAGAGTGCCCCC-----AAGGAAAG-GCTGA
Dcpe    CA--ACAATA-CACCGTTAATCCC-ACACTGGTGTGCCAA----C-AAGGAAAG-ATTGA
Dcti    AACATTAAACA--CCGTTAACCCC-ACACTGGTGTGCCAA-----CAAGGAAAG-ATTAA
Hehi    CAA---ACACAAACCGTTAACCCT-ACACAGGTGTGCACT-----TTAGGAAAG-ACTAA
Stam    CAACACA-----ACCGTTAACCCC-ACACAGGTGTGCCCCC-----CGGGAAAG-ACTA-
Hogi    CAAACATA---A-CCGTTAACCCT-ACACAGGCATGCATT-----TAAGGAAAG-ACCAA
Erzo    CAAAAAAAGA---TCGTTAACCCC-ACACAGGTGTGCCAAT-----AAGGAAAG-ACTAA
Hxot    CAAAAACCAA---CCGTTAAACCC-ACACAGGTGTGCCCCC-----AGGGAAAG-ACTAA
Core    CA--ACAACAA-ACCGTTAACCCC-ACACAGGTGTGCCCA-----CAGGGAAAG-ACTGA
Apve    CAAGAACT---GACCGTTAACCCC-ACACAGGTGTGCCCCT----A-GGGAAAG-ACTA-
Latj    CAA-CACAT--AACCGTTAACCCC-ACACTGGTGTGCTC----G--AAGGAAAG-ACTAA
Laja    CAATAAC-TTA--CCGTTAAACCC-ACACTGGTGTGCTCC------AAGGAAAG-ACTAA
```

```
Syja CAACAATA---AACCGTTAACCTT-ACACTAGAGTGCCAA-----CAAGGAAAG-ACTAA
Epme CAAACAATT--AACCGTTACCCCC-ACACAGGTGTGCTT----TT-AGGGAAAG-ACTAA
Grse CAACAA---CCAACCGTTACCCCT-ACACAGGTGTGCCCC-----AGAGAAAG-ACTAA
Clja CA-TTAATAAA--CCGTTAAACCT-ACACTGGAGTGCCCC-----ATGGGAAAG-ACTAA
Ogcy TC-AACACAAA-ACCGTTACCCCA-ACACTGGAGTGTGAA-----CAAGGAAAG-ATTAA
Plna CAA-ACTCT--TACCGTTAATCCC-ACACTGGTGTACTTTT-----AAGGAAAG-ACTAA
Lema CAAGCT--TTTAACCGTTAACCCC-ACACTGGTGTGCCCCT-----TGGGAAAG-ACTAA
Etzo CA-ACAGCCTA-ACCGTTATTCCC-ACACAGGCGTGCCCA-----CAAGGAAAG-ACTAA
Apse CA-CCCCC--TAACCGTTAACCTT-ACACCAGAGTGCACT----C-AAGGAAAG-ACTAA
Epde CAATAATT---AACCGTTAACCCC-ACACTGGTGTGCTTTT-----AAGGAAAG-ACTA-
Slja CACCCCCCCTAA-CCGTTTACCCC-ACACTGGAGTGCCTT-----TTAGGAAAG-ACTAA
Bsja CAGTAACCA---ACCGTTAACCCA-ACACCGGTGTGCCCTTTTTCAAAGGAAAG-ACTAA
Ecna TAA--AAAT--AAGCGTTAACCCC-ACACTGGTGAGC---A--TACAGGGAAAG-ACTAA
Cohi CAAAAT---TAAACCGTTAACCCT-ACAC-GGAGTGCTCTTTT---AAGGAAAG-ACTAA
Caar CTAAAAT----TACCGTTAATCCC-ACACTGGTGTGCC--TA---CTAGGAAAG-ACTAA
Came CAACCAAC----ACCGTTAACCCT-ACACTGGTGTGCTTAC-----CAGGAAAG-ACTA-
Mema CAAGACCC-T--ATCGTTAACCCC-ACACAGGTGTGCAC-----CCAAGGAAAG-ACTAA
Lenu CAACACATCC---CCGTCAACCCC-ACACAGGAGTGCCATTA----ATGGAAAG-ACTAA
Brja CAACAAGCAA---CCGTTAACCCT-ACACCGGTGTGCCCCC-----CAGGAAAG-ACTAA
Plma CAATAAACA---ACCGTTAACCCC-ACACTGGTGTGCCCCC----TGAGGAAAG-ACTA-
Emst CAATGA-GAA-A-CCGTTAACCCC-ACACTGGTGTGCCCAT-----GGGGAAAG-ACTAA
Ptti CAATTACACA--ACCGTTAACCCC-ACACTGGTGTGCACCC-----AAGGAAAG-ACTAA
Losu CAACCTCATT---CCGTTGACCCC-ACACTGGTGTGCAA-C----TAGGGAAAG-ACAAA
Geoy CAA--TGCAAC-ATCGTTAAACCC-ACACTGGAGTGCACC----CCCGGGAAAG-ACTAA
Dipi CAT--TAC-ACAACCGTTAACCTC-ACACTGGTGTGCCCC-----TAAGGAAAG-ACTAA
Pama CAAGACAA---AAGCGTTAACCCC-ACACTGGAGTGCTC-----TTAGGGAAAG-ACTAA
Leob CAATAACTA---ACCGTTGACCCC-ACACTGGTGTGCTTTT-----AAGGAAAG-ACTAA
Neba CAAAAATT-A---TCGTTAACCCT-ACACTGGTGTGTTTTT-----TAGGAAAG-ACTAA
Pdpl CACAACATTAACCCCGTTAAATCT-ACACCGACGTGCAAT----CCAGGGAAAG-ACTAA
Nimi CAATAC---TCAACCGTTGAACCC-ACACTGGTGTGCCCCC-----GAGGAAAG-ACTAA
Uptr CA--GCTCACCCACCGTTAACCCC-ACACTGGTGTGCCCA----C-AAGGAAAG-ACTAA
Pesc CAAACC---CACACCGTTAACCCC-ACACAGGCGTGCCTAA-----AAGGAAAG-ACTTA
Baar CACCAAATAC---CCGTTAGTCCC-ACACTGGAGTGCCCCCT----AAGGAAAG-ACTAA
Moar CAACAAACAA---CCGTTAAACCC-ACACTGGAGTGCTCTC-----AGGGAAAG-ACTAA
Toja -CATTC--TAACACCGTTAACCCT-ACACTGGCGTGCC-CC----TCGGGAAAG-ACTAA
Chau CA-ATAACT--A-CCGTTACCCCC-ACACTGGTGTGA-T----TTCAAGGAAAG-ACTAA
Chse CAGAAATT---AACCGTTAACCCT-ACACCGGTGTGCCCCC----TAGGGAAAG-ACTAA
Enar CAATAACT---AACCGTTAACCCC-ACACTGGCGTGCCCC-----TAGGGAAAG-ACTAA
Hpty CACAAA---TCAACCGTTAACCCC-ACACTGGCGTGCCCCC-----AAGGAAAG-ACTAA
Nana CACTAAA--TCAACCGTTTAACCC-ACACTGGTGTGCATAC-----TAGGAAAG-ACTAA
Mcst CAACAACACA-A-CCGTTACCCCC-ACACTGGCGTGCCCCT-----AAGGAAAG-ACTAA
Rhox CAAATACAAC-TACCGTTAACCCT-ACACCGGCGTGCTTAC-----AAGGAAAG-ACTAA
Opfa CATACAAC---GACCGTTAACCCC-ACACTGGTGTGCAC-----CCAAGGAAAG-ACTAA
Paar CAA----ATTCAAGCGTTAACCCC-ACACTGGAGTGCTTT-----TTAGGAAAG-ACTAA
Gozo CCACCATCT--AGCCGTTAACCCC-ACACAGGTGTGCCC-----TCCGGGAAAG-ACTAA
Ackr CA-AACCCCG---CCGTTAACCCC-ACACTGGAGTGCAAA-----CAAGGAAAG-ACTAA
Elev TAC--CGATT-AACCGTTAAACCT-ACACTGGTGTGCCAC-----CTGGGAAAG-ACTAA
Trdu TA-GTTCC--TCACCGTTAACCCC-ACACTGGTGTGCAAA----C-CGGGAAAG-ACTAA
Amoc CA-CACTTA--TACCGTTAATCCT-ACACTGGTGTGCCT-----ATAAGGAAAG-ACTAA
Hame CAAAAACAA---ACCGTTAACCCC-ACACTGGTGTATCTAA-----AGGGGAAG-GCTGA
Chso CCTAAAAT--TAACCGTTAACCCT-ACACTGGTGTGCCTAC-----AAGGAAAG-ACTAA
Lyto CAG---GAAATAACCGTTAACCCC-ACACAGGTGTGCCCA-----CAGGGAAAG-GCTAA
```

```
Encr    CAGGAA-TTA--ACCGTTAACCCC-ACACAGGTGTGCCCAC-----AGGGAAAG-GCTAA
Bvar    CAATAACACA---CCGTTAACCCC-ACACAGGCGTGCGCCT-----TAGGAAAG-ACTAA
Noco    CAACCA-CCA-A-CCGATACCCCA-ACACAGGTGTGCCCCT-----AGGGAAAA-ACTAA
Chsp    CA--AAACATA-ACCGTTACCCCT-ACACTGGCATGCCAC-----TAAGGAAAG-ACTAA
Arja    AAAAACTA-A---CCGTTACCCCT-ACACAGGTGTGCCCCC-----AGGGAAAG-ACTAA
Pase    CATCAATTA--AACCGTTAACCCC-ACACTGGTGTGCCT------TAAGGAAAG-ATTAA
Trel    CAAT--TAATCA-CCGTTGACCCC-ACACTGGAGAGCCTG-----CAAGGAAAG-ACAAA
Lifa    TAACTATTAA--ACCGTTTACCCC-ACACCGGAGTGCCACC-----CAGGGAAG-ACTAA
Acur    CATCTATTA--AACCGTTGATCTT-ACACTAGGTTACCT-----ATAAGGAAAG-ACTAA
Ampe    CAACTA---AAGACCGTTAACCCC-ACACTGGTGTGCCCCC-----AAGGAAAG-ACTGA
Urja    CACCCATT---A-CCGTTAACCCT-ACACAGGCATGCTTT-----TAAGGAAAG-ACTAA
Enet    CAC-TGCTAA---CCGTTAACCTT-ACACTAGAGTGCCTTT----AAAGGAAAG-ACTAA
Ptbr    TCA--CCCTGC-ACCGTTAACCTT-ACACTAGAATGACT-----ATAAGGAAAG-ACTAA
Safa    CACAGTCCAA---CCGTTAACCTT-ACACCAGAGTGCCCCC-----AAGGAAAG-ACTAA
Icae    CAATAA---ACAACCGTTAACCCC-ACACTGGTGTGCCCCT-----AAGGAAAG-ACTAA
Asmi    CTTATA--CCTTTGCATTACCCTA-ACACAAGCGTGCTTTC-----TAGGAAAG-ATTAA
Foal    CATAAA----TAACCGTTAACCCC-ACACAGGTGTGCCCAC-----AAGGAAAG-ACAAT
Drze    CAAAAT---TTTACCGTTAATCCT-ACACTGGTGTGTTAAA-----TGGGAAAG-ACTAA
Rhas    CA-ACAA-AAA--GCGTTAGCCCC-ACACAGGAGTGCCTC------CAGGAAAG-ACTAA
Elac    CATCAAACTA-A-GCGTTAACCCC-ACACAGGAGTGCCC-------CAGGAAAG-ACTAA
Kugu    CAATAACACCAAACCGTTAACCCC-ACACTGGTGTGCTAA-----CCAGGAAAG-ACTAA
Plor    CACATA--AACAACCGTTAACCCC-ACACTGGTGTGCCCCC-----AAGGAAAG-ACTAA
Sgun    TAC---AACCCAACCGTTAACCCC-ACACTGGTGTGCTCA-----AATGGAAAG-ACTAA
Zaco    CTACA----AAAACCGTTAACCCT-ACACAGGTGTGCCC-----ACAAGGAAAG-ACTAA
Zbfl    CATTAAA-TAA--CCGTTAACCCC-ACACTGGTGTGCCCC-----AAGGGAAAG-ACTAA
Spba    CCA--CCATCACACCGTTAACCCT-ACACTGGTATGCTCC-----CAAGGAAAG-ACTAA
Game    CA-ATAAGT--AACCGTTAACCCC-ACACTGGTGTGCCC-----CTAAGGAAAGTACTAA
Thth    CAA--AAA-ACAACCGTTAACCCC-ACACTGGTGTGCCATT-----AAGGAAAG-ACTAA
Xigl    CAGCTTAT---TACCGTTAACCCC-ACACTGGTGTGCCCCT-----AAGGAAAG-ACTA-
Hyja    CAATGA---ATAACCGTTAACCCA-ACACTGGTGTGCCTAT-----AAGGAAAG-ACTAA
Psan    CAATTGATTA---CCGTTAACCCA-ACACTGGTGTGCCCAT-----AAGGAAAG-ACTAA
Cupa    CA-ATTAATAA--CCGTTAACCCC-ACACTGGTGTGCCCC----T-AAGGAAAG-ACTAA
Mpch    CAACAA-T---AACCGTTAACCCC-ACACTGGTGTGCTAA-----AAAGGAAAG-ACTAA
Char    CAAGAA---ACAACCGTTAAGCCC-ACACTGGCGTGCCCCC-----AAGGAAAG-ACTAA
Pser    TAATAATATAAC-CGGTTAACCCC-ACWCTGGTATGCACCT-----CGGGAAAG-ACAAA
Prol    CAATAC---AACCCCGTTAACCCC-ACACTGGTGTGCCCCA-----GAGGAAAG-ACCCA
Plbi    CC-AATGTAAA-CCCGTTAACCCC-ACACTGGTGTGCCCA-----AAAGGAAAG-ACCAA
Calu    CACCCCCAA---CCCGTTGACCCC-ACACTGGCGTGCAT------GAAGGAAAG-ACTAA
Papa    CAAAAAAC---AACCGTTAACCCA-ACACAGGAGTACTATA--TTAAAGGAAAG-ACTAA
Sufr    CAAACAATTA-G-CCGTTGACCCC-ACACTGGAATTA-CCG--C--AAGGGAAA-GCTGA
Stci    CAGCCACC---CACCTTTACCCCC-ACACTGGCGTGCC-CA-----AAGGAAAA-ACTAA
Taru    TAACAC---TTTTCCGTTACCCCT-ACACTGGTGTGCCAAA-----TAGGAAAG-ACTAA
Rala    CAATAAACCC---CCGTTAACCCC-ACACTGGAATGCTTA-----ATAGGAAAG-ACTAA
                                     ** *
```

```
                        32                                33       34    C
Scca    AAGAAA-GTAAAGGAACTCGGCAAACA---------TAAACT-CCGCCTGTTTACCAA
Muma    AAGAAA-ATAAAGGAACTCGGCAAACAC---------AAACT-CCGCCTGTTTACCAA
Erca    AAGAGA-AAAAAGGAACTCGGCAAACC---------TGAGCC-CCGCCTGTTTACCAA
Pose    AAGAGA-AAAAAGGAACTCGGCAAACT---------CGAGCC-CCGCCTGTTTACCAA
Actr    AAGAAA-AAGAAGGAACTCGGCAAACA---------CGAGCC-TCGCCTGTTTACCAA
Scal    AAGAAA-AAGAAGGAACTCGGCAAACA---------CGAGCC-TCGCCTGTTTACCAA
Posp    AAGAAA-AAGAAGGAACTCGGCAAACA---------CGAGCC-TCGCCTGTTTACCAA
Atsp    AAGAAA-AAGAAGGAACTCGGCAACCC---------CGAGCC-TCGCCTGTTTACCAA
Leoc    AAGAAA-AAGAAGGAACTCGGCAACCC---------CGAGCC-TCGCCTGTTTACCAA
Amca    AAGAAA-AGAAAGGAACTCGGCAAACA---------CGAGCC-TCGCCTGTTTACCAA
Osbi    ATGAGGAAAGAAGGAACTCGGCAAACC---------AAGGTC-TCGCCTGTTTACCAA
Pabu    ATGGAG-AAAAAGGAACTCGGCAAACA---------CGGGCC-TCGCCTGTTTACCAA
Hial    AAGGAA-AAGAAGGAACTCGGCAAACA---------CGGGCC-TCGCCTGTTTACCAA
Elha    AAGAGA-CGGAAGGAACTCGGCAAACT---------CGAGCC-TCGCCTGTTTACCAA
Mlcy    AAGGAA-TAGAAGGAACTCGGCAAACA---------TGAGCC-CCGCCTGTTTACCAA
Algl    AGGGGA-GAGAAGGAACTCGGCAAACA---------TGAGCC-TCGCCTGTTTACCAA
Ptgi    AAGGGG-AAGAAGGAACTCGGCAAACA---------TGAGCC-TCGCCTGTTTACCAA
Alaf    AGGAGA-GAGAAGGAACTCGGCAAACA---------CGAGCC-TCGCCTGTTTACCAA
Nock    AGAAGA-GAGAAGGAACTCGGCAAACC---------CGAGCC-TCGCCTGTTTACCAA
Anja    AAAGGAGAAGAAGGAACTCGGCAAACA---------CAAACC-CCGCCTGTTTACCAA
Gyki    AAGGAA-GAGAAGGAACTCGGCAAAGC---------AAAGTC-CCGCCTGTTTACCAA
Syka    AAGGAG-AAGAAGGAACTCGGCAAACA---------CAAACC-CCGCCTGTTTACCAA
Opma    AAGGAG-AAGAAGGAACTCGGCAAACA---------TCAACC-CCGCCTGTTTACCAA
Comy    AAGGGG-AAGAAGGAACTCGGCAAACA---------CAAACC-TCGCCTGTTTACCAA
Sasp    AAGGGA-GGGAAGGAACTCGGCAAACA---------TATCCC-CCGCCTGTTTACCAA
Eupe    AAGGAG-AAGAAGGAACTCGGCAAACA---------CAAACC-CCGCCTGTTTACCAA
Enja    AAGAAA-AAAAAGGAACTCGGCAAACC---------CAGACC-TCGCCTGTTTACCAA
Same    AAGAAT-TAAAAGGAACTCGGCAAACT---------AGAACC-TCGCCTGTTTACCAA
Chch    AAGGGA-AGAAAGGAACTCGGCAAACA---------TAAGCC-TCGCCTGTTTACCAA
Grgr    GAGGGG-GGAAAGGAACTCGGCAAACA---------CAAGCC-TCGCCTGTTTACCAA
Caau    AAGAAA-AGGAAGGAACTCGGCAAACA---------CAAGCC-TCGCCTGTTTACCAA
Cyca    AAGAAA-AGGAAGGAACTCGGCAAACA---------CAAGCC-TCGCCTGTTTACCAA
Dare    AAGAAA-GGGAAGGAACTCGGCAAACA---------CAAGCC-TCGCCTGTTTACCAA
Cost    AAGAAA-GGGAAGGAACTCGGCAAACA---------CAAGCC-TCGCCTGTTTACCAA
Leec    AAGAAA-AGGAAGGAACTCGGCAAACA---------TAAGCC-TCGCCTGTTTACCAA
Crla    AAGAAA-AGGAAGGAACTCGGCAAACA---------TAAGCC-TCGCCTGTTTACCAA
Clmc    AAGAAA-AGGAAGGAACTCGGCAAACA---------CAAGCC-TCGCCTGTTTACCAA
Phin    AAAGGAGAGGAAGGAACTCGGCAAACA--------CCAAGCC-CCGCCTGTTTACCAA
Icpu    AAGAAA-AGGAAGGAACTCGGCAAACA---------TAAGCC-TCGCCTGTTTACCAA
Psto    AAGAAA-AGGAAGGAACTCGGCAAACACTTA-----CAAGCC-TCGCCTGTTTACCAA
Cora    AAGAAA-AGGAAGGAACTCGGCAAACA---------CAAGCC-TCGCCTGTTTACCAA
Eisp    AAGAAA-AGGAAGGAACTCGGCAAACA---------CAAGCC-TCGCCTGTTTACCAA
Apal    AAGAAA-AGGAAGGAACTCGGCAAACA---------CAAGCC-TCGCCTGTTTACCAA
Eslu    AAGAAA-GAGAAGGAACTCGGCAAACA---------CAAGCC-TCGCCTGTTTACCAA
Dape    AAGAAA-AAGAAGGAACTCGGCAAACA---------CAAGCC-TCGCCTGTTTACCAA
Glse    AAGGAG-AAGAAGGAACTCGGCAAACT---------CAAGCC-TCGCCTGTTTACCAA
Naar    AAAGAG-AAGAAGGAACTCGGCAAACC---------CAAGCC-TCGCCTGTTTACCAA
Baoc    AAAGAG-AAGAAGGAACTCGGCAAACC---------CAAGCC-TCGCCTGTTTACCAA
Opso    AAGGAG-AAGAAGGAACTCGGCAAATC---------CAAGCC-TCGCCTGTTTACCAA
Alte    AAGGAA-AAGAAGGAACTCGGCAAACA---------CAAGCC-TCGCCTGTTTACCAA
Plap    AAGGAA-AAGAAGGAACTCGGCAAACA---------CAAGCC-TCGCCTGTTTACCAA
```

```
Plal    AAGGGG-GAAAAGGAACTCGGCAAACA---------CAAGCC-TCGCCTGTTTACCAA
Sami    AAGGGG-GACAAGGAACTCGGCAAACA---------CAAGCC-TCGCCTGTTTACCAA
Rere    AAGGAA-GAGAAGGAACTCGGCAAACA---------TGAGCC-TCGCCTGTTTACCAA
Gama    AAGGAG-AAGAAGGAACTCGGCAAACG---------CAAGCC-TCGCCTGTTTACCAA
Onmy    AAGGAA-GAGAAGGAACTCGGCAAACA---------CAAGCC-TCGCCTGTTTACCAA
Sasa    AAGGAA-GAGAAGGAACTCGGCAAACA---------CAAGCC-TCGCCTGTTTACCAA
Cola    AAGGAA-GAGAAGGAACTCGGCAAACA---------CAAGCC-TCGCCTGTTTACCAA
Dita    AAGGGA-GAGAAGGAACTCGGCAAGCA---------CAAGCC-TCGCCTGTTTACCAA
Gogr    AAGGAA-GAGAAGGAACTCGGCAAACA---------TGAGCC-CCGCCTGTTTACCAA
Chsl    AAGGAA-AAAAAGGAACTCGGCAAACA---------TTGGCC-TCGCCTGTTTACCAA
Atja    AAGGAT-GAGAAGGAACTCGGCAAACA---------TAAGCC-TCGCCTGTTTACCAA
Iido    AAGGAC-AAGAAGGAACTCGGCAAACA---------CAAGCC-TCGCCTGTTTACCAA
Auja    AAGAGA-AGGAAGGAACTCGGCAAACC---------CCAGCC-CCGCCTGTTTACCAA
Chag    AAGATA-AGGAAGGAACTCGGCAAACA---------CAAGCC-TCGCCTGTTTACCAA
Hami    AGGGGG-AGGAAGGAACTCGGCAATCA---------AGAGCC-TCGCCTGTTTACCAA
Saun    AAAGAGAAGGAAGGAACTCGGCAAACCCA-------CGAGCC-CCGCCTGTTTACCAA
Nema    AATAAA-GGGAAGGAACTCGGCAAACA---------CAAGCC-TCGCCTGTTTACCAA
Disp    AATGAG-GGGAAGGAACTCGGCAAACA---------CAAGCC-TCGCCTGTTTACCAA
Myaf    ATTAAA-GGGAAGGAACTCGGCAAACA---------CAAGCC-TCGCCTGTTTACCAA
Lagu    AAGAGA-GAGAAGGAACTCGGCAAACC---------CAAGCC-CCGCCTGTTTACCAA
Trtr    AAGAGA-GGGAAGGAACTCGGCAAACA---------CTAGCC-CCGCCTGTTTACCAA
Zucr    AAGAGA-GGGAAGGAACTCGGCAAACA---------CTAGCC-CCGCCTGTTTACCAA
Pxja    AAGAAA-AGGAAGGAACTCGGCAAACA---------CAAGCC-TCGCCTGTTTACCAA
Pxlo    AAGAAA-AGGAAGGAACTCGGCAAACA---------CAAGCC-TCGCCTGTTTACCAA
Pctr    AAGAAA-AAGAAGGAACTCGGCAAACA---------CAAGCC-TCGCCTGTTTACCAA
Apsa    AAGAAA-AAGAAGGAACTCGGCAAACAC------CCCCAGCC-TCGCCTGTTTACCAA
Cabe    ATGAAG-AAAAAGGAACTCGGCAAACT---------AGGGCC-TCGCCTGTTTACCAA
Bzze    AAGAGA-AGGAAGGAACTCGGCAAGCA---------CAAGCC-TCGCCTGTTTACCAA
Siim    AAGGAA-GGGAAGGAACTCGGCAAACA---------AAAGCC-CCGCCTGTTTACCAA
Ctru    AAGAAA-AAGAAGGAATTCGGCAAACA---------CAAGCC-TCGCCTGTTTACCAA
Dpbr    AAGGAA-AAGAAGGAACTCAGCAAACA---------CAAGCC-TCGCCTGTTTACCAA
Caki    AAGGAA-AGAAAGGAACTCGGCAAACT---------CAAGCC-TCGCCTGTTTACCAA
Phja    AAGAGA-AGGAAGGAACTCGGCAAACA---------CAAGCC-TCGCCTGTTTACCAA
Brsp    AAAGGAGAAGAAGGAACTCGGCAAACC---------TAAGCC-CCGCCTGTTTACCAA
Gamo    AAGAGA-AGGAAGGAACTCGGCAAACA---------CAAGCC-TCGCCTGTTTACCAA
Lolo    AAGAGA-AGGAAGGAACTCGGCAAACA---------CAAGCC-TCGCCTGTTTACCAA
Batr    AAAGAAGAGGAAGGAACTCGGCAAACC---------ATAGCC-TCGCCTGTTTACCAA
Prmy    AAGAAA-ATAAAGGAACTCGGCAAACA---------TAAATC-CCGCCTGTTTACCAA
Loli    AAAGATGGGGAAGGAACTCGGCAAACACC-------CAAGCC-TCGCCTGTTTACCAA
Loam    AAGGGG-AAGAAGGAACTCGGCAAACACACC-----CAAGCC-TCGCCTGTTTACCAA
Chab    AAGAAA-GAGAAGGAACTCGGCAAACACAT------AAAGCC-TCGCCTGTTTACCAA
Chto    AAGAAA-GAGAAGGAACTCGGCAAACACAT------AAAGCC-TCGCCTGTTTACCAA
Majo    AAGAAA-AAGAAGGAACTCGGCAAATCAAA---T--GAAGCC-TCGCCTGTTTACCAA
Hlst    AAGGAA-GAGAAGGAACTCGGCAAATC---------CTAGCC-CCGCCTGTTTACCAA
Clpe    AAGAAA-GAGAAGGAACTCGGCAAACACAT------AAGGCC-TCGCCTGTTTACCAA
Mlmr    AAGAAA-GAGAAGGAACTCGGCAAACATACA----TTAAGCC-TCGCCTGTTTACCAA
Crcr    AAGGAA-AAGAAGGAACTCGGCAAACACACTC--CATAAGCC-TCGCCTGTTTACCAA
Muce    AAGGAA-AAGAAGGAACTCGGCAAACACACTC-CA-CAAGCC-TCGCCTGTTTACCAA
Bege    AAGAAA-AAGAAGGAACTCGGCAAAATTATC---CACAAGCC-TCGCCTGTTTACCAA
Mela    AAGAAA-AAGAAGGAACTCGGCAAACATA------ATTGGCC-TCGCCTGTTTACCAA
Hats    AAGAAA-AAGAAGGAACTCGGCAAAAATTCCT----CGAGCC-TCGCCTGTTTACCAA
Orla    AAGGAA-GAGAAGGAACTCGGCAAATATA------CCAAGCC-TCGCCTGTTTACCAA
```

Cosa	AAGAAA-AAGAAGGAACTCGGCAAACATAAAAC---CAAGCC-TTGCCTGTTTACCAA
Exsp	AAGGAG-AAGAAGGAACTCGGCAAACATATTT----ACGGCC-TCGCCTGTTTACCAA
Depa	AAGAAA-AAGAAGGAACTCGGCAAATA---------AAAGCC-TCGCCTGTTTACCAA
Rima	AAGGAG-CATAAGGAACTCGGCAAACACGCAC-A--CAAGCC-TCGCCTGTTTACCAA
Fuol	AAGGGG-AAGAAGGAACTCGGCAAATACCCAA-T--TAAGCC-TCGCCTGTTTACCAA
Gmaf	AAGGGG-GAGAAGGAACTCGGCAAACTAAACC----AAAGCC-TCGCCTGTTTACCAA
Xeei	AAAGAG-AAGAAGGAACTCGGCAAACTCTGCT---CTAAGCC-TCGCCTGTTTACCAA
Pros	AAGAGA-AAAAAGGAACTCGGCAAACA---------TAAGCC-TCGCCTGTTTACCAA
Scmi	AAGAGA-AAAAAGGAACTCGGCAAACA---------TAAGCC-TCGCCTGTTTACCAA
Rolo	AAGAAA-AAGAAGGAACTCGGCAAACA---------CAAGCC-TCGCCTGTTTACCAA
Cere	AAGGAA-AAGAAGGAACTCGGCAAACG---------CAAGCC-CCGCCTGTTTACCAA
Daga	AAGGAA-GAGAAGGAACTCGGCAAGCA---------AAAGCC-CCGCCTGTTTACCAA
Anco	AAGAAA-AAGAAGGAACTCGGCAAACC---------CAGGCC-TCGCCTGTTTACCAA
Dmve	AAAGAGAAAGAAGGAACTCGGCAAACC---------TGGGCT-CCGCCTGTTTACCAA
Dmar	AAGAAA-AAGAAGGAACTCGGCAAACC---------TGGGCT-CCGCCTGTTTACCAA
Anka	AAGAAA-GAGAAGGAACTCGGCAAACC---------CAGGCC-TCGCCTGTTTACCAA
Moja	AAGAAA-AAGAAGGAACTCGGCAAACC---------CAGGCC-TCGCCTGTTTACCAA
Hoja	AAGAAA-AAGAAGGAACTCGGCAAACC---------CAGGCC-TCGCCTGTTTACCAA
Bede	AAGAGA-AAAAAGGAACTCGGCAAACA---------CAAGCC-TCGCCTGTTTACCAA
Besp	AAGAGA-AAAAAGGAACTCGGCAAACA---------CAAGCC-TCGCCTGTTTACCAA
Mysp	AAGAAA-AAGAAGGAACTCGGCAAACA---------CAGGCC-TCGCCTGTTTACCAA
Osja	AAGAAA-AAGAAGGAACTCGGCAAACA---------CAAGCC-TCGCCTGTTTACCAA
Sgro	AAGAAT-AAGAAGGAACTCGGCAAACA---------CAAGCC-TCGCCTGTTTACCAA
Pzpa	AAAGAAAAAGAAGGAACTCGGCAAACC---------AAAGCC-TCGCCTGTTTACCAA
Zeja	AAGAAA-AAGAAGGAACTCGGCAAACC---------AAAGCC-TCGCCTGTTTACCAA
Znne	AAGAAA-AAGAAGGAACTCGGCAAACT--------ATCAGCC-TCGCCTGTTTACCAA
Zefa	AAGAAA-AAGAAGGAACTCGGCAAAC---------TAAAGCC-TCGCCTGTTTACCAA
Acni	AAGAAA-AAGAAGGAACTCGGCAAAC---------CAAAGCC-TCGCCTGTTTACCAA
Ncrh	AAGAAA-AAGAAGGAACTCGGCAAAC---------CAAAGCC-TCGCCTGTTTACCAA
Agca	AAGAAA-GAAAAGGAACTCGGCAAACACA-------CAAGCC-TCGCCTGTTTACCAA
Hydy	AAGGAA-AAGAAGGAACTCGGCAAACA---------TAAGCCCTCGCCTGTTTACCAA
Gsac	AAGAAA-GAGAAGGAACTCGGCAAACACAT------AAAGCC-TCGCCTGTTTACCAA
Pevo	AAGGAA-AAGAAGGAACTCGGCAAAC---A------CAAGCC-TCGCCTGTTTACCAA
Hiku	AAGGGT-GAGAAGGAACTCGGCAAACATAAT-AACACTAGCC-TCGCCTGTTTACCAA
Inpa	AAAAAA-GAAAAGGAACTCGGCAACCCCA------CCAAGCC-CCGCCTGTTTACCAA
Auch	AAGAAG-GAGAAGGAACTCGGCAAACACATAT-TCTGAAGCC-CCGCCTGTTTACCAA
Fico	AAGGGA-AAGAAGGAACTCGGCAAAACA-------CCAAGCC-TCGCCTGTTTACCAA
Macs	AAGAGA-GAGAAGGAACTCGGCAAACATATTT----GAAGCC-TCGCCTGTTTACCAA
Moal	ACCAAT-GAGAAGGAACTCGGCAAACACCT------CAGGCC-TCGCCTGTTTACCAA
Syma	AATAAAGAGAAGGGAATTCAGCAAACA---------TAGGCT-CCGCCTGTTTACCAA
Mafr	AAGAAA-AAGAAGGAACTCGGCAAACATACCTT--ATAAGCC-TCGCCTGTTTACCAA
Dcpe	AAGAAA-GAGAAGGAACTCGGCAAACA-------CCAAAGCC-TCGCCTGTTTACCAA
Dcti	AAGAAA-GAGAAGGAACTCGGCAAAC----AC---CTAAGCC-TCGCCTGTTTACCAA
Hehi	AAGAAA-GAGAAGGAACTCGGCAAACAAAT------CAAGCC-TCGCCTGTTTACCAA
Stam	AAAGAAAGAGAAGGAACTCGGCAAACACATTT----AAAGCC-TCGCCTGTTTACCAA
Hogi	AAGAAA-AAGAAGGAACTCGGCAAACA------CACCAAGCC-TCGCCTGTTTACCAA
Erzo	AAGAAA-GAGAAGGAACTCGGCAAACATAC------CAAGCC-TCGCCTGTTTACCAA
Hxot	AAGAAA-GAGAAGGAACTCGGCAAACACAT------AAAGCC-TCGCCTGTTTACCAA
Core	AAGAAA-GAGAAGGAACTCGGCAAACA---------CAAGCC-TCGCCTGTTTACCAA
Apve	AAAAAAAGAGAAGGAACTCGGCAAACACC-------TAAGCC-TCGCCTGTTTACCAA
Latj	AAGAAA-AAGAAGGAACTCGGCAAACA---------TAAGCC-TCGCCTGTTTACCAA
Laja	AAGAAA-AAGAAGGAACTCGGCAAACAC------ATGAAGCC-TCGCCTGTTTACCAA

```
Syja    AAGAAA-AAGAAGGAACTCGGCAAACA-------CATAAGCC-TCGCCTGTTTACCAA
Epme    AAGAAA-GAGAAGGAACTCGGCAAACACA------CTCAGCC-TCGCCTGTTTACCAA
Grse    AAGGAA-AAGAAGGAACTCGGCAAACACCCAT---TAAAGCC-TCGCCTGTTTACCAA
Clja    AAGAAG-GAGAAGGAACTCGGCAAACA---------CTGGCC-TCGCCTGTTTACCAA
Ogcy    AAGAAA-AAGAAGGAACTCGGCAAACATATCT-CTTAAAGCC-TCGCCTGTTTACCAA
Plna    AAGAAA-AAGAAGGAACTCGGCAAATATACCCT-ACTAGGCC-TCGCCTGTTTACCAA
Lema    AAGAAA-GAGAAGGAACTCGGCAAACACAT------GAAGCC-TCGCCTGTTTACCAA
Etzo    AAGAAA-GAGAAGGAACTCGGCAAACACA------TCAAGCC-TCGCCTGTTTACCAA
Apse    AAGAAA-AAGAAGGAACTCGGCAAACACTT------CTAGCC-TCGCCTGTTTACCAA
Epde    AAAGAAAAAGAAGGAACTCGGCAAACACA------TAAAGCC-TCGCCTGTTTACCAA
Slja    AAGAAA-AAGAAGGAACTCGGCAAACACC------TAGAGCC-TCGCCTGTTTACCAA
Bsja    AAGAAA-GAGAAGGAACTCGGCAAA----------TAAAGCC-TCGCCTGTTTACCAA
Ecna    AAGAAT-GAGAAGGAACTCGGCAAACAC------ATGAAGCC-TCGCCTGTTTACCAA
Cohi    AAGAAG-GAGAAGGAACTCGGCAAA----------ACAAGCC-TCGCCTGTTTACCAA
Caar    AAGAAA-GAGAAGGAACTCGGCAAACAAA----TTCCAAGCC-TCGCCTGTTTACCAA
Came    AAAGAAAGAGAAGGAACTCGGCAAACAT----TTCTCAAGCC-TCGCCTGTTTACCAA
Mema    AAGAAA-GAGAAGGAACTCGGCAAACA------CCTAAAACC-CCGCCTGTTTACCAA
Lenu    AAAAGA-AGGAAGGAACTCGGCAAACAAATCCACCTAAAGCC-TCGCCTGTTTACCAA
Brja    AAGAAG-GAGAAGGAACTCGGCAAACC-----AACCCCAGCC-TCGCCTGTTTACCAA
Plma    AAAGAAGAAGAAGGAACTCGGCAAACATA----ACTTAAGCC-TCGCCTGTTTACCAA
Emst    AGGAAA-GAGAAGGAACTCGGCAAACA------CATGAAGCC-TCGCCTGTTTACCAA
Ptti    AAGAAA-GAGAAGGAACTCGGCAAACAC-----ATAGAAGCC-TCGCCTGTTTACCAA
Losu    CAGAAA-GAGAAGGAACTCGGCAAACA--------TTTAGCC-TCGCCTGTTTACCAA
Geoy    AAGAAA-GAGAAGGAACTCGGCAAACAC------ATGAAGCC-TCGCCTGTTTACCAA
Dipi    AAGAAA-AAGAAGGAACTCGGCAAACACA------TGAAGCC-TCGCCTGTTTACCAA
Pama    AAGGGA-AGGAAGGAACTCGGCAAACACT--T----AAAGCC-TCGCCTGTTTACCAA
Leob    AAGAAA-GAGAAGGAACTCGGCAAACATC-------TGAGCC-TCGCCTGTTTACCAA
Neba    AAGAAA-AAGAAGGAACTCGGCAAAAAAC-------CTAGCC-TCGCCTGTTTACCAA
Pdpl    AAGAAA-AAGAAGGAACTCGGCAAACACTAAC----TAAGCC-TCGCCTGTTTACCAA
Nimi    CAGAAA-AAGAAGGAACTCGGCAAACACA------TAAAGCC-TCGCCTGTTTACCAA
Uptr    AAGAGA-AAGAAGGAACTCGGCAAATTAA------ATAAGCC-TCGCCTGTTTACCAA
Pesc    AAGAAA-GAGAAGGAACTCGGCAAACAC--------TATGCC-TCGCCTGTTTACCAA
Baar    AAGAAA-AAGAAGGAACTCGGCAAACACATAA---TTTAGTC-CCGCCTGTTTACCAA
Moar    AAGAAA-GAGAAGGAACTCGGCAAACACAT------AAAGCC-TCGCCTGTTTACCAA
Toja    AAGAAA-AAGAAGGAACTCGGCAAACACAT------GAAGCC-TCGCCTGTTTACCAA
Chau    AAGAAA-AAGAAGGAACTCGGCAAAT---------CAAGACC-CCGCCTGTTTACCAA
Chse    AAGAAA-GAAAAGGAACTCGGCAAACA------CACTTGGCC-TCGCCTGTTTACCAA
Enar    AAGAAAAAAGAAGGAACTCGGCAAACA------CATGAAGCC-TCGCCTGTTTACCAA
Hpty    AAGAAA-AAGAAGGAACTCGGCAAACAC------ACAAAGCC-TCGCCTGTTTACCAA
Nana    AAGAAA-AAGAAGGAACTCGGCAAACAC------ATAAAGCC-TCGCCTGTTTACCAA
Mcst    AAGAAA-AAGAAGGAACTCGGCAAACA------CATAAAGCC-TCGCCTGTTTACCAA
Rhox    AAGAGA-AAGAAGGAACTCGGCAAACAC-------ATTGGCC-TCGCCTGTTTACCAA
Opfa    AAGAAA-AAGAAGGAACTCGGCAAACAC--------AAAGCC-TCGCCTGTTTACCAA
Paar    AAGGAA-AAGAAGGAACTCGGCAAACCC--------ACAGCC-TCGCCTGTTTACCAA
Gozo    AAGAAA-GAGAAGGAACTCGGCAAACACA------TGAAGCC-TCGCCTGTTTACCAA
Ackr    AAGGGA-GAGAAGGAACTCGGCAAACA---------CATGCC-TCGCCTGTTTACCAA
Elev    AAAGAA-AAGAAGGAACTCGGCAAACA---------CAAGCC-CCGCCTGTTTACCAA
Trdu    AAGAAA-AAGAAGGAACTCGGCAAACA---------CAAGCC-TCGCCTGTTTACCAA
Amoc    AAGAAA-AAGAAGGAACTCGGCAAT--CATAA----TAAGCC-TCGCCTGTTTACCAA
Hame    AAGAGA-AAGAAGGAACTCGGCAAACACC------TCAAGCC-TCGCCTGTTTACCAA
Chso    AAAGGA-GAGAAGGAACTCGGCAAACATC-----CACAAGCC-TCGCCTGTTTACCAA
Lyto    AAGAAA-GAGAAGGAACTCGGCAAACACACA-----CAAGCC-TCGCCTGTTTACCAA
```

```
Encr    AAGAGA-GAGAAGGAACTCGGCAAACACA-----TCAAAGCC-TCGCCTGTTTACCAA
Bvar    AAGAAA-GAGAAGGAACTCGGCAAAC--------ATAAAGCC-TCGCCTGTTTACCAA
Noco    AAGGGG-GAGAAGGAACTCGGCAAACA------CATAAAGCC-TCGCCTGTTTACCAA
Chsp    AAGGGG-AAGAAGGAACTCGGCAAAC---------ATAAGCC-TCGCCTGTTTACCAA
Arja    AAGAAA-GAGAAGGAACTCGGCAAACA------CATTAAGCC-TCGCCTGTTTACCAA
Pase    AAGGAA-AAGAAGGAACTCGGCAAACT------TATCAAGCC-CCGCCTGTTTACCAA
Trel    AAGAAT-AAGAAGGAACTCGGCAAACTC------CCCTAGCC-CCGCCTGTTTACCAA
Lifa    AAGGAA-GAGAAGGAACTCGGCAAACAC------CTAAAGCC-TCGCCTGTTTACCAA
Acur    AAGGAA-GAGAAGGAACTAGGCAAA-------CACCTTAGCC-TCGCCTGTTTACCAA
Ampe    AAGAAA-AAGAAGGAACTCGGCAAACA------CATGAAGCC-TCGCCTGTTTACCAA
Urja    AAAAAG-AAGAAGGAACTCGGCAAACA-------CCCAAGCC-TCGCCTGTTTACCAA
Enet    AAGGGA-AAGAAGGAACTCGGCAAAC---------CCCAGCC-TCGCCTGTTTACCAA
Ptbr    AAGAGA-AAGAAGGAACTCGGCAAACTTTAT-----CAAGCC-TCGCCTGTTTACCAA
Safa    AAAGAG-AGGAAGGAACTCGGCAAG-------ATAACAAGCC-TCGCCTGTTTACCAA
Icae    AAGAAG-AAGAAGGAACTCGGCAAACATA------CCAAGCC-TCGCCTGTTTACCAA
Asmi    AAGAAA-AGGAAGGAACTCGGCAAAC---------ACAAGCC-TCGCCTGTTTACCAA
Foal    AAAGGT-TGGAAGGAACTCGGCAAAC---------CCAAGCC-TCGCCTGTTTACCAA
Drze    AAGAGA-AAGAAGGAACTCGGCAAATAT-------TGTAGCC-TCGCCTGTTTACCAA
Rhas    AGGAAA-AAGAAGGAACTCGGCAAA---------CATTAGCC-TCGCCTGTTTACCAA
Elac    AAGAAA-AAGAAGGAACTCGGCAAA---------CACTGGCC-TCGCCTGTTTACCAA
Kugu    AAGAAA-AAGAAGGAACTCGGCAAACA------CCTCTAGCC-CCG-CTGTTTACCAA
Plor    AAGAAAAGAGAAGGAACTCGGCAAACA-------TATACGCC-TCGCCTGTTTACCAA
Sgun    AAGAAA-GAGAAGGAACTCGGCAAACAC------ATGAAGCC-TCGCCTGTTTACCAA
Zaco    AAGAAA-GAGAAGGAACTCGGCAAACAC------ATGAAGCC-TCGCCTGTTTACCAA
Zbfl    AAGAAA-GAGAAGGAACTCGGCAAACAC------TACAAGCC-TCGCCTGTTTACCAA
Spba    AAGAAA-AAGAAGGAATTCAGCAAACCCA----TTACTAGCC-TCGCCTGTTTACCAA
Game    AAGAAA-AAGAAGGAACTCGGCAAACAC------ACCAAGCC-TCGCCTGTTTACCAA
Thth    AAGAAA-AAGAAGGAACTCGGCAAACA------TACCAAGCC-TCGCCTGTTTACCAA
Xigl    AAAGAAAGAGAAGGAACTCGGCAAACA------CATGAAGCCCTCGCCTGTTTACCAA
Hyja    AAGAAA-AAGAAGGAACTCGGCAAACACT------AGAAGCC-TCGCCTGTTTACCAA
Psan    AAGAAA-AAGAAGGAACTCGGCAAATA-------TTAAAGCC-TCGCCTGTTTACCAA
Cupa    AAGAAA-AAGAAGGAACTCGGCAAACAT----TTTCCAAGCC-TCGCCTGTTTACCAA
Mpch    AAGAAA-AAGAAGGAACTCGGCAAACA---------TAAGCC-TCGCCTGTTTACCAA
Char    AAGAAA-AAGAAGGAACTCGGCAAACACC------CTAAGCC-TCGCCTGTTTACCAA
Pser    AGGAAA-AAGAAGGAACTCGGCAAATACACAA----CAAGCC-TCGCCTGTTTACCAA
Prol    AGGGGG-GAGAAGGAACTCGGCAAACATACC----CCAAGCC-TCGCCTGTTTACCAA
Plbi    AGGGGG-GAGAAGGAACTCGGCAAACATACCC----CAAGCC-TCGCCTGTTTACCAA
Calu    AAGAGA-GAGAAGGAACTCGGCAAACAA------ATGAAGCC-TCGCCTGTTTACCAA
Papa    AAGGAC-GGGAAGGAACTCGGCAAATA--------CTTAGCC-TCGCCTGTTTACCAA
Sufr    AAGGGA-GAGAAGGAACTCGGCAAA---------CACAAGCC-TCGCCTGTTTACCAA
Stci    AAGAGA-AAGAAGGAACTCGGCAAACA---------CAAGCT-TCGCCTGTTTACCAA
Taru    AAGAAA-AAGAAGGAACTCGGCAAACT--------CAAAGCC-TCGCCTGTTTACCAA
Rala    AAGAAA-AAGAAGGAACTCGGCAAACATA-----CTCAAGCC-TCGCCTGTTTACCAA
                  * **** *   ****                    * ***********
```

```
      34'      35      ¦                    HVR                      ¦
Scca  AAACACCGCCTCTTGCTTA-------------------------------------------
Muma  AAACATCGCCTCTTGTT-A-AA----------------------------------------
Erca  AAACATCGCCTTCAGCTAAC------------------------------------------
Pose  AAACATCGCCTTCAGCTTT-------------------------------------------
Actr  AAACATCGCCTCTTGCAAA-------------------------------------------
Scal  AAACATCGCCTCTTGCAAACC-----------------------------------------
Posp  AAACATCGCCTCTTGCAAACAA----------------------------------------
Atsp  AAACATCGCCTCTTGCAAACACCA--------------------------------------
Leoc  AAACATCGCCTCTTGCNAACA-----------------------------------------
Amca  AAACATCGCCTCTTGCAATCC-----------------------------------------
Osbi  AAACATGGCCTCTTGCAAACCCCC--------------------------------------
Pabu  AAACATGGCCTCTTGC--AACC----------------------------------------
Hial  AAACATCGCCTCTTGCAAAAA-----------------------------------------
Elha  AAACATCGCCTCTTGCAAAACA----------------------------------------
Mlcy  AAACATCGCCTCTTGCGAACAA----------------------------------------
Algl  AAACATCGCCTCTTGCTAAAA-----------------------------------------
Ptgi  AAACATCGCCTCTTGCTAAA------------------------------------------
Alaf  AAACATCGCCTCTTGCTAAAAA----------------------------------------
Nock  AAACATCGCCTCTTGCTAAAA-----------------------------------------
Anja  AAACATCGCCTCTTGCTAACAA----------------------------------------
Gyki  AAACATCGCCTCTTGCTAATA-----------------------------------------
Syka  AAACATCGCCTCTTGCCAACAA----------------------------------------
Opma  AAACATCGCCTCTTGCTAAGA-----------------------------------------
Comy  AAACACCGCCTCCTGC-AAATA----------------------------------------
Sasp  AAACATCGCCTCTTGCTAATAA----------------------------------------
Eupe  AAACACCGCCTCTTGCAACAAC----------------------------------------
Enja  AAACATCGCCTCTTGCA--T------------------------------------------
Same  AAACATCGCCTCCTGCCACCAC----------------------------------------
Chch  AAACATCGCCTTCTGCCAA-AA----------------------------------------
Grgr  AAACATCGCCTCCTGCAAAAA-----------------------------------------
Caau  AAACATCGCCTCCTGCAACACA----------------------------------------
Cyca  AAACATCGCCTCCTGCAACA------------------------------------------
Dare  AAACATCGCCTCCTGACAT-------------------------------------------
Cost  AAACATCGCCTCCTGCA-AA------------------------------------------
Leec  AAACATCGCCTCCTGCAAACC-----------------------------------------
Crla  AAACATCGCCTCCTGCAAACC-----------------------------------------
Clmc  AAACATCGCCTCCCGCAAAAA-----------------------------------------
Phin  AAACATCGCCTCCTGCTAACCCTT--------------------------------------
Icpu  AAACATCGCCTCCTGCAAAAA-----------------------------------------
Psto  AAACATCGCCTCCTGCAAAAA-----------------------------------------
Cora  AAACATCGCCTCCCGCAAAAAT----------------------------------------
Eisp  AAACATCGCCTCCCGCAAAA------------------------------------------
Apal  AAACATCGCCTCCTGCAAAAAA----------------------------------------
Eslu  AAACACCGCCTCTTGT---CT-----------------------------------------
Dape  AAACATCGCCTCTTGC--CAAC----------------------------------------
Glse  AAACATCGCCTCTTGCAA---TCC--------------------------------------
Naar  AAACATCGCCTCTTGC-AAAT-----------------------------------------
Baoc  AAACATCGCCTCTTGCAAATTT----------------------------------------
Opso  AAACATCGCCTCTTGC---A------------------------------------------
Alte  AAACACCGCCTCCTGCAAATCA----------------------------------------
Plap  AAACACCGCCTCCTGCAAAT------------------------------------------
```

```
Plal    AAACATCGCCTCTTGATCACA-----------------------------------------
Sami    AAACATCGCCTCTTGA-GA--A----------------------------------------
Rere    AAACATCGCCTCTTGA--ATA-----------------------------------------
Gama    AAACATCGCCTCTTGGTA--------------------------------------------
Onmy    AAACATCGCCTCTTGC--AAAT----------------------------------------
Sasa    AAACATCGCCTCTTGTAAATC--A--------------------------------------
Cola    AAACATCGCCTCTTGCAAA--T----------------------------------------
Dita    AAACATCGCCTCTTGCTGAAC-----------------------------------------
Gogr    AAACATCGCCTCTTGCCTAAA-----------------------------------------
Chsl    AAACATCGCCTCTTGTAACACCCTCATAAGAGGTCCTGCCTGCCCAGTGACAATTGTTAAAC
Atja    AAACATCGCCTCTTGCGAACCA----------------------------------------
Iido    AAACATCGCCTCTTGC-AAAC-----------------------------------------
Auja    AAACATCGCCTCTTGCAAAATC----------------------------------------
Chag    AAACATCGCCTCTTGT--AAAC----------------------------------------
Hami    AAACATCGCCTCTTGC--AAAA----------------------------------------
Saun    AAACATCGCCTCTTGCAAGATC----------------------------------------
Nema    AAACATCGCCTCTTGCAAAAA-----------------------------------------
Disp    AAACATCGCCTCTTGCAAAAC-----------------------------------------
Myaf    AAACATCGCCTCTTGCAAAATG----------------------------------------
Lagu    AAACACCGCCTCTTGC-AA-------------------------------------------
Trtr    AAACATCACCTCTTGC-AAT-T----------------------------------------
Zucr    AAACATCACCTCTTGCAAG-------------------------------------------
Pxja    AAACATCGCCTCTTGT-A-TAT----------------------------------------
Pxlo    AAACATCGCCTCTTGT---A------------------------------------------
Pctr    AAACATCGCCTCTTGTAAC--A----------------------------------------
Apsa    AAACACCGCCTCTTGC-AAAAC----------------------------------------
Cabe    AAACATCGCCTCCCGC-GCA-C----------------------------------------
Bzze    AAACATCGCCTCTTGC-AAA-G----------------------------------------
Siim    AAACATCGCCTCTTGC-AAA-A----------------------------------------
Ctru    AAACATCGCCTCTTGCAAAAAT----------------------------------------
Dpbr    AAACATCGCCTCTTGCAAA--A----------------------------------------
Caki    AAACATCGCCTCTTGACCCAC-----------------------------------------
Phja    AAACATCGCCTCTTGCTTAAG-----------------------------------------
Brsp    AAACATCGCCTCTCGACTC-------------------------------------------
Gamo    AAACATCGCCTCTTGCTCC-------------------------------------------
Lolo    AAACATCGCCTCTTGAC---------------------------------------------
Batr    AAACATCGCTTCTTGCAC--------------------------------------------
Prmy    AAACATCGCTTCTTGA----------------------------------------------
Loli    AAACATCGCCTCTTGTACCCTA----------------------------------------
Loam    AAACATCGCCTCTTGTAATT------------------------------------------
Chab    AAACATCGCCTCTTGCATAAA-----------------------------------------
Chto    AAACATCGCCTCTTGCATAA------------------------------------------
Majo    AAACATCGCCTCTTGCCACAA-----------------------------------------
Hlst    AAACATCGCCTCTTGTAATA------------------------------------------
Clpe    AAACATCGCCTCTTGCAATCAG----------------------------------------
Mlmr    AAACATCGCCTCTTGCAATCA-----------------------------------------
Crcr    AAACATCGCCTCTTGTAAACC-----------------------------------------
Muce    AAACATCGCCTCTTGTTA--------------------------------------------
Bege    AAACATCGCCTCTTGTCA--------------------------------------------
Mela    AAACATCGCCTCTTGATCCCCT----------------------------------------
Hats    AAACATCGCCTCTTGTCAA-A-----------------------------------------
Orla    AAACATCGCCTCTTGTAAAG------------------------------------------
```

```
Cosa    AAACACCGCCTCTTGCAA--T-----------------------------------------
Exsp    AAACACCGCCTCTTGCTAAAA-----------------------------------------
Depa    AAACACCGCCTCTTGCAACTC-----------------------------------------
Rima    AAACATCGCCTCTTGTAAT-------------------------------------------
Fuol    AAACATCGCCTCTTGAATTAA-----------------------------------------
Gmaf    AAACATCGCCTTTTGAAC-C------------------------------------------
Xeei    AAACATCGCCTCTTGAAAATCC----------------------------------------
Pros    AAACATCGCCTCTTGTAGAC------------------------------------------
Scmi    AAACACCGCCTCTTGCCAACA-----------------------------------------
Rolo    AAACATCGCCTCTTGCAACAA-----------------------------------------
Cere    AAACATCGCCTCTTGT-AAA-A----------------------------------------
Daga    AAACATCGCCTCTTGCAGCAA-----------------------------------------
Anco    AAACATCGCCTCTTGCAAAA-ACA--------------------------------------
Dmve    AAACATCGCCTCTTGCAAACAA----------------------------------------
Dmar    AAACATCGCCTCTTGC-AACT-----------------------------------------
Anka    AAACATCGCCTCTTGCAAAAA-----------------------------------------
Moja    AAACATCGCCTCTTGCA-A-AA----------------------------------------
Hoja    AAACATCGCCTCTTGCAAA--A----------------------------------------
Bede    AAACATCGCCTCTTGCA---------------------------------------------
Besp    AAACATCGCCTCTTGC-AAAAC----------------------------------------
Mysp    AAACATCGCCTCTTGCAAAAA-----------------------------------------
Osja    AAACATCGCCTCTTGCA--A------------------------------------------
Sgro    AAACATCGCCTCTTGCAAAAA-----------------------------------------
Pzpa    AAACATCGCCTCTTGAACTAT-----------------------------------------
Zeja    AAACATCGCCTCTTGAATAAT-----------------------------------------
Znne    AAACATCGCCTCTTGA---AT-----------------------------------------
Zefa    AAACATCGCCTCTTGA-ATAAA----------------------------------------
Acni    AAACATCGCCTCTTGAAT--------------------------------------------
Ncrh    AAACATCGCCTCTTGAATA-------------------------------------------
Agca    AAACATCGCCTCTTGCAATCA-----------------------------------------
Hydy    AAACATCGCCTCTTGAAAC-------------------------------------------
Gsac    AAACATCGCCTCTTGTAT--TC----------------------------------------
Pevo    AAACATCGCCTCTTGC-AG-A-----------------------------------------
Hiku    AAACACCGCCTCTTGCATA-AC----------------------------------------
Inpa    AAACATCGCCTCTTGACT--------------------------------------------
Auch    AAACACCGCCTCTTGCTGAT------------------------------------------
Fico    AAACATCGCCTCTTGCTAAAAC----------------------------------------
Macs    AAACATCGCCTCTTGC--AAAA----------------------------------------
Moal    AAACATCACCTCCTGCAAACTT----------------------------------------
Syma    AAACATCGCCTCTTGCCCCCCC-C--------------------------------------
Mafr    AAACATCGCCTCTTGCAAC--A----------------------------------------
Dcpe    AAACATCGCCTCTTGC-AAAT-----------------------------------------
Dcti    AAACATCGCCTCTTGCAAAAT-----------------------------------------
Hehi    AAACATCGCCTCTTGCAAA-------------------------------------------
Stam    AAACATCGCCTCTTGCAAAAAT----------------------------------------
Hogi    AAACATCGCCTCTTGT--AAC-----------------------------------------
Erzo    AAACATCGCCTCTTGCAAAC-T----------------------------------------
Hxot    AAACATCGCCTCTTGCAAAC-T----------------------------------------
Core    AAACATCGCCTCTTGTATCAT-----------------------------------------
Apve    AAACATCGCCTCTTGCAAACCC----------------------------------------
Latj    AAACATCGCCTCTTGTACAACC----------------------------------------
Laja    AAACATCGCCTCTTGCAAAAC-----------------------------------------
```

```
Syja    AAACATCGCCTCTTGCAAAAA-----------------------------------------
Epme    AAACACCGCCTCTTGC-TAAAC----------------------------------------
Grse    AAACATCGCCTCTTGCGACAA-----------------------------------------
Clja    AAACACCGCCTCTTGCAA-AT-----------------------------------------
Ogcy    AAACATCGCCTCTTGTCCAC------------------------------------------
Plna    AAACATCGCCTCTTGCTT-TAC----------------------------------------
Lema    AAACATCGCCTCTTGCTGAAA-----------------------------------------
Etzo    AAACATCGCCTCTTGTAAGAC-----------------------------------------
Apse    AAACATCGCCTCTTGC---AA-----------------------------------------
Epde    AAACATCGCCTCTTGCAAA-AC----------------------------------------
Slja    AAACATCGCCTCTTGC--A-AA----------------------------------------
Bsja    AAACATCGCCTCTTGC-AAAAT----------------------------------------
Ecna    AAACATCGCCTCTTGCAAAAAT----------------------------------------
Cohi    AAACATCGCCTCTTGC-AAAAATA--------------------------------------
Caar    AAACATCGCCTCTTGCAAA--A----------------------------------------
Came    AAACATCGCCTCTTGCAAAACC----------------------------------------
Mema    AAACATCGCCTCTTGC-AAT-A----------------------------------------
Lenu    AAACATCGCCTCTTGC-TA--A----------------------------------------
Brja    AAACATCGCCTCTTGCCA---G----------------------------------------
Plma    AAACATCGCCTCTTGCAATG-C----------------------------------------
Emst    AAACATCGCCTCTTGC-AAAA-----------------------------------------
Ptti    AAACATCGCCTCTTGCAAAAT-----------------------------------------
Losu    AAACATCGCCTCTTGTAAGCAA----------------------------------------
Geoy    AAACATCGCCTCTTGCA--A------------------------------------------
Dipi    AAACATCGCCTCTTGCAAAAA-----------------------------------------
Pama    AAACATCGCCTCTTGC-AATAA----------------------------------------
Leob    AAACATCGCCTCTTGC-AAAAT----------------------------------------
Neba    AAACATCGCCTCTTGCAAA-------------------------------------------
Pdpl    AAACATCGCCTCTTGC-TA-------------------------------------------
Nimi    AAACATCGCCTCTTGCAATCA-----------------------------------------
Uptr    AAACATCGCCTCTTGC-A--A-----------------------------------------
Pesc    AAACATCGCCTCTTGCAAA-A-----------------------------------------
Baar    AAACACCGCCTTTTGCAAAT-CCA--------------------------------------
Moar    AAACATCGCCTCTTGCAAAAT-----------------------------------------
Toja    AAACATCGCCTCTTGTAAAC------------------------------------------
Chau    AAACACCGCCTCTTGC--AAAA----------------------------------------
Chse    AAACATCGCCTCTTGC--AAA-----------------------------------------
Enar    AAACATCGCCTCTTGCAAAAT-----------------------------------------
Hpty    AAACATCGCCTCTTGCAAA-A-----------------------------------------
Nana    AAACACCGCCTCTTGCAAAC------------------------------------------
Mcst    AAACATCGCCTCTTGC-AAAA-----------------------------------------
Rhox    AAACATCGCCTCTTGC-AAAGC----------------------------------------
Opfa    AAACATCGCCTCTTGC-AAA-A----------------------------------------
Paar    AAACACCGCCTCTTGCA--A------------------------------------------
Gozo    AAACATCGCCTCTTGC--AAAA----------------------------------------
Ackr    AAACATCGCCTCTTGCAAAAT-----------------------------------------
Elev    AAACATCGCCTCTTGAACCC------------------------------------------
Trdu    AAACATCGCCTCTTGAACC-------------------------------------------
Amoc    AAACATCGCCTCTTGA--AAAA----------------------------------------
Hame    AAACATCGCCTCTTGC-AAAAT----------------------------------------
Chso    AAACATCGCCTCTTGC-AAAAT----------------------------------------
Lyto    AAACATCGCCTCTTGCAA--------------------------------------------
```

```
Encr      AAACATCGCCTCTTGCAAACT-----------------------------------------
Bvar      AAACATCGCCTCTTGTAAATA-----------------------------------------
Noco      AAACATCGCCTCTTGC---AA-----------------------------------------
Chsp      AAACATCGCCTCTTGTACCCC-----------------------------------------
Arja      AAACATCGCCTCTTGTAGAAC-----------------------------------------
Pase      AAACACCGCCTCTTGC-AAA-A----------------------------------------
Trel      AAACATCGCCTCTTGCCCA-CC----------------------------------------
Lifa      AAACATCGCCTCCTGC-AATAC----------------------------------------
Acur      AAACACCGCCTCTTGAAT--------------------------------------------
Ampe      AAACATCGCCTCTTGCAAA-------------------------------------------
Urja      AAACACCGCCTCTTGT--AA------------------------------------------
Enet      AAACATCGCCTCTTGCATTTCT----------------------------------------
Ptbr      AAACATCGCCTCCTGAA--T------------------------------------------
Safa      AAACATCGCCTCCTGGACT--T----------------------------------------
Icae      AAACATCGCCTCTTGCAAAAG-----------------------------------------
Asmi      AAACACCGCCTCCTGC--ACT-----------------------------------------
Foal      AAACATCGCCTCTTGTACT-------------------------------------------
Drze      AAACATCGCCTCTTGC-AAAT-----------------------------------------
Rhas      AAACATCGCCTCTTGCAACAA-----------------------------------------
Elac      AAACATCGCCTCTTGAACCCT-----------------------------------------
Kugu      AAACACCGCCTCTTGCCCAAA-----------------------------------------
Plor      AAACATCGCCTCTTGCT-AAA-----------------------------------------
Sgun      AAACATCGCCTCTTGCAAAA------------------------------------------
Zaco      AAACATCGCCTCTTGCAAAA------------------------------------------
Zbfl      AAACATCGCCTCTTGCAAAAA-----------------------------------------
Spba      AAACATCGCCTCTTGA----------------------------------------------
Game      AAACATCGCCTCTTGC--AAAA----------------------------------------
Thth      AAACATCGCCTCTTGCAAAA------------------------------------------
Xigl      AAACATCGCCTCTTGCAAA-AC----------------------------------------
Hyja      AAACATCGCCTCTTGC-AAAAATA--------------------------------------
Psan      AAACATCGCCTCTTGC-AAAA-----------------------------------------
Cupa      AAACATCGCCTCTTGC-AAAA-----------------------------------------
Mpch      AAACATCGCCTCTTGCAAAAA-----------------------------------------
Char      AAACATCGCCTCTTGT-AAT--TT--------------------------------------
Pser      AAACATCGCCTCTTGCTAA-------------------------------------------
Prol      AAACATCGCCTCTTGCAAGAC-----------------------------------------
Plbi      AAACATCGCCTCTTGCATAA------------------------------------------
Calu      AAACATCGCCTCTTGCTAACTA----------------------------------------
Papa      AAACATCGCCTCTTGC-TTAT-----------------------------------------
Sufr      AAACATCGCCTCTTGCGAA-------------------------------------------
Stci      AAACATCGCCTCTTGA-AATC-----------------------------------------
Taru      AAACATCGCCTCTTGCTT-CA-----------------------------------------
Rala      AAACATCGCCTCTTGCAAA--A----------------------------------------
          *****   * *   *
```

```
                  ¦               HVR               ¦  35'       36      37
Scca    ---------------CC----------------ATAAGAGGTCCCGCCTGCCCTGTGAC
Muma    ---------------TT----------------ATAAGAGGTCCCGCCTGCCCTGTGAC
Erca    ------------CACAT----------------ATTGAAGGTCCTGCCTGCCCAGTGAC
Pose    ---------CC--ATGT----------------ATTGAAGGTCCTGCCTGCCCAGTGAC
Actr    ---------CCA-ATGT----------------ATTAGAGGTCCCGCCTGCCCTGTGAC
Scal    ---------AAT---GT----------------ATTAGAGGTCCCGCCTGCCCTGTGAC
Posp    ---------T-----GT----------------ATTAGAGGTCCCGCCTGCCCTGTGAC
Atsp    -------------AAGT----------------ATTAGAGGTCCCGCCTGCCCTGTGAC
Leoc    ---------CCN-AAGT----------------ATTAGAGGTCCCGCCTGCCCTGTGAC
Amca    ---------CC--AAGT----------------ATTAGAGGTCCCGCCTGCCCAGTGAA
Osbi    -------------AAAA----------------ATAAGAGGTCCAACCTGCCCAGTGAC
Pabu    ---------CAA---AC----------------ATAAGAGGTCCAGCCTGCCCAGTGAC
Hial    ---------CCA-AAGT----------------ATAAGAGGTCCCGCCTGCCCAGTGAC
Elha    ----------CA-AAAT----------------ATAAGAGGTCCCGCCTGCCCCGTGAC
Mlcy    ---------TCA-AAGT----------------ATAAGAGGTCCCGCCTGCCCCGTGAC
Algl    ---------ACA-AAGT----------------ATTAGAGGTCCCGCCTGCCCTGTGAA
Ptgi    ---------AACGAAGT----------------ATTAGAGGTCCCGCCTGCCCTGTGAC
Alaf    ----------AG-AAGT----------------ATTAGAGGTCCCGCCTGCCCAGTGAC
Nock    ---------AAG-AAGT----------------ATTAGAGGTACCGCCTGCCCAGTGAC
Anja    -----------AGGAAG----------------TCTAGAGGTCCCGCCTGCCCTGTGAC
Gyki    ---------ATG-AAGT----------------ATTAGAGGTCCCGCCTGCCCTGTGAC
Syka    ---------ATG-AAGT----------------ATTAGAGGTCCCGCCTGCCCTGTGAC
Opma    ---------AGA-ATGT----------------ATTAGAGGTCCCGCCTGCCCTGTGAC
Comy    ---------GA--ATGT----------------ATTGGAGGTCCTGCCTGCCCAGTGAC
Sasp    -----------TAATGT----------------ATAAGAGGTCTTGCCTGCCCAGTGAC
Eupe    -----------A-AAGT----------------ATAAGAGGTCCCGCCTGCCCAGTGAC
Enja    --------T-CCAAAGT----------------ATAAGAGGTCCCACCTGCCCTGTGAC
Same    ----------CA--ATT----------------ATAGGAGGTCCCGCCTGCCCTGTGAC
Chch    ---------TCG-ACGT----------------ATAGAAGGTCCTGCCTGCCCAGTGAC
Grgr    ---------TCG-ATGT----------------ATAGGAGGTCCCGCCTGCCCAATGAC
Caau    ---------ACC-AAGT----------------ATAGGAGGTCCAGCCTGCCCAGTGAC
Cyca    --------CAACCAAGT----------------ATAGGAGGTCCAGCCTGCCCAGTGAC
Dare    ---------C-C-CAAT----------------ATAGGAGGTCCAGCCTGCCCAGTGAC
Cost    --------ATTCAAGGT----------------ATAGGAGGTCCAGCCTGCCCAGTGAC
Leec    ---------CCA-ATGT----------------ATAGGAGGTCCAGCCTGCCCGGTGAC
Crla    ---------ACG-AAGT----------------ATAGGAGGTCCAGCCTGCCCAGTGAC
Clmc    ---------CCA-ATAT----------------ATGGGAGGTCTTACCTGCCCAGTGAC
Phin    -----------CAACGT----------------ATAGGAGGTCTTACCTGCCCAGTGAC
Icpu    ---------TCA-ACGT----------------ATAGGAGGTCTTGCCTGCCCAGTGAC
Psto    ---------TCA-ACGT----------------ATAGGAGGTCTTGCCTGCCCAGTGAC
Cora    ----------CA-ATGT----------------ATAGGAGGTCCTGCCTGCCCAGTGAC
Eisp    --------ATCA-ACAT----------------ATAGGAGGTCCTGCCTGCCCAGTGAA
Apal    ---------C--AATAC----------------ATAGGAGGTCCCGCCTGCCCACTGAC
Eslu    ---------CCC-AAAC----------------ATAAGAGGTCCCGCCTGCCCTGTGAC
Dape    -----------TAA-AC----------------ATAAGAGGTCCCGCCTGCCCTGTGAC
Glse    -------------AAGT----------------ATAAGAGGTCCCGCCTGCCCTGTGAC
Naar    ---------T-T-TAAT----------------ATAAGAGGTCCCGCCTGCCCTGTGAC
Baoc    -------------AAAC----------------ATAAGAGGTCCCGCCTGCCCTGTGAC
Opso    --------AATTAAAGT----------------ATAAGAGGTCCCGCCTGCCCTGTGAC
Alte    ------------A-AAC----------------ATAGGAGGTCCCGCCTGCCCTGTGAC
Plap    --------CAA---AAC----------------ATAGGAGGTCCCGCCTGCCCTGTGAC
```

```
Plal  ------------CACGC----------------ATAAGAGGTCCCGCCTGCCCGGTGAC
Sami  ---------CT--ATGC----------------ATAAGAAGTCCCGCCTGCCCGGTGAC
Rere  ----------TTT-TAC----------------ATAAGATGTCCCGCCTGCCCGGAGCC
Gama  ----------TTAACCT----------------ATAAGAGGTCCTGCCTGCCCTGTGAC
Onmy  ------------C-AAA----------------ACATAGAGGTCCGCCTGCCCTGTGAC
Sasa  -------------AAAC----------------ATAAGAGGTCCCGCCTGCCCTGTGAC
Cola  ----------CA-AAGC----------------ATAAGAGGTCCCGCCTGCCCTGTGAC
Dita  ---------CC--ATGA----------------ATAAGAGGTCCCGCCTGCCCTGTGAC
Gogr  ---------T----AAC----------------ATAAGAGGTCCTGCCTGCCCTGTGAC
Chsl  GGCCGCGGTATAATAACCGTGCTAAGGTACCTCATAAGAGGTCCTGCCTGCCCAGTGAC
Atja  ---------A-----GA----------------ATAAGAGGTCCCGCCTGCCCTGTGAC
Iido  ---------C---AAGA----------------ATAAGAGGTCCCGCCTGCCCTGTGAC
Auja  -----------A-AAAA----------------ATAAGAGGTCCCGCCTGCCCTGTGAC
Chag  -------------AAAG----------------ATAAGAGGTCCCGCCTGCCCAGTGAC
Hami  ---------TCA-AAGA----------------ATAAGAGGTCCCGCCTGCCCTGTGAC
Saun  ------------AAAGA----------------ATAAGAGGTCCCGCCTGCCCTGTGAC
Nema  -----------CGAAGA----------------ATAAGAGGTCCCGCCTGCCCTGTGAC
Disp  ---------G-A-ACAA----------------ATAAGAGGTCCTGCCTGCCCAGTGAC
Myaf  -----------A-ACGA----------------ATAAGAGGTCCAGCCTGCCCAGTGAC
Lagu  ---------TTT-GAGA----------------ATAAGAGGTCCCGCCTGCCCCGTGAC
Trtr  ---------TTA-ATGA----------------ATAAGAGGCCACGCCTGCCCGGTGAC
Zucr  ---------ATA-TTGT----------------ATAAGAGGCCACGCCTGCCCGGTGAC
Pxja  -----------TAA-AA----------------ATAAGAGGTCCCGCCTGCCCTGTGAC
Pxlo  ---------TATTAAAA----------------ATAAGAGGTCCCGCCTGCCCTGTGAC
Pctr  ----------C--CAAA----------------ATGAGAGGTCCCGCCTGCCCTGTGAC
Apsa  -------------AAGA----------------ATAAGAGGTCCCGCCTGCCCTGTGAC
Cabe  ------------ATGAA----------------ATGGGAGGTCCCGCCTGCCCCGTGAC
Bzze  ---------CTA-AAGA----------------ATAAGAGGTCCCGCCTGCCCTGTGAC
Siim  ---------CCA-AAGA----------------ATAAGAGGTCCGACCTGCCCAGTGAC
Ctru  -----------A-AAGA----------------ATAAGAGGTCCCGCCTGCCCTGTGAC
Dpbr  ---------CTA-AAGA----------------ATAAGAGGTCCCGCCTGCCCTGTGAC
Caki  -----------A-CTAT----------------ATAAGAGGTCTCGCCTGCCCAGTGAC
Phja  ---------CAA-A-GT----------------ATAAGAGGTCCCGCCTGCCCTGTGAC
Brsp  -------------TATC----------------ATGAGAGGTCCTGCCTGCCCAGTGAC
Gamo  ----------AA-AAAT----------------ATAAGAGGTCCCGCCTGCCCTGTGAC
Lolo  --------CCTAAAAAT----------------ATAAGAGGTCCCGCCTGCCCTGTGAC
Batr  ---------------AT----------------ATAAGAAGTCCTGCCTGCCCAGTGAG
Prmy  -------------CCTT----------------ATAAGAAGTCCCGTCTGCCCTGTGAT
Loli  --------------CAA----------------ATAAGAGGTCCTGCCTGCCCTGTGAC
Loam  --------AAT----AA----------------ATAAGAGGTCCTGCCTGCCCTGTGAC
Chab  -------------ACGA----------------ATAAGAGGTCCCGCCTGCCCAGTGAC
Chto  -----------A-ACGA----------------ATAAGAGGTCCCGCCTGCCCAGTGAC
Majo  ---------CTA-A-GA----------------ATAAGAGGTCCCGCCTGCCCTGTGAC
Hlst  ---------T---AAAT----------------ATAAGAGGTCCCGCCTGCCCTGTGAC
Clpe  ---------C-----GA----------------ATAAGAGGTCCCGCCTGCCCTGTGAC
Mlmr  ---------T-A-ATGA----------------ATAAGAGGCCCCGCCTGCCCTGTGAC
Crcr  -------------CCAC----------------ATAAGAGGTCCTGCCTGCCCAGTGAC
Muce  ----------ACCTCAC----------------ATAAGAGGTCCTGCCTGCCCAGTGAC
Bege  ---------CCA-AAAA----------------ATAAGAGGTCCCGCCTGCCCTGTGAC
Mela  -------------AAAT----------------ATAAGAGGTCCCGCCTGCCCTGTGAC
Hats  ---------C---AAGA----------------ATAAGAGGTCTCGCCTGCCCTGTGAC
Orla  ---------A--T-GAA----------------ATAAGAGGTACCGCCTGCCCTGTGAC
```

```
Cosa    ---------AA--ACGA----------------ATAAGAGGTCCCGCCTGCCCTGTGAC
Exsp    -------------AAGT----------------ATAAGAGGTCCCGCCTGCCCTGTGAC
Depa    -------------AAGT----------------ATAAGAGGTCCCGCCTGCCCTGTGAC
Rima    --------------AAC----------------ATAAGAGGTCCTGCCTGCCCAGTGAC
Fuol    -------------CTAC----------------ATAAGAGGTCCTGCCTGCCCTGTGAT
Gmaf    ---------ACC-CCTA----------------ATAAAAGGTCCTGCCTGCCCTGTGAA
Xeei    ---------C----TAT----------------ATAAGAGGTCCTGCCTGCCCCGTGAC
Pros    -----------A-AAAA----------------ATAAGAGGTCCCGCCTGCCCTGTGAC
Scmi    -----------A-A-AT----------------ATAAGAGGTCCCGCCTGCCCTGTGAC
Rolo    -----------TTA-AC----------------ATAAGAGGTCCCGCCTGCCCTGTGAC
Cere    -----------TAA-AA----------------ATAAGAGGTCCCACCTGCCCTGTGAC
Daga    ---------T---AAGA----------------ATAAGAGGTCCCGCCTGCCCTGTGAC
Anco    -------------ACAA----------------ATAAGAGGTCCCGCCTGCCCTGTGAC
Dmve    --------------TGT----------------ATAAGAGGTCCCGCCTGCCCTGTGAC
Dmar    ---------A---ACAT----------------ATAAGAGGTCCCGCCTGCCCTGTGAC
Anka    ---------T-A-ACAA----------------ATAAGAGGTCCCGCCTGCCCTGTGAC
Moja    ---------GCA-ACAA----------------ATAAGAGGTCCCGCCTGCCCTGTGAC
Hoja    ---------ACA-ACAA----------------ATAAGAGGTCCCGCCTGCCCTGTGAC
Bede    --------AAGCAAAAA----------------ATAAGAGGTCCCGCCTGCCCTGTGAC
Besp    ---------AA--A-AT----------------ATAAGAGGTCCCGCCTGCCCTGTGAC
Mysp    ---------CA--AAGA----------------ATAAGAGGTCCCGCCTGCCCCGTGAC
Osja    --------AAACAAAGA----------------ATAAGAGGTCCCGCCTGCCCTGTGAC
Sgro    ----------CA-AAGC----------------ATAAGAGGTCCCGCCTGCCCTGTGAC
Pzpa    -------------GTAT----------------ATAAGAGGTCCCGCCTGCCCTGTGAC
Zeja    ------------A-AAC----------------ATAAGAGGTCCCGCCTGCCCTGTGAC
Znne    ---------AAA-AAAC----------------ATAAGAGGTCCCGCCTGCCCTGTGAC
Zefa    -----------AA--AT----------------ATAAGAGGTCCCGCCTGCCCTGTGAC
Acni    ---------AAT-AAAT----------------ATAAGAGGTCCCGCCTGCCCTGTGAC
Ncrh    ---------ATA-A-AT----------------ATAAGAGGTCCCGCCTGCCCTGTGAC
Agca    -------------ATAA----------------ATAAGAGGTCCCGCCTGCCCTGTGAC
Hydy    ----------CTA-CTA----------------ATAAGAGGTCCCGCCTGCCCTGTGAC
Gsac    -----------A-ATAA----------------ATAAGAGGTCCCGCCTGCCCTGTGAC
Pevo    ---------ATC-GAAA----------------ATAAGAGGTCCAGCCTGCCCTGTGAC
Hiku    ---------ACA-ATGA----------------ATAAGAGGTCCCGCCTGCCCTGTGAC
Inpa    ---------CCC-GCCC----------------ATAAGAGGTCCCGCCTGCCCCGTGAC
Auch    --------AATTGAAGA----------------ATAAGAGGTCGAGCCTGCCCACTGAC
Fico    ---------CG--AAGA----------------ATAAGAGGTACTGCCTGCCCTGTGAC
Macs    ---------TTA-AGGA----------------ATAAGAGGTCCCGCCTGCCCTGTGAC
Moal    ---------TAT-GTAT----------------ATAGGAGGCATTGCCTGCCCAGTGAC
Syma    -------------CAAT----------------ATAAGAGGTCCCACCTGCCCCCTGAC
Mafr    ---------T--GACAT----------------ATAAGAGGTCCCGCCTGCCCCGTGAC
Dcpe    ---------TTA-AAGA----------------ATAAGAGGTCCTGCCTGCCCTGTGAC
Dcti    ---------TAA-A-GA----------------ATAAGAGGTCCTGCCTGCCCTGTGAC
Hehi    --------G-CTAAAGA----------------ATAAGAGGTCCCGCCTGCCCTGTGAC
Stam    ------------AACAA----------------ATAAGAGGTCCCGCCTGCCCTGTGAC
Hogi    ---------CAC-CAAA----------------ATAAGAGGTCCCGCCTGCCCTGTGAC
Erzo    ----------TA-AGAA----------------ATAAGAGGTCCTGCCTGCCCTGTGAC
Hxot    ----------CA-AAAA----------------ATAAGAGGTCCCGCCTGCCCTGTGAC
Core    -----------TAA-AA----------------ATAAGAGGTCCCGCCTGCCCTGTGAC
Apve    -------------AAAA----------------ATAAGAGGTCCCGCCTGCCCTGTGAC
Latj    ---------CAC-ACAA----------------ATAAGAGGTCCCGCCTGCCCAGTGAC
Laja    -----------T-ATGA----------------ATAAGAGGTCCCGCCTGCCCGGTGAC
```

```
Syja    ---------T-A-ATGA----------------ATAAGAGGTCCCGCCTGCCCAGTGAC
Epme    ---------CGA-AAGT----------------ATAAGAGGTCCCGCCTGCCCTGTGAC
Grse    -------------CAAC----------------ATAAGAGGTCCCGCCTGCCCTGTGAC
Clja    ---------TAA-T-GT----------------ATAAGAGGTCCCGCCTGCCCGGTGAC
Ogcy    -----------C-AAAA----------------ATAAGAGGTACTGCCTGCCCCGTGAC
Plna    ---------T----AAC----------------ATAAGAGGTCTCGCCTGCCCAGTGAC
Lema    ---------ACC-AAGA----------------ATAAGAGGTCCCGCCTGCCCTGTGAC
Etzo    ---------TTA-A-AA----------------ATAAGAGGTCCCGCCTGCCCTGTGAC
Apse    ---------TCT-AAGA----------------ATAAGAGGTCCCGCCTGCCCGGTGAC
Epde    -----------TAAAGA----------------ATAAGAGGTCCCGCCTGCCCTGTGAC
Slja    ---------TCA-ATAA----------------ATAAGAGGTCCTGCCTGCCCAGTGAC
Bsja    ---------TA--ACAA----------------ATAAGAGGTCCCGCCTGCCCTGTGAC
Ecna    ---------CA--AAGA----------------ATAAGAGGTCGCGCCTGCCCAGTGAC
Cohi    -------------AAGA----------------ATAAGAGGTCCCACCTGCCCGGTGAC
Caar    ---------CCA-AAGA----------------ATAAGAGGTCCCGCCTGCCCAGTGAC
Came    ------------AAAGA----------------ATAAGAGGTCCCGCCTGCCCGGTGAC
Mema    ---------CCA-AAGA----------------ATAAGAGGTCCCGCCTGCCCAGTGAC
Lenu    ----------CCC-AGA----------------ATAAGAGGTCCCACCTGCCCAGTGAC
Brja    ---------T--AGAAT----------------ATAAGAGGTCCCGCCTGCCCTGTGAC
Plma    ------------AAAAT----------------ATAAGAGGTCCAGCCTGCCCTGTGAC
Emst    ---------CTA-ATGA----------------ATAAGAGGTCCCGCCTGCCCTGTGAC
Ptti    ---------CA--ATGA----------------ATAAGAGGTCCCGCCTGCCCTGTGAC
Losu    ---------C-----AA----------------ATAAGAGGTCCCACCTGCCCAGTGAC
Geoy    ----------ACAACGA----------------ATAAGAGGTCCCGCCTGCCCATTGAC
Dipi    -----------CAATGA----------------ATAAGAGGTCCCGCCTGCCCCGTGAC
Pama    ---------TCG-ATGA----------------ATAAGAGGTCCCGCCTGCCCTGTGAC
Leob    ---------CA--ATGA----------------ATAAGAGGTCCCGCCTGCCCTGTGAC
Neba    ----------TT-ACGA----------------ATAAGAGGTCCCGCCTGCCCGGTGAC
Pdpl    ---------CAC-AAGA----------------ATAAGAGGTCCTGCCTGCCCAGTGAC
Nimi    -------------ACGA----------------ATAAGAGGTCCAGCCTGCCCAATGAC
Uptr    ---------ACT-AAGA----------------ATAAGAGGTCCCGCCTGCCCTGTGAC
Pesc    ---------GCA-ACAA----------------ATAAGAGGTCCTGCCTGCCCCGTGAC
Baar    -------------ACGA----------------ATAAGAGGTCTCACCTGCCCACTGAC
Moar    ----------TA-ATGA----------------ATAAGAGGTCCCGCCTGCCCTGTGAC
Toja    ----------CT-AAAA----------------ATAAGAGGTCCTGCCTGCCCAATGAC
Chau    ---------TTA-ACGA----------------ATAAGAGGTCCCGCCTGCCCTGTGAC
Chse    ---------TTA-ACAA----------------ATAAGAGGTCCCGCCTGCCCTGTGAC
Enar    ---------T-A-ATAA----------------ATAAGAGGTCCCGCCTGCCCTGTGAC
Hpty    ---------CTA-ATGA----------------ATAAGAGGTCCCGCCTGCCCTGTGAC
Nana    ------------AAAAA----------------ATAAGAGGTCTTGCCTGCCCTGTGAC
Mcst    ---------TTA-ATGA----------------ATAAGAGGTCCCGCCTGCCCTGTGAC
Rhox    ---------CA--AAGA----------------ATAAGAGGTCCCGCCTGCCCTGTGAC
Opfa    ---------CTG-AGGA----------------ATAAGAGGTCCCGCCTGCCCTGTGAC
Paar    --------AAACAAAAT----------------ATAAGAGGTCCCGCCTGCCCTGTGAC
Gozo    ----------CA-ACAT----------------ATAAGAGGTCCCGCCTGCCCTGTGAT
Ackr    ---------C-A-ACGA----------------ATAAGAGGTCCCGCCTGCCCTGTGAC
Elev    -----------CTAAAA----------------ATAAGAGGTCCCGCCTGCCCAGTGAC
Trdu    ---------CCT-AAGT----------------ATAAGAGGTCCCGCCTGCCCTGTGAC
Amoc    -----------AC-AGT----------------ATAAGAGGTCCCGCCTGCCCAGTGAC
Hame    ---------CA--ACGA----------------ATAAGAGGTCCCGCCTGCCCAGTGAC
Chso    ---------CA--ACAA----------------ATAAGAGGTCCCGCCTGCCCTGTGAC
Lyto    --------ACTTAATAA----------------ATAAGAGGTCCCGCCTGCCCTGTGAC
```

```
Encr      ---------TA--ATAA----------------ATAAGAGGTCCCGCCTGCCCTGTGAC
Bvar      ----------AA-TTAA----------------ATAAGAGGTCCCGCCTGCCCGGTGAC
Noco      ---------ATA-ATGA----------------ATAAGAGGTCCCGCCTGCCCTGTGAC
Chsp      ---------TAC-AAAT----------------ATAAGAGGTCCTGCCTGCCCCGTGAC
Arja      ---------TC--AAAA----------------ATAAGAGGTCCCGCCTGCCCTGTGAC
Pase      ---------TCA-ATGA----------------ATAAGAGGTCATGCCTGCCCTGTGAC
Trel      ---------CC----TG----------------ATAAGAGGTCCAGCCTGCCCTGTGAC
Lifa      ---------CT--T-GT----------------ATAGGAGGTCATGCCTGCCCCGTGAC
Acur      -----------TT-GAA----------------ATAAGAGGTCCAGCCTGCCCAGTGAC
Ampe      ---------GTA-ATGA----------------ATAAGAGGTCCCGCCTGCCCTGTGAC
Urja      ---------CCA-ATAA----------------ATAAGAGGTCCCACCTGCCCACTGAC
Enet      -----------A---GT----------------ATAAGAGGTCCCGCCTGCCCTGTGAC
Ptbr      --------A--CTAATA----------------ATAGGAGGTCCCGCCTGCCCCGTGAT
Safa      ---------ACA--AAT----------------ATAGGAGGTCCCGCCTGCCCTGTGAT
Icae      ---------C-A-AAGA----------------ATAAGAGGTCCAGCCTGCCCTGTGAC
Asmi      ---------CAA-CAGT----------------ATAGGAGGTCCTGCCTGCCCAGTGAC
Foal      -------------AAAA----------------ATAAGAGGTCCCGCCTGCCCAGTGAC
Drze      ---------T-T-AAGA----------------ATAAGAGGTCACGCCTGCCCGGTGAC
Rhas      ---------CAC-A-AT----------------ATAAGAGGTCCCGCCTGCCCTGTGAC
Elac      ---------CAA-TACT----------------ATAAGAGGTCCCGCCTGCCCTGTGAC
Kugu      -------------AAGA----------------ATAAGAGGTCCCGCCTGCCCTGTGAC
Plor      ---------CCC-ACAA----------------ATAAGAGGTCCCGCCTGCCCTGTGAC
Sgun      ----------TTAATGA----------------ATAAGAGGTCCCGCCTGCCCTGTGAC
Zaco      --------GC-A-ACGT----------------ATAAGAGGTCCCGCCTGCCCTGTGAC
Zbfl      ---------C-A-ACGA----------------ATAAGAGGTCCCGCCTGCCCTGTGAC
Spba      --------AAACCCAGT----------------ATAAGAGGTCTCGCCTGCCCAGTGAC
Game      ---------ATA-ACGA----------------ATAAGAGGTCCAGCCTGCCCTGTGAC
Thth      ---------ACAA-AGA----------------ATAAGAGGTCCAGCCTGCCCTGTGAC
Xigl      -----------CAAAGA----------------ATAAGAGGTCCCGCCTGCCCAGTGAC
Hyja      -------------AAGA----------------ATAAGAGGTCCAGCCTGCCCTGTGAC
Psan      ---------ACA-AAGA----------------ATAAGAGGTCTAGCCTGCCCTGTGAC
Cupa      ---------CGT-AAGA----------------ATAAGAGGTCCAGCCTGCCCTGTGAC
Mpch      ----------CTA-AGA----------------ATAAGAGGTCCCGCCTGCCCTGTGAC
Char      -------------AAAA----------------ATAAGAGGTCCCGCCTGCCCAGTGAC
Pser      ---------CC--AAAA----------------ATAAGAGGTCCAACCTGCCCAGTGAC
Prol      ---------A-C-AAGT----------------ATAAGAGGTCCCGCCTGCCCAGTGAC
Plbi      ---------CCA-CAGT----------------ATAAGAGGTCCCGCCTGCCCAGTGAC
Calu      ---------CTTGTAGA----------------ATAAGAGGTCTAGCCTGCCCAGTGAC
Papa      -----------G---AT----------------ATAAGAGGTCCTGCCTGCCCAATGAC
Sufr      ---------TCA-AAGA----------------ATAGGAGGTCCAGCCTGCCCTGTGAC
Stci      ---------C---CCAC----------------ATAAGAGGTCCCGCCTGCCCTGTGAC
Taru      -------------ATGA----------------ATAAGAGGTCACGCCTGCCCTGTGAC
Rala      ---------CTA-ATGA----------------ATAAGAGGTCCCGCCTGCCCTGTGAC
                                                  *     ******    *
```

```
                                 37'       38           38'    39
Scca    --AAT-----------------GTTCAAC-GGC-CGCGGTATT-TTGACCGTGCAAAGGTAG
Muma    AAT-------------------GTTCAAC-GGC-CGCGGTATT-TTGACCGTGCAAAGGTAG
Erca    TAAA------------------GTTTAAC-GGC-CGCGGTATC-TTGACCGTGCAAAGGTAG
Pose    --ATGA----------------GTTTAAC-GGC-CGCGGTATC-CTGACCGTGCAAAGGTAG
Actr    CAAAA-----------------GTTTAAC-GGC-CGCGGTATT-TTGACCGTGCGAAGGTAG
Scal    --CAAAA---------------GTTTAAC-GGC-CGCGGTATT-TTGACCGTGCGAAGGTAG
Posp    CAA-AA----------------GTTTAAC-GGC-CGCGGTATT-TTGACCGTGCGAAGGTAG
Atsp    T---TA----------------GTTTAAC-GGC-CGCGGTATT-TTGACCGTGCGAAGGTAG
Leoc    T-----------------T--AGTTTAAC-GGC-CGCGGTATT-TTGACCGTGCGAAGGTAG
Amca    --AAAT----------------TTTTAAC-GGC-CGCGGTATT-TTGACCGTGCGAAGGTAG
Osbi    TTCAAAA--------------AGTTAAAC-GGC-CGCGGTATT-TTAACCGTGCTAAGGTAG
Pabu    ATATA----------------AGTTAAAC-GGC-CGCAGTATT-TTAACCGCGCTAAGGTAG
Hial    --TCTGA---------------GTTAAAT-GGC-CGCGGTATT-TTAACCGTGCAAAGGTAG
Elha    CCAAAAA---------------GTTTAAC-GGC-CGCGGTATT-TTGACCGTGCGAAGGTAG
Mlcy    -TAACAA---------------GTTCAAC-GGC-CGCGGTATT-CTGACCGTGCGAAGGTAG
Algl    GTAAAA----------------ATTTAAC-GGC-CGCGGTATT-CTGACCGTGCAAAGGTAG
Ptgi    CAAGA-----------------GTTTAAC-GGC-CGCGGTATT-CTGACCGTGCGAAGGTAG
Alaf    A-TATAA---------------GTTAAAC-GGC-CGCGGTATT-CTGACCGTGCTAAGGTAG
Nock    A-AAAA----------------GTTAAAC-GGC-CGCGGTATT-CTGACCGTGCTAAGGTAG
Anja    CACAAA----------------GTTTAAC-GGC-CGCGGTATC-CTGACCGTGCGAAGGTAG
Gyki    -AGCAGT---------------GTTTAAC-GGC-CGCGGTATT-CTAACCGCGCGAAGGTAG
Syka    CAAAGT----------------GTTTAAC-GGC-CGCGGTATC-CTGACCGTGCGAAGGTAG
Opma    C-AATGT---------------GTTTAAC-GGC-CGCGGTATC-ATAACCGTGCGAAGGTAG
Comy    AA-TAG----------------GTTCAAC-GGC-CGCGGTATT-TTGACCGTGCAAAGGTAG
Sasp    CCGA------------------TTTCAAC-GGC-CGCGGTATT-ATGACCGTGCAAAGGTAG
Eupe    C--CG-----------------TTTCAAC-GGC-CGCGGTATT-ATGACCGTGCTAAGGTAG
Enja    TCTAA-----------------GTTTAAC-GGC-CGCGGTATT-CTAACCGTGCGAAGGTAG
Same    CAA-------------------TTTTAAC-GGC-CGCGGTATT-TTAACCGTGCGAAGGTAG
Chch    TATA-G----------------GTTCAAC-GGC-CGCGGTATT-TTGACCGTGCAAAGGTAG
Grgr    TAT-------------------GTTCAAT-GGC-CGCGGTATT-TTGACCGTGCTAAGGTAG
Caau    T-ACAA----------------GTTCAAC-GGC-CGCGGTATT-TTGACCGTGCAAAGGTAG
Cyca    TACAA-----------------GTTCAAC-GGC-CGCGGTATT-TTGACCGTGCAAAGGTAG
Dare    -A--ATA---------------GTTTAAC-GGC-CGCGGTATT-TTGACCGTGCAAAGGTAG
Cost    CACAA-----------------GTTCAAC-GGC-CGCGGTATT-TTGACCGTGCAAAGGTAG
Leec    T-ACAA----------------GTTTAAC-GGC-CGCGGTATT-TTGACCGTGCAAAGGTAG
Crla    C-TACAA---------------GTTCAAC-GGC-CGCTGTATT-TTGACCGTGCAAAGGTAG
Clmc    A-ACTA----------------GTTAAAC-GGC-CGCGGTATT-TTGACCGTGCTAAGGTAG
Phin    ATTCGT----------------GTTAAAC-GGC-CGCGGTATT-TTGACCGTGCTAAGGTAG
Icpu    -----AT---------------GTTAAAC-GGC-CGCGGTATT-TTGACCGTGCGAAGGTAG
Psto    ---AA-----------------GTTAAAC-GGC-CGCGGTATT-TTGACCGTGCGAAGGTAG
Cora    T--AT-----------------GTTGAAC-GGC-CGCGGTATT-TTGACCGTGCAAAGGTAG
Eisp    ---A------------------TTTCAAC-GGC-CGCGGTATT-TTGACCGTGCAAAGGTAG
Apal    TCT-GT----------------GTTTAAC-GGC-CGCGGTATT-TTGACCGTGCAAAGGTAG
Eslu    --TATAC---------------GTTTAAC-GGC-CGCGGTATT-TTAACCGTGCGAAGGTAG
Dape    TATGG-----------------GTTTAAC-GGC-CGCGGTATT-TTAACCGTGCGAAGGTAG
Glse    C---TTG--------------TGTTTAAC-GGC-CGCGGTATT-CTGACCGTGCGAAGGTAG
Naar    --TCTAA---------------GTTTAAC-GGC-CGCGGTATT-TTGACCGTGCGAAGGTAG
Baoc    T--ATAA---------------GTTTAAC-GGC-CGCGGTATT-TTGACCGTGCGAAGGTAG
Opso    CCTGG-----------------GTTTAAC-GGC-CGCGGTATC-TTGACCGTGCGAAGGTAG
Alte    --TCTAA---------------GTTTAAC-GGC-CGCGGTATT-TTGACCGTGCGAAGGTAG
Plap    TTTAA-----------------GTTTAAC-GGC-CGCGGTATT-TTGACCGTGCGAAGGTAG
```

```
Plal    CCTGG-----------------GTTAAAC-GGC-CGCGGTATT-TTAACCGTGCGAAGGTAG
Sami    CCCTGG----------------GTTAAAC-GGC-CGCGGTATT-TTGACCGTGCGAAGGTAG
Rere    -T-CTGG---------------GTTAAAC-GGC-CGCGGTATT-TTAACCGTGCGAAGGTAG
Gama    TATGA-----------------GTTTAAC-GGC-CGCGGTATT-TTGACCGTGCAAAGGTAG
Onmy    TATGG-----------------GTTTAAC-GGC-CGCGGTATT-TTGACCGTGCGAAGGTAG
Sasa    T---ATG--------------GGTTTAAC-GGC-CGCGGTATT-TTGACCGTGCGAAGGTAG
Cola    T--ATGG---------------GTTTAAC-GGC-CGCGGTATT-TTGACCGTGCGAAGGTAG
Dita    C-ACAA----------------GTTTAAC-GGC-CGCGGTATA-TTGACCGTGCAAAGGTAG
Gogr    CAC--GT---------------GTTTAAC-GGC-CGCGGTATT-TTAACCGTGCAAAGGTAG
Chsl    AATT------------------GTTAAAC-GGC-CGCGGTATA-ATAACCGTGCTAAGGTAG
Atja    --AATT----------------GTTTAAC-GGC-CGCGGTATT-TTGACCGTGCAAAGGTAG
Iido    AATTA-----------------GTTTAAC-GGC-CGCGGTATT-TTGACCGTGCAAAGGTAG
Auja    --CTAAG---------------GTTTAAC-GGC-CGCGGTATT-TTGACCGTGCGAAGGTAG
Chag    TTGTAT---------------AGTTTAAC-GGC-CGCGGTATT-TTGACCGTGCGAAGGTAG
Hami    TCGAG----------------AGTTTAAC-GGC-CGCGGTATC-TTGACCGTGCGAAGGTAG
Saun    TTAAGA----------------GTTTAAC-GGC-CGCGGTATC-TTGACCGTGCGAAGGTAG
Nema    TATAT-----------------GTTTAAC-GGC-CGCGGTATT-TTGACCGTGCGAAGGTAG
Disp    --TATAT---------------GTTTAAC-GGC-CGCGGTATT-CTGACCGTGCGAAGGTAG
Myaf    T--ATAC---------------GTTAAAC-GGC-CGCGGTATC-TTGACCGTGCAAAGGTAG
Lagu    AAT--A----------------GTTTAAC-GGC-CGCGGTATT-CTGACCGTGCGAAGGTAG
Trtr    C-----------------AAAAGTTTAAC-GGC-CGCGGTATT-ATGACCGTGCAAAGGTAG
Zucr    -TAGAA----------------GTTTAAC-GGC-CGCGGTATT-ATGACCGTGCAAAGGTAG
Pxja    T-----------------CTATGTTTAAC-GGC-CGCGGTATT-TTGACCGTGCGAAGGTAG
Pxlo    TCTAT-----------------GTTTAAC-GGC-CGCGGTATT-TTGACCGTGCGAAGGTAG
Pctr    AA--AT----------------GTTTAAC-GGC-CGCGGTATT-TTGACCGTGCGAAGGTAG
Apsa    AT-CTA----------------GTTTAAC-GGC-CGCGGTATT-TTGACCGTGCGAAGGTAG
Cabe    -----------------TATACGTTTAAC-GGC-CGCGGTATT-TTAACCGTGCAAAGGTAG
Bzze    TA-----------------TAAGTTTAAC-GGC-CGCGGTATT-TTGACCGTGCGAAGGTAG
Siim    T-----------------ATATGTTTAAC-GGC-CGCGGTATT-CTGACCGTGCGAAGGTAG
Ctru    --AATAC---------------GTTTAAC-GGC-CGCGGTATT-TTGACCGTGCGAAGGTAG
Dpbr    AATAT-----------------GTTTAAC-GGC-CGCGGTATT-TTGACCGTGCGAAGGTAG
Caki    --TAAAT---------------GTTAAAC-GGC-CGTGGTATC-TTAACCATGCGAAGGTAG
Phja    -GA--AA---------------GTTTAAC-GGC-CGCGGTATT-CTAACCGTGCGAAGGTAG
Brsp    TATAT-----------------GTTAAAC-GGC-CGTGGTATC-TTAACCATGCTAAGGTAG
Gamo    T--ATAA---------------GTTTAAC-GGC-CGCGGTATT-TTGACCGTGCGAAGGTAG
Lolo    TATAA-----------------GTTTAAC-GGC-CGCGGTATT-TTAACCGTGCGAAGGTAG
Batr    --CCA-----------------GTTCAAC-GGC-TGCAGTATC-ATGACTGTACTAAGGTAG
Prmy    -ATA------------------ATTTAAC-GGC-TGCAGTAAC-ACAACTGCACAAAGGTAG
Loli    -ACATA----------------GTTTAAC-GGC-CGCGGTATT-TTAACCGTGCGAAGGTAG
Loam    AATTA-----------------GTTCAAC-GGC-CGCGGTATT-TTGACCGTGCGAAGGTAG
Chab    -T-ACAA---------------GTTCAAC-GGC-CGCGGTATT-TTGACCGTGCGAAGGTAG
Chto    TAC--AA---------------GTTCAAC-GGC-CGCGGTATT-TTGACCGTGCGAAGGTAG
Majo    TCTAAGA---------------GTTTAAC-GGC-TGCGGTATT-TTGACCGTGCAAAGGTAG
Hlst    TGTTTAA---------------GTTTAAC-GGC-CGCGGTATT-TTGACCGTGCAAAGGTAG
Clpe    TAT-TA----------------GTTTAAC-GGC-CGCGGTATT-TTGACCGTGCGAAGGTAG
Mlmr    TAATTTA---------------GTTTAAC-GGC-CGCGGTATC-ATGACCGTGCAAAGGTAG
Crcr    C---CC---------------TGTTCAAC-GGC-CGCGGTATT-TTAACCGCGCAAAGGTAG
Muce    CCCT------------------GTTCAAC-GGC-CGCGGTATT-TTAACCGCGCAAAGGTAG
Bege    TATAT-----------------GTTTAAC-GGC-CGCGGTATT-TTGACCGTGCAAAGGTAG
Mela    T---ATA--------------CGTTTAAC-GGC-CGCGGTATC-TTGACCGTGCGAAGGTAG
Hats    T-----------------ATAAGTTTAAC-GGC-CGCGGTATT-TTAACCGTGCGAAGGTAG
Orla    --TAAAA---------------GTTTAAC-GGC-CGCGGTATT-TTGACCGTGCAAAGGTAG
```

```
Cosa    TC-TA-----------------GTTTAAC-GGC-CGCGGTATT-TTGACCGTGCAAAGGTAG
Exsp    A-AAAA----------------GTTTAAC-GGC-CGCGGTATC-TTGACCGTGCGAAGGTAG
Depa    -ACCA-----------------GTTTAAC-GGC-CGCGGTATT-TTAACCGTGCAAAGGTAG
Rima    -ACA-AT---------------GTTCAAC-GGC-CGCGGTATT-TTGACCGCGTGAAGGTAG
Fuol    ---AAT----------------ATTTAAC-GGC-CGCGGTATC-TTGACCGTGCAAAGGTAG
Gmaf    TTAA-A----------------TTTTAAC-GGC-CGCGGTATC-TTGACCGTGCAAAGGTAG
Xeei    TTTATA----------------GTTTAAC-GGC-CGCGGTATC-TTGACCGTGCAAAGGTAG
Pros    TAT--GT---------------GTTTAAC-GGC-CGCGGTATT-TTGACCGTGCAAAGGTAG
Scmi    T-ATAT----------------GTTTAAC-GGC-CGCGGTATT-TTGACCGTGCAAAGGTAG
Rolo    --TATAT---------------GTTTAAC-GGC-CGCGGTATT-TTGACCGTGCGAAGGTAG
Cere    ATATGTTTAACGTGTGACATATGTTTAAC-GGC-CGCGGTATT-TTGACCGTGCAAAGGTAG
Daga    ---ATCT---------------GTTTAAC-GGC-CGCGGTATT-CTGACCGTGCAAAGGTAG
Anco    T---ATA--------------TGTTTAAC-GGC-CGCGGTATT-TTGACCGTGCGAAGGTAG
Dmve    TA-ATT----------------GTTTAAC-GGC-CGCGGTATT-TTGACCGTGCAAAGGTAG
Dmar    AATT------------------GTTCAAC-GGC-CGCGGTATT-TTGACCGTGCGAAGGTAG
Anka    T-ATAA----------------GTTTAAC-GGC-CGCGGTATT-TTGACCGTGCGAAGGTAG
Moja    TATA-T----------------GTTTAAC-GGC-CGCGGTATT-TTGACCGTGCGAAGGTAG
Hoja    TATAT-----------------GTTTAAC-GGC-CGCGGTATT-TTGACCGTGCGAAGGTAG
Bede    TATAT-----------------GTTTAAC-GGC-CGCGGTATT-TTGACCGTGCGAAGGTAG
Besp    TA-TAT----------------GTTTAAC-GGC-CGCGGTATT-TTGACCGTGCGAAGGTAG
Mysp    T-ATAT----------------GTTTAAC-GGC-CGCGGTATT-TTGACCGTGCAAAGGTAG
Osja    TATAT-----------------GTTTAAC-GGC-CGCGGTATT-TTGACCGTGCAAAGGTAG
Sgro    -T-ATAT---------------GTTTAAC-GGC-CGCGGTATT-TTGACCGTGCAAAGGTAG
Pzpa    T-TTAA----------------GTTTAAC-GGC-CGCGGTATC-TTGACCGTGCGAAGGTAG
Zeja    --TTTAA---------------GTTTAAC-GGC-CGCGGTATT-TTGACCGTGCGAAGGTAG
Znne    -TTAAA----------------GTTTAAC-GGC-CGCGGTATT-TTGACCGTGCGAAGGTAG
Zefa    TT-AAA----------------GTTTAAC-GGC-CGCGGTATT-TTGACCGTGCAAAGGTAG
Acni    T-TTAA----------------GTTTAAC-GGC-CGCGGTATT-TTGACCGTGCGAAGGTAG
Ncrh    TTTAA-----------------GTTTAAC-GGC-CGCGGTATT-TTGACCGTGCGAAGGTAG
Agca    --TAAAT---------------GTTTAAC-GGC-CGCGGTATT-TTAACCGTGCAAAGGTAG
Hydy    T-ATA-----------------GTTTAAC-GGC-CGCGGTATT-TTGACCGTGCGAAGGTAG
Gsac    --TATAA---------------GTTTAAC-GGC-CGCGGTATT-TTGACCGTGCGAAGGTAG
Pevo    CAAA-AT---------------GTTTAAC-GGC-CGCGGTATT-TTGACCGTGCGAAGGTAG
Hiku    CATG-A----------------GTTTAAC-GGC-CGCGGTATT-TTGACCGTGCAAAGGTAG
Inpa    --AAT----------------AGTTTAAC-GGC-CGCGGTATT-CTAACCGTGCAAAGGTAG
Auch    TATAT-----------------GTTCAAC-GGC-CACGGTATT-TTGACCGTGCAAAGGTAG
Fico    CC-TAG----------------GTTTAAC-GGC-CGCGGTATT-TTGACCGTGCGAAGGTAG
Macs    TATAA-----------------GTTTAAC-GGC-CGCGGTATT-TTGACCGTGCGAAGGTAG
Moal    ---ACAT---------------GTTTAAC-GGC-CGCGGTATC-CTAACCGTGCAAAGGTAG
Syma    T---ATA--------------TGTTCAAC-GGC-CACGGTATC-CTAACCGTGCAAAGGTAG
Mafr    ACCCAA----------------GTTCAAC-GGC-CGCGGTATC-CTGACCGTGCAAAGGTAG
Dcpe    TAT--AA---------------GTTTAAC-GGC-CGCGGTATA-ATGACCGTGCGAAGGTAG
Dcti    --TATAA---------------GTTTAAC-GGC-CGCGGTATT-ATGACCGTGCGAAGGTAG
Hehi    TATTA-----------------GTTTAAC-GGC-CGCGGTATT-TTGACCGTGCAAAGGTAG
Stam    T-ATAA----------------GTTTAAC-GGC-CGCGGTATT-TTGACCGTGCGAAGGTAG
Hogi    T-ATTA----------------GTTTAAC-GGC-CGCGGTATT-TTGACCGTGCGAAGGTAG
Erzo    C--ATGA---------------GTTTAAC-GGC-CGCGGTATT-TTGACCGTGCGAAGGTAG
Hxot    T--ATTT---------------GTTTAAC-GGC-CGCGGTATT-TTGACCGTGCGAAGGTAG
Core    TAT--TT---------------GTTTAAC-GGC-CGCGGTATT-TTGACCGTGCGAAGGTAG
Apve    TATTAT----------------GTTTAAC-GGC-CGCGGTATT-TTGACCGTGCAAAGGTAG
Latj    AACTG-----------------GTTCAAC-GGC-CGCGGTATT-TTGACCGTGCGAAGGTAG
Laja    --TATAA---------------GTTCAAC-GGC-CGCGGTATT-TTGACCGTGCGAAGGTAG
```

Syja	T-ATAT----------------GTTCAAC-GGC-CGCGGTATT-TTAACCGTGCAAAGGTAG
Epme	TATAT-----------------GTTTAAC-GGC-CGCGGTATT-TTGACCGTGCGAAGGTAG
Grse	T---ATA--------------AGTTTAAC-GGC-CGCGGTATT-CTGACCGTGCGAAGGTAG
Clja	TAT--AT---------------GTTTAAC-GGC-CGCGGAACC-CTGACCGTGCGAAGGTAG
Ogcy	-CCT-GA---------------GTTTAAC-GGC-CGCGGTATT-TTGACCGTGCAAAGGTAG
Plna	TCATGA----------------GTTCAAC-GGC-CGCGGTATT-TTAACCGTGCAAAGGTAG
Lema	-T-ATAA---------------GTTTAAC-GGC-CGCGGTATT-TTGACCGTGCAAAGGTAG
Etzo	TAT--AT---------------GTTTAAC-GGC-CGCGGTATT-TTGACCGTGCGAAGGTAG
Apse	--TAT-T---------------GTTTAAC-GGC-CGCGGTACT-CTGACCGTGCGAAGGTAG
Epde	TATA-A----------------GTTTAAC-GGC-CGCGGTATT-TTGACCGTGCGAAGGTAG
Slja	CCTAGT----------------GTTCAAC-GGC-CGCGGTATCACTAACCGCGCGAAGGTAG
Bsja	TC-TAT----------------GTTTAAC-GGC-CGCGGTATA-CTGACCGTGCGAAGGTAG
Ecna	AATTA-----------------GTTTAAC-GGC-CGCGGTATT-TTGACCGTGCTAAGGTAG
Cohi	A---CC---------------AATTTAAC-GGC-CGCGGTATC-CTGACCGTGCGAAGGTAG
Caar	AATATG----------------GTTCAAC-GGC-CGCGGTATT-TTGACCGTGCAAAGGTAG
Came	T-ATAT----------------GTTTAAC-GGC-CGCGGTATT-TTGACCGTGCGAAGGTAG
Mema	TGA---------------TTCAGTTTAAC-GGC-CGCGGTATT-CTGACCGTGCAAAGGTAG
Lenu	TATAA-----------------GTTTAAC-GGC-CGCGGTATT-TTGACCGTGCAAAGGTAG
Brja	TAT-AT----------------GTTTAAC-GGC-CGCGGTATT-TTAACCGTGCGAAGGTAG
Plma	T-ATAT----------------GTTTAAC-GGC-CGCGGTATT-TTAACCGTGCGAAGGTAG
Emst	TATAA-----------------GTTTAAC-GGC-CGCGGTATT-TTGACCGTGCGAAGGTAG
Ptti	T-GAAA----------------GTTTAAC-GGC-CGCGGTATT-TTGACCGTGCGAAGGTAG
Losu	TAT-AT----------------GTTCAAC-GGC-CGCAGTATT-TTAACCGCGCGAAGGTAG
Geoy	TATAA-----------------GTTTAAC-GGC-CGCGGTATT-TTGACCGTGCAAAGGTAG
Dipi	CAAAG-----------------GTTTAAC-GGC-CGCGGTATT-TTGACCGTGCAAAGGTAG
Pama	T-----------------ATATGTTTAAC-GGC-CGCGGTATT-TTGACCGTGCGAAGGTAG
Leob	TA-TAT----------------GTTTAAC-GGC-CGCGGTATT-TTGACCGTGCGAAGGTAG
Neba	T--ATAT---------------GTTAAAC-GGC-CGCGGTATA-CTGACCGTGCCAAGGTAG
Pdpl	AACCCAA---------------GTTCAAC-GGC-CGCGGTATT-CTGACCGTGCGAAGGTAG
Nimi	C---ATG--------------TGTTTAAC-GGC-CGCGGTATT-TTGACCGTGCAAAGGTAG
Uptr	TGTA-A----------------GTTTAAC-GGC-CGCGGTACT-CTGACCGTGCGAAGGTAG
Pesc	-TATAT----------------GTTCAAC-GGC-CGCGGTATT-TTGACCGTGCAAAGGTAG
Baar	T---ACA--------------TGTTTAAC-GGC-CGCGGTATT-CTAACCGTGCGAAGGTAG
Moar	TA-TAA----------------GTTTAAC-GGC-CGCGGTATT-TTGACCGTGCGAAGGTAG
Toja	-ACACT----------------GTTCAAC-GGC-CGCGGTATT-TTGACCGTGCGAAGGTAG
Chau	-TATA----------------TGTTTAAC-GGC-CGCGGTATT-TTGACCGTGCGAAGGTAG
Chse	T-ATAC----------------GTTTAAC-GGC-CGCGGTATT-TTGACCGTGCAAAGGTAG
Enar	T-ATTA----------------GTTTAAC-GGC-CGCGGTATT-TTGACCGTGCGAAGGTAG
Hpty	-TATAA----------------GTTTAAC-GGC-CGCGGTATT-TTGACCGTGCGAAGGTAG
Nana	ATAAACT---------------GTTTAAC-GGC-CGCGGTATC-CTGACCGTGCAAAGGTAG
Mcst	TATAT-----------------GTTTAAC-GGC-CGCGGTATT-CTGACCGTGCGAAGGTAG
Rhox	TATA-T----------------GTTTAAC-GGC-CGCGGTATT-TTGACCGTGCGAAGGTAG
Opfa	T-----------------ATATGTTTAAC-GGC-CGCGGTATT-TTGACCGTGCAAAGGTAG
Paar	TATAC-----------------GTTTAAC-GGC-CGCGGTATC-CTGACCGTGCAAAGGTAG
Gozo	-AATA----------------AATTTAAC-GGC-CGCGGTATT-TTGACCGTGCGAAGGTAG
Ackr	TAT--AT---------------GTTTAAC-GGC-CGCGGTATT-TTGACCGTGCAAAGGTAG
Elev	GATGA-----------------GTTCAAC-GGC-CGCGGTATT-CTGACCGTGCAAAGGTAG
Trdu	--TATAA---------------GTTTAAC-GGC-CGCGGTATT-TTGACCGTGCAAAGGTAG
Amoc	AACATT----------------GTTTAAC-GGC-CGCGGTACT-TTGACCGTGCGAAGGTAG
Hame	TA-TAT----------------GTTTAAC-GGC-CGCGGTATT-TTGACCGTGCAAAGGTAG
Chso	TA-TAT----------------GTTTAAC-GGC-CGCGGTATT-TTGACCGTGCGAAGGTAG
Lyto	TATTA-----------------GTTTAAC-GGC-CGCGGTATT-TTGACCGTGCGAAGGTAG

```
Encr            T-ATTA----------------GTTTAAC-GGC-CGCGGTATT-TTGACCGTGCGAAGGTAG
Bvar            --TATAT---------------GTTCAAC-GGC-CGCGGTATT-TTAACTGCCCGAAGGTAG
Noco            -TATTA----------------GTTTAAC-GGC-CGCGGTATT-TTGACCGCCCGAAGGTAG
Chsp            CAC--GA---------------GTTTAAC-GGC-CGCGGTATT-TTGACCGTGCAAAGGTAG
Arja            T-ATTT----------------GTTTAAC-GGC-CGCGGTATT-TTGACCGTGCGAAGGTAG
Pase            -----------------GAAATGTTTAAC-GGC-CGCGGTATC-TTAACCGTGCAAAGGTAG
Trel            CAAA-A----------------GTTCAAC-GGC-CGCGGTATA-CTGACCGTGCGAAGGTAG
Lifa            TTTAGT----------------GTTTAAC-GGC-CGCGGTATC-TTGACCGTGCGAAGGTAG
Acur            TACTAA----------------GTTTAAC-GGC-CGCGGTATT-CTAACCGTGCGAAGGTAG
Ampe            -TCTAA----------------GTTTAAC-GGC-CGCGGTATT-TTGACCGTGCGAAGGTAG
Urja            T-ATAT----------------GTTTAAC-GGC-CGCGGTATT-TTAACCGTGCAAAGGTAG
Enet            --AC-AC---------------GTTTAAC-GGC-CGCGGTATT-CTGACCGTGCAAAGGTAG
Ptbr            TTTTA-----------------ATTTAAC-GGC-CGCGGTATC-CTAACCGTGCAAAGGTAG
Safa            CTAGA-----------------ATTTAAC-GGC-CGCGGTATT-TTGACCGTGCAAAGGTAG
Icae            --TATAT---------------GTTTAAC-GGC-CGCGGTATT-CTAACCGTGCGAAGGTAG
Asmi            -A-T--A---------------GTTTAAC-GGC-CGCGGTATT-TTGACCGTGCGAAGGTAG
Foal            -TTTAT----------------GTTTAAC-GGC-CGCGGTATT-TTAACCGTGCAAAGGTAG
Drze            -ATACAA---------------GTTTAAC-GGC-CGCGGTATC-CTAACCGTGCAAAGGTAG
Rhas            -GT--AA---------------GTTTAAC-GGC-CGCGGTATT-TTGACCGTGCGAAGGTAG
Elac            CAAAG-----------------GTTTAAC-GGC-CGCGGTATC-CTGACCGTGCGAAGGTAG
Kugu            TTACGA----------------GTTTAAC-GGC-CGCGGTATT-TTGACCGTGCAAAGGTAG
Plor            -T-ATAA---------------GTTTAAC-GGC-CGCGGTATT-TTAACCGTGCAAAGGTAG
Sgun            TATAT-----------------GTTTAAC-GGC-CGCGGTATT-TTGACCGTGCGAAGGTAG
Zaco            TATAA-----------------GTTCAAC-GGC-CGCGGTATT-TTGACCGTGCGAAGGTAG
Zbfl            --TTAAA---------------GTTTAAC-GGC-CGCGGTATT-TTGACCGTGCGAAGGTAG
Spba            TATAT-----------------GTTCAAC-GGC-CGCGGTATT-TTGACCGTGCAAAGGTAG
Game            TATAT-----------------GTTTAAC-GGC-CGCGGTATT-TTAACCGTGCGAAGGTAG
Thth            TATAT-----------------GTTTAAC-GGC-CGCGGTATT-TTAACCGTGCAAAGGTAG
Xigl            AATCTA----------------GTTCAAC-GGC-CGCGGTATT-TTGACCGTGCAAAGGTAG
Hyja            T---ATA--------------TGTTCAAC-GGC-CGCGGTATT-TTAACCGTGCGAAGGTAG
Psan            TATAT-----------------GTTTAAC-GGC-CGCGGTATT-TTAACCGTGCGAAGGTAG
Cupa            TAT--AT---------------GTTTAAC-GGC-CGCGGTATT-TTAACCGTGCGAAGGTAG
Mpch            A-AAAA----------------GTTTAAC-GGC-CGCGGTATT-TTGACCGTGCAAAGGTAG
Char            A---CTA--------------AGTTCAAC-GGC-CGCGGTATT-TTGACCGTGCAAAGGTAG
Pser            C-ATGT----------------GTTTAAC-GGC-CGCAGTATT-TTGACTGTGCGAAGGTAG
Prol            -ATCATA---------------GTTCAAC-GGC-CGCGGTATT-TTGACCGTGCAAAGGTAG
Plbi            AACT-TA---------------GTTCAAC-GGC-CGCGGTATT-TTGACCGTGCAAAGGTAG
Calu            AAT-AT----------------GTTCAAC-GGC-CGCAGTATT-TTGACTGTGCTAAGGTAG
Papa            ATACAT----------------GTTCAAT-GGC-CGCGGTATA-TTAACCGTGCGAAGGTAG
Sufr            TTTTAC----------------GTTTAAC-GGC-CGCGGTATT-TTGACCGTGCGAAGGTAG
Stci            TCTGG-----------------GTTTAAC-GGC-CGCGGTATC-TTAACCGTGCGAAGGTAG
Taru            T---ATA--------------TGTTTAAC-GGC-CGCGGTATT-TTGACCGTGCAAAGGTAG
Rala            TATAA-----------------GTTTAAC-GGC-CGCGGTATT-TTGACCGTGCGAAGGTAG
                                       ** **  ***     * *      **      ** ****
```

```
                    39'   40        40'          36'      33'
Scca    CGTAATCACTT-GTCTTTTAAATGAAGACCCGTATGAAAGGCACCACGAGAGTTTGACTG
Muma    CGTAATCATTT-GTCTTTTAAATGAAGACCCGTATGAAAGGCACCACGAGAGTTTAACTG
Erca    CGTAATCACTT-GTCCTTTAAATAGGGACTGGTATGAATGGCCAAACGAGGGCTCGACTG
Pose    CGTAATCACTT-GTCCTTTAAATGAGGACTGGTATGAATGGCCCCACGAGGGCTCAACTG
Actr    CGTAATCACTT-GTCTTTTAAATGAAGACCTGTATGAATGGCATAACGAGGGCTCAACTG
Scal    CGTAATCACTT-GTCTTTTAAATGAAGACCTGTATGAATGGCATTACGAGGGCTCAACTG
Posp    CGTAATCACTT-GTCTTTTAAATGAAGACCTGTATGAATGGCATAACGAGGGCTCGACTG
Atsp    CGTAATCACTT-GTCTTTTAAATGAAGACCGGTATGAATGGCATCACGAAGGCTCACCTG
Leoc    CGTAATCACTT-GTCTTTTAAATGAAGACCGGTATGAATGGCATCACGAGGGCTCACCTG
Amca    CGTAATCACTT-GTCTTTTAAATAAAGACCTGTATGAATGGCATCACGAGGGCTCATCTG
Osbi    CGTAATCACTT-GTCTTTTAAATGAAGACCCGTATGAAAGGTACCACGAAGACCTTACTG
Pabu    CGTAATCACTT-GTCTTTTAAATGAAGACCCGTATGAAAGGCACCACGAGGGCCCTACTG
Hial    CGTAATCACTT-GTCTTTTAAATGAAGACCCGTATGAATGGCATCACGAGGGCCCAGCTG
Elha    CGTAATCACTT-GTCTTTTAAATGAAGACCGGTATGAATGGCAACACGAGGGCTCAGCTG
Mlcy    CGTAATCACCT-GTCTTTTAAATGAAGACCTGTATGAAAGGCACCACGAGGGCTCAACTG
Algl    CGTAATCATTT-GTCTTTTAAATAAAGACCTGTATGAATGGCATCACGAGGGCTCGACTG
Ptgi    CGTAATCACTT-GTCTTTTAAATGGAGACCTGTATGAATGGCACCACGAGGGCTCGACTG
Alaf    CGTAATCACTT-GTCTTTTAAATGAAGACCCGTATGAACGGCATCACGAGGGCTCAACTG
Nock    CGTAATCACTT-GTCTTTTAAATGAAGACCCGTATGAATGGCATCACGAGGGCTCAGCTG
Anja    CGTAATCATTT-GTCTTTTAAATAGAGACCTGTATGAATGGCATAACGAGGGTTTAACTG
Gyki    CGTAATCACTT-GTCTTTTAAATGGAGACCTGTATGAATGGCATAACGAGGACTTAACTG
Syka    CGTAATCATTT-GTCTTTTAAATGAAGACCAGTATGAATGGCATCACGAGGGTTTAACTG
Opma    CGTAATCACTT-GTCTTTTAAATGGAGACCTGTATGAATGGCACCACGAGGGTTTAACTG
Comy    CGTAATCACCT-GTCTTTTAAATAAAGACCTGTATGAATGGCAAGACGAGGGTTTTACTG
Sasp    CGTAATCACTT-GTCTTTTAAATAAAGACCTGTATGAATGGCTTAACGAGGGGCTTACTG
Eupe    CACAATCACTT-GTCTTTTAAATAAAGACCTGTATGAATGGCACAACGAGGGATTAACTG
Enja    CGCAATCAATT-GCCTTTTAAATGAAGGCCTGTATGAATGGTATAACGAGGGTCTAGCTG
Same    CGCAATCAATT-GTCTTTTAAATGGAGACCTGTATGAATGGCATAACGAGGGTTCAACTG
Chch    CGCAATCACTT-GTCTCTTAAATAGAGACCTGTATGAATGGCACGACGAGGGCTTAACTG
Grgr    CGCAATCACTT-GTCGCTTAAATGGAGACCTGTATGAAGGGCGCCACGAGGGCTTGACTG
Caau    CGCAATCACTT-GTCTTTTAAATAGAGACCTGTATGAATGGCTAAACGAGGGCTTAACTG
Cyca    CGCAATCACTT-GTCTTTTAAATAGAGACCTGTATGAATGGCTAAACGAGGGCTTAACTG
Dare    CGCAATCACTC-GTCCTTTAAATAGGGACCTGTATGAATGGCCAAACGAGGGCTTAACTG
Cost    CGCAATCACTT-GTCTTTTAAATGAAGACCTGTATGAATGGCTAAACGAGGGCTTAGCTG
Leec    CGCAATCACTT-GTCTTTTAAATGAAGACCCGTATGAATGGCCAAACGAGGGCTTAACTG
Crla    CGCAATCACTT-GTCTTTTAAATAAAGACCTGTATGAATGGCCAAACGAGGGCTTAACTG
Clmc    CGCAATCACTT-GTCTTTTAAATGAAGACCTGTATGAATGGTGGAACGAGGGCTTAACTG
Phin    CGCAATCACTT-GTTTTTTAAATGAAGACCTGTATGAATGGTGAAACGAGGGCTTAACTG
Icpu    CGCAATCACTT-GTCTCTTAAATGGAGACCTGTATGAATGGTGGAACGAGGGCTTAACTG
Psto    CGCAATCACTT-GTCTTTTAAATGAAGACCTGTATGAATGGTGGAACGAGGGCTTAACTG
Cora    CGCAATCACTT-GTCTTTTAAATGAAGACCTGTATGAATGGTGGAACGAGGGCCTAACTG
Eisp    CGTAATCACTT-GTCTTTTAAATAAAGACCTGTATGAATGGCAAAACGAGGGCTTAACTG
Apal    CGCAATCACTT-GTTTCATAAATTGAAACCCGTATGAATGGCAAGACGAAGGCTTAACTG
Eslu    CGCAATCACTT-GTCTTTTAAATGAAGACCTGTATGAATGGCATCACGAGGGCTTAGCTG
Dape    CGCAATCACTT-GTCTTTTAAATGAAGACCTGTATGAATGGCATCACGAGGGCTTAGCTG
Glse    CGCAATCACTT-GTCTTTTAAATGAAGACCTGTATGAATGGCACGACGAGGGCTTAGCTG
Naar    CGTAATCACTT-GTCTTTTAAATGAAGACCCGTATGAATGGCACAACGAGGGCTTGACTG
Baoc    CGTAATCACTT-GTCTTTTAAATGAAGACCCGTATGAATGGCACGACGAGGGCTTAACTG
Opso    CGCAATCACTT-GTCTTTTAAATGAAGACCTGTATGAATGGCATAACGAGGGCTTAACTG
Alte    CGCAATCACTT-GTCTTTTAAATGAGGACCTGTATGAATGGCATCACGAGGGCTTAGCTG
Plap    CGCAATCACTT-GTCTTTTAAATGAGGACCTGTATGAATGGCATCACGAGGGCTTAGCTG
```

Plal	CGCAATCACTT-GTCCTTTAAATGAAGACCTGTATGAATGGCTAGACGAGGGCTTAACTG
Sami	CGCAATCACTT-GTCCTTTAAATGAGGACCTGTATGAATGGCTAGACGAGGGCTTAGCTG
Rere	CGCAATCACTT-GTCCTTTAAATGAAGACCTGTATGAATGGCTAGACGAGGGCTTGACTG
Gama	CGCAATCACTT-GTCTTTTAAATGAAGACCCGTATGAATGGCAAGACGAGGGCTAAGCTG
Onmy	CGCAATCACTT-GTCTTTTAAATGAAGACCTGTATGAATGGCATCACGAGGGCTTAGCTG
Sasa	CGCAATCACTT-GTCTTTTAAATGAAGACCTGTATGAATGGCATCACGAGGGCTTAGCTG
Cola	CGCAATCACTT-GTCTTTTAAATGAAGACCTGTATGAATGGCATCACGAGGGCTTAGCTG
Dita	CGCAATCACTT-GTCTTTTAAATGAAGACCCGTATGAAAGGCACGACGAGGGCTTAACTG
Gogr	CGCAATCACTT-GTCTTTTAAATAAAGACCCGTATGAATGGCATAACGAAGGCTCAACTG
Chsl	CGCAATCACTT-GTCTCCTAATTAGAGACCGGTATGAACGGCAAGACGAGGGTCTAACTG
Atja	CGCAATCACTT-GTCTTTTAAATGAAGACCTGTATGAACGGCATTACGAGGGCTTAGCTG
Iido	CGCAATCACTT-GTCTTTTAAATGAAGACCTGTATGAATGGCATTACGAGGGCTTAGCTG
Auja	CGCAATCACTT-GTCCTTTAAATGAGGACCTGTATGAATGGCACAACGAAGGCTTAACTG
Chag	CGCAATCACTT-GTCCTTTAAATGGGGACCTGTATGAATGGCACGACGAGGGCTTAGCTG
Hami	CGCAATCACTT-GTCCTTTAAATGAGGACCCGTATGAATGGCATAACGAGGGCTCAACTG
Saun	CGCAATCACTT-GTCCTTTAAATGAGGACCCGTATGAATGGCATAACGAGGGCTCAACTG
Nema	CGCAATCACTT-GTCTTTTAAATGGAGACCTGTATGAAAGGCACCACGAGGGCTTAACTG
Disp	CGCAATCACTT-GTCTCTTAAATGGAGACCTGTATGAAAGGCACGACGAGGGCTTAGCTG
Myaf	CGCAATAAATT-GTCTCTTAATTAGGGACTAGTATGAATGGCATCACGAGGGCTTAACTG
Lagu	CGCAATCACTT-GTCTTTTAAATGAAGACCTGTATGAATGGCACCACGAGGACTTAGCTG
Trtr	CGTAATCACTT-GTCTTTTAAATGGAGACCTGTATGAATGGCGAGACGAGGGCGAAACTG
Zucr	CGTAATCACTT-GTCTTTTAAATGGAGACCTGTATGAAAGGCGAGACGAGGGCAAAACTG
Pxja	CGCAATCACTT-GTCTTTTAAATGAAGACCCGTATGAATGGCATAACGAGGGCTTAACTG
Pxlo	CGCAATCACTT-GTCTTTTAAATGAAGACCCGTATGAATGGCATAACGAGGGCTTAACTG
Pctr	CGCAATCACTT-GTCTCTTAAATGGAGACCTGTATGAAAGGCATCACGAGGGCTTAGCTG
Apsa	CGTAATCACTT-GTCTCTTAAATAGGGACCTGTATGAATGGCAACACGAGGGCTTAACTG
Cabe	CGCAATCACTT-GTCTCTTAATTGGGGACCCGTATGAATGGCATAACGAGGGCCCAGCTG
Bzze	CGCAATCACTT-GTCTCTTAAATGGAGACCTGTATGAATGGCACGACGAGGGCTTAACTG
Siim	CGTAATCACTT-GTCTTTTAAATGAAGACCTGTATGAATGGCAAGACGAGGGCTTAACTG
Ctru	CGCAATCACTT-GTCTCTTAAATGGAGACCTGTATGAATGGCATAACGAGGGCTTAACTG
Dpbr	CGCAATCACTT-GTCTCTTAAATGGAGACCTGTATGAATGGCATAACGAGGGCTTAACTG
Caki	CGTAATCATTT-GTCTTTTAAATGAAGACCTGTATGAATGGCAAAACGAGGGCTTAACTG
Phja	CGTAATCACTT-GTTTTTTAAATGGAGACCAGTATGAATGGCATCACGAGGGCTTAACTG
Brsp	CGCAATCACTT-GTCTTTTAAATGAAGACCTGTATGAATGGCCCCACGAGGGCTTGACTG
Gamo	CGTAATCACTT-GTCTTTTAAATGAAGACCTGTATGAATGGCATCACGAGGGCTTAGCTG
Lolo	CGTAATCACTT-GTCTTTTAAATGAAGACCTGTATGAATGGCATCACGAGGGCTTAGCTG
Batr	CATAATCAAAT-GTCTTTTAATTGAAGACCGGTATCAAAGGCTTCACGAAGGCTCAACTG
Prmy	CGTAATCACTT-GTCTTTTAATTGAAGACCTGTATGAATGGCATCACGAGGATTTAACTG
Loli	CGTAATCACTT-GTCTTTTAAATGAGGACCAGTATGAATGGCAAAACGAGGGCTTAACTG
Loam	CGCAATCACTT-GTCTTTTAAATGAAGACCTGTATGAATGGCAAAACGAGGGCTTAGCTG
Chab	CGTAATCATTT-GTCTTTTAAATTAAGACCAGTATGAAAGGTGAGACGAGGGCTTAGCTG
Chto	CGTAATCATTT-GTCTTTTAAATTAAGACCAGTATGAAAGGTGAGACGAGGGCTTAGCTG
Majo	CGCAATCACTT-GTCTTTTAAATAAAGACCTGTATGAATGGCATGACGAGGGCTTAACTG
Hlst	CGCAACCACTT-GTCTTTTAAATAGAGACCTGTATGAATGGCATCACGAGGGCTAAACTG
Clpe	CGTAATCACTT-GTCTTTTAATTGGAGACCTGTATGAATGGCATAACGAGGGCTGAACTG
Mlmr	CGTAATCACTT-GTCTTTTAAATGGAGACCCGTATGAATGGCCTTACGAGGGCTTAGCTG
Crcr	CGCAATCACTT-GTCCCTTAAATGAGAACCAGTATGAATGGCTAGACGAGGGCTTAACTG
Muce	CGCAATCACTT-GTCCCTTAAATGAGAACCAGTATGAATGGCTAGACGAGGGCTTAACTG
Bege	CGCAATCACTT-GTCTTTTAAATGAAGACCCGTATGAATGGCATAACGAGGGCTTAACTG
Mela	CGCAATCACTT-GTCTTTTAAATGAAGACCTGTATGAATGGCACAACGAGGGCTCAACTG
Hats	CGCAATCACTT-GTCTTTTAAATGAAGACCCGTATGAATGGCACGACGAGGGCTTAACTG
Orla	CGCAATCACTT-GCCTTTTAAATGAGGGCCTGTATGAATGGCATAACGAGGGCTTAACTG

```
Cosa    CGCAATCACTT-GTCTTTTAAATGAAGACCTGTATGAATGGCATAACGAGGGCTTAACTG
Exsp    CGCAATCACTT-GTTTCTTAAATGGAGACCTGTATGAATGGCATGACGAGGGCCAACCTG
Depa    CGCAATCACTT-GTTTTTTAAATGAAGACCTGTATGAATGGCATAACGAGGGCTTAACTG
Rima    CGTAATCATTT-GTCTTTTAAATGAAGACCTGTATGAATGGCATAACGAGGGCTTAGCTG
Fuol    CGCAATCACTT-GTCTTTTAAATGAAGACCTGTATGAATGGCTAAACGAGGGCTAAACTG
Gmaf    CGTAATCACTT-GTCTTTTAAATGAAGACCCGTATGAATGGCAAAACGAGGGCTTAACTG
Xeei    CGCAATCACTT-GTCTTTTAAATGAAGACCTGTATGAATGGCAAAACGAGGGCTTAACTG
Pros    CGCAATCACTT-GTCTTTTAAATGAAGACCCGTATGAATGGCTTGACGAGGGCTTAGCTG
Scmi    CGCAATCACTT-GTCTTTTAAATGAAGACCCGTATGAATGGCTTGACGAGGGCTTAGCTG
Rolo    CGCAATCACTT-GTCTTTTAAATGAAGACCCGTATGAATGGCACAACGAGGGCTTAGCTG
Cere    CGCAATCACTT-GTCTTTTAAATGAAGACCTGTATGAATGGTATAACGAAGGCTTAGCTG
Daga    CGCAATCACTT-GTCTTTTAAATGAAGACCTGTATGAATGGCACGACGAGGGCTTAACTG
Anco    CGCAATCACTT-GTCTTTTAAATGAAGACCTGTATGAATGGCACGACGAGGGCCTAGCTG
Dmve    CGTAATCACTT-GTCTTTTAAATGAAGACCCGTATGAACGGCACGACGAAGGCCCAGCTG
Dmar    CGTAATCACTT-GTCTTTTAAATGAAGACCTGTATGAATGGCACGACGAAGGCCCAGCTG
Anka    CGCAATCACTT-GTCTTTTAAATGAAGACCCGTATGAATGGCACGACGAGGGCCTAACTG
Moja    CGCAATCACTT-GTCTTTTAAATGAAGACCCGTATGAATGGCACGACGAGGGCCTAACTG
Hoja    CGCAATCACTT-GTCTTTTAAATGAAGACCCGTATGAATGGCACGACGAGGGCCTAGCTG
Bede    CGCAATCACTT-GTCTTTTAAATGAAGACCCGTATGAATGGCATAACGAGGGCTTAGCTG
Besp    CGCAATCACTT-GTCTTTTAAATGAAGACCCGTATGAATGGCATAACGAGGGCTTAGCTG
Mysp    CGCAATCACTT-GTCTTTTAAATGAAGACCCGTATGAATGGCATAACGAGGGCCTAGCTG
Osja    CGCAATCACTT-GTCTTTTAAATGAAGACCCGTATGAATGGCATAACGAGGGCCTAACTG
Sgro    CGCAATCACTT-GTCTTTTAAATGAAGACCCGTATGAATGGCATAACGAGGGCTTAACTG
Pzpa    CGTAATCACTT-GTCTTTTAAATGGAGACCTGTATGAATGGCACCACGAGGGCTTAGCTG
Zeja    CGCAATCACTT-GTCTTTTAAATGGAGACCCGTATGAATGGCACCACGAGGGCTTAGCTG
Znne    CGCAATCACTT-GTCTTTTAAATGAAGACCCGTATGAATGGCACCACGAGGGCTTAGCTG
Zefa    CGCAATCACTT-GTCTTTTAAATGAAGACCTGTATGAATGGCATCACGAGGGCTTAGCTG
Acni    CGCAATCACTT-GTCTTTTAAATGAAGACCCGTATGAATGGCACCACGAGGGCTTAGCTG
Ncrh    CGCAATCACTT-GTCTTTTAAATGAAGACCCGTATGAATGGCACCACGAGGGCTTAGCTG
Agca    CGCAATCACTT-GTCTCTTAAATGGAGACCTGTATGAATGGCATAACGAGGGCTAAGCTG
Hydy    CGCAATCACTT-GTCTTTTAAATGGAGACCTGTATGAATGGCATAACGAGGGCTTAACTG
Gsac    CGCAATCACTT-GTCTTTTAAATGGAGACCTGTATGAATGGCATAACGAGGGCTTAGCTG
Pevo    CGCAATCACTT-GTCTTTTAAATGAAGACCTGTATGAATGGCACAACGAGGGCTTAGCTG
Hiku    CGCAATCACTT-GTCTTTTAAATAAAGACCTGTATGAATGGCATAACGAGGGCTAAACTG
Inpa    CGCAATCACTT-GTCTTTTAATTGAAGACCTGTATGAATGGCACAACGAGGGCTTAACTG
Auch    CGTAATCACTT-GTCTTTTAAATTGAGACCTGTATGAACGGAATAACGAGGGCTTAACTG
Fico    CGCAATCACTT-GTCTTTTAAATGAAGACCTGTATGAATGGCAAGACGAGGGCTTAACTG
Macs    CGCAATCACTT-GTCTTTTAAATGAAGACCTGTATGAATGGCACGACGAGGGCTTAACTG
Moal    CGTAATCACTT-GTCTTTTAATTGAAGACCTGTATGAATGGCATAACGAAGGCTTAACTG
Syma    CGCAATCATTT-GTCTTCTAATTAAAGACCTGTATGAACGGCATAACGAGAGCTTACCTG
Mafr    CGCAATCACTT-GTCTTTTAAATGAAGACCTGTATGAATGGCATAACGAAGGCTTAACTG
Dcpe    CGTAATCACTA-GTCTTTTAAATGAAGACCCGTATGAATGGCTTAACGAGGGCTTAACTG
Dcti    CGTAATCACTC-GTCTTTTAAATGAAGACCTGTATGAATGGCTTGACGAGGGCTTGACTG
Hehi    CGCAATCACTT-GTCTTTTAAATGAAGACCTGTATGAATGGCACAACGAGGGCTTAACTG
Stam    CGCAATCACTT-GTCTTTTAAATGAAGACCTGTATGAATGGCATAACGAGGGCTTAACTG
Hogi    CGCAATCACTT-GTCTTTTAAATGGAGACCTGTATGAATGGCATAACGAGGGCTTAACTG
Erzo    CGCAATCACTT-GTCTTTTAAATGAAGACCTGTATGAATGGCAAAACGAGGGCTTAACTG
Hxot    CGCAATCACTT-GTCTTTTAAATGGAGACCCGTATGAATGGCATAACGAGGGCTTAACTG
Core    CGCAATCACTT-GTCTTTTAAATGAAGACCCGTATGAATGGCATAACGAGGGCTTAGCTG
Apve    CGCAATCACTT-GTCTTTTAAATGAAGACCCGTATGAATGGCATAACGAGGGCTTAACTG
Latj    CGTAATCACTT-GTCTTTTAAATGAAGACCTGTATGAATGGCATGACGAGGGCTTAACTG
Laja    CGTAATCACCT-GTCTTTTAAATGGAGACCTGTATGAATGGCACGACGAGGGCTTGACTG
```

Syja	CGCAATCACTT-GTCCTTTAAATAGGGACCTGTATGAATGGCATAACGAGGGCTTAACTG
Epme	CGCAATCACTT-GTCTTTTAAATGGGGACCTGTATGAATGGCACAACGAGGGCTTGACTG
Grse	CGCAATCACTT-GTCTTTTAAATGGAGACCTGTATGAATGGCATAACGAGGGCTTAACTG
Clja	CGCAATCACTT-GTTTCTTAAATGGGGACCTGTATGAATGGCTTCACGAGGGCTTAACTG
Ogcy	CGCAATCATTT-GTCTTTTAAATAAAGACCCGTATGAATGGCAAGACGAGGGCTTAACTG
Plna	CGTAATCACTT-GTTTTTTAAATGAAGACCTGTATGAATGGCATAACGAGGGCTTAACTG
Lema	CGCAATCACTT-GTCTTTTAAATGAAGACCTGTATGAATGGCACAACGAGGGCTTAGCTG
Etzo	CGCAATCACTT-GTCTTTTAAATGGAGACCTGTATGAATGGCATAACGAGGGCTTAACTG
Apse	CGCAATCACTT-GTCTCTTAAATGGAGACCTGTATGAATGGCATGACGAGGGCTAAGCTG
Epde	CGCAATCACTT-GTCTTTTAAATGAAGACCTGTATGAATGGCACCACGAGGGCTTAGCTG
Slja	CGCAATCACTT-GCTCTTTAAATGGGGGCCCGTATGAATGGCGTGACGAGGGCTTGACTG
Bsja	CGCAATCACTT-GTCTTTTAAATGGAGACCTGTATGAATGGCATACCGAGGGCTTAACTG
Ecna	CGTAATCACTT-GTCTTTTAAATGAAGACCTGTATGAATGGTATGACGAGGGCTTAACTG
Cohi	CGTAATCATTT-GTCTTTTAAATGGGGACTTGTATGAATGGTATAACGAGGGCTTAACTG
Caar	CGTAATCACTT-GTCTTTTAAATGAAGACCTGTATGAATGGCATAACGAGGGCTTAACTG
Came	CGTAATCACTT-GTCTTTTAAATGGAGACCTGTATGAATGGCATAACGAGGGCTTAACTG
Mema	CGTAATCACTT-GTCTTTTAAATGGAGACCTGTATGAATGGCACAACGAGGGCTTAGCTG
Lenu	CACAATCACTT-GTCCTTTAAATGGGGACCTGTATGAATGGTATGACGAAGGCTAAGCTG
Brja	CGCAATCACTT-GTCTTTTAAATGAAGACCTGTATGAATGGCACGACGAGGGCTTAACTG
Plma	CGCAATCACTT-GTCTTTTAAATGGAGACCTGTATGAATGGCATAACGAGGGCTTAACTG
Emst	CGCAATCACTT-GTCTTTTAAATGGAGACCTGTATGAATGGCATAACGAGGGCTTAGCTG
Ptti	CGCAATCACTT-GTCTTTTAAATGGAGACCTGTATGAATGGCATAACGAGGGCTTAGCTG
Losu	CGCAATCACTT-GTCCTTTAAATGGGGACCAGTATGAATGGCATCACGAAGGCTTAACTG
Geoy	CGCAATCACTT-GTCCTTTAAATGAAGACCGGTATGAATGGCATCACGAGGGCTTTACTG
Dipi	CGCAATCACTT-GTCTCTTAAATGGGGACCCGTATGAATGGCATAACGAGGGCTTAGCTG
Pama	CGCAATCACTT-GTCTTTTAAATGGAGACCTGTATGAATGGCATCACGAGGGCTTAACTG
Leob	CGTAATCACTT-GTCTCTTAAATGGGGACCTGTATGAATGGCATAACGAGGGTTCAACTG
Neba	CGCAATCACTT-GCCCTTTAAATGGGGGCCCGTATGAATGGCATCACGAGGGCTAATCTG
Pdpl	CGTAATCACTT-GTCTTTTAAATGGAGACCTGTATGAATGGCAAGACGAGGGCTTAACTG
Nimi	CGCAATCACTT-GTCTTCTAAATAGAGACCCGTATGAACGGTAAAACGAGGGCTTAACTG
Uptr	CGCAATCACTT-GTCTTTTAAATGGAGACCTGTATGAATGGCATAACGAGGGCTTGACTG
Pesc	CGTAATCACTT-GTCTCTTAAATGGAGACCTGTATGAATGGCAAGACGAGGGCTCAACTG
Baar	CGAAATCATTT-GTCTTTTAAATGGGGACCAGTATGAACGGCACAACGAGGGCTTAGCTG
Moar	CGCAATCACTT-GTCTTTTAAATGAAGACCTGTATGAATGGCATAACGAGGGCTTAGCTG
Toja	CGTAATCACTT-GTCTTTTAAATGAAGACCTGTATGAATGGCAAGACGAGGGCTTAACTG
Chau	CGCAATCACTT-GTCTTTTAAATGAAGACCTGTATGAATGGCACAACGAGGGCCTAACTG
Chse	CGAAATCACTT-GTCTTTTAAATGGAGACCTGTATGAATGGCATAACGAGGGCTTAACTG
Enar	CGCAATCACTT-GTCTTTTAAATGGAGACCTGTATGAATGGCACGACGAGGGCTTAGCTG
Hpty	CGCAATCACTT-GTCTTTTAAATGGAGACCTGTATGAATGGCACGACGAGGGCTTAGCTG
Nana	CGCAATCACTT-GTCTTTTAAATGAAGACCTGTATGAATGGCATAACGAGGGCTAAGCTG
Mcst	CGCAATCACTT-GTCTTTTAAATGAAGACCCGTATGAATGGCATAACGAGGGCTTAACTG
Rhox	CGTAATCACTT-GTCTTTTAAATGGAGACCCGTATGAATGGCATGACGAGGGCTTAACTG
Opfa	CGCAATCACTT-GTCTTTTAAATGGAGACCCGTATGAATGGCATAACGAGGGCTTAACTG
Paar	CGCAATCACTT-GTCTTTTAAATGGAGACCTGTATGAATGGCATAACGAGGGCTTGACTG
Gozo	CGAAATCACTT-GTCTTTTAAATGAAGACCTGTATGAATGGCACGACGAGGGCTTAACTG
Ackr	CGCAATCACTT-GTCTTTTAAATGGAGACCCGTATGAATGGCATAACGAGGGCCTAACTG
Elev	CGCAATCAATT-GTCTTTTAAATGAAGACCTGTATGAATGGCATAACGAAGGCTTAACTG
Trdu	CGCAATCACTT-GTCTTTTAAATGAAGACCTGTATGAATGGCATAACGAGGGCTTAACTG
Amoc	CGCAATCACTT-GTCTTTTAAATGAAGACCTGTATGAATGGCACGACGAGGGCTTAACTG
Hame	CGCAATCACTT-GTCTTTTAAATGGAGACCTGTATGAATGGCATCACGAGGGCTTAACTG
Chso	CGCAATCACTT-GTCTTTTAAATGGAGACCTGTATGAATGGCACGACGAGGGCTTAACTG
Lyto	CGCAATCACTT-GTCTTTTAAATGAAGACCTGTATGAATGGCATAACGAGGGCTTAACTG

```
Encr    CGCAATCACTT-GTCTTTTAAATGAAGACCTGTATGAATGGCATAACGAGGGCTTAACTG
Bvar    CGAAATCACTT-GTCTTTTAAATGGGGACCTGTATGAATGGCATGACGAGGGCTTAACTG
Noco    CGCAATCACTT-GTCTCTTAAATGGAGACCTGTATGAATGGCACGACGAGGGCTTAGCTG
Chsp    CGCAATCACTT-GTCTCCTAAATGAAGACCTGTATGAATGGCAAGACGAGGGCATAACTG
Arja    CGCAATCACTT-GTCTTTTAAATGGAGACCCGTATGAATGGCATAACGAGGGCTTAGCTG
Pase    CGCAATCACTT-GTCTTTTAAATGGAGACCCGTATGAATGGCTAAACGAGGGCCTAACTG
Trel    CGCAATCACTT-GTCCTTTAAATGAAGACCTGTATGAATGGCATTACGAGGGCTAAGCTG
Lifa    CGTAATCACCT-GTCTTTTAAATGAAGACCTGTATGAATGGCCTCACGAGGGCTTTACTG
Acur    CGCAATCACTT-GTCTTTTAAATGAAGACCCGTATGAATGGTCAAACGAGGGCTTAACTG
Ampe    CGCAATCACTT-GTCTTTTAAATGGAGACCTGTATGAATGGCATAACGAGGGCTTAGCTG
Urja    CGCAATCACTT-GTCCTTTAAATGAAGACCTGTATGAACGGCACAACGAGGGCTTAACTG
Enet    CGCAATCACTT-GTCTTTTAAATAGAGACCTGTATGAATGGCACAACGAGGGCTTAACTG
Ptbr    CGCAATCACTT-GTCCTTTAAATAGGGACCTGTATGAATGGCACAACGAGGGCTTAACTG
Safa    CGCAATCACTT-GTCTTTTAAATGAAGACCTGTATGAATGGCATAACGAGGGCTTGACTG
Icae    CGCAATCACTT-GTCTTTTAAATGGAGACCTGTATGAATGGCATAACGAGGGCTTAACTG
Asmi    CGTAATCACTT-GTCTTTTAAATGAAGACCTGTATGAATGGCAAAACGAGGGCTTAACTG
Foal    CGTAATAACTT-GTCTTTTAAATGAAGACTGGTATGAATGGCATCACGAGGGCCTGACTG
Drze    CGCAATCACTT-GTCTTTTAAATGAAGACCAGTATGAACGGCACAACGAGGGCTACACTG
Rhas    CGCAATCACTT-GTCTTTTAAATGAAGACCTGTATGAATGGCATAACGAGGGCTAAGCTG
Elac    CGCAATCACTT-GTCTTTTAAATGGAGACCTGTATGAATGGCATAACGAGGGCTAAGCTG
Kugu    CGCAATCACTT-GTCTTTTAAATGAAGACCCGTATGAATGGCATAACGAGGGCTAAACTG
Plor    CGCAATCACTT-GTCCTTTAAATGAAGACCTGTATGAATGGCACGACGAGGGCTTGACTG
Sgun    CGCAATCACTT-GTCTTTTAAATGGAGACCTGTATGAATGGCATAACGAGGGCTTAGCTG
Zaco    CGCAATCACTT-GCCTTTTAAATGAAGGCCCGTATGAATGGCACGACGAGGGCTTAGCTG
Zbfl    CGCAATCACTT-GTCTTTTAAATGGAGACCTGTATGAATGGCACAACGAGGGCTTAACTG
Spba    CGTAATCACTT-GTCTTTTAAATGAAGACCCGTATGAATGGCGGAACGAGGGCTAAACTG
Game    CGCAATCACTT-GTCTTTTAAATGGAGACCTGTATGAATGGCATTACGAGGGCTTAACTG
Thth    CGCAATCACTT-GTCTTTTAAATGGAGACCTGTATGAATGGCATTACGAGGGCTTAACTG
Xigl    CGTAATCACTT-GTCTTTTAAATGAAGACCTGTATGAATGGCATAACGAGGGCTTAGCTG
Hyja    CGTAATCACTT-GTCTTTTAAATGGAGACCTGTATGAATGGCATTACGAGGGCTTAACTG
Psan    CGTAATCACTT-GTCTTTTAAATGGAGACCTGTATGAATGGCATTACGAGGGCTTAACTG
Cupa    CGCAATCACTT-GTCTTTTAAATGGAGACCTGTATGAATGGCATAACGAGGGCTTAACTG
Mpch    CGCAATCACTT-GTCTTTTAAATGAAGACCTGTATGAATGGCACGACGAGGGCTTGACTG
Char    CGCAATCACTT-GTCTTTTAAATGAAGACCCGTATGAATGGCATAACGAGGGCTTAACTG
Pser    CGTAATCACTT-GTCTTTTAAATGGAGACCTGTATGAACGGTAAAACGAGGGCTTAACTG
Prol    CGTAATCACTT-GTCTTTTAAATGAAGACCCGTATGAATGGCATAACGAGGGCTTAGCTG
Plbi    CGTAATCACTT-GTCTTTTAAATGAAGACCTGTATGAATGGCATAACGAGGGCTTAACTG
Calu    CGCAATCACTT-GTCTTTTAAATGAAGACCTGTATGAAAGGTATGACGAGGGCTTGACTG
Papa    CGCAATCACTT-GTCTTTTAAATGAAGACCCGTATGAATGGCAAGACGAGGGCTTAACTG
Sufr    CGCAATCACTT-GTCTCTTAAATGGGGACCTGTATGAATGGCATAACGAGGGTTTAACTG
Stci    CGTAATCACTT-GTCTCTTAAATAGAGACCTGTATGAATGGCATGACGAGGGCTTAGCTG
Taru    CGCAATCACTT-GTCCTTTAAATGTGGACCTGTATGAATGGCATAACGAGGGCTTAGCTG
Rala    CGCAATCACTT-GTCTTTTAAATGGAGACCTGTATGAATGGCACGACGAGGGCTTAACTG
        *  **  *    *     *** *     *  * ** ** **     ***        ***
```

```
          32'    31' 25'  A'   41        41'      42
Scca  TCTCTACTCTC-TAATCAATGAAATTGATCTTCTCGTGCAGAAGCGAGTATGATAACATAA
Muma  TCTCTATTTTC-TAATCAATGAAATTGATCTATTCGTGCAGAAGCGAATATAATAACATTA
Erca  TCTCTTTTCTC-CTGTCAATTAAACTGATCTACCCGTGCAGAAGCGGGTATAAAAACATAA
Pose  TCTCTTTTCTC-CTGTCAATTAAACTGATCTACCCGTGCAGAAGCGGGTATAAAAACATAA
Actr  TCTCCTTTTTC-CAGTCAGTGAAATTGACCTGCTCGTGCAGAGGCGAGCATAACCCCATAA
Scal  TCTCCTTTTTC-CAGTCAGTGAAATTGACCTGCTCGTGCAGAGGCGAGCATAACCCCATAA
Posp  TCTCCTTTTTC-CAGTCAGTGAAATTGACCTGCTCGTGCAGAGGCGAGCATAACCACATAA
Atsp  TCTCCTTTTTC-CAGTCAATGAAATTGATCTGCCCGTGCAGAAGCGGACATATAAACATAA
Leoc  TCTCCTTTTTC-CAGTCAATGAAATTGATCTGCCCGTGCAGAAGCGGACATGTAAACATAA
Amca  TCTCTCTTTTC-CAGTCAATGAAATTGATCTGCCCGTGCAGAAGCGGACATATGCCCATAA
Osbi  TCTCCTTCCTC-AAGTCAGTGAAATTGATCTATTCGTGCAGAAGCGAATATCACAACATAA
Pabu  TCTCCTTCCCC-AAGTCTATGAAATTGATCTGCCCGTGCAGAAGCGGACATAAATACATAA
Hial  TCTCCTTTTCC-CAGTCAATGAAATTGATCTGCCCGTGCAGAAGCGGGCATGTAAACATAA
Elha  TCTCCCGCCTC-CAGTCAATGAAATTGATCTGCCCGTGCAGAAGCGGACATATAACCATAA
Mlcy  TCTCCTACCCC-CAGTCAATGAAATTGATCTGCCCGTGCAGAAGCGGACATAAAAACATAA
Algl  TCTCCTATCCC-TAATCAGTGAAATTGATCTGTCCGTGCAGAAGCGGGCATAAAGACATAA
Ptgi  TCTCCTCCCCC-CAGTCAATGAAATTGATCTGCCCGTGCAGAAGCGGACATAAAAACATAA
Alaf  TCTCCTCTCCC-CAGTCAATGAAATTGATCTGCCCGTGCAGAAGCGGACATAAAAACATAA
Nock  TCTCCTCTCTT-CGGTCAATGAAATTGATCTGCCCGTGCAGAAGCGGACATAAACACATAA
Anja  TCTCCTTCCCC-TAGTCAATGAAATTGATCTGCCCGTGCAGAAGCGGACATAAATACATAA
Gyki  TCTCCTTTTCC-TGGTCAATGAAACTGATCTGCCCGTGCAGAAGCGGGCATGTTAACATAA
Syka  TCTCCTTCCCC-CAGTCAATGAAATTGATCTGCCCGTGCAGAAGCGGACATAAAAACATAA
Opma  TCTCCTTCCCC-CAGTCAATGAAATTGATCTGCCCGTGCAGAAGCGGACATAACAACATAA
Comy  TCTCCTCCCCC-CAGTCAATGAAATTGATCTACCCGTGCAGAAGCGGGTATAAAAACATAA
Sasp  TCTCCCCCCCC-CAGTCAATGAAATTGATCTACCCGTGCAGAAGCGGGTACAACCACATAA
Eupe  TCTCCTTCCCC-CAGTCAATGAAATTGATCTGCCCGTGCAGAAGCGAGCATAAACACATAA
Enja  TCTCTTTTTTC-TAGTCAGTTAAACTGATCTGTCCGTGCAGAAGCGGACATAAATATACAA
Same  TCTCTTTTTTC-CGGTCAATGAAACTGATCTGCCCGTGCAGAAGCGGGCATATTCACACAA
Chch  TCTCTCCTCCC-CAGTCAGTGAAATTGATCTGTCCGTGCAGAAGCGGACATTATACTGCAA
Grgr  TCTCCCTCCCC-CAGTCAGTGAAATTGATCTGTCCGTGCAGAAGCGGACATTACCCCGCAA
Caau  TCTCCCCTTTC-CAGTCAGTGAAATTGATCTACCCGTGCAGAAGCGGGTATAATAATACAA
Cyca  TCTCCCCTTTC-AAGTCAGTGAAATTGATCTACCCGTGCAGAAGCGGGTATAATACTACAA
Dare  TCTCCCCCATC-AAGTCAGTGAAATTGATCTATCCGTGCAGAAGCGGATATAATAATACAA
Cost  TCTCCCCTTTC-CAGTCAGTGAAATTGATCTGCCCGTGCAGAAGCGGACATAAAACTACAA
Leec  TCTCCCCTTTC-TAGTCAGTGAAATTGATCTACCCGTGCAGAAGCGGGTATAATAATACAA
Crla  TCTCCCCTTTC-AAGTCAGTGAAATTGATCTGCCCGTGCAGAAGCGGACATAAACCTACAA
Clmc  TCTCCCTTTTT-AGGTCAGTGAAATTGATCTGCCCGTGCAGAAGCGGGCATAAACATACAA
Phin  TCTCCCCCTCC-CAGTCAATGAAACTGATCTGTTCGTGCAGAAGCGAACATATACCTACAA
Icpu  TCTCCCCTTTC-AAGTCAATGAAATTGATCTGCCCGTGCAGAAGCGGACATATAAATACAA
Psto  TCTCCCCTTTC-AAGTCAATGAAATTGATCTGCCCGTGCAGAAGCGGACATAAAACTACAA
Cora  TCTCCCTTTTC-AGGTCAATGAAATTGATCTGCCCGTGCAGAAGCGGGTATACAAATACAA
Eisp  TCTCCCCTTTC-TAGTCAGTGAAATTGATCTGCCCGTGCAGAAGCGGACATAAAACTACAA
Apal  TCTCCCCTTTC-AGGTCAGTGAAATTGATCTGCCCGTGAAGAAGCGGACATACTTATACAA
Eslu  TCTCCTCTTTC-AAGTCAATGAAATTGATCTGCCCGTGCAGAAGCGGACATAAGAACATAA
Dape  TCTCCTTTTTC-AAGTCAATGAAATTGATCTGCCCGTGCAGAAGCGGACATAAATACATAA
Glse  TCTCCTTCTCC-CAGTCAATGAAATTGATCTCCCCGTGCAGAAGCGGGGATTAACCCATAA
Naar  TCTCCTTCTCT-TCGTCAATGAAATTGATCTTCCCGTGCAGAAGCGGGAATATATACGTAA
Baoc  TCTCCTTCTCT-TCGTCAATGAAATTGATCTCCCCGTGCAGAAGCGGGGATAAAAACATAA
Opso  TCTCCCTCTCC-TAGTCAATGAAATTGATCTCCCCGTGCAGAAGCGGGGATAGAACCATAA
Alte  TCTCCTTTTCC-AAGTCAATGAAATTGATCTACCCGTGCAGAAGCGGGTATACCCCTACAA
Plap  TCTCCTTTTCC-AAGTCAATGAAATTGATCTACCCGTGCAGAAGCGGGTATACCCCTACAA
```

```
Plal    TCTCCTCCCCC-CAGTCAATGAAATTGATCTCCCCGTGCAGAAGCGGGGATTCTCCCATAA
Sami    TCTCCTCCCCC-CAGTCAATGAAATTGATCTCCCCGTGCAGAAGCGGGGATTAACACATAA
Rere    TCTCCTCTCCC-CAGTCAGTGAAATTGATCTCCCCGTGCAGAAGCGGGGATTGTAGCATAA
Gama    TCTCCTTCTCC-AAGTCAGTGAAATTGATCTCCCCGTGCAGAAGCGGGGATTCCCTCATAA
Onmy    TCTCCTCTTCC-AAGTCAATGAAATTGATCTGCCCGTGCAGAAGCGGACATAAGCACATAA
Sasa    TCTCCTCTTCC-AAGTCAATGAAATTGATCTGCCCGTGCAGAAGCGGACATAAACACATAA
Cola    TCTCCTCTTCC-AAGTCAGTGAAATTGATCTGCCCGTGCAGAAGCGGACATAAGTACATAA
Dita    TCTCCTCTCCC-CAGTCTATGAAATTGATCCCCCCGTGCGGAAGCGGGGATCCCGATATAA
Gogr    TCTCCTCTCCC-TAGTCAATGAACTTGATCTCCCCGTGCAGAAGCGGGACTTTTCCCATAA
Chsl    TCTCCTTTCCC-CAGTCAATGAAATTGATCTTCCCGTGCAGAAGCGGGAATACCTATATAA
Atja    TCTCCTTATCC-CAGTCGATGAAATTGATCTCCCCGTGCAGAAGCGGGGATAACAACATAA
Iido    TCTCCTTGCCC-CAGTCAATGAAATTGATCTCCCCGTGCAGAAGCGGGGATAACACCATAA
Auja    TCTCCCCCCTC-CAGTCAATGAAATTGATCTCCTCGTGCAGAAGCGAGGATACCCCCATAA
Chag    TCTCCCCTATC-CTGTTAGTGAAATTGATCTCCCCGTGCAGAAGCGGGGATAACTACATAA
Hami    TCTCCCTACCC-CAGTCAATGAAATTGATCTTCCCGTGCAGAAGCGGGAATAACACCATAA
Saun    TCTCCCCCCTC-TAGTCAATGAAATTGATTTTCCCGTGCAGAAGCGGGAATGCTCCCATAA
Nema    TCTCCCCTTTA-AAGTCAATGAAATTGATCTCCCCGTGCAGAAGCGGGGATAGCCTCGTAA
Disp    TCTCCCCCTTA-AGGTCAATGAAATTGATCTCCCCGTGCAGAAGCGGGGATATACTCGTAA
Myaf    TCTCCCCTTTA-TAGTCAATGAAATTGATCTCCCCGTGCAGAAGCGGGGATACACTCGTAA
Lagu    TCTCCCCTCTC-CCGTCAGTAAAATTGACCTACCCGTGCAGAGGCGGGTACACCCCCACAA
Trtr    TCTCCCCCCTT-CGGTCAATGAAACTGATCTCCCCGTGCAGAAGCGGGGATACTCCCACAA
Zucr    TCTCCCTTCTC-ATGTCAATGAAACTGATTTCCCCGTGCAGAAGCGGGGATGCAGTCACAA
Pxja    TCTCCCCTTTC-TAGTCAATGAAATTGATCTCCCCGTGCAGAAGCGGGGATAAAAACATAA
Pxlo    TCTCCCCTTTC-TAGTCAATGAAATTGATCTCCCCGTGCAGAAGCGGGGATAAAAACATAA
Pctr    TCTCCTTTTTC-CAGTCAATGAAATTGATCTCCCCGTGCAGAAGCGGGGATGCGCACATAA
Apsa    TCTCCTTTTTC-CAGTCAGTGAAATTGATCTCCCCGTGCAGAAGCGGGGATAAAAACATAA
Cabe    TCTCTTTCTTC-GAGTCAATGAAATTGATCTTCCCGTGCAAAAGCGGGCATGAACACACGG
Bzze    TCTCCCTTCTC-AAGTCAATGAAATTGATCTCCCCGTGCAGAAGCGGGGATAAACACATAA
Siim    TCTCCCCTTTC-TGGTCAATGAAATTGATCCCCCCGTGCAGAAGCGGGGCTAGCCACATTA
Ctru    TCTCCTTTTTC-CAGTCAATGAAATTGATCTTCCCGTGCAGAAGCGGGAATTTATACATAA
Dpbr    TCTCCTTTTCC-CAGTCAATGAAATTGATCTTCCCGTGCAGAAGCGGGAATTAAAACATAA
Caki    TCTCCCTTCCC-CAGTCAATGAAATTGACCTCCCCGTGCAGAGGCGGGGATACAACTATAA
Phja    TCTCCCTTCTT-TAGTCAATGAAATTGACCTTCCCGTGCAGAGGCGGGAATAACTATATAA
Brsp    TCTCCTTCCCC-CAGTCAATGAAATTAAACTTCCCGTGCAGATGCGGGAATATGTACATAA
Gamo    TCTCCCATCTC-CAGTCAATGAAATTGACCTCCCCGTGCAGAGGCGGGGATAATTACATAA
Lolo    TCTCCCCTCTC-CAGTCAATGAAATTGACCTCCCCGTGCAGAGGCGGGGATAATTACATAA
Batr    TCTCCCCCCTC-CAGTCAATGAAATTGTCCTCTTCGTGAAAAGACGAAGATTAAAATATAA
Prmy    TCTCTATTTTC-TAATCAATAAAATTGTTCTCTTCGTGCAGAAACGAAGATACCCCTATAA
Loli    TCTCCCCCCTC-CAGTCAATGAAATTGATCTCCCCGTGAAGAAGCGGGGATACACCCATAA
Loam    TCTCCTCCCCC-CAGTCAATGAAATTGATCTCCCCGTGAAGAAGCGGGGATACTTACATAA
Chab    TCTCCTCCCTC-CAGTCAATGAAATTGATCCCCCCGTGCAGAAGCGGGGATTTTAACATAA
Chto    TCTCCTCCCTC-CAGTCAATGAAATTGATCCCCCCGTGCAGAAGCGGGGATTTTAACATAA
Majo    TCTCCTTTCCC-CGGTCAATGAAATTGATCTCCCCGTGAAGAAGCGGGGATATAACTATAA
Hlst    TCTCCTTTTCC-CAGTCAATGAAATTGATCTCCCCGTGAAGAAGCGGGGATAAGCTTATAA
Clpe    TCTCCTCTATC-TAGTCAGTGAAATTGATTTCCCCGTGCAGAAGCGGCCATAAACCCATAA
Mlmr    TCTCCTCTCTC-CAGTCAATGAAATTGATCTCCCCGTGCAGAAGCGGGGATGACTACATAA
Crcr    TCTCCTTTTCC-CAACCAATGAAATTGATCTTCCCGTGCAGAAGCGGGAATACTAACATAA
Muce    TCTCCTTTTCC-CAACCAATGAAATTGATCTTCCCGTGCAGAAGCGGGAATACCAACATAA
Bege    TCTCCTTTTTC-TAGTCAATGAAATTGATCTCCCCGTGCAGAAGCGGGGATACAACCATAA
Mela    TCTCCTTTTTC-CAGTCAATGAAATTGATCTCCCCGTGCAGAAGCGGGGATAAACCCATAA
Hats    TCTCCTTTTTC-TAGTCAATGAAATTGATCTCCCCGTGCAGAAGCGGGGATAAACCCATAA
Orla    TCTCCTCTCCC-TAGTCAATGAAATTGATCTCCCCGTGCAGAAGCGGGGATAAAAACATAA
```

Cosa	TCTCCTTTTTC-CAGTCAATGAAATTGATTTCTCCGTGCAGAAGCTGAGATATTTACATAA
Exsp	TCTCCTTCTCC-TAGTCAATGAAATTGATCTCCCCGTGCAGAAGCGGGGATAAGTACATAA
Depa	TCTCCTTTTTC-CAGTCAATGAAATTGATCTCCCCGTGCAGAAGCGGGGATAAACCCATAA
Rima	TCTCATCCTCC-CAGTCAATGAAATTGATCTCCCCGTGCAGAAGCGGGGATAAACACATAA
Fuol	TCTCCTTCCCT-AAGTCAATGAAATTGATCTTCCCGTGCAGAAGCGGGAATAAATACATAA
Gmaf	TCTCCTCCCCC-CAGTCAATGAAATTGATCCCCCCGTGCAGAAGCGGGGATAACAACATAA
Xeei	TCTCCTTCCCT-AAGTCAATGAAATTGATCTTCCCGTGCAGAAGCGGGAATACTCACATAA
Pros	TCTCTTTTCTC-CGGTCAATGAAATTGATCTCCCCGTGCAGAAGCGGGGATAATCGCATAA
Scmi	TCTCTTTTCTC-TAGTCAGTGAAATTGATCTCCCCGTGCAGAAGCGGGGATATTTACATAA
Rolo	TCTCCTTTTTC-TAGTCAATGAAATTGATCTCCCCGTGCAGAAGCGGGGATTATAACATAA
Cere	TCTCCTTTTCC-CAGTCAATGAAATTGATCTCCCCGTGCAGAAGCGGGGATAAAAACATAA
Daga	TCTCCTTTTCC-TAGTCAGTGAAATTGATCTTCCCGTGCAGAAGCGGGAATGACATCATAA
Anco	TCTCCTTTTTC-AGGTCAATGAAATTGATCTTCCCGTGCAGAAGCGGGAATAACTATATAA
Dmve	TCTCCTTTTTC-AGGTCAATGAAATTGATCTTCCCGTGCAGAAGCGGGAATGTTAACATAA
Dmar	TCTCCTTTTTC-AGGTCAGTGAAATTGATCTTCCCGTGCAGAAGCGGAAATGACAACATAA
Anka	TCTCCTCTTTC-AAGTCAATGAAATTGATCTTCCCGTGCAGAAGCGGGAATAGCCACATAA
Moja	TCTCCTTTTTC-AGGTCAATGAAATTGATCTTCCCGTGCAGAAGCGGGAATAACTACATAA
Hoja	TCTCCTTTTTC-AGGTCAATGAAATTGATCTTCCCGTGCAGAAGCGGGAATAAACACATAA
Bede	TCTCTTTTCTCCAAGTCAGTGAAATTGATCTCCCCGTGCAGAAGCGGGGATACAAACATAA
Besp	TCTCTTTTCTCCAAGTCAGTGAAATTGATCTCCCCGTGCAGAAGCGGGGATACAAACATAA
Mysp	TCTCCTTTTTC-CAGTCAATGAAATTAATCTCCCCGTGCAGAAGCGGGGATACAACCATAA
Osja	TCTCCTTTTTC-CAGTCAATGAAATTAATCCCCCCGTGCAGAAGCGGGGATAAATCCATAA
Sgro	TCTCCTTTTTC-CAGTCAATGAAATTGATCTCCCCGTGCAGAAGCGGGGATATAACCATAA
Pzpa	TCTCCTTTTTC-AAGTCAATGAAATTGATCTCCCCGTGCAGAAGCGGGGATAACTACATAA
Zeja	TCTCCTTTTTC-TAGTCAATGAAATTGATATCCCCGTGCAGAAGCGGGGATATTCACATAA
Znne	TCTCCTTTTTC-AAGTCAATGAAATTGATCTCCCCGTGCAGAAGCGGGGATGAATATATAA
Zefa	TCTCCTTTTTC-AAGTCAATGAAATTGATCTCCCCGTGCAGAAGCGGGGATAATAATATAA
Acni	TCTCCTTTTTC-AAGTCAATGAAATTGATCTCCCCGTGCAGAAGCGGGGATAATAACATAA
Ncrh	TCTCCTTTTTC-AAGTCAATGAAATTGATCTCCCCGTGCAGAAGCGGGGATAATAACATAA
Agca	TCTCCTCTTTC-AAGTCAATGAAATTGATTTCCCCGTGCAGAAGCGGGGATACCCACATAA
Hydy	TCTCCTTTTCC-CGGTCAATGAACTTGATCTCCCCGTGCAGAAGCGGGGATACTACCATAA
Gsac	TCTCCTTTTTC-CAGTCAATGAAATTGATCTCCCCGTGCAGAAGCGGGGATTACTACATAA
Pevo	TCTCCTTTCCC-CAGTCAATGAAATTGATCTCCCCGTGCAGAAGCGGGGATTTACCCATAA
Hiku	TCTCCTCACCC-CAGTTAATGAAATTGATCTTCCCGTGCAGAAGCGGGAATTAACACATAA
Inpa	TCTCCTCTTTT-TAGTCAATGAAATTGATCTCCCCGTGCAGAAGCGGGGCTACAAGCATAA
Auch	TCTCCTCCCTC-CAGTCAGTGAAATTGATCTCCCCGTGCAAAAGCGGGGATTCCCACATAA
Fico	TCTCCTCCCCC-CAGTCAATGAAATTGATCTTCCCGTGCAGAAGCGGGAATATGCCCATAA
Macs	TCTCCTTTCTC-CAGTCAATGAAATTGATCTCCCCGTGCAGAAGCGGGGATAAAGCCATAA
Moal	TCTCCTCTTTA-AAGTCAATAAAATTGATCTCCCCGTGCAGAAGCGGGGATTACACCATAA
Syma	TCTCCTCTTTA-AAGTCAATAAAATTGATCTCCCCGTGCAGAAGCGAGGATAAACACATAA
Mafr	TCTCCTTTCTC-TAGTCAATGAAATTGATCTCCCCGTGCAGAAGCGGGGATAACTCCATAA
Dcpe	TCTCCTCTTTC-AAATCAATGAAATTGATCTCCCCGTGCAGAAGCGGGGATATGCCCATAA
Dcti	TCTCCTCTTTT-AAATCAATGAAATTGATCTCCCCGTGCAGAAGCGGGGATATGACCATAA
Hehi	TCTCCTCTTTC-AAGTCAATGAAATTGATCTCCCCGTGCAGAAGCGGGGATATAAACATAA
Stam	TCTCCTCTTTC-AAGTCAATGAAATTGATCTCCCCGTGCAGAAGCGGGGATAACCTCATAA
Hogi	TCTCCTTTTTC-CGGTCAATGAAATTGATCTCCCCGTGCAGAAGCGGGGATACTAACATAA
Erzo	TCTCCTCTCTC-CAGTCAGTGAAATTGATCTTCCCGTGCAGAAGCGGGAATATATACATAA
Hxot	TCTCCTCTTTC-AGGTCAATGAAATTGATCTTCCCGTGCAGAAGCGGGAATATAAACATAA
Core	TCTCCTCTTTC-CAGTCAATGAACTTGATCTCCCCGTGCAGAAGCGGGGATATAACCATAA
Apve	TCTCCTCTTTC-AAGTCAATGAAATTGATCTTCCCGTGCAGAAGCGGGAATTTAAACATAA
Latj	TCTCCTTTTTC-AAGTCAATGAAATTGATCTCCCCGTGCAGAAGCGGGGATAAAAACATAA
Laja	TCTCCTTTTTC-CAGTCGATGAAATTGATCTCTCCGTGCAGAAGCGGGGATACCAACATAA

```
Syja  TCTCCTTTTTC-AAGTCAATGAAATTGATCCCCCCGTGCAGAAGCGGGGATAAGCACATAA
Epme  TCTCCTCTTTC-AAGTCAATGAAATTGATCTCCCCGTGCAGAAGCGGAGATAAACTCATAA
Grse  TCTCCTTTTCC-CAGTCAATGAAATTGATCTCCCCGTGCAGAAGCGGGGCTACACTCATAA
Clja  TCTCCTCCCTC-CAGTCAATGAAATTGATCTTCTCGTGAAGAAGCGAGAATTAAAACATAA
Ogcy  TCTCCTTTTTC-TAATCAATGAAATTGATCTTCCCGTGCAGAAGCGGGAATTAACCCATAA
Plna  TCTCCTTTTTC-CAGTCAATGAAATTGATCCTCCCGTGCAGAAGCGGGAATTCACACATAA
Lema  TCTCCTTTTTC-CAGTCAATGAAATTGATCTCCCCGTGCAGAAGCGAGGATAAAACCATAA
Etzo  TCTCCTCTTTC-AAGTCAATGAAATTGATCTCCCCGTGCAGAAGCGGGGATAATAACATAA
Apse  TCTCCTTTTTC-AAGTCAATGAAATTGATCTCCCCGTGCAGAAGCGGGGATATTTACATAA
Epde  TCTCCTTTTTC-AAGTCAATGAAATTGATCTCCCCGTGCAGAAGCGGGGATATACACATAA
Slja  TCTCCTTTTTC-AGGTCAATGAAATTGATCTCCCCGTGCAGAAGCGGGGATATTAACATAA
Bsja  TCTCCTCTTTC-AAGTCAATGAAATTGATCTCCCCGTGCAGAAGCGGGGATAAAACCATAA
Ecna  TCTCCTCACTC-TAGTCAATGAAATTGATCTTCCCGTGCAGAAGCGGGAATAAATACATAA
Cohi  TCTCCTCCCTC-TTGTCAATGAAATTGATCTCCCCGTGCAGAAGCGGGGATACTCTCATAA
Caar  TCTCCTCTCTC-CAGTCAATGAAATTGATCTCCCCGTGCAGAAGCGGGGATATTTACATAA
Came  TCTCCTCTTTC-CAGTCAATGAAATTGATCTCCCCGTGCAGAAGCGGGGATAAGTACATAA
Mema  TCTCCTCTTTC-AAGTCAATGAAATTGATCTCCCCGTGCAGAAGCGGGGATAACAACATAA
Lenu  TCTCCCCTCTT-AAGTCAATGAAATTGATCTCCCCGTGCAGAAGCGGGGATACAAACATAA
Brja  TCTCCTCCCTC-AAGTCAATGAAATTGATCTCCCCGTGCAGAAGCGGGGATGAGCCCATAA
Plma  TCTCCTTCCTC-AAGTCAATGAAATTGATCTCCCCGTGCAGAAGCGGGGATACACCCATAA
Emst  TCTCCTCTTTC-AAGTCAATGAAATTGATCTCCCCGTGCAGAAGCGGGGATATAACCATAA
Ptti  TCTCCTCTTTC-AAGTCAATGAAATTGATCTCCCCGTGCAGAAGCGGGGATACGCACATAA
Losu  TCTCCTCTTTC-AAGTCAATGAAATTGATCTCCCCGTGCAGAAGCGGGGATACTAACATAA
Geoy  TCTCCTTTTTC-CAGTCAATGAAATTGAGCTCTCCGTGCAGAAGCGGGGATCAAACCATAA
Dipi  TCTCCTTTTTC-AGGTCAATGAAATTGATCTCCCCGTGCAGAAGCGGGGATACCCACATAA
Pama  TCTCCCCTCCC-CAGTCAATGAAATTGATCTCCCCGTGCAGAAGCGGGGATAATACCATAA
Leob  TCTCCTCTCTC-AAGTCAGTTAAATTGATCTCTCCGTGCAGAAGCGGAGATAACACCATAA
Neba  TCTCCTTTTTC-CAGTCAATGAAATTGATCTCCCCGTGCAGAAGCGGGGATAAACCCATAA
Pdpl  TCTCCTTTTTC-AAGTCAGTGAAATTGATCTCCCCGTGCAGAAGCGGGGATAATTACATAA
Nimi  TCTCCTTTTTC-ATGTCAATGAAATTGATCTTCCCGTGCAGAAGCGGGAATCTCCCCATAA
Uptr  TCTCCTTTTTC-CAGTCAATGAAATTGATCTCCCCGTGCAGAAGCGGGGATAAGTACATAA
Pesc  TCTCCCCTCTC-TGGTCAATGAAATTGATCTCCCCGTGCAGAAGCGGGGATCCCCCCATAA
Baar  TCTCCTTTTTC-AAGTCAATGAAATTGATCTCCCCGTGCAGAAGCGGGGATATAAACATAA
Moar  TCTCCTCTTTC-AAGTCAATGAAATTGATCCCCCCGTGCAGAAGCGGGGATAATAACATAA
Toja  TCTCCTTTTTC-AAGTCAATGAAATTGATCTCCCCGTGCAGAAGCGGGGATCCCCCCATAA
Chau  TCTCCTTTTTC-AAGTCAATGAAATTGATCTTCCCGTGCAGAAGCGGAAATAACTACATAA
Chse  TCTCTTCTTTC-AAGCCAATGAAATTGATCTCCCCGTGCAGAAGCGGGGATACACACATAA
Enar  TCTCCTTTTTC-AGGTCAATGAAATTGATCTCCCCGTGCAGAAGCGGGGATAAATACATAA
Hpty  TCTCCTTTTTC-AAGTCAATGAAATTGATCTCCCCGTGCAGAAGCGGGGATACGCACATAA
Nana  TCTCCTTTTTC-CAGTCAATGAAATTGATCTCCCCGTGCAGAAGCGGGGATAACCACATAA
Mcst  TCTCCTTTTTC-AGGTCAATGAATTTGATCTTCCCGTGCAGAAGCGGGGATAAAACCATAA
Rhox  TCTCCTTTCTC-AAGTCAATGAAATTGATCTCCCCGTGCAGAAGCGGGGATAATACCATAA
Opfa  TCTCCTTTTTC-AAGTCAATGAAATTGATCTCCCCGTGCAGAAGCGGGGATAAAACCATAA
Paar  TCTCCTTTTCC-AAGTCAATGAAATTGATCTCCCCGTGCAGAAGCGGGGATAAAAATATAA
Gozo  TCTCCTTTTTC-AAGTCAATGAAATTGATCTCCCCGTGCAGAAGCGGGGATAAAAACATAA
Ackr  TCTCCTTTCCC-CAGTCAATGAAATTGATCTCCCCGTGCAGAAGCGGGGATCTTACCATAA
Elev  TCTCCTTTTCT-AAGTCAATGAAATTGATCTTCCCGTGCAGAAGCGGGAATAATAACATAA
Trdu  TCTCCTTTTTC-CAGTCAATGAAATTGATCCCCCCGTGCAGAAGCGGGGATACTAACATAA
Amoc  TCTCCTTTTTC-CAGTCAATGAAATTGATCTTCCCGTGCAGAAGCGGGAATAATACCATAA
Hame  TCTCCTTTCTC-AAGCCAATGAAATTGATCTCCCCGTGCAGAAGCGGGGATGAACCCATAA
Chso  TCTCCTTTCCT-AAGTCAATGAAATTGATCTCCCCGTGCAGAAGCGGGGATACACTCATAA
Lyto  TCTCCTCTTTC-CAGCCAATGAAGTTGATCTTCCCGTGCAGAAGCGGGAATACAAACATAA
```

```
Encr          TCTCCTCTCTC-CAGTCAATGAAATTGATCTTCCCGTGCAGAAGCGGGAATACAAACATAA
Bvar          TCTCCTTTTTC-AAGTCAATGAAATTGATCTCCCCGTGCAGAAGCGGGGATGCCCCCACAA
Noco          TCTCCTCCCCT-AAGTTAATGAAATTGATCTTTCCGTGCAGAAGCGGGAATAAACACATAA
Chsp          TCTCCTCCCCC-CAGTCAATGAAATTGATCTCCCCGTGCAGAAGCGGGGATAACCCCATCA
Arja          TCTCCTCTTTC-TAGTCAATGAAATTGATCTTTCCGTGCAGAAGCGGGAATAAAAACATAA
Pase          TCTCCTTTCCC-AAATCAGTGAAATTGATCTCTCCGTGCAGAAGCGGGGATAAAAACATAA
Trel          TCTCCTTATTC-CAGTCAATGAAATTGATCTCCCCGTGCAGAAGCGGGGGTAAGAAAATAA
Lifa          TCTCCTCTCCC-CAGTCAATGAAATTGATCTGCCCGTGCAGAAGCGGGCATTGAAACATTA
Acur          TCTCCTTTTTC-ATGTCAGTGAAATTGATCTCCCCGTGAAGAAGCGGGGGTGTTAATATAA
Ampe          TCTCCTTTCTC-TAGTCAATGAAATTGATCTCCCCGTGCAGAAGCGGGGATAGAACCATAA
Urja          TCTCCTTTTTT-AAGTCAATGAAATTGATTTTCCCGTGCAAAAGCGGGAATAAAACCATAA
Enet          TCTCCTTTCCC-CAGTCCATGAAATTGATCTTTCCGTGCAGAAGCGGAAATTCTAACATAA
Ptbr          TCTCCTTTCTC-CAGTCAATGAAATTGATTTCCCCGTGCAGAAGCGGGGATAAAACCATAA
Safa          TCTCCCCCTCC-CAGTCAGTGAAATTGATCTTTCCGTGCAGAAGCGGGAATACACACATAA
Icae          TCTCCTTTTTC-AAGTCAGTGAAATTGATCTCCCCGTGCAGAAGCGGGGATACACCCATAA
Asmi          TCTCCCTTTTT-TAATCAGTGAAATTGATCTCCCCGTGCAGAAGCGGGGATAAAATCATAA
Foal          TCTCCCACCCT-TAGTCAATTAAATTGATCTCCCCGTGCAGAAGCTGGGATACACACATTA
Drze          TCTCCTTTCTC-TAGTCAGTGAAATTGATCTCCCCGTGCAGAAGCGGGGATATACATATAA
Rhas          TCTCCCTTTCC-CGGTCAATGAAATTGATCTCCCCGTGCAGAAGCGGGGATAAACTCGTAA
Elac          TCTCCTTTTTC-CAGTCAATGAAATTGATCTGCCCGTGCAGAAGCGGGCATAAATACATAA
Kugu          TCTCCTTTCTC-AAGTCAATGAAATTAATCTTCCCGTGCAGAAGCGGGAATAACAACATAA
Plor          TCTCCTCTTTC-CAGTCAATGAAATTGATCTCCCCGTGCAGAAGCGGGGATTCTCCCATAA
Sgun          TCTCCTCTTTC-AAGTCAATGAAATTGATCTCCCCGTGCAGAAGCGGGGATAATCTCGTAA
Zaco          TCTCCTTTTTC-CAGTCAATGAAATTGATCCCCCCGTGCAGAAGCGGGGATCCAATCATAA
Zbfl          TCTCCTCTTTC-CAGTCAATGAAATTAATCTTCCCGTGCAGAAGCGGGAATATACTCATAA
Spba          TCTCCTTTTTC-AGGTCAATGAAATTGATCTCTCCGTGCAGAAGCGGAGATAACACCATAA
Game          TCTCCTTTTTC-AAGTCAGTGAAATTGATCTCCCCGTGCAGAAGCGGGGATATACCCATAA
Thth          TCTCCTTTTTC-TAGTCAGTGAAATTGATCTCCCCGTGCAGAAGCGGGGATGTAACCATAA
Xigl          TCTCCTCTTTC-CAGTCAATGAAATTGATCTCCCCGTGCAGAAGCGGGGATATAACCATAA
Hyja          TCTCCTTTTTC-AAGTCAATGAAATTGATCTCCCCGTGCAGAAGCGGGGATAATCCCATAA
Psan          TCTCCTTTTTC-AAGTCAATGAAATTGATCTCCCCGTGCAGAAGCGGGGATAATACCATAA
Cupa          TCTCCTTTTTC-AAGTCAATGAAATTGATCTCCCCGTGCAGAAGCGGGGATAAATCCATAA
Mpch          TCTCCTTTTTC-TAGTCAATGAAATTGATCTCCCCGTGCAGAAGCGGGGATAAGAACATAA
Char          TCTCCTTTTTC-AAGTCAATGAAATTGATCTCCCCGTGCAGAAGCGAGGATGACAACATAA
Pser          TCTCCTTTTTC-TAGTCAATGAAATTGATCCCCCCGTGCAGAAGCGGGGATACAAACATAA
Prol          TCTCCTTCCCC-CAGTCAATGAAATTGATCTCCCCGTGCAGAAGCGGGGATAACCTCATAA
Plbi          TCTCCTTCCCC-CAGTCAATGAAATTGATCTCCCCGTGCAGAAGCGGGGATAAAATCATAA
Calu          TCTCCTCTCTC-CGGTCAGTGAAATTGATCTCCCCGTGCAGAAGCGGGGATTCAAGCATAA
Papa          TCTCCCCCCCC-CAGTCAATGAAATTGATCTTTCCGTGCAGAAGCGGAAATGATAACATAA
Sufr          TCTCCTTTCCC-AAGTTAATGAAATTGATCCCCCCGTGCAGAAGCGGGGATAAGGACATAA
Stci          TCTCCTTTCTC-AAGTTAATGAAATTGACCTCCCCGTGCAGAGGCGGGGATATTACCATAA
Taru          TCTCCTTTCTC-AAGTCAATGAACTTGATCTCCCCGTGCAGAAGCGGGGATAAAACCATAA
Rala          TCTCCTTTTTC-TAGTCAATGAAACTGATCTCCCCGTGCAGAAGCGGGGATAACCCCATAA
              ****               * **  *        ***    *  *
```

```
              42          43      44         ¦          HVR         ¦
Scca  GACGAGAAGACCCTATGGAGCTTCAAATACATAAATTAAT------------TATGTACA
Muma  GACGAGAAGACCCTATGGAGCTTTAAACACTTAAGTTAAT------------TATGT--A
Erca  GACGAGAAGACCCTGTGGAGCTTTAGACCTAATCCAAAC------------ACT--ACAA
Pose  GACGAGAAGACCCTGTGGAGCTTTAGACTAAATCCAAA-------------TGACTACCA
Actr  GACGAGAAGACCCTATGGAGCTTAAAACACAAGATCAA-------------CTATGCTAT
Scal  GACGAGAAGACCCTATGGAGCTTAAAACACAAGATCAA-------------CTATGCTAT
Posp  GACGAGAAGACCCTATGGAGCTTAAAACACAAGATCAA-------------CTATGCTAT
Atsp  GACGAGAAGACCCTATGGAGCTTAAAACACAAGACCAA-------------CCACGCCAG
Leoc  GACGAGAAGACCCTATGGAGCTTAAAACACAAGACCAACCAC----G-CCAGTAACCC-A
Amca  GACGAGAAGACCCTATGGAGCTTTAAACAAAGAATCAA-------------CCATGCATA
Osbi  GACGAGAAGACCCTGTGGAGCTTAAGATGTTTATTTACTTGC--------GCTTAGT-AA
Pabu  GACGAGAAGACCCTATGGAGCTTTAGACATAAGTCAAT--------------CATGCCTA
Hial  GACGAGAAGACCCTATGGAGCTTAAGATATATGACCAA------------CC-GCGCTTA
Elha  GACGAGAAGACCCTGTGGAGCTTAAGACGTATGGCCAC-------------CCATGCCGA
Mlcy  GACGAGAAGACCCTGTGGAGCTTAAGGCACAAGGCCAA-------------CCACGCCAA
Algl  GACGAGAAGACCCTATGGAGCTTAAGATATAAGATCAC-------------ACGTACTTA
Ptgi  GACGAGAAGACCCTATGGAGCTTAAGACAAAAGGCCAAA-C------------ATGCCCA
Alaf  GACGAGAAGACCCTATGGAGCTTAAGACAAAAAGCCAA-------------ACATGTTAA
Nock  GACGAGAAGACCCTATGGAGCTTAAGACAAAAAGCCAA-------------TCATGTTTA
Anja  GACGAGAAGACCCTATGGAGCTTTAGACAAAAGATCAA-------------ACATGTA-A
Gyki  GACGAGAAGACCCTATGGAGCTTTAGGCAAATGATCAT-----------GTA-CACCAAA
Syka  GACGAGAAGACCCTATGGAGCTTTAGGCGAAAGTCAAA--------------CATGTACA
Opma  GACGAGAAGACCCTATGGAGCTTTAGGCTAAAGGCCCA------------AACATGTAAA
Comy  GACGAGAAGACCCTATGGAGCTTTAGGCACACATCAAA-------------T-ATGTAAA
Sasp  GACGAGAAGACCCTATGGAGCTTCAGACAAAACTAAAGCATTTTC-T-ATACTATGATAA
Eupe  GACGAAAAGACCCTATGGAGCTTTAGACAAAGTCAACA--------------TGCCTAAA
Enja  GACGAGAAGACCCTATGGAGCTTTAGACACTAGCCAACTG-------------TGAATAA
Same  GACGAGAAGACCCTATGGAGCTTTAGACACCAACCAAC--------------CACGAAAA
Chch  GACGAGAAGACCCTATGGAGCTTTAGACACAAGACCAAC-----------CATGTTA--A
Grgr  GACGAGAAGACCCTTTGGAGCTTAAAACATAAGGCCAA-------------CCACGT---
Caau  GACGAGAAGACCCTTTGGAGCTTAAGGTACAAAACTCA------------ACCACGTTAA
Cyca  GACGAGAAGACCCTTTGGAGCTTAAGGTACAAAACTCAACC------------ACGTTAA
Dare  GACGAGAAGACCCTTTGGAGCTTAAGGTACAAGATTTA-----------ATTTACATCAA
Cost  GACGAGAAGACCCTTTGGAGCTTAAGGTTCAAGTCCACCC-------------GCGTTAA
Leec  GACGAGAAGACCCTTTGGAGCTTAAGGTACAAACCCAC-------------CTATGTTAA
Crla  GACGAGAAGACCCTTTGGAGCTTAAGGTACAAACCCAA-------------CCACGT---
Clmc  GACGAGAAGACCCTTTGGAGCTTAAGACATAAAGCCAA-------------CTGTGTCAA
Phin  GACGAGAAGACCCTTTGGAGCTTTAGACAAAGGCCAAC--------------TATTCC-C
Icpu  GACGAGAAGACCCTTTGGAGCTTAAGATACAAGATCAT-------------CTATGT---
Psto  GACGAGAAGACCCTTTGGAGCTTAAGACTTAAGATCAA-------------CTATGT---
Cora  GACGAGAAGACCCTTTGGAGCTTAAGATTAAAAATCAA------------CT-ATGTCAA
Eisp  GACGAGAAGACCCTTTGGAGCTTAAGACCCCGGACCACCT-------------ATGTTAA
Apal  GACGAGAAGACCCTTTGGAGCTTAAGACTTAAACCAACT--------------GTGTAAC
Eslu  GACGAGAAGACCCTATGGAGCTTTAGACACCCGGCAGA-------------CCCTGT---
Dape  GACGAGAAGACCCTATGGAGCTTTAGACACTAGGCAGAC-------------CCTGTTAA
Glse  GACGAGAAGACCCTATGGAGCTTTAGACAAAAGGCAGA-------------CCACGTTTA
Naar  GTCGAGAAGACCCTATGGAGCTTTAGACAAAAGGCAGA------------CT-ACGTTAA
Baoc  GACGAGAAGACCCTATGGAGCTTTAGACAAAAGGCAGA-------------CTACGTTAA
Opso  GACGAGAAGACCCTATGGAGCTTTAGATAAAAGGTAGACC-------------ACGTTTT
Alte  GACGAGAAGACCCTGTGGAGCTTAAGATATAAGGTCAA------------CCACGTCAAA
Plap  GACGAGAAGACCCTGTGGAGCTTAAGACACAAGGTCAACC-------------ACGTCAA
```

```
Plal    GACGAGAAGACCCTATGGAGCTTTAGACACTAGACGGCCT-------------ACGTTAA
Sami    GACGAGAAGACCCTATGGAGCTTTAGACACTAGACAGC-------------CCACGTTAC
Rere    GACGAGAAGACCCTATGGAGCTTTAGACACTAGACAACC-------------CACGTCAA
Gama    GACGAGAAGACCCTATGGAGCTTTAGACGCTAGGAAGACC-------------ATGTTAA
Onmy    GACGAGAAGACCCTATGGAGCTTTAGACACCAGGCAGAT-------------CACGTCAA
Sasa    GACGAGAAGACCCTATGGAGCTTTAGACACCAGGCAGATCAC--------GTCAAGT-AA
Cola    GACGAGAAGACCCTATGGAGCTTTAGACACCAGGCAGA-------------TCACGTCAA
Dita    GACGAGAAGACCCCATGGAGCTTCAGACACTGGGCAAC-------------CCACCCCTG
Gogr    GACGAGAAGACCCCATGAAGCTTCAGACGCCAGTCAACC----------CCTCCCCC---
Chsl    GACGAGAAGACCCTGTGAAGCTTTAGACACTAGAAAGCC--------------CCACCAA
Atja    GACGAGAAGACCCTGTGGAGCTTAAGATTATAAGTAGA-------------CTATGTTAA
Iido    GACGAGAAGACCCTGTGGAGCTTTAAATTATAAGTAGA-------------CTAT-GTTA
Auja    GACGAGAAGACCCTATGGAGCTTTAGACATCAAACAGC-------------CCACGTAAA
Chag    GACGAGAAGACCCTGTGGAGCTTTAGACACTTAAGCGACC------------CAAATAGA
Hami    GACGAGAAGACCCTATGGAGCTTTAGACATCAAGCAGCC-------------TACAG-CA
Saun    GACGAGAAGACCCTATGGAGCTTTAGACATCAAGCAGC-------------CCACAAA-A
Nema    GACGAGAAGACCCTATGGAGCTTAAGACACAAGGCGGCCC-------------ACGTCAA
Disp    GACGAGAAGACCCTATGGAGCTTAAGACATAAAGCGAC-------------CCACGT---
Myaf    GACGAGAAGACCCTATGGAGCTTAAGACATAAAGCAAC-------------CCATGTTAA
Lagu    GACGAGAAGACCCTGTGGAGCTTTAGGCCCAAGAAAGAGGACA-----------------
Trtr    GACGAGAAGACCCTATGGAGCTTGAGACTTTGGAAAGCC-------------TGCCTT-A
Zucr    GACGAGAAGACCCTGTGGAGCTTTAGACTTTAGTGAGC------------TT-GCCCAAA
Pxja    GACGAGAAGACCCTATGGAGCTTGAAACCTATGATAGTA-------------CACGTC-A
Pxlo    GACGAGAAGACCCTATGGAGCTTGAAACCTATGATAGTGC-------------ACGTCAA
Pctr    GACGAGAAGACCCTATGGAGCTTAAGACCTCCCCCGAC-------------CCATGTGAC
Apsa    GACGAGAAGACCCTATGGAGCTTAAGACACCCCCTAAC-------------CTATGC-GA
Cabe    GACGAGAAGACCCTGTGGAGCTTGAAACAATTGGGGATT-------------CAGATC-A
Bzze    GACGAGAAGACCCTATGGAGCTTTAGACACCAAAGCAGAC------------CATGTT-A
Siim    GACGAGAAGACCCTATGGAGCTTCAGACACTAAGGAAGCC------------CA-GTCAA
Ctru    GACGAGAAGACCCTATGGAGCTTAAGATACTAAGACAG------------ATCATGTTAA
Dpbr    GACGAGAAGACCCTATGGAGCTTTAGATACTAAGATAGA-----------T-CATGT-TA
Caki    GACGAGAAGACCCTGTGGAGCTTTAGACTTAAAATAACC--------------CTAT---
Phja    GACGAGAAGACCCTGTGGAGCTTTAGACCTAAAATAAA-------------TCACAT---
Brsp    GACGAGAAGACCCTTTGGAGCTTCAGACCCTAAATAAG-------------TTATGTT-T
Gamo    GACGAGAAGACCCTATGGAGCTTTAGACCTAAAGTAAG-------------TCACGTTTA
Lolo    GACGAGAAGACCCTGTGGAGCTTTAGACCTAAGGTAAGTC-------------ACGTTTA
Batr    GACGAAAAGACCCCGTGGAGCTTTAGGACGCCTCAGAAAC--------------------
Prmy    GACGAGAAGACCCTGTGGAGCTTCAAGTAAACTAAGAG-------------------T--
Loli    GACGAGAAGACCCTATGGAGCTTTAGACACTAAGGCAGC------------TTACGTT-A
Loam    GACGAGAAGACCCTATGGAGCTTTAGACACCAAGGCAGATC------------ACGTTAA
Chab    GACGAGAAGACCCTGTGGAGCTTTAGACACTCGGGCAG------------ACTATGTCAA
Chto    GACGAGAAGACCCTGTGGAGCTTTAGACACTCGGGCAG------------ACTATGT---
Majo    GACGAGAAGACCCTGTGGAGCTTTAAACACCAGGTGAG------------CCCATGT---
Hlst    GACGAGAAGACCCTGTGGAGCTTTAAACAAGTAGCGGG-------------CCAT-GTCA
Clpe    TACGAGAAGACCCTATGGAGCTTTAGACCCAAGTGCAG-------------TCCTGTTTT
Mlmr    GACGAGAAGACCCTATGGAGCTTTAGACACTAGAACAG-----------ACT-ATATACA
Crcr    GACGAGAAGACCCTGCGGAGCTTTAGACGCCAGAACAG------------ATCACGTCA-
Muce    GACGAGAAGACCCTGCGGAGCTTTAGACGCCAGAACAG-ATC-----------ACGTCAA
Bege    GACGAGAAGACCCTGTGGAGCTTTAGACAACAGGGCAG------------ACCATGTCAA
Mela    GACGAGAAGACCCTGTGGAGCTTTAGACGAGAGCAGCTC--------------ACTTTCA
Hats    GACGAGAAGACCCTGTGGAGCTTTAGACAAAAGAACAG-AC-----------CATGTCAA
Orla    GACGAGAAGACCCTGTGGAGCTTTAGACCATAAATAGA------------CC-ATGTCAA
```

```
Cosa    GACGAGAAGACCCTATGGAGCTTAAGACGAAGGGCAGA-------------CCATGTTAA
Exsp    GACGAGAAGACCCTTTGGAGCTTTAGACTACAGGCAGA-------------CCATGCTTA
Depa    GACGAGAAGACCCTGTGGAGCTTTAGATAATATGATGG-AC----C-------ATGTTAA
Rima    GACGAAAAGACCCTGTGGAGCTTTAGATTACAGAGCAG----------------------
Fuol    GACGAGAAGACCCTATGGAGCTTCAGACACAAGGGCAT------------ATCATGT---
Gmaf    GACGAGAAGACCCTATGGAGCTTTAGACGCACTAGCAG------------ATCATAA---
Xeei    GACGAGAAGACCCTATGGAGCTTTAGACACAAGGACAG------------ATCATGTTAA
Pros    GACGAGAAGACCCTATGGAGCTTTAGACACAAAGTAGC-------------CCAAAC---
Scmi    GACGAGAAGACCCTATGAAGCTTTAGACGCAAAGCGAA-------------CCAAACCAC
Rolo    GACGAGAAGACCCTATGGAGCTTCAGACACAAAGCAGT-------------CCACTC---
Cere    GACGAGAAGACCCTATGGAGCTTAAGACAAAATAGTAG-TC-----------CATATA-A
Daga    GACGAGAAGACCCTGTGGAGCTTAAGACATGAGCAGAG-------------C-AAGCTGA
Anco    GACGAGAAGACCCTATGGAGCTTCAGACACAAAGCAGC-------------CCACGTTAA
Dmve    GACGAGAAGACCCTATGGAGCTTCAGACCCAAGGCAGC-------------CCACGTC-A
Dmar    GACGAGAAGACCCTATGGAGCTTCAGACATAGGGCAGC-------------TCAC-GTCA
Anka    GACGAGAAGACCCTATGGAGCTTCAGACACAAGGCAGC-------------TCATGTTAA
Moja    GACGAGAAGACCCTATGGAGCTTCAGACACAAAGCAGC------------C-CACGT--T
Hoja    GACGAGAAGACCCTATGGAGCTTCAGACACAAGGCAGC------------C-CACGT-TA
Bede    GACGAGAAGACCCTATGGAGCTTTAGACACAAAGCAGTC----C---------AGATTAA
Besp    GACGAGAAGACCCTATGGAGCTTTAGACACAAAGCAGC-------------CCA-AATTA
Mysp    GACGAGAAGACCCTGTGGAGCTTTAGACAAAAGACGGA-------------CCTTGCAAA
Osja    GACGAGAAGACCCTGTGGAGCTTTAGACATAAAGCGGAC------C-------ACATAAA
Sgro    GACGAGAAGACCCTGTGGAGCTTTAGACACAAGACAGA-------------CCATAAAGA
Pzpa    GACGAGAAGACCCTGTGGAGCTTTAGACCTAGTGCAGC-------------CCACAA--A
Zeja    GACGAGAAGACCCTATGGAGCTTTAGACCTAGTGCAGC-------------CCACGAAAA
Znne    GACGAGAAGACCCTATGGAGCTTTAGACCTAGTGAAGA-------------CCA--CGAA
Zefa    GACGAGAAGACCCTATGGAGCTTTAGACCTAATGCAGT-------------CCACGAATA
Acni    GACGAGAAGACCCTATGGAGCTTCAGACCTAGTGCAGA-------------CCACGAAAA
Ncrh    GACGAGAAGACCCTATGGAGCTTCAGACCTAGTGCAGA-------------TCACGA---
Agca    GACGAGAAGACCCTATGGAGCTTTAGACACAAGGTAAA-------------TTATGTTAA
Hydy    GACGAGAAGACCCTATGGAGCTTTAGACACTAGAGTAG------------ACCATGTTAA
Gsac    GACGAGAAGACCCTATGGAGCTTTAGACACTAAAGTGG------------ATCATGTCAA
Pevo    GACGAGAAGACCCTATGGAGCTTTAGACTACCAAGCAG------------ATTAAGT---
Hiku    GACGAGAAGACCCTGTGGAGCTTCAGACAATAGATGAA-T----------T-TATTA---
Inpa    GACGAGAAGACCCCGTGGAGCTTAAGACATAAAGCAAC--------------T-TGT-AA
Auch    GACGAGAAGACCCTATGGAGCTTTAGACACCCAACAGAC-----C--------AAGTAAA
Fico    GACGAGAAGACCCTATGGAGCTTTAGACATAAGACAGC-------------CTATGTTAA
Macs    GACGAGAAGACCCTATGGAGCTTTAGATATCAAAGCAG-CT-----------CGTGTTAA
Moal    GACGAGAAGACCCTGTGGAGCTTTAGACGCTAAAGCAG------------CCCACGAACT
Syma    GACGAGAAGACCCTGTGGAGCTTTAGGCACTAAAATAA----------------TGT-AC
Mafr    GACGAGAAGACCCTGTGGAGCTTTAGACACAAAGCAGA-------------TC-TGGCAA
Dcpe    GACGAGAAGACCCTGTGGAGCTTTAGACACAAGACAAA------------ATCACGT---
Dcti    GACGAGAAGACCCTGTGGAGCTTTAGACATAAGACAAA------------ATCACGT---
Hehi    GACGAGAAGACCCTATGGAGCTTTAGACACCAAAGAAG-ATC-----------CTGTCAA
Stam    GACGAGAAGACCCTATGGAGCTTTAGACACCAAAGGAG-A-----------TCATGTTTA
Hogi    GACGAGAAGACCCTATGGAGCTTTAGACGACAAGTCGG------------GTCATGTCAA
Erzo    GACGAGAAGACCCTATGGAGCTTTAGACACCAAAGCAG------------ATCACGTTAA
Hxot    GACGAGAAGACCCTATGGAGCTTTAGACACCAAGGCGG------------CCCACGTTAA
Core    GACGAGAAGACCCTATGGAGCTTTAGACACGAAAGCAG------------CCCACGT---
Apve    GACGAGAAGACCCTATGGAGCTTTAGACACCAAGGCAG-C-----------TTACGTT-A
Latj    GACGAGAAGACCCTATGGAGCTTTAGACACCAGGATAG-CT-----------CATGTTAA
Laja    GACGAGAAGACCCTATGGAGCTTTAGACGCCAGGACAG------------ACCATGT---
```

```
Syja    GACGAGAAGACCCTGTGGAGCTTTAGACACTAAAGCAG------------ATCAAGTTAA
Epme    GACGAGAAGACCCTATGGAGCTTTAGACACTAAAGCAG-GC-----------CA-GTATT
Grse    GACGAGAAGACCCTATGGAGCTTCAGACACCAAAGCAG------------ACCATAT-AC
Clja    GACGAGAAGACCCTATGGAGCTTTAAACACTCGAACAA-------------TTATGT---
Ogcy    GACGAGAAGACCCTGTGGAGCTTTAGGCAACAGAAAGG------------ATCTTGT---
Plna    GACGAGAAGACCCTATGAAGCTTTAGACACCAGAACAG------------CCCAGATCAA
Lema    GACGAGAAGACCCTATGGAGCTTTAGACGCCAGAATAA------------ATCATGTCAA
Etzo    GACGAGAAGACCCTATGAAGCTTTAGATATAAGACAGA-------------TCACGT---
Apse    GACGAGAAGACCCTATGGAGCTTAAGACACCAAAGCGG------------ACCATGT---
Epde    GACGAGAAGACCCTATGGAGCTTTAGACACCAAGACAG-A-----------TCATGTT-A
Slja    GACGAGAAGACCCTATGGAGCTTTAGACACTAAGGCAG-A----------C-CACGT--T
Bsja    GACGAGAAGACCCTATGGAGCTTTAGACACCAAGGCGG------------CCCACAGTTA
Ecna    GACGAGAAGACCCTATGGAGCTTTAGATACTAGAATAG-AC-----------CATGTTAA
Cohi    GACGAGAAGACCCTATGGAGCTTTAGATACTAAGGTAG------------ACCATATTCC
Caar    GACGAGAAGACCCTATGGAGCTTTAGACACCAAGACAG-C----------C-CATGT-TA
Came    GACGAGAAGACCCTATGGAGCTTTAGACACCAAGACAG-A-----------TCATGTT-A
Mema    GACGAGAAGACCCTATGGAGCTTTAGACACCCAGGACAG-CC----------CATGTTTA
Lenu    GACGAGAAGACCCTATGGAGCTTTAGACACCAGAACAG-A----------C-CACGT-TA
Brja    GACGAGAAGACCCTATGGAGCTTTAGACATACGGTATA-------------TCCCGTTAA
Plma    GACGAGAAGACCCTATGGAGCTTTAGACACCAAGGCAT-G-----------TCATGTT-A
Emst    GACGAGAAGACCCTATGGAGCTTTAGACACCAAAGCAG------------ATCAT-GTTA
Ptti    GACGAGAAGACCCTATGGAGCTTTAGACACCAAGGCAG------------ACCA-TGTTA
Losu    GACGAGAAGACCCTATGGAGCTTTAGATATTAAGCAGA-------------CTGTGTTAA
Geoy    GACGAGAAGACCCTATGGAGCTTTAGACTCAATGGGCAG-ACT----------ATGTTAA
Dipi    GACGAGAAGACCCTATGGAGCTTTAGACACCAAGACAG-ACC-----------ATGTTAA
Pama    GACGAGAAGACCCTATGGAGCTTTAGACGTCAGAGCAG-CC-----------CATGTA-A
Leob    GACGAGAAGACCCTATGGAGCTTTAGACACCAAGGTAG-A----------TTTATGTTTA
Neba    GACGAGAAGACCCTGTGGAGCTTTAGATACTAAGATTG------------ATC-CTCTTA
Pdpl    GACGAGAAGACCCTGTGGAGCTTTAGACGCAAGACAGA-------------CTATGT---
Nimi    GACGAGAAGACCCTATGGAGCTTTAGACACCTAGACAG------------ACCACGT-CA
Uptr    GACGAGAAGACCCTGTGGAGCTTTAGACACTAGGACAG------------ACCATGT---
Pesc    GACGAGAAGACCCTGTGGAGCTTTAGACACTAAAATAG-----------ACC-ATGTCCC
Baar    GACGAGAAGACCCTATGGAACTTCAGGCACTAAGACAG------------ACCATGTTAC
Moar    GACGAGAAGACCCTATGGAGCTTTAGACACCAAGGTAG------------ATCATGTTAA
Toja    GACGAGAAGACCCTATGGAGCTTTAGATGATGAAACAG------------ACCACGTCAA
Chau    GACGAGAAGACCCTATGGAGCTTTAGACAAAAGCAGGC--------------CATGT-TA
Chse    GACGAGAAGACCCTGTGGAGCTTTAGACACCAAGGCAG------------ATTATGTTAA
Enar    GACGAGAAGACCCTATGGAGCTTTAGACACCAAGGCAG------------TTTATGTTAA
Hpty    GACGAGAAGACCCTATGGAGCTTTAGACACTAAGACAG-----------ACC-ATGTTAA
Nana    GACGAGAAGACCCTATGGAGCTTTAGACACCAA-ACAG------------ATTTTGTAAA
Mcst    GACGAGAAGACCCTATGGAGCTTTAGACACCAAGACAG------------CTCAAAGTTA
Rhox    GACGAGAAGACCCTATGGAGCTTTAGACACAAGGCAGA-------------TCATGTTAA
Opfa    GACGAGAAGACCCTATGGAGCTTTAGACACCAAGGCAG-AT-----------CATGTT-A
Paar    GACGAGAAGACCCTATGGAGCTTTAGACACCAACGCAG--ATC----------ACGCTAC
Gozo    GACGAGAAGACCCTATGGAGCTTTAGACACCAGAATAG-AC-----------CATGT-TA
Ackr    GACGAGAAGACCCTATGGAGCTTTAGACACTAAAGCAG------------GTCACGT---
Elev    GACGAGAAGACCCTATGGAGCTTTAGACATAAGCAGACC--------------TTGTTAA
Trdu    GACGAGAAGACCCTATGGAGCTTTAGACGCCAGAACAG------------ACCATGT---
Amoc    GACGAGAAGACCCTGTGGAGCTTCAGACACTAAATCAG-CC-----------CTTGTCCA
Hame    GACGAGAAGACCCTATGGAGCTTTAGACACCAAAGCAG------------CTTACGTCAA
Chso    GACGAGAAGACCCTATGGAGCTTTAGACACTAAAACAG------------CTCATGTTAA
Lyto    GACGAGAAGACCCTATGAAGCTTTAGACACCAAGGCAG-ATC-----------ATGTTAA
```

```
Encr            GACGAGAAGACCCTATGGAGCTTTAGACACCAAGACAG------------ATCA-TGTTA
Bvar            GACGAGAAGACCCTATGAAGCTTTAGACACAAGGCAGC-------------CCCCGTTAA
Noco            GACGAGAAGACCCTATGAAGCTTTAGACGTAAGGTAGA-------------CCAGACTAC
Chsp            GACGAGAAGACCCTGTGGAGCTTTAGATACTAGAAAGA-------------CCATTT---
Arja            GACGAGAAGACCCTATGGAGCTTTAGACACCAAGACAG------------CTCATGTTAA
Pase            GACGAGAAGACCCTATGGAGCTTTAGACACTAAGATAG-CC-----------CCCCTC-C
Trel            GACGAGAAGACCCTATGGAGCTTTAGACAACAGAATAG-A----------C-CAT-----
Lifa            GACGAGAAGACCCTATGGAGCTTAAGACACAAAATAGCTC-------------GTACCAC
Acur            GACGAGAAGACCCTATGGAGCTTCAGGCACTAAGACAG-CT-----------CATGTCCC
Ampe            GACGAGAAGACCCTATGGAGCTTTAGACGCCAAGACAG-----------ACC-ATGTTAA
Urja            GACGAGAAGACCCTGTGGAGCTTAAGACACTAAGCAGCCC-------------ATGTTAC
Enet            GACGAGAAGACCCTGTGGAGCTTCAGACACCAAGGCAG------------CCCCCCCC--
Ptbr            GACGAGAAGACCCTGTGGAGCTTTAGACAAAAAATAAACT-------------TTGTTAA
Safa            GACGAGAAGACCCTGTGGAGCTTTAGACAGCAGACTGC-C----------C-TTACA-CA
Icae            GACGAGAAGACCCTATGGAGCTTTAGACACCAAGGCAT-----------ATC-ATGTTAA
Asmi            GACAAAAAGACCCTGTGGAGCTTTAAACACAAGAGAGG-AAT-------ACCCCCAACTC
Foal            GACGAGAAGACCCTATGGAGCTTTAGCCAAATTAGGCTT-----------AAGTTA-CTG
Drze            GACGAGAAGACCCTGTGGAGCTTTAGATGTAAAAGTAG-----------ACTAAATTAAT
Rhas            GACGAGAAGACCCTATGGAGCTTCAGACACCAGGACAG------------ACCATGT---
Elac            GACGAGAAGACCCTATGGAGCTTCAGACACCAGAACAG------------ACCGCACTGA
Kugu            GACGAGAAGACCCTATGGAGCTTTAGACAAAACACAGA-------------CTGCATCAA
Plor            GACGAGAAGACCCTATGGAGCTTTAGACACCAAGGCAG------------ACCATGTTAA
Sgun            GACGAGAAGACCCTATGGAGCTTTAGACACCAAAGCAGATC------------ATGTTAA
Zaco            GACGAGAAGACCCTATGGAGCTTTAGATACTAAGACAGATC------------ACGTCAC
Zbfl            GACGAGAAGACCCTATGGAGCTTTAGACACTAAGACGA------------ATCATGT---
Spba            GACGAGAAGACCCTGTGGAGCTTTAGGTAACAAAACAGATC------------GTGCCCA
Game            GACGAGAAGACCCTATGGAGCTTTAGACACCAAGGCATAT------------CATGTTAA
Thth            GACGAGAAGACCCTATGGAGCTTTAGACACCAAGGCATATC------------ATGTCAA
Xigl            GACGAGAAGACCCTATGGAGCTTTAGACGTCAAAGCAGA------------TCATGTT-A
Hyja            GACGAGAAGACCCTATGGAGCTTTAGACACCAAGGCAT------------ATCATGTTAC
Psan            GACGAGAAGACCCTATGGAGCTTTAGACACTAAGGCAT------------ATCAT-GTAA
Cupa            GACGAGAAGACCCTATGGAGCTTTAGACACCAAAGTAT------------ATCATGC---
Mpch            GACGAGAAGACCCTATGGAGCTTTAGACACCAAAACAG------------ATCTTGTTAA
Char            GACGAGAAGACCCTATGGAGCTTTAGACACCAGAGTAG------------CCCTTGTCAA
Pser            GACGAGAAGACCCTGTGGAGCTTTAGACGCCAGAGCAG------------ACCATGTTAA
Prol            GACGAGAAGACCCTATGGAGCTTTAGACGCAAGGGCAG-----------ATC-ATGTCAA
Plbi            GACGAGAAGACCCTATGGAGCTTTAGACACACAGGTGG------------ACCATGT---
Calu            GACGAGAAGACCCTGTGGAGCTTAAGACAACCGGGTAG------------CCCATGTCAA
Papa            GACGAGAAGACCCTGTGGAGCTTTAGACAGTTAACCGC-------------CCAT-AACA
Sufr            GACGAGAAGACCCTATGGAGCTTTAGGCAACAAGCAGA-------------CCGTACCA-
Stci            GACGAGAAGACCCTATGGAGCTTTAGGTGTAAAAACTG------------CTCAT-GT-A
Taru            GACGAGAAGACCCTATGGAGCTTTAGACAAAAAACAGC-------------CCCTGTCA-
Rala            GACGAGAAGACCCTATGGAGCTTTAGACACTAAAGTAG-A----------T-CATGT-TA
                  * * *******   * * ***
```

```
                HVR
Scca    TA---TTAATAAT--------CCCAGGACATAAACAAAAAAT-A-----TAATAC--TTCT
Muma    ACCATTTATTCCTC-----------AGGGTATAAACAAAATATAT-----AATACTTCT--
Erca    A---CCCTATAAAAAATATAG-------------GTAAAC-----CCTATGTTA-------
Pose    ACTA------TATTTT--A-------CCATATAGA-----TAAACA-CAGC-ATT-AT---
Actr    CAAGCCAAC--------TACCCACGGAAATAATA-----GCTAAAAGCATAATAGTACCCT
Scal    CAAGCCAACCACCTA------CGGAAATAAC---AGCTAAAAGCACA--ATAGTA--CTAT
Posp    CAAGCCAACACACCAA--CAGG-AAT-----AACAGCTATAAGCATAATAGTACCTCT---
Atsp    CAACCCAACTGTTCGAAAGAC-----ATAAAAAATATAAAG---CACAGTGATCCCT----
Leoc    ATTGCTCGAAAGAC----------------ATAAAAAATACAAAGCACAGTG---GTCTCT
Amca    CAAGTTTATCCCCCAA--CATG-AAATAAACAACTCAAGCA-CAT-----TGGCCTTC---
Osbi    --------AATT--AT-CAACT---AATTTTAAAGCC--------TAG----C-AACCCTA
Pabu    AACACTAT-AA--------------ATAAATAACA------AAGCAT-AA--TGA--CACT
Hial    -GTAACCCTAAACCCAAAAGGAAACAAAAG-----GCA----AAAGCGTAGCGGAAC--AT
Elha    GC--------AGCCCCAC---AACAAA---AAAGCGAACAAGCCTAATGGTACC------T
Mlcy    -GCACCACCCAA----------------AAGACACAA----GCAC--AAC-AGCCA--CTG
Algl    ATAGCTAAT--------GCCCGACAGGAATAAAA----G-CTAGTTTATAATGCGTTGGTT
Ptgi    GCAACCCCACTAAACAAAAGAAATAAAAGGAAAA-----CTAAGCCTAATGAAATGAT---
Alaf    ACGACCTATAAACCAATTTG-GGATAAAAAG---TACCTAAACCATATGCCA------TCT
Nock    A--CAACTAATAAACCGACCGGATTAAAAAGTAACTAAGCTCTG-TGATATA-------AT
Anja    AGAAACCAAGTTAACCAAAAGGAACACAAAGGCCACGAA-----ACCCGACGTAAACT---
Gyki    -CCCTCTCTTCA-----------TTCAAAGCCTG-----GGGAAACATAACGCTAAT----
Syka    AGGACCCGAAGCATTG--ATAAATAATAAAAGGCCTAAACCTA----ACCAAATGTAAAAT
Opma    GAAATTTCACATTAAA-----CAAAAGGAACAAAGCAAATTT-AAACTCAATG--TATTAA
Comy    A-AACCAAATCTTCCA--AATA-GGCCTAAAAAATGA-----GAAAAACAAGTATTAA---
Sasp    ACCG--CTAGG---AATACAA------------------ATGGACTCAATTGTGTCAA---
Eupe    AA--------ACCCCAAATG-ACCCAACAGAAC-AAAATGTTTATTAAACCCAACATGTCA
Enja    GCGAC-TGAACTGAGCAAGTC----------------CTAAATA-CCCGCAGCCTT---AT
Same    G-CGGCCCTAA---------------TTGGAGCCCCAAAC---------AAC-GTGATTCT
Chch    ACCACTTAAAACAA-----------AACAAGTGGAAACAG-----AGTGGTAAACCTT---
Grgr    -----TAAACAACTC------C---CCAAGGATAAG-----AACGGTATGGCAAGCA--CT
Caau    GCAA-------CTCAA--T---------------AAAAAATGAAAACTTTGTGGAATA---
Cyca    GCAA---CTCAA-TAAAAGC-------------------AAAAACCTTGTG-GACC-A---
Dare    -GCAAACC-AAT-----------------AAAAGTGATT----AAACTAGTAAAAG----A
Cost    ACAACTTACTAAATA-----AGA-AT-------------TAAA-CCTAGCAGAAAA-----
Leec    ACAACTTA----------TTAA---CAAAGTATAA----ACCTAGTAGAGA---------A
Crla    -----CAAGCAACC----------------ACATAAATGGCATAAAAT--TAGTGAGTA--
Clmc    AA-ACCCA----------AATCAAAAAGAAGCTAA----ACAAAACAGCAA--------AA
Phin    AACCCCTACA-----TTAAATA------GGACAAAA-----------CGAAATACCAC---
Icpu    -----CAAGGACTT--------------------------AGTCAAACTAAA-TAGCAC--
Psto    -----CAAG-AAT----------------CCAAAATAAAA---TTAAACTAAATAGCAA--
Cora    AT--------ATTTATC-----A---A---ATAAA---ACTAAAT--AGTAA---------
Eisp    TAAA---CCAGATTAATAA-----GA-------------TTAAACTAAATA-GCTT-A---
Apal    AGACTAATTACCTTTA--GT-----CA--AACTAAACCT--------------TGC-A---
Eslu    -----TAAATAACT-------------GAACTATCAGATTAAAACA--AAGCGGCCC--CT
Dape    GTAACCCTATA-------------------TTAACGGGTAAA-ACAA-AGC-GGCCC--CT
Glse    -------ACCCACCTCTT-------CT---ATAGGACTAAA---CACAGTGCCC----CCT
Naar    -AACCCCCTCAT----------------CACAGGACT-----AAACACAGTACCC-CC--T
Baoc    ----------AACTCCTA---A---ACCACCAG--GAAAAAACACAGT------AGCCCCT
Opso    AAACC-CTAA--ATCACAGGA-------------------CTAAACACATTGGCCCCT---
Alte    C---------AACCAAACTC---A---AAGGTATAA---ACAAAGTGTGAACAT-------
Plap    ACAA---CCAAACTCAAAGGC------------------ACAAACAAAGTGTAAGCAT---
```

Plal	GCTC--CCTACTCTAA------TGGGG-------------AAAAGCATTATAGCCCCC---
Sami	---ACCCCCTCCATTA------AG-A-GGGGAAAACATTGTG----------GCTCC---T
Rere	-ACACC------CTTAGTCT----------GCTAGGAGA----GAACATTGTGGCCCT--T
Gama	A---C-----AACCCCTTGTGCGGGA-------------GAGAACTTAGTGGCAC-CT---
Onmy	GCAACCTT-----GAA--------------TTAACAAGTAAAAACGC-AGTAGACCCC--T
Sasa	--------CC-----TTGAAT-TAACA---AGT--AAAAACG---CAGT-GACC----CCT
Cola	GC--------AACCTTGA---G---TTAACAAG---TAAAAACGCAGTGACCCCT------
Dita	ACCC-------CCTCG--G-----------CCACTGACGGGGACAATAACTGGCCTT---T
Gogr	-----TAACCTCTTC------TACCAAAAA-----GGGGGCTATAGGGA-GAGACCC--CC
Chsl	----CTACC-TATCTACCAGC------------------GTAAATCCTAGGGCCC-TT---
Atja	TAAA-------ACAAA--A------------CAAAGTAATACAAACCAAGTAGATACT---
Iido	A-TAC-GCC---------AAAATAAAGGACACTAA----ACCAAGTAG-A--------CAC
Auja	A---------CAATCA---CCAT---AAAGAGAACT----AACACAGTGAACCCTC-----
Chag	GCAAACTCACC--------------------ACAAATGAGCTGACTCCCGATGGTAAGACA
Hami	AGCAACCCCAT--------------------AAACGGCTAC-AAACAA-CGTTAA-CC--C
Saun	AACAACCCAT-----ACCA-----GTGGCCATAGAACAAC---------GTGGAATCC---
Nema	GTAC--CCTAATTTAA-AAC-----A-------------ATAAACAAAGTGTTACACC---
Disp	-----CAAAAACC--------------CCCTAA-AAGGAAATGAACAAAA--TGAGCT--T
Myaf	AT--------ACCCCCAA---TA-AAGGAACAAAACAAGATGGTCAT--------------
Lagu	------AGAGGACA-------------TCAAGCCAGAAAC--AGCC---ACAGCCTAT--C
Trtr	AGTAAATTGAT-------------------ATTACCTATTTAAACCCCGG-CATCCCT---
Zucr	-GTATAAACCCC-----------AACTATATAA---------AAACACGGCTACCCT----
Pxja	AACACCTCAAC--------------ATAAAGAAA-------AGAACTAAGTGTTTATC---
Pxlo	ACACC-TCAG--CACAAAGAA-------------------AAGAACTAAGTGTTTACC---
Pctr	ACACTCAA----CAAA--GGCA-GAA-----CTTAATGG--------------ACC-C---
Apsa	A-GATTCTTAAATCA---AAA-----A-GAAAGAGCTAAATAG----------GC--C---
Cabe	AACACCCCTCC--------------CAA-----GGGGAC--TA-GCACGCT-GACCCT---
Bzze	AACACCCCTGA-------------------TTATA-GGA--CTAAACTGATTGGGACC---
Siim	ACGAACCTAAA-------------------TCAACAAGA--TGACTATACTGAAGACC---
Ctru	A---------GACCCCT-TA---CAAAGGAGCA------AA-CTAAATGACCCC-------
Dpbr	AACAACCCTGACA---------------AA-----GGCAC-GAACTAAATGG-TCCC----
Caki	-----AAAATGAG--------CTT------TAATACAAGAACAAACTTAA--TGGGAC--T
Phja	-----TTAGCATAC---------------TATAATAACAGTAAAAATCTAGTGAAAAA--T
Brsp	AATATTCTG--CATCCAGTAAAAACTTAA-----------------------TAATTT---
Gamo	AC--------ATGC-TGT---GAT-AACAG---TAA---AAACTTAGTGATATT-------
Lolo	ATATACTA--GAATAACA-GT------------------AAAAACTTAGTGATATT-----
Batr	-----------T-AAAACA-------------------AACCACC----------------
Prmy	---------------------------------GTTTCA----AAATACACCAA-------
Loli	AAAATCCTG------CACCAAGGCCCTAAA----------------CTAAATAATTCC---
Loam	AACC---CCCGA-ACAAAGGA-------------A------TAAACAAGATGAAAG-----
Chab	-GTACCCCTAAT-------------AAAGGTGA----AAAC-----AGAGTATGCTA---C
Chto	-----CAAGTACCCC------T-AATAAAGGT---------GGAAACAGAGTATGCTA--C
Majo	-----CAAGCACC--------TCCAGTAA------GTAGAGATGAAACAAAATGAATA--C
Hlst	A--AC-ACA--------TTAAATAAGTGAAACAA------A---ATGGATA----------
Clpe	AACCCCCTTATACTAC--GCGG-GAACACAAC-----GA-------------GCC----CC
Mlmr	-CTAGTCTTAAC-----------ACAA--TACA---------GAACCAAAAAAGACC--CC
Crcr	--------A-ATACCTCCCTCA---AATAG------GTAACAA---CAAATGAA-C----C
Muce	ATACCTCTCTCAAACAG---------------------------GTAACAACAAATGAACC
Bege	TAT---AAT--------CT-GATTAAAGAACAAA-----ACCAATTGGAC---------CC
Mela	--------GCTTCCTC-TAATG---AAAGAATAAGAC--------TAATATGAATCC----
Hats	AAATCCCCCGG--------------ATAAGGGAA--AG-----AAC-TAATTAGA---CCC
Orla	-GGACAAC--AA-----------CAAATTACTC----AA-----ACAAATTGGCTA--C--

```
Cosa    TTAACCTAACC---CA--AATA-GATTAAACTAATTG-----G----------CTTTC---
Exsp    TTAG------ACCTAG--T-------TAAAGGA-----ACTAAGCC-AATTGGGTACC---
Depa    CTAAA-CCTTACTTAACAAGA-------------------ACAAACCAATTGGA----ACC
Rima    ------------CCT------T-AAT-------AAAA--------CTTTTTAAAGC--CCC
Fuol    -----TAAA-GTA--------------TTGATTTAAAACAATTAAAACAAATTGAA--TTC
Gmaf    -----AAAACATTCT------TTTCA-AAAAAATGA------AACC---AACT--GACCCC
Xeei    AACC-------CCTTA--T-------TTACA-----AGATAAAA--CCAA---TTGATTCC
Pros    -----TTCACCCCCA------A-AA------TTAAAATTGAGGGGACAC--AATGGCCCCC
Scmi    CC-TGCCC---------AAAAATCAAAAGGGGCA------ATAAAAT--------GGCACC
Rolo    -----CAAACACC--------CCAAAATAAAGGG----GCAAATCA-A----GTGGCCCCC
Cere    AGTAACTTCAA--------------ATAA-----AAAGT--TATATTT---TTT-GCCCCC
Daga    -ATGCTTTCAAT-----------T-----AAAAAAGTT----AAACTTGTGCCCCC-T--G
Anco    ---------TAACCTCCAAACA----AGGAACTAAA--------CTAAGTGA----TATCC
Dmve    AGCACCCCC------CGACAAAGGGTCAAA---------------CCAAG---TGAAGCTC
Dmar    AGCAC-CCC--------CCGATAAAGGG--CTAA-------------ACCAAGTGAACCCC
Anka    ---TAACC---------ATCAAACAAGAGAGTAA-----ACTA--------AATGAAAATC
Moja    AAGA---AACCCCA-----------AACAAGG-----AACTAAACT----AAGTGACGCCC
Hoja    ATAACTTTCAAAC--------------------AAGAAATAAAACTAA---GT-GGAACCC
Bede    GACT--CCAGAATTAAAACGG------------------AGCAATAAAATGACAATCC---
Besp    A--GACTCCAAAATTA--AAAC-GGAGCAATAAAATGAC--------------AACCC---
Mysp    ACAA-------CCCTG--A-------ACAGAGGACAAA-----ACA-CCAAAGGATCC---
Osja    ACAAC-CCAA--AATAAAGGA-------------------CAAAACATAGGGGATCCC---
Sgro    -ACAGTTAAAGAC-----------AAAAAACCA-------AACCCAAATGGACTT--TCTG
Pzpa    AATGCTTTAA-----AACAAAAAAC--AAACC-----CA----------ACGAGC-CC---
Zeja    A---------TACTTTTACA---CAAGAAAGAA---AAG---CC-------CAATGGCCCC
Znne    AAAA--GCT--------TTAAATTAAAAAAGAA-------A----ACCCCA--GTGATTTC
Zefa    A-TACTTTTGATTAT----AAA-AGAAA-----AGCTCAATGG--------------ATCC
Acni    A--TACTT----------TAAGACAAAAAAGAAA---AGC-CCA---------ATGGCCCC
Ncrh    -----AAAATACT--------TTAAGACAAAAAA------GA-AAAGCCCAATGACCC--C
Agca    T----------ACCCCTCC----ACATAAGA---GGATTAAACC-AA------ATAACCCT
Hydy    -TGAGCCT---------GAAC---AAAGGACTAA-----ACAGAATG---------GACCC
Gsac    T---------GACCCTAAAT---AAAG-GATT-----G----AACAAG-----ATGGAACC
Pevo    -----CAATCC--CC------TTAAA------TAAAAGACTAAACC---AAATAACAC--C
Hiku    AACAATTAACACCA-----------TATAAAAGGTATTTAAA-----ACAA-----TAACC
Inpa    CCCTCAGCCCA--------------ACAAAGGCCTTG-----AACTA-AACAAC----CCC
Auch    AATTAGTCTAGACCAACAGAC-------------AAGACT-----CGACTTGGACCCC---
Fico    A-CACCC-CCGATAA---GGCC-TCCAAA-CAAAATA-----G----------GC-CC---
Macs    TCACCTCCAAA--------------A-AAGGA-----GTAAA-ACAA-AAC-GA-A--CCC
Moal    A----------ACCTTGTC----ATAAACAAGTTATTAACC----AGTGA------AACCC
Syma    --------ACATTA----CACA---AACATTCA-------------------------TCT
Mafr    ACACTTA-----CACG--ACCC-AAGCTAAAAG-----A------------------ACCC
Dcpe    -----TCAGCAC-TT------CAACACAAAGAATAAAACTCTG------TGAGCACAC--T
Dcti    -----TCAACACT--------TCAAAATAAAGAATAAAAC---TTTG--TGAACA--CACT
Hehi    A---TAACCCCCAATAAGGGC-------------CTG-----AACT-AATGGAAT-CC---
Stam    AACACCCTTC-----AATAATAGGC-TAAACCAA---------------ATGAAT-CC---
Hogi    A--CAATC----------CTAAATACAGGACCAA----A-CAAAATGAAAG---------C
Erzo    CA--------CCCCCTTA---AT--ACCGG---ACTA----------AACTAAATGAAGCC
Hxot    AC--------ACCCCCCA---AT--AAGGG---ACTA----------AACCAAGTGAGCCC
Core    -----TAAGCACCC---------CCAAACAA------GGGACTAAACCAA-GTGGGCC--C
Apve    AGCACCCCCG-----ATAA--AGGACTAAA----------------CCAAGTAAACCC---
Latj    G-ACACCCTGA--------------ATAAAGGAC-TGAAC------TT-ATTGACC--ACC
Laja    -----CAAGCAAC--------CCCTGACAAGGGGTCA------AACCAAA--TGCACC--C
```

Syja	ACAACCCT---------AAAC---ACAGGGCCAA-----ACCAGACGAATA---------C
Epme	A-ATACCCCAA--------------ATATGAGG------CACGAATAA-ACTGAAT--TTC
Grse	--------CCACTCTC-CCACA---AAGAGAA-----AAAGC---AAAT-G----ATCCCC
Clja	-----TAAGGCCCC---------CTAAA-AAGGAATAAA------ACTTAAATACCA---C
Ogcy	-----CAAAAACCCT------A-AACAAAAAAAA-----AGAACTTATAGAAAACCC--CC
Plna	AAAC-------TTTCC--A-------TAA-----AAAGACTAAA--CCAACTGGCCCC---
Lema	-ATACCCCAAAC------------ACAGGGCCG----------AACTAAATGA---GC--A
Etzo	-----TAAACACTC---------CCTGAC------AAGGGACAAAACCAG-GTGAATC--C
Apse	-----TAAGTAACC--------CCATATAAGGGC------CTGAACC--TAGTGGCTA--C
Epde	AACACCCTTG-----AACAAAAGGACCAAA----------------CCAAATGAAACC---
Slja	AAAC---ATCCCCT-----------GTGAAAGGG-----TTAAACTGTATG-----GCCAC
Bsja	A-TAACACCAAAAC----AAAG-GACCAA-----ACAACACAAC---------A-CTC---
Ecna	T-AAACTCTAA---------------------ATTAAAGAATTGAACCT-AATG-GAATAC
Cohi	--------ATAACCCCTTGTTA---------AAGGAGTAAATC---TATGCTAA----TCC
Caar	AACACCCTGAACAC-------------------AACAGCCCAAACTTAATG----GCTTCC
Came	AACACCCCC------AATAAAGGCC-CAAACTTA---------------ATG---ACCACC
Mema	AACAACCCTAA--------------ATAAATGGA--TTAA------ACCAA---ATGAGAC
Lenu	AAAACCCCTAACA---------------CACAGAAGA-----AACTAAAT----GGATGAC
Brja	ACACCCCTGGACAAAG--GACT-TAACTAAAG--------GA---------------ACCA
Plma	AACATCCCTA-----GATAAAGGGC-TAAACC---AA---------------ATGAA-ATA
Emst	A--AC-ACC--------CCCGAATAAGGGGCT---AA------AC-----CAGATGAGACC
Ptti	AAAA------CCCTAA--A-------ACAAAA--------GACCAAACC-AAATGAAC-CC
Losu	GCACCCATTAAAGGAC--T----AAACTTAA-----C---GG--------------CCCC-
Geoy	ATACT-CTTA--GACAAAAGA----------------------CCGAACG-GTATAAACAC
Dipi	ACAC--CCCTAATCAA-AGGG----------------------CCAAACTAAATGGGTACC
Pama	AGCACCCCTTA--------------ACAAAGGA-----A--AAAACCAA---ATGAA-GCC
Leob	A-CCTTCTCCAACAA---GAAA-AGAAAA-----ACTAAATAG----------ACTCC---
Neba	AT--------ATATACAG---AAC-AAAGA---TAT---CCAAAT-------AAGGAATAC
Pdpl	-----CACTAAC-CC------CAAAATAAAGGGCAC------CAAACTAATAAGAC-C--C
Nimi	--------AAACCCCC-TAACA---AAGGACTGA------AC---TAAC-----TGAACCC
Uptr	-----TAAGCCTACC------CTTGATAAAGGACTAAAC------G---AAATGAGAC--C
Pesc	-TCCCCCAAGTA-----------TAAATGACA-----AAACT-----TAATG-ACCCC--C
Baar	--------CTGTCCCCTAAACA----AAGGGCCAAA--------CCTAA-----TGACCCC
Moar	-ACACCCCCAAACAA-GGGGCCAAACCAG-----------------------ATGAA-CCC
Toja	AGCAACCCTAAAT-----------AAGGGACTG----------AACT--AACTGGCCA--C
Chau	AGCACCCTCAA--------------ATAAGA------GGCT-AAACTC-AGTG--TT-CAC
Chse	A--CACTC----------CAGATCAACGGACCAA-----ACTAATTAATTA---------C
Enar	ATACCTTC---------TCAC---AAGGAGCCAA-----ACAAAATAATCC---------C
Hpty	-AGACCCCCAAA-----------CAAGGGGTT----------AAACCAAATGAACCC----
Nana	ACCTCCCCCCT-----------CATACGGGAA---CGAACAAAAAA-----------CACC
Mcst	A--GC-ACC--------CCTGAATAA--AGGACTAA------------ATGTATTGAAACC
Rhox	A-CACCCCTAAA--CA--AAG--GACTAAACCC---CGTGA----------------ACAC
Opfa	AGCACCCCAA---------------ATTAAAGGG-----CCAAACCAGAG-----GAGCCC
Paar	TTAC--CCCG--AATAAAGAA-------------T------AAAGCCAAA---CTGACTCC
Gozo	AACACCCCTAG--------------AAAA------CGGACT-AAACCA-A--ATGAG-ACC
Ackr	-----TAAAAGAATC------T-------AACTACAGA--ACAGAACTAAATGACAT---C
Elev	ACAC--CCCTACT-AA-AGGA-------------CTAAA-----CCAAA--TTG---ACCC
Trdu	-----TAAGCACCCC------TCAAATAAAGAACA-AA------ACT--CATTGACCC--C
Amoc	A-AATCCCAAT--------------TAAAGGACT------GAACACTA-GAGTG--AGACC
Hame	A-TACCCCTAAACA-----AAG-GACTAAAC-----TAATTAA--------------ACCC
Chso	A--ACCCCTCCCA--CAAGAGG-----------------CCAAACT----AGATG-ACCCC
Lyto	----TAACCCTGAATAAAGGC-------------TTA-----AACCAAGT-----GGAACC

Encr	ATAG------TCCTGA--A-------TAAAGGAGC-AAACC------AAG---TGGAA-TC
Bvar	A-------TAAATCTAATT---------AAAGGCCTAAAC---T-TA------CAAACCCC
Noco	AAAG--ACC--------CCCTAATAAGGGCACAA----AC-------CAAA--AGGACCCC
Chsp	-----CGCACATCC---------CCACATAACGG------GACAACCAAA--TGTAAACCC
Arja	GCAC-------CCTTG--A-------TAAAGGACTAAACCAA-----------ATGAGACC
Pase	CAACCCCTTAA--------------AGGGATACT--AGAGCT--------TGAAAGACCAC
Trel	GTCAATTAAACCCC-----------CACAACGGACTG-----AACTAA-----TTGGATCC
Lifa	--TACCTCACCA--CA--AAGA-GATCTAACCT----ACCGA--------------CCTC
Acur	TGCCTATTACT--------------AATAGAGGACAA-AC-----AA-AT----GAGACCT
Ampe	-ACACCTCCCCA-----------TAAGGAGCCC----A-----AACCAAA----TGGTCCC
Urja	AATTACCC----------CTTTACAAAGGATCAA-----ACTAAAT-------GTTTAGCC
Enet	C---------TACCAAAGCT---TGATTAACAGCT---CACACCCTATGG--CCATTCCCC
Ptbr	GCGAA-CTTAC-ATGAAAAGA-----------------ATAAACA-ATAAGAA---C-TTA
Safa	TAAACTCTTAATT---------------AACAAGCT------AAAGCCATAAGGA----CC
Icae	-ACACCCCCAA------------ACAAAGGGTT----AAA-----CCAAATGAACA-C--A
Asmi	-GCCTTTTTAGATT----------GCTAGACTA----TG-------TATTTTA-CCTC--C
Foal	---CTTTT---------------AAATTAAAAA---CTGTG-AAC-TAATTA-----AACC
Drze	-TTAATACTAAC-----------AACAGAAAC----------AAACAAATTA----GATAC
Rhas	-----TAAAACCCC---------TAAAACAAAGAC------TAAACCAAATGACTC---C
Elac	TTA---AAC--------CTTAATTAAAAGAACAA-----GCC---------AGACACCCCC
Kugu	ATAGATTA---------AAAATAAATAAACCTAA------ACCAA---------GCAATCC
Plor	-ACACACCCAAAC-----------ACGGAACCA----------AACTAAAT--GA---TAC
Sgun	A---CACCCTTAAATAAGAGA------------CTAAA-----CAA---GATGAAAC--C
Zaco	ACA-CCCC--TAAACAAAGGA----CTAAACCA---------------A--GTGAAAC--C
Zbfl	-----TAAGTACC--------TCTAAATAAAGAACCAAA-----CCAGA--TGAACC--TC
Spba	GCATA-CCTA--AATAAAGGA------------CTGA------GCCTAA----TGAAAAC
Game	ACACCCCCAGA-------------C-AAAGGGTTAA-A-----CCA-AAT----GAACCG
Thth	ACAC---CCCTAAACAAAGGA------------CTAAACCA-----AATG-----AATCA
Xigl	AACACCCCCA-----AATAAG-GGACCAAA----------CT--------AAATGACCCC
Hyja	--------CTATCCCTACACCC---TGGGAATTAAAC---C------TA----TTGAATCA
Psan	TCTAC-CCC--------TAAACCCTGAGGACTAA----AC--CT--------ATTGAAACA
Cupa	-----TAAACAC-CC------TTAAATAAAAGATTAAGCC---------A--AATGATCTA
Mpch	ATAACCCC---------AAAT---AAAGGAAAAA----ACC---------AAAAGAACAAC
Char	--------ACACCCC--CAGTA---AAAGGGCAAAAC---CA------A----AAGGACCC
Pser	ACAC-------CCCGA--A-------CTAAAGGAATAAAC--T--------GATTGGATGC
Prol	-ATACACCCAGC-----------TAAGGGCCCT----GAA-----CT--AA--ATGAAACC
Plbi	-----CAAGTACCCC------C-AGCTAAGGGTCCGAACT---A------AATGG--AGCC
Calu	GGATCCCACCTAAGGG--AA---AGAACTAATT---------------------GGACCC
Papa	AACAC-CAC--------CAAACAAAGCAAAGTGA--------------ACCAA-TGGTGCC
Sufr	------AGT--------AACCCTTATAAAGGA------ACTA-----AACATACGTAAACC
Stci	ATCAA-CCC--------CTAATAAAGGGA-CTTA----AC----------CTTATGACCAC
Taru	--------ATAAACCCTAAATA---AAGGGAATAAAC---CTAGTGA----------A-CC
Rala	AACACCCCAAATTT--------------AAAGGACTAAACC----AAA-----TG-AGCCC

44' 45 45' 46 46'

Scca	AATTTAACTATTTTTGGTTGGGGTGACC-AAGGGGAAA--AATGAATCCCCCTTA-TCGAC
Muma	AACTTAACTGTTTTTGGTTGGGGTGACC-GAGGGGGAA--AACAAATCCCCCTCA-TCGAT
Erca	TGGCTAAATGTCTTAGGTTGGGGCGACC-ACTGAGAAC--AAATAAACCTCAGAG-ATGAT
Pose	GGCCATAAAGTCTTAGGTTGGGGCGACC-ACTGAGAAC--AAATAATCCTCAGCG-ATGAT
Actr	GATCCTAATGTTTTCGGTTGGGGCGACC-ACGGAGGAC--AAAAAAGCCTCCATG-TCGA-
Scal	GATCCTAATGTTTTCGGTTGGGGCGACC-ACGGAGGAC--AAAAAAGCCTCCATG-TCGA-
Posp	GATCCTAATGTTTTCGGTTGGGGCGACC-ACGGAGGAC--AAAAAAGCCTCCATG-TCGA-
Atsp	GGTCCTAATGTTTTCGGTTGGGGCGACC-ACGGAGAAA--AACAAAACCTCCATG-TCGA-
Leoc	GGTCCTAATGTTTTCGGTTGGGGCGACC-ACGGAGAAAA-ATA-AAACCTCCACG-TCGAT
Amca	TGATTCTACGTTTTCGGTTGGGGCGACC-AAGGAGAAA--AACAAACCCTCCATG-TCGAC
Osbi	ACATACAACATCTTCGGTTGGGGCGACC-ATGGAGGAC--AAAAAAGCCTCCAAG-AAGAA
Pabu	GACCCACTTGTCTTCGGTTGGGGCGACC-AAGGAGGAAA-ACA-CAGCCTCCTAG-AAGAA
Hial	GGACTGAATATCTTCGGTTGGGGCGACC-GCGGAGGAA--AACAAAGCCTCCATG-TGGAC
Elha	GGCCACGATGTCTTCGGTTGGGGCGACC-GCGGAGGAA--AACCTAGCCTCCATG-TGGAC
Mlcy	GCCCCACATGCCTTCGGTTGGGGCGACC-GCGGAGAAA--AACAAAACCTCCACG-TGGAC
Algl	GATTGGATTGTCTTCGGTTGGGGCGACC-GCGGAGGAG--AAAAAAGCCTCCATG-TGGAG
Ptgi	GGCCGGAATGTCTTCGGTTGGGGCGACC-GCGGAGGAA--AAAACAGCCTCCATG-TGGAA
Alaf	GGCTAAAATGTCTTCGGTTGGGGCGACC-TCGGAGGAA--AAACAAGCCTCCGCA-TGGAC
Nock	GGCCAGAATGTCTTCGGTTGGGGCGACC-TCGGAGGAA--AAACAAGCCTCCGTG-TGGAC
Anja	GATCCAAATGTCTTCGGTTGGGGCGACC-ATGGGGGAG--AAAAAAGCCCCCACG-AGGAA
Gyki	GATCAATATGCCTTCGGTTGGGGCGACC-GTGGAGGAG--AGAAAAGCCTCCATG-TAGAA
Syka	GACTTATATGCCTTCGGTTGGGGCGACC-ATGGAGGAA--AACAAAGCCTCCACA-TGGAA
Opma	TGACCACATGCCTTCGGTTGGGGCGACC-ACGGGGGAA--AACAAAGCCCCCGAA-TGGAA
Comy	TGATTAATTGCCTTCGGTTGGGGCGACC-ACGGGGAAA--AATAAAACCCCCACA-TGGAA
Sasp	TTAGTGCATGTCTTCGGTTGGGGCGACC-ATGGCGGAT--AAAAAAGCCACCATA-AAGAT
Eupe	TTGACTAATGTCTTCGGTTGGGGCGACC-ATGGGGGAA--AAGAAAGCCCCCACA-CAGAC
Enja	GGTAATGTAGTCTTAGGTTGGGGCGACC-ACGGGAGAA--AGTAAAGCTCCCAAG-CAGAC
Same	GGCATAAGTGTCTTCGGTTGGGGCGACC-ACGGGAGATA-GCA-CAGCTCCCGAG-TGGA-
Chch	GGTCCGCCTGTCTTCGGTTGGGGCGACC-GCGGGGCAA--AGACAAACCCCCATG-TGGAA
Grgr	GGCCTGCCTGTTTTCGGTTGGGACGACC-GCGGGGGAG--AACCGAGCCCCCATG-TGGAG
Caau	TGAGATTTTACCTTCGGTTGGGGCGACC-ACGGAGGAA--AAAAAAGCCTCCAGG-TGGAC
Cyca	TGAGATTTTACCTTCGGTTGGGGCGACC-ACGGAGGAA--AGAAAAGCCTCCAGG-TGGAC
Dare	TAAACCTCTACCTTCGGTTGGGGCGACC-ACGGAGTAA--AAAACAACCTCCAAG-CGGAA
Cost	TGGAGCTTTACCTTCGGTTGGGGCGACC-ACGGAGAAA--AAATTATCCTCCGAG-TGGAT
Leec	TGAAGTTTTACCTTCGGTTGGGGCGACC-ATGGAGAAT--AAAAAATCCTCCAAG-TGGAA
Crla	GTGGAATTTTACTTCGGTTGGGGCGACC-ACGGAGAAA--AAAAGATCCTCCGAG-TGGAC
Clmc	CTGGCTGACGTCTTCGGTTGGGGCGACC-GCGGGGTAA--AACAAAACCCCCACG-TGGAA
Phin	CTGACCAAAGTCTTCGGTTGGGGCGACC-ATGGGGAAA--AACAAAACCCCCACG-TGGAA
Icpu	CTGATCCTAATCTTCAGTTGGGGCGACC-ACGGGAGAA--AATAAAGCTCCCACG-CGGAC
Psto	CTGATCCCTGTCTTCGGTTGGGGCGACC-GCGGGAGAA--AACAAAGCTCCCACG-CGGAC
Cora	CTGATCATAATCTTCGGTTGGGGCGACC-ACGGAATAA--AAACAAACTTCCACA-TGGAC
Eisp	TTGGTCCCTGTCTTCGGTTGGGGCGACC-GCGGAGGAA--AACAAAGCCTCCATG-TGGAA
Apal	CTGGCCTACGTCTTCGGTTGGGGCGACC-ACGGAGAAA--AACAAAGCCTCCGCG-CAGAA
Eslu	GGCCTACATGTCTTCGGTTGGGGCGACC-ACGGGGGAA--AACAAAGCCCCCACG-AGGAT
Dape	GGCCCACATGTCTTCGGTTGGGGCGACC-ATGGGGGAAA-ATA-AAGCCCCCACG-AGGAC
Glse	GACCTTAATGTCTTTGGTTGGGGCGACC-GCGGAGGAA--AACTAAGCCTCCATG-TGGAC
Naar	AGCCAACATGTCTTTGGTTGGGGCGACC-GCGGGGGAA--AATCAAGCCCCCATG-TGGAC
Baoc	CGCCAACATGTCTTCGGTTGGGGCGACC-GCGGGGGAA--AACCAAGCCCCCATG-TGGAA
Opso	AGCCTGAATGTCTTTGGTTGGGGCGACC-GCGGGGAAA--AATTAAACCCCCACG-TGGAT
Alte	GACCAACATGTCTTCGGTTGGGGCGACC-GCGGGGGAG--AAACAAGCCCCCACG-TGGAC
Plap	GACCAGCATGTCTTCGGTTGGGGCGACC-GCGGGGGAA--AAACAAGCCCCCACG-TGGAC

Plal	GTCTCTCCTGTCTTCGGTTGGGGCGACC-GCGGAGGA---CAAAAGCCTCCATG-TGGAC
Sami	GCCTCCCCTGTCTTCGGTTGGGGCGACC-GCGGAGGAT--AGCAAAGCCTCCTTG-TGGAC
Rere	GTCTCACCTGTCTTCGGTTGGGGCGACC-GCGGAGGAC--AAAA-AGCCTCCATG-TGGAC
Gama	ATCTTGACTGTCTTCGGTTGGGGCGACC-GCGGGGGAA--AAGACAGCCCCCATG-TGGAA
Onmy	AGCCCATATGTCTTTGGTTGGGGCGACC-GCGGGGGGAA-AATTAAGCCCCCACT-GTGGA
Sasa	AGCCCATATGTCTTTGGTTGGGGCGACC-GCGGGGGAA--AACAAAGCCCCCATG-TGGA-
Cola	AGCCCATATGTCTTTGGTTGGGGCGACC-GCGGGGGAA--AACAAAGCCCCCATG-TGGAC
Dita	CTCCCACTTGTCTTTGGTTGGGGCGACC-GAGGAGGAG--AAGA-AGCCTCCACA-CGGAC
Gogr	TTCCTAAGCGTCTTTGGTTGGGGCGACC-ACGGGGGAC----AAAAGCCCCCCCG-CGGAC
Chsl	AATCTACTTGTCTTTGGTTGGGGTGACC-ACGGAGGAA--AAATTAGCCTCCATG-AGGAC
Atja	GCTTTGAATATCTTTGGTTGGGGCGACC-ACGGGGGAA--AAGTAAGCCCCCATG-AGGAC
Iido	TACTTTAATATTTTTGGTTGGGGCGACC-ACGGGGGAC--AAATAAGCCCCCACG-AGGAC
Auja	TGTTGAAATGTCTTTGGTTGGGGCGACC-GTGGGAAAA--ACAA-AACACCCATG-TGGAC
Chag	CCGTTTAGTGTCTTTGGTTGGGGCGACC-GTGGGGAAAA-GAA-AAACCCCCATG-AGGAC
Hami	TGCTGAACTGTCTTCGGTTGGGGCGACC-GTGGGATAAA-ATT-AACCTCCCATG-AGGAC
Saun	TGCTGAACTGTCTTCGGTTGGGGCGACC-GTGGGATAT--AAAAAACCTCCCATG-AGGAC
Nema	CGCCGAAATGTCTTTGGTTGGGGCGACC-GCGGGGAAA--GAAAAAACCCCCACG-TGGAC
Disp	CGCTGAAATGTCTTCGGTTGGGGCGACC-GCGGGGAAA--AACAAAACCCCCACA-TGGAC
Myaf	TGCTGGGATGTTTTCGGTTGGGGCGACC-ACGGGGAAA--AACAAAACCCCCACA-TGGAC
Lagu	TTTCCCAATGCCTTTGGTTGGGGCGACC-ACGGAGATA--AAACAAACCCCCGCG-TGGAC
Trtr	TATCTAAATGTCTTTGGTTGGGGCGACC-GCGGAGACAA-ATC-TAACCCCCGCG-TGGAT
Zucr	CCCCTAAATGTCTTTGGTTGGGGCGACC-GCGGAGATA--AACTAAACCACCGCG-TGGAT
Pxja	TATCAAAGTGTTTTTGGTTGGGGCGACC-GCGGGGAAA--ACA-AAACCCCCATG-TGGAC
Pxlo	TATCAAAGTGTTTTTGGTTGGGGCGACC-GCGGGGAAA--A-CAAAACCCCCATG-TGGAC
Pctr	CGGGAAACTGTCTTTGGTTGGGGCGACC-GCGGGGGAT--GAAA-AGCCCCCATG-TGGAA
Apsa	TTAGTAAGTGTCTTTGGTTGGGGCGACC-ACGGGGGAC--AATAAAGCCCCCATG-CGGAA
Cabe	TCCTCATATGTTTTCGGTTGGGGCGACC-AAGGGATACA-ACG-GAACTCCCACG-TGGAT
Bzze	TGCCCTACTGTCTTTGGTTGGGGCGACC-GCGGGGAAAA-ACA-AAACCCCCACG-TGGAA
Siim	TTCCATAATGTCTTGGGTTGGGGCGACC-ACGGGGTAAA-AAA-CAACCCCCACG-TGGGA
Ctru	CGTCCTAATATCTTTGGTTGGGGCGACC-GCGGGGTAA--CATAAAACCCCCATG-TGGAG
Dpbr	TATCCTAATATCTTCGGTTGGGGCGACC-GCGGGGCAA--CACAAAACCCCCATG-TGGAA
Caki	TATTTAAATGTCTTTGGTTGGGGTGACC-ACGGGGCAA--AACATAACCCCCACG-AAGAT
Phja	TATTGAAGTGTCTTTGGTTGGGGCGACC-ATGGGGTAA--AACATAACCCCCATA-CGGAC
Brsp	TATTCAAGGGTCTTAGGTTGGGGCGACC-ACGGAGTAA--AATAAACCCTCCACG-AAGAA
Gamo	TACTGAAGTGTCTTTGGTTGGGGCGACC-GCGGGGTAA--AACACAACCCCCATG-TGGAC
Lolo	TATCGAAGTGTCTTTGGTTGGGGCGACC-GCGGGGTAA--AACACAACCCCCATG-TGGAC
Batr	TGAGCTTCTTCCTTTGGTTGGGGCGACC-ATGAGAAAT--AAAACAACTCTCATA-AGGCA
Prmy	CTTACGCTTACTTTCGGTTGGGGCGACC-GTGAAGAAC--AAAATAACCTTCATA-AGGAA
Loli	TGACCTGACGTCTTCGGTTGGGGCGACC-GCGGGGGAA--AGAAAAACCCCCACG-TGGAC
Loam	CTACCCTATGTCTTTGGTTGGGGCGACC-GCGGGGCAA--AAAGTACCCCCCATG-TGGAA
Chab	TGTCCCAATGTCTTCGGTTGGGGCGACC-ATGGGGAAA--TAAGAAACCCCCAAAGTGGAA
Chto	TGTCCCAATGTCTTCGGTTGGGGCGACC-ATGGGGAAA--TAAAAAACCCCCAAAGTGGAA
Majo	TCACCTAATGTTTTTGGTTGGGGCAACC-GCGGGGTAA--TAGAAAACCCCCGCG-TGGAA
Hlst	CCTCTTACAGTTTTTGGTTGGGGCGACC-GCGGGGTAA--CAGAAAACCCCCACG-TGGAA
Clpe	TGTCCTGGTGTCTTCGGTTGGGGCGACC-ACGGCGTAA--TAGATACCCCCCGCG-CGGAA
Mlmr	TGTTCCAATGTCTTCGGTTGGGGCGACC-ACGGGGCAA--TACAAAACCCCCGCG-CGGAA
Crcr	CTGTTCCACGTCTTAGGTTGGGGCGACC-ACGGTGAAC--AGAAAAACCCCCGCG-TGGAC
Muce	CTGTTCCACGTCTTAGGTTGGGGCGACC-ACGGTGAAC--AGAAAAACCCCCGCG-TGGAC
Bege	TGCCCCCATGTCTTTGGTTGGGGCGACC-GCGGGGGAA--CAAAAGCCCCCATG-TGGAG
Mela	TGCCCTAATGTCTTTGGTTGGGGCGACC-GCGGGGCAC--CAAAAACCCCCCACG-TGGAA
Hats	TGTTCTAATGTCTTTGGTTGGGGCGACC-ACGGGGAAAC-GAA-AATCCCCCATG-TGGAA
Orla	TGTTTTTATGTCTTTGGTTGGGGCGACC-GCGGGAAAA--TAAGAATCTCCCATG-AGGAT

```
Cosa    TGCCTTAATGTCTTTGGTTGGGGCGACC-GCGGGGAAA--TAAAAAACCCCCACG-TGGAA
Exsp    TGCCCTAATGTCTTCGGTTGGGGCGACC-ACGGGGAAA--CAAAGAACCCCCATG-AGGAG
Depa    CACCTTAATATCTTTGGTTGGGGCGACC-GCGGGGCAA--TAAAAAACCCCCATG-TGGAA
Rima    TGCTCATTTATCTTCGGTTGGGGCGACC-ACGGAGA-A--AACTTATCCTCCACG-CTGCA
Fuol    TGCCGCAATGTCTTTGGTTGGGGCGACC-GCGGGAAAA--AACCAAACCCCCGCG-CGGAA
Gmaf    TGCTTCAATGTCTTTGGTTGGGGCGACC-CCGGAGTAA--TAAAAAACCCCCGAG-CGGAC
Xeei    TGTCATGATGTCTTCGGTTGGGGCGACC-ACGGGGAAA--CAACCAACCCCCACG-TGGAG
Pros    TGCTCAAATGTCTTTGGTTGGGGCGACC-CTGGGGAAA--AACACAACCCCCACA-TGGAA
Scmi    CCGCTCAATGTCTTTGGTTGGGGCGACC-CTGGGGAAA--AACGAAACCCCCGCG-CGGAA
Rolo    TGCTCAAATGTCTTTGGTTGGGGCGACC-GCGGGGAAA--AATAAAACCCCCACG-TGGAA
Cere    TGCTTGAATGTCTTTGGTTGGGGCGACC-GCGGGGAAAT-AAA-TAACCCCCATG-TGGAT
Daga    CTTTTAAGTGTCTTTGGTTGGGGCGACC-GCGGGGAAA---GCAAAACCCCCATG-TGGAA
Anco    TGCCAGAATGTCTTTGGTTGGGGCGACC-GCGGGGAAA--AACCAAACCCCCATG-TGGAA
Dmve    TGCCCCAGTGTCTTTGGTTGGGGCGACC-GCGGGGAAA--AACTAAACCCCCATG-TAGAT
Dmar    TACCCCAATGTCTTTGGTTGGGGCGACC-ACGGGGAAA--AACCAAGCCCCCACG-CGGAC
Anka    TGCCAGAATGTCTTTGGTTGGGGCGACC-GCGGGGAAA--AGCAAAACCCCCATG-TGGAA
Moja    TGCCAGAATGTCTTTGGTTGGGGCGACC-GCGGGGAAA--GACTAAACCCCCATG-TGGAA
Hoja    TGCCAGAATGTCTTTGGTTGGGGCGACC-GCGGGGAAA--AACACAACCCCCATG-TGGAA
Bede    TGCTCAAATGTCTTTGGTTGGGGCGACC-ATGGGGAAA--AACAAAACCCCCACG-TGGAA
Besp    TGCTCAAATGTCTTTGGTTGGGGCGACC-ATGGGGAAA--AACGAAACCCCCACG-TGGAA
Mysp    CGCCCAAATGTCTTTGGTTGGGGCGACC-GCGGGGAAA--AATTAAACCCCCACG-TGGAA
Osja    CGCCCAAATGTCTTTGGTTGGGGCGACC-GCGGGGAAA--AACAAAACCCCCACG-TGGAA
Sgro    CCTGATTATGTCTTTGGTTGGGGCGACC-GCGGGGAAA--AACAAAACCCCCACG-TGGAA
Pzpa    TGCACAACTGTCTTTGGTTGGGGCGACC-GCGGGGAAA--AACAAAACCCCCACG-AGGAC
Zeja    CGCACAACTGTCTTTGGTTGGGGCGACC-GCGGGAAAA--AACAAAACCCCCACG-AGGAG
Znne    TCCACAACTGTCTTTGGTTGGGGCGACC-GCGGGGAAA--AACAAAACCCCCACG-AGGAT
Zefa    TGCACACCTGTCTTTGGTTGGGGCGACC-GCGGGGAAA--AATAAAACCCCCACG-AGGAT
Acni    TGCACAACTGTCTTTGGTTGGGGCGACC-GCGGGGAAA--AACAAAACCCCCACG-AGGAC
Ncrh    TGCACAACTGTCTTTGGTTGGGGCGACC-GCGGGGAAA--AACAAAACCCCCACG-AGGAC
Agca    TACCTCTATGTCTTTGGTTGGGGCGACC-GCGGGGAAA--TAAAAAACCCCCACG-TGGAA
Hydy    TCCCCTAATGTCTTTGGTTGGGGCGACC-GCGGGGTAC--TAAACAACCCCCACG-TGGAG
Gsac    CACCCTGATGTCTTTGGTTGGGGCGACC-GCGGGGAAG--TAAATAACCCCCATG-TGGAG
Pevo    TGCTTCCCTGTCTTTGGTTGGGGCGACC-ACGGGAAAA--AGAATAACTCCCGCG-TGGAA
Hiku    TCATCCTAAGTCTTTAGTTGGGGCGACC-GCGGAGCAA--AACAAAACCTCCGTG-AGGAA
Inpa    TGCCCAACAGTCTTCGGTTGGGGCGACC-GAGGGGTATG-ACA-CAACCCCCACG-CGGAA
Auch    TGCCTGGATGTCTTTGGTTGGGGCGACC-ACGGGGAAA--AATAAACCCCCCACG-TAGAA
Fico    TGCCTGAATGTCTTTGGTTGGGGCGACC-GCGGGGACA--TAAAGAACCCCCACG-TGGAA
Macs    TGTTTGAATATCTTTGGTTGGGGCGACC-GCGGGGAAAT-AAA-TAACCCCCACG-TGGAC
Moal    TGCTTCCCTGTCTTCGGTTGGGGCGACC-AGGAGAAAC--TAAAAAACTCTCCCG-AGGAG
Syma    TATTTCAATGCCTTCGGTTGGGGCGACC-AAGGAGAAT--TAACCAACCCCCACG-TGGAA
Mafr    TGCTCTAATGTCTTCGGTTGGGGCGACC-ACGGGGAAA--AAAACAACCCCCACG-CAGAC
Dcpe    TGCCTAAATGTCTTTGGTTGGGGCGACC-GCGGGGCAA--TAAACAACCCCCAAG-AGGAC
Dcti    TGCCTGAATGTCTTTGGTTGGGGCGACC-GCGGGGCAA--CAAACAACCCCCAAG-AGGAC
Hehi    TTCCCTAATGTCTTTGGTTGGGGCGACC-GCGGGGAAA--CAAAAAACCCCCACG-TGGAA
Stam    TCCCCTAATGTCTTTGGTTGGGGCGACC-GCGGGGAAA--GAAAAAACCCCCATG-TGGAA
Hogi    CAACTTAATGTCTTTGGTTGGGGCGACC-GCGGGGAAA--CAAAAAACCCCCATG-TGGAA
Erzo    TGCCCGAATGTCTTTGGTTGGGGCGACC-GCGGGGCAT--TAAAAAACCCCCACG-TGGAA
Hxot    CGCCCTAATGTCTTTGGTTGGGGCGACC-GCGGGGAAT--TACAAAACCCCCACG-TGGAA
Core    TGCCCTAATGTCTTTGGTTGGGGCGACC-GCGGGGAAT--TAAAGAACCCCCACG-TGGAA
Apve    TGCCCTAATGTCTTTGGTTGGGGCGACC-GCGGGGAAC--TAAATAACCCCCACG-TGGAA
Latj    TACTCCAATGTCTTCGGTTGGGGCAACT-ATGAGG-AAC-AAA-AACCCCTCACG-TGGAA
Laja    TGTCCCAATGTCTTTGGTTGGGGCGACC-GCGGGGAAC--TACAAAACCCCCACG-TGGAA
```

Syja	TGCCCGAATGTCTTTGGTTGGGGCGACC-ACGGGGAA---CACAAAACCCCGGCG-TGGAT
Epme	TGCCCTAATGTCTTAGGTTGGGGCGACC-GCGGAGCAAC-AAA-AAACCCCGGCA-AGGAC
Grse	TGCCCTAGTGTCTTCGGTTGGGGCGACC-GCGGAGAAA--AAAGAAACCCCGGCA-CGGAA
Clja	TGCCCCTGTGTTTTAGGTTGGGGCGACC-ACGGGGTAT--TAGATAACCCCGATG-TAGAA
Ogcy	CCCTTCTGTGCCTTTGGTTGGGGCGACC-GCGGGGACA--AAAATAACCCCGATG-TGGAA
Plna	TTTTCTAATGTCTTTGGTTGGGGCGACC-ATGGAGAAT--CATAAAGCCTCCACG-CGGAA
Lema	TTATTCCATGTCTTTGGTTGGGGCGACC-GCGGGGAAA--TAAAAAACCCCGACG-TGGAC
Etzo	TGTCCTAATGTCTTTGGTTGGGGCGACC-GCGGGGAAA--CAAAAAACCCCGACG-TGGAA
Apse	TGCCCGACTGTCTTTGGTTGGGGCGACC-ACGGAGAAA--AAGTAAACCCCGGTG-TGGAA
Epde	TGTCCGCATGTCTTTGGTTGGGGCGACC-GCGGGGAAA--CATAGAACCCCGACG-TGGAA
Slja	TGCCCTAATGTCTTCGGTTGGGGTGACC-GTGGGGGAA--AACAGAGCCCCGATG-CGGAA
Bsja	CGCCCTAATGTCTTTGGTTGGGGCGACC-GCGGGGTAG--TACAAAACTCCGGCG-TGGAA
Ecna	TATTTTTATATCTTCGGTTGGGGCGACC-ATGGAGAAAT-ATA-AAACCCCGACG-TGGAA
Cohi	TACCCCTATATCTTCGGTTGGGGCGACA-ATGGGGAAA--TAAAAATCCCCGATG-CGGAA
Caar	TGTCCTAATGTCTTCGGTTGGGGCGACC-ATGGGGAAG--CACAAAACCCCGATG-CGGAA
Came	TGTCCTAATGTCTTCGGTTGGGGCGACC-ATGGGGCAG--CACAAAACCCCGATG-TGGAT
Mema	CTGCCCTCTGTCTTCGGTTGGGGCGACC-ATGGGGAAAC-AAA-AAACCCCGACG-TGGAC
Lenu	TGTTCAAATGTCTTTGGTTGGGGCGACC-ACGGAGCAT--CAAAAAACCCCGGCG-TGGAA
Brja	TACCCCCCTGTCTTTGGTTGGGGCGACC-ACGGGGAAA--CAAAGAACCCCGATG-TGGAA
Plma	TGCCCTTATGTCTTTGGTTGGGGCGACC-GCGGGGAAA--TAAAAAACCCCGACG-TGGAA
Emst	TGCCCTAATGTCTTTGGTTGGGGCGACC-GCGGGGAAA--CACAAAACCCCGACG-TGGAA
Ptti	TGCCCTAATGTCTTTGGTTGGGGCGACC-GCGGGGAAA--CACAAAACCCCGACG-TGGAA
Losu	TGTCTTAATACCTTTGGTTGGGGCGACC-ATGGGGAA---TAAGAACCCCCGACG-TGGAC
Geoy	TGTCCCAATGTCTTTGGTTGGGGCGACC-CTGGGGAAC--AA-AAATCCCCGATG--AAGA
Dipi	TGTCCTAATGTCTTCGGTTGGGGCGACC-GCGGGGAAA--CACAAAACCCCGACG-TGGAA
Pama	TGCCCTAATGTCTTTGGTTGGGGCGACC-GCGGGGAAAT-ACA-AAACCCCGATG-TGGAG
Leob	TACCCTAATGTCTTTGGTTGGGGCGACC-GTGGGG-CA--CACAAAACCCCGACG-TGGAG
Neba	AATCCGACTATCTTTGGTTGGGGCGACC-GCGGGGACT--AAAAAAACCCCGATG-TGGAC
Pdpl	TGTTCCTCCGTCTTCGGTTGGGGCGACC-ATGGGGAAC--TAAATACCCCCGACG-CGGAC
Nimi	TGTCCTAGTGTCTTTGGTTGGGGCGACC-ACGGGGAAC--TACACAACCCCGGCG-TGGAA
Uptr	TGTTCTCATGTCTTTGGTTGGGGCGACC-GTGGGGTAA--CAAACAACCCCGATG-TGGAC
Pesc	TATCCTACTGTCTTCGGTTGGGGCGACC-GCGGAGAAA--CAAACATCCTCCACG-CGGAA
Baar	TGCTCTGATGCCTTAGGTTGGGGCGACC-CCGGGGAAT--AA-AAACCCCCGGCG-TGGAA
Moar	TGCCCTAATGTCTTTGGTTGGGGCGACC-GCGGGGAAA--TACAAAACCCCGATG-TGGAC
Toja	TGTTTTTATGTCTTCGGTTGGGGCGACC-ATGGGGAAG--TAAAAAACCTCCACG-TGGAA
Chau	TGCCCTAATGTCTTTGGTTGGGGTGACC-GCGGCGAAAT-AAA-TAACCCCGGTG-TAGAA
Chse	TGCCCTAATGTCTTAGGTTGGGGCGACC-GCGGGGAAA--TGAAAAACCCCGATG-TGGAA
Enar	TGCCCTAATGTCTTTGGTTGGGGCGACC-GCGGGGAAA--CAAAAAACCCCGATG-TGGAA
Hpty	TGCCCAAATGTCTTTGGTTGGGGCGACC-GCGGGGAAA--CATAAAACCCCGACG-TGGAA
Nana	TGTCCTAATGTCTTTGGTTGGGGCGACC-GCGGGGAAA--CAAAAAACCCCGACG-TGGAA
Mcst	TGCCCTAATGTCTTCGGTTGGGGCGACC-GCGGGGAAA--CAAATAACCCCGATG-TGGAA
Rhox	TGCCCTAATGTCTTTGGTTGGGGCGACC-ACGAGGAAA--A--AGAACCCCGGTG-CGGAC
Opfa	TGCCCTAATGTCTTTGGTTGGGGCGACC-GTGGGGAAAC-AAA-AAACCCCGATG-TGGAA
Paar	TGCCCTAATGTCTTTGGTTGGGGCGACC-GCGGGGAAA--TAGAGAACCCCGACG-TGGAA
Gozo	TATTCCAATGTCTTTGGTTGGGGCGACC-GCGGGGAAAC-AAA-AAACCCCGACG-TGGAA
Ackr	TGCCCTAATGTCTTTGGTTGGGGCGACC-CCGGAGCA---CAAAAACCCTCCGCG-TGGAA
Elev	TGTCTAAATGTCTTTGGTTGGGGCGACC-GCGGGGAAA--TAAAAAACCCCGATG-AGGAC
Trdu	TGTTCTAATGTCTTTGGTTGGGGCGACC-GCGGGGAAA--CAAAAAACCCCGATG-TGGAC
Amoc	TGATATAATGTCTTCGGTTGGGGCGACC-TCGGGGAAAG-AAA-AAACCCCGACG-CGGAG
Hame	TGCCCTAATGTCTTCGGTTGGGGCGACC-CCGGGGAAA--TAAGTAACCCCGATG-AGGAC
Chso	TGTCCTATTGTCTTTGGTTGGGGCGACC-ACGGGGCAA--CAAAAAACCCCGGCG-CGGAA
Lyto	TGCCCGAATGTCTTTGGTTGGGGCGACC-GCGGGGCAT--TGAAAAACCCCGACG-TGGAA

```
Encr    TGTCCTAATGTCTTTGGTTGGGCGACC-GCGGGGAAT--TAGAAAACCCCCACG-TGGAA
Bvar    TGCCCTTATGTCTTCGGTTGGGCGACC-GCGGGGTAA--AAATTAACCCCCACG-TGGAA
Noco    TACCCCGATGTCTTTGGTTGGGCGACC-GCGGGGAAA--GAAAAAACCCCCACG-TAGAA
Chsp    TTTCCTCATATCTTTGGTTGGGCGACC-ATGGGGAAA--CAAACAACCCCCGTA-TGGAT
Arja    TGTCCTAATGTCTTTGGTTGGGCGACC-GCGGGGAAT--TAAATAACCCCCACG-TGGAA
Pase    TATCCTAATGTCTTCGGTTGGGCGACC-GAGGGGGAAC-ACT-TAACCCCCATG-CAGAA
Trel    TGCTCGAATGTCTTCGGTTGGGCGACC-ACGGAGAAA--CAAGAAACCCCCGTA-TGGA-
Lifa    CTATTTAACGTCTTGGGTTGGGCGACC-AAGGGGAAA--AGAAAAACCCCCTTG-CGGAA
Acur    GTTTTACTTGCCTTTGGTTGGGCGACC-GCGGGGAAAT-AAA-AAACCCCCATG-AGGAT
Ampe    TGCCCTAGTGTCTTTGGTTGGGCGACC-GCGGGGAAA--CATAAAACCCCCATG-TGGAA
Urja    TGCCTTAGTATCTTCGGTTGGGCGACC-CCGAGGAAC--AAAAAAACCCCCGGG-TGGAC
Enet    TGCCTTATTGTCTTTGGTTGGGCGACC-GCGGAGTAA--CATTAAACCTCCACG-AGGAC
Ptbr    TTTTGAACTGTCTTCGGTTGGGCGACC-GCGGGGAAA--AATTTAACCCCCATG-AGGAA
Safa    CCGTTTCATGTCTTCGGTTGGGCGACC-GCGGGGAAA--TAAAAAACCCCCATG-TGGAA
Icae    TGCCCGTATGTCTTTGGTTGGGCGACC-GCGGGGAAA--TAAAAAACCCCCACG-TGGAC
Asmi    CTCTCCACTGTTTTCGGTTGGGCGACC-GCGGTGTAC--TAAATAACCCCCGCG-AGGAC
Foal    CCTTAAAATGTTTTTGGTTGGGCGACC-TTGGGAAAA--CAAGCAACTCCCATG-TCGAG
Drze    TACTTAGATATCTTTGGTTGGGCGACC-CTGGGGAAA--AAAAGAACCCCCATG-TGGAC
Rhas    TGTCCCCCTGTCTTTGGTTGGGCGACC-GCGGGGAAA--CAAAAAACCCCCACG-TGGAA
Elac    TGTTCTCCTGTCTTCGGTTGGGCGACC-GCGGGG-AA--CAAAAATCCCCCACG-TGGAA
Kugu    CTGTCCAATGTCTTTGGTTGGGCGACC-GCGGGGAAA--CAAAAAACCCCCACG-TGGAA
Plor    CTGCCAGATGTCTTTGGTTGGGCGACC-ACGGGGAA---CACGAAACCCCCGCG-CGGAA
Sgun    TGCCCTAATGTCTTTGGTTGGGCGACC-GCGGGGAAA--CACAAAACCCCCACG-TGGAA
Zaco    TGTCCTAATATCTTTGGTTGGGCGACC-GCGGGGAAA--TACAAAACCCCCATG-TGGAA
Zbfl    GCCCTATATGTCTTTGGTTGGGCGACC-GCGGGGAAA--TACAAAACCCCCACG-TGGAA
Spba    TGTTTTTATGCCTTCGGTTGGGCGACC-ATGGAGTAA--TAAAAATCCTCCAAG-TGAGA
Game    TGCCCTTATGTCTTTGGTTGGGCGACC-GCGGGGAAAT-AAA-AAACCCCCACG-TGGAA
Thth    TGCCCCCATGTCTTTGGTTGGGCGACC-GCGGGGAAA--TAAAAAACCCCCACG-TGGAA
Xigl    TGCTTTAATGTCTTCGGTTGGGCGACC-ATGGGGAAA--TAAAAAACCCCCACG-CGGAC
Hyja    TGCCCCTATGTCTTCGGTTGGGCGACC-ACGGGGAAA--TAAAGAACCCCCGCG-TGGAA
Psan    TGCCCCTATGTCTTCGGTTGGGCGACC-ACGGGGAAA--TAAAAAACCCCCGCG-TGGAG
Cupa    TACTCCTATGTCTTTGGTTGGGCGACC-GCGGGGAAA--TAAAAAACCCCCATG-TGGAA
Mpch    TGTCCTAATGTCTTTGGTTGGGCGACC-GCGGGGAAA--CAAACAACCCCCACG-TGGAC
Char    TACTCCCCTGTCTTTGGTTGGGCGACC-GCGGGGAA---TACAAAACCCCCATG-TGGAA
Pser    TGCTCCCATGTCTTCGGTTGGGCGACC-GTGAGGAAATTAAAAAACCCTCCACG-TGGAA
Prol    AGCCCTGATGTCTTCGGTTGGGCGACC-ATGGGG-AA--CACAAAACCCCCACG-TGGAA
Plbi    TGCCTTGATGTCTTCGGTTGGGCGACC-ATGGGG-AA--TACAAAACCCCCACG-TGGAA
Calu    TGCCCTAGTGTCTTAGGTTGGGCGACC-ATGGGGAA---CACCCAACCCCCATG-AGGAC
Papa    CGTCCCACTGTCTTCGGTTGGGCGACC-TTGGGGTAA--TATATAACCCCCACA-TGGAA
Sufr    TGTCCTAATGCCTTTGGTTGGGCGACC-GCGGGGAAA--CAAAAAACCCCCATG-TGGAA
Stci    AGTTTGGATGCCTTTGGTTGGGCGACC-GCGGGGAAA--CAAAAAACCCCCACG-TGGAC
Taru    TGTTTTAATGTCTTTGGTTGGGCGACC-GCGGGGTAA--CAAAAAACCCCCATG-TGGAA
Rala    TACCCTAATGTCTTTGGTTGGGCGACC-GCGGGGAAA--TAAAAAACCCCCATG-TGGAC
                    **  ******   **                  * *
```

```
                  ¦                HVR                                        ¦
Scca    --CGAGTATTCAAA------------------------------------TAC--------
Muma    --TGAGTA-CTCAGTAC----------------------------------TTG-------
Erca    --TGAAGCACAG--------------------------------------CTTT-------
Pose    --TGAAGCACAGC-------------------------------------TTT--------
Actr    --CGGGGGCACTGC-------------------------------------CCC-------
Scal    --CGGGGGCACTG-------------------------------------CCCC-------
Posp    --CGGGGGCACTG-------------------------------------CCCC-------
Atsp    --CTGGAGA--------------------------------------------TAGCCTCT
Leoc    --TGGAGATA--------------------------------------ACCTCTA-A----
Amca    --TAGGAA-TACTTTAA----------------------------TATAATTCCCAA----
Osbi    --ACTCGGGGAATAG--------------------------------------TCAAACAA
Pabu    --TTAAGATTAATTATCTTAA----------------------------------AGA---
Hial    --TGAGGGCCAAGCC--CTTAAA--------------------------------------
Elha    --CGGGGGGACTATTACTACC-------------------------------CCTAAA---
Mlcy    --CAAGGACA--CAGTCCT-----------------------------------CAAA---
Algl    --TGGGGTAATATTCCCTAA-------------------------------------A---
Ptgi    --TGGAACAA-----------------------------------CCCC------------
Alaf    --TGGGGGCA----CACCCCT----------------------------------AAA---
Nock    --TGGGGTTTATAA---------------------------------TTTACCCTAAA---
Anja    --CAGGGAC--------------------------------------TAA-TCCCTAA---
Gyki    --CGGGAAAACCC------TTAA--------------------------------------
Syka    --TGGGGA----------TATC------------------------------CCCTAA---
Opma    --CGAAAA--CATC------------------------------------TTCCTA-A---
Comy    --TGGGTA-AAATTA-----------------------------------TCCTAA-----
Sasp    --AAAGAATATAAGACTAA---------------------------------TCTTA----
Eupe    --AAGGA----TAAACTCCT------------------------------------A----
Enja    --CGGGAA-------------------------------AAC----------CCT------
Same    --TGGGGGAC------------------------------------ACCCT----------
Chch    --TGAA-C----------AAACCATT----------------------------TA-----
Grgr    --TGGGG---CAACCCTAG------------------------------------------
Caau    --CGGGAG-AAT--------------------------------------TTCCT------
Cyca    --TGGGAAAACC------------------------------------T---CCT------
Dare    --CGGG--CACAGC---CCTAAA--------------------------------------
Cost    --TGGGT-TAACAA------------------------------------A-CCT------
Leec    --TGGGATAA-----------------------------------A---TCC--T------
Crla    --TGAGCCAACA-----------------------------------AG---CTT------
Clmc    --TGGGAAA----------------------------------------ACTCCT------
Phin    --TAG----ACC------------------------------------ATTCTCT------
Icpu    --TGGGGC---AA--TCCCCCTAA-------------------------------------
Psto    --CGGGATAACA---------------------------------------TCCTA-----
Cora    --TGAGG--ACTA----ACCT---------------------------------TA-----
Eisp    --TGGGGCTATCGC------------------------------------CCC--------
Apal    --TAGGAA-AAATA-----------------------------------TTCC-T------
Eslu    --TAAGGAAAAC-----------------------------------CT--CCTT------
Dape    --TGAGGGTAATA--CCCTT-----------------------------------------
Glse    --CGGGAGAAC-----------------------------------------CCACTCCT-
Naar    --TGGGAAAACCTT--TCCTACA--------------------------------------
Baoc    --TGGAAAAACCTTTTCCAC-----------------------------------------
Opso    --TAGGAAAACCC------------------------------------TTCCC-------
Alte    --CGGGATTATTCCT----------------------------------------------
Plap    --CGGGATATC-----------------------------------------CC-------
```

```
Plal    --CAGGGTCACAGACC------------------------------------CC------
Sami    --CAAGGTTACGAA-----------------------------------C-CTCA-----
Rere    --TGAAGTAACTACCCTT-----------------------------------CA-----
Gama    --CGGGAGGAAATA-----------------------------------CTCCTA-----
Onmy    -CTGGGGGGCACTGCCCCA-----------------------------------C-----
Sasa    -CTGGGGGCACTG---------------------------------------CCA-----
Cola    --TGGGGGCACTG----CCCC------------------------------C--AC----
Dita    --TGGGAG-CACA-----------------------------------CATCCTAG----
Gogr    --TGAGGG---CACTGCCCCCCA-------------------------------------
Chsl    --TAGG-GCTTCTA-----------------------------------CCC--------
Atja    --TAGGAA-AACC------------------------------------TTCCTAA----
Iido    --CAGGAAA--ATCTTCCTA-----------------------------------A----
Auja    --CGAGGACAC---CTC-CTT---------------------------------AA----
Chag    --CTGGGGCGACCCCCCC------------------------------------AT----
Hami    --CGGAATAAACATTCC-A------------------------------------A----
Saun    --CGGGAAAAC-------------------------------------CTTCCCAA----
Nema    --CGGGGCTACACACC------------------------------------CTAA----
Disp    --CAGGGCTACCATA----------------------------------GCCTTA-----
Myaf    --CGGGGCCACTCAGCCCTA----------------------------------------
Lagu    --CGAGAG-------------------------------------ACCTCTCTTA-----
Trtr    --TGGGTA---------------------------------------CCCCCTA------
Zucr    --AGGGGGTAACC----CC----------------------------------TA-----
Pxja    --CGGGAATACT----------------------------------AATTCCTAC-----
Pxlo    --CGGGA--------------------------------ATACTAA--TTCCTAC-----
Pctr    --TAGAGA-AACAT----------------------------------CTC--TA-----
Apsa    --TAGGA--CAT-------------------------------------CTCCTT-----
Cabe    --TGGAGTACA-----------------------------------CTC---CA------
Bzze    --TGGGAGTAC-----------------------------------CCCTCCTA------
Siim    --TGGAATTAAA------------------------------GCACTTATTCCT------
Ctru    --CGGGGACAC--TATTCCCC---------------------------------A-----
Dpbr    --TGGAAG----------TACCACC-------------------------TCCCA-----
Caki    --CAGGGACATAAC-------------------------------------CCTA-----
Phja    --TGGGGAT--ACT--ATCCCTAA------------------------------------
Brsp    --GGGGCCTCTA----------------------------------TAAG----------
Gamo    --CGGGGATATTA---TCCCTAATA-----------------------------------
Lolo    --CGGGGATATTAT-----------------------------------CCCTA------
Batr    --TGGTA---------------------------------------CACACACC------
Prmy    --TAGAATAACCTT----------------------------------------------
Loli    --TGGGAGT--A----------------------------------CCTCTC--------
Loam    --AGGGAACACCCT---------------------------------T--CCT-------
Chab    --TGGAAACAGACCCTTTTC---------------------------------TAC----
Chto    --TGGAAACAGACCCTTTTTCTAC------------------------------------
Majo    --CGGGAGTACCC------------------------------------CTCCTAC----
Hlst    --TGGGGACACCTCC-CCTA-----------------------------------C----
Clpe    --TGAAAG-CACAC-----------------------------------CTTATTC----
Mlmr    --CAAGGACAAACC-------------------------------------TCCAC----
Crcr    --TGAGAGCACATATTCACAC--------------------------TCATTAATACTG-
Muce    --TGAGAGCACATACT-CACACTTATCAATACCGC-------------TTCT--------
Bege    --TGAGAGTACTTCTC------------------------------------CC------
Mela    --TAGGAGAACC-----------------------------------------CTCCCA-
Hats    --TGGAACCACAC------------------------------ACACACCT---------
Orla    --AGAGAAAACCCTATT----------------------------------CTT------
```

Cosa	--AAAGAG-CACTTT----------------------------------CTCTT------
Exsp	--CAGGAG-AACTTT---------------------------------TCTCCC------
Depa	--TAAGAGAATC-TTCT---------------------------------CTT-------
Rima	--CAAAACACCATGTTTTTAT---------------------------------------
Fuol	--TAAAAGCACAAT-----------------------------------CTTTTA---A-
Gmaf	--TGAAGACACT----------------------------------CTAGCTTCA---T-
Xeei	--AAGAAA-CACCTT-------------------------------ATGTTTCCA---A-
Pros	--CAGAAGAA----CTTCTTCCAC------------------------------------
Scmi	--CAGAAAACAC-----------------------------------TCCTTCCA---C-
Rolo	--TGAG-AGAACTT-----------------------------------CTCCCA---C-
Cere	--TGAGAGAAC-----------------------------------CTCTCCCA----A-
Daga	--CGAGAGAACATCTC-TT----------------------------------TA---A-
Anco	--AAGGAGGA--CTCCTCCT
Dmve	--AAGGAAT--A----------------------------------GCCCTCCTA-----
Dmar	--AAAGGAGTAACCCTCCTA--
Anka	--AAGGAGG-AC-----------------------------------TCCTCCTA-----
Moja	--AGGGAA----------GACTACCC------------------------TCCTA-----
Hoja	--AAGGAG----------GACACC--------------------------TCCTA-----
Bede	--TGGAAG-AACTT----------------------------------CTTCT-------
Besp	--CGGAAG-AACTTC-----------------------------------TTC-------
Mysp	--TGGGAG-AACA-----------------------------------TCCCCC------
Osja	--CAGGGAG--------------------------ACCCTC----------CCC------
Sgro	--TGAGAGCA---CCCCTCT---------------------------------TAC----
Pzpa	--TAGAAGTTT-----------------------------------ATACTCCTAT----
Zeja	--TGGAAGCAC--AAGCCACC---------------------------------CA----
Znne	--TGAGAGTTCAACTCCTAA--
Zefa	--TGAGAG-TTCTAC-----------------------------------TCCTAA----
Acni	--TGAGAGTTT------------------------------------TACTCCTAA----
Ncrh	--TGAGAGTTTTA------------------------------------CTCCTAA----
Agca	--AGGGAACAC---CCTTCCT---------------------------------AC----
Hydy	--TGGGAGTA----------------------------------C-CCCCTCCTGC----
Gsac	--TGGGAGCAC---CCCTCCT---------------------------------AC----
Pevo	--CGGAAACAC-----------------------------------CT--TTCTAC----
Hiku	--TGAGGT----------AAAAACCT---------------------------TAC----
Inpa	--TAAAACACCAGTTTTAC---
Auch	--TGGGAGCAACTTTCTCATACGTAC---------ATATTCTCTCATCCTCC--------
Fico	--TGGGAG-TAC---TT--------------------------------CTCCCA-----
Macs	--TGGGGGCACTTG-CCCCT----------------------------------A-----
Moal	--TAGGAAGAC---TCTTCCC---------------------------------A-----
Syma	--TAGAAGTACTC---------------------------------------CTTCTCA-
Mafr	--TAAGAG-GACAT----------------------------------CCTCT-------
Dcpe	--AGGGAG--TAA-----------------------------------ACTCC-------
Dcti	--AGGGAGTAACA------------------------------------CTCC-------
Hehi	--AGGGAGCACCC------------------------------------CTCC-------
Stam	--TGGGAGAAC------------------------------------CCCTCCTAA----
Hogi	--TGAAAGTAAGA----------------------------------TACTTTTAC----
Erzo	--TGGGAGCACCA----TCCT------------------------------CCTAC----
Hxot	--TGGGA--ACAC----CCCT------------------------------CCTAC----
Core	--CGGGA----ACACTTTTCCTAC------------------------------------
Apve	--CGGGAGAACC-----------------------------------CCCTCCTAC----
Latj	--TGGGAGCACTACAACCCACTATTATTT-------------TCTTCCCCTCCTGC----
Laja	--TGGGAACACCCT-------------------------------------CCTAC----

Syja	--AGGGAGCACCCCTCCCCCTTA---------------------TACTCCTCCTAC----
Epme	--CGAATGTACTACATTC------------------------------------AC----
Grse	--TAGGTACACTC---------------------------------------ATACCCAC
Clja	--TGGGAAA--ACTGACTGCCTAA------------------------------------
Ogcy	--GGGAAGATTAACTTCCTGA---------------------------------------
Plna	--TGGGAT-CAA----------------------------------ACCTTTCTAC----
Lema	--TGGAATTA--TGCTATTC---------------------------------CAC----
Etzo	--CGGGAG---AACTTCCTCCTAC------------------------------------
Apse	--CAGGGATAGC----------------------------------CC--CCCTTA----
Epde	--TGGGAGCACC------------------------------------CCTCCTAC----
Slja	--AGGACC----------ACCGTGTCCT---------------------------CTG--
Bsja	--CGAGAG-GACTTCC---------------------------------CTCTTAC----
Ecna	--TAAGAGAACTAACCCATAAT---------------------CAACCCTCTTAAA----
Cohi	--CGGGAAAATTT----------------------------------TATCTAAGAAAT-
Caar	--TAGGAGGACAACCCACTATCTTCCCCCTCC------------------TCCCA-----
Came	--TGGGAGAACAAACCCAAAACCCTTTTCC---------------CACACTCCCA-----
Mema	--TGGGGACACATACCTCCC----------------------ACCCCCTCCCCA------
Lenu	--CAGAAA----------CACACACCGT----------------------TTCTG-----
Brja	--GGGGAG-CACTG----------------------------------CTCC-TA-----
Plma	--TGGGAGCACT-----------------------------------GCTTCCTA-----
Emst	--TGGGAACACCCCCTCCTA--
Ptti	--TGAGAA-CACCTC----------------------------------CTCTCA-----
Losu	--TGGAGA-CCTACATCATCTTTTAACCAA----------------AGTCTAATA-----
Geoy	--CAGGGAG--------------------------AACATTTTTACTACTCCTA-----
Dipi	--AGGGAGCACAACCCT----------------------------------CCTA-----
Pama	--TAGGAATACA----------------------------------CATTCCTA------
Leob	--CAGGAG-CACAACTA--------------------------------CTCCCA-----
Neba	--CGAGAATAAAAGTTTTTCTTATT--------------------------CTTA-----
Pdpl	--TGGGAGCTTAATCCCATATTTCTTTCTTT-------------CTCCGCTCCCA-----
Nimi	--TGGAAGCATTT---------------------------------------TGCTGCCA
Uptr	--AGGGAGGACT----------------------------------CC--TCCTA-----
Pesc	--TAAGAGCACT-ACT-----------------------------------CTTA-----
Baar	--CTGGACCACCT--------------------------------------TATCCCTT-
Moar	--TGGGAATACCCCT------------------------------------CCTA-----
Toja	--TAGGAGCATTCAACCCTTTTTCTCTAAC---------------CCTCCTCCCA-----
Chau	--AGAGGGCTCACTCCCTC-----------------------------------T-----
Chse	--CGGGAGCAC------------------------------------CCCTCCCA-----
Enar	--TGAGAACA----------------------------------C-CCTCT--TA-----
Hpty	--TGGGAACACCCACT-----------------------------------CCTA-----
Nana	--CGGAGACACC-TTC----------------------------------CCCT------
Mcst	--TAGAGACACCCTCTCCTA--
Rhox	--TGGGGG-CATACC----------------------------------CCCCTA-----
Opfa	--TGGGAAAACC----------------------------------CTTTCCTA------
Paar	--TAGGA--------------------------------TTATTT----TCCTT------
Gozo	--TGGGAGTACTACTCCTA---
Ackr	--TGAGAAAAA---CTTCTT----------------------------------A-----
Elev	--TGGAAACACTTTTC-----------------------------------C-A------
Trdu	--CGGGAGCACA----------------------------------CTGCCCCA-----
Amoc	--CAGAAGTACCATACTTC-----------------------------------C-----
Hame	--TGGAAG-CA-TGC-----------------------------------TTCCA-----
Chso	--CAGAAA-CATTG-----------------------------------TTTCCA-----
Lyto	--TGGG-AGCACCC-----------------------------------CTCCT------

```
Encr      --TGGGAG-CACC-C----------------------------------CTCCTAC----
Bvar      --CGGGGGTATATACACCCCC---------------------------------CG----
Noco      --CAGGAGTACAACTCCTTA----------------------------------------
Chsp      --TGGGAGAA-AAATCTCTCCTAC------------------------------------
Arja      --TGGGAG-AACC-----------------------------------CCTCCTAC----
Pase      --GTGAAGGCC------------------------------------TCCTTCCAA----
Trel      ---GGGGA----------AAACTTCC---------------------------TCA----
Lifa      --TGGAAG-CTTTTT---------------------------------ACACCAATAAT-
Acur      ----AGGAAGTATTACTTCT----------------------------------AC----
Ampe      --TGGGAGCACCCTCT-C----------------------------------CTAC----
Urja      --TGGGACAGC------------------------------------ACACCCTAA----
Enet      --TGGAAACTCT-TCCATTCT---------------------------------AC----
Ptbr      --TGAGGAC--------------------------TTCATC---------CTCC------
Safa      --C---AT----------CATCACCTGT-----------------------TTTT-----
Icae      --TGGGAGCACTGCCT-----------------------------------CCTA-----
Asmi      --AGAAACCA--CTGTTTTT----------------------------------A-----
Foal      --AAAGAA--CTAA--TCTTT--------------------------------CA-----
Drze      --TGGGA-TACTTT-------------------------------------CCTA-----
Rhas      --CAGGGAT--ACC--CC--------------------------------CCTT------
Elac      --TGGGAACAACCT------------------------------------CCCT------
Kugu      --TAAGAG------------------------------------TACTCCTCTTT-----
Plor      --CGGAAGCA--CCTCCTTC---------------------------------TA-----
Sgun      --TGGG-ACTACCCG---------------------------------TCCTA-------
Zaco      --TGGGAATACCCCT----------------------------------CCT--------
Zbfl      --TGGGAACACCCC------------------------------------TCCT------
Spba      -GGGAGCACTTCACCCTTTTCTACCTCTACATCCACCCTTATCTAACCCTCCT-------
Game      --TGGGAGCACTAC-CTCTC----------------------------------------
Thth      --TGGGAGTACTAC----------------------------------CTCCT-------
Xigl      --TGGGAGCACTAAACCCC--TTCTTTACC---------------TCTCCTCCC------
Hyja      --TGGGAGCACTGA----------------------------------CTCCT-------
Psan      --TGGGAGTACAACTTCCTG----------------------------------------
Cupa      --TAGGGGC------------------------------------ACTGCCCCTA-----
Mpch      --CGAGAGCA----------------------------------T-ACTCT--TA-----
Char      --TGGGAGAACCC-----------------------------------CTCCTG------
Pser      --TGGGAGCAACAATCCCTCGAAGATCTTTCTCT------TC--TTTGTCTCCCAC----
Prol      --AAGGAGTACACC---CCTACATTCCTCT---------------CCTCC---TAC----
Plbi      --AGGGAGCACACCCCTAAGTTACTT--------------------CTTCTCCCGC----
Calu      --CGGGAGCACTGCAGGGAAAATCCCCCAGCCT-------TTGACCACCCTCCTA-----
Papa      --T-GAAGC--ATTTACCCACCAC--------------------ACCTGTCATCCA----
Sufr      --TGGGACCACCCGTCCCTC----------------------------------------
Stci      --TGGGACCCTACG-TCCTA-----------------------------------A----
Taru      --TGAAAACACC------------------------------------------------
Rala      --TGAGAG----------CACCCC--------------------------TCTTA-----
```

```
         HVR    ¦     47     D  47'            ¦        HVR        ¦
Scca    ----TTAAAAGTTAGAATTA-CAATTCTAACC-GATAAA------------------A-A
Muma    -------AAAATCAGAATAA-CAATTCTGATT-AATAAA------------------A-T
Erca    A-----ATTAACTAGAATGA-CAATTCTAAGT-ATCAGG------------------A-T
Pose    ----ATA-AGTTAAGAATGA-CAATTCAACAC-ATCAGG------------------A-C
Actr    -----TAAAACCTAGGGCGA-CAGCCCAAAGC-AACAGA------------------A--
Scal    -----TAAAACCTAGGGCGA-CAGCCCAAAGC-AACAGA------------------A-C
Posp    -----TAAAACCTAGGGCGA-CAGCCCAAAGC-AACAGA------------------A-T
Atsp    A------AAACCAAGAGCAA-CAACTCTAAGT-AACAG-------------------AAA
Leoc    --------AACCAAGAGCAA-CAACTCTAAGT-AACAGA--------------------A
Amca    --------AACTAAGAGCCA-CCACTCTAAGT-AACAGG------------------A-C
Osbi    TCCCTAAGAACCAAGAACCA-CAATTCCAAGT-AACAG-------------------AAA
Pabu    ---------ACCGAGAGCTA-CTGCTCTAAGC-AACAGA------------------A-A
Hial    ---------GCCAAGAGACA-CAACTCTAAGC-AACAGA------------------C-A
Elha    ---------GCCAAGAGACA-CACCTCTAAGC-AACAGA------------------A--
Mlcy    ---------ACCAAGAGACA-CAACTCCAAGC-AGCAGA--------------------A
Algl    ---------GCTGAGAGCTA-CAGCTCTAAGA-AGCAGA------------------A--
Ptgi    -----TAAAACTAAGAGAAA-CACCTCTAAGT-AACAGA------------------A-C
Alaf    ---------ACTGAGATACA-CAAATCTAAGT-AACAAA------------------A--
Nock    ---------ACTAAGAAATA-CAAATCTAAGT-AGCAAA------------------A--
Anja    ---------GCCAAGAGAGA-CATCTCTAAGC-AACAGA------------------AAA
Gyki    ---------GCTAAGACAAA-CAAGTCTAAGT-ATCAGA--------------------A
Syka    ---------ACCAAGAAAGA-CAATTCTAAGT-AACAGA------------------A-C
Opma    ---------ACCAAGAGACA-CATTTCCAAGT-AACAGA------------------A-A
Comy    ---------GCCAAGAAAAA-CAATTCTAAGC-AACAAA------------------A-T
Sasp    --------AACCAAGAAGTA-CACTTCAAGGC-AACAGA------------------A-A
Eupe    --------AACCAAGAAAAA-CACTTCTAAGC-AACAGA------------------A--
Enja    ------TAAGCCGAGAATTG-CAACTCTAAGC-CGCAAA------------------A-T
Same    ------AAAACCCAGAGCCA-CAGCTCTAAGT-CACAAA--------------------A
Chch    -------GAGCTAAGAGACA-CATCTCTAAGC-CACAGA------------------A-C
Grgr    --------AGCCCAGAGAGA-CACCTCTAAGC-CACAGA------------------C-A
Caau    ------AAAACTAAGAGAGA-CATCTCTAAGC-CACAGA------------------A-C
Cyca    ------AAAACCAAGAGAGA-CATCTCTAAGC-CACAGA------------------A-C
Dare    ---------ACCAAGAGAAA-CATCTCTAAGC-CTCAGA--------------------A
Cost    ------AAAACCAAGAAAGA-CATTTCTAAGC-CACAGA------------------A-A
Leec    ------AAAACTAAGAGAGA-CATCTCTAAGT-CACAGA------------------A--
Crla    ------AAAACTACGAAAAA-CATTTCTTTCT-CTCTAT------------------C-C
Clmc    ------AATACCAAGAGAGA-CATCTCTAAGT-CACAGA------------------A--
Phin    ------TTATCCGAGAGAAA-TATCTCTAAGT-CACAGA------------------ACA
Icpu    --------AACCAAGAGATA-CACCTCTAAGT-CGCAGA------------------A-T
Psto    -------AAACTAAGAGAGA-CATCTCTAAGT-CACAGA------------------A-C
Cora    -------AAACCAAGAAAGA-CATTTCTAAGT-CACAGA------------------A--
Eisp    ------AAAATTAAGAGGGA-CACCTCTAAAT-CGCAAA------------------A-C
Apal    ------TGAACCAAGAGAGA-CATCTCTAAGC-CACAGA------------------A-C
Eslu    ------ATAACCACGAGCGA-CAGCTCTAAGT-CTCAGA------------------A-C
Dape    ------ATAAGCATGAGCTA-CAGCTCTAAGC-ATCAGA--------------------A
Glse    ------AAAGTCAAGAGCCA-CAGCTCTAAGC-AACAG-------------------AAC
Naar    ---------GCCAAGAGCTA-CAACTCTAAGC-AACAGA--------------------A
Baoc    --------AGCCATGAGTTA-CAACTCTAAGC-AACAGA------------------A--
Opso    ------ACAACTAAGAGCTA-CGGCTCTAAGC-AACAGA------------------A-T
Alte    ------AAAGCCAAGAGGTA-CACCTCTAAGC-CTCAGA------------------A--
Plap    -----TAAAACCAAGAGGTA-CACCTCCAAGC-CTCAGA------------------A-C
```

```
Plal    ------ACAGCTGAGGGCTG-CGGCCCCAAGC-AACAGA------------------A-A
Sami    -------CACCTAAGGGCTG-CCGCCCTAAGC-AACAGA------------------A-A
Rere    -------CAGCTCAGAGCCG-CGGCTCTACGC-AACAGA--------------------A
Gama    -------GAGCTAAGAGTTA-CCACTCTAAGC-AACAGA------------------A-A
Onmy    -------CAGCCGAGAGCTA-CAGCTCTAAGC-ACCAGA--------------------A
Sasa    -------CAACCAAGAGTCA-CAACTCTAAGT-ACCAG-------------------AAT
Cola    --------AGCCGAGAGCTA-CAGCTCTAAGC-ACCAGA------------------A--
Dita    --------GACTGAGAGTTA-CATCTCTAAGG-AACAGA------------------A-C
Gogr    --------AGCTATGAGCCA-CAGCTCTAAGC-AACAGA------------------A-C
Chsl    ----CCACAGCCAAGGGCCC-CTAACCCAAGC-AACAGA------------------A-T
Atja    --------AACTAAGAGCCA-CAGCTCTAAGT-AACAGA------------------A-C
Iido    --------AACCAAGAGCTA-CAGCTCTAAGA-AACAGA------------------A--
Auja    --------AACCAAGAGCAA-CAGCTCTAAGT-ACCAGA------------------A--
Chag    --------AGCCGAGAGCCCACCACTCGAAGC-ATCAGA--------------------A
Hami    --------AACCAAGGGCTA-CCGCCCTAAGC-AACAGA------------------A-A
Saun    --------AACCAAGGGCTA-CCGCCCTAAGC-AACAGA------------------AAC
Nema    --------AACCGAGAGCCA-CAGCTCAAAGC-AGCAGA------------------A-C
Disp    -------GAACCCAGAGCCT-CAAGTCAAATC-AACAGT------------------A-C
Myaf    -------ATACATAGAGCCA-CAGGTCAAATC-AACAGT------------------A--
Lagu    -------GAACTAAGAGCCA-CCCCTCCAAGC-ACCAGA------------------A-C
Trtr    -------TAATCAAGAGCTA-CAGCTCTAAAT-ATCAGA--------------------A
Zucr    -------TAATTAAGAGCTA-CAGCTCTATAT-ATCAGG--------------------A
Pxja    -------AACCCTAGAGCTA-CCGCTCTAAGT-AACAGA--------------------A
Pxlo    -------AACCCTAGAGCTA-CCGCTCTAAGT-AACAGA------------------A-C
Pctr    -------AAACCCAGAGCCA-CTACTCCAAGT-AACAGA------------------A-C
Apsa    -------AACCTCAGAGCCA-CCGCTCCAAGT-AACAAA------------------A-C
Cabe    -------CAGCCAAGAGCCG-CTCGTTTAAGC-ACCAGA--------------------A
Bzze    -------AAACTGAGAGCCG-CAGCTCTAAGA-ATCAGA--------------------A
Siim    -------GAGCTAAGAGCTA-CCGCTCTAAGC-AGCAGA--------------------A
Ctru    -------AATCCAAGGGCGA-CAGCCCTAAGC-AACAGA------------------A--
Dpbr    -------AAACCAAGAGCCA-CAGCTCTAAGC-AACAGA------------------A-C
Caki    -------ACACTAAGAGTCA-CCCCTCTAAGT-AACAAA------------------A-T
Phja    --------CACCCAGAGCCT-CCGCTCCAAGT-AACAGA------------------A-T
Brsp    --CCCTAAATCCCCGATAAT-CACATCTAAGA-AACAGA------------------ATC
Gamo    --------CTCAGAGCCTTC-TTACTCCAAGT-AACAGA------------------A--
Lolo    -------ATACTCAGAGCCT-CTACTCCAAGT-AACAGA------------------A-A
Batr    ------TAAATCAAGAGCCA-CACCTCTCGTT-AATACA------------------AAA
Prmy    --------AAACACGAACAA-CAGTTCTAGTT-ATCAGA------------------A--
Loli    ----CTATCACCCCGAGCTA-CCGCTCTAATGTATCAGA------------------ACC
Loam    ------ATCACCCAGAGCTA-CCGCTCTAATT-ATCAGA------------------A-T
Chab    --------AACTGAGAGTAC-CCCCTCTAGCT-AACAGA--------------------A
Chto    --------AACTGAGAGTAC-CCCCTCTAGCT-AACAGA------------------A-T
Majo    --------AACTGAGAGCTG-CCGCTCTAATT-AACGGA------------------A-C
Hlst    --------AACTACGAACAA-CCTCTCTAATT-AACGGA------------------A--
Clpe    --------AACTGAGAGCTC-CCGCTCTAATT-AGCAGA------------------A-T
Mlmr    --------AAGCAAGAGCCC-CCGCTCTAACT-AACAGA--------------------A
Crcr    CTTCTCACAACCATGAGCTA-CAACTCTAAAT-AACAG-------------------AAC
Muce    -----CACAACCATGAGCTA-CAGCTCTAAAT-AACAGA------------------A-C
Bege    ------ACAACCAAGAGCGA-CAGCTCTAAGT-AACAGA------------------A--
Mela    -------CAACCAAGAGCGA-CAGCTCTAAGT-AACAG-------------------AAC
Hats    ---TCTACAACCGAGAGTTA-CAACTCTAAGC-AACAGA--------------------A
Orla    ------ATAACCAAGAGCTT-CCCCTCTAAGT-AACAGA--------------------A
```

```
Cosa    ------ACAACCCAGAGTGA-CAACTCTAAGT-AACAGA------------------A-C
Exsp    ------AGAACTAAGACCGA-CAGGTCTAAGT-ACCAGA------------------A-A
Depa    ------ATAACCAAGACTGA-CAAGTCTAAGC-ATCAGA------------------A-C
Rima    --------AGCCAAGACCGA-CAGCTCTAAGC-ACCAGA------------------A-T
Fuol    --------AACCAAGAGCTA-CAGCTCTAAGT-AACAGA------------------A-C
Gmaf    --------AATAAAGAGCCG-CAGCTCACAAA-ACCAGA------------------A-C
Xeei    --------ACCCAAGAGCCA-CAGCTCTAAGT-AGCAGA------------------A-T
Pros    --------AACCAAGGGCCC-CCGCTCTAAGT-AACAGA------------------A-C
Scmi    --------AACCAAGAGCCC-CCGCTCTAAGT-AACAGA------------------A--
Rolo    --------AACCAAGAGCCG-CCACTCTAAGT-AACAGA------------------A-C
Cere    --------AACCAAGAGTCA-CCACTCTAAGT-AACAGA--------------------A
Daga    --------AGCCGAGAACCC-CAGTTCTAGGC-AACAGA------------------A-T
Anco    ------ACAACCAAGAGCCA-CTGCTCTAAGT-AACAG-------------------AAT
Dmve    -------CAACCAAGAGTCA-CCACTCTAAGC-AACAGA------------------AAT
Dmar    -------CAACCAAGAGCCA-CCACTCTAAGT-AACAGA------------------A--
Anka    -------CAACCAAGAGCCA-CTACTCTAAGC-AACAGA------------------A--
Moja    -------CAGCCAAGAGCCA-CTGCTCTAAGT-AACAGA------------------A-T
Hoja    -------CAACCCAGAGCCA-CTGCTCTAAGT-AACAGA------------------A-C
Bede    ------ACAACCAAGAGTCG-CCACTCTAAGT-GACAGA------------------A-C
Besp    -----TACAACCAAGAGCCG-CCACTCCAAGT-GACAGA------------------A-T
Mysp    ------AAAACTAAGAGGTA-CCCCTCTAAGA-AGCAGA------------------A-C
Osja    ------AAAACTAAGAGATA-CCCCTCTAAGA-AGCAGA------------------A-C
Sgro    --------AACCGAGAGCTA-CATCTCTAAGA-AACAGT--------------------A
Pzpa    --------AACCAAGAGCCA-CGGCTCTAAGT-CTCAGA------------------AAT
Zeja    --------AGCCAAGAGCCA-CAGCTCTAAGC-ATCAGA------------------A--
Znne    --------AACCAAGAGCTA-CAGCTCTAAGA-AACAGA------------------A--
Zefa    --------AACCAAGAGCCA-CAGCTCTAAGT-AACAGA------------------A-A
Acni    --------AACCAAGAGCCA-CAGCTCTAAGT-ATCAGA------------------A--
Ncrh    --------AACCAAGAGCCA-CAGCTCTAAGT-ATCAGA------------------A-A
Agca    --------AAGTAAGAGCTC-CCGCTCTAACA-AACAAA------------------A--
Hydy    --------AGCCGAGAGTTA-CAGCTCTAATA-AACAGA------------------A--
Gsac    --------AGCTGAGAGTTA-CAACTCTAATA-AACAGA------------------A--
Pevo    --------AACCAAGAGCGA-CAGCTCTAAAG-AACAGA------------------A-C
Hiku    --------ACCCAAGAATGT-CATTTCTAAGT-ACCAAA------------------A-T
Inpa    --------AACCACGAGCCA-CAGCTCTAAGC-AACAGA------------------A-T
Auch    -----TTAAACCTAGAATGC-CAATTCAAAGT-AGCAGA------------------A-C
Fico    -------GAGCTAAGAGCTA-CGGCTCTAGGC-AACAGA------------------A-T
Macs    -------AAACCAAGAACTA-CAGTTCTAGTT-AACAGA--------------------A
Moal    -------AAACCAAGAGCCA-CACCTCTAATA-ATTAGA------------------A--
Syma    --------AACCAAGAGTTA-CAACTCTAACC-AATAG-------------------AAC
Mafr    -----TAAAAACAAGAGCCA-CCACTCCAAAA-AACAGA------------------A-C
Dcpe    -----TACACCCAAGAGCCA-CAGCTCTAATT-AACAGA------------------A-C
Dcti    -----TACAACCGAGAGCCA-CAGCTCTAATT-AACAGA------------------A-C
Hehi    -----TACAATTAAGAGCCG-CAGCTCTAATT-AACAGA------------------A-T
Stam    --------AACTAAGAGCTT-CTGCTCTAAGT-AACAGA------------------ACA
Hogi    --------AAATTTGAGCCA-CAGCTCTAATT-AACAGA------------------A--
Erzo    --------AGCCAAGAGCCA-CAGCTCTAGGC-AACAGA------------------A--
Hxot    --------AGCTAAGAGCTA-CAGCTCTAGTA-AACAGA------------------A--
Core    --------AACCAAGAGCTA-CAGCTCTAGAA-AACAGA------------------A-A
Apve    --------AGCTAAGAGCTA-CAGCTCTAGTA-AACAGA------------------ATT
Latj    --------AACTAAGAGCCA-CAGCTCTACCT-AACAGA------------------A-A
Laja    --------AACTGAGAGCTT-CCGCTCTAGTG-AACAGA------------------A-A
```

```
Syja    --------AATCAAGAGTCC-CCACTCTAATG-AACAGA------------------A--
Epme    --------AACCAAGAGCGA-CAGCTCTAATT-AACAGA------------------C-A
Grse    --------AACCAAGAGCCA-CAGCTCTAACT-ACCAG-------------------AAC
Clja    --------AACCAAGAGGGG-CACCTCAAAGG-AACAGA------------------A-T
Ogcy    --------ACCCAAGAGCCA-CAGCTCAAAAT-AACAGA------------------A-C
Plna    --------AACCAAGAATTA-CAATTCTAAGT-ATCAGA------------------A-C
Lema    --------AACTAAGAGCCC-CCGCTCTAGTT-AACAGA--------------------A
Etzo    --------AACTAAGAGCTA-CTGCTCTAATT-AACAGA------------------A-T
Apse    --------AACCCAGAGTGA-CAGCTCTAACA-AACAGA------------------A-T
Epde    --------AGCTAAGAGCTC-CCGCTCTAGCA-AACAGA------------------AAT
Slja    --------AACCAAGAGCTT-CCACTCTAATG-CCCAGA------------------A-T
Bsja    --------AGCTAAGAGCTA-CCGCTCTAAGA-AACAGA------------------A-T
Ecna    --------AACATAGAGCCA-CACCTCCAATT-AACAGA--------------------A
Cohi    TATTCTTACCATAAAGCTAC-TTCCCCAAATT-AAGAGTGACAGCTCTAAATTACAGTAT
Caar    -------CAAGCAAGAGTTA-CAACTCTAGCT-AACAGA------------------A-C
Came    -------CAAGCAAGAGCTA-CAACTCTAACT-AACAGA------------------ATC
Mema    -------CAAACAAGAGCTA-CAACTCTAACA-AACAGA--------------------A
Lenu    -------AGATTAAGAGCCC-CCACTCAAAGT-CCCAGA------------------A-T
Brja    -------CAACCAAGAGCCG-CAACTCTAGTG-AACAGA------------------A-C
Plma    -------CAACCAAGAGCCG-CAGCTCTAAAA-AACAGA------------------AAT
Emst    -------CAACTGAGAGCTC-CCGCTCTAGCG-AACAGA------------------A--
Ptti    -------CAACCAAGAGCTC-CCGCTCTAATA-AACAGA------------------A-T
Losu    -------AAACTAAGAGCTC-CCGCTCTAATA-CTCAGA------------------A-C
Geoy    -------CAAACAAGAACGA-CAGCTCTAGTT-AACAGA------------------A-A
Dipi    -------CAGCCAAGAGCTT-CCGCTCTACTA-AACAGA------------------A-C
Pama    -------AACCCAAGAGCTC-CCGCTCTAATG-AACAGA--------------------A
Leob    -------CAGCTAAGAGCTA-CCGCTCTAATA-AGCAGA------------------A-T
Neba    -------CAATCAAGAGTTA-CCACTCTAACT-AACAGA------------------A--
Pdpl    -------AAACCAAGAGCCA-CCACTCTAACT-AGCAGA------------------A-C
Nimi    -------CAACTAAGAGCTC-CCGCTCTAATA-AACAG-------------------AAT
Uptr    -------AAAGCAAGAACCG-CAATTCTAACT-AACAGA------------------A-T
Pesc    -------CAAACAAGATTTC-CCACTCTAACGTAACAGA--------------------A
Baar    --ATCCTAAATCAAGAGCCC-CCGCTCTACTG-AACAG-------------------AAA
Moar    -------CAACTAAGAGCTC-CCGCTCTAATA-AACAGA------------------A-T
Toja    -------GAATTAAGAGCTA-CAGCTCTAACT-AACAGA--------------------A
Chau    -------GAATCGAGAGCTT-CCGCTCAAACA-AACAGA------------------A-T
Chse    -------CAATCAAGAGTCC-CCACTCTAACA-AACAGA------------------A--
Enar    -------CAGCTGAGAGCTC-CCGCTCTAATA-AACAGA------------------A--
Hpty    -------CAACTAAGAGCTC-CCGCTCTAGTA-AACAGA--------------------A
Nana    -------GAAATAAGAGTTA-CAACTCTAACT-AGCAGA------------------A-C
Mcst    -------AAACAGAGAGCTC-CCGCTCTAATG-AACAGA------------------ACC
Rhox    -------TCACCAAGAGCTC-CCGCTCTAATG-ACCAGA------------------A-T
Opfa    -------AAACTAAGAGCCC-CCGCTCTAATA-AACAGA--------------------A
Paar    -------AAATCAAGAGCTA-CCGCTCTAAATCAACAGA------------------A-A
Gozo    -------AAACTAAGAGCTC-CCGCTCTAGAA-AACAGA------------------A-T
Ackr    -------GAAGCGCGAGCGC-CAGCTCTAACT-AACAGA------------------A-T
Elev    -------CAGCTAAGAGCTC-CCGTTCAAGGA-AACAGA------------------A-T
Trdu    -------CAACCCAGAGTTA-CAACTCCAAGT-AACAGA------------------A-C
Amoc    -------AAAATAAGAGCCA-CCGCTCAAAGT-ATCAGA--------------------A
Hame    -------AAACCAAGAGCCT-CTGCTCTAAGA-ATCAGA------------------A-C
Chso    -------GAACCAAGAGTTT-CCACTCTAAGT-AACAAG------------------ACC
Lyto    ------ACAACTAAGAGCCA-CAGCTCTAGTA-AACAGA------------------A-A
```

```
Encr    --------AACTAAGAGCTA-CAGCTCTAGTA-AACAGA------------------A-A
Bvar    --------AGTCAAGAATTA-CATCTCCAGGC-AGCAGA------------------A--
Noco    --------AACTCAGAGCCA-CAGCTCTAAGA-AACAAA------------------A--
Chsp    --------AACTTAGGGCCT-CCACCCTAAGT-CACAGA------------------A-C
Arja    --------AACTAAGAGCTA-CAGCTCTAGTA-AACAGA------------------A-C
Pase    --------AAACAAGAGCTA-CCGCTCTAATT-CTCAGA--------------------A
Trel    --------AACTGAGAGCGA-CAGCTCCAAGA-AGCAGA------------------A-C
Lifa    GTTTCCTAAAACACGAACAA-CCATTCTAGTT-ATCAGA------------------A-T
Acur    --------AATTAAGAGCCA-CCCCTCTAGTT-AACAGA--------------------A
Ampe    --------AACTAAGAGCTA-CAGCTCTAATG-AACAGA--------------------A
Urja    --------AACCAAGAGCCA-CCCCTCTAATC-AACAGA------------------A--
Enet    --------AACCCAGAGCAC-CCGCTCTAAGC-AACAGA------------------A--
Ptbr    -------GAACTTAGAGTGG-CAACTCAAAGT-TACAAA------------------A-C
Safa    -------GAACGAAGAGCGA-CAACTCAAAGT-ACCAGA------------------A-C
Icae    -------CAACCAAGAGCTG-CAGCTCTAGCA-AACAGA--------------------A
Asmi    -------CAGAGAAGAACCA-CAGTTCTTCCA-CACAAA--------------------A
Foal    -------AAACTAAGACAAA-CAAGTCCAGGT-CACAGA--------------------A
Drze    -------AAAGCTAGAACCC-TTATTCTAACT-AACAGA--------------------A
Rhas    -------AAAACCAGAGCCA-CAGCTCTAACA-AGCAGA------------------A-C
Elac    -------AAAAATAGGGCCA-CACCCCTAATA-AACAGA------------------A--
Kugu    -------AAACCAAGAGCCA-CAGCTCTAAAA-AACAGA------------------A--
Plor    -------CAACCAAGAGTTC-CCACTCCAATA-AACAGA--------------------A
Sgun    ------CAGCCATAGAGCTC-CCGCTCTAAGC-AACAGA------------------A-T
Zaco    ------ACAATCCAGAGCTC-CCGCTCTAACG-AACAGA------------------AAA
Zbfl    ------ACAACTCAGAGCTC-CCGCTCTAATA-AACAGA------------------A-C
Spba    ------AAAACCAAGAGCTA-CGACTCTAACT-AGCAGA------------------A-A
Game    ------ACAACCAAGAGCTG-CAGCTCTAATG-AACAGA--------------------A
Thth    ------ACAACCAAGAGCTG-CAGCTCTAAAG-AACAGA------------------A-T
Xigl    ------ACAACTAAGAGCTA-CAGCTCTAACT-AACAGA------------------ATA
Hyja    ------ACGACCAAGAGCTG-CAGCTCTAACG-AACAG-------------------AAT
Psan    -------CAACCAAGAGCTG-CAGCTCTAACA-AACAAA------------------A--
Cupa    -------CAACCAAGAGCTG-CAGCTCTAATA-AACAGA------------------A-T
Mpch    -------CAGCTAAGAGACA-CTACTCTAAGC-AACAGA------------------A--
Char    -------AAACCAAGAGCCA-CGGCTCTAAGC-AATAG-------------------AAC
Pser    --------AACTAAGAGCCA-CAGTTCTAAGC-AACAGA------------------A-T
Prol    --------AAGCTAGAGCGA-CAGCTCTAATC-AGCAGA--------------------A
Plbi    --------AAGCCAGAGCAA-CAGCTCTAACA-AGCAGA------------------A-A
Calu    -------GAAACCCGGGCTC-CGGCCCTAGTT-CACGAA------------------A-A
Papa    --------AAGTCAGAGCTAACAACTCTAACT-AACAGA------------------A--
Sufr    --------AAAAAAGAGCCC-CTGCTCTAAAA-AACAGA------------------A--
Stci    --------AAACAAGAGCAC-CCGCTCTAATT-AACAGA------------------A--
Taru    CTTTTTAAACCCAAGAGTCA-CCACTCTAGGA-TACAG-------------------AAC
Rala    -------CATCCTAGAGTTA-CCACTCTAATA-AACAGA------------------A-A
```

```
                                                48              48'     D'    43'
Scca      T-TTTATCG--AAAAAT---GACCCAGGATTTT-----CCTGATCAATGAACCAAGTTAC
Muma      A-TTTATCGA-A-AAAT---GACCCAGG-ATT-----TCCTGATCAATGAACCAAGTTAC
Erca      AC-CTG-TCATTAA------GATCCAGA-CTAA-----CCTGATCAACGAACCAAGTTAC
Pose      A-TCTGACAT-T-A--A---GATCCAGACTAAT------CTGATCAACGAACCAAGTTAC
Actr      CATCTGACGA-ACAAT----GACCCAGGC-T--A-AAGCCTGATCAACGAACCAAGTTAC
Scal      A-TCTGACG--AACAAT---GACCCAGGCCAAA----GCCTGATCAACGAACCAAGTTAC
Posp      A-TCTGACGA-A-CAAT---GATCCAGGCCCAGC-----CTGATCAACGAACCAAGTTAC
Atsp      -ATCTGAC--GATAA---ATGATCCAGACCTGT------CTGATCAACGGACCAAGTTAC
Leoc      AATCTGACGAT--AAAT---GACCCAGACC------TGTCTGATCAACGAACCAAGTTAC
Amca      A-CCTGACGA-A-CAAT---GACCCAGGCATACC-AAGCCTGATCAACGAACCAAGTTAC
Osbi      ATTCTGACTAACAA-----TGATCCGAACTT-------TTCGATCAACGAACCAAGTTAC
Pabu      ATTCTGACTAC--ACA----GATCCGAATT--------ATCGATTAACGAACCAAGTTAC
Hial      AATCTGACC--AAAAAT---GATCCGGGCCA----AAGCCCGATCAACGGACCAAGTTAC
Elha      CATCTGACCAAAAAT-----GATCCGGGCCAAA----GCCCGATCAACGAACCAAGTTAC
Mlcy      AATCTGACC--AAAAAG---ACCCAGGCCCAAAC-ACGCCTGATCAACGAACCAAGTTAC
Algl      TATCTGACCA-GAAAT----GATCCAGGC-C--A-AGGCCTGATCAACGAACCAAGTTAC
Ptgi      AT-CTGACCAAAAA--T---GATCCAGGCCGAA----GCCTGATCAACGAACCAAGTTAC
Alaf      ATTTTGACCAATAAT-----GACCCGGACCAA-----ATCCGATCAACGAACCAAGTTAC
Nock      AATTTGACCA-ATAAT----GACCCGGAATA-----AATCCGATCAACGAACCAAGTTAC
Anja      --TCTGACCAAAAGT-----GACCCAGAATACTA--ATTCTGATCAACGAACCAAGTTAC
Gyki      ATTCTGACT--AAATGT---GACCCAGGCC-----TAGCCTGATCAACGAACCTAGTTAC
Syka      A-TCTGACCA-A-AAAT---GACCCAGGACCTAG---TCCTGATCAATGAACCAAGTTAC
Opma      A-TCTGACC--AAACAT---GACCCGGGACAAAC-AAACCCGATCAATGAACCAAGTTAC
Comy      T-TTTGACCA-ATAAAC---GACCCAGGGACAAC-ATCCCTGAGCAATGAACCAAGTTAC
Sasp      AT-CTGACCAC-ATG-----ACCCAGGCCCATA----ACCTGATCAACGAACCAAGTTAC
Eupe      AATCTGACACAAGATCCG--GGTGCAATG-A------ACCCGATCAATGAACCAAGTTAC
Enja      T-TTTGAC-TGAAAT-----GATCCGGTTGAAA----AACCGATTAACGAACCAAGTTAC
Same      CATTTGACCAA--TAAT---GATCCGGCTT-----AATGCCGACCAACGGACCAAGTTAC
Chch      A-TCTGACCA-A-AAAT---GATCCGGA-CACCA---GACCGATCAACGAACCAAGTTAC
Grgr      CATCTGACC--AAACAT---GATCCGGCGTGCG----CGCCGAACAACGGACCAAGTTAC
Caau      A-TCTGACCA-A-ATAT---GATCCGGCTAATAC-AA-GCCGATCAACGAACCAAGTTAC
Cyca      AT-CTGACCAAATAT-----GATCCGGCTTAACACATAGCCGATCAACGAACCAAGTTAC
Dare      CACCTGACC--AAAGAC---GATCCGGCCTA----TAAGCCGATCAACGAACCAAGTTAC
Cost      AT-TTGACCAATAAT-----GATCCGGCCAA---TAAGACCGATCAGCGGACCAAGTTAC
Leec      ATTCTGACCA-ATTAT----GATCCGGCCACT----AGGCCGATCAACGAACCAAGTTAC
Crla      C-TCTGACC--AAATAT---GATCCGACCTATAA---AGTCGATCAACGAACCAAGTTAC
Clmc      CATCTGACCA---AAT----GATCCGGCCACA--T-AGACCGATCAACGGACCAAGTTAC
Phin      --TCTGACCA-TAAT-----GATCCGACCAATCG----GCCGATTAACAAACCAAGTTAC
Icpu      T-TCTGACC--AA-A-A---GATCCGGCTTCCT-----GCCGACCAACGGACCAAGTTAC
Psto      T-TCTGACC--AC-AAA---GATCCGGCACTAT-----GCCGATCAACGGACCAAGTTAC
Cora      TTTCTGACCACACA------GATCCGG-CAAAT----TGCCGATCAATGGACCAAGTTAC
Eisp      AT-TTGACCAA-AAA-----GATCCGGCCTCCCA---GGCCGATCAACGAACCAAGTTAC
Apal      A-TCTGACTA-A-AACA---GACCCGGGTATAAC-AC--CCGATTAACGAACCAAGTTAC
Eslu      T-TCTGACC--AAAAA----GATCCGACACC------AGTCGATCAACGGACCAAGTTAC
Dape      CTTCTGACCAA---AAT---GATCCGACAT-----AG--TCGATCAACGAACCAAGTTAC
Glse      -TTCTGACC--AAAA---ATGATCCGGTAACA-------CCGATCAACGAACCAAGTTAC
Naar      TTTCTGACC--AATA-T---GATCCGGCA------AATGCCGATCAACGGACCAAGTTAC
Baoc      TTTCTGACCAAAA-C-----GATCCGG--CATA----CGCCGATCAACGGACCAAGTTAC
Opso      T-TCTGACCAAAAAT-----GACCCGACATAAT----AATCGATCAACGAACCAAGTTAC
Alte      CATCTGACCATAAAT-----GATCCGG-CAAAA-----GCCGATCAACGAACCAAGTTAC
Plap      AT-CTGACCATAAAT-----GATCCGGCCAA-------GCCGATCAACGAACCAAGTTAC
```

```
Plal    AT-CTGACCAA-AAT-----GATCCGGCAAATT----TGCCGATTAACGGAACAAGTTAC
Sami    T-TCTGACCA-A--GAT---GATCCGGCGTAA-----CGCCGATTAACGGAACAAGTTAC
Rere    ATTCTGACC--AAAAAT---GATCCGGC----AC-AT-GCCGATTAACGGAACAAGTTAC
Gama    TT-CTGACCAGGAT------GATCCGGC--ATA-----GCCGATCAACGGACCCAGTTAC
Onmy    TATCTGACCAA--ATAT---GATCCGGCG-------AACGCATTCAACGGACCGAGTTAC
Sasa    -TTCTGACCAAAAA-----TGATCCGGCATCAC-----GCCGATCAACGGACCGAGTTAC
Cola    TTTCTGACCAGAAAT-----GATCCGG-CGAA-----CGCCGATCAACGGACCGAGTTAC
Dita    T-TCTGACTA-A---AT---GATCCGGCGCAC------GCCGATCGACGGACCGAGTTAC
Gogr    T-TCTGACC--AAAA-T---GATCCGGCCTGC------GCCGATCCACGAACCTAGTTAC
Chsl    AT-CTGACCATAAA------GATCCGGCTTAAC-----GCCGATTAACAAACCAAGTTAC
Atja    T-TCTGACCT-A-ACA----GATCCGGCCTTTTT-AAAGCCGATCAACGGACCGAGTTAC
Iido    CTTCTGACCT-ACCA-----GATCCGGCCTT--T--AAGCCGATCGACGGACCGAGTTAC
Auja    CTTCTGACCCAAAT------GATCCGG-CCAAA-----GCCGATCAACGAACCGAGTTAC
Chag    CCTCTGACCC---TAAT---GATCCGGCCCA----CCCGCCGATCAACGAACCGAGTTAC
Hami    CT-CTGACCTA--ACT----GATCCGGCCT-----ACCGCCGATCAACGGACCGAGTTAC
Saun    --TCTGACCTTC-AT-----GATCCGGCCTACCG---GCCGATCAACGGACCGAGTTAC
Nema    TT-CTGACCATTAAA-----GATCCGGCA---A----TGCCGATCAACGGACCGAGTTAC
Disp    T-TCTGACC--ACCAAG---GAGCCGGCAATG-------CCGATCAACGGACCGAGTTAC
Myaf    CTTCTGAC--CACCTAAA--GATCCGG--CATA----AGCCGATTAATGGACCTAGTTAC
Lagu    A-TCTGACC--ACCAAT---GACCCGGCCCT-------GCCGACAAACGAACCGAGTTAC
Trtr    GATCTGACCTT--TAAT---GATCCGGCC--------TGCCGATCAACGAACCGAGTTAC
Zucr    TTCCTGACT--TCTAAT---GATCCGGCC------TG--CCGATCAACGAACCGAGTTAC
Pxja    CATCTGACCTT--CAA----GATCCGGCAA------A-GCCGATCAACGGACCGAGTTAC
Pxlo    A-TCTGACCTTCAA------GATCCGGCAAA-------GCCGATCAACGGACCGAGTTAC
Pctr    A-TCTGACC--T-GTAC---GATCCGGCAAGG-------CCGATCAACGAACCGAGTTAC
Apsa    A-TTTGACCA-C-CA-----GATCCGGCAGTTCC--AAGCCGATCAACGAACCGAGTTAC
Cabe    TATCTGACCTA--TA-----GATCCGGCAA-------AGCCGATCAACGAACCAAGTTAC
Bzze    CTTCTGACCAT--AA-T---GATCCGGCAA-------AGCCGATCAACGGACCGAGTTAC
Siim    CTTCTGACCAT--AAAT---GATCCGGCAA-------AGCCGATCAACGAACCAAGTTAC
Ctru    CATCTGACCATCAA------GATCCGG-CAAAG------CCGATCAACGAACCGAGTTAC
Dpbr    A-TCTGACCA-G-CAA----GATCCGG--CAAA-----GCCGATCAACGAACCAAGTTAC
Caki    T-TTTGACTTTAAAAAT---GATCCGGTACAA-------CCGATTAACGAACCCAGTTAC
Phja    A-TCTGACC--T--TAC---GATCCGGTT-AC-------CCGATTAACGAACCGAGTTAC
Brsp    --TCTGACTTTATTA-----GATCCGGTTAATTA-----CCGACTAACGAACCAAGTTAC
Gamo    ATTCTGACCTTTTCT-----GATCCGG-TATA------ACCGATCAACGAACCGAGTTAC
Lolo    TT-CTGACTTTTC-T-----GATCCGG-------TATAACCGATCAACGAACCGAGTTAC
Batr    --TGTAACCTC--AA-----GACCCAATAACA------ATTGATCATTGAATTAAGTTAC
Prmy    TATCTGACCT-CTAA-----GACCCAGCA-------TGCCTGATTAATGAACCAAGTTAC
Loli    CTTCTGACCATAAAT-----GATCCGGCGCAAG------CCGATTAACGAAACAAGTTAC
Loam    AT-CTGACCAAAA-------GATCCGGCTAAA------GCCGATCAACGGACCGAGTTAC
Chab    CTTCTGACC--ACCAA----GACCCGGC-----A-A-GGCCGATCCACGGACCAAGTTAC
Chto    T-TCTGACC--ACCAA----GACCCGGCAAA-------GCCGATCCACGGACCAAGTTAC
Majo    A-TCCGACC--AACAAT---GATCCGGCC--AA-----GCCGATCAACGGACCGAGTTAC
Hlst    TATCCGACCA-ACAAT----GATCCGGTA------A--GCCGATCAACGAACCGAGTTAC
Clpe    T-TCTGACCT---TAAA---GATCCGGCA-------TGGCCGATCAACGGAACCAGTTAC
Mlmr    TTTCTGTCC--ATAA-A---GACCCGGCT------AAGGCCGATCAACGAACCGAGTTAC
Crcr    -TTCTGACCAAAAAA---ATGATCCGGC-AAT------GCCGATTAACGGACCAAGTTAC
Muce    TT-CTGACCAAAAAAAT---GATCCGG--CAAT-----GCCGATTAACGGACCAAGTTAC
Bege    TTTCTGACCA-TCAA-----GATCCGG---C--C--AAGCCGATCAACGAAACGAGTTAC
Mela    -CTCTGACCCTCA------AGATCCGGTTTA-------CCGATCAACGAAACAAGTTAC
Hats    ATTCTGACCAT--AA-T---GATCCGGCAC-------CGCCGATCAACGGAACGAGTTAC
Orla    CATCTGACC--TTTA-T---GATCCGGCC------TG-GCCGATCAACGGACCAAGTTAC
```

```
Cosa    T-TATGACCA-A-AAAT---GATCCGGCAATG-------CCGATCAACGAATCGAGTTAC
Exsp    T-TCTGACCA----GAA---GATCCGG-----CC--AAGCCGATCAACGGACCAAGTTAC
Depa    AT-TTGACCAAAAAT-----GATCCGGCA---A----TGCCGATTAACGAACCGAGTTAC
Rima    C-TCTGACC--CCAA-A---GATCCGGAC---A----CACCGATCAACGAACCAAGTTAC
Fuol    A-TCTGACC--TT-AAT---GATCCGGCA--GT-----GCCGATCAACGAACCGAGTTAC
Gmaf    T-TCTGACC--CT--AA---GATCCGACACA-------GTCGATCAACGGACCAAGTTAC
Xeei    T-TCTGACCT-T-ACA----GATCCGG-----T--ATTACCGATCAACGAACCAAGTTAC
Pros    C-TCTGACC--ATCAAT---GATCCGGTATA-------ACCGATCAACGGACCGAGTTAC
Scmi    CATCTGACCA-TTAAC----GATCCAGCAA--------GCTGATCAACGGACCAAGTTAC
Rolo    C-TCTGACC--AG-AAT---GATCCGG--CAAA-----GCCGATCAACGGACCGAGTTAC
Cere    CCTCTGACCAA--AAT----GATCCGGAAA-------TACCGATCAACGAACCGAGTTAC
Daga    ATTCTGACC--AA-AAC---GATCCGG-CTG-----AGGCCGATCAACGGACTGAGTTAC
Anco    -TTCTGAC---CAAA---ATGATCCGGCAATG-------CCGATCAACGGACCGAGTTAC
Dmve    --TCTGACCA-AAAT-----GATCCGGCAATG-------CCGATCCACGGACCGAGTTAC
Dmar    ATTCTGACCA-AAAT-----GATCCGGCA----A-C--GCCGATCCACGGACCGAGTTAC
Anka    CTTCTGACCA-TTA-T----GATCCGGCAGC-------GCCGATCAACGGACCGAGTTAC
Moja    T-TCTGACCA-A-CA-T---GATCCGGC-AAC------GCCGATCAACGGACCGAGTTAC
Hoja    T-TCTGACCA-A--TAT---GATCCGG--CAAC-----GCCGATCAACGGACCGAGTTAC
Bede    CT-CTGACCATAAAT-----GATCCGGT-------ACTACCGATCAACGAACCGAGTTAC
Besp    C-TCTGACCA-T-AAAT---GATCCGGTAC-------TACCGATCAACGAACCGAGTTAC
Mysp    A-TCTGACCA-T-AAAT---GATCCGGCACA-------GCCGATCAACGGACCGAGTTAC
Osja    A-TCTGACCATAAAT-----GATCCGGCACA-------GCCGATCAACGGACCGAGTTAC
Sgro    TATCTGACC--AAAAAT---GATCCGAC-----A-CTAGTCGATCAACGAACCGAGTTAC
Pzpa    --TCTGACCTACAAT-----GATCCGG--GCA------ACCGATCAACGAACCAAGTTAC
Zeja    ATTCTGACCTATAT------GATCCGG-GCAA-------CCGATCAACGAACCAAGTTAC
Znne    ATTCTGACCT--ATAT----GATCCGGG------A-A-ACCGATCAACGAACCGAGTTAC
Zefa    T-TCTGACC--T-AAAT---GATCCGGGCA-------A-CCGATCAACGAACCGAGTTAC
Acni    ATTCTGACCT-A-AAT----GATCCGGGC-------AA-CCGATCAACGAACCGAGTTAC
Ncrh    T-TCTGACC--TA-AAT---GATCCGGGC--AA------CCGATCAACGAACCGAGTTAC
Agca    TTTTTGACCAATA-A-----GATCCGG---CAA----CGCCGATCAACGGACCGAGTTAC
Hydy    ATTCTGACCA-AAAA-----GATCCGGCAAC-------GCCGATCAACGGACCGAGTTAC
Gsac    TTTCTGACCTAAAA------GATCCGG-C-AAT-----GCCGATCAACGGACCTAGTTAC
Pevo    T-TCTGACC--TTA-AT---GATCCGGCATC-------GCCGATCAACGAACCGAGTTAC
Hiku    A-TTTGACCC-----AT---GATCCGGC-AAC-----AGCCGATCAACGAACCTAGTTAC
Inpa    TT-CTGACCAA--ACA----GATCCGGC-------ACAGCCGATCAACGGACCAAGTTAC
Auch    CTTCTGATCTAATATT----GATCCGGCCT----CACGGCCGAACAACGGACCGAGTTAC
Fico    T-TCTGACCA-G-CAA----GATCCGGTA-------CATCCGATCAACGGACCGAGTTAC
Macs    TTTCTGACCAT---ACA---GATCCGGCAA-----AG--CCGATCAACGAACCGAGTTAC
Moal    CTTCTAACCATAATA-----GACCCGG---AAT----ACCCGATCTTCGAACAAAGTTAC
Syma    -TTCTAACCATTCAACACAAGACCCGGCAAC-------GCCGATCTACGAACTAAGTTAC
Mafr    T-TCTGAC---T-ACAA---GATCCGGCAATA-------CCGATCAACGAACCAAGTTAC
Dcpe    T-TCTGACC--ACA--C---GATCCGGCAAT-------GCCGATCAACGGACCGAGTTAC
Dcti    T-TCTGACC--AC--AC---GATCCGGCAAC-------GCCGATCAGCGGACCGAGTTAC
Hehi    AT-CTGACCAATAA------GATCCGGC--AAT-----GCCGATCAACGGACCGAGTTAC
Stam    --TCTGACCAGC-AA-----GATCCGGCAAT-------GCCGATCAACGGACCGAGTTAC
Hogi    TGTCTGACCA-G-CA-----GATCCGGCA-------AAGCCGATCAACGGACCCAGTTAC
Erzo    CTTCTGACCAGCAA------GATCCGG-C-AA-----TGCCGATCAACGGACCGAGTTAC
Hxot    ATTCTGACCAACAA------GATCCGG-CAA------CGCCGATCAACGGACCGAGTTAC
Core    T-TCTGACC--AACA-A---GATCCGGCA-AT------GCCGATCAACGGACCGAGTTAC
Apve    --TCTGACCA-ATAA-----GATCCGGC-AAT-----GCCGATCAACGGACCAAGTTAC
Latj    -TTCTGGCCAA--CAAT---GATCCGGCAA-------TGCTGATCAACGGACCAAGTTAC
Laja    T-TCTGACC---AACAA---GATCCGGCAACG-------CCGATTAACGGACCGAGTTAC
```

```
Syja    TTTCTGACCT-ACTA-----GATCCGGCAAC-------GCCGATCAACGGACCCAGTTAC
Epme    CTTCTGACCAA--TAA----GATCCGGCAA-------CGCCGATCAATGGACCAAGTTAC
Grse    -TTCTGACCAAC-------AGATCCGGCAAT-------GCCGATTAATGGACCAAGTTAC
Clja    T-TCTGACT--ATGCAC---GATCCGGCA-AT------GCCGATCTACGGACCAAGTTAC
Ogcy    T-TCTGACC--A-AA-A---GATCCGGCA---A----AGCCGATCAACGGACCAAGTTAC
Plna    T-TCTGACCT-C-TCGT---GATCCGG-----CC-AAAGCCGATCAACGGACCGAGTTAC
Lema    TTTCTGACC--AACAAA---GATCCGGC----AA-C--GCCGATCAACGGACCGAGTTAC
Etzo    T-TCTGACC--AATA-A---GATCCGGCA-AC------GCCGATCAACGGACCGAGTTAC
Apse    T-TCTGACC--AAGTAT---GATCCGGCAA-------AGCCGATCAACGGACCGAGTTAC
Epde    --TCTGACCAG-CAG-----GATCCGGCAATG-------CCGATCAACGGACCGAGTTAC
Slja    T-TCTGACTA-A-AAGT---GATCCGGCAAC-------GCCGATTAACGAACCAAGTTAC
Bsja    T-TCTGACC--T-ATTA---GATCCGGCTTA------CGCCGATCAACGGAACGAGTTAC
Ecna    CTTCTGACCAA--AAAT---GATCCGGC-------AATGCCGATCAACGGACCAAGTTAC
Cohi    -TTCTGACCATAAA-----TGATCCGGCATTT------GCCGATTAACGGACCAAGTTAC
Caar    T-TCTGACCC-T-ATAT---GATCCGGCTTC------TGCCGATCAACGGACCAAGTTAC
Came    --TCTGACCTCTTAC-----GATCCGGCTCTC------GCCGATCAACGGACCAAGTTAC
Mema    CTTCTGACCAA--AAAT---GATCCGGCAA-------CGCCGATCAACGGACCAAGTTAC
Lenu    A-TCTGACCA-A-CAAT---GATCCGGCAAC-------GCCGAACGACGGACCCAGTTAC
Brja    T-TCTGACCA-A-ATAT---GATCCGGCAGTG-------CCGATCAACGGACCGAGTTAC
Plma    --TCTGACCATA-AA-----GATCCGGCAAT-------GCCGATCAACGGACCGAGTTAC
Emst    TTTCTGACCA-ATAA-----GATCCGGCA-------ACGCCGATCAACGGACCGAGTTAC
Ptti    T-TCTGACCA-T-C-TA---GATCCGGCAAGA-------CCGATCAACGGACCGAGTTAC
Losu    ACTCTGACCT-A-ATAA---GATCCGGCAAG-------GCCGATCAACGGACCGAGTTAC
Geoy    AATCTGACTTTTTAA-----GATCCGGCATAC------GCCGATCAACGGACCGAGTTAC
Dipi    TT-CTGACCTTAA-------GATCCGGCA---A----AGCCGATCAACGGACCAAGTTAC
Pama    ATTCTGACCAA--TAA----GATCCGGCAC------ACGCCGATCAACGGACCGAGTTAC
Leob    T-TCTGACCA-A-AAAT---GATCCGACA-------ACGTCGATCAACGAACCGAGTTAC
Neba    CTTCTGACCTTAAGT-----GATCCGACAAA-------GTCGATCAACGGACCGAGTTAC
Pdpl    T-TCTGACC--AGAAAT---GATCCGGCAAC-------GCCGATCAACGGACCAAGTTAC
Nimi    -TTCTGACCAATAC-----AGATCCGGCAAA-------GCCGATCAACGAACCTAGTTAC
Uptr    T-TCTGACC--AAT-AA---GATCCGGCAAT-------GCCGACCAACGGACCGAGTTAC
Pesc    TTTCTGACC--T-TAT----GATCCGGCAA-------TGCCGATTAACGAACCAAGTTAC
Baar    -TTCTGACCTACTCA---ATGACCCGGCA-------ACGCCGATCAACGGACCAAGCTAC
Moar    T-TCTGACC--AACAA----GATCCGGCA-------AAGCCGATCAACGGACCGAGTTAC
Toja    TTTCTGACC--AAAAAT---GATCCGGC-------AGTGCCGATCAACGAATCAAGTTAC
Chau    AT-CTGACTAA--ACAA---GATCCGGC-------AATGCCGATCAACGGACCGAGTTAC
Chse    CCTCTGACCA-AAAAT----GATCCGGCA-------ATGCCGATCAACGGACCAAGTTAC
Enar    TTTCTGACCA-ATCA-----GATCCGGCAAC-------GCCGATCAACGGACCGAGTTAC
Hpty    ATTCTGACC--AATAA----GATCCGGCAG-----CG--CCGATCAACGGACCGAGTTAC
Nana    TT-CTGACCTT-AT------GATCCGGCAC------ACGCCGATCAACGAACCGAGTTAC
Mcst    TTTCTGACCA-GTAA-----GATCCGGCA-------GAGCCGATCAACGGACCAAGTTAC
Rhox    T-TCTGACCA-A--ACA---GATCCGGCATAG-------CCGATCAACGGACCGAGTTAC
Opfa    TTTCTGACCAA--TCA----GATCCGGCAA-------CGCCGATCAACGGACCGAGTTAC
Paar    T-TCTGACCAAACTA-----GATCCGGCAA-------AGCCGATCAACGGACCGAGTTAC
Gozo    TT-CTGACCAA--TAA----GATCCGGC-------AGCGCCGATCAACGAACCGAGTTAC
Ackr    T-TCTGACC--TCCCA----GACCCGGCACCCC-----GCCGATCAACGAACCGAGTTAC
Elev    AT-CTGACCAC--AA-----GATCCGGCA---A----TGCCGAACAACGGACCGAGTTAC
Trdu    T-TCTGACC--AA-TAA---GATCCGGCACATA-----GCCGATCAACGGACCGAGTTAC
Amoc    ATTTTGACCAACTATGT---GATCCGGCTT-----AACGCCGATCAACGGACCGAGTTAC
Hame    T-TCTGACC----ACCA---GATCCGGCAA-------AGCCGATCAACGGACCGAGTTAC
Chso    TACTTGACC--TTTGA----GATCCGGCAT------AAGCCGATCAACGGACCGAGTTAC
Lyto    TT-CTGACCAGCAA------GATCCGGC--AAT-----GCCGATCAACGGACCGAGTTAC
```

```
Encr    T-TCTGACCA-G-C-AA---GATCCGGCAATG-------CCGATCAACGGACCGAGTTAC
Bvar    TTTCTGACCA-TAA------GATCCGG---CAG----CGCCGATCAGCGAACCAAGTTAC
Noco    TTTTTGACCT--A-AA----GATCCGGC------A-ATGCCGATCAACGGACCGAGTTAC
Chsp    A-TCTGACC--CACATA---GATCCAGCACATC----AGCTGATCAACGAAACCAGTTAC
Arja    T-TCTGACCA-G-T-AA---GATCCGGCA-AT------GCCGATCAACGGACCGAGTTAC
Pase    CCTCTGACTTA--TAAT---GATCCGGCAA-------TGCCGATCAACGGACCAAGTTAC
Trel    T-TCTGACCA-T-A-AT---GATCCGGC-CA------AGCCGATTAATGGAACTAGTTAC
Lifa    T-TTTGACCT-A--AC----GACCCGGCAAAG-------CCGATCAACGAACCAAGTTAC
Acur    TATCTGACCCA---TA----GATCCAGCAA-----AG--CTGATCAACGGACCCAGTTAC
Ampe    TTTCTGACC--AACAA----GATCCGGCAA-----TG--CCGATCAACGGACCGAGTTAC
Urja    CTTCTGACCA-ATTA-----GATCCGGCA-------AAGCCGATTGACGAACCAAGTTAC
Enet    CTTCTGACCAAAACC-----GATCCGG-CGATT----TGCCGATCAACGAATCCAGTTAC
Ptbr    A-TTTGACCTAAGAT-----GATCCGGCTT-------AGCCGATCAACGAAACCAGTTAC
Safa    A-TCTGACCT-A-ATCT---GATCCGG--CTCAC---TGCCGATTAACGAAACCAGTTAC
Icae    TTTCTGACC--AGTAA----GATCCGG--CA-----ATGCCGATCAACGGACCGAGTTAC
Asmi    CTTTTGACC--ACCA-----GATCCGGC----AA-ATAGCCGATTAACGAACCAAGTTAC
Foal    CTTCTGACA--TAAT-----GATCCGGCC--------CGCCGATTAACGAAACGAGTTAC
Drze    ATTCTGACC--TTAT-----GATCCGGC-------AAAGCCGATTAACAGACCAAGTTAC
Rhas    T-TCTGACC--A-ACAA---GATCCGGCA-AC------GCCGATCAACGGACCGAGTTAC
Elac    AATCTGACCA-GAAAT----GACCCGG---C--A-A-AGCCGATCAACGAACCCAGTTAC
Kugu    TATCTGACCA-TAAA-----GATCCGGCATG-------ACCGATCAACGGACCGAGTTAC
Plor    TTTCTGACC--CATAT----GATCCGGC----AA-C--GCCGATCAACGGACCGAGTTAC
Sgun    TT-CTGACCAATAA------GATCCGG-CAAA------GCCGATCAACGGACCGAGTTAC
Zaco    T--CTGACCAATTA------GATCCGGCAG-------TGCCGATCAACGAACCCAGTTAC
Zbfl    T-TCTGACC--AATCAG----ATCCGGCAAAG-------CCGATCAACGGACCGAGTTAC
Spba    T-TCTGACCTAAATT-----GATCCGGCTAT-------GCCGATTAACGGACCAAGTTAC
Game    CTTCTGACCAA---TAA---GATCCGGCAA-----CG--CCGATCAACGGACCGAGTTAC
Thth    AT-CTGACCAA-TAA-----GATCCGGCAAC-------GCCGATCAACGGACCGAGTTAC
Xigl    --TCTGACCAATCAA-----GATCCGGCAATG-------CCGATCAACGGACCAAGTTAC
Hyja    -TTTTGACCAATAA-----AGATCCGGCAAC-------GCCGATCAACGGACCAAGTTAC
Psan    TTTTTGACCA-TAAACA---GATCCGGCA-------AAGCCGATTAACGGACCAAGTTAC
Cupa    T-TCTGACC--AATAA----GATCCGGCAAC-------GCCGATCAACGGACCGAGTTAC
Mpch    ATTCTGACCA-TTA------GATCCGGCAAT-------GCCGATCAACGGACCGAGTTAC
Char    -ATCTAACCA--AA-----AGATCCGGCATG-------GCCGATCAACGAACCGAGTTAC
Pser    A-TCTGACCA-A-TAGA---GATCCGGCAAA-------GCCGATTAACGAACCAAGTTAC
Prol    ATTCTGACC--AAAC-T---GATCCGGC-------AACGCCGATCAACGAACCAAGTTAC
Plbi    T-TCTGACC--AAAC-T---GATCCGGTA---A----AACCGATCAACGAACCAAGTTAC
Calu    A-TTCGACCT-A-TACT---GATCCGGCCCAA------GCCGATCAACGGACCAAGTTAC
Papa    CTTCTGACCC-ACCTT----GATCCGGC------A-ACGCCGATCAATGAACCAAGTTAC
Sufr    CATCTGAC---CTTAA----GATCCGG---C---A-AAGCCGATCAACGAACCGAGTTAC
Stci    CTTCTGACCT-TACT-----GACCCGGCA-----C-CCGCCGATCAACGGACCCAGTTAC
Taru    -ATCTGACCAATAA-----TGATCCGGCTAAA------GCCGATTAACGAACCGAGTTAC
Rala    T-TCTGACCT-A--TAT---GATCCGG--CAAT-----GCCGATCAACGGACCGAGTTAC
                                                      *    **  ***
```

```
             43'            49        50        50'        49'
Scca    CCTAGGGATAACAGCGCAATCCTT-TTTCAGAGTTCCT-ATCGACAAAAGGGTTTACGAC
Muma    CCTAGGGATAACAGCGCAATCCTT-TCTCAGAGTTCCT-ATCGAAGAAAGGGTTTACGAC
Erca    CCCAGGGATAACAGCGCAATCTTT-TCCAAGAGCCCAA-ATCGACGAAAAGGTTTACGAC
Pose    CCCAGGGATAACAGCGCAATCTTT-TCCAAGAGCCCGA-ATCGACGAAAAGGTTTACGAC
Actr    CCTAGGGATAACAGCGCAATCCTT-TCTAAGAGTCCAT-ATCGACGAAAGGGTTTACGAC
Scal    CCTAGGGATAACAGCGCAATCCTT-TCTGAGAGTCCAT-ATCGACGAAAGGGTTTACGAC
Posp    CCTAGGGATAACAGCGCAATCCTT-TCTAAGAGTCCAT-ATCGACGAAAGGGTTTACGAC
Atsp    CCTAGGGATAACAGCGCAATCCTT-TCCCAGAGTCCCT-ATCGACGAAAGGGTTTACGAC
Leoc    CCTAGGGATAACAGCGCAATCCTT-TCCCAGAGTTCCT-ATCGACGAAAGGGTTTACGAC
Amca    CCTAGGGATAACAGCGCAATCCTT-TCCCAGAGCCCAT-ATCGACGAAAGGGTTTACGAC
Osbi    CCCAGGGATAACAGCGCAATCCTT-TCCAAGAGCCCAT-ATCGCCGAAAGGGTTTACGAC
Pabu    CCTAGGGATAACAGCGCAATCCTT-TTTAAGAGCCCAT-ATCGACAAAAGGGTTTACGAC
Hial    CCTAGGGATAACAGCGCAATCCTT-TCCCAGAGTCCAT-ATCGACGAAAGGGTTTACGAC
Elha    CCCAGGGATAACAGCGCAATCCTT-TCCAAGAGTCCCTTATCGACGAAAGGGTTTACGAC
Mlcy    CCCAGGGATAACAGCGCAATCCTT-TCCAAGAGTCCAT-ATCGACGAAAGGGTTTACGAC
Algl    CCTAGGGATAACAGCGCAATCCTT-TCTCAGAGTTCAT-ATCGACGAAAGGGTTTACGAC
Ptgi    CCTAGGGATAACAGCGCAATCCTT-TCCCAGAGTCCAT-ATCGACGAAAGGGTTTACGAC
Alaf    CCTAGGGATAACAGCGCAATCCCT-TCCCAGAGTCCAT-ATCGACGAAGGGGTTTACGAC
Nock    CCTAGGGATAACAGCGCAATCCCT-TCCCAGAGTCCAT-ATCGACGAAGGGGTTTACGAC
Anja    CCTAGGGATAACAGCGCAATCCTT-TCCCAGAGTCCAT-ATCGACGAAAGGGTTTACGAC
Gyki    CCTAGGGATAACAGCGCAATCCCC-TCCCAGAGTCCGT-ATCGACGAGGGGGTTTACGAC
Syka    CCTAGGGATAACAGCGCAATCCTT-TCCCAGAGTCCAT-ATCGACGAAAGGGTTTACGAC
Opma    CCTAGGGATAACAGCGCAATCCTT-TCCCAGAGTCCCT-ATCGACGAAAGGGTTTACGAC
Comy    CCTAGGGATAACAGCGCAATCCTT-TCCCAGAGCCCAT-ATCAACGAAAGGGTTTACGAC
Sasp    CCTAGGGATAACAGCGCTATCCTC-TCCCAGAGTTCAT-ATCGACAAGAGGGTTTACGAC
Eupe    CCTAGGGATAACAGCGCTATCCTT-TCCCAGAGCCCAT-ATCGACGAAAGGGTTTACGAC
Enja    CCTAGGGATAACAGCGCAATCCTC-TCCCAGAGTCCCT-ATCGACGAGGGGGTTTACGAC
Same    CCTAGGGATAACAGCGCAATCCTC-TCCCAGAGTCCAT-ATCGACGAGGGGGTTTACGAC
Chch    CCTAGGGATAACAGCGCAATCCTC-TTCCAGAGTCCAT-ATCGACAAGAGGGTTTACGAC
Grgr    CCTAGGGATAACAGCGCAATCCCC-TCCCAGAGTCCCT-ATCGCCGAGGGGGTTTACGAC
Caau    CCTAGGGATAACAGCGCAATCCTC-TCCCAGAGTCCAT-ATCGACGAGGGGGTTTACGAC
Cyca    CCTAGGGATAACAGCGCAATCCTC-TCCCAGAGTCCAT-ATCGACGAGGGGGTTTACGAC
Dare    CCTAGGGATAACAGCGCAATCCTC-TCTAAGAGTCCAT-ATCGACGAGGGGGTTTACGAC
Cost    CCTAGGGATAACAGCGCAATCCTC-TCCCAGAGTCCAT-ATCGACGAGGGGGTTTACGAC
Leec    CCTAGGGATAACAGCGCAATCCCC-TCCGAGAGTTCAT-ATCGACGAGAGGGTTTACGAC
Crla    CCTAGGGATAACAGCGCAATCCTC-TCCCAGAGTCCAT-ATCGACGAGAGGGTTTACGAC
Clmc    CCTAGGGATAACAGCGCAATCCCC-TCCAAGAGTTCAT-ATCGACAAGGGGGTTTACGAC
Phin    CCTAGGGATAACAGCGCAATCCCC-TTCAAGAGTCCTT-ATCGACAAGGGGGTTTACGAC
Icpu    CCTAGGGATAACAGCGCAATCCCC-TTTCAGAGTCCAT-ATCGACAAGGGGGTTTACGAC
Psto    CCTAGGGATAACAGCGCAATCCCC-TTTAAGAGTCCAT-ATCGACAAGGGGGTTTACGAC
Cora    CCTAGGGATAACAGCGCAATCCCC-TTTTAGAGTTCCT-ATCGACAAGGGGGTTTACGAC
Eisp    CCTAGGGATAACAGCGCAATCCCC-TCCCAGAGTCCCT-ATCGACAAGGGGGTTTACGAC
Apal    CCTAGGGATAACAGCGCAATCCCC-TTCCAGAGCCCTT-ATCGACAAGGGGGTTTACGAC
Eslu    CCTAGGGATAACAGCGCAATCCCC-TCCCAGAGTCCCT-ATCGACGAGGGGGTTTACGAC
Dape    CCTAGGGATAACAGCGCAATCCTC-TCCCAGAGTCCCT-ATCGACGAGGGGGTTTACGAC
Glse    CCTAGGGATAACAGCGCAATCCCC-TCCCAGAGTCCCT-ATCGACAAGGGGGTTTACGAC
Naar    CCTAGGGATAACAGCGCAATCCCC-TCCCAGAGTCCCT-ATCGACAAGGGGGTTTACGAC
Baoc    CCTAGGGATAACAGCGCAATCCCC-TCCCAGAGTCCCT-ATCGACAAGGGGGTTTACGAC
Opso    CCTAGGGATAACAGCGCAATCCTC-TTCCAGAGCCCTT-ATCGACAAGGGGGTTTACGAC
Alte    CCCAGGGATAACAGCGCAATCCTC-TCCCAGAGTCCAT-ATCGACGAGGGGGTTTACGAC
Plap    CCCAGGGATAACAGCGCAATCCTC-TCCCAGAGTCCAT-ATCGACGAGGGGGTTTACGAC
```

Plal CCTAGGGATAACAGCGCAATCCTC-TCCCAGAGTCCCT-ATCGACGAGGGGTTTACGAC
Sami CCTAGGGATAACAGCGCAATCCTC-TCCCAGAGTCCCT-ATCGACGAGGGGTTTACGAC
Rere CCTAGGGATAACAGCGCAATCCTC-TCCCAGAGTCCCT-ATCGACGAGAGGTTTACGAC
Gama CCTAGGGATAACAGCGCAATCCCC-TCCCAGAGTCCCT-ATCGACGAGGGGTTTACGAC
Onmy CCTAGGGATAACAGCGCAATCCTC-TCCCAGAGTCCCT-ATCGACGAGGGGTTTACGAC
Sasa CCTAGGGATAACAGCGCAATCCTC-TCCCAGAGTCCCT-ATCGACGAGGGGTTTACGAC
Cola CCTAGGGATAACAGCGCAATCCTC-TCCCAGAGTCCCT-ATCGACGAGGGGTTTACGAC
Dita CCTGGGGATAACAGCGCAATCCTC-TCCCAGAGTCCCT-ATCGACGAGGGGTTTACGAC
Gogr CCTGGGGATAACAGCGCAATCCTC-TCCAAGAGCCCCT-ATCGACGAAAGGTTTACGAC
Chsl TCCAGGGATAACAGCGCAATCCCC-TCACAGAGCTCCT-ATCGACGAGGGGGTTACGAC
Atja CCCAGGGATAACAGCGCAATCTTC-TTTAAGAGCCCCT-ATCGACAAGAAGTTTACGAC
Iido CCCAGGGATAACAGCGCAATCTTC-TTTCAGAGCCCCT-ATCGACAAGAAGTTTACGAC
Auja CCTAGGGATAACAGCGCAATCCCC-TCCCAGAGCCCCT-ATCGACGAGAGGTTTACGAC
Chag CCCAGGGATAACAGCGCAATCCCC-TCCCAGAGCCCAT-ATCGACGAGGGGTTTACGAC
Hami CCTAGGGATAACAGCGCAATCCCC-TCCTAGAGCCCCC-ATCGACGAGAGGTTTACGAC
Saun CCTAGGGATAACAGCGCAATCCCC-TCCTAGAGCCCCC-ATCGACGAGAGGTTTACGAC
Nema CCTAGGGATAACAGCGCAATCCCC-TCCCAGAGCCCAT-ATCGACGAGGGGTTTACGAC
Disp CCTAGGGATAACAGCGCAATCCCC-TCCCAGAGCCCTT-ATCGACGAGGGGTTTACGAC
Myaf CCTAGGGATAACAGCGCAATCCCC-TCCAAGAGCCCTC-ATCGACGAGGGGTTTACGAC
Lagu CCCAGGGATAACAGCGCAATCCCC-TTCCAGAGCCCCT-ATCGACAAGGGGTTTACGAC
Trtr CCTAGGGATAACAGCGCAATCCTC-TTCTAGAGCCCCT-ATCGACGAGAGGTTTACGAC
Zucr CCTAGGGATAACAGCGCAATCCTC-TTTAAGAGCCCCT-ATCGACAAGAGGTTTACGAC
Pxja CCTAGGGATAACAGCGCAATCCTC-TCCCAGAGCCCAT-ATCGACGAGGGGTTTACGAC
Pxlo CCTAGGGATAACAGCGCAATCCTC-TCCCAGAGCCCAT-ATCGACGAGGGGTTTACGAC
Pctr CCTAGGGATAACAGCGCAATCCCC-TCCCAGAGCCCAT-ATCGACGAGGGGTTTACGAC
Apsa CCTAGGGATAACAGCGCAATCCCC-TCACAGAGCCCAT-ATCGACGAGGGGTTTACGAC
Cabe CCCAGGGATAACAGCGCAATCCTC-TTTTAGAGCCCTT-ATCGACGAGGGGTTTACGAC
Bzze CCTAGGGATAACAGCGCAATCCCC-TTCTAGAGTCCAT-ATCGACAAGGGGTTTACGAC
Siim CCTAGGGATAACAGCGCAATCCCC-TTTAAGAGTCCAT-ATCGACAAGGGGTTTACGAC
Ctru CCTAGGGATAACAGCGCAATCCCC-TTCTAGAGCCCAT-ATCGACAAGGGGTTTACGAC
Dpbr CCTAGGGATAACAGCGCAATCCCC-TTCTAGAGCCCAT-ATCGACAAGAGGTTTACGAC
Caki CCCAGGGATAACAGCGCAATCCCC-TCCCAGAGTCCTT-ATCGACGAGGGGTTTACGAC
Phja CCCAGGGATAACAGCGCAATCCCC-TCCCAAAGCCCTT-ATCGACGAGGGGTTTACGAC
Brsp CCTAGGGATAACAGCGCAATCCTC-TCCCAGAGCCCAT-ATCGACGAGGGGTTTACGAC
Gamo CCTAGGGATAACAGCGCAATCCCC-TCTCAGAGCCCAT-ATCGACGAGGGGTTTACGAC
Lolo CCCAGGGATAACAGCGCAATCCCC-TCTCAGAGCCCAT-ATCGACGAGGGGTTTACGAC
Batr CCCGGGGATAACAGCGCAATTTTC-TTTAAAAGACCAT-ATTGACAAGAAAGTTTACGAC
Prmy CCCAGGGATAACAGCGCAATTTTC-TTCAAGAGTCCTT-ATCGACAAGAAAGTTTACGAC
Loli CCTAGGGATAACAGCGCAATCCCC-TTTTAGAGACCCT-ATCGACAAGGAGTTTACGAC
Loam CCTAGGGATAACAGCGCAATCCCC-TTCTAGAGACCCT-ATCGACAAGGAGTTTACGAC
Chab CCCAGGGATAACAGCGCAATCCCC-TTTTAGAGACCCT-ATCGCCAAGGGGCTTACGAC
Chto CCCAGGGATAACAGCGCAATCCCc-TTTTAGAGACCCT-ATCGCCAAGGGGCTTACGAC
Majo CCCAGGGATAACAGCGCAATCCCC-TTTTAGAGACCTT-ATCGCCAAGGGGTTTACGAC
HLst CCCAGGGATAACAGCGCAATCCCC-TTCTAGAGACCCT-ATCGCCAAGGGGTTTACGAC
Clpe CCTAGGGATAACAGCGCAATCCCC-CTCAAGAGACCCT-ATCGACGGGGGGTTTACGAC
Mlmr CCTAGGGATAACAGCGCAATCCTC-TTCTAGAGACCCT-ATCGACAAGAGGTTTACGAC
Crcr CCCAGGGATAACAGCGCAATCCTC-TTTAAGAGCCCAT-ATCGACAAGAGGTTTACGAC
Muce CCCAGGGATAACAGCGCAATCCTC-TTTAAGAGCCCAT-ATCGACAAGAGGTTTACGAC
Bege CCCAGGGATAACAGCGCAATCCTC-TTTTAGAGCCCAT-ATCGACAAGAGGTTTACGAC
Mela CCCAGGGATAACAGCGCAATCCTC-TTCTAGAGACCTT-ATCAACAAGAGGTTTACGAC
Hats CCCAGGGATAACAGCGCAATCCTC-TTTTAGAGCCCAT-ATCGACAAGGGGTTTACGAC
Orla CCCAGGGATAACAGCGCAATCCTC-TTTTAGAGTCCAT-ATCGACAAGGGGTTTACGAC

Cosa	CCTAGGGATAACAGCGCAATCCCC-TCCTAGAGCCCAT-ATCGACGAGAGGGTTTACGAC
Exsp	CCTAGGGATAACAGCGCAATCCCC-TTTTAGAGCCCAT-ATCGACAAGGGGGTTTACGAC
Depa	CCTAGGGATAACAGCGCAATCCTC-TCCTAGAGACCCT-ATCGACGAGAGGGTTTACGAC
Rima	CCCAGGGATAACAGCGCAATCCCC-TTCTAGAGCCCCT-ATCGACAAGGGGGTTTACGAC
Fuol	CCTAGGGATAACAGCGCAATCCCC-TCTTAGAGTCCCT-ATCGACGAGGGGGTTTACGAC
Gmaf	CCTAGGGATAACAGCGCAATCCCC-TTTCAGAGCCCCT-ATCGACGAGGGGGTTTACGAC
Xeei	CCTAGGGATAACAGCGCAATCCTC-TTTTAGAGCCCAT-ATCGACAAGAGGGTTTACGAC
Pros	CCTAGGGATAACAGCGCAATCCCC-TCCCAGAGCCCAT-ATCGACGAGGGGGTTTACGAC
Scmi	TCTAGGGATAACAGCGCAATCCCC-TCCCAGAGCCCAT-ATCGACGAGGGGGTTTACGAC
Rolo	CCTAGGGATAACAGCGCAATCCCC-TTCCAGAGCCCAT-ATCGACAAGGGGGTTTACGAC
Cere	CCTAGGGATAACAGCGCAATCCCC-TTCCAGAGCCCAT-ATCGACAAGGGGGTTTACGAC
Daga	CCCAGGGATAACAGCGCAATCCCC-TTTGAGAGCCCAT-ATCGACAAGGGGGTTTACGAC
Anco	CCTAGGGATAACAGCGCAATCCTC-TTCCAGAGCCCAT-ATCGACAAGGGGGTTTACGAC
Dmve	CCTAGGGATAACAGCGCAATCCTC-TTCCAGAGCCCAT-ATCTACAAGAGGGTTTACGAC
Dmar	CCTAGGGATAACAGCGCAATCCTC-TTCCAGAGCCCAT-ATCTACAAGAGGGTTTACGAC
Anka	CCTAGGGATAACAGCGCAATCCTC-TTCCAGAGCCCAT-ATCGACAAGAGGGTTTACGAC
Moja	CCTAGGGATAACAGCGCAATCCTC-TTCCAGAGCCCAT-ATCGACAAGAGGGTTTACGAC
Hoja	CCTAGGGATAACAGCGCAATCCTC-TTCCAGAGCCCAT-ATCGACAAGAGGGTTTACGAC
Bede	CCTAGGGATAACAGCGCAATCCCC-TTCCAGAGCCCAT-ATCGACAAGGGGGTTTACGAC
Besp	CCTAGGGATAACAGCGCAATCCCC-TTCCAGAGCCCAT-ATCGACAAGGGGGTTTACGAC
Mysp	CCCAGGGATAACAGCGCAATCCCC-TCCCAGAGCCCAT-ATCGACGAGGGGGTTTACGAC
Osja	CCCAGGGATAACAGCGCAATCCCC-TCCCAGAGCCCAT-ATCGACGAGGGGGTTTACGAC
Sgro	CCCAGGGATAACAGCGCAATCCCC-TCCCAGAGCCCAT-ATCGACGAGGGGGTTTACGAC
Pzpa	CCCAGGGATAACAGCGCAATCCTC-TTACAGAGCCCAT-ATCGACAAGGGGGTTTACGAC
Zeja	CCTAGGGATAACAGCGCAATACCC-TTCCAGAGCCCAT-ATCGACAAGGGAGCTTACGAC
Znne	CCTAGGGATAACAGCGCAATCCCC-TTCCAGAGCCCAT-ATCGACAAGGGGGTTTACGAC
Zefa	CCTAGGGATAACAGCGCAATCCCC-TTCCAGAGCCCAT-ATCGACAAGGGGGTTTACGAC
Acni	CCTAGGGATAACAGCGCAATCCCC-TTCCAGAGCCCAT-ATCGACAAGGGGGTTTACGAC
Ncrh	CCTAGGGATAACAGCGCAATCCCC-TTCCAGAGCCCAT-ATCGACAAGGGGGTTTACGAC
Agca	CCTAGGGATAACAGCGCAATCCCC-TTTTAGAGCCCAT-ATCGACAAGGGGGTTTACGAC
Hydy	CCTAGGGATAACAGCGCAATCCTC-TTTTAGAGCCCAT-ATCGACAAGGGGGTTTACGAC
Gsac	CCTAGGGATAACAGCGCAATCCTC-TTTTAGAGCCCAT-ATCGACAAGGGGGTTTACGAC
Pevo	CCTAGGGATAACAGCGCAATCCTC-TTATAGAGTCCAT-ATCGACAAGGGGGTTTACGAC
Hiku	CCCAGGGATAACAGCGCAATTCTC-TTTGAGAGTCCCT-ATCGACAAGAGAGTTTACGAC
Inpa	CCCGGGGATAACAGCGCAATCCCC-TTTCAGAGCCCCT-ATCGACAAGGGGGTTTACGAC
Auch	CCTAGGGATAACAGCGCTATCCCC-TTACAGAGACCCT-ATCGACAAGGGGGTTTACGAC
Fico	CCTAGGGATAACAGCGCAATCCCCCTTCCAGAGTCCCT-ATCGACAAGGGGGTTTACGAC
Macs	CCTAGGGATAACAGCGCAATCCCC-TTCTAGAGCTCCT-ATCGACAAGGGGGTTTACGAC
Moal	CCCAGGGATAACAGCGCAATCCCC-TTTTAGAGCCCGT-ATCGACAAGGGGGTTTACGAC
Syma	CCCAGGGATAACAGCGCAATCCCC-TTTAAGAGCCCAT-ATCGACAAGGGGGTTTACGAC
Mafr	CCCAGGGATAACAGCGCAATCCCC-TTCAAGAGCCCAT-ATCGACAAGGGGGTTTACGAC
Dcpe	CCCAGGGATAACAGCGCAATCCCC-TTTTAGAGACCCT-ATCGACAAGGGGGTTTACGAC
Dcti	CCCAGGGATAACAGCGCAATCCCC-TTTTAGAGACCCT-ATCGACAAGGGGGTTTACGAC
Hehi	CCTAGGGATAACAGCGCAATCCCC-TTTTAGAGCCCAT-ATCGACAAGGGGGTTTACGAC
Stam	CCTAGGGATAACAGCGCAATCCCC-TTTTAGAGCCCAT-ATCGACAAGGGGGTTTACGAC
Hogi	CCTAGGGATAACAGCGCAATCCCC-TTTTAGAGCCCAT-ATCGACAAGGGGGTTTACGAC
Erzo	CCTAGGGATAACAGCGCAATCCTC-TTTTAGAGCCCAT-ATCGACAAGGGGGTTTACGAC
Hxot	CCTAGGGATAACAGCGCAATCCTC-TTTTAGAGCCCAT-ATCGACAAGGGGGTTTACGAC
Core	CCTAGGGATAACAGCGCAATCCTC-TTTTAGAGCCCAT-ATCGACAAGGGGGTTTACGAC
Apve	CCTAGGGATAACAGCGCAATCCTC-TTTTAGAGCCCAT-ATCGACAAGGGGGTTTACGAC
Latj	CCTAGGGATAACAGCGCAATCCCC-TTTTAGAGCCCAT-ATCGACAAGGGGGTTTACGAC
Laja	CCTAGGGATAACAGCGCAATCCCC-TTTTAGAGCCCAT-ATCGACAAGGGGGTTTACGAC

```
Syja    CCCAGGGATAACAGCGCAATCCCC-TTCTAGAGCCCAT-ATCGACAAGGGGGTTTACGAC
Epme    CCTAGGGATAACAGCGCAATCTCC-TCTTAGAGCCCAT-ATCAACGAGGAGGTTTACGAC
Grse    CCTAGGGATAACAGCGCAATCCCC-TTTTAGAGCCCAT-ATCGACAAGGGGGTTTACGAC
Clja    CCTAGGGATAACAGCGCAATCCTC-TCCAAGAGTCCAT-ATCGCCGAGTGGGTTTACGAC
Ogcy    CCCAGGGATAACAGCGCAATCCCC-TTCCAGAGGCCAT-ATCGACAAGGGGGTTTACGAC
Plna    TCTAGGGATAACAGCGCAATCTCC-TTCAAGAGCCCCT-ATCGACAAGGAGGTTTACGAC
Lema    CCTAGGGATAACAGCGCAATCCTC-TTTTAGAGCCCAT-ATCGACAAGAGGGTTTACGAC
Etzo    TCTAGGGATAACAGCGCAATCCCC-TTTTAGAGCCCAT-ATCGACAAGGGGGTTTACGAC
Apse    CCTAGGGATAACAGCGCAATCCCC-TTCTAGAGCCCAT-ATCGACAAGGGGGTTTACGAC
Epde    CCTAGGGATAACAGCGCAATCCCC-TTTTAGAGCCCAT-ATCGACAAGGGGGTTTACGAC
Slja    CCTAGGGATAACAGCGCAATCCTC-TTTTAGAGCTCAT-ATCGACAAGAGGGTTTACGAC
Bsja    CCTAGGGATAACAGCGCAATCCCC-TTTTAGAGCCCAT-ATCGACAAGGGGGTTTACGAC
Ecna    CCTAGGGATAACAGCGCAATCCCC-TTTTAGAGCCCTT-ATCAACAAGGGGGTTTACGAC
Cohi    CCTAGGGATAACAGCGCAATCCCC-TTTTAGAGCCCAC-ATCAACAAGGGGGTTTACGAC
Caar    CCTAGGGATAACAGCGCAATCCCC-TTTTAGAGCCCAT-ATCGACAAGGGGGTTTACGAC
Came    CCTAGGGATAACAGCGCAATCCCC-TTTTAGAGCCCAT-ATCGACAAGGGGGTTTACGAC
Mema    CCTAGGGATAACAGCGCAATCCCC-TTTTAGAGCCCAT-ATCGACAAGGGGGTTTACGAC
Lenu    CCTAGGGATAACAGCGCAATCCTC-TTCCAGAGTCCAT-ATCGACAAGAGGGTTTACGAC
Brja    CCTAGGGATAACAGCGCAATCCTC-TTTTAGAGCCCAT-ATCGACAAGAGGGTTTACGAC
Plma    CCTAGGGATAACAGCGCAATCCCC-TTTTAGAGCCCAT-ATCGACAAGGGGGTTTACGAC
Emst    CCTAGGGATAACAGCGCAATCCCC-TTTTAGAGCCCAT-ATCGACAAGGGGGTTTACGAC
Ptti    CCTAGGGATAACAGCGCAATCCCC-TTTTAGAGGCCAT-ATCGACAAGGGGGTTTACGAC
Losu    CCTAGGGATAACAGCGCAATCCCC-TTTTAGAGCCCAT-ATCGACAAGGGGGTTTACGAC
Geoy    CCTAGGGATAACAGCGCAATCCTC-TTTCAGAGCTCCT-ATCGACAAGAGGGTTTACGAC
Dipi    CCTAGGGATAACAGCGCAATCCCC-TTCTAGAGCCCAT-ATCGACAAGGGGGTTTACGAC
Pama    CCTAGGGATAACAGCGCAATCCCC-TTAAAGAGCCCTT-ATCGACAAGGGGGTTTACGAC
Leob    CCTAGGGATAACAGCGCAATCTCC-TTTTAGAGCCCAT-ATCGACAAGGAGGTTTACGAC
Neba    CCCAGGGATAACAGCGCAATCCCC-TTTAAGAGCTCTT-ATCGACAAGGGGGTTTACGAC
Pdpl    CCCAGGGATAACAGCGCAATCCCC-TTTTAGAGCCCAT-ATCAACAAGGGGGTTTACGAC
Nimi    CCTAGGGATAACAGCGCTATCCTC-TTTCAGAGCCCCC-ATCGACAAGAGGGTTTACGAC
Uptr    CCCAGGGATAACAGCGCAATCCCC-TCTAAGAGACCCT-ATCGATGAGAGGGTTTACGAC
Pesc    CCCAGGGATAACAGCGCAATCCTC-TCTTAGAGACCAT-ATCGACGAGTGGGTTTACGAC
Baar    CCTAGGGATAACAGCGCAATCCCC-TTCTAGAGCCCTT-ATCAACAAGGGGGTTTACGAC
Moar    CCTAGGGATAACAGCGCAATCCCC-TTTTAGAGCCCAT-ATCGACAAGGGGGTTTACGAC
Toja    CCTAGGGATAACAGCGCAATCCCC-TTTTAGAGCCCAT-ATCGACAAGGGGGTTTACGAC
Chau    CCTAGGGATAACAGCGCAATCCCC-TTCTAGAGCCCAT-ATCGACAAGGGGGTTTACGAC
Chse    CCCAGGGATAACAGCGCAATCCCC-TTTTAGAGCCCAT-ATCGACAAGGGGGTTTACGAC
Enar    CCTAGGGATAACAGCGCAATCCCC-TTTTAGAGCCCAT-ATCGACAAGGGGGTTTACGAC
Hpty    CCTAGGGATAACAGCGCAATCCCC-TTTTAGAGCCCAT-ATCGACAAGGGGGTTTACGAC
Nana    CCTAGGGATAACAGCGCAATCCTC-TTTTAGAGCCCAT-ATCGACAAGAGGGTTTACGAC
Mcst    CCTAGGGATAACAGCGCAATCCCC-TTTTAGAGCCCAT-ATCGACAAGGGGGTTTACGAC
Rhox    CCTAGGGATAACAGCGCAATCCCC-TTTTAGAGCCCAT-ATCGACAAGGGGGTTTACGAC
Opfa    CCTAGGGATAACAGCGCAATCCTC-TTTTAGAGCCCAT-ATCGACAAGAGGGTTTACGAC
Paar    CCTAGGGATAACAGCGCAATCCCC-TTTCAGAGCCCAT-ATCGACAAGGGGGTTTACGAC
Gozo    CCTAGGGATAACAGCGCAATCCCC-TTTTAGAGCCCAT-ATCGACAAGGGGGTTTACGAC
Ackr    CCTAGGGATAACAGCGCAATCCTC-TTTTAGAGACCAT-ATCGACAAGGGGGTTTACGAC
Elev    CCTAGGGATAACAGCGCAATCCCC-TTTAAGAGCCCAT-ATCGACAAGGGGGTTTACGAC
Trdu    CCTAGGGATAACAGCGCAATCCTC-TTTTAGAGCCCAT-ATCGACAAGAGGGTTTACGAC
Amoc    CCCAGGGATAACAGCGCAATCCTC-TTTTAGAGCCCAT-ATCGACAAGAGGGTTTACGAC
Hame    CCTAGGGATAACAGCGCAATCCCC-TTATAGAGTCCCT-ATCGACAAGGGGGTTTACGAC
Chso    CCTAGGGATAACAGCGCAATCTTC-TTCGAGAGTCCAT-ATCGACGAGAAGGTTTACGAC
Lyto    CCTAGGGATAACAGCGCAATCCTC-TTTTAGAGCCCAT-ATCGACAAGGGGGTTTACGAC
```

```
Encr    CCTAGGGATAACAGCGCAATCCTC-TTTTAGAGCCCAT-ATCGACAAGGGGGTTTACGAC
Bvar    TCTAGGGATAACAGCGCAATCCCC-TTACAGAGACCGC-ATCAACAAGGGGGTTTACGAC
Noco    TCTAGGGATAACAGCGCAATCCCC-TTTTAGAGACCAT-ATCAACAAGGGGGTTTACGAC
Chsp    CCCAGGGATAACAGCGCAATCCTA-TCTTAGAGCCCCT-ATCGAAGATGGGGTTTACGAC
Arja    CCTAGGGATAACAGCGCAATCCTC-TTTTAGAGCCCAT-ATCGACAAGGGGGTTTACGAC
Pase    CCTAGGGATAACAGCGCAATCCTC-TTTAAGAGACCCT-ATCGCCAAGAGGGTTTACGAC
Trel    CCTAGGGATAACAGCGCAATCCTC-TTCAAGAGTCCCT-ATCGACAAGCGGGTTTACGAC
Lifa    CCTAGGGATAACAGCGCAATCCCC-TCTTAGAGTTCAT-ATCGACGAGGGGGGTTACGAC
Acur    CCTAGGGATAACAGCGCAATCCCC-TTTTAGAGCCCCT-ATCGACAAGGGGGTTTACGAC
Ampe    CCTAGGGATAACAGCGCAATCCCC-TTTTAGAGCCCAT-ATCGACAAGGGGGTTTACGAC
Urja    CCCAGGGATAACAGCGCAATCCCC-TTTCAGAGCCCAT-ATCGACAAGGGGGCTTACGAC
Enet    CCCAGGGATAACAGCGCAATCCTC-TTCTAGAGCCCAT-ATCGACAAGGGGGTTTACGAC
Ptbr    CCCAGGGATAACAGCGCTATCCTT-TCCTAGAGTCCCT-ATCGACGAAAGGGTTTACGAC
Safa    CCCAGGGATAACAGCGCAATCCTC-TCCCAGAGTCCAT-ATCGACAAGAGGGTTTACGAC
Icae    CCTAGGGATAACAGCGCAATCCCC-TTTTAGAGCCCAT-ATCGACAAGGGGGTTTACGAC
Asmi    CCCAGGGATAACAGCGCAATCCCC-TTCCAGAGCCCAT-ATCGACGAGGGGGTTTACGAC
Foal    CCTAGGGATAACAGCGCAATCCTC-TTTTAGAGCCCAT-ATCGACAAGGGGGTTTACGAC
Drze    CCCAGGGATAACAGCGCAATCCTC-TTTTAGAGTCCCT-ATCGACAAGAGGGTTTACGAC
Rhas    CCTAGGGATAACAGCGCAATCCCC-TTTTAGAGCCCAT-ATCGACAAGGGGGTTTACGAC
Elac    CCTAGGGATAACAGCGCAATCCTC-TTTTAGAGCCCAT-ATCGACAAGAGGGTTTACGAC
Kugu    CCTAGGGATAACAGCGCAATCCCC-TTCTAGAGCCCAT-ATCGACAAGGGGGTTTACGAC
Plor    CCTAGGGATAACAGCGCAATCCCC-TTCTAGAGCCCAT-ATCGACAAGGGGGTTTACGAC
Sgun    CCTAGGGATAACAGCGCAATCCTC-TTTTAGAGCCCAT-ATCGACAAGAGGGTTTACGAC
Zaco    CCTAGGGATAACAGCGCAATCCCC-TTCTAGAGCCCCT-ATCGACAAGGGGGTTTACGAC
Zbfl    CCTAGGGATAACAGCGCAATCCCC-TTCTAGAGCCCAT-ATCGACAAGGGGGTTTACGAC
Spba    CCCAGGGATAACAGCGCAATCCTC-TTCTAGAGCCCAT-ATCGACAAGAGGGTTTACGAC
Game    CCTAGGGATAACAGCGCAATCCCC-TTTTAGAGCCCAT-ATCGACAAGGGGGTTTACGAC
Thth    CCTAGGGATAACAGCGCAATCCCC-TTTTAGAGCCCAT-ATCGACAAGGGGGTTTACGAC
Xigl    CCTAGGGATAACAGCGCAATCCCC-TTTTAGAGCCCAT-ATCGACAAGGGGGTTTACGAC
Hyja    CCTAGGGATAACAGCGCAATCCCC-TTTTAGAGCCCGT-ATCAACAAGGGGGTTTACGAC
Psan    CCTAGGGATAACAGCGCAATCCCC-TTTTAGAGCCCAT-ATCGACAAGGGGGTTTACGAC
Cupa    CCTAGGGATAACAGCGCAATCCCC-TTTTAGAGCCCAT-ATCGACAAGGGGGTTTACGAC
Mpch    CCTAGGGATAACAGCGCAATCCTC-TTTTAGAGTCCAT-ATCGACAAGAGGGTTTACGAC
Char    CCTAGGGATAACAGCGCAATCCCC-TTCTAGAGTCCAT-ATCGACAAGGGGGTTTACGAC
Pser    CCCAGGGATAACAGCGCAATCCCC-TTCTAGAGCCCAT-ATCGACAAGGGGGTTTACGAC
Prol    CCTAGGGATAACAGCGCAATCCCC-TTTTAGAGCCCAT-ATCGACAAGGGGGTTTACGAC
Plbi    CCTAGGGATAACAGCGCAATCCCC-TTTTAGAGCCCAT-ATCGACAAGGGGGTTTACGAC
Calu    CCCAGGGATAACAGCGCTATCCCC-TCCCAGAGCTCAT-ATCGACGAGGGGGTTTACGAC
Papa    CCCAGGGATAACAGCGCAATCCCC-TTATAGAGCCCAT-ATCAACAAGGGGGTTTACGAC
Sufr    CCTAGGGATAACAGCGCAATCCCC-TTACAGAGCCCAT-ATCGACAAGGGGGTTTACGAC
Stci    CCTAGGGATAACAGCGCAATCCTC-TTATAGAGCCCAT-ATCGACAAGGGGGTTTACGAC
Taru    CCTAGGGATAACAGCGCAATCCTC-TTTTAGAGTCCAT-ATCGACAAGAGGGTTTACGAC
Rala    CCTAGGGATAACAGCGCAATCCTC-TTTTAGAGCCCAT-ATCGACAAGAGGGTTTACGAC
         *  ************ **            **  *   **           * *******
```

```
                 51              52                 52'       53
Scca   CTCGATGTTGGATCAGGACATCCTAATGG-TGCAACC-GCTA-TTAAGGGTTCGTTTGTT
Muma   CTCGATGTTGGATCAGGACATCCTAATGG-TGCAACC-GCTA-TTAAGGGTTCGTTTGTT
Erca   CTCGATGTTGGATCAGGACATCCTAATGG-TGCAGCC-GCTA-TTAAGGGTTCGTTTGTT
Pose   CTCGATGTTGGATCAGGACATCCTAATGG-TGCAGCC-GCTA-TTAAGGGTTCGTTTGTT
Actr   CTCGATGTTGGATCAGGACATCCTAATGG-TGCAGCC-GCTA-TTAAGGGTTCGTTTGTT
Scal   CTCGATGTTGGATCAGGACATCCTAATGG-TGCAGCC-GCTA-TTAAGGGTTCGTTTGTT
Posp   CTCGATGTTGGATCAGGACATCCTAATGG-TGCAGCC-GCTA-TTAAGGGTTCGTTTGTT
Atsp   CTCGATGTTGGATCAGGACATCCTAATGG-TGCAGCC-GCTA-TTAAGGGTTCGTTTGTT
Leoc   CTCGATGTTGGATCAGGACATCCTAATGG-TGCAGCA-GCTA-TTAAGGGTTCGTTTGTT
Amca   CTCGATGTTGGATCAGGACATCCTAATGG-TGCAGCC-GCTA-TTAAGGGTTCGTTTGTT
Osbi   CTCGATGTTGGATCAGGACATCCTAATGG-CGAAAAT-TCTA-TTAAGGGTTCGTTTGTT
Pabu   CTCGATGTTGGATCAGGACATCCTAGTGG-CGAAAAT-TTTA-CTAAGGGTTCGTTTGTT
Hial   CTCGATGTTGGATCAGGACATCCTAATGG-CGCAGCA-GCTA-TTAAGGGTTCGTTTGTT
Elha   CTCGATGTTGGATCAGGACATCCTAATGG-TGCAGCC-GCTA-TTAAGGGTTCGTTTGTT
Mlcy   CTCGATGTTGGATCAGGACATCCTAATGG-TGCAGCC-GCTA-TTAAGGGTTCGTTTGTT
Algl   CTCGATGTTGGATCGGGACATCCTAATGG-TGCAGCC-GCTA-TTAAGGGTTCGTTTGTT
Ptgi   CTCGATGTTGGATCAGGACATCCTAATGG-TGCAGCC-GCTA-TTAAGGGTTCGTTTGTT
Alaf   CTCGATGTTGGATCAGGACATCCTAATGG-TGCAGCC-GCTA-TTAAGGGTTCGTTTGTT
Nock   CTCGATGTTGGATCAGGACATCCTAATGG-TGCAGCC-GCTA-TTAAGGGTTCGTTTGTT
Anja   CTCGATGTTGGATCAGGACATCCTAATGGGTGCAGCCCGCTA-TTAAGGGTTCGTTTGTT
Gyki   CTCGATGTTGGATCAGGACATCCTAATGG-TGCAGCC-GCTA-TTAAGGGTTCGTTTGTT
Syka   CTCGATGTTGGATCAGGACATCCTAATAG-TGCAGCC-GCTA-TTAAGGGTTCGTTTGTT
Opma   CTCGATGTTGGATCAGGACATCCTAATGG-TGAAGCC-GTTA-TTAAGGGTTCGTTTGTT
Comy   CTCGATGTTGGATCAGGGCATCCTAACGG-TGCAGCC-GCTG-TTAAGGGTTCGTTTGTT
Sasp   CTCGATGTTGGATCAGGACATCCTAATGG-TGCAGCC-GCTA-TTAAGGGTTCGTTTGTT
Eupe   CTCGATGTTGGATCAGGACATCCTAATGG-TGCAGCC-GCTA-TTAAGGGTTCGTTTGTT
Enja   CTCGATGTTGGATCAGGACATCCTAATGG-TGCAGCC-GCTA-TTAAGGGTTCGTTTGTT
Same   CTCGATGTTGGATCAGGACATCCTAATGG-TGCAGCC-GCTA-TTAAGGGTTCGTTTGTT
Chch   CTCGATGTTGGATCAGGACATCCTAATGG-TGCAGCC-GCTA-TTAAGGGTTCGTTTGTT
Grgr   CTCGATGTTGGATCAGGACATCCTAATGG-CGCAGCC-GCTA-TTAAGGGTTCGTTTGTT
Caau   CTCGATGTTGGATCAGGACATCCTAATGG-TGCAGCC-GCTA-TTAAGGGTTCGTTTGTT
Cyca   CTCGATGTTGGATCAGGACATCCTAATGG-TGCAGCC-GCTA-TTAAGGGTTCGTTTGTT
Dare   CTCGATGTTGGATCAGGACATCCTAATGG-TGCAGCC-GCTA-TTAAGGGTTCGTTTGTT
Cost   CTCGATGTTGGATCAGGACATCCTAATGG-TGCAGCC-GCTA-TTAAGGGTTCGTTTGTT
Leec   CTCGATGTTGGATCAGGACATCCTAATGG-TGCAGCC-GCTA-TTAAGGGTTCGTTTGTT
Crla   CTCGATGTTGGATCAGGACATCCTAATGG-TGCAGCC-GCTA-TTAAGGGTTCGTTTGTT
Clmc   CTCGATGTTGGATCAGGACATCCTAATGG-TGCAGCC-GCTA-TTAAGGGTTCGTTTGTT
Phin   CTCGATGTTGGATCAGGACATCCTAATGG-CGCAGCC-GCTA-TTAAGGGTTCGTTTGTT
Icpu   CTCGATGTTGGATCAGGACATCCTAATGG-TGCAGCC-GCTA-TTAAGGGTTCGTTTGTT
Psto   CTCGATGTTGGATCAGGACATCCTAATGG-TGCAGCC-GCTA-TTAAGGGTTCGTTTGTT
Cora   CTCGATGTTGGATCAGGACATCCTAATGG-TGCAGCC-GCTA-TTAAGGGTTCGTTTGTT
Eisp   CTCGATGTTGGATCAGGACATCCTATTGG-TGCAGCC-GCTA-TTAAGGGTTCGTTTGTT
Apal   CTCGATGTTGGATCAGGACATCCTAATGG-TGCAGCC-GCTA-TTAAGGGTTCGTTTGTT
Eslu   CTCGATGTTGGATCAGGACATCCTAATGG-TGCAGCC-GCTA-TTAAGGGTTCGTTTGTT
Dape   CTCGATGTTGGATCAGGACATCCTAATGG-TGCAGCC-GCTA-TTAAGGGTTCGTTTGTT
Glse   CTCGATGTTGGATCAGGACATCCTAATGG-TGCAACC-GCTA-TTAAGGGTTCGTTTGTT
Naar   CTCGATGTTGGATCAGGACATCCTAATGG-TGCAACC-GCTA-TTAAGGGTTCGTTTGTT
Baoc   CTCGATGTTGGATCAGGACATCCTAATGG-TGCAACC-GCTA-TTAAGGGTTCGTTTGTT
Opso   CTCGATGTTGGATCAGGACATCCTAATGG-TGCAGCC-GCTA-TTAAGGGTTCGTTTGTT
Alte   CTCGATGTTGGATCAGGACATCCTAATGG-TGCAGCC-GCTA-TTAAGGGTTCGTTTGTT
Plap   CTCGATGTTGGATCAGGACATCCTAATGG-TGCAGCC-GCTA-TTAAGGGTTCGTTTGTT
```

```
Plal    CTCGATGTTGGATCAGGACATCCTATTGG-TGCAGCC-GCTA-ATAAGGGTTCGTTTGTT
Sami    CTCGATGTTGGATCAGGACATCCTATTGG-TGCAGCC-GCTA-ATAAGGGTTCGTTTGTT
Rere    CTCGATGTTGGATCAGGACATCCTATTGG-TGCAGCC-GCTA-ATAAGGGTTCGTTTGTT
Gama    CTCGATGTTGGATCAGGACATCCTAATGG-TGCAGCC-GCTA-TTAAGGGTTCGTTTGTT
Onmy    CTCGATGTTGGATCAGGACATCCTAATGG-TGCAGCC-GCTA-TTAAGGGTTCGTTTGTT
Sasa    CTCGATGTTGGATCAGGACATCCTAATGG-TGCAGCC-GCTA-TTAAGGGTTCGTTTGTT
Cola    CTCGATGTTGGATCAGGACATCCTAATGG-TGCAGCC-GCTA-TTAAGGGTTCGTTTGTT
Dita    CTCGATGTTGGATCAGGACATCCCAGTGG-TGCAGCC-GCTA-CTAAGGGTTCGTTTGTT
Gogr    CTCGATGTTGGATCAGGACACCCCAATGG-TGCAACC-GCCA-TTAAAGGTTCGTTTGTT
Chsl    CTCGATGTTGGATCAGGGCATCCTAATGG-TGCAACC-GCTA-TTAAGGGTTCGTTTGTT
Atja    CTCGATGTTGGATCAGGACATCCTAATGG-TGCAACC-GCTA-TTAAGGGTTCGTTTGTT
Iido    CTCGATGTTGGATCAGGACATCCTAATGG-TGCAACC-GCTA-TTAAGGGTTCGTTTGTT
Auja    CTCGATGTTGGATCAGGACATCCTAATGG-TGCAGCC-GCTA-TTAAGGGTTCGTTTGTT
Chag    CTCGATGTTGGATCAGGACATCCTAATGG-TGCAGCC-GCTA-TTAAGGGTTCGTTTGTT
Hami    CTCGATGTTGGATCAGGACATCCTAATGG-TGCAGCC-GCTA-TTAAGGGTTCGTTTGTT
Saun    CTCGATGTTGGATCAGGACATCCTAATGG-TGCAGCC-GCTA-TTAAGGGTTCGTTTGTT
Nema    CTCGATGTTGGATCAGGACATCCTAATGG-TGCAGCC-GCTA-TTAAGGGTTCGTTTGTT
Disp    CTCGATGTTGGATCAGGACATCCTAATGG-TGCAGCC-GCTA-TTAAGGGTTCGTTTGTT
Myaf    CTCGATGTTGGATCAGGACATCCTGATGG-TGCAGCC-GCTA-TCAAGGGTTCGTTTGTT
Lagu    CTCGATGTTGGATCAGGATGTCCTAATGG-TGCAGCC-GCTA-TTAAGGGTTCGTTTGTT
Trtr    CTCGATGTTGGATCAGGATATCCTAATGG-TGCAGCC-GCTA-TTAAGGGTTCGTTTGTT
Zucr    CTCGATGTTGGATCAGGATATCCTAATGG-TGTAGCC-GCTA-TTAAAGGTTCGTTTGTT
Pxja    CTCGATGTTGGATCAGGACATCCTAATGG-TGCAGCC-GCTA-TTAAGGGTTCGTTTGTT
Pxlo    CTCGATGTTGGATCAGGACATCCTAATGG-TGCAGCC-GCTA-TTAAGGGTTCGTTTGTT
Pctr    CTCGATGTTGGATCAGGACATCCTAATGG-TGCAGCC-GCTA-TTAAGGGTTCGTTTGTT
Apsa    CTCGATGTTGGATCAGGACATCCTAATGG-TGCAGCC-GCTA-TTAAGGGTTCGTTTGTT
Cabe    CTCGATGTTGGATCGGGACTCCCTAATGG-TGCAGCC-GCTA-TTAAGGGTCTGTTTGTT
Bzze    CTCGATGTTGGATCAGGACATCCTAATGG-TGCAGCC-GCTA-TTAAGGGTTCGTTTGTT
Siim    CTCGATGTTGGATCAGGACATCCTAATGG-TGCAGCC-GCTA-TTAAGGGTTCGTTTGTT
Ctru    CTCGATGTTGGATCAGGACATCCTAATGG-TGCAGCC-GCTA-TTAAGGGTTCGTTTGTT
Dpbr    CTCGATGTTGGATCAGGACATCCTAACGG-TGCAGCC-GCTA-TTAAGGGTTCGTTTGTT
Caki    CTCGATGTTGGATCAGGACATCCTAATGG-TGCAGCC-GCTA-TTAAGGGTTCGTTTGTT
Phja    CTCGATGTTGGATCAGGACATCCTAATGG-TGCAGCC-GCTA-TTAAGGGTTCGTTTGTT
Brsp    CTCGATGTTGGATCAGGGCGTCCTAATGG-TGCAGCA-GCTA-TTAAGGGTTTGTTTGTT
Gamo    CTCGATGTTGGATCAGGACATCCTAATGG-TGCAGCC-GCTA-TTAAGGGTTCGTTTGTT
Lolo    CTCGATGTTGGATCAGGACATCCTAATGG-TGCAGCC-GCTA-TTAAGGGTTCGTTTGTT
Batr    CTCGATGTTGGATCAAGACACCCCAGTGG-TGCAGCA-GCTA-CTAATGGTTCGTTTGTT
Prmy    CTCGATGTTGGATCAGGACATCCCAATGG-TGCAACC-GTCA-TTAATGGTTCGTTTGTT
Loli    CTCGATGTTGGATCAGGACATCCTAATGG-TGCACCC-GCTA-TTAAGGGTTCGTTTGTT
Loam    CTCGATGTTGGATCAGGACATCCTAATGG-TGCAGCT-GCTA-TTAAGGGTTCGTTTGTT
Chab    CTCGATGTTGGATCAGGACATCCTAATGG-TGTAACC-GCTA-TTAAGGGTTCGTTTGTT
Chto    CTCGATGTTGGATCAGGACATCCTAATGG-TGTAACC-GCTA-TTAAGGGTTCGTTTGTT
Majo    CTCGATGTTGGATCAGGACATCCTAATGG-TGCAACC-GCTA-TTAAGGGTTCGTTTGTT
Hlst    CTCGATGTTGGATCAGGACATCCTAATGG-TGCAGCC-GCTA-TTAAGGGTTCGTTTGTT
Clpe    CTCGATGTTGGATCAGGACATCCTAATGG-TGCAGCC-GCTA-TTGAGGGTTCGTTTGTT
Mlmr    CTCGATGTTGGATCAGGACATCCTAATGG-TGCAGCC-GCTA-TTAAGGGTTTGTTTGTT
Crcr    CTCGATGTTGGATCAAGACATCCTAATGG-TGCAACC-GCTA-TTAAGGGTTCGTTTGTT
Muce    CTCGATGTTGGATCAAGACATCCTAATGG-TGCAACC-GCTA-TTAAGGGTTCGTTTGTT
Bege    CTCGATGTTGGATCAGGACATCCTAATGG-TGCAGCC-GCTA-TTAAGGGTTCGTTTGTT
Mela    CTCGATGTTGGATCAGGACATCCTAATGG-TGCAGCC-GCTA-TTAAGGGTTCGTTTGTT
Hats    CTCGATGTTGGATCAGGACATCCTAATGG-TGCAGCC-GCTA-TTAAGGGTTCGTTTGTT
Orla    CTCGATGTTGGATCAGGACATCCTAATGG-TGCAGCC-GCTA-TTAAGGGTTTGTTTGTT
```

Cosa CTCGATGTTGGATCAGGACATCCTAATGG-TGCAGCC-GCTA-TTAAGGGTTCGTTTGTT
Exsp CTCGATGTTGGATCAGGACATCCTAATGG-TGCAGCC-GCTA-TTAAGGGTTCGTTTGTT
Depa CTCGATGTTGGATCAGGACATCCTAATGG-CGCAGCC-GTTA-TTAAGGGTTCGTTTGTT
Rima CTCGATGTTGGATCAGGGCATCCTAATGG-CGCAACC-ACTA-TTAAGGGTTCGTTTGTT
Fuol CTCGATGTTGGATCAGGACATCCTAATGG-TGCAGCC-GCTG-TTTAGGGTTCGTTTGTT
Gmaf CTCGATGTTGGATCAGGACATCCTAATGG-TGCAGCC-GCTA-TTAAGGGCTCGTTTGTT
Xeei CTCGATGTTGGATCAGGACATCCTAATGG-TGCAGCC-GCTA-ATAAGGGTTCGTTTGTT
Pros CTCGATGTTGGATCAGGATATCCTACTGG-TGCAGCC-GCTA-TTAAGGGTTCGTTTGTT
Scmi CTCGATGTTGGATCAGGATATCCTACTGG-TGTAGCC-GCTA-TTAAGGGTTCGTTTGTT
Rolo CTCGATGTTGGATCAGGACATCCTAATGG-TGCAGCC-GCTA-TTAAGGGTTCGTTTGTT
Cere CTCGATGTTGGATCAGGACATCCTAATGG-TGCAGCC-GCTA-TTAAGGGTTCGTTTGTT
Daga CTCGATGTTGGATCAGGACATCCTAATGG-TGCAGCC-GCTA-TTAAGGGTTCGTTTGTT
Anco CTCGATGTTGGATCAGGACATCCTAATGG-TGCAGCC-GCTA-TTAAGGGTTCGTTTGTT
Dmve CTCGATGTTGGATCAGGACATCCTAACGG-TGCAGCC-GCTG-TTAAGGGTTCGTTTGTT
Dmar CTCGATGTTGGATCAGGACATCCTAACGG-TGCAACC-GCTG-TTAAGGGTTCGTTTGTT
Anka CTCGATGTTGGATCAGGACATCCTAATGG-TGCAGCC-GCTA-TTAAGGGTTCGTTTGTT
Moja CTCGATGTTGGATCAGGACATCCTAATGG-TGCAGCC-GCTA-TTAAGGGTTCGTTTGTT
Hoja CTCGATGTTGGATCAGGACATCCTAATGG-TGCAGCC-GCTA-TTAAGGGTTCGTTTGTT
Bede CTCGATGTTGGATCAGGACATCCTAATGG-TGCAGCC-GCTA-TTAAGGGTTCGTTTGTT
Besp CTCGATGTTGGATCAGGACATCCTAATGG-TGCAGCC-GCTA-TTAAGGGTTCGTTTGTT
Mysp CTCGATGTTGGATCAGGACATCCTAACGG-TGCAGCC-GCTG-TTAAGGGTTCGTTTGTT
Osja CTCGATGTTGGATCAGGACATCCTAACGG-TGCAGCC-GCTG-TTAAGGGTTCGTTTGTT
Sgro CTCGATGTTGGATCAGGACATCCTAATGG-TGCAACC-GCTA-TTAAGGGTTCGTTTGTT
Pzpa CTCGATGTTGGATCAGGACATCCTAATGG-TGCAGCC-GCTA-TTAAGGGTTCGTTTGTT
Zeja CTCGATGTTGGATCAGGACATCCTAATGG-TGCAGCC-GCTA-TTAAGGGTTCGTTTGTT
Znne CTCGATGTTGGATCAGGACATCCTAATGG-TGCAGCC-GCTA-TTAAGGGTTCGTTTGTT
Zefa CTCGATGTTGGATCAGGACATCCTAATGG-TGCAGCC-GCTA-TTAAGGGTTCGTTTGTT
Acni CTCGATGTTGGATCAGGACATCCTAATGG-TGCAGCC-GCTA-TTAAGGGTTCGTTTGTT
Ncrh CTCGATGTTGGATCAGGACATCCTAATGG-TGCAGCC-GCTA-TTAAGGGTTCGTTTGTT
Agca CTCGATGTTGGATCAGGACATCCTAATGG-TGCAGCC-GCTA-TTAAGGGTTCGTTTGTT
Hydy CTCGATGTTGGATCAGGACATCCTAATGG-TGCAGCC-GCTA-TTAAGGGTTCGTTTGTT
Gsac CTCGATGTTGGATCAGGACATCCTAATGG-TGCAGCC-GCTA-TTAAGGGTTCGTTTGTT
Pevo CTCGATGTTGGATCAGGGTATCCTAATGG-TGCAGCC-GCTA-TTAAGGGTTCGTTTGTT
Hiku CTCGATGTTGGATCAGGACATCCTAATGG-TGTAGCC-GCTA-TTAAGGGTTCGTTTGTT
Inpa CTCGATGTTGGATCAGGACATCCTAATGG-TGCAGCC-GCTA-TTAAGGGTTCGTTTGTT
Auch CTCGATGTTGGATCGGGACATCCTAATGG-CGCAGCC-GTTA-TTAAGGGTTCGTTTGTT
Fico CTCGATGTTGGATCAGGACATCCTAATGG-TGCAGCC-GCTA-TTAAGGGTTCGTTTGTT
Macs CTCGATGTTGGATCAGGACATCCTAATGG-TGCAGCC-GCTA-TTAAGGGTTTGTTTGTT
Moal CTCGATGTTGGATCAGGACACCCCAATGG-TGCAGCC-GCTA-TTAAAGGTTCGTTTGTT
Syma CTCGATGTTGGATCAGGACACCCCAATGG-TGCATCC-GTTA-TTAAAGGTTCGTTTGTT
Mafr CTCGATGTTGGATCAGGACATCCTACTGG-TGCAGCC-GCTA-TTAAGGGTTCGTTTGTT
Dcpe CTCGATGTTGGATCAGGACATCCTATTGG-CGCAGCC-GTCA-TTAAGGGTTCGTTTGTT
Dcti CTCGATGTTGGATCAGGACATCCTATTGG-CGCAGCC-GTCA-TTAAGGGTTCGTTTGTT
Hehi CTCGATGTTGGATCAGGACATCCTAATGG-TGCAGCC-GCTA-TTAAGGGTCCGTTTGTT
Stam CTCGATGTTGGATCAGGACATCCTAATGG-TGCAGCC-GCTA-TTAAGGGTTCGTTTGTT
Hogi CTCGATGTTGGATCAGGACATCCTAATGG-TGCAGCC-GCTA-TTAAGGGTTCGTTTGTT
Erzo CTCGATGTTGGATCAGGACATCCTAATGG-TGCAGCC-GCTA-TTAAGGGTTCGTTTGTT
Hxot CTCGATGTTGGATCAGGACATCCTAATGG-TGCAGCC-GCTA-TTAAGGGTTCGTTTGTT
Core CTCGATGTTGGATCAGGACATCCTAATGG-TGCAGCC-GCTA-TTAAGGGTTCGTTTGTT
Apve CTCGATGTTGGATCAGGACATCCTAATGG-TGCAGCC-GCTA-TTAAGGGTTCGTTTGTT
Latj CTCGATGTTGGATCAGGACATCCTAATGG-TGCAGCC-GCTA-TTAAGGGTTCGTTTGTT
Laja CTCGATGTTGGATCAGGACATCCTAATGG-TGCAGCC-GCTA-TTAAGGGTTCGTTTGTT

Syja	CTCGATGTTGGATCAGGACATCCTAATGG-TGCAGCC-GCTA-TTAAGGGTTCGTTTGTT
Epme	CTCGATGTTGGATCAGGACATCCTAATGG-TGCAGCC-GCTA-TTAAGGGTTCGTTTGTT
Grse	CTCGATGTTGGATCAGGACATCCTAATGG-TGCAGCC-GCTA-TTAAGGGCTCGTTTGTT
Clja	CTCGATGTTGGATCAGGACATCCTAATGG-TGCAGCC-GCTA-TTAAGGGTTCGTTTGTT
Ogcy	CTCGATGTTGGATCAGGACATCCTAATGG-TGCAGCC-GCTA-TTAAGGGTTCGTTTGTT
Plna	CTCGATGTTGGATCAGGACATCCTAATGG-CGCAGCC-GTTA-TTAAGGGTTCGTTTGTT
Lema	CTCGATGTTGGATCAGGACATCCTAATGG-TGCAGCC-GCTA-TTAAGGGTTCGTTTGTT
Etzo	CTCGATGTTGGATCAGGACATCCTAATGG-TGCAGCC-GCTA-TTAAGGGTTCGTTTGTT
Apse	CTCGATGTTGGATCAGGACATCCTATTGG-CGCAGCC-GCTA-TTAAGGGTTCGTTTGTT
Epde	CTCGATGTTGGATCAGGACATCCTAATGG-TGCAGCC-GCTA-TTAAGGGTTCGTTTGTT
Slja	CTCGATGTTGGATCAGGACATCCTAATGG-TGCAACC-ACTA-TTAAGGGTTCGTTTGTT
Bsja	CTCGATGTTGGATCAGGACATCCTAATGG-TGCAGCC-GCTA-TTAAGGGTTCGTTTGTT
Ecna	CTCGATGTTGGATCAGGACATCCTAATGG-TGCAACC-GCTA-TTAAGGGTTCGTTTGTT
Cohi	CTCGATGTTGGATCAGGACATCCTATTGG-TGCAGCC-GCTA-TTAAGGGTTCGTTTGTT
Caar	CTCGATGTTGGATCAGGACATCCTAATGG-TGCAGCC-GCTA-TTAAGGGTTCGTTTGTT
Came	CTCGATGTTGGATCAGGACATCCTAATGG-TGCAGCC-GCTA-TTAAGGGTTCGTTTGTT
Mema	CTCGATGTTGGATCAGGACATCCTAATGG-CGCAGCC-ACTA-TTAAGGGTTCGTTTGTT
Lenu	CTCGATGTTGGATCAGGGCATCCTAATGG-TGCAGCC-GCTA-TTAAGGGTTCGTTTGTT
Brja	CTCGATGTTGGATCAGGACATCCTAATGG-TGCAACC-GCTA-TTGAGGGTTCGTTTGTT
Plma	CTCGATGTTGGATCAGGACATCCTAATGG-TGCAGCC-GCTA-TTAAGGGTTCGTTTGTT
Emst	CTCGATGTTGGATCAGGACATCCTAATGG-TGCAGCC-GCTA-TTAAGGGTTCGTTTGTT
Ptti	CTCGATGTTGGATCAGGACATCCTAATGG-TGCAGCC-GCTA-TTAAGGGTTCGTTTGTT
Losu	CTCGATGTTGGATCAGGACATCCTAATGG-TGCAGCC-GCTA-TTAAGGGTTCGTTTGTT
Geoy	CTCGATGTTGGATCAGGACATCCTAATGG-TGCAGCC-GCTA-TTAAGGGTTCGTTTGTT
Dipi	CTCGATGTTGGATCAGGACATCCTAATGG-TGCAGCC-GCTA-TTAAGGGTTCGTTTGTT
Pama	CTCGATGTTGGATCAGGACATCCTAATGG-TGCAGCC-GCTA-TTAAGGGTTCGTTTGTT
Leob	CTCGATGTTGGATCAGGACATCCTAATGG-TGCAGCC-GCTA-TTAAGGGTTCGTTTGTT
Neba	CTCGATGTTGGATCAGGACATCCCATTGG-CGTAGAA-GCTA-TTAAGGGTTCGTTTGTT
Pdpl	CTCGATGTTGGATCAGGACATCCTAATGG-TGCAGCC-GCTA-TTAAGGGTTCGTTTGTT
Nimi	CTCGATGTTGGATCAGGACATCCTAATGG-TGCAACC-GCTA-TTAAGGGTTCGTTTGTT
Uptr	CTCGATGTTGGATCAGGACATCCTGATGG-TGCAGCC-GCTA-TTAAGGGTTCGTTTGTT
Pesc	CTCGATGTTGGATCAGGACATCCTAATGG-CGCAGCC-GCTA-TTAAGGGTTCGTTTGTT
Baar	CTCGATGTTGGATCAGGACATCCTAATGG-TGCAGCC-GCTAATTAAGGGTTCGTTTGTT
Moar	CTCGATGTTGGATCAGGACATCCTAATGG-TGCAGCC-GCTA-TTAAGGGTTCGTTTGTT
Toja	CTCGATGTTGGATCAGGACATCCTAATGG-TGCAGCC-GCTA-TTAAGGGTTCGTTTGTT
Chau	CTCGATGTTGGATCAGGACATCCTAATGG-TGCAGCC-GCTA-TTAAGGGTTCGTTTGTT
Chse	CTCGATGTTGGATCAGGACATCCTAATGG-TGCAGCC-GCTA-TTAAGGGTTCGTTTGTT
Enar	CTCGATGTTGGATCAGGACATCCTAATGG-TGCAGCC-GCTA-TTAAGGGTTCGTTTGTT
Hpty	CTCGATGTTGGATCAGGACATCCTAATGG-TGCAGCC-GCTA-TTGAGGGTTCGTTTGTT
Nana	CTCGATGTTGGATCAGGACATCCTAATGG-TGCAGCC-GCTA-TTAAGGGTTCGTTTGTT
Mcst	CTCGATGTTGGATCAGGACATCCTAATGG-AGCAGCC-ATTA-TTAAGGGTTCGTTTGTT
Rhox	CTCGATGTTGGATCAGGACATCCTAATGG-TGCAGCC-GCTA-TTAAGGGTTCGTTTGTT
Opfa	CTCGATGTTGGATCAGGACATCCTAATGG-TGCAGCC-GCTA-TTAAGGGTTCGTTTGTT
Paar	CTCGATGTTGGATCAGGACATCCTAATGG-TGCAGCC-GCTA-TTAAGGGTTCGTTTGTT
Gozo	CTCGATGTTGGATCAGGACATCCTAATGG-TGCAGCC-GCTA-TTAAGGGTTCGTTTGTT
Ackr	CTCGATGTTGGATCAGGACATCCTAATGG-TGCAGCC-GCTA-TTAAGGGTTCGTTTGTT
Elev	CTCGATGTTGGATCAGGACATCCTAATGG-TGCAACC-GCTA-TTAAGGGTTCGTTTGTT
Trdu	CTCGATGTTGGATCAGGACATCCTAATGG-TGCAGCC-GCTA-TTAAGGGTTCGTTTGTT
Amoc	CTCGATGTTGGATCAGGACATCCTAATGG-TGCAACC-GCTA-TTAAGGGTTCGTTTGTT
Hame	CTCGATGTTGGATCAGGACATCCTAATGG-TGCAGCC-GCTA-TTAAGGGTTCGTTTGTT
Chso	CTCGATGTTGGATCAGGACATCCTAATGG-TGCAGCC-GCTA-TTAAGGGTTCGTTTGTT
Lyto	CTCGATGTTGGATCAGGACATCCTAATGG-TGCAGCC-GCTA-TTAAGGGTTCGTTTGTT

```
Encr    CTCGATGTTGGATCAGGACATCCTAATGG-TGCAGCC-GCTA-TTAAGGGTTCGTTTGTT
Bvar    CTCGATGTTGGATCAGGACATCCCAGTAG-TGCAGCC-GCTA-TTAAGGGTTCGTTTGTT
Noco    CTCGATGTTGGATCAGGACATCCTATTGG-TGCAGCA-GCTA-TTAAGGGTTCGTTTGTT
Chsp    CTCGATGTTGGATCAGGACATCCTAATGG-TGCAACC-GCTA-TTAAGGGTTCGTTTGTT
Arja    CTCGATGTTGGATCAGGACATCCTAATGG-TGCAGCC-GCTA-TTAAGGGTTCGTTTGTT
Pase    CTCGATGTTGGATCAGGACATCCTAATGG-TGCAGCC-GCTA-TTAAGGGTTCGTTTGTT
Trel    CTCGATGTTGGATCAGGACATCCTACTGG-TGCAGCC-GCTA-TTAAGGGTTCGTTTGTT
Lifa    CTCGATGTTGGATCAGGACATCCTAATGG-TGCAGCC-ACTA-TTAAGGGTTCGTTTGTT
Acur    CTCGATGTTGGATCAGGACATCCTAATGG-TGCAGCC-GCTA-TTAAGGGTTCGTTTGTT
Ampe    CTCGATGTTGGATCAGGACATCCTAATGG-TGCAGCC-GCTA-TTAAGGGTTCGTTTGTT
Urja    CTCGATGTTGGATCAGGACATCCTAATGG-TGTAGCC-GCTA-TTAAGGGTTCGTTTGTT
Enet    CTCGATGTTGGATCAGGACATCCTAATGG-TGCAACC-GCTA-TTAAGGGTTCGTTTGTT
Ptbr    CTCGATGTTGGATCAGGACATCCTAATGG-TGCAACC-GCTA-TTAAGGGTTTGTTTGTT
Safa    CTCGATGTTGGATCAGGACATCCTAATGG-TGCAGCC-GCTA-TTAAGGGTTCGTTTGTT
Icae    CTCGATGTTGGATCAGGACATCCTAATGG-TGCAACC-GCTA-TTAAGGGTTCGTTTGTT
Asmi    CTCGATGTTGGATCAGGACATCCTAATGG-TGCAGCC-GCTA-TTAAGGGTTCGTTTGTT
Foal    CTCGATGTTGGATCAGGACATCCTAATGG-TGCAGCC-GCTG-TTAAGGGTTCGTTTGTT
Drze    CTCGATGTTGGATCAGGACATCCTAATGG-TGCAGCC-GCTA-TTAAGGGTTCGTTTGTT
Rhas    CTCGATGTTGGATCAGGACATCCTAATGG-TGCAGCC-GCTA-TTAAGGGTTCGTTTGTT
Elac    CTCGATGTTGGATCAGGACATCCTAATGG-TGCAGCC-GCTA-TTAAGGGTTCGTTTGTT
Kugu    CTCGATGTTGGATCAGGACATCCTAATGG-TGCAGCC-GCTA-TTAAGGGTTCGTTTGTT
Plor    CTCGATGTTGGATCAGGACATCCTAATGG-TGCAGCC-GCTA-TTAAGGGTTCGTTTGTT
Sgun    CTCGATGTTGGATCAGGACATCCTAATGG-TGCAGCC-GCTA-TTAAGGGTTCGTTTGTT
Zaco    CTCGATGTTGGATCAGGACATCCTAATGA-TGCAGCC-GTTA-TTAAGGGTTCGTTTGTT
Zbfl    CTCGATGTTGGATCAGGACATCCTAATGG-TGCAGCC-GCTA-TTAAGGGTTCGTTTGTT
Spba    CTCGATGTTGGATCAGGACATCCTAATGG-TGCAACC-GCTA-TTAAGGGTTCGTTTGTT
Game    CTCGATGTTGGATCAGGACATCCTAATGG-TGCAGCC-GCTA-TTAAGGGTTCGTTTGTT
Thth    CTCGATGTTGGATCAGGACATCCTAATGG-TGCAGCC-GCTG-TTAAGGGTTCGTTTGTT
Xigl    CTCGATGTTGGATCAGGACATCCTAATGG-TGCAGCC-GCTA-TTAAGGGTTCGTTTGTT
Hyja    CTCGATGTTGGATCAGGACATCCTAATGG-TGCAGCC-GCTA-TTGAGGGTTCGTTTGTT
Psan    CTCGATGTTGGATCAGGACATCCTAATGG-TGCAGCC-GCTA-TTAAGGGTTCGTTTGTT
Cupa    CTCGATGTTGGATCAGGACATCCTAATGG-TGCAGCC-GCTA-TTAAGGGTTCGTTTGTT
Mpch    CTCGATGTTGGATCAGGACATCCTAATGG-TGCAGCC-GCTA-TTAAGGGTTCGTTTGTT
Char    CTCGATGTTGGATCAGGACATCCTAATGG-TGCAGCC-GCTA-TTAAGGGTTCGTTTGTT
Pser    CTCGATGTTGGATCAGGACATCCTAATGG-CGCAGCC-GCTA-TTGAGGGTTCGTTTGTT
Prol    CTCGATGTTGGATCAGGACATCCTAATGG-TGCAGCC-GCTA-TTAAGGGTTCGTTTGTT
Plbi    CTCGATGTTGGATCAGGACATCCTAATGG-TGCAGCC-GCTA-TTAAGGGTTCGTTTGTT
Calu    CTCGATGTTGGATCAGGACATCCTAATGG-TGCAACC-GCTA-TTAAGGGTTCGTTTGTT
Papa    CTCGATGTTGGATCAGGACACCCCAATGG-TGCAACC-GCTA-TTAAAGGTTCGTTTGTT
Sufr    CTCGATGTTGGATCAGGATATCCCAATGG-TGCAGCC-GCTA-TTAAAGGTTCGTTTGTT
Stci    CTCGATGTTGGATCAGGATATCCTAATGG-TGCAGCC-GCTA-TTAAGGGTTCGTTTGTT
Taru    CTCGATGTTGGATCAGGACATCCTAATGG-TGCAGCC-GCTA-TTAAAGGTTCGTTTGTT
Rala    CTCGATGTTGGATCAGGACATCCTAATGG-TGCAGCC-GCTA-TTAAGGGTTCGTTTGTT
        **************  *    **     *  * *            * **   *******
```

```
                 53'             51'        C'   54        54'
Scca    CAACGATTAATAGTCCTACGTGATCTGAGTTCAGACCGGAGAAATCCAGGTCA-GTT
Muma    CAACGATTAATAGTCCTACGTGATCTGAGTTCAGACCGGAGAAATCCAGGTCA-GTT
Erca    CAACGATTAA-AGTCCTACGTGATCTGAGTTCAGACCGGAGTAATCCAGGTCA-GTT
Pose    CAACGATTAA-AGTCCTACGTGATCTGAGTTCAGACCGGAGTAATCCAGGTCA-GTT
Actr    CAACGATTAA-AGTCCTACGTGATCTGAGTTCAGACCGGAGTAATCCAGGTCA-GTT
Scal    CAACGATTAA-AGTCCTACGTGATCTGAGTTCAGACCGGAGTAATCCAGGTCA-GTT
Posp    CAACGATTAA-AGTCCTACGTGATCTGAGTTCAGACCGGAGTAATCCAGGTCA-GTT
Atsp    CAACGATTAA-AGTCCTACGTGATCTGAGTTCAGACCGGAGTAATCCAGGTCA-GTT
Leoc    CAACGATTAA-AGTCCTACGTGATCTGAGTTCAGACCGGAGTAATCCAGGTCA-GTT
Amca    CAACGATTAA-AGTCCTACGTGATCTGAGTTCAGACCGGAGTAATCCAGGTCA-GTT
Osbi    CAACGATTAA-AGTCCTACGTGATCTGAGTTCAGACCGGAGCAATCCAGGTCG-GTT
Pabu    CAACGATTAA-AGTCCTACGTGATCTGAGTTCAGACCGGAGAAATCCAGGTCA-GTT
Hial    CAACGATTAA-AGTCCTACGTGATCTGAGTTCAGACCGGAGTAATCCAGGTCA-GTT
Elha    CAACGATTAA-AGTCCTACGTGATCTGAGTTCAGACCGGAGCAATCCAGGTCG-GTT
Mlcy    CAACGATTAA-AGTCCTACGTGATCTGAGTTCAGACCGGAGCAATCCAGGTCA-GTT
Algl    CAACGATTAA-AGTCCTACGTGATCTGAGTTCAGACCGGAGTAATCCAGGTCG-GTT
Ptgi    CAACGATTAA-AGTCCTACGTGATCTGAGTTCAGACCGGAGAAATCCAGGTCG-GTT
Alaf    CAACGATTAA-AGTCCTACGTGATCTGAGTTCAGACCGGAGAAATCCAGGTCG-GTT
Nock    CAACGATTAA-AGTCCTACGTGATCTGAGTTCAGACCGGAGAAATCCAGGTCG-GTT
Anja    CAACGATTAATAGTCCTACGTGATCTGAGTTCAGACCGGAGTAATCCAGGTCG-GTT
Gyki    CAACGATTAATAGTCCTACGTGATCTGAGTTCAGACCGGAGTAATCCAGGTCG-GTT
Syka    CAACGATTAATAGTCCTACGTGATCTGAGTTCAGACCGGAGTAATCCAGGTCG-GTT
Opma    CAACGATTAACAGTCCTACGTGATCTGAGTTCAGACCGGAGCAATCCAGGTCG-GTT
Comy    CAACGATTAATAGCCCTACGTGATCTGAGTTCAGACCGGAGTAATCCAGGTCG-GTT
Sasp    CAACGATTAATAGTCCTACGTGATCTGAGTTCAGACCGGAGTAATCCAGGTCG-GTT
Eupe    CAACGATTAATAGTCCTACGTGATCTGAGTTCAGACCGGAGTAATCCAGGTCG-GTT
Enja    CAACGATTAA-AGTCCTACGTGATCTGAGTTCAGACCGGAGTAATCCAGGTCG-GTT
Same    CAACGATTAA-AGTCCTACGTGATCTGAGTTCAGACCGGAGTAATCCAGGTCA-GTT
Chch    CAACGATTAA-AGTCCTACGTGATCTGAGTTCAGACCGGAGTAATCCAGGTCA-GTT
Grgr    CAACGATTAA-AGTCCTACGTGATCTGAGTTCAGACCGGAGAAATCCAGGTCA-GTT
Caau    CAACGATTAA-AGTCCTACGTGATCTGAGTTCAGACCGGAGCAATCCAGGTCA-GTT
Cyca    CAACGATTAA-AGTCCTACGTGATCTGAGTTCAGACCGGAGCAATCCAGGTCA-GTT
Dare    CAACGATTAATAGTCCTACGTGATCTGAGTTCAGACCGGAGTAATCCAGGTCA-GTT
Cost    CAACGATTAA-AGTCCTACGTGATCTGAGTTCAGACCGGAGTAATCCAGGTCA-GTT
Leec    CAACGATTAA-AGTCCTACGTGATCTGAGTTCAGACCGGAGCAATCCAGGTCA-GTT
Crla    CAACGATTAA-AGTCCTACGTGATCTGAGTTCAGTCTGGTGTAATCCAGGTCA-GTT
Clmc    CAACGATTAA-AGTCCTACGTGATCTGAGTTCAGACCGGAGCAATCCAGGTCA-GTT
Phin    CAACGATTAA-AGTCCTACGTGATCTGAGTTCAGACCGGAGCAATCCAGGTCA-GTT
Icpu    CAACGATTAA-AGTCCTACGTGATCTGAGTTCAGACCGGAGCAATCCAGGTCA-GTT
Psto    CAACGATTAA-AGTCCTACGTGATCTGAGTTCAGACCGGAGCAATCCAGGTCA-GTT
Cora    CAACGATTAA-AGTCCTACGTGATCTGAGTTCAGACCGGAGCAATCCAGGTCA-GTT
Eisp    CAACGATTAA-AGTCCTACGTGATCTGAGTTCAGACCGGAGAAATCCAGGTCA-GTT
Apal    CAACGATTAA-AGTCCTACGTGATCTGAGTTCAGACCGGAGAAATCCAGGTCG-GTT
Eslu    CAACGATTAA-AGTCCTACGTGATCTGAGTTCAGACCGGAGTAATCCAGGTCA-GTT
Dape    CAACGATTAA-AGTCCTACGTGATCTGAGTTCAGACCGGAGTAATCCAGGTCA-GTT
Glse    CAACGATTAA-AGTCCTACGTGATCTGAGTTCAGACCGGAGTAATCCAGGTCA-GTT
Naar    CAACGATTAA-AGTCCTACGTGATCTGAGTTCAGACCGGAGTAATCCAGGTCA-GTT
Baoc    CAACGATTAA-AGTCCTACGTGATCTGAGTTCAGACCGGAGTAATCCAGGTCA-GTT
Opso    CAACGATTAA-AGTCCTACGTGATCTGAGTTCAGACCGGAGTAATCCAGGTCA-GTT
Alte    CAACGATTAA-AGTCCTACGTGATCTGAGTTCAGACCGGAGCAATCCAGGTCA-GTT
Plap    CAACGATTAA-AGTCCTACGTGATCTGAGTTCAGACCGGAGCAATCCAGGTCA-GTT
```

Plal	CAACGATTAA-AGTCCTACGTGATCTGAGTTCAGACCGGAGCAATCCAGGTCA-GTT
Sami	CAACGATTAA-AGTCCTACGTGATCTGAGTTCAGACCGGAGCAATCCAGGTCA-GTT
Rere	CAACGATTAA-AGTCCTACGTGATCTGAGTTCAGACCGGAGCAATCCAGGTCA-GTT
Gama	CAACGATTAA-AGTCCTACGTGATCTGAGTTCAGACCGGAGTAATCCAGGTCA-GTT
Onmy	CAACGATTAA-AGTCCTACGTGATCTGAGTTCAGACCGGAGTAATCCAGGTCA-GTT
Sasa	CAACGATTAA-AGTCCTACGTGATCTGAGTTCAGACCGGAGTAATCCAGGTCA-GTT
Cola	CAACGATTAA-AGTCCTACGTGATCTGAGTTCAGACCGGAGTAATCCAGGTCA-GTT
Dita	CAACGATTAA-AGTCCTACGTGATCTGAGTTCAGACCGGAGCAATCCAGGTCA-GTT
Gogr	CAACGATTAA-AGTCCTACGTGATCTGAGTTCAGACCGGAGCAATCCAGGTCA-GTT
Chsl	CAACGATTAA-AGCCCTACGTGATCTGAGTTCAGGCCGGAGTAATCCAGGCCA-GAT
Atja	CAACGATTAACAGTCCTACGTGATCTGAGTTCAGACCGGAGCAATCCAGGTCA-GTT
Iido	CAACGATTAATAGTCCTACGTGATCTGAGTTCAGACCGGAGTAATCCAGGTCA-GTT
Auja	CAACGATTAA-AGTCCTACGTGATCTGAGTTCAGACCGGAGCAATCCAGGTCAAGTT
Chag	CAACGATTAATAGTCCTACGCGATCTGAGTTCAGACCGGAGTAATCCAGGTCA-GTT
Hami	CAACGATTAA-AGTCCTACGTGATCTGAGTTCAGACCGGAGTAATCCAGGTCA-GTT
Saun	CAACGATTAA-AGTCCTACGTGATCTGAGTTCAGACCGGAGTAATCCAGGTCA-GTT
Nema	CAACGATTAA-AGTCCTACGTGATCTGAGTTCAGACCGGAGTAATCCAGGTCA-GTT
Disp	CAACGATTAA-AGTCCTACGCGATCTGAGTTCAGACCGGAGTAATCCAGGTCA-GTT
Myaf	CAACGATTAA-AGTCCTACGCGATCTGAGTTCAGACCGGAGTAATCCAGGTCA-GAT
Lagu	CAACGATTAA-AATCCTACGTGATCTGAGTTCAGACCGGAGTAATCCAGGTCA-GTT
Trtr	CAACGATTAA-AATCCTACGTGATCTGAGTTCAGACCGGAGAGATCCAGGTCA-GTT
Zucr	CAACGATTAA-AATCCTACGTGATCTGAGTTCAGACCGGAGAGATCCAGGTCA-GTT
Pxja	CAACGATTAA-AGTCCTACGTGATCTGAGTTCAGACCGGAGTAATCCAGGTCA-GTT
Pxlo	CAACGATTAA-AGTCCTACGTGATCTGAGTTCAGACCGGAGTAATCCAGGTCA-GTT
Pctr	CAACGATTAA-AGTCCTACGCGATCTGAGTTCAGACCGGAGCAATCCAGGTCA-GTT
Apsa	CAACGATTAA-AGTCCTACGTGATCTGAGTTCAGACCGGAGTAATCCAGGTCA-GTT
Cabe	CAACGGTTAA-AGTCCTACGTGATCTGAGTTCAGACCGGAGTAATCCAGGTCG-GTT
Bzze	CAACGATTAA-AGTCCTACGTGATCTGAGTTCAGACCGGAGTAATCCAGGTCA-GTT
Siim	CAACGATTAA-AGTCCTACGTGATCTGAGTTCAGACCGGAGTAATCCAGGTCG-GTT
Ctru	CAACGATTAA-AGTCCTACGTGATCTGAGTTCAGACCGGAGTAATCCAGGTCA-GTT
Dpbr	CAACGATTAA-AGTCCTACGTGATCTGAGTTCAGACCGGAGTAATCCAGGTCA-GTT
Caki	CAACGATTAA-AGTCCTACGTGATCTGAGTTCAGACCGGAGTAATCCAGGTCA-GTT
Phja	CAACGATTAA-AGTCCTACGTGATCTGAGTTCAGACCGGAGTAATCCAGGTCA-GTT
Brsp	CAACAATTAA-AGCCCTACGTGATCTGAGTTCAGACCGGAGAAATCCAGGTCG-GTT
Gamo	CAACGATTAA-AGTCCTACGTGATCTGAGTTCAGACCGGAGTAATCCAGGTCA-GTT
Lolo	CAACGATTAA-AGTCCTACGTGATCTGAGTTCAGACCGGAGTAATCCAGGTCA-GTT
Batr	CAACGATTAATAGTCTTACGTGATCTGAGTTCAGACCGGAGCAATCCAGGTCA-GTC
Prmy	CAACGATTAA-AGTCCTACGTGATCTGAGTTCAGACCGAAGCAATTCAGGTCA-GTT
Loli	CAACGATTAA-AGTCCTACGTGATCTGAGTTCAGACCGGAGTAATCCAGGTCG-GTT
Loam	CAACGATTAA-AGTCCTACGTGATCTGAGTTCAGACCGGAGTAATCCAGGTCA-GTT
Chab	CAACGATTAA-AGTCCTACGTGATCTGAGTTCAGACCGGAGCAATCCAGGTCA-GTT
Chto	CAACGATTAA-AGTCCTACGTGATCTGAGTTCAGACCGGAGCAATCCAGGTCA-GTT
Majo	CAACGATTAA-AGTCCTACGTGATCTGAGTTCAGACCGGAGTAATCCAGGTCA-GTT
Hlst	CAACGATTAA-AGTCCTACGTGATCTGAGTTCAGACCGGAGTAATCCAGGTCA-GTT
Clpe	CAACGATTAA-AGTCCTACGTGATCTGAGTTCAGACCGGAGTAATCCAGGTCG-GTT
Mlmr	CAACAATTAATAGTCCTACGTGATCTGAGTTCAGACCGGAGTAATCCAGGTCA-GTT
Crcr	CAACGATTAA-AGTCTTACGTGATCTGAGTTCAGACCGGAGTAATCCAGGTCA-GTT
Muce	CAACGATTAA-AGTCTTACGTGATCTGAGTTCAGACCGGAGTAATCCAGGTCA-GTT
Bege	CAACGATTAACAGTCCTACGTGATCTGAGTTCAGACCGGAGTAATCCAGGTCA-GTT
Mela	CAACGATTAA-AGTCCTACGTGATCTGAGTTCAGACCGGAGTAATCCAGGTCA-GTT
Hats	CAACGATTAA-AGTCCTACGTGATCTGAGTTCAGACCGGAGTAATCCAGGTCG-GTT
Orla	CAACAATTAA-AGTCCTACGTGATCTGAGTTCAGACCGGAGTAATCCAGGTCA-GTT

Cosa	CAACGATTAA-AGTCCTACGTGATCTGAGTTCAGACCGGAGTAATCCAGGTCA-GTT
Exsp	CAACGATTAA-AGTCCTACGTGATCTGAGTTCAGACCGGAGTAATCCAGGTCA-GTT
Depa	CAACGATTAA-AGTCCTACGTGATCTGAGTTCAGACCGGAGAAATCCAGGTCA-GTT
Rima	CAACGATTAA-AGCCCTACGTGATCTGAGTTCAGACCGGAGCAATCCAGGTCA-GTT
Fuol	CAACGATTAA-AGTCCTACGTGATCTGAGTTCAGACCGGAGTAATCCAGGTCA-GTT
Gmaf	CAACGATTAA-AGTCCTACGTGATCTGAGTTCAGACCGGAGTAATCCAGGTCA-GTT
Xeei	CAACGATTAA-AGTCCTACGTGATCTGAGTTCAGACCGGAGTAATCCAGGTCA-GTT
Pros	CAACGATTAA-AATCCTACGTGATCTGAGTTCAGACCGGAGTAATCCAGGTCA-GTT
Scmi	CAACGATTAA-AATCCTACGTGATCTGAGTTCAGACCGGAGTAATCCAGGTCA-GTT
Rolo	CAACGATTAA-AGTCCTACGTGATCTGAGTTCAGACCGGAGTAATCCAGGTCA-GTT
Cere	CAACGATTAA-AGTCCTACGTGATCTGAGTTCAGACCGGAGTAATCCAGGTCA-GTT
Daga	CAACGATTAA-AGTCCTACGTGATCTGAGTTCAGACCGGAGTAATCCAGGTCA-GTT
Anco	CAACGATTAA-AGTCCTACGTGATCTGAGTTCAGACCGGAGTAATCCAGGTCA-GTT
Dmve	CAACGATTAA-AGTCCTACGTGATCTGAGTTCAGACCGGAGTAATCCAGGTCA-GTT
Dmar	CAACGATTAA-AGTCCTACGTGATCTGAGTTCAGACCGGAGTAATCCAGGTCA-GTT
Anka	CAACGATTAA-AGTCCTACGTGATCTGAGTTCAGACCGGAGCAATCCAGGTCA-GTT
Moja	CAACGATTAA-AGTCCTACGTGATCTGAGTTCAGACCGGAGTAATCCAGGTCA-GTT
Hoja	CAACGATTAA-AGTCCTACGTGATCTGAGTTCAGACCGGAGTAATCCAGGTCA-GTT
Bede	CAACGATTAA-AGTCCTACGTGATCTGAGTTCAGACCGGAGTAATCCAGGTCA-GTT
Besp	CAACGATTAA-AGTCCTACGTGATCTGAGTTCAGACCGGAGTAATCCAGGTCA-GTT
Mysp	CAACGATTAA-AGTCCTACGTGATCTGAGTTCAGACCGGAGTAATCCAGGTCA-GTT
Osja	CAACGATTAA-AGTCCTACGTGATCTGAGTTCAGACCGGAGTAATCCAGGTCA-GTT
Sgro	CAACGATTAA-AGTCCTACGTGATCTGAGTTCAGACCGGAGTAATCCAGGTCA-GTT
Pzpa	CAACGATTAA-AGTCCTACGTGATCTGAGTTCAGACCGGAGCAATCCAGGTCA-GTT
Zeja	CAACGATTAA-AGTCCTACGTGATCTGAGTTCAGACCGGAGTAATCCAGGTCG-GTT
Znne	CAACGATTAA-AGTCCTACGTGATCTGAGTTCAGACCGGAGTAATCCAGGTCA-GTT
Zefa	CAACGATTAA-AGTCCTACGTGATCTGAGTTCAGACCGGAGTAATCCAGGTCA-GTT
Acni	CAACGATTAA-AGTCCTACGTGATCTGAGTTCAGACCGGAGTAATCCAGGTCA-GTT
Ncrh	CAACGATTAA-AGTCCTACGTGATCTGAGTTCAGACCGGAGTAATCCAGGTCA-GTT
Agca	CAACGATTAA-AGTCCTACGTGATCTGAGTTCAGACCGGAGTAATCCAGGTCA-GTT
Hydy	CAACGATTAA-AGTCCTACGTGATCTGAGTTCAGACCGGAGTAATCCAGGTCA-GTT
Gsac	CAACGATTAA-AGTCCTACGTGATCTGAGTTCAGACCGGAGTAATCCAGGTCA-GTT
Pevo	CAACGATTAA-AACCCTACGTGATCTGAGTTCAGACCGGAGTAATCCAGGTCA-GTT
Hiku	CAACGATTAA-AGTCCTACGTGATCTGAGTTCAGACCGGAGTAATCCAGGTCA-GTT
Inpa	CAACGATTAA-AGTCCTACGTGATCTGAGTTCAGACCGGAGCAATCCAGGTCA-GTT
Auch	CAACGATTAA-AGTCCCACGTGATCTGAGTTCAGACCGGAGTAATCCAGGTCG-GTT
Fico	CAACGATTAA-AGTCCTACGTGATCTGAGTTCAGACCGGAGTAATCCAGGTCA-GTT
Macs	CAACAATTAA-AGTCCTACGTGATCTGAGTTCAGACCGGAGTAATCCAGGTCA-GTT
Moal	CAACGATTAA-AGTCCTACGTGATCTGAGTTCAGACCGGAGTAATCCAGGTCA-GTT
Syma	CAACGATTAA-AGTCCTACGTGATCTGAGTTCAGACCGGAGTAATCCAGGTCA-GTT
Mafr	CAACGATTAA-AGTCCTACGTGATCTGAGTTCAGACCGGAGTAATCCAGGTCA-GTT
Dcpe	CAACGATTAATAGTCCTACGTGATCTGAGTTCAGACCGGAGTAATCCAGGTCA-GTT
Dcti	CAACGATTAATAGTCCTACGTGATCTGAGTTCAGACCGGAGTAATCCAGGTCA-GTT
Hehi	CAACGGTTAA-AGTCCTACGTGATCTGAGTTCAGACCGGAGTAATCCAGGTCA-GTT
Stam	CAACGATTAA-AGTCCTACGTGATCTGAGTTCAGACCGGAGTAATCCAGGTCA-GTT
Hogi	CAACGATTAA-AGTCCTACGTGATCTGAGTTCAGACCGGAGTAATCCAGGTCA-GTT
Erzo	CAACGATTAA-AGTCCTACGTGATCTGAGTTCAGACCGGAGTAATCCAGGTCA-GTT
Hxot	CAACGATTAA-AGTCCTACGTGATCTGAGTTCAGACCGGAGTAATCCAGGTCA-GTT
Core	CAACGATTAA-AGTCCTACGTGATCTGAGTTCAGACCGGAGTAATCCAGGTCA-GTT
Apve	CAACGATTAA-AGTCCTACGTGATCTGAGTTCAGACCGGAGTAATCCAGGTCA-GTT
Latj	CAACGATTAA-AGTCCTACGTGATCTGAGTTCAGACCGGAGTAATCCAGGTCA-GTT
Laja	CAACGATTAA-AGTCCTACGTGATCTGAGTTCAGACCGGAGCAATCCAGGTCA-GTT

Syja	CAACGATTAA-AGTCCTACGTGATCTGAGTTCAGACCGGAGTAATCCAGGTCG-GTT
Epme	CAACGATTAA-AGTCCTACGTGATCTGAGTTCAGACCGGAGTAATCCAGGTCG-GTT
Grse	CAACGATTAA-AGTCCTACGTGATCTGAGTTCAGACCGGAGTAATCCAGGTCA-GTT
Clja	CAACGATTAA-AGTCCTACGTGATCTGAGTTCAGACCGGAGTAATCCAGGTCG-GTT
Ogcy	CAACGATTAA-AGTCCTACGTGATCTGAGTTCAGACCGGAGTAATCCAGGTCA-GTT
Plna	CAACGATTAATAGTCCTACGTGATCTGAGTTCAGACCGGAGTAATCCAGGTCA-GTT
Lema	CAACGATTAA-AGTCCTACGTGATCTGAGTTCAGACCGGAGTAATCCAGGTCA-GTT
Etzo	CAACGATTAA-AGTCCTACGTGATCTGAGTTCAGACCGGAGTAATCCAGGTCA-GTT
Apse	CAACGATTAA-AGTCCTACGTGATCTGAGTTCAGACCGGAGTAATCCAGGTCA-GTT
Epde	CAACGATTAA-AGTCCTACGTGATCTGAGTTCAGACCGGAGAAATCCAGGTCA-GTT
Slja	CAACGATTAA-AGTCCTACGTGATCTGAGTTCAGACCGGAGTAATCCAGGTCG-GTT
Bsja	CAACGATTAA-AGTCCTACGTGATCTGAGTTCAGACCGGAGCAATCCAGGTCA-GCT
Ecna	CAACGATTAA-AGTCCTACGTGATCTGAGTTCAGACCGGAGTAATCCAGGTCA-GTT
Cohi	CAACGATTAA-AGTCCTACGTGATCTGAGTTCAGACCGGAGTAATCCAGGTCA-GTT
Caar	CAACGATTAA-AGTCCTACGTGATCTGAGTTCAGACCGGAGTAATCCAGGTCA-GTT
Came	CAACGATTAA-AGTCCTACGTGATCTGAGTTCAGACCGGAGTAATCCAGGTCA-GTT
Mema	CAACGATTAA-AGTCCTACGTGATCTGAGTTCAGACCGGAGTAATCCAGGTCA-GTT
Lenu	CAACGATTAA-AGCCCTACGTGATCTGAGTTCAGACCGGAGTAATCCAGGTCA-GTT
Brja	CAACGATTAA-AGTCCTACGTGATCTGAGTTCAGACCGGAGTAATCCAGGTCA-GTT
Plma	CAACGATTAA-AGTCCTACGTGATCTGAGTTCAGACCGGAGTAATCCAGGTCA-GTT
Emst	CAACGATTAA-AGTCCTACGTGATCTGAGTTCAGACCGGAGTAATCCAGGTCA-GTT
Ptti	CAACGATTAA-AGTCCTACGTGATCTGAGTTCAGACCGGAGTAATCCAGGTCA-GTT
Losu	CAACGATTAA-AGTCCTACGTGATCTGAGTTCAGACCGGAGTAATCCAGGTCA-GTT
Geoy	CAACGATTAACAGTCCTACGTGATCTGAGTTCAGACCGGAGTAATCCAGGTCA-GTT
Dipi	CAACGATTAA-AGTCCTACGTGATCTGAGTTCAGACCGGAGTAATCCAGGTCA-GTT
Pama	CAACGATTAA-AGTCCTACGTGATCTGAGTTCAGACCGGAGTAATCCAGGTCA-GTT
Leob	CAACGATTAA-AGTCCTACGTGATCTGAGTTCAGACCGGAGTAATCCAGGTCA-GTT
Neba	CAACGATTAA-AGTCCTACGTGATCTGAGTTCAGACCGGAGTAATCCAGGTCA-GTT
Pdpl	CAACGATTAA-AGTCCTACGTGATCTGAGTTCAGACCGGAGCAATCCAGGTCA-GTT
Nimi	CAACGATTAATAGTCCTACGTGATCTGAGTTCAGACCGGAGCAATCCAGGTCA-GTT
Uptr	CAACGATTAA-AGTCCTACGTGATCTGAGTTCAGACCGGAGTAATCCAGGTCA-GTT
Pesc	CAACGATTAA-AGTCCTACGTGATCTGAGTTCAGACCGGAGTAATCCAGGTCA-GTT
Baar	CAACGATTAA-AGTCCTACGTGATCTGAGTTCAGACCGGAGTAATCCAGGTCG-GTT
Moar	CAACGATTAA-AGTCCTACGTGATCTGAGTTCAGACCGGAGTAATCCAGGTCA-GTT
Toja	CAACGATTAA-AGTCCTACGTGATCTGAGTTCAGACCGGAGTAATCCAGGTCA-GTT
Chau	CAACGATTAA-AGTCCTACGTGATCTGAGTTCAGACCGGAGTAATCCAGGTCA-GTT
Chse	CAACGATTAA-AGTCCTACGTGATCTGAGTTCAGACCGGAGTAATCCAGGTCA-GTT
Enar	CAACGATTAA-AGTCCTACGTGATCTGAGTTCAGACCGGAGTAATCCAGGTCA-GTT
Hpty	CAACGATTAA-AGTCCTACGTGATCTGAGTTCAGACCGGAGTAATCCAGGTCA-GTT
Nana	CAACGATTAA-AGTCCTACGTGATCTGAGTTCAGACCGGAGTAATCCAGGTCA-GTT
Mcst	CAACGATTAA-AGTCCTACGTGATCTGAGTTCAGACCGGAGTAATCCAGGTCA-GTT
Rhox	CAACGATTAA-AGTCCTACGTGATCTGAGTTCAGACCGGAGTAATCCAGGTCA-GTT
Opfa	CAACGATTAA-AGTCCTACGTGATCTGAGTTCAGACCGGAGTAATCCAGGTCA-GTT
Paar	CAACGATTAA-AGTCCTACGTGATCTGAGTTCAGACCGGAGTAATCCAGGTCA-GTT
Gozo	CAACGATTAA-AGTCCTACGTGATCTGAGTTCAGACCGGAGTAATCCAGGTCA-GTT
Ackr	CAACGATTAACAGTCCTACGTGATCTGAGTTCAGACCGGAGTAATCCAGGTCA-GTT
Elev	CAACGATTAA-AGTCCTACGTGATCTGAGTTCAGACCGGAGTAATCCAGGTCA-GTT
Trdu	CAACGATTAA-AGTCCTACGTGATCTGAGTTCAGACCGGAGTAATCCAGGTCA-GTT
Amoc	CAACGATTAA-AGTCCTACGTGATCTGAGTTCAGACCGGAGCAATCCAGGTCA-GTT
Hame	CAACGATTAA-AGTCCTACGTGATCTGAGTTCAGACCGGAGTAATCCAGGTCA-GTT
Chso	CAACGATTAA-AGTCCTACGTGATCTGAGTTCAGACCGGAGCAATCCAGGTCA-GTT
Lyto	CAACGATTAA-AGTCCTACGTGATCTGAGTTCAGACCGGAGTAATCCAGGTCA-GTT

```
Encr    CAACGATTAA-AGTCCTACGTGATCTGAGTTCAGACCGGAGTAATCCAGGTCA-GTT
Bvar    CAACGATTAA-AGTCCTACGTGATCTGAGTTCAGACCGGAGAAATCCAGGTCA-GTT
Noco    CAACGATTAA-AGTCCTACGTGATCTGAGTTCAGACCGGAGTAATCCAGGTCA-GTT
Chsp    CAACGATTAA-AGTCCTACGTGATCTGAGTTCAGACCGGAGTAATCCAGGTCA-GTT
Arja    CAACGATTAA-AGTCCTACGTGATCTGAGTTCAGACCGGAGTAATCCAGGTCA-GTT
Pase    CAACGATTAATAGTCCTACGTGATCTGAGTTCAGACCGGAGTAATCCAGGTCA-GTT
Trel    CAACGATTAACAGTCCTACGTGATCTGAGTTCAGACCGGAGTAATCCAGGTCA-GTT
Lifa    CAACGATTAA-AGTCCTACGTGATCTGAGTTCAGACCGGAGTAATCCAGGTCA-GTT
Acur    CAACGATTAA-AGTCCTACGTGATCTGAGTTCAGACCGGAGTAATCCAGGTCG-GTT
Ampe    CAACGATTAA-AGTCCTACGTGATCTGAGTTCAGACCGGAGTAATCCAGGTCA-GTT
Urja    CAACGATTAA-AGTCCTACGTGATCTGAGTTCAGACCGGAGCAATCCAGGTCA-GTT
Enet    CAACGATTAACAGTCCTACGTGATCTGAGTTCAGACCGGAGTAATCCAGGTCA-GTT
Ptbr    CAACAATTAA-AGTCCTACGTGATCTGAGTTCAGACCGGAGTAATCCAGGTCA-GCT
Safa    CAACGATTAA-AGTCCTACGTGATCTGAGTTCAGACCGGAGTAATCCAGGTCA-GTT
Icae    CAACGATTAA-AGTCCTACGTGATCTGAGTTCAGACCGGAGTAATCCAGGTCA-GTT
Asmi    CAACGATTAA-AGTCCTACGTGATCTGAGTTCAGACCGGAGTAATCCAGGTCA-GTT
Foal    CAACGATTAA-AGTCCTACGTGATCTGAGTTCAGACCGGAGTAATCCAGGTCA-GTT
Drze    CAACGATTAATAGTCCTACGTGATCTGAGTTCAGACCGAAGCAATTCAGGTCA-GTT
Rhas    CAACGATTAA-AGTCCTACGTGATCTGAGTTCAGACCGGAGAAATCCAGGTCA-GTT
Elac    CAACGATTAA-AGTCCTACGTGATCTGAGTTCAGACCGGAGAAATCCAGGTCA-GTT
Kugu    CAACGATTAA-AGTCCTACGTGATCTGAGTTCAGACCGGAGCAATCCAGGTCA-GTT
Plor    CAACGATTAA-AGTCCTACGTGATCTGAGTTCAGACCGGAGTAATCCAGGTCA-GTT
Sgun    CAACGATTAA-AGTCCTACGTGATCTGAGTTCAGACCGGAGTAATCCAGGTCA-GTT
Zaco    CAACGATTAA-AGTCCTACGTGATCTGAGTTCAGACCGGAGTAATCCAGGTCA-GTT
Zbfl    CAACGATTAA-AGTCCTACGTGATCTGAGTTCAGACCGGAGTAATCCAGGTCA-GTT
Spba    CAACGATTAA-AGTCCTACGTGATCTGAGTTCAGACCGGAGTAATCCAGGTCG-GTT
Game    CAACGATTAA-AGTCCTACGTGATCTGAGTTCAGACCGGAGTAATCCAGGTCA-GTT
Thth    CAACGATTAA-AGTCCTACGTGATCTGAGTTCAGACCGGAGTAATCCAGGTCA-GTT
Xigl    CAACGATTAA-AGTCCTACGTGATCTGAGTTCAGACCGGAGTAATCCAGGTCA-GTT
Hyja    CAACGATTAA-AGTCCTACGTGATCTGAGTTCAGACCGGAGTAATCCAGGTCA-GTT
Psan    CAACGATTAA-AGTCCTACGTGATCTGAGTTCAGACCGGAGTAATCCAGGTCA-GTT
Cupa    CAACGATTAA-AGTCCTACGTGATCTGAGTTCAGACCGGAGTAATCCAGGTCA-GTT
Mpch    CAACGATTAA-AGTCCTACGTGATCTGAGTTCAGACCGGAGTAATCCAGGTCA-GTT
Char    CAACGATTAA-AGTCCTACGTGATCTGAGTTCAGACCGGAGTAATCCAGGTCA-GTT
Pser    CAACGATTAA-AGTCCTACGTGATCTGAGTTCAGACCGGAGTAATCCAGGTCG-GTT
Prol    CAACGATTAA-AGTCCTACGTGATCTGAGTTCAGACCGGAGTAATCCAGGTCA-GTT
Plbi    CAACGATTAA-AGTCCTACGTGATCTGAGTTCAGACCGGAGTAATCCAGGTCA-GTT
Calu    CAACGATTAA-AGTCCTACGTGATCTGAGTTCAGACCGGAGTAATCCAGGTCA-GTT
Papa    CAACGATTAA-AGTCCTACGTGATCTGAGTTCAGACCGGAGCAATCCAGGTCA-GTT
Sufr    CAACGATTAA-AATCCTACGTGATCTGAGTTCAGACCGGAGTAATCCAGGTCG-GTT
Stci    CAACGATTAA-AATCCTACGTGATCTGAGTTCAGACCGGAGTAATCCAGGTCG-GTT
Taru    CAACGATTAA-AGTCCTACGTGATCTGAGTTCAGACCGGAGTAATCCAGGTCA-GTT
Rala    CAACGATTAA-AGTCCTACGTGATCTGAGTTCAGACCGGAGTAATCCAGGTCA-GTT
        ****  ****    *  *** ************* * *  *  **  *** *  *
```

```
        ¦→               42'          ←¦          55
Scca    TCTA-------------------------TCTATG-AATTT-ATTTTTCCTAGTACGAAA
Muma    TCTA-------------------------TCTATG-AATAT-ACTTTTCCTAGTACGAAA
Erca    TCTA-------------------------TCTATG-AAGTT-ATTTATCCTAGTACGAAA
Pose    TCTA-------------------------TCTATG-AAGTT-ATTTATCCTAGTACGAAA
Actr    TCTA-------------------------TCTATG-CAGTG-ACCCTTCCTAGTACGAAA
Scal    TCTA-------------------------TCTATG-CAGTG-ACCCTTCCTAGTACGAAA
Posp    TCTA-------------------------TCTATG-CAGTG-ACCCTTCCTAGTACGAAA
Atsp    TCTA-------------------------TCTATG-CAATG-ACTCTTTCTAGTACGAAA
Leoc    TCTA-------------------------TCTATG-CAATG-ACTCTTTCTAGTACGAAA
Amca    TCTA-------------------------TCTATG-AAGTG-GTTCCCCCTAGTACGAAA
Osbi    TCTA-------------------------TCTATG-TAGCT-ACTCTTCCTAGTACGAAA
Pabu    TCTA-------------------------TCTATG-ACATG-ACCCTTCCCAGTACGAAA
Hial    TCTA-------------------------TCTATG-CAGTG-ACCCTTCCTAGTACGAAA
Elha    TCTA-------------------------TCTATG-CAGTA-ACCCTTCCTAGTACGAAA
Mlcy    TCTA-------------------------TCTATG-CAGTG-ACTCCCCCCAGTACGAAA
Algl    TCTA-------------------------TCTATG-AAGTG-ACTGTTCCTAGTACGAAA
Ptgi    TCTA-------------------------TCTATG-AAGTA-ATTTTTCCTAGTACGAAA
Alaf    TCTA-------------------------TCTATG-ATGTA-ACTTCTCCCAGTACGAAA
Nock    TCTA-------------------------TCTATG-ATGTA-ACTTCTCCTAGTACGAAA
Anja    TCTA-------------------------TCTATG-AACT--ACTTCTTCCAGTACGAAA
Gyki    TCTA-------------------------TCTATG-ATCTAACCTACCCCTAGTACGAAA
Syka    TCTA-------------------------TCTATG-AAATTACTTTTTCCCCGTACGAAA
Opma    TCTA-------------------------TCTATG-ACTTTACTTCTCCCCAGTACGAAA
Comy    TCTA-------------------------TCTATG-AAAGTGCCTTTTCCCAGTACGAAA
Sasp    TCTA-------------------------TCTATG-AAAAA-CCCACCCCCAGTACGAAA
Eupe    TCTA-------------------------TCTATG-T-CTA-CCCACCCCCAGTACGAAA
Enja    TCTA-------------------------TCTGTA-AAGCC-ATCACCCCTAGTACGAAA
Same    TCTA-------------------------TCTGTG-AAGCC-ATCCTTCCTAGTACGAAA
Chch    TCTA-------------------------TCTGCA-ATAGTTCTTTTTCCTAGTACGAAA
Grgr    TCTA-------------------------TCTGCT-AC-GCTACCTTTCCTAGTACGAAA
Caau    TCTA-------------------------TCTGTA-ACGCT-ACTTTTCCTAGTACGAAA
Cyca    TCTA-------------------------TCTGTA-AC-GCTACTTTTCCTAGTACGAAA
Dare    TCTA-------------------------TCTGTA-AAGCTATTTTTTCCTAGTACGAAA
Cost    TCTA-------------------------TCTGTA-ACGTC-ACTTTTCCTAGTACGAAA
Leec    TCTA-------------------------TCTGTA-AAGTC-GTTTTTCCTAGTACGAAA
Crla    TCTA-------------------------TCTGTA-AC-GTTACTTTTCCTAGTACGAAA
Clmc    TCTA-------------------------TCTGTATCAAGCCATTTTTCCTAGTACGAAA
Phin    TCTA-------------------------TCTGTA-ATGC-TATTTTTTCTAGTACGAAA
Icpu    TCTA-------------------------TCTGTA-ATG-CCACTTTCCCCAGTACGAAA
Psto    TCTA-------------------------TCTGTA-ATGCC-ACTTTTCCTAGTACGAAA
Cora    TCTA-------------------------TCTGTA-ACGCA-ATTTCTCCTAGTACGAAA
Eisp    TCTA-------------------------TCTGTA-ATGTT-ATTTCCTCTAGTACGAAA
Apal    TCTA-------------------------TCTGTA-ATGTC-ACTTCTCCTAGTACGAAA
Eslu    TCTA-------------------------TCTATG-AA-GTAATCTTTCCCAGTACGAAA
Dape    TCTA-------------------------TCTATG-CTATG-ATCTTCTTTAGTACGAAA
Glse    TCTA-------------------------TCTATG-ATGTA-ACTCTCCCTAGTACGAAA
Naar    TCTA-------------------------TCTATG-ATGTA-ACTTTCCCTAGTACGAAA
Baoc    TCTA-------------------------TCTATG-ATGTAATTTTTCCCTAGTACGAAA
Opso    TCTA-------------------------TCTATG--ACGTAACCTTCCCTAGTACGAAA
Alte    TCTA-------------------------TCTGTA--ACGCTACTTTTCCTAGTACGAAA
Plap    TCTA-------------------------TCTGTA-ACGCT-ACTTTTCCTAGTACGAAA
```

```
Plal   TCTA------------------------TCTATG-AAATG-GTCTCTCCCAGTACGAAA
Sami   TCTA------------------------TCTATG-AGA-TGATTCCCCCCAGTACGAAA
Rere   TCTA------------------------TCTATG-ACATA-CTCCTCCCCAGTACGAAA
Gama   TCTA------------------------TCTATG-ACGTG-ACCTTTTCTAGTACGAAA
Onmy   TCTA------------------------TCTATG-AAGTG-ATGTTTCCTAGTACGAAA
Sasa   TCTA------------------------TCTATG-AAGTG-ATGTTTCCTAGTACGAAA
Cola   TCTA------------------------TCTATG-AAGTG-ATGTTTCCTAGTACGAAA
Dita   TCTA------------------------TCTATA-AATGA-ATCTTCCCCAGTACGAAA
Gogr   TCTA------------------------TCTATT-AAG-TAGCCCTTCCCAGTACGAAA
Chsl   TCTA------------------------TCTATA-CCCC--ATCTTTCCCAGTACGAAA
Atja   TCTA------------------------TCTATG-ATGTA-GCTTACCCTAGTACGAAA
Iido   TCTA------------------------TCTATG-ATGTG-GTTTACCCTAGTACGAAA
Auja   TCTA------------------------TCTATG-ACCTTGATCTTCCCCAGTACGAAA
Chag   TCTA------------------------TCTATG-ACT-TGCTCTTCTCTAGTACGAAA
Hami   TCTA------------------------TCTATG-GTAATGCTCTTTTCCAGTACGAAA
Saun   TCTA------------------------TCTATG-GTCATGCTCTTTTCTAGTACGAAA
Nema   TCTA------------------------TCTACG-TAAT-GATCTCTTCTAGTACGAAA
Disp   TCTA------------------------TCTACG-CATTG-ATCTTTTCTAGTACGAAA
Myaf   TCTA------------------------TCTACG-CATTG-ATTTTCTCTAGTACGAAA
Lagu   TCTA------------------------TCTGTG-AT-ATGTTCTTTTCTAGTACGAAA
Trtr   TCTA------------------------TCTGTG-A-TGATATCTTTTCTAGTACGAAA
Zucr   TCTA------------------------TCTGTG-AAACT-ATCTTTTCTAGTACGAAA
Pxja   TCTA------------------------TCTATG-AATTTGACCTTTTCTATTACGAAA
Pxlo   TCTA------------------------TCTATG--AA-TTGACCTTTCTAGTACGAA-
Pctr   TCTA------------------------TCTATG-AAATG-CTCTCTTCCAGTACGAAA
Apsa   TCTA------------------------TCTATG-AT-TG-TTCTTTTCCAGTACGAAA
Cabe   TCTA------------------------TCTGTG-AAAACACCTCTGCCTTGTACGAAA
Bzze   TCTA------------------------TCTATG-AAACAGCCTTTTTCTAGTACGAAA
Siim   TCTA------------------------TCTATG-AAAATATCTTCTTCTAGTACGAAA
Ctru   TCTA------------------------TCTATG--AAGTGACCTTTTCTAGTACGAAA
Dpbr   TCTA------------------------TCTATG-AAGTG-ACCTTTTCTAGTACGAAA
Caki   TCTA------------------------TCTATA-AAAAG-TCTTTTCCCAGTACGAAA
Phja   TCTA------------------------TCTATA-AAG-AGCTTTTTTATAGTACGAAA
Brsp   TCTA------------------------TCTATG-AATT-GTTTTTCCCTAGTACGAAA
Gamo   TCTA------------------------TCTATGACAA-GCTCTTTTTCCAGTACGAAA
Lolo   TCTA------------------------TCTATG-ACAAG-CTCTTTTCCAGTACGAAA
Batr   TCTA------------------------TCTATA-ACATTGCTTTTTTCCAGTACGAAA
Prmy   TCTA------------------------TCTATA-TTTTA-TTTTTCTTCAGTACGAAA
Loli   TCTA------------------------TCTATG-ATGT-GATCTTTTCTAGTACGCAA
Loam   TCTA------------------------TCTATG-ACATG-ATCTTTTCTAGTACGCAA
Chab   TCTA------------------------TCTATG-GTGTG-ATCTTTTCTAGTACGCAA
Chto   TCTA------------------------TCTATG-GTGTG-ATCTTTTCTAGTACGCAA
Majo   TCTA------------------------TCTATA-ACATG-CTTTTTTCTAGTACGCAA
Hlst   TCTA------------------------TCTATA-ATATG-CTTTTTTCCAGTACGCAA
Clpe   TCTA------------------------TCTATG-ATATG-CTCTTTTCTAGTACGCAA
Mlmr   TCTA------------------------TCTATG-ATATG-CTCTTTTCTAGTACGCAA
Crcr   TCTA------------------------TCTATG-ATACA-TTCTCCTCCAGTACGAAA
Muce   TCTA------------------------TCTATG-ATACA-TTCTCCTCCAGTACGAAA
Bege   TCTA------------------------TCTATG-TAATG-ATTTCTTCTAGTACGAAA
Mela   TCTA------------------------TCTATG-TTATG-CTTTCTTCTAGTACGAAA
Hats   TCTA------------------------TCTATG-AAATG-ATCTCTTCTAGTACGAAA
Orla   TCTA------------------------TCTATG-ACATA-CTCCCTTCTAGTACGAAA
```

```
Cosa     TCTA-------------------------TCTATG-AAA-TGTCTTCCTCTAGTACGAAA
Exsp     TCTA-------------------------TCTATG-AGATG-TTCTCTTCTAGTACGAAA
Depa     TCTA-------------------------TCTATG-ATGTC-TCTTCCCCTAGTACGAAA
Rima     TCTA-------------------------TCTATG-ACCGTGCTTTCTTTCAGTACGAAA
Fuol     TCTA-------------------------TCTATG-AAATA-TTTTCTTCTAGTACGAAA
Gmaf     TCTA-------------------------TCTATG-AA-GTGTTTTCTTCCAGTACGAAA
Xeei     TCTA-------------------------TCTATG-AAATG-ATTTCTTCTAGTACGAAA
Pros     TCTA-------------------------TCTATG-GTGTAATCTCTTTCTAGTACGAAA
Scmi     TCTA-------------------------TCTATG-ATGTGATCTCTTTCTAGTACGAAA
Rolo     TCTA-------------------------TCTATG-ATATG-ATCTTTTCTAGTACGAAA
Cere     TCTA-------------------------TCTATG-GTATG-ATCTTTCCTAGTACGAAA
Daga     TCTA-------------------------TCTATG-GAGTG-ATCTTTTCTAGTACGAAA
Anco     TCTA-------------------------TCTATA-ATATG-ATCTTTTCTAGTACGAAA
Dmve     TCTA-------------------------TCTATG-ACAT-GCTCTTTTCCAGTACGAAA
Dmar     TCTA-------------------------TCTATG-CTGTG-CTCTTTTCTAGTACGAAA
Anka     TCTA-------------------------TCTATG-CTATG-ATCTTTTCTAGTACGAAA
Moja     TCTA-------------------------TCTATG-ATGTG-ATCTTTCCTAGTACGAAA
Hoja     TCTA-------------------------TCTATG-ATATG-ATCTTTTCTAGTACGAAA
Bede     TCTA-------------------------TCTATG-ATGTGATCTTCTTCTAGTACGAAA
Besp     TCTA-------------------------TCTATG-ATATGATCTTCTTCTAGTACGAAA
Mysp     TCTA-------------------------TCTATG-ACATG-TTCTTTTCTAGTACGAAA
Osja     TCTA-------------------------TCTATG--ACATGTTCTTTTCTAGTACGAAA
Sgro     TCTA-------------------------TCTATG-CTATG-TTCTTTTCCAGTACGAAA
Pzpa     TCTA-------------------------TCTATG-AAAT-GCTCTTTTCTAGTACGAAA
Zeja     TCTA-------------------------TCTATG--AAACATTCTTCTCCAGTACGAAA
Znne     TCTA-------------------------TCTATA-AAAAT-TTCCTCTCCAGTACGAAA
Zefa     TCTA-------------------------TCTATA-AGACGATTCCTCTCCAGTACGAAA
Acni     TCTA-------------------------TCTATG-AAAT-ATTCTTTTCCAGTACGAAA
Ncrh     TCTA-------------------------TCTATG-AAATA-TTCTTTTCCAGTACGAAA
Agca     TCTA-------------------------TCTATG--ACATGATCTTTTCTAGTACGAAA
Hydy     TCTA-------------------------TCTATG-CTGTG-CTCTTCTCTAGTACGAAA
Gsac     TCTA-------------------------TCTATG--CTGTGCTCTTCTCTAGTACGAAA
Pevo     TCTA-------------------------TCTATG-AATATGGCTTTTTCTAGTACGAAA
Hiku     TCTA-------------------------TCTATG-TATGTGGCCTTCTTCAGTACGAAA
Inpa     TCTA-------------------------TCTATG-ACA-CGCCTTTTCCCAGTACGAAA
Auch     TCTA-------------------------TCTATG-AATGTGCCCCTTCCTAGTACGAAA
Fico     TCTA-------------------------TCTATG-AAGC-ACCTTTCCCTAGTACGAAA
Macs     TCTA-------------------------TCTATG-AT-ATGCTCTTTTCTAGTACGAAA
Moal     TCTA-------------------------TCTATG--ATACAGTTTTTTCCAGTACGAAA
Syma     TCTA-------------------------TCTATG-TTATA-CTTTTTTCTAGTACGAAA
Mafr     TCTA-------------------------TCTATG-ATATG-ATCTTTTCCAGTACGAAA
Dcpe     TCTA-------------------------TCTATG-AA-GTGTTCTTTTCTAGTACGAAA
Dcti     TCTA-------------------------TCTATG-AAGTG-TTCTTTTCTAGTACGAAA
Hehi     TCTA-------------------------TCTATG-GTGTG-CTCTTTTCTAGTACGAAA
Stam     TCTA-------------------------TCTATG-ATAT-GATCTTTTCTAGTACGAAA
Hogi     TCTA-------------------------TCTATG-AAATTACGCTTTTCTAGTACGAAA
Erzo     TCTA-------------------------TCTATG-CTATG-CTCTTTTCTAGTACGAAA
Hxot     TCTA-------------------------TCTATG-ATATG-ATTTTTTCTAGTACGAAA
Core     TCTA-------------------------TCTATG-CTA-TGCTCTTTTCTAGTACGAAA
Apve     TCTA-------------------------TCTATG-CTAT-GATTTTTTCTAGTACGAAA
Latj     TCTA-------------------------TCTATG-CCCACGTTCTTTTTTAGTACGAAA
Laja     TCTA-------------------------TCTATG-ACATG-ATCTTCTCTAGTACGAAA
```

```
Syja    TCTA-------------------------TCTATT-GTATG-ACCTTCTCCAGTACGAAA
Epme    TCTA-------------------------TCTATG-TAT-TGATCTTTTCTAGTACGAAA
Grse    TCTA-------------------------TCTATG-ACATG-ATCTTTTCCAGTACGAAA
Clja    TCTA-------------------------TCTATG-ATA-TGACCTTTTCTAGTACGAAA
Ogcy    TCTA-------------------------TCTATG--TAATGCCTTTCTCTAGTACGAAA
Plna    TCTA-------------------------TCTATG-ATATG-ACTTTTTCTAGTACGAAA
Lema    TCTA-------------------------TCTATG-CAATG-ATTTTTTCTAGTACGAAA
Etzo    TCTA-------------------------TCTATG-ATA-TGATCTTCTCTAGTACGAAA
Apse    TCTA-------------------------TCTATG-AT-ATGTTTTTTTCTAGTACGAAA
Epde    TCTA-------------------------TCTATG-CTAT-GATCTTTTCTAGTACGAAA
Slja    TCTA-------------------------TCTATG-TTAT-GATCTTTTCTAGTACGAAA
Bsja    TCTA-------------------------TCTATG-ACAT-GAACTTTTCTAGTACGAAA
Ecna    TCTA-------------------------TCTATG-AAA-TAATCTTTTCTAGTACGAAA
Cohi    TCTA-------------------------TCTATG-TAGTA-TTCTTTTCTAGTACGAAA
Caar    TCTA-------------------------TCTATG-AAGCA-ATCTTTTCTAGTACGAAA
Came    TCTA-------------------------TCTATG-AAGTTAATCTTTTCTAGTACGAAA
Mema    TCTA-------------------------TCTATG-AAACG-ATCTTTTCCAGTACGAAA
Lenu    TCTA-------------------------TCTATG-ACCC-ACCCCCTTCCAGTACGAAA
Brja    TCTA-------------------------TCTATG-ATATG-TTTTTTTCTAGTACGAAA
Plma    TCTA-------------------------TCTATG--TTATGTTCTTTTCTAGTACGAAA
Emst    TCTA-------------------------TCTATG-ATATG-ATCTTTTCTAGTACGAAA
Ptti    TCTA-------------------------TCTATG-AAGTG-ATCTTTTCTAGTACGAAA
Losu    TCTA-------------------------TCTATG-ATATGATTCTTCCCCAGTACGAAA
Geoy    TCTA-------------------------TCTATG--AAGTATCCTTCTCTAGTACGAAA
Dipi    TCTA-------------------------TCTATG-ACA-TGATCTTTTCTAGTACGAAA
Pama    TCTA-------------------------TCTATG-A-TGTGACCTTTTCTAGTACGAAA
Leob    TCTA-------------------------TCTATG-ATA-CGACCTTTTCTAGTACGAAA
Neba    TCTA-------------------------TCTATG--TAATGTTTCCTTCTAGTACGAAA
Pdpl    TCTA-------------------------TCTATG-AC-GCAATCTTTTCTAGTACGAAA
Nimi    TCTA-------------------------TCTATG-CTTTA-ATCTTTTCTAGTACGAAA
Uptr    TCTA-------------------------TCTATG-AA-GCGATCTTTTCTAGTACGAAA
Pesc    TCTA-------------------------TCTATG--TGT-TACTTTTTCTAGTACGAAA
Baar    TCTA-------------------------TCTATG-ACATG-TTCTTCTCCAGTACGAAA
Moar    TCTA-------------------------TCTATGA--TATGATCTTTTCTAGTACGAAA
Toja    TCTA-------------------------TCTATG-TTATG-ATCTTTTCTAGTACGAAA
Chau    TCTA-------------------------TCTATG-ACA-TACTTTTTTCTAGTACGAAA
Chse    TCCA-------------------------TCTATG-AAAT-GTTCTTTTCCAGTACGAAA
Enar    TCTA-------------------------TCTATG-ACATG-ATCTTTTCTAGTACGAAA
Hpty    TCTA-------------------------TCTATG-ACATG-ATCTTTTCTAGTACGAAA
Nana    TCTA-------------------------TCTATG-ATACG-ATCTTTTCTAGTACGAAA
Mcst    TCTA-------------------------TCTATG-ACATG-AACTTTTCTAGTACGAAA
Rhox    TCTA-------------------------TCTATG-ACA-CGAGCTTCTCTAGTACGAAA
Opfa    TCTA-------------------------TCTATG-A-TATGATCTTTTCTAGTACGAAA
Paar    TCTA-------------------------TCTATA--ATGTGATCTTTTCTAGTACGAAA
Gozo    TCTA-------------------------TCTATG-ACA-TGATCTTTCCTAGTACGAAA
Ackr    TCTA-------------------------TCTATG-CAGT-GAGCTTTTCTAGTACGAAA
Elev    TCTA-------------------------TCTATG-AAAT-GATCTTTTCTAGTACGAAA
Trdu    TCTA-------------------------TCTATG-TC-ACGATCTTTTCTAGTACGAAA
Amoc    TCTA-------------------------TCTATG-ACAT-GAGCTTTTCTAGTACGAAA
Hame    TCTA-------------------------TCTATG-TTAT-GGCCTTTTCTAGTACGAAA
Chso    TCTA-------------------------TCTATG-A-CACCATTTTTTCTAGTACGAAA
Lyto    TCTA-------------------------TCTATG-CAGT-GCTTTTTTCTAGTACGAAA
```

```
Encr    TCTA-------------------------TCTATG-CTA-TGCTTTTTTCTAGTACGAAA
Bvar    TCTA-------------------------TCTATG--ATATGTTCTTCCCCAGTACGAGA
Noco    TCTA-------------------------TCTATG-ACATA-TTCTTTTCTAGTACGAAA
Chsp    TCTA-------------------------TCTATG-ATAATAATATTCTTTAGTACGAAA
Arja    TCTA-------------------------TCTATG-CTATG-ATCTTTTCTAGTACGAAA
Pase    TCTA-------------------------TCTATG-CCATA-CCCTCTCCTAGTACGAAA
Trel    TCTA-------------------------TCTATT-AAGTA-GCCCTCTCTAGTACGAAA
Lifa    TCTA-------------------------TCTATG-CCA-TGATCTTTTCTAGTACGAAA
Acur    TCTA-------------------------TCTATA-ACATTGTCCTTTTCTAGTACGAAA
Ampe    TCTA-------------------------TCTATG-ATAT-GCTCTTTCCTAGTACGAAA
Urja    TCTA-------------------------TCTATG-AAAT-GATTTTTTCCAGTACGAAA
Enet    TCTA-------------------------TCTATG--CCGTGCTTTTTTCTAGTACGAAA
Ptbr    TCTA-------------------------TCTATG--ATTAATTCTTTTCTAGTACGAAA
Safa    TCTA-------------------------TCTATG--CAATGTCCTTTTCCAGTACGAAA
Icae    TCTA-------------------------TCTATG-ACAT-GTTCTTCTCTAGTACGAAA
Asmi    TTTA-------------------------CCTATG-CAGCA-TCCTTTCCTAGTACGAAA
Foal    TCTA-------------------------TCTATG-ATTACCGCTTTTTCTAGTACGAAA
Drze    TCTA-------------------------TCTATA-AAATG-ATTTTTTCTAGTACGAAA
Rhas    TCTACCGGAGAAATCCAGGTCAGTTTCTATCTACG-AAA-TGTTCTTTTCTAGTACGAAA
Elac    TCTA-------------------------TCTATG-GAGTA-TTCTTTTCTAGTACGAAA
Kugu    TCTA-------------------------TCTATG-TAATG-ATCTTTTCTAGTACGAAA
Plor    TCTA-------------------------TCTATG-ATATG-ATTCTTTCTAGTACGAAA
Sgun    TCTA-------------------------TCTACG-ATGTG-ATCTTTTCTAGTACGAAA
Zaco    TCTA-------------------------TCTATG-ACATG-ATCTTTTCTAGTACGAAA
Zbfl    TCTA-------------------------TCTATG-ATTTG-ATCTTTTCTAGTACGAAA
Spba    TCTA-------------------------TCTATG-TAATTAACCTTTTCTAGTACGAAA
Game    TCTA-------------------------TCTATG-GTGTG-TTCTTTTCTAGTACGAAA
Thth    TCTA-------------------------TCTATG-ATATG-TTCTTTTCTAGTACGAAA
Xigl    TCTA-------------------------TCTATG-TTAT-GATCTTTTCTAGTACGAAA
Hyja    TCTA-------------------------TCTATG-ACATG-TTCTTTTCTAGTACGAAA
Psan    TCTA-------------------------TCTATG-TTGTG-TTTTTTTCTAGTACGAAA
Cupa    TCTA-------------------------TCTATG-CT-ATGTTCTTTTCTAGTACGAAA
Mpch    TCTA-------------------------TCTATG-CTATG-ATCTTTTCTAGTACGAAA
Char    TCTA-------------------------TCTATG-AGATG-TTTTTTTCTAGTACGAAA
Pser    TCTA-------------------------TCTATG-AAATG-ATCTTTTCTAGTACGAAA
Prol    TCTA-------------------------TCTATG-TGATG-ATCTTTTCTAGTACGAAA
Plbi    TCTA-------------------------TCTATG-AC-ATGATCTTTTCTAGTACGAAA
Calu    TCTA-------------------------TCTATG-GAGACGCTTTTTTCTAGTACGAAA
Papa    TCTA-------------------------CCTATG-GTGTT-ATCTTTTTTAGTACGAAA
Sufr    TCTA-------------------------TCTATG-ACGCAATCCTTTTTTAGTACGAAA
Stci    TCTA-------------------------TCTATG-ATGTA-ATTTATTTCAGTACGAAA
Taru    TCTA-------------------------TCTATG-AAGTA-CCTTTTTCCAGTACGAAA
Rala    TCTA-------------------------TCTATG-CTAT-GATCTTTTCTAGTACGAAA
           *                          **                     ****
```

```
                 55'         56            56'          57
Scca   GGA-CCGGAGAAAT--GGGGCCAATACCATT-GGCACGCCTC-ATTTTC-ATCTATTGAACCAA
Muma   GGA-CCGGAAAAGT--GGGGCCAATGCTA-CCAGCACGCCCC-ATTTTCA-TCTATTGAATAAA
Erca   GGA-TTGGATAAAT--AGGGCCTATATTAA-AAACACGCCCT-ACTTTAAC-CTGTTGAAACCA
Pose   GGA-TTGGATAAAT--GAGGCCTATAT-TTAAGACACGCCTC-CCT-TTAACCTGCTGAAGCCA
Actr   GGA-CCGGAAGGCT--GAGGCCAATGCTACA-AGTATGCCTC-ACCCCA-ACCTAGTGAAAACA
Scal   GGA-CCGGAAGGCT--GGGGCCAATGCTACA-AGTATGCCAC-ACCCCA-ACCTAATGAAGACA
Posp   GGA-CCGGAAGGCT--GGGGCCAATACTACA-AGCACGCCCC-ACC-CCAACCTAATGAAAACA
Atsp   GGA-CCGAAAGAAG--AGGGCCCATTCTCCA-AGTACGCCCT-ATCC-TCACCCACTGAACACA
Leoc   GGA-TCGAAAGAAG--GGGGCCTATTCTTCA-AGCACGCCCC-ATCCT-CATTCACTGAACACA
Amca   GGA-CCGGGAGAAC-AGAGGCCCATGC-TAAAAGTATGCCCT-ATC-CCCACCTAATGAAATCA
Osbi   GGA-CCGGAAGAAG-TGAAGCCAATATTCTA-AATACGCTTC-ATTC-CTACTTAATGAAAACA
Pabu   GGA-CCGGAAGAAA-TGAGGCCAATACTACA-AGCACGCCTC-ACCTC-TACCTGTTGAAAACA
Hial   GGA-CCGGAAGGAG-TGGGGCCAATTCTAAA-AGTACGCCCC-ACCCCCA-TCTGATGAAATCA
Elha   GGA-CCGGGAGGGA--GGGGCCCATGCCCACAAACAAGCCCC-ACCCCC-ATCCAATGAAAACA
Mlcy   GGA-CCGGGGAAAT--GGGGCCCATACCAAA-AGCACGCCCC-ACCCCC-ACCCGGCGAAAACA
Algl   GGA-CCGGAAAAGA--AGGGCCCATGCTACA-GGTAAGCCCC-CCTTCC-ATCTACTGTAGGCA
Ptgi   GGA-CCGGAAAAAG--GGGGCCAATACTGAA-AGCAAGCCCC-ACCCCC-ACCTGCTGAAACCA
Alaf   GGG-CCGGAGAAGT--GAGGCCCCTACTAACAAGCAAGCCTC-TCCCCC-ACCTGCTGAAAACA
Nock   GGG-CCGGAGAAGT--GAGGCCCCTACTAAA-AGCAAGCCTT-ACCCCC-ACCTGTTGAAAACA
Anja   GGA-CCGGAATGAA-GAGGGCCAATACTAAA-AGCAAGCCCT--ACCTCTACCTGCTGAAAGCA
Gyki   GGA-CCGGAGTAAG--GGGGCCCACGAGA-A-AACAAGCCCC-ACCCCA-ACCCGCTGAAACCA
Syka   GGA-CCGGAATAAA-GGGGGCCAATACTGAA-AGCAAGCCCC-ACC-TCTACCTACTGAAAACA
Opma   GGA-CCGGGAAAAG--AGGGCCCATACTAAA-AGCAAGCCCC-ACCTCA-ACCCCTTGAAGACA
Comy   GGG-CCGGAAAAGG--GGGGCCCATTC-TAAAAGCAAGCCCT-ACCACCCACTCTCTGAAAACA
Sasp   GGG-CCGGGGTGGG--AAGGCCCCTGTTAAA-GACAAGCCTT-ACCCACACCTACTGAAAAATA
Eupe   GGG-CCGGAGTGGG--GGGGCCCCTACTAAAAGCAAGCCCTA-CCTTC--ACCTGTTGAAATCA
Enja   GGA-CCGGAGTGAA--GGGGCCTATGCTAGA-AGCAAGCCCC-ATATC-TACCTGCTGAAATCA
Same   GGA-CCGGAGTAAT--GAGGCCTATGCCCCG-AGCACGCCTC-CCCCCA-ACCTGCTGAATACA
Chch   GGA-CCGGAAAAAG--GGGGCCTCCACCTCT-GATGCGCCCC-ACTTTTA-CCTGCTGACATCA
Grgr   GGA-CCGGGAAGAG--GGGGCCAATGCCAGA-GGCACGCCCC-GCCCCT-ACCTGATGAAGCCA
Caau   GGA-TCGGAAAAGA--GGGGCCAATGC-TCAAGGCACGCCCC-ACC-CCTAATTTATGAAAACA
Cyca   GGA-TCGGAAAAGG--GGGGCCCATACTTAA-AGCACGCCCC-ACCCCT-AATTTATGAAAACA
Dare   GGA-TCGGAAAAAG--GGGGCCCATACTAAA-AGCACGCCCC-ACCCTA-AATTTATGAATAAA
Cost   GGA-CCGGAAAAGA--GAGGCCCATGCCAAA-GGTACGCCTC-ACCCAT-AATTAATGAAAACA
Leec   GGA-CCGGAAAAGA--AAGGCCCATGCCAAA-GGCACGCCTT-ACCCCT-AATTAATGAAGACA
Crla   GGAACCGGAAAAGA--AGGGCCAACACTAAA-AGTGCGCCCT-ACCCCT-AATTAATGAAACCA
Clmc   GGA-CCGGAAAAAT--AAGGTCCATGCCAAA-AGCACACCTT-ACTCCA-ATTTGATGAAATCA
Phin   GGA-CCGAGAAAAT--GAGGCCCCTGCTTTA-AGCACGCCTC--CCTCTAACTTTATGAAACCA
Icpu   GGA-CCGGAAAAGG--GGGGCCCATACTATA-AGCACGCCCC-ACCCCA-ACTTAATGACATCA
Psto   GGA-CCGGAAAAAG--GGGGCCCATATTATT-AATACGCCCC-ACCACT-ATTTAATGCAACCA
Cora   GGA-CCGGAAAAAT--AGGGCCCATGTTTA-AAACAAGCCCT-TATCCA-ATCTAATGAAAACA
Eisp   GGA-CCGAAGAAAT--AAGGCCAATGCTATA-AGCACGCCTT-GCCCC-CACTTAATGAAATCA
Apal   GGA-CCGGAGAAGA--AGAGCCAATACTATA-AGCACGCCCT-ACT-ATAACTTGATGATACCA
Eslu   GGA-CCGGAAAGAT--GAGGCCCCTGC-AA-AAGTATGCCCC-ACCCCC-ACCTAATGAAAACA
Dape   GGA-CCAAGAAGAA--GAGGCCCCTGCTAAA-GGCACGCCTC-ACCCC-TACCTGTTGAAAACA
Glse   GGA-CCGGGAAAGA--GGGGCCCATGCTCTA-AGCATGCCCT-GCCC-CCACCTGATGAAAACA
Naar   GGA-CCGGGAAAGA--GAGGCCTATGCTCGA-GGCACGCCTC-ACCCCC-ACCTGATGAAGACA
Baoc   GGA-CCGGGAAGAA--GGGGCCCATGCTCA-AAGCATGCCTC-ACCCCT-ACCTGATGAAGACA
Opso   GGA-CCGGGAAGAG--GGGGCCCATGCCCA-AAGCATGCCTC-ACCCCC-TCCTGATGAAAACA
Alte   GGA-CCGGAAAAGG--AGGGCCCATGCTCCA-AGTACGCCCT-ACCCCG-ACCTGATGAAAACA
Plap   GGA-CCGGAAAAGA--GGGGCCCATACCC-CCGGTACGCCCC-ACCCCGA-CCTGATGAAGACA
```

Plal	GGA-CCGGAGAGAA--GAGGCCCATGCCC-AGGGCACGCCCC-ACCCTCACCTGCTGAAGGC-A
Sami	GGA-CCGGGGTAAA--GAGGCCCATGC-TCGAGGCACGCCCC-ACC-CTCACCTGCTGAAGGCA
Rere	GGA-CCGGGAAGAG--GGGGCCCATGCT-CGAGGCACACCCC-ACCCCC-ACTTGTTGAAATCA
Gama	GGA-CCGAAAAGGC--AGGGCCAATGCTC-TCGGTAAGCCCG-ACCCCCA-ACTGATGAAGGCA
Onmy	GGA-CCGGAAAGAA--GGGGCCCATGCTTGA-GGCACGCCCC-ACCCCC-ACCTGATGAAGGCA
Sasa	GGA-CCGGAAAGAA--GGGGCCCATGCTTAA-GGCACGCCCC-ATCC-CCACCTGATGAAGGCA
Cola	GGA-CCGGAAAGAA--GGGGCCCATGCTTA-AGGCACGCCCC-ACCCCC-ACCTGATGAAGGCA
Dita	GGA-CCGGGGAGAA--AAGGCCCATGC-CCCCGGTACGCCTT-GCC-CCTGCTCAATGAGGCCA
Gogr	GGG-CCGGAAGGAA--GAGGCCCATGCCCAT-CGCATGCCTT-CCCCCC-AACTATTGAAACCA
Chsl	GGG-CCGGAAAGTA--GAGGCCCATGCCCCC-GGTATGCCTC-CTCCCCATCCAGCTGAAACCA
Atja	GGA-CCGGGAAAAC--GGAGCCCATGC-TAAAAGCACACTCC-TCC-CCTACCTAATGAAAACA
Iido	GGA-CCGGGAAAAC--GGAGCCCATGCTAAA-AGCACGCTCC-TCCCCC-ACCTGATGAAGACA
Auja	GGA-CCGAGAAGAA--GGGGCCCCTGCTTA-TAGCAAGCCCC-ACCCCC-ACCTGAAGAAACCA
Chag	GGA-CCGAGAAGAA--AGGGCCTATGCCACC-GGCATGCCCT-ACCCCTACCTGTAGGAAAACA
Hami	GGA-CCGAAAAGGG--GGGGCCCATATCTCA-GATACGCCCC-GCCCC-CATCTGAGGAACCCA
Saun	GGA-CCGAAAAGAA--GGGGCCCATGCCCCA-GGCACGCCCC--ACCCCTATTTGAAGAAACCA
Nema	GGA-CCGAAAAGAA--GAAGCCACTGCCGAC-GGCACGCCTC-TCCCCCAACTAATGAATAC-A
Disp	GGA-CCGAAAAGAG--GGAGCCTCTATCTAT-GATACGCCCC-ACCCCC-TACTAATGACCACA
Myaf	GGA-CCGAGAATAA--TGAAGCCCCTGCCAGAGGTATGCTTC-ACTCCC-CACTAATGAATTCA
Lagu	GGA-CCGAAAAGAA--GAGGCCCATGCCCC-AAGCACGCCTC-ATCCTC-ACCTAATGAAGCCA
Trtr	GGA-CCGAAAAGAT--GGGGCCTATGCCTAA-GGCACGCCCC-CTCCCT-ACCCACTGAACTTA
Zucr	GGA-CCGAGAAGAT--GGGGTCCATGCTATA-GGTATACCCT-TTCCCC-ACCCACTGAACTTA
Pxja	GGA-CCGAAAAGGA--AGGGCCCATACCTAA-GGTACGCCCT-ACCCTT-ACCTAATGAAAACA
Pxlo	GGA-CCGAAAAGAA--GGGCCCATACCTAA--GGTACGCCTA---CCTT-ACCTAATGAAAACA
Pctr	GGG-CCGAAGAGAA--GAGGCCCATGCCAAG-AGCACGCCTC-CCC-CTTACCCAGTGAAAACA
Apsa	GGA-CCGAAAAGAA--GAGGCCTATGCTTA-ATGCACGCCTC-ACC-CTTACCCTATGAAATTA
Cabe	GGA-CCGGCAAAGG--GGGGTCAATGCTGCT-AGTACACCCC-GCCCCA-AATCGCTGACTACA
Bzze	GGA-CCGAAAAAGG--GAGGCCCATGCCCAA-AGTAAGCCTC-ACCCCC-ACCCTCTGAACGCA
Siim	GGA-CCGAAGAAGA--GGGGCCCATAATTTA-GATACGCCCC-GCCCCC-ACCTGCTGGTGCCC
Ctru	GGA-CCGAAAAGGA--GAGGCCCATACCAA-AGGCACGCCTC-CCCCTC-ACCTAGTGAAATAA
Dpbr	GGA-CCGAAAAGGA--GAGGCCCATACTAA-AAGCATGCCTC-CCC-CTCACCTAGTGAAATCA
Caki	GGA-CCTGAAAAGA--GGGGCCAATACTCCA-TGCACGCCCC-ACCCCCCAACCGCTGAGACCT
Phja	GGA-TCTAAAAGAG--GAGGCCCATACATTT-TGCACGCCTC-TCCCCC-AACCCCTGAAACCC
Brsp	GGA-ATGGGAAAAA--AAGGTCTATGTTAAA-GACACACCTT-TACCCC-CACCTCTGATAACC
Gamo	GGA-CCGGAAAAAG--GGGGCCTATGCCAA-AAGCACGCCCC-TCCCCT-AACCGCTGAAACCC
Lolo	GGA-CCGAAAAAAG--GGGGCCTATGCCTAA-AGCACGCCCC-TCCCCT-GACCGCTGACACCC
Batr	GGA-CCAAAAAAAG--AAGGCCCATAAGATC-CTCACGCCTT--ACAATTAGTGTTAGCTTCAA
Prmy	GGA-CAAAGAAAAG--AAGGCCAATGACTCC-ATTACGCCTT-ACCTTA-AGACACTAAATTAC
Loli	GGA-CCGAAAAGAA--GAGGCCCCTGTTACC-AACACGCCCC--TCTCTAACCTGATGCCACCA
Loam	GGA-CCGAAAAGAA--GAGGCCCCTGTAATAT-ACACGCCCC--TCCCTCACCTAATGAAATCA
Chab	GGA-CCGGGAAGAA--GGAGTCAATGTTTAT-AACACACCCC-ACCCTT-ACCTAATGAAAACA
Chto	GGA-CCGGGAAGAA--GGAGTCAATGTTTAT-AACACACCCC-ACCCTT-ACCTAATGAAAACA
Majo	GGA-CCGAAGAAAG--AGGGCCCATGCTTGA-AGCACGCCCT-CCCTCC-ATCTAGTGAATACA
Hlst	GGA-CCGAAAAAAG--GGGGCCAATGCTAAA-AGCACGCCTC-CTCCTC-ACTAAGTGATAACA
Clpe	GGA-CCGAGAAGAG--GGGGTCCATGTCCCA-TATACACCCC-GCC-CCCACCTAATGAAAACA
Mlmr	GGA-CCGAGAAGAG--GGGGCCCATGTTAAA-AATACGCCCC-TCCCCCCACCTAATGAAGACA
Crcr	GGA-CCGAGAAGAA--AAGGCCCATGCCAA--AATATGCCTT-ACCC-TTACCCAATGACACTA
Muce	GGA-CCGAGAAGAA--AAGGCCCATGCC-AA-AATATGCCTT-ACCCTTA-CCTAATGACACTA
Bege	GGA-CCGAAGAAAA--GAGGCCTATGCTCTC-AGTACGCCTC-CTCCTT-ACCTAATGAAGACA
Mela	GGA-CCGAAGAAAG--GGGGCCTCTGCTAAA-AGCACGCCCC-CTCC-TTATCTAATGAAAACA
Hats	GGA-CCGAAGAGAG--GAGGCCCATGCTCTA-AGTACGCCTC-ATCCC-CACCTAATGCAGCCA
Orla	GGA-CCGAAGAGGG--GGGGCCCATGGACC--ACTACGCCTC-AATCTC-ACCTTATGAAGAAA

```
Cosa GGA-CCGAAGAAGA--AAGGCCTATGC-TTAAAGCACGCCTT-ACC-CTCACCTGATGACACCC
Exsp GGA-CCGAAGAGAA--GAGGTCCATTC-TTTAGGAACACCTC-CCC-CTCACCTAATGAAACA
Depa GGA-CCGAAGAAGA--AAGGCCTATACTTA-AAGCAAGCCTT-ACCCTTACCTAATTGATACCC
Rima GGA-CCAAAGAAAA--AAGGCCCATCCCCAG-AGAACGCCTT-ACCCTT-ACCTGATGAAAACA
Fuol GGA-CCGAAGAAAG--GGGGCCAATGCTACA-AGCACGCCCT-ACCCTT-ATCTGCTGAAGGCA
Gmaf GGA-TCGAAGAAAA--AAGGCCTATGCTTAACAACATGCCTT-ACCCCT--CCTTATGAAACCA
Xeei GGA-CCGAAGAAAA--AAGGCCAATGC-TACAAGTACGCCTT-ATC-CCCATCTAATGAAAACA
Pros GGA-CCGAAGAGAA--AAGGCCCCTGCTAAA-GGCATACCTT-TCCCCC-ACCTAATGAAAGCA
Scmi GGA-CCGAAGAGAA--AAGGCCCCTGCTAAA-GGCACACCTT-CCCCCC-GCCTAATGAATTCA
Rolo GGA-CCGAAAAGAA--GAGGCCCATGCTTGA-AGTATGCCTC-GCCCCC-ACCTAGTGAAGACA
Cere GGA-CCGGAAAGAG--GGGGCCCCTGCTTTA-AGTAAGCCTC-ACCCCTA-CCTAGTGAAAACA
Daga GGA-CCGAGAAGGA--GAGGCCCATGCTACG-GGCACGCCTC-ACCCCCACCTAGATGAAGGCA
Anco GGA-CCGAAAAGAA--GAGGCCCATACTAAA-AGCACGCCTC-ACCC-CCACCTAATGAAAACA
Dmve GGA-CCGAAAAGAA--GGGGCCCATGCTTAA-AGCACGCCCC--ACCTCCCCTAATGAGAACA
Dmar GGA-CCGAAAAGAA--GAGGCCCATGCTTGA-AGCACGCCTC-CCCTCC-CCCTGATGAAAACA
Anka GGA-CCGAAAAGAA--GAGGCCTATGCCAAA-AGCACGCCTC-ACCCCC-ACCTAATGAAGACA
Moja GGA-CCGAAAAGAA--GAGGCCCATACCAA-AGGCACGCCTC-ACCCCCA-CCTAATGAAATCA
Hoja GGA-CCGAAAAGAA--GAGGCCCATACCAA-AAGCACGCCTC-GCC-CCCACCTAATGAAAACA
Bede GGA-CCGAAAAGAA--AAGGCCCATGCTCAT-GGCACGCCTT-ACCCCC-ACCTAATGAAAACA
Besp GGA-CCGGAAAGAA--AAGGCCCATGC-TCGAGGCACGCCTT-ACC-CCCACCTAATGAAAACA
Mysp GGA-CCGAAAAGAA--GAGGCCTATGC-TCACAGCACGCCTC-ACC-CTCACCTGATGAAACCA
Osja GGA-CCGAAAAGAA--GAGGCCTATGCTAAC-AGTATGCCTC-ACCCC-TACTTGATGAAACCA
Sgro GGA-CCGAAAAGAG--AGGGCCCATGCTAAA-AGTATGCCCT-TCCCCC-ACTTGATGAAAACA
Pzpa GGA-CCGAAAAGAA--AAGGCCCATGCCCCA-AGCATGCCTT--ACCCCTAACTACTGAAATCA
Zeja GGG-CCGAGAAGAA--AAGGCCCATGCTTC-AAGTACGCCTT-ACCTGT-AACTACTGAAACCA
Znne GGA-TCGAGAAGAA--AAGGCCCATGCTTCA-AGTACGCCTT-ACCCCT-AACTACTGAAACCA
Zefa GGA-CCGAGAAGAA--AAGGCCCATGC-TAAAAGTATGCCTT-ACC-CCTAACTACTGAAGCCA
Acni GGA-CCGAGAAGAA--AAGGCCCATGCTCCA-AGTATGCCTT-ACCCCC-AACTACTGAAACCA
Ncrh GGA-CCGAGAAGAA--AAGGCCCATGCTCCA-AGTATGCCTT-ACCCCC-AACTACTGAAACCA
Agca GGA-CCGAAAAGAG--GGGGCCCCTGCTT-AAGACATGCCTC-TCCCCC-ACCTGATGAAGACA
Hydy GGA-CCGAGAAGAG--GAGGCCTATACCCCT-GGCACGCCTC-CCCCCC-ACCTGATGAGTTTA
Gsac GGA-CCGAGAAGAG--GAGGCCTATACCAC-TGGTACGCCTC-TCCCTT-ACCTGTTGAATTTA
Pevo GGA-CCGAAAAAAG--GGGGCCCATGCTAAA-AGTACGCCCC-CTCCCC-ACCTAATGAAATCA
Hiku GGA-CCAAGAAGGA--TGGGCCCATGTTTTAAAACAAGCCCA-CTTTTT-AACACTTGAAGAAA
Inpa GGA-CCGAAAAAAG--GGGGCCCATGCTACA-AGCACGCCCC-ACCC-AAATCTGATGAAAACA
Auch GGA-CCGGACGGGG--AGAGCCCATCCCACA-TCGACGCCCT-TTCA-CCACCTGATGAAGCCA
Fico GGA-CCGAGAAAGT--AAGGCCCATGC-TTTCAGCACGCCTT-TCC-CCCACCTAATGAAGGCA
Macs GGA-CCGAAAAGAA--GAGGCCTATGCTCCA-GGCACGCCTC-TCC-CCCACTTACTGAAGACA
Moal GGA-CCAAAAAAAC--AAGGTCCCTGCCT-TAAGCACACCTT-ATTCTA-CCTCAATGAACCCA
Syma GGA-CCGAAAAAAG--AGGGCCCATACCTAA-AGCAAGCCCT-ACCC-CCAACCCCTGAAACAA
Mafr GGA-CCGAAAAGAA--GGGGCCCATGCTCAA-AGCACGCCCC-TCC-CCCACCTAATGAAAACA
Dcpe GGA-CCGTAAAGAA--GAGGCCCATACCC-CCCGCACGCCTC-CCCCTT-AGTTAATGATGTAA
Dcti GGA-CCGTAAAGAA--GAGGCCCATGCCTCC-CGCACGCCTC-CCCCTT-AATTAATGATATAA
Hehi GGA-CCGAAAAGAA--GAGGCCCATGCTCT-AAGCAAGCCTC-ACCCCC-ACCTAATGAAGACA
Stam GGA-CCGAAAAGAA--GAGGCCCATACCTCA-GGCACGCCTC--ACCCCCACCTAATGAAGACA
Hogi GGA-CCGAAAAGAG--GGGGCCCATGCTTAA-AGTACGCCTC-ACCCCT-ACTTAATGAAACCA
Erzo GGA-CCGAAAAGGA--GGGGCCTATACTGC-AAGTACGCCTC-ACCCTC-ACCTATTGAAATCA
Hxot GGA-CCGAAAAGAA--GAGGCCTATGCCAC-AGGCACGCCTC-CCCCCT-ACCTAATGAAGTCA
Core GGA-CCGAAAAGAA--GAGGCCCATGCTAGA-GGCACGCCTC-CCCCTC-ACCTAGTGAAGACA
Apve GGA-CCGAAAAGAA--GAGGCCCCTGCCAAA-GGTACGCCTC--CCCCCTACCTAATGAAACAA
Latj GGA-CCGAAAAGAA--AAGGCCCATGCCACA-AGTATGCCTT-ATCCCCA-CCCAATGAAAACA
Laja GGA-CCGAGAAGAA--GAGGCCCATGCTTAA-AGTACGCCTC--CCCCCGACCTGATGAAGACA
```

Syja	GGA-CCGAGAAGAA--GGAGCCCATATTTTA-AACACGCCCG-ACTCCT-ACCCAATGAAACA
Epme	GGA-CCGAAAAGAG--GGGGCCCATGTCCAA-AATATGCCTG-ACCCCC-ACCTGATGAAGCA
Grse	GGA-CCGAAAAGAA--GAGGCCAATGCTCCA-AGCACGCCTT-ACCC-CCACCTAATGAAACA
Clja	GGA-TCGAAAAGAG--GAGACCTCTACTCTA-AGTACGTCTC-ACCCTT-ACTGGGTGAAACA
Ogcy	GGA-CCGAAAAAGG--GAGGCCCATACTAAATAGCACGCCCC-CCCCCC-ATCTGATGAATA-G
Plna	GGA-CCGAAAAAAA--GGGGCCCATGC-TCAAAGTACGCCTC-AGC-CTCACCTAATGAAACA
Lema	GGA-CCGAAAAAAA--GAGGCCTATACTCCT-AGTATGCCTC-ACCCCC-ACCTAATGAAACA
Etzo	GGA-CCGAGAAGAA--GAGGCCTATGCCCTA-AGCACGCCTC-ACCCCC-ACCTACTGAAACA
Apse	GGA-CCGAAAAAAA--GAGGTCCATGCTAA-AAGCACACCTC-CCCCTC-ACCTAATGACACCA
Epde	GGA-CCGAAAAGAA--GAGGCCCATGCTTAA-GGTATGCCTC--ACCCCCACCTAATGAAGACA
Slja	GGA-CCGAAAAGGA--GAGGCCCATGCCAA-AAGCACACCTC-ACCCCCA-ACTAATGAATACT
Bsja	GGA-CCGAAAAGCG--GGAGCCCCTAT-TCCAAACACGCCCC-CCC-CCCACCTAATGAATCA
Ecna	GGA-CCGAAAAGAA--GAGGCCAATGCCA-A-AGCATGCCTC-CCCCTT-ACCTAATGAAACA
Cohi	GGA-CCGAAAAGAA--GAGGCCTATACTTCA-AGTACGCCTT-ATCC-CAATCTAATGAAACA
Caar	GGA-CCGAAAAGAA--GAGGCCCATACCTT-AGGCACGCCTC-CCC-CTTACCTAATGAACCA
Came	GGA-CCGAAAAGAA--GAGGCCTATACCCCA-GGCACGCCTC--ACCCTTACCTAATGAATCA
Mema	GGA-CCGAAAAGAA--GAGGCCCATGCCAAA-AGCACGCCTC-ACCC-TCACCTAATGAAGCA
Lenu	GGA-CCGAAGCGAG--GAGGCCCATGGCA-AAGCTAAGCCTC-ATC-CTCTTCTTATGAACCA
Brja	GGA-CCGGAAAGAA--GAGGCCCATGCTTGA-AGCACGCCTC-CCC-CTT-CCTAATGAAACA
Plma	GGA-CCGGGAAAGA-AGAGGCCAATACTCTA-GGCACGCCTC--ACCCCT-CCTAATGAAACA
Emst	GGA-CCGAAAAGAA--GAGGCCCATGCTCAA-GGTACGCCTC-ACCCCC-ACCTAATGAGACA
Ptti	GGA-CCGAAAAGAA--GAGGCCCCTAC-TTCAAGTACGCCTC-ACC-CCCACCTAATGAAACA
Losu	GGA-CCATGAAGAA--AAGGCCAATTCTTAA-AGTACGCCCT-ACC-CTTACTTATTGAAACA
Geoy	GGA-CCGAGAGGGA--GAGGTCTATACTTG-AAGCACACCTC-ACCCC-TACCTGCTGAAGTCA
Dipi	GGA-CCGAAAAGAA--GGGGCCCCTGCTCA-AAGTACGCCTC-TCCCT-TACCTAATGAAACA
Pama	GGA-CCGAAAAGGA--AAGGCCCCTGCCCAA-GGCACGCCTT-GCCCTCA-CCTGCTGAAGACA
Leob	GGA-CCGAAAAGGA--AAGGCCCCTAC-TTAAAGTACGCCTT-ACC-CTTACTTAATGACACCA
Neba	GGA-CCGAAATAAA--GGGGCCCATGCTTT-AAGTATGCCCC-TCCCTA-AATCAATGAATTA
Pdpl	GGA-CCGAAAAGTA--GGGGCCCCTACTACAAAGCACGCCCC-CCTCCA-ACCTAATGAACCA
Nimi	GGA-CCGAAAAGAA--AGGGCCCATACTTTA-AGCACGCCCT-ACCC-TTACCTGATGTAAGCA
Uptr	GGA-CCGAAAAGAA--GAGGCCAAAGCTCAA-AGTAAGCCTC-CCCCTA-ATTTATTGAAACA
Pesc	GGA-CCGAAAAGAT--AGAGCCCATGTTCTT-AGTACCCTCT-AATCTT-ACCTGATGCAAACA
Baar	GGA-CCAAGAAGAA--GAGGCCCATACTTAA-AGCACGCCTCTACCCCTCACCTAATGAAACA
Moar	GGA-CCGAAAAGAA--GAGGCCCATACCCTA-AGTACGCCTC-ACCCCC-ACCTACTGAATCA
Toja	GGA-CCGAAAAGAA--GAGGCCCATGCTCCA-AGCACGCCTC-ACCCTC-ACCTAAAGAAGCA
Chau	GGA-CCGGAAAGAG--GGGGCCCCTACCCCT-GGCACGCCCC-TCCCTC-ACCTTATGAGACCA
Chse	GGA-CCGAGAAGTA--GAGGCCCATGCTTCA-AGCACACCTC-CCCCTT-ACCTAATGATATCA
Enar	GGA-CCGAAGAGAA--GAGGCCCATTCTCCA-AGAACGCCTC-ACCCCT-ACCTAATGAAACA
Hpty	GGA-CCGAGAAGAA--GAGGCCCATGCTTAA-AGTACGCCTC-CCCCTC-ACCTAATGAGACA
Nana	GGA-CCGAAAAGAT--GGGGCCCATGCCAAA-GGTATGCCTC-CCCCTA-ACCTGATGAAACA
Mcst	GGA-CCGAAAAGAA--GAGGCCCATACTCAA-AGTACGCCTC-ACCCCC-ACCTAATGAAAAA
Rhox	GGA-CCGAGAAGAA--GAGGCCCATGC-CCAAAGTATGCCTC-CCC-CTAACCTAATGAGACA
Opfa	GGA-CCGAAAAGAA--GAGGCCCATATCCAA-GATACGCCTC-ACCCCT-ACCTAATGAAACA
Paar	GGA-CCGAAGAGAA--GAGGCCCATACTT-AAAGTACGCCTC-CCCCTC-ACTTAATGAACCA
Gozo	GGA-CCGGAAAGAG--GGGGCCCATGCTTTA-AGCACGCCTC-ACCCTC-ACCTACTGAAGCA
Ackr	GGA-CCGAAAAGAC--GGGGCCCCTGCCATA-CGCATGCCCC-CCCCTT-ACCTAATGAAGCA
Elev	GGA-CCGAAAAGAA--GAGGCCCATACTTA-AAGTAAGCCTC-ACCCA-CACCTAATGAAATA
Trdu	GGA-CCGAAAAGAA--GAGGCCCCTGTTC-CCAATATGCCTC-CCCCTC-ATCTATTGAATCA
Amoc	GGA-CCGAAAAGAA--GAGGCCAATACCTTA-AGCACGCCTC-CCCCCG-ACCTGATGAAACA
Hame	GGA-CCGAAAAGAA--GAGGCCAATGTT-AAAAATACACCTC-ACC-CTTACCTAGTGAATCA
Chso	GGA-CCGAAAAAAA--GAGGCCTAGCCT-AAAAGGATGCCTT-CCCCTCAT-CTAATGAGGCA
Lyto	GGA-CCGAAAAGAA--GAGGCCTATGCTT-AAGGTACGCCTC-CCCCTT-ACCTACTGAAGACA

```
Encr    GGA-CCGAGAAGAA--GAGGCCCATGCTCC-AGGCACGCCTC-CCC-CTTACCTAGTGAAAACA
Bvar    GGA-CCGAAAAGAA--GAAGTCAATATTT-TAAACACACTTC-ACCCCT-ACCCAATGAAGACA
Noco    GGA-CCGAAAAAAA--GGGGTCACTACACTA-TGTACACCCT-ACCCCT-ACCTAATGAAAACA
Chsp    GGA-CCAAGGATAA--GAAGCCCATGCTTAC-AGTACGCCCC-CTCCTC-A-CTATTGTATATA
Arja    GGA-CCGAAAAGAA--GAGGCCCCTGC-CTAAGGCACGCCTC-CCC-CCTACCTGTTGAAGTCA
Pase    GGA-TCGGAGAAGG--AAGGCCCCTGCTACA-CCCATGCCCT-ATCCTC-ATCTGCTGCCGTTC
Trel    GGA-CCGGGACAGC--AGGGCCAATGTTC-TCACCATGCCCC-CTCCTCA-CACACTGGCCACT
Lifa    GGA-CCGAAAAGAA--GAAGCCCACTCCA--AAGAGAGCTTC-ACC-CCCACCTGCTGAAATCA
Acur    GGA-CCGAAAAGGA--AAGGTCCATACTTTT-GGTACACCTG-CCCCTC-ACTTGATGAAAA-A
Ampe    GGA-CCGGAAAGAA--GAAGCCCATGCTATA-AGTATGCTTC-ACCCTC-ACCTAATGTAGATA
Urja    GGA-CCGAAAAAAC--GAGGCCCCTGCTCAA-AACACGCCCC-CCCCTT-A-TTAATGAAGCCA
Enet    GGA-CCGAAAAAAG--GGGGCCAATGTCTCAAGGCACGCCCC-ATCCCT-ACCCTCTGACACCC
Ptbr    GGA-TCGAAAAGAA--AAGGCCTATGATACC-TTCAAGCCTT-ATACTTAC-CTAATGAACCCA
Safa    GGA-CCGAAAAGAA--GAGGCCCCTGCCA-AAGGCACGCCTC-CCCACCCACCTAATGAAATCA
Icae    GGA-CCGAGAAGAA--GAGGCCAATGCTCAA-GGCACGCCTC-TCCCCT--CCTATTGAAAACA
Asmi    GGA-ATGGAAAGCA--GTGGCCCCTGCTCCA-AGCACGCCAC-CCTTTT-ACCCAATGAAAACA
Foal    GGA-CCGAAAAGGC--AAGGCCCATTCTTTC-AGTATGCCTC-CCCCCTTACCTGTTGAAATAA
Drze    GGA-CCGAAAAGAA--GGGGTCCATGCTAAT-AGTACACCCC-ACCCAT-AACTGTTGAAAGAA
Rhas    GGA-CCGAAAAGAA--GAGGCCCCTGCTAAA-AGCAAGCCCC-CTCCTG-ACCTGATGAAGTCA
Elac    GGA-CCGAAGAGAA--GAGGCCCCTGC-CAA-AGCAAGCCTC-ACCCCA-ACCTTAGGAAAACA
Kugu    GGA-CCGAAAAGAA--GAGGCCCATACTAAA-AGCACGCCTT-CCCCCT-ACCTAATGAAAACA
Plor    GGA-CCGAAAGAAA--GAGGCCCATGCTCCC-AGTATGCCTC-ACCCCC-ACCTGATGAAAACA
Sgun    GGA-CCGAAAAGAA--GAGGCCAATACC-ATCGGCACGCCTC-ACCCT-CACCTAATGAAACCA
Zaco    GGA-CCGAAAAGAA--GAGGTCCATACTAAA-AGTACACCTC-ACC-TTTACCTGCTGAAAACA
Zbfl    GGA-CCGAAAAGAA--GAGGTCCATGCCTAA-AGCACACCTC-ACCCCC-ACCTATTGAAAACA
Spba    GGA-CCGAAAAGGA--GGGGCCCATGCTAA-AGGCACGCCCC-ACCCTT-ACCCAATGAAAGCA
Game    GGA-CCGAAAAGAA--GAGGCCTATGCTCAA-AGCACGCCTC-ACCCCT--CCTATTGAAAACA
Thth    GGA-CCGAAAAGAA--GAGGCCAATGCTA-AAAGCACGCCTC-ACCCCT--CCTATTGAAAACA
Xigl    GGA-CCGAAAAGAA--GAGGCCCATGCTTGA-AGTACGCCTC--ACCCCCACCTAATGAAAGCA
Hyja    GGA-CCAAAAAGAA--GAGGCCAATGCTCAA-AGCACGCCTC-ACTC-CT-CCTATTGAAAACA
Psan    GGA-CCAAAAAGAA--GAGGCCTATACCCAA-AGCACGCCTC-ACTCCT--CCTATTGAAATCA
Cupa    GGA-CCGAAAAGAA--GAGGCCAATACTT-AAAGCACGCCTC-A-CCCC-TCCTAATGAAAACA
Mpch    GGA-CCGAAAAGAA--GGGGCCAATACTGAA-AGCACGCCCC-ACCCCT-ACCTAATGAAGACA
Char    GGA-CCGAAAAAAA--GAGGCCCATGCTATA-AGTATGCCTC-CCCC-TCACCTAATGAAATCA
Pser    GGA-CCGAAAAGAA--GAGGCCCATGC-TTAAAGTACGCCCC-ACC-TCTACCTAATGAAAACA
Prol    GGA-CCGAAAAGAA--GGGGCCCATGCTCTT-AGTACGCCTC-ACCCCC-ACCTAATGAAAAAA
Plbi    GGA-CCGAAAAGAA--GGGGCCCATGCTAAA-AGTACGCCTC-ACCCCC-ACCTAATGAAAAAA
Calu    GGA-CCGAAGAAAG--AGGGCCCATGCTTCCCAGTACGCCCT-ACC-CCCACCTGATGAAAACA
Papa    GGA-CCAAAGAGAA--GAGGCCCATTTCACA-GATACGCCTC-ACCCTC-AATTTGTGAATTAA
Sufr    GGA-CCAAAAAGAA--GAGGCCCCTGTTAAA-AACACGCCTT-ACCTCG-ACTCATTGAACCCA
Stci    GGA-CCAAAGAAAT--GAAGCCTCTGTTATT-AATAAGCCTC-ACTCCC-ACTTACTGACACCT
Taru    GGA-CCGAAAAAGA--GGGGCCAATGT-ACA-AACAAGCCCC-ACTC-TCACTTGCTGAATCCA
Rala    GGA-CCGAAAAGAA--GAGGCCCATGCTTC-AAGTAAGCCTC-CCC-CCCACCTAATGAAAACA
        **                  *
```

```
             57'                                         58          58'
Scca   -ACTAAAATAGATAA-----GAAAAGATTATCTA---------TT--GCCCAAGAAA-AGGGT
Muma   -ACTTAAATAGATAAG----AAAAGATC----A--CC------CACTGCCCAAAAAC-AGGGT
Erca   -AATCAAACAGATAAT----AAAGA-ACATATTTT----------AATCCAAGAAA-AGGAC
Pose   -GATCAAACAGATAAT----AAAGAACATATCCC-CA----------CCCTAGAACA-GGGTT
Actr   -ACTAAAATAGGTAAA----GGGGCACAAACCTCC------------CCCCTAGA-A-TAGGG
Scal   -ACTAAAATAGGTAA-----AGGGGCACAAATCC---------TCC-CCCCTAGAAT-AGGGC
Posp   -ACTAAAATAGGTAAA---GGGG-CACAAACCTT-TC----------CCCTAAAACA--GGGC
Atsp   -ACTAAAATGGAAAAG----AGGACATAACACCACA-----------GCCCCAAAAA-AGGGC
Leoc   -ACTAAAATGAAAAAG----AGGACATA--TCAT---------TAATACCCCAAAAA-AGGGT
Amca   -ACTAAAATAGGAAAA----AGGACACACCACCC-TT----------ATCCAAAACA-AGGAT
Osbi   -ACTAAAATAATAAAG----GAAA-GCACCTACTAA-----------CCCAGAGACA-ATGGT
Pabu   -ACTAAAACAA-TAAA----GGGGCATAACAAG-----------CTCGCTAAAAATA-ACAGC
Hial   -ACTAAAACAGA-AAG----GGGGAACAATAAA----------GCA-GCCCAAGATA-ATGGC
Elha   -ACTAAAAGGAAAAG----GGG--GTACAACGAA--------CACAGCCAAAGACA-ATGGC
Mlcy   -ACTAAAACGGAAAAG----GGGGCACAACCCACACC------ACAAGCCACAGATA-ATGGC
Algl   -AATAAAGTAGGAAAG----GAAGCACAATTG-TA-----------AGCCAGAGA-T-TATGG
Ptgi   -AATAAAACAGAGAAG----GGAGT-ACAATTAAAGC---------AACCGAAGATA-ACGGC
Alaf   -AATAAAACAGGAAAC----GGGGTCCAACACA----------AACAAACGGAAATA-ACGAA
Nock   -AATAAAACAGATAAC----GGGACGCAACAA------------ATAATCGAAAATA-ACGAA
Anja   -AATAAAACAGACAAA---GAG-GT--ATATTAAACC---------TACCAAAGATA-ATGGT
Gyki   -TATAAAGCAGATAAT----GGGGAAGACACA------------ACTGCCAGAGATA-ATGGC
Syka   -AATAAAATAGATAAA----GAGGTATACT-----TA------ATCGACCAAAAATA-ATGGT
Opma   -AATAAAAAGGATAA-----TGAGATAAACACTA---------CTT-ACCAAAGATA-ATGGT
Comy   -AATAAAGAAG-TAAG----GGTGAACA--TCCA-TT------A---GCCAAAGATT-ATGGC
Sasp   -AATAAAATAGGCATT----GGA----CACCCACACA---------ACCAAAGAATT-TTGGC
Eupe   -AAAGAAACAGATAAT----TAG--ACACCTA-----------CACAGCCATAGATA-ATGGT
Enja   -ACTAAAGCAGGTAAG----GATAA-GCAATCCA-AC---------GGCTCAAGAAA-AGAGC
Same   -ACTGAAGCATGTAAA----GGGGGGCACCCC-----------TCA-GCCCGAGAAA-ATGGT
Chch   -ACTAAAGCAGGCAAA----AAAGCTCAC---AA-CT------CCCAGCCAAAGACA-AAGGC
Grgr   -ACTAAACCGGACAAG----GGGGAGCAAA-ACT-----------CTGCCCTAGATATAGGGC
Caau   -AATAAATAAAATAAAGGGAGGGCCAAAACCCCA-GC---------TGGCCGAAATA-AGGAC
Cyca   -AATAAATAAAGTAAA----GGGAGAGCCAAAATCCC------AGCTGGCCAAAATA-AGGAT
Dare   -GATAAAAAAAATAAA----AGGAGAGCTAAAAC---------CTCTTCCCGAAATA-AGGGT
Cost   -ACTAAATTAGATAAA----TGGAGGACCTACAATTT---------AGGCAAAAATA-AAGCC
Leec   -ACTAAATTAAGTAAA----GGGGGGCCAAA-----C-------ACGTGCCAAAATA-AGGCC
Crla   -ACTAAATTAAGCAAA----GGT-AGAACCCCCT---------CTACTGCCAAAATA-AGGCC
Clmc   -ACTCAACCAAACAAA----AGAAAAGCACCAAA----------CCCACCCAAGATA-AGGGA
Phin   -ACTCAATTAAACAAA---AGAAGG--GCATAAA-TA--------AGCCCCGAGATA-AGAAA
Icpu   -ACTAAATTAAACAAA----GGGGGGCACAAT-C----------CCACATCAAGATA-AGATT
Psto   -ACTAAATTAAATAAT----GAGAGGGCATAACC---------CTAAAATCAAGATA-AGATT
Cora   -ACTAAAATAGATAAA----GGAAAGCATAAATA----------ACAAATTTAGATA-AAATT
Eisp   -ACTAAATTAAATAAG----GGCGG-AC-CCAATAGC---------CTCTCTAGATA-AGTGC
Apal   -AATCAATCAAACAAA---ATTA-AACACTCTAC------------CGCCCAAAAGA-AGGGG
Eslu   -ACTAAAATAGACAAG----GG--GGCACGCCAC---------AACTGCCGAAAAGA-ACCGC
Dape   -ACTAAAACAGGTAAG----GGGGCACATCCCT----------GT--GCCGAAAATT-ACTGC
Glse   -ACTAAAACAGAAAAG----GGGGTACACCCCAAAAA----------GCCCAAGAGA-ACGGC
Naar   -ACTTAAACAGATGAG----GGGGCACACCCAA----------ATGAGCCCAAGAGA-ATGGC
Baoc   -ACTAAAACAGAAAAA----GGG--GCACACCCAC--------TCGAGCCCGAGAGA-ACGGC
Opso   -ACTAAAACAGAAAAG----GGAAC-ACATCCCAGTG---------AGCCCGAGAGA-ATGGC
Alte   -ACTAAACCAGACAAG----GGGGCGCAATCCT-----------TCAGCCCTAGATA-AGGGC
Plap   -ACTAAACCAGACAAA----GGG---GCGCAATCCTC---------AGCCCTAGACA-AGGGC
```

```
Plal  -ACTAAAACAGACAAG----AGGGC-ATACTCAGGTG---------TGTCAGAGAGC-ATGAC
Sami  -ACTAAAACAGACAAG----AGGGCATAC-CCAG-GT------G--TGCCAAAGAGT-ATGGC
Rere  -ACTAAAACAAGTAAG----GGGGCATACCCAGC------------TCGCGTCAGAGA-TTGGC
Gama  -ACTAAATTAGACAAG----GGGGC-ACACCCTTG-C---------GACCCGAGATA-ATGGC
Onmy  -ACTAAAACAGACAAG----GGGGCACACCAAG----------AT-TGCCTAAAAGA-ACGGC
Sasa  -ACTAAAACAGAAAAG----GGGGCACACCAAGATT-----------GCCCAAAAGA-ACGGC
Cola  -ACTAAAACAGGCAAG----GGG--GCACACCAAG--------GTGTGCCTGAGATA-ACGGC
Dita  -ACTAAAATGGAAAAA----GGGGGGCAAGCCCG-GA-------GGCGCCCTAGAGA-AGGGC
Gogr  -ACTAAAATAGAAAAG----GGGTACCCCAAA-C-----------AAGCCCTAGATA-ATGGC
Chsl  -ACTTAAACAG--AGA----GGAGC-CC-CACGGC-T---------AGTCTAAGAAA-ATGAC
Atja  -ACTAAATTAGACAAG----GGGGTACAACCCT---T---------TGCCTAAGACA-AAGGC
Iido  -ACTAAATTAGACAAG----GGGGTACAACCCTTT-----------TGCCTAAGATA-AAGGC
Auja  -ACTAAAACAAGCAAG----AGGGCACGCCCTTTTG-------GCTTGTCAAAGAGA-ATGAC
Chag  -ACTAAAACAAGCAAG----AGGGCAAGCCCCGG---------CTATGTTAAAAATC-ATAAC
Hami  -ACTAAAGCAAAAAAC----AGGTCATACCCGAA----------CTTGTTTGAGAAA-ACAAC
Saun  -ACTAAAACAAGAAAA---AGG-AC--ATACCCGACA-------CTTGTTCAAGATA-ACAAC
Nema  -AATAAAATAGGAAAG----GGGGC-ATATCCTTCTT----------GCCCTAGAGA-AGGGC
Disp  -AATAAAATAGAAGG-------GGGACAAGATCC---------TGAAACCCAAGAGC-AGGGT
Myaf  -AATAAAATAGAAAGG----AAG--CAAAATC-----------CCAAGCCCAAGAAT-AGAGC
Lagu  -ACTAAAGCAGACAAG----AGGACATAAGAGGC-----------ATGCCAAAGAGC-ATGGT
Trtr  -ACTAAAGAGGATTAA----GGGGTACG-CCCAT---------GC--GCCAAAGAGC-ATGGC
Zucr  -GCTAAAGAGGCCTAA----GGGGGGTGCCCA------------TGCGCCAGAAAGC-ATGGC
Pxja  -ACTAAAATAGGTAAA----AGGGCATAACCCCT---------TTA-GCCACAGAAA-ATGGC
Pxlo  -ACTAAAATAGGTAAA----AGGGC-AAAACCCC-TA---------AGCCACAGAAA-ATGGC
Pctr  -ACTAAAGTGGACAAA---AGGG-CATGCCCCAG-TT------GG--GCCAAAGATA-ATGGC
Apsa  -ACTAAATAGGGCAAA----AGGGCATG--CCC--TC------CG-GGCCAAAATTA-ATGGC
Cabe  -ACTAAAGTGAGTAAT----GGGGCAGC--CC-C---------GTTAGCCGAAGAAT-ACGGC
Bzze  -ACTCAAGAGGACAAG----GGGGCATA--CT-A---------TAGTGCCCCAGAGA-ATGGC
Siim  -AATCAAGCAGCTAAC----GGGAACTA--TCAC---------CGCAAGTAGAGAGA-ATACT
Ctru  -ACTAAAATAGGCAAG-----AGGGCACGCCCC-----------CCTGCCTAAGAAA-ATGGC
Dpbr  -ACTAAAATAGGCAAG----AGGGCACGCC-----CC-------CCT-GCCTAAGAAA-ATGGC
Caki  -TATAAAACGGATGA-------GAGCACTTAATA---------ATTAAACGAAGATT-ACGGA
Phja  -AATAAAGTGGGCAAA----GAGGCTTAAAAA-T-----------TCGGCCCCGAGA-AGGCT
Brsp  -AATAAAAAGGATAAG---GGA-AC--ATGTTAAATT-------TACCTAGAAGGTA-GCAGA
Gamo  -AATAAAGCGGATAAG----GGG--GCTTAAAAA----------GACCCCAAAAAGA-ATGGG
Lolo  -AATAAAGCGGACAA-------GGGGGCTTAAAAAGA---------CCCCAAAAAGA-ATGGG
Batr  -ACTGCACACAACAAT---AAA-ACTAATAGTAAACC-------ACTCGTTCAGACA-ACTTC
Prmy  -CCTAAAATGTTTACA----AGGA-ATATTC--------------CCTCATTTAAAG-ACCCT
Loli  -ACTTAATCTGATAAT---AGA-GC--ACA--ACACT-------TCCCCAAAAGAGA-ATTGG
Loam  -ACTAAATTAGACAAG----AGGAC-ATACCTGTTCC---------AGCCAAAGAAA-ATGGC
Chab  -ACTAAAATAGGCAAA----AGGGCATTACACCC-----------CCGCCTGAAATA-ACGGC
Chto  -ACTAAAGTAGGCAAA----AGGGCATTGCACCC-----------CCGCCTGAAATA-ACGGC
Majo  -ACTAAACTAGACAA------GGAGGCATCACCC----------GCATGCCTTAGATATAAGGC
Hlst  -ACTCAACTTAATAAG----AGGACAT-CCTTAT--A---------TGCCTAAAAAC-AAGGC
Clpe  -ACTAAAATAGGCAAG---AGGG-CATACCTGTC-AC----------CCCATAAATA-ATGGC
Mlmr  -ACTAAAATAGGCAAT----AGGACATACCCTAT---------TTACGCCTGAGATA-ACGGC
Crcr  -ACTAAAATAGGCAAA----AGGGCGTCCCCTAAT------------GCTACAGATA-ATAGC
Muce  -ACTAAAATAGGCAAA----AGGGC-GTCCCCCAA-----------TGCTACAGATA-ATAGC
Bege  -ACTAAAATAGGCAAA----AGGGCATGCCCCCCC-----------AGCCTGAGATA-ACGGC
Mela  -ACTAAATAAGACAAA----AGGACGTACCCCCC------------CGTCTAAGAAA-ATGAC
Hats  -ACTAAACTAGGCAAG----AGGACATA--CCTC---------TTATGCCTTAGATA-ATGGC
Orla  -ACTAAATAAGACAAG----AGAATATATCT-------------TGAGTCATAGAAA-ATGAC
```

```
Cosa    -ACTAAAGCAGGCAAA----AGAACATAA-TACC-TT---------ATTCGAATAAA-ATGAT
Exsp    -ACTAAAATAGGCAAA----AGGACATATC-CTT-AA----------GCCGTAAAGA-ACGGC
Depa    -ACTCAAATAGGTAAA----AGAGG-ACATCATC-----------CTTCCAAAGATA-ATGGC
Rima    -ACTAAAACAGATAAA----AGAGCACAAC-TTT-------ACAGACGACTAAGATA-ACGTC
Fuol    -ACTAAAATAGATAA-------AAGGGTATATTT---------TTTAGCTAAAAATAATTAGC
Gmaf    -ACTTAATTAGGCAAA----AGG-CACACAAAAC-----------CCGCCAAAGATA-ATAGC
Xeei    -ACTAAAATAGACAAC----AGGACATAACCCCA------------CGCTATAAATA-ATAGC
Pros    -ACTAAATTAGGCAAG----GGGGTACATCCCTT-----------AAGCCCAAAAAT-AGGGC
Scmi    -ACTAAAATAGGAAAG----GGAGCACATCCCCT----------TAAGCCCAAAATT-AGGGC
Rolo    -ACTAAAGTAGGCAA-----GAGGGCATGC-CCC---------CCA-GCCTGAGAAC-ATGGC
Cere    -ACTTAAATAGGCCAG----AGGGCATA--CC-C---------TCTGGCCTAAAAGA-ACGGC
Daga    -ACTTAAGTAGGTTAA----AGGGCATG-CCCT----------TTA-GCCTAAAAGA-ACGGC
Anco    -ACTAAAATAGGCAAG----AGGGCATGCCCCTTAA-----------GCCTAAGAAA-ATGGC
Dmve    -ACTAAAATAGGCAAA---AG--GC--ATGCCCCCAC---------AGCCTCAGAAA-ACGGC
Dmar    -ACTAAATCAGGCAAA----AG-GCACACCTCCGGC----------AGCCCAAGAAA-AAGGC
Anka    -ACTAAAATAGGCAAG----AGGGCATAACCC-T----------AAAGCCCAAGAAA-ATGGC
Moja    -ACTAAAATAGGCAAG----AGGGCACA----AA-CC------CCCAGCCTAAAAAA-ACGGC
Hoja    -ACTAAAATAGGCAAG----AGGGCATACC-----CC------TTAAGCCTAAGAAA-ATGGC
Bede    -ACTAAAATAGGCAA-------GGGGGCATACCCCTA---------AGCCTAAGAAC-ATGGC
Besp    -ACTAAAGTAGGCAAG----GGGGCATA--CCCC-CT--------AAGCCTAAGAAC-ATGGC
Mysp    -ACTAAATCAGATAAG----AGGACATACCACCA-CA------TAAAGCCCACGAAA-ATGGC
Osja    -ACTAAAACAAACAAG----AGGGC-ATACCCTA-CG---------AGCCTCCGAGA-ACGGC
Sgro    -ACTAAAACAAACAAG----GGGGCATATCCCAC----------ATAGCCCTCGAGA-ATGGC
Pzpa    -AATAAAGAAGACAAA---GGG-AC--ATATTATCTT--------ATATTCTAGAAA-AGAAT
Zeja    -AATAAAGAAGATAAG----CAGGGTTATTTTT-----------ACTACTATAGACC-ACAGT
Znne    -AATAAAGAAGATAAA----AGGG-ATTACTAATT-----------TACTTAAGAAA-ACAGT
Zefa    -AATAAAGAAGGCAAG----AGGGTGTA--TTAA-TT--------ATACTTAAGAAA-ATAGT
Acni    -AATAAAGAAGGTAAA----GGGGGCTACTAC------------TCTACTTAAGAAA-ATAGT
Ncrh    -AATAAAGAAGGTAA------AGGGG-GCTACTA---------CTCTACTTAAGAAA-ATAGT
Agca    -ACTAAAACAGGCAAA----GGGGCATATCCTT----------AA-TGCCTGAGAGA-ATGGC
Hydy    -ACTGAAAAAGGTAAA----AGGGCATAACC-----C-------CGTGCCGAAGATT-ACGGC
Gsac    -ACTAAAACAGACAAA----AGGGCATACCCC------------CCTGCCGGAGATT-ACGGC
Pevo    -ACTAAAATAGACAAA----GGGACATAATCTTG------------CTTCTTAGAAT-AAGAC
Hiku    TATTAAAGGTTAAAAC----AACACAAG----AG-AC---------CCTAGAATATA-ACTAT
Inpa    -ACTTAATCAGACACA----AGGGCATACTCCC----------- TAGCCTGAGAAA-AAGAA
Auch    -TCTAAAACAGGTAA-------GGTGACACACACCCC---------CACGTCGAGAC-AAAGA
Fico    -ACTAAATTGGACAAG----GGGGAGCA--CCCA-TT------T---GCCCAAGATA-ATGGC
Macs    -ACTAAAATAAGTAAG----GGGGCATGCCCCT----------TT--GCCAAAGATA-ATGGC
Moal    -AATCA-ATTGACGTT----AGAAAGTAAC-TT----------AA-AATCTTAGACT-AAGAT
Syma    -ACTAAAGCGG-TATA----AGGATATATCCCTACAA----------ACCCAAGAGA-TTGGT
Mafr    -ACTAAATTAGGCATA---AGGG-CATCGTCC---CC------AT--GCCTAAGACT-AAGGC
Dcpe    -TCTAAATTAAACAAA----AGGACACCCCCCCC---------CCCGGCCGAAGACA-ACGGT
Dcti    -TCTAAATTAAACAA-----AAGGACACCCCCCC---------CCCCGCCGGAGACA-ACGGC
Hehi    -ACTAAAGTAGGCAAG----AGGGC-ATACCCCCC-G---------TGCCTGAGAGA-ACGGC
Stam    -ACTAAAATAGGCAAC---TGG-GC--ATGCCCCGCC----------GCCTAAGAAA-ACGGC
Hogi    -AATAAATTAAATGAC----GGGGGGAAACCT------------CACGCCTGAGAAA-ACGGC
Erzo    -ACTAAAATAGGTAAG----AGG--ACATACCA----------CCCAGCCTGAGAAA-ACGGC
Hxot    -ACTAAAGTAGGCAAA----AGG--GCATACCCT---------GTTTGCCTAAGAAA-AAGGC
Core    -ACTAAAATAGGCAAT----AGGGCATGCCCC-T-----------GTGCCTAAGAAA-AAGGC
Apve    -ACTAAAGTAGACAAA---A---GG--GCATGCCCTT-------ATGCCTGAGAAAA-AAGGC
Latj    -ACTAAACTGGATAAA----GGGACGTGCCATAA---------TTATGTCAGAGAGA-ATGAC
Laja    -ACTAAAACAGACAA-------AAGGGCATAACC---------CCGCGCCTGAAAAA-AAGGC
```

Syja	-ACTAAAATGGACAAA----GGAGCATACCCCCAACT------AATTACCGAAAATA-ACGGT
Epme	-ACTTAATCAGACAAG----GGGACAT-CCTCAT---------TTCTGTCCTAGAAA-ATGAC
Grse	-ACTAAAGCAGGCAAG----AGGGCATACCTCTAT------------GCCTGAGAAA-ATGGC
Clja	-ACTCAACTCCACAAA----AGGGCATAACTC-C-----------TTCTGTCGAGAC-CAGAC
Ogcy	-ACTAAAACAGGTAAG----GGGGCATA-C-TCC-----------CTTTTCAACCTG-AAAAA
Plna	-ACTAAAGTAGGTAAC----AGGCCATAACCTTA-------------ATGTCAAAAA-AAGAC
Lema	-ACTAAAATAGGCAAA----AGGGCATGCCCCT------------TTGCCGAAGATT-ACGGC
Etzo	-ACTAAAATAGGCAAG----AGGGCATGCCCC-C-----------CTGCCGGAGAAA-ACGGC
Apse	-ACTAAAATAGGTAAG----AG--AGCATACTCC---------T-TTGTCTAAGAGA-AAGAC
Epde	-ACTAAAATAGGCAAA---AGG-GC--ATGCCCCT-A---------TGCCTGAGAAA-ATGGC
Slja	-ACTAAAATAGAGAAG----GGGGCATA----AA-CC------CACAACTTGACACC-TGAGA
Bsja	-ACTAAAACAGGTAAA----AGGGCATA--CCCC-CT-------CC-GCCCAAGAAA-AAGGC
Ecna	-TCTAAATTAGGCAAA----AGGGTATACAAT-----------TAATGCCTAAGATA-AAGGC
Cohi	-TCTAAATTAGGTAAA----AGGACACAAAACTTCA-----------GCCTTAGAGA-AAGGT
Caar	-TCTAAACTAGGCAAA----AGGGTGCACC-----CC------CCGTGCCTAAGAGA-ACGGC
Came	-TCTAAACTAGGCAAG---AGG-GT--ACATTCCCCC--------ATGCCTAAGAGA-ATGGC
Mema	-TCTAAACTAGACAAG----AAGGCGTA--CC-C---------CTATGCCTGAGAGA-ATGGC
Lenu	-AATAAATAAGATAAG----GGGAAGACCC-----CC----CCCACCGCCC-AGAAC-AGGAC
Brja	-ACTAAAATAGGCAAG---AGGG-CATACCCACC-TC------CCACGCCTGAGATA-ATGGC
Plma	-ACTAAAATAGGCAAA---AGG-GC--ATATCCCTT--------CACGCCAAAGATA-ATGGC
Emst	-ACTAAAATAGGCAAA----AGGGCATA--CCCTG-C---------TGCCTGAGAAA-ATGGC
Ptti	-AATAAAATAGGCAAA----AGGGCACAACCCGT-AT----------GTCTGAAAGA-ACGAC
Losu	-ACTAAAACAAACAAA---AGGGACATAACACTT-TA----------CCTGAGAAA--AGAGT
Geoy	-ACTAAAGCAGGTTAA----GGGAA-ACATAATC-CC----------GCCTAAGAAA-ACGGC
Dipi	-ACTAAAATAGGTAAA----AGGGC-ATAACCC-----------CGTGCCTGAGAGA-ATGGC
Pama	-ACTAAAGCAGGCAAA----AGGGCACA--CCCC---------TCT-GCCTGAGAGA-ATGGC
Leob	-ACTAAAGTAAGCAAA----AGGGCGTA--CCCC-TT------TC-CGCCTAAGAAA-ACGGC
Neba	-ACTAAAATGAATAAC----AGG--GCATACCCC---------TGCTGCTTAAGAAA-ATAGC
Pdpl	-TCTAAATTAGACAAA-----GGAGGGCATCCAC---------CGATACCGAAGAGC-CCGGT
Nimi	-ACTAAAACAGATAAA----AGGACACATTTATTTGTAGGTACTACAGCCTAAGAAA-ATGGC
Uptr	-ACTAAAATAAATAAT----AGGGCGCAAACATT----------CCGACCTAAGATA-AAGGC
Pesc	-ACTAAATTAGGCAAG----GGACACTGAGTTT-------------AATCTGAGAGG-ACGAT
Baar	-ACTAAAATAGACAAG----AGGACATACCCCCCCC-----------GCCTAAGAAA-ATGGC
Moar	-ACTAAAATAGGCAAC----AGGGCATATCCCCCT------------GCCTGAGAAAATGGCA
Toja	-TCTAAACTAGGCAAA----AGGGCATGCCCT------------CGTGCCTAAGAAA-ATGGC
Chau	-ACTAAAATAGGCAAG----AGGGCATACTCAC-----------AGTAACCGAGAAA-ATGTT
Chse	-GCTCAAATAGGTAAA----AGGGCATAATTCC-----------TCTGCCATAGAAA-ATGGC
Enar	-ACTAAAATAGGCCAA----AGGGCATACCC-----C------CCGTTCCTAAGAAA-ACGGC
Hpty	-ACTAAAATAGGCAAA----AGGGCATGCCCCT-----------TGTGCCTAAGAAA-ATGGC
Nana	-ACTAAATTAGACAAA----AGGGCGTATCCCC-----------TGTGTCTAAGAAA-AAAGA
Mcst	-ACTAAAATAGGCAAA----AGGGCATGTCCCTTG-A---------TGCCTAAGAAA-ATGGC
Rhox	-ACTAAATTAGATAAT----AGGGCGTA--CAAC-CC------AC-AGCCAAAGACA-ATGGC
Opfa	-ACTAAAATAGCCAAA----AGGGCATATCCCAT---------CCCCGCCTGAGAAA-ACGGC
Paar	-AATAAATTAAACAAG----AGGGC-ATAAACCG-TA---------TGCCCAAGAAA-ATGGC
Gozo	-ACTAAAATAGGCCAA----AGGGCATAATTCA-----------CCCGCCAAAGAAA-ATGGC
Ackr	-ACTTAACTAGCTAAG----AGGGCACCCCCCCC------------ACCACGAAATC-AGTGA
Elev	-ACTAAACAAGGCTAG----AGGGC-ATGCCCC-CTA----------GCCTGAAATTTAAGGC
Trdu	-ACTAAAATAGATAAA----AG--GGCGTACCCC----------CTAGCCCTAGAAA-AGGGC
Amoc	-AATAAAACAGGTAAA----AGGGCATACTCCT----------CC-CGCCTAAGATA-ATAGC
Hame	-ACTAAAATAGGTAAA----AGGGCATG--TGCT-TC------CC-AGCCTAAGATT-ACGGC
Chso	-ACTAAACTAGCCAAG----AGGGGGTACCACCACC--------AAAGCCTGAGATC-AAGGC
Lyto	-ACTAAAGCAGGCAAA----AGGGC-AT-GCTGCC-C---------TGCCCAAGAAA-AAGGC

```
Encr    -ACTAAAATAGGCAAA----AGGGCATG-CCCTC-CT----------GCCTAAGATG-AAGGC
Bvar    -ACTAAAATTGGCAAG----GGGGCATGACCCT----------TATAGCCAAAGAAA-ATGGC
Noco    -ACTAAAATAGACAAG----GGGGTATGTTTTCCC-----------CGCCAGAGAAA-ATGGC
Chsp    -ATCAAAATAGACCAC----AGGACATAACAAAT-----------AGACCGAAGATC-TAAGT
Arja    -ACTAAAGTAGGCAAA----AGGG--CATGCCCT-T---------GTGCCTGAGAAA-AAGGC
Pase    -AATTAAACAGCCAAC----TGGATACC--CC-C-------TCCCCCATATGAGAGC-CATAT
Trel    -GCTAAAGTGAACAAG----GGGATATGTTTAAG-AC------CCACGCAGCAGAAA-ACTGT
Lifa    -ACTAAAATAGTTAAG----GGGGCATAAGTAAT-CC------A--AACCGCAGACA-ACGGT
Acur    -ACTAAAACAACCAAG----AGGACATACTATC------------TCCCCGTAGAAA-ATGGT
Ampe    -ACTAAAATAGGCAAA----AGGGCATGCCCCC-----------TATGCCTGAGAAA-ATGGC
Urja    -AATAAAATAAGCAAA----AGGGCATACCTT------------TCAGCCTGAGACT-AAGGC
Enet    -ACTAAAGAGGGTAAT----GAGACACCCGTCT------------CCGCCATAAAAT-ACGGC
Ptbr    -ACTTAATTAGATAAA----AGTAA-TT--TCAC-AC---------ACCCCTAGACT--AGGA
Safa    -ACTAAAATAGGCAAG----GGGACATCCT-----CC-------TAAGTTGAAGAAA-ATAAC
Icae    -ACTAAAATAGGCAAA----AGGGCATATCCCT----------TCACGCCCAAGATA-ATGGC
Asmi    -TCTCAATTGGATAAA----AATGCACACCCCTG--------CTTCTTCAAAAGAAA-ACTGC
Foal    -AATAAAATAGATAAA----AGGGTTAACTAACCC------------GCCATAAATT-ATGGC
Drze    -ACTTAAATGGATAAG----TGGGCATAATA------------CTCTGTCAAAAAAA-ATGAC
Rhas    -ACTAAAACAGGCAAA----AGGACATGACCC-C-----------CCGCCTAAGAGA-AAGGC
Elac    -AATAAAAAAGGCAAA----GGGGTACATCCTCCG-----------TGCCTGAGAAA-AAGGC
Kugu    -ACTAAAATAGATAAA----AGGGCATACCT---ACC--------AAACCTGAAAAA-ACGGC
Plor    -TCTAAAACAGGCAAG----AGGGCATACGCCC------------ATGCCTAAGAAA-ACGGC
Sgun    -ACTAAAATAGACAAA----AGGGC-ATATCCCTG-----------CGCCTGAGAAA-ATGGC
Zaco    -ACTAA-ATAGACAAA----AAGGCATA-CCCCTCCT---------ACCC-GAGAAA-ATGGG
Zbfl    -ACTAAAGTAGACAA-------AAGGGCATATTT---------CCCCGCCTGAGAAA-ATGGC
Spba    -TCTAAATTGGACAAA----AGGGC-ATAATTCT-TT-----TCCATGCCAAGACA--ACGGC
Game    -ACTAAAATAGGCAAA----AGGGCATACCCCT----------ACACGCCCAAGATA-ATGGC
Thth    -ACTAAAATAGGCAAA----AGGGC-ATACCCCTCCA---------CGCCCAAGATA-ATGGC
Xigl    -TCTAAACTAGGCAAG---AGG-GC--ATACCCTTCA---------TGCCTGAGAGA-ATGGC
Hyja    -ACTAAAATAGGCAAA----AGAGCATATCCCTTTAT----------GCCGGAGATACACGGT
Psan    -ACTAAAATAAATTAA----AGAGCATACCCTACCAG---------TGTCGGAGATAGACGGC
Cupa    -ACTAAAATAGGCAAA----AGGGTATACCCCTC---------CTACGCCCAAGATA-ATGGC
Mpch    -ACTTAATTAGGCAAG----GGGGCATACCT-----C-------TTTGCCTAAGAAA-ATGGC
Char    -ACTAAATTAGGTAAG----GGGGCATGACTCCTCCC----------GCCAAATATA-ATGGC
Pser    -TATCAACTAGGTAAA----AGG-CATATCCCCC-C-----------GCCCAAGAAA-ACGGC
Prol    -TCTAAACTAGGTAAA----AGG-GCATAACCAT---------CTGTGCCGAAGATA-ACGGC
Plbi    -TCTAAACTAGGCAAA----AGGGCATAACCATT-----------TTGCTGGAGATA-ACAGC
Calu    -ACTAAATCAGGCAAG---GAGG-CGCAACCCTC-CG----------CACTTAGAT---AGTG
Papa    -TCTAAACATA-TAAC----AGGGAGCGGTATCTC-----------AAGCGGAGATA-ACGCT
Sufr    -ACTCAAACGAACAAA----GAGATGCTTAACCAC------------GTTAAAGAGC-ATGGC
Stci    -AATAAAGTAAGCAAG----GGAGGACAACCTTTG-----------AGCCATAAATC-ATGGC
Taru    -GCTCAAGCAAATAAG----AGAGTACAAAACTAA------------GTCAAAGAAC-ATGAC
Rala    -ACTAAACTAGGTAAA----GGGGCATATA-----CC------CCT-GCCTGAGATA-ATGGC
              *
```

```
Scca    T--------------------------------------------------------
Muma    T--------------------------------------------------------
Erca    TA-------------------------------------------------------
Pose    A--------------------------------------------------------
Actr    CAA------------------------------------------------------
Scal    AA-------------------------------------------------------
Posp    AA-------------------------------------------------------
Atsp    CCA------------------------------------------------------
Leoc    ACA------------------------------------------------------
Amca    AA-------------------------------------------------------
Osbi    A--------------------------------------------------------
Pabu    AA-------------------------------------------------------
Hial    GC-------------------------------------------------------
Elha    GC-------------------------------------------------------
Mlcy    AC-------------------------------------------------------
Algl    TAC------------------------------------------------------
Ptgi    GC-------------------------------------------------------
Alaf    AC-------------------------------------------------------
Nock    AC-------------------------------------------------------
Anja    AT-------------------------------------------------------
Gyki    AT-------------------------------------------------------
Syka    AT-------------------------------------------------------
Opma    AG-------------------------------------------------------
Comy    AT-------------------------------------------------------
Sasp    GC-------------------------------------------------------
Eupe    ---------------------------------------------------------
Enja    CGTCTTATTTATTCGC-----------------------------------------
Same    TCGC-----------------------------------------------------
Chch    TT-------------------------------------------------------
Grgr    AT-------------------------------------------------------
Caau    AT-------------------------------------------------------
Cyca    ATACTGG--------------------------------------------------
Dare    T--------------------------------------------------------
Cost    AC-------------------------------------------------------
Leec    AC-------------------------------------------------------
Crla    AC-------------------------------------------------------
Clmc    CT-------------------------------------------------------
Phin    TAA------------------------------------------------------
Icpu    ---------------------------------------------------------
Psto    ---------------------------------------------------------
Cora    ---------------------------------------------------------
Eisp    T--------------------------------------------------------
Apal    ---------------------------------------------------------
Eslu    GC-------------------------------------------------------
Dape    GC-------------------------------------------------------
Glse    GC-------------------------------------------------------
Naar    GC-------------------------------------------------------
Baoc    GC-------------------------------------------------------
Opso    GC-------------------------------------------------------
Alte    AT-------------------------------------------------------
Plap    AT-------------------------------------------------------
```

```
Plal    ATCCTAGTGTGGCAGGGCTCGGGTAACAACCCACCCTGCTACCC-------------
Sami    ATCCTTGTGTGGCAGGATCTAGAAAGTCGTTCTGC----------------------
Rere    GCCCGCATCTCAACGTGGCGGTAACCGGCGTGCCGGGGCCTTCCGGCCTTGCCTTCT
Gama    GC-------------------------------------------------------
Onmy    GC-------------------------------------------------------
Sasa    GC-------------------------------------------------------
Cola    GC-------------------------------------------------------
Dita    T--------------------------------------------------------
Gogr    T--------------------------------------------------------
Chsl    A--------------------------------------------------------
Atja    AT-------------------------------------------------------
Iido    AT-------------------------------------------------------
Auja    AT-------------------------------------------------------
Chag    AT-------------------------------------------------------
Hami    AT-------------------------------------------------------
Saun    AT-------------------------------------------------------
Nema    AT-------------------------------------------------------
Disp    TTG------------------------------------------------------
Myaf    A--------------------------------------------------------
Lagu    TA-------------------------------------------------------
Trtr    CT-------------------------------------------------------
Zucr    CT-------------------------------------------------------
Pxja    AA-------------------------------------------------------
Pxlo    AA-------------------------------------------------------
Pctr    AA-------------------------------------------------------
Apsa    AT-------------------------------------------------------
Cabe    AC-------------------------------------------------------
Bzze    AT-------------------------------------------------------
Siim    AC-------------------------------------------------------
Ctru    AT-------------------------------------------------------
Dpbr    AT-------------------------------------------------------
Caki    AA-------------------------------------------------------
Phja    CGTTT----------------------------------------------------
Brsp    AGTTGGCAGATTGTTGAATATTCTGTAGCTCCTAA----------------------
Gamo    TGT------------------------------------------------------
Lolo    CGT------------------------------------------------------
Batr    TCCC-----------------------------------------------------
Prmy    ---------------------------------------------------------
Loli    AT-------------------------------------------------------
Loam    AT-------------------------------------------------------
Chab    AT-------------------------------------------------------
Chto    AT-------------------------------------------------------
Majo    TT-------------------------------------------------------
Hlst    TT-------------------------------------------------------
Clpe    AC-------------------------------------------------------
Mlmr    AT-------------------------------------------------------
Crcr    AT-------------------------------------------------------
Muce    AT-------------------------------------------------------
Bege    GT-------------------------------------------------------
Mela    AT-------------------------------------------------------
Hats    AAA------------------------------------------------------
Orla    AT-------------------------------------------------------
```

Cosa AT---
Exsp AT---
Depa TT---
Rima TT---
Fuol GT---
Gmaf T--
Xeei AA---
Pros GT---
Scmi CT---
Rolo AT---
Cere GT---
Daga GT---
Anco AT---
Dmve GT---
Dmar GT---
Anka AT---
Moja AT---
Hoja AT---
Bede AT---
Besp AT---
Mysp AA---
Osja AT---
Sgro AT---
Pzpa AT---
Zeja AT---
Znne AC---
Zefa AA---
Acni AT---
Ncrh AT---
Agca AT---
Hydy AT---
Gsac AT---
Pevo AT---
Hiku AT---
Inpa TTA--
Auch CCA--
Fico AT---
Macs AT---
Moal AT---
Syma AT---
Mafr CT---
Dcpe AG---
Dcti AG---
Hehi AT---
Stam AT---
Hogi AT---
Erzo AT---
Hxot AT---
Core AT---
Apve AT---
Latj AT---
Laja AT---

```
Syja    CATGTTAAAGTGG--------------------------------------------
Epme    AA-------------------------------------------------------
Grse    AT-------------------------------------------------------
Clja    AAT------------------------------------------------------
Ogcy    AAGCTT---------------------------------------------------
Plna    AA-------------------------------------------------------
Lema    AT-------------------------------------------------------
Etzo    AT-------------------------------------------------------
Apse    T--------------------------------------------------------
Epde    AT-------------------------------------------------------
Slja    TAACGGTAT------------------------------------------------
Bsja    AT-------------------------------------------------------
Ecna    AA-------------------------------------------------------
Cohi    ---------------------------------------------------------
Caar    AT-------------------------------------------------------
Came    AT-------------------------------------------------------
Mema    AT-------------------------------------------------------
Lenu    AT-------------------------------------------------------
Brja    AT-------------------------------------------------------
Plma    AT-------------------------------------------------------
Emst    AT-------------------------------------------------------
Ptti    AT-------------------------------------------------------
Losu    AC-------------------------------------------------------
Geoy    GT-------------------------------------------------------
Dipi    AT-------------------------------------------------------
Pama    TA-------------------------------------------------------
Leob    ATTCACCT-------------------------------------------------
Neba    ATT------------------------------------------------------
Pdpl    AACA-----------------------------------------------------
Nimi    TT-------------------------------------------------------
Uptr    AACC-----------------------------------------------------
Pesc    TC-------------------------------------------------------
Baar    AT-------------------------------------------------------
Moar    T--------------------------------------------------------
Toja    AT-------------------------------------------------------
Chau    AT-------------------------------------------------------
Chse    AA-------------------------------------------------------
Enar    AT-------------------------------------------------------
Hpty    AT-------------------------------------------------------
Nana    CAT------------------------------------------------------
Mcst    AT-------------------------------------------------------
Rhox    CT-------------------------------------------------------
Opfa    AT-------------------------------------------------------
Paar    AT-------------------------------------------------------
Gozo    AT-------------------------------------------------------
Ackr    TTA------------------------------------------------------
Elev    TT-------------------------------------------------------
Trdu    TT-------------------------------------------------------
Amoc    AT-------------------------------------------------------
Hame    CT-------------------------------------------------------
Chso    AC-------------------------------------------------------
Lyto    AT-------------------------------------------------------
```

```
Encr        AT-------------------------------------------------------
Bvar        AT-------------------------------------------------------
Noco        AC-------------------------------------------------------
Chsp        C--------------------------------------------------------
Arja        AT-------------------------------------------------------
Pase        AA-------------------------------------------------------
Trel        AA-------------------------------------------------------
Lifa        TCCT-----------------------------------------------------
Acur        AC-------------------------------------------------------
Ampe        AT-------------------------------------------------------
Urja        AC-------------------------------------------------------
Enet        TT-------------------------------------------------------
Ptbr        ---------------------------------------------------------
Safa        AT-------------------------------------------------------
Icae        AT-------------------------------------------------------
Asmi        T--------------------------------------------------------
Foal        TT-------------------------------------------------------
Drze        AT-------------------------------------------------------
Rhas        AT-------------------------------------------------------
Elac        AT-------------------------------------------------------
Kugu        AC-------------------------------------------------------
Plor        AT-------------------------------------------------------
Sgun        AT-------------------------------------------------------
Zaco        CC-------------------------------------------------------
Zbfl        AT-------------------------------------------------------
Spba        ACA------------------------------------------------------
Game        AT-------------------------------------------------------
Thth        AT-------------------------------------------------------
Xigl        AT-------------------------------------------------------
Hyja        ATA------------------------------------------------------
Psan        ATAC-----------------------------------------------------
Cupa        AT-------------------------------------------------------
Mpch        AT-------------------------------------------------------
Char        TT-------------------------------------------------------
Pser        AC-------------------------------------------------------
Prol        GT-------------------------------------------------------
Plbi        AA-------------------------------------------------------
Calu        T--------------------------------------------------------
Papa        TA-------------------------------------------------------
Sufr        AT-------------------------------------------------------
Stci        AT-------------------------------------------------------
Taru        AT-------------------------------------------------------
Rala        AT-------------------------------------------------------
```
