# Supplementary material for: Structure and variation of the mitochondrial genome of fishes
Source: BMC Genomics. 2016 Sep 7;17(1):719. doi: 10.1186/s12864-016-3054-y (PMC5015259; doi:10.1186/s12864-016-3054-y)
Supplement: Additional file 15: Figure S4-a. — Aligned nucleotide sequences of the conserved sequence blocks-D and -I in the control region (CR) in mt genomes of 182 fishes. Figure S4-b. Aligned nucleotide sequences of the conserved sequence blocks-II and -III in the control region (CR) in mt genomes of 182 fishes. (ZIP 87 kb) [file 12864_2016_3054_MOESM15_ESM.zip › Additional file 15 CSBs/Additional file15-a CSBD1.pdf]

Additional file 15: Figure S4–a. Aligned nucleotide sequences of the conserved sequence blocks-D and -I in the control region (CR) in mt genomes of 182 fishes.

	CSB-D		CSB-I
Scca	TGA----TCAAATCTG---GCAT-CTGATTAC-TGC------	Scca	T-AC---ATGC-CTCACTGT--ATCTGGC-ATAGGTTTAATTTATA----
Muma	TTGA---TCAAATCTG---GCAT-CTGATTAA-TGCTCGAAT	Muma	--AC---ATTA-TTCACTTT--ATCTGGC-ATAAATCTTATAT-------
Pose	AAAA---ATAACAGTC---TCGT-CCGGAGTGTAACCACCAT	Pose	-CTG---ATCATTCAACCAT--GAATGAC-ATACTTCTTTTATACCACTA
Actr	GAA----CTATTACTG---GCAT-CTGGTTCC-------TAT	Actr	---GAATAATG-AAT-GGTA--CAATGAC-ATATCCCTGATGTC-AC--A
Scal	GA-----CTATTACTG---GCAT-CTGGGTCC-------TAT	Scal	---GAGCAATG-AAT-GGTA--CAATGAC-ATACTTTAGACACCAC--AA
Posp	GAA----CTATTACTG---GC-T-CTGGGTC--------TAT	Posp	--C--TGAATG-AAT-GATT--TAATGAC-ATAAA-CATTATTACACTAC
Hial	GAA----CTATTACTG---GCAT-CTGGTTCC-------TAT	Hial	-CC--ACATTA-ATTTATCT--CAAGTAC-ATAAG--------GTTAT--
Elha	GAA----CTATTACTG---GCAT-CTGGTTCC-------TAC	Elha	-----ACATAA-TTATATAT--CAAGGAC-ATACACTATAATCATT----
Alaf	ACA----CTATTACTG---GCAT-CTGGTTCC-------TAT	Alaf	-T---GCATTA-ATCTCTAT--CAGGTGC-ATATAGCAGTACATC-----
Anja	GAA----CTATTACTG---GCAT-TTGGTTCC-------TAT	Anja	-CC--ACATTA-AAATATAT--CAAGTGC-ATAAC--------ACTTA--
Gyki	GAA----CTATTCCTG---GCAT-TTGGTTCC-------TAT	Gyki	-TT--GCATTA-GTTTGTAT--CAGGTGC-ATAAG-----GGTACTGT--
Opma	GAA----CTATTACTG---GCAT-TTGGTTCC-------TAT	Opma	-T---GCATTA-AGTTATAT--CAGGTGC-ATACAACATAACCGCTT---
Comy	CATA---GTATCTAT-----CAT-AGTATCTATCA-----TA	Comy	TTA--TTGTAACATACACAA-CAAACTTC-ATATGGAAGAAGACCACAAA
Enja	GAA----TTATTTCTG---GCCT-CTGGTTCC-------TTC	Enja	-T---GCATAA-GTG-ATTT--CAAGAGC-ATAAACTATCGTTATTTACT
Same	GAA----CTATTCCTG---GCAT-CTGGTTCC-------TAT	Same	-T---GCATAA-CTG-TTAT--CAGGTGC-ATACAAA-CAATCTCTTTAT
Grgr	GAA----CTATTACTG---GCAT-CTGGTTCC-------TAT	Grgr	-T--GGCATAC-ATCTACAT---ACGTGC-ATAGACTACAGAC-ATTACC
Caau	GAA----CTATTACTG---GCAT-CTGGTTCC-------TAT	Caau	-T---ACATAC-TTCTAACT--CAAGTGC-ATAACATATTCATCTCTTAT
Cyca	GAA----CTATTACTG---GCAT-CTGGTTCC-------TAT	Cyca	-AC----ATAC-TTTTATCT--CAAGTGC-ATAATATATCTATCTCTAGT
Dare	GAA----CTATTCCTG---GTAT-CTGGTTCA-------AAT	Dare	-ACTCACTAAC-TTTTAATT--CAAGTGC-ATAAAGTACT----------
Cost	GAA----CTATTACTG---GCAT-TTGGTTCC-------TAT	Cost	-T---GCATAT-ATAGAATT--CAGGTGC-ATAACCTATT-----CTTAT
Leec	GAA----YTATTACTG---GCAT-CTGATTCT-------CCT	Leec	-----ACATTA-GTTTATCT--CAAGTGC-ATAATACGTC-----CTTAC
Fola	GAA----CTATTACTG---GCAT-CTGGTTCC-------TAT	Fola	-T---ACATAT-GTTTATCT--CAGGTGC-ATAAGACTAT----TCCTGT
Clmc	GAA----CTATTACTG---GCAT-GTGGTTCC-------TAT	Clmc	-----ACATAC-ATTTATCT--CAAGAGC-ATACGGTACATTACTACTCC
Phin	GAA----TTATTACTG---GCAT-CTGGTTCC-------TAT	Phin	-CC--ACATAT-GTTTTTAT--CAAGTGC-ATAAACTATCAGTACCACCC
Icpu	GCA----CTATTACTG---GCAT-CTGGTTCC-------TAT	Icpu	-T---ACATAA-CTCTCTCT--CAAGTAC-ATA-ACTGATATTGC-----
Psto	GCA----CTATTACTG---GCAT-CTGGTTCC-------TAT	Psto	-CC--ACAGAT-ATGTATTT--CACGTGC-ATA-CCTTTACCCTT-----
Cora	GAA----CTATTACTG---GCAT-CTGGTTCC-------TAT	Cora	-----ACTATACGTTTATTT--CATGAGC-ATACATATTAT---------
Eisp	GAA----CTATTACTG---GCAT-CTGGTTCC-------TAT	Eisp	-C---ACATTA-ATCTCTTT--CAGGTGC-ATACTCTACT-----ATTCT
Eslu	GAA----TTATTACTT---GCAT-TTGGCTCC-------TTT	Eslu	--T--GCATAT-GTTTATAT--CATGTGC-ATAAGCCCTTAAGATTACC-
Dape	GAA----CTATTACTT---GCAT-CTGGTTCC-------TAT	Dape	--C---CATAA-TTTGTTTT--CTAGTGC-ATAAG---------CCTA--
Glse	GAT----CTATTCCTG---GCAT-TTGGTTCC-------TCT	Glse	--T--GCATAC-GTCTATCT--CAAGAGC-ATAATG-------TTTAT--
Naar	GAA----TTATTTCTG---GCAT-TTGGTTCC-------TTT	Naar	--T--GCATAT-CAGTATCT--CAAGTGC-ATAATG-------ATTTC--
Lioc	GAA----CTATTACTT---GCAT-CTGGTTCC-------TCG	Lioc	--T--GCATCT-CAGGATAT--CAAGTGC-ATAATGATTATGTCTTTA--
Opso	GAT----CTATTTCTG---GCAT-TGGGTTCC-------TTT	Opso	--C--GCATAT-TAGGATCT--CAAGTGC-ATAATG-------CTTTA--
Alte	GAA----TTATTACTG---GCAT-CTGGTTCC-------TAT	Alte	-CA--ACATTA-AATTATAT--CAAGTGC-ATAAGGTATGGCTCTAT---
Plap	GAA----CTATTACTG---GCAT-CTGGTTCC-------TAT	Plap	-TA--CCATTA-ATCTCTAT--CAAGTGC-ATAAAACATGGCCCTTT---
Plal	GAA----CTATTACTT---GCAT-TTGGTTCC-------TAT	Plal	--A--ACATAA-GT-GATAT--CATGTGC-ATAAAG--------GCTCTG

Species	CSB-D	Species	CSB-I
Sami	GAA----CTATTACTG---GCAT-TTGGTTCC-------TAT	Sami	-----ACATAA-GTG-ATAT--CAAGTGC-ATAAAG--AACCTGTCATTC
Rere	GAT----CTATTACTG---GCAT-CTGGTTCC-------TAT	Rere	-----ACATAA-TTG-ATAT--CAATTGC-ATATTA--CCCTCTCCGCCC
Gama	GAA----TTATTACTG---GCAT-CTGGTTCC-------TTC	Gama	--C--ACATAA-GT-GATAT--CAAGAGC-ATAAGACAATATTTTCAACC
Onmy	GAA----TTATTCCTG---GCAT-TTGGTTCC-------TAA	Onmy	-TC--ACATAC-TTGGATAT--CAAGTGC-ATAAGGT-CAA---------
Sasa	GAA----CTATTCCTG---GCAT-TTGGTTCC-------TAT	Sasa	-TC--ACATAC-TTGGATAT--CAAGTGC-ATAAGGT-TGATTTTTT---
Cola	GAA----TTATTACTT---GCAT-CTGGTTCC-------TAT	Cola	-T---GCATAA-TTG-ATAT--CAAGTGC-ATAAGGT-CAGTTTCTTTCC
Dita	GAA----CTATTCCTG---GCAT-TTGGTTCC-------TAT	Dita	--C--ACATAT-TTAGATAT--CAGGTGC-ATAAAG--------TCCTTG
Atja	GAA----CTATTCCTG---GCAT-TTGGTTCC-------TAT	Atja	--T--GCATAT-ATCTATTT--CATGTGC-ATAAGTAGTGCTTAATCAAT
Iido	GAA----CTATTCCTG---GCAT-TTGGTTCC-------TAT	Iido	--T--GCATAT-ATCTATTT--CATGTGC-ATAATGAGTGCTTAATCA--
Auja	GAA----CTATTTCTG---GCAT-CTGGTTCC-------TAT	Auja	-CC--ACAATT-GAAGATTT--CATGTGC-ATAACTATGATATTT-TT--
Nemi	GAA----CTATTTCTT---GCAT-TTGGCTCTA------CAT	Nemi	-TT--TCATA--ACTGATTT--CATGAGC-ATA-TATA------------
Pxja	GAA----CTATTCCTG---GCAT-TTGGTTCC-------TAC	Pxja	-TT--GCATAT-ATAGATTT--CAAGAGC-ATAAGGT-GTAATTATT---
Pxlo	GAA----CTATTCCTG---GCAT-TTGGTTCC-------TAT	Pxlo	-TT--GCATAT-ATAGATTT--CAAGAGC-ATAAGGTG------------
Apsa	GAA----CTATTCCTG---GCAT-TTGGTTCC-------TAT	Apsa	--T--GCATAA-TTGGATAT--CAAGAGC-ATAATAATTTATATTATTCT
Cabe	GAA----CTATTACTG---GCCA-CTGGTTCC-------TAT	Cabe	-----ACACATCAAAAGGTT--TAAAGAC-ATAAAGATATTATTA-----
Bzze	GAA----TTATTTCTG---GCCA-CGGGTTCC-------TCC	Bzze	-TT--ACAT-A-ACTTATAT--CAAGGAC-ATAAACT-------GTAT--
Ctru	GCA----CTATTCCTG---GCAT-TTGGCTCC-------TTC	Ctru	-TT--GCATAC-TTGGATCT--CACGGGC-ATATAAGAGATTGTTCTA--
Dpbr	GAA----CTATTCCTG---GCAT-TTGGTTCC-------TTC	Dpbr	-CT--ACACAC-TTAGATAT--CATGAGC-ATAATAGAT-TAATTATT--
Phja	GAA----TTTTTCCTG---GCAT-CTCTCCCT-------AGC	Phja	--T--ACATTA-TATTATTT--CAAGGGC-ATAACACAACTCCATTCG--
Gamo	AACA---CGTTTCCTG---GCTA-TTCTGCCT-------AGC	Gamo	--T--ACATTA-AAGTTTTT--CAAGAGC-ATAAG-GCTAAAAATTTTCT
Lolo	GCA----CTATTCCTG---GCAT-CCCTGCCT-------AGC	Lolo	--T--GCATAT-AAGTATAT--CAAGAGC-ATAATATGAGATTTTTCCTC
Prmy	GTAC---CTTTACCCA---ACATGCGGGGCGTATCTCTAAAG	Prmy	-TT--GCACAA--TATTTA---CTAGCAC-ATAATACTTGAGTCTTCATC
Loam	GAA----CTATTACTG---GCAT-CTGGTTCC-------TAT	Loam	-TT--ACATAA-GTGATTA---CAAGGAC-ATAATACACCA---------
Chab	GAA----TTATTCCTG---GCAT-CTGGTTCC-------TAC	Chab	-TT--ACATAT-ATTAATAT--CAAGAGC-ATAATAATACATAAATTA--
Chto	GAA----CTATTCCTG---GCAT-TTGGTTCC-------TAC	Chto	-TT--ACATAT-ATTAATAT--CAAGAGC-ATAATAATACATAAATTA--
Clpe	GAA----TTATTCCTG---GCAT-TTGGTTCC-------TAC	Clpe	--T--ACATAC--TCTATTT--CAAGGAC-ATAAAGGTGTGTCTATTT--
Muce	GAT----CTATTCCTG---GCAT-TTGGTTCC-------TAC	Muce	--T--ACATAA--CTAACAT--TACGAAC-ATAA-CATAATTAACTTTCC
Bega	GAA----TTATTCCTG---GCAT-TTGGTTCC-------TAT	Bega	--T--GCATTA--CTGAAGT--CAAGAGC-ATAAAG-----AGTGAGTTT
Mela	GAT----CTATTCCTG---GCAT-TTGGTTCC-------TAT	Mela	-TT--GCATAA--CTGATAT--CAAGTAC-ATAA-AGAGTTATTTTTT--
Hats	GAA----CTATTCCTG---GCAT-TTGGTTCC-------TAT	Hats	-TT--GCATAA--CTGATAT--CAAGAGC-ATAA-AGACTGATAATTT--
Orla	GAA----TTATTCCTG---GCAT-TTGGCTCTA------CAT	Orla	-TT--GCATAA--CTGATAT--CAAGAGC-ATAA-ATGGCCAAATGAA--
Cosa	GAA----TTATTCCTG---GCAT-TTGGTTCC-------TAT	Cosa	--T--GCATTA--CTGATAT--CAAGAGC-ATAA--TATGACTAG-TGTT
Exvo	GAA----TTATTCCTG---GCAT-TTGGTTCC-------TAT	Exvo	--T--GCATTA--CTGTTAT--CATGTGC-ATAACATA-TGAATTTTTC-
Depu	GAA----CTATTCCTG---GCAT-TTGGTTCC-------TAC	Depu	-TT--GCATAA--CTGATAT--CAAGAGC-ATAATATA------------
Rima	GAA----CTATTCCTG---GCAT-TTGGTTCC-------TAT	Rima	-----GCATAA--CTGATAT--CACGGGC-ATATCTCTCATGTTCGTT--
Fuol	GAA----TTATTCCTG---GCAT-TTGGTTCC-------TAT	Fuol	--T--ACATAA--CTGATAT--CAAGAGC-ATAAGG-----TGTGAAAAC
Gmaf	GAA----CTATTCCTG---GCAT-TTGGCTCC-------TAT	Gmaf	--T--GCATAA--CTGATAT--CAAGAGC-ATAAAGAATT-ATTAAACCT
Xeei	GAA----CTATTCCTT---GCAT-TTGGTTCC-------TAT	Xeei	--T--GCATAA--TTGATAT--CATGAGC-ATAATGAATGAAATATTCTC
Rolo	GCT----TTATTCCTG---ACAT-TTGGTTCC-------TAT	Rolo	-T---GCATCG-TTGTATCC--CACGAGC-ATAAACTATAATATTTT-CC

	CSB-D
Cere	GAA----CTATTCCCG---GCAT-TTGGTTCC-------TAT
Daga	GAA----CTATTCCTG---GCAT-TTGGCTCC-------TAT
Anco	GAA----TTATTACTG---GCAT-CTGGTTCC-------TAT
Moja	GAT----CTATTCCTG---GCAT-TTGGGTCC-------TAT
Hoja	GAT----CTATTCCTG---GCAT-TTGGTTCC-------TAT
Bede	GAA----CTATTTCTG---GCAT-TTGGTTCC-------TAT
Besp	GAT----CTATTTCTT---GCAT-TTGGTTCC-------TAT
Mybe	GAT----CTATTACTG---GCAT-CTGGTTCC-------TAT
Osja	GAA----CTATTCCTG---GCAT-TTGGTTCC-------TAT
Sgro	GAA----CTATTCCTG---GCAT-TTGGTTCC-------TAT
Pzpa	GCA----TTATTCCTG---GCAT-TTGGTTCC-------TAT
Zeja	GAA----TTATTCCTG---GCAT-TTGGCTCC-------TAT
Zefa	GAA----TTATTCCTG---GCAT-TTGGCTCC-------TTC
Acni	GAA----CTATTCCTG---GCAT-TTGGCTCC-------TAT
Ncrh	GAA----CTATTCCTG---GCAT-TTGGCTCC-------TAT
Agca	GAA----TTATTCCTG---GCAT-TTGGTTCC-------TAC
Hydy	GCA----CTATTCCCG---GCAT-TTGGTTCC-------TAC
Pevo	GAA----TTATTCCTG---GCAT-TTGGTTCC-------TAC
Hiku	GAA----TTATTCCTG---GCAT-CTC-CTCC-------TTT
Auch	GAA----CTATTATTGTAGAGATTACTATCAGTG----ATTT
Fico	GAA----CTATTCCTG---GCAT-TTGGTTCC-------TAC
Moal	GAA----TTATTCCTG---GCAT-TTGGTTCC-------TAT
Syma	GAA----TTATTCCTG---GCAT-TTGGTTCC-------TAT
Mafa	GAA----TTATTCCTG---GCAT-TTGGTTCC-------TAC
Dcpe	GAA----TTATTCCTG---GCAT-TTGGTTCC-------TAT
Dcti	GAA----TTATTCCTG---GCAT-TTGGTTCC-------TAT
Hehi	GAA----TTATTCCTG---GCAT-TTGGTTCC-------TAT
Stam	GAA----TTATTCCTG---GCAT-TTGGTTCC-------TAT
Hogi	GAA----TTATTCCTG---GCAT-TTGGTTCC-------TAC
Erzo	GAA----TTATTCCTG---GCAT-TTGGTTCC-------TAT
Hxot	GAA----CTATTCCTG---GCAT-TTGGTTCC-------TAC
Core	GCA----CTATTCCTG---GCAT-TTGGTTCC-------TAC
Latj	GAA----CTATTACTG---GCAT-TTGGTTCC-------TAT
Laja	GAA----CTATTCCTG---GCAT-TTGGTTCC-------TAC
Epme	GAA----TTATTCCTG---GCAT-TTGGTTCC-------TAC
Grse	GAA----TTATTCCTG---GCAT-TTGGTTCC-------TAT
Plna	GAA----TTATTACTG---GCAT-TTGGTTCC-------TAT
Lema	GAA----TTATTCCTG---GCAT-TTGGTTCC-------TAC
Apse	GCA----CTATTCCTG---GCAT-TTGGTTCC-------TAT

	CSB-I
Cere	-TT--GCATAT-TCTTATAT--CAAGAGC-ATAT-ATTGTGAGATTTT--
Daga	-TT--ACATAC-TACTCTAT--CAAGGAC-ATAACATTATGACAATTT--
Anco	-CC--ACAATT-AGGGATTT--CTAGTGC-ATAATAG-TTATTATTT---
Moja	--C--ACATTA-AGGGATAT--CAGGTGC-ATATCA-----ATTTT-TTT
Hoja	-TC--GCATTT-AGGGATAT--ATAGTGC-ATAATAA-TAATTATTT---
Bede	-T---GCATCC-TAGGATCT--CACGAGC-ATAAAGGATTAGTATTA-CT
Besp	-TT--GCATAC-TTGGATAT--CAAGAGC-ATAA-AGTATTAGTATTA--
Mybe	--T--GCATGT-AATGGATT--CAAGTGC-ATAATATATTTTTACTACC-
Osja	-CC--ACATAT-TAGGATAT--CATGTGC-ATAA-TATTTTTTATTTT--
Sgro	--C--ACATTA-TTAGATAT--CAAGTGC-ATAATAATTT---TTTTTTT
Pzpa	--T--ACATAT-TATAATAT--CAAGGAC-ATAAC-AATT--ACACCACT
Zeja	-AT--ACATAT-TATTATCT--CAAGGAC-ATAATAAGTGAAATTCCA--
Zefa	-TT--ACAGTT-GTTGATAT--CATGGAC-ATAA---CAGATAAATCT--
Acni	--T--ACATTT-AAGGATAT--CAAGGAC-ATAATAAATG---TCTTTCC
Ncrh	--T--ACATAT-GGGGATAT--CACGGAC-ATAATA-AATGTCTTTCC--
Agca	--T--ACATAT-TATAATAT--CAAGGAC-ATAATGTGTGAATTTTTT--
Hydy	--T--GCATAA-CAGTATAT--CAAGAGC-ATAATAGCTG-----CTCT-
Pevo	-----GCATAA-CTG-ATGT--CAAGAGC-ATAAAGT-CCACTTCGTTTC
Hiku	-TT--GCATAA-ATCTCTTT--CTAGAGC-ATAATAGAT--ACATTTT--
Auch	CCC--GGGTAC-TACTCGCA--AACGTTG-GTAAGGCGTAGCCCTTAAC-
Fico	-----GCATA--ACTGATTT--CAAGAGC-ATAATACT-CAGTGCTTTC-
Moal	-GT--ACATA--ACTGATAT--CACGAGC-ATAAGGTTACTGGTCTTT--
Syma	TTT--TCATA--ATTGGTAT--CATGAGC-ATA-ATAATTACTTTAAAA-
Mafa	--C--ACATAT-CTTGATAT--CAAGAGC-ATAAGGT---AATTATTT--
Dcpe	--T--ACATTT-ATCTCTAT--CAAGGAC-ATACACAATACTTTCTC---
Dcti	-TT--ACACTA-ATCTCTAT--CAGGGAC-ATAC---TACAATACTTA--
Hehi	-TT--ACATAT-TAGGTTAT--CAAGGAC-ATAAATAGTGA---------
Stam	--C--ACATAC-TGTAATAT--CAAGAGC-ATAAGTAGTGAGAATTTCT-
Hogi	--T--GCATA--CCTTATAT--CAAGAGC-ATATATAATGAATATTTACT
Erzo	-CC--ACATAC-TTGAATAT--CACGAGC-ATAAAT-------AGTGA--
Hxot	-TT--GCATAT-TAGGATAT--CAAGAGC-ATATATAGGGAAGTTCTA--
Core	-TT--GCATAA-CTGTATCT--CAAGAGC-ATAAAGAGTGATTTGTTC--
Latj	--C--GCATAC-TTGGATAT--CACGAGC-ATAACATACTGGTCTTTACT
Laja	--T--ACATAT-AGATATAT--CAAGGAC-ATAATG-----TGCGAGTAT
Epme	--T--GCATA--ACTGTTCT--CATGAGC-ATAAGTAATTGATTTACTCC
Grse	--T--ACATA--AAGAGGTA--TAAAGGC-ATAATATA-GTTATCATCA-
Plna	--T--GCATAA---CTGATA--TAAAGGC-ATAAATAGCTCAAATTTCCC
Lema	-CC--ACATAT-TAGGATAT--CAAGAGC-ATAAACTA------------
Apse	-AT--ACATAA-GG-GATAT--CAAGGAC-ATAACTAATGATATGATC--

	CSB-D		CSB-I
Epde	GAA----CTATTCCTG---GCAT-TTGGTTCC-------TAC	Epde	--C--ACATTA-AAGGATCT--CAAGAGC-ATAAAGTATGAGTATTTCC-
Sija	GAA----TTATTCCTG---GCAT-TTGGTTCC-------TAC	Sija	--C--ACATTA-AGTTATCT--CTAGTAC-ATAAAGTGGTGGTGATTTC-
Bsja	GAA----CTATTCCTG---GCAT-CTGGTTCC-------TAT	Bsja	-TT--GCATAT-TAGGATCT--CACGAGC-ATATATTAACG---------
Ecna	GAA----CTATTCCTG---GCAT-ATG-TTCC-------TAC	Ecna	-CC--ACATTA-TATGAAGT--CTAGTGC-ATAAGTATTTTACTTTTT--
Caar	GAA----CTATTCCTG---GCAT-TTGGTTCC-------TAT	Caar	-TT--GCATAT-TAGGATAT--CAAGTAC-ATAATAGAT-TAATCTTT--
Came	GAA----CTATTCCTG---GCAT-TTGGCTCC-------TAC	Came	-CC--ACATAT-TATTTTAT--CAAGAGC-ATAAGT--TAT---------
Mema	AACA---AGGCCCAC----CAACCTTCGACAGTCTGAAGTGA	Mema	----TGCATAT-TAGGATAT--CATGAGC-ATAATGAGTATGGTGTTTCT
Emst	GAA----CTATTCCTG---GCAT-TTGGTTCC-------TAT	Emst	-TT--GCATAT-TAGGATAT--CAAGAGC-ATAAATTA------------
Ptti	GAA----CTATTCCTG---GCAT-TTGGTTCC-------TAC	Ptti	--T--GCATAT-AGGTATCT--CATGAGC-ATAATGA----TGAGCTTCT
Losu	GAA----TTATTACTG---ACAT-TTGGTTAG-------TAT	Losu	-GATTACATAT-AATTATGT--CATGGAC-ATAATG----TTCTTATTC-
Geoy	TTTT---CTTTTTTAGGG-GGAT-TTCATTTA-------CAT	Geoy	CATAACTGAGT-TCTTGTTT--GGTGACC-ATA---TACTTCA-CT-ATG
Dipi	GAA----TTATTCCTG---GCAT-TTGGCTCC-------TAC	Dipi	-TT--GCATTA-GAGTTTT---CATGAGC-ATAAATAACAAGCAGGAA--
Pama	GAA----CTATTACTG---GCAA-CTGGTTCC-------TAT	Pama	--T--GCATA--ATTGAGTT--CAAGAGC-ATAATATGCTATTTTTTC--
Leob	GAA----TTATTACTG---GCAT-CTGGTTCC-------TAT	Leob	--T--GCATA--ACTGATAT--CAAGAGC-ATAAGGTGTGACCCTTACC-
Pdpl	GAA----CTATTCCTG---GCAT-TTGGTTCC-------TAT	Pdpl	------TAAAT-TAATGCTA--GGAAGAC-ATATTTATC--TAAAAGTTT
Nimi	GAA----CTATTCCTG---GCAT-TTGGTTCC-------TAT	Nimi	--C--ACATAT-TGATATAT--CAAGTGC-ATACACTATGACTTGTTTC-
Pesc	GGA-----CATTTTTG---ATGG--TGGCGCGCCCACGTCTC	Pesc	-----ACATAA--CTGATAT--CAAGGAC-ATAATGAAAGAATACCCCC-
Moar	GAA----CTATTCCTG---GCAT-TTGGTTCC-------TAT	Moar	-TT--GCATAA-AAGGATAT--CAAGAGC-ATAAACTGTTAATATTACT-
Toja	TAG----GTTTGCCTG---GCAT-TTGGTTCC-------TAT	Toja	--C--GCATAT-TAGGATAT--CAAGTAC-ATAATATACATATTCACA--
Chau	GAA----TTATTCCTG---GCAT-TTGGTTCC-------TAT	Chau	-TT--GCATTA-CACTATAT--CATGAGC-ATAAA-----TAGATATA--
Chse	GAA----C-ACTATCG---GCAT-CTGGTTCC-------TAT	Chse	-CA--CAATAG-T-TTACTC--CTCGAGC-ATAAGGCTT-G---------
Enar	GAA----TTATTCCTG---GCAT-TTGGTTCC-------TAC	Enar	--T--ACATAA-C-TGATTT--CAAGAGC-ATAAGGTATG-AATATTTCT
Hpty	GCA----CTATTCCTG---GCAT-TTGGTTCC-------TAC	Hpty	--T--GCATA--AGTGATAT--CAAGAGC-ATAAATTATGAATATTTCT-
Mcst	GAA----CTATTCCTG---GCAT-CTA-CTGT-------TAC	Mcst	-TT--GCATAA-C-TGATAT--CAAGAGC-ATAA-GTTGTGGTTATTT--
Rhox	GAA----TTATTCCTG---GCAT-TTGGTTCC-------TTC	Rhox	-TT--ACA-TA-ACTGATTT--CAAGTGC-ATAA---TAGTTGTATAT--
Opfa	GAA----CTATTCCTG---GCAT-TTGGTTCC-------TAT	Opfa	--T--GCATTA--ATGATAT--CAAGAGC-ATAAGTTATAAGTATCAA--
Paar	GAA----CTATTACTG---GCAT-TTGGTTCC-------TAT	Paar	-TT--ACATATTAACTAATT--CTCGGAC-ATAACATATTTGACTACACT
Gozo	GAA----CTATTACTG---GCAT-TTGGTTCC-------TAC	Gozo	--T--GCATAA-CCTTATAT--CATGAGC-ATAAGATATGGAACACTTCC
Ackr	GAA----TTATTCCTG---GCAT-TTGGTTCC-------TAT	Ackr	-TA--ATATAT-TATTATTT--CTAGGAC-ATAATGTA------------
Trdu	GAA----CTATTCCTG---GCAT-CTGGTTCC-------TAT	Trdu	--T--GCATA--ACTGATAT--CATGAGC-ATAAAGTTTAATCAGATATT
Amoc	GAA----CTATTCCTG---GCAT-TTGGTTCC-------TAT	Amoc	-TT--GCAT-A-ACTGATAT--CAAGAGC-ATAAATG----------A--
Hame	GAA----CTATTCCTG---GCAT-TTGGTTCC-------TAC	Hame	-TT--GCATAA-CT-GATAT--CAAGAGC-ATAATACTTTTACGTTTA--
Chso	ACA----CTATTACTG---GCAT-TTGGTTCC-------TAT	Chso	-TT--GCATTA-ATAGCTAT--CAAGAGC-ATA-ATGCTTACTTCCTACC
Lyto	GCA----CTATTCCCG---GCAT-TTGGTTCC-------GCA	Lyto	-TT--GCATAC-GTTAATAT--CAAGAGC-ATAAATAGTGCTTGTTCA--
Encr	GCA----CTATTCCTG---GCAT-TTGGTTCC-------TAC	Encr	-CT--GCATAT-CATAATCT--CAAGAGC-ATAAATAGTGCTTTTTCA--
Bvar	GAA----TTATTCCTG---ACAT-TTGGTTCC-------TAC	Bvar	TTT--TCATTT-TTAGAGTT--CAAGGAC-ATA-TAGGT-CAAAAATC--
Chsp	GAA----CTATTCCTG---GCAT-TTGGCTCC-------TAT	Chsp	-TA--ACATAA-ATG-ATTT--CAGGTGC-ATA--ATACATAAATTGC--
Arja	GAA----TTATTCCCG---GCAT-TTGGTTCC-------TAC	Arja	--T--GCATAC-TTCGATAT--CAAGAGC-ATAAAGAGTGAACTTTTACT
Lifa	GAA----TTATTCCTG---GCAT-TTGGTTCC-------TAC	Lifa	-TT--ACAT-A-ACTGATAT--CAAGTGC-ATACGTACATCCCCCTTA--

CSB-D

```
Acur    GAA----CTATTACTG---ACAT-CTGGTTCC-------TAC
Ampe    GAA----TTATTCCTG---GCAT-TTGGTTCC-------TAC
Enet    GAA----CTATTCCTG---GCAT-TTGGTTCC-------TAC
Ptbr    GAA----TTATTCCTG---GCAT-TTGGTTCC-------TAT
Safa    GAA----CTATTCCTG---GCAT-TTGGTTCC-------TAC
Asmi    GAA----TTATTACTG---GCCT-CTGGTTCC-------TAT
Drze    GAA----CTATTCCTG---GCAT-TTGGTTCC-------TAC
Rhas    GAA----CTATTCCTG---GCAT-TTGGTTCC-------TAT
Elac    GCA----CTATTCCTG---GCAT-TTGGTTCC-------TAT
Kugu    GAA----CTATTCCTG---GCAT-TTGGCTCTAC------AT
Plor    GAA----TTATTCCTG---GCAT-TTGGTTCC-------TAT
Sgun    GAA----TTATTCCTG---GCAT-TTGGTTCC-------TAC
Zaco    GAA----TTATTCCTG---GCAT-TTGGTTCC-------TAC
Zbfl    GAT----CTATTTCTG---GCAT-TTGGTTCC-------TAC
Spba    GAT----CTATTCCTG---GCAT-TTGGTTCCCTTTACCTAC
Game    GAA----TTATTCCTG---GCAT-TTGGTTCC-------TAC
Thth    GAA----CTATTCCTG---GCAT-CTGGTTCC-------TAC
Xigl    GAA----CTATTCCTG---GCAT-TTGGTTCC-------TAC
Hyja    GAA----TTATTCCTG---GCAT-TTGGTTCC-------TAT
Cupa    GAA----TTATTCCTG---GCAT-TTGGTTCC-------TAC
Mpch    GAA----TTATTTCTG---GCAT-TTGGTTCC-------TAT
Char    GAA----CTATTCCTG---GCAT-TTGGTTCC-------TAC
Prol    GAA----CTATTCCTG---GCAT-TTGGTTCC-------TAC
Calu    GAA----CTATTCCTG---GCAT-TTGGTTCC-------TAC
Papa    GAA----TTATTCCTG---GCAT-CTCCTTCA--------AT
Sufr    GAT----CTATTCCTG---GCAT-TTGGTTCC-------TAC
Taru    GAA----CTATTCCTG---GCATCTTGGTTCC-------TAC
Rala    GAA----CTATTCCTG---GCAT-TTGGTTCC-------TAT
```

CSB-I

```
Acur    TTA--ACATAT-ATA-ATTT--CAAGTAC-ATAATAGGT-CAAATATA--
Ampe    -TT--GCATAT-TAGGATCT--CATGAGC-ATAATGTGTACTTATCAA--
Enet    --T--GCATAA-CTGT-TAT--CATGAGC-ATAATGTACACTACCT----
Ptbr    -TA--GCATAA-TTT-ATAT--CAAGAGC-ATAATATATATATATTTC--
Safa    -TT--GCATAA-GT-GATTT--CAAGAGC-ATAAAGTAGGTAATACTT--
Asmi    --T--GCATA--ATTGATAT--CAAGAGC-ATAAGGGACAAAAATTTC--
Drze    -AT--ACATAG-CTGAT-AT--CATGGAC-ATAAAC--TAC---------
Rhas    --T--GCATAA-CT-GATCT--CAAGAGC-ATAAATACTGTTTATTTCTC
Elac    --T--GCATAA-CT-GATAT--CAAGAGC-ATAATTACTAATGGTTTCTC
Kugu    -TT-ATAAACA-TTGATTTT--AAATTGC-ATA---ACTGATTT------
Plor    -TT--GCATAT-TAGGATGT--CAGGAGC-ATAATAGATCATTATACA--
Sgun    --T--GCATAA-TTGGATAT--CAAGAGC-ATA---AGTATCTGATA---
Zaco    --T--TCATGCATTACTGATTTCAAGAGC-ATAACTCATGACTA-TT-AC
Zbfl    --T--ACATAA--CTGATAT--CATGGAC-ATAGTTCATGAAAATTTC--
Spba    AAG-TTCGAGT-GAATGGTG--AAAAGAC-ATAGACCT------------
Game    --C--ACATAC-TTGGATAT--CAAGAGC-ATAATA-ATGGTATTT----
Thth    --C--ACATAT-TTGGATAT--CATGAGC-ATAATGA----TAA---TTT
Xigl    --T--ACATAT-TTGGATAT--CAAGGAC-ATAAAGTAT-TTGTTCTT--
Hyja    --C--ACATAA-GAGAATAT--CAAGGGC-ATAA-GTACTAAA--TTACC
Cupa    -TA--ACATAT-TAGGATAT--CAAGAGC-ATAAATGAAATATTACTC--
Mpch    -TT--GCATAA-CTTAATAT--CAAGAGC-ATAAAGTATCAAGTAAAT--
Char    --T--GCATAA-TAGGTTAT--CAAGAGC-ATAAGATGAAATAATTTCC-
Prol    -TA--ACATTA-AAAGATAT--CAAGTGC-ATAAG--------GGTGG--
Calu    -CT--GCATTA-ATCTATAT--CAGGTGC-ATA--CAGTATTCTTTAT--
Papa    -GG--AAACAT-TCTTTAAA--CCATTAC-ATA---ACTGATATCAT---
Sufr    --G--ACATAA-ATCATCA---CCCGTAC-ATAAGGAATAATTCTTAA--
Taru    -TT--ACAT-T-ACTGATAT--CAAGGAC-ATAAATA----------A--
Rala    --C--ACATAA-GG-GATAT--CAAGGAC-ATAAT-ACTTTGATGTCACT
```
