# Supplementary material for: Structure and variation of the mitochondrial genome of fishes
Source: BMC Genomics. 2016 Sep 7;17(1):719. doi: 10.1186/s12864-016-3054-y (PMC5015259; doi:10.1186/s12864-016-3054-y)
Supplement: Additional file 15: Figure S4-a. — Aligned nucleotide sequences of the conserved sequence blocks-D and -I in the control region (CR) in mt genomes of 182 fishes. Figure S4-b. Aligned nucleotide sequences of the conserved sequence blocks-II and -III in the control region (CR) in mt genomes of 182 fishes. (ZIP 87 kb) [file 12864_2016_3054_MOESM15_ESM.zip › Additional file 15 CSBs/Additional file15-b CSB2-3.pdf]

**Additional file 15: Figure S4–b. Aligned nucleotide sequences of the conserved sequence blocks-II and -III in the control region (CR) in mt genomes of 182 fishes.**

```
                       CSB-II                                                      CSB-III
Scca   TTTGGTAAAAAC-CCCCC------------TCCCCC--TTAATATATA--CGG-------ACATC---------TCGAAAA--ACCCCA-AAAACGAG---------GGCCGACATATAT
Muma   TTTGGTAAAAAC-CCCCC------------TCCCCC--TTAATATACA--CGG-------TTATC---------TCGAAAA--ACCCCT-AAAACGAG---------GGCCGACGTATAT
Pose   -TAGCCTAAAAC-CCCC-------------TACCCCC-TTAAAAAAGACGTTT-TT----GGACTTTTTGGCCCTGTAAA---ACCCCG-AAAACAG---------GAAAGTGCCTAGAA
Actr   AT-CGA-CAAAC-CCCC-------------TACCCCC-TTATGTCGGACAGGCCTT--AT-ATTTCT-------TGTCAA---ACCCCA-AAAGCAG---------GACTGACTTGTCAT
Scal   GT-CGA-CAAAC-CCCC-------------TACCCCC-TTACGCCTGAC-GGTCTT--AT-ATTTCT-------TGTCAA---ACCCCA-AAAGCAG---------GACTGACCTATCAT
Posp   TCTCGA-CAAAC-CCCC-------------TACCCCC-TTACATTGAACAAGTTCT-TGTACTTTCC-------TGTCAA---ACCCCA-AAAGCAG---------GACTAACTCGC-AT
Hial   ----CGGCAAAC-CCCCC------------TACCCCCC-TACGCCGGACAAGTCCT--ATTTTATTC-------TGTCAA---ACCCCT-AAACCAGA--------A-AAGGCCGACCAG
Elha   GA-CGA-CAAAC-CCCCC------------TACCCCCC-TACGTCGAGACGACCCT--ATGAGTTCC-------TGTCAA---ACCCCCTAAACCAG---------GAACAGCCGACCAA
Alaf   TTTCGT-CAAAC-CCCCC------------TACCCCC-TACGCCCGGACTAGCCCA-TT-ACTTTCC-------TGACAA---ACCCCT-AAAC-AG---------GAAAAGGCCG--AC
Anja   -----GTCAAAC-CCCCC------------TACCCCCCTTACCCCTAACAACCTCA-TTATTTTCC--------TGTCAA---ACCCCT-AAACCAGG--------CCAAAGTCGAAAGA
Gyki   -----GTCAAAC-CCCCC------------TACCCCCCTTAATACTGGCAATTCCA-TCATA-TCT--------TGTCAA---ACCCCA-AAAGCAAG---------ATAAGCCGCACTA
Opma   ATTAGT-TAAAT-CCCCC------------TACCCCC-TCAGCCCAGACAACCTTA-TTCTAAGACA-------CCCTGA---TTTACA-AAATAGAG---------TAAAGAATAT-TC
Comy   TTTGTTTTAGAC-CCCCC-----------TTACCCCCGTATAAATATATTGAATAATTAATAATATTAT---TTAAACA---TTTACA-CTAACAC---------CCGCTCAACATACT
Enja   GCGCGA-CAAAC-CCCCT------------TACCCCC-T-ACGACCCAGACAAGCC-TATTTTTTTC-------TGTCAA---ACCCCG-AAACCAG---------GAAAGACCGG--AC
Same   GCGCGG-CAAAC-CCCCC------------TACCCCCC-TACGCTCTGCAAAGCCC--GGTA-TTCA-------TGTCAA---ACCCCAAAAAACCATGA-------GGGGACTCGGCGTG
Grgr   GCGCGT-TAAAC-CCCCC------------TACCCCCCAACG--CCCAGGGATCCT-TGTTACTCC--------TGCTAA---ACCCCG-AAATCAG---------GCAAGGCTCGA---
Caau   GCGCGA-CAAAC-CCCCC------------TACCCCC-T-ACGCTCAAAGAATCCT-GTT-ATCCT--------TGTCAA---ACCCCG-AAACCAA---------GGAGGACC-----C
Cyca   GCGCGA-CAAAC-CCCCT------------TACCCCC--TACGCTCAGCGAATCCT--GTTATCCT--------TGTCAA---ACCCCG-AAACCAA---------GGAGGACTCAA---
Dare   A------CAAAC-CCCCT------------TACCCCC-TTACGTCCAGCGATTCCT--GTTATCCT--------TGTCAA---ACCCCT-AAACCAAG--------GAAGGCCCGAGAAC
Cost   GCGCGA-CAAAC-CCCCC------------TACCCCCC-TACGCTGGGCGATTCCT--GTTTATCCT-------TGTCAA---ACCCCG-AAACCAA---------GGAAGACTCGA-CT
Leec   GCGCGT-TAAAC-CCCCC------------TACCCCC--TACGCTCAGTGATTCCT--GT-TTTCTT-------TGTCAA---ACCCCT-AAACCAA---------AGAAGGCTCGAC-T
Fola   GCGCGA-CAAAC-CCCCT------------TACCCCC-TTACACCTGGCAACTCCT--GT-ATCCT--------TGCTCAA--ACCCCG-AAACCAA---------GGAGGGCCTG--CC
Clmc   TCTCGG-TAAAC-CCCCCA-----------TACCCCCC--ACATCGAACAAGTC-T--AT-ATAATC-------TGTCAA---ACCCCAAAATCCAG---------ACAGACCCAGTCGA
Phin   ----GA-CAAAC-CCCCC------------TACCCCCCTACATCGAAAGAGTTCAT-ATAAT-TCC--------TGTCAA---ACCCC-AAAACCAGG--------TATGCCTCGACCAA
Icpu   GCGCGG-TAAAC-CCCCC------------TACCCCCC-A-TGCCGTACGAGTCCT--AATTAATCC-------TGTTAA---ACCCCT-AAACCAG---------GTAAGGCCCG--AT
Psto   GCGCGA-TAAAC-CCCCC------------TACCCCC-TAATGCCCAGCAAGTCCT--AAGTTATCC-------TGTTAA---ACCCCT-AAACCAG---------GTTAGGCCCG--AT
Cora   ACGCGG-TAAAC-CCCCC------------TACCCCC-AACGCCGAGAGAT-GCCT----GTTTAAATC-----TGTTAA---ACCCCT-AAACCAG---------ATTAGGCTCG--AT
Eisp   GCGCGA-CAAAC-CCCCC------------TACCCCC-T-ATGCCGGGCGGATCCT-GTTATTCC---------TGTCAA---ACCCCT-AAACCAG---------GTAAGACACG--AC
Eslu   --GCGA-CAAAC-CCCCC------------TACCCCCC-TACACTGG-GCGATCCT-TATTATCC---------TGTCAA---ACCCCG-AAACCAGGA-------GTCCCGCCTAATGC
Dape   --TCCT-AAAGC-CCCCC------------TACCCCCCAAAAATCTACGTAAGGGG-CACCACTTTTAGAACTAATTTACCTTAAAAAATATAAAACCCCAACATAAACTTGTTGCCCTC
Glse   ACGCGT-CAAAC-CCCCCCC----------TACCCCCCTACACCCTATGATTCTTA-AAAT--TCC--------TGTCAA---ACCCCA-AAACCAG---------GAAGAA-CTTCTA-
Naar   ACGCGT-CAAAC-CCCCC------------TACCCCCCTACACCCCGCAATTCTTA-CAAT--TCC--------TGTCAA---ACCCCG-AAACCAG---------GAAAGA-TTTGCA-
Lioc   GCGCGT-TAAAC-CCCCC------------TACCCCCCTACAACCCGCAATTCTTG-TTGTGCTCC--------TGTAAA---ACCCCA-AAACCAG---------GAAAGA-CTCGCA-
Opso   ATGCGT-CAAAC-CCCCCCC----------TACCCCCCCTACGCCCTACTGTCCTTATCAT--TCC--------TGTCAA---ACCCCG-AAACCAG---------GAAAAAACTCGTA-
Alte   GCGCGA-CAAAC-CCCCCC-----------TACCCCCCCTCGCCGAACAAGTC--T--TGTTCTTCC-------TGCCAA---ACCCCT-AAAACAG---------GTAAAGCTCG--AA
Plap   GCGCGT-CAAAC-CCCCC------------TACCCCC--TACGCCGAACAAGTC-C--TATTCTTCC-------TGCCAA---ACCCCT-AAAACAG---------GAAAGGCTCG--AA
Plal   ACGCGT-TAAAC-CCCCC------------TACCCCCCTT-TAGTCCTGACATTAC-TATTGTTTCT-------TGTTAA---ACCCCT-AAACCAAGAG------AG--TTACGACAAG
```

```
                  CSB-II                                                  CSB-III
Sami   ACGCGT-TAAAC-CCCCC-------------TACCCCCCTTTAGTCCTGACATTACT--ATTACTTCT-------TGTTAA---ACCCCT-AAACCAA---------GAAAGTGATGAGAA
Rere   ACCCGT-TAAAC-CCCCC-------------TACCCCC-TTAAGTCCCGACATAGCT--ATGACTTCT-------TGTTAA---ACCCCT-AAACCAA---------GGAAGCTGTTGTAA
Gama   GCGCGT-TAAAC-CCCCC-------------TACCCCCCTA-CACCCCTGGGATCAC-TATCGTTTCC-------TGTCAA---ACCCCT-AAACCAGGAG------AA-ATCCCTAAAAG
Onmy   GCGCGG-TAAAC-CCCCC-------------TACCCCCCTA--AGCTGAAAGATCCT-TA-TGTTCC--------TGTTAA---ACCCCT-AAACCAG---------GAAGTCTCAAATCA
Sasa   GCGCGG-CAAAC-CCCCC-------------TACCCCCCTACGCTGA--AGGATCCT-TA-T-ATTCC-------TGTCAA---ACCCCT-AAACCA----------GGAAGTCTCA--AA
Cola   GCGCGGCTAAAC-CCCCC-------------TACCCCCCTACGCTGA-GCGATCCTT--ATTA-TTCC-------TGTCAA---ACCCCA-AAACCA----------GGAAGTCTCGATAG
Dita   GCGCGT-CAAAC-CCCCC-------------TACCCCCCTA-CT-TACTAAGACCTT-TAATATTTCC-------TGTCAA---ACCCCA-AAAGCAGGAC------AAGATCTCGGGCGG
Atja   TACGGG-TAAAC-CCCCC-------------TACCCCCCTACACTCCTGACATCTC--TATGACTTC--------TGTAAAC--CCCCCGTAAAACAG----------AAAAGTGCCTAGT
Iido   TCGGG--TAAAC-CCCCC-------------TACCCCCCT-ACACTCCTGACATCGC-TATGACTTC--------TGTAAAC--CCCCCGTAAAACAG---------AAAAATGCCTAG--
Auja   GCGCG--TAAAC-CCCCC-------------TACCCCCC-TTCGCCCCTGAGATCAT-TATCATCC---------TGCAAAC--CCCCCGAGAAACAG---------GAAAACCTCTAGCG
Nemi   GGCCGT-CAAAC-CCCCC-------------TACCCCCCAAC-ACTCGAAAGATCCC-TATGACTCC--------TGCAAA---CCCCCG-AAAACAG---------GACGGACCCTAGAA
Pxja   GCGCGT-TAAAC-CCCCC-------------TACCCCCC-AACGCTCCTGGGATCAC-TA-TCATTCT-------TGTAAA---CCCCCCGTAAACAA---------GAAAAACCCT--AG
Pxlo   GCGCGT-TAAAC-CCCCC-------------TACCCCCCAAC-GCTCCTGGGATCGC-TATCATTCT--------TGTAAA---CCCCCCGTAAACAA---------GAAAAACCCTAG--
Apsa   CGCGTCTCAAAC--CCCC-------------TACCCCCTT-AACTTCAAAAAGTTT--CTAAATTCC--------TGCAAA---CCCCCCGGAAACAGG-------ATCAACCCTAAGAA-
Cabe   --TAGG-TATACCCCCC-------------TACCCCCCCGTGGGCCGAGACCA-GGCGAGGGGC--GAA-----TGCACAT--CAAGCCTCGCGTTTG---CTCGGCACAAGTTGTGTGA
Bzze   --GCGT-TAAACCCCCC-------------TACCCCCCCACACTCGTGAGATTGCT-AATACTCC---------TGAAAA---CCCCCCGGAAACAGG--------AAAACCTCTAGACA
Ctru   --GCGT-TAAAC-CCCCC-------------TACCCCCCTAC-ACTCCTGAGATCAC-TATCATCC---------TT-AAA---CCCCCCGGAAACAG---------GATTGAACCTCGAG
Dpbr   -------AAAAC-CCCCC-------------TACCCCCCTACTACTCCTGAGATCGC-TATCATCC---------TGAAAA---CCCCC-GCAAACAG---------GATTAAATCTCGAG
Phja   GGCCG--TAAACCCCCC-------------TACCCCCC--AGTTTTACGAGACTTG-TAAT--TCC--------TGCAAA---CCCCCCGGAAACAG---------AAAAATCCCCAAA-
Gamo   GTGCG--TAAACCCCCC-------------TCCCCCC--AGTTCTCCTGAGATTAC-TAATATTCC--------TGTAAA---CCCCCCGGAAACAG----------GAAAATCCCTAGA
Lolo   GCGCG--TAAATCCCCC-------------TACCCCCC-AGTTCTCCTGAGATTAC-TATGACTTCC-------TGTAAA---CCCCCCGGAAACAG----------GAAAACCTCGAGA
Prmy   TATTCG-TAAATCCCCC--------------AAACCCCCCTGAGCAGAGAAGTTTAAGTCACTAAAAATCCTCGTAAAATAAGTTTTCTAAGGCTTCGAGGCACGTATCATAAAAATATA-
Loam   ACACGC-AAAAC-CCCCC-------------CACCCCCTTT-ACTCGTAAGATAGT--TAATACTCC--------TGAAAAC--CCCCCGGAAAGCAG---------GAAAACCTCGAGTA
Chab   TATCGT-TAAAC-CCCCC-------------TACCCCCCTATACTCCTGAGATCAC--TAACAATCC--------TGCAAAC--CCCCCGG-AAACAG---------GTGGACCTCGAGTG
Chto   TATCGT-TAAAC-CCCCC-------------TACCCCCCTACACCCCTGAGATCAC--TAACAATCC--------TGCAAAC--CCCCCGG-AAACAG---------GTGGACCTCGAGTG
Clpe   GGTCG--TAAACCCCCCCC------------TACCCCCCCAACACTATTGAGATGCC-TAACGATCC--------TGCAAAC--CCCCCGG-AAACAG---------GAAGCTCTCGAG--
Muce   GGGCGGGAAAACCCCCC-------------TACCCCCCCTAACTCCTAAGATCAG--TGTCACTCC--------TGAAAAC--CCCCCGG-AAACAGG--------AAAACCTTAAGATC
Bega   GCGCGT-CAAAC-CCCCC-------------TACCCCCCTACACTCGTGAGATACT--TATTACTCC--------TGCAAAC--CCCCCGG-AAACAGGAA------AG--CCTCGACCAG
Mela   GCGCGT-TAAAC-CCCCC-------------TACCCCCCTACACTCGCGAGATCAC--TATCATTTC--------TGCAAAC--CCCCCGG-AAACAG---------AAAAACCTCGACTG
Hats   GCGGGT-TAAAC-CCCCCC------------TACCCCCCCAACACTCGCGAGATCCT-TATCACTCC--------TGTAAAC--CCCCCGGGAAACAG---------GAAAACCTCGACCA
Orla   GCGGG--CAAAC-CCCCC-------------TACCCCCCTATACTAGTAAGAGCTC--TATCATTCC--------TGCAAAC--CCCCCGG-AAACAG---------GAAAGCCCCTACTA
Cosa   ACGCGT-TAAAC-CCCCC-------------CACCCCCCTTA-CTCGTAAGATCCT--TATTATTTC--------TGCAAAC--CCCCTA--AAACAG----------AGAAGCTCCTACT
Exvo   G----GTCAAACCCCCC-------------TACCCCCCCAATACTCGTGAGATCCTTGT-GATTCC--------TGCAAAC--CCCCCTT-AAACAGG--------AGGATCCCTACGGT
Depu   TTTCGT-CAAACCCCCC-------------TACCCCCCCAC-ACTCGTAA-ATTCT-TATTACTCC--------TGCAAAC--CCCCCGG-AAACAG---------GAAAGCCTCTAC--
Rima   TAAAG--TAGAC-CCCCC-------------TACCCCCCTTAACTCCAGACATGTC--TGTCAAAATTGATAAAACACATT---TCACGGA-AAATAGAGGG-----GAAACTTTTTATTA
Fuol   AGGGGTTAAACC-CCCCC-------------TACCCCCCCAATACTCGAGACATCTT-TGTTACTCC--------TGCAAAC--CCCCCGG-AAACAGGAA------AG--AGCCTACTCG
Gmaf   ATGGGCCAAAAC-CCCCCC------------TCCCCCCTTAAACTCCTGAGATT-C--CATTGTGTC--------TGCAAAC--CCCCCGG-AAACAGAGC------AAATCTCCTAAGTT
Xeei   ACGCGT-TAAAC-CCCCCC------------TACCCCCCCA-CTTCTCGAGACATCATTATAAATCC--------TGCAAAC--CCCCCGGAAAACAGG--------AAAGCCTCTACCAG
Rolo   ACGCG--TAAACCCCCC-------------TACCCCCCAAATCTACTGAGATGAC--TATCATTCC--------TGCAAAC--CCCCCGG-AAACAG---------GAAAGCCCCT--AG
```

```
                  CSB-II                                                  CSB-III
Cere   GCGCGT-TAAAC-CCCCC------------TACCCCCCAACACTACTTACATGGC--CGTCACTTC--------TGTAAAC--CCCCCGG-AAACAG---------AAAAGCCTCTA---
Daga   ACGCGT-TAAACCCCCCC------------TACCCCCCCGGCAATACTAACATGAC-TGTCATTTC--------TGAAAAC--CCCCCGG-AAACAG---------AAAAGTCTCGA---
Anco   TTTCGG-GAAAC-CCCCC------------TACCCCCCAACACTCCTGACGTGGC--TA-TCATTCC-------TGGAAAC--CCCCCGG-AAACAG---------GAAAACCTCG--AC
Moja   TTTCGGGAAAAC-CCCCC------------TACCCCCTACTACTCCTGAGATCAC--TATCATTCC--------TGAAAAC--CCCCCGG-AAACAGGAA------AA--TCTCGAGTAG
Hoja   TTACGGGAAAAC-CCCCC------------TACCCCCCTACACCCCTGAGATCGT--TA-TCATTCC-------TGAAAAC--CCCCCGG-AAACAG---------GAAAATCTCG--AG
Bede   GCGCG--TAAAC-CCCCC------------TACCCCCCTACACCCCTGAGATCAC--TATTATTCC--------TGAAAAC--CCCCCGG-AAACAG---------GAAAACCTCT--AG
Besp   GCGCG--TAAAC-CCCCC------------TACCCCCCAACACCCCTGAGATCGC--TATTATTCC--------TGAAAAC--CCCCCGG-AAACAG---------GAAAACCTCTA---
Mybe   --GCGT-TAAACCCCCCC------------TACCCCCCTACACTCCTGAGATCCT--TATCACTCC--------TGCAAAC--CCCCCGG-AAACAGGA-------CAA--ACCTCAAA-
Osja   GCGCGT-CAAAC-CCCCC------------TACCCCCCTACACTCCTGAGATCTT--TATTA-CTCC-------TGCAAAC--CCCCCGG-AAACAG---------GAAAGTCTCAAGTG
Sgro   GCGCGT-TAAAC-CCCCC------------TACCCCCCTAACACTCCTAAGATCTA-TATTATTCC--------TGTAAAC--CCCCCGG-AAACAGG--------AAAACCTCGAG---
Pzpa   TCGCG--AAAACCCCCCCCC----------TACCCCCC----CT--------------------------CTTGAAAACC-CCCCCGG-AAACAAGAGCTAAAACTTGTAACTCTAAT
Zeja   TCGCGT-TAAACCCCCCC------------TACCCCCCTACACTCCTGAGATAAC--TATTAACTCC-------TGAAAC---CCCCCGG--AACAGGA-------AGAAACCCCAAGAA
Zefa   GCG----TAAACCCCCCC------------TACCCCCCCTTTCT---GAAGTACT--ATA---TCC--------TGTAAAC--CCCCCGG-AAACAG----------GAAAACCTAAGAA
Acni   TAGCG--TAAACCCCCCC------------TACCCCCCCAAACTCTTAAGATTAC--TATTACGCC--------TGAAAAC--CCCCCGG-AAACAGG--------CAAACCCTAAGAAA
Ncrh   TAGCG--TAAACCCCCCC------------TACCCCCCTACAACTCCTGAGATTAC-TATAACGCC--------TGTAAAC--CCCCCGG-AAACAG----------GCCAACCCCAAGA
Agca   GCGCGT-AAAAC-CCCCC------------TACCCCCCTACA-CTCCTGAGATCAC-TAACACTCC--------TGAAAAC--CCCCCGG-AAACAG----------GAAAACCTCGAGT
Hydy   --GCG-TAAAAC-CCCCC------------TACCCCCC-AAAACTCCTAGGATGCT-TAAGACTCC--------TGAAAAC--CCCCCGG-AAACAGGA---------AA--ACCCCTAG
Pevo   GCGCG--TAAAC-CCCCC------------TACCCCC-CTACCCTCGAGAATTATG-AA-GA-CTCC-------CGTAAAC--CCCCCGG-AAACA-----------GGAAACCCTCGAG
Hiku   A------AAAAC-CCCCC------------AACCCCCCTA-AGGCCCTAAAGTAA--CTAACAATTC-------AACAAA---ATCACTATAAACAA---------TAAAACCCCGTGAT
Auch   ---------------------------------------------------------------------------------------------------------------------------
Fico   G----CGTAAAACCCCCC------------TACCCCCCTAA-ACTCCTGAGATCATTAA-TACTCC--------TGCAAAC--CCCCCGG-AAACAGG--------AAAATCTCGGCTAA
Moal   GCGCG--TAAAC-CCCCC------------TACCCCCC-AATACTCCTAAGATCGT-TAGCATTC---------TG-AAAC---CCCCGG--AACAGAA-------AGTATCTCAAAGTA
Syma   CGGCGAAAAAAC-CCCCC------------TACCCCCT-AATACTCCTGAGATCTT-TATTACTCC--------TGAAAAC--CCCTCGG-AAACAGGA-------AAA---CCTCTAG-
Mafa   TCGCG--TAAAC-CCCCC------------TACCCCCCTACA-CTCCTGAGATCAC-TAACATTCC--------TGAAAAC--CCCCCGG-AAACAG---------GAAAACCTCTAGCA
Dcpe   GCGCG--TAAAC-CCCCC------------TACCCCCCTAAA-CTAGAGAGATCAC-TAACACTCC--------TGAAAAC--CCCCCGG-AAACAG---------GAAAACCTCTAC--
Dcti   GCGCG--TAAAC-CCCCC------------TACCCCCCTAAA-CTAGAGAGATCAT-TAACACTCC--------TGAAAAC--CCCCCGG-AAACAG---------GAAAACCTCTACTA
Hehi   TAGCG-TTAAAC-CCCCC------------TACCCCCCTAA-ACTCCTGAGATAAC-TAACGTTCC--------TGTAAAC--CCCCCGG-AAACAG---------GAAAACCTCGAGTC
Stam   --GCG-TAAAAC-CCCCC------------TACCCCCC-TAAACTCCTGAGATGTC-TATCACTCC--------TGAAAAC--CCCCCGG-AAACAGGA--------AG--ACCTCTAG-
Hogi   TTGCG-TAAAAC-CCCCC------------TACCCCCC-TAAACTCCTGAGATCAC-TAAGACTTC--------TGAAAAC--CCCC-GG-AAACAG----------AACAACCTCGAGT
Erzo   GGT----AAAAC-CCCCC------------TACCCCCCTAAACTCGTG-GGATCAC-TAAGACTCC--------TGAAAAC--CCCCCGG-AAACAGG--------AAAATCCCTAGTAG
Hxot   -------AAAAC-CCCCC------------TACCCCCCTAA-ACTCGTGAGATCAC-TAAGACTCC--------TGAAAAC--CCCCCGG-AAACAGG--------ACAACCTCTAGTAG
Core   -------TAAAC-CCCCC------------TACCCCCCTAA-CCTCCTGAGATCAC-TAACACTCC--------TGTAAAC--CCCCCGG-AAACAGG--------ACAACCTCTAGAAG
Latj   GC--GCGAAAACCCCCCC------------TAACCCCCCCTAAACTCCAGGGATCACTAACACTCC--------TGAAAAC--CCCCCGG-AAACAGG---------AAAATCCCTTGAT
Laja   CCGCG-TAAAAC-CCCCC------------TACCCCCTTA-AACTCCTAAGATCAT-TATCACTCC--------TGAAAAC--CCCCCGT-AAACAGGAA------GA--CCTCGAGTTG
Epme   TCGGT--TAAACCCCCCC------------TACCCCCCCTAAACTCCTGAGATCCT-TAACACTCC--------TGTAAAC--CCCCCG-GAAACAGG---------AAAAATCCCTAGT
Grse   C---GTATAAAC-CCCCC------------TACCCCCCTAA-ACTCGAGAGATCGCTAACTACTCC--------TGTAAAC--CCCCCAGAAAACAGG--------AAAACCCCGAGCAA
Plna   TCG-AGTTTATG-CCCCC------------TTACCCCCAGA-AGTCCTAAAACAACCATG--TTTT--------AATAAAC--ATCTGAAATAAAAT-T-------ACAGCCCCTAAAAC
Lema   GCGCG--TAAAC-CCCCC------------TACCCCCCTAT-ACTCCTAAGATGTC-TAACACTCC--------TGAAAAC--CCCCC-GGAAACAG---------GAAAACCCCTAGTA
Apse   GAGCG--TAAAC-CCCCC------------TACCCCCCTAATCTCCTGACTTGTC--TAACACTCC--------TGCAAAC--CCCCCGG-AAACAG---------GAAAGCCTCGAGAC
```

```
                    CSB-II                                                    CSB-III
Epde   --GCGT-TAAAC-CCCCC------------TACCCCCCAGTACCCCCGGGATCAC--TATCATTCC--------TGAAAAC--CCCCCGT-AAACAGGC---------GA--ACCCCGAG-
Sija   GCGAAGGAAAACCCCCCC------------TACCCCCCCG---TCCTTAAGTGGCT-AG-TACTCC--------TGAAAAC--CCCC-GG-AAACAGG---------AAAACTTTTTCGAT
Bsja   TCACGA-AAAAC-CCCCC------------TACCCCCCTAAGCCCCAGACATGTC--TAACACTCC--------TGAAAAC-CCCCCCGG-AAACAG----------GTAAACCTCTAATG
Ecna   C-----GTAAAC-CCCCC------------TACCCCCTA--AACTCCCAAAGAAA--TCA-TATTC--------TCGCAAA-CCCCCCGG-AAACGA----------GACCATTTC-TGTT
Caar   -GCGT--AAAAC-CCCCC------------TACCCCCCAAAACTCCTAAGATCGC--TATTATTCC--------TGAAAAC--CCCCCGG-AAACAG----------GA--AAGCCTCTAG
Came   TTGCGCGTAAAC-CCCCC------------TACCCCCCAAAACTCCTAAAATCGC--TATTTCTCC--------TGAAAAC--CCCCCGG-AAACAG----------GAAAGCCTCTAGAA
Mema   TTGCGCGTAAAACCCCCCC-----------TACCCCCCCTAAACCCCCAAGGATCGCTAT---------------TGAAAAC--CCCCCGG-AAACAG----------GAAAACCCCTAGAA
Emst   ACGCG-TAAAAC-CCCCC------------TACCCCCCTATACTCCTAAGATAGC--TAACACTCC--------TGAAAAC--CCCCCGG-AAACAG----------GAAAACCCCTAGTA
Ptti   TCGCG-TAAAAC-CCCCC------------TACCCCCCTACACTCCTAAGGTCTC--TAACAATCC--------TGAAAAC--CCCCCGT-AAACAGGAA-------AA--CCCCAAGCAA
Losu   T-TAATTTATAC-CCCCC------------CACCCCCCTAAACTTAGAATTGCCGCACCTCTCACCAACAACCACTATACATATAATATTACACATCACGTCGCAGTGCAATTACAATAT
Geoy   ---GCCTAAAAA-CCCCCC-----------TACCCCCCATTACTCCTAGAGTCTCTGTTGTCTCC---------TGAAAAC--CCCCAAA---ACAG----------GGC--AAGTCTC--
Dipi   GCGCG-TAAACC-CCCCC------------TACCCCCCCACACTCCTGAGATCAC--TAACACTCC--------TGCAAAC--CCCCCG-GAAACAG----------GAAAACCTCGAGTA
Pama   TCGT---TTTAC-CCCCC------------CACCCCCCTAAACTCCTAGGGTTCT--TAATACTTC--------TAAAGAC--CCCTAA-AAAACAG----------AAAAGTCTCAAT--
Leob   --T-CGCAAAAC-CCCCC------------TACCCCCCTACACTCCTAAGATCTC--TGCTAGTCC--------TGAAAAC--CCCCCGT-AAACAGGA--------AAGT--CCCTAGG-
Pdpl   ACTTATAAAGTTTCCCCC------------CACCCCCTTCAACTCCTGACTTGCC--TAATACTCC--------CGGAAAC--ATCTTATGTAACG-----------GACATGCCTGGACG
Nimi   --GCGTTAAACC-CCCCC------------TACCCCCCCAACACTCCTGAGATCTC-TAACACTCC--------TGTAAAC--CCCCCCGTAAACAGGA--------AGA---CCCCTGG-
Pesc   TTGCG-TTAAAC-CCCCC------------TACCCCCCTAAACTCCTAAGATCCT--TATTATCC---------TGCAAAC--CCCCCGGAAAACAG-----------GCGAATCTCGAGA
Moar   TTGCGCGTTAAACCCCCC------------TACCCCCCTATACTCCTGAGATCGC--TAACATTCC--------TGAAAAC---CCCCCGGAAACAG-------- GAAAACCTCTAGCA
Toja   TCGCGTTAAAAC-CCCCC------------TACCCCCCTAAAACTCCTGAGATAGC-TAACATTCC--------TGTAAAC---CCCCCGTAAACAG----------GAAA-TCTCTAGTC
Chau   -------AAACC-CCCCC------------TACCCCCCCAATACTCCTGAGATCGC-TATTATTCC--------TGTAAAC--CCCCCCGTAAACAG----------GA--AAGCCCCTAG
Chse   TTGTGCGAAACCCCCCCC------------TCCCCCCCCAACACTCGAAAGCTAAC-TAACATTCC--------TGAACCC--CCCCCGGAAAACAG----------GAACCCCTCGAGCG
Enar   TTTCGTCAAACC-CCCCC------------TACCCCCCCTTCACCCCCGAGATCAT-TAACACTCC--------TGCAAAC-CCCCCCAGGAAACAGG---------AAAATCTCTAGTGA
Hpty   --G-CGTAAAAC-CCCCC------------TACCCCCC-AATACTCCTGAGATCAC-TAACATTCC--------TGAAAAC---CCCCCGTAAACAGGA--------ATATAACCCCAAA-
Mcst   -GTGCGTAAAAC-CCCCC------------TACCCCCCTA-AACTCCTAGGATTAC-TAACACTCC--------TGAAAAC----CCCCGTAAACAG----------GAAAACCTCTAGCA
Rhox   -GCGCGTAAACC-CCCCC------------TACCCCCCTTC-ACTCGTGAGATTTT-AAACACTCC--------TGCAAAC---CCCCCGGAAACAG----------GAAAACCTCTA---
Opfa   -CGC-GTAAAAC-CCCCC------------TACCCCCCT-AAACTCCTGAGATCAC-TAACACTCC--------TGAAAAC----CCCCGAAAACAG----------GAAAACCTCTAG--
Paar   -ATTCTAAGTGC-CCCCCCCC---------TACCCCCCCCCA--------------------------------AAAAAAC--AAGGCCTAAAACAG--CA--TTTGTAACATTTTTTAT
Gozo   TCGCGTTAAACC-CCCCC------------TACCCCCCCGCCACTCCTGAGATCAC-TAATATTTC--------TGTAAAC--CCCCCCGTAAACAGA---------AAAGGCCCCAGGTG
Ackr   TCTC-GTTAAAC-CCCCC------------TACCCCCCTAA-ACTGCTGAGATGAC-TAATACTCC--------TGAAAAC--CCCCC-GGAAACAG----------GAAAGCCCCTGGCA
Trdu   GCG-CGTAAACCCCCCCCCCCC---------TACCCCCCCCAAACTCCTAAGATCTC-TAAGACTCC--------TGTAAAC--CCCCC-GGAAACAGGAAAAGCTCTAGAAGCGTTTTTT
Amoc   ------TTAACC-CCCCC-------------AACCCCCCCAAAACTCGTGAGATCCTTAATATACC---------TGCAAAC--CCCCCCGGAAGCAGG---------AAGAATCCTAAGAA
Hame   -------AACCC-CCCCA------------AACCCCCCCAAAACTACTGAGATATC-TAAGATTTC--------TGTGCAC--CCCCCCGGACACAG----------CG--AAATCCCAAA
Chso   -ATAAATCTTTGCCCCCC---------TTCCCCCCGCCCCAACTCACTATTAAATTCATCACCC-----GTCTCTAAAACAACTA--TTTTCCTAAG-------TTAAGCCCCCGTTTG
Lyto   -------AAAAC-CCCCC------------TACCCCCCTCAACTCGAGAGATCAC--TAAGACTCC--------TGAAAAC--CCCC--GGAAACAGG---------AAAACCTCTGGTAG
Encr   -------AAAAC-CCCCC------------TACCCCCCAAAACTCGAGAGATCAC--TAAGACTCC--------TGAAAAC--CCCC--GGAAACAGG---------AAAACCTCTAGTAG
Bvar   -----ACTAAATCCCCCC------------TACCCCCCTTACTTTCCCGACAAGC--CTAACACTA--------AAAATAC--CTTAA--AAAAATGG--------CA--CCAGTTTTGA
Chsp   -GCTACGTCCATCGCCCC-------------ACATTTATC--CTTTAATATA-TTTAAGCTAAATAAT--TAATTAAGACA---CTTAA--ACAGCTACGTCCATCGCCCCACATTTATC
Arja   TTGCG-TTAAAC-CCCCC------------TACCCCCCTAAACTCCAGGGATAAC--TAACACTCC--------TGCAAAC--CCCCCGG-AAACAGG----------ACACCCCCTAGAA
Lifa   ATGAAAACTTAG-CCCCC-----ATTTCTCCCCCCCCCCACCCTTTTTTTTCTACAAGCTACCGGTACCTGTAATGAAACAGATGCTCC-AAACCACCCATTCCAAGGAAGCTCTCAAAA
```

```
                                     CSB-II                                                        CSB-III
Acur    A-------AA--AGACGT-----------AAACCCCCCCC-CCCCCTTTTTAAAT--TT----TTAA--------GG------------------------------------------------
Ampe    -------TAAAC-CCCCC-----------TACCCCCCTAAACTCCTGAGATCAC--TAAGACTCC--------TGAAAAC---CCCCCGGAAACAGG--------TAGACCTCGAGTAG
Enet    GCGCG-TTAAAC-CCCCC-----------TACCCCCCTAAA-CTCCTGAGATCAC-TAACATTCC--------TGAAAAC---CCCCCGGAAACAG---------GAAAACCTCGAG--
Ptbr    G------CTTAC-CCCCC-----------TACCCCCCTT-TTTTTGGGGGGTGGGTT-CTTTTTTATTTAAC--AAGTAGATGCTGGATG-CTTCTAACTAGAGAAAAAAATTTTTTAT
Safa    -------AAATC-CCCCC-----------TACCCCCCTTTACTCCTGAGAT-CG--TATTGAACC--------TGAAAAC--CCCCCCGGAAACAG---------GT--AAACCTCTAG
Asmi    GAAT--TTTTAC-CCCCT-----------TACCCCCTTAAAGCCCCGACTTACC--ATTCAAATCG-------GTACTT----CTCAACTAGTCTA---------TAATTAAAAAAA--
Drze    GCGGGT-TAAAC-CCCCC-----------TACCCCCCTTCACTCCTGAGATGGC--TAACATTCC--------TAAAAAC--CCCCC-GGAAACAG---------GAAAACCTCTAGTG
Rhas    GCGCGT-TAAAC-CCCCC-----------TACCCCCCTAAACTCGTAAGCT-AC--TATTATTCC--------TGAAAAC--CCCCC-GTAAACAGG--------AAAGCCTCGAGCGG
Elac    GCGCGT-TAAAC-CCCCC-----------TACCCCCCTAAACTCGTAGGCT-AG--TATTATTCC--------TGAAAAC--CCCCC-GGAAACAGG--------AAAACCTCGAGTGG
Kugu    -------TTTACCCCCCC-----------TCCCCCCA-CGTTTTTAAACG---T--GATTCCTTTT-------CAAAGT---ATATA-GTATTAAT---------AAAAAACTTAAA--
Plor    CACGT--TAAAACCCCCC-----------TACCCCCCTACTCCCCCGAGATCGC--TTACACTCC--------TGAAAAC--CCCC--GTAAACAG---------GAAAACCTCGAACT
Sgun    GCGCG--TTAAACCCCCC-----------TACCCCCCTACACTCCCGAGATCAC--TATCATTCC--------TGAAAAC--CCCCC-GGAAACAG---------GAAAACCTCGAG--
Zaco    CTGCGCGTAAAACCCCCC-----------TACCCCCCAAAACTCCTGGGATGT--TATTTATTCC--------TGAAAAC--CCCCC-GTAAACAG---------GAAATCCCTTGGAT
Zbfl    GCGCG--TAAAC-CCCCC-----------TACCCCCCAAAACTCCTAAGATCGC--TATTATTCC--------TGAAAAC--CCCCC-GAAAACAG---------GAAAGCCCCTAG--
Spba    TGTTA--GCACT-CCACT-----------TATTACCCCTAAACTTTCCACCCCCT-CCTTTCATTT-------TGGTTT---ATAAACGCGCAAGA---------TAAACAGTTAAAAC
Game    GCGCGT-TAAAC-CCCCC-----------TACCCCCCTAAACTCGTGAGATACC--TAACATTCC--------TGCAAAC---CCCCTATAAACAG---------GAAAATCTCGAG--
Thth    GCGCGT-TAAAC-CCCCC-----------TACCCCCCTAAACTCGTGATATCAT--TAACACTCC--------TGTAAAC---CCCCCGTAAACAGGAA------AA--TCTCGAGTGG
Xigl    GCGT---AAAAC-CCCCC-----------TACCCCCCAAAACTCCAGAGATCAC--TAACACTCC--------TGAAAAC---CCCCCGGAAACAG---------GA--AAACCTCTAG
Hyja    AAACG-TTAAAC-CCCCC-----------TACCCCCCTAAACTCCTGAGATCTC--TAATACTCC--------TGCAAAC----CCCCATAAACAGA---------AAGATCCCG-AAT
Cupa    GCGCG-TTAAAC-CCCCC-----------TACCCCCCTAAACTCCTGAGATCAC--TAACA-TTCC-------TGAAAAC---CCCCCGTAAACAG---------GAAAATCTCAAGTG
Mpch    TCGCG-TTAAAC-CCCCC-----------TACCCCCCTAAACTCCTAAAAGGTA--TAACACTCC--------TGCAAAC---CCCCCGGAAACAG---------GAAACCCCCTAGAA
Char    --GGG-TTAAAC-CCCCC-----------TACCCCCCTAAACTACTGAGATCCT--TAACAATCC--------TGTAAAC---CCCCCGGAAACAGGA--------AA--ACCCCTAG-
Prol    GCGC---AAAACCCCCCC-----------CACCCCCCAAACTCCTA-AGGTTAT--CTATACTCC--------TGAAAAC---CCCCCGGAAACAGG--------AAAACCCCGAAGCA
Calu    T--AAGGTTTAG-CCCCC-----------TACCCCC-GTTACT----TGGGGAG--GGTTAAATCT-------TAAGTG---AGAAAGTATGAAAT---------TATTTTCAAGTTGC
Papa    -GG---CTATAT-CGCCG-----------TACCCCCTAAAACCCCCG--TTCTC--TGAATATGTA-------ACAAAC---ATAT--ACTAAACG---------CACACAATACATCA
Sufr    CTACG-TTATGCCCCCCT-----------TACCCCCCAACACTCCTAGGATCCC--TGACACTCC--------TGCCTGC-----CCCCATAACAGAA-------GTAAAACCCTAA--
Taru    TT-AACGTATACCCCCCC-----------TACCCCCCC---------------TAAAAAAT------ -AGGAGA---GACCTTTAAGTTTG-------------AACCAAGCTCTCCA
Rala    GTGCG-TAAAAC-CCCCC-----------TACCCCCCTAAACTACTGAGATGCC--TAACACTCC--------TGAAAAC--CCCCCG-TAAACAGG--------AACATCCCTAGCAG
```
