# Supplementary material for: Structure and variation of the mitochondrial genome of fishes
Source: BMC Genomics. 2016 Sep 7;17(1):719. doi: 10.1186/s12864-016-3054-y (PMC5015259; doi:10.1186/s12864-016-3054-y)
Supplement: Additional file 16: Figure S5. — Aligned nucleotide sequences of the origin of L-strand replication (blue and magenta letters) in the mt genomes of 250 fishes. (PDF 38 kb) [file 12864_2016_3054_MOESM16_ESM.pdf]

Additional file 16: Figure S5. Aligned nucleotide sequences of the origin of L-strand replication (blue and magenta letters) in mt genomes of 250 fishes.

Species name abbreviation followed by aligned sequences. See Additional file 1 for abbreviation of species name. Blue and magenta letters denote the stem and loop, respectively. Framed rectangle denotes conserved motif sequence. Underline indicates AA stem sequences of the tRNA-Asn gene.

```
             |→                          O_L                       ←|
Scca   ATTG--GAAAGAGGGCGGCGA-----GTTTTA-------------------TTGTTCCGCCCTCTTTC------------GAGGCCC
Muma   AGAT--CCGAAAGAGGGCGG------CGGAATGTTTTT----------------CCGCCCTCTTTCGG--------------GGCCC
Erca   GATT--CGAAGGAGGGCGG-------CCTTCGGTTTTT-----------------CCGCCCTCCTTCG-------------GGGCCA
Pose   GGAT--CCGAAAGGAGGGCGG-----CTTTGAGATTTT----------------CCGCCCTCCTTCGG--------------GGCCG
Actr   TAGA--TGAAGGAGGGCGG-------TCCCCCCTCCT------------------CCGCCCTCTTTCA-------------GGGCCG
Scal   TAGA--TGAAGGAGGGCGG-------TATGCCCTCCCCT----------------CCGCCCTCCTTCA-------------GAGCCG
Posp   TAGA--TGAAGGAGGGCGG-------TCCCTCCTATTT-----------------CCGACCTCCTTCA-------------GGGCCG
Atsp   TGCA--GAAGGAGGGCGGACGG----TTCCGTCCTC----------------CCTTCCGCCCTCCTTC------------GGGGCCG
Leoc   AGAT--GAAGGAGGGCGGA-------CGGTTCCGCCCTTTT--------------TCCGCCCTCCTTC------------GGGGCCG
Amca   GGTG--CTGAAAGAGGGCGG------CAAAGCTGCCGCT---------------CCGCCCTCTTTCGG--------------GGCCG
Osbi   GATG--GAAGGAGGGCGG--------CATTGCCTCACT------------------CCGCCCTCCTTC------------GGGGCCA
Pabu   GTAG--ATAGGAGGGCGG--------CGTCGGATTTT-------------------CCGCCCTCCTAT----------TCGGGGCCG
Hial   ATGA--GAAGGAGGGCGGGG------CCCTCCCCCT------------------CCCCGCCCTCCTTC------------GGGGCCG
Elha   ATTG--GGAGGAGGGCGG--------CGGCCCCTCACT------------------CCGCCCTCCTCC------------GGGGCCG
Mlcy   GGTG--GGGTGGAGGGCGG-------CGGATTTTTACT-----------------CCGCCCTCCATCC------------GGGGCCG
Algl   not found
Ptgi   TTGT--GAAGGAGGGCGG--------CCGAACACCCCCACT---------------CCGCCCTCCTTC------------GGGGCCG
Alaf   ATCA--GAAGGAGGGCGG--------CTTTCCCCTGGTT-----------------CCGCCCTCCTTC------------GGGGCCG
Nock   ATCG--GAAGGAAGGCGG--------CTTTCCCCTGGTT-----------------CCGCCTTCTTTC------------GGGGCCG
Anja   GTGG--ATGGGGGGGGGG--------TTACCCCCTTTC------------------CCCCCCCCCCAT---ATTGATTTCGAGGCCA
Gyki   AGAT--GAAGGGGGCAG---------AGGCCCC-------------------------CTCCCCCTTC------------GAAGCCG
Syka   AGAT--GGAGGAGGGCGG--------CAATTGCCCCCT------------------CCGCCCTCCTCC------------GGGGCCG
Opma   GATG--GAGGGGGCGGAG--------AAGCCCCCTCTCCTC----------CGCCAGAGCCAGCACTC------------TTTCGGG
Comy   AATA--CAGGGGGAGGGG--------CGCCCCCCCCCCT-----------------CCCCTCCCCCTG------------CTGTCCG
Sasp   AAGA--CCGGCGGGCGGGCA------TTTTA-------------ATGCCCGTTCCCGTTCCCCGCCGG-------------ATGCTG
Eupe   TGTT--CTGGCGGGGGGGGCG-----TATTTA--------------CGCCTAACCCGTTCCCCGCCAG-----------TCCCTCCG
Enja   GGAT--GAAAGGGGCGG---------CGGAGGCATTTT-------------------CCGCCCCTTTC------------GGGGCCG
Same   GGAT--GAAGAGGGGCGGCAG-----GCCCCTT---------------------CTCCGCCCCTCTTC------------GGGGCCG
Chch   AGAT--GAAAGGGGCGGA--------ATTAGCCTTGC-------------------TCCGCCCCTTTC------------GGGGCCG
Grgr   GAAA--TGAAGGGGGCGG--------CGCCGGACTCCGGCATCCCCCCT-------CCGCCCCCTTCA-------------GGGCCA
Caau   AGAT--GAAAAGGGCGGCAAA-----TTGAGTC---------------------TTTCCGCCCTTTTC------------GGGGCCG
Cyca   AGAT--GAAAAGGGCGG---------CAAATTGAGTCATT-----------------CCGCCCTTTTC------------GGGGCCG
Dare   AGAT--GAAAAGGGCGG---------CAACTTGGGCCATT-----------------CCGCCCTTTTC------------GGGGCCG
Cost   AGAT--GAAAGGGCGG----------CGAATCGGCTCATT------------------CCGCCCTTTC------------GGGGCCG
Leec   AGAT--GAAAGGGCGG----------CATCGGCCCATT--------------------CCGCCCTTTC------------GGGGCCG
Fola   GATG--GAAAGCGG------------GCAATCGCCTCATT------------------CCGCCCTTTC------------GGGGCCG
Clmc   ATGG--GAAAGGGGCGG---------CATAGTCTCTTT-------------------CCGCCCCTTTC------------GGGGCCG
Phin   AGAT--GAAAGGGGCGG---------CGGGAATTTT---------------------CCGCCCCTTTC------------GGGGCCG
Icpu   AGAT--GAAAGGGGCGGA--------GGGCGGGTTT--------------------TCCGCCCCTTTC------------GGGGCCA
Psto   AGAT--GAAGGGGGCGGAGGAA----AGT-----------------------TTTTTCCGCCCCTTTC------------GGGGCCG
Cora   AGAT--GAAAGGGGCGG---------ACGGACATTT--------------------TCCGCCCCTTTC------------GGGGCCG
Eisp   AGAT--GAAAGGGGCGG---------CGGAATTGTTT--------------------CCGCCCCTTTC------------GGGGCCG
Apal   AGAT--GAAAGGGGCGG---------CGGATTTTTT---------------------CCGCCCCTTTC------------GGGGCCG
Eslu   GGAT--GAAAGGGGGCG---------CGACTCCCCGGT-------------------CGCCCCCTTTC------------GGGGCCG
Dape   GATG--GAAAGGGGGCG---------CGAAGCCCTTT--------------------CGCCCCCTTTC------------GGGGCCG
Glse   AGAT--GAAGAAGGGCGGA-------CGGCCCTCTC-------------------TCCGCCCTTCTTC------------GGGGCCG
Naar   GGAT--GAAGAAGGGCGG--------CGACCCCCCCT-------------------CCGCCCTTCTTC------------GGGGCCG
Lioc   TCAC--CGAAGAAGGGCGGCAG----ATTGCT---------------------CTCCGCCCCTCTTCG-------------AGGCCA
Opso   AGAT--GAAGAAGGGCGG--------CGGTCCCTTCT-------------------CCGCCCTTCTTC------------GGGGCCG
Alte   GATG--GAAAGGGGCGG---------CTTCCCTTGTT--------------------CCGCCCCTTTC------------GGGGCCG
Plap   GATG--GAAAGGGGCGGCAGC-----CCGGCTC---------------------GTTCCGCCCCTTTC------------GGGGCCG
Plal   TAGT--AGGGGCGGCGGC--------CCC----------------------------GCCCCGCCCTT----------TCGGGGCCG
Sami   GGAT--GAGGGGGCGG----------CGGCCCCGCT----------------------CCGCCCTTTC------------GGGGCCG
Rere   GATG--AAGAGGGGCGG---------CGGCC-----------------------CCGCATGCCCTTTT----------TCGGGGCCG
Gama   AGAT--GAGAAGGGCGGCG-------GCCCT---------------------CGCAGCGCCCCTTTTC------------GGGGCCG
Onmy   AGAT--GAAAGGGGGCGGGGG-----CCCCC-----------------CCCCTCGCTCCGCCCCTTTC------------GGGGCCG
Sasa   AGAT--GAAAGGGGGCGGTGG-----CCCCA--------------------CCGCTCCGCCCCCTTTC------------GGGGCCG
```

```
Cola    GGAT--GAAAGGGGGCGGTGG-----CCCCA--------------------CCGCTCCGCCCCCTTTC------------GGGGCCA
Dita    AGAT--GAGAGGGGCGGCG-------GCCC------------------------CGCGCGCCCCTTTC------------GGGGCCG
Sigr    GATG--GGAAGGGGCGGCG-------GCCC------------------------CGCCCGCCTCTTTC------------GGGGCCG
Chsl    AGAG--GAAAGCGGCGGC--------CCGCA---------------------------GCTGCCTTTC------------GGGGCCG
Atja    AGAT--GAAAGGGGGCGGA-------TTGCCCCTGGT------------------TCCGCCCCCTTTC------------GGGGCCA
Iido    AGAT--GAAAGGGGGCGGA-------TTGCCCGCCGGT-----------------TCCGCCCCCTTTC------------GGGGCCA
Auja    AGAT--GAAAGGGGCGG---------CGGACCGCTCGGT------------------CCGCCCCTTTC------------GGGGCCG
Chag    AGAT--GAAAGGGGGCG---------CAATGCCCCGGCGCT----------------CGCCCCCTTTC------------GGGGCCG
Hami    GATA--GAAAGGGGCGG---------CATTCGCCCGTT-------------------CCGCCCCTTTC------------GAGGCCG
Saun    AGAT--CGAAAGGGCGG---------CTATTCTACTTGTT-----------------CCGCCCTTTCG-------------GGGCCG
Nema    GATG--GAAAGGGGGCGGCAGAACG-CCGCC---------------------CGTTCCGCCCCCTTTC------------GGGGCCA
Disp    AGAT--CAGAGGGGGCGG--------TAATCTTTACCGCT----------------CCGCCCCCTCTG--------------GGCCC
Myaf    TGCT--GGGGGGGGGGGGGG------RAWTTTNCCCCYTT-------------CCCCTCCCCCCCCCC------------CCCGCTC
Lagu    GTGG--GAGAGTGTGGGAATGGGGCTAGGGGGTGGGGGGGGGGCGT--TCCCCC----
                                           --ACCCCCCTCCCCCTCCCCCTGTGCCCCATTCCTATAACTTC--GAGGCCA
Trtr    TGTC--CGAAAGGGGGGCGGGG----CGG------------------------CCCCGCTCTTCCCCG------------CATCCCT
Zucr    GGAC--AAAGGGGCGGGG--------CGGCCCCGCTTTT-----------------CCCCGCCCCTTT------------AACCCCC
Pxja    GATG--GAAAGGGGGCGGAC------TATTAT----------------GTCCATTTTCGCCCCCTTTC------------GGGGCCG
Pxlo    GATG--GAAAGGGGGCGGAC------TTTTT------------------GTCCATCTCGCCCCCTTTC------------GGGGCCG
Pctr    GGTG--GGAAGGGGGCGG--------CGGCCCTTTT--------------------CCGCCCCCTTCC------------GGGGGCA
Apsa    GATG--GAAAGGGGGCGGA-------CGGCCCGCTTT------------------TCCGCCCCCTTTC------------GGGGCCG
Cabe    CGTA--GGCGAATGGCGGCGGT----CGAGATGTTTT--------------------ACCGCCGCCCC------------CCCCCCC
Bzze    ATCA--GAAAGGGGCGGCGGG-----TTTCCCGC----------------CCCTCCGCCGCCCCTTTC------------GGGACCG
Siim    GATG--GAAGGGGCGGA---------TAGGAATTGCTGTT------------------TCCGCCCTTC------------GGGGCCG
Ctru    GATT--GAAAGGGGCGGCCAGC----CCGCTC----------------------GCTCCGCCCCTTTC------------GGGGCCA
Dpbr    ATGG--GAAAGGGGCGG---------CAGGCCCGCTTGCT-----------------CCGCCCCTTTC------------GGGGCCA
Caki    TAGA--TGAAAGGGGGCG--------ACTGTCCGTTTTT-----------------CGCCCCCTTTCA-------------GGGCCG
Phja    TAGA--TGAAGGGGCGAA--------CAAATTCCCCCT------------------TTCGCCCCTTCA-------------GGGCCG
Brne    GTCA--GGGTGAAGGGTA--------TTTTG------------------------TACCCATTCACTT------------CACTATC
Gamo    TAGA--TGAAAGGGGGCGA-------CATTGCGCCCCT-----------------TCGCCCCCTTTCA-------------GGGCCG
Lolo    TAGA--TGAAAGGGGGCGA-------CATTGCGCCCCT-----------------TCGCCCCCTTTCA-------------GGGCCG
Batr    not found
Prmy    GGAT--CCGAGGGGGCGGA-------CCCCCCCCCT-------------------TCCGCCCCCTCGG------------GGGGCCC
Lose    AGAT--CGAAAGGGGCGGAA------ATTCCCGCCC------------------TTCCGCCCCTTTCG------------GGGCCCT
Loam    GATG--GAAAGGGGGCGGA-------TTATCCGCCGATT----------------TCCGCCCCCTTTC------------GGGGCCT
Chab    GATG--GAAAGGGCGGAC--------AGATCCG---------------------GTTTCCGCCCTTTC------------GGGGCCG
Chto    GATG--GAAAGGGCGGAC--------AGTCCG----------------------GTTTCCGCCCTTTC------------GGGGCCA
Majo    ATGA--AAGGGAGGGG----------TTCCTC--------------------------CCCCTCCCTT-----CCCTTTCAGGGCCG
Hlst    AAAA--TGCGGACGGCC---------AGCATCTTTCGG-------------------GGCCGTCCGCA----------AATTGGACG
Clpe    ATGT--GAAAGGGGCGGACGG-----CCCGT-----------------------CCTCCGCCCCTTTC------------GGGGCCA
Mlmr    GATG--GAAAGGGGGCGGA-------CAGGCCGCCGTT-----------------TCCGCCCCCTTTC------------GGGGCCA
Crcr    AGAG--GGGGGAGGGCGGA-------TGGGAATTTT-------------------TCCGCCCTGTTTC------------GGATCCG
Muce    AAGG--GGGGGAGGGCGGA-------TAGGATTTT--------------------TCCGCCCTGTTTC------------GGATCCG
Bege    GATT--GAAAGGGGCGGAT-------CGTCTCGTTTT------------------ATCCGCCCCTTTC------------GGGGCCG
Mela    AGAT--GAAAGGGGCGGAC-------GGGCGTGTTTT------------------GTCCGCCCCTTTC------------GGGGCCG
Hats    AGAT--GAAAGGGGGCGGAT------CGCCGCGTTTT-----------------ATCCGCCCCCTTTC------------GGGGCCG
Orla    GATG--GAAAGGGGGCGGA-------CGTCCCGCTTTT-----------------TCCGCCCCCTTTC------------GGGGCCG
Cosa    GATG--GAAAGGGGGCGGAT------CCGCTCGTTTT-----------------ATCCGCCCCCTTTC------------GGGGCCG
Exvo    GATT--GAAAGGGGGCGGAT------CGAAGTGTGTTT----------------ATCCGCCCCCTTTC------------GGGGCCG
Depa    GATG--GAAAGGGGGCGGA-------TCGGCCCGATTTA----------------TCCGCCCCCTTTC------------GGGGCCG
Rima    GATC--GAAGAGGGCGGAT-------CGGCTTCGTTTT-----------------ATCCGCCCTCTTT-----------CGGGGCCG
Fuol    GATT--GAAAAGAGGCGGAT------CGGACTCGTTTT----------------ATCCGCCTCTTTTC------------GGGGCCG
Gmaf    GATT--GAAAGGGAGCGGAT------CGTCGGATTTT-----------------ATCCGCTCCCTTTC------------GGAGCCG
Xeei    GATG--GAAAGGGGGCGGAT------CGGTCGATTTT-----------------ATCCGCCCCCTTTC------------GGGGCCG
Pros    TGTG--GAAAATGGCGGCATC-----CGGCCC----------------------GGTCCGCCATTTTC------------GGGGCCG
Scmi    GATG--GAAAATGGCGGCACC-----CTGCCC----------------------GGTCCGCCATTTTC------------GGGGCCG
Rolo    GATG--GAAAGGGGCGGCGGCC----ATTT------------------------GGTCCGCCCCTTTC------------GGGGCCG
Cere    GATG--GAAGGGGCGGGGATT-----CCGCTC-----------------------GGTCCGCCCCTTC------------GGGGCCG
Daga    GATG--GAAGGGGGCGG---------CGACCCTCGGT--------------------CCGCCCCATTC------------GGGACCG
Anco    GATG--GAAAGGGGCGGCGC------ATCCGGCTCG-------------------GTCCGCCCCTTTC------------GGGGCCG
Dmve    GGTT--CGAGAGGGGCGG--------CGACGCCGCCGGT-----------------CCGCCCCTCTCG-------------GGGCCG
Dmar    ATGG--GAAAGGGGCGGCGCC-----TGCGCC----------------------GGTCCGCCCCTTTC------------GGGGCCG
Anka    GATG--GAAAGGGGCGGCATT-----CCCGCTC---------------------GGTCCGCCCCTTTC------------GGGGCCC
Moja    GATG--GAAAGGGGCGGCGC------ATCCGGCTC-------------------GGTCCGCCCCTTTC------------GGGGCCG
Hoja    GATT--GAAAGGGGCGGCGTT-----CCAGCTT---------------------GGTCCGCCCCTTTC------------GGGGCCG
```

Bede	AGAT--GAAAGGGGCGGCGC------CCCCCT---------------------GCTCCGCCCCTTTC-----------GGGGCCG
Besp	AGAT--GAAAAGGGCGGCGGCC----ATCT-----------------------GGTCCGCCCTTTTC-----------GGGGCCG
Mybe	AGAT--GAAAGGGGGCGCGGAT----AATGTG---------------------GTCCGCCCCCTTTC-----------GGGGCCG
Osja	AGAT--GAAAGGGGGCG---------CAGAGGGGCGGTT------------------CGCCCCCTTTC-----------GGGGCCG
Sgro	AGAT--GAAAGGGGGCG---------CTTCATTCTTGATT-----------------CGCCCCCTTTC-----------GGGGCCG
Pzpa	GGAT--TAGAAAGGGAA---------CACCCTCT-----------------------TTCCCTTTCTA-------------GGCCA
Zeja	GGAT--TAGAAAGGGGGCAG------ACTGCCCC--------------------CTTCCCCCTTTCTA-------------GGCCA
Znne	GGAT--TAGAAAGGGGG---------CATTAGCCCCCTT------------------CCCCCTTTCTA-------------GGCCG
Zefa	GGAT--TAGAAAGGGGG---------CGGACGCCCCTTT------------------CCCCCTTTCTA-------------GGCCG
Acni	ATGA--GAAAGGGGG-----------CGGACGCCCCTTT--------------------CCCCCTTTC-----------AAGGCCA
Ncrh	ATGA--GAAAGGGGG-----------CGGACGCCCCTTT--------------------CCCCCTTTC-----------AAGGCCA
Agca	GATT--GAAAGGGGGCGGAT------TGCAAGTTGGATTT--------------ATCCGCCCCCTTTC-----------GGGGCCG
Hydy	TGAT--GAAAGGGGGCGGA-------ACCGTCCGCATTC-----------------TCCGCCCCCTTTC-----------GGGACCG
Gsac	CGAT--GAAAGGGGCGGAA-------CAGTCCGCATC-------------------TTCCGCCCCTTTC-----------GGGGTCG
Pevo	GTAG--ATGAAAGGGGGCGGAA----CCCCTCCCCCTT--------------TTCCGCCCGCTTTCGT-------------GGCCG
Hiku	AGAT--GAAAGGGGGCGGAA------TTTCCCGCCT-----------------TTCCGCCCCCTTTC-----------GGGGCCG
Inpa	ATGG--GAAGGGGGCGGAA-------GCTTT-------------------------TTTCGCCCCTTTC-----------GGGGCCG
Auch	AGTT--CGGAAGGGGCGG--------CAACCGTCCCT-------------------CCGCCCCTTCCG------------GGGCCA
Fico	AGAT--GAAAGGGGGCGGAAAG----CATCTGTTAT-----------------CTTCCGCCCCCTTTC-----------GGGGCCG
Macs	GATG--GAAAGGGGGCGGA-------AAACCCCCGCCTC-----------------TCCGCCCCCTTTC-----------GGGGCCG
Moal	GGAT--AAAAGAGGCGGA--------GGAATTTTT----------------------TCCGCCTCTTTT-----------GGGCCCG
Syma	AGAT--GAAAGGGGGCGGC-------CGAGGCGGAAATTT----------------GCCGCCCCCTTTC-----------GGGGCCG
Mafa	GATT--GAAAGGGGCGG---------GGGAGGATTTT----------------------CCGCCCCTTTC-----------GGGGCCG
Dcpe	ATCG--GAAGGGGGCGGGGA------CGCCCCCTCCTT----------------TCCCCGCCCCCTTC-----------GGGGCCG
Dcti	GTTG--GAAGGGGGCGGGG-------AAATTTTTCCCCCCGC-------------TCCCGCCCCCTTC-----------GGGGCCG
Hehi	ATTA--GAAAGGGGGCGG--------CATTCCGCCCGTT------------------CCGCCCCCTTTC-----------GGGGCCG
Stam	ATGG--GAAAGGGGGCGGA-------CAATTTTGCGCATC---------------TCCGCCCCCTTTC-----------GGGGCCG
Hogi	GATT--GAAAGGGGGCGGA-------CAGCTTTGTTTT-----------------TCCGCCCCCTTTC-----------GGGGCCG
Erzo	GATG--GAAAGGGGGCGGA-------CAAGGCTGCCAATC---------------TCCGCCCCCTTTC-----------GGGGCCG
Hxot	GATG--GAAAGGGAGCGGA-------ACAGTTTGCCCATC---------------TCCGCTCCCTTTC-----------GGGGTCG
Core	GATG--GAAAGGGAGCGGA-------CAGCATGCTCATT----------------TCCGCTCCCTTTC-----------GGGGTCG
Apve	GATA--GAAAGGGGGCGGA-------CAGCATGTCCATC----------------TCCGCCCCCTTTC-----------GGGGTCG
Ltjp	GATT--GAAAGGGGGCGGA-------TGAGCCCGTTGATT---------------TCCGCCCCCTTTC-----------GGGGCCG
Laja	GATG--GAGAGGGGGCGGA-------TTCCTCGTGGT------------------TCCGCCCCCTTTC-----------GGGGCCG
Syja	GATG--GAAAGGGGCGGA--------TTTTCTCGGT--------------------TCCGCCCCTTTC-----------GGGGCCG
Epme	GATT--GAAAGGGGGCGGGTGAA---TCTAAGTTATT--------------TTCACCCGCCCCCTTTC-----------GGGGCCG
Grse	AGGT--CGAAAGGGGCGGATG-----CGCCCGGCGGTT---------------TATCCGCCCCTTTCG------------GGGCCG
Clja	TGGA--CGAAAGGGAGCGGATCAA--CTCGGATT-----------------TTATCCGCTCCCTTTCG------------GGGCCG
Ogcy	GATG--GAAAGGGGCGGC--------TCCT----------------------------GCCCCCCTTTC-----------GGAGCCG
Plna	GATG--AAGGGGGCGGAT--------TACAATTGGTTTT------------------ATCCGCCCCCTT---------TCGGGGCCG
Lema	GATG--GAAAGGGGGCGGA-------CGGCTCATCGCCTTT--------------TCCGCCCCCTTTC-----------GGGGCCG
Etzo	ATGG--GAAAGGGGGCGGA-------CAGGCCCCTGTTT----------------TCCGCCCCCTTTC-----------GGGGCCG
Apse	GATG--GAAAGGGGCGGA--------CACCGCCGGTTT------------------TCCGCCCCTTTC-----------GGGGCCG
Epde	GATG--GAAAGGGGGCGGA-------CTGCCCCCGGATT----------------TCCGCCCCCTTTC-----------GGGGCCG
Sija	GATG--GAAAGGGGGCGGAT------CGGAATTTTTT----------------ATCCGCCCCCTTTC-----------GGGGTCG
Bsja	GATG--GAAAGGGGGCGGA-------TATTGAATATCCGCC--------------TCCGCCCCCTTTC-----------GGGGCCG
Ecna	AGAT--GAAAGGGGGCGGA-------CATTCATAAATGTT---------------TCCGCCCCCTTTC-----------GGGGCCG
Cohi	ATTG--ATGGGGCGGG----------CGGATTTTTT----------------------CCCGCCCCAT------TTTCCGGAGCCG
Caar	AGAT--GAAAGGGGGCGGAT------TGTGTTTGTATT---------------ATCCGCCCCCTTTC-----------GGGGCCG
Came	AGAT--GAAAGGGGGCGGAT------TGGATTCGTATT---------------ATCCGCCCCCTTTC-----------GGGGCCG
Mema	GATG--GAAAGGGGGCGGAT------CAATAAGTTGATT--------------ATCCGCCCCCTTTC-----------GGGGCCG
Lenu	GATT--GAAAGGGGCGGGT-------CAGAGTTTGTTTA----------------ACCCGCCCCTTTC-----------GGGGCCG
Brja	GGAT--GAGCGGAGCGGAAAGGA---AGTC-------------------TTTTTTCCGCTCCGTTC-----------GGGGCCG
Plma	AGAT--GAAAGGAGCGGAAA------AAGTCTGTTAC----------------TTTCCGCTCCTTTC-----------GGGGCCG
Emst	GATG--GAAAGGGGGCGGA-------CTATCCGCCGATT----------------TCCGCCCCCTTTC-----------GGGGCCG
Ptti	GATG--GAAAGGGGGCGGA-------TTATCTGTCTGATTT------------ATCCGCCCCCTTTC-----------GGGGCCG
Losu	ATAT--GAAGGGGCGGATA-------TGTCTTTTTTT-----------------TATCCGCCCCTTT----------CGGGGCCG
Geoy	ATTG--GAAGGGGGCGGA--------CTTCCGCTCGTT------------------TCCGCCCCCTTC-----------GGGGCCG
Dipi	GATG--GAAAGGGGGCGGA-------TCGTCTCTGTTTT----------------TCCGCCCCCTTTC-----------GGGGCCA
Pama	ATGG--GGAGGGGGCGGGTA------TAACCCCTCCT----------------TACCCGCCCCCTTC-----------GGGGCCG
Leob	GATG--GAAAGGGGGCGGA-------CTATTTAGGCGGT----------------TCCGCCCCCTTTC-----------GGGTCCG
Neba	AAAG--GAAAGGGGCGGGG-------CGTCTCGCCCCT-----------------CCCCGCCCCTTTC-----------GGGGCCG
Pdpl	GGTA--GATGGAAAGCGGGC------CAGAGAAGAGTTTT-------------GCCCGCTTTATATT----------CGGGTCCG
Nimi	AATG--GAAAGGGGGCGGG-------CTTTGCCCCCGCT-----------------CCCGCCCCCTTTC-----------GGGGCCG
Uptr	GATG--GAAAGGGGCGGGGGG-----CAT-----------------------TTTTCCGCCCCCTTTC-----------GGGGCCG

```
Pesc  GATG--GAAAGGGGCGGGGAG-----CCG------------------------CTTTCCGCCCCTTTC------------GGGGCCG
Baar  GATG--GAAAGGGGGCGGAT------TTTTTCCGGT------------------ATCCGCCCCCTTTC------------GGGGCCA
Moar  GATG--GAAAGGGGCGGAT-------TTATTTTTGATT-----------------ATCCGCCCCTTTC------------GGGGCCG
Toja  GATG--GAAAGGGGGCGGAT------TTATCTGTTGATC---------------ATCCGCCCCCTTTC------------GGGGCCG
Chau  GATT--GAAAGGGGGCGGA-------CTGGCCGCTTTT-----------------TCCGCCCCCTTTC------------GGGGCCG
Chse  GATG--GAAAGGGGGCGGA-------TGAATCTGTTGATT---------------TCCGCCCCCTTTC------------GGGGCCG
Enar  GATG--GAAAGGGGGCGGA-------AAATTGTTCCGATC---------------TCCGCCCCCTTTC------------GGGGCCA
Hpty  GATG--GAAAGGGGGCGGA-------CTTTTTTGGT-------------------TCCGCCCCCTTTC------------GGGGCCG
Nana  GATG--GAAAGGGGGCGGG-------CGGTCGCGGATT-----------------CCCGCCCCCTTTC------------GGGGCCG
Mcst  GATG--GAAAGGGGGCGGA-------CGGCCCCCCGCT-----------------TCCGCCCCCTTTC------------GGGGCCG
Rhox  GATG--GAAAGGGGGCGGA-------CCCTAAAGGGTTTA---------------TCCGCCCCCTTTC------------AGGGCCG
Opfa  TTGG--GAAAGGGGGCGGA-------TAGGTCCGCCCGGT---------------TCCGCCCCCTTTC------------GGGGCCG
Paar  GATG--GAAAGGGGCGGAA-------AAGGCCGATCC------------------TTCCGCCCCTTTC------------GGGGCCA
Gozo  AGAT--GAAAGGGGGCG---------CGCCCCCTTTTCGCCCCCTTT----------CGCCCCCTTTC------------GAGGCCA
Ackr  not found
Elev  AGAT--GAAAGGGGGCGGA-------CTCCCGCCCCT------------------TCCGCCCCCTTTC------------GGAGCCG
Trdu  ATTG--GAAAGGGGGCGGG-------CGGAGATTTT-------------------CCCGCCCCCTTTC------------GGGGCCG
Amoc  GATG--GAAAGGGGGCGGA-------CGGTTTTTT--------------------TCCGCCCCCTTTC------------GGGGCCA
Hame  GATG--AGGAATGGGGCGGAA-----CCGCCCGTT------------------TTCCGCCCCATTCCT---------TTCGGGGCCG
Chso  GATG--GAAAGGGGGCGGGC------CAATCATTGGCCCGCTT-----------GCCCGCCCCCTTTC------------GGGGCCG
Lyto  GGAT--GAGGGGGGGCGGA-------ACAGTCGGAAATC----------------TCCGCCCCCCTTC------------GGGATCG
Encr  GGAT--GAAAGGGGGCGGAA------CAGTTCGTAAATC---------------TTCCGCCCCCTTTC------------GGGATCG
Bvar  GATT--GGGGGGGGGGGAAA------AAAACCCCCC------------------TTTCCCCCCCCCCT----------CCTGGGCCG
Noco  ATGG--AAAGGGGGCGGA--------CAACCCGCCCT-------------------TCCGCCCCCTTT------------GGGGCCA
Chsp  AGAT--AAAAGGGGCGGGAG------ATCGG-----------------------TTTCCGCCCCTTTT------------GGGGCCA
Arja  GATG--GAAAGGGGGCGGA-------CAGCTTTGCCCATC---------------TCCGCCCCCTTTC------------GGGGTCG
Pase  ATGG--GGGGCGGAA-----------TTATT----------------------------TTTCGCCCC---AAAAAATTCGGGGCCG
Trel  AAGC--CGGGGGGGGGGGA-------CCCCCCCTT--------------------TCCCCCCCCCCCG----------ATCTGCCCG
Lifa  TGAA--AGGGGGCGGGGGAA------CGCCGCCCCTTT----------------TTCCCCCGCCCTTT-----------CAGAACCG
Acur  ATCG--AAGTGGGGGGGGGGGGG---CCCCCCCCCCACC-----------CCCCCACCCCCCCCATTT-----------CGGAGCCT
Ampe  AGAT--GAAAGGGGCGGA--------TTTTCCCCCGGTT-----------------TCCGCCCCTTTC------------GGGGCCA
Urja  not found
Enet  GTGT--GAAAGGGAGGCGGA------CGCGGAATTT------------------TCCGCCTCCCTTTC------------GGGGCCG
Ptbr  GATT--GAAAGAGGGCGGGG------CGGACT----------------------TTCCGCCCTCTTTC------------GGGGCCG
Safa  GATG--GAAAGGGGGCGGG-------CCGGATTT---------------------TCCGCCCCCTTTC------------GGGGCCG
Icae  ATGG--GAAAGGGGCGGA--------AAAGCCTTTTTCC-----------------TCCGCCCCTTTC------------GGGGCCA
Asmi  TAGA--TGGAAAAGGGGGGGGGG---CCCCCCCCTTT--------------TCCCCCCCCCTTTTCCA-------------GAACCG
Foal  AGAT--TGAAAGGGCGGA--------CAGCTCCCCCT-------------------TCCGCCCTTTCA-------------GGGCCG
Drze  ATGT--GAAAGGGGGCGGG-------AAGGTACCGCCTCCC--------------CCCGCCCCCTTTC------------GGGGCCG
Rhas  TGGA--AAAGGGGGCGGAGAG-----AATCTCTCCGA----------------TTCTCCGCCCCCTTT-----------CGGGGCCA
Elac  GATA--GAAAGGGGGCGGA-------CGGCCCGATTT------------------TCCGCCCCCTTTC------------GGGGCCG
Kugu  GATG--GAAAGGGGGCGG--------CCTTTTTTT---------------------CCGCCCCCTTTC------------GGGGCCG
Plor  GATT--GAAAGGGGGCGGA-------CAGTTTGTTCTGTT---------------TCCGCCCCCTTTC------------GGGGCCG
Sgun  GATG--GAAAGGGGGCGGA-------CTATCCGCGCGGT----------------TCCGCCCCCTTTC------------GGGGCCG
Zaco  GATG--GAAAGGGGGCGGG-------TCATATTCTGATTT---------------CCCGCCCCCTTTC------------GGGGCCG
Zbfl  AGAT--GAAAGGGGGCGGAC------ATATTTTTGGTC----------------GTCCGCCCCCTTTC------------GGGTCCA
Spba  GATG--GAAAGGGGGCGGA-------TTATTTATGATATT---------------TCCGCCCCCTTTC------------GGGGCCG
Game  ATGG--GAAAGGGGCGGA--------AATTTGCCCCC-------------------TCCGCCCCTTTC------------GGGGCCG
Thth  ATTG--GAAAGGGGCGGAA-------AAGTTTTTCC-------------------TTCCGCCCCTTTC------------GGGGCCG
Xigl  GATG--GAAAGAGGGCGGAC------AATTATATTGGCC---------------GTCCGCCCTCTTTC------------GGGGCCG
Hyja  GATG--GAAAGGGGCGGA--------AAATTTTTTTCC------------------TCCGCCCCTTTC------------GGGTCCG
Psan  GATG--GAAGAAGGGCGGAAA-----TCTTTTT--------------------TTTCCGCCCTTCTTC------------GGGCCCG
Cupa  GATG--GAAAGGGGCGGAA-------AAGTTTTTCC-------------------TTCCGCCCCTTTC------------GGGGCCG
Mpch  AGAT--CGAAAGGGGCGGA-------CGGAAATTTT-------------------TCCGCCCCTTTCG-------------GGGCCG
Char  GATG--GAAAGGGGGCGGAT------CGGCTTTTTTT-----------------ATCCGCCCCCTTTC------------GGGGCCG
Pser  ATAG--GAAGGGGGCGGGG-------CAGCCCCCTCCC----------------CCCTCGCCCCCTTC------------GGGGCCG
Prol  AGAT--GAAAGGGGGCGGAT------TGACCCGTCGATT---------------ATCCGCCCCCTTTC------------GGGGCCA
Plbi  AGAT--GAAAAGGGCGGAA-------CAAATCATTGATT----------------TTCCGCCCTTTTC------------GGGGCCG
Calu  GATG--AAGGGGCGGA----------CAGGCCCTCCC----------------------TCCGCCCTT------------GGGGGGG
Papa  not found
Sufr  GATT--GAAAGGGGGCGGATCAA---TTTTGTT-------------------TTATCCGCCCCCTTTC------------GGGGCCG
Stci  AGAT--GAAAGGGGCGGCC-------CCCCCCCCCCACT----------------GGCCGCCCCTTTC------------GGGGCCG
Taru  GATT--GAAAGGGGCGGA--------TCGTTTTCGTTT------------------TCCGCCCCTTTC------------GGGGCCG
Rala  GATG--GAAAGGGGGCGGA-------TTTGATTCTGATC----------------TCCGCCCCCTTTC------------GGGGCCG
```
